# Functional remodeling of gut microbiota and liver in laying hens as affected by fasting and refeeding after fasting

**DOI:** 10.5713/ab.24.0299

**Published:** 2024-10-28

**Authors:** Linjian Weng, Jingyi Zhang, Jianling Peng, Meng Ru, Haiping Liang, Qing Wei, Jiming Ruan, Ramlat Ali, Chao Yin, Jianzhen Huang

**Affiliations:** 1 College of Animal Science and Technology, Jiangxi Agricultural University, Nanchang 330045, China

**Keywords:** Energy Metabolism, Fasting, Gut Microbiota, Laying Hens, Metabolome, Transcriptome

## Abstract

**Objective:**

Animals will experience energy deprivation processes such as moulting, clutching, migration and long-distance transportation under natural survival conditions and in production practices, and the body will trigger a series of adaptive metabolic changes during these processes. Fasting and refeeding after fasting can induce remodeling of nutrients and energy metabolism. This study aims to investigate the mechanisms by which the gut microbiota and liver of poultry respond to energy deprivation under specific conditions.

**Methods:**

Ninety 252-day-old laying hens were randomly divided into 3 groups: (1) fed ad libitum (control group); (2) fasted from day 13 to day 17 (fasting group); (3) fasted from day 1 to day 5, then refed on a specific feeding way (refeeding group). After that, the serum, liver, jejunum tissues, and cecum contents were sampled and sent for metabolome, transcriptome, morphology, and 16S rDNA sequencing analyses, respectively.

**Results:**

Results showed that food deprivation not only observably decreased the body weight, liver index, and the villus height and villus/crypt ratio of jejunum, but also significantly changed the gut microbiota compositions, serum metabolic profiles, and the hepatic gene expression patterns of laying hens, whereas these changes were effectively reversed by the following refeeding operation. At the same time, metabolome combined transcriptome analysis revealed that both serum differential metabolites and hepatic differential expressed genes (DEGs) were consistently enriched in the lipid and amino metabolism pathways, and strong correlations were synchronously found between the differential metabolites and both of the differential gut microbial genera and DEGs, suggesting the crosstalks among gut, liver and their resulting serum metabolic products.

**Conclusion:**

The results suggested that the organism might coordinate to maintain metabolic homeostasis under energy deprivation through a combination of changes in gut microbial composition and hepatic gene expression.

## INTRODUCTION

Energy metabolism is one of the most basic features of animal life, which refers to the release, transfer, storage, and utilization of energy, accompanied by the material metabolism of carbohydrates, lipids, and proteins [1,2]. Animals have evolved to develop unique metabolic response systems in response to starvation, disease, stress, and other complexities that arise from changes in the external environment. Fasting is an energy-depleting process in animal bodies, with the conversion of glucose metabolism to lipid metabolism. Whereas, refeeding after fasting is an energy intake process, which causes a sharp rise in energy and leads to increases in blood glucose and lipid synthesis [3]. For example, Leveille et al [4] found that short periods of fasting could markedly decrease the fat production in chicken liver, while 1 h of refeeding would restore the decreased capacity. Approximately, a growing number of studies have shown that fasting and refeeding after fasting could induce the remodeling of energy metabolism thus leading to an improvement of metabolic levels in humans [5]. Therefore, exploring the mechanisms of energy metabolic remodeling induced by fasting and refeeding after fasting treatments might provide new ideas for us in improving human and animal health.

As a central energy metabolic organ, the liver is mainly responsible for the transference, transportation, and storage of a variety of nutrients recruited from the surrounding peripheral organs [6]. The portal vein, which receives most of the digestive metabolites and the gut microbiota products through the mesenteric veins, provides the major blood supply for the liver [7]. Hence, the crosstalk between the gut and liver, which was first proposed as the “gut-liver axis” by Marshall in 1998, is universally considered to be the vital bridge connecting energy metabolism and animal growth and development [8]. For example, Kong et al [9] found that fungicide thiram exposure not only significantly increased the liver index and lipid parameters in broiler chicks, but also altered their gut microbiota compositions which were positively correlated with the hepatic lipid metabolism-related parameters. However, despite growing evidence confirming that the gut microbiota and liver are strongly associated with energy homeostasis, the underlying mechanisms by which they regulate nutrients and energy metabolism in chickens remain poorly understood.

Multi-omics analysis methods including microbiomics, metabolomics, and transcriptomics have been widely applied to explore the regulatory roles of the gut microbiota and liver in animal metabolism during the last few years [10]. For instance, using an integrated analysis of the microbiome and transcriptome, Wu et al [11] found that supplementation of cholamine could reduce the diversity of the gut microbiota and induce the excess expression of *FABP1* and *APOA4* genes in the liver, leading to hepatic fat deposition and a reduction in egg production in laying hens. Analogously, by using the multi-omics analysis, Chen et al [12,13] proved that the main reason for high-fat diet-induced fat deposition in chickens is gut microbiota dysbiosis and the disturbed expression patterns of hepatic lipid metabolism-related genes. However, the energy metabolism in laying hens under the fasting and refeeding after fasting experimental model via multi-omics analysis has not been reported yet.

In this study, by using the morphology and ultra-high-performance liquid chromate-mass spectrometer/mass spectrometer (UHPLC-MS/MS) analyses, we are first aiming to investigate the metabolic changes of laying hens under the fasting and refeeding after fasting conditions. Meanwhile, based on 16S rDNA sequencing, RNA sequencing (RNA-seq), and multi-omics analysis, the potential regulatory mechanism of the gut microbiota and liver in chicken energy metabolism was explored. The findings of this study will provide us with a better understanding of the crosstalks between different organs in animal bodies and guide our practices in the future poultry industry.

## MATERIALS AND METHODS

### Animal care

All animal experiments were performed following the approval of the Institutional Animal Care and Use Committee (Jiangxi Agricultural University, Nanchang, Jiangxi, China, JXAULL-2024-05-01).

### Animals and experimental design

**Figure 1:**
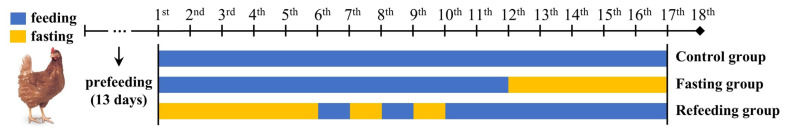
Animal experimental design. Blue squares indicate feeding periods and yellow squares indicate fasting periods.

Ninety 252-day-old Hyline brown hens were raised in the A-type cages at 18°C to 25°C under a lighting schedule of 16L:8D, fed at 8 a.m. and 5 p.m. with free access to drink water. Fasting begins at 8:00 a.m. on the first day and ends at 8:00 a.m. on the second day for one day of fasting, or two meals. After 13 days of acclimatization, chickens were randomly divided into 3 groups with 15 replicates in each group and 2 chickens in each replicate: (1) fed ad libitum (control group); (2) fasted for 5 days from day 13 to day 17 during the experiment (fasting group); (3) fasted for 5 days from day 1 to day 5, then refed ad libitum on day 6 and day 8 (fasted on day 7 and day 9), and fed daily from day 10 to day 17 during the experiment (refeeding group) (Figure 1).

### Sample collection

Sample collections for the control, fasting, and refeeding groups were conducted on day 18 morning, at the end of the experiment. Birds were weighed and then the blood was collected from the wing vein for non-targeted metabolomics analysis (n = 6, two serum samples from each replicate were mixed into one, and six samples were randomly selected from the fifteen replicates), followed by serum separation and then stored at −20°C. After the hens were sacrificed, the liver tissue samples were weighed for calculation of the liver index (the ratio of the liver weight to the body weight, n = 15, randomly selected one hen per replicate), and then stored at −80°C for the subsequent RNA-seq and quantitative real-time (qRT)-polymerase chain reaction (PCR) analyses (n = 4, using the 12 birds from which serum was collected as described above, the liver samples from each 3 birds were mixed into one). The jejunum segments (about 1 cm) were collected and fixed in 4% paraformaldehyde for intestinal morphological analysis (n = 3, three hens randomly selected from the six replicates described above), and the cecum contents were collected and stored at −80°C for the gut microbiota analysis via 16S rDNA sequencing (n = 6, same as the serum sample collection).

### Intestinal morphology

Jejunum tissues, fixed in 4% paraformaldehyde, were embedded in paraffin blocks and cut into 5 μm sections. Afterward, they were dewaxed and stained with hematoxylin and eosin (H&E) at Servicebio Technology Co., Ltd. (Wuhan, China). CaseViewer image viewing software (version 2.4) was used to measure the villus height and crypt depth of the jejunum and calculate the villus/crypt ratio. In each group, 3 sections were taken and scanned under the microscope, and in each tissue section, 3 areas of the field of view where the villi were flat were selected, and the heights of 6 villi and the depths of the corresponding 6 crypts were measured in each area, and the villus/crypt ratio was calculated accordingly.

### 16S rDNA sequencing analysis

The bacterial DNA of the cecum content was extracted using the HiPure stool DNA kits (Magen, Guangzhou, China), and its concentration and purity were detected by Nanorop 2000 (Thermo Fischer Scientific, Waltham, MA, USA). The 16S rRNA gene sequences of bacterial DNA samples were amplified by PCR using specific primers (341 F: CCTACGGGNGGCW GCAG; 806 R: GGACTACHVGGGTATCTAAT) that bind to the hypervariable region V3–V4. The products of the second round of amplification were purified using the AxyPrep DNA Gel Extraction Kit (Axygen Biosciences, Union city, CA, USA), and quantified using the ABI StepOnePlus Real-Time PCR System (Life Technologies, Foster city, CA, USA). Purified amplicons were pooled in equimolar and paired-end sequenced (PE250) on an Illumina platform according to the standard protocols at Gene Denovo Biotechnology Co., Ltd. (Guangzhou, China). Analyses of gut microbiota diversity, LEfSe, species composition, etc. were performed using QIIME (version 1.9.1), Muscle (version 3.8.31), FastTree (version 2.1), and R project packages.

### Non-targeted serum metabolomics analysis

UHPLC-MS/MS analyses were performed using a Vanquish UHPLC system (Thermo Fisher, Bremen, Germany) coupled with an Orbitrap Q ExactiveTM HF-X mass spectrometer (Thermo Fisher) at Gene Denovo Biotechnology Co., Ltd. The raw data files generated by UHPLC-MS/MS were processed using Compound Discoverer 3.1 (Thermo Fisher) to perform peak alignment, peak picking, and quantitation for each metabolite. Peak intensities were normalized to the total spectral intensity, and the normalized data was used to predict the molecular formula based on additive ions, molecular ion peaks, and fragment ions. Peaks were matched with the mzCloud, mzVaultand, and Mass List databases to obtain accurate qualitative and relative quantitative results. Analyses of principal component analysis (PCA), orthogonal projections to latent structures discriminant analysis (OPLS-DA), differential metabolite screening, and Kyoto encyclopedia of genes and genomes (KEGG) enrichment were conducted with R project packages.

### RNA-seq analysis and rna-seq data validation

Total RNA was extracted from liver tissues using the Trizol reagent (Life Technologies). Agarose gel electrophoresis and Nanorop Microspectrophotometer (Thermo Fisher) were used to assess the integrity and purity of total RNA. The mRNAs were separated from the total RNA, interrupted, and reverse-transcribed into the first cDNA. The second strand of cDNA was synthesized and purified using 1.8X Agencourt AMPure XP Beads. Purified cDNA was repaired at the end, and polyA was added and spliced. After fragment screening, PCR library enrichment, and purification, the sequence was performed using the Illumina sequencing platform at Gene Denovo Biotechnology Co., Ltd.. Quality control and screening of the raw sequencing data was performed using fastp (version 0.18.0), and data comparison was performed in Bowtie2 (version 2.2.8). The sequences obtained from double-end sequencing were then aligned to the chicken reference genome (Ensembl_release106) using HISAT (version 2.2.4). Analyses of PCA, differentially expressed genes (DEGs) screening, gene ontology (GO), and KEGG enrichment were carried out utilizing StringTie (version 1.3.1), RSEM (version 1.3.3), DESeq2 (version 1.20.0) and R project packages.

Six DEGs were randomly selected to validate the accuracy of the RNA-seq data by determining their expression levels through qRT-PCR. The total RNA of each sample in the three groups was reverse-transcribed into cDNA carried out on an A200 Gradient Thermal Cycler (LongGene, Hangzhou, China) using the PrimeScript RT Master Mix (TaKaRa, Dalian, China) reaction system: 5×PrimeScript RT Master Mix, 2 μL; total RNA (<500 ng); RNase Free dH_2_O, up to 10 μL, reacting at 37°C for 15 min and then at 85°C for 5 s. The primers for the 6 DEGs were produced by Sangon Biotech (Shanghai, China) (Supplementary Table S1). The qRT-PCR of these DEGs was performed on a StepOnePlus Real-Time PCR System (Thermo Fisher) using the TB Green Premix Ex TaqII (TaKaRa) reaction system: 2×TB Green Premix Ex Taq II, 5 μL; PCR forward primer (10 μM), 0.4 μL; PCR reverse primer (10 μM), 0.4 μL; 50×ROX Reference Dye, 0.2 μL; cDNA, 1 μL; sterilized water, 3 μL. And the reaction was executed in the following program: 95°C for 30 s; 40 cycles of 95°C for 5 s, and 60°C for 30 s. β-actin was selected as the housekeeping gene, and the relative expression levels of these DEGs were represented by the fold change (FC).

### Statistical analysis

The significance of the differences among groups was performed using the statistical software SPSS (version 26.0). One-way analysis of variance (ANOVA) was applied as a comparison of differences between multiple groups. The Tukey honestly significant difference test was used for post hoc comparisons assuming equal variance, and the Games-Howell test was used for post hoc comparisons not assuming equal variance. All data were presented as mean with standard error of mean. p<0.05 was used to indicate significant differences. The statistical results were plotted on the software GraphPad Prism (version 10.1.0).

## RESULTS

### Liver index and intestinal morphological analysis

**Figure 2:**
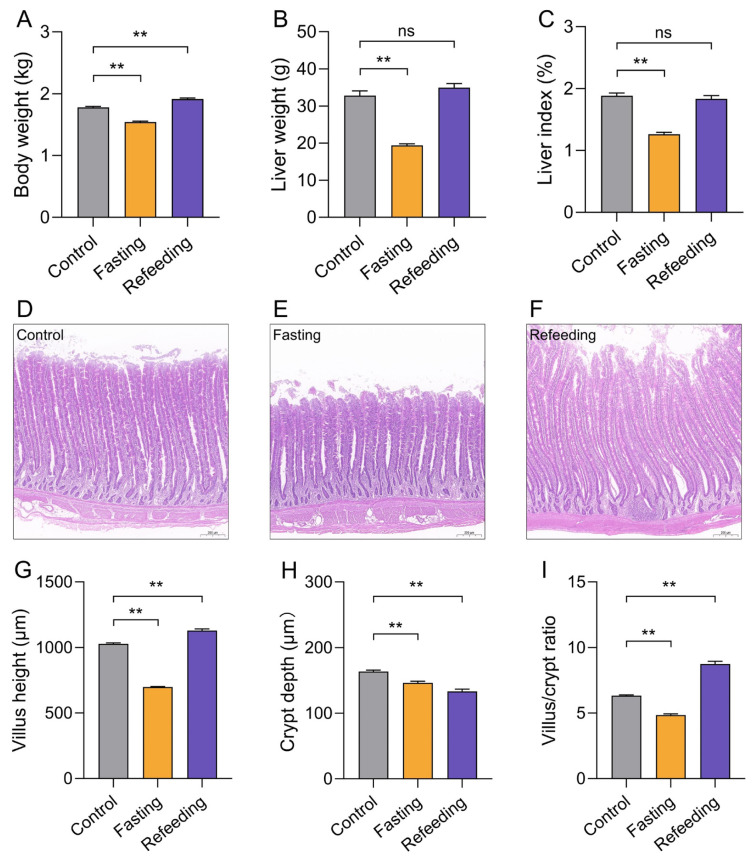
Effect of fasting and refeeding treatment on liver index (n = 15) and intestinal morphology of laying hens (n = 3). (A) Body weigh; (B) liver weight; (C) liver index; (D–F) H&E staining of the jejunum; (G) villus height; (H) crypt depth; (I) villus/crypt ratio. ns p>0.05, ** p<0.01.

As seen in Figure 2, at the end of the fasting treatment, the body weight, liver weight, and liver index of laying hens were considerably (p<0.01) lower than those in the control group, while these changes were remarkably (p<0.01) reversed by the refeeding treatment (Figures 2A–2C). The representative H&E staining sections of the jejunum are shown in Figures 2D–2F. Analogously, the villus height and crypt depth of jejunum were significantly (p<0.01) reduced in the fasting group laying hens, and refeeding treatment repaired the villus height and villus/crypt ratio of jejunum, when compared with the control group (Figures 2G–2I).

### Fasting and refeeding after fasting altered the composition of gut microbiota

**Figure 3:**
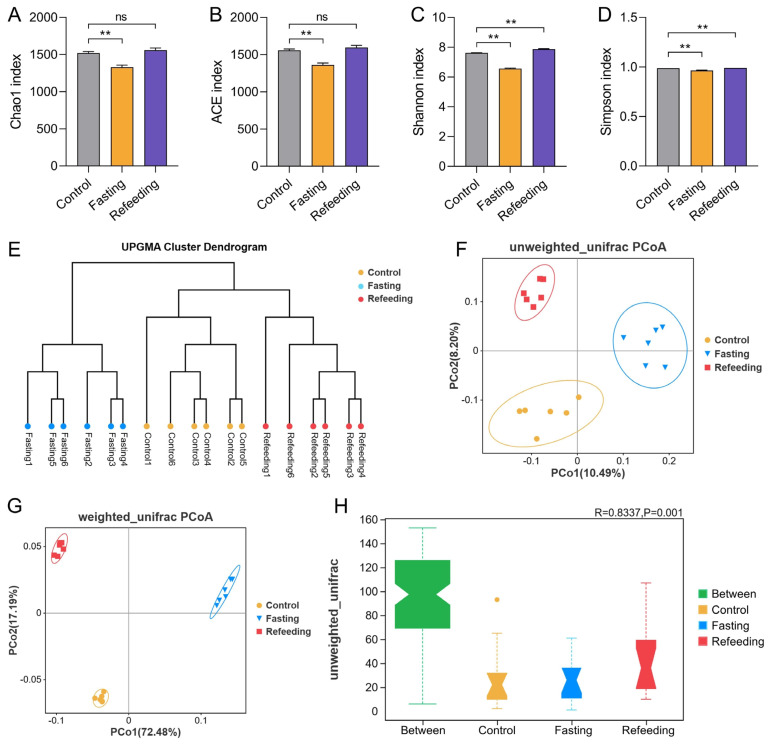
Effect of fasting and refeeding after fasting on diversity of gut microbiota. Alpha diversity indexes of gut microbial flora (n = 6): (A) Chao1; (B) Abundance-based coverage estimator (ACE); (C) Shannon; (D) Simpson. Beta diversity; (E) unweighted pair group method with arithmetic mean (UPGMA) cluster dendrogram according to the bray distance matrix; (F) unweighted unifrac-based and (G) weighted unifrac-based principal coordinates analysis (PCoA) plots; (H) analysis of similarities (Anosim) test based on the unweighted unifrac distance matrix. ns p>0.05, ** p<0.01.

16S rDNA sequencing was performed to determine the effect of fasting and refeeding after fasting treatments on the composition and levels of gut microbiota in chicken cecum contents. Four alpha diversity indexes (Chao1, ACE, Shannon, and Simpson) were applied to assess the richness and evenness of the bacterial community (Figures 3A–3D). The results showed that four indexes were significantly (p<0.01) lower in the fasting group than in the control group, while these decreases were restored after the refeeding treatment. Beta diversity was employed to evaluate the differences in bacterial community composition, including the unweighted pair group method with arithmetic mean (UPGMA) clustering analysis, principal coordinate analysis (PCoA), and the analysis of similarities (Anosim) test. As seen in Figure 3E, the control group was clearly separated from the fasting group, while clustered into the same group with the refeeding samples by the UPGMA clustering analysis (Figure 3E). At the same time, PCoA analysis and the Anosim test also pointed out that there were marked differences in the overall gut microbial flora among the three experimental groups of laying hens (Figures 3F–3H).

**Figure 4:**
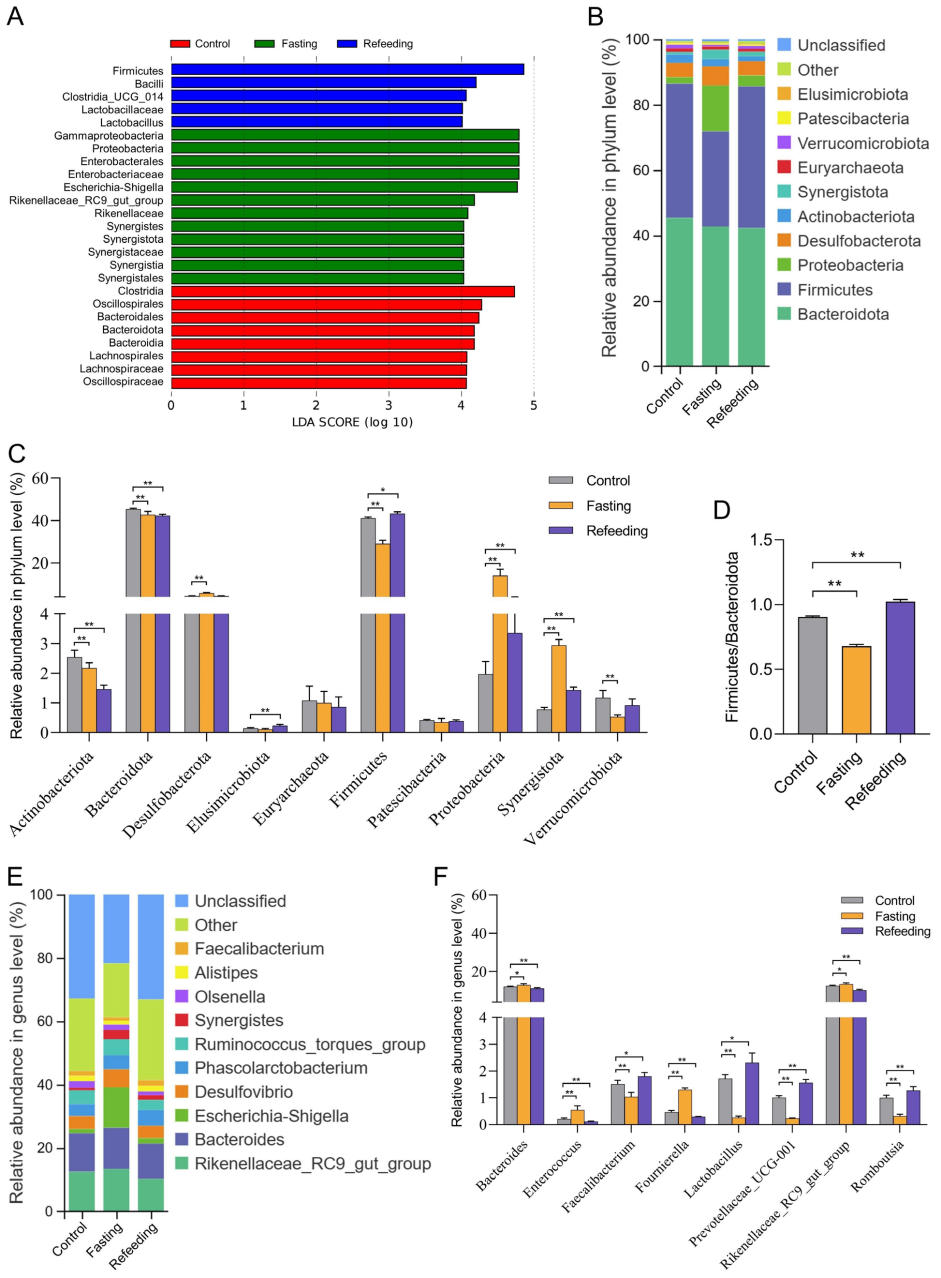
Differences in the taxonomic composition of cecum microbiota (n = 6). (A) Linear discriminant analysis (LDA score>4); (B) relative abundance in phylum level; (C) significant changes in relative abundance at the phylum level; (D) significant changes of Firmicutes/Bacteroidota ratio; (E) relative abundance in genus level; (F) significant changes in relative abundance at the genus level. * p<0.05, ** p<0.01.

Then, the linear discriminant analysis effect size (LEfSe) method was used to analyze the differences in flora between groups and identify the major flora specific in each group. As a result, the Bacteroidota phylum was enriched in the control group, while the Proteobacteria phylum, Synergistota phylum, *Rikenellaceae_RC9_gut_group* genus, *Escherichia-Shigella* genus, and *Synergistes* genus were enriched in the fasting group, and the Firmicutes phylum and *Lactobacillus* genus were enriched in the refeeding group, suggesting that fasting and refeeding after fasting recreated the gut microbial structure of laying hens (Figure 4A). Differences of the gut microbiota compositions in three groups at the phylum and genus levels were also resolved by the taxonomic analysis. At the phylum level, Bacteroidota and Firmicutes were the dominant phyla in the three groups (Figure 4B). Fasting notably (p<0.01) reduced the abundance of Actinobacteriota, Bacteroidota, Firmicutes, and Verrucomicrobiota, but considerably (p<0.01) raised the abundance of Desulfobacterota, Proteobacteria, and Synergistota. In the refeeding group, the abundance of Actinobacteriota and Bacteroidota were lower whereas Eludimicrobiota, Firmicutes, Proteobacteria, and Synergistota were higher than those of the control group (Figure 4C). Meanwhile, the Firmicutes/Bacteroidota (F/B) ratio was significantly (p<0.01) decreased after fasting whereas increased after refeeding treatment, when compared to the control group (Figure 4D). At the genus level, *Rikenellaceae_RC9_gut_group* and *Bacteroides* were the dominant genera in all groups, with an additional *Escherichia-Shigella* in the fasting group (Figure 4E). Moreover, when compared with control group individuals, fasting and refeeding after fasting visibly (p<0.05) remodeled the abundances of each genus, like *Bacteroides*, *Enterococcus*, *Faecalibacterium*, *Fournierella*, *Lactobacillus*, *Megamonas*, *Prevotellaceae_UCG-001*, *Rikenellaceae_RC9_gut_group*, *Romboutsia* and *Ruminococcus_torques_group* (Figure 4F; Supplementary Table S2).

### Fasting and refeeding after fasting changed the serum metabolic profiles

**Figure 5:**
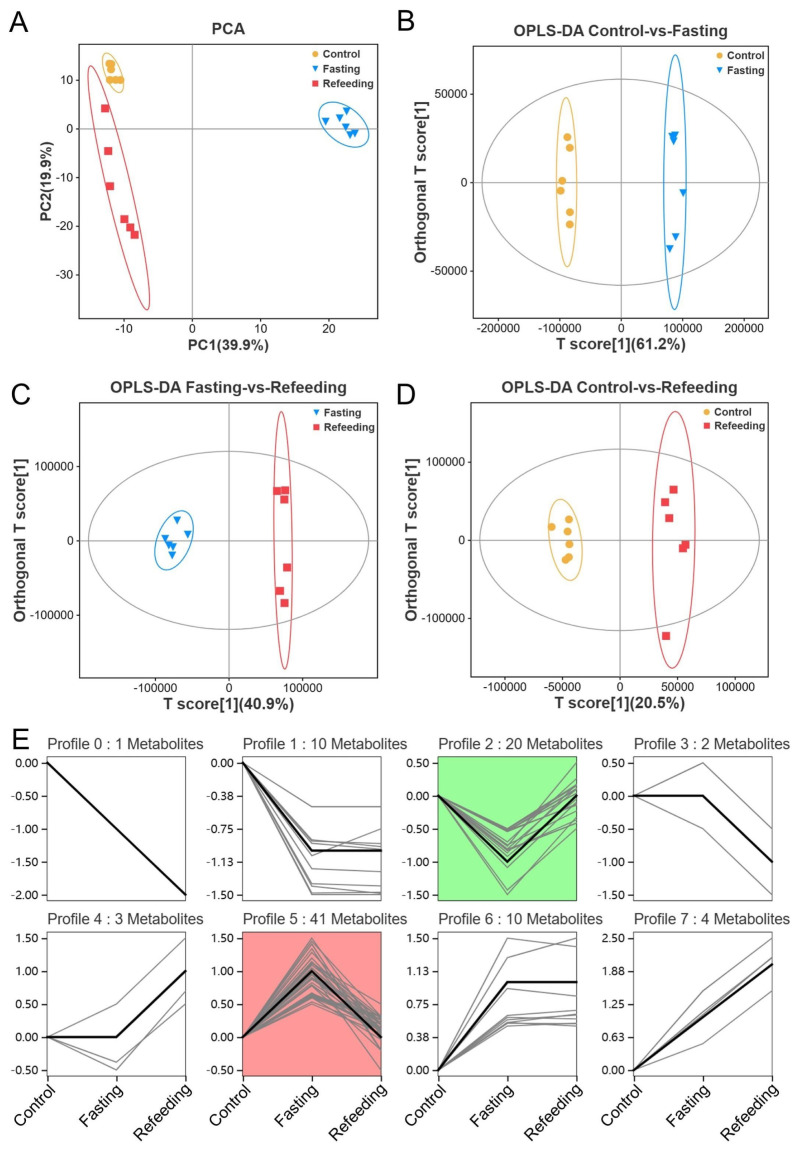
Changes of serum metabolic profiles (n = 6). (A) Principal components analysis (PCA); (B–D) orthogonal partial least squares-discriminant analysis (OPLS-DA); (E) series test of cluster of differential metabolites, green and red modules indicate significant enrichment.

**Figure 6:**
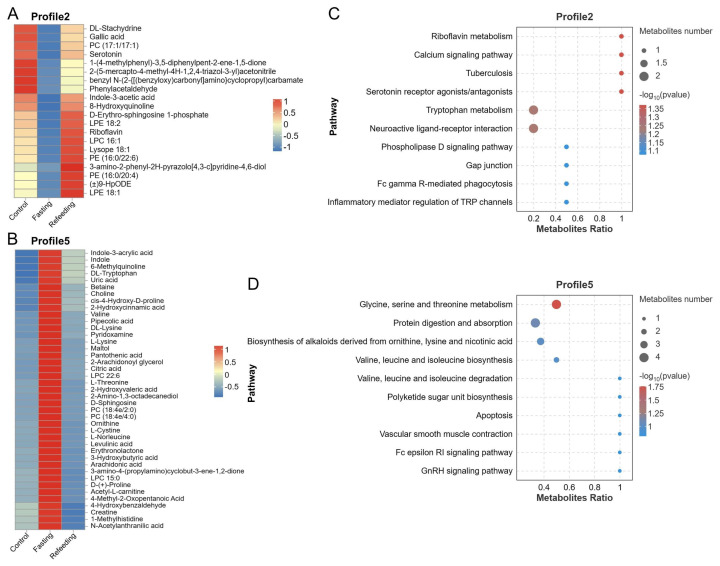
Enrichment analysis of metabolites in profile 2 and profile 5 (n = 6). (A,B) Heatmap of metabolites of profile 2 and profile 5; (C,D) KEGG pathway enrichment analysis of differential metabolites in profile 2 and profile 5. KEGG, Kyoto encyclopedia of genes and genomes.

Non-targeted metabolomic analysis was applied to identify the differential serum metabolites in each group. PCA and OPLS-DA analyses both revealed that the metabolic profiles in fasting and refeeding groups were significantly changed after the experimental treatments (Figures 5A–5D). 91 differential metabolites were filtered at variable importance in projection (VIP≥1, from OPLS-DA), FC upward and downward by 1.5 times and p<0.05. Series tests of clusters were performed to reveal the tendency of the abundances of these differential metabolites. And, the results showed that 20 and 41 differential metabolites were notably (p<0.05) enriched in the trends of profile 2 and profile 5, respectively (Figures 5E, 6A, 6B). They were markedly (p<0.01) altered after fasting but significantly mitigated by the refeeding treatment, including the significantly (p<0.01) decreased metabolites in the fasting group like riboflavin, D-erythro-sphingosine 1-phosphate, serotonin, indole-3-acetic acid, etc, and the increased metabolites like betaine, choline, creatine, L-threonine, L-cystine, etc. Moreover, KEGG pathway enrichment analysis demonstrated that the differential serum metabolites in profile 2 and profile 5 were notably (p<0.05) enriched in pathways including riboflavin metabolism, calcium signaling pathway, tuberculosis, serotonin receptor agonists/antagonists, and glycine, serine and threonine metabolism, respectively (Figures 6C–6D; Supplementary Table S3).

### Differential expression and enrichment analysis of genes in liver

**Figure 7:**
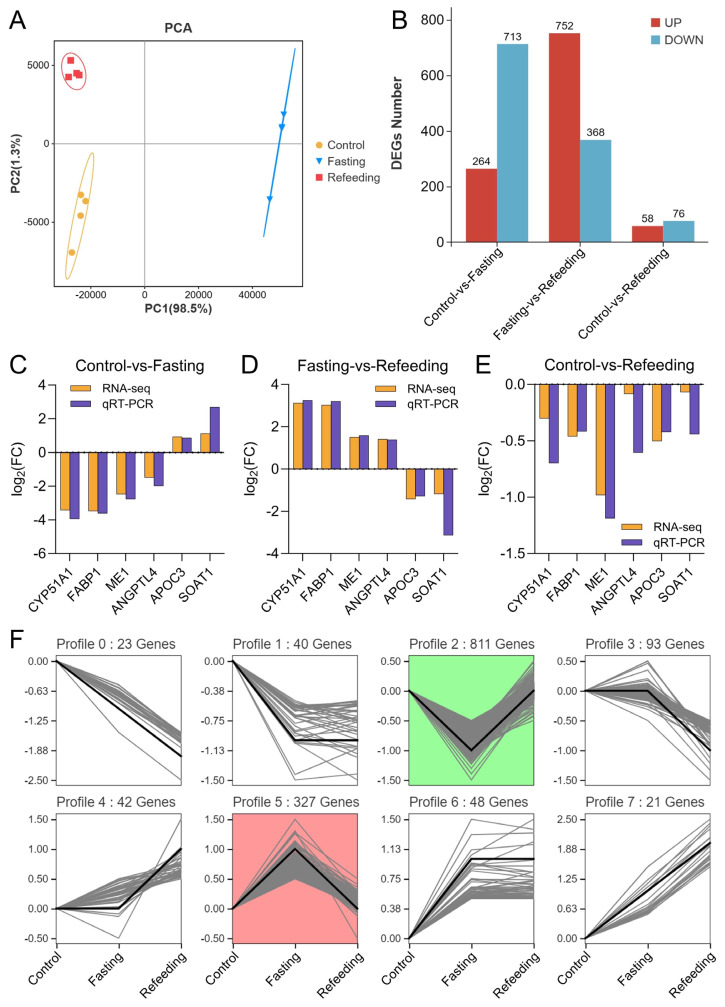
Changes of genes expression in liver (n = 4). (A–C) Log2(FC) of the 6 differentially expressed genes (DEGs) between the three groups; (D) PCA of samples in three groups; (E) number of DEGs; (F) series test of cluster of DEGs. PCA, principal component analysis; FC, fold change.

**Figure 8:**
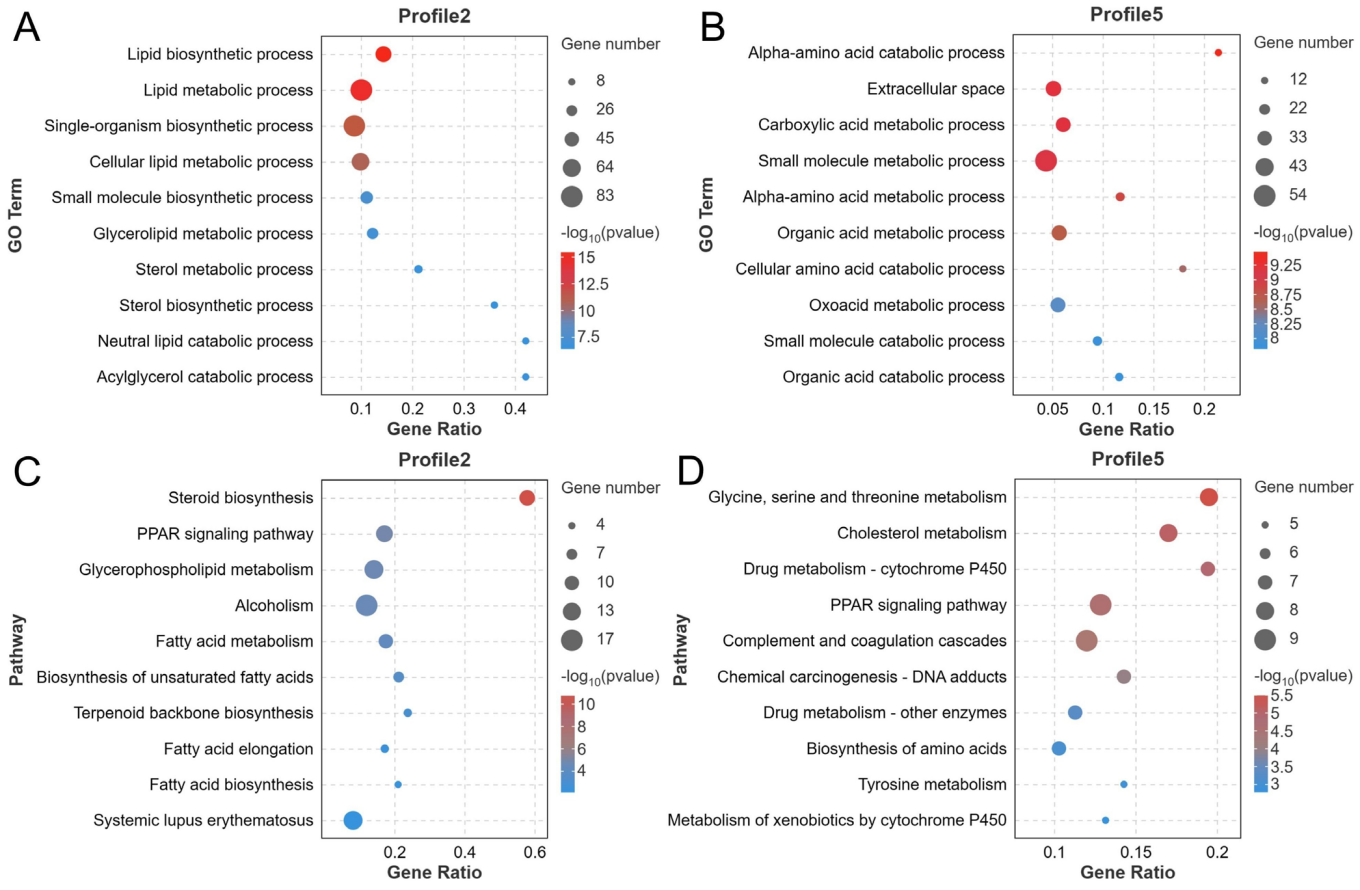
Enrichment analysis of DEGs in profile 2 and profile 5 (n = 4). (A–B) GO enrichment analysis of DEGs in profile 2 and profile 5; (C–D) KEGG pathway enrichment analysis of DEGs in profile 2 and profile 5. DEGs, differentially expressed genes; GO, gene ontology; KEGG, Kyoto encyclopedia of genes and genomes.

DEGs were identified by RNA-seq analysis and screened with a threshold of false discovery rate <0.05 and |log_2_(FC)|≥1. The PCA plot revealed a clear separation of samples between the three groups, indicating the dramatic changes in gene expression caused by fasting and refeeding after fasting operations (Figure 7A). A total of 1,405 DEGs were identified in three experimental groups, of which 977 and 134 DEGs were significantly changed in the fasting and refeeding groups, respectively, when compared with the control group, and 1,120 DEGs were markedly altered in the refeeding group when compared to the fasting group (Figure 7B). The accuracy of RNA-seq data was verified by checking the six randomly selected DEGs (*CYP51A1*, *FABP1*, *ME1*, *ANGPTL4*, *APOC3*, and *SOAT1*) expression levels using the qRT-PCR method (Figures 7C–7E). The series test of the cluster was performed to reveal the changing trends of 1,405 DEGs, and 811 and 327 DEGs were prominently (p<0.05) enriched in profile 2 and profile 5, respectively (Figure 7F; Supplementary Table S4), with the same changing patterns as the metabolic profiles mentioned above. Afterward, DEGs in profile 2 and profile 5 were classified through the GO and KEGG pathway enrichment analyses. The top 10 GO terms in profile 2 and profile 5 are shown in Figures 8A, 8B, the lipid metabolism-related processes like lipid biosynthesis, lipid metabolism, cellular lipid metabolism, glycerolipid metabolism, sterol metabolism, sterol biosynthesis, neutral lipid catabolism, and acylglycerol catabolism were mostly enriched in profile 2, and the processes including alpha-amino acid catabolism, carboxylic acid metabolism, alpha-amino acid metabolism, organic acid metabolism, cellular amino acid catabolism, oxoacid metabolism, and organic acid catabolism, which were largely associated with amino acid metabolism, were significantly enriched in profile 5. The functions of DEGs in profile 2 and profile 5 were further confirmed by KEGG enrichment analysis, which showed that most of the DEGs enriched in the top 10 pathways were closely tied to the lipid metabolism and the amino acid metabolism, respectively (Figures 8C, 8D; Supplementary Table S4).

### Association analysis of serum metabolites, gut microbiota and liver genes

**Figure 9:**
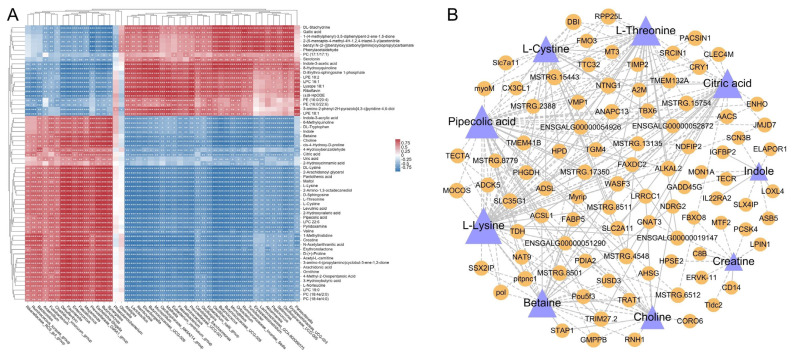
Differential serum metabolites associated with differential gut microbial genera and liver DEGs. (A) Correlation analysis between 61 metabolites (from the profile 2 and profile 5) and 49 differential gut microbial genera was performed using Pearson’s coefficient. Red and blue squares indicate positive and negative correlations between metabolites and genera, separately; (B) correlation analysis between 61 metabolites (from the profile 2 and profile 5) and 1183 DEGs (from the profile 2 and profile 5) was performed using Pearson’s coefficient. Purple triangles indicate metabolites, and yellow circles indicate genes. Positive and negative correlations are represented by solid and dashed lines, separately. DEGs, differentially expressed genes.

The correlation between 61 differential serum metabolites (from profile 2 and profile 5) and 47 differential gut flora was explored using the Pearson correlation analysis. In detail, as shown in Figure 9A, lipids and lipid-like molecules, including D-sphingosine, D-erythro-sphingosine 1-phosphate, acetyl-L-carnitine, 2-hydroxyvaleric acid, arachidonic acid, phosphatidylcholine (PC; 17:1/17:1), PC (18:4e/2:0), PC (18:4e/4:0), phos-phatidylethanolamine (PE; 16:0/20:4), PE (16:0/22:6), lysophosphatidylcholine (LPC) 15:0, LPC 16:1, LPC 22:6, lysophosphatidylethanolamine (LPE) 18:1, LPE 18:2 and lysope 18:1, showed strong correlations with 21 to 46 different genera. Organic acids and derivatives, including betaine, choline, valine, L-threonine, pantothenic acid, 1-methylhistidine, pipecolic acid, cis-4-hydroxy-D-proline, L-lysine, ornithine, creatine, L-cystine, L-norleucine, citric acid, 3-hydroxybutyric acid, 4-methyl-2-oxopentanoic acid and levulinic acid, were significantly (p<0.05) correlated with 42 to 45 genera. The other essential metabolites related to energy metabolism, like serotonin, indole, indole-3-acetic acid, and riboflavin, showed significant correlations with 35 to 43 genera.

Regulatory relationships between the serum metabolites and DEGs in chicken livers were also demonstrated by linking the 61 differential metabolites (from profile 2 and profile 5) and 1138 DEGs (from profile 2 and profile 5) (Supplementary Table S5). As represented in Figure 9B, the correlation network diagram showed us the relationship pairs of 9 amino acid metabolism-related metabolites (choline, L-threonine, creatine, betaine, L-lysine, indole, L-cystine, citric acid, and pipecolic acid) and their significantly correlated DEGs, revealing the highly significant (p<0.05) correlations between the serum metabolite changes and the gene expression alterations in chicken livers.

## DISCUSSION

Nutrients and energy metabolism are a focal point in livestock and poultry rearing, which has drawn much attention from scientific researchers and farmers concerned about animal health and economic interests. The gut microbiota and liver are the essential factors in modulating the nutrients and energy metabolism in animals. Numerous studies have shown that both inoculation with beneficial bacteria and dietary improvements can increase the nutrients and energy utilization of chickens through the gut-liver axis [14]. However, the potential mechanisms of the gut microbiota and liver on the nutrients and energy metabolism of laying hens are still not fully explored. To fill the research gaps on nutrients and energy metabolism in poultry, we utilized the fasting and refeeding after fasting experimental model and the multi-omics method in this study to help us reveal the effects and potential mechanisms of the gut microbiota and liver in regulating the organic metabolism in laying hens. The findings will provide us with new insights into the gut-liver axis-based mechanisms for nutrients and energy metabolism regulation in livestock and poultry production.

The intestine is the most direct site of energy and nutrient exchange between the animal body and the external environment. Previous studies have shown that the small intestine and cecum are two main parts of animal bodies that affect the digestion and absorption of nutrients [15]. On one hand, the small intestinal villus height and crypt depth are strongly correlated with material absorption [16]. For example, numerous studies have shown that an intestine villus height and crypt depth of chickens could be significantly reduced after fasting [17], but restored by the refeeding treatment [18]. On the other hand, the richness, evenness, and composition of the cecum microbiota are closely associated with organic metabolism. Multiple studies have proven that by altering the gut microbial composition, fasting and refeeding could notably affect the animal body’s metabolism [19]. For example, Firmicutes and Bacteroidota are the major bacterial phyla of animal gut microbiota, and the increase of F/B ratio may play an important role in strengthening energy metabolism and promoting the energy intake of the body [20]. At the genus level, changes in genera abundance would also influence the organismal metabolism. As an example, studies in rodents revealed that the abundance of intestinal *Rikenellaceae_RC9_gut_group* was significantly higher in mice fed a high-fat diet than those fed a low-fat diet [21]. While, after the addition of genistein into the maternal high-fat diet, the triglyceride and total cholesterol levels in their offspring rats were observably reduced, accompanied by a significant increase in the *Rikenellaceae_RC9_gut_group* abundance [22]. In the present study, alterations of villus height, F/B ratio, *Rikenellaceae_RC9_gut_group* genera and *Bacteroides* genera caused by fasting and refeeding treatments are consistent with those reported in previous studies. However, inconsistent with the prior research [23], the crypt depth is much less in the refeeding group than in the fasting group, suggesting that fasting may have caused some irreversible changes in the small intestinal structure of laying hens.

Through the bloodstream, the gut microbiota can synchronously interact with the liver and regulate the nutrient intake and energy metabolism of animals [24]. As reported previously, fasting could convert glucose metabolism into lipid and amino acid metabolism and induce extensive changes in the lipid and amino acid abundances in the blood of geese [3]. Zimmerman et al [25] revealed that a gradual increase in plasma concentrations of phenylalanine and lysine would take place in chicks when fasted for 3, 6, 12, or 24 hours. Jo et al [26] discovered that the serum threonine and lysine levels were significantly reduced when the mice were fed a high-fat diet. In humans, compared to the metabolically unhealthy obese populations, the metabolically healthy obese populations show significantly higher serum concentrations of choline, betaine, and D-sphingosine [27]. Similarly, in our study, Pearson’s correlation analysis also revealed strong associations between the gut microbial genera and the serum differential metabolites in laying hens under fasting and refeeding after fasting conditions. Furthermore, these differential metabolites are mostly lipids, organic acids and their derivatives, which are mainly enriched in lipid and amino acid metabolism-related KEGG pathways, such as glycine, serine and threonine metabolism, suggesting the existence of crosstalks between the gut microbiota and liver in chickens.

Nevertheless, the root of the alterations in serum metabolites may be attributed to the changes in the lipid and amino acid metabolism-related gene expressions in chicken livers. For instance, Park et al [28] found that knockout of the *Adh1* gene would increase the ethanol concentrations in the blood of mice, and the hyperuricemia model in mice could be established by knocking out the urate oxidase (*Uox*) gene [29]. In our study, the series test of cluster analysis revealed that most genetic expression in the livers of laying hens was remodeled post the fasting and refeeding experimental treatment, and most of this genetic expression was enriched in the lipid and amino metabolism-related biological processes or pathways including steroid biosynthesis, peroxisome proliferator-activated receptor (PPAR) signaling pathway and glycine, serine and threonine metabolism when using the GO and KEGG enrichment analyses. Among them, the glycine, serine and threonine production-related genes *SARDH*, *DMGDH*, *TDH*, etc. [30–32] in profile 5, and the depletion-related genes *AOC3* and *ALAS1* [33,34] in profile 2 were enriched in the glycine, serine and threonine metabolism pathway. And, the lipid synthesis-related genes *ME1*, *SCD*, *FABP1*, *ACSBG2*, etc. [35,36] in profile 2 and the lipid catabolism-related genes *LPL*, *ACSL1*, *ACAA1*, *APOA1*, etc. [37,38] in profile 5 were enriched in the PPAR signaling pathway, which plays important roles in regulating the hepatic lipid metabolism, inflammatory response and the cell growth and differentiation [39]. Additionally, correlation analysis in this study also presented the strong correlations between the hepatic DEGs and the serum metabolites (Supplementary Table S5), such as the amino acid metabolism-related metabolites and their candidate regulatory genes presented in Figure 9B, demonstrating a high degree of congruence between the changes of chicken liver and blood.

## CONCLUSION

In conclusion, by using the multi-omics approach, we noticed that fasting and refeeding after fasting induced remodeling of jejunal structure, gut microbiota composition, serum metabolic profiles, and hepatic metabolic functions in laying hens, and that differential genus, serum differential metabolites, and hepatic DEGs were all closely related to nutrients and energy metabolism. Therefore, we hypothesized that the body may coordinate to maintain metabolic homeostasis under energy deprivation through changes in gut microbiota composition and hepatic gene expression during post-fasting refeeding.

## SUPPLEMENTARY IMFORMATION

**Supplementary Table S1.** Primer sequences of qRT-PCR

**Supplementary Table S2.** The differential microbiota that were remodeled at the genus level

**Supplementary Table S3.** KEGG pathway enrichment of differential metabolites in profile 2 and profile 5

**Supplementary Table S4.** DEGs in profile 2 and 5 and their enriched KEGG pathways

**Supplementary Table S5.** Crosstalk between 61 differential metabolites and 1138 DEGs by Pearson’s correlation analysis
