# Supplementary material for: Functional remodeling of gut microbiota and liver in laying hens as affected by fasting and refeeding after fasting
Source: Anim Biosci. 2024 Oct 28;38(4):692–706. doi: 10.5713/ab.24.0299 (PMC11917430; doi:10.5713/ab.24.0299)
Supplement: Supplementary file 1 [file ab-24-0299-Supplementary-Table-1.pdf]

Table S1. Primer sequences of qRT-PCR

Gene	Forward primer sequences（5'-3'）	Reverse primer sequences（5'-3'）
CYP51A1	GAACCACCCTCCTCACATCCC	GAACACAGGTCCGTACTTGTCATAG
FABP1	TCAGATCCAGAAGGGTAAGGACATC	GGAGCCAGTAGTCACAGTAATCTTG
ME1	GAGACCTTGGCTGTTATGGAATGG	TCCAACATCACAGGTAGGCACTC
ANGPTL4	CCACACCAACCAGAGCCATAATG	GGAAGACACCGCTGCTTTGC
APOC3	TCCATCCTGCTCCTGCTCATC	CACCTTCTTCACCACCACTTCTG
SOAT1	ATTCCTCAGTTTCGCAACAGTAGTC	ACGCATTAAGCCAGCAGTGAAG
β-Actin	ACCCCAAAGCCAACAGAGAGAAG	TAACACCATCACCAGAGTCCATCAC
