# Supplementary material for: Functional remodeling of gut microbiota and liver in laying hens as affected by fasting and refeeding after fasting
Source: Anim Biosci. 2024 Oct 28;38(4):692–706. doi: 10.5713/ab.24.0299 (PMC11917430; doi:10.5713/ab.24.0299)
Supplement: Supplementary file 2 [file ab-24-0299-Supplementary-Table-2.pdf]

Table S2. The differential microbiota that were remodeled at the genus level.

Genus	C	F	R	p(C_vs_F)	p(C_vs_R)
Rikenellaceae_RC9_gut_group	12.53372	13.3863	10.26205	0.036543	6.73E-06
Bacteroides	12.118	12.95265	11.09522	0.027921	0.007632
Escherichia-Shigella	1.292783	12.8022	1.716033	1.52E-08	0.901661
Desulfovibrio	4.113	5.6929	3.933733	1.58E-08	0.396873
Phascolarctobacterium	3.750067	4.413933	4.941533	5.06E-05	3.71E-08
Ruminococcus_torques_group	4.3742	5.0896	3.249767	0.009398	0.000196
Synergistes	0.77045	2.931933	1.4313	0	1.20E-06
Olsenella	2.10975	1.632467	1.175867	0.00012	3.45E-08
Alistipes	1.6534	1.25695	1.7635	2.50E-06	0.100317
Faecalibacterium	1.514233	1.03205	1.802283	0.000154	0.012225
Lactobacillus	1.713583	0.26345	2.311533	3.88E-08	0.001052
Oscillibacter	1.479467	0.477983	1.438717	3.12E-11	0.74032
Prevotellaceae_UCG-001	0.999617	0.2314	1.560267	3.15E-10	2.57E-08
Romboutsia	0.99425	0.3162	1.26485	1.00E-07	0.002558
Erysipelatoclostridium	1.080517	0.648167	0.7582	1.81E-08	8.59E-07
Akkermansia	1.11455	0.486667	0.806467	1.00E-04	0.031385
Fournierella	0.463683	1.299017	0.291783	0	0.000197
Lachnospiraceae_GCA-900066575	0.697533	0.456917	0.536217	1.07E-06	0.000111
Butyricicoccus	0.735933	0.18255	0.6053	5.30E-13	0.000166
Prevotellaceae_Ga6A1_group	0.167833	0.697833	0.4328	7.86E-11	1.03E-06
Peptococcus	0.493783	0.247417	0.346483	1.05E-06	0.000348
Oscillospiraceae_NK4A214_group	0.317817	0.1849	0.46645	0.000613	0.000206
Oscillospiraceae_UCG-005	0.4378	0.195067	0.334067	7.17E-08	0.001152
Candidatus_Saccharimonas	0.331917	0.213117	0.321267	0.003766	0.934905
Enterococcus	0.208233	0.545167	0.112283	3.30E-05	0.195104
Parasutterella	0.205733	0.123667	0.502267	0.019802	3.51E-08
Megamonas	0.212483	0.362167	0.163133	0.000109	0.174805
Ruminococcaceae_Incertae_Sedis	0.32485	0.098967	0.257033	1.36E-08	0.007252
Turicibacter	0.331133	0.071767	0.261067	1.55E-07	0.04234
Butyricicoccaceae_UCG-008	0.344017	0.035033	0.277733	2.91E-11	0.003417
Eubacterium_hallii_group	0.2296	0.11415	0.246033	0.000664	0.777873
Anaerofilum	0.18355	0.2192	0.1833	0.028145	0.999773
Eisenbergiella	0.243917	0.045433	0.283667	6.20E-08	0.116476
Faecalicoccus	0.134933	0.3382	0.093833	5.40E-07	0.195221
Mailhella	0.17075	0.049417	0.2931	7.44E-07	6.69E-07
Oscillospira	0.18015	0.129883	0.193817	0.005483	0.581323
Butyricicoccaceae_UCG-009	0.186867	0.03385	0.2466	3.63E-07	0.006503
Sellimonas	0.20295	0.095667	0.1561	0.000128	0.063657
Monoglobus	0.176783	0.044517	0.173333	6.72E-06	0.979917
Ruminococcus	0.1284	0.043317	0.177267	0.000192	0.01807
Butyricimonas	0.049417	0.211983	0.070783	2.84E-12	0.030871
Erysipelotrichaceae_UCG-003	0.146333	0.034817	0.087617	1.86E-07	0.000322
Mucispirillum	0.103467	0.033917	0.129417	9.67E-06	0.04419
Fusobacterium	0.0164	0.15675	0.043967	0.004579	0.739273
Oribacterium	0.102917	0.01795	0.093283	1.36E-07	0.506389
Eubacterium_ventriosum_group	0.050433	0.0055	0.10875	8.15E-05	4.16E-06
Clostridium_innocuum_group	0.042633	0.074733	0.03725	0.02974	0.881239

p(F_vs_R)	Domain	Phylum	Class	Order	Family
1.22E-07	Bacteria	Bacteroidc	Bacteroidia	Bacteroida	Rikenellaceae
3.06E-05	Bacteria	Bacteroidc	Bacteroidia	Bacteroida	Bacteroidaceae
2.52E-08	Bacteria	Proteobac	Gammapro	Enterobact	Enterobacteriaceae
3.62E-09	Bacteria	Desulfoba	Desulfovib	Desulfovib	Desulfovibrionaceae
0.000529	Bacteria	Firmicutes	Negativicu	Acidaminc	Acidaminococcaceae
6.70E-07	Bacteria	Firmicutes	Clostridia	Lachnospi	Lachnospiraceae
1.63E-11	Bacteria	Synergisto	Synergistia	Synergista	Synergistaceae
0.00019	Bacteria	Actinobact	Coriobacte	Coriobacte	Atopobiaceae
1.09E-07	Bacteria	Bacteroidc	Bacteroidia	Bacteroida	Rikenellaceae
6.70E-07	Bacteria	Firmicutes	Clostridia	Oscillospir	Ruminococcaceae
3.17E-10	Bacteria	Firmicutes	Bacilli	Lactobacill	Lactobacillaceae
5.53E-11	Bacteria	Firmicutes	Clostridia	Oscillospir	Oscillospiraceae
0	Bacteria	Bacteroidc	Bacteroidia	Bacteroida	Prevotellaceae
1.01E-09	Bacteria	Firmicutes	Clostridia	Peptostrep	Peptostreptococcaceae
0.02434	Bacteria	Firmicutes	Bacilli	Erysipelotr	Erysipelatoclostridiaceae
0.025429	Bacteria	Verrucomi	Verrucomi	Verrucomi	Akkermansiaceae
0	Bacteria	Firmicutes	Clostridia	Oscillospir	Ruminococcaceae
0.032775	Bacteria	Firmicutes	Clostridia	Lachnospi	Lachnospiraceae
4.57E-11	Bacteria	Firmicutes	Clostridia	Oscillospir	Butyricicoccaceae
1.02E-06	Bacteria	Bacteroidc	Bacteroidia	Bacteroida	Prevotellaceae
0.009525	Bacteria	Firmicutes	Clostridia	Peptococc	Peptococcaceae
1.07E-07	Bacteria	Firmicutes	Clostridia	Oscillospir	Oscillospiraceae
6.43E-05	Bacteria	Firmicutes	Clostridia	Oscillospir	Oscillospiraceae
0.007598	Bacteria	Patescibac	Saccharim	Saccharim	Saccharimonadaceae
1.71E-06	Bacteria	Firmicutes	Bacilli	Lactobacill	Enterococcaceae
1.22E-09	Bacteria	Proteobac	Gammapro	Burkholde	Sutterellaceae
4.32E-06	Bacteria	Firmicutes	Negativicu	Veillonella	Selenomonadaceae
1.45E-06	Bacteria	Firmicutes	Clostridia	Oscillospir	Ruminococcaceae
7.97E-06	Bacteria	Firmicutes	Bacilli	Erysipelotr	Erysipelotrichaceae
9.09E-10	Bacteria	Firmicutes	Clostridia	Oscillospir	Butyricicoccaceae
0.000182	Bacteria	Firmicutes	Clostridia	Lachnospi	Lachnospiraceae
0.027052	Bacteria	Firmicutes	Clostridia	Oscillospir	Ruminococcaceae
5.22E-09	Bacteria	Firmicutes	Clostridia	Lachnospi	Lachnospiraceae
4.84E-08	Bacteria	Firmicutes	Bacilli	Erysipelotr	Erysipelotrichaceae
5.19E-11	Bacteria	Desulfoba	Desulfovib	Desulfovib	Desulfovibrionaceae
0.000737	Bacteria	Firmicutes	Clostridia	Oscillospir	Oscillospiraceae
4.41E-09	Bacteria	Firmicutes	Clostridia	Oscillospir	Butyricicoccaceae
0.015979	Bacteria	Firmicutes	Clostridia	Lachnospi	Lachnospiraceae
9.18E-06	Bacteria	Firmicutes	Clostridia	Monoglob	Monoglobaceae
1.06E-06	Bacteria	Firmicutes	Clostridia	Oscillospir	Ruminococcaceae
2.19E-11	Bacteria	Bacteroidc	Bacteroidia	Bacteroida	Marinifilaceae
0.000877	Bacteria	Firmicutes	Bacilli	Erysipelotr	Erysipelatoclostridiaceae
1.87E-07	Bacteria	Deferribac	Deferribac	Deferribac	Deferribacteraceae
0.020384	Bacteria	Fusobacte	Fusobacte	Fusobacte	Fusobacteriaceae
6.45E-07	Bacteria	Firmicutes	Clostridia	Lachnospi	Lachnospiraceae
2.31E-09	Bacteria	Firmicutes	Clostridia	Lachnospi	Lachnospiraceae
0.011506	Bacteria	Firmicutes	Bacilli	Erysipelotr	Erysipelotrichaceae
