# Supplementary material for: Functional remodeling of gut microbiota and liver in laying hens as affected by fasting and refeeding after fasting
Source: Anim Biosci. 2024 Oct 28;38(4):692–706. doi: 10.5713/ab.24.0299 (PMC11917430; doi:10.5713/ab.24.0299)
Supplement: Supplementary file 3 [file ab-24-0299-Supplementary-Table-3.pdf]

**Table S3.** KEGG pathway enrichment of differential metabolites in profile 2 and profile 5.

| Pathway | Pathway ID | Pvalue |
|---|---|---|
| Riboflavin metabolism | ko00740 | 0.042254 |
| Calcium signaling pathway | ko04020 | 0.042254 |
| Tuberculosis | ko05152 | 0.042254 |
| Serotonin receptor agonists/antagonists | ko07211 | 0.042254 |
| Tryptophan metabolism | ko00380 | 0.05772 |
| Neuroactive ligand-receptor interaction | ko04080 | 0.05772 |
| Phospholipase D signaling pathway | ko04072 | 0.083009 |
| Gap junction | ko04540 | 0.083009 |
| Fc gamma R-mediated phagocytosis | ko04666 | 0.083009 |
| Inflammatory mediator regulation of TRP channels | ko04750 | 0.083009 |
| Sphingolipid metabolism | ko00600 | 0.122308 |
| cAMP signaling pathway | ko04024 | 0.122308 |
| Sphingolipid signaling pathway | ko04071 | 0.122308 |
| Synaptic vesicle cycle | ko04721 | 0.122308 |
| Phenylalanine metabolism | ko00360 | 0.196708 |
| Serotonergic synapse | ko04726 | 0.231888 |
| Biosynthesis of phenylpropanoids | ko01061 | 0.265776 |
| Biosynthesis of plant hormones | ko01070 | 0.265776 |
| Taste transduction | ko04742 | 0.298408 |
| Vitamin digestion and absorption | ko04977 | 0.298408 |
| Microbial metabolism in diverse environments | ko01120 | 0.355181 |
| Biosynthesis of alkaloids derived from shikimate pathway | ko01063 | 0.360056 |
| Bile secretion | ko04976 | 0.389144 |
| Metabolic pathways | ko01100 | 0.557474 |
| Biosynthesis of secondary metabolites | ko01110 | 0.634566 |
| ABC transporters | ko02010 | 0.642972 |

| Qvalue | Metabolites |
|---|---|
| 0.215823 | Com_352_pos(Riboflavin) |
| 0.215823 | Com_215_pos(D-Erythro-sphingosine 1-phosphate) |
| 0.215823 | Com_215_pos(D-Erythro-sphingosine 1-phosphate) |
| 0.215823 | Com_203_pos(Serotonin) |
| 0.215823 | Com_471_pos(Indole-3-acetic acid);Com_203_pos(Serotonin) |
| 0.215823 | Com_215_pos(D-Erythro-sphingosine 1-phosphate);Com_203_pos(Serotonin) |
| 0.215823 | Com_215_pos(D-Erythro-sphingosine 1-phosphate) |
| 0.215823 | Com_203_pos(Serotonin) |
| 0.215823 | Com_215_pos(D-Erythro-sphingosine 1-phosphate) |
| 0.215823 | Com_203_pos(Serotonin) |
| 0.227144 | Com_215_pos(D-Erythro-sphingosine 1-phosphate) |
| 0.227144 | Com_203_pos(Serotonin) |
| 0.227144 | Com_215_pos(D-Erythro-sphingosine 1-phosphate) |
| 0.227144 | Com_203_pos(Serotonin) |
| 0.34096 | Com_155_neg(Phenylacetaldehyde) |
| 0.376819 | Com_203_pos(Serotonin) |
| 0.383898 | Com_89_neg(Gallic acid) |
| 0.383898 | Com_471_pos(Indole-3-acetic acid) |
| 0.38793 | Com_203_pos(Serotonin) |
| 0.38793 | Com_352_pos(Riboflavin) |
| 0.425521 | Com_89_neg(Gallic acid);Com_155_neg(Phenylacetaldehyde) |
| 0.425521 | Com_203_pos(Serotonin) |
| 0.439902 | Com_203_pos(Serotonin) |
| 0.60393 | Com_215_pos(D-Erythro-sphingosine 1-phosphate);Com_471_pos(Indole-3-acetic acid);[illegible] |
| 0.642972 | Com_352_pos(Riboflavin);Com_203_pos(Serotonin) |
| 0.642972 | Com_352_pos(Riboflavin) |

Com_352_pos(Riboflavin);Com_203_pos(Serotonin);Com_155_neg(Phenylacetaldehyde)
