# Supplementary material for: Functional remodeling of gut microbiota and liver in laying hens as affected by fasting and refeeding after fasting
Source: Anim Biosci. 2024 Oct 28;38(4):692–706. doi: 10.5713/ab.24.0299 (PMC11917430; doi:10.5713/ab.24.0299)
Supplement: Supplementary file 4 [file ab-24-0299-Supplementary-Table-4.pdf]

Table S4. DEGs in profile 2 and 5 and their enriched KEGG pathways.

id	Symbol	profile
ENSGALG00000000003	PANX2	2
ENSGALG00000000104	CRY1	2
ENSGALG00000000107	TRIM7.1	2
ENSGALG00000000226	TMEM9	2
ENSGALG00000000227	DPYSL2	2
ENSGALG00000000241	STARD4	2
ENSGALG00000000619	ANGPTL4	2
ENSGALG00000000645	Espn	2
ENSGALG00000000695	MFSD4A	2
ENSGALG00000000745	SLC26A9	2
ENSGALG00000000769	RAB7B	2
ENSGALG00000000802	DHODH	2
ENSGALG00000000950	MVB12B	2
ENSGALG00000001101	MBD3	2
ENSGALG00000001475	STMN1	2
ENSGALG00000001492	NDRG3	2
ENSGALG00000001642	GLT8D1	2
ENSGALG00000001749	ACSBG2	2
ENSGALG00000001790	METTL23	2
ENSGALG00000001863	VTG2	2
ENSGALG00000001918	DNAJB5	2
ENSGALG00000002098	GRIK3	2
ENSGALG00000002142	DGKQ	2
ENSGALG00000002249	AGO1	2
ENSGALG00000002362	MANF	2
ENSGALG00000002371	RUSC2	2
ENSGALG00000002437	DIPK1B	2
ENSGALG00000002445	KIAA0319L	2
ENSGALG00000002500	GMPPB	2
ENSGALG00000002519	SLC25A33	2
ENSGALG00000002579	RIMBP2	2
ENSGALG00000002707	CHRNB2	2
ENSGALG00000002742	TMEM132B	2
ENSGALG00000002775	FA2H	2
ENSGALG00000002802	PACSIN1	2
ENSGALG00000002845	CTNNA3	2
ENSGALG00000002899	AACS	2
ENSGALG00000002919	MON1A	2
ENSGALG00000002944	CPS1	2
ENSGALG00000003029	PLPP6	2
ENSGALG00000003081	SUCO	2
ENSGALG00000003099	PCTP	2
ENSGALG00000003103	MST1R	2
ENSGALG00000003147	TRPC4AP	2
ENSGALG00000003427	USP3	2
ENSGALG00000003446	PRLR	2
ENSGALG00000003456	CA12	2
ENSGALG00000003560	SLC6A2	2
ENSGALG00000003568	PPP1R16B	2
ENSGALG00000003575	Dnttip1	2
ENSGALG00000003805	PRKG1	2
ENSGALG00000003948	ALAS1	2
ENSGALG00000004055	C7orf50	2
ENSGALG00000004081	TMCO4	2
ENSGALG00000004106	DHCR7	2
ENSGALG00000004127	--	2

ENSGALG00000004155	MYOM3	2
ENSGALG00000004170	ADA	2
ENSGALG00000004216	TOR3A	2
ENSGALG00000004221	IL22RA1	2
ENSGALG00000004231	IFNLR1	2
ENSGALG00000004249	GRHL3	2
ENSGALG00000004268	NIPAL3	2
ENSGALG00000004282	RCAN3	2
ENSGALG00000004320	FAT2	2
ENSGALG00000004322	AHR	2
ENSGALG00000004341	Cryzl2	2
ENSGALG00000004373	KCNJ16	2
ENSGALG00000004410	ANAPC16	2
ENSGALG00000004424	SEC16B	2
ENSGALG00000004425	SCAMP1	2
ENSGALG00000004483	AHSA2	2
ENSGALG00000004498	SLC2A10	2
ENSGALG00000004505	CCDC137	2
ENSGALG00000004569	UNC5B	2
ENSGALG00000004590	CLCN6	2
ENSGALG00000004612	MTHFR	2
ENSGALG00000004631	DRAXIN	2
ENSGALG00000004657	FBXO2	2
ENSGALG00000004702	DYNC2I2	2
ENSGALG00000004729	SLC7A10	2
ENSGALG00000004782	TSEN15	2
ENSGALG00000004804	TGM3	2
ENSGALG00000004833	P3H1	2
ENSGALG00000004852	DNM1	2
ENSGALG00000004875	PEMT	2
ENSGALG00000004917	DOC2B	2
ENSGALG00000005043	ACACB	2
ENSGALG00000005160	VMP1	2
ENSGALG00000005215	CACNA1H	2
ENSGALG00000005284	LRRC39	2
ENSGALG00000005353	FAR1	2
ENSGALG00000005411	MEIOB	2
ENSGALG00000005418	FRRS1	2
ENSGALG00000005439	ACACA	2
ENSGALG00000005442	PALMD	2
ENSGALG00000005470	PLPPR5	2
ENSGALG00000005553	NLGN3	2
ENSGALG00000005580	TMEM56	2
ENSGALG00000005583	ALG14	2
ENSGALG00000005610	SLC44A3	2
ENSGALG00000005617	NTHL1	2
ENSGALG00000005648	Sesn3	2
ENSGALG00000005657	CRHR2	2
ENSGALG00000005696	ABHD6	2
ENSGALG00000005722	SEC31B	2
ENSGALG00000005739	SCD	2
ENSGALG00000005766	PKD2L1	2
ENSGALG00000005815	TMEM41B	2
ENSGALG00000005831	DNAI1	2
ENSGALG00000005839	ARID3C	2
ENSGALG00000005888	PGP	2
ENSGALG00000006054	CALCA	2
ENSGALG00000006076	RASGEF1C	2

ENSGALG00000006080	GPC4	2
ENSGALG00000006198	LSS	2
ENSGALG00000006217	S100B	2
ENSGALG00000006490	SCN3B	2
ENSGALG00000006521	TRPM5	2
ENSGALG00000006530	TSSC4	2
ENSGALG00000006534	PEX11A	2
ENSGALG00000006647	DUSP8	2
ENSGALG00000006649	TMEM41A	2
ENSGALG00000006662	BPIFB3	2
ENSGALG00000006689	ABHD2	2
ENSGALG00000006702	MFGE8	2
ENSGALG00000006723	IDI1	2
ENSGALG00000006783	PLOD2	2
ENSGALG00000006842	ACOT8	2
ENSGALG00000006872	PISD	2
ENSGALG00000006904	RNH1	2
ENSGALG00000006919	POF1B	2
ENSGALG00000007012	ARRDC4	2
ENSGALG00000007018	SLC26A11	2
ENSGALG00000007030	MFSD13A	2
ENSGALG00000007077	CPT1A	2
ENSGALG00000007109	APOA4	2
ENSGALG00000007127	FADS1	2
ENSGALG00000007178	FADS2	2
ENSGALG00000007191	TK1	2
ENSGALG00000007234	CLCN5	2
ENSGALG00000007242	SLITRK4	2
ENSGALG00000007267	MVD	2
ENSGALG00000007404	YIPF5	2
ENSGALG00000007492	YME1L1	2
ENSGALG00000007493	NSDHL	2
ENSGALG00000007507	MASTL	2
ENSGALG00000007508	HPSE2	2
ENSGALG00000007533	NPEPL1	2
ENSGALG00000007636	PCK1	2
ENSGALG00000007645	prom1a	2
ENSGALG00000007673	LRRC59	2
ENSGALG00000007723	NAT9	2
ENSGALG00000007778	PES1	2
ENSGALG00000007848	PTS	2
ENSGALG00000007955	SLC16A5	2
ENSGALG00000007993	DCX	2
ENSGALG00000008039	MFSD13A	2
ENSGALG00000008150	RASAL1	2
ENSGALG00000008226	NIF3L1	2
ENSGALG00000008262	RASGRF1	2
ENSGALG00000008297	SEMA4B	2
ENSGALG00000008326	SYTL2	2
ENSGALG00000008427	GNAT3	2
ENSGALG00000008434	SORCS3	2
ENSGALG00000008437	NET1	2
ENSGALG00000008462	CDK3	2
ENSGALG00000008539	ALG12	2
ENSGALG00000008601	AHSG	2
ENSGALG00000008604	TMEM255A	2
ENSGALG00000008752	ACOT1	2
ENSGALG00000008763	SSX2IP	2

ENSGALG00000008784	SPATA1	2
ENSGALG00000008795	GPAM	2
ENSGALG00000008850	TTLL7	2
ENSGALG00000008855	UGP2	2
ENSGALG00000008862	DNAJC10	2
ENSGALG00000008866	WDPCP	2
ENSGALG00000008903	ITPRID2	2
ENSGALG00000008930	B3GNT2	2
ENSGALG00000009016	SLX4IP	2
ENSGALG00000009024	CLIP4	2
ENSGALG00000009157	ECT2	2
ENSGALG00000009170	NCEH1	2
ENSGALG00000009312	RPL22L1	2
ENSGALG00000009365	CYP51A1	2
ENSGALG00000009415	SMOC1	2
ENSGALG00000009476	CDK6	2
ENSGALG00000009483	MARK1	2
ENSGALG00000009538	RDH12	2
ENSGALG00000009560	MSMO1	2
ENSGALG00000009680	PAQR7	2
ENSGALG00000009748	ASNS	2
ENSGALG00000009830	MGAT4D	2
ENSGALG00000009842	RASSF3	2
ENSGALG00000010009	TTC29	2
ENSGALG00000010013	EDNRA	2
ENSGALG00000010018	CTSEAL	2
ENSGALG00000010139	SSTR1	2
ENSGALG00000010229	ABCD4	2
ENSGALG00000010233	SYNDIG1L	2
ENSGALG00000010237	NPC2	2
ENSGALG00000010258	LTBP2	2
ENSGALG00000010293	RBP	2
ENSGALG00000010294	RPS6KL1	2
ENSGALG00000010301	EIF2B2	2
ENSGALG00000010391	MMRN1	2
ENSGALG00000010406	TMEM63C	2
ENSGALG00000010494	SLC5A9	2
ENSGALG00000010577	ARHGEF38	2
ENSGALG00000010641	SCCPDH	2
ENSGALG00000010643	ZYG11B	2
ENSGALG00000010703	DGLUCY	2
ENSGALG00000010708	ICA1	2
ENSGALG00000010764	FBXO8	2
ENSGALG00000010798	DHCR24	2
ENSGALG00000010825	AGR2	2
ENSGALG00000010837	ASB5	2
ENSGALG00000010978	ANGPTL3	2
ENSGALG00000011094	PDE4B	2
ENSGALG00000011113	SGIP1	2
ENSGALG00000011141	ITGB6	2
ENSGALG00000011169	PDCD2	2
ENSGALG00000011181	FKBP14	2
ENSGALG00000011254	SATB1	2
ENSGALG00000011319	ADGRL3	2
ENSGALG00000011335	NHEJ1	2
ENSGALG00000011347	IHH	2
ENSGALG00000011350	NEGR1	2
ENSGALG00000011560	PACRG	2

ENSGALG00000011616	NPFFR2	2
ENSGALG00000011657	EAF2	2
ENSGALG00000011808	CCR9	2
ENSGALG00000011809	GRIN2B	2
ENSGALG00000012045	slc12a8	2
ENSGALG00000012089	--	2
ENSGALG00000012106	SCTR	2
ENSGALG00000012111	TMEM37	2
ENSGALG00000012112	DBI	2
ENSGALG00000012185	PLA2G12A	2
ENSGALG00000012186	CASP6	2
ENSGALG00000012196	MCUB	2
ENSGALG00000012220	CDKN3	2
ENSGALG00000012254	KCNJ4	2
ENSGALG00000012322	KCTD16	2
ENSGALG00000012362	THSD7B	2
ENSGALG00000012377	HNMT	2
ENSGALG00000012414	GNPNAT1	2
ENSGALG00000012505	LRFN5	2
ENSGALG00000012586	GKAP1	2
ENSGALG00000012589	C9orf64	2
ENSGALG00000012748	ELOVL2	2
ENSGALG00000012812	SVOPL	2
ENSGALG00000012823	TRIM24	2
ENSGALG00000012847	Slc7a11	2
ENSGALG00000012877	CREB3L2	2
ENSGALG00000012882	KDSR	2
ENSGALG00000012886	--	2
ENSGALG00000012944	DENND5B	2
ENSGALG00000013001	CTNND2	2
ENSGALG00000013036	ATP6V1E1	2
ENSGALG00000013149	MOCOS	2
ENSGALG00000013511	ANKRA2	2
ENSGALG00000013569	SEC61B	2
ENSGALG00000013583	FAM114A1	2
ENSGALG00000013660	ZNF516	2
ENSGALG00000013743	ENPP7	2
ENSGALG00000013828	GALM	2
ENSGALG00000013830	PRELID3A	2
ENSGALG00000013848	MVK	2
ENSGALG00000014126	endou-a	2
ENSGALG00000014189	SULT4A1	2
ENSGALG00000014233	FBLN1	2
ENSGALG00000014261	UCHL1	2
ENSGALG00000014463	ACTN2	2
ENSGALG00000014464	MTR	2
ENSGALG00000014509	BST1	2
ENSGALG00000014516	CPEB2	2
ENSGALG00000014525	USP5	2
ENSGALG00000014581	BORCS8	2
ENSGALG00000014719	SETD9	2
ENSGALG00000014727	PDE4D	2
ENSGALG00000014813	HOMER1	2
ENSGALG00000014834	NCOA7	2
ENSGALG00000014906	MOCS2	2
ENSGALG00000014907	DCBLD1	2
ENSGALG00000014923	ARHGEF28	2
ENSGALG00000014935	GREB1L	2

ENSGALG00000014944	GCNT4	2
ENSGALG00000014948	HMGCR	2
ENSGALG00000014976	GATA6	2
ENSGALG00000015016	SLC22A15	2
ENSGALG00000015044	GTF3C6	2
ENSGALG00000015057	DDO	2
ENSGALG00000015134	APOV1	2
ENSGALG00000015136	ILDR1	2
ENSGALG00000015219	Selenoi	2
ENSGALG00000015234	clcC	2
ENSGALG00000015253	COL8A1	2
ENSGALG00000015263	TMEM30C	2
ENSGALG00000015333	PCGF3	2
ENSGALG00000015468	Tstd3	2
ENSGALG00000015540	RAD23B	2
ENSGALG00000015605	BACH2	2
ENSGALG00000015684	Dnajc25	2
ENSGALG00000015689	ECPAS	2
ENSGALG00000015721	SVEP1	2
ENSGALG00000015728	MUSK	2
ENSGALG00000015729	LPAR1	2
ENSGALG00000015768	ANKRD6	2
ENSGALG00000015795	ADAMTS5	2
ENSGALG00000015849	ME1	2
ENSGALG00000015935	SMYD1	2
ENSGALG00000015937	FABP1	2
ENSGALG00000016254	OTC	2
ENSGALG00000016296	GFRAL	2
ENSGALG00000016309	KLHL31	2
ENSGALG00000016412	MBOAT2	2
ENSGALG00000016415	MAP7D2	2
ENSGALG00000016430	PDHA2	2
ENSGALG00000016444	ODC1	2
ENSGALG00000016456	LPIN1	2
ENSGALG00000016475	Zp2	2
ENSGALG00000016476	TTC32	2
ENSGALG00000016491	APOB	2
ENSGALG00000016492	TDRD15	2
ENSGALG00000016511	ADGRG2	2
ENSGALG00000016560	SELENOI	2
ENSGALG00000016595	TRIM35	2
ENSGALG00000016610	PTRHD1	2
ENSGALG00000016665	FDFT1	2
ENSGALG00000016885	STK24	2
ENSGALG00000016899	DCT	2
ENSGALG00000016979	SLC25A30	2
ENSGALG00000017122	SGCG	2
ENSGALG00000017136	GJB6	2
ENSGALG00000017167	SLC35F2	2
ENSGALG00000017308	CHRDL2	2
ENSGALG00000017378	CRTAC1	2
ENSGALG00000019276	SLCO1C1	2
ENSGALG00000019738	FBXO47	2
ENSGALG00000020342	ABHD12	2
ENSGALG00000020538	SLC49A3	2
ENSGALG00000021039	HKDC1	2
ENSGALG00000021135	HAPLN3	2
ENSGALG00000021193	STARD5	2

ENSGALG00000021238	CYP2W1	2
ENSGALG00000021274	ENTPD8	2
ENSGALG00000021395	ABCA9	2
ENSGALG00000021451	RED3	2
ENSGALG00000021627	IFI27L2	2
ENSGALG00000021658	PAFAH2	2
ENSGALG00000021685	SERINC2	2
ENSGALG00000021686	--	2
ENSGALG00000022720	GJB2	2
ENSGALG00000022758	GGACT	2
ENSGALG00000023083	KIAA1958	2
ENSGALG00000023338	CBX2	2
ENSGALG00000023348	HPDL	2
ENSGALG00000023395	PLIN1	2
ENSGALG00000023517	AGPAT2	2
ENSGALG00000023626	NTN1	2
ENSGALG00000023691	ENTPD7	2
ENSGALG00000023740	HBZ	2
ENSGALG00000023760	CHIA	2
ENSGALG00000024047	MYCL	2
ENSGALG00000024295	MYCBP	2
ENSGALG00000024298	ADAMTSL5	2
ENSGALG00000024449	RAMP2	2
ENSGALG00000025738	RHOU	2
ENSGALG00000025743	CDR2	2
ENSGALG00000025886	SUSD3	2
ENSGALG00000026039	THEM4	2
ENSGALG00000026203	FAM174A	2
ENSGALG00000026214	LRTM2	2
ENSGALG00000026263	RGS8	2
ENSGALG00000026384	PCSK4	2
ENSGALG00000026460	myoM	2
ENSGALG00000026547	TPGS2	2
ENSGALG00000026598	NXNL2	2
ENSGALG00000026607	C15orf40	2
ENSGALG00000026809	SARS	2
ENSGALG00000026846	JMJD7	2
ENSGALG00000026957	SEMA4G	2
ENSGALG00000026973	KIF3C	2
ENSGALG00000027064	HIST1H3H	2
ENSGALG00000027070	TIMP2	2
ENSGALG00000027375	NR2C2AP	2
ENSGALG00000027561	GNG5	2
ENSGALG00000027571	H2B-I	2
ENSGALG00000027608	PIGC	2
ENSGALG00000027960	GRPR	2
ENSGALG00000028005	GADD45G	2
ENSGALG00000028135	--	2
ENSGALG00000028175	GJA9	2
ENSGALG00000028191	GLCE	2
ENSGALG00000028230	SUN2	2
ENSGALG00000028294	Vwa5b2	2
ENSGALG00000028376	FGF19	2
ENSGALG00000028560	OC3	2
ENSGALG00000028709	RNF144A	2
ENSGALG00000028822	RNF152	2
ENSGALG00000028880	FDPS	2
ENSGALG00000028949	CORO6	2

ENSGALG00000029015	TM6SF2	2
ENSGALG00000029033	Tldc2	2
ENSGALG00000029083	NXPH2	2
ENSGALG00000029102	PXYLP1	2
ENSGALG00000029235	CPNE4	2
ENSGALG00000029308	PNPLA3	2
ENSGALG00000029445	FADS6	2
ENSGALG00000029617	COL17A1	2
ENSGALG00000029766	ITGB5	2
ENSGALG00000029788	CCK	2
ENSGALG00000029857	Gimap1	2
ENSGALG00000029898	YKT6	2
ENSGALG00000029947	MMAB	2
ENSGALG00000030076	PCSK6	2
ENSGALG00000030151	LUZP2	2
ENSGALG00000030160	DRC7	2
ENSGALG00000030185	PTDSS1	2
ENSGALG00000030324	ELAPOR2	2
ENSGALG00000030357	ABL2	2
ENSGALG00000030511	SLC19A1	2
ENSGALG00000030661	STAT2	2
ENSGALG00000030801	CCKAR	2
ENSGALG00000030845	ENHO	2
ENSGALG00000030908	ATP2B2	2
ENSGALG00000030941	ELAPOR1	2
ENSGALG00000031067	TMEM132A	2
ENSGALG00000031159	HIST1H110	2
ENSGALG00000031164	WFDC2	2
ENSGALG00000031227	ELP6	2
ENSGALG00000031312	ANAPC13	2
ENSGALG00000031482	Pou5f3	2
ENSGALG00000031525	TSTA3	2
ENSGALG00000031570	WDR54	2
ENSGALG00000031754	KCNG2	2
ENSGALG00000031874	HIST1H101	2
ENSGALG00000031932	AGPAT3	2
ENSGALG00000032170	LCN15	2
ENSGALG00000032287	ATP2A2	2
ENSGALG00000032329	NPM3	2
ENSGALG00000032440	QPCT	2
ENSGALG00000032628	SRCIN1	2
ENSGALG00000032645	H2A-VIII	2
ENSGALG00000032889	RBFOX3	2
ENSGALG00000032975	UBE2QL1	2
ENSGALG00000033051	CAMK1D	2
ENSGALG00000033150	MIDN	2
ENSGALG00000033365	ALDH1A3	2
ENSGALG00000033461	hnmt	2
ENSGALG00000033541	FRMPD4	2
ENSGALG00000033591	Kazald1	2
ENSGALG00000033656	DQX1	2
ENSGALG00000033783	FER1L6	2
ENSGALG00000033867	PCOLCE	2
ENSGALG00000034107	TRIM63	2
ENSGALG00000034288	pclaf	2
ENSGALG00000034289	SLC41A3	2
ENSGALG00000034294	ATP6V0D2	2
ENSGALG00000034504	FAM20C	2

ENSGALG00000034655	ALDH18A1	2
ENSGALG00000034716	HEY2	2
ENSGALG00000034868	KRT7	2
ENSGALG00000034982	CYP11A1	2
ENSGALG00000035058	CAMKV	2
ENSGALG00000035060	FKBP11	2
ENSGALG00000035206	CNPY2	2
ENSGALG00000035239	GLCCI1	2
ENSGALG00000035244	H3-I	2
ENSGALG00000035478	FAM91A1	2
ENSGALG00000035626	DAD1	2
ENSGALG00000035675	--	2
ENSGALG00000035803	THRSP	2
ENSGALG00000035935	Unc13c	2
ENSGALG00000036021	MTMR7	2
ENSGALG00000036117	TENT5B	2
ENSGALG00000036234	RFWD3	2
ENSGALG00000036293	EBAG9	2
ENSGALG00000036492	DAGLA	2
ENSGALG00000036527	SYBU	2
ENSGALG00000036616	NUAK2	2
ENSGALG00000036754	CHKA	2
ENSGALG00000036787	HSD17B12	2
ENSGALG00000036836	SOSTDC1	2
ENSGALG00000036915	SQLE	2
ENSGALG00000037018	USP36	2
ENSGALG00000037050	FABP3	2
ENSGALG00000037065	SC5D	2
ENSGALG00000037253	CLEC4M	2
ENSGALG00000037261	RFXANK	2
ENSGALG00000037322	HIST1H46	2
ENSGALG00000037325	SERP1	2
ENSGALG00000037401	IDH3A	2
ENSGALG00000037467	NEU2	2
ENSGALG00000037479	IGSF21	2
ENSGALG00000037629	TRANK1	2
ENSGALG00000037665	LMBR1L	2
ENSGALG00000037769	NEBL	2
ENSGALG00000037773	ST3GAL1	2
ENSGALG00000037811	NRSN1	2
ENSGALG00000037852	HSD17B7	2
ENSGALG00000037935	RARA	2
ENSGALG00000037998	GLTP	2
ENSGALG00000038145	DPP7	2
ENSGALG00000038292	TMEM258	2
ENSGALG00000038532	--	2
ENSGALG00000038574	MYO15A	2
ENSGALG00000038666	FBXL12	2
ENSGALG00000038723	RPP25L	2
ENSGALG00000039354	VTG1	2
ENSGALG00000039536	C2H8ORF22	2
ENSGALG00000039538	CLDND1	2
ENSGALG00000040070	PDIA2	2
ENSGALG00000040269	SBSPON	2
ENSGALG00000040342	ADAMTS1	2
ENSGALG00000040363	ABHD4	2
ENSGALG00000040447	CDCP1	2
ENSGALG00000040484	SEC31A	2

ENSGALG00000040620	LSAMP	2
ENSGALG00000040730	RXRG	2
ENSGALG00000040857	TECTA	2
ENSGALG00000040896	FASN	2
ENSGALG00000040969	PTP4A3	2
ENSGALG00000041078	MID1IP1	2
ENSGALG00000041143	UMOD	2
ENSGALG00000041205	--	2
ENSGALG00000041238	NOS1AP	2
ENSGALG00000041280	FOSB	2
ENSGALG00000041296	SOX7	2
ENSGALG00000041372	Myrip	2
ENSGALG00000041456	SLC35G1	2
ENSGALG00000041510	DUOX2	2
ENSGALG00000041533	SLC11A2	2
ENSGALG00000041604	NPTXR	2
ENSGALG00000041687	SREBF2	2
ENSGALG00000041988	SIK1	2
ENSGALG00000042080	PGPEP1	2
ENSGALG00000042215	FAAP100	2
ENSGALG00000042275	esg1	2
ENSGALG00000042374	PDE11A	2
ENSGALG00000042491	H4-I	2
ENSGALG00000042511	PKDCC	2
ENSGALG00000042555	STAMBP	2
ENSGALG00000042706	TMC2	2
ENSGALG00000043087	MTF2	2
ENSGALG00000043336	COPZ1	2
ENSGALG00000043435	CARNS1	2
ENSGALG00000043451	DNAL4	2
ENSGALG00000043829	ext1c	2
ENSGALG00000043920	OTUD3	2
ENSGALG00000044251	RASSF7	2
ENSGALG00000044278	C1orf131	2
ENSGALG00000044464	TEPSIN	2
ENSGALG00000045127	slc12a8	2
ENSGALG00000045288	CAMK2N1	2
ENSGALG00000045557	MTTPL	2
ENSGALG00000045814	CRLF2	2
ENSGALG00000046210	SPTBN1	2
ENSGALG00000046412	Aoc3	2
ENSGALG00000046731	--	2
ENSGALG00000046757	ERVK-9	2
ENSGALG00000046789	pol	2
ENSGALG00000047027	ADCK5	2
ENSGALG00000047167	DNAJC30	2
ENSGALG00000047170	pol	2
ENSGALG00000047380	MR1	2
ENSGALG00000047464	FBXL22	2
ENSGALG00000047495	LRRC10	2
ENSGALG00000047687	SETD9	2
ENSGALG00000047720	KLHDC7A	2
ENSGALG00000047792	SELENOM	2
ENSGALG00000048020	STAG3	2
ENSGALG00000048035	GCNT2	2
ENSGALG00000048109	gag	2
ENSGALG00000048205	EBP	2
ENSGALG00000048223	FAM20C	2

ENSGALG00000048285	TCN2	2
ENSGALG00000048432	--	2
ENSGALG00000048599	Arhgap32	2
ENSGALG00000049232	POLR2A	2
ENSGALG00000049408	HIST1H2B8	2
ENSGALG00000049658	UTS2R	2
ENSGALG00000049751	H2B-I	2
ENSGALG00000050069	BMP1	2
ENSGALG00000050083	SYCP2L	2
ENSGALG00000050091	CLEC2B	2
ENSGALG00000050258	--	2
ENSGALG00000050267	CALU	2
ENSGALG00000050309	H2B-I	2
ENSGALG00000050420	CTNND2	2
ENSGALG00000050427	OSBPL10	2
ENSGALG00000050440	APOF	2
ENSGALG00000050491	SLC35E4	2
ENSGALG00000050520	pol	2
ENSGALG00000050668	Spata1	2
ENSGALG00000050676	Ctnnd2	2
ENSGALG00000050815	--	2
ENSGALG00000050857	PTPN20	2
ENSGALG00000050983	PRKG1	2
ENSGALG00000051123	pol	2
ENSGALG00000051159	--	2
ENSGALG00000051205	nicA	2
ENSGALG00000051251	H2B-I	2
ENSGALG00000051290	--	2
ENSGALG00000051325	H3-I	2
ENSGALG00000051398	TMEM14C	2
ENSGALG00000051466	NDFIP2	2
ENSGALG00000051550	ARMH4	2
ENSGALG00000051567	MRPL41	2
ENSGALG00000051779	PRORSD1P	2
ENSGALG00000052072	gag	2
ENSGALG00000052296	MEX3D	2
ENSGALG00000052328	CTNND2	2
ENSGALG00000052395	ERVK-11	2
ENSGALG00000052612	RPS27L	2
ENSGALG00000052768	LDLR	2
ENSGALG00000052786	Znf185	2
ENSGALG00000052797	EIF5A	2
ENSGALG00000052829	MAMDC4	2
ENSGALG00000052887	--	2
ENSGALG00000052986	Vwa5b2	2
ENSGALG00000053013	EPB41L4B	2
ENSGALG00000053043	CARHSP1	2
ENSGALG00000053112	FRMPD2	2
ENSGALG00000053164	gag-pol	2
ENSGALG00000053217	INPP5J	2
ENSGALG00000053245	VTG2	2
ENSGALG00000053262	HIST2H4B	2
ENSGALG00000053281	PKIA	2
ENSGALG00000053446	RED3	2
ENSGALG00000053659	--	2
ENSGALG00000053680	HIST1H46L2	2
ENSGALG00000053886	gag	2
ENSGALG00000053961	Ranbp17	2

ENSGALG00000054252	TM4SF1	2
ENSGALG00000054297	SND1	2
ENSGALG00000054319	ELOVL6	2
ENSGALG00000054546	ERVK-11	2
ENSGALG00000054619	GAB2	2
ENSGALG00000054746	--	2
ENSGALG00000054870	TXNL1	2
ENSGALG00000054926	--	2
ENSGALG00000055000	KCTD14	2
MSTRG.10121	--	2
MSTRG.10162	--	2
MSTRG.10409	--	2
MSTRG.1082	--	2
MSTRG.11802	TMEM221	2
MSTRG.11834	--	2
MSTRG.11955	--	2
MSTRG.12045	--	2
MSTRG.12567	--	2
MSTRG.12570	--	2
MSTRG.12629	--	2
MSTRG.12866	--	2
MSTRG.12923	--	2
MSTRG.13046	--	2
MSTRG.13135	--	2
MSTRG.13261	--	2
MSTRG.13407	--	2
MSTRG.13408	--	2
MSTRG.13439	--	2
MSTRG.13506	CGREF1	2
MSTRG.13526	--	2
MSTRG.13584	--	2
MSTRG.13975	APEX1	2
MSTRG.13977	PIP4P1	2
MSTRG.14201	ZNF541	2
MSTRG.14343	--	2
MSTRG.14577	SLC39A5	2
MSTRG.1468	--	2
MSTRG.14680	--	2
MSTRG.149	--	2
MSTRG.1502	gag	2
MSTRG.1503	gag	2
MSTRG.15162	--	2
MSTRG.15241	--	2
MSTRG.15443	--	2
MSTRG.15444	--	2
MSTRG.15507	--	2
MSTRG.15625	--	2
MSTRG.15732	--	2
MSTRG.15745	--	2
MSTRG.15754	--	2
MSTRG.16287	--	2
MSTRG.163	--	2
MSTRG.16398	Cdhr5	2
MSTRG.16433	B4GALNT4	2
MSTRG.16459	--	2
MSTRG.16504	gag	2
MSTRG.16505	--	2
MSTRG.1679	--	2

MSTRG.16903	--	2
MSTRG.16972	--	2
MSTRG.17021	--	2
MSTRG.17073	--	2
MSTRG.17162	--	2
MSTRG.17163	--	2
MSTRG.17389	DIO3	2
MSTRG.17623	--	2
MSTRG.17628	--	2
MSTRG.17677	--	2
MSTRG.177	--	2
MSTRG.17961	--	2
MSTRG.18132	FBXW4	2
MSTRG.18213	--	2
MSTRG.18216	--	2
MSTRG.1841	--	2
MSTRG.1843	--	2
MSTRG.1845	--	2
MSTRG.18495	UGT1A9	2
MSTRG.18742	LRP2	2
MSTRG.1890	gag	2
MSTRG.18923	--	2
MSTRG.19422	--	2
MSTRG.19463	VTG2	2
MSTRG.19484	--	2
MSTRG.19672	--	2
MSTRG.19854	--	2
MSTRG.20167	--	2
MSTRG.20356	--	2
MSTRG.20480	--	2
MSTRG.20491	--	2
MSTRG.20573	--	2
MSTRG.20656	--	2
MSTRG.21090	--	2
MSTRG.21091	--	2
MSTRG.21092	--	2
MSTRG.21123	--	2
MSTRG.21204	--	2
MSTRG.2126	--	2
MSTRG.21321	gag	2
MSTRG.21371	--	2
MSTRG.21394	gag-pol	2
MSTRG.21420	--	2
MSTRG.21536	--	2
MSTRG.21631	--	2
MSTRG.21653	--	2
MSTRG.2170	MYO16	2
MSTRG.2171	Myo16	2
MSTRG.2174	Irs2	2
MSTRG.21796	--	2
MSTRG.21822	--	2
MSTRG.2305	gag	2
MSTRG.2316	env	2
MSTRG.2387	--	2
MSTRG.2388	--	2
MSTRG.2391	--	2
MSTRG.2393	--	2
MSTRG.2403	--	2

MSTRG.2406	--	2
MSTRG.2409	--	2
MSTRG.2430	--	2
MSTRG.3156	--	2
MSTRG.3262	--	2
MSTRG.3473	--	2
MSTRG.4550	--	2
MSTRG.4608	--	2
MSTRG.4702	--	2
MSTRG.4813	--	2
MSTRG.4905	--	2
MSTRG.5038	--	2
MSTRG.5232	PERCC1	2
MSTRG.53	SND1	2
MSTRG.5319	--	2
MSTRG.6225	--	2
MSTRG.6227	--	2
MSTRG.6228	--	2
MSTRG.6499	--	2
MSTRG.6512	--	2
MSTRG.6546	--	2
MSTRG.6554	--	2
MSTRG.6555	--	2
MSTRG.7483	--	2
MSTRG.7572	--	2
MSTRG.7586	--	2
MSTRG.7811	--	2
MSTRG.8013	--	2
MSTRG.8034	--	2
MSTRG.8128	--	2
MSTRG.8248	--	2
MSTRG.835	--	2
MSTRG.8381	--	2
MSTRG.8471	--	2
MSTRG.8497	--	2
MSTRG.8499	Itpripl1	2
MSTRG.8501	--	2
MSTRG.8619	--	2
MSTRG.8779	--	2
MSTRG.88	--	2
MSTRG.8810	--	2
MSTRG.8874	gag	2
MSTRG.8904	--	2
MSTRG.8954	--	2
MSTRG.8955	--	2
MSTRG.8957	--	2
MSTRG.8985	gag	2
MSTRG.8986	gag	2
MSTRG.9006	--	2
MSTRG.9007	--	2
MSTRG.9165	--	2
MSTRG.9361	Fam110a	2
MSTRG.9410	--	2
MSTRG.9468	--	2
MSTRG.9524	--	2
MSTRG.9645	B3galt6	2
MSTRG.9774	--	2
MSTRG.9775	--	2

MSTRG.9835	--	2
ENSGALG00000000184	SLC27A6	5
ENSGALG00000000293	A2ML1	5
ENSGALG00000000309	Lad1	5
ENSGALG00000000378	SLC25A37	5
ENSGALG00000000498	ACE	5
ENSGALG00000000761	TSKU	5
ENSGALG00000001392	MMP23B	5
ENSGALG00000001531	FN3K	5
ENSGALG00000001565	C5	5
ENSGALG00000001697	ITIH3	5
ENSGALG00000001709	MUSTN1	5
ENSGALG00000001768	TENM2	5
ENSGALG00000002024	COMT	5
ENSGALG00000002116	TEN1	5
ENSGALG00000002431	CFH	5
ENSGALG00000002466	SLC2A5	5
ENSGALG00000002479	MAT1A	5
ENSGALG00000002549	RGS1	5
ENSGALG00000002594	TFPI	5
ENSGALG00000002728	SLC16A3	5
ENSGALG00000002790	ABLIM3	5
ENSGALG00000002855	SARDH	5
ENSGALG00000003015	SERPINF1	5
ENSGALG00000003022	Fmo5	5
ENSGALG00000003136	IKZF2	5
ENSGALG00000003212	TSPO2	5
ENSGALG00000003432	AGXT2	5
ENSGALG00000003537	SGK2	5
ENSGALG00000003553	ABCA12	5
ENSGALG00000003569	TMEM130	5
ENSGALG00000003578	FN1	5
ENSGALG00000003595	SARM1	5
ENSGALG00000003802	OTUD7A	5
ENSGALG00000003972	FAXDC2	5
ENSGALG00000004205	SOAT1	5
ENSGALG00000004230	LIPC	5
ENSGALG00000004343	HPD	5
ENSGALG00000004472	ASTN1	5
ENSGALG00000004491	DMGDH	5
ENSGALG00000004598	CUX2	5
ENSGALG00000004959	IRS1	5
ENSGALG00000005030	DOCK10	5
ENSGALG00000005204	GSTT1	5
ENSGALG00000005259	VIPR1	5
ENSGALG00000005263	SOX8	5
ENSGALG00000005408	BCO1	5
ENSGALG00000005472	NAT	5
ENSGALG00000005474	PNAT10	5
ENSGALG00000005540	MICAL2	5
ENSGALG00000005632	IRAG1	5
ENSGALG00000005776	TECR	5
ENSGALG00000005860	ACAA1	5
ENSGALG00000005977	BTBD8	5
ENSGALG00000006320	Slc2a9	5
ENSGALG00000006341	SLC25A48	5
ENSGALG00000006374	TBX6	5
ENSGALG00000006482	FAH	5

ENSGALG00000006724	GPC5	5
ENSGALG00000006812	TTC36	5
ENSGALG00000006864	COL24A1	5
ENSGALG00000006976	Bdh1	5
ENSGALG00000007014	PYROXD2	5
ENSGALG00000007114	APOA1	5
ENSGALG00000007132	ACOX2	5
ENSGALG00000007252	ANKDD1A	5
ENSGALG00000007478	SLC51A	5
ENSGALG00000007536	PHR	5
ENSGALG00000007710	zgc:110179	5
ENSGALG00000007728	Prodh	5
ENSGALG00000007814	ALPI	5
ENSGALG00000007839	NCAM1	5
ENSGALG00000008185	AOX1	5
ENSGALG00000008728	PTER	5
ENSGALG00000008780	CTBS	5
ENSGALG00000008859	WDR31	5
ENSGALG00000008912	ABCB1	5
ENSGALG00000008953	AASS	5
ENSGALG00000009002	CPED1	5
ENSGALG00000009050	CAPN3	5
ENSGALG00000009100	FSHR	5
ENSGALG00000009172	OSBPL6	5
ENSGALG00000009268	FGG	5
ENSGALG00000009479	SAMD9L	5
ENSGALG00000009545	SLC25A12	5
ENSGALG00000009700	PDK4	5
ENSGALG00000009740	RASGRP1	5
ENSGALG00000009848	LPGAT1	5
ENSGALG00000009880	INPP4B	5
ENSGALG00000009920	COCH	5
ENSGALG00000009926	HAAO	5
ENSGALG00000009947	PLEKHH2	5
ENSGALG00000009963	LYZ	5
ENSGALG00000010163	LGR5	5
ENSGALG00000010357	P2RY1	5
ENSGALG00000010628	ACSL1	5
ENSGALG00000010853	C8B	5
ENSGALG00000010857	DAB1	5
ENSGALG00000010889	HOOK1	5
ENSGALG00000011003	SLC35F3	5
ENSGALG00000011287	SULT	5
ENSGALG00000011314	LRRC3B	5
ENSGALG00000011320	TMCC3	5
ENSGALG00000011331	CTH	5
ENSGALG00000011376	ANKRD9	5
ENSGALG00000011391	AMN	5
ENSGALG00000011469	IGFBP2	5
ENSGALG00000011524	PPEF2	5
ENSGALG00000011571	AGPAT4	5
ENSGALG00000011684	STAP1	5
ENSGALG00000011687	AHNAK2	5
ENSGALG00000011894	CYP2D6	5
ENSGALG00000011957	TOB2	5
ENSGALG00000011994	SYNPO2	5
ENSGALG00000012034	ADSL	5
ENSGALG00000012420	CG-1B	5

ENSGALG00000012683	RNF144B	5
ENSGALG00000012704	MYLIP	5
ENSGALG00000012754	PAH	5
ENSGALG00000012755	IGF-I	5
ENSGALG00000012834	AKR1D1	5
ENSGALG00000013033	cmbl	5
ENSGALG00000013090	LOXL4	5
ENSGALG00000013100	GRB10	5
ENSGALG00000013124	FHOD3	5
ENSGALG00000013244	ABCC9	5
ENSGALG00000013356	IKBKE	5
ENSGALG00000013627	SLC7A2	5
ENSGALG00000013726	PAICS	5
ENSGALG00000013728	PPAT	5
ENSGALG00000013754	PLAGL1	5
ENSGALG00000013776	CEP135	5
ENSGALG00000013969	ALDH8A1	5
ENSGALG00000014128	A4GALT	5
ENSGALG00000014252	A2M	5
ENSGALG00000014412	CSTA	5
ENSGALG00000014616	MT3	5
ENSGALG00000014750	TRB	5
ENSGALG00000014821	THEMIS	5
ENSGALG00000014836	LPIN2	5
ENSGALG00000014840	C6	5
ENSGALG00000014872	FGF10	5
ENSGALG00000014950	SULT3A1	5
ENSGALG00000014971	SLC2A9	5
ENSGALG00000015034	ANKRD29	5
ENSGALG00000015040	SLC16A10	5
ENSGALG00000015358	MYH15	5
ENSGALG00000015362	TRAT1	5
ENSGALG00000015425	LPL	5
ENSGALG00000015492	PDZK1	5
ENSGALG00000015519	ROBO2	5
ENSGALG00000015624	VCAN	5
ENSGALG00000016027	CBR3	5
ENSGALG00000016036	DOP1B	5
ENSGALG00000016138	DSCAM	5
ENSGALG00000016164	ABCG1	5
ENSGALG00000016196	CBSL	5
ENSGALG00000016281	DMD	5
ENSGALG00000016287	NR0B1	5
ENSGALG00000016325	GSTA3	5
ENSGALG00000016364	ALKAL2	5
ENSGALG00000016446	ATP6V1C2	5
ENSGALG00000016558	VEGFD	5
ENSGALG00000016651	TDH	5
ENSGALG00000016690	CYP2AC1	5
ENSGALG00000016761	LYG2	5
ENSGALG00000017032	SLC25A15	5
ENSGALG00000017039	STOML3	5
ENSGALG00000017040	C4	5
ENSGALG00000017046	POSTN	5
ENSGALG00000017103	WASF3	5
ENSGALG00000017120	SACS	5
ENSGALG00000017199	MAML2	5
ENSGALG00000019147	--	5

ENSGALG00000019663	ACBD7	5
ENSGALG00000019768	ACSF2	5
ENSGALG00000019835	TRIM27.2	5
ENSGALG00000020391	SERPINA10	5
ENSGALG00000020688	CYP4A22	5
ENSGALG00000021340	CA9	5
ENSGALG00000021848	AVD	5
ENSGALG00000022750	GPR18	5
ENSGALG00000023122	SULT1B	5
ENSGALG00000023844	P2RY1	5
ENSGALG00000024085	IDO2	5
ENSGALG00000026313	RND3	5
ENSGALG00000026663	CX3CL1	5
ENSGALG00000027122	APPL2	5
ENSGALG00000027786	SOCS3	5
ENSGALG00000027793	SCN9A	5
ENSGALG00000027891	NREP	5
ENSGALG00000027908	CYP2U1	5
ENSGALG00000028256	CCL19	5
ENSGALG00000028284	PTX3	5
ENSGALG00000028341	MADCAM1	5
ENSGALG00000028407	GDF9	5
ENSGALG00000028451	MT4	5
ENSGALG00000028871	SLC38A3	5
ENSGALG00000028897	WDR25	5
ENSGALG00000028928	LCAT	5
ENSGALG00000029270	GATA3	5
ENSGALG00000029724	MTURN	5
ENSGALG00000029944	FAM222A	5
ENSGALG00000030025	FABP4	5
ENSGALG00000030031	TTPA	5
ENSGALG00000030038	C3	5
ENSGALG00000030121	SLC2A11	5
ENSGALG00000030251	ADCY8	5
ENSGALG00000030920	APOC3	5
ENSGALG00000031122	NTNG1	5
ENSGALG00000031158	OAT	5
ENSGALG00000031255	FGF1	5
ENSGALG00000031496	SPINK5	5
ENSGALG00000031593	TMSB15B	5
ENSGALG00000032231	C4	5
ENSGALG00000032746	ENPP2	5
ENSGALG00000032882	EVA1C	5
ENSGALG00000032903	RTN4RL2	5
ENSGALG00000033171	TGM4	5
ENSGALG00000033338	GPT2	5
ENSGALG00000033411	SLC26A2	5
ENSGALG00000033974	HGF	5
ENSGALG00000034081	AKT3	5
ENSGALG00000034140	ZNF395	5
ENSGALG00000034337	RHPN1	5
ENSGALG00000034438	GNB3	5
ENSGALG00000034478	CCL4	5
ENSGALG00000034507	CHST2	5
ENSGALG00000034616	INHBA	5
ENSGALG00000034737	GLIS2	5
ENSGALG00000034741	ETNPPL	5
ENSGALG00000035026	SLC22A4	5

ENSGALG00000035219	ALB	5
ENSGALG00000035903	FAM46A	5
ENSGALG00000036005	TIAM2	5
ENSGALG00000036086	TAGLN2	5
ENSGALG00000036190	AOC1	5
ENSGALG00000036742	GATSL2	5
ENSGALG00000037014	TSNARE1	5
ENSGALG00000037160	Smad7	5
ENSGALG00000037387	CLSTN2	5
ENSGALG00000037671	psuG	5
ENSGALG00000037780	PMEPA1	5
ENSGALG00000038242	CACNA2D2	5
ENSGALG00000038520	STRIP2	5
ENSGALG00000038652	Gsta3	5
ENSGALG00000038740	AMY2A	5
ENSGALG00000038923	Ces1e	5
ENSGALG00000039140	CD14	5
ENSGALG00000039239	SERPIND1	5
ENSGALG00000039474	ID4	5
ENSGALG00000039499	LRRCC1	5
ENSGALG00000039708	Pram1	5
ENSGALG00000040434	rab18b	5
ENSGALG00000040573	FMO3	5
ENSGALG00000040836	INSYN2A	5
ENSGALG00000040995	NEB	5
ENSGALG00000041258	msrA	5
ENSGALG00000041344	FABP5	5
ENSGALG00000041373	ARAP2	5
ENSGALG00000041491	ACKR4	5
ENSGALG00000041577	ITGA4	5
ENSGALG00000041680	KCNT2	5
ENSGALG00000043044	IL1R1	5
ENSGALG00000043582	LY6E	5
ENSGALG00000044763	GPR82	5
ENSGALG00000044996	TMEM71	5
ENSGALG00000046316	CFAP97D1	5
ENSGALG00000046639	CYP2AC2	5
ENSGALG00000046687	EPS8L3	5
ENSGALG00000047321	SARDH	5
ENSGALG00000047480	A2ML1	5
ENSGALG00000047632	Pc	5
ENSGALG00000047781	RAD9B	5
ENSGALG00000047821	--	5
ENSGALG00000047827	TMEM86A	5
ENSGALG00000048343	Ces1e	5
ENSGALG00000049068	ZNF831	5
ENSGALG00000049256	--	5
ENSGALG00000049755	IL22RA2	5
ENSGALG00000049966	Ufc1	5
ENSGALG00000050154	--	5
ENSGALG00000050176	SARM1	5
ENSGALG00000050840	APCDD1	5
ENSGALG00000051068	SIGLEC1	5
ENSGALG00000051203	Mas1	5
ENSGALG00000051274	B3GALT2	5
ENSGALG00000052388	METRN	5
ENSGALG00000052583	A2ML1	5
ENSGALG00000052872	--	5

ENSGALG00000052894	Ranbp2	5
ENSGALG00000052964	TOPAZ1	5
ENSGALG00000052991	DNAJC21	5
ENSGALG00000053140	NDRG2	5
ENSGALG00000053278	SUCNR1	5
ENSGALG00000053647	PFAS	5
ENSGALG00000053860	mas	5
ENSGALG00000054322	--	5
ENSGALG00000054442	ITIH3	5
ENSGALG00000054783	NDRG1	5
ENSGALG00000054856	ADH1	5
ENSGALG00000054981	F10	5
ENSGALG00000055021	GREM2	5
MSTRG.10101	--	5
MSTRG.11572	--	5
MSTRG.11633	Stap2	5
MSTRG.12291	--	5
MSTRG.13474	Klhl29	5
MSTRG.13819	--	5
MSTRG.14083	--	5
MSTRG.14987	--	5
MSTRG.15995	--	5
MSTRG.16661	--	5
MSTRG.17350	--	5
MSTRG.17721	--	5
MSTRG.19177	PHGDH	5
MSTRG.20377	--	5
MSTRG.20478	--	5
MSTRG.20494	AHNAK	5
MSTRG.20827	Plcg1	5
MSTRG.29	SHANK3	5
MSTRG.3009	--	5
MSTRG.3197	--	5
MSTRG.4548	--	5
MSTRG.5269	pitpnc1	5
MSTRG.8055	--	5
MSTRG.836	--	5
MSTRG.8511	--	5
