# Supplementary material for: Functional remodeling of gut microbiota and liver in laying hens as affected by fasting and refeeding after fasting
Source: Anim Biosci. 2024 Oct 28;38(4):692–706. doi: 10.5713/ab.24.0299 (PMC11917430; doi:10.5713/ab.24.0299)
Supplement: Supplementary file 5 [file ab-24-0299-Supplementary-Table-5.pdf]

Table S5. Crosstalk between 61 differential metabolites and 1138 DEGs by Pearson's correla

Var1	Metabolite_Name	Var2	Gene_Symbol
Com_413_pos	L-Cystine	ENSGALG00000040573	FMO3
Com_178_pos	Maltol	ENSGALG00000031122	NTNG1
Com_178_pos	Maltol	MSTRG.4548	--
Com_460_pos	3-amino-4-(propylamino)(	ENSGALG00000029944	FAM222A
Com_108_neg	LPE 18:2	ENSGALG00000052829	MAMDC4
Com_151_pos	Pyridoxamine	ENSGALG00000047827	TMEM86A
Com_130_neg	2-Hydroxyvaleric acid	ENSGALG00000012034	ADSL
Com_25_pos	2-Hydroxycinnamic acid	ENSGALG00000000184	SLC27A6
Com_155_neg	Phenylacetaldehyde	ENSGALG00000006217	S100B
Com_165_neg	(±)9-HpODE	ENSGALG00000000769	RAB7B
Com_55_pos	Valine	ENSGALG00000013244	ABCC9
Com_68_neg	PE (16:0/22:6)	MSTRG.3262	--
Com_482_pos	8-Hydroxyquinoline	ENSGALG00000037773	ST3GAL1
Com_413_pos	L-Cystine	ENSGALG00000014252	A2M
Com_692_pos	3-amino-2-phenyl-2H-py	MSTRG.6554	--
Com_252_pos	cis-4-Hydroxy-D-proline	ENSGALG00000005632	IRAG1
Com_89_neg	Gallic acid	ENSGALG00000007234	CLCN5
Com_203_pos	Serotonin	ENSGALG00000037467	NEU2
Com_413_pos	L-Cystine	ENSGALG00000026663	CX3CL1
Com_8_neg	4-Methyl-2-Oxopentanoic	MSTRG.8055	--
Com_40_pos	Choline	ENSGALG00000011684	STAP1
Com_130_neg	2-Hydroxyvaleric acid	ENSGALG00000040434	rab18b
Com_119_pos	DL-Stachydrine	ENSGALG00000021451	RED3
Com_16_neg	3-Hydroxybutyric acid	ENSGALG00000011894	CYP2D6
Com_108_neg	LPE 18:2	ENSGALG00000002249	AGO1
Com_130_neg	2-Hydroxyvaleric acid	MSTRG.3197	--
Com_89_neg	Gallic acid	ENSGALG00000041143	UMOD
Com_331_pos	L-Lysine	ENSGALG00000016364	ALKAL2
Com_108_neg	LPE 18:2	ENSGALG00000015721	SVEP1
Com_86_neg	Levulinic acid	ENSGALG00000007114	APOA1
Com_165_neg	(±)9-HpODE	ENSGALG00000003805	PRKG1
Com_55_pos	Valine	MSTRG.5269	pitpnc1
Com_588_pos	Ornithine	ENSGALG00000052964	TOPAZ1
Com_22_pos	Indole-3-acrylic acid	ENSGALG00000013090	LOXL4
Com_57_neg	LPC 16:1	ENSGALG00000052328	CTNND2
Com_588_pos	Ornithine	ENSGALG00000012755	IGF-I
Com_386_pos	2-Amino-1,3-octadecanec	ENSGALG00000008728	PTER
Com_97_pos	L-Threonine	MSTRG.8511	--
Com_386_pos	2-Amino-1,3-octadecanec	ENSGALG00000015034	ANKRD29
Com_151_neg	Lysope 18:1	MSTRG.17389	DIO3
Com_99_pos	Creatine	ENSGALG00000010853	C8B
Com_86_neg	Levulinic acid	ENSGALG00000004598	CUX2
Com_119_pos	DL-Stachydrine	ENSGALG00000011141	ITGB6
Com_68_neg	PE (16:0/22:6)	ENSGALG00000013830	PRELID3A
Com_413_pos	L-Cystine	ENSGALG00000052872	--
Com_588_pos	Ornithine	ENSGALG00000052583	A2ML1
Com_175_pos	Pantothenic acid	ENSGALG00000002790	ABLIM3
Com_413_pos	L-Cystine	ENSGALG00000014616	MT3
Com_311_pos	PC (18:4e/2:0)	ENSGALG00000005474	PNAT10
Com_108_neg	LPE 18:2	ENSGALG00000001642	GLT8D1
Com_16_neg	3-Hydroxybutyric acid	ENSGALG00000028451	MT4
Com_215_pos	D-Erythro-sphingosine 1-p	MSTRG.7811	--
Com_386_pos	2-Amino-1,3-octadecanec	ENSGALG00000002855	SARDH
Com_203_pos	Serotonin	ENSGALG00000036234	RFWD3
Com_40_pos	Choline	ENSGALG00000015362	TRAT1
Com_108_neg	LPE 18:2	ENSGALG00000037322	HIST1H46

Com_78_neg	Citric acid	ENSGALG00000003972	FAXDC2
Com_471_pos	Indole-3-acetic acid	MSTRG.8381	--
Com_588_pos	Ornithine	ENSGALG00000032882	EVA1C
Com_175_pos	Pantothenic acid	ENSGALG00000015425	LPL
Com_194_pos	Pipecolic acid	ENSGALG00000017103	WASF3
Com_471_pos	Indole-3-acetic acid	ENSGALG00000030151	LUZP2
Com_18_neg	Arachidonic acid	ENSGALG00000005030	DOCK10
Com_194_pos	Pipecolic acid	MSTRG.17350	--
Com_4_pos	PC (17:1/17:1)	ENSGALG00000026809	SARS
Com_203_pos	Serotonin	ENSGALG00000036915	SQLE
Com_86_neg	Levulinic acid	ENSGALG00000011524	PPEF2
Com_18_neg	Arachidonic acid	ENSGALG00000027122	APPL2
Com_25_pos	2-Hydroxycinnamic acid	ENSGALG00000039474	ID4
Com_108_neg	LPE 18:2	ENSGALG00000009312	RPL22L1
Com_152_pos	Acetyl-L-carnitine	ENSGALG00000037671	psuG
Com_76_neg	Erythronolactone	ENSGALG00000054981	F10
Com_97_pos	L-Threonine	ENSGALG00000004343	HPD
Com_588_pos	Ornithine	ENSGALG00000036190	AOC1
Com_362_pos	2-Arachidonoyl glycerol	ENSGALG00000030121	SLC2A11
Com_68_neg	PE (16:0/22:6)	ENSGALG00000029617	COL17A1
Com_264_pos	Indole	ENSGALG00000005776	TECR
Com_22_pos	Indole-3-acrylic acid	ENSGALG00000005776	TECR
Com_194_pos	Pipecolic acid	ENSGALG00000019835	TRIM27.2
Com_152_pos	Acetyl-L-carnitine	ENSGALG00000036086	TAGLN2
Com_120_neg	LPC 15:0	ENSGALG00000009479	SAMD9L
Com_17_pos	L-Norleucine	ENSGALG00000011524	PPEF2
Com_18_neg	Arachidonic acid	ENSGALG00000034616	INHBA
Com_151_neg	Lysope 18:1	MSTRG.17677	--
Com_192_pos	1-Methylhistidine	ENSGALG00000047632	Pc
Com_471_pos	Indole-3-acetic acid	ENSGALG00000026846	JMJD7
Com_471_pos	Indole-3-acetic acid	ENSGALG00000009476	CDK6
Com_362_pos	2-Arachidonoyl glycerol	ENSGALG00000016651	TDH
Com_80_pos	DL-Lysine	ENSGALG00000005263	SOX8
Com_18_neg	Arachidonic acid	MSTRG.10101	--
Com_386_pos	2-Amino-1,3-octadecanec	ENSGALG00000028341	MADCAM1
Com_471_pos	Indole-3-acetic acid	ENSGALG00000021686	--
Com_108_neg	LPE 18:2	MSTRG.7811	--
Com_352_pos	Riboflavin	MSTRG.21653	--
Com_215_pos	D-Erythro-sphingosine 1-ı	ENSGALG00000001642	GLT8D1
Com_16_neg	3-Hydroxybutyric acid	ENSGALG00000021848	AVD
Com_130_neg	2-Hydroxyvaleric acid	MSTRG.8511	--
Com_192_pos	1-Methylhistidine	ENSGALG00000002431	CFH
Com_8_neg	4-Methyl-2-Oxopentanoic	ENSGALG00000037671	psuG
Com_147_pos	D-Sphingosine	ENSGALG00000050154	--
Com_386_pos	2-Amino-1,3-octadecanec	ENSGALG00000016138	DSCAM
Com_24_neg	PE (16:0/20:4)	ENSGALG00000007242	SLITRK4
Com_165_neg	(±)9-HpODE	ENSGALG00000002742	TMEM132B
Com_152_pos	Acetyl-L-carnitine	ENSGALG00000035903	FAM46A
Com_24_neg	PE (16:0/20:4)	ENSGALG00000014727	PDE4D
Com_76_neg	Erythronolactone	MSTRG.14083	--
Com_252_pos	cis-4-Hydroxy-D-proline	ENSGALG00000001768	TENM2
Com_588_pos	Ornithine	ENSGALG00000028407	GDF9
Com_76_neg	Erythronolactone	ENSGALG00000008912	ABCB1
Com_151_pos	Pyridoxamine	ENSGALG00000039239	SERPIND1
Com_150_neg	benzyl N-(2-{[(benzyloxy)c	MSTRG.17162	--
Com_147_pos	D-Sphingosine	ENSGALG00000007839	NCAM1
Com_413_pos	L-Cystine	ENSGALG00000019147	--
Com_155_neg	Phenylacetaldehyde	ENSGALG00000011809	GRIN2B

Com_264_pos	Indole	ENSGALG00000013090	LOXL4
Com_352_pos	Riboflavin	ENSGALG00000026598	NXNL2
Com_440_pos	PC (18:4e/4:0)	ENSGALG00000005474	PNAT10
Com_215_pos	D-Erythro-sphingosine 1-p	ENSGALG00000002249	AGO1
Com_119_pos	DL-Stachydrine	MSTRG.15625	--
Com_57_neg	LPC 16:1	ENSGALG00000017136	GJB6
Com_92_pos	D-(+)-Proline	ENSGALG00000033338	GPT2
Com_97_pos	L-Threonine	ENSGALG00000006374	TBX6
Com_460_pos	3-amino-4-(propylamino)c	ENSGALG00000014836	LPIN2
Com_386_pos	2-Amino-1,3-octadecanec	ENSGALG00000044996	TMEM71
Com_53_neg	2-(5-mercapto-4-methyl-4	MSTRG.21420	--
Com_152_pos	Acetyl-L-carnitine	ENSGALG00000015492	PDZK1
Com_97_pos	L-Threonine	ENSGALG00000012034	ADSL
Com_440_pos	PC (18:4e/4:0)	ENSGALG00000007839	NCAM1
Com_413_pos	L-Cystine	MSTRG.19177	PHGDH
Com_215_pos	D-Erythro-sphingosine 1-p	ENSGALG00000052829	MAMDC4
Com_352_pos	Riboflavin	ENSGALG00000012106	SCTR
Com_24_neg	PE (16:0/20:4)	ENSGALG00000005284	LRRC39
Com_386_pos	2-Amino-1,3-octadecanec	ENSGALG00000041258	msrA
Com_97_pos	L-Threonine	ENSGALG00000011994	SYNPO2
Com_150_neg	benzyl N-(2-{[(benzyloxy)c	ENSGALG00000028560	OC3
Com_186_pos	4-Hydroxybenzaldehyde	ENSGALG00000006976	Bdh1
Com_588_pos	Ornithine	ENSGALG00000008185	AOX1
Com_97_pos	L-Threonine	ENSGALG00000040434	rab18b
Com_152_pos	Acetyl-L-carnitine	MSTRG.8055	--
Com_8_neg	4-Methyl-2-Oxopentanoic	ENSGALG00000017039	STOML3
Com_12_pos	Betaine	ENSGALG00000010628	ACSL1
Com_265_pos	6-Methylquinoline	ENSGALG00000049755	IL22RA2
Com_97_pos	L-Threonine	MSTRG.3197	--
Com_18_neg	Arachidonic acid	ENSGALG00000021848	AVD
Com_16_neg	3-Hydroxybutyric acid	MSTRG.10101	--
Com_151_pos	Pyridoxamine	ENSGALG00000034140	ZNF395
Com_413_pos	L-Cystine	ENSGALG00000033171	TGM4
Com_57_neg	LPC 16:1	ENSGALG00000029766	ITGB5
Com_331_pos	L-Lysine	ENSGALG00000031122	NTNG1
Com_460_pos	3-amino-4-(propylamino)c	ENSGALG00000016325	GSTA3
Com_331_pos	L-Lysine	MSTRG.4548	--
Com_471_pos	Indole-3-acetic acid	ENSGALG00000041456	SLC35G1
Com_130_neg	2-Hydroxyvaleric acid	ENSGALG00000017032	SLC25A15
Com_22_pos	Indole-3-acrylic acid	ENSGALG00000011469	IGFBP2
Com_57_neg	LPC 16:1	ENSGALG00000030160	DRC7
Com_215_pos	D-Erythro-sphingosine 1-p	ENSGALG00000015721	SVEP1
Com_362_pos	2-Arachidonoyl glycerol	ENSGALG00000017046	POSTN
Com_92_pos	D-(+)-Proline	ENSGALG00000015492	PDZK1
Com_17_pos	L-Norleucine	ENSGALG00000007114	APOA1
Com_56_neg	LPE 18:1	ENSGALG00000013743	ENPP7
Com_86_neg	Levulinic acid	ENSGALG00000021340	CA9
Com_352_pos	Riboflavin	ENSGALG00000021627	IFI27L2
Com_311_pos	PC (18:4e/2:0)	ENSGALG00000009740	RASGRP1
Com_362_pos	2-Arachidonoyl glycerol	ENSGALG00000016036	DOP1B
Com_460_pos	3-amino-4-(propylamino)c	ENSGALG00000016196	CBSL
Com_151_neg	Lysope 18:1	ENSGALG00000033541	FRMPD4
Com_78_neg	Citric acid	ENSGALG00000016651	TDH
Com_57_neg	LPC 16:1	ENSGALG00000026973	KIF3C
Com_413_pos	L-Cystine	ENSGALG00000036190	AOC1
Com_97_pos	L-Threonine	ENSGALG00000014412	CSTA
Com_178_pos	Maltol	ENSGALG00000016364	ALKAL2
Com_18_neg	Arachidonic acid	ENSGALG00000028451	MT4

Com_17_pos	L-Norleucine	ENSGALG00000004598	CUX2
Com_352_pos	Riboflavin	ENSGALG00000011335	NHEJ1
Com_192_pos	1-Methylhistidine	ENSGALG00000037780	PMEPA1
Com_151_neg	Lysope 18:1	ENSGALG00000026973	KIF3C
Com_76_neg	Erythronolactone	ENSGALG00000003537	SGK2
Com_57_neg	LPC 16:1	ENSGALG00000033541	FRMPD4
Com_99_pos	Creatine	ENSGALG00000039140	CD14
Com_151_pos	Pyridoxamine	ENSGALG00000006864	COL24A1
Com_78_neg	Citric acid	ENSGALG00000030121	SLC2A11
Com_171_neg	LPC 22:6	ENSGALG00000003972	FAXDC2
Com_57_neg	LPC 16:1	ENSGALG00000015234	clcC
Com_16_neg	3-Hydroxybutyric acid	MSTRG.29	SHANK3
Com_130_neg	2-Hydroxyvaleric acid	ENSGALG00000004343	HPD
Com_482_pos	8-Hydroxyquinoline	ENSGALG00000004341	Cryzl2
Com_413_pos	L-Cystine	ENSGALG00000013728	PPAT
Com_92_pos	D-(+)-Proline	ENSGALG00000035903	FAM46A
Com_24_neg	PE (16:0/20:4)	ENSGALG00000002742	TMEM132B
Com_215_pos	D-Erythro-sphingosine 1-[	ENSGALG00000037322	HIST1H46
Com_56_neg	LPE 18:1	ENSGALG00000007955	SLC16A5
Com_17_pos	L-Norleucine	ENSGALG00000031158	OAT
Com_120_neg	LPC 15:0	ENSGALG00000029944	FAM222A
Com_482_pos	8-Hydroxyquinoline	MSTRG.14577	SLC39A5
Com_55_pos	Valine	MSTRG.17350	--
Com_18_neg	Arachidonic acid	ENSGALG00000034081	AKT3
Com_68_neg	PE (16:0/22:6)	ENSGALG00000014719	SETD9
Com_362_pos	2-Arachidonoyl glycerol	ENSGALG00000003972	FAXDC2
Com_151_neg	Lysope 18:1	ENSGALG00000029766	ITGB5
Com_4_pos	PC (17:1/17:1)	ENSGALG00000041078	MID1IP1
Com_588_pos	Ornithine	ENSGALG00000052872	--
Com_150_neg	benzyl N-(2-{[(benzyloxy)c	MSTRG.8957	--
Com_215_pos	D-Erythro-sphingosine 1-[	ENSGALG00000009312	RPL22L1
Com_362_pos	2-Arachidonoyl glycerol	ENSGALG00000011003	SLC35F3
Com_18_neg	Arachidonic acid	ENSGALG00000011894	CYP2D6
Com_4_pos	PC (17:1/17:1)	MSTRG.10409	--
Com_413_pos	L-Cystine	ENSGALG00000012755	IGF-I
Com_76_neg	Erythronolactone	ENSGALG00000019663	ACBD7
Com_92_pos	D-(+)-Proline	ENSGALG00000036086	TAGLN2
Com_215_pos	D-Erythro-sphingosine 1-[	ENSGALG00000004702	DYNC2I2
Com_76_neg	Erythronolactone	ENSGALG00000005204	GSTT1
Com_311_pos	PC (18:4e/2:0)	ENSGALG00000004205	SOAT1
Com_482_pos	8-Hydroxyquinoline	ENSGALG00000006723	IDI1
Com_76_neg	Erythronolactone	ENSGALG00000009002	CPED1
Com_482_pos	8-Hydroxyquinoline	ENSGALG00000036836	SOSTDC1
Com_386_pos	2-Amino-1,3-octadecanec	ENSGALG00000014950	SULT3A1
Com_80_pos	DL-Lysine	ENSGALG00000003136	IKZF2
Com_80_pos	DL-Lysine	ENSGALG00000009963	LYZ
Com_147_pos	D-Sphingosine	ENSGALG00000041258	msrA
Com_194_pos	Pipecolic acid	MSTRG.5269	pitpnc1
Com_151_pos	Pyridoxamine	ENSGALG00000000761	TSKU
Com_8_neg	4-Methyl-2-Oxopentanoic	ENSGALG00000036086	TAGLN2
Com_178_pos	Maltol	ENSGALG00000016281	DMD
Com_68_neg	PE (16:0/22:6)	ENSGALG00000014516	CPEB2
Com_413_pos	L-Cystine	ENSGALG00000003212	TSPO2
Com_186_pos	4-Hydroxybenzaldehyde	ENSGALG00000016761	LYG2
Com_588_pos	Ornithine	ENSGALG00000014252	A2M
Com_80_pos	DL-Lysine	ENSGALG00000029724	MTURN
Com_215_pos	D-Erythro-sphingosine 1-[	ENSGALG00000016560	SELENOI
Com_178_pos	Maltol	ENSGALG00000038923	Ces1e

Com_413_pos	L-Cystine	ENSGALG00000012754	PAH
Com_17_pos	L-Norleucine	ENSGALG00000038520	STRIP2
Com_151_neg	Lysope 18:1	ENSGALG00000017136	GJB6
Com_482_pos	8-Hydroxyquinoline	ENSGALG00000012877	CREB3L2
Com_130_neg	2-Hydroxyvaleric acid	ENSGALG00000005472	NAT
Com_130_neg	2-Hydroxyvaleric acid	ENSGALG00000006374	TBX6
Com_588_pos	Ornithine	ENSGALG00000040573	FMO3
Com_120_neg	LPC 15:0	ENSGALG00000006482	FAH
Com_76_neg	Erythronolactone	ENSGALG00000017040	C4
Com_76_neg	Erythronolactone	ENSGALG00000003015	SERPINF1
Com_482_pos	8-Hydroxyquinoline	MSTRG.1502	gag
Com_192_pos	1-Methylhistidine	ENSGALG00000052388	METRN
Com_4_pos	PC (17:1/17:1)	ENSGALG00000050091	CLEC2B
Com_8_neg	4-Methyl-2-Oxopentanoic	ENSGALG00000035903	FAM46A
Com_215_pos	D-Erythro-sphingosine 1-p	ENSGALG00000038574	MYO15A
Com_386_pos	2-Amino-1,3-octadecanec	ENSGALG00000007728	Prodh
Com_130_neg	2-Hydroxyvaleric acid	ENSGALG00000011994	SYNPO2
Com_24_neg	PE (16:0/20:4)	ENSGALG00000003805	PRKG1
Com_588_pos	Ornithine	ENSGALG00000026663	CX3CL1
Com_460_pos	3-amino-4-(propylamino)c	ENSGALG00000008953	AASS
Com_194_pos	Pipecolic acid	ENSGALG00000013244	ABCC9
Com_194_pos	Pipecolic acid	ENSGALG00000017199	MAML2
Com_386_pos	2-Amino-1,3-octadecanec	ENSGALG00000038242	CACNA2D2
Com_208_neg	N-Acetylanthranilic acid	ENSGALG00000010163	LGR5
Com_68_neg	PE (16:0/22:6)	MSTRG.1843	--
Com_76_neg	Erythronolactone	ENSGALG00000048343	Ces1e
Com_331_pos	L-Lysine	ENSGALG00000034741	ETNPPL
Com_413_pos	L-Cystine	ENSGALG00000052964	TOPAZ1
Com_460_pos	3-amino-4-(propylamino)c	ENSGALG00000009479	SAMD9L
Com_147_pos	D-Sphingosine	ENSGALG00000016138	DSCAM
Com_386_pos	2-Amino-1,3-octadecanec	ENSGALG00000050154	--
Com_97_pos	L-Threonine	ENSGALG00000017032	SLC25A15
Com_24_neg	PE (16:0/20:4)	ENSGALG00000000769	RAB7B
Com_8_neg	4-Methyl-2-Oxopentanoic	ENSGALG00000015492	PDZK1
Com_331_pos	L-Lysine	ENSGALG00000026313	RND3
Com_147_pos	D-Sphingosine	ENSGALG00000028341	MADCAM1
Com_4_pos	PC (17:1/17:1)	ENSGALG00000027561	GNG5
Com_352_pos	Riboflavin	ENSGALG00000005553	NLGN3
Com_352_pos	Riboflavin	ENSGALG00000053680	HIST1H46L2
Com_17_pos	L-Norleucine	ENSGALG00000041680	KCNT2
Com_413_pos	L-Cystine	ENSGALG00000052583	A2ML1
Com_92_pos	D-(+)-Proline	ENSGALG00000041373	ARAP2
Com_16_neg	3-Hydroxybutyric acid	ENSGALG00000005030	DOCK10
Com_192_pos	1-Methylhistidine	ENSGALG00000020391	SERPINA10
Com_311_pos	PC (18:4e/2:0)	ENSGALG00000007839	NCAM1
Com_186_pos	4-Hydroxybenzaldehyde	ENSGALG00000006812	TTC36
Com_150_neg	benzyl N-(2-{[(benzyloxy)c	ENSGALG00000042275	esg1
Com_265_pos	6-Methylquinoline	ENSGALG00000011469	IGFBP2
Com_588_pos	Ornithine	ENSGALG00000014616	MT3
Com_4_pos	PC (17:1/17:1)	ENSGALG00000035803	THRSP
Com_16_neg	3-Hydroxybutyric acid	ENSGALG00000027122	APPL2
Com_130_neg	2-Hydroxyvaleric acid	ENSGALG00000014412	CSTA
Com_152_pos	Acetyl-L-carnitine	ENSGALG00000017039	STOML3
Com_471_pos	Indole-3-acetic acid	MSTRG.6512	--
Com_386_pos	2-Amino-1,3-octadecanec	ENSGALG00000013726	PAICS
Com_413_pos	L-Cystine	ENSGALG00000032882	EVA1C
Com_264_pos	Indole	ENSGALG00000011469	IGFBP2
Com_17_pos	L-Norleucine	ENSGALG00000021340	CA9

Com_171_neg	LPC 22:6	ENSGALG00000016651	TDH
Com_460_pos	3-amino-4-(propylamino)(	ENSGALG00000009880	INPP4B
Com_482_pos	8-Hydroxyquinoline	MSTRG.4702	--
Com_16_neg	3-Hydroxybutyric acid	ENSGALG00000034616	INHBA
Com_413_pos	L-Cystine	ENSGALG00000041680	KCNT2
Com_352_pos	Riboflavin	ENSGALG00000031874	HIST1H101
Com_22_pos	Indole-3-acrylic acid	ENSGALG00000049755	IL22RA2
Com_68_neg	PE (16:0/22:6)	ENSGALG00000050309	H2B-I
Com_147_pos	D-Sphingosine	ENSGALG00000002855	SARDH
Com_588_pos	Ornithine	ENSGALG00000002479	MAT1A
Com_68_neg	PE (16:0/22:6)	ENSGALG00000006054	CALCA
Com_471_pos	Indole-3-acetic acid	ENSGALG00000029445	FADS6
Com_76_neg	Erythronolactone	ENSGALG00000022750	GPR18
Com_55_pos	Valine	ENSGALG00000017103	WASF3
Com_18_neg	Arachidonic acid	ENSGALG00000054856	ADH1
Com_352_pos	Riboflavin	ENSGALG00000006534	PEX11A
Com_171_neg	LPC 22:6	ENSGALG00000030121	SLC2A11
Com_471_pos	Indole-3-acetic acid	MSTRG.14343	--
Com_203_pos	Serotonin	ENSGALG00000005215	CACNA1H
Com_120_neg	LPC 15:0	ENSGALG00000014836	LPIN2
Com_16_neg	3-Hydroxybutyric acid	ENSGALG00000002479	MAT1A
Com_440_pos	PC (18:4e/4:0)	ENSGALG00000050154	--
Com_151_neg	Lysope 18:1	ENSGALG00000052328	CTNND2
Com_119_pos	DL-Stachydrine	MSTRG.2171	Myo16
Com_192_pos	1-Methylhistidine	ENSGALG00000014821	THEMIS
Com_178_pos	Maltol	ENSGALG00000028928	LCAT
Com_89_neg	Gallic acid	ENSGALG00000043435	CARNS1
Com_99_pos	Creatine	MSTRG.12291	--
Com_175_pos	Pantothenic acid	ENSGALG00000054322	--
Com_147_pos	D-Sphingosine	ENSGALG00000015034	ANKRD29
Com_55_pos	Valine	ENSGALG00000019835	TRIM27.2
Com_86_neg	Levulinic acid	ENSGALG00000031158	OAT
Com_440_pos	PC (18:4e/4:0)	ENSGALG00000009740	RASGRP1
Com_147_pos	D-Sphingosine	ENSGALG00000008728	PTER
Com_165_neg	(±)9-HpODE	MSTRG.6546	--
Com_342_pos	1-(4-methylphenyl)-3,5-di	ENSGALG00000035060	FKBP11
Com_89_neg	Gallic acid	MSTRG.19672	--
Com_76_neg	Erythronolactone	ENSGALG00000002024	COMT
Com_482_pos	8-Hydroxyquinoline	MSTRG.149	--
Com_413_pos	L-Cystine	ENSGALG00000028407	GDF9
Com_194_pos	Pipecolic acid	MSTRG.17721	--
Com_150_neg	benzyl N-(2-{[(benzyloxy)c	ENSGALG00000015468	Tstd3
Com_147_pos	D-Sphingosine	ENSGALG00000005474	PNAT10
Com_119_pos	DL-Stachydrine	ENSGALG00000028880	FDPS
Com_12_pos	Betaine	ENSGALG00000039499	LRRCC1
Com_108_neg	LPE 18:2	ENSGALG00000004702	DYNC2I2
Com_25_pos	2-Hydroxycinnamic acid	ENSGALG00000001768	TENM2
Com_119_pos	DL-Stachydrine	ENSGALG00000051567	MRPL41
Com_86_neg	Levulinic acid	ENSGALG00000047321	SARDH
Com_92_pos	D-(+)-Proline	ENSGALG00000037671	psuG
Com_588_pos	Ornithine	ENSGALG00000019147	--
Com_120_neg	LPC 15:0	ENSGALG00000016325	GSTA3
Com_413_pos	L-Cystine	ENSGALG00000038520	STRIP2
Com_17_pos	L-Norleucine	ENSGALG00000012754	PAH
Com_97_pos	L-Threonine	ENSGALG00000005472	NAT
Com_460_pos	3-amino-4-(propylamino)(	ENSGALG00000027908	CYP2U1
Com_17_pos	L-Norleucine	ENSGALG00000003212	TSPO2
Com_86_neg	Levulinic acid	ENSGALG00000047480	A2ML1

Com_57_neg	LPC 16:1	MSTRG.13526	--
Com_482_pos	8-Hydroxyquinoline	ENSGALG00000004322	AHR
Com_89_neg	Gallic acid	ENSGALG00000054546	ERVK-11
Com_120_neg	LPC 15:0	ENSGALG00000011957	TOB2
Com_99_pos	Creatine	ENSGALG00000009172	OSBPL6
Com_12_pos	Betaine	ENSGALG00000053140	NDRG2
Com_352_pos	Riboflavin	ENSGALG00000006919	POF1B
Com_120_neg	LPC 15:0	ENSGALG00000016196	CBSL
Com_588_pos	Ornithine	MSTRG.19177	PHGDH
Com_108_neg	LPE 18:2	ENSGALG00000016560	SELENOI
Com_120_neg	LPC 15:0	ENSGALG00000051203	Mas1
Com_86_neg	Levulinic acid	ENSGALG00000038520	STRIP2
Com_165_neg	(±)9-HpODE	ENSGALG00000007242	SLITRK4
Com_40_pos	Choline	ENSGALG00000041344	FABP5
Com_482_pos	8-Hydroxyquinoline	ENSGALG00000038532	--
Com_440_pos	PC (18:4e/4:0)	ENSGALG00000004205	SOAT1
Com_89_neg	Gallic acid	ENSGALG00000024295	MYCBP
Com_413_pos	L-Cystine	ENSGALG00000008185	AOX1
Com_165_neg	(±)9-HpODE	ENSGALG00000014727	PDE4D
Com_18_neg	Arachidonic acid	ENSGALG00000012704	MYLIP
Com_331_pos	L-Lysine	ENSGALG00000016281	DMD
Com_471_pos	Indole-3-acetic acid	ENSGALG00000013149	MOCOS
Com_331_pos	L-Lysine	ENSGALG00000038923	Ces1e
Com_57_neg	LPC 16:1	MSTRG.10162	--
Com_482_pos	8-Hydroxyquinoline	MSTRG.1503	gag
Com_108_neg	LPE 18:2	ENSGALG00000038574	MYO15A
Com_362_pos	2-Arachidonoyl glycerol	ENSGALG00000005977	BTBD8
Com_57_neg	LPC 16:1	MSTRG.17389	DIO3
Com_108_neg	LPE 18:2	ENSGALG00000052395	ERVK-11
Com_588_pos	Ornithine	ENSGALG00000033171	TGM4
Com_68_neg	PE (16:0/22:6)	MSTRG.9524	--
Com_413_pos	L-Cystine	ENSGALG00000031158	OAT
Com_17_pos	L-Norleucine	ENSGALG00000013728	PPAT
Com_99_pos	Creatine	ENSGALG00000016761	LYG2
Com_152_pos	Acetyl-L-carnitine	ENSGALG00000033338	GPT2
Com_186_pos	4-Hydroxybenzaldehyde	ENSGALG00000049256	--
Com_331_pos	L-Lysine	ENSGALG00000037160	Smad7
Com_119_pos	DL-Stachydrine	ENSGALG00000044464	TEPSIN
Com_208_neg	N-Acetylanthranilic acid	ENSGALG00000013627	SLC7A2
Com_352_pos	Riboflavin	ENSGALG00000003446	PRLR
Com_151_pos	Pyridoxamine	ENSGALG00000003802	OTUD7A
Com_78_neg	Citric acid	ENSGALG00000017046	POSTN
Com_18_neg	Arachidonic acid	MSTRG.29	SHANK3
Com_165_neg	(±)9-HpODE	ENSGALG00000005284	LRRC39
Com_362_pos	2-Arachidonoyl glycerol	ENSGALG00000009700	PDK4
Com_4_pos	PC (17:1/17:1)	MSTRG.9361	Fam110a
Com_192_pos	1-Methylhistidine	ENSGALG00000013356	IKBKE
Com_178_pos	Maltol	ENSGALG00000034741	ETNPPL
Com_192_pos	1-Methylhistidine	ENSGALG00000030038	C3
Com_215_pos	D-Erythro-sphingosine 1-[illegible]	ENSGALG00000008539	ALG12
Com_86_neg	Levulinic acid	ENSGALG00000041680	KCNT2
Com_78_neg	Citric acid	ENSGALG00000016036	DOP1B
Com_92_pos	D-(+)-Proline	MSTRG.8055	--
Com_16_neg	3-Hydroxybutyric acid	ENSGALG00000034081	AKT3
Com_68_neg	PE (16:0/22:6)	MSTRG.21822	--
Com_194_pos	Pipecolic acid	ENSGALG00000046687	EPS8L3
Com_471_pos	Indole-3-acetic acid	MSTRG.8501	--
Com_86_neg	Levulinic acid	MSTRG.3009	--

Com_57_neg	LPC 16:1	MSTRG.17677	--
Com_178_pos	Maltol	ENSGALG00000026313	RND3
Com_588_pos	Ornithine	ENSGALG00000013728	PPAT
Com_92_pos	D-(+)-Proline	ENSGALG00000008780	CTBS
Com_186_pos	4-Hydroxybenzaldehyde	ENSGALG00000001697	ITIH3
Com_25_pos	2-Hydroxycinnamic acid	ENSGALG00000005632	IRAG1
Com_471_pos	Indole-3-acetic acid	ENSGALG00000016492	TDRD15
Com_17_pos	L-Norleucine	ENSGALG00000033171	TGM4
Com_460_pos	3-amino-4-(propylamino)c	ENSGALG00000006482	FAH
Com_99_pos	Creatine	ENSGALG00000034507	CHST2
Com_352_pos	Riboflavin	ENSGALG00000023083	KIAA1958
Com_252_pos	cis-4-Hydroxy-D-proline	ENSGALG00000000184	SLC27A6
Com_482_pos	8-Hydroxyquinoline	ENSGALG00000041533	SLC11A2
Com_588_pos	Ornithine	MSTRG.29	SHANK3
Com_471_pos	Indole-3-acetic acid	ENSGALG00000026607	C15orf40
Com_89_neg	Gallic acid	ENSGALG00000021039	HKDC1
Com_186_pos	4-Hydroxybenzaldehyde	ENSGALG00000039140	CD14
Com_264_pos	Indole	ENSGALG00000049755	IL22RA2
Com_108_neg	LPE 18:2	ENSGALG00000016456	LPIN1
Com_120_neg	LPC 15:0	ENSGALG00000008953	AASS
Com_89_neg	Gallic acid	ENSGALG00000014907	DCBLD1
Com_78_neg	Citric acid	ENSGALG00000011003	SLC35F3
Com_108_neg	LPE 18:2	ENSGALG00000043087	MTF2
Com_440_pos	PC (18:4e/4:0)	ENSGALG00000041258	msrA
Com_24_neg	PE (16:0/20:4)	ENSGALG00000023740	HBZ
Com_352_pos	Riboflavin	ENSGALG00000004833	P3H1
Com_17_pos	L-Norleucine	MSTRG.19177	PHGDH
Com_76_neg	Erythronolactone	MSTRG.11572	--
Com_386_pos	2-Amino-1,3-octadecanec	ENSGALG00000007839	NCAM1
Com_97_pos	L-Threonine	ENSGALG00000014750	TRB
Com_151_neg	Lysope 18:1	MSTRG.1845	--
Com_588_pos	Ornithine	ENSGALG00000003212	TSPO2
Com_352_pos	Riboflavin	MSTRG.13407	--
Com_331_pos	L-Lysine	ENSGALG00000028928	LCAT
Com_588_pos	Ornithine	ENSGALG00000012754	PAH
Com_265_pos	6-Methylquinoline	ENSGALG00000013090	LOXL4
Com_331_pos	L-Lysine	ENSGALG00000011314	LRRC3B
Com_76_neg	Erythronolactone	ENSGALG00000012704	MYLIP
Com_17_pos	L-Norleucine	ENSGALG00000019147	--
Com_331_pos	L-Lysine	ENSGALG00000011391	AMN
Com_311_pos	PC (18:4e/2:0)	ENSGALG00000050154	--
Com_342_pos	1-(4-methylphenyl)-3,5-di	ENSGALG00000012322	KCTD16
Com_147_pos	D-Sphingosine	ENSGALG00000044996	TMEM71
Com_17_pos	L-Norleucine	ENSGALG00000047321	SARDH
Com_342_pos	1-(4-methylphenyl)-3,5-di	ENSGALG00000017167	SLC35F2
Com_86_neg	Levulinic acid	ENSGALG00000012754	PAH
Com_16_neg	3-Hydroxybutyric acid	ENSGALG00000008185	AOX1
Com_120_neg	LPC 15:0	MSTRG.16661	--
Com_151_neg	Lysope 18:1	ENSGALG00000028175	GJA9
Com_86_neg	Levulinic acid	ENSGALG00000003212	TSPO2
Com_17_pos	L-Norleucine	ENSGALG00000047480	A2ML1
Com_150_neg	benzyl N-(2-{[(benzyloxy)c	ENSGALG00000008434	SORCS3
Com_208_neg	N-Acetylanthranilic acid	ENSGALG00000010889	HOOK1
Com_120_neg	LPC 15:0	ENSGALG00000009880	INPP4B
Com_92_pos	D-(+)-Proline	ENSGALG00000046639	CYP2AC2
Com_482_pos	8-Hydroxyquinoline	ENSGALG00000000645	Espn
Com_440_pos	PC (18:4e/4:0)	ENSGALG00000016138	DSCAM
Com_151_pos	Pyridoxamine	ENSGALG00000027891	NREP

Com_471_pos	Indole-3-acetic acid	ENSGALG00000051123	pol
Com_97_pos	L-Threonine	ENSGALG00000007252	ANKDD1A
Com_386_pos	2-Amino-1,3-octadecanec	ENSGALG00000030031	TTPA
Com_120_neg	LPC 15:0	ENSGALG00000030251	ADCY8
Com_108_neg	LPE 18:2	ENSGALG00000026384	PCSK4
Com_151_neg	Lysope 18:1	ENSGALG00000030160	DRC7
Com_440_pos	PC (18:4e/4:0)	ENSGALG00000028341	MADCAM1
Com_92_pos	D-(+)-Proline	ENSGALG00000005408	BCO1
Com_252_pos	cis-4-Hydroxy-D-proline	ENSGALG00000039474	ID4
Com_155_neg	Phenylacetaldehyde	ENSGALG00000005657	CRHR2
Com_55_pos	Valine	ENSGALG00000009963	LYZ
Com_55_pos	Valine	ENSGALG00000003136	IKZF2
Com_265_pos	6-Methylquinoline	ENSGALG00000005776	TECR
Com_55_pos	Valine	ENSGALG00000017199	MAML2
Com_192_pos	1-Methylhistidine	ENSGALG00000003569	TMEM130
Com_203_pos	Serotonin	ENSGALG00000032170	LCN15
Com_215_pos	D-Erythro-sphingosine 1-p	ENSGALG00000052395	ERVK-11
Com_175_pos	Pantothenic acid	ENSGALG00000011391	AMN
Com_8_neg	4-Methyl-2-Oxopentanoic	MSTRG.11572	--
Com_342_pos	1-(4-methylphenyl)-3,5-di	ENSGALG00000008150	RASAL1
Com_16_neg	3-Hydroxybutyric acid	ENSGALG00000028407	GDF9
Com_175_pos	Pantothenic acid	ENSGALG00000011314	LRRC3B
Com_208_neg	N-Acetylanthranilic acid	ENSGALG00000030038	C3
Com_208_neg	N-Acetylanthranilic acid	ENSGALG00000013356	IKBKE
Com_86_neg	Levulinic acid	ENSGALG00000013728	PPAT
Com_342_pos	1-(4-methylphenyl)-3,5-di	ENSGALG00000028709	RNF144A
Com_151_neg	Lysope 18:1	ENSGALG00000015234	clcC
Com_440_pos	PC (18:4e/4:0)	ENSGALG00000002855	SARDH
Com_17_pos	L-Norleucine	ENSGALG00000014616	MT3
Com_16_neg	3-Hydroxybutyric acid	ENSGALG00000054856	ADH1
Com_171_neg	LPC 22:6	ENSGALG00000017046	POSTN
Com_192_pos	1-Methylhistidine	ENSGALG00000013627	SLC7A2
Com_76_neg	Erythronolactone	ENSGALG00000054856	ADH1
Com_588_pos	Ornithine	ENSGALG00000041680	KCNT2
Com_68_neg	PE (16:0/22:6)	ENSGALG00000012089	--
Com_18_neg	Arachidonic acid	ENSGALG00000022750	GPR18
Com_18_neg	Arachidonic acid	ENSGALG00000002479	MAT1A
Com_413_pos	L-Cystine	ENSGALG00000002479	MAT1A
Com_4_pos	PC (17:1/17:1)	ENSGALG00000041687	SREBF2
Com_171_neg	LPC 22:6	ENSGALG00000016036	DOP1B
Com_342_pos	1-(4-methylphenyl)-3,5-di	ENSGALG00000009748	ASNS
Com_692_pos	3-amino-2-phenyl-2H-py	MSTRG.12567	--
Com_178_pos	Maltol	ENSGALG00000011287	SULT
Com_120_neg	LPC 15:0	ENSGALG00000027908	CYP2U1
Com_108_neg	LPE 18:2	ENSGALG00000008539	ALG12
Com_8_neg	4-Methyl-2-Oxopentanoic	ENSGALG00000033338	GPT2
Com_178_pos	Maltol	ENSGALG00000037160	Smad7
Com_440_pos	PC (18:4e/4:0)	ENSGALG00000015034	ANKRD29
Com_460_pos	3-amino-4-(propylamino)c	ENSGALG00000011957	TOB2
Com_97_pos	L-Threonine	ENSGALG00000009926	HAAO
Com_147_pos	D-Sphingosine	ENSGALG00000009740	RASGRP1
Com_440_pos	PC (18:4e/4:0)	ENSGALG00000008728	PTER
Com_151_pos	Pyridoxamine	ENSGALG00000034337	RHPN1
Com_17_pos	L-Norleucine	MSTRG.3009	--
Com_80_pos	DL-Lysine	MSTRG.836	--
Com_460_pos	3-amino-4-(propylamino)c	ENSGALG00000051203	Mas1
Com_152_pos	Acetyl-L-carnitine	ENSGALG00000041373	ARAP2
Com_108_neg	LPE 18:2	ENSGALG00000029033	Tldc2

Com_17_pos	L-Norleucine	ENSGALG00000026663	CX3CL1
Com_16_neg	3-Hydroxybutyric acid	ENSGALG00000032882	EVA1C
Com_86_neg	Levulinic acid	ENSGALG00000033171	TGM4
Com_92_pos	D-(+)-Proline	ENSGALG00000017039	STOML3
Com_471_pos	Indole-3-acetic acid	ENSGALG00000052072	gag
Com_57_neg	LPC 16:1	MSTRG.20491	--
Com_68_neg	PE (16:0/22:6)	MSTRG.17628	--
Com_86_neg	Levulinic acid	ENSGALG00000006320	Slc2a9
Com_108_neg	LPE 18:2	ENSGALG00000007508	HPSE2
Com_57_neg	LPC 16:1	ENSGALG00000007077	CPT1A
Com_215_pos	D-Erythro-sphingosine 1-p	ENSGALG00000016456	LPIN1
Com_147_pos	D-Sphingosine	ENSGALG00000014950	SULT3A1
Com_171_neg	LPC 22:6	ENSGALG00000011003	SLC35F3
Com_16_neg	3-Hydroxybutyric acid	ENSGALG00000052583	A2ML1
Com_130_neg	2-Hydroxyvaleric acid	ENSGALG00000014750	TRB
Com_89_neg	Gallic acid	ENSGALG00000052296	MEX3D
Com_18_neg	Arachidonic acid	ENSGALG00000048343	Ces1e
Com_17_pos	L-Norleucine	ENSGALG00000040573	FMO3
Com_352_pos	Riboflavin	MSTRG.21631	--
Com_194_pos	Pipecolic acid	ENSGALG00000035219	ALB
Com_215_pos	D-Erythro-sphingosine 1-p	ENSGALG00000043087	MTF2
Com_588_pos	Ornithine	ENSGALG00000038520	STRIP2
Com_55_pos	Valine	MSTRG.17721	--
Com_17_pos	L-Norleucine	ENSGALG00000014252	A2M
Com_86_neg	Levulinic acid	MSTRG.19177	PHGDH
Com_16_neg	3-Hydroxybutyric acid	ENSGALG00000052964	TOPAZ1
Com_175_pos	Pantothenic acid	ENSGALG00000037160	Smad7
Com_108_neg	LPE 18:2	ENSGALG00000008427	GNAT3
Com_203_pos	Serotonin	ENSGALG00000002845	CTNNA3
Com_147_pos	D-Sphingosine	ENSGALG00000004205	SOAT1
Com_16_neg	3-Hydroxybutyric acid	ENSGALG00000012704	MYLIP
Com_311_pos	PC (18:4e/2:0)	ENSGALG00000005472	NAT
Com_460_pos	3-amino-4-(propylamino)(	ENSGALG00000015040	SLC16A10
Com_482_pos	8-Hydroxyquinoline	ENSGALG00000014944	GCNT4
Com_147_pos	D-Sphingosine	ENSGALG00000007728	Prodh
Com_92_pos	D-(+)-Proline	ENSGALG00000010857	DAB1
Com_342_pos	1-(4-methylphenyl)-3,5-di	ENSGALG00000029947	MMAB
Com_24_neg	PE (16:0/20:4)	MSTRG.6546	--
Com_86_neg	Levulinic acid	ENSGALG00000019147	--
Com_352_pos	Riboflavin	ENSGALG00000016444	ODC1
Com_151_pos	Pyridoxamine	ENSGALG00000035219	ALB
Com_311_pos	PC (18:4e/2:0)	ENSGALG00000041258	msrA
Com_4_pos	PC (17:1/17:1)	MSTRG.14680	--
Com_155_neg	Phenylacetaldehyde	ENSGALG00000042275	esg1
Com_130_neg	2-Hydroxyvaleric acid	ENSGALG00000007252	ANKDD1A
Com_178_pos	Maltol	ENSGALG00000011314	LRRC3B
Com_17_pos	L-Norleucine	ENSGALG00000052872	--
Com_147_pos	D-Sphingosine	ENSGALG00000038242	CACNA2D2
Com_120_neg	LPC 15:0	ENSGALG00000000293	A2ML1
Com_178_pos	Maltol	ENSGALG00000011391	AMN
Com_208_neg	N-Acetylanthranilic acid	ENSGALG00000014821	THEMIS
Com_130_neg	2-Hydroxyvaleric acid	ENSGALG00000004205	SOAT1
Com_482_pos	8-Hydroxyquinoline	MSTRG.15507	--
Com_119_pos	DL-Stachydrine	ENSGALG00000029308	PNPLA3
Com_203_pos	Serotonin	ENSGALG00000001101	MBD3
Com_588_pos	Ornithine	ENSGALG00000031158	OAT
Com_203_pos	Serotonin	ENSGALG00000016979	SLC25A30
Com_18_neg	Arachidonic acid	ENSGALG00000009002	CPED1

Com_78_neg	Citric acid	ENSGALG00000005977	BTBD8
Com_203_pos	Serotonin	MSTRG.9006	--
Com_482_pos	8-Hydroxyquinoline	ENSGALG00000014813	HOMER1
Com_215_pos	D-Erythro-sphingosine 1-p	ENSGALG00000004590	CLCN6
Com_215_pos	D-Erythro-sphingosine 1-p	ENSGALG00000026384	PCSK4
Com_18_neg	Arachidonic acid	ENSGALG00000005204	GSTT1
Com_18_neg	Arachidonic acid	ENSGALG00000019663	ACBD7
Com_588_pos	Ornithine	ENSGALG00000011894	CYP2D6
Com_8_neg	4-Methyl-2-Oxopentanoic	ENSGALG00000002024	COMT
Com_203_pos	Serotonin	MSTRG.16505	--
Com_147_pos	D-Sphingosine	ENSGALG00000013726	PAICS
Com_16_neg	3-Hydroxybutyric acid	ENSGALG00000012755	IGF-I
Com_76_neg	Erythronolactone	ENSGALG00000034081	AKT3
Com_471_pos	Indole-3-acetic acid	ENSGALG00000027375	NR2C2AP
Com_413_pos	L-Cystine	ENSGALG00000011524	PPEF2
Com_311_pos	PC (18:4e/2:0)	ENSGALG00000016138	DSCAM
Com_208_neg	N-Acetylanthranilic acid	ENSGALG00000020391	SERPINA10
Com_482_pos	8-Hydroxyquinoline	ENSGALG00000014233	FBLN1
Com_186_pos	4-Hydroxybenzaldehyde	ENSGALG00000010853	C8B
Com_78_neg	Citric acid	ENSGALG00000009700	PDK4
Com_215_pos	D-Erythro-sphingosine 1-p	ENSGALG00000023348	HPDL
Com_311_pos	PC (18:4e/2:0)	ENSGALG00000028341	MADCAM1
Com_192_pos	1-Methylhistidine	ENSGALG00000015358	MYH15
Com_130_neg	2-Hydroxyvaleric acid	ENSGALG00000009740	RASGRP1
Com_18_neg	Arachidonic acid	ENSGALG00000003537	SGK2
Com_86_neg	Levulinic acid	ENSGALG00000014616	MT3
Com_192_pos	1-Methylhistidine	ENSGALG00000049966	Ufc1
Com_130_neg	2-Hydroxyvaleric acid	ENSGALG00000009926	HAAO
Com_55_pos	Valine	ENSGALG00000046687	EPS8L3
Com_588_pos	Ornithine	ENSGALG00000028451	MT4
Com_4_pos	PC (17:1/17:1)	ENSGALG00000011169	PDCD2
Com_152_pos	Acetyl-L-carnitine	MSTRG.11572	--
Com_413_pos	L-Cystine	MSTRG.29	SHANK3
Com_4_pos	PC (17:1/17:1)	ENSGALG00000053446	RED3
Com_311_pos	PC (18:4e/2:0)	ENSGALG00000017032	SLC25A15
Com_342_pos	1-(4-methylphenyl)-3,5-di	ENSGALG00000053281	PKIA
Com_68_neg	PE (16:0/22:6)	ENSGALG00000050491	SLC35E4
Com_460_pos	3-amino-4-(propylamino)(	MSTRG.16661	--
Com_24_neg	PE (16:0/20:4)	MSTRG.6227	--
Com_16_neg	3-Hydroxybutyric acid	ENSGALG00000036190	AOC1
Com_97_pos	L-Threonine	ENSGALG00000006320	Slc2a9
Com_311_pos	PC (18:4e/2:0)	ENSGALG00000002855	SARDH
Com_482_pos	8-Hydroxyquinoline	ENSGALG00000015044	GTF3C6
Com_460_pos	3-amino-4-(propylamino)(	ENSGALG00000030251	ADCY8
Com_120_neg	LPC 15:0	ENSGALG00000016164	ABCG1
Com_331_pos	L-Lysine	ENSGALG00000011287	SULT
Com_208_neg	N-Acetylanthranilic acid	ENSGALG00000052388	METRN
Com_386_pos	2-Amino-1,3-octadecanec	ENSGALG00000005474	PNAT10
Com_215_pos	D-Erythro-sphingosine 1-p	ENSGALG00000029033	Tldc2
Com_12_pos	Betaine	ENSGALG00000007014	PYROXD2
Com_151_neg	Lysope 18:1	MSTRG.13526	--
Com_86_neg	Levulinic acid	ENSGALG00000026663	CX3CL1
Com_12_pos	Betaine	ENSGALG00000011687	AHNAK2
Com_155_neg	Phenylacetaldehyde	MSTRG.8957	--
Com_171_neg	LPC 22:6	ENSGALG00000054322	--
Com_588_pos	Ornithine	ENSGALG00000021848	AVD
Com_152_pos	Acetyl-L-carnitine	ENSGALG00000008780	CTBS
Com_440_pos	PC (18:4e/4:0)	ENSGALG00000044996	TMEM71

Com_89_neg	Gallic acid	ENSGALG00000002362	MANF
Com_215_pos	D-Erythro-sphingosine 1-p	ENSGALG00000007508	HPSE2
Com_311_pos	PC (18:4e/2:0)	ENSGALG00000015034	ANKRD29
Com_18_neg	Arachidonic acid	ENSGALG00000008185	AOX1
Com_17_pos	L-Norleucine	ENSGALG00000006320	Slc2a9
Com_203_pos	Serotonin	ENSGALG00000000802	DHODH
Com_86_neg	Levulinic acid	ENSGALG00000009926	HAAO
Com_311_pos	PC (18:4e/2:0)	ENSGALG00000008728	PTER
Com_165_neg	(±)9-HpODE	ENSGALG00000012045	slc12a8
Com_8_neg	4-Methyl-2-Oxopentanoic	ENSGALG00000041373	ARAP2
Com_208_neg	N-Acetylanthranilic acid	ENSGALG00000017120	SACS
Com_86_neg	Levulinic acid	ENSGALG00000040573	FMO3
Com_471_pos	Indole-3-acetic acid	ENSGALG00000016491	APOB
Com_175_pos	Pantothenic acid	ENSGALG00000026313	RND3
Com_482_pos	8-Hydroxyquinoline	ENSGALG00000004425	SCAMP1
Com_80_pos	DL-Lysine	ENSGALG00000013244	ABCC9
Com_86_neg	Levulinic acid	ENSGALG00000014252	A2M
Com_151_neg	Lysope 18:1	MSTRG.10162	--
Com_482_pos	8-Hydroxyquinoline	MSTRG.8986	gag
Com_386_pos	2-Amino-1,3-octadecanec	ENSGALG00000043582	LY6E
Com_175_pos	Pantothenic acid	ENSGALG00000034741	ETNPPL
Com_215_pos	D-Erythro-sphingosine 1-p	ENSGALG00000008427	GNAT3
Com_165_neg	(±)9-HpODE	ENSGALG00000023740	HBZ
Com_21_pos	DL-Tryptophan	MSTRG.13474	Klhl29
Com_108_neg	LPE 18:2	ENSGALG00000010764	FBXO8
Com_92_pos	D-(+)-Proline	ENSGALG00000028871	SLC38A3
Com_8_neg	4-Methyl-2-Oxopentanoic	ENSGALG00000003015	SERPINF1
Com_8_neg	4-Methyl-2-Oxopentanoic	ENSGALG00000017040	C4
Com_413_pos	L-Cystine	ENSGALG00000007114	APOA1
Com_97_pos	L-Threonine	ENSGALG00000004205	SOAT1
Com_68_neg	PE (16:0/22:6)	MSTRG.9774	--
Com_89_neg	Gallic acid	ENSGALG00000029857	Gimap1
Com_18_neg	Arachidonic acid	ENSGALG00000028407	GDF9
Com_18_neg	Arachidonic acid	MSTRG.14083	--
Com_17_pos	L-Norleucine	ENSGALG00000036190	AOC1
Com_413_pos	L-Cystine	ENSGALG00000004598	CUX2
Com_99_pos	Creatine	ENSGALG00000006976	Bdh1
Com_86_neg	Levulinic acid	ENSGALG00000052872	--
Com_151_neg	Lysope 18:1	MSTRG.7586	--
Com_4_pos	PC (17:1/17:1)	ENSGALG00000044278	C1orf131
Com_80_pos	DL-Lysine	MSTRG.5269	pitpnc1
Com_68_neg	PE (16:0/22:6)	ENSGALG00000008462	CDK3
Com_440_pos	PC (18:4e/4:0)	ENSGALG00000005472	NAT
Com_482_pos	8-Hydroxyquinoline	ENSGALG00000006076	RASGEF1C
Com_171_neg	LPC 22:6	ENSGALG00000005977	BTBD8
Com_194_pos	Pipecolic acid	ENSGALG00000009963	LYZ
Com_194_pos	Pipecolic acid	ENSGALG00000003136	IKZF2
Com_16_neg	3-Hydroxybutyric acid	ENSGALG00000022750	GPR18
Com_588_pos	Ornithine	MSTRG.10101	--
Com_460_pos	3-amino-4-(propylamino)(	ENSGALG00000028871	SLC38A3
Com_12_pos	Betaine	ENSGALG00000009050	CAPN3
Com_4_pos	PC (17:1/17:1)	ENSGALG00000015937	FABP1
Com_86_neg	Levulinic acid	ENSGALG00000007252	ANKDD1A
Com_362_pos	2-Arachidonoyl glycerol	MSTRG.836	--
Com_120_neg	LPC 15:0	ENSGALG00000027793	SCN9A
Com_12_pos	Betaine	ENSGALG00000007710	zgc:110179
Com_471_pos	Indole-3-acetic acid	ENSGALG00000007778	PES1
Com_108_neg	LPE 18:2	ENSGALG00000004590	CLCN6

Com_76_neg	Erythronolactone	ENSGALG00000034616	INHBA
Com_152_pos	Acetyl-L-carnitine	ENSGALG00000046639	CYP2AC2
Com_24_neg	PE (16:0/20:4)	ENSGALG00000010494	SLC5A9
Com_4_pos	PC (17:1/17:1)	ENSGALG00000007178	FADS2
Com_151_pos	Pyridoxamine	ENSGALG00000046687	EPS8L3
Com_203_pos	Serotonin	ENSGALG00000004804	TGM3
Com_18_neg	Arachidonic acid	ENSGALG00000054981	F10
Com_25_pos	2-Hydroxycinnamic acid	ENSGALG00000036742	GATSL2
Com_97_pos	L-Threonine	ENSGALG00000009740	RASGRP1
Com_76_neg	Erythronolactone	ENSGALG00000027122	APPL2
Com_152_pos	Acetyl-L-carnitine	ENSGALG00000005408	BCO1
Com_17_pos	L-Norleucine	ENSGALG00000012755	IGF-I
Com_76_neg	Erythronolactone	ENSGALG00000005030	DOCK10
Com_171_neg	LPC 22:6	ENSGALG00000009700	PDK4
Com_18_neg	Arachidonic acid	ENSGALG00000032882	EVA1C
Com_119_pos	DL-Stachydrine	MSTRG.9007	--
Com_482_pos	8-Hydroxyquinoline	ENSGALG00000010229	ABCD4
Com_89_neg	Gallic acid	ENSGALG00000014261	UCHL1
Com_120_neg	LPC 15:0	ENSGALG00000015040	SLC16A10
Com_108_neg	LPE 18:2	ENSGALG00000023348	HPDL
Com_152_pos	Acetyl-L-carnitine	ENSGALG00000002024	COMT
Com_18_neg	Arachidonic acid	ENSGALG00000052583	A2ML1
Com_86_neg	Levulinic acid	ENSGALG00000014750	TRB
Com_16_neg	3-Hydroxybutyric acid	ENSGALG00000048343	Ces1e
Com_440_pos	PC (18:4e/4:0)	ENSGALG00000014950	SULT3A1
Com_130_neg	2-Hydroxyvaleric acid	ENSGALG00000006320	Slc2a9
Com_57_neg	LPC 16:1	MSTRG.1845	--
Com_311_pos	PC (18:4e/2:0)	MSTRG.3197	--
Com_482_pos	8-Hydroxyquinoline	MSTRG.2305	gag
Com_55_pos	Valine	ENSGALG00000005263	SOX8
Com_311_pos	PC (18:4e/2:0)	ENSGALG00000040434	rab18b
Com_151_pos	Pyridoxamine	ENSGALG00000013033	cmbl
Com_460_pos	3-amino-4-(propylamino)(	ENSGALG00000000293	A2ML1
Com_18_neg	Arachidonic acid	ENSGALG00000052964	TOPAZ1
Com_16_neg	3-Hydroxybutyric acid	ENSGALG00000052872	--
Com_25_pos	2-Hydroxycinnamic acid	ENSGALG00000014872	FGF10
Com_178_pos	Maltol	ENSGALG00000043582	LY6E
Com_311_pos	PC (18:4e/2:0)	ENSGALG00000012034	ADSL
Com_440_pos	PC (18:4e/4:0)	ENSGALG00000007728	Prodh
Com_89_neg	Gallic acid	ENSGALG00000015728	MUSK
Com_78_neg	Citric acid	ENSGALG00000054322	--
Com_57_neg	LPC 16:1	ENSGALG00000028175	GJA9
Com_186_pos	4-Hydroxybenzaldehyde	ENSGALG00000049966	Ufc1
Com_55_pos	Valine	ENSGALG00000035219	ALB
Com_440_pos	PC (18:4e/4:0)	ENSGALG00000017032	SLC25A15
Com_186_pos	4-Hydroxybenzaldehyde	ENSGALG00000015358	MYH15
Com_57_neg	LPC 16:1	MSTRG.20480	--
Com_76_neg	Erythronolactone	ENSGALG00000017039	STOML3
Com_482_pos	8-Hydroxyquinoline	ENSGALG00000042511	PKDCC
Com_57_neg	LPC 16:1	ENSGALG00000005442	PALMD
Com_97_pos	L-Threonine	MSTRG.3009	--
Com_17_pos	L-Norleucine	ENSGALG00000052964	TOPAZ1
Com_440_pos	PC (18:4e/4:0)	ENSGALG00000038242	CACNA2D2
Com_17_pos	L-Norleucine	ENSGALG00000009926	HAAO
Com_16_neg	3-Hydroxybutyric acid	ENSGALG00000014252	A2M
Com_692_pos	3-amino-2-phenyl-2H-py	ENSGALG00000017308	CHRDL2
Com_471_pos	Indole-3-acetic acid	ENSGALG00000043829	ext1c
Com_53_neg	2-(5-mercapto-4-methyl-	ENSGALG00000004373	KCNJ16

Com_342_pos	1-(4-methylphenyl)-3,5-di	ENSGALG00000001790	METTL23
Com_16_neg	3-Hydroxybutyric acid	ENSGALG00000009002	CPED1
Com_16_neg	3-Hydroxybutyric acid	ENSGALG00000040573	FMO3
Com_147_pos	D-Sphingosine	ENSGALG00000030031	TTPA
Com_8_neg	4-Methyl-2-Oxopentanoic	ENSGALG00000008780	CTBS
Com_471_pos	Indole-3-acetic acid	ENSGALG00000046731	--
Com_151_pos	Pyridoxamine	MSTRG.17721	--
Com_17_pos	L-Norleucine	ENSGALG00000052583	A2ML1
Com_68_neg	PE (16:0/22:6)	ENSGALG00000029102	PXYLP1
Com_16_neg	3-Hydroxybutyric acid	ENSGALG00000005204	GSTT1
Com_352_pos	Riboflavin	MSTRG.6228	--
Com_16_neg	3-Hydroxybutyric acid	ENSGALG00000019663	ACBD7
Com_311_pos	PC (18:4e/2:0)	ENSGALG00000044996	TMEM71
Com_215_pos	D-Erythro-sphingosine 1-p	ENSGALG00000010764	FBXO8
Com_352_pos	Riboflavin	ENSGALG00000047792	SELENOM
Com_17_pos	L-Norleucine	ENSGALG00000032882	EVA1C
Com_16_neg	3-Hydroxybutyric acid	ENSGALG00000026663	CX3CL1
Com_119_pos	DL-Stachydrine	ENSGALG00000052768	LDLR
Com_18_neg	Arachidonic acid	ENSGALG00000012755	IGF-I
Com_12_pos	Betaine	ENSGALG00000016690	CYP2AC1
Com_8_neg	4-Methyl-2-Oxopentanoic	ENSGALG00000008912	ABCB1
Com_413_pos	L-Cystine	ENSGALG00000021340	CA9
Com_440_pos	PC (18:4e/4:0)	ENSGALG00000013726	PAICS
Com_130_neg	2-Hydroxyvaleric acid	ENSGALG00000005474	PNAT10
Com_413_pos	L-Cystine	ENSGALG00000011894	CYP2D6
Com_215_pos	D-Erythro-sphingosine 1-p	MSTRG.19484	--
Com_151_neg	Lysope 18:1	ENSGALG00000005831	DNAI1
Com_68_neg	PE (16:0/22:6)	MSTRG.8904	--
Com_68_neg	PE (16:0/22:6)	ENSGALG00000013583	FAM114A1
Com_152_pos	Acetyl-L-carnitine	ENSGALG00000010857	DAB1
Com_86_neg	Levulinic acid	ENSGALG00000036190	AOC1
Com_215_pos	D-Erythro-sphingosine 1-p	ENSGALG00000005043	ACACB
Com_152_pos	Acetyl-L-carnitine	ENSGALG00000003015	SERPINF1
Com_152_pos	Acetyl-L-carnitine	ENSGALG00000017040	C4
Com_460_pos	3-amino-4-(propylamino)c	ENSGALG00000016164	ABCG1
Com_119_pos	DL-Stachydrine	MSTRG.7572	--
Com_16_neg	3-Hydroxybutyric acid	ENSGALG00000003537	SGK2
Com_588_pos	Ornithine	ENSGALG00000011524	PPEF2
Com_54_pos	Uric acid	ENSGALG00000038740	AMY2A
Com_215_pos	D-Erythro-sphingosine 1-p	ENSGALG00000003081	SUCO
Com_40_pos	Choline	ENSGALG00000034438	GNB3
Com_203_pos	Serotonin	ENSGALG00000037811	NRSN1
Com_460_pos	3-amino-4-(propylamino)c	ENSGALG00000010857	DAB1
Com_92_pos	D-(+)-Proline	ENSGALG00000015040	SLC16A10
Com_208_neg	N-Acetylanthranilic acid	ENSGALG00000030920	APOC3
Com_17_pos	L-Norleucine	ENSGALG00000007252	ANKDD1A
Com_588_pos	Ornithine	ENSGALG00000005030	DOCK10
Com_482_pos	8-Hydroxyquinoline	ENSGALG00000008866	WDPCP
Com_203_pos	Serotonin	ENSGALG00000002437	DIPK1B
Com_99_pos	Creatine	ENSGALG00000006812	TTC36
Com_16_neg	3-Hydroxybutyric acid	ENSGALG00000014616	MT3
Com_150_neg	benzyl N-(2-{[(benzyloxy)c	ENSGALG00000006217	S100B
Com_588_pos	Ornithine	ENSGALG00000027122	APPL2
Com_386_pos	2-Amino-1,3-octadecanec	ENSGALG00000009740	RASGRP1
Com_413_pos	L-Cystine	ENSGALG00000028451	MT4
Com_482_pos	8-Hydroxyquinoline	MSTRG.1082	--
Com_97_pos	L-Threonine	ENSGALG00000047480	A2ML1
Com_18_neg	Arachidonic acid	ENSGALG00000036190	AOC1

Com_80_pos	DL-Lysine	ENSGALG00000009700	PDK4
Com_311_pos	PC (18:4e/2:0)	MSTRG.8511	--
Com_17_pos	L-Norleucine	ENSGALG00000028407	GDF9
Com_4_pos	PC (17:1/17:1)	ENSGALG00000030941	ELAPOR1
Com_151_neg	Lysope 18:1	MSTRG.20491	--
Com_471_pos	Indole-3-acetic acid	ENSGALG00000000619	ANGPTL4
Com_588_pos	Ornithine	ENSGALG00000034616	INHBA
Com_151_neg	Lysope 18:1	ENSGALG00000007077	CPT1A
Com_86_neg	Levulinic acid	ENSGALG00000012755	IGF-I
Com_97_pos	L-Threonine	ENSGALG00000047321	SARDH
Com_119_pos	DL-Stachydrine	ENSGALG00000021193	STARD5
Com_482_pos	8-Hydroxyquinoline	ENSGALG00000012377	HNMT
Com_80_pos	DL-Lysine	MSTRG.17350	--
Com_76_neg	Erythronolactone	MSTRG.10101	--
Com_8_neg	4-Methyl-2-Oxopentanoic	ENSGALG00000046639	CYP2AC2
Com_68_neg	PE (16:0/22:6)	ENSGALG00000007645	prom1a
Com_120_neg	LPC 15:0	ENSGALG00000002594	TFPI
Com_342_pos	1-(4-methylphenyl)-3,5-di	ENSGALG00000004410	ANAPC16
Com_12_pos	Betaine	ENSGALG00000001531	FN3K
Com_203_pos	Serotonin	MSTRG.2403	--
Com_151_pos	Pyridoxamine	ENSGALG00000017199	MAML2
Com_8_neg	4-Methyl-2-Oxopentanoic	ENSGALG00000005408	BCO1
Com_68_neg	PE (16:0/22:6)	ENSGALG00000034107	TRIM63
Com_17_pos	L-Norleucine	ENSGALG00000014750	TRB
Com_120_neg	LPC 15:0	MSTRG.20377	--
Com_186_pos	4-Hydroxybenzaldehyde	MSTRG.12291	--
Com_413_pos	L-Cystine	ENSGALG00000021848	AVD
Com_80_pos	DL-Lysine	ENSGALG00000005977	BTBD8
Com_18_neg	Arachidonic acid	ENSGALG00000008912	ABCB1
Com_53_neg	2-(5-mercapto-4-methyl-	MSTRG.163	--
Com_56_neg	LPE 18:1	MSTRG.8034	--
Com_17_pos	L-Norleucine	ENSGALG00000008185	AOX1
Com_151_pos	Pyridoxamine	ENSGALG00000033411	SLC26A2
Com_386_pos	2-Amino-1,3-octadecanec	ENSGALG00000004205	SOAT1
Com_165_neg	(±)9-HpODE	ENSGALG00000043920	OTUD3
Com_92_pos	D-(+)-Proline	MSTRG.11572	--
Com_471_pos	Indole-3-acetic acid	MSTRG.19422	--
Com_352_pos	Riboflavin	ENSGALG00000030357	ABL2
Com_120_neg	LPC 15:0	ENSGALG00000028871	SLC38A3
Com_55_pos	Valine	ENSGALG00000029724	MTURN
Com_165_neg	(±)9-HpODE	MSTRG.6227	--
Com_460_pos	3-amino-4-(propylamino)(	ENSGALG00000027793	SCN9A
Com_130_neg	2-Hydroxyvaleric acid	MSTRG.3009	--
Com_471_pos	Indole-3-acetic acid	ENSGALG00000023517	AGPAT2
Com_471_pos	Indole-3-acetic acid	ENSGALG00000015684	Dnajc25
Com_311_pos	PC (18:4e/2:0)	ENSGALG00000014950	SULT3A1
Com_16_neg	3-Hydroxybutyric acid	ENSGALG00000019147	--
Com_57_neg	LPC 16:1	ENSGALG00000008903	ITPRID2
Com_440_pos	PC (18:4e/4:0)	MSTRG.3197	--
Com_331_pos	L-Lysine	ENSGALG00000043582	LY6E
Com_56_neg	LPE 18:1	ENSGALG00000007109	APOA4
Com_171_neg	LPC 22:6	ENSGALG00000015425	LPL
Com_175_pos	Pantothenic acid	ENSGALG00000016364	ALKAL2
Com_68_neg	PE (16:0/22:6)	ENSGALG00000022720	GJB2
Com_440_pos	PC (18:4e/4:0)	ENSGALG00000040434	rab18b
Com_16_neg	3-Hydroxybutyric acid	MSTRG.14083	--
Com_588_pos	Ornithine	ENSGALG00000007114	APOA1
Com_186_pos	4-Hydroxybenzaldehyde	ENSGALG00000009172	OSBPL6

Com_171_neg	LPC 22:6	ENSGALG00000002790	ABLIM3
Com_86_neg	Levulinic acid	ENSGALG00000052964	TOPAZ1
Com_16_neg	3-Hydroxybutyric acid	MSTRG.19177	PHGDH
Com_76_neg	Erythronolactone	ENSGALG00000021848	AVD
Com_147_pos	D-Sphingosine	ENSGALG00000005472	NAT
Com_440_pos	PC (18:4e/4:0)	ENSGALG00000012034	ADSL
Com_311_pos	PC (18:4e/2:0)	ENSGALG00000007728	Prodh
Com_588_pos	Ornithine	ENSGALG00000004598	CUX2
Com_186_pos	4-Hydroxybenzaldehyde	ENSGALG00000003569	TMEM130
Com_24_neg	PE (16:0/20:4)	ENSGALG00000012045	slc12a8
Com_76_neg	Erythronolactone	MSTRG.8055	--
Com_21_pos	DL-Tryptophan	ENSGALG00000001392	MMP23B
Com_119_pos	DL-Stachydrine	ENSGALG00000053043	CARHSP1
Com_86_neg	Levulinic acid	ENSGALG00000052583	A2ML1
Com_460_pos	3-amino-4-(propylamino)(	ENSGALG00000005408	BCO1
Com_97_pos	L-Threonine	ENSGALG00000005474	PNAT10
Com_471_pos	Indole-3-acetic acid	MSTRG.9165	--
Com_68_neg	PE (16:0/22:6)	ENSGALG00000025738	RHOU
Com_56_neg	LPE 18:1	ENSGALG00000026263	RGS8
Com_386_pos	2-Amino-1,3-octadecanec	ENSGALG00000011287	SULT
Com_155_neg	Phenylacetaldehyde	MSTRG.17162	--
Com_413_pos	L-Cystine	MSTRG.10101	--
Com_16_neg	3-Hydroxybutyric acid	ENSGALG00000054981	F10
Com_16_neg	3-Hydroxybutyric acid	ENSGALG00000033171	TGM4
Com_311_pos	PC (18:4e/2:0)	ENSGALG00000038242	CACNA2D2
Com_460_pos	3-amino-4-(propylamino)(	ENSGALG00000046639	CYP2AC2
Com_86_neg	Levulinic acid	ENSGALG00000032882	EVA1C
Com_119_pos	DL-Stachydrine	ENSGALG00000007636	PCK1
Com_53_neg	2-(5-mercapto-4-methyl-4	ENSGALG00000031164	WFDC2
Com_150_neg	benzyl N-(2-{[(benzyloxy)c	ENSGALG00000011809	GRIN2B
Com_56_neg	LPE 18:1	ENSGALG00000009830	MGAT4D
Com_108_neg	LPE 18:2	MSTRG.19484	--
Com_194_pos	Pipecolic acid	ENSGALG00000006864	COL24A1
Com_78_neg	Citric acid	MSTRG.836	--
Com_311_pos	PC (18:4e/2:0)	ENSGALG00000004343	HPD
Com_152_pos	Acetyl-L-carnitine	ENSGALG00000028871	SLC38A3
Com_208_neg	N-Acetylanthranilic acid	ENSGALG00000047632	Pc
Com_76_neg	Erythronolactone	ENSGALG00000028451	MT4
Com_471_pos	Indole-3-acetic acid	ENSGALG00000031754	KCNG2
Com_151_neg	Lysope 18:1	MSTRG.12866	--
Com_99_pos	Creatine	ENSGALG00000049256	--
Com_108_neg	LPE 18:2	ENSGALG00000005043	ACACB
Com_53_neg	2-(5-mercapto-4-methyl-4	MSTRG.6499	--
Com_208_neg	N-Acetylanthranilic acid	ENSGALG00000002431	CFH
Com_40_pos	Choline	ENSGALG00000055021	GREM2
Com_108_neg	LPE 18:2	ENSGALG00000003081	SUCO
Com_152_pos	Acetyl-L-carnitine	ENSGALG00000008912	ABCB1
Com_130_neg	2-Hydroxyvaleric acid	ENSGALG00000047480	A2ML1
Com_155_neg	Phenylacetaldehyde	ENSGALG00000028560	OC3
Com_57_neg	LPC 16:1	MSTRG.13046	--
Com_8_neg	4-Methyl-2-Oxopentanoic	ENSGALG00000010857	DAB1
Com_311_pos	PC (18:4e/2:0)	ENSGALG00000013726	PAICS
Com_588_pos	Ornithine	ENSGALG00000034081	AKT3
Com_80_pos	DL-Lysine	ENSGALG00000017103	WASF3
Com_331_pos	L-Lysine	ENSGALG00000002790	ABLIM3
Com_130_neg	2-Hydroxyvaleric acid	ENSGALG00000047321	SARDH
Com_18_neg	Arachidonic acid	ENSGALG00000052872	--
Com_8_neg	4-Methyl-2-Oxopentanoic	ENSGALG00000054981	F10

Com_16_neg	3-Hydroxybutyric acid	ENSGALG00000013728	PPAT
Com_99_pos	Creatine	ENSGALG00000001697	ITIH3
Com_178_pos	Maltol	ENSGALG00000030031	TTPA
Com_76_neg	Erythronolactone	ENSGALG00000011894	CYP2D6
Com_352_pos	Riboflavin	ENSGALG00000010708	ICA1
Com_331_pos	L-Lysine	ENSGALG00000015425	LPL
Com_194_pos	Pipecolic acid	ENSGALG00000034140	ZNF395
Com_119_pos	DL-Stachydrine	ENSGALG00000041238	NOS1AP
Com_482_pos	8-Hydroxyquinoline	MSTRG.16504	gag
Com_89_neg	Gallic acid	MSTRG.1841	--
Com_86_neg	Levulinic acid	ENSGALG00000028407	GDF9
Com_165_neg	(±)9-HpODE	ENSGALG00000010494	SLC5A9
Com_80_pos	DL-Lysine	ENSGALG00000019835	TRIM27.2
Com_186_pos	4-Hydroxybenzaldehyde	ENSGALG00000034507	CHST2
Com_413_pos	L-Cystine	ENSGALG00000047321	SARDH
Com_165_neg	(±)9-HpODE	ENSGALG00000029788	CCK
Com_194_pos	Pipecolic acid	ENSGALG00000005263	SOX8
Com_147_pos	D-Sphingosine	ENSGALG00000017032	SLC25A15
Com_482_pos	8-Hydroxyquinoline	ENSGALG00000000950	MVB12B
Com_76_neg	Erythronolactone	ENSGALG00000037671	psuG
Com_311_pos	PC (18:4e/2:0)	ENSGALG00000006374	TBX6
Com_99_pos	Creatine	ENSGALG00000012420	CG-1B
Com_413_pos	L-Cystine	ENSGALG00000047480	A2ML1
Com_342_pos	1-(4-methylphenyl)-3,5-di	ENSGALG00000011560	PACRG
Com_108_neg	LPE 18:2	ENSGALG00000006080	GPC4
Com_8_neg	4-Methyl-2-Oxopentanoic	MSTRG.14083	--
Com_18_neg	Arachidonic acid	ENSGALG00000014252	A2M
Com_208_neg	N-Acetylanthranilic acid	ENSGALG00000016446	ATP6V1C2
Com_440_pos	PC (18:4e/4:0)	MSTRG.8511	--
Com_460_pos	3-amino-4-(propylamino)c	ENSGALG00000008780	CTBS
Com_471_pos	Indole-3-acetic acid	ENSGALG00000051251	H2B-I
Com_203_pos	Serotonin	ENSGALG00000040969	PTP4A3
Com_4_pos	PC (17:1/17:1)	ENSGALG00000010837	ASB5
Com_192_pos	1-Methylhistidine	ENSGALG00000001697	ITIH3
Com_92_pos	D-(+)-Proline	ENSGALG00000002024	COMT
Com_18_neg	Arachidonic acid	ENSGALG00000040573	FMO3
Com_208_neg	N-Acetylanthranilic acid	MSTRG.20494	AHNAK
Com_311_pos	PC (18:4e/2:0)	ENSGALG00000011994	SYNPO2
Com_120_neg	LPC 15:0	ENSGALG00000032231	C4
Com_362_pos	2-Arachidonoyl glycerol	ENSGALG00000054322	--
Com_147_pos	D-Sphingosine	ENSGALG00000043582	LY6E
Com_440_pos	PC (18:4e/4:0)	ENSGALG00000030031	TTPA
Com_16_neg	3-Hydroxybutyric acid	ENSGALG00000003212	TSPO2
Com_18_neg	Arachidonic acid	ENSGALG00000017040	C4
Com_18_neg	Arachidonic acid	ENSGALG00000003015	SERPINF1
Com_692_pos	3-amino-2-phenyl-2H-py	ENSGALG00000016296	GFRAL
Com_86_neg	Levulinic acid	ENSGALG00000008185	AOX1
Com_4_pos	PC (17:1/17:1)	ENSGALG00000002919	MON1A
Com_16_neg	3-Hydroxybutyric acid	ENSGALG00000012754	PAH
Com_120_neg	LPC 15:0	ENSGALG00000010857	DAB1
Com_86_neg	Levulinic acid	ENSGALG00000014412	CSTA
Com_362_pos	2-Arachidonoyl glycerol	ENSGALG00000029724	MTURN
Com_18_neg	Arachidonic acid	ENSGALG00000026663	CX3CL1
Com_78_neg	Citric acid	ENSGALG00000015425	LPL
Com_57_neg	LPC 16:1	MSTRG.7586	--
Com_97_pos	L-Threonine	ENSGALG00000021340	CA9
Com_192_pos	1-Methylhistidine	ENSGALG00000049256	--
Com_78_neg	Citric acid	ENSGALG00000002790	ABLIM3

Com_471_pos	Indole-3-acetic acid	ENSGALG00000012748	ELOVL2
Com_471_pos	Indole-3-acetic acid	ENSGALG00000020538	SLC49A3
Com_130_neg	2-Hydroxyvaleric acid	ENSGALG00000007839	NCAM1
Com_588_pos	Ornithine	ENSGALG00000021340	CA9
Com_208_neg	N-Acetylanthranilic acid	ENSGALG00000037780	PMEPA1
Com_311_pos	PC (18:4e/2:0)	ENSGALG00000014412	CSTA
Com_471_pos	Indole-3-acetic acid	ENSGALG00000006702	MFGE8
Com_17_pos	L-Norleucine	ENSGALG00000002479	MAT1A
Com_21_pos	DL-Tryptophan	ENSGALG00000049755	IL22RA2
Com_151_pos	Pyridoxamine	ENSGALG00000019835	TRIM27.2
Com_482_pos	8-Hydroxyquinoline	MSTRG.15443	--
Com_54_pos	Uric acid	ENSGALG00000005776	TECR
Com_80_pos	DL-Lysine	ENSGALG00000011003	SLC35F3
Com_460_pos	3-amino-4-(propylamino)(	ENSGALG00000002594	TFPI
Com_119_pos	DL-Stachydrine	ENSGALG00000009415	SMOC1
Com_68_neg	PE (16:0/22:6)	MSTRG.12866	--
Com_18_neg	Arachidonic acid	ENSGALG00000014616	MT3
Com_203_pos	Serotonin	ENSGALG00000010798	DHCR24
Com_119_pos	DL-Stachydrine	ENSGALG00000008795	GPAM
Com_86_neg	Levulinic acid	ENSGALG00000011994	SYNPO2
Com_413_pos	L-Cystine	MSTRG.3009	--
Com_460_pos	3-amino-4-(propylamino)(	MSTRG.20377	--
Com_151_pos	Pyridoxamine	ENSGALG00000017103	WASF3
Com_413_pos	L-Cystine	ENSGALG00000005030	DOCK10
Com_12_pos	Betaine	ENSGALG00000055021	GREM2
Com_171_neg	LPC 22:6	MSTRG.836	--
Com_413_pos	L-Cystine	ENSGALG00000027122	APPL2
Com_175_pos	Pantothenic acid	ENSGALG00000031122	NTNG1
Com_175_pos	Pantothenic acid	MSTRG.4548	--
Com_86_neg	Levulinic acid	ENSGALG00000006374	TBX6
Com_92_pos	D-(+)-Proline	ENSGALG00000027908	CYP2U1
Com_56_neg	LPE 18:1	ENSGALG00000050069	BMP1
Com_165_neg	(±)9-HpODE	ENSGALG00000011181	FKBP14
Com_588_pos	Ornithine	ENSGALG00000054856	ADH1
Com_8_neg	4-Methyl-2-Oxopentanoic	ENSGALG00000003537	SGK2
Com_203_pos	Serotonin	ENSGALG00000003029	PLPP6
Com_413_pos	L-Cystine	ENSGALG00000034616	INHBA
Com_16_neg	3-Hydroxybutyric acid	ENSGALG00000041680	KCNT2
Com_80_pos	DL-Lysine	ENSGALG00000016036	DOP1B
Com_25_pos	2-Hydroxycinnamic acid	MSTRG.11633	Stap2
Com_482_pos	8-Hydroxyquinoline	ENSGALG00000038723	RPP25L
Com_54_pos	Uric acid	ENSGALG00000013090	LOXL4
Com_80_pos	DL-Lysine	ENSGALG00000017046	POSTN
Com_482_pos	8-Hydroxyquinoline	ENSGALG00000031525	TSTA3
Com_8_neg	4-Methyl-2-Oxopentanoic	ENSGALG00000028871	SLC38A3
Com_92_pos	D-(+)-Proline	ENSGALG00000003015	SERPINF1
Com_92_pos	D-(+)-Proline	ENSGALG00000017040	C4
Com_16_neg	3-Hydroxybutyric acid	ENSGALG00000008912	ABCB1
Com_440_pos	PC (18:4e/4:0)	ENSGALG00000004343	HPD
Com_18_neg	Arachidonic acid	ENSGALG00000002024	COMT
Com_152_pos	Acetyl-L-carnitine	ENSGALG00000015040	SLC16A10
Com_8_neg	4-Methyl-2-Oxopentanoic	ENSGALG00000019663	ACBD7
Com_208_neg	N-Acetylanthranilic acid	ENSGALG00000003432	AGXT2
Com_8_neg	4-Methyl-2-Oxopentanoic	ENSGALG00000005204	GSTT1
Com_151_neg	Lysope 18:1	MSTRG.20480	--
Com_178_pos	Maltol	ENSGALG00000002790	ABLIM3
Com_482_pos	8-Hydroxyquinoline	ENSGALG00000012112	DBI
Com_89_neg	Gallic acid	ENSGALG00000052887	--

Com_18_neg	Arachidonic acid	ENSGALG00000019147	--
Com_151_neg	Lysope 18:1	ENSGALG00000005442	PALMD
Com_120_neg	LPC 15:0	ENSGALG00000005408	BCO1
Com_194_pos	Pipecolic acid	ENSGALG00000047827	TMEM86A
Com_8_neg	4-Methyl-2-Oxopentanoic	ENSGALG00000009002	CPED1
Com_152_pos	Acetyl-L-carnitine	ENSGALG00000054981	F10
Com_692_pos	3-amino-2-phenyl-2H-py	MSTRG.18923	--
Com_331_pos	L-Lysine	ENSGALG00000030031	TTPA
Com_147_pos	D-Sphingosine	MSTRG.3197	--
Com_178_pos	Maltol	ENSGALG00000015425	LPL
Com_86_neg	Levulinic acid	ENSGALG00000004343	HPD
Com_147_pos	D-Sphingosine	ENSGALG00000040434	rab18b
Com_120_neg	LPC 15:0	ENSGALG00000046639	CYP2AC2
Com_92_pos	D-(+)-Proline	ENSGALG00000009880	INPP4B
Com_76_neg	Erythronolactone	ENSGALG00000036086	TAGLN2
Com_215_pos	D-Erythro-sphingosine 1-p	ENSGALG00000006080	GPC4
Com_192_pos	1-Methylhistidine	ENSGALG00000010163	LGR5
Com_56_neg	LPE 18:1	ENSGALG00000024298	ADAMTSL5
Com_18_neg	Arachidonic acid	MSTRG.19177	PHGDH
Com_16_neg	3-Hydroxybutyric acid	ENSGALG00000038520	STRIP2
Com_57_neg	LPC 16:1	MSTRG.13506	CGREF1
Com_175_pos	Pantothenic acid	ENSGALG00000003972	FAXDC2
Com_89_neg	Gallic acid	ENSGALG00000053043	CARHSP1
Com_151_neg	Lysope 18:1	ENSGALG00000034107	TRIM63
Com_194_pos	Pipecolic acid	ENSGALG00000029724	MTURN
Com_147_pos	D-Sphingosine	ENSGALG00000012034	ADSL
Com_352_pos	Riboflavin	MSTRG.18742	LRP2
Com_108_neg	LPE 18:2	ENSGALG00000031570	WDR54
Com_460_pos	3-amino-4-(propylamino)c	ENSGALG00000041373	ARAP2
Com_588_pos	Ornithine	ENSGALG00000012704	MYLIP
Com_192_pos	1-Methylhistidine	ENSGALG00000006812	TTC36
Com_252_pos	cis-4-Hydroxy-D-proline	ENSGALG00000036742	GATSL2
Com_130_neg	2-Hydroxyvaleric acid	ENSGALG00000021340	CA9
Com_76_neg	Erythronolactone	ENSGALG00000035903	FAM46A
Com_152_pos	Acetyl-L-carnitine	MSTRG.14083	--
Com_440_pos	PC (18:4e/4:0)	ENSGALG00000006374	TBX6
Com_97_pos	L-Threonine	ENSGALG00000004598	CUX2
Com_215_pos	D-Erythro-sphingosine 1-p	ENSGALG00000053217	INPP5J
Com_57_neg	LPC 16:1	ENSGALG00000005831	DNAI1
Com_386_pos	2-Amino-1,3-octadecanec	ENSGALG00000028928	LCAT
Com_17_pos	L-Norleucine	ENSGALG00000014412	CSTA
Com_18_neg	Arachidonic acid	ENSGALG00000033171	TGM4
Com_97_pos	L-Threonine	ENSGALG00000007839	NCAM1
Com_151_pos	Pyridoxamine	ENSGALG00000030025	FABP4
Com_24_neg	PE (16:0/20:4)	ENSGALG00000043920	OTUD3
Com_155_neg	Phenylacetaldehyde	ENSGALG00000015468	Tstd3
Com_108_neg	LPE 18:2	ENSGALG00000047720	KLHDC7A
Com_97_pos	L-Threonine	ENSGALG00000007114	APOA1
Com_440_pos	PC (18:4e/4:0)	ENSGALG00000011994	SYNPO2
Com_8_neg	4-Methyl-2-Oxopentanoic	ENSGALG00000048343	Ces1e
Com_80_pos	DL-Lysine	ENSGALG00000017199	MAML2
Com_311_pos	PC (18:4e/2:0)	ENSGALG00000030031	TTPA
Com_362_pos	2-Arachidonoyl glycerol	ENSGALG00000005263	SOX8
Com_215_pos	D-Erythro-sphingosine 1-p	MSTRG.8499	Itpripl1
Com_92_pos	D-(+)-Proline	ENSGALG00000008953	AASS
Com_16_neg	3-Hydroxybutyric acid	ENSGALG00000031158	OAT
Com_76_neg	Erythronolactone	ENSGALG00000015492	PDZK1
Com_76_neg	Erythronolactone	MSTRG.29	SHANK3

Com_17_pos	L-Norleucine	MSTRG.29	SHANK3
Com_151_pos	Pyridoxamine	MSTRG.17350	--
Com_178_pos	Maltol	ENSGALG00000013726	PAICS
Com_86_neg	Levulinic acid	ENSGALG00000002479	MAT1A
Com_68_neg	PE (16:0/22:6)	ENSGALG00000011094	PDE4B
Com_252_pos	cis-4-Hydroxy-D-proline	ENSGALG00000014872	FGF10
Com_471_pos	Indole-3-acetic acid	ENSGALG00000036754	CHKA
Com_151_neg	Lysope 18:1	ENSGALG00000013583	FAM114A1
Com_68_neg	PE (16:0/22:6)	ENSGALG00000005831	DNAI1
Com_55_pos	Valine	ENSGALG00000006864	COL24A1
Com_17_pos	L-Norleucine	ENSGALG00000011994	SYNPO2
Com_99_pos	Creatine	ENSGALG00000015624	VCAN
Com_151_pos	Pyridoxamine	ENSGALG00000009947	PLEKHH2
Com_18_neg	Arachidonic acid	ENSGALG00000013728	PPAT
Com_21_pos	DL-Tryptophan	ENSGALG00000011469	IGFBP2
Com_120_neg	LPC 15:0	ENSGALG00000008780	CTBS
Com_352_pos	Riboflavin	ENSGALG00000004569	UNC5B
Com_413_pos	L-Cystine	ENSGALG00000034081	AKT3
Com_460_pos	3-amino-4-(propylamino)(	ENSGALG00000032231	C4
Com_440_pos	PC (18:4e/4:0)	ENSGALG00000014412	CSTA
Com_482_pos	8-Hydroxyquinoline	ENSGALG00000011254	SATB1
Com_17_pos	L-Norleucine	ENSGALG00000006374	TBX6
Com_8_neg	4-Methyl-2-Oxopentanoic	ENSGALG00000022750	GPR18
Com_482_pos	8-Hydroxyquinoline	ENSGALG00000002802	PACSIN1
Com_178_pos	Maltol	ENSGALG00000038242	CACNA2D2
Com_151_neg	Lysope 18:1	ENSGALG00000029102	PXYLP1
Com_413_pos	L-Cystine	ENSGALG00000006320	Slc2a9
Com_55_pos	Valine	MSTRG.836	--
Com_471_pos	Indole-3-acetic acid	ENSGALG00000001492	NDRG3
Com_203_pos	Serotonin	ENSGALG00000050083	SYCP2L
Com_588_pos	Ornithine	ENSGALG00000047321	SARDH
Com_194_pos	Pipecolic acid	ENSGALG00000039239	SERPIND1
Com_86_neg	Levulinic acid	MSTRG.8511	--
Com_55_pos	Valine	ENSGALG00000034140	ZNF395
Com_18_neg	Arachidonic acid	MSTRG.11572	--
Com_178_pos	Maltol	ENSGALG00000007728	Prodh
Com_147_pos	D-Sphingosine	MSTRG.8511	--
Com_68_neg	PE (16:0/22:6)	ENSGALG00000010237	NPC2
Com_588_pos	Ornithine	ENSGALG00000047480	A2ML1
Com_130_neg	2-Hydroxyvaleric acid	ENSGALG00000050154	--
Com_152_pos	Acetyl-L-carnitine	ENSGALG00000003537	SGK2
Com_18_neg	Arachidonic acid	ENSGALG00000003212	TSPO2
Com_92_pos	D-(+)-Proline	ENSGALG00000008912	ABCB1
Com_16_neg	3-Hydroxybutyric acid	ENSGALG00000017040	C4
Com_16_neg	3-Hydroxybutyric acid	ENSGALG00000003015	SERPINF1
Com_24_neg	PE (16:0/20:4)	MSTRG.14201	ZNF541
Com_440_pos	PC (18:4e/4:0)	ENSGALG00000043582	LY6E
Com_18_neg	Arachidonic acid	ENSGALG00000012754	PAH
Com_151_neg	Lysope 18:1	ENSGALG00000008903	ITPRID2
Com_331_pos	L-Lysine	ENSGALG00000054322	--
Com_120_neg	LPC 15:0	ENSGALG00000002728	SLC16A3
Com_386_pos	2-Amino-1,3-octadecaned	ENSGALG00000038923	Ces1e
Com_108_neg	LPE 18:2	ENSGALG00000002371	RUSC2
Com_80_pos	DL-Lysine	MSTRG.17721	--
Com_386_pos	2-Amino-1,3-octadecaned	ENSGALG00000016281	DMD
Com_119_pos	DL-Stachydrine	ENSGALG00000006490	SCN3B
Com_175_pos	Pantothenic acid	ENSGALG00000016651	TDH
Com_342_pos	1-(4-methylphenyl)-3,5-di	MSTRG.21090	--

Com_97_pos	L-Threonine	ENSGALG00000011524	PPEF2
Com_186_pos	4-Hydroxybenzaldehyde	ENSGALG00000037780	PMEPA1
Com_178_pos	Maltol	ENSGALG00000014950	SULT3A1
Com_89_neg	Gallic acid	ENSGALG00000052768	LDLR
Com_68_neg	PE (16:0/22:6)	ENSGALG00000037629	TRANK1
Com_53_neg	2-(5-mercapto-4-methyl-	ENSGALG00000008434	SORCS3
Com_692_pos	3-amino-2-phenyl-2H-py	MSTRG.4608	--
Com_92_pos	D-(+)-Proline	ENSGALG00000016196	CBSL
Com_17_pos	L-Norleucine	ENSGALG00000004343	HPD
Com_8_neg	4-Methyl-2-Oxopentanoic	ENSGALG00000015040	SLC16A10
Com_203_pos	Serotonin	ENSGALG00000005766	PKD2L1
Com_175_pos	Pantothenic acid	ENSGALG00000030121	SLC2A11
Com_108_neg	LPE 18:2	ENSGALG00000024449	RAMP2
Com_482_pos	8-Hydroxyquinoline	ENSGALG00000051398	TMEM14C
Com_152_pos	Acetyl-L-carnitine	ENSGALG00000019663	ACBD7
Com_24_neg	PE (16:0/20:4)	ENSGALG00000029788	CCK
Com_152_pos	Acetyl-L-carnitine	ENSGALG00000005204	GSTT1
Com_215_pos	D-Erythro-sphingosine 1-p	ENSGALG00000010703	DGLUCY
Com_471_pos	Indole-3-acetic acid	ENSGALG00000007507	MASTL
Com_386_pos	2-Amino-1,3-octadecaned	ENSGALG00000005472	NAT
Com_130_neg	2-Hydroxyvaleric acid	ENSGALG00000004598	CUX2
Com_152_pos	Acetyl-L-carnitine	ENSGALG00000009002	CPED1
Com_471_pos	Indole-3-acetic acid	ENSGALG00000026957	SEMA4G
Com_78_neg	Citric acid	ENSGALG00000029724	MTURN
Com_362_pos	2-Arachidonoyl glycerol	ENSGALG00000015425	LPL
Com_92_pos	D-(+)-Proline	ENSGALG00000016325	GSTA3
Com_54_pos	Uric acid	ENSGALG00000011469	IGFBP2
Com_215_pos	D-Erythro-sphingosine 1-p	ENSGALG00000031570	WDR54
Com_21_pos	DL-Tryptophan	ENSGALG00000053860	mas
Com_362_pos	2-Arachidonoyl glycerol	ENSGALG00000002790	ABLIM3
Com_12_pos	Betaine	ENSGALG00000051274	B3GALT2
Com_130_neg	2-Hydroxyvaleric acid	ENSGALG00000007114	APOA1
Com_147_pos	D-Sphingosine	ENSGALG00000011287	SULT
Com_21_pos	DL-Tryptophan	ENSGALG00000008859	WDR31
Com_108_neg	LPE 18:2	ENSGALG00000053217	INPP5J
Com_471_pos	Indole-3-acetic acid	ENSGALG00000027608	PIGC
Com_57_neg	LPC 16:1	MSTRG.12866	--
Com_175_pos	Pantothenic acid	ENSGALG00000016281	DMD
Com_192_pos	1-Methylhistidine	ENSGALG00000006976	Bdh1
Com_588_pos	Ornithine	MSTRG.3009	--
Com_175_pos	Pantothenic acid	ENSGALG00000038923	Ces1e
Com_215_pos	D-Erythro-sphingosine 1-p	ENSGALG00000047720	KLHDC7A
Com_108_neg	LPE 18:2	MSTRG.8499	Itpripl1
Com_92_pos	D-(+)-Proline	ENSGALG00000014836	LPIN2
Com_18_neg	Arachidonic acid	ENSGALG00000041680	KCNT2
Com_151_neg	Lysope 18:1	MSTRG.13046	--
Com_588_pos	Ornithine	ENSGALG00000022750	GPR18
Com_76_neg	Erythronolactone	ENSGALG00000002479	MAT1A
Com_460_pos	3-amino-4-(propylamino)(	ENSGALG00000033338	GPT2
Com_165_neg	(±)9-HpODE	MSTRG.16972	--
Com_203_pos	Serotonin	MSTRG.21092	--
Com_86_neg	Levulinic acid	ENSGALG00000012034	ADSL
Com_86_neg	Levulinic acid	MSTRG.29	SHANK3
Com_203_pos	Serotonin	ENSGALG00000037261	RFXANK
Com_203_pos	Serotonin	ENSGALG00000037852	HSD17B7
Com_155_neg	Phenylacetaldehyde	ENSGALG00000008434	SORCS3
Com_413_pos	L-Cystine	ENSGALG00000054856	ADH1
Com_68_neg	PE (16:0/22:6)	MSTRG.7586	--

Com_151_pos	Pyridoxamine	ENSGALG00000013124	FHOD3
Com_80_pos	DL-Lysine	ENSGALG00000030121	SLC2A11
Com_152_pos	Acetyl-L-carnitine	ENSGALG00000048343	Ces1e
Com_40_pos	Choline	ENSGALG00000007710	zgc:110179
Com_4_pos	PC (17:1/17:1)	ENSGALG00000009016	SLX4IP
Com_16_neg	3-Hydroxybutyric acid	ENSGALG00000002024	COMT
Com_86_neg	Levulinic acid	ENSGALG00000040434	rab18b
Com_89_neg	Gallic acid	ENSGALG00000055000	KCTD14
Com_40_pos	Choline	ENSGALG00000009050	CAPN3
Com_80_pos	DL-Lysine	ENSGALG00000046687	EPS8L3
Com_147_pos	D-Sphingosine	ENSGALG00000004343	HPD
Com_12_pos	Betaine	MSTRG.15995	--
Com_331_pos	L-Lysine	ENSGALG00000013726	PAICS
Com_86_neg	Levulinic acid	MSTRG.3197	--
Com_119_pos	DL-Stachydrine	ENSGALG00000028005	GADD45G
Com_471_pos	Indole-3-acetic acid	ENSGALG00000008039	MFSD13A
Com_80_pos	DL-Lysine	ENSGALG00000016651	TDH
Com_151_neg	Lysope 18:1	MSTRG.9774	--
Com_252_pos	cis-4-Hydroxy-D-proline	ENSGALG00000034438	GNB3
Com_150_neg	benzyl N-(2-{[(benzyloxy)c	ENSGALG00000005657	CRHR2
Com_311_pos	PC (18:4e/2:0)	ENSGALG00000014750	TRB
Com_119_pos	DL-Stachydrine	ENSGALG00000014261	UCHL1
Com_482_pos	8-Hydroxyquinoline	ENSGALG00000001475	STMN1
Com_120_neg	LPC 15:0	ENSGALG00000041373	ARAP2
Com_24_neg	PE (16:0/20:4)	ENSGALG00000011808	CCR9
Com_89_neg	Gallic acid	MSTRG.9007	--
Com_186_pos	4-Hydroxybenzaldehyde	ENSGALG00000002431	CFH
Com_151_pos	Pyridoxamine	MSTRG.5269	pitpnc1
Com_194_pos	Pipecolic acid	ENSGALG00000000761	TSKU
Com_386_pos	2-Amino-1,3-octadecaneo	ENSGALG00000017032	SLC25A15
Com_97_pos	L-Threonine	ENSGALG00000050154	--
Com_588_pos	Ornithine	ENSGALG00000048343	Ces1e
Com_186_pos	4-Hydroxybenzaldehyde	ENSGALG00000047632	Pc
Com_178_pos	Maltol	ENSGALG00000044996	TMEM71
Com_215_pos	D-Erythro-sphingosine 1-p	ENSGALG00000004424	SEC16B
Com_152_pos	Acetyl-L-carnitine	ENSGALG00000027908	CYP2U1
Com_413_pos	L-Cystine	ENSGALG00000009926	HAAO
Com_24_neg	PE (16:0/20:4)	ENSGALG00000011181	FKBP14
Com_203_pos	Serotonin	ENSGALG00000014948	HMGCR
Com_352_pos	Riboflavin	MSTRG.1679	--
Com_192_pos	1-Methylhistidine	ENSGALG00000010889	HOOK1
Com_18_neg	Arachidonic acid	ENSGALG00000038520	STRIP2
Com_482_pos	8-Hydroxyquinoline	ENSGALG00000000104	CRY1
Com_17_pos	L-Norleucine	MSTRG.8511	--
Com_482_pos	8-Hydroxyquinoline	ENSGALG00000031067	TMEM132A
Com_331_pos	L-Lysine	ENSGALG00000038242	CACNA2D2
Com_152_pos	Acetyl-L-carnitine	ENSGALG00000022750	GPR18
Com_53_neg	2-(5-mercapto-4-methyl-4	ENSGALG00000012886	--
Com_55_pos	Valine	ENSGALG00000047827	TMEM86A
Com_130_neg	2-Hydroxyvaleric acid	ENSGALG00000041258	msrA
Com_215_pos	D-Erythro-sphingosine 1-p	ENSGALG00000036021	MTMR7
Com_130_neg	2-Hydroxyvaleric acid	ENSGALG00000011524	PPEF2
Com_352_pos	Riboflavin	ENSGALG00000010009	TTC29
Com_208_neg	N-Acetylanthranilic acid	ENSGALG00000038652	Gsta3
Com_12_pos	Betaine	ENSGALG00000034478	CCL4
Com_311_pos	PC (18:4e/2:0)	ENSGALG00000007252	ANKDD1A
Com_8_neg	4-Methyl-2-Oxopentanoic	ENSGALG00000012704	MYLIP
Com_108_neg	LPE 18:2	ENSGALG00000031932	AGPAT3

Com_331_pos	L-Lysine	ENSGALG00000007728	Prodh
Com_208_neg	N-Acetylanthranilic acid	ENSGALG00000003569	TMEM130
Com_215_pos	D-Erythro-sphingosine 1-p	ENSGALG00000002371	RUSC2
Com_147_pos	D-Sphingosine	ENSGALG00000006374	TBX6
Com_21_pos	DL-Tryptophan	ENSGALG00000013090	LOXL4
Com_151_pos	Pyridoxamine	ENSGALG00000013244	ABCC9
Com_99_pos	Creatine	ENSGALG00000049966	Ufc1
Com_413_pos	L-Cystine	ENSGALG00000012704	MYLIP
Com_17_pos	L-Norleucine	ENSGALG00000011894	CYP2D6
Com_99_pos	Creatine	ENSGALG00000015358	MYH15
Com_175_pos	Pantothenic acid	ENSGALG00000028928	LCAT
Com_53_neg	2-(5-mercapto-4-methyl-	ENSGALG00000045814	CRLF2
Com_482_pos	8-Hydroxyquinoline	MSTRG.15754	--
Com_40_pos	Choline	ENSGALG00000011687	AHNAK2
Com_178_pos	Maltol	ENSGALG00000054322	--
Com_4_pos	PC (17:1/17:1)	ENSGALG00000006198	LSS
Com_147_pos	D-Sphingosine	ENSGALG00000011994	SYNPO2
Com_215_pos	D-Erythro-sphingosine 1-p	ENSGALG00000024449	RAMP2
Com_311_pos	PC (18:4e/2:0)	ENSGALG00000043582	LY6E
Com_18_neg	Arachidonic acid	ENSGALG00000031158	OAT
Com_352_pos	Riboflavin	ENSGALG00000009483	MARK1
Com_108_neg	LPE 18:2	ENSGALG00000010703	DGLUCY
Com_171_neg	LPC 22:6	ENSGALG00000029724	MTURN
Com_588_pos	Ornithine	ENSGALG00000009002	CPED1
Com_331_pos	L-Lysine	ENSGALG00000014950	SULT3A1
Com_56_neg	LPE 18:1	ENSGALG00000039536	C2H8ORF22
Com_92_pos	D-(+)-Proline	ENSGALG00000054981	F10
Com_413_pos	L-Cystine	ENSGALG00000007252	ANKDD1A
Com_588_pos	Ornithine	ENSGALG00000005204	GSTT1
Com_588_pos	Ornithine	ENSGALG00000019663	ACBD7
Com_152_pos	Acetyl-L-carnitine	ENSGALG00000009880	INPP4B
Com_53_neg	2-(5-mercapto-4-methyl-	ENSGALG00000015468	Tstd3
Com_130_neg	2-Hydroxyvaleric acid	ENSGALG00000016138	DSCAM
Com_17_pos	L-Norleucine	ENSGALG00000028451	MT4
Com_165_neg	(±)9-HpODE	MSTRG.2409	--
Com_130_neg	2-Hydroxyvaleric acid	ENSGALG00000028341	MADCAM1
Com_78_neg	Citric acid	ENSGALG00000005263	SOX8
Com_21_pos	DL-Tryptophan	ENSGALG00000005776	TECR
Com_54_pos	Uric acid	ENSGALG00000049755	IL22RA2
Com_311_pos	PC (18:4e/2:0)	ENSGALG00000009926	HAAO
Com_203_pos	Serotonin	MSTRG.20656	--
Com_92_pos	D-(+)-Proline	ENSGALG00000029944	FAM222A
Com_120_neg	LPC 15:0	ENSGALG00000032746	ENPP2
Com_147_pos	D-Sphingosine	ENSGALG00000014412	CSTA
Com_92_pos	D-(+)-Proline	MSTRG.14083	--
Com_57_neg	LPC 16:1	MSTRG.10121	--
Com_460_pos	3-amino-4-(propylamino)c	ENSGALG00000002728	SLC16A3
Com_588_pos	Ornithine	ENSGALG00000003537	SGK2
Com_16_neg	3-Hydroxybutyric acid	ENSGALG00000011524	PPEF2
Com_8_neg	4-Methyl-2-Oxopentanoic	ENSGALG00000054856	ADH1
Com_203_pos	Serotonin	ENSGALG00000028376	FGF19
Com_413_pos	L-Cystine	ENSGALG00000014750	TRB
Com_215_pos	D-Erythro-sphingosine 1-p	ENSGALG00000006649	TMEM41A
Com_80_pos	DL-Lysine	ENSGALG00000003972	FAXDC2
Com_17_pos	L-Norleucine	ENSGALG00000021848	AVD
Com_17_pos	L-Norleucine	ENSGALG00000012034	ADSL
Com_130_neg	2-Hydroxyvaleric acid	ENSGALG00000002855	SARDH
Com_16_neg	3-Hydroxybutyric acid	MSTRG.11572	--

Com_76_neg	Erythronolactone	ENSGALG00000033338	GPT2
Com_152_pos	Acetyl-L-carnitine	ENSGALG00000008953	AASS
Com_588_pos	Ornithine	ENSGALG00000006320	Slc2a9
Com_120_neg	LPC 15:0	ENSGALG00000003578	FN1
Com_482_pos	8-Hydroxyquinoline	ENSGALG00000033051	CAMK1D
Com_4_pos	PC (17:1/17:1)	ENSGALG00000028005	GADD45G
Com_203_pos	Serotonin	ENSGALG00000037065	SC5D
Com_482_pos	8-Hydroxyquinoline	ENSGALG00000014525	USP5
Com_17_pos	L-Norleucine	ENSGALG00000040434	rab18b
Com_352_pos	Riboflavin	ENSGALG00000000003	PANX2
Com_86_neg	Levulinic acid	ENSGALG00000017032	SLC25A15
Com_119_pos	DL-Stachydrine	ENSGALG00000009016	SLX4IP
Com_342_pos	1-(4-methylphenyl)-3,5-di	ENSGALG00000045814	CRLF2
Com_17_pos	L-Norleucine	MSTRG.3197	--
Com_57_neg	LPC 16:1	ENSGALG00000042215	FAAP100
Com_130_neg	2-Hydroxyvaleric acid	ENSGALG00000015034	ANKRD29
Com_194_pos	Pipecolic acid	MSTRG.836	--
Com_21_pos	DL-Tryptophan	ENSGALG00000000498	ACE
Com_130_neg	2-Hydroxyvaleric acid	ENSGALG00000008728	PTER
Com_386_pos	2-Amino-1,3-octadecanec	MSTRG.4548	--
Com_386_pos	2-Amino-1,3-octadecanec	ENSGALG00000031122	NTNG1
Com_171_neg	LPC 22:6	ENSGALG00000011391	AMN
Com_362_pos	2-Arachidonoyl glycerol	ENSGALG00000003136	IKZF2
Com_362_pos	2-Arachidonoyl glycerol	ENSGALG00000009963	LYZ
Com_171_neg	LPC 22:6	ENSGALG00000011314	LRRC3B
Com_55_pos	Valine	ENSGALG00000039239	SERPIND1
Com_120_neg	LPC 15:0	ENSGALG00000016027	CBR3
Com_692_pos	3-amino-2-phenyl-2H-py	ENSGALG00000054619	GAB2
Com_151_neg	Lysope 18:1	MSTRG.13506	CGREF1
Com_386_pos	2-Amino-1,3-octadecanec	MSTRG.3197	--
Com_482_pos	8-Hydroxyquinoline	ENSGALG00000000226	TMEM9
Com_76_neg	Erythronolactone	ENSGALG00000008185	AOX1
Com_57_neg	LPC 16:1	ENSGALG00000034107	TRIM63
Com_386_pos	2-Amino-1,3-octadecanec	ENSGALG00000040434	rab18b
Com_342_pos	1-(4-methylphenyl)-3,5-di	ENSGALG00000012886	--
Com_215_pos	D-Erythro-sphingosine 1-p	ENSGALG00000011616	NPFFR2
Com_97_pos	L-Threonine	ENSGALG00000041258	msrA
Com_175_pos	Pantothenic acid	ENSGALG00000017046	POSTN
Com_108_neg	LPE 18:2	ENSGALG00000004424	SEC16B
Com_203_pos	Serotonin	ENSGALG00000040730	RXRG
Com_80_pos	DL-Lysine	ENSGALG00000035219	ALB
Com_175_pos	Pantothenic acid	ENSGALG00000016036	DOP1B
Com_440_pos	PC (18:4e/4:0)	ENSGALG00000014750	TRB
Com_386_pos	2-Amino-1,3-octadecanec	ENSGALG00000012034	ADSL
Com_108_neg	LPE 18:2	ENSGALG00000045127	slc12a8
Com_203_pos	Serotonin	ENSGALG00000006521	TRPM5
Com_56_neg	LPE 18:1	MSTRG.21123	--
Com_331_pos	L-Lysine	ENSGALG00000044996	TMEM71
Com_108_neg	LPE 18:2	ENSGALG00000036021	MTMR7
Com_120_neg	LPC 15:0	ENSGALG00000033338	GPT2
Com_178_pos	Maltol	ENSGALG00000008728	PTER
Com_8_neg	4-Methyl-2-Oxopentanoic	ENSGALG00000027908	CYP2U1
Com_89_neg	Gallic acid	ENSGALG00000029308	PNPLA3
Com_17_pos	L-Norleucine	MSTRG.10101	--
Com_165_neg	(±)9-HpODE	ENSGALG00000021238	CYP2W1
Com_215_pos	D-Erythro-sphingosine 1-p	ENSGALG00000031932	AGPAT3
Com_178_pos	Maltol	ENSGALG00000015034	ANKRD29
Com_151_pos	Pyridoxamine	ENSGALG00000028897	WDR25

Com_440_pos	PC (18:4e/4:0)	ENSGALG00000011287	SULT
Com_147_pos	D-Sphingosine	ENSGALG00000028928	LCAT
Com_92_pos	D-(+)-Proline	ENSGALG00000003537	SGK2
Com_151_pos	Pyridoxamine	ENSGALG00000029270	GATA3
Com_165_neg	(±)9-HpODE	MSTRG.14201	ZNF541
Com_97_pos	L-Threonine	ENSGALG00000031158	OAT
Com_76_neg	Erythronolactone	ENSGALG00000028407	GDF9
Com_99_pos	Creatine	ENSGALG00000051068	SIGLEC1
Com_588_pos	Ornithine	MSTRG.14083	--
Com_86_neg	Levulinic acid	ENSGALG00000011894	CYP2D6
Com_16_neg	3-Hydroxybutyric acid	ENSGALG00000007114	APOA1
Com_252_pos	cis-4-Hydroxy-D-proline	MSTRG.11633	Stap2
Com_178_pos	Maltol	ENSGALG00000002855	SARDH
Com_152_pos	Acetyl-L-carnitine	ENSGALG00000012704	MYLIP
Com_21_pos	DL-Tryptophan	ENSGALG00000002116	TEN1
Com_194_pos	Pipecolic acid	ENSGALG00000003802	OTUD7A
Com_175_pos	Pantothenic acid	ENSGALG00000011003	SLC35F3
Com_97_pos	L-Threonine	ENSGALG00000016138	DSCAM
Com_208_neg	N-Acetylanthranilic acid	ENSGALG00000015358	MYH15
Com_440_pos	PC (18:4e/4:0)	ENSGALG00000007252	ANKDD1A
Com_208_neg	N-Acetylanthranilic acid	ENSGALG00000049966	Ufc1
Com_57_neg	LPC 16:1	ENSGALG00000013583	FAM114A1
Com_151_neg	Lysope 18:1	ENSGALG00000012089	--
Com_97_pos	L-Threonine	ENSGALG00000028341	MADCAM1
Com_16_neg	3-Hydroxybutyric acid	ENSGALG00000004598	CUX2
Com_4_pos	PC (17:1/17:1)	MSTRG.8619	--
Com_99_pos	Creatine	ENSGALG00000003569	TMEM130
Com_152_pos	Acetyl-L-carnitine	ENSGALG00000016196	CBSL
Com_4_pos	PC (17:1/17:1)	ENSGALG00000006490	SCN3B
Com_86_neg	Levulinic acid	ENSGALG00000005472	NAT
Com_92_pos	D-(+)-Proline	ENSGALG00000019663	ACBD7
Com_171_neg	LPC 22:6	ENSGALG00000037160	Smad7
Com_92_pos	D-(+)-Proline	ENSGALG00000005204	GSTT1
Com_12_pos	Betaine	MSTRG.14987	--
Com_203_pos	Serotonin	ENSGALG00000037325	SERP1
Com_413_pos	L-Cystine	ENSGALG00000022750	GPR18
Com_171_neg	LPC 22:6	ENSGALG00000005263	SOX8
Com_588_pos	Ornithine	ENSGALG00000054981	F10
Com_120_neg	LPC 15:0	ENSGALG00000010357	P2RY1
Com_92_pos	D-(+)-Proline	ENSGALG00000009002	CPED1
Com_119_pos	DL-Stachydrine	ENSGALG00000052296	MEX3D
Com_86_neg	Levulinic acid	ENSGALG00000028451	MT4
Com_8_neg	4-Methyl-2-Oxopentanoic	ENSGALG00000034081	AKT3
Com_152_pos	Acetyl-L-carnitine	ENSGALG00000016325	GSTA3
Com_120_neg	LPC 15:0	ENSGALG00000005860	ACAA1
Com_178_pos	Maltol	ENSGALG00000028341	MADCAM1
Com_97_pos	L-Threonine	ENSGALG00000038520	STRIP2
Com_76_neg	Erythronolactone	ENSGALG00000032882	EVA1C
Com_57_neg	LPC 16:1	ENSGALG00000029102	PXYLP1
Com_482_pos	8-Hydroxyquinoline	ENSGALG00000033365	ALDH1A3
Com_8_neg	4-Methyl-2-Oxopentanoic	ENSGALG00000009880	INPP4B
Com_178_pos	Maltol	ENSGALG00000016138	DSCAM
Com_352_pos	Riboflavin	ENSGALG00000042215	FAAP100
Com_311_pos	PC (18:4e/2:0)	ENSGALG00000006320	Slc2a9
Com_352_pos	Riboflavin	ENSGALG00000010233	SYNDIG1L
Com_208_neg	N-Acetylanthranilic acid	ENSGALG00000023122	SULT1B
Com_215_pos	D-Erythro-sphingosine 1-p	ENSGALG00000033461	hnmt
Com_97_pos	L-Threonine	ENSGALG00000002855	SARDH

Com_55_pos	Valine	ENSGALG00000009700	PDK4
Com_482_pos	8-Hydroxyquinoline	MSTRG.21394	gag-pol
Com_76_neg	Erythronolactone	ENSGALG00000052583	A2ML1
Com_108_neg	LPE 18:2	ENSGALG00000006649	TMEM41A
Com_192_pos	1-Methylhistidine	ENSGALG00000017120	SACS
Com_215_pos	D-Erythro-sphingosine 1-p	ENSGALG00000015253	COL8A1
Com_86_neg	Levulinic acid	ENSGALG00000021848	AVD
Com_68_neg	PE (16:0/22:6)	ENSGALG00000050983	PRKG1
Com_97_pos	L-Threonine	ENSGALG00000015034	ANKRD29
Com_460_pos	3-amino-4-(propylamino)c	ENSGALG00000015492	PDZK1
Com_386_pos	2-Amino-1,3-octadecanec	MSTRG.8511	--
Com_440_pos	PC (18:4e/4:0)	ENSGALG00000009926	HAAO
Com_68_neg	PE (16:0/22:6)	ENSGALG00000028175	GJA9
Com_208_neg	N-Acetylanthranilic acid	ENSGALG00000012683	RNF144B
Com_152_pos	Acetyl-L-carnitine	ENSGALG00000014836	LPIN2
Com_76_neg	Erythronolactone	ENSGALG00000052964	TOPAZ1
Com_97_pos	L-Threonine	ENSGALG00000008728	PTER
Com_413_pos	L-Cystine	ENSGALG00000048343	Ces1e
Com_24_neg	PE (16:0/20:4)	MSTRG.16972	--
Com_588_pos	Ornithine	ENSGALG00000009926	HAAO
Com_152_pos	Acetyl-L-carnitine	ENSGALG00000054856	ADH1
Com_352_pos	Riboflavin	MSTRG.10121	--
Com_482_pos	8-Hydroxyquinoline	ENSGALG00000021658	PAFAH2
Com_17_pos	L-Norleucine	ENSGALG00000017032	SLC25A15
Com_55_pos	Valine	ENSGALG00000000761	TSKU
Com_55_pos	Valine	ENSGALG00000005977	BTBD8
Com_147_pos	D-Sphingosine	ENSGALG00000038923	Ces1e
Com_78_neg	Citric acid	ENSGALG00000011391	AMN
Com_92_pos	D-(+)-Proline	ENSGALG00000048343	Ces1e
Com_78_neg	Citric acid	ENSGALG00000011314	LRRC3B
Com_147_pos	D-Sphingosine	ENSGALG00000016281	DMD
Com_8_neg	4-Methyl-2-Oxopentanoic	ENSGALG00000008953	AASS
Com_97_pos	L-Threonine	ENSGALG00000041680	KCNT2
Com_178_pos	Maltol	ENSGALG00000041258	msrA
Com_68_neg	PE (16:0/22:6)	MSTRG.1845	--
Com_18_neg	Arachidonic acid	ENSGALG00000017039	STOML3
Com_460_pos	3-amino-4-(propylamino)c	ENSGALG00000035903	FAM46A
Com_175_pos	Pantothenic acid	ENSGALG00000011287	SULT
Com_186_pos	4-Hydroxybenzaldehyde	ENSGALG00000012420	CG-1B
Com_53_neg	2-(5-mercapto-4-methyl-.	ENSGALG00000028560	OC3
Com_203_pos	Serotonin	ENSGALG00000035206	CNPY2
Com_76_neg	Erythronolactone	ENSGALG00000041373	ARAP2
Com_194_pos	Pipecolic acid	ENSGALG00000027891	NREP
Com_165_neg	(±)9-HpODE	ENSGALG00000011808	CCR9
Com_108_neg	LPE 18:2	ENSGALG00000011616	NPFFR2
Com_130_neg	2-Hydroxyvaleric acid	ENSGALG00000044996	TMEM71
Com_4_pos	PC (17:1/17:1)	ENSGALG00000016665	FDFT1
Com_460_pos	3-amino-4-(propylamino)c	ENSGALG00000032746	ENPP2
Com_108_neg	LPE 18:2	MSTRG.8248	--
Com_482_pos	8-Hydroxyquinoline	MSTRG.5319	--
Com_203_pos	Serotonin	ENSGALG00000010301	EIF2B2
Com_208_neg	N-Acetylanthranilic acid	ENSGALG00000035026	SLC22A4
Com_215_pos	D-Erythro-sphingosine 1-p	ENSGALG00000045127	slc12a8
Com_130_neg	2-Hydroxyvaleric acid	ENSGALG00000031158	OAT
Com_460_pos	3-amino-4-(propylamino)c	ENSGALG00000036086	TAGLN2
Com_482_pos	8-Hydroxyquinoline	ENSGALG00000051779	PRORSD1P
Com_89_neg	Gallic acid	ENSGALG00000037401	IDH3A
Com_165_neg	(±)9-HpODE	ENSGALG00000033867	PCOLCE

Com_413_pos	L-Cystine	ENSGALG00000009002	CPED1
Com_92_pos	D-(+)-Proline	ENSGALG00000022750	GPR18
Com_265_pos	6-Methylquinoline	MSTRG.13474	Klhl29
Com_86_neg	Levulinic acid	MSTRG.10101	--
Com_588_pos	Ornithine	ENSGALG00000007252	ANKDD1A
Com_413_pos	L-Cystine	ENSGALG00000005204	GSTT1
Com_386_pos	2-Amino-1,3-octadecanec	ENSGALG00000016364	ALKAL2
Com_17_pos	L-Norleucine	ENSGALG00000005030	DOCK10
Com_413_pos	L-Cystine	ENSGALG00000019663	ACBD7
Com_76_neg	Erythronolactone	ENSGALG00000012755	IGF-I
Com_53_neg	2-(5-mercapto-4-methyl-·	MSTRG.17162	--
Com_460_pos	3-amino-4-(propylamino)(	ENSGALG00000003578	FN1
Com_17_pos	L-Norleucine	ENSGALG00000027122	APPL2
Com_331_pos	L-Lysine	ENSGALG00000008728	PTER
Com_192_pos	1-Methylhistidine	ENSGALG00000016761	LYG2
Com_331_pos	L-Lysine	ENSGALG00000015034	ANKRD29
Com_151_neg	Lysope 18:1	MSTRG.21822	--
Com_215_pos	D-Erythro-sphingosine 1-p	ENSGALG00000028230	SUN2
Com_16_neg	3-Hydroxybutyric acid	ENSGALG00000021340	CA9
Com_18_neg	Arachidonic acid	ENSGALG00000011524	PPEF2
Com_17_pos	L-Norleucine	ENSGALG00000034616	INHBA
Com_171_neg	LPC 22:6	ENSGALG00000026313	RND3
Com_460_pos	3-amino-4-(propylamino)(	ENSGALG00000016027	CBR3
Com_311_pos	PC (18:4e/2:0)	ENSGALG00000011287	SULT
Com_186_pos	4-Hydroxybenzaldehyde	ENSGALG00000052388	METRN
Com_8_neg	4-Methyl-2-Oxopentanoic	ENSGALG00000034616	INHBA
Com_78_neg	Citric acid	ENSGALG00000037160	Smad7
Com_57_neg	LPC 16:1	MSTRG.1679	--
Com_130_neg	2-Hydroxyvaleric acid	ENSGALG00000038520	STRIP2
Com_331_pos	L-Lysine	ENSGALG00000002855	SARDH
Com_97_pos	L-Threonine	ENSGALG00000012754	PAH
Com_194_pos	Pipecolic acid	ENSGALG00000034337	RHPN1
Com_413_pos	L-Cystine	ENSGALG00000003537	SGK2
Com_17_pos	L-Norleucine	ENSGALG00000005472	NAT
Com_57_neg	LPC 16:1	MSTRG.9774	--
Com_97_pos	L-Threonine	ENSGALG00000003212	TSPO2
Com_171_neg	LPC 22:6	ENSGALG00000034741	ETNPPL
Com_482_pos	8-Hydroxyquinoline	ENSGALG00000007404	YIPF5
Com_119_pos	DL-Stachydrine	ENSGALG00000014907	DCBLD1
Com_482_pos	8-Hydroxyquinoline	MSTRG.13261	--
Com_588_pos	Ornithine	ENSGALG00000014750	TRB
Com_76_neg	Erythronolactone	ENSGALG00000036190	AOC1
Com_151_neg	Lysope 18:1	MSTRG.9524	--
Com_8_neg	4-Methyl-2-Oxopentanoic	ENSGALG00000027122	APPL2
Com_386_pos	2-Amino-1,3-octadecanec	ENSGALG00000004343	HPD
Com_24_neg	PE (16:0/20:4)	MSTRG.21123	--
Com_8_neg	4-Methyl-2-Oxopentanoic	ENSGALG00000005030	DOCK10
Com_151_pos	Pyridoxamine	ENSGALG00000002466	SLC2A5
Com_8_neg	4-Methyl-2-Oxopentanoic	ENSGALG00000016196	CBSL
Com_119_pos	DL-Stachydrine	ENSGALG00000021039	HKDC1
Com_92_pos	D-(+)-Proline	ENSGALG00000009479	SAMD9L
Com_165_neg	(±)9-HpODE	ENSGALG00000048020	STAG3
Com_24_neg	PE (16:0/20:4)	MSTRG.2409	--
Com_108_neg	LPE 18:2	ENSGALG00000050520	pol
Com_155_neg	Phenylacetaldehyde	ENSGALG00000009157	ECT2
Com_108_neg	LPE 18:2	ENSGALG00000033461	hnmt
Com_208_neg	N-Acetylanthranilic acid	ENSGALG00000013100	GRB10
Com_331_pos	L-Lysine	ENSGALG00000028341	MADCAM1

Com_152_pos	Acetyl-L-carnitine	ENSGALG00000034081	AKT3
Com_8_neg	4-Methyl-2-Oxopentanoic	ENSGALG00000016325	GSTA3
Com_56_neg	LPE 18:1	ENSGALG00000054870	TXNL1
Com_152_pos	Acetyl-L-carnitine	ENSGALG00000029944	FAM222A
Com_331_pos	L-Lysine	ENSGALG00000016138	DSCAM
Com_215_pos	D-Erythro-sphingosine 1-p	ENSGALG00000016412	MBOAT2
Com_175_pos	Pantothenic acid	ENSGALG00000005977	BTBD8
Com_186_pos	4-Hydroxybenzaldehyde	ENSGALG00000020391	SERPINA10
Com_4_pos	PC (17:1/17:1)	ENSGALG00000035675	--
Com_588_pos	Ornithine	ENSGALG00000008912	ABCB1
Com_108_neg	LPE 18:2	ENSGALG00000015253	COL8A1
Com_342_pos	1-(4-methylphenyl)-3,5-di	MSTRG.13977	PIP4P1
Com_97_pos	L-Threonine	ENSGALG00000013728	PPAT
Com_311_pos	PC (18:4e/2:0)	MSTRG.3009	--
Com_78_neg	Citric acid	ENSGALG00000003136	IKZF2
Com_78_neg	Citric acid	ENSGALG00000009963	LYZ
Com_130_neg	2-Hydroxyvaleric acid	ENSGALG00000014950	SULT3A1
Com_12_pos	Betaine	ENSGALG00000041344	FABP5
Com_147_pos	D-Sphingosine	ENSGALG00000014750	TRB
Com_130_neg	2-Hydroxyvaleric acid	ENSGALG00000041680	KCNT2
Com_440_pos	PC (18:4e/4:0)	ENSGALG00000006320	Slc2a9
Com_151_pos	Pyridoxamine	MSTRG.20478	--
Com_4_pos	PC (17:1/17:1)	ENSGALG00000008795	GPAM
Com_40_pos	Choline	ENSGALG00000053140	NDRG2
Com_4_pos	PC (17:1/17:1)	ENSGALG00000009415	SMOC1
Com_413_pos	L-Cystine	ENSGALG00000014412	CSTA
Com_386_pos	2-Amino-1,3-octadecanec	ENSGALG00000006374	TBX6
Com_215_pos	D-Erythro-sphingosine 1-p	ENSGALG00000006783	PLOD2
Com_97_pos	L-Threonine	ENSGALG00000044996	TMEM71
Com_440_pos	PC (18:4e/4:0)	ENSGALG00000028928	LCAT
Com_8_neg	4-Methyl-2-Oxopentanoic	ENSGALG00000014836	LPIN2
Com_175_pos	Pantothenic acid	ENSGALG00000009700	PDK4
Com_203_pos	Serotonin	MSTRG.17623	--
Com_178_pos	Maltol	ENSGALG00000050154	--
Com_460_pos	3-amino-4-(propylamino)c	ENSGALG00000010357	P2RY1
Com_155_neg	Phenylacetaldehyde	ENSGALG00000015849	ME1
Com_186_pos	4-Hydroxybenzaldehyde	ENSGALG00000014821	THEMIS
Com_18_neg	Arachidonic acid	ENSGALG00000007114	APOA1
Com_130_neg	2-Hydroxyvaleric acid	ENSGALG00000007728	Prodh
Com_165_neg	(±)9-HpODE	MSTRG.20356	--
Com_460_pos	3-amino-4-(propylamino)c	ENSGALG00000005860	ACAA1
Com_203_pos	Serotonin	ENSGALG00000013569	SEC61B
Com_89_neg	Gallic acid	ENSGALG00000042706	TMC2
Com_108_neg	LPE 18:2	ENSGALG00000035239	GLCCI1
Com_386_pos	2-Amino-1,3-octadecanec	ENSGALG00000011994	SYNPO2
Com_99_pos	Creatine	ENSGALG00000004959	IRS1
Com_331_pos	L-Lysine	ENSGALG00000041258	msrA
Com_89_neg	Gallic acid	ENSGALG00000012220	CDKN3
Com_97_pos	L-Threonine	ENSGALG00000033171	TGM4
Com_413_pos	L-Cystine	MSTRG.14083	--
Com_147_pos	D-Sphingosine	ENSGALG00000007252	ANKDD1A
Com_76_neg	Erythronolactone	ENSGALG00000008780	CTBS
Com_18_neg	Arachidonic acid	ENSGALG00000004598	CUX2
Com_215_pos	D-Erythro-sphingosine 1-p	MSTRG.8248	--
Com_482_pos	8-Hydroxyquinoline	ENSGALG00000005610	SLC44A3
Com_130_neg	2-Hydroxyvaleric acid	ENSGALG00000038242	CACNA2D2
Com_18_neg	Arachidonic acid	MSTRG.8055	--
Com_192_pos	1-Methylhistidine	ENSGALG00000030920	APOC3

Com_151_neg	Lysope 18:1	ENSGALG00000050309	H2B-I
Com_460_pos	3-amino-4-(propylamino)(	ENSGALG00000037671	psuG
Com_108_neg	LPE 18:2	ENSGALG00000004498	SLC2A10
Com_119_pos	DL-Stachydrine	ENSGALG00000002919	MON1A
Com_25_pos	2-Hydroxycinnamic acid	ENSGALG00000034438	GNB3
Com_413_pos	L-Cystine	ENSGALG00000011994	SYNPO2
Com_120_neg	LPC 15:0	ENSGALG00000015492	PDZK1
Com_208_neg	N-Acetylanthranilic acid	ENSGALG00000011331	CTH
Com_692_pos	3-amino-2-phenyl-2H-py	ENSGALG00000026214	LRTM2
Com_55_pos	Valine	ENSGALG00000003802	OTUD7A
Com_17_pos	L-Norleucine	ENSGALG00000034081	AKT3
Com_86_neg	Levulinic acid	ENSGALG00000005030	DOCK10
Com_55_pos	Valine	ENSGALG00000011003	SLC35F3
Com_97_pos	L-Threonine	MSTRG.19177	PHGDH
Com_151_neg	Lysope 18:1	MSTRG.10121	--
Com_119_pos	DL-Stachydrine	ENSGALG00000010837	ASB5
Com_86_neg	Levulinic acid	ENSGALG00000027122	APPL2
Com_311_pos	PC (18:4e/2:0)	ENSGALG00000047480	A2ML1
Com_24_neg	PE (16:0/20:4)	ENSGALG00000021238	CYP2W1
Com_413_pos	L-Cystine	ENSGALG00000006374	TBX6
Com_413_pos	L-Cystine	ENSGALG00000054981	F10
Com_386_pos	2-Amino-1,3-octadecanec	ENSGALG00000014412	CSTA
Com_342_pos	1-(4-methylphenyl)-3,5-di	ENSGALG00000040896	FASN
Com_130_neg	2-Hydroxyvaleric acid	ENSGALG00000012754	PAH
Com_108_neg	LPE 18:2	ENSGALG00000028230	SUN2
Com_130_neg	2-Hydroxyvaleric acid	ENSGALG00000013726	PAICS
Com_311_pos	PC (18:4e/2:0)	ENSGALG00000047321	SARDH
Com_130_neg	2-Hydroxyvaleric acid	ENSGALG00000003212	TSPO2
Com_12_pos	Betaine	ENSGALG00000004230	LIPC
Com_86_neg	Levulinic acid	ENSGALG00000034616	INHBA
Com_97_pos	L-Threonine	ENSGALG00000019147	--
Com_78_neg	Citric acid	ENSGALG00000026313	RND3
Com_119_pos	DL-Stachydrine	MSTRG.19672	--
Com_471_pos	Indole-3-acetic acid	ENSGALG00000030845	ENHO
Com_482_pos	8-Hydroxyquinoline	ENSGALG00000007018	SLC26A11
Com_92_pos	D-(+)-Proline	ENSGALG00000012704	MYLIP
Com_78_neg	Citric acid	ENSGALG00000034741	ETNPPL
Com_120_neg	LPC 15:0	ENSGALG00000035903	FAM46A
Com_151_neg	Lysope 18:1	MSTRG.1843	--
Com_151_neg	Lysope 18:1	ENSGALG00000042215	FAAP100
Com_4_pos	PC (17:1/17:1)	ENSGALG00000041238	NOS1AP
Com_76_neg	Erythronolactone	ENSGALG00000052872	--
Com_147_pos	D-Sphingosine	ENSGALG00000009926	HAAO
Com_89_neg	Gallic acid	MSTRG.2171	Myo16
Com_186_pos	4-Hydroxybenzaldehyde	ENSGALG00000015624	VCAN
Com_55_pos	Valine	ENSGALG00000016036	DOP1B
Com_215_pos	D-Erythro-sphingosine 1-(	ENSGALG00000004127	--
Com_194_pos	Pipecolic acid	ENSGALG00000009700	PDK4
Com_55_pos	Valine	ENSGALG00000017046	POSTN
Com_4_pos	PC (17:1/17:1)	ENSGALG00000016610	PTRHD1
Com_120_neg	LPC 15:0	ENSGALG00000020688	CYP4A22
Com_152_pos	Acetyl-L-carnitine	ENSGALG00000034616	INHBA
Com_440_pos	PC (18:4e/4:0)	ENSGALG00000038923	Ces1e
Com_120_neg	LPC 15:0	ENSGALG00000036086	TAGLN2
Com_147_pos	D-Sphingosine	MSTRG.4548	--
Com_8_neg	4-Methyl-2-Oxopentanoic	MSTRG.10101	--
Com_76_neg	Erythronolactone	ENSGALG00000046639	CYP2AC2
Com_147_pos	D-Sphingosine	ENSGALG00000031122	NTNG1

Com_215_pos	D-Erythro-sphingosine 1-|	ENSGALG00000050520	pol
Com_352_pos	Riboflavin	ENSGALG00000008326	SYTL2
Com_57_neg	LPC 16:1	ENSGALG00000004569	UNC5B
Com_440_pos	PC (18:4e/4:0)	ENSGALG00000016281	DMD
Com_25_pos	2-Hydroxycinnamic acid	ENSGALG00000002549	RGS1
Com_18_neg	Arachidonic acid	ENSGALG00000037671	psuG
Com_16_neg	3-Hydroxybutyric acid	ENSGALG00000017039	STOML3
Com_130_neg	2-Hydroxyvaleric acid	ENSGALG00000013728	PPAT
Com_56_neg	LPE 18:1	ENSGALG00000016415	MAP7D2
Com_413_pos	L-Cystine	ENSGALG00000004343	HPD
Com_152_pos	Acetyl-L-carnitine	ENSGALG00000027122	APPL2
Com_97_pos	L-Threonine	ENSGALG00000014950	SULT3A1
Com_76_neg	Erythronolactone	ENSGALG00000005408	BCO1
Com_186_pos	4-Hydroxybenzaldehyde	ENSGALG00000013356	IKBKE
Com_152_pos	Acetyl-L-carnitine	ENSGALG00000005030	DOCK10
Com_460_pos	3-amino-4-(propylamino)(	MSTRG.8055	--
Com_16_neg	3-Hydroxybutyric acid	ENSGALG00000047321	SARDH
Com_186_pos	4-Hydroxybenzaldehyde	ENSGALG00000030038	C3
Com_76_neg	Erythronolactone	ENSGALG00000014252	A2M
Com_108_neg	LPE 18:2	ENSGALG00000016412	MBOAT2
Com_165_neg	(±)9-HpODE	ENSGALG00000002142	DGKQ
Com_165_neg	(±)9-HpODE	ENSGALG00000053164	gag-pol
Com_120_neg	LPC 15:0	ENSGALG00000003595	SARM1
Com_56_neg	LPE 18:1	ENSGALG00000011808	CCR9
Com_362_pos	2-Arachidonoyl glycerol	ENSGALG00000011391	AMN
Com_171_neg	LPC 22:6	ENSGALG00000003136	IKZF2
Com_171_neg	LPC 22:6	ENSGALG00000009963	LYZ
Com_194_pos	Pipecolic acid	ENSGALG00000005977	BTBD8
Com_76_neg	Erythronolactone	ENSGALG00000040573	FMO3
Com_362_pos	2-Arachidonoyl glycerol	ENSGALG00000011314	LRRC3B
Com_16_neg	3-Hydroxybutyric acid	ENSGALG00000047480	A2ML1
Com_4_pos	PC (17:1/17:1)	ENSGALG00000007636	PCK1
Com_151_pos	Pyridoxamine	ENSGALG00000041491	ACKR4
Com_588_pos	Ornithine	ENSGALG00000017040	C4
Com_588_pos	Ornithine	ENSGALG00000003015	SERPINF1
Com_53_neg	2-(5-mercapto-4-methyl-4	ENSGALG00000011560	PACRG
Com_97_pos	L-Threonine	ENSGALG00000014616	MT3
Com_92_pos	D-(+)-Proline	ENSGALG00000006482	FAH
Com_342_pos	1-(4-methylphenyl)-3,5-di	ENSGALG00000000227	DPYSL2
Com_8_neg	4-Methyl-2-Oxopentanoic	ENSGALG00000029944	FAM222A
Com_215_pos	D-Erythro-sphingosine 1-|	ENSGALG00000005722	SEC31B
Com_97_pos	L-Threonine	ENSGALG00000007728	Prodh
Com_76_neg	Erythronolactone	ENSGALG00000026663	CX3CL1
Com_12_pos	Betaine	ENSGALG00000041491	ACKR4
Com_55_pos	Valine	ENSGALG00000027891	NREP
Com_150_neg	benzyl N-(2-{[(benzyloxy)c	MSTRG.21420	--
Com_155_neg	Phenylacetaldehyde	ENSGALG00000010825	AGR2
Com_192_pos	1-Methylhistidine	ENSGALG00000039140	CD14
Com_264_pos	Indole	ENSGALG00000038740	AMY2A
Com_92_pos	D-(+)-Proline	ENSGALG00000054856	ADH1
Com_108_neg	LPE 18:2	ENSGALG00000006783	PLOD2
Com_130_neg	2-Hydroxyvaleric acid	ENSGALG00000033171	TGM4
Com_99_pos	Creatine	ENSGALG00000037780	PMEPA1
Com_108_neg	LPE 18:2	MSTRG.1890	gag
Com_440_pos	PC (18:4e/4:0)	MSTRG.3009	--
Com_151_neg	Lysope 18:1	ENSGALG00000014719	SETD9
Com_97_pos	L-Threonine	ENSGALG00000038242	CACNA2D2
Com_57_neg	LPC 16:1	ENSGALG00000012089	--

Com_151_pos	Pyridoxamine	ENSGALG00000009963	LYZ
Com_108_neg	LPE 18:2	ENSGALG00000010643	ZYG11B
Com_151_pos	Pyridoxamine	ENSGALG00000003136	IKZF2
Com_352_pos	Riboflavin	MSTRG.15241	--
Com_18_neg	Arachidonic acid	ENSGALG00000021340	CA9
Com_57_neg	LPC 16:1	MSTRG.18742	LRP2
Com_215_pos	D-Erythro-sphingosine 1-p	ENSGALG00000035239	GLCCI1
Com_8_neg	4-Methyl-2-Oxopentanoic	ENSGALG00000021848	AVD
Com_386_pos	2-Amino-1,3-octadecaned	ENSGALG00000034741	ETNPPL
Com_175_pos	Pantothenic acid	ENSGALG00000043582	LY6E
Com_208_neg	N-Acetylanthranilic acid	ENSGALG00000028256	CCL19
Com_97_pos	L-Threonine	ENSGALG00000026663	CX3CL1
Com_331_pos	L-Lysine	ENSGALG00000050154	--
Com_215_pos	D-Erythro-sphingosine 1-p	MSTRG.5038	--
Com_352_pos	Riboflavin	MSTRG.13506	CGREF1
Com_17_pos	L-Norleucine	ENSGALG00000054856	ADH1
Com_386_pos	2-Amino-1,3-octadecaned	ENSGALG00000026313	RND3
Com_130_neg	2-Hydroxyvaleric acid	MSTRG.19177	PHGDH
Com_311_pos	PC (18:4e/2:0)	ENSGALG00000028928	LCAT
Com_76_neg	Erythronolactone	ENSGALG00000014616	MT3
Com_342_pos	1-(4-methylphenyl)-3,5-di	MSTRG.6499	--
Com_265_pos	6-Methylquinoline	ENSGALG00000001392	MMP23B
Com_215_pos	D-Erythro-sphingosine 1-p	ENSGALG00000004498	SLC2A10
Com_97_pos	L-Threonine	ENSGALG00000040573	FMO3
Com_215_pos	D-Erythro-sphingosine 1-p	ENSGALG00000004657	FBXO2
Com_471_pos	Indole-3-acetic acid	MSTRG.13584	--
Com_482_pos	8-Hydroxyquinoline	MSTRG.21796	--
Com_482_pos	8-Hydroxyquinoline	ENSGALG00000026460	myoM
Com_97_pos	L-Threonine	ENSGALG00000013726	PAICS
Com_99_pos	Creatine	ENSGALG00000009920	COCH
Com_130_neg	2-Hydroxyvaleric acid	ENSGALG00000019147	--
Com_97_pos	L-Threonine	ENSGALG00000014252	A2M
Com_86_neg	Levulinic acid	ENSGALG00000034081	AKT3
Com_342_pos	1-(4-methylphenyl)-3,5-di	ENSGALG00000031164	WFDC2
Com_24_neg	PE (16:0/20:4)	ENSGALG00000033867	PCOLCE
Com_16_neg	3-Hydroxybutyric acid	MSTRG.3009	--
Com_8_neg	4-Methyl-2-Oxopentanoic	ENSGALG00000028451	MT4
Com_171_neg	LPC 22:6	ENSGALG00000016364	ALKAL2
Com_362_pos	2-Arachidonoyl glycerol	ENSGALG00000037160	Smad7
Com_80_pos	DL-Lysine	ENSGALG00000006864	COL24A1
Com_203_pos	Serotonin	ENSGALG00000004875	PEMT
Com_55_pos	Valine	ENSGALG00000034337	RHPN1
Com_120_neg	LPC 15:0	ENSGALG00000040836	INSYN2A
Com_151_pos	Pyridoxamine	ENSGALG00000004230	LIPC
Com_86_neg	Levulinic acid	ENSGALG00000004205	SOAT1
Com_482_pos	8-Hydroxyquinoline	ENSGALG00000012847	Slc7a11
Com_178_pos	Maltol	ENSGALG00000007839	NCAM1
Com_194_pos	Pipecolic acid	ENSGALG00000013033	cmbl
Com_482_pos	8-Hydroxyquinoline	ENSGALG00000005160	VMP1
Com_482_pos	8-Hydroxyquinoline	MSTRG.2388	--
Com_192_pos	1-Methylhistidine	ENSGALG00000016446	ATP6V1C2
Com_413_pos	L-Cystine	MSTRG.8511	--
Com_4_pos	PC (17:1/17:1)	ENSGALG00000021193	STARD5
Com_4_pos	PC (17:1/17:1)	ENSGALG00000029898	YKT6
Com_76_neg	Erythronolactone	ENSGALG00000010857	DAB1
Com_208_neg	N-Acetylanthranilic acid	ENSGALG00000001697	ITIH3
Com_215_pos	D-Erythro-sphingosine 1-p	ENSGALG00000050427	OSBPL10
Com_119_pos	DL-Stachydrine	ENSGALG00000030941	ELAPOR1

Com_440_pos	PC (18:4e/4:0)	ENSGALG00000047480	A2ML1
Com_588_pos	Ornithine	ENSGALG00000002024	COMT
Com_97_pos	L-Threonine	ENSGALG00000052872	--
Com_192_pos	1-Methylhistidine	MSTRG.20494	AHNAK
Com_203_pos	Serotonin	ENSGALG00000011657	EAF2
Com_53_neg	2-(5-mercapto-4-methyl-4	MSTRG.8957	--
Com_8_neg	4-Methyl-2-Oxopentanoic	ENSGALG00000011894	CYP2D6
Com_147_pos	D-Sphingosine	ENSGALG00000006320	Slc2a9
Com_56_neg	LPE 18:1	MSTRG.14201	ZNF541
Com_413_pos	L-Cystine	ENSGALG00000008912	ABCB1
Com_440_pos	PC (18:4e/4:0)	ENSGALG00000047321	SARDH
Com_108_neg	LPE 18:2	ENSGALG00000004127	--
Com_80_pos	DL-Lysine	ENSGALG00000034140	ZNF395
Com_76_neg	Erythronolactone	ENSGALG00000019147	--
Com_17_pos	L-Norleucine	ENSGALG00000012704	MYLIP
Com_186_pos	4-Hydroxybenzaldehyde	ENSGALG00000013627	SLC7A2
Com_99_pos	Creatine	ENSGALG00000002431	CFH
Com_120_neg	LPC 15:0	ENSGALG00000037671	psuG
Com_165_neg	(±)9-HpODE	MSTRG.21123	--
Com_203_pos	Serotonin	MSTRG.4813	--
Com_208_neg	N-Acetylanthranilic acid	ENSGALG00000049256	--
Com_4_pos	PC (17:1/17:1)	MSTRG.7572	--
Com_482_pos	8-Hydroxyquinoline	ENSGALG00000037935	RARA
Com_24_neg	PE (16:0/20:4)	ENSGALG00000048020	STAG3
Com_68_neg	PE (16:0/22:6)	ENSGALG00000010577	ARHGEF38
Com_588_pos	Ornithine	ENSGALG00000014412	CSTA
Com_99_pos	Creatine	ENSGALG00000047632	Pc
Com_18_neg	Arachidonic acid	ENSGALG00000036086	TAGLN2
Com_86_neg	Levulinic acid	ENSGALG00000009740	RASGRP1
Com_152_pos	Acetyl-L-carnitine	ENSGALG00000009479	SAMD9L
Com_362_pos	2-Arachidonoyl glycerol	ENSGALG00000013244	ABCC9
Com_130_neg	2-Hydroxyvaleric acid	ENSGALG00000014616	MT3
Com_311_pos	PC (18:4e/2:0)	ENSGALG00000021340	CA9
Com_76_neg	Erythronolactone	MSTRG.19177	PHGDH
Com_215_pos	D-Erythro-sphingosine 1-p	ENSGALG00000031159	HIST1H110
Com_92_pos	D-(+)-Proline	ENSGALG00000011957	TOB2
Com_482_pos	8-Hydroxyquinoline	ENSGALG00000003103	MST1R
Com_342_pos	1-(4-methylphenyl)-3,5-di	MSTRG.163	--
Com_152_pos	Acetyl-L-carnitine	MSTRG.10101	--
Com_352_pos	Riboflavin	ENSGALG00000013828	GALM
Com_56_neg	LPE 18:1	MSTRG.19854	--
Com_120_neg	LPC 15:0	ENSGALG00000027786	SOCS3
Com_92_pos	D-(+)-Proline	ENSGALG00000051203	Mas1
Com_311_pos	PC (18:4e/2:0)	ENSGALG00000038923	Ces1e
Com_92_pos	D-(+)-Proline	ENSGALG00000034081	AKT3
Com_16_neg	3-Hydroxybutyric acid	MSTRG.8055	--
Com_311_pos	PC (18:4e/2:0)	ENSGALG00000016281	DMD
Com_18_neg	Arachidonic acid	ENSGALG00000035903	FAM46A
Com_203_pos	Serotonin	ENSGALG00000009560	MSMO1
Com_460_pos	3-amino-4-(propylamino)c	ENSGALG00000017039	STOML3
Com_68_neg	PE (16:0/22:6)	MSTRG.17677	--
Com_108_neg	LPE 18:2	ENSGALG00000005722	SEC31B
Com_12_pos	Betaine	MSTRG.20478	--
Com_4_pos	PC (17:1/17:1)	ENSGALG00000038666	FBXL12
Com_130_neg	2-Hydroxyvaleric acid	ENSGALG00000030031	TTPA
Com_76_neg	Erythronolactone	ENSGALG00000033171	TGM4
Com_147_pos	D-Sphingosine	ENSGALG00000016364	ALKAL2
Com_57_neg	LPC 16:1	MSTRG.21822	--

Com_151_neg	Lysope 18:1	ENSGALG00000029617	COL17A1
Com_130_neg	2-Hydroxyvaleric acid	ENSGALG00000026663	CX3CL1
Com_362_pos	2-Arachidonoyl glycerol	MSTRG.5269	pitpnc1
Com_588_pos	Ornithine	ENSGALG00000011994	SYNPO2
Com_165_neg	(±)9-HpODE	ENSGALG00000024047	MYCL
Com_22_pos	Indole-3-acrylic acid	ENSGALG00000038740	AMY2A
Com_215_pos	D-Erythro-sphingosine 1-p	MSTRG.1890	gag
Com_215_pos	D-Erythro-sphingosine 1-p	ENSGALG00000010643	ZYG11B
Com_151_neg	Lysope 18:1	MSTRG.1679	--
Com_460_pos	3-amino-4-(propylamino)c	ENSGALG00000020688	CYP4A22
Com_18_neg	Arachidonic acid	ENSGALG00000015492	PDZK1
Com_386_pos	2-Amino-1,3-octadecanec	ENSGALG00000037160	Smad7
Com_130_neg	2-Hydroxyvaleric acid	ENSGALG00000040573	FMO3
Com_588_pos	Ornithine	ENSGALG00000006374	TBX6
Com_413_pos	L-Cystine	ENSGALG00000012034	ADSL
Com_24_neg	PE (16:0/20:4)	MSTRG.20356	--
Com_108_neg	LPE 18:2	MSTRG.5038	--
Com_57_neg	LPC 16:1	MSTRG.9524	--
Com_86_neg	Levulinic acid	ENSGALG00000054856	ADH1
Com_130_neg	2-Hydroxyvaleric acid	ENSGALG00000014252	A2M
Com_53_neg	2-(5-mercapto-4-methyl-4	ENSGALG00000042275	esg1
Com_194_pos	Pipecolic acid	ENSGALG00000011003	SLC35F3
Com_12_pos	Betaine	ENSGALG00000002466	SLC2A5
Com_120_neg	LPC 15:0	MSTRG.8055	--
Com_68_neg	PE (16:0/22:6)	MSTRG.17389	DIO3
Com_12_pos	Betaine	ENSGALG00000015362	TRAT1
Com_120_neg	LPC 15:0	ENSGALG00000031255	FGF1
Com_76_neg	Erythronolactone	ENSGALG00000013728	PPAT
Com_152_pos	Acetyl-L-carnitine	ENSGALG00000021848	AVD
Com_413_pos	L-Cystine	ENSGALG00000040434	rab18b
Com_108_neg	LPE 18:2	ENSGALG00000004657	FBXO2
Com_460_pos	3-amino-4-(propylamino)c	ENSGALG00000003595	SARM1
Com_413_pos	L-Cystine	MSTRG.3197	--
Com_362_pos	2-Arachidonoyl glycerol	ENSGALG00000026313	RND3
Com_192_pos	1-Methylhistidine	ENSGALG00000003432	AGXT2
Com_55_pos	Valine	ENSGALG00000030121	SLC2A11
Com_78_neg	Citric acid	ENSGALG00000016364	ALKAL2
Com_482_pos	8-Hydroxyquinoline	ENSGALG00000005815	TMEM41B
Com_440_pos	PC (18:4e/4:0)	MSTRG.4548	--
Com_440_pos	PC (18:4e/4:0)	ENSGALG00000031122	NTNG1
Com_108_neg	LPE 18:2	MSTRG.11834	--
Com_362_pos	2-Arachidonoyl glycerol	ENSGALG00000034741	ETNPPL
Com_24_neg	PE (16:0/20:4)	ENSGALG00000024298	ADAMTSL5
Com_16_neg	3-Hydroxybutyric acid	ENSGALG00000037671	psuG
Com_55_pos	Valine	ENSGALG00000016651	TDH
Com_130_neg	2-Hydroxyvaleric acid	ENSGALG00000052872	--
Com_17_pos	L-Norleucine	ENSGALG00000004205	SOAT1
Com_54_pos	Uric acid	ENSGALG00000053278	SUCNR1
Com_151_neg	Lysope 18:1	MSTRG.3262	--
Com_18_neg	Arachidonic acid	ENSGALG00000047321	SARDH
Com_352_pos	Riboflavin	MSTRG.13046	--
Com_471_pos	Indole-3-acetic acid	ENSGALG00000015768	ANKRD6
Com_89_neg	Gallic acid	ENSGALG00000048205	EBP
Com_194_pos	Pipecolic acid	ENSGALG00000016036	DOP1B
Com_471_pos	Indole-3-acetic acid	MSTRG.8810	--
Com_471_pos	Indole-3-acetic acid	MSTRG.13135	--
Com_588_pos	Ornithine	MSTRG.11572	--
Com_76_neg	Erythronolactone	ENSGALG00000028871	SLC38A3

Com_152_pos	Acetyl-L-carnitine	ENSGALG00000028451	MT4
Com_16_neg	3-Hydroxybutyric acid	ENSGALG00000006320	Slc2a9
Com_194_pos	Pipecolic acid	ENSGALG00000033411	SLC26A2
Com_12_pos	Betaine	ENSGALG00000011684	STAP1
Com_471_pos	Indole-3-acetic acid	ENSGALG00000002899	AACS
Com_194_pos	Pipecolic acid	ENSGALG00000017046	POSTN
Com_97_pos	L-Threonine	ENSGALG00000036190	AOC1
Com_18_neg	Arachidonic acid	ENSGALG00000047480	A2ML1
Com_588_pos	Ornithine	ENSGALG00000004343	HPD
Com_342_pos	1-(4-methylphenyl)-3,5-di	ENSGALG00000004373	KCNJ16
Com_108_neg	LPE 18:2	ENSGALG00000050427	OSBPL10
Com_386_pos	2-Amino-1,3-octadecanec	ENSGALG00000014750	TRB
Com_76_neg	Erythronolactone	ENSGALG00000003212	TSPO2
Com_203_pos	Serotonin	ENSGALG00000009365	CYP51A1
Com_22_pos	Indole-3-acrylic acid	MSTRG.13474	Klhl29
Com_331_pos	L-Lysine	ENSGALG00000007839	NCAM1
Com_208_neg	N-Acetylanthranilic acid	ENSGALG00000006812	TTC36
Com_76_neg	Erythronolactone	ENSGALG00000012754	PAH
Com_352_pos	Riboflavin	ENSGALG00000015219	Selenoi
Com_203_pos	Serotonin	ENSGALG00000030661	STAT2
Com_203_pos	Serotonin	ENSGALG00000004106	DHCR7
Com_192_pos	1-Methylhistidine	ENSGALG00000010853	C8B
Com_89_neg	Gallic acid	ENSGALG00000011141	ITGB6
Com_386_pos	2-Amino-1,3-octadecanec	ENSGALG00000011314	LRRC3B
Com_89_neg	Gallic acid	MSTRG.2126	--
Com_413_pos	L-Cystine	ENSGALG00000017040	C4
Com_413_pos	L-Cystine	ENSGALG00000003015	SERPINF1
Com_386_pos	2-Amino-1,3-octadecanec	ENSGALG00000011391	AMN
Com_175_pos	Pantothenic acid	MSTRG.836	--
Com_171_neg	LPC 22:6	ENSGALG00000031122	NTNG1
Com_171_neg	LPC 22:6	MSTRG.4548	--
Com_86_neg	Levulinic acid	ENSGALG00000012704	MYLIP
Com_80_pos	DL-Lysine	ENSGALG00000047827	TMEM86A
Com_311_pos	PC (18:4e/2:0)	ENSGALG00000004598	CUX2
Com_152_pos	Acetyl-L-carnitine	ENSGALG00000011894	CYP2D6
Com_92_pos	D-(+)-Proline	ENSGALG00000034616	INHBA
Com_147_pos	D-Sphingosine	MSTRG.3009	--
Com_92_pos	D-(+)-Proline	MSTRG.16661	--
Com_17_pos	L-Norleucine	ENSGALG00000009740	RASGRP1
Com_4_pos	PC (17:1/17:1)	MSTRG.7483	--
Com_108_neg	LPE 18:2	ENSGALG00000031159	HIST1H110
Com_89_neg	Gallic acid	ENSGALG00000021451	RED3
Com_97_pos	L-Threonine	ENSGALG00000012755	IGF-I
Com_151_neg	Lysope 18:1	ENSGALG00000013830	PRELID3A
Com_311_pos	PC (18:4e/2:0)	ENSGALG00000007114	APOA1
Com_175_pos	Pantothenic acid	ENSGALG00000030031	TTPA
Com_119_pos	DL-Stachydrine	ENSGALG00000007234	CLCN5
Com_57_neg	LPC 16:1	ENSGALG00000050309	H2B-I
Com_386_pos	2-Amino-1,3-octadecanec	ENSGALG00000007252	ANKDD1A
Com_203_pos	Serotonin	MSTRG.8954	--
Com_97_pos	L-Threonine	ENSGALG00000030031	TTPA
Com_92_pos	D-(+)-Proline	ENSGALG00000027122	APPL2
Com_92_pos	D-(+)-Proline	ENSGALG00000030251	ADCY8
Com_460_pos	3-amino-4-(propylamino)(	ENSGALG00000040836	INSYN2A
Com_92_pos	D-(+)-Proline	ENSGALG00000005030	DOCK10
Com_352_pos	Riboflavin	ENSGALG00000034868	KRT7
Com_17_pos	L-Norleucine	ENSGALG00000022750	GPR18
Com_24_neg	PE (16:0/20:4)	ENSGALG00000002142	DGKQ

Com_8_neg	4-Methyl-2-Oxopentanoic	ENSGALG00000009479	SAMD9L
Com_24_neg	PE (16:0/20:4)	ENSGALG00000053164	gag-pol
Com_331_pos	L-Lysine	ENSGALG00000003972	FAXDC2
Com_482_pos	8-Hydroxyquinoline	ENSGALG00000016476	TTC32
Com_471_pos	Indole-3-acetic acid	ENSGALG00000051466	NDFIP2
Com_352_pos	Riboflavin	ENSGALG00000008903	ITPRID2
Com_440_pos	PC (18:4e/4:0)	ENSGALG00000021340	CA9
Com_152_pos	Acetyl-L-carnitine	ENSGALG00000006482	FAH
Com_108_neg	LPE 18:2	ENSGALG00000053659	--
Com_186_pos	4-Hydroxybenzaldehyde	ENSGALG00000051068	SIGLEC1
Com_18_neg	Arachidonic acid	MSTRG.3009	--
Com_119_pos	DL-Stachydrine	ENSGALG00000041143	UMOD
Com_76_neg	Erythronolactone	ENSGALG00000041680	KCNT2
Com_57_neg	LPC 16:1	MSTRG.1843	--
Com_8_neg	4-Methyl-2-Oxopentanoic	MSTRG.29	SHANK3
Com_471_pos	Indole-3-acetic acid	ENSGALG00000023338	CBX2
Com_203_pos	Serotonin	MSTRG.16398	Cdhr5
Com_352_pos	Riboflavin	ENSGALG00000004268	NIPAL3
Com_17_pos	L-Norleucine	ENSGALG00000048343	Ces1e
Com_178_pos	Maltol	ENSGALG00000005474	PNAT10
Com_386_pos	2-Amino-1,3-octadecanec	ENSGALG00000009926	HAAO
Com_97_pos	L-Threonine	ENSGALG00000052964	TOPAZ1
Com_147_pos	D-Sphingosine	ENSGALG00000047480	A2ML1
Com_362_pos	2-Arachidonoyl glycerol	MSTRG.17350	--
Com_588_pos	Ornithine	MSTRG.8511	--
Com_215_pos	D-Erythro-sphingosine 1-p	MSTRG.8128	--
Com_413_pos	L-Cystine	ENSGALG00000017032	SLC25A15
Com_55_pos	Valine	ENSGALG00000003972	FAXDC2
Com_151_neg	Lysope 18:1	ENSGALG00000004569	UNC5B
Com_25_pos	2-Hydroxycinnamic acid	ENSGALG00000011571	AGPAT4
Com_151_pos	Pyridoxamine	MSTRG.14987	--
Com_460_pos	3-amino-4-(propylamino)(	ENSGALG00000027786	SOCS3
Com_208_neg	N-Acetylanthranilic acid	ENSGALG00000004491	DMGDH
Com_147_pos	D-Sphingosine	ENSGALG00000047321	SARDH
Com_413_pos	L-Cystine	ENSGALG00000002024	COMT
Com_151_pos	Pyridoxamine	ENSGALG00000005263	SOX8
Com_97_pos	L-Threonine	ENSGALG00000052583	A2ML1
Com_215_pos	D-Erythro-sphingosine 1-p	ENSGALG00000050668	Spata1
Com_86_neg	Levulinic acid	ENSGALG00000005474	PNAT10
Com_55_pos	Valine	ENSGALG00000013033	cmbl
Com_119_pos	DL-Stachydrine	ENSGALG00000011169	PDCD2
Com_120_neg	LPC 15:0	ENSGALG00000017039	STOML3
Com_215_pos	D-Erythro-sphingosine 1-p	MSTRG.11834	--
Com_130_neg	2-Hydroxyvaleric acid	ENSGALG00000036190	AOC1
Com_16_neg	3-Hydroxybutyric acid	ENSGALG00000036086	TAGLN2
Com_97_pos	L-Threonine	ENSGALG00000032882	EVA1C
Com_311_pos	PC (18:4e/2:0)	ENSGALG00000011524	PPEF2
Com_76_neg	Erythronolactone	ENSGALG00000038520	STRIP2
Com_80_pos	DL-Lysine	ENSGALG00000039239	SERPIND1
Com_12_pos	Betaine	ENSGALG00000029270	GATA3
Com_16_neg	3-Hydroxybutyric acid	ENSGALG00000009926	HAAO
Com_352_pos	Riboflavin	ENSGALG00000049232	POLR2A
Com_311_pos	PC (18:4e/2:0)	MSTRG.4548	--
Com_12_pos	Betaine	ENSGALG00000028897	WDR25
Com_311_pos	PC (18:4e/2:0)	ENSGALG00000031122	NTNG1
Com_16_neg	3-Hydroxybutyric acid	ENSGALG00000035903	FAM46A
Com_17_pos	L-Norleucine	ENSGALG00000009002	CPED1
Com_264_pos	Indole	MSTRG.13474	Klhl29

Com_78_neg	Citric acid	ENSGALG00000013244	ABCC9
Com_17_pos	L-Norleucine	ENSGALG00000005204	GSTT1
Com_78_neg	Citric acid	ENSGALG00000031122	NTNG1
Com_78_neg	Citric acid	MSTRG.4548	--
Com_17_pos	L-Norleucine	ENSGALG00000019663	ACBD7
Com_57_neg	LPC 16:1	ENSGALG00000014719	SETD9
Com_40_pos	Choline	ENSGALG00000010628	ACSL1
Com_130_neg	2-Hydroxyvaleric acid	ENSGALG00000043582	LY6E
Com_440_pos	PC (18:4e/4:0)	ENSGALG00000016364	ALKAL2
Com_130_neg	2-Hydroxyvaleric acid	ENSGALG00000012755	IGF-I
Com_151_neg	Lysope 18:1	MSTRG.18742	LRP2
Com_460_pos	3-amino-4-(propylamino)(	ENSGALG00000031255	FGF1
Com_57_neg	LPC 16:1	ENSGALG00000047792	SELENOM
Com_208_neg	N-Acetylanthranilic acid	ENSGALG00000006976	Bdh1
Com_76_neg	Erythronolactone	ENSGALG00000015040	SLC16A10
Com_68_neg	PE (16:0/22:6)	ENSGALG00000002944	CPS1
Com_40_pos	Choline	ENSGALG00000005632	IRAG1
Com_471_pos	Indole-3-acetic acid	ENSGALG00000027960	GRPR
Com_92_pos	D-(+)-Proline	ENSGALG00000000293	A2ML1
Com_68_neg	PE (16:0/22:6)	ENSGALG00000033541	FRMPD4
Com_252_pos	cis-4-Hydroxy-D-proline	ENSGALG00000011684	STAP1
Com_97_pos	L-Threonine	ENSGALG00000028407	GDF9
Com_331_pos	L-Lysine	ENSGALG00000016651	TDH
Com_89_neg	Gallic acid	MSTRG.15625	--
Com_76_neg	Erythronolactone	ENSGALG00000031158	OAT
Com_147_pos	D-Sphingosine	ENSGALG00000034741	ETNPPL
Com_16_neg	3-Hydroxybutyric acid	ENSGALG00000015492	PDZK1
Com_215_pos	D-Erythro-sphingosine 1-p	ENSGALG00000030511	SLC19A1
Com_68_neg	PE (16:0/22:6)	ENSGALG00000026973	KIF3C
Com_24_neg	PE (16:0/20:4)	ENSGALG00000009830	MGAT4D
Com_203_pos	Serotonin	MSTRG.20573	--
Com_331_pos	L-Lysine	ENSGALG00000030121	SLC2A11
Com_17_pos	L-Norleucine	ENSGALG00000003537	SGK2
Com_147_pos	D-Sphingosine	ENSGALG00000026313	RND3
Com_413_pos	L-Cystine	ENSGALG00000005472	NAT
Com_108_neg	LPE 18:2	ENSGALG00000007030	MFSD13A
Com_152_pos	Acetyl-L-carnitine	ENSGALG00000011957	TOB2
Com_78_neg	Citric acid	MSTRG.5269	pitpnc1
Com_352_pos	Riboflavin	ENSGALG00000005442	PALMD
Com_203_pos	Serotonin	ENSGALG00000005617	NTHL1
Com_440_pos	PC (18:4e/4:0)	ENSGALG00000004598	CUX2
Com_92_pos	D-(+)-Proline	MSTRG.10101	--
Com_86_neg	Levulinic acid	ENSGALG00000022750	GPR18
Com_352_pos	Riboflavin	MSTRG.20480	--
Com_265_pos	6-Methylquinoline	ENSGALG00000053860	mas
Com_89_neg	Gallic acid	ENSGALG00000007493	NSDHL
Com_24_neg	PE (16:0/20:4)	ENSGALG00000026263	RGS8
Com_16_neg	3-Hydroxybutyric acid	ENSGALG00000007252	ANKDD1A
Com_588_pos	Ornithine	ENSGALG00000012034	ADSL
Com_152_pos	Acetyl-L-carnitine	ENSGALG00000051203	Mas1
Com_68_neg	PE (16:0/22:6)	ENSGALG00000029766	ITGB5
Com_265_pos	6-Methylquinoline	ENSGALG00000008859	WDR31
Com_215_pos	D-Erythro-sphingosine 1-p	ENSGALG00000053659	--
Com_89_neg	Gallic acid	ENSGALG00000009538	RDH12
Com_440_pos	PC (18:4e/4:0)	ENSGALG00000007114	APOA1
Com_252_pos	cis-4-Hydroxy-D-proline	ENSGALG00000002549	RGS1
Com_120_neg	LPC 15:0	MSTRG.20827	Plcg1
Com_178_pos	Maltol	ENSGALG00000003972	FAXDC2

Com_482_pos	8-Hydroxyquinoline	ENSGALG00000052612	RPS27L
Com_252_pos	cis-4-Hydroxy-D-proline	ENSGALG00000015362	TRAT1
Com_97_pos	L-Threonine	ENSGALG00000008185	AOX1
Com_588_pos	Ornithine	ENSGALG00000040434	rab18b
Com_588_pos	Ornithine	MSTRG.3197	--
Com_4_pos	PC (17:1/17:1)	ENSGALG00000046412	Aoc3
Com_8_neg	4-Methyl-2-Oxopentanoic	ENSGALG00000002479	MAT1A
Com_203_pos	Serotonin	MSTRG.16903	--
Com_130_neg	2-Hydroxyvaleric acid	ENSGALG00000052964	TOPAZ1
Com_352_pos	Riboflavin	ENSGALG00000036527	SYBU
Com_362_pos	2-Arachidonoyl glycerol	ENSGALG00000017103	WASF3
Com_471_pos	Indole-3-acetic acid	MSTRG.21536	--
Com_24_neg	PE (16:0/20:4)	ENSGALG00000024047	MYCL
Com_68_neg	PE (16:0/22:6)	ENSGALG00000015795	ADAMTS5
Com_8_neg	4-Methyl-2-Oxopentanoic	ENSGALG00000006482	FAH
Com_108_neg	LPE 18:2	ENSGALG00000012589	C9orf64
Com_99_pos	Creatine	ENSGALG00000052388	METRN
Com_56_neg	LPE 18:1	ENSGALG00000040447	CDCP1
Com_24_neg	PE (16:0/20:4)	ENSGALG00000007109	APOA4
Com_18_neg	Arachidonic acid	ENSGALG00000033338	GPT2
Com_18_neg	Arachidonic acid	ENSGALG00000006320	Slc2a9
Com_471_pos	Indole-3-acetic acid	ENSGALG00000032628	SRCIN1
Com_362_pos	2-Arachidonoyl glycerol	ENSGALG00000016364	ALKAL2
Com_151_neg	Lysope 18:1	ENSGALG00000014516	CPEB2
Com_203_pos	Serotonin	ENSGALG00000032329	NPM3
Com_194_pos	Pipecolic acid	ENSGALG00000030121	SLC2A11
Com_16_neg	3-Hydroxybutyric acid	ENSGALG00000014750	TRB
Com_130_neg	2-Hydroxyvaleric acid	ENSGALG00000052583	A2ML1
Com_86_neg	Levulinic acid	ENSGALG00000048343	Ces1e
Com_203_pos	Serotonin	ENSGALG00000008604	TMEM255A
Com_68_neg	PE (16:0/22:6)	ENSGALG00000017136	GJB6
Com_362_pos	2-Arachidonoyl glycerol	ENSGALG00000019835	TRIM27.2
Com_203_pos	Serotonin	ENSGALG00000052786	Znf185
Com_152_pos	Acetyl-L-carnitine	MSTRG.29	SHANK3
Com_386_pos	2-Amino-1,3-octadecanec	ENSGALG00000006320	Slc2a9
Com_120_neg	LPC 15:0	ENSGALG00000005540	MICAL2
Com_413_pos	L-Cystine	MSTRG.11572	--
Com_192_pos	1-Methylhistidine	ENSGALG00000038652	Gsta3
Com_108_neg	LPE 18:2	MSTRG.8128	--
Com_194_pos	Pipecolic acid	ENSGALG00000016651	TDH
Com_24_neg	PE (16:0/20:4)	MSTRG.8034	--
Com_130_neg	2-Hydroxyvaleric acid	ENSGALG00000032882	EVA1C
Com_92_pos	D-(+)-Proline	ENSGALG00000016164	ABCG1
Com_119_pos	DL-Stachydrine	ENSGALG00000041687	SREBF2
Com_331_pos	L-Lysine	ENSGALG00000005474	PNAT10
Com_171_neg	LPC 22:6	ENSGALG00000016281	DMD
Com_108_neg	LPE 18:2	ENSGALG00000050668	Spata1
Com_471_pos	Indole-3-acetic acid	ENSGALG00000007673	LRRC59
Com_208_neg	N-Acetylanthranilic acid	ENSGALG00000032903	RTN4RL2
Com_92_pos	D-(+)-Proline	ENSGALG00000021848	AVD
Com_165_neg	(±)9-HpODE	ENSGALG00000013660	ZNF516
Com_53_neg	2-(5-mercapto-4-methyl-	ENSGALG00000029947	MMAB
Com_17_pos	L-Norleucine	ENSGALG00000005474	PNAT10
Com_40_pos	Choline	ENSGALG00000001768	TENM2
Com_120_neg	LPC 15:0	ENSGALG00000007814	ALPI
Com_175_pos	Pantothenic acid	ENSGALG00000013726	PAICS
Com_171_neg	LPC 22:6	ENSGALG00000038923	Ces1e
Com_55_pos	Valine	ENSGALG00000033411	SLC26A2

Com_471_pos	Indole-3-acetic acid	ENSGALG00000040342	ADAMTS1
Com_108_neg	LPE 18:2	ENSGALG00000004249	GRHL3
Com_80_pos	DL-Lysine	ENSGALG00000000761	TSKU
Com_151_pos	Pyridoxamine	ENSGALG00000029724	MTURN
Com_80_pos	DL-Lysine	ENSGALG00000054322	--
Com_99_pos	Creatine	ENSGALG00000020391	SERPINA10
Com_165_neg	(±)9-HpODE	ENSGALG00000024298	ADAMTSL5
Com_17_pos	L-Norleucine	MSTRG.14083	--
Com_482_pos	8-Hydroxyquinoline	ENSGALG00000035626	DAD1
Com_99_pos	Creatine	ENSGALG00000007478	SLC51A
Com_22_pos	Indole-3-acrylic acid	ENSGALG00000001392	MMP23B
Com_352_pos	Riboflavin	MSTRG.4550	--
Com_471_pos	Indole-3-acetic acid	ENSGALG00000037253	CLEC4M
Com_97_pos	L-Threonine	ENSGALG00000043582	LY6E
Com_99_pos	Creatine	ENSGALG00000028284	PTX3
Com_57_neg	LPC 16:1	ENSGALG00000029617	COL17A1
Com_171_neg	LPC 22:6	ENSGALG00000013244	ABCC9
Com_151_pos	Pyridoxamine	ENSGALG00000034478	CCL4
Com_175_pos	Pantothenic acid	ENSGALG00000038242	CACNA2D2
Com_194_pos	Pipecolic acid	ENSGALG00000030025	FABP4
Com_92_pos	D-(+)-Proline	ENSGALG00000028451	MT4
Com_86_neg	Levulinic acid	ENSGALG00000009002	CPED1
Com_208_neg	N-Acetylanthranilic acid	ENSGALG00000040995	NEB
Com_130_neg	2-Hydroxyvaleric acid	ENSGALG00000028407	GDF9
Com_86_neg	Levulinic acid	ENSGALG00000005204	GSTT1
Com_440_pos	PC (18:4e/4:0)	ENSGALG00000011524	PPEF2
Com_86_neg	Levulinic acid	ENSGALG00000019663	ACBD7
Com_147_pos	D-Sphingosine	ENSGALG00000021340	CA9
Com_17_pos	L-Norleucine	ENSGALG00000054981	F10
Com_99_pos	Creatine	ENSGALG00000014821	THEMIS
Com_175_pos	Pantothenic acid	ENSGALG00000007728	Prodh
Com_192_pos	1-Methylhistidine	MSTRG.12291	--
Com_165_neg	(±)9-HpODE	ENSGALG00000008930	B3GNT2
Com_186_pos	4-Hydroxybenzaldehyde	ENSGALG00000004959	IRS1
Com_460_pos	3-amino-4-(propylamino)(	MSTRG.11572	--
Com_151_neg	Lysope 18:1	ENSGALG00000006054	CALCA
Com_53_neg	2-(5-mercapto-4-methyl-4	ENSGALG00000009748	ASNS
Com_147_pos	D-Sphingosine	ENSGALG00000037160	Smad7
Com_108_neg	LPE 18:2	ENSGALG00000030511	SLC19A1
Com_155_neg	Phenylacetaldehyde	ENSGALG00000015935	SMYD1
Com_178_pos	Maltol	ENSGALG00000009740	RASGRP1
Com_92_pos	D-(+)-Proline	ENSGALG00000011894	CYP2D6
Com_108_neg	LPE 18:2	ENSGALG00000036117	TENT5B
Com_92_pos	D-(+)-Proline	ENSGALG00000027793	SCN9A
Com_171_neg	LPC 22:6	MSTRG.5269	pitpnc1
Com_152_pos	Acetyl-L-carnitine	MSTRG.16661	--
Com_311_pos	PC (18:4e/2:0)	ENSGALG00000016364	ALKAL2
Com_68_neg	PE (16:0/22:6)	ENSGALG00000052328	CTNND2
Com_194_pos	Pipecolic acid	ENSGALG00000009947	PLEKHH2
Com_215_pos	D-Erythro-sphingosine 1-p	ENSGALG00000007030	MFSD13A
Com_86_neg	Levulinic acid	ENSGALG00000003537	SGK2
Com_178_pos	Maltol	ENSGALG00000016651	TDH
Com_203_pos	Serotonin	ENSGALG00000023626	NTN1
Com_175_pos	Pantothenic acid	ENSGALG00000014950	SULT3A1
Com_482_pos	8-Hydroxyquinoline	ENSGALG00000008862	DNAJC10
Com_130_neg	2-Hydroxyvaleric acid	ENSGALG00000008185	AOX1
Com_151_pos	Pyridoxamine	MSTRG.15995	--
Com_108_neg	LPE 18:2	ENSGALG00000004917	DOC2B

Com_152_pos	Acetyl-L-carnitine	ENSGALG00000030251	ADCY8
Com_178_pos	Maltol	ENSGALG00000030121	SLC2A11
Com_471_pos	Indole-3-acetic acid	ENSGALG00000050420	CTNND2
Com_53_neg	2-(5-mercapto-4-methyl-4	ENSGALG00000008150	RASAL1
Com_12_pos	Betaine	ENSGALG00000013124	FHOD3
Com_192_pos	1-Methylhistidine	ENSGALG00000009172	OSBPL6
Com_8_neg	4-Methyl-2-Oxopentanoic	ENSGALG00000011957	TOB2
Com_57_neg	LPC 16:1	MSTRG.3262	--
Com_171_neg	LPC 22:6	ENSGALG00000028928	LCAT
Com_588_pos	Ornithine	ENSGALG00000017032	SLC25A15
Com_352_pos	Riboflavin	MSTRG.8013	--
Com_8_neg	4-Methyl-2-Oxopentanoic	ENSGALG00000051203	Mas1
Com_86_neg	Levulinic acid	ENSGALG00000007839	NCAM1
Com_4_pos	PC (17:1/17:1)	ENSGALG00000005888	PGP
Com_78_neg	Citric acid	MSTRG.17350	--
Com_175_pos	Pantothenic acid	ENSGALG00000029724	MTURN
Com_215_pos	D-Erythro-sphingosine 1-p	ENSGALG00000012589	C9orf64
Com_178_pos	Maltol	ENSGALG00000004205	SOAT1
Com_194_pos	Pipecolic acid	ENSGALG00000003972	FAXDC2
Com_165_neg	(±)9-HpODE	ENSGALG00000054252	TM4SF1
Com_40_pos	Choline	ENSGALG00000039499	LRRCC1
Com_18_neg	Arachidonic acid	ENSGALG00000009926	HAAO
Com_152_pos	Acetyl-L-carnitine	ENSGALG00000002479	MAT1A
Com_151_pos	Pyridoxamine	ENSGALG00000051274	B3GALT2
Com_78_neg	Citric acid	ENSGALG00000016281	DMD
Com_97_pos	L-Threonine	ENSGALG00000002479	MAT1A
Com_119_pos	DL-Stachydrine	ENSGALG00000043435	CARNS1
Com_119_pos	DL-Stachydrine	MSTRG.9361	Fam110a
Com_147_pos	D-Sphingosine	ENSGALG00000011314	LRRC3B
Com_147_pos	D-Sphingosine	ENSGALG00000011391	AMN
Com_108_neg	LPE 18:2	ENSGALG00000011113	SGIP1
Com_265_pos	6-Methylquinoline	ENSGALG00000038740	AMY2A
Com_78_neg	Citric acid	ENSGALG00000038923	Ces1e
Com_265_pos	6-Methylquinoline	ENSGALG00000000498	ACE
Com_588_pos	Ornithine	ENSGALG00000017039	STOML3
Com_8_neg	4-Methyl-2-Oxopentanoic	ENSGALG00000008185	AOX1
Com_4_pos	PC (17:1/17:1)	ENSGALG00000044464	TEPSIN
Com_57_neg	LPC 16:1	ENSGALG00000013830	PRELID3A
Com_482_pos	8-Hydroxyquinoline	ENSGALG00000015729	LPAR1
Com_18_neg	Arachidonic acid	ENSGALG00000041373	ARAP2
Com_215_pos	D-Erythro-sphingosine 1-p	ENSGALG00000004249	GRHL3
Com_155_neg	Phenylacetaldehyde	MSTRG.4905	--
Com_99_pos	Creatine	ENSGALG00000013356	IKBKE
Com_386_pos	2-Amino-1,3-octadecanec	MSTRG.3009	--
Com_311_pos	PC (18:4e/2:0)	ENSGALG00000031158	OAT
Com_99_pos	Creatine	ENSGALG00000030038	C3
Com_482_pos	8-Hydroxyquinoline	MSTRG.2387	--
Com_89_neg	Gallic acid	ENSGALG00000028880	FDPS
Com_460_pos	3-amino-4-(propylamino)c	MSTRG.20827	Plcg1
Com_86_neg	Levulinic acid	MSTRG.14083	--
Com_440_pos	PC (18:4e/4:0)	ENSGALG00000034741	ETNPPL
Com_89_neg	Gallic acid	ENSGALG00000051567	MRPL41
Com_362_pos	2-Arachidonoyl glycerol	ENSGALG00000031122	NTNG1
Com_362_pos	2-Arachidonoyl glycerol	MSTRG.4548	--
Com_203_pos	Serotonin	ENSGALG00000006842	ACOT8
Com_331_pos	L-Lysine	ENSGALG00000017046	POSTN
Com_203_pos	Serotonin	ENSGALG00000049751	H2B-I
Com_440_pos	PC (18:4e/4:0)	ENSGALG00000026313	RND3

Com_76_neg	Erythronolactone	ENSGALG00000027908	CYP2U1
Com_192_pos	1-Methylhistidine	ENSGALG00000034507	CHST2
Com_56_neg	LPE 18:1	ENSGALG00000005580	TMEM56
Com_342_pos	1-(4-methylphenyl)-3,5-di	MSTRG.17021	--
Com_264_pos	Indole	ENSGALG00000001392	MMP23B
Com_119_pos	DL-Stachydrine	ENSGALG00000054546	ERVK-11
Com_215_pos	D-Erythro-sphingosine 1-p	MSTRG.15745	--
Com_471_pos	Indole-3-acetic acid	ENSGALG00000037018	USP36
Com_147_pos	D-Sphingosine	ENSGALG00000004598	CUX2
Com_56_neg	LPE 18:1	ENSGALG00000010494	SLC5A9
Com_331_pos	L-Lysine	ENSGALG00000016036	DOP1B
Com_18_neg	Arachidonic acid	ENSGALG00000007252	ANKDD1A
Com_588_pos	Ornithine	ENSGALG00000005472	NAT
Com_8_neg	4-Methyl-2-Oxopentanoic	ENSGALG00000028407	GDF9
Com_362_pos	2-Arachidonoyl glycerol	ENSGALG00000017199	MAML2
Com_175_pos	Pantothenic acid	ENSGALG00000044996	TMEM71
Com_57_neg	LPC 16:1	ENSGALG00000016444	ODC1
Com_203_pos	Serotonin	ENSGALG00000034294	ATP6V0D2
Com_119_pos	DL-Stachydrine	ENSGALG00000024295	MYCBP
Com_86_neg	Levulinic acid	ENSGALG00000054981	F10
Com_147_pos	D-Sphingosine	ENSGALG00000007114	APOA1
Com_56_neg	LPE 18:1	ENSGALG00000053961	Ranbp17
Com_130_neg	2-Hydroxyvaleric acid	ENSGALG00000011287	SULT
Com_482_pos	8-Hydroxyquinoline	ENSGALG00000009680	PAQR7
Com_460_pos	3-amino-4-(propylamino)c	ENSGALG00000005540	MICAL2
Com_460_pos	3-amino-4-(propylamino)c	ENSGALG00000002024	COMT
Com_203_pos	Serotonin	ENSGALG00000040620	LSAMP
Com_192_pos	1-Methylhistidine	ENSGALG00000023122	SULT1B
Com_311_pos	PC (18:4e/2:0)	ENSGALG00000038520	STRIP2
Com_155_neg	Phenylacetaldehyde	ENSGALG00000016430	PDHA2
Com_76_neg	Erythronolactone	ENSGALG00000011524	PPEF2
Com_68_neg	PE (16:0/22:6)	MSTRG.2406	--
Com_80_pos	DL-Lysine	ENSGALG00000003802	OTUD7A
Com_265_pos	6-Methylquinoline	ENSGALG00000002116	TEN1
Com_194_pos	Pipecolic acid	ENSGALG00000013124	FHOD3
Com_460_pos	3-amino-4-(propylamino)c	ENSGALG00000007814	ALPI
Com_4_pos	PC (17:1/17:1)	ENSGALG00000051567	MRPL41
Com_215_pos	D-Erythro-sphingosine 1-p	ENSGALG00000036117	TENT5B
Com_16_neg	3-Hydroxybutyric acid	ENSGALG00000033338	GPT2
Com_57_neg	LPC 16:1	MSTRG.21631	--
Com_152_pos	Acetyl-L-carnitine	ENSGALG00000000293	A2ML1
Com_4_pos	PC (17:1/17:1)	ENSGALG00000028880	FDPS
Com_331_pos	L-Lysine	ENSGALG00000011003	SLC35F3
Com_192_pos	1-Methylhistidine	ENSGALG00000012683	RNF144B
Com_18_neg	Arachidonic acid	ENSGALG00000014750	TRB
Com_120_neg	LPC 15:0	ENSGALG00000000309	Lad1
Com_352_pos	Riboflavin	ENSGALG00000007077	CPT1A
Com_68_neg	PE (16:0/22:6)	ENSGALG00000007993	DCX
Com_482_pos	8-Hydroxyquinoline	ENSGALG00000010978	ANGPTL3
Com_12_pos	Betaine	ENSGALG00000009947	PLEKHH2
Com_386_pos	2-Amino-1,3-octadecaned	ENSGALG00000047480	A2ML1
Com_165_neg	(±)9-HpODE	ENSGALG00000009830	MGAT4D
Com_89_neg	Gallic acid	ENSGALG00000044464	TEPSIN
Com_352_pos	Riboflavin	MSTRG.20491	--
Com_331_pos	L-Lysine	ENSGALG00000009740	RASGRP1
Com_17_pos	L-Norleucine	ENSGALG00000008912	ABCB1
Com_215_pos	D-Erythro-sphingosine 1-p	ENSGALG00000004917	DOC2B
Com_186_pos	4-Hydroxybenzaldehyde	ENSGALG00000009920	COCH

Com_471_pos	Indole-3-acetic acid	ENSGALG00000031312	ANAPC13
Com_8_neg	4-Methyl-2-Oxopentanoic	ENSGALG00000032882	EVA1C
Com_76_neg	Erythronolactone	ENSGALG00000009880	INPP4B
Com_78_neg	Citric acid	ENSGALG00000017103	WASF3
Com_386_pos	2-Amino-1,3-octadecanec	ENSGALG00000047321	SARDH
Com_8_neg	4-Methyl-2-Oxopentanoic	MSTRG.16661	--
Com_692_pos	3-amino-2-phenyl-2H-py	ENSGALG00000037665	LMBR1L
Com_78_neg	Citric acid	ENSGALG00000028928	LCAT
Com_165_neg	(±)9-HpODE	ENSGALG00000026263	RGS8
Com_89_neg	Gallic acid	ENSGALG00000014189	SULT4A1
Com_482_pos	8-Hydroxyquinoline	ENSGALG00000016885	STK24
Com_8_neg	4-Methyl-2-Oxopentanoic	ENSGALG00000052583	A2ML1
Com_92_pos	D-(+)-Proline	ENSGALG00000002594	TFPI
Com_171_neg	LPC 22:6	MSTRG.17350	--
Com_311_pos	PC (18:4e/2:0)	ENSGALG00000041680	KCNT2
Com_8_neg	4-Methyl-2-Oxopentanoic	ENSGALG00000030251	ADCY8
Com_78_neg	Citric acid	ENSGALG00000019835	TRIM27.2
Com_17_pos	L-Norleucine	ENSGALG00000007839	NCAM1
Com_92_pos	D-(+)-Proline	MSTRG.20377	--
Com_8_neg	4-Methyl-2-Oxopentanoic	ENSGALG00000052964	TOPAZ1
Com_130_neg	2-Hydroxyvaleric acid	ENSGALG00000002479	MAT1A
Com_12_pos	Betaine	ENSGALG00000030025	FABP4
Com_362_pos	2-Arachidonoyl glycerol	MSTRG.17721	--
Com_192_pos	1-Methylhistidine	ENSGALG00000035026	SLC22A4
Com_165_neg	(±)9-HpODE	ENSGALG00000007109	APOA4
Com_175_pos	Pantothenic acid	ENSGALG00000005263	SOX8
Com_120_neg	LPC 15:0	MSTRG.11572	--
Com_331_pos	L-Lysine	ENSGALG00000004205	SOAT1
Com_119_pos	DL-Stachydrine	ENSGALG00000035803	THRSP
Com_68_neg	PE (16:0/22:6)	ENSGALG00000030160	DRC7
Com_151_neg	Lysope 18:1	ENSGALG00000047792	SELENOM
Com_97_pos	L-Threonine	MSTRG.29	SHANK3
Com_76_neg	Erythronolactone	ENSGALG00000008953	AASS
Com_56_neg	LPE 18:1	MSTRG.6227	--
Com_25_pos	2-Hydroxycinnamic acid	ENSGALG00000050840	APCDD1
Com_119_pos	DL-Stachydrine	ENSGALG00000027561	GNG5
Com_99_pos	Creatine	ENSGALG00000013627	SLC7A2
Com_215_pos	D-Erythro-sphingosine 1-p	ENSGALG00000011113	SGIP1
Com_147_pos	D-Sphingosine	ENSGALG00000011524	PPEF2
Com_92_pos	D-(+)-Proline	MSTRG.29	SHANK3
Com_692_pos	3-amino-2-phenyl-2H-py	ENSGALG00000046210	SPTBN1
Com_76_neg	Erythronolactone	ENSGALG00000007114	APOA1
Com_252_pos	cis-4-Hydroxy-D-proline	ENSGALG00000041344	FABP5
Com_16_neg	3-Hydroxybutyric acid	ENSGALG00000014412	CSTA
Com_165_neg	(±)9-HpODE	MSTRG.8034	--
Com_460_pos	3-amino-4-(propylamino)c	ENSGALG00000003015	SERPINF1
Com_460_pos	3-amino-4-(propylamino)c	ENSGALG00000017040	C4
Com_208_neg	N-Acetylanthranilic acid	ENSGALG00000016761	LYG2
Com_152_pos	Acetyl-L-carnitine	ENSGALG00000016164	ABCG1
Com_108_neg	LPE 18:2	ENSGALG00000032645	H2A-VIII
Com_208_neg	N-Acetylanthranilic acid	ENSGALG00000047821	--
Com_120_neg	LPC 15:0	ENSGALG00000001709	MUSTN1
Com_150_neg	benzyl N-(2-{[(benzyloxy)c	ENSGALG00000004373	KCNJ16
Com_80_pos	DL-Lysine	ENSGALG00000015425	LPL
Com_150_neg	benzyl N-(2-{[(benzyloxy)c	ENSGALG00000009157	ECT2
Com_24_neg	PE (16:0/20:4)	ENSGALG00000013660	ZNF516
Com_80_pos	DL-Lysine	ENSGALG00000027891	NREP
Com_471_pos	Indole-3-acetic acid	ENSGALG00000003948	ALAS1

Com_120_neg	LPC 15:0	ENSGALG00000001565	C5
Com_68_neg	PE (16:0/22:6)	ENSGALG00000015234	clcC
Com_89_neg	Gallic acid	ENSGALG00000012254	KCNJ4
Com_55_pos	Valine	ENSGALG00000030025	FABP4
Com_18_neg	Arachidonic acid	ENSGALG00000008780	CTBS
Com_76_neg	Erythronolactone	ENSGALG00000004598	CUX2
Com_440_pos	PC (18:4e/4:0)	ENSGALG00000037160	Smad7
Com_80_pos	DL-Lysine	ENSGALG00000002790	ABLIM3
Com_588_pos	Ornithine	MSTRG.8055	--
Com_152_pos	Acetyl-L-carnitine	ENSGALG00000008185	AOX1
Com_215_pos	D-Erythro-sphingosine 1-|	MSTRG.835	--
Com_86_neg	Levulinic acid	ENSGALG00000050154	--
Com_342_pos	1-(4-methylphenyl)-3,5-di	ENSGALG00000003099	PCTP
Com_252_pos	cis-4-Hydroxy-D-proline	ENSGALG00000011571	AGPAT4
Com_97_pos	L-Threonine	ENSGALG00000011287	SULT
Com_108_neg	LPE 18:2	MSTRG.15745	--
Com_352_pos	Riboflavin	MSTRG.18213	--
Com_311_pos	PC (18:4e/2:0)	ENSGALG00000012754	PAH
Com_8_neg	4-Methyl-2-Oxopentanoic	ENSGALG00000012755	IGF-I
Com_440_pos	PC (18:4e/4:0)	ENSGALG00000031158	OAT
Com_108_neg	LPE 18:2	MSTRG.15444	--
Com_54_pos	Uric acid	ENSGALG00000047781	RAD9B
Com_311_pos	PC (18:4e/2:0)	ENSGALG00000003212	TSPO2
Com_178_pos	Maltol	ENSGALG00000017046	POSTN
Com_471_pos	Indole-3-acetic acid	ENSGALG00000005470	PLPPR5
Com_16_neg	3-Hydroxybutyric acid	ENSGALG00000011994	SYNPO2
Com_311_pos	PC (18:4e/2:0)	ENSGALG00000034741	ETNPPL
Com_186_pos	4-Hydroxybenzaldehyde	ENSGALG00000010163	LGR5
Com_178_pos	Maltol	ENSGALG00000016036	DOP1B
Com_57_neg	LPC 16:1	MSTRG.13407	--
Com_171_neg	LPC 22:6	ENSGALG00000011287	SULT
Com_192_pos	1-Methylhistidine	ENSGALG00000013100	GRB10
Com_55_pos	Valine	ENSGALG00000009947	PLEKHH2
Com_311_pos	PC (18:4e/2:0)	ENSGALG00000026313	RND3
Com_413_pos	L-Cystine	ENSGALG00000004205	SOAT1
Com_16_neg	3-Hydroxybutyric acid	ENSGALG00000006374	TBX6
Com_150_neg	benzyl N-(2-{[(benzyloxy)c	ENSGALG00000015849	ME1
Com_152_pos	Acetyl-L-carnitine	ENSGALG00000028407	GDF9
Com_57_neg	LPC 16:1	ENSGALG00000014516	CPEB2
Com_175_pos	Pantothenic acid	ENSGALG00000008728	PTER
Com_151_neg	Lysope 18:1	MSTRG.17628	--
Com_175_pos	Pantothenic acid	ENSGALG00000015034	ANKRD29
Com_24_neg	PE (16:0/20:4)	ENSGALG00000008930	B3GNT2
Com_362_pos	2-Arachidonoyl glycerol	ENSGALG00000046687	EPS8L3
Com_53_neg	2-(5-mercapto-4-methyl-4	ENSGALG00000006217	S100B
Com_80_pos	DL-Lysine	ENSGALG00000034337	RHPN1
Com_40_pos	Choline	ENSGALG00000000184	SLC27A6
Com_8_neg	4-Methyl-2-Oxopentanoic	ENSGALG00000036190	AOC1
Com_108_neg	LPE 18:2	ENSGALG00000010406	TMEM63C
Com_386_pos	2-Amino-1,3-octadecanec	ENSGALG00000002790	ABLIM3
Com_151_pos	Pyridoxamine	MSTRG.836	--
Com_119_pos	DL-Stachydrine	ENSGALG00000041078	MID1IP1
Com_208_neg	N-Acetylanthranilic acid	ENSGALG00000019768	ACSF2
Com_440_pos	PC (18:4e/4:0)	ENSGALG00000038520	STRIP2
Com_152_pos	Acetyl-L-carnitine	ENSGALG00000027793	SCN9A
Com_171_neg	LPC 22:6	ENSGALG00000017103	WASF3
Com_311_pos	PC (18:4e/2:0)	ENSGALG00000013728	PPAT
Com_175_pos	Pantothenic acid	ENSGALG00000002855	SARDH

Com_76_neg	Erythronolactone	ENSGALG00000016196	CBSL
Com_86_neg	Levulinic acid	ENSGALG00000008912	ABCB1
Com_386_pos	2-Amino-1,3-octadecaneс	ENSGALG00000015425	LPL
Com_178_pos	Maltol	ENSGALG00000011003	SLC35F3
Com_17_pos	L-Norleucine	ENSGALG00000017040	C4
Com_17_pos	L-Norleucine	ENSGALG00000003015	SERPINF1
Com_18_neg	Arachidonic acid	ENSGALG00000046639	CYP2AC2
Com_8_neg	4-Methyl-2-Oxopentanoic	ENSGALG00000000293	A2ML1
Com_440_pos	PC (18:4e/4:0)	ENSGALG00000011314	LRRC3B
Com_440_pos	PC (18:4e/4:0)	ENSGALG00000011391	AMN
Com_588_pos	Ornithine	ENSGALG00000037671	psuG
Com_25_pos	2-Hydroxycinnamic acid	ENSGALG00000011684	STAP1
Com_171_neg	LPC 22:6	ENSGALG00000019835	TRIM27.2
Com_76_neg	Erythronolactone	ENSGALG00000016325	GSTA3
Com_18_neg	Arachidonic acid	ENSGALG00000005408	BCO1
Com_16_neg	3-Hydroxybutyric acid	ENSGALG00000041373	ARAP2
Com_413_pos	L-Cystine	ENSGALG00000009740	RASGRP1
Com_54_pos	Uric acid	MSTRG.13819	--
Com_152_pos	Acetyl-L-carnitine	ENSGALG00000032882	EVA1C
Com_21_pos	DL-Tryptophan	ENSGALG00000037014	TSNARE1
Com_413_pos	L-Cystine	ENSGALG00000017039	STOML3
Com_150_neg	benzyl N-(2-{[(benzyloxy)c	MSTRG.163	--
Com_215_pos	D-Erythro-sphingosine 1-ｐ	MSTRG.2430	--
Com_16_neg	3-Hydroxybutyric acid	ENSGALG00000004343	HPD
Com_362_pos	2-Arachidonoyl glycerol	ENSGALG00000016281	DMD
Com_130_neg	2-Hydroxyvaleric acid	MSTRG.29	SHANK3
Com_175_pos	Pantothenic acid	ENSGALG00000028341	MADCAM1
Com_57_neg	LPC 16:1	ENSGALG00000003446	PRLR
Com_120_neg	LPC 15:0	ENSGALG00000002024	COMT
Com_362_pos	2-Arachidonoyl glycerol	ENSGALG00000038923	Ces1e
Com_92_pos	D-(+)-Proline	ENSGALG00000032231	C4
Com_152_pos	Acetyl-L-carnitine	ENSGALG00000052583	A2ML1
Com_175_pos	Pantothenic acid	ENSGALG00000016138	DSCAM
Com_331_pos	L-Lysine	ENSGALG00000005977	BTBD8
Com_22_pos	Indole-3-acrylic acid	ENSGALG00000053860	mas
Com_194_pos	Pipecolic acid	ENSGALG00000028897	WDR25
Com_311_pos	PC (18:4e/2:0)	ENSGALG00000033171	TGM4
Com_120_neg	LPC 15:0	ENSGALG00000014840	C6
Com_22_pos	Indole-3-acrylic acid	ENSGALG00000008859	WDR31
Com_24_neg	PE (16:0/20:4)	ENSGALG00000054252	TM4SF1
Com_194_pos	Pipecolic acid	ENSGALG00000029270	GATA3
Com_440_pos	PC (18:4e/4:0)	ENSGALG00000041680	KCNT2
Com_76_neg	Erythronolactone	ENSGALG00000014836	LPIN2
Com_152_pos	Acetyl-L-carnitine	ENSGALG00000052964	TOPAZ1
Com_25_pos	2-Hydroxycinnamic acid	ENSGALG00000015362	TRAT1
Com_192_pos	1-Methylhistidine	ENSGALG00000011331	CTH
Com_92_pos	D-(+)-Proline	ENSGALG00000002479	MAT1A
Com_460_pos	3-amino-4-(propylamino)c	ENSGALG00000000309	Lad1
Com_386_pos	2-Amino-1,3-octadecaneс	ENSGALG00000021340	CA9
Com_56_neg	LPE 18:1	ENSGALG00000015605	BACH2
Com_352_pos	Riboflavin	MSTRG.10162	--
Com_57_neg	LPC 16:1	ENSGALG00000006054	CALCA
Com_40_pos	Choline	ENSGALG00000039474	ID4
Com_471_pos	Indole-3-acetic acid	ENSGALG00000022758	GGACT
Com_311_pos	PC (18:4e/2:0)	MSTRG.19177	PHGDH
Com_76_neg	Erythronolactone	ENSGALG00000021340	CA9
Com_130_neg	2-Hydroxyvaleric acid	ENSGALG00000028928	LCAT
Com_215_pos	D-Erythro-sphingosine 1-ｐ	ENSGALG00000032645	H2A-VIII

Com_331_pos	L-Lysine	ENSGALG00000009700	PDK4
Com_460_pos	3-amino-4-(propylamino)(	ENSGALG00000008912	ABCB1
Com_78_neg	Citric acid	ENSGALG00000017199	MAML2
Com_311_pos	PC (18:4e/2:0)	ENSGALG00000019147	--
Com_108_neg	LPE 18:2	MSTRG.835	--
Com_175_pos	Pantothenic acid	ENSGALG00000041258	msrA
Com_4_pos	PC (17:1/17:1)	MSTRG.15625	--
Com_8_neg	4-Methyl-2-Oxopentanoic	ENSGALG00000016164	ABCG1
Com_17_pos	L-Norleucine	ENSGALG00000050154	--
Com_352_pos	Riboflavin	MSTRG.13526	--
Com_471_pos	Indole-3-acetic acid	ENSGALG00000005739	SCD
Com_86_neg	Levulinic acid	ENSGALG00000041258	msrA
Com_203_pos	Serotonin	ENSGALG00000013848	MVK
Com_120_neg	LPC 15:0	ENSGALG00000033974	HGF
Com_151_neg	Lysope 18:1	ENSGALG00000050491	SLC35E4
Com_482_pos	8-Hydroxyquinoline	ENSGALG00000020342	ABHD12
Com_482_pos	8-Hydroxyquinoline	ENSGALG00000012882	KDSR
Com_150_neg	benzyl N-(2-{[(benzyloxy)c	ENSGALG00000031164	WFDC2
Com_215_pos	D-Erythro-sphingosine 1-p	MSTRG.15444	--
Com_53_neg	2-(5-mercapto-4-methyl-4	ENSGALG00000011809	GRIN2B
Com_482_pos	8-Hydroxyquinoline	ENSGALG00000043336	COPZ1
Com_17_pos	L-Norleucine	ENSGALG00000002024	COMT
Com_471_pos	Indole-3-acetic acid	ENSGALG00000012882	KDSR
Com_55_pos	Valine	ENSGALG00000013124	FHOD3
Com_311_pos	PC (18:4e/2:0)	ENSGALG00000037160	Smad7
Com_482_pos	8-Hydroxyquinoline	ENSGALG00000050676	Ctnnd2
Com_78_neg	Citric acid	ENSGALG00000011287	SULT
Com_155_neg	Phenylacetaldehyde	MSTRG.21420	--
Com_471_pos	Indole-3-acetic acid	ENSGALG00000042080	PGPEP1
Com_150_neg	benzyl N-(2-{[(benzyloxy)c	ENSGALG00000010825	AGR2
Com_150_neg	benzyl N-(2-{[(benzyloxy)c	MSTRG.6499	--
Com_482_pos	8-Hydroxyquinoline	ENSGALG00000005739	SCD
Com_68_neg	PE (16:0/22:6)	MSTRG.13526	--
Com_152_pos	Acetyl-L-carnitine	ENSGALG00000012755	IGF-I
Com_68_neg	PE (16:0/22:6)	ENSGALG00000047464	FBXL22
Com_440_pos	PC (18:4e/4:0)	ENSGALG00000012754	PAH
Com_165_neg	(±)9-HpODE	ENSGALG00000047464	FBXL22
Com_18_neg	Arachidonic acid	ENSGALG00000010857	DAB1
Com_97_pos	L-Threonine	ENSGALG00000011894	CYP2D6
Com_440_pos	PC (18:4e/4:0)	ENSGALG00000003212	TSPO2
Com_203_pos	Serotonin	ENSGALG00000003560	SLC6A2
Com_352_pos	Riboflavin	ENSGALG00000044251	RASSF7
Com_352_pos	Riboflavin	ENSGALG00000051550	ARMH4
Com_8_neg	4-Methyl-2-Oxopentanoic	ENSGALG00000052872	--
Com_362_pos	2-Arachidonoyl glycerol	ENSGALG00000028928	LCAT
Com_120_neg	LPC 15:0	ENSGALG00000003015	SERPINF1
Com_120_neg	LPC 15:0	ENSGALG00000017040	C4
Com_40_pos	Choline	ENSGALG00000007014	PYROXD2
Com_460_pos	3-amino-4-(propylamino)(	ENSGALG00000001709	MUSTN1
Com_482_pos	8-Hydroxyquinoline	ENSGALG00000022758	GGACT
Com_215_pos	D-Erythro-sphingosine 1-p	ENSGALG00000010406	TMEM63C
Com_86_neg	Levulinic acid	ENSGALG00000016138	DSCAM
Com_99_pos	Creatine	ENSGALG00000007536	PHR
Com_16_neg	3-Hydroxybutyric acid	MSTRG.8511	--
Com_460_pos	3-amino-4-(propylamino)(	ENSGALG00000001565	C5
Com_352_pos	Riboflavin	ENSGALG00000004852	DNM1
Com_68_neg	PE (16:0/22:6)	MSTRG.10162	--
Com_86_neg	Levulinic acid	ENSGALG00000028341	MADCAM1

Com_342_pos	1-(4-methylphenyl)-3,5-di	MSTRG.21420	--
Com_311_pos	PC (18:4e/2:0)	ENSGALG00000014616	MT3
Com_86_neg	Levulinic acid	ENSGALG00000017040	C4
Com_86_neg	Levulinic acid	ENSGALG00000003015	SERPINF1
Com_208_neg	N-Acetylanthranilic acid	ENSGALG00000039140	CD14
Com_56_neg	LPE 18:1	ENSGALG00000023740	HBZ
Com_152_pos	Acetyl-L-carnitine	ENSGALG00000036190	AOC1
Com_155_neg	Phenylacetaldehyde	ENSGALG00000050857	PTPN20
Com_97_pos	L-Threonine	ENSGALG00000028451	MT4
Com_178_pos	Maltol	ENSGALG00000005472	NAT
Com_18_neg	Arachidonic acid	ENSGALG00000014412	CSTA
Com_362_pos	2-Arachidonoyl glycerol	ENSGALG00000035219	ALB
Com_588_pos	Ornithine	ENSGALG00000036086	TAGLN2
Com_130_neg	2-Hydroxyvaleric acid	ENSGALG00000038923	Ces1e
Com_78_neg	Citric acid	MSTRG.17721	--
Com_8_neg	4-Methyl-2-Oxopentanoic	ENSGALG00000027793	SCN9A
Com_8_neg	4-Methyl-2-Oxopentanoic	ENSGALG00000014252	A2M
Com_99_pos	Creatine	ENSGALG00000013776	CEP135
Com_130_neg	2-Hydroxyvaleric acid	ENSGALG00000016281	DMD
Com_57_neg	LPC 16:1	ENSGALG00000053680	HIST1H46L2
Com_57_neg	LPC 16:1	ENSGALG00000005553	NLGN3
Com_440_pos	PC (18:4e/4:0)	ENSGALG00000013728	PPAT
Com_8_neg	4-Methyl-2-Oxopentanoic	ENSGALG00000040573	FMO3
Com_108_neg	LPE 18:2	ENSGALG00000014834	NCOA7
Com_192_pos	1-Methylhistidine	ENSGALG00000028256	CCL19
Com_16_neg	3-Hydroxybutyric acid	ENSGALG00000008780	CTBS
Com_108_neg	LPE 18:2	MSTRG.2430	--
Com_152_pos	Acetyl-L-carnitine	ENSGALG00000002594	TFPI
Com_86_neg	Levulinic acid	ENSGALG00000002855	SARDH
Com_151_neg	Lysope 18:1	ENSGALG00000008462	CDK3
Com_311_pos	PC (18:4e/2:0)	ENSGALG00000011314	LRRC3B
Com_108_neg	LPE 18:2	ENSGALG00000041205	--
Com_119_pos	DL-Stachydrine	ENSGALG00000002362	MANF
Com_588_pos	Ornithine	ENSGALG00000035903	FAM46A
Com_311_pos	PC (18:4e/2:0)	ENSGALG00000026663	CX3CL1
Com_311_pos	PC (18:4e/2:0)	ENSGALG00000011391	AMN
Com_76_neg	Erythronolactone	ENSGALG00000029944	FAM222A
Com_152_pos	Acetyl-L-carnitine	MSTRG.20377	--
Com_482_pos	8-Hydroxyquinoline	ENSGALG00000036293	EBAG9
Com_151_neg	Lysope 18:1	ENSGALG00000016444	ODC1
Com_8_neg	4-Methyl-2-Oxopentanoic	ENSGALG00000026663	CX3CL1
Com_386_pos	2-Amino-1,3-octadecanec	ENSGALG00000004598	CUX2
Com_264_pos	Indole	ENSGALG00000053860	mas
Com_12_pos	Betaine	ENSGALG00000033411	SLC26A2
Com_413_pos	L-Cystine	MSTRG.8055	--
Com_97_pos	L-Threonine	ENSGALG00000028928	LCAT
Com_97_pos	L-Threonine	ENSGALG00000021848	AVD
Com_86_neg	Levulinic acid	ENSGALG00000015034	ANKRD29
Com_471_pos	Indole-3-acetic acid	ENSGALG00000004505	CCDC137
Com_147_pos	D-Sphingosine	ENSGALG00000031158	OAT
Com_4_pos	PC (17:1/17:1)	ENSGALG00000040620	LSAMP
Com_264_pos	Indole	ENSGALG00000008859	WDR31
Com_86_neg	Levulinic acid	ENSGALG00000008728	PTER
Com_178_pos	Maltol	ENSGALG00000005977	BTBD8
Com_18_neg	Arachidonic acid	ENSGALG00000011994	SYNPO2
Com_311_pos	PC (18:4e/2:0)	ENSGALG00000040573	FMO3
Com_386_pos	2-Amino-1,3-octadecanec	ENSGALG00000007114	APOA1
Com_186_pos	4-Hydroxybenzaldehyde	ENSGALG00000010889	HOOK1

Com_311_pos	PC (18:4e/2:0)	ENSGALG00000014252	A2M
Com_440_pos	PC (18:4e/4:0)	ENSGALG00000033171	TGM4
Com_151_pos	Pyridoxamine	ENSGALG00000001531	FN3K
Com_151_neg	Lysope 18:1	MSTRG.21631	--
Com_588_pos	Ornithine	ENSGALG00000015492	PDZK1
Com_18_neg	Arachidonic acid	ENSGALG00000006374	TBX6
Com_352_pos	Riboflavin	ENSGALG00000019738	FBXO47
Com_171_neg	LPC 22:6	ENSGALG00000017199	MAML2
Com_471_pos	Indole-3-acetic acid	ENSGALG00000035935	Unc13c
Com_119_pos	DL-Stachydrine	ENSGALG00000029857	Gimap1
Com_175_pos	Pantothenic acid	ENSGALG00000003136	IKZF2
Com_203_pos	Serotonin	ENSGALG00000050440	APOF
Com_175_pos	Pantothenic acid	ENSGALG00000009963	LYZ
Com_8_neg	4-Methyl-2-Oxopentanoic	ENSGALG00000014616	MT3
Com_4_pos	PC (17:1/17:1)	ENSGALG00000021451	RED3
Com_352_pos	Riboflavin	ENSGALG00000000745	SLC26A9
Com_4_pos	PC (17:1/17:1)	ENSGALG00000006842	ACOT8
Com_178_pos	Maltol	ENSGALG00000009700	PDK4
Com_588_pos	Ornithine	ENSGALG00000004205	SOAT1
Com_175_pos	Pantothenic acid	ENSGALG00000050154	--
Com_194_pos	Pipecolic acid	ENSGALG00000002466	SLC2A5
Com_17_pos	L-Norleucine	ENSGALG00000041258	msrA
Com_203_pos	Serotonin	ENSGALG00000014509	BST1
Com_16_neg	3-Hydroxybutyric acid	ENSGALG00000012034	ADSL
Com_342_pos	1-(4-methylphenyl)-3,5-di	ENSGALG00000053112	FRMPD2
Com_17_pos	L-Norleucine	MSTRG.11572	--
Com_440_pos	PC (18:4e/4:0)	MSTRG.19177	PHGDH
Com_92_pos	D-(+)-Proline	ENSGALG00000008185	AOX1
Com_147_pos	D-Sphingosine	ENSGALG00000038520	STRIP2
Com_25_pos	2-Hydroxycinnamic acid	ENSGALG00000003553	ABCA12
Com_482_pos	8-Hydroxyquinoline	ENSGALG00000005470	PLPPR5
Com_22_pos	Indole-3-acrylic acid	ENSGALG00000000498	ACE
Com_311_pos	PC (18:4e/2:0)	ENSGALG00000052872	--
Com_178_pos	Maltol	ENSGALG00000017032	SLC25A15
Com_16_neg	3-Hydroxybutyric acid	ENSGALG00000046639	CYP2AC2
Com_215_pos	D-Erythro-sphingosine 1-p	MSTRG.12570	--
Com_86_neg	Levulinic acid	ENSGALG00000002024	COMT
Com_16_neg	3-Hydroxybutyric acid	ENSGALG00000040434	rab18b
Com_4_pos	PC (17:1/17:1)	ENSGALG00000011141	ITGB6
Com_482_pos	8-Hydroxyquinoline	ENSGALG00000041604	NPTXR
Com_460_pos	3-amino-4-(propylamino)(	ENSGALG00000014840	C6
Com_78_neg	Citric acid	ENSGALG00000046687	EPS8L3
Com_413_pos	L-Cystine	ENSGALG00000005474	PNAT10
Com_460_pos	3-amino-4-(propylamino)(	ENSGALG00000054981	F10
Com_130_neg	2-Hydroxyvaleric acid	ENSGALG00000011894	CYP2D6
Com_16_neg	3-Hydroxybutyric acid	MSTRG.3197	--
Com_440_pos	PC (18:4e/4:0)	ENSGALG00000019147	--
Com_97_pos	L-Threonine	MSTRG.10101	--
Com_16_neg	3-Hydroxybutyric acid	ENSGALG00000005408	BCO1
Com_76_neg	Erythronolactone	ENSGALG00000047321	SARDH
Com_171_neg	LPC 22:6	ENSGALG00000043582	LY6E
Com_18_neg	Arachidonic acid	ENSGALG00000028871	SLC38A3
Com_413_pos	L-Cystine	ENSGALG00000037671	psuG
Com_80_pos	DL-Lysine	ENSGALG00000013033	cmbl
Com_208_neg	N-Acetylanthranilic acid	ENSGALG00000041577	ITGA4
Com_352_pos	Riboflavin	ENSGALG00000015234	clcC
Com_18_neg	Arachidonic acid	ENSGALG00000004343	HPD
Com_76_neg	Erythronolactone	ENSGALG00000047480	A2ML1

Com_119_pos	DL-Stachydrine	ENSGALG00000026809	SARS
Com_482_pos	8-Hydroxyquinoline	ENSGALG00000003948	ALAS1
Com_194_pos	Pipecolic acid	MSTRG.20478	--
Com_89_neg	Gallic acid	ENSGALG00000009024	CLIP4
Com_108_neg	LPE 18:2	ENSGALG00000035478	FAM91A1
Com_151_pos	Pyridoxamine	ENSGALG00000016690	CYP2AC1
Com_25_pos	2-Hydroxycinnamic acid	ENSGALG00000031593	TMSB15B
Com_588_pos	Ornithine	ENSGALG00000009740	RASGRP1
Com_92_pos	D-(+)-Proline	ENSGALG00000028407	GDF9
Com_57_neg	LPC 16:1	ENSGALG00000011335	NHEJ1
Com_120_neg	LPC 15:0	ENSGALG00000008912	ABCB1
Com_17_pos	L-Norleucine	ENSGALG00000016138	DSCAM
Com_460_pos	3-amino-4-(propylamino)(	MSTRG.14083	--
Com_342_pos	1-(4-methylphenyl)-3,5-di	ENSGALG00000010139	SSTR1
Com_130_neg	2-Hydroxyvaleric acid	ENSGALG00000028451	MT4
Com_203_pos	Serotonin	ENSGALG00000005888	PGP
Com_482_pos	8-Hydroxyquinoline	ENSGALG00000021135	HAPLN3
Com_97_pos	L-Threonine	ENSGALG00000038923	Ces1e
Com_17_pos	L-Norleucine	ENSGALG00000028341	MADCAM1
Com_386_pos	2-Amino-1,3-octadecanec	ENSGALG00000054322	--
Com_92_pos	D-(+)-Proline	ENSGALG00000002728	SLC16A3
Com_8_neg	4-Methyl-2-Oxopentanoic	ENSGALG00000019147	--
Com_342_pos	1-(4-methylphenyl)-3,5-di	ENSGALG00000008437	NET1
Com_97_pos	L-Threonine	ENSGALG00000016281	DMD
Com_57_neg	LPC 16:1	ENSGALG00000021627	IFI27L2
Com_147_pos	D-Sphingosine	ENSGALG00000041680	KCNT2
Com_152_pos	Acetyl-L-carnitine	ENSGALG00000052872	--
Com_386_pos	2-Amino-1,3-octadecanec	ENSGALG00000011524	PPEF2
Com_208_neg	N-Acetylanthranilic acid	ENSGALG00000037387	CLSTN2
Com_460_pos	3-amino-4-(propylamino)(	ENSGALG00000033974	HGF
Com_352_pos	Riboflavin	ENSGALG00000030160	DRC7
Com_171_neg	LPC 22:6	MSTRG.17721	--
Com_22_pos	Indole-3-acrylic acid	ENSGALG00000002116	TEN1
Com_8_neg	4-Methyl-2-Oxopentanoic	MSTRG.19177	PHGDH
Com_55_pos	Valine	ENSGALG00000028897	WDR25
Com_151_neg	Lysope 18:1	MSTRG.8904	--
Com_119_pos	DL-Stachydrine	ENSGALG00000015728	MUSK
Com_55_pos	Valine	ENSGALG00000029270	GATA3
Com_186_pos	4-Hydroxybenzaldehyde	ENSGALG00000007478	SLC51A
Com_215_pos	D-Erythro-sphingosine 1-p	ENSGALG00000014834	NCOA7
Com_130_neg	2-Hydroxyvaleric acid	ENSGALG00000021848	AVD
Com_331_pos	L-Lysine	ENSGALG00000005472	NAT
Com_17_pos	L-Norleucine	ENSGALG00000002855	SARDH
Com_440_pos	PC (18:4e/4:0)	ENSGALG00000014616	MT3
Com_40_pos	Choline	ENSGALG00000016690	CYP2AC1
Com_186_pos	4-Hydroxybenzaldehyde	ENSGALG00000028284	PTX3
Com_92_pos	D-(+)-Proline	ENSGALG00000032882	EVA1C
Com_215_pos	D-Erythro-sphingosine 1-p	ENSGALG00000041205	--
Com_471_pos	Indole-3-acetic acid	ENSGALG00000003575	Dnttip1
Com_152_pos	Acetyl-L-carnitine	ENSGALG00000014252	A2M
Com_8_neg	4-Methyl-2-Oxopentanoic	ENSGALG00000033171	TGM4
Com_152_pos	Acetyl-L-carnitine	ENSGALG00000040573	FMO3
Com_471_pos	Indole-3-acetic acid	ENSGALG00000015263	TMEM30C
Com_152_pos	Acetyl-L-carnitine	ENSGALG00000032231	C4
Com_92_pos	D-(+)-Proline	ENSGALG00000052583	A2ML1
Com_8_neg	4-Methyl-2-Oxopentanoic	ENSGALG00000002594	TFPI
Com_17_pos	L-Norleucine	ENSGALG00000015034	ANKRD29
Com_12_pos	Betaine	ENSGALG00000034438	GNB3

Com_151_neg	Lysope 18:1	MSTRG.13407	--
Com_12_pos	Betaine	ENSGALG00000013033	cmbl
Com_482_pos	8-Hydroxyquinoline	ENSGALG00000031312	ANAPC13
Com_17_pos	L-Norleucine	ENSGALG00000008728	PTER
Com_76_neg	Erythronolactone	MSTRG.3009	--
Com_208_neg	N-Acetylanthranilic acid	ENSGALG00000007132	ACOX2
Com_68_neg	PE (16:0/22:6)	MSTRG.20491	--
Com_57_neg	LPC 16:1	MSTRG.17628	--
Com_471_pos	Indole-3-acetic acid	ENSGALG00000010978	ANGPTL3
Com_8_neg	4-Methyl-2-Oxopentanoic	MSTRG.20377	--
Com_68_neg	PE (16:0/22:6)	ENSGALG00000007077	CPT1A
Com_92_pos	D-(+)-Proline	ENSGALG00000052964	TOPAZ1
Com_152_pos	Acetyl-L-carnitine	ENSGALG00000026663	CX3CL1
Com_440_pos	PC (18:4e/4:0)	ENSGALG00000026663	CX3CL1
Com_151_neg	Lysope 18:1	ENSGALG00000007645	prom1a
Com_203_pos	Serotonin	ENSGALG00000023760	CHIA
Com_120_neg	LPC 15:0	ENSGALG00000024085	IDO2
Com_86_neg	Levulinic acid	ENSGALG00000044996	TMEM71
Com_16_neg	3-Hydroxybutyric acid	ENSGALG00000010857	DAB1
Com_147_pos	D-Sphingosine	ENSGALG00000012754	PAH
Com_460_pos	3-amino-4-(propylamino)(	ENSGALG00000003537	SGK2
Com_147_pos	D-Sphingosine	ENSGALG00000003212	TSPO2
Com_440_pos	PC (18:4e/4:0)	ENSGALG00000040573	FMO3
Com_8_neg	4-Methyl-2-Oxopentanoic	ENSGALG00000013728	PPAT
Com_362_pos	2-Arachidonoyl glycerol	ENSGALG00000011287	SULT
Com_482_pos	8-Hydroxyquinoline	ENSGALG00000003147	TRPC4AP
Com_147_pos	D-Sphingosine	ENSGALG00000002790	ABLIM3
Com_151_pos	Pyridoxamine	ENSGALG00000009700	PDK4
Com_471_pos	Indole-3-acetic acid	ENSGALG00000009680	PAQR7
Com_18_neg	Arachidonic acid	MSTRG.8511	--
Com_89_neg	Gallic acid	MSTRG.7572	--
Com_57_neg	LPC 16:1	MSTRG.21653	--
Com_440_pos	PC (18:4e/4:0)	ENSGALG00000014252	A2M
Com_40_pos	Choline	ENSGALG00000001531	FN3K
Com_311_pos	PC (18:4e/2:0)	ENSGALG00000036190	AOC1
Com_86_neg	Levulinic acid	MSTRG.11572	--
Com_130_neg	2-Hydroxyvaleric acid	MSTRG.10101	--
Com_252_pos	cis-4-Hydroxy-D-proline	ENSGALG00000050840	APCDD1
Com_208_neg	N-Acetylanthranilic acid	ENSGALG00000010853	C8B
Com_178_pos	Maltol	MSTRG.3197	--
Com_147_pos	D-Sphingosine	ENSGALG00000015425	LPL
Com_165_neg	(±)9-HpODE	ENSGALG00000007993	DCX
Com_171_neg	LPC 22:6	ENSGALG00000046687	EPS8L3
Com_152_pos	Acetyl-L-carnitine	ENSGALG00000014616	MT3
Com_178_pos	Maltol	ENSGALG00000040434	rab18b
Com_108_neg	LPE 18:2	MSTRG.12570	--
Com_25_pos	2-Hydroxycinnamic acid	ENSGALG00000041344	FABP5
Com_264_pos	Indole	ENSGALG00000000498	ACE
Com_16_neg	3-Hydroxybutyric acid	ENSGALG00000017032	SLC25A15
Com_155_neg	Phenylacetaldehyde	ENSGALG00000028135	--
Com_78_neg	Citric acid	ENSGALG00000043582	LY6E
Com_460_pos	3-amino-4-(propylamino)(	ENSGALG00000019663	ACBD7
Com_331_pos	L-Lysine	ENSGALG00000017032	SLC25A15
Com_178_pos	Maltol	ENSGALG00000012034	ADSL
Com_460_pos	3-amino-4-(propylamino)(	ENSGALG00000005204	GSTT1
Com_151_neg	Lysope 18:1	ENSGALG00000003446	PRLR
Com_130_neg	2-Hydroxyvaleric acid	MSTRG.4548	--
Com_151_neg	Lysope 18:1	ENSGALG00000022720	GJB2

Com_97_pos	L-Threonine	ENSGALG00000005030	DOCK10
Com_130_neg	2-Hydroxyvaleric acid	ENSGALG00000031122	NTNG1
Com_151_pos	Pyridoxamine	ENSGALG00000005977	BTBD8
Com_165_neg	(±)9-HpODE	MSTRG.2406	--
Com_97_pos	L-Threonine	ENSGALG00000027122	APPL2
Com_460_pos	3-amino-4-(propylamino)(	ENSGALG00000009002	CPED1
Com_482_pos	8-Hydroxyquinoline	ENSGALG00000046757	ERVK-9
Com_194_pos	Pipecolic acid	ENSGALG00000041491	ACKR4
Com_108_neg	LPE 18:2	ENSGALG00000037769	NEBL
Com_89_neg	Gallic acid	ENSGALG00000021193	STARD5
Com_147_pos	D-Sphingosine	ENSGALG00000013728	PPAT
Com_440_pos	PC (18:4e/4:0)	ENSGALG00000052872	--
Com_4_pos	PC (17:1/17:1)	ENSGALG00000032329	NPM3
Com_413_pos	L-Cystine	ENSGALG00000036086	TAGLN2
Com_92_pos	D-(+)-Proline	ENSGALG00000012755	IGF-I
Com_8_neg	4-Methyl-2-Oxopentanoic	ENSGALG00000003212	TSPO2
Com_80_pos	DL-Lysine	ENSGALG00000033411	SLC26A2
Com_215_pos	D-Erythro-sphingosine 1-p	ENSGALG00000035478	FAM91A1
Com_120_neg	LPC 15:0	ENSGALG00000009545	SLC25A12
Com_311_pos	PC (18:4e/2:0)	ENSGALG00000012755	IGF-I
Com_8_neg	4-Methyl-2-Oxopentanoic	ENSGALG00000012754	PAH
Com_24_neg	PE (16:0/20:4)	ENSGALG00000047464	FBXL22
Com_97_pos	L-Threonine	ENSGALG00000034616	INHBA
Com_55_pos	Valine	ENSGALG00000054322	--
Com_119_pos	DL-Stachydrine	MSTRG.10409	--
Com_78_neg	Citric acid	ENSGALG00000035219	ALB
Com_203_pos	Serotonin	MSTRG.21091	--
Com_203_pos	Serotonin	ENSGALG00000046412	Aoc3
Com_151_neg	Lysope 18:1	ENSGALG00000025738	RHOU
Com_24_neg	PE (16:0/20:4)	ENSGALG00000007955	SLC16A5
Com_413_pos	L-Cystine	ENSGALG00000035903	FAM46A
Com_175_pos	Pantothenic acid	ENSGALG00000007839	NCAM1
Com_352_pos	Riboflavin	ENSGALG00000037769	NEBL
Com_18_neg	Arachidonic acid	ENSGALG00000015040	SLC16A10
Com_471_pos	Indole-3-acetic acid	MSTRG.17073	--
Com_471_pos	Indole-3-acetic acid	ENSGALG00000036492	DAGLA
Com_186_pos	4-Hydroxybenzaldehyde	ENSGALG00000017120	SACS
Com_215_pos	D-Erythro-sphingosine 1-p	ENSGALG00000030801	CCKAR
Com_331_pos	L-Lysine	MSTRG.836	--
Com_152_pos	Acetyl-L-carnitine	ENSGALG00000019147	--
Com_147_pos	D-Sphingosine	ENSGALG00000033171	TGM4
Com_92_pos	D-(+)-Proline	ENSGALG00000036190	AOC1
Com_264_pos	Indole	ENSGALG00000002116	TEN1
Com_120_neg	LPC 15:0	ENSGALG00000043044	IL1R1
Com_120_neg	LPC 15:0	ENSGALG00000054981	F10
Com_57_neg	LPC 16:1	ENSGALG00000050491	SLC35E4
Com_151_pos	Pyridoxamine	ENSGALG00000007014	PYROXD2
Com_460_pos	3-amino-4-(propylamino)(	ENSGALG00000048343	Ces1e
Com_24_neg	PE (16:0/20:4)	ENSGALG00000013743	ENPP7
Com_76_neg	Erythronolactone	ENSGALG00000009479	SAMD9L
Com_86_neg	Levulinic acid	ENSGALG00000014950	SULT3A1
Com_413_pos	L-Cystine	ENSGALG00000015492	PDZK1
Com_119_pos	DL-Stachydrine	ENSGALG00000050091	CLEC2B
Com_56_neg	LPE 18:1	ENSGALG00000011319	ADGRL3
Com_152_pos	Acetyl-L-carnitine	MSTRG.19177	PHGDH
Com_18_neg	Arachidonic acid	ENSGALG00000012034	ADSL
Com_16_neg	3-Hydroxybutyric acid	ENSGALG00000005472	NAT
Com_147_pos	D-Sphingosine	MSTRG.19177	PHGDH

Com_192_pos	1-Methylhistidine	ENSGALG00000012420	CG-1B
Com_264_pos	Indole	ENSGALG00000053278	SUCNR1
Com_311_pos	PC (18:4e/2:0)	ENSGALG00000052964	TOPAZ1
Com_192_pos	1-Methylhistidine	ENSGALG00000004491	DMGDH
Com_18_neg	Arachidonic acid	ENSGALG00000040434	rab18b
Com_482_pos	8-Hydroxyquinoline	ENSGALG00000039538	CLDND1
Com_194_pos	Pipecolic acid	ENSGALG00000004230	LIPC
Com_92_pos	D-(+)-Proline	ENSGALG00000032746	ENPP2
Com_120_neg	LPC 15:0	MSTRG.14083	--
Com_18_neg	Arachidonic acid	MSTRG.3197	--
Com_68_neg	PE (16:0/22:6)	ENSGALG00000054252	TM4SF1
Com_86_neg	Levulinic acid	ENSGALG00000007728	Prodh
Com_8_neg	4-Methyl-2-Oxopentanoic	ENSGALG00000041680	KCNT2
Com_152_pos	Acetyl-L-carnitine	ENSGALG00000033171	TGM4
Com_56_neg	LPE 18:1	ENSGALG00000005284	LRRC39
Com_25_pos	2-Hydroxycinnamic acid	ENSGALG00000016558	VEGFD
Com_147_pos	D-Sphingosine	ENSGALG00000019147	--
Com_178_pos	Maltol	MSTRG.8511	--
Com_352_pos	Riboflavin	ENSGALG00000052328	CTNND2
Com_311_pos	PC (18:4e/2:0)	ENSGALG00000052583	A2ML1
Com_8_neg	4-Methyl-2-Oxopentanoic	ENSGALG00000032231	C4
Com_413_pos	L-Cystine	ENSGALG00000007839	NCAM1
Com_55_pos	Valine	ENSGALG00000002466	SLC2A5
Com_89_neg	Gallic acid	ENSGALG00000007636	PCK1
Com_17_pos	L-Norleucine	ENSGALG00000044996	TMEM71
Com_588_pos	Ornithine	ENSGALG00000005474	PNAT10
Com_16_neg	3-Hydroxybutyric acid	ENSGALG00000028871	SLC38A3
Com_171_neg	LPC 22:6	ENSGALG00000030031	TTPA
Com_588_pos	Ornithine	ENSGALG00000033338	GPT2
Com_460_pos	3-amino-4-(propylamino)c	ENSGALG00000022750	GPR18
Com_76_neg	Erythronolactone	ENSGALG00000006320	Slc2a9
Com_86_neg	Levulinic acid	ENSGALG00000038242	CACNA2D2
Com_311_pos	PC (18:4e/2:0)	ENSGALG00000032882	EVA1C
Com_92_pos	D-(+)-Proline	ENSGALG00000003578	FN1
Com_53_neg	2-(5-mercapto-4-methyl-4	ENSGALG00000005657	CRHR2
Com_97_pos	L-Threonine	MSTRG.4548	--
Com_130_neg	2-Hydroxyvaleric acid	ENSGALG00000005030	DOCK10
Com_97_pos	L-Threonine	ENSGALG00000031122	NTNG1
Com_203_pos	Serotonin	ENSGALG00000004612	MTHFR
Com_130_neg	2-Hydroxyvaleric acid	ENSGALG00000027122	APPL2
Com_56_neg	LPE 18:1	ENSGALG00000014727	PDE4D
Com_152_pos	Acetyl-L-carnitine	ENSGALG00000013728	PPAT
Com_92_pos	D-(+)-Proline	ENSGALG00000016027	CBR3
Com_151_neg	Lysope 18:1	ENSGALG00000053680	HIST1H46L2
Com_151_neg	Lysope 18:1	ENSGALG00000005553	NLGN3
Com_56_neg	LPE 18:1	ENSGALG00000007242	SLITRK4
Com_55_pos	Valine	MSTRG.20478	--
Com_80_pos	DL-Lysine	ENSGALG00000011391	AMN
Com_331_pos	L-Lysine	MSTRG.3197	--
Com_86_neg	Levulinic acid	ENSGALG00000013726	PAICS
Com_80_pos	DL-Lysine	ENSGALG00000011314	LRRC3B
Com_4_pos	PC (17:1/17:1)	MSTRG.2171	Myo16
Com_8_neg	4-Methyl-2-Oxopentanoic	ENSGALG00000038520	STRIP2
Com_57_neg	LPC 16:1	ENSGALG00000008462	CDK3
Com_482_pos	8-Hydroxyquinoline	ENSGALG00000037253	CLEC4M
Com_89_neg	Gallic acid	ENSGALG00000041238	NOS1AP
Com_331_pos	L-Lysine	ENSGALG00000040434	rab18b
Com_130_neg	2-Hydroxyvaleric acid	ENSGALG00000034616	INHBA

Com_97_pos	L-Threonine	ENSGALG00000034081	AKT3
Com_119_pos	DL-Stachydrine	MSTRG.1841	--
Com_208_neg	N-Acetylanthranilic acid	ENSGALG00000046316	CFAP97D1
Com_440_pos	PC (18:4e/4:0)	ENSGALG00000036190	AOC1
Com_471_pos	Indole-3-acetic acid	ENSGALG00000035626	DAD1
Com_215_pos	D-Erythro-sphingosine 1-|	ENSGALG00000037769	NEBL
Com_68_neg	PE (16:0/22:6)	ENSGALG00000008930	B3GNT2
Com_4_pos	PC (17:1/17:1)	MSTRG.16398	Cdhr5
Com_311_pos	PC (18:4e/2:0)	ENSGALG00000028407	GDF9
Com_150_neg	benzyl N-(2-{[(benzyloxy)c	ENSGALG00000012886	--
Com_331_pos	L-Lysine	ENSGALG00000012034	ADSL
Com_147_pos	D-Sphingosine	ENSGALG00000014616	MT3
Com_171_neg	LPC 22:6	ENSGALG00000035219	ALB
Com_460_pos	3-amino-4-(propylamino)(	ENSGALG00000024085	IDO2
Com_99_pos	Creatine	ENSGALG00000052991	DNAJC21
Com_482_pos	8-Hydroxyquinoline	ENSGALG00000021685	SERINC2
Com_120_neg	LPC 15:0	ENSGALG00000003537	SGK2
Com_386_pos	2-Amino-1,3-octadecanec	ENSGALG00000031158	OAT
Com_352_pos	Riboflavin	ENSGALG00000035478	FAM91A1
Com_152_pos	Acetyl-L-carnitine	ENSGALG00000003212	TSPO2
Com_150_neg	benzyl N-(2-{[(benzyloxy)c	ENSGALG00000045814	CRLF2
Com_482_pos	8-Hydroxyquinoline	MSTRG.11802	TMEM221
Com_152_pos	Acetyl-L-carnitine	ENSGALG00000012754	PAH
Com_57_neg	LPC 16:1	ENSGALG00000026598	NXNL2
Com_152_pos	Acetyl-L-carnitine	ENSGALG00000002728	SLC16A3
Com_352_pos	Riboflavin	ENSGALG00000017136	GJB6
Com_108_neg	LPE 18:2	ENSGALG00000030801	CCKAR
Com_8_neg	4-Methyl-2-Oxopentanoic	ENSGALG00000031158	OAT
Com_130_neg	2-Hydroxyvaleric acid	ENSGALG00000016364	ALKAL2
Com_440_pos	PC (18:4e/4:0)	ENSGALG00000012755	IGF-I
Com_92_pos	D-(+)-Proline	ENSGALG00000052872	--
Com_120_neg	LPC 15:0	ENSGALG00000016287	NR0B1
Com_482_pos	8-Hydroxyquinoline	ENSGALG00000032628	SRCIN1
Com_208_neg	N-Acetylanthranilic acid	ENSGALG00000015519	ROBO2
Com_147_pos	D-Sphingosine	ENSGALG00000026663	CX3CL1
Com_311_pos	PC (18:4e/2:0)	ENSGALG00000008185	AOX1
Com_151_pos	Pyridoxamine	ENSGALG00000011003	SLC35F3
Com_178_pos	Maltol	ENSGALG00000004343	HPD
Com_57_neg	LPC 16:1	ENSGALG00000012106	SCTR
Com_482_pos	8-Hydroxyquinoline	ENSGALG00000001749	ACSBG2
Com_68_neg	PE (16:0/22:6)	ENSGALG00000013660	ZNF516
Com_178_pos	Maltol	MSTRG.836	--
Com_120_neg	LPC 15:0	ENSGALG00000019663	ACBD7
Com_4_pos	PC (17:1/17:1)	MSTRG.8954	--
Com_120_neg	LPC 15:0	ENSGALG00000005204	GSTT1
Com_215_pos	D-Erythro-sphingosine 1-|	ENSGALG00000004231	IFNLR1
Com_17_pos	L-Norleucine	ENSGALG00000014950	SULT3A1
Com_147_pos	D-Sphingosine	ENSGALG00000040573	FMO3
Com_203_pos	Serotonin	ENSGALG00000049408	HIST1H2B8
Com_92_pos	D-(+)-Proline	ENSGALG00000010357	P2RY1
Com_203_pos	Serotonin	MSTRG.7483	--
Com_120_neg	LPC 15:0	ENSGALG00000009002	CPED1
Com_440_pos	PC (18:4e/4:0)	ENSGALG00000002790	ABLIM3
Com_12_pos	Betaine	ENSGALG00000034337	RHPN1
Com_80_pos	DL-Lysine	ENSGALG00000037160	Smad7
Com_362_pos	2-Arachidonoyl glycerol	ENSGALG00000006864	COL24A1
Com_252_pos	cis-4-Hydroxy-D-proline	ENSGALG00000055021	GREM2
Com_92_pos	D-(+)-Proline	ENSGALG00000005860	ACAA1

Com_386_pos	2-Amino-1,3-octadecaned	ENSGALG00000038520	STRIP2
Com_147_pos	D-Sphingosine	ENSGALG00000014252	A2M
Com_108_neg	LPE 18:2	ENSGALG00000000745	SLC26A9
Com_92_pos	D-(+)-Proline	ENSGALG00000014252	A2M
Com_352_pos	Riboflavin	ENSGALG00000029766	ITGB5
Com_99_pos	Creatine	ENSGALG00000010163	LGR5
Com_460_pos	3-amino-4-(propylamino)(	ENSGALG00000009545	SLC25A12
Com_440_pos	PC (18:4e/4:0)	ENSGALG00000015425	LPL
Com_53_neg	2-(5-mercapto-4-methyl-4	ENSGALG00000035060	FKBP11
Com_89_neg	Gallic acid	ENSGALG00000009415	SMOC1
Com_18_neg	Arachidonic acid	ENSGALG00000017032	SLC25A15
Com_68_neg	PE (16:0/22:6)	MSTRG.20480	--
Com_192_pos	1-Methylhistidine	ENSGALG00000032903	RTN4RL2
Com_22_pos	Indole-3-acrylic acid	ENSGALG00000053278	SUCNR1
Com_92_pos	D-(+)-Proline	ENSGALG00000040573	FMO3
Com_89_neg	Gallic acid	ENSGALG00000008795	GPAM
Com_108_neg	LPE 18:2	ENSGALG00000019738	FBXO47
Com_151_pos	Pyridoxamine	ENSGALG00000016036	DOP1B
Com_68_neg	PE (16:0/22:6)	ENSGALG00000005442	PALMD
Com_208_neg	N-Acetylanthranilic acid	ENSGALG00000011376	ANKRD9
Com_17_pos	L-Norleucine	ENSGALG00000007728	Prodh
Com_165_neg	(±)9-HpODE	ENSGALG00000015795	ADAMTS5
Com_186_pos	4-Hydroxybenzaldehyde	ENSGALG00000030920	APOC3
Com_76_neg	Erythronolactone	ENSGALG00000006482	FAH
Com_4_pos	PC (17:1/17:1)	ENSGALG00000004106	DHCR7
Com_4_pos	PC (17:1/17:1)	ENSGALG00000030661	STAT2
Com_78_neg	Citric acid	ENSGALG00000030031	TTPA
Com_151_pos	Pyridoxamine	ENSGALG00000017046	POSTN
Com_208_neg	N-Acetylanthranilic acid	ENSGALG00000013754	PLAGL1
Com_352_pos	Riboflavin	ENSGALG00000026973	KIF3C
Com_17_pos	L-Norleucine	ENSGALG00000017039	STOML3
Com_151_neg	Lysope 18:1	ENSGALG00000011335	NHEJ1
Com_54_pos	Uric acid	MSTRG.13474	Klhl29
Com_92_pos	D-(+)-Proline	ENSGALG00000026663	CX3CL1
Com_440_pos	PC (18:4e/4:0)	ENSGALG00000052964	TOPAZ1
Com_76_neg	Erythronolactone	ENSGALG00000009926	HAAO
Com_147_pos	D-Sphingosine	ENSGALG00000052872	--
Com_17_pos	L-Norleucine	ENSGALG00000038242	CACNA2D2
Com_331_pos	L-Lysine	MSTRG.8511	--
Com_352_pos	Riboflavin	ENSGALG00000033541	FRMPD4
Com_152_pos	Acetyl-L-carnitine	ENSGALG00000041680	KCNT2
Com_362_pos	2-Arachidonoyl glycerol	ENSGALG00000034140	ZNF395
Com_150_neg	benzyl N-(2-{[(benzyloxy)c	ENSGALG00000015935	SMYD1
Com_178_pos	Maltol	ENSGALG00000006374	TBX6
Com_151_neg	Lysope 18:1	ENSGALG00000021627	IFI27L2
Com_460_pos	3-amino-4-(propylamino)(	ENSGALG00000043044	IL1R1
Com_362_pos	2-Arachidonoyl glycerol	ENSGALG00000043582	LY6E
Com_440_pos	PC (18:4e/4:0)	ENSGALG00000052583	A2ML1
Com_147_pos	D-Sphingosine	ENSGALG00000054322	--
Com_352_pos	Riboflavin	ENSGALG00000041205	--
Com_120_neg	LPC 15:0	ENSGALG00000048343	Ces1e
Com_192_pos	1-Methylhistidine	ENSGALG00000040995	NEB
Com_97_pos	L-Threonine	ENSGALG00000054856	ADH1
Com_130_neg	2-Hydroxyvaleric acid	ENSGALG00000034081	AKT3
Com_386_pos	2-Amino-1,3-octadecaned	ENSGALG00000041680	KCNT2
Com_178_pos	Maltol	ENSGALG00000011994	SYNPO2
Com_352_pos	Riboflavin	ENSGALG00000014834	NCOA7
Com_12_pos	Betaine	ENSGALG00000027891	NREP

Com_55_pos	Valine	ENSGALG00000041491	ACKR4
Com_57_neg	LPC 16:1	MSTRG.8904	--
Com_16_neg	3-Hydroxybutyric acid	ENSGALG00000015040	SLC16A10
Com_460_pos	3-amino-4-(propylamino)(	ENSGALG00000012704	MYLIP
Com_588_pos	Ornithine	ENSGALG00000041373	ARAP2
Com_440_pos	PC (18:4e/4:0)	ENSGALG00000032882	EVA1C
Com_24_neg	PE (16:0/20:4)	ENSGALG00000007993	DCX
Com_89_neg	Gallic acid	ENSGALG00000031227	ELP6
Com_208_neg	N-Acetylanthranilic acid	MSTRG.12291	--
Com_92_pos	D-(+)-Proline	ENSGALG00000014616	MT3
Com_17_pos	L-Norleucine	ENSGALG00000013726	PAICS
Com_175_pos	Pantothenic acid	ENSGALG00000005474	PNAT10
Com_25_pos	2-Hydroxycinnamic acid	ENSGALG00000006724	GPC5
Com_194_pos	Pipecolic acid	ENSGALG00000054322	--
Com_192_pos	1-Methylhistidine	ENSGALG00000015624	VCAN
Com_119_pos	DL-Stachydrine	ENSGALG00000052887	--
Com_165_neg	(±)9-HpODE	ENSGALG00000002944	CPS1
Com_18_neg	Arachidonic acid	ENSGALG00000027908	CYP2U1
Com_24_neg	PE (16:0/20:4)	MSTRG.2406	--
Com_413_pos	L-Cystine	ENSGALG00000050154	--
Com_55_pos	Valine	ENSGALG00000015425	LPL
Com_152_pos	Acetyl-L-carnitine	ENSGALG00000038520	STRIP2
Com_151_neg	Lysope 18:1	ENSGALG00000011094	PDE4B
Com_108_neg	LPE 18:2	ENSGALG00000004852	DNM1
Com_252_pos	cis-4-Hydroxy-D-proline	ENSGALG00000003553	ABCA12
Com_76_neg	Erythronolactone	ENSGALG00000007252	ANKDD1A
Com_80_pos	DL-Lysine	ENSGALG00000030025	FABP4
Com_120_neg	LPC 15:0	ENSGALG00000022750	GPR18
Com_178_pos	Maltol	ENSGALG00000014412	CSTA
Com_18_neg	Arachidonic acid	ENSGALG00000005472	NAT
Com_97_pos	L-Threonine	ENSGALG00000016364	ALKAL2
Com_55_pos	Valine	ENSGALG00000002790	ABLIM3
Com_54_pos	Uric acid	ENSGALG00000006724	GPC5
Com_215_pos	D-Erythro-sphingosine 1-p	ENSGALG00000002519	SLC25A33
Com_108_neg	LPE 18:2	ENSGALG00000051550	ARMH4
Com_108_neg	LPE 18:2	ENSGALG00000044251	RASSF7
Com_208_neg	N-Acetylanthranilic acid	ENSGALG00000009172	OSBPL6
Com_57_neg	LPC 16:1	ENSGALG00000007645	prom1a
Com_171_neg	LPC 22:6	ENSGALG00000013726	PAICS
Com_440_pos	PC (18:4e/4:0)	ENSGALG00000028407	GDF9
Com_482_pos	8-Hydroxyquinoline	MSTRG.12923	--
Com_165_neg	(±)9-HpODE	ENSGALG00000007955	SLC16A5
Com_203_pos	Serotonin	ENSGALG00000038666	FBXL12
Com_342_pos	1-(4-methylphenyl)-3,5-di	ENSGALG00000021274	ENTPD8
Com_97_pos	L-Threonine	ENSGALG00000012704	MYLIP
Com_150_neg	benzyl N-(2-{[(benzyloxy)c	MSTRG.4905	--
Com_252_pos	cis-4-Hydroxy-D-proline	ENSGALG00000031593	TMSB15B
Com_386_pos	2-Amino-1,3-octadecanec	ENSGALG00000012754	PAH
Com_151_neg	Lysope 18:1	MSTRG.21653	--
Com_8_neg	4-Methyl-2-Oxopentanoic	ENSGALG00000002728	SLC16A3
Com_92_pos	D-(+)-Proline	ENSGALG00000019147	--
Com_386_pos	2-Amino-1,3-octadecanec	ENSGALG00000003212	TSPO2
Com_76_neg	Erythronolactone	ENSGALG00000014750	TRB
Com_460_pos	3-amino-4-(propylamino)(	ENSGALG00000054856	ADH1
Com_80_pos	DL-Lysine	ENSGALG00000026313	RND3
Com_203_pos	Serotonin	ENSGALG00000048109	gag
Com_152_pos	Acetyl-L-carnitine	ENSGALG00000031158	OAT
Com_80_pos	DL-Lysine	ENSGALG00000009947	PLEKHH2

Com_108_neg	LPE 18:2	ENSGALG00000004231	IFNLR1
Com_588_pos	Ornithine	ENSGALG00000007839	NCAM1
Com_194_pos	Pipecolic acid	MSTRG.14987	--
Com_55_pos	Valine	ENSGALG00000004230	LIPC
Com_175_pos	Pantothenic acid	ENSGALG00000013244	ABCC9
Com_171_neg	LPC 22:6	ENSGALG00000038242	CACNA2D2
Com_151_neg	Lysope 18:1	ENSGALG00000010237	NPC2
Com_413_pos	L-Cystine	ENSGALG00000033338	GPT2
Com_165_neg	(±)9-HpODE	ENSGALG00000013743	ENPP7
Com_80_pos	DL-Lysine	ENSGALG00000034741	ETNPPL
Com_331_pos	L-Lysine	ENSGALG00000004343	HPD
Com_18_neg	Arachidonic acid	ENSGALG00000009880	INPP4B
Com_215_pos	D-Erythro-sphingosine 1-p	ENSGALG00000050267	CALU
Com_86_neg	Levulinic acid	ENSGALG00000030031	TTPA
Com_215_pos	D-Erythro-sphingosine 1-p	ENSGALG00000000745	SLC26A9
Com_440_pos	PC (18:4e/4:0)	ENSGALG00000008185	AOX1
Com_92_pos	D-(+)-Proline	MSTRG.19177	PHGDH
Com_76_neg	Erythronolactone	ENSGALG00000011957	TOB2
Com_68_neg	PE (16:0/22:6)	ENSGALG00000008903	ITPRID2
Com_4_pos	PC (17:1/17:1)	MSTRG.4813	--
Com_482_pos	8-Hydroxyquinoline	ENSGALG00000005648	Sesn3
Com_57_neg	LPC 16:1	ENSGALG00000022720	GJB2
Com_482_pos	8-Hydroxyquinoline	ENSGALG00000051466	NDFIP2
Com_12_pos	Betaine	ENSGALG00000003802	OTUD7A
Com_171_neg	LPC 22:6	ENSGALG00000007728	Prodh
Com_692_pos	3-amino-2-phenyl-2H-py	ENSGALG00000051325	H3-I
Com_76_neg	Erythronolactone	ENSGALG00000051203	Mas1
Com_215_pos	D-Erythro-sphingosine 1-p	ENSGALG00000019738	FBXO47
Com_311_pos	PC (18:4e/2:0)	ENSGALG00000002479	MAT1A
Com_120_neg	LPC 15:0	ENSGALG00000044763	GPR82
Com_53_neg	2-(5-mercapto-4-methyl-	ENSGALG00000012322	KCTD16
Com_208_neg	N-Acetylanthranilic acid	ENSGALG00000050176	SARM1
Com_57_neg	LPC 16:1	ENSGALG00000031874	HIST1H101
Com_311_pos	PC (18:4e/2:0)	ENSGALG00000002790	ABLIM3
Com_152_pos	Acetyl-L-carnitine	ENSGALG00000032746	ENPP2
Com_151_neg	Lysope 18:1	ENSGALG00000037629	TRANK1
Com_150_neg	benzyl N-(2-{[(benzyloxy)c	ENSGALG00000016430	PDHA2
Com_53_neg	2-(5-mercapto-4-methyl-	ENSGALG00000017167	SLC35F2
Com_86_neg	Levulinic acid	ENSGALG00000017039	STOML3
Com_203_pos	Serotonin	ENSGALG00000007127	FADS1
Com_147_pos	D-Sphingosine	ENSGALG00000036190	AOC1
Com_386_pos	2-Amino-1,3-octadecanec	ENSGALG00000013728	PPAT
Com_92_pos	D-(+)-Proline	ENSGALG00000033171	TGM4
Com_175_pos	Pantothenic acid	MSTRG.5269	pitpnc1
Com_57_neg	LPC 16:1	ENSGALG00000006534	PEX11A
Com_130_neg	2-Hydroxyvaleric acid	ENSGALG00000054856	ADH1
Com_311_pos	PC (18:4e/2:0)	ENSGALG00000015425	LPL
Com_186_pos	4-Hydroxybenzaldehyde	ENSGALG00000007536	PHR
Com_203_pos	Serotonin	ENSGALG00000029898	YKT6
Com_460_pos	3-amino-4-(propylamino)c	ENSGALG00000016287	NR0B1
Com_57_neg	LPC 16:1	ENSGALG00000025738	RHOU
Com_331_pos	L-Lysine	ENSGALG00000029724	MTURN
Com_482_pos	8-Hydroxyquinoline	ENSGALG00000041296	SOX7
Com_171_neg	LPC 22:6	ENSGALG00000014950	SULT3A1
Com_208_neg	N-Acetylanthranilic acid	ENSGALG00000034507	CHST2
Com_362_pos	2-Arachidonoyl glycerol	ENSGALG00000047827	TMEM86A
Com_17_pos	L-Norleucine	MSTRG.8055	--
Com_18_neg	Arachidonic acid	ENSGALG00000008953	AASS

Com_152_pos	Acetyl-L-carnitine	ENSGALG00000003578	FN1
Com_208_neg	N-Acetylanthranilic acid	ENSGALG00000034737	GLIS2
Com_331_pos	L-Lysine	ENSGALG00000006374	TBX6
Com_21_pos	DL-Tryptophan	ENSGALG00000038740	AMY2A
Com_186_pos	4-Hydroxybenzaldehyde	ENSGALG00000016446	ATP6V1C2
Com_186_pos	4-Hydroxybenzaldehyde	ENSGALG00000013776	CEP135
Com_40_pos	Choline	ENSGALG00000036742	GATSL2
Com_482_pos	8-Hydroxyquinoline	ENSGALG00000002899	AACS
Com_147_pos	D-Sphingosine	ENSGALG00000012755	IGF-I
Com_331_pos	L-Lysine	ENSGALG00000011994	SYNPO2
Com_40_pos	Choline	ENSGALG00000051274	B3GALT2
Com_186_pos	4-Hydroxybenzaldehyde	MSTRG.20494	AHNAK
Com_386_pos	2-Amino-1,3-octadecanec	ENSGALG00000033171	TGM4
Com_92_pos	D-(+)-Proline	ENSGALG00000013728	PPAT
Com_588_pos	Ornithine	ENSGALG00000008780	CTBS
Com_152_pos	Acetyl-L-carnitine	ENSGALG00000016027	CBR3
Com_56_neg	LPE 18:1	ENSGALG00000002742	TMEM132B
Com_89_neg	Gallic acid	ENSGALG00000006490	SCN3B
Com_482_pos	8-Hydroxyquinoline	MSTRG.13135	--
Com_53_neg	2-(5-mercapto-4-methyl-	ENSGALG00000028709	RNF144A
Com_68_neg	PE (16:0/22:6)	MSTRG.13046	--
Com_120_neg	LPC 15:0	ENSGALG00000011320	TMCC3
Com_151_pos	Pyridoxamine	ENSGALG00000039499	LRRCC1
Com_57_neg	LPC 16:1	ENSGALG00000006919	POF1B
Com_130_neg	2-Hydroxyvaleric acid	ENSGALG00000034741	ETNPPL
Com_413_pos	L-Cystine	ENSGALG00000041258	msrA
Com_215_pos	D-Erythro-sphingosine 1-	ENSGALG00000004852	DNM1
Com_352_pos	Riboflavin	ENSGALG00000010406	TMEM63C
Com_130_neg	2-Hydroxyvaleric acid	ENSGALG00000026313	RND3
Com_119_pos	DL-Stachydrine	MSTRG.14680	--
Com_386_pos	2-Amino-1,3-octadecanec	MSTRG.19177	PHGDH
Com_108_neg	LPE 18:2	ENSGALG00000002519	SLC25A33
Com_78_neg	Citric acid	ENSGALG00000013726	PAICS
Com_215_pos	D-Erythro-sphingosine 1-	ENSGALG00000051550	ARMH4
Com_215_pos	D-Erythro-sphingosine 1-	ENSGALG00000044251	RASSF7
Com_130_neg	2-Hydroxyvaleric acid	ENSGALG00000012704	MYLIP
Com_99_pos	Creatine	ENSGALG00000010889	HOOK1
Com_40_pos	Choline	ENSGALG00000014872	FGF10
Com_8_neg	4-Methyl-2-Oxopentanoic	ENSGALG00000011524	PPEF2
Com_4_pos	PC (17:1/17:1)	ENSGALG00000029308	PNPLA3
Com_331_pos	L-Lysine	ENSGALG00000014412	CSTA
Com_352_pos	Riboflavin	MSTRG.17389	DIO3
Com_460_pos	3-amino-4-(propylamino)(	ENSGALG00000034081	AKT3
Com_92_pos	D-(+)-Proline	ENSGALG00000003212	TSPO2
Com_386_pos	2-Amino-1,3-octadecanec	ENSGALG00000019147	--
Com_92_pos	D-(+)-Proline	ENSGALG00000012754	PAH
Com_40_pos	Choline	MSTRG.15995	--
Com_80_pos	DL-Lysine	ENSGALG00000013124	FHOD3
Com_17_pos	L-Norleucine	ENSGALG00000037671	psuG
Com_151_pos	Pyridoxamine	ENSGALG00000030121	SLC2A11
Com_78_neg	Citric acid	ENSGALG00000006864	COL24A1
Com_155_neg	Phenylacetaldehyde	ENSGALG00000004373	KCNJ16
Com_120_neg	LPC 15:0	ENSGALG00000012704	MYLIP
Com_16_neg	3-Hydroxybutyric acid	ENSGALG00000004205	SOAT1
Com_78_neg	Citric acid	ENSGALG00000038242	CACNA2D2
Com_352_pos	Riboflavin	MSTRG.15444	--
Com_147_pos	D-Sphingosine	ENSGALG00000052964	TOPAZ1
Com_151_pos	Pyridoxamine	ENSGALG00000016651	TDH

Com_108_neg	LPE 18:2	MSTRG.18213	--
Com_413_pos	L-Cystine	ENSGALG00000016138	DSCAM
Com_208_neg	N-Acetylanthranilic acid	ENSGALG00000005259	VIPR1
Com_482_pos	8-Hydroxyquinoline	ENSGALG00000007848	PTS
Com_18_neg	Arachidonic acid	ENSGALG00000016196	CBSL
Com_56_neg	LPE 18:1	ENSGALG00000003805	PRKG1
Com_155_neg	Phenylacetaldehyde	ENSGALG00000006662	BPIFB3
Com_413_pos	L-Cystine	ENSGALG00000028341	MADCAM1
Com_208_neg	N-Acetylanthranilic acid	ENSGALG00000006341	SLC25A48
Com_76_neg	Erythronolactone	MSTRG.16661	--
Com_108_neg	LPE 18:2	ENSGALG00000050267	CALU
Com_588_pos	Ornithine	ENSGALG00000046639	CYP2AC2
Com_362_pos	2-Arachidonoyl glycerol	ENSGALG00000030031	TTPA
Com_17_pos	L-Norleucine	ENSGALG00000030031	TTPA
Com_92_pos	D-(+)-Proline	ENSGALG00000020688	CYP4A22
Com_147_pos	D-Sphingosine	ENSGALG00000052583	A2ML1
Com_171_neg	LPC 22:6	ENSGALG00000044996	TMEM71
Com_440_pos	PC (18:4e/4:0)	ENSGALG00000054322	--
Com_78_neg	Citric acid	ENSGALG00000007728	Prodh
Com_54_pos	Uric acid	ENSGALG00000016558	VEGFD
Com_152_pos	Acetyl-L-carnitine	ENSGALG00000010357	P2RY1
Com_175_pos	Pantothenic acid	ENSGALG00000009740	RASGRP1
Com_352_pos	Riboflavin	MSTRG.17677	--
Com_97_pos	L-Threonine	ENSGALG00000022750	GPR18
Com_311_pos	PC (18:4e/2:0)	MSTRG.29	SHANK3
Com_18_neg	Arachidonic acid	ENSGALG00000016325	GSTA3
Com_588_pos	Ornithine	ENSGALG00000005408	BCO1
Com_152_pos	Acetyl-L-carnitine	ENSGALG00000005860	ACAA1
Com_76_neg	Erythronolactone	ENSGALG00000030251	ADCY8
Com_362_pos	2-Arachidonoyl glycerol	ENSGALG00000039239	SERPIND1
Com_119_pos	DL-Stachydrine	ENSGALG00000055000	KCTD14
Com_203_pos	Serotonin	ENSGALG00000016610	PTRHD1
Com_78_neg	Citric acid	ENSGALG00000034140	ZNF395
Com_147_pos	D-Sphingosine	ENSGALG00000032882	EVA1C
Com_89_neg	Gallic acid	ENSGALG00000028005	GADD45G
Com_352_pos	Riboflavin	ENSGALG00000032645	H2A-VIII
Com_56_neg	LPE 18:1	ENSGALG00000000769	RAB7B
Com_192_pos	1-Methylhistidine	ENSGALG00000047821	--
Com_119_pos	DL-Stachydrine	ENSGALG00000053446	RED3
Com_57_neg	LPC 16:1	ENSGALG00000023083	KIAA1958
Com_40_pos	Choline	ENSGALG00000034478	CCL4
Com_482_pos	8-Hydroxyquinoline	MSTRG.21204	--
Com_54_pos	Uric acid	ENSGALG00000001392	MMP23B
Com_92_pos	D-(+)-Proline	ENSGALG00000003595	SARM1
Com_471_pos	Indole-3-acetic acid	ENSGALG00000003103	MST1R
Com_413_pos	L-Cystine	ENSGALG00000041373	ARAP2
Com_413_pos	L-Cystine	ENSGALG00000002855	SARDH
Com_24_neg	PE (16:0/20:4)	ENSGALG00000015795	ADAMTS5
Com_16_neg	3-Hydroxybutyric acid	ENSGALG00000009740	RASGRP1
Com_68_neg	PE (16:0/22:6)	ENSGALG00000024047	MYCL
Com_194_pos	Pipecolic acid	ENSGALG00000034478	CCL4
Com_78_neg	Citric acid	ENSGALG00000014950	SULT3A1
Com_386_pos	2-Amino-1,3-octadecanec	ENSGALG00000014616	MT3
Com_471_pos	Indole-3-acetic acid	ENSGALG00000037935	RARA
Com_16_neg	3-Hydroxybutyric acid	ENSGALG00000027908	CYP2U1
Com_186_pos	4-Hydroxybenzaldehyde	ENSGALG00000003432	AGXT2
Com_440_pos	PC (18:4e/4:0)	ENSGALG00000002479	MAT1A
Com_57_neg	LPC 16:1	ENSGALG00000004833	P3H1

Com_8_neg	4-Methyl-2-Oxopentanoic	ENSGALG00000032746	ENPP2
Com_151_neg	Lysope 18:1	ENSGALG00000026598	NXNL2
Com_18_neg	Arachidonic acid	ENSGALG00000014836	LPIN2
Com_86_neg	Levulinic acid	MSTRG.8055	--
Com_92_pos	D-(+)-Proline	ENSGALG00000041680	KCNT2
Com_413_pos	L-Cystine	ENSGALG00000015034	ANKRD29
Com_588_pos	Ornithine	ENSGALG00000050154	--
Com_120_neg	LPC 15:0	ENSGALG00000054856	ADH1
Com_413_pos	L-Cystine	ENSGALG00000008728	PTER
Com_331_pos	L-Lysine	ENSGALG00000005263	SOX8
Com_178_pos	Maltol	ENSGALG00000029724	MTURN
Com_97_pos	L-Threonine	ENSGALG00000048343	Ces1e
Com_8_neg	4-Methyl-2-Oxopentanoic	ENSGALG00000007114	APOA1
Com_175_pos	Pantothenic acid	ENSGALG00000004205	SOAT1
Com_252_pos	cis-4-Hydroxy-D-proline	ENSGALG00000016558	VEGFD
Com_12_pos	Betaine	ENSGALG00000000761	TSKU
Com_147_pos	D-Sphingosine	ENSGALG00000028407	GDF9
Com_175_pos	Pantothenic acid	MSTRG.17350	--
Com_151_neg	Lysope 18:1	ENSGALG00000012106	SCTR
Com_97_pos	L-Threonine	ENSGALG00000034741	ETNPPL
Com_8_neg	4-Methyl-2-Oxopentanoic	ENSGALG00000003578	FN1
Com_194_pos	Pipecolic acid	ENSGALG00000015425	LPL
Com_8_neg	4-Methyl-2-Oxopentanoic	ENSGALG00000004598	CUX2
Com_386_pos	2-Amino-1,3-octadecanec	ENSGALG00000026663	CX3CL1
Com_165_neg	(±)9-HpODE	ENSGALG00000010577	ARHGEF38
Com_471_pos	Indole-3-acetic acid	ENSGALG00000008226	NIF3L1
Com_97_pos	L-Threonine	ENSGALG00000026313	RND3
Com_194_pos	Pipecolic acid	MSTRG.15995	--
Com_194_pos	Pipecolic acid	ENSGALG00000002790	ABLIM3
Com_460_pos	3-amino-4-(propylamino)(	ENSGALG00000044763	GPR82
Com_203_pos	Serotonin	ENSGALG00000041372	Myrip
Com_155_neg	Phenylacetaldehyde	MSTRG.163	--
Com_471_pos	Indole-3-acetic acid	ENSGALG00000053886	gag
Com_471_pos	Indole-3-acetic acid	MSTRG.8955	--
Com_130_neg	2-Hydroxyvaleric acid	ENSGALG00000037160	Smad7
Com_386_pos	2-Amino-1,3-octadecanec	ENSGALG00000040573	FMO3
Com_24_neg	PE (16:0/20:4)	ENSGALG00000002944	CPS1
Com_8_neg	4-Methyl-2-Oxopentanoic	ENSGALG00000016027	CBR3
Com_482_pos	8-Hydroxyquinoline	ENSGALG00000016475	Zp2
Com_53_neg	2-(5-mercapto-4-methyl-	ENSGALG00000053281	PKIA
Com_192_pos	1-Methylhistidine	ENSGALG00000019768	ACSF2
Com_460_pos	3-amino-4-(propylamino)(	ENSGALG00000034616	INHBA
Com_4_pos	PC (17:1/17:1)	ENSGALG00000013569	SEC61B
Com_16_neg	3-Hydroxybutyric acid	ENSGALG00000009880	INPP4B
Com_215_pos	D-Erythro-sphingosine 1-p	ENSGALG00000008297	SEMA4B
Com_386_pos	2-Amino-1,3-octadecanec	ENSGALG00000014252	A2M
Com_86_neg	Levulinic acid	ENSGALG00000043582	LY6E
Com_92_pos	D-(+)-Proline	ENSGALG00000038520	STRIP2
Com_471_pos	Indole-3-acetic acid	MSTRG.21796	--
Com_147_pos	D-Sphingosine	ENSGALG00000008185	AOX1
Com_97_pos	L-Threonine	ENSGALG00000009002	CPED1
Com_119_pos	DL-Stachydrine	ENSGALG00000044278	C1orf131
Com_460_pos	3-amino-4-(propylamino)(	ENSGALG00000027122	APPL2
Com_92_pos	D-(+)-Proline	ENSGALG00000040836	INSYN2A
Com_460_pos	3-amino-4-(propylamino)(	ENSGALG00000005030	DOCK10
Com_252_pos	cis-4-Hydroxy-D-proline	ENSGALG00000007710	zgc:110179
Com_151_pos	Pyridoxamine	ENSGALG00000003972	FAXDC2
Com_80_pos	DL-Lysine	ENSGALG00000016364	ALKAL2

Com_97_pos	L-Threonine	ENSGALG00000005204	GSTT1
Com_252_pos	cis-4-Hydroxy-D-proline	ENSGALG00000009050	CAPN3
Com_99_pos	Creatine	ENSGALG00000054442	ITIH3
Com_97_pos	L-Threonine	ENSGALG00000019663	ACBD7
Com_171_neg	LPC 22:6	ENSGALG00000006864	COL24A1
Com_119_pos	DL-Stachydrine	ENSGALG00000015937	FABP1
Com_55_pos	Valine	MSTRG.14987	--
Com_68_neg	PE (16:0/22:6)	MSTRG.13506	CGREF1
Com_178_pos	Maltol	ENSGALG00000014750	TRB
Com_588_pos	Ornithine	ENSGALG00000010857	DAB1
Com_215_pos	D-Erythro-sphingosine 1-p	ENSGALG00000048285	TCN2
Com_482_pos	8-Hydroxyquinoline	MSTRG.8471	--
Com_86_neg	Levulinic acid	ENSGALG00000037671	psuG
Com_17_pos	L-Norleucine	ENSGALG00000036086	TAGLN2
Com_152_pos	Acetyl-L-carnitine	ENSGALG00000011524	PPEF2
Com_119_pos	DL-Stachydrine	ENSGALG00000007178	FADS2
Com_194_pos	Pipecolic acid	ENSGALG00000051274	B3GALT2
Com_386_pos	2-Amino-1,3-octadecanec	ENSGALG00000052872	--
Com_203_pos	Serotonin	ENSGALG00000035675	--
Com_192_pos	1-Methylhistidine	ENSGALG00000051068	SIGLEC1
Com_215_pos	D-Erythro-sphingosine 1-p	MSTRG.18213	--
Com_130_neg	2-Hydroxyvaleric acid	ENSGALG00000022750	GPR18
Com_471_pos	Indole-3-acetic acid	ENSGALG00000030185	PTDSS1
Com_78_neg	Citric acid	ENSGALG00000044996	TMEM71
Com_120_neg	LPC 15:0	ENSGALG00000006341	SLC25A48
Com_16_neg	3-Hydroxybutyric acid	ENSGALG00000008953	AASS
Com_76_neg	Erythronolactone	ENSGALG00000000293	A2ML1
Com_4_pos	PC (17:1/17:1)	MSTRG.9007	--
Com_460_pos	3-amino-4-(propylamino)c	ENSGALG00000011320	TMCC3
Com_92_pos	D-(+)-Proline	ENSGALG00000031158	OAT
Com_362_pos	2-Arachidonoyl glycerol	ENSGALG00000000761	TSKU
Com_17_pos	L-Norleucine	ENSGALG00000035903	FAM46A
Com_97_pos	L-Threonine	ENSGALG00000003537	SGK2
Com_120_neg	LPC 15:0	ENSGALG00000005259	VIPR1
Com_155_neg	Phenylacetaldehyde	ENSGALG00000031164	WFDC2
Com_171_neg	LPC 22:6	ENSGALG00000034140	ZNF395
Com_471_pos	Indole-3-acetic acid	ENSGALG00000021395	ABCA9
Com_151_pos	Pyridoxamine	ENSGALG00000010628	ACSL1
Com_76_neg	Erythronolactone	ENSGALG00000014412	CSTA
Com_56_neg	LPE 18:1	ENSGALG00000027571	H2B-I
Com_178_pos	Maltol	ENSGALG00000007252	ANKDD1A
Com_482_pos	8-Hydroxyquinoline	ENSGALG00000047495	LRRC10
Com_89_neg	Gallic acid	ENSGALG00000009016	SLX4IP
Com_203_pos	Serotonin	ENSGALG00000025886	SUSD3
Com_130_neg	2-Hydroxyvaleric acid	ENSGALG00000011314	LRRC3B
Com_130_neg	2-Hydroxyvaleric acid	ENSGALG00000011391	AMN
Com_155_neg	Phenylacetaldehyde	MSTRG.6499	--
Com_171_neg	LPC 22:6	ENSGALG00000008728	PTER
Com_78_neg	Citric acid	ENSGALG00000047827	TMEM86A
Com_171_neg	LPC 22:6	ENSGALG00000015034	ANKRD29
Com_265_pos	6-Methylquinoline	ENSGALG00000037014	TSNARE1
Com_471_pos	Indole-3-acetic acid	ENSGALG00000023691	ENTPD7
Com_8_neg	4-Methyl-2-Oxopentanoic	ENSGALG00000010357	P2RY1
Com_57_neg	LPC 16:1	ENSGALG00000011094	PDE4B
Com_352_pos	Riboflavin	ENSGALG00000011113	SGIP1
Com_150_neg	benzyl N-(2-{[(benzyloxy)c	ENSGALG00000011560	PACRG
Com_92_pos	D-(+)-Proline	ENSGALG00000027786	SOCS3
Com_264_pos	Indole	ENSGALG00000047781	RAD9B

Com_311_pos	PC (18:4e/2:0)	ENSGALG00000054322	--
Com_120_neg	LPC 15:0	ENSGALG00000034081	AKT3
Com_8_neg	4-Methyl-2-Oxopentanoic	ENSGALG00000005860	ACAA1
Com_17_pos	L-Norleucine	ENSGALG00000015492	PDZK1
Com_18_neg	Arachidonic acid	ENSGALG00000029944	FAM222A
Com_130_neg	2-Hydroxyvaleric acid	ENSGALG00000048343	Ces1e
Com_89_neg	Gallic acid	ENSGALG00000034655	ALDH18A1
Com_413_pos	L-Cystine	ENSGALG00000008780	CTBS
Com_252_pos	cis-4-Hydroxy-D-proline	ENSGALG00000011687	AHNAK2
Com_80_pos	DL-Lysine	ENSGALG00000028897	WDR25
Com_440_pos	PC (18:4e/4:0)	MSTRG.29	SHANK3
Com_25_pos	2-Hydroxycinnamic acid	ENSGALG00000055021	GREM2
Com_175_pos	Pantothenic acid	ENSGALG00000017103	WASF3
Com_80_pos	DL-Lysine	ENSGALG00000029270	GATA3
Com_171_neg	LPC 22:6	ENSGALG00000002855	SARDH
Com_12_pos	Betaine	ENSGALG00000039239	SERPIND1
Com_76_neg	Erythronolactone	ENSGALG00000011994	SYNPO2
Com_265_pos	6-Methylquinoline	ENSGALG00000053278	SUCNR1
Com_588_pos	Ornithine	ENSGALG00000041258	msrA
Com_99_pos	Creatine	ENSGALG00000017120	SACS
Com_8_neg	4-Methyl-2-Oxopentanoic	ENSGALG00000021340	CA9
Com_203_pos	Serotonin	ENSGALG00000008601	AHSG
Com_175_pos	Pantothenic acid	ENSGALG00000019835	TRIM27.2
Com_208_neg	N-Acetylanthranilic acid	ENSGALG00000011320	TMCC3
Com_68_neg	PE (16:0/22:6)	ENSGALG00000053164	gag-pol
Com_40_pos	Choline	MSTRG.14987	--
Com_68_neg	PE (16:0/22:6)	ENSGALG00000002142	DGKQ
Com_150_neg	benzyl N-(2-{[(benzyloxy)c	ENSGALG00000050857	PTPN20
Com_76_neg	Erythronolactone	ENSGALG00000006374	TBX6
Com_97_pos	L-Threonine	ENSGALG00000037160	Smad7
Com_108_neg	LPE 18:2	MSTRG.8013	--
Com_178_pos	Maltol	ENSGALG00000005263	SOX8
Com_178_pos	Maltol	ENSGALG00000009926	HAAO
Com_413_pos	L-Cystine	ENSGALG00000044996	TMEM71
Com_471_pos	Indole-3-acetic acid	ENSGALG00000007018	SLC26A11
Com_482_pos	8-Hydroxyquinoline	ENSGALG00000030845	ENHO
Com_152_pos	Acetyl-L-carnitine	ENSGALG00000007114	APOA1
Com_171_neg	LPC 22:6	ENSGALG00000028341	MADCAM1
Com_57_neg	LPC 16:1	ENSGALG00000010237	NPC2
Com_76_neg	Erythronolactone	ENSGALG00000016164	ABCG1
Com_362_pos	2-Arachidonoyl glycerol	ENSGALG00000013726	PAICS
Com_18_neg	Arachidonic acid	ENSGALG00000004205	SOAT1
Com_171_neg	LPC 22:6	ENSGALG00000016138	DSCAM
Com_54_pos	Uric acid	ENSGALG00000031593	TMSB15B
Com_352_pos	Riboflavin	ENSGALG00000004917	DOC2B
Com_151_neg	Lysope 18:1	ENSGALG00000050983	PRKG1
Com_203_pos	Serotonin	ENSGALG00000016665	FDFT1
Com_92_pos	D-(+)-Proline	ENSGALG00000031255	FGF1
Com_311_pos	PC (18:4e/2:0)	ENSGALG00000011894	CYP2D6
Com_4_pos	PC (17:1/17:1)	ENSGALG00000052768	LDLR
Com_130_neg	2-Hydroxyvaleric acid	ENSGALG00000009002	CPED1
Com_152_pos	Acetyl-L-carnitine	ENSGALG00000004598	CUX2
Com_120_neg	LPC 15:0	ENSGALG00000034737	GLIS2
Com_97_pos	L-Threonine	MSTRG.14083	--
Com_16_neg	3-Hydroxybutyric acid	ENSGALG00000016196	CBSL
Com_108_neg	LPE 18:2	ENSGALG00000008297	SEMA4B
Com_130_neg	2-Hydroxyvaleric acid	ENSGALG00000005204	GSTT1
Com_264_pos	Indole	MSTRG.13819	--

Com_130_neg	2-Hydroxyvaleric acid	ENSGALG00000019663	ACBD7
Com_17_pos	L-Norleucine	ENSGALG00000043582	LY6E
Com_352_pos	Riboflavin	ENSGALG00000036117	TENT5B
Com_588_pos	Ornithine	ENSGALG00000016138	DSCAM
Com_151_neg	Lysope 18:1	ENSGALG00000031874	HIST1H101
Com_57_neg	LPC 16:1	ENSGALG00000037629	TRANK1
Com_16_neg	3-Hydroxybutyric acid	ENSGALG00000016325	GSTA3
Com_588_pos	Ornithine	ENSGALG00000028341	MADCAM1
Com_252_pos	cis-4-Hydroxy-D-proline	ENSGALG00000006724	GPC5
Com_413_pos	L-Cystine	ENSGALG00000046639	CYP2AC2
Com_362_pos	2-Arachidonoyl glycerol	ENSGALG00000038242	CACNA2D2
Com_151_neg	Lysope 18:1	ENSGALG00000006534	PEX11A
Com_16_neg	3-Hydroxybutyric acid	ENSGALG00000005474	PNAT10
Com_588_pos	Ornithine	ENSGALG00000028871	SLC38A3
Com_460_pos	3-amino-4-(propylamino)(	MSTRG.10101	--
Com_471_pos	Indole-3-acetic acid	MSTRG.53	SND1
Com_54_pos	Uric acid	ENSGALG00000003553	ABCA12
Com_108_neg	LPE 18:2	ENSGALG00000048285	TCN2
Com_311_pos	PC (18:4e/2:0)	ENSGALG00000028451	MT4
Com_120_neg	LPC 15:0	ENSGALG00000050176	SARM1
Com_76_neg	Erythronolactone	ENSGALG00000004343	HPD
Com_97_pos	L-Threonine	ENSGALG00000054981	F10
Com_413_pos	L-Cystine	ENSGALG00000005408	BCO1
Com_208_neg	N-Acetylanthranilic acid	ENSGALG00000044763	GPR82
Com_386_pos	2-Amino-1,3-octadecanec	ENSGALG00000036190	AOC1
Com_86_neg	Levulinic acid	ENSGALG00000036086	TAGLN2
Com_215_pos	D-Erythro-sphingosine 1-p	ENSGALG00000012414	GNPNAT1
Com_18_neg	Arachidonic acid	ENSGALG00000009740	RASGRP1
Com_362_pos	2-Arachidonoyl glycerol	ENSGALG00000007728	Prodh
Com_130_neg	2-Hydroxyvaleric acid	ENSGALG00000003537	SGK2
Com_171_neg	LPC 22:6	ENSGALG00000041258	msrA
Com_331_pos	L-Lysine	ENSGALG00000014750	TRB
Com_53_neg	2-(5-mercapto-4-methyl--	ENSGALG00000001790	METTL23
Com_78_neg	Citric acid	ENSGALG00000039239	SERPIND1
Com_471_pos	Indole-3-acetic acid	ENSGALG00000014906	MOCS2
Com_588_pos	Ornithine	ENSGALG00000002855	SARDH
Com_147_pos	D-Sphingosine	ENSGALG00000002479	MAT1A
Com_97_pos	L-Threonine	ENSGALG00000011314	LRRC3B
Com_16_neg	3-Hydroxybutyric acid	ENSGALG00000014836	LPIN2
Com_97_pos	L-Threonine	ENSGALG00000011391	AMN
Com_108_neg	LPE 18:2	MSTRG.4550	--
Com_86_neg	Levulinic acid	ENSGALG00000035903	FAM46A
Com_311_pos	PC (18:4e/2:0)	ENSGALG00000021848	AVD
Com_215_pos	D-Erythro-sphingosine 1-p	ENSGALG00000040363	ABHD4
Com_76_neg	Erythronolactone	ENSGALG00000027793	SCN9A
Com_362_pos	2-Arachidonoyl glycerol	ENSGALG00000014950	SULT3A1
Com_80_pos	DL-Lysine	ENSGALG00000031122	NTNG1
Com_80_pos	DL-Lysine	MSTRG.4548	--
Com_55_pos	Valine	ENSGALG00000034478	CCL4
Com_152_pos	Acetyl-L-carnitine	ENSGALG00000020688	CYP4A22
Com_120_neg	LPC 15:0	ENSGALG00000034616	INHBA
Com_588_pos	Ornithine	ENSGALG00000015034	ANKRD29
Com_78_neg	Citric acid	ENSGALG00000008728	PTER
Com_171_neg	LPC 22:6	ENSGALG00000047827	TMEM86A
Com_352_pos	Riboflavin	ENSGALG00000004249	GRHL3
Com_12_pos	Betaine	ENSGALG00000047827	TMEM86A
Com_588_pos	Ornithine	ENSGALG00000008728	PTER
Com_78_neg	Citric acid	ENSGALG00000015034	ANKRD29

Com_386_pos	2-Amino-1,3-octadecanec	ENSGALG00000012755	IGF-I
Com_151_neg	Lysope 18:1	ENSGALG00000006919	POF1B
Com_68_neg	PE (16:0/22:6)	MSTRG.20356	--
Com_4_pos	PC (17:1/17:1)	ENSGALG00000037325	SERP1
Com_186_pos	4-Hydroxybenzaldehyde	ENSGALG00000038652	Gsta3
Com_120_neg	LPC 15:0	ENSGALG00000027122	APPL2
Com_331_pos	L-Lysine	ENSGALG00000007252	ANKDD1A
Com_120_neg	LPC 15:0	ENSGALG00000005030	DOCK10
Com_203_pos	Serotonin	MSTRG.8619	--
Com_413_pos	L-Cystine	ENSGALG00000014950	SULT3A1
Com_86_neg	Levulinic acid	ENSGALG00000015492	PDZK1
Com_186_pos	4-Hydroxybenzaldehyde	ENSGALG00000052991	DNAJC21
Com_99_pos	Creatine	ENSGALG00000054783	NDRG1
Com_460_pos	3-amino-4-(propylamino)(	ENSGALG00000021848	AVD
Com_22_pos	Indole-3-acrylic acid	ENSGALG00000047781	RAD9B
Com_78_neg	Citric acid	ENSGALG00000002855	SARDH
Com_40_pos	Choline	MSTRG.11633	Stap2
Com_152_pos	Acetyl-L-carnitine	ENSGALG00000003595	SARM1
Com_386_pos	2-Amino-1,3-octadecanec	ENSGALG00000003972	FAXDC2
Com_362_pos	2-Arachidonoyl glycerol	ENSGALG00000003802	OTUD7A
Com_352_pos	Riboflavin	ENSGALG00000012589	C9orf64
Com_119_pos	DL-Stachydrine	ENSGALG00000037401	IDH3A
Com_108_neg	LPE 18:2	ENSGALG00000036527	SYBU
Com_55_pos	Valine	MSTRG.15995	--
Com_413_pos	L-Cystine	ENSGALG00000007728	Prodh
Com_460_pos	3-amino-4-(propylamino)(	ENSGALG00000006341	SLC25A48
Com_215_pos	D-Erythro-sphingosine 1-p	MSTRG.8013	--
Com_78_neg	Citric acid	ENSGALG00000028341	MADCAM1
Com_460_pos	3-amino-4-(propylamino)(	ENSGALG00000005259	VIPR1
Com_460_pos	3-amino-4-(propylamino)(	ENSGALG00000028451	MT4
Com_130_neg	2-Hydroxyvaleric acid	MSTRG.14083	--
Com_4_pos	PC (17:1/17:1)	ENSGALG00000053043	CARHSP1
Com_311_pos	PC (18:4e/2:0)	MSTRG.10101	--
Com_471_pos	Indole-3-acetic acid	ENSGALG00000016511	ADGRG2
Com_178_pos	Maltol	ENSGALG00000006320	Slc2a9
Com_53_neg	2-(5-mercapto-4-methyl--	ENSGALG00000004410	ANAPC16
Com_192_pos	1-Methylhistidine	ENSGALG00000041577	ITGA4
Com_78_neg	Citric acid	ENSGALG00000016138	DSCAM
Com_152_pos	Acetyl-L-carnitine	ENSGALG00000021340	CA9
Com_413_pos	L-Cystine	ENSGALG00000038242	CACNA2D2
Com_24_neg	PE (16:0/20:4)	ENSGALG00000010577	ARHGEF38
Com_68_neg	PE (16:0/22:6)	ENSGALG00000048020	STAG3
Com_386_pos	2-Amino-1,3-octadecanec	ENSGALG00000052964	TOPAZ1
Com_482_pos	8-Hydroxyquinoline	ENSGALG00000019276	SLCO1C1
Com_76_neg	Erythronolactone	MSTRG.8511	--
Com_331_pos	L-Lysine	ENSGALG00000009926	HAAO
Com_413_pos	L-Cystine	ENSGALG00000010857	DAB1
Com_56_neg	LPE 18:1	MSTRG.6546	--
Com_331_pos	L-Lysine	ENSGALG00000003136	IKZF2
Com_80_pos	DL-Lysine	ENSGALG00000002466	SLC2A5
Com_331_pos	L-Lysine	ENSGALG00000009963	LYZ
Com_352_pos	Riboflavin	ENSGALG00000007030	MFSD13A
Com_192_pos	1-Methylhistidine	ENSGALG00000004959	IRS1
Com_386_pos	2-Amino-1,3-octadecanec	ENSGALG00000052583	A2ML1
Com_460_pos	3-amino-4-(propylamino)(	ENSGALG00000011894	CYP2D6
Com_22_pos	Indole-3-acrylic acid	MSTRG.13819	--
Com_151_neg	Lysope 18:1	ENSGALG00000023083	KIAA1958
Com_130_neg	2-Hydroxyvaleric acid	ENSGALG00000054981	F10

Com_86_neg	Levulinic acid	ENSGALG00000011287	SULT
Com_482_pos	8-Hydroxyquinoline	ENSGALG00000047027	ADCK5
Com_192_pos	1-Methylhistidine	ENSGALG00000037387	CLSTN2
Com_8_neg	4-Methyl-2-Oxopentanoic	ENSGALG00000047321	SARDH
Com_55_pos	Valine	ENSGALG00000051274	B3GALT2
Com_99_pos	Creatine	ENSGALG00000030920	APOC3
Com_413_pos	L-Cystine	ENSGALG00000013726	PAICS
Com_386_pos	2-Amino-1,3-octadecanec	ENSGALG00000032882	EVA1C
Com_440_pos	PC (18:4e/4:0)	ENSGALG00000011894	CYP2D6
Com_471_pos	Indole-3-acetic acid	MSTRG.5319	--
Com_120_neg	LPC 15:0	ENSGALG00000013754	PLAGL1
Com_175_pos	Pantothenic acid	ENSGALG00000017199	MAML2
Com_8_neg	4-Methyl-2-Oxopentanoic	ENSGALG00000047480	A2ML1
Com_4_pos	PC (17:1/17:1)	ENSGALG00000037065	SC5D
Com_362_pos	2-Arachidonoyl glycerol	ENSGALG00000044996	TMEM71
Com_54_pos	Uric acid	ENSGALG00000053860	mas
Com_152_pos	Acetyl-L-carnitine	ENSGALG00000040836	INSYN2A
Com_78_neg	Citric acid	ENSGALG00000041258	msrA
Com_120_neg	LPC 15:0	ENSGALG00000011376	ANKRD9
Com_78_neg	Citric acid	ENSGALG00000000761	TSKU
Com_151_neg	Lysope 18:1	ENSGALG00000004833	P3H1
Com_108_neg	LPE 18:2	ENSGALG00000012414	GNPNAT1
Com_54_pos	Uric acid	ENSGALG00000008859	WDR31
Com_215_pos	D-Erythro-sphingosine 1-p	MSTRG.18216	--
Com_80_pos	DL-Lysine	MSTRG.20478	--
Com_55_pos	Valine	ENSGALG00000011391	AMN
Com_342_pos	1-(4-methylphenyl)-3,5-di	ENSGALG00000052797	EIF5A
Com_16_neg	3-Hydroxybutyric acid	ENSGALG00000029944	FAM222A
Com_55_pos	Valine	ENSGALG00000011314	LRRC3B
Com_171_neg	LPC 22:6	ENSGALG00000039239	SERPIND1
Com_171_neg	LPC 22:6	ENSGALG00000050154	--
Com_352_pos	Riboflavin	MSTRG.1845	--
Com_147_pos	D-Sphingosine	MSTRG.29	SHANK3
Com_92_pos	D-(+)-Proline	ENSGALG00000011524	PPEF2
Com_108_neg	LPE 18:2	ENSGALG00000049232	POLR2A
Com_68_neg	PE (16:0/22:6)	ENSGALG00000033867	PCOLCE
Com_215_pos	D-Erythro-sphingosine 1-p	MSTRG.4550	--
Com_440_pos	PC (18:4e/4:0)	ENSGALG00000028451	MT4
Com_588_pos	Ornithine	ENSGALG00000015040	SLC16A10
Com_362_pos	2-Arachidonoyl glycerol	ENSGALG00000027891	NREP
Com_192_pos	1-Methylhistidine	ENSGALG00000007132	ACOX2
Com_108_neg	LPE 18:2	ENSGALG00000040363	ABHD4
Com_68_neg	PE (16:0/22:6)	MSTRG.10121	--
Com_460_pos	3-amino-4-(propylamino)c	ENSGALG00000034737	GLIS2
Com_97_pos	L-Threonine	ENSGALG00000008912	ABCB1
Com_386_pos	2-Amino-1,3-octadecanec	ENSGALG00000028407	GDF9
Com_352_pos	Riboflavin	ENSGALG00000028175	GJA9
Com_386_pos	2-Amino-1,3-octadecanec	ENSGALG00000016651	TDH
Com_21_pos	DL-Tryptophan	ENSGALG00000036005	TIAM2
Com_150_neg	benzyl N-(2-{[(benzyloxy)c	ENSGALG00000028135	--
Com_155_neg	Phenylacetaldehyde	MSTRG.6555	--
Com_120_neg	LPC 15:0	ENSGALG00000015519	ROBO2
Com_208_neg	N-Acetylanthranilic acid	ENSGALG00000016287	NR0B1
Com_12_pos	Betaine	ENSGALG00000034140	ZNF395
Com_18_neg	Arachidonic acid	ENSGALG00000009479	SAMD9L
Com_89_neg	Gallic acid	ENSGALG00000008855	UGP2
Com_386_pos	2-Amino-1,3-octadecanec	ENSGALG00000030121	SLC2A11
Com_8_neg	4-Methyl-2-Oxopentanoic	ENSGALG00000020688	CYP4A22

Com_119_pos	DL-Stachydrine	ENSGALG00000042706	TMC2
Com_76_neg	Erythronolactone	ENSGALG00000012034	ADSL
Com_440_pos	PC (18:4e/4:0)	ENSGALG00000021848	AVD
Com_120_neg	LPC 15:0	MSTRG.10101	--
Com_588_pos	Ornithine	ENSGALG00000044996	TMEM71
Com_17_pos	L-Norleucine	ENSGALG00000033338	GPT2
Com_175_pos	Pantothenic acid	ENSGALG00000005472	NAT
Com_68_neg	PE (16:0/22:6)	ENSGALG00000042215	FAAP100
Com_460_pos	3-amino-4-(propylamino)(	ENSGALG00000050176	SARM1
Com_76_neg	Erythronolactone	ENSGALG00000002594	TFPI
Com_152_pos	Acetyl-L-carnitine	ENSGALG00000027786	SOCS3
Com_119_pos	DL-Stachydrine	ENSGALG00000012220	CDKN3
Com_40_pos	Choline	ENSGALG00000004230	LIPC
Com_471_pos	Indole-3-acetic acid	ENSGALG00000033365	ALDH1A3
Com_203_pos	Serotonin	ENSGALG00000006198	LSS
Com_8_neg	4-Methyl-2-Oxopentanoic	MSTRG.3009	--
Com_108_neg	LPE 18:2	ENSGALG00000004268	NIPAL3
Com_57_neg	LPC 16:1	MSTRG.6228	--
Com_25_pos	2-Hydroxycinnamic acid	MSTRG.13819	--
Com_76_neg	Erythronolactone	ENSGALG00000040434	rab18b
Com_76_neg	Erythronolactone	MSTRG.20377	--
Com_92_pos	D-(+)-Proline	MSTRG.20827	Plcg1
Com_386_pos	2-Amino-1,3-octadecanec	ENSGALG00000008185	AOX1
Com_76_neg	Erythronolactone	MSTRG.3197	--
Com_89_neg	Gallic acid	MSTRG.9645	B3galt6
Com_175_pos	Pantothenic acid	MSTRG.17721	--
Com_165_neg	(±)9-HpODE	ENSGALG00000050983	PRKG1
Com_215_pos	D-Erythro-sphingosine 1-p	ENSGALG00000016254	OTC
Com_89_neg	Gallic acid	ENSGALG00000002919	MON1A
Com_215_pos	D-Erythro-sphingosine 1-p	ENSGALG00000036527	SYBU
Com_8_neg	4-Methyl-2-Oxopentanoic	ENSGALG00000003595	SARM1
Com_55_pos	Valine	ENSGALG00000037160	Smad7
Com_12_pos	Betaine	ENSGALG00000006864	COL24A1
Com_362_pos	2-Arachidonoyl glycerol	ENSGALG00000034337	RHPN1
Com_120_neg	LPC 15:0	ENSGALG00000046316	CFAP97D1
Com_18_neg	Arachidonic acid	ENSGALG00000005474	PNAT10
Com_89_neg	Gallic acid	ENSGALG00000010837	ASB5
Com_352_pos	Riboflavin	ENSGALG00000053659	--
Com_311_pos	PC (18:4e/2:0)	ENSGALG00000005030	DOCK10
Com_16_neg	3-Hydroxybutyric acid	ENSGALG00000007839	NCAM1
Com_471_pos	Indole-3-acetic acid	ENSGALG00000014126	endou-a
Com_311_pos	PC (18:4e/2:0)	ENSGALG00000027122	APPL2
Com_413_pos	L-Cystine	ENSGALG00000028871	SLC38A3
Com_4_pos	PC (17:1/17:1)	ENSGALG00000014948	HMGCR
Com_92_pos	D-(+)-Proline	ENSGALG00000007114	APOA1
Com_92_pos	D-(+)-Proline	ENSGALG00000005540	MICAL2
Com_331_pos	L-Lysine	ENSGALG00000006320	Slc2a9
Com_108_neg	LPE 18:2	ENSGALG00000034868	KRT7
Com_186_pos	4-Hydroxybenzaldehyde	ENSGALG00000023122	SULT1B
Com_311_pos	PC (18:4e/2:0)	ENSGALG00000034616	INHBA
Com_178_pos	Maltol	MSTRG.3009	--
Com_152_pos	Acetyl-L-carnitine	ENSGALG00000031255	FGF1
Com_440_pos	PC (18:4e/4:0)	MSTRG.10101	--
Com_120_neg	LPC 15:0	ENSGALG00000021848	AVD
Com_203_pos	Serotonin	MSTRG.8779	--
Com_92_pos	D-(+)-Proline	ENSGALG00000004598	CUX2
Com_178_pos	Maltol	ENSGALG00000003136	IKZF2
Com_92_pos	D-(+)-Proline	ENSGALG00000007814	ALPI

Com_178_pos	Maltol	ENSGALG00000009963	LYZ
Com_17_pos	L-Norleucine	ENSGALG00000011287	SULT
Com_215_pos	D-Erythro-sphingosine 1-p	ENSGALG00000005696	ABHD6
Com_80_pos	DL-Lysine	ENSGALG00000016281	DMD
Com_80_pos	DL-Lysine	ENSGALG00000038923	Ces1e
Com_186_pos	4-Hydroxybenzaldehyde	ENSGALG00000012683	RNF144B
Com_25_pos	2-Hydroxycinnamic acid	ENSGALG00000047781	RAD9B
Com_171_neg	LPC 22:6	ENSGALG00000000761	TSKU
Com_252_pos	cis-4-Hydroxy-D-proline	ENSGALG00000053140	NDRG2
Com_80_pos	DL-Lysine	ENSGALG00000041491	ACKR4
Com_40_pos	Choline	ENSGALG00000041491	ACKR4
Com_175_pos	Pantothenic acid	ENSGALG00000017032	SLC25A15
Com_192_pos	1-Methylhistidine	ENSGALG00000009920	COCH
Com_78_neg	Citric acid	ENSGALG00000050154	--
Com_108_neg	LPE 18:2	MSTRG.18216	--
Com_4_pos	PC (17:1/17:1)	ENSGALG00000037852	HSD17B7
Com_152_pos	Acetyl-L-carnitine	ENSGALG00000047321	SARDH
Com_4_pos	PC (17:1/17:1)	MSTRG.21092	--
Com_108_neg	LPE 18:2	ENSGALG00000015219	Selenoi
Com_120_neg	LPC 15:0	ENSGALG00000028451	MT4
Com_482_pos	8-Hydroxyquinoline	ENSGALG00000027070	TIMP2
Com_362_pos	2-Arachidonoyl glycerol	ENSGALG00000008728	PTER
Com_152_pos	Acetyl-L-carnitine	ENSGALG00000047480	A2ML1
Com_203_pos	Serotonin	ENSGALG00000040070	PDIA2
Com_130_neg	2-Hydroxyvaleric acid	ENSGALG00000008912	ABCB1
Com_68_neg	PE (16:0/22:6)	ENSGALG00000021238	CYP2W1
Com_89_neg	Gallic acid	ENSGALG00000026039	THEM4
Com_215_pos	D-Erythro-sphingosine 1-p	ENSGALG00000049232	POLR2A
Com_362_pos	2-Arachidonoyl glycerol	ENSGALG00000015034	ANKRD29
Com_8_neg	4-Methyl-2-Oxopentanoic	ENSGALG00000040836	INSYN2A
Com_175_pos	Pantothenic acid	ENSGALG00000046687	EPS8L3
Com_588_pos	Ornithine	ENSGALG00000014950	SULT3A1
Com_208_neg	N-Acetylanthranilic acid	ENSGALG00000012420	CG-1B
Com_215_pos	D-Erythro-sphingosine 1-p	ENSGALG00000032440	QPCT
Com_57_neg	LPC 16:1	ENSGALG00000030357	ABL2
Com_194_pos	Pipecolic acid	ENSGALG00000001531	FN3K
Com_99_pos	Creatine	ENSGALG00000016446	ATP6V1C2
Com_25_pos	2-Hydroxycinnamic acid	ENSGALG00000007710	zgc:110179
Com_25_pos	2-Hydroxycinnamic acid	ENSGALG00000009050	CAPN3
Com_54_pos	Uric acid	ENSGALG00000050840	APCDD1
Com_99_pos	Creatine	MSTRG.20494	AHNAK
Com_362_pos	2-Arachidonoyl glycerol	ENSGALG00000002855	SARDH
Com_208_neg	N-Acetylanthranilic acid	ENSGALG00000043044	IL1R1
Com_186_pos	4-Hydroxybenzaldehyde	ENSGALG00000035026	SLC22A4
Com_97_pos	L-Threonine	ENSGALG00000017040	C4
Com_97_pos	L-Threonine	ENSGALG00000003015	SERPINF1
Com_352_pos	Riboflavin	MSTRG.11834	--
Com_57_neg	LPC 16:1	ENSGALG00000050983	PRKG1
Com_178_pos	Maltol	ENSGALG00000047480	A2ML1
Com_120_neg	LPC 15:0	ENSGALG00000011894	CYP2D6
Com_588_pos	Ornithine	ENSGALG00000007728	Prodh
Com_78_neg	Citric acid	ENSGALG00000003802	OTUD7A
Com_178_pos	Maltol	ENSGALG00000047321	SARDH
Com_54_pos	Uric acid	ENSGALG00000000498	ACE
Com_460_pos	3-amino-4-(propylamino)(	MSTRG.29	SHANK3
Com_460_pos	3-amino-4-(propylamino)(	ENSGALG00000013754	PLAGL1
Com_155_neg	Phenylacetaldehyde	ENSGALG00000012886	--
Com_86_neg	Levulinic acid	ENSGALG00000033338	GPT2

Com_4_pos	PC (17:1/17:1)	ENSGALG00000005766	PKD2L1
Com_460_pos	3-amino-4-(propylamino)(	ENSGALG00000011376	ANKRD9
Com_413_pos	L-Cystine	ENSGALG00000030031	TTPA
Com_215_pos	D-Erythro-sphingosine 1-p	ENSGALG00000005418	FRRS1
Com_588_pos	Ornithine	ENSGALG00000038242	CACNA2D2
Com_215_pos	D-Erythro-sphingosine 1-p	ENSGALG00000004268	NIPAL3
Com_203_pos	Serotonin	ENSGALG00000051290	--
Com_362_pos	2-Arachidonoyl glycerol	ENSGALG00000028341	MADCAM1
Com_171_neg	LPC 22:6	ENSGALG00000007839	NCAM1
Com_55_pos	Valine	ENSGALG00000026313	RND3
Com_76_neg	Erythronolactone	ENSGALG00000017032	SLC25A15
Com_208_neg	N-Acetylanthranilic acid	ENSGALG00000009545	SLC25A12
Com_80_pos	DL-Lysine	ENSGALG00000004230	LIPC
Com_362_pos	2-Arachidonoyl glycerol	ENSGALG00000016138	DSCAM
Com_108_neg	LPE 18:2	ENSGALG00000016254	OTC
Com_155_neg	Phenylacetaldehyde	ENSGALG00000045814	CRLF2
Com_76_neg	Erythronolactone	ENSGALG00000032231	C4
Com_86_neg	Levulinic acid	ENSGALG00000028928	LCAT
Com_55_pos	Valine	ENSGALG00000034741	ETNPPL
Com_8_neg	4-Methyl-2-Oxopentanoic	ENSGALG00000027786	SOCS3
Com_18_neg	Arachidonic acid	ENSGALG00000006482	FAH
Com_80_pos	DL-Lysine	ENSGALG00000028928	LCAT
Com_22_pos	Indole-3-acrylic acid	ENSGALG00000037014	TSNARE1
Com_152_pos	Acetyl-L-carnitine	MSTRG.3009	--
Com_194_pos	Pipecolic acid	ENSGALG00000016690	CYP2AC1
Com_8_neg	4-Methyl-2-Oxopentanoic	ENSGALG00000006320	Slc2a9
Com_147_pos	D-Sphingosine	ENSGALG00000011894	CYP2D6
Com_151_neg	Lysope 18:1	ENSGALG00000010577	ARHGEF38
Com_192_pos	1-Methylhistidine	ENSGALG00000046316	CFAP97D1
Com_17_pos	L-Norleucine	ENSGALG00000041373	ARAP2
Com_25_pos	2-Hydroxycinnamic acid	ENSGALG00000011687	AHNAK2
Com_588_pos	Ornithine	ENSGALG00000013726	PAICS
Com_311_pos	PC (18:4e/2:0)	ENSGALG00000034081	AKT3
Com_471_pos	Indole-3-acetic acid	MSTRG.15754	--
Com_4_pos	PC (17:1/17:1)	ENSGALG00000050083	SYCP2L
Com_460_pos	3-amino-4-(propylamino)(	ENSGALG00000015519	ROBO2
Com_92_pos	D-(+)-Proline	ENSGALG00000021340	CA9
Com_386_pos	2-Amino-1,3-octadecanec	ENSGALG00000002479	MAT1A
Com_215_pos	D-Erythro-sphingosine 1-p	ENSGALG00000034868	KRT7
Com_68_neg	PE (16:0/22:6)	MSTRG.2409	--
Com_186_pos	4-Hydroxybenzaldehyde	ENSGALG00000013100	GRB10
Com_203_pos	Serotonin	ENSGALG00000028949	CORO6
Com_150_neg	benzyl N-(2-{[(benzyloxy)c	ENSGALG00000029947	MMAB
Com_54_pos	Uric acid	ENSGALG00000002116	TEN1
Com_89_neg	Gallic acid	ENSGALG00000030941	ELAPOR1
Com_108_neg	LPE 18:2	ENSGALG00000013828	GALM
Com_147_pos	D-Sphingosine	ENSGALG00000028451	MT4
Com_362_pos	2-Arachidonoyl glycerol	ENSGALG00000041258	msrA
Com_57_neg	LPC 16:1	ENSGALG00000010708	ICA1
Com_108_neg	LPE 18:2	ENSGALG00000005696	ABHD6
Com_440_pos	PC (18:4e/4:0)	ENSGALG00000005030	DOCK10
Com_151_pos	Pyridoxamine	ENSGALG00000053140	NDRG2
Com_194_pos	Pipecolic acid	ENSGALG00000011391	AMN
Com_440_pos	PC (18:4e/4:0)	ENSGALG00000027122	APPL2
Com_194_pos	Pipecolic acid	ENSGALG00000011314	LRRC3B
Com_331_pos	L-Lysine	MSTRG.3009	--
Com_40_pos	Choline	MSTRG.20478	--
Com_413_pos	L-Cystine	ENSGALG00000015040	SLC16A10

Com_471_pos	Indole-3-acetic acid	ENSGALG00000031067	TMEM132A
Com_97_pos	L-Threonine	ENSGALG00000002024	COMT
Com_192_pos	1-Methylhistidine	ENSGALG00000015519	ROBO2
Com_471_pos	Indole-3-acetic acid	ENSGALG00000000104	CRY1
Com_208_neg	N-Acetylanthranilic acid	ENSGALG00000024085	IDO2
Com_8_neg	4-Methyl-2-Oxopentanoic	ENSGALG00000031255	FGF1
Com_99_pos	Creatine	ENSGALG00000009100	FSHR
Com_68_neg	PE (16:0/22:6)	MSTRG.1679	--
Com_175_pos	Pantothenic acid	MSTRG.3197	--
Com_440_pos	PC (18:4e/4:0)	ENSGALG00000034616	INHBA
Com_16_neg	3-Hydroxybutyric acid	ENSGALG00000009479	SAMD9L
Com_588_pos	Ornithine	ENSGALG00000027908	CYP2U1
Com_175_pos	Pantothenic acid	ENSGALG00000040434	rab18b
Com_99_pos	Creatine	ENSGALG00000003432	AGXT2
Com_78_neg	Citric acid	ENSGALG00000027891	NREP
Com_386_pos	2-Amino-1,3-octadecanec	ENSGALG00000017046	POSTN
Com_460_pos	3-amino-4-(propylamino)(	ENSGALG00000046316	CFAP97D1
Com_120_neg	LPC 15:0	ENSGALG00000007132	ACOX2
Com_215_pos	D-Erythro-sphingosine 1-p	ENSGALG00000015219	Selenoi
Com_147_pos	D-Sphingosine	ENSGALG00000003972	FAXDC2
Com_76_neg	Erythronolactone	ENSGALG00000005472	NAT
Com_147_pos	D-Sphingosine	ENSGALG00000021848	AVD
Com_386_pos	2-Amino-1,3-octadecanec	ENSGALG00000016036	DOP1B
Com_175_pos	Pantothenic acid	ENSGALG00000012034	ADSL
Com_16_neg	3-Hydroxybutyric acid	ENSGALG00000050154	--
Com_130_neg	2-Hydroxyvaleric acid	ENSGALG00000017040	C4
Com_130_neg	2-Hydroxyvaleric acid	ENSGALG00000003015	SERPINF1
Com_482_pos	8-Hydroxyquinoline	ENSGALG00000008039	MFSD13A
Com_108_neg	LPE 18:2	ENSGALG00000032440	QPCT
Com_692_pos	3-amino-2-phenyl-2H-py	ENSGALG00000034982	CYP11A1
Com_40_pos	Choline	ENSGALG00000002466	SLC2A5
Com_86_neg	Levulinic acid	ENSGALG00000038923	Ces1e
Com_150_neg	benzyl N-(2-{[(benzyloxy)c	ENSGALG00000009748	ASNS
Com_192_pos	1-Methylhistidine	ENSGALG00000011376	ANKRD9
Com_86_neg	Levulinic acid	ENSGALG00000016281	DMD
Com_352_pos	Riboflavin	MSTRG.7586	--
Com_203_pos	Serotonin	ENSGALG00000005439	ACACA
Com_215_pos	D-Erythro-sphingosine 1-p	ENSGALG00000014935	GREB1L
Com_192_pos	1-Methylhistidine	ENSGALG00000013754	PLAGL1
Com_171_neg	LPC 22:6	ENSGALG00000003802	OTUD7A
Com_386_pos	2-Amino-1,3-octadecanec	ENSGALG00000011003	SLC35F3
Com_18_neg	Arachidonic acid	ENSGALG00000007839	NCAM1
Com_460_pos	3-amino-4-(propylamino)(	ENSGALG00000002479	MAT1A
Com_175_pos	Pantothenic acid	ENSGALG00000035219	ALB
Com_186_pos	4-Hydroxybenzaldehyde	ENSGALG00000011331	CTH
Com_92_pos	D-(+)-Proline	ENSGALG00000000309	Lad1
Com_120_neg	LPC 15:0	ENSGALG00000037387	CLSTN2
Com_331_pos	L-Lysine	ENSGALG00000047480	A2ML1
Com_150_neg	benzyl N-(2-{[(benzyloxy)c	ENSGALG00000008150	RASAL1
Com_588_pos	Ornithine	ENSGALG00000009880	INPP4B
Com_194_pos	Pipecolic acid	ENSGALG00000037160	Smad7
Com_78_neg	Citric acid	ENSGALG00000007839	NCAM1
Com_471_pos	Indole-3-acetic acid	ENSGALG00000035244	H3-I
Com_482_pos	8-Hydroxyquinoline	ENSGALG00000027608	PIGC
Com_78_neg	Citric acid	ENSGALG00000034337	RHPN1
Com_18_neg	Arachidonic acid	ENSGALG00000011957	TOB2
Com_108_neg	LPE 18:2	ENSGALG00000005418	FRRS1
Com_130_neg	2-Hydroxyvaleric acid	ENSGALG00000002790	ABLIM3

Com_331_pos	L-Lysine	ENSGALG00000047321	SARDH
Com_18_neg	Arachidonic acid	ENSGALG00000051203	Mas1
Com_147_pos	D-Sphingosine	MSTRG.10101	--
Com_362_pos	2-Arachidonoyl glycerol	ENSGALG00000013033	cmbl
Com_311_pos	PC (18:4e/2:0)	ENSGALG00000054856	ADH1
Com_130_neg	2-Hydroxyvaleric acid	ENSGALG00000015425	LPL
Com_68_neg	PE (16:0/22:6)	MSTRG.16972	--
Com_108_neg	LPE 18:2	MSTRG.15241	--
Com_186_pos	4-Hydroxybenzaldehyde	ENSGALG00000054442	ITIH3
Com_17_pos	L-Norleucine	ENSGALG00000028928	LCAT
Com_120_neg	LPC 15:0	ENSGALG00000041577	ITGA4
Com_352_pos	Riboflavin	ENSGALG00000010643	ZYG11B
Com_152_pos	Acetyl-L-carnitine	MSTRG.20827	Plcg1
Com_53_neg	2-(5-mercapto-4-methyl-	MSTRG.21090	--
Com_482_pos	8-Hydroxyquinoline	ENSGALG00000026957	SEMA4G
Com_352_pos	Riboflavin	MSTRG.1890	gag
Com_24_neg	PE (16:0/20:4)	ENSGALG00000050983	PRKG1
Com_56_neg	LPE 18:1	ENSGALG00000012045	slc12a8
Com_208_neg	N-Acetylanthranilic acid	ENSGALG00000015624	VCAN
Com_264_pos	Indole	ENSGALG00000037014	TSNARE1
Com_8_neg	4-Methyl-2-Oxopentanoic	ENSGALG00000009926	HAAO
Com_342_pos	1-(4-methylphenyl)-3,5-di	ENSGALG00000008434	SORCS3
Com_86_neg	Levulinic acid	ENSGALG00000041373	ARAP2
Com_265_pos	6-Methylquinoline	ENSGALG00000047781	RAD9B
Com_17_pos	L-Norleucine	ENSGALG00000008780	CTBS
Com_178_pos	Maltol	ENSGALG00000021340	CA9
Com_588_pos	Ornithine	ENSGALG00000008953	AASS
Com_119_pos	DL-Stachydrine	ENSGALG00000048205	EBP
Com_152_pos	Acetyl-L-carnitine	ENSGALG00000006320	Slc2a9
Com_4_pos	PC (17:1/17:1)	ENSGALG00000014261	UCHL1
Com_386_pos	2-Amino-1,3-octadecanec	MSTRG.29	SHANK3
Com_175_pos	Pantothenic acid	MSTRG.8511	--
Com_471_pos	Indole-3-acetic acid	ENSGALG00000027064	HIST1H3H
Com_130_neg	2-Hydroxyvaleric acid	ENSGALG00000002024	COMT
Com_215_pos	D-Erythro-sphingosine 1-p	ENSGALG00000013828	GALM
Com_120_neg	LPC 15:0	MSTRG.29	SHANK3
Com_152_pos	Acetyl-L-carnitine	ENSGALG00000005540	MICAL2
Com_362_pos	2-Arachidonoyl glycerol	ENSGALG00000050154	--
Com_440_pos	PC (18:4e/4:0)	ENSGALG00000034081	AKT3
Com_97_pos	L-Threonine	MSTRG.11572	--
Com_92_pos	D-(+)-Proline	ENSGALG00000001709	MUSTN1
Com_194_pos	Pipecolic acid	ENSGALG00000007014	PYROXD2
Com_147_pos	D-Sphingosine	ENSGALG00000016651	TDH
Com_119_pos	DL-Stachydrine	MSTRG.2126	--
Com_152_pos	Acetyl-L-carnitine	ENSGALG00000007814	ALPI
Com_92_pos	D-(+)-Proline	ENSGALG00000001565	C5
Com_4_pos	PC (17:1/17:1)	ENSGALG00000010798	DHCR24
Com_108_neg	LPE 18:2	ENSGALG00000008326	SYTL2
Com_165_neg	(±)9-HpODE	ENSGALG00000037629	TRANK1
Com_482_pos	8-Hydroxyquinoline	ENSGALG00000001492	NDRG3
Com_171_neg	LPC 22:6	ENSGALG00000027891	NREP
Com_147_pos	D-Sphingosine	ENSGALG00000030121	SLC2A11
Com_311_pos	PC (18:4e/2:0)	ENSGALG00000012704	MYLIP
Com_8_neg	4-Methyl-2-Oxopentanoic	ENSGALG00000007252	ANKDD1A
Com_151_neg	Lysope 18:1	MSTRG.6228	--
Com_471_pos	Indole-3-acetic acid	ENSGALG00000002802	PACSIN1
Com_55_pos	Valine	ENSGALG00000001531	FN3K
Com_342_pos	1-(4-methylphenyl)-3,5-di	ENSGALG00000007191	TK1

Com_471_pos	Indole-3-acetic acid	MSTRG.1468	--
Com_342_pos	1-(4-methylphenyl)-3,5-di	ENSGALG00000041510	DUOX2
Com_203_pos	Serotonin	ENSGALG00000033150	MIDN
Com_92_pos	D-(+)-Proline	ENSGALG00000047321	SARDH
Com_265_pos	6-Methylquinoline	MSTRG.13819	--
Com_16_neg	3-Hydroxybutyric acid	ENSGALG00000041258	msrA
Com_413_pos	L-Cystine	ENSGALG00000043582	LY6E
Com_68_neg	PE (16:0/22:6)	ENSGALG00000004569	UNC5B
Com_165_neg	(±)9-HpODE	ENSGALG00000010237	NPC2
Com_119_pos	DL-Stachydrine	ENSGALG00000006198	LSS
Com_92_pos	D-(+)-Proline	ENSGALG00000047480	A2ML1
Com_352_pos	Riboflavin	ENSGALG00000005831	DNAI1
Com_186_pos	4-Hydroxybenzaldehyde	ENSGALG00000028256	CCL19
Com_17_pos	L-Norleucine	ENSGALG00000046639	CYP2AC2
Com_215_pos	D-Erythro-sphingosine 1-p	ENSGALG00000004081	TMCO4
Com_342_pos	1-(4-methylphenyl)-3,5-di	ENSGALG00000047167	DNAJC30
Com_17_pos	L-Norleucine	ENSGALG00000038923	Ces1e
Com_588_pos	Ornithine	ENSGALG00000030031	TTPA
Com_16_neg	3-Hydroxybutyric acid	ENSGALG00000006482	FAH
Com_171_neg	LPC 22:6	ENSGALG00000005474	PNAT10
Com_17_pos	L-Norleucine	ENSGALG00000016281	DMD
Com_192_pos	1-Methylhistidine	ENSGALG00000050176	SARM1
Com_108_neg	LPE 18:2	ENSGALG00000014935	GREB1L
Com_80_pos	DL-Lysine	ENSGALG00000011287	SULT
Com_17_pos	L-Norleucine	ENSGALG00000005408	BCO1
Com_76_neg	Erythronolactone	ENSGALG00000002728	SLC16A3
Com_8_neg	4-Methyl-2-Oxopentanoic	ENSGALG00000014750	TRB
Com_155_neg	Phenylacetaldehyde	MSTRG.177	--
Com_97_pos	L-Threonine	ENSGALG00000002790	ABLIM3
Com_4_pos	PC (17:1/17:1)	ENSGALG00000040969	PTP4A3
Com_194_pos	Pipecolic acid	ENSGALG00000026313	RND3
Com_55_pos	Valine	ENSGALG00000016364	ALKAL2
Com_482_pos	8-Hydroxyquinoline	ENSGALG00000003427	USP3
Com_588_pos	Ornithine	ENSGALG00000016196	CBSL
Com_80_pos	DL-Lysine	MSTRG.14987	--
Com_203_pos	Serotonin	MSTRG.16433	B4GALNT4
Com_352_pos	Riboflavin	ENSGALG00000004498	SLC2A10
Com_171_neg	LPC 22:6	ENSGALG00000034337	RHPN1
Com_18_neg	Arachidonic acid	MSTRG.16661	--
Com_97_pos	L-Threonine	ENSGALG00000015425	LPL
Com_194_pos	Pipecolic acid	ENSGALG00000034741	ETNPPL
Com_16_neg	3-Hydroxybutyric acid	ENSGALG00000016138	DSCAM
Com_192_pos	1-Methylhistidine	ENSGALG00000034737	GLIS2
Com_55_pos	Valine	ENSGALG00000016690	CYP2AC1
Com_331_pos	L-Lysine	ENSGALG00000013244	ABCC9
Com_215_pos	D-Erythro-sphingosine 1-p	ENSGALG00000025743	CDR2
Com_460_pos	3-amino-4-(propylamino)c	ENSGALG00000007132	ACOX2
Com_588_pos	Ornithine	ENSGALG00000016325	GSTA3
Com_165_neg	(±)9-HpODE	ENSGALG00000011094	PDE4B
Com_16_neg	3-Hydroxybutyric acid	ENSGALG00000028341	MADCAM1
Com_175_pos	Pantothenic acid	ENSGALG00000004343	HPD
Com_40_pos	Choline	ENSGALG00000029270	GATA3
Com_18_neg	Arachidonic acid	ENSGALG00000030251	ADCY8
Com_89_neg	Gallic acid	MSTRG.13975	APEX1
Com_68_neg	PE (16:0/22:6)	MSTRG.18742	LRP2
Com_352_pos	Riboflavin	ENSGALG00000035239	GLCCI1
Com_40_pos	Choline	ENSGALG00000028897	WDR25
Com_215_pos	D-Erythro-sphingosine 1-p	MSTRG.15241	--

Com_147_pos	D-Sphingosine	ENSGALG00000005030	DOCK10
Com_362_pos	2-Arachidonoyl glycerol	ENSGALG00000033411	SLC26A2
Com_215_pos	D-Erythro-sphingosine 1-p	ENSGALG00000041988	SIK1
Com_151_neg	Lysope 18:1	ENSGALG00000002944	CPS1
Com_147_pos	D-Sphingosine	ENSGALG00000027122	APPL2
Com_460_pos	3-amino-4-(propylamino)c	ENSGALG00000008185	AOX1
Com_89_neg	Gallic acid	ENSGALG00000011169	PDCD2
Com_178_pos	Maltol	ENSGALG00000004598	CUX2
Com_264_pos	Indole	ENSGALG00000006724	GPC5
Com_342_pos	1-(4-methylphenyl)-3,5-di	MSTRG.18495	UGT1A9
Com_92_pos	D-(+)-Proline	MSTRG.3009	--
Com_147_pos	D-Sphingosine	ENSGALG00000034616	INHBA
Com_92_pos	D-(+)-Proline	ENSGALG00000014840	C6
Com_386_pos	2-Amino-1,3-octadecanec	ENSGALG00000005977	BTBD8
Com_86_neg	Levulinic acid	ENSGALG00000008780	CTBS
Com_12_pos	Betaine	ENSGALG00000005632	IRAG1
Com_8_neg	4-Methyl-2-Oxopentanoic	MSTRG.20827	Plcg1
Com_16_neg	3-Hydroxybutyric acid	ENSGALG00000002855	SARDH
Com_440_pos	PC (18:4e/4:0)	ENSGALG00000054856	ADH1
Com_331_pos	L-Lysine	MSTRG.5269	pitpnc1
Com_588_pos	Ornithine	ENSGALG00000014836	LPIN2
Com_130_neg	2-Hydroxyvaleric acid	MSTRG.11572	--
Com_178_pos	Maltol	ENSGALG00000007114	APOA1
Com_151_neg	Lysope 18:1	ENSGALG00000030357	ABL2
Com_252_pos	cis-4-Hydroxy-D-proline	MSTRG.13819	--
Com_440_pos	PC (18:4e/4:0)	ENSGALG00000003972	FAXDC2
Com_471_pos	Indole-3-acetic acid	ENSGALG00000012112	DBI
Com_413_pos	L-Cystine	ENSGALG00000027908	CYP2U1
Com_152_pos	Acetyl-L-carnitine	ENSGALG00000009926	HAAO
Com_12_pos	Betaine	ENSGALG00000035219	ALB
Com_18_neg	Arachidonic acid	ENSGALG00000050154	--
Com_342_pos	1-(4-methylphenyl)-3,5-di	ENSGALG00000015468	Tstd3
Com_24_neg	PE (16:0/20:4)	ENSGALG00000050069	BMP1
Com_120_neg	LPC 15:0	ENSGALG00000002479	MAT1A
Com_208_neg	N-Acetylanthranilic acid	ENSGALG00000033974	HGF
Com_150_neg	benzyl N-(2-{[(benzyloxy)c	ENSGALG00000006662	BPIFB3
Com_460_pos	3-amino-4-(propylamino)c	ENSGALG00000037387	CLSTN2
Com_331_pos	L-Lysine	ENSGALG00000021340	CA9
Com_16_neg	3-Hydroxybutyric acid	ENSGALG00000015034	ANKRD29
Com_53_neg	2-(5-mercapto-4-methyl-4	ENSGALG00000009157	ECT2
Com_16_neg	3-Hydroxybutyric acid	ENSGALG00000008728	PTER
Com_460_pos	3-amino-4-(propylamino)c	ENSGALG00000028407	GDF9
Com_175_pos	Pantothenic acid	ENSGALG00000006374	TBX6
Com_86_neg	Levulinic acid	MSTRG.4548	--
Com_86_neg	Levulinic acid	ENSGALG00000031122	NTNG1
Com_386_pos	2-Amino-1,3-octadecanec	ENSGALG00000009700	PDK4
Com_99_pos	Creatine	ENSGALG00000038652	Gsta3
Com_215_pos	D-Erythro-sphingosine 1-p	ENSGALG00000008326	SYTL2
Com_8_neg	4-Methyl-2-Oxopentanoic	ENSGALG00000005540	MICAL2
Com_352_pos	Riboflavin	ENSGALG00000050520	pol
Com_471_pos	Indole-3-acetic acid	ENSGALG00000038723	RPP25L
Com_151_neg	Lysope 18:1	ENSGALG00000015795	ADAMTS5
Com_89_neg	Gallic acid	ENSGALG00000038292	TMEM258
Com_175_pos	Pantothenic acid	ENSGALG00000011994	SYNPO2
Com_186_pos	4-Hydroxybenzaldehyde	ENSGALG00000054783	NDRG1
Com_460_pos	3-amino-4-(propylamino)c	ENSGALG00000041577	ITGA4
Com_17_pos	L-Norleucine	ENSGALG00000010857	DAB1
Com_92_pos	D-(+)-Proline	ENSGALG00000033974	HGF

Com_192_pos	1-Methylhistidine	ENSGALG00000005259	VIPR1
Com_8_neg	4-Methyl-2-Oxopentanoic	ENSGALG00000007814	ALPI
Com_192_pos	1-Methylhistidine	ENSGALG00000006341	SLC25A48
Com_16_neg	3-Hydroxybutyric acid	ENSGALG00000011957	TOB2
Com_208_neg	N-Acetylanthranilic acid	ENSGALG00000014840	C6
Com_56_neg	LPE 18:1	ENSGALG00000033783	FER1L6
Com_152_pos	Acetyl-L-carnitine	ENSGALG00000007252	ANKDD1A
Com_16_neg	3-Hydroxybutyric acid	ENSGALG00000051203	Mas1
Com_192_pos	1-Methylhistidine	ENSGALG00000007478	SLC51A
Com_413_pos	L-Cystine	ENSGALG00000009880	INPP4B
Com_78_neg	Citric acid	ENSGALG00000005474	PNAT10
Com_57_neg	LPC 16:1	ENSGALG00000010577	ARHGEF38
Com_53_neg	2-(5-mercapto-4-methyl-	ENSGALG00000015849	ME1
Com_352_pos	Riboflavin	MSTRG.12866	--
Com_108_neg	LPE 18:2	ENSGALG00000004081	TMCO4
Com_119_pos	DL-Stachydrine	ENSGALG00000007493	NSDHL
Com_252_pos	cis-4-Hydroxy-D-proline	ENSGALG00000047781	RAD9B
Com_460_pos	3-amino-4-(propylamino)	ENSGALG00000032882	EVA1C
Com_440_pos	PC (18:4e/4:0)	ENSGALG00000012704	MYLIP
Com_86_neg	Levulinic acid	ENSGALG00000046639	CYP2AC2
Com_192_pos	1-Methylhistidine	ENSGALG00000028284	PTX3
Com_25_pos	2-Hydroxycinnamic acid	ENSGALG00000053140	NDRG2
Com_119_pos	DL-Stachydrine	MSTRG.8619	--
Com_471_pos	Indole-3-acetic acid	MSTRG.15443	--
Com_311_pos	PC (18:4e/2:0)	ENSGALG00000022750	GPR18
Com_482_pos	8-Hydroxyquinoline	ENSGALG00000006702	MFGE8
Com_386_pos	2-Amino-1,3-octadecanec	ENSGALG00000011894	CYP2D6
Com_54_pos	Uric acid	ENSGALG00000011571	AGPAT4
Com_119_pos	DL-Stachydrine	ENSGALG00000009538	RDH12
Com_175_pos	Pantothenic acid	ENSGALG00000014412	CSTA
Com_362_pos	2-Arachidonoyl glycerol	ENSGALG00000007839	NCAM1
Com_68_neg	PE (16:0/22:6)	ENSGALG00000011181	FKBP14
Com_86_neg	Levulinic acid	ENSGALG00000005408	BCO1
Com_460_pos	3-amino-4-(propylamino)	ENSGALG00000052583	A2ML1
Com_4_pos	PC (17:1/17:1)	ENSGALG00000052296	MEX3D
Com_482_pos	8-Hydroxyquinoline	ENSGALG00000012748	ELOVL2
Com_12_pos	Betaine	ENSGALG00000001768	TENM2
Com_471_pos	Indole-3-acetic acid	ENSGALG00000023395	PLIN1
Com_178_pos	Maltol	ENSGALG00000011524	PPEF2
Com_78_neg	Citric acid	ENSGALG00000013033	cmbl
Com_18_neg	Arachidonic acid	ENSGALG00000000293	A2ML1
Com_56_neg	LPE 18:1	ENSGALG00000043920	OTUD3
Com_460_pos	3-amino-4-(propylamino)	ENSGALG00000052964	TOPAZ1
Com_151_neg	Lysope 18:1	ENSGALG00000010708	ICA1
Com_215_pos	D-Erythro-sphingosine 1-	ENSGALG00000051159	--
Com_152_pos	Acetyl-L-carnitine	ENSGALG00000014750	TRB
Com_151_pos	Pyridoxamine	ENSGALG00000011687	AHNAK2
Com_152_pos	Acetyl-L-carnitine	ENSGALG00000000309	Lad1
Com_120_neg	LPC 15:0	ENSGALG00000019768	ACSF2
Com_352_pos	Riboflavin	MSTRG.8248	--
Com_108_neg	LPE 18:2	ENSGALG00000025743	CDR2
Com_386_pos	2-Amino-1,3-octadecanec	ENSGALG00000028451	MT4
Com_413_pos	L-Cystine	ENSGALG00000008953	AASS
Com_151_pos	Pyridoxamine	ENSGALG00000054322	--
Com_130_neg	2-Hydroxyvaleric acid	ENSGALG00000054322	--
Com_311_pos	PC (18:4e/2:0)	ENSGALG00000048343	Ces1e
Com_108_neg	LPE 18:2	ENSGALG00000041988	SIK1
Com_147_pos	D-Sphingosine	ENSGALG00000034081	AKT3

Com_56_neg	LPE 18:1	ENSGALG00000040269	SBSPON
Com_178_pos	Maltol	ENSGALG00000013244	ABCC9
Com_89_neg	Gallic acid	ENSGALG00000041687	SREBF2
Com_440_pos	PC (18:4e/4:0)	ENSGALG00000016651	TDH
Com_252_pos	cis-4-Hydroxy-D-proline	ENSGALG00000010628	ACSL1
Com_56_neg	LPE 18:1	ENSGALG00000012362	THSD7B
Com_588_pos	Ornithine	ENSGALG00000029944	FAM222A
Com_55_pos	Valine	ENSGALG00000031122	NTNG1
Com_55_pos	Valine	MSTRG.4548	--
Com_80_pos	DL-Lysine	ENSGALG00000034478	CCL4
Com_471_pos	Indole-3-acetic acid	ENSGALG00000000950	MVB12B
Com_147_pos	D-Sphingosine	ENSGALG00000017046	POSTN
Com_76_neg	Erythronolactone	ENSGALG00000032746	ENPP2
Com_440_pos	PC (18:4e/4:0)	ENSGALG00000030121	SLC2A11
Com_89_neg	Gallic acid	MSTRG.88	--
Com_22_pos	Indole-3-acrylic acid	ENSGALG00000006724	GPC5
Com_386_pos	2-Amino-1,3-octadecanec	ENSGALG00000021848	AVD
Com_331_pos	L-Lysine	ENSGALG00000004598	CUX2
Com_147_pos	D-Sphingosine	ENSGALG00000016036	DOP1B
Com_55_pos	Valine	ENSGALG00000007014	PYROXD2
Com_18_neg	Arachidonic acid	ENSGALG00000041258	msrA
Com_57_neg	LPC 16:1	ENSGALG00000010009	TTC29
Com_171_neg	LPC 22:6	ENSGALG00000009740	RASGRP1
Com_92_pos	D-(+)-Proline	ENSGALG00000006320	Slc2a9
Com_178_pos	Maltol	MSTRG.5269	pitpnc1
Com_331_pos	L-Lysine	ENSGALG00000007114	APOA1
Com_460_pos	3-amino-4-(propylamino)(	ENSGALG00000012755	IGF-I
Com_76_neg	Erythronolactone	ENSGALG00000003578	FN1
Com_119_pos	DL-Stachydrine	ENSGALG00000016665	FDFT1
Com_588_pos	Ornithine	ENSGALG00000043582	LY6E
Com_17_pos	L-Norleucine	MSTRG.4548	--
Com_151_pos	Pyridoxamine	ENSGALG00000009050	CAPN3
Com_17_pos	L-Norleucine	ENSGALG00000031122	NTNG1
Com_208_neg	N-Acetylanthranilic acid	ENSGALG00000001565	C5
Com_342_pos	1-(4-methylphenyl)-3,5-di	ENSGALG00000032975	UBE2QL1
Com_151_pos	Pyridoxamine	ENSGALG00000007710	zgc:110179
Com_311_pos	PC (18:4e/2:0)	ENSGALG00000009002	CPED1
Com_108_neg	LPE 18:2	ENSGALG00000010233	SYNDIG1L
Com_311_pos	PC (18:4e/2:0)	ENSGALG00000003972	FAXDC2
Com_208_neg	N-Acetylanthranilic acid	ENSGALG00000001709	MUSTN1
Com_120_neg	LPC 15:0	ENSGALG00000047821	--
Com_18_neg	Arachidonic acid	ENSGALG00000016164	ABCG1
Com_331_pos	L-Lysine	MSTRG.17350	--
Com_152_pos	Acetyl-L-carnitine	ENSGALG00000001709	MUSTN1
Com_76_neg	Erythronolactone	ENSGALG00000004205	SOAT1
Com_40_pos	Choline	ENSGALG00000013124	FHOD3
Com_311_pos	PC (18:4e/2:0)	ENSGALG00000005204	GSTT1
Com_68_neg	PE (16:0/22:6)	ENSGALG00000029788	CCK
Com_80_pos	DL-Lysine	MSTRG.15995	--
Com_311_pos	PC (18:4e/2:0)	ENSGALG00000019663	ACBD7
Com_40_pos	Choline	ENSGALG00000002549	RGS1
Com_215_pos	D-Erythro-sphingosine 1-[	MSTRG.8985	gag
Com_12_pos	Betaine	ENSGALG00000046687	EPS8L3
Com_147_pos	D-Sphingosine	ENSGALG00000011003	SLC35F3
Com_57_neg	LPC 16:1	ENSGALG00000009483	MARK1
Com_16_neg	3-Hydroxybutyric acid	ENSGALG00000044996	TMEM71
Com_264_pos	Indole	ENSGALG00000016558	VEGFD
Com_152_pos	Acetyl-L-carnitine	ENSGALG00000001565	C5

Com_482_pos	8-Hydroxyquinoline	ENSGALG00000031754	KCNG2
Com_17_pos	L-Norleucine	ENSGALG00000028871	SLC38A3
Com_76_neg	Erythronolactone	ENSGALG00000016027	CBR3
Com_24_neg	PE (16:0/20:4)	ENSGALG00000037629	TRANK1
Com_155_neg	Phenylacetaldehyde	ENSGALG00000011560	PACRG
Com_86_neg	Levulinic acid	ENSGALG00000010857	DAB1
Com_208_neg	N-Acetylanthranilic acid	ENSGALG00000051068	SIGLEC1
Com_120_neg	LPC 15:0	ENSGALG00000008185	AOX1
Com_16_neg	3-Hydroxybutyric acid	MSTRG.16661	--
Com_25_pos	2-Hydroxycinnamic acid	ENSGALG00000053278	SUCNR1
Com_18_neg	Arachidonic acid	ENSGALG00000016138	DSCAM
Com_460_pos	3-amino-4-(propylamino)(	ENSGALG00000036190	AOC1
Com_53_neg	2-(5-mercapto-4-methyl-4	ENSGALG00000010825	AGR2
Com_171_neg	LPC 22:6	ENSGALG00000004205	SOAT1
Com_215_pos	D-Erythro-sphingosine 1-p	ENSGALG00000013511	ANKRA2
Com_413_pos	L-Cystine	ENSGALG00000016196	CBSL
Com_482_pos	8-Hydroxyquinoline	MSTRG.9165	--
Com_413_pos	L-Cystine	ENSGALG00000011287	SULT
Com_18_neg	Arachidonic acid	ENSGALG00000028341	MADCAM1
Com_386_pos	2-Amino-1,3-octadecaneo	MSTRG.10101	--
Com_16_neg	3-Hydroxybutyric acid	ENSGALG00000030251	ADCY8
Com_56_neg	LPE 18:1	ENSGALG00000029788	CCK
Com_86_neg	Levulinic acid	ENSGALG00000016364	ALKAL2
Com_311_pos	PC (18:4e/2:0)	ENSGALG00000003537	SGK2
Com_24_neg	PE (16:0/20:4)	ENSGALG00000010237	NPC2
Com_194_pos	Pipecolic acid	ENSGALG00000016364	ALKAL2
Com_471_pos	Indole-3-acetic acid	ENSGALG00000029083	NXPH2
Com_413_pos	L-Cystine	ENSGALG00000016325	GSTA3
Com_342_pos	1-(4-methylphenyl)-3,5-di	ENSGALG00000028560	OC3
Com_76_neg	Erythronolactone	ENSGALG00000009740	RASGRP1
Com_4_pos	PC (17:1/17:1)	ENSGALG00000014907	DCBLD1
Com_80_pos	DL-Lysine	ENSGALG00000043582	LY6E
Com_80_pos	DL-Lysine	ENSGALG00000051274	B3GALT2
Com_171_neg	LPC 22:6	ENSGALG00000013033	cmbl
Com_120_neg	LPC 15:0	ENSGALG00000028407	GDF9
Com_186_pos	4-Hydroxybenzaldehyde	ENSGALG00000004491	DMGDH
Com_4_pos	PC (17:1/17:1)	ENSGALG00000021039	HKDC1
Com_18_neg	Arachidonic acid	ENSGALG00000002855	SARDH
Com_97_pos	L-Threonine	ENSGALG00000054322	--
Com_99_pos	Creatine	ENSGALG00000023122	SULT1B
Com_155_neg	Phenylacetaldehyde	ENSGALG00000015057	DDO
Com_78_neg	Citric acid	ENSGALG00000033411	SLC26A2
Com_147_pos	D-Sphingosine	ENSGALG00000054856	ADH1
Com_108_neg	LPE 18:2	ENSGALG00000051159	--
Com_482_pos	8-Hydroxyquinoline	ENSGALG00000015684	Dnajc25
Com_352_pos	Riboflavin	ENSGALG00000045127	slc12a8
Com_165_neg	(±)9-HpODE	ENSGALG00000025738	RHOU
Com_440_pos	PC (18:4e/4:0)	ENSGALG00000022750	GPR18
Com_482_pos	8-Hydroxyquinoline	MSTRG.16287	--
Com_8_neg	4-Methyl-2-Oxopentanoic	ENSGALG00000014412	CSTA
Com_57_neg	LPC 16:1	ENSGALG00000000003	PANX2
Com_208_neg	N-Acetylanthranilic acid	ENSGALG00000000309	Lad1
Com_331_pos	L-Lysine	ENSGALG00000011524	PPEF2
Com_18_neg	Arachidonic acid	ENSGALG00000027793	SCN9A
Com_203_pos	Serotonin	MSTRG.8497	--
Com_413_pos	L-Cystine	ENSGALG00000014836	LPIN2
Com_18_neg	Arachidonic acid	ENSGALG00000015034	ANKRD29
Com_342_pos	1-(4-methylphenyl)-3,5-di	MSTRG.17162	--

Com_192_pos	1-Methylhistidine	ENSGALG00000011320	TMCC3
Com_18_neg	Arachidonic acid	ENSGALG00000008728	PTER
Com_119_pos	DL-Stachydrine	ENSGALG00000035675	--
Com_99_pos	Creatine	ENSGALG00000012683	RNF144B
Com_175_pos	Pantothenic acid	ENSGALG00000006864	COL24A1
Com_24_neg	PE (16:0/20:4)	ENSGALG00000011094	PDE4B
Com_89_neg	Gallic acid	MSTRG.9361	Fam110a
Com_76_neg	Erythronolactone	ENSGALG00000010357	P2RY1
Com_194_pos	Pipecolic acid	ENSGALG00000039499	LRRCC1
Com_8_neg	4-Methyl-2-Oxopentanoic	ENSGALG00000000309	Lad1
Com_203_pos	Serotonin	ENSGALG00000007178	FADS2
Com_151_neg	Lysope 18:1	MSTRG.2406	--
Com_471_pos	Indole-3-acetic acid	ENSGALG00000036616	NUAK2
Com_12_pos	Betaine	MSTRG.17721	--
Com_76_neg	Erythronolactone	ENSGALG00000005860	ACAA1
Com_352_pos	Riboflavin	ENSGALG00000034107	TRIM63
Com_165_neg	(±)9-HpODE	ENSGALG00000022720	GJB2
Com_362_pos	2-Arachidonoyl glycerol	ENSGALG00000030025	FABP4
Com_203_pos	Serotonin	ENSGALG00000015937	FABP1
Com_120_neg	LPC 15:0	ENSGALG00000032882	EVA1C
Com_108_neg	LPE 18:2	ENSGALG00000000003	PANX2
Com_215_pos	D-Erythro-sphingosine 1-p	MSTRG.15162	--
Com_203_pos	Serotonin	ENSGALG00000044278	C1orf131
Com_97_pos	L-Threonine	ENSGALG00000017039	STOML3
Com_440_pos	PC (18:4e/4:0)	ENSGALG00000048343	Ces1e
Com_8_neg	4-Methyl-2-Oxopentanoic	ENSGALG00000011994	SYNPO2
Com_16_neg	3-Hydroxybutyric acid	ENSGALG00000014950	SULT3A1
Com_151_neg	Lysope 18:1	ENSGALG00000007993	DCX
Com_120_neg	LPC 15:0	ENSGALG00000052583	A2ML1
Com_68_neg	PE (16:0/22:6)	ENSGALG00000043920	OTUD3
Com_78_neg	Citric acid	ENSGALG00000009740	RASGRP1
Com_152_pos	Acetyl-L-carnitine	ENSGALG00000014840	C6
Com_311_pos	PC (18:4e/2:0)	ENSGALG00000016651	TDH
Com_21_pos	DL-Tryptophan	ENSGALG00000053278	SUCNR1
Com_56_neg	LPE 18:1	ENSGALG00000004155	MYOM3
Com_311_pos	PC (18:4e/2:0)	MSTRG.14083	--
Com_331_pos	L-Lysine	ENSGALG00000017103	WASF3
Com_175_pos	Pantothenic acid	ENSGALG00000034140	ZNF395
Com_203_pos	Serotonin	ENSGALG00000001918	DNAJB5
Com_8_neg	4-Methyl-2-Oxopentanoic	ENSGALG00000006374	TBX6
Com_92_pos	D-(+)-Proline	ENSGALG00000009926	HAAO
Com_120_neg	LPC 15:0	ENSGALG00000052964	TOPAZ1
Com_165_neg	(±)9-HpODE	ENSGALG00000050069	BMP1
Com_56_neg	LPE 18:1	ENSGALG00000011181	FKBP14
Com_99_pos	Creatine	ENSGALG00000035026	SLC22A4
Com_311_pos	PC (18:4e/2:0)	ENSGALG00000030121	SLC2A11
Com_40_pos	Choline	ENSGALG00000009947	PLEKHH2
Com_92_pos	D-(+)-Proline	ENSGALG00000024085	IDO2
Com_460_pos	3-amino-4-(propylamino)c	ENSGALG00000019768	ACSF2
Com_147_pos	D-Sphingosine	ENSGALG00000012704	MYLIP
Com_362_pos	2-Arachidonoyl glycerol	ENSGALG00000009947	PLEKHH2
Com_215_pos	D-Erythro-sphingosine 1-p	ENSGALG00000010233	SYNDIG1L
Com_331_pos	L-Lysine	ENSGALG00000019835	TRIM27.2
Com_16_neg	3-Hydroxybutyric acid	ENSGALG00000007728	Prodh
Com_165_neg	(±)9-HpODE	ENSGALG00000007645	prom1a
Com_192_pos	1-Methylhistidine	ENSGALG00000044763	GPR82
Com_108_neg	LPE 18:2	MSTRG.8985	gag
Com_86_neg	Levulinic acid	ENSGALG00000028871	SLC38A3

Com_352_pos	Riboflavin	ENSGALG00000013583	FAM114A1
Com_311_pos	PC (18:4e/2:0)	ENSGALG00000054981	F10
Com_362_pos	2-Arachidonoyl glycerol	ENSGALG00000005474	PNAT10
Com_53_neg	2-(5-mercapto-4-methyl-	MSTRG.13977	PIP4P1
Com_22_pos	Indole-3-acrylic acid	ENSGALG00000016558	VEGFD
Com_16_neg	3-Hydroxybutyric acid	ENSGALG00000038242	CACNA2D2
Com_178_pos	Maltol	MSTRG.17350	--
Com_16_neg	3-Hydroxybutyric acid	ENSGALG00000000293	A2ML1
Com_460_pos	3-amino-4-(propylamino)	ENSGALG00000052872	--
Com_78_neg	Citric acid	ENSGALG00000004205	SOAT1
Com_482_pos	8-Hydroxyquinoline	ENSGALG00000038145	DPP7
Com_203_pos	Serotonin	ENSGALG00000000241	STARD4
Com_8_neg	4-Methyl-2-Oxopentanoic	ENSGALG00000001709	MUSTN1
Com_440_pos	PC (18:4e/4:0)	ENSGALG00000009002	CPED1
Com_68_neg	PE (16:0/22:6)	ENSGALG00000047792	SELENOM
Com_152_pos	Acetyl-L-carnitine	ENSGALG00000033974	HGF
Com_108_neg	LPE 18:2	ENSGALG00000013511	ANKRA2
Com_252_pos	cis-4-Hydroxy-D-proline	ENSGALG00000039499	LRRCC1
Com_57_neg	LPC 16:1	ENSGALG00000002944	CPS1
Com_8_neg	4-Methyl-2-Oxopentanoic	ENSGALG00000001565	C5
Com_440_pos	PC (18:4e/4:0)	ENSGALG00000005204	GSTT1
Com_108_neg	LPE 18:2	ENSGALG00000009483	MARK1
Com_40_pos	Choline	ENSGALG00000030025	FABP4
Com_440_pos	PC (18:4e/4:0)	ENSGALG00000019663	ACBD7
Com_12_pos	Betaine	ENSGALG00000017199	MAML2
Com_4_pos	PC (17:1/17:1)	MSTRG.19672	--
Com_352_pos	Riboflavin	ENSGALG00000029102	PXYLP1
Com_17_pos	L-Norleucine	ENSGALG00000016364	ALKAL2
Com_482_pos	8-Hydroxyquinoline	ENSGALG00000046731	--
Com_386_pos	2-Amino-1,3-octadecanec	ENSGALG00000005030	DOCK10
Com_24_neg	PE (16:0/20:4)	ENSGALG00000039536	C2H8ORF22
Com_8_neg	4-Methyl-2-Oxopentanoic	ENSGALG00000004343	HPD
Com_386_pos	2-Amino-1,3-octadecanec	ENSGALG00000027122	APPL2
Com_17_pos	L-Norleucine	ENSGALG00000015040	SLC16A10
Com_57_neg	LPC 16:1	ENSGALG00000010233	SYNDIG1L
Com_692_pos	3-amino-2-phenyl-2H-py	ENSGALG00000048599	Arhgap32
Com_92_pos	D-(+)-Proline	ENSGALG00000007252	ANKDD1A
Com_165_neg	(±)9-HpODE	MSTRG.8904	--
Com_119_pos	DL-Stachydrine	ENSGALG00000014189	SULT4A1
Com_16_neg	3-Hydroxybutyric acid	ENSGALG00000013726	PAICS
Com_120_neg	LPC 15:0	ENSGALG00000012755	IGF-I
Com_471_pos	Indole-3-acetic acid	ENSGALG00000042511	PKDCC
Com_460_pos	3-amino-4-(propylamino)	ENSGALG00000014252	A2M
Com_55_pos	Valine	ENSGALG00000016281	DMD
Com_203_pos	Serotonin	ENSGALG00000053446	RED3
Com_386_pos	2-Amino-1,3-octadecanec	ENSGALG00000034616	INHBA
Com_92_pos	D-(+)-Proline	ENSGALG00000009545	SLC25A12
Com_178_pos	Maltol	ENSGALG00000031158	OAT
Com_352_pos	Riboflavin	ENSGALG00000031932	AGPAT3
Com_55_pos	Valine	ENSGALG00000038923	Ces1e
Com_147_pos	D-Sphingosine	ENSGALG00000005977	BTBD8
Com_171_neg	LPC 22:6	ENSGALG00000033411	SLC26A2
Com_119_pos	DL-Stachydrine	ENSGALG00000016610	PTRHD1
Com_440_pos	PC (18:4e/4:0)	ENSGALG00000017046	POSTN
Com_460_pos	3-amino-4-(propylamino)	ENSGALG00000040573	FMO3
Com_108_neg	LPE 18:2	ENSGALG00000010009	TTC29
Com_186_pos	4-Hydroxybenzaldehyde	ENSGALG00000032903	RTN4RL2
Com_440_pos	PC (18:4e/4:0)	ENSGALG00000003537	SGK2

Com_99_pos	Creatine	ENSGALG00000013100	GRB10
Com_460_pos	3-amino-4-(propylamino)(	ENSGALG00000047821	--
Com_413_pos	L-Cystine	ENSGALG00000029944	FAM222A
Com_482_pos	8-Hydroxyquinoline	ENSGALG00000033656	DQX1
Com_440_pos	PC (18:4e/4:0)	ENSGALG00000016036	DOP1B
Com_175_pos	Pantothenic acid	ENSGALG00000014750	TRB
Com_99_pos	Creatine	ENSGALG00000013969	ALDH8A1
Com_151_pos	Pyridoxamine	ENSGALG00000015425	LPL
Com_460_pos	3-amino-4-(propylamino)(	ENSGALG00000026663	CX3CL1
Com_482_pos	8-Hydroxyquinoline	ENSGALG00000030908	ATP2B2
Com_89_neg	Gallic acid	ENSGALG00000035803	THRSP
Com_92_pos	D-(+)-Proline	ENSGALG00000014750	TRB
Com_194_pos	Pipecolic acid	ENSGALG00000031122	NTNG1
Com_194_pos	Pipecolic acid	MSTRG.4548	--
Com_4_pos	PC (17:1/17:1)	MSTRG.16505	--
Com_57_neg	LPC 16:1	ENSGALG00000015795	ADAMTS5
Com_264_pos	Indole	ENSGALG00000031593	TMSB15B
Com_386_pos	2-Amino-1,3-octadecanec	MSTRG.836	--
Com_147_pos	D-Sphingosine	ENSGALG00000009700	PDK4
Com_151_pos	Pyridoxamine	ENSGALG00000002790	ABLIM3
Com_120_neg	LPC 15:0	ENSGALG00000036190	AOC1
Com_186_pos	4-Hydroxybenzaldehyde	ENSGALG00000009100	FSHR
Com_203_pos	Serotonin	ENSGALG00000012505	LRFN5
Com_92_pos	D-(+)-Proline	ENSGALG00000043044	IL1R1
Com_89_neg	Gallic acid	ENSGALG00000027561	GNG5
Com_130_neg	2-Hydroxyvaleric acid	ENSGALG00000017039	STOML3
Com_53_neg	2-(5-mercapto-4-methyl-4	ENSGALG00000040896	FASN
Com_152_pos	Acetyl-L-carnitine	ENSGALG00000014412	CSTA
Com_588_pos	Ornithine	ENSGALG00000009479	SAMD9L
Com_16_neg	3-Hydroxybutyric acid	ENSGALG00000016164	ABCG1
Com_178_pos	Maltol	ENSGALG00000038520	STRIP2
Com_186_pos	4-Hydroxybenzaldehyde	ENSGALG00000040995	NEB
Com_18_neg	Arachidonic acid	ENSGALG00000044996	TMEM71
Com_4_pos	PC (17:1/17:1)	ENSGALG00000016979	SLC25A30
Com_440_pos	PC (18:4e/4:0)	ENSGALG00000011003	SLC35F3
Com_692_pos	3-amino-2-phenyl-2H-py	ENSGALG00000008850	TTLL7
Com_215_pos	D-Erythro-sphingosine 1-p	ENSGALG00000000003	PANX2
Com_108_neg	LPE 18:2	MSTRG.15162	--
Com_18_neg	Arachidonic acid	ENSGALG00000002594	TFPI
Com_471_pos	Indole-3-acetic acid	ENSGALG00000010229	ABCD4
Com_175_pos	Pantothenic acid	ENSGALG00000007252	ANKDD1A
Com_119_pos	DL-Stachydrine	ENSGALG00000012254	KCNJ4
Com_155_neg	Phenylacetaldehyde	ENSGALG00000050815	--
Com_120_neg	LPC 15:0	ENSGALG00000040995	NEB
Com_460_pos	3-amino-4-(propylamino)(	ENSGALG00000014616	MT3
Com_18_neg	Arachidonic acid	MSTRG.20377	--
Com_208_neg	N-Acetylanthranilic acid	ENSGALG00000004959	IRS1
Com_264_pos	Indole	ENSGALG00000003553	ABCA12
Com_203_pos	Serotonin	MSTRG.14680	--
Com_482_pos	8-Hydroxyquinoline	ENSGALG00000049658	UTS2R
Com_54_pos	Uric acid	ENSGALG00000002549	RGS1
Com_362_pos	2-Arachidonoyl glycerol	ENSGALG00000013124	FHOD3
Com_12_pos	Betaine	ENSGALG00000000184	SLC27A6
Com_97_pos	L-Threonine	MSTRG.8055	--
Com_413_pos	L-Cystine	ENSGALG00000028928	LCAT
Com_175_pos	Pantothenic acid	ENSGALG00000047827	TMEM86A
Com_76_neg	Erythronolactone	ENSGALG00000005474	PNAT10
Com_152_pos	Acetyl-L-carnitine	ENSGALG00000011994	SYNPO2

Com_8_neg	4-Methyl-2-Oxopentanoic	ENSGALG00000014840	C6
Com_178_pos	Maltol	ENSGALG00000017103	WASF3
Com_588_pos	Ornithine	ENSGALG00000011287	SULT
Com_471_pos	Indole-3-acetic acid	ENSGALG00000006076	RASGEF1C
Com_150_neg	benzyl N-(2-{[(benzyloxy)c	MSTRG.6555	--
Com_8_neg	4-Methyl-2-Oxopentanoic	MSTRG.8511	--
Com_99_pos	Creatine	ENSGALG00000011331	CTH
Com_80_pos	DL-Lysine	ENSGALG00000030031	TTPA
Com_152_pos	Acetyl-L-carnitine	ENSGALG00000006374	TBX6
Com_208_neg	N-Acetylanthranilic acid	ENSGALG00000007814	ALPI
Com_440_pos	PC (18:4e/4:0)	MSTRG.14083	--
Com_86_neg	Levulinic acid	ENSGALG00000034741	ETNPPL
Com_178_pos	Maltol	ENSGALG00000041680	KCNT2
Com_55_pos	Valine	ENSGALG00000028928	LCAT
Com_120_neg	LPC 15:0	ENSGALG00000032903	RTN4RL2
Com_178_pos	Maltol	ENSGALG00000019835	TRIM27.2
Com_86_neg	Levulinic acid	ENSGALG00000026313	RND3
Com_352_pos	Riboflavin	MSTRG.9774	--
Com_194_pos	Pipecolic acid	ENSGALG00000010628	ACSL1
Com_208_neg	N-Acetylanthranilic acid	ENSGALG00000005540	MICAL2
Com_53_neg	2-(5-mercapto-4-methyl-	ENSGALG00000000227	DPYSL2
Com_175_pos	Pantothenic acid	ENSGALG00000009926	HAAO
Com_147_pos	D-Sphingosine	ENSGALG00000022750	GPR18
Com_471_pos	Indole-3-acetic acid	ENSGALG00000008763	SSX2IP
Com_16_neg	3-Hydroxybutyric acid	ENSGALG00000027793	SCN9A
Com_215_pos	D-Erythro-sphingosine 1-p	ENSGALG00000009483	MARK1
Com_89_neg	Gallic acid	ENSGALG00000041078	MID1IP1
Com_352_pos	Riboflavin	ENSGALG00000024449	RAMP2
Com_460_pos	3-amino-4-(propylamino)c	ENSGALG00000019147	--
Com_386_pos	2-Amino-1,3-octadecanec	ENSGALG00000034081	AKT3
Com_119_pos	DL-Stachydrine	ENSGALG00000029898	YKT6
Com_165_neg	(±)9-HpODE	ENSGALG00000008462	CDK3
Com_440_pos	PC (18:4e/4:0)	ENSGALG00000054981	F10
Com_471_pos	Indole-3-acetic acid	ENSGALG00000004425	SCAMP1
Com_86_neg	Levulinic acid	ENSGALG00000015040	SLC16A10
Com_8_neg	4-Methyl-2-Oxopentanoic	ENSGALG00000033974	HGF
Com_311_pos	PC (18:4e/2:0)	ENSGALG00000008912	ABCB1
Com_192_pos	1-Methylhistidine	ENSGALG00000016287	NR0B1
Com_460_pos	3-amino-4-(propylamino)c	MSTRG.19177	PHGDH
Com_352_pos	Riboflavin	ENSGALG00000002371	RUSC2
Com_56_neg	LPE 18:1	ENSGALG00000008850	TTLL7
Com_331_pos	L-Lysine	ENSGALG00000017199	MAML2
Com_208_neg	N-Acetylanthranilic acid	MSTRG.20827	Plcg1
Com_151_neg	Lysope 18:1	ENSGALG00000010009	TTC29
Com_12_pos	Betaine	ENSGALG00000039474	ID4
Com_152_pos	Acetyl-L-carnitine	ENSGALG00000004343	HPD
Com_215_pos	D-Erythro-sphingosine 1-p	ENSGALG00000010009	TTC29
Com_97_pos	L-Threonine	ENSGALG00000037671	psuG
Com_89_neg	Gallic acid	ENSGALG00000005411	MEIOB
Com_18_neg	Arachidonic acid	ENSGALG00000014950	SULT3A1
Com_147_pos	D-Sphingosine	ENSGALG00000048343	Ces1e
Com_331_pos	L-Lysine	ENSGALG00000031158	OAT
Com_55_pos	Valine	ENSGALG00000039499	LRRCC1
Com_78_neg	Citric acid	ENSGALG00000030025	FABP4
Com_460_pos	3-amino-4-(propylamino)c	ENSGALG00000033171	TGM4
Com_24_neg	PE (16:0/20:4)	ENSGALG00000025738	RHOU
Com_178_pos	Maltol	ENSGALG00000012754	PAH
Com_413_pos	L-Cystine	ENSGALG00000038923	Ces1e

Com_40_pos	Choline	ENSGALG00000011571	AGPAT4
Com_120_neg	LPC 15:0	ENSGALG00000052872	--
Com_92_pos	D-(+)-Proline	ENSGALG00000016287	NR0B1
Com_68_neg	PE (16:0/22:6)	ENSGALG00000012045	slc12a8
Com_178_pos	Maltol	ENSGALG00000003212	TSPO2
Com_413_pos	L-Cystine	ENSGALG00000016281	DMD
Com_192_pos	1-Methylhistidine	ENSGALG00000007536	PHR
Com_311_pos	PC (18:4e/2:0)	ENSGALG00000017046	POSTN
Com_12_pos	Betaine	ENSGALG00000019835	TRIM27.2
Com_151_neg	Lysope 18:1	ENSGALG00000009483	MARK1
Com_151_neg	Lysope 18:1	ENSGALG00000047464	FBXL22
Com_265_pos	6-Methylquinoline	ENSGALG00000036005	TIAM2
Com_22_pos	Indole-3-acrylic acid	ENSGALG00000031593	TMSB15B
Com_18_neg	Arachidonic acid	ENSGALG00000007728	Prodh
Com_56_neg	LPE 18:1	MSTRG.16972	--
Com_362_pos	2-Arachidonoyl glycerol	ENSGALG00000009740	RASGRP1
Com_311_pos	PC (18:4e/2:0)	ENSGALG00000016036	DOP1B
Com_76_neg	Erythronolactone	ENSGALG00000020688	CYP4A22
Com_8_neg	4-Methyl-2-Oxopentanoic	ENSGALG00000012034	ADSL
Com_471_pos	Indole-3-acetic acid	ENSGALG00000002500	GMPPB
Com_175_pos	Pantothenic acid	ENSGALG00000039239	SERPIND1
Com_119_pos	DL-Stachydrine	ENSGALG00000038666	FBXL12
Com_12_pos	Betaine	ENSGALG00000017103	WASF3
Com_24_neg	PE (16:0/20:4)	ENSGALG00000022720	GJB2
Com_151_pos	Pyridoxamine	ENSGALG00000055021	GREM2
Com_18_neg	Arachidonic acid	ENSGALG00000038242	CACNA2D2
Com_130_neg	2-Hydroxyvaleric acid	MSTRG.8055	--
Com_8_neg	4-Methyl-2-Oxopentanoic	ENSGALG00000040434	rab18b
Com_192_pos	1-Methylhistidine	ENSGALG00000013776	CEP135
Com_331_pos	L-Lysine	ENSGALG00000038520	STRIP2
Com_342_pos	1-(4-methylphenyl)-3,5-di	MSTRG.8957	--
Com_155_neg	Phenylacetaldehyde	MSTRG.17163	--
Com_78_neg	Citric acid	ENSGALG00000009947	PLEKHH2
Com_120_neg	LPC 15:0	ENSGALG00000014252	A2M
Com_8_neg	4-Methyl-2-Oxopentanoic	MSTRG.3197	--
Com_460_pos	3-amino-4-(propylamino)(	ENSGALG00000013728	PPAT
Com_165_neg	(±)9-HpODE	ENSGALG00000050491	SLC35E4
Com_342_pos	1-(4-methylphenyl)-3,5-di	ENSGALG00000007267	MVD
Com_147_pos	D-Sphingosine	ENSGALG00000009002	CPED1
Com_76_neg	Erythronolactone	ENSGALG00000003595	SARM1
Com_99_pos	Creatine	ENSGALG00000028256	CCL19
Com_120_neg	LPC 15:0	ENSGALG00000040573	FMO3
Com_18_neg	Arachidonic acid	ENSGALG00000032231	C4
Com_16_neg	3-Hydroxybutyric acid	ENSGALG00000030031	TTPA
Com_22_pos	Indole-3-acrylic acid	ENSGALG00000003553	ABCA12
Com_588_pos	Ornithine	ENSGALG00000006482	FAH
Com_178_pos	Maltol	ENSGALG00000013728	PPAT
Com_147_pos	D-Sphingosine	ENSGALG00000005204	GSTT1
Com_147_pos	D-Sphingosine	ENSGALG00000019663	ACBD7
Com_311_pos	PC (18:4e/2:0)	ENSGALG00000011003	SLC35F3
Com_352_pos	Riboflavin	ENSGALG00000047720	KLHDC7A
Com_331_pos	L-Lysine	MSTRG.17721	--
Com_25_pos	2-Hydroxycinnamic acid	ENSGALG00000010628	ACSL1
Com_362_pos	2-Arachidonoyl glycerol	ENSGALG00000004205	SOAT1
Com_120_neg	LPC 15:0	ENSGALG00000026663	CX3CL1
Com_18_neg	Arachidonic acid	ENSGALG00000013726	PAICS
Com_265_pos	6-Methylquinoline	ENSGALG00000006724	GPC5
Com_152_pos	Acetyl-L-carnitine	ENSGALG00000024085	IDO2

Com_208_neg	N-Acetylanthranilic acid	ENSGALG00000009920	COCH
Com_86_neg	Levulinic acid	ENSGALG00000037160	Smad7
Com_17_pos	L-Norleucine	ENSGALG00000034741	ETNPPL
Com_171_neg	LPC 22:6	ENSGALG00000005472	NAT
Com_120_neg	LPC 15:0	ENSGALG00000004491	DMGDH
Com_24_neg	PE (16:0/20:4)	ENSGALG00000007645	prom1a
Com_151_neg	Lysope 18:1	ENSGALG00000000003	PANX2
Com_440_pos	PC (18:4e/4:0)	ENSGALG00000005977	BTBD8
Com_352_pos	Riboflavin	ENSGALG00000031570	WDR54
Com_386_pos	2-Amino-1,3-octadecanec	ENSGALG00000054856	ADH1
Com_460_pos	3-amino-4-(propylamino)(	ENSGALG00000003212	TSPO2
Com_17_pos	L-Norleucine	ENSGALG00000027908	CYP2U1
Com_68_neg	PE (16:0/22:6)	ENSGALG00000016444	ODC1
Com_147_pos	D-Sphingosine	ENSGALG00000003537	SGK2
Com_17_pos	L-Norleucine	ENSGALG00000026313	RND3
Com_460_pos	3-amino-4-(propylamino)(	ENSGALG00000012754	PAH
Com_331_pos	L-Lysine	ENSGALG00000041680	KCNT2
Com_152_pos	Acetyl-L-carnitine	MSTRG.8511	--
Com_471_pos	Indole-3-acetic acid	ENSGALG00000014944	GCNT4
Com_178_pos	Maltol	ENSGALG00000033171	TGM4
Com_89_neg	Gallic acid	ENSGALG00000037998	GLTP
Com_175_pos	Pantothenic acid	ENSGALG00000006320	Slc2a9
Com_57_neg	LPC 16:1	MSTRG.2406	--
Com_460_pos	3-amino-4-(propylamino)(	ENSGALG00000040995	NEB
Com_120_neg	LPC 15:0	ENSGALG00000014616	MT3
Com_194_pos	Pipecolic acid	ENSGALG00000016281	DMD
Com_692_pos	3-amino-2-phenyl-2H-py	ENSGALG00000004155	MYOM3
Com_57_neg	LPC 16:1	ENSGALG00000008326	SYTL2
Com_194_pos	Pipecolic acid	ENSGALG00000038923	Ces1e
Com_192_pos	1-Methylhistidine	ENSGALG00000043044	IL1R1
Com_40_pos	Choline	ENSGALG00000033411	SLC26A2
Com_362_pos	2-Arachidonoyl glycerol	ENSGALG00000028897	WDR25
Com_440_pos	PC (18:4e/4:0)	ENSGALG00000009700	PDK4
Com_130_neg	2-Hydroxyvaleric acid	ENSGALG00000037671	psuG
Com_68_neg	PE (16:0/22:6)	MSTRG.21631	--
Com_215_pos	D-Erythro-sphingosine 1-[	ENSGALG00000000695	MFSD4A
Com_362_pos	2-Arachidonoyl glycerol	ENSGALG00000029270	GATA3
Com_165_neg	(±)9-HpODE	ENSGALG00000039536	C2H8ORF22
Com_311_pos	PC (18:4e/2:0)	ENSGALG00000017040	C4
Com_311_pos	PC (18:4e/2:0)	ENSGALG00000003015	SERPINF1
Com_178_pos	Maltol	MSTRG.19177	PHGDH
Com_57_neg	LPC 16:1	ENSGALG00000007993	DCX
Com_24_neg	PE (16:0/20:4)	MSTRG.8904	--
Com_16_neg	3-Hydroxybutyric acid	ENSGALG00000002594	TFPI
Com_252_pos	cis-4-Hydroxy-D-proline	ENSGALG00000053278	SUCNR1
Com_413_pos	L-Cystine	ENSGALG00000009479	SAMD9L
Com_76_neg	Erythronolactone	ENSGALG00000040836	INSYN2A
Com_56_neg	LPE 18:1	MSTRG.2409	--
Com_342_pos	1-(4-methylphenyl)-3,5-di	ENSGALG00000042275	esg1
Com_152_pos	Acetyl-L-carnitine	ENSGALG00000009545	SLC25A12
Com_97_pos	L-Threonine	ENSGALG00000036086	TAGLN2
Com_178_pos	Maltol	ENSGALG00000019147	--
Com_171_neg	LPC 22:6	ENSGALG00000030025	FABP4
Com_178_pos	Maltol	ENSGALG00000017199	MAML2
Com_186_pos	4-Hydroxybenzaldehyde	ENSGALG00000047821	--
Com_16_neg	3-Hydroxybutyric acid	MSTRG.20377	--
Com_460_pos	3-amino-4-(propylamino)(	ENSGALG00000032903	RTN4RL2
Com_440_pos	PC (18:4e/4:0)	ENSGALG00000008912	ABCB1

Com_80_pos	DL-Lysine	ENSGALG00000001531	FN3K
Com_17_pos	L-Norleucine	ENSGALG00000009880	INPP4B
Com_155_neg	Phenylacetaldehyde	ENSGALG00000029947	MMAB
Com_203_pos	Serotonin	MSTRG.3473	--
Com_192_pos	1-Methylhistidine	ENSGALG00000009545	SLC25A12
Com_24_neg	PE (16:0/20:4)	ENSGALG00000054870	TXNL1
Com_119_pos	DL-Stachydrine	MSTRG.7483	--
Com_331_pos	L-Lysine	ENSGALG00000046687	EPS8L3
Com_86_neg	Levulinic acid	ENSGALG00000011314	LRRC3B
Com_80_pos	DL-Lysine	ENSGALG00000013726	PAICS
Com_4_pos	PC (17:1/17:1)	ENSGALG00000007234	CLCN5
Com_86_neg	Levulinic acid	ENSGALG00000011391	AMN
Com_12_pos	Betaine	MSTRG.17350	--
Com_386_pos	2-Amino-1,3-octadecanec	ENSGALG00000012704	MYLIP
Com_97_pos	L-Threonine	ENSGALG00000035903	FAM46A
Com_175_pos	Pantothenic acid	ENSGALG00000000761	TSKU
Com_352_pos	Riboflavin	ENSGALG00000012089	--
Com_165_neg	(±)9-HpODE	MSTRG.17628	--
Com_57_neg	LPC 16:1	MSTRG.15241	--
Com_203_pos	Serotonin	ENSGALG00000005839	ARID3C
Com_171_neg	LPC 22:6	ENSGALG00000017032	SLC25A15
Com_460_pos	3-amino-4-(propylamino)(	ENSGALG00000041680	KCNT2
Com_331_pos	L-Lysine	ENSGALG00000012754	PAH
Com_76_neg	Erythronolactone	ENSGALG00000007839	NCAM1
Com_203_pos	Serotonin	ENSGALG00000052986	Vwa5b2
Com_588_pos	Ornithine	ENSGALG00000028928	LCAT
Com_215_pos	D-Erythro-sphingosine 1-p	ENSGALG00000032287	ATP2A2
Com_120_neg	LPC 15:0	ENSGALG00000019147	--
Com_331_pos	L-Lysine	ENSGALG00000003212	TSPO2
Com_152_pos	Acetyl-L-carnitine	ENSGALG00000043044	IL1R1
Com_78_neg	Citric acid	ENSGALG00000013124	FHOD3
Com_147_pos	D-Sphingosine	MSTRG.14083	--
Com_92_pos	D-(+)-Proline	ENSGALG00000014412	CSTA
Com_55_pos	Valine	ENSGALG00000011287	SULT
Com_171_neg	LPC 22:6	ENSGALG00000009947	PLEKHH2
Com_588_pos	Ornithine	ENSGALG00000011957	TOB2
Com_150_neg	benzyl N-(2-{[(benzyloxy)c	ENSGALG00000035060	FKBP11
Com_8_neg	4-Methyl-2-Oxopentanoic	ENSGALG00000017032	SLC25A15
Com_80_pos	DL-Lysine	ENSGALG00000038242	CACNA2D2
Com_120_neg	LPC 15:0	MSTRG.19177	PHGDH
Com_97_pos	L-Threonine	ENSGALG00000015492	PDZK1
Com_152_pos	Acetyl-L-carnitine	ENSGALG00000012034	ADSL
Com_588_pos	Ornithine	ENSGALG00000051203	Mas1
Com_215_pos	D-Erythro-sphingosine 1-p	ENSGALG00000003568	PPP1R16B
Com_4_pos	PC (17:1/17:1)	ENSGALG00000041143	UMOD
Com_151_neg	Lysope 18:1	ENSGALG00000010233	SYNDIG1L
Com_17_pos	L-Norleucine	ENSGALG00000008953	AASS
Com_252_pos	cis-4-Hydroxy-D-proline	ENSGALG00000007014	PYROXD2
Com_76_neg	Erythronolactone	ENSGALG00000027786	SOCS3
Com_92_pos	D-(+)-Proline	ENSGALG00000044763	GPR82
Com_80_pos	DL-Lysine	ENSGALG00000007728	Prodh
Com_194_pos	Pipecolic acid	ENSGALG00000028928	LCAT
Com_152_pos	Acetyl-L-carnitine	ENSGALG00000040434	rab18b
Com_178_pos	Maltol	ENSGALG00000014616	MT3
Com_203_pos	Serotonin	ENSGALG00000002579	RIMBP2
Com_152_pos	Acetyl-L-carnitine	MSTRG.3197	--
Com_147_pos	D-Sphingosine	ENSGALG00000054981	F10
Com_80_pos	DL-Lysine	ENSGALG00000016690	CYP2AC1

Com_155_neg	Phenylacetaldehyde	ENSGALG00000009748	ASNS
Com_471_pos	Indole-3-acetic acid	ENSGALG00000000645	Espn
Com_192_pos	1-Methylhistidine	ENSGALG00000024085	IDO2
Com_53_neg	2-(5-mercapto-4-methyl-	ENSGALG00000015935	SMYD1
Com_120_neg	LPC 15:0	ENSGALG00000033171	TGM4
Com_186_pos	4-Hydroxybenzaldehyde	ENSGALG00000019768	ACSF2
Com_331_pos	L-Lysine	ENSGALG00000013728	PPAT
Com_55_pos	Valine	ENSGALG00000010628	ACSL1
Com_92_pos	D-(+)-Proline	ENSGALG00000011994	SYNPO2
Com_460_pos	3-amino-4-(propylamino)(	ENSGALG00000038520	STRIP2
Com_311_pos	PC (18:4e/2:0)	ENSGALG00000002024	COMT
Com_78_neg	Citric acid	ENSGALG00000005472	NAT
Com_178_pos	Maltol	MSTRG.17721	--
Com_386_pos	2-Amino-1,3-octadecanec	ENSGALG00000029724	MTURN
Com_17_pos	L-Norleucine	ENSGALG00000037160	Smad7
Com_4_pos	PC (17:1/17:1)	ENSGALG00000005215	CACNA1H
Com_203_pos	Serotonin	ENSGALG00000006530	TSSC4
Com_80_pos	DL-Lysine	ENSGALG00000014950	SULT3A1
Com_56_neg	LPE 18:1	ENSGALG00000021238	CYP2W1
Com_92_pos	D-(+)-Proline	ENSGALG00000006374	TBX6
Com_264_pos	Indole	ENSGALG00000050840	APCDD1
Com_86_neg	Levulinic acid	ENSGALG00000027908	CYP2U1
Com_89_neg	Gallic acid	ENSGALG00000026809	SARS
Com_155_neg	Phenylacetaldehyde	ENSGALG00000008150	RASAL1
Com_119_pos	DL-Stachydrine	ENSGALG00000009024	CLIP4
Com_208_neg	N-Acetylanthranilic acid	ENSGALG00000031255	FGF1
Com_8_neg	4-Methyl-2-Oxopentanoic	ENSGALG00000024085	IDO2
Com_178_pos	Maltol	ENSGALG00000026663	CX3CL1
Com_68_neg	PE (16:0/22:6)	MSTRG.13407	--
Com_352_pos	Riboflavin	ENSGALG00000006080	GPC4
Com_215_pos	D-Erythro-sphingosine 1-p	ENSGALG00000030076	PCSK6
Com_203_pos	Serotonin	ENSGALG00000010294	RPS6KL1
Com_147_pos	D-Sphingosine	MSTRG.836	--
Com_76_neg	Erythronolactone	ENSGALG00000031255	FGF1
Com_413_pos	L-Cystine	MSTRG.4548	--
Com_413_pos	L-Cystine	ENSGALG00000031122	NTNG1
Com_120_neg	LPC 15:0	ENSGALG00000013728	PPAT
Com_311_pos	PC (18:4e/2:0)	ENSGALG00000005977	BTBD8
Com_331_pos	L-Lysine	ENSGALG00000033171	TGM4
Com_178_pos	Maltol	ENSGALG00000040573	FMO3
Com_108_neg	LPE 18:2	ENSGALG00000000695	MFSD4A
Com_40_pos	Choline	ENSGALG00000013033	cmbl
Com_92_pos	D-(+)-Proline	ENSGALG00000011320	TMCC3
Com_130_neg	2-Hydroxyvaleric acid	ENSGALG00000036086	TAGLN2
Com_460_pos	3-amino-4-(propylamino)(	ENSGALG00000031158	OAT
Com_24_neg	PE (16:0/20:4)	ENSGALG00000008462	CDK3
Com_588_pos	Ornithine	ENSGALG00000038923	Ces1e
Com_175_pos	Pantothenic acid	MSTRG.3009	--
Com_178_pos	Maltol	ENSGALG00000014252	A2M
Com_108_neg	LPE 18:2	ENSGALG00000010708	ICA1
Com_588_pos	Ornithine	ENSGALG00000016281	DMD
Com_265_pos	6-Methylquinoline	ENSGALG00000016558	VEGFD
Com_482_pos	8-Hydroxyquinoline	ENSGALG00000026607	C15orf40
Com_8_neg	4-Methyl-2-Oxopentanoic	ENSGALG00000005472	NAT
Com_471_pos	Indole-3-acetic acid	ENSGALG00000041533	SLC11A2
Com_471_pos	Indole-3-acetic acid	ENSGALG00000054926	--
Com_57_neg	LPC 16:1	ENSGALG00000013828	GALM
Com_692_pos	3-amino-2-phenyl-2H-py	ENSGALG00000012362	THSD7B

Com_482_pos	8-Hydroxyquinoline	ENSGALG00000016492	TDRD15
Com_25_pos	2-Hydroxycinnamic acid	ENSGALG00000039499	LRRCC1
Com_331_pos	L-Lysine	MSTRG.19177	PHGDH
Com_130_neg	2-Hydroxyvaleric acid	ENSGALG00000035903	FAM46A
Com_311_pos	PC (18:4e/2:0)	ENSGALG00000009700	PDK4
Com_24_neg	PE (16:0/20:4)	ENSGALG00000016415	MAP7D2
Com_692_pos	3-amino-2-phenyl-2H-py	ENSGALG00000040269	SBSPON
Com_17_pos	L-Norleucine	ENSGALG00000016196	CBSL
Com_92_pos	D-(+)-Proline	ENSGALG00000004343	HPD
Com_460_pos	3-amino-4-(propylamino)c	ENSGALG00000004491	DMGDH
Com_86_neg	Levulinic acid	ENSGALG00000009880	INPP4B
Com_352_pos	Riboflavin	MSTRG.21822	--
Com_16_neg	3-Hydroxybutyric acid	ENSGALG00000032231	C4
Com_18_neg	Arachidonic acid	ENSGALG00000030031	TTPA
Com_440_pos	PC (18:4e/4:0)	ENSGALG00000017040	C4
Com_440_pos	PC (18:4e/4:0)	ENSGALG00000003015	SERPINF1
Com_208_neg	N-Acetylanthranilic acid	ENSGALG00000027786	SOCS3
Com_16_neg	3-Hydroxybutyric acid	ENSGALG00000043582	LY6E
Com_120_neg	LPC 15:0	ENSGALG00000003212	TSPO2
Com_17_pos	L-Norleucine	ENSGALG00000011314	LRRC3B
Com_53_neg	2-(5-mercapto-4-methyl-4	MSTRG.4905	--
Com_203_pos	Serotonin	ENSGALG00000050091	CLEC2B
Com_178_pos	Maltol	ENSGALG00000052872	--
Com_331_pos	L-Lysine	ENSGALG00000019147	--
Com_17_pos	L-Norleucine	ENSGALG00000011391	AMN
Com_8_neg	4-Methyl-2-Oxopentanoic	ENSGALG00000009545	SLC25A12
Com_120_neg	LPC 15:0	ENSGALG00000012754	PAH
Com_99_pos	Creatine	ENSGALG00000009268	FGG
Com_78_neg	Citric acid	ENSGALG00000017032	SLC25A15
Com_17_pos	L-Norleucine	ENSGALG00000016325	GSTA3
Com_18_neg	Arachidonic acid	ENSGALG00000002728	SLC16A3
Com_68_neg	PE (16:0/22:6)	ENSGALG00000003446	PRLR
Com_362_pos	2-Arachidonoyl glycerol	ENSGALG00000002466	SLC2A5
Com_171_neg	LPC 22:6	MSTRG.3197	--
Com_413_pos	L-Cystine	ENSGALG00000006482	FAH
Com_178_pos	Maltol	ENSGALG00000046687	EPS8L3
Com_152_pos	Acetyl-L-carnitine	ENSGALG00000016287	NR0B1
Com_171_neg	LPC 22:6	ENSGALG00000040434	rab18b
Com_108_neg	LPE 18:2	ENSGALG00000032287	ATP2A2
Com_171_neg	LPC 22:6	ENSGALG00000013124	FHOD3
Com_130_neg	2-Hydroxyvaleric acid	ENSGALG00000015492	PDZK1
Com_588_pos	Ornithine	MSTRG.16661	--
Com_352_pos	Riboflavin	MSTRG.9524	--
Com_171_neg	LPC 22:6	ENSGALG00000012034	ADSL
Com_331_pos	L-Lysine	ENSGALG00000035219	ALB
Com_215_pos	D-Erythro-sphingosine 1-p	ENSGALG00000002707	CHRNB2
Com_386_pos	2-Amino-1,3-octadecaned	ENSGALG00000022750	GPR18
Com_12_pos	Betaine	MSTRG.5269	pitpnc1
Com_119_pos	DL-Stachydrine	ENSGALG00000046412	Aoc3
Com_175_pos	Pantothenic acid	ENSGALG00000047480	A2ML1
Com_108_neg	LPE 18:2	ENSGALG00000003568	PPP1R16B
Com_76_neg	Erythronolactone	MSTRG.8986	gag
Com_55_pos	Valine	ENSGALG00000041533	SLC11A2
Com_130_neg	2-Hydroxyvaleric acid	ENSGALG00000032628	SRCIN1
Com_78_neg	Citric acid	ENSGALG00000003575	Dnttip1
Com_97_pos	L-Threonine	ENSGALG00000047687	SETD9
Com_352_pos	Riboflavin	ENSGALG00000034507	CHST2
Com_99_pos	Creatine	ENSGALG00000023083	KIAA1958

Com_55_pos	Valine	ENSGALG00000031482	Pou5f3
Com_8_neg	4-Methyl-2-Oxopentanoic	ENSGALG00000051779	PRORSD1P
Com_588_pos	Ornithine	MSTRG.12629	--
Com_194_pos	Pipecolic acid	ENSGALG00000007723	NAT9
Com_588_pos	Ornithine	MSTRG.20167	--
Com_386_pos	2-Amino-1,3-octadecaned	ENSGALG00000014976	GATA6
Com_40_pos	Choline	ENSGALG00000014948	HMGCR
Com_17_pos	L-Norleucine	ENSGALG00000015136	ILDR1
Com_386_pos	2-Amino-1,3-octadecaned	ENSGALG00000042555	STAMBP
Com_171_neg	LPC 22:6	ENSGALG00000026460	myoM
Com_186_pos	4-Hydroxybenzaldehyde	ENSGALG00000030076	PCSK6
Com_265_pos	6-Methylquinoline	MSTRG.4813	--
Com_413_pos	L-Cystine	ENSGALG00000012112	DBI
Com_120_neg	LPC 15:0	ENSGALG00000004081	TMCO4
Com_175_pos	Pantothenic acid	ENSGALG00000021135	HAPLN3
Com_252_pos	cis-4-Hydroxy-D-proline	ENSGALG00000023395	PLIN1
Com_97_pos	L-Threonine	MSTRG.6225	--
Com_25_pos	2-Hydroxycinnamic acid	ENSGALG00000002500	GMPPB
Com_54_pos	Uric acid	ENSGALG00000043435	CARNS1
Com_252_pos	cis-4-Hydroxy-D-proline	ENSGALG00000035675	--
Com_440_pos	PC (18:4e/4:0)	ENSGALG00000008039	MFSD13A
Com_99_pos	Creatine	ENSGALG00000004833	P3H1
Com_194_pos	Pipecolic acid	ENSGALG00000004425	SCAMP1
Com_99_pos	Creatine	ENSGALG00000051159	--
Com_22_pos	Indole-3-acrylic acid	ENSGALG00000037325	SERP1
Com_78_neg	Citric acid	ENSGALG00000005610	SLC44A3
Com_178_pos	Maltol	ENSGALG00000016491	APOB
Com_203_pos	Serotonin	ENSGALG00000009050	CAPN3
Com_152_pos	Acetyl-L-carnitine	ENSGALG00000004483	AHSA2
Com_8_neg	4-Methyl-2-Oxopentanoic	MSTRG.21371	--
Com_203_pos	Serotonin	ENSGALG00000007710	zgc:110179
Com_147_pos	D-Sphingosine	ENSGALG00000004782	TSEN15
Com_86_neg	Levulinic acid	ENSGALG00000007018	SLC26A11
Com_440_pos	PC (18:4e/4:0)	ENSGALG00000017122	SGCG
Com_178_pos	Maltol	ENSGALG00000027070	TIMP2
Com_460_pos	3-amino-4-(propylamino)c	ENSGALG00000016475	Zp2
Com_413_pos	L-Cystine	ENSGALG00000038723	RPP25L
Com_482_pos	8-Hydroxyquinoline	ENSGALG00000034616	INHBA
Com_86_neg	Levulinic acid	MSTRG.19463	VTG2
Com_40_pos	Choline	ENSGALG00000037852	HSD17B7
Com_130_neg	2-Hydroxyvaleric acid	ENSGALG00000037253	CLEC4M
Com_108_neg	LPE 18:2	ENSGALG00000019768	ACSF2
Com_12_pos	Betaine	ENSGALG00000004804	TGM3
Com_175_pos	Pantothenic acid	ENSGALG00000041604	NPTXR
Com_40_pos	Choline	MSTRG.21092	--
Com_331_pos	L-Lysine	ENSGALG00000043829	ext1c
Com_471_pos	Indole-3-acetic acid	ENSGALG00000010628	ACSL1
Com_588_pos	Ornithine	ENSGALG00000014581	BORCS8
Com_208_neg	N-Acetylanthranilic acid	ENSGALG00000010406	TMEM63C
Com_8_neg	4-Methyl-2-Oxopentanoic	ENSGALG00000007404	YIPF5
Com_92_pos	D-(+)-Proline	ENSGALG00000001863	VTG2
Com_120_neg	LPC 15:0	ENSGALG00000025743	CDR2
Com_76_neg	Erythronolactone	ENSGALG00000004631	DRAXIN
Com_8_neg	4-Methyl-2-Oxopentanoic	MSTRG.13261	--
Com_97_pos	L-Threonine	ENSGALG00000053245	VTG2
Com_80_pos	DL-Lysine	ENSGALG00000023395	PLIN1
Com_55_pos	Valine	ENSGALG00000000645	Espn
Com_331_pos	L-Lysine	ENSGALG00000047027	ADCK5

Com_482_pos	8-Hydroxyquinoline	ENSGALG00000027122	APPL2
Com_460_pos	3-amino-4-(propylamino)(	MSTRG.8985	gag
Com_120_neg	LPC 15:0	ENSGALG00000041988	SIK1
Com_482_pos	8-Hydroxyquinoline	ENSGALG00000005030	DOCK10
Com_386_pos	2-Amino-1,3-octadecanec	ENSGALG00000046731	--
Com_16_neg	3-Hydroxybutyric acid	ENSGALG00000014463	ACTN2
Com_40_pos	Choline	ENSGALG00000027960	GRPR
Com_186_pos	4-Hydroxybenzaldehyde	ENSGALG00000003568	PPP1R16B
Com_362_pos	2-Arachidonoyl glycerol	ENSGALG00000000226	TMEM9
Com_12_pos	Betaine	ENSGALG00000041456	SLC35G1
Com_92_pos	D-(+)-Proline	ENSGALG00000016476	TTC32
Com_460_pos	3-amino-4-(propylamino)(	MSTRG.8471	--
Com_194_pos	Pipecolic acid	ENSGALG00000006076	RASGEF1C
Com_386_pos	2-Amino-1,3-octadecanec	MSTRG.3156	--
Com_482_pos	8-Hydroxyquinoline	ENSGALG00000009963	LYZ
Com_482_pos	8-Hydroxyquinoline	ENSGALG00000003136	IKZF2
Com_171_neg	LPC 22:6	ENSGALG00000042080	PGPEP1
Com_17_pos	L-Norleucine	ENSGALG00000036787	HSD17B12
Com_80_pos	DL-Lysine	ENSGALG00000036836	SOSTDC1
Com_331_pos	L-Lysine	ENSGALG00000019276	SLCO1C1
Com_413_pos	L-Cystine	MSTRG.15443	--
Com_17_pos	L-Norleucine	MSTRG.5319	--
Com_460_pos	3-amino-4-(propylamino)(	ENSGALG00000013511	ANKRA2
Com_147_pos	D-Sphingosine	ENSGALG00000029235	CPNE4
Com_8_neg	4-Methyl-2-Oxopentanoic	ENSGALG00000012823	TRIM24
Com_76_neg	Erythronolactone	ENSGALG00000012586	GKAP1
Com_76_neg	Erythronolactone	MSTRG.2305	gag
Com_97_pos	L-Threonine	ENSGALG00000010018	CTSEAL
Com_208_neg	N-Acetylanthranilic acid	ENSGALG00000012586	GKAP1
Com_186_pos	4-Hydroxybenzaldehyde	ENSGALG00000032287	ATP2A2
Com_264_pos	Indole	ENSGALG00000037065	SC5D
Com_362_pos	2-Arachidonoyl glycerol	ENSGALG00000014525	USP5
Com_208_neg	N-Acetylanthranilic acid	MSTRG.15444	--
Com_362_pos	2-Arachidonoyl glycerol	ENSGALG00000033051	CAMK1D
Com_147_pos	D-Sphingosine	ENSGALG00000013036	ATP6V1E1
Com_57_neg	LPC 16:1	ENSGALG00000054442	ITIH3
Com_178_pos	Maltol	ENSGALG00000027375	NR2C2AP
Com_17_pos	L-Norleucine	ENSGALG00000047380	MR1
Com_8_neg	4-Methyl-2-Oxopentanoic	ENSGALG00000010391	MMRN1
Com_151_pos	Pyridoxamine	MSTRG.21796	--
Com_588_pos	Ornithine	ENSGALG00000004282	RCAN3
Com_78_neg	Citric acid	ENSGALG00000035935	Unc13c
Com_40_pos	Choline	ENSGALG00000005766	PKD2L1
Com_194_pos	Pipecolic acid	ENSGALG00000010229	ABCD4
Com_16_neg	3-Hydroxybutyric acid	MSTRG.1502	gag
Com_192_pos	1-Methylhistidine	ENSGALG00000010391	MMRN1
Com_460_pos	3-amino-4-(propylamino)(	ENSGALG00000047495	LRRC10
Com_440_pos	PC (18:4e/4:0)	ENSGALG00000026203	FAM174A
Com_175_pos	Pantothenic acid	ENSGALG00000036293	EBAG9
Com_80_pos	DL-Lysine	ENSGALG00000004341	Cryzl2
Com_17_pos	L-Norleucine	ENSGALG00000026547	TPGS2
Com_120_neg	LPC 15:0	ENSGALG00000019276	SLCO1C1
Com_588_pos	Ornithine	MSTRG.17961	--
Com_16_neg	3-Hydroxybutyric acid	ENSGALG00000012877	CREB3L2
Com_40_pos	Choline	MSTRG.21536	--
Com_78_neg	Citric acid	MSTRG.13261	--
Com_151_pos	Pyridoxamine	MSTRG.8497	--
Com_208_neg	N-Acetylanthranilic acid	ENSGALG00000004631	DRAXIN

Com_78_neg	Citric acid	ENSGALG00000007404	YIPF5
Com_108_neg	LPE 18:2	ENSGALG00000047821	--
Com_208_neg	N-Acetylanthranilic acid	ENSGALG00000032645	H2A-VIII
Com_55_pos	Valine	ENSGALG00000054926	--
Com_588_pos	Ornithine	ENSGALG00000042511	PKDCC
Com_97_pos	L-Threonine	ENSGALG00000031312	ANAPC13
Com_192_pos	1-Methylhistidine	ENSGALG00000012823	TRIM24
Com_17_pos	L-Norleucine	ENSGALG00000008262	RASGRF1
Com_78_neg	Citric acid	ENSGALG00000004505	CCDC137
Com_147_pos	D-Sphingosine	MSTRG.2316	env
Com_120_neg	LPC 15:0	ENSGALG00000047027	ADCK5
Com_16_neg	3-Hydroxybutyric acid	ENSGALG00000006723	IDI1
Com_18_neg	Arachidonic acid	ENSGALG00000037773	ST3GAL1
Com_252_pos	cis-4-Hydroxy-D-proline	ENSGALG00000016610	PTRHD1
Com_22_pos	Indole-3-acrylic acid	ENSGALG00000055000	KCTD14
Com_152_pos	Acetyl-L-carnitine	ENSGALG00000026460	myoM
Com_331_pos	L-Lysine	ENSGALG00000007778	PES1
Com_265_pos	6-Methylquinoline	ENSGALG00000030661	STAT2
Com_252_pos	cis-4-Hydroxy-D-proline	ENSGALG00000029083	NXPH2
Com_21_pos	DL-Tryptophan	ENSGALG00000046412	Aoc3
Com_265_pos	6-Methylquinoline	ENSGALG00000004106	DHCR7
Com_147_pos	D-Sphingosine	ENSGALG00000015134	APOV1
Com_8_neg	4-Methyl-2-Oxopentanoic	ENSGALG00000005610	SLC44A3
Com_186_pos	4-Hydroxybenzaldehyde	ENSGALG00000000695	MFSD4A
Com_413_pos	L-Cystine	ENSGALG00000000950	MVB12B
Com_362_pos	2-Arachidonoyl glycerol	ENSGALG00000036492	DAGLA
Com_25_pos	2-Hydroxycinnamic acid	ENSGALG00000006198	LSS
Com_147_pos	D-Sphingosine	ENSGALG00000001492	NDRG3
Com_265_pos	6-Methylquinoline	ENSGALG00000012220	CDKN3
Com_362_pos	2-Arachidonoyl glycerol	MSTRG.17073	--
Com_40_pos	Choline	ENSGALG00000050083	SYCP2L
Com_152_pos	Acetyl-L-carnitine	MSTRG.9835	--
Com_192_pos	1-Methylhistidine	MSTRG.18213	--
Com_40_pos	Choline	ENSGALG00000007673	LRRC59
Com_265_pos	6-Methylquinoline	ENSGALG00000042706	TMC2
Com_17_pos	L-Norleucine	ENSGALG00000033365	ALDH1A3
Com_99_pos	Creatine	ENSGALG00000041988	SIK1
Com_147_pos	D-Sphingosine	ENSGALG00000005353	FAR1
Com_152_pos	Acetyl-L-carnitine	ENSGALG00000012847	Slc7a11
Com_40_pos	Choline	ENSGALG00000040342	ADAMTS1
Com_16_neg	3-Hydroxybutyric acid	MSTRG.14577	SLC39A5
Com_460_pos	3-amino-4-(propylamino)(	MSTRG.15162	--
Com_99_pos	Creatine	ENSGALG00000025743	CDR2
Com_152_pos	Acetyl-L-carnitine	ENSGALG00000005160	VMP1
Com_413_pos	L-Cystine	ENSGALG00000007533	NPEPL1
Com_194_pos	Pipecolic acid	ENSGALG00000042511	PKDCC
Com_152_pos	Acetyl-L-carnitine	MSTRG.2388	--
Com_92_pos	D-(+)-Proline	ENSGALG00000007492	YME1L1
Com_78_neg	Citric acid	ENSGALG00000051779	PRORSD1P
Com_171_neg	LPC 22:6	ENSGALG00000005610	SLC44A3
Com_92_pos	D-(+)-Proline	ENSGALG00000010013	EDNRA
Com_151_pos	Pyridoxamine	ENSGALG00000037935	RARA
Com_151_pos	Pyridoxamine	ENSGALG00000001918	DNAJB5
Com_265_pos	6-Methylquinoline	MSTRG.8954	--
Com_55_pos	Valine	ENSGALG00000014944	GCNT4
Com_386_pos	2-Amino-1,3-octadecanec	MSTRG.2393	--
Com_386_pos	2-Amino-1,3-octadecanec	ENSGALG00000015684	Dnajc25
Com_12_pos	Betaine	ENSGALG00000037811	NRSN1

Com_192_pos	1-Methylhistidine	MSTRG.21371	--
Com_151_pos	Pyridoxamine	ENSGALG00000003103	MST1R
Com_92_pos	D-(+)-Proline	ENSGALG00000004221	IL22RA1
Com_92_pos	D-(+)-Proline	ENSGALG00000004320	FAT2
Com_588_pos	Ornithine	ENSGALG00000010229	ABCD4
Com_264_pos	Indole	ENSGALG00000037325	SERP1
Com_12_pos	Betaine	ENSGALG00000002437	DIPK1B
Com_120_neg	LPC 15:0	ENSGALG00000051159	--
Com_386_pos	2-Amino-1,3-octadecanec	MSTRG.15732	--
Com_76_neg	Erythronolactone	ENSGALG00000008866	WDPCP
Com_386_pos	2-Amino-1,3-octadecanec	ENSGALG00000013001	CTNND2
Com_175_pos	Pantothenic acid	ENSGALG00000050676	Ctnnd2
Com_178_pos	Maltol	ENSGALG00000052072	gag
Com_215_pos	D-Erythro-sphingosine 1-p	ENSGALG00000019768	ACSF2
Com_17_pos	L-Norleucine	MSTRG.19463	VTG2
Com_252_pos	cis-4-Hydroxy-D-proline	ENSGALG00000036616	NUAK2
Com_331_pos	L-Lysine	ENSGALG00000016491	APOB
Com_68_neg	PE (16:0/22:6)	ENSGALG00000049068	ZNF831
Com_76_neg	Erythronolactone	MSTRG.1082	--
Com_97_pos	L-Threonine	ENSGALG00000003948	ALAS1
Com_92_pos	D-(+)-Proline	MSTRG.5232	PERCC1
Com_175_pos	Pantothenic acid	ENSGALG00000043336	COPZ1
Com_152_pos	Acetyl-L-carnitine	MSTRG.21371	--
Com_147_pos	D-Sphingosine	ENSGALG00000026957	SEMA4G
Com_194_pos	Pipecolic acid	ENSGALG00000040857	TECTA
Com_99_pos	Creatine	ENSGALG00000004081	TMCO4
Com_175_pos	Pantothenic acid	ENSGALG00000020342	ABHD12
Com_151_pos	Pyridoxamine	ENSGALG00000000241	STARD4
Com_76_neg	Erythronolactone	ENSGALG00000012377	HNMT
Com_78_neg	Citric acid	ENSGALG00000021658	PAFAH2
Com_482_pos	8-Hydroxyquinoline	MSTRG.10101	--
Com_311_pos	PC (18:4e/2:0)	ENSGALG00000017122	SGCG
Com_264_pos	Indole	ENSGALG00000052887	--
Com_386_pos	2-Amino-1,3-octadecanec	MSTRG.9165	--
Com_78_neg	Citric acid	ENSGALG00000042080	PGPEP1
Com_12_pos	Betaine	MSTRG.6512	--
Com_471_pos	Indole-3-acetic acid	ENSGALG00000011287	SULT
Com_588_pos	Ornithine	ENSGALG00000006076	RASGEF1C
Com_12_pos	Betaine	MSTRG.2403	--
Com_92_pos	D-(+)-Proline	ENSGALG00000030324	ELAPOR2
Com_178_pos	Maltol	ENSGALG00000047027	ADCK5
Com_362_pos	2-Arachidonoyl glycerol	ENSGALG00000001475	STMN1
Com_8_neg	4-Methyl-2-Oxopentanoic	ENSGALG00000011347	IHH
Com_147_pos	D-Sphingosine	ENSGALG00000027608	PIGC
Com_18_neg	Arachidonic acid	ENSGALG00000045557	MTTPL
Com_76_neg	Erythronolactone	ENSGALG00000004055	C7orf50
Com_265_pos	6-Methylquinoline	MSTRG.16398	Cdhr5
Com_588_pos	Ornithine	MSTRG.2170	MYO16
Com_97_pos	L-Threonine	ENSGALG00000005470	PLPPR5
Com_86_neg	Levulinic acid	MSTRG.5319	--
Com_386_pos	2-Amino-1,3-octadecanec	ENSGALG00000028191	GLCE
Com_192_pos	1-Methylhistidine	MSTRG.9835	--
Com_92_pos	D-(+)-Proline	ENSGALG00000052612	RPS27L
Com_386_pos	2-Amino-1,3-octadecanec	ENSGALG00000031754	KCNG2
Com_78_neg	Citric acid	MSTRG.21394	gag-pol
Com_8_neg	4-Methyl-2-Oxopentanoic	ENSGALG00000034504	FAM20C
Com_178_pos	Maltol	ENSGALG00000019276	SLCO1C1
Com_40_pos	Choline	ENSGALG00000050420	CTNND2

Com_68_neg	PE (16:0/22:6)	ENSGALG00000031496	SPINK5
Com_80_pos	DL-Lysine	MSTRG.1468	--
Com_152_pos	Acetyl-L-carnitine	ENSGALG00000012823	TRIM24
Com_54_pos	Uric acid	ENSGALG00000016979	SLC25A30
Com_22_pos	Indole-3-acrylic acid	ENSGALG00000013569	SEC61B
Com_130_neg	2-Hydroxyvaleric acid	ENSGALG00000031312	ANAPC13
Com_362_pos	2-Arachidonoyl glycerol	ENSGALG00000015263	TMEM30C
Com_171_neg	LPC 22:6	MSTRG.13261	--
Com_8_neg	4-Methyl-2-Oxopentanoic	ENSGALG00000014923	ARHGEF28
Com_171_neg	LPC 22:6	ENSGALG00000007404	YIPF5
Com_252_pos	cis-4-Hydroxy-D-proline	ENSGALG00000029898	YKT6
Com_25_pos	2-Hydroxycinnamic acid	ENSGALG00000054926	--
Com_588_pos	Ornithine	ENSGALG00000004425	SCAMP1
Com_331_pos	L-Lysine	ENSGALG00000027375	NR2C2AP
Com_208_neg	N-Acetylanthranilic acid	ENSGALG00000015540	RAD23B
Com_215_pos	D-Erythro-sphingosine 1-p	ENSGALG00000047821	--
Com_362_pos	2-Arachidonoyl glycerol	ENSGALG00000003575	Dnttip1
Com_147_pos	D-Sphingosine	ENSGALG00000008039	MFSD13A
Com_152_pos	Acetyl-L-carnitine	ENSGALG00000010391	MMRN1
Com_178_pos	Maltol	ENSGALG00000051123	pol
Com_471_pos	Indole-3-acetic acid	ENSGALG00000039499	LRRCC1
Com_54_pos	Uric acid	MSTRG.16505	--
Com_120_neg	LPC 15:0	MSTRG.8985	gag
Com_194_pos	Pipecolic acid	ENSGALG00000031482	Pou5f3
Com_86_neg	Levulinic acid	ENSGALG00000042491	H4-I
Com_208_neg	N-Acetylanthranilic acid	ENSGALG00000011113	SGIP1
Com_482_pos	8-Hydroxyquinoline	ENSGALG00000021848	AVD
Com_265_pos	6-Methylquinoline	ENSGALG00000037401	IDH3A
Com_151_pos	Pyridoxamine	ENSGALG00000012505	LRFN5
Com_413_pos	L-Cystine	ENSGALG00000015689	ECPAS
Com_311_pos	PC (18:4e/2:0)	ENSGALG00000026203	FAM174A
Com_440_pos	PC (18:4e/4:0)	ENSGALG00000004782	TSEN15
Com_8_neg	4-Methyl-2-Oxopentanoic	ENSGALG00000002445	KIAA0319L
Com_80_pos	DL-Lysine	ENSGALG00000027064	HIST1H3H
Com_152_pos	Acetyl-L-carnitine	ENSGALG00000005815	TMEM41B
Com_120_neg	LPC 15:0	ENSGALG00000013511	ANKRA2
Com_331_pos	L-Lysine	ENSGALG00000047495	LRRC10
Com_76_neg	Erythronolactone	ENSGALG00000037479	IGSF21
Com_21_pos	DL-Tryptophan	ENSGALG00000012254	KCNJ4
Com_86_neg	Levulinic acid	ENSGALG00000033365	ALDH1A3
Com_55_pos	Valine	ENSGALG00000004425	SCAMP1
Com_25_pos	2-Hydroxycinnamic acid	MSTRG.8619	--
Com_8_neg	4-Methyl-2-Oxopentanoic	ENSGALG00000026460	myoM
Com_362_pos	2-Arachidonoyl glycerol	ENSGALG00000051398	TMEM14C
Com_80_pos	DL-Lysine	ENSGALG00000037773	ST3GAL1
Com_76_neg	Erythronolactone	ENSGALG00000032889	RBFOX3
Com_18_neg	Arachidonic acid	ENSGALG00000014463	ACTN2
Com_57_neg	LPC 16:1	ENSGALG00000054783	NDRG1
Com_151_neg	Lysope 18:1	ENSGALG00000009100	FSHR
Com_16_neg	3-Hydroxybutyric acid	ENSGALG00000012185	PLA2G12A
Com_171_neg	LPC 22:6	ENSGALG00000051779	PRORSD1P
Com_12_pos	Betaine	ENSGALG00000013149	MOCOS
Com_482_pos	8-Hydroxyquinoline	ENSGALG00000028451	MT4
Com_86_neg	Levulinic acid	ENSGALG00000006689	ABHD2
Com_120_neg	LPC 15:0	ENSGALG00000027070	TIMP2
Com_16_neg	3-Hydroxybutyric acid	ENSGALG00000039354	VTG1
Com_413_pos	L-Cystine	ENSGALG00000016595	TRIM35
Com_8_neg	4-Methyl-2-Oxopentanoic	ENSGALG00000012847	Slc7a11

Com_130_neg	2-Hydroxyvaleric acid	ENSGALG00000003948	ALAS1
Com_460_pos	3-amino-4-(propylamino)(	ENSGALG00000019276	SLCO1C1
Com_252_pos	cis-4-Hydroxy-D-proline	ENSGALG00000038666	FBXL12
Com_18_neg	Arachidonic acid	ENSGALG00000004341	Cryzl2
Com_40_pos	Choline	ENSGALG00000010798	DHCR24
Com_21_pos	DL-Tryptophan	MSTRG.7483	--
Com_440_pos	PC (18:4e/4:0)	ENSGALG00000029235	CPNE4
Com_192_pos	1-Methylhistidine	ENSGALG00000044251	RASSF7
Com_192_pos	1-Methylhistidine	ENSGALG00000051550	ARMH4
Com_8_neg	4-Methyl-2-Oxopentanoic	ENSGALG00000005160	VMP1
Com_40_pos	Choline	ENSGALG00000037018	USP36
Com_331_pos	L-Lysine	MSTRG.8471	--
Com_8_neg	4-Methyl-2-Oxopentanoic	MSTRG.2388	--
Com_208_neg	N-Acetylanthranilic acid	ENSGALG00000004917	DOC2B
Com_386_pos	2-Amino-1,3-octadecanec	ENSGALG00000012748	ELOVL2
Com_54_pos	Uric acid	ENSGALG00000041143	UMOD
Com_78_neg	Citric acid	ENSGALG00000000226	TMEM9
Com_97_pos	L-Threonine	ENSGALG00000022758	GGACT
Com_92_pos	D-(+)-Proline	ENSGALG00000004483	AHSA2
Com_440_pos	PC (18:4e/4:0)	ENSGALG00000013036	ATP6V1E1
Com_80_pos	DL-Lysine	ENSGALG00000035244	H3-I
Com_17_pos	L-Norleucine	MSTRG.15754	--
Com_192_pos	1-Methylhistidine	ENSGALG00000004852	DNM1
Com_99_pos	Creatine	ENSGALG00000014935	GREB1L
Com_192_pos	1-Methylhistidine	ENSGALG00000004483	AHSA2
Com_482_pos	8-Hydroxyquinoline	ENSGALG00000011894	CYP2D6
Com_55_pos	Valine	ENSGALG00000006076	RASGEF1C
Com_386_pos	2-Amino-1,3-octadecanec	ENSGALG00000006702	MFGE8
Com_460_pos	3-amino-4-(propylamino)(	ENSGALG00000047027	ADCK5
Com_76_neg	Erythronolactone	MSTRG.16504	gag
Com_413_pos	L-Cystine	ENSGALG00000048223	FAM20C
Com_208_neg	N-Acetylanthranilic acid	ENSGALG00000036117	TENT5B
Com_171_neg	LPC 22:6	ENSGALG00000021658	PAFAH2
Com_331_pos	L-Lysine	ENSGALG00000052072	gag
Com_130_neg	2-Hydroxyvaleric acid	ENSGALG00000005470	PLPPR5
Com_178_pos	Maltol	MSTRG.8501	--
Com_12_pos	Betaine	MSTRG.8501	--
Com_92_pos	D-(+)-Proline	ENSGALG00000008862	DNAJC10
Com_311_pos	PC (18:4e/2:0)	ENSGALG00000030845	ENHO
Com_97_pos	L-Threonine	ENSGALG00000005739	SCD
Com_78_neg	Citric acid	ENSGALG00000014525	USP5
Com_21_pos	DL-Tryptophan	ENSGALG00000014189	SULT4A1
Com_78_neg	Citric acid	ENSGALG00000033051	CAMK1D
Com_18_neg	Arachidonic acid	ENSGALG00000036836	SOSTDC1
Com_264_pos	Indole	MSTRG.1841	--
Com_331_pos	L-Lysine	ENSGALG00000016475	Zp2
Com_362_pos	2-Arachidonoyl glycerol	ENSGALG00000011254	SATB1
Com_22_pos	Indole-3-acrylic acid	ENSGALG00000052887	--
Com_55_pos	Valine	ENSGALG00000002500	GMPPB
Com_17_pos	L-Norleucine	ENSGALG00000031067	TMEM132A
Com_265_pos	6-Methylquinoline	ENSGALG00000032329	NPM3
Com_152_pos	Acetyl-L-carnitine	ENSGALG00000016476	TTC32
Com_97_pos	L-Threonine	ENSGALG00000012882	KDSR
Com_54_pos	Uric acid	ENSGALG00000007234	CLCN5
Com_120_neg	LPC 15:0	MSTRG.15162	--
Com_17_pos	L-Norleucine	ENSGALG00000000104	CRY1
Com_362_pos	2-Arachidonoyl glycerol	ENSGALG00000035935	Unc13c
Com_171_neg	LPC 22:6	MSTRG.21394	gag-pol

Com_16_neg	3-Hydroxybutyric acid	ENSGALG00000037773	ST3GAL1
Com_55_pos	Valine	ENSGALG00000010229	ABCD4
Com_108_neg	LPE 18:2	ENSGALG00000040995	NEB
Com_440_pos	PC (18:4e/4:0)	MSTRG.2316	env
Com_171_neg	LPC 22:6	ENSGALG00000037018	USP36
Com_413_pos	L-Cystine	ENSGALG00000042511	PKDCC
Com_25_pos	2-Hydroxycinnamic acid	ENSGALG00000031482	Pou5f3
Com_40_pos	Choline	ENSGALG00000040969	PTP4A3
Com_152_pos	Acetyl-L-carnitine	ENSGALG00000011347	IHH
Com_440_pos	PC (18:4e/4:0)	ENSGALG00000015134	APOV1
Com_215_pos	D-Erythro-sphingosine 1-p	ENSGALG00000009100	FSHR
Com_588_pos	Ornithine	ENSGALG00000014944	GCNT4
Com_86_neg	Levulinic acid	MSTRG.13439	--
Com_147_pos	D-Sphingosine	ENSGALG00000014976	GATA6
Com_25_pos	2-Hydroxycinnamic acid	ENSGALG00000016665	FDFT1
Com_147_pos	D-Sphingosine	ENSGALG00000042555	STAMBP
Com_194_pos	Pipecolic acid	ENSGALG00000054926	--
Com_178_pos	Maltol	ENSGALG00000013149	MOCOS
Com_362_pos	2-Arachidonoyl glycerol	ENSGALG00000004505	CCDC137
Com_440_pos	PC (18:4e/4:0)	ENSGALG00000005353	FAR1
Com_482_pos	8-Hydroxyquinoline	ENSGALG00000005263	SOX8
Com_194_pos	Pipecolic acid	ENSGALG00000000950	MVB12B
Com_152_pos	Acetyl-L-carnitine	ENSGALG00000034504	FAM20C
Com_264_pos	Indole	ENSGALG00000013569	SEC61B
Com_208_neg	N-Acetylanthranilic acid	ENSGALG00000004249	GRHL3
Com_252_pos	cis-4-Hydroxy-D-proline	ENSGALG00000008763	SSX2IP
Com_192_pos	1-Methylhistidine	ENSGALG00000019738	FBXO47
Com_108_neg	LPE 18:2	ENSGALG00000032903	RTN4RL2
Com_186_pos	4-Hydroxybenzaldehyde	ENSGALG00000010009	TTC29
Com_17_pos	L-Norleucine	ENSGALG00000042491	H4-I
Com_588_pos	Ornithine	ENSGALG00000007533	NPEPL1
Com_152_pos	Acetyl-L-carnitine	ENSGALG00000014923	ARHGEF28
Com_208_neg	N-Acetylanthranilic acid	MSTRG.2174	Irs2
Com_92_pos	D-(+)-Proline	ENSGALG00000015729	LPAR1
Com_119_pos	DL-Stachydrine	ENSGALG00000053278	SUCNR1
Com_192_pos	1-Methylhistidine	ENSGALG00000000745	SLC26A9
Com_175_pos	Pantothenic acid	ENSGALG00000016885	STK24
Com_331_pos	L-Lysine	ENSGALG00000051123	pol
Com_208_neg	N-Acetylanthranilic acid	ENSGALG00000045288	CAMK2N1
Com_92_pos	D-(+)-Proline	MSTRG.2387	--
Com_331_pos	L-Lysine	MSTRG.21204	--
Com_76_neg	Erythronolactone	ENSGALG00000051205	nicA
Com_55_pos	Valine	ENSGALG00000042511	PKDCC
Com_12_pos	Betaine	ENSGALG00000051123	pol
Com_413_pos	L-Cystine	ENSGALG00000010229	ABCD4
Com_203_pos	Serotonin	ENSGALG00000055021	GREM2
Com_252_pos	cis-4-Hydroxy-D-proline	MSTRG.7483	--
Com_55_pos	Valine	ENSGALG00000008763	SSX2IP
Com_18_neg	Arachidonic acid	MSTRG.4702	--
Com_21_pos	DL-Tryptophan	ENSGALG00000038666	FBXL12
Com_208_neg	N-Acetylanthranilic acid	ENSGALG00000012589	C9orf64
Com_152_pos	Acetyl-L-carnitine	ENSGALG00000002445	KIAA0319L
Com_25_pos	2-Hydroxycinnamic acid	ENSGALG00000040857	TECTA
Com_186_pos	4-Hydroxybenzaldehyde	ENSGALG00000009483	MARK1
Com_92_pos	D-(+)-Proline	MSTRG.9835	--
Com_178_pos	Maltol	ENSGALG00000047495	LRRC10
Com_12_pos	Betaine	ENSGALG00000003029	PLPP6
Com_8_neg	4-Methyl-2-Oxopentanoic	ENSGALG00000005815	TMEM41B

Com_311_pos	PC (18:4e/2:0)	ENSGALG00000004782	TSEN15
Com_17_pos	L-Norleucine	ENSGALG00000006689	ABHD2
Com_147_pos	D-Sphingosine	MSTRG.3156	--
Com_331_pos	L-Lysine	ENSGALG00000007848	PTS
Com_99_pos	Creatine	ENSGALG00000005418	FRRS1
Com_192_pos	1-Methylhistidine	ENSGALG00000030324	ELAPOR2
Com_99_pos	Creatine	MSTRG.6228	--
Com_130_neg	2-Hydroxyvaleric acid	ENSGALG00000022758	GGACT
Com_471_pos	Indole-3-acetic acid	ENSGALG00000028928	LCAT
Com_194_pos	Pipecolic acid	MSTRG.15443	--
Com_208_neg	N-Acetylanthranilic acid	ENSGALG00000012186	CASP6
Com_171_neg	LPC 22:6	ENSGALG00000050420	CTNND2
Com_76_neg	Erythronolactone	ENSGALG00000031525	TSTA3
Com_413_pos	L-Cystine	ENSGALG00000006076	RASGEF1C
Com_171_neg	LPC 22:6	ENSGALG00000000226	TMEM9
Com_192_pos	1-Methylhistidine	MSTRG.5232	PERCC1
Com_18_neg	Arachidonic acid	MSTRG.149	--
Com_362_pos	2-Arachidonoyl glycerol	ENSGALG00000042080	PGPEP1
Com_130_neg	2-Hydroxyvaleric acid	ENSGALG00000005739	SCD
Com_362_pos	2-Arachidonoyl glycerol	ENSGALG00000031525	TSTA3
Com_22_pos	Indole-3-acrylic acid	MSTRG.4813	--
Com_86_neg	Levulinic acid	MSTRG.15754	--
Com_78_neg	Citric acid	ENSGALG00000001475	STMN1
Com_208_neg	N-Acetylanthranilic acid	ENSGALG00000007030	MFSD13A
Com_130_neg	2-Hydroxyvaleric acid	ENSGALG00000014464	MTR
Com_252_pos	cis-4-Hydroxy-D-proline	ENSGALG00000002500	GMPPB
Com_178_pos	Maltol	MSTRG.8471	--
Com_25_pos	2-Hydroxycinnamic acid	ENSGALG00000035675	--
Com_178_pos	Maltol	MSTRG.6512	--
Com_171_neg	LPC 22:6	ENSGALG00000014525	USP5
Com_192_pos	1-Methylhistidine	ENSGALG00000004320	FAT2
Com_192_pos	1-Methylhistidine	ENSGALG00000004221	IL22RA1
Com_413_pos	L-Cystine	ENSGALG00000004170	ADA
Com_171_neg	LPC 22:6	ENSGALG00000033051	CAMK1D
Com_130_neg	2-Hydroxyvaleric acid	ENSGALG00000012882	KDSR
Com_311_pos	PC (18:4e/2:0)	ENSGALG00000029235	CPNE4
Com_99_pos	Creatine	ENSGALG00000032440	QPCT
Com_92_pos	D-(+)-Proline	MSTRG.21371	--
Com_588_pos	Ornithine	ENSGALG00000000645	Espn
Com_194_pos	Pipecolic acid	ENSGALG00000038723	RPP25L
Com_18_neg	Arachidonic acid	ENSGALG00000004322	AHR
Com_192_pos	1-Methylhistidine	ENSGALG00000010013	EDNRA
Com_311_pos	PC (18:4e/2:0)	ENSGALG00000013036	ATP6V1E1
Com_21_pos	DL-Tryptophan	ENSGALG00000029898	YKT6
Com_22_pos	Indole-3-acrylic acid	MSTRG.1841	--
Com_186_pos	4-Hydroxybenzaldehyde	MSTRG.15162	--
Com_192_pos	1-Methylhistidine	ENSGALG00000007492	YME1L1
Com_331_pos	L-Lysine	MSTRG.8501	--
Com_413_pos	L-Cystine	ENSGALG00000004425	SCAMP1
Com_86_neg	Levulinic acid	ENSGALG00000031067	TMEM132A
Com_175_pos	Pantothenic acid	MSTRG.2387	--
Com_186_pos	4-Hydroxybenzaldehyde	ENSGALG00000000003	PANX2
Com_12_pos	Betaine	ENSGALG00000052072	gag
Com_86_neg	Levulinic acid	ENSGALG00000000104	CRY1
Com_78_neg	Citric acid	ENSGALG00000037018	USP36
Com_92_pos	D-(+)-Proline	ENSGALG00000016885	STK24
Com_80_pos	DL-Lysine	MSTRG.14577	SLC39A5
Com_178_pos	Maltol	ENSGALG00000016475	Zp2

Com_175_pos	Pantothenic acid	ENSGALG00000015729	LPAR1
Com_460_pos	3-amino-4-(propylamino)(	ENSGALG00000027070	TIMP2
Com_215_pos	D-Erythro-sphingosine 1-p	ENSGALG00000040995	NEB
Com_440_pos	PC (18:4e/4:0)	ENSGALG00000030845	ENHO
Com_108_neg	LPE 18:2	ENSGALG00000009100	FSHR
Com_152_pos	Acetyl-L-carnitine	ENSGALG00000052612	RPS27L
Com_17_pos	L-Norleucine	ENSGALG00000002802	PACSIN1
Com_386_pos	2-Amino-1,3-octadecanec	ENSGALG00000001492	NDRG3
Com_17_pos	L-Norleucine	MSTRG.13439	--
Com_203_pos	Serotonin	ENSGALG00000038740	AMY2A
Com_18_neg	Arachidonic acid	MSTRG.1503	gag
Com_194_pos	Pipecolic acid	ENSGALG00000012112	DBI
Com_460_pos	3-amino-4-(propylamino)(	ENSGALG00000000695	MFSD4A
Com_8_neg	4-Methyl-2-Oxopentanoic	ENSGALG00000016476	TTC32
Com_588_pos	Ornithine	ENSGALG00000015689	ECPAS
Com_8_neg	4-Methyl-2-Oxopentanoic	ENSGALG00000033591	Kazald1
Com_16_neg	3-Hydroxybutyric acid	ENSGALG00000015333	PCGF3
Com_86_neg	Levulinic acid	ENSGALG00000015016	SLC22A15
Com_16_neg	3-Hydroxybutyric acid	ENSGALG00000029015	TM6SF2
Com_80_pos	DL-Lysine	ENSGALG00000006723	IDI1
Com_99_pos	Creatine	ENSGALG00000005696	ABHD6
Com_18_neg	Arachidonic acid	ENSGALG00000012185	PLA2G12A
Com_92_pos	D-(+)-Proline	ENSGALG00000012823	TRIM24
Com_311_pos	PC (18:4e/2:0)	MSTRG.2316	env
Com_130_neg	2-Hydroxyvaleric acid	ENSGALG00000054297	SND1
Com_171_neg	LPC 22:6	ENSGALG00000040342	ADAMTS1
Com_151_pos	Pyridoxamine	MSTRG.3473	--
Com_208_neg	N-Acetylanthranilic acid	MSTRG.11955	--
Com_18_neg	Arachidonic acid	ENSGALG00000039354	VTG1
Com_471_pos	Indole-3-acetic acid	ENSGALG00000038923	Ces1e
Com_265_pos	6-Methylquinoline	ENSGALG00000006842	ACOT8
Com_311_pos	PC (18:4e/2:0)	ENSGALG00000015134	APOV1
Com_331_pos	L-Lysine	ENSGALG00000013149	MOCOS
Com_331_pos	L-Lysine	ENSGALG00000041296	SOX7
Com_147_pos	D-Sphingosine	MSTRG.2393	--
Com_482_pos	8-Hydroxyquinoline	ENSGALG00000029724	MTURN
Com_171_neg	LPC 22:6	ENSGALG00000007673	LRRC59
Com_471_pos	Indole-3-acetic acid	ENSGALG00000016281	DMD
Com_215_pos	D-Erythro-sphingosine 1-p	ENSGALG00000032903	RTN4RL2
Com_80_pos	DL-Lysine	ENSGALG00000012877	CREB3L2
Com_482_pos	8-Hydroxyquinoline	MSTRG.29	SHANK3
Com_588_pos	Ornithine	ENSGALG00000041533	SLC11A2
Com_99_pos	Creatine	ENSGALG00000030357	ABL2
Com_151_pos	Pyridoxamine	ENSGALG00000035626	DAD1
Com_108_neg	LPE 18:2	ENSGALG00000004491	DMGDH
Com_16_neg	3-Hydroxybutyric acid	ENSGALG00000004341	Cryzl2
Com_78_neg	Citric acid	ENSGALG00000051398	TMEM14C
Com_92_pos	D-(+)-Proline	ENSGALG00000010391	MMRN1
Com_311_pos	PC (18:4e/2:0)	ENSGALG00000005353	FAR1
Com_151_pos	Pyridoxamine	ENSGALG00000005839	ARID3C
Com_147_pos	D-Sphingosine	MSTRG.15732	--
Com_80_pos	DL-Lysine	MSTRG.1502	gag
Com_147_pos	D-Sphingosine	ENSGALG00000013001	CTNND2
Com_151_pos	Pyridoxamine	ENSGALG00000052986	Vwa5b2
Com_588_pos	Ornithine	ENSGALG00000016595	TRIM35
Com_413_pos	L-Cystine	ENSGALG00000028822	RNF152
Com_16_neg	3-Hydroxybutyric acid	ENSGALG00000034716	HEY2
Com_386_pos	2-Amino-1,3-octadecanec	ENSGALG00000026957	SEMA4G

Com_76_neg	Erythronolactone	MSTRG.18132	FBXW4
Com_460_pos	3-amino-4-(propylamino)(	ENSGALG00000032287	ATP2A2
Com_80_pos	DL-Lysine	ENSGALG00000014126	endou-a
Com_265_pos	6-Methylquinoline	ENSGALG00000040620	LSAMP
Com_175_pos	Pantothenic acid	ENSGALG00000007507	MASTL
Com_331_pos	L-Lysine	ENSGALG00000005648	Sesn3
Com_178_pos	Maltol	MSTRG.21204	--
Com_99_pos	Creatine	ENSGALG00000016254	OTC
Com_175_pos	Pantothenic acid	ENSGALG00000008862	DNAJC10
Com_171_neg	LPC 22:6	MSTRG.21536	--
Com_76_neg	Erythronolactone	ENSGALG00000011254	SATB1
Com_186_pos	4-Hydroxybenzaldehyde	ENSGALG00000013511	ANKRA2
Com_264_pos	Indole	ENSGALG00000015728	MUSK
Com_130_neg	2-Hydroxyvaleric acid	ENSGALG00000010641	SCCPDH
Com_413_pos	L-Cystine	ENSGALG00000004729	SLC7A10
Com_16_neg	3-Hydroxybutyric acid	ENSGALG00000036836	SOSTDC1
Com_588_pos	Ornithine	ENSGALG00000048223	FAM20C
Com_76_neg	Erythronolactone	ENSGALG00000028294	Vwa5b2
Com_76_neg	Erythronolactone	ENSGALG00000004216	TOR3A
Com_12_pos	Betaine	ENSGALG00000027375	NR2C2AP
Com_386_pos	2-Amino-1,3-octadecanec	ENSGALG00000027608	PIGC
Com_362_pos	2-Arachidonoyl glycerol	MSTRG.16504	gag
Com_265_pos	6-Methylquinoline	ENSGALG00000055000	KCTD14
Com_25_pos	2-Hydroxycinnamic acid	ENSGALG00000016610	PTRHD1
Com_78_neg	Citric acid	ENSGALG00000050420	CTNND2
Com_460_pos	3-amino-4-(propylamino)(	ENSGALG00000003568	PPP1R16B
Com_208_neg	N-Acetylanthranilic acid	ENSGALG00000053659	--
Com_147_pos	D-Sphingosine	ENSGALG00000028191	GLCE
Com_97_pos	L-Threonine	ENSGALG00000014464	MTR
Com_22_pos	Indole-3-acrylic acid	ENSGALG00000030661	STAT2
Com_151_pos	Pyridoxamine	ENSGALG00000002579	RIMBP2
Com_22_pos	Indole-3-acrylic acid	ENSGALG00000004106	DHCR7
Com_186_pos	4-Hydroxybenzaldehyde	MSTRG.8985	gag
Com_97_pos	L-Threonine	ENSGALG00000010978	ANGPTL3
Com_178_pos	Maltol	ENSGALG00000007848	PTS
Com_4_pos	PC (17:1/17:1)	ENSGALG00000002549	RGS1
Com_171_neg	LPC 22:6	ENSGALG00000001475	STMN1
Com_192_pos	1-Methylhistidine	ENSGALG00000001863	VTG2
Com_186_pos	4-Hydroxybenzaldehyde	ENSGALG00000010233	SYNDIG1L
Com_78_neg	Citric acid	ENSGALG00000011254	SATB1
Com_331_pos	L-Lysine	MSTRG.12923	--
Com_252_pos	cis-4-Hydroxy-D-proline	ENSGALG00000046412	Aoc3
Com_440_pos	PC (18:4e/4:0)	ENSGALG00000014976	GATA6
Com_413_pos	L-Cystine	ENSGALG00000015136	ILDR1
Com_40_pos	Choline	ENSGALG00000042080	PGPEP1
Com_17_pos	L-Norleucine	ENSGALG00000012112	DBI
Com_21_pos	DL-Tryptophan	ENSGALG00000016610	PTRHD1
Com_151_pos	Pyridoxamine	ENSGALG00000006530	TSSC4
Com_264_pos	Indole	MSTRG.4813	--
Com_588_pos	Ornithine	ENSGALG00000038532	--
Com_440_pos	PC (18:4e/4:0)	ENSGALG00000042555	STAMBP
Com_194_pos	Pipecolic acid	ENSGALG00000002802	PACSIN1
Com_194_pos	Pipecolic acid	ENSGALG00000002500	GMPPB
Com_178_pos	Maltol	ENSGALG00000041456	SLC35G1
Com_192_pos	1-Methylhistidine	ENSGALG00000037769	NEBL
Com_413_pos	L-Cystine	ENSGALG00000014944	GCNT4
Com_120_neg	LPC 15:0	ENSGALG00000003427	USP3
Com_311_pos	PC (18:4e/2:0)	MSTRG.13135	--

Com_130_neg	2-Hydroxyvaleric acid	ENSGALG00000010293	RBP
Com_171_neg	LPC 22:6	ENSGALG00000027960	GRPR
Com_97_pos	L-Threonine	ENSGALG00000009680	PAQR7
Com_22_pos	Indole-3-acrylic acid	MSTRG.8954	--
Com_21_pos	DL-Tryptophan	ENSGALG00000009538	RDH12
Com_25_pos	2-Hydroxycinnamic acid	ENSGALG00000007723	NAT9
Com_311_pos	PC (18:4e/2:0)	ENSGALG00000002899	AACS
Com_386_pos	2-Amino-1,3-octadecanec	ENSGALG00000008039	MFSD13A
Com_331_pos	L-Lysine	MSTRG.6512	--
Com_76_neg	Erythronolactone	ENSGALG00000051398	TMEM14C
Com_86_neg	Levulinic acid	ENSGALG00000012196	MCUB
Com_471_pos	Indole-3-acetic acid	ENSGALG00000007014	PYROXD2
Com_151_pos	Pyridoxamine	ENSGALG00000010294	RPS6KL1
Com_86_neg	Levulinic acid	ENSGALG00000048432	--
Com_152_pos	Acetyl-L-carnitine	ENSGALG00000008862	DNAJC10
Com_21_pos	DL-Tryptophan	ENSGALG00000007493	NSDHL
Com_99_pos	Creatine	ENSGALG00000010708	ICA1
Com_55_pos	Valine	ENSGALG00000036616	NUAK2
Com_17_pos	L-Norleucine	ENSGALG00000038723	RPP25L
Com_460_pos	3-amino-4-(propylamino)(	ENSGALG00000030076	PCSK6
Com_99_pos	Creatine	MSTRG.18216	--
Com_8_neg	4-Methyl-2-Oxopentanoic	MSTRG.11955	--
Com_16_neg	3-Hydroxybutyric acid	MSTRG.12629	--
Com_16_neg	3-Hydroxybutyric acid	MSTRG.20167	--
Com_55_pos	Valine	ENSGALG00000000950	MVB12B
Com_12_pos	Betaine	ENSGALG00000016491	APOB
Com_86_neg	Levulinic acid	ENSGALG00000002802	PACSIN1
Com_97_pos	L-Threonine	ENSGALG00000054297	SND1
Com_175_pos	Pantothenic acid	ENSGALG00000036754	CHKA
Com_57_neg	LPC 16:1	ENSGALG00000009100	FSHR
Com_352_pos	Riboflavin	ENSGALG00000012420	CG-1B
Com_208_neg	N-Acetylanthranilic acid	ENSGALG00000033591	Kazald1
Com_192_pos	1-Methylhistidine	ENSGALG00000012944	DENND5B
Com_17_pos	L-Norleucine	ENSGALG00000015016	SLC22A15
Com_92_pos	D-(+)-Proline	ENSGALG00000011347	IHH
Com_16_neg	3-Hydroxybutyric acid	MSTRG.4702	--
Com_264_pos	Indole	ENSGALG00000029857	Gimap1
Com_78_neg	Citric acid	ENSGALG00000040342	ADAMTS1
Com_8_neg	4-Methyl-2-Oxopentanoic	ENSGALG00000052612	RPS27L
Com_186_pos	4-Hydroxybenzaldehyde	ENSGALG00000051159	--
Com_22_pos	Indole-3-acrylic acid	MSTRG.16398	Cdhr5
Com_78_neg	Citric acid	ENSGALG00000007673	LRRC59
Com_413_pos	L-Cystine	ENSGALG00000036787	HSD17B12
Com_208_neg	N-Acetylanthranilic acid	MSTRG.11834	--
Com_440_pos	PC (18:4e/4:0)	MSTRG.3156	--
Com_92_pos	D-(+)-Proline	ENSGALG00000034504	FAM20C
Com_152_pos	Acetyl-L-carnitine	ENSGALG00000033591	Kazald1
Com_17_pos	L-Norleucine	MSTRG.15443	--
Com_482_pos	8-Hydroxyquinoline	ENSGALG00000002479	MAT1A
Com_12_pos	Betaine	ENSGALG00000037261	RFXANK
Com_171_neg	LPC 22:6	ENSGALG00000051398	TMEM14C
Com_311_pos	PC (18:4e/2:0)	ENSGALG00000051466	NDFIP2
Com_215_pos	D-Erythro-sphingosine 1-|	ENSGALG00000004491	DMGDH
Com_80_pos	DL-Lysine	ENSGALG00000038532	--
Com_54_pos	Uric acid	MSTRG.19672	--
Com_194_pos	Pipecolic acid	ENSGALG00000008763	SSX2IP
Com_55_pos	Valine	ENSGALG00000029083	NXPH2
Com_178_pos	Maltol	ENSGALG00000041296	SOX7

Com_40_pos	Choline	ENSGALG00000004505	CCDC137
Com_175_pos	Pantothenic acid	ENSGALG00000052612	RPS27L
Com_16_neg	3-Hydroxybutyric acid	ENSGALG00000014581	BORCS8
Com_178_pos	Maltol	ENSGALG00000026846	JMJD7
Com_16_neg	3-Hydroxybutyric acid	MSTRG.149	--
Com_92_pos	D-(+)-Proline	ENSGALG00000014923	ARHGEF28
Com_97_pos	L-Threonine	ENSGALG00000010641	SCCPDH
Com_413_pos	L-Cystine	ENSGALG00000047380	MR1
Com_152_pos	Acetyl-L-carnitine	ENSGALG00000015729	LPAR1
Com_264_pos	Indole	ENSGALG00000002362	MANF
Com_12_pos	Betaine	ENSGALG00000007778	PES1
Com_78_neg	Citric acid	MSTRG.21536	--
Com_54_pos	Uric acid	ENSGALG00000040969	PTP4A3
Com_152_pos	Acetyl-L-carnitine	MSTRG.2387	--
Com_25_pos	2-Hydroxycinnamic acid	ENSGALG00000029898	YKT6
Com_171_neg	LPC 22:6	ENSGALG00000023338	CBX2
Com_252_pos	cis-4-Hydroxy-D-proline	ENSGALG00000054926	--
Com_55_pos	Valine	MSTRG.15443	--
Com_76_neg	Erythronolactone	ENSGALG00000001475	STMN1
Com_413_pos	L-Cystine	ENSGALG00000026547	TPGS2
Com_80_pos	DL-Lysine	ENSGALG00000016511	ADGRG2
Com_92_pos	D-(+)-Proline	ENSGALG00000020342	ABHD12
Com_151_pos	Pyridoxamine	ENSGALG00000009680	PAQR7
Com_40_pos	Choline	ENSGALG00000035935	Unc13c
Com_120_neg	LPC 15:0	ENSGALG00000000695	MFSD4A
Com_130_neg	2-Hydroxyvaleric acid	ENSGALG00000010978	ANGPTL3
Com_362_pos	2-Arachidonoyl glycerol	ENSGALG00000012377	HNMT
Com_178_pos	Maltol	MSTRG.8381	--
Com_16_neg	3-Hydroxybutyric acid	ENSGALG00000004322	AHR
Com_22_pos	Indole-3-acrylic acid	ENSGALG00000015728	MUSK
Com_92_pos	D-(+)-Proline	ENSGALG00000043336	COPZ1
Com_78_neg	Citric acid	ENSGALG00000031525	TSTA3
Com_178_pos	Maltol	ENSGALG00000005648	Sesn3
Com_92_pos	D-(+)-Proline	ENSGALG00000002445	KIAA0319L
Com_192_pos	1-Methylhistidine	ENSGALG00000054746	--
Com_460_pos	3-amino-4-(propylamino)(	ENSGALG00000002707	CHRNB2
Com_413_pos	L-Cystine	ENSGALG00000008262	RASGRF1
Com_588_pos	Ornithine	ENSGALG00000004170	ADA
Com_21_pos	DL-Tryptophan	ENSGALG00000035675	--
Com_8_neg	4-Methyl-2-Oxopentanoic	ENSGALG00000012186	CASP6
Com_331_pos	L-Lysine	ENSGALG00000001749	ACSBG2
Com_460_pos	3-amino-4-(propylamino)(	ENSGALG00000050258	--
Com_92_pos	D-(+)-Proline	ENSGALG00000050676	Ctnnd2
Com_215_pos	D-Erythro-sphingosine 1-p	ENSGALG00000054783	NDRG1
Com_362_pos	2-Arachidonoyl glycerol	MSTRG.1082	--
Com_413_pos	L-Cystine	ENSGALG00000000645	Espn
Com_460_pos	3-amino-4-(propylamino)(	ENSGALG00000008784	SPATA1
Com_264_pos	Indole	ENSGALG00000030661	STAT2
Com_264_pos	Indole	ENSGALG00000004106	DHCR7
Com_86_neg	Levulinic acid	MSTRG.21321	gag
Com_362_pos	2-Arachidonoyl glycerol	ENSGALG00000008866	WDPCP
Com_171_neg	LPC 22:6	ENSGALG00000011254	SATB1
Com_68_neg	PE (16:0/22:6)	ENSGALG00000000378	SLC25A37
Com_17_pos	L-Norleucine	ENSGALG00000000950	MVB12B
Com_151_pos	Pyridoxamine	ENSGALG00000010978	ANGPTL3
Com_97_pos	L-Threonine	ENSGALG00000010293	RBP
Com_16_neg	3-Hydroxybutyric acid	ENSGALG00000004282	RCAN3
Com_130_neg	2-Hydroxyvaleric acid	ENSGALG00000009680	PAQR7

Com_147_pos	D-Sphingosine	ENSGALG00000030845	ENHO
Com_55_pos	Valine	ENSGALG00000038723	RPP25L
Com_588_pos	Ornithine	MSTRG.1502	gag
Com_16_neg	3-Hydroxybutyric acid	MSTRG.1503	gag
Com_130_neg	2-Hydroxyvaleric acid	ENSGALG00000046789	pol
Com_252_pos	cis-4-Hydroxy-D-proline	ENSGALG00000005888	PGP
Com_192_pos	1-Methylhistidine	ENSGALG00000035478	FAM91A1
Com_18_neg	Arachidonic acid	ENSGALG00000015333	PCGF3
Com_86_neg	Levulinic acid	ENSGALG00000012112	DBI
Com_16_neg	3-Hydroxybutyric acid	MSTRG.17961	--
Com_331_pos	L-Lysine	MSTRG.11802	TMEM221
Com_80_pos	DL-Lysine	ENSGALG00000041533	SLC11A2
Com_18_neg	Arachidonic acid	ENSGALG00000029015	TM6SF2
Com_588_pos	Ornithine	ENSGALG00000012877	CREB3L2
Com_99_pos	Creatine	ENSGALG00000040363	ABHD4
Com_78_neg	Citric acid	ENSGALG00000027960	GRPR
Com_362_pos	2-Arachidonoyl glycerol	ENSGALG00000037018	USP36
Com_178_pos	Maltol	MSTRG.12923	--
Com_54_pos	Uric acid	ENSGALG00000010798	DHCR24
Com_331_pos	L-Lysine	ENSGALG00000021685	SERINC2
Com_8_neg	4-Methyl-2-Oxopentanoic	ENSGALG00000045288	CAMK2N1
Com_18_neg	Arachidonic acid	MSTRG.15507	--
Com_194_pos	Pipecolic acid	ENSGALG00000000104	CRY1
Com_120_neg	LPC 15:0	ENSGALG00000032287	ATP2A2
Com_25_pos	2-Hydroxycinnamic acid	ENSGALG00000038666	FBXL12
Com_194_pos	Pipecolic acid	ENSGALG00000031067	TMEM132A
Com_264_pos	Indole	MSTRG.8954	--
Com_8_neg	4-Methyl-2-Oxopentanoic	MSTRG.2174	Irs2
Com_331_pos	L-Lysine	ENSGALG00000041456	SLC35G1
Com_17_pos	L-Norleucine	ENSGALG00000012196	MCUB
Com_471_pos	Indole-3-acetic acid	MSTRG.4548	--
Com_471_pos	Indole-3-acetic acid	ENSGALG00000031122	NTNG1
Com_17_pos	L-Norleucine	ENSGALG00000048432	--
Com_588_pos	Ornithine	ENSGALG00000006723	IDI1
Com_440_pos	PC (18:4e/4:0)	MSTRG.2393	--
Com_311_pos	PC (18:4e/2:0)	ENSGALG00000014976	GATA6
Com_55_pos	Valine	ENSGALG00000012112	DBI
Com_18_neg	Arachidonic acid	ENSGALG00000014813	HOMER1
Com_311_pos	PC (18:4e/2:0)	ENSGALG00000042555	STAMBP
Com_86_neg	Levulinic acid	ENSGALG00000038723	RPP25L
Com_99_pos	Creatine	ENSGALG00000012414	GNPNAT1
Com_413_pos	L-Cystine	MSTRG.19463	VTG2
Com_440_pos	PC (18:4e/4:0)	MSTRG.15732	--
Com_460_pos	3-amino-4-(propylamino)c	ENSGALG00000042374	PDE11A
Com_440_pos	PC (18:4e/4:0)	MSTRG.13135	--
Com_440_pos	PC (18:4e/4:0)	ENSGALG00000013001	CTNND2
Com_12_pos	Betaine	ENSGALG00000043829	ext1c
Com_80_pos	DL-Lysine	ENSGALG00000014906	MOCS2
Com_120_neg	LPC 15:0	ENSGALG00000003568	PPP1R16B
Com_413_pos	L-Cystine	ENSGALG00000041533	SLC11A2
Com_152_pos	Acetyl-L-carnitine	ENSGALG00000016885	STK24
Com_68_neg	PE (16:0/22:6)	ENSGALG00000012834	AKR1D1
Com_8_neg	4-Methyl-2-Oxopentanoic	ENSGALG00000008862	DNAJC10
Com_440_pos	PC (18:4e/4:0)	ENSGALG00000002899	AACS
Com_186_pos	4-Hydroxybenzaldehyde	ENSGALG00000041988	SIK1
Com_18_neg	Arachidonic acid	ENSGALG00000034716	HEY2
Com_97_pos	L-Threonine	ENSGALG00000035626	DAD1
Com_192_pos	1-Methylhistidine	ENSGALG00000012111	TMEM37

Com_12_pos	Betaine	MSTRG.20656	--
Com_588_pos	Ornithine	MSTRG.14577	SLC39A5
Com_54_pos	Uric acid	ENSGALG00000021039	HKDC1
Com_194_pos	Pipecolic acid	MSTRG.15754	--
Com_18_neg	Arachidonic acid	ENSGALG00000014233	FBLN1
Com_171_neg	LPC 22:6	MSTRG.8810	--
Com_186_pos	4-Hydroxybenzaldehyde	ENSGALG00000025743	CDR2
Com_171_neg	LPC 22:6	ENSGALG00000015768	ANKRD6
Com_152_pos	Acetyl-L-carnitine	MSTRG.11955	--
Com_460_pos	3-amino-4-(propylamino)(	ENSGALG00000007012	ARRDC4
Com_175_pos	Pantothenic acid	ENSGALG00000020538	SLC49A3
Com_92_pos	D-(+)-Proline	ENSGALG00000036293	EBAG9
Com_265_pos	6-Methylquinoline	ENSGALG00000052887	--
Com_588_pos	Ornithine	ENSGALG00000028822	RNF152
Com_192_pos	1-Methylhistidine	ENSGALG00000053013	EPB41L4B
Com_54_pos	Uric acid	ENSGALG00000014907	DCBLD1
Com_80_pos	DL-Lysine	MSTRG.53	SND1
Com_22_pos	Indole-3-acrylic acid	ENSGALG00000029857	Gimap1
Com_86_neg	Levulinic acid	ENSGALG00000009170	NCEH1
Com_55_pos	Valine	ENSGALG00000023395	PLIN1
Com_80_pos	DL-Lysine	ENSGALG00000000645	Espn
Com_22_pos	Indole-3-acrylic acid	ENSGALG00000032329	NPM3
Com_21_pos	DL-Tryptophan	MSTRG.2126	--
Com_12_pos	Betaine	ENSGALG00000028376	FGF19
Com_471_pos	Indole-3-acetic acid	ENSGALG00000016690	CYP2AC1
Com_40_pos	Choline	MSTRG.16505	--
Com_86_neg	Levulinic acid	MSTRG.15443	--
Com_40_pos	Choline	ENSGALG00000003575	Dnttip1
Com_264_pos	Indole	MSTRG.16398	Cdhr5
Com_21_pos	DL-Tryptophan	ENSGALG00000016665	FDFT1
Com_460_pos	3-amino-4-(propylamino)(	ENSGALG00000003427	USP3
Com_252_pos	cis-4-Hydroxy-D-proline	ENSGALG00000031482	Pou5f3
Com_588_pos	Ornithine	ENSGALG00000004729	SLC7A10
Com_440_pos	PC (18:4e/4:0)	ENSGALG00000028191	GLCE
Com_130_neg	2-Hydroxyvaleric acid	MSTRG.13408	--
Com_40_pos	Choline	ENSGALG00000015263	TMEM30C
Com_76_neg	Erythronolactone	ENSGALG00000033051	CAMK1D
Com_40_pos	Choline	ENSGALG00000016979	SLC25A30
Com_76_neg	Erythronolactone	ENSGALG00000014525	USP5
Com_178_pos	Maltol	ENSGALG00000030151	LUZP2
Com_362_pos	2-Arachidonoyl glycerol	MSTRG.2305	gag
Com_78_neg	Citric acid	ENSGALG00000023338	CBX2
Com_18_neg	Arachidonic acid	ENSGALG00000015044	GTF3C6
Com_21_pos	DL-Tryptophan	ENSGALG00000048205	EBP
Com_192_pos	1-Methylhistidine	ENSGALG00000041205	--
Com_186_pos	4-Hydroxybenzaldehyde	ENSGALG00000004081	TMCO4
Com_311_pos	PC (18:4e/2:0)	MSTRG.3156	--
Com_120_neg	LPC 15:0	ENSGALG00000030076	PCSK6
Com_175_pos	Pantothenic acid	ENSGALG00000051251	H2B-I
Com_78_neg	Citric acid	MSTRG.16504	gag
Com_331_pos	L-Lysine	ENSGALG00000039538	CLDND1
Com_362_pos	2-Arachidonoyl glycerol	ENSGALG00000050420	CTNND2
Com_175_pos	Pantothenic acid	ENSGALG00000016476	TTC32
Com_386_pos	2-Amino-1,3-octadecanec	ENSGALG00000049658	UTS2R
Com_331_pos	L-Lysine	ENSGALG00000026846	JMJD7
Com_108_neg	LPE 18:2	ENSGALG00000028256	CCL19
Com_192_pos	1-Methylhistidine	ENSGALG00000014834	NCOA7
Com_22_pos	Indole-3-acrylic acid	ENSGALG00000002362	MANF

Com_440_pos	PC (18:4e/4:0)	ENSGALG00000051466	NDFIP2
Com_25_pos	2-Hydroxycinnamic acid	ENSGALG00000006904	RNH1
Com_76_neg	Erythronolactone	ENSGALG00000000226	TMEM9
Com_588_pos	Ornithine	ENSGALG00000015136	ILDR1
Com_208_neg	N-Acetylanthranilic acid	ENSGALG00000010643	ZYG11B
Com_482_pos	8-Hydroxyquinoline	ENSGALG00000008185	AOX1
Com_171_neg	LPC 22:6	ENSGALG00000031525	TSTA3
Com_178_pos	Maltol	ENSGALG00000009476	CDK6
Com_311_pos	PC (18:4e/2:0)	ENSGALG00000032628	SRCIN1
Com_12_pos	Betaine	ENSGALG00000000619	ANGPTL4
Com_413_pos	L-Cystine	ENSGALG00000038532	--
Com_8_neg	4-Methyl-2-Oxopentanoic	ENSGALG00000015729	LPAR1
Com_178_pos	Maltol	ENSGALG00000021686	--
Com_208_neg	N-Acetylanthranilic acid	MSTRG.1890	gag
Com_16_neg	3-Hydroxybutyric acid	MSTRG.2170	MYO16
Com_12_pos	Betaine	ENSGALG00000040730	RXRG
Com_8_neg	4-Methyl-2-Oxopentanoic	MSTRG.2387	--
Com_352_pos	Riboflavin	ENSGALG00000015624	VCAN
Com_92_pos	D-(+)-Proline	ENSGALG00000041604	NPTXR
Com_108_neg	LPE 18:2	ENSGALG00000054783	NDRG1
Com_97_pos	L-Threonine	ENSGALG00000046789	pol
Com_12_pos	Betaine	ENSGALG00000006521	TRPM5
Com_18_neg	Arachidonic acid	MSTRG.8986	gag
Com_331_pos	L-Lysine	MSTRG.8381	--
Com_471_pos	Indole-3-acetic acid	ENSGALG00000001531	FN3K
Com_55_pos	Valine	ENSGALG00000002802	PACSIN1
Com_460_pos	3-amino-4-(propylamino)(	ENSGALG00000034289	SLC41A3
Com_17_pos	L-Norleucine	MSTRG.21321	gag
Com_18_neg	Arachidonic acid	MSTRG.12629	--
Com_18_neg	Arachidonic acid	MSTRG.20167	--
Com_86_neg	Levulinic acid	ENSGALG00000000950	MVB12B
Com_482_pos	8-Hydroxyquinoline	MSTRG.836	--
Com_186_pos	4-Hydroxybenzaldehyde	ENSGALG00000008326	SYTL2
Com_178_pos	Maltol	ENSGALG00000001749	ACSBG2
Com_25_pos	2-Hydroxycinnamic acid	MSTRG.7483	--
Com_460_pos	3-amino-4-(propylamino)(	ENSGALG00000012812	SVOPL
Com_152_pos	Acetyl-L-carnitine	ENSGALG00000012186	CASP6
Com_386_pos	2-Amino-1,3-octadecanec	ENSGALG00000030908	ATP2B2
Com_252_pos	cis-4-Hydroxy-D-proline	ENSGALG00000040857	TECTA
Com_482_pos	8-Hydroxyquinoline	ENSGALG00000028407	GDF9
Com_80_pos	DL-Lysine	ENSGALG00000023691	ENTPD7
Com_386_pos	2-Amino-1,3-octadecanec	ENSGALG00000033656	DQX1
Com_12_pos	Betaine	MSTRG.19422	--
Com_194_pos	Pipecolic acid	ENSGALG00000036616	NUAK2
Com_92_pos	D-(+)-Proline	ENSGALG00000021135	HAPLN3
Com_208_neg	N-Acetylanthranilic acid	ENSGALG00000002445	KIAA0319L
Com_12_pos	Betaine	ENSGALG00000023517	AGPAT2
Com_311_pos	PC (18:4e/2:0)	ENSGALG00000037253	CLEC4M
Com_178_pos	Maltol	MSTRG.11802	TMEM221
Com_120_neg	LPC 15:0	MSTRG.16287	--
Com_21_pos	DL-Tryptophan	MSTRG.8619	--
Com_80_pos	DL-Lysine	ENSGALG00000021395	ABCA9
Com_130_neg	2-Hydroxyvaleric acid	ENSGALG00000035626	DAD1
Com_178_pos	Maltol	ENSGALG00000021685	SERINC2
Com_4_pos	PC (17:1/17:1)	ENSGALG00000002116	TEN1
Com_331_pos	L-Lysine	ENSGALG00000046757	ERVK-9
Com_588_pos	Ornithine	ENSGALG00000036787	HSD17B12
Com_152_pos	Acetyl-L-carnitine	ENSGALG00000045288	CAMK2N1

Com_151_neg	Lysope 18:1	ENSGALG00000013969	ALDH8A1
Com_362_pos	2-Arachidonoyl glycerol	ENSGALG00000040342	ADAMTS1
Com_362_pos	2-Arachidonoyl glycerol	MSTRG.8986	gag
Com_175_pos	Pantothenic acid	ENSGALG00000005815	TMEM41B
Com_8_neg	4-Methyl-2-Oxopentanoic	ENSGALG00000015540	RAD23B
Com_18_neg	Arachidonic acid	ENSGALG00000014581	BORCS8
Com_80_pos	DL-Lysine	ENSGALG00000014944	GCNT4
Com_460_pos	3-amino-4-(propylamino)(	MSTRG.2391	--
Com_120_neg	LPC 15:0	ENSGALG00000002707	CHRNB2
Com_80_pos	DL-Lysine	ENSGALG00000030185	PTDSS1
Com_152_pos	Acetyl-L-carnitine	MSTRG.2174	Irs2
Com_99_pos	Creatine	ENSGALG00000048285	TCN2
Com_208_neg	N-Acetylanthranilic acid	ENSGALG00000014923	ARHGEF28
Com_120_neg	LPC 15:0	ENSGALG00000050258	--
Com_362_pos	2-Arachidonoyl glycerol	ENSGALG00000007673	LRRC59
Com_215_pos	D-Erythro-sphingosine 1-p	ENSGALG00000054442	ITIH3
Com_54_pos	Uric acid	ENSGALG00000050083	SYCP2L
Com_40_pos	Choline	MSTRG.17073	--
Com_78_neg	Citric acid	MSTRG.8810	--
Com_120_neg	LPC 15:0	ENSGALG00000008784	SPATA1
Com_78_neg	Citric acid	ENSGALG00000015768	ANKRD6
Com_40_pos	Choline	ENSGALG00000036492	DAGLA
Com_186_pos	4-Hydroxybenzaldehyde	MSTRG.15241	--
Com_482_pos	8-Hydroxyquinoline	ENSGALG00000032882	EVA1C
Com_311_pos	PC (18:4e/2:0)	MSTRG.2393	--
Com_386_pos	2-Amino-1,3-octadecaned	ENSGALG00000038145	DPP7
Com_54_pos	Uric acid	ENSGALG00000052296	MEX3D
Com_460_pos	3-amino-4-(propylamino)(	MSTRG.8874	gag
Com_588_pos	Ornithine	ENSGALG00000047380	MR1
Com_8_neg	4-Methyl-2-Oxopentanoic	ENSGALG00000016885	STK24
Com_265_pos	6-Methylquinoline	MSTRG.1841	--
Com_208_neg	N-Acetylanthranilic acid	ENSGALG00000034504	FAM20C
Com_97_pos	L-Threonine	MSTRG.13408	--
Com_76_neg	Erythronolactone	MSTRG.21394	gag-pol
Com_194_pos	Pipecolic acid	ENSGALG00000029083	NXPH2
Com_99_pos	Creatine	ENSGALG00000008297	SEMA4B
Com_311_pos	PC (18:4e/2:0)	MSTRG.15732	--
Com_17_pos	L-Norleucine	ENSGALG00000009170	NCEH1
Com_482_pos	8-Hydroxyquinoline	ENSGALG00000052583	A2ML1
Com_311_pos	PC (18:4e/2:0)	ENSGALG00000013001	CTNND2
Com_471_pos	Indole-3-acetic acid	ENSGALG00000016364	ALKAL2
Com_4_pos	PC (17:1/17:1)	ENSGALG00000011571	AGPAT4
Com_331_pos	L-Lysine	ENSGALG00000003147	TRPC4AP
Com_460_pos	3-amino-4-(propylamino)(	ENSGALG00000040484	SEC31A
Com_362_pos	2-Arachidonoyl glycerol	ENSGALG00000015044	GTF3C6
Com_588_pos	Ornithine	ENSGALG00000026547	TPGS2
Com_264_pos	Indole	ENSGALG00000032329	NPM3
Com_18_neg	Arachidonic acid	MSTRG.2305	gag
Com_175_pos	Pantothenic acid	ENSGALG00000023517	AGPAT2
Com_362_pos	2-Arachidonoyl glycerol	MSTRG.21536	--
Com_108_neg	LPE 18:2	ENSGALG00000011331	CTH
Com_175_pos	Pantothenic acid	MSTRG.19422	--
Com_482_pos	8-Hydroxyquinoline	ENSGALG00000052964	TOPAZ1
Com_171_neg	LPC 22:6	MSTRG.13584	--
Com_208_neg	N-Acetylanthranilic acid	ENSGALG00000011347	IHH
Com_17_pos	L-Norleucine	ENSGALG00000042511	PKDCC
Com_92_pos	D-(+)-Proline	ENSGALG00000033591	Kazald1
Com_208_neg	N-Acetylanthranilic acid	ENSGALG00000004498	SLC2A10

Com_588_pos	Ornithine	ENSGALG00000008262	RASGRF1
Com_186_pos	4-Hydroxybenzaldehyde	ENSGALG00000014935	GREB1L
Com_54_pos	Uric acid	ENSGALG00000005766	PKD2L1
Com_76_neg	Erythronolactone	ENSGALG00000021658	PAFAH2
Com_78_neg	Citric acid	ENSGALG00000012377	HNMT
Com_18_neg	Arachidonic acid	ENSGALG00000004282	RCAN3
Com_194_pos	Pipecolic acid	ENSGALG00000033365	ALDH1A3
Com_171_neg	LPC 22:6	MSTRG.16504	gag
Com_151_pos	Pyridoxamine	ENSGALG00000012882	KDSR
Com_4_pos	PC (17:1/17:1)	ENSGALG00000000498	ACE
Com_80_pos	DL-Lysine	MSTRG.8955	--
Com_80_pos	DL-Lysine	ENSGALG00000053886	gag
Com_18_neg	Arachidonic acid	MSTRG.17961	--
Com_215_pos	D-Erythro-sphingosine 1-p	ENSGALG00000028256	CCL19
Com_92_pos	D-(+)-Proline	ENSGALG00000003147	TRPC4AP
Com_120_neg	LPC 15:0	ENSGALG00000042374	PDE11A
Com_331_pos	L-Lysine	ENSGALG00000030151	LUZP2
Com_78_neg	Citric acid	MSTRG.1082	--
Com_86_neg	Levulinic acid	ENSGALG00000017378	CRTAC1
Com_311_pos	PC (18:4e/2:0)	ENSGALG00000028191	GLCE
Com_80_pos	DL-Lysine	ENSGALG00000008226	NIF3L1
Com_12_pos	Betaine	ENSGALG00000035206	CNPY2
Com_22_pos	Indole-3-acrylic acid	ENSGALG00000006842	ACOT8
Com_208_neg	N-Acetylanthranilic acid	ENSGALG00000035239	GLCCI1
Com_413_pos	L-Cystine	MSTRG.1502	gag
Com_55_pos	Valine	MSTRG.1468	--
Com_151_pos	Pyridoxamine	ENSGALG00000005739	SCD
Com_362_pos	2-Arachidonoyl glycerol	ENSGALG00000014233	FBLN1
Com_78_neg	Citric acid	ENSGALG00000008866	WDPCP
Com_16_neg	3-Hydroxybutyric acid	MSTRG.15507	--
Com_178_pos	Maltol	ENSGALG00000039538	CLDND1
Com_120_neg	LPC 15:0	ENSGALG00000038145	DPP7
Com_413_pos	L-Cystine	ENSGALG00000012877	CREB3L2
Com_120_neg	LPC 15:0	ENSGALG00000007012	ARRDC4
Com_413_pos	L-Cystine	ENSGALG00000042491	H4-I
Com_152_pos	Acetyl-L-carnitine	ENSGALG00000020342	ABHD12
Com_12_pos	Betaine	ENSGALG00000010301	EIF2B2
Com_588_pos	Ornithine	ENSGALG00000037773	ST3GAL1
Com_175_pos	Pantothenic acid	ENSGALG00000000619	ANGPTL4
Com_460_pos	3-amino-4-(propylamino)c	ENSGALG00000009842	RASSF3
Com_16_neg	3-Hydroxybutyric acid	ENSGALG00000014813	HOMER1
Com_331_pos	L-Lysine	ENSGALG00000009476	CDK6
Com_265_pos	6-Methylquinoline	ENSGALG00000005888	PGP
Com_152_pos	Acetyl-L-carnitine	ENSGALG00000043336	COPZ1
Com_362_pos	2-Arachidonoyl glycerol	ENSGALG00000027960	GRPR
Com_331_pos	L-Lysine	ENSGALG00000021686	--
Com_76_neg	Erythronolactone	ENSGALG00000051779	PRORSD1P
Com_151_pos	Pyridoxamine	ENSGALG00000022758	GGACT
Com_151_pos	Pyridoxamine	ENSGALG00000006904	RNH1
Com_362_pos	2-Arachidonoyl glycerol	ENSGALG00000014813	HOMER1
Com_413_pos	L-Cystine	ENSGALG00000006723	IDI1
Com_460_pos	3-amino-4-(propylamino)c	ENSGALG00000006647	DUSP8
Com_152_pos	Acetyl-L-carnitine	ENSGALG00000050676	Ctnnd2
Com_12_pos	Betaine	ENSGALG00000051251	H2B-I
Com_92_pos	D-(+)-Proline	ENSGALG00000046757	ERVK-9
Com_147_pos	D-Sphingosine	MSTRG.13135	--
Com_192_pos	1-Methylhistidine	ENSGALG00000010406	TMEM63C
Com_17_pos	L-Norleucine	ENSGALG00000010229	ABCD4

Com_22_pos	Indole-3-acrylic acid	ENSGALG00000040620	LSAMP
Com_440_pos	PC (18:4e/4:0)	ENSGALG00000032628	SRCIN1
Com_54_pos	Uric acid	MSTRG.21092	--
Com_55_pos	Valine	ENSGALG00000000104	CRY1
Com_482_pos	8-Hydroxyquinoline	ENSGALG00000012755	IGF-I
Com_147_pos	D-Sphingosine	ENSGALG00000002899	AACS
Com_55_pos	Valine	ENSGALG00000031067	TMEM132A
Com_362_pos	2-Arachidonoyl glycerol	MSTRG.15507	--
Com_54_pos	Uric acid	ENSGALG00000037852	HSD17B7
Com_588_pos	Ornithine	MSTRG.19463	VTG2
Com_55_pos	Valine	ENSGALG00000027064	HIST1H3H
Com_16_neg	3-Hydroxybutyric acid	ENSGALG00000014233	FBLN1
Com_386_pos	2-Amino-1,3-octadecane(	MSTRG.16287	--
Com_186_pos	4-Hydroxybenzaldehyde	ENSGALG00000013828	GALM
Com_331_pos	L-Lysine	ENSGALG00000021135	HAPLN3
Com_413_pos	L-Cystine	ENSGALG00000006689	ABHD2
Com_413_pos	L-Cystine	MSTRG.14577	SLC39A5
Com_175_pos	Pantothenic acid	MSTRG.2388	--
Com_108_neg	LPE 18:2	ENSGALG00000013100	GRB10
Com_175_pos	Pantothenic acid	ENSGALG00000005160	VMP1
Com_120_neg	LPC 15:0	ENSGALG00000033656	DQX1
Com_192_pos	1-Methylhistidine	ENSGALG00000008752	ACOT1
Com_80_pos	DL-Lysine	ENSGALG00000004425	SCAMP1
Com_194_pos	Pipecolic acid	MSTRG.5319	--
Com_99_pos	Creatine	ENSGALG00000010009	TTC29
Com_16_neg	3-Hydroxybutyric acid	ENSGALG00000007533	NPEPL1
Com_208_neg	N-Acetylanthranilic acid	ENSGALG00000050520	pol
Com_460_pos	3-amino-4-(propylamino)(	MSTRG.9410	--
Com_264_pos	Indole	ENSGALG00000024295	MYCBP
Com_175_pos	Pantothenic acid	ENSGALG00000012847	Slc7a11
Com_17_pos	L-Norleucine	ENSGALG00000006076	RASGEF1C
Com_120_neg	LPC 15:0	ENSGALG00000030908	ATP2B2
Com_18_neg	Arachidonic acid	ENSGALG00000008866	WDPCP
Com_21_pos	DL-Tryptophan	ENSGALG00000006198	LSS
Com_55_pos	Valine	MSTRG.15754	--
Com_192_pos	1-Methylhistidine	MSTRG.15444	--
Com_12_pos	Betaine	ENSGALG00000020538	SLC49A3
Com_76_neg	Erythronolactone	ENSGALG00000007404	YIPF5
Com_8_neg	4-Methyl-2-Oxopentanoic	ENSGALG00000004631	DRAXIN
Com_25_pos	2-Hydroxycinnamic acid	ENSGALG00000046412	Aoc3
Com_76_neg	Erythronolactone	MSTRG.13261	--
Com_18_neg	Arachidonic acid	MSTRG.1082	--
Com_130_neg	2-Hydroxyvaleric acid	ENSGALG00000017122	SGCG
Com_482_pos	8-Hydroxyquinoline	ENSGALG00000036190	AOC1
Com_264_pos	Indole	ENSGALG00000054546	ERVK-11
Com_86_neg	Levulinic acid	ENSGALG00000002775	FA2H
Com_108_neg	LPE 18:2	ENSGALG00000054442	ITIH3
Com_54_pos	Uric acid	ENSGALG00000014948	HMGCR
Com_440_pos	PC (18:4e/4:0)	ENSGALG00000037253	CLEC4M
Com_16_neg	3-Hydroxybutyric acid	ENSGALG00000015044	GTF3C6
Com_175_pos	Pantothenic acid	ENSGALG00000026460	myoM
Com_194_pos	Pipecolic acid	ENSGALG00000023395	PLIN1
Com_178_pos	Maltol	ENSGALG00000046757	ERVK-9
Com_331_pos	L-Lysine	ENSGALG00000041604	NPTXR
Com_18_neg	Arachidonic acid	ENSGALG00000012377	HNMT
Com_99_pos	Creatine	ENSGALG00000009483	MARK1
Com_208_neg	N-Acetylanthranilic acid	ENSGALG00000010391	MMRN1
Com_175_pos	Pantothenic acid	ENSGALG00000043829	ext1c

Com_120_neg	LPC 15:0	ENSGALG00000034289	SLC41A3
Com_55_pos	Valine	ENSGALG00000035244	H3-I
Com_152_pos	Acetyl-L-carnitine	ENSGALG00000015540	RAD23B
Com_252_pos	cis-4-Hydroxy-D-proline	ENSGALG00000007723	NAT9
Com_68_neg	PE (16:0/22:6)	ENSGALG00000039708	Pram1
Com_147_pos	D-Sphingosine	ENSGALG00000051466	NDFIP2
Com_120_neg	LPC 15:0	ENSGALG00000012812	SVOPL
Com_186_pos	4-Hydroxybenzaldehyde	ENSGALG00000005418	FRRS1
Com_80_pos	DL-Lysine	ENSGALG00000006076	RASGEF1C
Com_192_pos	1-Methylhistidine	ENSGALG00000043451	DNAL4
Com_192_pos	1-Methylhistidine	ENSGALG00000032645	H2A-VIII
Com_76_neg	Erythronolactone	ENSGALG00000045557	MTTPL
Com_178_pos	Maltol	ENSGALG00000029445	FADS6
Com_92_pos	D-(+)-Proline	MSTRG.11955	--
Com_8_neg	4-Methyl-2-Oxopentanoic	ENSGALG00000012586	GKAP1
Com_18_neg	Arachidonic acid	MSTRG.2170	MYO16
Com_78_neg	Citric acid	MSTRG.2305	gag
Com_152_pos	Acetyl-L-carnitine	ENSGALG00000036293	EBAG9
Com_208_neg	N-Acetylanthranilic acid	ENSGALG00000012823	TRIM24
Com_86_neg	Levulinic acid	ENSGALG00000037050	FABP3
Com_17_pos	L-Norleucine	ENSGALG00000004425	SCAMP1
Com_120_neg	LPC 15:0	ENSGALG00000049658	UTS2R
Com_362_pos	2-Arachidonoyl glycerol	ENSGALG00000023338	CBX2
Com_178_pos	Maltol	MSTRG.14343	--
Com_92_pos	D-(+)-Proline	ENSGALG00000039538	CLDND1
Com_78_neg	Citric acid	MSTRG.13584	--
Com_215_pos	D-Erythro-sphingosine 1-p	ENSGALG00000011331	CTH
Com_68_neg	PE (16:0/22:6)	ENSGALG00000053647	PFAS
Com_203_pos	Serotonin	ENSGALG00000041344	FABP5
Com_192_pos	1-Methylhistidine	MSTRG.16459	--
Com_16_neg	3-Hydroxybutyric acid	MSTRG.8986	gag
Com_460_pos	3-amino-4-(propylamino)(	MSTRG.16459	--
Com_311_pos	PC (18:4e/2:0)	ENSGALG00000031312	ANAPC13
Com_264_pos	Indole	ENSGALG00000043435	CARNS1
Com_86_neg	Levulinic acid	ENSGALG00000042511	PKDCC
Com_178_pos	Maltol	ENSGALG00000003147	TRPC4AP
Com_171_neg	LPC 22:6	ENSGALG00000012377	HNMT
Com_460_pos	3-amino-4-(propylamino)(	MSTRG.16287	--
Com_413_pos	L-Cystine	MSTRG.13439	--
Com_386_pos	2-Amino-1,3-octadecanec	ENSGALG00000030845	ENHO
Com_108_neg	LPE 18:2	ENSGALG00000035026	SLC22A4
Com_460_pos	3-amino-4-(propylamino)(	ENSGALG00000043451	DNAL4
Com_151_pos	Pyridoxamine	ENSGALG00000005470	PLPPR5
Com_208_neg	N-Acetylanthranilic acid	MSTRG.8248	--
Com_80_pos	DL-Lysine	ENSGALG00000010229	ABCD4
Com_12_pos	Betaine	MSTRG.17623	--
Com_171_neg	LPC 22:6	MSTRG.1082	--
Com_186_pos	4-Hydroxybenzaldehyde	ENSGALG00000032440	QPCT
Com_120_neg	LPC 15:0	MSTRG.2391	--
Com_17_pos	L-Norleucine	ENSGALG00000017378	CRTAC1
Com_171_neg	LPC 22:6	ENSGALG00000008866	WDPCP
Com_130_neg	2-Hydroxyvaleric acid	ENSGALG00000026203	FAM174A
Com_331_pos	L-Lysine	ENSGALG00000036293	EBAG9
Com_264_pos	Indole	ENSGALG00000006842	ACOT8
Com_99_pos	Creatine	ENSGALG00000000003	PANX2
Com_186_pos	4-Hydroxybenzaldehyde	ENSGALG00000015219	Selenoi
Com_208_neg	N-Acetylanthranilic acid	MSTRG.21371	--
Com_120_neg	LPC 15:0	MSTRG.8874	gag

Com_76_neg	Erythronolactone	ENSGALG00000005610	SLC44A3
Com_151_pos	Pyridoxamine	ENSGALG00000003948	ALAS1
Com_265_pos	6-Methylquinoline	ENSGALG00000046412	Aoc3
Com_152_pos	Acetyl-L-carnitine	ENSGALG00000041604	NPTXR
Com_120_neg	LPC 15:0	ENSGALG00000040484	SEC31A
Com_192_pos	1-Methylhistidine	MSTRG.9410	--
Com_16_neg	3-Hydroxybutyric acid	ENSGALG00000015689	ECPAS
Com_460_pos	3-amino-4-(propylamino)(	ENSGALG00000008752	ACOT1
Com_21_pos	DL-Tryptophan	ENSGALG00000012220	CDKN3
Com_40_pos	Choline	ENSGALG00000005215	CACNA1H
Com_175_pos	Pantothenic acid	ENSGALG00000007778	PES1
Com_108_neg	LPE 18:2	ENSGALG00000012683	RNF144B
Com_8_neg	4-Methyl-2-Oxopentanoic	ENSGALG00000020342	ABHD12
Com_86_neg	Levulinic acid	ENSGALG00000010229	ABCD4
Com_54_pos	Uric acid	ENSGALG00000014261	UCHL1
Com_97_pos	L-Threonine	ENSGALG00000003103	MST1R
Com_186_pos	4-Hydroxybenzaldehyde	ENSGALG00000005696	ABHD6
Com_21_pos	DL-Tryptophan	ENSGALG00000042706	TMC2
Com_76_neg	Erythronolactone	ENSGALG00000014463	ACTN2
Com_4_pos	PC (17:1/17:1)	ENSGALG00000008859	WDR31
Com_264_pos	Indole	ENSGALG00000040620	LSAMP
Com_8_neg	4-Methyl-2-Oxopentanoic	ENSGALG00000043336	COPZ1
Com_311_pos	PC (18:4e/2:0)	ENSGALG00000003948	ALAS1
Com_97_pos	L-Threonine	ENSGALG00000037935	RARA
Com_4_pos	PC (17:1/17:1)	ENSGALG00000053860	mas
Com_92_pos	D-(+)-Proline	ENSGALG00000012186	CASP6
Com_265_pos	6-Methylquinoline	ENSGALG00000015728	MUSK
Com_80_pos	DL-Lysine	ENSGALG00000042511	PKDCC
Com_54_pos	Uric acid	ENSGALG00000037065	SC5D
Com_8_neg	4-Methyl-2-Oxopentanoic	ENSGALG00000050676	Ctnnd2
Com_92_pos	D-(+)-Proline	ENSGALG00000021685	SERINC2
Com_78_neg	Citric acid	MSTRG.8986	gag
Com_215_pos	D-Erythro-sphingosine 1-p	ENSGALG00000013100	GRB10
Com_99_pos	Creatine	ENSGALG00000050267	CALU
Com_108_neg	LPE 18:2	ENSGALG00000023122	SULT1B
Com_186_pos	4-Hydroxybenzaldehyde	ENSGALG00000034868	KRT7
Com_97_pos	L-Threonine	ENSGALG00000017122	SGCG
Com_192_pos	1-Methylhistidine	ENSGALG00000006647	DUSP8
Com_92_pos	D-(+)-Proline	MSTRG.11802	TMEM221
Com_588_pos	Ornithine	ENSGALG00000004341	Cryzl2
Com_86_neg	Levulinic acid	ENSGALG00000005583	ALG14
Com_16_neg	3-Hydroxybutyric acid	MSTRG.2305	gag
Com_152_pos	Acetyl-L-carnitine	ENSGALG00000021135	HAPLN3
Com_362_pos	2-Arachidonoyl glycerol	MSTRG.8810	--
Com_208_neg	N-Acetylanthranilic acid	MSTRG.9835	--
Com_362_pos	2-Arachidonoyl glycerol	ENSGALG00000015768	ANKRD6
Com_252_pos	cis-4-Hydroxy-D-proline	ENSGALG00000040620	LSAMP
Com_178_pos	Maltol	ENSGALG00000021135	HAPLN3
Com_86_neg	Levulinic acid	ENSGALG00000006076	RASGEF1C
Com_22_pos	Indole-3-acrylic acid	ENSGALG00000024295	MYCBP
Com_16_neg	3-Hydroxybutyric acid	ENSGALG00000016595	TRIM35
Com_192_pos	1-Methylhistidine	ENSGALG00000009842	RASSF3
Com_482_pos	8-Hydroxyquinoline	ENSGALG00000052872	--
Com_311_pos	PC (18:4e/2:0)	ENSGALG00000005470	PLPPR5
Com_352_pos	Riboflavin	ENSGALG00000051068	SIGLEC1
Com_460_pos	3-amino-4-(propylamino)(	ENSGALG00000038145	DPP7
Com_86_neg	Levulinic acid	ENSGALG00000006872	PISD
Com_471_pos	Indole-3-acetic acid	ENSGALG00000034741	ETNPPL

Com_25_pos	2-Hydroxycinnamic acid	ENSGALG00000005888	PGP
Com_18_neg	Arachidonic acid	MSTRG.16504	gag
Com_120_neg	LPC 15:0	ENSGALG00000009842	RASSF3
Com_17_pos	L-Norleucine	ENSGALG00000002775	FA2H
Com_331_pos	L-Lysine	ENSGALG00000050676	Ctnnd2
Com_92_pos	D-(+)-Proline	ENSGALG00000045288	CAMK2N1
Com_12_pos	Betaine	ENSGALG00000036754	CHKA
Com_186_pos	4-Hydroxybenzaldehyde	ENSGALG00000016254	OTC
Com_78_neg	Citric acid	ENSGALG00000015044	GTF3C6
Com_22_pos	Indole-3-acrylic acid	ENSGALG00000054546	ERVK-11
Com_471_pos	Indole-3-acetic acid	ENSGALG00000026313	RND3
Com_92_pos	D-(+)-Proline	ENSGALG00000001749	ACSBG2
Com_175_pos	Pantothenic acid	ENSGALG00000016491	APOB
Com_151_pos	Pyridoxamine	ENSGALG00000031312	ANAPC13
Com_147_pos	D-Sphingosine	ENSGALG00000049658	UTS2R
Com_178_pos	Maltol	ENSGALG00000016492	TDRD15
Com_92_pos	D-(+)-Proline	MSTRG.2174	Irs2
Com_17_pos	L-Norleucine	ENSGALG00000014944	GCNT4
Com_331_pos	L-Lysine	ENSGALG00000043336	COPZ1
Com_120_neg	LPC 15:0	ENSGALG00000006647	DUSP8
Com_152_pos	Acetyl-L-carnitine	ENSGALG00000004631	DRAXIN
Com_588_pos	Ornithine	ENSGALG00000036836	SOSTDC1
Com_16_neg	3-Hydroxybutyric acid	ENSGALG00000048223	FAM20C
Com_99_pos	Creatine	ENSGALG00000002519	SLC25A33
Com_175_pos	Pantothenic acid	ENSGALG00000005610	SLC44A3
Com_252_pos	cis-4-Hydroxy-D-proline	ENSGALG00000006842	ACOT8
Com_171_neg	LPC 22:6	MSTRG.2305	gag
Com_331_pos	L-Lysine	ENSGALG00000020342	ABHD12
Com_194_pos	Pipecolic acid	ENSGALG00000007018	SLC26A11
Com_178_pos	Maltol	ENSGALG00000026607	C15orf40
Com_8_neg	4-Methyl-2-Oxopentanoic	ENSGALG00000004055	C7orf50
Com_186_pos	4-Hydroxybenzaldehyde	ENSGALG00000004268	NIPAL3
Com_178_pos	Maltol	ENSGALG00000041604	NPTXR
Com_482_pos	8-Hydroxyquinoline	ENSGALG00000014252	A2M
Com_25_pos	2-Hydroxycinnamic acid	ENSGALG00000010294	RPS6KL1
Com_97_pos	L-Threonine	MSTRG.21796	--
Com_17_pos	L-Norleucine	ENSGALG00000037050	FABP3
Com_86_neg	Levulinic acid	ENSGALG00000004425	SCAMP1
Com_55_pos	Valine	ENSGALG00000033365	ALDH1A3
Com_86_neg	Levulinic acid	ENSGALG00000048035	GCNT2
Com_192_pos	1-Methylhistidine	ENSGALG00000011113	SGIP1
Com_482_pos	8-Hydroxyquinoline	ENSGALG00000040573	FMO3
Com_151_pos	Pyridoxamine	ENSGALG00000007723	NAT9
Com_86_neg	Levulinic acid	ENSGALG00000000107	TRIM7.1
Com_331_pos	L-Lysine	ENSGALG00000029445	FADS6
Com_460_pos	3-amino-4-(propylamino)(	ENSGALG00000033656	DQX1
Com_482_pos	8-Hydroxyquinoline	ENSGALG00000009700	PDK4
Com_99_pos	Creatine	ENSGALG00000010233	SYNDIG1L
Com_588_pos	Ornithine	ENSGALG00000042491	H4-I
Com_331_pos	L-Lysine	MSTRG.14343	--
Com_22_pos	Indole-3-acrylic acid	ENSGALG00000043435	CARNS1
Com_152_pos	Acetyl-L-carnitine	ENSGALG00000012586	GKAP1
Com_413_pos	L-Cystine	ENSGALG00000037773	ST3GAL1
Com_120_neg	LPC 15:0	MSTRG.9410	--
Com_192_pos	1-Methylhistidine	ENSGALG00000040484	SEC31A
Com_78_neg	Citric acid	ENSGALG00000014233	FBLN1
Com_8_neg	4-Methyl-2-Oxopentanoic	ENSGALG00000036293	EBAG9
Com_86_neg	Levulinic acid	ENSGALG00000054319	ELOVL6

Com_97_pos	L-Threonine	ENSGALG00000026203	FAM174A
Com_208_neg	N-Acetylanthranilic acid	ENSGALG00000045127	slc12a8
Com_194_pos	Pipecolic acid	MSTRG.1468	--
Com_215_pos	D-Erythro-sphingosine 1-|	ENSGALG00000035026	SLC22A4
Com_25_pos	2-Hydroxycinnamic acid	ENSGALG00000006530	TSSC4
Com_482_pos	8-Hydroxyquinoline	ENSGALG00000026663	CX3CL1
Com_460_pos	3-amino-4-(propylamino)(	ENSGALG00000030908	ATP2B2
Com_147_pos	D-Sphingosine	ENSGALG00000030908	ATP2B2
Com_147_pos	D-Sphingosine	ENSGALG00000032628	SRCIN1
Com_413_pos	L-Cystine	ENSGALG00000015016	SLC22A15
Com_192_pos	1-Methylhistidine	MSTRG.8874	gag
Com_265_pos	6-Methylquinoline	ENSGALG00000029857	Gimap1
Com_54_pos	Uric acid	ENSGALG00000037325	SERP1
Com_147_pos	D-Sphingosine	ENSGALG00000033656	DQX1
Com_440_pos	PC (18:4e/4:0)	ENSGALG00000031312	ANAPC13
Com_186_pos	4-Hydroxybenzaldehyde	ENSGALG00000049232	POLR2A
Com_78_neg	Citric acid	ENSGALG00000014813	HOMER1
Com_25_pos	2-Hydroxycinnamic acid	ENSGALG00000002579	RIMBP2
Com_152_pos	Acetyl-L-carnitine	ENSGALG00000003147	TRPC4AP
Com_482_pos	8-Hydroxyquinoline	ENSGALG00000005977	BTBD8
Com_18_neg	Arachidonic acid	ENSGALG00000007533	NPEPL1
Com_57_neg	LPC 16:1	ENSGALG00000013969	ALDH8A1
Com_175_pos	Pantothenic acid	ENSGALG00000027375	NR2C2AP
Com_362_pos	2-Arachidonoyl glycerol	MSTRG.1503	gag
Com_192_pos	1-Methylhistidine	MSTRG.2391	--
Com_186_pos	4-Hydroxybenzaldehyde	MSTRG.18216	--
Com_21_pos	DL-Tryptophan	ENSGALG00000037401	IDH3A
Com_588_pos	Ornithine	ENSGALG00000006689	ABHD2
Com_78_neg	Citric acid	MSTRG.15507	--
Com_130_neg	2-Hydroxyvaleric acid	ENSGALG00000003103	MST1R
Com_16_neg	3-Hydroxybutyric acid	ENSGALG00000008866	WDPCP
Com_482_pos	8-Hydroxyquinoline	ENSGALG00000014616	MT3
Com_178_pos	Maltol	ENSGALG00000036293	EBAG9
Com_192_pos	1-Methylhistidine	ENSGALG00000004917	DOC2B
Com_194_pos	Pipecolic acid	ENSGALG00000027064	HIST1H3H
Com_130_neg	2-Hydroxyvaleric acid	ENSGALG00000037935	RARA
Com_8_neg	4-Methyl-2-Oxopentanoic	ENSGALG00000037479	IGSF21
Com_16_neg	3-Hydroxybutyric acid	MSTRG.1082	--
Com_12_pos	Betaine	ENSGALG00000007507	MASTL
Com_460_pos	3-amino-4-(propylamino)(	ENSGALG00000049658	UTS2R
Com_130_neg	2-Hydroxyvaleric acid	ENSGALG00000004782	TSEN15
Com_76_neg	Erythronolactone	ENSGALG00000026460	myoM
Com_215_pos	D-Erythro-sphingosine 1-|	ENSGALG00000012683	RNF144B
Com_208_neg	N-Acetylanthranilic acid	ENSGALG00000004483	AHSA2
Com_588_pos	Ornithine	MSTRG.4702	--
Com_8_neg	4-Methyl-2-Oxopentanoic	ENSGALG00000032889	RBFOX3
Com_265_pos	6-Methylquinoline	ENSGALG00000002362	MANF
Com_311_pos	PC (18:4e/2:0)	ENSGALG00000022758	GGACT
Com_120_neg	LPC 15:0	MSTRG.16459	--
Com_252_pos	cis-4-Hydroxy-D-proline	ENSGALG00000006904	RNH1
Com_147_pos	D-Sphingosine	ENSGALG00000037253	CLEC4M
Com_362_pos	2-Arachidonoyl glycerol	ENSGALG00000004322	AHR
Com_55_pos	Valine	MSTRG.5319	--
Com_192_pos	1-Methylhistidine	ENSGALG00000036117	TENT5B
Com_16_neg	3-Hydroxybutyric acid	ENSGALG00000012377	HNMT
Com_175_pos	Pantothenic acid	MSTRG.13261	--
Com_86_neg	Levulinic acid	ENSGALG00000047687	SETD9
Com_175_pos	Pantothenic acid	ENSGALG00000007404	YIPF5

Com_152_pos	Acetyl-L-carnitine	ENSGALG00000046757	ERVK-9
Com_147_pos	D-Sphingosine	ENSGALG00000038145	DPP7
Com_12_pos	Betaine	ENSGALG00000004875	PEMT
Com_471_pos	Indole-3-acetic acid	ENSGALG00000051274	B3GALT2
Com_171_neg	LPC 22:6	MSTRG.8986	gag
Com_8_neg	4-Methyl-2-Oxopentanoic	ENSGALG00000041604	NPTXR
Com_25_pos	2-Hydroxycinnamic acid	ENSGALG00000052986	Vwa5b2
Com_25_pos	2-Hydroxycinnamic acid	ENSGALG00000005839	ARID3C
Com_120_neg	LPC 15:0	ENSGALG00000043451	DNAL4
Com_76_neg	Erythronolactone	ENSGALG00000012847	Slc7a11
Com_86_neg	Levulinic acid	MSTRG.6225	--
Com_215_pos	D-Erythro-sphingosine 1-p	ENSGALG00000023122	SULT1B
Com_192_pos	1-Methylhistidine	ENSGALG00000012812	SVOPL
Com_386_pos	2-Amino-1,3-octadecaned	ENSGALG00000003427	USP3
Com_55_pos	Valine	ENSGALG00000014126	endou-a
Com_311_pos	PC (18:4e/2:0)	ENSGALG00000005739	SCD
Com_76_neg	Erythronolactone	ENSGALG00000005160	VMP1
Com_76_neg	Erythronolactone	MSTRG.2388	--
Com_17_pos	L-Norleucine	ENSGALG00000005583	ALG14
Com_192_pos	1-Methylhistidine	ENSGALG00000034289	SLC41A3
Com_18_neg	Arachidonic acid	ENSGALG00000031525	TSTA3
Com_362_pos	2-Arachidonoyl glycerol	MSTRG.149	--
Com_17_pos	L-Norleucine	ENSGALG00000000645	Espn
Com_99_pos	Creatine	ENSGALG00000004231	IFNLR1
Com_265_pos	6-Methylquinoline	MSTRG.7483	--
Com_25_pos	2-Hydroxycinnamic acid	MSTRG.3473	--
Com_12_pos	Betaine	ENSGALG00000011657	EAF2
Com_92_pos	D-(+)-Proline	MSTRG.12923	--
Com_440_pos	PC (18:4e/4:0)	ENSGALG00000003948	ALAS1
Com_588_pos	Ornithine	MSTRG.149	--
Com_194_pos	Pipecolic acid	MSTRG.21796	--
Com_311_pos	PC (18:4e/2:0)	ENSGALG00000012882	KDSR
Com_130_neg	2-Hydroxyvaleric acid	ENSGALG00000029235	CPNE4
Com_194_pos	Pipecolic acid	ENSGALG00000035244	H3-I
Com_17_pos	L-Norleucine	ENSGALG00000006872	PISD
Com_471_pos	Indole-3-acetic acid	ENSGALG00000037160	Smad7
Com_171_neg	LPC 22:6	ENSGALG00000015044	GTF3C6
Com_97_pos	L-Threonine	ENSGALG00000007018	SLC26A11
Com_8_neg	4-Methyl-2-Oxopentanoic	ENSGALG00000021135	HAPLN3
Com_130_neg	2-Hydroxyvaleric acid	ENSGALG00000013036	ATP6V1E1
Com_482_pos	8-Hydroxyquinoline	ENSGALG00000019147	--
Com_151_neg	Lysope 18:1	ENSGALG00000009268	FGG
Com_186_pos	4-Hydroxybenzaldehyde	ENSGALG00000036527	SYBU
Com_362_pos	2-Arachidonoyl glycerol	MSTRG.13584	--
Com_130_neg	2-Hydroxyvaleric acid	MSTRG.21796	--
Com_175_pos	Pantothenic acid	ENSGALG00000051779	PRORSD1P
Com_86_neg	Levulinic acid	ENSGALG00000014944	GCNT4
Com_588_pos	Ornithine	MSTRG.13439	--
Com_471_pos	Indole-3-acetic acid	MSTRG.15995	--
Com_120_neg	LPC 15:0	ENSGALG00000008752	ACOT1
Com_588_pos	Ornithine	ENSGALG00000004322	AHR
Com_86_neg	Levulinic acid	ENSGALG00000053245	VTG2
Com_362_pos	2-Arachidonoyl glycerol	MSTRG.4702	--
Com_178_pos	Maltol	ENSGALG00000050676	Ctnnd2
Com_440_pos	PC (18:4e/4:0)	ENSGALG00000005470	PLPPR5
Com_175_pos	Pantothenic acid	ENSGALG00000052072	gag
Com_192_pos	1-Methylhistidine	ENSGALG00000004249	GRHL3
Com_16_neg	3-Hydroxybutyric acid	ENSGALG00000004170	ADA

Com_482_pos	8-Hydroxyquinoline	MSTRG.19177	PHGDH
Com_331_pos	L-Lysine	ENSGALG00000016492	TDRD15
Com_413_pos	L-Cystine	ENSGALG00000012196	MCUB
Com_92_pos	D-(+)-Proline	ENSGALG00000015540	RAD23B
Com_92_pos	D-(+)-Proline	ENSGALG00000005648	Sesn3
Com_413_pos	L-Cystine	ENSGALG00000048432	--
Com_178_pos	Maltol	ENSGALG00000043336	COPZ1
Com_215_pos	D-Erythro-sphingosine 1-[	ENSGALG00000052991	DNAJC21
Com_4_pos	PC (17:1/17:1)	ENSGALG00000050840	APCDD1
Com_17_pos	L-Norleucine	ENSGALG00000048035	GCNT2
Com_178_pos	Maltol	ENSGALG00000020342	ABHD12
Com_331_pos	L-Lysine	ENSGALG00000026607	C15orf40
Com_17_pos	L-Norleucine	ENSGALG00000000107	TRIM7.1
Com_208_neg	N-Acetylanthranilic acid	ENSGALG00000031932	AGPAT3
Com_12_pos	Betaine	ENSGALG00000009560	MSMO1
Com_171_neg	LPC 22:6	ENSGALG00000014233	FBLN1
Com_108_neg	LPE 18:2	ENSGALG00000038652	Gsta3
Com_152_pos	Acetyl-L-carnitine	ENSGALG00000004055	C7orf50
Com_152_pos	Acetyl-L-carnitine	ENSGALG00000039538	CLDND1
Com_588_pos	Ornithine	MSTRG.1503	gag
Com_17_pos	L-Norleucine	ENSGALG00000041533	SLC11A2
Com_130_neg	2-Hydroxyvaleric acid	MSTRG.2316	env
Com_386_pos	2-Amino-1,3-octadecanec	MSTRG.13135	--
Com_18_neg	Arachidonic acid	ENSGALG00000015689	ECPAS
Com_17_pos	L-Norleucine	ENSGALG00000054319	ELOVL6
Com_86_neg	Levulinic acid	ENSGALG00000010018	CTSEAL
Com_482_pos	8-Hydroxyquinoline	ENSGALG00000033171	TGM4
Com_130_neg	2-Hydroxyvaleric acid	ENSGALG00000015134	APOV1
Com_386_pos	2-Amino-1,3-octadecanec	ENSGALG00000002899	AACS
Com_186_pos	4-Hydroxybenzaldehyde	ENSGALG00000040363	ABHD4
Com_175_pos	Pantothenic acid	ENSGALG00000021658	PAFAH2
Com_460_pos	3-amino-4-(propylamino)(	ENSGALG00000053013	EPB41L4B
Com_76_neg	Erythronolactone	ENSGALG00000012185	PLA2G12A
Com_194_pos	Pipecolic acid	ENSGALG00000037935	RARA
Com_92_pos	D-(+)-Proline	ENSGALG00000041296	SOX7
Com_80_pos	DL-Lysine	ENSGALG00000000950	MVB12B
Com_192_pos	1-Methylhistidine	ENSGALG00000012589	C9orf64
Com_192_pos	1-Methylhistidine	ENSGALG00000007012	ARRDC4
Com_252_pos	cis-4-Hydroxy-D-proline	ENSGALG00000032329	NPM3
Com_147_pos	D-Sphingosine	MSTRG.16287	--
Com_471_pos	Indole-3-acetic acid	ENSGALG00000034478	CCL4
Com_171_neg	LPC 22:6	ENSGALG00000014813	HOMER1
Com_194_pos	Pipecolic acid	ENSGALG00000003103	MST1R
Com_76_neg	Erythronolactone	ENSGALG00000039354	VTG1
Com_130_neg	2-Hydroxyvaleric acid	ENSGALG00000005353	FAR1
Com_460_pos	3-amino-4-(propylamino)(	ENSGALG00000012111	TMEM37
Com_186_pos	4-Hydroxybenzaldehyde	MSTRG.4550	--
Com_264_pos	Indole	ENSGALG00000041143	UMOD
Com_151_pos	Pyridoxamine	ENSGALG00000037253	CLEC4M
Com_471_pos	Indole-3-acetic acid	ENSGALG00000011314	LRRC3B
Com_208_neg	N-Acetylanthranilic acid	ENSGALG00000030324	ELAPOR2
Com_471_pos	Indole-3-acetic acid	ENSGALG00000011391	AMN
Com_97_pos	L-Threonine	ENSGALG00000004782	TSEN15
Com_68_neg	PE (16:0/22:6)	ENSGALG00000004472	ASTN1
Com_171_neg	LPC 22:6	MSTRG.15507	--
Com_192_pos	1-Methylhistidine	ENSGALG00000042374	PDE11A
Com_175_pos	Pantothenic acid	MSTRG.21394	gag-pol
Com_186_pos	4-Hydroxybenzaldehyde	ENSGALG00000012414	GNPNAT1

Com_8_neg	4-Methyl-2-Oxopentanoic	ENSGALG00000051205	nicA
Com_203_pos	Serotonin	ENSGALG00000015362	TRAT1
Com_18_neg	Arachidonic acid	ENSGALG00000016595	TRIM35
Com_151_pos	Pyridoxamine	ENSGALG00000040857	TECTA
Com_413_pos	L-Cystine	ENSGALG00000004341	Cryzl2
Com_482_pos	8-Hydroxyquinoline	ENSGALG00000013728	PPAT
Com_8_neg	4-Methyl-2-Oxopentanoic	ENSGALG00000003147	TRPC4AP
Com_208_neg	N-Acetylanthranilic acid	MSTRG.5232	PERCC1
Com_16_neg	3-Hydroxybutyric acid	MSTRG.16504	gag
Com_331_pos	L-Lysine	ENSGALG00000016885	STK24
Com_192_pos	1-Methylhistidine	ENSGALG00000007030	MFSD13A
Com_175_pos	Pantothenic acid	ENSGALG00000051123	pol
Com_352_pos	Riboflavin	ENSGALG00000004959	IRS1
Com_18_neg	Arachidonic acid	ENSGALG00000011254	SATB1
Com_362_pos	2-Arachidonoyl glycerol	ENSGALG00000036836	SOSTDC1
Com_16_neg	3-Hydroxybutyric acid	ENSGALG00000028822	RNF152
Com_76_neg	Erythronolactone	ENSGALG00000005815	TMEM41B
Com_265_pos	6-Methylquinoline	ENSGALG00000038666	FBXL12
Com_264_pos	Indole	ENSGALG00000007234	CLCN5
Com_17_pos	L-Norleucine	ENSGALG00000047687	SETD9
Com_208_neg	N-Acetylanthranilic acid	ENSGALG00000004320	FAT2
Com_440_pos	PC (18:4e/4:0)	ENSGALG00000049658	UTS2R
Com_208_neg	N-Acetylanthranilic acid	ENSGALG00000004221	IL22RA1
Com_152_pos	Acetyl-L-carnitine	ENSGALG00000037479	IGSF21
Com_386_pos	2-Amino-1,3-octadecanec	ENSGALG00000051466	NDFIP2
Com_18_neg	Arachidonic acid	ENSGALG00000048223	FAM20C
Com_40_pos	Choline	ENSGALG00000008226	NIF3L1
Com_17_pos	L-Norleucine	ENSGALG00000038532	--
Com_119_pos	DL-Stachydrine	ENSGALG00000036005	TIAM2
Com_151_pos	Pyridoxamine	ENSGALG00000032628	SRCIN1
Com_208_neg	N-Acetylanthranilic acid	ENSGALG00000010013	EDNRA
Com_17_pos	L-Norleucine	MSTRG.6225	--
Com_97_pos	L-Threonine	ENSGALG00000029235	CPNE4
Com_152_pos	Acetyl-L-carnitine	ENSGALG00000032889	RBFOX3
Com_80_pos	DL-Lysine	MSTRG.15443	--
Com_40_pos	Choline	ENSGALG00000053886	gag
Com_482_pos	8-Hydroxyquinoline	ENSGALG00000011003	SLC35F3
Com_16_neg	3-Hydroxybutyric acid	ENSGALG00000004729	SLC7A10
Com_203_pos	Serotonin	ENSGALG00000011684	STAP1
Com_208_neg	N-Acetylanthranilic acid	ENSGALG00000007492	YME1L1
Com_22_pos	Indole-3-acrylic acid	ENSGALG00000005888	PGP
Com_40_pos	Choline	MSTRG.8955	--
Com_413_pos	L-Cystine	ENSGALG00000036836	SOSTDC1
Com_152_pos	Acetyl-L-carnitine	ENSGALG00000021685	SERINC2
Com_40_pos	Choline	ENSGALG00000037467	NEU2
Com_130_neg	2-Hydroxyvaleric acid	ENSGALG00000007018	SLC26A11
Com_55_pos	Valine	ENSGALG00000016511	ADGRG2
Com_440_pos	PC (18:4e/4:0)	ENSGALG00000022758	GGACT
Com_8_neg	4-Methyl-2-Oxopentanoic	ENSGALG00000046757	ERVK-9
Com_86_neg	Levulinic acid	ENSGALG00000000645	Espn
Com_97_pos	L-Threonine	ENSGALG00000013036	ATP6V1E1
Com_12_pos	Betaine	ENSGALG00000009365	CYP51A1
Com_192_pos	1-Methylhistidine	ENSGALG00000008784	SPATA1
Com_482_pos	8-Hydroxyquinoline	ENSGALG00000003212	TSPO2
Com_413_pos	L-Cystine	MSTRG.21321	gag
Com_152_pos	Acetyl-L-carnitine	MSTRG.11802	TMEM221
Com_192_pos	1-Methylhistidine	ENSGALG00000050258	--
Com_54_pos	Uric acid	ENSGALG00000013569	SEC61B

Com_482_pos	8-Hydroxyquinoline	ENSGALG00000012754	PAH
Com_362_pos	2-Arachidonoyl glycerol	ENSGALG00000004341	Cryzl2
Com_192_pos	1-Methylhistidine	ENSGALG00000002707	CHRNB2
Com_460_pos	3-amino-4-(propylamino)(	ENSGALG00000054746	--
Com_186_pos	4-Hydroxybenzaldehyde	MSTRG.8013	--
Com_55_pos	Valine	ENSGALG00000007018	SLC26A11
Com_440_pos	PC (18:4e/4:0)	ENSGALG00000005739	SCD
Com_99_pos	Creatine	ENSGALG00000030801	CCKAR
Com_440_pos	PC (18:4e/4:0)	ENSGALG00000030908	ATP2B2
Com_16_neg	3-Hydroxybutyric acid	ENSGALG00000015136	ILDR1
Com_92_pos	D-(+)-Proline	ENSGALG00000007848	PTS
Com_208_neg	N-Acetylanthranilic acid	ENSGALG00000024449	RAMP2
Com_151_pos	Pyridoxamine	ENSGALG00000031482	Pou5f3
Com_482_pos	8-Hydroxyquinoline	ENSGALG00000016036	DOP1B
Com_80_pos	DL-Lysine	ENSGALG00000038723	RPP25L
Com_440_pos	PC (18:4e/4:0)	ENSGALG00000033656	DQX1
Com_152_pos	Acetyl-L-carnitine	ENSGALG00000001749	ACSBG2
Com_17_pos	L-Norleucine	ENSGALG00000053245	VTG2
Com_18_neg	Arachidonic acid	ENSGALG00000051398	TMEM14C
Com_147_pos	D-Sphingosine	ENSGALG00000031312	ANAPC13
Com_588_pos	Ornithine	ENSGALG00000015016	SLC22A15
Com_440_pos	PC (18:4e/4:0)	ENSGALG00000012882	KDSR
Com_89_neg	Gallic acid	ENSGALG00000005776	TECR
Com_482_pos	8-Hydroxyquinoline	ENSGALG00000017046	POSTN
Com_92_pos	D-(+)-Proline	ENSGALG00000004631	DRAXIN
Com_80_pos	DL-Lysine	ENSGALG00000036492	DAGLA
Com_97_pos	L-Threonine	MSTRG.2316	env
Com_265_pos	6-Methylquinoline	ENSGALG00000029898	YKT6
Com_108_neg	LPE 18:2	ENSGALG00000052991	DNAJC21
Com_80_pos	DL-Lysine	MSTRG.17073	--
Com_208_neg	N-Acetylanthranilic acid	ENSGALG00000002371	RUSC2
Com_97_pos	L-Threonine	ENSGALG00000015134	APOV1
Com_175_pos	Pantothenic acid	ENSGALG00000000226	TMEM9
Com_40_pos	Choline	ENSGALG00000030185	PTDSS1
Com_175_pos	Pantothenic acid	MSTRG.8501	--
Com_99_pos	Creatine	ENSGALG00000008326	SYTL2
Com_215_pos	D-Erythro-sphingosine 1-p	ENSGALG00000038652	Gsta3
Com_92_pos	D-(+)-Proline	MSTRG.21204	--
Com_331_pos	L-Lysine	MSTRG.2387	--
Com_76_neg	Erythronolactone	ENSGALG00000016476	TTC32
Com_97_pos	L-Threonine	MSTRG.5319	--
Com_86_neg	Levulinic acid	ENSGALG00000041533	SLC11A2
Com_331_pos	L-Lysine	ENSGALG00000015729	LPAR1
Com_97_pos	L-Threonine	ENSGALG00000005353	FAR1
Com_80_pos	DL-Lysine	ENSGALG00000012112	DBI
Com_40_pos	Choline	ENSGALG00000021395	ABCA9
Com_17_pos	L-Norleucine	ENSGALG00000010018	CTSEAL
Com_78_neg	Citric acid	MSTRG.1503	gag
Com_40_pos	Choline	ENSGALG00000036234	RFWD3
Com_175_pos	Pantothenic acid	ENSGALG00000014525	USP5
Com_92_pos	D-(+)-Proline	ENSGALG00000012586	GKAP1
Com_175_pos	Pantothenic acid	ENSGALG00000033051	CAMK1D
Com_252_pos	cis-4-Hydroxy-D-proline	MSTRG.16398	Cdhr5
Com_8_neg	4-Methyl-2-Oxopentanoic	ENSGALG00000039538	CLDND1
Com_55_pos	Valine	ENSGALG00000014906	MOCS2
Com_40_pos	Choline	ENSGALG00000036915	SQLE
Com_460_pos	3-amino-4-(propylamino)(	ENSGALG00000012944	DENND5B
Com_4_pos	PC (17:1/17:1)	ENSGALG00000001392	MMP23B

Com_440_pos	PC (18:4e/4:0)	ENSGALG00000038145	DPP7
Com_40_pos	Choline	ENSGALG00000023691	ENTPD7
Com_413_pos	L-Cystine	MSTRG.4702	--
Com_89_neg	Gallic acid	ENSGALG00000013090	LOXL4
Com_413_pos	L-Cystine	ENSGALG00000009170	NCEH1
Com_482_pos	8-Hydroxyquinoline	ENSGALG00000041680	KCNT2
Com_22_pos	Indole-3-acrylic acid	ENSGALG00000041143	UMOD
Com_16_neg	3-Hydroxybutyric acid	ENSGALG00000031525	TSTA3
Com_192_pos	1-Methylhistidine	ENSGALG00000053659	--
Com_16_neg	3-Hydroxybutyric acid	ENSGALG00000036787	HSD17B12
Com_78_neg	Citric acid	ENSGALG00000004322	AHR
Com_175_pos	Pantothenic acid	ENSGALG00000013149	MOCOS
Com_192_pos	1-Methylhistidine	ENSGALG00000030076	PCSK6
Com_55_pos	Valine	MSTRG.53	SND1
Com_186_pos	4-Hydroxybenzaldehyde	ENSGALG00000048285	TCN2
Com_147_pos	D-Sphingosine	ENSGALG00000003948	ALAS1
Com_208_neg	N-Acetylanthranilic acid	ENSGALG00000001863	VTG2
Com_8_neg	4-Methyl-2-Oxopentanoic	MSTRG.18132	FBXW4
Com_311_pos	PC (18:4e/2:0)	ENSGALG00000010978	ANGPTL3
Com_99_pos	Creatine	MSTRG.15241	--
Com_18_neg	Arachidonic acid	ENSGALG00000001475	STMN1
Com_194_pos	Pipecolic acid	ENSGALG00000014126	endou-a
Com_471_pos	Indole-3-acetic acid	MSTRG.14987	--
Com_413_pos	L-Cystine	MSTRG.149	--
Com_152_pos	Acetyl-L-carnitine	ENSGALG00000051205	nicA
Com_78_neg	Citric acid	MSTRG.149	--
Com_16_neg	3-Hydroxybutyric acid	ENSGALG00000047380	MR1
Com_8_neg	4-Methyl-2-Oxopentanoic	ENSGALG00000028294	Vwa5b2
Com_17_pos	L-Norleucine	MSTRG.1502	gag
Com_8_neg	4-Methyl-2-Oxopentanoic	ENSGALG00000004216	TOR3A
Com_92_pos	D-(+)-Proline	ENSGALG00000016475	Zp2
Com_186_pos	4-Hydroxybenzaldehyde	ENSGALG00000008297	SEMA4B
Com_252_pos	cis-4-Hydroxy-D-proline	MSTRG.8954	--
Com_178_pos	Maltol	ENSGALG00000016885	STK24
Com_22_pos	Indole-3-acrylic acid	ENSGALG00000007234	CLCN5
Com_54_pos	Uric acid	ENSGALG00000053043	CARHSP1
Com_208_neg	N-Acetylanthranilic acid	ENSGALG00000047720	KLHDC7A
Com_16_neg	3-Hydroxybutyric acid	ENSGALG00000026547	TPGS2
Com_97_pos	L-Threonine	ENSGALG00000033365	ALDH1A3
Com_331_pos	L-Lysine	ENSGALG00000008862	DNAJC10
Com_55_pos	Valine	MSTRG.21796	--
Com_86_neg	Levulinic acid	ENSGALG00000038532	--
Com_18_neg	Arachidonic acid	ENSGALG00000004170	ADA
Com_25_pos	2-Hydroxycinnamic acid	ENSGALG00000012505	LRFN5
Com_120_neg	LPC 15:0	ENSGALG00000053013	EPB41L4B
Com_147_pos	D-Sphingosine	ENSGALG00000005470	PLPPR5
Com_17_pos	L-Norleucine	ENSGALG00000012877	CREB3L2
Com_482_pos	8-Hydroxyquinoline	ENSGALG00000038520	STRIP2
Com_460_pos	3-amino-4-(propylamino)(	ENSGALG00000001863	VTG2
Com_311_pos	PC (18:4e/2:0)	ENSGALG00000009680	PAQR7
Com_21_pos	DL-Tryptophan	ENSGALG00000055000	KCTD14
Com_413_pos	L-Cystine	ENSGALG00000004322	AHR
Com_22_pos	Indole-3-acrylic acid	ENSGALG00000046412	Aoc3
Com_352_pos	Riboflavin	ENSGALG00000009920	COCH
Com_120_neg	LPC 15:0	ENSGALG00000012111	TMEM37
Com_264_pos	Indole	ENSGALG00000005888	PGP
Com_16_neg	3-Hydroxybutyric acid	ENSGALG00000008262	RASGRF1
Com_151_pos	Pyridoxamine	ENSGALG00000054926	--

Com_311_pos	PC (18:4e/2:0)	ENSGALG00000049658	UTS2R
Com_588_pos	Ornithine	ENSGALG00000012196	MCUB
Com_80_pos	DL-Lysine	ENSGALG00000015263	TMEM30C
Com_588_pos	Ornithine	ENSGALG00000048432	--
Com_192_pos	1-Methylhistidine	ENSGALG00000003568	PPP1R16B
Com_17_pos	L-Norleucine	ENSGALG00000006723	IDI1
Com_208_neg	N-Acetylanthranilic acid	ENSGALG00000031570	WDR54
Com_78_neg	Citric acid	MSTRG.4702	--
Com_92_pos	D-(+)-Proline	MSTRG.8471	--
Com_152_pos	Acetyl-L-carnitine	MSTRG.12923	--
Com_76_neg	Erythronolactone	ENSGALG00000015333	PCGF3
Com_252_pos	cis-4-Hydroxy-D-proline	ENSGALG00000004106	DHCR7
Com_252_pos	cis-4-Hydroxy-D-proline	ENSGALG00000030661	STAT2
Com_8_neg	4-Methyl-2-Oxopentanoic	ENSGALG00000021685	SERINC2
Com_80_pos	DL-Lysine	ENSGALG00000003575	Dnttip1
Com_386_pos	2-Amino-1,3-octadecanec	ENSGALG00000032628	SRCIN1
Com_76_neg	Erythronolactone	ENSGALG00000029015	TM6SF2
Com_171_neg	LPC 22:6	ENSGALG00000007507	MASTL
Com_265_pos	6-Methylquinoline	ENSGALG00000016610	PTRHD1
Com_208_neg	N-Acetylanthranilic acid	ENSGALG00000012944	DENND5B
Com_80_pos	DL-Lysine	ENSGALG00000002802	PACSIN1
Com_130_neg	2-Hydroxyvaleric acid	ENSGALG00000014976	GATA6
Com_413_pos	L-Cystine	MSTRG.1503	gag
Com_151_pos	Pyridoxamine	ENSGALG00000051466	NDFIP2
Com_8_neg	4-Methyl-2-Oxopentanoic	MSTRG.11802	TMEM221
Com_40_pos	Choline	MSTRG.53	SND1
Com_588_pos	Ornithine	MSTRG.15507	--
Com_440_pos	PC (18:4e/4:0)	MSTRG.16287	--
Com_130_neg	2-Hydroxyvaleric acid	ENSGALG00000042555	STAMBP
Com_151_neg	Lysope 18:1	ENSGALG00000052894	Ranbp2
Com_192_pos	1-Methylhistidine	ENSGALG00000032287	ATP2A2
Com_386_pos	2-Amino-1,3-octadecanec	ENSGALG00000027070	TIMP2
Com_17_pos	L-Norleucine	MSTRG.14577	SLC39A5
Com_482_pos	8-Hydroxyquinoline	ENSGALG00000031158	OAT
Com_192_pos	1-Methylhistidine	MSTRG.11834	--
Com_25_pos	2-Hydroxycinnamic acid	ENSGALG00000040620	LSAMP
Com_130_neg	2-Hydroxyvaleric acid	MSTRG.5319	--
Com_588_pos	Ornithine	ENSGALG00000014813	HOMER1
Com_265_pos	6-Methylquinoline	ENSGALG00000024295	MYCBP
Com_12_pos	Betaine	MSTRG.20573	--
Com_55_pos	Valine	ENSGALG00000023691	ENTPD7
Com_108_neg	LPE 18:2	ENSGALG00000003432	AGXT2
Com_175_pos	Pantothenic acid	MSTRG.6512	--
Com_92_pos	D-(+)-Proline	ENSGALG00000047495	LRRC10
Com_40_pos	Choline	ENSGALG00000014906	MOCS2
Com_311_pos	PC (18:4e/2:0)	ENSGALG00000030908	ATP2B2
Com_12_pos	Betaine	ENSGALG00000005617	NTHL1
Com_8_neg	4-Methyl-2-Oxopentanoic	ENSGALG00000001749	ACSBG2
Com_152_pos	Acetyl-L-carnitine	ENSGALG00000005648	Sesn3
Com_55_pos	Valine	ENSGALG00000037935	RARA
Com_178_pos	Maltol	ENSGALG00000046731	--
Com_175_pos	Pantothenic acid	ENSGALG00000001475	STMN1
Com_362_pos	2-Arachidonoyl glycerol	ENSGALG00000037773	ST3GAL1
Com_25_pos	2-Hydroxycinnamic acid	ENSGALG00000000241	STARD4
Com_76_neg	Erythronolactone	ENSGALG00000034716	HEY2
Com_265_pos	6-Methylquinoline	ENSGALG00000054546	ERVK-11
Com_311_pos	PC (18:4e/2:0)	ENSGALG00000033656	DQX1
Com_55_pos	Valine	ENSGALG00000003103	MST1R

Com_55_pos	Valine	ENSGALG00000021395	ABCA9
Com_171_neg	LPC 22:6	MSTRG.1503	gag
Com_16_neg	3-Hydroxybutyric acid	MSTRG.19463	VTG2
Com_588_pos	Ornithine	ENSGALG00000014233	FBLN1
Com_386_pos	2-Amino-1,3-octadecanec	ENSGALG00000037253	CLEC4M
Com_92_pos	D-(+)-Proline	ENSGALG00000004055	C7orf50
Com_16_neg	3-Hydroxybutyric acid	ENSGALG00000011254	SATB1
Com_178_pos	Maltol	MSTRG.2387	--
Com_76_neg	Erythronolactone	ENSGALG00000052612	RPS27L
Com_18_neg	Arachidonic acid	ENSGALG00000028822	RNF152
Com_25_pos	2-Hydroxycinnamic acid	ENSGALG00000006842	ACOT8
Com_99_pos	Creatine	ENSGALG00000013828	GALM
Com_55_pos	Valine	ENSGALG00000030185	PTDSS1
Com_178_pos	Maltol	ENSGALG00000015729	LPAR1
Com_99_pos	Creatine	MSTRG.12570	--
Com_120_neg	LPC 15:0	ENSGALG00000054746	--
Com_12_pos	Betaine	MSTRG.16903	--
Com_252_pos	cis-4-Hydroxy-D-proline	ENSGALG00000010294	RPS6KL1
Com_192_pos	1-Methylhistidine	ENSGALG00000000695	MFSD4A
Com_147_pos	D-Sphingosine	ENSGALG00000003427	USP3
Com_151_pos	Pyridoxamine	ENSGALG00000002899	AACS
Com_152_pos	Acetyl-L-carnitine	ENSGALG00000041296	SOX7
Com_4_pos	PC (17:1/17:1)	ENSGALG00000003553	ABCA12
Com_208_neg	N-Acetylanthranilic acid	ENSGALG00000054746	--
Com_171_neg	LPC 22:6	ENSGALG00000004322	AHR
Com_18_neg	Arachidonic acid	ENSGALG00000004729	SLC7A10
Com_151_pos	Pyridoxamine	MSTRG.13135	--
Com_186_pos	4-Hydroxybenzaldehyde	MSTRG.18213	--
Com_25_pos	2-Hydroxycinnamic acid	ENSGALG00000001918	DNAJB5
Com_130_neg	2-Hydroxyvaleric acid	MSTRG.3156	--
Com_119_pos	DL-Stachydrine	ENSGALG00000038740	AMY2A
Com_54_pos	Uric acid	MSTRG.4813	--
Com_12_pos	Betaine	ENSGALG00000008604	TMEM255A
Com_147_pos	D-Sphingosine	ENSGALG00000022758	GGACT
Com_78_neg	Citric acid	ENSGALG00000036836	SOSTDC1
Com_18_neg	Arachidonic acid	ENSGALG00000033051	CAMK1D
Com_265_pos	6-Methylquinoline	ENSGALG00000043435	CARNS1
Com_12_pos	Betaine	ENSGALG00000052786	Znf185
Com_18_neg	Arachidonic acid	ENSGALG00000014525	USP5
Com_57_neg	LPC 16:1	ENSGALG00000009268	FGG
Com_252_pos	cis-4-Hydroxy-D-proline	ENSGALG00000006530	TSSC4
Com_460_pos	3-amino-4-(propylamino)(	ENSGALG00000007492	YME1L1
Com_171_neg	LPC 22:6	ENSGALG00000036754	CHKA
Com_588_pos	Ornithine	ENSGALG00000015044	GTF3C6
Com_311_pos	PC (18:4e/2:0)	ENSGALG00000038145	DPP7
Com_460_pos	3-amino-4-(propylamino)(	ENSGALG00000010013	EDNRA
Com_130_neg	2-Hydroxyvaleric acid	ENSGALG00000033365	ALDH1A3
Com_331_pos	L-Lysine	ENSGALG00000052612	RPS27L
Com_171_neg	LPC 22:6	MSTRG.149	--
Com_54_pos	Uric acid	ENSGALG00000052768	LDLR
Com_588_pos	Ornithine	MSTRG.21321	gag
Com_120_neg	LPC 15:0	ENSGALG00000028191	GLCE
Com_147_pos	D-Sphingosine	ENSGALG00000005739	SCD
Com_4_pos	PC (17:1/17:1)	ENSGALG00000031593	TMSB15B
Com_460_pos	3-amino-4-(propylamino)(	ENSGALG00000004221	IL22RA1
Com_460_pos	3-amino-4-(propylamino)(	ENSGALG00000004320	FAT2
Com_413_pos	L-Cystine	ENSGALG00000017378	CRTAC1
Com_18_neg	Arachidonic acid	ENSGALG00000000226	TMEM9

Com_18_neg	Arachidonic acid	ENSGALG00000015136	ILDR1
Com_194_pos	Pipecolic acid	ENSGALG00000016511	ADGRG2
Com_80_pos	DL-Lysine	ENSGALG00000035935	Unc13c
Com_86_neg	Levulinic acid	MSTRG.1502	gag
Com_252_pos	cis-4-Hydroxy-D-proline	ENSGALG00000002579	RIMBP2
Com_40_pos	Choline	ENSGALG00000016511	ADGRG2
Com_152_pos	Acetyl-L-carnitine	MSTRG.18132	FBXW4
Com_16_neg	3-Hydroxybutyric acid	ENSGALG00000051398	TMEM14C
Com_55_pos	Valine	MSTRG.8955	--
Com_55_pos	Valine	ENSGALG00000053886	gag
Com_78_neg	Citric acid	ENSGALG00000004341	Cryzl2
Com_147_pos	D-Sphingosine	ENSGALG00000012882	KDSR
Com_86_neg	Levulinic acid	ENSGALG00000012877	CREB3L2
Com_89_neg	Gallic acid	ENSGALG00000011469	IGFBP2
Com_440_pos	PC (18:4e/4:0)	ENSGALG00000010978	ANGPTL3
Com_175_pos	Pantothenic acid	ENSGALG00000051398	TMEM14C
Com_460_pos	3-amino-4-(propylamino)(	MSTRG.5232	PERCC1
Com_92_pos	D-(+)-Proline	ENSGALG00000037479	IGSF21
Com_482_pos	8-Hydroxyquinoline	ENSGALG00000030121	SLC2A11
Com_265_pos	6-Methylquinoline	ENSGALG00000035675	--
Com_588_pos	Ornithine	MSTRG.8986	gag
Com_55_pos	Valine	ENSGALG00000008226	NIF3L1
Com_120_neg	LPC 15:0	ENSGALG00000012944	DENND5B
Com_152_pos	Acetyl-L-carnitine	ENSGALG00000028294	Vwa5b2
Com_252_pos	cis-4-Hydroxy-D-proline	MSTRG.4813	--
Com_152_pos	Acetyl-L-carnitine	ENSGALG00000004216	TOR3A
Com_25_pos	2-Hydroxycinnamic acid	MSTRG.8497	--
Com_76_neg	Erythronolactone	MSTRG.12629	--
Com_178_pos	Maltol	ENSGALG00000008862	DNAJC10
Com_92_pos	D-(+)-Proline	ENSGALG00000032889	RBFOX3
Com_76_neg	Erythronolactone	MSTRG.20167	--
Com_80_pos	DL-Lysine	ENSGALG00000004505	CCDC137
Com_97_pos	L-Threonine	ENSGALG00000014976	GATA6
Com_171_neg	LPC 22:6	MSTRG.4702	--
Com_264_pos	Indole	ENSGALG00000046412	Aoc3
Com_482_pos	8-Hydroxyquinoline	ENSGALG00000016651	TDH
Com_86_neg	Levulinic acid	ENSGALG00000006723	IDI1
Com_97_pos	L-Threonine	ENSGALG00000042555	STAMBP
Com_120_neg	LPC 15:0	ENSGALG00000013001	CTNND2
Com_108_neg	LPE 18:2	MSTRG.20494	AHNAK
Com_120_neg	LPC 15:0	MSTRG.15732	--
Com_460_pos	3-amino-4-(propylamino)(	ENSGALG00000030324	ELAPOR2
Com_78_neg	Citric acid	ENSGALG00000007507	MASTL
Com_215_pos	D-Erythro-sphingosine 1-p	ENSGALG00000013776	CEP135
Com_208_neg	N-Acetylanthranilic acid	ENSGALG00000012111	TMEM37
Com_440_pos	PC (18:4e/4:0)	ENSGALG00000009680	PAQR7
Com_252_pos	cis-4-Hydroxy-D-proline	ENSGALG00000052986	Vwa5b2
Com_108_neg	LPE 18:2	ENSGALG00000016446	ATP6V1C2
Com_178_pos	Maltol	ENSGALG00000015684	Dnajc25
Com_252_pos	cis-4-Hydroxy-D-proline	ENSGALG00000005839	ARID3C
Com_68_neg	PE (16:0/22:6)	ENSGALG00000014971	SLC2A9
Com_21_pos	DL-Tryptophan	ENSGALG00000007178	FADS2
Com_208_neg	N-Acetylanthranilic acid	ENSGALG00000006080	GPC4
Com_120_neg	LPC 15:0	MSTRG.2393	--
Com_311_pos	PC (18:4e/2:0)	ENSGALG00000035626	DAD1
Com_97_pos	L-Threonine	MSTRG.15754	--
Com_264_pos	Indole	MSTRG.19672	--
Com_208_neg	N-Acetylanthranilic acid	ENSGALG00000053013	EPB41L4B

Com_194_pos	Pipecolic acid	ENSGALG00000035626	DAD1
Com_86_neg	Levulinic acid	MSTRG.14577	SLC39A5
Com_8_neg	4-Methyl-2-Oxopentanoic	MSTRG.12923	--
Com_80_pos	DL-Lysine	ENSGALG00000000104	CRY1
Com_21_pos	DL-Tryptophan	ENSGALG00000015937	FABP1
Com_80_pos	DL-Lysine	ENSGALG00000031067	TMEM132A
Com_99_pos	Creatine	ENSGALG00000015219	Selenoi
Com_152_pos	Acetyl-L-carnitine	ENSGALG00000007848	PTS
Com_22_pos	Indole-3-acrylic acid	MSTRG.7483	--
Com_252_pos	cis-4-Hydroxy-D-proline	MSTRG.3473	--
Com_215_pos	D-Erythro-sphingosine 1-p	ENSGALG00000007536	PHR
Com_21_pos	DL-Tryptophan	ENSGALG00000044278	C1orf131
Com_215_pos	D-Erythro-sphingosine 1-p	ENSGALG00000003432	AGXT2
Com_18_neg	Arachidonic acid	ENSGALG00000036787	HSD17B12
Com_12_pos	Betaine	ENSGALG00000023626	NTN1
Com_588_pos	Ornithine	ENSGALG00000009170	NCEH1
Com_175_pos	Pantothenic acid	ENSGALG00000041456	SLC35G1
Com_76_neg	Erythronolactone	ENSGALG00000014581	BORCS8
Com_130_neg	2-Hydroxyvaleric acid	MSTRG.2393	--
Com_175_pos	Pantothenic acid	ENSGALG00000011254	SATB1
Com_76_neg	Erythronolactone	ENSGALG00000008862	DNAJC10
Com_120_neg	LPC 15:0	ENSGALG00000001863	VTG2
Com_311_pos	PC (18:4e/2:0)	MSTRG.16287	--
Com_203_pos	Serotonin	ENSGALG00000053278	SUCNR1
Com_194_pos	Pipecolic acid	ENSGALG00000014906	MOCS2
Com_97_pos	L-Threonine	ENSGALG00000031067	TMEM132A
Com_178_pos	Maltol	MSTRG.9165	--
Com_54_pos	Uric acid	MSTRG.9007	--
Com_97_pos	L-Threonine	ENSGALG00000000104	CRY1
Com_413_pos	L-Cystine	ENSGALG00000002775	FA2H
Com_130_neg	2-Hydroxyvaleric acid	MSTRG.15732	--
Com_130_neg	2-Hydroxyvaleric acid	ENSGALG00000013001	CTNND2
Com_386_pos	2-Amino-1,3-octadecanec	ENSGALG00000047027	ADCK5
Com_151_neg	Lysope 18:1	ENSGALG00000023844	P2RY1
Com_16_neg	3-Hydroxybutyric acid	ENSGALG00000001475	STMN1
Com_152_pos	Acetyl-L-carnitine	MSTRG.21204	--
Com_80_pos	DL-Lysine	MSTRG.15754	--
Com_331_pos	L-Lysine	ENSGALG00000046731	--
Com_4_pos	PC (17:1/17:1)	MSTRG.13474	Klhl29
Com_18_neg	Arachidonic acid	ENSGALG00000047380	MR1
Com_8_neg	4-Methyl-2-Oxopentanoic	ENSGALG00000005648	Sesn3
Com_97_pos	L-Threonine	MSTRG.3156	--
Com_18_neg	Arachidonic acid	MSTRG.21394	gag-pol
Com_21_pos	DL-Tryptophan	ENSGALG00000052887	--
Com_186_pos	4-Hydroxybenzaldehyde	ENSGALG00000050267	CALU
Com_54_pos	Uric acid	ENSGALG00000030661	STAT2
Com_99_pos	Creatine	ENSGALG00000034868	KRT7
Com_54_pos	Uric acid	ENSGALG00000004106	DHCR7
Com_192_pos	1-Methylhistidine	ENSGALG00000010643	ZYG11B
Com_194_pos	Pipecolic acid	MSTRG.53	SND1
Com_178_pos	Maltol	ENSGALG00000031754	KCNG2
Com_18_neg	Arachidonic acid	ENSGALG00000026547	TPGS2
Com_192_pos	1-Methylhistidine	MSTRG.1890	gag
Com_386_pos	2-Amino-1,3-octadecanec	ENSGALG00000019276	SLCO1C1
Com_588_pos	Ornithine	MSTRG.2305	gag
Com_413_pos	L-Cystine	ENSGALG00000037050	FABP3
Com_80_pos	DL-Lysine	ENSGALG00000042080	PGPEP1
Com_171_neg	LPC 22:6	ENSGALG00000036836	SOSTDC1

Com_265_pos	6-Methylquinoline	ENSGALG00000016665	FDFT1
Com_471_pos	Indole-3-acetic acid	ENSGALG00000004230	LIPC
Com_18_neg	Arachidonic acid	ENSGALG00000008262	RASGRF1
Com_78_neg	Citric acid	ENSGALG00000036754	CHKA
Com_18_neg	Arachidonic acid	ENSGALG00000021658	PAFAH2
Com_130_neg	2-Hydroxyvaleric acid	ENSGALG00000028191	GLCE
Com_76_neg	Erythronolactone	ENSGALG00000004282	RCAN3
Com_17_pos	L-Norleucine	ENSGALG00000037773	ST3GAL1
Com_8_neg	4-Methyl-2-Oxopentanoic	ENSGALG00000041296	SOX7
Com_151_pos	Pyridoxamine	ENSGALG00000002500	GMPPB
Com_54_pos	Uric acid	MSTRG.8954	--
Com_76_neg	Erythronolactone	MSTRG.17961	--
Com_25_pos	2-Hydroxycinnamic acid	ENSGALG00000032329	NPM3
Com_92_pos	D-(+)-Proline	ENSGALG00000019276	SLCO1C1
Com_171_neg	LPC 22:6	ENSGALG00000020538	SLC49A3
Com_186_pos	4-Hydroxybenzaldehyde	ENSGALG00000002519	SLC25A33
Com_76_neg	Erythronolactone	ENSGALG00000015729	LPAR1
Com_186_pos	4-Hydroxybenzaldehyde	ENSGALG00000044251	RASSF7
Com_120_neg	LPC 15:0	MSTRG.3156	--
Com_99_pos	Creatine	ENSGALG00000004268	NIPAL3
Com_171_neg	LPC 22:6	ENSGALG00000004341	Cryzl2
Com_186_pos	4-Hydroxybenzaldehyde	ENSGALG00000051550	ARMH4
Com_362_pos	2-Arachidonoyl glycerol	MSTRG.14577	SLC39A5
Com_331_pos	L-Lysine	ENSGALG00000016476	TTC32
Com_178_pos	Maltol	ENSGALG00000052612	RPS27L
Com_76_neg	Erythronolactone	MSTRG.2387	--
Com_386_pos	2-Amino-1,3-octadecanec	ENSGALG00000031312	ANAPC13
Com_482_pos	8-Hydroxyquinoline	ENSGALG00000003972	FAXDC2
Com_175_pos	Pantothenic acid	ENSGALG00000026846	JMJD7
Com_460_pos	3-amino-4-(propylamino)(	ENSGALG00000004483	AHSA2
Com_92_pos	D-(+)-Proline	ENSGALG00000051205	nicA
Com_21_pos	DL-Tryptophan	ENSGALG00000053446	RED3
Com_40_pos	Choline	ENSGALG00000014126	endou-a
Com_186_pos	4-Hydroxybenzaldehyde	ENSGALG00000004852	DNM1
Com_152_pos	Acetyl-L-carnitine	ENSGALG00000016475	Zp2
Com_92_pos	D-(+)-Proline	ENSGALG00000047027	ADCK5
Com_413_pos	L-Cystine	MSTRG.15507	--
Com_18_neg	Arachidonic acid	ENSGALG00000051779	PRORSD1P
Com_12_pos	Betaine	ENSGALG00000049751	H2B-I
Com_264_pos	Indole	ENSGALG00000021039	HKDC1
Com_16_neg	3-Hydroxybutyric acid	ENSGALG00000042491	H4-I
Com_89_neg	Gallic acid	ENSGALG00000049755	IL22RA2
Com_99_pos	Creatine	MSTRG.2430	--
Com_362_pos	2-Arachidonoyl glycerol	ENSGALG00000006723	IDI1
Com_413_pos	L-Cystine	ENSGALG00000014813	HOMER1
Com_130_neg	2-Hydroxyvaleric acid	MSTRG.15754	--
Com_86_neg	Levulinic acid	ENSGALG00000014464	MTR
Com_151_neg	Lysope 18:1	ENSGALG00000014128	A4GALT
Com_175_pos	Pantothenic acid	MSTRG.8381	--
Com_264_pos	Indole	ENSGALG00000014907	DCBLD1
Com_215_pos	D-Erythro-sphingosine 1-p	MSTRG.20494	AHNAK
Com_108_neg	LPE 18:2	ENSGALG00000013776	CEP135
Com_22_pos	Indole-3-acrylic acid	ENSGALG00000038666	FBXL12
Com_18_neg	Arachidonic acid	MSTRG.19463	VTG2
Com_171_neg	LPC 22:6	ENSGALG00000051251	H2B-I
Com_12_pos	Betaine	ENSGALG00000034294	ATP6V0D2
Com_215_pos	D-Erythro-sphingosine 1-p	ENSGALG00000016446	ATP6V1C2
Com_54_pos	Uric acid	MSTRG.16398	Cdhr5

Com_152_pos	Acetyl-L-carnitine	MSTRG.8471	--
Com_362_pos	2-Arachidonoyl glycerol	ENSGALG00000012877	CREB3L2
Com_120_neg	LPC 15:0	ENSGALG00000007492	YME1L1
Com_194_pos	Pipecolic acid	ENSGALG00000023691	ENTPD7
Com_175_pos	Pantothenic acid	ENSGALG00000031525	TSTA3
Com_482_pos	8-Hydroxyquinoline	ENSGALG00000011524	PPEF2
Com_99_pos	Creatine	ENSGALG00000049232	POLR2A
Com_120_neg	LPC 15:0	ENSGALG00000010013	EDNRA
Com_471_pos	Indole-3-acetic acid	ENSGALG00000041491	ACKR4
Com_97_pos	L-Threonine	MSTRG.2393	--
Com_413_pos	L-Cystine	ENSGALG00000014233	FBLN1
Com_78_neg	Citric acid	ENSGALG00000037773	ST3GAL1
Com_178_pos	Maltol	ENSGALG00000012748	ELOVL2
Com_362_pos	2-Arachidonoyl glycerol	MSTRG.1502	gag
Com_22_pos	Indole-3-acrylic acid	MSTRG.19672	--
Com_460_pos	3-amino-4-(propylamino)(	ENSGALG00000028191	GLCE
Com_331_pos	L-Lysine	ENSGALG00000015684	Dnajc25
Com_151_pos	Pyridoxamine	ENSGALG00000008763	SSX2IP
Com_440_pos	PC (18:4e/4:0)	ENSGALG00000003427	USP3
Com_265_pos	6-Methylquinoline	MSTRG.8619	--
Com_130_neg	2-Hydroxyvaleric acid	ENSGALG00000031067	TMEM132A
Com_16_neg	3-Hydroxybutyric acid	ENSGALG00000006689	ABHD2
Com_120_neg	LPC 15:0	ENSGALG00000004221	IL22RA1
Com_413_pos	L-Cystine	ENSGALG00000005583	ALG14
Com_120_neg	LPC 15:0	ENSGALG00000004320	FAT2
Com_108_neg	LPE 18:2	ENSGALG00000007536	PHR
Com_130_neg	2-Hydroxyvaleric acid	ENSGALG00000000104	CRY1
Com_588_pos	Ornithine	ENSGALG00000008866	WDPCP
Com_194_pos	Pipecolic acid	ENSGALG00000021395	ABCA9
Com_97_pos	L-Threonine	MSTRG.15732	--
Com_178_pos	Maltol	ENSGALG00000006702	MFGE8
Com_97_pos	L-Threonine	ENSGALG00000013001	CTNND2
Com_120_neg	LPC 15:0	ENSGALG00000042555	STAMBP
Com_264_pos	Indole	MSTRG.7483	--
Com_18_neg	Arachidonic acid	ENSGALG00000007404	YIPF5
Com_16_neg	3-Hydroxybutyric acid	ENSGALG00000033051	CAMK1D
Com_18_neg	Arachidonic acid	MSTRG.13261	--
Com_588_pos	Ornithine	MSTRG.1082	--
Com_120_neg	LPC 15:0	ENSGALG00000014976	GATA6
Com_16_neg	3-Hydroxybutyric acid	ENSGALG00000014525	USP5
Com_4_pos	PC (17:1/17:1)	ENSGALG00000016558	VEGFD
Com_440_pos	PC (18:4e/4:0)	ENSGALG00000035626	DAD1
Com_413_pos	L-Cystine	ENSGALG00000006872	PISD
Com_386_pos	2-Amino-1,3-octadecaned	ENSGALG00000003948	ALAS1
Com_21_pos	DL-Tryptophan	MSTRG.14680	--
Com_192_pos	1-Methylhistidine	ENSGALG00000004498	SLC2A10
Com_194_pos	Pipecolic acid	ENSGALG00000030185	PTDSS1
Com_120_neg	LPC 15:0	MSTRG.5232	PERCC1
Com_8_neg	4-Methyl-2-Oxopentanoic	ENSGALG00000007848	PTS
Com_152_pos	Acetyl-L-carnitine	ENSGALG00000047495	LRRC10
Com_108_neg	LPE 18:2	ENSGALG00000030920	APOC3
Com_588_pos	Ornithine	ENSGALG00000012377	HNMT
Com_331_pos	L-Lysine	ENSGALG00000005815	TMEM41B
Com_194_pos	Pipecolic acid	ENSGALG00000009680	PAQR7
Com_186_pos	4-Hydroxybenzaldehyde	ENSGALG00000019738	FBXO47
Com_76_neg	Erythronolactone	ENSGALG00000016885	STK24
Com_413_pos	L-Cystine	ENSGALG00000015044	GTF3C6
Com_12_pos	Betaine	MSTRG.13584	--

Com_97_pos	L-Threonine	ENSGALG00000002802	PACSIN1
Com_86_neg	Levulinic acid	ENSGALG00000054297	SND1
Com_16_neg	3-Hydroxybutyric acid	ENSGALG00000000226	TMEM9
Com_331_pos	L-Lysine	MSTRG.9165	--
Com_460_pos	3-amino-4-(propylamino)(	ENSGALG00000013001	CTNND2
Com_192_pos	1-Methylhistidine	ENSGALG00000035239	GLCCI1
Com_460_pos	3-amino-4-(propylamino)(	MSTRG.15732	--
Com_120_neg	LPC 15:0	ENSGALG00000030324	ELAPOR2
Com_186_pos	4-Hydroxybenzaldehyde	ENSGALG00000000745	SLC26A9
Com_22_pos	Indole-3-acrylic acid	ENSGALG00000029898	YKT6
Com_76_neg	Erythronolactone	MSTRG.2170	MYO16
Com_21_pos	DL-Tryptophan	MSTRG.1841	--
Com_386_pos	2-Amino-1,3-octadecanec	ENSGALG00000005470	PLPPR5
Com_97_pos	L-Threonine	ENSGALG00000028191	GLCE
Com_588_pos	Ornithine	ENSGALG00000017378	CRTAC1
Com_8_neg	4-Methyl-2-Oxopentanoic	MSTRG.21204	--
Com_186_pos	4-Hydroxybenzaldehyde	ENSGALG00000004231	IFNLR1
Com_252_pos	cis-4-Hydroxy-D-proline	ENSGALG00000013569	SEC61B
Com_151_pos	Pyridoxamine	ENSGALG00000030845	ENHO
Com_460_pos	3-amino-4-(propylamino)(	MSTRG.2393	--
Com_147_pos	D-Sphingosine	ENSGALG00000010978	ANGPTL3
Com_413_pos	L-Cystine	ENSGALG00000048035	GCNT2
Com_194_pos	Pipecolic acid	ENSGALG00000010978	ANGPTL3
Com_99_pos	Creatine	MSTRG.835	--
Com_54_pos	Uric acid	ENSGALG00000029308	PNPLA3
Com_413_pos	L-Cystine	ENSGALG00000000107	TRIM7.1
Com_331_pos	L-Lysine	ENSGALG00000031754	KCNG2
Com_460_pos	3-amino-4-(propylamino)(	MSTRG.9835	--
Com_86_neg	Levulinic acid	ENSGALG00000037773	ST3GAL1
Com_413_pos	L-Cystine	MSTRG.8986	gag
Com_413_pos	L-Cystine	ENSGALG00000054319	ELOVL6
Com_80_pos	DL-Lysine	ENSGALG00000033365	ALDH1A3
Com_194_pos	Pipecolic acid	MSTRG.8955	--
Com_57_neg	LPC 16:1	ENSGALG00000052894	Ranbp2
Com_78_neg	Citric acid	ENSGALG00000020538	SLC49A3
Com_99_pos	Creatine	ENSGALG00000036527	SYBU
Com_194_pos	Pipecolic acid	ENSGALG00000053886	gag
Com_25_pos	2-Hydroxycinnamic acid	MSTRG.16398	Cdhr5
Com_16_neg	3-Hydroxybutyric acid	MSTRG.13439	--
Com_471_pos	Indole-3-acetic acid	ENSGALG00000002790	ABLIM3
Com_147_pos	D-Sphingosine	ENSGALG00000009680	PAQR7
Com_482_pos	8-Hydroxyquinoline	ENSGALG00000007114	APOA1
Com_86_neg	Levulinic acid	ENSGALG00000010641	SCCPDH
Com_194_pos	Pipecolic acid	ENSGALG00000008226	NIF3L1
Com_151_neg	Lysope 18:1	ENSGALG00000003022	Fmo5
Com_147_pos	D-Sphingosine	ENSGALG00000027070	TIMP2
Com_265_pos	6-Methylquinoline	ENSGALG00000041143	UMOD
Com_471_pos	Indole-3-acetic acid	ENSGALG00000015425	LPL
Com_175_pos	Pantothenic acid	ENSGALG00000030151	LUZP2
Com_208_neg	N-Acetylanthranilic acid	ENSGALG00000008752	ACOT1
Com_40_pos	Choline	ENSGALG00000032170	LCN15
Com_482_pos	8-Hydroxyquinoline	ENSGALG00000004598	CUX2
Com_362_pos	2-Arachidonoyl glycerol	ENSGALG00000007507	MASTL
Com_18_neg	Arachidonic acid	ENSGALG00000005610	SLC44A3
Com_171_neg	LPC 22:6	ENSGALG00000023517	AGPAT2
Com_192_pos	1-Methylhistidine	ENSGALG00000050520	pol
Com_92_pos	D-(+)-Proline	MSTRG.18132	FBXW4
Com_171_neg	LPC 22:6	MSTRG.19422	--

Com_178_pos	Maltol	ENSGALG00000016476	TTC32
Com_17_pos	L-Norleucine	ENSGALG00000004341	Cryzl2
Com_22_pos	Indole-3-acrylic acid	ENSGALG00000021039	HKDC1
Com_264_pos	Indole	ENSGALG00000052296	MEX3D
Com_460_pos	3-amino-4-(propylamino)(	MSTRG.21371	--
Com_78_neg	Citric acid	ENSGALG00000051251	H2B-I
Com_175_pos	Pantothenic acid	ENSGALG00000009476	CDK6
Com_175_pos	Pantothenic acid	MSTRG.16504	gag
Com_386_pos	2-Amino-1,3-octadecanec	ENSGALG00000047495	LRRC10
Com_17_pos	L-Norleucine	ENSGALG00000014464	MTR
Com_92_pos	D-(+)-Proline	ENSGALG00000028294	Vwa5b2
Com_92_pos	D-(+)-Proline	ENSGALG00000004216	TOR3A
Com_55_pos	Valine	ENSGALG00000035626	DAD1
Com_175_pos	Pantothenic acid	ENSGALG00000021686	--
Com_8_neg	4-Methyl-2-Oxopentanoic	ENSGALG00000016475	Zp2
Com_25_pos	2-Hydroxycinnamic acid	MSTRG.16433	B4GALNT4
Com_22_pos	Indole-3-acrylic acid	ENSGALG00000014907	DCBLD1
Com_192_pos	1-Methylhistidine	MSTRG.15162	--
Com_413_pos	L-Cystine	ENSGALG00000047687	SETD9
Com_54_pos	Uric acid	ENSGALG00000032329	NPM3
Com_25_pos	2-Hydroxycinnamic acid	MSTRG.8954	--
Com_264_pos	Indole	ENSGALG00000038666	FBXL12
Com_265_pos	6-Methylquinoline	ENSGALG00000007234	CLCN5
Com_352_pos	Riboflavin	ENSGALG00000007478	SLC51A
Com_86_neg	Levulinic acid	ENSGALG00000010293	RBP
Com_471_pos	Indole-3-acetic acid	MSTRG.20478	--
Com_171_neg	LPC 22:6	ENSGALG00000037773	ST3GAL1
Com_92_pos	D-(+)-Proline	ENSGALG00000027070	TIMP2
Com_99_pos	Creatine	MSTRG.4550	--
Com_362_pos	2-Arachidonoyl glycerol	ENSGALG00000038532	--
Com_252_pos	cis-4-Hydroxy-D-proline	ENSGALG00000012505	LRFN5
Com_588_pos	Ornithine	ENSGALG00000002775	FA2H
Com_16_neg	3-Hydroxybutyric acid	MSTRG.21394	gag-pol
Com_352_pos	Riboflavin	ENSGALG00000028284	PTX3
Com_208_neg	N-Acetylanthranilic acid	ENSGALG00000043451	DNAL4
Com_97_pos	L-Threonine	ENSGALG00000012112	DBI
Com_413_pos	L-Cystine	MSTRG.6225	--
Com_80_pos	DL-Lysine	MSTRG.5319	--
Com_17_pos	L-Norleucine	ENSGALG00000036836	SOSTDC1
Com_171_neg	LPC 22:6	ENSGALG00000000619	ANGPTL4
Com_331_pos	L-Lysine	MSTRG.2388	--
Com_8_neg	4-Methyl-2-Oxopentanoic	MSTRG.8471	--
Com_331_pos	L-Lysine	ENSGALG00000005160	VMP1
Com_460_pos	3-amino-4-(propylamino)(	MSTRG.3156	--
Com_386_pos	2-Amino-1,3-octadecanec	MSTRG.8471	--
Com_386_pos	2-Amino-1,3-octadecanec	ENSGALG00000022758	GGACT
Com_208_neg	N-Acetylanthranilic acid	MSTRG.16459	--
Com_331_pos	L-Lysine	ENSGALG00000012748	ELOVL2
Com_265_pos	6-Methylquinoline	ENSGALG00000006198	LSS
Com_120_neg	LPC 15:0	ENSGALG00000004483	AHSA2
Com_331_pos	L-Lysine	ENSGALG00000012847	Slc7a11
Com_413_pos	L-Cystine	MSTRG.2305	gag
Com_16_neg	3-Hydroxybutyric acid	ENSGALG00000021658	PAFAH2
Com_130_neg	2-Hydroxyvaleric acid	ENSGALG00000002802	PACSIN1
Com_25_pos	2-Hydroxycinnamic acid	ENSGALG00000004106	DHCR7
Com_588_pos	Ornithine	ENSGALG00000037050	FABP3
Com_25_pos	2-Hydroxycinnamic acid	ENSGALG00000030661	STAT2
Com_588_pos	Ornithine	MSTRG.16504	gag

Com_460_pos	3-amino-4-(propylamino)(	ENSGALG00000012823	TRIM24
Com_25_pos	2-Hydroxycinnamic acid	ENSGALG00000033150	MIDN
Com_22_pos	Indole-3-acrylic acid	ENSGALG00000016610	PTRHD1
Com_331_pos	L-Lysine	ENSGALG00000006702	MFGE8
Com_215_pos	D-Erythro-sphingosine 1-p	ENSGALG00000030920	APOC3
Com_97_pos	L-Threonine	ENSGALG00000038723	RPP25L
Com_471_pos	Indole-3-acetic acid	ENSGALG00000002466	SLC2A5
Com_192_pos	1-Methylhistidine	MSTRG.8248	--
Com_311_pos	PC (18:4e/2:0)	ENSGALG00000003427	USP3
Com_386_pos	2-Amino-1,3-octadecaned	ENSGALG00000005739	SCD
Com_331_pos	L-Lysine	ENSGALG00000026460	myoM
Com_40_pos	Choline	ENSGALG00000002845	CTNNA3
Com_8_neg	4-Methyl-2-Oxopentanoic	ENSGALG00000045557	MTTPL
Com_17_pos	L-Norleucine	ENSGALG00000054297	SND1
Com_460_pos	3-amino-4-(propylamino)(	ENSGALG00000010391	MMRN1
Com_413_pos	L-Cystine	ENSGALG00000053245	VTG2
Com_386_pos	2-Amino-1,3-octadecaned	ENSGALG00000016475	Zp2
Com_362_pos	2-Arachidonoyl glycerol	ENSGALG00000036754	CHKA
Com_178_pos	Maltol	ENSGALG00000005815	TMEM41B
Com_8_neg	4-Methyl-2-Oxopentanoic	ENSGALG00000047495	LRRC10
Com_386_pos	2-Amino-1,3-octadecaned	ENSGALG00000012882	KDSR
Com_99_pos	Creatine	MSTRG.15745	--
Com_178_pos	Maltol	ENSGALG00000001492	NDRG3
Com_264_pos	Indole	ENSGALG00000029898	YKT6
Com_208_neg	N-Acetylanthranilic acid	ENSGALG00000010764	FBXO8
Com_16_neg	3-Hydroxybutyric acid	ENSGALG00000051779	PRORSD1P
Com_252_pos	cis-4-Hydroxy-D-proline	ENSGALG00000000241	STARD4
Com_40_pos	Choline	ENSGALG00000035244	H3-I
Com_12_pos	Betaine	ENSGALG00000015768	ANKRD6
Com_18_neg	Arachidonic acid	ENSGALG00000042491	H4-I
Com_12_pos	Betaine	MSTRG.8810	--
Com_362_pos	2-Arachidonoyl glycerol	ENSGALG00000041533	SLC11A2
Com_97_pos	L-Threonine	MSTRG.15443	--
Com_192_pos	1-Methylhistidine	ENSGALG00000013511	ANKRA2
Com_208_neg	N-Acetylanthranilic acid	MSTRG.9410	--
Com_80_pos	DL-Lysine	ENSGALG00000037018	USP36
Com_99_pos	Creatine	MSTRG.8013	--
Com_4_pos	PC (17:1/17:1)	ENSGALG00000006724	GPC5
Com_40_pos	Choline	ENSGALG00000001101	MBD3
Com_78_neg	Citric acid	MSTRG.14577	SLC39A5
Com_413_pos	L-Cystine	ENSGALG00000010018	CTSEAL
Com_186_pos	4-Hydroxybenzaldehyde	ENSGALG00000030801	CCKAR
Com_460_pos	3-amino-4-(propylamino)(	ENSGALG00000042555	STAMBP
Com_151_pos	Pyridoxamine	ENSGALG00000036616	NUAK2
Com_40_pos	Choline	MSTRG.9006	--
Com_171_neg	LPC 22:6	ENSGALG00000043829	ext1c
Com_108_neg	LPE 18:2	ENSGALG00000017120	SACS
Com_460_pos	3-amino-4-(propylamino)(	ENSGALG00000014976	GATA6
Com_192_pos	1-Methylhistidine	MSTRG.8985	gag
Com_311_pos	PC (18:4e/2:0)	ENSGALG00000003103	MST1R
Com_17_pos	L-Norleucine	ENSGALG00000010641	SCCPDH
Com_16_neg	3-Hydroxybutyric acid	ENSGALG00000015016	SLC22A15
Com_482_pos	8-Hydroxyquinoline	ENSGALG00000021340	CA9
Com_17_pos	L-Norleucine	MSTRG.4702	--
Com_78_neg	Citric acid	ENSGALG00000006723	IDI1
Com_76_neg	Erythronolactone	ENSGALG00000007533	NPEPL1
Com_311_pos	PC (18:4e/2:0)	ENSGALG00000037935	RARA
Com_152_pos	Acetyl-L-carnitine	ENSGALG00000019276	SLCO1C1

Com_78_neg	Citric acid	ENSGALG00000023517	AGPAT2
Com_252_pos	cis-4-Hydroxy-D-proline	ENSGALG00000001918	DNAJB5
Com_208_neg	N-Acetylanthranilic acid	ENSGALG00000006647	DUSP8
Com_78_neg	Citric acid	MSTRG.19422	--
Com_18_neg	Arachidonic acid	ENSGALG00000006689	ABHD2
Com_120_neg	LPC 15:0	ENSGALG00000005353	FAR1
Com_12_pos	Betaine	ENSGALG00000013848	MVK
Com_178_pos	Maltol	ENSGALG00000026957	SEMA4G
Com_16_neg	3-Hydroxybutyric acid	ENSGALG00000007404	YIPF5
Com_16_neg	3-Hydroxybutyric acid	MSTRG.13261	--
Com_175_pos	Pantothenic acid	ENSGALG00000012377	HNMT
Com_386_pos	2-Amino-1,3-octadecanec	MSTRG.21204	--
Com_208_neg	N-Acetylanthranilic acid	ENSGALG00000009842	RASSF3
Com_78_neg	Citric acid	ENSGALG00000012877	CREB3L2
Com_57_neg	LPC 16:1	ENSGALG00000023844	P2RY1
Com_40_pos	Choline	ENSGALG00000027064	HIST1H3H
Com_186_pos	4-Hydroxybenzaldehyde	ENSGALG00000037769	NEBL
Com_76_neg	Erythronolactone	ENSGALG00000020342	ABHD12
Com_86_neg	Levulinic acid	ENSGALG00000004341	Cryzl2
Com_152_pos	Acetyl-L-carnitine	ENSGALG00000047027	ADCK5
Com_588_pos	Ornithine	ENSGALG00000005583	ALG14
Com_18_neg	Arachidonic acid	ENSGALG00000026460	myoM
Com_147_pos	D-Sphingosine	ENSGALG00000035626	DAD1
Com_413_pos	L-Cystine	ENSGALG00000008866	WDPCP
Com_120_neg	LPC 15:0	ENSGALG00000015134	APOV1
Com_8_neg	4-Methyl-2-Oxopentanoic	ENSGALG00000014463	ACTN2
Com_86_neg	Levulinic acid	ENSGALG00000046789	pol
Com_78_neg	Citric acid	MSTRG.1502	gag
Com_22_pos	Indole-3-acrylic acid	ENSGALG00000052296	MEX3D
Com_76_neg	Erythronolactone	ENSGALG00000043336	COPZ1
Com_130_neg	2-Hydroxyvaleric acid	ENSGALG00000012112	DBI
Com_147_pos	D-Sphingosine	ENSGALG00000047027	ADCK5
Com_175_pos	Pantothenic acid	MSTRG.1082	--
Com_120_neg	LPC 15:0	MSTRG.2316	env
Com_362_pos	2-Arachidonoyl glycerol	ENSGALG00000000645	Espn
Com_17_pos	L-Norleucine	MSTRG.149	--
Com_413_pos	L-Cystine	MSTRG.1082	--
Com_252_pos	cis-4-Hydroxy-D-proline	ENSGALG00000037325	SERP1
Com_178_pos	Maltol	ENSGALG00000027608	PIGC
Com_151_pos	Pyridoxamine	ENSGALG00000029083	NXPH2
Com_588_pos	Ornithine	ENSGALG00000006872	PISD
Com_120_neg	LPC 15:0	MSTRG.9835	--
Com_76_neg	Erythronolactone	ENSGALG00000050676	Ctnnd2
Com_97_pos	L-Threonine	ENSGALG00000000950	MVB12B
Com_175_pos	Pantothenic acid	ENSGALG00000008866	WDPCP
Com_17_pos	L-Norleucine	ENSGALG00000010293	RBP
Com_21_pos	DL-Tryptophan	ENSGALG00000015728	MUSK
Com_18_neg	Arachidonic acid	ENSGALG00000012847	Slc7a11
Com_413_pos	L-Cystine	ENSGALG00000012377	HNMT
Com_386_pos	2-Amino-1,3-octadecanec	ENSGALG00000007848	PTS
Com_22_pos	Indole-3-acrylic acid	ENSGALG00000035675	--
Com_78_neg	Citric acid	ENSGALG00000000619	ANGPTL4
Com_18_neg	Arachidonic acid	ENSGALG00000005160	VMP1
Com_147_pos	D-Sphingosine	ENSGALG00000019276	SLCO1C1
Com_25_pos	2-Hydroxycinnamic acid	ENSGALG00000005439	ACACA
Com_18_neg	Arachidonic acid	MSTRG.2388	--
Com_588_pos	Ornithine	ENSGALG00000031525	TSTA3
Com_12_pos	Betaine	ENSGALG00000003560	SLC6A2

Com_25_pos	2-Hydroxycinnamic acid	MSTRG.4813	--
Com_86_neg	Levulinic acid	ENSGALG00000036836	SOSTDC1
Com_252_pos	cis-4-Hydroxy-D-proline	MSTRG.8497	--
Com_17_pos	L-Norleucine	ENSGALG00000004322	AHR
Com_40_pos	Choline	MSTRG.1468	--
Com_130_neg	2-Hydroxyvaleric acid	ENSGALG00000038723	RPP25L
Com_460_pos	3-amino-4-(propylamino)(	ENSGALG00000011347	IHH
Com_55_pos	Valine	ENSGALG00000009680	PAQR7
Com_80_pos	DL-Lysine	ENSGALG00000050420	CTNND2
Com_194_pos	Pipecolic acid	ENSGALG00000012882	KDSR
Com_311_pos	PC (18:4e/2:0)	MSTRG.21796	--
Com_151_neg	Lysope 18:1	ENSGALG00000009848	LPGAT1
Com_12_pos	Betaine	ENSGALG00000023338	CBX2
Com_40_pos	Choline	ENSGALG00000000802	DHODH
Com_171_neg	LPC 22:6	ENSGALG00000007778	PES1
Com_120_neg	LPC 15:0	ENSGALG00000013036	ATP6V1E1
Com_208_neg	N-Acetylanthranilic acid	ENSGALG00000040484	SEC31A
Com_588_pos	Ornithine	ENSGALG00000048035	GCNT2
Com_178_pos	Maltol	MSTRG.2388	--
Com_178_pos	Maltol	ENSGALG00000005160	VMP1
Com_192_pos	1-Methylhistidine	ENSGALG00000045127	slc12a8
Com_192_pos	1-Methylhistidine	ENSGALG00000051159	--
Com_588_pos	Ornithine	ENSGALG00000000107	TRIM7.1
Com_54_pos	Uric acid	ENSGALG00000006842	ACOT8
Com_178_pos	Maltol	ENSGALG00000008039	MFSD13A
Com_460_pos	3-amino-4-(propylamino)(	ENSGALG00000034504	FAM20C
Com_194_pos	Pipecolic acid	ENSGALG00000005739	SCD
Com_264_pos	Indole	ENSGALG00000016610	PTRHD1
Com_120_neg	LPC 15:0	ENSGALG00000029235	CPNE4
Com_178_pos	Maltol	ENSGALG00000012847	Slc7a11
Com_18_neg	Arachidonic acid	MSTRG.13439	--
Com_17_pos	L-Norleucine	MSTRG.1503	gag
Com_208_neg	N-Acetylanthranilic acid	MSTRG.8874	gag
Com_120_neg	LPC 15:0	MSTRG.21371	--
Com_588_pos	Ornithine	ENSGALG00000054319	ELOVL6
Com_55_pos	Valine	ENSGALG00000010978	ANGPTL3
Com_80_pos	DL-Lysine	ENSGALG00000007018	SLC26A11
Com_440_pos	PC (18:4e/4:0)	ENSGALG00000027070	TIMP2
Com_130_neg	2-Hydroxyvaleric acid	MSTRG.15443	--
Com_460_pos	3-amino-4-(propylamino)(	ENSGALG00000014923	ARHGEF28
Com_194_pos	Pipecolic acid	ENSGALG00000022758	GGACT
Com_16_neg	3-Hydroxybutyric acid	ENSGALG00000005610	SLC44A3
Com_208_neg	N-Acetylanthranilic acid	MSTRG.2391	--
Com_178_pos	Maltol	ENSGALG00000026460	myoM
Com_331_pos	L-Lysine	ENSGALG00000005610	SLC44A3
Com_215_pos	D-Erythro-sphingosine 1-p	ENSGALG00000028284	PTX3
Com_362_pos	2-Arachidonoyl glycerol	ENSGALG00000020538	SLC49A3
Com_264_pos	Indole	ENSGALG00000014261	UCHL1
Com_57_neg	LPC 16:1	ENSGALG00000014128	A4GALT
Com_86_neg	Levulinic acid	MSTRG.13408	--
Com_152_pos	Acetyl-L-carnitine	ENSGALG00000045557	MTTPL
Com_16_neg	3-Hydroxybutyric acid	ENSGALG00000012196	MCUB
Com_54_pos	Uric acid	ENSGALG00000040620	LSAMP
Com_16_neg	3-Hydroxybutyric acid	ENSGALG00000048432	--
Com_215_pos	D-Erythro-sphingosine 1-p	ENSGALG00000007478	SLC51A
Com_171_neg	LPC 22:6	MSTRG.14577	SLC39A5
Com_76_neg	Erythronolactone	ENSGALG00000036293	EBAG9
Com_208_neg	N-Acetylanthranilic acid	ENSGALG00000008427	GNAT3

Com_76_neg	Erythronolactone	ENSGALG00000015689	ECPAS
Com_331_pos	L-Lysine	ENSGALG00000001492	NDRG3
Com_25_pos	2-Hydroxycinnamic acid	ENSGALG00000028949	CORO6
Com_55_pos	Valine	ENSGALG00000036492	DAGLA
Com_55_pos	Valine	MSTRG.17073	--
Com_78_neg	Citric acid	ENSGALG00000043829	ext1c
Com_471_pos	Indole-3-acetic acid	ENSGALG00000029270	GATA3
Com_175_pos	Pantothenic acid	ENSGALG00000029445	FADS6
Com_120_neg	LPC 15:0	ENSGALG00000004782	TSEN15
Com_460_pos	3-amino-4-(propylamino)(	ENSGALG00000002445	KIAA0319L
Com_171_neg	LPC 22:6	ENSGALG00000016491	APOB
Com_252_pos	cis-4-Hydroxy-D-proline	ENSGALG00000037065	SC5D
Com_471_pos	Indole-3-acetic acid	ENSGALG00000028897	WDR25
Com_215_pos	D-Erythro-sphingosine 1-p	ENSGALG00000017120	SACS
Com_175_pos	Pantothenic acid	MSTRG.14343	--
Com_120_neg	LPC 15:0	ENSGALG00000012823	TRIM24
Com_21_pos	DL-Tryptophan	ENSGALG00000029857	Gimap1
Com_208_neg	N-Acetylanthranilic acid	ENSGALG00000007508	HPSE2
Com_171_neg	LPC 22:6	ENSGALG00000006723	IDI1
Com_175_pos	Pantothenic acid	MSTRG.2305	gag
Com_40_pos	Choline	ENSGALG00000004804	TGM3
Com_471_pos	Indole-3-acetic acid	ENSGALG00000054322	--
Com_86_neg	Levulinic acid	MSTRG.4702	--
Com_22_pos	Indole-3-acrylic acid	ENSGALG00000016665	FDFT1
Com_386_pos	2-Amino-1,3-octadecanec	ENSGALG00000041296	SOX7
Com_80_pos	DL-Lysine	ENSGALG00000040342	ADAMTS1
Com_208_neg	N-Acetylanthranilic acid	ENSGALG00000012812	SVOPL
Com_12_pos	Betaine	ENSGALG00000050440	APOF
Com_362_pos	2-Arachidonoyl glycerol	ENSGALG00000051251	H2B-I
Com_588_pos	Ornithine	ENSGALG00000047687	SETD9
Com_362_pos	2-Arachidonoyl glycerol	ENSGALG00000014944	GCNT4
Com_208_neg	N-Acetylanthranilic acid	ENSGALG00000029033	Tldc2
Com_54_pos	Uric acid	MSTRG.2171	Myo16
Com_208_neg	N-Acetylanthranilic acid	ENSGALG00000034289	SLC41A3
Com_80_pos	DL-Lysine	ENSGALG00000007673	LRRC59
Com_120_neg	LPC 15:0	ENSGALG00000010391	MMRN1
Com_12_pos	Betaine	ENSGALG00000027960	GRPR
Com_171_neg	LPC 22:6	ENSGALG00000012877	CREB3L2
Com_76_neg	Erythronolactone	ENSGALG00000016595	TRIM35
Com_186_pos	4-Hydroxybenzaldehyde	ENSGALG00000035478	FAM91A1
Com_12_pos	Betaine	ENSGALG00000014509	BST1
Com_588_pos	Ornithine	MSTRG.6225	--
Com_130_neg	2-Hydroxyvaleric acid	ENSGALG00000000950	MVB12B
Com_151_pos	Pyridoxamine	ENSGALG00000023395	PLIN1
Com_25_pos	2-Hydroxycinnamic acid	ENSGALG00000051290	--
Com_440_pos	PC (18:4e/4:0)	ENSGALG00000003103	MST1R
Com_8_neg	4-Methyl-2-Oxopentanoic	ENSGALG00000019276	SLCO1C1
Com_78_neg	Citric acid	ENSGALG00000038532	--
Com_171_neg	LPC 22:6	MSTRG.1502	gag
Com_440_pos	PC (18:4e/4:0)	ENSGALG00000037935	RARA
Com_17_pos	L-Norleucine	ENSGALG00000046789	pol
Com_331_pos	L-Lysine	ENSGALG00000026957	SEMA4G
Com_588_pos	Ornithine	ENSGALG00000011254	SATB1
Com_21_pos	DL-Tryptophan	ENSGALG00000050091	CLEC2B
Com_21_pos	DL-Tryptophan	ENSGALG00000002362	MANF
Com_18_neg	Arachidonic acid	ENSGALG00000005815	TMEM41B
Com_86_neg	Levulinic acid	MSTRG.149	--
Com_386_pos	2-Amino-1,3-octadecanec	ENSGALG00000005648	Sesn3

Com_76_neg	Erythronolactone	ENSGALG00000041604	NPTXR
Com_186_pos	4-Hydroxybenzaldehyde	MSTRG.12570	--
Com_413_pos	L-Cystine	MSTRG.16504	gag
Com_76_neg	Erythronolactone	ENSGALG00000048223	FAM20C
Com_311_pos	PC (18:4e/2:0)	ENSGALG00000007018	SLC26A11
Com_8_neg	4-Methyl-2-Oxopentanoic	ENSGALG00000047027	ADCK5
Com_80_pos	DL-Lysine	MSTRG.21536	--
Com_482_pos	8-Hydroxyquinoline	ENSGALG00000047321	SARDH
Com_331_pos	L-Lysine	MSTRG.13261	--
Com_152_pos	Acetyl-L-carnitine	ENSGALG00000014463	ACTN2
Com_331_pos	L-Lysine	ENSGALG00000007404	YIPF5
Com_331_pos	L-Lysine	ENSGALG00000027608	PIGC
Com_171_neg	LPC 22:6	ENSGALG00000027375	NR2C2AP
Com_99_pos	Creatine	MSTRG.18213	--
Com_152_pos	Acetyl-L-carnitine	ENSGALG00000027070	TIMP2
Com_482_pos	8-Hydroxyquinoline	ENSGALG00000047480	A2ML1
Com_264_pos	Indole	ENSGALG00000035675	--
Com_386_pos	2-Amino-1,3-octadecanec	ENSGALG00000010978	ANGPTL3
Com_192_pos	1-Methylhistidine	ENSGALG00000041988	SIK1
Com_86_neg	Levulinic acid	ENSGALG00000004322	AHR
Com_588_pos	Ornithine	ENSGALG00000053245	VTG2
Com_192_pos	1-Methylhistidine	ENSGALG00000031932	AGPAT3
Com_12_pos	Betaine	MSTRG.21536	--
Com_80_pos	DL-Lysine	MSTRG.21796	--
Com_194_pos	Pipecolic acid	ENSGALG00000005470	PLPPR5
Com_78_neg	Citric acid	ENSGALG00000007778	PES1
Com_192_pos	1-Methylhistidine	ENSGALG00000025743	CDR2
Com_99_pos	Creatine	ENSGALG00000030511	SLC19A1
Com_76_neg	Erythronolactone	ENSGALG00000021135	HAPLN3
Com_208_neg	N-Acetylanthranilic acid	ENSGALG00000026384	PCSK4
Com_386_pos	2-Amino-1,3-octadecanec	MSTRG.12923	--
Com_25_pos	2-Hydroxycinnamic acid	ENSGALG00000040070	PDIA2
Com_21_pos	DL-Tryptophan	MSTRG.10409	--
Com_460_pos	3-amino-4-(propylamino)(	ENSGALG00000005353	FAR1
Com_16_neg	3-Hydroxybutyric acid	MSTRG.21321	gag
Com_57_neg	LPC 16:1	ENSGALG00000003022	Fmo5
Com_108_neg	LPE 18:2	ENSGALG00000010889	HOOK1
Com_386_pos	2-Amino-1,3-octadecanec	ENSGALG00000009680	PAQR7
Com_86_neg	Levulinic acid	MSTRG.1503	gag
Com_92_pos	D-(+)-Proline	ENSGALG00000003427	USP3
Com_55_pos	Valine	ENSGALG00000015263	TMEM30C
Com_175_pos	Pantothenic acid	MSTRG.8986	gag
Com_208_neg	N-Acetylanthranilic acid	ENSGALG00000007012	ARRDC4
Com_588_pos	Ornithine	ENSGALG00000051398	TMEM14C
Com_265_pos	6-Methylquinoline	MSTRG.19672	--
Com_460_pos	3-amino-4-(propylamino)(	ENSGALG00000015134	APOV1
Com_18_neg	Arachidonic acid	ENSGALG00000015016	SLC22A15
Com_588_pos	Ornithine	ENSGALG00000010018	CTSEAL
Com_331_pos	L-Lysine	ENSGALG00000008039	MFSD13A
Com_22_pos	Indole-3-acrylic acid	MSTRG.8619	--
Com_78_neg	Citric acid	ENSGALG00000041533	SLC11A2
Com_440_pos	PC (18:4e/4:0)	MSTRG.21796	--
Com_252_pos	cis-4-Hydroxy-D-proline	ENSGALG00000014948	HMGCR
Com_80_pos	DL-Lysine	ENSGALG00000027960	GRPR
Com_460_pos	3-amino-4-(propylamino)(	MSTRG.2316	env
Com_55_pos	Valine	ENSGALG00000003575	Dnttip1
Com_194_pos	Pipecolic acid	ENSGALG00000003948	ALAS1
Com_203_pos	Serotonin	ENSGALG00000047781	RAD9B

Com_12_pos	Betaine	ENSGALG00000007673	LRRC59
Com_331_pos	L-Lysine	ENSGALG00000051779	PRORSD1P
Com_40_pos	Choline	ENSGALG00000023395	PLIN1
Com_208_neg	N-Acetylanthranilic acid	ENSGALG00000042374	PDE11A
Com_12_pos	Betaine	ENSGALG00000040342	ADAMTS1
Com_108_neg	LPE 18:2	ENSGALG00000028284	PTX3
Com_186_pos	4-Hydroxybenzaldehyde	ENSGALG00000041205	--
Com_192_pos	1-Methylhistidine	ENSGALG00000004081	TMCO4
Com_175_pos	Pantothenic acid	ENSGALG00000016492	TDRD15
Com_17_pos	L-Norleucine	MSTRG.13408	--
Com_25_pos	2-Hydroxycinnamic acid	MSTRG.8779	--
Com_108_neg	LPE 18:2	ENSGALG00000007478	SLC51A
Com_18_neg	Arachidonic acid	ENSGALG00000016476	TTC32
Com_22_pos	Indole-3-acrylic acid	ENSGALG00000014261	UCHL1
Com_186_pos	4-Hydroxybenzaldehyde	ENSGALG00000014834	NCOA7
Com_16_neg	3-Hydroxybutyric acid	ENSGALG00000026460	myoM
Com_178_pos	Maltol	ENSGALG00000005610	SLC44A3
Com_175_pos	Pantothenic acid	ENSGALG00000015044	GTF3C6
Com_203_pos	Serotonin	ENSGALG00000034438	GNB3
Com_147_pos	D-Sphingosine	ENSGALG00000047495	LRRC10
Com_78_neg	Citric acid	ENSGALG00000016491	APOB
Com_175_pos	Pantothenic acid	ENSGALG00000026607	C15orf40
Com_151_neg	Lysope 18:1	ENSGALG00000049068	ZNF831
Com_40_pos	Choline	ENSGALG00000037811	NRSN1
Com_311_pos	PC (18:4e/2:0)	ENSGALG00000027070	TIMP2
Com_8_neg	4-Methyl-2-Oxopentanoic	ENSGALG00000012185	PLA2G12A
Com_362_pos	2-Arachidonoyl glycerol	ENSGALG00000023517	AGPAT2
Com_120_neg	LPC 15:0	ENSGALG00000011347	IHH
Com_208_neg	N-Acetylanthranilic acid	ENSGALG00000043087	MTF2
Com_482_pos	8-Hydroxyquinoline	MSTRG.3009	--
Com_97_pos	L-Threonine	ENSGALG00000042511	PKDCC
Com_362_pos	2-Arachidonoyl glycerol	MSTRG.19422	--
Com_440_pos	PC (18:4e/4:0)	ENSGALG00000047027	ADCK5
Com_362_pos	2-Arachidonoyl glycerol	ENSGALG00000004425	SCAMP1
Com_80_pos	DL-Lysine	ENSGALG00000037935	RARA
Com_40_pos	Choline	ENSGALG00000002437	DIPK1B
Com_8_neg	4-Methyl-2-Oxopentanoic	ENSGALG00000039354	VTG1
Com_208_neg	N-Acetylanthranilic acid	ENSGALG00000016456	LPIN1
Com_99_pos	Creatine	ENSGALG00000050668	Spata1
Com_252_pos	cis-4-Hydroxy-D-proline	ENSGALG00000037852	HSD17B7
Com_460_pos	3-amino-4-(propylamino)c	ENSGALG00000013036	ATP6V1E1
Com_12_pos	Betaine	ENSGALG00000023760	CHIA
Com_331_pos	L-Lysine	ENSGALG00000021658	PAFAH2
Com_80_pos	DL-Lysine	ENSGALG00000003103	MST1R
Com_171_neg	LPC 22:6	ENSGALG00000052072	gag
Com_16_neg	3-Hydroxybutyric acid	ENSGALG00000012847	Slc7a11
Com_252_pos	cis-4-Hydroxy-D-proline	MSTRG.21092	--
Com_25_pos	2-Hydroxycinnamic acid	ENSGALG00000013569	SEC61B
Com_120_neg	LPC 15:0	ENSGALG00000034504	FAM20C
Com_99_pos	Creatine	MSTRG.8128	--
Com_460_pos	3-amino-4-(propylamino)c	ENSGALG00000029235	CPNE4
Com_16_neg	3-Hydroxybutyric acid	ENSGALG00000005160	VMP1
Com_16_neg	3-Hydroxybutyric acid	MSTRG.2388	--
Com_440_pos	PC (18:4e/4:0)	ENSGALG00000019276	SLCO1C1
Com_16_neg	3-Hydroxybutyric acid	ENSGALG00000009170	NCEH1
Com_264_pos	Indole	ENSGALG00000016665	FDFT1
Com_120_neg	LPC 15:0	ENSGALG00000026203	FAM174A
Com_78_neg	Citric acid	ENSGALG00000000645	Espn

Com_147_pos	D-Sphingosine	MSTRG.8471	--
Com_208_neg	N-Acetylanthranilic acid	ENSGALG00000008784	SPATA1
Com_171_neg	LPC 22:6	ENSGALG00000038532	--
Com_175_pos	Pantothenic acid	ENSGALG00000014233	FBLN1
Com_76_neg	Erythronolactone	ENSGALG00000003147	TRPC4AP
Com_413_pos	L-Cystine	ENSGALG00000031525	TSTA3
Com_203_pos	Serotonin	MSTRG.13819	--
Com_331_pos	L-Lysine	MSTRG.21394	gag-pol
Com_208_neg	N-Acetylanthranilic acid	ENSGALG00000050258	--
Com_120_neg	LPC 15:0	ENSGALG00000014923	ARHGEF28
Com_40_pos	Choline	MSTRG.2403	--
Com_194_pos	Pipecolic acid	ENSGALG00000031312	ANAPC13
Com_208_neg	N-Acetylanthranilic acid	ENSGALG00000002707	CHRNB2
Com_588_pos	Ornithine	ENSGALG00000001475	STMN1
Com_362_pos	2-Arachidonoyl glycerol	ENSGALG00000000619	ANGPTL4
Com_362_pos	2-Arachidonoyl glycerol	ENSGALG00000006076	RASGEF1C
Com_151_neg	Lysope 18:1	ENSGALG00000031496	SPINK5
Com_151_pos	Pyridoxamine	ENSGALG00000008039	MFSD13A
Com_80_pos	DL-Lysine	ENSGALG00000023338	CBX2
Com_175_pos	Pantothenic acid	ENSGALG00000014813	HOMER1
Com_192_pos	1-Methylhistidine	ENSGALG00000024449	RAMP2
Com_12_pos	Betaine	ENSGALG00000050420	CTNND2
Com_97_pos	L-Threonine	ENSGALG00000010229	ABCD4
Com_147_pos	D-Sphingosine	ENSGALG00000016475	Zp2
Com_76_neg	Erythronolactone	ENSGALG00000004170	ADA
Com_78_neg	Citric acid	ENSGALG00000027375	NR2C2AP
Com_471_pos	Indole-3-acetic acid	ENSGALG00000013124	FHOD3
Com_460_pos	3-amino-4-(propylamino)(	ENSGALG00000004782	TSEN15
Com_120_neg	LPC 15:0	ENSGALG00000002445	KIAA0319L
Com_55_pos	Valine	ENSGALG00000012882	KDSR
Com_130_neg	2-Hydroxyvaleric acid	ENSGALG00000049658	UTS2R
Com_76_neg	Erythronolactone	ENSGALG00000046757	ERVK-9
Com_252_pos	cis-4-Hydroxy-D-proline	ENSGALG00000005766	PKD2L1
Com_175_pos	Pantothenic acid	MSTRG.15507	--
Com_208_neg	N-Acetylanthranilic acid	ENSGALG00000052395	ERVK-11
Com_386_pos	2-Amino-1,3-octadecanec	ENSGALG00000001749	ACSBG2
Com_17_pos	L-Norleucine	MSTRG.15507	--
Com_55_pos	Valine	ENSGALG00000035935	Unc13c
Com_192_pos	1-Methylhistidine	ENSGALG00000002371	RUSC2
Com_265_pos	6-Methylquinoline	ENSGALG00000021039	HKDC1
Com_178_pos	Maltol	MSTRG.13261	--
Com_18_neg	Arachidonic acid	ENSGALG00000012196	MCUB
Com_362_pos	2-Arachidonoyl glycerol	ENSGALG00000010229	ABCD4
Com_151_pos	Pyridoxamine	MSTRG.1468	--
Com_55_pos	Valine	ENSGALG00000005739	SCD
Com_18_neg	Arachidonic acid	ENSGALG00000048432	--
Com_178_pos	Maltol	ENSGALG00000007404	YIPF5
Com_12_pos	Betaine	MSTRG.21091	--
Com_440_pos	PC (18:4e/4:0)	ENSGALG00000007018	SLC26A11
Com_86_neg	Levulinic acid	ENSGALG00000017122	SGCG
Com_17_pos	L-Norleucine	ENSGALG00000014813	HOMER1
Com_252_pos	cis-4-Hydroxy-D-proline	MSTRG.16433	B4GALNT4
Com_265_pos	6-Methylquinoline	ENSGALG00000014907	DCBLD1
Com_171_neg	LPC 22:6	ENSGALG00000051123	pol
Com_8_neg	4-Methyl-2-Oxopentanoic	ENSGALG00000027070	TIMP2
Com_151_pos	Pyridoxamine	ENSGALG00000027608	PIGC
Com_97_pos	L-Threonine	ENSGALG00000006076	RASGEF1C
Com_120_neg	LPC 15:0	ENSGALG00000017122	SGCG

Com_311_pos	PC (18:4e/2:0)	MSTRG.5319	--
Com_386_pos	2-Amino-1,3-octadecanec	MSTRG.11802	TMEM221
Com_215_pos	D-Erythro-sphingosine 1-p	ENSGALG00000010889	HOOK1
Com_55_pos	Valine	ENSGALG00000004505	CCDC137
Com_55_pos	Valine	ENSGALG00000022758	GGACT
Com_40_pos	Choline	ENSGALG00000029083	NXPH2
Com_21_pos	DL-Tryptophan	ENSGALG00000026809	SARS
Com_99_pos	Creatine	ENSGALG00000044251	RASSF7
Com_99_pos	Creatine	ENSGALG00000051550	ARMH4
Com_171_neg	LPC 22:6	ENSGALG00000041533	SLC11A2
Com_130_neg	2-Hydroxyvaleric acid	ENSGALG00000030908	ATP2B2
Com_386_pos	2-Amino-1,3-octadecanec	ENSGALG00000021685	SERINC2
Com_17_pos	L-Norleucine	ENSGALG00000014233	FBLN1
Com_460_pos	3-amino-4-(propylamino)(	ENSGALG00000033591	Kazald1
Com_252_pos	cis-4-Hydroxy-D-proline	ENSGALG00000050083	SYCP2L
Com_352_pos	Riboflavin	ENSGALG00000007536	PHR
Com_151_pos	Pyridoxamine	ENSGALG00000026957	SEMA4G
Com_99_pos	Creatine	ENSGALG00000004852	DNM1
Com_192_pos	1-Methylhistidine	ENSGALG00000014935	GREB1L
Com_130_neg	2-Hydroxyvaleric acid	ENSGALG00000033656	DQX1
Com_194_pos	Pipecolic acid	ENSGALG00000036492	DAGLA
Com_151_pos	Pyridoxamine	ENSGALG00000027064	HIST1H3H
Com_264_pos	Indole	MSTRG.8619	--
Com_362_pos	2-Arachidonoyl glycerol	ENSGALG00000043829	ext1c
Com_194_pos	Pipecolic acid	MSTRG.17073	--
Com_22_pos	Indole-3-acrylic acid	ENSGALG00000006198	LSS
Com_130_neg	2-Hydroxyvaleric acid	ENSGALG00000042511	PKDCC
Com_186_pos	4-Hydroxybenzaldehyde	MSTRG.2430	--
Com_208_neg	N-Acetylanthranilic acid	ENSGALG00000030076	PCSK6
Com_178_pos	Maltol	ENSGALG00000051779	PRORSD1P
Com_147_pos	D-Sphingosine	MSTRG.21204	--
Com_252_pos	cis-4-Hydroxy-D-proline	ENSGALG00000033150	MIDN
Com_331_pos	L-Lysine	ENSGALG00000000226	TMEM9
Com_386_pos	2-Amino-1,3-octadecanec	ENSGALG00000035626	DAD1
Com_97_pos	L-Threonine	ENSGALG00000004425	SCAMP1
Com_147_pos	D-Sphingosine	ENSGALG00000003103	MST1R
Com_362_pos	2-Arachidonoyl glycerol	ENSGALG00000042511	PKDCC
Com_92_pos	D-(+)-Proline	ENSGALG00000045557	MTTPL
Com_352_pos	Riboflavin	ENSGALG00000013776	CEP135
Com_78_neg	Citric acid	ENSGALG00000014944	GCNT4
Com_18_neg	Arachidonic acid	ENSGALG00000052612	RPS27L
Com_147_pos	D-Sphingosine	ENSGALG00000037935	RARA
Com_76_neg	Erythronolactone	ENSGALG00000028822	RNF152
Com_16_neg	3-Hydroxybutyric acid	ENSGALG00000005815	TMEM41B
Com_76_neg	Erythronolactone	ENSGALG00000039538	CLDND1
Com_12_pos	Betaine	ENSGALG00000037018	USP36
Com_17_pos	L-Norleucine	ENSGALG00000015044	GTF3C6
Com_78_neg	Citric acid	ENSGALG00000052072	gag
Com_80_pos	DL-Lysine	MSTRG.8810	--
Com_86_neg	Levulinic acid	ENSGALG00000026203	FAM174A
Com_80_pos	DL-Lysine	ENSGALG00000015768	ANKRD6
Com_40_pos	Choline	ENSGALG00000036616	NUAK2
Com_331_pos	L-Lysine	ENSGALG00000014525	USP5
Com_413_pos	L-Cystine	ENSGALG00000011254	SATB1
Com_147_pos	D-Sphingosine	ENSGALG00000007848	PTS
Com_331_pos	L-Lysine	ENSGALG00000033051	CAMK1D
Com_192_pos	1-Methylhistidine	ENSGALG00000047720	KLHDC7A
Com_152_pos	Acetyl-L-carnitine	ENSGALG00000012185	PLA2G12A

Com_76_neg	Erythronolactone	ENSGALG00000004729	SLC7A10
Com_130_neg	2-Hydroxyvaleric acid	ENSGALG00000038145	DPP7
Com_171_neg	LPC 22:6	ENSGALG00000000645	Espn
Com_482_pos	8-Hydroxyquinoline	ENSGALG00000006320	Slc2a9
Com_57_neg	LPC 16:1	ENSGALG00000009848	LPGAT1
Com_151_pos	Pyridoxamine	ENSGALG00000001492	NDRG3
Com_186_pos	4-Hydroxybenzaldehyde	ENSGALG00000010406	TMEM63C
Com_152_pos	Acetyl-L-carnitine	ENSGALG00000039354	VTG1
Com_151_pos	Pyridoxamine	ENSGALG00000035244	H3-I
Com_311_pos	PC (18:4e/2:0)	ENSGALG00000033365	ALDH1A3
Com_588_pos	Ornithine	ENSGALG00000033051	CAMK1D
Com_178_pos	Maltol	ENSGALG00000021658	PAFAH2
Com_588_pos	Ornithine	ENSGALG00000014525	USP5
Com_311_pos	PC (18:4e/2:0)	ENSGALG00000047027	ADCK5
Com_171_neg	LPC 22:6	MSTRG.8501	--
Com_54_pos	Uric acid	ENSGALG00000011141	ITGB6
Com_208_neg	N-Acetylanthranilic acid	ENSGALG00000003568	PPP1R16B
Com_17_pos	L-Norleucine	MSTRG.8986	gag
Com_192_pos	1-Methylhistidine	ENSGALG00000031570	WDR54
Com_55_pos	Valine	ENSGALG00000042080	PGPEP1
Com_130_neg	2-Hydroxyvaleric acid	ENSGALG00000010229	ABCD4
Com_12_pos	Betaine	ENSGALG00000004612	MTHFR
Com_18_neg	Arachidonic acid	MSTRG.21321	gag
Com_97_pos	L-Threonine	ENSGALG00000049658	UTS2R
Com_99_pos	Creatine	ENSGALG00000019738	FBXO47
Com_386_pos	2-Amino-1,3-octadecanec	ENSGALG00000039538	CLDND1
Com_311_pos	PC (18:4e/2:0)	ENSGALG00000019276	SLCO1C1
Com_588_pos	Ornithine	ENSGALG00000000226	TMEM9
Com_471_pos	Indole-3-acetic acid	ENSGALG00000009947	PLEKHH2
Com_76_neg	Erythronolactone	ENSGALG00000015136	ILDR1
Com_178_pos	Maltol	MSTRG.21394	gag-pol
Com_54_pos	Uric acid	ENSGALG00000021451	RED3
Com_108_neg	LPE 18:2	ENSGALG00000010163	LGR5
Com_99_pos	Creatine	ENSGALG00000000745	SLC26A9
Com_208_neg	N-Acetylanthranilic acid	ENSGALG00000032287	ATP2A2
Com_362_pos	2-Arachidonoyl glycerol	ENSGALG00000007778	PES1
Com_186_pos	4-Hydroxybenzaldehyde	MSTRG.15444	--
Com_16_neg	3-Hydroxybutyric acid	ENSGALG00000017378	CRTAC1
Com_55_pos	Valine	ENSGALG00000005470	PLPPR5
Com_147_pos	D-Sphingosine	MSTRG.21796	--
Com_186_pos	4-Hydroxybenzaldehyde	MSTRG.835	--
Com_413_pos	L-Cystine	ENSGALG00000051398	TMEM14C
Com_86_neg	Levulinic acid	MSTRG.15507	--
Com_130_neg	2-Hydroxyvaleric acid	ENSGALG00000006076	RASGEF1C
Com_171_neg	LPC 22:6	ENSGALG00000013149	MOCOS
Com_92_pos	D-(+)-Proline	ENSGALG00000014463	ACTN2
Com_16_neg	3-Hydroxybutyric acid	ENSGALG00000016476	TTC32
Com_17_pos	L-Norleucine	ENSGALG00000017122	SGCG
Com_194_pos	Pipecolic acid	ENSGALG00000015263	TMEM30C
Com_78_neg	Citric acid	ENSGALG00000051123	pol
Com_265_pos	6-Methylquinoline	ENSGALG00000052296	MEX3D
Com_86_neg	Levulinic acid	ENSGALG00000014813	HOMER1
Com_8_neg	4-Methyl-2-Oxopentanoic	ENSGALG00000015333	PCGF3
Com_76_neg	Erythronolactone	ENSGALG00000021685	SERINC2
Com_99_pos	Creatine	ENSGALG00000031159	HIST1H110
Com_413_pos	L-Cystine	ENSGALG00000014464	MTR
Com_97_pos	L-Threonine	ENSGALG00000030908	ATP2B2
Com_8_neg	4-Methyl-2-Oxopentanoic	ENSGALG00000029015	TM6SF2

Com_192_pos	1-Methylhistidine	ENSGALG00000005418	FRRS1
Com_25_pos	2-Hydroxycinnamic acid	ENSGALG00000037325	SERP1
Com_460_pos	3-amino-4-(propylamino)(	ENSGALG00000026203	FAM174A
Com_471_pos	Indole-3-acetic acid	ENSGALG00000030025	FABP4
Com_440_pos	PC (18:4e/4:0)	ENSGALG00000047495	LRRC10
Com_186_pos	4-Hydroxybenzaldehyde	ENSGALG00000032645	H2A-VIII
Com_460_pos	3-amino-4-(propylamino)(	MSTRG.11955	--
Com_194_pos	Pipecolic acid	ENSGALG00000003575	Dnttip1
Com_55_pos	Valine	ENSGALG00000003948	ALAS1
Com_76_neg	Erythronolactone	MSTRG.11802	TMEM221
Com_97_pos	L-Threonine	ENSGALG00000033656	DQX1
Com_194_pos	Pipecolic acid	ENSGALG00000037253	CLEC4M
Com_252_pos	cis-4-Hydroxy-D-proline	ENSGALG00000005439	ACACA
Com_86_neg	Levulinic acid	ENSGALG00000014233	FBLN1
Com_92_pos	D-(+)-Proline	MSTRG.16287	--
Com_208_neg	N-Acetylanthranilic acid	ENSGALG00000000695	MFSD4A
Com_147_pos	D-Sphingosine	ENSGALG00000041296	SOX7
Com_152_pos	Acetyl-L-carnitine	ENSGALG00000003427	USP3
Com_130_neg	2-Hydroxyvaleric acid	MSTRG.16287	--
Com_362_pos	2-Arachidonoyl glycerol	ENSGALG00000016491	APOB
Com_130_neg	2-Hydroxyvaleric acid	ENSGALG00000004425	SCAMP1
Com_97_pos	L-Threonine	ENSGALG00000014944	GCNT4
Com_76_neg	Erythronolactone	ENSGALG00000036787	HSD17B12
Com_21_pos	DL-Tryptophan	ENSGALG00000024295	MYCBP
Com_18_neg	Arachidonic acid	ENSGALG00000009170	NCEH1
Com_76_neg	Erythronolactone	ENSGALG00000001749	ACSBG2
Com_99_pos	Creatine	ENSGALG00000050427	OSBPL10
Com_440_pos	PC (18:4e/4:0)	MSTRG.5319	--
Com_386_pos	2-Amino-1,3-octadecaned	ENSGALG00000046757	ERVK-9
Com_78_neg	Citric acid	ENSGALG00000004425	SCAMP1
Com_12_pos	Betaine	ENSGALG00000049408	HIST1H2B8
Com_18_neg	Arachidonic acid	ENSGALG00000008862	DNAJC10
Com_264_pos	Indole	ENSGALG00000006198	LSS
Com_40_pos	Choline	ENSGALG00000003029	PLPP6
Com_8_neg	4-Methyl-2-Oxopentanoic	ENSGALG00000034716	HEY2
Com_17_pos	L-Norleucine	MSTRG.2305	gag
Com_440_pos	PC (18:4e/4:0)	MSTRG.8471	--
Com_192_pos	1-Methylhistidine	ENSGALG00000032440	QPCT
Com_171_neg	LPC 22:6	ENSGALG00000014944	GCNT4
Com_21_pos	DL-Tryptophan	ENSGALG00000054546	ERVK-11
Com_264_pos	Indole	ENSGALG00000053043	CARHSP1
Com_331_pos	L-Lysine	ENSGALG00000001475	STMN1
Com_252_pos	cis-4-Hydroxy-D-proline	ENSGALG00000010798	DHCR24
Com_76_neg	Erythronolactone	ENSGALG00000047380	MR1
Com_147_pos	D-Sphingosine	ENSGALG00000005648	Sesn3
Com_97_pos	L-Threonine	ENSGALG00000038145	DPP7
Com_16_neg	3-Hydroxybutyric acid	ENSGALG00000002775	FA2H
Com_194_pos	Pipecolic acid	ENSGALG00000032628	SRCIN1
Com_588_pos	Ornithine	MSTRG.21394	gag-pol
Com_178_pos	Maltol	ENSGALG00000000226	TMEM9
Com_86_neg	Levulinic acid	ENSGALG00000015044	GTF3C6
Com_413_pos	L-Cystine	ENSGALG00000054297	SND1
Com_215_pos	D-Erythro-sphingosine 1-p	ENSGALG00000009920	COCH
Com_54_pos	Uric acid	ENSGALG00000005888	PGP
Com_17_pos	L-Norleucine	ENSGALG00000026203	FAM174A
Com_413_pos	L-Cystine	ENSGALG00000001475	STMN1
Com_76_neg	Erythronolactone	ENSGALG00000026547	TPGS2
Com_440_pos	PC (18:4e/4:0)	ENSGALG00000016475	Zp2

Com_386_pos	2-Amino-1,3-octadecaned	ENSGALG00000003147	TRPC4AP
Com_99_pos	Creatine	ENSGALG00000004657	FBXO2
Com_460_pos	3-amino-4-(propylamino)c	ENSGALG00000017122	SGCG
Com_55_pos	Valine	ENSGALG00000031312	ANAPC13
Com_178_pos	Maltol	ENSGALG00000030845	ENHO
Com_78_neg	Citric acid	ENSGALG00000006076	RASGEF1C
Com_78_neg	Citric acid	MSTRG.8501	--
Com_171_neg	LPC 22:6	MSTRG.6512	--
Com_482_pos	8-Hydroxyquinoline	ENSGALG00000009926	HAAO
Com_25_pos	2-Hydroxycinnamic acid	ENSGALG00000008601	AHSG
Com_76_neg	Erythronolactone	ENSGALG00000008262	RASGRF1
Com_178_pos	Maltol	ENSGALG00000014525	USP5
Com_120_neg	LPC 15:0	ENSGALG00000033591	Kazald1
Com_588_pos	Ornithine	ENSGALG00000021658	PAFAH2
Com_192_pos	1-Methylhistidine	ENSGALG00000005696	ABHD6
Com_178_pos	Maltol	ENSGALG00000033051	CAMK1D
Com_99_pos	Creatine	MSTRG.5038	--
Com_252_pos	cis-4-Hydroxy-D-proline	ENSGALG00000028949	CORO6
Com_16_neg	3-Hydroxybutyric acid	ENSGALG00000037050	FABP3
Com_21_pos	DL-Tryptophan	ENSGALG00000043435	CARNS1
Com_80_pos	DL-Lysine	MSTRG.13584	--
Com_460_pos	3-amino-4-(propylamino)c	ENSGALG00000012186	CASP6
Com_86_neg	Levulinic acid	ENSGALG00000004782	TSEN15
Com_147_pos	D-Sphingosine	ENSGALG00000007018	SLC26A11
Com_86_neg	Levulinic acid	MSTRG.8986	gag
Com_147_pos	D-Sphingosine	MSTRG.12923	--
Com_362_pos	2-Arachidonoyl glycerol	ENSGALG00000027375	NR2C2AP
Com_175_pos	Pantothenic acid	MSTRG.1503	gag
Com_25_pos	2-Hydroxycinnamic acid	ENSGALG00000037065	SC5D
Com_54_pos	Uric acid	MSTRG.15625	--
Com_18_neg	Arachidonic acid	ENSGALG00000015729	LPAR1
Com_440_pos	PC (18:4e/4:0)	ENSGALG00000033365	ALDH1A3
Com_120_neg	LPC 15:0	MSTRG.13408	--
Com_215_pos	D-Erythro-sphingosine 1-p	ENSGALG00000010163	LGR5
Com_192_pos	1-Methylhistidine	ENSGALG00000006080	GPC4
Com_413_pos	L-Cystine	ENSGALG00000010641	SCCPDH
Com_194_pos	Pipecolic acid	ENSGALG00000035935	Unc13c
Com_252_pos	cis-4-Hydroxy-D-proline	ENSGALG00000040969	PTP4A3
Com_18_neg	Arachidonic acid	MSTRG.2387	--
Com_78_neg	Citric acid	ENSGALG00000010229	ABCD4
Com_119_pos	DL-Stachydrine	ENSGALG00000037014	TSNARE1
Com_92_pos	D-(+)-Proline	ENSGALG00000038145	DPP7
Com_186_pos	4-Hydroxybenzaldehyde	MSTRG.15745	--
Com_99_pos	Creatine	ENSGALG00000005722	SEC31B
Com_311_pos	PC (18:4e/2:0)	MSTRG.15754	--
Com_175_pos	Pantothenic acid	ENSGALG00000004322	AHR
Com_151_pos	Pyridoxamine	ENSGALG00000006702	MFGE8
Com_78_neg	Citric acid	ENSGALG00000013149	MOCOS
Com_25_pos	2-Hydroxycinnamic acid	ENSGALG00000025886	SUSD3
Com_588_pos	Ornithine	ENSGALG00000051779	PRORSD1P
Com_8_neg	4-Methyl-2-Oxopentanoic	MSTRG.12629	--
Com_8_neg	4-Methyl-2-Oxopentanoic	MSTRG.20167	--
Com_460_pos	3-amino-4-(propylamino)c	ENSGALG00000045288	CAMK2N1
Com_57_neg	LPC 16:1	ENSGALG00000049068	ZNF831
Com_192_pos	1-Methylhistidine	ENSGALG00000016254	OTC
Com_252_pos	cis-4-Hydroxy-D-proline	ENSGALG00000051290	--
Com_151_pos	Pyridoxamine	ENSGALG00000012748	ELOVL2
Com_460_pos	3-amino-4-(propylamino)c	MSTRG.2174	Irs2

Com_331_pos	L-Lysine	ENSGALG00000051398	TMEM14C
Com_86_neg	Levulinic acid	ENSGALG00000029235	CPNE4
Com_194_pos	Pipecolic acid	ENSGALG00000004505	CCDC137
Com_482_pos	8-Hydroxyquinoline	ENSGALG00000007252	ANKDD1A
Com_362_pos	2-Arachidonoyl glycerol	ENSGALG00000000950	MVB12B
Com_440_pos	PC (18:4e/4:0)	MSTRG.21204	--
Com_40_pos	Choline	ENSGALG00000008763	SSX2IP
Com_17_pos	L-Norleucine	ENSGALG00000008866	WDPCP
Com_76_neg	Erythronolactone	MSTRG.19463	VTG2
Com_21_pos	DL-Tryptophan	ENSGALG00000041078	MID1IP1
Com_97_pos	L-Threonine	ENSGALG00000000645	Espn
Com_86_neg	Levulinic acid	ENSGALG00000013036	ATP6V1E1
Com_175_pos	Pantothenic acid	MSTRG.149	--
Com_17_pos	L-Norleucine	MSTRG.1082	--
Com_16_neg	3-Hydroxybutyric acid	ENSGALG00000052612	RPS27L
Com_97_pos	L-Threonine	MSTRG.16287	--
Com_311_pos	PC (18:4e/2:0)	ENSGALG00000031067	TMEM132A
Com_130_neg	2-Hydroxyvaleric acid	ENSGALG00000014944	GCNT4
Com_311_pos	PC (18:4e/2:0)	ENSGALG00000000104	CRY1
Com_413_pos	L-Cystine	ENSGALG00000010293	RBP
Com_482_pos	8-Hydroxyquinoline	ENSGALG00000054322	--
Com_265_pos	6-Methylquinoline	ENSGALG00000007178	FADS2
Com_76_neg	Erythronolactone	MSTRG.12923	--
Com_186_pos	4-Hydroxybenzaldehyde	ENSGALG00000011113	SGIP1
Com_152_pos	Acetyl-L-carnitine	ENSGALG00000015333	PCGF3
Com_386_pos	2-Amino-1,3-octadecanec	ENSGALG00000021135	HAPLN3
Com_17_pos	L-Norleucine	ENSGALG00000012377	HNMT
Com_152_pos	Acetyl-L-carnitine	ENSGALG00000029015	TM6SF2
Com_99_pos	Creatine	ENSGALG00000004127	--
Com_80_pos	DL-Lysine	ENSGALG00000035626	DAD1
Com_92_pos	D-(+)-Proline	ENSGALG00000033656	DQX1
Com_440_pos	PC (18:4e/4:0)	ENSGALG00000007848	PTS
Com_265_pos	6-Methylquinoline	ENSGALG00000015937	FABP1
Com_4_pos	PC (17:1/17:1)	MSTRG.13819	--
Com_78_neg	Citric acid	ENSGALG00000042511	PKDCC
Com_171_neg	LPC 22:6	ENSGALG00000004425	SCAMP1
Com_92_pos	D-(+)-Proline	ENSGALG00000030908	ATP2B2
Com_311_pos	PC (18:4e/2:0)	ENSGALG00000047495	LRRC10
Com_8_neg	4-Methyl-2-Oxopentanoic	ENSGALG00000014581	BORCS8
Com_588_pos	Ornithine	ENSGALG00000007404	YIPF5
Com_12_pos	Betaine	ENSGALG00000048109	gag
Com_265_pos	6-Methylquinoline	ENSGALG00000044278	C1orf131
Com_588_pos	Ornithine	MSTRG.13261	--
Com_264_pos	Indole	ENSGALG00000052768	LDLR
Com_482_pos	8-Hydroxyquinoline	ENSGALG00000014750	TRB
Com_208_neg	N-Acetylanthranilic acid	ENSGALG00000009312	RPL22L1
Com_99_pos	Creatine	ENSGALG00000037769	NEBL
Com_175_pos	Pantothenic acid	MSTRG.4702	--
Com_16_neg	3-Hydroxybutyric acid	ENSGALG00000005583	ALG14
Com_362_pos	2-Arachidonoyl glycerol	ENSGALG00000052072	gag
Com_57_neg	LPC 16:1	ENSGALG00000031496	SPINK5
Com_86_neg	Levulinic acid	MSTRG.2305	gag
Com_252_pos	cis-4-Hydroxy-D-proline	ENSGALG00000040070	PDIA2
Com_86_neg	Levulinic acid	MSTRG.2316	env
Com_151_pos	Pyridoxamine	ENSGALG00000014126	endou-a
Com_8_neg	4-Methyl-2-Oxopentanoic	ENSGALG00000003427	USP3
Com_120_neg	LPC 15:0	ENSGALG00000046789	pol
Com_208_neg	N-Acetylanthranilic acid	ENSGALG00000037322	HIST1H46

Com_413_pos	L-Cystine	ENSGALG00000033051	CAMK1D
Com_86_neg	Levulinic acid	ENSGALG00000015134	APOV1
Com_331_pos	L-Lysine	ENSGALG00000011254	SATB1
Com_413_pos	L-Cystine	ENSGALG00000014525	USP5
Com_16_neg	3-Hydroxybutyric acid	ENSGALG00000006872	PISD
Com_386_pos	2-Amino-1,3-octadecanec	ENSGALG00000041604	NPTXR
Com_76_neg	Erythronolactone	ENSGALG00000005648	Sesn3
Com_18_neg	Arachidonic acid	ENSGALG00000016885	STK24
Com_22_pos	Indole-3-acrylic acid	ENSGALG00000053043	CARHSP1
Com_25_pos	2-Hydroxycinnamic acid	ENSGALG00000041372	Myrip
Com_362_pos	2-Arachidonoyl glycerol	MSTRG.15443	--
Com_152_pos	Acetyl-L-carnitine	ENSGALG00000034716	HEY2
Com_97_pos	L-Threonine	ENSGALG00000041533	SLC11A2
Com_25_pos	2-Hydroxycinnamic acid	ENSGALG00000014948	HMGCR
Com_86_neg	Levulinic acid	ENSGALG00000005353	FAR1
Com_192_pos	1-Methylhistidine	MSTRG.18216	--
Com_108_neg	LPE 18:2	ENSGALG00000009920	COCH
Com_311_pos	PC (18:4e/2:0)	MSTRG.8471	--
Com_175_pos	Pantothenic acid	ENSGALG00000046731	--
Com_178_pos	Maltol	ENSGALG00000001475	STMN1
Com_120_neg	LPC 15:0	MSTRG.11955	--
Com_54_pos	Uric acid	ENSGALG00000046412	Aoc3
Com_186_pos	4-Hydroxybenzaldehyde	ENSGALG00000004917	DOC2B
Com_40_pos	Choline	ENSGALG00000002500	GMPPB
Com_18_neg	Arachidonic acid	ENSGALG00000017378	CRTAC1
Com_12_pos	Betaine	ENSGALG00000007127	FADS1
Com_413_pos	L-Cystine	ENSGALG00000000226	TMEM9
Com_171_neg	LPC 22:6	ENSGALG00000006076	RASGEF1C
Com_92_pos	D-(+)-Proline	ENSGALG00000049658	UTS2R
Com_78_neg	Citric acid	MSTRG.6512	--
Com_12_pos	Betaine	ENSGALG00000042080	PGPEP1
Com_147_pos	D-Sphingosine	ENSGALG00000001749	ACSBG2
Com_194_pos	Pipecolic acid	ENSGALG00000042080	PGPEP1
Com_252_pos	cis-4-Hydroxy-D-proline	MSTRG.8779	--
Com_208_neg	N-Acetylanthranilic acid	ENSGALG00000015721	SVEP1
Com_265_pos	6-Methylquinoline	ENSGALG00000014261	UCHL1
Com_171_neg	LPC 22:6	ENSGALG00000041456	SLC35G1
Com_92_pos	D-(+)-Proline	ENSGALG00000012185	PLA2G12A
Com_8_neg	4-Methyl-2-Oxopentanoic	ENSGALG00000004282	RCAN3
Com_21_pos	DL-Tryptophan	ENSGALG00000027561	GNG5
Com_76_neg	Erythronolactone	ENSGALG00000041296	SOX7
Com_151_pos	Pyridoxamine	ENSGALG00000031754	KCNG2
Com_186_pos	4-Hydroxybenzaldehyde	ENSGALG00000036117	TENT5B
Com_16_neg	3-Hydroxybutyric acid	ENSGALG00000048035	GCNT2
Com_92_pos	D-(+)-Proline	ENSGALG00000039354	VTG1
Com_4_pos	PC (17:1/17:1)	ENSGALG00000047781	RAD9B
Com_17_pos	L-Norleucine	ENSGALG00000004782	TSEN15
Com_331_pos	L-Lysine	ENSGALG00000030845	ENHO
Com_16_neg	3-Hydroxybutyric acid	ENSGALG00000000107	TRIM7.1
Com_8_neg	4-Methyl-2-Oxopentanoic	MSTRG.17961	--
Com_215_pos	D-Erythro-sphingosine 1-[	ENSGALG00000004959	IRS1
Com_21_pos	DL-Tryptophan	ENSGALG00000035803	THRSP
Com_194_pos	Pipecolic acid	ENSGALG00000051466	NDFIP2
Com_362_pos	2-Arachidonoyl glycerol	ENSGALG00000038723	RPP25L
Com_311_pos	PC (18:4e/2:0)	ENSGALG00000016475	Zp2
Com_147_pos	D-Sphingosine	MSTRG.11802	TMEM221
Com_25_pos	2-Hydroxycinnamic acid	ENSGALG00000037852	HSD17B7
Com_16_neg	3-Hydroxybutyric acid	ENSGALG00000054319	ELOVL6

Com_171_neg	LPC 22:6	ENSGALG00000010229	ABCD4
Com_25_pos	2-Hydroxycinnamic acid	MSTRG.21092	--
Com_151_pos	Pyridoxamine	MSTRG.9165	--
Com_208_neg	N-Acetylanthranilic acid	ENSGALG00000052829	MAMDC4
Com_99_pos	Creatine	ENSGALG00000006783	PLOD2
Com_588_pos	Ornithine	ENSGALG00000014464	MTR
Com_55_pos	Valine	ENSGALG00000037018	USP36
Com_147_pos	D-Sphingosine	ENSGALG00000021685	SERINC2
Com_386_pos	2-Amino-1,3-octadecanec	ENSGALG00000036293	EBAG9
Com_362_pos	2-Arachidonoyl glycerol	ENSGALG00000051123	pol
Com_588_pos	Ornithine	ENSGALG00000005610	SLC44A3
Com_265_pos	6-Methylquinoline	ENSGALG00000053446	RED3
Com_440_pos	PC (18:4e/4:0)	ENSGALG00000041296	SOX7
Com_264_pos	Indole	MSTRG.9007	--
Com_175_pos	Pantothenic acid	ENSGALG00000036836	SOSTDC1
Com_130_neg	2-Hydroxyvaleric acid	ENSGALG00000000645	Espn
Com_208_neg	N-Acetylanthranilic acid	ENSGALG00000002249	AGO1
Com_362_pos	2-Arachidonoyl glycerol	ENSGALG00000012112	DBI
Com_16_neg	3-Hydroxybutyric acid	ENSGALG00000008862	DNAJC10
Com_97_pos	L-Threonine	ENSGALG00000038532	--
Com_147_pos	D-Sphingosine	MSTRG.5319	--
Com_55_pos	Valine	ENSGALG00000037253	CLEC4M
Com_17_pos	L-Norleucine	ENSGALG00000029235	CPNE4
Com_99_pos	Creatine	ENSGALG00000016412	MBOAT2
Com_86_neg	Levulinic acid	ENSGALG00000008866	WDPCP
Com_386_pos	2-Amino-1,3-octadecanec	ENSGALG00000003103	MST1R
Com_413_pos	L-Cystine	ENSGALG00000046789	pol
Com_151_pos	Pyridoxamine	ENSGALG00000015684	Dnajc25
Com_17_pos	L-Norleucine	ENSGALG00000013036	ATP6V1E1
Com_152_pos	Acetyl-L-carnitine	MSTRG.12629	--
Com_152_pos	Acetyl-L-carnitine	MSTRG.20167	--
Com_186_pos	4-Hydroxybenzaldehyde	ENSGALG00000004249	GRHL3
Com_40_pos	Choline	ENSGALG00000037261	RFXANK
Com_440_pos	PC (18:4e/4:0)	MSTRG.15754	--
Com_460_pos	3-amino-4-(propylamino)(	ENSGALG00000015540	RAD23B
Com_86_neg	Levulinic acid	MSTRG.1082	--
Com_178_pos	Maltol	ENSGALG00000051398	TMEM14C
Com_386_pos	2-Amino-1,3-octadecanec	ENSGALG00000037935	RARA
Com_17_pos	L-Norleucine	MSTRG.16504	gag
Com_120_neg	LPC 15:0	ENSGALG00000012186	CASP6
Com_440_pos	PC (18:4e/4:0)	ENSGALG00000005648	Sesn3
Com_18_neg	Arachidonic acid	ENSGALG00000002775	FA2H
Com_194_pos	Pipecolic acid	ENSGALG00000002899	AACS
Com_471_pos	Indole-3-acetic acid	ENSGALG00000033411	SLC26A2
Com_175_pos	Pantothenic acid	ENSGALG00000004341	Cryzl2
Com_12_pos	Betaine	ENSGALG00000004505	CCDC137
Com_208_neg	N-Acetylanthranilic acid	ENSGALG00000001642	GLT8D1
Com_194_pos	Pipecolic acid	MSTRG.13135	--
Com_171_neg	LPC 22:6	ENSGALG00000026846	JMJD7
Com_86_neg	Levulinic acid	ENSGALG00000012377	HNMT
Com_25_pos	2-Hydroxycinnamic acid	ENSGALG00000005766	PKD2L1
Com_16_neg	3-Hydroxybutyric acid	ENSGALG00000047687	SETD9
Com_171_neg	LPC 22:6	ENSGALG00000042511	PKDCC
Com_352_pos	Riboflavin	ENSGALG00000052991	DNAJC21
Com_311_pos	PC (18:4e/2:0)	MSTRG.21204	--
Com_311_pos	PC (18:4e/2:0)	ENSGALG00000002802	PACSIN1
Com_413_pos	L-Cystine	MSTRG.21394	gag-pol
Com_331_pos	L-Lysine	ENSGALG00000031525	TSTA3

Com_588_pos	Ornithine	ENSGALG00000054297	SND1
Com_460_pos	3-amino-4-(propylamino)(	MSTRG.13408	--
Com_99_pos	Creatine	ENSGALG00000035478	FAM91A1
Com_16_neg	3-Hydroxybutyric acid	MSTRG.6225	--
Com_152_pos	Acetyl-L-carnitine	MSTRG.16287	--
Com_120_neg	LPC 15:0	ENSGALG00000010293	RBP
Com_130_neg	2-Hydroxyvaleric acid	ENSGALG00000003427	USP3
Com_440_pos	PC (18:4e/4:0)	ENSGALG00000031067	TMEM132A
Com_175_pos	Pantothenic acid	ENSGALG00000015684	Dnajc25
Com_192_pos	1-Methylhistidine	ENSGALG00000040363	ABHD4
Com_440_pos	PC (18:4e/4:0)	ENSGALG00000000104	CRY1
Com_18_neg	Arachidonic acid	ENSGALG00000037050	FABP3
Com_12_pos	Betaine	ENSGALG00000035935	Unc13c
Com_76_neg	Erythronolactone	ENSGALG00000007848	PTS
Com_22_pos	Indole-3-acrylic acid	ENSGALG00000052768	LDLR
Com_55_pos	Valine	ENSGALG00000032628	SRCIN1
Com_186_pos	4-Hydroxybenzaldehyde	ENSGALG00000012589	C9orf64
Com_130_neg	2-Hydroxyvaleric acid	ENSGALG00000041533	SLC11A2
Com_17_pos	L-Norleucine	MSTRG.2316	env
Com_171_neg	LPC 22:6	MSTRG.8381	--
Com_265_pos	6-Methylquinoline	MSTRG.14680	--
Com_151_neg	Lysope 18:1	ENSGALG00000000378	SLC25A37
Com_120_neg	LPC 15:0	ENSGALG00000045288	CAMK2N1
Com_208_neg	N-Acetylanthranilic acid	MSTRG.7811	--
Com_17_pos	L-Norleucine	ENSGALG00000015134	APOV1
Com_151_pos	Pyridoxamine	ENSGALG00000016511	ADGRG2
Com_440_pos	PC (18:4e/4:0)	MSTRG.12923	--
Com_386_pos	2-Amino-1,3-octadecanec	ENSGALG00000050676	Ctnnd2
Com_99_pos	Creatine	ENSGALG00000028230	SUN2
Com_152_pos	Acetyl-L-carnitine	ENSGALG00000014581	BORCS8
Com_311_pos	PC (18:4e/2:0)	ENSGALG00000007848	PTS
Com_413_pos	L-Cystine	ENSGALG00000021658	PAFAH2
Com_147_pos	D-Sphingosine	ENSGALG00000039538	CLDND1
Com_80_pos	DL-Lysine	ENSGALG00000009680	PAQR7
Com_120_neg	LPC 15:0	MSTRG.2174	Irs2
Com_55_pos	Valine	ENSGALG00000050420	CTNND2
Com_386_pos	2-Amino-1,3-octadecanec	ENSGALG00000043336	COPZ1
Com_362_pos	2-Arachidonoyl glycerol	MSTRG.8501	--
Com_16_neg	3-Hydroxybutyric acid	ENSGALG00000015729	LPAR1
Com_54_pos	Uric acid	ENSGALG00000028880	FDPS
Com_25_pos	2-Hydroxycinnamic acid	ENSGALG00000050083	SYCP2L
Com_8_neg	4-Methyl-2-Oxopentanoic	MSTRG.2170	MYO16
Com_147_pos	D-Sphingosine	ENSGALG00000033365	ALDH1A3
Com_192_pos	1-Methylhistidine	ENSGALG00000012414	GNPNAT1
Com_54_pos	Uric acid	ENSGALG00000051567	MRPL41
Com_17_pos	L-Norleucine	ENSGALG00000005353	FAR1
Com_16_neg	3-Hydroxybutyric acid	MSTRG.2387	--
Com_178_pos	Maltol	ENSGALG00000011254	SATB1
Com_386_pos	2-Amino-1,3-octadecanec	ENSGALG00000020342	ABHD12
Com_76_neg	Erythronolactone	MSTRG.21204	--
Com_386_pos	2-Amino-1,3-octadecanec	MSTRG.21796	--
Com_175_pos	Pantothenic acid	MSTRG.9165	--
Com_16_neg	3-Hydroxybutyric acid	ENSGALG00000053245	VTG2
Com_78_neg	Citric acid	ENSGALG00000041456	SLC35G1
Com_178_pos	Maltol	MSTRG.13135	--
Com_588_pos	Ornithine	ENSGALG00000010641	SCCPDH
Com_120_neg	LPC 15:0	ENSGALG00000010641	SCCPDH
Com_80_pos	DL-Lysine	ENSGALG00000010978	ANGPTL3

Com_186_pos	4-Hydroxybenzaldehyde	ENSGALG00000007030	MFSD13A
Com_413_pos	L-Cystine	MSTRG.13408	--
Com_178_pos	Maltol	ENSGALG00000002899	AACS
Com_76_neg	Erythronolactone	ENSGALG00000042491	H4-I
Com_186_pos	4-Hydroxybenzaldehyde	ENSGALG00000030511	SLC19A1
Com_21_pos	DL-Tryptophan	MSTRG.9361	Fam110a
Com_362_pos	2-Arachidonoyl glycerol	ENSGALG00000002802	PACSIN1
Com_413_pos	L-Cystine	ENSGALG00000051779	PRORSD1P
Com_108_neg	LPE 18:2	ENSGALG00000004959	IRS1
Com_175_pos	Pantothenic acid	ENSGALG00000031754	KCNG2
Com_151_neg	Lysope 18:1	ENSGALG00000012834	AKR1D1
Com_362_pos	2-Arachidonoyl glycerol	ENSGALG00000013149	MOCOS
Com_152_pos	Acetyl-L-carnitine	ENSGALG00000004282	RCAN3
Com_208_neg	N-Acetylanthranilic acid	MSTRG.15162	--
Com_16_neg	3-Hydroxybutyric acid	ENSGALG00000010018	CTSEAL
Com_460_pos	3-amino-4-(propylamino)(	ENSGALG00000046789	pol
Com_54_pos	Uric acid	MSTRG.7483	--
Com_25_pos	2-Hydroxycinnamic acid	ENSGALG00000007127	FADS1
Com_151_pos	Pyridoxamine	ENSGALG00000046731	--
Com_152_pos	Acetyl-L-carnitine	MSTRG.17961	--
Com_130_neg	2-Hydroxyvaleric acid	ENSGALG00000038532	--
Com_78_neg	Citric acid	ENSGALG00000000950	MVB12B
Com_21_pos	DL-Tryptophan	ENSGALG00000041143	UMOD
Com_76_neg	Erythronolactone	ENSGALG00000006689	ABHD2
Com_40_pos	Choline	MSTRG.20656	--
Com_588_pos	Ornithine	ENSGALG00000010293	RBP
Com_99_pos	Creatine	ENSGALG00000041205	--
Com_12_pos	Betaine	ENSGALG00000041372	Myrip
Com_54_pos	Uric acid	ENSGALG00000044464	TEPSIN
Com_97_pos	L-Threonine	MSTRG.1502	gag
Com_311_pos	PC (18:4e/2:0)	ENSGALG00000012112	DBI
Com_120_neg	LPC 15:0	ENSGALG00000054297	SND1
Com_151_pos	Pyridoxamine	ENSGALG00000014906	MOCS2
Com_152_pos	Acetyl-L-carnitine	ENSGALG00000038145	DPP7
Com_147_pos	D-Sphingosine	ENSGALG00000046757	ERVK-9
Com_22_pos	Indole-3-acrylic acid	MSTRG.9007	--
Com_99_pos	Creatine	ENSGALG00000015253	COL8A1
Com_99_pos	Creatine	ENSGALG00000014834	NCOA7
Com_18_neg	Arachidonic acid	ENSGALG00000020342	ABHD12
Com_18_neg	Arachidonic acid	ENSGALG00000005583	ALG14
Com_588_pos	Ornithine	ENSGALG00000026460	myoM
Com_17_pos	L-Norleucine	ENSGALG00000031525	TSTA3
Com_97_pos	L-Threonine	ENSGALG00000012877	CREB3L2
Com_55_pos	Valine	ENSGALG00000040342	ADAMTS1
Com_76_neg	Erythronolactone	ENSGALG00000016475	Zp2
Com_40_pos	Choline	ENSGALG00000028376	FGF19
Com_178_pos	Maltol	ENSGALG00000051466	NDFIP2
Com_18_neg	Arachidonic acid	ENSGALG00000043336	COPZ1
Com_413_pos	L-Cystine	ENSGALG00000007404	YIPF5
Com_413_pos	L-Cystine	MSTRG.13261	--
Com_55_pos	Valine	ENSGALG00000007673	LRRC59
Com_12_pos	Betaine	ENSGALG00000003575	Dnttip1
Com_18_neg	Arachidonic acid	ENSGALG00000006872	PISD
Com_18_neg	Arachidonic acid	ENSGALG00000050676	Ctnnd2
Com_460_pos	3-amino-4-(propylamino)(	ENSGALG00000004631	DRAXIN
Com_99_pos	Creatine	ENSGALG00000033461	hnmt
Com_311_pos	PC (18:4e/2:0)	ENSGALG00000041296	SOX7
Com_86_neg	Levulinic acid	ENSGALG00000014976	GATA6

Com_151_pos	Pyridoxamine	MSTRG.53	SND1
Com_16_neg	3-Hydroxybutyric acid	ENSGALG00000016885	STK24
Com_25_pos	2-Hydroxycinnamic acid	ENSGALG00000048109	gag
Com_186_pos	4-Hydroxybenzaldehyde	ENSGALG00000050668	Spata1
Com_331_pos	L-Lysine	MSTRG.16504	gag
Com_97_pos	L-Threonine	ENSGALG00000006723	IDI1
Com_482_pos	8-Hydroxyquinoline	ENSGALG00000015425	LPL
Com_86_neg	Levulinic acid	MSTRG.16504	gag
Com_588_pos	Ornithine	ENSGALG00000012847	Slc7a11
Com_78_neg	Citric acid	ENSGALG00000026846	JMJD7
Com_86_neg	Levulinic acid	ENSGALG00000042555	STAMBP
Com_12_pos	Betaine	ENSGALG00000015263	TMEM30C
Com_21_pos	DL-Tryptophan	ENSGALG00000007234	CLCN5
Com_264_pos	Indole	ENSGALG00000029308	PNPLA3
Com_311_pos	PC (18:4e/2:0)	ENSGALG00000038723	RPP25L
Com_97_pos	L-Threonine	ENSGALG00000003427	USP3
Com_471_pos	Indole-3-acetic acid	ENSGALG00000013033	cmbl
Com_186_pos	4-Hydroxybenzaldehyde	MSTRG.8128	--
Com_588_pos	Ornithine	ENSGALG00000005160	VMP1
Com_171_neg	LPC 22:6	ENSGALG00000030151	LUZP2
Com_588_pos	Ornithine	MSTRG.2388	--
Com_482_pos	8-Hydroxyquinoline	ENSGALG00000002790	ABLIM3
Com_147_pos	D-Sphingosine	ENSGALG00000003147	TRPC4AP
Com_76_neg	Erythronolactone	MSTRG.8471	--
Com_92_pos	D-(+)-Proline	ENSGALG00000015333	PCGF3
Com_40_pos	Choline	ENSGALG00000054926	--
Com_92_pos	D-(+)-Proline	ENSGALG00000029015	TM6SF2
Com_440_pos	PC (18:4e/4:0)	ENSGALG00000001749	ACSBG2
Com_152_pos	Acetyl-L-carnitine	ENSGALG00000033656	DQX1
Com_55_pos	Valine	MSTRG.21536	--
Com_97_pos	L-Threonine	MSTRG.14577	SLC39A5
Com_192_pos	1-Methylhistidine	ENSGALG00000010764	FBXO8
Com_460_pos	3-amino-4-(propylamino)(	ENSGALG00000012586	GKAP1
Com_78_neg	Citric acid	MSTRG.15443	--
Com_78_neg	Citric acid	MSTRG.8381	--
Com_311_pos	PC (18:4e/2:0)	ENSGALG00000005648	Sesn3
Com_18_neg	Arachidonic acid	ENSGALG00000048035	GCNT2
Com_178_pos	Maltol	ENSGALG00000031525	TSTA3
Com_152_pos	Acetyl-L-carnitine	ENSGALG00000030908	ATP2B2
Com_171_neg	LPC 22:6	ENSGALG00000009476	CDK6
Com_208_neg	N-Acetylanthranilic acid	ENSGALG00000013511	ANKRA2
Com_386_pos	2-Amino-1,3-octadecaned	ENSGALG00000007018	SLC26A11
Com_18_neg	Arachidonic acid	ENSGALG00000000107	TRIM7.1
Com_171_neg	LPC 22:6	ENSGALG00000021686	--
Com_40_pos	Choline	ENSGALG00000040730	RXRG
Com_120_neg	LPC 15:0	ENSGALG00000014464	MTR
Com_175_pos	Pantothenic acid	ENSGALG00000012748	ELOVL2
Com_440_pos	PC (18:4e/4:0)	ENSGALG00000002802	PACSIN1
Com_76_neg	Erythronolactone	MSTRG.13439	--
Com_311_pos	PC (18:4e/2:0)	MSTRG.15443	--
Com_362_pos	2-Arachidonoyl glycerol	MSTRG.6512	--
Com_8_neg	4-Methyl-2-Oxopentanoic	MSTRG.16287	--
Com_40_pos	Choline	ENSGALG00000006521	TRPM5
Com_215_pos	D-Erythro-sphingosine 1-p	ENSGALG00000051068	SIGLEC1
Com_18_neg	Arachidonic acid	ENSGALG00000054319	ELOVL6
Com_482_pos	8-Hydroxyquinoline	ENSGALG00000014412	CSTA
Com_440_pos	PC (18:4e/4:0)	MSTRG.11802	TMEM221
Com_175_pos	Pantothenic acid	ENSGALG00000006702	MFGE8

Com_12_pos	Betaine	ENSGALG00000025886	SUSD3
Com_175_pos	Pantothenic acid	ENSGALG00000037773	ST3GAL1
Com_186_pos	4-Hydroxybenzaldehyde	ENSGALG00000053659	--
Com_76_neg	Erythronolactone	ENSGALG00000047495	LRRC10
Com_208_neg	N-Acetylanthranilic acid	MSTRG.8985	gag
Com_440_pos	PC (18:4e/4:0)	ENSGALG00000021685	SERINC2
Com_203_pos	Serotonin	ENSGALG00000006724	GPC5
Com_120_neg	LPC 15:0	ENSGALG00000015540	RAD23B
Com_192_pos	1-Methylhistidine	ENSGALG00000048285	TCN2
Com_92_pos	D-(+)-Proline	ENSGALG00000034716	HEY2
Com_78_neg	Citric acid	ENSGALG00000038723	RPP25L
Com_86_neg	Levulinic acid	MSTRG.3156	--
Com_311_pos	PC (18:4e/2:0)	MSTRG.12923	--
Com_55_pos	Valine	ENSGALG00000051466	NDFIP2
Com_152_pos	Acetyl-L-carnitine	MSTRG.2170	MYO16
Com_18_neg	Arachidonic acid	ENSGALG00000036293	EBAG9
Com_21_pos	DL-Tryptophan	ENSGALG00000041687	SREBF2
Com_152_pos	Acetyl-L-carnitine	ENSGALG00000049658	UTS2R
Com_55_pos	Valine	ENSGALG00000027960	GRPR
Com_54_pos	Uric acid	ENSGALG00000038666	FBXL12
Com_413_pos	L-Cystine	ENSGALG00000005610	SLC44A3
Com_194_pos	Pipecolic acid	ENSGALG00000037018	USP36
Com_99_pos	Creatine	ENSGALG00000011616	NPFFR2
Com_331_pos	L-Lysine	MSTRG.13135	--
Com_25_pos	2-Hydroxycinnamic acid	ENSGALG00000010798	DHCR24
Com_192_pos	1-Methylhistidine	ENSGALG00000008297	SEMA4B
Com_482_pos	8-Hydroxyquinoline	ENSGALG00000011994	SYNPO2
Com_151_pos	Pyridoxamine	ENSGALG00000023691	ENTPD7
Com_331_pos	L-Lysine	ENSGALG00000002899	AACS
Com_460_pos	3-amino-4-(propylamino)(	ENSGALG00000010293	RBP
Com_362_pos	2-Arachidonoyl glycerol	ENSGALG00000000104	CRY1
Com_12_pos	Betaine	ENSGALG00000008601	AHSG
Com_362_pos	2-Arachidonoyl glycerol	ENSGALG00000031067	TMEM132A
Com_147_pos	D-Sphingosine	MSTRG.15754	--
Com_78_neg	Citric acid	ENSGALG00000012112	DBI
Com_130_neg	2-Hydroxyvaleric acid	MSTRG.1502	gag
Com_8_neg	4-Methyl-2-Oxopentanoic	ENSGALG00000007533	NPEPL1
Com_588_pos	Ornithine	ENSGALG00000046789	pol
Com_147_pos	D-Sphingosine	ENSGALG00000021135	HAPLN3
Com_171_neg	LPC 22:6	ENSGALG00000000950	MVB12B
Com_17_pos	L-Norleucine	ENSGALG00000011254	SATB1
Com_482_pos	8-Hydroxyquinoline	ENSGALG00000006374	TBX6
Com_151_pos	Pyridoxamine	ENSGALG00000021395	ABCA9
Com_18_neg	Arachidonic acid	ENSGALG00000047687	SETD9
Com_311_pos	PC (18:4e/2:0)	ENSGALG00000000950	MVB12B
Com_130_neg	2-Hydroxyvaleric acid	ENSGALG00000012877	CREB3L2
Com_12_pos	Betaine	MSTRG.17073	--
Com_252_pos	cis-4-Hydroxy-D-proline	ENSGALG00000008601	AHSG
Com_12_pos	Betaine	ENSGALG00000036492	DAGLA
Com_151_pos	Pyridoxamine	ENSGALG00000030185	PTDSS1
Com_18_neg	Arachidonic acid	MSTRG.6225	--
Com_362_pos	2-Arachidonoyl glycerol	MSTRG.15754	--
Com_99_pos	Creatine	ENSGALG00000006649	TMEM41A
Com_147_pos	D-Sphingosine	ENSGALG00000031067	TMEM132A
Com_130_neg	2-Hydroxyvaleric acid	ENSGALG00000006723	IDI1
Com_86_neg	Levulinic acid	ENSGALG00000031525	TSTA3
Com_55_pos	Valine	ENSGALG00000002899	AACS
Com_147_pos	D-Sphingosine	ENSGALG00000000104	CRY1

Com_252_pos	cis-4-Hydroxy-D-proline	MSTRG.16505	--
Com_55_pos	Valine	MSTRG.13135	--
Com_386_pos	2-Amino-1,3-octadecaned	ENSGALG00000016885	STK24
Com_588_pos	Ornithine	ENSGALG00000005815	TMEM41B
Com_460_pos	3-amino-4-(propylamino)	ENSGALG00000010641	SCCPDH
Com_331_pos	L-Lysine	ENSGALG00000012377	HNMT
Com_18_neg	Arachidonic acid	ENSGALG00000041604	NPTXR
Com_25_pos	2-Hydroxycinnamic acid	ENSGALG00000049408	HIST1H2B8
Com_208_neg	N-Acetylanthranilic acid	ENSGALG00000051159	--
Com_147_pos	D-Sphingosine	ENSGALG00000041604	NPTXR
Com_8_neg	4-Methyl-2-Oxopentanoic	ENSGALG00000038145	DPP7
Com_40_pos	Choline	ENSGALG00000031482	Pou5f3
Com_186_pos	4-Hydroxybenzaldehyde	MSTRG.11834	--
Com_54_pos	Uric acid	ENSGALG00000029898	YKT6
Com_25_pos	2-Hydroxycinnamic acid	ENSGALG00000040969	PTP4A3
Com_440_pos	PC (18:4e/4:0)	ENSGALG00000039538	CLDND1
Com_17_pos	L-Norleucine	ENSGALG00000014976	GATA6
Com_471_pos	Indole-3-acetic acid	ENSGALG00000003972	FAXDC2
Com_440_pos	PC (18:4e/4:0)	ENSGALG00000012112	DBI
Com_331_pos	L-Lysine	ENSGALG00000051466	NDFIP2
Com_17_pos	L-Norleucine	ENSGALG00000042555	STAMBP
Com_78_neg	Citric acid	ENSGALG00000030151	LUZP2
Com_331_pos	L-Lysine	MSTRG.1082	--
Com_92_pos	D-(+)-Proline	MSTRG.12629	--
Com_92_pos	D-(+)-Proline	MSTRG.20167	--
Com_252_pos	cis-4-Hydroxy-D-proline	ENSGALG00000016979	SLC25A30
Com_130_neg	2-Hydroxyvaleric acid	MSTRG.14577	SLC39A5
Com_194_pos	Pipecolic acid	ENSGALG00000030845	ENHO
Com_252_pos	cis-4-Hydroxy-D-proline	ENSGALG00000025886	SUSD3
Com_22_pos	Indole-3-acrylic acid	ENSGALG00000029308	PNPLA3
Com_331_pos	L-Lysine	ENSGALG00000008866	WDPCP
Com_178_pos	Maltol	MSTRG.16504	gag
Com_482_pos	8-Hydroxyquinoline	ENSGALG00000004343	HPD
Com_99_pos	Creatine	ENSGALG00000010406	TMEM63C
Com_18_neg	Arachidonic acid	ENSGALG00000053245	VTG2
Com_17_pos	L-Norleucine	ENSGALG00000051398	TMEM14C
Com_55_pos	Valine	ENSGALG00000023338	CBX2
Com_171_neg	LPC 22:6	MSTRG.15443	--
Com_460_pos	3-amino-4-(propylamino)	ENSGALG00000004055	C7orf50
Com_86_neg	Levulinic acid	MSTRG.2393	--
Com_178_pos	Maltol	ENSGALG00000032628	SRCIN1
Com_40_pos	Choline	ENSGALG00000035206	CNPY2
Com_78_neg	Citric acid	ENSGALG00000009476	CDK6
Com_194_pos	Pipecolic acid	ENSGALG00000050420	CTNND2
Com_80_pos	DL-Lysine	ENSGALG00000012882	KDSR
Com_413_pos	L-Cystine	ENSGALG00000017122	SGCG
Com_18_neg	Arachidonic acid	ENSGALG00000021135	HAPLN3
Com_208_neg	N-Acetylanthranilic acid	ENSGALG00000004702	DYNC2I2
Com_151_pos	Pyridoxamine	MSTRG.8955	--
Com_78_neg	Citric acid	ENSGALG00000021686	--
Com_151_pos	Pyridoxamine	ENSGALG00000053886	gag
Com_440_pos	PC (18:4e/4:0)	ENSGALG00000038723	RPP25L
Com_192_pos	1-Methylhistidine	ENSGALG00000008427	GNAT3
Com_86_neg	Levulinic acid	MSTRG.15732	--
Com_86_neg	Levulinic acid	ENSGALG00000013001	CTNND2
Com_588_pos	Ornithine	MSTRG.13408	--
Com_460_pos	3-amino-4-(propylamino)	ENSGALG00000054297	SND1
Com_151_pos	Pyridoxamine	ENSGALG00000008226	NIF3L1

Com_40_pos	Choline	ENSGALG00000010301	EIF2B2
Com_8_neg	4-Methyl-2-Oxopentanoic	ENSGALG00000033656	DQX1
Com_22_pos	Indole-3-acrylic acid	ENSGALG00000007178	FADS2
Com_80_pos	DL-Lysine	ENSGALG00000005739	SCD
Com_362_pos	2-Arachidonoyl glycerol	ENSGALG00000041456	SLC35G1
Com_108_neg	LPE 18:2	ENSGALG00000051068	SIGLEC1
Com_311_pos	PC (18:4e/2:0)	ENSGALG00000001749	ACSBG2
Com_18_neg	Arachidonic acid	ENSGALG00000010018	CTSEAL
Com_76_neg	Erythronolactone	ENSGALG00000015016	SLC22A15
Com_208_neg	N-Acetylanthranilic acid	ENSGALG00000016560	SELENOI
Com_8_neg	4-Methyl-2-Oxopentanoic	ENSGALG00000030908	ATP2B2
Com_92_pos	D-(+)-Proline	ENSGALG00000014581	BORCS8
Com_192_pos	1-Methylhistidine	ENSGALG00000007508	HPSE2
Com_22_pos	Indole-3-acrylic acid	ENSGALG00000015937	FABP1
Com_99_pos	Creatine	MSTRG.15444	--
Com_78_neg	Citric acid	ENSGALG00000002802	PACSIN1
Com_147_pos	D-Sphingosine	ENSGALG00000036293	EBAG9
Com_171_neg	LPC 22:6	ENSGALG00000038723	RPP25L
Com_120_neg	LPC 15:0	ENSGALG00000004631	DRAXIN
Com_22_pos	Indole-3-acrylic acid	ENSGALG00000044278	C1orf131
Com_99_pos	Creatine	ENSGALG00000036021	MTMR7
Com_40_pos	Choline	ENSGALG00000040857	TECTA
Com_80_pos	DL-Lysine	ENSGALG00000022758	GGACT
Com_192_pos	1-Methylhistidine	ENSGALG00000029033	Tldc2
Com_130_neg	2-Hydroxyvaleric acid	ENSGALG00000027070	TIMP2
Com_17_pos	L-Norleucine	MSTRG.3156	--
Com_21_pos	DL-Tryptophan	ENSGALG00000011169	PDCD2
Com_440_pos	PC (18:4e/4:0)	MSTRG.15443	--
Com_208_neg	N-Acetylanthranilic acid	ENSGALG00000038574	MYO15A
Com_588_pos	Ornithine	ENSGALG00000016476	TTC32
Com_8_neg	4-Methyl-2-Oxopentanoic	ENSGALG00000015689	ECPAS
Com_178_pos	Maltol	ENSGALG00000037253	CLEC4M
Com_25_pos	2-Hydroxycinnamic acid	ENSGALG00000004612	MTHFR
Com_386_pos	2-Amino-1,3-octadecanec	MSTRG.5319	--
Com_16_neg	3-Hydroxybutyric acid	ENSGALG00000020342	ABHD12
Com_311_pos	PC (18:4e/2:0)	MSTRG.11802	TMEM221
Com_440_pos	PC (18:4e/4:0)	ENSGALG00000046757	ERVK-9
Com_86_neg	Levulinic acid	ENSGALG00000028191	GLCE
Com_186_pos	4-Hydroxybenzaldehyde	ENSGALG00000031159	HIST1H110
Com_386_pos	2-Amino-1,3-octadecanec	MSTRG.2387	--
Com_99_pos	Creatine	ENSGALG00000004424	SEC16B
Com_386_pos	2-Amino-1,3-octadecanec	ENSGALG00000015729	LPAR1
Com_99_pos	Creatine	ENSGALG00000032645	H2A-VIII
Com_16_neg	3-Hydroxybutyric acid	ENSGALG00000043336	COPZ1
Com_311_pos	PC (18:4e/2:0)	ENSGALG00000021685	SERINC2
Com_265_pos	6-Methylquinoline	ENSGALG00000053043	CARHSP1
Com_252_pos	cis-4-Hydroxy-D-proline	ENSGALG00000041372	Myrip
Com_171_neg	LPC 22:6	ENSGALG00000012112	DBI
Com_16_neg	3-Hydroxybutyric acid	ENSGALG00000050676	Ctnnd2
Com_120_neg	LPC 15:0	ENSGALG00000012586	GKAP1
Com_175_pos	Pantothenic acid	ENSGALG00000001492	NDRG3
Com_413_pos	L-Cystine	ENSGALG00000026460	myoM
Com_460_pos	3-amino-4-(propylamino)(	ENSGALG00000037479	IGSF21
Com_108_neg	LPE 18:2	ENSGALG00000013627	SLC7A2
Com_8_neg	4-Methyl-2-Oxopentanoic	ENSGALG00000049658	UTS2R
Com_97_pos	L-Threonine	ENSGALG00000037773	ST3GAL1
Com_413_pos	L-Cystine	ENSGALG00000026203	FAM174A
Com_17_pos	L-Norleucine	ENSGALG00000001475	STMN1

Com_460_pos	3-amino-4-(propylamino)(	ENSGALG00000032889	RBFOX3
Com_460_pos	3-amino-4-(propylamino)(	ENSGALG00000014464	MTR
Com_151_neg	Lysope 18:1	ENSGALG00000039708	Pram1
Com_86_neg	Levulinic acid	ENSGALG00000011254	SATB1
Com_352_pos	Riboflavin	ENSGALG00000014971	SLC2A9
Com_92_pos	D-(+)-Proline	ENSGALG00000004282	RCAN3
Com_194_pos	Pipecolic acid	ENSGALG00000040342	ADAMTS1
Com_440_pos	PC (18:4e/4:0)	ENSGALG00000003147	TRPC4AP
Com_203_pos	Serotonin	ENSGALG00000016558	VEGFD
Com_331_pos	L-Lysine	MSTRG.2305	gag
Com_8_neg	4-Methyl-2-Oxopentanoic	ENSGALG00000016595	TRIM35
Com_413_pos	L-Cystine	ENSGALG00000012847	Slc7a11
Com_186_pos	4-Hydroxybenzaldehyde	ENSGALG00000050427	OSBPL10
Com_92_pos	D-(+)-Proline	MSTRG.17961	--
Com_194_pos	Pipecolic acid	ENSGALG00000007673	LRRC59
Com_55_pos	Valine	MSTRG.8810	--
Com_208_neg	N-Acetylanthranilic acid	ENSGALG00000041988	SIK1
Com_57_neg	LPC 16:1	ENSGALG00000000378	SLC25A37
Com_18_neg	Arachidonic acid	ENSGALG00000003147	TRPC4AP
Com_55_pos	Valine	ENSGALG00000015768	ANKRD6
Com_413_pos	L-Cystine	ENSGALG00000005160	VMP1
Com_54_pos	Uric acid	ENSGALG00000016610	PTRHD1
Com_152_pos	Acetyl-L-carnitine	ENSGALG00000007533	NPEPL1
Com_76_neg	Erythronolactone	ENSGALG00000019276	SLCO1C1
Com_413_pos	L-Cystine	MSTRG.2388	--
Com_482_pos	8-Hydroxyquinoline	MSTRG.8511	--
Com_175_pos	Pantothenic acid	MSTRG.14577	SLC39A5
Com_151_neg	Lysope 18:1	ENSGALG00000053647	PFAS
Com_471_pos	Indole-3-acetic acid	ENSGALG00000016651	TDH
Com_362_pos	2-Arachidonoyl glycerol	ENSGALG00000026846	JMJD7
Com_208_neg	N-Acetylanthranilic acid	ENSGALG00000025743	CDR2
Com_192_pos	1-Methylhistidine	ENSGALG00000026384	PCSK4
Com_171_neg	LPC 22:6	ENSGALG00000029445	FADS6
Com_440_pos	PC (18:4e/4:0)	ENSGALG00000000950	MVB12B
Com_147_pos	D-Sphingosine	ENSGALG00000050676	Ctnnd2
Com_471_pos	Indole-3-acetic acid	ENSGALG00000030121	SLC2A11
Com_8_neg	4-Methyl-2-Oxopentanoic	ENSGALG00000048223	FAM20C
Com_171_neg	LPC 22:6	MSTRG.14343	--
Com_147_pos	D-Sphingosine	ENSGALG00000002802	PACSIN1
Com_265_pos	6-Methylquinoline	ENSGALG00000050091	CLEC2B
Com_76_neg	Erythronolactone	ENSGALG00000047027	ADCK5
Com_471_pos	Indole-3-acetic acid	ENSGALG00000034337	RHPN1
Com_147_pos	D-Sphingosine	ENSGALG00000043336	COPZ1
Com_175_pos	Pantothenic acid	ENSGALG00000006723	IDI1
Com_175_pos	Pantothenic acid	ENSGALG00000026957	SEMA4G
Com_386_pos	2-Amino-1,3-octadecanec	ENSGALG00000033365	ALDH1A3
Com_194_pos	Pipecolic acid	MSTRG.21536	--
Com_178_pos	Maltol	ENSGALG00000012377	HNMT
Com_22_pos	Indole-3-acrylic acid	ENSGALG00000053446	RED3
Com_362_pos	2-Arachidonoyl glycerol	MSTRG.8381	--
Com_147_pos	D-Sphingosine	ENSGALG00000020342	ABHD12
Com_186_pos	4-Hydroxybenzaldehyde	ENSGALG00000004657	FBXO2
Com_386_pos	2-Amino-1,3-octadecanec	ENSGALG00000008862	DNAJC10
Com_18_neg	Arachidonic acid	ENSGALG00000046757	ERVK-9
Com_40_pos	Choline	MSTRG.17623	--
Com_86_neg	Levulinic acid	ENSGALG00000051398	TMEM14C
Com_178_pos	Maltol	MSTRG.1082	--
Com_362_pos	2-Arachidonoyl glycerol	ENSGALG00000033365	ALDH1A3

Com_76_neg	Erythronolactone	ENSGALG00000012196	MCUB
Com_175_pos	Pantothenic acid	ENSGALG00000012877	CREB3L2
Com_186_pos	4-Hydroxybenzaldehyde	MSTRG.5038	--
Com_76_neg	Erythronolactone	ENSGALG00000048432	--
Com_208_neg	N-Acetylanthranilic acid	ENSGALG00000004081	TMCO4
Com_175_pos	Pantothenic acid	ENSGALG00000027608	PIGC
Com_17_pos	L-Norleucine	MSTRG.2393	--
Com_311_pos	PC (18:4e/2:0)	ENSGALG00000039538	CLDND1
Com_16_neg	3-Hydroxybutyric acid	ENSGALG00000036293	EBAG9
Com_57_neg	LPC 16:1	ENSGALG00000012834	AKR1D1
Com_178_pos	Maltol	ENSGALG00000008866	WDPCP
Com_80_pos	DL-Lysine	ENSGALG00000005470	PLPPR5
Com_99_pos	Creatine	ENSGALG00000010703	DGLUCY
Com_175_pos	Pantothenic acid	MSTRG.1502	gag
Com_192_pos	1-Methylhistidine	ENSGALG00000050267	CALU
Com_25_pos	2-Hydroxycinnamic acid	MSTRG.21091	--
Com_352_pos	Riboflavin	ENSGALG00000054442	ITIH3
Com_17_pos	L-Norleucine	MSTRG.15732	--
Com_17_pos	L-Norleucine	ENSGALG00000013001	CTNND2
Com_186_pos	4-Hydroxybenzaldehyde	ENSGALG00000010643	ZYG11B
Com_265_pos	6-Methylquinoline	MSTRG.10409	--
Com_331_pos	L-Lysine	ENSGALG00000032628	SRCIN1
Com_192_pos	1-Methylhistidine	ENSGALG00000043087	MTF2
Com_186_pos	4-Hydroxybenzaldehyde	MSTRG.1890	gag
Com_264_pos	Indole	MSTRG.2171	Myo16
Com_80_pos	DL-Lysine	ENSGALG00000007507	MASTL
Com_97_pos	L-Threonine	ENSGALG00000027070	TIMP2
Com_192_pos	1-Methylhistidine	ENSGALG00000016456	LPIN1
Com_171_neg	LPC 22:6	ENSGALG00000002802	PACSIN1
Com_264_pos	Indole	ENSGALG00000007178	FADS2
Com_311_pos	PC (18:4e/2:0)	ENSGALG00000042511	PKDCC
Com_21_pos	DL-Tryptophan	MSTRG.19672	--
Com_186_pos	4-Hydroxybenzaldehyde	ENSGALG00000005722	SEC31B
Com_440_pos	PC (18:4e/4:0)	ENSGALG00000021135	HAPLN3
Com_194_pos	Pipecolic acid	ENSGALG00000027960	GRPR
Com_80_pos	DL-Lysine	ENSGALG00000003948	ALAS1
Com_471_pos	Indole-3-acetic acid	ENSGALG00000027891	NREP
Com_331_pos	L-Lysine	MSTRG.8986	gag
Com_264_pos	Indole	ENSGALG00000015937	FABP1
Com_175_pos	Pantothenic acid	ENSGALG00000008039	MFSD13A
Com_78_neg	Citric acid	ENSGALG00000000104	CRY1
Com_264_pos	Indole	ENSGALG00000044278	C1orf131
Com_265_pos	6-Methylquinoline	ENSGALG00000052768	LDLR
Com_78_neg	Citric acid	ENSGALG00000031067	TMEM132A
Com_482_pos	8-Hydroxyquinoline	ENSGALG00000012034	ADSL
Com_22_pos	Indole-3-acrylic acid	MSTRG.14680	--
Com_17_pos	L-Norleucine	ENSGALG00000028191	GLCE
Com_130_neg	2-Hydroxyvaleric acid	ENSGALG00000037773	ST3GAL1
Com_108_neg	LPE 18:2	ENSGALG00000030038	C3
Com_192_pos	1-Methylhistidine	ENSGALG00000002519	SLC25A33
Com_17_pos	L-Norleucine	ENSGALG00000033051	CAMK1D
Com_108_neg	LPE 18:2	ENSGALG00000013356	IKBKE
Com_92_pos	D-(+)-Proline	MSTRG.2170	MYO16
Com_17_pos	L-Norleucine	ENSGALG00000014525	USP5
Com_588_pos	Ornithine	ENSGALG00000052612	RPS27L
Com_460_pos	3-amino-4-(propylamino)(	ENSGALG00000051205	nicA
Com_482_pos	8-Hydroxyquinoline	ENSGALG00000040434	rab18b
Com_120_neg	LPC 15:0	ENSGALG00000004055	C7orf50

Com_18_neg	Arachidonic acid	ENSGALG00000039538	CLDND1
Com_16_neg	3-Hydroxybutyric acid	ENSGALG00000041604	NPTXR
Com_331_pos	L-Lysine	ENSGALG00000037253	CLEC4M
Com_482_pos	8-Hydroxyquinoline	MSTRG.3197	--
Com_331_pos	L-Lysine	ENSGALG00000015044	GTF3C6
Com_362_pos	2-Arachidonoyl glycerol	MSTRG.5319	--
Com_440_pos	PC (18:4e/4:0)	ENSGALG00000041604	NPTXR
Com_152_pos	Acetyl-L-carnitine	ENSGALG00000015689	ECPAS
Com_99_pos	Creatine	ENSGALG00000011113	SGIP1
Com_215_pos	D-Erythro-sphingosine 1-p	ENSGALG00000013627	SLC7A2
Com_54_pos	Uric acid	ENSGALG00000035675	--
Com_186_pos	4-Hydroxybenzaldehyde	ENSGALG00000004127	--
Com_147_pos	D-Sphingosine	ENSGALG00000012112	DBI
Com_130_neg	2-Hydroxyvaleric acid	ENSGALG00000047027	ADCK5
Com_86_neg	Levulinic acid	ENSGALG00000001475	STMN1
Com_413_pos	L-Cystine	ENSGALG00000005815	TMEM41B
Com_78_neg	Citric acid	MSTRG.15754	--
Com_311_pos	PC (18:4e/2:0)	ENSGALG00000046757	ERVK-9
Com_215_pos	D-Erythro-sphingosine 1-p	ENSGALG00000015624	VCAN
Com_208_neg	N-Acetylanthranilic acid	ENSGALG00000008539	ALG12
Com_17_pos	L-Norleucine	ENSGALG00000000226	TMEM9
Com_4_pos	PC (17:1/17:1)	ENSGALG00000053278	SUCNR1
Com_25_pos	2-Hydroxycinnamic acid	ENSGALG00000023760	CHIA
Com_311_pos	PC (18:4e/2:0)	ENSGALG00000010229	ABCD4
Com_192_pos	1-Methylhistidine	ENSGALG00000052395	ERVK-11
Com_171_neg	LPC 22:6	ENSGALG00000016492	TDRD15
Com_40_pos	Choline	ENSGALG00000007723	NAT9
Com_130_neg	2-Hydroxyvaleric acid	ENSGALG00000019276	SLCO1C1
Com_151_pos	Pyridoxamine	ENSGALG00000026607	C15orf40
Com_16_neg	3-Hydroxybutyric acid	ENSGALG00000021135	HAPLN3
Com_78_neg	Citric acid	ENSGALG00000029445	FADS6
Com_362_pos	2-Arachidonoyl glycerol	ENSGALG00000030151	LUZP2
Com_413_pos	L-Cystine	ENSGALG00000004782	TSEN15
Com_178_pos	Maltol	MSTRG.2305	gag
Com_80_pos	DL-Lysine	ENSGALG00000031312	ANAPC13
Com_171_neg	LPC 22:6	ENSGALG00000026607	C15orf40
Com_76_neg	Erythronolactone	MSTRG.21321	gag
Com_147_pos	D-Sphingosine	ENSGALG00000038723	RPP25L
Com_99_pos	Creatine	MSTRG.8499	Itpripl1
Com_55_pos	Valine	ENSGALG00000030845	ENHO
Com_78_neg	Citric acid	MSTRG.14343	--
Com_152_pos	Acetyl-L-carnitine	ENSGALG00000016595	TRIM35
Com_331_pos	L-Lysine	ENSGALG00000014233	FBLN1
Com_151_pos	Pyridoxamine	ENSGALG00000016492	TDRD15
Com_80_pos	DL-Lysine	ENSGALG00000036754	CHKA
Com_12_pos	Betaine	MSTRG.8779	--
Com_386_pos	2-Amino-1,3-octadecanec	ENSGALG00000052612	RPS27L
Com_178_pos	Maltol	ENSGALG00000031312	ANAPC13
Com_92_pos	D-(+)-Proline	ENSGALG00000028191	GLCE
Com_311_pos	PC (18:4e/2:0)	ENSGALG00000003147	TRPC4AP
Com_588_pos	Ornithine	ENSGALG00000017122	SGCG
Com_252_pos	cis-4-Hydroxy-D-proline	ENSGALG00000007127	FADS1
Com_97_pos	L-Threonine	ENSGALG00000004341	Cryzl2
Com_55_pos	Valine	MSTRG.13584	--
Com_8_neg	4-Methyl-2-Oxopentanoic	ENSGALG00000004170	ADA
Com_194_pos	Pipecolic acid	ENSGALG00000023338	CBX2
Com_311_pos	PC (18:4e/2:0)	ENSGALG00000006076	RASGEF1C
Com_362_pos	2-Arachidonoyl glycerol	ENSGALG00000009476	CDK6

Com_99_pos	Creatine	ENSGALG00000004917	DOC2B
Com_99_pos	Creatine	ENSGALG00000053217	INPP5J
Com_362_pos	2-Arachidonoyl glycerol	ENSGALG00000021686	--
Com_471_pos	Indole-3-acetic acid	ENSGALG00000003802	OTUD7A
Com_120_neg	LPC 15:0	ENSGALG00000037479	IGSF21
Com_440_pos	PC (18:4e/4:0)	ENSGALG00000036293	EBAG9
Com_152_pos	Acetyl-L-carnitine	ENSGALG00000048223	FAM20C
Com_264_pos	Indole	ENSGALG00000053446	RED3
Com_186_pos	4-Hydroxybenzaldehyde	ENSGALG00000004498	SLC2A10
Com_331_pos	L-Lysine	ENSGALG00000014813	HOMER1
Com_208_neg	N-Acetylanthranilic acid	ENSGALG00000014935	GREB1L
Com_265_pos	6-Methylquinoline	MSTRG.9007	--
Com_99_pos	Creatine	ENSGALG00000036117	TENT5B
Com_120_neg	LPC 15:0	ENSGALG00000032889	RBFOX3
Com_175_pos	Pantothenic acid	ENSGALG00000038532	--
Com_21_pos	DL-Tryptophan	ENSGALG00000021039	HKDC1
Com_147_pos	D-Sphingosine	MSTRG.15443	--
Com_331_pos	L-Lysine	MSTRG.15507	--
Com_413_pos	L-Cystine	ENSGALG00000029235	CPNE4
Com_12_pos	Betaine	ENSGALG00000040070	PDIA2
Com_18_neg	Arachidonic acid	ENSGALG00000021685	SERINC2
Com_76_neg	Erythronolactone	ENSGALG00000027070	TIMP2
Com_92_pos	D-(+)-Proline	ENSGALG00000013001	CTNND2
Com_92_pos	D-(+)-Proline	MSTRG.15732	--
Com_97_pos	L-Threonine	ENSGALG00000036836	SOSTDC1
Com_186_pos	4-Hydroxybenzaldehyde	ENSGALG00000035239	GLCCI1
Com_413_pos	L-Cystine	ENSGALG00000013036	ATP6V1E1
Com_18_neg	Arachidonic acid	MSTRG.11802	TMEM221
Com_21_pos	DL-Tryptophan	ENSGALG00000014907	DCBLD1
Com_252_pos	cis-4-Hydroxy-D-proline	ENSGALG00000048109	gag
Com_413_pos	L-Cystine	ENSGALG00000016476	TTC32
Com_108_neg	LPE 18:2	ENSGALG00000014821	THEMIS
Com_21_pos	DL-Tryptophan	ENSGALG00000030941	ELAPOR1
Com_311_pos	PC (18:4e/2:0)	ENSGALG00000004425	SCAMP1
Com_386_pos	2-Amino-1,3-octadecanec	MSTRG.15754	--
Com_16_neg	3-Hydroxybutyric acid	ENSGALG00000014464	MTR
Com_92_pos	D-(+)-Proline	MSTRG.2393	--
Com_186_pos	4-Hydroxybenzaldehyde	ENSGALG00000006783	PLOD2
Com_203_pos	Serotonin	ENSGALG00000031593	TMSB15B
Com_192_pos	1-Methylhistidine	ENSGALG00000004231	IFNLR1
Com_54_pos	Uric acid	ENSGALG00000016665	FDFT1
Com_265_pos	6-Methylquinoline	ENSGALG00000026809	SARS
Com_22_pos	Indole-3-acrylic acid	MSTRG.2171	Myo16
Com_252_pos	cis-4-Hydroxy-D-proline	ENSGALG00000005215	CACNA1H
Com_178_pos	Maltol	ENSGALG00000003948	ALAS1
Com_17_pos	L-Norleucine	MSTRG.21394	gag-pol
Com_171_neg	LPC 22:6	ENSGALG00000000104	CRY1
Com_76_neg	Erythronolactone	ENSGALG00000009170	NCEH1
Com_18_neg	Arachidonic acid	ENSGALG00000001749	ACSBG2
Com_54_pos	Uric acid	MSTRG.7572	--
Com_171_neg	LPC 22:6	ENSGALG00000031067	TMEM132A
Com_588_pos	Ornithine	ENSGALG00000026203	FAM174A
Com_588_pos	Ornithine	ENSGALG00000008862	DNAJC10
Com_16_neg	3-Hydroxybutyric acid	ENSGALG00000003147	TRPC4AP
Com_40_pos	Choline	ENSGALG00000004875	PEMT
Com_186_pos	4-Hydroxybenzaldehyde	ENSGALG00000016412	MBOAT2
Com_178_pos	Maltol	MSTRG.8986	gag
Com_386_pos	2-Amino-1,3-octadecanec	ENSGALG00000031067	TMEM132A

Com_108_neg	LPE 18:2	ENSGALG00000020391	SERPINA10
Com_386_pos	2-Amino-1,3-octadecanec	ENSGALG00000000104	CRY1
Com_413_pos	L-Cystine	MSTRG.2316	env
Com_203_pos	Serotonin	ENSGALG00000003553	ABCA12
Com_215_pos	D-Erythro-sphingosine 1-p	ENSGALG00000030038	C3
Com_440_pos	PC (18:4e/4:0)	ENSGALG00000042511	PKDCC
Com_12_pos	Betaine	ENSGALG00000051290	--
Com_264_pos	Indole	MSTRG.14680	--
Com_97_pos	L-Threonine	ENSGALG00000047027	ADCK5
Com_215_pos	D-Erythro-sphingosine 1-p	ENSGALG00000013356	IKBKE
Com_99_pos	Creatine	ENSGALG00000004249	GRHL3
Com_86_neg	Levulinic acid	ENSGALG00000033051	CAMK1D
Com_8_neg	4-Methyl-2-Oxopentanoic	ENSGALG00000028822	RNF152
Com_311_pos	PC (18:4e/2:0)	ENSGALG00000021135	HAPLN3
Com_413_pos	L-Cystine	ENSGALG00000015134	APOV1
Com_147_pos	D-Sphingosine	ENSGALG00000016885	STK24
Com_17_pos	L-Norleucine	ENSGALG00000021658	PAFAH2
Com_86_neg	Levulinic acid	ENSGALG00000014525	USP5
Com_25_pos	2-Hydroxycinnamic acid	ENSGALG00000014509	BST1
Com_147_pos	D-Sphingosine	ENSGALG00000000950	MVB12B
Com_482_pos	8-Hydroxyquinoline	ENSGALG00000017032	SLC25A15
Com_471_pos	Indole-3-acetic acid	ENSGALG00000017046	POSTN
Com_440_pos	PC (18:4e/4:0)	ENSGALG00000050676	Ctnnd2
Com_178_pos	Maltol	ENSGALG00000005470	PLPPR5
Com_40_pos	Choline	ENSGALG00000011657	EAF2
Com_460_pos	3-amino-4-(propylamino)c	MSTRG.18132	FBXW4
Com_175_pos	Pantothenic acid	ENSGALG00000041533	SLC11A2
Com_186_pos	4-Hydroxybenzaldehyde	ENSGALG00000050520	pol
Com_171_neg	LPC 22:6	MSTRG.15754	--
Com_440_pos	PC (18:4e/4:0)	ENSGALG00000043336	COPZ1
Com_413_pos	L-Cystine	ENSGALG00000005353	FAR1
Com_194_pos	Pipecolic acid	MSTRG.8810	--
Com_25_pos	2-Hydroxycinnamic acid	ENSGALG00000050440	APOF
Com_54_pos	Uric acid	ENSGALG00000021193	STARD5
Com_471_pos	Indole-3-acetic acid	ENSGALG00000016036	DOP1B
Com_194_pos	Pipecolic acid	ENSGALG00000015768	ANKRD6
Com_352_pos	Riboflavin	ENSGALG00000054783	NDRG1
Com_8_neg	4-Methyl-2-Oxopentanoic	ENSGALG00000004729	SLC7A10
Com_97_pos	L-Threonine	ENSGALG00000019276	SLCO1C1
Com_16_neg	3-Hydroxybutyric acid	ENSGALG00000046757	ERVK-9
Com_440_pos	PC (18:4e/4:0)	ENSGALG00000020342	ABHD12
Com_108_neg	LPE 18:2	ENSGALG00000015624	VCAN
Com_16_neg	3-Hydroxybutyric acid	ENSGALG00000054297	SND1
Com_178_pos	Maltol	ENSGALG00000015044	GTF3C6
Com_86_neg	Levulinic acid	ENSGALG00000000226	TMEM9
Com_460_pos	3-amino-4-(propylamino)c	ENSGALG00000028294	Vwa5b2
Com_78_neg	Citric acid	ENSGALG00000016492	TDRD15
Com_460_pos	3-amino-4-(propylamino)c	ENSGALG00000004216	TOR3A
Com_151_pos	Pyridoxamine	MSTRG.14343	--
Com_97_pos	L-Threonine	MSTRG.4702	--
Com_151_pos	Pyridoxamine	ENSGALG00000029445	FADS6
Com_108_neg	LPE 18:2	ENSGALG00000052388	METRN
Com_311_pos	PC (18:4e/2:0)	ENSGALG00000041604	NPTXR
Com_99_pos	Creatine	ENSGALG00000012589	C9orf64
Com_130_neg	2-Hydroxyvaleric acid	ENSGALG00000004341	Cryzl2
Com_78_neg	Citric acid	ENSGALG00000026607	C15orf40
Com_12_pos	Betaine	ENSGALG00000028949	CORO6
Com_17_pos	L-Norleucine	ENSGALG00000051779	PRORSD1P

Com_120_neg	LPC 15:0	ENSGALG00000010018	CTSEAL
Com_186_pos	4-Hydroxybenzaldehyde	ENSGALG00000028230	SUN2
Com_92_pos	D-(+)-Proline	ENSGALG00000007533	NPEPL1
Com_208_neg	N-Acetylanthranilic acid	ENSGALG00000005418	FRRS1
Com_8_neg	4-Methyl-2-Oxopentanoic	ENSGALG00000015136	ILDR1
Com_362_pos	2-Arachidonoyl glycerol	ENSGALG00000007018	SLC26A11
Com_471_pos	Indole-3-acetic acid	ENSGALG00000011003	SLC35F3
Com_588_pos	Ornithine	ENSGALG00000015729	LPAR1
Com_440_pos	PC (18:4e/4:0)	ENSGALG00000010229	ABCD4
Com_54_pos	Uric acid	MSTRG.8619	--
Com_588_pos	Ornithine	MSTRG.2387	--
Com_78_neg	Citric acid	ENSGALG00000033365	ALDH1A3
Com_40_pos	Choline	ENSGALG00000009560	MSMO1
Com_92_pos	D-(+)-Proline	MSTRG.3156	--
Com_80_pos	DL-Lysine	ENSGALG00000020538	SLC49A3
Com_178_pos	Maltol	ENSGALG00000014233	FBLN1
Com_386_pos	2-Amino-1,3-octadecaned	ENSGALG00000016476	TTC32
Com_331_pos	L-Lysine	ENSGALG00000031312	ANAPC13
Com_120_neg	LPC 15:0	ENSGALG00000051205	nicA
Com_97_pos	L-Threonine	MSTRG.149	--
Com_16_neg	3-Hydroxybutyric acid	ENSGALG00000010641	SCCPDH
Com_175_pos	Pantothenic acid	ENSGALG00000000645	Espn
Com_130_neg	2-Hydroxyvaleric acid	ENSGALG00000036836	SOSTDC1
Com_99_pos	Creatine	ENSGALG00000007030	MFSD13A
Com_120_neg	LPC 15:0	ENSGALG00000053245	VTG2
Com_152_pos	Acetyl-L-carnitine	ENSGALG00000004170	ADA
Com_151_neg	Lysope 18:1	ENSGALG00000004472	ASTN1
Com_186_pos	4-Hydroxybenzaldehyde	MSTRG.8248	--
Com_311_pos	PC (18:4e/2:0)	ENSGALG00000014944	GCNT4
Com_25_pos	2-Hydroxycinnamic acid	MSTRG.16505	--
Com_440_pos	PC (18:4e/4:0)	ENSGALG00000006076	RASGEF1C
Com_147_pos	D-Sphingosine	MSTRG.2387	--
Com_482_pos	8-Hydroxyquinoline	ENSGALG00000005472	NAT
Com_178_pos	Maltol	ENSGALG00000014813	HOMER1
Com_208_neg	N-Acetylanthranilic acid	ENSGALG00000032440	QPCT
Com_21_pos	DL-Tryptophan	ENSGALG00000052296	MEX3D
Com_252_pos	cis-4-Hydroxy-D-proline	ENSGALG00000049408	HIST1H2B8
Com_147_pos	D-Sphingosine	ENSGALG00000015729	LPAR1
Com_17_pos	L-Norleucine	ENSGALG00000007404	YIPF5
Com_215_pos	D-Erythro-sphingosine 1-p	ENSGALG00000012420	CG-1B
Com_17_pos	L-Norleucine	MSTRG.13261	--
Com_97_pos	L-Threonine	ENSGALG00000004322	AHR
Com_352_pos	Riboflavin	ENSGALG00000004472	ASTN1
Com_311_pos	PC (18:4e/2:0)	ENSGALG00000036293	EBAG9
Com_16_neg	3-Hydroxybutyric acid	ENSGALG00000039538	CLDND1
Com_215_pos	D-Erythro-sphingosine 1-p	ENSGALG00000014821	THEMIS
Com_178_pos	Maltol	MSTRG.15507	--
Com_57_neg	LPC 16:1	ENSGALG00000039708	Pram1
Com_482_pos	8-Hydroxyquinoline	ENSGALG00000011391	AMN
Com_80_pos	DL-Lysine	ENSGALG00000051251	H2B-I
Com_482_pos	8-Hydroxyquinoline	ENSGALG00000011314	LRRC3B
Com_25_pos	2-Hydroxycinnamic acid	ENSGALG00000016979	SLC25A30
Com_80_pos	DL-Lysine	ENSGALG00000037253	CLEC4M
Com_18_neg	Arachidonic acid	MSTRG.12923	--
Com_130_neg	2-Hydroxyvaleric acid	ENSGALG00000047495	LRRC10
Com_54_pos	Uric acid	ENSGALG00000007636	PCK1
Com_25_pos	2-Hydroxycinnamic acid	ENSGALG00000003560	SLC6A2
Com_265_pos	6-Methylquinoline	ENSGALG00000029308	PNPLA3

Com_12_pos	Betaine	ENSGALG00000005439	ACACA
Com_413_pos	L-Cystine	ENSGALG00000052612	RPS27L
Com_208_neg	N-Acetylanthranilic acid	ENSGALG00000004590	CLCN6
Com_264_pos	Indole	ENSGALG00000011141	ITGB6
Com_178_pos	Maltol	ENSGALG00000022758	GGACT
Com_16_neg	3-Hydroxybutyric acid	ENSGALG00000010293	RBP
Com_8_neg	4-Methyl-2-Oxopentanoic	ENSGALG00000036787	HSD17B12
Com_120_neg	LPC 15:0	MSTRG.6225	--
Com_57_neg	LPC 16:1	ENSGALG00000053647	PFAS
Com_97_pos	L-Threonine	MSTRG.1503	gag
Com_471_pos	Indole-3-acetic acid	ENSGALG00000000761	TSKU
Com_40_pos	Choline	ENSGALG00000006904	RNH1
Com_86_neg	Levulinic acid	MSTRG.21394	gag-pol
Com_92_pos	D-(+)-Proline	ENSGALG00000042555	STAMBP
Com_192_pos	1-Methylhistidine	ENSGALG00000030801	CCKAR
Com_186_pos	4-Hydroxybenzaldehyde	ENSGALG00000015253	COL8A1
Com_21_pos	DL-Tryptophan	ENSGALG00000010837	ASB5
Com_440_pos	PC (18:4e/4:0)	ENSGALG00000004425	SCAMP1
Com_92_pos	D-(+)-Proline	ENSGALG00000014976	GATA6
Com_78_neg	Citric acid	MSTRG.5319	--
Com_120_neg	LPC 15:0	ENSGALG00000047687	SETD9
Com_208_neg	N-Acetylanthranilic acid	ENSGALG00000005696	ABHD6
Com_12_pos	Betaine	ENSGALG00000008226	NIF3L1
Com_264_pos	Indole	ENSGALG00000021451	RED3
Com_178_pos	Maltol	ENSGALG00000005739	SCD
Com_331_pos	L-Lysine	ENSGALG00000003948	ALAS1
Com_215_pos	D-Erythro-sphingosine 1-p	ENSGALG00000020391	SERPINA10
Com_194_pos	Pipecolic acid	ENSGALG00000008039	MFSD13A
Com_208_neg	N-Acetylanthranilic acid	ENSGALG00000023348	HPDL
Com_21_pos	DL-Tryptophan	ENSGALG00000002919	MON1A
Com_130_neg	2-Hydroxyvaleric acid	MSTRG.8471	--
Com_8_neg	4-Methyl-2-Oxopentanoic	ENSGALG00000047380	MR1
Com_18_neg	Arachidonic acid	ENSGALG00000005648	Sesn3
Com_12_pos	Betaine	ENSGALG00000053886	gag
Com_186_pos	4-Hydroxybenzaldehyde	ENSGALG00000033461	hnmt
Com_386_pos	2-Amino-1,3-octadecanec	ENSGALG00000005815	TMEM41B
Com_12_pos	Betaine	MSTRG.8955	--
Com_588_pos	Ornithine	ENSGALG00000016885	STK24
Com_86_neg	Levulinic acid	ENSGALG00000021658	PAFAH2
Com_25_pos	2-Hydroxycinnamic acid	ENSGALG00000013848	MVK
Com_54_pos	Uric acid	ENSGALG00000041238	NOS1AP
Com_386_pos	2-Amino-1,3-octadecanec	ENSGALG00000002802	PACSIN1
Com_178_pos	Maltol	ENSGALG00000012882	KDSR
Com_40_pos	Choline	ENSGALG00000009365	CYP51A1
Com_8_neg	4-Methyl-2-Oxopentanoic	ENSGALG00000026547	TPGS2
Com_588_pos	Ornithine	ENSGALG00000004782	TSEN15
Com_80_pos	DL-Lysine	ENSGALG00000032628	SRCIN1
Com_130_neg	2-Hydroxyvaleric acid	MSTRG.4702	--
Com_76_neg	Erythronolactone	ENSGALG00000017378	CRTAC1
Com_147_pos	D-Sphingosine	ENSGALG00000008862	DNAJC10
Com_152_pos	Acetyl-L-carnitine	ENSGALG00000028822	RNF152
Com_362_pos	2-Arachidonoyl glycerol	ENSGALG00000029445	FADS6
Com_92_pos	D-(+)-Proline	ENSGALG00000015689	ECPAS
Com_331_pos	L-Lysine	ENSGALG00000005470	PLPPR5
Com_362_pos	2-Arachidonoyl glycerol	MSTRG.21796	--
Com_8_neg	4-Methyl-2-Oxopentanoic	ENSGALG00000008262	RASGRF1
Com_362_pos	2-Arachidonoyl glycerol	MSTRG.14343	--
Com_130_neg	2-Hydroxyvaleric acid	ENSGALG00000016475	Zp2

Com_252_pos	cis-4-Hydroxy-D-proline	ENSGALG00000004612	MTHFR
Com_18_neg	Arachidonic acid	ENSGALG00000041296	SOX7
Com_311_pos	PC (18:4e/2:0)	ENSGALG00000050676	Ctnnd2
Com_4_pos	PC (17:1/17:1)	ENSGALG00000049755	IL22RA2
Com_482_pos	8-Hydroxyquinoline	ENSGALG00000037160	Smad7
Com_215_pos	D-Erythro-sphingosine 1-|	ENSGALG00000052388	METRN
Com_208_neg	N-Acetylanthranilic acid	ENSGALG00000016254	OTC
Com_17_pos	L-Norleucine	ENSGALG00000005610	SLC44A3
Com_89_neg	Gallic acid	ENSGALG00000036005	TIAM2
Com_152_pos	Acetyl-L-carnitine	ENSGALG00000004729	SLC7A10
Com_194_pos	Pipecolic acid	ENSGALG00000027608	PIGC
Com_16_neg	3-Hydroxybutyric acid	ENSGALG00000021685	SERINC2
Com_311_pos	PC (18:4e/2:0)	ENSGALG00000043336	COPZ1
Com_194_pos	Pipecolic acid	MSTRG.13584	--
Com_175_pos	Pantothenic acid	ENSGALG00000014944	GCNT4
Com_86_neg	Levulinic acid	ENSGALG00000051779	PRORSD1P
Com_120_neg	LPC 15:0	ENSGALG00000054319	ELOVL6
Com_16_neg	3-Hydroxybutyric acid	MSTRG.11802	TMEM221
Com_311_pos	PC (18:4e/2:0)	ENSGALG00000020342	ABHD12
Com_130_neg	2-Hydroxyvaleric acid	MSTRG.149	--
Com_171_neg	LPC 22:6	ENSGALG00000033365	ALDH1A3
Com_588_pos	Ornithine	ENSGALG00000029235	CPNE4
Com_18_neg	Arachidonic acid	ENSGALG00000014464	MTR
Com_99_pos	Creatine	ENSGALG00000053659	--
Com_331_pos	L-Lysine	MSTRG.1503	gag
Com_311_pos	PC (18:4e/2:0)	ENSGALG00000000645	Espn
Com_12_pos	Betaine	ENSGALG00000030185	PTDSS1
Com_120_neg	LPC 15:0	ENSGALG00000000107	TRIM7.1
Com_194_pos	Pipecolic acid	ENSGALG00000026957	SEMA4G
Com_92_pos	D-(+)-Proline	ENSGALG00000016595	TRIM35
Com_120_neg	LPC 15:0	ENSGALG00000048035	GCNT2
Com_151_pos	Pyridoxamine	ENSGALG00000036492	DAGLA
Com_588_pos	Ornithine	ENSGALG00000013036	ATP6V1E1
Com_152_pos	Acetyl-L-carnitine	ENSGALG00000028191	GLCE
Com_151_pos	Pyridoxamine	MSTRG.17073	--
Com_54_pos	Uric acid	ENSGALG00000006198	LSS
Com_152_pos	Acetyl-L-carnitine	ENSGALG00000015136	ILDR1
Com_12_pos	Betaine	ENSGALG00000021395	ABCA9
Com_120_neg	LPC 15:0	MSTRG.18132	FBXW4
Com_16_neg	3-Hydroxybutyric acid	ENSGALG00000001749	ACSBG2
Com_130_neg	2-Hydroxyvaleric acid	ENSGALG00000004322	AHR
Com_186_pos	4-Hydroxybenzaldehyde	ENSGALG00000045127	slc12a8
Com_80_pos	DL-Lysine	ENSGALG00000023517	AGPAT2
Com_331_pos	L-Lysine	ENSGALG00000004322	AHR
Com_192_pos	1-Methylhistidine	ENSGALG00000009312	RPL22L1
Com_92_pos	D-(+)-Proline	ENSGALG00000048223	FAM20C
Com_80_pos	DL-Lysine	MSTRG.19422	--
Com_119_pos	DL-Stachydrine	ENSGALG00000005776	TECR
Com_8_neg	4-Methyl-2-Oxopentanoic	MSTRG.19463	VTG2
Com_186_pos	4-Hydroxybenzaldehyde	ENSGALG00000011616	NPFFR2
Com_12_pos	Betaine	ENSGALG00000023691	ENTPD7
Com_97_pos	L-Threonine	ENSGALG00000047495	LRRC10
Com_12_pos	Betaine	ENSGALG00000033150	MIDN
Com_362_pos	2-Arachidonoyl glycerol	ENSGALG00000037935	RARA
Com_54_pos	Uric acid	ENSGALG00000009415	SMOC1
Com_120_neg	LPC 15:0	ENSGALG00000028294	Vwa5b2
Com_120_neg	LPC 15:0	ENSGALG00000004216	TOR3A
Com_108_neg	LPE 18:2	ENSGALG00000012420	CG-1B

Com_86_neg	Levulinic acid	ENSGALG00000007404	YIPF5
Com_54_pos	Uric acid	ENSGALG00000008795	GPAM
Com_413_pos	L-Cystine	ENSGALG00000008862	DNAJC10
Com_192_pos	1-Methylhistidine	ENSGALG00000037322	HIST1H46
Com_22_pos	Indole-3-acrylic acid	ENSGALG00000050091	CLEC2B
Com_147_pos	D-Sphingosine	ENSGALG00000042511	PKDCC
Com_86_neg	Levulinic acid	MSTRG.13261	--
Com_362_pos	2-Arachidonoyl glycerol	ENSGALG00000003103	MST1R
Com_265_pos	6-Methylquinoline	ENSGALG00000041078	MID1IP1
Com_130_neg	2-Hydroxyvaleric acid	MSTRG.21204	--
Com_440_pos	PC (18:4e/4:0)	ENSGALG00000016885	STK24
Com_120_neg	LPC 15:0	ENSGALG00000006872	PISD
Com_76_neg	Erythronolactone	ENSGALG00000002775	FA2H
Com_471_pos	Indole-3-acetic acid	ENSGALG00000005977	BTBD8
Com_413_pos	L-Cystine	ENSGALG00000014976	GATA6
Com_130_neg	2-Hydroxyvaleric acid	MSTRG.1503	gag
Com_152_pos	Acetyl-L-carnitine	ENSGALG00000013001	CTNND2
Com_152_pos	Acetyl-L-carnitine	MSTRG.15732	--
Com_588_pos	Ornithine	MSTRG.2316	env
Com_16_neg	3-Hydroxybutyric acid	ENSGALG00000046789	pol
Com_413_pos	L-Cystine	ENSGALG00000042555	STAMBP
Com_331_pos	L-Lysine	MSTRG.149	--
Com_460_pos	3-amino-4-(propylamino)c	ENSGALG00000010018	CTSEAL
Com_471_pos	Indole-3-acetic acid	ENSGALG00000039239	SERPIND1
Com_386_pos	2-Amino-1,3-octadecanec	ENSGALG00000012112	DBI
Com_588_pos	Ornithine	ENSGALG00000015134	APOV1
Com_89_neg	Gallic acid	MSTRG.13474	Klhl29
Com_208_neg	N-Acetylanthranilic acid	MSTRG.18216	--
Com_440_pos	PC (18:4e/4:0)	ENSGALG00000014944	GCNT4
Com_120_neg	LPC 15:0	ENSGALG00000005583	ALG14
Com_194_pos	Pipecolic acid	ENSGALG00000001492	NDRG3
Com_152_pos	Acetyl-L-carnitine	MSTRG.2393	--
Com_311_pos	PC (18:4e/2:0)	ENSGALG00000041533	SLC11A2
Com_18_neg	Arachidonic acid	ENSGALG00000054297	SND1
Com_22_pos	Indole-3-acrylic acid	ENSGALG00000011141	ITGB6
Com_97_pos	L-Threonine	MSTRG.8471	--
Com_588_pos	Ornithine	ENSGALG00000005353	FAR1
Com_171_neg	LPC 22:6	MSTRG.5319	--
Com_386_pos	2-Amino-1,3-octadecanec	MSTRG.2388	--
Com_130_neg	2-Hydroxyvaleric acid	ENSGALG00000007848	PTS
Com_386_pos	2-Amino-1,3-octadecanec	ENSGALG00000005160	VMP1
Com_192_pos	1-Methylhistidine	ENSGALG00000015721	SVEP1
Com_80_pos	DL-Lysine	ENSGALG00000000619	ANGPTL4
Com_331_pos	L-Lysine	ENSGALG00000022758	GGACT
Com_76_neg	Erythronolactone	ENSGALG00000037050	FABP3
Com_12_pos	Betaine	MSTRG.16433	B4GALNT4
Com_119_pos	DL-Stachydrine	ENSGALG00000013090	LOXL4
Com_151_pos	Pyridoxamine	ENSGALG00000021686	--
Com_186_pos	4-Hydroxybenzaldehyde	ENSGALG00000006649	TMEM41A
Com_18_neg	Arachidonic acid	ENSGALG00000007848	PTS
Com_264_pos	Indole	MSTRG.15625	--
Com_76_neg	Erythronolactone	ENSGALG00000003427	USP3
Com_471_pos	Indole-3-acetic acid	ENSGALG00000009700	PDK4
Com_386_pos	2-Amino-1,3-octadecanec	ENSGALG00000012847	Slc7a11
Com_151_pos	Pyridoxamine	ENSGALG00000009476	CDK6
Com_252_pos	cis-4-Hydroxy-D-proline	MSTRG.21091	--
Com_99_pos	Creatine	ENSGALG00000003081	SUCO
Com_22_pos	Indole-3-acrylic acid	MSTRG.10409	--

Com_152_pos	Acetyl-L-carnitine	ENSGALG00000036787	HSD17B12
Com_386_pos	2-Amino-1,3-octadecanec	ENSGALG00000038723	RPP25L
Com_460_pos	3-amino-4-(propylamino)(	ENSGALG00000053245	VTG2
Com_22_pos	Indole-3-acrylic acid	ENSGALG00000021451	RED3
Com_99_pos	Creatine	ENSGALG00000005043	ACACB
Com_331_pos	L-Lysine	MSTRG.4702	--
Com_147_pos	D-Sphingosine	ENSGALG00000052612	RPS27L
Com_99_pos	Creatine	MSTRG.11834	--
Com_252_pos	cis-4-Hydroxy-D-proline	ENSGALG00000037467	NEU2
Com_99_pos	Creatine	MSTRG.19484	--
Com_362_pos	2-Arachidonoyl glycerol	ENSGALG00000016492	TDRD15
Com_331_pos	L-Lysine	ENSGALG00000005739	SCD
Com_147_pos	D-Sphingosine	ENSGALG00000010229	ABCD4
Com_97_pos	L-Threonine	ENSGALG00000016475	Zp2
Com_386_pos	2-Amino-1,3-octadecanec	ENSGALG00000026460	myoM
Com_151_pos	Pyridoxamine	ENSGALG00000030151	LUZP2
Com_192_pos	1-Methylhistidine	ENSGALG00000052829	MAMDC4
Com_18_neg	Arachidonic acid	MSTRG.21204	--
Com_362_pos	2-Arachidonoyl glycerol	ENSGALG00000026607	C15orf40
Com_152_pos	Acetyl-L-carnitine	ENSGALG00000047380	MR1
Com_413_pos	L-Cystine	ENSGALG00000015729	LPAR1
Com_331_pos	L-Lysine	ENSGALG00000012882	KDSR
Com_18_neg	Arachidonic acid	ENSGALG00000010641	SCCPDH
Com_482_pos	8-Hydroxyquinoline	ENSGALG00000026313	RND3
Com_413_pos	L-Cystine	MSTRG.2387	--
Com_175_pos	Pantothenic acid	ENSGALG00000004425	SCAMP1
Com_413_pos	L-Cystine	MSTRG.3156	--
Com_151_pos	Pyridoxamine	ENSGALG00000015263	TMEM30C
Com_152_pos	Acetyl-L-carnitine	ENSGALG00000026547	TPGS2
Com_78_neg	Citric acid	ENSGALG00000007018	SLC26A11
Com_192_pos	1-Methylhistidine	ENSGALG00000002249	AGO1
Com_203_pos	Serotonin	ENSGALG00000005632	IRAG1
Com_21_pos	DL-Tryptophan	ENSGALG00000014261	UCHL1
Com_12_pos	Betaine	MSTRG.53	SND1
Com_386_pos	2-Amino-1,3-octadecanec	MSTRG.15443	--
Com_482_pos	8-Hydroxyquinoline	ENSGALG00000034741	ETNPPL
Com_203_pos	Serotonin	ENSGALG00000050840	APCDD1
Com_265_pos	6-Methylquinoline	ENSGALG00000027561	GNG5
Com_86_neg	Levulinic acid	ENSGALG00000005610	SLC44A3
Com_151_pos	Pyridoxamine	ENSGALG00000003575	Dnttip1
Com_192_pos	1-Methylhistidine	MSTRG.12570	--
Com_460_pos	3-amino-4-(propylamino)(	MSTRG.6225	--
Com_147_pos	D-Sphingosine	ENSGALG00000006076	RASGEF1C
Com_16_neg	3-Hydroxybutyric acid	MSTRG.13408	--
Com_440_pos	PC (18:4e/4:0)	MSTRG.2387	--
Com_152_pos	Acetyl-L-carnitine	ENSGALG00000008262	RASGRF1
Com_311_pos	PC (18:4e/2:0)	ENSGALG00000038532	--
Com_4_pos	PC (17:1/17:1)	ENSGALG00000011469	IGFBP2
Com_80_pos	DL-Lysine	ENSGALG00000051466	NDFIP2
Com_440_pos	PC (18:4e/4:0)	ENSGALG00000015729	LPAR1
Com_265_pos	6-Methylquinoline	ENSGALG00000035803	THRSP
Com_40_pos	Choline	MSTRG.20573	--
Com_17_pos	L-Norleucine	ENSGALG00000026460	myoM
Com_460_pos	3-amino-4-(propylamino)(	ENSGALG00000047687	SETD9
Com_175_pos	Pantothenic acid	ENSGALG00000030845	ENHO
Com_12_pos	Betaine	ENSGALG00000014906	MOCS2
Com_16_neg	3-Hydroxybutyric acid	MSTRG.12923	--
Com_40_pos	Choline	ENSGALG00000005617	NTHL1

Com_80_pos	DL-Lysine	ENSGALG00000043829	ext1c
Com_252_pos	cis-4-Hydroxy-D-proline	ENSGALG00000023760	CHIA
Com_192_pos	1-Methylhistidine	ENSGALG00000001642	GLT8D1
Com_171_neg	LPC 22:6	ENSGALG00000046731	--
Com_86_neg	Levulinic acid	ENSGALG00000049658	UTS2R
Com_120_neg	LPC 15:0	ENSGALG00000037050	FABP3
Com_186_pos	4-Hydroxybenzaldehyde	ENSGALG00000031932	AGPAT3
Com_186_pos	4-Hydroxybenzaldehyde	ENSGALG00000036021	MTMR7
Com_175_pos	Pantothenic acid	ENSGALG00000006076	RASGEF1C
Com_18_neg	Arachidonic acid	ENSGALG00000010293	RBP
Com_178_pos	Maltol	MSTRG.1503	gag
Com_152_pos	Acetyl-L-carnitine	MSTRG.3156	--
Com_17_pos	L-Norleucine	ENSGALG00000012847	Slc7a11
Com_252_pos	cis-4-Hydroxy-D-proline	ENSGALG00000036234	RFWD3
Com_92_pos	D-(+)-Proline	ENSGALG00000005353	FAR1
Com_471_pos	Indole-3-acetic acid	ENSGALG00000047827	TMEM86A
Com_17_pos	L-Norleucine	ENSGALG00000005160	VMP1
Com_588_pos	Ornithine	ENSGALG00000020342	ABHD12
Com_97_pos	L-Threonine	MSTRG.21204	--
Com_17_pos	L-Norleucine	MSTRG.2388	--
Com_76_neg	Erythronolactone	ENSGALG00000005583	ALG14
Com_130_neg	2-Hydroxyvaleric acid	ENSGALG00000041296	SOX7
Com_252_pos	cis-4-Hydroxy-D-proline	ENSGALG00000036915	SQLE
Com_8_neg	4-Methyl-2-Oxopentanoic	ENSGALG00000028191	GLCE
Com_208_neg	N-Acetylanthranilic acid	ENSGALG00000040363	ABHD4
Com_440_pos	PC (18:4e/4:0)	ENSGALG00000000645	Espn
Com_18_neg	Arachidonic acid	ENSGALG00000016475	Zp2
Com_186_pos	4-Hydroxybenzaldehyde	ENSGALG00000004424	SEC16B
Com_40_pos	Choline	MSTRG.16903	--
Com_147_pos	D-Sphingosine	ENSGALG00000004425	SCAMP1
Com_331_pos	L-Lysine	ENSGALG00000036836	SOSTDC1
Com_588_pos	Ornithine	ENSGALG00000043336	COPZ1
Com_208_neg	N-Acetylanthranilic acid	MSTRG.19484	--
Com_92_pos	D-(+)-Proline	ENSGALG00000015134	APOV1
Com_108_neg	LPE 18:2	ENSGALG00000047632	Pc
Com_92_pos	D-(+)-Proline	ENSGALG00000004170	ADA
Com_120_neg	LPC 15:0	ENSGALG00000002775	FA2H
Com_97_pos	L-Threonine	MSTRG.15507	--
Com_76_neg	Erythronolactone	ENSGALG00000006872	PISD
Com_92_pos	D-(+)-Proline	MSTRG.2316	env
Com_208_neg	N-Acetylanthranilic acid	ENSGALG00000005043	ACACB
Com_588_pos	Ornithine	ENSGALG00000050676	Ctnnd2
Com_16_neg	3-Hydroxybutyric acid	ENSGALG00000005648	Sesn3
Com_192_pos	1-Methylhistidine	MSTRG.7811	--
Com_178_pos	Maltol	ENSGALG00000004322	AHR
Com_386_pos	2-Amino-1,3-octadecanec	ENSGALG00000000950	MVB12B
Com_108_neg	LPE 18:2	ENSGALG00000002431	CFH
Com_352_pos	Riboflavin	ENSGALG00000009100	FSHR
Com_208_neg	N-Acetylanthranilic acid	ENSGALG00000003081	SUCO
Com_175_pos	Pantothenic acid	ENSGALG00000010229	ABCD4
Com_264_pos	Indole	ENSGALG00000050091	CLEC2B
Com_40_pos	Choline	ENSGALG00000008604	TMEM255A
Com_97_pos	L-Threonine	ENSGALG00000014813	HOMER1
Com_80_pos	DL-Lysine	ENSGALG00000002899	AACS
Com_152_pos	Acetyl-L-carnitine	MSTRG.19463	VTG2
Com_40_pos	Choline	ENSGALG00000052786	Znf185
Com_86_neg	Levulinic acid	ENSGALG00000030908	ATP2B2
Com_178_pos	Maltol	ENSGALG00000010978	ANGPTL3

Com_97_pos	L-Threonine	ENSGALG00000007848	PTS
Com_460_pos	3-amino-4-(propylamino)(	ENSGALG00000054319	ELOVL6
Com_208_neg	N-Acetylanthranilic acid	ENSGALG00000012414	GNPNAT1
Com_80_pos	DL-Lysine	MSTRG.13135	--
Com_413_pos	L-Cystine	ENSGALG00000016885	STK24
Com_130_neg	2-Hydroxyvaleric acid	ENSGALG00000005648	Sesn3
Com_18_neg	Arachidonic acid	MSTRG.8471	--
Com_119_pos	DL-Stachydrine	ENSGALG00000002116	TEN1
Com_440_pos	PC (18:4e/4:0)	ENSGALG00000008862	DNAJC10
Com_86_neg	Levulinic acid	ENSGALG00000033656	DQX1
Com_55_pos	Valine	ENSGALG00000008039	MFSD13A
Com_331_pos	L-Lysine	ENSGALG00000004341	Cryzl2
Com_460_pos	3-amino-4-(propylamino)(	ENSGALG00000000107	TRIM7.1
Com_8_neg	4-Methyl-2-Oxopentanoic	ENSGALG00000013001	CTNND2
Com_203_pos	Serotonin	ENSGALG00000001768	TENM2
Com_178_pos	Maltol	MSTRG.149	--
Com_8_neg	4-Methyl-2-Oxopentanoic	MSTRG.15732	--
Com_460_pos	3-amino-4-(propylamino)(	ENSGALG00000048035	GCNT2
Com_311_pos	PC (18:4e/2:0)	ENSGALG00000016885	STK24
Com_16_neg	3-Hydroxybutyric acid	ENSGALG00000041296	SOX7
Com_413_pos	L-Cystine	MSTRG.2393	--
Com_97_pos	L-Threonine	ENSGALG00000014233	FBLN1
Com_25_pos	2-Hydroxycinnamic acid	ENSGALG00000034294	ATP6V0D2
Com_76_neg	Erythronolactone	ENSGALG00000048035	GCNT2
Com_151_pos	Pyridoxamine	MSTRG.8381	--
Com_12_pos	Betaine	ENSGALG00000016511	ADGRG2
Com_178_pos	Maltol	ENSGALG00000009680	PAQR7
Com_76_neg	Erythronolactone	ENSGALG00000000107	TRIM7.1
Com_8_neg	4-Methyl-2-Oxopentanoic	MSTRG.2393	--
Com_152_pos	Acetyl-L-carnitine	ENSGALG00000042555	STAMBP
Com_92_pos	D-(+)-Proline	ENSGALG00000013036	ATP6V1E1
Com_22_pos	Indole-3-acrylic acid	MSTRG.15625	--
Com_352_pos	Riboflavin	ENSGALG00000053647	PFAS
Com_413_pos	L-Cystine	MSTRG.15732	--
Com_413_pos	L-Cystine	ENSGALG00000013001	CTNND2
Com_152_pos	Acetyl-L-carnitine	ENSGALG00000014976	GATA6
Com_264_pos	Indole	MSTRG.10409	--
Com_78_neg	Citric acid	MSTRG.21796	--
Com_440_pos	PC (18:4e/4:0)	ENSGALG00000041533	SLC11A2
Com_80_pos	DL-Lysine	ENSGALG00000007778	PES1
Com_151_pos	Pyridoxamine	ENSGALG00000035935	Unc13c
Com_92_pos	D-(+)-Proline	ENSGALG00000029235	CPNE4
Com_25_pos	2-Hydroxycinnamic acid	ENSGALG00000049751	H2B-I
Com_76_neg	Erythronolactone	ENSGALG00000054319	ELOVL6
Com_22_pos	Indole-3-acrylic acid	ENSGALG00000026809	SARS
Com_265_pos	6-Methylquinoline	MSTRG.2171	Myo16
Com_18_neg	Arachidonic acid	ENSGALG00000047495	LRRC10
Com_130_neg	2-Hydroxyvaleric acid	MSTRG.12923	--
Com_54_pos	Uric acid	ENSGALG00000006490	SCN3B
Com_194_pos	Pipecolic acid	ENSGALG00000006702	MFGE8
Com_25_pos	2-Hydroxycinnamic acid	ENSGALG00000005215	CACNA1H
Com_151_neg	Lysope 18:1	ENSGALG00000014971	SLC2A9
Com_151_pos	Pyridoxamine	ENSGALG00000026846	JMJD7
Com_147_pos	D-Sphingosine	ENSGALG00000016476	TTC32
Com_352_pos	Riboflavin	ENSGALG00000039708	Pram1
Com_178_pos	Maltol	MSTRG.4702	--
Com_175_pos	Pantothenic acid	ENSGALG00000042511	PKDCC
Com_57_neg	LPC 16:1	ENSGALG00000004472	ASTN1

Com_55_pos	Valine	ENSGALG00000027608	PIGC
Com_460_pos	3-amino-4-(propylamino)(	ENSGALG00000006872	PISD
Com_86_neg	Levulinic acid	ENSGALG00000038145	DPP7
Com_194_pos	Pipecolic acid	ENSGALG00000012748	ELOVL2
Com_171_neg	LPC 22:6	ENSGALG00000007018	SLC26A11
Com_97_pos	L-Threonine	ENSGALG00000015044	GTF3C6
Com_386_pos	2-Amino-1,3-octadecanec	ENSGALG00000005610	SLC44A3
Com_151_pos	Pyridoxamine	ENSGALG00000004505	CCDC137
Com_8_neg	4-Methyl-2-Oxopentanoic	ENSGALG00000042491	H4-I
Com_588_pos	Ornithine	ENSGALG00000036293	EBAG9
Com_92_pos	D-(+)-Proline	ENSGALG00000028822	RNF152
Com_171_neg	LPC 22:6	ENSGALG00000015684	Dnajc25
Com_120_neg	LPC 15:0	ENSGALG00000017378	CRTAC1
Com_460_pos	3-amino-4-(propylamino)(	ENSGALG00000005583	ALG14
Com_186_pos	4-Hydroxybenzaldehyde	ENSGALG00000010703	DGLUCY
Com_119_pos	DL-Stachydrine	ENSGALG00000000498	ACE
Com_413_pos	L-Cystine	ENSGALG00000028191	GLCE
Com_265_pos	6-Methylquinoline	MSTRG.9361	Fam110a
Com_311_pos	PC (18:4e/2:0)	MSTRG.1502	gag
Com_55_pos	Valine	ENSGALG00000026957	SEMA4G
Com_119_pos	DL-Stachydrine	ENSGALG00000011469	IGFBP2
Com_21_pos	DL-Tryptophan	ENSGALG00000009016	SLX4IP
Com_252_pos	cis-4-Hydroxy-D-proline	ENSGALG00000014509	BST1
Com_99_pos	Creatine	ENSGALG00000010643	ZYG11B
Com_186_pos	4-Hydroxybenzaldehyde	ENSGALG00000024449	RAMP2
Com_92_pos	D-(+)-Proline	ENSGALG00000004782	TSEN15
Com_99_pos	Creatine	MSTRG.1890	gag
Com_311_pos	PC (18:4e/2:0)	ENSGALG00000012877	CREB3L2
Com_92_pos	D-(+)-Proline	ENSGALG00000004729	SLC7A10
Com_108_neg	LPE 18:2	ENSGALG00000037780	PMEPA1
Com_86_neg	Levulinic acid	ENSGALG00000026460	myoM
Com_252_pos	cis-4-Hydroxy-D-proline	ENSGALG00000050440	APOF
Com_80_pos	DL-Lysine	ENSGALG00000016491	APOB
Com_40_pos	Choline	ENSGALG00000023626	NTN1
Com_78_neg	Citric acid	ENSGALG00000046731	--
Com_18_neg	Arachidonic acid	ENSGALG00000046789	pol
Com_97_pos	L-Threonine	MSTRG.8986	gag
Com_17_pos	L-Norleucine	ENSGALG00000005815	TMEM41B
Com_76_neg	Erythronolactone	ENSGALG00000047687	SETD9
Com_186_pos	4-Hydroxybenzaldehyde	ENSGALG00000002371	RUSC2
Com_8_neg	4-Methyl-2-Oxopentanoic	ENSGALG00000006689	ABHD2
Com_97_pos	L-Threonine	ENSGALG00000041296	SOX7
Com_17_pos	L-Norleucine	ENSGALG00000049658	UTS2R
Com_78_neg	Citric acid	ENSGALG00000037935	RARA
Com_311_pos	PC (18:4e/2:0)	ENSGALG00000006723	IDI1
Com_147_pos	D-Sphingosine	ENSGALG00000014944	GCNT4
Com_171_neg	LPC 22:6	MSTRG.9165	--
Com_588_pos	Ornithine	ENSGALG00000014976	GATA6
Com_311_pos	PC (18:4e/2:0)	MSTRG.2387	--
Com_482_pos	8-Hydroxyquinoline	ENSGALG00000004205	SOAT1
Com_440_pos	PC (18:4e/4:0)	ENSGALG00000038532	--
Com_78_neg	Citric acid	ENSGALG00000003103	MST1R
Com_130_neg	2-Hydroxyvaleric acid	MSTRG.15507	--
Com_86_neg	Levulinic acid	ENSGALG00000012847	Slc7a11
Com_311_pos	PC (18:4e/2:0)	ENSGALG00000015729	LPAR1
Com_588_pos	Ornithine	ENSGALG00000042555	STAMBP
Com_76_neg	Erythronolactone	MSTRG.6225	--
Com_86_neg	Levulinic acid	ENSGALG00000005160	VMP1

Com_92_pos	D-(+)-Proline	ENSGALG00000015136	ILDR1
Com_86_neg	Levulinic acid	MSTRG.2388	--
Com_16_neg	3-Hydroxybutyric acid	ENSGALG00000007848	PTS
Com_130_neg	2-Hydroxyvaleric acid	ENSGALG00000014813	HOMER1
Com_264_pos	Indole	ENSGALG00000028880	FDPS
Com_471_pos	Indole-3-acetic acid	ENSGALG00000034140	ZNF395
Com_40_pos	Choline	ENSGALG00000010294	RPS6KL1
Com_264_pos	Indole	ENSGALG00000051567	MRPL41
Com_215_pos	D-Erythro-sphingosine 1-p	ENSGALG00000047632	Pc
Com_171_neg	LPC 22:6	ENSGALG00000031754	KCNG2
Com_311_pos	PC (18:4e/2:0)	MSTRG.14577	SLC39A5
Com_588_pos	Ornithine	ENSGALG00000041604	NPTXR
Com_8_neg	4-Methyl-2-Oxopentanoic	MSTRG.3156	--
Com_151_pos	Pyridoxamine	ENSGALG00000041456	SLC35G1
Com_55_pos	Valine	ENSGALG00000001492	NDRG3
Com_97_pos	L-Threonine	ENSGALG00000005648	Sesn3
Com_147_pos	D-Sphingosine	ENSGALG00000005815	TMEM41B
Com_99_pos	Creatine	ENSGALG00000023348	HPDL
Com_25_pos	2-Hydroxycinnamic acid	ENSGALG00000023626	NTN1
Com_54_pos	Uric acid	ENSGALG00000028005	GADD45G
Com_215_pos	D-Erythro-sphingosine 1-p	ENSGALG00000002431	CFH
Com_440_pos	PC (18:4e/4:0)	ENSGALG00000052612	RPS27L
Com_178_pos	Maltol	ENSGALG00000036836	SOSTDC1
Com_4_pos	PC (17:1/17:1)	ENSGALG00000013090	LOXL4
Com_89_neg	Gallic acid	ENSGALG00000001392	MMP23B
Com_17_pos	L-Norleucine	ENSGALG00000030908	ATP2B2
Com_40_pos	Choline	ENSGALG00000006530	TSSC4
Com_151_pos	Pyridoxamine	ENSGALG00000042080	PGPEP1
Com_130_neg	2-Hydroxyvaleric acid	ENSGALG00000014233	FBLN1
Com_16_neg	3-Hydroxybutyric acid	MSTRG.21204	--
Com_386_pos	2-Amino-1,3-octadecanec	MSTRG.13261	--
Com_192_pos	1-Methylhistidine	MSTRG.2430	--
Com_386_pos	2-Amino-1,3-octadecanec	ENSGALG00000007404	YIPF5
Com_86_neg	Levulinic acid	MSTRG.16287	--
Com_76_neg	Erythronolactone	ENSGALG00000053245	VTG2
Com_208_neg	N-Acetylanthranilic acid	ENSGALG00000048285	TCN2
Com_17_pos	L-Norleucine	ENSGALG00000033656	DQX1
Com_194_pos	Pipecolic acid	ENSGALG00000031754	KCNG2
Com_482_pos	8-Hydroxyquinoline	ENSGALG00000009740	RASGRP1
Com_460_pos	3-amino-4-(propylamino)(	ENSGALG00000045557	MTTPL
Com_331_pos	L-Lysine	ENSGALG00000010978	ANGPTL3
Com_471_pos	Indole-3-acetic acid	ENSGALG00000006864	COL24A1
Com_80_pos	DL-Lysine	ENSGALG00000027375	NR2C2AP
Com_99_pos	Creatine	ENSGALG00000004590	CLCN6
Com_588_pos	Ornithine	ENSGALG00000021135	HAPLN3
Com_460_pos	3-amino-4-(propylamino)(	ENSGALG00000037050	FABP3
Com_40_pos	Choline	ENSGALG00000002579	RIMBP2
Com_130_neg	2-Hydroxyvaleric acid	ENSGALG00000001749	ACSBG2
Com_97_pos	L-Threonine	MSTRG.12923	--
Com_178_pos	Maltol	ENSGALG00000004341	Cryzl2
Com_186_pos	4-Hydroxybenzaldehyde	MSTRG.8499	Itpripl1
Com_171_neg	LPC 22:6	MSTRG.21796	--
Com_588_pos	Ornithine	MSTRG.3156	--
Com_8_neg	4-Methyl-2-Oxopentanoic	MSTRG.13439	--
Com_18_neg	Arachidonic acid	MSTRG.13408	--
Com_76_neg	Erythronolactone	MSTRG.16287	--
Com_194_pos	Pipecolic acid	MSTRG.9165	--
Com_17_pos	L-Norleucine	ENSGALG00000016476	TTC32

Com_12_pos	Betaine	ENSGALG00000014126	endou-a
Com_208_neg	N-Acetylanthranilic acid	ENSGALG00000008297	SEMA4B
Com_16_neg	3-Hydroxybutyric acid	ENSGALG00000017122	SGCG
Com_12_pos	Betaine	MSTRG.8497	--
Com_4_pos	PC (17:1/17:1)	ENSGALG00000005776	TECR
Com_311_pos	PC (18:4e/2:0)	ENSGALG00000008862	DNAJC10
Com_186_pos	4-Hydroxybenzaldehyde	ENSGALG00000047720	KLHDC7A
Com_252_pos	cis-4-Hydroxy-D-proline	ENSGALG00000003560	SLC6A2
Com_97_pos	L-Threonine	MSTRG.2305	gag
Com_76_neg	Erythronolactone	ENSGALG00000010018	CTSEAL
Com_92_pos	D-(+)-Proline	ENSGALG00000036787	HSD17B12
Com_40_pos	Choline	ENSGALG00000049751	H2B-I
Com_130_neg	2-Hydroxyvaleric acid	ENSGALG00000015044	GTF3C6
Com_482_pos	8-Hydroxyquinoline	ENSGALG00000016364	ALKAL2
Com_331_pos	L-Lysine	ENSGALG00000009680	PAQR7
Com_264_pos	Indole	ENSGALG00000044464	TEPSIN
Com_186_pos	4-Hydroxybenzaldehyde	ENSGALG00000053217	INPP5J
Com_264_pos	Indole	ENSGALG00000026809	SARS
Com_460_pos	3-amino-4-(propylamino)(	ENSGALG00000002775	FA2H
Com_8_neg	4-Methyl-2-Oxopentanoic	ENSGALG00000042555	STAMBP
Com_130_neg	2-Hydroxyvaleric acid	MSTRG.11802	TMEM221
Com_8_neg	4-Methyl-2-Oxopentanoic	ENSGALG00000014976	GATA6
Com_99_pos	Creatine	ENSGALG00000004498	SLC2A10
Com_386_pos	2-Amino-1,3-octadecaned	ENSGALG00000051779	PRORSD1P
Com_331_pos	L-Lysine	ENSGALG00000037773	ST3GAL1
Com_17_pos	L-Norleucine	ENSGALG00000038145	DPP7
Com_40_pos	Choline	ENSGALG00000034294	ATP6V0D2
Com_186_pos	4-Hydroxybenzaldehyde	ENSGALG00000031570	WDR54
Com_178_pos	Maltol	ENSGALG00000035626	DAD1
Com_78_neg	Citric acid	ENSGALG00000015684	Dnajc25
Com_130_neg	2-Hydroxyvaleric acid	ENSGALG00000021685	SERINC2
Com_55_pos	Valine	ENSGALG00000007507	MASTL
Com_40_pos	Choline	ENSGALG00000052986	Vwa5b2
Com_40_pos	Choline	ENSGALG00000005839	ARID3C
Com_92_pos	D-(+)-Proline	ENSGALG00000047380	MR1
Com_194_pos	Pipecolic acid	ENSGALG00000015684	Dnajc25
Com_265_pos	6-Methylquinoline	ENSGALG00000041687	SREBF2
Com_16_neg	3-Hydroxybutyric acid	ENSGALG00000016475	Zp2
Com_21_pos	DL-Tryptophan	ENSGALG00000028005	GADD45G
Com_147_pos	D-Sphingosine	ENSGALG00000000645	Espn
Com_130_neg	2-Hydroxyvaleric acid	MSTRG.8986	gag
Com_99_pos	Creatine	ENSGALG00000035239	GLCCI1
Com_92_pos	D-(+)-Proline	ENSGALG00000026547	TPGS2
Com_252_pos	cis-4-Hydroxy-D-proline	ENSGALG00000013848	MVK
Com_171_neg	LPC 22:6	ENSGALG00000012748	ELOVL2
Com_386_pos	2-Amino-1,3-octadecaned	ENSGALG00000042511	PKDCC
Com_25_pos	2-Hydroxycinnamic acid	ENSGALG00000052786	Znf185
Com_152_pos	Acetyl-L-carnitine	ENSGALG00000042491	H4-I
Com_413_pos	L-Cystine	ENSGALG00000020342	ABHD12
Com_40_pos	Choline	MSTRG.3473	--
Com_362_pos	2-Arachidonoyl glycerol	ENSGALG00000035626	DAD1
Com_25_pos	2-Hydroxycinnamic acid	ENSGALG00000008604	TMEM255A
Com_12_pos	Betaine	ENSGALG00000001918	DNAJB5
Com_215_pos	D-Erythro-sphingosine 1-p	ENSGALG00000037780	PMEPA1
Com_119_pos	DL-Stachydrine	ENSGALG00000049755	IL22RA2
Com_175_pos	Pantothenic acid	MSTRG.13135	--
Com_171_neg	LPC 22:6	ENSGALG00000006702	MFGE8
Com_413_pos	L-Cystine	ENSGALG00000043336	COPZ1

Com_192_pos	1-Methylhistidine	MSTRG.835	--
Com_92_pos	D-(+)-Proline	ENSGALG00000008262	RASGRF1
Com_460_pos	3-amino-4-(propylamino)(	ENSGALG00000014463	ACTN2
Com_175_pos	Pantothenic acid	ENSGALG00000002899	AACS
Com_171_neg	LPC 22:6	ENSGALG00000037935	RARA
Com_386_pos	2-Amino-1,3-octadecanec	ENSGALG00000021658	PAFAH2
Com_413_pos	L-Cystine	ENSGALG00000050676	Ctnnd2
Com_86_neg	Levulinic acid	ENSGALG00000005815	TMEM41B
Com_78_neg	Citric acid	MSTRG.9165	--
Com_440_pos	PC (18:4e/4:0)	MSTRG.1502	gag
Com_16_neg	3-Hydroxybutyric acid	MSTRG.8471	--
Com_171_neg	LPC 22:6	ENSGALG00000003103	MST1R
Com_25_pos	2-Hydroxycinnamic acid	MSTRG.16903	--
Com_80_pos	DL-Lysine	ENSGALG00000052072	gag
Com_16_neg	3-Hydroxybutyric acid	ENSGALG00000026203	FAM174A
Com_120_neg	LPC 15:0	ENSGALG00000009170	NCEH1
Com_92_pos	D-(+)-Proline	ENSGALG00000026203	FAM174A
Com_588_pos	Ornithine	ENSGALG00000003147	TRPC4AP
Com_147_pos	D-Sphingosine	MSTRG.2388	--
Com_440_pos	PC (18:4e/4:0)	ENSGALG00000012877	CREB3L2
Com_18_neg	Arachidonic acid	ENSGALG00000019276	SLCO1C1
Com_147_pos	D-Sphingosine	ENSGALG00000005160	VMP1
Com_471_pos	Indole-3-acetic acid	MSTRG.836	--
Com_152_pos	Acetyl-L-carnitine	ENSGALG00000006689	ABHD2
Com_147_pos	D-Sphingosine	ENSGALG00000012847	Slc7a11
Com_78_neg	Citric acid	ENSGALG00000031754	KCNG2
Com_386_pos	2-Amino-1,3-octadecanec	MSTRG.21394	gag-pol
Com_192_pos	1-Methylhistidine	ENSGALG00000004702	DYNC2I2
Com_119_pos	DL-Stachydrine	ENSGALG00000008859	WDR31
Com_152_pos	Acetyl-L-carnitine	ENSGALG00000005353	FAR1
Com_12_pos	Betaine	ENSGALG00000000241	STARD4
Com_76_neg	Erythronolactone	ENSGALG00000038145	DPP7
Com_119_pos	DL-Stachydrine	ENSGALG00000053860	mas
Com_22_pos	Indole-3-acrylic acid	ENSGALG00000028880	FDPS
Com_440_pos	PC (18:4e/4:0)	ENSGALG00000006723	IDI1
Com_588_pos	Ornithine	MSTRG.2393	--
Com_54_pos	Uric acid	ENSGALG00000009016	SLX4IP
Com_25_pos	2-Hydroxycinnamic acid	ENSGALG00000005617	NTHL1
Com_151_pos	Pyridoxamine	MSTRG.6512	--
Com_460_pos	3-amino-4-(propylamino)(	ENSGALG00000017378	CRTAC1
Com_22_pos	Indole-3-acrylic acid	ENSGALG00000051567	MRPL41
Com_18_neg	Arachidonic acid	ENSGALG00000047027	ADCK5
Com_147_pos	D-Sphingosine	ENSGALG00000041533	SLC11A2
Com_99_pos	Creatine	ENSGALG00000050520	pol
Com_152_pos	Acetyl-L-carnitine	ENSGALG00000015134	APOV1
Com_25_pos	2-Hydroxycinnamic acid	MSTRG.20573	--
Com_16_neg	3-Hydroxybutyric acid	ENSGALG00000047495	LRRC10
Com_386_pos	2-Amino-1,3-octadecanec	ENSGALG00000010229	ABCD4
Com_147_pos	D-Sphingosine	ENSGALG00000026460	myoM
Com_97_pos	L-Threonine	ENSGALG00000008866	WDPCP
Com_152_pos	Acetyl-L-carnitine	MSTRG.2316	env
Com_175_pos	Pantothenic acid	ENSGALG00000000950	MVB12B
Com_588_pos	Ornithine	MSTRG.15732	--
Com_588_pos	Ornithine	ENSGALG00000013001	CTNND2
Com_588_pos	Ornithine	ENSGALG00000046757	ERVK-9
Com_192_pos	1-Methylhistidine	ENSGALG00000016560	SELENOI
Com_80_pos	DL-Lysine	ENSGALG00000030845	ENHO
Com_130_neg	2-Hydroxyvaleric acid	ENSGALG00000039538	CLDND1

Com_55_pos	Valine	ENSGALG00000036754	CHKA
Com_97_pos	L-Threonine	MSTRG.1082	--
Com_440_pos	PC (18:4e/4:0)	ENSGALG00000016476	TTC32
Com_97_pos	L-Threonine	ENSGALG00000001749	ACSBG2
Com_17_pos	L-Norleucine	MSTRG.16287	--
Com_175_pos	Pantothenic acid	ENSGALG00000051466	NDFIP2
Com_440_pos	PC (18:4e/4:0)	MSTRG.14577	SLC39A5
Com_92_pos	D-(+)-Proline	MSTRG.19463	VTG2
Com_97_pos	L-Threonine	ENSGALG00000012377	HNMT
Com_21_pos	DL-Tryptophan	ENSGALG00000006490	SCN3B
Com_311_pos	PC (18:4e/2:0)	ENSGALG00000052612	RPS27L
Com_192_pos	1-Methylhistidine	ENSGALG00000038574	MYO15A
Com_130_neg	2-Hydroxyvaleric acid	MSTRG.2305	gag
Com_22_pos	Indole-3-acrylic acid	ENSGALG00000041078	MID1IP1
Com_55_pos	Valine	ENSGALG00000006702	MFGE8
Com_386_pos	2-Amino-1,3-octadecanec	ENSGALG00000006076	RASGEF1C
Com_76_neg	Erythronolactone	ENSGALG00000033656	DQX1
Com_413_pos	L-Cystine	ENSGALG00000036293	EBAG9
Com_97_pos	L-Threonine	MSTRG.11802	TMEM221
Com_86_neg	Levulinic acid	ENSGALG00000016476	TTC32
Com_92_pos	D-(+)-Proline	ENSGALG00000017122	SGCG
Com_8_neg	4-Methyl-2-Oxopentanoic	ENSGALG00000015016	SLC22A15
Com_80_pos	DL-Lysine	ENSGALG00000051123	pol
Com_194_pos	Pipecolic acid	ENSGALG00000046731	--
Com_55_pos	Valine	ENSGALG00000012748	ELOVL2
Com_588_pos	Ornithine	ENSGALG00000028191	GLCE
Com_120_neg	LPC 15:0	MSTRG.21321	gag
Com_152_pos	Acetyl-L-carnitine	ENSGALG00000013036	ATP6V1E1
Com_76_neg	Erythronolactone	ENSGALG00000030908	ATP2B2
Com_97_pos	L-Threonine	ENSGALG00000021685	SERINC2
Com_17_pos	L-Norleucine	ENSGALG00000052612	RPS27L
Com_12_pos	Betaine	ENSGALG00000012505	LRFN5
Com_152_pos	Acetyl-L-carnitine	ENSGALG00000029235	CPNE4
Com_152_pos	Acetyl-L-carnitine	MSTRG.13439	--
Com_265_pos	6-Methylquinoline	ENSGALG00000011141	ITGB6
Com_22_pos	Indole-3-acrylic acid	ENSGALG00000044464	TEPSIN
Com_311_pos	PC (18:4e/2:0)	ENSGALG00000037773	ST3GAL1
Com_175_pos	Pantothenic acid	MSTRG.15443	--
Com_208_neg	N-Acetylanthranilic acid	ENSGALG00000053217	INPP5J
Com_147_pos	D-Sphingosine	ENSGALG00000038532	--
Com_99_pos	Creatine	MSTRG.8248	--
Com_352_pos	Riboflavin	ENSGALG00000012834	AKR1D1
Com_265_pos	6-Methylquinoline	ENSGALG00000011169	PDCD2
Com_362_pos	2-Arachidonoyl glycerol	ENSGALG00000046731	--
Com_386_pos	2-Amino-1,3-octadecanec	ENSGALG00000004425	SCAMP1
Com_178_pos	Maltol	ENSGALG00000037773	ST3GAL1
Com_265_pos	6-Methylquinoline	ENSGALG00000021451	RED3
Com_78_neg	Citric acid	ENSGALG00000012748	ELOVL2
Com_386_pos	2-Amino-1,3-octadecanec	ENSGALG00000000226	TMEM9
Com_331_pos	L-Lysine	ENSGALG00000035626	DAD1
Com_186_pos	4-Hydroxybenzaldehyde	ENSGALG00000006080	GPC4
Com_203_pos	Serotonin	ENSGALG00000011571	AGPAT4
Com_120_neg	LPC 15:0	ENSGALG00000045557	MTTPL
Com_25_pos	2-Hydroxycinnamic acid	ENSGALG00000037467	NEU2
Com_588_pos	Ornithine	ENSGALG00000039538	CLDND1
Com_130_neg	2-Hydroxyvaleric acid	ENSGALG00000046757	ERVK-9
Com_151_pos	Pyridoxamine	ENSGALG00000013149	MOCOS
Com_208_neg	N-Acetylanthranilic acid	MSTRG.8499	Itpripl1

Com_21_pos	DL-Tryptophan	ENSGALG00000053043	CARHSP1
Com_192_pos	1-Methylhistidine	MSTRG.15745	--
Com_78_neg	Citric acid	ENSGALG00000006702	MFGE8
Com_76_neg	Erythronolactone	ENSGALG00000049658	UTS2R
Com_482_pos	8-Hydroxyquinoline	ENSGALG00000031122	NTNG1
Com_482_pos	8-Hydroxyquinoline	MSTRG.4548	--
Com_215_pos	D-Erythro-sphingosine 1-p	ENSGALG00000034507	CHST2
Com_440_pos	PC (18:4e/4:0)	ENSGALG00000005815	TMEM41B
Com_203_pos	Serotonin	ENSGALG00000000184	SLC27A6
Com_152_pos	Acetyl-L-carnitine	ENSGALG00000004782	TSEN15
Com_413_pos	L-Cystine	ENSGALG00000041604	NPTXR
Com_482_pos	8-Hydroxyquinoline	ENSGALG00000005474	PNAT10
Com_386_pos	2-Amino-1,3-octadecanec	ENSGALG00000014525	USP5
Com_386_pos	2-Amino-1,3-octadecanec	ENSGALG00000033051	CAMK1D
Com_175_pos	Pantothenic acid	ENSGALG00000038723	RPP25L
Com_99_pos	Creatine	ENSGALG00000008539	ALG12
Com_22_pos	Indole-3-acrylic acid	ENSGALG00000027561	GNG5
Com_130_neg	2-Hydroxyvaleric acid	ENSGALG00000003147	TRPC4AP
Com_362_pos	2-Arachidonoyl glycerol	ENSGALG00000009680	PAQR7
Com_352_pos	Riboflavin	ENSGALG00000000378	SLC25A37
Com_22_pos	Indole-3-acrylic acid	ENSGALG00000035803	THRSP
Com_80_pos	DL-Lysine	MSTRG.8501	--
Com_130_neg	2-Hydroxyvaleric acid	ENSGALG00000008866	WDPCP
Com_151_pos	Pyridoxamine	MSTRG.8501	--
Com_97_pos	L-Threonine	ENSGALG00000039538	CLDND1
Com_331_pos	L-Lysine	MSTRG.14577	SLC39A5
Com_208_neg	N-Acetylanthranilic acid	ENSGALG00000050267	CALU
Com_130_neg	2-Hydroxyvaleric acid	MSTRG.1082	--
Com_57_neg	LPC 16:1	ENSGALG00000014971	SLC2A9
Com_18_neg	Arachidonic acid	ENSGALG00000017122	SGCG
Com_413_pos	L-Cystine	ENSGALG00000021135	HAPLN3
Com_175_pos	Pantothenic acid	ENSGALG00000012112	DBI
Com_171_neg	LPC 22:6	ENSGALG00000001492	NDRG3
Com_16_neg	3-Hydroxybutyric acid	ENSGALG00000004782	TSEN15
Com_55_pos	Valine	ENSGALG00000031754	KCNG2
Com_12_pos	Betaine	ENSGALG00000035244	H3-I
Com_8_neg	4-Methyl-2-Oxopentanoic	ENSGALG00000005353	FAR1
Com_130_neg	2-Hydroxyvaleric acid	ENSGALG00000012377	HNMT
Com_362_pos	2-Arachidonoyl glycerol	ENSGALG00000010978	ANGPTL3
Com_120_neg	LPC 15:0	ENSGALG00000048432	--
Com_120_neg	LPC 15:0	ENSGALG00000012196	MCUB
Com_25_pos	2-Hydroxycinnamic acid	ENSGALG00000036234	RFWD3
Com_331_pos	L-Lysine	ENSGALG00000006723	IDI1
Com_8_neg	4-Methyl-2-Oxopentanoic	ENSGALG00000012196	MCUB
Com_97_pos	L-Threonine	MSTRG.16504	gag
Com_8_neg	4-Methyl-2-Oxopentanoic	ENSGALG00000048432	--
Com_147_pos	D-Sphingosine	ENSGALG00000005610	SLC44A3
Com_8_neg	4-Methyl-2-Oxopentanoic	ENSGALG00000015134	APOV1
Com_25_pos	2-Hydroxycinnamic acid	ENSGALG00000036915	SQLE
Com_120_neg	LPC 15:0	ENSGALG00000014463	ACTN2
Com_55_pos	Valine	MSTRG.9165	--
Com_108_neg	LPE 18:2	ENSGALG00000003569	TMEM130
Com_86_neg	Levulinic acid	ENSGALG00000003427	USP3
Com_151_pos	Pyridoxamine	ENSGALG00000037018	USP36
Com_55_pos	Valine	ENSGALG00000020538	SLC49A3
Com_203_pos	Serotonin	ENSGALG00000039474	ID4
Com_8_neg	4-Methyl-2-Oxopentanoic	MSTRG.2316	env
Com_588_pos	Ornithine	ENSGALG00000021685	SERINC2

Com_80_pos	DL-Lysine	ENSGALG00000013149	MOCOS
Com_18_neg	Arachidonic acid	ENSGALG00000027070	TIMP2
Com_215_pos	D-Erythro-sphingosine 1-p	ENSGALG00000009172	OSBPL6
Com_460_pos	3-amino-4-(propylamino)c	ENSGALG00000009170	NCEH1
Com_331_pos	L-Lysine	ENSGALG00000012877	CREB3L2
Com_588_pos	Ornithine	MSTRG.11802	TMEM221
Com_208_neg	N-Acetylanthranilic acid	ENSGALG00000002519	SLC25A33
Com_17_pos	L-Norleucine	ENSGALG00000008862	DNAJC10
Com_252_pos	cis-4-Hydroxy-D-proline	ENSGALG00000032170	LCN15
Com_311_pos	PC (18:4e/2:0)	ENSGALG00000016476	TTC32
Com_16_neg	3-Hydroxybutyric acid	ENSGALG00000029235	CPNE4
Com_264_pos	Indole	ENSGALG00000041078	MID1IP1
Com_331_pos	L-Lysine	MSTRG.1502	gag
Com_25_pos	2-Hydroxycinnamic acid	ENSGALG00000009365	CYP51A1
Com_362_pos	2-Arachidonoyl glycerol	ENSGALG00000015684	Dnajc25
Com_208_neg	N-Acetylanthranilic acid	ENSGALG00000010703	DGLUCY
Com_40_pos	Choline	ENSGALG00000013848	MVK
Com_16_neg	3-Hydroxybutyric acid	ENSGALG00000013036	ATP6V1E1
Com_194_pos	Pipecolic acid	ENSGALG00000007507	MASTL
Com_171_neg	LPC 22:6	ENSGALG00000026957	SEMA4G
Com_175_pos	Pantothenic acid	ENSGALG00000032628	SRCIN1
Com_460_pos	3-amino-4-(propylamino)c	ENSGALG00000012185	PLA2G12A
Com_12_pos	Betaine	ENSGALG00000027064	HIST1H3H
Com_86_neg	Levulinic acid	ENSGALG00000052612	RPS27L
Com_55_pos	Valine	ENSGALG00000015684	Dnajc25
Com_386_pos	2-Amino-1,3-octadecanec	ENSGALG00000014944	GCNT4
Com_192_pos	1-Methylhistidine	ENSGALG00000008539	ALG12
Com_588_pos	Ornithine	ENSGALG00000001749	ACSBG2
Com_460_pos	3-amino-4-(propylamino)c	ENSGALG00000039354	VTG1
Com_147_pos	D-Sphingosine	MSTRG.1502	gag
Com_152_pos	Acetyl-L-carnitine	ENSGALG00000015016	SLC22A15
Com_18_neg	Arachidonic acid	ENSGALG00000026203	FAM174A
Com_55_pos	Valine	ENSGALG00000051251	H2B-I
Com_99_pos	Creatine	ENSGALG00000045127	slc12a8
Com_252_pos	cis-4-Hydroxy-D-proline	ENSGALG00000034294	ATP6V0D2
Com_8_neg	4-Methyl-2-Oxopentanoic	ENSGALG00000013036	ATP6V1E1
Com_130_neg	2-Hydroxyvaleric acid	ENSGALG00000021135	HAPLN3
Com_97_pos	L-Threonine	ENSGALG00000046757	ERVK-9
Com_78_neg	Citric acid	ENSGALG00000035626	DAD1
Com_215_pos	D-Erythro-sphingosine 1-p	MSTRG.12291	--
Com_16_neg	3-Hydroxybutyric acid	ENSGALG00000019276	SLCO1C1
Com_171_neg	LPC 22:6	ENSGALG00000027608	PIGC
Com_151_pos	Pyridoxamine	ENSGALG00000051123	pol
Com_440_pos	PC (18:4e/4:0)	MSTRG.2388	--
Com_147_pos	D-Sphingosine	ENSGALG00000012877	CREB3L2
Com_21_pos	DL-Tryptophan	ENSGALG00000052768	LDLR
Com_440_pos	PC (18:4e/4:0)	ENSGALG00000005160	VMP1
Com_40_pos	Choline	ENSGALG00000012505	LRFN5
Com_8_neg	4-Methyl-2-Oxopentanoic	ENSGALG00000029235	CPNE4
Com_265_pos	6-Methylquinoline	MSTRG.15625	--
Com_440_pos	PC (18:4e/4:0)	ENSGALG00000012847	Slc7a11
Com_362_pos	2-Arachidonoyl glycerol	MSTRG.9165	--
Com_413_pos	L-Cystine	ENSGALG00000003147	TRPC4AP
Com_252_pos	cis-4-Hydroxy-D-proline	ENSGALG00000049751	H2B-I
Com_21_pos	DL-Tryptophan	ENSGALG00000008795	GPAM
Com_40_pos	Choline	ENSGALG00000003560	SLC6A2
Com_440_pos	PC (18:4e/4:0)	ENSGALG00000037773	ST3GAL1
Com_386_pos	2-Amino-1,3-octadecanec	ENSGALG00000001475	STMN1

Com_21_pos	DL-Tryptophan	ENSGALG00000009415	SMOC1
Com_16_neg	3-Hydroxybutyric acid	MSTRG.2316	env
Com_16_neg	3-Hydroxybutyric acid	ENSGALG00000047027	ADCK5
Com_147_pos	D-Sphingosine	ENSGALG00000006723	IDI1
Com_108_neg	LPE 18:2	ENSGALG00000034507	CHST2
Com_12_pos	Betaine	MSTRG.1468	--
Com_16_neg	3-Hydroxybutyric acid	ENSGALG00000015134	APOV1
Com_175_pos	Pantothenic acid	ENSGALG00000002802	PACSIN1
Com_89_neg	Gallic acid	ENSGALG00000053860	mas
Com_92_pos	D-(+)-Proline	ENSGALG00000042491	H4-I
Com_175_pos	Pantothenic acid	ENSGALG00000037253	CLEC4M
Com_440_pos	PC (18:4e/4:0)	ENSGALG00000026460	myoM
Com_97_pos	L-Threonine	ENSGALG00000003147	TRPC4AP
Com_130_neg	2-Hydroxyvaleric acid	ENSGALG00000041604	NPTXR
Com_147_pos	D-Sphingosine	MSTRG.13261	--
Com_89_neg	Gallic acid	ENSGALG00000008859	WDR31
Com_362_pos	2-Arachidonoyl glycerol	ENSGALG00000031754	KCNG2
Com_311_pos	PC (18:4e/2:0)	ENSGALG00000004341	Cryzl2
Com_147_pos	D-Sphingosine	ENSGALG00000007404	YIPF5
Com_17_pos	L-Norleucine	ENSGALG00000015729	LPAR1
Com_460_pos	3-amino-4-(propylamino)(	MSTRG.21321	gag
Com_16_neg	3-Hydroxybutyric acid	ENSGALG00000005353	FAR1
Com_17_pos	L-Norleucine	MSTRG.2387	--
Com_151_pos	Pyridoxamine	ENSGALG00000050420	CTNND2
Com_171_neg	LPC 22:6	ENSGALG00000008039	MFSD13A
Com_147_pos	D-Sphingosine	MSTRG.14577	SLC39A5
Com_413_pos	L-Cystine	ENSGALG00000046757	ERVK-9
Com_8_neg	4-Methyl-2-Oxopentanoic	ENSGALG00000004782	TSEN15
Com_22_pos	Indole-3-acrylic acid	MSTRG.9361	Fam110a
Com_311_pos	PC (18:4e/2:0)	ENSGALG00000005815	TMEM41B
Com_252_pos	cis-4-Hydroxy-D-proline	ENSGALG00000002845	CTNNA3
Com_80_pos	DL-Lysine	MSTRG.6512	--
Com_186_pos	4-Hydroxybenzaldehyde	ENSGALG00000003081	SUCO
Com_264_pos	Indole	ENSGALG00000027561	GNG5
Com_8_neg	4-Methyl-2-Oxopentanoic	MSTRG.21321	gag
Com_120_neg	LPC 15:0	ENSGALG00000015016	SLC22A15
Com_152_pos	Acetyl-L-carnitine	ENSGALG00000026203	FAM174A
Com_54_pos	Uric acid	ENSGALG00000007178	FADS2
Com_186_pos	4-Hydroxybenzaldehyde	ENSGALG00000005043	ACACB
Com_78_neg	Citric acid	ENSGALG00000001492	NDRG3
Com_25_pos	2-Hydroxycinnamic acid	ENSGALG00000009560	MSMO1
Com_264_pos	Indole	ENSGALG00000035803	THRSP
Com_194_pos	Pipecolic acid	ENSGALG00000036754	CHKA
Com_186_pos	4-Hydroxybenzaldehyde	MSTRG.19484	--
Com_92_pos	D-(+)-Proline	ENSGALG00000006689	ABHD2
Com_54_pos	Uric acid	ENSGALG00000015937	FABP1
Com_208_neg	N-Acetylanthranilic acid	ENSGALG00000004424	SEC16B
Com_311_pos	PC (18:4e/2:0)	ENSGALG00000036836	SOSTDC1
Com_130_neg	2-Hydroxyvaleric acid	MSTRG.16504	gag
Com_97_pos	L-Threonine	ENSGALG00000031525	TSTA3
Com_92_pos	D-(+)-Proline	MSTRG.13408	--
Com_40_pos	Choline	ENSGALG00000000241	STARD4
Com_208_neg	N-Acetylanthranilic acid	ENSGALG00000004231	IFNLR1
Com_54_pos	Uric acid	ENSGALG00000044278	C1orf131
Com_99_pos	Creatine	ENSGALG00000038574	MYO15A
Com_178_pos	Maltol	MSTRG.14577	SLC39A5
Com_151_pos	Pyridoxamine	ENSGALG00000052072	gag
Com_252_pos	cis-4-Hydroxy-D-proline	ENSGALG00000001101	MBD3

Com_147_pos	D-Sphingosine	ENSGALG00000051779	PRORSD1P
Com_208_neg	N-Acetylanthranilic acid	ENSGALG00000036021	MTMR7
Com_252_pos	cis-4-Hydroxy-D-proline	MSTRG.9006	--
Com_178_pos	Maltol	ENSGALG00000003103	MST1R
Com_21_pos	DL-Tryptophan	MSTRG.9007	--
Com_215_pos	D-Erythro-sphingosine 1-p	ENSGALG00000003569	TMEM130
Com_130_neg	2-Hydroxyvaleric acid	ENSGALG00000036293	EBAG9
Com_17_pos	L-Norleucine	ENSGALG00000003427	USP3
Com_99_pos	Creatine	ENSGALG00000016560	SELENOI
Com_55_pos	Valine	ENSGALG00000046731	--
Com_386_pos	2-Amino-1,3-octadecanec	ENSGALG00000051398	TMEM14C
Com_178_pos	Maltol	ENSGALG00000037935	RARA
Com_21_pos	DL-Tryptophan	ENSGALG00000041238	NOS1AP
Com_178_pos	Maltol	ENSGALG00000006723	IDI1
Com_108_neg	LPE 18:2	ENSGALG00000009172	OSBPL6
Com_386_pos	2-Amino-1,3-octadecanec	ENSGALG00000000645	Espn
Com_252_pos	cis-4-Hydroxy-D-proline	ENSGALG00000023626	NTN1
Com_152_pos	Acetyl-L-carnitine	ENSGALG00000012196	MCUB
Com_331_pos	L-Lysine	ENSGALG00000038532	--
Com_152_pos	Acetyl-L-carnitine	ENSGALG00000048432	--
Com_54_pos	Uric acid	ENSGALG00000002919	MON1A
Com_40_pos	Choline	ENSGALG00000050440	APOF
Com_86_neg	Levulinic acid	ENSGALG00000008862	DNAJC10
Com_40_pos	Choline	ENSGALG00000001918	DNAJB5
Com_55_pos	Valine	ENSGALG00000023517	AGPAT2
Com_108_neg	LPE 18:2	ENSGALG00000015358	MYH15
Com_25_pos	2-Hydroxycinnamic acid	ENSGALG00000011657	EAF2
Com_12_pos	Betaine	MSTRG.3473	--
Com_482_pos	8-Hydroxyquinoline	ENSGALG00000016281	DMD
Com_588_pos	Ornithine	MSTRG.12923	--
Com_78_neg	Citric acid	ENSGALG00000026957	SEMA4G
Com_471_pos	Indole-3-acetic acid	ENSGALG00000029724	MTURN
Com_108_neg	LPE 18:2	ENSGALG00000049966	Ufc1
Com_265_pos	6-Methylquinoline	ENSGALG00000030941	ELAPOR1
Com_55_pos	Valine	MSTRG.19422	--
Com_99_pos	Creatine	ENSGALG00000004702	DYNC2I2
Com_482_pos	8-Hydroxyquinoline	ENSGALG00000038923	Ces1e
Com_97_pos	L-Threonine	ENSGALG00000021135	HAPLN3
Com_178_pos	Maltol	ENSGALG00000012877	CREB3L2
Com_40_pos	Choline	ENSGALG00000014509	BST1
Com_151_pos	Pyridoxamine	ENSGALG00000040342	ADAMTS1
Com_54_pos	Uric acid	ENSGALG00000010837	ASB5
Com_471_pos	Indole-3-acetic acid	ENSGALG00000035219	ALB
Com_413_pos	L-Cystine	ENSGALG00000039538	CLDND1
Com_147_pos	D-Sphingosine	ENSGALG00000021658	PAFAH2
Com_99_pos	Creatine	ENSGALG00000031932	AGPAT3
Com_152_pos	Acetyl-L-carnitine	ENSGALG00000017122	SGCG
Com_17_pos	L-Norleucine	ENSGALG00000016885	STK24
Com_151_pos	Pyridoxamine	ENSGALG00000007673	LRRC59
Com_362_pos	2-Arachidonoyl glycerol	ENSGALG00000012748	ELOVL2
Com_178_pos	Maltol	MSTRG.1502	gag
Com_8_neg	4-Methyl-2-Oxopentanoic	ENSGALG00000009170	NCEH1
Com_192_pos	1-Methylhistidine	ENSGALG00000030511	SLC19A1
Com_12_pos	Betaine	ENSGALG00000005839	ARID3C
Com_171_neg	LPC 22:6	ENSGALG00000035626	DAD1
Com_12_pos	Betaine	ENSGALG00000052986	Vwa5b2
Com_25_pos	2-Hydroxycinnamic acid	ENSGALG00000004875	PEMT
Com_78_neg	Citric acid	ENSGALG00000027608	PIGC

Com_482_pos	8-Hydroxyquinoline	ENSGALG00000007839	NCAM1
Com_92_pos	D-(+)-Proline	MSTRG.13439	--
Com_362_pos	2-Arachidonoyl glycerol	ENSGALG00000006702	MFGE8
Com_108_neg	LPE 18:2	MSTRG.12291	--
Com_352_pos	Riboflavin	ENSGALG00000013969	ALDH8A1
Com_21_pos	DL-Tryptophan	ENSGALG00000007636	PCK1
Com_460_pos	3-amino-4-(propylamino)c	ENSGALG00000048432	--
Com_460_pos	3-amino-4-(propylamino)c	ENSGALG00000012196	MCUB
Com_147_pos	D-Sphingosine	MSTRG.21394	gag-pol
Com_92_pos	D-(+)-Proline	ENSGALG00000046789	pol
Com_588_pos	Ornithine	ENSGALG00000005648	Sesn3
Com_311_pos	PC (18:4e/2:0)	MSTRG.4702	--
Com_97_pos	L-Threonine	ENSGALG00000041604	NPTXR
Com_386_pos	2-Amino-1,3-octadecanec	ENSGALG00000011254	SATB1
Com_119_pos	DL-Stachydrine	ENSGALG00000001392	MMP23B
Com_55_pos	Valine	ENSGALG00000000619	ANGPTL4
Com_40_pos	Choline	MSTRG.8497	--
Com_151_pos	Pyridoxamine	MSTRG.21536	--
Com_54_pos	Uric acid	ENSGALG00000053446	RED3
Com_18_neg	Arachidonic acid	ENSGALG00000004782	TSEN15
Com_386_pos	2-Amino-1,3-octadecanec	ENSGALG00000041533	SLC11A2
Com_178_pos	Maltol	MSTRG.21796	--
Com_208_neg	N-Acetylanthranilic acid	ENSGALG00000006649	TMEM41A
Com_151_pos	Pyridoxamine	ENSGALG00000027375	NR2C2AP
Com_311_pos	PC (18:4e/2:0)	MSTRG.2388	--
Com_130_neg	2-Hydroxyvaleric acid	ENSGALG00000050676	Ctnnd2
Com_311_pos	PC (18:4e/2:0)	ENSGALG00000005160	VMP1
Com_78_neg	Citric acid	ENSGALG00000009680	PAQR7
Com_12_pos	Betaine	ENSGALG00000002579	RIMBP2
Com_252_pos	cis-4-Hydroxy-D-proline	ENSGALG00000000802	DHODH
Com_130_neg	2-Hydroxyvaleric acid	ENSGALG00000043336	COPZ1
Com_86_neg	Levulinic acid	ENSGALG00000015729	LPAR1
Com_362_pos	2-Arachidonoyl glycerol	ENSGALG00000012882	KDSR
Com_78_neg	Citric acid	ENSGALG00000008039	MFSD13A
Com_311_pos	PC (18:4e/2:0)	ENSGALG00000012847	Slc7a11
Com_331_pos	L-Lysine	ENSGALG00000041533	SLC11A2
Com_175_pos	Pantothenic acid	ENSGALG00000000104	CRY1
Com_86_neg	Levulinic acid	MSTRG.2387	--
Com_175_pos	Pantothenic acid	ENSGALG00000031067	TMEM132A
Com_588_pos	Ornithine	ENSGALG00000041296	SOX7
Com_130_neg	2-Hydroxyvaleric acid	ENSGALG00000020342	ABHD12
Com_22_pos	Indole-3-acrylic acid	ENSGALG00000041687	SREBF2
Com_440_pos	PC (18:4e/4:0)	ENSGALG00000005610	SLC44A3
Com_120_neg	LPC 15:0	ENSGALG00000012185	PLA2G12A
Com_311_pos	PC (18:4e/2:0)	MSTRG.149	--
Com_97_pos	L-Threonine	ENSGALG00000011254	SATB1
Com_130_neg	2-Hydroxyvaleric acid	ENSGALG00000031525	TSTA3
Com_362_pos	2-Arachidonoyl glycerol	ENSGALG00000005739	SCD
Com_12_pos	Betaine	ENSGALG00000006530	TSSC4
Com_16_neg	3-Hydroxybutyric acid	ENSGALG00000027070	TIMP2
Com_192_pos	1-Methylhistidine	ENSGALG00000050668	Spata1
Com_80_pos	DL-Lysine	ENSGALG00000041456	SLC35G1
Com_120_neg	LPC 15:0	ENSGALG00000039354	VTG1
Com_264_pos	Indole	MSTRG.9361	Fam110a
Com_252_pos	cis-4-Hydroxy-D-proline	ENSGALG00000052786	Znf185
Com_311_pos	PC (18:4e/2:0)	ENSGALG00000026460	myoM
Com_78_neg	Citric acid	ENSGALG00000010978	ANGPTL3
Com_252_pos	cis-4-Hydroxy-D-proline	ENSGALG00000008604	TMEM255A

Com_440_pos	PC (18:4e/4:0)	ENSGALG00000004341	Cryzl2
Com_192_pos	1-Methylhistidine	MSTRG.8128	--
Com_120_neg	LPC 15:0	MSTRG.13439	--
Com_18_neg	Arachidonic acid	ENSGALG00000029235	CPNE4
Com_413_pos	L-Cystine	ENSGALG00000021685	SERINC2
Com_12_pos	Betaine	ENSGALG00000023395	PLIN1
Com_208_neg	N-Acetylanthranilic acid	ENSGALG00000011616	NPFFR2
Com_76_neg	Erythronolactone	ENSGALG00000014464	MTR
Com_460_pos	3-amino-4-(propylamino)(	ENSGALG00000015333	PCGF3
Com_482_pos	8-Hydroxyquinoline	ENSGALG00000028928	LCAT
Com_264_pos	Indole	MSTRG.7572	--
Com_151_pos	Pyridoxamine	ENSGALG00000027960	GRPR
Com_311_pos	PC (18:4e/2:0)	ENSGALG00000004322	AHR
Com_175_pos	Pantothenic acid	MSTRG.15754	--
Com_18_neg	Arachidonic acid	ENSGALG00000013036	ATP6V1E1
Com_460_pos	3-amino-4-(propylamino)(	ENSGALG00000029015	TM6SF2
Com_12_pos	Betaine	ENSGALG00000010294	RPS6KL1
Com_152_pos	Acetyl-L-carnitine	MSTRG.21321	gag
Com_362_pos	2-Arachidonoyl glycerol	ENSGALG00000022758	GGACT
Com_413_pos	L-Cystine	MSTRG.11802	TMEM221
Com_8_neg	4-Methyl-2-Oxopentanoic	ENSGALG00000026203	FAM174A
Com_97_pos	L-Threonine	ENSGALG00000036293	EBAG9
Com_194_pos	Pipecolic acid	ENSGALG00000020538	SLC49A3
Com_252_pos	cis-4-Hydroxy-D-proline	MSTRG.16903	--
Com_413_pos	L-Cystine	ENSGALG00000049658	UTS2R
Com_186_pos	4-Hydroxybenzaldehyde	ENSGALG00000010764	FBXO8
Com_192_pos	1-Methylhistidine	ENSGALG00000004590	CLCN6
Com_89_neg	Gallic acid	ENSGALG00000000498	ACE
Com_54_pos	Uric acid	MSTRG.14680	--
Com_203_pos	Serotonin	ENSGALG00000002549	RGS1
Com_175_pos	Pantothenic acid	ENSGALG00000031312	ANAPC13
Com_55_pos	Valine	ENSGALG00000043829	ext1c
Com_151_pos	Pyridoxamine	ENSGALG00000016491	APOB
Com_21_pos	DL-Tryptophan	ENSGALG00000021193	STARD5
Com_386_pos	2-Amino-1,3-octadecanec	ENSGALG00000038532	--
Com_208_neg	N-Acetylanthranilic acid	ENSGALG00000030801	CCKAR
Com_440_pos	PC (18:4e/4:0)	ENSGALG00000036836	SOSTDC1
Com_331_pos	L-Lysine	ENSGALG00000000645	Espn
Com_311_pos	PC (18:4e/2:0)	MSTRG.1503	gag
Com_40_pos	Choline	ENSGALG00000023760	CHIA
Com_413_pos	L-Cystine	ENSGALG00000001749	ACSBG2
Com_252_pos	cis-4-Hydroxy-D-proline	ENSGALG00000004804	TGM3
Com_265_pos	6-Methylquinoline	ENSGALG00000028880	FDPS
Com_99_pos	Creatine	ENSGALG00000024449	RAMP2
Com_147_pos	D-Sphingosine	ENSGALG00000000226	TMEM9
Com_264_pos	Indole	ENSGALG00000021193	STARD5
Com_252_pos	cis-4-Hydroxy-D-proline	ENSGALG00000005617	NTHL1
Com_265_pos	6-Methylquinoline	ENSGALG00000051567	MRPL41
Com_331_pos	L-Lysine	ENSGALG00000003103	MST1R
Com_97_pos	L-Threonine	ENSGALG00000051398	TMEM14C
Com_192_pos	1-Methylhistidine	ENSGALG00000023348	HPDL
Com_252_pos	cis-4-Hydroxy-D-proline	MSTRG.20573	--
Com_460_pos	3-amino-4-(propylamino)(	ENSGALG00000034716	HEY2
Com_215_pos	D-Erythro-sphingosine 1-p	ENSGALG00000015358	MYH15
Com_331_pos	L-Lysine	ENSGALG00000037935	RARA
Com_18_neg	Arachidonic acid	MSTRG.2316	env
Com_147_pos	D-Sphingosine	ENSGALG00000037773	ST3GAL1
Com_215_pos	D-Erythro-sphingosine 1-p	ENSGALG00000049966	Ufc1

Com_194_pos	Pipecolic acid	ENSGALG00000051251	H2B-I
Com_460_pos	3-amino-4-(propylamino)(	ENSGALG00000015016	SLC22A15
Com_18_neg	Arachidonic acid	ENSGALG00000015134	APOV1
Com_99_pos	Creatine	ENSGALG00000002371	RUSC2
Com_413_pos	L-Cystine	ENSGALG00000030908	ATP2B2
Com_178_pos	Maltol	ENSGALG00000038532	--
Com_21_pos	DL-Tryptophan	ENSGALG00000029308	PNPLA3
Com_147_pos	D-Sphingosine	ENSGALG00000014525	USP5
Com_21_pos	DL-Tryptophan	MSTRG.7572	--
Com_16_neg	3-Hydroxybutyric acid	ENSGALG00000014976	GATA6
Com_86_neg	Levulinic acid	ENSGALG00000016885	STK24
Com_147_pos	D-Sphingosine	ENSGALG00000033051	CAMK1D
Com_76_neg	Erythronolactone	ENSGALG00000054297	SND1
Com_471_pos	Indole-3-acetic acid	ENSGALG00000005263	SOX8
Com_440_pos	PC (18:4e/4:0)	MSTRG.13261	--
Com_16_neg	3-Hydroxybutyric acid	ENSGALG00000042555	STAMBP
Com_413_pos	L-Cystine	ENSGALG00000033656	DQX1
Com_80_pos	DL-Lysine	ENSGALG00000026846	JMJD7
Com_440_pos	PC (18:4e/4:0)	ENSGALG00000007404	YIPF5
Com_18_neg	Arachidonic acid	ENSGALG00000005353	FAR1
Com_194_pos	Pipecolic acid	ENSGALG00000026607	C15orf40
Com_386_pos	2-Amino-1,3-octadecanec	ENSGALG00000031525	TSTA3
Com_178_pos	Maltol	ENSGALG00000007018	SLC26A11
Com_86_neg	Levulinic acid	ENSGALG00000027070	TIMP2
Com_120_neg	LPC 15:0	ENSGALG00000006689	ABHD2
Com_92_pos	D-(+)-Proline	ENSGALG00000010293	RBP
Com_588_pos	Ornithine	ENSGALG00000007848	PTS
Com_18_neg	Arachidonic acid	ENSGALG00000003427	USP3
Com_265_pos	6-Methylquinoline	ENSGALG00000010837	ASB5
Com_8_neg	4-Methyl-2-Oxopentanoic	ENSGALG00000017122	SGCG
Com_151_pos	Pyridoxamine	ENSGALG00000007778	PES1
Com_194_pos	Pipecolic acid	ENSGALG00000016492	TDRD15
Com_92_pos	D-(+)-Proline	ENSGALG00000015016	SLC22A15
Com_89_neg	Gallic acid	ENSGALG00000002116	TEN1
Com_54_pos	Uric acid	ENSGALG00000030941	ELAPOR1
Com_152_pos	Acetyl-L-carnitine	ENSGALG00000009170	NCEH1
Com_208_neg	N-Acetylanthranilic acid	ENSGALG00000033461	hnmt
Com_80_pos	DL-Lysine	ENSGALG00000008039	MFSD13A
Com_97_pos	L-Threonine	ENSGALG00000050676	Ctnnd2
Com_186_pos	4-Hydroxybenzaldehyde	ENSGALG00000023348	HPDL
Com_471_pos	Indole-3-acetic acid	ENSGALG00000046687	EPS8L3
Com_215_pos	D-Erythro-sphingosine 1-p	ENSGALG00000010853	C8B
Com_151_pos	Pyridoxamine	ENSGALG00000023338	CBX2
Com_265_pos	6-Methylquinoline	ENSGALG00000002919	MON1A
Com_80_pos	DL-Lysine	MSTRG.8381	--
Com_175_pos	Pantothenic acid	ENSGALG00000003948	ALAS1
Com_97_pos	L-Threonine	ENSGALG00000043336	COPZ1
Com_171_neg	LPC 22:6	ENSGALG00000009680	PAQR7
Com_265_pos	6-Methylquinoline	ENSGALG00000044464	TEPSIN
Com_8_neg	4-Methyl-2-Oxopentanoic	ENSGALG00000017378	CRTAC1
Com_588_pos	Ornithine	MSTRG.21204	--
Com_208_neg	N-Acetylanthranilic acid	ENSGALG00000015253	COL8A1
Com_97_pos	L-Threonine	ENSGALG00000020342	ABHD12
Com_40_pos	Choline	MSTRG.21091	--
Com_130_neg	2-Hydroxyvaleric acid	ENSGALG00000011254	SATB1
Com_120_neg	LPC 15:0	ENSGALG00000042491	H4-I
Com_4_pos	PC (17:1/17:1)	ENSGALG00000038740	AMY2A
Com_76_neg	Erythronolactone	ENSGALG00000010641	SCCPDH

Com_55_pos	Valine	ENSGALG00000007778	PES1
Com_331_pos	L-Lysine	MSTRG.21796	--
Com_413_pos	L-Cystine	ENSGALG00000038145	DPP7
Com_362_pos	2-Arachidonoyl glycerol	ENSGALG00000005470	PLPPR5
Com_440_pos	PC (18:4e/4:0)	MSTRG.4702	--
Com_440_pos	PC (18:4e/4:0)	ENSGALG00000051779	PRORSD1P
Com_362_pos	2-Arachidonoyl glycerol	ENSGALG00000001492	NDRG3
Com_22_pos	Indole-3-acrylic acid	ENSGALG00000011169	PDCD2
Com_186_pos	4-Hydroxybenzaldehyde	ENSGALG00000004590	CLCN6
Com_92_pos	D-(+)-Proline	ENSGALG00000010641	SCCPDH
Com_97_pos	L-Threonine	ENSGALG00000001475	STMN1
Com_175_pos	Pantothenic acid	ENSGALG00000005470	PLPPR5
Com_25_pos	2-Hydroxycinnamic acid	MSTRG.17623	--
Com_12_pos	Betaine	ENSGALG00000029083	NXPH2
Com_80_pos	DL-Lysine	ENSGALG00000027608	PIGC
Com_171_neg	LPC 22:6	ENSGALG00000010978	ANGPTL3
Com_178_pos	Maltol	ENSGALG00000041533	SLC11A2
Com_264_pos	Indole	ENSGALG00000041687	SREBF2
Com_264_pos	Indole	ENSGALG00000007636	PCK1
Com_16_neg	3-Hydroxybutyric acid	MSTRG.3156	--
Com_482_pos	8-Hydroxyquinoline	ENSGALG00000050154	--
Com_460_pos	3-amino-4-(propylamino)(	MSTRG.12629	--
Com_460_pos	3-amino-4-(propylamino)(	MSTRG.20167	--
Com_17_pos	L-Norleucine	ENSGALG00000020342	ABHD12
Com_99_pos	Creatine	ENSGALG00000047720	KLHDC7A
Com_362_pos	2-Arachidonoyl glycerol	ENSGALG00000003948	ALAS1
Com_17_pos	L-Norleucine	ENSGALG00000043336	COPZ1
Com_331_pos	L-Lysine	ENSGALG00000014944	GCNT4
Com_80_pos	DL-Lysine	ENSGALG00000026957	SEMA4G
Com_311_pos	PC (18:4e/2:0)	ENSGALG00000005610	SLC44A3
Com_440_pos	PC (18:4e/4:0)	MSTRG.149	--
Com_17_pos	L-Norleucine	ENSGALG00000050676	Ctnnd2
Com_22_pos	Indole-3-acrylic acid	MSTRG.7572	--
Com_76_neg	Erythronolactone	ENSGALG00000010293	RBP
Com_440_pos	PC (18:4e/4:0)	ENSGALG00000021658	PAFAH2
Com_413_pos	L-Cystine	MSTRG.12923	--
Com_152_pos	Acetyl-L-carnitine	MSTRG.13408	--
Com_252_pos	cis-4-Hydroxy-D-proline	ENSGALG00000037811	NRSN1
Com_99_pos	Creatine	ENSGALG00000031570	WDR54
Com_386_pos	2-Amino-1,3-octadecanec	MSTRG.1502	gag
Com_186_pos	4-Hydroxybenzaldehyde	ENSGALG00000008427	GNAT3
Com_130_neg	2-Hydroxyvaleric acid	ENSGALG00000051398	TMEM14C
Com_55_pos	Valine	ENSGALG00000016491	APOB
Com_151_pos	Pyridoxamine	ENSGALG00000043829	ext1c
Com_92_pos	D-(+)-Proline	ENSGALG00000054297	SND1
Com_252_pos	cis-4-Hydroxy-D-proline	ENSGALG00000002437	DIPK1B
Com_362_pos	2-Arachidonoyl glycerol	ENSGALG00000026957	SEMA4G
Com_588_pos	Ornithine	ENSGALG00000016475	Zp2
Com_194_pos	Pipecolic acid	ENSGALG00000023517	AGPAT2
Com_386_pos	2-Amino-1,3-octadecanec	ENSGALG00000012877	CREB3L2
Com_264_pos	Indole	ENSGALG00000041238	NOS1AP
Com_440_pos	PC (18:4e/4:0)	ENSGALG00000004322	AHR
Com_12_pos	Betaine	ENSGALG00000036616	NUAK2
Com_471_pos	Indole-3-acetic acid	MSTRG.17721	--
Com_194_pos	Pipecolic acid	MSTRG.19422	--
Com_460_pos	3-amino-4-(propylamino)(	ENSGALG00000014581	BORCS8
Com_440_pos	PC (18:4e/4:0)	MSTRG.21394	gag-pol
Com_208_neg	N-Acetylanthranilic acid	ENSGALG00000028230	SUN2

Com_186_pos	4-Hydroxybenzaldehyde	ENSGALG00000007508	HPSE2
Com_99_pos	Creatine	MSTRG.7811	--
Com_8_neg	4-Methyl-2-Oxopentanoic	ENSGALG00000002775	FA2H
Com_147_pos	D-Sphingosine	ENSGALG00000001475	STMN1
Com_151_pos	Pyridoxamine	MSTRG.8810	--
Com_22_pos	Indole-3-acrylic acid	ENSGALG00000021193	STARD5
Com_175_pos	Pantothenic acid	ENSGALG00000033365	ALDH1A3
Com_178_pos	Maltol	ENSGALG00000000645	Espn
Com_151_pos	Pyridoxamine	ENSGALG00000015768	ANKRD6
Com_386_pos	2-Amino-1,3-octadecaned	ENSGALG00000006723	IDI1
Com_362_pos	2-Arachidonoyl glycerol	ENSGALG00000027608	PIGC
Com_386_pos	2-Amino-1,3-octadecaned	MSTRG.16504	gag
Com_92_pos	D-(+)-Proline	ENSGALG00000012196	MCUB
Com_186_pos	4-Hydroxybenzaldehyde	ENSGALG00000029033	Tldc2
Com_413_pos	L-Cystine	ENSGALG00000005648	Sesn3
Com_92_pos	D-(+)-Proline	ENSGALG00000048432	--
Com_130_neg	2-Hydroxyvaleric acid	ENSGALG00000016885	STK24
Com_252_pos	cis-4-Hydroxy-D-proline	MSTRG.2403	--
Com_440_pos	PC (18:4e/4:0)	MSTRG.1503	gag
Com_588_pos	Ornithine	MSTRG.8471	--
Com_194_pos	Pipecolic acid	MSTRG.14343	--
Com_460_pos	3-amino-4-(propylamino)c	MSTRG.13439	--
Com_80_pos	DL-Lysine	ENSGALG00000001492	NDRG3
Com_413_pos	L-Cystine	MSTRG.16287	--
Com_192_pos	1-Methylhistidine	ENSGALG00000031159	HIST1H110
Com_194_pos	Pipecolic acid	ENSGALG00000029445	FADS6
Com_40_pos	Choline	ENSGALG00000004612	MTHFR
Com_120_neg	LPC 15:0	ENSGALG00000015333	PCGF3
Com_80_pos	DL-Lysine	ENSGALG00000030151	LUZP2
Com_362_pos	2-Arachidonoyl glycerol	ENSGALG00000031312	ANAPC13
Com_8_neg	4-Methyl-2-Oxopentanoic	ENSGALG00000037050	FABP3
Com_120_neg	LPC 15:0	ENSGALG00000029015	TM6SF2
Com_17_pos	L-Norleucine	ENSGALG00000027070	TIMP2
Com_386_pos	2-Amino-1,3-octadecaned	MSTRG.14577	SLC39A5
Com_352_pos	Riboflavin	ENSGALG00000031496	SPINK5
Com_194_pos	Pipecolic acid	ENSGALG00000000619	ANGPTL4
Com_99_pos	Creatine	ENSGALG00000001642	GLT8D1
Com_25_pos	2-Hydroxycinnamic acid	ENSGALG00000032170	LCN15
Com_208_neg	N-Acetylanthranilic acid	MSTRG.12570	--
Com_331_pos	L-Lysine	ENSGALG00000007018	SLC26A11
Com_413_pos	L-Cystine	ENSGALG00000041296	SOX7
Com_119_pos	DL-Stachydrine	MSTRG.13474	Klhl29
Com_16_neg	3-Hydroxybutyric acid	MSTRG.2393	--
Com_92_pos	D-(+)-Proline	ENSGALG00000014464	MTR
Com_17_pos	L-Norleucine	ENSGALG00000036293	EBAG9
Com_175_pos	Pantothenic acid	ENSGALG00000022758	GGACT
Com_252_pos	cis-4-Hydroxy-D-proline	ENSGALG00000009365	CYP51A1
Com_55_pos	Valine	ENSGALG00000027375	NR2C2AP
Com_108_neg	LPE 18:2	ENSGALG00000010853	C8B
Com_80_pos	DL-Lysine	ENSGALG00000009476	CDK6
Com_460_pos	3-amino-4-(propylamino)c	ENSGALG00000004282	RCAN3
Com_78_neg	Citric acid	ENSGALG00000012882	KDSR
Com_362_pos	2-Arachidonoyl glycerol	ENSGALG00000008039	MFSD13A
Com_311_pos	PC (18:4e/2:0)	MSTRG.13261	--
Com_147_pos	D-Sphingosine	ENSGALG00000004341	Cryzl2
Com_311_pos	PC (18:4e/2:0)	ENSGALG00000007404	YIPF5
Com_80_pos	DL-Lysine	ENSGALG00000021686	--
Com_588_pos	Ornithine	ENSGALG00000047495	LRRC10

Com_208_neg	N-Acetylanthranilic acid	ENSGALG00000016412	MBOAT2
Com_151_pos	Pyridoxamine	ENSGALG00000000619	ANGPTL4
Com_152_pos	Acetyl-L-carnitine	ENSGALG00000046789	pol
Com_16_neg	3-Hydroxybutyric acid	MSTRG.15732	--
Com_16_neg	3-Hydroxybutyric acid	ENSGALG00000013001	CTNND2
Com_25_pos	2-Hydroxycinnamic acid	ENSGALG00000010301	EIF2B2
Com_460_pos	3-amino-4-(propylamino)(	MSTRG.17961	--
Com_192_pos	1-Methylhistidine	ENSGALG00000050427	OSBPL10
Com_86_neg	Levulinic acid	ENSGALG00000047027	ADCK5
Com_130_neg	2-Hydroxyvaleric acid	ENSGALG00000001475	STMN1
Com_482_pos	8-Hydroxyquinoline	ENSGALG00000011287	SULT
Com_97_pos	L-Threonine	ENSGALG00000033051	CAMK1D
Com_97_pos	L-Threonine	ENSGALG00000014525	USP5
Com_99_pos	Creatine	ENSGALG00000002249	AGO1
Com_78_neg	Citric acid	ENSGALG00000005739	SCD
Com_175_pos	Pantothenic acid	ENSGALG00000005739	SCD
Com_40_pos	Choline	MSTRG.16433	B4GALNT4
Com_120_neg	LPC 15:0	ENSGALG00000034716	HEY2
Com_178_pos	Maltol	MSTRG.5319	--
Com_264_pos	Indole	ENSGALG00000009415	SMOC1
Com_25_pos	2-Hydroxycinnamic acid	ENSGALG00000035206	CNPY2
Com_471_pos	Indole-3-acetic acid	ENSGALG00000017199	MAML2
Com_264_pos	Indole	ENSGALG00000008795	GPAM
Com_175_pos	Pantothenic acid	MSTRG.5319	--
Com_186_pos	4-Hydroxybenzaldehyde	ENSGALG00000026384	PCSK4
Com_152_pos	Acetyl-L-carnitine	ENSGALG00000017378	CRTAC1
Com_588_pos	Ornithine	ENSGALG00000049658	UTS2R
Com_86_neg	Levulinic acid	ENSGALG00000019276	SLCO1C1
Com_264_pos	Indole	ENSGALG00000011169	PDCD2
Com_175_pos	Pantothenic acid	ENSGALG00000012882	KDSR
Com_352_pos	Riboflavin	ENSGALG00000049068	ZNF831
Com_208_neg	N-Acetylanthranilic acid	ENSGALG00000006783	PLOD2
Com_99_pos	Creatine	ENSGALG00000052829	MAMDC4
Com_147_pos	D-Sphingosine	ENSGALG00000051398	TMEM14C
Com_147_pos	D-Sphingosine	ENSGALG00000036836	SOSTDC1
Com_331_pos	L-Lysine	ENSGALG00000004425	SCAMP1
Com_78_neg	Citric acid	ENSGALG00000022758	GGACT
Com_97_pos	L-Threonine	ENSGALG00000000226	TMEM9
Com_192_pos	1-Methylhistidine	MSTRG.19484	--
Com_482_pos	8-Hydroxyquinoline	ENSGALG00000041258	msrA
Com_460_pos	3-amino-4-(propylamino)(	ENSGALG00000006689	ABHD2
Com_440_pos	PC (18:4e/4:0)	ENSGALG00000000226	TMEM9
Com_22_pos	Indole-3-acrylic acid	ENSGALG00000007636	PCK1
Com_192_pos	1-Methylhistidine	ENSGALG00000004657	FBXO2
Com_151_pos	Pyridoxamine	MSTRG.19422	--
Com_86_neg	Levulinic acid	ENSGALG00000020342	ABHD12
Com_16_neg	3-Hydroxybutyric acid	ENSGALG00000028191	GLCE
Com_192_pos	1-Methylhistidine	ENSGALG00000005043	ACACB
Com_130_neg	2-Hydroxyvaleric acid	MSTRG.2387	--
Com_311_pos	PC (18:4e/2:0)	ENSGALG00000051779	PRORSD1P
Com_151_pos	Pyridoxamine	ENSGALG00000023517	AGPAT2
Com_76_neg	Erythronolactone	ENSGALG00000046789	pol
Com_192_pos	1-Methylhistidine	ENSGALG00000003081	SUCO
Com_311_pos	PC (18:4e/2:0)	MSTRG.15507	--
Com_130_neg	2-Hydroxyvaleric acid	ENSGALG00000015729	LPAR1
Com_86_neg	Levulinic acid	ENSGALG00000043336	COPZ1
Com_40_pos	Choline	ENSGALG00000049408	HIST1H2B8
Com_194_pos	Pipecolic acid	ENSGALG00000043829	ext1c

Com_17_pos	L-Norleucine	ENSGALG00000041604	NPTXR
Com_40_pos	Choline	ENSGALG00000033150	MIDN
Com_192_pos	1-Methylhistidine	MSTRG.5038	--
Com_86_neg	Levulinic acid	ENSGALG00000050676	Ctnnd2
Com_99_pos	Creatine	ENSGALG00000015721	SVEP1
Com_18_neg	Arachidonic acid	ENSGALG00000014976	GATA6
Com_311_pos	PC (18:4e/2:0)	ENSGALG00000014813	HOMER1
Com_440_pos	PC (18:4e/4:0)	ENSGALG00000014525	USP5
Com_440_pos	PC (18:4e/4:0)	ENSGALG00000033051	CAMK1D
Com_178_pos	Maltol	ENSGALG00000014944	GCNT4
Com_18_neg	Arachidonic acid	ENSGALG00000042555	STAMBP
Com_92_pos	D-(+)-Proline	MSTRG.21321	gag
Com_8_neg	4-Methyl-2-Oxopentanoic	ENSGALG00000005583	ALG14
Com_25_pos	2-Hydroxycinnamic acid	ENSGALG00000002845	CTNNA3
Com_588_pos	Ornithine	ENSGALG00000030908	ATP2B2
Com_76_neg	Erythronolactone	ENSGALG00000028191	GLCE
Com_331_pos	L-Lysine	ENSGALG00000006076	RASGEF1C
Com_16_neg	3-Hydroxybutyric acid	ENSGALG00000003427	USP3
Com_460_pos	3-amino-4-(propylamino)(	ENSGALG00000042491	H4-I
Com_186_pos	4-Hydroxybenzaldehyde	ENSGALG00000043087	MTF2
Com_99_pos	Creatine	ENSGALG00000006080	GPC4
Com_588_pos	Ornithine	ENSGALG00000033656	DQX1
Com_55_pos	Valine	ENSGALG00000052072	gag
Com_215_pos	D-Erythro-sphingosine 1-p	ENSGALG00000039140	CD14
Com_8_neg	4-Methyl-2-Oxopentanoic	ENSGALG00000006872	PISD
Com_252_pos	cis-4-Hydroxy-D-proline	ENSGALG00000009560	MSMO1
Com_8_neg	4-Methyl-2-Oxopentanoic	MSTRG.13408	--
Com_22_pos	Indole-3-acrylic acid	ENSGALG00000041238	NOS1AP
Com_97_pos	L-Threonine	ENSGALG00000016885	STK24
Com_186_pos	4-Hydroxybenzaldehyde	ENSGALG00000016456	LPIN1
Com_482_pos	8-Hydroxyquinoline	ENSGALG00000016138	DSCAM
Com_311_pos	PC (18:4e/2:0)	ENSGALG00000014233	FBLN1
Com_178_pos	Maltol	ENSGALG00000033365	ALDH1A3
Com_311_pos	PC (18:4e/2:0)	ENSGALG00000021658	PAFAH2
Com_413_pos	L-Cystine	ENSGALG00000007848	PTS
Com_386_pos	2-Amino-1,3-octadecanec	ENSGALG00000012377	HNMT
Com_147_pos	D-Sphingosine	ENSGALG00000011254	SATB1
Com_192_pos	1-Methylhistidine	ENSGALG00000005722	SEC31B
Com_17_pos	L-Norleucine	ENSGALG00000021135	HAPLN3
Com_99_pos	Creatine	ENSGALG00000037322	HIST1H46
Com_482_pos	8-Hydroxyquinoline	ENSGALG00000028341	MADCAM1
Com_171_neg	LPC 22:6	ENSGALG00000030845	ENHO
Com_120_neg	LPC 15:0	MSTRG.19463	VTG2
Com_55_pos	Valine	ENSGALG00000026607	C15orf40
Com_25_pos	2-Hydroxycinnamic acid	ENSGALG00000001101	MBD3
Com_120_neg	LPC 15:0	MSTRG.12629	--
Com_120_neg	LPC 15:0	MSTRG.20167	--
Com_99_pos	Creatine	ENSGALG00000009312	RPL22L1
Com_386_pos	2-Amino-1,3-octadecanec	MSTRG.1082	--
Com_54_pos	Uric acid	ENSGALG00000011169	PDCD2
Com_108_neg	LPE 18:2	ENSGALG00000001697	ITIH3
Com_331_pos	L-Lysine	ENSGALG00000010229	ABCD4
Com_55_pos	Valine	ENSGALG00000016492	TDRD15
Com_152_pos	Acetyl-L-carnitine	ENSGALG00000002775	FA2H
Com_25_pos	2-Hydroxycinnamic acid	MSTRG.9006	--
Com_76_neg	Erythronolactone	ENSGALG00000013001	CTNND2
Com_386_pos	2-Amino-1,3-octadecanec	ENSGALG00000008866	WDPCP
Com_186_pos	4-Hydroxybenzaldehyde	ENSGALG00000008539	ALG12

Com_76_neg	Erythronolactone	MSTRG.15732	--
Com_311_pos	PC (18:4e/2:0)	MSTRG.21394	gag-pol
Com_460_pos	3-amino-4-(propylamino)(	MSTRG.2170	MYO16
Com_413_pos	L-Cystine	MSTRG.21204	--
Com_147_pos	D-Sphingosine	MSTRG.4702	--
Com_151_pos	Pyridoxamine	MSTRG.13584	--
Com_208_neg	N-Acetylanthranilic acid	ENSGALG00000004127	--
Com_8_neg	4-Methyl-2-Oxopentanoic	ENSGALG00000048035	GCNT2
Com_130_neg	2-Hydroxyvaleric acid	ENSGALG00000008862	DNAJC10
Com_8_neg	4-Methyl-2-Oxopentanoic	ENSGALG00000000107	TRIM7.1
Com_76_neg	Erythronolactone	MSTRG.2393	--
Com_482_pos	8-Hydroxyquinoline	ENSGALG00000002855	SARDH
Com_311_pos	PC (18:4e/2:0)	ENSGALG00000015044	GTF3C6
Com_588_pos	Ornithine	ENSGALG00000038145	DPP7
Com_18_neg	Arachidonic acid	MSTRG.3156	--
Com_76_neg	Erythronolactone	MSTRG.13408	--
Com_97_pos	L-Threonine	MSTRG.21394	gag-pol
Com_18_neg	Arachidonic acid	MSTRG.16287	--
Com_171_neg	LPC 22:6	ENSGALG00000012882	KDSR
Com_130_neg	2-Hydroxyvaleric acid	ENSGALG00000033051	CAMK1D
Com_152_pos	Acetyl-L-carnitine	ENSGALG00000010293	RBP
Com_108_neg	LPE 18:2	ENSGALG00000049256	--
Com_192_pos	1-Methylhistidine	ENSGALG00000004127	--
Com_252_pos	cis-4-Hydroxy-D-proline	ENSGALG00000011657	EAF2
Com_86_neg	Levulinic acid	ENSGALG00000036293	EBAG9
Com_130_neg	2-Hydroxyvaleric acid	ENSGALG00000014525	USP5
Com_8_neg	4-Methyl-2-Oxopentanoic	ENSGALG00000054319	ELOVL6
Com_352_pos	Riboflavin	ENSGALG00000009268	FGG
Com_152_pos	Acetyl-L-carnitine	ENSGALG00000037050	FABP3
Com_22_pos	Indole-3-acrylic acid	ENSGALG00000030941	ELAPOR1
Com_78_neg	Citric acid	ENSGALG00000005470	PLPPR5
Com_194_pos	Pipecolic acid	ENSGALG00000007778	PES1
Com_25_pos	2-Hydroxycinnamic acid	ENSGALG00000006521	TRPM5
Com_92_pos	D-(+)-Proline	ENSGALG00000009170	NCEH1
Com_482_pos	8-Hydroxyquinoline	ENSGALG00000015034	ANKRD29
Com_471_pos	Indole-3-acetic acid	ENSGALG00000003136	IKZF2
Com_21_pos	DL-Tryptophan	MSTRG.2171	Myo16
Com_482_pos	8-Hydroxyquinoline	ENSGALG00000008728	PTER
Com_471_pos	Indole-3-acetic acid	ENSGALG00000009963	LYZ
Com_186_pos	4-Hydroxybenzaldehyde	ENSGALG00000052395	ERVK-11
Com_171_neg	LPC 22:6	ENSGALG00000005739	SCD
Com_120_neg	LPC 15:0	ENSGALG00000014581	BORCS8
Com_25_pos	2-Hydroxycinnamic acid	ENSGALG00000040730	RXRG
Com_80_pos	DL-Lysine	ENSGALG00000006702	MFGE8
Com_17_pos	L-Norleucine	ENSGALG00000047027	ADCK5
Com_147_pos	D-Sphingosine	MSTRG.149	--
Com_12_pos	Betaine	ENSGALG00000008763	SSX2IP
Com_97_pos	L-Threonine	ENSGALG00000021658	PAFAH2
Com_252_pos	cis-4-Hydroxy-D-proline	ENSGALG00000004875	PEMT
Com_120_neg	LPC 15:0	ENSGALG00000008262	RASGRF1
Com_130_neg	2-Hydroxyvaleric acid	ENSGALG00000000226	TMEM9
Com_55_pos	Valine	ENSGALG00000051123	pol
Com_311_pos	PC (18:4e/2:0)	MSTRG.8986	gag
Com_80_pos	DL-Lysine	ENSGALG00000012748	ELOVL2
Com_208_neg	N-Acetylanthranilic acid	ENSGALG00000005722	SEC31B
Com_12_pos	Betaine	ENSGALG00000006904	RNH1
Com_8_neg	4-Methyl-2-Oxopentanoic	ENSGALG00000046789	pol
Com_331_pos	L-Lysine	ENSGALG00000042511	PKDCC

Com_22_pos	Indole-3-acrylic acid	ENSGALG00000009415	SMOC1
Com_151_pos	Pyridoxamine	ENSGALG00000051251	H2B-I
Com_78_neg	Citric acid	ENSGALG00000003948	ALAS1
Com_171_neg	LPC 22:6	ENSGALG00000022758	GGACT
Com_97_pos	L-Threonine	MSTRG.2387	--
Com_22_pos	Indole-3-acrylic acid	ENSGALG00000008795	GPAM
Com_89_neg	Gallic acid	ENSGALG00000037014	TSNARE1
Com_17_pos	L-Norleucine	ENSGALG00000019276	SLCO1C1
Com_120_neg	LPC 15:0	ENSGALG00000026547	TPGS2
Com_54_pos	Uric acid	ENSGALG00000050091	CLEC2B
Com_331_pos	L-Lysine	MSTRG.5319	--
Com_97_pos	L-Threonine	ENSGALG00000015729	LPAR1
Com_40_pos	Choline	ENSGALG00000005439	ACACA
Com_147_pos	D-Sphingosine	ENSGALG00000004322	AHR
Com_362_pos	2-Arachidonoyl glycerol	ENSGALG00000037253	CLEC4M
Com_152_pos	Acetyl-L-carnitine	ENSGALG00000010641	SCCPDH
Com_17_pos	L-Norleucine	ENSGALG00000003147	TRPC4AP
Com_265_pos	6-Methylquinoline	ENSGALG00000009016	SLX4IP
Com_120_neg	LPC 15:0	ENSGALG00000047380	MR1
Com_413_pos	L-Cystine	ENSGALG00000016475	Zp2
Com_40_pos	Choline	ENSGALG00000048109	gag
Com_386_pos	2-Amino-1,3-octadecanec	ENSGALG00000037773	ST3GAL1
Com_178_pos	Maltol	ENSGALG00000004425	SCAMP1
Com_440_pos	PC (18:4e/4:0)	ENSGALG00000001475	STMN1
Com_8_neg	4-Methyl-2-Oxopentanoic	ENSGALG00000047687	SETD9
Com_25_pos	2-Hydroxycinnamic acid	ENSGALG00000000802	DHODH
Com_194_pos	Pipecolic acid	ENSGALG00000016491	APOB
Com_97_pos	L-Threonine	ENSGALG00000051779	PRORSD1P
Com_208_neg	N-Acetylanthranilic acid	MSTRG.5038	--
Com_252_pos	cis-4-Hydroxy-D-proline	ENSGALG00000003029	PLPP6
Com_86_neg	Levulinic acid	ENSGALG00000041604	NPTXR
Com_25_pos	2-Hydroxycinnamic acid	ENSGALG00000028376	FGF19
Com_120_neg	LPC 15:0	ENSGALG00000004282	RCAN3
Com_208_neg	N-Acetylanthranilic acid	MSTRG.2430	--
Com_147_pos	D-Sphingosine	MSTRG.1503	gag
Com_8_neg	4-Methyl-2-Oxopentanoic	MSTRG.6225	--
Com_175_pos	Pantothenic acid	ENSGALG00000007018	SLC26A11
Com_120_neg	LPC 15:0	ENSGALG00000036787	HSD17B12
Com_194_pos	Pipecolic acid	ENSGALG00000021686	--
Com_151_pos	Pyridoxamine	ENSGALG00000020538	SLC49A3
Com_147_pos	D-Sphingosine	ENSGALG00000031525	TSTA3
Com_208_neg	N-Acetylanthranilic acid	ENSGALG00000004657	FBXO2
Com_386_pos	2-Amino-1,3-octadecanec	MSTRG.2305	gag
Com_311_pos	PC (18:4e/2:0)	ENSGALG00000000226	TMEM9
Com_588_pos	Ornithine	ENSGALG00000019276	SLCO1C1
Com_194_pos	Pipecolic acid	ENSGALG00000009476	CDK6
Com_120_neg	LPC 15:0	MSTRG.17961	--
Com_17_pos	L-Norleucine	ENSGALG00000046757	ERVK-9
Com_54_pos	Uric acid	MSTRG.10409	--
Com_413_pos	L-Cystine	MSTRG.8471	--
Com_471_pos	Indole-3-acetic acid	ENSGALG00000019835	TRIM27.2
Com_25_pos	2-Hydroxycinnamic acid	MSTRG.20656	--
Com_440_pos	PC (18:4e/4:0)	MSTRG.15507	--
Com_588_pos	Ornithine	MSTRG.16287	--
Com_192_pos	1-Methylhistidine	ENSGALG00000006783	PLOD2
Com_76_neg	Erythronolactone	MSTRG.3156	--
Com_264_pos	Indole	ENSGALG00000006490	SCN3B
Com_108_neg	LPE 18:2	ENSGALG00000039140	CD14

Com_352_pos	Riboflavin	ENSGALG00000009848	LPGAT1
Com_362_pos	2-Arachidonoyl glycerol	ENSGALG00000032628	SRCIN1
Com_152_pos	Acetyl-L-carnitine	ENSGALG00000054297	SND1
Com_18_neg	Arachidonic acid	ENSGALG00000038145	DPP7
Com_12_pos	Betaine	ENSGALG00000002500	GMPPB
Com_18_neg	Arachidonic acid	MSTRG.2393	--
Com_40_pos	Choline	ENSGALG00000007127	FADS1
Com_55_pos	Valine	MSTRG.14343	--
Com_78_neg	Citric acid	ENSGALG00000030845	ENHO
Com_588_pos	Ornithine	ENSGALG00000047027	ADCK5
Com_440_pos	PC (18:4e/4:0)	ENSGALG00000014813	HOMER1
Com_311_pos	PC (18:4e/2:0)	ENSGALG00000014525	USP5
Com_152_pos	Acetyl-L-carnitine	ENSGALG00000005583	ALG14
Com_178_pos	Maltol	ENSGALG00000006076	RASGEF1C
Com_194_pos	Pipecolic acid	ENSGALG00000030151	LUZP2
Com_86_neg	Levulinic acid	ENSGALG00000021135	HAPLN3
Com_311_pos	PC (18:4e/2:0)	ENSGALG00000033051	CAMK1D
Com_471_pos	Indole-3-acetic acid	ENSGALG00000017103	WASF3
Com_80_pos	DL-Lysine	ENSGALG00000029445	FADS6
Com_55_pos	Valine	ENSGALG00000029445	FADS6
Com_78_neg	Citric acid	ENSGALG00000031312	ANAPC13
Com_86_neg	Levulinic acid	ENSGALG00000047495	LRRC10
Com_54_pos	Uric acid	ENSGALG00000041687	SREBF2
Com_18_neg	Arachidonic acid	MSTRG.15732	--
Com_80_pos	DL-Lysine	MSTRG.14343	--
Com_40_pos	Choline	ENSGALG00000028949	CORO6
Com_18_neg	Arachidonic acid	ENSGALG00000013001	CTNND2
Com_55_pos	Valine	MSTRG.8501	--
Com_152_pos	Acetyl-L-carnitine	ENSGALG00000006872	PISD
Com_97_pos	L-Threonine	ENSGALG00000007404	YIPF5
Com_331_pos	L-Lysine	ENSGALG00000033365	ALDH1A3
Com_97_pos	L-Threonine	ENSGALG00000008862	DNAJC10
Com_130_neg	2-Hydroxyvaleric acid	ENSGALG00000052612	RPS27L
Com_215_pos	D-Erythro-sphingosine 1-|	ENSGALG00000001697	ITIH3
Com_97_pos	L-Threonine	MSTRG.13261	--
Com_208_neg	N-Acetylanthranilic acid	ENSGALG00000050427	OSBPL10
Com_8_neg	4-Methyl-2-Oxopentanoic	ENSGALG00000053245	VTG2
Com_311_pos	PC (18:4e/2:0)	MSTRG.2305	gag
Com_192_pos	1-Methylhistidine	ENSGALG00000016412	MBOAT2
Com_413_pos	L-Cystine	ENSGALG00000047495	LRRC10
Com_108_neg	LPE 18:2	ENSGALG00000006812	TTC36
Com_130_neg	2-Hydroxyvaleric acid	MSTRG.21394	gag-pol
Com_440_pos	PC (18:4e/4:0)	ENSGALG00000014233	FBLN1
Com_80_pos	DL-Lysine	ENSGALG00000031754	KCNG2
Com_194_pos	Pipecolic acid	ENSGALG00000027375	NR2C2AP
Com_25_pos	2-Hydroxycinnamic acid	ENSGALG00000004804	TGM3
Com_413_pos	L-Cystine	ENSGALG00000003427	USP3
Com_203_pos	Serotonin	ENSGALG00000036742	GATSL2
Com_178_pos	Maltol	ENSGALG00000010229	ABCD4
Com_178_pos	Maltol	MSTRG.15754	--
Com_175_pos	Pantothenic acid	ENSGALG00000010978	ANGPTL3
Com_440_pos	PC (18:4e/4:0)	ENSGALG00000051398	TMEM14C
Com_18_neg	Arachidonic acid	ENSGALG00000033656	DQX1
Com_120_neg	LPC 15:0	ENSGALG00000015136	ILDR1
Com_86_neg	Levulinic acid	MSTRG.8471	--
Com_215_pos	D-Erythro-sphingosine 1-|	ENSGALG00000049256	--
Com_171_neg	LPC 22:6	ENSGALG00000005470	PLPPR5
Com_80_pos	DL-Lysine	MSTRG.9165	--

Com_130_neg	2-Hydroxyvaleric acid	ENSGALG00000021658	PAFAH2
Com_8_neg	4-Methyl-2-Oxopentanoic	ENSGALG00000010018	CTSEAL
Com_18_neg	Arachidonic acid	ENSGALG00000028191	GLCE
Com_152_pos	Acetyl-L-carnitine	ENSGALG00000048035	GCNT2
Com_18_neg	Arachidonic acid	ENSGALG00000030908	ATP2B2
Com_208_neg	N-Acetylanthranilic acid	ENSGALG00000031159	HIST1H110
Com_152_pos	Acetyl-L-carnitine	ENSGALG00000000107	TRIM7.1
Com_55_pos	Valine	ENSGALG00000013149	MOCOS
Com_264_pos	Indole	ENSGALG00000030941	ELAPOR1
Com_76_neg	Erythronolactone	ENSGALG00000042555	STAMBP
Com_40_pos	Choline	ENSGALG00000051290	--
Com_152_pos	Acetyl-L-carnitine	ENSGALG00000014464	MTR
Com_186_pos	4-Hydroxybenzaldehyde	ENSGALG00000038574	MYO15A
Com_208_neg	N-Acetylanthranilic acid	MSTRG.835	--
Com_17_pos	L-Norleucine	ENSGALG00000039538	CLDND1
Com_76_neg	Erythronolactone	ENSGALG00000014976	GATA6
Com_8_neg	4-Methyl-2-Oxopentanoic	ENSGALG00000010293	RBP
Com_440_pos	PC (18:4e/4:0)	ENSGALG00000015044	GTF3C6
Com_482_pos	8-Hydroxyquinoline	ENSGALG00000043582	LY6E
Com_152_pos	Acetyl-L-carnitine	ENSGALG00000054319	ELOVL6
Com_175_pos	Pantothenic acid	ENSGALG00000009680	PAQR7
Com_386_pos	2-Amino-1,3-octadecanec	MSTRG.8986	gag
Com_178_pos	Maltol	ENSGALG00000031067	TMEM132A
Com_460_pos	3-amino-4-(propylamino)(	MSTRG.19463	VTG2
Com_178_pos	Maltol	ENSGALG00000000104	CRY1
Com_86_neg	Levulinic acid	ENSGALG00000016475	Zp2
Com_171_neg	LPC 22:6	ENSGALG00000003948	ALAS1
Com_460_pos	3-amino-4-(propylamino)(	ENSGALG00000007533	NPEPL1
Com_203_pos	Serotonin	ENSGALG00000014872	FGF10
Com_22_pos	Indole-3-acrylic acid	ENSGALG00000010837	ASB5
Com_120_neg	LPC 15:0	ENSGALG00000004729	SLC7A10
Com_192_pos	1-Methylhistidine	ENSGALG00000028230	SUN2
Com_482_pos	8-Hydroxyquinoline	ENSGALG00000044996	TMEM71
Com_186_pos	4-Hydroxybenzaldehyde	ENSGALG00000016560	SELENOI
Com_215_pos	D-Erythro-sphingosine 1-p	ENSGALG00000016761	LYG2
Com_264_pos	Indole	ENSGALG00000028005	GADD45G
Com_80_pos	DL-Lysine	ENSGALG00000015684	Dnajc25
Com_22_pos	Indole-3-acrylic acid	ENSGALG00000002919	MON1A
Com_130_neg	2-Hydroxyvaleric acid	ENSGALG00000051779	PRORSD1P
Com_175_pos	Pantothenic acid	MSTRG.21796	--
Com_120_neg	LPC 15:0	MSTRG.2170	MYO16
Com_265_pos	6-Methylquinoline	ENSGALG00000028005	GADD45G
Com_86_neg	Levulinic acid	ENSGALG00000003147	TRPC4AP
Com_178_pos	Maltol	ENSGALG00000042511	PKDCC
Com_92_pos	D-(+)-Proline	ENSGALG00000017378	CRTAC1
Com_120_neg	LPC 15:0	ENSGALG00000028822	RNF152
Com_18_neg	Arachidonic acid	ENSGALG00000049658	UTS2R
Com_440_pos	PC (18:4e/4:0)	MSTRG.8986	gag
Com_440_pos	PC (18:4e/4:0)	ENSGALG00000011254	SATB1
Com_186_pos	4-Hydroxybenzaldehyde	ENSGALG00000004702	DYNC2I2
Com_97_pos	L-Threonine	ENSGALG00000005610	SLC44A3
Com_386_pos	2-Amino-1,3-octadecanec	ENSGALG00000015044	GTF3C6
Com_147_pos	D-Sphingosine	MSTRG.16504	gag
Com_25_pos	2-Hydroxycinnamic acid	ENSGALG00000037261	RFXANK
Com_8_neg	4-Methyl-2-Oxopentanoic	ENSGALG00000010641	SCCPDH
Com_40_pos	Choline	ENSGALG00000040070	PDIA2
Com_21_pos	DL-Tryptophan	ENSGALG00000044464	TEPSIN
Com_194_pos	Pipecolic acid	ENSGALG00000052072	gag

Com_311_pos	PC (18:4e/2:0)	ENSGALG00000008866	WDPCP
Com_252_pos	cis-4-Hydroxy-D-proline	MSTRG.17623	--
Com_194_pos	Pipecolic acid	MSTRG.8381	--
Com_152_pos	Acetyl-L-carnitine	ENSGALG00000047687	SETD9
Com_311_pos	PC (18:4e/2:0)	MSTRG.1082	--
Com_86_neg	Levulinic acid	ENSGALG00000046757	ERVK-9
Com_471_pos	Indole-3-acetic acid	MSTRG.17350	--
Com_311_pos	PC (18:4e/2:0)	ENSGALG00000001475	STMN1
Com_460_pos	3-amino-4-(propylamino)(	ENSGALG00000008262	RASGRF1
Com_151_pos	Pyridoxamine	ENSGALG00000036754	CHKA
Com_80_pos	DL-Lysine	ENSGALG00000016492	TDRD15
Com_152_pos	Acetyl-L-carnitine	MSTRG.6225	--
Com_54_pos	Uric acid	ENSGALG00000026809	SARS
Com_130_neg	2-Hydroxyvaleric acid	ENSGALG00000007404	YIPF5
Com_311_pos	PC (18:4e/2:0)	ENSGALG00000012377	HNMT
Com_16_neg	3-Hydroxybutyric acid	MSTRG.16287	--
Com_97_pos	L-Threonine	ENSGALG00000052612	RPS27L
Com_130_neg	2-Hydroxyvaleric acid	MSTRG.13261	--
Com_76_neg	Erythronolactone	ENSGALG00000017122	SGCG
Com_386_pos	2-Amino-1,3-octadecanec	ENSGALG00000014233	FBLN1
Com_22_pos	Indole-3-acrylic acid	ENSGALG00000006490	SCN3B
Com_86_neg	Levulinic acid	MSTRG.21204	--
Com_17_pos	L-Norleucine	ENSGALG00000021685	SERINC2
Com_171_neg	LPC 22:6	ENSGALG00000031312	ANAPC13
Com_80_pos	DL-Lysine	ENSGALG00000026607	C15orf40
Com_40_pos	Choline	MSTRG.8779	--
Com_386_pos	2-Amino-1,3-octadecanec	ENSGALG00000004341	Cryzl2
Com_194_pos	Pipecolic acid	ENSGALG00000026846	JMJD7
Com_460_pos	3-amino-4-(propylamino)(	ENSGALG00000026547	TPGS2
Com_171_neg	LPC 22:6	MSTRG.13135	--
Com_55_pos	Valine	MSTRG.6512	--
Com_54_pos	Uric acid	MSTRG.9361	Fam110a
Com_17_pos	L-Norleucine	ENSGALG00000047495	LRRC10
Com_265_pos	6-Methylquinoline	MSTRG.7572	--
Com_17_pos	L-Norleucine	MSTRG.11802	TMEM221
Com_171_neg	LPC 22:6	ENSGALG00000002899	AACS
Com_175_pos	Pantothenic acid	ENSGALG00000037935	RARA
Com_8_neg	4-Methyl-2-Oxopentanoic	ENSGALG00000054297	SND1
Com_192_pos	1-Methylhistidine	ENSGALG00000015253	COL8A1
Com_460_pos	3-amino-4-(propylamino)(	ENSGALG00000047380	MR1
Com_25_pos	2-Hydroxycinnamic acid	ENSGALG00000037811	NRSN1
Com_12_pos	Betaine	ENSGALG00000007723	NAT9
Com_175_pos	Pantothenic acid	ENSGALG00000003103	MST1R
Com_215_pos	D-Erythro-sphingosine 1-p	ENSGALG00000006812	TTC36
Com_386_pos	2-Amino-1,3-octadecanec	ENSGALG00000014813	HOMER1
Com_25_pos	2-Hydroxycinnamic acid	ENSGALG00000002437	DIPK1B
Com_331_pos	L-Lysine	ENSGALG00000000950	MVB12B
Com_86_neg	Levulinic acid	ENSGALG00000007848	PTS
Com_130_neg	2-Hydroxyvaleric acid	ENSGALG00000016476	TTC32
Com_21_pos	DL-Tryptophan	ENSGALG00000051567	MRPL41
Com_362_pos	2-Arachidonoyl glycerol	ENSGALG00000051466	NDFIP2
Com_192_pos	1-Methylhistidine	ENSGALG00000033461	hnmt
Com_192_pos	1-Methylhistidine	ENSGALG00000053217	INPP5J
Com_21_pos	DL-Tryptophan	ENSGALG00000028880	FDPS
Com_588_pos	Ornithine	ENSGALG00000027070	TIMP2
Com_92_pos	D-(+)-Proline	ENSGALG00000002775	FA2H
Com_120_neg	LPC 15:0	ENSGALG00000004170	ADA
Com_386_pos	2-Amino-1,3-octadecanec	MSTRG.15507	--

Com_265_pos	6-Methylquinoline	ENSGALG00000006490	SCN3B
Com_17_pos	L-Norleucine	ENSGALG00000001749	ACSBG2
Com_152_pos	Acetyl-L-carnitine	ENSGALG00000053245	VTG2
Com_386_pos	2-Amino-1,3-octadecanec	ENSGALG00000036836	SOSTDC1
Com_460_pos	3-amino-4-(propylamino)(	ENSGALG00000036787	HSD17B12
Com_108_neg	LPE 18:2	ENSGALG00000006976	Bdh1
Com_482_pos	8-Hydroxyquinoline	ENSGALG00000014950	SULT3A1
Com_265_pos	6-Methylquinoline	ENSGALG00000021193	STARD5
Com_440_pos	PC (18:4e/4:0)	MSTRG.2305	gag
Com_17_pos	L-Norleucine	MSTRG.8471	--
Com_460_pos	3-amino-4-(propylamino)(	ENSGALG00000015689	ECPAS
Com_40_pos	Choline	ENSGALG00000041372	Myrip
Com_192_pos	1-Methylhistidine	MSTRG.8499	Itpripl1
Com_208_neg	N-Acetylanthranilic acid	MSTRG.15745	--
Com_194_pos	Pipecolic acid	ENSGALG00000051123	pol
Com_80_pos	DL-Lysine	ENSGALG00000046731	--
Com_331_pos	L-Lysine	MSTRG.15754	--
Com_25_pos	2-Hydroxycinnamic acid	MSTRG.2403	--
Com_352_pos	Riboflavin	ENSGALG00000052894	Ranbp2
Com_92_pos	D-(+)-Proline	ENSGALG00000037050	FABP3
Com_99_pos	Creatine	ENSGALG00000010764	FBXO8
Com_86_neg	Levulinic acid	ENSGALG00000039538	CLDND1
Com_352_pos	Riboflavin	ENSGALG00000003022	Fmo5
Com_311_pos	PC (18:4e/2:0)	ENSGALG00000051398	TMEM14C
Com_21_pos	DL-Tryptophan	ENSGALG00000011141	ITGB6
Com_482_pos	8-Hydroxyquinoline	ENSGALG00000007728	Prodh
Com_78_neg	Citric acid	ENSGALG00000037253	CLEC4M
Com_152_pos	Acetyl-L-carnitine	ENSGALG00000010018	CTSEAL
Com_99_pos	Creatine	ENSGALG00000052395	ERVK-11
Com_76_neg	Erythronolactone	ENSGALG00000026203	FAM174A
Com_171_neg	LPC 22:6	ENSGALG00000051466	NDFIP2
Com_252_pos	cis-4-Hydroxy-D-proline	ENSGALG00000037261	RFXANK
Com_17_pos	L-Norleucine	ENSGALG00000016475	Zp2
Com_151_pos	Pyridoxamine	ENSGALG00000007507	MASTL
Com_440_pos	PC (18:4e/4:0)	ENSGALG00000031525	TSTA3
Com_264_pos	Indole	ENSGALG00000009016	SLX4IP
Com_8_neg	4-Methyl-2-Oxopentanoic	ENSGALG00000014464	MTR
Com_108_neg	LPE 18:2	ENSGALG00000016761	LYG2
Com_130_neg	2-Hydroxyvaleric acid	ENSGALG00000005610	SLC44A3
Com_264_pos	Indole	ENSGALG00000010837	ASB5
Com_194_pos	Pipecolic acid	ENSGALG00000041456	SLC35G1
Com_482_pos	8-Hydroxyquinoline	ENSGALG00000038242	CACNA2D2
Com_331_pos	L-Lysine	ENSGALG00000031067	TMEM132A
Com_178_pos	Maltol	ENSGALG00000002802	PACSIN1
Com_22_pos	Indole-3-acrylic acid	ENSGALG00000028005	GADD45G
Com_331_pos	L-Lysine	ENSGALG00000000104	CRY1
Com_147_pos	D-Sphingosine	MSTRG.15507	--
Com_331_pos	L-Lysine	MSTRG.15443	--
Com_460_pos	3-amino-4-(propylamino)(	ENSGALG00000016595	TRIM35
Com_21_pos	DL-Tryptophan	ENSGALG00000021451	RED3
Com_413_pos	L-Cystine	ENSGALG00000019276	SLCO1C1
Com_362_pos	2-Arachidonoyl glycerol	ENSGALG00000002899	AACS
Com_16_neg	3-Hydroxybutyric acid	ENSGALG00000038145	DPP7
Com_147_pos	D-Sphingosine	ENSGALG00000012377	HNMT
Com_362_pos	2-Arachidonoyl glycerol	MSTRG.13135	--
Com_264_pos	Indole	ENSGALG00000002919	MON1A
Com_12_pos	Betaine	ENSGALG00000054926	--
Com_252_pos	cis-4-Hydroxy-D-proline	ENSGALG00000010301	EIF2B2

Com_147_pos	D-Sphingosine	ENSGALG00000014813	HOMER1
Com_55_pos	Valine	ENSGALG00000021686	--
Com_175_pos	Pantothenic acid	ENSGALG00000035626	DAD1
Com_55_pos	Valine	ENSGALG00000009476	CDK6
Com_460_pos	3-amino-4-(propylamino)(	ENSGALG00000015136	ILDR1
Com_147_pos	D-Sphingosine	MSTRG.1082	--
Com_130_neg	2-Hydroxyvaleric acid	ENSGALG00000005815	TMEM41B
Com_413_pos	L-Cystine	ENSGALG00000047027	ADCK5
Com_252_pos	cis-4-Hydroxy-D-proline	ENSGALG00000035206	CNPY2
Com_460_pos	3-amino-4-(propylamino)(	ENSGALG00000048223	FAM20C
Com_186_pos	4-Hydroxybenzaldehyde	ENSGALG00000009312	RPL22L1
Com_147_pos	D-Sphingosine	ENSGALG00000008866	WDPCP
Com_78_neg	Citric acid	ENSGALG00000032628	SRCIN1
Com_97_pos	L-Threonine	ENSGALG00000026460	myoM
Com_482_pos	8-Hydroxyquinoline	ENSGALG00000013726	PAICS
Com_192_pos	1-Methylhistidine	ENSGALG00000011616	NPFFR2
Com_208_neg	N-Acetylanthranilic acid	MSTRG.8128	--
Com_362_pos	2-Arachidonoyl glycerol	ENSGALG00000030845	ENHO
Com_386_pos	2-Amino-1,3-octadecanec	MSTRG.4702	--
Com_147_pos	D-Sphingosine	ENSGALG00000014233	FBLN1
Com_186_pos	4-Hydroxybenzaldehyde	ENSGALG00000037322	HIST1H46
Com_331_pos	L-Lysine	ENSGALG00000038723	RPP25L
Com_311_pos	PC (18:4e/2:0)	ENSGALG00000011254	SATB1
Com_55_pos	Valine	ENSGALG00000030151	LUZP2
Com_86_neg	Levulinic acid	ENSGALG00000041296	SOX7
Com_40_pos	Choline	ENSGALG00000025886	SUSD3
Com_208_neg	N-Acetylanthranilic acid	ENSGALG00000050668	Spata1
Com_311_pos	PC (18:4e/2:0)	MSTRG.16504	gag
Com_99_pos	Creatine	ENSGALG00000016456	LPIN1
Com_265_pos	6-Methylquinoline	ENSGALG00000007636	PCK1
Com_92_pos	D-(+)-Proline	ENSGALG00000010018	CTSEAL
Com_194_pos	Pipecolic acid	MSTRG.8501	--
Com_16_neg	3-Hydroxybutyric acid	ENSGALG00000033656	DQX1
Com_97_pos	L-Threonine	ENSGALG00000012847	Slc7a11
Com_99_pos	Creatine	ENSGALG00000043087	MTF2
Com_120_neg	LPC 15:0	ENSGALG00000007533	NPEPL1
Com_460_pos	3-amino-4-(propylamino)(	ENSGALG00000004729	SLC7A10
Com_54_pos	Uric acid	ENSGALG00000035803	THRSP
Com_588_pos	Ornithine	ENSGALG00000003427	USP3
Com_78_neg	Citric acid	MSTRG.13135	--
Com_97_pos	L-Threonine	ENSGALG00000005160	VMP1
Com_17_pos	L-Norleucine	MSTRG.21204	--
Com_86_neg	Levulinic acid	ENSGALG00000021685	SERINC2
Com_440_pos	PC (18:4e/4:0)	ENSGALG00000008866	WDPCP
Com_97_pos	L-Threonine	MSTRG.2388	--
Com_16_neg	3-Hydroxybutyric acid	ENSGALG00000030908	ATP2B2
Com_78_neg	Citric acid	ENSGALG00000002899	AACS
Com_76_neg	Erythronolactone	ENSGALG00000005353	FAR1
Com_55_pos	Valine	ENSGALG00000041456	SLC35G1
Com_54_pos	Uric acid	ENSGALG00000027561	GNG5
Com_192_pos	1-Methylhistidine	ENSGALG00000010703	DGLUCY
Com_440_pos	PC (18:4e/4:0)	MSTRG.1082	--
Com_97_pos	L-Threonine	ENSGALG00000016476	TTC32
Com_86_neg	Levulinic acid	MSTRG.11802	TMEM221
Com_92_pos	D-(+)-Proline	ENSGALG00000005583	ALG14
Com_331_pos	L-Lysine	ENSGALG00000012112	DBI
Com_186_pos	4-Hydroxybenzaldehyde	ENSGALG00000015721	SVEP1
Com_460_pos	3-amino-4-(propylamino)(	ENSGALG00000028822	RNF152

Com_386_pos	2-Amino-1,3-octadecanec	MSTRG.149	--
Com_86_neg	Levulinic acid	ENSGALG00000005648	Sesn3
Com_471_pos	Indole-3-acetic acid	MSTRG.5269	pitpnc1
Com_192_pos	1-Methylhistidine	ENSGALG00000006649	TMEM41A
Com_17_pos	L-Norleucine	MSTRG.12923	--
Com_76_neg	Erythronolactone	ENSGALG00000015134	APOV1
Com_147_pos	D-Sphingosine	ENSGALG00000015044	GTF3C6
Com_482_pos	8-Hydroxyquinoline	ENSGALG00000030031	TTPA
Com_440_pos	PC (18:4e/4:0)	ENSGALG00000012377	HNMT
Com_92_pos	D-(+)-Proline	ENSGALG00000053245	VTG2
Com_92_pos	D-(+)-Proline	ENSGALG00000006872	PISD
Com_76_neg	Erythronolactone	MSTRG.2316	env
Com_40_pos	Choline	ENSGALG00000008601	AHSG
Com_17_pos	L-Norleucine	ENSGALG00000007848	PTS
Com_265_pos	6-Methylquinoline	ENSGALG00000041238	NOS1AP
Com_352_pos	Riboflavin	ENSGALG00000014128	A4GALT
Com_194_pos	Pipecolic acid	ENSGALG00000013149	MOCOS
Com_86_neg	Levulinic acid	ENSGALG00000001749	ACSBG2
Com_215_pos	D-Erythro-sphingosine 1-p	ENSGALG00000006976	Bdh1
Com_178_pos	Maltol	ENSGALG00000000950	MVB12B
Com_386_pos	2-Amino-1,3-octadecanec	ENSGALG00000004322	AHR
Com_120_neg	LPC 15:0	ENSGALG00000048223	FAM20C
Com_186_pos	4-Hydroxybenzaldehyde	ENSGALG00000052829	MAMDC4
Com_178_pos	Maltol	ENSGALG00000012112	DBI
Com_252_pos	cis-4-Hydroxy-D-proline	MSTRG.20656	--
Com_16_neg	3-Hydroxybutyric acid	ENSGALG00000049658	UTS2R
Com_99_pos	Creatine	ENSGALG00000026384	PCSK4
Com_208_neg	N-Acetylanthranilic acid	ENSGALG00000030511	SLC19A1
Com_471_pos	Indole-3-acetic acid	ENSGALG00000013244	ABCC9
Com_147_pos	D-Sphingosine	MSTRG.8986	gag
Com_86_neg	Levulinic acid	MSTRG.12923	--
Com_17_pos	L-Norleucine	ENSGALG00000005648	Sesn3
Com_440_pos	PC (18:4e/4:0)	MSTRG.16504	gag
Com_186_pos	4-Hydroxybenzaldehyde	ENSGALG00000002249	AGO1
Com_171_neg	LPC 22:6	ENSGALG00000037253	CLEC4M
Com_92_pos	D-(+)-Proline	ENSGALG00000048035	GCNT2
Com_78_neg	Citric acid	ENSGALG00000051466	NDFIP2
Com_120_neg	LPC 15:0	ENSGALG00000016595	TRIM35
Com_203_pos	Serotonin	MSTRG.11633	Stap2
Com_252_pos	cis-4-Hydroxy-D-proline	ENSGALG00000028376	FGF19
Com_386_pos	2-Amino-1,3-octadecanec	MSTRG.1503	gag
Com_92_pos	D-(+)-Proline	ENSGALG00000000107	TRIM7.1
Com_92_pos	D-(+)-Proline	MSTRG.6225	--
Com_76_neg	Erythronolactone	ENSGALG00000013036	ATP6V1E1
Com_99_pos	Creatine	ENSGALG00000008427	GNAT3
Com_25_pos	2-Hydroxycinnamic acid	ENSGALG00000003029	PLPP6
Com_147_pos	D-Sphingosine	MSTRG.2305	gag
Com_413_pos	L-Cystine	ENSGALG00000027070	TIMP2
Com_12_pos	Betaine	ENSGALG00000040857	TECTA
Com_12_pos	Betaine	ENSGALG00000031482	Pou5f3
Com_178_pos	Maltol	ENSGALG00000038723	RPP25L
Com_76_neg	Erythronolactone	ENSGALG00000029235	CPNE4
Com_92_pos	D-(+)-Proline	ENSGALG00000054319	ELOVL6
Com_92_pos	D-(+)-Proline	ENSGALG00000047687	SETD9
Com_22_pos	Indole-3-acrylic acid	ENSGALG00000009016	SLX4IP
Com_21_pos	DL-Tryptophan	MSTRG.15625	--
Com_194_pos	Pipecolic acid	MSTRG.6512	--
Com_55_pos	Valine	ENSGALG00000026846	JMJD7

Com_17_pos	L-Norleucine	ENSGALG00000041296	SOX7
Com_99_pos	Creatine	ENSGALG00000007508	HPSE2
Com_130_neg	2-Hydroxyvaleric acid	MSTRG.2388	--
Com_186_pos	4-Hydroxybenzaldehyde	MSTRG.7811	--
Com_97_pos	L-Threonine	ENSGALG00000005815	TMEM41B
Com_130_neg	2-Hydroxyvaleric acid	ENSGALG00000005160	VMP1
Com_252_pos	cis-4-Hydroxy-D-proline	ENSGALG00000006521	TRPM5
Com_54_pos	Uric acid	ENSGALG00000041078	MID1IP1
Com_352_pos	Riboflavin	ENSGALG00000023844	P2RY1
Com_186_pos	4-Hydroxybenzaldehyde	ENSGALG00000001642	GLT8D1
Com_265_pos	6-Methylquinoline	ENSGALG00000008795	GPAM
Com_76_neg	Erythronolactone	ENSGALG00000004782	TSEN15
Com_130_neg	2-Hydroxyvaleric acid	ENSGALG00000012847	Slc7a11
Com_460_pos	3-amino-4-(propylamino)(	ENSGALG00000004170	ADA
Com_130_neg	2-Hydroxyvaleric acid	ENSGALG00000026460	myoM
Com_192_pos	1-Methylhistidine	ENSGALG00000036021	MTMR7
Com_265_pos	6-Methylquinoline	ENSGALG00000009415	SMOC1
Com_331_pos	L-Lysine	ENSGALG00000002802	PACSIN1
Com_252_pos	cis-4-Hydroxy-D-proline	ENSGALG00000040730	RXRG
Com_99_pos	Creatine	ENSGALG00000029033	Tldc2
Com_192_pos	1-Methylhistidine	ENSGALG00000004424	SEC16B
Com_178_pos	Maltol	MSTRG.15443	--
Com_55_pos	Valine	MSTRG.8381	--
Com_311_pos	PC (18:4e/2:0)	ENSGALG00000031525	TSTA3
Com_120_neg	LPC 15:0	ENSGALG00000015689	ECPAS
Com_171_neg	LPC 22:6	ENSGALG00000032628	SRCIN1

tion analysis.

cor	p_value	Metabolite_pathway	Gene_pathway	Gene_GO Component
1	1.51703E-05	ko01100//Metabolic p	ko00982//Drug	-
0.999999999	2.28911E-05	ko01110//Biosynthesis	ko04514//Cell a	GO:0005886//plasma m
0.999999999	2.83106E-05	ko01110//Biosynthesis	-	-
0.999999999	3.411E-05	-	-	-
0.999999998	3.66857E-05	-	-	GO:0016020//membran
0.999999998	4.05587E-05	ko01100//Metabolic p	-	GO:0016021//integral c
0.999999997	4.83123E-05	-	ko01100//Metab	GO:0005829//cytosol;G
0.999999997	5.07119E-05	ko01110//Biosynthesis	ko04931//Insulir	GO:0016021//integral c
0.999999996	5.363E-05	ko01100//Metabolic p	-	GO:0001726//ruffle;GO:
0.999999995	6.13214E-05	-	ko05132//Salmc	GO:0005764//lysosome;
0.999999994	7.11099E-05	-	ko02010//ABC t	GO:0005886//plasma m
0.999999993	7.52854E-05	-	-	-
0.99999999	8.7798E-05	-	ko01100//Metab	GO:0005576//extracellu
0.99999999	8.93358E-05	ko01100//Metabolic p	-	GO:0005576//extracellu
0.999999987	0.000102541	-	-	-
0.999999982	0.000122342	ko01100//Metabolic p	ko04022//cGMP	-
0.999999979	0.0001295	ko01120//Microbial m	-	GO:0000139//Golgi mer
0.999999978	0.000133511	ko01100//Metabolic p	ko01100//Metab	GO:0005829//cytosol;G
0.999999978	0.000134884	ko01100//Metabolic p	ko04060//Cytok	GO:0005576//extracellu
0.99999997	0.000155665	ko01100//Metabolic p	-	-
0.99999997	0.000156412	ko01100//Metabolic p	-	GO:0005634//nucleus;G
0.999999966	0.000166024	-	-	-
0.999999965	0.000169143	-	-	-
0.999999954	0.000192306	-	ko04726//Serot	GO:0005739//mitochon
0.999999954	0.000192616	-	ko04361//Axon	GO:0005737//cytoplasm
0.999999952	0.000197401	-	-	-
0.999999951	0.000200212	ko01120//Microbial m	-	-
0.999999945	0.000210278	ko01100//Metabolic p	-	-
0.999999944	0.000212539	-	-	GO:0005615//extracellu
0.999999941	0.000219551	-	ko05143//Africa	GO:0005576//extracellu
0.999999927	0.000243118	-	ko04714//Therm	-
0.999999926	0.000244181	-	-	-
0.999999915	0.000262006	-	-	-
0.999999908	0.000272593	-	-	GO:0016020//membran
0.999999908	0.000273016	-	ko04310//Wnt s	GO:0005634//nucleus;G
0.999999903	0.000280929	-	ko05200//Pathw	GO:0005576//extracellu
0.999999899	0.00028563	-	-	-
0.99999989	0.000298491	ko01100//Metabolic p	-	-
0.999999876	0.000317325	-	-	-
0.999999861	0.000335371	-	-	GO:0031090//organelle
0.999999857	0.000341042	ko01100//Metabolic p	-	GO:0005579//membran
0.999999851	0.000346954	-	-	GO:0005634//nucleus
0.999999845	0.000354305	-	ko04151//PI3K-/	GO:0005654//nucleopla
0.999999832	0.000369182	-	-	GO:0005758//mitochon
0.99999983	0.000370969	ko01100//Metabolic p	-	-
0.99999982	0.000381964	-	-	-
0.99999979	0.000412587	ko01100//Metabolic p	-	GO:0001725//stress fibe
0.999999772	0.00042968	ko01100//Metabolic p	-	-
0.999999764	0.000436939	-	ko01100//Metab	-
0.999999734	0.000464162	-	-	-
0.999999727	0.000470743	-	-	GO:0005634//nucleus;G
0.999999725	0.000472268	ko01100//Metabolic p	-	GO:0044424//intracellul
0.99999972	0.000476164	-	ko01100//Metab	GO:0005737//cytoplasm
0.999999717	0.000478651	ko01100//Metabolic p	-	GO:0005634//nucleus;G
0.999999709	0.000485709	ko01100//Metabolic p	-	GO:0005886//plasma m
0.999999698	0.000494854	-	ko05203//Viral c	GO:0000786//nucleosor

0.999999698	0.000494901	ko01100//Metabolic p	-	GO:0005737//cytoplasm
0.999999694	0.000497733	ko01100//Metabolic p	-	-
0.999999687	0.000503465	-	-	GO:0005576//extracellu
0.999999685	0.000505489	ko01100//Metabolic p	ko05010//Alzhei	GO:0005576//extracellu
0.999999684	0.000506377	ko01100//Metabolic p	ko05130//Patho	GO:0005856//cytoskelet
0.999999659	0.000525413	ko01100//Metabolic p	-	-
0.999999651	0.000532046	ko01100//Metabolic p	-	GO:0005634//nucleus;G
0.999999641	0.000539461	ko01100//Metabolic p	-	-
0.999999634	0.000545028	-	ko00970//Amino	GO:0005737//cytoplasm
0.999999626	0.000550517	ko01100//Metabolic p	ko01100//Metab	GO:0016021//integral co
0.999999615	0.000558839	-	ko04745//Photo	-
0.999999607	0.00056451	ko01100//Metabolic p	-	GO:0001726//ruffle;GO:
0.999999577	0.000585276	ko01110//Biosynthesis	ko04550//Signal	GO:0005634//nucleus;G
0.999999575	0.000586815	-	ko03010//Ribos	GO:0005840//ribosome
0.999999566	0.00059282	-	ko01100//Metab	-
0.999999547	0.000605739	-	ko04610//Comp	GO:0005615//extracellu
0.999999533	0.000615266	ko01100//Metabolic p	ko01100//Metab	GO:0000139//Golgi mer
0.99999953	0.000617447	-	ko01100//Metab	GO:0005615//extracellu
0.999999527	0.000619018	-	-	GO:0016021//integral co
0.999999512	0.0006292	-	-	GO:0005576//extracellu
0.999999492	0.000641547	ko01100//Metabolic p	ko01100//Metab	-
0.999999483	0.000647217	-	ko01100//Metab	-
0.999999463	0.000659609	ko01100//Metabolic p	-	GO:0005737//cytoplasm
0.999999454	0.000665429	-	-	-
0.999999449	0.000668387	-	-	-
0.999999435	0.000676732	-	ko04745//Photo	-
0.99999943	0.000679855	ko01100//Metabolic p	ko04060//Cytok	GO:0005576//extracellu
0.999999393	0.000701636	-	-	-
0.999999392	0.000702152	ko01100//Metabolic p	ko01100//Metab	GO:0005737//cytoplasm
0.99999937	0.000714568	ko01100//Metabolic p	-	GO:0005634//nucleus;G
0.999999364	0.00071783	ko01100//Metabolic p	ko05200//Pathw	GO:0000307//cyclin-dep
0.999999343	0.000729659	-	ko01100//Metab	-
0.999999338	0.000732528	-	-	GO:0005634//nucleus;G
0.999999333	0.00073548	ko01100//Metabolic p	-	-
0.999999316	0.000744865	-	ko04514//Cell ad	GO:0016020//membran
0.999999307	0.000749245	ko01100//Metabolic p	-	GO:0005634//nucleus;G
0.999999307	0.000749745	-	-	GO:0044424//intracellul
0.999999306	0.000749968	ko01100//Metabolic p	-	-
0.999999291	0.000757851	ko01100//Metabolic p	-	-
0.999999281	0.000763175	-	-	-
0.99999927	0.000769131	-	-	-
0.999999264	0.000772314	ko01100//Metabolic p	ko05150//Staphy	-
0.999999206	0.000802412	ko01100//Metabolic p	ko01100//Metab	-
0.999999205	0.000802559	ko01100//Metabolic p	-	-
0.999999197	0.000806975	-	-	GO:0005886//plasma m
0.999999174	0.00081845	-	-	GO:0016021//integral co
0.999999145	0.000832416	-	-	GO:0016020//membran
0.999999115	0.000847134	-	-	GO:0005737//cytoplasm
0.999999084	0.00086169	-	ko01100//Metab	GO:0005829//cytosol;GO
0.999999057	0.000874304	-	-	-
0.999999019	0.000891603	ko01100//Metabolic p	-	GO:0005886//plasma m
0.999999005	0.000897949	-	ko04060//Cytok	-
0.999998962	0.000917207	-	ko04976//Bile se	GO:0005654//nucleopla
0.999998939	0.000927239	ko01100//Metabolic p	ko04610//Comp	GO:0005615//extracellu
0.999998937	0.000928043	-	-	-
0.999998896	0.000946126	ko01100//Metabolic p	ko04514//Cell ad	GO:0005829//cytosol;GO
0.999998808	0.000982768	ko01100//Metabolic p	ko05200//Pathw	-
0.999998759	0.001003053	ko01100//Metabolic p	ko04080//Neuro	GO:0005886//plasma m

0.999998726	0.00101617	ko01100//Metabolic p	-	GO:0016020//membran
0.999998723	0.001017455	ko01100//Metabolic p	-	-
0.999998714	0.001021007	-	ko01100//Metak	-
0.999998693	0.001029398	ko01100//Metabolic p	ko04361//Axon	GO:0005737//cytoplasm
0.999998689	0.001030677	-	ko03013//Nucle	-
0.999998666	0.00103995	-	-	GO:0005886//plasma m
0.999998625	0.001055638	-	ko01100//Metak	-
0.999998547	0.001085268	ko01100//Metabolic p	-	GO:0005634//nucleus
0.99999853	0.001091531	-	ko01100//Metak	-
0.999998508	0.001099738	-	-	GO:0005739//mitochon
0.999998498	0.001103312	-	-	-
0.999998473	0.001112708	-	-	GO:0005886//plasma m
0.999998464	0.001115934	ko01100//Metabolic p	ko01100//Metak	GO:0005829//cytosol;G(
0.999998438	0.001125084	-	ko04514//Cell a(	GO:0005829//cytosol;G(
0.999998423	0.001130469	ko01100//Metabolic p	ko01100//Metak	GO:0031988//membran
0.999998267	0.001185327	ko01100//Metabolic p	-	GO:0016020//membran
0.999998261	0.00118709	ko01100//Metabolic p	ko04080//Neurc	GO:0005881//cytoplasm
0.999998226	0.001199167	-	-	-
0.999998197	0.001208993	-	-	-
0.999998178	0.001215349	ko01100//Metabolic p	-	GO:0001725//stress fibe
0.999998173	0.001217024	-	-	GO:0005576//extracellu
0.999998165	0.001219427	ko01100//Metabolic p	ko01100//Metak	-
0.999998132	0.001230381	-	ko01100//Metak	-
0.999998122	0.001233645	ko01100//Metabolic p	-	-
0.999998104	0.001239568	-	-	-
0.999998104	0.001239686	ko01100//Metabolic p	-	GO:0005886//plasma m
0.999998067	0.001251713	ko01100//Metabolic p	ko01100//Metak	GO:0005739//mitochon
0.999998044	0.001259246	-	ko04630//JAK-S	GO:0005829//cytosol
0.999998026	0.001265023	ko01100//Metabolic p	-	-
0.999997978	0.00128016	ko01100//Metabolic p	-	-
0.99999789	0.001307855	-	-	-
0.999997795	0.001337037	ko01100//Metabolic p	-	GO:0005634//nucleus;G
0.999997738	0.00135403	ko01100//Metabolic p	-	GO:0005737//cytoplasm
0.999997679	0.001371674	-	ko04151//PI3K-/	GO:0008305//integrin c
0.999997656	0.001378294	ko01100//Metabolic p	ko04514//Cell a(	GO:0005886//plasma m
0.999997651	0.001379727	-	ko01100//Metak	GO:0005615//extracellu
0.999997638	0.001383713	ko01100//Metabolic p	-	-
0.999997593	0.001396869	ko01100//Metabolic p	-	GO:0005789//endoplasr
0.999997564	0.001405099	-	-	GO:0016020//membran
0.999997527	0.001415914	-	-	GO:0005576//extracellu
0.99999749	0.001426237	-	-	GO:0005737//cytoplasm
0.999997461	0.001434552	ko01100//Metabolic p	-	GO:0005615//extracellu
0.99999743	0.001443383	-	-	GO:0005802//trans-Gol
0.999997429	0.00144351	-	-	GO:0005886//plasma m
0.999997388	0.001455122	-	ko05143//Africa	GO:0005576//extracellu
0.999997339	0.001468578	-	ko01100//Metak	GO:0005794//Golgi app
0.999997317	0.001474809	-	ko01100//Metak	GO:0005886//plasma m
0.999997276	0.001486008	ko01100//Metabolic p	-	GO:0016021//integral c
0.999997196	0.001507631	-	ko05200//Pathw	GO:0005794//Golgi app
0.999997154	0.001518921	-	-	GO:0005768//endosom
0.999997135	0.001523894	-	ko01100//Metak	GO:0005737//cytoplasm
0.999997134	0.00152413	-	-	GO:0005856//cytoskelet
0.999997073	0.001540392	ko01100//Metabolic p	ko01100//Metak	-
0.999997072	0.001540441	-	-	-
0.999997028	0.001551972	ko01100//Metabolic p	ko01100//Metak	GO:0005615//extracellu
0.999997015	0.001555431	ko01100//Metabolic p	-	GO:0001533//cornified
0.999996976	0.00156568	ko01110//Biosynthesis	-	-
0.999996949	0.001572592	ko01100//Metabolic p	-	GO:0005634//nucleus;G

0.99999691	0.001582525	-	-	GO:0005634//nucleus
0.99999684	0.001600389	ko01100//Metabolic p	-	GO:0001650//fibrillar ce
0.999996829	0.001603255	ko01100//Metabolic p	-	-
0.999996793	0.001612395	-	-	-
0.999996757	0.001621318	-	ko04151//PI3K-/	GO:0005654//nucleopla
0.999996727	0.001628706	-	-	GO:0005856//cytoskelet
0.9999967	0.001635605	ko01100//Metabolic p	ko04010//MAPK	-
0.999996655	0.001646611	ko01100//Metabolic p	ko04974//Protei	-
0.999996637	0.001651033	ko01100//Metabolic p	-	GO:0016021//integral co
0.999996565	0.001668523	-	-	GO:0005737//cytoplasm
0.999996558	0.001670266	-	-	GO:0016020//membran
0.999996527	0.001677771	-	-	GO:0016020//membran
0.999996506	0.001682888	-	ko01100//Metab	GO:0000139//Golgi mer
0.999996441	0.001698571	-	-	-
0.999996427	0.001701738	ko01100//Metabolic p	ko01100//Metab	-
0.999996396	0.001709084	-	-	GO:0005737//cytoplasm
0.999996394	0.001709632	-	-	GO:0016020//membran
0.999996364	0.001716867	ko01100//Metabolic p	ko05203//Viral c	GO:0000786//nucleosor
0.999996355	0.00171893	-	-	-
0.999996322	0.00172665	-	ko01100//Metab	GO:0005654//nucleopla
0.999996261	0.001740832	-	-	-
0.999996205	0.001753872	-	-	GO:0016020//membran
0.999996199	0.001755156	-	-	-
0.999996184	0.00175879	ko01100//Metabolic p	ko05200//Pathw	-
0.999996154	0.001765725	-	-	-
0.999996112	0.00177515	-	-	GO:0005737//cytoplasm
0.999996086	0.001781163	-	ko04151//PI3K-/	GO:0008305//integrin co
0.999996049	0.001789507	-	-	GO:0005634//nucleus;G
0.99999601	0.001798451	-	-	-
0.999995976	0.001806069	-	-	-
0.999995964	0.001808828	ko01100//Metabolic p	ko03010//Ribos	GO:0005840//ribosome
0.999995868	0.001830082	-	-	GO:0016020//membran
0.999995773	0.001851029	ko01100//Metabolic p	ko04726//Seroto	GO:0005739//mitochon
0.999995686	0.001869905	-	-	-
0.9999956	0.001888491	ko01100//Metabolic p	ko05200//Pathw	GO:0005576//extracellu
0.999995599	0.001888654	-	-	-
0.999995589	0.001890789	-	-	-
0.999995564	0.001896233	ko01100//Metabolic p	-	GO:0005737//cytoplasm
0.999995534	0.001902651	-	ko01100//Metab	-
0.999995514	0.0019068	-	ko04979//Chole	GO:0005783//endoplasr
0.999995281	0.001955685	-	ko01100//Metab	-
0.99999526	0.001960192	-	-	GO:0005783//endoplasr
0.999995106	0.001991715	-	-	GO:0005576//extracellu
0.999995	0.002013082	-	-	-
0.999994988	0.002015538	-	-	-
0.99999495	0.0020232	-	ko04970//Saliva	GO:0005576//extracellu
0.999994817	0.002049646	ko01100//Metabolic p	-	-
0.999994813	0.002050436	ko01100//Metabolic p	-	-
0.999994808	0.002051433	ko01100//Metabolic p	-	GO:0005576//extracellu
0.999994761	0.002060662	ko01100//Metabolic p	-	-
0.999994748	0.00206325	ko01110//Biosynthesis	ko05414//Dilate	-
0.999994731	0.002066539	-	ko04114//Oocyt	GO:0005634//nucleus;G
0.999994725	0.002067796	ko01100//Metabolic p	ko04080//Neuro	GO:0005783//endoplasr
0.999994695	0.002073616	ko01100//Metabolic p	-	-
0.999994662	0.002080084	-	-	GO:0005576//extracellu
0.999994647	0.002083077	-	-	GO:0005737//cytoplasm
0.999994598	0.002092543	ko01100//Metabolic p	ko01100//Metab	GO:0016020//membran
0.99999455	0.002101886	ko01110//Biosynthesis	ko00983//Drug	-

0.999994518	0.002107922	ko01100//Metabolic p	ko01100//Metak	-
0.999994518	0.002108056	-	-	GO:0005737//cytoplasm
0.999994492	0.002112886	-	-	GO:0005886//plasma m
0.999994479	0.002115432	-	ko04151//PI3K-/	GO:0005634//nucleus;G
0.999994456	0.002119872	-	ko01100//Metak	-
0.999994282	0.002152889	-	-	GO:0005634//nucleus
0.999994275	0.002154249	-	ko00982//Drug	-
0.999994179	0.002172252	-	ko01100//Metak	-
0.999994071	0.002192273	-	ko05322//Syster	GO:0005576//extracellu
0.999994068	0.002192775	-	ko04310//Wnt s	GO:0005576//extracellu
0.999994055	0.002195138	-	-	-
0.999993927	0.002218783	ko01100//Metabolic p	-	GO:0005615//extracellu
0.999993872	0.002228801	-	-	-
0.999993797	0.002242366	ko01100//Metabolic p	-	GO:0005737//cytoplasm
0.999993788	0.002243908	ko01100//Metabolic p	-	GO:0005856//cytoskelet
0.999993767	0.002247733	-	ko01100//Metak	-
0.99999357	0.002282971	-	-	GO:0001725//stress fibe
0.99999348	0.002298931	-	ko04714//Therm	-
0.999993449	0.002304304	-	ko04060//Cytok	GO:0005576//extracellu
0.999993389	0.002314846	-	ko01100//Metak	GO:0005739//mitochon
0.999993095	0.002365727	ko01100//Metabolic p	ko02010//ABC ti	GO:0005886//plasma m
0.999993078	0.002368751	ko01100//Metabolic p	ko05165//Huma	GO:0005634//nucleus;G
0.999992909	0.002397482	-	ko04010//MAPK	-
0.999992802	0.002415391	ko01063//Biosynthesis	ko04310//Wnt s	GO:0005654//nucleopla
0.999992778	0.002419534	-	-	-
0.99999277	0.002420805	-	ko00983//Drug	-
0.999992761	0.002422304	ko01100//Metabolic p	ko01100//Metak	-
0.999992707	0.002431425	ko01100//Metabolic p	-	-
0.999992635	0.002443329	-	-	-
0.999992585	0.002451665	ko01100//Metabolic p	-	GO:0005886//plasma m
0.999992558	0.002456081	-	-	-
0.999992457	0.002472721	ko01100//Metabolic p	-	GO:0016020//membran
0.999992408	0.002480727	-	ko05132//Salmc	GO:0005764//lysosome;
0.99999224	0.002507941	ko01100//Metabolic p	-	GO:0005886//plasma m
0.999992223	0.002510678	ko01100//Metabolic p	-	-
0.999992204	0.002513774	ko01100//Metabolic p	ko04514//Cell a	GO:0016020//membran
0.999992195	0.002515231	-	ko05200//Pathw	GO:0005834//heterotrin
0.999992133	0.002525161	ko01100//Metabolic p	ko04514//Cell a	GO:0005886//plasma m
0.99999211	0.002528931	ko01100//Metabolic p	ko05203//Viral c	GO:0000786//nucleosor
0.999991985	0.002548889	-	-	GO:0005886//plasma m
0.999991969	0.002551383	ko01100//Metabolic p	-	-
0.999991877	0.002566039	-	ko04144//Endoc	-
0.999991817	0.002575381	-	-	GO:0005634//nucleus;G
0.999991777	0.002581726	ko01100//Metabolic p	-	GO:0005615//extracellu
0.999991769	0.00258303	-	ko04514//Cell a	GO:0005829//cytosol;G
0.999991733	0.002588555	ko01100//Metabolic p	-	-
0.999991712	0.002591928	-	-	GO:0005634//nucleus
0.999991689	0.002595463	-	-	GO:0005576//extracellu
0.999991666	0.002599099	-	-	-
0.999991634	0.002604071	-	-	GO:0005634//nucleus;G
0.99999161	0.002607845	-	-	GO:0001726//ruffle;GO:
0.999991512	0.002623052	-	-	GO:0001533//cornified
0.999991435	0.002634919	-	-	GO:0005886//plasma m
0.999991386	0.002642442	ko01100//Metabolic p	-	-
0.999991192	0.002672014	-	ko01100//Metak	-
0.999991186	0.002672885	ko01100//Metabolic p	-	GO:0005576//extracellu
0.999990975	0.002704678	ko01100//Metabolic p	-	GO:0005576//extracellu
0.999990937	0.00271038	-	ko01100//Metak	GO:0005886//plasma m

0.999990913	0.002714014	-	ko01100//Metak	-
0.999990908	0.002714681	-	ko01100//Metak	-
0.999990878	0.002719144	-	-	-
0.999990851	0.002723191	-	ko04060//Cytok	GO:0005576//extracellu
0.999990772	0.002734905	ko01100//Metabolic p	-	GO:0005886//plasma m
0.999990679	0.002748717	ko01100//Metabolic p	-	GO:0000786//nucleosor
0.999990656	0.002752132	-	ko04630//JAK-S	GO:0005829//cytosol
0.999990518	0.002772293	-	ko05203//Viral c	GO:0000786//nucleosor
0.999990448	0.002782475	ko01100//Metabolic p	ko01100//Metak	GO:0005737//cytoplasm
0.999990423	0.002786206	-	ko01100//Metak	GO:0048269//methionir
0.99999042	0.00278664	-	ko04080//Neurc	GO:0005576//extracellu
0.999990388	0.002791329	ko01100//Metabolic p	-	-
0.999990379	0.002792558	-	-	GO:0016021//integral c
0.999990321	0.002800995	-	ko05130//Patho	GO:0005856//cytoskelet
0.999990229	0.002814213	ko01100//Metabolic p	ko01100//Metak	-
0.999990167	0.002823212	ko01100//Metabolic p	ko04146//Perox	GO:0005777//peroxison
0.999990157	0.002824654	-	-	GO:0016021//integral c
0.999990042	0.002840998	ko01100//Metabolic p	-	-
0.999990035	0.002842046	ko01100//Metabolic p	ko04020//Calciu	GO:0001518//voltage-g
0.999989863	0.002866474	-	ko01100//Metak	-
0.999989816	0.002873076	-	ko01100//Metak	GO:0048269//methionir
0.999989811	0.002873768	-	-	-
0.999989768	0.00287982	-	ko04310//Wnt s	GO:0005634//nucleus;G
0.999989714	0.002887532	-	-	-
0.999989621	0.002900499	ko01100//Metabolic p	-	GO:0005634//nucleus;G
0.99998957	0.002907665	ko01110//Biosynthesis	ko00564//Glycer	GO:0005615//extracellu
0.99998955	0.00291045	ko01120//Microbial m	ko01100//Metak	-
0.99998947	0.002921462	ko01100//Metabolic p	-	-
0.999989398	0.002931485	ko01100//Metabolic p	-	GO:0005884//actin filam
0.999989327	0.002941315	ko01100//Metabolic p	-	-
0.999989233	0.002954226	-	-	GO:0005737//cytoplasm
0.999989175	0.00296222	-	ko01100//Metak	GO:0005654//nucleopla
0.99998915	0.002965578	-	ko05200//Pathw	GO:0005794//Golgi app
0.999989096	0.002973009	ko01100//Metabolic p	-	-
0.999989094	0.002973289	-	-	-
0.999988842	0.003007328	-	-	-
0.999988785	0.003015	ko01120//Microbial m	-	-
0.999988765	0.003017692	-	ko01100//Metak	GO:0043231//intracellul
0.999988714	0.003024644	-	-	-
0.999988392	0.003067368	ko01100//Metabolic p	ko04060//Cytok	-
0.999988389	0.003067793	ko01100//Metabolic p	-	-
0.999988348	0.003073275	-	-	-
0.999988204	0.003092217	ko01100//Metabolic p	ko01100//Metak	-
0.999988154	0.003098742	-	ko01100//Metak	GO:0005654//nucleopla
0.999988126	0.003102347	ko01100//Metabolic p	-	GO:0005813//centrosor
0.999988004	0.003118246	-	-	GO:0005737//cytoplasm
0.999987963	0.003123637	ko01110//Biosynthesis	-	GO:0005886//plasma m
0.999987961	0.003123911	-	-	GO:0005739//mitochon
0.999987934	0.00312739	-	ko01100//Metak	GO:0005739//mitochon
0.999987766	0.003149038	-	ko01100//Metak	-
0.999987742	0.003152187	-	ko05200//Pathw	-
0.999987722	0.003154669	-	ko01100//Metak	GO:0005615//extracellu
0.999987558	0.003175738	ko01100//Metabolic p	-	GO:0005737//cytoplasm
0.999987557	0.003175872	-	ko01100//Metak	-
0.999987465	0.003187493	ko01100//Metabolic p	ko01100//Metak	-
0.999987245	0.003215413	-	ko01100//Metak	-
0.99998724	0.003215998	-	ko04080//Neurc	GO:0005783//endoplasr
0.999987222	0.00321834	-	-	-

0.999987143	0.003228296	-	-	-
0.99998706	0.003238643	-	ko04934//Cushi	-
0.999987017	0.003244034	ko01120//Microbial m	-	-
0.99998691	0.003257305	-	ko03018//RNA c	GO:0005829//cytosol
0.999986909	0.003257512	ko01100//Metabolic p	-	GO:0005789//endoplasr
0.999986868	0.003262567	ko01100//Metabolic p	-	-
0.999986746	0.00327774	ko01100//Metabolic p	-	GO:0005884//actin filam
0.999986575	0.003298837	-	ko01100//Metak	GO:0005737//cytoplasm
0.999986566	0.003299888	-	ko01100//Metak	GO:0031988//membran
0.999986446	0.003314556	-	ko01100//Metak	GO:0016020//membran
0.999986294	0.003333128	-	-	GO:0016021//integral co
0.999986207	0.003343627	-	-	GO:0005737//cytoplasm
0.999986068	0.003360498	-	-	GO:0016021//integral co
0.999986059	0.003361517	ko01100//Metabolic p	ko03320//PPAR	-
0.999986038	0.003364149	-	-	-
0.999986033	0.003364747	-	ko04979//Chole	GO:0005783//endoplasr
0.999985854	0.00338614	ko01120//Microbial m	-	-
0.99998574	0.003399801	ko01100//Metabolic p	ko01100//Metak	-
0.999985707	0.003403738	-	ko01100//Metak	GO:0005829//cytosol;G(
0.999985633	0.003412599	ko01100//Metabolic p	ko04979//Chole	GO:0005856//cytoskelet
0.999985582	0.003418653	ko01100//Metabolic p	ko05414//Dilate	-
0.999985335	0.003447816	ko01100//Metabolic p	ko00790//Folate	-
0.999985254	0.003457289	ko01100//Metabolic p	ko00983//Drug	-
0.999985229	0.003460244	-	-	-
0.999985184	0.003465425	-	-	-
0.99998518	0.003465921	-	-	GO:0005856//cytoskelet
0.999984994	0.003487566	-	-	GO:0005654//nucleopla
0.999984989	0.003488207	-	-	GO:0031090//organelle
0.999984837	0.003505836	-	-	-
0.999984684	0.00352345	-	-	GO:0005737//cytoplasm
0.999984472	0.003547804	-	-	-
0.99998439	0.003557145	ko01100//Metabolic p	ko01100//Metak	GO:0005654//nucleopla
0.99998417	0.003582056	-	ko01100//Metak	-
0.99998406	0.003594462	ko01100//Metabolic p	-	-
0.999983906	0.003611856	-	ko01100//Metak	-
0.999983731	0.003631461	ko01100//Metabolic p	-	-
0.999983543	0.003652356	ko01100//Metabolic p	ko04390//Hippo	GO:0005667//transcript
0.999983495	0.00365761	-	-	GO:0005794//Golgi app
0.999983321	0.003676848	ko01063//Biosynthesis	-	GO:0005886//plasma m
0.999983222	0.003687777	ko01100//Metabolic p	ko04151//PI3K-/	GO:0009986//cell surfac
0.99998304	0.003707767	ko01100//Metabolic p	-	-
0.999982988	0.003713434	ko01100//Metabolic p	-	GO:0005802//trans-Gol
0.999982917	0.003721106	ko01100//Metabolic p	-	GO:0016020//membran
0.999982732	0.003741215	-	-	-
0.999982656	0.003749438	-	-	-
0.999982575	0.003758191	-	-	GO:0043231//intracellul
0.999982482	0.003768237	ko01100//Metabolic p	ko05165//Huma	GO:0005634//nucleus;G
0.999982394	0.003777706	ko01110//Biosynthesis	ko01100//Metak	-
0.999982358	0.003781556	ko01100//Metabolic p	ko04080//Neurc	GO:0005576//extracellu
0.999982356	0.003781735	ko01100//Metabolic p	ko01100//Metak	GO:0005788//endoplasr
0.999982331	0.00378446	-	-	GO:0005886//plasma m
0.999982289	0.003788972	ko01100//Metabolic p	-	GO:0005768//endosom
0.999982225	0.003795785	-	-	-
0.999982165	0.003802125	-	ko05200//Pathw	-
0.999982161	0.003802565	-	-	-
0.999982046	0.003814877	ko01100//Metabolic p	-	GO:0005737//cytoplasm
0.999981997	0.003820011	ko01100//Metabolic p	-	-
0.999981939	0.003826176	-	-	-

0.999981671	0.003854472	-	-	-
0.99998156	0.003866081	ko01110//Biosynthesis	-	-
0.999981512	0.003871157	-	ko01100//Metak	-
0.999981501	0.003872265	-	-	-
0.999981452	0.003877442	ko01100//Metabolic p	-	-
0.999981304	0.003892898	ko01110//Biosynthesis	ko04022//cGMP	-
0.999981303	0.003892988	ko01100//Metabolic p	-	-
0.999980948	0.003929764	-	-	GO:0005737//cytoplasm
0.999980779	0.003947194	-	ko01100//Metak	-
0.999980662	0.003959172	ko01100//Metabolic p	ko00533//Glycos	GO:0000139//Golgi mer
0.999980635	0.003961927	ko01100//Metabolic p	-	-
0.999980609	0.003964527	ko01100//Metabolic p	ko04931//Insulir	GO:0016021//integral c
0.999980445	0.003981294	-	ko04142//Lysos	GO:0016020//membran
0.999980443	0.00398151	-	-	GO:0016020//membran
0.999980315	0.003994488	ko01100//Metabolic p	-	GO:0005737//cytoplasm
0.999980308	0.003995177	ko01120//Microbial m	ko01100//Metak	GO:0005739//mitochon
0.999979939	0.004032473	ko01100//Metabolic p	ko04010//MAPK	-
0.999979855	0.004040895	ko01100//Metabolic p	ko04630//JAK-S	GO:0005829//cytosol
0.999979771	0.004049351	-	ko01100//Metak	GO:0005783//endoplasr
0.999979365	0.004089788	-	ko01100//Metak	GO:0005739//mitochon
0.999979264	0.004099775	ko01120//Microbial m	-	-
0.99997926	0.004100134	ko01100//Metabolic p	-	GO:0016020//membran
0.999979156	0.00411043	-	-	GO:0005634//nucleus;G
0.99997905	0.004120856	-	-	-
0.999978991	0.004126625	-	-	GO:0005833//hemoglok
0.999978836	0.00414188	ko01100//Metabolic p	-	GO:0005783//endoplasr
0.999978719	0.004153326	-	ko01100//Metak	GO:0031988//membran
0.99997868	0.004157137	-	ko04640//Hema	GO:0031226//intrinsic c
0.999978188	0.004204765	-	ko04514//Cell a	GO:0005829//cytosol;G
0.999978122	0.004211121	ko01100//Metabolic p	ko04020//Calciu	GO:0005886//plasma m
0.999977961	0.004226605	-	ko03013//Nucle	-
0.99997785	0.004237215	-	ko04080//Neurc	GO:0005783//endoplasr
0.999977798	0.004242214	ko01100//Metabolic p	-	-
0.999977579	0.004263068	ko01100//Metabolic p	ko00564//Glycer	GO:0005615//extracellu
0.999977429	0.004277342	-	ko01100//Metak	-
0.999977359	0.004283971	-	-	GO:0016020//membran
0.999977313	0.004288319	ko01100//Metabolic p	-	-
0.999977255	0.004293764	-	ko04979//Chole	GO:0005856//cytoskelet
0.999977178	0.004301026	-	ko05200//Pathw	-
0.999977082	0.004310107	ko01100//Metabolic p	-	GO:0005615//extracellu
0.999976851	0.004331715	-	-	-
0.999976783	0.004338115	-	-	GO:0042734//presynapt
0.999976565	0.004358377	ko01100//Metabolic p	-	GO:0005739//mitochon
0.999976516	0.00436296	-	ko01100//Metak	GO:0005739//mitochon
0.999976124	0.004399218	-	-	GO:0016021//integral c
0.999975991	0.004411443	-	ko01100//Metak	-
0.999975801	0.0044289	-	ko01100//Metak	-
0.999975676	0.004440276	-	-	-
0.999975556	0.004451289	-	-	GO:0005922//connexon
0.999975553	0.004451569	-	ko04080//Neurc	GO:0005783//endoplasr
0.999975527	0.004453911	-	-	-
0.999975317	0.004472964	-	-	GO:0014069//postsynap
0.999975238	0.004480121	ko01063//Biosynthesis	-	GO:0005737//cytoplasm
0.999975133	0.004489623	-	ko01100//Metak	-
0.9999751	0.004492562	-	-	-
0.99997504	0.004497951	-	-	GO:0005737//cytoplasm
0.999974763	0.004522874	-	-	GO:0005886//plasma m
0.999974719	0.004526833	ko01100//Metabolic p	-	-

0.999974595	0.004537948	ko01100//Metabolic p	-	-
0.999974457	0.004550249	ko01100//Metabolic p	-	-
0.999974317	0.004562677	-	-	-
0.999974269	0.004566931	-	ko01100//Metab	GO:0005886//plasma m
0.999974165	0.004576115	-	-	-
0.999974132	0.004579073	-	-	GO:0005737//cytoplasm
0.999974065	0.004584984	-	ko04514//Cell ac	GO:0016020//membran
0.999973922	0.004597597	-	ko01100//Metab	-
0.999973889	0.004600516	ko01100//Metabolic p	ko04550//Signal	GO:0005634//nucleus;G
0.99997346	0.004638157	ko01100//Metabolic p	ko04080//Neuro	GO:0016020//membran
0.999973371	0.004645924	-	ko04970//Saliva	GO:0005576//extracellu
0.999973283	0.004653587	-	-	-
0.999973226	0.004658594	-	ko01100//Metab	-
0.999973171	0.004663368	-	ko05165//Huma	GO:0005634//nucleus;G
0.999973139	0.004666103	ko01100//Metabolic p	-	GO:0005794//Golgi app
0.999972591	0.00471352	ko01100//Metabolic p	-	-
0.999972424	0.004727849	ko01100//Metabolic p	-	-
0.999972186	0.004748164	ko01100//Metabolic p	-	GO:0005615//extracellu
0.999972183	0.004748454	ko01100//Metabolic p	ko04640//Hema	GO:0031226//intrinsic c
0.999972056	0.004759277	-	-	-
0.999972032	0.004761333	-	ko04060//Cytok	-
0.99997193	0.004769951	ko01100//Metabolic p	-	-
0.999971751	0.004785183	ko01063//Biosynthesis	ko04080//Neuro	GO:0005576//extracellu
0.999971593	0.004798502	ko01063//Biosynthesis	ko05165//Huma	GO:0005634//nucleus;G
0.999971367	0.004817627	-	ko01100//Metab	-
0.999971363	0.004817905	-	-	GO:0005794//Golgi app
0.999971301	0.004823102	-	-	GO:0016020//membran
0.999970936	0.004853685	-	ko01100//Metab	GO:0005737//cytoplasm
0.999970931	0.004854114	-	-	-
0.99997089	0.004857549	-	ko01100//Metab	-
0.999970535	0.004887056	-	-	GO:0005802//trans-Gol
0.999970501	0.004889891	ko01100//Metabolic p	-	GO:0005886//plasma m
0.999970474	0.004892149	-	ko01100//Metab	-
0.999970327	0.004904325	-	-	GO:0005886//plasma m
0.999970318	0.004905042	-	-	-
0.999970212	0.004913804	ko01100//Metabolic p	-	GO:0016021//integral c
0.99997018	0.004916411	ko01100//Metabolic p	ko01100//Metab	GO:0048269//methionir
0.999969703	0.004955625	ko01100//Metabolic p	ko01100//Metab	GO:0048269//methionir
0.999969661	0.004959071	-	-	GO:0005634//nucleus;G
0.999969617	0.004962593	-	-	GO:0005768//endosome
0.999969406	0.004979821	-	ko01100//Metab	GO:0005829//cytosol
0.999969398	0.004980494	-	ko04974//Protei	GO:0005924//cell-subst
0.999969288	0.00498939	ko01110//Biosynthesis	-	-
0.999969277	0.004990356	-	ko01100//Metab	-
0.999969111	0.005003748	-	ko01100//Metab	GO:0005788//endoplasr
0.99996907	0.005007088	ko01100//Metabolic p	ko01100//Metab	-
0.999969062	0.005007758	ko01110//Biosynthesis	ko04390//Hippo	GO:0005667//transcript
0.999969003	0.005012525	-	-	-
0.999968759	0.005032247	-	ko03018//RNA c	GO:0005829//cytosol
0.999968725	0.005034924	ko01100//Metabolic p	ko01100//Metab	GO:0005737//cytoplasm
0.999968702	0.005036787	ko01100//Metabolic p	ko05200//Pathw	GO:0005794//Golgi app
0.99996861	0.005044219	-	-	-
0.999968527	0.005050878	ko01100//Metabolic p	-	-
0.999968391	0.005061747	-	-	-
0.999967935	0.005098117	-	-	-
0.99996781	0.00510807	-	-	GO:0016021//integral c
0.999967631	0.005122257	-	ko04144//Endoc	-
0.999967431	0.005138031	-	-	-

0.999967293	0.00514891	-	ko04060//Cytok	GO:0005576//extracellu
0.999967205	0.005155817	-	-	GO:0005576//extracellu
0.999967084	0.005165335	-	-	GO:0005737//cytoplasm
0.999966755	0.005191137	-	-	GO:0005886//plasma m
0.999966584	0.005204453	ko01100//Metabolic p	-	-
0.999966436	0.005215944	-	-	-
0.99996635	0.005222664	-	-	-
0.999966135	0.005239293	-	-	GO:0016020//membran
0.999966109	0.005241279	-	ko01100//Metab	GO:0016020//membran
0.999965983	0.005251045	-	ko04714//Therm	GO:0005739//mitochon
0.999965719	0.005271364	ko01100//Metabolic p	ko01100//Metab	GO:0005783//endoplası
0.999965714	0.005271721	ko01100//Metabolic p	-	-
0.999965688	0.005273755	-	-	GO:0016020//membran
0.999965642	0.005277318	-	-	-
0.999965623	0.005278742	-	ko04020//Calciu	GO:0005886//plasma m
0.999965543	0.005284885	ko01120//Microbial m	-	GO:0005634//nucleus;G
0.999965534	0.005285558	ko01100//Metabolic p	ko00983//Drug	-
0.999965359	0.005298965	-	ko00982//Drug	-
0.999965196	0.005311402	ko01100//Metabolic p	-	-
0.99996502	0.005324847	ko01100//Metabolic p	ko04918//Thyro	GO:0005576//extracellu
0.99996492	0.005332444	ko01100//Metabolic p	-	GO:0005634//nucleus;G
0.999964753	0.005345158	-	-	GO:0005737//cytoplasm
0.999964525	0.00536241	-	-	-
0.999964383	0.00537313	-	-	GO:0005576//extracellu
0.999964173	0.005388896	-	ko01100//Metab	GO:0031988//membran
0.999964062	0.005397276	-	-	-
0.999963947	0.005405915	ko01100//Metabolic p	ko04390//Hippo	GO:0005667//transcript
0.99996377	0.005419144	-	ko04742//Taste	GO:0005737//cytoplasm
0.999963655	0.005427734	ko01100//Metabolic p	ko05200//Pathw	GO:0005829//cytosol;G
0.999963545	0.005435956	ko01100//Metabolic p	ko04979//Chole	GO:0005783//endoplası
0.999963276	0.005455934	-	ko04979//Chole	GO:0005856//cytoskelet
0.999963238	0.005458816	-	ko01100//Metab	-
0.999963087	0.005470007	-	ko04919//Thyro	GO:0005887//integral c
0.999962901	0.005483721	-	ko01100//Metab	GO:0016020//membran
0.999962594	0.005506372	ko01100//Metabolic p	ko01100//Metab	-
0.99996258	0.005507459	-	ko05017//Spino	GO:0043231//intracellul
0.999962559	0.005508977	-	ko01100//Metab	-
0.999962472	0.005515338	-	-	-
0.999962182	0.005536597	-	ko05200//Pathw	-
0.999962111	0.005541826	ko01100//Metabolic p	ko01100//Metab	GO:0005737//cytoplasm
0.99996162	0.005577625	ko01100//Metabolic p	ko04918//Thyro	GO:0005576//extracellu
0.999961604	0.005578803	-	-	-
0.999961542	0.005583259	-	-	-
0.999961083	0.005616503	ko01100//Metabolic p	-	GO:0005634//nucleus
0.999961064	0.00561787	-	-	-
0.999960705	0.005643722	ko01110//Biosynthesis	-	-
0.999960551	0.005654763	-	-	-
0.999960532	0.005656122	ko01100//Metabolic p	ko04010//MAPK	-
0.999960474	0.005660252	-	-	GO:0005576//extracellu
0.999960401	0.005665509	ko01110//Biosynthesis	-	GO:0005615//extracellu
0.999960391	0.00566624	ko01063//Biosynthesis	-	GO:0005634//nucleus;G
0.999960312	0.005671888	-	ko04979//Chole	GO:0005783//endoplası
0.999960228	0.005677883	-	-	-
0.999960143	0.005683958	-	ko01100//Metab	GO:0005737//cytoplasm
0.999959996	0.00569437	ko01100//Metabolic p	-	GO:0016581//NuRD cor
0.999959543	0.005726564	-	ko01100//Metab	GO:0005654//nucleopla
0.999959439	0.005733906	ko01100//Metabolic p	-	GO:0005739//mitochon
0.999959265	0.00574617	ko01100//Metabolic p	-	GO:0005783//endoplası

0.999959103	0.005757617	ko01100//Metabolic p	-	GO:0005654//nucleopla
0.999958909	0.005771231	ko01100//Metabolic p	-	-
0.999958865	0.005774305	-	ko04068//FoxO	GO:0005737//cytoplasm
0.999958769	0.005781041	ko01100//Metabolic p	-	GO:0016020//membran
0.999958525	0.005798128	ko01100//Metabolic p	-	-
0.999958445	0.005803712	ko01100//Metabolic p	ko01100//Metab	-
0.999958245	0.005817708	ko01100//Metabolic p	-	-
0.999957757	0.005851588	-	ko04726//Serot	GO:0005739//mitochon
0.999957231	0.005887898	ko01100//Metabolic p	ko01100//Metab	GO:0043231//intracellul
0.999957085	0.005897952	ko01100//Metabolic p	-	-
0.999956608	0.005930654	ko01100//Metabolic p	ko01100//Metab	-
0.999956468	0.00594021	-	ko05200//Pathw	GO:0005576//extracellu
0.99995636	0.005947573	-	ko05200//Pathw	-
0.999956187	0.005959331	ko01100//Metabolic p	-	GO:0005654//nucleopla
0.99995617	0.005960526	ko01100//Metabolic p	ko04745//Photo	-
0.999955871	0.005980821	-	-	GO:0005886//plasma m
0.999955809	0.005985012	ko01063//Biosynthesis	-	GO:0005615//extracellu
0.999955806	0.005985227	-	-	GO:0005576//extracellu
0.999955452	0.00600912	ko01100//Metabolic p	-	GO:0005579//membran
0.999955298	0.006019489	ko01100//Metabolic p	-	-
0.999955214	0.006025151	ko01100//Metabolic p	-	GO:0005739//mitochon
0.999954949	0.00604293	-	ko04514//Cell a	GO:0016020//membran
0.999954628	0.006064446	ko01100//Metabolic p	ko05414//Dilate	GO:0016459//myosin co
0.999954529	0.006071057	-	ko05200//Pathw	GO:0005794//Golgi app
0.999954319	0.006085045	ko01100//Metabolic p	ko04151//PI3K-/	GO:0005654//nucleopla
0.999954249	0.006089685	-	-	-
0.999954242	0.006090171	ko01100//Metabolic p	-	-
0.999954056	0.006102546	-	ko01100//Metab	GO:0005737//cytoplasm
0.999953951	0.006109494	-	-	GO:0005737//cytoplasm
0.999953641	0.006130025	-	-	GO:0005634//nucleus;G
0.999953437	0.006143544	-	ko00564//Glyce	GO:0005634//nucleus;G
0.999953434	0.006143686	-	ko04640//Hema	GO:0031226//intrinsic c
0.999953325	0.00615093	ko01100//Metabolic p	-	GO:0016020//membran
0.99995324	0.006156509	-	-	-
0.99995298	0.006173589	-	-	GO:0016020//membran
0.999952836	0.00618305	-	-	GO:0005634//nucleus;G
0.999952617	0.006197405	-	-	GO:0005794//Golgi app
0.999952344	0.006215218	-	-	-
0.999952304	0.006217833	-	-	-
0.999951396	0.006276729	-	ko01100//Metab	GO:0005615//extracellu
0.999951076	0.006297331	ko01100//Metabolic p	-	GO:0016020//membran
0.999950854	0.006311632	-	ko01100//Metab	GO:0005737//cytoplasm
0.99995055	0.00633109	-	-	GO:0000127//transcript
0.999950382	0.006341873	-	ko01100//Metab	GO:0005886//plasma m
0.999950366	0.006342888	-	ko02010//ABC t	GO:0005739//mitochon
0.999950336	0.006344792	ko01100//Metabolic p	-	-
0.999950287	0.006347956	ko01063//Biosynthesis	-	GO:0005615//extracellu
0.999950241	0.006350857	-	ko01100//Metab	-
0.999950097	0.006360045	ko01100//Metabolic p	-	-
0.999949858	0.006375284	ko01100//Metabolic p	-	GO:0005759//mitochon
0.999949766	0.006381132	-	-	-
0.999949713	0.006384481	-	ko04060//Cytok	GO:0005576//extracellu
0.999949431	0.006402333	ko01100//Metabolic p	ko05132//Salmo	-
0.999949431	0.006402362	ko01100//Metabolic p	-	-
0.999949131	0.006421289	-	-	GO:0005884//actin filam
0.999949113	0.006422457	-	-	-
0.999949017	0.006428483	-	-	-
0.999949	0.006429587	-	-	GO:0005739//mitochon

0.999948641	0.006452158	ko01120//Microbial m	-	GO:0005788//endoplasr
0.999948464	0.006463292	ko01100//Metabolic p	ko01100//Metab	GO:0016020//membran
0.999948349	0.006470471	-	-	-
0.999948321	0.006472236	ko01100//Metabolic p	ko01100//Metab	-
0.999948279	0.006474863	-	-	GO:0016020//membran
0.999947998	0.006492428	ko01100//Metabolic p	ko01100//Metab	GO:0005737//cytoplasm
0.999947849	0.006501699	-	ko01100//Metab	GO:0005737//cytoplasm
0.999947842	0.006502165	-	-	-
0.999947718	0.006509876	-	-	GO:0016020//membran
0.999947596	0.006517489	ko01100//Metabolic p	ko04144//Endoc	-
0.999947394	0.006530027	ko01063//Biosynthesis	-	GO:0005634//nucleus;G
0.999947321	0.006534535	-	ko00982//Drug	-
0.999947267	0.00653793	ko01100//Metabolic p	ko04979//Chole	GO:0005737//cytoplasm
0.999947111	0.006547592	ko01100//Metabolic p	-	-
0.999946934	0.006558519	-	-	GO:0005802//trans-Gol
0.999946293	0.006598015	-	ko02010//ABC t	GO:0005886//plasma m
0.999946119	0.006608701	-	-	GO:0005576//extracellu
0.999946047	0.00661308	-	-	-
0.999946032	0.006614017	-	-	-
0.999945728	0.00663264	-	-	-
0.999945673	0.006635967	ko01100//Metabolic p	ko01100//Metab	-
0.999945588	0.006641157	ko01100//Metabolic p	ko04742//Taste	GO:0005737//cytoplasm
0.999945136	0.006668674	-	-	GO:0005833//hemoglob
0.999945094	0.006671275	-	-	-
0.999944478	0.006708545	-	-	GO:0000151//ubiquitin
0.999944464	0.006709389	-	ko04724//Glutar	GO:0005886//plasma m
0.999944408	0.006712815	ko01100//Metabolic p	ko04310//Wnt s	GO:0005576//extracellu
0.999944399	0.006713317	ko01100//Metabolic p	ko05322//Syster	GO:0005576//extracellu
0.999943974	0.006738916	ko01100//Metabolic p	ko05143//Africa	GO:0005576//extracellu
0.999943965	0.006739509	ko01100//Metabolic p	ko04979//Chole	GO:0005783//endoplasr
0.999943406	0.006773043	-	-	-
0.999943132	0.006789419	ko01120//Microbial m	-	GO:0016020//membran
0.999942876	0.006804668	ko01100//Metabolic p	ko04060//Cytok	-
0.999942415	0.006832059	ko01100//Metabolic p	-	-
0.999942353	0.006835767	-	ko01100//Metab	GO:0005615//extracellu
0.999941836	0.006866319	ko01100//Metabolic p	-	GO:0005634//nucleus
0.999941477	0.006887505	ko01100//Metabolic p	ko01100//Metab	-
0.999941429	0.006890334	-	-	-
0.999941322	0.006896611	-	-	-
0.999941185	0.006904651	-	-	-
0.999941037	0.006913306	-	-	-
0.999941035	0.006913458	-	-	GO:0000307//cyclin-dep
0.999940978	0.006916763	-	ko01100//Metab	-
0.999940854	0.006924035	-	-	-
0.999940731	0.006931238	-	-	GO:0005654//nucleopla
0.999940572	0.006940542	ko01100//Metabolic p	ko04970//Saliva	GO:0005576//extracellu
0.999940441	0.006948204	ko01100//Metabolic p	-	-
0.999940287	0.00695714	-	-	GO:0016021//integral co
0.999940116	0.006967137	-	-	-
0.999940063	0.006970201	-	ko04724//Glutar	GO:0005886//plasma m
0.999940062	0.006970241	ko01100//Metabolic p	-	GO:0005634//nucleus;G
0.999939885	0.00698055	-	ko03320//PPAR	GO:0005634//nucleus;G
0.999939784	0.006986374	-	-	-
0.999939765	0.006987513	-	-	-
0.999939755	0.006988071	-	ko04742//Taste	GO:0001518//voltage-g
0.999939661	0.006993508	ko01100//Metabolic p	-	-
0.999939497	0.007003041	ko01100//Metabolic p	-	GO:0005654//nucleopla
0.999939497	0.007003054	-	-	GO:0016020//membran

0.999939091	0.007026507	-	ko04060//Cytok	GO:0005576//extracellu
0.999938704	0.00704878	-	-	-
0.9999384	0.007066257	-	-	GO:0016020//membran
0.999938339	0.007069738	-	ko01100//Metak	GO:0005789//endoplasn
0.999938027	0.007087595	ko01100//Metabolic p	-	GO:0005737//cytoplasm
0.999937997	0.007089308	ko01100//Metabolic p	-	-
0.999937799	0.007100624	ko01100//Metabolic p	ko04610//Comp	GO:0005615//extracellu
0.999937752	0.007103282	ko01110//Biosynthesis	ko04150//mTOF	GO:0005829//cytosol;G(
0.99993713	0.007138679	ko01100//Metabolic p	ko05200//Pathw	GO:0005794//Golgi app
0.999937074	0.007141853	-	-	GO:0001726//ruffle;GO:
0.999936863	0.007153815	-	ko01100//Metak	-
0.999936537	0.007172285	-	ko05200//Pathw	GO:0005576//extracellu
0.999936501	0.007174317	-	-	GO:0005634//nucleus;G
0.999936168	0.007193111	-	-	-
0.999936061	0.007199152	ko01100//Metabolic p	-	GO:0005576//extracellu
0.999936031	0.007200825	-	-	-
0.9999359	0.007208174	-	ko04146//Perox	GO:0005765//lysosomal
0.999935391	0.007236784	ko01120//Microbial m	ko05012//Parkin	GO:0005654//nucleopla
0.999935245	0.007244949	-	ko04919//Thyro	GO:0005887//integral co
0.999935205	0.007247164	-	-	GO:0005739//mitochon
0.99993456	0.007283131	-	ko01100//Metak	GO:0043231//intracellul
0.999933884	0.007320653	ko01100//Metabolic p	-	-
0.999933797	0.007325502	-	ko04020//Calciu	GO:0005886//plasma m
0.999933735	0.007328893	-	ko00983//Drug	-
0.999933481	0.007342931	-	-	-
0.999933082	0.007364952	-	-	GO:0016020//membran
0.999932818	0.007379441	-	ko03013//Nucle	-
0.999932785	0.007381287	-	-	-
0.999932721	0.00738477	-	-	-
0.999932413	0.007401653	-	-	GO:0005634//nucleus;G
0.999932212	0.007412664	-	-	-
0.999931998	0.007424328	ko01100//Metabolic p	-	-
0.999931799	0.007435194	-	-	GO:0005576//extracellu
0.9999317	0.007440611	ko01100//Metabolic p	-	-
0.999931385	0.007457732	-	-	-
0.999931289	0.007462941	ko01110//Biosynthesis	ko05200//Pathw	GO:0005576//extracellu
0.999930904	0.007483858	ko01110//Biosynthesis	-	-
0.999930042	0.007530376	-	ko01100//Metak	GO:0005829//cytosol;G(
0.999929162	0.007577582	-	ko01100//Metak	-
0.999929049	0.007583647	ko01120//Microbial m	-	GO:0043235//receptor c
0.999928838	0.00759491	ko01100//Metabolic p	-	GO:0005884//actin filam
0.999928665	0.007604125	-	-	GO:0005922//connexon
0.99992846	0.007615026	ko01100//Metabolic p	-	-
0.999928377	0.007619464	-	ko04918//Thyro	GO:0005576//extracellu
0.99992815	0.007631535	-	-	GO:0016020//membran
0.999927976	0.007640751	ko01100//Metabolic p	ko05414//Dilate	GO:0016459//myosin co
0.999927672	0.007656879	-	-	-
0.999927501	0.007665904	-	-	GO:0005886//plasma m
0.999926788	0.007703504	-	-	GO:0005576//extracellu
0.99992678	0.007703932	-	-	GO:0005737//cytoplasm
0.999926656	0.007710447	ko01100//Metabolic p	-	-
0.999926565	0.00771522	-	-	-
0.999926335	0.007727331	-	ko04010//MAPK	-
0.999926145	0.00773727	-	ko01100//Metak	GO:0005737//cytoplasm
0.999926105	0.007739365	-	-	GO:0005576//extracellu
0.999926067	0.007741367	-	-	-
0.999926019	0.007743891	ko01100//Metabolic p	ko01100//Metak	GO:0005783//endoplasn
0.999925946	0.007747703	-	ko04971//Gastri	GO:0005886//plasma m

0.999925481	0.007771966	-	-	GO:0005634//nucleus;G
0.999925144	0.007789505	-	-	GO:0005783//endoplasr
0.999924682	0.007813531	-	ko00982//Drug	-
0.999924532	0.007821317	ko01100//Metabolic p	-	-
0.999924485	0.007823716	ko01100//Metabolic p	-	-
0.999924404	0.007827955	ko01100//Metabolic p	-	-
0.999924274	0.007834679	ko01100//Metabolic p	-	-
0.999924264	0.007835177	-	-	-
0.999924188	0.007839136	-	-	GO:0005794//Golgi app
0.999924034	0.007847047	-	ko01100//Metak	-
0.999923778	0.007860296	ko01100//Metabolic p	-	-
0.999923763	0.007861044	-	-	-
0.999923249	0.007887533	-	-	GO:0005739//mitochon
0.999922409	0.007930558	ko01100//Metabolic p	-	GO:0000151//ubiquitin
0.999922242	0.007939079	ko01100//Metabolic p	-	GO:0005783//endoplasr
0.999921897	0.007956679	-	-	GO:0005576//extracellu
0.999921761	0.007963585	-	ko04060//Cytok	GO:0005576//extracellu
0.999921667	0.007968362	-	ko04144//Endoc	-
0.999921369	0.007983546	ko01100//Metabolic p	ko05200//Pathw	GO:0005576//extracellu
0.99992133	0.007985495	ko01100//Metabolic p	-	-
0.999921273	0.007988383	ko01100//Metabolic p	ko04976//Bile se	GO:0005654//nucleopla
0.999921159	0.007994174	ko01100//Metabolic p	ko01100//Metak	GO:0005886//plasma m
0.999921007	0.008001863	-	ko01100//Metak	-
0.999920735	0.008015627	-	ko01100//Metak	-
0.999920629	0.008021007	ko01100//Metabolic p	ko04726//Serotc	GO:0005739//mitochon
0.999920462	0.008029427	ko01100//Metabolic p	-	-
0.999920172	0.008044066	-	ko05016//Huntir	GO:0005929//cilium;GO
0.999919906	0.008057474	-	-	-
0.99991983	0.008061273	-	-	GO:0005654//nucleopla
0.999919782	0.008063677	-	ko05017//Spino	GO:0043231//intracellul
0.99991963	0.008071338	-	ko01100//Metak	GO:0005615//extracellu
0.999919372	0.00808427	ko01100//Metabolic p	ko01100//Metak	-
0.999918897	0.008108048	-	ko04310//Wnt s	GO:0005576//extracellu
0.999918887	0.00810855	-	ko05322//Syster	GO:0005576//extracellu
0.999918701	0.008117831	-	ko02010//ABC tı	GO:0005739//mitochon
0.999918566	0.008124594	-	-	-
0.99991849	0.00812838	-	ko04151//PI3K-/	GO:0005654//nucleopla
0.999918458	0.008129945	-	ko04745//Photo	-
0.999918417	0.008132008	ko01100//Metabolic p	ko01100//Metak	-
0.999918221	0.008141753	ko01100//Metabolic p	-	GO:0005791//rough enc
0.999918206	0.008142518	ko01100//Metabolic p	ko05200//Pathw	GO:0005834//heterotrin
0.999917614	0.008171945	ko01100//Metabolic p	-	GO:0016020//membran
0.99991761	0.008172131	-	ko05017//Spino	GO:0043231//intracellul
0.999916853	0.008209583	-	ko04919//Thyro	GO:0005887//integral c
0.999916829	0.008210752	ko01063//Biosynthesis	ko03320//PPAR	GO:0005576//extracellu
0.999916603	0.008221945	-	-	-
0.999916344	0.008234663	-	-	GO:0005634//nucleus;G
0.999916197	0.008241909	-	-	GO:0097541//axonemal
0.999916016	0.00825079	ko01100//Metabolic p	-	-
0.999915897	0.008256633	ko01100//Metabolic p	-	-
0.999915862	0.008258381	-	-	-
0.999915787	0.008262061	-	-	GO:0001726//ruffle;GO:
0.999915683	0.008267127	-	-	GO:0001726//ruffle;GO:
0.999915105	0.008295427	-	ko05200//Pathw	GO:0005794//Golgi app
0.999915023	0.008299444	ko01100//Metabolic p	-	GO:0005634//nucleus;G
0.999914792	0.0083107	-	-	-
0.999914637	0.008318283	ko01100//Metabolic p	-	-
0.9999146	0.008320064	ko01100//Metabolic p	ko01100//Metak	GO:0005615//extracellu

0.999914269	0.008336192	-	-	-
0.999914029	0.008347819	-	-	-
0.999913961	0.008351163	-	ko04060//Cytok	-
0.999913646	0.008366404	-	-	GO:0005764//lysosome;
0.999913597	0.00836878	-	-	-
0.999913462	0.008375313	ko01100//Metabolic p	ko03320//PPAR	GO:0005576//extracellu
0.999913314	0.008382472	-	ko04060//Cytok	GO:0005576//extracellu
0.999912871	0.008403881	-	ko04714//Therm	GO:0005739//mitochon
0.999912788	0.008407856	-	ko05200//Pathw	GO:0005576//extracellu
0.99991276	0.008409233	ko01100//Metabolic p	ko01100//Metab	GO:0005739//mitochon
0.999912641	0.008414985	-	-	-
0.999912484	0.008422512	-	ko01100//Metab	GO:0005654//nucleopla
0.999912447	0.008424281	-	-	-
0.999912082	0.008441843	-	-	-
0.999912037	0.008444013	ko01100//Metabolic p	-	-
0.999911883	0.008451385	-	ko05202//Transc	GO:0016021//integral co
0.999911272	0.008480622	-	ko04610//Comp	GO:0005576//extracellu
0.999910916	0.008497614	-	-	GO:0000776//kinetocho
0.999910725	0.008506722	ko01100//Metabolic p	-	-
0.999910377	0.008523321	ko01100//Metabolic p	-	-
0.999910158	0.008533721	ko01100//Metabolic p	ko05165//Huma	GO:0005634//nucleus;G
0.999909835	0.008549048	ko01100//Metabolic p	ko01100//Metab	-
0.999909808	0.008550331	-	-	GO:0005737//cytoplasm
0.999909581	0.008561073	-	ko04020//Calciu	GO:0005886//plasma m
0.999909377	0.008570707	-	-	-
0.999908979	0.00858954	ko01100//Metabolic p	-	-
0.999908929	0.008591877	ko01100//Metabolic p	-	-
0.999908798	0.008598064	-	-	GO:0005654//nucleopla
0.999908256	0.00862357	ko01100//Metabolic p	ko04976//Bile se	GO:0005654//nucleopla
0.999908085	0.008631617	-	-	-
0.999907784	0.008645715	-	-	-
0.999906974	0.008683595	-	ko01100//Metab	-
0.999906879	0.008688053	ko01100//Metabolic p	-	GO:0016020//membran
0.999906739	0.008694596	-	ko04979//Chole	GO:0005783//endoplasm
0.999906653	0.008698575	-	-	GO:0005737//cytoplasm
0.999906625	0.008699904	-	ko04640//Hema	GO:0031226//intrinsic co
0.999905859	0.008735516	ko01100//Metabolic p	-	-
0.999905813	0.008737647	ko01100//Metabolic p	ko04014//Ras sig	-
0.999905651	0.008745143	-	ko04724//Glutar	GO:0005886//plasma m
0.999905499	0.008752202	-	-	GO:0005737//cytoplasm
0.999905333	0.008759881	-	-	-
0.999905265	0.008763013	-	ko04742//Taste	GO:0001518//voltage-g
0.999904939	0.008778069	-	-	-
0.999904917	0.008779105	ko01100//Metabolic p	ko01100//Metab	GO:0005783//endoplasm
0.999904725	0.008787944	ko01100//Metabolic p	-	-
0.999904445	0.008800877	-	-	-
0.999904215	0.008811469	-	ko05200//Pathw	-
0.999903774	0.008831718	-	-	GO:0005654//nucleopla
0.99990361	0.008839234	-	-	-
0.999903609	0.008839261	ko01100//Metabolic p	-	-
0.999903524	0.008843157	-	-	GO:0005576//extracellu
0.999903434	0.008847285	-	ko05010//Alzhei	GO:0005576//extracellu
0.999903419	0.008847993	ko01100//Metabolic p	-	-
0.999903032	0.008865708	-	ko05131//Shigel	GO:0005922//connexon
0.999902924	0.008870611	-	-	-
0.99990282	0.008875394	-	-	-
0.999902097	0.008908336	-	ko05143//Africa	GO:0005576//extracellu
0.999901717	0.00892559	ko01100//Metabolic p	-	GO:0005789//endoplasm

0.999901396	0.008940187	-	-	GO:0001725//stress fibe
0.999901162	0.00895079	-	-	-
0.999900977	0.00895917	-	ko01100//Metab	GO:0031988//membran
0.999900371	0.008986522	-	-	-
0.999900339	0.008987973	ko01100//Metabolic p	ko01100//Metab	-
0.999900331	0.008988322	-	ko01100//Metab	GO:0005829//cytosol;G
0.999899281	0.009035529	-	ko01100//Metab	-
0.999899277	0.009035738	-	-	GO:0005634//nucleus
0.999899202	0.009039094	ko01100//Metabolic p	-	GO:0005794//Golgi app
0.999898916	0.009051924	-	-	GO:0016020//membran
0.999898707	0.009061255	-	-	-
0.999898689	0.009062057	-	-	GO:0031012//extracellu
0.999898643	0.009064117	-	-	GO:0000177//cytoplasm
0.999898495	0.009070748	-	-	-
0.999898243	0.009081992	-	ko01100//Metab	-
0.999898215	0.009083249	ko01100//Metabolic p	ko01100//Metab	-
0.999897812	0.009101212	ko01100//Metabolic p	-	-
0.999897738	0.009104506	-	-	-
0.999897706	0.009105935	-	-	-
0.999897229	0.009127108	-	-	-
0.999897018	0.009136474	ko01100//Metabolic p	-	-
0.999897017	0.009136556	ko01100//Metabolic p	-	-
0.99989685	0.009143959	-	ko04610//Comp	GO:0005615//extracellu
0.999895973	0.009182732	-	-	GO:0005737//cytoplasm
0.999895915	0.009185278	-	ko04010//MAPK	-
0.999895876	0.009187028	-	-	-
0.999895757	0.00919225	-	-	GO:0005576//extracellu
0.999895525	0.009202477	-	ko01100//Metab	GO:0005783//endoplasm
0.999895386	0.009208595	-	-	GO:0005576//extracellu
0.999895321	0.009211484	-	ko04080//Neuro	GO:0005886//plasma m
0.999895055	0.00922317	-	ko01100//Metab	-
0.99989441	0.00925144	-	-	-
0.99989431	0.009255861	ko01100//Metabolic p	ko04974//Protei	-
0.999894271	0.009257564	ko01100//Metabolic p	-	-
0.999894179	0.009261576	-	ko01100//Metab	GO:0000139//Golgi mer
0.999894087	0.009265607	-	ko04724//Glutar	GO:0005886//plasma m
0.999894012	0.009268891	ko01063//Biosynthesis	ko01100//Metab	GO:0005737//cytoplasm
0.999893781	0.009278955	-	-	GO:0005634//nucleus;G
0.999893502	0.009291151	ko01100//Metabolic p	-	GO:0008076//voltage-g
0.999893414	0.009294977	-	-	-
0.99989331	0.009299539	ko01100//Metabolic p	-	-
0.999893155	0.009306283	-	ko01100//Metab	-
0.999892551	0.009332568	-	-	-
0.999892401	0.009339053	ko01063//Biosynthesis	ko05150//Staphy	-
0.999892331	0.009342101	ko01100//Metabolic p	ko04350//TGF-b	-
0.999891831	0.009363767	-	-	GO:0005791//rough end
0.999891372	0.009383616	-	ko04976//Bile se	GO:0005654//nucleopla
0.999891319	0.009385904	-	-	-
0.999890401	0.009425455	ko01100//Metabolic p	-	GO:0005576//extracellu
0.999889628	0.009458626	-	-	-
0.999889622	0.009458909	ko01100//Metabolic p	ko05017//Spino	GO:0043231//intracellul
0.999889601	0.00945981	-	ko01100//Metab	-
0.999889563	0.009461407	-	ko05200//Pathw	-
0.99988936	0.00947012	-	ko05130//Patho	GO:0005856//cytoskelet
0.999889343	0.009470857	ko01100//Metabolic p	-	GO:0001725//stress fibe
0.999889202	0.009476855	-	ko01100//Metab	GO:0005739//mitochon
0.999888636	0.009501067	ko01100//Metabolic p	-	-
0.999888395	0.009511329	ko01100//Metabolic p	ko04610//Comp	GO:0005615//extracellu

0.999887946	0.009530439	-	ko01100//Metab	-
0.999887591	0.00954552	ko01100//Metabolic p	-	-
0.999887395	0.009553821	ko01110//Biosynthesis	-	-
0.999887311	0.009557392	-	ko04726//Seroto	GO:0005739//mitochon
0.999887193	0.009562399	ko01100//Metabolic p	-	GO:0000139//Golgi mem
0.999887161	0.00956376	ko01100//Metabolic p	ko05010//Alzhei	GO:0005576//extracellu
0.999887122	0.009565435	ko01100//Metabolic p	-	GO:0005634//nucleus;G
0.999887106	0.009566079	-	ko04713//Circac	-
0.99988673	0.009581994	-	-	-
0.999886642	0.009585745	ko01120//Microbial m	-	-
0.999886618	0.009586733	-	ko04060//Cytok	-
0.999886107	0.009608306	-	-	GO:0016020//membran
0.999885751	0.009623351	-	-	GO:0005737//cytoplasm
0.999885658	0.00962725	ko01100//Metabolic p	ko00533//Glycos	GO:0000139//Golgi mem
0.999885194	0.009646755	ko01100//Metabolic p	ko01100//Metab	GO:0005739//mitochon
0.999884917	0.009658393	-	ko04080//Neuro	GO:0005576//extracellu
0.999884013	0.00969627	ko01100//Metabolic p	-	GO:0005634//nucleus;G
0.999883858	0.009702745	ko01100//Metabolic p	-	GO:0016020//membran
0.999883787	0.009705686	-	ko04144//Endoc	GO:0000813//ESCRT I c
0.999883732	0.009708003	-	ko01100//Metab	-
0.999883166	0.009731577	-	-	GO:0005634//nucleus
0.999883038	0.009736906	ko01100//Metabolic p	-	GO:0005615//extracellu
0.999883019	0.009737705	ko01100//Metabolic p	-	-
0.999882742	0.009749252	-	-	GO:0002177//manchett
0.999882662	0.009752578	-	ko04310//Wnt s	GO:0009897//external s
0.999882003	0.009779894	ko01100//Metabolic p	-	-
0.999881936	0.0097827	ko01100//Metabolic p	-	GO:0005576//extracellu
0.999881833	0.009786972	ko01063//Biosynthesis	ko01100//Metab	GO:0033180//proton-tr
0.999881378	0.009805765	-	-	-
0.999881341	0.009807324	-	-	-
0.999881303	0.009808863	ko01100//Metabolic p	ko05203//Viral c	GO:0000786//nucleosor
0.999881071	0.009818451	ko01100//Metabolic p	-	GO:0005634//nucleus;G
0.999880932	0.009824204	-	-	GO:0016020//membran
0.999880846	0.009827755	ko01100//Metabolic p	-	-
0.999880564	0.009839349	-	ko01100//Metab	GO:0043231//intracellul
0.999880139	0.009856866	ko01100//Metabolic p	ko00982//Drug	-
0.999880057	0.009860232	ko01063//Biosynthesis	ko05132//Salmo	GO:0005737//cytoplasm
0.999880022	0.009861659	-	-	GO:0001725//stress fibe
0.999879986	0.009863131	-	ko05322//Syster	GO:0005576//extracellu
0.999879942	0.009864962	-	-	GO:0005884//actin filam
0.9998793	0.00989128	ko01100//Metabolic p	-	-
0.99987927	0.009892526	-	-	-
0.999879173	0.009896497	-	ko04080//Neuro	GO:0005783//endoplasr
0.999879121	0.009898636	ko01100//Metabolic p	ko05322//Syster	GO:0005576//extracellu
0.999879109	0.009899138	ko01100//Metabolic p	ko04310//Wnt s	GO:0005576//extracellu
0.99987863	0.009918694	-	-	GO:0005654//nucleopla
0.999878619	0.009919166	-	ko01100//Metab	-
0.999878199	0.009936303	-	-	GO:0035658//Mon1-Cc
0.999878191	0.009936623	-	ko01100//Metab	-
0.999877935	0.009947073	-	ko05017//Spino	GO:0043231//intracellul
0.999877096	0.009981192	-	-	GO:0001533//cornified
0.999876569	0.010002553	-	-	GO:0005737//cytoplasm
0.999876462	0.010006921	ko01100//Metabolic p	ko04060//Cytok	GO:0005576//extracellu
0.999876116	0.010020906	ko01100//Metabolic p	ko05010//Alzhei	GO:0005576//extracellu
0.999875409	0.010049447	-	-	-
0.999875103	0.010061814	ko01100//Metabolic p	ko01100//Metab	GO:0005886//plasma m
0.999874806	0.010073736	ko01100//Metabolic p	-	-
0.999873809	0.010113808	ko01100//Metabolic p	-	GO:0001725//stress fibe

0.999873741	0.010116532	ko01100//Metabolic p	ko01100//Metab	GO:0005783//endoplasr
0.999872707	0.010157843	ko01100//Metabolic p	-	-
0.99987261	0.010161718	-	ko04514//Cell a	GO:0005829//cytosol;G
0.999872563	0.010163594	-	ko01100//Metab	GO:0005886//plasma m
0.999872403	0.010169994	ko01063//Biosynthesis	-	-
0.999871605	0.01020174	-	-	GO:0001533//cornified
0.999871448	0.010207962	ko01100//Metabolic p	-	GO:0005615//extracellu
0.999870655	0.010239419	-	ko01100//Metab	GO:0048269//methionir
0.999870631	0.010240333	-	ko04630//JAK-S	GO:0005829//cytosol
0.999870568	0.010242863	ko01100//Metabolic p	-	GO:0005737//cytoplasm
0.999870426	0.010248477	-	-	-
0.999870356	0.010251228	ko01100//Metabolic p	ko01100//Metab	-
0.999870247	0.010255547	-	-	GO:0016020//membran
0.999870246	0.010255564	-	ko04610//Comp	GO:0005576//extracellu
0.999869606	0.010280834	-	-	-
0.999869157	0.010298514	-	-	-
0.999869076	0.010301716	ko01100//Metabolic p	-	-
0.999868842	0.010310921	ko01100//Metabolic p	ko01100//Metab	GO:0005634//nucleus;G
0.999868681	0.010317241	-	ko01100//Metab	GO:0005739//mitochon
0.999868578	0.010321274	-	-	GO:0001725//stress fibe
0.99986796	0.010345541	ko01100//Metabolic p	-	-
0.999867957	0.01034565	-	-	-
0.999866666	0.010396095	ko01100//Metabolic p	ko05130//Patho	GO:0005856//cytoskelet
0.999866461	0.010404082	ko01100//Metabolic p	-	GO:0005634//nucleus;G
0.99986643	0.010405297	ko01100//Metabolic p	ko04350//TGF-b	-
0.999865765	0.010431185	-	-	-
0.999865626	0.010436546	ko01100//Metabolic p	-	GO:0001726//ruffle;GO:
0.999865626	0.010436564	ko01100//Metabolic p	ko04514//Cell a	GO:0005886//plasma m
0.999865486	0.010441984	ko01100//Metabolic p	-	-
0.999865245	0.010451355	-	-	GO:0005634//nucleus
0.999864914	0.010464176	-	ko01100//Metab	-
0.999864552	0.010478175	-	-	-
0.99986408	0.010496421	-	-	-
0.999863551	0.01051683	-	ko01100//Metab	-
0.99986329	0.010526908	ko01100//Metabolic p	ko04151//PI3K-/	GO:0005654//nucleopla
0.999863255	0.010528232	ko01100//Metabolic p	-	GO:0016020//membran
0.99986264	0.010551892	ko01100//Metabolic p	ko04060//Cytok	GO:0005576//extracellu
0.999862335	0.010563606	-	-	GO:0005886//plasma m
0.999862254	0.010566709	-	-	GO:0005768//endosom
0.999862184	0.010569408	ko01110//Biosynthesis	-	GO:0016020//membran
0.999862049	0.010574579	-	ko03013//Nucle	-
0.999860708	0.010625852	ko01100//Metabolic p	-	GO:0016020//membran
0.999860278	0.010642247	-	-	GO:0005802//trans-Gol
0.999860077	0.010649882	-	ko01100//Metab	-
0.999859789	0.01066084	ko01100//Metabolic p	ko04724//Glutar	GO:0005886//plasma m
0.999859699	0.010664266	-	ko04310//Wnt s	GO:0005576//extracellu
0.999859686	0.010664768	-	ko05322//Syster	GO:0005576//extracellu
0.999859629	0.010666905	-	ko04976//Bile se	GO:0005654//nucleopla
0.999858241	0.010719523	-	ko01100//Metab	GO:0000139//Golgi mer
0.999858121	0.010724055	ko01100//Metabolic p	ko01100//Metab	GO:0043231//intracellul
0.999857014	0.010765801	-	ko04919//Thyro	GO:0005887//integral c
0.999856258	0.010794245	ko01100//Metabolic p	-	-
0.999856089	0.010800582	ko01063//Biosynthesis	ko01100//Metab	GO:0005739//mitochon
0.999855885	0.010808241	ko01100//Metabolic p	ko01100//Metab	-
0.999855846	0.010809715	-	-	-
0.999855404	0.01082626	ko01110//Biosynthesis	-	GO:0001725//stress fibe
0.999855187	0.010834374	-	ko03320//PPAR	GO:0005783//endoplasr
0.999855032	0.010840196	ko01120//Microbial m	-	-

0.999854641	0.010854804	ko01100//Metabolic p	ko05200//Pathw	-
0.999854588	0.010856768	-	-	GO:0005737//cytoplasm
0.999854584	0.010856935	-	ko01100//Metab	-
0.99985445	0.010861913	ko01100//Metabolic p	-	GO:0016021//integral c
0.999854346	0.010865783	ko01100//Metabolic p	-	GO:0005783//endoplasm
0.999853251	0.010906562	-	ko04610//Comp	GO:0005615//extracellu
0.999853237	0.010907076	-	-	-
0.999853179	0.010909224	ko01100//Metabolic p	-	-
0.999853147	0.010910443	ko01100//Metabolic p	-	-
0.999852912	0.010919163	ko01110//Biosynthesis	ko05010//Alzhei	GO:0005576//extracellu
0.999852853	0.010921357	-	ko01100//Metab	GO:0000139//Golgi mer
0.999852301	0.01094182	ko01100//Metabolic p	-	-
0.999851756	0.01096197	-	-	-
0.999851677	0.010964909	-	ko01100//Metab	-
0.99985164	0.010966252	-	-	-
0.999851415	0.010974591	ko01100//Metabolic p	ko04310//Wnt s	GO:0009897//external s
0.999851211	0.01098213	ko01100//Metabolic p	ko04310//Wnt s	GO:0005654//nucleopla
0.999850743	0.010999354	-	-	-
0.999850658	0.011002505	ko01100//Metabolic p	ko01100//Metab	GO:0031988//membran
0.999850605	0.011004439	-	-	GO:0005737//cytoplasm
0.999850396	0.011012138	-	-	-
0.999850147	0.011021297	ko01100//Metabolic p	-	GO:0005737//cytoplasm
0.999849663	0.011039092	ko01120//Microbial m	-	GO:0000177//cytoplasm
0.999849552	0.011043161	-	-	GO:0005737//cytoplasm
0.999849453	0.011046819	ko01100//Metabolic p	-	GO:0005737//cytoplasm
0.999849106	0.011059532	ko01100//Metabolic p	ko01100//Metab	GO:0005829//cytosol;GO
0.999847655	0.011112584	ko01100//Metabolic p	ko04918//Thyro	GO:0043231//intracellul
0.999847631	0.011113449	-	-	GO:0031982//vesicle
0.999847628	0.011113551	-	ko04144//Endoc	-
0.999847583	0.011115216	-	ko04979//Chole	GO:0005856//cytoskelet
0.999847543	0.011116642	ko01100//Metabolic p	-	-
0.999847492	0.011118521	ko01100//Metabolic p	ko04150//mTOR	GO:0005829//cytosol;GO
0.999847192	0.011129436	-	ko01100//Metab	GO:0005886//plasma m
0.999846683	0.011147957	-	-	GO:0005737//cytoplasm
0.999845935	0.011175127	-	-	-
0.999845538	0.011189524	-	-	GO:0005634//nucleus
0.999845534	0.011189669	ko01100//Metabolic p	-	GO:0005634//nucleus
0.999845421	0.011193749	ko01100//Metabolic p	ko01100//Metab	GO:0048471//perinuclea
0.999845334	0.011196902	-	ko05016//Huntin	GO:0005929//cilium;GO
0.999845004	0.011208833	-	ko00564//Glycer	GO:0005615//extracellu
0.999844785	0.011216763	-	-	GO:0001533//cornified
0.999844527	0.011226067	ko01100//Metabolic p	-	GO:0005737//cytoplasm
0.999844437	0.01122934	ko01100//Metabolic p	ko04514//Cell ad	GO:0005829//cytosol;GO
0.999844367	0.01123187	ko01100//Metabolic p	ko03320//PPAR	GO:0005634//nucleus;G
0.999844124	0.011240623	-	-	GO:0005737//cytoplasm
0.999842983	0.011281706	ko01100//Metabolic p	-	-
0.999842201	0.011309743	-	-	-
0.999841997	0.011317072	ko01100//Metabolic p	ko05143//Africa	GO:0005576//extracellu
0.999841926	0.011319605	-	-	GO:0001725//stress fibe
0.999841736	0.011326395	ko01100//Metabolic p	ko00983//Drug	-
0.999841566	0.011332493	-	ko05165//Huma	GO:0005634//nucleus;G
0.999841063	0.011350473	-	-	-
0.999840989	0.011353102	-	-	GO:0005634//nucleus;G
0.999840939	0.011354889	ko01100//Metabolic p	-	-
0.999840663	0.011364744	-	ko01100//Metab	GO:0005739//mitochon
0.99984007	0.011385846	-	ko01100//Metab	GO:0005654//nucleopla
0.999839292	0.011413531	-	-	GO:0005886//plasma m
0.999838899	0.011427469	-	-	GO:0016020//membran

0.999838694	0.011434724	-	-	GO:0016020//membran
0.999838491	0.011441934	ko01100//Metabolic p	-	-
0.999838419	0.011444484	ko01110//Biosynthesis	ko01100//Metak	-
0.999837556	0.01147499	-	ko01100//Metak	GO:0048269//methionir
0.999837549	0.011475244	-	ko01100//Metak	-
0.999837466	0.01147818	ko01100//Metabolic p	ko05200//Pathw	GO:0005576//extracellu
0.999837017	0.011494035	ko01100//Metabolic p	ko01100//Metak	GO:0005829//cytosol
0.999835932	0.011532218	-	-	GO:0005654//nucleopla
0.999835442	0.011549425	-	ko05016//Huntir	GO:0005929//cilium;GO
0.999835412	0.011550479	-	ko04974//Protei	-
0.999835231	0.011556845	-	-	GO:0001725//stress fibe
0.999835059	0.011562854	ko01100//Metabolic p	ko04514//Cell a	-
0.99983479	0.011572297	ko01100//Metabolic p	-	GO:0005654//nucleopla
0.999834748	0.011573774	ko01100//Metabolic p	ko01100//Metak	-
0.999834668	0.01157655	-	-	GO:0005576//extracellu
0.999834505	0.011582267	-	-	-
0.999834011	0.011599554	ko01100//Metabolic p	-	GO:0016021//integral c
0.999833114	0.011630826	ko01100//Metabolic p	ko05200//Pathw	-
0.999832906	0.011638073	-	ko05322//Syster	GO:0005576//extracellu
0.999832285	0.011659687	-	-	GO:0001533//cornified
0.999831696	0.011680153	-	-	GO:0005634//nucleus;G
0.999831501	0.011686926	-	-	GO:0005634//nucleus
0.999831177	0.011698149	ko01100//Metabolic p	-	GO:0016021//integral c
0.999830676	0.011715498	-	-	GO:0005737//cytoplasm
0.999830574	0.011719016	ko01110//Biosynthesis	ko04010//MAPK	-
0.999829551	0.011754356	-	-	GO:0005794//Golgi app
0.999829426	0.011758658	ko01100//Metabolic p	-	GO:0016020//membran
0.999829177	0.011767242	-	-	-
0.99982826	0.011798776	ko01100//Metabolic p	-	GO:0005737//cytoplasm
0.999827788	0.011814978	ko01100//Metabolic p	-	GO:0000779//condense
0.999827753	0.011816174	-	ko01100//Metak	GO:0005739//mitochon
0.999827358	0.011829711	ko01100//Metabolic p	ko04610//Comp	GO:0005615//extracellu
0.999827201	0.011835114	-	-	-
0.999826472	0.011860052	-	-	GO:0005634//nucleus;G
0.999826371	0.0118635	ko01100//Metabolic p	ko04640//Hema	GO:0031226//intrinsic c
0.999826217	0.011868765	ko01110//Biosynthesis	ko01100//Metak	-
0.999825976	0.011876975	ko01100//Metabolic p	-	-
0.999825472	0.011894182	-	ko04142//Lysos	GO:0005615//extracellu
0.999825092	0.011907125	-	-	-
0.999824995	0.011910403	-	-	-
0.99982465	0.011922141	-	ko04151//PI3K-	GO:0005654//nucleopla
0.999824129	0.011939832	ko01100//Metabolic p	ko04080//Neurc	GO:0005783//endoplası
0.999824129	0.011939834	-	ko04976//Bile se	GO:0005654//nucleopla
0.999824066	0.011941971	-	ko05322//Syster	GO:0005576//extracellu
0.999824052	0.011942473	-	ko04310//Wnt s	GO:0005576//extracellu
0.999823838	0.011949701	-	-	GO:0043231//intracellul
0.999823461	0.011962489	-	-	-
0.999822945	0.011979959	ko01100//Metabolic p	ko01100//Metak	-
0.999822809	0.011984554	-	-	GO:0005654//nucleopla
0.999822656	0.011989755	ko01100//Metabolic p	-	GO:0005884//actin filam
0.99982208	0.012009211	-	ko05230//Centra	GO:0005886//plasma m
0.99982192	0.012014612	-	ko00983//Drug	-
0.999821824	0.012017817	-	-	GO:0005737//cytoplasm
0.999821417	0.012031535	-	-	-
0.999820772	0.012053248	-	ko05414//Dilate	-
0.999820698	0.012055751	-	-	GO:0001518//voltage-g
0.999820369	0.012066787	ko01100//Metabolic p	ko01100//Metak	-
0.999819819	0.01208527	-	-	-

0.999819515	0.012095462	ko01100//Metabolic p	ko04745//Photo	-
0.999819321	0.012101942	ko01100//Metabolic p	-	-
0.999819277	0.012103416	ko01110//Biosynthesis	-	-
0.999818338	0.012134847	ko01120//Microbial m	ko04144//Endoc	-
0.999818065	0.012143959	-	-	-
0.999817845	0.012151287	-	-	GO:0014069//postsynap
0.999817799	0.012152827	-	-	GO:0044464//cell part
0.999817713	0.012155695	-	ko01100//Metak	GO:0005737//cytoplasm
0.999817676	0.012156928	-	ko01100//Metak	GO:0000139//Golgi mer
0.999817553	0.012161033	ko01100//Metabolic p	ko04919//Thyro	GO:0005887//integral co
0.999817147	0.012174558	ko01100//Metabolic p	ko04742//Taste	GO:0005783//endoplasr
0.99981706	0.012177428	ko01100//Metabolic p	-	GO:0016021//integral co
0.999817036	0.012178254	-	ko04270//Vascu	GO:0005737//cytoplasm
0.999816727	0.012188531	-	-	GO:0016020//membran
0.999816698	0.012189477	-	-	-
0.999816368	0.012200441	-	ko04080//Neuro	GO:0005576//extracellu
0.999816277	0.012203474	-	ko01100//Metak	-
0.999815668	0.012223691	ko01100//Metabolic p	ko01100//Metak	-
0.999815448	0.012230991	ko01100//Metabolic p	-	GO:0005634//nucleus;G
0.999814976	0.012246612	-	ko01100//Metak	-
0.999814653	0.012257291	-	-	GO:0005634//nucleus
0.99981454	0.012261015	-	-	GO:0005783//endoplasr
0.999814371	0.012266617	ko01100//Metabolic p	ko04360//Axon	-
0.99981419	0.012272604	ko01100//Metabolic p	-	GO:0005737//cytoplasm
0.999813634	0.012290957	-	ko05010//Alzhei	GO:0005576//extracellu
0.999813363	0.012299863	-	ko01100//Metak	GO:0005615//extracellu
0.999812923	0.012314359	ko01100//Metabolic p	-	GO:0005576//extracellu
0.999812282	0.012335462	ko01100//Metabolic p	-	GO:0031982//vesicle
0.999811779	0.012351965	-	-	-
0.999810806	0.01238386	-	-	GO:0001725//stress fibe
0.999810803	0.012383942	ko01100//Metabolic p	ko01100//Metak	GO:0000139//Golgi mer
0.99981078	0.012384694	-	ko05143//Africa	GO:0005576//extracellu
0.999810748	0.012385748	ko01100//Metabolic p	-	-
0.999810359	0.012398485	-	-	-
0.99980983	0.012415762	-	ko01100//Metak	GO:0048471//perinuclea
0.999808885	0.01244657	ko01100//Metabolic p	ko01100//Metak	GO:0000506//glycosylpl
0.999808847	0.012447814	-	-	-
0.999807952	0.012476923	ko01100//Metabolic p	ko05414//Dilate	-
0.999807679	0.01248577	ko01100//Metabolic p	ko01100//Metak	-
0.999806779	0.01251496	-	-	-
0.99980676	0.012515559	ko01100//Metabolic p	ko00983//Drug	-
0.99980626	0.012531757	ko01100//Metabolic p	-	-
0.999804861	0.012576902	-	-	-
0.999804515	0.012588058	-	ko01100//Metak	-
0.999803928	0.012606942	ko01100//Metabolic p	-	GO:0005886//plasma m
0.999803788	0.012611462	-	-	-
0.999803633	0.012616421	-	-	GO:0016021//integral co
0.999803435	0.012622774	-	ko01100//Metak	GO:0048269//methionir
0.999803399	0.012623952	-	ko01100//Metak	-
0.999802946	0.012638491	-	-	-
0.999802776	0.01264393	ko01100//Metabolic p	-	-
0.999802507	0.012652557	-	ko01100//Metak	GO:0005829//cytosol;G(
0.999801953	0.012670295	-	-	GO:0016020//membran
0.999801819	0.012674572	ko01100//Metabolic p	-	GO:0005634//nucleus;G
0.99980163	0.012680611	ko01100//Metabolic p	ko01100//Metak	GO:0005789//endoplasr
0.999801606	0.012681395	ko01100//Metabolic p	-	GO:0014069//postsynap
0.999801454	0.01268625	ko01100//Metabolic p	ko01100//Metak	-
0.999801121	0.012696881	-	-	-

0.999801096	0.012697664	ko01100//Metabolic p	-	GO:0005737//cytoplasm
0.999800877	0.012704648	-	-	GO:0016021//integral c
0.999800345	0.012721627	-	ko00983//Drug	-
0.999799331	0.012753889	ko01100//Metabolic p	-	-
0.99979913	0.012760279	-	-	-
0.999798906	0.01276739	-	ko01100//Metab	GO:0043231//intracellul
0.999798815	0.012770268	-	-	-
0.999798634	0.012776013	ko01120//Microbial m	-	-
0.999798598	0.012777157	ko01100//Metabolic p	-	GO:0005634//nucleus;G
0.999798552	0.012778619	-	-	GO:0005737//cytoplasm
0.99979817	0.012790732	ko01100//Metabolic p	ko01100//Metab	GO:0000139//Golgi mem
0.999797904	0.012799156	ko01100//Metabolic p	-	-
0.999797881	0.012799886	ko01100//Metabolic p	ko01100//Metab	-
0.999797825	0.012801646	-	-	-
0.99979778	0.012803097	-	ko05200//Pathw	GO:0005634//nucleus;G
0.99979748	0.012812585	ko01100//Metabolic p	-	GO:0016020//membran
0.999797394	0.012815289	-	ko01100//Metab	-
0.999797231	0.012820448	-	-	-
0.999797202	0.012821354	ko01100//Metabolic p	ko05200//Pathw	GO:0005834//heterotrim
0.999796403	0.012846588	-	ko04080//Neuro	GO:0016020//membran
0.99979606	0.012857431	-	ko04020//Calciu	GO:0005886//plasma m
0.999795774	0.012866425	-	ko05012//Parkin	GO:0005654//nucleopla
0.999795165	0.012885609	-	ko04010//MAPK	GO:0005737//cytoplasm
0.999795073	0.012888493	-	ko04144//Endoc	-
0.999794829	0.012896155	-	ko04060//Cytok	GO:0009986//cell surfac
0.999794631	0.012902384	ko01120//Microbial m	-	-
0.999793659	0.012932883	ko01100//Metabolic p	ko05150//Staphy	-
0.99979302	0.012952908	ko01100//Metabolic p	-	-
0.999792988	0.012953905	ko01100//Metabolic p	-	GO:0005576//extracellu
0.999792749	0.012961384	-	-	GO:0016020//membran
0.999792216	0.012978024	ko01100//Metabolic p	-	-
0.999791891	0.012988175	-	ko00983//Drug	-
0.999791414	0.013003045	ko01100//Metabolic p	ko01100//Metab	GO:0005737//cytoplasm
0.999790974	0.01301676	ko01110//Biosynthesis	-	GO:0005739//mitochon
0.999790913	0.013018644	ko01100//Metabolic p	-	GO:0043231//intracellul
0.999790857	0.013020394	-	ko01100//Metab	-
0.999790836	0.013021064	ko01100//Metabolic p	ko01100//Metab	GO:0005737//cytoplasm
0.999790276	0.013038469	-	-	-
0.999790259	0.013039013	ko01100//Metabolic p	ko01100//Metab	GO:0005778//peroxison
0.999790035	0.013045966	ko01100//Metabolic p	-	-
0.999790006	0.013046859	ko01100//Metabolic p	-	GO:0005737//cytoplasm
0.999789977	0.013047774	ko01100//Metabolic p	-	GO:0005737//cytoplasm
0.999789733	0.013055349	-	ko04710//Circac	GO:0005634//nucleus;G
0.999789239	0.013070685	-	-	-
0.999789213	0.013071487	-	-	-
0.999789118	0.013074418	ko01100//Metabolic p	ko04010//MAPK	-
0.999788506	0.013093381	-	-	GO:0016021//integral c
0.999786482	0.013155899	-	ko04530//Tight	GO:0016020//membran
0.999786461	0.013156531	-	-	GO:0016021//integral c
0.99978643	0.013157491	-	-	-
0.99978632	0.013160882	ko01100//Metabolic p	ko01100//Metab	GO:0005737//cytoplasm
0.999786248	0.013163084	-	ko04745//Photo	-
0.999785917	0.013173293	ko01100//Metabolic p	-	GO:0036126//sperm flag
0.999785877	0.013174526	ko01063//Biosynthesis	ko01100//Metab	-
0.999785193	0.013195533	ko01100//Metabolic p	ko04060//Cytok	GO:0005576//extracellu
0.99978516	0.013196558	-	-	-
0.999785069	0.013199354	ko01100//Metabolic p	ko04979//Chole	GO:0005856//cytoskelet
0.99978479	0.013207916	-	ko01100//Metab	GO:0005635//nuclear er

0.99978426	0.013224168	ko01100//Metabolic p	ko01100//Metak	-
0.999783977	0.013232842	ko01063//Biosynthesis	-	GO:0005794//Golgi app
0.999783749	0.01323983	ko01100//Metabolic p	-	GO:0005737//cytoplasm
0.999783065	0.013260734	ko01100//Metabolic p	-	GO:0005634//nucleus
0.999782924	0.013265058	-	-	GO:0016020//membran
0.999782821	0.013268199	ko01100//Metabolic p	ko02010//ABC ti	GO:0005886//plasma m
0.999782333	0.013283104	ko01100//Metabolic p	-	-
0.999782283	0.013284635	ko01100//Metabolic p	ko04979//Chole	GO:0005856//cytoskelet
0.999781621	0.013304802	-	ko04726//Seroto	GO:0005739//mitochon
0.999781489	0.013308829	ko01100//Metabolic p	ko05414//Dilate	GO:0016459//myosin co
0.999781078	0.013321339	ko01100//Metabolic p	ko00564//Glycer	GO:0005615//extracellu
0.999780712	0.013332462	-	ko04060//Cytok	GO:0016020//membran
0.999780368	0.013342935	-	-	-
0.999780297	0.013345065	ko01100//Metabolic p	ko05132//Salmo	-
0.999780294	0.013345158	ko01110//Biosynthesis	-	GO:0005884//actin filam
0.999779138	0.013380241	-	ko01100//Metak	GO:0005789//endoplasm
0.999778788	0.013390815	ko01100//Metabolic p	-	GO:0001725//stress fibe
0.999778476	0.013400267	ko01100//Metabolic p	ko04270//Vascu	GO:0005737//cytoplasm
0.999777809	0.013420436	-	-	-
0.999777519	0.013429181	ko01100//Metabolic p	ko01100//Metak	GO:0005654//nucleopla
0.999777274	0.013436564	ko01100//Metabolic p	-	GO:0005737//cytoplasm
0.999776971	0.013445704	-	ko01100//Metak	-
0.999776954	0.013446225	-	-	GO:0005737//cytoplasm
0.999776869	0.013448787	-	-	GO:0005783//endoplasm
0.999776536	0.013458819	ko01100//Metabolic p	-	-
0.999776465	0.013460954	-	-	-
0.999776405	0.013462779	-	ko04610//Comp	GO:0005615//extracellu
0.999774975	0.01350574	ko01100//Metabolic p	-	-
0.999774956	0.013506329	-	ko01100//Metak	-
0.999774489	0.013520325	-	-	-
0.999774462	0.013521126	-	ko01100//Metak	-
0.999773465	0.013550977	-	-	-
0.99977318	0.013559509	-	-	GO:0005886//plasma m
0.999772385	0.013583239	-	-	GO:0005634//nucleus;G
0.999772226	0.013588009	-	-	-
0.999771097	0.013621618	-	ko04514//Cell ac	GO:0016020//membran
0.999771046	0.013623153	ko01100//Metabolic p	-	GO:0005634//nucleus;G
0.99977049	0.013639682	-	ko01100//Metak	-
0.999770123	0.013650576	ko01100//Metabolic p	ko04630//JAK-S	GO:0005829//cytosol
0.99976909	0.013681234	-	ko01100//Metak	GO:0005737//cytoplasm
0.999768985	0.013684321	ko01100//Metabolic p	-	GO:0044424//intracellul
0.999767992	0.0137137	-	-	-
0.999767617	0.013724793	-	ko00565//Ether	GO:0005615//extracellu
0.99976741	0.013730896	ko01100//Metabolic p	-	GO:0001533//cornified
0.999767395	0.013731345	-	-	-
0.999765925	0.013774652	-	-	-
0.999765602	0.013784153	-	ko05230//Centra	GO:0005886//plasma m
0.999765483	0.013787661	-	ko04151//PI3K-/	GO:0005654//nucleopla
0.99976543	0.013789227	-	ko04745//Photo	-
0.99976514	0.01379774	ko01100//Metabolic p	ko01100//Metak	-
0.999763713	0.013839606	ko01100//Metabolic p	ko05200//Pathw	GO:0005576//extracellu
0.999763533	0.013844867	ko01100//Metabolic p	ko04020//Calciu	GO:0005886//plasma m
0.999763155	0.013855934	ko01100//Metabolic p	-	-
0.999762989	0.01386078	-	-	GO:0005737//cytoplasm
0.99976248	0.013875671	-	-	-
0.999762053	0.013888128	-	ko01100//Metak	GO:0005829//cytosol;G(
0.999761978	0.01389032	-	ko01100//Metak	GO:0005737//cytoplasm
0.999761412	0.013906835	-	ko04640//Hema	GO:0031226//intrinsic c

0.999761211	0.013912679	-	ko01100//Metal	-
0.999760927	0.013920961	-	ko01100//Metal	GO:0005739//mitochon
0.999760682	0.013928077	-	-	GO:0016020//membran
0.99976056	0.013931643	-	ko05200//Pathw	GO:0005576//extracellu
0.999759473	0.013963214	-	ko04020//Calciu	GO:0005634//nucleus;G
0.999759276	0.013968957	-	ko05200//Pathw	GO:0005634//nucleus;G
0.999759098	0.013974098	ko01100//Metabolic p	ko01100//Metal	GO:0005789//endoplasr
0.999758647	0.013987188	-	-	-
0.999758003	0.014005839	-	-	-
0.999757992	0.01400616	ko01100//Metabolic p	-	GO:0005737//cytoplasm
0.999757882	0.014009344	-	-	GO:0016020//membran
0.999757798	0.014011774	-	-	-
0.999757236	0.014028004	-	ko04060//Cytok	GO:0016020//membran
0.999756917	0.014037216	-	-	-
0.999756824	0.014039918	-	ko03460//Fanco	GO:0000785//chromatir
0.999756503	0.014049159	-	-	-
0.999756063	0.014061859	ko01100//Metabolic p	-	-
0.999755678	0.014072951	-	ko05142//Chaga	GO:0016020//membran
0.999755404	0.014080853	-	-	-
0.999755149	0.014088187	-	-	-
0.99975496	0.014093607	-	ko04514//Cell a	GO:0005886//plasma m
0.999754705	0.014100938	-	-	GO:0005615//extracellu
0.999754697	0.014101168	-	-	-
0.999754431	0.01410883	-	ko04970//Saliva	GO:0005576//extracellu
0.999753947	0.014122725	-	-	-
0.999753891	0.014124329	-	ko04610//Comp	GO:0005615//extracellu
0.999753719	0.01412927	-	ko01100//Metal	-
0.999752496	0.014164298	-	-	GO:0005737//cytoplasm
0.999752473	0.014164974	-	-	-
0.999752329	0.014169083	-	-	-
0.999752245	0.014171493	-	-	GO:0005764//lysosome;
0.999751996	0.014178598	-	ko01100//Metal	-
0.999751387	0.014195997	-	-	GO:0005737//cytoplasm
0.999751231	0.01420046	-	-	-
0.999751087	0.014204568	-	ko04530//Tight	GO:0016020//membran
0.999750368	0.014225071	ko01100//Metabolic p	ko04080//Neuro	GO:0005886//plasma m
0.999750366	0.014225112	ko01100//Metabolic p	-	-
0.99974985	0.014239829	ko01100//Metabolic p	-	GO:0005802//trans-Gol
0.999749821	0.014240657	-	-	GO:0043231//intracellul
0.999748546	0.014276889	ko01100//Metabolic p	ko05200//Pathw	GO:0005634//nucleus
0.999748134	0.014288589	-	ko04918//Thyro	GO:0005576//extracellu
0.999747189	0.014315367	ko01100//Metabolic p	-	GO:0005768//endosom
0.999747188	0.014315377	-	ko04020//Calciu	GO:0005886//plasma m
0.99974709	0.014318171	-	ko01100//Metal	GO:0005829//cytosol;G
0.999746559	0.014333173	-	-	GO:0016020//membran
0.999746033	0.014348052	ko01100//Metabolic p	ko04742//Taste	GO:0016020//membran
0.999745401	0.014365882	-	-	-
0.999745179	0.014372163	ko01100//Metabolic p	-	GO:0005739//mitochon
0.999744798	0.014382895	-	ko01100//Metal	GO:0005737//cytoplasm
0.99974423	0.014398894	-	ko01100//Metal	-
0.999744115	0.014402128	ko01110//Biosynthesis	-	-
0.999743635	0.014415627	ko01100//Metabolic p	ko01100//Metal	-
0.999743506	0.014419251	ko01120//Microbial m	ko01100//Metal	GO:0005737//cytoplasm
0.999743467	0.014420351	-	-	-
0.999743346	0.01442376	-	-	-
0.999743126	0.014429929	ko01100//Metabolic p	ko01100//Metal	GO:0005635//nuclear er
0.999742988	0.014433823	ko01110//Biosynthesis	-	-
0.999742634	0.014443753	ko01100//Metabolic p	-	-

0.999742163	0.014456957	-	-	-
0.999741788	0.014467472	ko01100//Metabolic p	ko00564//Glycer	GO:0005615//extracellu
0.999741399	0.014478359	-	ko04151//PI3K-/	GO:0005654//nucleopla
0.999740971	0.014490353	ko01100//Metabolic p	ko04659//Th17 (	GO:0000785//chromatir
0.999740921	0.014491749	-	-	GO:0043231//intracellul
0.999740667	0.014498843	ko01100//Metabolic p	ko01100//Metak	GO:0005654//nucleopla
0.999740231	0.014511031	-	ko04060//Cytok	-
0.999739752	0.014524415	ko01100//Metabolic p	ko04514//Cell ac	GO:0005769//early endo
0.999739384	0.014534676	-	-	-
0.999739179	0.014540372	-	ko04726//Seroto	GO:0005739//mitochon
0.999738201	0.014567617	-	ko05143//Africa	GO:0005576//extracellu
0.999737589	0.014584647	ko01100//Metabolic p	-	GO:0016020//membran
0.9997373	0.014592662	ko01110//Biosynthesis	ko01100//Metak	GO:0005737//cytoplasm
0.999737231	0.014594587	-	ko04979//Chole	GO:0005856//cytoskelet
0.999736731	0.014608451	-	-	GO:0000781//chromoso
0.999736667	0.014610239	ko01100//Metabolic p	-	-
0.999736079	0.014626529	ko01100//Metabolic p	-	GO:0016020//membran
0.999736058	0.01462713	ko01100//Metabolic p	-	GO:0005886//plasma m
0.999735911	0.014631184	ko01063//Biosynthesis	ko05414//Dilate	GO:0016459//myosin co
0.999735069	0.014654505	-	-	-
0.999734982	0.014656909	ko01063//Biosynthesis	-	-
0.999733963	0.014685054	-	-	GO:0005654//nucleopla
0.99973384	0.014688449	-	-	-
0.999733812	0.01468924	ko01100//Metabolic p	ko04514//Cell ac	GO:0016020//membran
0.999733602	0.01469502	-	-	GO:0005634//nucleus
0.999733589	0.014695376	-	-	-
0.999733161	0.014707172	ko01100//Metabolic p	-	GO:0005794//Golgi app
0.999732989	0.014711913	-	ko01100//Metak	GO:0005737//cytoplasm
0.99973283	0.014716303	-	-	GO:0001518//voltage-g
0.999732546	0.014724116	-	ko01100//Metak	-
0.999731762	0.014745695	-	-	-
0.999731289	0.014758689	-	ko04390//Hippo	GO:0005667//transcript
0.999731252	0.014759691	-	ko01100//Metak	-
0.999730971	0.01476742	ko01100//Metabolic p	-	-
0.999730656	0.014776054	ko01100//Metabolic p	-	GO:0005783//endoplasr
0.999730299	0.014785841	ko01100//Metabolic p	-	GO:0016021//integral co
0.9997299	0.014796774	-	-	GO:0005634//nucleus;G
0.999729664	0.014803241	-	ko04610//Comp	GO:0005615//extracellu
0.999729332	0.014812336	-	ko04080//Neuro	GO:0005886//plasma m
0.999729153	0.014817233	-	-	GO:0005783//endoplasr
0.999729113	0.014818324	-	-	GO:0005634//nucleus;G
0.999729095	0.01481881	-	-	GO:0005634//nucleus;G
0.999727838	0.014853163	ko01100//Metabolic p	ko05200//Pathw	-
0.999727731	0.014856081	-	ko01100//Metak	GO:0005615//extracellu
0.999727658	0.014858071	-	ko01100//Metak	GO:0005777//peroxison
0.999727537	0.014861363	ko01110//Biosynthesis	ko04514//Cell ac	GO:0016020//membran
0.999726844	0.01488025	ko01100//Metabolic p	-	GO:0005737//cytoplasm
0.999725916	0.014905514	-	-	GO:0005576//extracellu
0.999725854	0.014907192	-	-	GO:0005794//Golgi app
0.999725752	0.014909978	-	ko01100//Metak	GO:0005737//cytoplasm
0.999725517	0.014916359	ko01100//Metabolic p	ko01100//Metak	-
0.999725255	0.014923473	ko01110//Biosynthesis	-	GO:0005886//plasma m
0.99972463	0.014940427	ko01100//Metabolic p	ko03460//Fanco	GO:0000785//chromatir
0.999724512	0.01494364	-	-	GO:0016020//membran
0.999724248	0.014950797	ko01100//Metabolic p	-	GO:0005794//Golgi app
0.999724022	0.014956921	ko01063//Biosynthesis	-	-
0.999724004	0.014957401	ko01100//Metabolic p	ko01100//Metak	-
0.999723985	0.014957941	ko01100//Metabolic p	ko01100//Metak	GO:0005737//cytoplasm

0.999722233	0.015005316	-	-	-
0.999722037	0.015010635	-	-	-
0.99972143	0.015027016	-	-	-
0.999719538	0.015077947	-	-	-
0.999718838	0.015096766	ko01100//Metabolic p	-	GO:0005634//nucleus;G
0.999718522	0.015105231	ko01100//Metabolic p	-	-
0.999718298	0.015111242	-	-	-
0.999718121	0.015116002	-	ko04714//Therm	-
0.999718092	0.015116781	ko01100//Metabolic p	-	-
0.999717856	0.0151231	-	-	GO:0005886//plasma m
0.999717389	0.015135615	-	-	-
0.999717256	0.015139181	-	ko01100//Metab	GO:0005737//cytoplasm
0.999717143	0.015142203	-	-	GO:0005922//connexon
0.999717119	0.015142825	ko01063//Biosynthesis	-	GO:0005737//cytoplasm
0.999717065	0.015144276	-	ko01100//Metab	-
0.999716964	0.015146974	-	-	-
0.999716908	0.015148475	ko01100//Metabolic p	-	-
0.999716567	0.015157594	ko01100//Metabolic p	ko00983//Drug	-
0.999715709	0.01518054	-	-	-
0.999715336	0.015190483	-	ko01100//Metab	GO:0005737//cytoplasm
0.999715243	0.015192972	-	ko01100//Metab	-
0.999714766	0.015205692	ko01100//Metabolic p	-	-
0.999714212	0.015220454	-	ko01100//Metab	-
0.999713293	0.015244915	-	-	GO:0016020//membran
0.999713157	0.015248523	-	-	GO:0005576//extracellu
0.999712454	0.015267189	-	-	GO:0005654//nucleopla
0.999712226	0.015273251	ko01100//Metabolic p	ko00983//Drug	-
0.999712176	0.015274559	ko01100//Metabolic p	-	GO:0005615//extracellu
0.999712053	0.015277845	-	ko00983//Drug	-
0.999711355	0.015296347	ko01100//Metabolic p	-	-
0.999710768	0.015311887	ko01100//Metabolic p	ko05414//Dilate	-
0.999710605	0.015316194	ko01100//Metabolic p	ko01100//Metab	GO:0005739//mitochon
0.999710421	0.015321083	ko01100//Metabolic p	-	GO:0005886//plasma m
0.999710254	0.015325491	ko01110//Biosynthesis	-	-
0.999708687	0.015366886	-	ko03013//Nucle	-
0.999708483	0.015372267	ko01100//Metabolic p	-	GO:0005886//plasma m
0.99970786	0.015388674	-	-	GO:0005737//cytoplasm
0.999707313	0.015403063	ko01100//Metabolic p	-	-
0.99970724	0.015404984	ko01100//Metabolic p	-	GO:0005615//extracellu
0.999707155	0.015407228	-	-	GO:0005576//extracellu
0.999707125	0.015408022	ko01100//Metabolic p	-	-
0.999706552	0.01542308	-	ko04144//Endoc	-
0.999706315	0.015429305	ko01100//Metabolic p	-	-
0.999705977	0.015438204	-	ko04060//Cytok	GO:0009986//cell surfac
0.999705638	0.015447084	-	ko04080//Neuro	GO:0005886//plasma m
0.999704908	0.015466221	-	-	GO:0005739//mitochon
0.999704385	0.015479927	-	ko01100//Metab	GO:0005783//endoplasr
0.999703628	0.015499736	-	ko00565//Ether	GO:0005615//extracellu
0.999702793	0.015521572	-	-	-
0.999702633	0.01552575	-	-	-
0.999702605	0.015526469	ko01100//Metabolic p	ko03013//Nucle	-
0.99970162	0.015552151	ko01063//Biosynthesis	ko05231//Cholir	GO:0005739//mitochon
0.999701504	0.015555187	ko01100//Metabolic p	-	GO:0016020//membran
0.999701071	0.015566465	-	ko01100//Metab	GO:0005654//nucleopla
0.999700921	0.015570379	-	-	-
0.999700819	0.015573031	-	-	-
0.999700002	0.01559428	ko01120//Microbial m	ko01100//Metab	GO:0005739//mitochon
0.999699323	0.015611916	-	-	GO:0005615//extracellu

0.999699081	0.015618207	ko01100//Metabolic p	-	GO:0005783//endoplasr
0.99969787	0.015649599	-	-	GO:0016021//integral c
0.999697763	0.015652363	-	-	-
0.999697626	0.015655922	-	-	-
0.999696882	0.015675159	-	-	-
0.999696859	0.015675748	ko01100//Metabolic p	ko01100//Metab	-
0.99969661	0.015682178	-	-	-
0.99969639	0.015687876	-	-	GO:0005634//nucleus;G
0.999696318	0.015689745	ko01100//Metabolic p	-	-
0.999696311	0.015689908	-	ko05200//Pathw	GO:0005576//extracellu
0.999696067	0.015696209	-	-	-
0.999695665	0.015706585	-	ko05200//Pathw	GO:0005576//extracellu
0.999695132	0.015720341	-	-	GO:0001726//ruffle;GO:
0.999693688	0.015757531	ko01100//Metabolic p	-	-
0.99969286	0.015778813	ko01100//Metabolic p	-	-
0.999692455	0.015789225	ko01100//Metabolic p	-	-
0.999692388	0.015790927	-	-	-
0.99969192	0.015802936	ko01100//Metabolic p	-	GO:0000781//chromoso
0.999691142	0.015822875	-	ko01100//Metab	GO:0005886//plasma m
0.999690764	0.015832562	ko01100//Metabolic p	ko04745//Photo	-
0.999690642	0.015835686	-	ko04060//Cytok	GO:0005576//extracellu
0.99968811	0.015900366	-	-	-
0.999687959	0.015904212	-	ko01100//Metab	-
0.999687539	0.015914904	-	-	-
0.999687183	0.01592398	ko01100//Metabolic p	-	GO:0005615//extracellu
0.999686864	0.015932098	ko01100//Metabolic p	ko04060//Cytok	GO:0005576//extracellu
0.999686856	0.01593231	ko01100//Metabolic p	ko04390//Hippo	GO:0005667//transcript
0.999686774	0.015934379	-	-	-
0.999686244	0.015947872	-	-	GO:0005737//cytoplasm
0.999686236	0.015948065	ko01100//Metabolic p	ko01100//Metab	GO:0005737//cytoplasm
0.999686236	0.015948066	ko01100//Metabolic p	ko01100//Metab	-
0.999686028	0.01595335	ko01100//Metabolic p	-	-
0.999685881	0.015957081	ko01100//Metabolic p	ko04151//PI3K-/	GO:0005654//nucleopla
0.999685779	0.015959687	-	ko01100//Metab	-
0.999685243	0.015973284	-	-	-
0.999684655	0.015988192	ko01100//Metabolic p	ko04080//Neuro	GO:0005783//endoplasr
0.999684634	0.015988741	-	ko01100//Metab	-
0.999684478	0.015992697	-	-	GO:0005654//nucleopla
0.999684054	0.016003434	-	-	-
0.999683767	0.016010688	-	-	-
0.999683625	0.016014287	-	ko04020//Calciu	GO:0005886//plasma m
0.999683145	0.016026427	-	ko01100//Metab	GO:0005615//extracellu
0.999682383	0.016045687	-	-	-
0.999682314	0.016047443	ko01100//Metabolic p	-	GO:0001726//ruffle;GO:
0.999682237	0.016049372	-	ko01100//Metab	GO:0000139//Golgi mer
0.999681265	0.016073908	-	-	-
0.999681027	0.016079907	ko01100//Metabolic p	-	GO:0005634//nucleus;G
0.999680556	0.016091775	ko01100//Metabolic p	ko04973//Carbo	GO:0005886//plasma m
0.999679946	0.016107146	ko01100//Metabolic p	ko01100//Metab	GO:0005737//cytoplasm
0.99967991	0.016108032	-	ko01100//Metab	GO:0005739//mitochon
0.999679319	0.016122919	-	-	-
0.999679222	0.016125355	-	ko04114//Oocyt	-
0.999679035	0.016130057	-	-	-
0.999678959	0.016131958	-	-	-
0.999677237	0.016175164	ko01100//Metabolic p	-	GO:0005634//nucleus;G
0.999677067	0.016179414	-	ko01100//Metab	-
0.999676567	0.016191952	ko01063//Biosynthesis	ko04150//mTOR	GO:0032991//macromo
0.999675575	0.016216766	ko01100//Metabolic p	ko04514//Cell a	GO:0016020//membran

0.999674308	0.016248396	-	ko05200//Pathw	-
0.999674191	0.016251314	ko01100//Metabolic p	ko01100//Metab	GO:0005615//extracellu
0.999674004	0.016255969	-	-	-
0.999673445	0.016269918	-	-	-
0.999673085	0.016278876	ko01100//Metabolic p	-	GO:0005886//plasma m
0.999673021	0.016280468	ko01100//Metabolic p	ko01100//Metab	GO:0005783//endoplasr
0.999672879	0.016284012	ko01100//Metabolic p	-	GO:0005654//nucleopla
0.999672762	0.016286923	ko01100//Metabolic p	-	GO:0005615//extracellu
0.999672014	0.016305524	-	-	-
0.999671182	0.016326187	-	ko04976//Bile se	GO:0005654//nucleopla
0.999671139	0.016327244	-	-	-
0.999670615	0.016340268	-	ko04070//Phosp	GO:0000323//lytic vacu
0.999670051	0.01635425	ko01100//Metabolic p	ko01100//Metab	-
0.99966995	0.016356757	-	-	-
0.999669366	0.016371219	ko01100//Metabolic p	-	-
0.999669056	0.016378881	ko01100//Metabolic p	ko04970//Saliva	GO:0005576//extracellu
0.999669029	0.016379565	-	-	-
0.999668773	0.016385881	ko01100//Metabolic p	ko03320//PPAR	-
0.999668745	0.016386587	ko01100//Metabolic p	ko04020//Calciu	GO:0005886//plasma m
0.999668659	0.016388705	-	-	GO:0005886//plasma m
0.999668138	0.016401587	-	-	GO:0016020//membran
0.999666947	0.016430993	ko01100//Metabolic p	-	-
0.999665981	0.016454813	-	ko01100//Metab	GO:0005739//mitochon
0.999664761	0.016484831	ko01100//Metabolic p	-	-
0.999664501	0.016491219	-	-	-
0.999664121	0.016500558	ko01100//Metabolic p	-	GO:0001533//cornified
0.999663355	0.016519373	-	-	GO:0005634//nucleus
0.999662948	0.016529348	ko01100//Metabolic p	ko01100//Metab	-
0.999662765	0.016533843	ko01100//Metabolic p	-	GO:0005739//mitochon
0.999662567	0.016538682	-	ko00564//Glycer	GO:0005615//extracellu
0.999662534	0.016539509	ko01100//Metabolic p	ko01100//Metab	-
0.999662273	0.016545885	ko01100//Metabolic p	-	-
0.999662091	0.016550362	ko01100//Metabolic p	-	-
0.999661183	0.016572579	ko01110//Biosynthesis	-	-
0.999660582	0.016587279	-	ko04080//Neuro	GO:0005886//plasma m
0.999660291	0.016594388	ko01100//Metabolic p	ko01100//Metab	-
0.999659827	0.016605696	ko01100//Metabolic p	-	GO:0005634//nucleus;G
0.999659612	0.016610952	ko01100//Metabolic p	ko05143//Africa	GO:0005576//extracellu
0.999659478	0.016614217	-	ko01100//Metab	-
0.999658756	0.016631818	-	-	-
0.999658707	0.016633013	-	ko01100//Metab	GO:0005777//peroxison
0.999658691	0.016633406	ko01100//Metabolic p	ko04145//Phago	GO:0005783//endoplasr
0.999658621	0.016635128	ko01120//Microbial m	-	GO:0005887//integral c
0.999658069	0.016648567	-	-	-
0.999658032	0.016649455	-	-	GO:0001725//stress fibe
0.999657862	0.016653592	ko01100//Metabolic p	ko04151//PI3K-,	GO:0005654//nucleopla
0.99965674	0.016680894	ko01100//Metabolic p	-	-
0.999656251	0.016692773	ko01120//Microbial m	-	GO:0005829//cytosol;G
0.999655872	0.016701958	ko01100//Metabolic p	-	GO:0005737//cytoplasm
0.999655784	0.016704095	ko01100//Metabolic p	-	-
0.999654893	0.016725715	ko01100//Metabolic p	-	-
0.999654744	0.016729306	-	-	-
0.999654371	0.016738355	ko01100//Metabolic p	-	GO:0005634//nucleus
0.999654155	0.016743585	ko01100//Metabolic p	-	-
0.999654102	0.016744871	-	ko05231//Cholir	-
0.999653312	0.016763966	-	ko04010//MAPK	-
0.999653161	0.016767618	ko01100//Metabolic p	-	-
0.999652753	0.016777491	ko01100//Metabolic p	ko03320//PPAR	GO:0005576//extracellu

0.999650941	0.016821199	-	ko05203//Viral c	GO:0000786//nucleosor
0.999650633	0.016828628	-	ko01100//Metab	-
0.999650551	0.016830597	-	-	GO:0005829//cytosol;G(
0.999650337	0.01683575	-	-	GO:0035658//Mon1-Cc
0.999650302	0.016836593	ko01110//Biosynthesis	ko05200//Pathw	GO:0005834//heterotrin
0.999650134	0.016840639	ko01100//Metabolic p	-	GO:0001725//stress fibe
0.999647745	0.016898042	-	-	GO:0005886//plasma m
0.999647629	0.016900836	ko01063//Biosynthesis	ko01100//Metab	-
0.999647616	0.016901143	-	-	-
0.999647461	0.016904856	-	-	-
0.999647054	0.016914621	-	ko05200//Pathw	-
0.999646685	0.016923447	-	-	GO:0005634//nucleus;G
0.999646634	0.016924672	-	-	GO:0016020//membran
0.999646599	0.01692552	ko01100//Metabolic p	ko01100//Metab	GO:0031988//membran
0.999646516	0.016927489	-	-	-
0.999645666	0.016947849	-	-	GO:0016020//membran
0.999645328	0.016955912	-	-	GO:0001726//ruffle;GO:
0.999644965	0.016964593	-	-	-
0.999644914	0.016965808	-	-	-
0.999644709	0.016970721	ko01100//Metabolic p	-	GO:0005634//nucleus
0.999644627	0.01697266	ko01100//Metabolic p	ko04610//Comp	GO:0005615//extracellu
0.99964392	0.016989536	-	-	GO:0001533//cornified
0.99964298	0.017011965	-	ko01100//Metab	GO:0005794//Golgi app
0.999642823	0.017015688	-	ko01100//Metab	-
0.999642435	0.017024949	-	-	GO:0000781//chromoso
0.999641865	0.017038498	-	ko01100//Metab	-
0.999641148	0.017055543	-	ko01100//Metab	GO:0005739//mitochon
0.999641137	0.017055814	-	ko04080//Neuro	GO:0005783//endoplasr
0.99964093	0.017060729	ko01100//Metabolic p	ko01100//Metab	GO:0005615//extracellu
0.999640487	0.017071257	-	ko04060//Cytok	GO:0005576//extracellu
0.999640404	0.01707322	ko01100//Metabolic p	ko05200//Pathw	-
0.999640372	0.017073988	ko01100//Metabolic p	-	-
0.999639773	0.017088209	-	-	-
0.999639038	0.017105636	ko01100//Metabolic p	-	-
0.999638619	0.017115538	-	-	GO:0005654//nucleopla
0.999637129	0.017150805	-	ko04979//Chole	GO:0005856//cytoskelet
0.99963664	0.017162362	ko01100//Metabolic p	ko01100//Metab	-
0.999636587	0.017163616	-	-	GO:0005737//cytoplasm
0.999636148	0.017173957	-	-	-
0.999635352	0.017192754	-	ko03460//Fanco	GO:0000785//chromatir
0.999634791	0.017205974	-	ko04713//Circac	-
0.999634729	0.01720743	-	-	-
0.999634603	0.01721039	ko01100//Metabolic p	ko01100//Metab	GO:0005737//cytoplasm
0.999634379	0.017215677	ko01120//Microbial m	-	-
0.99963373	0.017230932	ko01100//Metabolic p	ko04514//Cell ac	-
0.999633522	0.017235834	-	-	GO:0005768//endosom
0.999630848	0.017298598	ko01100//Metabolic p	-	GO:0016021//integral c
0.999630791	0.017299934	ko01100//Metabolic p	-	-
0.999630303	0.017311372	-	-	GO:0005802//trans-Gol
0.999630233	0.017313017	-	-	-
0.999629697	0.017325543	-	-	-
0.999629621	0.01732733	-	ko04060//Cytok	GO:0005576//extracellu
0.999628888	0.017344461	-	ko00983//Drug	-
0.999628852	0.017345321	-	-	-
0.999628787	0.017346827	ko01100//Metabolic p	-	-
0.999628761	0.017347433	ko01100//Metabolic p	-	-
0.999628668	0.017349603	-	-	-
0.999628555	0.017352246	ko01100//Metabolic p	ko04514//Cell ac	GO:0005886//plasma m

0.999628481	0.017353971	ko01100//Metabolic p	-	-
0.999627343	0.017380534	ko01100//Metabolic p	-	-
0.999627332	0.017380791	-	-	GO:0016021//integral c
0.999627233	0.017383097	-	ko05414//Dilate	-
0.999625952	0.017412938	ko01110//Biosynthesis	-	GO:0005829//cytosol;G
0.999625891	0.017414365	ko01100//Metabolic p	ko01100//Metab	-
0.999625838	0.017415602	-	-	GO:0005886//plasma m
0.999625569	0.017421872	-	ko01100//Metab	-
0.999625364	0.01742662	-	-	GO:0015630//microtubu
0.999624758	0.017440722	ko01100//Metabolic p	ko01100//Metab	GO:0000139//Golgi mer
0.999624674	0.017442676	-	-	GO:0001726//ruffle;GO:
0.99962448	0.017447187	ko01100//Metabolic p	-	-
0.999624159	0.017454638	-	ko01100//Metab	-
0.999623349	0.017473434	ko01100//Metabolic p	ko05165//Huma	GO:0005634//nucleus;G
0.999623276	0.01747514	-	-	GO:0005634//nucleus;G
0.999623265	0.017475375	-	-	-
0.999623262	0.017475456	-	ko01100//Metab	GO:0005739//mitochon
0.999622775	0.017486753	ko01100//Metabolic p	ko04080//Neuro	GO:0005576//extracellu
0.999622675	0.017489063	-	-	GO:0005576//extracellu
0.999622096	0.017502481	-	ko01100//Metab	GO:0005783//endoplasr
0.999620949	0.017529014	-	ko01100//Metab	GO:0005634//nucleus;G
0.999620787	0.017532756	-	-	-
0.999620548	0.017538291	-	-	GO:0030425//dendrite
0.999620317	0.017543635	-	ko04060//Cytok	GO:0009986//cell surfac
0.999620275	0.017544611	-	-	GO:0005615//extracellu
0.999620265	0.01754484	-	-	-
0.999619933	0.017552503	-	ko04970//Saliva	GO:0005576//extracellu
0.99961953	0.017561806	ko01100//Metabolic p	-	GO:0005654//nucleopla
0.999619468	0.017563229	-	ko00982//Drug	-
0.999619331	0.017566398	-	-	-
0.999619331	0.017566407	-	-	-
0.999619193	0.017569577	-	ko01100//Metab	GO:0005783//endoplasr
0.99961784	0.017600768	ko01100//Metabolic p	ko04060//Cytok	GO:0009897//external s
0.999617819	0.017601253	-	ko05322//Syster	GO:0005576//extracellu
0.999617797	0.017601755	-	ko04310//Wnt s	GO:0005576//extracellu
0.999617386	0.017611214	-	-	GO:0002177//manchett
0.99961673	0.017626308	ko01100//Metabolic p	-	-
0.999616709	0.017626784	-	ko01100//Metab	-
0.999616255	0.017637231	-	-	GO:0005737//cytoplasm
0.999615039	0.01766515	ko01100//Metabolic p	-	-
0.999614785	0.017670979	ko01100//Metabolic p	ko04141//Protei	GO:0030120//vesicle co
0.999614312	0.017681838	ko01100//Metabolic p	ko01100//Metab	-
0.999612939	0.017713283	-	ko04060//Cytok	GO:0005576//extracellu
0.999612488	0.01772359	ko01100//Metabolic p	ko04060//Cytok	GO:0009897//external s
0.999612474	0.017723922	-	-	-
0.999612314	0.017727563	-	-	-
0.999611892	0.017737226	ko01100//Metabolic p	-	GO:0005576//extracellu
0.999611872	0.01773767	ko01100//Metabolic p	ko04010//MAPK	-
0.999611696	0.01774169	ko01100//Metabolic p	ko01100//Metab	-
0.999611368	0.01774919	-	ko01100//Metab	-
0.999611273	0.017751362	-	ko01100//Metab	-
0.999610475	0.017769579	-	-	GO:0005737//cytoplasm
0.999610455	0.01777002	ko01100//Metabolic p	-	-
0.999608642	0.017811333	-	-	-
0.999608494	0.017814703	-	-	-
0.999607919	0.017827766	-	-	-
0.999607751	0.017831588	ko01100//Metabolic p	ko04010//MAPK	-
0.999607325	0.017841286	-	-	-

0.999607249	0.017843014	ko01100//Metabolic p	ko04970//Saliva	GO:0005576//extracellu
0.99960698	0.01784912	-	-	GO:0031462//Cul2-RIN(
0.999606911	0.017850676	ko01100//Metabolic p	-	-
0.999606456	0.017861015	ko01100//Metabolic p	-	-
0.999606227	0.01786621	ko01100//Metabolic p	ko01100//Metak	GO:0005886//plasma m
0.999606158	0.017867761	-	ko04918//Thyro	GO:0043231//intracellul
0.999606034	0.01787058	ko01100//Metabolic p	-	-
0.999605084	0.017892113	ko01100//Metabolic p	-	-
0.999604992	0.017894204	-	ko01100//Metak	-
0.999604845	0.017897531	ko01100//Metabolic p	-	-
0.999604254	0.017910908	ko01063//Biosynthesis	ko04060//Cytok	GO:0005576//extracellu
0.999603804	0.017921104	ko01100//Metabolic p	ko04060//Cytok	GO:0005576//extracellu
0.999603499	0.017927982	ko01100//Metabolic p	-	-
0.999602154	0.01795838	ko01100//Metabolic p	-	-
0.999601718	0.017968207	ko01100//Metabolic p	-	-
0.999601637	0.017970044	-	ko01100//Metak	-
0.999601081	0.017982579	-	-	-
0.999600612	0.017993141	-	ko01100//Metak	GO:0031988//membran
0.999600457	0.017996628	-	ko00564//Glycei	GO:0005615//extracellu
0.999599949	0.018008079	-	-	-
0.999599068	0.018027899	-	-	-
0.999598389	0.018043144	-	-	GO:0031012//extracellu
0.999597968	0.01805261	ko01100//Metabolic p	-	GO:0005829//cytosol;G(
0.999597141	0.018071159	ko01100//Metabolic p	ko00982//Drug	-
0.999596819	0.018078384	ko01100//Metabolic p	ko04141//Protei	GO:0005737//cytoplasm
0.999596488	0.018085802	ko01100//Metabolic p	-	-
0.99959596	0.018097634	-	-	-
0.999595752	0.018102303	-	-	GO:0005737//cytoplasm
0.999595581	0.01810612	ko01100//Metabolic p	ko01100//Metak	-
0.999594435	0.018131756	ko01100//Metabolic p	-	GO:0031012//extracellu
0.999594029	0.018140842	-	ko05200//Pathw	-
0.999593828	0.018145324	ko01100//Metabolic p	-	GO:0005576//extracellu
0.99959361	0.018150191	-	ko05200//Pathw	-
0.999593535	0.018151871	-	-	GO:0005576//extracellu
0.999593441	0.018153964	-	-	GO:0005615//extracellu
0.999592533	0.018174242	-	-	-
0.999592071	0.018184545	ko01100//Metabolic p	-	GO:0005634//nucleus;G
0.999591342	0.018200767	-	-	-
0.999591271	0.018202361	-	ko04390//Hippo	GO:0005667//transcript
0.999590496	0.018219604	-	ko04974//Protei	-
0.999589388	0.018244235	ko01100//Metabolic p	ko01100//Metak	GO:0005739//mitochon
0.99958922	0.018247968	-	-	-
0.999589178	0.018248901	-	-	-
0.999588515	0.018263628	ko01100//Metabolic p	ko01100//Metak	GO:0005615//extracellu
0.999587951	0.018276132	-	ko04979//Chole	GO:0005783//endoplasi
0.999587639	0.018283063	-	ko04216//Ferrop	GO:0016020//membran
0.999585914	0.018321263	ko01110//Biosynthesis	ko04514//Cell a	GO:0005829//cytosol;G(
0.999585664	0.0183268	ko01100//Metabolic p	-	-
0.999585102	0.018339229	-	ko04140//Autop	GO:0000407//pre-autop
0.999584555	0.018351295	-	-	-
0.999584446	0.018353711	ko01100//Metabolic p	ko01100//Metak	GO:0033180//proton-tr
0.999584411	0.018354479	ko01100//Metabolic p	-	-
0.999584294	0.018357069	-	-	-
0.999584269	0.018357626	-	ko04130//SNAR	GO:0005737//cytoplasm
0.999583957	0.0183645	-	ko05017//Spino	GO:0043231//intracellul
0.999582597	0.018394494	ko01063//Biosynthesis	-	-
0.999582357	0.018399783	ko01100//Metabolic p	-	-
0.999582091	0.01840565	-	-	GO:0005764//lysosome;

0.999581324	0.018422539	-	-	-
0.999581136	0.018426672	-	ko01100//Metab	GO:0043231//intracellul
0.999581123	0.018426957	ko01100//Metabolic p	-	-
0.999581122	0.018426971	ko01100//Metabolic p	ko05132//Salmo	GO:0005737//cytoplasm
0.999581008	0.018429483	ko01100//Metabolic p	-	GO:0005634//nucleus;G
0.99958097	0.01843032	-	-	-
0.999579484	0.018462982	ko01100//Metabolic p	ko04726//Seroto	GO:0005739//mitochon
0.999579036	0.018472796	ko01100//Metabolic p	-	GO:0016020//membran
0.999578248	0.018490089	-	-	GO:0043231//intracellul
0.999577996	0.018495606	ko01100//Metabolic p	ko04976//Bile se	GO:0005654//nucleopla
0.99957718	0.01851349	-	ko01100//Metab	GO:0005739//mitochon
0.999576854	0.018520611	-	-	GO:0016021//integral co
0.999576463	0.018529177	-	-	GO:0005634//nucleus;G
0.999574999	0.018561167	-	ko05200//Pathw	-
0.999574667	0.01856843	-	ko04979//Chole	GO:0005856//cytoskelet
0.999573445	0.018595088	ko01100//Metabolic p	-	GO:0005886//plasma m
0.999573175	0.018600961	ko01100//Metabolic p	ko05150//Staphy	-
0.999573055	0.01860357	-	ko01100//Metab	-
0.999572487	0.018615957	-	-	-
0.99957246	0.018616537	ko01100//Metabolic p	-	-
0.99957136	0.018640475	ko01063//Biosynthesis	-	-
0.999571039	0.01864746	-	-	-
0.999570575	0.018657535	-	ko05200//Pathw	GO:0000785//chromatin
0.999570121	0.018667403	-	ko04114//Oocyt	-
0.999570011	0.018669784	-	-	GO:0005737//cytoplasm
0.999570002	0.018669977	-	-	GO:0001533//cornified
0.999569949	0.018671123	ko01100//Metabolic p	ko01100//Metab	GO:0005737//cytoplasm
0.999569881	0.018672615	ko01100//Metabolic p	-	-
0.999569757	0.018675302	-	ko05200//Pathw	GO:0005794//Golgi app
0.99956958	0.018679137	-	-	-
0.999569372	0.018683645	-	ko02010//ABC tı	GO:0005886//plasma m
0.999568898	0.01869393	-	-	-
0.999568243	0.018708124	-	ko01100//Metab	GO:0005886//plasma m
0.999568209	0.018708868	-	ko01100//Metab	GO:0031988//membran
0.999568105	0.018711123	ko01100//Metabolic p	-	GO:0000786//nucleosor
0.999568072	0.018711837	-	ko03018//RNA c	GO:0005829//cytosol
0.999567689	0.018720124	-	-	GO:0001725//stress fibe
0.999567286	0.01872885	-	-	-
0.999566648	0.018742666	-	-	-
0.999565647	0.0187643	ko01100//Metabolic p	ko01100//Metab	-
0.999565043	0.018777333	-	-	-
0.999564788	0.018782836	-	ko05168//Herpe	-
0.999564565	0.01878766	-	-	GO:0016021//integral co
0.999563881	0.018802408	-	ko00983//Drug	-
0.999563779	0.018804613	-	ko05200//Pathw	-
0.999563484	0.018810953	-	-	-
0.999562087	0.018841044	-	ko05414//Dilate	-
0.99956147	0.01885432	ko01100//Metabolic p	-	GO:0005737//cytoplasm
0.999560966	0.018865134	ko01100//Metabolic p	ko01100//Metab	-
0.999560706	0.018870726	-	-	GO:0005886//plasma m
0.999559722	0.018891856	-	-	-
0.999559669	0.018892992	-	ko04141//Protei	GO:0030120//vesicle co
0.999559652	0.018893364	ko01100//Metabolic p	-	-
0.999558824	0.018911106	-	-	-
0.999557981	0.018929161	-	-	-
0.999557829	0.018932429	-	-	GO:0005737//cytoplasm
0.999557437	0.018940818	ko01100//Metabolic p	-	-
0.999557299	0.018943763	-	-	-

0.999556339	0.018964291	-	-	GO:0005576//extracellu
0.999555195	0.018988725	-	ko04060//Cytok	GO:0005576//extracellu
0.999554717	0.018998936	-	-	-
0.999554196	0.019010058	-	-	GO:0001725//stress fibe
0.999553681	0.019021018	-	-	GO:0005654//nucleopla
0.999553239	0.019030453	-	ko01100//Metak	-
0.999553103	0.019033346	ko01100//Metabolic p	-	-
0.999551327	0.019071133	ko01100//Metabolic p	-	GO:0031462//Cul2-RIN
0.99955057	0.019087215	-	-	-
0.999549945	0.019100485	-	-	-
0.99954903	0.019119894	ko01100//Metabolic p	-	GO:0005886//plasma m
0.999548824	0.019124256	-	ko04390//Hippo	GO:0005667//transcript
0.999548138	0.01913878	-	ko00982//Drug	-
0.999548074	0.01914014	-	-	GO:0005634//nucleus
0.999546572	0.019171922	ko01100//Metabolic p	ko01100//Metak	GO:0005829//cytosol;G
0.99954648	0.019173866	-	-	-
0.999546171	0.019180393	-	-	-
0.999545313	0.019198523	-	-	-
0.999544977	0.019205615	-	ko01100//Metak	-
0.99954463	0.019212946	-	-	GO:0005576//extracellu
0.999544476	0.019216179	-	-	GO:0005634//nucleus
0.999544329	0.019219289	ko01100//Metabolic p	-	GO:0016020//membran
0.999543698	0.019232582	ko01100//Metabolic p	ko04973//Carbo	GO:0005886//plasma m
0.999542857	0.019250317	-	-	-
0.999542486	0.01925812	-	-	GO:0031090//organelle
0.999542316	0.019261689	ko01100//Metabolic p	-	GO:0005886//plasma m
0.99954211	0.019266036	-	ko05200//Pathw	GO:0005576//extracellu
0.999541439	0.019280137	-	ko01100//Metak	-
0.999541096	0.019287345	-	-	-
0.999540988	0.019289634	ko01100//Metabolic p	-	-
0.999540475	0.019300397	-	ko04141//Protei	GO:0005737//cytoplasm
0.999539864	0.019313233	-	-	GO:0030425//dendrite
0.999539493	0.019321011	ko01100//Metabolic p	-	-
0.999538395	0.019344039	-	-	-
0.999537283	0.01936732	ko01100//Metabolic p	ko01100//Metak	GO:0005739//mitochon
0.999536975	0.019373773	-	-	GO:0016021//integral c
0.999536945	0.019374388	ko01100//Metabolic p	-	-
0.999535333	0.019408088	-	-	GO:0005783//endoplasr
0.999534857	0.019418037	-	-	-
0.999534597	0.019423456	-	ko04514//Cell a	GO:0005886//plasma m
0.999534326	0.019429117	-	ko01100//Metak	-
0.999534168	0.019432413	-	ko01100//Metak	-
0.999533783	0.019440437	-	-	-
0.999532955	0.0194577	-	ko01100//Metak	-
0.999531672	0.019484414	-	ko01100//Metak	-
0.999531183	0.019494579	-	-	-
0.999530359	0.019511703	-	ko04979//Chole	GO:0005783//endoplasr
0.999530247	0.019514031	ko01100//Metabolic p	ko04024//cAMP	GO:0016021//integral c
0.999530046	0.019518206	-	-	-
0.999530018	0.019518791	ko01100//Metabolic p	ko01100//Metak	GO:0005739//mitochon
0.999529877	0.019521719	ko01100//Metabolic p	-	-
0.999529825	0.019522795	ko01100//Metabolic p	-	GO:0005634//nucleus;G
0.999529545	0.019528605	ko01120//Microbial m	ko01100//Metak	GO:0016021//integral c
0.999529456	0.019530451	ko01100//Metabolic p	-	GO:0005768//endosom
0.999529083	0.019538199	ko01100//Metabolic p	-	-
0.99952848	0.019550696	ko01100//Metabolic p	-	-
0.999527736	0.019566116	-	ko04640//Hema	GO:0031226//intrinsic c
0.999527721	0.01956643	-	ko04724//Glutar	GO:0005886//plasma m

0.999527076	0.019579778	-	-	GO:0005634//nucleus;G
0.99952671	0.019587359	-	-	GO:0016020//membran
0.999526557	0.019590525	ko01100//Metabolic p	-	GO:0016020//membran
0.999526535	0.019590985	ko01100//Metabolic p	-	GO:0005634//nucleus;G
0.999526105	0.019599872	ko01100//Metabolic p	ko01100//Metak	-
0.999525809	0.019605989	ko01100//Metabolic p	-	GO:0005802//trans-Gol
0.999525714	0.019607961	ko01100//Metabolic p	ko01100//Metak	GO:0005615//extracellu
0.999525628	0.019609742	ko01100//Metabolic p	-	-
0.999525609	0.019610141	-	ko01100//Metak	GO:0000139//Golgi mer
0.999525482	0.019612764	-	ko04971//Gastri	GO:0005886//plasma m
0.999525045	0.019621796	-	-	-
0.99952391	0.019645226	-	ko04020//Calciu	GO:0005886//plasma m
0.999523863	0.019646195	-	ko04080//Neurc	GO:0005783//endoplasr
0.99952376	0.019648311	ko01100//Metabolic p	ko01100//Metak	-
0.999523012	0.01966374	-	-	-
0.999522385	0.019676666	ko01100//Metabolic p	ko04514//Cell a	GO:0005829//cytosol;G(
0.999522059	0.01968338	ko01063//Biosynthesis	-	-
0.999521916	0.019686321	-	ko01100//Metak	-
0.999521856	0.01968756	ko01100//Metabolic p	ko01100//Metak	GO:0016020//membran
0.999521002	0.019705131	ko01100//Metabolic p	ko05200//Pathw	GO:0005634//nucleus;G
0.999520878	0.019707677	ko01100//Metabolic p	ko01100//Metak	GO:0005640//nuclear ou
0.999520555	0.019714317	ko01100//Metabolic p	-	GO:0005579//membran
0.999518872	0.019748904	ko01120//Microbial m	ko04151//PI3K-/	GO:0005654//nucleopla
0.99951832	0.01976022	-	-	-
0.999518261	0.019761435	ko01120//Microbial m	-	-
0.999517811	0.019770672	ko01100//Metabolic p	ko05322//Syster	GO:0005576//extracellu
0.999517786	0.019771174	ko01100//Metabolic p	ko04310//Wnt s	GO:0005576//extracellu
0.999517258	0.019782007	-	-	GO:0005615//extracellu
0.999517162	0.019783959	ko01100//Metabolic p	-	-
0.9995169	0.019789338	-	ko04514//Cell a	GO:0005886//plasma m
0.999516635	0.019794758	-	-	-
0.999516184	0.019804	-	ko04979//Chole	GO:0005856//cytoskelet
0.999515125	0.019825656	-	-	GO:0016021//integral c
0.99951462	0.019835979	-	-	GO:0005634//nucleus
0.999513531	0.019858215	-	ko04726//Serot	GO:0005739//mitochon
0.999512289	0.019883548	-	ko04060//Cytok	GO:0005576//extracellu
0.999512173	0.019885913	ko01100//Metabolic p	-	-
0.999511737	0.019894808	-	-	-
0.999510948	0.019910873	-	ko05200//Pathw	GO:0005794//Golgi app
0.999510032	0.019929507	-	-	-
0.999509854	0.019933136	-	-	GO:0000786//nucleosor
0.999509808	0.019934065	ko01120//Microbial m	-	-
0.999509296	0.019944479	ko01100//Metabolic p	ko05200//Pathw	GO:0005576//extracellu
0.9995084	0.019962673	-	-	GO:0005758//mitochon
0.999508365	0.019963382	-	ko05143//Africa	GO:0005576//extracellu
0.999508163	0.019967494	ko01100//Metabolic p	-	-
0.999507857	0.019973708	-	-	GO:0000139//Golgi mer
0.999507841	0.019974035	-	ko05203//Viral c	GO:0000786//nucleosor
0.999507332	0.019984354	-	-	-
0.999506999	0.019991099	ko01100//Metabolic p	-	-
0.999506719	0.019996783	ko01100//Metabolic p	-	-
0.999506615	0.019998893	-	-	GO:0001726//ruffle;GO:
0.999505501	0.020021463	-	ko01100//Metak	GO:0005886//plasma m
0.999505383	0.020023843	-	-	-
0.999505012	0.020031358	-	-	GO:0005634//nucleus;G
0.999504252	0.020046721	ko01100//Metabolic p	-	GO:0045095//keratin fila
0.999503119	0.020069635	-	-	GO:0016021//integral c
0.999503048	0.020071062	-	ko01100//Metak	GO:0005634//nucleus;G

0.999502884	0.020074369	ko01100//Metabolic p	-	-
0.999502863	0.020074804	-	-	-
0.999502627	0.020079567	ko01100//Metabolic p	-	GO:0005737//cytoplasm
0.999501792	0.020096414	-	-	-
0.999501514	0.020102024	ko01100//Metabolic p	-	GO:0005737//cytoplasm
0.9994992	0.020148627	ko01100//Metabolic p	-	GO:0005654//nucleopla
0.999498332	0.02016607	-	ko01100//Metab	GO:0005886//plasma m
0.99949749	0.020183001	-	ko01100//Metab	-
0.99949702	0.020192437	-	-	-
0.999497017	0.020192493	ko01100//Metabolic p	ko04514//Cell a	GO:0005769//early end
0.999495767	0.020217577	ko01100//Metabolic p	-	-
0.999491476	0.020303421	-	-	-
0.999490981	0.020313304	-	-	GO:0005886//plasma m
0.999490305	0.020326793	-	-	-
0.99948999	0.020333059	ko01100//Metabolic p	-	GO:0016020//membran
0.999488026	0.020372175	ko01100//Metabolic p	-	GO:0000791//euchroma
0.99948495	0.020433291	ko01100//Metabolic p	-	GO:0031224//intrinsic c
0.999484731	0.020437642	ko01100//Metabolic p	-	GO:0016021//integral c
0.999484542	0.020441389	-	ko00983//Drug	-
0.999483232	0.020467355	ko01110//Biosynthesis	ko01100//Metab	-
0.999483147	0.02046903	-	ko01100//Metab	GO:0005737//cytoplasm
0.999482218	0.020487414	ko01100//Metabolic p	-	-
0.999481898	0.020493749	ko01100//Metabolic p	-	-
0.999481081	0.02050991	-	-	-
0.999480373	0.020523898	-	-	-
0.999480231	0.0205267	ko01100//Metabolic p	-	-
0.999480129	0.020528709	ko01100//Metabolic p	-	GO:0016020//membran
0.999480069	0.020529904	-	-	GO:0005737//cytoplasm
0.99947988	0.020533627	-	-	GO:0016021//integral c
0.999479822	0.020534772	ko01110//Biosynthesis	ko01100//Metab	GO:0005741//mitochon
0.999478699	0.020556937	ko01100//Metabolic p	-	-
0.999478656	0.020557778	-	ko05168//Herpe	-
0.999477318	0.02058415	ko01063//Biosynthesis	ko01100//Metab	GO:0005739//mitochon
0.99947729	0.020584699	ko01100//Metabolic p	ko01100//Metab	GO:0005739//mitochon
0.999476711	0.020596091	ko01100//Metabolic p	ko01100//Metab	GO:0043231//intracellul
0.999476576	0.020598742	ko01100//Metabolic p	-	GO:0005634//nucleus;G
0.999476138	0.020607371	ko01100//Metabolic p	-	-
0.999476088	0.020608361	ko01100//Metabolic p	-	-
0.999475502	0.020619872	-	ko01100//Metab	-
0.999475424	0.020621417	-	-	-
0.999475063	0.02062851	-	ko00564//Glyce	GO:0005634//nucleus;G
0.999474189	0.020645669	-	-	GO:0005886//plasma m
0.999473911	0.020651131	ko01100//Metabolic p	ko01100//Metab	-
0.999472664	0.020675582	-	ko01100//Metab	GO:0005615//extracellu
0.999470603	0.02071595	-	-	-
0.999469943	0.020728873	ko01100//Metabolic p	-	GO:0005576//extracellu
0.999469283	0.020741772	-	ko04745//Photo	-
0.99946865	0.020754137	-	-	GO:0005737//cytoplasm
0.999466635	0.020793454	-	ko04610//Comp	GO:0005615//extracellu
0.999464553	0.020834005	ko01100//Metabolic p	ko04659//Th17	GO:0000785//chromatin
0.999463743	0.020849765	-	ko01100//Metab	GO:0005737//cytoplasm
0.999463647	0.020851632	ko01100//Metabolic p	ko05016//Huntir	-
0.999462393	0.020875983	-	-	-
0.999462155	0.020880604	ko01100//Metabolic p	-	-
0.999462114	0.020881403	-	ko04514//Cell a	GO:0005886//plasma m
0.999461277	0.020897655	-	-	GO:0005737//cytoplasm
0.999461052	0.020902001	-	-	GO:0005783//endoplasr
0.999458445	0.020952504	ko01100//Metabolic p	-	-

0.999458384	0.020953696	ko01100//Metabolic p	ko02010//ABC tı	GO:0005886//plasma m
0.999458081	0.020959543	-	ko01100//Metab	-
0.999457905	0.02096296	ko01100//Metabolic p	ko04514//Cell a	GO:0005886//plasma m
0.999457624	0.020968379	ko01100//Metabolic p	-	-
0.999457357	0.020973539	-	-	-
0.999456992	0.020980603	-	-	-
0.999456033	0.020999111	ko01100//Metabolic p	ko01100//Metab	GO:0005739//mitochon
0.999456033	0.020999124	-	-	-
0.999455364	0.021012027	-	-	-
0.99945536	0.021012101	-	ko05200//Pathw	GO:0005576//extracellu
0.99945492	0.021020597	-	ko04918//Thyro	GO:0043231//intracellul
0.999453862	0.021040979	-	ko05200//Pathw	GO:0005576//extracellu
0.999453847	0.021041265	-	-	GO:0005783//endoplasr
0.999453264	0.021052509	ko01063//Biosynthesis	ko01100//Metab	-
0.99945253	0.021066624	-	ko04919//Thyro	GO:0005887//integral c
0.999452329	0.021070498	-	ko01100//Metab	GO:0005730//nucleolus
0.999451512	0.021086214	ko01100//Metabolic p	ko04022//cGMP	-
0.999450232	0.021110798	ko01100//Metabolic p	ko04080//Neurc	GO:0005886//plasma m
0.999450025	0.021114784	-	-	GO:0005576//extracellu
0.999449877	0.021117621	-	-	GO:0005856//cytoskelet
0.999449738	0.021120285	ko01100//Metabolic p	-	GO:0005634//nucleus;G
0.999449578	0.021123357	ko01100//Metabolic p	ko04060//Cytok	-
0.999449489	0.021125058	ko01100//Metabolic p	ko01100//Metab	-
0.999449029	0.021133886	ko01120//Microbial m	ko03013//Nucle	-
0.999448943	0.021135544	-	ko01100//Metab	GO:0005654//nucleopla
0.99944804	0.021152844	ko01100//Metabolic p	ko01100//Metab	-
0.999447498	0.021163229	-	-	GO:0005886//plasma m
0.999447163	0.021169649	ko01100//Metabolic p	ko01523//Antifo	GO:0005886//plasma m
0.999445269	0.021205886	-	-	-
0.999444707	0.021216621	-	ko01100//Metab	-
0.999443874	0.021232535	ko01100//Metabolic p	-	-
0.999443708	0.021235699	ko01100//Metabolic p	-	GO:0016021//integral c
0.999443437	0.021240875	-	ko04151//PI3K-,	GO:0005654//nucleopla
0.999443419	0.021241218	ko01100//Metabolic p	-	-
0.9994433	0.021243482	ko01100//Metabolic p	ko01100//Metab	-
0.999442764	0.021253719	-	-	GO:0016021//integral c
0.999442012	0.021268055	-	ko03018//RNA c	GO:0005829//cytosol
0.999441963	0.021268987	ko01100//Metabolic p	-	-
0.999441573	0.021276413	ko01100//Metabolic p	-	GO:0005737//cytoplasm
0.999440761	0.021291883	ko01100//Metabolic p	ko03410//Base e	GO:0005634//nucleus;G
0.999440654	0.021293925	-	-	GO:0005634//nucleus
0.999440393	0.021298883	-	-	-
0.999440061	0.021305206	-	-	GO:0016021//integral c
0.999439101	0.021323466	ko01100//Metabolic p	-	-
0.999438597	0.021333052	-	-	-
0.999438572	0.021333509	ko01120//Microbial m	ko01100//Metab	GO:0005783//endoplasr
0.999438554	0.021333855	-	-	-
0.999438523	0.021334441	-	-	-
0.99943816	0.021341342	-	ko01100//Metab	GO:0005829//cytosol;G
0.999438027	0.021343878	-	-	GO:0016021//integral c
0.999436405	0.021374654	-	ko04151//PI3K-,	GO:0008305//integrin c
0.999436146	0.021379572	-	-	-
0.999434305	0.02141445	ko01100//Metabolic p	-	-
0.999434285	0.02141482	ko01120//Microbial m	ko01100//Metab	-
0.999433941	0.021421328	-	ko05143//Africa	GO:0005576//extracellu
0.999433579	0.021428178	ko01100//Metabolic p	-	GO:0005829//cytosol;G
0.999433551	0.021428719	-	ko01100//Metab	GO:0016020//membran
0.99943322	0.02143497	ko01110//Biosynthesis	-	GO:0005737//cytoplasm

0.99943275	0.021443861	-	ko03010//Ribos	GO:0005634//nucleus;G
0.999432447	0.021449581	ko01100//Metabolic p	-	GO:0005886//plasma m
0.999432119	0.021455789	ko01100//Metabolic p	ko01100//Metab	-
0.999431946	0.021459053	-	-	-
0.999430284	0.02149043	-	-	-
0.999429293	0.021509098	-	ko01100//Metab	-
0.999428271	0.021528364	ko01100//Metabolic p	ko01100//Metab	GO:0048269//methionir
0.999427988	0.021533685	ko01100//Metabolic p	-	-
0.999426853	0.021555035	-	-	-
0.999426845	0.021555196	ko01100//Metabolic p	-	GO:0005874//microtubu
0.999426815	0.021555749	-	ko05130//Patho	GO:0005856//cytoskelet
0.999426526	0.021561194	ko01100//Metabolic p	-	-
0.999426426	0.021563066	-	-	GO:0005654//nucleopla
0.999426333	0.021564822	-	-	GO:0005615//extracellu
0.999425619	0.021578234	ko01100//Metabolic p	ko01100//Metab	-
0.999425299	0.021584249	-	-	-
0.999424883	0.021592058	ko01100//Metabolic p	-	GO:0005615//extracellu
0.999424853	0.021592626	-	-	-
0.999424639	0.021596634	-	-	GO:0005576//extracellu
0.999423445	0.021619041	ko01100//Metabolic p	ko01100//Metab	-
0.999422823	0.021630694	ko01100//Metabolic p	-	GO:0016020//membran
0.999422153	0.021643246	ko01100//Metabolic p	-	GO:0005737//cytoplasm
0.999422089	0.021644439	-	-	-
0.999421257	0.02166003	-	ko04114//Oocyt	GO:0005634//nucleus;G
0.999421007	0.021664699	ko01100//Metabolic p	-	GO:0005730//nucleolus
0.99942081	0.02166839	ko01100//Metabolic p	-	GO:0016021//integral c
0.999420533	0.021673569	-	ko04020//Calciu	GO:0005886//plasma m
0.999420457	0.021674993	-	-	-
0.999420352	0.02167696	-	ko00983//Drug	-
0.99941995	0.021684479	ko01100//Metabolic p	-	-
0.999418778	0.021706377	-	-	GO:0005886//plasma m
0.999418638	0.021708981	-	-	GO:0005737//cytoplasm
0.999418101	0.021719	ko01100//Metabolic p	-	GO:0015629//actin cyto
0.999417603	0.021728292	-	-	GO:0016020//membran
0.999417435	0.021731436	-	-	GO:0016020//membran
0.99941738	0.021732456	-	-	GO:0005634//nucleus
0.999417215	0.021735536	ko01100//Metabolic p	ko04640//Hema	GO:0031226//intrinsic c
0.999416908	0.021741265	ko01100//Metabolic p	ko01100//Metab	-
0.999416508	0.021748713	-	-	-
0.999414881	0.021779031	ko01100//Metabolic p	ko01100//Metab	-
0.999414072	0.021794075	-	-	-
0.999413942	0.021796494	-	-	GO:0005576//extracellu
0.999413892	0.02179742	-	ko02010//ABC t	GO:0005739//mitochon
0.999413055	0.021812982	-	-	GO:0005634//nucleus;G
0.999412529	0.021822757	ko01100//Metabolic p	ko01100//Metab	-
0.999412155	0.021829697	-	ko05414//Dilate	-
0.999412119	0.021830374	-	-	-
0.999411821	0.021835903	ko01100//Metabolic p	-	GO:0005783//endoplas
0.999411559	0.021840774	ko01063//Biosynthesis	-	-
0.999411408	0.021843563	-	-	-
0.999411049	0.021850227	-	ko04714//Therm	GO:0005634//nucleus
0.999410981	0.021851489	-	ko01100//Metab	-
0.999410768	0.021855443	-	ko01100//Metab	-
0.999410766	0.021855475	ko01100//Metabolic p	-	GO:0005886//plasma m
0.999410664	0.021857376	-	ko01100//Metab	-
0.999410622	0.021858157	ko01100//Metabolic p	ko01100//Metab	-
0.999410073	0.021868333	-	ko00983//Drug	-
0.999409166	0.021885142	-	-	GO:0016020//membran

0.99940822	0.021902652	ko01100//Metabolic p	-	GO:0005604//basement
0.999407794	0.021910529	-	-	GO:0005634//nucleus;G
0.999407409	0.021917647	-	-	GO:0005576//extracellu
0.999405697	0.021949291	ko01100//Metabolic p	-	GO:0005737//cytoplasm
0.999405627	0.021950592	-	-	GO:0005884//actin filam
0.999405388	0.021955001	ko01100//Metabolic p	-	GO:0005615//extracellu
0.999403899	0.021982485	-	-	-
0.999403605	0.02198789	-	-	-
0.999403194	0.021995467	-	ko01100//Metab	GO:0005783//endoplasm
0.999401709	0.022022832	ko01100//Metabolic p	ko04976//Bile se	GO:0005789//endoplasm
0.999399986	0.022054521	-	-	GO:0031012//extracellu
0.999399849	0.022057029	ko01100//Metabolic p	-	-
0.999399795	0.02205803	ko01100//Metabolic p	ko05152//Tuber	GO:0009897//external s
0.99939932	0.022066746	ko01100//Metabolic p	-	-
0.999398529	0.022081274	ko01100//Metabolic p	-	GO:0005615//extracellu
0.999396575	0.022117127	-	-	GO:0005576//extracellu
0.999396019	0.022127317	-	ko02010//ABC tı	GO:0005886//plasma m
0.999395936	0.022128825	ko01100//Metabolic p	ko04060//Cytok	GO:0005576//extracellu
0.999395725	0.022132689	ko01100//Metabolic p	ko04010//MAPK	-
0.999395635	0.022134342	ko01100//Metabolic p	ko03320//PPAR	GO:0005634//nucleus;G
0.999395545	0.022135995	-	-	GO:0005634//nucleus;G
0.999395459	0.022137572	-	-	GO:0005783//endoplasm
0.999393468	0.022173993	ko01063//Biosynthesis	-	GO:0005737//cytoplasm
0.999392539	0.022190978	-	ko04060//Cytok	-
0.999392312	0.022195113	-	ko01100//Metab	-
0.99939206	0.022199718	-	ko04745//Photo	-
0.999391546	0.02220911	-	-	-
0.999390001	0.02223728	ko01100//Metabolic p	ko01100//Metab	GO:0005886//plasma m
0.999388949	0.022256455	-	ko04610//Comp	GO:0005615//extracellu
0.999387998	0.022273774	ko01100//Metabolic p	-	GO:0005634//nucleus;G
0.999387522	0.022282438	ko01100//Metabolic p	ko01100//Metab	-
0.999386845	0.022294737	ko01100//Metabolic p	-	-
0.999386238	0.022305777	-	ko01100//Metab	GO:0016020//membran
0.999385363	0.02232167	ko01100//Metabolic p	ko04151//PI3K-/	GO:0005654//nucleopla
0.999382175	0.022379494	-	ko04640//Hema	GO:0031226//intrinsic c
0.99938214	0.022380131	-	ko04080//Neuro	GO:0005576//extracellu
0.999382111	0.022380646	-	ko01100//Metab	GO:0005829//cytosol
0.999381987	0.022382896	ko01100//Metabolic p	ko04390//Hippo	GO:0005667//transcript
0.999381503	0.022391662	-	ko01523//Antifo	GO:0005886//plasma m
0.999380633	0.022407401	ko01100//Metabolic p	ko01100//Metab	GO:0005634//nucleus;G
0.999380383	0.022411925	ko01110//Biosynthesis	ko05200//Pathw	GO:0005794//Golgi app
0.999380245	0.022414433	-	ko04726//Seroto	GO:0005739//mitochon
0.999379155	0.022434119	-	-	GO:0005634//nucleus;G
0.999378686	0.022442603	-	ko04742//Taste	GO:0001518//voltage-g
0.999378686	0.022442609	-	-	-
0.999378219	0.022451026	-	-	-
0.99937717	0.022469974	-	-	-
0.999376985	0.022473311	-	ko04310//Wnt s	GO:0005634//nucleus;G
0.999376904	0.022474769	ko01100//Metabolic p	-	GO:0005654//nucleopla
0.99937685	0.022475732	ko01100//Metabolic p	-	GO:0016021//integral c
0.999376811	0.022476446	-	ko04151//PI3K-/	GO:0005654//nucleopla
0.999376588	0.022480461	ko01110//Biosynthesis	ko01100//Metab	-
0.999374885	0.022511151	ko01100//Metabolic p	ko04360//Axon	GO:0005576//extracellu
0.999374555	0.02251709	ko01100//Metabolic p	-	-
0.999374472	0.02251858	-	ko04141//Protei	GO:0005783//endoplasm
0.999374204	0.022523411	-	ko01100//Metab	-
0.999374105	0.022525201	ko01100//Metabolic p	-	-
0.999371683	0.022568733	-	-	GO:0005737//cytoplasm

0.999371185	0.022577681	-	ko01100//Metak	GO:0005886//plasma m
0.999370437	0.022591102	ko01110//Biosynthesis	-	GO:0016021//integral co
0.999370332	0.022592986	ko01100//Metabolic p	ko04310//Wnt s	-
0.999369875	0.02260119	-	-	-
0.999368452	0.022626693	ko01100//Metabolic p	-	GO:0005737//cytoplasm
0.999368224	0.022630787	ko01100//Metabolic p	-	GO:0005789//endoplasm
0.999366408	0.022663288	ko01100//Metabolic p	ko03018//RNA c	GO:0005829//cytosol
0.999365974	0.022671042	-	-	-
0.999365803	0.022674112	-	ko00564//Glycer	GO:0005615//extracellu
0.999364459	0.022698129	-	-	GO:0016020//membran
0.999363667	0.022712266	ko01100//Metabolic p	-	-
0.999362162	0.022739111	ko01100//Metabolic p	-	GO:0016021//integral co
0.999360655	0.022765963	-	ko04514//Cell ac	GO:0005829//cytosol;GC
0.999360379	0.022770877	-	ko01100//Metak	-
0.999359868	0.022779962	ko01100//Metabolic p	-	-
0.999358798	0.022798999	ko01100//Metabolic p	-	GO:0005737//cytoplasm
0.999358389	0.022806262	ko01100//Metabolic p	-	-
0.999358118	0.022811094	ko01110//Biosynthesis	ko04979//Chole	GO:0005783//endoplasm
0.999357362	0.022824522	ko01100//Metabolic p	-	GO:0005737//cytoplasm
0.99935596	0.022849411	-	-	GO:0005887//integral co
0.999355941	0.022849745	ko01100//Metabolic p	-	GO:0005813//centrosom
0.999353495	0.0228931	ko01100//Metabolic p	ko01100//Metak	GO:0005737//cytoplasm
0.999351771	0.022923597	-	ko01100//Metak	GO:0048269//methionir
0.99935082	0.022940415	ko01100//Metabolic p	ko01100//Metak	GO:0000139//Golgi mem
0.999347255	0.023003319	ko01100//Metabolic p	ko05414//Dilate	-
0.999346784	0.023011613	ko01100//Metabolic p	ko01100//Metak	GO:0048269//methionir
0.999346668	0.023013659	-	ko01100//Metak	-
0.999346657	0.023013862	-	-	GO:0043231//intracellul
0.999346373	0.023018859	ko01100//Metabolic p	-	-
0.999345135	0.023040647	ko01100//Metabolic p	-	GO:0005615//extracellu
0.999345088	0.023041471	-	-	GO:0005737//cytoplasm
0.999345068	0.023041831	-	ko01100//Metak	-
0.999345061	0.023041955	ko01100//Metabolic p	ko00983//Drug	-
0.999344374	0.023054038	-	ko05142//Chaga	GO:0016020//membran
0.999343188	0.023074884	-	-	GO:0005886//plasma m
0.999342658	0.023084189	ko01100//Metabolic p	ko01100//Metak	-
0.999340934	0.023114443	-	-	GO:0005794//Golgi app
0.999340873	0.023115509	-	-	GO:0005758//mitochon
0.999340557	0.023121045	-	ko05200//Pathw	GO:0005737//cytoplasm
0.999340078	0.023129443	ko01100//Metabolic p	ko04144//Endoc	-
0.999339902	0.023132542	ko01100//Metabolic p	-	GO:0005634//nucleus;G
0.999339808	0.023134176	ko01100//Metabolic p	-	-
0.99933939	0.023141512	ko01100//Metabolic p	ko05165//Huma	GO:0005634//nucleus;G
0.999339216	0.023144552	-	-	-
0.999339182	0.023145153	-	ko01100//Metak	GO:0005654//nucleopla
0.999338629	0.023154831	ko01100//Metabolic p	ko04080//Neuro	GO:0005576//extracellu
0.999338573	0.023155805	-	-	-
0.999335935	0.023201951	ko01120//Microbial m	ko01100//Metak	GO:0005654//nucleopla
0.999335837	0.023203661	-	ko01100//Metak	GO:0016020//membran
0.999334703	0.02322346	-	-	-
0.999334669	0.023224054	-	ko01100//Metak	-
0.999334494	0.02322712	ko01120//Microbial m	-	GO:0005739//mitochon
0.999334156	0.023233011	-	ko04514//Cell ac	GO:0005886//plasma m
0.999333845	0.02323843	-	-	-
0.99933378	0.023239562	ko01100//Metabolic p	ko01100//Metak	GO:0005777//peroxison
0.99933042	0.0232981	ko01100//Metabolic p	-	GO:0005802//trans-Gol
0.999329667	0.023311199	ko01100//Metabolic p	ko05203//Viral c	GO:0000786//nucleosor
0.999329597	0.023312428	-	-	-

0.999329091	0.023321217	-	ko01100//Metak	-
0.999328445	0.023332447	ko01100//Metabolic p	ko00533//Glycos	GO:0000139//Golgi mer
0.99932826	0.023335651	-	-	GO:0016021//integral co
0.999328061	0.023339109	-	-	-
0.999327821	0.023343285	ko01100//Metabolic p	-	GO:0031012//extracellu
0.999327593	0.023347243	-	-	-
0.999327229	0.023353553	ko01100//Metabolic p	-	-
0.999326766	0.023361591	ko01100//Metabolic p	-	GO:0005654//nucleopla
0.999326562	0.023365135	ko01100//Metabolic p	-	GO:0005634//nucleus
0.999326078	0.023373533	-	-	GO:0016020//membran
0.999326072	0.023373638	ko01100//Metabolic p	-	GO:0005768//endosom
0.999325833	0.023377776	ko01100//Metabolic p	-	-
0.999323806	0.023412901	-	ko01100//Metak	-
0.999323591	0.023416621	ko01100//Metabolic p	ko04060//Cytok	-
0.999323504	0.023418123	-	ko05165//Huma	GO:0005634//nucleus;G
0.999322793	0.023430434	ko01100//Metabolic p	-	GO:0005739//mitochon
0.999322326	0.023438519	-	ko01100//Metak	GO:0005737//cytoplasm
0.999320714	0.023466372	ko01100//Metabolic p	ko01100//Metak	GO:0005769//early endo
0.999319384	0.023489349	-	-	-
0.999319228	0.023492025	-	ko04610//Comp	GO:0005615//extracellu
0.999319199	0.023492538	ko01100//Metabolic p	ko05143//Africa	GO:0005576//extracellu
0.999319154	0.023493308	-	-	GO:0005634//nucleus;G
0.999319138	0.023493592	-	-	-
0.999318416	0.023506043	-	-	GO:0016021//integral co
0.999318337	0.023507398	-	-	GO:0005634//nucleus
0.999317668	0.023518938	-	ko01100//Metak	GO:0043231//intracellul
0.99931745	0.023522686	ko01100//Metabolic p	-	GO:0005829//cytosol;G(
0.999317394	0.02352366	ko01100//Metabolic p	-	-
0.999317226	0.02352656	-	-	GO:0005737//cytoplasm
0.99931705	0.02352959	ko01100//Metabolic p	ko01100//Metak	GO:0005737//cytoplasm
0.999316508	0.023538925	-	ko04745//Photo	-
0.999315606	0.023554447	-	-	-
0.999314471	0.023573981	-	-	-
0.999313566	0.023589538	-	-	GO:0000781//chromosc
0.999312949	0.023600136	ko01100//Metabolic p	-	GO:0005737//cytoplasm
0.999311074	0.023632318	-	ko01100//Metak	-
0.999310151	0.023648143	-	-	GO:0005739//mitochon
0.999309685	0.023656132	ko01100//Metabolic p	-	GO:0005634//nucleus;G
0.999309321	0.023662377	-	ko01100//Metak	-
0.999308937	0.023668943	-	-	-
0.999308817	0.023671002	-	-	GO:0005576//extracellu
0.999308682	0.023673311	-	ko01100//Metak	GO:0005654//nucleopla
0.999308011	0.0236848	ko01100//Metabolic p	-	GO:0016020//membran
0.999306564	0.023709564	ko01100//Metabolic p	-	GO:0005737//cytoplasm
0.999306134	0.023716904	ko01100//Metabolic p	ko04020//Calciu	GO:0005886//plasma m
0.999305952	0.023720016	-	-	-
0.999305409	0.0237293	ko01100//Metabolic p	ko04714//Therm	GO:0005739//mitochon
0.999304829	0.023739203	-	-	GO:0005737//cytoplasm
0.999304626	0.023742676	-	-	GO:0005615//extracellu
0.999304076	0.02375206	ko01100//Metabolic p	-	GO:0005654//nucleopla
0.999304057	0.023752388	-	-	-
0.999303689	0.023758669	-	ko01100//Metak	-
0.999303562	0.023760819	ko01120//Microbial m	-	GO:0005794//Golgi app
0.999303353	0.023764401	ko01100//Metabolic p	-	-
0.999303181	0.023767327	ko01100//Metabolic p	ko05200//Pathw	GO:0005794//Golgi app
0.999302473	0.0237794	-	ko04976//Bile se	GO:0005654//nucleopla
0.999301807	0.023790746	ko01100//Metabolic p	-	GO:0005737//cytoplasm
0.999301274	0.023799834	ko01100//Metabolic p	-	GO:0031012//extracellu

0.999300893	0.023806326	ko01100//Metabolic p	ko04120//Ubiqu	GO:0005680//anaphase
0.999300612	0.023811105	ko01100//Metabolic p	-	GO:0005576//extracellu
0.999299975	0.023821949	-	ko01100//Metab	-
0.999299748	0.023825801	ko01100//Metabolic p	ko05130//Patho	GO:0005856//cytoskelet
0.999298717	0.023843339	-	ko01100//Metab	GO:0005739//mitochon
0.999298546	0.023846258	ko01100//Metabolic p	-	-
0.999298523	0.023846643	-	-	GO:0005789//endoplasr
0.999298459	0.023847734	ko01100//Metabolic p	ko00564//Glycer	GO:0005615//extracellu
0.999296801	0.023875904	-	-	-
0.999296161	0.023886759	ko01120//Microbial m	-	GO:0005739//mitochon
0.999295195	0.023903141	-	-	GO:0005634//nucleus;G
0.999293457	0.023932606	ko01100//Metabolic p	-	-
0.999293307	0.023935154	-	ko04610//Comp	GO:0005576//extracellu
0.999292218	0.023953583	-	-	-
0.999291402	0.023967393	-	-	GO:0005886//plasma m
0.999291075	0.023972914	ko01100//Metabolic p	ko01100//Metab	GO:0005886//plasma m
0.999290713	0.023979032	ko01100//Metabolic p	-	GO:0005737//cytoplasm
0.999289382	0.024001534	-	ko04514//Cell a	GO:0005829//cytosol;G
0.999287978	0.02402524	-	-	-
0.999286357	0.024052564	ko01100//Metabolic p	-	-
0.999284774	0.024079235	-	ko01100//Metab	GO:0048269//methionir
0.999283987	0.024092487	ko01100//Metabolic p	ko03320//PPAR	GO:0005634//nucleus;G
0.999282519	0.024117165	-	-	-
0.999282417	0.02411889	ko01100//Metabolic p	ko05231//Cholir	GO:0005739//mitochon
0.999281238	0.024138682	-	-	GO:0005576//extracellu
0.999280591	0.024149548	ko01100//Metabolic p	-	GO:0005634//nucleus;G
0.9992803	0.024154436	-	ko04640//Hema	GO:0031226//intrinsic c
0.999279581	0.024166497	ko01100//Metabolic p	ko04979//Chole	GO:0005783//endoplasr
0.999279493	0.024167982	-	-	GO:0005634//nucleus;G
0.999279219	0.024172565	-	-	GO:0005737//cytoplasm
0.999277935	0.024194102	-	-	GO:0005783//endoplasr
0.99927717	0.024206918	ko01100//Metabolic p	-	GO:0016020//membran
0.999276282	0.024221784	-	ko01100//Metab	GO:0005739//mitochon
0.999276271	0.024221957	-	-	-
0.999274327	0.024254481	ko01110//Biosynthesis	-	GO:0005886//plasma m
0.999274187	0.024256822	-	ko05200//Pathw	GO:0005834//heterotrin
0.999273807	0.024263166	ko01100//Metabolic p	-	GO:0005886//plasma m
0.999273788	0.024263485	ko01100//Metabolic p	-	GO:0005737//cytoplasm
0.999273342	0.024270928	ko01100//Metabolic p	ko04745//Photo	-
0.999272529	0.02428451	-	-	GO:0016020//membran
0.999271833	0.024296116	-	ko05017//Spino	GO:0005886//plasma m
0.999270562	0.024317315	-	ko05143//Africa	GO:0005576//extracellu
0.999270078	0.024325389	ko01100//Metabolic p	ko03320//PPAR	-
0.999269846	0.024329259	-	-	GO:0001533//cornified
0.999269434	0.024336124	-	-	-
0.999268969	0.024343856	-	ko04310//Wnt s	GO:0005576//extracellu
0.999268939	0.024344357	-	ko05322//Syster	GO:0005576//extracellu
0.999268868	0.024345552	ko01063//Biosynthesis	-	-
0.999268382	0.024353638	-	ko02010//ABC t	GO:0005739//mitochon
0.999268179	0.024357013	-	ko05322//Syster	GO:0000786//nucleosor
0.999268109	0.02435818	ko01063//Biosynthesis	-	-
0.999267933	0.024361113	-	-	GO:0005634//nucleus
0.999267281	0.024371954	-	ko04971//Gastri	GO:0005886//plasma m
0.999267003	0.024376587	-	ko05010//Alzhei	GO:0005576//extracellu
0.999266581	0.024383594	-	-	GO:0005634//nucleus;G
0.999266059	0.024392276	-	ko04714//Therm	GO:0005634//nucleus
0.999266012	0.024393047	-	-	-
0.999265671	0.024398717	ko01100//Metabolic p	ko01100//Metab	GO:0005654//nucleopla

0.999264742	0.024414149	-	ko04080//Neurc	GO:0005576//extracellu
0.999264595	0.024416593	-	-	GO:0016020//membran
0.999264313	0.024421271	ko01120//Microbial m	ko04921//Oxyto	GO:0016020//membran
0.99926385	0.02442896	-	ko03320//PPAR	GO:0005634//nucleus;G
0.999263445	0.024435669	ko01100//Metabolic p	-	-
0.9992629	0.024444718	-	-	GO:0005634//nucleus
0.999262334	0.024454106	-	ko04390//Hippc	GO:0005667//transcript
0.999261405	0.024469489	-	-	GO:0001725//stress fibe
0.99926136	0.024470235	-	-	-
0.999260806	0.024479421	-	ko01100//Metab	-
0.999259156	0.024506724	ko01100//Metabolic p	-	-
0.999258677	0.024514648	-	-	-
0.999256875	0.024544435	-	-	-
0.999256537	0.024550011	ko01100//Metabolic p	ko01100//Metab	GO:0005741//mitochon
0.999255858	0.024561214	ko01100//Metabolic p	-	-
0.999254989	0.024575566	-	-	-
0.999254079	0.024590566	ko01100//Metabolic p	-	-
0.999253848	0.024594376	-	ko01100//Metab	-
0.99925378	0.024595499	ko01100//Metabolic p	ko05200//Pathw	GO:0005576//extracellu
0.999253319	0.0246031	-	ko01100//Metab	GO:0005654//nucleopla
0.999252839	0.024611003	-	-	-
0.999251814	0.02462788	ko01100//Metabolic p	ko04218//Cellul	GO:0030896//checkpoir
0.999251412	0.024634502	-	ko04080//Neurc	GO:0005783//endoplasr
0.999250256	0.024653503	ko01110//Biosynthesis	-	GO:0005802//trans-Gol
0.999250161	0.024655076	ko01100//Metabolic p	-	-
0.999249293	0.02466934	-	-	GO:0001725//stress fibe
0.999248522	0.024682	-	ko01100//Metab	-
0.999248198	0.024687327	ko01100//Metabolic p	ko04310//Wnt s	GO:0005654//nucleopla
0.999245656	0.024729041	ko01110//Biosynthesis	-	GO:0005768//endosom
0.999245101	0.024738131	-	-	-
0.99924402	0.024755837	-	-	-
0.999243846	0.024758691	ko01100//Metabolic p	ko04150//mTOR	GO:0032991//macromo
0.999243192	0.024769387	-	-	GO:0005654//nucleopla
0.999243132	0.024770375	-	-	-
0.999241596	0.024795498	ko01100//Metabolic p	ko04979//Chole	GO:0005783//endoplasr
0.999241356	0.024799422	-	-	GO:0005634//nucleus
0.999241148	0.024802819	-	ko01100//Metab	-
0.999240595	0.024811854	-	ko04060//Cytok	-
0.999240533	0.024812866	-	ko04114//Oocyt	GO:0005634//nucleus;G
0.999240354	0.024815801	ko01100//Metabolic p	-	-
0.999240332	0.024816155	-	-	-
0.999238412	0.024847496	ko01100//Metabolic p	-	-
0.999238392	0.024847826	-	ko01100//Metab	GO:0016020//membran
0.999237385	0.024864249	-	-	GO:0005737//cytoplasm
0.999236031	0.024886312	-	-	GO:0001726//ruffle;GO:
0.999234141	0.024917092	-	-	-
0.999233445	0.0249284	ko01100//Metabolic p	ko04931//Insulir	GO:0016021//integral c
0.999233223	0.024932017	ko01100//Metabolic p	ko01100//Metab	GO:0005615//extracellu
0.999232675	0.024940932	-	-	GO:0005886//plasma m
0.999232562	0.024942758	-	-	GO:0001725//stress fibe
0.999231234	0.024964331	ko01100//Metabolic p	-	-
0.999230112	0.024982546	-	-	GO:0005634//nucleus;G
0.999230079	0.024983085	ko01063//Biosynthesis	-	-
0.999229991	0.024984506	-	-	GO:0005737//cytoplasm
0.999229109	0.024998821	-	ko04742//Taste	GO:0001518//voltage-g
0.999229072	0.024999422	-	ko05130//Patho	GO:0005856//cytoskelet
0.999229002	0.02500056	-	ko01100//Metab	-
0.999228646	0.025006335	ko01100//Metabolic p	ko01100//Metab	GO:0005737//cytoplasm

0.999228251	0.025012736	-	ko01100//Metak	GO:0005737//cytoplasm
0.999228113	0.025014971	-	ko04976//Bile se	GO:0005654//nucleopla
0.999226836	0.025035661	-	ko05010//Alzhei	GO:0005576//extracellu
0.999226555	0.025040202	ko01110//Biosynthesis	-	GO:0016020//membran
0.999225674	0.025054467	-	ko05322//Syster	GO:0005576//extracellu
0.999225643	0.025054968	-	ko04310//Wnt s	GO:0005576//extracellu
0.999225581	0.025055966	ko01100//Metabolic p	-	-
0.999224946	0.025066235	ko01100//Metabolic p	-	GO:0005576//extracellu
0.999223472	0.025090069	-	-	-
0.999222123	0.025111856	-	-	GO:0005615//extracellu
0.999221805	0.025116982	-	ko01100//Metak	-
0.999220656	0.025135524	ko01110//Biosynthesis	-	GO:0005634//nucleus;G
0.999219593	0.025152653	-	-	GO:0005737//cytoplasm
0.99921933	0.025156904	-	ko01100//Metak	GO:0005615//extracellu
0.999219075	0.025161001	ko01100//Metabolic p	ko01100//Metak	-
0.999218344	0.025172778	-	ko04144//Endoc	-
0.999216985	0.025194667	ko01100//Metabolic p	ko05200//Pathw	GO:0005794//Golgi app
0.999216879	0.025196362	ko01100//Metabolic p	-	-
0.999216259	0.025206337	-	-	GO:0005576//extracellu
0.9992152	0.025223369	-	-	GO:0016020//membran
0.999213897	0.025244303	ko01100//Metabolic p	-	GO:0005886//plasma m
0.999213176	0.025255868	-	-	-
0.999212863	0.0252609	ko01100//Metabolic p	-	-
0.999212332	0.025269423	-	ko01100//Metak	GO:0000139//Golgi mer
0.999212086	0.02527337	-	ko05414//Dilate	-
0.999212013	0.02527454	-	-	GO:0016020//membran
0.999211982	0.025275037	ko01100//Metabolic p	ko04514//Cell a	GO:0016020//membran
0.999210888	0.025292568	-	ko04151//PI3K-/	GO:0009986//cell surfac
0.999210806	0.025293881	-	ko01100//Metak	GO:0043231//intracellul
0.999209675	0.025312006	-	ko00983//Drug	-
0.999209322	0.025317663	-	ko05322//Syster	GO:0005576//extracellu
0.999208686	0.025327839	-	-	-
0.999208105	0.025337146	ko01100//Metabolic p	-	GO:0005886//plasma m
0.999207784	0.025342283	ko01100//Metabolic p	-	GO:0005654//nucleopla
0.999207649	0.02534443	-	-	-
0.999207537	0.025346225	ko01100//Metabolic p	-	-
0.999207409	0.025348268	-	-	GO:0005737//cytoplasm
0.999207061	0.025353837	-	-	GO:0005576//extracellu
0.999204738	0.02539095	-	-	-
0.999204706	0.02539146	-	-	GO:0005887//integral c
0.999204621	0.025392825	ko01100//Metabolic p	ko04659//Th17	GO:0000785//chromatir
0.999202583	0.025425339	-	-	GO:0005886//plasma m
0.999201343	0.025445099	-	ko01100//Metak	-
0.999201174	0.025447797	-	-	-
0.999200105	0.02546482	ko01110//Biosynthesis	-	GO:0005886//plasma m
0.999199932	0.025467574	ko01100//Metabolic p	ko01100//Metak	-
0.999199163	0.025479815	-	ko01100//Metak	GO:0048269//methionir
0.999198211	0.025494958	-	-	-
0.99919815	0.025495919	-	ko01100//Metak	GO:0005886//plasma m
0.999197889	0.025500073	-	-	GO:0005634//nucleus;G
0.999196629	0.025520101	ko01100//Metabolic p	-	-
0.999195818	0.025532967	-	ko04080//Neurc	GO:0005576//extracellu
0.999193838	0.025564388	ko01100//Metabolic p	ko04550//Signal	GO:0005634//nucleus;G
0.999193399	0.02557135	ko01100//Metabolic p	-	-
0.999193369	0.025571829	-	ko01100//Metak	GO:0031988//membran
0.999193322	0.025572573	-	ko01100//Metak	GO:0005886//plasma m
0.999193149	0.025575316	-	ko00564//Glycei	GO:0005615//extracellu
0.999192915	0.025579026	ko01100//Metabolic p	ko05322//Syster	GO:0000786//nucleosor

0.999191329	0.025604155	ko01100//Metabolic p	-	-
0.999190364	0.025619424	-	ko04976//Bile se	GO:0005654//nucleopla
0.999186013	0.025688174	ko01100//Metabolic p	ko05165//Huma	GO:0005634//nucleus;G
0.999184025	0.02571953	-	ko05200//Pathw	-
0.999183441	0.025728737	-	-	-
0.999182779	0.025739164	ko01100//Metabolic p	-	-
0.999182639	0.025741376	-	ko03013//Nucle	-
0.999182163	0.025748871	ko01100//Metabolic p	ko02010//ABC ti	GO:0005739//mitochon
0.999182077	0.025750218	-	-	-
0.999181961	0.025752049	ko01100//Metabolic p	-	-
0.999181899	0.025753014	ko01100//Metabolic p	ko01100//Metab	-
0.999181345	0.025761735	-	-	-
0.999181338	0.025761844	ko01100//Metabolic p	ko01100//Metab	GO:0005737//cytoplasm
0.99918038	0.025776919	-	ko05200//Pathw	-
0.999179491	0.025790896	-	-	GO:0005794//Golgi app
0.999179308	0.025793774	-	-	-
0.999176967	0.02583054	-	ko01100//Metab	GO:0005783//endoplasr
0.99917682	0.025832847	-	-	GO:0005576//extracellu
0.999176809	0.025833016	ko01100//Metabolic p	-	-
0.999176636	0.025835735	-	ko04080//Neuro	GO:0005886//plasma m
0.999174847	0.025863794	-	-	GO:0000139//Golgi mer
0.99917382	0.025879886	-	ko01100//Metab	GO:0043231//intracellul
0.999173311	0.025887856	ko01100//Metabolic p	ko01100//Metab	GO:0005783//endoplasr
0.999172871	0.025894754	-	-	GO:0005737//cytoplasm
0.999171765	0.025912052	-	ko04390//Hippo	GO:0005667//transcript
0.999170935	0.025925039	-	ko04310//Wnt s	GO:0005634//nucleus;G
0.999170652	0.025929458	ko01100//Metabolic p	-	-
0.999170234	0.025935994	ko01100//Metabolic p	-	-
0.999169874	0.025941621	ko01100//Metabolic p	-	GO:0005829//cytosol
0.999169616	0.025945657	-	-	GO:0005576//extracellu
0.999168901	0.025956819	-	-	-
0.999168353	0.025965382	-	ko01100//Metab	-
0.999167761	0.025974623	-	-	-
0.999166729	0.025990731	-	ko05200//Pathw	GO:0005576//extracellu
0.999166674	0.025991577	-	-	GO:0005634//nucleus;G
0.999162775	0.026052322	-	ko01100//Metab	-
0.999162698	0.02605353	-	-	GO:0005634//nucleus;G
0.999161583	0.026070862	ko01100//Metabolic p	ko05017//Spino	GO:0043231//intracellul
0.999161189	0.026076995	ko01100//Metabolic p	ko04726//Seroto	GO:0005739//mitochon
0.999160195	0.026092448	-	ko04080//Neuro	GO:0005783//endoplasr
0.999159482	0.026103513	ko01100//Metabolic p	ko04721//Synap	GO:0005887//integral co
0.999159459	0.02610388	ko01100//Metabolic p	-	GO:0034451//centriolar
0.999159079	0.026109781	ko01100//Metabolic p	-	GO:0016020//membran
0.99915887	0.02611302	ko01100//Metabolic p	-	-
0.999158563	0.026117785	-	ko00564//Glycer	GO:0005615//extracellu
0.999158498	0.026118798	-	ko04310//Wnt s	GO:0005576//extracellu
0.999158466	0.0261193	-	ko05322//Syster	GO:0005576//extracellu
0.999158248	0.026122682	ko01100//Metabolic p	-	GO:0005759//mitochon
0.999157386	0.026136056	-	-	GO:0005634//nucleus
0.999156677	0.026147047	-	-	-
0.999155651	0.026162945	ko01100//Metabolic p	-	GO:0005886//plasma m
0.999155599	0.026163753	-	-	GO:0005886//plasma m
0.999155142	0.026170831	ko01100//Metabolic p	-	-
0.999154345	0.02618318	-	-	-
0.999153963	0.026189091	-	ko04080//Neuro	GO:0005576//extracellu
0.999153049	0.026203241	ko01100//Metabolic p	ko04144//Endoc	GO:0001917//photorece
0.999152833	0.026206571	-	-	-
0.999151586	0.026225863	-	ko04514//Cell ac	GO:0016020//membran

0.99914956	0.026257155	-	-	-
0.999148558	0.026272618	-	-	-
0.999147429	0.026290037	-	ko05322//Syster	GO:0005576//extracellu
0.999147397	0.026290539	-	ko04310//Wnt s	GO:0005576//extracellu
0.999146497	0.026304409	ko01063//Biosynthesis	ko04010//MAPK	-
0.999145929	0.026313165	-	-	GO:0005833//hemoglok
0.999145014	0.02632725	-	ko01100//Metak	GO:0005615//extracellu
0.999144413	0.026336513	ko01100//Metabolic p	-	GO:0034451//centriolar
0.999143183	0.026355433	ko01100//Metabolic p	-	GO:0005634//nucleus;G
0.999142684	0.02636311	ko01110//Biosynthesis	ko01100//Metak	-
0.999142067	0.026372594	ko01100//Metabolic p	-	GO:0001533//cornified
0.999141961	0.026374219	-	ko04918//Thyro	GO:0005576//extracellu
0.999141895	0.026375231	-	-	-
0.999141514	0.026381096	-	ko00983//Drug	-
0.999141116	0.026387216	ko01100//Metabolic p	-	-
0.99914067	0.026394054	ko01100//Metabolic p	ko04742//Taste	GO:0001518//voltage-g
0.999140631	0.026394654	ko01100//Metabolic p	-	GO:0005576//extracellu
0.999139679	0.02640927	ko01100//Metabolic p	-	GO:0005813//centrosor
0.999138998	0.026419732	-	ko05414//Dilate	-
0.999136932	0.026451414	-	ko05203//Viral c	GO:0000786//nucleosor
0.999136686	0.026455184	-	ko04514//Cell a	GO:0005886//plasma m
0.999136469	0.026458506	-	ko01100//Metak	-
0.999135796	0.026468819	ko01100//Metabolic p	ko00982//Drug	-
0.999135319	0.026476123	-	-	-
0.999135219	0.026477647	ko01100//Metabolic p	ko04060//Cytok	GO:0005576//extracellu
0.999135131	0.026479004	-	-	-
0.999134875	0.026482913	-	-	-
0.999134323	0.026491372	-	ko04610//Comp	GO:0005576//extracellu
0.999134114	0.026494564	-	ko01100//Metak	GO:0005737//cytoplasm
0.999133305	0.026506949	-	-	GO:0000307//cyclin-dep
0.999130617	0.026548016	-	-	-
0.99913042	0.026551031	-	-	-
0.999130136	0.026555367	-	-	GO:0005788//endoplasr
0.999130033	0.026556936	-	-	GO:0005737//cytoplasm
0.999129347	0.026567414	-	ko04060//Cytok	GO:0005576//extracellu
0.99912919	0.026569803	-	-	GO:0005615//extracellu
0.999129129	0.026570741	-	-	-
0.999128426	0.026581457	-	-	-
0.999127969	0.02658843	-	-	-
0.999127777	0.026591355	-	ko01100//Metak	GO:0005737//cytoplasm
0.999125971	0.026618874	ko01100//Metabolic p	ko04060//Cytok	GO:0005576//extracellu
0.999125649	0.026623774	-	-	GO:0005634//nucleus
0.999125031	0.026633193	ko01100//Metabolic p	-	-
0.999124826	0.026636305	ko01100//Metabolic p	-	GO:0016020//membran
0.999124606	0.026639654	ko01100//Metabolic p	-	-
0.99912439	0.026642938	ko01100//Metabolic p	ko00564//Glycer	GO:0005615//extracellu
0.999124066	0.026647865	ko01100//Metabolic p	-	-
0.999123702	0.026653404	-	-	-
0.999123455	0.026657172	ko01100//Metabolic p	-	GO:0001650//fibrillar ce
0.999122327	0.026674309	ko01100//Metabolic p	ko01100//Metak	GO:0005654//nucleopla
0.999122299	0.026674736	-	-	GO:0005829//cytosol;G(
0.999121972	0.026679713	ko01100//Metabolic p	-	-
0.999121617	0.026685098	-	-	-
0.999120789	0.026697685	ko01110//Biosynthesis	-	GO:0005654//nucleopla
0.999119801	0.026712675	ko01100//Metabolic p	-	GO:0001725//stress fibe
0.999119485	0.026717468	-	ko00982//Drug	-
0.999117262	0.026751177	-	ko05143//Africa	GO:0005576//extracellu
0.999117204	0.026752056	ko01100//Metabolic p	-	GO:0005737//cytoplasm

0.999114591	0.026791634	-	-	GO:0005576//extracellu
0.999113627	0.026806214	-	-	GO:0005737//cytoplasm
0.999112872	0.026817635	ko01100//Metabolic p	-	-
0.999112597	0.026821779	-	-	-
0.999112549	0.026822511	-	-	GO:0005886//plasma m
0.999111209	0.026842757	ko01100//Metabolic p	-	GO:0005634//nucleus
0.999111102	0.026844369	ko01100//Metabolic p	-	-
0.999109948	0.026861795	-	ko05165//Huma	GO:0005634//nucleus;G
0.999109566	0.026867557	ko01100//Metabolic p	ko04721//Synap	-
0.999107904	0.026892628	-	-	GO:0016020//membran
0.999107573	0.026897614	ko01100//Metabolic p	-	-
0.999107531	0.026898242	ko01100//Metabolic p	-	-
0.999107065	0.026905277	ko01100//Metabolic p	ko04970//Saliva	GO:0005576//extracellu
0.999106508	0.026913669	ko01100//Metabolic p	-	-
0.999104679	0.026941197	-	-	-
0.999104204	0.026948343	ko01100//Metabolic p	ko04978//Miner	GO:0009986//cell surfac
0.999103571	0.02695786	-	ko01100//Metab	GO:0005777//peroxison
0.999103459	0.026959558	ko01110//Biosynthesis	-	-
0.999103102	0.026964917	-	ko04979//Chole	GO:0005783//endoplasm
0.999101682	0.026986252	ko01100//Metabolic p	-	-
0.99910115	0.026994247	ko01100//Metabolic p	ko04973//Carbo	GO:0005886//plasma m
0.999100947	0.026997306	-	-	-
0.999100808	0.02699938	ko01100//Metabolic p	ko01100//Metab	GO:0001931//uropod;G
0.999100726	0.027000623	-	ko01100//Metab	GO:0005829//cytosol;G
0.999100481	0.027004301	-	-	-
0.999099479	0.02701933	-	ko04640//Hema	GO:0031226//intrinsic c
0.999098783	0.027029776	-	ko01100//Metab	GO:0031988//membran
0.999098392	0.027035639	-	ko01100//Metab	-
0.999097053	0.027055716	ko01100//Metabolic p	-	GO:0005737//cytoplasm
0.999096757	0.027060145	ko01110//Biosynthesis	ko02010//ABC t	GO:0000139//Golgi mer
0.999096545	0.02706332	-	-	-
0.999096405	0.027065416	-	ko05142//Chaga	GO:0016020//membran
0.999095881	0.027073267	-	-	-
0.999095573	0.027077882	ko01110//Biosynthesis	-	GO:0016020//membran
0.999094142	0.027099301	-	-	-
0.999093384	0.027110627	ko01100//Metabolic p	-	-
0.999093061	0.027115456	-	ko01100//Metab	GO:0043231//intracellul
0.999092869	0.027118335	-	-	-
0.999092332	0.027126358	-	ko04151//PI3K-/	GO:0005654//nucleopla
0.999092314	0.027126622	-	-	-
0.99909217	0.027128779	-	-	GO:0005576//extracellu
0.999091801	0.0271343	ko01100//Metabolic p	-	GO:0005737//cytoplasm
0.99909147	0.027139237	ko01100//Metabolic p	ko01100//Metab	-
0.99909126	0.027142369	-	ko04610//Comp	GO:0005615//extracellu
0.99909111	0.027144617	-	ko04726//Serot	GO:0005739//mitochon
0.999090769	0.027149712	-	-	-
0.999088908	0.027177476	-	ko05200//Pathw	-
0.999087898	0.027192545	ko01100//Metabolic p	-	-
0.999087107	0.027204336	-	ko01100//Metab	-
0.999085709	0.027225154	-	ko01100//Metab	GO:0005739//mitochon
0.99908402	0.027250305	-	-	-
0.999082507	0.027272793	ko01100//Metabolic p	ko04724//Glutar	GO:0005886//plasma m
0.999081592	0.027286402	ko01100//Metabolic p	ko01100//Metab	-
0.999081313	0.027290542	-	-	-
0.999081175	0.027292589	ko01063//Biosynthesis	ko04151//PI3K-/	GO:0005886//plasma m
0.999079997	0.027310079	ko01100//Metabolic p	-	GO:0016020//membran
0.999079817	0.027312758	ko01100//Metabolic p	ko01100//Metab	GO:0000139//Golgi mer
0.999079591	0.027316104	-	-	-

0.999079526	0.027317082	-	ko00970//Aminc	GO:0005737//cytoplasm
0.999079351	0.02731968	-	ko01100//Metat	GO:0005654//nucleopla
0.999078421	0.027333465	ko01100//Metabolic p	-	-
0.999078233	0.027336252	ko01120//Microbial m	-	GO:0043231//intracellul
0.999078164	0.027337287	-	-	GO:0005802//trans-Gol
0.999078057	0.027338863	ko01100//Metabolic p	-	-
0.999077326	0.027349711	ko01110//Biosynthesis	-	GO:0005737//cytoplasm
0.999076356	0.027364086	-	ko05200//Pathw	GO:0005794//Golgi app
0.999076087	0.027368071	-	ko04060//Cytok	-
0.999075284	0.027379956	-	-	GO:0001650//fibrillar ce
0.999074311	0.027394366	-	ko04976//Bile se	GO:0005654//nucleopla
0.999073976	0.027399324	-	-	GO:0005886//plasma m
0.999073191	0.027410934	-	-	-
0.999073059	0.027412885	-	ko04080//Neurc	GO:0005886//plasma m
0.999072371	0.027423054	-	-	GO:0005634//nucleus;G
0.999072135	0.027426545	ko01100//Metabolic p	ko01100//Metat	-
0.999071309	0.027438746	-	-	-
0.999070634	0.027448717	ko01100//Metabolic p	ko00983//Drug	-
0.999069773	0.027461434	-	ko04514//Cell ad	GO:0016020//membran
0.999069758	0.027461656	-	-	GO:0005884//actin filam
0.999069617	0.027463743	-	ko05230//Centra	GO:0005886//plasma m
0.999069413	0.027466757	ko01100//Metabolic p	ko05200//Pathw	-
0.999068223	0.027484309	-	-	-
0.999068017	0.027487353	ko01100//Metabolic p	ko05414//Dilate	-
0.999067543	0.027494337	-	-	GO:0016021//integral co
0.999067393	0.027496549	ko01100//Metabolic p	-	GO:0005886//plasma m
0.999066599	0.027508253	-	-	-
0.999065152	0.027529568	-	ko04745//Photo	-
0.999064593	0.027537809	ko01063//Biosynthesis	-	GO:0009986//cell surfac
0.999063638	0.027551861	-	ko05200//Pathw	-
0.999063485	0.027554108	ko01100//Metabolic p	-	GO:0005737//cytoplasm
0.999063028	0.027560837	-	-	-
0.999060301	0.027600916	-	-	GO:0000781//chromoso
0.999059379	0.027614458	ko01100//Metabolic p	ko01100//Metat	GO:0031988//membran
0.999057581	0.027640842	-	-	-
0.999056891	0.027650965	-	-	-
0.999054441	0.027686856	-	-	GO:0043235//receptor c
0.999054401	0.027687442	-	ko04659//Th17 c	GO:0000785//chromatir
0.999054164	0.02769091	ko01100//Metabolic p	ko04976//Bile se	GO:0005789//endoplasr
0.999053671	0.027698136	ko01100//Metabolic p	-	-
0.999052485	0.027715486	-	-	-
0.999052278	0.027718513	ko01100//Metabolic p	ko01100//Metat	-
0.999051483	0.027730135	-	ko01100//Metat	GO:0005737//cytoplasm
0.999051454	0.027730565	-	-	-
0.999051295	0.027732893	ko01100//Metabolic p	-	-
0.999050168	0.027749353	ko01100//Metabolic p	-	GO:0005615//extracellu
0.999049264	0.027762555	-	-	GO:0005576//extracellu
0.999048546	0.027773044	ko01100//Metabolic p	-	-
0.999048388	0.027775353	ko01100//Metabolic p	-	GO:0005634//nucleus
0.999047392	0.027789886	-	-	GO:0005576//extracellu
0.99904409	0.02783802	ko01100//Metabolic p	-	GO:0005737//cytoplasm
0.999042301	0.027864052	-	ko00982//Drug	-
0.999042258	0.027864676	ko01100//Metabolic p	-	GO:0016020//membran
0.999041625	0.027873881	-	ko05322//Syster	GO:0005576//extracellu
0.999040926	0.027884057	-	-	-
0.99904075	0.027886604	ko01100//Metabolic p	ko04610//Comp	GO:0005576//extracellu
0.999040587	0.027888975	-	-	-
0.999040523	0.027889916	ko01100//Metabolic p	ko05200//Pathw	GO:0005834//heterotrin

0.99904045	0.027890967	-	-	-
0.999039827	0.02790003	ko01100//Metabolic p	-	-
0.999038998	0.02791207	-	ko04120//Ubiqu	GO:0005680//anaphase
0.999038406	0.027920669	-	-	-
0.999038181	0.02792394	-	-	-
0.999038056	0.02792575	ko01063//Biosynthesis	ko01100//Metak	GO:0005777//peroxison
0.999035539	0.027962271	-	-	-
0.999035075	0.027968991	-	-	-
0.999034611	0.02797572	ko01100//Metabolic p	-	GO:0005615//extracellu
0.999034544	0.02797669	ko01100//Metabolic p	-	-
0.999033116	0.027997373	-	ko04714//Therm	GO:0005739//mitochon
0.999032657	0.028004014	-	-	-
0.99903196	0.028014106	-	ko04060//Cytok	GO:0005576//extracellu
0.999031182	0.02802536	-	ko04060//Cytok	GO:0005576//extracellu
0.999029833	0.028044876	-	ko05202//Transc	GO:0016021//integral c
0.999029767	0.028045824	ko01100//Metabolic p	ko01100//Metak	GO:0005576//extracellu
0.99902932	0.028052285	-	ko01100//Metak	-
0.999028062	0.028070466	-	-	GO:0005739//mitochon
0.999025032	0.028114197	-	ko05017//Spino	GO:0043231//intracellul
0.999024384	0.028123532	ko01100//Metabolic p	ko01100//Metak	-
0.999021995	0.028157949	-	ko04151//PI3K-/	GO:0005654//nucleopla
0.999021599	0.028163658	ko01100//Metabolic p	ko04080//Neurc	GO:0005783//endoplasr
0.999020782	0.028175415	-	ko00982//Drug	-
0.999020065	0.028185727	ko01100//Metabolic p	ko01100//Metak	-
0.999019107	0.02819951	-	-	-
0.999019048	0.028200357	-	-	GO:0031464//Cul4A-RII
0.999018975	0.028201398	ko01100//Metabolic p	-	GO:0001725//stress fibe
0.999018905	0.028202406	ko01100//Metabolic p	-	-
0.999018213	0.028212353	ko01100//Metabolic p	-	GO:0016021//integral c
0.999017227	0.028226515	ko01100//Metabolic p	-	-
0.999017138	0.028227803	ko01120//Microbial m	-	-
0.999016958	0.028230377	-	-	-
0.999015621	0.02824958	-	-	GO:0005576//extracellu
0.999015304	0.02825412	ko01100//Metabolic p	-	-
0.999015294	0.02825427	-	ko01100//Metak	GO:0005615//extracellu
0.99901525	0.028254901	-	ko04640//Hema	GO:0031226//intrinsic c
0.999014883	0.028260166	-	-	-
0.999014217	0.028269721	ko01100//Metabolic p	-	GO:0005886//plasma m
0.999013426	0.028281055	ko01063//Biosynthesis	-	GO:0005579//membran
0.999013111	0.028285581	ko01110//Biosynthesis	-	-
0.999012502	0.0282943	ko01100//Metabolic p	ko05010//Alzhei	GO:0005576//extracellu
0.999011692	0.028305905	-	-	GO:0005737//cytoplasm
0.999011551	0.028307921	-	-	GO:0005737//cytoplasm
0.999011483	0.028308902	-	-	-
0.99901092	0.028316958	ko01110//Biosynthesis	-	-
0.999009824	0.028332641	-	-	-
0.999009266	0.028340628	ko01110//Biosynthesis	ko03320//PPAR	-
0.999008319	0.028354179	ko01100//Metabolic p	ko05142//Chaga	GO:0016020//membran
0.999008093	0.02835741	-	-	GO:0016020//membran
0.999004425	0.028409793	ko01100//Metabolic p	-	-
0.999003435	0.028423926	ko01100//Metabolic p	-	-
0.999003339	0.028425285	-	-	-
0.999002778	0.028433285	ko01100//Metabolic p	-	GO:0016020//membran
0.999002681	0.028434669	ko01110//Biosynthesis	ko01100//Metak	GO:0005829//cytosol;G
0.999002358	0.028439281	-	ko01100//Metak	-
0.999001928	0.028445404	-	ko04151//PI3K-/	GO:0009986//cell surfac
0.999001278	0.028454671	-	-	-
0.99900096	0.0284592	-	ko05131//Shigel	GO:0005922//connexon

0.999000899	0.02846007	ko01100//Metabolic p	-	GO:0005634//nucleus;G
0.999000898	0.028460091	-	ko04514//Cell a	GO:0005886//plasma m
0.999000604	0.028464278	ko01100//Metabolic p	-	GO:0005654//nucleopla
0.99899875	0.028490661	-	-	-
0.998998619	0.028492535	ko01100//Metabolic p	-	GO:0001726//ruffle;GO:
0.998998317	0.028496823	-	-	GO:0005783//endoplasr
0.99899823	0.028498062	-	-	-
0.998997866	0.02850324	ko01100//Metabolic p	ko04060//Cytok	GO:0009897//external s
0.99899746	0.028509023	-	-	GO:0005737//cytoplasm
0.998996815	0.028518194	ko01120//Microbial m	-	-
0.998996004	0.028529716	ko01100//Metabolic p	ko01100//Metab	-
0.998995899	0.028531213	-	-	-
0.998995792	0.028532723	-	-	GO:0005730//nucleolus
0.998994953	0.028544651	ko01100//Metabolic p	-	-
0.998994791	0.028546949	-	ko05200//Pathw	GO:0005576//extracellu
0.99899445	0.028551785	ko01100//Metabolic p	ko04080//Neuro	GO:0005783//endoplasr
0.998994276	0.028554267	-	-	GO:0016020//membran
0.998993921	0.0285593	ko01100//Metabolic p	-	GO:0005802//trans-Gol
0.998991932	0.028587522	-	-	GO:0005739//mitochon
0.998991702	0.028590789	-	ko05200//Pathw	GO:0005576//extracellu
0.998991623	0.028591912	ko01100//Metabolic p	ko01100//Metab	-
0.998991364	0.028595578	-	-	GO:0005634//nucleus;G
0.998990496	0.02860788	ko01100//Metabolic p	ko04060//Cytok	GO:0005576//extracellu
0.998989661	0.028619716	-	-	GO:0005884//actin filam
0.99898809	0.028641958	-	-	-
0.998987927	0.02864427	ko01100//Metabolic p	ko04918//Thyro	GO:0005576//extracellu
0.998987369	0.028652156	ko01100//Metabolic p	-	-
0.998984812	0.028688324	ko01100//Metabolic p	ko01100//Metab	-
0.998984127	0.028697998	-	-	-
0.998982508	0.02872086	-	-	-
0.998982119	0.028726356	ko01100//Metabolic p	-	GO:0005737//cytoplasm
0.998981511	0.028734937	ko01100//Metabolic p	ko04514//Cell a	GO:0005829//cytosol;G(
0.998979436	0.028764187	ko01100//Metabolic p	-	GO:0005737//cytoplasm
0.998978812	0.028772986	ko01100//Metabolic p	ko04919//Thyro	GO:0005887//integral c
0.998978106	0.028782929	ko01100//Metabolic p	-	-
0.998976758	0.028801911	ko01100//Metabolic p	-	GO:0005886//plasma m
0.998976754	0.028801963	ko01100//Metabolic p	-	GO:0005634//nucleus;G
0.998975883	0.028814234	ko01100//Metabolic p	ko04080//Neuro	GO:0005654//nucleopla
0.998973892	0.02884223	ko01100//Metabolic p	-	-
0.998972486	0.02886199	-	ko05200//Pathw	-
0.998971387	0.028877424	ko01100//Metabolic p	-	GO:0005737//cytoplasm
0.998970956	0.028883467	-	ko01100//Metab	GO:0005615//extracellu
0.998970513	0.028889679	ko01100//Metabolic p	-	GO:0000781//chromoso
0.99896949	0.028904034	-	ko05130//Patho	-
0.998968544	0.028917311	-	ko04610//Comp	GO:0005615//extracellu
0.998966658	0.028943733	-	-	GO:0005794//Golgi app
0.998966277	0.028949074	ko01100//Metabolic p	-	GO:0005759//mitochon
0.99896568	0.028957435	-	ko00983//Drug	-
0.998964695	0.028971212	-	ko01100//Metab	GO:0005794//Golgi app
0.99896407	0.02897996	-	-	-
0.998963795	0.02898381	-	-	-
0.998963214	0.02899193	ko01100//Metabolic p	-	GO:0005886//plasma m
0.998962576	0.029000855	-	-	-
0.998962014	0.029008713	-	-	GO:0016020//membran
0.998961944	0.029009691	-	ko01100//Metab	GO:0031988//membran
0.998959491	0.029043958	ko01100//Metabolic p	ko01100//Metab	GO:0005829//cytosol;G(
0.998957468	0.029072183	-	ko01100//Metab	-
0.998955401	0.029100985	ko01100//Metabolic p	ko01100//Metab	GO:0031988//membran

0.998954741	0.029110181	ko01100//Metabolic p	-	GO:0005615//extracellu
0.998953769	0.029123713	ko01100//Metabolic p	ko04024//cAMP	GO:0016021//integral co
0.99895305	0.029133723	-	-	-
0.998951816	0.029150889	ko01100//Metabolic p	ko01100//Metab	GO:0005739//mitochon
0.998951041	0.02916167	ko01100//Metabolic p	-	-
0.998950749	0.029165731	-	-	GO:0016020//membran
0.998950722	0.0291661	ko01100//Metabolic p	ko01100//Metab	GO:0005615//extracellu
0.99894977	0.029179325	-	ko00565//Ether	GO:0005615//extracellu
0.998949299	0.029185877	-	-	-
0.998948783	0.029193047	ko01100//Metabolic p	-	-
0.998948592	0.029195696	-	-	GO:0005887//integral co
0.998946952	0.029218461	-	ko01100//Metab	-
0.998946921	0.029218895	ko01100//Metabolic p	-	GO:0005886//plasma m
0.998945886	0.029233252	-	-	GO:0005737//cytoplasm
0.998945354	0.029240624	-	-	-
0.998945185	0.029242966	ko01110//Biosynthesis	ko05200//Pathw	GO:0005576//extracellu
0.998944773	0.029248686	ko01100//Metabolic p	ko05200//Pathw	-
0.998944525	0.029252113	ko01110//Biosynthesis	-	-
0.998944435	0.029253361	ko01100//Metabolic p	ko04310//Wnt s	GO:0005634//nucleus;G
0.998944412	0.029253681	-	-	-
0.998943298	0.029269113	ko01100//Metabolic p	ko05322//Systen	GO:0005576//extracellu
0.998942127	0.029285328	ko01100//Metabolic p	ko04514//Cell a	GO:0005829//cytosol;G
0.998941872	0.029288864	-	ko04973//Carbo	GO:0005886//plasma m
0.998940656	0.029305685	ko01120//Microbial m	ko01100//Metab	GO:0005783//endoplasm
0.998940631	0.029306037	-	-	GO:0005739//mitochon
0.998940442	0.029308656	-	ko01100//Metab	-
0.998939901	0.029316128	-	ko04724//Glutar	GO:0005886//plasma m
0.998939602	0.029320268	-	-	-
0.998939502	0.029321658	-	ko01100//Metab	-
0.998938957	0.029329189	-	-	GO:0016021//integral co
0.998938388	0.029337057	-	-	GO:0016020//membran
0.998936132	0.029368211	-	ko04010//MAPK	-
0.998935627	0.029375182	-	-	GO:0005576//extracellu
0.99893483	0.029386175	-	ko05200//Pathw	GO:0005576//extracellu
0.998928685	0.029470839	-	ko04080//Neuro	GO:0016020//membran
0.998924942	0.029522293	ko01100//Metabolic p	-	-
0.998924548	0.029527692	-	-	GO:0005634//nucleus;G
0.998924547	0.029527712	ko01100//Metabolic p	ko04514//Cell a	GO:0005886//plasma m
0.998922523	0.029555494	ko01100//Metabolic p	ko01100//Metab	GO:0005829//cytosol
0.998922183	0.029560156	-	-	GO:0001726//ruffle;GO:
0.998920874	0.029578101	-	ko01100//Metab	GO:0005829//cytosol;G
0.998920665	0.02958096	-	ko01100//Metab	-
0.998920458	0.029583802	-	ko01100//Metab	-
0.998918965	0.02960425	-	ko05203//Viral c	GO:0000786//nucleosor
0.99891869	0.02960802	-	ko04514//Cell a	GO:0005886//plasma m
0.998917717	0.029621341	-	-	GO:0016021//integral co
0.998917224	0.029628082	-	-	-
0.998917067	0.02963024	-	-	GO:0005615//extracellu
0.998916281	0.029640983	ko01100//Metabolic p	-	-
0.998916153	0.029642743	-	ko01100//Metab	-
0.998915474	0.029652028	-	-	-
0.998914921	0.029659586	-	-	-
0.998914911	0.029659728	ko01100//Metabolic p	-	GO:0005737//cytoplasm
0.998914906	0.029659785	-	-	GO:0000307//cyclin-de
0.998914864	0.029660366	-	ko05152//Tuber	GO:0009897//external s
0.998914211	0.029669288	ko01120//Microbial m	ko04713//Circad	-
0.998913986	0.029672361	ko01100//Metabolic p	-	-
0.998913756	0.029675501	-	ko04060//Cytok	GO:0005576//extracellu

0.998912928	0.029686815	ko01100//Metabolic p	ko05200//Pathw	-
0.998912771	0.029688954	-	-	-
0.998911068	0.029712212	ko01063//Biosynthesis	-	-
0.998911067	0.029712217	-	ko01100//Metab	GO:0005615//extracellu
0.998910282	0.029722929	ko01100//Metabolic p	ko01100//Metab	GO:0005783//endoplasn
0.998909688	0.029731037	ko01100//Metabolic p	-	GO:0005737//cytoplasm
0.998909079	0.02973933	-	ko01100//Metab	GO:0016020//membran
0.998907259	0.029764131	-	-	GO:0031224//intrinsic c
0.998906853	0.029769666	-	ko04060//Cytok	-
0.998906083	0.02978015	-	ko04530//Tight	GO:0016020//membran
0.998905354	0.029790072	ko01100//Metabolic p	ko01100//Metab	GO:0005829//cytosol;G(
0.998904494	0.029801774	ko01100//Metabolic p	-	-
0.998903309	0.029817891	-	ko04918//Thyro	GO:0005576//extracellu
0.998902622	0.029827227	-	ko01100//Metab	-
0.998901009	0.029849145	ko01100//Metabolic p	-	-
0.998898634	0.029881385	-	-	GO:0016020//membran
0.998894835	0.029932891	-	ko04151//PI3K-/	GO:0005654//nucleopla
0.998894831	0.029932949	-	ko01100//Metab	GO:0005654//nucleopla
0.998894611	0.029935924	ko01100//Metabolic p	-	GO:0005802//trans-Gol
0.998893792	0.029947018	-	ko04080//Neuro	GO:0005783//endoplasn
0.998893076	0.029956714	-	ko04060//Cytok	GO:0016020//membran
0.998892872	0.029959467	-	-	-
0.998890826	0.029987144	-	ko01100//Metab	-
0.998890038	0.0299978	-	-	-
0.998888397	0.030019961	-	ko05230//Centra	GO:0005886//plasma m
0.998888373	0.030020295	ko01100//Metabolic p	-	GO:0005886//plasma m
0.998887191	0.030036247	-	ko04080//Neuro	GO:0005654//nucleopla
0.998886829	0.030041134	ko01100//Metabolic p	ko01100//Metab	GO:0005654//nucleopla
0.998886271	0.030048662	-	-	-
0.998886266	0.030048735	-	ko05200//Pathw	GO:0005576//extracellu
0.998885099	0.030064471	-	-	-
0.998884971	0.030066208	-	ko04927//Cortis	GO:0005634//nucleus;G
0.998884307	0.030075151	-	-	GO:0005737//cytoplasm
0.998883389	0.030087534	ko01063//Biosynthesis	ko04360//Axon	GO:0016020//membran
0.998882718	0.03009657	ko01100//Metabolic p	ko04060//Cytok	GO:0005576//extracellu
0.998882307	0.030102099	-	ko01100//Metab	-
0.998880847	0.030121761	ko01100//Metabolic p	-	GO:0016020//membran
0.998877568	0.03016587	ko01110//Biosynthesis	ko01100//Metab	GO:0000139//Golgi mer
0.998877451	0.030167435	-	ko04080//Neuro	GO:0005881//cytoplasm
0.99887573	0.030190562	-	ko01100//Metab	GO:0005737//cytoplasm
0.998875408	0.03019488	-	ko04714//Therm	GO:0005634//nucleus
0.998875203	0.030197632	ko01110//Biosynthesis	-	-
0.99887501	0.030200227	-	-	-
0.998874556	0.030206323	-	-	-
0.998873967	0.030214223	-	ko01100//Metab	-
0.998873645	0.030218551	ko01100//Metabolic p	ko04060//Cytok	GO:0005887//integral c
0.998873583	0.030219381	-	-	-
0.998871551	0.030246624	ko01100//Metabolic p	ko00982//Drug	-
0.998871177	0.030251637	ko01100//Metabolic p	ko05203//Viral c	GO:0000786//nucleosor
0.99887004	0.030266868	-	ko04080//Neuro	GO:0005886//plasma m
0.998869962	0.030267915	ko01100//Metabolic p	-	-
0.998869675	0.030271765	-	-	GO:0005783//endoplasn
0.998869612	0.030272607	-	-	GO:0001725//stress fibe
0.998869547	0.030273479	ko01100//Metabolic p	-	-
0.998868463	0.030287991	-	ko04390//Hippo	GO:0005667//transcript
0.998867175	0.030305233	-	ko04974//Protei	-
0.998867119	0.030305974	ko01100//Metabolic p	ko04350//TGF-b	-
0.998866624	0.030312603	-	ko01100//Metab	GO:0005777//peroxison

0.998866493	0.030314355	-	-	GO:0005737//cytoplasm
0.998866011	0.03032079	ko01100//Metabolic p	-	GO:0005576//extracellu
0.998865707	0.030324867	-	ko04978//Miner	GO:0009986//cell surfac
0.998864118	0.030346104	-	-	GO:0005576//extracellu
0.998863675	0.030352018	ko01100//Metabolic p	ko04151//PI3K-/	GO:0008305//integrin c
0.998863421	0.030355405	ko01100//Metabolic p	ko04310//Wnt s	GO:0005654//nucleopla
0.998862893	0.030362464	-	-	GO:0005739//mitochon
0.998862665	0.03036551	-	ko05010//Alzhei	GO:0005576//extracellu
0.998862493	0.030367794	-	-	-
0.998861276	0.030384043	ko01120//Microbial m	-	-
0.998860024	0.030400746	ko01100//Metabolic p	-	GO:0016020//membran
0.99885984	0.030403206	-	-	-
0.998859517	0.030407513	ko01100//Metabolic p	-	-
0.998859144	0.030412476	-	ko04024//cAMP	GO:0016021//integral c
0.99885856	0.030420269	-	ko00982//Drug	-
0.998858546	0.03042045	ko01120//Microbial m	ko01100//Metab	GO:0005739//mitochon
0.998857916	0.030428841	-	-	-
0.99885761	0.030432923	ko01100//Metabolic p	-	GO:0005768//endosom
0.998856308	0.03045026	-	-	GO:0005737//cytoplasm
0.998856269	0.03045078	ko01063//Biosynthesis	-	GO:0005829//cytosol;G
0.998856025	0.030454032	-	ko01100//Metab	-
0.998854052	0.030480285	-	-	GO:0005615//extracellu
0.998853872	0.030482688	ko01100//Metabolic p	ko03320//PPAR	GO:0005576//extracellu
0.998853786	0.030483824	-	ko01100//Metab	-
0.998853341	0.030489745	-	ko01100//Metab	GO:0005640//nuclear o
0.998853149	0.030492291	-	ko05200//Pathw	GO:0005634//nucleus;G
0.998853029	0.030493889	ko01100//Metabolic p	-	-
0.998851933	0.030508461	ko01100//Metabolic p	-	GO:0005802//trans-Gol
0.998851724	0.030511235	ko01063//Biosynthesis	-	-
0.998851005	0.030520786	ko01100//Metabolic p	-	-
0.998850455	0.030528098	-	-	GO:0005886//plasma m
0.998850101	0.030532792	-	-	GO:0001650//fibrillar ce
0.998847887	0.030562185	ko01100//Metabolic p	-	-
0.998847273	0.030570324	-	ko04060//Cytok	GO:0005576//extracellu
0.998845663	0.03059167	-	-	-
0.998845075	0.030599463	-	ko01100//Metab	GO:0005737//cytoplasm
0.998844852	0.030602423	ko01100//Metabolic p	-	-
0.998844749	0.030603782	-	ko04010//MAPK	-
0.998844467	0.030607515	ko01100//Metabolic p	-	-
0.998844351	0.030609051	ko01100//Metabolic p	-	GO:0005856//cytoskelet
0.998843968	0.030614127	-	-	GO:0005886//plasma m
0.998843917	0.030614807	-	-	GO:0005634//nucleus;G
0.998843839	0.030615832	-	ko01100//Metab	GO:0005634//nucleus;G
0.998842326	0.030635871	ko01110//Biosynthesis	-	GO:0005634//nucleus
0.998841471	0.030647173	-	-	GO:0016021//integral c
0.998839066	0.030678976	-	ko05130//Patho	-
0.998837931	0.030693978	-	-	-
0.998836594	0.030711627	-	-	-
0.998835938	0.030720295	ko01100//Metabolic p	-	GO:0005884//actin filan
0.998835795	0.030722179	ko01100//Metabolic p	-	-
0.998835022	0.030732377	-	ko00983//Drug	-
0.998834389	0.030740732	ko01100//Metabolic p	-	GO:0005737//cytoplasm
0.998834274	0.030742238	ko01100//Metabolic p	ko01100//Metab	-
0.998833349	0.030754436	-	ko05200//Pathw	-
0.998833292	0.030755188	-	-	GO:0005886//plasma m
0.998832476	0.030765953	ko01110//Biosynthesis	-	GO:0001725//stress fibe
0.998830112	0.030797087	ko01100//Metabolic p	-	-
0.998830079	0.030797524	ko01100//Metabolic p	-	-

0.998830053	0.030797857	-	ko04060//Cytok	GO:0009897//external s
0.998829602	0.030803801	-	-	-
0.99882865	0.030816322	-	ko04919//Thyro	GO:0005887//integral co
0.99882758	0.030830394	-	ko04979//Chole	GO:0005856//cytoskelet
0.998827454	0.030832059	-	ko04144//Endoc	-
0.998827373	0.030833129	-	-	GO:0005576//extracellu
0.998826245	0.030847953	-	-	GO:0005737//cytoplasm
0.998826203	0.030848504	ko01120//Microbial m	-	GO:0005634//nucleus;G
0.998825216	0.030861475	ko01063//Biosynthesis	-	-
0.998824938	0.03086512	-	-	-
0.998823934	0.030878314	-	ko01100//Metab	-
0.998823727	0.030881028	ko01100//Metabolic p	ko01100//Metab	-
0.998821348	0.030912245	ko01110//Biosynthesis	-	GO:0046658//anchored
0.998821189	0.030914334	ko01100//Metabolic p	-	GO:0005884//actin filam
0.998819527	0.030936129	ko01100//Metabolic p	ko04514//Cell ad	-
0.998818971	0.030943405	-	-	-
0.998816589	0.030974609	-	ko01100//Metab	GO:0005730//nucleolus
0.998812538	0.03102758	ko01100//Metabolic p	ko01100//Metab	-
0.998812146	0.031032709	-	-	-
0.998812046	0.031034013	ko01100//Metabolic p	-	-
0.99881115	0.031045712	-	ko05010//Alzhei	GO:0005576//extracellu
0.998810442	0.03105496	-	-	GO:0005737//cytoplasm
0.998809387	0.031068735	-	ko01100//Metab	-
0.998809292	0.031069969	-	ko04144//Endoc	GO:0001917//photorece
0.998808877	0.031075384	ko01100//Metabolic p	ko02010//ABC tr	GO:0000139//Golgi mem
0.998808206	0.031084139	-	-	-
0.998807137	0.031098085	-	ko03320//PPAR	GO:0005634//nucleus;G
0.998806673	0.031104131	-	-	GO:0016021//integral co
0.998806527	0.031106034	ko01110//Biosynthesis	-	GO:0001533//cornified
0.998805799	0.031115518	ko01100//Metabolic p	ko01100//Metab	-
0.99880574	0.031116284	ko01100//Metabolic p	-	-
0.998804026	0.031138614	-	-	GO:0001725//stress fibe
0.998803897	0.031140298	ko01100//Metabolic p	-	GO:0046658//anchored
0.998802822	0.031154292	ko01100//Metabolic p	-	GO:0031966//mitochon
0.998802119	0.031163429	-	-	GO:0016020//membran
0.998801666	0.03116933	-	-	GO:0034451//centriolar
0.998799497	0.031197525	ko01063//Biosynthesis	-	GO:0005789//endoplasm
0.998799483	0.031197712	-	ko05202//Transc	GO:0016021//integral co
0.998798466	0.031210931	-	ko01100//Metab	-
0.998797181	0.031227613	-	ko04060//Cytok	-
0.998794863	0.031257691	-	-	-
0.998794461	0.031262909	-	-	-
0.998792655	0.031286317	ko01100//Metabolic p	-	-
0.998789854	0.031322605	-	ko01100//Metab	-
0.998788461	0.031340623	ko01100//Metabolic p	ko04979//Chole	GO:0005856//cytoskelet
0.998788308	0.031342607	-	-	-
0.99878658	0.03136495	ko01100//Metabolic p	-	GO:0005737//cytoplasm
0.998785248	0.031382171	-	ko01100//Metab	-
0.998785167	0.031383213	-	-	-
0.99878269	0.031415194	ko01100//Metabolic p	ko05230//Centra	GO:0005886//plasma m
0.998782457	0.031418208	-	ko05200//Pathw	-
0.99878214	0.031422298	-	ko04080//Neuro	GO:0005783//endoplasm
0.998782065	0.031423266	-	ko04020//Calciu	GO:0005886//plasma m
0.998781637	0.03142878	-	ko01100//Metab	-
0.998781568	0.031429669	-	-	-
0.998781122	0.031435431	ko01100//Metabolic p	-	-
0.998781049	0.031436367	-	ko01100//Metab	GO:0005654//nucleopla
0.998780883	0.031438512	-	-	GO:0005654//nucleopla

0.998780724	0.031440564	-	ko04060//Cytok	GO:0005887//integral co
0.998779624	0.031454747	-	ko04514//Cell ad	GO:0005829//cytosol;GO
0.998779262	0.031459409	ko01100//Metabolic p	-	-
0.99877916	0.031460718	-	ko01100//Metab	GO:0005615//extracellu
0.998777657	0.031480091	ko01100//Metabolic p	ko02010//ABC tr	GO:0005886//plasma m
0.99877724	0.031485463	-	ko04010//MAPK	-
0.998777068	0.031487673	-	ko04142//Lysoso	GO:0005615//extracellu
0.998776803	0.031491078	ko01100//Metabolic p	ko01100//Metab	-
0.99877508	0.031513261	-	ko01100//Metab	GO:0005794//Golgi app
0.998774708	0.031518043	-	ko01100//Metab	-
0.998774457	0.031521272	ko01100//Metabolic p	ko01100//Metab	GO:0000139//Golgi mem
0.99877391	0.031528312	ko01100//Metabolic p	ko01100//Metab	-
0.998773539	0.031533085	ko01100//Metabolic p	-	GO:0005576//extracellu
0.998773514	0.031533406	-	-	-
0.998772465	0.03154688	ko01100//Metabolic p	ko04978//Miner	GO:0009986//cell surfac
0.998771441	0.031560045	-	ko01100//Metab	-
0.998770984	0.031565908	-	ko01100//Metab	GO:0031988//membran
0.998770753	0.031568878	-	ko03018//RNA d	GO:0005829//cytosol
0.998770039	0.031578045	-	-	GO:0005654//nucleopla
0.998769818	0.031580885	-	-	-
0.998769123	0.031589816	-	ko04211//Longe	GO:0005634//nucleus
0.998767391	0.031612036	-	ko05131//Shigel	GO:0005922//connexon
0.998767053	0.031616372	-	-	GO:0005737//cytoplasm
0.998767036	0.031616591	ko01100//Metabolic p	-	-
0.998765583	0.031635212	-	ko01100//Metab	-
0.998765253	0.031639447	-	ko05202//Transc	GO:0000786//nucleosom
0.998764843	0.031644701	-	-	GO:0016021//integral co
0.998764362	0.031650854	ko01100//Metabolic p	-	-
0.99876381	0.031657923	-	ko01100//Metab	GO:0048269//methionin
0.998762208	0.031678433	-	-	GO:0016021//integral co
0.998760634	0.031698581	-	-	GO:0042734//presynapt
0.998760416	0.031701363	ko01063//Biosynthesis	-	GO:0030425//dendrite
0.99875825	0.031729062	-	-	GO:0000786//nucleosom
0.998758133	0.031730554	-	-	GO:0001725//stress fibe
0.998757742	0.031735543	-	ko00565//Ether	GO:0005615//extracellu
0.998757593	0.03173745	-	-	-
0.998757548	0.031738021	-	ko01100//Metab	GO:0005737//cytoplasm
0.998755852	0.031759684	-	-	GO:0016021//integral co
0.99875554	0.031763668	-	-	GO:0005886//plasma m
0.99875452	0.031776682	ko01100//Metabolic p	ko01100//Metab	GO:0016021//integral co
0.998753992	0.031783426	ko01100//Metabolic p	ko01100//Metab	GO:0005615//extracellu
0.998753605	0.031788355	-	ko01100//Metab	-
0.998753518	0.03178947	-	-	GO:0005737//cytoplasm
0.998753054	0.031795382	ko01100//Metabolic p	-	-
0.998752413	0.031803557	-	ko04146//Peroxi	GO:0005777//peroxisom
0.998751919	0.03180986	-	ko01100//Metab	-
0.998750852	0.031823456	-	ko05010//Alzhei	GO:0005576//extracellu
0.998749639	0.031838909	ko01100//Metabolic p	-	-
0.998749569	0.031839796	ko01100//Metabolic p	ko04130//SNAR	GO:0005737//cytoplasm
0.998749463	0.03184115	-	ko04927//Cortis	GO:0005634//nucleus;G
0.998748702	0.031850834	-	-	-
0.998748196	0.03185727	ko01100//Metabolic p	-	GO:0005737//cytoplasm
0.998748087	0.031858664	-	-	GO:0005634//nucleus;G
0.998747207	0.031869864	-	-	-
0.998744901	0.031899186	ko01063//Biosynthesis	ko00533//Glycos	GO:0000139//Golgi mem
0.998743949	0.031911285	-	-	GO:0016021//integral co
0.998742991	0.031923449	-	-	-
0.998742621	0.031928147	ko01100//Metabolic p	ko01100//Metab	GO:0005739//mitochon

0.998741499	0.031942393	-	ko05200//Pathw	GO:0005576//extracellu
0.998739402	0.031968998	ko01063//Biosynthesis	-	-
0.998737645	0.031991274	ko01100//Metabolic p	-	GO:0005634//nucleus
0.998735147	0.032022918	-	ko01100//Metab	-
0.998732303	0.032058908	ko01100//Metabolic p	ko01100//Metab	GO:0033180//proton-tr
0.998730845	0.032077348	ko01100//Metabolic p	-	GO:0005813//centrosor
0.998730445	0.032082393	ko01100//Metabolic p	ko04150//mTOR	GO:0005829//cytosol;G
0.998727585	0.032118524	-	ko01100//Metab	-
0.998727472	0.032119945	ko01100//Metabolic p	ko05200//Pathw	GO:0005576//extracellu
0.998727361	0.032121355	ko01100//Metabolic p	-	GO:0001725//stress fibe
0.99872657	0.03213134	ko01100//Metabolic p	ko01100//Metab	GO:0000139//Golgi mer
0.998726504	0.032132168	ko01100//Metabolic p	ko05132//Salmo	GO:0005737//cytoplasm
0.998726195	0.032136063	-	-	GO:0005737//cytoplasm
0.998726107	0.032137178	-	ko01100//Metab	-
0.998726019	0.032138286	-	-	-
0.998725882	0.03214002	-	ko01100//Metab	-
0.998725136	0.032149422	-	-	GO:0016020//membran
0.99872438	0.03215896	ko01120//Microbial m	-	GO:0001518//voltage-g
0.998723686	0.0321677	-	-	-
0.99872284	0.032178371	-	-	GO:0005794//Golgi app
0.998720729	0.032204953	-	-	-
0.99872049	0.032207964	-	-	GO:0005783//endoplasr
0.998719374	0.032222011	ko01100//Metabolic p	-	GO:0005813//centrosor
0.998716506	0.032258085	-	-	GO:0005884//actin filan
0.998716298	0.032260688	-	ko01100//Metab	-
0.998714674	0.0322811	ko01100//Metabolic p	-	-
0.998713807	0.032291983	ko01100//Metabolic p	ko04144//Endoc	GO:0001917//photorece
0.998710596	0.032332278	ko01100//Metabolic p	-	GO:0005886//plasma m
0.998709257	0.032349063	-	-	-
0.998708759	0.032355312	-	-	-
0.998708414	0.032359625	-	ko01100//Metab	GO:0031988//membran
0.998707083	0.032376305	-	-	GO:0031966//mitochon
0.998706424	0.032384552	ko01100//Metabolic p	ko01100//Metab	-
0.998706353	0.032385442	ko01100//Metabolic p	-	GO:0016020//membran
0.998705882	0.032391343	ko01100//Metabolic p	-	GO:0034451//centriolar
0.998704531	0.032408245	-	ko04979//Chole	GO:0005856//cytoskelet
0.998703581	0.032420135	ko01100//Metabolic p	-	GO:0005737//cytoplasm
0.998701827	0.032442052	ko01100//Metabolic p	ko05200//Pathw	GO:0005576//extracellu
0.99870163	0.032444515	ko01100//Metabolic p	ko04745//Photo	-
0.99870071	0.032456011	-	ko01100//Metab	GO:0005737//cytoplasm
0.998700276	0.032461437	ko01100//Metabolic p	-	GO:0001533//cornified
0.998699706	0.032468552	ko01100//Metabolic p	-	GO:0031090//organelle
0.998698453	0.032484203	-	ko05200//Pathw	-
0.998696927	0.032503236	-	ko04080//Neuro	GO:0005783//endoplasr
0.9986966	0.032507325	-	ko05200//Pathw	-
0.998693709	0.032543362	-	ko01100//Metab	-
0.998693453	0.032546554	ko01100//Metabolic p	-	-
0.998692062	0.032563879	-	-	GO:0005737//cytoplasm
0.998691554	0.032570196	-	ko01100//Metab	-
0.998691501	0.032570862	ko01100//Metabolic p	-	GO:0016021//integral c
0.998691145	0.032575285	ko01100//Metabolic p	ko04974//Protei	-
0.998690735	0.032580385	ko01100//Metabolic p	ko04971//Gastri	GO:0005886//plasma m
0.99868873	0.032605337	-	ko04979//Chole	GO:0005856//cytoskelet
0.998687213	0.032624199	-	ko04979//Chole	GO:0005783//endoplasr
0.998684404	0.032659084	ko01100//Metabolic p	ko04010//MAPK	-
0.998684153	0.032662207	ko01100//Metabolic p	-	-
0.998684098	0.032662879	ko01100//Metabolic p	-	-
0.998682598	0.032681503	ko01100//Metabolic p	ko01100//Metab	-

0.998682506	0.032682644	-	-	-
0.998682467	0.032683119	ko01100//Metabolic p	-	GO:0005886//plasma m
0.998682088	0.032687824	ko01063//Biosynthesis	ko04080//Neurc	GO:0016020//membran
0.998680615	0.032706088	-	ko01100//Metab	-
0.998679566	0.032719099	ko01100//Metabolic p	ko01100//Metab	GO:0005737//cytoplasm
0.998677982	0.032738721	-	ko04714//Therm	-
0.998677533	0.032744276	ko01100//Metabolic p	-	-
0.998677456	0.032745228	ko01100//Metabolic p	ko04514//Cell ad	GO:0016020//membran
0.998676991	0.032750989	ko01063//Biosynthesis	-	-
0.998676922	0.032751849	-	-	-
0.998676659	0.032755098	-	-	GO:0005576//extracellu
0.998676378	0.032758583	-	-	-
0.998675945	0.032763941	-	-	-
0.998675538	0.032768976	-	-	-
0.998674641	0.032780075	-	-	-
0.998674417	0.032782837	ko01100//Metabolic p	-	-
0.998674387	0.032783207	-	-	GO:0005739//mitochon
0.998673716	0.032791505	-	-	GO:0005884//actin filam
0.998672314	0.032808834	ko01100//Metabolic p	ko01100//Metab	-
0.998672254	0.032809577	ko01100//Metabolic p	ko05200//Pathw	GO:0005576//extracellu
0.998671161	0.032823086	-	ko04080//Neurc	GO:0005886//plasma m
0.998670958	0.032825598	ko01100//Metabolic p	ko05200//Pathw	GO:0005794//Golgi app
0.998670211	0.032834817	ko01100//Metabolic p	-	-
0.998669643	0.032841829	ko01100//Metabolic p	-	GO:0016021//integral co
0.99866872	0.032853228	-	-	GO:0016020//membran
0.998667906	0.032863267	ko01100//Metabolic p	ko01100//Metab	GO:0005615//extracellu
0.998667878	0.032863618	-	ko01100//Metab	-
0.998667456	0.032868821	-	ko01100//Metab	GO:0005777//peroxison
0.998666671	0.032878504	-	ko01100//Metab	GO:0005886//plasma m
0.998666624	0.032879083	-	ko04610//Comp	GO:0005615//extracellu
0.998666613	0.032879222	-	-	-
0.998666192	0.032884405	ko01100//Metabolic p	-	-
0.998666156	0.032884858	ko01100//Metabolic p	-	GO:0005634//nucleus;G
0.998664575	0.032904339	ko01100//Metabolic p	-	GO:0005576//extracellu
0.998664416	0.032906306	ko01120//Microbial m	ko05200//Pathw	GO:0005634//nucleus;G
0.998663613	0.032916197	ko01100//Metabolic p	ko05322//Syster	GO:0000786//nucleosor
0.998663262	0.032920517	-	ko05132//Salmc	GO:0005764//lysosome;
0.998662905	0.032924919	ko01100//Metabolic p	-	-
0.998662609	0.032928562	-	-	-
0.998661495	0.032942272	-	-	-
0.998661442	0.03294293	ko01100//Metabolic p	ko04060//Cytok	GO:0005576//extracellu
0.998661352	0.032944032	-	-	-
0.998660626	0.032952966	ko01100//Metabolic p	-	GO:0031012//extracellu
0.998657385	0.032992823	-	-	GO:0030425//dendrite
0.998656942	0.032998272	ko01100//Metabolic p	-	GO:0001725//stress fibe
0.998656681	0.033001479	ko01100//Metabolic p	ko04144//Endoc	-
0.998655667	0.03301393	ko01100//Metabolic p	ko01100//Metab	GO:0005737//cytoplasm
0.998654983	0.033022334	-	-	GO:0005615//extracellu
0.998654898	0.033023368	-	ko05200//Pathw	GO:0005794//Golgi app
0.99865484	0.033024089	-	-	GO:0005654//nucleopla
0.998654253	0.033031297	ko01100//Metabolic p	ko04060//Cytok	GO:0005576//extracellu
0.998653259	0.033043485	ko01100//Metabolic p	-	-
0.998651879	0.033060414	-	-	-
0.998651843	0.033060862	ko01100//Metabolic p	ko05200//Pathw	GO:0000785//chromatin
0.998651023	0.033070915	-	ko01100//Metab	-
0.998650892	0.033072517	ko01100//Metabolic p	ko01100//Metab	GO:0005739//mitochon
0.998647354	0.03311587	-	ko01100//Metab	GO:0048269//methionin
0.998646835	0.033122225	-	-	GO:0005783//endoplasr

0.998646136	0.033130776	ko01100//Metabolic p	ko00565//Ether	GO:0005615//extracellu
0.998644513	0.033150636	-	-	-
0.998644445	0.033151462	ko01100//Metabolic p	ko01100//Metab	-
0.998643827	0.03315902	-	-	-
0.998642901	0.033170345	-	-	GO:0005886//plasma m
0.998642703	0.033172769	ko01100//Metabolic p	-	-
0.998640193	0.033203432	-	-	-
0.998640169	0.033203722	-	ko01100//Metab	-
0.998640109	0.033204463	ko01100//Metabolic p	-	-
0.998639834	0.033207819	ko01100//Metabolic p	-	GO:0005634//nucleus;G
0.998639436	0.033212673	ko01110//Biosynthesis	-	GO:0005737//cytoplasm
0.998639362	0.033213583	ko01100//Metabolic p	ko00983//Drug	-
0.998638598	0.033222905	ko01100//Metabolic p	ko05143//Africa	GO:0005576//extracellu
0.998638445	0.033224767	ko01100//Metabolic p	ko04979//Chole	GO:0005783//endoplasi
0.998635704	0.033258205	ko01100//Metabolic p	ko05200//Pathw	GO:0005576//extracellu
0.998634496	0.033272924	ko01100//Metabolic p	-	GO:0005576//extracellu
0.99863237	0.033298822	ko01100//Metabolic p	ko04060//Cytok	-
0.998631752	0.033306357	ko01100//Metabolic p	-	-
0.998630608	0.033320271	-	ko04080//Neuro	GO:0005881//cytoplasm
0.998629948	0.03332831	ko01100//Metabolic p	ko01100//Metab	-
0.998629182	0.033337626	ko01100//Metabolic p	ko05200//Pathw	GO:0005576//extracellu
0.99862896	0.033340329	ko01100//Metabolic p	ko05010//Alzhei	GO:0005576//extracellu
0.998628139	0.033350308	ko01100//Metabolic p	-	GO:0005634//nucleus
0.998627736	0.033355209	-	ko04060//Cytok	GO:0005576//extracellu
0.998626081	0.033375324	-	-	GO:0005737//cytoplasm
0.998625095	0.033387292	ko01100//Metabolic p	-	GO:0005634//nucleus;G
0.998622674	0.033416684	ko01100//Metabolic p	-	-
0.998621768	0.033427673	ko01100//Metabolic p	-	-
0.99862131	0.033433232	ko01100//Metabolic p	-	GO:0001725//stress fibe
0.998619648	0.033453375	-	-	GO:0016021//integral c
0.998619578	0.033454226	ko01100//Metabolic p	-	GO:0030864//cortical ac
0.998618747	0.033464299	ko01100//Metabolic p	-	-
0.998618088	0.033472285	ko01100//Metabolic p	-	GO:0016020//membran
0.998617368	0.03348101	ko01100//Metabolic p	-	-
0.998616564	0.03349074	-	ko04390//Hippo	GO:0005667//transcript
0.998615364	0.033505264	-	ko00982//Drug	-
0.998614422	0.033516657	-	ko01100//Metab	GO:0005730//nucleolus
0.998612885	0.033535252	ko01100//Metabolic p	ko01100//Metab	-
0.998612777	0.033536554	-	-	-
0.998612201	0.033543516	-	-	GO:0005634//nucleus;G
0.99861168	0.033549823	ko01100//Metabolic p	-	-
0.998610578	0.033563138	-	ko04060//Cytok	GO:0005576//extracellu
0.998610505	0.033564016	-	ko04145//Phago	GO:0005783//endoplasi
0.998609873	0.033571647	-	ko01100//Metab	-
0.998609688	0.033573885	ko01100//Metabolic p	ko04360//Axon	GO:0005886//plasma m
0.998609229	0.033579429	-	-	GO:0005576//extracellu
0.998607246	0.033603369	-	-	-
0.998606598	0.033611178	-	-	GO:0005737//cytoplasm
0.998605804	0.033620762	ko01100//Metabolic p	-	-
0.998604934	0.033631255	ko01100//Metabolic p	ko01100//Metab	-
0.99860137	0.033674195	ko01100//Metabolic p	-	GO:0005783//endoplasi
0.998601161	0.033676704	-	-	-
0.998601014	0.033678483	-	-	GO:0001726//ruffle;GO:
0.99859894	0.033703433	-	-	-
0.998598316	0.033710948	-	-	GO:0005634//nucleus;G
0.998597749	0.033717762	ko01100//Metabolic p	-	-
0.998596981	0.033726994	ko01100//Metabolic p	-	GO:0005737//cytoplasm
0.998596726	0.033730069	-	-	-

0.998596587	0.033731737	ko01100//Metabolic p	ko01100//Metab	-
0.998595814	0.033741029	ko01100//Metabolic p	-	GO:0005634//nucleus;G
0.998595532	0.033744412	ko01100//Metabolic p	-	-
0.998595422	0.033745733	ko01100//Metabolic p	-	-
0.998595158	0.033748906	-	ko04974//Protei	-
0.99859485	0.033752603	-	ko03320//PPAR	GO:0005634//nucleus;G
0.998594732	0.033754026	-	-	-
0.998594362	0.033758465	-	-	-
0.998594091	0.033761724	ko01110//Biosynthesis	ko04020//Calciu	GO:0005886//plasma m
0.998593112	0.033773479	-	ko05017//Spino	GO:0043231//intracellul
0.998592768	0.033777612	ko01100//Metabolic p	-	-
0.998591081	0.033797859	-	-	-
0.998590421	0.033805767	-	ko01100//Metab	-
0.99858853	0.033828445	-	-	-
0.998587587	0.033839748	-	ko04745//Photo	-
0.998587416	0.033841792	-	ko01100//Metab	GO:0005789//endoplasm
0.998587325	0.033842887	ko01100//Metabolic p	ko01100//Metab	GO:0000139//Golgi mer
0.998585808	0.033861062	-	-	-
0.998583231	0.033891899	ko01100//Metabolic p	-	-
0.998582747	0.03389769	ko01100//Metabolic p	ko04514//Cell a	GO:0005769//early endo
0.998582165	0.033904657	ko01100//Metabolic p	-	-
0.998581764	0.033909451	-	-	GO:0016021//integral co
0.998577961	0.033954898	ko01100//Metabolic p	ko01100//Metab	GO:0005783//endoplasm
0.998577799	0.033956829	ko01100//Metabolic p	-	GO:0005739//mitochon
0.998577084	0.033965367	-	-	-
0.998576571	0.033971482	-	ko01100//Metab	GO:0005739//mitochon
0.998576543	0.033971825	-	-	GO:0005576//extracellu
0.998576454	0.033972878	-	-	-
0.998575614	0.033982906	-	-	GO:0005783//endoplasm
0.998574803	0.033992585	-	ko01100//Metab	GO:0005654//nucleopla
0.998573906	0.034003277	-	-	GO:0005576//extracellu
0.99857333	0.03401015	-	-	GO:0005737//cytoplasm
0.998573085	0.034013069	ko01100//Metabolic p	ko04151//PI3K-/	GO:0005654//nucleopla
0.998571788	0.034028532	-	ko04080//Neuro	GO:0016020//membran
0.998570718	0.034041277	ko01100//Metabolic p	-	GO:0005576//extracellu
0.998569273	0.034058479	-	-	GO:0005634//nucleus;G
0.998568715	0.034065127	ko01100//Metabolic p	ko02010//ABC tr	GO:0016021//integral co
0.998568083	0.034072644	ko01100//Metabolic p	ko01100//Metab	GO:0005739//mitochon
0.998567553	0.034078957	-	-	GO:0001533//cornified
0.998566308	0.034093762	-	ko05203//Viral c	GO:0000786//nucleosor
0.998565712	0.034100852	ko01110//Biosynthesis	-	-
0.998565026	0.034109007	-	-	GO:0005634//nucleus;G
0.998564523	0.034114983	ko01120//Microbial m	-	-
0.998563774	0.034123884	ko01100//Metabolic p	-	GO:0005886//plasma m
0.998563537	0.034126704	-	-	-
0.998561703	0.034148491	-	-	GO:0005615//extracellu
0.998560291	0.03416525	ko01100//Metabolic p	-	-
0.998560011	0.034168575	-	-	-
0.998558935	0.034181337	ko01100//Metabolic p	-	GO:0016021//integral co
0.998557339	0.03420027	-	-	-
0.998556986	0.034204456	-	-	GO:0016020//membran
0.998556788	0.034206794	ko01100//Metabolic p	-	GO:0030659//cytoplasm
0.998555816	0.034218319	ko01100//Metabolic p	ko04080//Neuro	GO:0005886//plasma m
0.998555542	0.034221571	-	ko01100//Metab	-
0.998554683	0.034231739	ko01100//Metabolic p	-	GO:0005737//cytoplasm
0.998554369	0.034235466	-	-	GO:0002177//manchett
0.998554208	0.034237368	-	ko05168//Herpe	-
0.998554192	0.034237561	ko01100//Metabolic p	ko04218//Cellula	GO:0030896//checkpoir

0.998553187	0.034249452	-	-	GO:0005884//actin filam
0.998552369	0.034259146	-	ko05200//Pathw	-
0.998551954	0.034264054	ko01100//Metabolic p	ko01100//Metak	GO:0005777//peroxison
0.998550967	0.034275724	-	-	GO:0005886//plasma m
0.998550851	0.034277103	ko01100//Metabolic p	-	-
0.998550504	0.034281204	-	ko00983//Drug	-
0.99854855	0.03430431	ko01120//Microbial m	ko01100//Metak	GO:0005737//cytoplasm
0.998548263	0.034307705	ko01100//Metabolic p	-	-
0.998548159	0.034308937	ko01100//Metabolic p	ko05132//Salmc	-
0.998548071	0.034309967	-	-	-
0.998547969	0.034311174	-	-	GO:0016020//membran
0.99854712	0.034321213	ko01110//Biosynthesis	ko04350//TGF-k	-
0.998544496	0.034352196	ko01100//Metabolic p	ko05130//Patho	GO:0005856//cytoskelet
0.998544126	0.034356567	-	ko04659//Th17	GO:0000785//chromatir
0.99854391	0.034359109	-	ko01100//Metak	GO:0005737//cytoplasm
0.998540688	0.034397118	ko01100//Metabolic p	ko04610//Comp	GO:0005615//extracellu
0.998538828	0.034419038	-	-	GO:0001725//stress fibe
0.998538419	0.034423854	-	ko04024//cAMP	GO:0016021//integral co
0.998536154	0.03445052	-	-	-
0.998534495	0.034470041	ko01100//Metabolic p	-	GO:0005634//nucleus;G
0.998533805	0.034478163	ko01100//Metabolic p	ko01100//Metak	GO:0005886//plasma m
0.998533137	0.034486019	ko01100//Metabolic p	-	GO:0005615//extracellu
0.998531485	0.034505427	ko01100//Metabolic p	-	GO:0005737//cytoplasm
0.998531233	0.034508392	ko01063//Biosynthesis	-	GO:0005783//endoplasr
0.998530896	0.034512351	-	-	-
0.998530686	0.034514818	ko01100//Metabolic p	-	-
0.998530578	0.034516094	-	ko01100//Metak	GO:0005634//nucleus;G
0.998528121	0.034544944	-	-	GO:0034451//centriolar
0.998527765	0.03454912	-	-	GO:0005634//nucleus
0.998526977	0.034558362	ko01100//Metabolic p	ko04390//Hippc	GO:0005667//transcript
0.998526757	0.034560944	-	-	-
0.998526563	0.034563222	ko01110//Biosynthesis	-	GO:0005634//nucleus;G
0.998524661	0.034585528	ko01110//Biosynthesis	ko01100//Metak	GO:0005737//cytoplasm
0.998524294	0.034589831	ko01100//Metabolic p	-	GO:0005739//mitochon
0.998523183	0.034602858	ko01100//Metabolic p	-	GO:0005654//nucleopla
0.998522338	0.03461276	-	-	-
0.998521878	0.034618138	-	ko05143//Africa	GO:0005576//extracellu
0.998521053	0.03462781	-	ko04514//Cell a	GO:0016020//membran
0.998519968	0.034640509	-	ko04142//Lysos	GO:0005615//extracellu
0.998518776	0.034654461	-	ko02010//ABC t	GO:0005739//mitochon
0.998518764	0.034654603	-	ko01100//Metak	-
0.998517658	0.034667534	ko01100//Metabolic p	ko04979//Chole	GO:0005783//endoplasr
0.998515744	0.03468992	-	-	GO:0005886//plasma m
0.998514639	0.034702832	ko01100//Metabolic p	-	GO:0005737//cytoplasm
0.998514498	0.034704477	ko01100//Metabolic p	-	GO:0005737//cytoplasm
0.998514069	0.034709493	-	ko04714//Therm	-
0.998513384	0.034717496	ko01100//Metabolic p	ko01100//Metak	GO:0005783//endoplasr
0.998513121	0.034720568	-	ko05200//Pathw	GO:0005576//extracellu
0.998512886	0.034723305	-	ko04726//Serotc	GO:0005739//mitochon
0.998511421	0.034740415	-	ko04144//Endoc	-
0.998511301	0.034741816	-	-	GO:0005783//endoplasr
0.998510981	0.034745541	-	-	GO:0005634//nucleus
0.998510826	0.034747358	-	-	-
0.998509735	0.034760083	ko01100//Metabolic p	-	-
0.998509534	0.034762434	-	ko01100//Metak	GO:0005737//cytoplasm
0.998506663	0.034795898	-	ko04360//Axon	GO:0005886//plasma m
0.998506366	0.034799358	-	ko01100//Metak	-
0.998505793	0.034806044	ko01100//Metabolic p	-	-

0.998505165	0.034813354	-	-	-
0.998502968	0.034838939	-	-	-
0.998502955	0.034839091	ko01100//Metabolic p	-	GO:0005634//nucleus;G
0.998501799	0.034852538	-	-	GO:0005886//plasma m
0.998499274	0.034881898	-	-	GO:0000786//nucleosor
0.998498553	0.034890287	-	-	-
0.998497148	0.034906602	-	ko01100//Metak	GO:0005615//extracellu
0.998496456	0.034914648	-	ko04514//Cell a	GO:0016020//membran
0.99849535	0.034927484	ko01100//Metabolic p	-	GO:0046658//anchored
0.998495306	0.034928002	ko01100//Metabolic p	-	-
0.998495208	0.034929135	-	ko04010//MAPK	-
0.998492859	0.034956393	-	ko04146//Perox	GO:0005777//peroxison
0.998491864	0.034967938	-	ko01100//Metak	-
0.998491219	0.034975409	-	ko04724//Glutar	GO:0005886//plasma m
0.998490955	0.034978473	-	-	-
0.998490856	0.034979617	ko01100//Metabolic p	-	-
0.998489754	0.034992398	ko01100//Metabolic p	ko02010//ABC tı	GO:0000139//Golgi mer
0.99848913	0.034999625	-	-	-
0.998488947	0.035001742	-	-	GO:0005634//nucleus;G
0.998487803	0.035014992	-	-	GO:0030425//dendrite
0.998487447	0.035019121	-	ko01100//Metak	GO:0000139//Golgi mer
0.998486624	0.035028649	ko01100//Metabolic p	ko04610//Comp	GO:0005615//extracellu
0.998486244	0.035033037	ko01100//Metabolic p	ko01100//Metak	-
0.998485822	0.035037923	ko01063//Biosynthesis	-	GO:0016021//integral c
0.998485464	0.035042066	-	ko01100//Metak	GO:0005615//extracellu
0.998483567	0.035064016	-	-	-
0.998483368	0.035066317	ko01100//Metabolic p	ko01100//Metak	-
0.998483334	0.035066703	ko01100//Metabolic p	ko05200//Pathw	GO:0005794//Golgi app
0.998482281	0.035078885	-	ko01100//Metak	-
0.998482125	0.035080691	-	ko04151//PI3K-,	GO:0005654//nucleopla
0.998481151	0.035091938	-	-	-
0.998478971	0.035117127	ko01100//Metabolic p	ko04020//Calciu	GO:0005886//plasma m
0.998477645	0.035132432	-	-	GO:0005634//nucleus;G
0.998476197	0.035149135	ko01100//Metabolic p	ko04610//Comp	GO:0005615//extracellu
0.99847449	0.035168832	ko01100//Metabolic p	ko01100//Metak	GO:0005654//nucleopla
0.99847323	0.035183349	-	ko01100//Metak	GO:0005737//cytoplasm
0.998472906	0.035187079	ko01100//Metabolic p	ko01100//Metak	GO:0048269//methionir
0.998472278	0.035194325	ko01100//Metabolic p	-	-
0.998472237	0.035194797	-	ko01100//Metak	-
0.998470386	0.035216113	ko01100//Metabolic p	-	GO:0005615//extracellu
0.99847038	0.035216182	-	-	-
0.998467814	0.035245721	-	-	GO:0005737//cytoplasm
0.998463599	0.035294174	-	-	-
0.998463193	0.035298835	ko01100//Metabolic p	ko04923//Regul	GO:0005654//nucleopla
0.998463123	0.035299644	-	ko04742//Taste	GO:0001518//voltage-g
0.998461913	0.035313536	-	-	-
0.998461469	0.035318641	-	ko04514//Cell a	GO:0005886//plasma m
0.998460997	0.03532406	-	-	-
0.998460835	0.035325914	-	ko04060//Cytok	GO:0005576//extracellu
0.998459931	0.035336293	-	-	-
0.998459775	0.03533808	-	ko04060//Cytok	GO:0005576//extracellu
0.998459417	0.035342188	-	-	-
0.998459416	0.035342197	ko01100//Metabolic p	-	-
0.998458304	0.035354958	-	-	GO:0016021//integral c
0.99845763	0.035362682	ko01100//Metabolic p	-	GO:0005634//nucleus;G
0.998457435	0.035364916	ko01100//Metabolic p	-	GO:0016021//integral c
0.998456653	0.035373883	-	-	-
0.998456653	0.035373891	ko01100//Metabolic p	-	-

0.998456243	0.035378584	-	ko05200//Pathw	GO:0005576//extracellu
0.998453421	0.035410921	-	-	GO:0005884//actin filam
0.998453214	0.03541329	-	-	-
0.998452508	0.035421368	-	-	GO:0005783//endoplasr
0.998450315	0.035446462	ko01100//Metabolic p	ko01100//Metab	-
0.998449707	0.035453425	-	-	GO:0001726//ruffle;GO:
0.998449459	0.035456255	ko01100//Metabolic p	-	-
0.998446867	0.03548589	-	-	GO:0005634//nucleus;G
0.998445453	0.035502046	ko01100//Metabolic p	-	-
0.998445354	0.035503175	ko01100//Metabolic p	-	-
0.998444643	0.035511295	-	-	GO:0005886//plasma m
0.998444123	0.035517223	ko01100//Metabolic p	-	-
0.998444107	0.03551741	ko01100//Metabolic p	-	GO:0005634//nucleus;G
0.998443604	0.035523153	-	-	-
0.998443326	0.035526325	-	ko04218//Cellula	GO:0030896//checkpoir
0.998442765	0.03553273	ko01100//Metabolic p	ko01100//Metab	GO:0005737//cytoplasm
0.998441381	0.035548519	ko01100//Metabolic p	-	GO:0016020//membran
0.998441335	0.035549041	-	-	GO:0030425//dendrite
0.998441123	0.035551468	-	-	GO:0005737//cytoplasm
0.998431627	0.035659611	-	-	-
0.998429045	0.035688961	ko01100//Metabolic p	-	-
0.998428294	0.035697489	-	ko01100//Metab	GO:0005739//mitochon
0.998426487	0.035718014	-	-	GO:0005874//microtubu
0.99842611	0.03572229	-	-	-
0.998424741	0.035737826	ko01100//Metabolic p	ko01100//Metab	-
0.998424522	0.035740309	-	-	-
0.998420761	0.035782957	ko01100//Metabolic p	-	-
0.99841913	0.035801432	ko01100//Metabolic p	ko04514//Cell ad	GO:0016020//membran
0.99841895	0.035803474	-	ko04080//Neuro	GO:0016020//membran
0.99841788	0.035815585	-	-	GO:0005634//nucleus;G
0.99841681	0.035827705	-	-	-
0.998416062	0.03583617	-	-	GO:0000177//cytoplasm
0.998415824	0.035838854	-	-	-
0.998415548	0.035841988	ko01100//Metabolic p	-	GO:0005829//cytosol;GO
0.998415022	0.035847934	ko01110//Biosynthesis	-	GO:0016020//membran
0.998414125	0.035858081	-	-	GO:0000776//kinetocho
0.998414014	0.035859328	ko01100//Metabolic p	ko04151//PI3K-A	GO:0005886//plasma m
0.998413642	0.035863541	ko01100//Metabolic p	-	GO:0005886//plasma m
0.99841277	0.035873396	-	ko01100//Metab	GO:0005886//plasma m
0.998411515	0.035887576	ko01100//Metabolic p	ko04010//MAPK	-
0.998408877	0.035917372	-	-	GO:0005737//cytoplasm
0.998408666	0.035919753	-	ko04114//Oocyt	-
0.99840851	0.035921519	-	-	-
0.998408175	0.035925301	-	ko04919//Thyro	GO:0016020//membran
0.998407504	0.035932878	-	-	-
0.99840679	0.03594093	ko01100//Metabolic p	ko01100//Metab	GO:0005737//cytoplasm
0.998406616	0.035942899	ko01100//Metabolic p	ko05017//Spino	GO:0043231//intracellul
0.998405531	0.035955128	-	-	-
0.998405464	0.035955885	ko01100//Metabolic p	-	-
0.998405278	0.035957989	-	ko04973//Carbo	GO:0005886//plasma m
0.998404785	0.035963547	ko01100//Metabolic p	ko04970//Saliva	GO:0005576//extracellu
0.998399819	0.036019491	ko01100//Metabolic p	-	GO:0016021//integral co
0.998399164	0.036026867	ko01100//Metabolic p	ko04151//PI3K-A	GO:0005654//nucleopla
0.998397866	0.036041476	-	-	-
0.998393192	0.036094022	-	ko04726//Seroto	GO:0005739//mitochon
0.998393122	0.036094807	-	-	-
0.998393095	0.036095108	-	-	-
0.998392992	0.03609627	-	ko04610//Comp	GO:0005615//extracellu

0.998392852	0.036097837	-	-	-
0.998392342	0.03610357	-	-	-
0.998392255	0.036104547	ko01100//Metabolic p	-	GO:0009986//cell surfac
0.998389922	0.036130744	ko01100//Metabolic p	ko01100//Metab	GO:0005739//mitochon
0.998389319	0.036137505	-	ko01100//Metab	GO:0000139//Golgi mer
0.998388137	0.036150766	ko01100//Metabolic p	ko03320//PPAR	GO:0005576//extracellu
0.998387126	0.036162108	ko01100//Metabolic p	ko01100//Metab	-
0.998387048	0.036162978	-	-	GO:0005576//extracellu
0.998385418	0.036181252	-	ko04726//Seroto	GO:0005739//mitochon
0.998384401	0.036192646	ko01100//Metabolic p	-	-
0.998383288	0.036205121	-	-	-
0.998382444	0.036214569	ko01100//Metabolic p	ko05165//Huma	GO:0005634//nucleus;G
0.998381808	0.036221695	ko01100//Metabolic p	-	-
0.998381662	0.036223324	-	ko01100//Metab	GO:0005789//endoplası
0.998381344	0.03622688	-	-	GO:0005739//mitochon
0.998379915	0.036242875	ko01100//Metabolic p	-	-
0.998378415	0.036259651	-	-	-
0.998377887	0.03626556	ko01100//Metabolic p	-	-
0.998377886	0.036265576	-	-	GO:0005829//cytosol;G(
0.998377192	0.036273328	ko01100//Metabolic p	-	GO:0005576//extracellu
0.998377037	0.036275061	-	-	GO:0005783//endoplasr
0.99837585	0.03628833	-	ko01100//Metab	-
0.998375755	0.036289395	ko01100//Metabolic p	-	-
0.998375488	0.036292378	ko01100//Metabolic p	-	-
0.998375056	0.036297207	-	-	-
0.998374862	0.036299365	-	-	GO:0005615//extracellu
0.99837361	0.036313356	-	-	GO:0005643//nuclear p
0.998372975	0.036320438	-	-	-
0.998372911	0.036321152	-	-	-
0.998372768	0.036322756	-	ko04610//Comp	GO:0005615//extracellu
0.99837131	0.036339026	-	-	-
0.998369449	0.036359786	ko01100//Metabolic p	ko03013//Nucle	-
0.998367422	0.036382384	ko01100//Metabolic p	-	GO:0016020//membran
0.998366204	0.036395966	-	ko04745//Photo	-
0.998363904	0.036421578	-	ko05016//Huntir	-
0.998362861	0.036433192	-	-	GO:0005615//extracellu
0.998362411	0.036438195	ko01100//Metabolic p	-	-
0.998360479	0.036459689	-	-	GO:0005634//nucleus;G
0.998359048	0.036475603	-	ko04919//Thyro	GO:0005887//integral c
0.998358771	0.036478677	-	-	-
0.998357529	0.036492488	ko01100//Metabolic p	ko01100//Metab	GO:0005777//peroxison
0.998354976	0.036520848	-	ko04923//Regul	GO:0005654//nucleopla
0.998354964	0.03652098	-	-	-
0.998354845	0.0365223	-	-	-
0.998352205	0.036551594	ko01100//Metabolic p	ko04976//Bile se	GO:0005654//nucleopla
0.998351676	0.036557462	-	ko04060//Cytok	-
0.998349241	0.03658447	ko01100//Metabolic p	-	GO:0005922//connexon
0.998348114	0.036596959	-	ko01100//Metab	-
0.998347031	0.03660895	-	-	GO:0005737//cytoplasm
0.998346194	0.036618224	-	-	-
0.99834528	0.036628348	ko01100//Metabolic p	-	-
0.998345237	0.036628822	-	ko04360//Axon	GO:0016020//membran
0.99834331	0.036650148	ko01063//Biosynthesis	ko04927//Cortis	GO:0005634//nucleus;G
0.998342293	0.036661395	ko01100//Metabolic p	-	GO:0005634//nucleus;G
0.998340039	0.036686322	ko01100//Metabolic p	-	-
0.998339736	0.036689666	ko01120//Microbial m	ko01100//Metab	-
0.998338113	0.0367076	-	-	GO:0016021//integral c
0.998335947	0.036731526	ko01100//Metabolic p	-	-

0.99833533	0.036738337	-	-	GO:0005887//integral co
0.998334244	0.036750321	-	ko01100//Metab	GO:0005829//cytosol;GO
0.998334081	0.036752121	-	-	-
0.998333963	0.036753415	-	-	-
0.998333434	0.036759251	-	-	GO:0005739//mitochon
0.998332018	0.036774872	-	ko01100//Metab	-
0.998331845	0.036776783	ko01100//Metabolic p	ko01100//Metab	-
0.998330987	0.036786245	-	ko03460//Fanco	GO:0000785//chromatin
0.998330652	0.036789935	-	-	GO:0030425//dendrite
0.998330447	0.036792195	-	ko04610//Comp	GO:0005576//extracellu
0.998330321	0.036793585	-	ko05168//Herpe	-
0.998330103	0.036795982	-	-	GO:0005829//cytosol;GO
0.998329001	0.036808127	ko01100//Metabolic p	ko01100//Metab	GO:0005615//extracellu
0.998328974	0.036808419	ko01100//Metabolic p	ko01100//Metab	GO:0005737//cytoplasm
0.998328179	0.036817181	ko01100//Metabolic p	ko01100//Metab	GO:0005789//endoplasm
0.998327058	0.03682953	ko01100//Metabolic p	-	-
0.998326509	0.036835569	-	-	GO:0016021//integral co
0.998326048	0.036840641	-	-	-
0.998324636	0.036856181	ko01110//Biosynthesis	-	-
0.998323559	0.036868033	-	-	-
0.998322263	0.03688228	-	-	-
0.998322175	0.036883251	-	ko01100//Metab	GO:0016020//membran
0.998321571	0.036889894	-	ko01100//Metab	-
0.998320705	0.03689941	-	-	-
0.998320248	0.036904428	ko01120//Microbial m	ko01100//Metab	GO:0000139//Golgi mem
0.998319412	0.036913611	ko01100//Metabolic p	-	-
0.998318002	0.036929106	-	ko04714//Therm	-
0.998317449	0.036935176	ko01100//Metabolic p	ko01100//Metab	GO:0005739//mitochon
0.998317104	0.036938959	ko01120//Microbial m	-	GO:0035658//Mon1-Cc
0.998317007	0.036940027	ko01100//Metabolic p	-	GO:0005874//microtubu
0.99831662	0.036944273	ko01100//Metabolic p	-	GO:0030425//dendrite
0.99831545	0.036957116	-	ko04390//Hippo	GO:0005667//transcript
0.998314187	0.036970968	ko01100//Metabolic p	ko04974//Protei	-
0.998311291	0.037002722	-	-	-
0.998311161	0.037004144	-	-	-
0.998310511	0.037011273	ko01100//Metabolic p	ko01100//Metab	-
0.998306877	0.037051058	ko01120//Microbial m	-	GO:0016020//membran
0.998304161	0.037080773	ko01100//Metabolic p	-	-
0.998301819	0.03710638	-	-	GO:0005634//nucleus;G
0.998301119	0.037114029	-	ko04514//Cell ad	GO:0005829//cytosol;GO
0.998299828	0.037128129	ko01100//Metabolic p	-	-
0.998298847	0.037138844	-	-	GO:0001726//ruffle;GO:
0.998298299	0.037144829	ko01100//Metabolic p	ko04724//Glutar	GO:0005886//plasma m
0.998297055	0.037158409	-	ko01100//Metab	GO:0005778//peroxison
0.998295593	0.037174356	-	ko05143//Africa	GO:0005576//extracellu
0.998294435	0.037186988	-	-	GO:0005634//nucleus
0.998292935	0.037203337	ko01100//Metabolic p	-	GO:0016020//membran
0.998290811	0.037226489	-	-	GO:0045095//keratin fila
0.998290593	0.037228857	ko01100//Metabolic p	-	-
0.998288267	0.03725419	-	ko04060//Cytok	GO:0005576//extracellu
0.998287636	0.03726105	ko01110//Biosynthesis	-	-
0.99828619	0.037276786	-	ko05200//Pathw	GO:0005576//extracellu
0.99828435	0.037296801	-	-	-
0.998284231	0.037298095	-	-	-
0.99828392	0.037301475	ko01100//Metabolic p	-	-
0.998283894	0.037301759	-	-	GO:0005634//nucleus
0.998283017	0.037311287	ko01110//Biosynthesis	-	-
0.99828296	0.037311908	-	ko01100//Metab	-

0.998282312	0.03731895	ko01110//Biosynthesis	ko04970//Saliva	GO:0005576//extracellu
0.998280981	0.037333407	-	-	-
0.998280962	0.037333615	ko01100//Metabolic p	ko04723//Retrog	GO:0005739//mitochon
0.998278625	0.037359	-	ko05414//Dilate	-
0.998275063	0.037397636	-	ko00983//Drug	-
0.998273484	0.037414761	ko01100//Metabolic p	-	GO:0005737//cytoplasm
0.99827257	0.037424663	ko01110//Biosynthesis	ko04218//Cellula	GO:0030896//checkpoir
0.998270512	0.03744695	-	-	GO:0005576//extracellu
0.998270351	0.037448703	ko01100//Metabolic p	-	-
0.998268662	0.037466982	-	ko04060//Cytok	GO:0009897//external s
0.998268292	0.037470987	ko01100//Metabolic p	ko04060//Cytok	GO:0009897//external s
0.998266391	0.037491555	ko01100//Metabolic p	-	GO:0016020//membran
0.998265145	0.037505031	ko01100//Metabolic p	-	GO:0031012//extracellu
0.99826444	0.037512647	ko01100//Metabolic p	-	-
0.998264279	0.037514391	-	-	-
0.998264055	0.037516811	-	ko01100//Metak	GO:0005789//endoplasr
0.998263207	0.037525977	-	ko01100//Metak	GO:0005739//mitochon
0.99826066	0.037553492	-	-	-
0.998257681	0.03758565	-	ko01100//Metak	GO:0016020//membran
0.998257229	0.037590527	-	-	GO:0005634//nucleus;G
0.998255581	0.037608298	-	-	-
0.998255215	0.037612248	-	-	-
0.998254781	0.037616927	-	-	-
0.998254758	0.037617174	ko01100//Metabolic p	-	-
0.998254568	0.037619216	-	ko04976//Bile se	GO:0005654//nucleopla
0.998254371	0.037621348	-	-	-
0.998253776	0.037627751	ko01120//Microbial m	ko04151//PI3K-/	GO:0005739//mitochon
0.998252306	0.037643592	ko01100//Metabolic p	ko05016//Huntir	-
0.998252274	0.037643942	-	-	-
0.998251258	0.037654884	ko01100//Metabolic p	-	-
0.998250718	0.037660695	ko01100//Metabolic p	-	GO:0005737//cytoplasm
0.998249613	0.037672594	-	-	-
0.998249211	0.03767692	ko01063//Biosynthesis	-	GO:0005615//extracellu
0.998246832	0.037702517	ko01100//Metabolic p	-	-
0.998245393	0.037717992	-	ko04014//Ras sig	-
0.998245196	0.037720107	ko01100//Metabolic p	-	-
0.998244556	0.037726986	ko01100//Metabolic p	ko01100//Metak	GO:0033180//proton-tr
0.998243997	0.037733001	ko01110//Biosynthesis	-	-
0.998241831	0.037756268	ko01110//Biosynthesis	-	GO:0005634//nucleus;G
0.998237938	0.037798062	ko01100//Metabolic p	-	GO:0005886//plasma m
0.998237734	0.037800246	ko01100//Metabolic p	ko05132//Salmo	GO:0005737//cytoplasm
0.998237498	0.037802782	-	ko01100//Metak	GO:0005737//cytoplasm
0.998236608	0.037812322	ko01063//Biosynthesis	ko05130//Patho	-
0.998235511	0.037824087	ko01100//Metabolic p	ko05231//Cholir	GO:0005739//mitochon
0.998235271	0.03782666	ko01100//Metabolic p	ko05322//Syster	GO:0005576//extracellu
0.998235224	0.037827162	ko01100//Metabolic p	ko04310//Wnt s	GO:0005576//extracellu
0.998233645	0.037844093	ko01100//Metabolic p	ko01100//Metak	-
0.998231942	0.037862329	-	ko04714//Therm	-
0.99823133	0.037868886	ko01110//Biosynthesis	-	-
0.998231323	0.037868965	-	ko04726//Serotc	GO:0005739//mitochon
0.998227746	0.037907246	-	ko01100//Metak	-
0.99822565	0.037929662	ko01100//Metabolic p	-	-
0.998222827	0.037959837	ko01110//Biosynthesis	ko01100//Metak	GO:0005739//mitochon
0.99822245	0.03796386	ko01100//Metabolic p	ko05142//Chaga	GO:0016020//membran
0.998222428	0.0379641	-	-	GO:0016020//membran
0.998220933	0.037980063	-	-	-
0.998220135	0.037988581	ko01100//Metabolic p	ko04530//Tight	GO:0016020//membran
0.998218087	0.038010443	-	ko01100//Metak	-

0.998216922	0.038022865	-	ko04742//Taste	GO:0005783//endoplasr
0.998215266	0.038040518	-	-	GO:0005829//cytosol;G(
0.998214117	0.038052771	ko01100//Metabolic p	-	-
0.998213885	0.038055242	ko01100//Metabolic p	-	-
0.99821372	0.038056995	-	ko04010//MAPK	-
0.998213665	0.038057582	ko01100//Metabolic p	-	GO:0016021//integral c
0.998212492	0.038070081	ko01100//Metabolic p	-	GO:0016020//membran
0.998212361	0.038071483	-	ko04514//Cell a	GO:0016020//membran
0.998210837	0.038087711	-	ko04514//Cell a	GO:0005829//cytosol;G(
0.998209796	0.038098793	-	-	-
0.998209015	0.038107108	-	-	GO:0016020//membran
0.998206972	0.038128834	ko01063//Biosynthesis	-	GO:0005739//mitochon
0.998206878	0.038129842	-	ko01100//Metab	GO:0005615//extracellu
0.998206525	0.038133593	-	-	GO:0005886//plasma m
0.998204305	0.038157189	-	ko01100//Metab	GO:0005739//mitochon
0.998203557	0.038165145	ko01100//Metabolic p	ko04060//Cytok	GO:0016020//membran
0.998202657	0.038174704	-	ko05322//Syster	GO:0005576//extracellu
0.9982022	0.038179561	-	ko00564//Glycer	GO:0005615//extracellu
0.998201483	0.038187168	-	ko01100//Metab	-
0.998201328	0.038188818	ko01100//Metabolic p	ko05168//Herpe	-
0.998201199	0.038190187	ko01100//Metabolic p	ko01100//Metab	-
0.998199953	0.038203415	-	ko00564//Glycer	GO:0005615//extracellu
0.998198783	0.038215834	-	-	GO:0016020//membran
0.998197942	0.038224763	-	-	-
0.998196379	0.038241335	ko01100//Metabolic p	-	-
0.998196255	0.038242647	ko01100//Metabolic p	-	GO:0016020//membran
0.99819533	0.038252461	ko01100//Metabolic p	ko04726//Seroto	GO:0005739//mitochon
0.99819431	0.038263275	-	-	GO:0005737//cytoplasm
0.99819283	0.038278951	ko01100//Metabolic p	-	-
0.998192233	0.038285273	-	ko04144//Endoc	-
0.998188559	0.038324176	ko01110//Biosynthesis	ko05132//Salmo	-
0.998187864	0.038331527	-	ko01100//Metab	-
0.998187713	0.038333124	-	ko05200//Pathw	-
0.998183709	0.038375461	ko01100//Metabolic p	-	-
0.998183048	0.038382444	-	-	GO:0000779//condense
0.99818103	0.038403764	-	ko04360//Axon	GO:0016020//membran
0.998178581	0.038429614	-	ko01100//Metab	GO:0005886//plasma m
0.998177054	0.038445719	-	ko01100//Metab	GO:0048269//methionir
0.99817679	0.038448502	ko01100//Metabolic p	-	GO:0045095//keratin fil
0.998175975	0.038457099	-	-	-
0.998175331	0.038463888	ko01100//Metabolic p	ko04150//mTOF	GO:0032991//macromo
0.998175308	0.038464139	ko01100//Metabolic p	-	-
0.998174207	0.03847574	-	ko01100//Metab	-
0.998171965	0.038499361	ko01100//Metabolic p	-	GO:0000781//chromoso
0.998171064	0.038508859	ko01120//Microbial m	-	GO:0005764//lysosome;
0.998171059	0.03850891	-	ko01100//Metab	-
0.99816897	0.038530899	ko01100//Metabolic p	-	GO:0005634//nucleus;G
0.998168522	0.038535611	-	-	-
0.998167844	0.038542744	-	-	GO:0000139//Golgi mer
0.99816662	0.038555628	-	ko04723//Retro	GO:0005739//mitochon
0.998165793	0.038564326	-	-	GO:0005634//nucleus;G
0.998163643	0.038586925	ko01100//Metabolic p	-	-
0.998162971	0.038593982	ko01100//Metabolic p	-	GO:0005615//extracellu
0.998162704	0.038596791	-	-	GO:0001726//ruffle;GO:
0.998160897	0.03861577	ko01100//Metabolic p	-	-
0.998160832	0.038616453	ko01100//Metabolic p	-	-
0.998158517	0.038640762	ko01100//Metabolic p	-	-
0.998158111	0.038645023	ko01100//Metabolic p	ko04919//Thyro	GO:0005887//integral c

0.998157931	0.038646909	ko01100//Metabolic p	-	-
0.998157438	0.038652079	ko01100//Metabolic p	ko01100//Metab	GO:0043231//intracellul
0.998157229	0.038654272	ko01100//Metabolic p	ko04360//Axon	GO:0016020//membran
0.998156393	0.038663048	ko01100//Metabolic p	ko04710//Circad	GO:0005634//nucleus;G
0.998156295	0.038664071	ko01063//Biosynthesis	ko01100//Metab	-
0.998155537	0.038672019	ko01100//Metabolic p	ko05200//Pathw	GO:0005576//extracellu
0.998155417	0.038673283	ko01100//Metabolic p	ko04080//Neuro	GO:0009986//cell surfac
0.998154709	0.038680707	-	-	-
0.998152939	0.038699254	ko01100//Metabolic p	-	-
0.99815171	0.038712136	-	ko04060//Cytok	GO:0005576//extracellu
0.998150037	0.038729658	-	-	-
0.998149985	0.038730197	-	ko01100//Metab	-
0.998149944	0.038730631	ko01100//Metabolic p	-	-
0.998148992	0.038740595	ko01100//Metabolic p	ko01100//Metab	GO:0005739//mitochon
0.998148215	0.038748728	ko01100//Metabolic p	-	-
0.998146182	0.038770001	-	-	GO:0005802//trans-Gol
0.998145313	0.038779086	-	-	-
0.998144211	0.038790606	-	ko01100//Metab	GO:0005777//peroxison
0.998142579	0.038807663	ko01100//Metabolic p	ko01100//Metab	GO:0016020//membran
0.998142346	0.038810107	ko01100//Metabolic p	-	GO:0005737//cytoplasm
0.998141219	0.038821881	-	ko01100//Metab	-
0.99814108	0.038823331	ko01100//Metabolic p	-	-
0.998138953	0.038845539	-	-	GO:0005768//endosom
0.998138685	0.038848343	ko01100//Metabolic p	ko01100//Metab	GO:0005829//cytosol;G
0.998137308	0.038862714	-	-	-
0.998134281	0.038894282	-	ko05322//Syster	GO:0005576//extracellu
0.998134233	0.038894784	-	ko04310//Wnt s	GO:0005576//extracellu
0.998133175	0.038905812	-	-	GO:0016020//membran
0.998131379	0.03892453	-	-	-
0.998129883	0.038940116	-	ko01100//Metab	GO:0005739//mitochon
0.998126053	0.03897998	ko01100//Metabolic p	ko04973//Carbo	GO:0005886//plasma m
0.998125538	0.03898534	-	ko00983//Drug	-
0.998123657	0.039004897	-	ko01100//Metab	GO:0005829//cytosol
0.998122443	0.039017518	ko01100//Metabolic p	-	GO:0005829//cytosol;G
0.998121822	0.039023976	-	ko05414//Dilate	-
0.998121262	0.039029792	ko01100//Metabolic p	-	-
0.998120555	0.039037143	ko01100//Metabolic p	ko01100//Metab	GO:0001650//fibrillar ce
0.998118844	0.039054908	ko01100//Metabolic p	-	-
0.998116622	0.039077974	ko01100//Metabolic p	-	-
0.998114183	0.039103283	-	-	-
0.998109028	0.0391567	-	-	GO:0016020//membran
0.998108964	0.039157364	ko01100//Metabolic p	ko04514//Cell a	GO:0005829//cytosol;G
0.998108767	0.039159404	-	ko01100//Metab	GO:0048269//methionir
0.99810768	0.039170665	ko01100//Metabolic p	ko04918//Thyro	GO:0005576//extracellu
0.998107476	0.039172771	ko01100//Metabolic p	ko01100//Metab	-
0.998107305	0.039174548	-	-	-
0.998106918	0.039178547	-	-	GO:0009986//cell surfac
0.998102497	0.039224289	ko01100//Metabolic p	-	-
0.998102385	0.039225441	-	-	-
0.998101854	0.039230929	-	ko01100//Metab	-
0.998099841	0.039251733	ko01100//Metabolic p	ko04390//Hippo	GO:0005667//transcript
0.998098912	0.039261332	ko01100//Metabolic p	ko04514//Cell a	GO:0005829//cytosol;G
0.998098719	0.03926333	ko01100//Metabolic p	ko05202//Trans	GO:0000786//nucleosor
0.998097896	0.039271826	-	ko01100//Metab	GO:0000506//glycosylpl
0.998097804	0.039272773	ko01100//Metabolic p	-	-
0.998097565	0.039275241	ko01100//Metabolic p	ko03018//RNA c	GO:0005829//cytosol
0.99809737	0.039277255	-	-	-
0.998094271	0.039309242	-	-	GO:0001725//stress fibe

0.99809369	0.03931524	ko01100//Metabolic p	ko01100//Metab	GO:0005739//mitochon
0.998090215	0.039351064	ko01100//Metabolic p	-	GO:0016021//integral c
0.99808857	0.039368011	ko01100//Metabolic p	-	-
0.998087778	0.039376172	-	-	-
0.998086576	0.039388548	-	ko01100//Metab	-
0.998085256	0.039402144	-	ko05010//Alzhei	GO:0005576//extracellu
0.998084821	0.039406616	-	-	-
0.998084279	0.039412195	-	-	-
0.99808425	0.039412491	ko01100//Metabolic p	-	-
0.998083993	0.039415132	-	ko00564//Glycer	GO:0005615//extracellu
0.998083154	0.039423766	-	ko04151//PI3K-/	GO:0005886//plasma m
0.998083123	0.03942409	ko01100//Metabolic p	-	GO:0031462//Cul2-RIN
0.998081627	0.039439468	-	ko01100//Metab	GO:0016020//membran
0.998081018	0.039445736	-	-	-
0.99808043	0.039451779	-	ko04360//Axon	-
0.998079447	0.039461878	ko01100//Metabolic p	-	-
0.998078545	0.039471154	-	ko04714//Therm	-
0.998076543	0.039491715	-	-	GO:0016020//membran
0.998075457	0.039502867	ko01063//Biosynthesis	ko04514//Cell ad	-
0.998075288	0.039504597	ko01100//Metabolic p	-	GO:0016020//membran
0.998075244	0.039505053	ko01100//Metabolic p	ko01100//Metab	GO:0005737//cytoplasm
0.998074591	0.039511754	-	-	GO:0014069//postsynap
0.998073705	0.039520844	-	ko04144//Endoc	-
0.998072062	0.039537702	-	ko04218//Cellula	GO:0030896//checkpoir
0.998066814	0.0395915	-	-	-
0.998064771	0.039612417	ko01110//Biosynthesis	ko01100//Metab	GO:0005886//plasma m
0.998062979	0.039630764	-	ko01100//Metab	GO:0005739//mitochon
0.998062876	0.039631814	-	ko01100//Metab	GO:0016021//integral c
0.998062283	0.03963788	-	-	GO:0016020//membran
0.998062225	0.039638478	-	ko05012//Parkin	GO:0005654//nucleopla
0.998061976	0.039641023	-	-	GO:0016020//membran
0.998059555	0.039665786	ko01100//Metabolic p	-	-
0.998056168	0.039700395	ko01100//Metabolic p	ko05202//Transc	GO:0000786//nucleosor
0.998054278	0.039719701	-	ko01100//Metab	GO:0043231//intracellul
0.998053179	0.039730923	ko01100//Metabolic p	ko01100//Metab	-
0.998052383	0.039739042	-	-	GO:0016020//membran
0.998051975	0.039743205	-	-	GO:0005634//nucleus
0.998048103	0.039782699	-	-	-
0.998047282	0.039791071	-	ko05200//Pathw	-
0.998047237	0.039791524	ko01100//Metabolic p	ko04640//Hema	GO:0031226//intrinsic c
0.99804487	0.039815645	-	-	GO:0005634//nucleus
0.998041344	0.039851546	ko01100//Metabolic p	-	GO:0005759//mitochon
0.998040945	0.039855598	ko01100//Metabolic p	ko01100//Metab	-
0.998040056	0.039864644	-	-	-
0.998039714	0.039868126	-	ko01100//Metab	-
0.998039659	0.039868681	-	ko04080//Neuro	GO:0005576//extracellu
0.998037907	0.039886502	-	ko01100//Metab	GO:0005634//nucleus;G
0.9980373	0.039892676	-	-	-
0.998036466	0.039901148	-	-	-
0.998034648	0.03991962	-	-	GO:0005737//cytoplasm
0.99803438	0.03992235	-	-	-
0.998030057	0.039966239	ko01100//Metabolic p	-	GO:0016021//integral c
0.998028017	0.039986933	-	ko04979//Chole	GO:0005856//cytoskelet
0.998027741	0.039989729	ko01100//Metabolic p	-	-
0.998027372	0.039993477	-	-	-
0.998026443	0.040002899	ko01100//Metabolic p	-	GO:0005737//cytoplasm
0.998025276	0.040014724	-	-	-
0.998024924	0.040018287	-	ko01100//Metab	GO:0005737//cytoplasm

0.998023788	0.0400298	ko01100//Metabolic p	-	-
0.998022745	0.040040363	-	ko04013//MAPK	-
0.998019542	0.040072794	ko01100//Metabolic p	-	GO:0005634//nucleus;G
0.998018613	0.040082195	-	ko01100//Metab	GO:0005739//mitochon
0.998016241	0.040106185	-	-	-
0.998015884	0.040109802	-	-	-
0.998014605	0.040122734	ko01100//Metabolic p	-	-
0.998014171	0.040127118	-	-	GO:0016021//integral c
0.998011814	0.040150926	-	ko04142//Lysos	GO:0005615//extracellu
0.998011679	0.040152294	-	ko01100//Metab	GO:0005789//endoplasr
0.998009614	0.040173145	-	-	-
0.998009208	0.040177247	ko01100//Metabolic p	ko05016//Huntir	GO:0005929//cilium;GO
0.998008653	0.040182844	ko01100//Metabolic p	ko04060//Cytok	GO:0005576//extracellu
0.998005783	0.040211796	-	-	-
0.998005762	0.040212015	ko01100//Metabolic p	-	-
0.998005648	0.040213164	-	-	GO:0005743//mitochon
0.99800488	0.040220911	-	ko00983//Drug	-
0.998004753	0.04022219	-	-	-
0.998003629	0.040233522	-	ko01100//Metab	-
0.998003601	0.040233802	-	ko01100//Metab	-
0.998001046	0.040259547	-	ko05414//Dilate	-
0.998000197	0.040268102	ko01100//Metabolic p	-	GO:0030425//dendrite
0.997999321	0.040276921	-	-	-
0.997998505	0.040285139	-	-	-
0.997995355	0.040316831	-	ko01100//Metab	-
0.997994962	0.040320784	-	ko05230//Centra	GO:0005886//plasma m
0.99799416	0.040328857	ko01100//Metabolic p	ko04020//Calciu	GO:0005886//plasma m
0.997993086	0.040339652	ko01100//Metabolic p	-	-
0.997989383	0.040376863	ko01100//Metabolic p	-	GO:0001725//stress fibe
0.997989173	0.040378971	-	-	GO:0005634//nucleus;G
0.997987735	0.040393411	ko01100//Metabolic p	-	-
0.997987159	0.040399194	-	-	-
0.997985061	0.040420248	-	-	GO:0000785//chromatir
0.997984915	0.040421716	-	ko01100//Metab	GO:0005737//cytoplasm
0.997984772	0.040423151	-	-	-
0.997983883	0.040432072	ko01100//Metabolic p	ko01100//Metab	GO:0000139//Golgi mer
0.997982832	0.040442613	ko01100//Metabolic p	-	GO:0005829//cytosol;G(
0.997982455	0.040446395	-	-	-
0.997981276	0.040458211	ko01100//Metabolic p	-	-
0.997980123	0.040469766	ko01100//Metabolic p	ko05010//Alzhei	GO:0005576//extracellu
0.997978924	0.040481785	ko01100//Metabolic p	ko01100//Metab	-
0.997975925	0.04051182	-	-	GO:0005886//plasma m
0.997973535	0.040535737	ko01100//Metabolic p	-	-
0.997973513	0.040535952	-	-	-
0.997973272	0.040538362	ko01100//Metabolic p	ko02010//ABC tı	GO:0005886//plasma m
0.997971163	0.040559457	ko01100//Metabolic p	-	GO:0005737//cytoplasm
0.997970554	0.040565548	-	ko01100//Metab	GO:0005777//peroxison
0.99797052	0.040565884	-	ko01100//Metab	GO:0005615//extracellu
0.997970122	0.040569864	-	ko01100//Metab	-
0.997969716	0.040573929	-	ko04514//Cell a	GO:0016020//membran
0.997969154	0.040579543	ko01100//Metabolic p	ko01100//Metab	GO:0000139//Golgi mer
0.997968968	0.040581403	ko01100//Metabolic p	ko04659//Th17	GO:0000785//chromatir
0.997968621	0.040584867	ko01100//Metabolic p	ko01100//Metab	GO:0005886//plasma m
0.997968251	0.040588569	ko01120//Microbial m	ko03410//Base e	-
0.997965696	0.040614088	-	ko04918//Thyro	GO:0043231//intracellul
0.997964639	0.040624643	ko01100//Metabolic p	-	-
0.997964302	0.040628002	ko01100//Metabolic p	-	-
0.997963681	0.040634208	ko01100//Metabolic p	-	-

0.997963547	0.040635536	ko01100//Metabolic p	-	GO:0005634//nucleus;G
0.997963111	0.040639897	-	-	GO:0016020//membran
0.997963101	0.040639994	ko01100//Metabolic p	ko04922//Glucag	GO:0005634//nucleus;G
0.997960696	0.04066399	-	ko01100//Metab	GO:0005730//nucleolus
0.997960293	0.040668001	ko01100//Metabolic p	-	GO:0001726//ruffle;GO:
0.997955555	0.040715229	-	ko01100//Metab	-
0.997953899	0.040731718	ko01120//Microbial m	ko00564//Glycer	GO:0005634//nucleus;G
0.99795304	0.040740272	ko01110//Biosynthesis	-	GO:0005634//nucleus
0.997952065	0.04074998	ko01100//Metabolic p	-	GO:0046658//anchored
0.997950905	0.040761522	-	ko01100//Metab	-
0.997948948	0.040780981	-	-	-
0.997948711	0.040783346	ko01100//Metabolic p	ko04060//Cytok	GO:0005576//extracellu
0.997946194	0.040808369	-	-	GO:0005576//extracellu
0.997945608	0.040814183	-	-	GO:0005654//nucleopla
0.997944311	0.04082707	-	-	-
0.997943653	0.040833612	ko01100//Metabolic p	ko04022//cGMP	-
0.997943543	0.040834701	ko01100//Metabolic p	ko01100//Metab	GO:0016020//membran
0.997942745	0.040842631	-	ko01100//Metab	GO:0005737//cytoplasm
0.997942356	0.040846494	-	ko01100//Metab	-
0.997941634	0.040853653	ko01100//Metabolic p	-	-
0.997941592	0.040854079	-	ko01100//Metab	-
0.997941081	0.040859146	-	ko04640//Hema	GO:0031226//intrinsic c
0.997940222	0.040867675	ko01110//Biosynthesis	ko05143//Africa	GO:0005576//extracellu
0.997939904	0.040870828	-	ko04014//Ras sig	-
0.997939844	0.04087142	ko01100//Metabolic p	-	-
0.997938847	0.040881317	-	-	GO:0005737//cytoplasm
0.997938574	0.040884022	ko01100//Metabolic p	ko03320//PPAR	GO:0005783//endoplasm
0.997937002	0.040899616	ko01100//Metabolic p	ko01100//Metab	-
0.997936934	0.040900286	-	ko01100//Metab	GO:0005737//cytoplasm
0.997936763	0.040901983	ko01100//Metabolic p	ko04918//Thyro	GO:0005576//extracellu
0.997936353	0.040906049	ko01100//Metabolic p	-	-
0.997935809	0.040911443	-	-	-
0.997935151	0.040917966	-	-	-
0.997933498	0.040934347	-	ko01100//Metab	GO:0048269//methionin
0.997932984	0.040939437	ko01063//Biosynthesis	ko05200//Pathw	-
0.997931644	0.040952707	-	-	-
0.997931565	0.040953489	-	-	GO:0009986//cell surfac
0.997930118	0.04096782	ko01100//Metabolic p	ko01100//Metab	GO:0005886//plasma m
0.997926717	0.04100147	-	-	-
0.997926073	0.041007846	-	-	GO:0005634//nucleus;G
0.997923512	0.041033164	-	-	-
0.997922045	0.041047661	-	ko04060//Cytok	-
0.997921854	0.041049544	ko01100//Metabolic p	-	GO:0005634//nucleus
0.997920906	0.041058916	-	-	-
0.997920357	0.041064335	-	ko04514//Cell ad	GO:0005886//plasma m
0.99791917	0.041076056	-	-	-
0.997915271	0.04111454	ko01100//Metabolic p	ko01100//Metab	-
0.997915256	0.041114689	ko01100//Metabolic p	-	-
0.997912847	0.041138438	ko01100//Metabolic p	-	GO:0005634//nucleus
0.997912562	0.041141252	ko01100//Metabolic p	-	-
0.997912302	0.041143817	ko01100//Metabolic p	ko03013//Nucle	-
0.997910831	0.041158313	-	-	GO:0005615//extracellu
0.99790974	0.041169058	ko01120//Microbial m	-	GO:0005575//cellular_co
0.997908667	0.041179626	ko01100//Metabolic p	-	GO:0001725//stress fibe
0.997908072	0.041185488	ko01100//Metabolic p	-	GO:0005634//nucleus;G
0.997906729	0.041198709	-	ko04151//PI3K-/	GO:0005886//plasma m
0.997903886	0.041226693	-	ko05017//Spino	GO:0043231//intracellul
0.997903402	0.041231451	-	ko05200//Pathw	-

0.997901052	0.041254562	ko01100//Metabolic p	ko04080//Neurc	GO:0016020//membran
0.997900157	0.041263358	ko01100//Metabolic p	ko01100//Metak	-
0.997894622	0.041317727	ko01100//Metabolic p	-	-
0.997894535	0.041318576	-	ko03018//RNA c	GO:0005829//cytosol
0.997890056	0.041362519	ko01063//Biosynthesis	-	GO:0005576//extracellu
0.997889562	0.041367361	-	-	GO:0016021//integral c
0.997887767	0.041384962	-	-	-
0.997886803	0.041394399	-	-	GO:0016021//integral c
0.997886629	0.041396107	ko01100//Metabolic p	ko04976//Bile se	GO:0005789//endoplasr
0.997886196	0.041400348	ko01100//Metabolic p	ko01100//Metak	-
0.997885474	0.041407423	ko01100//Metabolic p	ko01100//Metak	-
0.997884587	0.041416111	-	-	GO:0005737//cytoplasm
0.997883467	0.041427071	-	ko01100//Metak	-
0.997883356	0.041428158	ko01100//Metabolic p	-	-
0.997882757	0.041434028	-	-	-
0.997882482	0.041436718	-	ko01100//Metak	GO:0005783//endoplasr
0.997882157	0.041439903	ko01100//Metabolic p	ko04218//Cellul	GO:0030896//checkpoir
0.997881927	0.041442145	-	-	GO:0005576//extracellu
0.997881648	0.04144488	-	ko04979//Chole	GO:0005856//cytoskelet
0.997881394	0.041447367	-	-	-
0.99788066	0.04145455	ko01100//Metabolic p	-	GO:0005615//extracellu
0.9978797	0.041463942	ko01110//Biosynthesis	-	-
0.997879343	0.04146743	-	-	-
0.997879088	0.04146992	ko01100//Metabolic p	-	-
0.997877225	0.041488139	-	-	GO:0016021//integral c
0.997874944	0.041510434	-	-	GO:0005615//extracellu
0.997874876	0.041511101	-	ko04726//Serotc	GO:0005739//mitochon
0.997874193	0.041517771	ko01100//Metabolic p	ko01100//Metak	GO:0005741//mitochon
0.997874166	0.041518029	-	ko01100//Metak	-
0.997873995	0.041519707	ko01100//Metabolic p	-	GO:0001533//cornified
0.997872799	0.041531383	-	ko04514//Cell a	GO:0005829//cytosol;G
0.997871027	0.041548687	-	-	-
0.997870646	0.041552402	-	ko01100//Metak	-
0.997869494	0.041563647	-	-	-
0.997866754	0.041590378	-	-	GO:0005634//nucleus;G
0.997865575	0.041601865	-	ko01100//Metak	GO:0005783//endoplasr
0.997865472	0.041602873	ko01100//Metabolic p	-	GO:0005886//plasma m
0.997862601	0.041630853	ko01100//Metabolic p	ko04714//Therm	GO:0005811//lipid parti
0.997861039	0.041646066	ko01110//Biosynthesis	ko04745//Photo	-
0.997861023	0.041646223	ko01100//Metabolic p	-	-
0.997857739	0.041678188	ko01100//Metabolic p	-	GO:0005576//extracellu
0.997857511	0.041680414	-	-	GO:0005737//cytoplasm
0.997857183	0.041683604	-	-	-
0.997855952	0.04169558	-	-	GO:0000139//Golgi mer
0.99785592	0.041695888	ko01100//Metabolic p	-	-
0.99785302	0.041724089	-	ko04020//Calciu	GO:0005886//plasma m
0.997852752	0.041726691	ko01100//Metabolic p	ko05132//Salmc	-
0.997852333	0.041730766	-	-	-
0.997852075	0.041733271	-	-	-
0.997850185	0.041751638	ko01100//Metabolic p	-	-
0.997847113	0.04178147	-	-	GO:0005737//cytoplasm
0.997846281	0.041789538	-	-	GO:0005634//nucleus;G
0.997845184	0.041800183	ko01100//Metabolic p	ko01100//Metak	GO:0005739//mitochon
0.997843471	0.041816806	ko01100//Metabolic p	-	GO:0005884//actin filam
0.997842302	0.04182814	-	-	GO:0005884//actin filam
0.997839026	0.041859892	-	ko00983//Drug	-
0.997838808	0.041862007	-	ko04922//Gluca	GO:0005634//nucleus;G
0.99783878	0.04186228	ko01100//Metabolic p	ko05200//Pathw	-

0.997836525	0.041884118	-	-	-
0.997835529	0.041893764	ko01110//Biosynthesis	ko02010//ABC tı	GO:0005886//plasma m
0.997833212	0.041916191	ko01120//Microbial m	-	GO:0005634//nucleus;G
0.997832114	0.041926808	-	ko01100//Metak	-
0.997828373	0.041962983	ko01100//Metabolic p	ko01100//Metak	GO:0005739//mitochon
0.997826724	0.041978917	-	-	-
0.997826641	0.04197972	-	-	-
0.997825808	0.041987765	-	ko04514//Cell ad	GO:0005886//plasma m
0.997825247	0.041993185	-	-	-
0.997825055	0.041995039	-	ko04060//Cytok	GO:0005576//extracellu
0.997823225	0.04201271	ko01100//Metabolic p	ko04144//Endoc	GO:0000813//ESCRT I c
0.997821575	0.04202864	ko01100//Metabolic p	-	GO:0005802//trans-Gol
0.997820774	0.042036366	-	ko00565//Ether	GO:0005615//extracellu
0.997820662	0.042037449	-	-	GO:0016021//integral c
0.997820555	0.04203848	ko01120//Microbial m	-	-
0.997820528	0.042038743	-	-	GO:0046658//anchored
0.997816045	0.04208197	-	-	-
0.997814622	0.042095675	ko01100//Metabolic p	-	GO:0005634//nucleus
0.99781374	0.042104178	ko01100//Metabolic p	-	GO:0005768//endosom
0.997809379	0.042146163	-	-	GO:0005759//mitochon
0.997808654	0.042153137	ko01100//Metabolic p	-	-
0.997808602	0.042153638	-	-	GO:0036126//sperm flag
0.997806031	0.042178372	-	ko05200//Pathw	GO:0005794//Golgi app
0.997804395	0.042194097	-	-	GO:0016020//membran
0.997802839	0.042209056	ko01110//Biosynthesis	-	-
0.997801379	0.042223078	ko01100//Metabolic p	ko05143//Africa	GO:0005576//extracellu
0.997801019	0.042226539	-	ko05200//Pathw	GO:0005576//extracellu
0.997799282	0.042243216	-	ko05200//Pathw	GO:0005576//extracellu
0.997798369	0.04225198	-	ko01100//Metak	GO:0005783//endoplasr
0.997794182	0.042292153	-	-	-
0.997793939	0.042294487	-	-	-
0.997793927	0.042294599	ko01100//Metabolic p	-	GO:0005634//nucleus;G
0.997793374	0.042299906	-	ko04514//Cell ad	GO:0005886//plasma m
0.997793134	0.042302207	ko01063//Biosynthesis	ko04080//Neurc	GO:0005576//extracellu
0.997791844	0.042314567	-	ko04120//Ubiqu	GO:0005634//nucleus
0.9977915	0.042317866	ko01100//Metabolic p	-	-
0.997791225	0.042320504	-	-	GO:0005783//endoplasr
0.997791026	0.042322414	-	-	GO:0005794//Golgi app
0.997789267	0.042339264	-	-	GO:0005737//cytoplasm
0.997787599	0.042355242	ko01063//Biosynthesis	-	GO:0005634//nucleus
0.997787292	0.042358176	-	-	-
0.997787016	0.042360824	ko01100//Metabolic p	ko02010//ABC tı	GO:0005739//mitochon
0.997786619	0.042364627	ko01100//Metabolic p	-	-
0.997785863	0.042371863	-	-	GO:0005634//nucleus
0.99778565	0.042373897	-	ko04979//Chole	GO:0005783//endoplasr
0.99778563	0.042374091	ko01100//Metabolic p	-	GO:0005737//cytoplasm
0.997785217	0.042378046	-	ko01100//Metak	-
0.997784311	0.042386715	-	ko04080//Neurc	GO:0005576//extracellu
0.99778382	0.042391415	-	-	-
0.997783754	0.042392043	-	-	-
0.997783753	0.04239205	ko01100//Metabolic p	-	GO:0005829//cytosol;G(
0.997781859	0.042410173	ko01100//Metabolic p	-	-
0.997781673	0.042411953	ko01100//Metabolic p	-	GO:0005737//cytoplasm
0.997781318	0.04241534	ko01100//Metabolic p	-	GO:0016020//membran
0.997781154	0.042416909	-	-	GO:0005737//cytoplasm
0.997780984	0.042418532	-	-	GO:0005739//mitochon
0.997780908	0.042419259	ko01100//Metabolic p	ko05200//Pathw	GO:0005576//extracellu
0.997780319	0.042424898	-	ko04080//Neurc	GO:0005576//extracellu

0.997780073	0.042427245	-	-	GO:0008076//voltage-g
0.997779929	0.042428623	-	ko04724//Glutar	GO:0005886//plasma m
0.99777865	0.042440843	-	ko01100//Metab	-
0.997778404	0.042443197	-	-	-
0.997778331	0.042443897	ko01100//Metabolic p	-	GO:0002177//manchett
0.997776408	0.042462264	-	ko05017//Spino	GO:0043231//intracellul
0.997776182	0.042464428	ko01063//Biosynthesis	ko04514//Cell a	GO:0005769//early endo
0.997773486	0.042490171	-	ko01100//Metab	-
0.997772294	0.042501546	-	-	-
0.997768419	0.042538511	ko01110//Biosynthesis	ko04024//cAMP	GO:0016021//integral co
0.997766673	0.042555155	ko01100//Metabolic p	-	GO:0005886//plasma m
0.997765843	0.042563057	-	ko01100//Metab	GO:0005615//extracellu
0.997765124	0.042569908	-	-	GO:0005576//extracellu
0.997764323	0.042577541	-	ko04979//Chole	GO:0005783//endoplasr
0.997763911	0.042581468	ko01100//Metabolic p	-	GO:0005634//nucleus;G
0.997762896	0.042591135	ko01100//Metabolic p	ko01100//Metab	GO:0005737//cytoplasm
0.99776016	0.042617184	-	-	-
0.997760158	0.042617202	ko01100//Metabolic p	-	-
0.997760151	0.042617265	ko01100//Metabolic p	ko04514//Cell a	GO:0016020//membran
0.997759165	0.04262665	-	-	-
0.997759002	0.042628202	-	ko01100//Metab	GO:0005886//plasma m
0.997757737	0.042640232	-	ko04080//Neuro	GO:0005576//extracellu
0.997756405	0.042652907	-	-	-
0.997755724	0.042659379	-	ko04151//PI3K-/	GO:0005654//nucleopla
0.997752189	0.042692974	-	ko04142//Lysos	GO:0005615//extracellu
0.997752101	0.042693811	ko01100//Metabolic p	-	-
0.997750207	0.042711797	ko01100//Metabolic p	-	-
0.997747731	0.042735303	ko01100//Metabolic p	ko01100//Metab	GO:0005615//extracellu
0.997744317	0.042767694	-	-	GO:0005576//extracellu
0.99774375	0.042773066	-	ko05200//Pathw	GO:0005794//Golgi app
0.997743495	0.042775487	-	-	-
0.997743061	0.042779607	-	-	-
0.997740209	0.042806629	-	ko01100//Metab	GO:0000139//Golgi mer
0.997738815	0.042819844	-	-	-
0.997738523	0.042822604	-	ko04060//Cytok	-
0.997734987	0.042856086	ko01100//Metabolic p	ko01100//Metab	GO:0005739//mitochon
0.99773245	0.042880085	-	ko01100//Metab	GO:0005739//mitochon
0.997731828	0.042885966	ko01100//Metabolic p	ko01100//Metab	GO:0005737//cytoplasm
0.997730793	0.042895761	ko01100//Metabolic p	-	GO:0005884//actin filan
0.997730668	0.042896935	ko01100//Metabolic p	-	-
0.997729866	0.042904518	ko01100//Metabolic p	ko01100//Metab	GO:0005829//cytosol
0.997729292	0.042909948	ko01100//Metabolic p	-	GO:0016020//membran
0.997728471	0.042917704	ko01100//Metabolic p	ko01100//Metab	-
0.99772845	0.042917902	-	-	-
0.997727122	0.042930452	-	-	-
0.997726108	0.042940037	ko01100//Metabolic p	-	GO:0016020//membran
0.997726048	0.042940601	-	-	-
0.997725467	0.042946085	-	-	GO:0016021//integral co
0.997724492	0.04295529	-	-	-
0.997721393	0.042984547	ko01100//Metabolic p	-	GO:0001533//cornified
0.997721185	0.042986505	-	-	GO:0005737//cytoplasm
0.997720143	0.04299634	ko01063//Biosynthesis	-	-
0.997719599	0.043001468	ko01100//Metabolic p	ko04745//Photo	-
0.997719118	0.043006007	ko01100//Metabolic p	ko04742//Taste	GO:0001518//voltage-g
0.99771857	0.043011174	ko01100//Metabolic p	-	-
0.997717263	0.043023498	ko01100//Metabolic p	ko01100//Metab	-
0.997715002	0.043044805	ko01100//Metabolic p	-	-
0.997713742	0.043056675	-	-	-

0.997711783	0.043075131	ko01100//Metabolic p	-	GO:0005783//endoplasr
0.997711637	0.0430765	ko01100//Metabolic p	-	-
0.997711523	0.043077577	-	-	-
0.997710964	0.043082839	ko01100//Metabolic p	-	GO:0005737//cytoplasm
0.997708962	0.04310168	ko01100//Metabolic p	ko04974//Protei	-
0.997707875	0.043111912	-	ko01100//Metab	-
0.997707326	0.043117071	ko01120//Microbial m	-	GO:0043231//intracellul
0.997706599	0.043123909	-	ko04080//Neuro	GO:0005886//plasma m
0.997706538	0.043124483	ko01100//Metabolic p	-	GO:0005813//centrosor
0.997706377	0.043125998	ko01100//Metabolic p	-	-
0.997706198	0.043127684	ko01100//Metabolic p	ko01100//Metab	GO:0005789//endoplasr
0.997704044	0.043147938	-	-	-
0.997703076	0.043157031	ko01100//Metabolic p	-	-
0.997702863	0.043159037	ko01100//Metabolic p	-	-
0.997701734	0.043169644	-	ko01100//Metab	GO:0005777//peroxison
0.997701021	0.043176342	ko01100//Metabolic p	-	GO:0005737//cytoplasm
0.997700696	0.043179399	-	ko05131//Shigel	GO:0005922//connexon
0.997700236	0.043183714	-	ko03320//PPAR	GO:0005634//nucleus;G
0.997696705	0.043216872	ko01100//Metabolic p	ko03320//PPAR	GO:0005634//nucleus;G
0.997696682	0.043217087	-	-	GO:0005576//extracellu
0.997691355	0.04326705	-	-	GO:0005737//cytoplasm
0.99769082	0.043272063	ko01100//Metabolic p	-	-
0.99768861	0.043292772	ko01100//Metabolic p	-	-
0.997687808	0.043300291	ko01100//Metabolic p	-	GO:0005886//plasma m
0.997685934	0.043317839	-	ko00983//Drug	-
0.997685209	0.043324628	ko01100//Metabolic p	-	GO:0001725//stress fibe
0.997684435	0.043331876	-	-	-
0.997684347	0.043332694	-	-	GO:0005737//cytoplasm
0.997683717	0.043338589	-	-	-
0.997682868	0.043346533	-	-	GO:0005737//cytoplasm
0.997682285	0.043351993	ko01100//Metabolic p	ko05200//Pathw	GO:0005794//Golgi app
0.997680939	0.043364586	-	-	GO:0005576//extracellu
0.997678782	0.043384754	-	ko01100//Metab	-
0.997676622	0.043404941	-	ko04024//cAMP	GO:0016021//integral co
0.997676557	0.043405551	-	-	GO:0031430//M band
0.997676467	0.043406393	-	-	-
0.997676031	0.043410466	ko01100//Metabolic p	ko05130//Patho	GO:0005856//cytoskelet
0.997675947	0.043411253	ko01100//Metabolic p	-	GO:0005634//nucleus;G
0.997671771	0.04345025	ko01100//Metabolic p	-	-
0.997671293	0.04345471	ko01100//Metabolic p	-	GO:0005634//nucleus
0.997671101	0.043456504	-	ko01100//Metab	GO:0005737//cytoplasm
0.997670882	0.043458546	-	-	-
0.997670725	0.043460014	-	-	-
0.997668769	0.04347826	-	-	-
0.997667279	0.043492165	ko01100//Metabolic p	ko05231//Cholir	GO:0005739//mitochon
0.997666932	0.043495395	-	-	GO:0016021//integral co
0.997666496	0.043499458	ko01100//Metabolic p	-	GO:0005654//nucleopla
0.997665707	0.043506817	-	ko01100//Metab	-
0.997665557	0.043508213	-	-	-
0.997664712	0.043516089	ko01100//Metabolic p	ko04979//Chole	GO:0005856//cytoskelet
0.997663848	0.043524141	-	-	GO:0005654//nucleopla
0.997661671	0.043544427	ko01100//Metabolic p	-	GO:0005794//Golgi app
0.997659602	0.043563698	ko01100//Metabolic p	-	GO:0005737//cytoplasm
0.997659298	0.043566528	-	ko01100//Metab	-
0.997656376	0.043593723	-	ko05202//Transc	GO:0016021//integral co
0.9976552	0.043604662	ko01100//Metabolic p	-	GO:0016021//integral co
0.99765224	0.043632186	-	-	-
0.997648796	0.043664194	-	ko04724//Glutar	GO:0005886//plasma m

0.997648666	0.043665399	ko01100//Metabolic p	-	GO:0005654//nucleopla
0.997647637	0.043674958	-	ko04610//Comp	GO:0005615//extracellu
0.997647366	0.043677474	-	ko01100//Metab	-
0.99764486	0.043700735	-	ko04070//Phosp	GO:0000323//lytic vacu
0.997644075	0.043708022	-	ko05200//Pathw	GO:0005576//extracellu
0.997643185	0.043716277	-	ko04010//MAPK	-
0.997642781	0.04372003	ko01110//Biosynthesis	-	-
0.99764262	0.043721523	-	-	GO:0005576//extracellu
0.99764019	0.043744061	-	-	-
0.997639424	0.043751162	ko01100//Metabolic p	ko04979//Chole	GO:0005783//endoplasr
0.997638367	0.043760962	-	-	GO:0005794//Golgi app
0.997638116	0.043763282	ko01100//Metabolic p	-	GO:0005737//cytoplasm
0.997637705	0.043767096	ko01100//Metabolic p	-	GO:0005634//nucleus
0.997636479	0.043778451	-	-	GO:0005783//endoplasr
0.997635493	0.043787593	-	-	GO:0005783//endoplasr
0.997635484	0.043787669	-	ko05200//Pathw	-
0.997633777	0.043803482	-	-	GO:0005634//nucleus;G
0.997632682	0.043813617	ko01100//Metabolic p	-	GO:0005813//centrosor
0.997632336	0.043816826	-	ko01100//Metab	GO:0005730//nucleolus
0.997631979	0.043820131	ko01100//Metabolic p	ko04080//Neuro	GO:0005576//extracellu
0.997630265	0.043835993	-	ko01100//Metab	-
0.997630194	0.043836646	-	-	GO:0005737//cytoplasm
0.997629844	0.043839885	ko01100//Metabolic p	ko03320//PPAR	GO:0005634//nucleus;G
0.997628752	0.043849989	-	-	-
0.997627877	0.043858079	ko01100//Metabolic p	ko05165//Huma	GO:0005634//nucleus;G
0.997627641	0.043860262	-	-	-
0.997624691	0.043887537	ko01100//Metabolic p	-	GO:0005794//Golgi app
0.997624589	0.043888477	-	-	-
0.997624376	0.043890441	-	-	-
0.997623972	0.043894175	-	-	GO:0005634//nucleus;G
0.997623261	0.043900744	-	-	-
0.997620667	0.043924711	ko01100//Metabolic p	ko01100//Metab	GO:0000139//Golgi mer
0.997620458	0.04392664	-	-	GO:0001726//ruffle;GO:
0.997620222	0.043928817	-	ko04919//Thyro	GO:0005887//integral c
0.99761997	0.043931141	-	-	GO:0005794//Golgi app
0.997619855	0.043932206	-	-	GO:0001650//fibrillar ce
0.997618883	0.043941179	-	-	-
0.997613848	0.043987633	-	-	-
0.997613595	0.043989968	-	-	GO:0005739//mitochon
0.997613503	0.043990809	-	ko01100//Metab	-
0.997612346	0.044001481	-	ko05200//Pathw	GO:0005576//extracellu
0.997610891	0.044014892	ko01100//Metabolic p	-	GO:0005576//extracellu
0.997609718	0.044025694	-	-	GO:0005576//extracellu
0.997609454	0.044028124	-	ko05414//Dilate	-
0.997608066	0.044040913	ko01100//Metabolic p	-	-
0.99760795	0.044041985	-	ko04060//Cytok	GO:0005576//extracellu
0.997607942	0.044042054	-	-	GO:0005739//mitochon
0.997607139	0.044049447	ko01110//Biosynthesis	ko01100//Metab	GO:0005654//nucleopla
0.997605418	0.044065294	ko01100//Metabolic p	ko01100//Metab	GO:0005635//nuclear er
0.997605259	0.044066761	-	ko00983//Drug	-
0.9976046	0.044072823	ko01100//Metabolic p	-	GO:0005654//nucleopla
0.997603432	0.044083569	-	-	GO:0016020//membran
0.997603269	0.044085071	-	-	-
0.997601662	0.04409985	-	-	GO:0005802//trans-Gol
0.997601661	0.044099859	-	ko00982//Drug	-
0.997601655	0.044099917	-	-	GO:0036126//sperm flag
0.997600264	0.04411271	ko01100//Metabolic p	-	-
0.997599762	0.044117325	-	ko04151//PI3K-/	GO:0005654//nucleopla

0.997598169	0.044131966	ko01100//Metabolic p	ko04150//mTOF	GO:0032991//macromo
0.997598044	0.044133118	-	-	-
0.9975963	0.04414914	ko01100//Metabolic p	-	-
0.997595226	0.044159008	-	-	-
0.997593443	0.044175388	-	-	GO:0005768//endosom
0.997593442	0.044175397	ko01100//Metabolic p	ko04020//Calciu	GO:0005886//plasma m
0.997590534	0.044202088	ko01100//Metabolic p	ko01100//Metak	-
0.997586095	0.044242801	ko01100//Metabolic p	ko05010//Alzhei	GO:0005576//extracellu
0.997585319	0.044249914	-	ko04060//Cytok	GO:0005576//extracellu
0.997585261	0.04425045	-	ko04020//Calciu	GO:0016020//membran
0.997582997	0.044271191	ko01120//Microbial m	-	GO:0005634//nucleus;G
0.997582002	0.044280307	-	ko04020//Calciu	GO:0005886//plasma m
0.997581776	0.044282383	ko01100//Metabolic p	ko04514//Cell a	GO:0005886//plasma m
0.997581184	0.044287802	ko01100//Metabolic p	-	-
0.99757991	0.04429947	-	-	-
0.997578634	0.04431115	-	-	GO:0005615//extracellu
0.997578485	0.044312513	ko01100//Metabolic p	-	GO:0005737//cytoplasm
0.997578308	0.04431413	-	-	-
0.997576061	0.044334695	ko01100//Metabolic p	-	-
0.997575951	0.044335704	ko01100//Metabolic p	-	GO:0001725//stress fibe
0.9975757	0.044337999	-	ko01100//Metak	GO:0005615//extracellu
0.997575332	0.044341361	ko01100//Metabolic p	ko04080//Neurc	GO:0009986//cell surfac
0.997574465	0.044349292	ko01100//Metabolic p	-	GO:0009986//cell surfac
0.997573451	0.044358566	-	ko05130//Patho	-
0.997573291	0.044360031	ko01120//Microbial m	ko05200//Pathw	GO:0005834//heterotrin
0.997572429	0.044367913	-	-	GO:0005886//plasma m
0.997571935	0.044372432	-	ko01100//Metak	GO:0005794//Golgi app
0.997571131	0.04437978	-	-	GO:0001533//cornified
0.997570128	0.044388939	-	-	-
0.997568462	0.044404159	-	ko02010//ABC t	GO:0005739//mitochon
0.997565539	0.044430853	ko01110//Biosynthesis	-	GO:0005737//cytoplasm
0.997563888	0.044445929	ko01100//Metabolic p	-	GO:0005737//cytoplasm
0.997562141	0.044461868	ko01100//Metabolic p	-	GO:0005739//mitochon
0.99756196	0.044463516	-	-	GO:0005739//mitochon
0.997559435	0.044486549	-	-	GO:0016020//membran
0.997559196	0.04448872	-	-	-
0.997559159	0.044489063	ko01100//Metabolic p	-	GO:0005737//cytoplasm
0.997558609	0.044494076	-	-	-
0.997558117	0.044498557	ko01100//Metabolic p	ko04610//Comp	GO:0005576//extracellu
0.997556837	0.044510223	ko01100//Metabolic p	ko04146//Perox	GO:0005765//lysosomal
0.997556365	0.044514525	ko01100//Metabolic p	-	-
0.997555273	0.04452448	-	ko04921//Oxyto	GO:0016020//membran
0.997554688	0.044529807	ko01100//Metabolic p	-	GO:0005856//cytoskelet
0.997553309	0.044542363	-	-	GO:0005737//cytoplasm
0.997553052	0.044544709	-	-	-
0.997548224	0.044588643	ko01100//Metabolic p	-	-
0.997547679	0.044593606	ko01063//Biosynthesis	ko04151//PI3K-/	GO:0005654//nucleopla
0.997546747	0.04460208	ko01100//Metabolic p	ko02010//ABC t	GO:0000139//Golgi mer
0.997545418	0.044614164	ko01100//Metabolic p	-	-
0.997545212	0.044616039	-	ko04080//Neurc	GO:0005886//plasma m
0.997542619	0.044639605	ko01100//Metabolic p	-	GO:0005829//cytosol;G
0.997541529	0.044649508	-	-	GO:0005737//cytoplasm
0.997538634	0.044675798	ko01100//Metabolic p	ko04931//Insulir	GO:0016021//integral c
0.997536448	0.044695643	ko01100//Metabolic p	-	-
0.997536086	0.044698926	ko01100//Metabolic p	ko00564//Glycer	GO:0005615//extracellu
0.997535116	0.044707732	ko01100//Metabolic p	-	GO:0016021//integral c
0.997534024	0.044717636	-	ko01100//Metak	-
0.997533779	0.044719861	-	-	GO:0001725//stress fibe

0.997529371	0.044759819	ko01100//Metabolic p	-	GO:0005576//extracellu
0.997528704	0.044765869	ko01110//Biosynthesis	ko05130//Patho	GO:0005856//cytoskelet
0.997526413	0.044786621	-	-	-
0.997525558	0.044794361	ko01100//Metabolic p	-	-
0.997520872	0.044836779	-	-	-
0.997520685	0.044838468	ko01100//Metabolic p	-	-
0.997520422	0.044840849	ko01100//Metabolic p	ko01100//Metak	-
0.997519457	0.04484957	-	-	-
0.997519416	0.044849943	-	-	GO:0005634//nucleus
0.997518417	0.04485898	ko01063//Biosynthesis	ko01100//Metak	-
0.997517824	0.04486434	-	-	-
0.997517758	0.044864933	-	ko01100//Metak	-
0.997517011	0.044871686	ko01110//Biosynthesis	-	GO:0005886//plasma m
0.997516917	0.04487254	-	ko00564//Glycer	GO:0005615//extracellu
0.99751658	0.044875582	-	-	-
0.997511763	0.044919101	ko01110//Biosynthesis	-	GO:0005737//cytoplasm
0.997507974	0.044953307	-	-	-
0.997507938	0.044953629	ko01100//Metabolic p	-	-
0.997505556	0.044975116	ko01100//Metabolic p	ko01100//Metak	GO:0005739//mitochon
0.997504582	0.0449839	ko01063//Biosynthesis	-	GO:0005634//nucleus
0.997503052	0.044997697	-	-	GO:0005737//cytoplasm
0.997502885	0.044999201	ko01100//Metabolic p	ko01100//Metak	GO:0005737//cytoplasm
0.997500877	0.045017295	ko01100//Metabolic p	-	GO:0016021//integral c
0.997500752	0.045018423	ko01100//Metabolic p	ko04520//Adher	GO:0031252//cell leadir
0.997497319	0.045049342	-	ko04742//Taste	GO:0001518//voltage-g
0.997496285	0.045058659	ko01100//Metabolic p	-	GO:0005737//cytoplasm
0.997493274	0.045085755	ko01120//Microbial m	-	GO:0005634//nucleus;G
0.997492251	0.045094956	ko01100//Metabolic p	ko04270//Vascu	GO:0005737//cytoplasm
0.997491935	0.045097798	-	ko05200//Pathw	-
0.997489364	0.04512092	-	ko05200//Pathw	-
0.997488389	0.045129679	-	ko04130//SNAR	GO:0005737//cytoplasm
0.99748817	0.04513165	-	-	GO:0000307//cyclin-de
0.99748803	0.045132905	-	ko04610//Comp	GO:0005615//extracellu
0.997485028	0.045159878	ko01100//Metabolic p	-	GO:0005802//trans-Gol
0.997484526	0.045164388	-	ko04919//Thyro	GO:0005887//integral c
0.997482464	0.045182901	ko01100//Metabolic p	ko05200//Pathw	-
0.997480793	0.045197904	-	ko04976//Bile se	GO:0005654//nucleopla
0.997478677	0.045216886	ko01100//Metabolic p	ko04927//Cortis	GO:0005634//nucleus;G
0.997475487	0.045245498	-	ko01100//Metak	GO:0031988//membran
0.997474383	0.045255393	ko01100//Metabolic p	-	GO:0005737//cytoplasm
0.997473028	0.045267535	-	-	-
0.997472436	0.04527284	ko01100//Metabolic p	ko05165//Huma	GO:0005634//nucleus;G
0.997470784	0.045287637	ko01063//Biosynthesis	ko01100//Metak	GO:0016020//membran
0.99746868	0.045306474	-	-	GO:0036126//sperm fla
0.997468087	0.045311786	ko01100//Metabolic p	ko04550//Signal	GO:0005634//nucleus;G
0.997467176	0.045319944	-	ko01100//Metak	GO:0000139//Golgi mer
0.997466954	0.04532193	ko01100//Metabolic p	-	GO:0036126//sperm fla
0.997464667	0.04534239	ko01100//Metabolic p	ko01100//Metak	-
0.997463199	0.045355525	ko01120//Microbial m	-	GO:0005737//cytoplasm
0.997460997	0.045375211	ko01100//Metabolic p	-	-
0.997459449	0.045389049	ko01100//Metabolic p	ko00983//Drug	-
0.99745768	0.04540485	ko01100//Metabolic p	ko01100//Metak	GO:0005654//nucleopla
0.997456085	0.0454191	-	-	GO:0005813//centrosor
0.997452202	0.045453766	ko01100//Metabolic p	ko03320//PPAR	GO:0005634//nucleus;G
0.997450488	0.04546906	-	-	GO:0005737//cytoplasm
0.997448964	0.045482649	-	-	-
0.997447168	0.045498669	ko01110//Biosynthesis	ko01100//Metak	-
0.997446491	0.045504705	ko01100//Metabolic p	ko00983//Drug	-

0.997445461	0.045513883	ko01100//Metabolic p	ko01100//Metak	GO:0005741//mitochon
0.997444886	0.045519003	-	-	-
0.997444691	0.04552074	-	ko04927//Cortis	GO:0005634//nucleus;G
0.997443065	0.045535232	-	-	GO:0016020//membran
0.997442665	0.045538796	ko01110//Biosynthesis	ko04080//Neurc	GO:0005783//endoplasr
0.997442154	0.045543342	ko01100//Metabolic p	ko05414//Dilate	-
0.997442069	0.045544106	ko01100//Metabolic p	-	-
0.997440531	0.045557796	-	-	GO:0005802//trans-Gol
0.997439473	0.04556722	ko01100//Metabolic p	-	GO:0005737//cytoplasm
0.997439189	0.045569745	-	-	GO:0005737//cytoplasm
0.997437467	0.045585069	-	-	GO:0005634//nucleus;G
0.997436909	0.045590037	-	-	GO:0005737//cytoplasm
0.997435646	0.045601277	-	-	GO:0005737//cytoplasm
0.99743468	0.045609863	ko01100//Metabolic p	ko01100//Metak	-
0.997433503	0.04562033	-	-	-
0.99743331	0.045622044	-	ko05200//Pathw	GO:0005794//Golgi app
0.99743204	0.045633334	-	-	GO:0005768//endosom
0.997431615	0.045637116	-	-	-
0.9974295	0.045655911	ko01100//Metabolic p	ko01100//Metak	GO:0005829//cytosol;G
0.997429436	0.04565648	ko01100//Metabolic p	ko01100//Metak	GO:0005737//cytoplasm
0.997427291	0.04567553	ko01100//Metabolic p	ko04610//Comp	GO:0005615//extracellu
0.997426432	0.045683159	-	-	-
0.99742223	0.045720452	ko01100//Metabolic p	ko05130//Patho	GO:0005856//cytoskelet
0.997422118	0.045721447	-	ko05131//Shigel	GO:0005922//connexon
0.997421193	0.045729654	ko01100//Metabolic p	ko04350//TGF-b	-
0.997417815	0.045759612	ko01100//Metabolic p	ko04010//MAPK	-
0.997417403	0.045763264	-	-	-
0.997416234	0.045773623	ko01100//Metabolic p	-	-
0.997415227	0.045782545	ko01100//Metabolic p	-	GO:0005813//centrosor
0.997414808	0.045786256	ko01100//Metabolic p	-	GO:0005737//cytoplasm
0.997414297	0.045790786	-	-	-
0.997414119	0.045792356	ko01100//Metabolic p	-	-
0.997413912	0.045794193	ko01100//Metabolic p	-	GO:0005654//nucleopla
0.997413185	0.045800636	-	-	GO:0005576//extracellu
0.997412692	0.045805	ko01100//Metabolic p	-	-
0.997411363	0.045816768	-	ko01100//Metak	-
0.997407868	0.045847702	-	-	GO:0005794//Golgi app
0.997407843	0.045847923	-	ko01100//Metak	GO:0005829//cytosol
0.997407646	0.045849661	ko01100//Metabolic p	-	GO:0005783//endoplasr
0.997407623	0.045849864	-	-	GO:0030425//dendrite
0.997407504	0.045850922	ko01100//Metabolic p	ko04060//Cytok	GO:0005576//extracellu
0.997404804	0.045874802	-	ko00982//Drug	-
0.997404095	0.045881066	ko01100//Metabolic p	ko05322//Syster	GO:0005576//extracellu
0.997404049	0.045881472	-	-	-
0.997402989	0.045890843	-	ko02010//ABC tı	GO:0000139//Golgi mer
0.997402767	0.045892804	-	ko01100//Metak	-
0.997401404	0.045904853	ko01110//Biosynthesis	ko01100//Metak	-
0.997401138	0.045907202	ko01100//Metabolic p	ko01100//Metak	-
0.997399554	0.045921199	ko01100//Metabolic p	-	-
0.997396916	0.045944496	-	-	GO:0016020//membran
0.997394767	0.045963467	ko01100//Metabolic p	-	-
0.997393813	0.045971882	ko01100//Metabolic p	-	-
0.997393094	0.045978223	ko01110//Biosynthesis	ko01100//Metak	GO:0005739//mitochon
0.997388219	0.046021214	-	ko04979//Chole	GO:0005783//endoplasr
0.997387806	0.046024856	-	ko04060//Cytok	GO:0005576//extracellu
0.997386752	0.046034144	ko01100//Metabolic p	ko01100//Metak	-
0.997384938	0.046050121	-	-	GO:0046658//anchored
0.997383472	0.046063035	-	ko01100//Metak	-

0.99738248	0.046071769	ko01063//Biosynthesis	-	GO:0031012//extracellu
0.997379843	0.046094985	-	ko04390//Hippo	GO:0005667//transcript
0.997379216	0.046100504	-	ko01100//Metab	-
0.997375913	0.046129557	-	ko01100//Metab	-
0.997375611	0.046132206	-	ko01100//Metab	GO:0005739//mitochon
0.997375206	0.046135771	-	ko05202//Transc	GO:0016021//integral co
0.9973748	0.046139341	-	-	GO:0005737//cytoplasm
0.997374266	0.046144033	-	-	GO:0005654//nucleopla
0.997372477	0.046159761	ko01100//Metabolic p	-	GO:0031982//vesicle
0.997370589	0.046176343	-	ko01100//Metab	-
0.997369851	0.046182826	-	ko04080//Neuro	GO:0005783//endoplasm
0.997369785	0.046183411	-	ko01100//Metab	-
0.997369621	0.046184846	-	ko01100//Metab	GO:0005737//cytoplasm
0.997369201	0.046188535	ko01100//Metabolic p	ko04151//PI3K-A	GO:0005654//nucleopla
0.997369162	0.046188878	-	-	-
0.997365281	0.046222952	-	ko01100//Metab	-
0.997364809	0.046227089	ko01100//Metabolic p	-	GO:0005886//plasma m
0.997364056	0.046233701	-	-	-
0.997363945	0.046234675	ko01100//Metabolic p	ko01100//Metab	GO:0016020//membran
0.997361906	0.046252561	ko01110//Biosynthesis	-	GO:0005737//cytoplasm
0.99736174	0.046254014	ko01120//Microbial m	-	GO:0005737//cytoplasm
0.997360874	0.046261607	ko01100//Metabolic p	-	GO:0016020//membran
0.997356406	0.046300774	-	-	-
0.997354518	0.046317305	-	-	GO:0005737//cytoplasm
0.99735425	0.046319652	-	-	-
0.997353898	0.046322742	ko01100//Metabolic p	ko05414//Dilate	-
0.997350703	0.046350704	-	-	GO:0031430//M band
0.997349541	0.046360879	-	-	-
0.997349484	0.046361378	ko01100//Metabolic p	ko00983//Drug	-
0.997347462	0.04637906	ko01100//Metabolic p	ko05130//Patho	-
0.997346932	0.046383703	ko01100//Metabolic p	-	GO:0016020//membran
0.997345571	0.046395597	-	-	-
0.997344392	0.046405905	-	-	-
0.997343922	0.046410011	-	ko01100//Metab	-
0.997343321	0.046415271	-	-	-
0.997342804	0.046419783	ko01100//Metabolic p	-	-
0.997340239	0.046442197	-	ko04659//Th17	GO:0000785//chromatin
0.997340171	0.046442792	-	-	-
0.997336714	0.04647297	-	ko05322//Syster	GO:0005576//extracellu
0.997336657	0.046473472	-	ko04310//Wnt s	GO:0005576//extracellu
0.997336353	0.046476123	ko01110//Biosynthesis	ko01100//Metab	GO:0031988//membran
0.997335275	0.04648553	-	-	GO:0005737//cytoplasm
0.997330213	0.046529682	-	-	-
0.997328812	0.046541893	-	ko04610//Comp	GO:0005576//extracellu
0.997327452	0.046553751	ko01100//Metabolic p	ko04024//cAMP	GO:0016021//integral co
0.997326923	0.046558359	ko01100//Metabolic p	-	-
0.99732668	0.046560474	-	-	-
0.997325604	0.046569848	-	-	-
0.997324823	0.046576646	-	-	GO:0005634//nucleus
0.99732234	0.046598272	-	-	GO:0005739//mitochon
0.997322068	0.046600639	ko01100//Metabolic p	-	-
0.997319404	0.046623823	ko01110//Biosynthesis	ko05200//Pathw	-
0.997318994	0.046627387	-	ko03320//PPAR	GO:0005634//nucleus;G
0.997318896	0.046628242	ko01110//Biosynthesis	ko05165//Huma	GO:0005634//nucleus;G
0.997318681	0.046630116	ko01100//Metabolic p	-	-
0.997318466	0.046631978	-	-	-
0.997316334	0.046650524	-	-	-
0.997315721	0.04665585	-	ko04976//Bile se	GO:0005654//nucleopla

0.9973125	0.046683849	-	-	-
0.997312466	0.046684143	-	ko01100//Metab	-
0.997312463	0.046684171	ko01100//Metabolic p	ko01100//Metab	-
0.997311674	0.04669103	ko01100//Metabolic p	-	-
0.997311151	0.046695572	ko01100//Metabolic p	-	GO:0005739//mitochon
0.997311129	0.046695759	-	-	-
0.997310461	0.04670156	-	-	-
0.997308457	0.046718966	ko01100//Metabolic p	-	GO:0005737//cytoplasm
0.997307077	0.046730948	-	-	-
0.997306007	0.046740233	-	ko01100//Metab	-
0.99730537	0.046745762	-	-	GO:0000139//Golgi mer
0.997304567	0.046752736	-	-	GO:0005615//extracellu
0.997303004	0.046766291	ko01100//Metabolic p	-	-
0.997302031	0.046774729	-	ko04979//Chole	GO:0005856//cytoskelet
0.997301153	0.046782344	ko01100//Metabolic p	-	GO:0005737//cytoplasm
0.997299148	0.046799724	ko01100//Metabolic p	-	GO:0005576//extracellu
0.99729662	0.04682163	ko01100//Metabolic p	-	-
0.997296526	0.046822444	-	-	-
0.997294343	0.04684136	-	-	-
0.997294046	0.046843929	ko01100//Metabolic p	-	GO:0005634//nucleus;G
0.997294	0.046844329	-	-	GO:0016020//membran
0.997293352	0.046849935	-	-	GO:0005886//plasma m
0.997292874	0.046854072	ko01100//Metabolic p	ko01100//Metab	-
0.997291759	0.046863727	-	ko04514//Cell a	GO:0005829//cytosol;G
0.997291586	0.046865229	ko01100//Metabolic p	-	-
0.997291226	0.046868346	-	ko00564//Glycer	GO:0005615//extracellu
0.997291162	0.046868897	ko01100//Metabolic p	ko05010//Alzhei	GO:0005783//endoplasr
0.997290718	0.04687274	-	ko05200//Pathw	-
0.997288238	0.046894198	ko01100//Metabolic p	ko04080//Neuro	GO:0005783//endoplasr
0.997285858	0.046914784	-	ko05130//Patho	-
0.997285305	0.04691956	ko01100//Metabolic p	-	GO:0005737//cytoplasm
0.997283455	0.046935549	ko01100//Metabolic p	-	-
0.997283404	0.046935997	-	-	GO:0001533//cornified
0.99728129	0.046954264	-	-	-
0.997279721	0.046967814	-	-	GO:0005654//nucleopla
0.997278558	0.046977858	-	ko03018//RNA d	GO:0005829//cytosol
0.997276915	0.046992046	-	-	-
0.997274522	0.047012699	ko01100//Metabolic p	-	GO:0016020//membran
0.997274282	0.047014765	-	ko04010//MAPK	-
0.997273624	0.04702044	-	ko01100//Metab	GO:0031988//membran
0.997270438	0.047047918	ko01100//Metabolic p	-	GO:0005886//plasma m
0.997270064	0.047051144	-	ko01100//Metab	GO:0005829//cytosol;G
0.99726977	0.047053681	-	-	GO:0016021//integral c
0.997268846	0.047061643	ko01100//Metabolic p	-	GO:0005634//nucleus;G
0.997267241	0.047075474	-	-	-
0.997266255	0.047083978	-	-	GO:0005794//Golgi app
0.997266255	0.047083978	-	ko01100//Metab	GO:0005739//mitochon
0.997265956	0.047086554	ko01100//Metabolic p	-	GO:0005759//mitochon
0.997265044	0.047094408	-	ko05168//Herpe	-
0.997260566	0.047132965	-	-	GO:0016021//integral c
0.997256899	0.047164515	-	ko01100//Metab	-
0.997256591	0.047167157	ko01100//Metabolic p	ko00564//Glycer	GO:0005615//extracellu
0.997256394	0.047168856	-	-	-
0.997255457	0.047176912	ko01110//Biosynthesis	-	-
0.997252801	0.047199747	ko01100//Metabolic p	-	GO:0005737//cytoplasm
0.997252744	0.047200233	-	-	-
0.997252293	0.047204114	ko01100//Metabolic p	ko04610//Comp	GO:0005615//extracellu
0.997252181	0.047205077	-	-	-

0.99725122	0.047213328	ko01100//Metabolic p	ko01100//Metat	GO:0005829//cytosol
0.997250392	0.047220445	ko01100//Metabolic p	-	GO:0005737//cytoplasm
0.997249185	0.04723081	ko01100//Metabolic p	ko01100//Metat	-
0.997248106	0.047240083	-	ko01100//Metat	GO:0005634//nucleus;G
0.997247649	0.047244002	-	-	GO:0005737//cytoplasm
0.997246366	0.04725502	ko01100//Metabolic p	-	-
0.997245756	0.047260256	ko01100//Metabolic p	ko01100//Metat	-
0.997244652	0.047269733	-	ko01100//Metat	GO:0005739//mitochon
0.997243912	0.047276079	-	-	GO:0001725//stress fibe
0.9972422	0.047290768	-	-	GO:0005737//cytoplasm
0.997241311	0.047298389	-	ko01100//Metat	GO:0043231//intracellul
0.997240753	0.047303178	ko01100//Metabolic p	ko01100//Metat	-
0.997237941	0.047327284	ko01110//Biosynthesis	-	-
0.997237721	0.047329171	-	-	GO:0005737//cytoplasm
0.99723756	0.047330556	-	ko04390//Hippc	GO:0005667//transcript
0.997234663	0.047355376	-	ko04020//Calciu	GO:0001518//voltage-g
0.997231053	0.047386286	ko01100//Metabolic p	-	-
0.997229549	0.047399166	-	-	-
0.997228797	0.047405599	-	-	-
0.997228731	0.04740616	-	-	GO:0005634//nucleus
0.997228546	0.047407743	ko01100//Metabolic p	-	GO:0005886//plasma m
0.997227233	0.047418981	-	ko01100//Metat	-
0.99722708	0.047420291	ko01120//Microbial m	ko00970//Aminc	GO:0005737//cytoplasm
0.997225492	0.047433872	ko01100//Metabolic p	-	-
0.997224838	0.047439461	-	-	GO:0043231//intracellul
0.997223568	0.047450319	ko01063//Biosynthesis	ko05200//Pathw	GO:0005576//extracellu
0.997222639	0.047458267	ko01100//Metabolic p	ko01100//Metat	-
0.997221066	0.047471707	ko01110//Biosynthesis	ko04060//Cytok	GO:0005576//extracellu
0.997219574	0.047484458	-	-	-
0.997215338	0.047520632	ko01100//Metabolic p	ko04310//Wnt s	GO:0009897//external s
0.997214068	0.047531473	ko01100//Metabolic p	-	GO:0005615//extracellu
0.997210251	0.047564034	ko01100//Metabolic p	-	-
0.997209227	0.04757277	ko01100//Metabolic p	-	-
0.997208659	0.047577609	-	ko05200//Pathw	GO:0005576//extracellu
0.997208581	0.047578281	ko01100//Metabolic p	-	-
0.997207945	0.0475837	ko01100//Metabolic p	ko04514//Cell ac	GO:0005886//plasma m
0.997207005	0.04759171	-	ko01100//Metat	-
0.9972058	0.047601979	-	-	GO:0005654//nucleopla
0.997205098	0.047607964	ko01100//Metabolic p	-	GO:0005737//cytoplasm
0.997203479	0.047621762	ko01110//Biosynthesis	ko00982//Drug	-
0.997201126	0.047641796	-	-	-
0.997200465	0.047647428	ko01100//Metabolic p	-	-
0.997198695	0.047662496	-	-	GO:0005783//endoplasr
0.997198017	0.047668261	-	-	-
0.997197557	0.047672174	-	ko01100//Metat	GO:0005654//nucleopla
0.997197378	0.047673698	-	-	GO:0000307//cyclin-de
0.997197328	0.047674125	-	ko00983//Drug	-
0.997197258	0.047674724	ko01100//Metabolic p	-	-
0.997194765	0.047695927	ko01110//Biosynthesis	-	GO:0005576//extracellu
0.997193015	0.047710811	-	-	GO:0000139//Golgi mer
0.997192786	0.047712761	-	ko05414//Dilate	-
0.997192005	0.0477194	-	ko05200//Pathw	GO:0005576//extracellu
0.997191475	0.047723908	-	-	GO:0005737//cytoplasm
0.997191055	0.047727471	ko01100//Metabolic p	ko01100//Metat	-
0.997189922	0.047737103	ko01100//Metabolic p	ko04142//Lysos	GO:0016020//membran
0.997189252	0.047742799	ko01100//Metabolic p	-	-
0.997189034	0.047744645	-	ko01100//Metat	-
0.997185185	0.047777338	-	-	-

0.997179521	0.047825408	-	-	-
0.997179115	0.047828856	ko01110//Biosynthesis	-	GO:0005813//centrosor
0.9971788	0.047831526	ko01100//Metabolic p	ko01100//Metab	GO:0031988//membran
0.997176625	0.047849966	-	-	GO:0005737//cytoplasm
0.997174987	0.047863852	-	-	-
0.997174685	0.04786641	-	-	GO:0015630//microtubu
0.997174009	0.047872137	-	-	-
0.99717368	0.047874929	-	ko01100//Metab	GO:0005737//cytoplasm
0.997173534	0.047876162	-	ko01100//Metab	GO:0000139//Golgi mer
0.997169876	0.047907148	-	ko01100//Metab	GO:0005739//mitochon
0.997168392	0.047919714	-	ko01100//Metab	-
0.997167873	0.047924108	ko01100//Metabolic p	-	-
0.997167838	0.047924402	-	ko05322//Syster	GO:0005576//extracellu
0.99716779	0.047924807	ko01100//Metabolic p	-	-
0.997167069	0.047930917	-	ko05322//Syster	GO:0005576//extracellu
0.997167009	0.047931418	-	ko04310//Wnt s	GO:0005576//extracellu
0.997166761	0.04793352	ko01063//Biosynthesis	ko05168//Herpe	-
0.997164644	0.047951435	-	-	-
0.997163895	0.047957768	-	ko04080//Neuro	GO:0005783//endoplasr
0.99716286	0.047966519	-	-	-
0.99716282	0.047966858	-	-	-
0.997162612	0.047968621	ko01100//Metabolic p	-	-
0.997161555	0.04797756	ko01110//Biosynthesis	-	-
0.997161358	0.047979226	ko01100//Metabolic p	ko05200//Pathw	-
0.997160284	0.047988306	-	-	GO:0005615//extracellu
0.997159669	0.047993504	ko01100//Metabolic p	-	GO:0005739//mitochon
0.997159149	0.047997894	-	ko01100//Metab	-
0.997158576	0.048002743	ko01100//Metabolic p	ko05150//Staphy	GO:0005577//fibrinoger
0.997156776	0.048017951	ko01100//Metabolic p	-	GO:0016020//membran
0.99715664	0.048019097	-	ko01100//Metab	GO:0005615//extracellu
0.997155687	0.048027147	ko01100//Metabolic p	ko05230//Centra	GO:0005886//plasma m
0.997154296	0.048038895	-	ko04151//PI3K-/	GO:0009986//cell surfac
0.997153737	0.048043619	-	ko04973//Carbo	GO:0005886//plasma m
0.997152741	0.048052028	-	-	-
0.997151533	0.048062223	ko01100//Metabolic p	ko01100//Metab	-
0.997150094	0.048074369	ko01110//Biosynthesis	-	GO:0005737//cytoplasm
0.997149787	0.048076958	-	ko04927//Cortis	GO:0005634//nucleus;G
0.997149023	0.048083405	-	-	-
0.997148133	0.04809091	-	ko05010//Alzhei	GO:0005783//endoplasr
0.997147864	0.048093181	-	-	GO:0005737//cytoplasm
0.997145213	0.04811554	-	-	GO:0005886//plasma m
0.997139839	0.048160828	-	-	-
0.997137697	0.048178868	ko01100//Metabolic p	-	-
0.997135054	0.048201117	-	ko01100//Metab	GO:0005829//cytosol;G(
0.997131747	0.048228936	ko01100//Metabolic p	ko04918//Thyro	GO:0005576//extracellu
0.997127482	0.048264798	ko01100//Metabolic p	ko04080//Neuro	GO:0016021//integral c
0.997126157	0.048275934	-	-	GO:0016021//integral c
0.997125999	0.048277265	ko01100//Metabolic p	-	-
0.997125536	0.048281151	-	ko01100//Metab	-
0.997125369	0.048282559	ko01100//Metabolic p	-	-
0.997125238	0.048283656	-	-	GO:0005634//nucleus;G
-0.997125926	0.048277875	-	-	-
-0.997126069	0.048276679	-	ko04142//Lysoso	GO:0016020//membran
-0.997127454	0.048265034	-	-	GO:0005737//cytoplasm
-0.997127984	0.048260583	ko01100//Metabolic p	-	GO:0005634//nucleus
-0.997129429	0.048248437	ko01100//Metabolic p	-	-
-0.997130637	0.048238273	ko01100//Metabolic p	ko00533//Glycos	GO:0000139//Golgi mer
-0.997130965	0.048235518	ko01100//Metabolic p	-	-

-0.997131238	0.048233222	-	ko04550//Signal	GO:0005634//nucleus;G
-0.99713228	0.048224452	ko01100//Metabolic p	-	-
-0.997132511	0.048222514	-	-	-
-0.997132945	0.048218865	ko01100//Metabolic p	-	GO:0032991//macromo
-0.997133131	0.048217296	-	-	-
-0.9971333	0.048215872	-	-	GO:0005634//nucleus;G
-0.997133444	0.048214665	ko01100//Metabolic p	ko01100//Metab	GO:0005778//peroxison
-0.997138821	0.048169404	-	-	GO:0032991//macromo
-0.997139066	0.048167339	-	ko04144//Endoc	GO:0005654//nucleopla
-0.997139718	0.048161849	-	-	GO:0005737//cytoplasm
-0.997139798	0.048161173	ko01100//Metabolic p	-	GO:0005615//extracellu
-0.997141209	0.048149288	-	-	-
-0.997141377	0.048147867	ko01100//Metabolic p	ko03320//PPAR	GO:0005783//endoplasn
-0.99714153	0.048146577	-	-	-
-0.997141656	0.048145517	ko01100//Metabolic p	-	-
-0.99714214	0.048141441	ko01100//Metabolic p	ko04714//Therm	GO:0005811//lipid parti
-0.997142417	0.048139109	ko01100//Metabolic p	-	-
-0.997143373	0.048131053	ko01110//Biosynthesis	ko01100//Metab	GO:0005737//cytoplasm
-0.997143838	0.048127132	ko01100//Metabolic p	ko01100//Metab	-
-0.99714662	0.048103678	ko01100//Metabolic p	-	-
-0.997151908	0.04805906	-	-	GO:0016020//membran
-0.997152322	0.048055565	ko01100//Metabolic p	-	GO:0005783//endoplasn
-0.997159602	0.047994072	ko01100//Metabolic p	-	GO:0005802//trans-Gol
-0.99715965	0.047993666	ko01100//Metabolic p	-	-
-0.997161456	0.047978394	-	-	GO:0005783//endoplasn
-0.997161498	0.047978038	ko01100//Metabolic p	ko05231//Cholir	-
-0.99716325	0.047963228	ko01110//Biosynthesis	ko04979//Chole	GO:0005737//cytoplasm
-0.997164481	0.047952808	ko01100//Metabolic p	-	GO:0005634//nucleus;G
-0.99716471	0.047950876	-	-	-
-0.997165205	0.047946686	ko01100//Metabolic p	-	-
-0.997167231	0.047929541	ko01100//Metabolic p	-	-
-0.997168811	0.047916167	ko01100//Metabolic p	-	-
-0.997169579	0.047909666	-	-	GO:0005654//nucleopla
-0.997169829	0.04790755	-	-	GO:0005886//plasma m
-0.997170555	0.047901396	ko01110//Biosynthesis	-	-
-0.997171668	0.047891969	-	-	-
-0.997172129	0.047888072	ko01100//Metabolic p	ko03013//Nucle	-
-0.997174806	0.047865385	-	ko04060//Cytok	GO:0005576//extracellu
-0.997175514	0.047859386	-	-	-
-0.997175882	0.047856263	ko01100//Metabolic p	ko01100//Metab	GO:0005789//endoplasn
-0.997176592	0.04785025	-	ko05152//Tuber	GO:0009897//external s
-0.997176936	0.047847334	-	-	-
-0.997178538	0.047833741	ko01100//Metabolic p	-	-
-0.997178579	0.047833394	ko01100//Metabolic p	-	-
-0.997180208	0.047819582	ko01100//Metabolic p	-	-
-0.997180891	0.047813786	ko01100//Metabolic p	ko01100//Metab	GO:0005783//endoplasn
-0.997181419	0.04780931	ko01100//Metabolic p	ko01100//Metab	GO:0005739//mitochon
-0.997181586	0.047807889	-	-	-
-0.997181905	0.047805181	ko01063//Biosynthesis	-	GO:0005886//plasma m
-0.997181952	0.047804786	ko01100//Metabolic p	-	GO:0005654//nucleopla
-0.997182208	0.047802612	-	-	-
-0.997182618	0.047799135	-	-	GO:0005737//cytoplasm
-0.9971833	0.047793345	-	-	GO:0005576//extracellu
-0.997184071	0.047786795	ko01100//Metabolic p	-	-
-0.997185595	0.04777386	ko01100//Metabolic p	-	-
-0.99718718	0.047760401	-	ko04714//Therm	GO:0005811//lipid parti
-0.997187224	0.047760021	-	-	GO:0005737//cytoplasm
-0.99718816	0.047752071	ko01100//Metabolic p	-	-

-0.997188399	0.04775004	-	-	GO:0001726//ruffle;GO:
-0.997191539	0.047723361	-	-	-
-0.997192099	0.047718598	-	ko04922//Gluca	GO:0005634//nucleus;G
-0.99719222	0.047717575	-	-	GO:0005634//nucleus;G
-0.997192659	0.047713841	-	-	-
-0.997193623	0.047705644	-	ko05412//Arrhyt	GO:0005886//plasma m
-0.997194566	0.047697624	ko01100//Metabolic p	ko04080//Neurc	GO:0005886//plasma m
-0.997195304	0.047691343	ko01100//Metabolic p	-	GO:0005634//nucleus;G
-0.997197259	0.047674711	-	-	GO:0005764//lysosome;
-0.9971985	0.047664155	ko01100//Metabolic p	-	GO:0005789//endoplasr
-0.997199867	0.047652519	-	-	-
-0.997202433	0.047630664	-	-	-
-0.997202681	0.047628555	ko01100//Metabolic p	-	-
-0.997203697	0.047619904	-	-	-
-0.997204619	0.047612048	-	ko04970//Saliva	GO:0005576//extracellu
-0.997205518	0.047604386	-	-	-
-0.997205977	0.047600472	-	-	GO:0005829//cytosol
-0.99720757	0.047586899	-	ko01100//Metab	GO:0009923//fatty acid
-0.997208313	0.047580563	-	-	GO:0005576//extracellu
-0.997209106	0.047573802	ko01100//Metabolic p	ko04919//Thyro	GO:0016020//membran
-0.997210493	0.04756197	ko01100//Metabolic p	-	-
-0.997211258	0.047555449	-	-	-
-0.997211654	0.047552066	-	-	GO:0005634//nucleus;G
-0.997212798	0.04754231	ko01100//Metabolic p	-	-
-0.997214096	0.047531231	ko01100//Metabolic p	-	GO:0000791//euchroma
-0.997214429	0.04752839	-	-	GO:0005794//Golgi app
-0.99721692	0.047507122	-	-	-
-0.997217102	0.047505569	ko01100//Metabolic p	ko04142//Lysos	-
-0.997217808	0.047499539	ko01063//Biosynthesis	-	GO:0005794//Golgi app
-0.997217919	0.047498597	ko01100//Metabolic p	ko05010//Alzhei	GO:0005783//endoplasr
-0.997218739	0.047491586	ko01100//Metabolic p	ko01100//Metab	GO:0005789//endoplasr
-0.997218878	0.047490406	-	-	-
-0.997220651	0.047475252	ko01063//Biosynthesis	-	-
-0.997221683	0.047466432	-	ko04020//Calciu	GO:0005634//nucleus;G
-0.997221772	0.047465675	ko01100//Metabolic p	ko01100//Metab	GO:0033178//proton-tr
-0.99722555	0.047433378	-	-	-
-0.997231247	0.047384629	ko01110//Biosynthesis	-	GO:0005654//nucleopla
-0.997232922	0.047370289	-	ko05165//Huma	-
-0.997233353	0.047366598	ko01100//Metabolic p	-	GO:0031091//platelet al
-0.997234423	0.047357428	ko01100//Metabolic p	-	-
-0.997234666	0.047355352	-	-	GO:0005634//nucleus;G
-0.997234965	0.047352786	ko01100//Metabolic p	ko04721//Synap	-
-0.997235266	0.04735021	ko01100//Metabolic p	ko04742//Taste	GO:0005783//endoplasr
-0.997235942	0.047344416	ko01100//Metabolic p	ko04146//Peroxi	GO:0005765//lysosomal
-0.997236769	0.047337333	-	-	-
-0.997238502	0.047322479	ko01100//Metabolic p	-	GO:0031091//platelet al
-0.997238848	0.047319516	-	-	GO:0005634//nucleus;G
-0.997240464	0.047305655	-	-	-
-0.997241683	0.047295202	ko01100//Metabolic p	-	-
-0.99724259	0.047287419	-	-	-
-0.997242932	0.047284491	-	-	-
-0.997243669	0.047278164	-	ko04919//Thyro	GO:0016020//membran
-0.997244432	0.047271615	-	-	-
-0.997246062	0.047257627	-	ko04151//PI3K-/	GO:0005634//nucleus;G
-0.997247273	0.047247227	ko01100//Metabolic p	-	-
-0.997247666	0.047243854	ko01100//Metabolic p	-	-
-0.997248554	0.047236233	ko01100//Metabolic p	-	-
-0.997248746	0.047234584	ko01063//Biosynthesis	-	GO:0005576//extracellu

-0.997249761	0.047225864	ko01100//Metabolic p	-	GO:0005654//nucleopla
-0.997250161	0.047222429	-	-	-
-0.997250297	0.047221262	ko01063//Biosynthesis	ko05322//Syster	GO:0000786//nucleosor
-0.997252396	0.047203222	-	-	-
-0.997254352	0.047186417	-	-	GO:0005576//extracellu
-0.99725631	0.047169575	ko01100//Metabolic p	ko04120//Ubiqu	GO:0005680//anaphase
-0.997257674	0.047157846	ko01100//Metabolic p	-	GO:0000791//euchroma
-0.997258962	0.047146763	-	ko04010//MAPK	-
-0.997259469	0.047142401	ko01100//Metabolic p	-	GO:0001650//fibrillar ce
-0.997263657	0.04710635	ko01100//Metabolic p	-	-
-0.997264407	0.047099895	-	-	-
-0.997264641	0.04709788	-	ko01100//Metak	-
-0.997264658	0.047097731	ko01100//Metabolic p	ko01100//Metak	GO:0005576//extracellu
-0.997264838	0.047096184	ko01100//Metabolic p	-	-
-0.997265062	0.04709425	-	-	-
-0.997265508	0.047090412	-	-	GO:0005737//cytoplasm
-0.997267536	0.047072936	ko01100//Metabolic p	-	GO:0005654//nucleopla
-0.997268956	0.047060694	-	ko05200//Pathw	GO:0005634//nucleus;G
-0.997268979	0.047060496	ko01100//Metabolic p	-	-
-0.997269201	0.047058588	-	ko01100//Metak	-
-0.997269252	0.047058148	-	ko01100//Metak	GO:0005640//nuclear ou
-0.99726957	0.047055404	ko01100//Metabolic p	-	GO:0034361//very-low-
-0.997269894	0.047052612	ko01100//Metabolic p	ko05231//Cholir	-
-0.997270257	0.047049482	ko01100//Metabolic p	-	-
-0.99727377	0.047019179	ko01100//Metabolic p	ko04144//Endoc	GO:0000813//ESCRT I co
-0.997274013	0.047017089	-	-	GO:0005886//plasma m
-0.997274403	0.047013721	ko01110//Biosynthesis	ko01100//Metak	GO:0005789//endoplasr
-0.997275801	0.047001659	ko01100//Metabolic p	-	GO:0005737//cytoplasm
-0.997276036	0.046999632	-	-	GO:0005829//cytosol;G(
-0.997276212	0.046998107	-	-	-
-0.997277079	0.04699063	ko01100//Metabolic p	-	GO:0000779//condense
-0.997277958	0.046983037	-	-	-
-0.997278292	0.046980154	ko01100//Metabolic p	-	-
-0.997279176	0.046972518	ko01100//Metabolic p	-	GO:0005783//endoplasr
-0.99728271	0.046941987	-	-	GO:0005887//integral co
-0.997282978	0.046939677	-	ko01100//Metak	GO:0005737//cytoplasm
-0.997283198	0.046937772	ko01100//Metabolic p	ko04922//Gluca	GO:0005634//nucleus;G
-0.997283432	0.046935748	ko01100//Metabolic p	ko04146//Perox	GO:0005777//peroxison
-0.997286451	0.046909652	-	ko04216//Ferror	GO:0016020//membran
-0.9972869	0.04690577	ko01100//Metabolic p	-	GO:0005604//basement
-0.997288022	0.046896066	-	-	GO:0016020//membran
-0.99729202	0.046861471	-	-	-
-0.997292509	0.046857235	ko01100//Metabolic p	-	GO:0005737//cytoplasm
-0.997292942	0.046853487	-	ko04140//Autop	GO:0000407//pre-autop
-0.997293328	0.046850148	ko01100//Metabolic p	-	GO:0005737//cytoplasm
-0.99729345	0.046849086	ko01100//Metabolic p	-	GO:0005576//extracellu
-0.997294336	0.04684142	-	-	-
-0.997294433	0.046840581	-	-	GO:0005739//mitochon
-0.997298401	0.046806198	ko01100//Metabolic p	-	-
-0.997298607	0.046804417	-	ko05231//Cholir	-
-0.99729917	0.046799535	-	ko05200//Pathw	GO:0005886//plasma m
-0.997299402	0.046797527	ko01100//Metabolic p	ko05200//Pathw	GO:0000785//chromatir
-0.997299444	0.046797157	ko01100//Metabolic p	-	-
-0.997302031	0.046774726	-	-	-
-0.997302086	0.046774252	-	ko01100//Metak	GO:0016020//membran
-0.997303125	0.046765239	-	-	-
-0.997304438	0.046753852	-	-	-
-0.997304755	0.046751105	ko01100//Metabolic p	-	GO:0016020//membran

-0.997305759	0.046742391	ko01100//Metabolic p	-	-
-0.997306617	0.046734938	ko01100//Metabolic p	-	GO:0001725//stress fibe
-0.997309295	0.046711689	-	ko04060//Cytok	GO:0005887//integral c
-0.997309887	0.046706546	-	-	GO:0005886//plasma m
-0.997311667	0.046691086	-	ko04146//Perox	GO:0005765//lysosomal
-0.997311835	0.04668963	ko01100//Metabolic p	-	GO:0005783//endoplasi
-0.997313834	0.04667226	ko01100//Metabolic p	-	-
-0.997314933	0.046662703	-	-	-
-0.997315323	0.046659316	-	-	-
-0.997316396	0.046649983	-	-	GO:0097541//axonemal
-0.99731644	0.046649605	-	ko04310//Wnt s	GO:0005634//nucleus;G
-0.997318486	0.046631811	ko01100//Metabolic p	ko04310//Wnt s	GO:0005634//nucleus;G
-0.997318722	0.046629751	ko01110//Biosynthesis	-	-
-0.997319232	0.046625321	ko01100//Metabolic p	-	-
-0.997319405	0.046623815	-	-	-
-0.997320388	0.046615262	ko01100//Metabolic p	-	-
-0.997321242	0.046607825	ko01100//Metabolic p	ko04979//Chole	GO:0005737//cytoplasm
-0.997321362	0.046606786	-	-	-
-0.997324301	0.046581193	-	-	-
-0.997324762	0.046577184	ko01100//Metabolic p	ko01100//Metak	GO:0005654//nucleopla
-0.997325058	0.0465746	-	-	-
-0.997325522	0.046570565	ko01100//Metabolic p	-	GO:0000139//Golgi mer
-0.997327715	0.046551453	-	-	-
-0.997329739	0.046533818	ko01100//Metabolic p	ko04360//Axon	-
-0.997331368	0.046519615	ko01100//Metabolic p	-	GO:0005576//extracellu
-0.997332494	0.046509792	ko01100//Metabolic p	-	-
-0.997333554	0.046500546	ko01100//Metabolic p	-	-
-0.997335436	0.046484125	ko01100//Metabolic p	-	GO:0005737//cytoplasm
-0.997337126	0.04646938	-	ko01100//Metak	GO:0005654//nucleopla
-0.997338931	0.04645362	ko01100//Metabolic p	ko01100//Metak	-
-0.99733934	0.04645005	-	-	-
-0.997339391	0.046449603	-	-	GO:0005886//plasma m
-0.997339666	0.046447196	ko01100//Metabolic p	-	-
-0.997340423	0.046440584	-	-	-
-0.997341995	0.04642685	ko01100//Metabolic p	-	GO:0005829//cytosol
-0.997342942	0.046418581	ko01100//Metabolic p	-	-
-0.997343387	0.046414688	ko01100//Metabolic p	-	-
-0.997344273	0.046406948	-	-	-
-0.997345099	0.046399729	ko01100//Metabolic p	-	-
-0.997345204	0.046398808	-	-	-
-0.997345449	0.046396668	ko01110//Biosynthesis	-	-
-0.997346346	0.046388827	-	ko04010//MAPK	GO:0005737//cytoplasm
-0.997346978	0.046383301	ko01100//Metabolic p	ko05205//Prote	GO:0005576//extracellu
-0.997350342	0.046353865	ko01100//Metabolic p	ko01100//Metak	GO:0000506//glycosylpl
-0.997350378	0.04635355	ko01100//Metabolic p	-	GO:0005783//endoplasi
-0.997352642	0.046333734	-	-	-
-0.997352779	0.046332534	-	-	GO:0031224//intrinsic c
-0.997353994	0.046321899	-	-	-
-0.997354116	0.046320825	ko01100//Metabolic p	-	-
-0.997354225	0.046319878	-	-	-
-0.997354832	0.046314557	-	ko01100//Metak	GO:0005794//Golgi app
-0.99735526	0.046310807	ko01100//Metabolic p	-	-
-0.997355915	0.046305071	-	ko03010//Ribos	GO:0005634//nucleus;G
-0.997362124	0.046250645	-	-	GO:0008076//voltage-g
-0.997362905	0.046243801	ko01100//Metabolic p	-	-
-0.997364272	0.046231806	ko01100//Metabolic p	-	GO:0005794//Golgi app
-0.9973658	0.046218399	ko01110//Biosynthesis	ko04919//Thyro	GO:0016020//membran
-0.997366137	0.046215435	ko01100//Metabolic p	ko04310//Wnt s	-

-0.997371882	0.046164986	-	-	-
-0.997372524	0.046159349	-	-	-
-0.99737518	0.046135999	-	-	GO:0000791//euchroma
-0.997376761	0.046122096	ko01100//Metabolic p	-	GO:0005739//mitochon
-0.997376881	0.046121042	-	ko04145//Phago	GO:0005783//endoplasr
-0.997379051	0.046101953	-	ko04120//Ubiqu	GO:0005680//anaphase
-0.997381562	0.046079854	-	-	GO:0016020//membran
-0.997382655	0.046070233	-	-	-
-0.997383439	0.046063324	ko01100//Metabolic p	ko05135//Yersin	-
-0.997384698	0.046052242	-	-	GO:0005654//nucleopla
-0.997384773	0.046051576	ko01100//Metabolic p	ko04130//SNAR	GO:0005737//cytoplasm
-0.99738555	0.046044734	ko01110//Biosynthesis	-	-
-0.997385925	0.046041431	-	-	GO:0005802//trans-Gol
-0.99738731	0.046029226	ko01100//Metabolic p	-	GO:0005654//nucleopla
-0.997389736	0.046007842	ko01063//Biosynthesis	ko04141//Protei	GO:0005634//nucleus;G
-0.997390578	0.046000416	ko01100//Metabolic p	-	-
-0.997391699	0.045990531	-	-	GO:0005634//nucleus
-0.997392003	0.045987851	ko01100//Metabolic p	-	GO:0016020//membran
-0.997393871	0.045971365	-	-	GO:0031091//platelet al
-0.997394792	0.045963246	ko01110//Biosynthesis	-	-
-0.997395309	0.045958677	ko01100//Metabolic p	-	GO:0005813//centrosor
-0.99739538	0.04595805	ko01100//Metabolic p	-	-
-0.997396471	0.045948419	-	-	-
-0.997397583	0.045938605	ko01100//Metabolic p	ko04550//Signal	GO:0005634//nucleus;G
-0.997399798	0.045919041	-	ko05203//Viral c	GO:0000786//nucleosor
-0.997401306	0.045905721	ko01063//Biosynthesis	-	GO:0005737//cytoplasm
-0.997401345	0.04590537	-	-	-
-0.997401824	0.045901139	-	ko01100//Metak	GO:0005739//mitochon
-0.997402166	0.045898115	ko01100//Metabolic p	-	GO:0009986//cell surfac
-0.997407448	0.045851411	ko01100//Metabolic p	-	GO:0005634//nucleus;G
-0.997407867	0.045847709	-	-	-
-0.997408178	0.045844957	-	-	-
-0.997409452	0.045833681	ko01100//Metabolic p	-	GO:0005730//nucleolus
-0.997409874	0.045829944	-	ko05202//Transc	GO:0000786//nucleosor
-0.997414992	0.045784627	-	-	GO:0005783//endoplasr
-0.997415839	0.045777123	-	-	GO:0005634//nucleus;G
-0.997418052	0.045757508	ko01100//Metabolic p	-	GO:0005634//nucleus;G
-0.997419224	0.045747119	-	-	GO:0031362//anchored
-0.997419232	0.045747043	-	ko04921//Oxyto	GO:0016020//membran
-0.997424072	0.045704106	-	ko01100//Metak	GO:0005737//cytoplasm
-0.997424596	0.045699454	-	-	GO:0005802//trans-Gol
-0.997424694	0.045698586	ko01110//Biosynthesis	-	-
-0.997425078	0.045695179	ko01100//Metabolic p	-	GO:0005737//cytoplasm
-0.997425464	0.045691749	-	-	GO:0016020//membran
-0.997427165	0.045676646	-	ko01100//Metak	GO:0005576//extracellu
-0.997428233	0.045667164	-	-	GO:0005634//nucleus;G
-0.99742878	0.045662308	ko01100//Metabolic p	ko05412//Arrhyt	GO:0005886//plasma m
-0.997428997	0.04566038	-	-	GO:0005634//nucleus;G
-0.997429339	0.045657344	-	ko04080//Neuro	GO:0009986//cell surfac
-0.997430003	0.045651442	-	ko01100//Metak	GO:0005576//extracellu
-0.997432126	0.045632576	-	-	-
-0.997434304	0.045613207	ko01100//Metabolic p	ko00790//Folate	-
-0.997434334	0.045612938	-	-	GO:0005634//nucleus;G
-0.997435061	0.045606473	-	-	GO:0001669//acrosoma
-0.997436332	0.045595167	-	-	-
-0.997438278	0.045577852	-	-	-
-0.997445376	0.045514643	ko01100//Metabolic p	-	-
-0.997445401	0.045514419	ko01100//Metabolic p	ko04216//Ferrop	GO:0016020//membran

-0.997445946	0.045509563	-	ko01100//Metak	GO:0005654//nucleopla
-0.997446657	0.045503222	-	ko04919//Thyro	GO:0016020//membran
-0.997447232	0.045498096	ko01100//Metabolic p	-	-
-0.997448481	0.045486959	ko01100//Metabolic p	-	-
-0.997448524	0.045486572	ko01100//Metabolic p	ko01100//Metak	GO:0005634//nucleus;G
-0.997449374	0.045478997	-	-	-
-0.997450259	0.0454711	-	-	-
-0.997450737	0.045466841	ko01100//Metabolic p	-	GO:0034451//centriolar
-0.997451398	0.045460939	ko01100//Metabolic p	-	GO:0016020//membran
-0.997451699	0.045458254	ko01100//Metabolic p	ko04140//Autop	GO:0000407//pre-autop
-0.997452979	0.04544683	ko01100//Metabolic p	-	GO:0005654//nucleopla
-0.997453032	0.04544636	ko01100//Metabolic p	-	-
-0.997453051	0.045446187	ko01100//Metabolic p	-	-
-0.99745453	0.045432982	ko01063//Biosynthesis	-	GO:0005737//cytoplasm
-0.997455395	0.045425264	-	ko01100//Metak	GO:0005783//endoplasr
-0.997456332	0.045416894	ko01100//Metabolic p	-	-
-0.997457702	0.04540466	ko01100//Metabolic p	-	GO:0005764//lysosome;
-0.997457714	0.045404552	ko01100//Metabolic p	-	-
-0.997458821	0.045394658	-	-	-
-0.997458843	0.045394465	-	ko01100//Metak	GO:0033178//proton-tr
-0.99745902	0.045392878	-	ko05202//Transc	GO:0000786//nucleosor
-0.997461285	0.045372634	-	-	-
-0.997461862	0.04536748	ko01100//Metabolic p	ko04144//Endoc	GO:0001917//photorece
-0.997463516	0.045352686	ko01100//Metabolic p	-	-
-0.997464603	0.045342969	ko01100//Metabolic p	-	-
-0.997465549	0.0453345	-	ko04726//Seroto	GO:0005739//mitochon
-0.997465612	0.045333938	-	-	-
-0.997465624	0.045333834	-	-	GO:0005615//extracellu
-0.997466616	0.045324953	-	-	-
-0.997468298	0.045309898	-	-	-
-0.99746873	0.045306033	ko01100//Metabolic p	-	GO:0005794//Golgi app
-0.997469586	0.045298368	ko01063//Biosynthesis	-	GO:0005634//nucleus;G
-0.997471637	0.045279999	-	ko01100//Metak	-
-0.997472267	0.045274348	ko01100//Metabolic p	-	-
-0.997474627	0.045253203	-	-	-
-0.997475508	0.045245309	ko01110//Biosynthesis	-	-
-0.997475987	0.045241012	ko01100//Metabolic p	-	-
-0.997477176	0.045230353	-	ko04141//Protei	GO:0005783//endoplasr
-0.997477889	0.045223956	-	-	-
-0.997478008	0.045222887	ko01100//Metabolic p	ko01100//Metak	-
-0.99747829	0.045220354	ko01100//Metabolic p	-	-
-0.997479163	0.045212531	-	-	GO:0005739//mitochon
-0.997480962	0.045196381	ko01100//Metabolic p	ko04020//Calciu	GO:0005634//nucleus;G
-0.997481248	0.045193814	ko01100//Metabolic p	-	GO:0005576//extracellu
-0.997481367	0.045192745	ko01100//Metabolic p	-	-
-0.997482224	0.045185055	ko01100//Metabolic p	-	-
-0.997482412	0.045183371	-	-	GO:0005634//nucleus;G
-0.997485189	0.045158433	-	-	-
-0.99748981	0.045116904	-	ko01100//Metak	GO:0005737//cytoplasm
-0.997491558	0.045101186	-	-	-
-0.997491565	0.045101126	-	-	GO:0005730//nucleolus
-0.997492101	0.045096301	-	-	-
-0.997493019	0.045088045	ko01100//Metabolic p	ko01100//Metak	GO:0005783//endoplasr
-0.997493115	0.045087181	ko01100//Metabolic p	-	GO:0000139//Golgi mer
-0.997493188	0.045086529	-	-	-
-0.997493352	0.045085048	-	ko04710//Circac	GO:0005634//nucleus;G
-0.997493609	0.045082735	-	ko04721//Synap	-
-0.997495005	0.04507018	-	-	-

-0.997496758	0.045054396	-	ko01100//Metab	GO:0005576//extracellu
-0.997497269	0.045049799	-	ko04146//Perox	GO:0005765//lysosomal
-0.997498552	0.045038242	-	-	GO:0005737//cytoplasm
-0.997498896	0.04503514	-	-	-
-0.997500528	0.045020441	-	-	GO:0005654//nucleopla
-0.99750091	0.045016998	ko01100//Metabolic p	-	GO:0005576//extracellu
-0.997501162	0.045014734	ko01110//Biosynthesis	ko04550//Signal	GO:0005634//nucleus;G
-0.997503451	0.044994103	ko01100//Metabolic p	-	GO:0005634//nucleus;G
-0.99750412	0.044988068	-	ko05205//Protec	GO:0005576//extracellu
-0.99750455	0.044984194	-	-	GO:0034361//very-low-
-0.997506006	0.04497106	ko01100//Metabolic p	ko04080//Neuro	GO:0009986//cell surfac
-0.997506497	0.044966634	-	ko01100//Metab	GO:0016020//membran
-0.997506824	0.044963679	-	-	-
-0.997507539	0.044957233	ko01100//Metabolic p	-	GO:0005634//nucleus;G
-0.997512324	0.044914036	ko01110//Biosynthesis	ko01100//Metab	GO:0005783//endoplasn
-0.997512915	0.0449087	ko01100//Metabolic p	ko04144//Endoc	GO:0005654//nucleopla
-0.997512926	0.044908605	ko01100//Metabolic p	-	-
-0.997516853	0.044873114	ko01110//Biosynthesis	ko00790//Folate	-
-0.997516938	0.04487235	-	-	GO:0001650//fibrillar ce
-0.997517802	0.044864538	-	ko04146//Perox	GO:0005777//peroxison
-0.997518711	0.04485632	-	-	GO:0005634//nucleus;G
-0.997519752	0.044846904	ko01100//Metabolic p	ko04144//Endoc	GO:0000813//ESCRT I co
-0.997520894	0.044836574	-	-	GO:0005794//Golgi app
-0.997521369	0.044832278	ko01100//Metabolic p	ko04145//Phago	GO:0005783//endoplasn
-0.99752772	0.044774778	ko01063//Biosynthesis	-	GO:0005634//nucleus;G
-0.997530028	0.04475387	ko01100//Metabolic p	ko04520//Adher	GO:0031252//cell leadir
-0.997533063	0.044726352	ko01100//Metabolic p	-	-
-0.997535414	0.044705023	-	-	-
-0.997536824	0.04469223	ko01100//Metabolic p	-	GO:0036126//sperm flag
-0.997537789	0.04468347	-	ko05203//Viral c	GO:0000786//nucleosor
-0.997538091	0.044680728	-	-	GO:0005737//cytoplasm
-0.997539483	0.044668091	-	ko05135//Yersin	-
-0.997541697	0.044647984	ko01063//Biosynthesis	ko05010//Alzhei	-
-0.997543908	0.044627888	-	ko05200//Pathw	GO:0005737//cytoplasm
-0.997543951	0.044627504	-	ko04024//cAMP	GO:0016021//integral co
-0.997544515	0.044622377	ko01100//Metabolic p	ko04978//Miner	GO:0009986//cell surfac
-0.997545886	0.044609913	ko01100//Metabolic p	-	GO:0005634//nucleus;G
-0.997546113	0.044607843	ko01100//Metabolic p	-	-
-0.997547219	0.044597792	ko01063//Biosynthesis	-	GO:0014069//postsynap
-0.997547731	0.044593128	-	-	-
-0.997547797	0.044592533	ko01100//Metabolic p	-	-
-0.997550177	0.044570873	-	-	-
-0.99755198	0.044554468	-	-	GO:0005576//extracellu
-0.997555427	0.044523076	ko01100//Metabolic p	-	-
-0.997555581	0.044521667	ko01100//Metabolic p	ko04146//Perox	GO:0005765//lysosomal
-0.997556011	0.044517753	ko01100//Metabolic p	ko04350//TGF-b	-
-0.997560186	0.044479695	ko01100//Metabolic p	-	-
-0.997560279	0.044478847	-	ko04520//Adher	GO:0031252//cell leadir
-0.997561646	0.044466385	ko01100//Metabolic p	-	-
-0.99756228	0.044460596	-	-	-
-0.997563606	0.044448498	ko01063//Biosynthesis	-	-
-0.997564707	0.044438449	-	-	GO:0005730//nucleolus
-0.997565225	0.044433724	ko01110//Biosynthesis	-	GO:0005576//extracellu
-0.997565747	0.044428959	ko01100//Metabolic p	-	GO:0005737//cytoplasm
-0.997565981	0.044426819	-	-	-
-0.997568687	0.044402105	ko01110//Biosynthesis	-	GO:0005634//nucleus;G
-0.997569485	0.044394817	ko01100//Metabolic p	-	GO:0016020//membran
-0.997570079	0.044389394	ko01100//Metabolic p	-	GO:0005783//endoplasn

-0.997570339	0.04438701	-	-	-
-0.997572102	0.044370902	-	-	GO:0001669//acrosoma
-0.997573156	0.044361264	ko01100//Metabolic p	-	-
-0.997573886	0.044354589	ko01100//Metabolic p	ko01100//Metab	-
-0.997574058	0.04435302	ko01100//Metabolic p	-	-
-0.99757561	0.044338819	ko01100//Metabolic p	-	-
-0.997575793	0.044337149	ko01100//Metabolic p	-	-
-0.997575817	0.04433693	-	-	-
-0.99757625	0.044332963	ko01100//Metabolic p	ko00564//Glycer	GO:0005615//extracellu
-0.997579403	0.044304113	ko01100//Metabolic p	-	-
-0.99758225	0.044278038	ko01063//Biosynthesis	ko04210//Apopt	GO:0005634//nucleus;G
-0.997585109	0.044251837	-	ko04310//Wnt s	-
-0.997586181	0.04424201	-	ko01100//Metab	-
-0.99758667	0.044237528	ko01100//Metabolic p	-	-
-0.997587378	0.044231039	-	-	GO:0005764//lysosome;
-0.997594789	0.044163027	ko01100//Metabolic p	-	-
-0.997595022	0.044160886	ko01100//Metabolic p	-	-
-0.997595467	0.044156799	-	-	GO:0005829//cytosol
-0.997595634	0.044155266	-	ko01100//Metab	-
-0.997595869	0.044153101	-	ko01100//Metab	-
-0.997597523	0.04413791	-	-	-
-0.997597615	0.044137063	-	-	-
-0.997599604	0.044118776	ko01100//Metabolic p	ko04010//MAPK	GO:0005737//cytoplasm
-0.997599692	0.044117968	ko01063//Biosynthesis	-	GO:0016021//integral co
-0.997599776	0.0441172	-	ko01100//Metab	GO:0005829//cytosol
-0.997599927	0.044115813	ko01100//Metabolic p	ko01100//Metab	GO:0005737//cytoplasm
-0.997602629	0.044090957	ko01110//Biosynthesis	-	-
-0.997602903	0.044088439	ko01110//Biosynthesis	-	-
-0.997605152	0.04406774	ko01110//Biosynthesis	-	-
-0.997607434	0.044046733	-	-	-
-0.997609134	0.044031081	ko01100//Metabolic p	-	GO:0005886//plasma m
-0.997609692	0.044025938	ko01100//Metabolic p	ko04060//Cytok	GO:0005887//integral co
-0.997609776	0.044025166	ko01100//Metabolic p	ko01100//Metab	GO:0005737//cytoplasm
-0.997610037	0.04402276	-	ko04020//Calciu	GO:0005634//nucleus;G
-0.99761029	0.044020424	-	ko01100//Metab	GO:0005783//endoplasr
-0.997611079	0.044013154	-	-	-
-0.997612474	0.044000295	ko01100//Metabolic p	-	-
-0.997613023	0.043995235	-	-	-
-0.997614582	0.043980864	-	-	GO:0005737//cytoplasm
-0.997614891	0.043978011	ko01100//Metabolic p	ko03013//Nucle	-
-0.997618265	0.043946886	ko01100//Metabolic p	ko04934//Cushi	-
-0.997619217	0.043938092	ko01100//Metabolic p	ko05200//Pathw	GO:0005886//plasma m
-0.997619388	0.043936519	-	ko01100//Metab	GO:0033178//proton-tr
-0.997622572	0.043907116	-	ko04130//SNAR	GO:0005737//cytoplasm
-0.997622911	0.043903982	-	-	-
-0.997623151	0.043901763	ko01100//Metabolic p	-	-
-0.997623662	0.043897046	ko01100//Metabolic p	-	GO:0005739//mitochon
-0.997624434	0.043889906	ko01100//Metabolic p	-	-
-0.99762637	0.043872012	ko01100//Metabolic p	-	GO:0005802//trans-Gol
-0.997627062	0.043865615	-	-	-
-0.997627391	0.043862577	ko01100//Metabolic p	-	-
-0.997627738	0.043859363	ko01100//Metabolic p	-	GO:0005737//cytoplasm
-0.99762804	0.04385657	ko01100//Metabolic p	-	-
-0.997628807	0.043849477	-	ko04710//Circad	GO:0005634//nucleus;G
-0.997629094	0.04384682	ko01100//Metabolic p	-	GO:0005654//nucleopla
-0.997629205	0.043845792	-	-	GO:0005634//nucleus;G
-0.997630375	0.043834976	-	-	GO:0016020//membran
-0.99763095	0.043829652	ko01110//Biosynthesis	-	-

-0.997631148	0.043827817	ko01100//Metabolic p	ko05200//Pathw	GO:0005737//cytoplasm
-0.997631968	0.043820225	-	-	-
-0.9976324	0.043816229	ko01100//Metabolic p	-	GO:0005737//cytoplasm
-0.997637822	0.043766009	-	-	-
-0.997639652	0.043749047	-	ko04080//Neuro	GO:0009986//cell surfac
-0.997639673	0.043748854	-	ko03010//Ribos	GO:0005634//nucleus;G
-0.997640067	0.043745197	-	-	GO:0005737//cytoplasm
-0.997640302	0.04374302	-	-	GO:0005737//cytoplasm
-0.99764191	0.043728108	-	-	-
-0.997642353	0.043723995	ko01100//Metabolic p	ko01100//Metab	-
-0.997642773	0.043720105	ko01100//Metabolic p	-	-
-0.997642976	0.043718216	ko01100//Metabolic p	ko03320//PPAR	GO:0005783//endoplasr
-0.997643458	0.043713751	-	-	-
-0.997644824	0.043701069	ko01100//Metabolic p	-	-
-0.997646879	0.043681991	-	-	GO:0005634//nucleus;G
-0.997647633	0.043674995	ko01100//Metabolic p	-	GO:0005576//extracellu
-0.997647646	0.043674875	-	ko04550//Signal	GO:0005634//nucleus;G
-0.997647796	0.043673474	-	-	GO:0016021//integral c
-0.997650595	0.043647478	-	-	GO:0005789//endoplasr
-0.997652135	0.043633163	-	ko01100//Metab	-
-0.997652325	0.043631393	ko01100//Metabolic p	ko04723//Retro	GO:0005739//mitochon
-0.99765483	0.043608107	ko01100//Metabolic p	ko01100//Metab	GO:0005576//extracellu
-0.997657874	0.043579781	-	-	GO:0000791//euchroma
-0.997658152	0.043577193	-	-	-
-0.997658175	0.043576977	-	ko05203//Viral c	-
-0.997659838	0.043561502	-	-	GO:0005604//basement
-0.997660388	0.043556377	ko01100//Metabolic p	-	-
-0.997661353	0.043547392	ko01063//Biosynthesis	-	-
-0.997662735	0.043534516	ko01100//Metabolic p	-	-
-0.997663522	0.043527184	ko01100//Metabolic p	ko00983//Drug	-
-0.997663621	0.043526263	-	ko01100//Metab	GO:0005777//peroxison
-0.997663622	0.043526247	-	-	GO:0034361//very-low-
-0.997664538	0.043517711	ko01100//Metabolic p	ko00790//Folate	-
-0.99766567	0.043507165	ko01100//Metabolic p	-	GO:0005634//nucleus;G
-0.99766573	0.043506599	ko01100//Metabolic p	-	-
-0.997665819	0.043505771	-	-	GO:0005737//cytoplasm
-0.997667001	0.043494754	-	-	GO:0005783//endoplasr
-0.997667666	0.043488548	ko01100//Metabolic p	ko05414//Dilate	-
-0.99766826	0.04348301	ko01100//Metabolic p	-	-
-0.997669289	0.043473416	-	ko04151//PI3K-/	GO:0005634//nucleus;G
-0.997670252	0.043464423	-	-	GO:0016020//membran
-0.997670276	0.043464206	-	ko04142//Lysos	GO:0016020//membran
-0.997670748	0.043459799	ko01100//Metabolic p	ko04014//Ras si	-
-0.99767077	0.043459595	ko01100//Metabolic p	ko01100//Metab	GO:0005783//endoplasr
-0.997671969	0.043448399	-	ko01100//Metab	GO:0005739//mitochon
-0.997672481	0.043443624	-	-	-
-0.997674828	0.043421698	ko01100//Metabolic p	-	GO:0016020//membran
-0.99767553	0.043415148	-	-	GO:0031091//platelet al
-0.997676445	0.043406591	-	ko04146//Perox	GO:0005777//peroxison
-0.997676779	0.043403478	ko01100//Metabolic p	-	GO:0005634//nucleus;G
-0.997677078	0.043400677	ko01100//Metabolic p	-	-
-0.997677824	0.043393709	-	-	-
-0.997678117	0.043390965	ko01100//Metabolic p	ko04310//Wnt s	GO:0005634//nucleus;G
-0.997679057	0.043382178	ko01100//Metabolic p	-	-
-0.997683008	0.043345224	-	-	-
-0.997686332	0.043314111	ko01100//Metabolic p	ko04150//mTOF	GO:0005764//lysosome;
-0.997687726	0.043301058	-	ko05200//Pathw	GO:0005634//nucleus;G
-0.997690488	0.043275179	-	ko04360//Axon	-

-0.997691608	0.043264683	-	-	GO:0044424//intracellul
-0.997691612	0.043264637	-	ko05010//Alzhei	GO:0005783//endoplasr
-0.997692355	0.043257677	-	-	-
-0.997693905	0.043243139	-	-	GO:0005829//cytosol;G(
-0.997693961	0.043242615	ko01100//Metabolic p	-	GO:0005634//nucleus;G
-0.997694419	0.043238317	ko01100//Metabolic p	ko04211//Longe	GO:0005634//nucleus
-0.997694546	0.04323713	ko01110//Biosynthesis	-	-
-0.997694991	0.043232953	ko01100//Metabolic p	ko01100//Metak	GO:0005739//mitochon
-0.997695801	0.043225351	ko01100//Metabolic p	ko04141//Protei	GO:0005783//endoplasr
-0.997696367	0.043220044	-	-	-
-0.997697252	0.043211739	-	-	GO:0005634//nucleus;G
-0.997697312	0.043211168	ko01100//Metabolic p	-	GO:0005634//nucleus;G
-0.997699498	0.043190648	ko01100//Metabolic p	-	GO:0043235//receptor c
-0.997702149	0.04316575	-	-	GO:0005811//lipid parti
-0.997702618	0.043161343	ko01100//Metabolic p	-	GO:0016020//membran
-0.997703774	0.043150479	-	-	GO:0005576//extracellu
-0.997705248	0.043136614	-	-	GO:0005794//Golgi app
-0.997705632	0.04313301	-	-	-
-0.997706336	0.043126383	-	-	GO:0005783//endoplasr
-0.997708961	0.043101692	ko01100//Metabolic p	-	GO:0005654//nucleopla
-0.997709648	0.043095226	-	ko01100//Metak	GO:0000506//glycosylpl
-0.997710712	0.043085212	-	-	-
-0.99771096	0.043082872	-	-	-
-0.997711165	0.043080945	ko01110//Biosynthesis	-	-
-0.997711455	0.043078216	ko01100//Metabolic p	ko04310//Wnt s	-
-0.997712127	0.043071891	-	-	GO:0005634//nucleus;G
-0.997713741	0.043056686	ko01063//Biosynthesis	-	-
-0.997713823	0.043055917	ko01100//Metabolic p	ko01100//Metak	GO:0005794//Golgi app
-0.997714496	0.043049579	ko01100//Metabolic p	ko01100//Metak	GO:0005829//cytosol
-0.997714523	0.043049316	-	ko05200//Pathw	GO:0005634//nucleus;G
-0.997714699	0.04304766	ko01100//Metabolic p	-	GO:0005737//cytoplasm
-0.997714794	0.04304677	-	ko01100//Metak	GO:0005640//nuclear ou
-0.997715526	0.043039873	ko01100//Metabolic p	-	-
-0.997719736	0.043000181	ko01100//Metabolic p	-	GO:0005615//extracellu
-0.997719841	0.042999186	ko01110//Biosynthesis	ko01100//Metak	-
-0.997721766	0.042981024	-	-	GO:0005829//cytosol;G(
-0.997725566	0.042945154	-	ko04010//MAPK	GO:0005737//cytoplasm
-0.997726639	0.042935015	ko01100//Metabolic p	-	-
-0.997728786	0.042914727	ko01100//Metabolic p	-	GO:0005794//Golgi app
-0.997728935	0.04291332	ko01100//Metabolic p	-	GO:0005634//nucleus;G
-0.997729689	0.042906192	ko01100//Metabolic p	-	-
-0.997730333	0.042900104	ko01100//Metabolic p	ko01100//Metak	-
-0.997731822	0.042886023	-	-	GO:0005634//nucleus;G
-0.997731866	0.042885609	ko01100//Metabolic p	-	GO:0032991//macromo
-0.997733854	0.0428668	ko01100//Metabolic p	-	GO:0005829//cytosol
-0.997734143	0.042864073	-	ko03320//PPAR	GO:0005783//endoplasr
-0.997734308	0.042862508	-	-	-
-0.997734455	0.042861121	ko01100//Metabolic p	-	-
-0.99773572	0.042849147	ko01100//Metabolic p	-	-
-0.99773594	0.042847061	-	-	-
-0.997736951	0.04283749	-	ko04144//Endoc	GO:0005654//nucleopla
-0.997736993	0.042837092	ko01100//Metabolic p	-	GO:0005737//cytoplasm
-0.997738557	0.042822286	ko01100//Metabolic p	ko01100//Metak	GO:0005737//cytoplasm
-0.997738569	0.042822167	ko01110//Biosynthesis	-	GO:0005789//endoplasr
-0.99774022	0.042806534	ko01100//Metabolic p	-	GO:0005737//cytoplasm
-0.997741203	0.042797214	ko01100//Metabolic p	ko01100//Metak	GO:0016020//membran
-0.99774268	0.042783217	-	-	GO:0000785//chromatir
-0.997743136	0.042778895	-	-	-

-0.997743408	0.042776317	-	ko04144//Endoc	-
-0.997744111	0.042769648	-	ko04080//Neuro	GO:0005886//plasma m
-0.997744754	0.042763548	ko01100//Metabolic p	-	GO:0016021//integral co
-0.997744775	0.042763348	-	-	-
-0.997747174	0.042740592	-	ko01100//Metab	-
-0.997747818	0.042734474	ko01110//Biosynthesis	-	GO:0032991//macromo
-0.997748319	0.04272972	-	ko01100//Metab	-
-0.997748373	0.042729211	-	-	GO:0016020//membran
-0.99775015	0.042712337	ko01100//Metabolic p	-	-
-0.997751095	0.042703361	-	-	GO:0016020//membran
-0.997752565	0.042689404	-	-	GO:0005654//nucleopla
-0.99775295	0.042685739	ko01100//Metabolic p	-	GO:0005759//mitochon
-0.997753199	0.042683373	ko01100//Metabolic p	-	-
-0.997753735	0.042678284	-	-	-
-0.997754171	0.042674136	-	ko04141//Protei	GO:0005783//endoplasr
-0.997755734	0.042659281	-	ko01100//Metab	GO:0005783//endoplasr
-0.997758282	0.042635046	ko01100//Metabolic p	-	GO:0000139//Golgi mer
-0.997760131	0.042617455	-	-	-
-0.997761516	0.042604278	-	ko03013//Nucle	-
-0.997761748	0.042602061	-	-	GO:0005615//extracellu
-0.997762999	0.042590156	ko01100//Metabolic p	-	-
-0.997764596	0.042574946	ko01100//Metabolic p	-	-
-0.997765825	0.042563233	-	-	-
-0.997766373	0.042558015	-	-	-
-0.997766974	0.042552287	-	ko04144//Endoc	GO:0000813//ESCRT I co
-0.997770035	0.042523094	ko01100//Metabolic p	ko04979//Chole	GO:0005737//cytoplasm
-0.997771447	0.042509626	-	-	GO:0005737//cytoplasm
-0.997771475	0.042509356	ko01100//Metabolic p	ko05203//Viral c	-
-0.997771863	0.042505659	ko01100//Metabolic p	ko01100//Metab	GO:0005829//cytosol
-0.997771983	0.042504508	-	ko04080//Neuro	GO:0009986//cell surfac
-0.997776589	0.042460539	ko01100//Metabolic p	-	GO:0005615//extracellu
-0.99777797	0.042447343	ko01063//Biosynthesis	-	GO:0005576//extracellu
-0.997778162	0.042445511	ko01100//Metabolic p	-	-
-0.997778958	0.042437903	-	-	GO:0016021//integral co
-0.997779591	0.04243185	-	ko05205//Protec	GO:0005576//extracellu
-0.997780512	0.04242305	-	-	-
-0.997783297	0.042396419	ko01100//Metabolic p	-	GO:0016020//membran
-0.997784189	0.042387881	ko01100//Metabolic p	-	GO:0005604//basement
-0.997787768	0.042353621	ko01100//Metabolic p	ko03010//Ribos	GO:0005634//nucleus;G
-0.997790694	0.042325588	ko01100//Metabolic p	-	-
-0.997791157	0.042321157	-	-	GO:0031224//intrinsic co
-0.997791159	0.042321133	ko01100//Metabolic p	-	GO:0005783//endoplasr
-0.99779304	0.042303105	ko01100//Metabolic p	ko01100//Metab	GO:0009923//fatty acid
-0.997794056	0.042293366	ko01063//Biosynthesis	ko01100//Metab	-
-0.997794401	0.042290054	-	-	-
-0.997795412	0.042280356	-	-	GO:0005794//Golgi app
-0.997795474	0.042279763	-	-	GO:0005576//extracellu
-0.99779564	0.042278175	-	-	-
-0.997796584	0.042269118	-	ko01100//Metab	GO:0048269//methionir
-0.997798734	0.042248478	ko01100//Metabolic p	-	GO:0005634//nucleus;G
-0.997798776	0.042248077	-	-	GO:0016020//membran
-0.997800912	0.042227567	-	-	GO:0005737//cytoplasm
-0.997801035	0.042226386	ko01100//Metabolic p	ko01100//Metab	GO:0005739//mitochon
-0.99780121	0.042224699	-	-	-
-0.997803606	0.042201682	ko01100//Metabolic p	-	-
-0.997805422	0.04218423	ko01100//Metabolic p	ko04520//Adher	GO:0031252//cell leadir
-0.997806671	0.042172221	-	-	-
-0.997808797	0.042151762	ko01110//Biosynthesis	-	GO:0005634//nucleus;G

-0.997808851	0.042151249	ko01100//Metabolic p	-	GO:0001650//fibrillar ce
-0.997808915	0.042150633	ko01100//Metabolic p	ko03010//Ribos	GO:0005634//nucleus;G
-0.997809125	0.042148607	-	-	-
-0.997810033	0.042139866	ko01110//Biosynthesis	-	GO:0005634//nucleus;G
-0.997812351	0.042117551	-	-	-
-0.997812941	0.042111873	-	ko05135//Yersin	-
-0.997814368	0.042098128	ko01100//Metabolic p	-	GO:0005811//lipid parti
-0.997815575	0.042086494	ko01100//Metabolic p	ko05165//Huma	-
-0.997817113	0.04207167	-	ko05200//Pathw	GO:0005737//cytoplasm
-0.997818411	0.042059158	ko01100//Metabolic p	-	GO:0005788//endoplasm
-0.997818533	0.042057982	ko01100//Metabolic p	-	GO:0005654//nucleopla
-0.997819731	0.042046423	ko01100//Metabolic p	-	-
-0.997820651	0.042037551	ko01100//Metabolic p	-	GO:0005634//nucleus;G
-0.997820718	0.04203691	-	-	-
-0.997820777	0.042036336	ko01110//Biosynthesis	ko04130//SNAR	GO:0005737//cytoplasm
-0.997821327	0.042031026	-	-	GO:0000791//euchroma
-0.997821486	0.042029495	ko01100//Metabolic p	-	-
-0.997823558	0.042009496	-	-	-
-0.997823891	0.042006283	-	ko04010//MAPK	GO:0005737//cytoplasm
-0.997824469	0.042000696	ko01100//Metabolic p	-	-
-0.997827488	0.041971537	-	-	GO:0005829//cytosol;G
-0.997829183	0.041955159	-	-	-
-0.997829818	0.041949019	ko01100//Metabolic p	-	GO:0016021//integral c
-0.997830661	0.041940865	ko01100//Metabolic p	ko04721//Synap	-
-0.997830874	0.041938809	-	-	-
-0.99783152	0.04193256	-	-	GO:0005615//extracellu
-0.997832226	0.04192573	-	ko01100//Metab	GO:0005654//nucleopla
-0.997832505	0.041923031	ko01110//Biosynthesis	-	-
-0.997834518	0.041903551	-	ko04934//Cushi	-
-0.99783469	0.041901884	-	-	GO:0043235//receptor c
-0.99783642	0.041885139	-	-	GO:0000139//Golgi mem
-0.997836636	0.041883049	ko01100//Metabolic p	ko01100//Metab	-
-0.99783665	0.041882914	ko01110//Biosynthesis	ko04211//Longe	GO:0005634//nucleus
-0.99783672	0.041882231	-	-	GO:0005730//nucleolus
-0.997836947	0.041880029	ko01100//Metabolic p	ko03460//Fanco	-
-0.997838113	0.041868736	-	ko04080//Neuro	GO:0016021//integral c
-0.997838709	0.041862969	ko01100//Metabolic p	ko04010//MAPK	-
-0.997839454	0.041855746	-	ko01100//Metab	GO:0005737//cytoplasm
-0.99783953	0.041855014	-	-	-
-0.997840635	0.041844301	ko01100//Metabolic p	ko04210//Apopt	GO:0005634//nucleus;G
-0.997841175	0.041839064	ko01100//Metabolic p	ko01100//Metab	GO:0005737//cytoplasm
-0.997841878	0.041832251	-	-	GO:0016020//membran
-0.99784274	0.041823894	-	ko04310//Wnt s	GO:0005634//nucleus;G
-0.997843638	0.041815188	ko01100//Metabolic p	-	GO:0005634//nucleus;G
-0.997843769	0.041813918	-	-	-
-0.997844024	0.041811445	ko01100//Metabolic p	-	GO:0005737//cytoplasm
-0.997846466	0.04178775	-	-	-
-0.997849267	0.041760553	ko01100//Metabolic p	ko05200//Pathw	GO:0005634//nucleus;G
-0.997849529	0.041758007	ko01100//Metabolic p	ko01100//Metab	GO:0005640//nuclear ou
-0.997849703	0.041756314	-	-	-
-0.997850855	0.041745127	-	-	GO:0097541//axonemal
-0.997851414	0.041739699	-	-	GO:0005634//nucleus;G
-0.997851736	0.041736567	-	-	GO:0005739//mitochon
-0.997851858	0.041735385	-	ko04144//Endoc	GO:0000813//ESCRT I c
-0.997854224	0.041712386	ko01100//Metabolic p	-	GO:0005615//extracellu
-0.997854603	0.041708696	ko01100//Metabolic p	ko04144//Endoc	-
-0.997855901	0.041696071	-	-	GO:0005634//nucleus;G
-0.997855916	0.041695926	-	-	GO:0016021//integral c

-0.997856032	0.041694799	ko01100//Metabolic p	-	-
-0.997857204	0.041683394	-	ko03013//Nucle	-
-0.997857754	0.041678051	-	-	-
-0.997857885	0.04167677	-	-	-
-0.997860612	0.041650222	-	-	-
-0.997861834	0.041638325	ko01100//Metabolic p	ko01100//Metab	-
-0.997862196	0.041634797	ko01100//Metabolic p	-	GO:0005802//trans-Gol
-0.99786253	0.04163154	ko01100//Metabolic p	ko04550//Signal	GO:0005634//nucleus;G
-0.997862842	0.041628502	-	ko03320//PPAR	GO:0005783//endoplasm
-0.997864502	0.041612334	-	-	-
-0.997864949	0.041607968	ko01100//Metabolic p	-	-
-0.997864992	0.041607554	-	ko04142//Lysos	GO:0016020//membran
-0.997865342	0.041604143	ko01100//Metabolic p	-	GO:0005789//endoplasm
-0.997865937	0.041598345	-	ko04151//PI3K-/	GO:0005634//nucleus;G
-0.997866114	0.041596613	ko01100//Metabolic p	ko04923//Regul	GO:0005654//nucleopla
-0.997866174	0.041596027	ko01100//Metabolic p	ko04080//Neuro	GO:0005886//plasma m
-0.997868149	0.041576769	-	-	GO:0005654//nucleopla
-0.997870811	0.041550789	ko01110//Biosynthesis	-	-
-0.997871396	0.041545082	ko01100//Metabolic p	ko01100//Metab	GO:0005634//nucleus;G
-0.997872952	0.041529886	ko01100//Metabolic p	-	GO:0016020//membran
-0.997873499	0.041524546	ko01100//Metabolic p	-	GO:0014069//postsynap
-0.997875229	0.041507646	ko01100//Metabolic p	-	-
-0.997876294	0.041497241	ko01100//Metabolic p	ko04710//Circad	GO:0005634//nucleus;G
-0.997877066	0.041489694	-	ko05010//Alzhei	GO:0005783//endoplasm
-0.997877765	0.041482857	ko01110//Biosynthesis	-	-
-0.997877945	0.041481103	ko01100//Metabolic p	-	-
-0.997878611	0.041474585	ko01100//Metabolic p	-	-
-0.997878635	0.041474354	ko01100//Metabolic p	ko05010//Alzhei	-
-0.997879411	0.041466764	ko01100//Metabolic p	-	GO:0005789//endoplasm
-0.997880733	0.041453833	-	-	GO:0005654//nucleopla
-0.997880756	0.041453609	ko01100//Metabolic p	-	-
-0.99788131	0.041448189	ko01100//Metabolic p	ko04514//Cell ad	GO:0005886//plasma m
-0.997881869	0.041442713	-	-	-
-0.99788229	0.041438598	-	ko01100//Metab	-
-0.997882618	0.04143539	-	-	-
-0.997883365	0.041428077	-	-	GO:0005634//nucleus;G
-0.997883822	0.041423599	-	ko03320//PPAR	GO:0005783//endoplasm
-0.997885086	0.041411224	ko01100//Metabolic p	ko04068//FoxO	GO:0005737//cytoplasm
-0.997888319	0.041379544	-	ko04144//Endoc	GO:0005654//nucleopla
-0.997889425	0.041368707	-	ko03013//Nucle	-
-0.997889895	0.041364094	ko01100//Metabolic p	ko01100//Metab	-
-0.99789235	0.041340021	ko01100//Metabolic p	-	-
-0.997893425	0.041329467	-	-	-
-0.997894214	0.041321726	-	ko01100//Metab	-
-0.997894293	0.041320949	-	-	-
-0.997894415	0.041319756	-	ko04310//Wnt s	GO:0005634//nucleus;G
-0.997894682	0.041317132	ko01100//Metabolic p	ko01100//Metab	GO:0005783//endoplasm
-0.997896592	0.04129838	-	ko01100//Metab	GO:0005654//nucleopla
-0.997896738	0.041296949	-	-	GO:0005634//nucleus;G
-0.997896958	0.041294787	ko01100//Metabolic p	ko04142//Lysos	GO:0016020//membran
-0.997897489	0.041289574	-	-	GO:0005634//nucleus;G
-0.997897588	0.041288596	-	ko01100//Metab	GO:0005829//cytosol
-0.997898575	0.041278903	ko01100//Metabolic p	ko04141//Protei	GO:0005783//endoplasm
-0.997899301	0.041271773	-	ko01100//Metab	-
-0.997899512	0.041269694	ko01100//Metabolic p	ko04922//Gluca	GO:0005634//nucleus;G
-0.99790073	0.041257723	ko01100//Metabolic p	ko05200//Pathw	GO:0005634//nucleus;G
-0.997901213	0.041252972	ko01100//Metabolic p	ko01100//Metab	GO:0005783//endoplasm
-0.997902391	0.041241395	ko01100//Metabolic p	-	GO:0016021//integral c

-0.997902662	0.041238729	ko01100//Metabolic p	-	GO:0044424//intracellul
-0.99790286	0.041236785	-	-	GO:0016020//membran
-0.997904413	0.041221504	ko01100//Metabolic p	ko01100//Metab	GO:0005739//mitochon
-0.997905617	0.041209655	ko01100//Metabolic p	-	-
-0.997906567	0.041200303	ko01100//Metabolic p	-	GO:0005576//extracellu
-0.997906898	0.04119705	-	-	-
-0.9979077	0.041189157	ko01100//Metabolic p	-	GO:0005737//cytoplasm
-0.997908462	0.041181646	-	-	GO:0005634//nucleus;G
-0.997908658	0.041179714	-	-	-
-0.997908854	0.041177792	-	-	GO:0005768//endosome
-0.997909699	0.041169468	ko01100//Metabolic p	-	-
-0.997910609	0.041160503	-	-	-
-0.997911973	0.041147055	-	-	-
-0.997912213	0.041144692	-	ko04150//mTOR	GO:0005764//lysosome;
-0.997913305	0.04113393	ko01100//Metabolic p	ko04530//Tight	GO:0005856//cytoskelet
-0.997915031	0.041116907	ko01100//Metabolic p	-	-
-0.997915815	0.041109166	-	-	-
-0.997915968	0.041107656	-	-	GO:0016020//membran
-0.997916679	0.041100645	-	ko04934//Cushi	GO:0016020//membran
-0.997917628	0.041091276	-	ko04714//Therm	GO:0005811//lipid parti
-0.997917667	0.041090896	-	-	GO:0005737//cytoplasm
-0.997917783	0.041089748	-	-	GO:0005730//nucleolus
-0.997918041	0.041087207	-	-	-
-0.997918422	0.041083444	ko01100//Metabolic p	ko05200//Pathw	GO:0005576//extracellu
-0.997919224	0.041075529	ko01100//Metabolic p	-	-
-0.997919419	0.041073604	ko01100//Metabolic p	-	-
-0.997922557	0.041042605	-	-	-
-0.997923522	0.041033068	ko01100//Metabolic p	-	GO:0005634//nucleus
-0.99792359	0.041032393	ko01100//Metabolic p	-	GO:0031224//intrinsic c
-0.997923891	0.041029417	-	ko01100//Metab	GO:0005783//endoplasm
-0.99792603	0.041008274	-	-	GO:0000785//chromatin
-0.997926917	0.040999495	ko01100//Metabolic p	ko04550//Signal	GO:0005634//nucleus;G
-0.997927682	0.040991924	-	-	GO:0016020//membran
-0.997928412	0.040984708	-	ko01100//Metab	GO:0005794//Golgi app
-0.997931417	0.040954961	-	-	-
-0.997932549	0.040943745	ko01100//Metabolic p	-	GO:0016020//membran
-0.99793407	0.040928678	-	ko04020//Calciu	GO:0005634//nucleus;G
-0.997935999	0.040909558	ko01100//Metabolic p	-	GO:0005739//mitochon
-0.997936489	0.040904705	-	-	-
-0.997936975	0.040899885	ko01110//Biosynthesis	-	-
-0.997938174	0.040887988	-	-	-
-0.997941256	0.040857405	ko01100//Metabolic p	-	GO:0000791//euchroma
-0.997941555	0.04085444	ko01100//Metabolic p	-	GO:0000127//transcript
-0.997941562	0.040854377	-	ko01100//Metab	GO:0016021//integral c
-0.997942149	0.040848541	ko01100//Metabolic p	-	-
-0.997942837	0.040841714	ko01100//Metabolic p	-	-
-0.997943804	0.040832108	-	-	-
-0.997944306	0.040827119	-	-	GO:0005615//extracellu
-0.997944974	0.040820488	ko01100//Metabolic p	ko05203//Viral c	GO:0000786//nucleoson
-0.99794551	0.040815161	ko01100//Metabolic p	-	-
-0.997945604	0.040814232	ko01100//Metabolic p	-	GO:0016020//membran
-0.997946214	0.040808164	-	ko04310//Wnt s	-
-0.997946715	0.040803185	ko01100//Metabolic p	-	-
-0.997947776	0.04079264	-	ko04080//Neuro	GO:0005886//plasma m
-0.997948598	0.040784463	ko01100//Metabolic p	-	GO:0005634//nucleus;G
-0.997949534	0.040775157	-	ko04060//Cytok	GO:0005576//extracellu
-0.997949687	0.040773633	ko01100//Metabolic p	-	-
-0.997950013	0.040770394	-	-	GO:0005788//endoplasm

-0.997950091	0.040769621	-	-	GO:0005737//cytoplasm
-0.997950737	0.040763186	ko01110//Biosynthesis	-	GO:0005654//nucleopla
-0.997955036	0.040720399	-	-	GO:0005764//lysosome;
-0.997955458	0.04071619	-	-	GO:0032991//macromo
-0.997955741	0.040713369	ko01063//Biosynthesis	-	GO:0031462//Cul2-RIN(
-0.997955749	0.040713294	-	ko01100//Metab	-
-0.997956137	0.040709428	-	ko01100//Metab	-
-0.997956334	0.040707468	ko01110//Biosynthesis	ko05200//Pathw	GO:0000307//cyclin-dep
-0.997958453	0.040686346	-	-	GO:0005737//cytoplasm
-0.997958517	0.04068571	ko01100//Metabolic p	ko03320//PPAR	GO:0005576//extracellu
-0.997959326	0.040677642	ko01100//Metabolic p	-	-
-0.997959447	0.040676437	ko01100//Metabolic p	ko05200//Pathw	GO:0005737//cytoplasm
-0.997959486	0.040676053	ko01110//Biosynthesis	-	GO:0005634//nucleus;G
-0.997959533	0.040675582	ko01063//Biosynthesis	-	-
-0.997960833	0.040662617	-	-	-
-0.997962483	0.040646161	ko01100//Metabolic p	ko05200//Pathw	GO:0005634//nucleus
-0.997962932	0.040641677	ko01100//Metabolic p	-	-
-0.997963642	0.040634592	ko01100//Metabolic p	ko04514//Cell ad	-
-0.997964872	0.040622311	-	-	-
-0.99796779	0.040593175	-	-	GO:0005634//nucleus;G
-0.997968848	0.0405826	ko01100//Metabolic p	-	-
-0.997969609	0.040574998	ko01100//Metabolic p	ko04742//Taste	GO:0016020//membran
-0.997969957	0.040571512	ko01100//Metabolic p	-	-
-0.997970346	0.040567628	ko01100//Metabolic p	-	-
-0.997971679	0.040554301	ko01100//Metabolic p	-	-
-0.997972861	0.040542475	-	-	GO:0005737//cytoplasm
-0.997974886	0.040522219	-	-	GO:0005886//plasma m
-0.997975033	0.040520743	-	-	-
-0.997975118	0.040519897	ko01100//Metabolic p	-	-
-0.997975639	0.040514679	ko01100//Metabolic p	-	-
-0.997977124	0.040499814	-	ko04144//Endoc	GO:0000813//ESCRT I co
-0.997978031	0.040490731	-	-	-
-0.997978604	0.040484989	ko01100//Metabolic p	-	-
-0.997978736	0.040483661	ko01110//Biosynthesis	ko01100//Metab	GO:0005737//cytoplasm
-0.997980653	0.040464456	ko01110//Biosynthesis	-	-
-0.997981026	0.040460718	-	-	-
-0.997982188	0.040449068	-	ko04210//Apopt	GO:0005634//nucleus;G
-0.997984383	0.04042705	-	ko04020//Calciu	GO:0016020//membran
-0.997985237	0.040418485	ko01100//Metabolic p	-	GO:0005576//extracellu
-0.997988985	0.040380861	-	ko04060//Cytok	-
-0.997993415	0.040336343	-	-	GO:0030659//cytoplasm
-0.997993488	0.040335608	-	-	-
-0.997994493	0.040325508	ko01100//Metabolic p	-	-
-0.997994758	0.040322837	ko01100//Metabolic p	-	-
-0.997996016	0.040310187	-	-	-
-0.997998155	0.040288657	ko01063//Biosynthesis	-	GO:0005730//nucleolus
-0.997998825	0.040281918	ko01100//Metabolic p	ko01100//Metab	GO:0005783//endoplasm
-0.997999853	0.040271562	-	ko05152//Tuber	GO:0009897//external s
-0.998001739	0.040252566	ko01110//Biosynthesis	-	-
-0.998002175	0.040248175	-	-	-
-0.998002503	0.040244867	-	-	-
-0.998007481	0.040194676	-	ko02010//ABC tr	GO:0016021//integral co
-0.998008405	0.04018535	-	ko01100//Metab	GO:0005783//endoplasm
-0.998009481	0.040174484	ko01110//Biosynthesis	-	GO:0016020//membran
-0.99801112	0.040157941	-	-	GO:0000781//chromoso
-0.998012246	0.040146564	ko01100//Metabolic p	-	-
-0.998013521	0.040133685	-	ko01100//Metab	GO:0009923//fatty acid
-0.998013953	0.040129314	-	-	GO:0014069//postsynap

-0.99801403	0.040128538	-	ko01100//Metab	-
-0.99801509	0.04011783	-	-	GO:0005604//basement
-0.998015148	0.040117236	-	-	-
-0.998015383	0.04011486	ko01100//Metabolic p	-	GO:0005783//endoplasr
-0.998015419	0.040114496	ko01100//Metabolic p	ko04141//Protei	GO:0005634//nucleus;G
-0.998016332	0.040105272	ko01100//Metabolic p	-	-
-0.998016346	0.040105127	-	ko01100//Metab	GO:0016020//membran
-0.998017382	0.040094649	-	-	-
-0.998017467	0.040093794	-	ko04080//Neuro	GO:0016021//integral co
-0.998018391	0.040084446	-	ko01100//Metab	GO:0005783//endoplasr
-0.998018917	0.040079121	-	ko05010//Alzhei	-
-0.998019286	0.04007539	ko01100//Metabolic p	-	-
-0.998020903	0.040059014	ko01063//Biosynthesis	ko05135//Yersin	-
-0.998021072	0.040057308	-	-	GO:0016020//membran
-0.998021687	0.040051081	-	-	GO:0005783//endoplasr
-0.998022565	0.04004219	ko01100//Metabolic p	-	-
-0.99802268	0.040041025	ko01100//Metabolic p	-	GO:0000779//condense
-0.998024213	0.040025492	ko01100//Metabolic p	-	-
-0.998024417	0.040023429	ko01100//Metabolic p	-	-
-0.998025465	0.040012808	-	-	-
-0.998025937	0.040008025	ko01100//Metabolic p	-	GO:0005634//nucleus;G
-0.998026086	0.040006511	ko01100//Metabolic p	-	GO:0005886//plasma m
-0.998026284	0.040004509	ko01100//Metabolic p	-	-
-0.998028072	0.039986378	-	-	GO:0005576//extracellu
-0.998028953	0.039977443	-	-	-
-0.998032882	0.039937563	-	-	GO:0005794//Golgi app
-0.99803345	0.039931797	ko01100//Metabolic p	-	GO:0005634//nucleus;G
-0.998033745	0.039928797	-	-	-
-0.998034899	0.039917075	-	ko05165//Huma	-
-0.998037136	0.039894341	ko01100//Metabolic p	-	GO:0005634//nucleus;G
-0.998037307	0.039892604	-	-	-
-0.998037511	0.039890532	ko01063//Biosynthesis	-	GO:0005794//Golgi app
-0.998037825	0.03988734	ko01100//Metabolic p	-	-
-0.998038423	0.039881258	-	-	-
-0.998038782	0.039877603	ko01100//Metabolic p	-	-
-0.998039366	0.039871662	ko01100//Metabolic p	ko04360//Axon	GO:0005886//plasma m
-0.99803938	0.039871521	-	-	-
-0.998040014	0.039865074	-	ko04934//Cushir	GO:0016020//membran
-0.998040033	0.039864876	-	-	-
-0.998040335	0.039861809	-	ko04310//Wnt s	GO:0005634//nucleus;G
-0.99804055	0.039859618	ko01100//Metabolic p	-	-
-0.998040592	0.039859191	-	ko01100//Metab	GO:0005741//mitochon
-0.998041608	0.039848858	ko01100//Metabolic p	-	GO:0031464//Cul4A-RII
-0.998042643	0.03983832	-	ko04141//Protei	GO:0005737//cytoplasm
-0.998043037	0.039834308	-	-	GO:0000127//transcript
-0.998043335	0.039831277	-	-	-
-0.998046309	0.039800985	ko01100//Metabolic p	-	GO:0005730//nucleolus
-0.998046331	0.039800759	ko01100//Metabolic p	-	-
-0.998047315	0.03979073	ko01100//Metabolic p	ko01100//Metab	GO:0005783//endoplasr
-0.998048724	0.039776372	-	-	-
-0.99804983	0.039765085	-	ko01100//Metab	-
-0.99805159	0.03974714	ko01100//Metabolic p	-	-
-0.998051807	0.039744919	-	-	-
-0.998051833	0.039744652	-	-	-
-0.998052384	0.039739038	ko01063//Biosynthesis	ko05205//Proteo	GO:0005576//extracellu
-0.998052955	0.039733203	-	-	GO:0005576//extracellu
-0.998053901	0.039723545	-	-	GO:0005576//extracellu
-0.998056711	0.039694846	ko01063//Biosynthesis	-	GO:0005829//cytosol;G(

-0.998056838	0.03969355	-	ko04010//MAPK	-
-0.998057713	0.039684607	ko01100//Metabolic p	-	-
-0.998058023	0.039681445	ko01100//Metabolic p	ko04742//Taste	GO:0005783//endoplasr
-0.998059002	0.039671439	-	ko01100//Metab	-
-0.998060543	0.039655679	ko01100//Metabolic p	ko01100//Metab	GO:0005654//nucleopla
-0.998060831	0.039652736	ko01100//Metabolic p	-	GO:0005634//nucleus;G
-0.998061821	0.039642612	ko01100//Metabolic p	ko01100//Metab	GO:0005737//cytoplasm
-0.998061926	0.039641539	-	-	-
-0.998063589	0.039624522	ko01100//Metabolic p	ko01100//Metab	GO:0005783//endoplasr
-0.998063792	0.039622441	-	ko05142//Chaga	GO:0016020//membran
-0.998064953	0.039610559	-	-	-
-0.998065805	0.039601833	-	-	GO:0016020//membran
-0.99806901	0.039568999	ko01100//Metabolic p	-	-
-0.998070556	0.039553144	ko01100//Metabolic p	ko04060//Cytok	GO:0005576//extracellu
-0.998071002	0.039548576	-	-	GO:0031464//Cul4A-RII
-0.998071177	0.039546784	-	ko01100//Metab	-
-0.998071401	0.039544482	ko01100//Metabolic p	-	-
-0.998071461	0.039543867	ko01100//Metabolic p	-	-
-0.998071568	0.039542774	-	-	-
-0.998073129	0.039526761	-	ko01100//Metab	GO:0005794//Golgi app
-0.998074096	0.03951684	-	-	GO:0005634//nucleus;G
-0.998074272	0.039515028	ko01100//Metabolic p	-	-
-0.998074286	0.039514886	-	ko01100//Metab	GO:0005777//peroxison
-0.998074488	0.039512816	ko01063//Biosynthesis	-	-
-0.998074895	0.039508632	ko01100//Metabolic p	-	-
-0.998076688	0.039490224	-	-	-
-0.998076741	0.03948968	ko01100//Metabolic p	ko01100//Metab	-
-0.998076861	0.039488445	-	-	GO:0005576//extracellu
-0.998078163	0.039475076	ko01100//Metabolic p	-	GO:0097541//axonemal
-0.998079211	0.039464311	-	-	-
-0.998079744	0.039458829	ko01110//Biosynthesis	-	GO:0016020//membran
-0.998081332	0.039442503	-	-	GO:0005794//Golgi app
-0.998082653	0.039428926	ko01100//Metabolic p	ko04151//PI3K-/	GO:0005634//nucleus;G
-0.998085187	0.03940285	-	-	GO:0005768//endosom
-0.998085495	0.039399675	ko01100//Metabolic p	ko05203//Viral c	GO:0000786//nucleosor
-0.998085567	0.039398941	-	-	-
-0.998085796	0.03939658	ko01100//Metabolic p	ko03013//Nucle	-
-0.998085938	0.039395115	-	ko01100//Metab	GO:0005576//extracellu
-0.998086733	0.039386938	ko01100//Metabolic p	ko03320//PPAR	GO:0005576//extracellu
-0.998088163	0.039372205	-	-	GO:0005829//cytosol;G
-0.998088582	0.039367889	-	ko04068//FoxO	GO:0005737//cytoplasm
-0.998090118	0.039352066	ko01100//Metabolic p	ko05200//Pathw	GO:0000307//cyclin-de
-0.998091358	0.03933928	-	ko01100//Metab	-
-0.998092363	0.039328921	-	-	GO:0000139//Golgi mer
-0.998092649	0.039325976	-	ko04080//Neuro	GO:0005886//plasma m
-0.998093165	0.03932065	ko01100//Metabolic p	-	GO:0005634//nucleus;G
-0.998093339	0.039318862	-	-	-
-0.99809439	0.039308015	ko01100//Metabolic p	-	-
-0.998096361	0.039287681	ko01100//Metabolic p	-	GO:0005654//nucleopla
-0.998097344	0.039277524	-	ko04068//FoxO	GO:0005737//cytoplasm
-0.998098153	0.039269179	ko01100//Metabolic p	ko01100//Metab	-
-0.998098268	0.039267983	-	ko04010//MAPK	-
-0.998098298	0.039267676	-	ko04310//Wnt s	GO:0005634//nucleus;G
-0.9980998	0.03925216	ko01100//Metabolic p	ko05203//Viral c	GO:0000786//nucleosor
-0.998099925	0.039250871	-	-	-
-0.998100034	0.039249739	ko01100//Metabolic p	-	-
-0.998101128	0.039238443	ko01100//Metabolic p	-	GO:0005886//plasma m
-0.998101183	0.039237872	-	ko04146//Perox	GO:0005765//lysosomal

-0.998101774	0.039231761	-	-	GO:0005829//cytosol;G(
-0.998102099	0.039228399	-	-	GO:0005737//cytoplasm
-0.998103678	0.039212073	ko01100//Metabolic p	-	-
-0.998104592	0.039202624	-	ko04710//Circac	GO:0005634//nucleus;G
-0.998104654	0.039201984	-	ko05200//Pathw	GO:0005576//extracellu
-0.998104791	0.039200563	ko01100//Metabolic p	ko01100//Metak	-
-0.998106151	0.039186485	-	-	-
-0.998106672	0.039181101	-	-	-
-0.998107223	0.039175392	ko01100//Metabolic p	ko01100//Metak	GO:0005789//endoplasr
-0.998107686	0.039170602	-	-	-
-0.998108631	0.039160819	-	ko05202//Transc	GO:0000786//nucleosor
-0.998109003	0.039156968	-	-	GO:0005576//extracellu
-0.998111423	0.03913189	-	-	-
-0.998114381	0.039101223	ko01100//Metabolic p	ko01100//Metak	-
-0.998115728	0.039087247	ko01100//Metabolic p	-	-
-0.998115742	0.039087108	ko01100//Metabolic p	-	GO:0001669//acrosoma
-0.998117644	0.039067365	ko01100//Metabolic p	-	GO:0016020//membran
-0.99811854	0.039058067	ko01100//Metabolic p	-	-
-0.99811872	0.039056201	-	ko04150//mTOF	GO:0032991//macromo
-0.998119702	0.039046	ko01100//Metabolic p	ko04140//Autop	GO:0000407//pre-autor
-0.99811985	0.039044457	-	-	-
-0.99812068	0.03903584	ko01100//Metabolic p	ko01100//Metak	-
-0.99812121	0.039030329	-	-	GO:0005802//trans-Gol
-0.998121546	0.03902684	ko01100//Metabolic p	-	-
-0.998121805	0.039024152	ko01100//Metabolic p	-	GO:0036126//sperm fla
-0.998122065	0.039021446	-	-	GO:0005737//cytoplasm
-0.998124493	0.038996207	ko01063//Biosynthesis	-	-
-0.998124504	0.038996092	-	-	-
-0.998124788	0.03899314	ko01100//Metabolic p	-	-
-0.998125106	0.038989835	ko01100//Metabolic p	ko04216//Ferrop	GO:0016020//membran
-0.998128575	0.038953734	-	-	-
-0.998128644	0.038953015	-	ko04020//Calciu	GO:0016020//membran
-0.998129546	0.038943621	ko01100//Metabolic p	-	GO:0097541//axonemal
-0.99813088	0.038929731	-	ko01100//Metak	GO:0005789//endoplasr
-0.99813229	0.038915038	-	-	-
-0.998132916	0.038908513	ko01100//Metabolic p	-	-
-0.998133428	0.03890318	ko01100//Metabolic p	-	-
-0.99813381	0.038899195	-	-	GO:0005654//nucleopla
-0.998134907	0.038887754	ko01100//Metabolic p	-	GO:0005576//extracellu
-0.998135184	0.038884865	ko01110//Biosynthesis	ko01100//Metak	-
-0.998135535	0.038881205	-	-	-
-0.998136146	0.03887483	ko01100//Metabolic p	-	-
-0.998136522	0.038870915	-	-	GO:0005886//plasma m
-0.998137044	0.038865466	-	ko01100//Metak	GO:0005615//extracellu
-0.998138427	0.038851034	ko01100//Metabolic p	-	-
-0.99814086	0.038825629	-	-	GO:0005783//endoplasr
-0.998141382	0.038820177	-	-	-
-0.998141687	0.038816989	ko01100//Metabolic p	ko01100//Metak	GO:0005778//peroxisom
-0.99814201	0.038813615	-	ko05152//Tuber	GO:0009897//external s
-0.99814225	0.038811105	-	-	GO:0000127//transcript
-0.998142444	0.038809075	ko01100//Metabolic p	-	GO:0005737//cytoplasm
-0.998143632	0.038796659	ko01100//Metabolic p	ko04714//Therm	GO:0005811//lipid parti
-0.998144158	0.038791161	ko01110//Biosynthesis	-	-
-0.998145692	0.038775123	ko01100//Metabolic p	-	-
-0.998146849	0.038763017	ko01100//Metabolic p	ko01100//Metak	GO:0005654//nucleopla
-0.998147054	0.038760881	ko01100//Metabolic p	-	GO:0005737//cytoplasm
-0.998147545	0.03875574	ko01063//Biosynthesis	-	GO:0031091//platelet al
-0.998147566	0.038755516	ko01100//Metabolic p	ko01100//Metak	GO:0005783//endoplasr

-0.998148354	0.038747277	-	-	GO:0005886//plasma m
-0.9981506	0.038723753	-	ko05202//Transc	GO:0000786//nucleosor
-0.998151029	0.038719264	-	ko04141//Protei	GO:0005634//nucleus;G
-0.998151032	0.038719234	ko01100//Metabolic p	-	GO:0032991//macromo
-0.998151643	0.038712832	-	-	GO:0005886//plasma m
-0.99815302	0.038698411	ko01100//Metabolic p	-	GO:0005737//cytoplasm
-0.998154225	0.038685775	-	-	-
-0.998154305	0.038684941	ko01100//Metabolic p	-	-
-0.998156224	0.038664813	-	-	-
-0.998156559	0.038661301	ko01100//Metabolic p	ko05016//Huntin	GO:0005737//cytoplasm
-0.998157205	0.038654524	ko01100//Metabolic p	ko05322//Systen	GO:0000786//nucleosor
-0.998157904	0.038647187	-	-	GO:0005783//endoplasn
-0.998159164	0.038633969	ko01110//Biosynthesis	-	-
-0.998160161	0.038623496	-	-	-
-0.998160228	0.0386228	ko01100//Metabolic p	-	GO:0005794//Golgi app
-0.998160563	0.038619282	ko01100//Metabolic p	-	-
-0.998160691	0.038617937	ko01100//Metabolic p	-	-
-0.998161991	0.038604285	-	-	-
-0.998163245	0.038591107	ko01063//Biosynthesis	-	GO:0000791//euchroma
-0.998163294	0.03859059	-	ko03320//PPAR	GO:0005615//extracellu
-0.99816352	0.038588217	-	-	GO:0005802//trans-Gol
-0.998163595	0.038587426	-	ko04080//Neuro	GO:0005886//plasma m
-0.998163602	0.038587354	-	-	GO:0000791//euchroma
-0.998163893	0.0385843	ko01110//Biosynthesis	-	-
-0.998163997	0.038583202	-	-	GO:0016020//membran
-0.998165155	0.038571031	ko01100//Metabolic p	-	-
-0.998167813	0.038543072	ko01100//Metabolic p	ko01100//Metab	-
-0.998167875	0.038542423	-	ko01100//Metab	-
-0.998168374	0.038537168	ko01100//Metabolic p	ko03320//PPAR	-
-0.99816884	0.038532264	ko01100//Metabolic p	-	-
-0.998169229	0.038528177	-	-	-
-0.998169457	0.038525773	-	-	-
-0.998169695	0.038523265	-	ko04120//Ubiqu	GO:0005680//anaphase
-0.998170248	0.03851745	ko01100//Metabolic p	ko01100//Metab	-
-0.998172129	0.038497632	-	-	GO:0005576//extracellu
-0.998172526	0.038493455	ko01110//Biosynthesis	-	GO:0031464//Cul4A-RIN
-0.998173608	0.038482058	-	ko01100//Metab	GO:0005654//nucleopla
-0.998174445	0.038473233	-	-	-
-0.998177188	0.038444314	ko01100//Metabolic p	-	-
-0.99817796	0.03843616	-	-	-
-0.998179833	0.0384164	-	ko05231//Cholin	GO:0005739//mitochon
-0.998181695	0.038396736	-	ko05016//Huntin	GO:0005737//cytoplasm
-0.998182168	0.038391742	ko01100//Metabolic p	-	-
-0.998182728	0.038385821	ko01063//Biosynthesis	-	-
-0.998183216	0.038380674	-	ko04146//Perox	GO:0005765//lysosomal
-0.998183971	0.038372688	ko01100//Metabolic p	-	-
-0.998184202	0.038370245	-	-	-
-0.998187799	0.038332217	ko01100//Metabolic p	-	-
-0.998188981	0.038319707	-	-	-
-0.998190162	0.038307203	-	-	-
-0.998190705	0.038301454	-	-	GO:0097541//axonemal
-0.998193767	0.038269021	-	-	-
-0.998196794	0.038236931	ko01100//Metabolic p	-	-
-0.998197813	0.038226122	ko01100//Metabolic p	ko01100//Metab	GO:0005777//peroxison
-0.998201096	0.038191285	ko01100//Metabolic p	-	GO:0005737//cytoplasm
-0.99820235	0.038177964	ko01100//Metabolic p	ko01100//Metab	GO:0016020//membran
-0.998202568	0.038175653	ko01063//Biosynthesis	-	-
-0.998204619	0.038153854	-	-	-

-0.998205262	0.038147021	-	ko05231//Cholir	-
-0.998206357	0.038135383	ko01100//Metabolic p	ko01100//Metak	GO:0005654//nucleopla
-0.998211796	0.038077501	-	ko01100//Metak	-
-0.998212867	0.038066093	-	-	-
-0.998213121	0.038063378	-	ko04141//Protei	GO:0005737//cytoplasm
-0.998213256	0.038061945	ko01100//Metabolic p	-	-
-0.998216937	0.03802271	-	-	GO:0005634//nucleus;G
-0.998216985	0.038022196	-	ko01100//Metak	-
-0.998217327	0.038018545	-	-	GO:0005829//cytosol;G(
-0.998217406	0.038017698	ko01100//Metabolic p	ko04020//Calciu	GO:0001518//voltage-g
-0.998217691	0.038014666	ko01100//Metabolic p	-	GO:0005654//nucleopla
-0.998218402	0.038007074	-	-	GO:0005737//cytoplasm
-0.998218718	0.038003708	ko01100//Metabolic p	-	-
-0.99821885	0.038002302	-	ko04146//Peroxi	GO:0005765//lysosomal
-0.998220949	0.037979897	ko01100//Metabolic p	ko05012//Parkin	GO:0005654//nucleopla
-0.998221161	0.037977629	ko01100//Metabolic p	-	GO:0001725//stress fibe
-0.998222501	0.037963315	ko01100//Metabolic p	ko04723//Retro(	GO:0005739//mitochon
-0.998222727	0.0379609	-	-	GO:0005887//integral c(
-0.998223191	0.037955946	-	ko05412//Arrhyt	GO:0005886//plasma m
-0.998223937	0.037947975	-	-	-
-0.998224403	0.037942998	ko01100//Metabolic p	-	GO:0005829//cytosol;G(
-0.998225274	0.037933689	ko01100//Metabolic p	-	GO:0000139//Golgi mer
-0.998225537	0.037930875	-	ko01100//Metak	GO:0005654//nucleopla
-0.998227018	0.03791504	ko01100//Metabolic p	ko05200//Pathw	GO:0000785//chromatir
-0.998228288	0.037901455	-	-	-
-0.998229092	0.03789285	-	ko04210//Apopt	GO:0005634//nucleus;G
-0.998229311	0.037890507	-	-	GO:0043235//receptor (
-0.998229793	0.037885343	-	-	GO:0005576//extracellu
-0.998230114	0.037881904	ko01100//Metabolic p	ko01100//Metak	GO:0005789//endoplasr
-0.998230998	0.037872443	ko01100//Metabolic p	ko04310//Wnt s	GO:0005634//nucleus;G
-0.998231455	0.037867548	-	-	GO:0016020//membran
-0.998233356	0.037847184	ko01100//Metabolic p	-	-
-0.998234569	0.037834188	ko01100//Metabolic p	ko04150//mTOF	GO:0032991//macromo
-0.998234879	0.037830863	ko01100//Metabolic p	-	GO:0005576//extracellu
-0.998235783	0.03782117	-	-	-
-0.998236004	0.037818803	ko01100//Metabolic p	-	GO:0045095//keratin fila
-0.99823745	0.037803294	ko01100//Metabolic p	-	GO:0005886//plasma m
-0.998238684	0.037790054	ko01100//Metabolic p	ko04010//MAPK	-
-0.998238739	0.037789466	-	-	-
-0.998239216	0.037784342	-	-	-
-0.9982403	0.037772712	-	ko01100//Metak	-
-0.998241724	0.037757424	-	-	-
-0.998242045	0.03775397	-	-	-
-0.9982421	0.037753378	-	-	-
-0.998242967	0.037744069	ko01063//Biosynthesis	-	-
-0.998243534	0.037737974	-	-	GO:0005634//nucleus;G
-0.99824386	0.037734466	ko01100//Metabolic p	-	GO:0005829//cytosol;G(
-0.998244104	0.037731844	ko01110//Biosynthesis	-	-
-0.998245377	0.037718163	-	-	-
-0.998246659	0.037704377	-	-	-
-0.998248373	0.037685942	-	-	-
-0.998248383	0.037685832	ko01100//Metabolic p	-	GO:0005829//cytosol;G(
-0.99824851	0.037684462	-	-	-
-0.998249435	0.037674515	-	-	-
-0.998249572	0.037673031	ko01100//Metabolic p	ko04514//Cell a(	GO:0005769//early end(
-0.998250081	0.03766756	-	-	GO:0005794//Golgi app
-0.99825044	0.037663693	-	ko01100//Metak	GO:0005634//nucleus;G
-0.998251935	0.037647592	ko01100//Metabolic p	ko01100//Metak	-

-0.998254209	0.037623086	ko01110//Biosynthesis	ko01100//Metab	-
-0.998256023	0.037603536	ko01100//Metabolic p	-	-
-0.998256604	0.037597262	-	-	GO:0005829//cytosol;G(
-0.998257272	0.037590059	-	-	GO:0005783//endoplasr
-0.998258803	0.03757354	ko01100//Metabolic p	ko04310//Wnt s	GO:0005634//nucleus;G
-0.998258844	0.037573096	-	-	GO:0014069//postsynap
-0.99825941	0.037566988	ko01100//Metabolic p	ko01100//Metab	GO:0005829//cytosol
-0.998259606	0.037564875	ko01100//Metabolic p	ko01100//Metab	GO:0005739//mitochon
-0.998259663	0.037564257	ko01100//Metabolic p	-	GO:0000127//transcript
-0.998259847	0.037562271	-	-	-
-0.99826013	0.037559217	ko01100//Metabolic p	-	-
-0.998260208	0.03755837	-	ko01100//Metab	GO:0005737//cytoplasm
-0.998261025	0.037549554	ko01100//Metabolic p	ko04979//Chole	GO:0005737//cytoplasm
-0.998261632	0.037542992	ko01100//Metabolic p	ko04120//Ubiqu	GO:0005680//anaphase
-0.998262465	0.037534	ko01100//Metabolic p	ko04080//Neuro	GO:0005886//plasma m
-0.998262621	0.03753231	ko01110//Biosynthesis	-	-
-0.998263492	0.037522904	-	ko05010//Alzhei	-
-0.998264369	0.03751342	-	ko01100//Metab	GO:0016020//membran
-0.998264473	0.037512295	ko01100//Metabolic p	-	GO:0000139//Golgi mer
-0.998266254	0.037493041	-	ko04010//MAPK	-
-0.998266302	0.037492522	-	-	GO:0005576//extracellu
-0.998266424	0.037491197	-	-	GO:0005576//extracellu
-0.998267706	0.037477332	-	-	GO:0005794//Golgi app
-0.99827004	0.03745207	ko01100//Metabolic p	-	GO:0031966//mitochon
-0.998270079	0.037451643	ko01100//Metabolic p	ko05231//Cholir	-
-0.998270107	0.037451341	ko01100//Metabolic p	ko01100//Metab	GO:0005777//peroxison
-0.998270756	0.037444316	-	-	-
-0.998270944	0.037442275	ko01100//Metabolic p	-	-
-0.998271426	0.037437052	ko01100//Metabolic p	-	GO:0005654//nucleopla
-0.998272003	0.03743081	ko01110//Biosynthesis	-	GO:0005737//cytoplasm
-0.998272249	0.037428144	ko01100//Metabolic p	-	-
-0.998272273	0.037427882	ko01100//Metabolic p	-	GO:0016021//integral co
-0.998273026	0.03741972	ko01110//Biosynthesis	-	-
-0.998274584	0.037402829	-	-	GO:0005576//extracellu
-0.998277842	0.037367494	ko01110//Biosynthesis	-	-
-0.99827898	0.037355139	ko01100//Metabolic p	-	-
-0.998278991	0.037355019	-	ko03320//PPAR	GO:0005615//extracellu
-0.99827921	0.037352647	-	-	GO:0005802//trans-Gol
-0.998279638	0.037347995	-	ko01100//Metab	GO:0005737//cytoplasm
-0.998280297	0.037340837	-	ko01100//Metab	GO:0005794//Golgi app
-0.998280468	0.037338982	ko01100//Metabolic p	-	GO:0005737//cytoplasm
-0.998281418	0.037328663	-	ko00982//Drug	-
-0.998282548	0.037316393	ko01100//Metabolic p	-	GO:0032991//macromo
-0.998283339	0.037307789	-	-	GO:0005737//cytoplasm
-0.998286027	0.037278567	ko01100//Metabolic p	-	-
-0.998286859	0.037269515	-	-	-
-0.998288408	0.037252656	-	-	-
-0.99828896	0.037246649	ko01100//Metabolic p	-	GO:0005794//Golgi app
-0.998290465	0.037230256	-	ko05203//Viral c	GO:0000786//nucleosor
-0.99829059	0.037228897	ko01100//Metabolic p	-	-
-0.998290609	0.037228687	-	ko01100//Metab	-
-0.998290712	0.037227567	-	-	GO:0005794//Golgi app
-0.998290884	0.037225695	ko01100//Metabolic p	ko01100//Metab	GO:0005576//extracellu
-0.998291301	0.03722115	-	-	-
-0.998291432	0.037219717	ko01100//Metabolic p	ko04141//Protei	GO:0005737//cytoplasm
-0.998291554	0.037218394	ko01100//Metabolic p	-	GO:0005576//extracellu
-0.998292411	0.037209052	ko01100//Metabolic p	-	-
-0.99829274	0.03720547	-	ko01100//Metab	GO:0005783//endoplasr

-0.998293113	0.037201399	ko01100//Metabolic p	-	-
-0.998293478	0.037197422	ko01063//Biosynthesis	-	GO:0016020//membran
-0.998293645	0.037195607	ko01100//Metabolic p	-	-
-0.998293756	0.037194387	ko01100//Metabolic p	ko05231//Cholir	GO:0005739//mitochon
-0.998294182	0.037189746	ko01110//Biosynthesis	-	-
-0.998295203	0.037178609	-	ko04060//Cytok	GO:0005576//extracellu
-0.998295252	0.037178073	-	ko04020//Calciu	GO:0016020//membran
-0.998296138	0.037168411	ko01100//Metabolic p	ko04020//Calciu	GO:0016020//membran
-0.998297167	0.03715719	ko01100//Metabolic p	-	GO:0005737//cytoplasm
-0.998297449	0.037154109	ko01100//Metabolic p	-	GO:0016021//integral co
-0.998299727	0.03712924	ko01100//Metabolic p	-	-
-0.998302743	0.037096278	-	-	GO:0016020//membran
-0.998304237	0.037079949	ko01100//Metabolic p	-	GO:0005783//endoplasr
-0.998304509	0.037076969	ko01100//Metabolic p	-	-
-0.998305574	0.037065319	-	ko04120//Ubiqu	GO:0005680//anaphase
-0.998310272	0.037013892	ko01100//Metabolic p	ko05016//Huntir	-
-0.998310857	0.037007472	ko01100//Metabolic p	ko04068//FoxO	GO:0005737//cytoplasm
-0.998311247	0.037003207	ko01110//Biosynthesis	-	GO:0005737//cytoplasm
-0.998312236	0.036992358	-	-	GO:0031464//Cul4A-RII
-0.99831238	0.036990784	-	-	GO:0005654//nucleopla
-0.998313536	0.036978111	ko01100//Metabolic p	-	GO:0005737//cytoplasm
-0.998313756	0.036975702	-	ko01100//Metak	-
-0.998314188	0.036970956	ko01100//Metabolic p	-	GO:0005654//nucleopla
-0.998314399	0.036968643	-	-	-
-0.998314878	0.036963388	ko01100//Metabolic p	-	-
-0.998318642	0.036922078	ko01100//Metabolic p	-	-
-0.998318826	0.036920052	-	ko01100//Metak	GO:0005739//mitochon
-0.998319041	0.036917688	-	-	GO:0001669//acrosoma
-0.998319646	0.03691105	ko01100//Metabolic p	-	-
-0.99831974	0.036910007	-	-	GO:0001725//stress fibe
-0.998320625	0.036900286	-	-	GO:0097541//axonemal
-0.998322124	0.036883813	-	-	-
-0.998322332	0.036881528	ko01110//Biosynthesis	-	-
-0.998323722	0.036866244	ko01100//Metabolic p	-	GO:0005737//cytoplasm
-0.998323725	0.036866201	ko01100//Metabolic p	ko05202//Transc	GO:0000786//nucleosor
-0.998325433	0.036847418	-	ko05200//Pathw	GO:0000785//chromatir
-0.998325968	0.036841528	ko01100//Metabolic p	-	GO:0031362//anchored
-0.998326879	0.036831495	-	-	-
-0.998327012	0.036830033	ko01100//Metabolic p	-	GO:0005634//nucleus;G
-0.998328605	0.036812484	-	ko04080//Neurc	GO:0005886//plasma m
-0.998328983	0.036808322	-	-	-
-0.998330683	0.036789589	-	-	GO:0005737//cytoplasm
-0.998331094	0.036785061	ko01100//Metabolic p	-	GO:0005737//cytoplasm
-0.998331895	0.03677623	ko01063//Biosynthesis	-	-
-0.998333025	0.036763768	-	-	-
-0.998333224	0.036761573	ko01100//Metabolic p	-	GO:0005634//nucleus;G
-0.998333456	0.036759017	-	-	GO:0005788//endoplasr
-0.998333526	0.036758242	-	-	-
-0.998334198	0.036750831	-	-	-
-0.998334459	0.036747947	ko01100//Metabolic p	-	GO:0005654//nucleopla
-0.998334961	0.036742405	ko01100//Metabolic p	ko05152//Tuber	GO:0009897//external s
-0.99833501	0.036741861	-	ko04934//Cushir	-
-0.998335884	0.036732223	-	-	-
-0.998335937	0.03673163	ko01100//Metabolic p	-	GO:0005634//nucleus;G
-0.998337019	0.036719682	-	ko01100//Metak	GO:0005654//nucleopla
-0.998337221	0.036717459	ko01100//Metabolic p	-	-
-0.998337732	0.036711814	-	-	-
-0.998338849	0.036699468	ko01100//Metabolic p	-	GO:0005654//nucleopla

-0.998339285	0.036694653	-	-	-
-0.998340708	0.036678923	ko01100//Metabolic p	-	GO:0005794//Golgi app
-0.998340718	0.036678815	ko01100//Metabolic p	ko01100//Metab	GO:0005739//mitochon
-0.998340875	0.036677081	ko01100//Metabolic p	ko01100//Metab	GO:0000139//Golgi mer
-0.998341193	0.036673563	-	-	-
-0.998341437	0.036670861	ko01100//Metabolic p	-	-
-0.998341634	0.036668689	ko01110//Biosynthesis	-	-
-0.998343559	0.036647389	ko01110//Biosynthesis	-	GO:0005634//nucleus;G
-0.998345872	0.036621794	-	ko05016//Huntir	GO:0005737//cytoplasm
-0.998347042	0.036608829	-	ko04216//Ferrop	GO:0016020//membran
-0.998347615	0.036602486	-	-	-
-0.998347915	0.036599157	ko01100//Metabolic p	-	-
-0.998348081	0.036597319	ko01100//Metabolic p	-	-
-0.998348124	0.036596849	-	-	GO:0000785//chromatir
-0.998348872	0.036588553	-	-	-
-0.998349953	0.036576577	-	ko01100//Metab	-
-0.998352109	0.036552664	-	ko04140//Autop	GO:0000407//pre-autop
-0.998353196	0.036540597	-	-	-
-0.998353508	0.036537141	-	ko01100//Metab	-
-0.998353627	0.036535817	ko01100//Metabolic p	-	GO:0005886//plasma m
-0.998353642	0.036535647	ko01100//Metabolic p	ko01100//Metab	-
-0.998354344	0.036527862	-	-	-
-0.998354363	0.03652765	-	-	GO:0005737//cytoplasm
-0.998355383	0.036516329	ko01100//Metabolic p	ko04060//Cytok	GO:0005887//integral c
-0.998357041	0.03649791	-	-	-
-0.998357349	0.03649449	ko01110//Biosynthesis	-	-
-0.998357432	0.036493567	ko01100//Metabolic p	-	GO:0005634//nucleus;G
-0.998357641	0.036491242	-	-	-
-0.998359288	0.036472928	-	ko01100//Metab	GO:0005654//nucleopla
-0.998360607	0.036458269	-	-	-
-0.998360905	0.036454956	ko01100//Metabolic p	-	-
-0.998362093	0.036441736	-	ko01100//Metab	GO:0005783//endoplasi
-0.998362746	0.036434466	-	-	-
-0.998363225	0.036429136	ko01100//Metabolic p	ko05202//Transc	GO:0000786//nucleosor
-0.998363316	0.036428122	-	ko01100//Metab	GO:0005634//nucleus;G
-0.998364267	0.03641754	ko01100//Metabolic p	ko04390//Hippo	GO:0005667//transcript
-0.998366682	0.036390635	-	-	GO:0000127//transcript
-0.99836826	0.036373043	ko01100//Metabolic p	-	GO:0005654//nucleopla
-0.998369543	0.036358737	ko01100//Metabolic p	-	-
-0.998369625	0.036357831	-	ko01100//Metab	GO:0033178//proton-tr
-0.998372054	0.036330725	-	ko05200//Pathw	-
-0.998372308	0.036327884	-	ko05150//Staphy	GO:0005577//fibrinoger
-0.998373881	0.036310327	ko01100//Metabolic p	-	GO:0005874//microtubu
-0.998374718	0.03630098	-	-	-
-0.998375923	0.036287517	-	-	-
-0.998376613	0.036279802	ko01100//Metabolic p	-	-
-0.998376788	0.036277849	-	ko01100//Metab	GO:0016020//membran
-0.998377052	0.036274894	-	-	-
-0.998378217	0.036261867	ko01100//Metabolic p	-	-
-0.998379524	0.036247254	-	ko01100//Metab	-
-0.998379791	0.03624427	-	ko04934//Cushi	-
-0.998380419	0.036237237	-	-	-
-0.998381748	0.036222363	-	-	-
-0.998382125	0.036218138	ko01110//Biosynthesis	ko04310//Wnt s	GO:0005634//nucleus;G
-0.998382266	0.036216569	-	-	-
-0.998382309	0.036216078	ko01100//Metabolic p	-	-
-0.998383027	0.036208039	ko01100//Metabolic p	-	GO:0005634//nucleus;G
-0.998384061	0.036196465	-	ko01100//Metab	GO:0005737//cytoplasm

-0.99838526	0.036183025	-	ko01100//Metab	GO:0031988//membran
-0.998385806	0.036176907	ko01100//Metabolic p	-	-
-0.998386419	0.036170039	ko01100//Metabolic p	-	GO:0005654//nucleopla
-0.998387042	0.036163046	-	ko04141//Protei	GO:0005634//nucleus;G
-0.998387393	0.036159117	-	ko04211//Longe	GO:0005634//nucleus
-0.99838741	0.036158918	ko01100//Metabolic p	-	-
-0.998387591	0.036156892	ko01110//Biosynthesis	-	GO:0000139//Golgi mer
-0.99838848	0.036146923	ko01100//Metabolic p	-	-
-0.998389143	0.036139481	-	-	GO:0005886//plasma m
-0.998392191	0.036105266	-	ko01100//Metab	GO:0005794//Golgi app
-0.998393828	0.036086872	ko01110//Biosynthesis	-	-
-0.998394849	0.036075407	ko01100//Metabolic p	-	GO:0005737//cytoplasm
-0.998395132	0.036072218	-	-	GO:0005737//cytoplasm
-0.998395137	0.036072165	ko01063//Biosynthesis	ko01100//Metab	GO:0005635//nuclear er
-0.998396404	0.036057915	ko01100//Metabolic p	ko01100//Metab	-
-0.998397573	0.036044772	-	-	GO:0005576//extracellu
-0.998398106	0.036038775	-	ko01100//Metab	-
-0.998398627	0.036032911	-	-	-
-0.998399154	0.036026984	-	-	GO:0016020//membran
-0.998399997	0.036017488	-	-	-
-0.998400574	0.036010992	-	ko04142//Lysos	GO:0016020//membran
-0.998401683	0.035998505	-	-	-
-0.99840234	0.0359911	-	-	-
-0.998403381	0.035979374	ko01100//Metabolic p	-	GO:0005634//nucleus;G
-0.998404221	0.035969899	-	ko01100//Metab	GO:0005783//endoplasr
-0.998404306	0.035968945	-	ko04142//Lysos	-
-0.998405147	0.035959463	-	-	GO:0005737//cytoplasm
-0.998406202	0.035947559	-	-	GO:0034361//very-low-
-0.998406702	0.035941924	-	ko01100//Metab	-
-0.998407888	0.035928535	ko01100//Metabolic p	ko04923//Regul	GO:0005654//nucleopla
-0.998408004	0.035927225	ko01100//Metabolic p	ko01100//Metab	-
-0.998408281	0.035924107	-	ko04530//Tight	GO:0005856//cytoskelet
-0.998410261	0.035901745	-	ko01100//Metab	GO:0005576//extracellu
-0.998410853	0.035895055	ko01100//Metabolic p	ko05200//Pathw	GO:0000785//chromatir
-0.998411277	0.035890269	-	-	GO:0005634//nucleus;G
-0.998411906	0.035883162	-	ko04144//Endoc	GO:0000813//ESCRT I c
-0.99841203	0.03588176	ko01100//Metabolic p	-	-
-0.998412164	0.035880245	ko01100//Metabolic p	-	GO:0005768//endosom
-0.998412497	0.035876479	ko01100//Metabolic p	-	GO:0005730//nucleolus
-0.998412783	0.035873251	ko01100//Metabolic p	-	-
-0.998413469	0.035865491	ko01100//Metabolic p	ko04060//Cytok	GO:0005576//extracellu
-0.998416267	0.035833851	-	ko04068//FoxO	GO:0005737//cytoplasm
-0.998416389	0.035832466	ko01100//Metabolic p	-	GO:0001725//stress fibe
-0.99841677	0.035828154	-	-	-
-0.998416792	0.035827903	-	ko04146//Perox	GO:0005777//peroxison
-0.998417787	0.035816642	-	-	GO:0016021//integral c
-0.998418507	0.035808495	ko01100//Metabolic p	-	-
-0.99841862	0.035807212	ko01100//Metabolic p	-	-
-0.998419725	0.035794696	ko01100//Metabolic p	ko05152//Tuber	GO:0009897//external s
-0.998420883	0.035781576	ko01100//Metabolic p	-	-
-0.998421721	0.03577208	ko01063//Biosynthesis	-	-
-0.998422805	0.035759789	ko01100//Metabolic p	-	GO:0005615//extracellu
-0.998424488	0.035740701	ko01100//Metabolic p	-	-
-0.998424667	0.035738663	-	-	GO:0005768//endosom
-0.998424776	0.035737428	-	-	-
-0.998424875	0.03573631	ko01100//Metabolic p	ko01100//Metab	-
-0.99842654	0.035717406	ko01100//Metabolic p	-	-
-0.998428424	0.035696016	ko01100//Metabolic p	ko01100//Metab	-

-0.998431128	0.035665282	ko01100//Metabolic p	-	-
-0.998431473	0.03566136	ko01100//Metabolic p	-	GO:0005886//plasma m
-0.998433122	0.035642607	ko01100//Metabolic p	-	-
-0.998435359	0.035617143	ko01100//Metabolic p	-	GO:0005576//extracellu
-0.998435554	0.035614922	ko01100//Metabolic p	-	-
-0.998435832	0.035611755	-	ko01100//Metab	-
-0.998437117	0.035597126	ko01100//Metabolic p	-	GO:0031464//Cul4A-RII
-0.998437191	0.035596288	ko01063//Biosynthesis	-	-
-0.998440357	0.035560201	-	-	-
-0.998441107	0.035551642	ko01100//Metabolic p	-	GO:0005634//nucleus;G
-0.998441143	0.03555123	ko01100//Metabolic p	-	GO:0016021//integral c
-0.998441289	0.035549572	ko01100//Metabolic p	-	-
-0.99844179	0.035543853	ko01100//Metabolic p	ko04151//PI3K-/	GO:0005654//nucleopla
-0.998445161	0.035505376	ko01100//Metabolic p	-	GO:0005634//nucleus;G
-0.998446075	0.035494933	-	-	GO:0005576//extracellu
-0.998446909	0.03548541	-	ko04150//mTOR	GO:0005764//lysosome;
-0.998447049	0.035483804	-	-	GO:0005783//endoplasr
-0.998447425	0.035479509	-	-	-
-0.998447601	0.0354775	ko01100//Metabolic p	-	GO:0000139//Golgi mer
-0.998447711	0.035476243	-	-	-
-0.998448752	0.035464342	ko01063//Biosynthesis	-	GO:0005886//plasma m
-0.998448888	0.03546279	-	ko04080//Neurc	GO:0005886//plasma m
-0.998449202	0.035459199	ko01063//Biosynthesis	ko04060//Cytok	GO:0005887//integral c
-0.99845033	0.035446296	-	-	GO:0031362//anchored
-0.9984509	0.035439772	-	-	GO:0005737//cytoplasm
-0.998451405	0.035433997	ko01100//Metabolic p	-	GO:0005794//Golgi app
-0.998452529	0.035421129	ko01100//Metabolic p	-	GO:0005634//nucleus;G
-0.998454911	0.035393847	-	-	-
-0.998455562	0.035386387	-	-	GO:0005737//cytoplasm
-0.998456127	0.035379911	ko01100//Metabolic p	-	GO:0005737//cytoplasm
-0.998456874	0.035371353	ko01063//Biosynthesis	ko05200//Pathw	GO:0005886//plasma m
-0.998457261	0.035366915	-	-	-
-0.998457267	0.035366844	ko01100//Metabolic p	-	-
-0.998457311	0.035366341	-	-	GO:0005634//nucleus;G
-0.998459575	0.035340371	-	-	-
-0.998459944	0.035336137	ko01100//Metabolic p	-	GO:0016020//membran
-0.998460192	0.035333301	-	-	GO:0016020//membran
-0.998460249	0.035332642	-	-	GO:0016020//membran
-0.998460299	0.035332064	ko01100//Metabolic p	-	GO:0005634//nucleus;G
-0.998460452	0.035330307	ko01063//Biosynthesis	-	GO:0005739//mitochon
-0.998460662	0.035327902	-	ko01100//Metab	-
-0.998460705	0.035327411	ko01100//Metabolic p	-	-
-0.998461195	0.035321778	ko01100//Metabolic p	-	GO:0005576//extracellu
-0.998462105	0.03531133	-	-	GO:0016020//membran
-0.998462294	0.035309162	ko01100//Metabolic p	ko01100//Metab	GO:0005829//cytosol;G
-0.99846262	0.035305422	-	-	GO:0005654//nucleopla
-0.998462882	0.035302412	-	-	GO:0005829//cytosol;G
-0.998463066	0.035300295	-	-	-
-0.998463142	0.03529942	ko01100//Metabolic p	-	-
-0.998463781	0.035292079	-	-	GO:0005737//cytoplasm
-0.998463944	0.035290209	ko01100//Metabolic p	ko01100//Metab	GO:0033178//proton-tr
-0.99846529	0.035274738	ko01100//Metabolic p	ko01100//Metab	-
-0.998465678	0.035270287	ko01100//Metabolic p	-	-
-0.998467816	0.035245697	-	ko04080//Neurc	GO:0005783//endoplasr
-0.998468576	0.035236949	ko01100//Metabolic p	-	-
-0.998468898	0.035233248	-	-	-
-0.998469546	0.035225786	ko01100//Metabolic p	-	GO:0016020//membran
-0.998469823	0.035222597	ko01100//Metabolic p	ko04145//Phagc	GO:0005783//endoplasr

-0.998471301	0.035205571	-	ko01100//Metak	-
-0.99847163	0.035201789	-	-	-
-0.998472714	0.035189301	ko01100//Metabolic p	ko04080//Neurc	GO:0016021//integral c
-0.998473694	0.035178008	-	ko03460//Fanco	-
-0.99847584	0.035153257	ko01100//Metabolic p	-	-
-0.998476778	0.035142434	-	-	GO:0005654//nucleopla
-0.99847884	0.035118631	-	ko01100//Metak	-
-0.998479414	0.035112012	ko01100//Metabolic p	ko04080//Neurc	GO:0005654//nucleopla
-0.998480696	0.035097201	-	ko04020//Calciu	GO:0016020//membran
-0.998484181	0.035056908	-	-	GO:0032991//macromo
-0.998485397	0.035042845	-	ko01100//Metak	-
-0.998485427	0.035042503	ko01063//Biosynthesis	ko04270//Vascu	GO:0005737//cytoplasm
-0.998485977	0.035036133	ko01100//Metabolic p	ko04550//Signal	GO:0005634//nucleus;G
-0.998487186	0.035022139	-	-	GO:0005768//endosome
-0.998487866	0.035014269	-	ko03013//Nucle	-
-0.9984886	0.035005759	-	-	-
-0.998488912	0.035002153	-	ko01100//Metak	GO:0005737//cytoplasm
-0.998488954	0.035001666	-	-	-
-0.998489357	0.034996998	ko01100//Metabolic p	-	GO:0016020//membran
-0.998489606	0.034994109	ko01100//Metabolic p	ko04120//Ubiqu	GO:0005680//anaphase
-0.998490419	0.03498469	-	-	GO:0016021//integral c
-0.998490496	0.034983789	-	ko01100//Metak	GO:0005783//endoplasi
-0.998492078	0.034965454	ko01120//Microbial m	ko01100//Metak	-
-0.998493703	0.034946601	-	-	GO:0005802//trans-Gol
-0.99849459	0.034936304	-	-	GO:0005576//extracellu
-0.998495008	0.034931459	-	-	GO:0005886//plasma m
-0.998495057	0.034930884	ko01100//Metabolic p	-	-
-0.998495476	0.034926029	-	ko04130//SNAR	GO:0005737//cytoplasm
-0.998495572	0.03492491	-	-	-
-0.998496643	0.034912477	-	-	-
-0.99849926	0.034882066	ko01063//Biosynthesis	-	GO:0005737//cytoplasm
-0.998499443	0.034879938	ko01100//Metabolic p	-	GO:0034361//very-low-
-0.998499587	0.034878265	ko01100//Metabolic p	-	GO:0005764//lysosome;
-0.998501714	0.034853523	ko01100//Metabolic p	ko01100//Metak	GO:0005783//endoplasi
-0.998503595	0.034831636	ko01100//Metabolic p	-	-
-0.99850486	0.034816911	ko01100//Metabolic p	-	-
-0.998504872	0.034816762	ko01100//Metabolic p	ko01100//Metak	-
-0.998505891	0.034804901	-	-	-
-0.998505942	0.034804306	ko01100//Metabolic p	-	-
-0.998506699	0.034795478	-	-	-
-0.998507748	0.034783255	ko01100//Metabolic p	-	-
-0.99850842	0.034775422	-	ko04142//Lysos	GO:0016020//membran
-0.998508924	0.034769546	ko01100//Metabolic p	ko05200//Pathw	GO:0005737//cytoplasm
-0.998509718	0.034760282	ko01100//Metabolic p	ko04146//Perox	GO:0005777//peroxison
-0.998510216	0.034754474	-	ko03320//PPAR	GO:0005783//endoplasi
-0.998511174	0.034743294	ko01100//Metabolic p	ko02010//ABC t	GO:0016021//integral c
-0.998512024	0.034733375	-	ko04142//Lysos	-
-0.998515002	0.034698592	ko01100//Metabolic p	-	-
-0.998515138	0.034697	ko01100//Metabolic p	-	GO:0005634//nucleus;G
-0.998515398	0.034693959	ko01100//Metabolic p	-	-
-0.998517332	0.034671349	-	-	GO:0005794//Golgi app
-0.998517449	0.034669986	ko01100//Metabolic p	ko04020//Calciu	GO:0005634//nucleus;G
-0.998519578	0.034645071	ko01100//Metabolic p	-	GO:0031224//intrinsic c
-0.998520716	0.034631752	ko01100//Metabolic p	-	GO:0016020//membran
-0.998520929	0.034629255	-	ko01100//Metak	GO:0005654//nucleopla
-0.998521281	0.034625135	ko01100//Metabolic p	ko01100//Metak	GO:0016021//integral c
-0.998522358	0.034612526	-	-	-
-0.998522441	0.034611547	-	-	GO:0031012//extracellu

-0.998522768	0.034607714	-	-	GO:0005794//Golgi app
-0.998523288	0.034601627	ko01100//Metabolic p	-	GO:0030659//cytoplasm
-0.998523909	0.034594349	ko01100//Metabolic p	-	-
-0.998524209	0.03459083	ko01120//Microbial m	-	GO:0016020//membran
-0.998525024	0.03458128	ko01100//Metabolic p	ko04934//Cushi	GO:0016020//membran
-0.998525253	0.034578588	-	-	GO:0005886//plasma m
-0.998530377	0.034518449	-	-	-
-0.998532601	0.034492312	-	ko01100//Metab	-
-0.998532802	0.034489948	ko01100//Metabolic p	-	-
-0.998534124	0.034474403	-	ko01100//Metab	GO:0009923//fatty acid
-0.998534345	0.03447181	ko01100//Metabolic p	ko04934//Cushi	-
-0.998535396	0.034459441	ko01100//Metabolic p	ko00790//Folate	-
-0.998535691	0.034455976	ko01100//Metabolic p	-	GO:0005615//extracellu
-0.998537044	0.034440041	-	-	-
-0.998539823	0.034407312	ko01100//Metabolic p	-	-
-0.998540297	0.034401719	ko01100//Metabolic p	ko01100//Metab	GO:0005654//nucleopla
-0.998543133	0.034368276	ko01063//Biosynthesis	-	-
-0.998543912	0.034359093	ko01100//Metabolic p	-	GO:0044424//intracellul
-0.998544354	0.034353872	-	-	GO:0005615//extracellu
-0.998545831	0.034336431	ko01100//Metabolic p	-	-
-0.998548921	0.034299921	ko01100//Metabolic p	ko04010//MAPK	GO:0005737//cytoplasm
-0.998549428	0.034293935	ko01100//Metabolic p	-	-
-0.998549456	0.034293603	ko01100//Metabolic p	-	-
-0.998549858	0.03428885	ko01100//Metabolic p	-	-
-0.998551447	0.03427005	-	-	-
-0.998552481	0.03425781	ko01100//Metabolic p	-	-
-0.998552483	0.034257793	-	ko05165//Huma	-
-0.998555048	0.034227419	ko01100//Metabolic p	-	-
-0.998555266	0.034224837	-	-	-
-0.998555607	0.034220793	ko01100//Metabolic p	-	GO:0005783//endoplasi
-0.998556317	0.034212379	-	-	-
-0.998557059	0.034203584	ko01100//Metabolic p	ko04360//Axon	GO:0005886//plasma m
-0.998557119	0.034202879	ko01100//Metabolic p	-	-
-0.998557679	0.034196239	ko01110//Biosynthesis	-	GO:0005634//nucleus;G
-0.998558311	0.034188736	-	-	GO:0000139//Golgi mer
-0.998559251	0.03417759	ko01100//Metabolic p	-	GO:0000177//cytoplasm
-0.998559554	0.034173993	ko01063//Biosynthesis	-	-
-0.998559723	0.034171995	-	-	-
-0.998560103	0.034167483	ko01100//Metabolic p	ko01100//Metab	GO:0005737//cytoplasm
-0.998560137	0.034167081	ko01100//Metabolic p	ko04141//Protei	GO:0005783//endoplasi
-0.998560705	0.034160339	-	-	-
-0.998560878	0.034158277	-	-	-
-0.998561312	0.03415313	ko01100//Metabolic p	ko01100//Metab	GO:0005737//cytoplasm
-0.998561344	0.034152751	ko01110//Biosynthesis	-	GO:0009986//cell surfac
-0.998561646	0.034149165	-	ko04530//Tight	GO:0005856//cytoskelet
-0.998561966	0.034145359	ko01100//Metabolic p	-	-
-0.998561986	0.034145131	-	ko04151//PI3K-/	GO:0005634//nucleus;G
-0.998562607	0.034137755	-	-	GO:0005737//cytoplasm
-0.998563847	0.034123022	-	-	-
-0.998564334	0.034117238	-	-	GO:0016021//integral c
-0.998565634	0.034101785	-	-	-
-0.998567898	0.03407485	ko01100//Metabolic p	ko04934//Cushi	-
-0.998568631	0.034066123	-	ko01100//Metab	-
-0.998568668	0.03406569	ko01100//Metabolic p	-	GO:0031012//extracellu
-0.998570682	0.0340417	-	-	GO:0016021//integral c
-0.998570897	0.034039139	ko01100//Metabolic p	ko01100//Metab	-
-0.998571306	0.034034268	-	ko04010//MAPK	-
-0.998573667	0.034006133	ko01100//Metabolic p	-	-

-0.998573775	0.034004844	-	ko04080//Neuro	GO:0005886//plasma m
-0.998574129	0.034000619	-	-	GO:0005654//nucleopla
-0.998574665	0.033994224	-	-	GO:0016020//membran
-0.998575062	0.033989499	-	-	-
-0.998575343	0.033986146	ko01100//Metabolic p	-	GO:0005634//nucleus;G
-0.998575406	0.033985384	-	ko01100//Metab	-
-0.998576051	0.033977698	ko01063//Biosynthesis	-	GO:0031982//vesicle
-0.998578177	0.033952311	ko01100//Metabolic p	-	-
-0.998578281	0.033951074	-	-	-
-0.998579624	0.033935024	-	-	-
-0.998580448	0.033925178	-	ko04550//Signal	GO:0005634//nucleus;G
-0.998580927	0.033919457	ko01100//Metabolic p	ko01100//Metab	GO:0005640//nuclear ou
-0.99858114	0.033916911	ko01100//Metabolic p	ko05200//Pathw	GO:0005634//nucleus;G
-0.998581208	0.033916097	ko01100//Metabolic p	-	GO:0016020//membran
-0.998582144	0.033904902	-	-	GO:0005634//nucleus
-0.998582675	0.03389855	-	-	GO:0005737//cytoplasm
-0.99858274	0.03389778	-	-	GO:0005789//endoplasm
-0.998583403	0.033889841	-	-	GO:0005634//nucleus;G
-0.998584107	0.03388142	-	-	-
-0.998584328	0.033878772	ko01063//Biosynthesis	-	-
-0.998584781	0.03387335	-	-	GO:0005737//cytoplasm
-0.998586782	0.033849389	-	-	GO:0005634//nucleus;G
-0.998586892	0.033848068	ko01100//Metabolic p	-	-
-0.998587675	0.03383869	ko01100//Metabolic p	-	GO:0005737//cytoplasm
-0.998587732	0.033838015	ko01100//Metabolic p	-	-
-0.9985885	0.033828804	ko01100//Metabolic p	-	-
-0.998590483	0.03380503	-	-	-
-0.998590732	0.033802041	-	-	-
-0.998590831	0.033800856	-	ko04144//Endoc	GO:0005654//nucleopla
-0.99859107	0.033797984	-	ko03013//Nucle	-
-0.998591452	0.0337934	ko01100//Metabolic p	ko05010//Alzhei	GO:0005783//endoplasm
-0.998592161	0.033784898	-	-	-
-0.998592271	0.033783571	-	-	GO:0016020//membran
-0.998594539	0.033756348	-	ko01100//Metab	GO:0005654//nucleopla
-0.998597012	0.033726628	ko01100//Metabolic p	ko01100//Metab	-
-0.998597627	0.033719226	ko01110//Biosynthesis	-	GO:0005829//cytosol;G(
-0.998597926	0.033715633	-	-	-
-0.99859851	0.033708607	-	ko04068//FoxO	GO:0005737//cytoplasm
-0.998599808	0.033692999	-	-	-
-0.998600014	0.033690515	ko01100//Metabolic p	-	-
-0.998601949	0.033667218	-	-	GO:0030659//cytoplasm
-0.998602147	0.033664831	-	ko01100//Metab	GO:0005739//mitochon
-0.998603041	0.033654066	ko01100//Metabolic p	-	-
-0.998604214	0.033639926	-	-	GO:0005634//nucleus;G
-0.998604242	0.033639589	ko01100//Metabolic p	ko01100//Metab	GO:0005654//nucleopla
-0.99860427	0.033639255	-	ko04020//Calciu	GO:0016020//membran
-0.998604941	0.033631167	ko01100//Metabolic p	ko03410//Base e	GO:0005634//nucleus;G
-0.998606951	0.03360692	ko01100//Metabolic p	ko01100//Metab	GO:0005737//cytoplasm
-0.998607284	0.0336029	-	ko04211//Longe	GO:0005634//nucleus
-0.998607488	0.033600438	-	ko05200//Pathw	GO:0000785//chromatin
-0.998607745	0.033597343	ko01110//Biosynthesis	-	-
-0.998608156	0.03359238	ko01100//Metabolic p	ko04010//MAPK	GO:0005737//cytoplasm
-0.998608269	0.033591016	-	ko01100//Metab	GO:0005576//extracellu
-0.998610279	0.033566741	ko01110//Biosynthesis	-	GO:0005737//cytoplasm
-0.998611552	0.03355136	-	ko05200//Pathw	GO:0005634//nucleus;G
-0.998611591	0.033550893	-	-	-
-0.998611846	0.033547812	-	-	-
-0.99861267	0.033537849	-	-	GO:0001725//stress fibe

-0.998613687	0.033525551	-	ko02010//ABC tı	GO:0016021//integral cı
-0.998613735	0.03352497	-	-	-
-0.998614864	0.03351132	-	-	-
-0.99861599	0.033497686	-	-	GO:0005576//extracellu
-0.99861714	0.033483766	-	ko05152//Tuber	GO:0009897//external s
-0.998617724	0.033476693	-	-	-
-0.998618933	0.033462041	-	-	GO:0005634//nucleus;G
-0.998620017	0.033448904	ko01110//Biosynthesis	-	-
-0.998620089	0.033448031	-	ko03010//Ribos	GO:0005634//nucleus;G
-0.998620581	0.033442075	ko01100//Metabolic p	ko04150//mTOF	GO:0005764//lysosome;
-0.998621073	0.033436102	ko01110//Biosynthesis	ko01100//Metab	GO:0005777//peroxison
-0.998621317	0.033433145	ko01100//Metabolic p	ko01100//Metab	-
-0.998622786	0.033415322	-	ko01100//Metab	GO:0005783//endoplasn
-0.998622883	0.033414144	ko01110//Biosynthesis	ko05200//Pathw	GO:0005737//cytoplasm
-0.998623356	0.033408405	ko01100//Metabolic p	-	-
-0.998623796	0.033403065	-	ko03460//Fanco	-
-0.998624925	0.033389364	ko01100//Metabolic p	-	-
-0.998627979	0.033352254	ko01100//Metabolic p	-	-
-0.998628634	0.033344285	ko01100//Metabolic p	-	-
-0.998629134	0.033338209	ko01100//Metabolic p	-	GO:0000785//chromatin
-0.998629271	0.033336538	ko01100//Metabolic p	ko01100//Metab	-
-0.998629476	0.033334051	-	-	GO:0005634//nucleus;G
-0.998629495	0.033333818	-	ko02010//ABC tı	GO:0000139//Golgi mer
-0.998631182	0.03331329	ko01063//Biosynthesis	ko03460//Fanco	-
-0.998632422	0.033298189	-	ko04934//Cushi	-
-0.998633152	0.033289307	ko01100//Metabolic p	-	GO:0016020//membran
-0.998633311	0.033287362	ko01100//Metabolic p	-	-
-0.99863433	0.033274957	ko01100//Metabolic p	-	-
-0.998636073	0.033253709	ko01110//Biosynthesis	-	-
-0.998636097	0.03325342	-	-	-
-0.998636748	0.033245481	-	ko01100//Metab	-
-0.998637241	0.033239466	ko01100//Metabolic p	-	-
-0.998637314	0.033238571	ko01100//Metabolic p	-	-
-0.998638091	0.033229086	ko01100//Metabolic p	-	-
-0.998638436	0.033224882	ko01100//Metabolic p	-	GO:0005576//extracellu
-0.998638646	0.033222315	ko01100//Metabolic p	ko04020//Calciu	GO:0005634//nucleus;G
-0.998639056	0.033217309	-	ko01100//Metab	-
-0.998640143	0.033204049	ko01100//Metabolic p	-	GO:0015629//actin cyto
-0.99864061	0.033198342	ko01100//Metabolic p	-	-
-0.998642516	0.033175047	-	ko05150//Staph	GO:0005577//fibrinoger
-0.998642561	0.033174506	ko01100//Metabolic p	-	-
-0.998643666	0.033160991	-	-	GO:0005739//mitochon
-0.998644329	0.033152886	-	ko01100//Metab	GO:0005829//cytosol
-0.998644416	0.033151823	-	-	GO:0000127//transcript
-0.998644584	0.033149767	-	-	GO:0005794//Golgi app
-0.998647021	0.033119945	-	ko05200//Pathw	GO:0005886//plasma m
-0.998648661	0.033099861	-	ko01100//Metab	GO:0005737//cytoplasm
-0.998649273	0.033092363	ko01100//Metabolic p	ko03010//Ribos	GO:0005634//nucleus;G
-0.99864994	0.033084189	-	-	-
-0.998650132	0.033081835	ko01100//Metabolic p	ko04144//Endoc	-
-0.998651299	0.033067529	-	-	-
-0.998651464	0.033065509	-	ko01100//Metab	GO:0005794//Golgi app
-0.998652939	0.033047421	ko01100//Metabolic p	ko01100//Metab	-
-0.998653197	0.033044251	-	-	GO:0005737//cytoplasm
-0.998654187	0.033032099	-	ko04060//Cytok	GO:0005887//integral cı
-0.998654606	0.033026956	-	-	GO:0005886//plasma m
-0.998654895	0.033023409	ko01100//Metabolic p	-	-
-0.998655658	0.033014036	ko01100//Metabolic p	-	GO:0005764//lysosome;

-0.998655696	0.033013573	ko01100//Metabolic p	-	GO:0032991//macromo
-0.998656166	0.033007794	ko01100//Metabolic p	-	GO:0005829//cytosol;G(
-0.998657037	0.032997105	-	ko04721//Synap	-
-0.998657674	0.032989266	-	-	-
-0.99865778	0.032987967	ko01100//Metabolic p	-	GO:0005737//cytoplasm
-0.998659531	0.032966433	ko01100//Metabolic p	-	GO:0005829//cytosol;G(
-0.998659741	0.03296386	-	-	GO:0044424//intracellul
-0.99866057	0.032953663	-	-	GO:0016020//membran
-0.998661563	0.032941434	-	-	-
-0.998662272	0.032932708	-	-	GO:0016020//membran
-0.998662351	0.032931738	ko01100//Metabolic p	-	-
-0.998663906	0.032912579	ko01100//Metabolic p	ko01100//Metak	GO:0005783//endoplasr
-0.998664152	0.03290956	-	ko04151//PI3K-/	GO:0005634//nucleus;G
-0.998664739	0.032902322	ko01120//Microbial m	-	GO:0005576//extracellu
-0.998665258	0.032895925	-	-	GO:0005615//extracellu
-0.998665308	0.032895303	ko01100//Metabolic p	-	GO:0016020//membran
-0.998665332	0.03289501	-	-	-
-0.998665732	0.032890078	-	-	GO:0031362//anchored
-0.998666209	0.0328842	-	-	GO:0016021//integral c
-0.998667042	0.032873927	-	-	-
-0.99866745	0.032868895	-	-	-
-0.998669166	0.032847715	-	-	GO:0005634//nucleus;G
-0.998669987	0.032837584	-	-	-
-0.998670424	0.032832187	-	-	-
-0.998670738	0.032828317	ko01100//Metabolic p	-	-
-0.998670961	0.03282556	-	-	GO:0005783//endoplasr
-0.998671845	0.032814633	ko01110//Biosynthesis	-	-
-0.998671934	0.032813535	-	-	-
-0.998672084	0.032811678	ko01110//Biosynthesis	ko04141//Protei	GO:0005783//endoplasr
-0.99867221	0.032810123	-	-	GO:0005634//nucleus;G
-0.998672356	0.032808317	-	-	-
-0.998674103	0.03278672	-	-	GO:0001650//fibrillar ce
-0.998674504	0.032781767	ko01100//Metabolic p	-	GO:0005634//nucleus;G
-0.998674752	0.03277869	-	-	-
-0.99867486	0.03277736	ko01100//Metabolic p	ko01100//Metak	-
-0.998675167	0.032773559	-	ko01100//Metak	-
-0.998677086	0.032749813	-	ko01100//Metak	-
-0.998678425	0.032733234	ko01100//Metabolic p	ko04144//Endoc	GO:0005654//nucleopla
-0.998678649	0.03273046	-	ko04310//Wnt s	GO:0005634//nucleus;G
-0.998679131	0.032724481	-	ko05132//Salmc	GO:0005737//cytoplasm
-0.998679432	0.032720749	-	-	-
-0.998679556	0.032719218	-	-	-
-0.998679798	0.03271622	ko01100//Metabolic p	-	GO:0005634//nucleus;G
-0.998680538	0.032707048	ko01100//Metabolic p	-	GO:0005813//centrosor
-0.99868315	0.032674656	ko01063//Biosynthesis	-	GO:0016021//integral c
-0.998684387	0.032659292	-	-	GO:0016021//integral c
-0.998684858	0.03265345	ko01100//Metabolic p	-	-
-0.998685037	0.032651221	-	ko01100//Metak	GO:0033180//proton-tr
-0.998686154	0.032637354	ko01110//Biosynthesis	-	-
-0.998686573	0.03263215	ko01100//Metabolic p	-	GO:0005634//nucleus;G
-0.998687282	0.032623336	-	-	GO:0005886//plasma m
-0.998687612	0.032619229	-	ko01100//Metak	GO:0005789//endoplasr
-0.998687806	0.032616827	ko01063//Biosynthesis	ko04310//Wnt s	GO:0009897//external s
-0.998687967	0.032614827	-	-	-
-0.998688623	0.032606662	-	ko01100//Metak	GO:0005783//endoplasr
-0.998689124	0.03260044	ko01100//Metabolic p	-	-
-0.998689802	0.032592	ko01100//Metabolic p	-	-
-0.998691795	0.032567191	ko01063//Biosynthesis	ko04530//Tight	GO:0005856//cytoskelet

-0.998692604	0.032557123	ko01100//Metabolic p	ko01100//Metat	GO:0005783//endoplasr
-0.998693336	0.032548	-	-	GO:0016020//membran
-0.998693995	0.032539792	ko01100//Metabolic p	-	-
-0.9986945	0.032533499	-	ko04710//Circac	GO:0005634//nucleus;G
-0.998694778	0.03253004	-	ko03320//PPAR	GO:0005634//nucleus;G
-0.998695795	0.032517361	-	-	-
-0.998696394	0.032509886	ko01100//Metabolic p	ko01100//Metat	GO:0016020//membran
-0.998698258	0.032486627	-	ko01100//Metat	-
-0.998698266	0.032486532	-	-	-
-0.998698849	0.03247925	ko01100//Metabolic p	-	-
-0.998699702	0.032468609	ko01100//Metabolic p	-	-
-0.99870086	0.032454141	-	-	-
-0.998701766	0.032442818	ko01100//Metabolic p	ko01100//Metat	GO:0005739//mitochon
-0.998702706	0.032431068	ko01100//Metabolic p	ko01100//Metat	GO:0009923//fatty acid
-0.998704239	0.032411899	ko01100//Metabolic p	ko04360//Axon	GO:0005576//extracellu
-0.998704242	0.03241186	-	ko04934//Cushi	GO:0016020//membran
-0.998704511	0.032408493	ko01100//Metabolic p	-	GO:0005789//endoplasr
-0.998705277	0.032398909	-	-	-
-0.99870529	0.032398755	-	-	-
-0.998706235	0.032386925	ko01100//Metabolic p	-	GO:0005634//nucleus;G
-0.998707322	0.032373313	-	ko04141//Protei	GO:0005783//endoplasr
-0.998709336	0.032348079	-	-	-
-0.998709654	0.032344094	-	-	-
-0.998709823	0.032341971	ko01100//Metabolic p	ko04024//cAMP	GO:0016021//integral c
-0.998710408	0.032334638	ko01100//Metabolic p	ko01100//Metat	GO:0005654//nucleopla
-0.998710858	0.032328992	ko01100//Metabolic p	-	-
-0.998711249	0.032324086	ko01110//Biosynthesis	-	-
-0.998712029	0.032314297	ko01100//Metabolic p	-	-
-0.998712144	0.032312854	ko01100//Metabolic p	ko04710//Circac	GO:0005634//nucleus;G
-0.99871267	0.032306264	ko01100//Metabolic p	-	GO:0005783//endoplasr
-0.99871374	0.032292833	-	-	-
-0.998714513	0.032283121	-	ko04310//Wnt s	GO:0005634//nucleus;G
-0.998714748	0.03228017	-	-	-
-0.998716097	0.032263213	-	ko04080//Neurc	GO:0016021//integral c
-0.998716625	0.032256586	-	ko04010//MAPK	GO:0005737//cytoplasm
-0.998717253	0.032248684	-	-	-
-0.998717474	0.032245913	-	-	-
-0.99871779	0.03224194	ko01100//Metabolic p	-	-
-0.998719473	0.032220766	-	-	-
-0.998719974	0.032214458	ko01100//Metabolic p	ko05165//Huma	-
-0.998720514	0.032207667	ko01100//Metabolic p	ko04211//Longe	GO:0005634//nucleus
-0.99872225	0.032185798	ko01100//Metabolic p	-	-
-0.998723116	0.032174895	ko01100//Metabolic p	-	-
-0.998723824	0.032165968	-	-	-
-0.998724076	0.032162785	ko01100//Metabolic p	-	GO:0005576//extracellu
-0.998725021	0.032150872	ko01100//Metabolic p	ko05200//Pathw	GO:0005634//nucleus;G
-0.998725033	0.032150725	ko01100//Metabolic p	-	GO:0045095//keratin fil
-0.998725223	0.032148325	ko01100//Metabolic p	ko01100//Metat	GO:0005640//nuclear o
-0.998725358	0.03214663	ko01100//Metabolic p	-	GO:0031462//Cul2-RIN
-0.998725453	0.032145424	ko01100//Metabolic p	-	-
-0.998726347	0.032134147	ko01110//Biosynthesis	-	GO:0008076//voltage-g
-0.998726782	0.03212866	ko01100//Metabolic p	-	-
-0.998728352	0.032108843	ko01100//Metabolic p	-	-
-0.998728901	0.032101901	-	ko04919//Thyro	GO:0016020//membran
-0.998729199	0.032098142	-	-	-
-0.998731329	0.032071225	ko01100//Metabolic p	ko03320//PPAR	GO:0005615//extracellu
-0.998731333	0.032071169	-	-	GO:0005829//cytosol
-0.998732908	0.032051261	-	-	GO:0005576//extracellu

-0.998733139	0.03204833	-	ko01100//Metab	GO:0005783//endoplasr
-0.998736933	0.032000294	ko01100//Metabolic p	ko01100//Metab	GO:0005615//extracellu
-0.998737672	0.031990933	ko01100//Metabolic p	ko04010//MAPK	-
-0.998738593	0.031979264	ko01100//Metabolic p	ko01100//Metab	GO:0005829//cytosol
-0.998739711	0.031965076	ko01100//Metabolic p	ko01100//Metab	-
-0.998741052	0.031948073	-	ko01100//Metab	GO:0005794//Golgi app
-0.998741186	0.031946373	-	-	GO:0005634//nucleus;G
-0.998741538	0.031941901	-	ko01100//Metab	GO:0005576//extracellu
-0.998741781	0.031938819	ko01100//Metabolic p	-	GO:0005634//nucleus;G
-0.998743277	0.031919814	ko01100//Metabolic p	ko01100//Metab	GO:0005737//cytoplasm
-0.998747596	0.031864903	ko01100//Metabolic p	-	-
-0.998747775	0.031862636	-	-	-
-0.998747884	0.03186124	ko01110//Biosynthesis	-	GO:0005730//nucleolus
-0.998750838	0.031823632	-	ko04919//Thyro	GO:0016020//membran
-0.998751382	0.031816694	-	-	-
-0.998753947	0.031783992	ko01100//Metabolic p	-	GO:0031966//mitochon
-0.998754977	0.031770847	-	ko05200//Pathw	GO:0005737//cytoplasm
-0.998755699	0.031761644	ko01100//Metabolic p	-	GO:0034451//centriolar
-0.998755815	0.031760162	-	-	-
-0.998755843	0.031759804	ko01100//Metabolic p	-	GO:0016021//integral c
-0.998755975	0.031758116	-	-	-
-0.998756161	0.031755742	ko01100//Metabolic p	-	GO:0016020//membran
-0.998756662	0.031749346	-	-	GO:0016020//membran
-0.998757009	0.031744915	ko01100//Metabolic p	-	-
-0.998757631	0.03173696	ko01110//Biosynthesis	ko03010//Ribos	GO:0005634//nucleus;G
-0.9987577	0.031736087	-	-	-
-0.998757748	0.03173547	-	ko04120//Ubiqu	GO:0005680//anaphase
-0.998758327	0.031728068	-	-	GO:0005737//cytoplasm
-0.998758474	0.031726193	ko01100//Metabolic p	-	GO:0005634//nucleus;G
-0.998759344	0.031715068	-	-	-
-0.998759441	0.031713832	-	-	-
-0.998760054	0.031705999	-	-	-
-0.998762063	0.031680292	ko01100//Metabolic p	-	-
-0.99876347	0.031662283	ko01100//Metabolic p	ko04144//Endoc	GO:0001917//photorece
-0.998763948	0.031656162	-	-	-
-0.998764791	0.031645363	-	-	-
-0.998765552	0.03163561	ko01100//Metabolic p	-	-
-0.998767355	0.031612499	ko01100//Metabolic p	-	-
-0.998767405	0.031611851	ko01100//Metabolic p	ko05203//Viral c	GO:0000786//nucleosor
-0.998767407	0.031611823	ko01100//Metabolic p	ko01100//Metab	GO:0005739//mitochon
-0.99877059	0.031570974	-	ko05203//Viral c	GO:0000786//nucleosor
-0.998770969	0.031566105	ko01120//Microbial m	ko04630//JAK-S	GO:0005829//cytosol
-0.998771547	0.031558677	ko01100//Metabolic p	-	-
-0.998772415	0.031547533	-	ko01100//Metab	-
-0.998773064	0.031539188	ko01100//Metabolic p	ko04068//FoxO	GO:0005737//cytoplasm
-0.998773559	0.031532818	-	-	-
-0.998775104	0.031512956	-	ko01100//Metab	GO:0005829//cytosol
-0.998775309	0.031510311	-	ko01100//Metab	GO:0016020//membran
-0.998775383	0.031509358	ko01100//Metabolic p	-	-
-0.998775549	0.031507225	ko01100//Metabolic p	-	-
-0.998775919	0.031502468	ko01100//Metabolic p	ko05132//Salmo	GO:0005737//cytoplasm
-0.998777273	0.031485035	-	-	GO:0005813//centrosor
-0.998778585	0.031468131	-	-	-
-0.998778596	0.031467985	ko01100//Metabolic p	-	-
-0.998778617	0.031467714	-	ko05203//Viral c	GO:0000786//nucleosor
-0.998779474	0.031456677	ko01100//Metabolic p	ko01100//Metab	GO:0005769//early endo
-0.998781604	0.031429208	ko01100//Metabolic p	ko01100//Metab	GO:0033180//proton-tr
-0.998782108	0.031422712	ko01100//Metabolic p	-	GO:0031224//intrinsic c

-0.998784266	0.031394856	-	-	-
-0.998784813	0.031387786	-	ko04151//PI3K-/	GO:0005634//nucleus;G
-0.998784947	0.031386049	-	-	GO:0005739//mitochon
-0.998785988	0.031372601	ko01100//Metabolic p	-	GO:0030659//cytoplasm
-0.998787222	0.031356654	ko01100//Metabolic p	ko01100//Metab	-
-0.998787507	0.031352967	-	ko04745//Photo	-
-0.99878806	0.031345814	ko01100//Metabolic p	ko05016//Huntir	-
-0.998788123	0.031345002	-	ko05200//Pathw	GO:0005886//plasma m
-0.998788708	0.031337434	ko01100//Metabolic p	ko04060//Cytok	GO:0009897//external s
-0.998789195	0.031331133	ko01100//Metabolic p	-	-
-0.998789416	0.031328266	ko01100//Metabolic p	-	GO:0005576//extracellu
-0.99878998	0.031320965	ko01100//Metabolic p	ko01100//Metab	GO:0005576//extracellu
-0.998790923	0.031308766	ko01110//Biosynthesis	ko01100//Metab	GO:0005783//endoplasn
-0.998790976	0.03130808	-	-	-
-0.998791349	0.031303237	-	-	-
-0.998792328	0.031290566	-	ko01100//Metab	GO:0005794//Golgi app
-0.998792992	0.031281951	ko01100//Metabolic p	-	-
-0.998793007	0.031281757	ko01100//Metabolic p	ko04520//Adher	GO:0031252//cell leadir
-0.998794146	0.031267	-	-	GO:0000785//chromatir
-0.998794394	0.031263779	-	-	-
-0.99879458	0.031261371	-	-	-
-0.998794808	0.031258407	-	-	GO:0001669//acrosoma
-0.998794905	0.031257157	-	ko04060//Cytok	GO:0005887//integral co
-0.998795198	0.031253347	ko01100//Metabolic p	ko01100//Metab	-
-0.998795301	0.031252014	-	-	GO:0005886//plasma m
-0.998795719	0.031246596	-	-	-
-0.998795824	0.031245232	-	ko04710//Circac	GO:0005634//nucleus;G
-0.998796149	0.031241004	-	-	GO:0097541//axonemal
-0.998796925	0.031230934	ko01100//Metabolic p	ko02010//ABC tı	GO:0016021//integral co
-0.998797366	0.031225211	ko01100//Metabolic p	-	-
-0.998797972	0.031217336	ko01110//Biosynthesis	-	GO:0005615//extracellu
-0.998798114	0.0312155	ko01100//Metabolic p	ko04310//Wnt s	GO:0005634//nucleus;G
-0.998798327	0.031212726	-	ko04144//Endoc	GO:0005654//nucleopla
-0.998799479	0.031197769	ko01100//Metabolic p	-	-
-0.998799858	0.031192833	ko01100//Metabolic p	-	GO:0005654//nucleopla
-0.998800924	0.03117898	-	ko04020//Calciu	GO:0005634//nucleus;G
-0.998801242	0.031174842	ko01100//Metabolic p	-	-
-0.998801444	0.031172213	-	-	-
-0.998802061	0.031164193	-	-	GO:0005634//nucleus;G
-0.998802767	0.031155007	-	-	-
-0.998803075	0.031150997	-	ko05200//Pathw	GO:0005576//extracellu
-0.99880325	0.031148716	-	ko01100//Metab	GO:0005783//endoplasn
-0.998803587	0.031144327	ko01100//Metabolic p	ko01100//Metab	GO:0005634//nucleus;G
-0.998803683	0.031143079	-	ko01100//Metab	GO:0005654//nucleopla
-0.998804476	0.031132749	-	-	-
-0.998804833	0.031128108	ko01100//Metabolic p	-	GO:0005829//cytosol;G(
-0.998805401	0.031120704	ko01100//Metabolic p	ko01100//Metab	GO:0005783//endoplasn
-0.99880545	0.031120068	-	-	-
-0.99880765	0.031091395	ko01100//Metabolic p	ko01100//Metab	-
-0.998808239	0.031083708	-	-	GO:0005634//nucleus;G
-0.998808907	0.031075001	-	ko03320//PPAR	GO:0005576//extracellu
-0.998810025	0.031060401	-	ko01100//Metab	GO:0005654//nucleopla
-0.998810317	0.031056589	ko01100//Metabolic p	-	GO:0005783//endoplasn
-0.998811086	0.031046547	ko01100//Metabolic p	-	GO:0016021//integral co
-0.99881303	0.031021155	ko01100//Metabolic p	-	-
-0.998815508	0.030988751	-	-	GO:0005634//nucleus;G
-0.998815993	0.030982403	ko01100//Metabolic p	-	GO:0000127//transcript
-0.998816542	0.030975221	ko01100//Metabolic p	-	-

-0.998816711	0.030973003	ko01100//Metabolic p	-	GO:0005737//cytoplasm
-0.998816732	0.030972733	-	ko05203//Viral c	-
-0.998816887	0.030970701	-	-	GO:0005764//lysosome;
-0.998817041	0.030968683	ko01100//Metabolic p	-	-
-0.998818047	0.030955518	-	ko04310//Wnt s	GO:0005634//nucleus;G
-0.998818767	0.030946078	ko01100//Metabolic p	-	-
-0.998818788	0.030945807	-	-	-
-0.998818905	0.030944276	-	-	-
-0.998820972	0.03091718	ko01100//Metabolic p	ko04978//Miner	GO:0009986//cell surfac
-0.998821165	0.030914651	-	ko04130//SNAR	GO:0005737//cytoplasm
-0.998821297	0.030912919	-	-	-
-0.998821404	0.030911517	-	-	-
-0.998823294	0.03088672	-	-	-
-0.998823771	0.030880452	ko01100//Metabolic p	ko01100//Metab	GO:0005794//Golgi app
-0.998825786	0.030853989	-	-	-
-0.998825827	0.030853451	ko01100//Metabolic p	-	-
-0.998826222	0.030848251	ko01100//Metabolic p	ko04060//Cytok	GO:0005887//integral c
-0.998826455	0.030845186	ko01100//Metabolic p	ko04145//Phago	GO:0005783//endoplasm
-0.998826675	0.030842302	ko01100//Metabolic p	-	-
-0.998826859	0.030839884	-	-	-
-0.998828012	0.030824715	ko01100//Metabolic p	-	GO:0005615//extracellu
-0.998828259	0.030821472	ko01100//Metabolic p	ko01100//Metab	GO:0005794//Golgi app
-0.998829137	0.030809914	ko01100//Metabolic p	-	GO:0005615//extracellu
-0.998829549	0.030804502	ko01100//Metabolic p	-	-
-0.998830086	0.03079743	ko01100//Metabolic p	ko01100//Metab	GO:0005737//cytoplasm
-0.99883077	0.030788424	ko01100//Metabolic p	-	GO:0005737//cytoplasm
-0.998831505	0.030778744	ko01100//Metabolic p	-	GO:0008076//voltage-g
-0.998833896	0.030747229	-	-	-
-0.998836996	0.03070633	-	ko01100//Metab	GO:0005576//extracellu
-0.998837515	0.030699476	ko01100//Metabolic p	-	-
-0.998838527	0.030686105	ko01100//Metabolic p	ko01100//Metab	GO:0005783//endoplasm
-0.998839074	0.03067887	-	ko01100//Metab	GO:0005737//cytoplasm
-0.998841498	0.030646816	ko01100//Metabolic p	-	-
-0.998841625	0.030645148	-	ko03013//Nucle	-
-0.998841781	0.030643073	ko01100//Metabolic p	-	-
-0.998841844	0.030642249	ko01100//Metabolic p	-	GO:0005874//microtubu
-0.998842158	0.030638091	ko01100//Metabolic p	-	GO:0016020//membran
-0.998842782	0.030629831	ko01110//Biosynthesis	-	GO:0031224//intrinsic c
-0.998843856	0.030615612	-	-	-
-0.998845107	0.030599038	ko01100//Metabolic p	-	GO:0001725//stress fibe
-0.998845934	0.030588082	ko01100//Metabolic p	-	GO:0016021//integral c
-0.998846953	0.030574577	-	ko05143//Africa	GO:0005576//extracellu
-0.998847938	0.030561505	-	-	GO:0005811//lipid parti
-0.998848572	0.030553098	ko01100//Metabolic p	-	GO:0005634//nucleus;G
-0.998850417	0.030528601	-	ko00982//Drug	-
-0.998850593	0.030526259	ko01100//Metabolic p	-	-
-0.998852038	0.030507071	-	-	-
-0.998852108	0.030506135	ko01100//Metabolic p	ko05010//Alzhei	GO:0005576//extracellu
-0.998853607	0.030486211	ko01100//Metabolic p	-	-
-0.998854893	0.030469102	ko01063//Biosynthesis	ko01100//Metab	-
-0.998855416	0.030462132	ko01100//Metabolic p	-	-
-0.99885654	0.030447174	-	-	GO:0005634//nucleus
-0.998856616	0.030446169	-	-	GO:0005634//nucleus;G
-0.998857029	0.030440658	ko01100//Metabolic p	ko05231//Cholir	-
-0.998857232	0.030437956	-	ko01100//Metab	GO:0005783//endoplasm
-0.998857869	0.030429468	ko01100//Metabolic p	-	-
-0.998859507	0.030407642	-	-	GO:0044424//intracellul
-0.998860502	0.030394366	-	-	-

-0.998860866	0.030389512	ko01110//Biosynthesis	-	-
-0.998865238	0.030331128	-	-	-
-0.998865842	0.03032306	-	ko01100//Metab	GO:0005739//mitochon
-0.998865912	0.030322116	ko01100//Metabolic p	-	GO:0005634//nucleus;G
-0.998866396	0.030315645	-	-	-
-0.998868007	0.030294093	ko01100//Metabolic p	ko05203//Viral c	GO:0000786//nucleosor
-0.998868029	0.030293795	ko01100//Metabolic p	ko05200//Pathw	GO:0000307//cyclin-de
-0.998868405	0.030288765	ko01100//Metabolic p	-	-
-0.998868641	0.030285607	-	-	GO:0005634//nucleus;G
-0.998869255	0.030277385	-	ko01100//Metab	GO:0005829//cytosol
-0.998869361	0.030275969	-	-	-
-0.998869856	0.030269342	-	-	GO:0005783//endoplasr
-0.998870366	0.030262506	-	ko01100//Metab	GO:0005783//endoplasr
-0.998870375	0.030262379	ko01100//Metabolic p	-	GO:0005634//nucleus;G
-0.998870484	0.030260929	ko01100//Metabolic p	-	-
-0.998872379	0.030235532	ko01110//Biosynthesis	ko01100//Metab	GO:0000139//Golgi mer
-0.998873651	0.030218462	-	-	-
-0.998875283	0.030196566	ko01100//Metabolic p	-	-
-0.998875589	0.030192449	ko01100//Metabolic p	-	-
-0.998875674	0.030191303	ko01100//Metabolic p	-	GO:0005730//nucleolus
-0.998875947	0.03018764	ko01110//Biosynthesis	-	-
-0.998876563	0.030179368	ko01100//Metabolic p	-	-
-0.998876713	0.030177359	-	-	GO:0000139//Golgi mer
-0.998876917	0.030174613	ko01100//Metabolic p	ko04976//Bile se	GO:0005789//endoplasr
-0.998877106	0.030172073	-	ko04144//Endoc	-
-0.998877434	0.030167659	ko01100//Metabolic p	-	-
-0.998878946	0.030147344	-	ko01100//Metab	GO:0005576//extracellu
-0.998879444	0.030140635	-	-	-
-0.998879461	0.030140417	ko01100//Metabolic p	-	-
-0.998879561	0.03013907	-	-	-
-0.998879676	0.030137512	ko01100//Metabolic p	-	GO:0009986//cell surfac
-0.998879726	0.030136845	-	-	GO:0005783//endoplasr
-0.998880119	0.03013156	-	-	-
-0.998881262	0.030116171	ko01100//Metabolic p	-	GO:0005615//extracellu
-0.998882867	0.030094562	ko01063//Biosynthesis	ko05016//Huntir	GO:0005737//cytoplasm
-0.998883066	0.030091879	ko01100//Metabolic p	ko03320//PPAR	GO:0005783//endoplasr
-0.998883716	0.030083121	ko01100//Metabolic p	-	-
-0.998885201	0.030063098	-	-	-
-0.998887063	0.030037983	-	-	GO:0005576//extracellu
-0.998887346	0.030034164	-	ko03320//PPAR	GO:0005576//extracellu
-0.99888989	0.029999796	ko01100//Metabolic p	-	-
-0.998889903	0.029999624	ko01100//Metabolic p	-	-
-0.998890783	0.02998773	ko01100//Metabolic p	ko04140//Autop	GO:0000407//pre-autop
-0.998890968	0.029985219	-	-	-
-0.998891764	0.029974459	-	-	-
-0.998892061	0.029970446	-	-	-
-0.998892424	0.029965525	ko01063//Biosynthesis	-	-
-0.998893323	0.029953364	ko01100//Metabolic p	ko01100//Metab	GO:0005783//endoplasr
-0.998893672	0.029948644	-	ko01100//Metab	GO:0005789//endoplasr
-0.998894301	0.029940126	-	-	-
-0.998894933	0.029931565	ko01100//Metabolic p	ko04216//Ferrop	GO:0016020//membran
-0.998895143	0.029928723	ko01100//Metabolic p	-	-
-0.998895658	0.029921741	-	ko01100//Metab	-
-0.998896865	0.029905381	-	-	GO:0005737//cytoplasm
-0.998896951	0.029904217	ko01110//Biosynthesis	ko01100//Metab	GO:0005640//nuclear ou
-0.998897129	0.029901806	-	ko03320//PPAR	GO:0005615//extracellu
-0.998897139	0.029901671	ko01110//Biosynthesis	ko05200//Pathw	GO:0005634//nucleus;G
-0.998897194	0.029900919	-	-	-

-0.998897248	0.029900191	-	-	GO:0000791//euchroma
-0.998899013	0.029876254	ko01110//Biosynthesis	-	GO:0005634//nucleus;G
-0.99889947	0.029870043	-	-	-
-0.998900068	0.029861933	ko01100//Metabolic p	-	GO:0005615//extracellu
-0.998900726	0.029852988	ko01100//Metabolic p	ko03320//PPAR	GO:0005576//extracellu
-0.998902265	0.029832084	ko01100//Metabolic p	ko03013//Nucle	-
-0.998902533	0.029828441	ko01100//Metabolic p	ko04973//Carbo	GO:0005886//plasma m
-0.998903222	0.029819082	ko01100//Metabolic p	-	-
-0.998903959	0.029809053	-	-	GO:0000785//chromatin
-0.998905449	0.029788782	-	ko01100//Metab	-
-0.998908238	0.029750805	ko01100//Metabolic p	-	GO:0005737//cytoplasm
-0.998908449	0.029747918	ko01100//Metabolic p	ko05200//Pathw	GO:0005829//cytosol;G
-0.998908913	0.029741597	ko01100//Metabolic p	-	GO:0005783//endoplasm
-0.998909238	0.029737162	-	ko05203//Viral c	-
-0.998909356	0.029735558	-	-	GO:0031091//platelet al
-0.998910653	0.029717872	ko01100//Metabolic p	-	-
-0.998910999	0.029713154	-	-	-
-0.998911287	0.029709213	-	ko01100//Metab	GO:0005829//cytosol
-0.998911876	0.029701187	ko01110//Biosynthesis	-	GO:0005783//endoplasm
-0.998912806	0.029688476	ko01100//Metabolic p	-	GO:0005634//nucleus;G
-0.998915334	0.02965394	-	ko01100//Metab	GO:0005783//endoplasm
-0.998915525	0.029651331	ko01100//Metabolic p	-	-
-0.998917338	0.029626521	ko01110//Biosynthesis	-	GO:0005737//cytoplasm
-0.998917385	0.029625888	ko01100//Metabolic p	ko04130//SNAR	GO:0005737//cytoplasm
-0.998921261	0.029572794	ko01063//Biosynthesis	-	GO:0000151//ubiquitin
-0.998921526	0.029569164	-	-	-
-0.998922814	0.029551502	ko01100//Metabolic p	-	GO:0005737//cytoplasm
-0.998923281	0.029545091	ko01100//Metabolic p	ko05202//Transc	GO:0000786//nucleosom
-0.998923781	0.029538228	ko01100//Metabolic p	-	GO:0005634//nucleus;G
-0.998924552	0.029527639	ko01100//Metabolic p	ko05203//Viral c	GO:0000786//nucleosom
-0.998924903	0.029522824	ko01100//Metabolic p	-	-
-0.998924968	0.029521924	-	ko04142//Lysos	GO:0016020//membran
-0.998926129	0.029505981	ko01100//Metabolic p	-	-
-0.99892613	0.029505971	ko01100//Metabolic p	-	GO:0005634//nucleus;G
-0.998926913	0.029495206	ko01063//Biosynthesis	-	-
-0.998927209	0.029491139	-	-	GO:0005654//nucleopla
-0.998927642	0.029485179	ko01100//Metabolic p	-	-
-0.998927894	0.029481718	-	-	GO:0046658//anchored
-0.998927926	0.029481281	ko01100//Metabolic p	-	GO:0016581//NuRD cor
-0.99892807	0.029479295	ko01100//Metabolic p	-	GO:0016020//membran
-0.99893023	0.02944958	ko01100//Metabolic p	ko04142//Lysos	-
-0.99893064	0.029443934	ko01100//Metabolic p	ko04080//Neuro	GO:0005654//nucleopla
-0.998931086	0.029437784	-	ko04144//Endoc	GO:0005654//nucleopla
-0.998932351	0.029420365	ko01100//Metabolic p	-	-
-0.998933508	0.029404421	ko01100//Metabolic p	-	-
-0.998933629	0.029402742	-	ko01100//Metab	GO:0005783//endoplasm
-0.998934243	0.029394276	-	-	GO:0005634//nucleus;G
-0.998934607	0.029389251	-	-	GO:0005634//nucleus;G
-0.99893856	0.029334676	ko01100//Metabolic p	-	-
-0.998938803	0.029331319	-	-	GO:0001725//stress fibe
-0.998939192	0.029325934	-	-	GO:0005811//lipid parti
-0.99893923	0.029325408	-	-	GO:0016021//integral c
-0.998939671	0.029319319	-	ko01100//Metab	GO:0005886//plasma m
-0.998940304	0.029310554	-	-	-
-0.998942694	0.029277482	ko01100//Metabolic p	ko01100//Metab	-
-0.998943108	0.029271749	-	-	GO:0005737//cytoplasm
-0.998943326	0.02926873	-	ko05200//Pathw	GO:0000785//chromatin
-0.998943421	0.029267414	-	ko04919//Thyro	GO:0016020//membran

-0.998943643	0.029264334	ko01100//Metabolic p	ko01100//Metak	GO:0005783//endoplasr
-0.998945509	0.02923847	ko01100//Metabolic p	-	-
-0.998946602	0.029223315	ko01063//Biosynthesis	ko04010//MAPK	-
-0.998946787	0.029220745	ko01100//Metabolic p	-	-
-0.998947196	0.029215071	ko01100//Metabolic p	-	GO:0001669//acrosoma
-0.998949313	0.029185678	-	ko04146//Perox	GO:0005777//peroxison
-0.998951074	0.029161205	ko01100//Metabolic p	ko01100//Metak	GO:0005737//cytoplasm
-0.998951256	0.029158681	ko01110//Biosynthesis	ko04360//Axon	-
-0.998951916	0.029149498	-	-	GO:0005654//nucleopla
-0.998953209	0.029131507	-	-	-
-0.998953369	0.029129284	ko01100//Metabolic p	ko01100//Metak	GO:0005654//nucleopla
-0.998953991	0.029120632	-	-	-
-0.998954101	0.029119093	ko01063//Biosynthesis	-	GO:0005829//cytosol;G(
-0.998954199	0.029117735	ko01100//Metabolic p	ko04151//PI3K-/	GO:0005634//nucleus;G
-0.998954727	0.029110377	-	ko04080//Neuro	GO:0016021//integral c
-0.998954896	0.029108026	ko01100//Metabolic p	ko05202//Transc	GO:0000786//nucleosor
-0.998955376	0.029101337	ko01100//Metabolic p	-	GO:0005737//cytoplasm
-0.998955607	0.029098118	-	-	-
-0.998955791	0.029095557	-	-	-
-0.998956251	0.029089145	-	-	-
-0.998956625	0.029083927	-	ko01100//Metak	-
-0.998956676	0.029083226	ko01100//Metabolic p	-	GO:0005737//cytoplasm
-0.998957086	0.029077506	ko01100//Metabolic p	ko01100//Metak	GO:0005783//endoplasr
-0.99895751	0.029071584	ko01100//Metabolic p	-	GO:0097541//axonemal
-0.998957909	0.029066022	-	-	GO:0034361//very-low-
-0.998959032	0.029050355	ko01100//Metabolic p	ko05412//Arrhyt	GO:0005886//plasma m
-0.998959346	0.029045977	-	-	-
-0.998959915	0.029038028	ko01100//Metabolic p	-	-
-0.99896025	0.029033352	-	-	GO:0005634//nucleus;G
-0.998960626	0.029028098	-	-	GO:0000139//Golgi mer
-0.998960901	0.029024257	-	ko03320//PPAR	GO:0005783//endoplasr
-0.998961097	0.029021531	ko01100//Metabolic p	-	-
-0.998961387	0.029017471	ko01100//Metabolic p	-	-
-0.998961559	0.029015076	-	-	-
-0.99896226	0.029005267	-	-	GO:0005737//cytoplasm
-0.998962276	0.029005055	-	-	-
-0.998962437	0.029002793	ko01100//Metabolic p	-	-
-0.998963507	0.028987833	ko01100//Metabolic p	-	GO:0005783//endoplasr
-0.998964158	0.028978728	ko01110//Biosynthesis	ko01100//Metak	GO:0000506//glycosylpl
-0.998964415	0.028975131	ko01100//Metabolic p	-	-
-0.998964431	0.028974908	-	ko01100//Metak	GO:0005634//nucleus;G
-0.998964619	0.028972287	-	-	-
-0.998965007	0.028966853	-	ko04310//Wnt s	GO:0005634//nucleus;G
-0.998965269	0.028963191	ko01100//Metabolic p	ko04144//Endoc	GO:0000813//ESCRT I c
-0.998966305	0.02894868	ko01100//Metabolic p	-	GO:0097541//axonemal
-0.998967174	0.028936502	-	ko04144//Endoc	-
-0.998969106	0.028909419	-	-	GO:0043235//receptor c
-0.998969602	0.028902466	ko01100//Metabolic p	ko04216//Ferrop	GO:0016020//membran
-0.998970421	0.028890981	ko01100//Metabolic p	ko01100//Metak	GO:0005654//nucleopla
-0.998971012	0.028882688	-	ko01100//Metak	-
-0.998972446	0.028862549	-	-	-
-0.998972589	0.028860543	ko01100//Metabolic p	ko03320//PPAR	GO:0005576//extracellu
-0.998973602	0.028846301	ko01100//Metabolic p	ko04140//Autop	GO:0000407//pre-autop
-0.998973818	0.028843262	ko01100//Metabolic p	ko04919//Thyro	GO:0016020//membran
-0.998974008	0.028840602	ko01110//Biosynthesis	ko01100//Metak	GO:0001650//fibrillar ce
-0.998974461	0.028834234	ko01100//Metabolic p	-	-
-0.998974546	0.02883303	-	ko01100//Metak	-
-0.998975506	0.028819537	ko01100//Metabolic p	ko04721//Synap	GO:0005887//integral c

-0.998975965	0.028813077	ko01110//Biosynthesis	-	-
-0.998976723	0.028802413	-	-	GO:0005576//extracellu
-0.998976937	0.028799394	ko01100//Metabolic p	-	-
-0.998977529	0.028791056	-	ko04934//Cushi	-
-0.998978412	0.028778621	ko01100//Metabolic p	-	-
-0.998979417	0.028764462	-	ko03013//Nucle	-
-0.998980282	0.02875226	-	ko05205//Prote	GO:0005576//extracellu
-0.998980306	0.02875193	-	-	GO:0016021//integral c
-0.998982389	0.028722534	-	ko04310//Wnt s	-
-0.998982424	0.02872205	ko01100//Metabolic p	ko01100//Metab	GO:0005783//endoplasr
-0.99898336	0.028708829	-	-	-
-0.998983919	0.028700938	-	ko00564//Glycer	GO:0005737//cytoplasm
-0.998984775	0.028688848	ko01100//Metabolic p	-	GO:0000791//euchroma
-0.998985173	0.028683224	ko01100//Metabolic p	ko01100//Metab	GO:0005737//cytoplasm
-0.998986681	0.028661892	-	-	GO:0005654//nucleopla
-0.998987115	0.028655751	-	ko01100//Metab	GO:0033178//proton-tr
-0.998987311	0.028652978	ko01063//Biosynthesis	ko04141//Protei	GO:0005737//cytoplasm
-0.998987377	0.028652052	-	ko01100//Metab	GO:0005794//Golgi app
-0.998987918	0.028644393	ko01110//Biosynthesis	-	-
-0.99898877	0.028632327	ko01110//Biosynthesis	ko04140//Autop	GO:0000407//pre-autop
-0.998988886	0.028630684	ko01100//Metabolic p	-	GO:0016020//membran
-0.998989613	0.028620391	ko01100//Metabolic p	-	-
-0.998989711	0.028619004	-	-	GO:0005737//cytoplasm
-0.998989892	0.028616441	ko01100//Metabolic p	ko01100//Metab	GO:0005777//peroxison
-0.998990155	0.028612713	ko01110//Biosynthesis	-	GO:0016020//membran
-0.998990998	0.028600766	-	-	GO:0005794//Golgi app
-0.998991954	0.028587208	ko01100//Metabolic p	ko01100//Metab	-
-0.998992372	0.028581279	ko01100//Metabolic p	-	-
-0.998992525	0.028579116	-	-	-
-0.998992733	0.028576162	ko01110//Biosynthesis	ko04216//Ferrop	GO:0016020//membran
-0.998993007	0.028572277	ko01100//Metabolic p	-	-
-0.998993571	0.028564274	-	-	-
-0.998993696	0.028562501	ko01063//Biosynthesis	-	-
-0.998995231	0.028540703	-	-	-
-0.998996921	0.028516686	-	ko01100//Metab	GO:0005783//endoplasr
-0.998997018	0.028515297	-	-	GO:0005615//extracellu
-0.998999969	0.028473309	-	-	GO:0005654//nucleopla
-0.999001252	0.028455049	-	-	-
-0.999002423	0.02843836	-	-	-
-0.999002849	0.028432284	-	ko05135//Yersin	-
-0.999004723	0.028405543	ko01100//Metabolic p	-	-
-0.999005299	0.028397323	-	ko05231//Cholir	-
-0.999005346	0.028396649	ko01063//Biosynthesis	-	-
-0.999005434	0.028395402	ko01110//Biosynthesis	-	GO:0005737//cytoplasm
-0.999005576	0.028393372	ko01100//Metabolic p	ko05231//Cholir	-
-0.999006578	0.028379052	ko01100//Metabolic p	-	GO:0005615//extracellu
-0.999007	0.028373021	-	-	-
-0.999007197	0.028370216	ko01100//Metabolic p	ko05012//Parkin	GO:0005654//nucleopla
-0.999008088	0.028357475	-	ko01100//Metab	GO:0016020//membran
-0.999008561	0.028350717	-	-	-
-0.999008865	0.028346364	-	-	GO:0005783//endoplasr
-0.999009217	0.028341338	-	-	GO:0005654//nucleopla
-0.999009777	0.028333316	ko01100//Metabolic p	-	GO:0005829//cytosol;G(
-0.999009994	0.028330217	-	-	-
-0.999010665	0.02832061	ko01100//Metabolic p	ko04976//Bile se	GO:0005789//endoplasr
-0.999011708	0.028305673	-	-	GO:0016020//membran
-0.999011863	0.028303462	-	-	-
-0.999013263	0.028283393	ko01063//Biosynthesis	ko04742//Taste	GO:0005737//cytoplasm

-0.999013987	0.028273012	-	-	GO:0005634//nucleus;G
-0.999014119	0.028271119	ko01100//Metabolic p	-	GO:0005737//cytoplasm
-0.999014365	0.028267599	ko01110//Biosynthesis	-	-
-0.999014732	0.028262334	-	-	GO:0005886//plasma m
-0.999016055	0.028243352	-	-	-
-0.999017046	0.02822912	ko01100//Metabolic p	ko01100//Metak	GO:0005783//endoplasr
-0.999017192	0.028227019	ko01100//Metabolic p	ko04659//Th17	GO:0000785//chromatir
-0.99901766	0.028220296	ko01100//Metabolic p	-	-
-0.999018707	0.028205259	-	-	-
-0.999018889	0.028202641	-	-	GO:0005730//nucleolus
-0.999019296	0.02819678	-	ko04979//Chole	GO:0005737//cytoplasm
-0.999020054	0.028185878	ko01100//Metabolic p	ko01100//Metak	GO:0005789//endoplasr
-0.999020434	0.028180419	ko01100//Metabolic p	-	-
-0.999021001	0.028172263	ko01100//Metabolic p	-	GO:0005634//nucleus;G
-0.999021114	0.028170627	ko01100//Metabolic p	-	-
-0.999024265	0.028125249	-	-	GO:0000791//euchroma
-0.999024963	0.028115191	-	-	GO:0016020//membran
-0.999025633	0.028105528	ko01063//Biosynthesis	ko01100//Metak	GO:0016020//membran
-0.999025748	0.02810386	-	ko01100//Metak	-
-0.999026602	0.028091542	ko01100//Metabolic p	-	-
-0.999026962	0.028086343	ko01100//Metabolic p	-	-
-0.999027392	0.02808014	ko01100//Metabolic p	-	GO:0005884//actin filam
-0.999027749	0.028074984	-	-	-
-0.999030381	0.028036952	-	ko01100//Metak	GO:0005783//endoplasr
-0.999030498	0.028035264	-	-	GO:0005634//nucleus;G
-0.99903071	0.0280322	-	-	GO:0005604//basement
-0.999030821	0.02803058	ko01063//Biosynthesis	-	-
-0.999031221	0.028024807	ko01100//Metabolic p	-	-
-0.999031274	0.028024041	-	ko05203//Viral c	GO:0000786//nucleosor
-0.999031344	0.028023029	-	-	-
-0.999031588	0.028019497	-	ko01100//Metak	GO:0016020//membran
-0.999032777	0.028002281	ko01063//Biosynthesis	-	-
-0.999032865	0.028001005	ko01100//Metabolic p	-	-
-0.999035069	0.027969079	ko01063//Biosynthesis	-	GO:0005886//plasma m
-0.999035319	0.027965452	-	-	GO:0005783//endoplasr
-0.999035653	0.027960616	-	-	GO:0031091//platelet al
-0.999036369	0.027950226	ko01100//Metabolic p	ko04080//Neurc	GO:0005886//plasma m
-0.999036791	0.027944113	-	ko04151//PI3K-/	GO:0005634//nucleus;G
-0.999037333	0.027936244	-	-	-
-0.999037791	0.0279296	ko01100//Metabolic p	-	GO:0005802//trans-Gol
-0.999038199	0.027923669	ko01100//Metabolic p	ko01100//Metak	GO:0001931//uropod;G
-0.999038886	0.027913702	-	-	-
-0.999040134	0.027895569	-	ko04144//Endoc	GO:0000813//ESCRT I c
-0.999040229	0.027894187	ko01100//Metabolic p	ko04714//Therm	GO:0005811//lipid parti
-0.999041649	0.02787354	ko01110//Biosynthesis	-	GO:0016020//membran
-0.99904166	0.027873373	-	-	GO:0001725//stress fibe
-0.999041742	0.027872181	ko01100//Metabolic p	ko04919//Thyro	GO:0016020//membran
-0.99904196	0.027869018	ko01100//Metabolic p	-	-
-0.999042277	0.027864407	-	-	-
-0.999045959	0.027810783	-	ko05200//Pathw	GO:0000785//chromatir
-0.999045985	0.027810406	-	-	-
-0.999046474	0.027803278	ko01100//Metabolic p	ko04360//Axon	-
-0.999046509	0.02780276	-	-	GO:0005634//nucleus;G
-0.999048186	0.027778292	-	-	-
-0.999048211	0.02777793	-	-	GO:0005788//endoplasr
-0.999048245	0.027777441	ko01100//Metabolic p	-	GO:0005783//endoplasr
-0.99904879	0.027769484	-	-	-
-0.999049	0.027766416	-	ko04211//Longe	GO:0005634//nucleus

-0.999049078	0.02776527	-	-	-
-0.999050786	0.027740327	ko01100//Metabolic p	-	-
-0.99905139	0.027731499	ko01100//Metabolic p	-	-
-0.999051654	0.027727634	-	-	GO:0005794//Golgi app
-0.999051716	0.027726734	-	-	GO:0005654//nucleopla
-0.999053959	0.027693913	ko01100//Metabolic p	-	-
-0.999054176	0.027690742	-	-	-
-0.999055814	0.027666738	-	ko01100//Metak	GO:0005739//mitochon
-0.99905633	0.027659189	ko01100//Metabolic p	-	-
-0.999056607	0.027655123	-	ko05412//Arrhyt	GO:0005886//plasma m
-0.999057557	0.027641198	ko01100//Metabolic p	-	GO:0005654//nucleopla
-0.999058775	0.027623325	ko01100//Metabolic p	ko01100//Metak	GO:0000506//glycosylpl
-0.999059125	0.027618182	-	-	GO:0005654//nucleopla
-0.999059895	0.027606879	ko01100//Metabolic p	-	-
-0.999061424	0.027584417	-	-	-
-0.999062011	0.027575788	-	-	-
-0.999062147	0.027573786	ko01100//Metabolic p	-	-
-0.999062672	0.027566076	-	-	GO:0005615//extracellu
-0.999062779	0.027564497	ko01100//Metabolic p	ko04922//Gluca	GO:0005634//nucleus;G
-0.999063392	0.027555485	-	ko04934//Cushi	-
-0.99906387	0.027548452	-	-	-
-0.999066791	0.027505426	ko01100//Metabolic p	ko01100//Metak	GO:0005635//nuclear e
-0.999067171	0.027499829	ko01100//Metabolic p	-	-
-0.999067755	0.027491214	-	-	-
-0.999067887	0.02748927	ko01100//Metabolic p	-	-
-0.999067955	0.02748827	ko01100//Metabolic p	-	GO:0005654//nucleopla
-0.999068247	0.02748396	ko01100//Metabolic p	-	GO:0005737//cytoplasm
-0.999069367	0.027467427	ko01100//Metabolic p	ko01523//Antifo	GO:0005886//plasma m
-0.999070335	0.027453147	-	-	-
-0.9990712	0.027440364	ko01063//Biosynthesis	-	-
-0.999071611	0.027434291	-	-	-
-0.999072535	0.027420634	ko01110//Biosynthesis	-	-
-0.999072619	0.027419395	-	-	-
-0.999073204	0.027410736	-	ko04146//Perox	GO:0005777//peroxison
-0.999073372	0.027408247	-	-	-
-0.999075567	0.027375765	-	ko00982//Drug	-
-0.999077686	0.02734437	-	-	GO:0005737//cytoplasm
-0.999078692	0.027329443	-	-	GO:0016021//integral c
-0.999078742	0.027328703	-	-	-
-0.999078744	0.027328685	-	-	GO:0000785//chromatir
-0.999078985	0.027325099	-	-	GO:0016020//membran
-0.999079276	0.027320789	ko01100//Metabolic p	-	-
-0.999079766	0.027313506	ko01063//Biosynthesis	-	GO:0005768//endosom
-0.999081055	0.027294381	-	-	GO:0016020//membran
-0.999081224	0.027291859	-	-	-
-0.999081277	0.02729108	-	-	GO:0034361//very-low-
-0.999081883	0.027282073	ko01100//Metabolic p	-	GO:0016021//integral c
-0.999082012	0.027280161	-	ko04142//Lysos	-
-0.999083549	0.02725731	ko01100//Metabolic p	-	GO:0016020//membran
-0.999083879	0.027252402	-	-	-
-0.999083914	0.027251873	ko01100//Metabolic p	ko04142//Lysos	GO:0016020//membran
-0.999083981	0.027250883	-	-	-
-0.999083987	0.027250793	ko01100//Metabolic p	ko01100//Metak	GO:0005778//peroxison
-0.999084689	0.027240346	-	ko04080//Neurc	GO:0005886//plasma m
-0.999084703	0.027240134	-	-	-
-0.999084996	0.027235777	-	-	GO:0005634//nucleus
-0.999085188	0.027232911	ko01100//Metabolic p	ko01100//Metak	GO:0005654//nucleopla
-0.99908551	0.027228123	ko01100//Metabolic p	ko04218//Cellul	GO:0030896//checkpoir

-0.999085712	0.02722512	ko01100//Metabolic p	-	GO:0005783//endoplasr
-0.999085953	0.027221532	ko01100//Metabolic p	-	-
-0.999088902	0.027177568	ko01100//Metabolic p	ko04714//Therm	GO:0005811//lipid parti
-0.999089438	0.027169572	ko01063//Biosynthesis	ko01100//Metab	-
-0.999090189	0.027158372	ko01100//Metabolic p	-	GO:0005604//basement
-0.999090278	0.027157039	-	-	GO:0005615//extracellu
-0.999091195	0.027143344	ko01100//Metabolic p	-	-
-0.999091652	0.027136517	ko01100//Metabolic p	-	-
-0.999092849	0.027118637	ko01100//Metabolic p	-	-
-0.999093082	0.027115146	-	-	-
-0.999093765	0.027104934	ko01110//Biosynthesis	-	-
-0.999094189	0.027098597	-	ko04976//Bile se	GO:0005789//endoplasr
-0.999094822	0.027089116	ko01100//Metabolic p	-	-
-0.999095334	0.027081453	-	ko05012//Parkin	GO:0005654//nucleopla
-0.999096203	0.027068436	ko01100//Metabolic p	-	-
-0.999098108	0.027039891	-	-	GO:0005737//cytoplasm
-0.999098237	0.027037969	ko01110//Biosynthesis	ko05231//Cholir	-
-0.999098244	0.027037861	ko01100//Metabolic p	-	GO:0000127//transcript
-0.999098559	0.027033134	ko01100//Metabolic p	ko05200//Pathw	GO:0005834//heterotrin
-0.99909897	0.027026968	ko01100//Metabolic p	-	GO:0005634//nucleus;G
-0.999099224	0.027023159	ko01100//Metabolic p	ko04979//Chole	GO:0005737//cytoplasm
-0.999099625	0.027017137	ko01100//Metabolic p	-	GO:0005737//cytoplasm
-0.999099881	0.027013295	-	-	-
-0.99910052	0.027003707	ko01100//Metabolic p	-	GO:0016020//membran
-0.99910096	0.026997103	-	-	-
-0.999101023	0.026996154	ko01100//Metabolic p	ko01100//Metab	GO:0005576//extracellu
-0.999101148	0.026994283	-	ko01100//Metab	GO:0005783//endoplasr
-0.999102277	0.026977318	-	ko05205//Proteo	GO:0005576//extracellu
-0.999102453	0.02697468	ko01063//Biosynthesis	-	GO:0005634//nucleus;G
-0.9991029	0.026967952	-	-	-
-0.999103362	0.026961009	ko01100//Metabolic p	-	GO:0005576//extracellu
-0.999104048	0.026950694	-	-	-
-0.999104073	0.026950321	-	-	-
-0.999104446	0.0269447	-	-	GO:0005802//trans-Gol
-0.999105336	0.026931313	-	ko05200//Pathw	GO:0000785//chromatir
-0.999105764	0.026924862	ko01100//Metabolic p	-	-
-0.999105917	0.026922563	ko01100//Metabolic p	-	-
-0.999106512	0.0269136	ko01063//Biosynthesis	ko01100//Metab	GO:0005783//endoplasr
-0.999107007	0.026906138	ko01100//Metabolic p	-	-
-0.99910792	0.026892391	ko01100//Metabolic p	ko01100//Metab	GO:0005789//endoplasr
-0.999108688	0.026880809	-	ko01100//Metab	GO:0033178//proton-tr
-0.999108925	0.026877226	ko01100//Metabolic p	ko01100//Metab	GO:0005576//extracellu
-0.999109474	0.026868955	ko01100//Metabolic p	ko01100//Metab	-
-0.999109489	0.026868724	-	-	GO:0001725//stress fibe
-0.999109848	0.026863304	-	-	-
-0.999110125	0.026859131	-	ko04216//Ferrop	GO:0016020//membran
-0.999110351	0.02685571	ko01100//Metabolic p	-	-
-0.999112057	0.026829946	ko01110//Biosynthesis	ko04145//Phago	GO:0005783//endoplasr
-0.99911233	0.026825824	-	-	GO:0005794//Golgi app
-0.999112419	0.026824478	ko01100//Metabolic p	-	-
-0.999113762	0.026804174	-	-	-
-0.999113842	0.026802966	-	ko04140//Autop	GO:0000407//pre-autop
-0.999114639	0.026790899	-	-	-
-0.999115884	0.026772052	-	ko04919//Thyro	GO:0016020//membran
-0.99911717	0.026752579	-	ko04934//Cushi	GO:0016020//membran
-0.99911746	0.026748189	ko01100//Metabolic p	ko01100//Metab	GO:0005783//endoplasr
-0.999117699	0.026744561	-	-	-
-0.999118315	0.026735215	ko01100//Metabolic p	-	GO:0005737//cytoplasm

-0.999119594	0.02671582	ko01100//Metabolic p	-	-
-0.999120402	0.026703548	ko01063//Biosynthesis	-	-
-0.999120939	0.026695397	-	-	-
-0.999121163	0.026691998	ko01100//Metabolic p	-	GO:0005576//extracellu
-0.999121194	0.026691535	-	-	GO:0031464//Cul4A-RII
-0.999123031	0.026663611	ko01100//Metabolic p	ko01100//Metak	-
-0.999123292	0.02665964	ko01100//Metabolic p	-	-
-0.999123325	0.026659136	ko01100//Metabolic p	-	-
-0.999123331	0.026659047	ko01063//Biosynthesis	-	GO:0016020//membran
-0.999123443	0.026657341	-	ko05135//Yersin	-
-0.999123773	0.026652331	ko01100//Metabolic p	-	-
-0.999124549	0.02664052	ko01100//Metabolic p	ko04120//Ubiqu	GO:0005680//anaphase
-0.999125729	0.026622562	ko01063//Biosynthesis	ko04080//Neurc	GO:0016021//integral c
-0.999127387	0.026597304	-	ko04010//MAPK	GO:0005737//cytoplasm
-0.999127834	0.026590491	-	ko03320//PPAR	GO:0005576//extracellu
-0.999128575	0.026579183	-	-	-
-0.999129079	0.026571495	-	-	-
-0.999130537	0.02654925	ko01100//Metabolic p	-	GO:0016020//membran
-0.999133646	0.026501724	-	-	GO:0000791//euchroma
-0.999134995	0.026481077	ko01100//Metabolic p	ko04068//FoxO	GO:0005737//cytoplasm
-0.999135342	0.026475765	ko01100//Metabolic p	ko04270//Vascu	GO:0005737//cytoplasm
-0.999135847	0.026468037	ko01100//Metabolic p	ko04310//Wnt s	-
-0.999136001	0.026465679	ko01100//Metabolic p	ko04146//Peroxi	GO:0005765//lysosomal
-0.99913673	0.026454514	ko01100//Metabolic p	-	-
-0.999137235	0.026446767	-	ko01100//Metak	GO:0005737//cytoplasm
-0.999137379	0.026444561	ko01100//Metabolic p	-	GO:0005654//nucleopla
-0.999138046	0.02643433	ko01100//Metabolic p	-	GO:0005737//cytoplasm
-0.999138308	0.026430317	-	-	-
-0.999138479	0.026427699	-	-	GO:0005730//nucleolus
-0.999138496	0.026427432	-	ko01100//Metak	GO:0005783//endoplasi
-0.999138579	0.026426156	-	ko04080//Neurc	GO:0005886//plasma m
-0.999140685	0.02639383	-	-	-
-0.999141173	0.026386337	ko01100//Metabolic p	ko04742//Taste	GO:0005783//endoplasi
-0.999141282	0.026384655	ko01100//Metabolic p	-	-
-0.99914223	0.026370085	ko01063//Biosynthesis	-	-
-0.99914242	0.026367163	-	ko01100//Metak	GO:0005737//cytoplasm
-0.999143418	0.026351816	-	-	-
-0.999144967	0.02632798	-	ko04721//Synap	-
-0.999145788	0.026315328	ko01100//Metabolic p	-	GO:0005737//cytoplasm
-0.999146025	0.026311682	-	ko01100//Metak	GO:0005739//mitochon
-0.999146537	0.026303786	ko01110//Biosynthesis	-	-
-0.999146913	0.026298003	ko01100//Metabolic p	-	GO:0005654//nucleopla
-0.999147104	0.026295045	-	ko04146//Peroxi	GO:0005765//lysosomal
-0.999147228	0.026293135	ko01100//Metabolic p	-	-
-0.999147264	0.026292591	-	ko01100//Metak	-
-0.999147634	0.026286882	ko01100//Metabolic p	-	-
-0.999147704	0.026285795	ko01110//Biosynthesis	-	GO:0005654//nucleopla
-0.99914867	0.026270893	ko01100//Metabolic p	-	-
-0.999148807	0.026268787	-	-	GO:0005654//nucleopla
-0.999148944	0.026266671	-	-	GO:0005886//plasma m
-0.999149674	0.026255393	-	ko04068//FoxO	GO:0005737//cytoplasm
-0.999151946	0.026220292	ko01100//Metabolic p	ko01100//Metak	GO:0000139//Golgi mer
-0.9991528	0.026207084	-	-	-
-0.999153465	0.026196798	-	-	-
-0.999153957	0.026189184	ko01100//Metabolic p	-	-
-0.999154341	0.026183236	ko01100//Metabolic p	ko01100//Metak	GO:0000506//glycosylpl
-0.999154451	0.02618154	ko01100//Metabolic p	-	-
-0.999156959	0.026142666	-	-	GO:0005886//plasma m

-0.999157328	0.026136945	-	-	-
-0.999157385	0.026136068	-	-	-
-0.999158269	0.026122357	ko01100//Metabolic p	-	GO:0005737//cytoplasm
-0.999158575	0.026117595	-	-	GO:0001650//fibrillar ce
-0.999159005	0.026110926	-	-	-
-0.999159926	0.026096624	ko01100//Metabolic p	-	-
-0.999160061	0.026094519	-	ko00970//Amino	GO:0005737//cytoplasm
-0.999160123	0.026093565	ko01100//Metabolic p	-	GO:0034451//centriolar
-0.999160503	0.026087664	ko01100//Metabolic p	-	GO:0016020//membran
-0.999161108	0.026078252	-	ko04142//Lysoso	GO:0016020//membran
-0.999162245	0.026060567	-	ko04020//Calciu	GO:0016020//membran
-0.999162411	0.026057986	-	-	GO:0016020//membran
-0.99916328	0.026044472	-	-	GO:0005576//extracellu
-0.999163313	0.026043955	-	-	GO:0005576//extracellu
-0.999164417	0.026026758	ko01100//Metabolic p	-	GO:0000779//condense
-0.999164427	0.026026615	ko01100//Metabolic p	-	-
-0.999165924	0.026003283	ko01100//Metabolic p	ko04360//Axon	-
-0.999166506	0.025994205	ko01100//Metabolic p	ko04144//Endoc	GO:0001917//photorece
-0.999167454	0.02597941	ko01100//Metabolic p	-	-
-0.999168113	0.025969124	-	-	-
-0.999168204	0.025967717	ko01100//Metabolic p	-	GO:0005886//plasma m
-0.999168459	0.025963729	ko01100//Metabolic p	ko05202//Transc	GO:0000786//nucleosor
-0.999168465	0.025963638	ko01100//Metabolic p	-	-
-0.999168757	0.025959069	-	ko01100//Metab	GO:0005783//endoplasn
-0.999169419	0.025948735	ko01100//Metabolic p	-	-
-0.999170153	0.025937267	-	ko01100//Metab	GO:0005789//endoplasn
-0.999172958	0.025893388	-	-	GO:0005576//extracellu
-0.999173136	0.025890599	ko01100//Metabolic p	-	-
-0.999173223	0.025889237	ko01063//Biosynthesis	-	GO:0005615//extracellu
-0.999174698	0.025866129	ko01110//Biosynthesis	-	-
-0.999174962	0.025861992	ko01100//Metabolic p	-	-
-0.999175024	0.025861014	ko01100//Metabolic p	-	GO:0005634//nucleus;G
-0.999177639	0.025819994	ko01100//Metabolic p	-	GO:0005764//lysosome;
-0.999177711	0.025818867	-	ko01100//Metab	GO:0005783//endoplasn
-0.999177892	0.025816023	ko01100//Metabolic p	-	GO:0005802//trans-Gol
-0.999178774	0.025802163	ko01100//Metabolic p	-	GO:0001725//stress fibe
-0.99917893	0.025799714	-	-	GO:0005576//extracellu
-0.999179539	0.025790146	-	-	GO:0005783//endoplasn
-0.999179664	0.025788175	ko01100//Metabolic p	-	GO:0005813//centrosor
-0.999182126	0.025749446	ko01100//Metabolic p	ko01100//Metab	GO:0016020//membran
-0.99918262	0.025741668	ko01100//Metabolic p	ko03010//Riboso	GO:0005634//nucleus;G
-0.999182753	0.025739574	ko01100//Metabolic p	ko05200//Pathw	GO:0000785//chromatir
-0.999182998	0.025735712	-	ko04150//mTOR	GO:0005764//lysosome;
-0.9991831	0.025734106	-	-	GO:0005783//endoplasn
-0.999183604	0.025726162	-	-	GO:0016020//membran
-0.9991853	0.025699432	ko01100//Metabolic p	-	GO:0005654//nucleopla
-0.999185352	0.025698609	-	-	GO:0000127//transcript
-0.999185918	0.025689683	ko01100//Metabolic p	-	-
-0.999187307	0.025667748	-	-	-
-0.999187495	0.025664776	-	-	-
-0.999188282	0.025652344	-	-	GO:0005634//nucleus;G
-0.999188342	0.02565139	ko01100//Metabolic p	-	-
-0.999189336	0.025635689	ko01100//Metabolic p	-	-
-0.999189484	0.02563334	ko01100//Metabolic p	-	GO:0005634//nucleus;G
-0.999190072	0.025624049	ko01100//Metabolic p	ko01100//Metab	-
-0.999190851	0.025611715	ko01100//Metabolic p	ko04020//Calciu	GO:0005634//nucleus;G
-0.999191133	0.025607254	ko01100//Metabolic p	-	-
-0.999191533	0.025600922	-	ko01100//Metab	GO:0005576//extracellu

-0.999192668	0.025582944	-	-	GO:0016020//membran
-0.999193416	0.025571079	-	-	GO:0005794//Golgi app
-0.999194014	0.025561594	-	-	GO:0005737//cytoplasm
-0.999194441	0.025554835	-	-	GO:0016020//membran
-0.999194865	0.025548102	-	ko00564//Glycer	GO:0005737//cytoplasm
-0.999195663	0.025535442	ko01100//Metabolic p	-	GO:0005737//cytoplasm
-0.999195801	0.025533246	ko01100//Metabolic p	-	GO:0005886//plasma m
-0.999196173	0.025527331	-	-	-
-0.999196215	0.025526664	ko01100//Metabolic p	ko05202//Transc	GO:0000786//nucleosor
-0.999196561	0.025521173	-	ko01100//Metab	GO:0005737//cytoplasm
-0.999196654	0.025519698	-	ko04020//Calciu	GO:0005634//nucleus;G
-0.999197041	0.025513552	ko01110//Biosynthesis	ko01100//Metab	-
-0.999198162	0.025495725	-	-	-
-0.999198373	0.025492375	-	-	-
-0.999199223	0.025478862	-	-	-
-0.999199919	0.025467778	ko01100//Metabolic p	ko04151//PI3K-/	GO:0005654//nucleopla
-0.999202955	0.025419407	ko01063//Biosynthesis	-	GO:0005634//nucleus;G
-0.999203189	0.025415681	-	-	-
-0.999203485	0.025410959	ko01100//Metabolic p	-	GO:0031982//vesicle
-0.999204043	0.025402044	-	-	GO:0005829//cytosol
-0.999204293	0.025398057	-	ko04146//Peroxi	GO:0005765//lysosomal
-0.999206203	0.025367555	ko01100//Metabolic p	ko01100//Metab	GO:0005829//cytosol
-0.999206368	0.025364912	ko01100//Metabolic p	-	-
-0.999206767	0.025358534	ko01100//Metabolic p	ko04080//Neuro	GO:0005886//plasma m
-0.999207109	0.025353076	ko01100//Metabolic p	-	-
-0.999207781	0.025342331	-	-	GO:0016020//membran
-0.999209544	0.025314106	-	ko04919//Thyro	GO:0016020//membran
-0.999209712	0.025311419	-	-	GO:0005764//lysosome;
-0.999209865	0.025308963	ko01100//Metabolic p	-	GO:0005654//nucleopla
-0.999209974	0.02530721	-	-	GO:0032991//macromo
-0.999210192	0.025303733	ko01110//Biosynthesis	-	-
-0.999211509	0.025282616	ko01100//Metabolic p	-	-
-0.999211695	0.02527964	-	ko04310//Wnt s	GO:0005654//nucleopla
-0.999213598	0.025249102	ko01100//Metabolic p	ko04978//Miner	GO:0009986//cell surfac
-0.999214995	0.025226661	ko01063//Biosynthesis	ko05010//Alzhei	GO:0005783//endoplasr
-0.99921552	0.025218219	-	-	GO:0005654//nucleopla
-0.999216447	0.025203316	ko01100//Metabolic p	-	-
-0.999216982	0.025194707	-	-	-
-0.999216985	0.025194653	-	-	-
-0.999217916	0.025179673	ko01100//Metabolic p	-	-
-0.9992206	0.025136423	ko01100//Metabolic p	-	-
-0.999221311	0.025124962	ko01100//Metabolic p	-	GO:0016020//membran
-0.999221851	0.025116245	-	-	-
-0.999221995	0.025113918	-	-	-
-0.999222444	0.025106667	-	ko00790//Folate	-
-0.999222925	0.025098905	-	ko05412//Arrhyt	GO:0005886//plasma m
-0.99922621	0.025045781	-	-	-
-0.999227117	0.0250311	-	-	GO:0005886//plasma m
-0.999227155	0.025030482	ko01100//Metabolic p	-	GO:0016020//membran
-0.999227606	0.025023177	ko01100//Metabolic p	-	-
-0.99922768	0.025021975	-	-	GO:0005634//nucleus;G
-0.999227813	0.025019823	-	ko04068//FoxO	GO:0005737//cytoplasm
-0.999227828	0.025019587	ko01100//Metabolic p	ko04550//Signal	GO:0005634//nucleus;G
-0.999228388	0.025010507	-	-	GO:0016020//membran
-0.999228487	0.025008901	ko01100//Metabolic p	-	GO:0000786//nucleosor
-0.999229431	0.024993591	ko01100//Metabolic p	ko01100//Metab	GO:0005829//cytosol
-0.999229471	0.024992945	ko01100//Metabolic p	ko04020//Calciu	GO:0016020//membran
-0.999229518	0.02499219	ko01100//Metabolic p	-	GO:0005789//endoplasr

-0.999230285	0.024979744	ko01100//Metabolic p	-	-
-0.999230725	0.024972594	ko01110//Biosynthesis	-	GO:0005783//endoplasr
-0.999230909	0.024969619	-	-	-
-0.999230975	0.024968536	ko01100//Metabolic p	ko03320//PPAR	GO:0005634//nucleus;G
-0.999231762	0.024955758	-	-	GO:0005634//nucleus;G
-0.999232158	0.024949327	ko01100//Metabolic p	ko05322//Syster	GO:0000786//nucleosor
-0.999232492	0.024943906	-	-	-
-0.999232661	0.024941159	ko01100//Metabolic p	-	GO:0005634//nucleus
-0.999232837	0.024938293	-	ko01100//Metab	GO:0005654//nucleopla
-0.999233198	0.024932425	-	-	-
-0.999235099	0.024901503	ko01100//Metabolic p	-	-
-0.999235668	0.024892224	ko01100//Metabolic p	ko05152//Tuber	GO:0009897//external s
-0.999239768	0.024825363	ko01100//Metabolic p	ko01100//Metab	GO:0001650//fibrillar ce
-0.999240776	0.024808901	-	-	GO:0005576//extracellu
-0.99924171	0.024793643	-	-	-
-0.999242694	0.024777547	ko01063//Biosynthesis	-	-
-0.99924275	0.024776625	ko01100//Metabolic p	-	GO:0005634//nucleus;G
-0.999243004	0.024772467	-	-	GO:0000785//chromatir
-0.999243436	0.024765406	-	-	-
-0.999244187	0.024753108	-	ko04979//Chole	GO:0005737//cytoplasm
-0.999244474	0.024748402	-	-	GO:0005802//trans-Gol
-0.999244912	0.024741226	ko01100//Metabolic p	ko01100//Metab	GO:0016020//membran
-0.99924592	0.024724706	-	ko01100//Metab	GO:0009923//fatty acid
-0.9992467	0.024711912	-	-	-
-0.999246863	0.024709243	ko01100//Metabolic p	ko04934//Cushir	GO:0016020//membran
-0.999247345	0.02470133	-	ko01100//Metab	GO:0005737//cytoplasm
-0.999247575	0.024697561	ko01100//Metabolic p	-	-
-0.999248705	0.024678999	-	-	-
-0.999248969	0.024674663	-	-	-
-0.99924897	0.024674648	ko01100//Metabolic p	-	GO:0005802//trans-Gol
-0.999249167	0.024671412	ko01100//Metabolic p	ko05203//Viral c	GO:0000786//nucleosor
-0.999249438	0.02466695	ko01100//Metabolic p	ko04141//Protei	GO:0005783//endoplasr
-0.99925056	0.024648503	ko01100//Metabolic p	ko01100//Metab	GO:0005789//endoplasr
-0.999250626	0.024647419	ko01100//Metabolic p	-	GO:0016020//membran
-0.999250727	0.02464577	ko01100//Metabolic p	ko05200//Pathw	GO:0005634//nucleus;G
-0.999250778	0.024644928	-	-	-
-0.999250797	0.02464461	-	-	-
-0.999251866	0.02462702	ko01100//Metabolic p	-	-
-0.999254973	0.024575824	-	ko01100//Metab	GO:0016020//membran
-0.999255338	0.024569806	-	-	-
-0.999255453	0.024567908	ko01100//Metabolic p	-	GO:0000177//cytoplasm
-0.9992575	0.02453411	ko01100//Metabolic p	ko04010//MAPK	GO:0005737//cytoplasm
-0.99925819	0.0245227	ko01100//Metabolic p	ko01100//Metab	GO:0005634//nucleus;G
-0.999259073	0.024508095	-	ko05165//Huma	-
-0.999259093	0.024507777	ko01100//Metabolic p	ko04211//Longe	GO:0005634//nucleus
-0.999259354	0.024503457	ko01100//Metabolic p	-	GO:0005794//Golgi app
-0.999260918	0.024477563	-	-	GO:0005783//endoplasr
-0.999260925	0.024477439	ko01100//Metabolic p	-	GO:0005737//cytoplasm
-0.999261237	0.024472278	-	-	-
-0.999261701	0.024464591	ko01110//Biosynthesis	-	GO:0005764//lysosome;
-0.999261795	0.024463038	-	-	GO:0000127//transcript
-0.999262378	0.024453367	ko01100//Metabolic p	ko05203//Viral c	-
-0.999263815	0.024429533	ko01100//Metabolic p	-	GO:0031012//extracellu
-0.99926382	0.024429457	ko01100//Metabolic p	ko01100//Metab	-
-0.999263835	0.024429205	-	-	-
-0.999263915	0.024427884	ko01100//Metabolic p	ko04010//MAPK	GO:0005737//cytoplasm
-0.999264251	0.024422297	-	-	-
-0.999266598	0.024383305	-	-	-

-0.99926698	0.024376957	-	-	GO:0031464//Cul4A-RII
-0.999267028	0.024376162	ko01100//Metabolic p	ko04141//Protei	GO:0005737//cytoplasm
-0.999267535	0.024367724	-	-	GO:0005886//plasma m
-0.999268846	0.024345903	-	ko04120//Ubiqu	GO:0005680//anaphase
-0.999270422	0.024319662	ko01110//Biosynthesis	-	-
-0.999271053	0.024309132	ko01100//Metabolic p	-	-
-0.999271286	0.024305241	ko01100//Metabolic p	-	-
-0.999271523	0.024301292	-	-	-
-0.999272054	0.024292429	-	ko01100//Metab	GO:0005737//cytoplasm
-0.999272231	0.024289479	ko01110//Biosynthesis	-	GO:0005615//extracellu
-0.999272525	0.02428457	-	ko04010//MAPK	-
-0.999272782	0.024280286	ko01110//Biosynthesis	-	-
-0.999273457	0.024269013	-	-	GO:0005576//extracellu
-0.999273849	0.024262459	-	ko01100//Metab	-
-0.999274109	0.024258118	ko01100//Metabolic p	ko04723//Retrog	GO:0005739//mitochon
-0.999274217	0.024256313	ko01110//Biosynthesis	ko04020//Calciu	GO:0005634//nucleus;G
-0.999274226	0.024256158	ko01100//Metabolic p	-	-
-0.999274454	0.02425236	ko01100//Metabolic p	-	-
-0.999275042	0.024242524	-	ko03320//PPAR	GO:0005615//extracellu
-0.999275419	0.024236222	-	ko01100//Metab	-
-0.999276666	0.02421535	-	-	-
-0.999276791	0.02421326	-	ko04210//Apopt	GO:0005634//nucleus;G
-0.999277339	0.024204078	-	-	-
-0.999277727	0.024197577	ko01100//Metabolic p	-	GO:0005654//nucleopla
-0.999278769	0.024180111	-	-	-
-0.999279035	0.024175652	ko01100//Metabolic p	-	-
-0.999279104	0.024174509	-	-	GO:0005654//nucleopla
-0.999279241	0.024172196	ko01100//Metabolic p	-	-
-0.999279334	0.024170639	ko01110//Biosynthesis	ko01100//Metab	GO:0005789//endoplasr
-0.999284562	0.024082796	ko01100//Metabolic p	ko03013//Nucle	-
-0.99928565	0.024064484	ko01100//Metabolic p	ko05200//Pathw	GO:0005737//cytoplasm
-0.999285724	0.024063227	-	ko01100//Metab	GO:0005737//cytoplasm
-0.999285998	0.02405862	-	-	-
-0.999286057	0.024057627	ko01100//Metabolic p	ko04310//Wnt s	GO:0005654//nucleopla
-0.999286504	0.024050089	ko01100//Metabolic p	ko04310//Wnt s	GO:0009897//external s
-0.999286976	0.02404214	ko01100//Metabolic p	-	GO:0005811//lipid parti
-0.999287496	0.024033363	ko01100//Metabolic p	ko04721//Synap	-
-0.999287682	0.024030231	ko01100//Metabolic p	-	GO:0005634//nucleus;G
-0.999287712	0.024029724	ko01100//Metabolic p	-	-
-0.999287992	0.024024993	ko01100//Metabolic p	ko04146//Peroxi	GO:0005765//lysosomal
-0.999289425	0.024000806	-	-	GO:0016020//membran
-0.999290185	0.023987971	-	-	GO:0005794//Golgi app
-0.999290464	0.023983253	ko01100//Metabolic p	-	-
-0.999291321	0.023968757	ko01100//Metabolic p	ko04141//Protei	GO:0030120//vesicle co
-0.999292186	0.02395413	-	-	-
-0.999292701	0.023945415	ko01100//Metabolic p	ko04934//Cushi	-
-0.999292747	0.023944628	ko01100//Metabolic p	-	GO:0005615//extracellu
-0.999293431	0.023933045	ko01100//Metabolic p	ko00790//Folate	-
-0.999293768	0.023927344	ko01110//Biosynthesis	-	GO:0005886//plasma m
-0.999294798	0.023909882	-	-	-
-0.999294912	0.023907944	ko01100//Metabolic p	-	-
-0.99929522	0.023902726	ko01100//Metabolic p	-	-
-0.999295763	0.023893506	-	-	GO:0014069//postsynap
-0.99929771	0.023860459	-	-	-
-0.999297756	0.023859678	ko01100//Metabolic p	ko01100//Metab	GO:0005739//mitochon
-0.999297837	0.023858301	ko01100//Metabolic p	-	GO:0016020//membran
-0.999298137	0.023853197	ko01100//Metabolic p	ko01100//Metab	GO:0005783//endoplasr
-0.999298719	0.023843314	-	ko05010//Alzhei	-

-0.999299088	0.023837032	ko01100//Metabolic p	-	GO:0016020//membran
-0.999299489	0.023830221	-	-	-
-0.999299914	0.023822978	ko01100//Metabolic p	-	GO:0001650//fibrillar ce
-0.999300809	0.023807754	-	-	-
-0.999301409	0.023797532	-	ko04144//Endoc	GO:0000813//ESCRT I co
-0.999301805	0.023790783	-	-	-
-0.999301851	0.023789998	ko01100//Metabolic p	ko04520//Adher	GO:0031252//cell leadir
-0.999301981	0.02378779	-	-	GO:0097541//axonemal
-0.999303515	0.023761622	-	-	-
-0.999303611	0.023759983	-	-	GO:0005634//nucleus;G
-0.999303877	0.023755456	ko01100//Metabolic p	-	GO:0005737//cytoplasm
-0.999303986	0.023753586	-	ko01100//Metak	GO:0033178//proton-tr
-0.999305285	0.023731415	ko01100//Metabolic p	-	-
-0.999306012	0.023718999	-	-	-
-0.99930722	0.023698333	-	ko03010//Ribos	GO:0005634//nucleus;G
-0.999307252	0.023697785	ko01100//Metabolic p	-	-
-0.999308135	0.023682683	-	-	-
-0.999308665	0.023673604	-	ko01100//Metak	GO:0016020//membran
-0.999309077	0.023666544	-	ko04710//Circac	GO:0005634//nucleus;G
-0.999309885	0.023652707	ko01100//Metabolic p	ko04144//Endoc	-
-0.999310728	0.023638256	-	-	GO:0005884//actin filam
-0.999310735	0.023638141	-	ko01100//Metak	GO:0005789//endoplasr
-0.999310964	0.023634201	-	-	-
-0.999310989	0.023633785	ko01100//Metabolic p	-	GO:0005737//cytoplasm
-0.999311538	0.023624355	-	ko04550//Signal	GO:0005634//nucleus;G
-0.999312063	0.023615346	-	-	-
-0.999312538	0.023607187	-	ko01100//Metak	GO:0005654//nucleopla
-0.999313134	0.023596957	-	-	GO:0005789//endoplasr
-0.999313168	0.023596376	ko01100//Metabolic p	-	GO:0016021//integral co
-0.999313342	0.023593381	-	ko01100//Metak	GO:0005783//endoplasr
-0.999313543	0.023589925	-	-	-
-0.9993157	0.023552839	-	ko01100//Metak	-
-0.999315925	0.023548953	-	ko03320//PPAR	GO:0005634//nucleus;G
-0.999316574	0.023537782	-	-	-
-0.999317046	0.023529662	ko01100//Metabolic p	-	GO:0005576//extracellu
-0.999318707	0.023501027	-	-	GO:0005802//trans-Gol
-0.999318854	0.023498483	-	ko04020//Calciu	GO:0016020//membran
-0.999318893	0.023497811	-	-	GO:0005634//nucleus;G
-0.999319153	0.023493319	ko01100//Metabolic p	-	-
-0.999319333	0.023490216	-	-	GO:0005654//nucleopla
-0.999319484	0.023487618	ko01100//Metabolic p	-	-
-0.999320327	0.023473054	-	-	-
-0.999320375	0.023472225	-	-	-
-0.99932038	0.023472153	ko01100//Metabolic p	ko04144//Endoc	-
-0.999320584	0.023468626	-	ko04020//Calciu	GO:0005886//plasma m
-0.9993216	0.023451064	ko01063//Biosynthesis	ko03010//Ribos	GO:0005840//ribosome
-0.99932263	0.023433259	ko01100//Metabolic p	-	GO:0005737//cytoplasm
-0.999323054	0.023425916	ko01100//Metabolic p	-	-
-0.999323128	0.023424646	-	ko01100//Metak	-
-0.999323417	0.023419631	-	-	-
-0.999323473	0.023418659	-	-	-
-0.999324011	0.023409358	-	-	-
-0.99932424	0.023405394	ko01100//Metabolic p	-	-
-0.999324882	0.023394261	-	-	-
-0.999325044	0.023391463	ko01100//Metabolic p	-	-
-0.999325864	0.023377234	ko01100//Metabolic p	-	GO:0000785//chromatir
-0.999326664	0.02336336	-	-	-
-0.99932691	0.023359103	ko01063//Biosynthesis	ko05203//Viral c	GO:0000786//nucleosor

-0.999327418	0.023350279	ko01100//Metabolic p	ko04020//Calciu	GO:0005634//nucleus;G
-0.999327819	0.023343315	-	-	GO:0034361//very-low-
-0.999328663	0.023328654	ko01100//Metabolic p	-	GO:0005634//nucleus;G
-0.999328798	0.023326306	ko01100//Metabolic p	-	-
-0.999329413	0.023315626	-	ko01100//Metab	GO:0005634//nucleus;G
-0.999330126	0.023303222	-	-	-
-0.999330192	0.023302077	-	ko04211//Longe	GO:0005634//nucleus
-0.999331323	0.023282388	ko01100//Metabolic p	-	GO:0005634//nucleus;G
-0.999331509	0.023279145	-	-	GO:0000177//cytoplasm
-0.999332741	0.023257686	ko01110//Biosynthesis	-	GO:0030864//cortical ac
-0.99933291	0.023254742	-	-	-
-0.999333151	0.023250537	-	ko05200//Pathw	GO:0005634//nucleus;G
-0.999333824	0.023238799	ko01100//Metabolic p	ko04142//Lysos	GO:0016020//membran
-0.99933401	0.023235554	ko01110//Biosynthesis	ko01100//Metab	GO:0005778//peroxison
-0.999334692	0.023223659	-	ko04146//Perox	GO:0005777//peroxison
-0.99933508	0.023216881	ko01100//Metabolic p	-	-
-0.999335616	0.02320752	-	-	GO:0031012//extracellu
-0.99933681	0.023186664	-	-	-
-0.999336981	0.02318367	ko01100//Metabolic p	-	-
-0.999337265	0.023178707	ko01110//Biosynthesis	ko04010//MAPK	GO:0005737//cytoplasm
-0.999337822	0.023168964	-	-	-
-0.999337895	0.023167678	ko01100//Metabolic p	ko01100//Metab	-
-0.999338274	0.023161047	ko01100//Metabolic p	-	GO:0005737//cytoplasm
-0.999338794	0.023151941	ko01100//Metabolic p	ko01100//Metab	GO:0005737//cytoplasm
-0.999338827	0.023151372	ko01100//Metabolic p	-	-
-0.999339112	0.023146368	ko01100//Metabolic p	ko01100//Metab	GO:0016021//integral co
-0.999339362	0.023142	ko01100//Metabolic p	-	GO:0005764//lysosome;
-0.999339732	0.02313551	-	-	-
-0.999339882	0.023132894	-	ko04080//Neuro	GO:0005886//plasma m
-0.99934018	0.023127671	ko01100//Metabolic p	-	-
-0.999340651	0.023119402	ko01100//Metabolic p	-	GO:0005829//cytosol
-0.999341271	0.023108523	ko01100//Metabolic p	ko01100//Metab	GO:0005737//cytoplasm
-0.999341334	0.023107427	ko01100//Metabolic p	-	GO:0005829//cytosol
-0.999342344	0.023089695	ko01100//Metabolic p	-	-
-0.999343079	0.023076788	ko01063//Biosynthesis	-	GO:0005615//extracellu
-0.999343461	0.023070075	-	ko05012//Parkin	GO:0005654//nucleopla
-0.999344278	0.023055719	-	-	GO:0005789//endoplasn
-0.999344905	0.023044704	-	ko01100//Metab	GO:0005576//extracellu
-0.999345127	0.023040782	ko01100//Metabolic p	-	GO:0005634//nucleus;G
-0.999345498	0.023034259	-	ko05200//Pathw	GO:0005834//heterotrin
-0.999345557	0.023033228	-	-	GO:0005634//nucleus;G
-0.999345864	0.023027817	ko01100//Metabolic p	-	GO:0008076//voltage-g
-0.999345943	0.023026433	ko01100//Metabolic p	-	GO:0005634//nucleus;G
-0.999347854	0.022992771	-	ko01100//Metab	GO:0005794//Golgi app
-0.999349081	0.022971113	-	-	-
-0.999349184	0.022969299	-	ko04218//Cellul	GO:0030896//checkpoir
-0.999349229	0.022968507	-	-	-
-0.99934947	0.022964259	ko01100//Metabolic p	-	-
-0.999349727	0.022959723	-	-	GO:0005737//cytoplasm
-0.999349878	0.022957045	ko01100//Metabolic p	-	-
-0.9993502	0.02295137	ko01100//Metabolic p	ko04151//PI3K-/	GO:0005654//nucleopla
-0.999350537	0.022945419	-	-	GO:0005634//nucleus;G
-0.999351057	0.022936218	ko01100//Metabolic p	-	GO:0005737//cytoplasm
-0.999351486	0.022928639	-	ko03013//Nucle	-
-0.999351672	0.022925358	-	-	-
-0.999354379	0.022877428	ko01100//Metabolic p	-	-
-0.999354395	0.022877151	ko01110//Biosynthesis	ko01100//Metab	GO:0005789//endoplasn
-0.999355509	0.022857404	-	ko01100//Metab	GO:0005783//endoplasn

-0.999355849	0.022851372	-	ko04146//Perox	GO:0005765//lysosomal
-0.999356463	0.02284047	ko01110//Biosynthesis	-	-
-0.999356609	0.022837878	ko01100//Metabolic p	-	-
-0.99935719	0.022827563	ko01063//Biosynthesis	-	GO:0016020//membran
-0.999357215	0.022827126	ko01100//Metabolic p	ko01100//Metak	-
-0.999357381	0.022824171	-	ko01100//Metak	GO:0005829//cytosol
-0.999357503	0.022822014	-	-	GO:0005654//nucleopla
-0.999358778	0.022799346	ko01100//Metabolic p	-	GO:0016020//membran
-0.999360707	0.02276503	-	-	-
-0.999361375	0.022753126	-	-	-
-0.999362222	0.022738042	-	ko05231//Cholir	-
-0.999362958	0.022724912	-	-	-
-0.999364051	0.022705415	-	-	GO:0005634//nucleus;G
-0.999364095	0.022704616	ko01100//Metabolic p	-	-
-0.999364439	0.022698487	ko01100//Metabolic p	-	GO:0005576//extracellu
-0.999365035	0.022687835	-	-	GO:0005737//cytoplasm
-0.999365941	0.022671633	ko01063//Biosynthesis	ko04361//Axon	GO:0005737//cytoplasm
-0.999366097	0.022668844	-	ko03320//PPAR	GO:0005783//endoplası
-0.999368624	0.022623615	-	ko04141//Protei	GO:0005783//endoplası
-0.999368733	0.022621654	ko01100//Metabolic p	-	-
-0.999369507	0.022607789	ko01100//Metabolic p	-	-
-0.999370075	0.022597607	-	ko05152//Tuber	GO:0009897//external s
-0.99937024	0.02259465	-	-	-
-0.999371154	0.022578245	ko01100//Metabolic p	ko01100//Metak	GO:0005783//endoplası
-0.999372602	0.022552219	-	-	GO:0097541//axonemal
-0.999373086	0.022543524	-	-	GO:0001725//stress fibe
-0.999374026	0.022526612	ko01100//Metabolic p	-	-
-0.999374137	0.02252461	ko01100//Metabolic p	-	-
-0.999374504	0.022518015	-	ko01100//Metak	GO:0033178//proton-tr
-0.999374798	0.022512712	-	-	-
-0.999375088	0.022507494	-	-	-
-0.999375346	0.022502842	ko01100//Metabolic p	-	GO:0005634//nucleus;G
-0.999375444	0.02250108	ko01100//Metabolic p	-	GO:0005634//nucleus;G
-0.999375716	0.022496184	-	-	-
-0.999376422	0.022483456	-	ko04141//Protei	GO:0005634//nucleus;G
-0.999376424	0.022483428	-	-	-
-0.999376523	0.02248163	ko01110//Biosynthesis	-	GO:0016020//membran
-0.999376562	0.022480934	-	ko05200//Pathw	GO:0000785//chromatir
-0.999378403	0.022447705	-	-	-
-0.999378923	0.022438318	-	ko04210//Apopt	GO:0005634//nucleus;G
-0.99937902	0.022436567	-	ko04211//Longe	GO:0005634//nucleus
-0.999379149	0.022434228	ko01100//Metabolic p	-	GO:0005783//endoplası
-0.999379158	0.022434066	ko01100//Metabolic p	ko01100//Metak	-
-0.999379676	0.022424718	ko01100//Metabolic p	-	GO:0016020//membran
-0.999380747	0.022405342	ko01100//Metabolic p	-	-
-0.999380829	0.022403851	ko01100//Metabolic p	-	GO:0001650//fibrillar ce
-0.999381037	0.022400087	ko01063//Biosynthesis	-	-
-0.999381877	0.02238489	ko01100//Metabolic p	-	-
-0.99938251	0.022373419	-	-	GO:0005634//nucleus;G
-0.99938261	0.022371616	-	ko01100//Metak	GO:0005654//nucleopla
-0.999382638	0.022371098	ko01110//Biosynthesis	ko04742//Taste	GO:0005783//endoplası
-0.999383044	0.022363748	-	-	-
-0.999383469	0.022356041	-	-	GO:0005576//extracellu
-0.999383896	0.0223483	ko01100//Metabolic p	-	-
-0.999384748	0.022332836	-	-	-
-0.999385086	0.022326693	-	-	GO:0005737//cytoplasm
-0.999386399	0.022302859	ko01100//Metabolic p	-	-
-0.999386645	0.022298384	ko01100//Metabolic p	ko01100//Metak	-

-0.999387439	0.022283948	-	ko05203//Viral c	-
-0.999387453	0.022283678	-	-	-
-0.999388671	0.022261522	ko01100//Metabolic p	-	GO:0005802//trans-Gol
-0.999389061	0.02225442	-	-	-
-0.999389993	0.022237425	-	-	-
-0.999390002	0.022237264	-	ko04144//Endoc	-
-0.999390381	0.022230365	-	-	GO:0000785//chromatir
-0.999390689	0.022224736	-	-	-
-0.999390747	0.02222368	ko01100//Metabolic p	-	-
-0.999390766	0.022223338	ko01100//Metabolic p	ko04923//Regul	GO:0005654//nucleopla
-0.999391574	0.022208597	-	ko04710//Circac	GO:0005634//nucleus;G
-0.999392089	0.022199189	ko01100//Metabolic p	ko03320//PPAR	GO:0005615//extracellu
-0.999392402	0.022193467	ko01100//Metabolic p	ko04721//Synap	-
-0.999392822	0.022185804	-	ko01100//Metak	-
-0.999392954	0.02218339	-	ko04144//Endoc	-
-0.999392985	0.022182822	-	-	GO:0005737//cytoplasm
-0.999393327	0.022176563	ko01100//Metabolic p	-	-
-0.999393622	0.022171177	-	ko04142//Lysos	GO:0016020//membran
-0.999394305	0.02215869	-	-	-
-0.99939442	0.022156584	-	-	-
-0.999394689	0.022151662	-	-	-
-0.999395158	0.022143075	-	-	GO:0005739//mitochon
-0.999396496	0.022118564	-	-	GO:0014069//postsynap
-0.999396718	0.022114504	ko01063//Biosynthesis	-	GO:0044424//intracellul
-0.999397087	0.022107744	-	-	GO:0034361//very-low-
-0.999397219	0.022105322	ko01100//Metabolic p	-	GO:0005829//cytosol;G
-0.999397267	0.022104442	-	-	-
-0.999397419	0.02210164	-	ko04310//Wnt s	GO:0005634//nucleus;G
-0.99939747	0.022100714	ko01100//Metabolic p	-	GO:0000781//chromoso
-0.999397613	0.022098086	-	-	-
-0.999397787	0.022094892	-	ko01100//Metak	-
-0.999397888	0.02209304	ko01100//Metabolic p	ko01100//Metak	-
-0.999398398	0.022083692	ko01100//Metabolic p	-	GO:0016020//membran
-0.999398446	0.022082805	-	-	GO:0016021//integral c
-0.999399232	0.022068371	-	ko05010//Alzhei	-
-0.999400046	0.02205341	-	ko04310//Wnt s	-
-0.999400754	0.022040394	-	-	GO:0000139//Golgi mer
-0.999401037	0.022035189	-	-	-
-0.9994018	0.022021149	-	ko05200//Pathw	GO:0005737//cytoplasm
-0.999402149	0.02201473	ko01100//Metabolic p	ko01100//Metak	GO:0005654//nucleopla
-0.999402323	0.022011518	ko01110//Biosynthesis	-	GO:0000779//condense
-0.999402551	0.022007329	ko01100//Metabolic p	-	-
-0.999403382	0.021992017	ko01100//Metabolic p	ko01100//Metak	GO:0005737//cytoplasm
-0.999403447	0.021990819	ko01100//Metabolic p	ko01100//Metak	-
-0.999403515	0.021989562	ko01100//Metabolic p	-	GO:0005739//mitochon
-0.999403595	0.021988088	-	ko04146//Perox	GO:0005777//peroxison
-0.999403687	0.021986389	-	-	-
-0.999404399	0.021973251	ko01110//Biosynthesis	-	GO:0005634//nucleus;G
-0.999404555	0.021970374	-	-	-
-0.999405775	0.021947861	-	-	-
-0.999407226	0.021921034	-	-	-
-0.999407801	0.021910413	ko01100//Metabolic p	-	-
-0.999408948	0.02188917	-	-	-
-0.99940933	0.021882098	ko01100//Metabolic p	-	GO:0005789//endoplasr
-0.999409735	0.021874602	ko01110//Biosynthesis	-	-
-0.999409836	0.02187272	-	-	GO:0005811//lipid parti
-0.999411178	0.021847832	-	-	GO:0005811//lipid parti
-0.999411268	0.021846172	-	-	GO:0005615//extracellu

-0.999411275	0.021846033	ko01100//Metabolic p	-	GO:0016021//integral co
-0.999412066	0.021831351	ko01100//Metabolic p	-	-
-0.999412385	0.021825426	ko01110//Biosynthesis	ko01100//Metab	-
-0.999412609	0.021821276	-	ko05203//Viral c	GO:0000786//nucleosor
-0.999413788	0.021799349	ko01100//Metabolic p	ko01523//Antifo	GO:0005886//plasma m
-0.999414221	0.021791299	-	-	GO:0043231//intracellul
-0.999414414	0.02178772	-	-	GO:0005737//cytoplasm
-0.999416951	0.021740463	ko01100//Metabolic p	-	-
-0.999417546	0.021729357	-	ko04151//PI3K-/	GO:0005654//nucleopla
-0.999418022	0.021720474	ko01100//Metabolic p	-	GO:0008076//voltage-g
-0.999419381	0.021695105	-	ko01100//Metab	GO:0005829//cytosol
-0.999421098	0.021662994	-	ko00790//Folate	-
-0.99942203	0.02164555	-	-	GO:0005634//nucleus;G
-0.999422869	0.021629827	ko01063//Biosynthesis	-	-
-0.999423347	0.021620879	-	ko04142//Lysos	-
-0.999425077	0.021588418	-	-	-
-0.999425094	0.021588087	ko01100//Metabolic p	-	-
-0.999425518	0.021580142	ko01110//Biosynthesis	ko01100//Metab	GO:0016021//integral co
-0.999426344	0.021564621	ko01100//Metabolic p	-	-
-0.999426493	0.021561813	-	-	-
-0.999426907	0.021554032	-	-	-
-0.999428318	0.021527481	ko01100//Metabolic p	ko04144//Endoc	GO:0000813//ESCRT I co
-0.999428397	0.021525984	-	-	-
-0.999429314	0.021508709	-	-	GO:0001669//acrosoma
-0.999430236	0.021491331	ko01100//Metabolic p	-	GO:0044424//intracellul
-0.999430662	0.021483288	-	ko04144//Endoc	-
-0.999431087	0.021475266	ko01100//Metabolic p	-	-
-0.999431429	0.021468823	ko01100//Metabolic p	-	GO:0030864//cortical ac
-0.999432115	0.021455863	ko01100//Metabolic p	-	GO:0005794//Golgi app
-0.999432285	0.021452643	ko01100//Metabolic p	-	-
-0.999432659	0.021445569	-	ko03320//PPAR	GO:0005783//endoplasr
-0.999433134	0.021436604	-	ko05203//Viral c	-
-0.999433368	0.021432166	ko01100//Metabolic p	ko01100//Metab	GO:0005654//nucleopla
-0.99943339	0.021431753	-	-	GO:0005794//Golgi app
-0.999434221	0.021416023	ko01100//Metabolic p	-	-
-0.99943423	0.021415853	-	-	-
-0.999434909	0.021403009	ko01100//Metabolic p	-	-
-0.999435049	0.021400358	ko01100//Metabolic p	-	-
-0.999435503	0.021391755	ko01100//Metabolic p	-	-
-0.999436054	0.021381311	ko01100//Metabolic p	ko01100//Metab	-
-0.999436091	0.021380609	-	-	GO:0005737//cytoplasm
-0.999436133	0.021379816	-	ko01100//Metab	-
-0.999436496	0.021372937	ko01100//Metabolic p	ko04151//PI3K-/	GO:0005634//nucleus;G
-0.999437015	0.021363075	-	-	GO:0005604//basement
-0.999437423	0.021355339	-	-	-
-0.999438439	0.021336046	ko01100//Metabolic p	ko05200//Pathw	GO:0005576//extracellu
-0.999439111	0.021323274	ko01110//Biosynthesis	-	GO:0005737//cytoplasm
-0.999439192	0.021321736	ko01100//Metabolic p	-	GO:0000139//Golgi mer
-0.999439241	0.021320796	ko01100//Metabolic p	-	GO:0005654//nucleopla
-0.999440187	0.021302806	ko01100//Metabolic p	-	-
-0.999440528	0.021296327	-	-	GO:0005783//endoplasr
-0.999441087	0.02128567	ko01100//Metabolic p	-	GO:0005634//nucleus
-0.99944179	0.021272291	ko01100//Metabolic p	ko01100//Metab	GO:0005634//nucleus;G
-0.999442409	0.02126049	ko01100//Metabolic p	ko04310//Wnt s	GO:0005634//nucleus;G
-0.999442607	0.021256714	-	-	GO:0005576//extracellu
-0.999442687	0.021255179	ko01100//Metabolic p	ko01100//Metab	-
-0.999443092	0.021247468	-	-	GO:0005634//nucleus;G
-0.999443213	0.021245144	-	-	GO:0005634//nucleus;G

-0.999443328	0.021242952	ko01100//Metabolic p	-	-
-0.999443533	0.021239053	-	-	GO:0005634//nucleus;G
-0.999443541	0.021238891	ko01110//Biosynthesis	-	-
-0.999443585	0.02123806	ko01100//Metabolic p	-	-
-0.999443981	0.021230495	ko01100//Metabolic p	-	-
-0.999444887	0.02121319	ko01100//Metabolic p	ko01100//Metab	-
-0.999444935	0.021212261	-	ko05010//Alzhei	GO:0005576//extracellu
-0.999444942	0.021212134	-	-	-
-0.999445585	0.021199849	-	ko04216//Ferrop	GO:0016020//membran
-0.999445587	0.021199797	ko01100//Metabolic p	-	GO:0005634//nucleus;G
-0.999445754	0.021196611	-	ko04144//Endoc	GO:0005654//nucleopla
-0.999445768	0.021196347	ko01100//Metabolic p	-	GO:0016020//membran
-0.999445772	0.021196271	-	-	GO:0000139//Golgi mer
-0.999446217	0.021187749	ko01100//Metabolic p	ko01100//Metab	GO:0005737//cytoplasm
-0.999446321	0.021185774	-	ko03013//Nucle	-
-0.999447524	0.021162743	ko01100//Metabolic p	-	GO:0000785//chromatir
-0.999447615	0.021160993	ko01100//Metabolic p	-	-
-0.999447855	0.0211564	ko01100//Metabolic p	-	-
-0.999448518	0.021143684	-	ko04140//Autop	GO:0000407//pre-autop
-0.999449053	0.021133437	-	-	-
-0.999449147	0.021131618	-	-	-
-0.999449786	0.021119358	-	-	GO:0001725//stress fibe
-0.999449841	0.021118318	ko01100//Metabolic p	-	GO:0031464//Cul4A-RII
-0.999451105	0.021094033	-	-	-
-0.999452452	0.021068137	-	ko04550//Signal	GO:0005634//nucleus;G
-0.999452582	0.021065623	ko01100//Metabolic p	-	-
-0.999453875	0.02104074	-	-	GO:0005789//endoplasr
-0.999454053	0.021037314	-	ko01100//Metab	GO:0005737//cytoplasm
-0.99945424	0.021033708	-	-	-
-0.999454867	0.021021617	-	-	-
-0.999455398	0.021011377	ko01100//Metabolic p	-	GO:0016020//membran
-0.999455674	0.021006055	ko01100//Metabolic p	-	GO:0000151//ubiquitin
-0.999456414	0.020991759	-	-	GO:0005794//Golgi app
-0.99945678	0.02098469	ko01100//Metabolic p	-	-
-0.99945687	0.020982962	ko01100//Metabolic p	-	-
-0.999457094	0.02097862	-	ko04211//Longe	GO:0005634//nucleus
-0.999458604	0.020949436	ko01100//Metabolic p	ko01100//Metab	GO:0005794//Golgi app
-0.999458937	0.020942981	ko01110//Biosynthesis	ko01100//Metab	-
-0.999458974	0.020942265	-	ko04020//Calciu	GO:0016020//membran
-0.999459039	0.020941021	-	ko05200//Pathw	GO:0000307//cyclin-dep
-0.999459131	0.020939232	ko01063//Biosynthesis	-	GO:0005634//nucleus;G
-0.999459146	0.020938938	-	-	GO:0005654//nucleopla
-0.99946031	0.020916387	ko01100//Metabolic p	-	GO:0005737//cytoplasm
-0.99946066	0.020909605	-	-	GO:0005634//nucleus;G
-0.999461219	0.020898763	ko01100//Metabolic p	ko05200//Pathw	GO:0005634//nucleus
-0.999461342	0.020896381	-	ko01100//Metab	GO:0005829//cytosol
-0.999461409	0.020895093	ko01100//Metabolic p	ko01100//Metab	GO:0005783//endoplasr
-0.999462766	0.020868746	-	-	GO:0005737//cytoplasm
-0.999462912	0.020865915	-	-	-
-0.999463233	0.020859672	-	-	-
-0.999463339	0.02085762	-	-	-
-0.999464132	0.020842193	ko01100//Metabolic p	-	-
-0.999464882	0.0208276	ko01100//Metabolic p	ko04742//Taste	GO:0016020//membran
-0.99946516	0.020822193	ko01100//Metabolic p	ko04514//Cell a	GO:0005769//early endo
-0.999465577	0.020814069	ko01100//Metabolic p	ko01100//Metab	GO:0005783//endoplasr
-0.999465635	0.020812936	-	-	GO:0001533//cornified
-0.99946598	0.020806219	-	-	-
-0.999466111	0.020803663	ko01100//Metabolic p	-	GO:0005615//extracellu

-0.999466342	0.020799166	ko01100//Metabolic p	-	GO:0005886//plasma m
-0.999466578	0.02079457	ko01100//Metabolic p	ko01100//Metak	GO:0005576//extracellu
-0.999467081	0.020784751	ko01100//Metabolic p	-	-
-0.999467177	0.020782885	-	-	GO:0005634//nucleus;G
-0.999467943	0.020767937	ko01063//Biosynthesis	-	-
-0.99946998	0.020728137	-	-	GO:0016020//membran
-0.999470616	0.020715705	ko01100//Metabolic p	-	GO:0046658//anchored
-0.999470983	0.020708514	-	ko04141//Protei	GO:0005634//nucleus;G
-0.99947131	0.020702115	ko01100//Metabolic p	-	-
-0.999471708	0.020694319	-	ko05200//Pathw	GO:0005634//nucleus;G
-0.999473531	0.020658588	ko01100//Metabolic p	ko03013//Nucle	-
-0.99947401	0.020649175	-	-	-
-0.999474147	0.020646496	-	-	-
-0.999474396	0.020641601	-	-	GO:0005737//cytoplasm
-0.999475898	0.020612096	-	-	-
-0.99947666	0.020597099	ko01100//Metabolic p	-	-
-0.999476999	0.020590419	-	-	GO:0005634//nucleus;G
-0.999477697	0.020576676	-	ko04080//Neuro	GO:0005886//plasma m
-0.999477974	0.020571221	-	ko04080//Neuro	GO:0005886//plasma m
-0.999478052	0.020569686	ko01100//Metabolic p	-	-
-0.999478106	0.020568622	ko01100//Metabolic p	ko05231//Cholir	-
-0.999480196	0.020527397	ko01100//Metabolic p	-	GO:0005654//nucleopla
-0.999480426	0.020522849	ko01100//Metabolic p	ko04080//Neuro	GO:0005886//plasma m
-0.999480611	0.020519199	ko01100//Metabolic p	-	-
-0.999481205	0.020507461	ko01110//Biosynthesis	ko01100//Metak	GO:0005634//nucleus;G
-0.999481664	0.020498387	ko01100//Metabolic p	ko04360//Axon	GO:0005886//plasma m
-0.999482954	0.020472854	-	-	GO:0001725//stress fibe
-0.999483092	0.020470129	ko01100//Metabolic p	-	GO:0030659//cytoplasm
-0.999483097	0.020470023	ko01100//Metabolic p	ko01100//Metak	-
-0.999483486	0.020462322	-	ko04144//Endoc	-
-0.999484215	0.020447869	-	ko04710//Circac	GO:0005634//nucleus;G
-0.999484762	0.02043703	ko01100//Metabolic p	-	GO:0005615//extracellu
-0.999485029	0.020431731	-	-	-
-0.999485369	0.020424974	ko01100//Metabolic p	-	-
-0.999486688	0.020398793	ko01100//Metabolic p	ko03320//PPAR	GO:0005783//endoplasr
-0.999487381	0.020385022	-	-	-
-0.999488329	0.020366158	ko01100//Metabolic p	-	GO:0005737//cytoplasm
-0.999488779	0.020357192	-	-	-
-0.999488804	0.020356707	ko01100//Metabolic p	-	-
-0.999488947	0.02035386	-	ko04144//Endoc	GO:0000813//ESCRT I co
-0.999489163	0.020349546	-	-	GO:0005634//nucleus;G
-0.999489503	0.020342773	-	-	GO:0005634//nucleus
-0.999490221	0.020328461	ko01100//Metabolic p	ko02010//ABC tr	GO:0016021//integral co
-0.999490625	0.020320412	ko01100//Metabolic p	-	-
-0.999490802	0.020316881	-	ko04144//Endoc	GO:0000813//ESCRT I co
-0.999491381	0.020305316	-	ko04151//PI3K-/	GO:0005634//nucleus;G
-0.999492744	0.020278094	ko01100//Metabolic p	-	-
-0.999492937	0.02027424	ko01100//Metabolic p	-	GO:0005615//extracellu
-0.999493693	0.020259113	ko01100//Metabolic p	-	GO:0005886//plasma m
-0.999495734	0.020218232	ko01100//Metabolic p	ko01100//Metak	GO:0005783//endoplasr
-0.999496091	0.020211085	ko01100//Metabolic p	-	-
-0.99949862	0.020160283	-	-	-
-0.999498947	0.020153712	ko01100//Metabolic p	-	-
-0.999498956	0.020153526	ko01100//Metabolic p	-	-
-0.999499352	0.020145569	-	ko01100//Metak	-
-0.999499418	0.020144246	-	ko01100//Metak	-
-0.999499656	0.020139449	-	ko01100//Metak	-
-0.999499758	0.020137388	ko01100//Metabolic p	ko04710//Circac	GO:0005634//nucleus;G

-0.999501131	0.020109732	ko01100//Metabolic p	-	-
-0.999502096	0.020090273	-	-	-
-0.999502618	0.020079741	-	-	GO:0005634//nucleus;G
-0.999502862	0.020074824	-	-	GO:0005783//endoplasr
-0.999502957	0.02007289	-	-	GO:0005811//lipid parti
-0.99950305	0.020071013	ko01100//Metabolic p	ko01100//Metat	GO:0005654//nucleopla
-0.99950365	0.020058908	ko01100//Metabolic p	-	-
-0.999503838	0.020055097	ko01110//Biosynthesis	ko05203//Viral c	GO:0000786//nucleosor
-0.99950391	0.020053653	ko01063//Biosynthesis	-	-
-0.999504358	0.020044583	ko01100//Metabolic p	-	-
-0.999504757	0.02003652	ko01100//Metabolic p	-	GO:0005794//Golgi app
-0.999504801	0.020035622	ko01100//Metabolic p	ko04550//Signal	GO:0005634//nucleus;G
-0.999505502	0.020021431	ko01100//Metabolic p	ko01100//Metat	-
-0.99950576	0.020016206	ko01100//Metabolic p	ko04130//SNAR	GO:0005737//cytoplasm
-0.99950582	0.020014992	ko01110//Biosynthesis	-	GO:0005634//nucleus;G
-0.999505944	0.020012482	-	-	GO:0016020//membran
-0.999506088	0.020009573	-	-	GO:0005634//nucleus;G
-0.999507037	0.019990328	ko01100//Metabolic p	-	GO:0005737//cytoplasm
-0.999507171	0.019987623	-	ko03320//PPAR	GO:0005783//endoplasr
-0.999508144	0.019967871	ko01100//Metabolic p	-	GO:0005737//cytoplasm
-0.999508481	0.01996104	-	ko04144//Endoc	GO:0005654//nucleopla
-0.999508541	0.019959816	ko01100//Metabolic p	-	-
-0.999508571	0.019959201	ko01100//Metabolic p	-	-
-0.999508705	0.019956494	-	-	-
-0.999508961	0.019951276	-	-	-
-0.999509237	0.019945686	ko01100//Metabolic p	-	GO:0005739//mitochon
-0.999509332	0.019943755	-	-	GO:0016020//membran
-0.999509525	0.01993983	ko01100//Metabolic p	-	-
-0.999510888	0.019912104	ko01100//Metabolic p	-	GO:0005886//plasma m
-0.999511532	0.019898985	-	ko01100//Metat	GO:0005737//cytoplasm
-0.999511953	0.01989041	ko01100//Metabolic p	-	GO:0097541//axonemal
-0.999512704	0.019875092	ko01110//Biosynthesis	-	-
-0.999512818	0.019872771	-	ko01100//Metat	GO:0000139//Golgi mer
-0.999513191	0.019865167	ko01100//Metabolic p	-	GO:0005886//plasma m
-0.999514138	0.019845835	ko01100//Metabolic p	-	-
-0.999514366	0.019841168	-	-	GO:0016020//membran
-0.999514785	0.019832599	-	-	GO:0000791//euchroma
-0.999515838	0.019811069	-	-	-
-0.999516521	0.019797103	-	-	-
-0.999516647	0.01979451	-	-	-
-0.999517255	0.019782052	ko01110//Biosynthesis	-	GO:0005737//cytoplasm
-0.999517959	0.01976763	ko01100//Metabolic p	-	-
-0.99951797	0.0197674	ko01100//Metabolic p	ko05200//Pathw	GO:0000307//cyclin-de
-0.99951839	0.019758792	ko01100//Metabolic p	ko04310//Wnt s	-
-0.999518413	0.019758308	-	ko01100//Metat	GO:0005783//endoplasr
-0.99951895	0.019747305	ko01100//Metabolic p	-	GO:0005886//plasma m
-0.999518975	0.019746784	ko01100//Metabolic p	-	-
-0.999519013	0.019746003	ko01063//Biosynthesis	-	GO:0005737//cytoplasm
-0.999519094	0.019744344	ko01100//Metabolic p	-	-
-0.999519501	0.019735984	ko01100//Metabolic p	-	GO:0005634//nucleus;G
-0.999519519	0.019735619	ko01100//Metabolic p	-	GO:0016020//membran
-0.999519898	0.019727828	-	ko03013//Nucle	-
-0.999520442	0.019716654	ko01100//Metabolic p	ko04742//Taste	GO:0005737//cytoplasm
-0.999521806	0.019688588	-	-	-
-0.999522277	0.019678877	-	ko04310//Wnt s	GO:0005634//nucleus;G
-0.9995231	0.019661932	-	-	-
-0.999523113	0.019661662	-	ko05203//Viral c	-
-0.999523648	0.019650626	ko01100//Metabolic p	-	GO:0005634//nucleus;G

-0.999523718	0.019649182	ko01100//Metabolic p	ko03013//Nucle	-
-0.999524237	0.019638475	ko01100//Metabolic p	-	-
-0.999524804	0.019626764	-	ko01100//Metak	GO:0005789//endoplasr
-0.999524964	0.019623466	-	ko01100//Metak	-
-0.999525516	0.019612047	-	-	GO:0005789//endoplasr
-0.99952609	0.019600179	-	ko04514//Cell a	GO:0005769//early endo
-0.999527096	0.019579367	-	ko01100//Metak	GO:0005737//cytoplasm
-0.999527184	0.019577544	ko01100//Metabolic p	ko04142//Lysos	-
-0.999527273	0.01957571	-	-	GO:0016021//integral c
-0.999528528	0.019549693	ko01063//Biosynthesis	ko01100//Metak	GO:0016020//membran
-0.999528657	0.019547033	ko01100//Metabolic p	ko04020//Calciu	GO:0016020//membran
-0.999528906	0.019541868	-	-	-
-0.999529054	0.01953879	ko01100//Metabolic p	ko01100//Metak	GO:0016020//membran
-0.999529113	0.019537575	-	ko03320//PPAR	GO:0005634//nucleus;G
-0.999529225	0.019535238	ko01100//Metabolic p	-	-
-0.999530072	0.019517669	ko01100//Metabolic p	-	GO:0005737//cytoplasm
-0.999530615	0.019506391	ko01100//Metabolic p	-	-
-0.999531645	0.019484967	-	ko03013//Nucle	-
-0.999531799	0.019481772	-	-	GO:0005576//extracellu
-0.999532764	0.019461676	-	-	-
-0.999532909	0.01945866	ko01100//Metabolic p	ko01100//Metak	GO:0005737//cytoplasm
-0.999533103	0.019454612	ko01100//Metabolic p	-	GO:0005576//extracellu
-0.999533718	0.019441801	-	-	-
-0.999534018	0.019435542	ko01100//Metabolic p	-	-
-0.999534839	0.019418415	-	-	-
-0.999535069	0.019413604	-	-	-
-0.999535436	0.019405947	-	ko00564//Glycer	GO:0005634//nucleus;G
-0.999535638	0.019401725	-	-	-
-0.9995358	0.019398328	ko01063//Biosynthesis	-	GO:0005856//cytoskelet
-0.999536366	0.019386499	-	-	-
-0.999537278	0.019367421	ko01100//Metabolic p	-	GO:0005634//nucleus;G
-0.999537286	0.019367268	ko01110//Biosynthesis	ko05152//Tuber	GO:0009897//external s
-0.999537683	0.019358954	ko01110//Biosynthesis	ko01100//Metak	GO:0005829//cytosol
-0.999538151	0.01934915	-	-	-
-0.999538186	0.01934842	-	-	-
-0.999538193	0.019348272	-	-	-
-0.999538358	0.019344814	-	-	-
-0.999538405	0.019343828	-	ko01100//Metak	GO:0005794//Golgi app
-0.999538548	0.019340823	ko01100//Metabolic p	-	GO:0000786//nucleosor
-0.99953895	0.019332406	-	-	-
-0.999539712	0.019316422	ko01100//Metabolic p	-	GO:0043231//intracellul
-0.999540606	0.019297645	-	ko05200//Pathw	GO:0005737//cytoplasm
-0.999541386	0.019281249	ko01100//Metabolic p	ko05322//Syster	GO:0000786//nucleosor
-0.999541522	0.019278401	-	-	GO:0000139//Golgi mer
-0.999541912	0.01927019	-	-	GO:0016020//membran
-0.999542028	0.019267767	-	-	GO:0000177//cytoplasm
-0.99954323	0.019242447	ko01100//Metabolic p	-	GO:0030864//cortical ac
-0.99954405	0.019225172	-	ko03320//PPAR	GO:0005783//endoplasr
-0.99954443	0.019217155	-	ko04310//Wnt s	GO:0005634//nucleus;G
-0.999544446	0.019216817	-	-	GO:0005794//Golgi app
-0.999544634	0.019212848	ko01100//Metabolic p	-	GO:0005737//cytoplasm
-0.999544713	0.01921119	ko01100//Metabolic p	-	GO:0005737//cytoplasm
-0.999544746	0.019210488	-	-	GO:0031362//anchored
-0.99954584	0.019187401	-	-	GO:0005886//plasma m
-0.999546122	0.019181443	ko01100//Metabolic p	ko04080//Neuro	GO:0005886//plasma m
-0.999546677	0.019169707	ko01100//Metabolic p	ko01100//Metak	GO:0005576//extracellu
-0.999547825	0.019145411	ko01100//Metabolic p	-	-
-0.999547888	0.01914409	-	ko04010//MAPK	GO:0005737//cytoplasm

-0.999548528	0.019130533	-	-	GO:0005634//nucleus;G
-0.999548957	0.019121439	-	ko01100//Metab	GO:0005829//cytosol
-0.999549056	0.019119341	-	-	GO:0005886//plasma m
-0.999549309	0.019113975	-	-	GO:0005634//nucleus;G
-0.99954981	0.019103336	ko01100//Metabolic p	-	GO:0005886//plasma m
-0.99955047	0.019089332	-	-	GO:0005634//nucleus;G
-0.999551453	0.019068458	ko01100//Metabolic p	-	GO:0005604//basement
-0.999552456	0.019047108	-	-	GO:0031464//Cul4A-RII
-0.999552488	0.019046426	ko01100//Metabolic p	ko05200//Pathw	GO:0005576//extracellu
-0.999553106	0.019033271	ko01100//Metabolic p	-	-
-0.999553229	0.019030654	ko01100//Metabolic p	-	-
-0.99955324	0.01903043	ko01100//Metabolic p	ko04216//Ferrop	GO:0016020//membran
-0.999553284	0.019029483	ko01100//Metabolic p	-	-
-0.999554405	0.019005595	-	-	-
-0.999554587	0.019001709	ko01100//Metabolic p	-	GO:0005783//endoplasr
-0.999554732	0.018998623	-	-	-
-0.999554772	0.018997758	ko01063//Biosynthesis	ko04922//Gluca	GO:0005634//nucleus;G
-0.999555125	0.018990239	-	-	GO:0005739//mitochon
-0.999555362	0.018985172	ko01100//Metabolic p	-	GO:0031464//Cul4A-RII
-0.999555453	0.018983219	-	-	GO:0005634//nucleus;G
-0.999555873	0.018974265	ko01100//Metabolic p	ko04140//Autop	GO:0000407//pre-autop
-0.999555997	0.018971598	ko01100//Metabolic p	-	-
-0.999556029	0.018970926	-	-	GO:0005737//cytoplasm
-0.999556232	0.018966591	-	ko04919//Thyro	GO:0016020//membran
-0.999556437	0.018962198	ko01100//Metabolic p	-	-
-0.999556586	0.018959014	-	-	-
-0.999556872	0.018952899	ko01100//Metabolic p	-	GO:0016020//membran
-0.999557058	0.018948932	-	ko01100//Metab	-
-0.999557249	0.018944837	ko01100//Metabolic p	ko01100//Metab	-
-0.999557954	0.018929746	-	-	GO:0005634//nucleus;G
-0.999558539	0.018917221	ko01063//Biosynthesis	-	GO:0005737//cytoplasm
-0.999560571	0.018873626	ko01100//Metabolic p	-	-
-0.999560855	0.018867522	-	-	-
-0.999561255	0.018858935	-	ko04144//Endoc	GO:0000813//ESCRT I co
-0.999561996	0.018843	ko01100//Metabolic p	ko04310//Wnt s	GO:0005634//nucleus;G
-0.999562405	0.018834196	ko01100//Metabolic p	-	GO:0016021//integral co
-0.99956297	0.018822044	ko01100//Metabolic p	-	GO:0005794//Golgi app
-0.999563164	0.018817853	-	-	-
-0.999564107	0.018797537	ko01100//Metabolic p	-	GO:0005737//cytoplasm
-0.999564122	0.018797204	-	-	-
-0.999564534	0.018788322	-	-	-
-0.99956457	0.018787544	ko01100//Metabolic p	-	-
-0.999564838	0.018781755	ko01100//Metabolic p	-	GO:0000139//Golgi mer
-0.999566258	0.018751086	ko01100//Metabolic p	ko01100//Metab	-
-0.999566539	0.018745008	ko01100//Metabolic p	ko04360//Axon	-
-0.999567077	0.018733377	-	ko01100//Metab	GO:0005737//cytoplasm
-0.999567372	0.018727	ko01100//Metabolic p	-	-
-0.999567898	0.01871561	ko01110//Biosynthesis	ko01100//Metab	GO:0005654//nucleopla
-0.999567994	0.018713534	-	-	-
-0.999568022	0.018712911	-	-	-
-0.999568077	0.018711735	ko01100//Metabolic p	-	-
-0.999568245	0.018708084	ko01100//Metabolic p	ko04141//Protei	GO:0005737//cytoplasm
-0.999568841	0.01869518	-	ko04141//Protei	GO:0005783//endoplasr
-0.999569196	0.018687467	ko01100//Metabolic p	-	-
-0.999572058	0.01862529	ko01100//Metabolic p	-	-
-0.999572962	0.018605597	-	-	GO:0016020//membran
-0.999573045	0.018603798	ko01110//Biosynthesis	-	-
-0.999573529	0.01859324	-	ko01100//Metab	GO:0005737//cytoplasm

-0.999573603	0.01859164	-	-	GO:0005654//nucleopla
-0.999573617	0.018591339	ko01100//Metabolic p	ko04151//PI3K-/	GO:0005634//nucleus;G
-0.999573766	0.01858808	ko01100//Metabolic p	-	-
-0.999574113	0.018580519	-	-	-
-0.999574605	0.018569779	ko01063//Biosynthesis	-	-
-0.999574821	0.018565054	ko01100//Metabolic p	ko01100//Metak	GO:0000506//glycosylpl
-0.999575101	0.01855894	-	-	-
-0.999575303	0.018554535	-	-	GO:0016020//membran
-0.999575338	0.018553764	-	-	-
-0.999575864	0.018542269	-	ko01100//Metak	GO:0005829//cytosol
-0.999576196	0.018535007	ko01110//Biosynthesis	-	GO:0097541//axonemal
-0.99957663	0.018525528	-	-	-
-0.999576815	0.018521469	ko01100//Metabolic p	ko01100//Metak	-
-0.999577265	0.018511633	ko01100//Metabolic p	-	-
-0.999579729	0.018457588	ko01100//Metabolic p	-	GO:0005576//extracellu
-0.999579819	0.018455616	ko01110//Biosynthesis	-	-
-0.999579937	0.018453033	ko01100//Metabolic p	-	-
-0.999579937	0.018453017	-	-	-
-0.999580379	0.018443306	-	ko04310//Wnt s	GO:0005634//nucleus;G
-0.999580464	0.018441433	ko01100//Metabolic p	-	GO:0031462//Cul2-RIN(
-0.999580607	0.018438308	-	-	-
-0.999581137	0.01842665	ko01100//Metabolic p	-	GO:0005737//cytoplasm
-0.999581987	0.018407941	ko01100//Metabolic p	-	GO:0005634//nucleus;G
-0.999582182	0.018403646	ko01100//Metabolic p	-	-
-0.999582741	0.018391324	ko01100//Metabolic p	-	-
-0.999584137	0.018360539	-	-	GO:0005634//nucleus;G
-0.999584578	0.018350793	ko01100//Metabolic p	-	-
-0.999584756	0.018346862	ko01100//Metabolic p	ko01100//Metak	GO:0005783//endoplasr
-0.999584883	0.018344048	-	-	GO:0005737//cytoplasm
-0.999585157	0.018338	ko01100//Metabolic p	ko01100//Metak	GO:0005789//endoplasr
-0.999586211	0.0183147	-	-	GO:0005576//extracellu
-0.999586388	0.018310772	-	-	-
-0.999586844	0.018300679	ko01100//Metabolic p	ko04141//Protei	GO:0030120//vesicle co
-0.999587529	0.018285497	-	-	-
-0.99958793	0.018276604	ko01100//Metabolic p	ko04080//Neurc	GO:0005886//plasma m
-0.999588265	0.018269168	-	ko01100//Metak	GO:0005654//nucleopla
-0.999588521	0.018263499	ko01100//Metabolic p	-	-
-0.999588565	0.018262519	ko01100//Metabolic p	-	-
-0.999589182	0.018248812	ko01100//Metabolic p	ko03320//PPAR	GO:0005634//nucleus;G
-0.99959142	0.01819904	ko01100//Metabolic p	-	GO:0016020//membran
-0.999592372	0.018177818	ko01100//Metabolic p	ko04710//Circac	GO:0005634//nucleus;G
-0.999592592	0.018172913	ko01100//Metabolic p	-	-
-0.999592633	0.018172012	-	ko04144//Endoc	-
-0.999593096	0.01816168	ko01100//Metabolic p	-	-
-0.999593996	0.018141571	-	ko01100//Metak	GO:0005829//cytosol;G(
-0.999594054	0.018140284	-	-	-
-0.999595486	0.018108258	-	ko01100//Metak	GO:0005794//Golgi app
-0.999595761	0.018102085	-	ko01100//Metak	GO:0005576//extracellu
-0.999596789	0.018079066	-	ko04080//Neurc	GO:0005576//extracellu
-0.999596801	0.018078795	ko01100//Metabolic p	-	GO:0031966//mitochon
-0.99959735	0.018066485	-	ko04020//Calciu	GO:0005634//nucleus;G
-0.999597383	0.018065747	-	ko05165//Huma	GO:0005634//nucleus;G
-0.999597822	0.018055879	-	-	-
-0.999598418	0.018042511	-	-	-
-0.999598572	0.018039051	-	ko03010//Ribos(	GO:0005634//nucleus;G
-0.999598786	0.018034242	-	-	-
-0.999599247	0.01802386	-	-	-
-0.999599323	0.018022161	-	-	-

-0.999599428	0.018019799	ko01100//Metabolic p	-	GO:0016020//membran
-0.999599616	0.018015572	-	-	-
-0.999599781	0.018011865	ko01100//Metabolic p	ko05152//Tuber	GO:0009897//external s
-0.999600641	0.017992482	-	-	-
-0.999601213	0.017979591	ko01100//Metabolic p	-	GO:0000127//transcript
-0.999601308	0.017977468	-	-	-
-0.999601489	0.017973373	-	-	-
-0.999601542	0.017972189	-	-	GO:0005634//nucleus;G
-0.999601829	0.017965707	ko01100//Metabolic p	-	GO:0005737//cytoplasm
-0.999601843	0.017965388	ko01100//Metabolic p	-	GO:0005886//plasma m
-0.9996019	0.017964104	ko01100//Metabolic p	-	-
-0.999603485	0.017928298	ko01100//Metabolic p	-	GO:0016021//integral c
-0.999604011	0.017916413	ko01100//Metabolic p	ko03320//PPAR	GO:0005783//endoplasr
-0.999604131	0.017913687	-	-	-
-0.99960436	0.017908519	-	ko04010//MAPK	GO:0005737//cytoplasm
-0.999604497	0.017905405	ko01100//Metabolic p	-	GO:0005783//endoplasr
-0.999605167	0.017890232	ko01100//Metabolic p	-	-
-0.999605316	0.017886867	-	-	-
-0.999606473	0.017860632	ko01100//Metabolic p	ko04514//Cell a	-
-0.999606478	0.017860501	ko01063//Biosynthesis	ko01100//Metab	GO:0005788//endoplasr
-0.99960658	0.017858206	-	-	GO:0005764//lysosome;
-0.999606701	0.017855451	-	ko04024//cAMP	GO:0016021//integral c
-0.999606973	0.017849283	ko01110//Biosynthesis	ko01100//Metab	GO:0005576//extracellu
-0.999608289	0.017819369	-	ko04146//Perox	GO:0005765//lysosomal
-0.999608993	0.017803346	ko01100//Metabolic p	-	-
-0.999610637	0.017765863	-	-	-
-0.999611098	0.017755362	ko01100//Metabolic p	-	GO:0032991//macromo
-0.999611971	0.017735418	-	ko04919//Thyro	GO:0016020//membran
-0.999612157	0.017731154	ko01100//Metabolic p	-	GO:0005737//cytoplasm
-0.999613368	0.017703449	-	-	-
-0.999613785	0.017693901	ko01100//Metabolic p	-	-
-0.999613966	0.017689765	-	-	-
-0.999614186	0.017684713	ko01100//Metabolic p	-	-
-0.999614485	0.017677869	ko01110//Biosynthesis	-	-
-0.999614532	0.017676778	-	ko04120//Ubiqu	GO:0005680//anaphase
-0.999615073	0.017664363	-	-	GO:0005737//cytoplasm
-0.999615327	0.01765855	-	-	-
-0.999615411	0.017656618	ko01100//Metabolic p	ko03013//Nucle	-
-0.999615583	0.017652667	ko01100//Metabolic p	-	-
-0.999615908	0.017645212	-	-	-
-0.99961595	0.017644231	ko01100//Metabolic p	-	-
-0.999616334	0.017635421	-	-	-
-0.999616407	0.017633728	ko01100//Metabolic p	-	GO:0005576//extracellu
-0.999616585	0.017629654	ko01100//Metabolic p	-	-
-0.999616849	0.017623583	-	ko01100//Metab	GO:0005829//cytosol
-0.999616936	0.017621575	ko01100//Metabolic p	-	-
-0.999616984	0.017620462	-	ko03010//Ribos	GO:0005634//nucleus;G
-0.999617049	0.017618972	ko01110//Biosynthesis	ko04120//Ubiqu	GO:0005680//anaphase
-0.999617397	0.017610976	-	ko01100//Metab	GO:0005794//Golgi app
-0.999618344	0.017589162	-	-	GO:0031464//Cul4A-RIN
-0.999618833	0.017577886	-	-	GO:0005886//plasma m
-0.999619396	0.017564902	ko01100//Metabolic p	ko01100//Metab	GO:0016021//integral c
-0.999619654	0.017558934	ko01100//Metabolic p	-	-
-0.999620205	0.017546225	-	-	-
-0.999620423	0.017541177	ko01100//Metabolic p	ko01100//Metab	GO:0005737//cytoplasm
-0.999620561	0.017537982	ko01100//Metabolic p	-	GO:0000791//euchroma
-0.99962068	0.01753523	-	-	-
-0.999622317	0.017497349	-	ko05200//Pathw	GO:0000307//cyclin-dep

-0.999622507	0.017492968	ko01100//Metabolic p	-	GO:0005737//cytoplasm
-0.999622569	0.017491527	ko01100//Metabolic p	ko01100//Metab	GO:0048471//perinucle
-0.999623672	0.017465933	-	-	GO:0005634//nucleus;G
-0.999624598	0.017444433	ko01100//Metabolic p	-	-
-0.999624981	0.017435546	-	-	GO:0031362//anchored
-0.999624996	0.017435181	-	-	-
-0.999625356	0.017426811	-	-	GO:0005794//Golgi app
-0.999625444	0.017424771	ko01100//Metabolic p	-	-
-0.999625524	0.017422911	ko01100//Metabolic p	-	GO:0005829//cytosol;G
-0.999625528	0.017422807	ko01100//Metabolic p	ko04068//FoxO	GO:0005737//cytoplasm
-0.999625964	0.017412672	ko01063//Biosynthesis	-	-
-0.999626316	0.017404475	-	-	-
-0.999628294	0.017358355	ko01100//Metabolic p	-	GO:0005634//nucleus;G
-0.999628412	0.017355591	-	-	GO:0005634//nucleus;G
-0.999628967	0.017342623	ko01100//Metabolic p	-	-
-0.999629482	0.017330595	-	ko01100//Metab	GO:0005739//mitochon
-0.999629485	0.017330516	ko01100//Metabolic p	-	-
-0.999629662	0.017326384	ko01100//Metabolic p	-	-
-0.999630325	0.017310856	ko01100//Metabolic p	-	-
-0.999630538	0.017305876	ko01100//Metabolic p	-	-
-0.999630612	0.017304144	ko01100//Metabolic p	-	GO:0016020//membran
-0.999631488	0.017283594	-	-	-
-0.999631815	0.017275928	-	ko04310//Wnt s	GO:0005634//nucleus;G
-0.999632229	0.017266217	-	-	-
-0.999632247	0.01726579	ko01100//Metabolic p	-	GO:0005576//extracellu
-0.999633307	0.017240881	ko01100//Metabolic p	-	-
-0.999633591	0.017234221	ko01100//Metabolic p	ko01100//Metab	GO:0033178//proton-tr
-0.999633937	0.017226062	ko01100//Metabolic p	-	-
-0.99963394	0.017225997	-	-	-
-0.99963404	0.017223652	ko01100//Metabolic p	-	-
-0.999634319	0.017217079	ko01100//Metabolic p	-	-
-0.999635129	0.017198009	-	-	GO:0005634//nucleus;G
-0.999635762	0.017183087	-	-	GO:0005764//lysosome;
-0.999636328	0.017169714	-	-	GO:0005802//trans-Gol
-0.999636471	0.017166335	-	-	-
-0.999636533	0.017164889	-	ko01100//Metab	GO:0005829//cytosol
-0.999636727	0.017160295	-	-	-
-0.99963678	0.017159048	ko01100//Metabolic p	ko01100//Metab	-
-0.999637029	0.017153171	ko01100//Metabolic p	-	GO:0005737//cytoplasm
-0.999637457	0.017143054	ko01100//Metabolic p	ko04060//Cytok	GO:0005887//integral c
-0.999637649	0.017138507	ko01100//Metabolic p	ko01100//Metab	GO:0005783//endoplasr
-0.999638708	0.017113431	-	ko00970//Amino	GO:0005737//cytoplasm
-0.999639167	0.01710256	-	-	-
-0.999641221	0.017053826	ko01100//Metabolic p	ko04020//Calciu	GO:0001518//voltage-g
-0.999642366	0.017026581	ko01110//Biosynthesis	ko01100//Metab	GO:0005654//nucleopla
-0.999642682	0.017019064	-	-	-
-0.999643306	0.017004197	-	ko04710//Circac	GO:0005634//nucleus;G
-0.999643361	0.017002881	-	ko04934//Cushi	GO:0016020//membran
-0.999643693	0.016994967	ko01100//Metabolic p	ko01100//Metab	GO:0005737//cytoplasm
-0.999643948	0.016988879	ko01100//Metabolic p	-	-
-0.999643982	0.016988058	-	-	-
-0.999644488	0.016975991	-	-	-
-0.999644976	0.016964333	-	ko04141//Protei	GO:0005783//endoplasr
-0.999645917	0.016941837	-	-	GO:0031464//Cul4A-RI
-0.999646352	0.016931417	ko01100//Metabolic p	ko01100//Metab	GO:0005739//mitochon
-0.999647239	0.016910167	ko01100//Metabolic p	ko01100//Metab	GO:0005783//endoplasr
-0.999647367	0.016907116	ko01110//Biosynthesis	-	-
-0.999647877	0.016894887	-	-	-

-0.999648529	0.016879237	-	-	GO:0005615//extracellu
-0.999648549	0.016878748	-	ko04710//Circac	GO:0005634//nucleus;G
-0.999648709	0.016874895	ko01100//Metabolic p	-	-
-0.999649179	0.016863604	ko01100//Metabolic p	ko02010//ABC tı	GO:0000139//Golgi mer
-0.999649452	0.016857053	ko01100//Metabolic p	ko04080//Neurc	GO:0005576//extracellu
-0.999649464	0.016856753	-	-	GO:0005576//extracellu
-0.999649622	0.016852969	ko01100//Metabolic p	-	GO:0016020//membran
-0.999649682	0.016851521	ko01100//Metabolic p	-	-
-0.999649909	0.016846065	ko01100//Metabolic p	-	-
-0.999650006	0.016843734	ko01100//Metabolic p	ko05165//Huma	GO:0005634//nucleus;G
-0.999650378	0.016834764	ko01100//Metabolic p	-	GO:0005634//nucleus;G
-0.999650538	0.016830914	-	ko04020//Calciu	GO:0005634//nucleus;G
-0.999650571	0.016830122	ko01100//Metabolic p	ko04150//mTOF	GO:0005764//lysosome;
-0.999650678	0.01682755	-	-	-
-0.999650827	0.01682395	ko01100//Metabolic p	-	GO:0034361//very-low-
-0.999650945	0.016821102	ko01100//Metabolic p	-	GO:0005634//nucleus;G
-0.999651437	0.016809245	-	ko01100//Metak	-
-0.999651533	0.01680694	-	-	-
-0.999651703	0.01680284	ko01110//Biosynthesis	ko01100//Metak	GO:0001931//uropod;G
-0.999652329	0.016787725	ko01100//Metabolic p	ko04144//Endoc	GO:0000813//ESCRT I c
-0.999652451	0.016784784	-	-	GO:0016020//membran
-0.999652989	0.016771795	ko01100//Metabolic p	-	GO:0005802//trans-Gol
-0.999652989	0.016771791	-	ko04310//Wnt s	GO:0005634//nucleus;G
-0.999653054	0.016770222	ko01110//Biosynthesis	-	-
-0.999654048	0.016746169	ko01100//Metabolic p	-	GO:0005634//nucleus;G
-0.999654796	0.016728052	-	-	GO:0044424//intracellul
-0.999654902	0.016725478	ko01100//Metabolic p	ko04142//Lysos	GO:0016020//membran
-0.999654952	0.016724271	ko01100//Metabolic p	-	-
-0.999655268	0.01671661	-	-	-
-0.999655518	0.016710545	-	-	GO:0000139//Golgi mer
-0.999655776	0.016704293	ko01100//Metabolic p	ko04146//Perox	GO:0005777//peroxison
-0.999655788	0.016704006	ko01100//Metabolic p	-	-
-0.999655883	0.016701702	ko01110//Biosynthesis	-	-
-0.999656015	0.016698488	ko01100//Metabolic p	-	-
-0.999656107	0.016696257	ko01100//Metabolic p	-	GO:0005768//endosom
-0.999656422	0.016688602	ko01100//Metabolic p	-	GO:0005634//nucleus;G
-0.999656775	0.016680035	ko01100//Metabolic p	-	GO:0005634//nucleus;G
-0.999656885	0.016677354	ko01100//Metabolic p	-	GO:0016020//membran
-0.999657279	0.016667796	ko01100//Metabolic p	ko04919//Thyro	GO:0016020//membran
-0.999658251	0.016644132	-	-	-
-0.999658399	0.016640525	-	-	-
-0.999658477	0.016638618	-	ko04514//Cell a	-
-0.99965905	0.016624666	-	ko05203//Viral c	-
-0.999659069	0.016624188	ko01110//Biosynthesis	-	GO:0000127//transcript
-0.999659133	0.016622635	-	-	GO:0005764//lysosome;
-0.999660209	0.016596379	-	-	-
-0.999660378	0.016592242	ko01100//Metabolic p	-	-
-0.99966048	0.016589752	-	-	GO:0005783//endoplasr
-0.999660975	0.016577664	ko01100//Metabolic p	-	-
-0.99966258	0.016538361	ko01100//Metabolic p	-	-
-0.999663003	0.016527994	ko01100//Metabolic p	-	-
-0.99966348	0.016516293	-	-	GO:0005615//extracellu
-0.999663516	0.016515427	-	-	-
-0.999663798	0.016508485	ko01100//Metabolic p	-	-
-0.999664497	0.016491313	-	-	-
-0.999664521	0.016490741	ko01100//Metabolic p	-	GO:0005737//cytoplasm
-0.999665814	0.01645891	ko01100//Metabolic p	-	-
-0.999665905	0.016456668	-	-	-

-0.999666566	0.016440392	-	ko04142//Lysoso	-
-0.99966688	0.016432636	ko01100//Metabolic p	-	GO:0000781//chromoso
-0.999667607	0.016414708	-	-	GO:0005737//cytoplasm
-0.999667676	0.016413006	ko01063//Biosynthesis	-	-
-0.999668137	0.01640162	ko01100//Metabolic p	-	GO:0032991//macromo
-0.999668701	0.01638768	-	-	GO:0005654//nucleopla
-0.999668805	0.016385096	ko01100//Metabolic p	-	GO:0016020//membran
-0.999669743	0.016361867	-	ko05200//Pathw	GO:0005737//cytoplasm
-0.999669761	0.016361422	-	ko04146//Peroxi	GO:0005765//lysosomal
-0.999670063	0.016353957	ko01100//Metabolic p	-	-
-0.999671145	0.016327107	-	-	-
-0.999671303	0.016323189	ko01100//Metabolic p	ko01100//Metab	GO:0005737//cytoplasm
-0.999671813	0.016310517	ko01100//Metabolic p	ko01100//Metab	-
-0.99967201	0.01630563	-	-	-
-0.999672743	0.016287392	-	-	-
-0.999673107	0.016278325	ko01110//Biosynthesis	-	GO:0005576//extracellu
-0.99967332	0.016273014	-	-	-
-0.999673699	0.016263569	ko01100//Metabolic p	ko04120//Ubiqu	GO:0005680//anaphase
-0.999673871	0.0162593	-	-	-
-0.99967493	0.016232861	ko01100//Metabolic p	-	-
-0.999675708	0.016213439	-	-	GO:0005811//lipid parti
-0.999675892	0.01620882	ko01100//Metabolic p	-	GO:0005737//cytoplasm
-0.999676318	0.016198168	-	-	GO:0005576//extracellu
-0.999677126	0.016177955	ko01100//Metabolic p	-	GO:0016021//integral co
-0.999677359	0.0161721	-	-	-
-0.999678402	0.016145944	-	ko01100//Metab	GO:0005737//cytoplasm
-0.999678433	0.016145172	-	-	GO:0005768//endosome
-0.999679678	0.016113885	ko01100//Metabolic p	-	-
-0.999680431	0.016094916	-	ko01100//Metab	GO:0016020//membran
-0.999680448	0.016094493	ko01110//Biosynthesis	-	-
-0.999681131	0.016077284	-	-	-
-0.999681271	0.016073766	ko01100//Metabolic p	-	-
-0.99968142	0.016070012	-	ko01100//Metab	-
-0.999681523	0.016067404	ko01110//Biosynthesis	ko04068//FoxO	GO:0005737//cytoplasm
-0.999681805	0.016060281	ko01063//Biosynthesis	-	-
-0.999682573	0.016040887	-	-	GO:0005634//nucleus;G
-0.999682614	0.016039858	ko01100//Metabolic p	ko05203//Viral c	GO:0000786//nucleosor
-0.999682648	0.016039006	ko01100//Metabolic p	ko05200//Pathw	GO:0005737//cytoplasm
-0.999682727	0.016037002	-	-	GO:0005654//nucleopla
-0.999682819	0.016034684	ko01100//Metabolic p	-	GO:0005615//extracellu
-0.999683438	0.016019011	-	-	-
-0.999683444	0.016018862	ko01100//Metabolic p	ko04934//Cushi	-
-0.999684662	0.015988009	ko01100//Metabolic p	-	GO:0005768//endosome
-0.999685087	0.015977235	-	-	-
-0.999685118	0.015976464	-	-	GO:0016020//membran
-0.999685136	0.015975996	ko01100//Metabolic p	-	GO:0005634//nucleus;G
-0.999685334	0.015970981	ko01110//Biosynthesis	-	-
-0.99968551	0.015966504	-	-	GO:0005886//plasma m
-0.999685821	0.015958608	-	-	GO:0005615//extracellu
-0.999686616	0.015938412	-	ko05203//Viral c	GO:0000786//nucleosor
-0.999686678	0.01593682	-	-	-
-0.999686929	0.015930447	ko01110//Biosynthesis	-	GO:0005739//mitochon
-0.999687006	0.015928482	-	ko05152//Tuber	GO:0009897//external s
-0.999687031	0.015927839	ko01100//Metabolic p	-	-
-0.999687374	0.015919123	-	-	GO:0005634//nucleus;G
-0.999687693	0.015910996	ko01100//Metabolic p	ko01100//Metab	GO:0005783//endoplasr
-0.999687851	0.015906973	ko01110//Biosynthesis	ko04721//Synap	GO:0005887//integral co
-0.99968861	0.015887608	-	ko01100//Metab	GO:0005737//cytoplasm

-0.999688677	0.015885907	ko01100//Metabolic p	ko01100//Metat	GO:0001650//fibrillar ce
-0.999689315	0.015869632	ko01100//Metabolic p	ko03010//Ribos	GO:0005634//nucleus;G
-0.999689645	0.015861195	ko01063//Biosynthesis	-	GO:0016020//membran
-0.999689766	0.015858096	ko01100//Metabolic p	ko04151//PI3K-,	GO:0005654//nucleopla
-0.999689928	0.015853948	ko01110//Biosynthesis	-	-
-0.999691098	0.015824006	-	ko04144//Endoc	-
-0.999691289	0.015819115	ko01100//Metabolic p	ko01100//Metat	GO:0009923//fatty acid
-0.999691768	0.015806851	-	-	-
-0.999692187	0.015796095	-	ko01100//Metat	-
-0.999692343	0.01579208	ko01100//Metabolic p	-	-
-0.999692499	0.015788099	ko01100//Metabolic p	-	GO:0005576//extracellu
-0.999692655	0.015784075	ko01100//Metabolic p	-	GO:0005654//nucleopla
-0.999692678	0.015783493	-	-	-
-0.999693662	0.015758194	-	ko04144//Endoc	GO:0005654//nucleopla
-0.999694418	0.015738737	ko01100//Metabolic p	ko04080//Neuro	GO:0005654//nucleopla
-0.999694566	0.015734931	ko01100//Metabolic p	-	-
-0.99969494	0.015725286	-	-	GO:0016020//membran
-0.999695465	0.015711767	-	-	GO:0005802//trans-Gol
-0.999695546	0.015709661	-	-	GO:0005634//nucleus;G
-0.999695633	0.015707417	ko01100//Metabolic p	-	-
-0.999696016	0.015697523	-	-	-
-0.999696254	0.015691379	ko01063//Biosynthesis	ko04723//Retro	GO:0005739//mitochon
-0.999696937	0.015673731	ko01100//Metabolic p	-	GO:0005634//nucleus;G
-0.999696968	0.015672935	ko01100//Metabolic p	-	-
-0.999696993	0.015672284	ko01110//Biosynthesis	ko01100//Metat	-
-0.999697036	0.015671178	ko01100//Metabolic p	ko01100//Metat	GO:0005654//nucleopla
-0.999697575	0.015657224	ko01100//Metabolic p	-	GO:0005615//extracellu
-0.999697979	0.015646778	ko01100//Metabolic p	-	GO:0016020//membran
-0.999699124	0.015617085	ko01063//Biosynthesis	-	GO:0005739//mitochon
-0.999699274	0.015613187	-	-	GO:0035658//Mon1-Cc
-0.999699475	0.015607976	-	-	-
-0.999699685	0.015602505	ko01100//Metabolic p	ko05165//Huma	-
-0.999699947	0.015595714	ko01100//Metabolic p	ko04211//Longe	GO:0005634//nucleus
-0.999700215	0.015588739	ko01100//Metabolic p	-	GO:0016020//membran
-0.999700278	0.0155871	ko01100//Metabolic p	ko01100//Metat	-
-0.999700371	0.015584688	-	-	GO:0005783//endoplasr
-0.99970055	0.015580013	ko01100//Metabolic p	-	-
-0.99970056	0.015579772	-	-	GO:0005634//nucleus;G
-0.999700794	0.015573674	-	ko01100//Metat	-
-0.999701116	0.015565304	ko01110//Biosynthesis	ko01100//Metat	GO:0005737//cytoplasm
-0.999701803	0.015547394	ko01100//Metabolic p	ko04713//Circac	-
-0.999702129	0.015538897	-	-	GO:0005737//cytoplasm
-0.999702185	0.015537442	ko01110//Biosynthesis	ko01100//Metat	GO:0005783//endoplasr
-0.999702572	0.01552734	ko01100//Metabolic p	ko01100//Metat	-
-0.999702979	0.015516707	ko01100//Metabolic p	-	-
-0.999703033	0.015515293	-	-	-
-0.999703094	0.015513697	-	-	GO:0005737//cytoplasm
-0.999704736	0.015470739	-	-	-
-0.999705717	0.01544501	-	-	-
-0.99970604	0.015436541	ko01100//Metabolic p	ko04141//Protei	GO:0005783//endoplasr
-0.999706103	0.015434889	-	ko04150//mTOR	GO:0005764//lysosome;
-0.999706523	0.01542385	-	-	-
-0.999706823	0.015415971	-	-	GO:0005634//nucleus;G
-0.999706867	0.015414819	ko01100//Metabolic p	-	-
-0.999707218	0.015405584	-	-	-
-0.999708228	0.01537898	ko01100//Metabolic p	ko04010//MAPK	-
-0.99970841	0.01537418	-	-	-
-0.999709453	0.01534667	-	-	-

-0.999709564	0.015343715	ko01100//Metabolic p	ko01100//Metak	GO:0005829//cytosol
-0.999710202	0.015326866	ko01100//Metabolic p	-	GO:0005634//nucleus;G
-0.999710694	0.015313844	-	ko04310//Wnt s	GO:0005634//nucleus;G
-0.999710871	0.015309157	-	ko04630//JAK-S	GO:0005829//cytosol
-0.999711185	0.015300857	-	ko04390//Hippc	GO:0005667//transcript
-0.999711433	0.01529428	ko01100//Metabolic p	-	GO:0005615//extracellu
-0.999711483	0.01529294	ko01063//Biosynthesis	ko01100//Metak	GO:0005739//mitochon
-0.999711789	0.015284828	-	ko05231//Cholir	-
-0.999711852	0.015283178	ko01120//Microbial m	-	GO:0005737//cytoplasm
-0.999711891	0.015282121	-	-	GO:0016020//membran
-0.999711943	0.015280764	ko01100//Metabolic p	ko01100//Metak	GO:0000506//glycosylpl
-0.999712694	0.015260809	-	-	GO:0016020//membran
-0.999713003	0.015252598	-	-	GO:0000139//Golgi mer
-0.999713041	0.015251608	ko01100//Metabolic p	-	-
-0.999714114	0.015223051	ko01100//Metabolic p	ko01100//Metak	GO:0016020//membran
-0.999714188	0.015221097	-	-	-
-0.999714834	0.015203867	-	ko01100//Metak	GO:0005783//endoplasr
-0.999715627	0.015182727	-	-	-
-0.999715632	0.015182579	-	-	-
-0.999716281	0.01516524	-	-	-
-0.999716867	0.015149568	-	ko01100//Metak	GO:0005737//cytoplasm
-0.999717171	0.015141436	-	-	-
-0.999717914	0.015121554	ko01100//Metabolic p	ko01100//Metak	GO:0005829//cytosol
-0.999718096	0.015116673	ko01100//Metabolic p	-	-
-0.999718198	0.015113926	ko01100//Metabolic p	-	-
-0.999718376	0.015109147	-	-	GO:0005737//cytoplasm
-0.999718489	0.015106125	ko01100//Metabolic p	ko01100//Metak	GO:0005783//endoplasr
-0.999718659	0.015101548	-	-	GO:0005737//cytoplasm
-0.999718687	0.015100811	ko01100//Metabolic p	ko04360//Axon	-
-0.999719491	0.015079204	-	-	-
-0.999719889	0.0150685	-	ko01100//Metak	GO:0005794//Golgi app
-0.99972001	0.015065245	ko01100//Metabolic p	-	GO:0005886//plasma m
-0.999720027	0.015064802	-	ko01100//Metak	GO:0033178//proton-tr
-0.9997204	0.015054759	-	ko01100//Metak	GO:0005794//Golgi app
-0.999720715	0.015046263	ko01100//Metabolic p	-	-
-0.999720992	0.015038822	ko01100//Metabolic p	ko01100//Metak	GO:0005789//endoplasr
-0.999722194	0.015006387	-	-	GO:0032991//macromo
-0.999722582	0.014995896	ko01100//Metabolic p	ko02010//ABC ti	GO:0016021//integral co
-0.999724163	0.01495311	-	-	GO:0044424//intracellul
-0.999724217	0.014951632	-	ko01100//Metak	GO:0005737//cytoplasm
-0.999724232	0.01495124	-	ko04934//Cushii	-
-0.999725181	0.014925487	ko01100//Metabolic p	-	GO:0016020//membran
-0.9997258	0.014908653	-	ko01100//Metak	GO:0005783//endoplasr
-0.999726591	0.014887144	ko01100//Metabolic p	ko04934//Cushii	-
-0.999726695	0.014884326	ko01100//Metabolic p	ko03010//Ribosc	GO:0005840//ribosome
-0.999727199	0.014870593	-	-	GO:0005794//Golgi app
-0.999727401	0.014865064	-	-	-
-0.999727505	0.014862245	-	ko01100//Metak	-
-0.999727733	0.014856032	ko01100//Metabolic p	-	-
-0.999727779	0.014854771	ko01100//Metabolic p	ko04080//Neurc	GO:0005886//plasma m
-0.999727799	0.014854229	ko01100//Metabolic p	-	GO:0030659//cytoplasm
-0.999727899	0.014851502	ko01100//Metabolic p	-	GO:0005634//nucleus;G
-0.999727944	0.014850256	ko01100//Metabolic p	-	GO:0005634//nucleus;G
-0.999728112	0.014845683	-	ko05200//Pathw	GO:0000785//chromatir
-0.999728589	0.014832639	ko01100//Metabolic p	-	-
-0.999728999	0.014821437	-	-	-
-0.999729241	0.01481481	-	-	GO:0005783//endoplasr
-0.99972932	0.014812671	-	-	GO:0005615//extracellu

-0.99972973	0.014801431	-	-	GO:0005654//nucleopla
-0.99972992	0.014796232	ko01100//Metabolic p	ko01100//Metak	GO:0005739//mitochon
-0.999729968	0.014794914	ko01100//Metabolic p	ko04141//Protei	GO:0005783//endoplasr
-0.999730061	0.014792364	ko01100//Metabolic p	ko05203//Viral c	GO:0000786//nucleosor
-0.9997303	0.014785827	-	-	-
-0.99973031	0.014785543	ko01100//Metabolic p	-	GO:0005576//extracellu
-0.999730387	0.01478344	-	-	-
-0.999730399	0.014783094	-	-	GO:0001725//stress fibe
-0.999730552	0.014778896	-	-	GO:0005634//nucleus;G
-0.999731454	0.014754148	-	-	-
-0.999731609	0.014749892	-	-	GO:0005634//nucleus;G
-0.999731764	0.014745644	-	ko01100//Metak	GO:0005634//nucleus;G
-0.99973241	0.014727865	-	-	GO:0005783//endoplasr
-0.999732419	0.014727613	ko01100//Metabolic p	-	GO:0005654//nucleopla
-0.999732486	0.014725779	ko01100//Metabolic p	-	GO:0005634//nucleus;G
-0.999732534	0.014724459	-	-	-
-0.999732706	0.014719711	-	ko04310//Wnt s	GO:0005634//nucleus;G
-0.999733059	0.014709999	-	-	-
-0.999733223	0.014705476	-	-	-
-0.999733497	0.014697911	-	-	-
-0.999734246	0.014677246	ko01100//Metabolic p	ko04144//Endoc	GO:0005654//nucleopla
-0.999734395	0.014673145	ko01100//Metabolic p	-	-
-0.999734673	0.014665449	-	ko04142//Lysosc	-
-0.999734729	0.014663905	ko01100//Metabolic p	ko04610//Comp	GO:0005615//extracellu
-0.999734951	0.014657774	-	ko03320//PPAR	GO:0005783//endoplasr
-0.999735068	0.01465453	-	-	GO:0034361//very-low-
-0.999735069	0.014654496	ko01120//Microbial m	-	-
-0.999735227	0.014650142	ko01063//Biosynthesis	-	-
-0.999735703	0.01463697	-	ko01100//Metak	GO:0016020//membran
-0.999735715	0.014636625	-	ko01100//Metak	-
-0.999735847	0.01463297	ko01100//Metabolic p	-	GO:0005737//cytoplasm
-0.999736889	0.014604077	-	-	-
-0.999737306	0.014592489	-	ko04142//Lysosc	GO:0016020//membran
-0.999737708	0.014581331	ko01100//Metabolic p	ko05203//Viral c	-
-0.999738139	0.014569333	-	ko04151//PI3K-/	GO:0005654//nucleopla
-0.99973918	0.014540354	ko01100//Metabolic p	-	-
-0.999739377	0.014534874	-	ko04146//Peroxi	GO:0005777//peroxison
-0.999739415	0.014533796	-	-	-
-0.999739627	0.014527895	-	-	-
-0.999740046	0.014516204	-	ko01100//Metak	-
-0.999740059	0.014515829	-	ko04140//Autop	GO:0000407//pre-autop
-0.999740266	0.014510049	ko01100//Metabolic p	-	GO:0005615//extracellu
-0.999740452	0.014504862	-	ko03320//PPAR	GO:0005576//extracellu
-0.999740678	0.014498546	ko01100//Metabolic p	-	-
-0.999740882	0.014492826	-	ko03320//PPAR	GO:0005615//extracellu
-0.999740948	0.014490978	ko01100//Metabolic p	ko01100//Metak	GO:0000139//Golgi mer
-0.999741068	0.014487621	-	-	GO:0016020//membran
-0.999741129	0.014485911	ko01100//Metabolic p	-	GO:0005634//nucleus;G
-0.999741139	0.014485634	ko01100//Metabolic p	-	-
-0.999741361	0.014479442	ko01100//Metabolic p	ko01100//Metak	-
-0.999741587	0.014473114	ko01100//Metabolic p	ko03013//Nucle	-
-0.999741639	0.014471644	-	-	GO:0000785//chromatir
-0.99974185	0.01446574	ko01100//Metabolic p	-	-
-0.999742067	0.014459664	-	ko04216//Ferrop	GO:0016020//membran
-0.999742251	0.014454495	ko01100//Metabolic p	ko05200//Pathw	GO:0000307//cyclin-dep
-0.999742754	0.014440377	ko01100//Metabolic p	-	-
-0.999742784	0.014439531	ko01100//Metabolic p	-	GO:0005791//rough enc
-0.999743233	0.01442693	-	-	-

-0.999743342	0.014423883	-	ko01100//Metak	GO:0009923//fatty acid
-0.999744263	0.014397979	-	ko03013//Nucle	-
-0.999744292	0.014397158	-	-	-
-0.999744753	0.014384171	-	-	-
-0.999744828	0.014382047	ko01100//Metabolic p	ko01100//Metak	-
-0.999745339	0.014367645	ko01100//Metabolic p	-	-
-0.999745545	0.014361822	ko01100//Metabolic p	ko03010//Ribos	GO:0005634//nucleus;G
-0.999745845	0.014353353	ko01100//Metabolic p	ko01100//Metak	-
-0.999746131	0.01434529	ko01100//Metabolic p	ko01100//Metak	GO:0005829//cytosol;G
-0.99974677	0.014327204	ko01100//Metabolic p	-	-
-0.999746948	0.01432219	-	-	-
-0.999747135	0.014316881	ko01100//Metabolic p	ko01100//Metak	-
-0.999748076	0.014290213	ko01100//Metabolic p	ko04146//Perox	GO:0005765//lysosomal
-0.99974847	0.014279049	ko01100//Metabolic p	-	-
-0.999748475	0.014278904	-	-	GO:0005737//cytoplasm
-0.999749067	0.014262079	ko01100//Metabolic p	-	-
-0.999749111	0.014260825	ko01100//Metabolic p	-	GO:0016020//membran
-0.999749791	0.014241498	ko01100//Metabolic p	-	-
-0.999750522	0.01422069	-	-	GO:0005737//cytoplasm
-0.999750992	0.014207272	-	ko05165//Huma	-
-0.999751511	0.014192448	ko01100//Metabolic p	ko05200//Pathw	GO:0005737//cytoplasm
-0.999751876	0.014182039	ko01100//Metabolic p	ko01100//Metak	GO:0005783//endoplasi
-0.999752293	0.014170104	ko01100//Metabolic p	-	GO:0005811//lipid parti
-0.999752675	0.014159179	-	-	-
-0.999752727	0.014157688	ko01100//Metabolic p	-	-
-0.999753057	0.014148253	ko01100//Metabolic p	-	GO:0005802//trans-Gol
-0.9997537	0.01412981	ko01100//Metabolic p	-	-
-0.999753763	0.01412801	ko01100//Metabolic p	-	GO:0016020//membran
-0.99975399	0.014121474	-	-	-
-0.999754124	0.014117628	ko01100//Metabolic p	-	GO:0005654//nucleopla
-0.999754568	0.014104895	ko01100//Metabolic p	ko04361//Axon	GO:0005737//cytoplasm
-0.999755105	0.014089438	ko01100//Metabolic p	ko04022//cGMP	-
-0.999755121	0.014088988	-	ko05012//Parkin	GO:0005654//nucleopla
-0.999755384	0.014081406	ko01100//Metabolic p	-	-
-0.999755715	0.014071876	-	-	-
-0.999755753	0.014070805	-	ko01100//Metak	-
-0.999756199	0.014057941	ko01100//Metabolic p	-	GO:0005886//plasma m
-0.999756364	0.014053172	-	ko05200//Pathw	GO:0005834//heterotrin
-0.9997565	0.014049257	-	ko05231//Cholir	-
-0.999756866	0.014038687	ko01100//Metabolic p	-	GO:0005634//nucleus
-0.999756989	0.01403513	ko01100//Metabolic p	-	-
-0.999757101	0.014031908	-	-	-
-0.999757995	0.014006074	ko01100//Metabolic p	-	-
-0.999758113	0.01400265	-	-	-
-0.999758116	0.014002556	-	-	-
-0.999758766	0.013983747	-	ko04010//MAPK	-
-0.999759055	0.013975344	-	-	-
-0.999759138	0.01397294	-	-	GO:0005576//extracellu
-0.999759154	0.013972476	-	-	GO:0005737//cytoplasm
-0.999759315	0.013967796	-	ko05200//Pathw	GO:0005737//cytoplasm
-0.999759435	0.013964332	-	-	GO:0005634//nucleus;G
-0.999760165	0.013943117	ko01100//Metabolic p	-	-
-0.999760706	0.013927395	-	-	GO:0005737//cytoplasm
-0.999760871	0.013922581	-	-	-
-0.999761441	0.013905989	ko01100//Metabolic p	-	-
-0.999761914	0.013892191	ko01100//Metabolic p	ko01100//Metak	GO:0005654//nucleopla
-0.999762177	0.013884504	-	-	-
-0.999762202	0.013883769	ko01100//Metabolic p	ko03410//Base e	GO:0005634//nucleus;G

-0.999762556	0.013873439	-	ko01100//Metab	GO:0005783//endoplasm
-0.999763903	0.013834044	ko01100//Metabolic p	ko01100//Metab	GO:0005576//extracellu
-0.999763926	0.013833349	ko01100//Metabolic p	-	-
-0.99976401	0.013830896	-	-	-
-0.999764317	0.013821911	-	ko04080//Neuro	GO:0005886//plasma m
-0.999764425	0.013818747	-	ko03320//PPAR	GO:0005615//extracellu
-0.999765055	0.013800229	ko01100//Metabolic p	ko01100//Metab	GO:0005635//nuclear er
-0.999765384	0.013790581	ko01100//Metabolic p	ko01100//Metab	GO:0005737//cytoplasm
-0.999765651	0.013782737	ko01100//Metabolic p	-	-
-0.999765721	0.013780671	ko01100//Metabolic p	ko04144//Endoc	-
-0.999766473	0.013758523	ko01110//Biosynthesis	-	-
-0.999766783	0.013749412	-	-	-
-0.999766877	0.013746635	-	ko04216//Ferrop	GO:0016020//membran
-0.999767335	0.013733128	ko01100//Metabolic p	-	GO:0005634//nucleus;G
-0.999767402	0.013731146	-	ko04146//Perox	GO:0005777//peroxisom
-0.999768587	0.013696107	ko01100//Metabolic p	-	GO:0016021//integral co
-0.999768778	0.01369047	-	ko04140//Autop	GO:0000407//pre-autop
-0.999768823	0.013689139	-	-	-
-0.999768911	0.013686527	ko01100//Metabolic p	-	-
-0.999769185	0.013678404	-	-	-
-0.999769302	0.013674948	-	ko01100//Metab	-
-0.99976951	0.01366878	-	-	GO:0005634//nucleus;G
-0.999769763	0.013661263	ko01100//Metabolic p	ko01100//Metab	GO:0016021//integral co
-0.999769822	0.013659526	ko01100//Metabolic p	ko01100//Metab	GO:0005794//Golgi app
-0.99976992	0.013656599	ko01063//Biosynthesis	ko04923//Regul	GO:0005654//nucleopla
-0.999770102	0.0136512	-	-	GO:0005737//cytoplasm
-0.999770177	0.013648976	ko01100//Metabolic p	-	-
-0.999770198	0.013648344	ko01100//Metabolic p	-	GO:0043231//intracellul
-0.999770413	0.013641966	ko01100//Metabolic p	-	-
-0.999770461	0.013640558	ko01100//Metabolic p	-	GO:0005802//trans-Gol
-0.999770472	0.013640216	ko01100//Metabolic p	-	GO:0005576//extracellu
-0.999771181	0.013619119	-	-	GO:0000139//Golgi mer
-0.999771393	0.013612809	ko01063//Biosynthesis	-	-
-0.999771438	0.01361149	-	-	GO:0034361//very-low-
-0.999771979	0.013595358	-	ko01100//Metab	GO:0005737//cytoplasm
-0.999772168	0.013589726	-	ko01100//Metab	GO:0005737//cytoplasm
-0.99977237	0.013583708	-	-	GO:0005783//endoplasm
-0.999772507	0.013579622	ko01100//Metabolic p	-	-
-0.999772965	0.013565928	-	ko01100//Metab	GO:0005634//nucleus;G
-0.999773145	0.013560544	-	-	-
-0.999773232	0.013557966	ko01063//Biosynthesis	ko01100//Metab	-
-0.999773235	0.013557873	-	ko04310//Wnt s	GO:0005634//nucleus;G
-0.999773419	0.013552379	-	ko04211//Longe	GO:0005634//nucleus
-0.999773573	0.013547765	ko01100//Metabolic p	-	GO:0044424//intracellul
-0.999774108	0.013531741	ko01110//Biosynthesis	ko04934//Cushin	-
-0.999774197	0.013529086	-	ko04144//Endoc	GO:0000813//ESCRT I co
-0.999774327	0.013525196	-	ko05150//Staphy	-
-0.999774361	0.013524163	ko01100//Metabolic p	ko04080//Neuro	GO:0009986//cell surfac
-0.99977515	0.013500483	ko01063//Biosynthesis	-	GO:0005791//rough end
-0.999775213	0.013498598	ko01100//Metabolic p	ko04146//Perox	GO:0005765//lysosomal
-0.999775264	0.013497063	ko01100//Metabolic p	-	-
-0.99977546	0.013491173	ko01100//Metabolic p	-	-
-0.999775726	0.0134832	ko01100//Metabolic p	ko04068//FoxO	GO:0005737//cytoplasm
-0.999776154	0.013470324	-	ko01100//Metab	-
-0.99977647	0.013460799	-	-	-
-0.999776608	0.013456651	ko01100//Metabolic p	-	GO:0015629//actin cyto
-0.999776619	0.013456322	-	ko04020//Calciu	GO:0016020//membran
-0.999776843	0.013449578	ko01110//Biosynthesis	-	GO:0005615//extracellu

-0.999776876	0.013448583	ko01100//Metabolic p	ko01100//Metab	-
-0.999777528	0.013428924	-	ko01100//Metab	GO:0005783//endoplasr
-0.999777688	0.013424081	ko01063//Biosynthesis	ko01100//Metab	-
-0.999777785	0.013421148	-	-	-
-0.999778143	0.013410352	ko01100//Metabolic p	-	GO:0005634//nucleus;G
-0.999778487	0.013399932	-	ko04211//Longe	GO:0005634//nucleus
-0.999778892	0.01338767	ko01100//Metabolic p	-	-
-0.999778951	0.013385888	-	-	GO:0000781//chromosc
-0.99977963	0.013365331	-	ko04141//Protei	GO:0005783//endoplasr
-0.999779644	0.01336488	-	-	-
-0.999780064	0.013352161	-	-	GO:0016020//membran
-0.999780231	0.013347072	ko01100//Metabolic p	-	-
-0.999780905	0.013326606	-	-	GO:0005737//cytoplasm
-0.999780975	0.013324478	ko01100//Metabolic p	ko04310//Wnt s	GO:0005634//nucleus;G
-0.999781116	0.013320177	ko01100//Metabolic p	-	GO:0005886//plasma m
-0.999781196	0.013317742	ko01110//Biosynthesis	-	-
-0.999781294	0.013314767	ko01100//Metabolic p	-	-
-0.99978199	0.013293558	-	ko01100//Metab	GO:0005794//Golgi app
-0.999782043	0.013291946	-	-	GO:0005634//nucleus;G
-0.999782319	0.013283531	-	-	GO:0005634//nucleus;G
-0.999782593	0.013275145	ko01100//Metabolic p	-	-
-0.999782687	0.013272278	ko01100//Metabolic p	-	GO:0005576//extracellu
-0.999782767	0.013269832	ko01110//Biosynthesis	ko01100//Metab	GO:0005769//early endo
-0.999783643	0.013243073	-	ko01100//Metab	GO:0005794//Golgi app
-0.999783778	0.013238933	ko01100//Metabolic p	-	-
-0.999784427	0.013219035	ko01100//Metabolic p	-	GO:0005829//cytosol;GO
-0.999784626	0.013212945	ko01110//Biosynthesis	-	GO:0016021//integral co
-0.999784721	0.013210025	-	-	GO:0005737//cytoplasm
-0.99978476	0.013208844	ko01100//Metabolic p	-	-
-0.999784983	0.013201976	-	ko04144//Endoc	GO:0005654//nucleopla
-0.999785008	0.013201219	-	ko01100//Metab	GO:0033178//proton-tr
-0.999785557	0.013184351	-	ko03013//Nucle	-
-0.99978556	0.013184249	ko01100//Metabolic p	ko01100//Metab	-
-0.999786049	0.013169223	ko01100//Metabolic p	-	-
-0.999786364	0.013159511	ko01100//Metabolic p	ko04310//Wnt s	GO:0005634//nucleus;G
-0.999786561	0.013153443	-	-	GO:0005634//nucleus;G
-0.999787057	0.013138167	ko01100//Metabolic p	-	-
-0.999787142	0.013135533	ko01100//Metabolic p	-	-
-0.999787174	0.013134542	-	ko04142//Lysos	GO:0016020//membran
-0.999787237	0.013132589	-	-	GO:0005654//nucleopla
-0.999787293	0.013130891	ko01100//Metabolic p	ko04721//Synap	-
-0.999787497	0.013124584	-	-	-
-0.999787818	0.013114659	ko01110//Biosynthesis	ko05203//Viral c	GO:0000786//nucleosor
-0.999788043	0.013107706	-	ko01100//Metab	GO:0005783//endoplasr
-0.999788226	0.013102054	-	ko00970//Amino	GO:0005737//cytoplasm
-0.999788577	0.013091182	-	-	-
-0.99978905	0.013076523	ko01100//Metabolic p	-	GO:0005634//nucleus;G
-0.999789331	0.013067807	-	-	-
-0.999789656	0.013057722	ko01100//Metabolic p	-	GO:0001518//voltage-g
-0.999790158	0.013042156	ko01100//Metabolic p	-	GO:0005615//extracellu
-0.999790272	0.013038587	ko01110//Biosynthesis	ko04020//Calciu	GO:0001518//voltage-g
-0.999790554	0.013029845	-	-	GO:0005886//plasma m
-0.999790802	0.013022098	ko01100//Metabolic p	-	GO:0005634//nucleus;G
-0.999791051	0.013014375	ko01100//Metabolic p	-	-
-0.999791068	0.01301384	ko01100//Metabolic p	-	GO:0005886//plasma m
-0.999791119	0.013012243	ko01110//Biosynthesis	-	-
-0.999791407	0.013003267	ko01100//Metabolic p	-	GO:0005576//extracellu
-0.999791758	0.012992336	-	-	GO:0005768//endosom

-0.999791956	0.012986147	-	ko01100//Metak	GO:0000506//glycosylpl
-0.99979245	0.012970702	-	ko01100//Metak	GO:0005634//nucleus;G
-0.999792574	0.012966834	-	-	GO:0005794//Golgi app
-0.999793089	0.012950725	ko01100//Metabolic p	ko01100//Metak	GO:0005783//endoplasr
-0.999793304	0.012944007	-	-	GO:0005654//nucleopla
-0.999793865	0.012926415	ko01100//Metabolic p	-	GO:0000127//transcript
-0.999794023	0.012921471	-	ko05231//Cholir	-
-0.999794054	0.012920506	ko01100//Metabolic p	-	GO:0001650//fibrillar ce
-0.999794207	0.012915686	ko01100//Metabolic p	ko05203//Viral c	GO:0000786//nucleosor
-0.999794882	0.012894482	-	-	-
-0.999795385	0.012878672	-	ko04150//mTOF	GO:0005764//lysosome;
-0.999795632	0.012870907	-	-	-
-0.99979577	0.012866563	-	-	-
-0.999795925	0.012861683	-	ko01100//Metak	-
-0.999796188	0.012853391	ko01100//Metabolic p	ko01100//Metak	-
-0.999796283	0.012850388	-	ko05142//Chaga	GO:0016020//membran
-0.999797104	0.012824463	ko01100//Metabolic p	ko01100//Metak	GO:0005794//Golgi app
-0.999797555	0.012810212	-	-	GO:0043231//intracellul
-0.999797677	0.012806334	-	-	-
-0.999797682	0.012806194	-	ko04360//Axon	-
-0.999797905	0.012799114	-	-	GO:0005576//extracellu
-0.999798218	0.012789211	-	-	-
-0.999798269	0.012787601	ko01100//Metabolic p	ko01100//Metak	GO:0001931//uropod;G
-0.999798718	0.012773355	ko01100//Metabolic p	-	GO:0031462//Cul2-RIN(
-0.999798806	0.012770568	ko01100//Metabolic p	ko04270//Vascu	GO:0005737//cytoplasm
-0.99979943	0.012750727	-	-	-
-0.999799907	0.012735568	ko01100//Metabolic p	-	-
-0.999800188	0.012726628	-	ko04151//PI3K-/	GO:0005634//nucleus;G
-0.999800211	0.012725904	-	-	GO:0016020//membran
-0.999801203	0.012694255	-	-	-
-0.999801279	0.012691825	-	-	GO:0005737//cytoplasm
-0.999801447	0.012686463	ko01100//Metabolic p	-	-
-0.999802041	0.012667478	-	ko04979//Chole	GO:0005737//cytoplasm
-0.999802134	0.012664501	ko01100//Metabolic p	ko04360//Axon	GO:0005576//extracellu
-0.99980236	0.012657274	ko01100//Metabolic p	-	-
-0.999802444	0.012654576	ko01100//Metabolic p	-	-
-0.99980279	0.012643488	ko01100//Metabolic p	-	-
-0.999803472	0.012621611	-	-	GO:0005783//endoplasr
-0.999803707	0.01261405	-	-	-
-0.999803829	0.012610131	ko01100//Metabolic p	-	GO:0005737//cytoplasm
-0.999804047	0.012603118	ko01100//Metabolic p	-	GO:0001669//acrosoma
-0.999804108	0.012601159	ko01100//Metabolic p	-	GO:0005634//nucleus;G
-0.999804569	0.01258634	-	ko04080//Neurc	GO:0005886//plasma m
-0.999804901	0.012575632	ko01100//Metabolic p	ko05200//Pathw	GO:0000785//chromatir
-0.999805172	0.012566881	-	ko01100//Metak	-
-0.999805207	0.01256576	ko01100//Metabolic p	ko01100//Metak	GO:0016020//membran
-0.999805459	0.012557639	-	-	-
-0.999805498	0.012556359	-	-	GO:0005634//nucleus;G
-0.999805862	0.01254461	-	-	-
-0.999806685	0.012517996	-	ko04979//Chole	GO:0005783//endoplasr
-0.999806704	0.012517397	-	-	-
-0.999806838	0.012513043	ko01100//Metabolic p	-	GO:0001725//stress fibe
-0.99980687	0.012512	-	-	-
-0.999806899	0.012511065	-	ko04216//Ferrop	GO:0016020//membran
-0.999806937	0.01250985	-	ko05200//Pathw	GO:0005737//cytoplasm
-0.999806999	0.012507826	-	ko04144//Endoc	GO:0005654//nucleopla
-0.999807095	0.012504722	-	-	-
-0.999808629	0.012454899	-	ko04140//Autop	GO:0000407//pre-autop

-0.999808774	0.01245017	-	-	GO:0032991//macromo
-0.999809	0.012442833	-	-	-
-0.999809206	0.012436107	-	ko01100//Metak	-
-0.999809835	0.012415578	-	ko04068//FoxO	GO:0005737//cytoplasm
-0.999810158	0.012405049	ko01100//Metabolic p	ko01100//Metak	GO:0005654//nucleopla
-0.999810324	0.012399629	ko01100//Metabolic p	-	GO:0005634//nucleus;G
-0.999810667	0.012388382	ko01100//Metabolic p	-	-
-0.999810927	0.012379881	ko01100//Metabolic p	-	GO:0005739//mitochon
-0.999811127	0.012373345	ko01100//Metabolic p	ko01100//Metak	GO:0005737//cytoplasm
-0.999811299	0.0123677	-	-	GO:0008076//voltage-g
-0.999811379	0.012365067	-	-	GO:0016020//membran
-0.999811647	0.012356291	-	-	-
-0.999811711	0.012354179	ko01100//Metabolic p	-	-
-0.99981215	0.012339797	ko01100//Metabolic p	-	GO:0005789//endoplasr
-0.999812194	0.012338353	-	-	GO:0005737//cytoplasm
-0.999812377	0.012332311	ko01100//Metabolic p	ko04211//Longe	GO:0005634//nucleus
-0.999812568	0.012326049	ko01100//Metabolic p	-	GO:0005783//endoplasr
-0.999812663	0.012322929	ko01100//Metabolic p	-	GO:0005739//mitochon
-0.999812916	0.01231461	ko01110//Biosynthesis	ko04360//Axon	GO:0005576//extracellu
-0.999813044	0.012310376	ko01100//Metabolic p	ko05200//Pathw	GO:0005634//nucleus;G
-0.999813263	0.012303183	ko01100//Metabolic p	ko05150//Staph	-
-0.999813644	0.012290613	-	ko03010//Ribos	GO:0005634//nucleus;G
-0.99981382	0.012284814	ko01110//Biosynthesis	-	GO:0005576//extracellu
-0.999813831	0.012284432	-	-	GO:0016020//membran
-0.999814459	0.012263715	ko01120//Microbial m	-	GO:0031012//extracellu
-0.999815756	0.012220751	-	ko04020//Calciu	GO:0016020//membran
-0.999816061	0.012210634	ko01100//Metabolic p	-	-
-0.999816232	0.012204955	ko01100//Metabolic p	-	GO:0005829//cytosol
-0.999816241	0.012204657	-	-	GO:0005576//extracellu
-0.999816437	0.012198163	-	-	-
-0.999816764	0.012187288	-	-	-
-0.999816821	0.012185402	ko01100//Metabolic p	-	-
-0.999817305	0.012169297	-	-	GO:0005654//nucleopla
-0.999817549	0.012161162	-	-	-
-0.999818199	0.012139473	-	-	-
-0.999818322	0.012135376	ko01063//Biosynthesis	-	-
-0.999818503	0.012129309	-	-	-
-0.999818622	0.012125345	ko01100//Metabolic p	-	GO:0008076//voltage-g
-0.999818817	0.012118826	-	ko05200//Pathw	GO:0005794//Golgi app
-0.999819064	0.012110557	-	-	GO:0005783//endoplasr
-0.999819553	0.012094175	ko01100//Metabolic p	-	GO:0005615//extracellu
-0.999819676	0.012090055	ko01100//Metabolic p	ko04974//Protei	-
-0.999819711	0.01208888	-	-	GO:0005654//nucleopla
-0.999820011	0.012078819	ko01100//Metabolic p	-	GO:0016020//membran
-0.999821042	0.012044167	-	-	-
-0.999821053	0.012043805	-	ko03320//PPAR	GO:0005615//extracellu
-0.999821638	0.012024095	ko01100//Metabolic p	-	GO:0005737//cytoplasm
-0.999822332	0.012000679	-	ko01100//Metak	GO:0005737//cytoplasm
-0.999822347	0.012000186	ko01100//Metabolic p	-	-
-0.999822599	0.011991669	ko01110//Biosynthesis	-	-
-0.999822808	0.011984589	ko01100//Metabolic p	-	-
-0.999823478	0.011961911	-	-	-
-0.999823523	0.011960391	-	-	-
-0.999823525	0.011960324	ko01100//Metabolic p	-	-
-0.999823555	0.011959315	ko01100//Metabolic p	-	-
-0.999824224	0.011936602	-	-	-
-0.99982426	0.011935406	ko01100//Metabolic p	-	-
-0.999824322	0.011933285	-	-	-

-0.999824334	0.011932894	ko01100//Metabolic p	-	-
-0.99982437	0.011931649	ko01063//Biosynthesis	ko04360//Axon	GO:0005886//plasma m
-0.999824754	0.011918604	-	-	GO:0005886//plasma m
-0.999824952	0.011911876	ko01100//Metabolic p	-	-
-0.999825013	0.011909809	-	ko01100//Metab	-
-0.999825084	0.011907384	-	ko04141//Protei	GO:0005783//endoplasr
-0.99982524	0.011902057	ko01100//Metabolic p	-	-
-0.999825543	0.011891733	ko01100//Metabolic p	ko04721//Synap	GO:0005887//integral c
-0.9998261	0.011872735	ko01100//Metabolic p	-	-
-0.999826146	0.011871181	-	ko04142//Lysos	-
-0.999826249	0.011867665	-	ko01100//Metab	GO:0009923//fatty acid
-0.999826343	0.011864453	ko01100//Metabolic p	ko05203//Viral c	GO:0000786//nucleosor
-0.999826508	0.011858794	-	-	GO:0000127//transcript
-0.999826509	0.011858779	-	-	-
-0.999826545	0.011857542	ko01100//Metabolic p	-	GO:0016021//integral c
-0.999826877	0.011846181	ko01100//Metabolic p	-	GO:0005794//Golgi app
-0.999827541	0.011823449	ko01100//Metabolic p	ko01100//Metab	GO:0048471//perinuclea
-0.999827837	0.01181329	ko01100//Metabolic p	ko00970//Amino	GO:0005737//cytoplasm
-0.999827969	0.011808765	-	-	GO:0005783//endoplasr
-0.999828028	0.011806744	ko01100//Metabolic p	ko04144//Endoc	GO:0005654//nucleopla
-0.999829109	0.011769584	-	-	-
-0.999829439	0.011758211	ko01100//Metabolic p	-	GO:0005634//nucleus;G
-0.999829537	0.011754833	ko01100//Metabolic p	-	GO:0005829//cytosol;G
-0.999829688	0.011749631	-	-	-
-0.999830074	0.011736299	ko01100//Metabolic p	ko01100//Metab	GO:0005576//extracellu
-0.99983022	0.011731263	-	-	GO:0005794//Golgi app
-0.999830856	0.011709279	ko01100//Metabolic p	ko01100//Metab	GO:0005769//early endo
-0.999830957	0.011705762	ko01100//Metabolic p	-	GO:0031982//vesicle
-0.999831055	0.011702369	ko01110//Biosynthesis	ko01100//Metab	GO:0005783//endoplasr
-0.999831202	0.011697285	ko01100//Metabolic p	-	-
-0.999831369	0.011691502	-	-	GO:0016020//membran
-0.999831371	0.011691414	-	-	GO:0005634//nucleus;G
-0.999831424	0.011689578	ko01100//Metabolic p	-	-
-0.999832038	0.011668278	ko01100//Metabolic p	-	GO:0005634//nucleus;G
-0.999832533	0.011651055	-	ko05165//Huma	-
-0.999833364	0.011622138	ko01100//Metabolic p	-	-
-0.999833731	0.011609332	-	-	GO:0005634//nucleus;G
-0.999833836	0.011605641	-	-	-
-0.999834555	0.011580534	-	ko05200//Pathw	GO:0005634//nucleus;G
-0.99983457	0.01157999	ko01100//Metabolic p	-	GO:0005737//cytoplasm
-0.999834688	0.011575866	-	-	-
-0.999834775	0.011572802	ko01100//Metabolic p	-	-
-0.999834991	0.011565257	-	-	-
-0.999835424	0.011550065	ko01100//Metabolic p	ko01100//Metab	GO:0005737//cytoplasm
-0.999835645	0.011542319	-	ko01100//Metab	GO:0005783//endoplasr
-0.999836083	0.011526904	-	-	GO:0005576//extracellu
-0.99983621	0.01152246	ko01110//Biosynthesis	-	GO:0015629//actin cyto
-0.999836267	0.011520453	-	ko05203//Viral c	GO:0000786//nucleosor
-0.999836288	0.011519719	ko01100//Metabolic p	-	-
-0.999836411	0.011515378	ko01100//Metabolic p	-	-
-0.999836628	0.011507751	-	ko01100//Metab	GO:0005783//endoplasr
-0.99983719	0.011487938	ko01110//Biosynthesis	-	-
-0.999837618	0.0114728	ko01100//Metabolic p	-	-
-0.999837634	0.011472242	ko01100//Metabolic p	-	-
-0.999837899	0.011462896	-	ko04630//JAK-S	GO:0005829//cytosol
-0.999837954	0.011460928	ko01100//Metabolic p	-	-
-0.999838238	0.011450889	-	-	GO:0005615//extracellu
-0.999838272	0.011449699	ko01100//Metabolic p	-	GO:0000139//Golgi mer

-0.999838793	0.011431226	ko01100//Metabolic p	-	-
-0.999838897	0.011427529	-	ko04010//MAPK	-
-0.999839129	0.011419315	-	ko05412//Arrhyt	GO:0005886//plasma m
-0.999839342	0.011411753	ko01100//Metabolic p	ko01100//Metab	-
-0.999839616	0.011402011	-	ko05200//Pathw	GO:0000785//chromatin
-0.999839755	0.011397054	-	ko01100//Metab	-
-0.999839997	0.011388454	ko01100//Metabolic p	ko04310//Wnt s	GO:0005634//nucleus;G
-0.999840065	0.01138604	-	-	GO:0005783//endoplasm
-0.999840122	0.011384017	ko01100//Metabolic p	-	-
-0.999841121	0.011348387	-	-	-
-0.999841234	0.011344335	-	-	-
-0.999841372	0.011339422	-	-	GO:0001725//stress fibe
-0.999841436	0.011337145	ko01110//Biosynthesis	-	-
-0.999841524	0.011334002	-	-	-
-0.999842007	0.01131671	-	-	-
-0.99984223	0.011308692	-	ko04934//Cushin	GO:0016020//membran
-0.999842751	0.011290029	-	-	-
-0.999842959	0.011282556	-	-	GO:0031464//Cul4A-RIN
-0.999843329	0.011269256	ko01100//Metabolic p	-	-
-0.999843345	0.011268681	-	ko04151//PI3K-A	GO:0005634//nucleus;G
-0.99984358	0.011260228	ko01100//Metabolic p	ko04919//Thyro	GO:0016020//membran
-0.999843664	0.01125719	ko01100//Metabolic p	ko04140//Autop	GO:0000407//pre-autop
-0.999844483	0.011227666	ko01100//Metabolic p	-	-
-0.999845031	0.011207886	-	-	GO:0001669//acrosoma
-0.99984522	0.011201024	ko01100//Metabolic p	ko04216//Ferrop	GO:0016020//membran
-0.999845412	0.011194078	ko01100//Metabolic p	-	GO:0008076//voltage-g
-0.999845601	0.011187235	-	-	-
-0.999845821	0.011179264	ko01100//Metabolic p	-	GO:0005737//cytoplasm
-0.999845913	0.011175922	-	-	-
-0.999845941	0.011174928	-	ko04146//Peroxi	GO:0005777//peroxison
-0.999846358	0.011159768	ko01100//Metabolic p	-	GO:0005737//cytoplasm
-0.999847151	0.01113093	-	-	GO:0005794//Golgi app
-0.999847193	0.011129402	-	-	-
-0.999847553	0.011116286	-	ko01100//Metab	GO:0005654//nucleopla
-0.999847755	0.011108934	-	ko01100//Metab	-
-0.999847843	0.011105726	-	-	-
-0.999847953	0.011101698	ko01100//Metabolic p	-	-
-0.999848127	0.011095343	ko01110//Biosynthesis	ko03410//Base e	GO:0005634//nucleus;G
-0.999848158	0.011094224	ko01100//Metabolic p	-	-
-0.999848229	0.011091621	-	-	-
-0.999848243	0.011091117	-	-	GO:0005739//mitochon
-0.999848493	0.01108196	ko01100//Metabolic p	-	-
-0.999849002	0.011063333	ko01100//Metabolic p	ko04142//Lysoso	GO:0016020//membran
-0.999849197	0.011056193	ko01100//Metabolic p	-	-
-0.999849222	0.011055272	-	-	GO:0034361//very-low-
-0.999849747	0.011035994	ko01110//Biosynthesis	-	-
-0.999849824	0.011033188	-	-	GO:0005634//nucleus;G
-0.999849868	0.011031573	-	ko04146//Peroxi	GO:0005765//lysosomal
-0.999850175	0.011020264	ko01100//Metabolic p	-	GO:0005737//cytoplasm
-0.999850302	0.011015596	ko01100//Metabolic p	-	GO:0097541//axonemal
-0.999850609	0.011004327	-	-	-
-0.999850696	0.011001086	ko01100//Metabolic p	ko04144//Endoc	GO:0000813//ESCRT I c
-0.999850731	0.010999803	-	-	-
-0.999850995	0.010990092	-	ko04310//Wnt s	GO:0005634//nucleus;G
-0.999851137	0.01098485	-	-	-
-0.999851188	0.010982954	ko01100//Metabolic p	ko01100//Metab	GO:0016020//membran
-0.999851374	0.010976088	-	-	-
-0.999851381	0.010975847	-	-	GO:0016020//membran

-0.999851959	0.010954458	-	ko01100//Metal	GO:0005829//cytosol
-0.999852166	0.010946805	ko01100//Metabolic p	-	-
-0.999852264	0.010943165	-	-	-
-0.999852537	0.010933058	ko01100//Metabolic p	ko01100//Metal	GO:0005737//cytoplasm
-0.999852739	0.010925591	-	-	-
-0.999853169	0.0109096	ko01100//Metabolic p	-	GO:0005737//cytoplasm
-0.999853236	0.010907121	-	-	GO:0016020//membran
-0.999853304	0.010904581	-	-	-
-0.999855171	0.010834993	ko01100//Metabolic p	ko01100//Metal	GO:0005654//nucleopla
-0.999855219	0.010833188	-	-	GO:0001518//voltage-g
-0.999855233	0.010832666	-	ko03010//Ribos	GO:0005634//nucleus;G
-0.999855262	0.010831589	ko01100//Metabolic p	-	GO:0005856//cytoskelet
-0.999855968	0.010805113	-	-	-
-0.999856969	0.010767518	-	-	GO:0005634//nucleus;G
-0.999857499	0.010747539	-	-	GO:0005615//extracellu
-0.999857502	0.010747435	-	-	-
-0.999857887	0.010732885	-	-	-
-0.999858095	0.010725063	ko01100//Metabolic p	-	-
-0.999858705	0.010701962	ko01100//Metabolic p	-	-
-0.999858817	0.010697714	-	-	-
-0.99985907	0.010688134	-	-	GO:0005886//plasma m
-0.999859545	0.01067012	ko01100//Metabolic p	-	GO:0016021//integral co
-0.999859614	0.010667496	-	-	-
-0.999859754	0.010662149	ko01100//Metabolic p	-	-
-0.999859913	0.010656108	-	ko01100//Metal	GO:0005783//endoplasr
-0.999859941	0.010655044	-	ko01100//Metal	GO:0005794//Golgi app
-0.999859994	0.010653023	-	-	-
-0.999860205	0.010645001	-	ko01100//Metal	GO:0033178//proton-tr
-0.999860299	0.010641442	-	ko04020//Calciu	GO:0016020//membran
-0.999860759	0.01062388	ko01100//Metabolic p	-	GO:0016020//membran
-0.999861755	0.010585837	-	ko03010//Ribos	GO:0005634//nucleus;G
-0.99986207	0.010573758	ko01100//Metabolic p	-	GO:0009986//cell surfac
-0.999862211	0.010568366	-	-	-
-0.999862296	0.010565092	-	-	-
-0.999862482	0.010557955	-	ko04151//PI3K-/	GO:0005654//nucleopla
-0.999862496	0.010557418	-	-	GO:0005794//Golgi app
-0.999863381	0.010523397	-	ko01100//Metal	GO:0005576//extracellu
-0.999865066	0.010458295	ko01100//Metabolic p	-	-
-0.999865319	0.010448487	ko01063//Biosynthesis	ko01100//Metal	GO:0048471//perinucle
-0.999865378	0.010446188	ko01100//Metabolic p	-	-
-0.999865388	0.010445807	ko01100//Metabolic p	-	-
-0.999865587	0.010438076	ko01100//Metabolic p	ko01100//Metal	GO:0005829//cytosol
-0.999865927	0.010424859	-	ko00564//Glyce	GO:0005634//nucleus;G
-0.999866894	0.010387223	-	-	-
-0.999867029	0.010381918	-	-	GO:0005802//trans-Gol
-0.999867056	0.010380896	ko01110//Biosynthesis	ko01100//Metal	GO:0005576//extracellu
-0.999867263	0.010372794	-	-	-
-0.999867368	0.010368698	ko01100//Metabolic p	ko01100//Metal	GO:0005783//endoplasr
-0.999867895	0.010348093	-	-	GO:0005764//lysosome;
-0.999867923	0.010346966	ko01100//Metabolic p	ko01100//Metal	GO:0005783//endoplasr
-0.999867976	0.010344892	ko01100//Metabolic p	ko04310//Wnt s	GO:0009897//external s
-0.999868146	0.010338231	ko01100//Metabolic p	ko01100//Metal	GO:0005741//mitochon
-0.999868213	0.010335614	-	-	GO:0005783//endoplasr
-0.999868355	0.010330051	ko01110//Biosynthesis	ko01100//Metal	GO:0005829//cytosol;G
-0.999868683	0.010317182	-	-	GO:0016020//membran
-0.999868912	0.010308179	-	-	-
-0.999869403	0.01028885	ko01100//Metabolic p	ko00790//Folate	-
-0.999869441	0.010287347	ko01063//Biosynthesis	-	-

-0.999869458	0.01028668	-	-	GO:0000177//cytoplasm
-0.999869677	0.010278056	ko01100//Metabolic p	-	-
-0.999869697	0.010277267	ko01100//Metabolic p	-	GO:0005615//extracellu
-0.999869732	0.010275853	-	ko04080//Neuro	GO:0005886//plasma m
-0.999869876	0.010270207	-	ko04514//Cell a	GO:0005886//plasma m
-0.999870013	0.010264788	-	-	-
-0.999870211	0.01025695	ko01100//Metabolic p	ko00533//Glycos	GO:0000139//Golgi mer
-0.999870265	0.010254839	-	-	GO:0005783//endoplasr
-0.999870457	0.010247252	ko01100//Metabolic p	ko04931//Insulir	GO:0016021//integral co
-0.999871787	0.010194509	-	-	-
-0.999871979	0.010186871	ko01100//Metabolic p	-	-
-0.999872296	0.010174256	-	ko01100//Metab	-
-0.999872558	0.010163788	-	-	-
-0.999873159	0.010139815	-	ko04020//Calciu	GO:0005634//nucleus;G
-0.999873349	0.010132193	ko01100//Metabolic p	ko03013//Nucle	-
-0.999874663	0.010079512	ko01100//Metabolic p	ko01100//Metab	GO:0005788//endoplasr
-0.999875599	0.010041794	-	ko05200//Pathw	GO:0005834//heterotrin
-0.999876374	0.010010474	-	-	GO:0031464//Cul4A-RII
-0.999876702	0.009997175	-	-	GO:0016021//integral co
-0.999876876	0.009990106	ko01100//Metabolic p	-	GO:0005739//mitochon
-0.999877791	0.009952954	-	-	GO:0005634//nucleus;G
-0.999877874	0.00994956	-	-	-
-0.999877913	0.009947975	-	-	GO:0097541//axonemal
-0.99987868	0.009916655	ko01100//Metabolic p	-	-
-0.999878886	0.009908226	ko01100//Metabolic p	-	GO:0016020//membran
-0.999879219	0.009894629	ko01100//Metabolic p	-	GO:0016020//membran
-0.999879311	0.009890849	ko01063//Biosynthesis	-	GO:0005576//extracellu
-0.999879595	0.009879184	-	-	-
-0.999879648	0.009877008	-	-	GO:0005886//plasma m
-0.999879691	0.009875269	ko01100//Metabolic p	-	GO:0005886//plasma m
-0.999879703	0.009874748	ko01100//Metabolic p	-	-
-0.999879761	0.009872398	ko01100//Metabolic p	ko03320//PPAR	GO:0005783//endoplasr
-0.999880061	0.009860074	-	-	GO:0005737//cytoplasm
-0.99988016	0.009856011	-	-	-
-0.999880774	0.009830727	-	-	GO:0008076//voltage-g
-0.999881574	0.009797693	ko01100//Metabolic p	ko05202//Transc	GO:0000786//nucleosor
-0.999882008	0.009779696	ko01100//Metabolic p	ko04146//Peroxi	GO:0005777//peroxison
-0.999882305	0.009767371	-	ko01100//Metab	GO:0005654//nucleopla
-0.99988247	0.009760542	-	-	GO:0005615//extracellu
-0.999883179	0.009731053	-	-	-
-0.999883446	0.009719933	-	-	GO:0005654//nucleopla
-0.999883495	0.009717889	ko01110//Biosynthesis	-	GO:0005634//nucleus;G
-0.999884095	0.009692816	ko01100//Metabolic p	ko01100//Metab	-
-0.999884257	0.009686049	ko01100//Metabolic p	-	GO:0005654//nucleopla
-0.999884509	0.009675511	ko01100//Metabolic p	-	-
-0.999884523	0.009674929	ko01100//Metabolic p	-	-
-0.999884811	0.009662832	ko01100//Metabolic p	ko05231//Cholir	-
-0.999884878	0.00966004	ko01100//Metabolic p	-	GO:0034361//very-low-
-0.999885212	0.009646024	ko01110//Biosynthesis	ko01100//Metab	GO:0016021//integral co
-0.999885251	0.009644373	-	ko05412//Arrhyt	GO:0005886//plasma m
-0.999885336	0.009640789	-	-	-
-0.999885559	0.009631408	-	-	GO:0005794//Golgi app
-0.999885685	0.00962612	-	-	GO:0000785//chromatir
-0.999885713	0.009624925	ko01100//Metabolic p	-	GO:0005654//nucleopla
-0.999885871	0.009618267	-	-	-
-0.999886037	0.009611264	ko01100//Metabolic p	ko04550//Signal	GO:0005634//nucleus;G
-0.999886089	0.009609094	ko01100//Metabolic p	-	-
-0.999886268	0.009601527	-	-	GO:0016020//membran

-0.99988684	0.009577364	-	ko00790//Folate	-
-0.999886843	0.009577231	ko01100//Metabolic p	-	-
-0.999887361	0.009555289	ko01100//Metabolic p	-	GO:0005789//endoplasr
-0.999887868	0.00953375	-	ko04934//Cushi	GO:0016020//membran
-0.999887884	0.009533069	ko01100//Metabolic p	ko04151//PI3K-/	GO:0005634//nucleus;G
-0.99988811	0.009523445	-	-	-
-0.999888378	0.009512056	ko01063//Biosynthesis	-	GO:0031966//mitochon
-0.9998884	0.009511119	-	ko04141//Protei	GO:0005783//endoplasr
-0.999888701	0.00949826	ko01100//Metabolic p	-	-
-0.999889007	0.009485219	-	-	-
-0.999889078	0.009482155	-	-	-
-0.999889158	0.009478755	ko01100//Metabolic p	-	GO:0005634//nucleus;G
-0.999889751	0.009453363	ko01100//Metabolic p	-	-
-0.999889788	0.009451771	ko01110//Biosynthesis	ko01100//Metab	-
-0.99989036	0.009427234	-	-	-
-0.999890562	0.009418545	ko01063//Biosynthesis	ko01100//Metab	-
-0.999890672	0.009413808	ko01100//Metabolic p	ko01100//Metab	GO:0005737//cytoplasm
-0.999890864	0.00940552	-	ko01100//Metab	GO:0033178//proton-tr
-0.999891066	0.009396797	ko01100//Metabolic p	-	GO:0005634//nucleus;G
-0.999891172	0.009392234	-	ko04360//Axon	-
-0.999891724	0.009368379	ko01100//Metabolic p	-	GO:0005737//cytoplasm
-0.9998918	0.009365114	-	ko01100//Metab	GO:0005576//extracellu
-0.999891903	0.009360628	ko01100//Metabolic p	ko05202//Trans	GO:0000786//nucleosor
-0.999892143	0.009350267	-	ko03010//Ribos	GO:0005634//nucleus;G
-0.999892667	0.009327521	-	-	-
-0.999893136	0.009307121	-	ko01100//Metab	GO:0016020//membran
-0.999893442	0.009293763	ko01100//Metabolic p	ko01100//Metab	GO:0005788//endoplasr
-0.999893475	0.00929235	-	ko01100//Metab	GO:0005737//cytoplasm
-0.999893494	0.009291523	-	-	-
-0.999893822	0.009277177	ko01100//Metabolic p	-	-
-0.999893875	0.009274887	-	-	GO:0016021//integral c
-0.999893909	0.009273375	ko01100//Metabolic p	-	-
-0.999894003	0.009269287	-	ko05203//Viral c	GO:0000786//nucleosor
-0.999894274	0.009257409	ko01100//Metabolic p	-	GO:0016020//membran
-0.999894339	0.009254593	ko01100//Metabolic p	ko01100//Metab	GO:0005769//early endo
-0.999894449	0.009249768	ko01100//Metabolic p	ko01100//Metab	GO:0033178//proton-tr
-0.999894469	0.009248862	-	-	-
-0.999894659	0.009240557	ko01100//Metabolic p	-	-
-0.999894724	0.0092377	ko01100//Metabolic p	ko01100//Metab	GO:0005783//endoplasr
-0.999895144	0.009219239	ko01100//Metabolic p	-	-
-0.999895198	0.009216893	-	ko04919//Thyro	GO:0016020//membran
-0.999895302	0.00921228	-	ko01100//Metab	GO:0000506//glycosylpl
-0.99989561	0.009198718	ko01100//Metabolic p	-	-
-0.999895626	0.009198046	-	-	-
-0.999895639	0.009197471	ko01100//Metabolic p	ko04151//PI3K-/	GO:0005634//nucleus;G
-0.999895787	0.009190925	-	ko04144//Endoc	-
-0.999895899	0.00918598	-	ko04140//Autop	GO:0000407//pre-autop
-0.999896179	0.00917364	ko01100//Metabolic p	-	GO:0009986//cell surfac
-0.99989619	0.009173133	ko01100//Metabolic p	-	-
-0.999896194	0.009172973	-	ko03013//Nucle	-
-0.999897168	0.009129815	-	ko04216//Ferrop	GO:0016020//membran
-0.999897525	0.009113966	-	-	-
-0.999897544	0.009113136	ko01100//Metabolic p	-	GO:0031464//Cul4A-RII
-0.999897852	0.009099419	ko01100//Metabolic p	ko05203//Viral c	GO:0000786//nucleosor
-0.999897958	0.009094678	-	ko01100//Metab	GO:0005739//mitochon
-0.999898464	0.009072139	ko01100//Metabolic p	ko04721//Synap	GO:0005887//integral c
-0.999898613	0.009065451	-	ko01100//Metab	GO:0005576//extracellu
-0.999898686	0.009062209	-	ko04010//MAPK	GO:0005737//cytoplasm

-0.999898774	0.009058271	-	-	-
-0.999899044	0.009046194	-	-	-
-0.999899212	0.009038624	-	-	-
-0.999899232	0.009037724	ko01100//Metabolic p	ko01100//Metab	-
-0.999899295	0.009034937	-	ko00533//Glycos	GO:0000139//Golgi mer
-0.999899377	0.009031223	ko01100//Metabolic p	-	-
-0.999900177	0.008995248	-	-	GO:0034361//very-low-
-0.999900266	0.008991274	ko01100//Metabolic p	-	GO:0005737//cytoplasm
-0.999900653	0.008973807	ko01120//Microbial m	-	-
-0.999900865	0.008964236	-	ko05203//Viral c	GO:0000786//nucleosor
-0.9999011	0.008953595	ko01100//Metabolic p	ko05152//Tuber	GO:0009897//external s
-0.9999012	0.008949055	-	-	GO:0005737//cytoplasm
-0.999901337	0.008942852	ko01100//Metabolic p	-	GO:0031464//Cul4A-RII
-0.999901472	0.008936739	-	-	-
-0.99990165	0.008928648	ko01100//Metabolic p	-	-
-0.99990168	0.008927287	ko01120//Microbial m	-	-
-0.999901752	0.008924027	-	-	GO:0008076//voltage-g
-0.999902003	0.008912625	-	-	-
-0.999902046	0.008910658	ko01100//Metabolic p	-	GO:0005654//nucleopla
-0.99990209	0.008908654	-	ko05200//Pathw	GO:0005737//cytoplasm
-0.999902761	0.008878081	-	-	-
-0.999902815	0.008875592	-	ko04146//Perox	GO:0005777//peroxison
-0.999902853	0.008873894	-	-	-
-0.999903237	0.00885632	ko01100//Metabolic p	ko04310//Wnt s	-
-0.999903457	0.008846266	-	-	GO:0016020//membran
-0.999903682	0.008835911	ko01100//Metabolic p	-	GO:0016020//membran
-0.999904128	0.008815431	ko01100//Metabolic p	-	-
-0.999904479	0.008799277	ko01100//Metabolic p	-	-
-0.999904489	0.008798834	-	-	GO:0043231//intracellul
-0.999904531	0.008796893	-	-	GO:0005783//endoplasr
-0.99990481	0.008784046	ko01100//Metabolic p	ko05200//Pathw	GO:0005829//cytosol;G(
-0.999905071	0.00877199	-	-	-
-0.999905083	0.008771453	ko01100//Metabolic p	-	GO:0005791//rough enc
-0.999905481	0.008753031	ko01100//Metabolic p	ko05200//Pathw	GO:0005834//heterotrin
-0.999905482	0.008752959	ko01100//Metabolic p	-	-
-0.999905851	0.008735863	-	-	GO:0016021//integral co
-0.999905895	0.008733811	-	-	-
-0.999906014	0.008728319	ko01100//Metabolic p	ko01100//Metab	GO:0005789//endoplasr
-0.999906323	0.008713969	ko01100//Metabolic p	ko01100//Metab	-
-0.999906913	0.008686453	ko01100//Metabolic p	-	GO:0005737//cytoplasm
-0.999907296	0.008668594	ko01110//Biosynthesis	ko01100//Metab	-
-0.99990739	0.008664191	ko01100//Metabolic p	-	GO:0005634//nucleus;G
-0.999907483	0.008659841	ko01100//Metabolic p	ko01100//Metab	GO:0005829//cytosol
-0.999907498	0.008659126	ko01100//Metabolic p	-	-
-0.999907657	0.008651668	-	-	GO:0001669//acrosoma
-0.999907925	0.00863913	ko01100//Metabolic p	ko03320//PPAR	GO:0005634//nucleus;G
-0.999908256	0.008623592	ko01063//Biosynthesis	-	GO:0043231//intracellul
-0.999908343	0.00861948	-	-	GO:0005576//extracellu
-0.999908589	0.00860789	-	-	-
-0.999908595	0.008607623	ko01100//Metabolic p	ko01100//Metab	-
-0.99990867	0.008604088	-	-	-
-0.999909019	0.00858763	ko01100//Metabolic p	-	GO:0005737//cytoplasm
-0.999909259	0.008576315	ko01063//Biosynthesis	ko04060//Cytok	GO:0005887//integral co
-0.999909535	0.008563231	ko01100//Metabolic p	-	-
-0.99990999	0.008541686	ko01100//Metabolic p	-	GO:0005856//cytoskelet
-0.999910042	0.008539226	ko01110//Biosynthesis	-	GO:0016020//membran
-0.99991019	0.008532213	ko01100//Metabolic p	-	-
-0.999910501	0.008517409	ko01100//Metabolic p	-	GO:0016581//NuRD cor

-0.999911055	0.008490992	ko01100//Metabolic p	-	-
-0.999911257	0.008481354	ko01063//Biosynthesis	ko01100//Metab	GO:0005737//cytoplasm
-0.999912109	0.008440548	ko01100//Metabolic p	-	-
-0.99991239	0.008427026	ko01110//Biosynthesis	-	GO:0001725//stress fibe
-0.999912466	0.008423388	-	-	-
-0.999912757	0.008409394	ko01100//Metabolic p	-	GO:0005794//Golgi app
-0.999912981	0.008398547	-	-	-
-0.999913147	0.00839055	-	-	GO:0000785//chromatin
-0.999913152	0.008390321	ko01100//Metabolic p	ko01100//Metab	GO:0016020//membran
-0.999913623	0.008367531	-	-	-
-0.999913673	0.008365132	-	-	GO:0016020//membran
-0.999913687	0.008364436	ko01110//Biosynthesis	ko05200//Pathw	GO:0000785//chromatin
-0.999914118	0.008343516	-	ko04713//Circac	-
-0.999914244	0.008337413	ko01110//Biosynthesis	ko01100//Metab	-
-0.999914329	0.008333276	-	-	GO:0005789//endoplasr
-0.999914574	0.008321351	-	-	GO:0005737//cytoplasm
-0.999915024	0.008299371	ko01100//Metabolic p	ko04360//Axon	GO:0005576//extracellu
-0.9999152	0.008290817	-	-	GO:0005654//nucleopla
-0.999915332	0.008284353	ko01100//Metabolic p	-	-
-0.999915427	0.008279696	-	-	-
-0.999915467	0.008277723	ko01100//Metabolic p	-	GO:0035658//Mon1-Cc
-0.999915474	0.00827741	ko01100//Metabolic p	-	-
-0.999915512	0.008275548	-	ko04141//Protei	GO:0005783//endoplasr
-0.999915531	0.008274598	ko01100//Metabolic p	-	-
-0.999916245	0.008239529	-	ko01100//Metab	GO:0005783//endoplasr
-0.999916377	0.008233065	-	ko05414//Dilate	GO:0016459//myosin cc
-0.999916379	0.008232943	ko01110//Biosynthesis	-	GO:0005634//nucleus;G
-0.999916398	0.00823202	ko01100//Metabolic p	-	-
-0.999916442	0.008229848	-	ko05414//Dilate	-
-0.999916536	0.008225222	-	-	-
-0.99991667	0.008218612	ko01100//Metabolic p	ko04360//Axon	-
-0.999916791	0.008212625	ko01100//Metabolic p	-	GO:0005737//cytoplasm
-0.999916899	0.00820734	-	-	-
-0.999917007	0.008201999	-	-	GO:0005764//lysosome;
-0.999917129	0.008195939	-	-	-
-0.999917168	0.008194011	ko01100//Metabolic p	-	GO:0005737//cytoplasm
-0.999917225	0.008191212	-	ko00983//Drug	-
-0.999917426	0.008181241	ko01100//Metabolic p	-	-
-0.999917498	0.008177666	ko01110//Biosynthesis	ko04151//PI3K-/	GO:0005634//nucleus;G
-0.999917527	0.008176272	ko01100//Metabolic p	ko01100//Metab	GO:0001931//uropod;G
-0.999917734	0.008165986	ko01100//Metabolic p	-	GO:0005604//basement
-0.999917741	0.008165624	ko01100//Metabolic p	-	GO:0016020//membran
-0.999917874	0.008159041	ko01100//Metabolic p	ko04918//Thyro	GO:0005576//extracellu
-0.999918101	0.008147762	ko01100//Metabolic p	-	GO:0016020//membran
-0.999918288	0.008138415	ko01100//Metabolic p	ko01100//Metab	-
-0.999918414	0.008132151	ko01100//Metabolic p	ko01100//Metab	GO:0005635//nuclear er
-0.999918419	0.008131916	-	-	GO:0005886//plasma m
-0.999918526	0.008126558	-	-	GO:0005634//nucleus;G
-0.999919073	0.008099237	ko01100//Metabolic p	-	GO:0005783//endoplasr
-0.999919085	0.008098647	-	ko01100//Metab	GO:0005783//endoplasr
-0.999919099	0.00809796	ko01110//Biosynthesis	-	-
-0.999919112	0.00809729	ko01100//Metabolic p	ko04934//Cushi	GO:0016020//membran
-0.999919175	0.008094152	ko01100//Metabolic p	ko01523//Antifo	GO:0005886//plasma m
-0.999919475	0.00807912	ko01100//Metabolic p	-	GO:0005634//nucleus;G
-0.999919774	0.008064079	-	ko01100//Metab	GO:0005783//endoplasr
-0.999919899	0.00805782	ko01100//Metabolic p	-	-
-0.9999201	0.008047695	ko01110//Biosynthesis	ko01100//Metab	GO:0005739//mitochon
-0.999920279	0.008038659	ko01100//Metabolic p	ko01100//Metab	GO:0000506//glycosylpl

-0.999920487	0.008028165	-	ko04514//Cell a	GO:0005829//cytosol;G
-0.999920869	0.008008874	-	-	-
-0.999920902	0.008007216	-	-	GO:0005615//extracellu
-0.999921099	0.007997226	-	-	-
-0.999921136	0.007995357	ko01100//Metabolic p	ko01100//Metab	-
-0.99992144	0.007979914	-	ko01100//Metab	GO:0005783//endoplasi
-0.999921908	0.007956111	-	-	-
-0.999922126	0.007944991	-	-	GO:0005654//nucleopla
-0.999922447	0.007928595	ko01100//Metabolic p	-	-
-0.999922834	0.007908828	-	-	-
-0.99992314	0.007893097	-	ko04211//Longe	GO:0005634//nucleus
-0.999923161	0.007892051	-	-	-
-0.999923607	0.007869117	ko01100//Metabolic p	-	-
-0.999923846	0.007856753	-	-	GO:0005634//nucleus;G
-0.999924181	0.007839493	-	-	GO:0031012//extracellu
-0.999924253	0.007835737	-	ko03320//PPAR	GO:0005576//extracellu
-0.999924257	0.007835522	ko01100//Metabolic p	-	-
-0.99992447	0.007824528	ko01100//Metabolic p	-	-
-0.999924652	0.007815089	ko01100//Metabolic p	-	-
-0.999924698	0.007812676	ko01100//Metabolic p	-	-
-0.999924852	0.007804693	-	ko04142//Lysos	GO:0016020//membran
-0.999924855	0.007804536	ko01110//Biosynthesis	-	-
-0.999925206	0.007786302	ko01063//Biosynthesis	-	-
-0.999925378	0.007777335	ko01100//Metabolic p	-	GO:0005654//nucleopla
-0.999926091	0.0077401	-	-	-
-0.999926185	0.007735156	-	ko04310//Wnt s	GO:0005634//nucleus;G
-0.999926321	0.007728033	-	ko04140//Autop	GO:0000407//pre-autop
-0.999926339	0.007727124	ko01100//Metabolic p	-	GO:0016021//integral c
-0.999926411	0.007723303	ko01100//Metabolic p	-	GO:0005737//cytoplasm
-0.999926487	0.007719352	ko01100//Metabolic p	ko01100//Metab	GO:0005737//cytoplasm
-0.99992735	0.00767391	-	-	GO:0000139//Golgi mer
-0.999927365	0.007673083	-	ko05200//Pathw	GO:0005737//cytoplasm
-0.999927373	0.007672678	-	ko01100//Metab	GO:0005783//endoplasi
-0.999927374	0.007672645	ko01100//Metabolic p	-	GO:0016020//membran
-0.999927388	0.007671868	-	ko04216//Ferrop	GO:0016020//membran
-0.999927476	0.007667207	ko01100//Metabolic p	ko04142//Lysos	GO:0016020//membran
-0.999927775	0.007651423	ko01100//Metabolic p	ko04710//Circac	GO:0005634//nucleus;G
-0.999928022	0.007638323	-	-	-
-0.999928079	0.007635284	ko01100//Metabolic p	-	-
-0.999928287	0.007624249	-	-	GO:0005634//nucleus;G
-0.999928669	0.007603891	-	-	-
-0.999928781	0.007597954	-	-	GO:0005634//nucleus;G
-0.999928899	0.007591622	-	ko05231//Cholir	-
-0.999928926	0.007590172	-	ko01100//Metab	GO:0005576//extracellu
-0.999928994	0.007586552	-	-	-
-0.999929166	0.007577352	ko01100//Metabolic p	-	GO:0005634//nucleus;G
-0.999929863	0.007540001	-	ko01100//Metab	-
-0.999929903	0.007537836	-	ko01100//Metab	-
-0.999929923	0.007536764	ko01100//Metabolic p	-	-
-0.999929976	0.007533896	-	-	-
-0.999929996	0.007532863	ko01100//Metabolic p	-	-
-0.999930115	0.007526417	-	-	GO:0005789//endoplasi
-0.999930298	0.007516581	-	-	-
-0.999930419	0.007510071	ko01100//Metabolic p	-	GO:0043231//intracellul
-0.999930471	0.007507221	ko01100//Metabolic p	-	GO:0015629//actin cyto
-0.99993077	0.007491108	-	-	GO:0005737//cytoplasm
-0.999930781	0.007490491	ko01100//Metabolic p	-	GO:0005615//extracellu
-0.999931109	0.007472699	ko01100//Metabolic p	-	-

-0.999931441	0.007454678	-	-	-
-0.999931505	0.007451203	ko01100//Metabolic p	-	-
-0.999931607	0.007445658	-	-	-
-0.999931733	0.00743882	ko01100//Metabolic p	-	-
-0.999931856	0.007432108	ko01100//Metabolic p	-	GO:0016020//membran
-0.999931891	0.00743017	ko01100//Metabolic p	ko04714//Therm	GO:0005811//lipid parti
-0.99993213	0.007417165	ko01063//Biosynthesis	ko04080//Neuro	GO:0005886//plasma m
-0.999932166	0.007415192	-	ko01100//Metal	GO:0005829//cytosol
-0.999932652	0.007388547	-	ko04550//Signal	GO:0005634//nucleus;G
-0.999932709	0.007385433	-	ko00564//Glycer	GO:0005615//extracellu
-0.999932823	0.007379198	ko01100//Metabolic p	-	-
-0.999932915	0.007374132	ko01100//Metabolic p	ko04080//Neuro	GO:0005886//plasma m
-0.999932944	0.007372552	-	ko04934//Cushi	-
-0.999933102	0.007363837	ko01100//Metabolic p	-	-
-0.999933132	0.007362185	ko01100//Metabolic p	ko01100//Metal	GO:0033178//proton-tr
-0.999933151	0.00736115	-	-	GO:0005789//endoplas
-0.99993319	0.007359016	ko01100//Metabolic p	-	-
-0.999933213	0.007357727	-	-	-
-0.999933241	0.007356171	-	-	-
-0.99993328	0.007354026	ko01100//Metabolic p	-	-
-0.99993356	0.007338579	ko01100//Metabolic p	-	-
-0.999933699	0.007330925	ko01100//Metabolic p	-	-
-0.99993383	0.007323649	ko01100//Metabolic p	-	-
-0.999933862	0.007321906	ko01100//Metabolic p	-	-
-0.999934211	0.007302546	ko01100//Metabolic p	ko04080//Neuro	GO:0005886//plasma m
-0.999934241	0.007300858	ko01100//Metabolic p	-	GO:0000151//ubiquitin
-0.999934357	0.007294456	ko01100//Metabolic p	-	GO:0016020//membran
-0.999935104	0.007252821	ko01120//Microbial m	ko05142//Chaga	GO:0016020//membran
-0.9999353	0.007241839	ko01100//Metabolic p	-	-
-0.999935754	0.007216398	ko01100//Metabolic p	-	GO:0005829//cytosol;G
-0.999935951	0.007205298	ko01100//Metabolic p	ko04120//Ubiqu	GO:0005680//anaphase
-0.999935969	0.007204314	-	ko01100//Metal	GO:0005783//endoplas
-0.999936068	0.007198736	ko01100//Metabolic p	ko04979//Chole	GO:0005737//cytoplasm
-0.99993618	0.007192422	-	-	-
-0.999936267	0.007187548	-	-	-
-0.999936542	0.007171998	ko01063//Biosynthesis	ko04080//Neuro	GO:0005654//nucleopla
-0.999936727	0.007161533	-	-	GO:0005576//extracellu
-0.999936921	0.00715055	ko01100//Metabolic p	-	GO:0005737//cytoplasm
-0.999937005	0.007145771	-	-	-
-0.999937286	0.007129828	ko01100//Metabolic p	ko01100//Metal	GO:0005576//extracellu
-0.999937407	0.007122931	ko01100//Metabolic p	ko01100//Metal	GO:0005737//cytoplasm
-0.999937416	0.007122471	ko01100//Metabolic p	-	-
-0.999937724	0.007104908	-	ko01100//Metal	GO:0005654//nucleopla
-0.999937766	0.007102489	ko01100//Metabolic p	ko04270//Vascu	GO:0005737//cytoplasm
-0.999937994	0.007089454	ko01100//Metabolic p	-	GO:0005764//lysosome;
-0.999938006	0.007088807	ko01100//Metabolic p	-	-
-0.999938158	0.007080103	ko01100//Metabolic p	ko03410//Base e	GO:0005634//nucleus;G
-0.999938164	0.00707974	-	-	GO:0005739//mitochon
-0.999938306	0.007071623	ko01100//Metabolic p	-	GO:0001725//stress fibe
-0.999938352	0.007068974	ko01100//Metabolic p	-	GO:0016020//membran
-0.999938677	0.007050346	ko01100//Metabolic p	-	GO:0005739//mitochon
-0.99993919	0.007020755	ko01100//Metabolic p	-	-
-0.999939295	0.007014729	-	ko05200//Pathw	GO:0005634//nucleus;G
-0.999939358	0.007011052	ko01100//Metabolic p	ko05414//Dilate	GO:0016459//myosin co
-0.999939393	0.007009034	ko01100//Metabolic p	ko05200//Pathw	GO:0000785//chromatir
-0.9999395	0.007002859	ko01100//Metabolic p	-	-
-0.999939649	0.006994241	ko01100//Metabolic p	ko01100//Metal	GO:0005576//extracellu
-0.999939802	0.006985327	ko01100//Metabolic p	-	-

-0.999939986	0.00697467	ko01100//Metabolic p	ko05203//Viral c	GO:0000786//nucleosor
-0.999940222	0.006960921	-	-	GO:0016021//integral c
-0.999940377	0.006951913	ko01100//Metabolic p	-	GO:0034361//very-low-
-0.999940546	0.006942053	ko01100//Metabolic p	-	GO:0005737//cytoplasm
-0.999940633	0.006936957	ko01100//Metabolic p	ko04020//Calciu	GO:0016020//membran
-0.99994077	0.00692895	ko01110//Biosynthesis	-	-
-0.999941153	0.006906521	-	ko01100//Metab	GO:0005737//cytoplasm
-0.999941176	0.006905149	ko01100//Metabolic p	-	-
-0.99994123	0.006902031	-	-	-
-0.999941314	0.006897078	-	-	GO:0005634//nucleus;G
-0.999941417	0.006890987	-	-	GO:0005634//nucleus;G
-0.999941584	0.006881175	ko01100//Metabolic p	ko04020//Calciu	GO:0005634//nucleus;G
-0.999941689	0.006874968	-	ko05203//Viral c	-
-0.999941908	0.006862076	ko01100//Metabolic p	-	GO:0005634//nucleus;G
-0.999941986	0.006857439	-	-	-
-0.999942137	0.006848545	-	ko04144//Endoc	GO:0005654//nucleopla
-0.999942188	0.006845514	ko01100//Metabolic p	-	-
-0.999942212	0.006844116	-	-	GO:0005634//nucleus;G
-0.99994229	0.006839448	-	-	GO:0005654//nucleopla
-0.999942412	0.006832257	ko01100//Metabolic p	ko04146//Peroxi	GO:0005777//peroxison
-0.999942472	0.006828682	ko01100//Metabolic p	-	GO:0005737//cytoplasm
-0.999942509	0.006826483	-	ko01100//Metab	-
-0.999942577	0.00682244	ko01110//Biosynthesis	-	GO:0005654//nucleopla
-0.999942716	0.00681417	-	-	-
-0.999942906	0.006802864	-	-	GO:0001669//acrosoma
-0.999943244	0.006782732	-	ko04144//Endoc	-
-0.999943342	0.006776825	-	ko01100//Metab	-
-0.999943535	0.006765281	ko01100//Metabolic p	-	GO:0000785//chromatir
-0.999943887	0.006744199	-	-	GO:0016020//membran
-0.999944012	0.006736684	ko01100//Metabolic p	-	GO:0005886//plasma m
-0.999944062	0.006733625	ko01100//Metabolic p	-	GO:0005654//nucleopla
-0.999944169	0.006727182	ko01100//Metabolic p	-	-
-0.999944311	0.006718669	-	-	GO:0016021//integral c
-0.999944333	0.006717321	ko01120//Microbial m	-	GO:0000781//chromosc
-0.99994449	0.006707823	ko01100//Metabolic p	-	GO:0005764//lysosome;
-0.999944586	0.006702058	-	ko04934//Cushi	GO:0016020//membran
-0.99994487	0.006684835	ko01063//Biosynthesis	ko01100//Metab	-
-0.9999449	0.006683036	-	-	GO:0016020//membran
-0.999945155	0.006667534	ko01100//Metabolic p	ko04310//Wnt s	GO:0005634//nucleus;G
-0.999945364	0.006654851	ko01100//Metabolic p	-	GO:0005739//mitochon
-0.999945458	0.006649071	ko01100//Metabolic p	-	GO:0005737//cytoplasm
-0.999945626	0.006638819	ko01100//Metabolic p	-	GO:0005579//membran
-0.999945681	0.00663551	ko01100//Metabolic p	-	GO:0000791//euchroma
-0.999945736	0.0066321	-	-	GO:0035658//Mon1-Cc
-0.999945815	0.006627281	-	-	-
-0.99994605	0.006612908	ko01100//Metabolic p	ko01100//Metab	GO:0005654//nucleopla
-0.999946158	0.006606289	ko01100//Metabolic p	-	GO:0000139//Golgi mer
-0.999947015	0.006553503	-	-	GO:0016021//integral c
-0.999947136	0.00654604	-	-	GO:0005794//Golgi app
-0.999947242	0.006539419	ko01100//Metabolic p	-	-
-0.999947251	0.006538881	-	-	-
-0.999947281	0.006537005	ko01063//Biosynthesis	-	-
-0.999947293	0.006536269	ko01100//Metabolic p	-	-
-0.999947499	0.006523495	ko01100//Metabolic p	-	-
-0.99994772	0.00650973	-	-	GO:0005634//nucleus;G
-0.999948032	0.006490296	-	ko05203//Viral c	GO:0000786//nucleosor
-0.999948302	0.006473428	-	ko01100//Metab	-
-0.999948457	0.006463741	-	-	GO:0005811//lipid parti

-0.999948461	0.006463465	-	-	GO:0005654//nucleopla
-0.999948689	0.006449133	ko01100//Metabolic p	-	-
-0.999948716	0.006447469	ko01100//Metabolic p	-	GO:0005794//Golgi app
-0.999948836	0.006439898	-	-	-
-0.999948928	0.006434104	-	-	-
-0.999949155	0.006419782	-	-	-
-0.999949209	0.006416402	-	-	GO:0005737//cytoplasm
-0.999949255	0.006413482	-	ko00564//Glycer	GO:0005634//nucleus;G
-0.999949298	0.006410741	ko01100//Metabolic p	-	GO:0016020//membran
-0.999949574	0.0063933	-	-	GO:0005811//lipid parti
-0.999949911	0.006371896	ko01100//Metabolic p	ko04010//MAPK	GO:0005737//cytoplasm
-0.999950152	0.006356548	ko01100//Metabolic p	-	-
-0.999950195	0.006353821	ko01110//Biosynthesis	-	-
-0.999950267	0.006349226	ko01100//Metabolic p	-	-
-0.99995077	0.006317022	-	ko01100//Metab	GO:0000506//glycosylpl
-0.999950772	0.006316869	-	-	GO:0005615//extracellu
-0.999950851	0.006311805	ko01110//Biosynthesis	ko04142//Lysos	GO:0016020//membran
-0.999950892	0.006309191	ko01100//Metabolic p	-	GO:0005634//nucleus;G
-0.999951014	0.006301315	ko01100//Metabolic p	ko01100//Metab	GO:0005783//endoplasr
-0.999951018	0.006301109	-	-	-
-0.999951353	0.00627948	-	-	-
-0.999951393	0.006276904	-	-	-
-0.999951474	0.006271686	-	-	-
-0.999952026	0.006235925	-	-	-
-0.999952056	0.006233979	ko01100//Metabolic p	-	-
-0.999952828	0.006183539	-	ko01100//Metab	GO:0005654//nucleopla
-0.999953097	0.006165905	-	-	GO:0000139//Golgi mer
-0.999953114	0.00616478	ko01100//Metabolic p	ko01100//Metab	GO:0016020//membran
-0.999953535	0.006137069	-	ko04360//Axon	-
-0.999953586	0.006133676	-	ko05231//Cholir	-
-0.999953663	0.006128605	-	-	-
-0.999954024	0.00610466	-	ko04310//Wnt s	GO:0005634//nucleus;G
-0.999954238	0.006090434	-	-	-
-0.99995448	0.006074308	-	ko04144//Endoc	-
-0.999954587	0.006067205	-	ko01100//Metab	-
-0.999954757	0.006055802	ko01100//Metabolic p	-	-
-0.999954876	0.006047871	-	-	-
-0.999954995	0.006039835	ko01100//Metabolic p	-	GO:0016020//membran
-0.999955027	0.006037684	ko01100//Metabolic p	-	GO:0031982//vesicle
-0.999955312	0.006018538	-	-	-
-0.999955417	0.006011457	ko01100//Metabolic p	ko04742//Taste	GO:0005737//cytoplasm
-0.999955567	0.006001352	-	-	GO:0016020//membran
-0.999955611	0.005998353	-	ko04979//Chole	GO:0005737//cytoplasm
-0.999955694	0.005992775	ko01100//Metabolic p	ko01100//Metab	GO:0005783//endoplasr
-0.999955852	0.005982072	-	ko05203//Viral c	-
-0.999956163	0.005960989	ko01100//Metabolic p	-	-
-0.999956345	0.005948561	-	ko04360//Axon	-
-0.999956378	0.005946359	-	-	-
-0.999956399	0.005944911	ko01100//Metabolic p	ko01100//Metab	GO:0005783//endoplasr
-0.999956488	0.005938832	-	ko04151//PI3K-/	GO:0005634//nucleus;G
-0.999956504	0.005937712	ko01100//Metabolic p	ko04713//Circac	-
-0.999956842	0.005914606	-	ko04934//Cushi	-
-0.999956997	0.005903992	ko01100//Metabolic p	-	-
-0.999957026	0.005901986	ko01100//Metabolic p	-	-
-0.999957036	0.005901322	ko01100//Metabolic p	-	-
-0.999957603	0.005862279	-	-	-
-0.999957673	0.005857386	-	-	-
-0.999957934	0.0058393	ko01063//Biosynthesis	-	GO:0000781//chromoso

-0.999958016	0.005833593	ko01100//Metabolic p	ko01100//Metal	GO:0016020//membran
-0.999958133	0.00582551	ko01100//Metabolic p	-	GO:0044424//intracellul
-0.999958179	0.005822275	ko01100//Metabolic p	-	GO:0005783//endoplasr
-0.999958448	0.00580357	ko01100//Metabolic p	ko04010//MAPK	GO:0005737//cytoplasm
-0.999958477	0.005801534	ko01100//Metabolic p	-	-
-0.999958498	0.005800043	-	-	-
-0.999958544	0.005796794	ko01100//Metabolic p	ko01100//Metal	GO:0005737//cytoplasm
-0.999958568	0.005795147	ko01110//Biosynthesis	-	GO:0005737//cytoplasm
-0.999958697	0.00578613	ko01100//Metabolic p	-	GO:0005634//nucleus;G
-0.999958797	0.005779085	-	ko01100//Metal	-
-0.999958947	0.005768608	-	ko01100//Metal	GO:0000506//glycosylpl
-0.999959089	0.005758594	-	-	-
-0.999959429	0.005734599	-	-	GO:0005654//nucleopla
-0.999959489	0.005730345	ko01100//Metabolic p	-	-
-0.999959584	0.005723678	ko01100//Metabolic p	ko04211//Longe	GO:0005634//nucleus
-0.999959586	0.005723479	-	-	-
-0.999959731	0.005713258	-	-	GO:0005634//nucleus;G
-0.999960079	0.005688459	ko01100//Metabolic p	-	-
-0.999960088	0.005687824	-	-	-
-0.999960127	0.005685054	-	-	-
-0.999960265	0.005675192	ko01100//Metabolic p	-	-
-0.999960328	0.005670716	-	-	-
-0.999960349	0.005669228	-	-	GO:0005737//cytoplasm
-0.999960732	0.005641797	ko01100//Metabolic p	-	-
-0.999960818	0.005635626	ko01100//Metabolic p	-	GO:0000786//nucleosor
-0.999960958	0.005625522	ko01100//Metabolic p	-	-
-0.999961032	0.005620157	ko01100//Metabolic p	ko01100//Metal	GO:0005829//cytosol
-0.999961123	0.005613605	-	ko04550//Signal	GO:0005634//nucleus;G
-0.999961254	0.005604135	-	-	-
-0.999961434	0.005591148	-	ko04120//Ubiqu	GO:0005680//anaphase
-0.999961488	0.005587236	ko01100//Metabolic p	ko03320//PPAR	GO:0005615//extracellu
-0.999961502	0.005586208	-	-	GO:0005789//endoplasr
-0.999961607	0.005578599	-	-	-
-0.999961625	0.005577272	-	-	GO:0016020//membran
-0.999961839	0.005561686	ko01100//Metabolic p	-	-
-0.999962121	0.00554112	ko01100//Metabolic p	ko03320//PPAR	GO:0005576//extracellu
-0.999962137	0.005539926	ko01100//Metabolic p	-	-
-0.999962911	0.005483021	ko01110//Biosynthesis	-	-
-0.999963109	0.005468391	ko01063//Biosynthesis	-	-
-0.999963127	0.005467037	ko01100//Metabolic p	-	GO:0005654//nucleopla
-0.999963291	0.005454829	ko01100//Metabolic p	-	GO:0005634//nucleus;G
-0.999963374	0.005448712	-	-	-
-0.999963404	0.005446444	-	-	-
-0.999963466	0.005441849	-	ko01100//Metal	GO:0005829//cytosol
-0.999963474	0.005441269	-	-	-
-0.999963487	0.005440275	ko01100//Metabolic p	-	-
-0.999963537	0.005436532	ko01100//Metabolic p	ko01100//Metal	-
-0.999963762	0.005419755	-	-	GO:0005654//nucleopla
-0.999963801	0.005416806	-	-	GO:0005579//membran
-0.999963869	0.005411719	-	ko05200//Pathw	GO:0000307//cyclin-de
-0.999963896	0.005409742	-	-	GO:0005634//nucleus;G
-0.999963991	0.005402627	ko01100//Metabolic p	ko01100//Metal	GO:0005783//endoplasr
-0.999963991	0.005402593	-	-	GO:0016020//membran
-0.999964032	0.005399492	-	-	-
-0.999964245	0.005383468	ko01100//Metabolic p	-	-
-0.999964272	0.005381501	-	-	GO:0005654//nucleopla
-0.999964287	0.005380303	-	-	GO:0005634//nucleus;G
-0.999964372	0.005373906	-	-	GO:0005634//nucleus;G

-0.999964533	0.005361768	ko01063//Biosynthesis	ko01100//Metab	GO:0005783//endoplasr
-0.999964539	0.005361353	ko01100//Metabolic p	ko03320//PPAR	GO:0005576//extracellu
-0.999964654	0.00535261	-	-	-
-0.999964814	0.005340522	-	-	-
-0.999964941	0.00533081	-	ko04310//Wnt s	GO:0005634//nucleus;G
-0.999964953	0.005329929	ko01110//Biosynthesis	ko03013//Nucle	-
-0.999965005	0.005326005	-	-	-
-0.999965027	0.005324286	ko01100//Metabolic p	-	-
-0.999965222	0.005309442	-	-	-
-0.99996529	0.005304275	-	ko04010//MAPK	GO:0005737//cytoplasm
-0.999965297	0.005303709	-	-	-
-0.99996542	0.005294291	ko01100//Metabolic p	ko04020//Calciu	GO:0005634//nucleus;G
-0.999965733	0.005270317	ko01100//Metabolic p	-	-
-0.999965758	0.00526838	ko01100//Metabolic p	ko04361//Axon	GO:0005737//cytoplasm
-0.999965766	0.005267785	ko01100//Metabolic p	ko01100//Metab	-
-0.999965885	0.005258611	ko01100//Metabolic p	ko01100//Metab	-
-0.999965913	0.00525642	ko01100//Metabolic p	ko01100//Metab	GO:0000139//Golgi mer
-0.999966128	0.005239787	-	ko05200//Pathw	GO:0005634//nucleus;G
-0.999966221	0.005232652	ko01110//Biosynthesis	-	-
-0.999966346	0.005222957	ko01100//Metabolic p	-	-
-0.999966493	0.005211482	ko01110//Biosynthesis	-	-
-0.999966603	0.005202944	ko01100//Metabolic p	ko05165//Huma	GO:0005634//nucleus;G
-0.999966813	0.005186551	ko01100//Metabolic p	ko01100//Metab	GO:0005739//mitochon
-0.999966884	0.005181022	ko01100//Metabolic p	-	-
-0.999967045	0.005168429	ko01100//Metabolic p	-	-
-0.999967353	0.005144187	-	-	-
-0.999967493	0.005133127	-	ko04080//Neuro	GO:0005886//plasma m
-0.999967518	0.005131173	-	ko04919//Thyro	GO:0016020//membran
-0.9999676	0.005124718	ko01100//Metabolic p	ko00564//Glycer	GO:0005634//nucleus;G
-0.999967612	0.005123769	ko01100//Metabolic p	ko01100//Metab	GO:0005783//endoplasr
-0.999967661	0.005119886	ko01100//Metabolic p	-	-
-0.999967749	0.005112887	ko01063//Biosynthesis	ko01100//Metab	-
-0.999967755	0.00511245	ko01100//Metabolic p	-	GO:0016020//membran
-0.99996783	0.005106492	ko01100//Metabolic p	-	GO:0016020//membran
-0.999968033	0.005090324	ko01100//Metabolic p	-	GO:0005576//extracellu
-0.999968038	0.005089983	ko01100//Metabolic p	-	GO:0005802//trans-Gol
-0.999968086	0.00508612	ko01100//Metabolic p	-	-
-0.999968087	0.005086012	ko01100//Metabolic p	-	GO:0005764//lysosome;
-0.999968587	0.005046071	ko01100//Metabolic p	-	-
-0.999968757	0.005032393	-	-	-
-0.999968812	0.005027922	-	-	GO:0001669//acrosoma
-0.999968932	0.005018244	-	-	GO:0005764//lysosome;
-0.999969003	0.005012551	-	ko01100//Metab	GO:0005783//endoplasr
-0.999969122	0.005002887	ko01100//Metabolic p	ko04141//Protei	GO:0005737//cytoplasm
-0.999969143	0.00500115	ko01100//Metabolic p	-	-
-0.999969153	0.005000354	-	-	-
-0.99996921	0.004995762	-	ko01100//Metab	GO:0005794//Golgi app
-0.999969266	0.004991228	ko01100//Metabolic p	ko01100//Metab	-
-0.999969577	0.004965922	-	-	-
-0.999969627	0.004961835	-	-	-
-0.999969679	0.004957561	ko01100//Metabolic p	ko01100//Metab	GO:0005783//endoplasr
-0.999969793	0.004948213	-	-	-
-0.99996997	0.004933744	ko01100//Metabolic p	-	GO:0005791//rough enc
-0.999969975	0.004933312	-	-	-
-0.999970001	0.004931162	-	ko05200//Pathw	GO:0005737//cytoplasm
-0.999970011	0.004930334	-	-	GO:0000139//Golgi mer
-0.999970088	0.004924015	ko01100//Metabolic p	ko05203//Viral c	GO:0000786//nucleosor
-0.999970262	0.004909697	ko01100//Metabolic p	ko01100//Metab	GO:0005783//endoplasr

-0.999970342	0.004903077	-	-	-
-0.999970414	0.004897142	ko01100//Metabolic p	-	GO:0005634//nucleus;G
-0.999970586	0.004882883	ko01100//Metabolic p	-	-
-0.999970752	0.004869089	-	ko04310//Wnt s	GO:0005634//nucleus;G
-0.999970822	0.004863226	ko01100//Metabolic p	-	GO:0005615//extracellu
-0.999970936	0.004853743	ko01100//Metabolic p	-	GO:0005634//nucleus;G
-0.999971137	0.00483689	-	ko04068//FoxO	GO:0005737//cytoplasm
-0.999971172	0.004833939	-	-	-
-0.999971458	0.004809965	-	ko04020//Calciu	GO:0005634//nucleus;G
-0.999971465	0.004809377	ko01110//Biosynthesis	ko01100//Metab	GO:0016020//membran
-0.999971514	0.004805209	ko01100//Metabolic p	ko04144//Endoc	GO:0005654//nucleopla
-0.999971558	0.004801509	-	-	-
-0.999971937	0.004769357	ko01100//Metabolic p	ko01100//Metab	-
-0.999971944	0.004768807	ko01110//Biosynthesis	ko05200//Pathw	GO:0005829//cytosol;G
-0.999971959	0.004767537	-	ko04020//Calciu	GO:0016020//membran
-0.999972119	0.004753936	-	ko01100//Metab	GO:0005794//Golgi app
-0.999972463	0.004724466	ko01100//Metabolic p	-	-
-0.999972493	0.004721946	-	-	GO:0000785//chromatin
-0.999972569	0.004715354	-	ko05203//Viral c	GO:0000786//nucleosor
-0.999972716	0.004702744	ko01100//Metabolic p	-	GO:0005634//nucleus;G
-0.999973016	0.004676814	ko01100//Metabolic p	ko04310//Wnt s	GO:0009897//external s
-0.999973024	0.004676095	-	-	-
-0.999973153	0.004664877	-	-	-
-0.999973185	0.004662173	ko01100//Metabolic p	ko04010//MAPK	-
-0.999973206	0.004660338	ko01100//Metabolic p	ko01100//Metab	GO:0005634//nucleus;G
-0.999973286	0.004653355	ko01100//Metabolic p	ko01100//Metab	-
-0.999973294	0.004652638	ko01100//Metabolic p	-	-
-0.999973336	0.004648949	-	ko04713//Circac	-
-0.999973374	0.004645636	ko01100//Metabolic p	-	GO:0005634//nucleus;G
-0.99997342	0.004641665	ko01100//Metabolic p	ko01100//Metab	GO:0005783//endoplasr
-0.999973549	0.004630375	-	-	GO:0005886//plasma m
-0.999973599	0.004625969	-	-	GO:0005576//extracellu
-0.999973703	0.004616879	ko01110//Biosynthesis	ko01100//Metab	GO:0005737//cytoplasm
-0.99997379	0.004609258	-	ko01100//Metab	-
-0.999973811	0.004607405	ko01100//Metabolic p	ko01100//Metab	-
-0.999973905	0.004599112	-	ko01100//Metab	GO:0005654//nucleopla
-0.999973916	0.004598114	ko01100//Metabolic p	-	GO:0005634//nucleus;G
-0.999973946	0.004595482	ko01100//Metabolic p	ko04141//Protei	GO:0030120//vesicle co
-0.999973998	0.004590953	-	-	-
-0.999974111	0.004580911	ko01100//Metabolic p	ko05203//Viral c	GO:0000786//nucleosor
-0.999974254	0.004568265	-	ko04514//Cell a	GO:0016020//membran
-0.999974423	0.004553215	-	-	-
-0.99997446	0.004549951	-	-	-
-0.999974638	0.004534065	-	-	GO:0005737//cytoplasm
-0.999974994	0.00450217	ko01110//Biosynthesis	-	GO:0016581//NuRD cor
-0.999974996	0.004501962	-	-	-
-0.999975054	0.004496744	-	-	-
-0.99997514	0.00448895	ko01100//Metabolic p	ko03010//Ribos	GO:0005840//ribosome
-0.999975158	0.0044873	-	-	-
-0.999975184	0.004484963	ko01100//Metabolic p	ko00564//Glycer	GO:0005634//nucleus;G
-0.999975352	0.004469755	-	-	-
-0.999975676	0.004440328	ko01100//Metabolic p	ko04146//Perox	GO:0005765//lysosomal
-0.999975761	0.004432564	-	-	-
-0.999975821	0.004427042	-	-	GO:0005783//endoplasr
-0.99997584	0.004425309	ko01110//Biosynthesis	-	-
-0.99997591	0.004418888	-	ko04310//Wnt s	GO:0005634//nucleus;G
-0.999975914	0.004418509	-	-	GO:0097541//axonemal
-0.999975991	0.004411434	ko01100//Metabolic p	ko01100//Metab	GO:0005788//endoplasr

-0.999976016	0.004409176	-	-	-
-0.999976122	0.004399439	-	-	-
-0.999976372	0.004376289	-	-	-
-0.999976446	0.004369462	ko01100//Metabolic p	-	-
-0.999976517	0.004362895	ko01100//Metabolic p	-	-
-0.999976665	0.004349136	ko01100//Metabolic p	-	-
-0.999976724	0.004343638	ko01063//Biosynthesis	-	GO:0016021//integral co
-0.99997679	0.004337482	ko01100//Metabolic p	ko01100//Metab	GO:0005794//Golgi app
-0.999976883	0.004328696	-	ko04141//Protei	GO:0005783//endoplasn
-0.999977142	0.004304434	ko01100//Metabolic p	-	GO:0005737//cytoplasm
-0.999977154	0.004303254	-	-	-
-0.999977194	0.004299564	-	ko01100//Metab	GO:0005737//cytoplasm
-0.9999774	0.004280106	-	-	GO:0000127//transcript
-0.999977421	0.00427805	-	-	GO:0005794//Golgi app
-0.999977635	0.004257774	ko01100//Metabolic p	-	-
-0.999977685	0.004252952	-	-	-
-0.999977749	0.00424687	ko01100//Metabolic p	-	-
-0.999977923	0.00423024	ko01100//Metabolic p	-	-
-0.999977936	0.004229005	-	ko01100//Metab	GO:0005783//endoplasn
-0.99997796	0.004226669	-	ko04020//Calciu	GO:0005634//nucleus;G
-0.999977962	0.004226515	-	ko04144//Endoc	-
-0.999977991	0.004223774	-	-	-
-0.999977998	0.004223101	ko01100//Metabolic p	-	GO:0016021//integral co
-0.999978054	0.004217703	ko01100//Metabolic p	-	GO:0005634//nucleus;G
-0.999978179	0.004205698	-	-	-
-0.99997821	0.004202696	-	-	-
-0.999978216	0.004202116	ko01100//Metabolic p	ko01100//Metab	GO:0005783//endoplasn
-0.999978292	0.004194703	ko01100//Metabolic p	ko05150//Staphy	GO:0005577//fibrinoger
-0.99997832	0.004192003	-	ko03320//PPAR	GO:0005615//extracellu
-0.999978335	0.004190622	-	-	GO:0005764//lysosome;
-0.999978549	0.004169847	ko01100//Metabolic p	-	-
-0.999978559	0.004168847	ko01100//Metabolic p	-	GO:0005654//nucleopla
-0.999978737	0.004151511	ko01110//Biosynthesis	ko04742//Taste	GO:0016020//membran
-0.999978795	0.00414584	-	ko04934//Cushin	GO:0016020//membran
-0.999978848	0.004140724	-	-	-
-0.99997912	0.004114011	ko01100//Metabolic p	-	-
-0.999979159	0.004110095	-	-	-
-0.99997917	0.00410903	-	-	-
-0.999979197	0.004106348	ko01100//Metabolic p	ko04970//Saliva	GO:0005576//extracellu
-0.99997928	0.00409815	ko01100//Metabolic p	-	-
-0.999979321	0.004094163	-	ko01100//Metab	-
-0.999979389	0.004087336	-	-	-
-0.99997946	0.004080348	ko01110//Biosynthesis	ko05200//Pathw	GO:0005634//nucleus
-0.999979479	0.004078414	-	-	GO:0005615//extracellu
-0.999979525	0.004073871	-	-	-
-0.99997969	0.004057396	ko01100//Metabolic p	-	-
-0.999979838	0.0040426	ko01100//Metabolic p	ko04520//Adher	GO:0031252//cell leadir
-0.999979893	0.004037051	ko01100//Metabolic p	ko01100//Metab	-
-0.999979939	0.004032455	ko01100//Metabolic p	ko01100//Metab	GO:0005739//mitochon
-0.999979993	0.004027003	-	ko04010//MAPK	-
-0.999980079	0.00401839	-	-	GO:0005764//lysosome;
-0.999980277	0.003998371	-	-	-
-0.999980289	0.003997178	-	-	-
-0.999980389	0.003986983	-	ko01100//Metab	GO:0005783//endoplasn
-0.999980544	0.003971257	ko01063//Biosynthesis	ko04141//Protei	GO:0030120//vesicle co
-0.999980621	0.003963323	ko01100//Metabolic p	-	GO:0005654//nucleopla
-0.999980679	0.003957377	ko01100//Metabolic p	-	-
-0.9999808	0.003944997	ko01100//Metabolic p	-	GO:0005576//extracellu

-0.999980905	0.003934194	-	-	-
-0.999980967	0.003927802	ko01100//Metabolic p	ko05203//Viral c	GO:0000786//nucleosor
-0.999981105	0.003913487	ko01100//Metabolic p	ko01100//Metab	GO:0005654//nucleopla
-0.999981115	0.003912499	-	-	-
-0.999981252	0.0038983	ko01100//Metabolic p	-	-
-0.999981257	0.003897787	-	ko01100//Metab	GO:0005739//mitochon
-0.999981259	0.003897597	ko01120//Microbial m	-	GO:0016020//membran
-0.999981278	0.003895602	-	ko04919//Thyro	GO:0016020//membran
-0.999981339	0.003889276	-	-	-
-0.999981357	0.003887382	ko01100//Metabolic p	-	-
-0.999981454	0.003877249	ko01100//Metabolic p	-	-
-0.999981585	0.00386354	ko01100//Metabolic p	ko05200//Pathw	GO:0005737//cytoplasm
-0.999981604	0.003861491	ko01100//Metabolic p	ko01100//Metab	GO:0001650//fibrillar ce
-0.999981776	0.003843396	ko01100//Metabolic p	ko04934//Cushi	-
-0.999981781	0.003842852	-	ko05152//Tuber	GO:0009897//external s
-0.999981836	0.003837082	-	-	GO:0005811//lipid parti
-0.999981909	0.003829342	-	-	GO:0031464//Cul4A-RI
-0.999982109	0.003808124	-	-	-
-0.999982153	0.003803477	-	ko05165//Huma	-
-0.999982401	0.00377694	ko01100//Metabolic p	-	-
-0.999982742	0.003740221	ko01100//Metabolic p	-	-
-0.999982784	0.003735602	-	ko01100//Metab	GO:0005576//extracellu
-0.999982794	0.00373458	ko01110//Biosynthesis	-	GO:0005802//trans-Gol
-0.999982814	0.00373236	-	ko04010//MAPK	GO:0005737//cytoplasm
-0.999983033	0.003708459	ko01100//Metabolic p	-	-
-0.999983073	0.003704113	ko01110//Biosynthesis	ko01100//Metab	GO:0005737//cytoplasm
-0.999983077	0.003703736	ko01100//Metabolic p	ko04979//Chole	GO:0005737//cytoplasm
-0.999983252	0.003684474	ko01100//Metabolic p	-	-
-0.999983258	0.003683856	ko01063//Biosynthesis	-	-
-0.999983261	0.003683547	ko01100//Metabolic p	-	GO:0016020//membran
-0.999983406	0.003667506	-	-	-
-0.999983626	0.003643066	ko01110//Biosynthesis	ko05200//Pathw	GO:0005576//extracellu
-0.999983701	0.0036348	-	-	GO:0005634//nucleus;G
-0.999983845	0.003618664	ko01063//Biosynthesis	-	-
-0.999983863	0.003616614	ko01100//Metabolic p	-	-
-0.999984019	0.003599132	ko01100//Metabolic p	-	-
-0.999984089	0.003591233	ko01100//Metabolic p	-	GO:0005654//nucleopla
-0.999984128	0.003586867	-	ko01100//Metab	GO:0009923//fatty acid
-0.999984158	0.003583439	ko01100//Metabolic p	-	GO:0005634//nucleus;G
-0.999984199	0.003578823	ko01100//Metabolic p	-	-
-0.999984296	0.003567843	ko01100//Metabolic p	ko01100//Metab	-
-0.999984331	0.003563852	ko01063//Biosynthesis	ko04141//Protei	GO:0005737//cytoplasm
-0.999984353	0.003561371	-	-	-
-0.999984362	0.003560298	-	-	GO:0005764//lysosome;
-0.999984386	0.003557611	-	ko04919//Thyro	GO:0016020//membran
-0.999984435	0.003552023	ko01100//Metabolic p	ko05200//Pathw	GO:0000307//cyclin-de
-0.999984443	0.003551063	-	-	-
-0.999984613	0.003531636	-	-	-
-0.99998464	0.003528485	ko01100//Metabolic p	-	-
-0.999984752	0.003515634	ko01100//Metabolic p	-	-
-0.999984941	0.003493803	ko01100//Metabolic p	-	GO:0005737//cytoplasm
-0.999984993	0.003487781	ko01110//Biosynthesis	-	GO:0044424//intracellul
-0.999985099	0.003475366	-	-	-
-0.999985125	0.003472377	-	-	-
-0.999985283	0.003453851	ko01100//Metabolic p	ko01100//Metab	-
-0.999985328	0.003448589	-	-	-
-0.999985333	0.003448041	ko01100//Metabolic p	-	GO:0001518//voltage-g
-0.9999854	0.00344016	-	ko04010//MAPK	-

-0.999985467	0.003432243	ko01100//Metabolic p	ko00564//Glycer	GO:0005737//cytoplasm
-0.999985502	0.003428068	-	-	GO:0005737//cytoplasm
-0.999985521	0.003425855	-	ko05203//Viral c	-
-0.999985532	0.003424567	ko01100//Metabolic p	-	GO:0005794//Golgi app
-0.9999857	0.003404543	ko01100//Metabolic p	ko01100//Metak	GO:0005737//cytoplasm
-0.999985712	0.003403109	ko01100//Metabolic p	-	-
-0.999985747	0.00339897	ko01100//Metabolic p	ko01100//Metak	GO:0016021//integral c
-0.999985901	0.003380575	-	-	-
-0.999985909	0.003379594	ko01100//Metabolic p	-	-
-0.999985911	0.003379343	-	-	-
-0.999985915	0.003378943	-	ko04068//FoxO	GO:0005737//cytoplasm
-0.999985939	0.003375992	-	-	-
-0.999985955	0.003374125	-	ko01100//Metak	-
-0.999985997	0.003369063	ko01110//Biosynthesis	-	-
-0.999986075	0.003359607	ko01100//Metabolic p	-	-
-0.99998611	0.003355382	-	-	-
-0.999986138	0.003352019	-	ko04020//Calciu	GO:0005634//nucleus;G
-0.999986233	0.003340571	ko01100//Metabolic p	ko05130//Patho	GO:0005856//cytoskelet
-0.999986252	0.00333822	-	-	-
-0.999986312	0.003330905	-	-	-
-0.999986393	0.003321097	ko01100//Metabolic p	ko04120//Ubiqu	GO:0005680//anaphase
-0.999986444	0.003314879	-	-	GO:0005634//nucleus;G
-0.999986561	0.003300491	ko01100//Metabolic p	-	GO:0005634//nucleus;G
-0.999986588	0.003297187	ko01100//Metabolic p	-	-
-0.999986658	0.00328855	-	-	-
-0.999986659	0.003288488	ko01100//Metabolic p	-	-
-0.999986667	0.003287475	ko01100//Metabolic p	ko04310//Wnt s	GO:0005634//nucleus;G
-0.999986724	0.003280435	-	-	-
-0.999986848	0.003265105	-	ko01100//Metak	GO:0005634//nucleus;G
-0.99998685	0.003264808	ko01100//Metabolic p	-	GO:0005654//nucleopla
-0.999986877	0.003261477	ko01100//Metabolic p	ko01100//Metak	GO:0005737//cytoplasm
-0.99998688	0.003261075	ko01100//Metabolic p	ko04141//Protei	GO:0005783//endoplasr
-0.999986937	0.003253978	-	ko03010//Ribos	GO:0005634//nucleus;G
-0.999986987	0.003247742	ko01100//Metabolic p	-	-
-0.999986995	0.003246817	ko01100//Metabolic p	-	-
-0.99998703	0.003242453	ko01063//Biosynthesis	-	-
-0.999987098	0.003233882	ko01100//Metabolic p	-	-
-0.999987157	0.003226425	-	-	-
-0.999987328	0.00320497	ko01100//Metabolic p	ko01100//Metak	GO:0005783//endoplasr
-0.999987331	0.003204486	ko01100//Metabolic p	-	GO:0005634//nucleus;G
-0.999987518	0.003180869	-	-	-
-0.99998753	0.003179249	-	-	-
-0.999987618	0.003168022	-	-	GO:0005576//extracellu
-0.999987668	0.003161603	-	-	GO:0008076//voltage-g
-0.999987951	0.003125137	ko01100//Metabolic p	-	GO:0005654//nucleopla
-0.999988089	0.003107232	ko01110//Biosynthesis	-	-
-0.999988092	0.003106755	ko01100//Metabolic p	-	GO:0000785//chromatir
-0.999988196	0.003093259	ko01100//Metabolic p	ko04150//mTOF	GO:0005829//cytosol;G(
-0.999988259	0.003084925	ko01110//Biosynthesis	ko04146//Peroxi	GO:0005765//lysosomal
-0.999988525	0.003049837	ko01110//Biosynthesis	-	-
-0.999988629	0.003035905	ko01100//Metabolic p	-	GO:0005615//extracellu
-0.999988634	0.003035282	-	-	GO:0016020//membran
-0.9999887	0.003026522	ko01100//Metabolic p	-	-
-0.999988864	0.003004362	-	-	GO:0032991//macromo
-0.999988869	0.003003731	-	-	-
-0.999988884	0.003001761	ko01100//Metabolic p	-	-
-0.999988925	0.002996226	-	-	-
-0.999989105	0.002971664	-	-	-

-0.999989122	0.00296943	-	ko01100//Metab	-
-0.99998915	0.002965591	ko01100//Metabolic p	ko04142//Lysoso	-
-0.999989246	0.002952427	ko01100//Metabolic p	ko01100//Metab	GO:0005794//Golgi app
-0.99998932	0.00294225	-	ko01100//Metab	GO:0005794//Golgi app
-0.999989372	0.00293508	ko01100//Metabolic p	ko04020//Calciu	GO:0016020//membran
-0.999989401	0.002931113	ko01063//Biosynthesis	-	GO:0000786//nucleosor
-0.999989559	0.002909202	-	-	GO:0005737//cytoplasm
-0.999989566	0.002908239	-	ko00790//Folate	-
-0.999989611	0.002901858	ko01100//Metabolic p	-	GO:0005764//lysosome;
-0.999989616	0.002901153	-	ko04144//Endoc	GO:0005654//nucleopla
-0.999989664	0.002894429	ko01100//Metabolic p	-	GO:0016020//membran
-0.999989727	0.002885631	-	ko01100//Metab	GO:0005829//cytosol
-0.999989813	0.002873608	ko01100//Metabolic p	-	GO:0005856//cytoskelet
-0.999989877	0.002864488	ko01063//Biosynthesis	-	-
-0.999989881	0.002863968	-	-	GO:0016020//membran
-0.999989961	0.00285262	-	-	GO:0005634//nucleus;G
-0.99999011	0.002831282	ko01100//Metabolic p	ko04144//Endoc	-
-0.999990174	0.002822159	-	-	GO:0000127//transcript
-0.999990264	0.002809241	-	-	-
-0.99999028	0.002806883	-	ko01100//Metab	GO:0005783//endoplasm
-0.999990333	0.002799271	ko01100//Metabolic p	-	GO:0016021//integral co
-0.999990393	0.002790618	-	-	-
-0.999990477	0.002778389	ko01110//Biosynthesis	-	-
-0.9999905	0.002775008	-	-	-
-0.999990587	0.00276225	ko01110//Biosynthesis	ko04710//Circac	GO:0005634//nucleus;G
-0.999990721	0.002742426	-	-	-
-0.999990739	0.002739866	-	ko01100//Metab	GO:0005654//nucleopla
-0.999990771	0.002735118	-	-	GO:0005737//cytoplasm
-0.999990781	0.002733599	ko01100//Metabolic p	ko05200//Pathw	GO:0005576//extracellu
-0.999990786	0.002732821	-	-	GO:0016020//membran
-0.999990815	0.002728628	-	-	GO:0016020//membran
-0.999990823	0.002727439	ko01100//Metabolic p	-	GO:0000781//chromosc
-0.999990848	0.002723662	-	-	GO:0005739//mitochon
-0.999990858	0.002722243	ko01100//Metabolic p	ko01100//Metab	GO:0016020//membran
-0.999990984	0.002703316	ko01100//Metabolic p	-	-
-0.999991002	0.002700695	ko01100//Metabolic p	ko05200//Pathw	GO:0005634//nucleus;G
-0.999991281	0.002658396	-	-	-
-0.999991527	0.002620722	-	-	GO:0035658//Mon1-Cc
-0.999991552	0.002616853	-	-	-
-0.999991601	0.002609138	ko01100//Metabolic p	-	-
-0.999991652	0.002601346	-	-	-
-0.999991664	0.002599446	-	ko05200//Pathw	GO:0005634//nucleus;G
-0.9999917	0.002593771	-	-	GO:0031464//Cul4A-RII
-0.999991727	0.002589594	ko01110//Biosynthesis	-	GO:0005576//extracellu
-0.999991737	0.002587969	-	-	-
-0.999991814	0.002575861	-	ko04150//mTOF	GO:0005764//lysosome;
-0.999991855	0.00256949	ko01100//Metabolic p	ko04080//Neurc	GO:0005886//plasma m
-0.999992045	0.002539231	-	-	-
-0.999992123	0.002526904	-	-	GO:0005634//nucleus;G
-0.999992129	0.002525933	ko01100//Metabolic p	-	GO:0005737//cytoplasm
-0.999992211	0.002512634	ko01100//Metabolic p	ko05231//Cholir	-
-0.999992242	0.00250769	-	-	GO:0000127//transcript
-0.99999229	0.002499955	ko01100//Metabolic p	-	-
-0.999992424	0.002478032	ko01110//Biosynthesis	-	GO:0005634//nucleus;G
-0.999992644	0.00244185	ko01100//Metabolic p	-	GO:0005811//lipid parti
-0.999992646	0.002441522	ko01100//Metabolic p	-	-
-0.999992685	0.002435047	-	-	GO:0005794//Golgi app
-0.999993069	0.002370259	ko01100//Metabolic p	-	-

-0.999993075	0.002369287	-	-	GO:0097541//axonemal
-0.999993253	0.002338582	ko01100//Metabolic p	-	-
-0.999993265	0.002336461	ko01100//Metabolic p	-	-
-0.999993398	0.002313227	-	-	-
-0.999993471	0.002300496	-	-	-
-0.999993496	0.002296066	-	-	-
-0.999993504	0.002294732	ko01100//Metabolic p	-	-
-0.999993618	0.002274413	-	ko04010//MAPK	GO:0005737//cytoplasm
-0.999993743	0.002252061	-	ko04010//MAPK	-
-0.999993795	0.002242631	ko01100//Metabolic p	ko01100//Metab	GO:0005829//cytosol
-0.999993829	0.002236561	-	-	-
-0.999994008	0.002203899	-	-	-
-0.999994009	0.002203609	ko01100//Metabolic p	ko00970//Amino	GO:0005737//cytoplasm
-0.999994044	0.002197187	-	-	GO:0005654//nucleopla
-0.99999409	0.002188683	-	ko01100//Metab	GO:0005654//nucleopla
-0.9999941	0.002186905	-	-	-
-0.999994103	0.002186357	ko01100//Metabolic p	ko03010//Ribos	GO:0005634//nucleus;G
-0.999994141	0.002179196	-	-	-
-0.999994196	0.002168906	-	-	GO:0005886//plasma m
-0.999994234	0.002161827	-	-	GO:0005576//extracellu
-0.999994248	0.002159277	-	-	GO:0001518//voltage-g
-0.999994298	0.002149903	-	-	-
-0.999994306	0.002148314	-	-	GO:0016020//membran
-0.999994311	0.002147476	-	ko04120//Ubiqu	GO:0005680//anaphase
-0.999994376	0.00213506	-	-	GO:0005737//cytoplasm
-0.999994425	0.002125823	ko01100//Metabolic p	-	-
-0.99999443	0.002124829	-	-	-
-0.999994457	0.002119626	ko01100//Metabolic p	-	GO:0005634//nucleus;G
-0.999994485	0.002114333	-	-	-
-0.999994517	0.002108154	-	-	-
-0.999994545	0.002102865	-	-	-
-0.999994561	0.002099611	ko01100//Metabolic p	-	GO:0043231//intracellul
-0.999994666	0.002079308	-	-	GO:0005634//nucleus;G
-0.999994667	0.002079057	-	-	-
-0.999994713	0.002070232	-	-	-
-0.99999477	0.002058979	-	ko01100//Metab	-
-0.999994819	0.002049237	ko01100//Metabolic p	ko05200//Pathw	GO:0000785//chromatir
-0.999994913	0.002030622	ko01100//Metabolic p	ko05203//Viral c	-
-0.999994917	0.002029734	ko01100//Metabolic p	-	-
-0.999994923	0.002028535	-	ko05165//Huma	-
-0.999994943	0.002024595	ko01110//Biosynthesis	-	GO:0016020//membran
-0.999995104	0.001992036	ko01100//Metabolic p	-	GO:0032991//macromo
-0.999995131	0.001986648	ko01100//Metabolic p	-	GO:0001725//stress fibe
-0.999995266	0.001958855	ko01100//Metabolic p	-	-
-0.999995304	0.001950906	-	ko04068//FoxO	GO:0005737//cytoplasm
-0.999995329	0.00194575	ko01110//Biosynthesis	-	-
-0.999995343	0.001942815	ko01100//Metabolic p	ko04144//Endoc	GO:0000813//ESCRT I c
-0.99999549	0.00191196	-	ko01100//Metab	-
-0.999995516	0.00190653	-	-	-
-0.99999554	0.001901348	-	-	GO:0005739//mitochon
-0.999995608	0.001886846	-	-	GO:0005737//cytoplasm
-0.999995631	0.001881903	ko01100//Metabolic p	ko01100//Metab	-
-0.999995631	0.001881748	ko01100//Metabolic p	ko01100//Metab	GO:0048471//perinucle
-0.999995657	0.001876179	-	ko01100//Metab	GO:0005654//nucleopla
-0.999995665	0.001874615	-	-	-
-0.999995682	0.001870824	-	-	GO:0005783//endoplasi
-0.99999571	0.001864806	-	ko01100//Metab	GO:0005737//cytoplasm
-0.999995757	0.001854483	-	-	-

-0.999995768	0.0018521	-	-	GO:0001518//voltage-g
-0.999995827	0.001839136	-	ko01100//Metal	GO:0005737//cytoplasm
-0.999995829	0.00183865	-	-	-
-0.999995861	0.001831684	-	-	GO:0005576//extracellu
-0.99999595	0.001811925	-	ko01100//Metal	GO:0009923//fatty acid
-0.999995951	0.00181174	-	ko01100//Metal	-
-0.999995957	0.001810318	-	-	-
-0.999996053	0.001788665	-	-	-
-0.999996142	0.001768478	-	-	-
-0.999996143	0.00176816	-	-	-
-0.99999628	0.001736381	-	-	GO:0005634//nucleus;G
-0.999996344	0.001721425	ko01100//Metabolic p	-	GO:0030864//cortical ac
-0.999996348	0.001720608	ko01100//Metabolic p	-	-
-0.999996387	0.001711317	ko01063//Biosynthesis	-	-
-0.999996419	0.001703754	ko01100//Metabolic p	-	-
-0.999996441	0.001698407	-	-	-
-0.999996458	0.001694434	ko01100//Metabolic p	-	-
-0.999996546	0.001673219	ko01110//Biosynthesis	-	-
-0.999996581	0.001664803	ko01100//Metabolic p	ko03013//Nucle	-
-0.999996699	0.001635785	-	ko03320//PPAR	GO:0005615//extracellu
-0.999996711	0.00163278	ko01100//Metabolic p	-	GO:0000151//ubiquitin
-0.999996729	0.001628397	-	-	GO:0016020//membran
-0.999996824	0.00160458	ko01100//Metabolic p	ko00982//Drug	-
-0.999996931	0.001577336	-	-	GO:0016020//membran
-0.999996932	0.001576868	-	ko04151//PI3K-/	GO:0005654//nucleopla
-0.999996937	0.001575667	-	ko01100//Metal	-
-0.999996948	0.001572801	ko01100//Metabolic p	ko05152//Tuber	GO:0009897//external s
-0.999996958	0.001570358	-	ko04142//Lysos	-
-0.999996959	0.001569929	ko01100//Metabolic p	-	-
-0.999996971	0.001567012	-	-	-
-0.99999701	0.001556826	-	-	GO:0005737//cytoplasm
-0.999997085	0.001537207	ko01100//Metabolic p	-	GO:0005634//nucleus;G
-0.999997199	0.001506855	-	-	-
-0.999997203	0.001505675	ko01100//Metabolic p	-	GO:0005634//nucleus;G
-0.999997237	0.001496634	-	ko01100//Metal	-
-0.999997254	0.001492017	ko01100//Metabolic p	-	-
-0.99999726	0.001490399	ko01100//Metabolic p	ko01100//Metal	GO:0005829//cytosol
-0.999997293	0.001481303	-	-	-
-0.999997424	0.001445012	-	ko05231//Cholir	-
-0.999997427	0.001444058	ko01100//Metabolic p	-	GO:0016020//membran
-0.999997451	0.001437325	ko01100//Metabolic p	-	GO:0005789//endoplasr
-0.999997492	0.001425917	-	ko04010//MAPK	-
-0.999997502	0.001422986	ko01100//Metabolic p	-	-
-0.999997504	0.001422399	ko01110//Biosynthesis	-	GO:0005737//cytoplasm
-0.999997541	0.001411931	-	ko05200//Pathw	GO:0005634//nucleus;G
-0.999997558	0.001406848	ko01100//Metabolic p	ko04710//Circac	GO:0005634//nucleus;G
-0.999997568	0.001404156	ko01100//Metabolic p	-	-
-0.999997582	0.001400025	ko01100//Metabolic p	-	-
-0.999997583	0.001399614	-	-	-
-0.999997611	0.001391706	-	-	-
-0.999997623	0.001388192	ko01100//Metabolic p	ko04919//Thyro	GO:0016020//membran
-0.999997635	0.001384694	-	ko01100//Metal	-
-0.999997646	0.001381232	-	-	GO:0005794//Golgi app
-0.999997783	0.001340473	ko01100//Metabolic p	ko01100//Metal	GO:0005654//nucleopla
-0.9999978	0.001335518	-	-	-
-0.999997811	0.001331959	ko01100//Metabolic p	-	GO:0035658//Mon1-Cc
-0.999997856	0.001318225	ko01100//Metabolic p	-	-
-0.999997868	0.00131469	ko01100//Metabolic p	ko03013//Nucle	-

-0.99999789	0.001307734	ko01100//Metabolic p	ko04068//FoxO	GO:0005737//cytoplasm
-0.999997951	0.001288822	-	-	GO:0005634//nucleus;G
-0.999997951	0.001288695	ko01100//Metabolic p	ko01100//Metab	GO:0005783//endoplasm
-0.999998049	0.001257406	-	ko05200//Pathw	GO:0000307//cyclin-dep
-0.999998135	0.00122942	-	-	GO:0032991//macromo
-0.999998138	0.001228661	ko01100//Metabolic p	-	-
-0.999998169	0.001218205	-	-	GO:0005783//endoplasm
-0.999998194	0.001209923	ko01100//Metabolic p	-	-
-0.999998235	0.001196243	ko01100//Metabolic p	-	-
-0.99999825	0.001191003	-	-	GO:0005794//Golgi app
-0.999998285	0.001179129	ko01100//Metabolic p	ko03010//Ribos	GO:0005840//ribosome
-0.99999834	0.00115987	ko01100//Metabolic p	-	GO:0097541//axonemal
-0.999998346	0.001158016	ko01100//Metabolic p	-	GO:0005737//cytoplasm
-0.999998354	0.001155201	ko01100//Metabolic p	-	GO:0005737//cytoplasm
-0.999998364	0.001151385	-	ko01100//Metab	-
-0.99999837	0.001149574	ko01100//Metabolic p	ko04080//Neuro	GO:0005886//plasma m
-0.999998465	0.001115536	ko01063//Biosynthesis	-	-
-0.999998481	0.001109542	-	-	-
-0.999998496	0.001104255	-	-	-
-0.999998516	0.001096812	ko01100//Metabolic p	-	GO:0005576//extracellu
-0.999998542	0.001087167	ko01100//Metabolic p	ko05203//Viral c	GO:0000786//nucleosom
-0.999998577	0.001073922	ko01100//Metabolic p	ko03013//Nucle	-
-0.99999859	0.001068958	-	-	GO:0005634//nucleus;G
-0.999998601	0.00106499	-	-	-
-0.999998602	0.001064536	-	-	GO:0005634//nucleus;G
-0.999998635	0.001051768	ko01100//Metabolic p	-	GO:0005886//plasma m
-0.999998681	0.001033875	ko01063//Biosynthesis	-	-
-0.999998693	0.001029202	-	-	-
-0.9999987	0.001026414	ko01100//Metabolic p	ko01100//Metab	GO:0005783//endoplasm
-0.999998763	0.001001174	-	ko01100//Metab	GO:0005783//endoplasm
-0.999998801	0.000985859	-	ko04142//Lysos	-
-0.999998801	0.000985817	ko01100//Metabolic p	-	-
-0.999998807	0.000983187	-	-	-
-0.999998829	0.000974441	ko01100//Metabolic p	ko04216//Ferrop	GO:0016020//membran
-0.99999885	0.000965334	ko01100//Metabolic p	-	GO:0005634//nucleus;G
-0.999998863	0.000960176	-	-	GO:0005737//cytoplasm
-0.999998878	0.000953686	-	-	GO:0016020//membran
-0.999998897	0.000945491	ko01100//Metabolic p	-	GO:0005634//nucleus;G
-0.999998916	0.000937336	-	-	GO:0000785//chromatin
-0.999998923	0.000934533	ko01100//Metabolic p	-	-
-0.99999896	0.000918276	ko01100//Metabolic p	ko04140//Autop	GO:0000407//pre-autop
-0.999998969	0.000914333	-	-	-
-0.999998972	0.000912743	-	-	GO:0016020//membran
-0.999998975	0.00091134	-	-	GO:0097541//axonemal
-0.999998987	0.00090621	ko01100//Metabolic p	-	-
-0.999999019	0.000891744	-	ko04020//Calciu	GO:0016020//membran
-0.999999033	0.000885357	ko01100//Metabolic p	ko01100//Metab	-
-0.999999057	0.000874106	-	ko04146//Perox	GO:0005777//peroxisom
-0.999999093	0.000857292	-	-	GO:0005789//endoplasm
-0.999999095	0.000856651	ko01100//Metabolic p	ko05200//Pathw	GO:0005834//heterotrim
-0.999999105	0.000851806	ko01100//Metabolic p	ko01100//Metab	-
-0.999999124	0.000842549	-	-	-
-0.999999132	0.000838909	ko01100//Metabolic p	-	-
-0.999999141	0.000834661	-	-	-
-0.999999175	0.000817907	-	ko01100//Metab	-
-0.999999182	0.000814127	ko01100//Metabolic p	ko03320//PPAR	GO:0005783//endoplasm
-0.999999201	0.000804852	ko01100//Metabolic p	-	GO:0005615//extracellu
-0.999999209	0.000800918	-	ko04150//mTOR	GO:0005764//lysosome;

-0.999999213	0.000798756	-	-	-
-0.999999219	0.000795688	-	ko04211//Longe	GO:0005634//nucleus
-0.999999242	0.000783758	ko01100//Metabolic p	-	-
-0.999999249	0.000780437	ko01100//Metabolic p	-	-
-0.999999265	0.000772008	-	-	-
-0.999999298	0.000754449	-	-	GO:0034361//very-low-
-0.999999304	0.000750949	ko01100//Metabolic p	-	GO:0000127//transcript
-0.999999326	0.000739278	-	-	-
-0.999999341	0.000730737	-	ko01100//Metak	GO:0005654//nucleopla
-0.999999365	0.000717568	-	-	-
-0.99999938	0.000708888	-	ko01100//Metak	GO:0005634//nucleus;G
-0.999999389	0.000703504	-	-	-
-0.999999413	0.000689632	ko01100//Metabolic p	-	GO:0005615//extracellu
-0.999999436	0.000676389	-	ko01100//Metak	-
-0.999999499	0.000637571	-	ko04713//Circac	-
-0.999999521	0.00062287	ko01100//Metabolic p	ko01100//Metak	GO:0016020//membran
-0.999999535	0.000613622	ko01100//Metabolic p	ko00790//Folate	-
-0.999999551	0.000603566	-	ko01100//Metak	GO:0005737//cytoplasm
-0.999999571	0.000589727	ko01100//Metabolic p	ko01100//Metak	-
-0.999999574	0.000587412	ko01110//Biosynthesis	ko04144//Endoc	GO:0000813//ESCRT I c
-0.999999578	0.000584757	-	ko04934//Cushi	-
-0.999999579	0.000583939	-	-	GO:0005794//Golgi app
-0.999999619	0.000555628	ko01100//Metabolic p	-	GO:0016020//membran
-0.999999639	0.000541276	ko01110//Biosynthesis	ko03320//PPAR	GO:0005783//endoplasr
-0.999999657	0.000527459	ko01100//Metabolic p	-	GO:0044424//intracellul
-0.999999658	0.000526155	-	ko04080//Neurc	GO:0005886//plasma m
-0.999999692	0.00049965	ko01100//Metabolic p	-	-
-0.999999724	0.000472587	ko01063//Biosynthesis	ko01523//Antifo	GO:0005886//plasma m
-0.999999729	0.000468466	ko01100//Metabolic p	ko02010//ABC tı	GO:0005886//plasma m
-0.99999973	0.000468022	ko01100//Metabolic p	-	-
-0.999999735	0.000463563	-	-	-
-0.999999761	0.000439883	-	ko04211//Longe	GO:0005634//nucleus
-0.999999773	0.000428745	-	-	-
-0.999999803	0.000399698	ko01100//Metabolic p	ko04361//Axon	GO:0005737//cytoplasm
-0.999999803	0.000399179	-	ko05152//Tuber	GO:0009897//external s
-0.999999816	0.000386032	-	ko01100//Metak	GO:0005794//Golgi app
-0.999999819	0.000383205	ko01100//Metabolic p	-	GO:0005737//cytoplasm
-0.999999826	0.000375328	-	-	-
-0.999999828	0.000372867	ko01100//Metabolic p	-	GO:0016020//membran
-0.999999829	0.000372174	ko01100//Metabolic p	ko05200//Pathw	GO:0005576//extracellu
-0.999999842	0.000357975	-	-	-
-0.999999846	0.000352984	-	-	GO:0005737//cytoplasm
-0.999999847	0.000352319	-	-	-
-0.999999854	0.000344178	-	ko01100//Metak	GO:0033178//proton-tr
-0.999999855	0.000343379	ko01100//Metabolic p	ko04742//Taste	GO:0005737//cytoplasm
-0.999999864	0.000331692	ko01110//Biosynthesis	-	GO:0016020//membran
-0.999999887	0.000302731	ko01100//Metabolic p	-	-
-0.999999893	0.000294805	ko01100//Metabolic p	-	-
-0.999999894	0.000292785	ko01100//Metabolic p	-	GO:0005576//extracellu
-0.999999898	0.000288225	ko01100//Metabolic p	ko04550//Signal	GO:0005634//nucleus;G
-0.999999902	0.000281481	ko01110//Biosynthesis	ko03013//Nucle	-
-0.999999912	0.000267543	-	-	-
-0.999999922	0.000250665	-	ko01100//Metak	GO:0005783//endoplasr
-0.999999927	0.000242991	-	-	-
-0.999999949	0.000203253	-	-	-
-0.999999955	0.000191886	-	ko03013//Nucle	-
-0.999999955	0.000191752	ko01100//Metabolic p	-	-
-0.999999962	0.000174991	-	-	GO:0005634//nucleus;G

-0.999999964	0.000171035	-	-	GO:0005634//nucleus;G
-0.999999966	0.000165514	ko01100//Metabolic p	ko01100//Metab	GO:0016020//membran
-0.999999968	0.000161412	-	-	-
-0.999999969	0.000157432	ko01100//Metabolic p	-	GO:0044424//intracellul
-0.999999972	0.000150583	ko01100//Metabolic p	-	GO:0005783//endoplasn
-0.999999972	0.000149345	-	ko04140//Autop	GO:0000407//pre-autop
-0.999999977	0.000136272	ko01100//Metabolic p	ko04742//Taste	GO:0016020//membran
-0.999999979	0.000130927	ko01100//Metabolic p	-	GO:0005634//nucleus;G
-0.999999979	0.000130032	ko01100//Metabolic p	ko04080//Neuro	GO:0016021//integral co
-0.99999998	0.000128152	ko01100//Metabolic p	-	-
-0.999999984	0.00011359	-	ko01100//Metab	GO:0005739//mitochon
-0.999999986	0.000106314	-	-	-
-0.999999989	9.31801E-05	-	ko04216//Ferrop	GO:0016020//membran
-0.99999999	8.98637E-05	-	ko01100//Metab	GO:0005737//cytoplasm
-0.999999991	8.75799E-05	-	-	GO:0005737//cytoplasm
-0.999999991	8.53845E-05	ko01100//Metabolic p	ko01100//Metab	GO:0005737//cytoplasm
-0.999999993	7.71841E-05	-	-	-
-0.999999994	6.69966E-05	ko01100//Metabolic p	-	GO:0005737//cytoplasm
-0.999999995	6.51091E-05	ko01100//Metabolic p	ko05200//Pathw	GO:0005634//nucleus
-0.999999995	6.22668E-05	ko01100//Metabolic p	-	-
-0.999999996	5.68534E-05	ko01100//Metabolic p	-	GO:0043231//intracellul
-0.999999998	4.46218E-05	ko01110//Biosynthesis	-	-
-0.999999998	4.18434E-05	-	-	-
-0.999999998	3.86871E-05	-	ko01100//Metab	-
-0.999999998	3.85611E-05	-	-	GO:0005634//nucleus;G
-1	1.56051E-05	-	-	GO:0005737//cytoplasm

Gene_GO Function	Gene_GO Process
GO:0004499//N,N-d	-
GO:0050839//cell ad	GO:0007409//axonogenesis;GO:0010975//regulation of neuron projection
-	-
-	-
GO:0005515//protei	-
-	-
GO:0003824//catalyt	GO:0006167//AMP biosynthetic process;GO:0006177//GMP biosynthetic p
GO:0004467//long-c	GO:0015908//fatty acid transport
GO:0005509//calciur	GO:0007611//learning or memory;GO:0007613//memory;GO:0043123//pc
GO:0003924//GTPas	GO:0032755//positive regulation of interleukin-6 production;GO:0034144
GO:0005267//potass	GO:0006813//potassium ion transport;GO:0033198//response to ATP;GO:
-	-
GO:0003836//beta-g	GO:0002319//memory B cell differentiation;GO:0006054//N-acetylneuram
-	-
-	-
-	-
GO:0000166//nucleo	GO:0003014//renal system process;GO:0006811//ion transport;GO:000682
GO:0004308//exo-al	GO:0006516//glycoprotein catabolic process;GO:0006689//ganglioside ca
GO:0008009//chemo	GO:0006954//inflammatory response;GO:0006955//immune response;GO
-	-
GO:0001784//phosp	GO:0007169//transmembrane receptor protein tyrosine kinase signaling p
GO:0003924//GTPas	-
GO:0000166//nucleo	-
GO:0004497//mono	GO:0006805//xenobiotic metabolic process;GO:0008202//steroid metabol
GO:0000993//RNA p	GO:0000956//nuclear-transcribed mRNA catabolic process;GO:0010501//
-	-
-	-
GO:0030298//recept	GO:0010976//positive regulation of neuron projection development;GO:0
GO:0003682//chrom	GO:0001945//lymph vessel development;GO:0003017//lymph circulation;
GO:0001540//beta-a	GO:0001932//regulation of protein phosphorylation;GO:0001935//endoth
GO:0004672//protei	GO:0006468//protein phosphorylation
-	-
-	GO:0007286//spermatid development;GO:0048137//spermatocyte division
GO:0003909//DNA li	-
GO:0005515//protei	GO:0007043//cell-cell junction assembly;GO:0007155//cell adhesion;GO:0
GO:0005158//insulin	GO:0001775//cell activation;GO:0007405//neuroblast proliferation;GO:000
GO:0008270//zinc io	GO:0009056//catabolic process;GO:0030855//epithelial cell differentiation
-	-
GO:0005515//protei	-
GO:0016491//oxidor	GO:0009058//biosynthetic process;GO:0009792//embryo development en
GO:0005515//protei	GO:0006955//immune response;GO:0006956//complement activation
GO:0000977//RNA p	GO:0000122//negative regulation of transcription from RNA polymerase I
GO:0005178//integri	GO:0000902//cell morphogenesis;GO:0006954//inflammatory response;G
GO:1990050//phosp	GO:0015914//phospholipid transport
-	-
-	-
GO:0003779//actin b	GO:0006351//transcription, DNA-templated;GO:0030032//lamellipodium
GO:0046872//metal	GO:0006875//cellular metal ion homeostasis
GO:0016407//acetylt	-
GO:0016757//transfe	-
GO:0008270//zinc io	GO:0006882//cellular zinc ion homeostasis;GO:0010273//detoxification of
GO:0003677//DNA b	GO:0010467//gene expression
GO:0008480//sarcos	GO:1901053//sarcosine catabolic process
GO:0002039//p53 bi	GO:0000724//double-strand break repair via homologous recombination;
-	GO:0001920//negative regulation of receptor recycling;GO:0050850//posi
GO:0003677//DNA b	GO:0006334//nucleosome assembly;GO:0006352//DNA-templated transc

GO:0000254//C-4 m	GO:0001934//positive regulation of protein phosphorylation;GO:0008610/
-	-
GO:0008201//hepari	-
GO:0004465//lipopr	GO:0006629//lipid metabolic process;GO:0006631//fatty acid metabolic p
GO:0003779//actin b	GO:0007010//cytoskeleton organization;GO:0008360//regulation of cell sh
-	-
GO:0005085//guany	GO:0007264//small GTPase mediated signal transduction;GO:0030334//re
-	-
GO:0000166//nucleo	GO:0000122//negative regulation of transcription from RNA polymerase I
GO:0004506//squale	GO:0016126//sterol biosynthetic process;GO:0042127//regulation of cell p
GO:0004721//phosp	GO:0010801//negative regulation of peptidyl-threonine phosphorylation;
GO:0001786//phosp	GO:0002024//diet induced thermogenesis;GO:0006606//protein import in
GO:0046983//protei	GO:0000082//G1/S transition of mitotic cell cycle;GO:0000122//negative r
GO:0003735//structu	GO:0006412//translation
GO:0004730//pseud	GO:0001522//pseudouridine synthesis;GO:0006796//phosphate-containin
GO:0004252//serine	GO:0006508//proteolysis
GO:0003868//4-hyd	GO:0006572//tyrosine catabolic process;GO:0009072//aromatic amino aci
GO:0005507//coppe	GO:0009308//amine metabolic process;GO:0009445//putrescine metaboli
GO:0022857//transm	GO:0055085//transmembrane transport
GO:0005201//extrac	GO:0030198//extracellular matrix organization;GO:0031581//hemidesmos
GO:0016627//oxidor	GO:0006629//lipid metabolic process
GO:0016627//oxidor	GO:0006629//lipid metabolic process
GO:0005515//protei	GO:0010468//regulation of gene expression;GO:0016567//protein ubiquit
GO:0005515//protei	GO:0030855//epithelial cell differentiation
-	-
GO:0004721//phosp	GO:0010801//negative regulation of peptidyl-threonine phosphorylation;
GO:0005102//recept	GO:0001541//ovarian follicle development;GO:0001707//mesoderm forma
-	-
GO:0000166//nucleo	GO:0006090//pyruvate metabolic process;GO:0006094//gluconeogenesis
GO:0004175//endop	GO:0018126//protein hydroxylation
GO:0000166//nucleo	GO:0001954//positive regulation of cell-matrix adhesion;GO:0003323//typ
GO:0003824//catalyt	GO:0006567//threonine catabolic process
GO:0000978//RNA p	GO:0001649//osteoblast differentiation;GO:0001755//neural crest cell mig
-	-
GO:0098640//integri	GO:0007155//cell adhesion;GO:0007229//integrin-mediated signaling pat
GO:0004862//cAMP	GO:0006469//negative regulation of protein kinase activity;GO:0051338//r
GO:0003677//DNA b	GO:0010467//gene expression
-	-
GO:0016757//transfe	-
GO:0009374//biotin	-
-	-
-	-
GO:0004730//pseud	GO:0001522//pseudouridine synthesis;GO:0006796//phosphate-containin
-	-
GO:0005515//protei	GO:0007155//cell adhesion;GO:0007156//homophilic cell adhesion via pla
GO:0005515//protei	GO:0007409//axonogenesis;GO:0050807//regulation of synapse organizat
-	-
GO:1990817//RNA a	GO:0009617//response to bacterium;GO:0030278//regulation of ossificatio
GO:0004114//3',5'-c	GO:0007165//signal transduction;GO:0032729//positive regulation of inte
-	-
GO:0005509//calciur	GO:0007165//signal transduction
GO:0008083//growt	-
GO:0005524//ATP bi	GO:0000086//G2/M transition of mitotic cell cycle;GO:0045332//phosphol
GO:0004867//serine	GO:0007596//blood coagulation
-	-
GO:0005515//protei	GO:0007155//cell adhesion
GO:0005515//protei	-
GO:0004970//ionotr	GO:0006811//ion transport;GO:0045471//response to ethanol;GO:009755

GO:0003909//DNA li	-
-	GO:0007600//sensory perception;GO:0007601//visual perception;GO:0007
GO:0016407//acetylt	-
GO:0000993//RNA p	GO:0000956//nuclear-transcribed mRNA catabolic process;GO:0010501//
-	-
GO:0005243//gap ju	GO:0007154//cell communication;GO:0007267//cell-cell signaling;GO:000
GO:0003824//catalyt	GO:0006103//2-oxoglutarate metabolic process;GO:0009058//biosyntheti
GO:0003700//transc	GO:0006355//regulation of transcription, DNA-templated
GO:0008195//phosp	GO:0006796//phosphate-containing compound metabolic process;GO:00
-	-
-	-
GO:0005515//protei	GO:0015879//carnitine transport;GO:0032414//positive regulation of ion t
GO:0003824//catalyt	GO:0006167//AMP biosynthetic process;GO:0006177//GMP biosynthetic p
GO:0005515//protei	GO:0007155//cell adhesion
GO:0000166//nucleo	GO:0006563//L-serine metabolic process;GO:0006790//sulfur compound
GO:0005515//protei	-
GO:0004888//transm	GO:0007165//signal transduction;GO:0007166//cell surface receptor signa
GO:0005515//protei	-
GO:0008113//peptid	-
GO:0005515//protei	-
GO:0005509//calciur	GO:0032571//response to vitamin K;GO:0060348//bone development;GO:
GO:0016491//oxidor	-
GO:0005506//iron ic	-
GO:0003924//GTPas	-
-	-
-	GO:0007165//signal transduction
GO:0004467//long-c	GO:0001676//long-chain fatty acid metabolic process;GO:0008610//lipid
GO:0005515//protei	GO:0042509//regulation of tyrosine phosphorylation of STAT protein;GO:
-	-
GO:0009374//biotin	-
-	-
GO:0000978//RNA p	GO:0006357//regulation of transcription from RNA polymerase II promote
GO:0003810//protei	GO:0018149//peptide cross-linking
GO:0038023//signali	GO:0007160//cell-matrix adhesion;GO:0007179//transforming growth fac
GO:0050839//cell ad	GO:0007409//axonogenesis;GO:0010975//regulation of neuron projection
GO:0004364//glutatl	GO:0006749//glutathione metabolic process;GO:0006805//xenobiotic met
-	-
-	GO:0051480//regulation of cytosolic calcium ion concentration;GO:19900
GO:0000064//L-orni	GO:1990575//mitochondrial L-ornithine transmembrane transport
GO:0005520//insulin	GO:0042104//positive regulation of activated T cell proliferation
-	GO:0007283//spermatogenesis;GO:0007288//sperm axoneme assembly;G
GO:0003682//chrom	GO:0001945//lymph vessel development;GO:0003017//lymph circulation;
GO:0005515//protei	GO:0007155//cell adhesion;GO:0008593//regulation of Notch signaling pa
GO:0005515//protei	GO:0015879//carnitine transport;GO:0032414//positive regulation of ion t
GO:0001540//beta-a	GO:0001932//regulation of protein phosphorylation;GO:0001935//endoth
GO:0003824//catalyt	GO:0006684//sphingomyelin metabolic process;GO:0006685//sphingomy
GO:0004089//carbor	GO:0002009//morphogenesis of an epithelium;GO:0046903//secretion
-	-
GO:0005085//guany	GO:0001934//positive regulation of protein phosphorylation;GO:0002437
-	GO:0006895//Golgi to endosome transport;GO:0015031//protein transpor
GO:0004122//cystatl	GO:0001958//endochondral ossification;GO:0001974//blood vessel remoc
GO:0005515//protei	-
GO:0003824//catalyt	GO:0006567//threonine catabolic process
GO:0003777//microt	GO:0007018//microtubule-based movement;GO:0072384//organelle tran
GO:0005507//coppe	GO:0009308//amine metabolic process;GO:0009445//putrescine metaboli
GO:0002020//protea	GO:0010466//negative regulation of peptidase activity;GO:0018149//pept
GO:0030298//recept	GO:0010976//positive regulation of neuron projection development;GO:0
GO:0008270//zinc ic	GO:0006882//cellular zinc ion homeostasis;GO:0010273//detoxification of

GO:0000977//RNA p	GO:0000122//negative regulation of transcription from RNA polymerase I
GO:0045027//DNA e	GO:0006302//double-strand break repair;GO:0006303//double-strand bre
GO:0070412//R-SMA	GO:0010991//negative regulation of SMAD protein complex assembly;GO
GO:0003777//microt	GO:0007018//microtubule-based movement;GO:0072384//organelle tran
GO:0004672//protei	GO:0006468//protein phosphorylation
GO:0005515//protei	-
GO:0005515//protei	-
GO:0005201//extrac	GO:0002244//hematopoietic progenitor cell differentiation
GO:0022857//transm	GO:0055085//transmembrane transport
GO:0000254//C-4 m	GO:0001934//positive regulation of protein phosphorylation;GO:0008610/
GO:0015108//chloric	GO:1902476//chloride transmembrane transport
GO:0035254//glutan	GO:0001662//behavioral fear response;GO:0006884//cell volume homeos
GO:0003868//4-hyd	GO:0006572//tyrosine catabolic process;GO:0009072//aromatic amino aci
GO:0016491//oxidor	-
GO:0004044//amido	GO:0006177//GMP biosynthetic process;GO:0006189//'de novo' IMP bios
GO:1990817//RNA a	GO:0009617//response to bacterium;GO:0030278//regulation of ossificati
-	-
GO:0003677//DNA b	GO:0006334//nucleosome assembly;GO:0006352//DNA-templated transc
GO:0022857//transm	GO:0055085//transmembrane transport
GO:0003824//catalyt	GO:0010121//arginine catabolic process to proline via ornithine;GO:0019
-	-
-	GO:0009267//cellular response to starvation;GO:0030001//metal ion trans
-	-
GO:0004672//protei	GO:0000002//mitochondrial genome maintenance;GO:0001938//positive
GO:0005515//protei	-
GO:0000254//C-4 m	GO:0001934//positive regulation of protein phosphorylation;GO:0008610/
GO:0038023//signali	GO:0007160//cell-matrix adhesion;GO:0007179//transforming growth fac
GO:0008022//protei	GO:0007026//negative regulation of microtubule depolymerization;GO:00
-	-
-	-
GO:0003735//structu	GO:0006412//translation
-	GO:0015888//thiamine transport
GO:0004497//mono	GO:0006805//xenobiotic metabolic process;GO:0008202//steroid metabol
-	-
GO:0005158//insulin	GO:0001775//cell activation;GO:0007405//neuroblast proliferation;GO:000
GO:0000062//fatty-a	-
GO:0005515//protei	GO:0030855//epithelial cell differentiation
GO:0005515//protei	GO:0035721//intraciliary retrograde transport;GO:0060271//cilium morph
GO:0005515//protei	GO:0006749//glutathione metabolic process
GO:0000062//fatty-a	GO:0008203//cholesterol metabolic process;GO:0010742//macrophage de
GO:0004452//isoper	GO:0008299//isoprenoid biosynthetic process
-	-
GO:0036122//BMP b	GO:0007389//pattern specification process;GO:0010454//negative regulat
GO:0008146//sulfotr	-
-	-
GO:0003796//lysozy	GO:0008152//metabolic process;GO:0016998//cell wall macromolecule ca
GO:0008113//peptid	-
-	-
GO:0005515//protei	GO:0003431//growth plate cartilage chondrocyte development;GO:00082
GO:0005515//protei	GO:0030855//epithelial cell differentiation
GO:0005515//protei	GO:0051017//actin filament bundle assembly
GO:0003676//nuclei	GO:0006417//regulation of translation;GO:0032869//cellular response to i
GO:0015485//choles	GO:0032367//intracellular cholesterol transport;GO:0034389//lipid particle
GO:0003796//lysozy	GO:0009253//peptidoglycan catabolic process;GO:0016998//cell wall mac
-	-
-	GO:0032088//negative regulation of NF-kappaB transcription factor activi
GO:0004307//ethan	GO:0006646//phosphatidylethanolamine biosynthetic process;GO:00086
-	-

GO:0004497//mono	GO:0006559//L-phenylalanine catabolic process;GO:0006571//tyrosine bi
-	GO:0007010//cytoskeleton organization;GO:0008360//regulation of cell sh
GO:0005243//gap ju	GO:0007154//cell communication;GO:0007267//cell-cell signaling;GO:000
GO:0000976//transc	GO:0002062//chondrocyte differentiation;GO:0006355//regulation of tran
GO:0016407//acetylt	-
GO:0003700//transc	GO:0006355//regulation of transcription, DNA-templated
GO:0004499//N,N-d	-
GO:0003824//catalyt	GO:0006527//arginine catabolic process;GO:0009072//aromatic amino aci
GO:0004866//endop	-
GO:0004867//serine	GO:0016525//negative regulation of angiogenesis;GO:0050769//positive r
-	-
-	GO:0010001//glial cell differentiation;GO:0050772//positive regulation of
-	-
GO:1990817//RNA a	GO:0009617//response to bacterium;GO:0030278//regulation of ossificati
-	-
GO:0004657//prolin	GO:0006562//proline catabolic process
GO:0005515//protei	-
GO:0004672//protei	GO:0006468//protein phosphorylation
GO:0008009//chemo	GO:0006954//inflammatory response;GO:0006955//immune response;GO
GO:0016491//oxidor	GO:0019477//L-lysine catabolic process
GO:0005267//potass	GO:0006813//potassium ion transport;GO:0033198//response to ATP;GO:
GO:0003713//transc	GO:0007219//Notch signaling pathway;GO:0045944//positive regulation o
-	-
GO:0004888//transm	GO:0007186//G-protein coupled receptor signaling pathway;GO:0009994
-	-
-	-
GO:0003824//catalyt	-
-	GO:0007286//spermatid development;GO:0048137//spermatocyte divisio
-	-
GO:0005515//protei	GO:0007155//cell adhesion;GO:0007156//homophilic cell adhesion via pla
-	-
GO:0000064//L-orni	GO:1990575//mitochondrial L-ornithine transmembrane transport
GO:0003924//GTPas	GO:0032755//positive regulation of interleukin-6 production;GO:0034144
GO:0005515//protei	GO:0015879//carnitine transport;GO:0032414//positive regulation of ion t
GO:0003924//GTPas	-
GO:0098640//integri	GO:0007155//cell adhesion;GO:0007229//integrin-mediated signaling pat
GO:0031681//G-pro	GO:0007186//G-protein coupled receptor signaling pathway
GO:0038023//signali	GO:0007155//cell adhesion;GO:0007158//neuron cell-cell adhesion;GO:00
GO:0003677//DNA b	GO:0006334//nucleosome assembly;GO:0006352//DNA-templated transc
GO:0070089//chlori	GO:0006813//potassium ion transport;GO:0097623//potassium ion export
-	-
GO:0005096//GTPas	GO:0007165//signal transduction;GO:0043547//positive regulation of GTP
GO:0005085//guany	GO:0007264//small GTPase mediated signal transduction;GO:0030334//re
GO:0004867//serine	GO:0007596//blood coagulation
GO:0005515//protei	GO:0007155//cell adhesion
GO:0005515//protei	GO:0006570//tyrosine metabolic process;GO:0007613//memory;GO:0008
GO:0005515//protei	GO:0006355//regulation of transcription, DNA-templated
GO:0005520//insulin	GO:0042104//positive regulation of activated T cell proliferation
GO:0046872//metal	GO:0006875//cellular metal ion homeostasis
-	GO:0046890//regulation of lipid biosynthetic process
GO:0001786//phosp	GO:0002024//diet induced thermogenesis;GO:0006606//protein import in
GO:0002020//protea	GO:0010466//negative regulation of peptidase activity;GO:0018149//pept
-	GO:0007165//signal transduction
-	-
GO:0004639//phosp	GO:0006164//purine nucleotide biosynthetic process;GO:0006189//'de no
GO:0008201//hepari	-
GO:0005520//insulin	GO:0042104//positive regulation of activated T cell proliferation
GO:0004089//carbor	GO:0002009//morphogenesis of an epithelium;GO:0046903//secretion

GO:0003824//catalyt	GO:0006567//threonine catabolic process
GO:0016316//phosp	-
-	-
GO:0005102//recept	GO:0001541//ovarian follicle development;GO:0001707//mesoderm forma
GO:0070089//chloric	GO:0006813//potassium ion transport;GO:0097623//potassium ion export
GO:0003677//DNA b	GO:0006334//nucleosome assembly
GO:0005515//protei	GO:0042509//regulation of tyrosine phosphorylation of STAT protein;GO:
GO:0003677//DNA b	GO:0006334//nucleosome assembly;GO:0042742//defense response to ba
GO:0008480//sarcos	GO:1901053//sarcosine catabolic process
GO:0004478//methi	GO:0006556//S-adenosylmethionine biosynthetic process;GO:0009087//m
GO:0005179//hormo	-
-	GO:0006629//lipid metabolic process
GO:0004930//G-pro	GO:0002300//CD8-positive, alpha-beta intraepithelial T cell differentiatior
GO:0003779//actin b	GO:0007010//cytoskeleton organization;GO:0008360//regulation of cell sh
GO:0008270//zinc io	-
-	GO:0016559//peroxisome fission;GO:0044375//regulation of peroxisome
GO:0022857//transm	GO:0055085//transmembrane transport
-	-
GO:0005216//ion ch	GO:0006811//ion transport;GO:0019228//neuronal action potential;GO:00
GO:0008195//phosp	GO:0006796//phosphate-containing compound metabolic process;GO:00
GO:0004478//methi	GO:0006556//S-adenosylmethionine biosynthetic process;GO:0009087//m
-	-
GO:0005515//protei	GO:0007043//cell-cell junction assembly;GO:0007155//cell adhesion;GO:0
-	-
-	GO:0043368//positive T cell selection;GO:0043383//negative T cell selectio
GO:0003847//1-alky	GO:0006629//lipid metabolic process;GO:0006644//phospholipid metabo
GO:0005524//ATP bi	GO:0035499//carnosine biosynthetic process
-	-
-	GO:0008154//actin polymerization or depolymerization;GO:0030048//acti
GO:0005515//protei	-
GO:0005515//protei	GO:0010468//regulation of gene expression;GO:0016567//protein ubiquit
GO:0003824//catalyt	GO:0010121//arginine catabolic process to proline via ornithine;GO:0019
GO:0005085//guany	GO:0001934//positive regulation of protein phosphorylation;GO:0002437
GO:0008270//zinc io	GO:0009056//catabolic process;GO:0030855//epithelial cell differentiation
-	-
GO:0003755//peptid	-
-	-
GO:0000287//magne	GO:0006584//catecholamine metabolic process;GO:0016036//cellular resp
-	-
GO:0008083//growt	-
-	-
-	-
GO:0016407//acetylt	-
GO:0016765//transfe	GO:0008299//isoprenoid biosynthetic process
GO:0005515//protei	-
GO:0005515//protei	GO:0035721//intraciliary retrograde transport;GO:0060271//cilium morph
GO:0005509//calciur	GO:0007165//signal transduction
GO:0003735//structu	GO:0006412//translation
GO:0004047//amino	GO:0019464//glycine decarboxylation via glycine cleavage system
GO:0004730//pseud	GO:0001522//pseudouridine synthesis;GO:0006796//phosphate-containin
GO:0005515//protei	-
GO:0004364//glutat	GO:0006749//glutathione metabolic process;GO:0006805//xenobiotic met
-	GO:0007010//cytoskeleton organization;GO:0008360//regulation of cell sh
GO:0004497//mono	GO:0006559//L-phenylalanine catabolic process;GO:0006571//tyrosine bi
GO:0016407//acetylt	-
GO:0004497//mono	GO:0097267//omega-hydroxylase P450 pathway
GO:0015485//choles	GO:0032367//intracellular cholesterol transport;GO:0034389//lipid particle
GO:0004866//endop	GO:0010951//negative regulation of endopeptidase activity

-	-
GO:0005515//protei	GO:0006355//regulation of transcription, DNA-templated;GO:0006805//x
GO:0003676//nuclei	GO:0006278//RNA-dependent DNA replication;GO:0015074//DNA integr
GO:0042809//vitami	GO:0008285//negative regulation of cell proliferation;GO:0010468//regula
GO:0008289//lipid b	GO:0032374//regulation of cholesterol transport
-	-
GO:0051015//actin f	GO:0003382//epithelial cell morphogenesis;GO:0007015//actin filament o
GO:0004122//cystat	GO:0001958//endochondral ossification;GO:0001974//blood vessel remo
GO:0000166//nucleo	GO:0006563//L-serine metabolic process;GO:0006790//sulfur compound
GO:0004307//ethan	GO:0006646//phosphatidylethanolamine biosynthetic process;GO:00086
GO:0004930//G-pro	GO:0007186//G-protein coupled receptor signaling pathway
-	GO:0007010//cytoskeleton organization;GO:0008360//regulation of cell s
GO:0005515//protei	GO:0007409//axonogenesis;GO:0050807//regulation of synapse organiza
GO:0008289//lipid b	-
GO:0003779//actin b	GO:0051017//actin filament bundle assembly
GO:0000062//fatty-a	GO:0008203//cholesterol metabolic process;GO:0010742//macrophage d
GO:0003713//transc	GO:0006355//regulation of transcription, DNA-templated
GO:0005506//iron io	-
GO:0004114//3',5'-c	GO:0007165//signal transduction;GO:0032729//positive regulation of inte
GO:0004842//ubiqui	GO:0006511//ubiquitin-dependent protein catabolic process;GO:0007399
GO:0005515//protei	GO:0051017//actin filament bundle assembly
GO:0003824//catalyt	GO:0006777//Mo-molybdopterin cofactor biosynthetic process;GO:00435
-	-
-	-
-	-
-	-
GO:0005515//protei	-
GO:0016491//oxidor	GO:0009058//biosynthetic process;GO:0009792//embryo development e
GO:0003676//nuclei	GO:0006278//RNA-dependent DNA replication;GO:0044260//cellular ma
GO:0003810//protei	GO:0018149//peptide cross-linking
-	-
GO:0003824//catalyt	GO:0010121//arginine catabolic process to proline via ornithine;GO:0019
GO:0004044//amido	GO:0006177//GMP biosynthetic process;GO:0006189//'de novo' IMP bios
GO:0003796//lysozy	GO:0009253//peptidoglycan catabolic process;GO:0016998//cell wall ma
GO:0003824//catalyt	GO:0006103//2-oxoglutarate metabolic process;GO:0009058//biosyntheti
GO:0003924//GTPas	-
GO:0005515//protei	GO:0006355//regulation of transcription, DNA-templated;GO:0007179//t
-	-
GO:0022857//transm	GO:0006865//amino acid transport;GO:0055085//transmembrane transpo
GO:0004896//cytoki	GO:0007171//activation of transmembrane receptor protein tyrosine kina
GO:0003677//DNA b	GO:0035871//protein K11-linked deubiquitination
GO:0005515//protei	GO:0007155//cell adhesion;GO:0008593//regulation of Notch signaling p
GO:0035254//glutam	GO:0001662//behavioral fear response;GO:0006884//cell volume homeos
GO:0005515//protei	-
GO:0004672//protei	GO:0006885//regulation of pH;GO:0008286//insulin receptor signaling pa
GO:0005488//bindin	-
GO:0000166//nucleo	GO:0006468//protein phosphorylation;GO:0006955//immune response;G
GO:0003824//catalyt	-
GO:0004866//endop	GO:0001934//positive regulation of protein phosphorylation;GO:0006954
GO:0016757//transfe	GO:0006488//dolichol-linked oligosaccharide biosynthetic process
GO:0070089//chlori	GO:0006813//potassium ion transport;GO:0097623//potassium ion export
-	GO:0006895//Golgi to endosome transport;GO:0015031//protein transpo
-	-
GO:0004672//protei	GO:0000002//mitochondrial genome maintenance;GO:0001938//positive
-	-
GO:0005515//protei	GO:0042634//regulation of hair cycle
-	-
-	-

-	-
GO:0003924//GTPas	-
GO:0004044//amido	GO:0006177//GMP biosynthetic process;GO:0006189//'de novo' IMP biosy
GO:0004553//hydrol	GO:0005975//carbohydrate metabolic process
-	-
-	-
-	-
GO:0003810//protei	GO:0018149//peptide cross-linking
GO:0003824//catalyt	GO:0006527//arginine catabolic process;GO:0009072//aromatic amino aci
GO:0001517//N-ace	GO:0005975//carbohydrate metabolic process;GO:0006044//N-acetylgluc
-	-
GO:0004467//long-c	GO:0015908//fatty acid transport
GO:0046873//metal	GO:0030001//metal ion transport
GO:0035254//glutan	GO:0001662//behavioral fear response;GO:0006884//cell volume homeos
-	-
GO:0004340//glucok	GO:0001678//cellular glucose homeostasis;GO:0005975//carbohydrate me
GO:0005515//protei	-
GO:0005515//protei	GO:0042509//regulation of tyrosine phosphorylation of STAT protein;GO:
GO:0008195//phosp	GO:0006357//regulation of transcription from RNA polymerase II promote
GO:0016491//oxidor	GO:0019477//L-lysine catabolic process
-	-
-	GO:0015888//thiamine transport
GO:0000977//RNA p	GO:0000122//negative regulation of transcription from RNA polymerase I
GO:0008113//peptid	-
GO:0004601//peroxi	GO:0015671//oxygen transport;GO:0042744//hydrogen peroxide cataboli
GO:0005506//iron ic	GO:0006457//protein folding;GO:0018126//protein hydroxylation;GO:001
GO:0000166//nucleo	GO:0006563//L-serine metabolic process;GO:0006790//sulfur compound
-	GO:0030193//regulation of blood coagulation;GO:0044763//single-organ
GO:0005515//protei	GO:0007155//cell adhesion
-	GO:0007166//cell surface receptor signaling pathway
-	-
GO:0015485//choles	GO:0032367//intracellular cholesterol transport;GO:0034389//lipid particle
-	-
GO:0003847//1-alky	GO:0006629//lipid metabolic process;GO:0006644//phospholipid metabo
GO:0004497//monoo	GO:0006559//L-phenylalanine catabolic process;GO:0006571//tyrosine bi
GO:0003909//DNA li	-
GO:0005515//protei	-
GO:0004842//ubiqui	GO:0006511//ubiquitin-dependent protein catabolic process;GO:0007399
GO:0005515//protei	-
GO:0005102//recept	GO:0006898//receptor-mediated endocytosis;GO:0008104//protein locali
-	-
GO:0005515//protei	GO:0008277//regulation of G-protein coupled receptor protein signaling
-	-
GO:0004047//amino	GO:0019464//glycine decarboxylation via glycine cleavage system
GO:0022857//transm	GO:0055085//transmembrane transport
GO:0004497//monoo	GO:0006559//L-phenylalanine catabolic process;GO:0006571//tyrosine bi
GO:0005506//iron ic	-
-	-
-	GO:0007154//cell communication
GO:0015485//choles	GO:0032367//intracellular cholesterol transport;GO:0034389//lipid particle
GO:0004866//endop	GO:0010951//negative regulation of endopeptidase activity
GO:0005515//protei	GO:0007612//learning;GO:0007613//memory;GO:1900452//regulation of
GO:0003779//actin b	GO:0007030//Golgi organization;GO:0007032//endosome organization;G
GO:0016316//phosp	-
GO:0004497//monoo	-
GO:0003779//actin b	GO:0051017//actin filament bundle assembly
GO:0005515//protei	GO:0007155//cell adhesion;GO:0007156//homophilic cell adhesion via pla
-	GO:0017015//regulation of transforming growth factor beta receptor sign

GO:0003676//nuclei	GO:0006278//RNA-dependent DNA replication;GO:0006310//DNA recom
GO:0005515//protei	GO:0007165//signal transduction
GO:0005546//phosp	GO:0009636//response to toxic substance;GO:0042360//vitamin E metabo
GO:0004016//adeny	GO:0006171//cAMP biosynthetic process;GO:0007613//memory;GO:0007
GO:0004252//serine	GO:0006508//proteolysis;GO:0016485//protein processing
-	GO:0007283//spermatogenesis;GO:0007288//sperm axoneme assembly;G
GO:0098640//integri	GO:0007155//cell adhesion;GO:0007229//integrin-mediated signaling pat
GO:0003834//beta-c	GO:0001523//retinoid metabolic process;GO:0016121//carotene catabolic
GO:0046983//protei	GO:0000082//G1/S transition of mitotic cell cycle;GO:0000122//negative r
GO:0004888//transm	GO:0007166//cell surface receptor signaling pathway;GO:0007186//G-pro
GO:0003796//lysozy	GO:0008152//metabolic process;GO:0016998//cell wall macromolecule ca
-	-
GO:0016627//oxidor	GO:0006629//lipid metabolic process
GO:0003713//transc	GO:0007219//Notch signaling pathway;GO:0045944//positive regulation c
-	-
GO:0036094//small r	-
GO:0003676//nuclei	GO:0006278//RNA-dependent DNA replication;GO:0044260//cellular mac
GO:0005102//recept	GO:0006898//receptor-mediated endocytosis;GO:0008104//protein locali
-	GO:0030193//regulation of blood coagulation;GO:0044763//single-organ
GO:0005096//GTPas	GO:0007165//signal transduction;GO:0035556//intracellular signal transdu
GO:0008083//growtl	-
GO:0005515//protei	-
GO:0004866//endop	GO:0001934//positive regulation of protein phosphorylation;GO:0006954/
GO:0000166//nucleo	GO:0006468//protein phosphorylation;GO:0006955//immune response;G(
GO:0004044//amido	GO:0006177//GMP biosynthetic process;GO:0006189//'de novo' IMP bios
GO:0004842//ubiqui	GO:0016567//protein ubiquitination
GO:0015108//chloric	GO:1902476//chloride transmembrane transport
GO:0008480//sarcos	GO:1901053//sarcosine catabolic process
GO:0046872//metal	GO:0006875//cellular metal ion homeostasis
GO:0008270//zinc ic	-
GO:0005515//protei	GO:0007155//cell adhesion;GO:0008593//regulation of Notch signaling pa
GO:0022857//transm	GO:0006865//amino acid transport;GO:0055085//transmembrane transpo
GO:0008270//zinc ic	-
GO:0070089//chloric	GO:0006813//potassium ion transport;GO:0097623//potassium ion export
-	-
GO:0004930//G-pro	GO:0002300//CD8-positive, alpha-beta intraepithelial T cell differentiatior
GO:0004478//methi	GO:0006556//S-adenosylmethionine biosynthetic process;GO:0009087//n
GO:0004478//methi	GO:0006556//S-adenosylmethionine biosynthetic process;GO:0009087//n
GO:0000976//transc	GO:0000122//negative regulation of transcription from RNA polymerase I
-	GO:0006895//Golgi to endosome transport;GO:0015031//protein transpo
GO:0000166//nucleo	GO:0006529//asparagine biosynthetic process;GO:0006541//glutamine m
GO:0017048//Rho G	GO:0001667//ameboidal-type cell migration;GO:0007265//Ras protein sig
GO:0008146//sulfotr	-
GO:0004497//mono	GO:0097267//omega-hydroxylase P450 pathway
GO:0016757//transfe	GO:0006488//dolichol-linked oligosaccharide biosynthetic process
GO:0003824//cataly	GO:0006103//2-oxoglutarate metabolic process;GO:0009058//biosyntheti
GO:0005515//protei	GO:0006355//regulation of transcription, DNA-templated;GO:0007179//tr
GO:0005515//protei	-
GO:0042809//vitami	GO:0008285//negative regulation of cell proliferation;GO:0010468//regula
GO:0000334//3-hyd	GO:0009435//NAD biosynthetic process;GO:0010043//response to zinc io
GO:0005085//guany	GO:0001934//positive regulation of protein phosphorylation;GO:0002437/
GO:0008270//zinc ic	GO:0009056//catabolic process;GO:0030855//epithelial cell differentiation
GO:0005515//protei	GO:0007165//signal transduction
-	-
-	-
GO:0004930//G-pro	GO:0007186//G-protein coupled receptor signaling pathway
GO:0005096//GTPas	GO:0007165//signal transduction;GO:0043547//positive regulation of GTP
-	-

GO:0008009//chemo	GO:0006954//inflammatory response;GO:0006955//immune response;GO
GO:0008201//hepari	-
GO:0003810//protei	GO:0018149//peptide cross-linking
-	GO:0007165//signal transduction
GO:0003676//nuclei	GO:0006508//proteolysis;GO:0016032//viral process
-	-
-	-
GO:0022857//transm	GO:0055085//transmembrane transport
GO:0016798//hydrol	GO:0008284//positive regulation of cell proliferation;GO:0030198//extrace
GO:0004095//carniti	GO:0001676//long-chain fatty acid metabolic process;GO:0006629//lipid
GO:0008195//phosp	GO:0006357//regulation of transcription from RNA polymerase II promote
GO:0008146//sulfotr	-
-	GO:0015888//thiamine transport
-	-
-	GO:0007166//cell surface receptor signaling pathway
GO:0003676//nuclei	-
-	-
GO:0004499//N,N-d	-
-	-
GO:0003677//DNA b	GO:0009267//cellular response to starvation;GO:0043066//negative regula
GO:0000977//RNA p	GO:0000122//negative regulation of transcription from RNA polymerase I
-	GO:0007010//cytoskeleton organization;GO:0008360//regulation of cell sh
-	-
-	-
GO:0000166//nucleo	GO:0006563//L-serine metabolic process;GO:0006790//sulfur compound
-	GO:0007286//spermatid development;GO:0048137//spermatocyte divisio
GO:0005515//protei	GO:0006355//regulation of transcription, DNA-templated;GO:0007179//tr
GO:0003924//GTPas	GO:0007165//signal transduction;GO:0007186//G-protein coupled recept
GO:0008013//beta-c	GO:0007015//actin filament organization;GO:0007155//cell adhesion;GO:
GO:0000062//fatty-a	GO:0008203//cholesterol metabolic process;GO:0010742//macrophage de
GO:0004842//ubiqui	GO:0006511//ubiquitin-dependent protein catabolic process;GO:0007399
GO:0016407//acetylt	-
GO:0015173//aroma	GO:0006590//thyroid hormone generation;GO:0055085//transmembrane
GO:0016757//transfe	GO:0002121//inter-male aggressive behavior;GO:0042403//thyroid horm
GO:0004657//prolin	GO:0006562//proline catabolic process
-	GO:0001764//neuron migration;GO:0007162//negative regulation of cell a
GO:0000166//nucleo	-
-	-
GO:0005515//protei	-
GO:0003824//catalyt	GO:0001822//kidney development;GO:0006596//polyamine biosynthetic p
GO:0003677//DNA b	GO:0009267//cellular response to starvation;GO:0043066//negative regula
GO:0008113//peptid	-
-	-
GO:0005515//protei	GO:0006355//regulation of transcription, DNA-templated
GO:0005515//protei	GO:0007165//signal transduction
GO:0005515//protei	-
-	-
-	-
GO:0004866//endop	-
GO:0005102//recept	GO:0006898//receptor-mediated endocytosis;GO:0008104//protein locali
-	GO:0043368//positive T cell selection;GO:0043383//negative T cell selectio
GO:0000062//fatty-a	GO:0008203//cholesterol metabolic process;GO:0010742//macrophage de
-	-
GO:0003841//1-acyl	GO:0001676//long-chain fatty acid metabolic process;GO:0006629//lipid
GO:0003677//DNA b	-
GO:0003824//catalyt	GO:0010121//arginine catabolic process to proline via ornithine;GO:0019
-	GO:0006839//mitochondrial transport
-	-

GO:0005515//protei	-
-	-
GO:0035256//G-pro	GO:0003009//skeletal muscle contraction;GO:0007216//G-protein couple
GO:0005247//voltag	GO:0006821//chloride transport;GO:0055085//transmembrane transport
GO:0004252//serine	GO:0006508//proteolysis;GO:0016485//protein processing
GO:0005515//protei	GO:0006749//glutathione metabolic process
GO:0000062//fatty-a	-
GO:0004497//mono	GO:0006805//xenobiotic metabolic process;GO:0008202//steroid metabol
GO:0000287//magn	GO:0006584//catecholamine metabolic process;GO:0016036//cellular resp
-	-
GO:0004639//phosp	GO:0006164//purine nucleotide biosynthetic process;GO:0006189//'de no
GO:0005158//insulin	GO:0001775//cell activation;GO:0007405//neuroblast proliferation;GO:000
GO:0004672//protei	GO:0000002//mitochondrial genome maintenance;GO:0001938//positive
-	-
GO:0004721//phosp	GO:0010801//negative regulation of peptidyl-threonine phosphorylation;
GO:0005515//protei	GO:0007155//cell adhesion;GO:0007156//homophilic cell adhesion via pla
GO:0004867//serine	GO:0007596//blood coagulation
GO:0001968//fibron	GO:0001933//negative regulation of protein phosphorylation;GO:000716
GO:0005515//protei	GO:0006955//immune response;GO:0006956//complement activation
GO:0004672//protei	GO:0006885//regulation of pH;GO:0008286//insulin receptor signaling pa
GO:0003868//4-hyd	GO:0009072//aromatic amino acid family metabolic process
GO:0098640//integri	GO:0007155//cell adhesion;GO:0007229//integrin-mediated signaling pat
GO:0003774//motor	-
GO:0005085//guany	GO:0001934//positive regulation of protein phosphorylation;GO:0002437
GO:0004672//protei	GO:0006468//protein phosphorylation
GO:0046872//metal	GO:0006875//cellular metal ion homeostasis
GO:0061657//UFM1	GO:0071569//protein ufmylation
GO:0000334//3-hyd	GO:0009435//NAD biosynthetic process;GO:0010043//response to zinc io
GO:0005515//protei	GO:0042634//regulation of hair cycle
GO:0008270//zinc ic	GO:0006882//cellular zinc ion homeostasis;GO:0010273//detoxification of
GO:0003824//cataly	GO:0006650//glycerophospholipid metabolic process;GO:0007416//synap
-	GO:0030193//regulation of blood coagulation;GO:0044763//single-organ
GO:0035254//glutan	GO:0001662//behavioral fear response;GO:0006884//cell volume homeos
GO:0016491//oxidor	-
GO:0000064//L-orni	GO:1990575//mitochondrial L-ornithine transmembrane transport
GO:0004860//protei	GO:0006469//negative regulation of protein kinase activity;GO:2000480//
GO:0015297//antipo	GO:0055085//transmembrane transport
-	-
-	-
GO:0005507//coppe	GO:0009308//amine metabolic process;GO:0009445//putrescine metaboli
GO:0022857//transm	GO:0055085//transmembrane transport
GO:0008480//sarcos	GO:1901053//sarcosine catabolic process
GO:0003677//DNA b	GO:0006383//transcription from RNA polymerase III promoter
GO:0004016//adeny	GO:0006171//cAMP biosynthetic process;GO:0007613//memory;GO:0007
GO:0005524//ATP bi	GO:0008203//cholesterol metabolic process;GO:0010033//response to or
GO:0008146//sulfotr	-
-	GO:0010001//glial cell differentiation;GO:0050772//positive regulation of
GO:0016407//acetylt	-
-	-
GO:0016491//oxidor	GO:0007005//mitochondrion organization
-	-
GO:0008009//chemo	GO:0006954//inflammatory response;GO:0006955//immune response;GO
GO:0005515//protei	-
-	-
-	GO:0008154//actin polymerization or depolymerization;GO:0030048//acti
GO:0009374//biotin	-
GO:0004553//hydrol	GO:0005975//carbohydrate metabolic process
-	-

-	-
GO:0016798//hydrol	GO:0008284//positive regulation of cell proliferation;GO:0030198//extrace
GO:0005515//protei	-
GO:0005506//iron io	-
GO:0022857//transm	GO:0055085//transmembrane transport
GO:0003824//catalyt	GO:0006207//'de novo' pyrimidine nucleobase biosynthetic process
GO:0000334//3-hyd	GO:0009435//NAD biosynthetic process;GO:0010043//response to zinc io
GO:0008270//zinc io	GO:0009056//catabolic process;GO:0030855//epithelial cell differentiation
-	GO:0055085//transmembrane transport
GO:0005096//GTPas	GO:0007165//signal transduction;GO:0043547//positive regulation of GTP
GO:0005515//protei	GO:0090084//negative regulation of inclusion body assembly
GO:0004499//N,N-d	-
GO:0005319//lipid tr	GO:0006629//lipid metabolic process;GO:0006642//triglyceride mobilizati
GO:0003924//GTPas	-
-	GO:0006887//exocytosis;GO:0015031//protein transport
GO:0005267//potass	GO:0006813//potassium ion transport;GO:0033198//response to ATP;GO:
-	-
-	-
GO:0005515//protei	GO:0044763//single-organism cellular process
-	-
GO:0003824//catalyt	-
GO:0003924//GTPas	GO:0007165//signal transduction;GO:0007186//G-protein coupled recept
GO:0004601//peroxi	GO:0015671//oxygen transport;GO:0042744//hydrogen peroxide cataboli
-	-
GO:0005085//guany	GO:0006511//ubiquitin-dependent protein catabolic process;GO:0032012
GO:0005290//L-histi	GO:0006867//asparagine transport;GO:0006868//glutamine transport;GO:
GO:0004867//serine	GO:0016525//negative regulation of angiogenesis;GO:0050769//positive r
GO:0004866//endop	-
GO:0001540//beta-a	GO:0001932//regulation of protein phosphorylation;GO:0001935//endoth
GO:0000062//fatty-a	GO:0008203//cholesterol metabolic process;GO:0010742//macrophage de
-	-
GO:0000166//nucleo	-
GO:0008083//growt	-
-	-
GO:0005507//coppe	GO:0009308//amine metabolic process;GO:0009445//putrescine metaboli
GO:0000977//RNA p	GO:0000122//negative regulation of transcription from RNA polymerase I
GO:0016491//oxidor	-
-	-
-	-
-	-
-	-
GO:0000166//nucleo	GO:0006468//protein phosphorylation;GO:0006974//cellular response to I
GO:0016407//acetylt	-
GO:0005085//guany	GO:0007264//small GTPase mediated signal transduction
GO:0005515//protei	-
GO:0003796//lysozy	GO:0008152//metabolic process;GO:0016998//cell wall macromolecule ca
-	-
GO:0004930//G-pro	GO:0002300//CD8-positive, alpha-beta intraepithelial T cell differentiation
-	-
GO:0005290//L-histi	GO:0006867//asparagine transport;GO:0006868//glutamine transport;GO:
GO:0003824//catalyt	GO:0006508//proteolysis;GO:0012501//programmed cell death;GO:00147
GO:0003682//chrom	GO:0015908//fatty acid transport;GO:0043066//negative regulation of apo
GO:0005515//protei	GO:0007165//signal transduction
-	-
GO:0005216//ion ch	GO:0006811//ion transport;GO:0006814//sodium ion transport;GO:005508
GO:0003924//GTPas	-
-	GO:0000463//maturation of LSU-rRNA from tricistronic rRNA transcript (S
GO:0005247//voltag	GO:0006821//chloride transport;GO:0055085//transmembrane transport

GO:0005102//recept	GO:0001541//ovarian follicle development;GO:0001707//mesoderm forma
GO:0004497//mono	-
GO:0022857//transm	GO:0055085//transmembrane transport
GO:0016491//oxidor	GO:0006629//lipid metabolic process;GO:0006631//fatty acid metabolic p
GO:0005515//protei	GO:0042634//regulation of hair cycle
GO:0003810//protei	GO:0018149//peptide cross-linking
GO:0004252//serine	GO:0006508//proteolysis
GO:0042802//identic	GO:1904262//negative regulation of TORC1 signaling
GO:0005085//guany	GO:0001934//positive regulation of protein phosphorylation;GO:0002437
GO:0001786//phosp	GO:0002024//diet induced thermogenesis;GO:0006606//protein import in
GO:0003834//beta-	GO:0001523//retinoid metabolic process;GO:0016121//carotene catabolic
GO:0005158//insulin	GO:0001775//cell activation;GO:0007405//neuroblast proliferation;GO:000
GO:0005085//guany	GO:0007264//small GTPase mediated signal transduction;GO:0030334//re
GO:0004672//protei	GO:0006885//regulation of pH;GO:0008286//insulin receptor signaling pa
GO:0008201//hepari	-
-	-
GO:0005524//ATP bi	GO:0009235//cobalamin metabolic process;GO:0015889//cobalamin trans
GO:0004197//cystei	GO:0002931//response to ischemia;GO:0006508//proteolysis;GO:0006511
GO:0015173//aroma	GO:0006590//thyroid hormone generation;GO:0055085//transmembrane
GO:0003868//4-hyd	GO:0009072//aromatic amino acid family metabolic process
GO:0000287//magne	GO:0006584//catecholamine metabolic process;GO:0016036//cellular res
-	-
-	GO:0007166//cell surface receptor signaling pathway
-	-
GO:0008146//sulfotr	-
GO:0022857//transm	GO:0055085//transmembrane transport
-	-
-	-
-	-
GO:0000978//RNA p	GO:0001649//osteoblast differentiation;GO:0001755//neural crest cell mig
GO:0003924//GTPas	-
GO:0016787//hydrol	-
GO:0004866//endop	-
-	GO:0007286//spermatid development;GO:0048137//spermatocyte divisio
-	-
GO:0005104//fibrob	GO:0000132//establishment of mitotic spindle orientation;GO:0001656//
-	-
GO:0003824//catalyt	GO:0006167//AMP biosynthetic process;GO:0006177//GMP biosynthetic
GO:0004657//prolin	GO:0006562//proline catabolic process
GO:0004672//protei	GO:0001934//positive regulation of protein phosphorylation;GO:0006468
-	GO:0008154//actin polymerization or depolymerization;GO:0030048//acti
-	GO:0007154//cell communication
GO:0061657//UFM1	GO:0071569//protein ufmylation
GO:0003677//DNA	GO:0009267//cellular response to starvation;GO:0043066//negative regul
GO:0000064//L-orni	GO:1990575//mitochondrial L-ornithine transmembrane transport
GO:0003774//motor	-
-	-
-	GO:0007165//signal transduction
GO:0004672//protei	GO:0001501//skeletal system development;GO:0006468//protein phosph
-	GO:0008360//regulation of cell shape
-	-
-	GO:0007286//spermatid development;GO:0048137//spermatocyte divisio
-	-
GO:0000334//3-hyd	GO:0009435//NAD biosynthetic process;GO:0010043//response to zinc io
-	-
GO:0005515//protei	-
GO:0015020//glucur	GO:0006486//protein glycosylation;GO:0015012//heparan sulfate proteog
GO:0005242//inwar	GO:0006813//potassium ion transport

GO:0031072//heat sl	GO:0045944//positive regulation of transcription from RNA polymerase II
-	-
GO:0004499//N,N-d	-
GO:0005546//phosp	GO:0009636//response to toxic substance;GO:0042360//vitamin E metabo
GO:0004553//hydrol	GO:0005975//carbohydrate metabolic process
-	-
-	-
-	-
GO:0016791//phosp	GO:0006024//glycosaminoglycan biosynthetic process;GO:0010909//posit
GO:0005515//protei	GO:0006749//glutathione metabolic process
-	-
GO:0000062//fatty-a	-
-	-
GO:0005085//guany	GO:0006511//ubiquitin-dependent protein catabolic process;GO:0032012
-	GO:0010269//response to selenium ion;GO:0035264//multicellular organis
GO:0008201//hepari	-
GO:0008009//chemo	GO:0006954//inflammatory response;GO:0006955//immune response;GO
-	-
GO:0005158//insulin	GO:0001775//cell activation;GO:0007405//neuroblast proliferation;GO:000
GO:0004497//mono	-
GO:0005524//ATP bi	GO:0000086//G2/M transition of mitotic cell cycle;GO:0045332//phosphol
GO:0004089//carbo	GO:0002009//morphogenesis of an epithelium;GO:0046903//secretion
GO:0004639//phosp	GO:0006164//purine nucleotide biosynthetic process;GO:0006189//'de no
GO:0016407//acetylt	-
GO:0004497//mono	GO:0006805//xenobiotic metabolic process;GO:0008202//steroid metabol
-	-
GO:0005515//protei	GO:0003341//cilium movement;GO:0007368//determination of left/right s
-	-
-	-
-	GO:0001764//neuron migration;GO:0007162//negative regulation of cell a
GO:0005507//coppe	GO:0009308//amine metabolic process;GO:0009445//putrescine metaboli
GO:0003989//acetyl-	GO:0006633//fatty acid biosynthetic process;GO:0044281//small molecule
GO:0004867//serine	GO:0016525//negative regulation of angiogenesis;GO:0050769//positive r
GO:0004866//endop	-
GO:0005524//ATP bi	GO:0008203//cholesterol metabolic process;GO:0010033//response to org
-	-
GO:0004672//protei	GO:0006468//protein phosphorylation
GO:0004721//phosp	GO:0010801//negative regulation of peptidyl-threonine phosphorylation;
GO:0003824//catalyt	GO:0005975//carbohydrate metabolic process
-	GO:0032967//positive regulation of collagen biosynthetic process;GO:004
GO:0005515//protei	GO:0006884//cell volume homeostasis;GO:0007165//signal transduction;
-	GO:0007399//nervous system development
-	GO:0001764//neuron migration;GO:0007162//negative regulation of cell a
GO:0015173//aroma	GO:0006590//thyroid hormone generation;GO:0055085//transmembrane
GO:0008289//lipid b	GO:0006869//lipid transport;GO:0042157//lipoprotein metabolic process
GO:0005515//protei	GO:0007165//signal transduction
GO:0005085//guany	GO:0007264//small GTPase mediated signal transduction;GO:0030334//re
GO:0005515//protei	GO:0001822//kidney development;GO:0002093//auditory receptor cell mo
-	-
GO:0005515//protei	GO:0006570//tyrosine metabolic process;GO:0007613//memory;GO:0008
GO:0046872//metal	GO:0006875//cellular metal ion homeostasis
GO:0005509//calciu	GO:0007611//learning or memory;GO:0007613//memory;GO:0043123//po
GO:0001786//phosp	GO:0002024//diet induced thermogenesis;GO:0006606//protein import in
GO:0005085//guany	GO:0001934//positive regulation of protein phosphorylation;GO:0002437
GO:0008270//zinc io	GO:0006882//cellular zinc ion homeostasis;GO:0010273//detoxification of
-	-
GO:0004866//endop	GO:0010951//negative regulation of endopeptidase activity
GO:0005507//coppe	GO:0009308//amine metabolic process;GO:0009445//putrescine metaboli

GO:0004672//protei	GO:0006885//regulation of pH;GO:0008286//insulin receptor signaling pa
-	-
GO:0008083//growtl	-
-	GO:0000045//autophagosome assembly;GO:0009267//cellular response t
-	-
GO:0004857//enzym	GO:0001525//angiogenesis;GO:0043066//negative regulation of apoptoti
GO:0005102//recept	GO:0001541//ovarian follicle development;GO:0001707//mesoderm form
GO:0004095//carniti	GO:0001676//long-chain fatty acid metabolic process;GO:0006629//lipid
GO:0005158//insulin	GO:0001775//cell activation;GO:0007405//neuroblast proliferation;GO:000
GO:0004047//amino	GO:0019464//glycine decarboxylation via glycine cleavage system
GO:0008289//lipid b	GO:0070508//cholesterol import
GO:0008170//N-me	GO:0001692//histamine metabolic process;GO:0001695//histamine catab
-	-
-	-
GO:0004497//mono	-
-	-
GO:0004867//serine	GO:0010466//negative regulation of peptidase activity;GO:0030195//nega
-	GO:0016567//protein ubiquitination
-	GO:0030389//fructosamine metabolic process;GO:0030855//epithelial cell
-	-
GO:0003713//transc	GO:0007219//Notch signaling pathway;GO:0045944//positive regulation
GO:0003834//beta-	GO:0001523//retinoid metabolic process;GO:0016121//carotene catabolic
GO:0008270//zinc ic	-
-	GO:0007166//cell surface receptor signaling pathway
-	-
-	-
GO:0009374//biotin	-
GO:0005515//protei	-
GO:0005524//ATP b	GO:0000086//G2/M transition of mitotic cell cycle;GO:0045332//phosphol
-	-
-	-
GO:0005506//iron ic	-
GO:0008271//secon	GO:0008272//sulfate transport;GO:0055085//transmembrane transport
GO:0000062//fatty-a	GO:0008203//cholesterol metabolic process;GO:0010742//macrophage de
GO:0004843//thiol-	GO:0035871//protein K11-linked deubiquitination;GO:0044313//protein K
-	GO:0030193//regulation of blood coagulation;GO:0044763//single-organ
-	-
GO:0000287//magn	GO:0006468//protein phosphorylation;GO:0007204//positive regulation o
GO:0005290//L-histi	GO:0006867//asparagine transport;GO:0006868//glutamine transport;GO:
-	GO:0032088//negative regulation of NF-kappaB transcription factor activi
-	-
GO:0005216//ion ch	GO:0006811//ion transport;GO:0006814//sodium ion transport;GO:00550
-	-
GO:0003841//1-acyl	GO:0001819//positive regulation of cytokine production;GO:0006654//ph
-	-
GO:0008146//sulfotr	-
GO:0005515//protei	-
GO:0003779//actin b	-
-	-
-	-
GO:0005507//coppe	GO:0002227//innate immune response in mucosa;GO:0006869//lipid tran
GO:0004465//lipopr	GO:0006629//lipid metabolic process;GO:0006631//fatty acid metabolic p
GO:0030298//recept	GO:0010976//positive regulation of neuron projection development;GO:0
-	GO:0007154//cell communication
GO:0003924//GTPas	-
-	-
GO:0001540//beta-	GO:0001932//regulation of protein phosphorylation;GO:0001935//endoth
GO:0008289//lipid b	GO:0032374//regulation of cholesterol transport

GO:0003779//actin b	GO:0006351//transcription, DNA-templated;GO:0030032//lamellipodium
-	GO:0007286//spermatid development;GO:0048137//spermatocyte divisio
GO:0000166//nucleo	GO:0006563//L-serine metabolic process;GO:0006790//sulfur compound
GO:0009374//biotin	-
GO:0016407//acetylt	-
GO:0003824//catalyt	GO:0006167//AMP biosynthetic process;GO:0006177//GMP biosynthetic p
GO:0004657//prolin	GO:0006562//proline catabolic process
GO:0000977//RNA p	GO:0000122//negative regulation of transcription from RNA polymerase I
-	-
-	GO:0055085//transmembrane transport
-	-
GO:0004222//metall	GO:0000003//reproduction;GO:0006508//proteolysis
GO:0003676//nuclei	GO:0043488//regulation of mRNA stability
-	-
GO:0003834//beta-c	GO:0001523//retinoid metabolic process;GO:0016121//carotene catabolic
GO:0016407//acetylt	-
-	-
GO:0003924//GTPas	GO:0000082//G1/S transition of mitotic cell cycle;GO:0007010//cytoskelet
GO:0005096//GTPas	GO:0007186//G-protein coupled receptor signaling pathway;GO:0043547
GO:0008146//sulfotr	-
-	-
-	-
GO:0004252//serine	GO:0006508//proteolysis
GO:0003810//protei	GO:0018149//peptide cross-linking
-	-
GO:0004497//mono	-
GO:0008201//hepari	-
GO:0000287//magne	GO:0006006//glucose metabolic process;GO:0006094//gluconeogenesis;G
GO:0004867//serine	-
GO:0004970//ionotr	GO:0006811//ion transport;GO:0045471//response to ethanol;GO:009755
-	-
-	-
GO:0005201//extrac	GO:0002244//hematopoietic progenitor cell differentiation
-	-
GO:0003868//4-hyd	GO:0006572//tyrosine catabolic process;GO:0009072//aromatic amino aci
GO:0005290//L-histi	GO:0006867//asparagine transport;GO:0006868//glutamine transport;GO:
GO:0000166//nucleo	GO:0006090//pyruvate metabolic process;GO:0006094//gluconeogenesis
GO:0008270//zinc io	GO:0006882//cellular zinc ion homeostasis;GO:0010273//detoxification of
GO:0005216//ion ch	GO:0006811//ion transport;GO:0006813//potassium ion transport;GO:003
-	-
GO:0003924//GTPas	-
GO:0003989//acetyl-	GO:0006633//fatty acid biosynthetic process;GO:0044281//small molecule
-	-
-	-
-	-
-	GO:0032967//positive regulation of collagen biosynthetic process;GO:004
GO:0005524//ATP bi	GO:0000086//G2/M transition of mitotic cell cycle;GO:0045332//phosphol
GO:0004866//endop	GO:0010951//negative regulation of endopeptidase activity
GO:0005509//calciu	GO:0032571//response to vitamin K;GO:0060348//bone development;GO:
-	-
-	GO:0001764//neuron migration;GO:0007162//negative regulation of cell a
GO:0004639//phosp	GO:0006164//purine nucleotide biosynthetic process;GO:0006189//'de no
GO:0004672//protei	GO:0000002//mitochondrial genome maintenance;GO:0001938//positive
GO:0003779//actin b	GO:0007010//cytoskeleton organization;GO:0008360//regulation of cell sh
GO:0003779//actin b	GO:0006351//transcription, DNA-templated;GO:0030032//lamellipodium
GO:0004047//amino	GO:0019464//glycine decarboxylation via glycine cleavage system
-	-
GO:0004252//serine	GO:0006508//proteolysis

GO:0004044//amido	GO:0006177//GMP biosynthetic process;GO:0006189//'de novo' IMP bios
-	-
GO:0005546//phosp	GO:0009636//response to toxic substance;GO:0042360//vitamin E metabo
GO:0004497//mono	GO:0006805//xenobiotic metabolic process;GO:0008202//steroid metabol
GO:0019904//protei	GO:0050796//regulation of insulin secretion
GO:0004465//lipopr	GO:0006629//lipid metabolic process;GO:0006631//fatty acid metabolic p
GO:0000978//RNA p	GO:0006357//regulation of transcription from RNA polymerase II promote
-	-
-	-
-	-
GO:0008083//growtl	-
GO:0022857//transm	GO:0055085//transmembrane transport
GO:0005515//protei	GO:0010468//regulation of gene expression;GO:0016567//protein ubiquit
GO:0001517//N-ace	GO:0005975//carbohydrate metabolic process;GO:0006044//N-acetylgluc
GO:0004047//amino	GO:0019464//glycine decarboxylation via glycine cleavage system
GO:0005179//hormc	-
GO:0000978//RNA p	GO:0001649//osteoblast differentiation;GO:0001755//neural crest cell mig
GO:0000064//L-orni	GO:1990575//mitochondrial L-ornithine transmembrane transport
GO:0008289//lipid b	GO:0015031//protein transport;GO:0019075//virus maturation;GO:004205
GO:0004730//pseud	GO:0001522//pseudouridine synthesis;GO:0006796//phosphate-containir
GO:0003700//transc	GO:0006355//regulation of transcription, DNA-templated
GO:0030246//carbol	-
GO:0004866//endop	GO:0010951//negative regulation of endopeptidase activity
GO:0001664//G-pro	GO:0007286//spermatid development;GO:0034613//cellular protein locali
-	GO:0009966//regulation of signal transduction;GO:0016055//Wnt signalin
-	-
-	-
GO:0015078//hydro	GO:0030177//positive regulation of Wnt signaling pathway;GO:1902600//
-	-
GO:0004553//hydrol	GO:0005975//carbohydrate metabolic process
GO:0003677//DNA k	GO:0006334//nucleosome assembly;GO:0042742//defense response to ba
GO:0008138//protei	GO:0006355//regulation of transcription, DNA-templated;GO:0006470//p
GO:0005515//protei	GO:0016567//protein ubiquitination;GO:0035556//intracellular signal trans
-	-
GO:0000287//magne	GO:0006584//catecholamine metabolic process;GO:0016036//cellular resp
GO:0004499//N,N-d	-
-	-
GO:0005515//protei	-
GO:0004866//endop	-
-	GO:0008154//actin polymerization or depolymerization;GO:0030048//acti
-	-
GO:0005546//phosp	GO:0009636//response to toxic substance;GO:0042360//vitamin E metabo
GO:0015485//choles	GO:0032367//intracellular cholesterol transport;GO:0034389//lipid particle
GO:0004866//endop	-
GO:0004867//serine	GO:0016525//negative regulation of angiogenesis;GO:0050769//positive r
GO:0016167//glial c	GO:0002023//reduction of food intake in response to dietary excess;GO:0
GO:0005506//iron ic	-
GO:0005085//guany	GO:0006623//protein targeting to vacuole;GO:0009306//protein secretion
GO:0004497//mono	GO:0006559//L-phenylalanine catabolic process;GO:0006571//tyrosine bi
-	GO:0001764//neuron migration;GO:0007162//negative regulation of cell a
GO:0002020//protea	GO:0010466//negative regulation of peptidase activity;GO:0018149//pept
-	GO:0032088//negative regulation of NF-kappaB transcription factor activi
GO:0008009//chemc	GO:0006954//inflammatory response;GO:0006955//immune response;GO
GO:0004465//lipopr	GO:0006629//lipid metabolic process;GO:0006631//fatty acid metabolic p
-	-
GO:0004089//carbor	GO:0002009//morphogenesis of an epithelium;GO:0046903//secretion
GO:0003924//GTPas	-
GO:0003779//actin k	GO:0006351//transcription, DNA-templated;GO:0030032//lamellipodium

GO:0009922//fatty a	GO:0000038//very long-chain fatty acid metabolic process;GO:0006629//l
GO:0022857//transm	GO:0055085//transmembrane transport
GO:0005515//protei	GO:0007155//cell adhesion
GO:0004089//carbor	GO:0002009//morphogenesis of an epithelium;GO:0046903//secretion
GO:0070412//R-SM/	GO:0010991//negative regulation of SMAD protein complex assembly;GO
GO:0002020//protea	GO:0010466//negative regulation of peptidase activity;GO:0018149//pept
GO:0001786//phosp	GO:0006910//phagocytosis, recognition;GO:0006911//phagocytosis, engu
GO:0004478//methic	GO:0006556//S-adenosylmethionine biosynthetic process;GO:0009087//m
GO:0005515//protei	GO:0042509//regulation of tyrosine phosphorylation of STAT protein;GO:
GO:0005515//protei	GO:0010468//regulation of gene expression;GO:0016567//protein ubiquit
-	-
GO:0016627//oxidor	GO:0006629//lipid metabolic process
-	GO:0015888//thiamine transport
GO:0004867//serine	GO:0010466//negative regulation of peptidase activity;GO:0030195//nega
GO:0005509//calciur	GO:0001654//eye development;GO:0045667//regulation of osteoblast diff
-	-
GO:0046872//metal	GO:0006875//cellular metal ion homeostasis
GO:0000246//delta2	GO:0006695//cholesterol biosynthetic process;GO:0006979//response to
GO:0004366//glycer	GO:0001817//regulation of cytokine production;GO:0006631//fatty acid m
GO:0005515//protei	-
-	-
-	-
GO:0003779//actin b	GO:0007010//cytoskeleton organization;GO:0008360//regulation of cell sh
GO:0005085//guany	GO:0007264//small GTPase mediated signal transduction;GO:0030334//re
-	-
-	-
GO:0001786//phosp	GO:0002024//diet induced thermogenesis;GO:0006606//protein import in
GO:0050839//cell ad	GO:0007409//axonogenesis;GO:0010975//regulation of neuron projection
-	-
GO:0003700//transc	GO:0006355//regulation of transcription, DNA-templated
GO:0004497//mono	GO:0097267//omega-hydroxylase P450 pathway
-	-
GO:0003755//peptid	-
GO:0008270//zinc io	-
GO:0004672//protei	GO:0006468//protein phosphorylation
GO:0008195//phosp	GO:0006720//isoprenoid metabolic process;GO:0018342//protein prenyla
GO:0005102//recept	GO:0001541//ovarian follicle development;GO:0001707//mesoderm form
GO:0070089//chloric	GO:0006813//potassium ion transport;GO:0097623//potassium ion export
-	GO:0006895//Golgi to endosome transport;GO:0015031//protein transpor
-	-
GO:0003676//nuclei	-
GO:0003909//DNA li	-
GO:0005515//protei	GO:0007155//cell adhesion;GO:0008593//regulation of Notch signaling pa
GO:0003824//catalyt	GO:0009226//nucleotide-sugar biosynthetic process;GO:0010595//positiv
GO:0005290//L-histi	GO:0006867//asparagine transport;GO:0006868//glutamine transport;GO:
GO:0004867//serine	GO:0016525//negative regulation of angiogenesis;GO:0050769//positive r
GO:0004866//endop	-
GO:0005524//ATP bi	GO:0000086//G2/M transition of mitotic cell cycle;GO:0045332//phosphol
GO:0003868//4-hyd	GO:0006572//tyrosine catabolic process;GO:0009072//aromatic amino aci
GO:0000287//magne	GO:0006584//catecholamine metabolic process;GO:0016036//cellular resp
GO:0015173//aroma	GO:0006590//thyroid hormone generation;GO:0055085//transmembrane
GO:0000062//fatty-a	-
GO:0003824//catalyt	GO:0009436//glyoxylate catabolic process;GO:0019265//glycine biosynthe
GO:0005515//protei	GO:0006749//glutathione metabolic process
-	-
GO:0003779//actin b	GO:0006351//transcription, DNA-templated;GO:0030032//lamellipodium
GO:0000062//fatty-a	GO:0036151//phosphatidylcholine acyl-chain remodeling;GO:1903060//n
GO:0016491//oxidor	-

GO:0005515//protei	-
-	GO:0008360//regulation of cell shape
GO:0003834//beta-c	GO:0001523//retinoid metabolic process;GO:0016121//carotene catabolic
-	-
-	-
GO:0004252//serine	GO:0006508//proteolysis
-	-
GO:0005546//phosp	GO:0009636//response to toxic substance;GO:0042360//vitamin E metabo
-	-
GO:0004465//lipopr	GO:0006629//lipid metabolic process;GO:0006631//fatty acid metabolic p
GO:0003868//4-hyd	GO:0006572//tyrosine catabolic process;GO:0009072//aromatic amino aci
GO:0003924//GTPas	-
GO:0004497//mono	-
GO:0016316//phosp	-
GO:0005515//protei	GO:0030855//epithelial cell differentiation
-	GO:0009966//regulation of signal transduction;GO:0016055//Wnt signalin
GO:0004888//transm	GO:0007186//G-protein coupled receptor signaling pathway;GO:0009994
GO:0005515//protei	-
GO:0000166//nucleo	GO:0006563//L-serine metabolic process;GO:0006790//sulfur compound
-	GO:0007010//cytoskeleton organization;GO:0008360//regulation of cell sh
-	GO:0001558//regulation of cell growth;GO:0008283//cell proliferation;GO
GO:0000254//C-4 m	GO:0001934//positive regulation of protein phosphorylation;GO:0008610/
GO:0003676//nuclei	GO:0043488//regulation of mRNA stability
GO:0008270//zinc io	-
-	GO:0032088//negative regulation of NF-kappaB transcription factor activi
GO:0003824//catalyt	GO:0006167//AMP biosynthetic process;GO:0006177//GMP biosynthetic p
GO:0003707//steroic	GO:0009755//hormone-mediated signaling pathway
GO:0005515//protei	GO:0002091//negative regulation of receptor internalization;GO:0042058/
GO:0005096//GTPas	GO:0007165//signal transduction;GO:0043547//positive regulation of GTP
GO:0004842//ubiqui	GO:0006511//ubiquitin-dependent protein catabolic process;GO:0007399
GO:0005515//protei	GO:0006570//tyrosine metabolic process;GO:0007613//memory;GO:0008
GO:0042802//identic	GO:1904262//negative regulation of TORC1 signaling
GO:0004089//carbor	GO:0002009//morphogenesis of an epithelium;GO:0046903//secretion
GO:1990817//RNA a	GO:0009617//response to bacterium;GO:0030278//regulation of ossificati
-	-
GO:0003700//transc	GO:0006355//regulation of transcription, DNA-templated
GO:0000977//RNA p	GO:0000122//negative regulation of transcription from RNA polymerase I
GO:0004439//phosp	GO:0007420//brain development;GO:0046855//inositol phosphate depho
GO:0005515//protei	GO:0003341//cilium movement;GO:0007368//determination of left/right s
GO:0003847//1-alky	GO:0006629//lipid metabolic process;GO:0006644//phospholipid metabo
GO:0002020//protea	GO:0010466//negative regulation of peptidase activity;GO:0018149//pept
GO:0003810//protei	GO:0018149//peptide cross-linking
GO:0005515//protei	GO:0007155//cell adhesion
GO:0005324//long-c	GO:0006469//negative regulation of protein kinase activity;GO:0009617//r
GO:0004843//thiol-c	GO:0035871//protein K11-linked deubiquitination;GO:0044313//protein K
-	-
GO:0005515//protei	-
GO:0001540//beta-a	GO:0001932//regulation of protein phosphorylation;GO:0001935//endoth
GO:0005515//protei	-
-	-
GO:0003713//transc	GO:0007219//Notch signaling pathway;GO:0045944//positive regulation c
GO:0005546//phosp	GO:0009636//response to toxic substance;GO:0042360//vitamin E metabo
GO:0000978//RNA p	GO:0001649//osteoblast differentiation;GO:0001755//neural crest cell mig
-	-
GO:0016491//oxidor	GO:0019477//L-lysine catabolic process
GO:0003824//catalyt	GO:0010121//arginine catabolic process to proline via ornithine;GO:0019
GO:0005515//protei	GO:0015879//carnitine transport;GO:0032414//positive regulation of ion t
GO:0035254//glutan	GO:0001662//behavioral fear response;GO:0006884//cell volume homeos

GO:0035254//glutan	GO:0001662//behavioral fear response;GO:0006884//cell volume homeos
-	-
GO:0004639//phosp	GO:0006164//purine nucleotide biosynthetic process;GO:0006189//'de no
GO:0004478//methio	GO:0006556//S-adenosylmethionine biosynthetic process;GO:0009087//m
GO:0004114//3',5'-c	GO:0006198//cAMP catabolic process;GO:0007165//signal transduction
GO:0005104//fibrob	GO:0000132//establishment of mitotic spindle orientation;GO:0001656//m
GO:0004103//cholin	GO:0006646//phosphatidylethanolamine biosynthetic process;GO:000665
-	-
GO:0005515//protei	GO:0003341//cilium movement;GO:0007368//determination of left/right s
GO:0005201//extrac	GO:0002244//hematopoietic progenitor cell differentiation
GO:0005515//protei	-
GO:0005509//calciur	GO:0007155//cell adhesion
GO:0003779//actin b	GO:0030835//negative regulation of actin filament depolymerization
GO:0004044//amido	GO:0006177//GMP biosynthetic process;GO:0006189//'de novo' IMP bios
GO:0005520//insulin	GO:0042104//positive regulation of activated T cell proliferation
GO:0004553//hydrol	GO:0005975//carbohydrate metabolic process
GO:0005042//netrin	GO:0007165//signal transduction;GO:0014068//positive regulation of pho
GO:0004672//protei	GO:0000002//mitochondrial genome maintenance;GO:0001938//positive
GO:0004866//endop	-
GO:0002020//protea	GO:0010466//negative regulation of peptidase activity;GO:0018149//pept
GO:0000977//RNA p	GO:0000122//negative regulation of transcription from RNA polymerase I
GO:0003700//transc	GO:0006355//regulation of transcription, DNA-templated
GO:0004930//G-pro	GO:0002300//CD8-positive, alpha-beta intraepithelial T cell differentiation
GO:0005515//protei	GO:0007015//actin filament organization;GO:0045806//negative regulatio
-	-
GO:0016791//phosp	GO:0006024//glycosaminoglycan biosynthetic process;GO:0010909//posit
GO:0022857//transm	GO:0055085//transmembrane transport
-	-
-	-
-	-
GO:0004047//amino	GO:0019464//glycine decarboxylation via glycine cleavage system
GO:0004867//serine	GO:0007596//blood coagulation
-	-
GO:0000978//RNA p	GO:0006357//regulation of transcription from RNA polymerase II promote
-	GO:0030193//regulation of blood coagulation;GO:0044763//single-organ
GO:0004657//prolin	GO:0006562//proline catabolic process
-	-
GO:0015485//choles	GO:0009615//response to virus;GO:0030301//cholesterol transport;GO:00
GO:0004866//endop	GO:0010951//negative regulation of endopeptidase activity
-	-
GO:0004672//protei	GO:0006468//protein phosphorylation
GO:0015485//choles	GO:0032367//intracellular cholesterol transport;GO:0034389//lipid particle
GO:0005524//ATP bi	GO:0000086//G2/M transition of mitotic cell cycle;GO:0045332//phosphol
GO:0004866//endop	-
GO:0004867//serine	GO:0016525//negative regulation of angiogenesis;GO:0050769//positive r
GO:0003676//nuclei	GO:0009987//cellular process;GO:0044699//single-organism process
-	-
GO:0004497//monoo	GO:0006559//L-phenylalanine catabolic process;GO:0006571//tyrosine bi
GO:0003779//actin b	-
-	GO:0008154//actin polymerization or depolymerization;GO:0030048//acti
GO:0008028//monoc	GO:0015718//monocarboxylic acid transport;GO:0055085//transmembran
-	-
GO:0005515//protei	-
-	-
GO:0005515//protei	GO:0051017//actin filament bundle assembly
GO:0017080//sodiur	GO:0006814//sodium ion transport;GO:0010460//positive regulation of he
GO:0003824//catalyt	GO:0006567//threonine catabolic process
-	-

GO:0004721//phosp	GO:0010801//negative regulation of peptidyl-threonine phosphorylation;
GO:0070412//R-SM/	GO:0010991//negative regulation of SMAD protein complex assembly;GO
GO:0008146//sulfotr	-
-	-
GO:0005515//protei	-
GO:0005515//protei	GO:0007612//learning;GO:0007613//memory;GO:1900452//regulation of
GO:0005515//protei	GO:0001667//ameboidal-type cell migration;GO:0007498//mesoderm dev
GO:0004122//cystatl	GO:0001958//endochondral ossification;GO:0001974//blood vessel remoc
GO:0003868//4-hyd	GO:0006572//tyrosine catabolic process;GO:0009072//aromatic amino aci
GO:0015173//aroma	GO:0006590//thyroid hormone generation;GO:0055085//transmembrane
GO:0005227//calciur	GO:0001581//detection of chemical stimulus involved in sensory perceptic
GO:0022857//transm	GO:0055085//transmembrane transport
GO:0001605//adrenc	GO:0001525//angiogenesis;GO:0001570//vasculogenesis;GO:0002040//sp
-	GO:0070453//regulation of heme biosynthetic process
GO:0000062//fatty-a	-
GO:0005179//hormc	-
GO:0005515//protei	GO:0006749//glutathione metabolic process
-	-
GO:0004672//protei	GO:0000086//G2/M transition of mitotic cell cycle;GO:0000278//mitotic ce
GO:0016407//acetylt	-
GO:0000977//RNA p	GO:0000122//negative regulation of transcription from RNA polymerase I
-	-
GO:0005515//protei	-
-	GO:0032088//negative regulation of NF-kappaB transcription factor activi
GO:0004465//lipopr	GO:0006629//lipid metabolic process;GO:0006631//fatty acid metabolic p
GO:0004364//glutatl	GO:0006749//glutathione metabolic process;GO:0006805//xenobiotic met
GO:0005520//insulin	GO:0042104//positive regulation of activated T cell proliferation
GO:0005515//protei	GO:0002091//negative regulation of receptor internalization;GO:0042058/
GO:0016740//transfe	-
GO:0003779//actin b	GO:0006351//transcription, DNA-templated;GO:0030032//lamellipodium
GO:0008194//UDP-g	GO:0006486//protein glycosylation;GO:0006682//galactosylceramide bios
GO:0001540//beta-a	GO:0001932//regulation of protein phosphorylation;GO:0001935//endoth
GO:0008146//sulfotr	-
GO:0005515//protei	-
GO:0004439//phosp	GO:0007420//brain development;GO:0046855//inositol phosphate depho
-	GO:0006506//GPI anchor biosynthetic process
-	-
GO:0005515//protei	GO:0051017//actin filament bundle assembly
GO:0016491//oxidor	-
-	-
-	-
GO:0005515//protei	-
-	-
GO:0008195//phosp	GO:0006796//phosphate-containing compound metabolic process;GO:00
GO:0070089//chloric	GO:0006813//potassium ion transport;GO:0097623//potassium ion export
-	-
GO:0004930//G-pro	GO:0002300//CD8-positive, alpha-beta intraepithelial T cell differentiatior
GO:0004478//methic	GO:0006556//S-adenosylmethionine biosynthetic process;GO:0009087//n
GO:0003824//catalyt	GO:0006103//2-oxoglutarate metabolic process;GO:0009058//biosyntheti
-	-
-	-
GO:0003824//catalyt	GO:0006167//AMP biosynthetic process;GO:0006177//GMP biosynthetic p
GO:0035254//glutan	GO:0001662//behavioral fear response;GO:0006884//cell volume homeos
GO:0000977//RNA p	GO:0007265//Ras protein signal transduction;GO:0045944//positive regula
GO:0000253//3-ketc	GO:0006695//cholesterol biosynthetic process;GO:0006703//estrogen bio
GO:0005515//protei	GO:0007612//learning;GO:0007613//memory;GO:1900452//regulation of
GO:0008270//zinc ic	-
-	-

GO:0051015//actin f	GO:0030866//cortical actin cytoskeleton organization;GO:0045214//sarcor
GO:0022857//transm	GO:0055085//transmembrane transport
-	-
GO:0003924//GTPas	-
-	-
GO:0000287//magne	GO:0006584//catecholamine metabolic process;GO:0016036//cellular resp
GO:0003924//GTPas	-
GO:0005515//protei	GO:0051260//protein homooligomerization
GO:0003824//catalyt	GO:0006508//proteolysis;GO:0012501//programmed cell death;GO:00147
GO:0005515//protei	GO:0042634//regulation of hair cycle
GO:0003868//4-hyd	GO:0006572//tyrosine catabolic process;GO:0009072//aromatic amino aci
-	-
GO:0004639//phosp	GO:0006164//purine nucleotide biosynthetic process;GO:0006189//'de no
-	-
-	GO:0043065//positive regulation of apoptotic process;GO:0046330//posit
-	-
GO:0003824//catalyt	GO:0006567//threonine catabolic process
-	-
GO:0005515//protei	GO:0006884//cell volume homeostasis;GO:0007165//signal transduction;G
GO:0004888//transm	GO:0007166//cell surface receptor signaling pathway;GO:0007186//G-prc
-	GO:0007166//cell surface receptor signaling pathway
GO:0004197//cystei	GO:0002931//response to ischemia;GO:0006508//proteolysis;GO:0006511
GO:0005515//protei	GO:0000281//mitotic cytokinesis;GO:0007019//microtubule depolymeriza
GO:0005096//GTPas	GO:0007165//signal transduction;GO:0043547//positive regulation of GTP
GO:0004930//G-pro	GO:0002305//CD8-positive, gamma-delta intraepithelial T cell differentiat
-	-
-	-
-	-
GO:0005515//protei	GO:0003431//growth plate cartilage chondrocyte development;GO:00082
GO:0000064//L-orni	GO:1990575//mitochondrial L-ornithine transmembrane transport
-	-
-	-
GO:0000166//nucleo	GO:0006090//pyruvate metabolic process;GO:0006094//gluconeogenesis
-	-
-	GO:0006888//ER to Golgi vesicle-mediated transport;GO:0006914//autop
GO:0004497//mono	GO:0097267//omega-hydroxylase P450 pathway
GO:0000334//3-hyd	GO:0009435//NAD biosynthetic process;GO:0010043//response to zinc io
GO:0003755//peptid	-
GO:0004420//hydro	GO:0006695//cholesterol biosynthetic process;GO:0008299//isoprenoid b
-	-
GO:0003779//actin b	GO:0007030//Golgi organization;GO:0007032//endosome organization;G
-	GO:0007010//cytoskeleton organization;GO:0008360//regulation of cell sh
GO:0003677//DNA b	GO:0009646//response to absence of light;GO:0018298//protein-chromo
-	-
-	-
-	-
GO:0004930//G-pro	GO:0002300//CD8-positive, alpha-beta intraepithelial T cell differentiation
-	-
-	-
GO:0008113//peptid	-
GO:0004725//protei	GO:0016311//dephosphorylation;GO:0046855//inositol phosphate depho
GO:0004721//phosp	GO:0010801//negative regulation of peptidyl-threonine phosphorylation;
GO:0005515//protei	GO:0003341//cilium movement;GO:0044782//cilium organization
GO:0004364//glutat	GO:0006749//glutathione metabolic process;GO:0006805//xenobiotic met
GO:0005125//cytoki	GO:0002232//leukocyte chemotaxis involved in inflammatory response;GC
GO:0005515//protei	GO:0007165//signal transduction
GO:0004842//ubiqui	GO:0006511//ubiquitin-dependent protein catabolic process;GO:0007399
GO:0003841//1-acyl	-

GO:0004657//prolin	GO:0006562//proline catabolic process
-	-
GO:0005515//protei	-
GO:0003700//transc	GO:0006355//regulation of transcription, DNA-templated
GO:0003909//DNA li	-
GO:0005267//potass	GO:0006813//potassium ion transport;GO:0033198//response to ATP;GO:
GO:0061657//UFM1	GO:0071569//protein ufmylation
GO:0004842//ubiqui	GO:0006511//ubiquitin-dependent protein catabolic process;GO:0007399
GO:0004497//mono	GO:0006805//xenobiotic metabolic process;GO:0008202//steroid metabol
GO:0003774//motor	-
GO:0003847//1-alky	GO:0006629//lipid metabolic process;GO:0006644//phospholipid metabo
GO:0004896//cytoki	GO:0008284//positive regulation of cell proliferation;GO:0019221//cytokir
-	-
GO:0005515//protei	-
-	GO:0008154//actin polymerization or depolymerization;GO:0030048//acti
GO:0000250//lanost	GO:0006694//steroid biosynthetic process;GO:0006695//cholesterol biosy
GO:0005515//protei	-
GO:0001605//adren	GO:0001525//angiogenesis;GO:0001570//vasculogenesis;GO:0002040//sp
-	-
GO:0003824//cataly	GO:0010121//arginine catabolic process to proline via ornithine;GO:0019
GO:0000287//magn	GO:0000226//microtubule cytoskeleton organization;GO:0006468//protei
-	-
-	GO:0032088//negative regulation of NF-kappaB transcription factor activi
-	-
GO:0008146//sulfotr	-
-	GO:0007275//multicellular organism development;GO:0030154//cell differ
GO:0004252//serine	GO:0006508//proteolysis
GO:0005515//protei	GO:0007165//signal transduction
GO:0005515//protei	GO:0006749//glutathione metabolic process
GO:0000062//fatty-a	-
GO:0016316//phosp	-
-	-
GO:0005515//protei	GO:0007155//cell adhesion;GO:0007156//homophilic cell adhesion via pla
GO:0008270//zinc io	GO:0006882//cellular zinc ion homeostasis;GO:0010273//detoxification of
-	-
GO:0098640//integri	GO:0007155//cell adhesion;GO:0007229//integrin-mediated signaling pat
GO:0000978//RNA p	GO:0001649//osteoblast differentiation;GO:0001755//neural crest cell mig
GO:0016627//oxidor	GO:0006629//lipid metabolic process
GO:0005515//protei	GO:0042509//regulation of tyrosine phosphorylation of STAT protein;GO:
GO:0000334//3-hyd	GO:0009435//NAD biosynthetic process;GO:0010043//response to zinc io
-	GO:0009987//cellular process
-	-
GO:0003676//nuclei	GO:0006644//phospholipid metabolic process;GO:0006935//chemotaxis;G
GO:0002020//protea	GO:0010466//negative regulation of peptidase activity;GO:0018149//pept
-	-
-	-
GO:0008028//mono	GO:0015718//monocarboxylic acid transport;GO:0055085//transmembran
GO:0004672//protei	GO:0006468//protein phosphorylation
GO:0004721//phosp	GO:0010801//negative regulation of peptidyl-threonine phosphorylation;
GO:0008270//zinc io	-
GO:0005104//fibrob	GO:0001755//neural crest cell migration;GO:0001934//positive regulation
-	GO:0007166//cell surface receptor signaling pathway
-	-
GO:0000254//C-4 m	GO:0001934//positive regulation of protein phosphorylation;GO:0008610/
GO:0009374//biotin	-
GO:0003824//cataly	GO:0006167//AMP biosynthetic process;GO:0006177//GMP biosynthetic p
GO:0008480//sarcos	GO:1901053//sarcosine catabolic process
-	GO:0030193//regulation of blood coagulation;GO:0044763//single-organ

GO:0003824//catalyt	GO:0006103//2-oxoglutarate metabolic process;GO:0009058//biosyntheti
GO:0016491//oxidor	GO:0019477//L-lysine catabolic process
GO:0022857//transm	GO:0055085//transmembrane transport
GO:0002020//protea	GO:0001932//regulation of protein phosphorylation;GO:0007044//cell-sul
GO:0000166//nucleo	GO:0006468//protein phosphorylation;GO:0010976//positive regulation o
-	GO:0043065//positive regulation of apoptotic process;GO:0046330//posit
GO:0000248//C-5 st	GO:0008610//lipid biosynthetic process;GO:0016126//sterol biosynthetic p
GO:0004843//thiol-c	GO:0006511//ubiquitin-dependent protein catabolic process;GO:0016579
GO:0003924//GTPas	-
GO:0015267//chann	GO:0002931//response to ischemia;GO:0006812//cation transport;GO:003
GO:0000064//L-orni	GO:1990575//mitochondrial L-ornithine transmembrane transport
-	-
GO:0004896//cytoki	GO:0008284//positive regulation of cell proliferation;GO:0019221//cytokir
-	-
-	GO:0006513//protein monoubiquitination;GO:0036297//interstrand cross-
GO:0005515//protei	-
-	-
GO:0008237//metall	GO:0006508//proteolysis
GO:0008270//zinc io	GO:0009056//catabolic process;GO:0030855//epithelial cell differentiation
-	-
GO:0050839//cell ad	GO:0007409//axonogenesis;GO:0010975//regulation of neuron projection
GO:0005102//recept	GO:0006898//receptor-mediated endocytosis;GO:0008104//protein locali
-	-
GO:0003796//lysozy	GO:0008152//metabolic process;GO:0016998//cell wall macromolecule ca
GO:0005515//protei	-
GO:0004867//serine	GO:0007596//blood coagulation
GO:0004090//carbor	GO:0006805//xenobiotic metabolic process;GO:0030855//epithelial cell di
GO:0005068//transm	GO:0008284//positive regulation of cell proliferation;GO:0030316//osteoc
-	GO:0001558//regulation of cell growth;GO:0008283//cell proliferation;GO
-	-
-	GO:0007042//lysosomal lumen acidification;GO:0042176//regulation of pr
GO:0005506//iron io	-
GO:0008270//zinc io	-
GO:0003924//GTPas	-
-	-
GO:0004930//G-pro	GO:0007186//G-protein coupled receptor signaling pathway;GO:0043408
GO:0008113//peptid	-
GO:0005515//protei	GO:0007155//cell adhesion;GO:0008593//regulation of Notch signaling pa
-	GO:0006888//ER to Golgi vesicle-mediated transport;GO:0006914//autop
GO:0003677//DNA b	GO:0006355//regulation of transcription, DNA-templated;GO:0045944//p
GO:0003677//DNA b	GO:0009267//cellular response to starvation;GO:0043066//negative regula
-	GO:0006895//Golgi to endosome transport;GO:0015031//protein transpor
-	GO:0007166//cell surface receptor signaling pathway
GO:0003824//catalyt	GO:0006167//AMP biosynthetic process;GO:0006177//GMP biosynthetic p
GO:0015377//cation	GO:0006811//ion transport;GO:0006884//cell volume homeostasis;GO:005
GO:0005216//ion ch	GO:0006811//ion transport;GO:0006812//cation transport;GO:0034220//io
-	-
-	-
GO:0004725//protei	GO:0016311//dephosphorylation;GO:0046855//inositol phosphate depho
GO:0003824//catalyt	GO:0006103//2-oxoglutarate metabolic process;GO:0009058//biosyntheti
GO:0008270//zinc io	GO:0009056//catabolic process;GO:0030855//epithelial cell differentiation
GO:0004497//mono	GO:0097267//omega-hydroxylase P450 pathway
GO:0003841//1-acyl	GO:0001676//long-chain fatty acid metabolic process;GO:0006629//lipid
-	-
GO:0004497//mono	-
GO:0003841//1-acyl	-
GO:0005515//protei	-
GO:0005515//protei	-

GO:0008146//sulfotr	-
GO:0003847//1-alky	GO:0006629//lipid metabolic process;GO:0006644//phospholipid metabo
GO:0004672//protei	GO:0006468//protein phosphorylation
GO:0000976//transc	GO:0000122//negative regulation of transcription from RNA polymerase I
GO:0003676//nuclei	GO:0009987//cellular process;GO:0044699//single-organism process
GO:0003824//catalyt	GO:0010121//arginine catabolic process to proline via ornithine;GO:0019
GO:0008083//growt	-
GO:0046790//virion	GO:0075512//clathrin-mediated endocytosis of virus by host cell
-	-
GO:0004497//mono	GO:0006805//xenobiotic metabolic process;GO:0008202//steroid metabol
GO:0001540//beta-	GO:0001932//regulation of protein phosphorylation;GO:0001935//endoth
-	-
GO:0008480//sarcos	GO:1901053//sarcosine catabolic process
GO:0004842//ubiqui	GO:0006511//ubiquitin-dependent protein catabolic process;GO:0007399
GO:0003697//single-	-
GO:0003677//DNA	GO:0035871//protein K11-linked deubiquitination
-	GO:0015888//thiamine transport
GO:0005515//protei	GO:0007155//cell adhesion;GO:0007156//homophilic cell adhesion via pla
GO:0003774//motor	-
GO:0005515//protei	GO:0007165//signal transduction
GO:0061657//UFM1	GO:0071569//protein ufmylation
-	-
-	-
GO:0098640//integri	GO:0007155//cell adhesion;GO:0007229//integrin-mediated signaling pat
GO:0000977//RNA p	GO:0000122//negative regulation of transcription from RNA polymerase I
-	-
-	-
GO:0004122//cystat	GO:0001958//endochondral ossification;GO:0001974//blood vessel remo
GO:0017080//sodiu	GO:0006814//sodium ion transport;GO:0010460//positive regulation of he
GO:0016407//acetyl	-
GO:0000062//fatty-	-
GO:0005515//protei	GO:0006355//regulation of transcription, DNA-templated;GO:0007179//tr
GO:0005515//protei	GO:0006749//glutathione metabolic process
-	-
-	GO:0001501//skeletal system development;GO:0006006//glucose metabo
GO:0004930//G-pro	GO:0002300//CD8-positive, alpha-beta intraepithelial T cell differentiatio
GO:0000978//RNA p	GO:0001649//osteoblast differentiation;GO:0001755//neural crest cell mig
GO:0004252//serine	GO:0006508//proteolysis
GO:0000166//nucleo	GO:0007165//signal transduction;GO:0007186//G-protein coupled recept
-	-
GO:0003676//nuclei	-
GO:0008270//zinc io	GO:0006882//cellular zinc ion homeostasis;GO:0010273//detoxification of
GO:0004672//protei	GO:0000002//mitochondrial genome maintenance;GO:0001938//positive
GO:0004364//glutat	GO:0006749//glutathione metabolic process;GO:0006805//xenobiotic me
GO:0003988//acetyl-	GO:0000038//very long-chain fatty acid metabolic process;GO:0006635//
GO:0098640//integri	GO:0007155//cell adhesion;GO:0007229//integrin-mediated signaling pat
-	GO:0007010//cytoskeleton organization;GO:0008360//regulation of cell s
GO:0008201//hepari	-
GO:0016791//phosp	GO:0006024//glycosaminoglycan biosynthetic process;GO:0010909//posit
GO:0001758//retinal	GO:0002072//optic cup morphogenesis involved in camera-type eye deve
GO:0016316//phosp	-
GO:0005515//protei	GO:0007155//cell adhesion;GO:0007156//homophilic cell adhesion via pla
-	GO:0006513//protein monoubiquitination;GO:0036297//interstrand cross-
GO:0022857//transm	GO:0055085//transmembrane transport
-	-
GO:0008146//sulfotr	GO:0006805//xenobiotic metabolic process;GO:0042403//thyroid hormon
GO:0008168//methy	GO:0032259//methylation;GO:0035498//carnosine metabolic process
GO:0008480//sarcos	GO:1901053//sarcosine catabolic process

GO:0004672//protei	GO:0006885//regulation of pH;GO:0008286//insulin receptor signaling pa
-	-
-	-
-	-
GO:0005515//protei	GO:0090084//negative regulation of inclusion body assembly
GO:0005515//protei	GO:0035987//endodermal cell differentiation
GO:0009374//biotin	-
-	-
GO:0005515//protei	-
GO:0005515//protei	GO:0015879//carnitine transport;GO:0032414//positive regulation of ion t
-	-
GO:0000334//3-hyd	GO:0009435//NAD biosynthetic process;GO:0010043//response to zinc io
-	GO:0007154//cell communication
GO:0004842//ubiqui	GO:0006511//ubiquitin-dependent protein catabolic process;GO:0016567
GO:0008195//phosp	GO:0006796//phosphate-containing compound metabolic process;GO:00
-	GO:0007286//spermatid development;GO:0048137//spermatocyte divisio
GO:0008270//zinc ic	GO:0009056//catabolic process;GO:0030855//epithelial cell differentiation
-	-
-	-
GO:0000334//3-hyd	GO:0009435//NAD biosynthetic process;GO:0010043//response to zinc io
GO:0008270//zinc ic	-
-	-
GO:0003847//1-alky	GO:0016042//lipid catabolic process
GO:0000064//L-orni	GO:1990575//mitochondrial L-ornithine transmembrane transport
GO:0005515//protei	GO:0003431//growth plate cartilage chondrocyte development;GO:00082
GO:0005515//protei	-
-	-
GO:0005102//recept	GO:0006898//receptor-mediated endocytosis;GO:0008104//protein locali
-	-
GO:0005515//protei	-
GO:0005515//protei	GO:0051017//actin filament bundle assembly
GO:0016491//oxidor	GO:0019477//L-lysine catabolic process
GO:0070089//chloric	GO:0006813//potassium ion transport;GO:0097623//potassium ion export
GO:0008113//peptid	-
-	-
-	GO:0007165//signal transduction
GO:1990817//RNA a	GO:0009617//response to bacterium;GO:0030278//regulation of ossificati
GO:0008146//sulfotr	-
GO:0030246//carbol	-
GO:0005509//calciur	GO:0032571//response to vitamin K;GO:0060348//bone development;GO:
-	-
GO:0005096//GTPas	GO:0007165//signal transduction;GO:0043547//positive regulation of GTP
-	GO:0017015//regulation of transforming growth factor beta receptor sign
GO:0004930//G-pro	GO:0002305//CD8-positive, gamma-delta intraepithelial T cell differentiat
GO:0004930//G-pro	GO:0007186//G-protein coupled receptor signaling pathway;GO:0043408
-	-
GO:0004310//farnes	GO:0008610//lipid biosynthetic process;GO:0009058//biosynthetic proces
GO:0003676//nuclei	GO:0006644//phospholipid metabolic process;GO:0006935//chemotaxis;G
-	-
-	-
-	GO:0044237//cellular metabolic process
GO:0015171//amino	GO:0006641//triglyceride metabolic process;GO:0009437//carnitine metal
GO:0015377//cation	GO:0006811//ion transport;GO:0006884//cell volume homeostasis;GO:005
GO:0003824//catalyt	GO:0010121//arginine catabolic process to proline via ornithine;GO:0019
GO:0005515//protei	GO:0030855//epithelial cell differentiation
GO:0002161//amino	-
GO:0000287//magn	GO:0006099//tricarboxylic acid cycle
GO:0005518//collag	GO:0010952//positive regulation of peptidase activity

-	-
GO:0004930//G-pro	GO:0002300//CD8-positive, alpha-beta intraepithelial T cell differentiatior
-	-
-	-
GO:0005515//protei	GO:0007165//signal transduction
GO:0005515//protei	GO:0006749//glutathione metabolic process
GO:0030298//recept	GO:0010976//positive regulation of neuron projection development;GO:0
GO:0005085//guany	GO:0007264//small GTPase mediated signal transduction;GO:0030334//re
GO:0000062//fatty-a	-
GO:0005158//insulin	GO:0001775//cell activation;GO:0007405//neuroblast proliferation;GO:000
-	-
GO:0002020//protea	GO:0001932//regulation of protein phosphorylation;GO:0007044//cell-sul
GO:0001786//phosp	GO:0002024//diet induced thermogenesis;GO:0006606//protein import in
GO:0008270//zinc ic	GO:0009056//catabolic process;GO:0030855//epithelial cell differentiation
GO:0003796//lysozy	GO:0009253//peptidoglycan catabolic process;GO:0016998//cell wall mac
GO:0005515//protei	-
-	-
-	GO:0021817//nucleokinesis involved in cell motility in cerebral cortex radi
GO:0004089//carbor	GO:0002009//morphogenesis of an epithelium;GO:0046903//secretion
GO:0004721//phosp	GO:0010801//negative regulation of peptidyl-threonine phosphorylation;
GO:0005102//recept	GO:0001541//ovarian follicle development;GO:0001707//mesoderm forma
GO:0003924//GTPas	-
GO:0004090//carbor	GO:0006805//xenobiotic metabolic process;GO:0030855//epithelial cell di
GO:0008146//sulfotr	-
-	GO:0010001//glial cell differentiation;GO:0050772//positive regulation of
GO:0005102//recept	GO:0001541//ovarian follicle development;GO:0001707//mesoderm forma
GO:0005515//protei	GO:0006355//regulation of transcription, DNA-templated;GO:0007179//tr
-	-
-	GO:0007010//cytoskeleton organization;GO:0008360//regulation of cell sh
GO:0008480//sarcos	GO:1901053//sarcosine catabolic process
GO:0004497//monoo	GO:0006559//L-phenylalanine catabolic process;GO:0006571//tyrosine bi
GO:0005515//protei	GO:0007165//signal transduction
GO:0004672//protei	GO:0006468//protein phosphorylation
GO:0016407//acetylt	-
-	-
GO:0015485//choles	GO:0032367//intracellular cholesterol transport;GO:0034389//lipid particle
GO:0003824//catalyt	-
-	GO:0030070//insulin processing;GO:0060628//regulation of ER to Golgi ve
-	-
-	-
-	GO:0007166//cell surface receptor signaling pathway
GO:0005507//coppe	GO:0009308//amine metabolic process;GO:0009445//putrescine metaboli
-	-
GO:0001786//phosp	GO:0002024//diet induced thermogenesis;GO:0006606//protein import in
GO:0003868//4-hyd	GO:0006572//tyrosine catabolic process;GO:0009072//aromatic amino aci
-	-
GO:0005085//guany	GO:0007264//small GTPase mediated signal transduction;GO:0030334//re
GO:0005353//fructo	GO:0003044//regulation of systemic arterial blood pressure mediated by a
GO:0004122//cystatl	GO:0001958//endochondral ossification;GO:0001974//blood vessel remoc
GO:0004340//glucok	GO:0001678//cellular glucose homeostasis;GO:0005975//carbohydrate me
-	-
-	-
-	-
GO:0003676//nuclei	GO:0015074//DNA integration;GO:0044260//cellular macromolecule meta
GO:0005085//guany	GO:0000281//mitotic cytokinesis;GO:0000902//cell morphogenesis;GO:00
GO:0008168//methy	GO:0032259//methylation;GO:0035498//carnosine metabolic process
GO:0005158//insulin	GO:0007165//signal transduction;GO:0010467//gene expression;GO:0030
GO:0098640//integri	GO:0007155//cell adhesion;GO:0007229//integrin-mediated signaling pat

GO:0004672//protei	GO:0000002//mitochondrial genome maintenance;GO:0001938//positive
GO:0004364//glutat	GO:0006749//glutathione metabolic process;GO:0006805//xenobiotic me
-	-
-	-
GO:0005515//protei	GO:0007155//cell adhesion;GO:0007156//homophilic cell adhesion via pla
GO:0003841//1-acyl	GO:0006629//lipid metabolic process;GO:0006644//phospholipid metabo
GO:0005515//protei	-
GO:0004867//serine	GO:0007596//blood coagulation
-	-
GO:0005524//ATP bi	GO:0000086//G2/M transition of mitotic cell cycle;GO:0045332//phosphol
GO:0005515//protei	GO:0035987//endodermal cell differentiation
GO:0008138//protei	-
GO:0004044//amido	GO:0006177//GMP biosynthetic process;GO:0006189//'de novo' IMP bios
-	-
-	-
GO:0003796//lysozy	GO:0008152//metabolic process;GO:0016998//cell wall macromolecule ca
GO:0008146//sulfotr	-
GO:0008289//lipid b	-
-	GO:0007166//cell surface receptor signaling pathway
GO:0070089//chlori	GO:0006813//potassium ion transport;GO:0097623//potassium ion export
GO:0022857//transm	GO:0055085//transmembrane transport
-	-
GO:0004366//glycer	GO:0001817//regulation of cytokine production;GO:0006631//fatty acid m
-	-
GO:0005509//calciu	GO:0001654//eye development;GO:0045667//regulation of osteoblast diff
GO:0002020//protea	GO:0010466//negative regulation of peptidase activity;GO:0018149//pept
GO:0003700//transc	GO:0006355//regulation of transcription, DNA-templated
GO:0005506//iron ic	GO:0001666//response to hypoxia;GO:0017185//peptidyl-lysine hydroxyla
-	-
GO:0003847//1-alky	GO:0006629//lipid metabolic process;GO:0006644//phospholipid metabo
GO:0008195//phosp	GO:0006796//phosphate-containing compound metabolic process;GO:00
GO:0004672//protei	GO:0006885//regulation of pH;GO:0008286//insulin receptor signaling pa
-	-
-	-
GO:0000166//nucleo	GO:0007165//signal transduction;GO:0007186//G-protein coupled recept
GO:0004470//malic	GO:0006108//malate metabolic process;GO:1902031//regulation of NADP
-	GO:0043368//positive T cell selection;GO:0043383//negative T cell selectio
GO:0001540//beta-a	GO:0001932//regulation of protein phosphorylation;GO:0001935//endoth
GO:0004657//prolin	GO:0006562//proline catabolic process
-	-
GO:0003988//acetyl-	GO:0000038//very long-chain fatty acid metabolic process;GO:0006635//f
GO:0043022//riboso	GO:0006886//intracellular protein transport;GO:0030433//ER-associated u
-	GO:0050910//detection of mechanical stimulus involved in sensory percep
-	-
GO:0005515//protei	-
GO:0001784//phosp	GO:0008286//insulin receptor signaling pathway;GO:0010907//positive re
GO:0008113//peptid	-
GO:0004721//phosp	GO:0006470//protein dephosphorylation;GO:0016311//dephosphorylatio
GO:0003810//protei	GO:0018149//peptide cross-linking
-	-
GO:0005515//protei	GO:0007165//signal transduction
GO:0004553//hydrol	GO:0005975//carbohydrate metabolic process
GO:0000977//RNA p	GO:0000122//negative regulation of transcription from RNA polymerase I
-	-
GO:0022857//transm	GO:0055085//transmembrane transport
-	-
-	-
GO:0008289//lipid b	GO:0006869//lipid transport;GO:0042157//lipoprotein metabolic process

GO:0003677//DNA b	GO:0006334//nucleosome assembly;GO:0042742//defense response to ba
GO:0004730//pseud	GO:0001522//pseudouridine synthesis;GO:0006796//phosphate-containin
GO:0022857//transm	GO:0010628//positive regulation of gene expression;GO:0010629//negativ
GO:0005085//guany	GO:0006623//protein targeting to vacuole;GO:0009306//protein secretion
GO:0005515//protei	GO:0006884//cell volume homeostasis;GO:0007165//signal transduction;G
GO:0005515//protei	-
GO:0005515//protei	GO:0015879//carnitine transport;GO:0032414//positive regulation of ion t
GO:0003824//catalyt	GO:0018272//protein-pyridoxal-5-phosphate linkage via peptidyl-N6-py
GO:0005515//protei	GO:0051965//positive regulation of synapse assembly
GO:0003677//DNA b	GO:0035871//protein K11-linked deubiquitination
GO:0004672//protei	GO:0000002//mitochondrial genome maintenance;GO:0001938//positive
GO:0005085//guany	GO:0007264//small GTPase mediated signal transduction;GO:0030334//re
-	GO:0015888//thiamine transport
GO:0000166//nucleo	GO:0006563//L-serine metabolic process;GO:0006790//sulfur compound
-	-
GO:0005515//protei	GO:0016567//protein ubiquitination;GO:0035556//intracellular signal trans
GO:0001786//phosp	GO:0002024//diet induced thermogenesis;GO:0006606//protein import in
GO:0004866//endop	GO:0010951//negative regulation of endopeptidase activity
GO:0004497//mono	-
GO:0003700//transc	GO:0006355//regulation of transcription, DNA-templated
GO:0004252//serine	GO:0006508//proteolysis
GO:0002020//protea	GO:0010466//negative regulation of peptidase activity;GO:0018149//pept
GO:0004312//fatty a	GO:0002068//glandular epithelial cell development;GO:0006633//fatty aci
GO:0004497//mono	GO:0006559//L-phenylalanine catabolic process;GO:0006571//tyrosine bi
-	GO:0021817//nucleokinesis involved in cell motility in cerebral cortex radi
GO:0004639//phosp	GO:0006164//purine nucleotide biosynthetic process;GO:0006189//'de no
GO:0004047//amino	GO:0019464//glycine decarboxylation via glycine cleavage system
GO:0015485//choles	GO:0032367//intracellular cholesterol transport;GO:0034389//lipid particle
GO:0004806//triglyc	GO:0006629//lipid metabolic process;GO:0006633//fatty acid biosynthetic
GO:0005102//recept	GO:0001541//ovarian follicle development;GO:0001707//mesoderm form
GO:0005515//protei	-
GO:0003924//GTPas	-
-	-
-	-
GO:0008271//secon	GO:0008272//sulfate transport;GO:0055085//transmembrane transport
GO:0004842//ubiqui	GO:0006511//ubiquitin-dependent protein catabolic process;GO:0007399
GO:0003824//catalyt	-
GO:1990817//RNA a	GO:0009617//response to bacterium;GO:0030278//regulation of ossificati
-	-
-	GO:0006513//protein monoubiquitination;GO:0036297//interstrand cross-
-	-
-	-
GO:0000334//3-hyd	GO:0009435//NAD biosynthetic process;GO:0010043//response to zinc io
-	-
GO:0005509//calciur	GO:0007155//cell adhesion
-	GO:0006895//Golgi to endosome transport;GO:0015031//protein transpo
GO:0004930//G-pro	GO:0007186//G-protein coupled receptor signaling pathway
GO:0004672//protei	GO:0006885//regulation of pH;GO:0008286//insulin receptor signaling pa
GO:0005515//protei	GO:0007155//cell adhesion;GO:0008593//regulation of Notch signaling pa
GO:0004045//amino	-
GO:0004497//mono	-
GO:0005102//recept	GO:0001541//ovarian follicle development;GO:0001707//mesoderm form
-	-
GO:0005515//protei	GO:0030855//epithelial cell differentiation
-	-
-	-
GO:0004497//mono	-
GO:0050839//cell ad	GO:0007409//axonogenesis;GO:0010975//regulation of neuron projectior

GO:0003676//nuclei	GO:0015074//DNA integration;GO:0044260//cellular macromolecule meta
GO:0031267//small (	GO:0006886//intracellular protein transport
GO:0005042//netrin	GO:0007165//signal transduction;GO:0014068//positive regulation of pho
GO:0005515//protei	GO:0051017//actin filament bundle assembly
GO:0001965//G-pro	GO:0007165//signal transduction;GO:0007186//G-protein coupled recept
GO:0004730//pseud	GO:0001522//pseudouridine synthesis;GO:0006796//phosphate-containir
-	GO:0007165//signal transduction
GO:0004044//amido	GO:0006177//GMP biosynthetic process;GO:0006189//'de novo' IMP bios
-	GO:0000226//microtubule cytoskeleton organization
GO:0003868//4-hyd	GO:0006572//tyrosine catabolic process;GO:0009072//aromatic amino aci
GO:0001786//phosp	GO:0002024//diet induced thermogenesis;GO:0006606//protein import in
GO:0008146//sulfotr	-
GO:0003834//beta-(	GO:0001523//retinoid metabolic process;GO:0016121//carotene catabolic
GO:0000166//nucleo	GO:0006468//protein phosphorylation;GO:0006955//immune response;G(
GO:0005085//guany	GO:0007264//small GTPase mediated signal transduction;GO:0030334//re
-	-
GO:0004047//amino	GO:0019464//glycine decarboxylation via glycine cleavage system
GO:0004866//endop	GO:0001934//positive regulation of protein phosphorylation;GO:0006954/
-	-
GO:0003841//1-acyl	GO:0006629//lipid metabolic process;GO:0006644//phospholipid metabo
GO:0003951//NAD+	GO:0006111//regulation of gluconeogenesis;GO:0006357//regulation of t
GO:0003676//nuclei	GO:0006278//RNA-dependent DNA replication;GO:0015074//DNA integra
GO:0035591//signali	GO:0034128//negative regulation of MyD88-independent toll-like recepto
GO:0004930//G-pro	GO:0002305//CD8-positive, gamma-delta intraepithelial T cell differentiat
GO:0005102//recept	GO:0006898//receptor-mediated endocytosis;GO:0008104//protein locali
-	-
GO:0003796//lysozy	GO:0008152//metabolic process;GO:0016998//cell wall macromolecule ca
GO:0005515//protei	-
GO:0004499//N,N-d	-
GO:0005515//protei	-
GO:0004866//endop	GO:0010951//negative regulation of endopeptidase activity
GO:0000287//magne	GO:0006006//glucose metabolic process;GO:0006094//gluconeogenesis;G
GO:0004930//G-pro	GO:0006897//endocytosis;GO:0006935//chemotaxis;GO:0006955//immun
GO:0004866//endop	-
GO:0004867//serine	GO:0016525//negative regulation of angiogenesis;GO:0050769//positive r
GO:0001664//G-pro	GO:0007286//spermatid development;GO:0034613//cellular protein locali
GO:0046872//metal	GO:0006875//cellular metal ion homeostasis
GO:0003824//catalyt	GO:0006527//arginine catabolic process;GO:0009072//aromatic amino aci
GO:0008017//microt	GO:0007010//cytoskeleton organization;GO:0007399//nervous system dev
-	-
GO:0005515//protei	GO:0006888//ER to Golgi vesicle-mediated transport
GO:0004657//prolin	GO:0006562//proline catabolic process
GO:0008009//chemo	GO:0006954//inflammatory response;GO:0006955//immune response;GO
GO:0004930//G-pro	GO:0006897//endocytosis;GO:0006935//chemotaxis;GO:0006955//immun
-	GO:0017015//regulation of transforming growth factor beta receptor sign
-	-
GO:0002162//dystro	GO:0010628//positive regulation of gene expression;GO:0010811//positiv
GO:0005515//protei	-
GO:0003824//catalyt	GO:0005975//carbohydrate metabolic process
GO:0008270//zinc ic	-
GO:0005506//iron ic	GO:0001666//response to hypoxia;GO:0017185//peptidyl-lysine hydroxyla
GO:0003810//protei	GO:0018149//peptide cross-linking
GO:0070412//R-SM/	GO:0010991//negative regulation of SMAD protein complex assembly;GO
-	-
-	-
GO:0005515//protei	-
-	-
-	-

GO:0003796//lysozy	GO:0008152//metabolic process;GO:0016998//cell wall macromolecule ca
-	-
-	-
-	-
GO:0004089//carbor	GO:0002009//morphogenesis of an epithelium;GO:0046903//secretion
GO:0003707//steroic	GO:0009755//hormone-mediated signaling pathway
-	-
GO:0009374//biotin	-
GO:0003824//catalyt	-
-	-
GO:0008009//chemc	GO:0006955//immune response
GO:0008009//chemc	GO:0006954//inflammatory response;GO:0006955//immune response;GO
-	-
-	-
-	GO:0001558//regulation of cell growth;GO:0008283//cell proliferation;GO
GO:0008270//zinc ic	-
GO:0003924//GTPas	-
GO:0000166//nuclec	GO:0006563//L-serine metabolic process;GO:0006790//sulfur compound
GO:0003847//1-alky	GO:0006629//lipid metabolic process;GO:0006644//phospholipid metabo
GO:0046872//metal	GO:0006875//cellular metal ion homeostasis
-	-
GO:0004222//metall	GO:0000003//reproduction;GO:0006508//proteolysis
GO:0022857//transm	GO:0010628//positive regulation of gene expression;GO:0010629//negativ
GO:0004499//N,N-d	-
GO:0001540//beta-a	GO:0006511//ubiquitin-dependent protein catabolic process;GO:0006516
-	-
-	-
GO:0005085//guany	GO:0050790//regulation of catalytic activity
GO:0004639//phosp	GO:0006164//purine nucleotide biosynthetic process;GO:0006189//'de no
GO:0005518//collag	GO:0007605//sensory perception of sound;GO:0008360//regulation of cel
GO:0005515//protei	-
-	-
GO:0004672//protei	GO:0000002//mitochondrial genome maintenance;GO:0001938//positive
GO:0004867//serine	-
GO:0005518//collag	GO:0010952//positive regulation of peptidase activity
-	-
GO:0008270//zinc ic	GO:0006882//cellular zinc ion homeostasis;GO:0010273//detoxification of
GO:0030298//recept	GO:0010976//positive regulation of neuron projection development;GO:0
GO:0005515//protei	GO:0006355//regulation of transcription, DNA-templated;GO:0007179//tr
GO:0005201//extrac	GO:0002244//hematopoietic progenitor cell differentiation
GO:0008757//S-ade	GO:0006656//phosphatidylcholine biosynthetic process;GO:0006686//sph
GO:0005515//protei	GO:0007165//signal transduction
-	-
GO:0004806//triglyc	GO:0006629//lipid metabolic process;GO:0006633//fatty acid biosynthetic
GO:0000062//fatty-a	GO:0008203//cholesterol metabolic process;GO:0010742//macrophage de
GO:0022857//transm	GO:0055085//transmembrane transport
GO:0005515//protei	GO:0007155//cell adhesion
GO:0016787//hydrol	-
GO:0017128//phosp	GO:0000045//autophagosome assembly;GO:0006914//autophagy;GO:001
-	-
GO:0015078//hydrog	GO:0030177//positive regulation of Wnt signaling pathway;GO:1902600//
-	-
GO:0008289//lipid b	GO:0070508//cholesterol import
GO:0005484//SNAP	GO:0006888//ER to Golgi vesicle-mediated transport;GO:0006903//vesicle
-	GO:0001764//neuron migration;GO:0007162//negative regulation of cell a
-	-
GO:0001786//phosp	-
-	GO:0000045//autophagosome assembly;GO:0009267//cellular response to

GO:0004866//endop	GO:0010951//negative regulation of endopeptidase activity
GO:0000287//magne	GO:0006584//catecholamine metabolic process;GO:0016036//cellular resp
-	-
-	-
-	GO:0006355//regulation of transcription, DNA-templated;GO:0034243//re
-	-
GO:0004497//mono	GO:0006805//xenobiotic metabolic process;GO:0008202//steroid metabol
GO:0022857//transm	GO:0055085//transmembrane transport
GO:0003676//nuclei	GO:0009987//cellular process;GO:0044699//single-organism process
GO:0005524//ATP bi	GO:0000086//G2/M transition of mitotic cell cycle;GO:0045332//phosphol
GO:0004047//amino	GO:0019464//glycine decarboxylation via glycine cleavage system
GO:0004930//G-pro	GO:0007186//G-protein coupled receptor signaling pathway
GO:0000978//RNA p	GO:0006357//regulation of transcription from RNA polymerase II promote
GO:0005515//protei	-
GO:0004842//ubiqui	GO:0006511//ubiquitin-dependent protein catabolic process;GO:0007399
GO:0022857//transm	GO:0006865//amino acid transport;GO:0055085//transmembrane transpo
-	-
GO:0004730//pseud	GO:0001522//pseudouridine synthesis;GO:0006796//phosphate-containin
-	-
-	-
GO:0003924//GTPas	-
-	-
GO:0000976//transc	GO:0000122//negative regulation of transcription from RNA polymerase I
-	-
GO:0005085//guany	-
GO:0002020//protea	GO:0010466//negative regulation of peptidase activity;GO:0018149//pept
GO:0000166//nucleo	GO:0006090//pyruvate metabolic process;GO:0006094//gluconeogenesis
GO:0005515//protei	GO:0030855//epithelial cell differentiation
GO:0005085//guany	GO:0001934//positive regulation of protein phosphorylation;GO:0002437/
-	-
GO:0005267//potass	GO:0006813//potassium ion transport;GO:0033198//response to ATP;GO:
GO:0046872//metal	GO:0006875//cellular metal ion homeostasis
GO:0004089//carbo	GO:0002009//morphogenesis of an epithelium;GO:0046903//secretion
GO:0000166//nucleo	GO:0006563//L-serine metabolic process;GO:0006790//sulfur compound
GO:0003677//DNA b	GO:0006334//nucleosome assembly;GO:0016584//nucleosome positionin
GO:0042809//vitami	GO:0008285//negative regulation of cell proliferation;GO:0010468//regula
GO:0004672//protei	GO:0006468//protein phosphorylation;GO:0007169//transmembrane rece
-	-
-	-
GO:0003824//catalyt	GO:0005975//carbohydrate metabolic process;GO:0006006//glucose meta
-	-
GO:0001784//phosp	GO:0001932//regulation of protein phosphorylation;GO:0007259//JAK-ST
GO:0004930//G-pro	GO:0007186//G-protein coupled receptor signaling pathway
-	-
GO:0004672//protei	GO:0000002//mitochondrial genome maintenance;GO:0001938//positive
-	-
GO:0005515//protei	GO:0051017//actin filament bundle assembly
GO:1990817//RNA a	GO:0009617//response to bacterium;GO:0030278//regulation of ossificati
GO:0005506//iron ic	GO:0008610//lipid biosynthetic process
-	GO:0007165//signal transduction
-	-
GO:0005515//protei	GO:0006888//ER to Golgi vesicle-mediated transport
-	-
GO:0005515//protei	-
GO:0005546//phosp	GO:0009636//response to toxic substance;GO:0042360//vitamin E metabo
GO:0003810//protei	GO:0018149//peptide cross-linking
GO:0030298//recept	GO:0010976//positive regulation of neuron projection development;GO:0
-	-

GO:0005201//extrac	GO:0030198//extracellular matrix organization;GO:0031581//hemidesmos
GO:0008009//chemc	GO:0006954//inflammatory response;GO:0006955//immune response;GO
-	-
GO:0005515//protei	-
GO:0003700//transc	GO:0006355//regulation of transcription, DNA-templated;GO:0045607//re
GO:0003824//catalyt	GO:0005975//carbohydrate metabolic process
-	-
-	-
-	-
GO:0004497//mono	-
GO:0005515//protei	GO:0015879//carnitine transport;GO:0032414//positive regulation of ion t
GO:0005515//protei	GO:0006355//regulation of transcription, DNA-templated;GO:0007179//tr
GO:0004499//N,N-d	-
GO:0003700//transc	GO:0006355//regulation of transcription, DNA-templated
GO:0003824//catalyt	GO:0006167//AMP biosynthetic process;GO:0006177//GMP biosynthetic p
-	-
-	-
-	-
GO:0008270//zinc ic	-
-	-
GO:0005515//protei	GO:0006355//regulation of transcription, DNA-templated
-	GO:0015888//thiamine transport
GO:0005353//fructo	GO:0003044//regulation of systemic arterial blood pressure mediated by a
-	-
GO:0016491//oxidor	GO:0009058//biosynthetic process;GO:0009792//embryo development en
-	GO:0001920//negative regulation of receptor recycling;GO:0050850//posi
GO:0005104//fibrob	GO:0001525//angiogenesis;GO:0001759//organ induction;GO:0001934//p
GO:0004044//amido	GO:0006177//GMP biosynthetic process;GO:0006189//'de novo' IMP bios
GO:0009374//biotin	-
GO:0003924//GTPas	-
GO:0001540//beta-a	GO:0006511//ubiquitin-dependent protein catabolic process;GO:0006516
GO:0035591//signali	GO:0034128//negative regulation of MyD88-independent toll-like recepto
-	-
GO:0003924//GTPas	-
GO:0003824//catalyt	GO:0009436//glyoxylate catabolic process;GO:0019265//glycine biosynthe
GO:0022857//transm	GO:0055085//transmembrane transport
GO:0030298//recept	GO:0010976//positive regulation of neuron projection development;GO:0
GO:0017128//phosp	GO:0000045//autophagosome assembly;GO:0006869//lipid transport;GO:
-	-
GO:0050839//cell ad	GO:0007409//axonogenesis;GO:0010975//regulation of neuron projection
-	-
GO:0003824//catalyt	-
GO:0005515//protei	-
GO:0004730//pseud	GO:0001522//pseudouridine synthesis;GO:0006796//phosphate-containin
GO:0003824//catalyt	GO:0006567//threonine catabolic process
-	-
GO:0000062//fatty-a	GO:0008203//cholesterol metabolic process;GO:0010742//macrophage de
GO:0004930//G-pro	GO:0002001//renin secretion into blood stream;GO:0002281//macrophag
-	-
GO:0004047//amino	GO:0019464//glycine decarboxylation via glycine cleavage system
-	-
GO:0005515//protei	GO:0046330//positive regulation of JNK cascade;GO:0090090//negative re
GO:0047750//choles	GO:0016125//sterol metabolic process
-	GO:0006895//Golgi to endosome transport;GO:0015031//protein transpo
-	-
-	-
-	GO:0030193//regulation of blood coagulation;GO:0044763//single-organ
GO:0005290//L-histi	GO:0006867//asparagine transport;GO:0006868//glutamine transport;GO:

GO:0008270//zinc ic	GO:0006882//cellular zinc ion homeostasis;GO:0010273//detoxification of
GO:0022857//transm	GO:0055085//transmembrane transport
GO:0008271//secon	GO:0008272//sulfate transport;GO:0055085//transmembrane transport
GO:0001784//phosp	GO:0007169//transmembrane receptor protein tyrosine kinase signaling p
GO:0030729//acetoa	GO:0006629//lipid metabolic process
GO:0005515//protei	GO:0007155//cell adhesion;GO:0008593//regulation of Notch signaling pa
GO:0005507//coppe	GO:0009308//amine metabolic process;GO:0009445//putrescine metaboli
GO:0004866//endop	GO:0010951//negative regulation of endopeptidase activity
GO:0003868//4-hyd	GO:0006572//tyrosine catabolic process;GO:0009072//aromatic amino aci
GO:0005242//inwarc	GO:0006813//potassium ion transport
GO:0001786//phosp	-
-	GO:0007166//cell surface receptor signaling pathway
GO:0015485//choles	GO:0032367//intracellular cholesterol transport;GO:0034389//lipid particle
GO:0004497//mono	-
-	-
GO:0005515//protei	GO:0007155//cell adhesion
GO:0005515//protei	GO:0006570//tyrosine metabolic process;GO:0007613//memory;GO:0008
GO:0004497//mono	GO:0006559//L-phenylalanine catabolic process;GO:0006571//tyrosine bi
GO:0016780//phosp	GO:0008654//phospholipid biosynthetic process
GO:0000978//RNA p	GO:0001932//regulation of protein phosphorylation;GO:0006355//regulat
GO:0016628//oxidor	GO:0006695//cholesterol biosynthetic process;GO:0016126//sterol biosyn
GO:0005515//protei	GO:0006955//immune response;GO:0006956//complement activation
GO:0005178//integri	GO:0000902//cell morphogenesis;GO:0006954//inflammatory response;G
GO:0005515//protei	-
-	-
GO:0004866//endop	-
GO:0004867//serine	GO:0016525//negative regulation of angiogenesis;GO:0050769//positive r
GO:0005102//recept	GO:0006898//receptor-mediated endocytosis;GO:0008104//protein locali
-	-
GO:0050839//cell ad	GO:0007409//axonogenesis;GO:0010975//regulation of neuron projection
-	-
GO:0004842//ubiqui	GO:0006511//ubiquitin-dependent protein catabolic process;GO:0007399
-	-
GO:0000977//RNA p	GO:0000122//negative regulation of transcription from RNA polymerase I
GO:0004497//mono	GO:0006805//xenobiotic metabolic process;GO:0008202//steroid metabol
GO:0005102//recept	GO:0001541//ovarian follicle development;GO:0001707//mesoderm forma
-	-
-	-
GO:0005085//guany	GO:0001934//positive regulation of protein phosphorylation;GO:0002437/
-	-
GO:0003677//DNA b	GO:0006334//nucleosome assembly;GO:0016584//nucleosome positionin
GO:0000166//nucleo	-
GO:0005158//insulin	GO:0001775//cell activation;GO:0007405//neuroblast proliferation;GO:000
GO:1990050//phosp	GO:0015914//phospholipid transport
GO:0001540//beta-a	GO:0001932//regulation of protein phosphorylation;GO:0001935//endoth
GO:0005546//phosp	GO:0009636//response to toxic substance;GO:0042360//vitamin E metabo
GO:0000166//nucleo	GO:0003014//renal system process;GO:0006811//ion transport;GO:00068
GO:0003677//DNA b	GO:0006334//nucleosome assembly;GO:0042742//defense response to ba
GO:0005515//protei	GO:0007165//signal transduction
-	-
GO:0005546//phosp	GO:0009636//response to toxic substance;GO:0042360//vitamin E metabo
GO:0001786//phosp	GO:0002024//diet induced thermogenesis;GO:0006606//protein import in
GO:0004016//adeny	GO:0006171//cAMP biosynthetic process;GO:0007613//memory;GO:0007
-	-
GO:0005085//guany	GO:0007264//small GTPase mediated signal transduction;GO:0030334//re
-	-
GO:0004930//G-pro	GO:0002300//CD8-positive, alpha-beta intraepithelial T cell differentiation
GO:0003951//NAD+	GO:0006111//regulation of gluconeogenesis;GO:0006357//regulation of t

-	-
GO:0003676//nuclei	GO:0006278//RNA-dependent DNA replication;GO:0015074//DNA integr
GO:0000254//C-4 m	GO:0001934//positive regulation of protein phosphorylation;GO:0008610/
GO:0005515//protei	-
GO:0050699//WW d	GO:0007034//vacuolar transport;GO:0010629//negative regulation of gen
GO:0003779//actin b	-
GO:0004089//carbo	GO:0002009//morphogenesis of an epithelium;GO:0046903//secretion
GO:0003824//catalyt	GO:0006527//arginine catabolic process;GO:0009072//aromatic amino aci
-	-
GO:0046790//virion	GO:0075512//clathrin-mediated endocytosis of virus by host cell
-	-
-	-
GO:0070089//chlori	GO:0006813//potassium ion transport;GO:0097623//potassium ion export
-	-
GO:0035254//glutan	GO:0001662//behavioral fear response;GO:0006884//cell volume homeos
GO:0003682//chrom	GO:0000122//negative regulation of transcription from RNA polymerase I
GO:0005515//protei	GO:0098742//cell-cell adhesion via plasma-membrane adhesion molecul
GO:0015095//magne	GO:0015693//magnesium ion transport
-	-
GO:0016407//acetylt	-
GO:0000334//3-hyd	GO:0009435//NAD biosynthetic process;GO:0010043//response to zinc io
-	GO:0007286//spermatid development;GO:0048137//spermatocyte divisio
GO:0004866//endop	GO:0010951//negative regulation of endopeptidase activity
-	-
-	-
-	-
GO:0000064//L-orni	GO:1990575//mitochondrial L-ornithine transmembrane transport
GO:0000254//C-4 m	GO:0001934//positive regulation of protein phosphorylation;GO:0008610/
GO:0005042//netrin	GO:0007165//signal transduction;GO:0014068//positive regulation of pho
GO:0003841//1-acyl	GO:0006644//phospholipid metabolic process
-	-
GO:0001784//phosp	GO:0001932//regulation of protein phosphorylation;GO:0007259//JAK-ST
GO:0005515//protei	GO:0042426//choline catabolic process
GO:0004047//amino	GO:0019464//glycine decarboxylation via glycine cleavage system
GO:0000287//magne	GO:0006584//catecholamine metabolic process;GO:0016036//cellular resp
GO:0000978//RNA p	GO:0001649//osteoblast differentiation;GO:0001755//neural crest cell mig
-	-
-	-
GO:0016407//acetylt	-
GO:0016787//hydrol	-
GO:0003824//catalyt	GO:0006650//glycerophospholipid metabolic process;GO:0007416//synap
-	GO:0007165//signal transduction
-	-
GO:0005507//coppe	GO:0009308//amine metabolic process;GO:0009445//putrescine metaboli
GO:0005515//protei	GO:0030855//epithelial cell differentiation
GO:0008201//hepari	-
GO:0004721//phosp	GO:0010801//negative regulation of peptidyl-threonine phosphorylation;
-	GO:0007010//cytoskeleton organization;GO:0008360//regulation of cell sh
GO:0004867//serine	GO:0007596//blood coagulation
GO:0000976//transc	GO:0000122//negative regulation of transcription from RNA polymerase I
GO:0000334//3-hyd	GO:0009435//NAD biosynthetic process;GO:0010043//response to zinc io
GO:0003677//DNA b	GO:0006351//transcription, DNA-templated
-	-
GO:0005515//protei	-
GO:0050839//cell ad	GO:0007409//axonogenesis;GO:0010975//regulation of neuron projectio
GO:1990817//RNA a	GO:0009617//response to bacterium;GO:0030278//regulation of ossificati
-	-
-	-

GO:0005267//potass	GO:0006813//potassium ion transport;GO:0033198//response to ATP;GO:
GO:0005515//protei	GO:0006749//glutathione metabolic process
GO:0050839//cell ad	GO:0007409//axonogenesis;GO:0010975//regulation of neuron projection
-	-
GO:0000062//fatty-a	-
GO:0005515//protei	-
GO:0004467//long-c	GO:0001676//long-chain fatty acid metabolic process;GO:0008610//lipid
-	-
GO:0030298//recept	GO:0010976//positive regulation of neuron projection development;GO:0
GO:0005158//insulin	GO:0001775//cell activation;GO:0007405//neuroblast proliferation;GO:000
GO:0003707//steroic	GO:0009755//hormone-mediated signaling pathway
GO:0005104//fibrob	GO:0001525//angiogenesis;GO:0001759//organ induction;GO:0001934//p
-	GO:0010269//response to selenium ion;GO:0035264//multicellular organis
GO:0016491//oxidor	-
GO:0015173//aroma	GO:0006590//thyroid hormone generation;GO:0055085//transmembrane
GO:0004087//carbar	GO:0006207//'de novo' pyrimidine nucleobase biosynthetic process;GO:0
-	-
GO:0004930//G-pro	GO:0007186//G-protein coupled receptor signaling pathway;GO:0007200
GO:0004866//endop	-
GO:0005515//protei	-
GO:0001784//phosp	GO:0007169//transmembrane receptor protein tyrosine kinase signaling p
GO:0008083//growt	-
GO:0003824//catalyt	GO:0006567//threonine catabolic process
-	-
GO:0003824//catalyt	GO:0010121//arginine catabolic process to proline via ornithine;GO:0019
GO:0003824//catalyt	-
GO:0005515//protei	GO:0015879//carnitine transport;GO:0032414//positive regulation of ion t
GO:0008517//folic a	GO:0015884//folic acid transport;GO:0051180//vitamin transport;GO:0051
GO:0003777//microt	GO:0007018//microtubule-based movement;GO:0072384//organelle tran
-	-
-	-
GO:0022857//transm	GO:0055085//transmembrane transport
GO:0004672//protei	GO:0006468//protein phosphorylation
GO:0003924//GTPas	-
GO:0016407//acetylt	-
GO:0015293//sympo	GO:0008643//carbohydrate transport
GO:0042809//vitami	GO:0008285//negative regulation of cell proliferation;GO:0010468//regula
-	-
-	GO:0008360//regulation of cell shape
GO:0000703//oxidiz	GO:0006281//DNA repair;GO:0006284//base-excision repair;GO:0006285/
GO:0000977//RNA p	GO:0000122//negative regulation of transcription from RNA polymerase I
-	-
GO:0004930//G-pro	GO:0002300//CD8-positive, alpha-beta intraepithelial T cell differentiation
-	-
GO:0016740//transfe	-
GO:0003854//3-beta	GO:0006694//steroid biosynthetic process
GO:0005096//GTPas	GO:0007186//G-protein coupled receptor signaling pathway;GO:0043547
GO:0005515//protei	GO:0007165//signal transduction
GO:0003824//catalyt	GO:0006167//AMP biosynthetic process;GO:0006177//GMP biosynthetic
GO:0004930//G-pro	GO:0007186//G-protein coupled receptor signaling pathway
GO:0038023//signali	GO:0007160//cell-matrix adhesion;GO:0007179//transforming growth fac
GO:0005515//protei	-
-	-
GO:0016491//oxidor	-
GO:0001540//beta-a	GO:0001932//regulation of protein phosphorylation;GO:0001935//endoth
GO:0001965//G-pro	GO:0007165//signal transduction;GO:0007186//G-protein coupled recept
GO:0004629//phosp	GO:0002431//Fc receptor mediated stimulatory signaling pathway;GO:000
GO:0000254//C-4 m	GO:0001934//positive regulation of protein phosphorylation;GO:0008610/

GO:0003735//structu	GO:0006412//translation;GO:0006919//activation of cysteine-type endope
-	GO:0001920//negative regulation of receptor recycling;GO:0050850//posi
GO:0005506//iron ic	-
GO:0003924//GTPas	-
-	-
GO:0005507//coppe	GO:0007601//visual perception;GO:0009308//amine metabolic process
GO:0004478//methic	GO:0006556//S-adenosylmethionine biosynthetic process;GO:0009087//m
-	-
-	GO:0007286//spermatid development;GO:0048137//spermatocyte divisior
GO:0017075//syntax	-
GO:0003779//actin b	GO:0007010//cytoskeleton organization;GO:0008360//regulation of cell sh
-	-
GO:0003700//transc	GO:0006355//regulation of transcription, DNA-templated;GO:0045607//re
GO:0004175//endop	GO:0006508//proteolysis;GO:0007520//myoblast fusion;GO:0022617//extr
GO:0003824//catalyt	GO:0006527//arginine catabolic process;GO:0009072//aromatic amino aci
-	-
-	GO:0010001//glial cell differentiation;GO:0050772//positive regulation of
-	-
GO:0005507//coppe	GO:0002227//innate immune response in mucosa;GO:0006869//lipid trans
GO:0003824//catalyt	GO:0006103//2-oxoglutarate metabolic process;GO:0009058//biosyntheti
GO:0022857//transm	GO:0055085//transmembrane transport
GO:0019901//protei	GO:0030334//regulation of cell migration;GO:0034446//substrate adhesio
GO:0030298//recept	GO:0010976//positive regulation of neuron projection development;GO:0
GO:0003676//nuclei	GO:0006417//regulation of translation;GO:0032869//cellular response to i
-	GO:0006364//rRNA processing;GO:0009303//rRNA transcription
GO:0022857//transm	GO:0055085//transmembrane transport
-	GO:0007166//cell surface receptor signaling pathway
-	-
-	-
-	GO:0009617//response to bacterium
GO:0005243//gap ju	GO:0007154//cell communication;GO:0007267//cell-cell signaling;GO:000
GO:0005515//protei	GO:0010468//regulation of gene expression;GO:0016567//protein ubiquit
GO:0051015//actin f	-
GO:0035254//glutan	GO:0001662//behavioral fear response;GO:0006884//cell volume homeos
GO:0022857//transm	GO:0055085//transmembrane transport
GO:0003779//actin b	GO:0010735//positive regulation of transcription via serum response elem
-	GO:0030193//regulation of blood coagulation;GO:0044763//single-organ
GO:0004364//glutatl	GO:0006749//glutathione metabolic process;GO:0006805//xenobiotic met
-	-
GO:0003824//catalyt	GO:0006567//threonine catabolic process
-	-
GO:0008201//hepari	-
GO:0005524//ATP bi	GO:0008203//cholesterol metabolic process;GO:0010033//response to org
GO:0000976//transc	GO:0000122//negative regulation of transcription from RNA polymerase I
GO:0016407//acetylt	-
GO:0005515//protei	GO:0051017//actin filament bundle assembly
-	-
GO:0005515//protei	-
GO:0005515//protei	-
GO:0009374//biotin	-
GO:0000987//core p	GO:0009409//response to cold;GO:0045893//positive regulation of transc
GO:0000166//nucleo	-
GO:0016407//acetylt	-
GO:0005509//calciur	GO:0007165//signal transduction
GO:0003824//catalyt	-
GO:0004639//phosp	GO:0006164//purine nucleotide biosynthetic process;GO:0006189//'de no
-	-
GO:0008271//secon	GO:0008272//sulfate transport;GO:0055085//transmembrane transport

GO:0004222//metall	GO:0001542//ovulation from ovarian follicle;GO:0001822//kidney develop
GO:0000978//RNA p	GO:0001736//establishment of planar polarity;GO:0001843//neural tube c
GO:0005515//protei	GO:0003431//growth plate cartilage chondrocyte development;GO:00082
-	GO:0032088//negative regulation of NF-kappaB transcription factor activi
-	GO:0008154//actin polymerization or depolymerization;GO:0030048//acti
GO:0004867//serine	GO:0007596//blood coagulation
GO:0005515//protei	-
-	-
GO:0008047//enzym	GO:0006486//protein glycosylation;GO:0006487//protein N-linked glycos
GO:0015125//bile ac	GO:0015721//bile acid and bile salt transport;GO:0032782//bile acid secre
GO:0004222//metall	GO:0000003//reproduction;GO:0006508//proteolysis
-	-
GO:0030246//carbol	GO:0006897//endocytosis
-	-
GO:0001849//compl	GO:0001878//response to yeast;GO:0008228//opsonization;GO:0030198//
GO:0005201//extrac	GO:0030198//extracellular matrix organization;GO:0031581//hemidesmos
GO:0005267//potass	GO:0006813//potassium ion transport;GO:0033198//response to ATP;GO:
GO:0005125//cytoki	GO:0002232//leukocyte chemotaxis involved in inflammatory response;GC
-	-
GO:0005324//long-c	GO:0006469//negative regulation of protein kinase activity;GO:0009617//
GO:0008270//zinc ic	GO:0006882//cellular zinc ion homeostasis;GO:0010273//detoxification of
-	-
GO:0003779//actin k	GO:0071691//cardiac muscle thin filament assembly
GO:0008083//growtl	-
GO:0005515//protei	GO:0006749//glutathione metabolic process
GO:0004721//phosp	GO:0010801//negative regulation of peptidyl-threonine phosphorylation;
GO:0000062//fatty-a	-
GO:0004089//carbor	GO:0002009//morphogenesis of an epithelium;GO:0046903//secretion
GO:0004252//serine	GO:0006508//proteolysis
-	GO:0043368//positive T cell selection;GO:0043383//negative T cell selectic
GO:0004657//prolin	GO:0006562//proline catabolic process
-	-
GO:0008532//N-ace	GO:0006486//protein glycosylation;GO:0007411//axon guidance;GO:0007
GO:0001784//phosp	GO:0008286//insulin receptor signaling pathway;GO:0010907//positive re
-	GO:0030193//regulation of blood coagulation;GO:0044763//single-organ
GO:0005179//hormc	-
GO:0000166//nucleo	GO:0006529//asparagine biosynthetic process;GO:0006541//glutamine m
GO:0005515//protei	GO:0006355//regulation of transcription, DNA-templated;GO:0007179//tr
GO:0008517//folic a	GO:0015884//folic acid transport;GO:0051180//vitamin transport;GO:0051
GO:0003714//transc	GO:0006338//chromatin remodeling;GO:0007507//heart development;GC
GO:0005085//guany	GO:0001934//positive regulation of protein phosphorylation;GO:0002437/
GO:0004497//monoo	GO:0006805//xenobiotic metabolic process;GO:0008202//steroid metabol
GO:0004652//polynu	GO:0008285//negative regulation of cell proliferation;GO:0043066//negat
GO:0005216//ion ch	GO:0006811//ion transport;GO:0006814//sodium ion transport;GO:00550
-	-
-	-
GO:0030298//recept	GO:0010976//positive regulation of neuron projection development;GO:0
GO:0005515//protei	GO:0007043//cell-cell junction assembly;GO:0007155//cell adhesion;GO:0
GO:0003779//actin k	GO:0030835//negative regulation of actin filament depolymerization
GO:0015293//sympo	GO:0008643//carbohydrate transport
GO:0004672//protei	GO:0006468//protein phosphorylation
GO:0003824//catalyt	GO:0006567//threonine catabolic process
GO:0005515//protei	GO:0001764//neuron migration;GO:0007097//nuclear migration;GO:0007
GO:0008146//sulfotr	-
GO:0001671//ATPas	GO:0001933//negative regulation of protein phosphorylation;GO:003043
GO:0005506//iron ic	-
-	-
GO:0005509//calciur	GO:0007268//synaptic transmission;GO:0008104//protein localization;GO:

GO:0004016//adeny	GO:0006171//cAMP biosynthetic process;GO:0007613//memory;GO:0007(
GO:0022857//transm	GO:0055085//transmembrane transport
-	GO:0098609//cell-cell adhesion
GO:0005096//GTPas	GO:0007165//signal transduction;GO:0035556//intracellular signal transdu
GO:0051015//actin f	GO:0030866//cortical actin cytoskeleton organization;GO:0045214//sarcor
GO:0008289//lipid b	GO:0032374//regulation of cholesterol transport
GO:0042809//vitami	GO:0008285//negative regulation of cell proliferation;GO:0010468//regula
-	-
GO:0003847//1-alky	GO:0006629//lipid metabolic process;GO:0006644//phospholipid metabo
GO:0000064//L-orni	GO:1990575//mitochondrial L-ornithine transmembrane transport
-	-
GO:0004930//G-pro	GO:0007186//G-protein coupled receptor signaling pathway
GO:0005515//protei	GO:0007155//cell adhesion
GO:0000287//magne	GO:0006114//glycerol biosynthetic process;GO:0006650//glycerophospho
-	-
-	GO:0032088//negative regulation of NF-kappaB transcription factor activi
-	-
GO:0000062//fatty-a	GO:0008203//cholesterol metabolic process;GO:0010742//macrophage de
GO:0000254//C-4 m	GO:0001934//positive regulation of protein phosphorylation;GO:0008610/
-	-
GO:0005515//protei	-
GO:0000334//3-hyd	GO:0009435//NAD biosynthetic process;GO:0010043//response to zinc io
GO:0004478//methi	GO:0006556//S-adenosylmethionine biosynthetic process;GO:0009087//m
GO:0008194//UDP-g	GO:0006486//protein glycosylation;GO:0006682//galactosylceramide bios
GO:0005515//protei	GO:0051017//actin filament bundle assembly
GO:0004478//methi	GO:0006556//S-adenosylmethionine biosynthetic process;GO:0009087//m
GO:0005524//ATP bi	GO:0035499//carnosine biosynthetic process
GO:0005488//bindin	-
GO:0005515//protei	-
GO:0005102//recept	GO:0006898//receptor-mediated endocytosis;GO:0008104//protein locali
GO:0005543//phosp	GO:0048260//positive regulation of receptor-mediated endocytosis;GO:0(
GO:0003824//catalyt	GO:0005975//carbohydrate metabolic process
-	-
GO:0008237//metall	GO:0006508//proteolysis
-	GO:0007165//signal transduction
GO:0005506//iron io	-
-	-
GO:1990050//phosp	GO:0015914//phospholipid transport
GO:0004930//G-pro	GO:0007165//signal transduction;GO:0007186//G-protein coupled recept
GO:0005096//GTPas	GO:0007165//signal transduction;GO:0043547//positive regulation of GTP
GO:0000978//RNA p	GO:0001736//establishment of planar polarity;GO:0001843//neural tube c
-	-
GO:0000166//nucleo	GO:0006468//protein phosphorylation;GO:0006955//immune response;G(
-	-
GO:0003824//catalyt	GO:0010121//arginine catabolic process to proline via ornithine;GO:00195
GO:0004866//endop	GO:0001934//positive regulation of protein phosphorylation;GO:0006954/
-	-
GO:0016765//transfe	GO:0008299//isoprenoid biosynthetic process
GO:0004629//phosp	GO:0002431//Fc receptor mediated stimulatory signaling pathway;GO:000
-	-
GO:0003824//catalyt	-
GO:0003735//structu	GO:0006412//translation
GO:0050839//cell ad	GO:0007409//axonogenesis;GO:0010975//regulation of neuron projection
-	-
GO:0016290//palmit	GO:0006637//acyl-CoA metabolic process;GO:0016559//peroxisome fissio
GO:0005515//protei	GO:0007155//cell adhesion;GO:0008593//regulation of Notch signaling pa
GO:0003677//DNA b	GO:0006334//nucleosome assembly;GO:0042742//defense response to ba
GO:0003924//GTPas	-

GO:0004497//mono	GO:0097267//omega-hydroxylase P450 pathway
GO:0001517//N-ace	GO:0005975//carbohydrate metabolic process;GO:0006044//N-acetylgluc
-	-
-	-
GO:0004222//metall	GO:0000003//reproduction;GO:0006508//proteolysis
GO:0003676//nuclei	GO:0006278//RNA-dependent DNA replication;GO:0015074//DNA integr
-	-
GO:0004843//thiol-	GO:0006511//ubiquitin-dependent protein catabolic process;GO:0007000
GO:0000977//RNA p	GO:0000122//negative regulation of transcription from RNA polymerase I
GO:0022857//transm	GO:0055085//transmembrane transport
-	GO:0006895//Golgi to endosome transport;GO:0015031//protein transpo
GO:0005515//protei	GO:0007165//signal transduction
GO:0016407//acetylt	-
GO:0008083//growtl	-
GO:0003713//transc	GO:0007219//Notch signaling pathway;GO:0045944//positive regulation c
-	-
GO:0003824//catalyt	GO:0001822//kidney development;GO:0006596//polyamine biosynthetic
GO:0015078//hydro	GO:0006811//ion transport;GO:0007034//vacuolar transport;GO:0007035/
GO:0003713//transc	GO:0006355//regulation of transcription, DNA-templated
GO:0004252//serine	GO:0006508//proteolysis
GO:0001540//beta-	GO:0001932//regulation of protein phosphorylation;GO:0001935//endoth
GO:0005049//nucle	GO:0006611//protein export from nucleus;GO:0051169//nuclear transport
GO:0008146//sulfotr	-
-	-
GO:0003779//actin b	GO:0010735//positive regulation of transcription via serum response elem
GO:0000287//magne	GO:0006584//catecholamine metabolic process;GO:0016036//cellular resp
-	GO:0007155//cell adhesion
GO:0008146//sulfotr	GO:0006805//xenobiotic metabolic process;GO:0042403//thyroid hormon
-	GO:0007010//cytoskeleton organization;GO:0008360//regulation of cell sh
GO:0004738//pyruva	GO:0006086//acetyl-CoA biosynthetic process from pyruvate;GO:000609
GO:0004721//phosp	GO:0010801//negative regulation of peptidyl-threonine phosphorylation;
-	-
GO:0003677//DNA b	GO:0035871//protein K11-linked deubiquitination
GO:0003697//single	-
GO:0051015//actin f	GO:0030866//cortical actin cytoskeleton organization;GO:0045214//sarco
GO:0003824//catalyt	-
GO:0003735//structu	GO:0006412//translation
GO:0004652//polynu	GO:0008285//negative regulation of cell proliferation;GO:0043066//negat
GO:0003824//catalyt	GO:0006103//2-oxoglutarate metabolic process;GO:0009058//biosyntheti
-	-
GO:0004866//endop	-
GO:0016765//transfe	GO:0008299//isoprenoid biosynthetic process
-	GO:0015888//thiamine transport
GO:0004842//ubiqui	GO:0006511//ubiquitin-dependent protein catabolic process;GO:0016567
-	GO:0007166//cell surface receptor signaling pathway
GO:0005198//structu	-
GO:0004095//carniti	GO:0001676//long-chain fatty acid metabolic process;GO:0006629//lipid
-	GO:0001764//neuron migration;GO:0007420//brain development;GO:002
GO:0004857//enzym	GO:0006071//glycerol metabolic process;GO:0006631//fatty acid metabol
GO:0003779//actin b	GO:0030835//negative regulation of actin filament depolymerization
GO:0004866//endop	GO:0010951//negative regulation of endopeptidase activity
-	-
-	-
-	-
GO:0005085//guany	GO:0001934//positive regulation of protein phosphorylation;GO:0002437
GO:0005524//ATP bi	GO:0000086//G2/M transition of mitotic cell cycle;GO:0045332//phosphol
GO:0005509//calciu	GO:0007268//synaptic transmission;GO:0008104//protein localization;GO:
GO:0005518//collag	GO:0007605//sensory perception of sound;GO:0008360//regulation of cel

-	GO:0070979//protein K11-linked ubiquitination
GO:0008201//hepari	-
GO:0016316//phosp	-
GO:0003779//actin b	GO:0007010//cytoskeleton organization;GO:0008360//regulation of cell sh
GO:0004047//amino	GO:0019464//glycine decarboxylation via glycine cleavage system
-	-
GO:0004888//transm	GO:0006898//receptor-mediated endocytosis;GO:0007165//signal transdu
GO:0003847//1-alky	GO:0006629//lipid metabolic process;GO:0006644//phospholipid metabo
GO:0005096//GTPas	GO:0007186//G-protein coupled receptor signaling pathway;GO:0043547
GO:0008146//sulfotr	GO:0006790//sulfur compound metabolic process
GO:0004672//protei	GO:0006468//protein phosphorylation;GO:0008631//intrinsic apoptotic si
-	-
GO:0004867//serine	GO:0010466//negative regulation of peptidase activity;GO:0030195//nega
-	-
GO:0070089//chloric	GO:0006813//potassium ion transport;GO:0097623//potassium ion export
GO:0004016//adeny	GO:0006171//cAMP biosynthetic process;GO:0007613//memory;GO:0007(
GO:0005515//protei	GO:0010468//regulation of gene expression;GO:0016567//protein ubiquit
GO:0005515//protei	GO:0007155//cell adhesion
-	-
-	GO:0007286//spermatid development;GO:0048137//spermatocyte divisio
GO:0004478//methi	GO:0006556//S-adenosylmethionine biosynthetic process;GO:0009087//m
GO:0005324//long-c	GO:0006469//negative regulation of protein kinase activity;GO:0009617//r
-	-
GO:0015171//amino	GO:0006641//triglyceride metabolic process;GO:0009437//carnitine metab
GO:0005507//coppe	GO:0002227//innate immune response in mucosa;GO:0006869//lipid trans
GO:0000978//RNA p	GO:0001649//osteoblast differentiation;GO:0001755//neural crest cell mig
-	GO:0030193//regulation of blood coagulation;GO:0044763//single-organ
GO:0000062//fatty-a	GO:0008203//cholesterol metabolic process;GO:0010742//macrophage de
-	GO:0046890//regulation of lipid biosynthetic process
-	GO:0007283//spermatogenesis;GO:0007288//sperm axoneme assembly;G
-	GO:0010269//response to selenium ion;GO:0035264//multicellular organis
GO:0035254//glutan	GO:0001662//behavioral fear response;GO:0006884//cell volume homeos
GO:0016491//oxidor	GO:0019477//L-lysine catabolic process
-	-
GO:0017147//Wnt-p	GO:0030178//negative regulation of Wnt signaling pathway
GO:0031681//G-pro	GO:0007186//G-protein coupled receptor signaling pathway
GO:0022857//transm	GO:0006865//amino acid transport;GO:0055085//transmembrane transpo
GO:0005543//phosp	GO:0048260//positive regulation of receptor-mediated endocytosis;GO:00
GO:0004721//phosp	GO:0010801//negative regulation of peptidyl-threonine phosphorylation;
GO:0035254//glutan	GO:0001662//behavioral fear response;GO:0006884//cell volume homeos
GO:0005515//protei	GO:0030036//actin cytoskeleton organization;GO:0072659//protein locali
GO:0001540//beta-a	GO:0001932//regulation of protein phosphorylation;GO:0001935//endoth
GO:0008289//lipid b	-
GO:0002020//protea	GO:0010466//negative regulation of peptidase activity;GO:0018149//pept
-	-
GO:0004867//serine	GO:0016525//negative regulation of angiogenesis;GO:0050769//positive r
GO:0004866//endop	-
GO:0003796//lysozy	GO:0009253//peptidoglycan catabolic process;GO:0016998//cell wall mac
GO:0005524//ATP bi	GO:0008203//cholesterol metabolic process;GO:0010033//response to org
GO:0003677//DNA b	-
-	-
GO:0003674//molec	GO:0002062//chondrocyte differentiation;GO:0008150//biological_proces
GO:0005242//inward	GO:0006813//potassium ion transport
GO:0004465//lipopro	GO:0006629//lipid metabolic process;GO:0006631//fatty acid metabolic p
GO:0005085//guany	GO:0000281//mitotic cytokinesis;GO:0000902//cell morphogenesis;GO:00
GO:0000987//core p	GO:0009409//response to cold;GO:0045893//positive regulation of transc
-	GO:0017015//regulation of transforming growth factor beta receptor sign
GO:0003824//cataly	GO:0006778//porphyrin-containing compound metabolic process;GO:000

GO:0004866//endop	GO:0010575//positive regulation of vascular endothelial growth factor pro
GO:0015108//chloric	GO:1902476//chloride transmembrane transport
GO:0005242//inwarc	GO:0006813//potassium ion transport
GO:0005324//long-c	GO:0006469//negative regulation of protein kinase activity;GO:0009617//r
GO:0004553//hydrol	GO:0005975//carbohydrate metabolic process
GO:0000977//RNA p	GO:0000122//negative regulation of transcription from RNA polymerase I
GO:0005515//protei	GO:0006355//regulation of transcription, DNA-templated;GO:0007179//tr
GO:0003779//actin b	GO:0006351//transcription, DNA-templated;GO:0030032//lamellipodium
-	-
GO:0005506//iron ic	-
-	-
-	-
GO:0008289//lipid b	GO:0015914//phospholipid transport
GO:0003841//1-acyl	GO:0006644//phospholipid metabolic process
GO:0008146//sulfotr	-
-	-
-	-
GO:0004497//mono	GO:0006559//L-phenylalanine catabolic process;GO:0006571//tyrosine bi
GO:0005158//insulin	GO:0001775//cell activation;GO:0007405//neuroblast proliferation;GO:000
GO:0003824//catalyt	GO:0010121//arginine catabolic process to proline via ornithine;GO:0019
-	-
-	GO:0000077//DNA damage checkpoint
GO:0015485//choles	GO:0032367//intracellular cholesterol transport;GO:0034389//lipid particle
GO:0005515//protei	GO:0007155//cell adhesion;GO:0008593//regulation of Notch signaling pa
GO:0042577//lipid p	GO:0006644//phospholipid metabolic process
GO:0005515//protei	-
GO:0003824//catalyt	-
GO:0004888//transm	GO:0007186//G-protein coupled receptor signaling pathway;GO:0009994
-	GO:0006895//Golgi to endosome transport;GO:0015031//protein transpor
-	-
GO:0008146//sulfotr	-
GO:0005158//insulin	GO:0007165//signal transduction;GO:0010467//gene expression;GO:0030
GO:0003779//actin b	GO:0030835//negative regulation of actin filament depolymerization
GO:0003924//GTPas	-
GO:0000062//fatty-a	GO:0008203//cholesterol metabolic process;GO:0010742//macrophage de
GO:0003700//transc	GO:0006355//regulation of transcription, DNA-templated
GO:0004470//malic	GO:0006108//malate metabolic process;GO:1902031//regulation of NADP
GO:0008083//growtl	-
GO:0003676//nuclei	GO:0006417//regulation of translation;GO:0032869//cellular response to i
GO:0008270//zinc ic	GO:0009056//catabolic process;GO:0030855//epithelial cell differentiation
-	-
GO:0005515//protei	-
GO:0008532//N-ace	GO:0006486//protein glycosylation;GO:0007411//axon guidance;GO:0007
GO:0005515//protei	GO:0042634//regulation of hair cycle
GO:0005509//calciur	GO:0007611//learning or memory;GO:0007613//memory;GO:0043123//po
GO:0005515//protei	GO:0007165//signal transduction
GO:0004467//long-c	GO:0015908//fatty acid transport
GO:0005507//coppe	GO:0009308//amine metabolic process;GO:0009445//putrescine metaboli
GO:0005227//calciur	GO:0006812//cation transport
GO:0003779//actin b	GO:0006351//transcription, DNA-templated;GO:0030032//lamellipodium
-	-
GO:0008022//protei	GO:0007026//negative regulation of microtubule depolymerization;GO:00
GO:0031956//mediu	-
-	GO:0007010//cytoskeleton organization;GO:0008360//regulation of cell sh
GO:0005216//ion ch	GO:0006811//ion transport;GO:0006814//sodium ion transport;GO:0055
GO:0003779//actin b	GO:0007010//cytoskeleton organization;GO:0008360//regulation of cell sh
GO:0004044//amido	GO:0006177//GMP biosynthetic process;GO:0006189//'de novo' IMP bios
GO:0008480//sarcos	GO:1901053//sarcosine catabolic process

GO:0004122//cystatl	GO:0001958//endochondral ossification;GO:0001974//blood vessel remoc
GO:0005524//ATP bi	GO:0000086//G2/M transition of mitotic cell cycle;GO:0045332//phosphol
GO:0004465//lipopr	GO:0006629//lipid metabolic process;GO:0006631//fatty acid metabolic p
-	GO:0015888//thiamine transport
GO:0004866//endop	-
GO:0004867//serine	GO:0016525//negative regulation of angiogenesis;GO:0050769//positive r
GO:0004497//mono	-
GO:0004866//endop	-
GO:0005515//protei	-
GO:0005102//recept	GO:0006898//receptor-mediated endocytosis;GO:0008104//protein locali
GO:0004730//pseud	GO:0001522//pseudouridine synthesis;GO:0006796//phosphate-containir
GO:0001784//phosp	GO:0007169//transmembrane receptor protein tyrosine kinase signaling p
GO:0005515//protei	GO:0010468//regulation of gene expression;GO:0016567//protein ubiquit
GO:0004364//glutat	GO:0006749//glutathione metabolic process;GO:0006805//xenobiotic met
GO:0003834//beta-c	GO:0001523//retinoid metabolic process;GO:0016121//carotene catabolic
GO:0005096//GTPas	GO:0007165//signal transduction;GO:0043547//positive regulation of GTP
GO:0005085//guany	GO:0001934//positive regulation of protein phosphorylation;GO:0002437/
-	-
GO:0008201//hepari	-
-	GO:0016192//vesicle-mediated transport
-	GO:0007165//signal transduction
-	-
-	-
GO:0003868//4-hyd	GO:0006572//tyrosine catabolic process;GO:0009072//aromatic amino aci
GO:0005515//protei	GO:0051017//actin filament bundle assembly
GO:0035254//glutan	GO:0001662//behavioral fear response;GO:0006884//cell volume homeos
GO:0098640//integri	GO:0007155//cell adhesion;GO:0007229//integrin-mediated signaling pat
GO:0004896//cytoki	GO:0007171//activation of transmembrane receptor protein tyrosine kinas
GO:0000287//magne	GO:0006584//catecholamine metabolic process;GO:0016036//cellular resp
-	-
GO:0004866//endop	-
-	-
GO:0005515//protei	GO:0007155//cell adhesion;GO:0007156//homophilic cell adhesion via pla
GO:0005515//protei	-
GO:0016740//transfe	-
GO:0005515//protei	-
GO:0003810//protei	GO:0018149//peptide cross-linking
GO:0005515//protei	GO:0002376//immune system process;GO:0006955//immune response;G(
GO:0005515//protei	-
-	-
GO:0000976//transc	GO:0000122//negative regulation of transcription from RNA polymerase I
GO:0070089//chloric	GO:0006813//potassium ion transport;GO:0097623//potassium ion export
GO:0008195//phosp	GO:0006796//phosphate-containing compound metabolic process;GO:00
-	GO:0007286//spermatid development;GO:0048137//spermatocyte divisio
-	GO:0001920//negative regulation of receptor recycling;GO:0050850//posi
GO:0003824//catalyt	GO:0018272//protein-pyridoxal-5-phosphate linkage via peptidyl-N6-py
GO:0004478//methi	GO:0006556//S-adenosylmethionine biosynthetic process;GO:0009087//n
GO:0005198//structu	-
GO:0004089//carbo	GO:0002009//morphogenesis of an epithelium;GO:0046903//secretion
GO:0000978//RNA p	GO:0000122//negative regulation of transcription from RNA polymerase I
-	-
GO:0005179//hormo	-
GO:0046983//protei	GO:0000082//G1/S transition of mitotic cell cycle;GO:0000122//negative r
-	-
GO:0000166//nucle	GO:0006563//L-serine metabolic process;GO:0006790//sulfur compound
GO:0004089//carbo	GO:0002009//morphogenesis of an epithelium;GO:0046903//secretion
GO:0003847//1-alky	GO:0006629//lipid metabolic process;GO:0006644//phospholipid metabo
GO:0003677//DNA b	-

GO:0004672//protei	GO:0006885//regulation of pH;GO:0008286//insulin receptor signaling pa
GO:0005524//ATP bi	GO:0000086//G2/M transition of mitotic cell cycle;GO:0045332//phosphol
GO:0003713//transc	GO:0007219//Notch signaling pathway;GO:0045944//positive regulation c
GO:0005515//protei	-
-	-
GO:0008113//peptid	-
-	-
GO:0005524//ATP bi	GO:0008203//cholesterol metabolic process;GO:0010033//response to org
-	-
-	-
GO:0016717//oxidor	GO:0006629//lipid metabolic process
GO:0008113//peptid	-
GO:0004496//meval	GO:0006695//cholesterol biosynthetic process;GO:0008299//isoprenoid b
GO:0004252//serine	GO:0000902//cell morphogenesis;GO:0001889//liver development;GO:00
GO:0015297//antipo	GO:0055085//transmembrane transport
-	-
GO:0016491//oxidor	GO:0006666//3-keto-sphinganine metabolic process;GO:0030148//sphing
GO:0004867//serine	-
-	-
GO:0004970//ionotr	GO:0006811//ion transport;GO:0045471//response to ethanol;GO:009755
-	GO:0006886//intracellular protein transport;GO:0006890//retrograde vesic
GO:0000287//magne	GO:0006584//catecholamine metabolic process;GO:0016036//cellular resp
GO:0016491//oxidor	GO:0006666//3-keto-sphinganine metabolic process;GO:0030148//sphing
GO:0051015//actin f	GO:0030866//cortical actin cytoskeleton organization;GO:0045214//sarco
GO:0005515//protei	GO:0006355//regulation of transcription, DNA-templated;GO:0007179//tr
GO:0045296//cadhe	GO:0007043//cell-cell junction assembly;GO:0060997//dendritic spine mc
GO:0008146//sulfotr	-
-	-
GO:0008233//peptid	GO:0006508//proteolysis
GO:0002162//dystro	GO:0010628//positive regulation of gene expression;GO:0010811//positiv
-	-
GO:0016717//oxidor	GO:0006629//lipid metabolic process
-	-
GO:0005158//insulin	GO:0001775//cell activation;GO:0007405//neuroblast proliferation;GO:000
GO:0005515//protei	GO:0000086//G2/M transition of mitotic cell cycle;GO:0031146//SCF-depe
GO:0004497//mono	GO:0006559//L-phenylalanine catabolic process;GO:0006571//tyrosine bi
GO:0005515//protei	GO:0000086//G2/M transition of mitotic cell cycle;GO:0031146//SCF-depe
-	GO:0001764//neuron migration;GO:0007162//negative regulation of cell a
GO:0004497//mono	GO:0006805//xenobiotic metabolic process;GO:0008202//steroid metabol
GO:0015485//choles	GO:0032367//intracellular cholesterol transport;GO:0034389//lipid particle
GO:0003779//actin b	GO:0006836//neurotransmitter transport;GO:0015844//monoamine transp
-	GO:0007165//signal transduction;GO:0070507//regulation of microtubule
-	-
-	-
GO:0003847//1-alky	GO:0006629//lipid metabolic process;GO:0006644//phospholipid metabo
GO:0004867//serine	GO:0016525//negative regulation of angiogenesis;GO:0050769//positive r
GO:0004866//endop	-
GO:0016491//oxidor	GO:0007005//mitochondrion organization
GO:0003674//molec	GO:0002062//chondrocyte differentiation;GO:0008150//biological_proces
-	-
GO:0005227//calciur	GO:0006812//cation transport
GO:0005515//protei	GO:0007155//cell adhesion;GO:0007156//homophilic cell adhesion via pla
GO:0003677//DNA b	GO:0006281//DNA repair;GO:0006974//cellular response to DNA damage
-	-
GO:0004866//endop	GO:0010575//positive regulation of vascular endothelial growth factor pro
GO:0000166//nuclec	GO:0006897//endocytosis;GO:0006898//receptor-mediated endocytosis;
-	-
GO:0098640//integri	GO:0007155//cell adhesion;GO:0007229//integrin-mediated signaling pat

-	-
GO:0046872//metal	GO:0006875//cellular metal ion homeostasis
GO:0004866//endop	-
GO:0004867//serine	GO:0016525//negative regulation of angiogenesis;GO:0050769//positive r
GO:0005515//protei	-
GO:0004601//peroxi	GO:0015671//oxygen transport;GO:0042744//hydrogen peroxide cataboli
GO:0005507//coppe	GO:0009308//amine metabolic process;GO:0009445//putrescine metaboli
GO:0004725//protei	GO:0006470//protein dephosphorylation;GO:0016311//dephosphorylatio
GO:0008270//zinc ic	GO:0006882//cellular zinc ion homeostasis;GO:0010273//detoxification of
GO:0016407//acetylt	-
GO:0002020//protea	GO:0010466//negative regulation of peptidase activity;GO:0018149//pept
GO:0003677//DNA b	GO:0009267//cellular response to starvation;GO:0043066//negative regula
GO:0005515//protei	GO:0030855//epithelial cell differentiation
-	-
-	-
GO:0005216//ion ch	GO:0006811//ion transport;GO:0006814//sodium ion transport;GO:00550
-	-
GO:0008022//protei	GO:0007099//centriole replication;GO:0010457//centriole-centriole cohes
GO:0005515//protei	GO:0051017//actin filament bundle assembly
GO:0003677//DNA b	GO:0006334//nucleosome assembly;GO:0006352//DNA-templated transc
GO:0038023//signali	GO:0007155//cell adhesion;GO:0007158//neuron cell-cell adhesion;GO:00
GO:0004044//amido	GO:0006177//GMP biosynthetic process;GO:0006189//'de novo' IMP bios
GO:0004499//N,N-d	-
GO:0016922//ligand	GO:0045944//positive regulation of transcription from RNA polymerase II
GO:0008009//chemo	GO:0006955//immune response
GO:0004553//hydrol	GO:0005975//carbohydrate metabolic process
-	-
GO:0004867//serine	GO:0010466//negative regulation of peptidase activity;GO:0030195//nega
GO:0008480//sarcos	GO:1901053//sarcosine catabolic process
GO:0000166//nucleo	GO:0006468//protein phosphorylation;GO:0006974//cellular response to I
GO:0005515//protei	-
-	-
-	-
GO:1990817//RNA a	GO:0009617//response to bacterium;GO:0030278//regulation of ossificati
GO:0008009//chemo	GO:0006954//inflammatory response;GO:0006955//immune response;GO
GO:0005102//recept	GO:0006898//receptor-mediated endocytosis;GO:0008104//protein locali
-	-
-	-
-	-
GO:0003824//catalyt	GO:0001822//kidney development;GO:0006596//polyamine biosynthetic p
GO:0008009//chemo	GO:0006954//inflammatory response;GO:0006955//immune response;GO
GO:0000977//RNA p	GO:0000122//negative regulation of transcription from RNA polymerase I
GO:0016740//transfe	-
GO:0008271//secon	GO:0008272//sulfate transport;GO:0055085//transmembrane transport
-	-
GO:0003847//1-alky	GO:0006629//lipid metabolic process;GO:0006644//phospholipid metabo
GO:0009374//biotin	-
GO:0005515//protei	-
-	-
GO:0003824//catalyt	GO:0010121//arginine catabolic process to proline via ornithine;GO:0019
-	GO:0007155//cell adhesion
GO:0005515//protei	-
GO:0008270//zinc ic	GO:0009056//catabolic process;GO:0030855//epithelial cell differentiation
GO:0005515//protei	-
GO:0005515//protei	-
GO:0004499//N,N-d	-
GO:0001540//beta-a	GO:0001932//regulation of protein phosphorylation;GO:0001935//endoth
GO:0003779//actin b	GO:0007030//Golgi organization;GO:0007032//endosome organization;G

-	-
GO:0003810//protei	GO:0018149//peptide cross-linking
-	GO:0030389//fructosamine metabolic process;GO:0030855//epithelial cell
-	-
GO:0005515//protei	GO:0015879//carnitine transport;GO:0032414//positive regulation of ion t
GO:0003700//transc	GO:0006355//regulation of transcription, DNA-templated
GO:0005515//protei	-
GO:0003713//transc	GO:0007219//Notch signaling pathway;GO:0045944//positive regulation c
GO:0005509//calciur	GO:0007268//synaptic transmission;GO:0035556//intracellular signal trans
GO:0000166//nucleo	-
-	-
-	-
GO:0003796//lysozy	GO:0008152//metabolic process;GO:0016998//cell wall macromolecule ca
GO:0046872//metal	GO:0006875//cellular metal ion homeostasis
GO:0000166//nucleo	-
GO:0005254//chloric	GO:0006811//ion transport;GO:0006820//anion transport;GO:0006821//ch
GO:0016290//palmit	GO:0006637//acyl-CoA metabolic process;GO:0016559//peroxisome fissic
GO:0004672//protei	GO:0006885//regulation of pH;GO:0008286//insulin receptor signaling pa
GO:0000062//fatty-a	GO:0008203//cholesterol metabolic process;GO:0010742//macrophage de
-	-
GO:0005353//fructos	GO:0003044//regulation of systemic arterial blood pressure mediated by a
GO:0008113//peptid	-
GO:0003953//NAD+	GO:0001952//regulation of cell-matrix adhesion;GO:0008284//positive reg
GO:0003824//catalyt	GO:0006167//AMP biosynthetic process;GO:0006177//GMP biosynthetic p
GO:0005515//protei	GO:0070830//bicellular tight junction assembly
-	GO:0030193//regulation of blood coagulation;GO:0044763//single-organ
GO:0000166//nucleo	GO:0006563//L-serine metabolic process;GO:0006790//sulfur compound
GO:0005506//iron ic	-
-	GO:0007010//cytoskeleton organization;GO:0008360//regulation of cell sh
GO:0005102//recept	GO:0003336//corneocyte desquamation;GO:0006672//ceramide metaboli
GO:0042577//lipid p	GO:0006644//phospholipid metabolic process
GO:0008237//metall	GO:0006508//proteolysis
-	-
GO:0000064//L-orni	GO:1990575//mitochondrial L-ornithine transmembrane transport
GO:0004497//monoo	-
-	-
GO:0000287//magne	GO:0006584//catecholamine metabolic process;GO:0016036//cellular resp
GO:0003924//GTPas	-
GO:0005178//integri	GO:0000902//cell morphogenesis;GO:0006954//inflammatory response;G
-	-
GO:0005515//protei	GO:0002376//immune system process;GO:0006955//immune response;GC
GO:0005515//protei	GO:0042634//regulation of hair cycle
GO:0016407//acetylt	-
GO:0004252//serine	GO:0006508//proteolysis
GO:0004497//monoo	GO:0006805//xenobiotic metabolic process;GO:0008202//steroid metabol
-	-
GO:0005515//protei	-
-	-
GO:0003834//beta-c	GO:0001523//retinoid metabolic process;GO:0016121//carotene catabolic
GO:0004047//amino	GO:0019464//glycine decarboxylation via glycine cleavage system
-	-
GO:0005290//L-histi	GO:0006867//asparagine transport;GO:0006868//glutamine transport;GO:
GO:0004730//pseud	GO:0001522//pseudouridine synthesis;GO:0006796//phosphate-containir
GO:0016787//hydrol	-
GO:0019960//C-X3-	GO:0003366//cell-matrix adhesion involved in ameboidal cell migration;G
GO:0015108//chloric	GO:1902476//chloride transmembrane transport
GO:0003868//4-hyd	GO:0006572//tyrosine catabolic process;GO:0009072//aromatic amino aci
GO:0004866//endop	GO:0010951//negative regulation of endopeptidase activity

GO:0000166//nucleo	GO:0000122//negative regulation of transcription from RNA polymerase I
GO:0003824//catalyt	GO:0006778//porphyrin-containing compound metabolic process;GO:000
-	-
GO:0005515//protei	-
-	GO:0006886//intracellular protein transport
GO:0004497//mono	-
GO:0003779//actin b	GO:0007015//actin filament organization;GO:0030334//regulation of cell r
GO:0005085//guany	GO:0001934//positive regulation of protein phosphorylation;GO:0002437/
GO:0008083//growt	-
GO:0045027//DNA e	GO:0006302//double-strand break repair;GO:0006303//double-strand bre
GO:0005524//ATP bi	GO:0000086//G2/M transition of mitotic cell cycle;GO:0045332//phosphol
GO:0005515//protei	GO:0007155//cell adhesion;GO:0007156//homophilic cell adhesion via pla
-	-
GO:0004930//G-pro	GO:0007186//G-protein coupled receptor signaling pathway;GO:0007215
GO:0008270//zinc io	GO:0006882//cellular zinc ion homeostasis;GO:0010273//detoxification of
GO:0000287//magne	GO:0006114//glycerol biosynthetic process;GO:0006650//glycerophospho
GO:0005540//hyalur	GO:0007155//cell adhesion
-	-
GO:0098640//integri	GO:0007155//cell adhesion;GO:0007229//integrin-mediated signaling pat
-	GO:0008154//actin polymerization or depolymerization;GO:0030048//acti
GO:0008028//mono	GO:0015718//monocarboxylic acid transport;GO:0055085//transmembran
GO:0005515//protei	-
GO:0005085//guany	GO:0035025//positive regulation of Rho protein signal transduction;GO:0
GO:0005515//protei	GO:0051017//actin filament bundle assembly
-	-
GO:0070089//chlori	GO:0006813//potassium ion transport;GO:0097623//potassium ion export
-	-
GO:0004721//phosp	GO:0010801//negative regulation of peptidyl-threonine phosphorylation;
GO:0005509//calciu	GO:0007156//homophilic cell adhesion via plasma membrane adhesion m
GO:0004252//serine	GO:0000902//cell morphogenesis;GO:0001889//liver development;GO:000
-	GO:0007283//spermatogenesis;GO:0007288//sperm axoneme assembly;G
-	-
GO:0003697//single-	-
GO:0000166//nucleo	GO:0006563//L-serine metabolic process;GO:0006790//sulfur compound
GO:0005515//protei	-
-	-
GO:0004672//protei	GO:0001934//positive regulation of protein phosphorylation;GO:0006468/
GO:0000976//transc	GO:0000122//negative regulation of transcription from RNA polymerase I
GO:0015125//bile ac	GO:0015721//bile acid and bile salt transport;GO:0032782//bile acid secre
GO:0016922//ligand	GO:0045944//positive regulation of transcription from RNA polymerase II
GO:0009374//biotin	-
GO:0016407//acetylt	-
GO:0008480//sarcos	GO:1901053//sarcosine catabolic process
GO:0046872//metal	GO:0006875//cellular metal ion homeostasis
GO:0004497//mono	-
GO:0001849//compl	GO:0001878//response to yeast;GO:0008228//opsonization;GO:0030198/
GO:0008201//hepari	-
-	-
GO:0003677//DNA b	-
-	-
GO:0003810//protei	GO:0018149//peptide cross-linking
GO:0004499//N,N-d	-
-	-
GO:0004866//endop	-
-	-
GO:0004867//serine	GO:0010466//negative regulation of peptidase activity;GO:0030195//nega
GO:0005515//protei	-
GO:0005515//protei	GO:0006884//cell volume homeostasis;GO:0007165//signal transduction;

-	-
GO:0016787//hydrol	-
-	GO:0070979//protein K11-linked ubiquitination
GO:0008270//zinc ic	GO:0009056//catabolic process;GO:0030855//epithelial cell differentiation
-	-
GO:0003997//acyl-C	GO:0006631//fatty acid metabolic process;GO:0006635//fatty acid beta-o
-	-
-	-
GO:0004857//enzym	GO:0006071//glycerol metabolic process;GO:0006631//fatty acid metabol
-	-
GO:0004095//carniti	GO:0001676//long-chain fatty acid metabolic process;GO:0006629//lipid r
-	GO:0007286//spermatid development;GO:0048137//spermatocyte divisior
GO:0008009//chemc	GO:0006954//inflammatory response;GO:0006955//immune response;GO
GO:0008009//chemc	GO:0006954//inflammatory response;GO:0006955//immune response;GO
-	-
GO:0004553//hydrol	GO:0005975//carbohydrate metabolic process
GO:0020037//heme	GO:0019441//tryptophan catabolic process to kynurenine
-	-
-	GO:0001764//neuron migration;GO:0007162//negative regulation of cell a
GO:0004497//mono	GO:0006559//L-phenylalanine catabolic process;GO:0006571//tyrosine bi
GO:0004672//protei	GO:0006468//protein phosphorylation
GO:0015485//choles	GO:0032367//intracellular cholesterol transport;GO:0034389//lipid particle
GO:0004499//N,N-d	-
GO:0004044//amido	GO:0006177//GMP biosynthetic process;GO:0006189//'de novo' IMP bios
GO:0008146//sulfotr	-
GO:0019902//phosp	GO:0006511//ubiquitin-dependent protein catabolic process;GO:0016567
GO:0003779//actin b	GO:0006351//transcription, DNA-templated;GO:0030032//lamellipodium
GO:0004672//protei	GO:0006885//regulation of pH;GO:0008286//insulin receptor signaling pa
-	-
-	-
-	-
-	-
-	-
-	GO:0030389//fructosamine metabolic process;GO:0030855//epithelial cell
GO:0005507//coppe	GO:0009308//amine metabolic process;GO:0009445//putrescine metaboli
-	GO:0030193//regulation of blood coagulation;GO:0044763//single-organ
-	-
GO:0017147//Wnt-p	GO:0030178//negative regulation of Wnt signaling pathway
GO:0005515//protei	GO:0006955//immune response;GO:0006956//complement activation
-	-
GO:0004465//lipopr	GO:0006629//lipid metabolic process;GO:0006631//fatty acid metabolic p
-	GO:0001764//neuron migration;GO:0007420//brain development;GO:002
GO:0005515//protei	GO:0042634//regulation of hair cycle
GO:0046872//metal	GO:0006875//cellular metal ion homeostasis
GO:0003924//GTPas	-
-	-
GO:0008289//lipid b	-
GO:0008237//metall	GO:0006508//proteolysis
GO:0000064//L-orni	GO:1990575//mitochondrial L-ornithine transmembrane transport
-	-
-	-
GO:0000062//fatty-a	-
GO:0000064//L-orni	GO:1990575//mitochondrial L-ornithine transmembrane transport
GO:0003824//catalyt	GO:0006167//AMP biosynthetic process;GO:0006177//GMP biosynthetic p
GO:0005515//protei	GO:0006749//glutathione metabolic process
GO:0004896//cytoki	GO:0007171//activation of transmembrane receptor protein tyrosine kinas
-	-
-	GO:0007154//cell communication

GO:0005085//guany	GO:0007264//small GTPase mediated signal transduction;GO:0030334//re
GO:0050839//cell ad	GO:0007409//axonogenesis;GO:0010975//regulation of neuron projection
GO:0005515//protei	-
-	-
GO:0001786//phosp	GO:0002024//diet induced thermogenesis;GO:0006606//protein import in
-	-
GO:0003676//nuclei	GO:0006508//proteolysis;GO:0008152//metabolic process;GO:0016032//v
GO:0004930//G-pro	GO:0006897//endocytosis;GO:0006935//chemotaxis;GO:0006955//immun
GO:0005515//protei	-
GO:0008289//lipid b	GO:0070508//cholesterol import
GO:0004044//amido	GO:0006177//GMP biosynthetic process;GO:0006189//'de novo' IMP bios
-	-
-	GO:0006364//rRNA processing;GO:0009303//rRNA transcription
GO:0005515//protei	GO:0030855//epithelial cell differentiation
GO:0005158//insulin	GO:0001775//cell activation;GO:0007405//neuroblast proliferation;GO:000
GO:0015485//choles	GO:0032367//intracellular cholesterol transport;GO:0034389//lipid particle
GO:0008271//second	GO:0008272//sulfate transport;GO:0055085//transmembrane transport
-	GO:0006886//intracellular protein transport
GO:0005313//L-glut	GO:0015810//aspartate transport;GO:0015813//L-glutamate transport;GO
GO:0005158//insulin	GO:0001775//cell activation;GO:0007405//neuroblast proliferation;GO:000
GO:0004497//mono	GO:0006559//L-phenylalanine catabolic process;GO:0006571//tyrosine bi
GO:0005515//protei	GO:0000086//G2/M transition of mitotic cell cycle;GO:0031146//SCF-depe
GO:0005102//recept	GO:0001541//ovarian follicle development;GO:0001707//mesoderm forma
-	GO:0008154//actin polymerization or depolymerization;GO:0030048//acti
-	-
GO:0003677//DNA b	GO:0009267//cellular response to starvation;GO:0043066//negative regula
-	-
GO:0005507//coppe	GO:0007601//visual perception;GO:0009308//amine metabolic process
GO:0003924//GTPas	GO:0000082//G1/S transition of mitotic cell cycle;GO:0007010//cytoskelet
GO:0022857//transm	GO:0055085//transmembrane transport
GO:1990817//RNA a	GO:0009617//response to bacterium;GO:0030278//regulation of ossificati
GO:0005515//protei	GO:0007155//cell adhesion
GO:0005515//protei	-
GO:0015173//aroma	GO:0006590//thyroid hormone generation;GO:0055085//transmembrane
-	-
GO:0016787//hydrol	GO:0006629//lipid metabolic process;GO:0007216//G-protein coupled gl
GO:0005515//protei	GO:0090084//negative regulation of inclusion body assembly
GO:0004930//G-pro	GO:0001764//neuron migration;GO:0007186//G-protein coupled recepto
-	-
GO:0005515//protei	-
GO:0003810//protei	GO:0018149//peptide cross-linking
GO:0005507//coppe	GO:0009308//amine metabolic process;GO:0009445//putrescine metaboli
GO:0003697//single-	-
GO:0003953//NAD+	GO:0007165//signal transduction
GO:0004252//serine	GO:0006508//proteolysis
GO:0015297//antipo	GO:0055085//transmembrane transport
GO:0016491//oxidor	GO:0007005//mitochondrion organization
-	-
GO:0003824//catalyt	GO:0006684//sphingomyelin metabolic process;GO:0006685//sphingomy
-	-
GO:0008146//sulfotr	-
GO:0005515//protei	GO:0015879//carnitine transport;GO:0032414//positive regulation of ion t
-	-
GO:0004888//transm	GO:0007166//cell surface receptor signaling pathway;GO:0007186//G-pro
GO:0000166//nucleo	GO:0006563//L-serine metabolic process;GO:0006790//sulfur compound
GO:0003824//catalyt	GO:0006167//AMP biosynthetic process;GO:0006177//GMP biosynthetic p
GO:0016407//acetylt	-
GO:0000166//nucleo	GO:0006563//L-serine metabolic process;GO:0006790//sulfur compound

GO:0030246//carbol	-
GO:0004930//G-pro	GO:0002001//renin secretion into blood stream;GO:0002281//macrophag
-	GO:0007286//spermatid development;GO:0048137//spermatocyte divisio
GO:0005515//protei	GO:0042426//choline catabolic process
GO:0003924//GTPas	-
-	-
GO:0004806//triglyc	GO:0006629//lipid metabolic process;GO:0006633//fatty acid biosynthetic
GO:0003676//nuclei	GO:0006644//phospholipid metabolic process;GO:0006935//chemotaxis;G
-	-
-	-
-	-
GO:0004657//prolin	GO:0006562//proline catabolic process
GO:0070089//chloric	GO:0006813//potassium ion transport;GO:0097623//potassium ion export
GO:0003810//protei	GO:0018149//peptide cross-linking
GO:0005515//protei	-
GO:0005172//vascul	GO:0008283//cell proliferation;GO:0008284//positive regulation of cell pro
GO:0005515//protei	-
-	-
GO:0005515//protei	GO:0007043//cell-cell junction assembly;GO:0007155//cell adhesion;GO:0
-	-
GO:0004866//endop	-
GO:0005515//protei	GO:0007155//cell adhesion
GO:0005353//fructo	GO:0003044//regulation of systemic arterial blood pressure mediated by a
GO:0000287//magne	GO:0006006//glucose metabolic process;GO:0006094//gluconeogenesis;G
-	-
GO:0016407//acetylt	-
GO:0005290//L-histi	GO:0006867//asparagine transport;GO:0006868//glutamine transport;GO:
GO:0005546//phosp	GO:0009636//response to toxic substance;GO:0042360//vitamin E metabo
GO:0003824//catalyt	GO:0006103//2-oxoglutarate metabolic process;GO:0009058//biosyntheti
GO:0004930//G-pro	GO:0002300//CD8-positive, alpha-beta intraepithelial T cell differentiatio
GO:0022857//transm	GO:0055085//transmembrane transport
-	-
GO:0008201//hepari	-
GO:0002020//protea	GO:0001932//regulation of protein phosphorylation;GO:0007044//cell-sul
GO:0004888//transm	GO:0007166//cell surface receptor signaling pathway;GO:0007186//G-pro
-	-
GO:0005085//guany	GO:0007264//small GTPase mediated signal transduction;GO:0030334//re
GO:0050839//cell ad	GO:0007409//axonogenesis;GO:0010975//regulation of neuron projectio
GO:0004489//methy	GO:0001843//neural tube closure;GO:0006555//methionine metabolic pro
GO:0001786//phosp	GO:0002024//diet induced thermogenesis;GO:0006606//protein import in
GO:0004114//3',5'-c	GO:0007165//signal transduction;GO:0032729//positive regulation of inte
GO:0004044//amido	GO:0006177//GMP biosynthetic process;GO:0006189//'de novo' IMP bios
GO:0004090//carbor	GO:0006805//xenobiotic metabolic process;GO:0030855//epithelial cell di
GO:0003677//DNA b	GO:0006334//nucleosome assembly;GO:0006352//DNA-templated transc
GO:0038023//signali	GO:0007155//cell adhesion;GO:0007158//neuron cell-cell adhesion;GO:00
GO:0005515//protei	GO:0007409//axonogenesis;GO:0050807//regulation of synapse organiza
-	-
GO:0005102//recept	GO:0006898//receptor-mediated endocytosis;GO:0008104//protein locali
-	-
GO:0004639//phosp	GO:0006164//purine nucleotide biosynthetic process;GO:0006189//'de no
GO:0005515//protei	-
-	-
-	GO:0007010//cytoskeleton organization;GO:0008360//regulation of cell sh
GO:0000166//nucleo	GO:0006468//protein phosphorylation;GO:0006974//cellular response to I
GO:0030246//carbol	GO:0006897//endocytosis
-	-
GO:0003924//GTPas	-
GO:0005102//recept	GO:0001541//ovarian follicle development;GO:0001707//mesoderm forma

GO:0004672//protei	GO:0000002//mitochondrial genome maintenance;GO:0001938//positive
-	-
-	GO:0007288//sperm axoneme assembly
GO:0005507//coppe	GO:0009308//amine metabolic process;GO:0009445//putrescine metaboli
GO:0008047//enzym	GO:0006486//protein glycosylation;GO:0006487//protein N-linked glycos
GO:0005515//protei	-
GO:0008532//N-ace	GO:0006486//protein glycosylation;GO:0007411//axon guidance;GO:0007
GO:0005515//protei	GO:0098742//cell-cell adhesion via plasma-membrane adhesion molecul
GO:0008083//growt	-
-	-
GO:0003824//catalyt	GO:0006167//AMP biosynthetic process;GO:0006177//GMP biosynthetic p
GO:0046872//metal	GO:0006875//cellular metal ion homeostasis
GO:0003677//DNA b	GO:0009267//cellular response to starvation;GO:0043066//negative regul
GO:0020037//heme	GO:0019441//tryptophan catabolic process to kynurenine
-	-
-	GO:0006658//phosphatidylserine metabolic process;GO:0006665//sphing
GO:0004672//protei	GO:0006468//protein phosphorylation
GO:0003824//catalyt	GO:0010121//arginine catabolic process to proline via ornithine;GO:0019
-	GO:0006886//intracellular protein transport
GO:0015485//choles	GO:0032367//intracellular cholesterol transport;GO:0034389//lipid particl
GO:0004896//cytoki	GO:0008284//positive regulation of cell proliferation;GO:0019221//cytoki
-	-
GO:0004497//mono	GO:0006559//L-phenylalanine catabolic process;GO:0006571//tyrosine bi
-	GO:0007600//sensory perception;GO:0007601//visual perception;GO:0007
GO:0008028//mono	GO:0015718//monocarboxylic acid transport;GO:0055085//transmembran
GO:0005243//gap ju	GO:0007154//cell communication;GO:0007267//cell-cell signaling;GO:000
GO:0004930//G-pro	GO:0001764//neuron migration;GO:0007186//G-protein coupled recepto
GO:0003824//catalyt	GO:0010121//arginine catabolic process to proline via ornithine;GO:0019
GO:0030298//recept	GO:0010976//positive regulation of neuron projection development;GO:0
GO:0005158//insulin	GO:0001775//cell activation;GO:0007405//neuroblast proliferation;GO:000
-	-
GO:0003714//transc	GO:0008104//protein localization;GO:0008406//gonad development;GO:
GO:0019901//protei	GO:0030334//regulation of cell migration;GO:0034446//substrate adhesio
GO:0005515//protei	-
GO:0008009//chemo	GO:0006954//inflammatory response;GO:0006955//immune response;GO
GO:0005506//iron io	-
-	GO:0015888//thiamine transport
GO:0003868//4-hyd	GO:0006572//tyrosine catabolic process;GO:0009072//aromatic amino aci
GO:0004888//transm	GO:0007165//signal transduction;GO:0007166//cell surface receptor signa
GO:0004467//long-	GO:0006631//fatty acid metabolic process
GO:0000987//core p	GO:0009409//response to cold;GO:0045893//positive regulation of transc
-	-
GO:0000062//fatty-a	-
-	-
GO:0005515//protei	GO:0006749//glutathione metabolic process
GO:0004896//cytoki	GO:0019221//cytokine-mediated signaling pathway;GO:0034342//respon
GO:0008146//sulfotr	-
GO:0004499//N,N-d	-
GO:0003677//DNA b	GO:0006334//nucleosome assembly
GO:0000166//nucleo	GO:0007165//signal transduction;GO:0007186//G-protein coupled recept
-	-
-	-
GO:0003779//actin b	GO:0006351//transcription, DNA-templated;GO:0030032//lamellipodium
GO:0005515//protei	GO:0007165//signal transduction
GO:0005515//protei	GO:0006355//regulation of transcription, DNA-templated;GO:0007179//tr
GO:0005201//extrac	GO:0002244//hematopoietic progenitor cell differentiation
-	-
GO:0003988//acetyl-	GO:0000038//very long-chain fatty acid metabolic process;GO:0006635//

-	GO:0007010//cytoskeleton organization;GO:0008360//regulation of cell sh
-	-
GO:0005254//chloric	GO:0006811//ion transport;GO:0006820//anion transport;GO:0006821//ch
-	-
GO:0038023//signali	GO:0007160//cell-matrix adhesion;GO:0007179//transforming growth fac
GO:0004888//transm	GO:0007186//G-protein coupled receptor signaling pathway;GO:0009994
GO:0005313//L-glut	GO:0015810//aspartate transport;GO:0015813//L-glutamate transport;GC
GO:0004465//lipopr	GO:0006629//lipid metabolic process;GO:0006631//fatty acid metabolic p
GO:0003755//peptid	-
GO:0005509//calciur	GO:0001654//eye development;GO:0045667//regulation of osteoblast diff
GO:0000064//L-orni	GO:1990575//mitochondrial L-ornithine transmembrane transport
-	-
GO:0005515//protei	-
GO:0004930//G-pro	GO:0002001//renin secretion into blood stream;GO:0002281//macrophag
GO:0004499//N,N-d	-
GO:0004366//glycer	GO:0001817//regulation of cytokine production;GO:0006631//fatty acid m
GO:0005515//protei	-
-	GO:0006895//Golgi to endosome transport;GO:0015031//protein transpor
-	GO:0008360//regulation of cell shape
GO:0005515//protei	GO:0006878//cellular copper ion homeostasis;GO:0016567//protein ubiqu
GO:0004657//proline	GO:0006562//proline catabolic process
GO:0004175//endop	GO:0006508//proteolysis;GO:0007520//myoblast fusion;GO:0022617//extr
GO:0008289//lipid b	GO:0006869//lipid transport;GO:0042157//lipoprotein metabolic process
GO:0003824//catalyt	GO:0006527//arginine catabolic process;GO:0009072//aromatic amino aci
GO:0016628//oxidor	GO:0006695//cholesterol biosynthetic process;GO:0016126//sterol biosyn
GO:0000978//RNA p	GO:0001932//regulation of protein phosphorylation;GO:0006355//regulat
GO:0005546//phosp	GO:0009636//response to toxic substance;GO:0042360//vitamin E metabo
GO:0005515//protei	GO:0007155//cell adhesion;GO:0008593//regulation of Notch signaling pa
GO:0005515//protei	-
GO:0003777//microt	GO:0007018//microtubule-based movement;GO:0072384//organelle tran
-	GO:0007165//signal transduction
GO:0045027//DNA e	GO:0006302//double-strand break repair;GO:0006303//double-strand bre
-	-
GO:0008009//chemo	GO:0006954//inflammatory response;GO:0006955//immune response;GO
-	GO:0007286//spermatid development;GO:0048137//spermatocyte divisior
GO:0000334//3-hyd	GO:0009435//NAD biosynthetic process;GO:0010043//response to zinc io
-	-
-	-
-	-
GO:0005515//protei	-
GO:0070089//chloric	GO:0006813//potassium ion transport;GO:0097623//potassium ion export
GO:0000978//RNA p	GO:0006357//regulation of transcription from RNA polymerase II promote
GO:0003714//transc	GO:0006338//chromatin remodeling;GO:0007507//heart development;GC
GO:0003700//transc	GO:0006355//regulation of transcription, DNA-templated
-	-
GO:0003953//NAD+	GO:0007165//signal transduction
-	-
-	-
-	GO:0008154//actin polymerization or depolymerization;GO:0030048//acti
-	-
-	-
GO:0003779//actin b	GO:0071691//cardiac muscle thin filament assembly
GO:0008270//zinc ic	-
GO:0004672//protei	GO:0000002//mitochondrial genome maintenance;GO:0001938//positive
GO:0070089//chloric	GO:0006813//potassium ion transport;GO:0097623//potassium ion export
GO:0005515//protei	-
GO:0016922//ligand	GO:0045944//positive regulation of transcription from RNA polymerase II
-	GO:0017015//regulation of transforming growth factor beta receptor sign

GO:0004930//G-pro	GO:0006897//endocytosis;GO:0006935//chemotaxis;GO:0006955//immun
-	-
GO:0015173//aroma	GO:0006590//thyroid hormone generation;GO:0055085//transmembrane
GO:0004842//ubiqui	GO:0006511//ubiquitin-dependent protein catabolic process;GO:0007399
GO:0005096//GTPas	GO:0007165//signal transduction;GO:0043547//positive regulation of GTP
GO:0008201//hepari	-
-	GO:0001764//neuron migration;GO:0007420//brain development;GO:002
-	GO:0002098//tRNA wobble uridine modification;GO:0030335//positive re
-	-
GO:0046872//metal	GO:0006875//cellular metal ion homeostasis
GO:0004639//phosp	GO:0006164//purine nucleotide biosynthetic process;GO:0006189//'de no
GO:0016407//acetylt	-
-	GO:0009966//regulation of signal transduction
-	GO:0008154//actin polymerization or depolymerization;GO:0030048//acti
GO:0005509//calciur	GO:0007155//cell adhesion
GO:0016491//oxidor	-
GO:0004087//carbar	GO:0006207//'de novo' pyrimidine nucleobase biosynthetic process;GO:0(
GO:0004497//monoo	GO:0097267//omega-hydroxylase P450 pathway
-	-
-	-
GO:0004465//lipopr	GO:0006629//lipid metabolic process;GO:0006631//fatty acid metabolic p
-	GO:0007010//cytoskeleton organization;GO:0008360//regulation of cell sh
GO:0004114//3',5'-c	GO:0006198//cAMP catabolic process;GO:0007165//signal transduction
GO:0000166//nucleo	GO:0006897//endocytosis;GO:0006898//receptor-mediated endocytosis;(
GO:0005102//recept	GO:0003336//corneocyte desquamation;GO:0006672//ceramide metaboli
GO:0005515//protei	GO:0007165//signal transduction
GO:0005324//long-c	GO:0006469//negative regulation of protein kinase activity;GO:0009617//r
GO:0004930//G-pro	GO:0002300//CD8-positive, alpha-beta intraepithelial T cell differentiatior
GO:0002020//protea	GO:0010466//negative regulation of peptidase activity;GO:0018149//pept
GO:0016407//acetylt	-
GO:0030298//recept	GO:0010976//positive regulation of neuron projection development;GO:0
GO:0003779//actin b	GO:0006351//transcription, DNA-templated;GO:0030032//lamellipodium
-	GO:0009966//regulation of signal transduction
GO:0015218//pyrimi	GO:0000002//mitochondrial genome maintenance;GO:0002082//regulatio
-	-
-	GO:0007165//signal transduction;GO:0070507//regulation of microtubule
GO:0008289//lipid b	GO:0032374//regulation of cholesterol transport
-	-
GO:0004639//phosp	GO:0006164//purine nucleotide biosynthetic process;GO:0006189//'de no
GO:0008083//growtl	-
-	-
GO:0022857//transm	GO:0055085//transmembrane transport
GO:0005515//protei	-
GO:0016787//hydrol	-
GO:0004842//ubiqui	GO:0006511//ubiquitin-dependent protein catabolic process;GO:0007399
-	-
GO:0003779//actin b	GO:0007015//actin filament organization;GO:0030334//regulation of cell r
GO:0004497//monoo	GO:0006559//L-phenylalanine catabolic process;GO:0006571//tyrosine bi
-	-
GO:0008028//monoo	GO:0015718//monocarboxylic acid transport;GO:0055085//transmembran
GO:0005515//protei	-
GO:0015485//choles	GO:0032367//intracellular cholesterol transport;GO:0034389//lipid particle
-	GO:0007166//cell surface receptor signaling pathway
GO:0008270//zinc io	-
GO:0003924//GTPas	-
GO:0003676//nuclei	GO:0006508//proteolysis;GO:0016032//viral process
GO:0003824//catalyt	GO:0010121//arginine catabolic process to proline via ornithine;GO:0019
GO:0003779//actin b	GO:0030835//negative regulation of actin filament depolymerization

GO:0004896//cytoki	GO:0019221//cytokine-mediated signaling pathway;GO:0034342//respons
GO:0005515//protei	GO:0007155//cell adhesion
-	-
GO:0004806//triglyc	GO:0006629//lipid metabolic process;GO:0006633//fatty acid biosynthetic
GO:0005267//potass	GO:0006813//potassium ion transport;GO:0033198//response to ATP;GO:
-	-
GO:0015485//choles	GO:0009615//response to virus;GO:0030301//cholesterol transport;GO:00
GO:0003824//catalyt	GO:0006103//2-oxoglutarate metabolic process;GO:0009058//biosyntheti
GO:0003824//catalyt	GO:0006684//sphingomyelin metabolic process;GO:0006685//sphingomy
GO:0003824//catalyt	-
GO:0003868//4-hyd	GO:0006572//tyrosine catabolic process;GO:0009072//aromatic amino aci
GO:0016316//phosp	-
GO:0005509//calciur	-
GO:0005546//phosp	GO:0009636//response to toxic substance;GO:0042360//vitamin E metabo
GO:0005254//chloric	GO:0006811//ion transport;GO:0006820//anion transport;GO:0006821//cl
GO:0005506//iron ic	-
GO:0000166//nucleo	GO:0006563//L-serine metabolic process;GO:0006790//sulfur compound
GO:0042809//vitami	GO:0008285//negative regulation of cell proliferation;GO:0010468//regula
GO:0003779//actin k	-
-	-
-	GO:1901031//regulation of response to reactive oxygen species
-	GO:0007154//cell communication
GO:0050699//WW d	GO:0007034//vacuolar transport;GO:0010629//negative regulation of gen
GO:0003677//DNA k	GO:0035871//protein K11-linked deubiquitination
GO:0004657//prolin	GO:0006562//proline catabolic process
GO:0003677//DNA k	-
GO:0004930//G-pro	GO:0007186//G-protein coupled receptor signaling pathway
GO:0005515//protei	-
GO:0004478//methi	GO:0006556//S-adenosylmethionine biosynthetic process;GO:0009087//m
GO:0004930//G-pro	GO:0007186//G-protein coupled receptor signaling pathway
GO:0005515//protei	GO:0008277//regulation of G-protein coupled receptor protein signaling
GO:0003953//NAD+	GO:0007165//signal transduction;GO:0034128//negative regulation of My
GO:0003677//DNA k	GO:0006334//nucleosome assembly
GO:0003779//actin k	GO:0006351//transcription, DNA-templated;GO:0030032//lamellipodium
GO:0003676//nuclei	GO:0006644//phospholipid metabolic process;GO:0006935//chemotaxis;G
GO:0005515//protei	-
GO:0004738//pyruva	GO:0006086//acetyl-CoA biosynthetic process from pyruvate;GO:000609
GO:0022857//transm	GO:0055085//transmembrane transport
-	GO:0007165//signal transduction
GO:0016491//oxidor	GO:0006629//lipid metabolic process
GO:0005507//coppe	GO:0009308//amine metabolic process;GO:0009445//putrescine metaboli
GO:0004044//amido	GO:0006177//GMP biosynthetic process;GO:0006189//'de novo' IMP bios
GO:0003810//protei	GO:0018149//peptide cross-linking
-	-
-	GO:0016559//peroxisome fission;GO:0044375//regulation of peroxisome s
GO:0008270//zinc ic	-
GO:0004465//lipopro	GO:0006629//lipid metabolic process;GO:0006631//fatty acid metabolic p
GO:0003677//DNA k	GO:0006281//DNA repair;GO:0006974//cellular response to DNA damage
GO:0005484//SNAP	GO:0006888//ER to Golgi vesicle-mediated transport;GO:0006903//vesicle
GO:0003714//transc	GO:0008104//protein localization;GO:0008406//gonad development;GO:0
GO:0003924//GTPas	GO:0000082//G1/S transition of mitotic cell cycle;GO:0007010//cytoskelet
-	GO:0032088//negative regulation of NF-kappaB transcription factor activi
GO:0000976//transc	GO:0006357//regulation of transcription from RNA polymerase II promote
GO:0008146//sulfotr	-
GO:0001517//N-ace	GO:0005975//carbohydrate metabolic process;GO:0006044//N-acetylgluc
-	-
-	-
GO:0016491//oxidor	GO:0019477//L-lysine catabolic process

GO:0002020//protea	GO:0001932//regulation of protein phosphorylation;GO:0007044//cell-su
-	GO:0006355//regulation of transcription, DNA-templated
GO:0003700//transc	GO:0006355//regulation of transcription, DNA-templated
GO:0003824//catalyt	GO:0005975//carbohydrate metabolic process
GO:0015078//hydro	GO:0030177//positive regulation of Wnt signaling pathway;GO:1902600//
GO:0008022//protei	GO:0007099//centriole replication;GO:0010457//centriole-centriole cohes
GO:0042802//identic	GO:1904262//negative regulation of TORC1 signaling
GO:0030729//acetoa	GO:0006629//lipid metabolic process
GO:0005158//insulin	GO:0001775//cell activation;GO:0007405//neuroblast proliferation;GO:00
GO:0005515//protei	-
GO:0008194//UDP-	GO:0006486//protein glycosylation;GO:0006682//galactosylceramide bios
-	-
GO:0003810//protei	GO:0018149//peptide cross-linking
GO:0004044//amido	GO:0006177//GMP biosynthetic process;GO:0006189//'de novo' IMP bios
GO:0004553//hydrol	GO:0005975//carbohydrate metabolic process
GO:0004090//carbo	GO:0006805//xenobiotic metabolic process;GO:0030855//epithelial cell di
-	-
GO:0017080//sodiu	GO:0006814//sodium ion transport;GO:0010460//positive regulation of h
-	-
GO:0004842//ubiqui	GO:0016567//protein ubiquitination
-	-
GO:0042802//identic	-
GO:0005515//protei	-
GO:0051015//actin f	GO:0003382//epithelial cell morphogenesis;GO:0007015//actin filament o
GO:0003824//catalyt	-
GO:0008113//peptid	-
GO:0000166//nucleo	GO:0006897//endocytosis;GO:0006898//receptor-mediated endocytosis;
GO:0005227//calciu	GO:0006812//cation transport
GO:0003924//GTPas	-
-	-
GO:0000166//nucleo	GO:0006563//L-serine metabolic process;GO:0006790//sulfur compound
GO:0015218//pyrimi	GO:0000002//mitochondrial genome maintenance;GO:0002082//regulati
GO:0004639//phosp	GO:0006164//purine nucleotide biosynthetic process;GO:0006189//'de no
-	-
-	GO:0007165//signal transduction;GO:0070507//regulation of microtubule
GO:0004842//ubiqui	GO:0006511//ubiquitin-dependent protein catabolic process;GO:0007399
GO:0003779//actin b	GO:0007030//Golgi organization;GO:0007032//endosome organization;G
GO:0005104//fibrob	GO:0000132//establishment of mitotic spindle orientation;GO:0001656//
GO:0004721//phosp	GO:0010801//negative regulation of peptidyl-threonine phosphorylation;
GO:0003841//1-acyl	GO:0001676//long-chain fatty acid metabolic process;GO:0006629//lipid
GO:0002020//protea	GO:0010466//negative regulation of peptidase activity;GO:0018149//pept
GO:0016491//oxido	GO:0009058//biosynthetic process;GO:0009792//embryo development e
GO:0004672//protei	GO:0000002//mitochondrial genome maintenance;GO:0001938//positive
GO:0015485//choles	GO:0032367//intracellular cholesterol transport;GO:0034389//lipid particl
GO:0005515//protei	-
GO:0004497//mono	GO:0006559//L-phenylalanine catabolic process;GO:0006571//tyrosine bi
-	-
GO:0051015//actin f	GO:0030866//cortical actin cytoskeleton organization;GO:0045214//sarco
GO:0004730//pseud	GO:0001522//pseudouridine synthesis;GO:0006796//phosphate-containi
GO:0022857//trans	GO:0055085//transmembrane transport
GO:0005201//extrac	GO:0002244//hematopoietic progenitor cell differentiation
GO:0005242//inwar	GO:0006813//potassium ion transport
GO:0004842//ubiqui	GO:0006511//ubiquitin-dependent protein catabolic process;GO:0007399
GO:0000062//fatty-	GO:0008203//cholesterol metabolic process;GO:0010742//macrophage d
-	-
-	-
-	GO:0007286//spermatid development;GO:0048137//spermatocyte divisio
GO:0003824//catalyt	GO:0006567//threonine catabolic process

-	-
GO:0005515//protei	GO:0007155//cell adhesion;GO:0007156//homophilic cell adhesion via pla
GO:0004888//transm	GO:0007166//cell surface receptor signaling pathway;GO:0007186//G-pro
GO:0003874//6-pyru	GO:0006729//tetrahydrobiopterin biosynthetic process
GO:0004122//cystatl	GO:0001958//endochondral ossification;GO:0001974//blood vessel remoc
GO:0004672//protei	GO:0006468//protein phosphorylation
GO:0008289//lipid b	-
GO:0098640//integri	GO:0007155//cell adhesion;GO:0007229//integrin-mediated signaling pat
-	GO:0055085//transmembrane transport
-	-
GO:0005509//calciur	-
GO:0004497//monoo	-
GO:0005546//phosp	GO:0009636//response to toxic substance;GO:0042360//vitamin E metabc
GO:0005546//phosp	GO:0009636//response to toxic substance;GO:0042360//vitamin E metabc
GO:0004497//monoo	-
-	-
-	-
-	GO:0008154//actin polymerization or depolymerization;GO:0030048//acti
GO:0004657//proline	GO:0006562//proline catabolic process
GO:0005172//vascul	GO:0008283//cell proliferation;GO:0008284//positive regulation of cell pro
GO:0000166//nucleo	GO:0007165//signal transduction;GO:0007186//G-protein coupled recept
GO:0005085//guany	GO:0001934//positive regulation of protein phosphorylation;GO:0002437/
-	-
GO:0004930//G-pro	GO:0002300//CD8-positive, alpha-beta intraepithelial T cell differentiatior
GO:0035254//glutan	GO:0001662//behavioral fear response;GO:0006884//cell volume homeos
GO:0004364//glutatl	GO:0006749//glutathione metabolic process;GO:0006805//xenobiotic met
GO:0003834//beta-c	GO:0001523//retinoid metabolic process;GO:0016121//carotene catabolic
GO:0003988//acetyl-	GO:0000038//very long-chain fatty acid metabolic process;GO:0006635//f
GO:0004016//adeny	GO:0006171//cAMP biosynthetic process;GO:0007613//memory;GO:0007
GO:0004867//serine	GO:0007596//blood coagulation
GO:0005515//protei	GO:0051260//protein homooligomerization
GO:0004045//amino	-
GO:0000978//RNA p	GO:0006357//regulation of transcription from RNA polymerase II promote
GO:0008201//hepari	-
-	GO:0043065//positive regulation of apoptotic process;GO:0046330//posit
GO:0003677//DNA b	-
GO:0003924//GTPas	GO:0032755//positive regulation of interleukin-6 production;GO:0034144
-	-
GO:0016491//oxidor	-
-	-
GO:0005125//cytoki	GO:0002232//leukocyte chemotaxis involved in inflammatory response;GC
-	-
GO:0004222//metall	GO:0000003//reproduction;GO:0006508//proteolysis
GO:0035591//signali	GO:0034128//negative regulation of MyD88-independent toll-like recepto
GO:0004672//protei	GO:0006468//protein phosphorylation;GO:0007169//transmembrane rece
GO:0005096//GTPas	GO:0007165//signal transduction;GO:0043547//positive regulation of GTP
GO:0008480//sarcos	GO:1901053//sarcosine catabolic process
GO:0004175//endop	GO:0006508//proteolysis;GO:0007520//myoblast fusion;GO:0022617//extr
GO:0005085//guany	GO:0001934//positive regulation of protein phosphorylation;GO:0002437/
GO:0003700//transc	GO:0006355//regulation of transcription, DNA-templated;GO:0045607//re
GO:0005125//cytoki	GO:0002232//leukocyte chemotaxis involved in inflammatory response;GC
GO:0008146//sulfotr	-
GO:0046872//metal	GO:0006875//cellular metal ion homeostasis
GO:0000976//transc	GO:0000122//negative regulation of transcription from RNA polymerase I
GO:0004497//monoo	GO:0097267//omega-hydroxylase P450 pathway
GO:0003824//catalyt	GO:0009436//glyoxylate catabolic process;GO:0019265//glycine biosynthe
GO:0004478//methic	GO:0006556//S-adenosylmethionine biosynthetic process;GO:0009087//m
GO:0005506//iron ic	GO:0006457//protein folding;GO:0018126//protein hydroxylation;GO:001

GO:0003676//nuclei	GO:0006644//phospholipid metabolic process;GO:0006935//chemotaxis;G
-	GO:0007600//sensory perception;GO:0007601//visual perception;GO:0007
GO:0008195//phosp	GO:0006796//phosphate-containing compound metabolic process;GO:00
-	-
GO:0070089//chloric	GO:0006813//potassium ion transport;GO:0097623//potassium ion export
GO:0005515//protei	-
-	-
GO:0008270//zinc ic	-
GO:0008270//zinc ic	GO:0009056//catabolic process;GO:0030855//epithelial cell differentiation
GO:0000978//RNA p	GO:0001649//osteoblast differentiation;GO:0001755//neural crest cell mig
-	GO:0032088//negative regulation of NF-kappaB transcription factor activi
-	-
GO:0001540//beta-a	GO:0001932//regulation of protein phosphorylation;GO:0001935//endoth
GO:0000062//fatty-a	GO:0008203//cholesterol metabolic process;GO:0010742//macrophage de
GO:0005172//vascul	GO:0008283//cell proliferation;GO:0008284//positive regulation of cell pro
GO:0005515//protei	GO:0003431//growth plate cartilage chondrocyte development;GO:00082
GO:0008083//growtl	-
-	-
GO:0004888//transm	GO:0007165//signal transduction;GO:0007166//cell surface receptor signa
GO:0003824//catalyt	-
GO:0002020//protea	GO:0001932//regulation of protein phosphorylation;GO:0007044//cell-sul
GO:0004465//lipopro	GO:0006629//lipid metabolic process;GO:0006631//fatty acid metabolic p
GO:0000977//RNA p	GO:0000122//negative regulation of transcription from RNA polymerase I
GO:0008009//chemo	GO:0006954//inflammatory response;GO:0006955//immune response;GO
GO:0005085//guany	-
GO:0042802//identic	GO:0030182//neuron differentiation;GO:0045893//positive regulation of tr
GO:0003924//GTPas	-
-	-
GO:0003779//actin b	GO:0006351//transcription, DNA-templated;GO:0030032//lamellipodium
GO:0004930//G-pro	GO:0007186//G-protein coupled receptor signaling pathway
GO:0003779//actin b	-
-	-
GO:0003676//nuclei	GO:0006508//proteolysis;GO:0016032//viral process
-	-
GO:0005515//protei	GO:0006355//regulation of transcription, DNA-templated;GO:0007179//tr
GO:0004499//N,N-d	-
GO:0004087//carbar	GO:0006207//'de novo' pyrimidine nucleobase biosynthetic process;GO:0
GO:0004090//carbor	GO:0006805//xenobiotic metabolic process;GO:0030855//epithelial cell di
-	-
GO:0004860//protei	GO:0006469//negative regulation of protein kinase activity;GO:2000480//r
GO:0031956//mediu	-
GO:0005102//recept	GO:0001541//ovarian follicle development;GO:0001707//mesoderm forma
GO:0043022//riboso	GO:0006886//intracellular protein transport;GO:0030433//ER-associated u
GO:0016316//phosp	-
GO:0005515//protei	-
-	-
-	-
-	GO:0007010//cytoskeleton organization;GO:0008360//regulation of cell sh
-	-
GO:0005506//iron ic	-
-	-
-	-
GO:0001786//phosp	GO:0002024//diet induced thermogenesis;GO:0006606//protein import in
-	-
GO:0005085//guany	GO:0007264//small GTPase mediated signal transduction;GO:0030334//re
GO:0003924//GTPas	-
GO:0000254//C-4 m	GO:0001934//positive regulation of protein phosphorylation;GO:0008610/
GO:0030298//recept	GO:0010976//positive regulation of neuron projection development;GO:0

GO:0005515//protei	GO:0006749//glutathione metabolic process
GO:0003824//catalyt	GO:0006508//proteolysis;GO:0012501//programmed cell death;GO:00147
GO:0004867//serine	GO:0010951//negative regulation of endopeptidase activity;GO:0030212/
GO:0000062//fatty-a	-
GO:0005201//extrac	GO:0002244//hematopoietic progenitor cell differentiation
GO:0003682//chrom	GO:0015908//fatty acid transport;GO:0043066//negative regulation of apo
-	-
-	GO:0001558//regulation of cell growth;GO:0008283//cell proliferation;GO
-	GO:0007166//cell surface receptor signaling pathway
-	GO:0001764//neuron migration;GO:0007162//negative regulation of cell
GO:0031419//cobala	GO:0015889//cobalamin transport
-	-
GO:0004730//pseud	GO:0001522//pseudouridine synthesis;GO:0006796//phosphate-containin
GO:0005515//protei	GO:0030855//epithelial cell differentiation
GO:0004721//phosp	GO:0010801//negative regulation of peptidyl-threonine phosphorylation;
GO:0016491//oxidor	GO:0006629//lipid metabolic process;GO:0006631//fatty acid metabolic p
GO:0008194//UDP-	GO:0006486//protein glycosylation;GO:0006682//galactosylceramide bios
-	-
-	-
GO:0046790//virion	GO:0075512//clathrin-mediated endocytosis of virus by host cell
-	-
GO:0004930//G-pro	GO:0002300//CD8-positive, alpha-beta intraepithelial T cell differentiatio
GO:0016740//transf	GO:0006629//lipid metabolic process;GO:0006659//phosphatidylserine bi
-	-
-	GO:0055085//transmembrane transport
GO:0016491//oxidor	GO:0019477//L-lysine catabolic process
GO:0004866//endop	-
-	-
GO:0042802//identic	-
GO:0003824//catalyt	GO:0010121//arginine catabolic process to proline via ornithine;GO:0019
GO:0005515//protei	GO:0003431//growth plate cartilage chondrocyte development;GO:00082
GO:1990817//RNA a	GO:0009617//response to bacterium;GO:0030278//regulation of ossificati
GO:0004672//protei	GO:0006468//protein phosphorylation
GO:0004888//transm	GO:0007166//cell surface receptor signaling pathway;GO:0007186//G-pro
GO:0004867//serine	-
GO:0000978//RNA p	GO:0006357//regulation of transcription from RNA polymerase II promot
GO:0005524//ATP b	GO:0055085//transmembrane transport
GO:0004467//long-	GO:0001676//long-chain fatty acid metabolic process;GO:0008610//lipid
GO:0002020//protea	GO:0010466//negative regulation of peptidase activity;GO:0018149//pept
GO:0003677//DNA	GO:0006334//nucleosome assembly;GO:0042742//defense response to ba
GO:0005515//protei	GO:0007165//signal transduction
GO:0003779//actin	GO:0055013//cardiac muscle cell development
-	-
-	-
GO:0005515//protei	-
GO:0005102//recept	GO:0006898//receptor-mediated endocytosis;GO:0008104//protein locali
-	-
GO:0008270//zinc io	GO:0009056//catabolic process;GO:0030855//epithelial cell differentiation
-	-
GO:0005515//protei	-
-	GO:0016192//vesicle-mediated transport
GO:0003924//GTPas	GO:0006254//CTP catabolic process;GO:0034656//nucleobase-containing
GO:0000166//nucle	GO:0007165//signal transduction;GO:0007186//G-protein coupled recept
GO:0004114//3',5'-c	GO:0006198//cAMP catabolic process;GO:0007165//signal transduction
GO:0005543//phosp	GO:0048260//positive regulation of receptor-mediated endocytosis;GO:0
GO:0001664//G-pro	GO:0007286//spermatid development;GO:0034613//cellular protein locali
GO:0001784//phosp	GO:0001932//regulation of protein phosphorylation;GO:0007259//JAK-ST
-	GO:0000077//DNA damage checkpoint

-	GO:0008154//actin polymerization or depolymerization;GO:0030048//acti
GO:0004672//protei	GO:0000002//mitochondrial genome maintenance;GO:0001938//positive
GO:0003988//acetyl-	GO:0000038//very long-chain fatty acid metabolic process;GO:0006635//f
GO:0005515//protei	GO:0015879//carnitine transport;GO:0032414//positive regulation of ion t
-	-
-	-
GO:0003824//catalyt	GO:0006536//glutamate metabolic process;GO:0006561//proline biosynth
GO:0004553//hydrol	GO:0005975//carbohydrate metabolic process
GO:0005515//protei	-
GO:0005515//protei	-
GO:0035254//glutan	GO:0001662//behavioral fear response;GO:0006884//cell volume homeos
-	-
GO:0003779//actin b	GO:0007010//cytoskeleton organization;GO:0008360//regulation of cell sh
GO:0000976//transc	GO:0000122//negative regulation of transcription from RNA polymerase I
GO:0008480//sarcos	GO:1901053//sarcosine catabolic process
GO:0004867//serine	GO:0007596//blood coagulation
GO:0005515//protei	-
GO:0004930//G-pro	GO:0002001//renin secretion into blood stream;GO:0002281//macrophag
GO:0008113//peptid	-
GO:0005515//protei	GO:0090084//negative regulation of inclusion body assembly
GO:0004089//carbor	GO:0002009//morphogenesis of an epithelium;GO:0046903//secretion
GO:0004869//cystein	GO:0001503//ossification;GO:0006953//acute-phase response;GO:00305C
GO:0005515//protei	GO:0010468//regulation of gene expression;GO:0016567//protein ubiquit
GO:0042802//identic	-
GO:0003676//nucleic	GO:0006278//RNA-dependent DNA replication;GO:0015074//DNA integra
-	-
GO:0003951//NAD+	GO:0006111//regulation of gluconeogenesis;GO:0006357//regulation of t
GO:0004725//protei	GO:0006470//protein dephosphorylation;GO:0016311//dephosphorylatio
GO:0003700//transc	GO:0006355//regulation of transcription, DNA-templated
GO:0005515//protei	GO:0006355//regulation of transcription, DNA-templated;GO:0007179//tr
-	-
GO:0000978//RNA p	GO:0001649//osteoblast differentiation;GO:0001755//neural crest cell mig
GO:0000334//3-hyd	GO:0009435//NAD biosynthetic process;GO:0010043//response to zinc io
-	-
GO:0008271//secon	GO:0008272//sulfate transport;GO:0055085//transmembrane transport
-	-
GO:0001540//beta-a	GO:0001932//regulation of protein phosphorylation;GO:0001935//endoth
GO:0098640//integri	GO:0007155//cell adhesion;GO:0007229//integrin-mediated signaling pat
GO:0015485//choles	GO:0009615//response to virus;GO:0030301//cholesterol transport;GO:00
GO:0005524//ATP bi	GO:0008203//cholesterol metabolic process;GO:0010033//response to or
GO:0004639//phosp	GO:0006164//purine nucleotide biosynthetic process;GO:0006189//'de no
GO:0000062//fatty-a	GO:0008203//cholesterol metabolic process;GO:0010742//macrophage de
GO:0005515//protei	GO:0007155//cell adhesion;GO:0007156//homophilic cell adhesion via pla
GO:0003779//actin b	GO:0007015//actin filament organization;GO:0030334//regulation of cell r
GO:0005509//calciur	GO:0007268//synaptic transmission;GO:0008104//protein localization;GO:
-	-
GO:0004310//farnes	GO:0008610//lipid biosynthetic process;GO:0009058//biosynthetic proces
GO:0005104//fibrob	GO:0001525//angiogenesis;GO:0001759//organ induction;GO:0001934//p
GO:0004497//mono	GO:0006805//xenobiotic metabolic process;GO:0008202//steroid metabol
-	-
-	-
GO:0000977//RNA p	GO:0000122//negative regulation of transcription from RNA polymerase I
-	GO:0006355//regulation of transcription, DNA-templated
-	-
GO:0004122//cystatl	GO:0001958//endochondral ossification;GO:0001974//blood vessel remoc
GO:0005515//protei	-
GO:0005515//protei	GO:0006749//glutathione metabolic process
-	-

GO:0000062//fatty-a	-
-	-
GO:0004652//polynu	GO:0008285//negative regulation of cell proliferation;GO:0043066//negat
GO:0005515//protei	GO:0007155//cell adhesion;GO:0007156//homophilic cell adhesion via pla
GO:0003677//DNA b	GO:0006334//nucleosome assembly
GO:0005515//protei	-
GO:0004364//glutath	GO:0006749//glutathione metabolic process;GO:0006805//xenobiotic met
GO:0098640//integri	GO:0007155//cell adhesion;GO:0007229//integrin-mediated signaling pat
-	GO:0009966//regulation of signal transduction
GO:0004497//mono	-
-	-
-	GO:0016559//peroxisome fission;GO:0044375//regulation of peroxisome s
GO:0016407//acetylt	-
GO:0005290//L-histi	GO:0006867//asparagine transport;GO:0006868//glutamine transport;GO:
-	-
-	-
GO:0005102//recept	GO:0003336//corneocyte desquamation;GO:0006672//ceramide metaboli
GO:0031419//cobala	GO:0015889//cobalamin transport
GO:0008270//zinc ic	GO:0006882//cellular zinc ion homeostasis;GO:0010273//detoxification of
GO:0003953//NAD+	GO:0007165//signal transduction;GO:0034128//negative regulation of My
GO:0003868//4-hyd	GO:0006572//tyrosine catabolic process;GO:0009072//aromatic amino aci
GO:0004252//serine	GO:0006508//proteolysis
GO:0003834//beta-c	GO:0001523//retinoid metabolic process;GO:0016121//carotene catabolic
GO:0004930//G-pro	GO:0007186//G-protein coupled receptor signaling pathway
GO:0005507//coppe	GO:0009308//amine metabolic process;GO:0009445//putrescine metaboli
GO:0005515//protei	GO:0030855//epithelial cell differentiation
GO:0004343//glucos	GO:0006048//UDP-N-acetylglucosamine biosynthetic process
GO:0005085//guany	GO:0001934//positive regulation of protein phosphorylation;GO:0002437/
GO:0004657//prolin	GO:0006562//proline catabolic process
GO:0004672//protei	GO:0006468//protein phosphorylation
GO:0008113//peptid	-
-	GO:0007166//cell surface receptor signaling pathway
GO:0031072//heat sl	GO:0045944//positive regulation of transcription from RNA polymerase II
GO:0004867//serine	GO:0007596//blood coagulation
GO:0016740//transfe	GO:0006777//Mo-molybdopterin cofactor biosynthetic process
GO:0008480//sarcos	GO:1901053//sarcosine catabolic process
GO:0004478//methio	GO:0006556//S-adenosylmethionine biosynthetic process;GO:0009087//m
GO:0005515//protei	-
GO:0008195//phosp	GO:0006796//phosphate-containing compound metabolic process;GO:00
GO:0005102//recept	GO:0006898//receptor-mediated endocytosis;GO:0008104//protein locali
-	-
GO:1990817//RNA a	GO:0009617//response to bacterium;GO:0030278//regulation of ossificati
GO:0009374//biotin	-
GO:0003841//1-acyl	GO:0006654//phosphatidic acid biosynthetic process;GO:0010891//negati
GO:0005216//ion ch	GO:0006811//ion transport;GO:0006814//sodium ion transport;GO:00550
GO:0008146//sulfotr	-
GO:0050839//cell ad	GO:0007409//axonogenesis;GO:0010975//regulation of neuron projection
-	-
GO:0005125//cytoki	GO:0002232//leukocyte chemotaxis involved in inflammatory response;GO
GO:0004497//mono	-
GO:0005102//recept	GO:0001541//ovarian follicle development;GO:0001707//mesoderm forma
GO:0005515//protei	-
GO:0008270//zinc ic	GO:0009056//catabolic process;GO:0030855//epithelial cell differentiation
-	-
GO:0000978//RNA p	GO:0001736//establishment of planar polarity;GO:0001843//neural tube c
-	-
GO:0008270//zinc ic	GO:0009056//catabolic process;GO:0030855//epithelial cell differentiation
GO:0005515//protei	-

GO:0005158//insulin	GO:0001775//cell activation;GO:0007405//neuroblast proliferation;GO:000
GO:0051015//actin f	GO:0003382//epithelial cell morphogenesis;GO:0007015//actin filament o
-	-
-	GO:0001501//skeletal system development;GO:0006006//glucose metabo
GO:0004364//glutatl	GO:0006749//glutathione metabolic process;GO:0006805//xenobiotic met
GO:0001786//phosp	GO:0002024//diet induced thermogenesis;GO:0006606//protein import in
GO:0005515//protei	GO:0007165//signal transduction
GO:0005085//guany	GO:0007264//small GTPase mediated signal transduction;GO:0030334//re
-	-
GO:0008146//sulfotr	-
GO:0005515//protei	GO:0015879//carnitine transport;GO:0032414//positive regulation of ion t
-	-
GO:0008017//microt	GO:0008285//negative regulation of cell proliferation;GO:0030330//DNA (
GO:0009374//biotin	-
-	GO:0000077//DNA damage checkpoint
GO:0008480//sarcos	GO:1901053//sarcosine catabolic process
-	-
GO:0035591//signali	GO:0034128//negative regulation of MyD88-independent toll-like recepto
GO:0000254//C-4 m	GO:0001934//positive regulation of protein phosphorylation;GO:0008610/
GO:0003677//DNA b	GO:0035871//protein K11-linked deubiquitination
-	-
GO:0000287//magne	GO:0006099//tricarboxylic acid cycle
GO:0017075//syntax	-
-	-
GO:0004657//prolin	GO:0006562//proline catabolic process
-	GO:0055085//transmembrane transport
-	-
GO:0098640//integri	GO:0007155//cell adhesion;GO:0007229//integrin-mediated signaling pat
GO:0004888//transm	GO:0007166//cell surface receptor signaling pathway;GO:0007186//G-pro
GO:0008270//zinc ic	GO:0006882//cellular zinc ion homeostasis;GO:0010273//detoxification of
-	-
GO:0003676//nuclei	GO:0043488//regulation of mRNA stability
-	-
GO:0004888//transm	GO:0007166//cell surface receptor signaling pathway;GO:0007186//G-pro
GO:0022857//transm	GO:0055085//transmembrane transport
-	GO:0016567//protein ubiquitination
GO:0019960//C-X3-	GO:0003366//cell-matrix adhesion involved in ameboidal cell migration;G
GO:0005515//protei	GO:0007155//cell adhesion;GO:0007156//homophilic cell adhesion via pla
GO:0004089//carbor	GO:0002009//morphogenesis of an epithelium;GO:0046903//secretion
-	-
GO:0005085//guany	-
-	-
-	GO:0007286//spermatid development;GO:0048137//spermatocyte divisio
GO:0005515//protei	GO:0055085//transmembrane transport
-	-
GO:0000334//3-hyd	GO:0009435//NAD biosynthetic process;GO:0010043//response to zinc io
-	GO:0001764//neuron migration;GO:0007162//negative regulation of cell a
-	-
-	-
GO:0005353//fructo:	GO:0003044//regulation of systemic arterial blood pressure mediated by a
GO:0003796//lysozy	GO:0008152//metabolic process;GO:0016998//cell wall macromolecule ca
GO:0015293//sympo	GO:0008643//carbohydrate transport
GO:0001784//phosp	GO:0008286//insulin receptor signaling pathway;GO:0010907//positive re
-	-
GO:0004497//mono	GO:0006805//xenobiotic metabolic process;GO:0008202//steroid metabol
-	-
-	-
GO:0004252//serine	GO:0006508//proteolysis

GO:0008146//sulfotr	-
-	-
GO:0005509//calciur	GO:0007156//homophilic cell adhesion via plasma membrane adhesion m
GO:0004047//amino	GO:0019464//glycine decarboxylation via glycine cleavage system
GO:0008194//UDP-(	GO:0006486//protein glycosylation;GO:0006682//galactosylceramide bios
GO:0008289//lipid b	GO:0006869//lipid transport;GO:0042157//lipoprotein metabolic process
GO:0004639//phosp	GO:0006164//purine nucleotide biosynthetic process;GO:0006189//'de no
GO:0008201//hepari	-
GO:0004497//monoo	GO:0006805//xenobiotic metabolic process;GO:0008202//steroid metabol
-	-
GO:0005515//protei	-
GO:0003713//transc	GO:0007219//Notch signaling pathway;GO:0045944//positive regulation c
GO:0004866//endop	GO:0010951//negative regulation of endopeptidase activity
GO:0000248//C-5 st	GO:0008610//lipid biosynthetic process;GO:0016126//sterol biosynthetic p
-	-
GO:0016740//transfe	-
-	-
GO:0008113//peptid	-
GO:0005515//protei	GO:0006878//cellular copper ion homeostasis;GO:0016567//protein ubiqu
GO:0005515//protei	GO:0003431//growth plate cartilage chondrocyte development;GO:00082
GO:0005506//iron ic	GO:0006457//protein folding;GO:0018126//protein hydroxylation;GO:001
GO:0004343//glucos	GO:0006048//UDP-N-acetylglucosamine biosynthetic process
GO:0005515//protei	-
-	-
-	-
GO:0005102//recept	GO:0006898//receptor-mediated endocytosis;GO:0008104//protein locali
GO:0003723//RNA b	GO:0006412//translation;GO:0006414//translational elongation;GO:00150
-	-
GO:0005515//protei	-
GO:0004867//serine	GO:0007596//blood coagulation
-	-
-	-
GO:0035254//glutan	GO:0001662//behavioral fear response;GO:0006884//cell volume homeos
GO:0004721//phosp	GO:0010801//negative regulation of peptidyl-threonine phosphorylation;
GO:0003677//DNA b	GO:0006351//transcription, DNA-templated
GO:0005518//collag	GO:0010952//positive regulation of peptidase activity
-	-
GO:0008270//zinc ic	GO:0006882//cellular zinc ion homeostasis;GO:0010273//detoxification of
GO:0015173//aroma	GO:0006590//thyroid hormone generation;GO:0055085//transmembrane
-	GO:0017015//regulation of transforming growth factor beta receptor sign
GO:0003997//acyl-C	GO:0006631//fatty acid metabolic process;GO:0006635//fatty acid beta-o
GO:0003841//1-acyl	GO:0006654//phosphatidic acid biosynthetic process;GO:0010891//negati
-	-
-	GO:0006355//regulation of transcription, DNA-templated
GO:0005524//ATP bi	GO:0000086//G2/M transition of mitotic cell cycle;GO:0045332//phosphol
GO:0008083//growtl	-
-	GO:0007154//cell communication
GO:0003824//catalyt	GO:0006567//threonine catabolic process
GO:0005085//guany	GO:0007165//signal transduction;GO:0007264//small GTPase mediated si
-	-
-	-
GO:0005515//protei	-
GO:0003714//transc	GO:0008104//protein localization;GO:0008406//gonad development;GO:0
GO:0000978//RNA p	GO:0006357//regulation of transcription from RNA polymerase II promote
-	-
GO:0003983//UTP:gl	GO:0005978//glycogen biosynthetic process;GO:0006011//UDP-glucose r
GO:0022857//transm	GO:0055085//transmembrane transport
GO:0004497//monoo	-

-	GO:0050910//detection of mechanical stimulus involved in sensory percep
GO:0003824//catalyt	GO:0006167//AMP biosynthetic process;GO:0006177//GMP biosynthetic p
GO:0009374//biotin	-
-	-
-	-
GO:0003824//catalyt	GO:0006103//2-oxoglutarate metabolic process;GO:0009058//biosyntheti
GO:0016407//acetylt	-
-	GO:0006513//protein monoubiquitination;GO:0036297//interstrand cross-
GO:0003953//NAD+	GO:0007165//signal transduction;GO:0034128//negative regulation of My
GO:0004867//serine	GO:0010466//negative regulation of peptidase activity;GO:0030195//nega
GO:0001784//phosp	GO:0001932//regulation of protein phosphorylation;GO:0007259//JAK-ST
GO:0004721//phosp	GO:0006470//protein dephosphorylation;GO:0016311//dephosphorylatio
GO:0004806//triglyc	GO:0006629//lipid metabolic process;GO:0006633//fatty acid biosynthetic
GO:0001758//retinal	GO:0002072//optic cup morphogenesis involved in camera-type eye deve
GO:0000250//lanost	GO:0006694//steroid biosynthetic process;GO:0006695//cholesterol biosy
-	-
GO:0015095//magne	GO:0015693//magnesium ion transport
-	-
-	-
GO:0003924//GTPas	-
-	-
GO:0004629//phosp	GO:0002431//Fc receptor mediated stimulatory signaling pathway;GO:000
GO:0005506//iron ic	-
-	-
GO:0035250//UDP-g	GO:0006024//glycosaminoglycan biosynthetic process;GO:0006464//cellu
-	-
-	-
GO:0004585//ornithi	GO:0000050//urea cycle;GO:0006520//cellular amino acid metabolic proc
GO:0005085//guany	GO:0006623//protein targeting to vacuole;GO:0009306//protein secretion
GO:0017075//syntax	-
GO:0035591//signali	GO:0034128//negative regulation of MyD88-independent toll-like recepto
GO:0005515//protei	GO:0006355//regulation of transcription, DNA-templated;GO:0007179//tr
GO:0005201//extrac	GO:0002244//hematopoietic progenitor cell differentiation
GO:0005515//protei	GO:0007165//signal transduction
-	GO:0007288//sperm axoneme assembly
GO:0016407//acetylt	-
GO:0005515//protei	GO:0016567//protein ubiquitination;GO:0035556//intracellular signal trans
-	-
GO:0005085//guany	GO:0007264//small GTPase mediated signal transduction;GO:0030334//re
GO:0005515//protei	GO:0007155//cell adhesion
GO:0004521//endori	-
GO:0001786//phosp	GO:0002024//diet induced thermogenesis;GO:0006606//protein import in
GO:0005290//L-histi	GO:0006867//asparagine transport;GO:0006868//glutamine transport;GO:
GO:0004420//hydrox	GO:0006695//cholesterol biosynthetic process;GO:0008299//isoprenoid b
GO:0001540//beta-a	GO:0001932//regulation of protein phosphorylation;GO:0001935//endoth
GO:0003779//actin b	GO:0010735//positive regulation of transcription via serum response elem
GO:0022857//transm	GO:0055085//transmembrane transport
-	-
GO:0008146//sulfotr	GO:0006805//xenobiotic metabolic process;GO:0042403//thyroid hormon
GO:0005102//recept	GO:0001541//ovarian follicle development;GO:0001707//mesoderm forma
-	-
GO:0005104//fibrob	GO:0001525//angiogenesis;GO:0001759//organ induction;GO:0001934//p
-	-
GO:0009374//biotin	-
-	-
GO:0000977//RNA p	GO:0000122//negative regulation of transcription from RNA polymerase I
-	-
GO:0003824//catalyt	-

GO:0003796//lysozy	GO:0008152//metabolic process;GO:0016998//cell wall macromolecule ca
GO:0008146//sulfotr	-
GO:0004620//phosp	GO:0009395//phospholipid catabolic process;GO:0030336//negative regu
GO:0005515//protei	GO:0051017//actin filament bundle assembly
-	-
GO:0004842//ubiqui	GO:0006511//ubiquitin-dependent protein catabolic process;GO:0016567
-	GO:0000077//DNA damage checkpoint
GO:0005515//protei	GO:0003431//growth plate cartilage chondrocyte development;GO:00082
-	-
GO:0004930//G-pro	GO:0006897//endocytosis;GO:0006935//chemotaxis;GO:0006955//immun
GO:0004930//G-pro	GO:0006897//endocytosis;GO:0006935//chemotaxis;GO:0006955//immun
GO:0000064//L-orni	GO:1990575//mitochondrial L-ornithine transmembrane transport
GO:0005518//collag	GO:0007605//sensory perception of sound;GO:0008360//regulation of cel
-	-
-	-
GO:0000253//3-ketc	GO:0006695//cholesterol biosynthetic process;GO:0006703//estrogen bio
GO:0004047//amino	GO:0019464//glycine decarboxylation via glycine cleavage system
-	-
GO:0016780//phosp	GO:0008654//phospholipid biosynthetic process
GO:0008270//zinc ic	GO:0006882//cellular zinc ion homeostasis;GO:0010273//detoxification of
GO:0002020//protea	GO:0010951//negative regulation of endopeptidase activity
GO:0008270//zinc ic	GO:0009056//catabolic process;GO:0030855//epithelial cell differentiation
GO:0004866//endop	GO:0010951//negative regulation of endopeptidase activity
GO:0003756//protei	-
GO:0005524//ATP bi	GO:0000086//G2/M transition of mitotic cell cycle;GO:0045332//phosphol
GO:0004497//mono	-
GO:0016290//palmit	GO:0006631//fatty acid metabolic process;GO:0043491//protein kinase B
GO:0003677//DNA b	GO:0006351//transcription, DNA-templated
GO:0005515//protei	-
-	-
GO:0005515//protei	GO:0042634//regulation of hair cycle
GO:0008146//sulfotr	-
GO:0030246//carbol	-
GO:0008235//metall	GO:0017186//peptidyl-pyroglutamic acid biosynthetic process, using glut
GO:0000287//magne	GO:0006468//protein phosphorylation;GO:0007204//positive regulation o
-	GO:0030389//fructosamine metabolic process;GO:0030855//epithelial cell
GO:0015078//hydro	GO:0030177//positive regulation of Wnt signaling pathway;GO:1902600//
GO:0003924//GTPas	-
GO:0003824//catalyt	GO:0006508//proteolysis;GO:0012501//programmed cell death;GO:00147
GO:0017147//Wnt-p	GO:0030178//negative regulation of Wnt signaling pathway
-	-
GO:0008480//sarcos	GO:1901053//sarcosine catabolic process
GO:0003953//NAD+	GO:0007165//signal transduction
GO:0015171//amino	GO:0006641//triglyceride metabolic process;GO:0009437//carnitine metal
GO:0004866//endop	-
GO:0004867//serine	GO:0016525//negative regulation of angiogenesis;GO:0050769//positive r
-	-
-	-
GO:0004866//endop	GO:0010951//negative regulation of endopeptidase activity
GO:0004497//mono	GO:0006805//xenobiotic metabolic process;GO:0008202//steroid metabol
GO:0004657//proline	GO:0006562//proline catabolic process
GO:0003677//DNA b	GO:0035871//protein K11-linked deubiquitination
GO:0004047//amino	GO:0019464//glycine decarboxylation via glycine cleavage system
GO:0008237//metall	GO:0006508//proteolysis
GO:0035254//glutan	GO:0001662//behavioral fear response;GO:0006884//cell volume homeos
GO:0005515//protei	-
-	-
GO:0003824//catalyt	GO:0006103//2-oxoglutarate metabolic process;GO:0009058//biosyntheti

GO:0005227//calciur	GO:0001581//detection of chemical stimulus involved in sensory perceptic
GO:0005515//protei	GO:0006878//cellular copper ion homeostasis;GO:0016567//protein ubiqu
GO:0005546//phosp	GO:0009636//response to toxic substance;GO:0042360//vitamin E metabc
-	-
-	-
GO:0015095//magne	GO:0015693//magnesium ion transport
-	-
GO:0098640//integri	GO:0007155//cell adhesion;GO:0007229//integrin-mediated signaling pat
GO:0005515//protei	GO:0007155//cell adhesion
GO:0003924//GTPas	-
GO:0000064//L-orni	GO:1990575//mitochondrial L-ornithine transmembrane transport
GO:0005313//L-glut	GO:0015810//aspartate transport;GO:0015813//L-glutamate transport;GC
GO:0004806//triglyc	GO:0006629//lipid metabolic process;GO:0006633//fatty acid biosynthetic
GO:0005515//protei	GO:0007155//cell adhesion;GO:0007156//homophilic cell adhesion via pla
GO:0004585//ornith	GO:0000050//urea cycle;GO:0006520//cellular amino acid metabolic proc
GO:0004896//cytoki	GO:0008284//positive regulation of cell proliferation;GO:0019221//cytokir
GO:0004866//endop	-
GO:0003847//1-alky	GO:0006629//lipid metabolic process;GO:0006644//phospholipid metabo
GO:0003824//catalyt	-
GO:0001784//phosp	GO:0001932//regulation of protein phosphorylation;GO:0007259//JAK-ST
GO:0003824//catalyt	GO:0006527//arginine catabolic process;GO:0009072//aromatic amino aci
GO:0003847//1-alky	GO:0006629//lipid metabolic process;GO:0006644//phospholipid metabo
-	GO:0016192//vesicle-mediated transport
-	-
GO:0004497//mono	-
GO:0022857//transm	GO:0055085//transmembrane transport
GO:0004497//mono	GO:0006805//xenobiotic metabolic process;GO:0008202//steroid metabol
GO:0005085//guany	-
-	GO:0007288//sperm axoneme assembly
GO:0005096//GTPas	GO:0007165//signal transduction;GO:0043547//positive regulation of GTP
GO:0005515//protei	-
GO:0004639//phosp	GO:0006164//purine nucleotide biosynthetic process;GO:0006189//'de no
GO:0004672//protei	GO:0000002//mitochondrial genome maintenance;GO:0001938//positive
-	-
-	-
GO:0005515//protei	-
GO:0004089//carbor	GO:0002009//morphogenesis of an epithelium;GO:0046903//secretion
GO:0004478//methic	GO:0006556//S-adenosylmethionine biosynthetic process;GO:0009087//n
-	-
-	-
GO:0005158//insulin	GO:0007165//signal transduction;GO:0010467//gene expression;GO:0030:
GO:0005515//protei	-
GO:0000166//nuclec	-
GO:0003697//single-	-
-	GO:0000045//autophagosome assembly;GO:0009267//cellular response tc
GO:0003824//catalyt	GO:0005975//carbohydrate metabolic process;GO:0006006//glucose meta
GO:0008270//zinc ic	GO:0006882//cellular zinc ion homeostasis;GO:0010273//detoxification of
GO:0008113//peptid	-
GO:0019904//protei	GO:0050796//regulation of insulin secretion
GO:0004620//phosp	GO:0009395//phospholipid catabolic process;GO:0030336//negative regu
GO:0005085//guany	GO:0007264//small GTPase mediated signal transduction;GO:0030334//re
-	-
GO:0005102//recept	GO:0006898//receptor-mediated endocytosis;GO:0008104//protein locali:
GO:0001786//phosp	GO:0002024//diet induced thermogenesis;GO:0006606//protein import in
GO:0005515//protei	-
-	-
-	-
GO:0015173//aroma	GO:0006590//thyroid hormone generation;GO:0055085//transmembrane

-	-
GO:0000287//magn	GO:0006584//catecholamine metabolic process;GO:0016036//cellular resp
GO:0005515//protei	-
GO:0003677//DNA b	GO:0009646//response to absence of light;GO:0018298//protein-chromo
GO:0020037//heme	GO:0019441//tryptophan catabolic process to kynurenine
GO:0005104//fibrob	GO:0001525//angiogenesis;GO:0001759//organ induction;GO:0001934//p
GO:0004930//G-pro	GO:0001541//ovarian follicle development;GO:0001545//primary ovarian
-	-
-	-
GO:0005102//recept	GO:0001541//ovarian follicle development;GO:0001707//mesoderm form
-	-
GO:0004497//mono	GO:0097267//omega-hydroxylase P450 pathway
GO:0003924//GTPas	-
GO:0003824//cataly	GO:0009436//glyoxylate catabolic process;GO:0019265//glycine biosynthe
-	GO:0017015//regulation of transforming growth factor beta receptor sign
GO:0005515//protei	GO:0007155//cell adhesion;GO:0008593//regulation of Notch signaling pa
-	GO:0007288//sperm axoneme assembly
GO:0003997//acyl-C	GO:0006631//fatty acid metabolic process;GO:0006635//fatty acid beta-o
GO:0016780//phosp	GO:0008654//phospholipid biosynthetic process
GO:0000254//C-4 m	GO:0001934//positive regulation of protein phosphorylation;GO:0008610/
GO:0016407//acetylt	-
GO:0009374//biotin	-
-	GO:0006895//Golgi to endosome transport;GO:0015031//protein transpo
GO:0003824//cataly	GO:0006167//AMP biosynthetic process;GO:0006177//GMP biosynthetic
-	-
GO:0004866//endop	-
GO:0004867//serine	GO:0016525//negative regulation of angiogenesis;GO:0050769//positive
-	-
GO:0008235//metall	GO:0017186//peptidyl-pyroglutamic acid biosynthetic process, using glut
GO:0004497//mono	GO:0006700//C21-steroid hormone biosynthetic process;GO:0008203//ch
GO:0005353//fructo	GO:0003044//regulation of systemic arterial blood pressure mediated by
-	-
GO:0000166//nucleo	GO:0006529//asparagine biosynthetic process;GO:0006541//glutamine m
GO:0005515//protei	GO:0006878//cellular copper ion homeostasis;GO:0016567//protein ubiqu
GO:0005515//protei	GO:0051017//actin filament bundle assembly
-	-
GO:0003989//acetyl	GO:0001894//tissue homeostasis;GO:0006084//acetyl-CoA metabolic pro
-	GO:0001656//metanephros development;GO:0001658//branching involve
GO:0005515//protei	-
GO:0003677//DNA b	GO:0035871//protein K11-linked deubiquitination
-	GO:0015888//thiamine transport
GO:0005515//protei	GO:0007155//cell adhesion
GO:0004478//methi	GO:0006556//S-adenosylmethionine biosynthetic process;GO:0009087//m
GO:0003677//DNA b	GO:0009267//cellular response to starvation;GO:0043066//negative regul
GO:0003824//cataly	GO:0018272//protein-pyridoxal-5-phosphate linkage via peptidyl-N6-py
GO:0005198//struct	-
GO:0005509//calciu	GO:0007156//homophilic cell adhesion via plasma membrane adhesion m
GO:0004866//endop	GO:0010951//negative regulation of endopeptidase activity
GO:0005096//GTPas	GO:0007165//signal transduction;GO:0035556//intracellular signal transd
GO:0016316//phosp	-
GO:0005515//protei	GO:0006355//regulation of transcription, DNA-templated;GO:0007179//tr
GO:0005515//protei	GO:0007155//cell adhesion
GO:0003677//DNA b	-
-	GO:0006506//GPI anchor biosynthetic process
GO:0005515//protei	GO:0007165//signal transduction
GO:0042809//vitami	GO:0008285//negative regulation of cell proliferation;GO:0010468//regul
-	-
GO:0003779//actin b	GO:0006351//transcription, DNA-templated;GO:0030032//lamellipodium

GO:0004047//amino	GO:0019464//glycine decarboxylation via glycine cleavage system
GO:0004930//G-pro	GO:0007186//G-protein coupled receptor signaling pathway
-	-
GO:0016787//hydrol	-
GO:0008270//zinc ic	-
GO:0004465//lipopr	GO:0006629//lipid metabolic process;GO:0006631//fatty acid metabolic p
-	-
-	-
GO:0004867//serine	GO:0010951//negative regulation of endopeptidase activity;GO:0030212//
GO:0003847//1-alky	GO:0006629//lipid metabolic process;GO:0006644//phospholipid metabo
GO:0019960//C-X3-	GO:0003366//cell-matrix adhesion involved in ameboidal cell migration;G
-	-
GO:0004629//phosp	GO:0002431//Fc receptor mediated stimulatory signaling pathway;GO:000
-	-
GO:0005515//protei	-
-	-
-	-
-	GO:0055085//transmembrane transport
GO:0005509//calciu	GO:0007155//cell adhesion
-	GO:0016192//vesicle-mediated transport
GO:0000334//3-hyd	GO:0009435//NAD biosynthetic process;GO:0010043//response to zinc io
GO:0005515//protei	GO:0007612//learning;GO:0007613//memory;GO:1900452//regulation of
GO:0005096//GTPas	GO:0007165//signal transduction;GO:0043547//positive regulation of GTP
-	GO:0000077//DNA damage checkpoint
GO:0004553//hydrol	GO:0005975//carbohydrate metabolic process
GO:0004089//carbor	GO:0002009//morphogenesis of an epithelium;GO:0046903//secretion
GO:0016491//oxidor	GO:0019477//L-lysine catabolic process
GO:0047750//choles	GO:0016125//sterol metabolic process
GO:0022857//transm	GO:0055085//transmembrane transport
GO:0004197//cystei	GO:0002931//response to ischemia;GO:0006508//proteolysis;GO:0006511
GO:0035254//glutan	GO:0001662//behavioral fear response;GO:0006884//cell volume homeos
-	-
GO:0003677//DNA b	-
GO:0000287//magne	GO:0006584//catecholamine metabolic process;GO:0016036//cellular resp
GO:0003824//catalyt	GO:0005975//carbohydrate metabolic process;GO:0006006//glucose meta
GO:0035254//glutan	GO:0001662//behavioral fear response;GO:0006884//cell volume homeos
GO:0003779//actin b	GO:0010735//positive regulation of transcription via serum response elem
-	-
GO:0004672//protei	GO:0000002//mitochondrial genome maintenance;GO:0001938//positive
-	GO:0030193//regulation of blood coagulation;GO:0044763//single-organ
GO:0003674//molec	GO:0002062//chondrocyte differentiation;GO:0008150//biological_proces
GO:0016491//oxidor	GO:0007005//mitochondrion organization
GO:0003824//catalyt	GO:0006567//threonine catabolic process
-	-
GO:0003824//catalyt	-
GO:0004866//endop	GO:0010575//positive regulation of vascular endothelial growth factor prc
GO:0000246//delta2	GO:0006695//cholesterol biosynthetic process;GO:0006979//response to c
GO:0031267//small (	GO:0006886//intracellular protein transport
GO:0005515//protei	-
-	-
-	GO:0017015//regulation of transforming growth factor beta receptor sign
GO:0022857//transm	GO:0055085//transmembrane transport
GO:0004842//ubiqui	GO:0006511//ubiquitin-dependent protein catabolic process;GO:0007399
GO:0005515//protei	GO:0007165//signal transduction
-	-
GO:0005515//protei	GO:0007015//actin filament organization;GO:0045806//negative regulatio
-	GO:0030389//fructosamine metabolic process;GO:0030855//epithelial cell
GO:0000166//nucleo	GO:0009157//deoxyribonucleoside monophosphate biosynthetic process;

-	-
GO:0004601//peroxi	GO:0006979//response to oxidative stress;GO:0050665//hydrogen peroxic
GO:0005515//protei	-
GO:0004047//amino	GO:0019464//glycine decarboxylation via glycine cleavage system
-	-
GO:0008113//peptid	-
-	-
GO:0005042//netrin	GO:0007165//signal transduction;GO:0014068//positive regulation of pho
GO:0015485//choles	GO:0009615//response to virus;GO:0030301//cholesterol transport;GO:00
GO:0000250//lanost	GO:0006694//steroid biosynthetic process;GO:0006695//cholesterol biosy
GO:0004866//endop	GO:0010951//negative regulation of endopeptidase activity
GO:0005515//protei	GO:0003341//cilium movement;GO:0007368//determination of left/right s
GO:0008009//chemo	GO:0006955//immune response
GO:0004497//mono	-
-	-
-	GO:0007420//brain development
-	-
GO:0005546//phosp	GO:0009636//response to toxic substance;GO:0042360//vitamin E metabo
GO:0003824//catalyt	GO:0006527//arginine catabolic process;GO:0009072//aromatic amino aci
GO:0016407//acetylt	-
GO:0005515//protei	GO:0051017//actin filament bundle assembly
GO:0003953//NAD+	GO:0007165//signal transduction;GO:0034128//negative regulation of My
-	GO:0001656//metanephros development;GO:0001658//branching involve
GO:0008146//sulfotr	-
GO:0003834//beta-c	GO:0001523//retinoid metabolic process;GO:0016121//carotene catabolic
GO:0008028//mono	GO:0015718//monocarboxylic acid transport;GO:0055085//transmembran
-	GO:0007166//cell surface receptor signaling pathway
-	-
GO:0003779//actin b	GO:0006351//transcription, DNA-templated;GO:0030032//lamellipodium
GO:0008138//protei	GO:0006355//regulation of transcription, DNA-templated;GO:0006470//p
GO:0003924//GTPas	-
GO:0030298//recept	GO:0010976//positive regulation of neuron projection development;GO:0
GO:0004843//thiol-c	GO:0000278//mitotic cell cycle;GO:0006281//DNA repair;GO:0006511//ub
GO:0004122//cystatl	GO:0001958//endochondral ossification;GO:0001974//blood vessel remoc
-	-
GO:0008194//UDP-g	-
GO:0022857//transm	GO:0010628//positive regulation of gene expression;GO:0010629//negativ
GO:0005515//protei	GO:0007165//signal transduction
-	-
GO:0004465//lipopro	GO:0006629//lipid metabolic process;GO:0006631//fatty acid metabolic p
GO:0003824//catalyt	-
GO:0005515//protei	GO:0007155//cell adhesion;GO:0007156//homophilic cell adhesion via pla
-	GO:0006355//regulation of transcription, DNA-templated
GO:0004497//mono	-
GO:0005267//potass	GO:0006813//potassium ion transport;GO:0033198//response to ATP;GO:
-	-
GO:0003997//acyl-C	GO:0006631//fatty acid metabolic process;GO:0006635//fatty acid beta-o
GO:0004364//glutatl	GO:0006749//glutathione metabolic process;GO:0006805//xenobiotic met
GO:0004114//3',5'-c	GO:0006198//cAMP catabolic process;GO:0007165//signal transduction
GO:0098640//integri	GO:0007155//cell adhesion;GO:0007229//integrin-mediated signaling pat
GO:0003868//4-hyd	GO:0006572//tyrosine catabolic process;GO:0009072//aromatic amino aci
GO:0000976//transc	GO:0000122//negative regulation of transcription from RNA polymerase I
GO:0004016//adeny	GO:0006171//cAMP biosynthetic process;GO:0007613//memory;GO:0007
-	-
GO:0003707//steroi	GO:0009755//hormone-mediated signaling pathway
-	-
GO:0005515//protei	-
-	-

GO:0005085//guany	GO:0007264//small GTPase mediated signal transduction;GO:0030334//re
GO:0008271//second	GO:0008272//sulfate transport;GO:0055085//transmembrane transport
GO:0000287//magne	GO:0006468//protein phosphorylation;GO:0007346//regulation of mitotic
GO:0004087//carbar	GO:0006207//'de novo' pyrimidine nucleobase biosynthetic process;GO:0(
GO:0001786//phosp	GO:0002024//diet induced thermogenesis;GO:0006606//protein import in
GO:0005506//iron ic	-
GO:0003824//catalyt	GO:0006650//glycerophospholipid metabolic process;GO:0007416//synap
GO:0000977//RNA p	GO:0000122//negative regulation of transcription from RNA polymerase I
-	GO:0009966//regulation of signal transduction
-	-
-	-
GO:0005102//recept	GO:0001541//ovarian follicle development;GO:0001707//mesoderm forma
GO:0005515//protei	GO:0002376//immune system process;GO:0006955//immune response;G(
GO:0005515//protei	-
GO:0004553//hydrol	GO:0005975//carbohydrate metabolic process
-	-
GO:0004629//phosp	GO:0002431//Fc receptor mediated stimulatory signaling pathway;GO:000
GO:0008480//sarcos	GO:1901053//sarcosine catabolic process
GO:0008270//zinc ic	-
-	-
GO:0008195//phosp	GO:0006796//phosphate-containing compound metabolic process;GO:00
-	GO:0030193//regulation of blood coagulation;GO:0044763//single-organ
GO:0001540//beta-a	GO:0001932//regulation of protein phosphorylation;GO:0001935//endoth
GO:0000287//magne	GO:0006468//protein phosphorylation;GO:0007204//positive regulation o
-	-
GO:0000254//C-4 m	GO:0001934//positive regulation of protein phosphorylation;GO:0008610/
GO:0000062//fatty-a	GO:0036151//phosphatidylcholine acyl-chain remodeling;GO:1903060//n
GO:0004497//mono	GO:0097267//omega-hydroxylase P450 pathway
GO:0000334//3-hyd	GO:0009435//NAD biosynthetic process;GO:0010043//response to zinc io
GO:0003677//DNA b	GO:0009267//cellular response to starvation;GO:0043066//negative regula
-	-
-	-
-	-
GO:0004478//methic	GO:0006556//S-adenosylmethionine biosynthetic process;GO:0009087//n
GO:0004252//serine	GO:0000902//cell morphogenesis;GO:0001889//liver development;GO:000
GO:0008289//lipid b	-
GO:0005509//calciur	GO:0007156//homophilic cell adhesion via plasma membrane adhesion m
GO:0004089//carbor	GO:0002009//morphogenesis of an epithelium;GO:0046903//secretion
GO:0005515//protei	-
GO:0005085//guany	GO:0000281//mitotic cytokinesis;GO:0000902//cell morphogenesis;GO:00
GO:0008270//zinc ic	GO:0009056//catabolic process;GO:0030855//epithelial cell differentiation
GO:0008083//growtl	-
GO:0003700//transc	GO:0006355//regulation of transcription, DNA-templated
-	-
GO:0050839//cell ad	GO:0007409//axonogenesis;GO:0010975//regulation of neuron projectior
GO:0004672//protei	GO:0006885//regulation of pH;GO:0008286//insulin receptor signaling pa
GO:0004364//glutatl	GO:0006749//glutathione metabolic process;GO:0006805//xenobiotic met
GO:0031267//small (	GO:0006886//intracellular protein transport
GO:0003779//actin b	GO:0010735//positive regulation of transcription via serum response elem
GO:0003676//nuclei	GO:0015074//DNA integration;GO:0044260//cellular macromolecule meta
GO:0003676//nuclei	-
GO:0004175//endop	GO:0006508//proteolysis;GO:0007520//myoblast fusion;GO:0022617//extr
GO:0003674//molec	GO:0006486//protein glycosylation;GO:0006487//protein N-linked glycos
GO:0005515//protei	-
GO:0008017//micro1	GO:0008285//negative regulation of cell proliferation;GO:0030330//DNA (
GO:0019960//C-X3-	GO:0003366//cell-matrix adhesion involved in ameboidal cell migration;G
-	GO:0001764//neuron migration;GO:0007162//negative regulation of cell a
GO:0004252//serine	GO:0000902//cell morphogenesis;GO:0001889//liver development;GO:000

GO:0004888//transm	GO:0007166//cell surface receptor signaling pathway;GO:0007186//G-pro
GO:0003824//catalyt	-
-	GO:0055085//transmembrane transport
GO:0042809//vitami	GO:0008285//negative regulation of cell proliferation;GO:0010468//regula
GO:0005515//protei	GO:0002376//immune system process;GO:0006955//immune response;G(
-	GO:0009617//response to bacterium
GO:0005515//protei	GO:0007165//signal transduction
GO:0004930//G-pro	GO:0007186//G-protein coupled receptor signaling pathway
GO:0015125//bile ac	GO:0015721//bile acid and bile salt transport;GO:0032782//bile acid secre
GO:0016316//phosp	-
GO:0016407//acetylt	-
GO:0005085//guany	-
GO:0004470//malic	GO:0006108//malate metabolic process;GO:1902031//regulation of NADF
-	-
-	-
GO:0003854//3-beta	GO:0006694//steroid biosynthetic process
-	GO:0000077//DNA damage checkpoint
GO:0008201//hepari	-
GO:0004842//ubiqui	GO:0006511//ubiquitin-dependent protein catabolic process;GO:0007399
GO:0004497//mono	-
GO:0001849//compl	GO:0001878//response to yeast;GO:0008228//opsonization;GO:0030198//
-	-
-	-
-	-
GO:0004930//G-pro	GO:0002300//CD8-positive, alpha-beta intraepithelial T cell differentiatior
GO:0001786//phosp	GO:0006910//phagocytosis, recognition;GO:0006911//phagocytosis, engu
GO:0004497//mono	GO:0006805//xenobiotic metabolic process;GO:0008202//steroid metabol
GO:0003841//1-acyl	GO:0006644//phospholipid metabolic process
GO:0016491//oxidor	-
GO:0002020//protea	GO:0010466//negative regulation of peptidase activity;GO:0018149//pept
GO:0005515//protei	GO:0007155//cell adhesion
GO:0003755//peptid	-
GO:0003834//beta-	GO:0001523//retinoid metabolic process;GO:0016121//carotene catabolic
-	-
GO:0003676//nuclei	-
GO:0009922//fatty a	GO:0000038//very long-chain fatty acid metabolic process;GO:0006629//l
GO:0005509//calciur	GO:0007165//signal transduction
-	GO:0006629//lipid metabolic process
GO:0004721//phosp	GO:0010801//negative regulation of peptidyl-threonine phosphorylation;
GO:0016787//hydrol	-
GO:0004866//endop	-
GO:0004843//thiol-	GO:0035871//protein K11-linked deubiquitination;GO:0044313//protein K
-	GO:0007286//spermatid development;GO:0048137//spermatocyte divisior
GO:0019904//protei	GO:0050796//regulation of insulin secretion
GO:0005515//protei	-
-	GO:0007166//cell surface receptor signaling pathway
GO:0005515//protei	-
GO:0005198//structu	-
GO:0031956//mediu	-
-	-
-	-
GO:0008270//zinc ic	GO:0006882//cellular zinc ion homeostasis;GO:0010273//detoxification of
GO:0016491//oxidor	GO:0019477//L-lysine catabolic process
-	GO:0008154//actin polymerization or depolymerization;GO:0030048//acti
-	GO:0008154//actin polymerization or depolymerization;GO:0030048//acti
-	-
GO:0000287//magne	GO:0006468//protein phosphorylation;GO:0007346//regulation of mitotic
GO:0004672//protei	GO:0000002//mitochondrial genome maintenance;GO:0001938//positive

-	-
GO:0005267//potass	GO:0006813//potassium ion transport;GO:0033198//response to ATP;GO:
GO:0000976//transc	GO:0000122//negative regulation of transcription from RNA polymerase I
GO:0003824//catalyt	GO:0006567//threonine catabolic process
GO:0004467//long-c	GO:0001676//long-chain fatty acid metabolic process;GO:0008610//lipid I
-	-
-	-
GO:0050839//cell ad	GO:0007409//axonogenesis;GO:0010975//regulation of neuron projection
-	-
GO:0005125//cytoki	GO:0002232//leukocyte chemotaxis involved in inflammatory response;GC
GO:0008289//lipid b	GO:0015031//protein transport;GO:0019075//virus maturation;GO:004205
GO:0005515//protei	GO:0007155//cell adhesion;GO:0008593//regulation of Notch signaling pa
GO:0003676//nuclei	GO:0006644//phospholipid metabolic process;GO:0006935//chemotaxis;G
GO:0022857//transm	GO:0055085//transmembrane transport
-	-
-	GO:0009966//regulation of signal transduction
GO:0009374//biotin	-
GO:0000977//RNA p	GO:0000122//negative regulation of transcription from RNA polymerase I
-	GO:0006895//Golgi to endosome transport;GO:0015031//protein transpor
GO:0016491//oxidor	GO:0007005//mitochondrion organization
GO:0008113//peptid	-
GO:0005515//protei	GO:0003341//cilium movement;GO:0044782//cilium organization
GO:0005085//guany	GO:0001934//positive regulation of protein phosphorylation;GO:0002437/
GO:0022857//transm	GO:0055085//transmembrane transport
-	-
GO:0001540//beta-a	GO:0001932//regulation of protein phosphorylation;GO:0001935//endoth
GO:0005158//insulin	GO:0001775//cell activation;GO:0007405//neuroblast proliferation;GO:000
GO:0002020//protea	GO:0001932//regulation of protein phosphorylation;GO:0007044//cell-sul
GO:0004310//farnes	GO:0008610//lipid biosynthetic process;GO:0009058//biosynthetic proces
-	-
-	-
GO:0003824//catalyt	GO:0006508//proteolysis;GO:0012501//programmed cell death;GO:00147
GO:0050839//cell ad	GO:0007409//axonogenesis;GO:0010975//regulation of neuron projection
GO:0004866//endop	GO:0010575//positive regulation of vascular endothelial growth factor pro
GO:0061631//ubiqui	GO:0000209//protein polyubiquitination
GO:0003924//GTPas	-
-	-
-	-
GO:0000254//C-4 m	GO:0001934//positive regulation of protein phosphorylation;GO:0008610/
GO:0003674//molec	GO:0002062//chondrocyte differentiation;GO:0008150//biological_proces
-	-
GO:0005524//ATP bi	GO:0008203//cholesterol metabolic process;GO:0010033//response to org
-	-
GO:0003674//molec	GO:0002062//chondrocyte differentiation;GO:0008150//biological_proces
GO:0000062//fatty-a	GO:0008203//cholesterol metabolic process;GO:0010742//macrophage de
GO:0051015//actin f	GO:0030866//cortical actin cytoskeleton organization;GO:0045214//sarcor
GO:0005515//protei	GO:0006749//glutathione metabolic process
GO:0005179//hormo	-
-	-
GO:0000062//fatty-a	-
GO:0001965//G-pro	GO:0007165//signal transduction;GO:0007186//G-protein coupled recept
-	GO:0009987//cellular process;GO:0044699//single-organism process
GO:0005515//protei	GO:0042634//regulation of hair cycle
-	GO:0015888//thiamine transport
GO:0000287//magne	GO:0000226//microtubule cytoskeleton organization;GO:0006468//protein
-	-
GO:0005172//vascul	GO:0008283//cell proliferation;GO:0008284//positive regulation of cell pro
GO:0004866//endop	GO:0010575//positive regulation of vascular endothelial growth factor pro

GO:0005216//ion ch	GO:0006811//ion transport;GO:0006813//potassium ion transport;GO:003
GO:0005290//L-histi	GO:0006867//asparagine transport;GO:0006868//glutamine transport;GO:
GO:0004090//carbor	GO:0006805//xenobiotic metabolic process;GO:0030855//epithelial cell di
GO:0005515//protei	-
GO:0001664//G-pro	GO:0007286//spermatid development;GO:0034613//cellular protein locali
-	GO:0001764//neuron migration;GO:0007162//negative regulation of cell a
GO:0046790//virion	GO:0075512//clathrin-mediated endocytosis of virus by host cell
GO:0005506//iron ic	-
-	-
GO:0004930//G-pro	GO:0002001//renin secretion into blood stream;GO:0002281//macrophag
GO:0005515//protei	GO:0007155//cell adhesion;GO:0007156//homophilic cell adhesion via pla
GO:0005507//coppe	GO:0009308//amine metabolic process;GO:0009445//putrescine metaboli
GO:0002162//dystro	GO:0010628//positive regulation of gene expression;GO:0010811//positiv
GO:0000062//fatty-a	GO:0008203//cholesterol metabolic process;GO:0010742//macrophage de
GO:0005515//protei	GO:0043254//regulation of protein complex assembly
GO:0004122//cystatl	GO:0001958//endochondral ossification;GO:0001974//blood vessel remod
-	-
GO:0008146//sulfotr	-
GO:0098640//integri	GO:0007155//cell adhesion;GO:0007229//integrin-mediated signaling pat
-	-
GO:0004016//adeny	GO:0006171//cAMP biosynthetic process;GO:0007613//memory;GO:0007
GO:0005179//hormc	-
GO:0030298//recept	GO:0010976//positive regulation of neuron projection development;GO:0
GO:0004672//protei	GO:0006468//protein phosphorylation
GO:0015485//choles	GO:0009615//response to virus;GO:0030301//cholesterol transport;GO:00
GO:0030298//recept	GO:0010976//positive regulation of neuron projection development;GO:0
-	-
GO:0004364//glutatl	GO:0006749//glutathione metabolic process;GO:0006805//xenobiotic met
GO:0005509//calciur	GO:0032571//response to vitamin K;GO:0060348//bone development;GO:
GO:0005085//guany	GO:0001934//positive regulation of protein phosphorylation;GO:0002437/
-	-
-	-
GO:0008194//UDP-g	GO:0006486//protein glycosylation;GO:0006682//galactosylceramide bios
GO:0016787//hydrol	-
GO:0008083//growtl	-
GO:0005515//protei	GO:0042426//choline catabolic process
GO:0004340//glucok	GO:0001678//cellular glucose homeostasis;GO:0005975//carbohydrate me
GO:0008480//sarcos	GO:1901053//sarcosine catabolic process
-	GO:0008154//actin polymerization or depolymerization;GO:0030048//acti
GO:0008146//sulfotr	GO:0006805//xenobiotic metabolic process;GO:0042403//thyroid hormon
GO:0003884//D-ami	GO:0006531//aspartate metabolic process;GO:0007320//insemination;GO
GO:0008271//secon	GO:0008272//sulfate transport;GO:0055085//transmembrane transport
GO:0008270//zinc ic	-
GO:0005515//protei	-
-	-
GO:0015377//cation	GO:0006811//ion transport;GO:0006884//cell volume homeostasis;GO:005
GO:0003924//GTPas	GO:0000082//G1/S transition of mitotic cell cycle;GO:0007010//cytoskelet
GO:0004930//G-pro	GO:0002300//CD8-positive, alpha-beta intraepithelial T cell differentiatior
-	-
GO:0002020//protea	GO:0010466//negative regulation of peptidase activity;GO:0018149//pept
GO:0015267//chann	GO:0002931//response to ischemia;GO:0006812//cation transport;GO:003
GO:0005198//structu	-
GO:0004721//phosp	GO:0010801//negative regulation of peptidyl-threonine phosphorylation;
GO:0005216//ion ch	GO:0006811//ion transport;GO:0006814//sodium ion transport;GO:00550
-	-
GO:0008195//phosp	GO:0006796//phosphate-containing compound metabolic process;GO:00
GO:0005515//protei	-
-	-

GO:0042802//identic	-
GO:0008270//zinc ic	GO:0009056//catabolic process;GO:0030855//epithelial cell differentiation
-	-
GO:0004842//ubiqui	GO:0006511//ubiquitin-dependent protein catabolic process;GO:0016567
GO:0005201//extrac	GO:0002244//hematopoietic progenitor cell differentiation
GO:0004114//3',5'-c	GO:0006198//cAMP catabolic process;GO:0007165//signal transduction
GO:0005488//bindin	-
GO:0000166//nucle	GO:0007165//signal transduction;GO:0007186//G-protein coupled recept
GO:0005515//protei	-
GO:0005198//structu	-
GO:0016491//oxidor	GO:0006629//lipid metabolic process;GO:0006631//fatty acid metabolic p
-	-
GO:0000287//magn	GO:0006468//protein phosphorylation;GO:0030036//actin cytoskeleton or
-	-
GO:0003988//acetyl-	GO:0000038//very long-chain fatty acid metabolic process;GO:0006635//
GO:0008270//zinc ic	-
-	GO:0007154//cell communication
GO:0005324//long-c	GO:0006469//negative regulation of protein kinase activity;GO:0009617//
GO:0003682//chrom	GO:0015908//fatty acid transport;GO:0043066//negative regulation of apo
GO:0008201//hepari	-
GO:0015267//chann	GO:0002931//response to ischemia;GO:0006812//cation transport;GO:003
-	-
-	-
-	GO:0007165//signal transduction
-	-
GO:0005515//protei	-
GO:0008146//sulfotr	-
-	GO:0001764//neuron migration;GO:0007420//brain development;GO:002
-	-
GO:0004843//thiol-c	GO:0035871//protein K11-linked deubiquitination;GO:0044313//protein K
GO:0005085//guany	GO:0001934//positive regulation of protein phosphorylation;GO:0002437/
GO:0005515//protei	GO:0002376//immune system process;GO:0006955//immune response;G
GO:0003824//catalyt	GO:0006567//threonine catabolic process
GO:0004930//G-pro	GO:0002001//renin secretion into blood stream;GO:0002281//macrophag
GO:0005515//protei	GO:0006936//muscle contraction
-	-
GO:0003779//actin b	GO:0007010//cytoskeleton organization;GO:0008360//regulation of cell sh
GO:0000978//RNA p	GO:0006357//regulation of transcription from RNA polymerase II promote
GO:0051082//unfold	GO:0006457//protein folding
GO:0003700//transc	GO:0006355//regulation of transcription, DNA-templated
GO:0000334//3-hyd	GO:0009435//NAD biosynthetic process;GO:0010043//response to zinc io
-	GO:0007286//spermatid development;GO:0048137//spermatocyte divisio
-	-
GO:0003755//peptid	-
GO:0015171//amino	GO:0006641//triglyceride metabolic process;GO:0009437//carnitine meta
GO:0022857//transm	GO:0055085//transmembrane transport
GO:0003779//actin b	GO:0030835//negative regulation of actin filament depolymerization
GO:0020037//heme	GO:0019441//tryptophan catabolic process to kynurenine
GO:0031956//mediu	-
GO:0004842//ubiqui	GO:0006511//ubiquitin-dependent protein catabolic process;GO:0007399
GO:0003779//actin b	GO:0030835//negative regulation of actin filament depolymerization
-	-
GO:0005515//protei	GO:0010468//regulation of gene expression;GO:0016567//protein ubiquit
GO:0004657//prolin	GO:0006562//proline catabolic process
-	-
GO:0004930//G-pro	GO:0007186//G-protein coupled receptor signaling pathway
-	GO:0009987//cellular process;GO:0044699//single-organism process
GO:0005290//L-histi	GO:0006867//asparagine transport;GO:0006868//glutamine transport;GO:

-	-
GO:0004252//serine	GO:0006508//proteolysis
GO:0016407//acetylt	-
GO:0008138//protei	-
GO:0005172//vascul	GO:0008283//cell proliferation;GO:0008284//positive regulation of cell pro
-	-
-	-
GO:0004866//endop	-
-	-
GO:0000062//fatty-a	GO:0008203//cholesterol metabolic process;GO:0010742//macrophage de
GO:0008236//serine	GO:0006508//proteolysis
GO:0008289//lipid b	GO:0010873//positive regulation of cholesterol esterification;GO:0010879/
GO:0003674//molec	GO:0002062//chondrocyte differentiation;GO:0008150//biological_process
-	-
-	GO:0010269//response to selenium ion;GO:0035264//multicellular organis
GO:0004252//serine	GO:0000902//cell morphogenesis;GO:0001889//liver development;GO:000
GO:0005515//protei	GO:0043254//regulation of protein complex assembly
GO:0005515//protei	-
GO:0004087//carbar	GO:0006207//'de novo' pyrimidine nucleobase biosynthetic process;GO:00
GO:0004866//endop	GO:0010575//positive regulation of vascular endothelial growth factor pro
GO:0005515//protei	GO:0006749//glutathione metabolic process
GO:0000287//magne	GO:0000226//microtubule cytoskeleton organization;GO:0006468//protein
GO:0005324//long-c	GO:0006469//negative regulation of protein kinase activity;GO:0009617//r
GO:0000062//fatty-a	-
GO:0003713//transc	GO:0007219//Notch signaling pathway;GO:0045944//positive regulation o
-	-
GO:0016791//phosp	GO:0006024//glycosaminoglycan biosynthetic process;GO:0010909//posit
GO:0030298//recept	GO:0010976//positive regulation of neuron projection development;GO:0
-	-
GO:0005085//guany	GO:0007264//small GTPase mediated signal transduction;GO:0030334//re
-	GO:0007275//multicellular organism development;GO:0030154//cell differ
GO:0003868//4-hyd	GO:0006572//tyrosine catabolic process;GO:0009072//aromatic amino aci
GO:0001786//phosp	GO:0002024//diet induced thermogenesis;GO:0006606//protein import in
GO:0015173//aroma	GO:0006590//thyroid hormone generation;GO:0055085//transmembrane
-	-
GO:0005096//GTPas	GO:0007264//small GTPase mediated signal transduction;GO:0050790//re
GO:0005515//protei	GO:0007165//signal transduction
-	-
GO:0008146//sulfotr	GO:0006790//sulfur compound metabolic process
GO:0004639//phosp	GO:0006164//purine nucleotide biosynthetic process;GO:0006189//'de no
GO:0005158//insulin	GO:0001775//cell activation;GO:0007405//neuroblast proliferation;GO:000
GO:0004672//protei	GO:0001501//skeletal system development;GO:0006468//protein phospho
-	-
GO:0005515//protei	GO:0051017//actin filament bundle assembly
GO:0016491//oxidor	-
GO:0005102//recept	GO:0001541//ovarian follicle development;GO:0001707//mesoderm forma
GO:0005313//L-glut	GO:0015810//aspartate transport;GO:0015813//L-glutamate transport;GO
GO:0003824//catalyt	GO:0010121//arginine catabolic process to proline via ornithine;GO:0019
GO:0003841//1-acyl	-
-	-
GO:0005515//protei	-
GO:0008271//secon	GO:0008272//sulfate transport;GO:0055085//transmembrane transport
GO:0004045//amino	-
GO:0005515//protei	GO:0007155//cell adhesion;GO:0008593//regulation of Notch signaling pa
GO:0004499//N,N-d	-
GO:0005515//protei	GO:0003341//cilium movement;GO:0044782//cilium organization
GO:0005515//protei	-
GO:0004672//protei	GO:0006468//protein phosphorylation

GO:0005158//insulin	GO:0007165//signal transduction;GO:0010467//gene expression;GO:0030
-	-
-	-
GO:0004386//helicas	-
-	GO:0006895//Golgi to endosome transport;GO:0015031//protein transpo
-	GO:0007166//cell surface receptor signaling pathway
GO:0001758//retinal	GO:0042573//retinoic acid metabolic process;GO:0042574//retinal metabo
GO:0004465//lipopr	GO:0006629//lipid metabolic process;GO:0006631//fatty acid metabolic p
GO:0008009//chemo	GO:0006954//inflammatory response;GO:0006955//immune response;GO
GO:0000166//nucleo	GO:0070588//calcium ion transmembrane transport
-	GO:0046890//regulation of lipid biosynthetic process
-	GO:0007166//cell surface receptor signaling pathway
GO:0050839//cell ad	GO:0007409//axonogenesis;GO:0010975//regulation of neuron projectio
-	-
-	-
GO:0004175//endop	GO:0006508//proteolysis;GO:0007520//myoblast fusion;GO:0022617//extr
GO:0003779//actin b	GO:0007015//actin filament organization;GO:0030334//regulation of cell
-	-
GO:0004672//protei	GO:0006885//regulation of pH;GO:0008286//insulin receptor signaling pa
GO:0003779//actin b	GO:0006351//transcription, DNA-templated;GO:0030032//lamellipodium
GO:0005507//coppe	GO:0009308//amine metabolic process;GO:0009445//putrescine metaboli
GO:0004930//G-pro	GO:0001541//ovarian follicle development;GO:0001545//primary ovarian
GO:0005515//protei	GO:0043031//negative regulation of macrophage activation;GO:0050728/
GO:0003953//NAD+	GO:0007165//signal transduction
GO:0031681//G-pro	GO:0007186//G-protein coupled receptor signaling pathway
-	GO:0007165//signal transduction
GO:0004312//fatty a	GO:0002068//glandular epithelial cell development;GO:0006633//fatty aci
GO:0002020//protea	GO:0010466//negative regulation of peptidase activity;GO:0018149//pept
-	-
GO:0005524//ATP bi	GO:0008203//cholesterol metabolic process;GO:0010033//response to or
-	GO:0007010//cytoskeleton organization;GO:0008360//regulation of cell sh
GO:0003779//actin b	GO:0071691//cardiac muscle thin filament assembly
-	-
-	GO:0006839//mitochondrial transport
-	GO:0015888//thiamine transport
GO:0043014//alpha-	GO:0006464//cellular protein modification process;GO:0018095//protein
GO:0015267//chann	GO:0002931//response to ischemia;GO:0006812//cation transport;GO:003
-	-
GO:0004867//serine	GO:0010466//negative regulation of peptidase activity;GO:0030195//nega
GO:0005524//ATP bi	GO:0009235//cobalamin metabolic process;GO:0015889//cobalamin trans
GO:0005515//protei	GO:0007165//signal transduction
GO:0005242//inwar	GO:0006813//potassium ion transport
-	-
GO:0003779//actin b	GO:0071691//cardiac muscle thin filament assembly
GO:0046872//metal	GO:0006875//cellular metal ion homeostasis
-	-
GO:0001784//phosp	GO:0008286//insulin receptor signaling pathway;GO:0010907//positive re
GO:0005102//recept	GO:0003336//corneocyte desquamation;GO:0006672//ceramide metaboli
-	-
GO:0001604//urote	GO:0007165//signal transduction;GO:0007186//G-protein coupled recept
GO:0001965//G-pro	GO:0007165//signal transduction;GO:0007186//G-protein coupled recept
GO:0051015//actin f	GO:0030866//cortical actin cytoskeleton organization;GO:0045214//sarco
GO:0004467//long-	GO:0015908//fatty acid transport
-	-
GO:0003847//1-alky	GO:0006629//lipid metabolic process;GO:0006644//phospholipid metabo
-	-
GO:0016407//acetyl	-
GO:0005515//protei	-

GO:0005515//protei	GO:0002376//immune system process;GO:0006955//immune response;GC
GO:0003779//actin b	GO:0007010//cytoskeleton organization;GO:0008360//regulation of cell sh
GO:0008146//sulfotr	-
GO:0005085//guany	GO:0007264//small GTPase mediated signal transduction
-	-
-	-
GO:0003824//catalyt	GO:0018272//protein-pyridoxal-5-phosphate linkage via peptidyl-N6-py
GO:0005546//phosp	GO:0009636//response to toxic substance;GO:0042360//vitamin E metabo
GO:0003700//transc	GO:0006355//regulation of transcription, DNA-templated
GO:0003824//catalyt	-
-	-
GO:0003824//catalyt	-
GO:0070089//chloric	GO:0006813//potassium ion transport;GO:0097623//potassium ion export
GO:0003847//1-alky	GO:0006629//lipid metabolic process;GO:0006644//phospholipid metabo
GO:0005515//protei	-
GO:0005515//protei	GO:0010468//regulation of gene expression;GO:0016567//protein ubiquit
GO:0003924//GTPas	-
-	-
GO:0004467//long-c	GO:0001676//long-chain fatty acid metabolic process;GO:0008610//lipid b
GO:0003779//actin b	GO:0010735//positive regulation of transcription via serum response elem
GO:0008017//microt	GO:0007010//cytoskeleton organization;GO:0007399//nervous system dev
GO:0000334//3-hyd	GO:0009435//NAD biosynthetic process;GO:0010043//response to zinc io
GO:0004930//G-pro	GO:0002300//CD8-positive, alpha-beta intraepithelial T cell differentiation
-	GO:0007098//centrosome cycle;GO:0035020//regulation of Rac protein si
GO:0005216//ion ch	GO:0006811//ion transport;GO:0006814//sodium ion transport;GO:00550
GO:0000287//magne	GO:0000226//microtubule cytoskeleton organization;GO:0006468//protei
GO:0008022//protei	GO:0007026//negative regulation of microtubule depolymerization;GO:00
GO:0001605//adren	GO:0001525//angiogenesis;GO:0001570//vasculogenesis;GO:0002040//sp
GO:0005515//protei	-
GO:0004672//protei	GO:0000002//mitochondrial genome maintenance;GO:0001938//positive
GO:0005484//SNAP	GO:0006888//ER to Golgi vesicle-mediated transport;GO:0006903//vesicle
GO:0000166//nucleo	GO:0006468//protein phosphorylation;GO:0006974//cellular response to I
GO:0004252//serine	GO:0006508//proteolysis
-	GO:0006887//exocytosis;GO:0015031//protein transport
GO:0015173//aroma	GO:0006590//thyroid hormone generation;GO:0055085//transmembrane
GO:0004252//serine	GO:0000902//cell morphogenesis;GO:0001889//liver development;GO:000
GO:0005524//ATP bi	GO:0000086//G2/M transition of mitotic cell cycle;GO:0045332//phosphol
GO:0003714//transc	GO:0008104//protein localization;GO:0008406//gonad development;GO:0
GO:0000166//nucleo	GO:0006563//L-serine metabolic process;GO:0006790//sulfur compound
GO:0005515//protei	-
GO:0043014//alpha-	GO:0006464//cellular protein modification process;GO:0018095//protein p
GO:0003713//transc	GO:0007219//Notch signaling pathway;GO:0045944//positive regulation o
GO:0004629//phosp	GO:0002431//Fc receptor mediated stimulatory signaling pathway;GO:000
GO:0005515//protei	GO:0003341//cilium movement;GO:0044782//cilium organization
GO:0046983//protei	GO:0000082//G1/S transition of mitotic cell cycle;GO:0000122//negative r
GO:0003868//4-hyd	GO:0006572//tyrosine catabolic process;GO:0009072//aromatic amino aci
GO:0005515//protei	GO:0003341//cilium movement;GO:0044782//cilium organization
GO:0004730//pseud	GO:0001522//pseudouridine synthesis;GO:0006796//phosphate-containin
GO:0003682//chrom	GO:0000712//resolution of meiotic recombination intermediates;GO:0000
GO:0008146//sulfotr	-
-	-
GO:0003824//catalyt	GO:0010121//arginine catabolic process to proline via ornithine;GO:0019
GO:0005515//protei	-
GO:0005324//long-c	GO:0006469//negative regulation of protein kinase activity;GO:0009617//r
GO:0003810//protei	GO:0018149//peptide cross-linking
GO:0003924//GTPas	GO:0000082//G1/S transition of mitotic cell cycle;GO:0007010//cytoskelet
GO:0004497//mono	GO:0006559//L-phenylalanine catabolic process;GO:0006571//tyrosine bi
-	-

GO:0003841//1-acyl	GO:0006644//phospholipid metabolic process
-	-
GO:0003714//transc	GO:0008104//protein localization;GO:0008406//gonad development;GO:0
-	GO:0055085//transmembrane transport
GO:0015485//choles	GO:0032367//intracellular cholesterol transport;GO:0034389//lipid particle
GO:0005515//protei	GO:0051017//actin filament bundle assembly
GO:0003677//DNA b	GO:0006281//DNA repair;GO:0006974//cellular response to DNA damage
GO:0005515//protei	GO:0007155//cell adhesion;GO:0008593//regulation of Notch signaling pa
GO:0005515//protei	GO:0010468//regulation of gene expression;GO:0016567//protein ubiquit
GO:0000287//magne	GO:0000226//microtubule cytoskeleton organization;GO:0006468//protei
GO:0005515//protei	GO:0000086//G2/M transition of mitotic cell cycle;GO:0031146//SCF-depe
GO:0005085//guany	GO:0007165//signal transduction;GO:0007264//small GTPase mediated si
GO:0003779//actin b	GO:0007015//actin filament organization;GO:0030334//regulation of cell r
GO:0004657//prolin	GO:0006562//proline catabolic process
-	-
GO:0005085//guany	GO:0001934//positive regulation of protein phosphorylation;GO:0002437/
-	GO:0006895//Golgi to endosome transport;GO:0015031//protein transpo
GO:0004497//mono	-
GO:0003824//catalyt	GO:0006167//AMP biosynthetic process;GO:0006177//GMP biosynthetic
GO:0004475//mann	GO:0006486//protein glycosylation;GO:0009058//biosynthetic process;GC
GO:0004867//serine	GO:0007596//blood coagulation
GO:0005515//protei	-
GO:0003779//actin b	GO:0007010//cytoskeleton organization;GO:0008360//regulation of cell sh
-	GO:0007154//cell communication
-	-
-	-
-	-
GO:0003924//GTPas	-
GO:0008022//protei	GO:0007099//centriole replication;GO:0010457//centriole-centriole cohes
-	GO:0007010//cytoskeleton organization;GO:0008360//regulation of cell sh
-	-
-	-
GO:0003779//actin b	GO:0030835//negative regulation of actin filament depolymerization
-	-
-	-
GO:0004044//amido	GO:0006177//GMP biosynthetic process;GO:0006189//'de novo' IMP bios
GO:0015297//antipo	GO:0055085//transmembrane transport
GO:0004163//dipho	GO:0019287//isopentenyl diphosphate biosynthetic process, mevalonate
-	-
GO:0035591//signali	GO:0034128//negative regulation of MyD88-independent toll-like recept
GO:0008009//chem	GO:0006955//immune response
GO:0004499//N,N-d	-
GO:0004866//endop	-
GO:0005546//phosp	GO:0009636//response to toxic substance;GO:0042360//vitamin E metab
GO:0005102//recept	GO:0003336//corneocyte desquamation;GO:0006672//ceramide metaboli
GO:0003824//catalyt	GO:0006527//arginine catabolic process;GO:0009072//aromatic amino aci
GO:0004044//amido	GO:0006177//GMP biosynthetic process;GO:0006189//'de novo' IMP bios
GO:0005515//protei	GO:0006749//glutathione metabolic process
GO:0000062//fatty-a	-
-	GO:0015888//thiamine transport
GO:0005515//protei	-
-	-
GO:0004467//long-	GO:0001676//long-chain fatty acid metabolic process;GO:0008610//lipid
GO:0000062//fatty-a	GO:0008203//cholesterol metabolic process;GO:0010742//macrophage de
GO:0008009//chem	GO:0006954//inflammatory response;GO:0006955//immune response;GO
GO:0004639//phosp	GO:0006164//purine nucleotide biosynthetic process;GO:0006189//'de no
-	GO:0009966//regulation of signal transduction
GO:0020037//heme	GO:0019441//tryptophan catabolic process to kynurenine

GO:0005518//collag	GO:0007605//sensory perception of sound;GO:0008360//regulation of cel
GO:0005515//protei	GO:0006355//regulation of transcription, DNA-templated;GO:0007179//tr
GO:0003824//catalyt	-
GO:0016407//acetylt	-
GO:0005515//protei	GO:0042426//choline catabolic process
-	-
GO:0015267//chann	GO:0002931//response to ischemia;GO:0006812//cation transport;GO:003
GO:0005515//protei	-
GO:0005515//protei	GO:0002091//negative regulation of receptor internalization;GO:0042058/
GO:0008270//zinc ic	-
GO:0015485//choles	GO:0032367//intracellular cholesterol transport;GO:0034389//lipid particle
GO:0004497//mono	GO:0097267//omega-hydroxylase P450 pathway
GO:0003824//catalyt	GO:0001822//kidney development;GO:0006596//polyamine biosynthetic p
GO:0004672//protei	GO:0006468//protein phosphorylation
GO:0003924//GTPas	-
GO:0004497//mono	GO:0006559//L-phenylalanine catabolic process;GO:0006571//tyrosine bi
GO:0070089//chloric	GO:0006813//potassium ion transport;GO:0097623//potassium ion export
-	-
GO:0016757//transfe	GO:0002121//inter-male aggressive behavior;GO:0042403//thyroid horm
GO:0003810//protei	GO:0018149//peptide cross-linking
GO:0008289//lipid b	-
GO:0022857//transm	GO:0055085//transmembrane transport
-	-
GO:0003779//actin b	GO:0071691//cardiac muscle thin filament assembly
GO:0046872//metal	GO:0006875//cellular metal ion homeostasis
GO:0005515//protei	GO:0051017//actin filament bundle assembly
GO:0005515//protei	GO:0006936//muscle contraction
GO:0031267//small	GO:0006886//intracellular protein transport
-	-
GO:0003953//NAD+	GO:0007165//signal transduction
GO:0008271//secon	GO:0008272//sulfate transport;GO:0055085//transmembrane transport
GO:0005515//protei	-
GO:0004672//protei	GO:0006885//regulation of pH;GO:0008286//insulin receptor signaling pa
GO:0004730//pseud	GO:0001522//pseudouridine synthesis;GO:0006796//phosphate-containir
-	-
-	-
GO:0000976//transc	GO:0000122//negative regulation of transcription from RNA polymerase I
-	GO:0007275//multicellular organism development;GO:0030154//cell differ
GO:0004866//endop	-
GO:0004867//serine	GO:0016525//negative regulation of angiogenesis;GO:0050769//positive r
GO:0000166//nucleo	GO:0006563//L-serine metabolic process;GO:0006790//sulfur compound
-	GO:0001764//neuron migration;GO:0007420//brain development;GO:002
-	-
GO:0004867//serine	GO:0010466//negative regulation of peptidase activity;GO:0030195//nega
GO:0004930//G-pro	GO:0002001//renin secretion into blood stream;GO:0002281//macrophag
-	-
-	-
-	-
GO:0005515//protei	GO:0006355//regulation of transcription, DNA-templated
GO:0005313//L-glut	GO:0015810//aspartate transport;GO:0015813//L-glutamate transport;GC
GO:0005515//protei	GO:0030855//epithelial cell differentiation
GO:0005515//protei	-
GO:0005324//long-c	GO:0006469//negative regulation of protein kinase activity;GO:0009617//r
GO:0003713//transc	GO:0007219//Notch signaling pathway;GO:0045944//positive regulation c
-	-
-	-
GO:0005515//protei	-
GO:0005524//ATP bi	GO:0000086//G2/M transition of mitotic cell cycle;GO:0045332//phosphol

-	GO:0030389//fructosamine metabolic process;GO:0030855//epithelial cell
GO:0016316//phosp	-
GO:0000166//nucleo	-
GO:0003824//catalyt	GO:0044710//single-organism metabolic process
GO:0005313//L-glut	GO:0015810//aspartate transport;GO:0015813//L-glutamate transport;GC
-	-
-	-
GO:0005515//protei	GO:0042634//regulation of hair cycle
GO:0005515//protei	-
GO:0004639//phosp	GO:0006164//purine nucleotide biosynthetic process;GO:0006189//'de no
GO:0000166//nucleo	GO:0003014//renal system process;GO:0006811//ion transport;GO:000682
GO:0005102//recept	GO:0006898//receptor-mediated endocytosis;GO:0008104//protein locali
-	-
GO:0004842//ubiqui	GO:0006511//ubiquitin-dependent protein catabolic process;GO:0007399
GO:1990817//RNA a	GO:0009617//response to bacterium;GO:0030278//regulation of ossificati
GO:0005515//protei	GO:0003431//growth plate cartilage chondrocyte development;GO:00082
-	-
-	-
-	-
GO:0003677//DNA b	GO:0045944//positive regulation of transcription from RNA polymerase II
GO:0000064//L-orni	GO:1990575//mitochondrial L-ornithine transmembrane transport
GO:0070089//chloric	GO:0006813//potassium ion transport;GO:0097623//potassium ion export
GO:0004497//mono	GO:0006559//L-phenylalanine catabolic process;GO:0006571//tyrosine bi
GO:0005515//protei	GO:0007155//cell adhesion
-	-
GO:0003847//1-alky	GO:0006629//lipid metabolic process;GO:0006644//phospholipid metabo
GO:0000166//nucleo	GO:0000045//autophagosome assembly;GO:0002026//regulation of the fo
GO:0005515//protei	-
GO:0015485//choles	GO:0032367//intracellular cholesterol transport;GO:0034389//lipid particle
GO:0003953//NAD+	GO:0007165//signal transduction
GO:0051015//actin f	GO:0030866//cortical actin cytoskeleton organization;GO:0045214//sarcor
-	-
GO:0002020//protea	GO:0010466//negative regulation of peptidase activity;GO:0018149//pept
GO:0008146//sulfotr	-
GO:0003779//actin b	GO:0030835//negative regulation of actin filament depolymerization
GO:0042809//vitami	GO:0008285//negative regulation of cell proliferation;GO:0010468//regula
GO:0003755//peptid	-
GO:0000064//L-orni	GO:1990575//mitochondrial L-ornithine transmembrane transport
-	-
GO:0000166//nucleo	GO:0006563//L-serine metabolic process;GO:0006790//sulfur compound
GO:0005515//protei	GO:0015879//carnitine transport;GO:0032414//positive regulation of ion t
GO:0003824//catalyt	GO:0006167//AMP biosynthetic process;GO:0006177//GMP biosynthetic p
GO:0004930//G-pro	GO:0007186//G-protein coupled receptor signaling pathway
GO:0005515//protei	GO:0001938//positive regulation of endothelial cell proliferation;GO:0014
-	-
-	-
GO:0016491//oxidor	GO:0019477//L-lysine catabolic process
GO:0016491//oxidor	GO:0007005//mitochondrion organization
GO:0001784//phosp	GO:0001932//regulation of protein phosphorylation;GO:0007259//JAK-ST
GO:0004930//G-pro	GO:0007186//G-protein coupled receptor signaling pathway
GO:0004657//prolin	GO:0006562//proline catabolic process
GO:0003847//1-alky	GO:0006629//lipid metabolic process;GO:0006644//phospholipid metabo
GO:0003924//GTPas	-
GO:0046872//metal	GO:0006875//cellular metal ion homeostasis
GO:0005515//protei	-
-	-
GO:0004252//serine	GO:0006508//proteolysis
GO:0004497//mono	-

GO:0000166//nucleo	GO:0006529//asparagine biosynthetic process;GO:0006541//glutamine m
GO:0003779//actin b	GO:0051017//actin filament bundle assembly
GO:0020037//heme	GO:0019441//tryptophan catabolic process to kynurenine
GO:0003714//transc	GO:0006338//chromatin remodeling;GO:0007507//heart development;GC
GO:0003810//protei	GO:0018149//peptide cross-linking
GO:0031956//mediu	-
GO:0004044//amido	GO:0006177//GMP biosynthetic process;GO:0006189//'de novo' IMP bios
GO:0004467//long-c	GO:0001676//long-chain fatty acid metabolic process;GO:0008610//lipid
GO:0005515//protei	-
-	GO:0007010//cytoskeleton organization;GO:0008360//regulation of cell sh
GO:0000287//magne	GO:0006584//catecholamine metabolic process;GO:0016036//cellular resp
GO:0016407//acetylt	-
-	-
-	GO:0032088//negative regulation of NF-kappaB transcription factor activi
GO:0005515//protei	GO:0006355//regulation of transcription, DNA-templated;GO:0007179//tr
GO:0005216//ion ch	GO:0006811//ion transport;GO:0019228//neuronal action potential;GO:00
-	-
GO:0008146//sulfotr	-
GO:0004497//mono	-
GO:0003700//transc	GO:0006355//regulation of transcription, DNA-templated
GO:0017147//Wnt-p	GO:0030178//negative regulation of Wnt signaling pathway
GO:0004497//mono	GO:0097267//omega-hydroxylase P450 pathway
GO:0000166//nucleo	GO:0000122//negative regulation of transcription from RNA polymerase I
GO:0005096//GTPas	GO:0007165//signal transduction;GO:0035556//intracellular signal transdu
GO:0005515//protei	-
GO:0005104//fibrob	GO:0001525//angiogenesis;GO:0001759//organ induction;GO:0001934//p
GO:0020037//heme	GO:0019441//tryptophan catabolic process to kynurenine
GO:0008009//chemo	GO:0006954//inflammatory response;GO:0006955//immune response;GO
-	-
-	GO:0009966//regulation of signal transduction;GO:0016055//Wnt signalin
GO:0004175//endop	GO:0006508//proteolysis;GO:0007354//zygotic determination of anterior/
GO:0004672//protei	GO:0006468//protein phosphorylation
-	-
GO:0005104//fibrob	GO:0001525//angiogenesis;GO:0001759//organ induction;GO:0001934//p
-	-
GO:0050839//cell ad	GO:0007409//axonogenesis;GO:0010975//regulation of neuron projectior
GO:0004044//amido	GO:0006177//GMP biosynthetic process;GO:0006189//'de novo' IMP bios
GO:0005515//protei	-
GO:0003810//protei	GO:0018149//peptide cross-linking
GO:0004499//N,N-d	-
-	-
GO:0016787//hydrol	-
GO:0042802//identic	-
GO:0005515//protei	GO:0030855//epithelial cell differentiation
GO:0003824//catalyt	GO:0010121//arginine catabolic process to proline via ornithine;GO:0019
GO:0000166//nucleo	GO:0006468//protein phosphorylation;GO:0006974//cellular response to I
-	-
-	-
-	-
GO:0019904//protei	GO:0050796//regulation of insulin secretion
GO:0005515//protei	GO:0051017//actin filament bundle assembly
GO:0005172//vascul	GO:0008283//cell proliferation;GO:0008284//positive regulation of cell pro
-	-
GO:0016407//acetylt	-
GO:0046873//metal	GO:0030001//metal ion transport
GO:0016491//oxidor	-
GO:0003824//catalyt	GO:0005975//carbohydrate metabolic process;GO:0006006//glucose meta
-	-

-	-
GO:0005515//protei	-
GO:0000166//nucleo	GO:0006563//L-serine metabolic process;GO:0006790//sulfur compound
GO:1990817//RNA a	GO:0009617//response to bacterium;GO:0030278//regulation of ossificati
GO:0004672//protei	GO:0006885//regulation of pH;GO:0008286//insulin receptor signaling pa
-	GO:0000226//microtubule cytoskeleton organization
-	-
GO:0004122//cystatl	GO:0001958//endochondral ossification;GO:0001974//blood vessel remoc
GO:0003868//4-hyd	GO:0006572//tyrosine catabolic process;GO:0009072//aromatic amino aci
GO:0005515//protei	GO:0042426//choline catabolic process
GO:0016316//phosp	-
-	-
GO:0004866//endop	-
GO:0005546//phosp	GO:0009636//response to toxic substance;GO:0042360//vitamin E metabo
GO:0004866//endop	-
GO:0004867//serine	GO:0016525//negative regulation of angiogenesis;GO:0050769//positive r
GO:0001784//phosp	GO:0001932//regulation of protein phosphorylation;GO:0007259//JAK-ST
-	-
GO:0015485//choles	GO:0032367//intracellular cholesterol transport;GO:0034389//lipid particle
GO:0005515//protei	-
-	-
-	-
-	-
GO:0005515//protei	-
GO:0005102//recept	GO:0006898//receptor-mediated endocytosis;GO:0008104//protein locali:
GO:0005313//L-glut	GO:0015810//aspartate transport;GO:0015813//L-glutamate transport;GC
GO:0004497//mono	GO:0006559//L-phenylalanine catabolic process;GO:0006571//tyrosine bi
GO:0005102//recept	GO:0007160//cell-matrix adhesion;GO:0007596//blood coagulation;GO:0(
GO:0000064//L-orni	GO:1990575//mitochondrial L-ornithine transmembrane transport
GO:0004364//glutatl	GO:0006749//glutathione metabolic process;GO:0006805//xenobiotic met
GO:0008028//mono	GO:0015718//monocarboxylic acid transport;GO:0055085//transmembran
GO:0004896//cytoki	GO:0007171//activation of transmembrane receptor protein tyrosine kina
GO:0005353//fructo	GO:0003044//regulation of systemic arterial blood pressure mediated by a
-	-
GO:0003824//catalyt	GO:0006527//arginine catabolic process;GO:0009072//aromatic amino aci
GO:0005515//protei	GO:0042634//regulation of hair cycle
GO:0003714//transc	GO:0008104//protein localization;GO:0008406//gonad development;GO:0
GO:0003924//GTPas	-
GO:0000166//nucleo	GO:0000045//autophagosome assembly;GO:0002026//regulation of the fo
GO:0051015//actin f	GO:0030866//cortical actin cytoskeleton organization;GO:0045214//sarco
GO:0005515//protei	GO:0015879//carnitine transport;GO:0032414//positive regulation of ion t
-	-
-	-
GO:0003824//catalyt	GO:0006167//AMP biosynthetic process;GO:0006177//GMP biosynthetic p
GO:0003677//DNA b	GO:0009267//cellular response to starvation;GO:0043066//negative regula
GO:0004888//transm	GO:0006811//ion transport;GO:0034220//ion transmembrane transport
GO:0004930//G-pro	GO:0002300//CD8-positive, alpha-beta intraepithelial T cell differentiatior
-	-
GO:0005507//coppe	GO:0007601//visual perception;GO:0009308//amine metabolic process
GO:0004866//endop	GO:0010951//negative regulation of endopeptidase activity
GO:0005515//protei	GO:0001938//positive regulation of endothelial cell proliferation;GO:0014
GO:0005515//protei	GO:0044763//single-organism cellular process
GO:0046873//metal	GO:0030001//metal ion transport
GO:0019901//protei	GO:0030334//regulation of cell migration;GO:0034446//substrate adhesio
GO:0003677//DNA b	-
-	-
GO:0001517//N-ace	GO:0005975//carbohydrate metabolic process;GO:0006044//N-acetylgluc
-	-

GO:0000978//RNA p	GO:0006355//regulation of transcription, DNA-templated;GO:0006357//re
GO:0002161//amino	-
-	-
GO:0008080//N-ace	-
-	-
GO:0000976//transc	GO:0000122//negative regulation of transcription from RNA polymerase I
GO:0004420//hydro:	GO:0006695//cholesterol biosynthetic process;GO:0008299//isoprenoid b
GO:0042802//identic	-
GO:0005515//protei	GO:0000281//mitotic cytokinesis;GO:0014067//negative regulation of pho
GO:0005085//guany	GO:0050790//regulation of catalytic activity
GO:0004175//endop	GO:0006508//proteolysis;GO:0007354//zygotic determination of anterior/
-	-
GO:0000062//fatty-a	GO:0036151//phosphatidylcholine acyl-chain remodeling;GO:1903060//n
-	-
GO:0005540//hyalur	GO:0007155//cell adhesion
-	GO:0006629//lipid metabolic process
-	-
GO:0004475//mann	GO:0006486//protein glycosylation;GO:0009058//biosynthetic process;GC
GO:0005524//ATP bi	GO:0035499//carnosine biosynthetic process
-	-
-	-
GO:0005506//iron ic	GO:0006457//protein folding;GO:0018126//protein hydroxylation;GO:001
-	GO:0006887//exocytosis;GO:0015031//protein transport
GO:0005515//protei	-
-	GO:0001501//skeletal system development;GO:0006006//glucose metabo
GO:0022857//transm	GO:0055085//transmembrane transport
GO:0005319//lipid tr	GO:0006629//lipid metabolic process;GO:0006642//triglyceride mobilizati
GO:0003824//catalyt	GO:0006508//proteolysis;GO:0012501//programmed cell death;GO:00147
GO:0001671//ATPas	-
-	-
GO:0003924//GTPas	-
GO:0003676//nuclei	GO:0006388//tRNA splicing, via endonucleolytic cleavage and ligation
GO:0008271//secon	GO:0008272//sulfate transport;GO:0055085//transmembrane transport
-	-
GO:0002020//protea	GO:0010951//negative regulation of endopeptidase activity
-	-
GO:0003676//nuclei	-
GO:0005102//recept	GO:0001541//ovarian follicle development;GO:0001707//mesoderm form
GO:0022892//substr	GO:0044765//single-organism transport
GO:0000253//3-ketc	GO:0006695//cholesterol biosynthetic process;GO:0006703//estrogen bio
GO:0030246//carbol	GO:0006897//endocytosis
GO:0031956//mediu	-
GO:0003810//protei	GO:0018149//peptide cross-linking
-	-
-	-
GO:0015020//glucur	GO:0006486//protein glycosylation;GO:0015012//heparan sulfate proteog
GO:0004467//long-c	GO:0001676//long-chain fatty acid metabolic process;GO:0008610//lipid l
-	GO:0007507//heart development
GO:0005227//calciu	GO:0006812//cation transport
-	GO:0030070//insulin processing;GO:0060628//regulation of ER to Golgi ve
GO:0005319//lipid tr	GO:0006869//lipid transport
-	-
-	GO:0007411//axon guidance;GO:0010977//negative regulation of neuron
-	-
GO:0005319//lipid tr	GO:0006869//lipid transport
-	GO:0006629//lipid metabolic process
GO:0003779//actin b	GO:0051017//actin filament bundle assembly
-	-

GO:0001786//phosp	GO:0002024//diet induced thermogenesis;GO:0006606//protein import in
-	GO:0009987//cellular process;GO:0044699//single-organism process
GO:0000287//magne	GO:0006468//protein phosphorylation;GO:0007346//regulation of mitotic
GO:0005085//guany	GO:0007264//small GTPase mediated signal transduction;GO:0030334//re
-	-
GO:0005509//calciur	GO:0006936//muscle contraction;GO:0030035//microspike assembly;GO:0
GO:0004930//G-pro	GO:0007186//G-protein coupled receptor signaling pathway;GO:0007200
GO:0005515//protei	GO:0001938//positive regulation of endothelial cell proliferation;GO:0014
-	GO:0007042//lysosomal lumen acidification;GO:0042176//regulation of pr
-	GO:0051480//regulation of cytosolic calcium ion concentration;GO:19900
GO:0005515//protei	-
-	-
GO:0005085//guany	GO:0007264//small GTPase mediated signal transduction
-	-
GO:0003796//lysozy	GO:0008152//metabolic process;GO:0016998//cell wall macromolecule ca
-	-
GO:0008233//peptid	GO:0006508//proteolysis
GO:0001968//fibron	GO:0010811//positive regulation of cell-substrate adhesion;GO:0030198//
GO:0036122//BMP b	GO:0007389//pattern specification process;GO:0010454//negative regulat
GO:0005515//protei	GO:0055085//transmembrane transport
-	-
-	-
GO:0005515//protei	GO:0043254//regulation of protein complex assembly
-	-
GO:0002039//p53 bi	GO:0006468//protein phosphorylation;GO:0008285//negative regulation c
GO:0042802//identic	GO:0007165//signal transduction;GO:0046628//positive regulation of insu
-	-
GO:0004190//aspart	GO:0006508//proteolysis;GO:0019538//protein metabolic process
GO:0042802//identic	GO:0007165//signal transduction;GO:0046628//positive regulation of insu
GO:0000166//nucleo	GO:0000045//autophagosome assembly;GO:0002026//regulation of the fo
GO:0000248//C-5 st	GO:0008610//lipid biosynthetic process;GO:0016126//sterol biosynthetic p
GO:0004843//thiol-c	GO:0006511//ubiquitin-dependent protein catabolic process;GO:0016579
-	-
GO:0000166//nucleo	GO:0006468//protein phosphorylation;GO:0010976//positive regulation o
GO:0046961//proton	GO:0006811//ion transport;GO:1902600//hydrogen ion transmembrane tr
GO:0004867//serine	GO:0010951//negative regulation of endopeptidase activity;GO:0030212//
-	-
-	-
GO:0005515//protei	GO:0007596//blood coagulation;GO:0033627//cell adhesion mediated by
-	-
GO:0003676//nuclei	GO:0019722//calcium-mediated signaling;GO:0050790//regulation of cata
GO:0005509//calciur	GO:0007268//synaptic transmission;GO:0035556//intracellular signal trans
GO:0005227//calciur	GO:0001581//detection of chemical stimulus involved in sensory perceptio
GO:0005524//ATP bi	GO:0009235//cobalamin metabolic process;GO:0015889//cobalamin trans
-	-
GO:0005515//protei	GO:0007596//blood coagulation;GO:0033627//cell adhesion mediated by
GO:0003779//actin b	GO:0055013//cardiac muscle cell development
-	-
-	-
GO:0016491//oxidor	-
-	GO:0018095//protein polyglutamylation
GO:0005515//protei	GO:0055085//transmembrane transport
-	-
GO:0000976//transc	GO:0002062//chondrocyte differentiation;GO:0006355//regulation of tran
-	-
-	-
-	-
-	GO:0007411//axon guidance;GO:0010977//negative regulation of neuron

-	GO:0030070//insulin processing;GO:0060628//regulation of ER to Golgi ve
-	-
GO:0003677//DNA b	-
GO:0016491//oxidor	-
GO:0004672//protei	GO:0001501//skeletal system development;GO:0006468//protein phospho
-	GO:0070979//protein K11-linked ubiquitination
GO:0002039//p53 bi	GO:0006468//protein phosphorylation;GO:0008285//negative regulation c
GO:0005085//guany	GO:0007264//small GTPase mediated signal transduction;GO:0034976//re
-	-
-	-
-	-
GO:0004452//isoper	GO:0008299//isoprenoid biosynthetic process
GO:0003836//beta-g	GO:0002319//memory B cell differentiation;GO:0006054//N-acetylneuram
GO:0004045//amino	-
GO:0005515//protei	GO:0051260//protein homooligomerization
GO:0005085//guany	GO:0050790//regulation of catalytic activity
-	GO:0000463//maturation of LSU-rRNA from tricistronic rRNA transcript (S
GO:0000978//RNA p	GO:0001932//regulation of protein phosphorylation;GO:0006355//regulat
-	-
GO:0005507//coppe	GO:0007601//visual perception;GO:0009308//amine metabolic process
GO:0016628//oxidor	GO:0006695//cholesterol biosynthetic process;GO:0016126//sterol biosyn
GO:0004857//enzym	GO:0006629//lipid metabolic process;GO:0043086//negative regulation of
GO:0022857//transm	GO:0055085//transmembrane transport
-	-
GO:0008289//lipid b	GO:0015031//protein transport;GO:0019075//virus maturation;GO:004205
GO:0016787//hydrol	GO:0006629//lipid metabolic process;GO:0007216//G-protein coupled glu
GO:0000250//lanost	GO:0006694//steroid biosynthetic process;GO:0006695//cholesterol biosy
-	-
GO:0004721//phosp	GO:0006470//protein dephosphorylation;GO:0016311//dephosphorylation
-	-
-	-
-	-
-	-
GO:0005515//protei	-
-	GO:0050910//detection of mechanical stimulus involved in sensory percep
GO:0001758//retinal	GO:0002072//optic cup morphogenesis involved in camera-type eye deve
GO:0000287//magne	GO:0006468//protein phosphorylation;GO:0007346//regulation of mitotic
GO:0016491//oxidor	GO:0006629//lipid metabolic process;GO:0008611//ether lipid biosyntheti
GO:0022857//transm	GO:0055085//transmembrane transport
GO:0004222//metall	GO:0001542//ovulation from ovarian follicle;GO:0001822//kidney develop
-	GO:0009267//cellular response to starvation;GO:0030001//metal ion trans
-	-
-	-
GO:0017128//phosp	GO:0000045//autophagosome assembly;GO:0006914//autophagy;GO:001
GO:0030145//manga	GO:0006508//proteolysis;GO:0019538//protein metabolic process
GO:0004672//protei	GO:0001501//skeletal system development;GO:0006468//protein phospho
-	-
GO:0004176//ATP-d	GO:0006508//proteolysis;GO:0006515//misfolded or incompletely synthes
GO:0002161//amino	-
GO:0022857//transm	GO:0055085//transmembrane transport
GO:0004930//G-pro	GO:0000278//mitotic cell cycle;GO:0001525//angiogenesis;GO:0001569//
GO:0000976//transc	GO:0000122//negative regulation of transcription from RNA polymerase I
GO:0051082//unfold	GO:0006457//protein folding
-	-
GO:0016757//transfe	GO:0002121//inter-male aggressive behavior;GO:0042403//thyroid horm
-	-
-	-
-	GO:0007399//nervous system development

-	-
GO:0004672//protei	GO:0006468//protein phosphorylation;GO:0007169//transmembrane rece
GO:0004896//cytoki	GO:0019221//cytokine-mediated signaling pathway;GO:0050829//defens
GO:0005509//calciu	GO:0007155//cell adhesion;GO:0007156//homophilic cell adhesion via pla
GO:0005524//ATP b	GO:0009235//cobalamin metabolic process;GO:0015889//cobalamin trans
-	GO:0001501//skeletal system development;GO:0006006//glucose metabo
-	-
GO:0005515//protei	-
GO:0043169//cation	GO:0007165//signal transduction;GO:0022603//regulation of anatomical s
GO:0005515//protei	GO:0001822//kidney development;GO:0002093//auditory receptor cell m
GO:0005515//protei	GO:0007043//cell-cell junction assembly;GO:0007155//cell adhesion;GO:0
GO:0045296//cadhe	GO:0007043//cell-cell junction assembly;GO:0060997//dendritic spine mo
GO:0003676//nuclei	GO:0006508//proteolysis;GO:0016032//viral process
GO:0031956//mediu	-
GO:0022892//substr	GO:0044765//single-organism transport
GO:0000287//magn	GO:0006468//protein phosphorylation;GO:0030036//actin cytoskeleton or
GO:0005319//lipid tr	GO:0006629//lipid metabolic process;GO:0006642//triglyceride mobilizati
-	-
-	-
GO:0003824//catalyt	GO:0006778//porphyrin-containing compound metabolic process;GO:00
-	-
-	GO:0006886//intracellular protein transport;GO:0006890//retrograde vesi
-	-
GO:0005515//protei	-
GO:0005201//extrac	GO:0007160//cell-matrix adhesion;GO:0007605//sensory perception of so
-	-
-	-
GO:0008289//lipid b	GO:0010873//positive regulation of cholesterol esterification;GO:0010879/
GO:0008170//N-me	GO:0001692//histamine metabolic process;GO:0001695//histamine catabo
GO:0003847//1-alky	GO:0016042//lipid catabolic process
-	-
-	-
GO:0016491//oxidor	-
-	-
GO:0008233//peptid	GO:0006508//proteolysis
-	-
GO:0008146//sulfotr	-
GO:0005085//guany	GO:0007264//small GTPase mediated signal transduction
-	-
-	GO:0030513//positive regulation of BMP signaling pathway
-	-
GO:0005515//protei	GO:0000281//mitotic cytokinesis;GO:0007019//microtubule depolymeriza
GO:0005113//patche	GO:0001501//skeletal system development;GO:0001503//ossification;GO:0
-	GO:0006506//GPI anchor biosynthetic process
GO:0005319//lipid tr	GO:0006869//lipid transport;GO:0015914//phospholipid transport;GO:004
-	-
GO:0005515//protei	GO:0098742//cell-cell adhesion via plasma-membrane adhesion molecul
-	-
GO:0042577//lipid p	GO:0006644//phospholipid metabolic process
-	-
GO:0005509//calciu	GO:0015012//heparan sulfate proteoglycan biosynthetic process;GO:0030
-	-
GO:0003735//structu	GO:0006412//translation;GO:0006919//activation of cysteine-type endope
GO:0005216//ion ch	GO:0006811//ion transport;GO:0006813//potassium ion transport;GO:003
-	-
GO:0004674//protei	GO:0006468//protein phosphorylation
GO:0005515//protei	GO:0055085//transmembrane transport
-	GO:0098609//cell-cell adhesion

GO:0005515//protei	-
-	-
GO:0002039//p53 bi	GO:0006468//protein phosphorylation;GO:0008285//negative regulation
-	GO:0006839//mitochondrial transport
GO:0043022//riboso	GO:0006886//intracellular protein transport;GO:0030433//ER-associated
-	GO:0070979//protein K11-linked ubiquitination
-	-
-	-
GO:0005085//guany	GO:0035556//intracellular signal transduction
-	GO:0030070//insulin processing;GO:0060628//regulation of ER to Golgi ve
GO:0005484//SNAP	GO:0006888//ER to Golgi vesicle-mediated transport;GO:0006903//vesicle
GO:0016491//oxidor	-
-	GO:0006887//exocytosis;GO:0015031//protein transport
-	-
GO:0000976//transc	GO:0006289//nucleotide-excision repair;GO:0006974//cellular response to
-	-
GO:0003677//DNA b	-
-	-
GO:0005515//protei	GO:0007596//blood coagulation;GO:0033627//cell adhesion mediated by
GO:0003676//nuclei	GO:0006278//RNA-dependent DNA replication;GO:0006310//DNA recom
GO:0005515//protei	-
-	-
-	GO:0009987//cellular process;GO:0044699//single-organism process
GO:0000978//RNA p	GO:0006355//regulation of transcription, DNA-templated;GO:0006357//re
GO:0003677//DNA b	GO:0006334//nucleosome assembly;GO:0006352//DNA-templated transc
GO:0005543//phosp	GO:0048260//positive regulation of receptor-mediated endocytosis;GO:0
GO:0009374//biotin	-
GO:0000287//magne	GO:0006099//tricarboxylic acid cycle
GO:0005515//protei	GO:0043031//negative regulation of macrophage activation;GO:0050728/
GO:0060090//bindin	GO:0030433//ER-associated ubiquitin-dependent protein catabolic proce
-	-
GO:0003676//nuclei	GO:0006388//tRNA splicing, via endonucleolytic cleavage and ligation
GO:0005515//protei	-
GO:0003677//DNA b	-
GO:0017128//phosp	GO:0000045//autophagosome assembly;GO:0006869//lipid transport;GO:
GO:0005515//protei	GO:0043254//regulation of protein complex assembly
GO:0003779//actin b	GO:0055013//cardiac muscle cell development
-	GO:0060074//synapse maturation
GO:0005242//inwarc	GO:0006813//potassium ion transport
GO:0001758//retinal	GO:0002072//optic cup morphogenesis involved in camera-type eye deve
-	GO:0006887//exocytosis;GO:0015031//protein transport
-	-
GO:0005085//guany	GO:0050790//regulation of catalytic activity
-	GO:0070453//regulation of heme biosynthetic process
GO:0003836//beta-	GO:0002319//memory B cell differentiation;GO:0006054//N-acetylneuram
GO:0003676//nuclei	GO:0043484//regulation of RNA splicing
GO:0005509//calciur	GO:0006936//muscle contraction;GO:0030035//microspike assembly;GO:
GO:0008017//microt	GO:0008285//negative regulation of cell proliferation;GO:0030330//DNA
GO:0004930//G-pro	GO:0001541//ovarian follicle development;GO:0001545//primary ovarian
GO:0004623//phosp	GO:0006644//phospholipid metabolic process;GO:0016042//lipid cataboli
GO:0002161//amino	-
GO:0003824//catalyt	GO:0006777//Mo-molybdopterin cofactor biosynthetic process;GO:00435
GO:0008270//zinc ic	GO:0006882//cellular zinc ion homeostasis;GO:0010273//detoxification of
GO:0003707//steroic	GO:0007340//acrosome reaction;GO:0009611//response to wounding;GO
GO:0002020//protea	GO:0010951//negative regulation of endopeptidase activity
GO:0005319//lipid tr	GO:0006869//lipid transport
GO:0005515//protei	GO:0043065//positive regulation of apoptotic process;GO:0044790//nega
GO:0022857//transn	GO:0055085//transmembrane transport

GO:0003824//catalyt	GO:0006778//porphyrin-containing compound metabolic process;GO:000
GO:0005515//protei	GO:0055085//transmembrane transport
GO:0005515//protei	-
GO:0016491//oxidor	-
GO:0000246//delta2	GO:0006695//cholesterol biosynthetic process;GO:0006979//response to
-	-
-	-
-	GO:0007165//signal transduction;GO:0070507//regulation of microtubule
-	-
GO:0017128//phosp	GO:0000045//autophagosome assembly;GO:0006914//autophagy;GO:001
GO:0004843//thiol-c	GO:0006511//ubiquitin-dependent protein catabolic process;GO:0007000
-	-
-	-
GO:0005509//calciur	GO:0007268//synaptic transmission;GO:0008104//protein localization;GO:
GO:0009922//fatty a	GO:0000038//very long-chain fatty acid metabolic process;GO:0006629//l
-	-
-	GO:0007042//lysosomal lumen acidification;GO:0042176//regulation of pr
-	-
GO:0001671//ATPas	-
GO:0046961//protor	GO:0006811//ion transport;GO:1902600//hydrogen ion transmembrane tr
GO:0003677//DNA b	-
-	-
GO:0000166//nucleo	GO:0006897//endocytosis;GO:0006898//receptor-mediated endocytosis;G
-	GO:0001656//metanephros development;GO:0001658//branching involve
GO:0001671//ATPas	-
GO:0004497//mono	GO:0006805//xenobiotic metabolic process;GO:0008202//steroid metabol
GO:0005085//guany	GO:0007264//small GTPase mediated signal transduction
GO:0001786//phosp	GO:0006910//phagocytosis, recognition;GO:0006911//phagocytosis, engu
-	-
-	-
GO:0004674//protei	GO:0006468//protein phosphorylation
GO:0004652//polynu	GO:0008285//negative regulation of cell proliferation;GO:0043066//negat
GO:0003847//1-alky	GO:0016042//lipid catabolic process
GO:0003676//nuclei	GO:0006508//proteolysis;GO:0016032//viral process
GO:0042577//lipid p	GO:0006644//phospholipid metabolic process
-	-
-	-
GO:0001671//ATPas	GO:0001933//negative regulation of protein phosphorylation;GO:003043
-	-
GO:0016717//oxidor	GO:0006629//lipid metabolic process
GO:0004843//thiol-c	GO:0006511//ubiquitin-dependent protein catabolic process;GO:0016579
GO:0008146//sulfotr	GO:0006790//sulfur compound metabolic process
GO:0000166//nucleo	GO:0006468//protein phosphorylation;GO:0010976//positive regulation o
GO:0036122//BMP b	GO:0007389//pattern specification process;GO:0010454//negative regulat
-	-
-	-
GO:0000977//RNA p	GO:0000122//negative regulation of transcription from RNA polymerase I
GO:0016491//oxidor	-
GO:0004475//mann	GO:0006486//protein glycosylation;GO:0009058//biosynthetic process;GC
-	-
-	GO:0006364//rRNA processing;GO:0009303//rRNA transcription
GO:0005515//protei	-
GO:0016491//oxidor	GO:0006666//3-keto-sphinganine metabolic process;GO:0030148//sphin
GO:0000166//nucleo	GO:0003014//renal system process;GO:0006811//ion transport;GO:00068
-	-
GO:0003677//DNA b	GO:0009646//response to absence of light;GO:0018298//protein-chromo
GO:0005509//calciur	GO:0007268//synaptic transmission;GO:0035556//intracellular signal trans
-	-

GO:0003836//beta-	GO:0002319//memory B cell differentiation;GO:0006054//N-acetylneuram
GO:0005524//ATP b	GO:0009235//cobalamin metabolic process;GO:0015889//cobalamin trans
GO:0003779//actin b	GO:0071691//cardiac muscle thin filament assembly
-	-
GO:0004843//thiol-	GO:0006511//ubiquitin-dependent protein catabolic process;GO:0007000
GO:0004672//protei	GO:0001501//skeletal system development;GO:0006468//protein phospho
GO:0000978//RNA p	GO:0006355//regulation of transcription, DNA-templated;GO:0006357//re
GO:0008138//protei	GO:0006355//regulation of transcription, DNA-templated;GO:0006470//p
GO:0005113//patche	GO:0001501//skeletal system development;GO:0001503//ossification;GO:
GO:0004857//enzym	GO:0006629//lipid metabolic process;GO:0043086//negative regulation of
GO:0004930//G-pro	GO:0001541//ovarian follicle development;GO:0001545//primary ovarian
GO:0016757//transfe	GO:0002121//inter-male aggressive behavior;GO:0042403//thyroid horm
-	-
GO:0000976//transc	GO:0000122//negative regulation of transcription from RNA polymerase I
GO:0004310//farnes	GO:0008610//lipid biosynthetic process;GO:0009058//biosynthetic proces
GO:0005515//protei	GO:0000281//mitotic cytokinesis;GO:0014067//negative regulation of pho
GO:0016491//oxidor	-
GO:0003824//catalyt	GO:0006777//Mo-molybdopterin cofactor biosynthetic process;GO:00435
-	-
GO:0016491//oxidor	GO:0006629//lipid metabolic process;GO:0008611//ether lipid biosyntheti
GO:0000978//RNA p	GO:0001649//osteoblast differentiation;GO:0001755//neural crest cell mig
GO:0008289//lipid b	GO:0015031//protein transport;GO:0019075//virus maturation;GO:004205
GO:0004674//protei	GO:0006468//protein phosphorylation
GO:0043022//riboso	GO:0006886//intracellular protein transport;GO:0030433//ER-associated u
GO:0000978//RNA p	GO:0001736//establishment of planar polarity;GO:0001843//neural tube c
-	GO:0007098//centrosome cycle;GO:0035020//regulation of Rac protein si
GO:0005515//protei	-
GO:0005515//protei	-
GO:0005515//protei	GO:0003341//cilium movement;GO:0044782//cilium organization
GO:0003677//DNA b	GO:0006334//nucleosome assembly;GO:0006352//DNA-templated transc
GO:0030145//manga	GO:0006508//proteolysis;GO:0019538//protein metabolic process
GO:0005085//guany	GO:0035556//intracellular signal transduction
-	-
GO:0004930//G-pro	GO:0007165//signal transduction;GO:0007186//G-protein coupled recept
GO:0004930//G-pro	GO:0002001//renin secretion into blood stream;GO:0002281//macrophag
GO:0005254//chloric	GO:0006811//ion transport;GO:0006820//anion transport;GO:0006821//ch
GO:0004672//protei	GO:0006468//protein phosphorylation;GO:0008631//intrinsic apoptotic si
GO:0003676//nuclei	GO:0006278//RNA-dependent DNA replication;GO:0006310//DNA recom
GO:0004860//protei	GO:0007616//long-term memory;GO:0050729//positive regulation of infla
-	-
-	-
-	-
GO:0004672//protei	GO:0001501//skeletal system development;GO:0006468//protein phospho
GO:0003676//nuclei	GO:0006278//RNA-dependent DNA replication;GO:0006310//DNA recom
GO:0005524//ATP b	GO:0009235//cobalamin metabolic process;GO:0015889//cobalamin trans
-	-
-	-
-	GO:0007098//centrosome cycle;GO:0035020//regulation of Rac protein si
-	-
GO:0005515//protei	-
-	-
GO:0005515//protei	-
GO:0005201//extrac	GO:0007160//cell-matrix adhesion;GO:0007605//sensory perception of sc
GO:0000287//magne	GO:0000226//microtubule cytoskeleton organization;GO:0006468//protei
-	-
GO:0003779//actin b	GO:0055013//cardiac muscle cell development
GO:0008195//phosp	GO:0006720//isoprenoid metabolic process;GO:0018342//protein prenyla
GO:0017128//phosp	GO:0000045//autophagosome assembly;GO:0006869//lipid transport;GO:

GO:0003676//nuclei	GO:0006388//tRNA splicing, via endonucleolytic cleavage and ligation
GO:0003707//steroic	GO:0007340//acrosome reaction;GO:0009611//response to wounding;GO
-	-
GO:0003874//6-pyru	GO:0006729//tetrahydrobiopterin biosynthetic process
-	-
-	GO:0030513//positive regulation of BMP signaling pathway
-	-
-	-
GO:0003847//1-alky	GO:0006629//lipid metabolic process;GO:0006644//phospholipid metabo
-	-
GO:0004175//endop	GO:0002218//activation of innate immune response;GO:0006508//proteol
-	GO:0098609//cell-cell adhesion
GO:0003824//catalyt	GO:0009226//nucleotide-sugar biosynthetic process;GO:0010595//positiv
GO:0005085//guany	GO:0007264//small GTPase mediated signal transduction
-	GO:0007042//lysosomal lumen acidification;GO:0042176//regulation of pr
-	-
-	-
GO:0008233//peptid	GO:0006508//proteolysis
GO:0016717//oxidor	GO:0006629//lipid metabolic process
GO:0003824//catalyt	GO:0009226//nucleotide-sugar biosynthetic process;GO:0010595//positiv
-	-
-	-
GO:0005515//protei	GO:0000281//mitotic cytokinesis;GO:0007019//microtubule depolymeriza
GO:0015293//sympo	GO:0008643//carbohydrate transport
GO:0008270//zinc io	GO:0009086//methionine biosynthetic process;GO:0009235//cobalamin m
GO:0004475//mann	GO:0006486//protein glycosylation;GO:0009058//biosynthetic process;GC
-	-
-	-
-	-
GO:0004843//thiol-c	GO:0006511//ubiquitin-dependent protein catabolic process;GO:0016579
GO:0005509//calciur	GO:0007155//cell adhesion;GO:0007156//homophilic cell adhesion via pla
GO:0004896//cytoki	GO:0019221//cytokine-mediated signaling pathway;GO:0050829//defense
GO:0004000//adeno	GO:0000255//allantoin metabolic process;GO:0001666//response to hypo
GO:0000166//nucleo	GO:0006468//protein phosphorylation;GO:0010976//positive regulation o
GO:0016491//oxidor	GO:0006666//3-keto-sphinganine metabolic process;GO:0030148//sphing
-	-
GO:0008235//metall	GO:0017186//peptidyl-pyroglutamic acid biosynthetic process, using glut
-	-
GO:0003779//actin b	GO:0051017//actin filament bundle assembly
GO:0003676//nuclei	-
GO:0005515//protei	GO:0006355//regulation of transcription, DNA-templated;GO:0006805//x
GO:0004930//G-pro	GO:0000278//mitotic cell cycle;GO:0001525//angiogenesis;GO:0001569//p
GO:0046961//protor	GO:0006811//ion transport;GO:1902600//hydrogen ion transmembrane tr
GO:0005484//SNAP	GO:0006888//ER to Golgi vesicle-mediated transport;GO:0006903//vesicle
-	-
-	-
GO:0004176//ATP-d	GO:0006508//proteolysis;GO:0006515//misfolded or incompletely synthes
-	-
-	GO:0006887//exocytosis;GO:0015031//protein transport
-	-
-	-
GO:0015267//chann	GO:0002931//response to ischemia;GO:0006812//cation transport;GO:003
GO:0003676//nuclei	GO:0006508//proteolysis;GO:0016032//viral process
GO:0003677//DNA b	GO:0009646//response to absence of light;GO:0018298//protein-chromo
GO:0004843//thiol-c	GO:0006511//ubiquitin-dependent protein catabolic process;GO:0007000
GO:0004672//protei	GO:0006468//protein phosphorylation;GO:0008631//intrinsic apoptotic si
-	GO:0009267//cellular response to starvation;GO:0030001//metal ion trans
-	-

GO:0004930//G-pro	GO:0007165//signal transduction;GO:0007186//G-protein coupled recept
GO:0002020//protea	GO:0010951//negative regulation of endopeptidase activity
GO:0003779//actin b	GO:0071691//cardiac muscle thin filament assembly
-	-
GO:0004930//G-pro	GO:0001541//ovarian follicle development;GO:0001545//primary ovarian
GO:0003735//structu	GO:0006412//translation;GO:0006919//activation of cysteine-type endope
GO:0005515//protei	GO:0007015//actin filament organization;GO:0045806//negative regulatio
-	-
-	-
GO:0003824//catalyt	GO:0005975//carbohydrate metabolic process
-	-
GO:0000062//fatty-a	GO:0036151//phosphatidylcholine acyl-chain remodeling;GO:1903060//n
-	-
GO:0005515//protei	-
GO:0060090//bindin	GO:0030433//ER-associated ubiquitin-dependent protein catabolic proce
GO:0005515//protei	GO:0001558//regulation of cell growth
-	-
GO:0022857//transm	GO:0055085//transmembrane transport
GO:0042802//identic	GO:0019216//regulation of lipid metabolic process;GO:0055088//lipid hor
GO:0004452//isoper	GO:0008299//isoprenoid biosynthetic process
GO:0004620//phosp	GO:0009395//phospholipid catabolic process;GO:0030336//negative regu
GO:0004623//phosp	GO:0006644//phospholipid metabolic process;GO:0016042//lipid cataboli
GO:0002039//p53 bi	GO:0006468//protein phosphorylation;GO:0008285//negative regulation
-	-
-	-
GO:0004222//metall	GO:0001542//ovulation from ovarian follicle;GO:0001822//kidney develop
GO:0003824//catalyt	GO:0044710//single-organism metabolic process
-	-
GO:0005319//lipid tr	GO:0006869//lipid transport
-	-
GO:0016290//palmit	GO:0006637//acyl-CoA metabolic process;GO:0016559//peroxisome fissio
GO:0004857//enzym	GO:0006629//lipid metabolic process;GO:0043086//negative regulation o
GO:0003824//catalyt	GO:0006777//Mo-molybdopterin cofactor biosynthetic process;GO:00435
GO:0000976//transc	GO:0006357//regulation of transcription from RNA polymerase II promote
-	-
-	GO:0032088//negative regulation of NF-kappaB transcription factor activi
GO:0005515//protei	-
GO:0005515//protei	GO:0051017//actin filament bundle assembly
GO:0005515//protei	-
GO:0000976//transc	GO:0002062//chondrocyte differentiation;GO:0006355//regulation of tran
GO:0035254//glutan	GO:0001662//behavioral fear response;GO:0006884//cell volume homeos
GO:0046873//metal	GO:0030001//metal ion transport
GO:0000287//magne	GO:0006468//protein phosphorylation;GO:0007204//positive regulation o
GO:0008047//enzym	GO:0006486//protein glycosylation;GO:0006487//protein N-linked glycos
GO:0005515//protei	GO:0042426//choline catabolic process
GO:0016491//oxidor	-
-	GO:0070453//regulation of heme biosynthetic process
GO:0005515//protei	GO:0007596//blood coagulation;GO:0033627//cell adhesion mediated by
GO:0016491//oxidor	GO:0006629//lipid metabolic process;GO:0008611//ether lipid biosyntheti
GO:0003677//DNA b	GO:0045944//positive regulation of transcription from RNA polymerase II
GO:0043169//cation	GO:0007165//signal transduction;GO:0022603//regulation of anatomical s
-	-
GO:0005515//protei	GO:0007043//cell-cell junction assembly;GO:0007155//cell adhesion;GO:0
-	-
GO:0005515//protei	GO:0043065//positive regulation of apoptotic process;GO:0044790//nega
GO:0004842//ubiqui	GO:0000209//protein polyubiquitination;GO:0006915//apoptotic process;
GO:0000981//RNA p	GO:0000122//negative regulation of transcription from RNA polymerase I
GO:0005515//protei	-

-	GO:0019941//modification-dependent protein catabolic process;GO:0030
GO:0000166//nucleo	GO:0000045//autophagosome assembly;GO:0002026//regulation of the fo
GO:0004521//endori	-
-	GO:0007155//cell adhesion
GO:0004672//protei	GO:0000086//G2/M transition of mitotic cell cycle;GO:0000278//mitotic ce
-	GO:1901031//regulation of response to reactive oxygen species
-	-
GO:0004585//ornith	GO:0000050//urea cycle;GO:0006520//cellular amino acid metabolic proc
GO:0001671//ATPas	GO:0001933//negative regulation of protein phosphorylation;GO:003043
-	-
GO:0000977//RNA p	GO:0000122//negative regulation of transcription from RNA polymerase I
GO:0005515//protei	GO:0043254//regulation of protein complex assembly
GO:0004672//protei	GO:0001934//positive regulation of protein phosphorylation;GO:0006468/
GO:0016491//oxidor	GO:0009247//glycolipid biosynthetic process
GO:0015175//neutra	GO:0015804//neutral amino acid transport;GO:0042941//D-alanine transp
GO:0036122//BMP b	GO:0007389//pattern specification process;GO:0010454//negative regulat
GO:0004674//protei	GO:0006468//protein phosphorylation
-	-
GO:0005524//ATP bi	-
-	-
-	GO:0006506//GPI anchor biosynthetic process
-	-
GO:0005515//protei	GO:0051260//protein homooligomerization
GO:0004045//amino	-
-	GO:0098609//cell-cell adhesion
GO:0005515//protei	GO:0001938//positive regulation of endothelial cell proliferation;GO:0014
-	-
GO:0005509//calciu	GO:0015012//heparan sulfate proteoglycan biosynthetic process;GO:0030
GO:0008270//zinc io	GO:0009086//methionine biosynthetic process;GO:0009235//cobalamin m
GO:0000978//RNA p	GO:0001932//regulation of protein phosphorylation;GO:0006355//regulat
GO:0005515//protei	-
GO:0016628//oxidor	GO:0006695//cholesterol biosynthetic process;GO:0016126//sterol biosyn
-	GO:0009987//cellular process;GO:0044699//single-organism process
GO:0004857//enzym	GO:0006071//glycerol metabolic process;GO:0006631//fatty acid metabol
GO:0003874//6-pyru	GO:0006729//tetrahydrobiopterin biosynthetic process
GO:0001965//G-pro	GO:0007165//signal transduction;GO:0007186//G-protein coupled recept
GO:0005515//protei	GO:0000281//mitotic cytokinesis;GO:0007019//microtubule depolymeriza
GO:0005319//lipid tr	GO:0006869//lipid transport
-	-
GO:0000977//RNA p	GO:0000122//negative regulation of transcription from RNA polymerase I
-	-
GO:0005507//coppe	GO:0007601//visual perception;GO:0009308//amine metabolic process
GO:0000976//transc	GO:0000122//negative regulation of transcription from RNA polymerase I
GO:0042802//identic	-
GO:0008233//peptid	GO:0006508//proteolysis
GO:0000062//fatty-a	GO:0036151//phosphatidylcholine acyl-chain remodeling;GO:1903060//n
GO:0004045//amino	-
-	-
-	-
GO:0003779//actin b	GO:0051017//actin filament bundle assembly
GO:0005515//protei	GO:0000281//mitotic cytokinesis;GO:0014067//negative regulation of pho
GO:0005515//protei	GO:0007015//actin filament organization;GO:0045806//negative regulatio
GO:0004475//manno	GO:0006486//protein glycosylation;GO:0009058//biosynthetic process;GC
-	GO:0051480//regulation of cytosolic calcium ion concentration;GO:19900
GO:0005515//protei	-
GO:0016757//transfe	GO:0002121//inter-male aggressive behavior;GO:0042403//thyroid horm
GO:0004843//thiol-(	GO:0000278//mitotic cell cycle;GO:0006281//DNA repair;GO:0006511//ub
-	-

-	-
GO:0004930//G-pro	GO:0007186//G-protein coupled receptor signaling pathway;GO:0007200
-	-
-	-
GO:0016491//oxidor	-
GO:0008080//N-ace	-
GO:0030729//acetoa	GO:0006629//lipid metabolic process
-	-
-	-
-	GO:0070453//regulation of heme biosynthetic process
GO:0019855//calciur	GO:0006851//mitochondrial calcium ion transport;GO:0051560//mitochor
GO:0016491//oxidor	GO:0007005//mitochondrion organization
GO:0004672//protei	GO:0006468//protein phosphorylation
-	-
GO:0001671//ATPas	GO:0001933//negative regulation of protein phosphorylation;GO:003043
GO:0003854//3-beta	GO:0006694//steroid biosynthetic process
GO:0019904//protei	GO:0050796//regulation of insulin secretion
GO:0000287//magne	GO:0006468//protein phosphorylation;GO:0030036//actin cytoskeleton or
GO:0003676//nucleic	-
GO:0004175//endop	GO:0006508//proteolysis;GO:0007354//zygotic determination of anterior/
-	-
-	-
-	-
-	-
GO:0008289//lipid b	GO:0015031//protein transport;GO:0019075//virus maturation;GO:004205
GO:0005319//lipid tr	GO:0006629//lipid metabolic process;GO:0006642//triglyceride mobilizati
GO:0005515//protei	GO:0007015//actin filament organization;GO:0045806//negative regulatio
-	-
GO:0004103//cholin	GO:0006646//phosphatidylethanolamine biosynthetic process;GO:000665
GO:0004930//G-pro	GO:0001541//ovarian follicle development;GO:0001545//primary ovarian
GO:0030246//carbol	-
GO:0005515//protei	GO:0001558//regulation of cell growth
GO:0005085//guany	-
GO:0022857//transm	GO:0055085//transmembrane transport
GO:0005113//patche	GO:0001501//skeletal system development;GO:0001503//ossification;GO:(
-	-
GO:0000166//nucleo	-
GO:0004222//metall	GO:0001542//ovulation from ovarian follicle;GO:0001822//kidney develop
GO:0003735//structu	GO:0006412//translation;GO:0006919//activation of cysteine-type endope
GO:0005515//protei	-
GO:0005515//protei	GO:0098742//cell-cell adhesion via plasma-membrane adhesion molecule
GO:0005515//protei	-
GO:0001968//fibron	GO:0010811//positive regulation of cell-substrate adhesion;GO:0030198//
-	-
-	-
GO:0004674//protei	GO:0006468//protein phosphorylation
GO:0005515//protei	GO:0001558//regulation of cell growth
-	-
GO:0004478//methio	GO:0006556//S-adenosylmethionine biosynthetic process;GO:0009087//n
GO:0000977//RNA p	GO:0007265//Ras protein signal transduction;GO:0045944//positive regul
-	GO:0070453//regulation of heme biosynthetic process
GO:0050699//WW d	GO:0007034//vacuolar transport;GO:0010629//negative regulation of gen
GO:0005515//protei	GO:0042426//choline catabolic process
GO:0003779//actin b	GO:0051017//actin filament bundle assembly
-	-
-	GO:0007098//centrosome cycle;GO:0035020//regulation of Rac protein si
-	-
GO:0000976//transc	GO:0006357//regulation of transcription from RNA polymerase II promote

-	-
GO:0003735//structu	GO:0006412//translation;GO:0006919//activation of cysteine-type endope
-	GO:0007507//heart development
GO:0004175//endop	GO:0018126//protein hydroxylation
-	-
GO:0005085//guany	GO:0035556//intracellular signal transduction
GO:0016491//oxidor	GO:0009247//glycolipid biosynthetic process
-	-
GO:0004930//G-pro	GO:0007165//signal transduction;GO:0007186//G-protein coupled recept
-	-
-	GO:0000463//maturation of LSU-rRNA from tricistronic rRNA transcript (S
-	-
GO:0008138//protei	GO:0006355//regulation of transcription, DNA-templated;GO:0006470//p
-	-
GO:0005484//SNAP	GO:0006888//ER to Golgi vesicle-mediated transport;GO:0006903//vesicle
GO:0003682//chrom	GO:0000122//negative regulation of transcription from RNA polymerase I
GO:0016491//oxidor	-
-	-
GO:0005515//protei	GO:0000281//mitotic cytokinesis;GO:0007019//microtubule depolymeriza
-	GO:0018095//protein polyglutamylation
GO:0004888//transm	GO:0007166//cell surface receptor signaling pathway;GO:0007186//G-pro
-	-
-	-
GO:0005509//calciur	GO:0007268//synaptic transmission;GO:0035556//intracellular signal trans
-	-
GO:0004857//enzym	GO:0006071//glycerol metabolic process;GO:0006631//fatty acid metabol
GO:0008170//N-me	GO:0001692//histamine metabolic process;GO:0001695//histamine catabo
-	-
GO:0005515//protei	GO:0006355//regulation of transcription, DNA-templated;GO:0006805//x
GO:0004672//protei	GO:0001934//positive regulation of protein phosphorylation;GO:0006468/
-	GO:0006886//intracellular protein transport;GO:0006890//retrograde vesi
GO:0003824//catalyt	GO:0009226//nucleotide-sugar biosynthetic process;GO:0010595//positiv
-	GO:1901031//regulation of response to reactive oxygen species
GO:0005515//protei	-
-	-
GO:0004888//transm	GO:0006811//ion transport;GO:0034220//ion transmembrane transport
GO:0005085//guany	GO:0007264//small GTPase mediated signal transduction;GO:0034976//re
GO:0004000//adeno	GO:0000255//allantoin metabolic process;GO:0001666//response to hypo
-	-
GO:0004175//endop	GO:0002218//activation of innate immune response;GO:0006508//proteol
GO:0004467//long-c	GO:0006631//fatty acid metabolic process
-	-
GO:0045296//cadhe	GO:0007043//cell-cell junction assembly;GO:0060997//dendritic spine mo
GO:0008017//microt	GO:0008285//negative regulation of cell proliferation;GO:0030330//DNA c
-	-
GO:0003779//actin b	GO:0051017//actin filament bundle assembly
-	-
GO:0000978//RNA p	GO:0001932//regulation of protein phosphorylation;GO:0006355//regulat
GO:0016628//oxidor	GO:0006695//cholesterol biosynthetic process;GO:0016126//sterol biosyn
-	-
GO:0005515//protei	GO:0001822//kidney development;GO:0002093//auditory receptor cell mo
GO:0000977//RNA p	GO:0000122//negative regulation of transcription from RNA polymerase I
GO:0005381//iron io	GO:0046985//positive regulation of hemoglobin biosynthetic process;GO:
GO:0008289//lipid b	GO:0015031//protein transport;GO:0019075//virus maturation;GO:004205
GO:0004857//enzym	GO:0006071//glycerol metabolic process;GO:0006631//fatty acid metabol
-	-
GO:0003676//nuclei	GO:0019722//calcium-mediated signaling;GO:0050790//regulation of cata
-	-

-	-
GO:0003676//nuclei	-
-	-
-	-
GO:0003676//nuclei	GO:0006508//proteolysis
GO:0000287//magne	GO:0006114//glycerol biosynthetic process;GO:0006650//glycerophospho
-	GO:0006886//intracellular protein transport
-	-
GO:0000062//fatty-a	GO:0036151//phosphatidylcholine acyl-chain remodeling;GO:1903060//n
-	-
-	-
GO:0046873//metal	GO:0030001//metal ion transport
GO:0042802//identic	GO:0019216//regulation of lipid metabolic process;GO:0055088//lipid hor
GO:0000976//transc	GO:0002062//chondrocyte differentiation;GO:0006355//regulation of tran
GO:0003841//1-acyl	GO:0006654//phosphatidic acid biosynthetic process;GO:0010891//negati
GO:0004930//G-pro	GO:0007186//G-protein coupled receptor signaling pathway;GO:0007200
GO:0004843//thiol-c	GO:0006511//ubiquitin-dependent protein catabolic process;GO:0007000
-	-
GO:0000246//delta2	GO:0006695//cholesterol biosynthetic process;GO:0006979//response to
-	GO:0006658//phosphatidylserine metabolic process;GO:0006665//sphing
GO:0004860//protei	GO:0007616//long-term memory;GO:0050729//positive regulation of infla
-	-
GO:0003677//DNA b	GO:0009646//response to absence of light;GO:0018298//protein-chromo
GO:0000166//nucleo	GO:0000045//autophagosome assembly;GO:0002026//regulation of the fo
GO:0005515//protei	-
-	-
-	-
-	-
-	GO:0051480//regulation of cytosolic calcium ion concentration;GO:19900
GO:0019855//calciur	GO:0006851//mitochondrial calcium ion transport;GO:0051560//mitochon
-	-
GO:0050839//cell ad	GO:0007409//axonogenesis;GO:0010975//regulation of neuron projection
-	-
GO:0004452//isoper	GO:0008299//isoprenoid biosynthetic process
-	-
GO:0000976//transc	GO:0000122//negative regulation of transcription from RNA polymerase I
GO:0000062//fatty-a	GO:0036151//phosphatidylcholine acyl-chain remodeling;GO:1903060//n
GO:0035256//G-pro	GO:0003009//skeletal muscle contraction;GO:0007216//G-protein couple
GO:0005515//protei	GO:0000281//mitotic cytokinesis;GO:0014067//negative regulation of pho
GO:0003676//nuclei	-
GO:0004343//glucos	GO:0006048//UDP-N-acetylglucosamine biosynthetic process
GO:0022892//substr	GO:0044765//single-organism transport
GO:0043169//cation	GO:0007165//signal transduction;GO:0022603//regulation of anatomical s
GO:0004114//3',5'-c	GO:0007165//signal transduction
-	-
GO:0005515//protei	GO:0007043//cell-cell junction assembly;GO:0007155//cell adhesion;GO:0
GO:0015020//glucur	GO:0006486//protein glycosylation;GO:0015012//heparan sulfate proteog
GO:0016740//transfe	GO:0006777//Mo-molybdopterin cofactor biosynthetic process
GO:0005515//protei	GO:0001938//positive regulation of endothelial cell proliferation;GO:0014
GO:0046873//metal	GO:0030001//metal ion transport
GO:0004672//protei	GO:0006468//protein phosphorylation;GO:0008631//intrinsic apoptotic si
GO:0004032//alditol	GO:0006699//bile acid biosynthetic process;GO:0006707//cholesterol cata
GO:0001671//ATPas	GO:0001933//negative regulation of protein phosphorylation;GO:003043
GO:0030729//acetoa	GO:0006629//lipid metabolic process
GO:0000287//magne	GO:0006468//protein phosphorylation;GO:0007346//regulation of mitotic
GO:0000981//RNA p	GO:0000122//negative regulation of transcription from RNA polymerase I
GO:0008047//enzym	GO:0006486//protein glycosylation;GO:0006487//protein N-linked glycos
GO:0005244//voltag	-

-	GO:0009987//cellular process
-	GO:0009267//cellular response to starvation;GO:0030001//metal ion trans
GO:0004340//glucok	GO:0001678//cellular glucose homeostasis;GO:0005975//carbohydrate me
-	-
GO:0001968//fibron	GO:0001933//negative regulation of protein phosphorylation;GO:0007162
-	-
-	-
GO:0005515//protei	GO:0046330//positive regulation of JNK cascade;GO:0090090//negative re
-	-
GO:1990756//protei	GO:0015031//protein transport;GO:0016567//protein ubiquitination;GO:00
GO:0022857//transm	GO:0055085//transmembrane transport
-	-
GO:0016491//oxidor	-
GO:0004842//ubiqui	GO:0000209//protein polyubiquitination;GO:0006915//apoptotic process;
-	GO:0031032//actomyosin structure organization
-	-
-	-
GO:0000166//nucleo	-
GO:0016787//hydrol	GO:0006470//protein dephosphorylation;GO:0006805//xenobiotic metabo
-	GO:0006629//lipid metabolic process
GO:0003779//actin b	GO:0051017//actin filament bundle assembly
-	GO:0006364//rRNA processing;GO:0009303//rRNA transcription
-	-
GO:0005104//fibrob	GO:0001755//neural crest cell migration;GO:0001934//positive regulation
GO:0004497//mono	-
-	-
-	-
GO:0003677//DNA b	-
GO:0005515//protei	GO:0098742//cell-cell adhesion via plasma-membrane adhesion molecule
GO:0004310//farnes	GO:0008610//lipid biosynthetic process;GO:0009058//biosynthetic proces
GO:0004843//thiol-c	GO:0000278//mitotic cell cycle;GO:0006281//DNA repair;GO:0006511//ub
GO:0000978//RNA p	GO:0006355//regulation of transcription, DNA-templated;GO:0006357//re
GO:0015175//neutra	GO:0015804//neutral amino acid transport;GO:0042941//D-alanine transp
GO:0005509//calciur	GO:0015012//heparan sulfate proteoglycan biosynthetic process;GO:0030
-	-
-	-
GO:0000166//nucleo	GO:0006468//protein phosphorylation;GO:0010976//positive regulation o
-	GO:0006839//mitochondrial transport
GO:0004843//thiol-c	GO:0006511//ubiquitin-dependent protein catabolic process;GO:0016579
-	-
-	-
GO:0003682//chrom	GO:0000122//negative regulation of transcription from RNA polymerase I
GO:0003677//DNA b	GO:0006383//transcription from RNA polymerase III promoter
GO:0047750//choles	GO:0016125//sterol metabolic process
-	-
-	-
-	-
GO:0004175//endop	GO:0006508//proteolysis;GO:0007354//zygotic determination of anterior/
GO:0003677//DNA b	GO:0006334//nucleosome assembly;GO:0042742//defense response to ba
-	-
-	-
-	GO:0098609//cell-cell adhesion
GO:0005515//protei	-
GO:0001604//uroter	GO:0007165//signal transduction;GO:0007186//G-protein coupled recept
GO:0004175//endop	GO:0018126//protein hydroxylation
GO:0008009//chemo	GO:0006955//immune response
GO:0016922//ligand	GO:0045944//positive regulation of transcription from RNA polymerase II
-	-

GO:0050699//WW d	GO:0007034//vacuolar transport;GO:0010629//negative regulation of gen
GO:0005515//protei	GO:0045765//regulation of angiogenesis
-	GO:0007042//lysosomal lumen acidification;GO:0042176//regulation of pr
GO:0042802//identic	-
-	-
GO:0005506//iron ic	-
GO:0003824//catalyt	GO:0009226//nucleotide-sugar biosynthetic process;GO:0010595//positiv
GO:0000166//nucleo	GO:0001954//positive regulation of cell-matrix adhesion;GO:0003323//typ
GO:0019901//protei	GO:0030334//regulation of cell migration;GO:0034446//substrate adhesio
GO:0004857//enzym	GO:0001525//angiogenesis;GO:0043066//negative regulation of apoptotic
GO:0003779//actin b	GO:0051017//actin filament bundle assembly
GO:0004930//G-pro	GO:0007165//signal transduction;GO:0007186//G-protein coupled recept
GO:0004862//cAMP	GO:0006469//negative regulation of protein kinase activity;GO:0051338//r
-	-
-	-
GO:0003677//DNA b	GO:0006355//regulation of transcription, DNA-templated;GO:0045944//p
-	-
GO:0005509//calciur	GO:0007155//cell adhesion
-	-
GO:0008017//microt	GO:0008285//negative regulation of cell proliferation;GO:0030330//DNA d
GO:0003676//nuclei	GO:0006508//proteolysis
GO:0005216//ion ch	GO:0006811//ion transport;GO:0006812//cation transport;GO:0034220//ic
GO:0005515//protei	GO:0044763//single-organism cellular process
-	-
-	GO:0030389//fructosamine metabolic process;GO:0030855//epithelial cell
GO:0005515//protei	GO:0007015//actin filament organization;GO:0045806//negative regulatio
GO:0008324//cation	GO:0006812//cation transport
-	-
-	-
-	-
GO:0008289//lipid b	GO:0015031//protein transport;GO:0019075//virus maturation;GO:004205
-	-
GO:0031267//small (	GO:0006886//intracellular protein transport
GO:0004467//long-c	GO:0006631//fatty acid metabolic process
-	-
GO:0022857//transm	GO:0055085//transmembrane transport
GO:0004175//endop	GO:0002218//activation of innate immune response;GO:0006508//proteol
GO:0000166//nucleo	GO:0070588//calcium ion transmembrane transport
GO:0005201//extrac	GO:0007160//cell-matrix adhesion;GO:0007605//sensory perception of sc
GO:0008083//growtl	-
GO:0003924//GTPas	GO:0006254//CTP catabolic process;GO:0034656//nucleobase-containing
GO:0004386//helicas	-
-	-
GO:0000287//magne	GO:0006468//protein phosphorylation;GO:0030036//actin cytoskeleton or
GO:0005540//hyalur	GO:0007155//cell adhesion
GO:0005515//protei	-
GO:0003841//1-acyl	GO:0001819//positive regulation of cytokine production;GO:0006654//ph
GO:0030246//carbol	GO:0006897//endocytosis
-	-
-	-
-	-
GO:0005524//ATP bi	GO:0055085//transmembrane transport
GO:0008047//enzym	GO:0006486//protein glycosylation;GO:0006487//protein N-linked glycosy
-	GO:0006658//phosphatidylserine metabolic process;GO:0006665//sphingo
GO:0003697//single-	-
GO:0003676//nuclei	GO:0006508//proteolysis;GO:0008152//metabolic process;GO:0016032//v
GO:0001968//fibron	GO:0010811//positive regulation of cell-substrate adhesion;GO:0030198//
GO:0004860//protei	GO:0007616//long-term memory;GO:0050729//positive regulation of infla

GO:0001758//retinal	GO:0042573//retinoic acid metabolic process;GO:0042574//retinal metabo
GO:0004222//metall	GO:0001542//ovulation from ovarian follicle;GO:0001822//kidney develop
GO:0005515//protei	GO:0044763//single-organism cellular process
GO:0017128//phosp	GO:0000045//autophagosome assembly;GO:0006869//lipid transport;GO:
GO:0000976//transc	GO:0006289//nucleotide-excision repair;GO:0006974//cellular response to
-	GO:0007507//heart development
GO:0016757//transfe	GO:0002121//inter-male aggressive behavior;GO:0042403//thyroid horm
-	-
GO:0004888//transm	GO:0006811//ion transport;GO:0034220//ion transmembrane transport
GO:0016740//transfe	GO:0006629//lipid metabolic process;GO:0006659//phosphatidylserine bi
-	-
GO:0031419//cobala	GO:0015889//cobalamin transport
GO:0005085//guany	GO:0035556//intracellular signal transduction
-	-
GO:0005515//protei	-
GO:0004867//serine	GO:0010951//negative regulation of endopeptidase activity;GO:0030212//
-	-
-	-
-	-
-	-
GO:0005515//protei	GO:0046330//positive regulation of JNK cascade;GO:0090090//negative re
GO:0016787//hydrol	GO:0006629//lipid metabolic process;GO:0007216//G-protein coupled gl
-	-
GO:0008201//hepari	-
-	-
GO:0008236//serine	GO:0006508//proteolysis
GO:0003676//nuclei	-
-	-
-	-
GO:0004672//protei	GO:0006468//protein phosphorylation;GO:0008631//intrinsic apoptotic si
-	-
GO:0004674//protei	GO:0006468//protein phosphorylation
-	-
-	-
-	-
GO:0005515//protei	-
GO:0043169//cation	GO:0007165//signal transduction;GO:0022603//regulation of anatomical s
GO:0016787//hydrol	GO:0006470//protein dephosphorylation;GO:0006805//xenobiotic metabo
-	-
GO:0005515//protei	GO:0007043//cell-cell junction assembly;GO:0007155//cell adhesion;GO:0
GO:0030298//recept	GO:0010976//positive regulation of neuron projection development;GO:0
GO:0003841//1-acyl	GO:0006644//phospholipid metabolic process
GO:0019902//phosp	GO:0006511//ubiquitin-dependent protein catabolic process;GO:0016567
GO:0005515//protei	GO:0006888//ER to Golgi vesicle-mediated transport;GO:0016192//vesicle
GO:0003677//DNA b	GO:0006383//transcription from RNA polymerase III promoter
-	GO:0018095//protein polyglutamylation
-	GO:0006364//rRNA processing;GO:0009303//rRNA transcription
-	-
GO:0003841//1-acyl	GO:0001819//positive regulation of cytokine production;GO:0006654//ph
-	-
GO:0003824//catalyt	GO:0018272//protein-pyridoxal-5-phosphate linkage via peptidyl-N6-py
-	-
-	GO:0007286//spermatid development;GO:0048137//spermatocyte divisio
-	-
GO:0005113//patche	GO:0001501//skeletal system development;GO:0001503//ossification;GO:0
GO:0004672//protei	GO:0001501//skeletal system development;GO:0006468//protein phospho
GO:0005515//protei	GO:0001558//regulation of cell growth
GO:0022857//transm	GO:0010628//positive regulation of gene expression;GO:0010629//negati

GO:0005085//guany	GO:0007264//small GTPase mediated signal transduction;GO:0034976//re
-	GO:0001656//metanephros development;GO:0001658//branching involve
GO:0005227//calciu	GO:0001581//detection of chemical stimulus involved in sensory percepti
GO:0003847//1-alky	GO:0016042//lipid catabolic process
GO:0008170//N-me	GO:0001692//histamine metabolic process;GO:0001695//histamine catab
GO:0003676//nucle	GO:0019722//calcium-mediated signaling;GO:0050790//regulation of cat
GO:0001758//retinal	GO:0002072//optic cup morphogenesis involved in camera-type eye deve
-	-
GO:0016491//oxido	GO:0006666//3-keto-sphinganine metabolic process;GO:0030148//sphin
GO:0008237//metall	GO:0006508//proteolysis
-	-
GO:0003676//nucle	GO:0006508//proteolysis;GO:0016032//viral process
-	-
GO:0008009//chem	GO:0006955//immune response
GO:0019902//phosp	GO:0006511//ubiquitin-dependent protein catabolic process;GO:0016567
GO:0004114//3',5'-c	GO:0007165//signal transduction
-	-
-	-
GO:0005509//calciu	-
GO:0005509//calciu	GO:0015012//heparan sulfate proteoglycan biosynthetic process;GO:0030
GO:0042802//identi	GO:0030182//neuron differentiation;GO:0045893//positive regulation of t
-	-
GO:0016290//palmit	GO:0006637//acyl-CoA metabolic process;GO:0016559//peroxisome fissi
-	-
-	-
-	-
GO:0016717//oxido	GO:0006629//lipid metabolic process
GO:0001968//fibron	GO:0001933//negative regulation of protein phosphorylation;GO:0007162
GO:0005515//protei	GO:0001822//kidney development;GO:0002093//auditory receptor cell m
-	-
-	-
GO:0008236//serine	GO:0006508//proteolysis
GO:0000976//transc	GO:0002062//chondrocyte differentiation;GO:0006355//regulation of tran
GO:1990756//protei	GO:0015031//protein transport;GO:0016567//protein ubiquitination;GO:0
GO:0003677//DNA b	GO:0006334//nucleosome assembly;GO:0006352//DNA-templated transc
-	-
-	GO:0044237//cellular metabolic process
GO:0003836//beta-	GO:0002319//memory B cell differentiation;GO:0006054//N-acetylneuram
GO:0004857//enzym	GO:0001525//angiogenesis;GO:0043066//negative regulation of apoptoti
GO:0042802//identi	GO:0007165//signal transduction;GO:0042981//regulation of apoptotic pr
GO:0035256//G-pro	GO:0003009//skeletal muscle contraction;GO:0007216//G-protein couple
GO:0000166//nucleo	GO:0001954//positive regulation of cell-matrix adhesion;GO:0003323//ty
GO:0000287//magn	GO:0006114//glycerol biosynthetic process;GO:0006650//glycerophosph
-	GO:0006886//intracellular protein transport;GO:0006890//retrograde vesi
GO:0004930//G-pro	GO:0007186//G-protein coupled receptor signaling pathway;GO:0007200
GO:0004862//cAMP	GO:0006469//negative regulation of protein kinase activity;GO:0051338//
GO:0002161//amino	-
-	-
GO:0005515//protei	GO:0045765//regulation of angiogenesis
GO:0035256//G-pro	GO:0003009//skeletal muscle contraction;GO:0007216//G-protein couple
GO:0004452//isoper	GO:0008299//isoprenoid biosynthetic process
GO:0004725//protei	GO:0006470//protein dephosphorylation;GO:0016311//dephosphorylatio
GO:0045296//cadhe	GO:0007043//cell-cell junction assembly;GO:0060997//dendritic spine mo
GO:0003677//DNA b	GO:0006334//nucleosome assembly;GO:0042742//defense response to ba
GO:0003676//nucle	GO:0006508//proteolysis;GO:0008152//metabolic process;GO:0016032//v
-	-
GO:0005227//calciu	GO:0006812//cation transport
GO:0005524//ATP bi	GO:0009235//cobalamin metabolic process;GO:0015889//cobalamin trans

-	GO:0007155//cell adhesion
GO:0019901//protei	GO:0030334//regulation of cell migration;GO:0034446//substrate adhesio
-	-
GO:0003677//DNA b	GO:0009646//response to absence of light;GO:0018298//protein-chromo
GO:0005158//insulin	GO:0001775//cell activation;GO:0007405//neuroblast proliferation;GO:000
GO:0030729//acetoa	GO:0006629//lipid metabolic process
-	-
-	-
GO:0000253//3-keto	GO:0006695//cholesterol biosynthetic process;GO:0006703//estrogen bio
GO:0022892//substra	GO:0044765//single-organism transport
GO:0003677//DNA b	-
GO:0001968//fibron	GO:0001933//negative regulation of protein phosphorylation;GO:000716
-	-
GO:0003824//catalyt	GO:0005975//carbohydrate metabolic process;GO:0006006//glucose meta
GO:0005540//hyalur	GO:0007155//cell adhesion
GO:0003707//steroic	GO:0007340//acrosome reaction;GO:0009611//response to wounding;GO
-	GO:0009267//cellular response to starvation;GO:0030001//metal ion trans
-	-
GO:0005158//insulin	GO:0007165//signal transduction;GO:0010467//gene expression;GO:0030
GO:0017128//phosp	GO:0000045//autophagosome assembly;GO:0006914//autophagy;GO:001
GO:0004386//helicas	-
GO:0016790//thioles	GO:0006637//acyl-CoA metabolic process
-	GO:0006887//exocytosis;GO:0015031//protein transport
-	-
GO:0005515//protei	GO:0003341//cilium movement;GO:0044782//cilium organization
GO:0030145//manga	GO:0006508//proteolysis;GO:0019538//protein metabolic process
GO:0003676//nuclei	GO:0015074//DNA integration;GO:0044260//cellular macromolecule meta
-	-
GO:0003713//transc	GO:0006355//regulation of transcription, DNA-templated
GO:0022857//transm	GO:0055085//transmembrane transport
GO:0005085//guany	GO:0007264//small GTPase mediated signal transduction
GO:0000166//nucleo	GO:0070588//calcium ion transmembrane transport
GO:0005515//protei	GO:0001822//kidney development;GO:0002093//auditory receptor cell m
GO:0000250//lanost	GO:0006694//steroid biosynthetic process;GO:0006695//cholesterol biosy
-	-
-	-
GO:0022857//transm	GO:0055085//transmembrane transport
-	GO:0030070//insulin processing;GO:0060628//regulation of ER to Golgi ve
-	GO:0007411//axon guidance;GO:0010977//negative regulation of neuron
GO:0005507//coppe	GO:0007601//visual perception;GO:0009308//amine metabolic process
-	-
-	-
-	-
GO:0005507//coppe	GO:0009308//amine metabolic process;GO:0009445//putrescine metaboli
GO:0003676//nuclei	GO:0006278//RNA-dependent DNA replication;GO:0015074//DNA integr
GO:0005506//iron io	GO:0001949//sebaceous gland cell differentiation;GO:0006629//lipid meta
GO:0004867//serine	GO:0010951//negative regulation of endopeptidase activity;GO:0030212/
GO:0004420//hydro	GO:0006695//cholesterol biosynthetic process;GO:0008299//isoprenoid b
GO:0030246//carbo	GO:0006897//endocytosis
GO:0003677//DNA b	GO:0006383//transcription from RNA polymerase III promoter
GO:0005085//guany	GO:0050790//regulation of catalytic activity
-	GO:0006629//lipid metabolic process
GO:0003676//nuclei	GO:0006508//proteolysis;GO:0008152//metabolic process;GO:0016032//v
-	-
GO:0008170//N-me	GO:0001692//histamine metabolic process;GO:0001695//histamine catab
GO:0000287//magn	GO:0000226//microtubule cytoskeleton organization;GO:0006468//protei
GO:0005515//protei	GO:0007596//blood coagulation;GO:0033627//cell adhesion mediated by
GO:0015020//glucur	GO:0006486//protein glycosylation;GO:0015012//heparan sulfate proteog

GO:0008324//cation	GO:0006812//cation transport
GO:0003677//DNA b	-
GO:0000976//transc	GO:0006289//nucleotide-excision repair;GO:0006974//cellular response to
GO:0008080//N-ace	-
GO:0005515//protei	GO:0007229//integrin-mediated signaling pathway
GO:0050699//WW d	GO:0007034//vacuolar transport;GO:0010629//negative regulation of gen
GO:0022857//transm	GO:0055085//transmembrane transport
-	-
GO:0005085//guany	GO:0007264//small GTPase mediated signal transduction
GO:0042802//identic	GO:0007017//microtubule-based process
GO:0003677//DNA b	-
GO:0005319//lipid tr	GO:0006869//lipid transport;GO:0015914//phospholipid transport;GO:004
-	GO:0006629//lipid metabolic process
-	-
GO:0042802//identic	GO:0007165//signal transduction;GO:0046628//positive regulation of insu
-	-
-	-
-	-
GO:0002039//p53 bi	GO:0006468//protein phosphorylation;GO:0008285//negative regulation o
GO:0005504//fatty a	GO:0015908//fatty acid transport;GO:0015909//long-chain fatty acid trans
-	GO:0006887//exocytosis;GO:0015031//protein transport
GO:0001604//uroten	GO:0007165//signal transduction;GO:0007186//G-protein coupled recept
GO:0003682//chrom	GO:0000122//negative regulation of transcription from RNA polymerase I
-	-
-	-
-	-
GO:0003824//catalyt	GO:0018272//protein-pyridoxal-5-phosphate linkage via peptidyl-N6-py
-	-
GO:0008289//lipid b	-
-	-
GO:0005515//protei	GO:0044763//single-organism cellular process
-	-
-	GO:0070979//protein K11-linked ubiquitination
GO:0005524//ATP bi	GO:0035499//carnosine biosynthetic process
GO:0004672//protei	GO:0001501//skeletal system development;GO:0006468//protein phospho
GO:0019902//phosp	GO:0006511//ubiquitin-dependent protein catabolic process;GO:0016567
GO:0008170//N-me	GO:0001692//histamine metabolic process;GO:0001695//histamine catabo
-	-
-	-
-	-
GO:0015171//amino	GO:0006641//triglyceride metabolic process;GO:0009437//carnitine metab
GO:0042802//identic	GO:0007017//microtubule-based process
GO:0042577//lipid p	GO:0006644//phospholipid metabolic process
-	-
GO:0005524//ATP bi	GO:0009235//cobalamin metabolic process;GO:0015889//cobalamin trans
-	-
-	-
GO:0008235//metall	GO:0017186//peptidyl-pyroglutamic acid biosynthetic process, using glut
-	-
GO:0005509//calciur	-
GO:0005515//protei	GO:0001822//kidney development;GO:0002093//auditory receptor cell mo
-	-
-	-
GO:0016290//palmit	GO:0006637//acyl-CoA metabolic process;GO:0016559//peroxisome fissio
GO:0015267//chann	GO:0002931//response to ischemia;GO:0006812//cation transport;GO:003
GO:0016780//phosp	GO:0008654//phospholipid biosynthetic process
-	-
-	-

GO:0022857//transm	GO:0055085//transmembrane transport
GO:0003824//catalyt	GO:0006778//porphyrin-containing compound metabolic process;GO:000
GO:0005507//coppe	GO:0007601//visual perception;GO:0009308//amine metabolic process
-	-
GO:0005515//protei	GO:0006888//ER to Golgi vesicle-mediated transport;GO:0016192//vesicle
-	-
GO:0060090//bindin	GO:0030433//ER-associated ubiquitin-dependent protein catabolic proce
GO:0016790//thioles	GO:0006637//acyl-CoA metabolic process
GO:0004721//phosp	GO:0006470//protein dephosphorylation;GO:0016311//dephosphorylatio
GO:0005216//ion ch	GO:0006811//ion transport;GO:0019228//neuronal action potential;GO:00
-	GO:0000463//maturation of LSU-rRNA from tricistronic rRNA transcript (S
GO:0004842//ubiqui	GO:0006511//ubiquitin-dependent protein catabolic process;GO:0016567
-	-
GO:0005524//ATP bi	GO:0009235//cobalamin metabolic process;GO:0015889//cobalamin trans
GO:0004197//cystei	GO:0002931//response to ischemia;GO:0006508//proteolysis;GO:0006511
GO:0004672//protei	GO:0006468//protein phosphorylation;GO:0007169//transmembrane rece
GO:0004620//phosp	GO:0009395//phospholipid catabolic process;GO:0030336//negative regu
-	GO:0050910//detection of mechanical stimulus involved in sensory percep
GO:0005509//calciu	GO:0006936//muscle contraction;GO:0030035//microspike assembly;GO:0
GO:0005515//protei	-
-	GO:0007155//cell adhesion
-	GO:0006886//intracellular protein transport;GO:0006890//retrograde vesi
GO:0003824//catalyt	GO:0006778//porphyrin-containing compound metabolic process;GO:000
GO:0000976//transc	GO:0000122//negative regulation of transcription from RNA polymerase I
GO:0016740//transfe	-
GO:0004175//endop	GO:0002218//activation of innate immune response;GO:0006508//proteol
GO:0004672//protei	GO:0001934//positive regulation of protein phosphorylation;GO:0006468/
GO:0004672//protei	GO:0001501//skeletal system development;GO:0006468//protein phospho
GO:0000248//C-5 st	GO:0008610//lipid biosynthetic process;GO:0016126//sterol biosynthetic
GO:0045296//cadhe	GO:0007043//cell-cell junction assembly;GO:0060997//dendritic spine mo
-	GO:0006658//phosphatidylserine metabolic process;GO:0006665//sphing
GO:0005515//protei	GO:0044763//single-organism cellular process
GO:0005158//insulin	GO:0007165//signal transduction;GO:0010467//gene expression;GO:0030
GO:0005509//calciu	-
GO:0008146//sulfotr	GO:0006805//xenobiotic metabolic process;GO:0042403//thyroid hormon
-	-
-	-
GO:0004725//protei	GO:0006470//protein dephosphorylation;GO:0016311//dephosphorylatio
-	-
GO:0016491//oxidor	-
-	GO:0006488//dolichol-linked oligosaccharide biosynthetic process
-	-
GO:0005540//hyalur	GO:0007155//cell adhesion
-	-
-	-
GO:0005515//protei	GO:0046330//positive regulation of JNK cascade;GO:0090090//negative re
-	GO:0007155//cell adhesion
GO:0005540//hyalur	GO:0007155//cell adhesion
GO:0005085//guany	GO:0007264//small GTPase mediated signal transduction
GO:0003713//transc	GO:0006355//regulation of transcription, DNA-templated
GO:0005515//protei	GO:0043065//positive regulation of apoptotic process;GO:0044790//nega
GO:0042802//identic	GO:0007165//signal transduction;GO:0042981//regulation of apoptotic pr
-	-
GO:0042577//lipid p	GO:0006644//phospholipid metabolic process
GO:0046790//virion	GO:0075512//clathrin-mediated endocytosis of virus by host cell
GO:0008236//serine	GO:0006508//proteolysis
GO:0004609//phosp	GO:0006646//phosphatidylethanolamine biosynthetic process;GO:00086
GO:0003824//catalyt	-

GO:0000287//magne	GO:0006114//glycerol biosynthetic process;GO:0006650//glycerophospho
-	-
GO:0042802//identic	GO:0007165//signal transduction;GO:0042981//regulation of apoptotic pr
GO:0005506//iron io	GO:0001949//sebaceous gland cell differentiation;GO:0006629//lipid meta
GO:0045296//cadhe	GO:0007043//cell-cell junction assembly;GO:0060997//dendritic spine mo
GO:0004860//protei	GO:0007616//long-term memory;GO:0050729//positive regulation of infla
GO:0004103//cholin	GO:0006646//phosphatidylethanolamine biosynthetic process;GO:000665
GO:0004585//ornithi	GO:0000050//urea cycle;GO:0006520//cellular amino acid metabolic proc
GO:0003677//DNA b	GO:0006383//transcription from RNA polymerase III promoter
GO:0003676//nuclei	GO:0006278//RNA-dependent DNA replication;GO:0015074//DNA integra
GO:0003924//GTPas	-
GO:0004467//long-c	GO:0006631//fatty acid metabolic process
GO:0005319//lipid tr	GO:0006629//lipid metabolic process;GO:0006642//triglyceride mobilizati
-	GO:0070979//protein K11-linked ubiquitination
GO:0001604//uroter	GO:0007165//signal transduction;GO:0007186//G-protein coupled recept
-	-
-	-
GO:0016757//transfe	GO:0002121//inter-male aggressive behavior;GO:0042403//thyroid horm
-	GO:0006886//intracellular protein transport;GO:0006890//retrograde vesi
GO:0004725//protei	GO:0006470//protein dephosphorylation;GO:0016311//dephosphorylation
-	GO:0007411//axon guidance;GO:0010977//negative regulation of neuron
GO:0036122//BMP b	GO:0007389//pattern specification process;GO:0010454//negative regulat
GO:0004674//protei	GO:0006468//protein phosphorylation
GO:0015218//pyrimi	GO:0000002//mitochondrial genome maintenance;GO:0002082//regulatio
GO:0022857//transm	GO:0055085//transmembrane transport
GO:0016290//palmit	GO:0006637//acyl-CoA metabolic process;GO:0016559//peroxisome fissio
-	-
-	-
GO:0008271//secon	GO:0008272//sulfate transport;GO:0055085//transmembrane transport
-	-
-	-
GO:0015095//magne	GO:0015693//magnesium ion transport
-	-
-	-
GO:0004672//protei	GO:0006468//protein phosphorylation
-	-
GO:0005504//fatty a	GO:0015908//fatty acid transport;GO:0015909//long-chain fatty acid trans
-	GO:0006887//exocytosis;GO:0015031//protein transport
GO:0001758//retinal	GO:0002072//optic cup morphogenesis involved in camera-type eye deve
GO:0008109//N-ace	GO:0006486//protein glycosylation;GO:0007179//transforming growth fac
GO:0005543//phosp	GO:0048260//positive regulation of receptor-mediated endocytosis;GO:0(
GO:0004499//N,N-d	-
GO:0008080//N-ace	-
GO:0005515//protei	GO:0016567//protein ubiquitination
-	GO:0006629//lipid metabolic process
GO:0004386//helica	-
GO:0004672//protei	GO:0006885//regulation of pH;GO:0008286//insulin receptor signaling pa
-	-
GO:0003677//DNA b	GO:0006334//nucleosome assembly;GO:0006352//DNA-templated transc
-	-
GO:0005524//ATP bi	GO:0035499//carnosine biosynthetic process
GO:0042802//identic	GO:0007165//signal transduction;GO:0046628//positive regulation of insu
GO:0003836//beta-g	GO:0002319//memory B cell differentiation;GO:0006054//N-acetylneuram
-	-
GO:0005515//protei	GO:0006888//ER to Golgi vesicle-mediated transport;GO:0016192//vesicle
GO:0001968//fibron	GO:0001933//negative regulation of protein phosphorylation;GO:0007162
-	-
GO:0009922//fatty a	GO:0019367//fatty acid elongation, saturated fatty acid;GO:0042761//very

-	-
GO:0015377//cation	GO:0006811//ion transport;GO:0006884//cell volume homeostasis;GO:005
-	-
GO:0015171//amino	GO:0006641//triglyceride metabolic process;GO:0009437//carnitine metab
-	-
GO:0008009//chemo	GO:0006954//inflammatory response;GO:0006955//immune response;GO
GO:0000166//nucleo	GO:0070588//calcium ion transmembrane transport
GO:0000166//nucleo	GO:0070588//calcium ion transmembrane transport
GO:0019901//protei	GO:0030334//regulation of cell migration;GO:0034446//substrate adhesio
GO:0022857//transm	GO:0055085//transmembrane transport
-	-
GO:0000166//nucleo	-
-	GO:0001501//skeletal system development;GO:0006006//glucose metabo
GO:0004386//helicas	-
-	GO:0070979//protein K11-linked ubiquitination
GO:0003677//DNA b	GO:0006351//transcription, DNA-templated
GO:0035256//G-pro	GO:0003009//skeletal muscle contraction;GO:0007216//G-protein couplec
GO:0005515//protei	-
GO:0019902//phosp	GO:0006511//ubiquitin-dependent protein catabolic process;GO:0016567
GO:0005515//protei	-
GO:0030145//manga	GO:0006508//proteolysis;GO:0019538//protein metabolic process
GO:0001758//retinal	GO:0042573//retinoic acid metabolic process;GO:0042574//retinal metabo
-	-
-	-
-	-
-	-
GO:0000287//magne	GO:0006099//tricarboxylic acid cycle
GO:0003707//steroic	GO:0007340//acrosome reaction;GO:0009611//response to wounding;GO
-	-
GO:0004672//protei	GO:0006468//protein phosphorylation;GO:0007169//transmembrane rece
GO:0005515//protei	GO:0001822//kidney development;GO:0002093//auditory receptor cell mo
GO:0046872//metal	GO:0006875//cellular metal ion homeostasis
-	-
GO:0005509//calciur	GO:0007268//synaptic transmission;GO:0008104//protein localization;GO:
GO:0003677//DNA b	-
GO:0000976//transc	GO:0000122//negative regulation of transcription from RNA polymerase I
-	GO:0060074//synapse maturation
-	-
GO:0004672//protei	GO:0000086//G2/M transition of mitotic cell cycle;GO:0000278//mitotic ce
GO:0001604//uroter	GO:0007165//signal transduction;GO:0007186//G-protein coupled recept
GO:0003676//nuclei	GO:0006388//tRNA splicing, via endonucleolytic cleavage and ligation
GO:0005085//guany	GO:0050790//regulation of catalytic activity
GO:0004842//ubiqui	GO:0006511//ubiquitin-dependent protein catabolic process;GO:0016567
GO:0001671//ATPas	-
-	-
GO:0003676//nuclei	GO:0043484//regulation of RNA splicing
-	-
-	-
-	-
GO:0005515//protei	GO:0045765//regulation of angiogenesis
GO:0030246//carbol	GO:0006897//endocytosis
GO:0005515//protei	GO:0006355//regulation of transcription, DNA-templated;GO:0006805//xe
-	-
GO:0004652//polynu	GO:0008285//negative regulation of cell proliferation;GO:0043066//negat
GO:0008170//N-me	GO:0001692//histamine metabolic process;GO:0001695//histamine catabo
-	-
-	-
-	GO:0030070//insulin processing;GO:0060628//regulation of ER to Golgi ve

GO:0003676//nuclei	GO:0006508//proteolysis;GO:0008152//metabolic process;GO:0016032//v
GO:0008236//serine	GO:0006508//proteolysis
GO:0008757//S-ade	GO:0006656//phosphatidylcholine biosynthetic process;GO:0006686//sph
GO:0008194//UDP-(	GO:0006486//protein glycosylation;GO:0006682//galactosylceramide bios
GO:0005515//protei	GO:0044763//single-organism cellular process
-	-
-	-
GO:0003677//DNA b	GO:0045944//positive regulation of transcription from RNA polymerase II
GO:0042802//identic	GO:0007017//microtubule-based process
GO:0022857//transm	GO:0055085//transmembrane transport
-	-
GO:0008146//sulfotr	GO:0006805//xenobiotic metabolic process;GO:0042403//thyroid hormon
GO:0022857//transm	GO:0055085//transmembrane transport
GO:0004843//thiol-c	GO:0000278//mitotic cell cycle;GO:0006281//DNA repair;GO:0006511//ub
GO:0004521//endori	-
GO:0016717//oxidor	GO:0006629//lipid metabolic process
GO:0017128//phosp	GO:0000045//autophagosome assembly;GO:0006914//autophagy;GO:001
-	-
-	GO:0006488//dolichol-linked oligosaccharide biosynthetic process
GO:0008324//cation	GO:0006812//cation transport
GO:0003824//catalyt	GO:0009226//nucleotide-sugar biosynthetic process;GO:0010595//positiv
-	-
GO:0003779//actin b	GO:0051017//actin filament bundle assembly
GO:0004896//cytokir	GO:0019221//cytokine-mediated signaling pathway;GO:0034342//respons
-	-
GO:0003824//catalyt	GO:0044710//single-organism metabolic process
-	GO:0006355//regulation of transcription, DNA-templated;GO:0034243//re
-	-
GO:0003824//catalyt	GO:0006778//porphyrin-containing compound metabolic process;GO:000
-	-
-	-
GO:0016491//oxidor	GO:0006666//3-keto-sphinganine metabolic process;GO:0030148//sphing
-	-
GO:0003677//DNA b	-
GO:0004609//phosp	GO:0006646//phosphatidylethanolamine biosynthetic process;GO:000865
GO:0005515//protei	GO:0006355//regulation of transcription, DNA-templated;GO:0007179//tr
GO:0003677//DNA b	GO:0006383//transcription from RNA polymerase III promoter
GO:0008271//secon	GO:0008272//sulfate transport;GO:0055085//transmembrane transport
GO:0005540//hyalur	GO:0007155//cell adhesion
GO:0046961//protor	GO:0006811//ion transport;GO:1902600//hydrogen ion transmembrane tr
GO:0005515//protei	-
GO:0005102//recept	GO:0007160//cell-matrix adhesion;GO:0007596//blood coagulation;GO:00
GO:0017075//syntax	-
-	-
-	-
GO:0002161//amino	-
GO:0016757//transfe	GO:0002121//inter-male aggressive behavior;GO:0042403//thyroid horm
-	-
-	-
GO:0016790//thioles	GO:0006637//acyl-CoA metabolic process
GO:0005515//protei	GO:0006355//regulation of transcription, DNA-templated;GO:0006805//x
GO:0005319//lipid tr	GO:0006869//lipid transport
-	-
GO:0045296//cadhe	GO:0007043//cell-cell junction assembly;GO:0060997//dendritic spine mc
GO:0042577//lipid p	GO:0006644//phospholipid metabolic process
GO:0003676//nuclei	GO:0006508//proteolysis;GO:0016032//viral process
GO:0000978//RNA p	GO:0001736//establishment of planar polarity;GO:0001843//neural tube c
GO:0004000//adeno	GO:0000255//allantoin metabolic process;GO:0001666//response to hypo

GO:0000166//nucleo	GO:0006563//L-serine metabolic process;GO:0006790//sulfur compound
-	-
GO:0019855//calciur	GO:0006851//mitochondrial calcium ion transport;GO:0051560//mitochor
GO:0000976//transc	GO:0006289//nucleotide-excision repair;GO:0006974//cellular response to
-	GO:1901031//regulation of response to reactive oxygen species
-	-
-	GO:0006886//intracellular protein transport;GO:0006890//retrograde vesic
-	-
GO:0017147//Wnt-p	GO:0030178//negative regulation of Wnt signaling pathway
GO:0008109//N-ace	GO:0006486//protein glycosylation;GO:0007179//transforming growth fac
-	-
-	-
GO:0005515//protei	GO:0016567//protein ubiquitination
GO:0003841//1-acyl	-
GO:0005506//iron io	GO:0008610//lipid biosynthetic process
GO:0001968//fibron	GO:0001933//negative regulation of protein phosphorylation;GO:0007162
GO:0004364//glutath	GO:0006749//glutathione metabolic process;GO:0006805//xenobiotic met
-	-
-	-
-	-
GO:0046873//metal	GO:0030001//metal ion transport
-	-
-	-
GO:0060090//bindin	GO:0030433//ER-associated ubiquitin-dependent protein catabolic proce
GO:0009922//fatty a	GO:0019367//fatty acid elongation, saturated fatty acid;GO:0042761//very
GO:0004190//aspart	GO:0006508//proteolysis;GO:0019538//protein metabolic process
GO:0003810//protei	GO:0018149//peptide cross-linking
GO:0004857//enzym	GO:0006629//lipid metabolic process;GO:0043086//negative regulation of
GO:0030729//acetoa	GO:0006629//lipid metabolic process
GO:0003841//1-acyl	GO:0006654//phosphatidic acid biosynthetic process;GO:0010891//negati
GO:0003847//1-alky	GO:0016042//lipid catabolic process
-	GO:0031032//actomyosin structure organization
GO:0004623//phosp	GO:0006644//phospholipid metabolic process;GO:0016042//lipid cataboli
GO:0000976//transc	GO:0000122//negative regulation of transcription from RNA polymerase I
GO:0000976//transc	GO:0006357//regulation of transcription from RNA polymerase II promote
GO:0008289//lipid b	GO:0015031//protein transport;GO:0019075//virus maturation;GO:004205
-	-
GO:1990756//protei	GO:0015031//protein transport;GO:0016567//protein ubiquitination;GO:0(
-	GO:0006364//rRNA processing;GO:0009303//rRNA transcription
-	-
GO:0005125//cytokii	GO:0002232//leukocyte chemotaxis involved in inflammatory response;GC
GO:0035256//G-pro	GO:0003009//skeletal muscle contraction;GO:0007216//G-protein couplec
GO:0004672//protei	GO:0006468//protein phosphorylation;GO:0007169//transmembrane rece
GO:0005319//lipid tr	GO:0006869//lipid transport
GO:0016491//oxidor	GO:0006629//lipid metabolic process;GO:0008611//ether lipid biosyntheti
GO:0005244//voltag	-
-	-
-	-
GO:0030246//carbol	GO:0006897//endocytosis
GO:0005515//protei	-
-	GO:0030513//positive regulation of BMP signaling pathway
GO:0005102//recept	GO:0006898//receptor-mediated endocytosis;GO:0008104//protein locali:
GO:0003676//nuclei	GO:0006388//tRNA splicing, via endonucleolytic cleavage and ligation
GO:0005515//protei	GO:0001764//neuron migration;GO:0007158//neuron cell-cell adhesion;G
-	-
GO:0004114//3',5'-c	GO:0007165//signal transduction
-	-
GO:0004343//glucos	GO:0006048//UDP-N-acetylglucosamine biosynthetic process

-	-
-	GO:0001920//negative regulation of receptor recycling;GO:0050850//posi
GO:0005515//protei	GO:0043065//positive regulation of apoptotic process;GO:0044790//nega
GO:0005201//extrac	GO:0007160//cell-matrix adhesion;GO:0007605//sensory perception of sc
GO:0016491//oxidor	-
GO:0004044//amido	GO:0006177//GMP biosynthetic process;GO:0006189//'de novo' IMP bios
GO:0019902//phosp	GO:0006511//ubiquitin-dependent protein catabolic process;GO:0016567
-	-
-	-
GO:0004672//protei	GO:0006468//protein phosphorylation;GO:0008631//intrinsic apoptotic si
GO:0015293//sympo	GO:0008643//carbohydrate transport
GO:0003676//nuclei	GO:0006278//RNA-dependent DNA replication;GO:0006310//DNA recom
GO:0001784//phosp	GO:0008286//insulin receptor signaling pathway;GO:0010907//positive re
GO:0000977//RNA p	GO:0000122//negative regulation of transcription from RNA polymerase I
GO:0036122//BMP b	GO:0007389//pattern specification process;GO:0010454//negative regulat
GO:0004842//ubiqui	GO:0000209//protein polyubiquitination;GO:0006915//apoptotic process;
GO:0017128//phosp	GO:0000045//autophagosome assembly;GO:0006869//lipid transport;GO:
GO:0005515//protei	-
GO:0000166//nucleo	GO:0003014//renal system process;GO:0006811//ion transport;GO:000682
-	-
GO:0005509//calciu	GO:0007155//cell adhesion;GO:0007156//homophilic cell adhesion via pla
GO:0001604//urote	GO:0007165//signal transduction;GO:0007186//G-protein coupled recept
GO:0004896//cytoki	GO:0019221//cytokine-mediated signaling pathway;GO:0050829//defense
-	GO:0060074//synapse maturation
GO:0050699//WW d	GO:0007034//vacuolar transport;GO:0010629//negative regulation of gen
GO:0004674//protei	GO:0006468//protein phosphorylation
GO:0042802//identi	GO:0030182//neuron differentiation;GO:0045893//positive regulation of t
GO:0003779//actin b	GO:0051017//actin filament bundle assembly
GO:0005085//guany	GO:0007165//signal transduction;GO:0007264//small GTPase mediated si
GO:0019901//protei	GO:0030334//regulation of cell migration;GO:0034446//substrate adhesio
GO:0004930//G-pro	GO:0000278//mitotic cell cycle;GO:0001525//angiogenesis;GO:0001569//
-	-
-	-
GO:0003676//nuclei	GO:0043484//regulation of RNA splicing
-	-
GO:0003676//nuclei	GO:0006508//proteolysis;GO:0016032//viral process
-	GO:0015888//thiamine transport
GO:0015175//neutra	GO:0015804//neutral amino acid transport;GO:0042941//D-alanine transp
GO:0001784//phosp	GO:0007169//transmembrane receptor protein tyrosine kinase signaling p
GO:0004176//ATP-d	GO:0006508//proteolysis;GO:0006515//misfolded or incompletely synthes
GO:0000287//magne	GO:0006114//glycerol biosynthetic process;GO:0006650//glycerophospho
-	-
GO:0036122//BMP b	GO:0007389//pattern specification process;GO:0010454//negative regulat
-	GO:0006658//phosphatidylserine metabolic process;GO:0006665//sphing
GO:0004308//exo-al	GO:0006516//glycoprotein catabolic process;GO:0006689//ganglioside ca
GO:0008271//secon	GO:0008272//sulfate transport;GO:0055085//transmembrane transport
GO:0004888//transm	GO:0007166//cell surface receptor signaling pathway;GO:0007186//G-pro
-	-
GO:0003676//nuclei	GO:0006508//proteolysis;GO:0008152//metabolic process;GO:0016032//v
GO:0003779//actin b	GO:0051017//actin filament bundle assembly
GO:0046961//protor	GO:0006811//ion transport;GO:1902600//hydrogen ion transmembrane tr
GO:0004497//mono	-
-	-
GO:0015485//choles	GO:0032367//intracellular cholesterol transport;GO:0034389//lipid particle
-	-
-	-
-	-
GO:0043022//riboso	GO:0006886//intracellular protein transport;GO:0030433//ER-associated

GO:0004497//monoo	GO:0006559//L-phenylalanine catabolic process;GO:0006571//tyrosine bi
GO:0016491//oxidor	-
GO:0004888//transm	GO:0006811//ion transport;GO:0034220//ion transmembrane transport
-	-
-	-
GO:0008271//secon	GO:0008272//sulfate transport;GO:0055085//transmembrane transport
GO:0016717//oxidor	GO:0006629//lipid metabolic process
GO:0004930//G-pro	GO:0001764//neuron migration;GO:0007186//G-protein coupled recepto
GO:0000166//nucleo	GO:0070588//calcium ion transmembrane transport
GO:0042802//identic	-
GO:0003874//6-pyru	GO:0006729//tetrahydrobiopterin biosynthetic process
GO:0001605//adren	GO:0001525//angiogenesis;GO:0001570//vasculogenesis;GO:0002040//sp
GO:0000978//RNA p	GO:0006355//regulation of transcription, DNA-templated;GO:0006357//re
-	GO:0006895//Golgi to endosome transport;GO:0015031//protein transpo
GO:0003676//nuclei	-
GO:0004386//helica	-
GO:0004467//long-c	GO:0006631//fatty acid metabolic process
GO:0005319//lipid tr	GO:0006869//lipid transport
-	GO:0070453//regulation of heme biosynthetic process
-	GO:0070979//protein K11-linked ubiquitination
GO:0022857//transm	GO:0055085//transmembrane transport
GO:0016491//oxidor	GO:0006666//3-keto-sphinganine metabolic process;GO:0030148//sphin
GO:0016627//oxidor	GO:0006629//lipid metabolic process
GO:0005515//protei	GO:0007155//cell adhesion;GO:0008593//regulation of Notch signaling pa
-	GO:0007411//axon guidance;GO:0010977//negative regulation of neuron
GO:0016787//hydrol	GO:0006629//lipid metabolic process;GO:0007216//G-protein coupled glu
-	-
GO:0005484//SNAP	GO:0006888//ER to Golgi vesicle-mediated transport;GO:0006903//vesicle
-	-
-	-
GO:0005515//protei	-
GO:0004857//enzym	GO:0006629//lipid metabolic process;GO:0043086//negative regulation o
-	GO:0007042//lysosomal lumen acidification;GO:0042176//regulation of pr
GO:0016740//transfe	GO:0006629//lipid metabolic process;GO:0006659//phosphatidylserine bi
-	-
GO:0031267//small	GO:0006886//intracellular protein transport
GO:0004364//glutat	GO:0006749//glutathione metabolic process;GO:0006805//xenobiotic me
-	-
-	-
GO:0005515//protei	-
-	-
GO:0046873//metal	GO:0030001//metal ion transport
GO:0004930//G-pro	GO:0007165//signal transduction;GO:0007186//G-protein coupled recept
GO:0016491//oxidor	GO:0006629//lipid metabolic process;GO:0008611//ether lipid biosyntheti
GO:0000062//fatty-a	GO:0036151//phosphatidylcholine acyl-chain remodeling;GO:1903060//n
GO:0005524//ATP bi	GO:0055085//transmembrane transport
GO:0004190//aspart	GO:0006508//proteolysis;GO:0019538//protein metabolic process
-	-
GO:0002039//p53 bi	GO:0000724//double-strand break repair via homologous recombination;
GO:0004843//thiol-c	GO:0006511//ubiquitin-dependent protein catabolic process;GO:0016579
GO:0042802//identic	GO:0007165//signal transduction;GO:0046628//positive regulation of insu
GO:0000166//nucleo	GO:0006468//protein phosphorylation;GO:0010976//positive regulation o
GO:0005515//protei	GO:0098742//cell-cell adhesion via plasma-membrane adhesion molecul
-	-
GO:0016740//transfe	GO:0006777//Mo-molybdopterin cofactor biosynthetic process
GO:0004506//squale	GO:0016126//sterol biosynthetic process;GO:0042127//regulation of cell p
GO:0005085//guany	-
GO:0004222//metall	GO:0000003//reproduction;GO:0006508//proteolysis

GO:0008236//serine	GO:0006508//proteolysis
GO:0003924//GTPas	GO:0006254//CTP catabolic process;GO:0034656//nucleobase-containing
-	-
GO:0003909//DNA li	-
GO:0016787//hydrol	GO:0006470//protein dephosphorylation;GO:0006805//xenobiotic metabo
GO:0070089//chloric	GO:0006813//potassium ion transport;GO:0097623//potassium ion export
-	-
GO:0003824//catalyt	GO:0009226//nucleotide-sugar biosynthetic process;GO:0010595//positiv
-	-
GO:0001968//fibron	GO:0010811//positive regulation of cell-substrate adhesion;GO:0030198//
GO:0005515//protei	GO:0006355//regulation of transcription, DNA-templated;GO:0006805//x
GO:0003824//catalyt	GO:0006777//Mo-molybdopterin cofactor biosynthetic process;GO:00435
GO:0004175//endop	GO:0006508//proteolysis;GO:0007354//zygotic determination of anterior/
-	-
GO:0031419//cobala	GO:0015889//cobalamin transport
GO:0003824//catalyt	GO:0006778//porphyrin-containing compound metabolic process;GO:000
GO:0005319//lipid tr	GO:0006869//lipid transport
-	GO:0019941//modification-dependent protein catabolic process;GO:0030
GO:0004857//enzym	GO:0006071//glycerol metabolic process;GO:0006631//fatty acid metabol
-	-
GO:0005515//protei	GO:0000281//mitotic cytokinesis;GO:0007019//microtubule depolymeriza
GO:0004521//endori	-
-	-
-	-
-	-
-	-
-	-
-	-
-	-
GO:0005524//ATP bi	-
-	-
GO:0005515//protei	-
-	-
GO:0004672//protei	GO:0006468//protein phosphorylation;GO:0008631//intrinsic apoptotic si
GO:0000166//nucleo	GO:0003014//renal system process;GO:0006811//ion transport;GO:00068
GO:0003676//nuclei	GO:0043488//regulation of mRNA stability
GO:0005515//protei	-
-	GO:0018095//protein polyglutamylation
GO:0001758//retinal	GO:0002072//optic cup morphogenesis involved in camera-type eye deve
GO:0001671//ATPas	GO:0001933//negative regulation of protein phosphorylation;GO:003043
-	-
GO:0003779//actin b	GO:0051017//actin filament bundle assembly
GO:0004000//adeno	GO:0000255//allantoin metabolic process;GO:0001666//response to hypo
GO:0005515//protei	GO:0043031//negative regulation of macrophage activation;GO:0050728/
-	GO:0031032//actomyosin structure organization
GO:0042577//lipid p	GO:0006644//phospholipid metabolic process
GO:0000976//transc	GO:0002062//chondrocyte differentiation;GO:0006355//regulation of tran
-	GO:0007010//cytoskeleton organization;GO:0008360//regulation of cell sh
GO:0005319//lipid tr	GO:0006869//lipid transport
-	-
GO:0005515//protei	GO:0051260//protein homooligomerization
GO:0005515//protei	GO:0006355//regulation of transcription, DNA-templated;GO:0006805//x
GO:0005507//coppe	GO:0007601//visual perception;GO:0009308//amine metabolic process
GO:0005518//collag	GO:0007605//sensory perception of sound;GO:0008360//regulation of cel
GO:0005244//voltag	-
GO:0000287//magn	GO:0006114//glycerol biosynthetic process;GO:0006650//glycerophospho
GO:0005085//guany	GO:0007264//small GTPase mediated signal transduction;GO:0034976//re
GO:0016491//oxidor	-

GO:0001604//uroter	GO:0007165//signal transduction;GO:0007186//G-protein coupled recept
GO:0019855//calciur	GO:0006851//mitochondrial calcium ion transport;GO:0051560//mitochor
-	-
-	-
GO:0005515//protei	GO:0001938//positive regulation of endothelial cell proliferation;GO:0014
GO:0004452//isoper	GO:0008299//isoprenoid biosynthetic process
GO:0005515//protei	GO:0002091//negative regulation of receptor internalization;GO:0042058
-	-
-	-
-	-
-	-
GO:0016628//oxidor	GO:0006695//cholesterol biosynthetic process;GO:0016126//sterol biosyn
GO:0000978//RNA p	GO:0001932//regulation of protein phosphorylation;GO:0006355//regulat
-	GO:0006658//phosphatidylserine metabolic process;GO:0006665//sphing
GO:0003677//DNA b	-
GO:0019901//protei	GO:0030334//regulation of cell migration;GO:0034446//substrate adhesio
GO:0042802//identic	GO:0019216//regulation of lipid metabolic process;GO:0055088//lipid hor
GO:0004672//protei	GO:0000086//G2/M transition of mitotic cell cycle;GO:0000278//mitotic ce
GO:0004045//amino	-
GO:0005085//guany	-
GO:0005515//protei	GO:0007015//actin filament organization;GO:0045806//negative regulatio
GO:0000976//transc	GO:0000122//negative regulation of transcription from RNA polymerase I
-	-
GO:0050699//WW d	GO:0007034//vacuolar transport;GO:0010629//negative regulation of gen
-	-
-	-
-	-
-	-
GO:0005515//protei	GO:0000281//mitotic cytokinesis;GO:0014067//negative regulation of pho
-	GO:0006606//protein import into nucleus
GO:0000166//nucleo	GO:0000045//autophagosome assembly;GO:0002026//regulation of the fo
GO:0002020//protea	GO:0010951//negative regulation of endopeptidase activity
-	GO:0009267//cellular response to starvation;GO:0030001//metal ion trans
GO:0003824//catalyt	GO:0010121//arginine catabolic process to proline via ornithine;GO:0019
-	-
-	GO:0007155//cell adhesion
-	-
GO:0035256//G-pro	GO:0003009//skeletal muscle contraction;GO:0007216//G-protein couple
GO:0003713//transc	GO:0006355//regulation of transcription, DNA-templated
-	-
GO:0003924//GTPas	GO:0006254//CTP catabolic process;GO:0034656//nucleobase-containing
GO:0003824//catalyt	GO:0009436//glyoxylate catabolic process;GO:0019265//glycine biosynthe
-	-
GO:0003779//actin b	GO:0055013//cardiac muscle cell development
GO:0016740//transfe	GO:0006777//Mo-molybdopterin cofactor biosynthetic process
GO:0000166//nucleo	GO:0070588//calcium ion transmembrane transport
GO:0000703//oxidiz	GO:0006281//DNA repair;GO:0006284//base-excision repair;GO:0006285
GO:0004467//long-	GO:0006631//fatty acid metabolic process
-	GO:1901031//regulation of response to reactive oxygen species
GO:0000976//transc	GO:0000122//negative regulation of transcription from RNA polymerase I
-	-
GO:0005515//protei	GO:0000281//mitotic cytokinesis;GO:0007019//microtubule depolymeriza
GO:0003836//beta-	GO:0002319//memory B cell differentiation;GO:0006054//N-acetylneuram
GO:0008289//lipid b	GO:0010873//positive regulation of cholesterol esterification;GO:0010879
GO:0000981//RNA p	GO:0000122//negative regulation of transcription from RNA polymerase I
GO:0003676//nuclei	GO:0006278//RNA-dependent DNA replication;GO:0015074//DNA integra
GO:0004386//helica	-
GO:0004672//protei	GO:0006468//protein phosphorylation;GO:0007169//transmembrane rece

GO:0005524//ATP bi	GO:0055085//transmembrane transport
-	-
GO:0022892//substra	GO:0044765//single-organism transport
GO:0001968//fibron	GO:0001933//negative regulation of protein phosphorylation;GO:000716
GO:0030246//carbol	GO:0006897//endocytosis
-	-
GO:0000977//RNA p	GO:0000122//negative regulation of transcription from RNA polymerase I
-	-
GO:0003735//structu	GO:0006412//translation;GO:0006919//activation of cysteine-type endope
GO:0004842//ubiqui	GO:0000209//protein polyubiquitination;GO:0006915//apoptotic process;
GO:0016290//palmit	GO:0006637//acyl-CoA metabolic process;GO:0016559//peroxisome fissic
GO:0003824//catalyt	GO:0005975//carbohydrate metabolic process;GO:0006006//glucose meta
GO:0016740//transfe	GO:0006629//lipid metabolic process;GO:0006659//phosphatidylserine bi
GO:0004930//G-pro	GO:0007165//signal transduction;GO:0007186//G-protein coupled recept
-	-
-	-
-	-
GO:0004672//protei	GO:0006468//protein phosphorylation
-	-
GO:0004843//thiol-c	GO:0000278//mitotic cell cycle;GO:0006281//DNA repair;GO:0006511//ub
GO:0030729//acetoa	GO:0006629//lipid metabolic process
GO:0000976//transc	GO:0006357//regulation of transcription from RNA polymerase II promote
GO:0005102//recept	GO:0003336//corneocyte desquamation;GO:0006672//ceramide metaboli
-	-
GO:0005515//protei	GO:0006355//regulation of transcription, DNA-templated;GO:0006805//x
GO:0015175//neutra	GO:0015804//neutral amino acid transport;GO:0042941//D-alanine transp
-	-
-	-
GO:0051082//unfold	GO:0006457//protein folding
-	-
GO:0003824//catalyt	GO:0005975//carbohydrate metabolic process
-	-
-	GO:0009617//response to bacterium
-	-
GO:0036122//BMP b	GO:0007389//pattern specification process;GO:0010454//negative regulat
GO:0000166//nucleo	GO:0006468//protein phosphorylation;GO:0010976//positive regulation o
GO:0005524//ATP bi	GO:0035499//carnosine biosynthetic process
GO:0051015//actin f	-
GO:0004843//thiol-c	GO:0006511//ubiquitin-dependent protein catabolic process;GO:0016579
GO:0005102//recept	GO:0007160//cell-matrix adhesion;GO:0007596//blood coagulation;GO:0
-	-
GO:0004176//ATP-d	GO:0006508//proteolysis;GO:0006515//misfolded or incompletely synthes
GO:0004103//cholin	GO:0006646//phosphatidylethanolamine biosynthetic process;GO:000665
GO:0003677//DNA b	GO:0006383//transcription from RNA polymerase III promoter
GO:0008236//serine	GO:0006508//proteolysis
GO:0004930//G-pro	GO:0000278//mitotic cell cycle;GO:0001525//angiogenesis;GO:0001569//
GO:0001758//retinal	GO:0002072//optic cup morphogenesis involved in camera-type eye deve
GO:0003735//structu	GO:0006412//translation;GO:0006919//activation of cysteine-type endope
-	-
-	-
-	-
GO:0005509//calciur	GO:0015012//heparan sulfate proteoglycan biosynthetic process;GO:0030
GO:0016717//oxidor	GO:0006629//lipid metabolic process
GO:0003779//actin b	GO:0007015//actin filament organization;GO:0030334//regulation of cell
GO:0004896//cytoki	GO:0019221//cytokine-mediated signaling pathway;GO:0050829//defens
GO:0005509//calciur	GO:0007155//cell adhesion;GO:0007156//homophilic cell adhesion via pla
GO:0005509//calciur	-
-	GO:0007042//lysosomal lumen acidification;GO:0042176//regulation of pr

GO:0042802//identic	-
GO:0004888//transm	GO:0007166//cell surface receptor signaling pathway;GO:0007186//G-pro
GO:0005509//calciur	GO:0007268//synaptic transmission;GO:0035556//intracellular signal trans
-	-
GO:0005515//protei	-
GO:0004888//transm	GO:0007166//cell surface receptor signaling pathway;GO:0007186//G-pro
-	GO:0019941//modification-dependent protein catabolic process;GO:0030
-	GO:0070453//regulation of heme biosynthetic process
-	-
GO:0003676//nuclei	GO:0006508//proteolysis;GO:0016032//viral process
GO:0016491//oxidor	-
GO:0016491//oxidor	GO:0006666//3-keto-sphinganine metabolic process;GO:0030148//sphing
GO:0000976//transc	GO:0002062//chondrocyte differentiation;GO:0006355//regulation of tran
GO:0005520//insulin	GO:0042104//positive regulation of activated T cell proliferation
GO:0004857//enzym	GO:0006071//glycerol metabolic process;GO:0006631//fatty acid metabol
-	GO:0070453//regulation of heme biosynthetic process
-	-
-	GO:0060074//synapse maturation
GO:0022857//transm	GO:0055085//transmembrane transport
-	-
GO:0005515//protei	GO:0044763//single-organism cellular process
GO:0042802//identic	GO:0030182//neuron differentiation;GO:0045893//positive regulation of t
GO:0005085//guany	-
-	-
-	-
GO:0005524//ATP bi	-
-	-
-	-
GO:0001671//ATPas	GO:0001933//negative regulation of protein phosphorylation;GO:003043
GO:0003676//nuclei	GO:0043484//regulation of RNA splicing
-	-
-	-
GO:0000976//transc	GO:0000122//negative regulation of transcription from RNA polymerase I
-	-
GO:0005507//coppe	GO:0007601//visual perception;GO:0009308//amine metabolic process
GO:0003824//catalyt	GO:0006567//threonine catabolic process
GO:0004452//isoper	GO:0008299//isoprenoid biosynthetic process
GO:0005515//protei	GO:0000281//mitotic cytokinesis;GO:0014067//negative regulation of pho
GO:0005515//protei	GO:0007043//cell-cell junction assembly;GO:0007155//cell adhesion;GO:0
-	-
GO:0043169//cation	GO:0007165//signal transduction;GO:0022603//regulation of anatomical s
-	GO:0030513//positive regulation of BMP signaling pathway
GO:0004672//protei	GO:0000086//G2/M transition of mitotic cell cycle;GO:0000278//mitotic ce
GO:0008022//protei	GO:0007099//centriole replication;GO:0010457//centriole-centriole cohes
GO:0005244//voltag	-
-	-
-	-
GO:0015078//hydro	GO:0030177//positive regulation of Wnt signaling pathway;GO:1902600//
-	-
GO:0003677//DNA b	GO:0045944//positive regulation of transcription from RNA polymerase II
GO:0005355//glucos	GO:0015747//urate transport;GO:0046415//urate metabolic process;GO:0
GO:0016491//oxidor	GO:0006629//lipid metabolic process;GO:0006631//fatty acid metabolic p
-	GO:0009966//regulation of signal transduction;GO:0016055//Wnt signalin
-	-
GO:0008047//enzym	GO:0006486//protein glycosylation;GO:0006487//protein N-linked glycos
-	-
-	-
-	GO:0031032//actomyosin structure organization

GO:0008047//enzym	GO:0006486//protein glycosylation;GO:0006487//protein N-linked glycosy
-	GO:0009267//cellular response to starvation;GO:0030001//metal ion trans
-	-
GO:0003677//DNA b	GO:0009646//response to absence of light;GO:0018298//protein-chromo
GO:0003682//chrom	GO:0015908//fatty acid transport;GO:0043066//negative regulation of apo
-	-
GO:0016780//phosp	GO:0008654//phospholipid biosynthetic process
GO:0003874//6-pyru	GO:0006729//tetrahydrobiopterin biosynthetic process
-	-
GO:0003824//catalyt	GO:0044710//single-organism metabolic process
GO:0003677//DNA b	GO:0006281//DNA repair;GO:0006974//cellular response to DNA damage
-	-
GO:0003824//catalyt	GO:0009436//glyoxylate catabolic process;GO:0019265//glycine biosynthe
GO:0001968//fibron	GO:0010811//positive regulation of cell-substrate adhesion;GO:0030198//
GO:0005515//protei	GO:0001764//neuron migration;GO:0007097//nuclear migration;GO:0007
GO:0016787//hydrol	GO:0006470//protein dephosphorylation;GO:0006805//xenobiotic metabo
-	GO:0051480//regulation of cytosolic calcium ion concentration;GO:19900
-	GO:0007507//heart development
-	-
GO:0000977//RNA p	GO:0000122//negative regulation of transcription from RNA polymerase I
GO:0001671//ATPas	GO:0001933//negative regulation of protein phosphorylation;GO:003043
GO:0005319//lipid tr	GO:0006869//lipid transport
-	-
GO:0004930//G-pro	GO:0002001//renin secretion into blood stream;GO:0002281//macrophag
GO:0016740//transf	GO:0006777//Mo-molybdopterin cofactor biosynthetic process
-	-
-	-
-	-
GO:0003677//DNA b	GO:0009646//response to absence of light;GO:0018298//protein-chromo
GO:0005506//iron ic	GO:0001949//sebaceous gland cell differentiation;GO:0006629//lipid meta
GO:0043169//cation	GO:0007165//signal transduction;GO:0022603//regulation of anatomical s
GO:0005515//protei	GO:0007043//cell-cell junction assembly;GO:0007155//cell adhesion;GO:0
-	-
GO:0004930//G-pro	GO:0007186//G-protein coupled receptor signaling pathway
GO:0005515//protei	GO:0000281//mitotic cytokinesis;GO:0007019//microtubule depolymeriza
-	-
-	-
-	-
-	-
-	-
-	GO:1901031//regulation of response to reactive oxygen species
-	-
-	-
GO:0016491//oxidor	-
GO:0005509//calciur	-
GO:0000978//RNA p	GO:0001932//regulation of protein phosphorylation;GO:0006355//regulat
-	-
GO:0016628//oxidor	GO:0006695//cholesterol biosynthetic process;GO:0016126//sterol biosyn
-	-
-	-
GO:0005216//ion ch	GO:0006811//ion transport;GO:0006813//potassium ion transport;GO:003
-	GO:0018095//protein polyglutamylation
-	-
GO:0005515//protei	GO:0055085//transmembrane transport
-	-
GO:0005504//fatty a	GO:0015908//fatty acid transport;GO:0015909//long-chain fatty acid trans
GO:0008233//peptid	GO:0006508//proteolysis
GO:0036122//BMP b	GO:0007389//pattern specification process;GO:0010454//negative regulat

GO:0004310//farnes	GO:0008610//lipid biosynthetic process;GO:0009058//biosynthetic proces
GO:0004806//triglyc	GO:0006629//lipid metabolic process;GO:0006633//fatty acid biosynthetic
GO:0005085//guany	GO:0007264//small GTPase mediated signal transduction;GO:0034976//re
GO:0004103//cholin	GO:0006646//phosphatidylethanolamine biosynthetic process;GO:000665
GO:0003847//1-alky	GO:0016042//lipid catabolic process
GO:0005509//calciur	GO:0015012//heparan sulfate proteoglycan biosynthetic process;GO:0030
GO:0003676//nuclei	GO:0019722//calcium-mediated signaling;GO:0050790//regulation of cat
GO:0003836//beta-g	GO:0002319//memory B cell differentiation;GO:0006054//N-acetylneuram
GO:0000976//transc	GO:0006357//regulation of transcription from RNA polymerase II promote
GO:0004475//mann	GO:0006486//protein glycosylation;GO:0009058//biosynthetic process;GC
-	-
-	-
-	GO:0006364//rRNA processing;GO:0009303//rRNA transcription
GO:0005515//protei	GO:0055085//transmembrane transport
GO:0022857//transm	GO:0055085//transmembrane transport
GO:0015218//pyrimi	GO:0000002//mitochondrial genome maintenance;GO:0002082//regulatic
GO:0004930//G-pro	GO:0007165//signal transduction;GO:0007186//G-protein coupled recept
-	GO:0007165//signal transduction;GO:0070507//regulation of microtubule
-	-
GO:0015095//magn	GO:0015693//magnesium ion transport
GO:0016491//oxidor	-
-	-
-	GO:0009267//cellular response to starvation;GO:0030001//metal ion trans
GO:0005515//protei	-
GO:0003735//structu	GO:0006412//translation;GO:0006919//activation of cysteine-type endope
-	-
-	GO:0070979//protein K11-linked ubiquitination
GO:0000254//C-4 m	GO:0001934//positive regulation of protein phosphorylation;GO:0008610/
GO:0004175//endop	GO:0018126//protein hydroxylation
GO:0001671//ATPas	-
-	-
GO:0016491//oxidor	-
GO:0004521//endori	-
GO:0000166//nucle	GO:0006897//endocytosis;GO:0006898//receptor-mediated endocytosis;(
-	-
-	-
-	-
GO:0002161//amino	-
GO:0003677//DNA b	GO:0006334//nucleosome assembly;GO:0042742//defense response to ba
GO:0004340//gluco	GO:0001678//cellular glucose homeostasis;GO:0005975//carbohydrate me
GO:0003677//DNA b	GO:0006334//nucleosome assembly;GO:0006352//DNA-templated transc
GO:0005515//protei	GO:0042509//regulation of tyrosine phosphorylation of STAT protein;GO:(
-	-
GO:0004452//isoper	GO:0008299//isoprenoid biosynthetic process
GO:0035256//G-pro	GO:0003009//skeletal muscle contraction;GO:0007216//G-protein couple
-	-
GO:0008270//zinc ic	GO:0009086//methionine biosynthetic process;GO:0009235//cobalamin m
GO:0008378//galact	GO:0001576//globoside biosynthetic process;GO:0007009//plasma memb
-	-
-	-
-	-
GO:0008022//protei	GO:0007099//centriole replication;GO:0010457//centriole-centriole cohes
GO:0005515//protei	-
GO:0022892//substr	GO:0044765//single-organism transport
GO:0003677//DNA b	GO:0006334//nucleosome assembly;GO:0042742//defense response to ba
GO:0015078//hydro	GO:0006811//ion transport;GO:0007034//vacuolar transport;GO:0007035/
GO:0015078//hydro	GO:0030177//positive regulation of Wnt signaling pathway;GO:1902600//
GO:0005515//protei	GO:0098742//cell-cell adhesion via plasma-membrane adhesion molecul

-	-
GO:0000976//transc	GO:0002062//chondrocyte differentiation;GO:0006355//regulation of tran
GO:0004176//ATP-d	GO:0006508//proteolysis;GO:0006515//misfolded or incompletely synthes
GO:0003924//GTPas	GO:0006254//CTP catabolic process;GO:0034656//nucleobase-containing
GO:0003824//catalyt	GO:0009226//nucleotide-sugar biosynthetic process;GO:0010595//positiv
GO:0004721//phosp	GO:0010801//negative regulation of peptidyl-threonine phosphorylation;
GO:0003677//DNA b	GO:0006351//transcription, DNA-templated
GO:0004930//G-pro	GO:0000278//mitotic cell cycle;GO:0001525//angiogenesis;GO:0001569//
GO:0004930//G-pro	GO:0006897//endocytosis;GO:0006935//chemotaxis;GO:0006955//immun
-	-
GO:0001968//fibron	GO:0001933//negative regulation of protein phosphorylation;GO:0007162
GO:0003836//beta-g	GO:0002319//memory B cell differentiation;GO:0006054//N-acetylneuram
GO:0009922//fatty a	GO:0000038//very long-chain fatty acid metabolic process;GO:0006629//l
-	-
-	-
GO:0005509//calciur	GO:0015012//heparan sulfate proteoglycan biosynthetic process;GO:0030
-	-
-	GO:0007098//centrosome cycle;GO:0035020//regulation of Rac protein si
GO:0004843//thiol-c	GO:0000278//mitotic cell cycle;GO:0006281//DNA repair;GO:0006511//ub
-	-
-	-
GO:0003707//steroic	GO:0007340//acrosome reaction;GO:0009611//response to wounding;GO
GO:0004896//cytoki	GO:0019221//cytokine-mediated signaling pathway;GO:0050829//defense
-	GO:0006488//dolichol-linked oligosaccharide biosynthetic process
GO:0005509//calciur	GO:0007155//cell adhesion;GO:0007156//homophilic cell adhesion via pla
GO:0003677//DNA b	GO:0006281//DNA repair;GO:0006974//cellular response to DNA damage
GO:0003677//DNA b	GO:0009646//response to absence of light;GO:0018298//protein-chromo
GO:0005515//protei	GO:0001822//kidney development;GO:0002093//auditory receptor cell mo
GO:0005524//ATP bi	GO:0055085//transmembrane transport
GO:0043169//cation	GO:0007165//signal transduction;GO:0022603//regulation of anatomical s
GO:0001786//phosp	GO:0006910//phagocytosis, recognition;GO:0006911//phagocytosis, engu
GO:0005515//protei	GO:0007043//cell-cell junction assembly;GO:0007155//cell adhesion;GO:0
GO:0005515//protei	GO:0000281//mitotic cytokinesis;GO:0014067//negative regulation of pho
-	-
-	GO:0030070//insulin processing;GO:0060628//regulation of ER to Golgi ve
GO:0000166//nucleo	GO:0006468//protein phosphorylation;GO:0010976//positive regulation o
-	-
-	-
GO:0000976//transc	GO:0000122//negative regulation of transcription from RNA polymerase I
GO:0004843//thiol-c	GO:0006511//ubiquitin-dependent protein catabolic process;GO:0016579
GO:0005172//vascul	GO:0008283//cell proliferation;GO:0008284//positive regulation of cell pro
GO:0008047//enzym	GO:0006486//protein glycosylation;GO:0006487//protein N-linked glycos
GO:0004609//phosp	GO:0006646//phosphatidylethanolamine biosynthetic process;GO:000865
GO:0003824//catalyt	GO:0006778//porphyrin-containing compound metabolic process;GO:000
-	-
GO:0022857//transm	GO:0010628//positive regulation of gene expression;GO:0010629//negativ
GO:0016740//transfe	GO:0006629//lipid metabolic process;GO:0006659//phosphatidylserine bi
-	-
GO:0003874//6-pyru	GO:0006729//tetrahydrobiopterin biosynthetic process
GO:0003779//actin b	GO:0055013//cardiac muscle cell development
GO:0008289//lipid b	GO:0006869//lipid transport;GO:0042157//lipoprotein metabolic process
GO:0008170//N-me	GO:0001692//histamine metabolic process;GO:0001695//histamine catabo
GO:0017128//phosp	GO:0000045//autophagosome assembly;GO:0006869//lipid transport;GO:
-	-
GO:0005515//protei	-
GO:0004672//protei	GO:0006468//protein phosphorylation;GO:0008631//intrinsic apoptotic si
GO:0003677//DNA b	GO:0006383//transcription from RNA polymerase III promoter
-	-

GO:0005515//protei	GO:0007015//actin filament organization;GO:0045806//negative regulatio
-	-
-	GO:0007042//lysosomal lumen acidification;GO:0042176//regulation of pr
-	-
GO:0005515//protei	GO:0007043//cell-cell junction assembly;GO:0007155//cell adhesion;GO:0
-	-
GO:0043169//cation	GO:0007165//signal transduction;GO:0022603//regulation of anatomical s
-	GO:0030513//positive regulation of BMP signaling pathway
GO:0005254//chloric	GO:0006811//ion transport;GO:0006820//anion transport;GO:0006821//ch
GO:0005484//SNAP	GO:0006888//ER to Golgi vesicle-mediated transport;GO:0006903//vesicle
-	-
-	-
GO:0042577//lipid p	GO:0006644//phospholipid metabolic process
GO:0005509//calciur	GO:0015012//heparan sulfate proteoglycan biosynthetic process;GO:0030
GO:0005509//calciur	-
-	-
GO:0004896//cytoki	GO:0019221//cytokine-mediated signaling pathway;GO:0034342//respons
GO:0043022//riboso	GO:0006886//intracellular protein transport;GO:0030433//ER-associated u
-	-
-	-
GO:0004857//enzym	GO:0006071//glycerol metabolic process;GO:0006631//fatty acid metabol
GO:0008109//N-ace	GO:0006486//protein glycosylation;GO:0007179//transforming growth fac
GO:0004857//enzym	GO:0006071//glycerol metabolic process;GO:0006631//fatty acid metabol
-	-
GO:0003841//1-acyl	GO:0001676//long-chain fatty acid metabolic process;GO:0006629//lipid r
GO:0005515//protei	GO:0016567//protein ubiquitination
GO:0005216//ion ch	GO:0006811//ion transport;GO:0006813//potassium ion transport;GO:003
-	-
GO:0003836//beta-g	GO:0002319//memory B cell differentiation;GO:0006054//N-acetylneuram
GO:0005515//protei	GO:0044763//single-organism cellular process
GO:0009922//fatty a	GO:0019367//fatty acid elongation, saturated fatty acid;GO:0042761//very
GO:0001758//retinal	GO:0002072//optic cup morphogenesis involved in camera-type eye deve
-	-
-	GO:0006606//protein import into nucleus
GO:0022857//transm	GO:0055085//transmembrane transport
GO:0017075//syntax	-
GO:0003676//nuclei	GO:0006508//proteolysis;GO:0016032//viral process
GO:0005515//protei	GO:0098742//cell-cell adhesion via plasma-membrane adhesion molecule
-	-
GO:0003779//actin b	GO:0006351//transcription, DNA-templated;GO:0030032//lamellipodium
-	-
GO:0001540//beta-a	GO:0001932//regulation of protein phosphorylation;GO:0001935//endoth
GO:0016491//oxidor	GO:0009247//glycolipid biosynthetic process
GO:0042802//identic	GO:0030182//neuron differentiation;GO:0045893//positive regulation of tr
GO:0004499//N,N-d	-
GO:0002020//protea	GO:0010951//negative regulation of endopeptidase activity
-	-
GO:0004465//lipopr	GO:0006629//lipid metabolic process;GO:0006631//fatty acid metabolic p
-	-
GO:0016790//thioles	GO:0006637//acyl-CoA metabolic process
GO:0036094//small r	-
GO:0000977//RNA p	GO:0000122//negative regulation of transcription from RNA polymerase I
GO:0004672//protei	GO:0000086//G2/M transition of mitotic cell cycle;GO:0000278//mitotic ce
GO:0022857//transm	GO:0055085//transmembrane transport
GO:0003841//1-acyl	GO:0001819//positive regulation of cytokine production;GO:0006654//ph
GO:0003676//nuclei	GO:0015074//DNA integration;GO:0044260//cellular macromolecule meta
-	GO:0019941//modification-dependent protein catabolic process;GO:0030
-	-

GO:0005515//protei	-
GO:0016491//oxidor	-
GO:0004340//glucok	GO:0001678//cellular glucose homeostasis;GO:0005975//carbohydrate me
GO:0003676//nuclei	-
-	-
GO:0003677//DNA b	GO:0006334//nucleosome assembly;GO:0042742//defense response to ba
GO:0000166//nucleo	GO:0001954//positive regulation of cell-matrix adhesion;GO:0003323//typ
-	-
GO:0003779//actin b	GO:0055013//cardiac muscle cell development
GO:0008270//zinc io	GO:0009086//methionine biosynthetic process;GO:0009235//cobalamin m
-	-
GO:0005524//ATP bi	-
GO:0008047//enzym	GO:0006486//protein glycosylation;GO:0006487//protein N-linked glycosy
GO:0004862//cAMP	GO:0006469//negative regulation of protein kinase activity;GO:0051338//r
-	-
GO:0008194//UDP-g	-
-	-
-	-
-	-
-	GO:0006364//rRNA processing;GO:0009303//rRNA transcription
-	-
GO:0005515//protei	-
GO:0000166//nucleo	GO:0003014//renal system process;GO:0006811//ion transport;GO:000682
GO:0015125//bile ac	GO:0015721//bile acid and bile salt transport;GO:0032782//bile acid secre
-	-
-	-
GO:0003836//beta-g	GO:0002319//memory B cell differentiation;GO:0006054//N-acetylneuram
GO:0002020//protea	GO:0010951//negative regulation of endopeptidase activity
-	-
GO:0003779//actin b	GO:0051017//actin filament bundle assembly
GO:0005515//protei	GO:0043031//negative regulation of macrophage activation;GO:0050728/
GO:0005506//iron io	GO:0001949//sebaceous gland cell differentiation;GO:0006629//lipid meta
-	-
GO:0001849//compl	GO:0001878//response to yeast;GO:0008228//opsonization;GO:0030198//
GO:0042802//identic	GO:0007017//microtubule-based process
GO:0000062//fatty-a	GO:0036151//phosphatidylcholine acyl-chain remodeling;GO:1903060//n
-	-
-	-
GO:0036122//BMP b	GO:0007389//pattern specification process;GO:0010454//negative regulat
GO:0004857//enzym	GO:0001525//angiogenesis;GO:0043066//negative regulation of apoptoti
-	-
-	-
GO:0017128//phosp	GO:0000045//autophagosome assembly;GO:0006914//autophagy;GO:001
-	-
-	-
-	-
-	-
GO:0009922//fatty a	GO:0000038//very long-chain fatty acid metabolic process;GO:0006629//l
GO:0000250//lanost	GO:0006694//steroid biosynthetic process;GO:0006695//cholesterol biosy
GO:0001671//ATPas	-
GO:0022857//transm	GO:0055085//transmembrane transport
-	-
GO:0003847//1-alky	GO:0016042//lipid catabolic process
GO:0005515//protei	GO:0007015//actin filament organization;GO:0045806//negative regulatio
GO:0016628//oxidor	GO:0006695//cholesterol biosynthetic process;GO:0016126//sterol biosyn
GO:0005504//fatty a	GO:0015908//fatty acid transport;GO:0015909//long-chain fatty acid trans
GO:0000978//RNA p	GO:0001932//regulation of protein phosphorylation;GO:0006355//regulat
-	-

GO:0002039//p53 bi	GO:0006468//protein phosphorylation;GO:0008285//negative regulation c
GO:0005515//protei	-
GO:0004045//amino	-
GO:0001786//phosp	GO:0006910//phagocytosis, recognition;GO:0006911//phagocytosis, engu
GO:0008289//lipid b	GO:0006869//lipid transport;GO:0042157//lipoprotein metabolic process
GO:0003676//nuclei	-
GO:0005353//fructo	GO:0003044//regulation of systemic arterial blood pressure mediated by a
-	-
GO:0004843//thiol-c	GO:0000278//mitotic cell cycle;GO:0006281//DNA repair;GO:0006511//ub
GO:0016717//oxidor	GO:0006629//lipid metabolic process
GO:0005085//guany	GO:0050790//regulation of catalytic activity
GO:0008013//beta-c	GO:0007015//actin filament organization;GO:0007155//cell adhesion;GO:(
GO:0005319//lipid tr	GO:0006869//lipid transport;GO:0015914//phospholipid transport;GO:004
-	-
GO:0005515//protei	GO:0007596//blood coagulation;GO:0033627//cell adhesion mediated by
GO:0005319//lipid tr	GO:0006869//lipid transport
-	-
GO:0004103//cholin	GO:0006646//phosphatidylethanolamine biosynthetic process;GO:000665
GO:0017128//phosp	GO:0000045//autophagosome assembly;GO:0006869//lipid transport;GO:
GO:0003779//actin b	GO:0055013//cardiac muscle cell development
GO:0016491//oxidor	GO:0006666//3-keto-sphinganine metabolic process;GO:0030148//sphing
-	-
-	-
GO:0005484//SNAP	GO:0006888//ER to Golgi vesicle-mediated transport;GO:0006903//vesicle
GO:0005085//guany	GO:0006511//ubiquitin-dependent protein catabolic process;GO:0032012
GO:0002161//amino	-
GO:0008289//lipid b	GO:0010873//positive regulation of cholesterol esterification;GO:0010879/
GO:0003677//DNA b	-
GO:0005515//protei	GO:0046330//positive regulation of JNK cascade;GO:0090090//negative re
GO:0003677//DNA b	GO:0006334//nucleosome assembly;GO:0006352//DNA-templated transc
-	-
GO:0046873//metal	GO:0030001//metal ion transport
-	-
GO:0005515//protei	GO:0043254//regulation of protein complex assembly
-	-
GO:0004843//thiol-c	GO:0006511//ubiquitin-dependent protein catabolic process;GO:0007000
-	-
-	GO:0009966//regulation of signal transduction
GO:0003677//DNA b	-
-	GO:0009267//cellular response to starvation;GO:0030001//metal ion trans
GO:0004190//aspart	GO:0006508//proteolysis;GO:0019538//protein metabolic process
GO:0004930//G-pro	GO:0001764//neuron migration;GO:0007186//G-protein coupled recepto
GO:0005515//protei	GO:0000281//mitotic cytokinesis;GO:0014067//negative regulation of pho
GO:0000287//magne	GO:0006468//protein phosphorylation;GO:0030036//actin cytoskeleton or
-	-
GO:0015020//glucur	GO:0006486//protein glycosylation;GO:0015012//heparan sulfate proteog
GO:0005515//protei	GO:0090084//negative regulation of inclusion body assembly
GO:0000976//transc	GO:0000122//negative regulation of transcription from RNA polymerase I
-	GO:0009987//cellular process;GO:0044699//single-organism process
GO:0004672//protei	GO:0006468//protein phosphorylation;GO:0007169//transmembrane rece
GO:0016491//oxidor	GO:0009247//glycolipid biosynthetic process
GO:0022857//transm	GO:0055085//transmembrane transport
GO:0004089//carbo	GO:0002009//morphogenesis of an epithelium;GO:0046903//secretion
-	-
GO:0004452//isoper	GO:0008299//isoprenoid biosynthetic process
GO:0030145//mang	GO:0006508//proteolysis;GO:0019538//protein metabolic process
GO:0000976//transc	GO:0000122//negative regulation of transcription from RNA polymerase I
GO:0005515//protei	GO:0055085//transmembrane transport

GO:0003841//1-acyl	GO:0001819//positive regulation of cytokine production;GO:0006654//ph
GO:0051082//unfold	GO:0006457//protein folding
GO:0004725//protei	GO:0006470//protein dephosphorylation;GO:0016311//dephosphorylatio
-	-
GO:0003707//steroi	GO:0007340//acrosome reaction;GO:0009611//response to wounding;GO
GO:0016491//oxidor	GO:0006629//lipid metabolic process;GO:0008611//ether lipid biosyntheti
GO:0004496//meval	GO:0006695//cholesterol biosynthetic process;GO:0008299//isoprenoid b
GO:0005515//protei	-
-	GO:0030070//insulin processing;GO:0060628//regulation of ER to Golgi ve
-	-
GO:0008170//N-me	GO:0001692//histamine metabolic process;GO:0001695//histamine catab
-	-
GO:0042802//identi	GO:0007165//signal transduction;GO:0042981//regulation of apoptotic pr
GO:0000976//transc	GO:0002062//chondrocyte differentiation;GO:0006355//regulation of tran
GO:0004930//G-pro	GO:0007186//G-protein coupled receptor signaling pathway
GO:0003677//DNA b	-
GO:0005515//protei	-
-	-
GO:0016491//oxidor	-
-	-
-	GO:0006488//dolichol-linked oligosaccharide biosynthetic process
GO:0005085//guany	GO:0050790//regulation of catalytic activity
GO:0008047//enzym	GO:0006486//protein glycosylation;GO:0006487//protein N-linked glycos
GO:0005515//protei	GO:0001822//kidney development;GO:0002093//auditory receptor cell mo
GO:0004857//enzym	GO:0006629//lipid metabolic process;GO:0043086//negative regulation o
GO:0005509//calciu	GO:0006936//muscle contraction;GO:0030035//microspike assembly;GO:
GO:0003676//nuclei	GO:0006508//proteolysis
-	-
GO:0003676//nuclei	-
-	GO:0006886//intracellular protein transport;GO:0006890//retrograde vesi
GO:0000062//fatty-a	GO:0036151//phosphatidylcholine acyl-chain remodeling;GO:1903060//n
-	-
-	-
-	-
GO:0003779//actin b	GO:0051017//actin filament bundle assembly
-	-
-	-
-	GO:0001501//skeletal system development;GO:0006006//glucose metabo
-	GO:0006506//GPI anchor biosynthetic process
-	-
GO:0004609//phosp	GO:0006646//phosphatidylethanolamine biosynthetic process;GO:000865
-	-
GO:0045296//cadhe	GO:0007043//cell-cell junction assembly;GO:0060997//dendritic spine mo
GO:0008289//lipid b	GO:0015031//protein transport;GO:0019075//virus maturation;GO:004205
GO:0005515//protei	GO:0001822//kidney development;GO:0002093//auditory receptor cell mo
-	-
GO:0004672//protei	GO:0001934//positive regulation of protein phosphorylation;GO:0006468/
GO:0022857//transm	GO:0055085//transmembrane transport
GO:0008170//N-me	GO:0001692//histamine metabolic process;GO:0001695//histamine catab
GO:0003874//6-pyr	GO:0006729//tetrahydrobiopterin biosynthetic process
-	-
GO:0004857//enzym	GO:0001525//angiogenesis;GO:0043066//negative regulation of apoptoti
GO:0017128//phosp	GO:0000045//autophagosome assembly;GO:0006914//autophagy;GO:001
GO:0005515//protei	GO:0055085//transmembrane transport
GO:0003989//acetyl-	GO:0001894//tissue homeostasis;GO:0006084//acetyl-CoA metabolic pro
-	-
GO:0003824//cataly	GO:0009226//nucleotide-sugar biosynthetic process;GO:0010595//positiv
GO:0003779//actin b	GO:0006836//neurotransmitter transport;GO:0015844//monoamine trans

-	-
GO:0036122//BMP b	GO:0007389//pattern specification process;GO:0010454//negative regulat
-	-
GO:0005515//protei	GO:0006355//regulation of transcription, DNA-templated;GO:0006805//x
-	-
GO:0003676//nuclei	-
GO:0005113//patche	GO:0001501//skeletal system development;GO:0001503//ossification;GO:(
-	-
-	GO:0098609//cell-cell adhesion
GO:0016491//oxidor	GO:0006666//3-keto-sphinganine metabolic process;GO:0030148//sphing
-	-
GO:0016740//transfe	GO:0045723//positive regulation of fatty acid biosynthetic process
GO:0003682//chrom	GO:0000122//negative regulation of transcription from RNA polymerase I
GO:0003824//catalyt	GO:0006207//'de novo' pyrimidine nucleobase biosynthetic process
-	GO:0000463//maturation of LSU-rRNA from tricistronic rRNA transcript (S
GO:0046961//protor	GO:0006811//ion transport;GO:1902600//hydrogen ion transmembrane tr
GO:0005515//protei	GO:0006888//ER to Golgi vesicle-mediated transport;GO:0016192//vesicle
GO:0008109//N-ace	GO:0006486//protein glycosylation;GO:0007179//transforming growth fac
-	-
GO:0017128//phosp	GO:0000045//autophagosome assembly;GO:0006914//autophagy;GO:001
GO:0015377//cation	GO:0006811//ion transport;GO:0006884//cell volume homeostasis;GO:005
GO:0005515//protei	-
GO:0005515//protei	GO:0016567//protein ubiquitination
GO:0016290//palmit	GO:0006637//acyl-CoA metabolic process;GO:0016559//peroxisome fissic
-	-
GO:0004674//protei	GO:0006468//protein phosphorylation
GO:0016717//oxidor	GO:0006629//lipid metabolic process
GO:0004045//amino	-
-	-
GO:0022857//transm	GO:0055085//transmembrane transport
-	-
-	-
-	-
-	-
GO:0009922//fatty a	GO:0019367//fatty acid elongation, saturated fatty acid;GO:0042761//very
GO:0004857//enzym	GO:0006071//glycerol metabolic process;GO:0006631//fatty acid metabol
GO:0008271//secon	GO:0008272//sulfate transport;GO:0055085//transmembrane transport
GO:0002020//protea	GO:0010951//negative regulation of endopeptidase activity
-	-
GO:0005085//guany	GO:0035556//intracellular signal transduction
-	-
GO:0022857//transm	GO:0055085//transmembrane transport
-	-
GO:0005085//guany	GO:0050790//regulation of catalytic activity
GO:0022857//transm	GO:0055085//transmembrane transport
GO:0001849//compl	GO:0001878//response to yeast;GO:0008228//opsonization;GO:0030198//
GO:0022857//transm	GO:0055085//transmembrane transport
GO:0004197//cystei	GO:0002931//response to ischemia;GO:0006508//proteolysis;GO:0006511
GO:0008378//galact	GO:0001576//globoside biosynthetic process;GO:0007009//plasma memb
-	-
GO:0005319//lipid tr	GO:0006869//lipid transport;GO:0015914//phospholipid transport;GO:004
GO:0019855//calciu	GO:0006851//mitochondrial calcium ion transport;GO:0051560//mitochor
-	GO:0007155//cell adhesion
-	-
GO:0015125//bile ac	GO:0015721//bile acid and bile salt transport;GO:0032782//bile acid secre
-	GO:0009267//cellular response to starvation;GO:0030001//metal ion trans
-	-
GO:0003924//GTPas	GO:0007165//signal transduction;GO:0007186//G-protein coupled recept

GO:0060090//bindin	GO:0030433//ER-associated ubiquitin-dependent protein catabolic proce
-	-
GO:0005515//protei	-
GO:0016787//hydrol	GO:0006629//lipid metabolic process;GO:0007216//G-protein coupled glu
-	-
GO:0015020//glucur	GO:0006486//protein glycosylation;GO:0015012//heparan sulfate proteog
GO:0000976//transc	GO:0000122//negative regulation of transcription from RNA polymerase I
-	GO:0006629//lipid metabolic process
GO:0003676//nuclei	GO:0006388//tRNA splicing, via endonucleolytic cleavage and ligation
GO:0005515//protei	-
GO:0005319//lipid tr	GO:0006629//lipid metabolic process;GO:0006642//triglyceride mobilizati
GO:0000248//C-5 st	GO:0008610//lipid biosynthetic process;GO:0016126//sterol biosynthetic p
GO:0005515//protei	-
GO:0005515//protei	GO:0090084//negative regulation of inclusion body assembly
-	-
GO:0002039//p53 bi	GO:0006468//protein phosphorylation;GO:0008285//negative regulation c
GO:0000166//nucleo	-
GO:0016798//hydrol	GO:0008284//positive regulation of cell proliferation;GO:0030198//extrace
GO:0004452//isoper	GO:0008299//isoprenoid biosynthetic process
-	-
GO:0003810//protei	GO:0018149//peptide cross-linking
-	GO:0008154//actin polymerization or depolymerization;GO:0030048//acti
-	-
GO:0004310//farnes	GO:0008610//lipid biosynthetic process;GO:0009058//biosynthetic proces
GO:0000976//transc	GO:0006357//regulation of transcription from RNA polymerase II promote
GO:0004222//metall	GO:0001542//ovulation from ovarian follicle;GO:0001822//kidney develop
GO:0022857//transm	GO:0055085//transmembrane transport
-	-
GO:0003677//DNA b	GO:0006334//nucleosome assembly;GO:0042742//defense response to ba
-	-
GO:0016757//transfe	GO:0002121//inter-male aggressive behavior;GO:0042403//thyroid hormo
-	-
-	-
GO:0008324//cation	GO:0006812//cation transport
GO:0005515//protei	-
GO:0005515//protei	GO:0007596//blood coagulation;GO:0033627//cell adhesion mediated by
GO:0004930//G-pro	GO:0007186//G-protein coupled receptor signaling pathway;GO:0007200
GO:0000976//transc	GO:0002062//chondrocyte differentiation;GO:0006355//regulation of tran
GO:0005515//protei	GO:0043065//positive regulation of apoptotic process;GO:0044790//nega
-	GO:0006886//intracellular protein transport
GO:0003953//NAD+	GO:0001952//regulation of cell-matrix adhesion;GO:0008284//positive re
-	-
GO:0008289//lipid b	GO:0015031//protein transport;GO:0019075//virus maturation;GO:004205
-	GO:0006629//lipid metabolic process
-	-
GO:0004672//protei	GO:0006468//protein phosphorylation;GO:0007169//transmembrane rece
GO:0005515//protei	GO:0055085//transmembrane transport
GO:0003779//actin b	GO:0051017//actin filament bundle assembly
-	-
GO:0000976//transc	GO:0000122//negative regulation of transcription from RNA polymerase I
GO:0003676//nuclei	GO:0006508//proteolysis
GO:0005515//protei	-
GO:0000977//RNA p	GO:0000122//negative regulation of transcription from RNA polymerase I
-	-
-	-
GO:0017128//phosp	GO:0000045//autophagosome assembly;GO:0006869//lipid transport;GO:
-	-
-	GO:1901031//regulation of response to reactive oxygen species

-	-
-	-
-	-
GO:0004674//protei	GO:0006468//protein phosphorylation
GO:0008271//secon	GO:0008272//sulfate transport;GO:0055085//transmembrane transport
-	-
-	-
GO:0004047//amino	GO:0019464//glycine decarboxylation via glycine cleavage system
-	-
GO:0005509//calciu	GO:0006936//muscle contraction;GO:0030035//microspike assembly;GO:0
-	GO:0030070//insulin processing;GO:0060628//regulation of ER to Golgi ve
-	GO:0006506//GPI anchor biosynthetic process
-	-
-	-
GO:0002020//protea	GO:0010951//negative regulation of endopeptidase activity
GO:0004866//endop	GO:0010951//negative regulation of endopeptidase activity
-	-
GO:0004857//enzym	GO:0006071//glycerol metabolic process;GO:0006631//fatty acid metabol
GO:0000287//magn	GO:0006468//protein phosphorylation;GO:0007346//regulation of mitotic
GO:0005515//protei	GO:0006355//regulation of transcription, DNA-templated;GO:0006805//x
GO:0005319//lipid t	GO:0006869//lipid transport
GO:0003841//1-acyl	-
-	-
-	-
GO:0042577//lipid p	GO:0006644//phospholipid metabolic process
-	GO:0000463//maturation of LSU-rRNA from tricistronic rRNA transcript (S
-	-
GO:0008517//folic a	GO:0015884//folic acid transport;GO:0051180//vitamin transport;GO:0051
GO:0005540//hyalur	GO:0007155//cell adhesion
GO:0004252//serine	GO:0006508//proteolysis;GO:0016485//protein processing
-	-
GO:0003756//protei	-
-	-
GO:0016491//oxidor	GO:0006629//lipid metabolic process;GO:0008611//ether lipid biosyntheti
-	-
GO:0004499//N,N-d	-
GO:0003779//actin b	GO:0007030//Golgi organization;GO:0007032//endosome organization;G
-	-
-	-
GO:0004843//thiol-	GO:0000278//mitotic cell cycle;GO:0006281//DNA repair;GO:0006511//ub
-	-
GO:0005515//protei	GO:0044763//single-organism cellular process
GO:1990756//protei	GO:0015031//protein transport;GO:0016567//protein ubiquitination;GO:0
-	GO:0070453//regulation of heme biosynthetic process
-	-
GO:0004857//enzym	GO:0006629//lipid metabolic process;GO:0043086//negative regulation o
GO:0022857//transm	GO:0055085//transmembrane transport
GO:0004190//aspart	GO:0006508//proteolysis;GO:0019538//protein metabolic process
-	-
-	-
GO:0046873//metal	GO:0030001//metal ion transport
-	-
GO:0004420//hydro	GO:0006695//cholesterol biosynthetic process;GO:0008299//isoprenoid b
GO:0004930//G-pro	GO:0007186//G-protein coupled receptor signaling pathway;GO:0007200
-	-
GO:0003677//DNA b	-
GO:0003824//catalyt	GO:0006778//porphyrin-containing compound metabolic process;GO:000
-	GO:0000077//DNA damage checkpoint

GO:0005515//protei	-
GO:0002161//amino	-
-	GO:0006629//lipid metabolic process
GO:0004114//3',5'-c	GO:0007165//signal transduction
GO:0004222//metall	GO:0001542//ovulation from ovarian follicle;GO:0001822//kidney develop
GO:0001849//compl	GO:0001878//response to yeast;GO:0008228//opsonization;GO:0030198//
-	-
-	-
-	-
-	-
-	-
GO:0015125//bile ac	GO:0015721//bile acid and bile salt transport;GO:0032782//bile acid secre
GO:0005515//protei	-
GO:0004197//cystei	GO:0002931//response to ischemia;GO:0006508//proteolysis;GO:0006511
GO:0016922//ligand	GO:0045944//positive regulation of transcription from RNA polymerase II
GO:0005085//guany	GO:0050790//regulation of catalytic activity
GO:0022857//transm	GO:0055085//transmembrane transport
GO:0003677//DNA b	GO:0006383//transcription from RNA polymerase III promoter
GO:0005515//protei	GO:0006884//cell volume homeostasis;GO:0007165//signal transduction;(
GO:0003779//actin b	GO:0055013//cardiac muscle cell development
GO:0005319//lipid tr	GO:0006629//lipid metabolic process;GO:0006642//triglyceride mobilizati
-	-
-	-
-	GO:0007399//nervous system development
GO:0002020//protea	GO:0010951//negative regulation of endopeptidase activity
GO:0004623//phosp	GO:0006644//phospholipid metabolic process;GO:0016042//lipid cataboli
GO:0003841//1-acyl	GO:0001819//positive regulation of cytokine production;GO:0006654//ph
GO:0005113//patche	GO:0001501//skeletal system development;GO:0001503//ossification;GO:(
GO:0000977//RNA p	GO:0000122//negative regulation of transcription from RNA polymerase I
-	-
GO:0004672//protei	GO:0001501//skeletal system development;GO:0006468//protein phospho
-	-
-	-
-	GO:0006887//exocytosis;GO:0015031//protein transport
GO:0000976//transc	GO:0000122//negative regulation of transcription from RNA polymerase I
-	-
GO:0005319//lipid tr	GO:0006869//lipid transport
GO:0008195//phosp	GO:0006357//regulation of transcription from RNA polymerase II promote
-	-
GO:0000253//3-ketc	GO:0006695//cholesterol biosynthetic process;GO:0006703//estrogen bio
GO:0046961//protor	GO:0006811//ion transport;GO:1902600//hydrogen ion transmembrane tr
GO:0004553//hydrol	GO:0005975//carbohydrate metabolic process
GO:0003847//1-alky	GO:0016042//lipid catabolic process
GO:0004672//protei	GO:0006468//protein phosphorylation;GO:0007169//transmembrane rece
GO:0003676//nuclei	GO:0006508//proteolysis;GO:0016032//viral process
GO:0022857//transm	GO:0055085//transmembrane transport
-	-
GO:0043022//riboso	GO:0006886//intracellular protein transport;GO:0030433//ER-associated u
GO:0004674//protei	GO:0006468//protein phosphorylation
-	-
-	-
GO:0017128//phosp	GO:0000045//autophagosome assembly;GO:0006914//autophagy;GO:001
-	-
GO:0005515//protei	GO:0055085//transmembrane transport
GO:0016787//hydrol	GO:0006470//protein dephosphorylation;GO:0006805//xenobiotic metabo
GO:0004310//farnes	GO:0008610//lipid biosynthetic process;GO:0009058//biosynthetic proces
-	-
GO:0003779//actin b	GO:0051017//actin filament bundle assembly

-	-
-	-
GO:0003779//actin b	GO:0051017//actin filament bundle assembly
GO:0001968//fibron	GO:0001933//negative regulation of protein phosphorylation;GO:000716
GO:0019902//phosp	GO:0006511//ubiquitin-dependent protein catabolic process;GO:0016567
GO:0003824//catalyt	GO:0009226//nucleotide-sugar biosynthetic process;GO:0010595//positiv
-	-
-	-
-	-
GO:0005085//guany	GO:0035556//intracellular signal transduction
-	-
-	GO:0070979//protein K11-linked ubiquitination
GO:0004888//transm	GO:0006811//ion transport;GO:0034220//ion transmembrane transport
GO:0005515//protei	GO:0000281//mitotic cytokinesis;GO:0007019//microtubule depolymeriza
GO:0004857//enzym	GO:0001525//angiogenesis;GO:0043066//negative regulation of apoptoti
GO:0005085//guany	GO:0007264//small GTPase mediated signal transduction
GO:0005515//protei	-
-	-
GO:0003682//chrom	GO:0000122//negative regulation of transcription from RNA polymerase I
GO:0035256//G-pro	GO:0003009//skeletal muscle contraction;GO:0007216//G-protein couple
GO:0001605//adren	GO:0001525//angiogenesis;GO:0001570//vasculogenesis;GO:0002040//sp
-	GO:0098609//cell-cell adhesion
GO:0005524//ATP b	GO:0009235//cobalamin metabolic process;GO:0015889//cobalamin trans
-	-
GO:0004000//adeno	GO:0000255//allantoin metabolic process;GO:0001666//response to hypo
-	-
GO:0051015//actin f	GO:0030866//cortical actin cytoskeleton organization;GO:0045214//sarco
GO:0003676//nuclei	GO:0006388//tRNA splicing, via endonucleolytic cleavage and ligation
GO:0005515//protei	-
GO:0016491//oxidor	GO:0006666//3-keto-sphinganine metabolic process;GO:0030148//sphin
GO:0001604//uroter	GO:0007165//signal transduction;GO:0007186//G-protein coupled recept
GO:0003676//nuclei	GO:0006508//proteolysis;GO:0008152//metabolic process;GO:0016032//v
GO:0005227//calciu	GO:0001581//detection of chemical stimulus involved in sensory percepti
-	-
GO:0003676//nuclei	GO:0006278//RNA-dependent DNA replication;GO:0044260//cellular ma
GO:0004467//long-	GO:0006631//fatty acid metabolic process
-	-
GO:0005509//calciu	GO:0007268//synaptic transmission;GO:0035556//intracellular signal trans
GO:0005515//protei	-
GO:0004340//gluco	GO:0001678//cellular glucose homeostasis;GO:0005975//carbohydrate m
-	-
GO:0019855//calciu	GO:0006851//mitochondrial calcium ion transport;GO:0051560//mitochon
GO:0005524//ATP b	GO:0009235//cobalamin metabolic process;GO:0015889//cobalamin trans
-	-
GO:0016717//oxidor	GO:0006629//lipid metabolic process
-	-
-	GO:0030070//insulin processing;GO:0060628//regulation of ER to Golgi v
-	-
GO:0008271//secon	GO:0008272//sulfate transport;GO:0055085//transmembrane transport
-	-
GO:0035256//G-pro	GO:0003009//skeletal muscle contraction;GO:0007216//G-protein couple
GO:0008194//UDP-	-
-	-
GO:0003676//nuclei	GO:0006278//RNA-dependent DNA replication;GO:0006310//DNA recom
GO:0002020//protea	GO:0010951//negative regulation of endopeptidase activity
-	GO:0006506//GPI anchor biosynthetic process
GO:0005085//guany	GO:0007264//small GTPase mediated signal transduction
-	-

-	-
-	-
GO:0003779//actin b	GO:0007030//Golgi organization;GO:0007032//endosome organization;G
-	-
-	-
-	-
GO:0000166//nucleo	GO:0000122//negative regulation of transcription from RNA polymerase I
-	GO:0007165//signal transduction;GO:0070507//regulation of microtubule
-	-
GO:0046873//metal	GO:0030001//metal ion transport
GO:0000166//nucleo	GO:0070588//calcium ion transmembrane transport
-	GO:0006658//phosphatidylserine metabolic process;GO:0006665//sphing
GO:0001968//fibron	GO:0001933//negative regulation of protein phosphorylation;GO:0007162
GO:0005515//protei	GO:0001558//regulation of cell growth
-	-
GO:0003677//DNA b	GO:0006281//DNA repair;GO:0006974//cellular response to DNA damage
GO:0005515//protei	-
GO:0000166//nucleo	GO:0006897//endocytosis;GO:0006898//receptor-mediated endocytosis;G
-	GO:0001656//metanephros development;GO:0001658//branching involve
GO:0004386//helicas	-
GO:0016787//hydrol	GO:0006629//lipid metabolic process;GO:0007216//G-protein coupled glu
GO:0003677//DNA b	-
-	-
GO:0015020//glucur	GO:0006486//protein glycosylation;GO:0015012//heparan sulfate proteog
-	-
GO:0000250//lanost	GO:0006694//steroid biosynthetic process;GO:0006695//cholesterol biosy
GO:0004672//protei	GO:0001501//skeletal system development;GO:0006468//protein phospho
-	-
GO:0004175//endop	GO:0006508//proteolysis;GO:0007354//zygotic determination of anterior/
GO:0002161//amino	-
-	-
GO:0005515//protei	-
-	GO:0007042//lysosomal lumen acidification;GO:0042176//regulation of pr
GO:0008047//enzym	GO:0006486//protein glycosylation;GO:0006487//protein N-linked glycos
-	GO:0006887//exocytosis;GO:0015031//protein transport
GO:0004672//protei	GO:0006468//protein phosphorylation;GO:0007169//transmembrane rece
GO:0004672//protei	GO:0001501//skeletal system development;GO:0006468//protein phospho
GO:0005319//lipid tr	GO:0006869//lipid transport;GO:0015914//phospholipid transport;GO:004
GO:0008022//protei	GO:0007099//centriole replication;GO:0010457//centriole-centriole cohes
GO:0016757//transfe	GO:0002121//inter-male aggressive behavior;GO:0042403//thyroid horm
GO:0003735//structu	GO:0006412//translation;GO:0006919//activation of cysteine-type endope
GO:0000976//transc	GO:0000122//negative regulation of transcription from RNA polymerase I
GO:0004842//ubiqui	GO:0000209//protein polyubiquitination;GO:0006915//apoptotic process;
GO:0017128//phosp	GO:0000045//autophagosome assembly;GO:0006869//lipid transport;GO:
-	-
GO:0004843//thiol-c	GO:0006511//ubiquitin-dependent protein catabolic process;GO:0007000
GO:0003677//DNA b	GO:0006383//transcription from RNA polymerase III promoter
GO:0003676//nuclei	GO:0006508//proteolysis;GO:0016032//viral process
-	-
-	-
GO:0005515//protei	GO:0046330//positive regulation of JNK cascade;GO:0090090//negative re
GO:0000287//magne	GO:0006468//protein phosphorylation;GO:0030036//actin cytoskeleton or
GO:0004843//thiol-c	GO:0006511//ubiquitin-dependent protein catabolic process;GO:0016579
GO:0000977//RNA p	GO:0000122//negative regulation of transcription from RNA polymerase I
GO:0003874//6-pyru	GO:0006729//tetrahydrobiopterin biosynthetic process
GO:0000166//nucleo	GO:0006468//protein phosphorylation;GO:0010976//positive regulation o
GO:0005515//protei	-
GO:0004623//phosp	GO:0006644//phospholipid metabolic process;GO:0016042//lipid cataboli

GO:0015175//neutra	GO:0015804//neutral amino acid transport;GO:0042941//D-alanine transp
GO:0008236//serine	GO:0006508//proteolysis
GO:0003779//actin b	GO:0051017//actin filament bundle assembly
GO:0022857//transm	GO:0055085//transmembrane transport
GO:0016740//transfe	GO:0045723//positive regulation of fatty acid biosynthetic process
-	-
GO:0005227//calciu	GO:0006812//cation transport
GO:0005319//lipid tr	GO:0006869//lipid transport
GO:0003677//DNA b	-
GO:0001758//retinal	GO:0002072//optic cup morphogenesis involved in camera-type eye deve
GO:0000166//nucleo	GO:0006468//protein phosphorylation;GO:0010976//positive regulation o
GO:0003847//1-alky	GO:0016042//lipid catabolic process
GO:0004843//thiol-c	GO:0006511//ubiquitin-dependent protein catabolic process;GO:0016579
-	-
-	-
GO:0005178//integri	GO:0000902//cell morphogenesis;GO:0006954//inflammatory response;G
GO:0005515//protei	GO:0001938//positive regulation of endothelial cell proliferation;GO:0014
GO:0005515//protei	GO:0044763//single-organism cellular process
GO:0005515//protei	GO:0002091//negative regulation of receptor internalization;GO:0042058/
GO:0008233//peptid	GO:0006508//proteolysis
GO:0005524//ATP bi	GO:0009235//cobalamin metabolic process;GO:0015889//cobalamin trans
GO:0004489//methy	GO:0001843//neural tube closure;GO:0006555//methionine metabolic pro
-	-
GO:0001604//uroter	GO:0007165//signal transduction;GO:0007186//G-protein coupled recept
GO:0005515//protei	-
-	-
GO:0005515//protei	GO:0055085//transmembrane transport
-	GO:0007042//lysosomal lumen acidification;GO:0042176//regulation of pr
GO:0003779//actin b	GO:0030835//negative regulation of actin filament depolymerization
GO:0042802//identic	-
-	-
GO:0000166//nucleo	-
GO:0004888//transm	GO:0007186//G-protein coupled receptor signaling pathway;GO:0009994
GO:0005254//chloric	GO:0006811//ion transport;GO:0006820//anion transport;GO:0006821//ch
GO:0000166//nucleo	GO:0000045//autophagosome assembly;GO:0002026//regulation of the fo
-	GO:0000463//maturation of LSU-rRNA from tricistronic rRNA transcript (S
-	-
GO:0005509//calciu	-
GO:0042577//lipid p	GO:0006644//phospholipid metabolic process
-	-
-	-
-	GO:0070453//regulation of heme biosynthetic process
-	-
GO:0005085//guany	GO:0007264//small GTPase mediated signal transduction
GO:0003824//catalyt	GO:0006777//Mo-molybdopterin cofactor biosynthetic process;GO:00435
GO:0005509//calciu	GO:0006936//muscle contraction;GO:0030035//microspike assembly;GO:0
GO:0005515//protei	-
-	-
-	-
GO:0003676//nuclei	GO:0006278//RNA-dependent DNA replication;GO:0006310//DNA recom
GO:0003676//nuclei	-
GO:0035256//G-pro	GO:0003009//skeletal muscle contraction;GO:0007216//G-protein couple
-	-
-	GO:0006658//phosphatidylserine metabolic process;GO:0006665//sphing
GO:0003677//DNA b	GO:0006334//nucleosome assembly;GO:0016584//nucleosome positionin
GO:0008270//zinc io	GO:0009086//methionine biosynthetic process;GO:0009235//cobalamin m
GO:0000166//nucleo	GO:0070588//calcium ion transmembrane transport
GO:0042802//identic	GO:0019216//regulation of lipid metabolic process;GO:0055088//lipid hor

-	-
-	GO:0001501//skeletal system development;GO:0006006//glucose metabo
-	-
GO:0005324//long-c	GO:0006469//negative regulation of protein kinase activity;GO:0009617//r
GO:0003779//actin b	GO:0055013//cardiac muscle cell development
GO:0003677//DNA b	-
-	-
GO:0003677//DNA b	-
GO:0003824//catalyt	GO:0006778//porphyrin-containing compound metabolic process;GO:000
-	-
GO:0004386//helicas	-
GO:0030246//carboh	GO:0006897//endocytosis
GO:0003989//acetyl-	GO:0001894//tissue homeostasis;GO:0006084//acetyl-CoA metabolic proc
GO:0001968//fibron	GO:0001933//negative regulation of protein phosphorylation;GO:0007162
-	-
-	-
GO:0000976//transc	GO:0006357//regulation of transcription from RNA polymerase II promote
GO:0004843//thiol-c	GO:0000278//mitotic cell cycle;GO:0006281//DNA repair;GO:0006511//ub
-	-
GO:0005319//lipid tr	GO:0006629//lipid metabolic process;GO:0006642//triglyceride mobilizati
-	GO:0006887//exocytosis;GO:0015031//protein transport
GO:0016757//transfe	GO:0002121//inter-male aggressive behavior;GO:0042403//thyroid horm
GO:0001968//fibron	GO:0010811//positive regulation of cell-substrate adhesion;GO:0030198//
GO:0003713//transc	GO:0006355//regulation of transcription, DNA-templated
GO:0016787//hydrol	GO:0006470//protein dephosphorylation;GO:0006805//xenobiotic metab
GO:0004467//long-c	GO:0006631//fatty acid metabolic process
GO:0001786//phosp	-
-	-
GO:0003676//nuclei	GO:0006508//proteolysis;GO:0008152//metabolic process;GO:0016032//v
-	GO:0006887//exocytosis;GO:0015031//protein transport
GO:0003677//DNA b	GO:0006334//nucleosome assembly
GO:0001671//ATPas	GO:0001933//negative regulation of protein phosphorylation;GO:003043
GO:0000250//lanost	GO:0006694//steroid biosynthetic process;GO:0006695//cholesterol biosy
GO:0008195//phosp	GO:0006720//isoprenoid metabolic process;GO:0018342//protein prenyla
GO:0000981//RNA p	GO:0000122//negative regulation of transcription from RNA polymerase I
-	-
-	-
GO:0008235//metall	GO:0017186//peptidyl-pyroglutamic acid biosynthetic process, using glut
GO:0016757//transfe	GO:0002121//inter-male aggressive behavior;GO:0042403//thyroid horm
GO:0003676//nuclei	GO:0006278//RNA-dependent DNA replication;GO:0015074//DNA integr
GO:0003676//nuclei	GO:0043488//regulation of mRNA stability
GO:0005515//protei	GO:0000281//mitotic cytokinesis;GO:0007019//microtubule depolymeriza
GO:0000246//delta2	GO:0006695//cholesterol biosynthetic process;GO:0006979//response to
-	-
-	GO:1901031//regulation of response to reactive oxygen species
GO:0008236//serine	GO:0006508//proteolysis
GO:0005506//iron ic	GO:0001949//sebaceous gland cell differentiation;GO:0006629//lipid met
GO:0019901//protei	GO:0030334//regulation of cell migration;GO:0034446//substrate adhesio
-	-
-	GO:0007042//lysosomal lumen acidification;GO:0042176//regulation of pr
GO:0003677//DNA b	GO:0006383//transcription from RNA polymerase III promoter
-	-
GO:0005518//collag	GO:0007605//sensory perception of sound;GO:0008360//regulation of cel
GO:0000287//magne	GO:0006114//glycerol biosynthetic process;GO:0006650//glycerophosph
-	-
GO:0005515//protei	GO:0000281//mitotic cytokinesis;GO:0007019//microtubule depolymeriza
-	GO:0018095//protein polyglutamylation
-	-

GO:0019902//phosp	GO:0006511//ubiquitin-dependent protein catabolic process;GO:0016567
GO:0001540//beta-	GO:0006511//ubiquitin-dependent protein catabolic process;GO:0006516
-	-
-	GO:0070979//protein K11-linked ubiquitination
-	-
GO:0005085//guany	GO:0007264//small GTPase mediated signal transduction
-	-
-	-
GO:0000334//3-hyd	GO:0009435//NAD biosynthetic process;GO:0010043//response to zinc io
GO:0004869//cystei	GO:0001503//ossification;GO:0006953//acute-phase response;GO:00305
GO:0005085//guany	GO:0007264//small GTPase mediated signal transduction;GO:0034976//re
GO:0004843//thiol-	GO:0006511//ubiquitin-dependent protein catabolic process;GO:0016579
GO:0005515//protei	GO:0001558//regulation of cell growth
GO:0003847//1-alky	GO:0016042//lipid catabolic process
GO:0004620//phosp	GO:0009395//phospholipid catabolic process;GO:0030336//negative regu
GO:0000166//nucle	GO:0006468//protein phosphorylation;GO:0010976//positive regulation o
-	-
GO:0005515//protei	-
GO:0005504//fatty a	GO:0015908//fatty acid transport;GO:0015909//long-chain fatty acid trans
GO:0005524//ATP b	GO:0035499//carnosine biosynthetic process
-	-
GO:0004175//endop	GO:0002218//activation of innate immune response;GO:0006508//proteol
GO:0003676//nuclei	GO:0006388//tRNA splicing, via endonucleolytic cleavage and ligation
GO:0008271//secon	GO:0008272//sulfate transport;GO:0055085//transmembrane transport
GO:0005515//protei	GO:0044763//single-organism cellular process
-	-
-	-
-	-
GO:0000248//C-5 st	GO:0008610//lipid biosynthetic process;GO:0016126//sterol biosynthetic
-	-
GO:0004930//G-pro	GO:0007165//signal transduction;GO:0007186//G-protein coupled recept
GO:0001758//retinal	GO:0002072//optic cup morphogenesis involved in camera-type eye dev
-	-
GO:0004888//transm	GO:0007186//G-protein coupled receptor signaling pathway;GO:0009994
-	GO:0009966//regulation of signal transduction;GO:0016055//Wnt signalin
GO:0016491//oxido	GO:0009247//glycolipid biosynthetic process
GO:0005509//calciu	GO:0007268//synaptic transmission;GO:0035556//intracellular signal trans
GO:0008138//protei	GO:0006355//regulation of transcription, DNA-templated;GO:0006470//p
-	-
GO:0005524//ATP b	GO:0009235//cobalamin metabolic process;GO:0015889//cobalamin trans
-	GO:0016192//vesicle-mediated transport
GO:0008236//serine	GO:0006508//proteolysis
-	-
GO:0005515//protei	GO:0006888//ER to Golgi vesicle-mediated transport
-	-
GO:0005515//protei	GO:0006355//regulation of transcription, DNA-templated;GO:0006805//x
GO:0001786//phosp	GO:0006910//phagocytosis, recognition;GO:0006911//phagocytosis, engu
GO:0003824//cataly	GO:0006777//Mo-molybdopterin cofactor biosynthetic process;GO:00435
-	-
GO:0002161//amino	-
-	-
-	-
GO:0004860//protei	GO:0007616//long-term memory;GO:0050729//positive regulation of infl
-	-
GO:0004585//ornith	GO:0000050//urea cycle;GO:0006520//cellular amino acid metabolic proc
-	-
GO:0009922//fatty a	GO:0000038//very long-chain fatty acid metabolic process;GO:0006629//l
-	-

-	GO:0070453//regulation of heme biosynthetic process
-	-
-	-
GO:0005515//protei	GO:0007165//signal transduction
GO:0008289//lipid b	GO:0015031//protein transport;GO:0019075//virus maturation;GO:004205
-	-
-	GO:0007098//centrosome cycle;GO:0035020//regulation of Rac protein si
GO:0005515//protei	GO:0001822//kidney development;GO:0002093//auditory receptor cell m
GO:0022892//substr	GO:0044765//single-organism transport
GO:0008022//protei	GO:0007026//negative regulation of microtubule depolymerization;GO:00
GO:0003779//actin b	GO:0051017//actin filament bundle assembly
GO:0046961//proton	GO:0006811//ion transport;GO:1902600//hydrogen ion transmembrane tr
-	-
-	-
GO:0003735//structu	GO:0006412//translation;GO:0006919//activation of cysteine-type endope
-	-
-	-
GO:0016757//transfe	GO:0002121//inter-male aggressive behavior;GO:0042403//thyroid horm
GO:0003677//DNA b	GO:0009646//response to absence of light;GO:0018298//protein-chromo
-	-
-	GO:0008154//actin polymerization or depolymerization;GO:0030048//acti
GO:0016491//oxidor	GO:0006629//lipid metabolic process;GO:0006631//fatty acid metabolic p
-	-
GO:0005543//phosp	GO:0048260//positive regulation of receptor-mediated endocytosis;GO:0
-	-
GO:0005540//hyalur	GO:0007155//cell adhesion
GO:0008170//N-me	GO:0001692//histamine metabolic process;GO:0001695//histamine catab
GO:0042802//identi	GO:0019216//regulation of lipid metabolic process;GO:0055088//lipid ho
GO:0004930//G-pro	GO:0007186//G-protein coupled receptor signaling pathway
GO:0008047//enzym	GO:0006486//protein glycosylation;GO:0006487//protein N-linked glyco
GO:0004386//helica	-
GO:0003874//6-pyr	GO:0006729//tetrahydrobiopterin biosynthetic process
GO:0003682//chrom	GO:0015908//fatty acid transport;GO:0043066//negative regulation of ap
-	-
GO:0004672//protei	GO:0001501//skeletal system development;GO:0006468//protein phosph
-	GO:0006887//exocytosis;GO:0015031//protein transport
GO:0000166//nucle	GO:0070588//calcium ion transmembrane transport
GO:0003779//actin b	GO:0055013//cardiac muscle cell development
-	GO:0007507//heart development
-	GO:0030070//insulin processing;GO:0060628//regulation of ER to Golgi v
GO:0003676//nuclei	GO:0006508//proteolysis;GO:0016032//viral process
-	-
-	-
-	-
-	GO:0007166//cell surface receptor signaling pathway
GO:0003735//structu	GO:0006412//translation
GO:0005515//protei	-
-	-
-	GO:0006488//dolichol-linked oligosaccharide biosynthetic process
GO:0003676//nuclei	GO:0006508//proteolysis;GO:0016032//viral process
GO:0005515//protei	-
-	-
GO:0003756//protei	-
-	-
GO:0004521//endori	-
GO:0004843//thiol-c	GO:0000278//mitotic cell cycle;GO:0006281//DNA repair;GO:0006511//ub
GO:0003676//nuclei	GO:0006508//proteolysis
GO:0003677//DNA b	GO:0006334//nucleosome assembly;GO:0006352//DNA-templated transc

GO:0000166//nucleo	GO:0006468//protein phosphorylation;GO:0010976//positive regulation o
GO:0004857//enzym	GO:0006629//lipid metabolic process;GO:0043086//negative regulation of
GO:0000977//RNA p	GO:0000122//negative regulation of transcription from RNA polymerase I
GO:0004843//thiol-d	GO:0006511//ubiquitin-dependent protein catabolic process;GO:0016579
GO:0004609//phosp	GO:0006646//phosphatidylethanolamine biosynthetic process;GO:000865
-	-
-	GO:1901031//regulation of response to reactive oxygen species
GO:0004672//protei	GO:0006468//protein phosphorylation;GO:0008631//intrinsic apoptotic sig
GO:0003676//nuclei	GO:0043488//regulation of mRNA stability
GO:0003779//actin b	-
-	-
GO:0000981//RNA p	GO:0000122//negative regulation of transcription from RNA polymerase I
GO:0046873//metal	GO:0030001//metal ion transport
GO:0004420//hydrox	GO:0006695//cholesterol biosynthetic process;GO:0008299//isoprenoid b
GO:0016491//oxidor	GO:0006629//lipid metabolic process;GO:0008611//ether lipid biosyntheti
-	-
GO:0005518//collag	GO:0007605//sensory perception of sound;GO:0008360//regulation of cel
-	-
-	-
GO:0005515//protei	GO:0000281//mitotic cytokinesis;GO:0007019//microtubule depolymeriza
-	-
GO:0005507//coppe	GO:0007601//visual perception;GO:0009308//amine metabolic process
GO:0005509//calciur	GO:0007268//synaptic transmission;GO:0008104//protein localization;GO:
GO:0004475//manno	GO:0006486//protein glycosylation;GO:0009058//biosynthetic process;GC
GO:0005509//calciur	-
GO:0016491//oxidor	GO:0006629//lipid metabolic process
-	GO:0007042//lysosomal lumen acidification;GO:0042176//regulation of pr
GO:0005085//guany	GO:0007264//small GTPase mediated signal transduction
GO:0001604//uroter	GO:0007165//signal transduction;GO:0007186//G-protein coupled recept
-	-
GO:0008233//peptid	GO:0006508//proteolysis
GO:0004467//long-c	GO:0006631//fatty acid metabolic process
GO:0008233//peptid	GO:0006508//proteolysis
-	-
GO:0003682//chrom	GO:0001945//lymph vessel development;GO:0003017//lymph circulation;(
GO:0004197//cystein	GO:0002931//response to ischemia;GO:0006508//proteolysis;GO:0006511
-	GO:0051480//regulation of cytosolic calcium ion concentration;GO:19900
GO:0004623//phosp	GO:0006644//phospholipid metabolic process;GO:0016042//lipid catabol
GO:0003676//nuclei	GO:0019722//calcium-mediated signaling;GO:0050790//regulation of cat
GO:0031681//G-pro	GO:0007186//G-protein coupled receptor signaling pathway
GO:0000976//transc	GO:0006357//regulation of transcription from RNA polymerase II promote
GO:0005216//ion ch	GO:0006811//ion transport;GO:0006813//potassium ion transport;GO:003
GO:0004652//polynu	GO:0008285//negative regulation of cell proliferation;GO:0043066//negat
GO:0008109//N-ace	GO:0006486//protein glycosylation;GO:0007179//transforming growth fac
GO:0005319//lipid tr	GO:0006869//lipid transport
-	GO:0000077//DNA damage checkpoint
GO:0003676//nuclei	GO:0006388//tRNA splicing, via endonucleolytic cleavage and ligation
-	-
GO:0005515//protei	GO:0016567//protein ubiquitination
-	-
GO:0001784//phosp	GO:0008286//insulin receptor signaling pathway;GO:0010907//positive re
-	GO:0046890//regulation of lipid biosynthetic process
GO:0050699//WW d	GO:0007034//vacuolar transport;GO:0010629//negative regulation of gen
GO:0003676//nuclei	-
-	-
-	-
GO:0000253//3-keto	GO:0006695//cholesterol biosynthetic process;GO:0006703//estrogen bio
GO:0009922//fatty a	GO:0019367//fatty acid elongation, saturated fatty acid;GO:0042761//very

GO:0005524//ATP bi	GO:0009235//cobalamin metabolic process;GO:0015889//cobalamin trans
-	-
-	-
GO:0005515//protei	-
GO:0005506//iron ic	GO:0001666//response to hypoxia;GO:0017185//peptidyl-lysine hydroxyla
GO:0008270//zinc ic	GO:0009086//methionine biosynthetic process;GO:0009235//cobalamin m
GO:0004843//thiol-c	GO:0006511//ubiquitin-dependent protein catabolic process;GO:0007000
-	GO:0006658//phosphatidylserine metabolic process;GO:0006665//sphingo
-	-
GO:0003676//nuclei	GO:0006278//RNA-dependent DNA replication;GO:0006310//DNA recom
GO:0022857//transm	GO:0055085//transmembrane transport
GO:0016491//oxidor	-
GO:0000976//transc	GO:0006357//regulation of transcription from RNA polymerase II promote
-	-
GO:0036122//BMP b	GO:0007389//pattern specification process;GO:0010454//negative regulat
GO:0003779//actin b	GO:0051017//actin filament bundle assembly
GO:0000993//RNA p	GO:0000956//nuclear-transcribed mRNA catabolic process;GO:0010501//
GO:0000062//fatty-a	GO:0036151//phosphatidylcholine acyl-chain remodeling;GO:1903060//n
GO:0001671//ATPas	GO:0001933//negative regulation of protein phosphorylation;GO:0030433
GO:0003779//actin b	GO:0051017//actin filament bundle assembly
-	-
GO:0030246//carbol	GO:0006897//endocytosis
-	-
GO:0003841//1-acyl	GO:0006629//lipid metabolic process;GO:0006644//phospholipid metabo
GO:0005515//protei	GO:0001822//kidney development;GO:0002093//auditory receptor cell mo
GO:0004672//protei	GO:0006468//protein phosphorylation;GO:0007169//transmembrane rece
GO:0003676//nuclei	GO:0006508//proteolysis
-	-
GO:0046961//protor	GO:0006811//ion transport;GO:1902600//hydrogen ion transmembrane tr
-	-
-	-
GO:0000978//RNA p	GO:0001736//establishment of planar polarity;GO:0001843//neural tube c
GO:0000977//RNA p	GO:0007265//Ras protein signal transduction;GO:0045944//positive regula
-	-
GO:0000976//transc	GO:0006289//nucleotide-excision repair;GO:0006974//cellular response to
-	-
-	GO:0070453//regulation of heme biosynthetic process
GO:0000976//transc	GO:0000122//negative regulation of transcription from RNA polymerase I
-	-
GO:0004175//endop	GO:0002218//activation of innate immune response;GO:0006508//proteol
-	GO:1901031//regulation of response to reactive oxygen species
GO:0005506//iron ic	GO:0001949//sebaceous gland cell differentiation;GO:0006629//lipid meta
GO:0030729//acetoa	GO:0006629//lipid metabolic process
GO:0008271//secon	GO:0008272//sulfate transport;GO:0055085//transmembrane transport
GO:0016491//oxidor	-
-	-
GO:0016757//transfe	-
-	-
GO:0004175//endop	GO:0018126//protein hydroxylation
GO:0008170//N-me	GO:0001692//histamine metabolic process;GO:0001695//histamine catabo
GO:0005227//calciur	GO:0001581//detection of chemical stimulus involved in sensory perceptio
-	-
GO:0004672//protei	GO:0001501//skeletal system development;GO:0006468//protein phospho
-	-
-	-
GO:0005515//protei	GO:0007015//actin filament organization;GO:0045806//negative regulatio
-	-
GO:0003824//catalyt	GO:0009226//nucleotide-sugar biosynthetic process;GO:0010595//positiv

-	-
-	-
-	GO:0006886//intracellular protein transport
-	-
-	-
-	-
GO:0004843//thiol-c	GO:0000278//mitotic cell cycle;GO:0006281//DNA repair;GO:0006511//ub
-	-
-	-
GO:0003841//1-acyl	GO:0006654//phosphatidic acid biosynthetic process;GO:0010891//negati
GO:0003677//DNA b	GO:0009646//response to absence of light;GO:0018298//protein-chromo
GO:0005504//fatty a	GO:0015908//fatty acid transport;GO:0015909//long-chain fatty acid trans
GO:0005509//calciur	GO:0007268//synaptic transmission;GO:0035556//intracellular signal trans
GO:0003874//6-pyru	GO:0006729//tetrahydrobiopterin biosynthetic process
-	-
GO:0019901//protei	GO:0030334//regulation of cell migration;GO:0034446//substrate adhesio
-	-
GO:0046873//metal	GO:0030001//metal ion transport
-	-
-	-
-	-
GO:0005381//iron ic	GO:0046985//positive regulation of hemoglobin biosynthetic process;GO:
GO:0004860//protei	GO:0007616//long-term memory;GO:0050729//positive regulation of infla
GO:0003677//DNA b	GO:0010467//gene expression
GO:0004857//enzym	GO:0006629//lipid metabolic process;GO:0043086//negative regulation of
GO:0004888//transm	GO:0007166//cell surface receptor signaling pathway;GO:0007186//G-pro
-	-
GO:0045296//cadhe	GO:0007043//cell-cell junction assembly;GO:0060997//dendritic spine mo
-	GO:0021817//nucleokinesis involved in cell motility in cerebral cortex radi
-	GO:0007507//heart development
GO:0003874//6-pyru	GO:0006729//tetrahydrobiopterin biosynthetic process
GO:0003847//1-alky	GO:0016042//lipid catabolic process
-	-
-	-
-	-
-	GO:0098609//cell-cell adhesion
-	GO:0006886//intracellular protein transport;GO:0006890//retrograde vesi
-	-
GO:0004930//G-pro	GO:0007165//signal transduction;GO:0007186//G-protein coupled recept
GO:0016765//transfe	GO:0008299//isoprenoid biosynthetic process
-	-
-	-
GO:0001758//retinal	GO:0002072//optic cup morphogenesis involved in camera-type eye deve
GO:0004343//glucos	GO:0006048//UDP-N-acetylglucosamine biosynthetic process
GO:0003735//structu	GO:0006412//translation
GO:0016491//oxidor	GO:0006629//lipid metabolic process;GO:0008611//ether lipid biosyntheti
-	-
GO:0000977//RNA p	GO:0000122//negative regulation of transcription from RNA polymerase I
-	-
-	-
-	-
-	-
GO:0005319//lipid tr	GO:0006869//lipid transport
-	GO:0051480//regulation of cytosolic calcium ion concentration;GO:19900
-	-
GO:0016491//oxidor	GO:0009247//glycolipid biosynthetic process
GO:0016491//oxidor	GO:0009247//glycolipid biosynthetic process
GO:0004857//enzym	GO:0006071//glycerol metabolic process;GO:0006631//fatty acid metabol

GO:0015293//sympo	GO:0008643//carbohydrate transport
-	-
GO:0030729//acetoa	GO:0006629//lipid metabolic process
GO:0003677//DNA b	GO:0006334//nucleosome assembly;GO:0006352//DNA-templated transc
GO:0008517//folic a	GO:0015884//folic acid transport;GO:0051180//vitamin transport;GO:0051
GO:0005488//bindin	-
GO:0005515//protei	GO:0007015//actin filament organization;GO:0045806//negative regulatio
GO:0002161//amino	-
GO:0001784//phosp	GO:0008286//insulin receptor signaling pathway;GO:0010907//positive re
GO:0005216//ion ch	GO:0006811//ion transport;GO:0006813//potassium ion transport;GO:003
GO:0004032//alditol	GO:0006699//bile acid biosynthetic process;GO:0006707//cholesterol cata
GO:0003824//catalyt	GO:0006777//Mo-molybdopterin cofactor biosynthetic process;GO:00435
GO:0003676//nuclei	GO:0019722//calcium-mediated signaling;GO:0050790//regulation of cat
-	-
GO:0004190//aspart	GO:0006508//proteolysis;GO:0019538//protein metabolic process
GO:0003676//nuclei	GO:0006508//proteolysis
-	-
GO:0016491//oxidor	GO:0006629//lipid metabolic process
-	-
-	-
GO:0003779//actin b	GO:0051017//actin filament bundle assembly
GO:0008289//lipid b	GO:0015031//protein transport;GO:0019075//virus maturation;GO:004205
-	-
GO:0003707//steroic	GO:0007340//acrosome reaction;GO:0009611//response to wounding;GO
-	GO:0009987//cellular process
-	-
-	-
GO:0003779//actin b	-
-	-
-	-
GO:0000062//fatty-a	GO:0036151//phosphatidylcholine acyl-chain remodeling;GO:1903060//n
-	-
GO:0016740//transfe	GO:0006777//Mo-molybdopterin cofactor biosynthetic process
GO:0008236//serine	GO:0006508//proteolysis
GO:0003676//nuclei	GO:0006508//proteolysis;GO:0008152//metabolic process;GO:0016032//v
-	-
GO:0005515//protei	GO:0035987//endodermal cell differentiation
GO:0016922//ligand	GO:0045944//positive regulation of transcription from RNA polymerase II
-	-
-	GO:0006488//dolichol-linked oligosaccharide biosynthetic process
GO:0005085//guany	GO:0050790//regulation of catalytic activity
GO:0003824//catalyt	GO:0009226//nucleotide-sugar biosynthetic process;GO:0010595//positiv
GO:0000976//transc	GO:0002062//chondrocyte differentiation;GO:0006355//regulation of tran
GO:0004222//metall	GO:0001542//ovulation from ovarian follicle;GO:0001822//kidney develop
-	-
GO:0005104//fibrob	GO:0001755//neural crest cell migration;GO:0001934//positive regulation
GO:0050699//WW d	GO:0007034//vacuolar transport;GO:0010629//negative regulation of gen
-	GO:0006886//intracellular protein transport;GO:0006890//retrograde vesi
-	GO:0030070//insulin processing;GO:0060628//regulation of ER to Golgi ve
-	-
GO:0005515//protei	-
GO:0003677//DNA b	-
GO:0004609//phosp	GO:0006646//phosphatidylethanolamine biosynthetic process;GO:00086
GO:0045296//cadhe	GO:0007043//cell-cell junction assembly;GO:0060997//dendritic spine mo
-	GO:0007411//axon guidance;GO:0010977//negative regulation of neuron
GO:0008168//methy	GO:0032259//methylation;GO:0035498//carnosine metabolic process
GO:0000976//transc	GO:0006357//regulation of transcription from RNA polymerase II promote
GO:0000976//transc	GO:0000122//negative regulation of transcription from RNA polymerase I

-	-
GO:0004672//protei	GO:0006468//protein phosphorylation;GO:0008631//intrinsic apoptotic si
GO:0003676//nuclei	GO:0006508//proteolysis;GO:0016032//viral process
-	-
-	-
GO:0004452//isoper	GO:0008299//isoprenoid biosynthetic process
GO:0004465//lipopr	GO:0006629//lipid metabolic process;GO:0006631//fatty acid metabolic p
-	-
GO:0022857//transm	GO:0055085//transmembrane transport
GO:0004175//endop	GO:0018126//protein hydroxylation
GO:0005515//protei	GO:0000281//mitotic cytokinesis;GO:0014067//negative regulation of pho
-	-
GO:0000166//nucleo	GO:0003014//renal system process;GO:0006811//ion transport;GO:00068
GO:0003841//1-acyl	GO:0001676//long-chain fatty acid metabolic process;GO:0006629//lipid
GO:0003676//nuclei	-
GO:0004843//thiol-c	GO:0000278//mitotic cell cycle;GO:0006281//DNA repair;GO:0006511//ub
GO:0016787//hydrol	-
-	-
GO:0017128//phosp	GO:0000045//autophagosome assembly;GO:0006914//autophagy;GO:001
-	-
-	-
GO:0003779//actin b	GO:0006351//transcription, DNA-templated;GO:0030032//lamellipodium
GO:0019902//phosp	GO:0006511//ubiquitin-dependent protein catabolic process;GO:0016567
-	-
-	-
GO:0016491//oxidor	-
GO:0042802//identic	GO:0019216//regulation of lipid metabolic process;GO:0055088//lipid hor
GO:0004467//long-c	GO:0006631//fatty acid metabolic process
GO:0004386//helicas	-
-	-
-	GO:0009267//cellular response to starvation;GO:0030001//metal ion trans
GO:0005085//guany	GO:0006511//ubiquitin-dependent protein catabolic process;GO:0032012
GO:0042802//identic	GO:0007165//signal transduction;GO:0046628//positive regulation of insu
-	-
-	-
-	GO:1901031//regulation of response to reactive oxygen species
GO:0008109//N-ace	GO:0006486//protein glycosylation;GO:0007179//transforming growth fac
GO:0003824//catalyt	GO:0009226//nucleotide-sugar biosynthetic process;GO:0010595//positiv
GO:0000166//nucleo	GO:0070588//calcium ion transmembrane transport
GO:0000166//nucleo	GO:0001954//positive regulation of cell-matrix adhesion;GO:0003323//typ
GO:0005515//protei	GO:0043254//regulation of protein complex assembly
GO:0008271//secon	GO:0008272//sulfate transport;GO:0055085//transmembrane transport
GO:0005515//protei	GO:0016567//protein ubiquitination
GO:0004862//cAMP	GO:0006469//negative regulation of protein kinase activity;GO:0051338//
GO:0003677//DNA b	GO:0006355//regulation of transcription, DNA-templated;GO:0045944//p
GO:0008270//zinc io	GO:0009086//methionine biosynthetic process;GO:0009235//cobalamin m
GO:0009922//fatty a	GO:0000038//very long-chain fatty acid metabolic process;GO:0006629//l
GO:0005515//protei	GO:0007015//actin filament organization;GO:0045806//negative regulatio
-	-
-	-
-	-
-	-
GO:0005216//ion ch	GO:0006811//ion transport;GO:0006812//cation transport;GO:0034220//io
GO:0046790//virion	GO:0075512//clathrin-mediated endocytosis of virus by host cell
GO:0009922//fatty a	GO:0019367//fatty acid elongation, saturated fatty acid;GO:0042761//very
GO:0002020//protea	GO:0010466//negative regulation of peptidase activity;GO:0018149//pept
-	-
GO:0001786//phosp	GO:0006910//phagocytosis, recognition;GO:0006911//phagocytosis, engu

-	-
GO:0003836//beta-(	GO:0002319//memory B cell differentiation;GO:0006054//N-acetylneuram
-	-
GO:0003779//actin b	GO:0055013//cardiac muscle cell development
-	GO:0009987//cellular process;GO:0044699//single-organism process
-	GO:0006658//phosphatidylserine metabolic process;GO:0006665//sphing(
-	GO:0009966//regulation of signal transduction
GO:0000976//transc	GO:0006289//nucleotide-excision repair;GO:0006974//cellular response to
GO:0031419//cobala	GO:0015889//cobalamin transport
GO:0000981//RNA p	GO:0000122//negative regulation of transcription from RNA polymerase I
GO:0003676//nuclei	-
-	-
-	-
GO:0050699//WW d	GO:0007034//vacuolar transport;GO:0010629//negative regulation of gen
-	-
-	-
GO:0000976//transc	GO:0000122//negative regulation of transcription from RNA polymerase I
GO:0001604//uroter	GO:0007165//signal transduction;GO:0007186//G-protein coupled recept
GO:0004930//G-pro	GO:0007186//G-protein coupled receptor signaling pathway;GO:0007200
GO:0005515//protei	-
GO:0022857//transm	GO:0055085//transmembrane transport
GO:0004843//thiol-(	GO:0006511//ubiquitin-dependent protein catabolic process;GO:0007000
GO:0004930//G-pro	GO:0007186//G-protein coupled receptor signaling pathway;GO:0043408
-	-
GO:0000246//delta2	GO:0006695//cholesterol biosynthetic process;GO:0006979//response to (
GO:0005515//protei	-
GO:0005515//protei	-
GO:0003924//GTPas	GO:0006254//CTP catabolic process;GO:0034656//nucleobase-containing
GO:0030729//acetoa	GO:0006629//lipid metabolic process
-	-
GO:0003677//DNA b	GO:0009646//response to absence of light;GO:0018298//protein-chromo
GO:0004869//cystein	GO:0001503//ossification;GO:0006953//acute-phase response;GO:00305C
-	-
-	-
GO:0000062//fatty-a	GO:0036151//phosphatidylcholine acyl-chain remodeling;GO:1903060//n
-	-
GO:0030145//manga	GO:0006508//proteolysis;GO:0019538//protein metabolic process
GO:0003676//nuclei	GO:0006508//proteolysis
GO:0005540//hyalur	GO:0007155//cell adhesion
GO:0008289//lipid b	GO:0015031//protein transport;GO:0019075//virus maturation;GO:004205
GO:0000977//RNA p	GO:0000122//negative regulation of transcription from RNA polymerase I
GO:0003700//transc	GO:0006355//regulation of transcription, DNA-templated
GO:0005524//ATP bi	GO:0055085//transmembrane transport
-	-
GO:0008289//lipid b	GO:0015031//protein transport;GO:0019075//virus maturation;GO:004205
GO:0000976//transc	GO:0002062//chondrocyte differentiation;GO:0006355//regulation of tran
-	-
GO:0004869//cystein	GO:0001503//ossification;GO:0006953//acute-phase response;GO:00305C
GO:0016787//hydrol	GO:0006629//lipid metabolic process;GO:0007216//G-protein coupled glu
GO:0016740//transfe	GO:0006629//lipid metabolic process;GO:0006659//phosphatidylserine bi
-	-
-	-
-	-
-	-
GO:0004452//isoper	GO:0008299//isoprenoid biosynthetic process
GO:0003824//catalyt	GO:0009226//nucleotide-sugar biosynthetic process;GO:0010595//positiv
GO:0030729//acetoa	GO:0006629//lipid metabolic process
GO:0003677//DNA b	GO:0009646//response to absence of light;GO:0018298//protein-chromo

-	-
-	-
GO:0004672//protei	GO:0006468//protein phosphorylation;GO:0008631//intrinsic apoptotic si
GO:0017128//phosp	GO:0000045//autophagosome assembly;GO:0006869//lipid transport;GO:
GO:0016491//oxidor	GO:0009247//glycolipid biosynthetic process
GO:0008170//N-me	GO:0001692//histamine metabolic process;GO:0001695//histamine catab
-	-
GO:0003677//DNA b	GO:0006334//nucleosome assembly
GO:0005515//protei	-
-	-
GO:0008236//serine	GO:0006508//proteolysis
GO:0000978//RNA p	GO:0006355//regulation of transcription, DNA-templated;GO:0006357//re
-	-
GO:0005484//SNAP	GO:0006888//ER to Golgi vesicle-mediated transport;GO:0006903//vesicle
GO:0008138//protei	GO:0006355//regulation of transcription, DNA-templated;GO:0006470//p
-	-
GO:0000976//transc	GO:0000122//negative regulation of transcription from RNA polymerase I
GO:0000254//C-4 m	GO:0001934//positive regulation of protein phosphorylation;GO:0008610/
GO:0000062//fatty-a	GO:0036151//phosphatidylcholine acyl-chain remodeling;GO:1903060//n
GO:0050699//WW d	GO:0007034//vacuolar transport;GO:0010629//negative regulation of gen
GO:0005515//protei	GO:0000281//mitotic cytokinesis;GO:0014067//negative regulation of pho
-	-
-	-
-	-
-	-
-	GO:0006839//mitochondrial transport
-	GO:0009267//cellular response to starvation;GO:0030001//metal ion trans
-	-
-	-
GO:0003841//1-acyl	GO:0001676//long-chain fatty acid metabolic process;GO:0006629//lipid
GO:0005515//protei	GO:0001822//kidney development;GO:0002093//auditory receptor cell m
-	-
GO:0003868//4-hyd	GO:0006572//tyrosine catabolic process;GO:0009072//aromatic amino aci
GO:0005227//calciu	GO:0006812//cation transport
GO:0005319//lipid tr	GO:0006869//lipid transport
-	GO:0070453//regulation of heme biosynthetic process
GO:0003682//chrom	GO:0000122//negative regulation of transcription from RNA polymerase I
-	-
-	-
-	-
GO:0019901//protei	GO:0030334//regulation of cell migration;GO:0034446//substrate adhesio
-	-
GO:0000166//nucleo	GO:0001954//positive regulation of cell-matrix adhesion;GO:0003323//ty
-	GO:0098609//cell-cell adhesion
GO:0016491//oxidor	GO:0006666//3-keto-sphinganine metabolic process;GO:0030148//sphin
-	-
GO:0005540//hyalur	GO:0007155//cell adhesion
GO:0005515//protei	GO:0035721//intraciliary retrograde transport;GO:0060271//cilium morph
-	-
GO:0004862//cAMP	GO:0006469//negative regulation of protein kinase activity;GO:0051338//
GO:0003676//nuclei	GO:0006508//proteolysis;GO:0016032//viral process
GO:0003676//nuclei	-
GO:0003924//GTPas	GO:0007165//signal transduction;GO:0007186//G-protein coupled recept
GO:0043169//cation	GO:0007165//signal transduction;GO:0022603//regulation of anatomical
GO:0005515//protei	GO:0007043//cell-cell junction assembly;GO:0007155//cell adhesion;GO:
-	-
-	-
GO:0042802//identi	GO:0030182//neuron differentiation;GO:0045893//positive regulation of t

-	GO:0044237//cellular metabolic process
GO:0004386//helicas	-
GO:0016491//oxidor	GO:0006629//lipid metabolic process;GO:0006631//fatty acid metabolic p
GO:0016717//oxidor	GO:0006629//lipid metabolic process
-	GO:0051480//regulation of cytosolic calcium ion concentration;GO:19900
GO:0046790//virion	GO:0075512//clathrin-mediated endocytosis of virus by host cell
GO:0004467//long-c	GO:0006631//fatty acid metabolic process
GO:0004190//aspart	GO:0006508//proteolysis;GO:0019538//protein metabolic process
GO:0022857//transm	GO:0055085//transmembrane transport
GO:0004307//ethano	GO:0006646//phosphatidylethanolamine biosynthetic process;GO:00086
GO:0000166//nucleo	GO:0070588//calcium ion transmembrane transport
-	GO:0007507//heart development
GO:0016798//hydrol	GO:0008284//positive regulation of cell proliferation;GO:0030198//extrace
GO:0003682//chrom	GO:0015908//fatty acid transport;GO:0043066//negative regulation of apo
-	-
GO:0005515//protei	GO:0007015//actin filament organization;GO:0045806//negative regulatio
-	-
GO:0003676//nuclei	-
-	GO:0007411//axon guidance;GO:0010977//negative regulation of neuron
-	-
GO:0004725//protei	GO:0016311//dephosphorylation;GO:0046855//inositol phosphate depho
GO:0005201//extrac	GO:0007160//cell-matrix adhesion;GO:0007605//sensory perception of sc
-	-
-	-
GO:0002020//protea	GO:0010951//negative regulation of endopeptidase activity
-	-
GO:0003824//catalyt	GO:0006650//glycerophospholipid metabolic process;GO:0007416//synap
-	-
-	-
GO:0005515//protei	-
GO:0060090//bindin	GO:0030433//ER-associated ubiquitin-dependent protein catabolic proce
GO:0030246//carboh	GO:0006897//endocytosis
GO:0004489//methy	GO:0001843//neural tube closure;GO:0006555//methionine metabolic pro
-	-
-	-
-	-
GO:0003676//nuclei	GO:0006508//proteolysis;GO:0008152//metabolic process;GO:0016032//v
GO:0005509//calciu	GO:0015012//heparan sulfate proteoglycan biosynthetic process;GO:0030
GO:0003677//DNA b	GO:0006334//nucleosome assembly;GO:0016584//nucleosome positionin
-	-
-	GO:0006888//ER to Golgi vesicle-mediated transport;GO:0006914//autop
GO:0004930//G-pro	GO:0007165//signal transduction;GO:0007186//G-protein coupled recept
GO:0003677//DNA b	-
-	GO:0006886//intracellular protein transport;GO:0006890//retrograde vesi
-	GO:0006658//phosphatidylserine metabolic process;GO:0006665//sphing
GO:0003676//nuclei	GO:0043488//regulation of mRNA stability
GO:0003779//actin b	-
GO:0000062//fatty-a	GO:0036151//phosphatidylcholine acyl-chain remodeling;GO:1903060//n
GO:0045296//cadhe	GO:0007043//cell-cell junction assembly;GO:0060997//dendritic spine mo
GO:0042802//identic	GO:0007165//signal transduction;GO:0046628//positive regulation of insu
-	-
GO:0005085//guany	GO:0050790//regulation of catalytic activity
-	GO:0060074//synapse maturation
GO:0022857//transm	GO:0006865//amino acid transport;GO:0055085//transmembrane transpo
GO:0001604//uroter	GO:0007165//signal transduction;GO:0007186//G-protein coupled recept
GO:0003836//beta-g	GO:0002319//memory B cell differentiation;GO:0006054//N-acetylneuram
-	-
GO:0005515//protei	GO:0000281//mitotic cytokinesis;GO:0007019//microtubule depolymeriza

GO:0003676//nuclei	GO:0043484//regulation of RNA splicing
GO:0008270//zinc ic	GO:0009086//methionine biosynthetic process;GO:0009235//cobalamin m
GO:0005515//protei	GO:0007229//integrin-mediated signaling pathway
GO:0000977//RNA p	GO:0000122//negative regulation of transcription from RNA polymerase I
GO:0005355//glucos	GO:0015747//urate transport;GO:0046415//urate metabolic process;GO:0
GO:0003676//nuclei	GO:0019722//calcium-mediated signaling;GO:0050790//regulation of cata
GO:0004222//metall	GO:0001542//ovulation from ovarian follicle;GO:0001822//kidney develop
GO:0019902//phosp	GO:0006511//ubiquitin-dependent protein catabolic process;GO:0016567
GO:0005172//vascul	GO:0008283//cell proliferation;GO:0008284//positive regulation of cell pro
-	-
GO:0005515//protei	GO:0043065//positive regulation of apoptotic process;GO:0044790//nega
GO:0022857//transm	GO:0055085//transmembrane transport
GO:0001786//phosp	-
-	-
GO:0005515//protei	-
-	-
GO:0000287//magne	GO:0006468//protein phosphorylation;GO:0007346//regulation of mitotic
GO:0005381//iron ic	GO:0046985//positive regulation of hemoglobin biosynthetic process;GO:
GO:0019902//phosp	GO:0006511//ubiquitin-dependent protein catabolic process;GO:0016567
GO:0005515//protei	GO:0046330//positive regulation of JNK cascade;GO:0090090//negative re
GO:0017128//phosp	GO:0000045//autophagosome assembly;GO:0006914//autophagy;GO:001
GO:0004045//amino	-
GO:0030145//manga	GO:0006508//proteolysis;GO:0019538//protein metabolic process
GO:0005515//protei	GO:0055085//transmembrane transport
-	-
-	-
-	GO:0009267//cellular response to starvation;GO:0030001//metal ion trans
-	-
GO:0003824//catalyt	GO:0006567//threonine catabolic process
GO:0004175//endop	GO:0018126//protein hydroxylation
-	-
GO:0004252//serine	GO:0006508//proteolysis;GO:0016485//protein processing
-	GO:0006629//lipid metabolic process
GO:0008289//lipid b	GO:0015031//protein transport;GO:0019075//virus maturation;GO:004205
GO:0045296//cadhe	GO:0007043//cell-cell junction assembly;GO:0060997//dendritic spine mo
GO:0022857//transm	GO:0055085//transmembrane transport
GO:0004674//protei	GO:0006468//protein phosphorylation
-	-
GO:0005515//protei	GO:0007015//actin filament organization;GO:0045806//negative regulatio
-	-
-	-
GO:0005515//protei	GO:0007165//signal transduction
-	GO:0006886//intracellular protein transport;GO:0006890//retrograde vesic
GO:0004452//isoper	GO:0008299//isoprenoid biosynthetic process
GO:0005515//protei	-
GO:0001758//retinal	GO:0002072//optic cup morphogenesis involved in camera-type eye deve
-	-
GO:0008170//N-me	GO:0001692//histamine metabolic process;GO:0001695//histamine catabo
GO:0016491//oxidor	-
-	-
-	-
GO:0001540//beta-a	GO:0006511//ubiquitin-dependent protein catabolic process;GO:0006516
GO:0001671//ATPas	GO:0001933//negative regulation of protein phosphorylation;GO:0030433
GO:0003676//nuclei	GO:0006508//proteolysis;GO:0008152//metabolic process;GO:0016032//v
-	-
-	GO:0070453//regulation of heme biosynthetic process
-	-
GO:0001758//retinal	GO:0002072//optic cup morphogenesis involved in camera-type eye deve

GO:0019855//calciur	GO:0006851//mitochondrial calcium ion transport;GO:0051560//mitochor
GO:0000976//transc	GO:0002062//chondrocyte differentiation;GO:0006355//regulation of tran
-	-
-	-
-	-
-	GO:0006506//GPI anchor biosynthetic process
-	-
-	-
-	-
GO:0004032//alditol	GO:0006699//bile acid biosynthetic process;GO:0006707//cholesterol cata
GO:0005515//protei	GO:0001822//kidney development;GO:0002093//auditory receptor cell mo
GO:0042577//lipid p	GO:0006644//phospholipid metabolic process
-	-
-	-
GO:0005509//calciur	-
-	-
GO:0004867//serine	GO:0010951//negative regulation of endopeptidase activity;GO:0030212//
GO:0043169//cation	GO:0007165//signal transduction;GO:0022603//regulation of anatomical s
GO:0005515//protei	GO:0007043//cell-cell junction assembly;GO:0007155//cell adhesion;GO:0
-	-
-	-
GO:0019901//protei	GO:0030334//regulation of cell migration;GO:0034446//substrate adhesio
GO:0000977//RNA p	GO:0000122//negative regulation of transcription from RNA polymerase I
-	-
-	-
GO:0004672//protei	GO:0000086//G2/M transition of mitotic cell cycle;GO:0000278//mitotic ce
GO:0002020//protea	GO:0010951//negative regulation of endopeptidase activity
GO:0008195//phosp	GO:0006357//regulation of transcription from RNA polymerase II promote
GO:0005515//protei	GO:0007015//actin filament organization;GO:0045806//negative regulatio
GO:0016491//oxidor	GO:0006629//lipid metabolic process;GO:0006631//fatty acid metabolic p
GO:0004672//protei	GO:0001501//skeletal system development;GO:0006468//protein phospho
-	-
GO:0005515//protei	GO:0006888//ER to Golgi vesicle-mediated transport
GO:0005540//hyalur	GO:0007155//cell adhesion
GO:0004930//G-pro	GO:0007186//G-protein coupled receptor signaling pathway;GO:0007200
GO:0003824//catalyt	GO:0006778//porphyrin-containing compound metabolic process;GO:000
-	GO:0017015//regulation of transforming growth factor beta receptor sign
GO:0005515//protei	GO:0044763//single-organism cellular process
GO:0003682//chrom	GO:0015908//fatty acid transport;GO:0043066//negative regulation of apo
-	-
GO:0003677//DNA b	GO:0009646//response to absence of light;GO:0018298//protein-chromo
-	-
-	-
-	-
GO:0003824//catalyt	GO:0006167//AMP biosynthetic process;GO:0006177//GMP biosynthetic p
-	-
GO:0005509//calciur	GO:0015012//heparan sulfate proteoglycan biosynthetic process;GO:0030
GO:0003836//beta-g	GO:0002319//memory B cell differentiation;GO:0006054//N-acetylneuram
GO:0004866//endop	GO:0001934//positive regulation of protein phosphorylation;GO:0006954/
GO:0015218//pyrimi	GO:0000002//mitochondrial genome maintenance;GO:0002082//regulatic
GO:0000166//nucleo	GO:0006468//protein phosphorylation;GO:0010976//positive regulation o
GO:0000166//nucleo	GO:0006468//protein phosphorylation;GO:0006955//immune response;G(
-	-
GO:0004843//thiol-c	GO:0006511//ubiquitin-dependent protein catabolic process;GO:0016579
GO:0003735//structu	GO:0006412//translation;GO:0006919//activation of cysteine-type endope
-	-
GO:0003924//GTPas	-
-	-

-	-
-	-
GO:0030246//carbol	GO:0006897//endocytosis
-	-
GO:0003677//DNA b	GO:0006383//transcription from RNA polymerase III promoter
-	-
-	-
GO:0060090//bindin	GO:0030433//ER-associated ubiquitin-dependent protein catabolic proce
GO:0005543//phosp	GO:0048260//positive regulation of receptor-mediated endocytosis;GO:0
GO:0022857//transm	GO:0006865//amino acid transport;GO:0055085//transmembrane transpo
-	-
GO:0004930//G-pro	GO:0007186//G-protein coupled receptor signaling pathway
GO:0000062//fatty-a	GO:0036151//phosphatidylcholine acyl-chain remodeling;GO:1903060//n
-	-
GO:0005515//protei	GO:0000281//mitotic cytokinesis;GO:0007019//microtubule depolymeriza
GO:0017128//phosp	GO:0000045//autophagosome assembly;GO:0006869//lipid transport;GO:
-	-
GO:0003676//nuclei	GO:0006508//proteolysis;GO:0008152//metabolic process;GO:0016032//v
GO:0005509//calciur	GO:0007155//cell adhesion
GO:0016757//transfe	GO:0006488//dolichol-linked oligosaccharide biosynthetic process
-	GO:0007042//lysosomal lumen acidification;GO:0042176//regulation of pr
GO:0004930//G-pro	GO:0002001//renin secretion into blood stream;GO:0002281//macrophag
GO:0004553//hydrol	GO:0005975//carbohydrate metabolic process
GO:0005524//ATP bi	GO:0009235//cobalamin metabolic process;GO:0015889//cobalamin trans
GO:0003676//nuclei	GO:0006278//RNA-dependent DNA replication;GO:0044260//cellular mac
-	-
GO:0008080//N-ace	-
GO:0005515//protei	GO:0055085//transmembrane transport
-	-
GO:0005540//hyalur	GO:0007155//cell adhesion
-	GO:0006629//lipid metabolic process
-	-
GO:0003676//nuclei	GO:0006388//tRNA splicing, via endonucleolytic cleavage and ligation
-	-
-	GO:0070979//protein K11-linked ubiquitination
-	-
-	-
GO:0003676//nuclei	-
-	-
-	-
-	-
GO:0005515//protei	GO:0043065//positive regulation of apoptotic process;GO:0044790//nega
GO:0001968//fibron	GO:0001933//negative regulation of protein phosphorylation;GO:0007162
-	-
GO:0004103//cholin	GO:0006646//phosphatidylethanolamine biosynthetic process;GO:000665
-	-
GO:0003735//structu	GO:0006412//translation;GO:0006919//activation of cysteine-type endope
-	GO:0070979//protein K11-linked ubiquitination
GO:0005509//calciur	GO:0015012//heparan sulfate proteoglycan biosynthetic process;GO:0030
GO:0019902//phosp	GO:0006511//ubiquitin-dependent protein catabolic process;GO:0016567
-	-
GO:0016491//oxidor	GO:0006629//lipid metabolic process
GO:0016491//oxidor	-
-	-
GO:0004000//adeno	GO:0000255//allantoin metabolic process;GO:0001666//response to hypo
GO:0003682//chrom	GO:0000122//negative regulation of transcription from RNA polymerase I
GO:0005085//guany	GO:0007264//small GTPase mediated signal transduction
GO:0000166//nucleo	GO:0001954//positive regulation of cell-matrix adhesion;GO:0003323//ty

GO:0005509//calciur	GO:0007268//synaptic transmission;GO:0008104//protein localization;GO:
GO:0004439//phosp	GO:0007420//brain development;GO:0046855//inositol phosphate depho
GO:0004862//cAMP	GO:0006469//negative regulation of protein kinase activity;GO:0051338//r
GO:0003677//DNA b	GO:0035871//protein K11-linked deubiquitination
-	GO:0060074//synapse maturation
-	-
GO:0004674//protei	GO:0006468//protein phosphorylation
GO:0016491//oxidor	-
GO:0022857//transm	GO:0010628//positive regulation of gene expression;GO:0010629//negativ
GO:0035256//G-pro	GO:0003009//skeletal muscle contraction;GO:0007216//G-protein couplec
-	GO:0001656//metanephros development;GO:0001658//branching involve
-	-
GO:0004652//polynu	GO:0008285//negative regulation of cell proliferation;GO:0043066//negat
GO:0003676//nuclei	GO:0043484//regulation of RNA splicing
GO:0003779//actin b	GO:0051017//actin filament bundle assembly
GO:0004340//glucok	GO:0001678//cellular glucose homeostasis;GO:0005975//carbohydrate me
-	-
-	-
-	-
GO:0003756//protei	-
-	GO:0006658//phosphatidylserine metabolic process;GO:0006665//sphing
GO:0002020//protea	GO:0010951//negative regulation of endopeptidase activity
GO:0005515//protei	GO:0007043//cell-cell junction assembly;GO:0007155//cell adhesion;GO:0
GO:0043169//cation	GO:0007165//signal transduction;GO:0022603//regulation of anatomical s
GO:0036122//BMP b	GO:0007389//pattern specification process;GO:0010454//negative regulat
-	-
GO:0046961//protor	GO:0006811//ion transport;GO:1902600//hydrogen ion transmembrane tr
-	-
-	-
GO:0003676//nuclei	GO:0006508//proteolysis;GO:0016032//viral process
GO:0005515//protei	-
-	GO:0043368//positive T cell selection;GO:0043383//negative T cell selectic
-	GO:0000045//autophagosome assembly;GO:0009267//cellular response t
-	GO:0006887//exocytosis;GO:0015031//protein transport
-	-
GO:0008270//zinc ic	GO:0009086//methionine biosynthetic process;GO:0009235//cobalamin m
-	-
GO:0005506//iron ic	GO:0001666//response to hypoxia;GO:0017185//peptidyl-lysine hydroxyla
GO:0003779//actin b	GO:0007015//actin filament organization;GO:0030334//regulation of cell r
GO:0004896//cytoki	GO:0019221//cytokine-mediated signaling pathway;GO:0034342//respons
GO:0004310//farnes	GO:0008610//lipid biosynthetic process;GO:0009058//biosynthetic proces
GO:0000166//nucleo	GO:0000122//negative regulation of transcription from RNA polymerase I
-	-
GO:0005216//ion ch	GO:0006811//ion transport;GO:0019228//neuronal action potential;GO:00
GO:0003824//catalyt	GO:0006778//porphyrin-containing compound metabolic process;GO:000
-	-
GO:0003677//DNA b	GO:0009646//response to absence of light;GO:0018298//protein-chromo
GO:0016787//hydrol	GO:0006470//protein dephosphorylation;GO:0006805//xenobiotic metabo
GO:0004467//long-c	GO:0006631//fatty acid metabolic process
-	-
-	-
-	-
GO:0001671//ATPas	GO:0001933//negative regulation of protein phosphorylation;GO:003043
GO:0019902//phosp	GO:0006511//ubiquitin-dependent protein catabolic process;GO:0016567
GO:0008757//S-ade	GO:0006656//phosphatidylcholine biosynthetic process;GO:0006686//sph
GO:0003841//1-acyl	GO:0006629//lipid metabolic process;GO:0006644//phospholipid metabo
GO:0005515//protei	GO:0044763//single-organism cellular process
-	-

GO:0004867//serine	GO:0007596//blood coagulation
GO:0003677//DNA b	GO:0009646//response to absence of light;GO:0018298//protein-chromo
-	-
GO:0005102//recept	GO:0003336//corneocyte desquamation;GO:0006672//ceramide metaboli
GO:0004866//endop	GO:0001934//positive regulation of protein phosphorylation;GO:0006954/
GO:0004672//protei	GO:0001501//skeletal system development;GO:0006468//protein phospho
-	-
-	-
-	-
GO:0000166//nucleo	GO:0006468//protein phosphorylation;GO:0006955//immune response;G
GO:0000978//RNA p	GO:0001736//establishment of planar polarity;GO:0001843//neural tube c
GO:0000166//nucleo	GO:0006468//protein phosphorylation;GO:0010976//positive regulation o
GO:0004842//ubiqui	GO:0000209//protein polyubiquitination;GO:0006915//apoptotic process;
GO:0005540//hyalur	GO:0007155//cell adhesion
GO:0004857//enzym	GO:0006629//lipid metabolic process;GO:0043086//negative regulation o
GO:0004672//protei	GO:0006468//protein phosphorylation;GO:0008631//intrinsic apoptotic si
GO:0003847//1-alky	GO:0016042//lipid catabolic process
GO:0004843//thiol-	GO:0006511//ubiquitin-dependent protein catabolic process;GO:0016579
GO:0003953//NAD+	GO:0001952//regulation of cell-matrix adhesion;GO:0008284//positive re
GO:0008289//lipid b	GO:0015031//protein transport;GO:0019075//virus maturation;GO:004205
GO:0000064//L-orni	GO:1990575//mitochondrial L-ornithine transmembrane transport
GO:0005515//protei	GO:0007155//cell adhesion;GO:0008593//regulation of Notch signaling p
GO:0045296//cadhe	GO:0007043//cell-cell junction assembly;GO:0060997//dendritic spine mo
GO:0042577//lipid p	GO:0006644//phospholipid metabolic process
-	GO:0006355//regulation of transcription, DNA-templated;GO:0034243//re
-	GO:0019941//modification-dependent protein catabolic process;GO:0030
GO:0046873//metal	GO:0030001//metal ion transport
GO:0003676//nuclei	GO:0015074//DNA integration;GO:0044260//cellular macromolecule meta
-	-
-	GO:0006886//intracellular protein transport;GO:0006890//retrograde vesi
GO:0016491//oxidor	GO:0006629//lipid metabolic process;GO:0008611//ether lipid biosyntheti
-	-
-	-
GO:0008289//lipid b	GO:0070508//cholesterol import
-	GO:0006895//Golgi to endosome transport;GO:0015031//protein transpo
GO:0005515//protei	GO:0046330//positive regulation of JNK cascade;GO:0090090//negative re
GO:0008017//microt	GO:0008285//negative regulation of cell proliferation;GO:0030330//DNA
GO:0015175//neutra	GO:0015804//neutral amino acid transport;GO:0042941//D-alanine transp
GO:0005515//protei	GO:0055085//transmembrane transport
GO:0003676//nuclei	GO:0006508//proteolysis;GO:0008152//metabolic process;GO:0016032//v
-	-
GO:0005509//calciur	GO:0007155//cell adhesion
-	-
GO:0003677//DNA b	GO:0006383//transcription from RNA polymerase III promoter
-	GO:0007042//lysosomal lumen acidification;GO:0042176//regulation of p
-	-
-	-
GO:0005524//ATP bi	-
-	-
-	-
-	GO:0006629//lipid metabolic process
-	GO:0010001//glial cell differentiation;GO:0050772//positive regulation of
-	-
-	-
GO:0016491//oxidor	-
-	-
GO:0005515//protei	-
GO:0002161//amino	-

GO:0004190//aspart	GO:0006508//proteolysis;GO:0019538//protein metabolic process
-	GO:0021817//nucleokinesis involved in cell motility in cerebral cortex radi
GO:0030145//manga	GO:0006508//proteolysis;GO:0019538//protein metabolic process
-	-
GO:0042802//identic	-
GO:0008271//secon	GO:0008272//sulfate transport;GO:0055085//transmembrane transport
-	GO:0015888//thiamine transport
GO:0004930//G-pro	GO:0007165//signal transduction;GO:0007186//G-protein coupled recept
GO:0005524//ATP bi	GO:0009235//cobalamin metabolic process;GO:0015889//cobalamin trans
-	-
-	-
GO:0001758//retinal	GO:0002072//optic cup morphogenesis involved in camera-type eye deve
GO:0005506//iron io	GO:0008610//lipid biosynthetic process
-	-
GO:0022857//transm	GO:0055085//transmembrane transport
GO:0001968//fibron	GO:0001933//negative regulation of protein phosphorylation;GO:000716
GO:0005515//protei	-
-	GO:0070979//protein K11-linked ubiquitination
-	-
-	-
GO:0016491//oxidor	GO:0009247//glycolipid biosynthetic process
GO:0003779//actin b	GO:0051017//actin filament bundle assembly
GO:0036122//BMP b	GO:0007389//pattern specification process;GO:0010454//negative regulat
GO:0015293//sympo	GO:0008643//carbohydrate transport
GO:0005319//lipid tr	GO:0006869//lipid transport
GO:0004000//adeno	GO:0000255//allantoin metabolic process;GO:0001666//response to hypo
GO:0005515//protei	GO:0001764//neuron migration;GO:0007158//neuron cell-cell adhesion;G
-	-
GO:0016757//transfe	GO:0002121//inter-male aggressive behavior;GO:0042403//thyroid horm
-	-
GO:0005085//guany	GO:0007264//small GTPase mediated signal transduction
-	-
GO:0016407//acetylt	-
GO:0035256//G-pro	GO:0003009//skeletal muscle contraction;GO:0007216//G-protein couple
GO:0008235//metall	GO:0017186//peptidyl-pyroglutamic acid biosynthetic process, using glut
GO:0003676//nuclei	-
GO:0003677//DNA b	GO:0006334//nucleosome assembly
GO:0004930//G-pro	GO:0007165//signal transduction;GO:0007186//G-protein coupled recept
-	GO:0030070//insulin processing;GO:0060628//regulation of ER to Golgi ve
GO:0030246//carboh	-
-	-
GO:0005515//protei	GO:0006355//regulation of transcription, DNA-templated;GO:0006805//x
GO:0005515//protei	GO:0001764//neuron migration;GO:0007158//neuron cell-cell adhesion;G
-	-
-	-
-	GO:0043368//positive T cell selection;GO:0043383//negative T cell selectio
-	-
GO:0005515//protei	GO:0007229//integrin-mediated signaling pathway
GO:0005102//recept	GO:0006898//receptor-mediated endocytosis;GO:0008104//protein locali
GO:0003677//DNA b	GO:0006334//nucleosome assembly;GO:0042742//defense response to ba
GO:0005515//protei	-
-	GO:0006839//mitochondrial transport
GO:0030246//carboh	GO:0006897//endocytosis
-	-
GO:0003779//actin b	GO:0055013//cardiac muscle cell development
GO:0000287//magne	GO:0006006//glucose metabolic process;GO:0006094//gluconeogenesis;G
GO:0003779//actin b	GO:0006836//neurotransmitter transport;GO:0015844//monoamine transp
GO:0003841//1-acyl	GO:0001676//long-chain fatty acid metabolic process;GO:0006629//lipid

GO:0003989//acetyl-	GO:0001894//tissue homeostasis;GO:0006084//acetyl-CoA metabolic proc
GO:0003735//structu	GO:0006412//translation;GO:0006919//activation of cysteine-type endope
GO:0005247//voltag	GO:0006821//chloride transport;GO:0055085//transmembrane transport
GO:0005178//integri	GO:0000902//cell morphogenesis;GO:0006954//inflammatory response;G
-	-
-	-
GO:0001968//fibron	GO:0010811//positive regulation of cell-substrate adhesion;GO:0030198//
-	-
-	-
-	-
GO:0005515//protei	GO:0003431//growth plate cartilage chondrocyte development;GO:00082
GO:0005515//protei	GO:0045765//regulation of angiogenesis
-	-
GO:0005515//protei	GO:0000281//mitotic cytokinesis;GO:0014067//negative regulation of pho
GO:0004930//G-pro	GO:0001764//neuron migration;GO:0007186//G-protein coupled recepto
GO:0005515//protei	GO:0035987//endodermal cell differentiation
GO:0005515//protei	GO:0016567//protein ubiquitination;GO:0035556//intracellular signal tran
-	GO:0006887//exocytosis;GO:0015031//protein transport
GO:0000976//transc	GO:0000122//negative regulation of transcription from RNA polymerase I
-	-
-	-
GO:0004620//phosp	GO:0009395//phospholipid catabolic process;GO:0030336//negative regu
GO:0042802//identic	GO:0030182//neuron differentiation;GO:0045893//positive regulation of t
GO:0000166//nucleo	-
GO:0016717//oxidor	GO:0006629//lipid metabolic process
GO:0003824//catalyt	GO:0006778//porphyrin-containing compound metabolic process;GO:000
GO:0004867//serine	GO:0007596//blood coagulation
-	-
GO:0003868//4-hyd	GO:0009072//aromatic amino acid family metabolic process
GO:0005085//guany	GO:0006623//protein targeting to vacuole;GO:0009306//protein secretion
-	-
-	-
-	GO:1901031//regulation of response to reactive oxygen species
GO:0003676//nuclei	GO:0006508//proteolysis;GO:0016032//viral process
GO:0008168//methy	GO:0032259//methylation;GO:0035498//carnosine metabolic process
GO:0017128//phosp	GO:0000045//autophagosome assembly;GO:0006869//lipid transport;GO:
-	-
GO:0004672//protei	GO:0006468//protein phosphorylation;GO:0008631//intrinsic apoptotic si
GO:0003847//1-alky	GO:0016042//lipid catabolic process
GO:0004496//meval	GO:0006695//cholesterol biosynthetic process;GO:0008299//isoprenoid b
-	-
GO:0005515//protei	GO:0007015//actin filament organization;GO:0045806//negative regulatio
GO:0016491//oxidor	GO:0006666//3-keto-sphinganine metabolic process;GO:0030148//sphin
GO:0004497//mono	-
-	GO:0018095//protein polyglutamylation
GO:0003676//nuclei	GO:0006388//tRNA splicing, via endonucleolytic cleavage and ligation
GO:0019901//protei	GO:0030334//regulation of cell migration;GO:0034446//substrate adhesio
-	-
GO:0005509//calciu	-
GO:0001671//ATPas	GO:0001933//negative regulation of protein phosphorylation;GO:003043
GO:0004842//ubiqui	GO:0000209//protein polyubiquitination;GO:0006915//apoptotic process;
-	GO:0006629//lipid metabolic process
GO:0060090//bindin	GO:0030433//ER-associated ubiquitin-dependent protein catabolic proce
GO:0042577//lipid p	GO:0006644//phospholipid metabolic process
-	-
GO:0005085//guany	GO:0007264//small GTPase mediated signal transduction;GO:0034976//re
-	-
-	-

GO:0004489//methy	GO:0001843//neural tube closure;GO:0006555//methionine metabolic prc
GO:0000976//transc	GO:0006357//regulation of transcription from RNA polymerase II promote
GO:0045296//cadhe	GO:0007043//cell-cell junction assembly;GO:0060997//dendritic spine mc
GO:0005515//protei	GO:0042509//regulation of tyrosine phosphorylation of STAT protein;GO:
GO:0005515//protei	GO:0006355//regulation of transcription, DNA-templated;GO:0007179//tr
-	GO:0010001//glial cell differentiation;GO:0050772//positive regulation of
GO:0004585//ornithi	GO:0000050//urea cycle;GO:0006520//cellular amino acid metabolic proc
GO:0022857//transm	GO:0055085//transmembrane transport
GO:0005085//guany	GO:0007165//signal transduction;GO:0007264//small GTPase mediated si
GO:0015175//neutra	GO:0015804//neutral amino acid transport;GO:0042941//D-alanine transp
-	GO:0006506//GPI anchor biosynthetic process
-	GO:0006658//phosphatidylserine metabolic process;GO:0006665//sphing
-	GO:0006886//intracellular protein transport;GO:0006890//retrograde vesi
-	-
GO:0016757//transfe	GO:0002121//inter-male aggressive behavior;GO:0042403//thyroid horm
GO:0002161//amino	-
GO:0009922//fatty a	GO:0019367//fatty acid elongation, saturated fatty acid;GO:0042761//very
-	-
-	-
-	-
GO:0001758//retinal	GO:0002072//optic cup morphogenesis involved in camera-type eye deve
-	-
GO:0008270//zinc ic	GO:0009086//methionine biosynthetic process;GO:0009235//cobalamin m
-	-
-	-
GO:0003779//actin b	GO:0051017//actin filament bundle assembly
GO:0016740//transfe	GO:0006629//lipid metabolic process;GO:0006659//phosphatidylserine bi
GO:0005515//protei	GO:0016567//protein ubiquitination
GO:0005515//protei	-
GO:0005515//protei	GO:0043065//positive regulation of apoptotic process;GO:0044790//nega
GO:0008109//N-ace	GO:0006486//protein glycosylation;GO:0007179//transforming growth fac
GO:0016787//hydrol	GO:0006629//lipid metabolic process;GO:0007216//G-protein coupled gl
GO:0046961//protor	GO:0006811//ion transport;GO:1902600//hydrogen ion transmembrane tr
GO:0005509//calciur	GO:0015012//heparan sulfate proteoglycan biosynthetic process;GO:0030
-	-
GO:0000250//lanost	GO:0006694//steroid biosynthetic process;GO:0006695//cholesterol biosy
GO:0042802//identic	-
GO:0005524//ATP bi	GO:0055085//transmembrane transport
-	GO:0019941//modification-dependent protein catabolic process;GO:0030
GO:0004467//long-c	GO:0006631//fatty acid metabolic process
GO:0005515//protei	GO:0006355//regulation of transcription, DNA-templated;GO:0006805//x
GO:0015377//cation	GO:0006811//ion transport;GO:0006884//cell volume homeostasis;GO:005
GO:0003841//1-acyl	GO:0001819//positive regulation of cytokine production;GO:0006654//ph
GO:0005515//protei	GO:0006355//regulation of transcription, DNA-templated;GO:0006805//x
GO:0003735//structu	GO:0006412//translation
GO:0004674//protei	GO:0006468//protein phosphorylation
-	-
GO:0016627//oxidor	GO:0006629//lipid metabolic process
GO:0022892//substr	GO:0044765//single-organism transport
GO:0004930//G-pro	GO:0007186//G-protein coupled receptor signaling pathway;GO:0043408
GO:0003924//GTPas	GO:0006254//CTP catabolic process;GO:0034656//nucleobase-containing
GO:0003779//actin b	GO:0055013//cardiac muscle cell development
GO:0005515//protei	-
GO:0000976//transc	GO:0000122//negative regulation of transcription from RNA polymerase I
GO:0005509//calciur	GO:0001654//eye development;GO:0045667//regulation of osteoblast diff
-	-
GO:0005524//ATP bi	-
GO:0030246//carbol	-

-	GO:0030070//insulin processing;GO:0060628//regulation of ER to Golgi ve
GO:0004366//glycer	GO:0001817//regulation of cytokine production;GO:0006631//fatty acid m
GO:0001671//ATPas	GO:0001933//negative regulation of protein phosphorylation;GO:003043
GO:0003677//DNA b	GO:0006334//nucleosome assembly;GO:0006352//DNA-templated transc
-	-
GO:0004672//protei	GO:0001501//skeletal system development;GO:0006468//protein phospho
-	-
GO:0004672//protei	GO:0006468//protein phosphorylation;GO:0007169//transmembrane rece
GO:0008022//protei	GO:0007026//negative regulation of microtubule depolymerization;GO:00
-	-
GO:0004672//protei	GO:0006468//protein phosphorylation;GO:0008631//intrinsic apoptotic si
GO:0004609//phosp	GO:0006646//phosphatidylethanolamine biosynthetic process;GO:000865
GO:0005506//iron ic	GO:0001949//sebaceous gland cell differentiation;GO:0006629//lipid meta
GO:0005515//protei	-
GO:0000976//transc	GO:0000122//negative regulation of transcription from RNA polymerase I
-	-
GO:0005515//protei	GO:0007043//cell-cell junction assembly;GO:0007155//cell adhesion;GO:0
GO:0043169//cation	GO:0007165//signal transduction;GO:0022603//regulation of anatomical s
-	-
GO:0003676//nuclei	GO:0006508//proteolysis
GO:0005515//protei	GO:0000281//mitotic cytokinesis;GO:0014067//negative regulation of pho
-	-
GO:0004190//aspart	GO:0006508//proteolysis;GO:0019538//protein metabolic process
GO:0004867//serine	GO:0007596//blood coagulation
GO:0000062//fatty-a	GO:0036151//phosphatidylcholine acyl-chain remodeling;GO:1903060//n
GO:0004857//enzym	GO:0006629//lipid metabolic process;GO:0043086//negative regulation o
-	-
-	-
GO:0016757//transfe	GO:0002121//inter-male aggressive behavior;GO:0042403//thyroid hormo
-	GO:0006488//dolichol-linked oligosaccharide biosynthetic process
-	-
-	-
GO:0046873//metal	GO:0030001//metal ion transport
-	-
GO:0005178//integri	GO:0000902//cell morphogenesis;GO:0006954//inflammatory response;G
-	-
GO:0016491//oxidor	GO:0006629//lipid metabolic process;GO:0008611//ether lipid biosyntheti
-	-
-	-
GO:0003874//6-pyru	GO:0006729//tetrahydrobiopterin biosynthetic process
GO:0017128//phosp	GO:0000045//autophagosome assembly;GO:0006914//autophagy;GO:001
GO:0003682//chrom	GO:0001945//lymph vessel development;GO:0003017//lymph circulation;
GO:0004857//enzym	GO:0001525//angiogenesis;GO:0043066//negative regulation of apoptoti
-	-
GO:0005504//fatty a	GO:0015908//fatty acid transport;GO:0015909//long-chain fatty acid trans
GO:0008194//UDP-g	-
GO:0003909//DNA li	-
GO:0004862//cAMP	GO:0006469//negative regulation of protein kinase activity;GO:0051338//r
-	-
GO:0003874//6-pyru	GO:0006729//tetrahydrobiopterin biosynthetic process
-	-
GO:0004843//thiol-c	GO:0000278//mitotic cell cycle;GO:0006281//DNA repair;GO:0006511//ub
GO:0004672//protei	GO:0006885//regulation of pH;GO:0008286//insulin receptor signaling pa
GO:0022857//transm	GO:0055085//transmembrane transport
GO:0000166//nucleo	GO:0001954//positive regulation of cell-matrix adhesion;GO:0003323//typ
-	-
-	GO:0032967//positive regulation of collagen biosynthetic process;GO:004
-	-

GO:0001968//fibron	GO:0010811//positive regulation of cell-substrate adhesion;GO:0030198//
GO:0003676//nuclei	-
GO:0005319//lipid tr	GO:0006869//lipid transport
GO:0000166//nucleo	-
GO:0003989//acetyl-	GO:0006633//fatty acid biosynthetic process;GO:0044281//small molecule
-	-
GO:0003735//structu	GO:0006412//translation;GO:0006919//activation of cysteine-type endope
-	-
GO:0004308//exo-al	GO:0006516//glycoprotein catabolic process;GO:0006689//ganglioside ca
-	-
-	-
GO:0016717//oxidor	GO:0006629//lipid metabolic process
GO:0005524//ATP bi	GO:0009235//cobalamin metabolic process;GO:0015889//cobalamin trans
-	-
GO:0005085//guany	GO:0050790//regulation of catalytic activity
-	-
GO:0005515//protei	-
-	-
-	-
-	-
GO:0004930//G-pro	GO:0007165//signal transduction;GO:0007186//G-protein coupled recept
GO:0016491//oxidor	GO:0006666//3-keto-sphinganine metabolic process;GO:0030148//sphing
GO:0016491//oxidor	GO:0009247//glycolipid biosynthetic process
GO:0003924//GTPas	-
-	-
-	GO:0006887//exocytosis;GO:0015031//protein transport
-	-
-	-
-	GO:0018095//protein polyglutamylation
GO:0008271//secon	GO:0008272//sulfate transport;GO:0055085//transmembrane transport
GO:0000993//RNA p	GO:0000956//nuclear-transcribed mRNA catabolic process;GO:0010501//
-	-
GO:0004197//cystei	GO:0002931//response to ischemia;GO:0006508//proteolysis;GO:0006511
-	-
-	-
GO:0003824//catalyt	-
GO:0017147//Wnt-p	GO:0030178//negative regulation of Wnt signaling pathway
GO:0031681//G-pro	GO:0007186//G-protein coupled receptor signaling pathway
GO:0022857//transm	GO:0055085//transmembrane transport
GO:0003677//DNA b	-
-	-
-	-
GO:0005085//guany	GO:0007264//small GTPase mediated signal transduction
-	-
-	-
GO:0005085//guany	GO:0007264//small GTPase mediated signal transduction;GO:0034976//re
GO:0003779//actin b	GO:0051017//actin filament bundle assembly
GO:0005520//insulin	GO:0042104//positive regulation of activated T cell proliferation
GO:0050699//WW d	GO:0007034//vacuolar transport;GO:0010629//negative regulation of gen
GO:0004930//G-pro	GO:0007165//signal transduction;GO:0007186//G-protein coupled recept
-	GO:0046890//regulation of lipid biosynthetic process
-	-
GO:0005085//guany	GO:0050790//regulation of catalytic activity
-	-
-	-
GO:0016740//transfe	GO:0006777//Mo-molybdopterin cofactor biosynthetic process
-	-
GO:0000703//oxidiz	GO:0006281//DNA repair;GO:0006284//base-excision repair;GO:0006285/

GO:0015020//glucur	GO:0006486//protein glycosylation;GO:0015012//heparan sulfate proteog
GO:0004553//hydrol	GO:0005975//carbohydrate metabolic process
GO:0016757//transfe	-
-	-
GO:0001604//uroter	GO:0007165//signal transduction;GO:0007186//G-protein coupled recept
GO:0005504//fatty a	GO:0015908//fatty acid transport;GO:0015909//long-chain fatty acid trans
GO:0003841//1-acyl	-
GO:0004725//protei	GO:0016311//dephosphorylation;GO:0046855//inositol phosphate depho
GO:0005085//guany	GO:0007264//small GTPase mediated signal transduction
-	-
-	-
-	-
GO:0022857//transm	GO:0055085//transmembrane transport
GO:0002039//p53 bi	GO:0000724//double-strand break repair via homologous recombination;
GO:0016491//oxidor	GO:0006629//lipid metabolic process;GO:0008611//ether lipid biosyntheti
-	-
GO:0017128//phosp	GO:0000045//autophagosome assembly;GO:0006914//autophagy;GO:001
-	-
-	-
-	-
-	GO:0006488//dolichol-linked oligosaccharide biosynthetic process
GO:0000976//transc	GO:0006357//regulation of transcription from RNA polymerase II promote
GO:0004506//squale	GO:0016126//sterol biosynthetic process;GO:0042127//regulation of cell p
GO:0005509//calciur	GO:0015012//heparan sulfate proteoglycan biosynthetic process;GO:0030
GO:0003841//1-acyl	GO:0006654//phosphatidic acid biosynthetic process;GO:0010891//negati
GO:0003779//actin b	GO:0051017//actin filament bundle assembly
-	-
-	GO:0006888//ER to Golgi vesicle-mediated transport;GO:0006914//autop
-	-
-	GO:0006887//exocytosis;GO:0015031//protein transport
GO:0036122//BMP b	GO:0007389//pattern specification process;GO:0010454//negative regulat
-	GO:0006886//intracellular protein transport;GO:0006890//retrograde vesi
-	-
GO:0004857//enzym	GO:0006629//lipid metabolic process;GO:0043086//negative regulation of
GO:0000166//nucleo	GO:0006090//pyruvate metabolic process;GO:0006094//gluconeogenesis
GO:0004000//adeno	GO:0000255//allantoin metabolic process;GO:0001666//response to hypo
GO:0005506//iron io	GO:0001949//sebaceous gland cell differentiation;GO:0006629//lipid meta
-	-
GO:0004609//phosp	GO:0006646//phosphatidylethanolamine biosynthetic process;GO:000865
-	-
GO:0003989//acetyl-	GO:0006633//fatty acid biosynthetic process;GO:0044281//small molecule
GO:0045296//cadhe	GO:0007043//cell-cell junction assembly;GO:0060997//dendritic spine mo
-	GO:1901031//regulation of response to reactive oxygen species
GO:0003677//DNA b	GO:0010467//gene expression
GO:0005515//protei	GO:0006355//regulation of transcription, DNA-templated;GO:0006805//xe
GO:0008289//lipid b	GO:0015031//protein transport;GO:0019075//virus maturation;GO:004205
-	-
GO:0004930//G-pro	GO:0001541//ovarian follicle development;GO:0001545//primary ovarian
-	GO:0032967//positive regulation of collagen biosynthetic process;GO:004
GO:0005524//ATP bi	GO:0009235//cobalamin metabolic process;GO:0015889//cobalamin trans
-	-
-	GO:0009617//response to bacterium
GO:0035256//G-pro	GO:0003009//skeletal muscle contraction;GO:0007216//G-protein couple
GO:0030729//acetoa	GO:0006629//lipid metabolic process
GO:0022892//substr	GO:0044765//single-organism transport
GO:0051015//actin f	-
GO:0000166//nucleo	GO:0070588//calcium ion transmembrane transport
GO:0004857//enzym	GO:0006071//glycerol metabolic process;GO:0006631//fatty acid metabol

GO:0003874//6-pyru	GO:0006729//tetrahydrobiopterin biosynthetic process
GO:0009922//fatty a	GO:0019367//fatty acid elongation, saturated fatty acid;GO:0042761//very
GO:0004343//glucos	GO:0006048//UDP-N-acetylglucosamine biosynthetic process
-	-
GO:0004672//protei	GO:0006468//protein phosphorylation;GO:0008631//intrinsic apoptotic si
-	GO:1901031//regulation of response to reactive oxygen species
-	-
GO:0003697//single-	-
GO:0001671//ATPas	GO:0001933//negative regulation of protein phosphorylation;GO:003043
GO:0004386//helica	-
-	-
GO:0016491//oxidor	-
GO:0005515//protei	GO:0016567//protein ubiquitination
GO:0005515//protei	GO:0007043//cell-cell junction assembly;GO:0007155//cell adhesion;GO:0
GO:0005509//calciur	GO:0007165//signal transduction
-	-
GO:0043169//cation	GO:0007165//signal transduction;GO:0022603//regulation of anatomical s
GO:0008109//N-ace	GO:0006486//protein glycosylation;GO:0007179//transforming growth fac
GO:0004672//protei	GO:0006468//protein phosphorylation;GO:0008631//intrinsic apoptotic si
GO:0000976//transc	GO:0006357//regulation of transcription from RNA polymerase II promote
-	-
GO:0001968//fibron	GO:0001933//negative regulation of protein phosphorylation;GO:000716
GO:0015078//hydro	GO:0006811//ion transport;GO:0007034//vacuolar transport;GO:0007035/
GO:0008109//N-ace	GO:0006486//protein glycosylation;GO:0007179//transforming growth fac
-	-
GO:0004888//transm	GO:0007166//cell surface receptor signaling pathway;GO:0007186//G-pro
-	-
GO:0005515//protei	GO:0016567//protein ubiquitination
-	-
GO:0005515//protei	GO:0000281//mitotic cytokinesis;GO:0014067//negative regulation of pho
GO:0046961//protor	GO:0006811//ion transport;GO:1902600//hydrogen ion transmembrane tr
-	-
-	-
GO:0043169//cation	GO:0007165//signal transduction;GO:0022603//regulation of anatomical s
GO:0005515//protei	GO:0007043//cell-cell junction assembly;GO:0007155//cell adhesion;GO:0
GO:0000976//transc	GO:0000122//negative regulation of transcription from RNA polymerase I
-	-
-	-
GO:0046873//metal	GO:0030001//metal ion transport
-	GO:0000463//maturation of LSU-rRNA from tricistronic rRNA transcript (S
GO:0005509//calciur	GO:0007268//synaptic transmission;GO:0035556//intracellular signal trans
-	-
GO:0003677//DNA b	GO:0006334//nucleosome assembly;GO:0042742//defense response to ba
GO:0009922//fatty a	GO:0019367//fatty acid elongation, saturated fatty acid;GO:0042761//very
GO:0000166//nucleo	GO:0000122//negative regulation of transcription from RNA polymerase I
-	-
GO:0003779//actin b	GO:0055013//cardiac muscle cell development
-	-
GO:0017080//sodiur	GO:0006814//sodium ion transport;GO:0010460//positive regulation of he
GO:0001786//phosp	GO:0006910//phagocytosis, recognition;GO:0006911//phagocytosis, engu
GO:0005216//ion ch	GO:0006811//ion transport;GO:0019228//neuronal action potential;GO:00
GO:0005355//glucos	GO:0015747//urate transport;GO:0046415//urate metabolic process;GO:0
GO:0004175//endop	GO:0018126//protein hydroxylation
GO:0005515//protei	-
GO:0005515//protei	GO:0007229//integrin-mediated signaling pathway
-	-
GO:0004672//protei	GO:0001501//skeletal system development;GO:0006468//protein phospho
GO:0005515//protei	GO:0001764//neuron migration;GO:0007158//neuron cell-cell adhesion;G

-	GO:0006506//GPI anchor biosynthetic process
GO:0004609//phosp	GO:0006646//phosphatidylethanolamine biosynthetic process;GO:000865
GO:0008236//serine	GO:0006508//proteolysis
GO:0009922//fatty a	GO:0000038//very long-chain fatty acid metabolic process;GO:0006629//l
GO:0008271//secon	GO:0008272//sulfate transport;GO:0055085//transmembrane transport
GO:0003677//DNA b	GO:0006383//transcription from RNA polymerase III promoter
GO:0022857//transm	GO:0055085//transmembrane transport
-	-
GO:0003677//DNA b	GO:0006334//nucleosome assembly;GO:0006352//DNA-templated transc
-	-
GO:0004842//ubiqui	GO:0000209//protein polyubiquitination;GO:0006915//apoptotic process;
-	-
GO:0005509//calciu	-
-	GO:0006488//dolichol-linked oligosaccharide biosynthetic process
-	-
GO:0008237//metall	GO:0006508//proteolysis
GO:0005509//calciu	GO:0015012//heparan sulfate proteoglycan biosynthetic process;GO:0030
GO:0005488//bindin	-
-	-
GO:0005515//protei	-
GO:0005520//insulin	GO:0042104//positive regulation of activated T cell proliferation
-	-
GO:0003953//NAD+	GO:0001952//regulation of cell-matrix adhesion;GO:0008284//positive re
-	-
GO:0001605//adren	GO:0001525//angiogenesis;GO:0001570//vasculogenesis;GO:0002040//sp
GO:0003676//nuclei	GO:0006388//tRNA splicing, via endonucleolytic cleavage and ligation
-	-
GO:0000976//transc	GO:0002062//chondrocyte differentiation;GO:0006355//regulation of tran
GO:0015175//neutra	GO:0015804//neutral amino acid transport;GO:0042941//D-alanine transp
GO:0070412//R-SM	GO:0010991//negative regulation of SMAD protein complex assembly;GO
GO:0005085//guany	GO:0050790//regulation of catalytic activity
-	-
GO:0005319//lipid t	GO:0006629//lipid metabolic process;GO:0006642//triglyceride mobilizati
GO:0005515//protei	GO:0001764//neuron migration;GO:0007097//nuclear migration;GO:0007
-	-
GO:0003676//nuclei	GO:0006508//proteolysis
GO:0005515//protei	GO:0044763//single-organism cellular process
GO:0017128//phosp	GO:0000045//autophagosome assembly;GO:0006869//lipid transport;GO:
-	-
GO:0005515//protei	-
GO:0003707//steroi	GO:0007340//acrosome reaction;GO:0009611//response to wounding;GO
GO:0000976//transc	GO:0006357//regulation of transcription from RNA polymerase II promote
GO:0001604//uroter	GO:0007165//signal transduction;GO:0007186//G-protein coupled recept
GO:0000976//transc	GO:0000122//negative regulation of transcription from RNA polymerase I
GO:0004452//isoper	GO:0008299//isoprenoid biosynthetic process
GO:0016757//transf	GO:0002121//inter-male aggressive behavior;GO:0042403//thyroid horm
-	-
GO:0000976//transc	GO:0000122//negative regulation of transcription from RNA polymerase I
-	-
GO:0000062//fatty-	GO:0008203//cholesterol metabolic process;GO:0010742//macrophage de
GO:0003779//actin b	GO:0051017//actin filament bundle assembly
GO:0004672//protei	GO:0006468//protein phosphorylation;GO:0007169//transmembrane rece
-	-
GO:0022857//transm	GO:0055085//transmembrane transport
GO:0004930//G-pro	GO:0007165//signal transduction;GO:0007186//G-protein coupled recept
GO:0005515//protei	GO:0000281//mitotic cytokinesis;GO:0014067//negative regulation of pho
-	-
GO:0017128//phosp	GO:0000045//autophagosome assembly;GO:0006914//autophagy;GO:001

GO:0042802//identic	-
-	-
GO:0003874//6-pyru	GO:0006729//tetrahydrobiopterin biosynthetic process
GO:0035256//G-pro	GO:0003009//skeletal muscle contraction;GO:0007216//G-protein couple
GO:0016765//transfe	GO:0008299//isoprenoid biosynthetic process
GO:0000978//RNA p	GO:0006357//regulation of transcription from RNA polymerase II promote
GO:0004672//protei	GO:0006468//protein phosphorylation
GO:0003735//structu	GO:0006412//translation
GO:0000166//nucleo	GO:0006090//pyruvate metabolic process;GO:0006094//gluconeogenesis
GO:0005216//ion ch	GO:0006811//ion transport;GO:0006813//potassium ion transport;GO:003
-	GO:0009267//cellular response to starvation;GO:0030001//metal ion trans
-	-
-	-
-	GO:0051480//regulation of cytosolic calcium ion concentration;GO:19900
-	-
-	GO:1901031//regulation of response to reactive oxygen species
GO:0017128//phosp	GO:0000045//autophagosome assembly;GO:0006869//lipid transport;GO:
GO:0003868//4-hyd	GO:0009072//aromatic amino acid family metabolic process
GO:0005515//protei	GO:0001764//neuron migration;GO:0007097//nuclear migration;GO:0007
-	GO:0043065//positive regulation of apoptotic process;GO:0046330//posit
-	-
GO:0003735//structu	GO:0006412//translation;GO:0006919//activation of cysteine-type endope
GO:0036122//BMP b	GO:0007389//pattern specification process;GO:0010454//negative regulat
GO:0003909//DNA li	-
GO:0004222//metall	GO:0000003//reproduction;GO:0006508//proteolysis
GO:0000166//nucleo	GO:0070588//calcium ion transmembrane transport
-	-
GO:0008233//peptid	GO:0006508//proteolysis
GO:0001968//fibron	GO:0001933//negative regulation of protein phosphorylation;GO:0007162
-	-
-	-
-	-
-	GO:0030070//insulin processing;GO:0060628//regulation of ER to Golgi ve
-	-
GO:0005319//lipid tr	GO:0006869//lipid transport
GO:0031419//cobala	GO:0015889//cobalamin transport
GO:0004386//helicas	-
GO:0005216//ion ch	GO:0006811//ion transport;GO:0006813//potassium ion transport;GO:003
GO:0005085//guany	GO:0001934//positive regulation of protein phosphorylation;GO:0002437/
GO:0005319//lipid tr	GO:0006869//lipid transport;GO:0015914//phospholipid transport;GO:004
GO:0004857//enzym	GO:0006071//glycerol metabolic process;GO:0006631//fatty acid metabol
GO:0005201//extrac	GO:0002244//hematopoietic progenitor cell differentiation
-	-
GO:0005247//voltag	GO:0006821//chloride transport;GO:0055085//transmembrane transport
GO:0005540//hyalur	GO:0007155//cell adhesion
GO:0005504//fatty a	GO:0015908//fatty acid transport;GO:0015909//long-chain fatty acid trans
GO:0005515//protei	-
GO:0004467//long-c	GO:0006631//fatty acid metabolic process
-	-
GO:0016491//oxidor	-
-	-
-	-
-	-
-	-
-	-
-	-
-	-
GO:0005515//protei	-

GO:0004521//endori	-
GO:0005515//protei	-
-	-
-	-
GO:0016627//oxidor	GO:0006629//lipid metabolic process
GO:0001671//ATPas	GO:0001933//negative regulation of protein phosphorylation;GO:003043
GO:0005515//protei	-
GO:0003779//actin b	GO:0006836//neurotransmitter transport;GO:0015844//monoamine transp
-	-
GO:0004190//aspart	GO:0006508//proteolysis;GO:0019538//protein metabolic process
GO:0001968//fibron	GO:0010811//positive regulation of cell-substrate adhesion;GO:0030198//
GO:0003677//DNA b	GO:0006334//nucleosome assembly;GO:0042742//defense response to ba
GO:0003677//DNA b	GO:0006383//transcription from RNA polymerase III promoter
GO:0030298//recept	GO:0010976//positive regulation of neuron projection development;GO:0
-	-
-	-
GO:0004439//phosp	GO:0007420//brain development;GO:0046855//inositol phosphate depho
GO:0000166//nucle	GO:0000122//negative regulation of transcription from RNA polymerase I
GO:0005506//iron ic	GO:0001949//sebaceous gland cell differentiation;GO:0006629//lipid meta
GO:0005515//protei	GO:0000281//mitotic cytokinesis;GO:0014067//negative regulation of pho
-	-
GO:0000976//transc	GO:0000122//negative regulation of transcription from RNA polymerase I
GO:0022857//transm	GO:0010628//positive regulation of gene expression;GO:0010629//negati
GO:0002161//amino	-
GO:0003836//beta-g	GO:0002319//memory B cell differentiation;GO:0006054//N-acetylneuram
GO:0008236//serine	GO:0006508//proteolysis
GO:0015078//hydro	GO:0006811//ion transport;GO:0007034//vacuolar transport;GO:0007035/
GO:0005515//protei	GO:0002091//negative regulation of receptor internalization;GO:0042058/
GO:0008047//enzym	GO:0006486//protein glycosylation;GO:0006487//protein N-linked glycos
-	-
-	GO:0006658//phosphatidylserine metabolic process;GO:0006665//sphing
GO:0004672//protei	GO:0000086//G2/M transition of mitotic cell cycle;GO:0000278//mitotic ce
-	-
GO:0003677//DNA b	GO:0045944//positive regulation of transcription from RNA polymerase II
-	-
-	-
GO:0000976//transc	GO:0000122//negative regulation of transcription from RNA polymerase I
-	-
-	GO:0043065//positive regulation of apoptotic process;GO:0046330//posit
GO:0003779//actin b	GO:0051017//actin filament bundle assembly
GO:0005515//protei	GO:0044763//single-organism cellular process
-	-
-	GO:0018095//protein polyglutamylation
GO:0004496//meval	GO:0006695//cholesterol biosynthetic process;GO:0008299//isoprenoid b
GO:0009922//fatty a	GO:0000038//very long-chain fatty acid metabolic process;GO:0006629//l
GO:0004672//protei	GO:0001501//skeletal system development;GO:0006468//protein phosph
GO:0051015//actin f	-
GO:0003677//DNA b	GO:0006334//nucleosome assembly;GO:0006352//DNA-templated transc
-	-
GO:0003824//cataly	GO:0044710//single-organism metabolic process
GO:0008047//enzym	GO:0006486//protein glycosylation;GO:0006487//protein N-linked glycos
-	GO:0009617//response to bacterium
GO:0051082//unfold	GO:0006457//protein folding
GO:0070412//R-SM	GO:0010991//negative regulation of SMAD protein complex assembly;GO
GO:0005515//protei	GO:0042509//regulation of tyrosine phosphorylation of STAT protein;GO:
-	-
GO:0001786//phosp	GO:0006910//phagocytosis, recognition;GO:0006911//phagocytosis, engu
-	GO:0006886//intracellular protein transport;GO:0006890//retrograde vesi

-	-
GO:0005085//guany	GO:0007264//small GTPase mediated signal transduction;GO:0034976//re
GO:0005509//calciur	GO:0006936//muscle contraction;GO:0030035//microspike assembly;GO:
GO:0030729//acetoa	GO:0006629//lipid metabolic process
GO:0000976//transc	GO:0000122//negative regulation of transcription from RNA polymerase I
GO:0003847//1-alky	GO:0016042//lipid catabolic process
GO:0045296//cadhe	GO:0007043//cell-cell junction assembly;GO:0060997//dendritic spine mc
GO:0017128//phosp	GO:0000045//autophagosome assembly;GO:0006869//lipid transport;GO:
-	-
-	-
-	-
GO:0004672//protei	GO:0006468//protein phosphorylation;GO:0007169//transmembrane rece
-	-
GO:0003676//nuclei	GO:0006508//proteolysis;GO:0016032//viral process
-	-
GO:0016787//hydrol	GO:0006470//protein dephosphorylation;GO:0006805//xenobiotic metabo
-	-
GO:0019902//phosp	GO:0006511//ubiquitin-dependent protein catabolic process;GO:0016567
-	-
GO:0000976//transc	GO:0002062//chondrocyte differentiation;GO:0006355//regulation of tran
GO:0005515//protei	GO:0055085//transmembrane transport
GO:0017128//phosp	GO:0000045//autophagosome assembly;GO:0006914//autophagy;GO:001
-	-
GO:0003707//steroi	GO:0007340//acrosome reaction;GO:0009611//response to wounding;GO
GO:0022857//transm	GO:0055085//transmembrane transport
GO:0005216//ion ch	GO:0006811//ion transport;GO:0006813//potassium ion transport;GO:003
-	-
GO:0005515//protei	GO:0035721//intraciliary retrograde transport;GO:0060271//cilium morph
GO:0005515//protei	-
GO:0016491//oxidor	GO:0006629//lipid metabolic process;GO:0008611//ether lipid biosyntheti
GO:0008289//lipid b	GO:0010873//positive regulation of cholesterol esterification;GO:0010879/
GO:0008236//serine	GO:0006508//proteolysis
GO:0016740//transfe	-
GO:0016765//transfe	GO:0008299//isoprenoid biosynthetic process
GO:0004452//isoper	GO:0008299//isoprenoid biosynthetic process
-	-
-	-
GO:0000703//oxidiz	GO:0006281//DNA repair;GO:0006284//base-excision repair;GO:0006285/
-	-
GO:0005509//calciur	-
GO:0003735//structu	GO:0006412//translation
-	-
GO:0046873//metal	GO:0030001//metal ion transport
GO:0003676//nuclei	GO:0015074//DNA integration;GO:0044260//cellular macromolecule meta
GO:0004857//enzym	GO:0006629//lipid metabolic process;GO:0043086//negative regulation o
-	-
GO:0003779//actin b	GO:0055013//cardiac muscle cell development
GO:0005524//ATP bi	GO:0009235//cobalamin metabolic process;GO:0015889//cobalamin trans
GO:0005085//guany	GO:0050790//regulation of catalytic activity
GO:0005515//protei	GO:0001822//kidney development;GO:0002093//auditory receptor cell mo
-	-
GO:0008289//lipid b	GO:0015031//protein transport;GO:0019075//virus maturation;GO:004205
GO:0043169//cation	GO:0007165//signal transduction;GO:0022603//regulation of anatomical s
GO:0005515//protei	GO:0007043//cell-cell junction assembly;GO:0007155//cell adhesion;GO:0
GO:0003676//nuclei	GO:0006508//proteolysis;GO:0008152//metabolic process;GO:0016032//v
GO:0004307//ethan	GO:0006646//phosphatidylethanolamine biosynthetic process;GO:00865
-	-
-	-

GO:0004103//cholin	GO:0006646//phosphatidylethanolamine biosynthetic process;GO:000665
-	-
GO:0005515//protei	-
GO:0004467//long-c	GO:0006631//fatty acid metabolic process
-	-
GO:0050699//WW d	GO:0007034//vacuolar transport;GO:0010629//negative regulation of gen
-	GO:0009267//cellular response to starvation;GO:0030001//metal ion trans
GO:0022892//substr	GO:0044765//single-organism transport
GO:0008170//N-me	GO:0001692//histamine metabolic process;GO:0001695//histamine catabo
GO:0017080//sodiur	GO:0006814//sodium ion transport;GO:0010460//positive regulation of he
GO:0003735//structu	GO:0006412//translation;GO:0006919//activation of cysteine-type endope
-	-
-	-
GO:0008022//protei	GO:0007026//negative regulation of microtubule depolymerization;GO:00
GO:0001786//phosp	GO:0006910//phagocytosis, recognition;GO:0006911//phagocytosis, engu
GO:0005085//guany	GO:0007264//small GTPase mediated signal transduction
GO:0004386//helicas	-
-	-
-	-
GO:0005515//protei	-
-	-
GO:0022857//transm	GO:0055085//transmembrane transport
GO:0003676//nuclei	GO:0006278//RNA-dependent DNA replication;GO:0006310//DNA recom
-	-
GO:0009922//fatty a	GO:0000038//very long-chain fatty acid metabolic process;GO:0006629//l
GO:0005509//calciur	GO:0015012//heparan sulfate proteoglycan biosynthetic process;GO:0030
-	-
GO:0046961//protor	GO:0006811//ion transport;GO:1902600//hydrogen ion transmembrane tr
GO:0000166//nucleo	GO:0070588//calcium ion transmembrane transport
-	GO:0006658//phosphatidylserine metabolic process;GO:0006665//sphing
GO:0003735//structu	GO:0006412//translation;GO:0006919//activation of cysteine-type endope
GO:0005515//protei	GO:0043031//negative regulation of macrophage activation;GO:0050728/
-	-
-	-
GO:0005178//integri	GO:0000902//cell morphogenesis;GO:0006954//inflammatory response;G
-	-
GO:0003836//beta-g	GO:0002319//memory B cell differentiation;GO:0006054//N-acetylneuram
-	-
GO:0004439//phosp	GO:0007420//brain development;GO:0046855//inositol phosphate depho
GO:0003779//actin b	GO:0051017//actin filament bundle assembly
-	-
GO:0004032//alditol	GO:0006699//bile acid biosynthetic process;GO:0006707//cholesterol cata
GO:0003824//catalyt	GO:0006650//glycerophospholipid metabolic process;GO:0007416//synap
-	-
-	GO:0006887//exocytosis;GO:0015031//protein transport
GO:0003836//beta-g	GO:0002319//memory B cell differentiation;GO:0006054//N-acetylneuram
GO:0000166//nucleo	-
GO:0009922//fatty a	GO:0000038//very long-chain fatty acid metabolic process;GO:0006629//l
-	GO:0007042//lysosomal lumen acidification;GO:0042176//regulation of pr
GO:0008047//enzym	GO:0006486//protein glycosylation;GO:0006487//protein N-linked glycos
-	GO:0009966//regulation of signal transduction;GO:0016055//Wnt signalin
GO:0003841//1-acyl	GO:0006644//phospholipid metabolic process
GO:0005319//lipid tr	GO:0006869//lipid transport;GO:0015914//phospholipid transport;GO:004
GO:0004308//exo-al	GO:0006516//glycoprotein catabolic process;GO:0006689//ganglioside ca
-	-
GO:0003676//nuclei	GO:0006508//proteolysis;GO:0008152//metabolic process;GO:0016032//v
GO:0003824//catalyt	GO:0006777//Mo-molybdopterin cofactor biosynthetic process;GO:00435
-	-

GO:0003676//nuclei	GO:0043488//regulation of mRNA stability
-	-
GO:0001786//phosp	GO:0006910//phagocytosis, recognition;GO:0006911//phagocytosis, engu
GO:0001604//uroter	GO:0007165//signal transduction;GO:0007186//G-protein coupled recept
GO:0050839//cell ad	GO:0007409//axonogenesis;GO:0010975//regulation of neuron projection
-	-
GO:0001517//N-ace	GO:0005975//carbohydrate metabolic process;GO:0006044//N-acetylgluc
GO:0017128//phosp	GO:0000045//autophagosome assembly;GO:0006869//lipid transport;GO:
GO:0004467//long-	GO:0015908//fatty acid transport
GO:0003676//nuclei	GO:0006388//tRNA splicing, via endonucleolytic cleavage and ligation
-	-
GO:0016407//acetylt	-
GO:0004843//thiol-	GO:0006511//ubiquitin-dependent protein catabolic process;GO:0016579
GO:0000166//nucleo	GO:0006468//protein phosphorylation;GO:0010976//positive regulation o
GO:0003676//nuclei	-
GO:0016757//transfe	GO:0006488//dolichol-linked oligosaccharide biosynthetic process
GO:0031681//G-pro	GO:0007186//G-protein coupled receptor signaling pathway
GO:0019902//phosp	GO:0006511//ubiquitin-dependent protein catabolic process;GO:0016567
-	-
GO:0005381//iron io	GO:0046985//positive regulation of hemoglobin biosynthetic process;GO:
-	GO:0046890//regulation of lipid biosynthetic process
-	-
GO:0005515//protei	GO:0001822//kidney development;GO:0002093//auditory receptor cell mo
-	-
-	-
-	GO:0009267//cellular response to starvation;GO:0030001//metal ion trans
GO:0005509//calciur	-
-	-
GO:0005355//glucos	GO:0015747//urate transport;GO:0046415//urate metabolic process;GO:0
-	-
GO:0005540//hyalur	GO:0007155//cell adhesion
GO:0000062//fatty-a	GO:0036151//phosphatidylcholine acyl-chain remodeling;GO:1903060//n
-	-
GO:0003676//nuclei	GO:0006388//tRNA splicing, via endonucleolytic cleavage and ligation
GO:0005216//ion ch	GO:0006811//ion transport;GO:0006813//potassium ion transport;GO:003
GO:0003677//DNA b	-
GO:0016491//oxidor	GO:0006629//lipid metabolic process;GO:0008611//ether lipid biosyntheti
GO:0008170//N-me	GO:0001692//histamine metabolic process;GO:0001695//histamine catabo
GO:0004857//enzym	GO:0006071//glycerol metabolic process;GO:0006631//fatty acid metabol
-	-
GO:0019855//calciur	GO:0006851//mitochondrial calcium ion transport;GO:0051560//mitochor
GO:0002039//p53 bi	GO:0000724//double-strand break repair via homologous recombination;
GO:0004452//isoper	GO:0008299//isoprenoid biosynthetic process
GO:0019855//calciur	GO:0006851//mitochondrial calcium ion transport;GO:0051560//mitochor
-	-
-	-
GO:0022857//transm	GO:0055085//transmembrane transport
GO:0004857//enzym	GO:0006629//lipid metabolic process;GO:0043086//negative regulation of
GO:0004506//squale	GO:0016126//sterol biosynthetic process;GO:0042127//regulation of cell p
GO:0005509//calciur	GO:0006936//muscle contraction;GO:0030035//microspike assembly;GO:0
-	-
-	-
GO:0004843//thiol-	GO:0000278//mitotic cell cycle;GO:0006281//DNA repair;GO:0006511//ub
GO:0004843//thiol-	GO:0006511//ubiquitin-dependent protein catabolic process;GO:0007000
GO:0022857//transm	GO:0055085//transmembrane transport
GO:0046983//protei	GO:0000082//G1/S transition of mitotic cell cycle;GO:0000122//negative r
-	-
-	GO:0006658//phosphatidylserine metabolic process;GO:0006665//sphing

GO:0003824//catalyt	GO:0006777//Mo-molybdopterin cofactor biosynthetic process;GO:00435
GO:0002020//protea	GO:0010951//negative regulation of endopeptidase activity
GO:0008289//lipid b	GO:0032374//regulation of cholesterol transport
GO:0016787//hydrol	GO:0006470//protein dephosphorylation;GO:0006805//xenobiotic metabo
GO:0000976//transc	GO:0002062//chondrocyte differentiation;GO:0006355//regulation of tran
-	-
GO:0015218//pyrimi	GO:0000002//mitochondrial genome maintenance;GO:0002082//regulatic
GO:0001671//ATPas	GO:0001933//negative regulation of protein phosphorylation;GO:003043
GO:0036094//small r	-
GO:0005515//protei	-
-	-
GO:0008022//protei	GO:0007026//negative regulation of microtubule depolymerization;GO:00
-	-
GO:0004497//monoo	-
-	-
-	-
GO:0004496//meval	GO:0006695//cholesterol biosynthetic process;GO:0008299//isoprenoid b
GO:0046961//proton	GO:0006811//ion transport;GO:1902600//hydrogen ion transmembrane tr
GO:0004672//protei	GO:0000086//G2/M transition of mitotic cell cycle;GO:0000278//mitotic ce
GO:0005515//protei	-
GO:0019901//protei	GO:0030334//regulation of cell migration;GO:0034446//substrate adhesio
GO:0004623//phosp	GO:0006644//phospholipid metabolic process;GO:0016042//lipid cataboli
GO:0003677//DNA b	-
GO:0003735//structu	GO:0006412//translation;GO:0006919//activation of cysteine-type endope
-	-
GO:0016757//transfe	GO:0002121//inter-male aggressive behavior;GO:0042403//thyroid horm
GO:0016757//transfe	GO:0006488//dolichol-linked oligosaccharide biosynthetic process
GO:0004467//long-c	GO:0006631//fatty acid metabolic process
GO:0005319//lipid tr	GO:0006869//lipid transport
-	-
GO:0022857//transm	GO:0055085//transmembrane transport
-	-
GO:0003677//DNA b	GO:0006334//nucleosome assembly;GO:0042742//defense response to ba
GO:0015377//cation	GO:0006811//ion transport;GO:0006884//cell volume homeostasis;GO:005
GO:0015078//hydrog	GO:0006811//ion transport;GO:0007034//vacuolar transport;GO:0007035/
GO:0046961//proton	GO:0006811//ion transport;GO:1902600//hydrogen ion transmembrane tr
GO:0005540//hyalur	GO:0007155//cell adhesion
GO:0003676//nuclei	GO:0006508//proteolysis;GO:0008152//metabolic process;GO:0016032//v
GO:0008047//enzym	GO:0006486//protein glycosylation;GO:0006487//protein N-linked glycos
-	-
GO:0005515//protei	GO:0055085//transmembrane transport
-	GO:0006506//GPI anchor biosynthetic process
GO:0003676//nuclei	GO:0006278//RNA-dependent DNA replication;GO:0006310//DNA recom
-	-
GO:0000976//transc	GO:0002062//chondrocyte differentiation;GO:0006355//regulation of tran
-	-
GO:0017128//phosp	GO:0000045//autophagosome assembly;GO:0006914//autophagy;GO:001
GO:0005515//protei	GO:0043031//negative regulation of macrophage activation;GO:0050728/
-	-
-	-
GO:0022857//transm	GO:0055085//transmembrane transport
-	-
GO:0019902//phosp	GO:0006511//ubiquitin-dependent protein catabolic process;GO:0016567
GO:0003677//DNA b	GO:0006334//nucleosome assembly;GO:0042742//defense response to ba
GO:0004366//glycer	GO:0001817//regulation of cytokine production;GO:0006631//fatty acid m
GO:0003779//actin b	GO:0006836//neurotransmitter transport;GO:0015844//monoamine transp
GO:0003836//beta-g	GO:0002319//memory B cell differentiation;GO:0006054//N-acetylneuram
GO:0005515//protei	GO:0000281//mitotic cytokinesis;GO:0007019//microtubule depolymeriza

GO:0005509//calciur	GO:0001654//eye development;GO:0045667//regulation of osteoblast diff
-	-
-	-
GO:0004452//isoper	GO:0008299//isoprenoid biosynthetic process
GO:0001517//N-ace	GO:0005975//carbohydrate metabolic process;GO:0006044//N-acetylgluc
-	-
GO:0004857//enzym	GO:0006629//lipid metabolic process;GO:0043086//negative regulation of
GO:0005515//protei	GO:0007015//actin filament organization;GO:0045806//negative regulatio
GO:0016740//transfe	-
GO:0003677//DNA b	GO:0006334//nucleosome assembly;GO:0006352//DNA-templated transc
GO:0030246//carbol	GO:0006897//endocytosis
GO:0005085//guany	GO:0050790//regulation of catalytic activity
GO:0019902//phosp	GO:0006511//ubiquitin-dependent protein catabolic process;GO:0016567
-	-
-	-
GO:0005515//protei	-
GO:0005216//ion ch	GO:0006811//ion transport;GO:0006813//potassium ion transport;GO:003
GO:0016491//oxidor	-
-	GO:0030070//insulin processing;GO:0060628//regulation of ER to Golgi ve
GO:0004930//G-pro	GO:0007165//signal transduction;GO:0007186//G-protein coupled recept
-	-
GO:0016491//oxidor	GO:0006629//lipid metabolic process;GO:0008611//ether lipid biosyntheti
-	-
-	GO:0098609//cell-cell adhesion
-	-
-	GO:0009267//cellular response to starvation;GO:0030001//metal ion trans
GO:0003676//nuclei	GO:0006508//proteolysis;GO:0008152//metabolic process;GO:0016032//v
GO:0003676//nuclei	GO:0006388//tRNA splicing, via endonucleolytic cleavage and ligation
GO:0005488//bindin	-
GO:0017128//phosp	GO:0000045//autophagosome assembly;GO:0006869//lipid transport;GO:
GO:0008013//beta-c	GO:0007015//actin filament organization;GO:0007155//cell adhesion;GO:0
-	-
-	GO:0032967//positive regulation of collagen biosynthetic process;GO:004
GO:0031681//G-pro	GO:0007186//G-protein coupled receptor signaling pathway
-	-
GO:0022857//transm	GO:0055085//transmembrane transport
-	-
GO:0016491//oxidor	GO:0006629//lipid metabolic process;GO:0006631//fatty acid metabolic p
GO:0003989//acetyl-	GO:0006633//fatty acid biosynthetic process;GO:0044281//small molecule
-	-
GO:0005506//iron ic	GO:0008610//lipid biosynthetic process
-	GO:0046890//regulation of lipid biosynthetic process
GO:0004103//cholin	GO:0006646//phosphatidylethanolamine biosynthetic process;GO:000665
-	-
GO:0003707//steroic	GO:0007340//acrosome reaction;GO:0009611//response to wounding;GO
GO:0003682//chrom	GO:0015908//fatty acid transport;GO:0043066//negative regulation of apo
-	GO:0006888//ER to Golgi vesicle-mediated transport;GO:0006914//autop
GO:0036122//BMP b	GO:0007389//pattern specification process;GO:0010454//negative regulat
-	-
GO:0003824//catalyt	GO:0009226//nucleotide-sugar biosynthetic process;GO:0010595//positiv
-	-
GO:0008289//lipid b	GO:0010873//positive regulation of cholesterol esterification;GO:0010879/
GO:0004896//cytokii	GO:0019221//cytokine-mediated signaling pathway;GO:0034342//respons
-	-
-	-
-	GO:0009267//cellular response to starvation;GO:0030001//metal ion trans
GO:0003676//nuclei	GO:0006508//proteolysis;GO:0016032//viral process
GO:0003677//DNA b	-

GO:0002161//amino	-
GO:0004725//protei	GO:0016311//dephosphorylation;GO:0046855//inositol phosphate depho
-	-
GO:0004672//protei	GO:0006468//protein phosphorylation;GO:0007169//transmembrane rece
-	-
-	-
-	-
GO:0004843//thiol-c	GO:0000278//mitotic cell cycle;GO:0006281//DNA repair;GO:0006511//ub
GO:0004307//ethan	GO:0006646//phosphatidylethanolamine biosynthetic process;GO:000865
-	-
-	GO:0070453//regulation of heme biosynthetic process
GO:0000976//transc	GO:0000122//negative regulation of transcription from RNA polymerase I
-	-
GO:0004452//isoper	GO:0008299//isoprenoid biosynthetic process
GO:0008289//lipid b	GO:0032374//regulation of cholesterol transport
GO:0003779//actin b	GO:0051017//actin filament bundle assembly
GO:0005515//protei	GO:0001764//neuron migration;GO:0007097//nuclear migration;GO:0007
GO:0019855//calciu	GO:0006851//mitochondrial calcium ion transport;GO:0051560//mitochor
GO:0003779//actin b	GO:0051017//actin filament bundle assembly
-	-
GO:0005085//guany	GO:0006623//protein targeting to vacuole;GO:0009306//protein secretion
-	-
GO:0001671//ATPas	GO:0001933//negative regulation of protein phosphorylation;GO:003043
GO:0051082//unfold	GO:0006457//protein folding
GO:0003841//1-acyl	GO:0001819//positive regulation of cytokine production;GO:0006654//ph
GO:0003774//motor	-
-	GO:0006355//regulation of transcription, DNA-templated;GO:0034243//re
GO:0003824//catalyt	GO:0044710//single-organism metabolic process
GO:0005515//protei	GO:0051017//actin filament bundle assembly
-	-
GO:0005515//protei	-
-	GO:0032088//negative regulation of NF-kappaB transcription factor activi
GO:0061657//UFM1	GO:0071569//protein ufmylation
-	GO:0000045//autophagosome assembly;GO:0009267//cellular response t
-	-
GO:0005515//protei	GO:0035721//intraciliary retrograde transport;GO:0060271//cilium morph
-	-
GO:0005540//hyalur	GO:0007155//cell adhesion
GO:0000976//transc	GO:0002062//chondrocyte differentiation;GO:0006355//regulation of tran
GO:0003953//NAD+	GO:0001952//regulation of cell-matrix adhesion;GO:0008284//positive re
GO:0004222//metall	GO:0001542//ovulation from ovarian follicle;GO:0001822//kidney develop
GO:0005515//protei	GO:0016567//protein ubiquitination;GO:0035556//intracellular signal tran
GO:0003677//DNA b	GO:0009267//cellular response to starvation;GO:0043066//negative regula
-	-
GO:0003847//1-alky	GO:0016042//lipid catabolic process
GO:0003841//1-acyl	-
-	-
GO:0004672//protei	GO:0006468//protein phosphorylation;GO:0008631//intrinsic apoptotic si
GO:0005515//protei	-
GO:0009922//fatty a	GO:0000038//very long-chain fatty acid metabolic process;GO:0006629//l
-	-
GO:0016787//hydrol	GO:0006470//protein dephosphorylation;GO:0006805//xenobiotic metab
GO:0008517//folic a	GO:0015884//folic acid transport;GO:0051180//vitamin transport;GO:0051
GO:0003677//DNA b	GO:0045944//positive regulation of transcription from RNA polymerase II
GO:0008047//enzym	GO:0006486//protein glycosylation;GO:0006487//protein N-linked glycos
-	-
GO:0008757//S-ade	GO:0006656//phosphatidylcholine biosynthetic process;GO:0006686//sph
-	GO:0006506//GPI anchor biosynthetic process

GO:0005515//protei	GO:0007155//cell adhesion
-	-
GO:0001786//phosp	GO:0006910//phagocytosis, recognition;GO:0006911//phagocytosis, engu
-	-
GO:0001758//retinal	GO:0042573//retinoic acid metabolic process;GO:0042574//retinal metabo
GO:0000287//magne	GO:0006006//glucose metabolic process;GO:0006094//gluconeogenesis;G
-	-
GO:0019855//calciur	GO:0006851//mitochondrial calcium ion transport;GO:0051560//mitochor
-	-
GO:0003676//nuclei	GO:0006508//proteolysis
-	GO:1901031//regulation of response to reactive oxygen species
-	-
-	-
GO:0000977//RNA p	GO:0000122//negative regulation of transcription from RNA polymerase I
GO:0004222//metall	GO:0000003//reproduction;GO:0006508//proteolysis
GO:0004857//enzym	GO:0001525//angiogenesis;GO:0043066//negative regulation of apoptoti
-	-
-	-
GO:0016491//oxidor	-
GO:0003676//nuclei	GO:0006388//tRNA splicing, via endonucleolytic cleavage and ligation
GO:0046873//metal	GO:0030001//metal ion transport
-	-
-	-
-	-
-	-
GO:0045296//cadhe	GO:0007043//cell-cell junction assembly;GO:0060997//dendritic spine mc
GO:0017128//phosp	GO:0000045//autophagosome assembly;GO:0006914//autophagy;GO:001
-	-
GO:0005515//protei	-
GO:0003824//catalyt	GO:0006207//'de novo' pyrimidine nucleobase biosynthetic process
-	GO:0006886//intracellular protein transport;GO:0006890//retrograde vesi
GO:0004930//G-pro	GO:0007165//signal transduction;GO:0007186//G-protein coupled recept
GO:0016491//oxidor	GO:0006666//3-keto-sphinganine metabolic process;GO:0030148//sphin
-	-
GO:0022857//transm	GO:0055085//transmembrane transport
GO:0046873//metal	GO:0030001//metal ion transport
GO:0003677//DNA b	GO:0009646//response to absence of light;GO:0018298//protein-chromo
-	-
-	-
GO:0000976//transc	GO:0006357//regulation of transcription from RNA polymerase II promote
-	-
GO:0000976//transc	GO:0000122//negative regulation of transcription from RNA polymerase I
GO:0022857//transm	GO:0055085//transmembrane transport
GO:0004623//phosp	GO:0006644//phospholipid metabolic process;GO:0016042//lipid catabo
-	-
GO:0000977//RNA p	GO:0000122//negative regulation of transcription from RNA polymerase I
GO:0003824//catalyt	GO:0009226//nucleotide-sugar biosynthetic process;GO:0010595//positiv
GO:0016717//oxidor	GO:0006629//lipid metabolic process
-	-
GO:0002020//protea	GO:0010951//negative regulation of endopeptidase activity
-	-
-	GO:0051480//regulation of cytosolic calcium ion concentration;GO:19900
GO:0005319//lipid tr	GO:0006869//lipid transport
GO:0005488//bindin	-
GO:0051015//actin f	-
GO:0005085//guany	GO:0050790//regulation of catalytic activity
GO:0004857//enzym	GO:0006071//glycerol metabolic process;GO:0006631//fatty acid metabol
-	GO:0009617//response to bacterium

GO:0016491//oxidor	-
-	-
-	-
-	-
-	GO:0006658//phosphatidylserine metabolic process;GO:0006665//sphing
-	GO:0006629//lipid metabolic process
GO:0004930//G-pro	GO:0007186//G-protein coupled receptor signaling pathway;GO:0043408
GO:0008270//zinc ic	GO:0009086//methionine biosynthetic process;GO:0009235//cobalamin m
-	-
GO:0003847//1-alky	GO:0006629//lipid metabolic process;GO:0006644//phospholipid metabo
-	-
GO:0004930//G-pro	GO:0007186//G-protein coupled receptor signaling pathway;GO:0007200
GO:0005515//protei	GO:0006355//regulation of transcription, DNA-templated;GO:0006805//x
-	-
GO:0046961//protor	GO:0006811//ion transport;GO:1902600//hydrogen ion transmembrane tr
GO:0042802//identic	GO:0019216//regulation of lipid metabolic process;GO:0055088//lipid hor
GO:0004672//protei	GO:0006468//protein phosphorylation
-	-
-	-
-	-
-	-
-	-
GO:0022857//transm	GO:0055085//transmembrane transport
-	-
GO:0001604//uroter	GO:0007165//signal transduction;GO:0007186//G-protein coupled recept
GO:0005085//guany	GO:0006511//ubiquitin-dependent protein catabolic process;GO:0032012
GO:0005247//voltag	GO:0006821//chloride transport;GO:0055085//transmembrane transport
GO:0008237//metall	GO:0006508//proteolysis
-	-
GO:0001965//G-pro	GO:0007165//signal transduction;GO:0007186//G-protein coupled recept
-	GO:0070979//protein K11-linked ubiquitination
GO:0015020//glucur	GO:0006486//protein glycosylation;GO:0015012//heparan sulfate proteog
GO:0005319//lipid tr	GO:0006629//lipid metabolic process;GO:0006642//triglyceride mobilizati
GO:0008289//lipid b	GO:0070508//cholesterol import
GO:0003779//actin b	GO:0051017//actin filament bundle assembly
GO:0004930//G-pro	GO:0001764//neuron migration;GO:0007186//G-protein coupled recepto
GO:0036122//BMP b	GO:0007389//pattern specification process;GO:0010454//negative regulat
GO:0003779//actin b	GO:0051017//actin filament bundle assembly
-	-
GO:0004553//hydrol	GO:0005975//carbohydrate metabolic process
GO:0004467//long-c	GO:0006631//fatty acid metabolic process
GO:0003810//protei	GO:0018149//peptide cross-linking
GO:0016765//transfe	GO:0008299//isoprenoid biosynthetic process
GO:0001605//adrend	GO:0001525//angiogenesis;GO:0001570//vasculogenesis;GO:0002040//sp
-	GO:0007042//lysosomal lumen acidification;GO:0042176//regulation of pr
GO:0008289//lipid b	GO:0070508//cholesterol import
GO:0000703//oxidize	GO:0006281//DNA repair;GO:0006284//base-excision repair;GO:0006285/
GO:0003735//structu	GO:0006412//translation
GO:0004672//protei	GO:0006468//protein phosphorylation;GO:0007169//transmembrane rece
-	GO:0070453//regulation of heme biosynthetic process
GO:0003868//4-hyd	GO:0009072//aromatic amino acid family metabolic process
-	-
GO:0000981//RNA p	GO:0000122//negative regulation of transcription from RNA polymerase I
GO:0003774//motor	-
GO:0000976//transc	GO:0000122//negative regulation of transcription from RNA polymerase I
-	-
GO:0003836//beta-g	GO:0002319//memory B cell differentiation;GO:0006054//N-acetylneuram
GO:0061657//UFM1	GO:0071569//protein ufmylation

GO:0003677//DNA b	GO:0006334//nucleosome assembly;GO:0042742//defense response to ba
GO:0022857//transm	GO:0055085//transmembrane transport
GO:0004857//enzym	GO:0006629//lipid metabolic process;GO:0043086//negative regulation of
GO:0005515//protei	-
GO:0000166//nucleo	GO:0070588//calcium ion transmembrane transport
GO:0003779//actin b	GO:0051017//actin filament bundle assembly
GO:0003841//1-acyl	GO:0001676//long-chain fatty acid metabolic process;GO:0006629//lipid
GO:0004843//thiol-c	GO:0006511//ubiquitin-dependent protein catabolic process;GO:0016579
-	-
GO:0000976//transc	GO:0000122//negative regulation of transcription from RNA polymerase I
GO:0004672//protei	GO:0006468//protein phosphorylation;GO:0008631//intrinsic apoptotic sig
GO:0000166//nucleo	GO:0006468//protein phosphorylation;GO:0010976//positive regulation o
-	-
GO:0000978//RNA p	GO:0001649//osteoblast differentiation;GO:0001755//neural crest cell mig
-	-
GO:0005515//protei	GO:0000281//mitotic cytokinesis;GO:0014067//negative regulation of pho
GO:0004386//helica	-
GO:0004175//endop	GO:0018126//protein hydroxylation
-	GO:0030070//insulin processing;GO:0060628//regulation of ER to Golgi ve
GO:0016491//oxidor	GO:0006629//lipid metabolic process;GO:0008611//ether lipid biosyntheti
-	-
GO:0003824//catalyt	GO:0009226//nucleotide-sugar biosynthetic process;GO:0010595//positiv
GO:0008271//secon	GO:0008272//sulfate transport;GO:0055085//transmembrane transport
GO:0002020//protea	GO:0010951//negative regulation of endopeptidase activity
GO:0003707//steroi	GO:0007340//acrosome reaction;GO:0009611//response to wounding;GO
-	-
GO:0003874//6-pyru	GO:0006729//tetrahydrobiopterin biosynthetic process
GO:0004843//thiol-c	GO:0000278//mitotic cell cycle;GO:0006281//DNA repair;GO:0006511//ub
GO:0005515//protei	GO:0016567//protein ubiquitination;GO:0035556//intracellular signal trans
-	-
-	GO:0000463//maturation of LSU-rRNA from tricistronic rRNA transcript (S
-	-
GO:0022857//transm	GO:0055085//transmembrane transport
GO:0003697//single-	-
-	GO:0000045//autophagosome assembly;GO:0009267//cellular response to
GO:0016787//hydrol	GO:0006470//protein dephosphorylation;GO:0006805//xenobiotic metabo
GO:0008168//methy	GO:0032259//methylation;GO:0035498//carnosine metabolic process
-	-
GO:0045296//cadhe	GO:0007043//cell-cell junction assembly;GO:0060997//dendritic spine mo
GO:0003868//4-hyd	GO:0009072//aromatic amino acid family metabolic process
GO:0005515//protei	GO:0042634//regulation of hair cycle
GO:0005515//protei	GO:0006955//immune response;GO:0006956//complement activation
GO:0003682//chrom	GO:0000122//negative regulation of transcription from RNA polymerase I
GO:0005085//guany	GO:0006623//protein targeting to vacuole;GO:0009306//protein secretion
-	-
GO:0003824//catalyt	GO:0006778//porphyrin-containing compound metabolic process;GO:000
-	GO:0006886//intracellular protein transport;GO:0006890//retrograde vesic
-	-
-	-
GO:0005509//calciur	-
-	-
GO:0005515//protei	GO:0035987//endodermal cell differentiation
-	-
-	-
GO:0000977//RNA p	GO:0000122//negative regulation of transcription from RNA polymerase I
GO:0003677//DNA b	GO:0006334//nucleosome assembly;GO:0006352//DNA-templated transc
GO:0003824//catalyt	GO:0005975//carbohydrate metabolic process
GO:0016491//oxidor	GO:0009247//glycolipid biosynthetic process

-	GO:0000463//maturation of LSU-rRNA from tricistronic rRNA transcript (S
-	-
GO:0008236//serine	GO:0006508//proteolysis
GO:0042577//lipid p	GO:0006644//phospholipid metabolic process
-	-
GO:0002161//amino	-
-	-
GO:0003824//catalyt	GO:0006650//glycerophospholipid metabolic process;GO:0007416//synap
GO:0005247//voltag	GO:0006821//chloride transport;GO:0055085//transmembrane transport
GO:0016491//oxidor	GO:0009247//glycolipid biosynthetic process
GO:0005515//protei	GO:0000281//mitotic cytokinesis;GO:0007019//microtubule depolymeriza
GO:0042577//lipid p	GO:0006644//phospholipid metabolic process
-	-
-	-
-	GO:0006506//GPI anchor biosynthetic process
GO:0004857//enzym	GO:0006071//glycerol metabolic process;GO:0006631//fatty acid metabol
GO:0046873//metal	GO:0030001//metal ion transport
GO:0000976//transc	GO:0000122//negative regulation of transcription from RNA polymerase I
GO:0000287//magne	GO:0006006//glucose metabolic process;GO:0006094//gluconeogenesis;G
-	-
-	-
-	-
-	-
-	-
GO:0005515//protei	-
GO:0003824//catalyt	GO:0006778//porphyrin-containing compound metabolic process;GO:000
-	GO:0006886//intracellular protein transport;GO:0006890//retrograde vesi
GO:0016757//transfe	GO:0002121//inter-male aggressive behavior;GO:0042403//thyroid horm
GO:0005515//protei	-
GO:0022857//transm	GO:0055085//transmembrane transport
-	-
GO:0045296//cadhe	GO:0007043//cell-cell junction assembly;GO:0060997//dendritic spine mo
-	-
-	-
GO:0003847//1-alky	GO:0016042//lipid catabolic process
-	-
-	-
-	GO:0007399//nervous system development
GO:0005515//protei	GO:0002091//negative regulation of receptor internalization;GO:0042058/
-	-
GO:0003924//GTPas	GO:0007165//signal transduction;GO:0007186//G-protein coupled recept
-	GO:0070453//regulation of heme biosynthetic process
GO:0005319//lipid tr	GO:0006629//lipid metabolic process;GO:0006642//triglyceride mobilizati
GO:0015020//glucur	GO:0006486//protein glycosylation;GO:0015012//heparan sulfate proteog
-	-
-	-
GO:0005515//protei	-
-	-
GO:0003841//1-acyl	GO:0001819//positive regulation of cytokine production;GO:0006654//ph
GO:0000976//transc	GO:0002062//chondrocyte differentiation;GO:0006355//regulation of tran
-	-
GO:0005515//protei	GO:0006355//regulation of transcription, DNA-templated;GO:0006805//x
GO:0000287//magne	GO:0006468//protein phosphorylation;GO:0030036//actin cytoskeleton or
-	-
-	-
-	GO:0007507//heart development
-	-
-	GO:0021817//nucleokinesis involved in cell motility in cerebral cortex radi

GO:0016798//hydrol	GO:0008284//positive regulation of cell proliferation;GO:0030198//extrace
GO:0003677//DNA b	GO:0010467//gene expression
GO:0005506//iron ic	GO:0001949//sebaceous gland cell differentiation;GO:0006629//lipid meta
GO:0005515//protei	GO:0000281//mitotic cytokinesis;GO:0007019//microtubule depolymeriza
-	-
GO:0008289//lipid b	GO:0070508//cholesterol import
GO:0001758//retinal	GO:0002072//optic cup morphogenesis involved in camera-type eye deve
GO:0003779//actin b	GO:0051017//actin filament bundle assembly
GO:0005515//protei	GO:0046330//positive regulation of JNK cascade;GO:0090090//negative re
GO:0004452//isoper	GO:0008299//isoprenoid biosynthetic process
-	GO:0006506//GPI anchor biosynthetic process
-	-
GO:0019855//calciur	GO:0006851//mitochondrial calcium ion transport;GO:0051560//mitochor
-	-
-	GO:1901031//regulation of response to reactive oxygen species
-	-
GO:0004672//protei	GO:0006468//protein phosphorylation;GO:0008631//intrinsic apoptotic si
-	-
-	-
-	-
-	-
-	-
-	-
-	-
GO:0003677//DNA b	GO:0006334//nucleosome assembly;GO:0016584//nucleosome positionin
-	GO:0006629//lipid metabolic process
GO:0004489//methy	GO:0001843//neural tube closure;GO:0006555//methionine metabolic pro
-	-
-	-
-	GO:0070979//protein K11-linked ubiquitination
GO:0005504//fatty a	GO:0015908//fatty acid transport;GO:0015909//long-chain fatty acid trans
GO:0042802//identic	GO:0019216//regulation of lipid metabolic process;GO:0055088//lipid hor
GO:0002020//protea	GO:0010951//negative regulation of endopeptidase activity
-	GO:0009267//cellular response to starvation;GO:0030001//metal ion trans
GO:0005515//protei	-
GO:0004857//enzym	GO:0001525//angiogenesis;GO:0043066//negative regulation of apoptoti
GO:0016757//transfe	-
GO:0036094//small r	-
-	-
GO:0008271//secon	GO:0008272//sulfate transport;GO:0055085//transmembrane transport
GO:0000976//transc	GO:0006357//regulation of transcription from RNA polymerase II promote
-	-
-	-
GO:0008270//zinc ic	GO:0009086//methionine biosynthetic process;GO:0009235//cobalamin m
-	-
-	-
GO:0004497//mono	-
-	-
GO:0005515//protei	GO:0006955//immune response;GO:0006956//complement activation
GO:0000166//nucleo	GO:0001954//positive regulation of cell-matrix adhesion;GO:0003323//typ
GO:0003676//nuclei	GO:0019722//calcium-mediated signaling;GO:0050790//regulation of cata
GO:0016491//oxidor	GO:0006666//3-keto-sphinganine metabolic process;GO:0030148//sphing
-	-
-	-
GO:0016491//oxidor	-
-	GO:0030070//insulin processing;GO:0060628//regulation of ER to Golgi ve
GO:0004862//cAMP	GO:0006469//negative regulation of protein kinase activity;GO:0051338//r
GO:0003779//actin b	GO:0055013//cardiac muscle cell development

GO:0003841//1-acyl	GO:0006629//lipid metabolic process;GO:0006644//phospholipid metabo
GO:0004857//enzym	GO:0001525//angiogenesis;GO:0043066//negative regulation of apoptotic
GO:0003676//nuclei	GO:0006508//proteolysis
GO:0043169//cation	GO:0007165//signal transduction;GO:0022603//regulation of anatomical s
GO:0005515//protei	GO:0007043//cell-cell junction assembly;GO:0007155//cell adhesion;GO:0
-	GO:0044237//cellular metabolic process
-	-
GO:0001786//phosp	-
-	-
GO:0005515//protei	GO:0000281//mitotic cytokinesis;GO:0007019//microtubule depolymerizat
GO:0008146//sulfotr	-
GO:0000166//nucleo	GO:0006468//protein phosphorylation;GO:0010976//positive regulation o
GO:0004843//thiol-c	GO:0006511//ubiquitin-dependent protein catabolic process;GO:0016579
GO:0000993//RNA p	GO:0000956//nuclear-transcribed mRNA catabolic process;GO:0010501//
GO:0016717//oxidor	GO:0006629//lipid metabolic process
GO:0016717//oxidor	GO:0006629//lipid metabolic process
GO:0008194//UDP-g	-
GO:0000981//RNA p	GO:0000122//negative regulation of transcription from RNA polymerase I
-	-
GO:0005509//calcium	GO:0001654//eye development;GO:0045667//regulation of osteoblast diff
-	-
GO:0003713//transc	GO:0007219//Notch signaling pathway;GO:0045944//positive regulation o
GO:0004366//glycer	GO:0001817//regulation of cytokine production;GO:0006631//fatty acid m
-	-
GO:0004252//serine	GO:0006508//proteolysis;GO:0016485//protein processing
GO:0005509//calcium	-
GO:0001604//uroten	GO:0007165//signal transduction;GO:0007186//G-protein coupled recept
GO:0005515//protei	GO:0055085//transmembrane transport
GO:0003824//catalyt	GO:0006650//glycerophospholipid metabolic process;GO:0007416//synap
GO:0016491//oxidor	GO:0006666//3-keto-sphinganine metabolic process;GO:0030148//sphing
-	-
GO:0005506//iron io	GO:0001666//response to hypoxia;GO:0017185//peptidyl-lysine hydroxyla
GO:0005515//protei	-
-	GO:0070453//regulation of heme biosynthetic process
GO:0036122//BMP b	GO:0007389//pattern specification process;GO:0010454//negative regulat
-	GO:0006887//exocytosis;GO:0015031//protein transport
-	-
-	GO:0007042//lysosomal lumen acidification;GO:0042176//regulation of pr
-	-
GO:0008113//peptid	-
GO:0003707//steroid	GO:0007340//acrosome reaction;GO:0009611//response to wounding;GO
-	GO:0007042//lysosomal lumen acidification;GO:0042176//regulation of pr
GO:0000287//magne	GO:0006006//glucose metabolic process;GO:0006094//gluconeogenesis;G
GO:0001540//beta-a	GO:0006511//ubiquitin-dependent protein catabolic process;GO:0006516
-	-
-	-
GO:0005509//calcium	GO:0015012//heparan sulfate proteoglycan biosynthetic process;GO:0030
GO:0003989//acetyl-	GO:0006633//fatty acid biosynthetic process;GO:0044281//small molecule
-	-
GO:0002161//amino	-
GO:0003841//1-acyl	GO:0001819//positive regulation of cytokine production;GO:0006654//ph
GO:0003676//nuclei	GO:0006508//proteolysis
-	GO:0032967//positive regulation of collagen biosynthetic process;GO:004
-	-
GO:0004930//G-pro	GO:0007165//signal transduction;GO:0007186//G-protein coupled recept
-	GO:0006886//intracellular protein transport;GO:0006890//retrograde vesic
GO:0003677//DNA b	GO:0006334//nucleosome assembly
GO:0015020//glucur	GO:0006486//protein glycosylation;GO:0015012//heparan sulfate proteog

-	-
GO:0005515//protei	-
-	-
GO:0045296//cadhe	GO:0007043//cell-cell junction assembly;GO:0060997//dendritic spine mo
GO:0003682//chrom	GO:0001945//lymph vessel development;GO:0003017//lymph circulation;(
GO:0000976//transc	GO:0000122//negative regulation of transcription from RNA polymerase I
GO:0035256//G-pro	GO:0003009//skeletal muscle contraction;GO:0007216//G-protein couple
GO:0004843//thiol-(	GO:0006511//ubiquitin-dependent protein catabolic process;GO:0016579
GO:0000166//nucle	GO:0006468//protein phosphorylation;GO:0010976//positive regulation o
GO:0016757//transfe	GO:0002121//inter-male aggressive behavior;GO:0042403//thyroid horm
GO:0005515//protei	GO:0000281//mitotic cytokinesis;GO:0014067//negative regulation of pho
-	-
-	GO:0006488//dolichol-linked oligosaccharide biosynthetic process
GO:0008013//beta-(	GO:0007015//actin filament organization;GO:0007155//cell adhesion;GO:(
GO:0000166//nucle	GO:0070588//calcium ion transmembrane transport
GO:0005509//calciu	GO:0015012//heparan sulfate proteoglycan biosynthetic process;GO:0030
GO:0005085//guany	GO:0007264//small GTPase mediated signal transduction
GO:0004843//thiol-(	GO:0000278//mitotic cell cycle;GO:0006281//DNA repair;GO:0006511//ub
GO:0003677//DNA b	GO:0006334//nucleosome assembly;GO:0006352//DNA-templated transc
GO:0000977//RNA p	GO:0000122//negative regulation of transcription from RNA polymerase I
-	GO:0009966//regulation of signal transduction;GO:0016055//Wnt signalin
GO:0004386//helica	-
GO:0003676//nuclei	GO:0006508//proteolysis;GO:0016032//viral process
GO:0005515//protei	-
GO:0004609//phosp	GO:0006646//phosphatidylethanolamine biosynthetic process;GO:00865
GO:0005506//iron ic	GO:0008610//lipid biosynthetic process
-	-
-	-
GO:0004672//protei	GO:0006468//protein phosphorylation;GO:0008631//intrinsic apoptotic si
GO:0008195//phosp	GO:0006357//regulation of transcription from RNA polymerase II promote
GO:0005515//protei	GO:0007155//cell adhesion;GO:0007156//homophilic cell adhesion via pla
GO:0001968//fibron	GO:0001933//negative regulation of protein phosphorylation;GO:000716
GO:0001758//retinal	GO:0002072//optic cup morphogenesis involved in camera-type eye deve
GO:0003847//1-alky	GO:0016042//lipid catabolic process
GO:0003874//6-pyru	GO:0006729//tetrahydrobiopterin biosynthetic process
GO:0008170//N-me	GO:0001692//histamine metabolic process;GO:0001695//histamine catab
GO:0000977//RNA p	GO:0000122//negative regulation of transcription from RNA polymerase I
GO:0005515//protei	GO:0006888//ER to Golgi vesicle-mediated transport
GO:0005540//hyalur	GO:0007155//cell adhesion
GO:0003677//DNA b	GO:0006334//nucleosome assembly;GO:0006352//DNA-templated transc
GO:0098640//integri	GO:0007155//cell adhesion;GO:0007229//integrin-mediated signaling pat
-	-
GO:0022892//substr	GO:0044765//single-organism transport
-	-
GO:0003677//DNA b	-
-	-
-	-
GO:0003735//structu	GO:0006412//translation
-	-
GO:0003824//cataly	GO:0006650//glycerophospholipid metabolic process;GO:0007416//synap
-	-
GO:0005524//ATP bi	GO:0009235//cobalamin metabolic process;GO:0015889//cobalamin trans
-	-
GO:0005506//iron ic	GO:0001949//sebaceous gland cell differentiation;GO:0006629//lipid meta
-	-
GO:0005515//protei	GO:0007043//cell-cell junction assembly;GO:0007155//cell adhesion;GO:(
GO:0005515//protei	GO:0001822//kidney development;GO:0002093//auditory receptor cell m
GO:0016757//transfe	GO:0006488//dolichol-linked oligosaccharide biosynthetic process

GO:0043169//cation	GO:0007165//signal transduction;GO:0022603//regulation of anatomical s
-	-
-	-
-	-
-	-
-	-
GO:0004930//G-pro	GO:0007186//G-protein coupled receptor signaling pathway
GO:0008109//N-ace	GO:0006486//protein glycosylation;GO:0007179//transforming growth fac
GO:0001671//ATPas	GO:0001933//negative regulation of protein phosphorylation;GO:003043
GO:0005515//protei	GO:0016567//protein ubiquitination
-	-
GO:0008480//sarcos	GO:1901053//sarcosine catabolic process
GO:0003677//DNA b	GO:0006383//transcription from RNA polymerase III promoter
GO:0008236//serine	GO:0006508//proteolysis
-	-
-	-
-	-
-	-
GO:0016491//oxidor	GO:0006666//3-keto-sphinganine metabolic process;GO:0030148//sphing
GO:0000166//nucleo	GO:0006468//protein phosphorylation;GO:0010976//positive regulation o
-	-
GO:0003924//GTPas	-
GO:0004930//G-pro	GO:0007186//G-protein coupled receptor signaling pathway
-	GO:0006355//regulation of transcription, DNA-templated;GO:0034243//re
-	-
GO:0004843//thiol-c	GO:0006511//ubiquitin-dependent protein catabolic process;GO:0016579
GO:0009922//fatty a	GO:0019367//fatty acid elongation, saturated fatty acid;GO:0042761//very
GO:0005102//recept	GO:0007160//cell-matrix adhesion;GO:0007596//blood coagulation;GO:00
GO:0005504//fatty a	GO:0015908//fatty acid transport;GO:0015909//long-chain fatty acid trans
-	GO:0000045//autophagosome assembly;GO:0009267//cellular response to
GO:0042577//lipid p	GO:0006644//phospholipid metabolic process
-	GO:0000463//maturation of LSU-rRNA from tricistronic rRNA transcript (S
GO:0005216//ion ch	GO:0006811//ion transport;GO:0006812//cation transport;GO:0034220//io
GO:0016787//hydrol	GO:0006470//protein dephosphorylation;GO:0006805//xenobiotic metabo
GO:0005515//protei	-
-	-
-	-
GO:0008270//zinc io	GO:0009056//catabolic process;GO:0030855//epithelial cell differentiation
GO:0003796//lysozy	GO:0008152//metabolic process;GO:0016998//cell wall macromolecule ca
GO:0003676//nuclei	GO:0006278//RNA-dependent DNA replication;GO:0044260//cellular mac
GO:0016717//oxidor	GO:0006629//lipid metabolic process
-	GO:0007507//heart development
GO:0003677//DNA b	GO:0006355//regulation of transcription, DNA-templated;GO:0045944//p
GO:0001786//phosp	GO:0006910//phagocytosis, recognition;GO:0006911//phagocytosis, engu
-	-
-	-
-	GO:0007098//centrosome cycle;GO:0035020//regulation of Rac protein si
GO:0003847//1-alky	GO:0016042//lipid catabolic process
GO:0008757//S-ade	GO:0006656//phosphatidylcholine biosynthetic process;GO:0006686//sph
GO:0005085//guany	GO:0007264//small GTPase mediated signal transduction;GO:0034976//re
-	GO:0007042//lysosomal lumen acidification;GO:0042176//regulation of pr
GO:0003676//nuclei	GO:0006278//RNA-dependent DNA replication;GO:0006310//DNA recom
GO:0005515//protei	GO:0044763//single-organism cellular process
GO:0009922//fatty a	GO:0000038//very long-chain fatty acid metabolic process;GO:0006629//l
GO:0005515//protei	GO:0006888//ER to Golgi vesicle-mediated transport
GO:0005515//protei	GO:0045765//regulation of angiogenesis
GO:0003676//nuclei	GO:0006508//proteolysis
GO:0004672//protei	GO:0001501//skeletal system development;GO:0006468//protein phospho

GO:0005509//calciur	GO:0001654//eye development;GO:0045667//regulation of osteoblast diff
GO:0003677//DNA k	GO:0006334//nucleosome assembly;GO:0042742//defense response to ba
GO:0003824//catalyt	GO:0006778//porphyrin-containing compound metabolic process;GO:000(
-	-
-	-
GO:0004366//glycer	GO:0001817//regulation of cytokine production;GO:0006631//fatty acid m
-	GO:0016192//vesicle-mediated transport
GO:0005515//protei	GO:0055085//transmembrane transport
-	GO:0018095//protein polyglutamylation
-	-
-	-
GO:0004930//G-pro	GO:0007165//signal transduction;GO:0007186//G-protein coupled recept
GO:0003989//acetyl-	GO:0001894//tissue homeostasis;GO:0006084//acetyl-CoA metabolic pro
GO:0005515//protei	GO:0006355//regulation of transcription, DNA-templated;GO:0006805//x
GO:0030246//carbol	GO:0006897//endocytosis
GO:0016491//oxidor	GO:0009247//glycolipid biosynthetic process
GO:0019902//phosp	GO:0006511//ubiquitin-dependent protein catabolic process;GO:0016567
-	-
-	-
-	-
GO:0003676//nuclei	GO:0006508//proteolysis;GO:0016032//viral process
GO:0003836//beta-(	GO:0002319//memory B cell differentiation;GO:0006054//N-acetylneuram
-	GO:0006887//exocytosis;GO:0015031//protein transport
GO:0005515//protei	GO:0000281//mitotic cytokinesis;GO:0007019//microtubule depolymeriza
-	-
GO:0003824//catalyt	GO:0006207//'de novo' pyrimidine nucleobase biosynthetic process
GO:0005319//lipid tr	GO:0006629//lipid metabolic process;GO:0006642//triglyceride mobilizati
GO:0002161//amino	-
-	-
GO:0008195//phosp	GO:0006720//isoprenoid metabolic process;GO:0018342//protein prenyla
-	-
GO:0005104//fibrob	GO:0001755//neural crest cell migration;GO:0001934//positive regulation
GO:0003676//nuclei	GO:0019722//calcium-mediated signaling;GO:0050790//regulation of cat
-	-
-	-
-	-
GO:0008271//secon	GO:0008272//sulfate transport;GO:0055085//transmembrane transport
GO:0001968//fibron	GO:0010811//positive regulation of cell-substrate adhesion;GO:0030198//
GO:0004862//cAMP	GO:0006469//negative regulation of protein kinase activity;GO:0051338//r
GO:0022857//transm	GO:0055085//transmembrane transport
GO:0003824//catalyt	GO:0009226//nucleotide-sugar biosynthetic process;GO:0010595//positiv
GO:0001540//beta-a	GO:0006511//ubiquitin-dependent protein catabolic process;GO:0006516
-	-
-	GO:0007042//lysosomal lumen acidification;GO:0042176//regulation of pr
GO:0005515//protei	GO:0055085//transmembrane transport
GO:0000166//nucle	GO:0001954//positive regulation of cell-matrix adhesion;GO:0003323//typ
-	-
GO:0003676//nuclei	GO:0006508//proteolysis;GO:0008152//metabolic process;GO:0016032//v
-	-
-	-
GO:0005515//protei	GO:0010468//regulation of gene expression;GO:0016567//protein ubiquit
-	GO:0009987//cellular process
-	-
-	-
GO:0005506//iron io	GO:0001666//response to hypoxia;GO:0017185//peptidyl-lysine hydroxyla
-	-
GO:0017080//sodiur	GO:0006814//sodium ion transport;GO:0010460//positive regulation of he
GO:0005515//protei	-

GO:0016740//transfe	GO:0045723//positive regulation of fatty acid biosynthetic process
GO:0019901//protei	GO:0030334//regulation of cell migration;GO:0034446//substrate adhesio
-	-
GO:0008236//serine	GO:0006508//proteolysis
GO:0004475//mann	GO:0006486//protein glycosylation;GO:0009058//biosynthetic process;GC
-	-
GO:0016491//oxidor	GO:0006629//lipid metabolic process
-	-
-	-
-	-
GO:0035256//G-pro	GO:0003009//skeletal muscle contraction;GO:0007216//G-protein couple
GO:0004843//thiol-c	GO:0006511//ubiquitin-dependent protein catabolic process;GO:0016579
-	GO:0006488//dolichol-linked oligosaccharide biosynthetic process
GO:0005085//guany	GO:0007264//small GTPase mediated signal transduction
-	-
GO:0005540//hyalur	GO:0007155//cell adhesion
GO:0000166//nucleo	GO:0006468//protein phosphorylation;GO:0010976//positive regulation o
GO:0003779//actin b	GO:0007010//cytoskeleton organization;GO:0008360//regulation of cell sh
-	GO:0006629//lipid metabolic process
-	GO:0006629//lipid metabolic process
-	GO:0070979//protein K11-linked ubiquitination
GO:0003779//actin b	GO:0055013//cardiac muscle cell development
GO:0000976//transc	GO:0000122//negative regulation of transcription from RNA polymerase I
GO:0043169//cation	GO:0007165//signal transduction;GO:0022603//regulation of anatomical s
-	-
GO:0005515//protei	-
GO:0005515//protei	GO:0007043//cell-cell junction assembly;GO:0007155//cell adhesion;GO:0
-	-
GO:0004609//phosp	GO:0006646//phosphatidylethanolamine biosynthetic process;GO:000865
-	GO:0030070//insulin processing;GO:0060628//regulation of ER to Golgi ve
GO:0001758//retinal	GO:0002072//optic cup morphogenesis involved in camera-type eye deve
GO:0001671//ATPas	GO:0001933//negative regulation of protein phosphorylation;GO:003043
GO:0003735//structu	GO:0006412//translation;GO:0006919//activation of cysteine-type endope
-	-
-	-
GO:0001786//phosp	-
GO:0005319//lipid tr	GO:0006869//lipid transport
-	-
GO:0003841//1-acyl	GO:0006629//lipid metabolic process;GO:0006644//phospholipid metabo
GO:0003779//actin b	GO:0055013//cardiac muscle cell development
GO:0005515//protei	GO:0006570//tyrosine metabolic process;GO:0007613//memory;GO:0008
-	-
GO:0001968//fibron	GO:0001933//negative regulation of protein phosphorylation;GO:0007162
GO:0005216//ion ch	GO:0006811//ion transport;GO:0006813//potassium ion transport;GO:003
-	-
GO:0003810//protei	GO:0018149//peptide cross-linking
GO:0004843//thiol-c	GO:0000278//mitotic cell cycle;GO:0006281//DNA repair;GO:0006511//ub
GO:0042802//identic	GO:1904262//negative regulation of TORC1 signaling
GO:0005524//ATP bi	GO:0009235//cobalamin metabolic process;GO:0015889//cobalamin trans
-	-
GO:0004857//enzym	GO:0006071//glycerol metabolic process;GO:0006631//fatty acid metabol
-	GO:0070453//regulation of heme biosynthetic process
GO:0004386//helicas	-
GO:0042802//identic	-
-	-
GO:0003924//GTPas	-
GO:0042577//lipid p	GO:0006644//phospholipid metabolic process
-	-

GO:0003847//1-alky	GO:0016042//lipid catabolic process
GO:0004190//aspart	GO:0006508//proteolysis;GO:0019538//protein metabolic process
GO:0005509//calciur	GO:0015012//heparan sulfate proteoglycan biosynthetic process;GO:0030
GO:0008109//N-ace	GO:0006486//protein glycosylation;GO:0007179//transforming growth fac
GO:0000166//nucleo	GO:0070588//calcium ion transmembrane transport
GO:0003677//DNA b	GO:0006334//nucleosome assembly;GO:0016584//nucleosome positionin
GO:0005515//protei	GO:0016567//protein ubiquitination
GO:0003824//catalyt	GO:0006777//Mo-molybdopterin cofactor biosynthetic process;GO:00435
-	GO:0000045//autophagosome assembly;GO:0009267//cellular response to
GO:0005515//protei	GO:0000281//mitotic cytokinesis;GO:0014067//negative regulation of pho
-	-
GO:0008270//zinc ic	GO:0009086//methionine biosynthetic process;GO:0009235//cobalamin m
-	-
-	-
-	-
GO:0000976//transc	GO:0000122//negative regulation of transcription from RNA polymerase I
-	-
GO:0003677//DNA b	GO:0006383//transcription from RNA polymerase III promoter
-	-
GO:0009922//fatty a	GO:0019367//fatty acid elongation, saturated fatty acid;GO:0042761//very
-	-
GO:0005515//protei	GO:0044763//single-organism cellular process
-	-
GO:0022892//substr	GO:0044765//single-organism transport
GO:0003677//DNA b	GO:0009646//response to absence of light;GO:0018298//protein-chromo
-	-
GO:0003824//catalyt	GO:0006778//porphyrin-containing compound metabolic process;GO:000
GO:0030145//manga	GO:0006508//proteolysis;GO:0019538//protein metabolic process
GO:0005104//fibrob	GO:0000132//establishment of mitotic spindle orientation;GO:0001656//m
GO:0005515//protei	GO:0016567//protein ubiquitination;GO:0035556//intracellular signal tran
GO:0015175//neutra	GO:0015804//neutral amino acid transport;GO:0042941//D-alanine transp
-	GO:0021817//nucleokinesis involved in cell motility in cerebral cortex radi
-	-
GO:0004307//ethano	GO:0006646//phosphatidylethanolamine biosynthetic process;GO:000865
GO:0003796//lysozy	GO:0009253//peptidoglycan catabolic process;GO:0016998//cell wall mac
-	GO:0043065//positive regulation of apoptotic process;GO:0046330//posit
-	-
GO:0005085//guany	GO:0006623//protein targeting to vacuole;GO:0009306//protein secretion
GO:0002161//amino	-
-	-
-	-
-	GO:0043065//positive regulation of apoptotic process;GO:0046330//posit
GO:0019902//phosp	GO:0006511//ubiquitin-dependent protein catabolic process;GO:0016567
GO:0004672//protei	GO:0001501//skeletal system development;GO:0006468//protein phospho
GO:0005509//calciur	-
GO:0004842//ubiqui	GO:0000209//protein polyubiquitination;GO:0006915//apoptotic process;
GO:0001604//uroter	GO:0007165//signal transduction;GO:0007186//G-protein coupled recept
GO:0005515//protei	GO:0044763//single-organism cellular process
GO:0000977//RNA p	GO:0000122//negative regulation of transcription from RNA polymerase I
GO:0005515//protei	GO:0035721//intraciliary retrograde transport;GO:0060271//cilium morph
GO:0022857//transm	GO:0055085//transmembrane transport
GO:0003677//DNA b	GO:0006383//transcription from RNA polymerase III promoter
-	-
GO:0000977//RNA p	GO:0007265//Ras protein signal transduction;GO:0045944//positive regul
GO:0016491//oxidor	GO:0009247//glycolipid biosynthetic process
GO:0003756//protei	-
-	-
GO:0003676//nuclei	GO:0006508//proteolysis;GO:0016032//viral process

GO:0005515//protei	GO:0001822//kidney development;GO:0002093//auditory receptor cell mo
-	-
-	-
-	-
-	-
GO:0003676//nuclei	GO:0006508//proteolysis;GO:0008152//metabolic process;GO:0016032//v
-	-
GO:0005515//protei	GO:0000281//mitotic cytokinesis;GO:0007019//microtubule depolymeriza
GO:0005085//guany	GO:0007264//small GTPase mediated signal transduction;GO:0034976//re
GO:0004103//cholin	GO:0006646//phosphatidylethanolamine biosynthetic process;GO:000665
-	-
-	-
GO:0000166//nucleo	GO:0000122//negative regulation of transcription from RNA polymerase I
-	GO:0030070//insulin processing;GO:0060628//regulation of ER to Golgi ve
GO:0008170//N-me	GO:0001692//histamine metabolic process;GO:0001695//histamine catabo
-	-
GO:0003735//structu	GO:0006412//translation;GO:0006919//activation of cysteine-type endope
-	-
-	-
GO:0001968//fibron	GO:0001933//negative regulation of protein phosphorylation;GO:0007162
GO:0017080//sodiur	GO:0006814//sodium ion transport;GO:0010460//positive regulation of he
-	-
-	GO:0006658//phosphatidylserine metabolic process;GO:0006665//sphingo
-	GO:0070979//protein K11-linked ubiquitination
-	-
-	-
GO:0016491//oxidor	-
GO:0004175//endop	GO:0018126//protein hydroxylation
-	GO:0018095//protein polyglutamylation
-	-
-	-
GO:0005488//bindin	-
GO:0003779//actin b	GO:0055013//cardiac muscle cell development
-	-
-	-
GO:0030729//acetoa	GO:0006629//lipid metabolic process
GO:0000976//transc	GO:0000122//negative regulation of transcription from RNA polymerase I
-	-
GO:0005515//protei	GO:0035987//endodermal cell differentiation
-	-
-	GO:0007399//nervous system development
GO:0008080//N-ace	-
GO:0004672//protei	GO:0006468//protein phosphorylation;GO:0007169//transmembrane rece
GO:0005515//protei	GO:0006570//tyrosine metabolic process;GO:0007613//memory;GO:0008
GO:0035256//G-pro	GO:0003009//skeletal muscle contraction;GO:0007216//G-protein couple
-	-
GO:0008289//lipid b	GO:0015031//protein transport;GO:0019075//virus maturation;GO:004205
GO:0003874//6-pyru	GO:0006729//tetrahydrobiopterin biosynthetic process
GO:0005515//protei	-
GO:0003735//structu	GO:0006412//translation
GO:0050699//WW d	GO:0007034//vacuolar transport;GO:0010629//negative regulation of gen
GO:0008168//methy	GO:0032259//methylation;GO:0035498//carnosine metabolic process
GO:0004439//phosp	GO:0007420//brain development;GO:0046855//inositol phosphate depho
GO:0016765//transfe	GO:0008299//isoprenoid biosynthetic process
GO:0002020//protea	GO:0010951//negative regulation of endopeptidase activity
GO:0005506//iron ic	GO:0001949//sebaceous gland cell differentiation;GO:0006629//lipid meta
GO:0004000//adeno	GO:0000255//allantoin metabolic process;GO:0001666//response to hypo
-	-

GO:0017080//sodiur	GO:0006814//sodium ion transport;GO:0010460//positive regulation of he
GO:0004467//long-(	GO:0006631//fatty acid metabolic process
GO:0005319//lipid tr	GO:0006869//lipid transport
GO:0036122//BMP b	GO:0007389//pattern specification process;GO:0010454//negative regulat
GO:0001968//fibron	GO:0010811//positive regulation of cell-substrate adhesion;GO:0030198//
GO:0016491//oxidor	-
GO:0008146//sulfotr	-
GO:0008289//lipid b	GO:0070508//cholesterol import
-	-
-	-
GO:0060090//bindin	GO:0030433//ER-associated ubiquitin-dependent protein catabolic proce
GO:0003779//actin b	-
-	-
-	-
GO:0003676//nuclei	GO:0006278//RNA-dependent DNA replication;GO:0006310//DNA recom
-	-
-	-
-	-
-	GO:0006606//protein import into nucleus
GO:0005504//fatty a	GO:0015908//fatty acid transport;GO:0015909//long-chain fatty acid trans
GO:0005085//guany	GO:0006511//ubiquitin-dependent protein catabolic process;GO:0032012
-	-
GO:0004499//N,N-d	-
-	GO:0070453//regulation of heme biosynthetic process
GO:0005178//integri	GO:0000902//cell morphogenesis;GO:0006954//inflammatory response;G
GO:0004657//proline	GO:0006562//proline catabolic process
GO:0030246//carbol	GO:0006897//endocytosis
GO:0004190//aspart	GO:0006508//proteolysis;GO:0019538//protein metabolic process
GO:0003676//nuclei	GO:0006278//RNA-dependent DNA replication;GO:0044260//cellular mac
-	-
GO:0050699//WW d	GO:0007034//vacuolar transport;GO:0010629//negative regulation of gen
GO:0000977//RNA p	GO:0007265//Ras protein signal transduction;GO:0045944//positive regul
-	-
GO:0004672//protei	GO:0000086//G2/M transition of mitotic cell cycle;GO:0000278//mitotic ce
GO:0003824//catalyt	GO:0009226//nucleotide-sugar biosynthetic process;GO:0010595//positiv
-	-
GO:0008270//zinc ic	GO:0009086//methionine biosynthetic process;GO:0009235//cobalamin m
GO:0003796//lysozy	GO:0009253//peptidoglycan catabolic process;GO:0016998//cell wall mac
GO:0022857//transm	GO:0055085//transmembrane transport
GO:0005515//protei	GO:0016567//protein ubiquitination;GO:0035556//intracellular signal trans
-	GO:0051480//regulation of cytosolic calcium ion concentration;GO:19900
-	-
-	-
GO:0005515//protei	GO:0007015//actin filament organization;GO:0045806//negative regulatio
-	GO:0043065//positive regulation of apoptotic process;GO:0046330//posit
GO:0003677//DNA b	GO:0009646//response to absence of light;GO:0018298//protein-chromo
-	-
-	-
GO:0005515//protei	GO:0043065//positive regulation of apoptotic process;GO:0044790//nega
GO:0000166//nucleo	-
GO:0005515//protei	GO:0055085//transmembrane transport
GO:0030729//acetoa	GO:0006629//lipid metabolic process
GO:0008236//serine	GO:0006508//proteolysis
GO:0008170//N-me	GO:0001692//histamine metabolic process;GO:0001695//histamine catabo
-	-
GO:0005085//guany	GO:0006623//protein targeting to vacuole;GO:0009306//protein secretion
GO:0016491//oxidor	-
-	GO:0044237//cellular metabolic process

GO:0035256//G-pro	GO:0003009//skeletal muscle contraction;GO:0007216//G-protein couplec
GO:0004862//cAMP-	GO:0006469//negative regulation of protein kinase activity;GO:0051338//r
GO:0008047//enzym	GO:0006486//protein glycosylation;GO:0006487//protein N-linked glycosy
GO:0000166//nucleo	GO:0001954//positive regulation of cell-matrix adhesion;GO:0003323//typ
GO:0042802//identic	-
-	-
GO:0017128//phosp	GO:0000045//autophagosome assembly;GO:0006869//lipid transport;GO:
-	-
-	-
GO:0004674//protei	GO:0006468//protein phosphorylation
GO:0003735//structu	GO:0006412//translation
GO:0005515//protei	GO:0001822//kidney development;GO:0002093//auditory receptor cell mo
GO:0019901//protei	GO:0030334//regulation of cell migration;GO:0034446//substrate adhesio
GO:0005085//guany	GO:0050790//regulation of catalytic activity
GO:0004639//phosp	GO:0006164//purine nucleotide biosynthetic process;GO:0006189//'de no
GO:0004930//G-pro	GO:0007186//G-protein coupled receptor signaling pathway;GO:0043408
-	-
-	-
-	-
GO:0001968//fibron	GO:0001933//negative regulation of protein phosphorylation;GO:0007162
GO:0003677//DNA b	GO:0006334//nucleosome assembly;GO:0006352//DNA-templated transc
GO:0003676//nuclei	-
GO:0000977//RNA p	GO:0000122//negative regulation of transcription from RNA polymerase I
-	-
GO:0000976//transc	GO:0006357//regulation of transcription from RNA polymerase II promote
-	-
-	-
-	-
GO:0008195//phosp	GO:0006357//regulation of transcription from RNA polymerase II promote
GO:0000287//magne	GO:0006006//glucose metabolic process;GO:0006094//gluconeogenesis;G
GO:0004190//aspart	GO:0006508//proteolysis;GO:0019538//protein metabolic process
-	-
GO:0004386//helicas	-
GO:0022857//transm	GO:0055085//transmembrane transport
GO:0000977//RNA p	GO:0000122//negative regulation of transcription from RNA polymerase I
GO:0030145//manga	GO:0006508//proteolysis;GO:0019538//protein metabolic process
GO:0015175//neutra	GO:0015804//neutral amino acid transport;GO:0042941//D-alanine transp
-	GO:0046890//regulation of lipid biosynthetic process
GO:0004843//thiol-c	GO:0000278//mitotic cell cycle;GO:0006281//DNA repair;GO:0006511//ub
-	-
GO:0017128//phosp	GO:0000045//autophagosome assembly;GO:0006914//autophagy;GO:001
-	-
-	GO:0006658//phosphatidylserine metabolic process;GO:0006665//sphingo
GO:0005515//protei	GO:0001822//kidney development;GO:0002093//auditory receptor cell mo
-	-
GO:0000166//nucleo	GO:0070588//calcium ion transmembrane transport
GO:0030729//acetoa	GO:0006629//lipid metabolic process
GO:0016491//oxidor	GO:0006629//lipid metabolic process;GO:0008611//ether lipid biosyntheti
-	GO:0051480//regulation of cytosolic calcium ion concentration;GO:19900
GO:0031681//G-pro	GO:0007186//G-protein coupled receptor signaling pathway
-	-
-	-
GO:0005515//protei	-
-	-
-	GO:0006488//dolichol-linked oligosaccharide biosynthetic process
GO:0000062//fatty-a	GO:0036151//phosphatidylcholine acyl-chain remodeling;GO:1903060//n
GO:0003682//chrom	GO:0001945//lymph vessel development;GO:0003017//lymph circulation;G
GO:0004842//ubiqui	GO:0000209//protein polyubiquitination;GO:0006915//apoptotic process;G

-	-
-	GO:1901031//regulation of response to reactive oxygen species
-	-
-	-
-	-
GO:0004857//enzym	GO:0006629//lipid metabolic process;GO:0043086//negative regulation of
GO:0003677//DNA b	GO:0006383//transcription from RNA polymerase III promoter
GO:0005546//phosp	GO:0009636//response to toxic substance;GO:0042360//vitamin E metabo
GO:0008170//N-me	GO:0001692//histamine metabolic process;GO:0001695//histamine catabo
GO:0005319//lipid tr	GO:0006869//lipid transport
GO:0004609//phosp	GO:0006646//phosphatidylethanolamine biosynthetic process;GO:000865
-	-
GO:0004869//cystein	GO:0001503//ossification;GO:0006953//acute-phase response;GO:003050
GO:0003874//6-pyru	GO:0006729//tetrahydrobiopterin biosynthetic process
-	-
GO:0008378//galact	GO:0001576//globoside biosynthetic process;GO:0007009//plasma memb
GO:0003824//catalyt	GO:0006777//Mo-molybdopterin cofactor biosynthetic process;GO:00435
GO:0004467//long-c	GO:0006631//fatty acid metabolic process
GO:0016491//oxidor	-
GO:0008289//lipid b	GO:0015031//protein transport;GO:0019075//virus maturation;GO:004205
GO:0005515//protei	GO:0006355//regulation of transcription, DNA-templated;GO:0006805//x
GO:0004674//protei	GO:0006468//protein phosphorylation
GO:0005515//protei	-
GO:0000062//fatty-a	GO:0036151//phosphatidylcholine acyl-chain remodeling;GO:1903060//n
-	GO:0009987//cellular process
GO:0001604//uroter	GO:0007165//signal transduction;GO:0007186//G-protein coupled recept
GO:0004252//serine	GO:0006508//proteolysis;GO:0016485//protein processing
GO:0008517//folic a	GO:0015884//folic acid transport;GO:0051180//vitamin transport;GO:0051
GO:0005267//potass	GO:0006813//potassium ion transport;GO:0033198//response to ATP;GO:
GO:0005515//protei	GO:0044763//single-organism cellular process
-	-
-	GO:1901031//regulation of response to reactive oxygen species
-	-
GO:0000993//RNA p	GO:0000956//nuclear-transcribed mRNA catabolic process;GO:0010501//
GO:0030246//carbol	GO:0006897//endocytosis
GO:0008109//N-ace	GO:0006486//protein glycosylation;GO:0007179//transforming growth fac
GO:0050699//WW d	GO:0007034//vacuolar transport;GO:0010629//negative regulation of gen
GO:0005515//protei	GO:0043065//positive regulation of apoptotic process;GO:0044790//nega
-	-
GO:0005104//fibrob	GO:0001755//neural crest cell migration;GO:0001934//positive regulation
-	-
GO:0005515//protei	GO:0016567//protein ubiquitination
-	-
GO:0046961//protor	GO:0006811//ion transport;GO:1902600//hydrogen ion transmembrane tr
GO:0003924//GTPas	GO:0007165//signal transduction;GO:0007186//G-protein coupled recept
GO:0008195//phosp	GO:0006720//isoprenoid metabolic process;GO:0018342//protein prenyla
-	-
GO:0002020//protea	GO:0010951//negative regulation of endopeptidase activity
GO:0005201//extrac	GO:0007160//cell-matrix adhesion;GO:0007605//sensory perception of sc
GO:0000978//RNA p	GO:0006355//regulation of transcription, DNA-templated;GO:0006357//re
GO:0003676//nuclei	-
-	-
GO:0009922//fatty a	GO:0019367//fatty acid elongation, saturated fatty acid;GO:0042761//very
-	-
-	-
-	-
-	-
GO:0004175//endop	GO:0018126//protein hydroxylation

GO:0000976//transc	GO:0006357//regulation of transcription from RNA polymerase II promote
GO:0016798//hydrol	GO:0008284//positive regulation of cell proliferation;GO:0030198//extrace
-	-
GO:0003677//DNA b	GO:0010467//gene expression
GO:0017128//phosp	GO:0000045//autophagosome assembly;GO:0006869//lipid transport;GO:
GO:0017128//phosp	GO:0000045//autophagosome assembly;GO:0006914//autophagy;GO:001
GO:0005216//ion ch	GO:0006811//ion transport;GO:0006812//cation transport;GO:0034220//ic
GO:0008022//protei	GO:0007026//negative regulation of microtubule depolymerization;GO:00
GO:0004930//G-pro	GO:0007186//G-protein coupled receptor signaling pathway
GO:0016757//transfe	-
GO:0004366//glycer	GO:0001817//regulation of cytokine production;GO:0006631//fatty acid m
GO:0003676//nuclei	GO:0006388//tRNA splicing, via endonucleolytic cleavage and ligation
GO:0022857//transm	GO:0055085//transmembrane transport
GO:0004000//adeno	GO:0000255//allantoin metabolic process;GO:0001666//response to hypo
GO:0005085//guany	GO:0050790//regulation of catalytic activity
GO:0004725//protei	GO:0016311//dephosphorylation;GO:0046855//inositol phosphate depho
GO:0005509//calciur	GO:0001654//eye development;GO:0045667//regulation of osteoblast diff
GO:0005515//protei	GO:0007015//actin filament organization;GO:0045806//negative regulatio
GO:0003677//DNA b	GO:0006355//regulation of transcription, DNA-templated;GO:0045944//p
-	-
-	GO:0006888//ER to Golgi vesicle-mediated transport;GO:0006914//autop
-	-
-	-
GO:0003824//catalyt	GO:0009226//nucleotide-sugar biosynthetic process;GO:0010595//positiv
GO:0060090//bindin	GO:0030433//ER-associated ubiquitin-dependent protein catabolic proce
GO:0019901//protei	GO:0030334//regulation of cell migration;GO:0034446//substrate adhesio

ı development;GO:0050804//modulation of synaptic transmission;GO:0099560//synaptic membrar

ɔrocess;GO:0006189//'de novo' IMP biosynthetic process;GO:0009152//purine ribonucleotide bios

ɔsitive regulation of I-kappaB kinase/NF-kappaB signaling;GO:0048168//regulation of neuronal sy
//negative regulation of toll-like receptor 4 signaling pathway;GO:0034164//negative regulation o
0045776//negative regulation of blood pressure;GO:0051607//defense response to virus;GO:0055

ıinate metabolic process;GO:0006468//protein phosphorylation;GO:0006486//protein glycosylatio

21//chloride transport;GO:0034220//ion transmembrane transport;GO:0055085//transmembrane t
tabolic process;GO:0009313//oligosaccharide catabolic process;GO:0051692//cellular oligosaccha

ɔathway;GO:0009617//response to bacterium;GO:0010628//positive regulation of gene expression

lic process;GO:0008210//estrogen metabolic process;GO:0009804//coumarin metabolic process;G
RNA secondary structure unwinding;GO:0016525//negative regulation of angiogenesis;GO:003105

070374//positive regulation of ERK1 and ERK2 cascade;GO:0070378//positive regulation of ERK5 c
GO:0008544//epidermis development;GO:0010467//gene expression;GO:0036303//lymph vessel m
ıelial cell proliferation;GO:0002719//negative regulation of cytokine production involved in immun

)8283//cell proliferation;GO:0008284//positive regulation of cell proliferation;GO:0009408//respon

ıding in birth or egg hatching;GO:0019336//phenol-containing compound catabolic process;GO:0

l promoter;GO:0006355//regulation of transcription, DNA-templated;GO:0006357//regulation of t
O:0006955//immune response;GO:0007155//cell adhesion;GO:0007160//cell-matrix adhesion;GO:

assembly;GO:0030036//actin cytoskeleton organization;GO:0045944//positive regulation of transc

copper ion;GO:0032496//response to lipopolysaccharide;GO:0070555//response to interleukin-1;

;GO:0006974//cellular response to DNA damage stimulus;GO:0010212//response to ionizing radia
tive regulation of calcium-mediated signaling;GO:0050862//positive regulation of T cell receptor

//lipid biosynthetic process;GO:0016126//sterol biosynthetic process;GO:0045654//positive regulat

rocess;GO:0006633//fatty acid biosynthetic process;GO:0010886//positive regulation of cholesterc

I promoter;GO:0006418//tRNA aminoacylation for protein translation;GO:0006434//seryl-tRNA am

GO:0043409//negative regulation of MAPK cascade;GO:0050906//detection of stimulus involved ir
to nucleus;GO:0007049//cell cycle;GO:0007179//transforming growth factor beta receptor signalir
egulation of transcription from RNA polymerase II promoter;GO:0001649//osteoblast differentiatic

ng compound metabolic process;GO:0008152//metabolic process;GO:0016310//phosphorylation;G

c process;GO:0035874//cellular response to copper ion starvation;GO:0046677//response to antib

GO:0043409//negative regulation of MAPK cascade;GO:0050906//detection of stimulus involved ir
ation;GO:0002244//hematopoietic progenitor cell differentiation;GO:0006357//regulation of transc

pe B pancreatic cell development;GO:0006468//protein phosphorylation;GO:0009615//response tc

gration;GO:0006355//regulation of transcription, DNA-templated;GO:0007165//signal transduction

regulation of transferase activity;GO:2000480//negative regulation of cAMP-dependent protein kir

ng compound metabolic process;GO:0008152//metabolic process;GO:0016310//phosphorylation;G

asma membrane adhesion molecules;GO:0007162//negative regulation of cell adhesion;GO:00073

on;GO:0030501//positive regulation of bone mineralization;GO:0045669//positive regulation of os
rferon-gamma production;GO:0032743//positive regulation of interleukin-2 production;GO:00327

ipid translocation;GO:0047484//regulation of response to osmotic stress;GO:0055085//transmemb

RNA secondary structure unwinding;GO:0016525//negative regulation of angiogenesis;GO:00310[illegible]

ransmembrane transporter activity;GO:1904064//positive regulation of cation transmembrane tra[illegible]
process;GO:0006189//'de novo' IMP biosynthetic process;GO:0009152//purine ribonucleotide bios[illegible]

metabolic process;GO:0007417//central nervous system development;GO:0009064//glutamine fa[illegible]

aling pathway;GO:0007186//G-protein coupled receptor signaling pathway;GO:0007188//adenylat[illegible]

biosynthetic process;GO:0015908//fatty acid transport;GO:0033211//adiponectin-activated signali

tor beta receptor signaling pathway;GO:0007229//integrin-mediated signaling pathway;GO:00164
development;GO:0050804//modulation of synaptic transmission;GO:0099560//synaptic membra[illegible]

GO:0008544//epidermis development;GO:0010467//gene expression;GO:0036303//lymph vessel [illegible]
athway;GO:0009888//tissue development;GO:0030198//extracellular matrix organization;GO:0071[illegible]
ransmembrane transporter activity;GO:1904064//positive regulation of cation transmembrane tra[illegible]
elial cell proliferation;GO:0002719//negative regulation of cytokine production involved in immun
elin catabolic process;GO:0008156//negative regulation of DNA replication;GO:0008285//negative

//inflammatory response to antigenic stimulus;GO:0007264//small GTPase mediated signal transdu

deling;GO:0006535//cysteine biosynthetic process from serine;GO:0006801//superoxide metabolic

c process;GO:0035874//cellular response to copper ion starvation;GO:0046677//response to antib
ide cross-linking;GO:0030216//keratinocyte differentiation;GO:0045861//negative regulation of pr
070374//positive regulation of ERK1 and ERK2 cascade;GO:0070378//positive regulation of ERK5 [illegible]
copper ion;GO:0032496//response to lipopolysaccharide;GO:0070555//response to interleukin-1;

l promoter;GO:0006355//regulation of transcription, DNA-templated;GO:0006357//regulation of t
eak repair via nonhomologous end joining;GO:0010212//response to ionizing radiation;GO:00301
:0030512//negative regulation of transforming growth factor beta receptor signaling pathway

//lipid biosynthetic process;GO:0016126//sterol biosynthetic process;GO:0045654//positive regulat

tasis;GO:0007611//learning or memory;GO:0007625//grooming behavior;GO:0007626//locomotor

ynthetic process;GO:0009113//purine nucleobase biosynthetic process;GO:0009116//nucleoside m
on;GO:0030501//positive regulation of bone mineralization;GO:0045669//positive regulation of os

544//arginine catabolic process to glutamate;GO:0055129//L-proline biosynthetic process

sport;GO:0033120//positive regulation of RNA splicing;GO:0044767//single-organism developmen

regulation of endothelial cell proliferation;GO:0006468//protein phosphorylation;GO:0007165//sig

//lipid biosynthetic process;GO:0016126//sterol biosynthetic process;GO:0045654//positive regulat
tor beta receptor signaling pathway;GO:0007229//integrin-mediated signaling pathway;GO:00164
045723//positive regulation of fatty acid biosynthetic process;GO:0051258//protein polymerization

lic process;GO:0008210//estrogen metabolic process;GO:0009804//coumarin metabolic process;G

08283//cell proliferation;GO:0008284//positive regulation of cell proliferation;GO:0009408//respon

erived foam cell differentiation;GO:0010878//cholesterol storage;GO:0033344//cholesterol efflux;G

ion of cell fate commitment;GO:0016055//Wnt signaling pathway;GO:0030178//negative regulatic

tabolic process;GO:0019835//cytolysis;GO:0031640//killing of cells of other organism;GO:0042742

03//cholesterol metabolic process;GO:0010468//regulation of gene expression;GO:0010977//nega

nsulin stimulus;GO:0071456//cellular response to hypoxia;GO:2000766//negative regulation of cyt
e organization;GO:0043353//enucleate erythrocyte differentiation;GO:0098739//import across plas

ty;GO:0045654//positive regulation of megakaryocyte differentiation;GO:0046330//positive regula

osynthetic process;GO:0009072//aromatic amino acid family metabolic process;GO:0019293//tyros

scription, DNA-templated;GO:0006888//ER to Golgi vesicle-mediated transport;GO:0030968//end

egulation of neurogenesis;GO:0060770//negative regulation of epithelial cell proliferation involve

on;GO:0030501//positive regulation of bone mineralization;GO:0045669//positive regulation of os

0045776//negative regulation of blood pressure;GO:0051607//defense response to virus;GO:0055

//oocyte differentiation;GO:0042127//regulation of cell proliferation;GO:0048839//inner ear devel

asma membrane adhesion molecules;GO:0007162//negative regulation of cell adhesion;GO:00073

//negative regulation of toll-like receptor 4 signaling pathway;GO:0034164//negative regulation o
ransmembrane transporter activity;GO:1904064//positive regulation of cation transmembrane tran

)07268//synaptic transmission;GO:0007416//synapse assembly;GO:0007612//learning;GO:0030534

542//visual learning;GO:0010801//negative regulation of peptidyl-threonine phosphorylation;GO:

to nucleus;GO:0007049//cell cycle;GO:0007179//transforming growth factor beta receptor signali
ide cross-linking;GO:0030216//keratinocyte differentiation;GO:0045861//negative regulation of pr

ation;GO:0002244//hematopoietic progenitor cell differentiation;GO:0006357//regulation of transc

n;GO:0002305//CD8-positive, gamma-delta intraepithelial T cell differentiation;GO:0002689//nega

032342//aldosterone biosynthetic process;GO:0032870//cellular response to hormone stimulus;GO

lic process;GO:0006656//phosphatidylcholine biosynthetic process;GO:0008203//cholesterol meta

544//arginine catabolic process to glutamate;GO:0055129//L-proline biosynthetic process
//inflammatory response to antigenic stimulus;GO:0007264//small GTPase mediated signal transdu

ponse to phosphate starvation;GO:0032259//methylation;GO:0042135//neurotransmitter catabolic

ng compound metabolic process;GO:0008152//metabolic process;GO:0016310//phosphorylation;G

osynthetic process;GO:0009072//aromatic amino acid family metabolic process;GO:0019293//tyros

e organization;GO:0043353//enucleate erythrocyte differentiation;GO:0098739//import across plas

ation;GO:0090305//nucleic acid phosphodiester bond hydrolysis;GO:0090502//RNA phosphodiest
ation of gene expression;GO:0045671//negative regulation of osteoclast differentiation;GO:004577

rganization;GO:0030036//actin cytoskeleton organization;GO:0070830//bicellular tight junction as
deling;GO:0006535//cysteine biosynthetic process from serine;GO:0006801//superoxide metabolic
metabolic process;GO:0007417//central nervous system development;GO:0009064//glutamine far

erived foam cell differentiation;GO:0010878//cholesterol storage;GO:0033344//cholesterol efflux;G

rferon-gamma production;GO:0032743//positive regulation of interleukin-2 production;GO:00327
)//nervous system development;GO:0010977//negative regulation of neuron projection developm

ding in birth or egg hatching;GO:0019336//phenol-containing compound catabolic process;GO:0
cromolecule metabolic process;GO:0090502//RNA phosphodiester bond hydrolysis, endonucleolyt

544//arginine catabolic process to glutamate;GO:0055129//L-proline biosynthetic process
ynthetic process;GO:0009113//purine nucleobase biosynthetic process;GO:0009116//nucleoside m

se activity;GO:0038161//prolactin signaling pathway;GO:0042976//activation of Janus kinase activit

athway;GO:0009888//tissue development;GO:0030198//extracellular matrix organization;GO:00713
tasis;GO:0007611//learning or memory;GO:0007625//grooming behavior;GO:0007626//locomotor

thway;GO:0009267//cellular response to starvation;GO:0010510//regulation of acetyl-CoA biosynt

O:0007252//I-kappaB phosphorylation;GO:0008630//intrinsic apoptotic signaling pathway in respo

//inflammatory response;GO:0006956//complement activation;GO:0010575//positive regulation of

regulation of endothelial cell proliferation;GO:0006468//protein phosphorylation;GO:0007165//sig

ynthetic process;GO:0009113//purine nucleobase biosynthetic process;GO:0009116//nucleoside m

:osamine metabolic process;GO:0006790//sulfur compound metabolic process;GO:1903238//posit

tasis;GO:0007611//learning or memory;GO:0007625//grooming behavior;GO:0007626//locomotor

etabolic process;GO:0006096//glycolytic process;GO:0051156//glucose 6-phosphate metabolic pr

er;GO:0009062//fatty acid catabolic process;GO:0019432//triglyceride biosynthetic process

I promoter;GO:0007379//segment specification;GO:0019827//stem cell population maintenance;G

9511//peptidyl-proline hydroxylation;GO:0032963//collagen metabolic process;GO:0050708//regu
metabolic process;GO:0007417//central nervous system development;GO:0009064//glutamine far

e organization;GO:0043353//enucleate erythrocyte differentiation;GO:0098739//import across plas

lic process;GO:0006656//phosphatidylcholine biosynthetic process;GO:0008203//cholesterol meta
osynthetic process;GO:0009072//aromatic amino acid family metabolic process;GO:0019293//tyros

)//nervous system development;GO:0010977//negative regulation of neuron projection developme

zation;GO:0009235//cobalamin metabolic process;GO:0015889//cobalamin transport;GO:0043001

osynthetic process;GO:0009072//aromatic amino acid family metabolic process;GO:0019293//tyros

e organization;GO:0043353//enucleate erythrocyte differentiation;GO:0098739//import across plas

O:0007040//lysosome organization;GO:0007286//spermatid development;GO:0008333//endosom

asma membrane adhesion molecules;GO:0007162//negative regulation of cell adhesion;GO:00073

ıbination;GO:0006508//proteolysis;GO:0008152//metabolic process;GO:0015074//DNA integration

olic process;GO:0051180//vitamin transport;GO:0090212//negative regulation of establishment of b
616//long-term memory;GO:0007626//locomotory behavior;GO:0009190//cyclic nucleotide biosyr

: process;GO:0042574//retinal metabolic process;GO:1901810//beta-carotene metabolic process
egulation of transcription from RNA polymerase II promoter;GO:0001649//osteoblast differentiatic

tabolic process;GO:0019835//cytolysis;GO:0031640//killing of cells of other organism;GO:0042742

cromolecule metabolic process;GO:0090502//RNA phosphodiester bond hydrolysis, endonucleolyt
zation;GO:0009235//cobalamin metabolic process;GO:0015889//cobalamin transport;GO:0043001

ıction;GO:0043087//regulation of GTPase activity;GO:0046580//negative regulation of Ras protein

//inflammatory response;GO:0006956//complement activation;GO:0010575//positive regulation of
O:0007252//I-kappaB phosphorylation;GO:0008630//intrinsic apoptotic signaling pathway in respo
ynthetic process;GO:0009113//purine nucleobase biosynthetic process;GO:0009116//nucleoside m

athway;GO:0009888//tissue development;GO:0030198//extracellular matrix organization;GO:0071З

ı;GO:0002305//CD8-positive, gamma-delta intraepithelial T cell differentiation;GO:0002689//nega

I promoter;GO:0006355//regulation of transcription, DNA-templated;GO:0009267//cellular respor

etabolic process;GO:0008652//cellular amino acid biosynthetic process;GO:0042149//cellular respo
ınal transduction;GO:0008105//asymmetric protein localization;GO:0008595//anterior/posterior ax

ation of gene expression;GO:0045671//negative regulation of osteoclast differentiation;GO:004577
n;GO:0019805//quinolinate biosynthetic process;GO:0046686//response to cadmium ion;GO:0070
//inflammatory response to antigenic stimulus;GO:0007264//small GTPase mediated signal transdu

metabolic process;GO:0006631//fatty acid metabolic process;GO:0006635//fatty acid beta-oxidati
er;GO:0009062//fatty acid catabolic process;GO:0019432//triglyceride biosynthetic process

I promoter;GO:0007379//segment specification;GO:0019827//stem cell population maintenance;G

metabolic process;GO:0007417//central nervous system development;GO:0009064//glutamine fa

or signaling pathway;GO:0007188//adenylate cyclase-modulating G-protein coupled receptor sig
0086073//bundle of His cell-Purkinje myocyte adhesion involved in cell communication;GO:00860
erived foam cell differentiation;GO:0010878//cholesterol storage;GO:0033344//cholesterol efflux;G
//nervous system development;GO:0010977//negative regulation of neuron projection developm

transport;GO:0070327//thyroid hormone transport;GO:0070460//thyroid-stimulating hormone se
one metabolic process;GO:0048729//tissue morphogenesis;GO:0048872//homeostasis of number

adhesion;GO:0007264//small GTPase mediated signal transduction;GO:0007628//adult walking be

process;GO:0008284//positive regulation of cell proliferation;GO:0009615//response to virus;GO:0

zation;GO:0009235//cobalamin metabolic process;GO:0015889//cobalamin transport;GO:0043001

erived foam cell differentiation;GO:0010878//cholesterol storage;GO:0033344//cholesterol efflux;G

metabolic process;GO:0006650//glycerophospholipid metabolic process;GO:0006654//phosphatid

544//arginine catabolic process to glutamate;GO:0055129//L-proline biosynthetic process

d glutamate receptor signaling pathway;GO:0048148//behavioral response to cocaine;GO:004874[illegible]

lic process;GO:0008210//estrogen metabolic process;GO:0009804//coumarin metabolic process;G[illegible]
ponse to phosphate starvation;GO:0032259//methylation;GO:0042135//neurotransmitter catabolic

)8283//cell proliferation;GO:0008284//positive regulation of cell proliferation;GO:0009408//respon
regulation of endothelial cell proliferation;GO:0006468//protein phosphorylation;GO:0007165//sig

GO:0043409//negative regulation of MAPK cascade;GO:0050906//detection of stimulus involved i[illegible]
ısma membrane adhesion molecules;GO:0007162//negative regulation of cell adhesion;GO:00073[illegible]

2//negative regulation of cell adhesion;GO:0010952//positive regulation of peptidase activity;GO:0

thway;GO:0009267//cellular response to starvation;GO:0010510//regulation of acetyl-CoA biosynt

//inflammatory response to antigenic stimulus;GO:0007264//small GTPase mediated signal transdu

n;GO:0019805//quinolinate biosynthetic process;GO:0046686//response to cadmium ion;GO:0070

copper ion;GO:0032496//response to lipopolysaccharide;GO:0070555//response to interleukin-1;
ɔse assembly;GO:0008611//ether lipid biosynthetic process;GO:0021587//cerebellum morphogene

tasis;GO:0007611//learning or memory;GO:0007625//grooming behavior;GO:0007626//locomotor

c process;GO:0035874//cellular response to copper ion starvation;GO:0046677//response to antib

616//long-term memory;GO:0007626//locomotory behavior;GO:0009190//cyclic nucleotide biosyı
ganic substance;GO:0010872//regulation of cholesterol esterification;GO:0010875//positive regula

n;GO:0019805//quinolinate biosynthetic process;GO:0046686//response to cadmium ion;GO:0070

on;GO:0006869//lipid transport;GO:0007283//spermatogenesis;GO:0007399//nervous system deve

0045776//negative regulation of blood pressure;GO:0051607//defense response to virus;GO:0055

or signaling pathway;GO:0007188//adenylate cyclase-modulating G-protein coupled receptor sig

regulation of neurogenesis;GO:0060770//negative regulation of epithelial cell proliferation involve

elial cell proliferation;GO:0002719//negative regulation of cytokine production involved in immun
erived foam cell differentiation;GO:0010878//cholesterol storage;GO:0033344//cholesterol efflux;G

c process;GO:0035874//cellular response to copper ion starvation;GO:0046677//response to antib
I promoter;GO:0006355//regulation of transcription, DNA-templated;GO:0006357//regulation of t

DNA damage stimulus;GO:0045023//G0 to G1 transition;GO:0045746//negative regulation of Notc

tabolic process;GO:0019835//cytolysis;GO:0031640//killing of cells of other organism;GO:0042742

n;GO:0002305//CD8-positive, gamma-delta intraepithelial T cell differentiation;GO:0002689//nega

18//positive regulation of satellite cell activation involved in skeletal muscle regeneration;GO:0030
optotic process;GO:0043154//negative regulation of cysteine-type endopeptidase activity involved

SU-rRNA, 5.8S rRNA, LSU-rRNA);GO:0000466//maturation of 5.8S rRNA from tricistronic rRNA tra

ation;GO:0002244//hematopoietic progenitor cell differentiation;GO:0006357//regulation of transc

rocess;GO:0006633//fatty acid biosynthetic process;GO:0006636//unsaturated fatty acid biosynthe

//inflammatory response to antigenic stimulus;GO:0007264//small GTPase mediated signal transdu
to nucleus;GO:0007049//cell cycle;GO:0007179//transforming growth factor beta receptor signalir
: process;GO:0042574//retinal metabolic process;GO:1901810//beta-carotene metabolic process
)8283//cell proliferation;GO:0008284//positive regulation of cell proliferation;GO:0009408//respon

thway;GO:0009267//cellular response to starvation;GO:0010510//regulation of acetyl-CoA biosynt

sport;GO:0055085//transmembrane transport;GO:1990830//cellular response to leukemia inhibitor
//ubiquitin-dependent protein catabolic process;GO:0007409//axonogenesis;GO:0007412//axon t
transport;GO:0070327//thyroid hormone transport;GO:0070460//thyroid-stimulating hormone se

onse to phosphate starvation;GO:0032259//methylation;GO:0042135//neurotransmitter catabolic

ration;GO:0006355//regulation of transcription, DNA-templated;GO:0007165//signal transduction

netanephros development;GO:0001759//organ induction;GO:0001823//mesonephros developmer

orocess;GO:0006189//'de novo' IMP biosynthetic process;GO:0009152//purine ribonucleotide bios

orylation;GO:0018108//peptidyl-tyrosine phosphorylation;GO:0030282//bone mineralization;GO:0

n;GO:0019805//quinolinate biosynthetic process;GO:0046686//response to cadmium ion;GO:0070

)lic process;GO:0051180//vitamin transport;GO:0090212//negative regulation of establishment of l

:ive regulation of heparan sulfate proteoglycan biosynthetic process;GO:0050650//chondroitin sulf

sm growth;GO:0035934//corticosterone secretion;GO:0042445//hormone metabolic process;GO:0(

)8283//cell proliferation;GO:0008284//positive regulation of cell proliferation;GO:0009408//respon

ipid translocation;GO:0047484//regulation of response to osmotic stress;GO:0055085//transmemk

lic process;GO:0008210//estrogen metabolic process;GO:0009804//coumarin metabolic process;G(

adhesion;GO:0007264//small GTPase mediated signal transduction;GO:0007628//adult walking bel
c process;GO:0035874//cellular response to copper ion starvation;GO:0046677//response to antib

regulation of neurogenesis;GO:0060770//negative regulation of epithelial cell proliferation involve

ganic substance;GO:0010872//regulation of cholesterol esterification;GO:0010875//positive regula

GO:0043409//negative regulation of MAPK cascade;GO:0050906//detection of stimulus involved i

5669//positive regulation of osteoblast differentiation;GO:0046850//regulation of bone remodelin
GO:0010468//regulation of gene expression;GO:0010906//regulation of glucose metabolic process

adhesion;GO:0007264//small GTPase mediated signal transduction;GO:0007628//adult walking bel
transport;GO:0070327//thyroid hormone transport;GO:0070460//thyroid-stimulating hormone se

orphogenesis;GO:0007224//smoothened signaling pathway;GO:0007399//nervous system develop

542//visual learning;GO:0010801//negative regulation of peptidyl-threonine phosphorylation;GO:(

ositive regulation of I-kappaB kinase/NF-kappaB signaling;GO:0048168//regulation of neuronal sy
to nucleus;GO:0007049//cell cycle;GO:0007179//transforming growth factor beta receptor signalir
//inflammatory response to antigenic stimulus;GO:0007264//small GTPase mediated signal transdu
copper ion;GO:0032496//response to lipopolysaccharide;GO:0070555//response to interleukin-1;

c process;GO:0035874//cellular response to copper ion starvation;GO:0046677//response to antib

thway;GO:0009267//cellular response to starvation;GO:0010510//regulation of acetyl-CoA biosynt

ɔ starvation;GO:0044090//positive regulation of vacuole organization;GO:2000786//positive regula

c process;GO:0043335//protein unfolding;GO:0051005//negative regulation of lipoprotein lipase a
ation;GO:0002244//hematopoietic progenitor cell differentiation;GO:0006357//regulation of transc
metabolic process;GO:0006631//fatty acid metabolic process;GO:0006635//fatty acid beta-oxidati
)8283//cell proliferation;GO:0008284//positive regulation of cell proliferation;GO:0009408//respon

ative regulation of blood coagulation;GO:0071383//cellular response to steroid hormone stimulus

: process;GO:0042574//retinal metabolic process;GO:1901810//beta-carotene metabolic process

ipid translocation;GO:0047484//regulation of response to osmotic stress;GO:0055085//transmemb

erived foam cell differentiation;GO:0010878//cholesterol storage;GO:0033344//cholesterol efflux;G
(6-linked deubiquitination;GO:0050821//protein stabilization;GO:0051898//negative regulation of

f cytosolic calcium ion concentration;GO:0010863//positive regulation of phospholipase C activity;

ty;GO:0045654//positive regulation of megakaryocyte differentiation;GO:0046330//positive regula

sport;GO:0006982//response to lipid hydroperoxide;GO:0007159//leukocyte cell-cell adhesion;GO
rocess;GO:0006633//fatty acid biosynthetic process;GO:0010886//positive regulation of cholester
070374//positive regulation of ERK1 and ERK2 cascade;GO:0070378//positive regulation of ERK5 c

ıelial cell proliferation;GO:0002719//negative regulation of cytokine production involved in immun

assembly;GO:0030036//actin cytoskeleton organization;GO:0045944//positive regulation of transc

metabolic process;GO:0007417//central nervous system development;GO:0009064//glutamine far

process;GO:0006189//'de novo' IMP biosynthetic process;GO:0009152//purine ribonucleotide bios

I promoter;GO:0006355//regulation of transcription, DNA-templated;GO:0006357//regulation of t

: process;GO:0042574//retinal metabolic process;GO:1901810//beta-carotene metabolic process

on organization;GO:0008360//regulation of cell shape;GO:0016601//Rac protein signal transductic

GO:0006107//oxaloacetate metabolic process;GO:0006629//lipid metabolic process;GO:0009617//r

copper ion;GO:0032496//response to lipopolysaccharide;GO:0070555//response to interleukin-1;
4765//regulation of ion transmembrane transport;GO:0051260//protein homooligomerization;GO

5669//positive regulation of osteoblast differentiation;GO:0046850//regulation of bone remodelin
ipid translocation;GO:0047484//regulation of response to osmotic stress;GO:0055085//transmemb

adhesion;GO:0007264//small GTPase mediated signal transduction;GO:0007628//adult walking bel

regulation of endothelial cell proliferation;GO:0006468//protein phosphorylation;GO:0007165//sig

assembly;GO:0030036//actin cytoskeleton organization;GO:0045944//positive regulation of transc

ynthetic process;GO:0009113//purine nucleobase biosynthetic process;GO:0009116//nucleoside m

lic process;GO:0051180//vitamin transport;GO:0090212//negative regulation of establishment of b
lic process;GO:0008210//estrogen metabolic process;GO:0009804//coumarin metabolic process;G

rocess;GO:0006633//fatty acid biosynthetic process;GO:0010886//positive regulation of cholestero

cosamine metabolic process;GO:0006790//sulfur compound metabolic process;GO:1903238//posit

gration;GO:0006355//regulation of transcription, DNA-templated;GO:0007165//signal transduction

58//regulation of epidermal growth factor receptor signaling pathway;GO:0046755//viral budding
ng compound metabolic process;GO:0008152//metabolic process;GO:0016310//phosphorylation;G

rotein dephosphorylation;GO:0016311//dephosphorylation;GO:0043542//endothelial cell migratio

ponse to phosphate starvation;GO:0032259//methylation;GO:0042135//neurotransmitter catabolic

lic process;GO:0051180//vitamin transport;GO:0090212//negative regulation of establishment of b
e organization;GO:0043353//enucleate erythrocyte differentiation;GO:0098739//import across plas

regulation of neurogenesis;GO:0060770//negative regulation of epithelial cell proliferation involve
031098//stress-activated protein kinase signaling cascade;GO:0035860//glial cell-derived neurotro

osynthetic process;GO:0009072//aromatic amino acid family metabolic process;GO:0019293//tyros
adhesion;GO:0007264//small GTPase mediated signal transduction;GO:0007628//adult walking beh
ide cross-linking;GO:0030216//keratinocyte differentiation;GO:0045861//negative regulation of pr
ty;GO:0045654//positive regulation of megakaryocyte differentiation;GO:0046330//positive regula

rocess;GO:0006633//fatty acid biosynthetic process;GO:0010886//positive regulation of cholestero

assembly;GO:0030036//actin cytoskeleton organization;GO:0045944//positive regulation of transc

lipid metabolic process;GO:0006631//fatty acid metabolic process;GO:0006633//fatty acid biosynth

:0030512//negative regulation of transforming growth factor beta receptor signaling pathway
ide cross-linking;GO:0030216//keratinocyte differentiation;GO:0045861//negative regulation of pr
ulfment;GO:0043277//apoptotic cell clearance;GO:0050766//positive regulation of phagocytosis

ative regulation of blood coagulation;GO:0071383//cellular response to steroid hormone stimulus

oxidative stress;GO:0008104//protein localization;GO:0008203//cholesterol metabolic process;GO:
netabolic process;GO:0006637//acyl-CoA metabolic process;GO:0006641//triglyceride metabolic p

to nucleus;GO:0007049//cell cycle;GO:0007179//transforming growth factor beta receptor signalir
n development;GO:0050804//modulation of synaptic transmission;GO:0099560//synaptic membrar

tion;GO:0033383//geranyl diphosphate metabolic process;GO:0045339//farnesyl diphosphate cata
ation;GO:0002244//hematopoietic progenitor cell differentiation;GO:0006357//regulation of transc

athway;GO:0009888//tissue development;GO:0030198//extracellular matrix organization;GO:0071
e regulation of endothelial cell migration;GO:0019673//GDP-mannose metabolic process;GO:004

regulation of neurogenesis;GO:0060770//negative regulation of epithelial cell proliferation involve

ipid translocation;GO:0047484//regulation of response to osmotic stress;GO:0055085//transmemb

ponse to phosphate starvation;GO:0032259//methylation;GO:0042135//neurotransmitter catabolic
transport;GO:0070327//thyroid hormone transport;GO:0070460//thyroid-stimulating hormone se

etic process, by transamination of glyoxylate;GO:0019481//L-alanine catabolic process, by transam

assembly;GO:0030036//actin cytoskeleton organization;GO:0045944//positive regulation of transc
egative regulation of protein lipidation;GO:2001140//positive regulation of phospholipid transport

: process;GO:0042574//retinal metabolic process;GO:1901810//beta-carotene metabolic process

olic process;GO:0051180//vitamin transport;GO:0090212//negative regulation of establishment of b

rocess;GO:0006633//fatty acid biosynthetic process;GO:0010886//positive regulation of cholestero

//oocyte differentiation;GO:0042127//regulation of cell proliferation;GO:0048839//inner ear develo

metabolic process;GO:0007417//central nervous system development;GO:0009064//glutamine far

//lipid biosynthetic process;GO:0016126//sterol biosynthetic process;GO:0045654//positive regulat

ty;GO:0045654//positive regulation of megakaryocyte differentiation;GO:0046330//positive regula
process;GO:0006189//'de novo' IMP biosynthetic process;GO:0009152//purine ribonucleotide bios

//regulation of epidermal growth factor receptor signaling pathway;GO:0043408//regulation of MA

)//nervous system development;GO:0010977//negative regulation of neuron projection developme
542//visual learning;GO:0010801//negative regulation of peptidyl-threonine phosphorylation;GO:0

on;GO:0030501//positive regulation of bone mineralization;GO:0045669//positive regulation of os

I promoter;GO:0006355//regulation of transcription, DNA-templated;GO:0006357//regulation of t
sphorylation;GO:0046856//phosphatidylinositol dephosphorylation;GO:0048488//synaptic vesicle

lic process;GO:0006656//phosphatidylcholine biosynthetic process;GO:0008203//cholesterol meta
ide cross-linking;GO:0030216//keratinocyte differentiation;GO:0045861//negative regulation of pr

response to bacterium;GO:0015909//long-chain fatty acid transport;GO:0042632//cholesterol hom
(6-linked deubiquitination;GO:0050821//protein stabilization;GO:0051898//negative regulation of

nelial cell proliferation;GO:0002719//negative regulation of cytokine production involved in immun

olic process;GO:0051180//vitamin transport;GO:0090212//negative regulation of establishment of b
gration;GO:0006355//regulation of transcription, DNA-templated;GO:0007165//signal transduction

544//arginine catabolic process to glutamate;GO:0055129//L-proline biosynthetic process
transmembrane transporter activity;GO:1904064//positive regulation of cation transmembrane tran
tasis;GO:0007611//learning or memory;GO:0007625//grooming behavior;GO:0007626//locomotor

tasis;GO:0007611//learning or memory;GO:0007625//grooming behavior;GO:0007626//locomotor

netanephros development;GO:0001759//organ induction;GO:0001823//mesonephros developmen

ynthetic process;GO:0009113//purine nucleobase biosynthetic process;GO:0009116//nucleoside m

sphatidylinositol 3-kinase signaling;GO:0033564//anterior/posterior axon guidance;GO:0038007//
regulation of endothelial cell proliferation;GO:0006468//protein phosphorylation;GO:0007165//sig

ide cross-linking;GO:0030216//keratinocyte differentiation;GO:0045861//negative regulation of pr

n;GO:0002305//CD8-positive, gamma-delta intraepithelial T cell differentiation;GO:0002689//nega
n of endocytosis;GO:0048488//synaptic vesicle endocytosis;GO:0048666//neuron development;GC

tive regulation of heparan sulfate proteoglycan biosynthetic process;GO:0050650//chondroitin sulf

32366//intracellular sterol transport;GO:0032367//intracellular cholesterol transport;GO:0033344//

e organization;GO:0043353//enucleate erythrocyte differentiation;GO:0098739//import across plas
ipid translocation;GO:0047484//regulation of response to osmotic stress;GO:0055085//transmemb

regulation of neurogenesis;GO:0060770//negative regulation of epithelial cell proliferation involve

osynthetic process;GO:0009072//aromatic amino acid family metabolic process;GO:0019293//tyros

eart rate;GO:0010765//positive regulation of sodium ion transport;GO:0035725//sodium ion transr

GO:0043409//negative regulation of MAPK cascade;GO:0050906//detection of stimulus involved i
:0030512//negative regulation of transforming growth factor beta receptor signaling pathway

velopment;GO:0008105//asymmetric protein localization;GO:0009887//organ morphogenesis;GO:
deling;GO:0006535//cysteine biosynthetic process from serine;GO:0006801//superoxide metabolic

transport;GO:0070327//thyroid hormone transport;GO:0070460//thyroid-stimulating hormone se
on of sour taste;GO:0006812//cation transport;GO:0007224//smoothened signaling pathway;GO:0

routing angiogenesis;GO:0006816//calcium ion transport;GO:0006886//intracellular protein transp

ell cycle;GO:0006468//protein phosphorylation;GO:0007147//female meiosis II;GO:0032515//negat

l promoter;GO:0006355//regulation of transcription, DNA-templated;GO:0006357//regulation of t

ty;GO:0045654//positive regulation of megakaryocyte differentiation;GO:0046330//positive regula
rocess;GO:0006633//fatty acid biosynthetic process;GO:0010886//positive regulation of cholesterc

'/regulation of epidermal growth factor receptor signaling pathway;GO:0043408//regulation of M

assembly;GO:0030036//actin cytoskeleton organization;GO:0045944//positive regulation of transc

ielial cell proliferation;GO:0002719//negative regulation of cytokine production involved in immun

sphorylation;GO:0046856//phosphatidylinositol dephosphorylation;GO:0048488//synaptic vesicle

i;GO:0002305//CD8-positive, gamma-delta intraepithelial T cell differentiation;GO:0002689//nega

process;GO:0006189//'de novo' IMP biosynthetic process;GO:0009152//purine ribonucleotide bios
tasis;GO:0007611//learning or memory;GO:0007625//grooming behavior;GO:0007626//locomotor

mere organization;GO:0051639//actin filament network formation;GO:0055003//cardiac myofibril a

ponse to phosphate starvation;GO:0032259//methylation;GO:0042135//neurotransmitter catabolic

18//positive regulation of satellite cell activation involved in skeletal muscle regeneration;GO:0030

ive regulation of JNK cascade;GO:0051726//regulation of cell cycle;GO:1900745//positive regulatio

GO:0010468//regulation of gene expression;GO:0010906//regulation of glucose metabolic process

//ubiquitin-dependent protein catabolic process;GO:0007409//axonogenesis;GO:0007412//axon t
tion;GO:0007052//mitotic spindle organization;GO:0007409//axonogenesis;GO:0009615//response

tion;GO:0006935//chemotaxis;GO:0007186//G-protein coupled receptor signaling pathway

03//cholesterol metabolic process;GO:0010468//regulation of gene expression;GO:0010977//nega

hagy;GO:0007029//endoplasmic reticulum organization;GO:0007031//peroxisome organization;G

n;GO:0019805//quinolinate biosynthetic process;GO:0046686//response to cadmium ion;GO:0070

iosynthetic process;GO:0008542//visual learning;GO:0015936//coenzyme A metabolic process;GO

O:0007040//lysosome organization;GO:0007286//spermatid development;GO:0008333//endosom

phore linkage;GO:0032922//circadian regulation of gene expression;GO:0043153//entrainment of

n;GO:0002305//CD8-positive, gamma-delta intraepithelial T cell differentiation;GO:0002689//nega

GO:0043409//negative regulation of MAPK cascade;GO:0050906//detection of stimulus involved in

O:0002269//leukocyte activation involved in inflammatory response;GO:0002548//monocyte chem

0//nervous system development;GO:0010977//negative regulation of neuron projection developme

0045776//negative regulation of blood pressure;GO:0051607//defense response to virus;GO:0055[illegible]

[illegible]//nervous system development;GO:0010977//negative regulation of neuron projection developm[illegible]
lic process;GO:0008210//estrogen metabolic process;GO:0009804//coumarin metabolic process;G[illegible]

lic process;GO:0006656//phosphatidylcholine biosynthetic process;GO:0008203//cholesterol meta[illegible]
ne-mediated signaling pathway;GO:0032754//positive regulation of interleukin-5 production;GO:0[illegible]

nthetic process;GO:0016104//triterpenoid biosynthetic process;GO:0031647//regulation of protein[illegible]

routing angiogenesis;GO:0006816//calcium ion transport;GO:0006886//intracellular protein transp[illegible]

544//arginine catabolic process to glutamate;GO:0055129//L-proline biosynthetic process
n phosphorylation;GO:0010628//positive regulation of gene expression;GO:0010629//negative reg[illegible]

ty;GO:0045654//positive regulation of megakaryocyte differentiation;GO:0046330//positive regula[illegible]

asma membrane adhesion molecules;GO:0007162//negative regulation of cell adhesion;GO:00073[illegible]
copper ion;GO:0032496//response to lipopolysaccharide;GO:0070555//response to interleukin-1;

ration;GO:0006355//regulation of transcription, DNA-templated;GO:0007165//signal transduction

n;GO:0019805//quinolinate biosynthetic process;GO:0046686//response to cadmium ion;GO:0070

GO:0006955//immune response;GO:0009395//phospholipid catabolic process;GO:0010634//positiv[illegible]
ide cross-linking;GO:0030216//keratinocyte differentiation;GO:0045861//negative regulation of pr[illegible]

GO:0043409//negative regulation of MAPK cascade;GO:0050906//detection of stimulus involved i[illegible]

of protein phosphorylation;GO:0007507//heart development;GO:0008284//positive regulation of

//lipid biosynthetic process;GO:0016126//sterol biosynthetic process;GO:0045654//positive regula[illegible]

process;GO:0006189//'de novo' IMP biosynthetic process;GO:0009152//purine ribonucleotide bios[illegible]

bstrate junction assembly;GO:0007155//cell adhesion;GO:0007160//cell-matrix adhesion;GO:0007[illegible]
f neuron projection development;GO:0018105//peptidyl-serine phosphorylation;GO:0032793//po
ive regulation of JNK cascade;GO:0051726//regulation of cell cycle;GO:1900745//positive regulatic

)//protein deubiquitination;GO:0032436//positive regulation of proteasomal ubiquitin-dependent

32732//positive regulation of interleukin-1 production;GO:0055085//transmembrane transport

ne-mediated signaling pathway;GO:0032754//positive regulation of interleukin-5 production;GO:0

n development;GO:0050804//modulation of synaptic transmission;GO:0099560//synaptic membrar
zation;GO:0009235//cobalamin metabolic process;GO:0015889//cobalamin transport;GO:0043001

tabolic process;GO:0019835//cytolysis;GO:0031640//killing of cells of other organism;GO:0042742

fferentiation;GO:0042373//vitamin K metabolic process;GO:2000379//positive regulation of reactiv

rotein catabolic process;GO:0048388//endosomal lumen acidification;GO:0070070//proton-transp

//regulation of MAPK cascade;GO:0045761//regulation of adenylate cyclase activity;GO:2000479//

athway;GO:0009888//tissue development;GO:0030198//extracellular matrix organization;GO:0071[illegible]
hagy;GO:0007029//endoplasmic reticulum organization;GO:0007031//peroxisome organization;G[illegible]
ositive regulation of transcription from RNA polymerase II promoter;GO:0048384//retinoic acid re[illegible]

process;GO:0006189//'de novo' IMP biosynthetic process;GO:0009152//purine ribonucleotide bios[illegible]
55064//chloride ion homeostasis;GO:0055075//potassium ion homeostasis;GO:0055085//transmen
on transmembrane transport;GO:0050909//sensory perception of taste;GO:0055085//transmembra

metabolic process;GO:0006650//glycerophospholipid metabolic process;GO:0006654//phosphatid

lic process;GO:0006656//phosphatidylcholine biosynthetic process;GO:0008203//cholesterol meta

l promoter;GO:0000902//cell morphogenesis;GO:0001709//cell fate determination;GO:0001764//n

544//arginine catabolic process to glutamate;GO:0055129//L-proline biosynthetic process

lic process;GO:0008210//estrogen metabolic process;GO:0009804//coumarin metabolic process;G
ıelial cell proliferation;GO:0002719//negative regulation of cytokine production involved in immun

)//nervous system development;GO:0010977//negative regulation of neuron projection developm

ısma membrane adhesion molecules;GO:0007162//negative regulation of cell adhesion;GO:00073

l promoter;GO:0006355//regulation of transcription, DNA-templated;GO:0006357//regulation of t

deling;GO:0006535//cysteine biosynthetic process from serine;GO:0006801//superoxide metabolic
eart rate;GO:0010765//positive regulation of sodium ion transport;GO:0035725//sodium ion transp

lic process;GO:0009791//post-embryonic development;GO:0010259//multicellular organism aging
ı;GO:0002305//CD8-positive, gamma-delta intraepithelial T cell differentiation;GO:0002689//nega
ration;GO:0006355//regulation of transcription, DNA-templated;GO:0007165//signal transduction

or signaling pathway;GO:0007200//phospholipase C-activating G-protein coupled receptor signal

copper ion;GO:0032496//response to lipopolysaccharide;GO:0070555//response to interleukin-1;
regulation of endothelial cell proliferation;GO:0006468//protein phosphorylation;GO:0007165//sig

fatty acid beta-oxidation;GO:0008206//bile acid metabolic process;GO:0010124//phenylacetate ca

tive regulation of heparan sulfate proteoglycan biosynthetic process;GO:0050650//chondroitin sulf
elopment;GO:0002138//retinoic acid biosynthetic process;GO:0006081//cellular aldehyde metabol

ısma membrane adhesion molecules;GO:0007162//negative regulation of cell adhesion;GO:00073

thway;GO:0009267//cellular response to starvation;GO:0010510//regulation of acetyl-CoA biosynt

ransmembrane transporter activity;GO:1904064//positive regulation of cation transmembrane trar

n;GO:0019805//quinolinate biosynthetic process;GO:0046686//response to cadmium ion;GO:0070

n;GO:0019805//quinolinate biosynthetic process;GO:0046686//response to cadmium ion;GO:0070

03//cholesterol metabolic process;GO:0010468//regulation of gene expression;GO:0010977//nega

zation;GO:0009235//cobalamin metabolic process;GO:0015889//cobalamin transport;GO:0043001

on;GO:0030501//positive regulation of bone mineralization;GO:0045669//positive regulation of os

ion;GO:0006935//chemotaxis;GO:0007186//G-protein coupled receptor signaling pathway
//regulation of MAPK cascade;GO:0045761//regulation of adenylate cyclase activity;GO:2000479//

GO:0006955//immune response;GO:0009395//phospholipid catabolic process;GO:0010634//positiv

olic process;GO:0015697//quaternary ammonium group transport;GO:0015879//carnitine transpo
55064//chloride ion homeostasis;GO:0055075//potassium ion homeostasis;GO:0055085//transmen
544//arginine catabolic process to glutamate;GO:0055129//L-proline biosynthetic process

n;GO:0002305//CD8-positive, gamma-delta intraepithelial T cell differentiation;GO:0002689//nega

070374//positive regulation of ERK1 and ERK2 cascade;GO:0070378//positive regulation of ERK5 c

)8283//cell proliferation;GO:0008284//positive regulation of cell proliferation;GO:0009408//respon

bstrate junction assembly;GO:0007155//cell adhesion;GO:0007160//cell-matrix adhesion;GO:0007:
ito nucleus;GO:0007049//cell cycle;GO:0007179//transforming growth factor beta receptor signalir

al glia guided migration;GO:0030335//positive regulation of cell migration;GO:0031022//nuclear n

GO:0043409//negative regulation of MAPK cascade;GO:0050906//detection of stimulus involved ir
ation;GO:0002244//hematopoietic progenitor cell differentiation;GO:0006357//regulation of transc

fferentiation;GO:0042373//vitamin K metabolic process;GO:2000379//positive regulation of reactiv

ation;GO:0002244//hematopoietic progenitor cell differentiation;GO:0006357//regulation of transc

osynthetic process;GO:0009072//aromatic amino acid family metabolic process;GO:0019293//tyros

e organization;GO:0043353//enucleate erythrocyte differentiation;GO:0098739//import across plas

c process;GO:0035874//cellular response to copper ion starvation;GO:0046677//response to antib

ito nucleus;GO:0007049//cell cycle;GO:0007179//transforming growth factor beta receptor signalir

a chemical signal;GO:0009750//response to fructose;GO:0015755//fructose transport;GO:0055085/
deling;GO:0006535//cysteine biosynthetic process from serine;GO:0006801//superoxide metabolic
etabolic process;GO:0006096//glycolytic process;GO:0051156//glucose 6-phosphate metabolic pr

07399//nervous system development;GO:0032147//activation of protein kinase activity;GO:00324€

178//negative regulation of Wnt signaling pathway;GO:0030949//positive regulation of vascular er

regulation of endothelial cell proliferation;GO:0006468//protein phosphorylation;GO:0007165//sig

asma membrane adhesion molecules;GO:0007162//negative regulation of cell adhesion;GO:00073
lic process;GO:0008654//phospholipid biosynthetic process;GO:0030258//lipid modification;GO:00

ipid translocation;GO:0047484//regulation of response to osmotic stress;GO:0055085//transmemb

ynthetic process;GO:0009113//purine nucleobase biosynthetic process;GO:0009116//nucleoside m

tabolic process;GO:0019835//cytolysis;GO:0031640//killing of cells of other organism;GO:0042742

netabolic process;GO:0006637//acyl-CoA metabolic process;GO:0006641//triglyceride metabolic p

ide cross-linking;GO:0030216//keratinocyte differentiation;GO:0045861//negative regulation of pr

lic process;GO:0006656//phosphatidylcholine biosynthetic process;GO:0008203//cholesterol meta

thway;GO:0009267//cellular response to starvation;GO:0010510//regulation of acetyl-CoA biosynt

or signaling pathway;GO:0007200//phospholipase C-activating G-protein coupled receptor signal

elial cell proliferation;GO:0002719//negative regulation of cytokine production involved in immun

fatty acid beta-oxidation;GO:0008206//bile acid metabolic process;GO:0010124//phenylacetate ca
ubiquitin-dependent protein catabolic process;GO:0030970//retrograde protein transport, ER to cy

gulation of glucose metabolic process;GO:0014065//phosphatidylinositol 3-kinase signaling;GO:00

l promoter;GO:0006355//regulation of transcription, DNA-templated;GO:0006357//regulation of t

ıg compound metabolic process;GO:0008152//metabolic process;GO:0016310//phosphorylation;G
ve regulation of gene expression;GO:0030511//positive regulation of transforming growth factor b

GO:0010468//regulation of gene expression;GO:0010906//regulation of glucose metabolic process

ransmembrane transporter activity;GO:1904064//positive regulation of cation transmembrane tran
ridoxal phosphate-L-lysine;GO:0019343//cysteine biosynthetic process via cystathionine;GO:0019

regulation of endothelial cell proliferation;GO:0006468//protein phosphorylation;GO:0007165//sig

metabolic process;GO:0007417//central nervous system development;GO:0009064//glutamine fan

ıto nucleus;GO:0007049//cell cycle;GO:0007179//transforming growth factor beta receptor signalir

ide cross-linking;GO:0030216//keratinocyte differentiation;GO:0045861//negative regulation of pr
d biosynthetic process;GO:0006954//inflammatory response;GO:0008611//ether lipid biosynthetic
osynthetic process;GO:0009072//aromatic amino acid family metabolic process;GO:0019293//tyros
al glia guided migration;GO:0030335//positive regulation of cell migration;GO:0031022//nuclear n

e organization;GO:0043353//enucleate erythrocyte differentiation;GO:0098739//import across plas
process;GO:0008203//cholesterol metabolic process;GO:0019433//triglyceride catabolic process;G
ation;GO:0002244//hematopoietic progenitor cell differentiation;GO:0006357//regulation of transc

)//nervous system development;GO:0010977//negative regulation of neuron projection developme

on;GO:0030501//positive regulation of bone mineralization;GO:0045669//positive regulation of os

n;GO:0019805//quinolinate biosynthetic process;GO:0046686//response to cadmium ion;GO:0070

thway;GO:0009267//cellular response to starvation;GO:0010510//regulation of acetyl-CoA biosynt
athway;GO:0009888//tissue development;GO:0030198//extracellular matrix organization;GO:0071

ation;GO:0002244//hematopoietic progenitor cell differentiation;GO:0006357//regulation of transc

ı development;GO:0050804//modulation of synaptic transmission;GO:0099560//synaptic membran

sphatidylinositol 3-kinase signaling;GO:0033564//anterior/posterior axon guidance;GO:0038007//

or signaling pathway;GO:0008277//regulation of G-protein coupled receptor protein signaling pat
ng compound metabolic process;GO:0008152//metabolic process;GO:0016310//phosphorylation;G

ynthetic process;GO:0009113//purine nucleobase biosynthetic process;GO:0009116//nucleoside m

nto nucleus;GO:0007049//cell cycle;GO:0007179//transforming growth factor beta receptor signalir

: process;GO:0042574//retinal metabolic process;GO:1901810//beta-carotene metabolic process
O:0007252//I-kappaB phosphorylation;GO:0008630//intrinsic apoptotic signaling pathway in respo

//inflammatory response;GO:0006956//complement activation;GO:0010575//positive regulation of

lic process;GO:0008654//phospholipid biosynthetic process;GO:0030258//lipid modification;GO:00
ranscription from RNA polymerase II promoter;GO:0006654//phosphatidic acid biosynthetic proce
ation;GO:0044260//cellular macromolecule metabolic process;GO:0090305//nucleic acid phospho
or signaling pathway;GO:0048678//response to axon injury;GO:1901214//regulation of neuron dea
tion;GO:0006935//chemotaxis;GO:0007186//G-protein coupled receptor signaling pathway
zation;GO:0009235//cobalamin metabolic process;GO:0015889//cobalamin transport;GO:0043001

tabolic process;GO:0019835//cytolysis;GO:0031640//killing of cells of other organism;GO:0042742

GO:0006107//oxaloacetate metabolic process;GO:0006629//lipid metabolic process;GO:0009617//r
e response;GO:0007186//G-protein coupled receptor signaling pathway;GO:0007204//positive reg

regulation of neurogenesis;GO:0060770//negative regulation of epithelial cell proliferation involve

velopment;GO:0007411//axon guidance;GO:0007420//brain development;GO:0010975//regulation

e response;GO:0007186//G-protein coupled receptor signaling pathway;GO:0007204//positive reg

e regulation of cell-substrate adhesion;GO:0045742//positive regulation of epidermal growth fact

:0030512//negative regulation of transforming growth factor beta receptor signaling pathway

tabolic process;GO:0019835//cytolysis;GO:0031640//killing of cells of other organism;GO:0042742

metabolic process;GO:0007417//central nervous system development;GO:0009064//glutamine far
lic process;GO:0006656//phosphatidylcholine biosynthetic process;GO:0008203//cholesterol meta

ve regulation of gene expression;GO:0030511//positive regulation of transforming growth factor b

//glycoprotein catabolic process;GO:0016567//protein ubiquitination;GO:0030433//ER-associated

l shape;GO:0042742//defense response to bacterium;GO:0045089//positive regulation of innate ir

regulation of endothelial cell proliferation;GO:0006468//protein phosphorylation;GO:0007165//sig

copper ion;GO:0032496//response to lipopolysaccharide;GO:0070555//response to interleukin-1;
070374//positive regulation of ERK1 and ERK2 cascade;GO:0070378//positive regulation of ERK5 c

process;GO:0008203//cholesterol metabolic process;GO:0019433//triglyceride catabolic process;(
erived foam cell differentiation;GO:0010878//cholesterol storage;GO:0033344//cholesterol efflux;G

6240//autophagosome docking;GO:0034329//cell junction assembly;GO:0042953//lipoprotein tra

targeting;GO:0006904//vesicle docking involved in exocytosis;GO:0016192//vesicle-mediated tra
adhesion;GO:0007264//small GTPase mediated signal transduction;GO:0007628//adult walking bel

starvation;GO:0044090//positive regulation of vacuole organization;GO:2000786//positive regula

ɔonse to phosphate starvation;GO:0032259//methylation;GO:0042135//neurotransmitter catabolic

egulation of transcription elongation from RNA polymerase II promoter;GO:0045893//positive regu

lic process;GO:0008210//estrogen metabolic process;GO:0009804//coumarin metabolic process;G

ipid translocation;GO:0047484//regulation of response to osmotic stress;GO:0055085//transmemb

)//nervous system development;GO:0010977//negative regulation of neuron projection developm

ıg compound metabolic process;GO:0008152//metabolic process;GO:0016310//phosphorylation;G

I promoter;GO:0001657//ureteric bud development;GO:0001843//neural tube closure;GO:000206

ide cross-linking;GO:0030216//keratinocyte differentiation;GO:0045861//negative regulation of pr

//inflammatory response to antigenic stimulus;GO:0007264//small GTPase mediated signal transdu

0045776//negative regulation of blood pressure;GO:0051607//defense response to virus;GO:0055

metabolic process;GO:0007417//central nervous system development;GO:0009064//glutamine far
g;GO:0030261//chromosome condensation;GO:0045910//negative regulation of DNA recombinat
ation of gene expression;GO:0045671//negative regulation of osteoclast differentiation;GO:004577
ptor protein tyrosine kinase signaling pathway;GO:0009615//response to virus;GO:0043406//posit

abolic process;GO:0006012//galactose metabolic process;GO:0019318//hexose metabolic process;

AT cascade;GO:0009968//negative regulation of signal transduction;GO:0035556//intracellular sig

regulation of endothelial cell proliferation;GO:0006468//protein phosphorylation;GO:0007165//sig

on;GO:0030501//positive regulation of bone mineralization;GO:0045669//positive regulation of os

lic process;GO:0051180//vitamin transport;GO:0090212//negative regulation of establishment of

070374//positive regulation of ERK1 and ERK2 cascade;GO:0070378//positive regulation of ERK5 c

ransmembrane transporter activity;GO:1904064//positive regulation of cation transmembrane trar

process;GO:0006189//'de novo' IMP biosynthetic process;GO:0009152//purine ribonucleotide bios

a chemical signal;GO:0009750//response to fructose;GO:0015755//fructose transport;GO:0055085/

ding in birth or egg hatching;GO:0019336//phenol-containing compound catabolic process;GO:0
tive regulation of calcium-mediated signaling;GO:0050862//positive regulation of T cell receptor s
positive regulation of protein phosphorylation;GO:0003347//epicardial cell to mesenchymal cell tra
ynthetic process;GO:0009113//purine nucleobase biosynthetic process;GO:0009116//nucleoside m

//glycoprotein catabolic process;GO:0016567//protein ubiquitination;GO:0030433//ER-associated
or signaling pathway;GO:0048678//response to axon injury;GO:1901214//regulation of neuron dea

etic process, by transamination of glyoxylate;GO:0019481//L-alanine catabolic process, by transam

070374//positive regulation of ERK1 and ERK2 cascade;GO:0070378//positive regulation of ERK5 c
0006914//autophagy;GO:0007399//nervous system development;GO:0017121//phospholipid scra

development;GO:0050804//modulation of synaptic transmission;GO:0099560//synaptic membrar

g compound metabolic process;GO:0008152//metabolic process;GO:0016310//phosphorylation;G

erived foam cell differentiation;GO:0010878//cholesterol storage;GO:0033344//cholesterol efflux;G
e activation involved in immune response;GO:0007186//G-protein coupled receptor signaling pat

egulation of canonical Wnt signaling pathway;GO:2000096//positive regulation of Wnt signaling p

copper ion;GO:0032496//response to lipopolysaccharide;GO:0070555//response to interleukin-1;

pathway;GO:0009617//response to bacterium;GO:0010628//positive regulation of gene expression;

athway;GO:0009888//tissue development;GO:0030198//extracellular matrix organization;GO:0071
c process;GO:0035874//cellular response to copper ion starvation;GO:0046677//response to antib

e organization;GO:0043353//enucleate erythrocyte differentiation;GO:0098739//import across plas

542//visual learning;GO:0010801//negative regulation of peptidyl-threonine phosphorylation;GO:0
osynthetic process;GO:0009072//aromatic amino acid family metabolic process;GO:0019293//tyros

tion of transcription, DNA-templated;GO:0006357//regulation of transcription from RNA polymera

O:0006955//immune response;GO:0007155//cell adhesion;GO:0007160//cell-matrix adhesion;GO:

regulation of neurogenesis;GO:0060770//negative regulation of epithelial cell proliferation involve
zation;GO:0009235//cobalamin metabolic process;GO:0015889//cobalamin transport;GO:0043001

n development;GO:0050804//modulation of synaptic transmission;GO:0099560//synaptic membran

//nervous system development;GO:0010977//negative regulation of neuron projection developm

I promoter;GO:0006355//regulation of transcription, DNA-templated;GO:0006357//regulation of t
lic process;GO:0008210//estrogen metabolic process;GO:0009804//coumarin metabolic process;G
ation;GO:0002244//hematopoietic progenitor cell differentiation;GO:0006357//regulation of transc

//inflammatory response to antigenic stimulus;GO:0007264//small GTPase mediated signal transdu

g;GO:0030261//chromosome condensation;GO:0045910//negative regulation of DNA recombinat

08283//cell proliferation;GO:0008284//positive regulation of cell proliferation;GO:0009408//respon

helial cell proliferation;GO:0002719//negative regulation of cytokine production involved in immun
olic process;GO:0051180//vitamin transport;GO:0090212//negative regulation of establishment of b
21//chloride transport;GO:0034220//ion transmembrane transport;GO:0055085//transmembrane t

olic process;GO:0051180//vitamin transport;GO:0090212//negative regulation of establishment of b
nto nucleus;GO:0007049//cell cycle;GO:0007179//transforming growth factor beta receptor signalin
616//long-term memory;GO:0007626//locomotory behavior;GO:0009190//cyclic nucleotide biosyn

n;GO:0002305//CD8-positive, gamma-delta intraepithelial T cell differentiation;GO:0002689//nega
ranscription from RNA polymerase II promoter;GO:0006654//phosphatidic acid biosynthetic proce

ation;GO:0044260//cellular macromolecule metabolic process;GO:0090305//nucleic acid phospho
//lipid biosynthetic process;GO:0016126//sterol biosynthetic process;GO:0045654//positive regulat

e expression;GO:0030001//metal ion transport;GO:0031398//positive regulation of protein ubiquit

tasis;GO:0007611//learning or memory;GO:0007625//grooming behavior;GO:0007626//locomotor

n;GO:0019805//quinolinate biosynthetic process;GO:0046686//response to cadmium ion;GO:0070

//lipid biosynthetic process;GO:0016126//sterol biosynthetic process;GO:0045654//positive regulat
sphatidylinositol 3-kinase signaling;GO:0033564//anterior/posterior axon guidance;GO:0038007//

'AT cascade;GO:0009968//negative regulation of signal transduction;GO:0035556//intracellular sig

ponse to phosphate starvation;GO:0032259//methylation;GO:0042135//neurotransmitter catabolic
gration;GO:0006355//regulation of transcription, DNA-templated;GO:0007165//signal transduction

ose assembly;GO:0008611//ether lipid biosynthetic process;GO:0021587//cerebellum morphogene

c process;GO:0035874//cellular response to copper ion starvation;GO:0046677//response to antib

GO:0043409//negative regulation of MAPK cascade;GO:0050906//detection of stimulus involved i

I promoter;GO:0000902//cell morphogenesis;GO:0001709//cell fate determination;GO:0001764//n
n;GO:0019805//quinolinate biosynthetic process;GO:0046686//response to cadmium ion;GO:0070

n development;GO:0050804//modulation of synaptic transmission;GO:0099560//synaptic membrar
on;GO:0030501//positive regulation of bone mineralization;GO:0045669//positive regulation of os

0045776//negative regulation of blood pressure;GO:0051607//defense response to virus;GO:0055(

ı development;GO:0050804//modulation of synaptic transmission;GO:0099560//synaptic membrar

biosynthetic process;GO:0015908//fatty acid transport;GO:0033211//adiponectin-activated signali

070374//positive regulation of ERK1 and ERK2 cascade;GO:0070378//positive regulation of ERK5 c
)8283//cell proliferation;GO:0008284//positive regulation of cell proliferation;GO:0009408//respon

ɔositive regulation of protein phosphorylation;GO:0003347//epicardial cell to mesenchymal cell tra
sm growth;GO:0035934//corticosterone secretion;GO:0042445//hormone metabolic process;GO:0(

transport;GO:0070327//thyroid hormone transport;GO:0070460//thyroid-stimulating hormone se
)06541//glutamine metabolic process;GO:0006807//nitrogen compound metabolic process;GO:00

//phospholipase C-activating G-protein coupled receptor signaling pathway;GO:0035176//social l

ɔathway;GO:0009617//response to bacterium;GO:0010628//positive regulation of gene expression;

544//arginine catabolic process to glutamate;GO:0055129//L-proline biosynthetic process

ransmembrane transporter activity;GO:1904064//positive regulation of cation transmembrane trar
.958//methotrexate transport;GO:0098838//reduced folate transmembrane transport;GO:1904447/

ation of gene expression;GO:0045671//negative regulation of osteoclast differentiation;GO:004577

//base-excision repair, AP site formation;GO:0006296//nucleotide-excision repair, DNA incision, 5'
I promoter;GO:0006355//regulation of transcription, DNA-templated;GO:0006357//regulation of t

ı;GO:0002305//CD8-positive, gamma-delta intraepithelial T cell differentiation;GO:0002689//nega

ɔrocess;GO:0006189//'de novo' IMP biosynthetic process;GO:0009152//purine ribonucleotide bios

tor beta receptor signaling pathway;GO:0007229//integrin-mediated signaling pathway;GO:00164

ıelial cell proliferation;GO:0002719//negative regulation of cytokine production involved in immun
or signaling pathway;GO:0008277//regulation of G-protein coupled receptor protein signaling pat
)6952//defense response;GO:0007599//hemostasis;GO:0010524//positive regulation of calcium ior
//lipid biosynthetic process;GO:0016126//sterol biosynthetic process;GO:0045654//positive regulat

ɛptidase activity involved in apoptotic process;GO:0006978//DNA damage response, signal transdı
tive regulation of calcium-mediated signaling;GO:0050862//positive regulation of T cell receptor s

sport;GO:0006982//response to lipid hydroperoxide;GO:0007159//leukocyte cell-cell adhesion;GO

n-dependent cell spreading;GO:0061001//regulation of dendritic spine morphogenesis;GO:00610
070374//positive regulation of ERK1 and ERK2 cascade;GO:0070378//positive regulation of ERK5 c
nsulin stimulus;GO:0071456//cellular response to hypoxia;GO:2000766//negative regulation of cyt

tasis;GO:0007611//learning or memory;GO:0007625//grooming behavior;GO:0007626//locomotor

ganic substance;GO:0010872//regulation of cholesterol esterification;GO:0010875//positive regula
l promoter;GO:0006355//regulation of transcription, DNA-templated;GO:0009267//cellular respor

oment;GO:0006508//proteolysis;GO:0016525//negative regulation of angiogenesis;GO:0060347//h
losure;GO:0007389//pattern specification process;GO:0007398//ectoderm development;GO:00074
03//cholesterol metabolic process;GO:0010468//regulation of gene expression;GO:0010977//nega
ty;GO:0045654//positive regulation of megakaryocyte differentiation;GO:0046330//positive regula

ylation;GO:0006915//apoptotic process;GO:0031647//regulation of protein stability;GO:0050790//

/extracellular matrix organization;GO:0044869//negative regulation by host of viral exo-alpha-siali

0045776//negative regulation of blood pressure;GO:0051607//defense response to virus;GO:0055
):0002269//leukocyte activation involved in inflammatory response;GO:0002548//monocyte chem

response to bacterium;GO:0015909//long-chain fatty acid transport;GO:0042632//cholesterol hom
copper ion;GO:0032496//response to lipopolysaccharide;GO:0070555//response to interleukin-1;

GO:0043409//negative regulation of MAPK cascade;GO:0050906//detection of stimulus involved i

608//sensory perception of smell;GO:0030311//poly-N-acetyllactosamine biosynthetic process;G
gulation of glucose metabolic process;GO:0014065//phosphatidylinositol 3-kinase signaling;GO:0

etabolic process;GO:0008652//cellular amino acid biosynthetic process;GO:0042149//cellular resp

.958//methotrexate transport;GO:0098838//reduced folate transmembrane transport;GO:1904447/
):0010831//positive regulation of myotube differentiation;GO:0034968//histone lysine methylation
//inflammatory response to antigenic stimulus;GO:0007264//small GTPase mediated signal transdu
lic process;GO:0008210//estrogen metabolic process;GO:0009804//coumarin metabolic process;G
ive regulation of apoptotic process;GO:0045727//positive regulation of translation;GO:0045786//n

070374//positive regulation of ERK1 and ERK2 cascade;GO:0070378//positive regulation of ERK5 c

409//axonogenesis;GO:0007411//axon guidance;GO:0008284//positive regulation of cell proliferat

3//ER-associated ubiquitin-dependent protein catabolic process;GO:0032781//positive regulation

0017158//regulation of calcium ion-dependent exocytosis;GO:0032024//positive regulation of ins

616//long-term memory;GO:0007626//locomotory behavior;GO:0009190//cyclic nucleotide biosyı

uction;GO:0043087//regulation of GTPase activity;GO:0046580//negative regulation of Ras protein
mere organization;GO:0051639//actin filament network formation;GO:0055003//cardiac myofibril a

ation of gene expression;GO:0045671//negative regulation of osteoclast differentiation;GO:004577

lic process;GO:0006656//phosphatidylcholine biosynthetic process;GO:0008203//cholesterol meta

plipid metabolic process;GO:0016311//dephosphorylation;GO:0035335//peptidyl-tyrosine dephosp

ty;GO:0045654//positive regulation of megakaryocyte differentiation;GO:0046330//positive regula

erived foam cell differentiation;GO:0010878//cholesterol storage;GO:0033344//cholesterol efflux;G
//lipid biosynthetic process;GO:0016126//sterol biosynthetic process;GO:0045654//positive regulat

n;GO:0019805//quinolinate biosynthetic process;GO:0046686//response to cadmium ion;GO:0070

zation;GO:0009235//cobalamin metabolic process;GO:0015889//cobalamin transport;GO:0043001

or signaling pathway;GO:0007189//adenylate cyclase-activating G-protein coupled receptor signa

losure;GO:0007389//pattern specification process;GO:0007398//ectoderm development;GO:00074

O:0007252//I-kappaB phosphorylation;GO:0008630//intrinsic apoptotic signaling pathway in respo

544//arginine catabolic process to glutamate;GO:0055129//L-proline biosynthetic process
//inflammatory response;GO:0006956//complement activation;GO:0010575//positive regulation of

06952//defense response;GO:0007599//hemostasis;GO:0010524//positive regulation of calcium ior

n development;GO:0050804//modulation of synaptic transmission;GO:0099560//synaptic membrar

on;GO:0043649//dicarboxylic acid catabolic process;GO:0045225//negative regulation of CD4 bios
athway;GO:0009888//tissue development;GO:0030198//extracellular matrix organization;GO:0071

:osamine metabolic process;GO:0006790//sulfur compound metabolic process;GO:1903238//posit

ation;GO:0090305//nucleic acid phosphodiester bond hydrolysis;GO:0090502//RNA phosphodiest

)//nucleolus organization;GO:0016579//protein deubiquitination;GO:0050821//protein stabilizatior
l promoter;GO:0006355//regulation of transcription, DNA-templated;GO:0006357//regulation of t

process;GO:0008284//positive regulation of cell proliferation;GO:0009615//response to virus;GO:0(

ielial cell proliferation;GO:0002719//negative regulation of cytokine production involved in immun

ponse to phosphate starvation;GO:0032259//methylation;GO:0042135//neurotransmitter catabolic

GO:0043409//negative regulation of MAPK cascade;GO:0050906//detection of stimulus involved ir

mere organization;GO:0051639//actin filament network formation;GO:0055003//cardiac myofibril a

ive regulation of apoptotic process;GO:0045727//positive regulation of translation;GO:0045786//n

metabolic process;GO:0006631//fatty acid metabolic process;GO:0006635//fatty acid beta-oxidatio
1766//hippocampus development;GO:0021819//layer formation in cerebral cortex;GO:0021860//p
ic process;GO:0006644//phospholipid metabolic process;GO:0007160//cell-matrix adhesion;GO:0(

//inflammatory response to antigenic stimulus;GO:0007264//small GTPase mediated signal transdu
ipid translocation;GO:0047484//regulation of response to osmotic stress;GO:0055085//transmemb
0017158//regulation of calcium ion-dependent exocytosis;GO:0032024//positive regulation of ins
l shape;GO:0042742//defense response to bacterium;GO:0045089//positive regulation of innate ir

uction;GO:0030217//T cell differentiation;GO:0042098//T cell proliferation;GO:0060218//hematopo
lic process;GO:0006656//phosphatidylcholine biosynthetic process;GO:0008203//cholesterol meta

gnaling pathway in response to oxidative stress;GO:0009267//cellular response to starvation;GO:00

ative regulation of blood coagulation;GO:0071383//cellular response to steroid hormone stimulus

616//long-term memory;GO:0007626//locomotory behavior;GO:0009190//cyclic nucleotide biosyn

response to bacterium;GO:0015909//long-chain fatty acid transport;GO:0042632//cholesterol hom

bolic process;GO:0015697//quaternary ammonium group transport;GO:0015879//carnitine transpo
sport;GO:0006982//response to lipid hydroperoxide;GO:0007159//leukocyte cell-cell adhesion;GO
gration;GO:0006355//regulation of transcription, DNA-templated;GO:0007165//signal transduction

erived foam cell differentiation;GO:0010878//cholesterol storage;GO:0033344//cholesterol efflux;G

sm growth;GO:0035934//corticosterone secretion;GO:0042445//hormone metabolic process;GO:00
tasis;GO:0007611//learning or memory;GO:0007625//grooming behavior;GO:0007626//locomotor

GO:0043409//negative regulation of MAPK cascade;GO:0050906//detection of stimulus involved in
tasis;GO:0007611//learning or memory;GO:0007625//grooming behavior;GO:0007626//locomotor

helial cell proliferation;GO:0002719//negative regulation of cytokine production involved in immun

ide cross-linking;GO:0030216//keratinocyte differentiation;GO:0045861//negative regulation of pr

regulation of neurogenesis;GO:0060770//negative regulation of epithelial cell proliferation involve

ganic substance;GO:0010872//regulation of cholesterol esterification;GO:0010875//positive regula

s;GO:0010628//positive regulation of gene expression;GO:0032332//positive regulation of chondr

rocess;GO:0006633//fatty acid biosynthetic process;GO:0010886//positive regulation of cholester
07399//nervous system development;GO:0032147//activation of protein kinase activity;GO:00324

06782//protoporphyrinogen IX biosynthetic process;GO:0006783//heme biosynthetic process;GO:0

oduction;GO:0010760//negative regulation of macrophage chemotaxis;GO:0032722//positive regu

response to bacterium;GO:0015909//long-chain fatty acid transport;GO:0042632//cholesterol hom

l promoter;GO:0006355//regulation of transcription, DNA-templated;GO:0006357//regulation of t

assembly;GO:0030036//actin cytoskeleton organization;GO:0045944//positive regulation of transc

osynthetic process;GO:0009072//aromatic amino acid family metabolic process;GO:0019293//tyros
08283//cell proliferation;GO:0008284//positive regulation of cell proliferation;GO:0009408//respon
544//arginine catabolic process to glutamate;GO:0055129//L-proline biosynthetic process

e organization;GO:0043353//enucleate erythrocyte differentiation;GO:0098739//import across plas
athway;GO:0009888//tissue development;GO:0030198//extracellular matrix organization;GO:00713

//oocyte differentiation;GO:0042127//regulation of cell proliferation;GO:0048839//inner ear devel

178//negative regulation of Wnt signaling pathway;GO:0030949//positive regulation of vascular en

erived foam cell differentiation;GO:0010878//cholesterol storage;GO:0033344//cholesterol efflux;G

nsulin stimulus;GO:0071456//cellular response to hypoxia;GO:2000766//negative regulation of cyt

608//sensory perception of smell;GO:0030311//poly-N-acetyllactosamine biosynthetic process;GO

ositive regulation of I-kappaB kinase/NF-kappaB signaling;GO:0048168//regulation of neuronal sy

c process;GO:0035874//cellular response to copper ion starvation;GO:0046677//response to antib

assembly;GO:0030036//actin cytoskeleton organization;GO:0045944//positive regulation of transc

045723//positive regulation of fatty acid biosynthetic process;GO:0051258//protein polymerization

ynthetic process;GO:0009113//purine nucleobase biosynthetic process;GO:0009116//nucleoside m

deling;GO:0006535//cysteine biosynthetic process from serine;GO:0006801//superoxide metabolic
ipid translocation;GO:0047484//regulation of response to osmotic stress;GO:0055085//transmemb
rocess;GO:0006633//fatty acid biosynthetic process;GO:0010886//positive regulation of choleste

regulation of neurogenesis;GO:0060770//negative regulation of epithelial cell proliferation involve

zation;GO:0009235//cobalamin metabolic process;GO:0015889//cobalamin transport;GO:0043001
ng compound metabolic process;GO:0008152//metabolic process;GO:0016310//phosphorylation;G
pathway;GO:0009617//response to bacterium;GO:0010628//positive regulation of gene expression

process;GO:0042574//retinal metabolic process;GO:1901810//beta-carotene metabolic process

//inflammatory response to antigenic stimulus;GO:0007264//small GTPase mediated signal transdu

tasis;GO:0007611//learning or memory;GO:0007625//grooming behavior;GO:0007626//locomotor

se activity;GO:0038161//prolactin signaling pathway;GO:0042976//activation of Janus kinase activit
ponse to phosphate starvation;GO:0032259//methylation;GO:0042135//neurotransmitter catabolic

asma membrane adhesion molecules;GO:0007162//negative regulation of cell adhesion;GO:00073

O:0006956//complement activation;GO:0006958//complement activation, classical pathway;GO:00

I promoter;GO:0000902//cell morphogenesis;GO:0001709//cell fate determination;GO:0001764//n

tive regulation of calcium-mediated signaling;GO:0050862//positive regulation of T cell receptor s
ridoxal phosphate-L-lysine;GO:0019343//cysteine biosynthetic process via cystathionine;GO:0019

I promoter;GO:0006355//regulation of transcription, DNA-templated;GO:0051170//nuclear import

egulation of transcription from RNA polymerase II promoter;GO:0001649//osteoblast differentiatio

metabolic process;GO:0007417//central nervous system development;GO:0009064//glutamine fa

lic process;GO:0006656//phosphatidylcholine biosynthetic process;GO:0008203//cholesterol meta

thway;GO:0009267//cellular response to starvation;GO:0010510//regulation of acetyl-CoA biosynt
ipid translocation;GO:0047484//regulation of response to osmotic stress;GO:0055085//transmemb

ganic substance;GO:0010872//regulation of cholesterol esterification;GO:0010875//positive regula

)6508//proteolysis;GO:0014068//positive regulation of phosphatidylinositol 3-kinase signaling;GO

cle-mediated transport, Golgi to ER;GO:0015031//protein transport;GO:0016192//vesicle-mediate
ponse to phosphate starvation;GO:0032259//methylation;GO:0042135//neurotransmitter catabolic

mere organization;GO:0051639//actin filament network formation;GO:0055003//cardiac myofibril

e regulation of cell-substrate adhesion;GO:0045742//positive regulation of epidermal growth fact

)8283//cell proliferation;GO:0008284//positive regulation of cell proliferation;GO:0009408//respon
endent proteasomal ubiquitin-dependent protein catabolic process;GO:0043153//entrainment of
osynthetic process;GO:0009072//aromatic amino acid family metabolic process;GO:0019293//tyros
endent proteasomal ubiquitin-dependent protein catabolic process;GO:0043153//entrainment of
adhesion;GO:0007264//small GTPase mediated signal transduction;GO:0007628//adult walking be
lic process;GO:0008210//estrogen metabolic process;GO:0009804//coumarin metabolic process;G
organization;GO:0043353//enucleate erythrocyte differentiation;GO:0098739//import across plas
port;GO:0048265//response to pain;GO:0051620//norepinephrine uptake;GO:0055085//transmem

lic process;GO:0006656//phosphatidylcholine biosynthetic process;GO:0008203//cholesterol meta
regulation of neurogenesis;GO:0060770//negative regulation of epithelial cell proliferation involve

s;GO:0010628//positive regulation of gene expression;GO:0032332//positive regulation of chondr

asma membrane adhesion molecules;GO:0007162//negative regulation of cell adhesion;GO:00073

duction;GO:0010760//negative regulation of macrophage chemotaxis;GO:0032722//positive regu
GO:0007032//endosome organization;GO:0016185//synaptic vesicle budding from presynaptic en

regulation of neurogenesis;GO:0060770//negative regulation of epithelial cell proliferation involve

c process;GO:0035874//cellular response to copper ion starvation;GO:0046677//response to antib

copper ion;GO:0032496//response to lipopolysaccharide;GO:0070555//response to interleukin-1;

ide cross-linking;GO:0030216//keratinocyte differentiation;GO:0045861//negative regulation of pr

ion;GO:1902857//positive regulation of nonmotile primary cilium assembly;GO:1904951//positive

007268//synaptic transmission;GO:0007416//synapse assembly;GO:0007612//learning;GO:0030534
ynthetic process;GO:0009113//purine nucleobase biosynthetic process;GO:0009116//nucleoside m

promoter;GO:1900408//negative regulation of cellular response to oxidative stress;GO:1902083//r

ative regulation of blood coagulation;GO:0071383//cellular response to steroid hormone stimulus

DNA damage stimulus;GO:0045023//G0 to G1 transition;GO:0045746//negative regulation of Notc

on;GO:0030501//positive regulation of bone mineralization;GO:0045669//positive regulation of os

zation;GO:0009235//cobalamin metabolic process;GO:0015889//cobalamin transport;GO:0043001

process;GO:0008284//positive regulation of cell proliferation;GO:0009615//response to virus;GO:0

l promoter;GO:0006355//regulation of transcription, DNA-templated;GO:0006357//regulation of t

lic process;GO:0006656//phosphatidylcholine biosynthetic process;GO:0008203//cholesterol meta

544//arginine catabolic process to glutamate;GO:0055129//L-proline biosynthetic process

helial cell proliferation;GO:0002719//negative regulation of cytokine production involved in immun
O:0007040//lysosome organization;GO:0007286//spermatid development;GO:0008333//endosom

ransmembrane transporter activity;GO:1904064//positive regulation of cation transmembrane trar

tabolic process;GO:0019835//cytolysis;GO:0031640//killing of cells of other organism;GO:0042742

nloride transport;GO:0008272//sulfate transport;GO:0010628//positive regulation of gene expressi
on;GO:0043649//dicarboxylic acid catabolic process;GO:0045225//negative regulation of CD4 bios
thway;GO:0009267//cellular response to starvation;GO:0010510//regulation of acetyl-CoA biosynt
erived foam cell differentiation;GO:0010878//cholesterol storage;GO:0033344//cholesterol efflux;G

a chemical signal;GO:0009750//response to fructose;GO:0015755//fructose transport;GO:0055085/

gulation of cell proliferation;GO:0032956//regulation of actin cytoskeleton organization;GO:00507
process;GO:0006189//'de novo' IMP biosynthetic process;GO:0009152//purine ribonucleotide bios

metabolic process;GO:0007417//central nervous system development;GO:0009064//glutamine far

c process;GO:0006869//lipid transport;GO:0006886//intracellular protein transport;GO:0010875//p

ponse to phosphate starvation;GO:0032259//methylation;GO:0042135//neurotransmitter catabolic

O:0006955//immune response;GO:0007155//cell adhesion;GO:0007160//cell-matrix adhesion;GO:

O:0006956//complement activation;GO:0006958//complement activation, classical pathway;GO:00

lic process;GO:0008210//estrogen metabolic process;GO:0009804//coumarin metabolic process;G

: process;GO:0042574//retinal metabolic process;GO:1901810//beta-carotene metabolic process

ng compound metabolic process;GO:0008152//metabolic process;GO:0016310//phosphorylation;G

O:0007155//cell adhesion;GO:0007159//leukocyte cell-cell adhesion;GO:0007160//cell-matrix adh

I promoter;GO:0006418//tRNA aminoacylation for protein translation;GO:0006434//seryl-tRNA am
)6782//protoporphyrinogen IX biosynthetic process;GO:0006783//heme biosynthetic process;GO:(

//inflammatory response to antigenic stimulus;GO:0007264//small GTPase mediated signal transdu

eak repair via nonhomologous end joining;GO:0010212//response to ionizing radiation;GO:00301
ipid translocation;GO:0047484//regulation of response to osmotic stress;GO:0055085//transmemb
asma membrane adhesion molecules;GO:0007162//negative regulation of cell adhesion;GO:00073

//glutamate receptor signaling pathway;GO:0007218//neuropeptide signaling pathway;GO:199083
copper ion;GO:0032496//response to lipopolysaccharide;GO:0070555//response to interleukin-1;
lipid metabolic process;GO:0016311//dephosphorylation;GO:0035335//peptidyl-tyrosine dephos

)35556//intracellular signal transduction;GO:0043065//positive regulation of apoptotic process;GC

GO:0043409//negative regulation of MAPK cascade;GO:0050906//detection of stimulus involved i
nolecules;GO:0050806//positive regulation of synaptic transmission;GO:0051965//positive regulatic
)6508//proteolysis;GO:0014068//positive regulation of phosphatidylinositol 3-kinase signaling;GO

metabolic process;GO:0007417//central nervous system development;GO:0009064//glutamine fa

I promoter;GO:0000902//cell morphogenesis;GO:0001709//cell fate determination;GO:0001764//n

promoter;GO:1900408//negative regulation of cellular response to oxidative stress;GO:1902083//

/extracellular matrix organization;GO:0044869//negative regulation by host of viral exo-alpha-siali

ative regulation of blood coagulation;GO:0071383//cellular response to steroid hormone stimulus

GO:0010468//regulation of gene expression;GO:0010906//regulation of glucose metabolic process

xidation;GO:0010942//positive regulation of cell death;GO:0033540//fatty acid beta-oxidation usir

ic process;GO:0006644//phospholipid metabolic process;GO:0007160//cell-matrix adhesion;GO:00

metabolic process;GO:0006631//fatty acid metabolic process;GO:0006635//fatty acid beta-oxidati

adhesion;GO:0007264//small GTPase mediated signal transduction;GO:0007628//adult walking bel
osynthetic process;GO:0009072//aromatic amino acid family metabolic process;GO:0019293//tyros

e organization;GO:0043353//enucleate erythrocyte differentiation;GO:0098739//import across plas

ynthetic process;GO:0009113//purine nucleobase biosynthetic process;GO:0009116//nucleoside m

assembly;GO:0030036//actin cytoskeleton organization;GO:0045944//positive regulation of transc
thway;GO:0009267//cellular response to starvation;GO:0010510//regulation of acetyl-CoA biosynt

c process;GO:0035874//cellular response to copper ion starvation;GO:0046677//response to antib

rocess;GO:0006633//fatty acid biosynthetic process;GO:0010886//positive regulation of cholesterc
1766//hippocampus development;GO:0021819//layer formation in cerebral cortex;GO:0021860//p

process;GO:0006189//'de novo' IMP biosynthetic process;GO:0009152//purine ribonucleotide bios

se activity;GO:0038161//prolactin signaling pathway;GO:0042976//activation of Janus kinase activit

ı development;GO:0050804//modulation of synaptic transmission;GO:0099560//synaptic membrar

ıto nucleus;GO:0007049//cell cycle;GO:0007179//transforming growth factor beta receptor signalir

e response;GO:0007186//G-protein coupled receptor signaling pathway;GO:0007204//positive re

ynthetic process;GO:0009113//purine nucleobase biosynthetic process;GO:0009116//nucleoside m

)8283//cell proliferation;GO:0008284//positive regulation of cell proliferation;GO:0009408//respon
e organization;GO:0043353//enucleate erythrocyte differentiation;GO:0098739//import across plas

):0043490//malate-aspartate shuttle;GO:0051592//response to calcium ion;GO:0055085//transmer
)8283//cell proliferation;GO:0008284//positive regulation of cell proliferation;GO:0009408//respon
osynthetic process;GO:0009072//aromatic amino acid family metabolic process;GO:0019293//tyros
endent proteasomal ubiquitin-dependent protein catabolic process;GO:0043153//entrainment of
ation;GO:0002244//hematopoietic progenitor cell differentiation;GO:0006357//regulation of transc

on organization;GO:0008360//regulation of cell shape;GO:0016601//Rac protein signal transductic

on;GO:0030501//positive regulation of bone mineralization;GO:0045669//positive regulation of os

transport;GO:0070327//thyroid hormone transport;GO:0070460//thyroid-stimulating hormone se

ıtamate receptor signaling pathway;GO:0007405//neuroblast proliferation;GO:0019369//arachidor

r signaling pathway;GO:0007409//axonogenesis;GO:0030900//forebrain development;GO:003818

c process;GO:0035874//cellular response to copper ion starvation;GO:0046677//response to antib

elin catabolic process;GO:0008156//negative regulation of DNA replication;GO:0008285//negative

ransmembrane transporter activity;GO:1904064//positive regulation of cation transmembrane trar

metabolic process;GO:0007417//central nervous system development;GO:0009064//glutamine far
process;GO:0006189//'de novo' IMP biosynthetic process;GO:0009152//purine ribonucleotide bios

metabolic process;GO:0007417//central nervous system development;GO:0009064//glutamine far

e activation involved in immune response;GO:0007186//G-protein coupled receptor signaling pat

: process;GO:0008203//cholesterol metabolic process;GO:0019433//triglyceride catabolic process;(
GO:0006955//immune response;GO:0009395//phospholipid catabolic process;GO:0010634//positiv

oliferation;GO:0009617//response to bacterium;GO:0048010//vascular endothelial growth factor re

a chemical signal;GO:0009750//response to fructose;GO:0015755//fructose transport;GO:0055085/
GO:0006107//oxaloacetate metabolic process;GO:0006629//lipid metabolic process;GO:0009617//r

olic process;GO:0051180//vitamin transport;GO:0090212//negative regulation of establishment of b

n;GO:0002305//CD8-positive, gamma-delta intraepithelial T cell differentiation;GO:0002689//nega

bstrate junction assembly;GO:0007155//cell adhesion;GO:0007160//cell-matrix adhesion;GO:0007

n development;GO:0050804//modulation of synaptic transmission;GO:0099560//synaptic membrar
ocess;GO:0009086//methionine biosynthetic process;GO:0031060//regulation of histone methylatic
nto nucleus;GO:0007049//cell cycle;GO:0007179//transforming growth factor beta receptor signalir
rferon-gamma production;GO:0032743//positive regulation of interleukin-2 production;GO:00327
ynthetic process;GO:0009113//purine nucleobase biosynthetic process;GO:0009116//nucleoside m
fferentiation;GO:0042373//vitamin K metabolic process;GO:2000379//positive regulation of reactiv

007268//synaptic transmission;GO:0007416//synapse assembly;GO:0007612//learning;GO:0030534

zation;GO:0009235//cobalamin metabolic process;GO:0015889//cobalamin transport;GO:0043001

DNA damage stimulus;GO:0045023//G0 to G1 transition;GO:0045746//negative regulation of Notc

ation;GO:0002244//hematopoietic progenitor cell differentiation;GO:0006357//regulation of transc

regulation of endothelial cell proliferation;GO:0006468//protein phosphorylation;GO:0007165//sig

c process;GO:0035874//cellular response to copper ion starvation;GO:0046677//response to antib
ylation;GO:0006915//apoptotic process;GO:0031647//regulation of protein stability;GO:0050790//

608//sensory perception of smell;GO:0030311//poly-N-acetyllactosamine biosynthetic process;GC

process;GO:0006189//'de novo' IMP biosynthetic process;GO:0009152//purine ribonucleotide bios

544//arginine catabolic process to glutamate;GO:0055129//L-proline biosynthetic process

e organization;GO:0043353//enucleate erythrocyte differentiation;GO:0098739//import across plas
ne-mediated signaling pathway;GO:0032754//positive regulation of interleukin-5 production;GO:0

osynthetic process;GO:0009072//aromatic amino acid family metabolic process;GO:0019293//tyros

r signaling pathway;GO:0007409//axonogenesis;GO:0030900//forebrain development;GO:003818
544//arginine catabolic process to glutamate;GO:0055129//L-proline biosynthetic process
070374//positive regulation of ERK1 and ERK2 cascade;GO:0070378//positive regulation of ERK5 c
08283//cell proliferation;GO:0008284//positive regulation of cell proliferation;GO:0009408//respon

0008584//male gonad development;GO:0010894//negative regulation of steroid biosynthetic proc
n-dependent cell spreading;GO:0061001//regulation of dendritic spine morphogenesis;GO:00610

aling pathway;GO:0007186//G-protein coupled receptor signaling pathway;GO:0007188//adenylat

or signaling pathway;GO:0007200//phospholipase C-activating G-protein coupled receptor signal

assembly;GO:0030036//actin cytoskeleton organization;GO:0045944//positive regulation of transc

fatty acid beta-oxidation;GO:0008206//bile acid metabolic process;GO:0010124//phenylacetate ca

hloride transport;GO:0008272//sulfate transport;GO:0010628//positive regulation of gene expressi

tor beta receptor signaling pathway;GO:0007229//integrin-mediated signaling pathway;GO:00164
//oocyte differentiation;GO:0042127//regulation of cell proliferation;GO:0048839//inner ear devel
):0043490//malate-aspartate shuttle;GO:0051592//response to calcium ion;GO:0055085//transmer
rocess;GO:0006633//fatty acid biosynthetic process;GO:0010886//positive regulation of cholesterc

e activation involved in immune response;GO:0007186//G-protein coupled receptor signaling pat

netabolic process;GO:0006637//acyl-CoA metabolic process;GO:0006641//triglyceride metabolic p

uitination;GO:0043161//proteasome-mediated ubiquitin-dependent protein catabolic process

tion of transcription, DNA-templated;GO:0006357//regulation of transcription from RNA polymera
olic process;GO:0051180//vitamin transport;GO:0090212//negative regulation of establishment of b
athway;GO:0009888//tissue development;GO:0030198//extracellular matrix organization;GO:00713

eak repair via nonhomologous end joining;GO:0010212//response to ionizing radiation;GO:00301

n;GO:0019805//quinolinate biosynthetic process;GO:0046686//response to cadmium ion;GO:0070

):0010831//positive regulation of myotube differentiation;GO:0034968//histone lysine methylation

regulation of endothelial cell proliferation;GO:0006468//protein phosphorylation;GO:0007165//sig

promoter;GO:1900408//negative regulation of cellular response to oxidative stress;GO:1902083//r

e response;GO:0007186//G-protein coupled receptor signaling pathway;GO:0007204//positive re[illegible]

transport;GO:0070327//thyroid hormone transport;GO:0070460//thyroid-stimulating hormone se[illegible]
[illegible]//nervous system development;GO:0010977//negative regulation of neuron projection developm[illegible]

1766//hippocampus development;GO:0021819//layer formation in cerebral cortex;GO:0021860//p[illegible]

006541//glutamine metabolic process;GO:0006807//nitrogen compound metabolic process;GO:00[illegible]

rocess;GO:0006633//fatty acid biosynthetic process;GO:0010886//positive regulation of cholester[illegible]

[illegible]O:0007032//endosome organization;GO:0016185//synaptic vesicle budding from presynaptic en[illegible]
c process;GO:0006869//lipid transport;GO:0006886//intracellular protein transport;GO:0010875//p[illegible]

response to bacterium;GO:0015909//long-chain fatty acid transport;GO:0042632//cholesterol hom[illegible]
[illegible];GO:0002305//CD8-positive, gamma-delta intraepithelial T cell differentiation;GO:0002689//nega[illegible]
ide cross-linking;GO:0030216//keratinocyte differentiation;GO:0045861//negative regulation of pr[illegible]

070374//positive regulation of ERK1 and ERK2 cascade;GO:0070378//positive regulation of ERK5 c[illegible]
assembly;GO:0030036//actin cytoskeleton organization;GO:0045944//positive regulation of transc[illegible]

on of oxidative phosphorylation;GO:0006390//transcription from mitochondrial promoter;GO:0006[illegible]

[illegible]//nervous system development;GO:0010977//negative regulation of neuron projection developm[illegible]

osynthetic process;GO:0009072//aromatic amino acid family metabolic process;GO:0019293//tyro[illegible]

[illegible] organization;GO:0043353//enucleate erythrocyte differentiation;GO:0098739//import across plas[illegible]

[illegible]44//arginine catabolic process to glutamate;GO:0055129//L-proline biosynthetic process

: process;GO:0008203//cholesterol metabolic process;GO:0019433//triglyceride catabolic process;(
0045776//negative regulation of blood pressure;GO:0051607//defense response to virus;GO:0055(

32366//intracellular sterol transport;GO:0032367//intracellular cholesterol transport;GO:0033344//

elin catabolic process;GO:0008156//negative regulation of DNA replication;GO:0008285//negative

olic process;GO:0051180//vitamin transport;GO:0090212//negative regulation of establishment of b
hloride transport;GO:0008272//sulfate transport;GO:0010628//positive regulation of gene expressio

metabolic process;GO:0007417//central nervous system development;GO:0009064//glutamine far
ation of gene expression;GO:0045671//negative regulation of osteoclast differentiation;GO:004577

e expression;GO:0030001//metal ion transport;GO:0031398//positive regulation of protein ubiquit

D88-independent toll-like receptor signaling pathway;GO:0048678//response to axon injury;GO:1

assembly;GO:0030036//actin cytoskeleton organization;GO:0045944//positive regulation of transc
GO:0006955//immune response;GO:0009395//phospholipid catabolic process;GO:0010634//positiv

c process;GO:0035874//cellular response to copper ion starvation;GO:0046677//response to antib
ynthetic process;GO:0009113//purine nucleobase biosynthetic process;GO:0009116//nucleoside m

rocess;GO:0006633//fatty acid biosynthetic process;GO:0010886//positive regulation of cholesterc

e targeting;GO:0006904//vesicle docking involved in exocytosis;GO:0016192//vesicle-mediated tra
0008584//male gonad development;GO:0010894//negative regulation of steroid biosynthetic proc
on organization;GO:0008360//regulation of cell shape;GO:0016601//Rac protein signal transductic
ty;GO:0045654//positive regulation of megakaryocyte differentiation;GO:0046330//positive regula

cosamine metabolic process;GO:0006790//sulfur compound metabolic process;GO:1903238//posit

bstrate junction assembly;GO:0007155//cell adhesion;GO:0007160//cell-matrix adhesion;GO:0007[illegible]

ion;GO:1902857//positive regulation of nonmotile primary cilium assembly;GO:1904951//positive

)8283//cell proliferation;GO:0008284//positive regulation of cell proliferation;GO:0009408//respon

ynthetic process;GO:0009113//purine nucleobase biosynthetic process;GO:0009116//nucleoside m

fferentiation;GO:0042373//vitamin K metabolic process;GO:2000379//positive regulation of reactiv

eart rate;GO:0010765//positive regulation of sodium ion transport;GO:0035725//sodium ion transp

rganization;GO:0030036//actin cytoskeleton organization;GO:0070830//bicellular tight junction as

GO:0007032//endosome organization;GO:0016185//synaptic vesicle budding from presynaptic end

metabolic process;GO:0007417//central nervous system development;GO:0009064//glutamine far
on of oxidative phosphorylation;GO:0006390//transcription from mitochondrial promoter;GO:0006

)//nervous system development;GO:0010977//negative regulation of neuron projection developm
O:0007040//lysosome organization;GO:0007286//spermatid development;GO:0008333//endosom
netanephros development;GO:0001759//organ induction;GO:0001823//mesonephros developmen
GO:0043409//negative regulation of MAPK cascade;GO:0050906//detection of stimulus involved in
metabolic process;GO:0006650//glycerophospholipid metabolic process;GO:0006654//phosphatid
ide cross-linking;GO:0030216//keratinocyte differentiation;GO:0045861//negative regulation of pr
iding in birth or egg hatching;GO:0019336//phenol-containing compound catabolic process;GO:0
regulation of endothelial cell proliferation;GO:0006468//protein phosphorylation;GO:0007165//sig
e organization;GO:0043353//enucleate erythrocyte differentiation;GO:0098739//import across plas

osynthetic process;GO:0009072//aromatic amino acid family metabolic process;GO:0019293//tyros

mere organization;GO:0051639//actin filament network formation;GO:0055003//cardiac myofibril a
ng compound metabolic process;GO:0008152//metabolic process;GO:0016310//phosphorylation;G

)//nervous system development;GO:0010977//negative regulation of neuron projection developm
erived foam cell differentiation;GO:0010878//cholesterol storage;GO:0033344//cholesterol efflux;G

ısma membrane adhesion molecules;GO:0007162//negative regulation of cell adhesion;GO:00073
ɔtein coupled receptor signaling pathway;GO:0007187//G-protein coupled receptor signaling path

deling;GO:0006535//cysteine biosynthetic process from serine;GO:0006801//superoxide metabolic

ɔlic process;GO:0051180//vitamin transport;GO:0090212//negative regulation of establishment of l
ɔlic process;GO:0051180//vitamin transport;GO:0090212//negative regulation of establishment of l

ɔliferation;GO:0009617//response to bacterium;GO:0048010//vascular endothelial growth factor re
or signaling pathway;GO:0007200//phospholipase C-activating G-protein coupled receptor signal
'/inflammatory response to antigenic stimulus;GO:0007264//small GTPase mediated signal transdu

ı;GO:0002305//CD8-positive, gamma-delta intraepithelial T cell differentiation;GO:0002689//nega
tasis;GO:0007611//learning or memory;GO:0007625//grooming behavior;GO:0007626//locomotor

: process;GO:0042574//retinal metabolic process;GO:1901810//beta-carotene metabolic process
fatty acid beta-oxidation;GO:0008206//bile acid metabolic process;GO:0010124//phenylacetate ca
616//long-term memory;GO:0007626//locomotory behavior;GO:0009190//cyclic nucleotide biosyr

ive regulation of JNK cascade;GO:0051726//regulation of cell cycle;GO:1900745//positive regulatic

//negative regulation of toll-like receptor 4 signaling pathway;GO:0034164//negative regulation o

):0002269//leukocyte activation involved in inflammatory response;GO:0002548//monocyte chem

ɔr signaling pathway;GO:0048678//response to axon injury;GO:1901214//regulation of neuron dea
ɛptor protein tyrosine kinase signaling pathway;GO:0009615//response to virus;GO:0043406//posit

'/inflammatory response to antigenic stimulus;GO:0007264//small GTPase mediated signal transdu

):0002269//leukocyte activation involved in inflammatory response;GO:0002548//monocyte chem

I promoter;GO:0001657//ureteric bud development;GO:0001843//neural tube closure;GO:000206

ɛtic process, by transamination of glyoxylate;GO:0019481//L-alanine catabolic process, by transam

9511//peptidyl-proline hydroxylation;GO:0032963//collagen metabolic process;GO:0050708//regu

;O:0006955//immune response;GO:0009395//phospholipid catabolic process;GO:0010634//positiv

jration;GO:0006355//regulation of transcription, DNA-templated;GO:0007165//signal transduction
ty;GO:0045654//positive regulation of megakaryocyte differentiation;GO:0046330//positive regula

ıelial cell proliferation;GO:0002719//negative regulation of cytokine production involved in immun
erived foam cell differentiation;GO:0010878//cholesterol storage;GO:0033344//cholesterol efflux;G
oliferation;GO:0009617//response to bacterium;GO:0048010//vascular endothelial growth factor re
03//cholesterol metabolic process;GO:0010468//regulation of gene expression;GO:0010977//nega

ıling pathway;GO:0007186//G-protein coupled receptor signaling pathway;GO:0007188//adenylat

bstrate junction assembly;GO:0007155//cell adhesion;GO:0007160//cell-matrix adhesion;GO:0007
rocess;GO:0006633//fatty acid biosynthetic process;GO:0010886//positive regulation of cholesterc
l promoter;GO:0006355//regulation of transcription, DNA-templated;GO:0006357//regulation of t

ranscription, DNA-templated;GO:1903507//negative regulation of nucleic acid-templated transcri

assembly;GO:0030036//actin cytoskeleton organization;GO:0045944//positive regulation of transc

006541//glutamine metabolic process;GO:0006807//nitrogen compound metabolic process;GO:00
fferentiation;GO:0042373//vitamin K metabolic process;GO:2000379//positive regulation of reactiv

ation;GO:0002244//hematopoietic progenitor cell differentiation;GO:0006357//regulation of transc
ubiquitin-dependent protein catabolic process;GO:0030970//retrograde protein transport, ER to cy

ıto nucleus;GO:0007049//cell cycle;GO:0007179//transforming growth factor beta receptor signalir

//lipid biosynthetic process;GO:0016126//sterol biosynthetic process;GO:0045654//positive regulat
070374//positive regulation of ERK1 and ERK2 cascade;GO:0070378//positive regulation of ERK5 c

18//positive regulation of satellite cell activation involved in skeletal muscle regeneration;GO:0030

ɔptotic process;GO:0043154//negative regulation of cysteine-type endopeptidase activity involvec

adhesion;GO:0007264//small GTPase mediated signal transduction;GO:0007628//adult walking bel

ıg compound metabolic process;GO:0008152//metabolic process;GO:0016310//phosphorylation;G

GO:0043409//negative regulation of MAPK cascade;GO:0050906//detection of stimulus involved iı
rocess;GO:0006633//fatty acid biosynthetic process;GO:0006636//unsaturated fatty acid biosynthe

ı;GO:0002305//CD8-positive, gamma-delta intraepithelial T cell differentiation;GO:0002689//nega

544//arginine catabolic process to glutamate;GO:0055129//L-proline biosynthetic process
03//cholesterol metabolic process;GO:0010468//regulation of gene expression;GO:0010977//nega
on;GO:0030501//positive regulation of bone mineralization;GO:0045669//positive regulation of os

ɔtein coupled receptor signaling pathway;GO:0007187//G-protein coupled receptor signaling path

biosynthetic process;GO:0015908//fatty acid transport;GO:0033211//adiponectin-activated signali
ide cross-linking;GO:0030216//keratinocyte differentiation;GO:0045861//negative regulation of pr

zation;GO:0009235//cobalamin metabolic process;GO:0015889//cobalamin transport;GO:0043001

ı small molecule catabolic process;GO:0046039//GTP metabolic process;GO:0046052//UTP catabol
or signaling pathway;GO:0007200//phospholipase C-activating G-protein coupled receptor signal

AT cascade;GO:0009968//negative regulation of signal transduction;GO:0035556//intracellular sig

regulation of endothelial cell proliferation;GO:0006468//protein phosphorylation;GO:0007165//sig
fatty acid beta-oxidation;GO:0008206//bile acid metabolic process;GO:0010124//phenylacetate ca
ransmembrane transporter activity;GO:1904064//positive regulation of cation transmembrane trar

ıetic process;GO:0006592//ornithine biosynthetic process;GO:0019240//citrulline biosynthetic proc

tasis;GO:0007611//learning or memory;GO:0007625//grooming behavior;GO:0007626//locomotor

I promoter;GO:0000902//cell morphogenesis;GO:0001709//cell fate determination;GO:0001764//n

e activation involved in immune response;GO:0007186//G-protein coupled receptor signaling pat

)2//negative regulation of bone mineralization;GO:0050727//regulation of inflammatory response;

ation;GO:0044260//cellular macromolecule metabolic process;GO:0090305//nucleic acid phospho

ranscription from RNA polymerase II promoter;GO:0006654//phosphatidic acid biosynthetic proce

ration;GO:0006355//regulation of transcription, DNA-templated;GO:0007165//signal transduction
n;GO:0019805//quinolinate biosynthetic process;GO:0046686//response to cadmium ion;GO:0070

ıelial cell proliferation;GO:0002719//negative regulation of cytokine production involved in immun

32366//intracellular sterol transport;GO:0032367//intracellular cholesterol transport;GO:0033344//
ganic substance;GO:0010872//regulation of cholesterol esterification;GO:0010875//positive regula

erived foam cell differentiation;GO:0010878//cholesterol storage;GO:0033344//cholesterol efflux;G
ısma membrane adhesion molecules;GO:0007162//negative regulation of cell adhesion;GO:00073

0017158//regulation of calcium ion-dependent exocytosis;GO:0032024//positive regulation of ins

ɔositive regulation of protein phosphorylation;GO:0003347//epicardial cell to mesenchymal cell tra
lic process;GO:0008210//estrogen metabolic process;GO:0009804//coumarin metabolic process;G

I promoter;GO:0006355//regulation of transcription, DNA-templated;GO:0006357//regulation of t

deling;GO:0006535//cysteine biosynthetic process from serine;GO:0006801//superoxide metabolic

ive regulation of apoptotic process;GO:0045727//positive regulation of translation;GO:0045786//n
asma membrane adhesion molecules;GO:0007162//negative regulation of cell adhesion;GO:00073[illegible]

c process;GO:0006869//lipid transport;GO:0006886//intracellular protein transport;GO:0010875//p

copper ion;GO:0032496//response to lipopolysaccharide;GO:0070555//response to interleukin-1;
D88-independent toll-like receptor signaling pathway;GO:0048678//response to axon injury;GO:1

process;GO:0042574//retinal metabolic process;GO:1901810//beta-carotene metabolic process

c process;GO:0035874//cellular response to copper ion starvation;GO:0046677//response to antib

//inflammatory response to antigenic stimulus;GO:0007264//small GTPase mediated signal transdu

zation;GO:0009235//cobalamin metabolic process;GO:0015889//cobalamin transport;GO:0043001

on;GO:0030501//positive regulation of bone mineralization;GO:0045669//positive regulation of os

ive regulation of sequestering of triglyceride;GO:0010898//positive regulation of triglyceride catab

development;GO:0050804//modulation of synaptic transmission;GO:0099560//synaptic membrar

):0002269//leukocyte activation involved in inflammatory response;GO:0002548//monocyte chem

ation;GO:0002244//hematopoietic progenitor cell differentiation;GO:0006357//regulation of transc

losure;GO:0007389//pattern specification process;GO:0007398//ectoderm development;GO:00074

)8283//cell proliferation;GO:0008284//positive regulation of cell proliferation;GO:0009408//respon
rganization;GO:0030036//actin cytoskeleton organization;GO:0070830//bicellular tight junction as

lic process;GO:0009791//post-embryonic development;GO:0010259//multicellular organism aging

ito nucleus;GO:0007049//cell cycle;GO:0007179//transforming growth factor beta receptor signalir

ransmembrane transporter activity;GO:1904064//positive regulation of cation transmembrane trar

damage response, signal transduction by p53 class mediator;GO:0032287//peripheral nervous syst

or signaling pathway;GO:0048678//response to axon injury;GO:1901214//regulation of neuron dea
//lipid biosynthetic process;GO:0016126//sterol biosynthetic process;GO:0045654//positive regulat

otein coupled receptor signaling pathway;GO:0007187//G-protein coupled receptor signaling path
copper ion;GO:0032496//response to lipopolysaccharide;GO:0070555//response to interleukin-1;

iO:0007155//cell adhesion;GO:0007159//leukocyte cell-cell adhesion;GO:0007160//cell-matrix adh
isma membrane adhesion molecules;GO:0007162//negative regulation of cell adhesion;GO:00073

n;GO:0019805//quinolinate biosynthetic process;GO:0046686//response to cadmium ion;GO:0070
adhesion;GO:0007264//small GTPase mediated signal transduction;GO:0007628//adult walking bel

a chemical signal;GO:0009750//response to fructose;GO:0015755//fructose transport;GO:0055085/
tabolic process;GO:0019835//cytolysis;GO:0031640//killing of cells of other organism;GO:0042742

gulation of glucose metabolic process;GO:0014065//phosphatidylinositol 3-kinase signaling;GO:0

lic process;GO:0008210//estrogen metabolic process;GO:0009804//coumarin metabolic process;G

nolecules;GO:0050806//positive regulation of synaptic transmission;GO:0051965//positive regulatic

lic process;GO:0008210//estrogen metabolic process;GO:0009804//coumarin metabolic process;G

uitination;GO:0043161//proteasome-mediated ubiquitin-dependent protein catabolic process
03//cholesterol metabolic process;GO:0010468//regulation of gene expression;GO:0010977//nega
9511//peptidyl-proline hydroxylation;GO:0032963//collagen metabolic process;GO:0050708//regu

zation;GO:0009235//cobalamin metabolic process;GO:0015889//cobalamin transport;GO:0043001
31//protein transport;GO:0045901//positive regulation of translational elongation;GO:0045905//p

tasis;GO:0007611//learning or memory;GO:0007625//grooming behavior;GO:0007626//locomotor
GO:0043409//negative regulation of MAPK cascade;GO:0050906//detection of stimulus involved i

copper ion;GO:0032496//response to lipopolysaccharide;GO:0070555//response to interleukin-1;
transport;GO:0070327//thyroid hormone transport;GO:0070460//thyroid-stimulating hormone se

xidation;GO:0010942//positive regulation of cell death;GO:0033540//fatty acid beta-oxidation usi
ive regulation of sequestering of triglyceride;GO:0010898//positive regulation of triglyceride catab

ipid translocation;GO:0047484//regulation of response to osmotic stress;GO:0055085//transmemb

gnal transduction;GO:0035556//intracellular signal transduction;GO:0090630//activation of GTPase

)008584//male gonad development;GO:0010894//negative regulation of steroid biosynthetic proc

process;GO:0006189//'de novo' IMP biosynthetic process;GO:0009152//purine ribonucleotide bios

D88-independent toll-like receptor signaling pathway;GO:0048678//response to axon injury;GO:1
ative regulation of blood coagulation;GO:0071383//cellular response to steroid hormone stimulus
AT cascade;GO:0009968//negative regulation of signal transduction;GO:0035556//intracellular sig

process;GO:0008203//cholesterol metabolic process;GO:0019433//triglyceride catabolic process;
elopment;GO:0002138//retinoic acid biosynthetic process;GO:0006081//cellular aldehyde metabol
nthetic process;GO:0016104//triterpenoid biosynthetic process;GO:0031647//regulation of protein

)6952//defense response;GO:0007599//hemostasis;GO:0010524//positive regulation of calcium ion

lar protein modification process;GO:0006790//sulfur compound metabolic process;GO:0030166//p

ess;GO:0006593//ornithine catabolic process;GO:0019240//citrulline biosynthetic process;GO:0097

or signaling pathway;GO:0048678//response to axon injury;GO:1901214//regulation of neuron dea

to nucleus;GO:0007049//cell cycle;GO:0007179//transforming growth factor beta receptor signalir

iosynthetic process;GO:0008542//visual learning;GO:0015936//coenzyme A metabolic process;GO
ielial cell proliferation;GO:0002719//negative regulation of cytokine production involved in immun

ation;GO:0002244//hematopoietic progenitor cell differentiation;GO:0006357//regulation of transc

positive regulation of protein phosphorylation;GO:0003347//epicardial cell to mesenchymal cell tra

l promoter;GO:0006355//regulation of transcription, DNA-templated;GO:0006357//regulation of t

tabolic process;GO:0019835//cytolysis;GO:0031640//killing of cells of other organism;GO:0042742

lation of cell migration;GO:0046464//acylglycerol catabolic process;GO:0046889//positive regulati

03//cholesterol metabolic process;GO:0010468//regulation of gene expression;GO:0010977//nega

e response;GO:0007186//G-protein coupled receptor signaling pathway;GO:0007204//positive reg

e response;GO:0007186//G-protein coupled receptor signaling pathway;GO:0007204//positive reg

l shape;GO:0042742//defense response to bacterium;GO:0045089//positive regulation of innate ir

copper ion;GO:0032496//response to lipopolysaccharide;GO:0070555//response to interleukin-1;

ipid translocation;GO:0047484//regulation of response to osmotic stress;GO:0055085//transmemb

signaling;GO:1902108//regulation of mitochondrial membrane permeability involved in apoptotic

f cytosolic calcium ion concentration;GO:0010863//positive regulation of phospholipase C activity;

18//positive regulation of satellite cell activation involved in skeletal muscle regeneration;GO:0030

olic process;GO:0015697//quaternary ammonium group transport;GO:0015879//carnitine transpo

regulation of neurogenesis;GO:0060770//negative regulation of epithelial cell proliferation involve

lic process;GO:0008210//estrogen metabolic process;GO:0009804//coumarin metabolic process;G

tasis;GO:0007611//learning or memory;GO:0007625//grooming behavior;GO:0007626//locomotor

on of sour taste;GO:0006812//cation transport;GO:0007224//smoothened signaling pathway;GO:0
uitination;GO:0043161//proteasome-mediated ubiquitin-dependent protein catabolic process
olic process;GO:0051180//vitamin transport;GO:0090212//negative regulation of establishment of b

):0043490//malate-aspartate shuttle;GO:0051592//response to calcium ion;GO:0055085//transmem
: process;GO:0008203//cholesterol metabolic process;GO:0019433//triglyceride catabolic process;G
asma membrane adhesion molecules;GO:0007162//negative regulation of cell adhesion;GO:00073
ess;GO:0006593//ornithine catabolic process;GO:0019240//citrulline biosynthetic process;GO:0097
ne-mediated signaling pathway;GO:0032754//positive regulation of interleukin-5 production;GO:0

lic process;GO:0006656//phosphatidylcholine biosynthetic process;GO:0008203//cholesterol meta

'AT cascade;GO:0009968//negative regulation of signal transduction;GO:0035556//intracellular sig

lic process;GO:0006656//phosphatidylcholine biosynthetic process;GO:0008203//cholesterol meta

lic process;GO:0008210//estrogen metabolic process;GO:0009804//coumarin metabolic process;G

regulation of endothelial cell proliferation;GO:0006468//protein phosphorylation;GO:0007165//sig

178//negative regulation of Wnt signaling pathway;GO:0030949//positive regulation of vascular e

o starvation;GO:0044090//positive regulation of vacuole organization;GO:2000786//positive regula
abolic process;GO:0006012//galactose metabolic process;GO:0019318//hexose metabolic process;
copper ion;GO:0032496//response to lipopolysaccharide;GO:0070555//response to interleukin-1;

lation of cell migration;GO:0046464//acylglycerol catabolic process;GO:0046889//positive regulati

zation;GO:0009235//cobalamin metabolic process;GO:0015889//cobalamin transport;GO:0043001
nto nucleus;GO:0007049//cell cycle;GO:0007179//transforming growth factor beta receptor signalin

transport;GO:0070327//thyroid hormone transport;GO:0070460//thyroid-stimulating hormone se

ponse to phosphate starvation;GO:0032259//methylation;GO:0042135//neurotransmitter catabolic

phore linkage;GO:0032922//circadian regulation of gene expression;GO:0043153//entrainment of

positive regulation of protein phosphorylation;GO:0003347//epicardial cell to mesenchymal cell tra
follicle growth;GO:0001932//regulation of protein phosphorylation;GO:0003073//regulation of sys

ation;GO:0002244//hematopoietic progenitor cell differentiation;GO:0006357//regulation of transc

etic process, by transamination of glyoxylate;GO:0019481//L-alanine catabolic process, by transam

athway;GO:0009888//tissue development;GO:0030198//extracellular matrix organization;GO:00713

xidation;GO:0010942//positive regulation of cell death;GO:0033540//fatty acid beta-oxidation usin

//lipid biosynthetic process;GO:0016126//sterol biosynthetic process;GO:0045654//positive regulat

process;GO:0006189//'de novo' IMP biosynthetic process;GO:0009152//purine ribonucleotide bios

regulation of neurogenesis;GO:0060770//negative regulation of epithelial cell proliferation involve

a chemical signal;GO:0009750//response to fructose;GO:0015755//fructose transport;GO:0055085/

etabolic process;GO:0008652//cellular amino acid biosynthetic process;GO:0042149//cellular respo
uitination;GO:0043161//proteasome-mediated ubiquitin-dependent protein catabolic process

cess;GO:0006629//lipid metabolic process;GO:0006633//fatty acid biosynthetic process;GO:001953
ed in ureteric bud morphogenesis;GO:0001822//kidney development;GO:0003231//cardiac ventricl

ridoxal phosphate-L-lysine;GO:0019343//cysteine biosynthetic process via cystathionine;GO:0019

nolecules;GO:0050806//positive regulation of synaptic transmission;GO:0051965//positive regulatio

uction;GO:0043087//regulation of GTPase activity;GO:0046580//negative regulation of Ras protein

ation of gene expression;GO:0045671//negative regulation of osteoclast differentiation;GO:004577

assembly;GO:0030036//actin cytoskeleton organization;GO:0045944//positive regulation of transc

rocess;GO:0006633//fatty acid biosynthetic process;GO:0010886//positive regulation of cholesterc

lic process;GO:0006656//phosphatidylcholine biosynthetic process;GO:0008203//cholesterol meta
O:0007155//cell adhesion;GO:0007159//leukocyte cell-cell adhesion;GO:0007160//cell-matrix adh

)6952//defense response;GO:0007599//hemostasis;GO:0010524//positive regulation of calcium ior

n;GO:0019805//quinolinate biosynthetic process;GO:0046686//response to cadmium ion;GO:0070

//ubiquitin-dependent protein catabolic process;GO:0007409//axonogenesis;GO:0007412//axon t
tasis;GO:0007611//learning or memory;GO:0007625//grooming behavior;GO:0007626//locomotor

oonse to phosphate starvation;GO:0032259//methylation;GO:0042135//neurotransmitter catabolic
abolic process;GO:0006012//galactose metabolic process;GO:0019318//hexose metabolic process;
tasis;GO:0007611//learning or memory;GO:0007625//grooming behavior;GO:0007626//locomotor

regulation of endothelial cell proliferation;GO:0006468//protein phosphorylation;GO:0007165//sic

s;GO:0010628//positive regulation of gene expression;GO:0032332//positive regulation of chondr

oduction;GO:0010760//negative regulation of macrophage chemotaxis;GO:0032722//positive regu
oxidative stress;GO:0008104//protein localization;GO:0008203//cholesterol metabolic process;GO:

)//nervous system development;GO:0010977//negative regulation of neuron projection developm

n of endocytosis;GO:0048488//synaptic vesicle endocytosis;GO:0048666//neuron development;G

GO:0016310//phosphorylation;GO:0046104//thymidine metabolic process;GO:0051289//protein h

sphatidylinositol 3-kinase signaling;GO:0033564//anterior/posterior axon guidance;GO:0038007//
32366//intracellular sterol transport;GO:0032367//intracellular cholesterol transport;GO:0033344//
nthetic process;GO:0016104//triterpenoid biosynthetic process;GO:0031647//regulation of protein

lic process;GO:0051180//vitamin transport;GO:0090212//negative regulation of establishment of b

D88-independent toll-like receptor signaling pathway;GO:0048678//response to axon injury;GO:1
d in ureteric bud morphogenesis;GO:0001822//kidney development;GO:0003231//cardiac ventricl

process;GO:0042574//retinal metabolic process;GO:1901810//beta-carotene metabolic process

assembly;GO:0030036//actin cytoskeleton organization;GO:0045944//positive regulation of transc
rotein dephosphorylation;GO:0016311//dephosphorylation;GO:0043542//endothelial cell migratio

070374//positive regulation of ERK1 and ERK2 cascade;GO:0070378//positive regulation of ERK5 c
iquitin-dependent protein catabolic process;GO:0016578//histone deubiquitination;GO:0016579//
deling;GO:0006535//cysteine biosynthetic process from serine;GO:0006801//superoxide metabolic

ve regulation of gene expression;GO:0030511//positive regulation of transforming growth factor b

rocess;GO:0006633//fatty acid biosynthetic process;GO:0010886//positive regulation of cholester

sma membrane adhesion molecules;GO:0007162//negative regulation of cell adhesion;GO:00073

0045776//negative regulation of blood pressure;GO:0051607//defense response to virus;GO:0055

xidation;GO:0010942//positive regulation of cell death;GO:0033540//fatty acid beta-oxidation usi

I promoter;GO:0000902//cell morphogenesis;GO:0001709//cell fate determination;GO:0001764//n
616//long-term memory;GO:0007626//locomotory behavior;GO:0009190//cyclic nucleotide biosy

cell cycle;GO:0010830//regulation of myotube differentiation;GO:0010868//negative regulation o
006541//glutamine metabolic process;GO:0006807//nitrogen compound metabolic process;GO:00
to nucleus;GO:0007049//cell cycle;GO:0007179//transforming growth factor beta receptor signalir

se assembly;GO:0008611//ether lipid biosynthetic process;GO:0021587//cerebellum morphogene
l promoter;GO:0006355//regulation of transcription, DNA-templated;GO:0006357//regulation of t

ation;GO:0002244//hematopoietic progenitor cell differentiation;GO:0006357//regulation of transc
):0006956//complement activation;GO:0006958//complement activation, classical pathway;GO:00

)6952//defense response;GO:0007599//hemostasis;GO:0010524//positive regulation of calcium ior

ielial cell proliferation;GO:0002719//negative regulation of cytokine production involved in immun
f cytosolic calcium ion concentration;GO:0010863//positive regulation of phospholipase C activity

'/lipid biosynthetic process;GO:0016126//sterol biosynthetic process;GO:0045654//positive regulat
egative regulation of protein lipidation;GO:2001140//positive regulation of phospholipid transpor

n;GO:0019805//quinolinate biosynthetic process;GO:0046686//response to cadmium ion;GO:0070

)6508//proteolysis;GO:0014068//positive regulation of phosphatidylinositol 3-kinase signaling;GO

nolecules;GO:0050806//positive regulation of synaptic transmission;GO:0051965//positive regulatic

07399//nervous system development;GO:0032147//activation of protein kinase activity;GO:003246

n development;GO:0050804//modulation of synaptic transmission;GO:0099560//synaptic membrar
thway;GO:0009267//cellular response to starvation;GO:0010510//regulation of acetyl-CoA biosynt

damage response, signal transduction by p53 class mediator;GO:0032287//peripheral nervous syst
O:0007155//cell adhesion;GO:0007159//leukocyte cell-cell adhesion;GO:0007160//cell-matrix adh
adhesion;GO:0007264//small GTPase mediated signal transduction;GO:0007628//adult walking bel
)6508//proteolysis;GO:0014068//positive regulation of phosphatidylinositol 3-kinase signaling;GO

otein coupled receptor signaling pathway;GO:0007187//G-protein coupled receptor signaling path

ation of gene expression;GO:0045671//negative regulation of osteoclast differentiation;GO:004577
):0006956//complement activation;GO:0006958//complement activation, classical pathway;GO:00

)//nervous system development;GO:0010977//negative regulation of neuron projection developm

/extracellular matrix organization;GO:0044869//negative regulation by host of viral exo-alpha-siali

ı;GO:0002305//CD8-positive, gamma-delta intraepithelial T cell differentiation;GO:0002689//nega
ılfment;GO:0043277//apoptotic cell clearance;GO:0050766//positive regulation of phagocytosis
lic process;GO:0008210//estrogen metabolic process;GO:0009804//coumarin metabolic process;G

ide cross-linking;GO:0030216//keratinocyte differentiation;GO:0045861//negative regulation of pr

: process;GO:0042574//retinal metabolic process;GO:1901810//beta-carotene metabolic process

lipid metabolic process;GO:0006631//fatty acid metabolic process;GO:0006633//fatty acid biosynth

GO:0043409//negative regulation of MAPK cascade;GO:0050906//detection of stimulus involved i

(6-linked deubiquitination;GO:0050821//protein stabilization;GO:0051898//negative regulation of

copper ion;GO:0032496//response to lipopolysaccharide;GO:0070555//response to interleukin-1;

cell cycle;GO:0010830//regulation of myotube differentiation;GO:0010868//negative regulation o
regulation of endothelial cell proliferation;GO:0006468//protein phosphorylation;GO:0007165//sig

0045776//negative regulation of blood pressure;GO:0051607//defense response to virus;GO:0055[illegible]
l promoter;GO:0006355//regulation of transcription, DNA-templated;GO:0009267//cellular respor[illegible]

biosynthetic process;GO:0015908//fatty acid transport;GO:0033211//adiponectin-activated signali[illegible]

ı development;GO:0050804//modulation of synaptic transmission;GO:0099560//synaptic membrar[illegible]

):0002269//leukocyte activation involved in inflammatory response;GO:0002548//monocyte chem[illegible]
i8//regulation of epidermal growth factor receptor signaling pathway;GO:0046755//viral budding
athway;GO:0009888//tissue development;GO:0030198//extracellular matrix organization;GO:0071[illegible]
GO:0006955//immune response;GO:0009395//phospholipid catabolic process;GO:0010634//positiv[illegible]

l promoter;GO:0006355//regulation of transcription, DNA-templated;GO:0006357//regulation of t[illegible]

'//inflammatory response to antigenic stimulus;GO:0007264//small GTPase mediated signal transdu[illegible]

ıelial cell proliferation;GO:0002719//negative regulation of cytokine production involved in immun[illegible]
08283//cell proliferation;GO:0008284//positive regulation of cell proliferation;GO:0009408//respon[illegible]
bstrate junction assembly;GO:0007155//cell adhesion;GO:0007160//cell-matrix adhesion;GO:0007[illegible]

18//positive regulation of satellite cell activation involved in skeletal muscle regeneration;GO:0030[illegible]
ı development;GO:0050804//modulation of synaptic transmission;GO:0099560//synaptic membrar[illegible]
oduction;GO:0010760//negative regulation of macrophage chemotaxis;GO:0032722//positive regu[illegible]

'//lipid biosynthetic process;GO:0016126//sterol biosynthetic process;GO:0045654//positive regulat[illegible]
s;GO:0010628//positive regulation of gene expression;GO:0032332//positive regulation of chondr[illegible]

ganic substance;GO:0010872//regulation of cholesterol esterification;GO:0010875//positive regula[illegible]

s;GO:0010628//positive regulation of gene expression;GO:0032332//positive regulation of chondr[illegible]
erived foam cell differentiation;GO:0010878//cholesterol storage;GO:0033344//cholesterol efflux;G[illegible]
mere organization;GO:0051639//actin filament network formation;GO:0055003//cardiac myofibril [illegible]

or signaling pathway;GO:0008277//regulation of G-protein coupled receptor protein signaling pa[illegible]

n phosphorylation;GO:0010628//positive regulation of gene expression;GO:0010629//negative reg[illegible]

oliferation;GO:0009617//response to bacterium;GO:0048010//vascular endothelial growth factor re[illegible]
oduction;GO:0010760//negative regulation of macrophage chemotaxis;GO:0032722//positive regu[illegible]

4765//regulation of ion transmembrane transport;GO:0051260//protein homooligomerization;GO

fferentiation;GO:0042373//vitamin K metabolic process;GO:2000379//positive regulation of reactiv

adhesion;GO:0007264//small GTPase mediated signal transduction;GO:0007628//adult walking bel

e activation involved in immune response;GO:0007186//G-protein coupled receptor signaling pat
asma membrane adhesion molecules;GO:0007162//negative regulation of cell adhesion;GO:00073
c process;GO:0035874//cellular response to copper ion starvation;GO:0046677//response to antib
e regulation of cell-substrate adhesion;GO:0045742//positive regulation of epidermal growth fact
erived foam cell differentiation;GO:0010878//cholesterol storage;GO:0033344//cholesterol efflux;G

deling;GO:0006535//cysteine biosynthetic process from serine;GO:0006801//superoxide metabolic

616//long-term memory;GO:0007626//locomotory behavior;GO:0009190//cyclic nucleotide biosy

070374//positive regulation of ERK1 and ERK2 cascade;GO:0070378//positive regulation of ERK5 c

32366//intracellular sterol transport;GO:0032367//intracellular cholesterol transport;GO:0033344//
070374//positive regulation of ERK1 and ERK2 cascade;GO:0070378//positive regulation of ERK5 c

//inflammatory response to antigenic stimulus;GO:0007264//small GTPase mediated signal transdu

etabolic process;GO:0006096//glycolytic process;GO:0051156//glucose 6-phosphate metabolic pr

:0007625//grooming behavior;GO:0019478//D-amino acid catabolic process;GO:0042445//hormo

55064//chloride ion homeostasis;GO:0055075//potassium ion homeostasis;GO:0055085//transmen
on organization;GO:0008360//regulation of cell shape;GO:0016601//Rac protein signal transductic
n;GO:0002305//CD8-positive, gamma-delta intraepithelial T cell differentiation;GO:0002689//nega

ide cross-linking;GO:0030216//keratinocyte differentiation;GO:0045861//negative regulation of pr
32732//positive regulation of interleukin-1 production;GO:0055085//transmembrane transport

GO:0043409//negative regulation of MAPK cascade;GO:0050906//detection of stimulus involved i

or signaling pathway;GO:0007200//phospholipase C-activating G-protein coupled receptor signal

rocess;GO:0006633//fatty acid biosynthetic process;GO:0006636//unsaturated fatty acid biosynthe

ganization;GO:0042149//cellular response to glucose starvation;GO:0043066//negative regulation

fatty acid beta-oxidation;GO:0008206//bile acid metabolic process;GO:0010124//phenylacetate ca

response to bacterium;GO:0015909//long-chain fatty acid transport;GO:0042632//cholesterol hon
ptotic process;GO:0043154//negative regulation of cysteine-type endopeptidase activity involvec

2732//positive regulation of interleukin-1 production;GO:0055085//transmembrane transport

1766//hippocampus development;GO:0021819//layer formation in cerebral cortex;GO:0021860//p

6-linked deubiquitination;GO:0050821//protein stabilization;GO:0051898//negative regulation of
//inflammatory response to antigenic stimulus;GO:0007264//small GTPase mediated signal transdu
:0006956//complement activation;GO:0006958//complement activation, classical pathway;GO:00

e activation involved in immune response;GO:0007186//G-protein coupled receptor signaling pat

n;GO:0019805//quinolinate biosynthetic process;GO:0046686//response to cadmium ion;GO:0070

olic process;GO:0015697//quaternary ammonium group transport;GO:0015879//carnitine transpo

//nervous system development;GO:0010977//negative regulation of neuron projection developm

oliferation;GO:0009617//response to bacterium;GO:0048010//vascular endothelial growth factor re

erived foam cell differentiation;GO:0010878//cholesterol storage;GO:0033344//cholesterol efflux;G

//cholesterol transport involved in cholesterol storage;GO:0032367//intracellular cholesterol transp
s;GO:0010628//positive regulation of gene expression;GO:0032332//positive regulation of chondr

sm growth;GO:0035934//corticosterone secretion;GO:0042445//hormone metabolic process;GO:00
06508//proteolysis;GO:0014068//positive regulation of phosphatidylinositol 3-kinase signaling;GO

006541//glutamine metabolic process;GO:0006807//nitrogen compound metabolic process;GO:00
oduction;GO:0010760//negative regulation of macrophage chemotaxis;GO:0032722//positive regu

n phosphorylation;GO:0010628//positive regulation of gene expression;GO:0010629//negative reg
response to bacterium;GO:0015909//long-chain fatty acid transport;GO:0042632//cholesterol hom

tive regulation of heparan sulfate proteoglycan biosynthetic process;GO:0050650//chondroitin sulf
070374//positive regulation of ERK1 and ERK2 cascade;GO:0070378//positive regulation of ERK5 c

to nucleus;GO:0007049//cell cycle;GO:0007179//transforming growth factor beta receptor signalir
transport;GO:0070327//thyroid hormone transport;GO:0070460//thyroid-stimulating hormone se

08283//cell proliferation;GO:0008284//positive regulation of cell proliferation;GO:0009408//respon
orylation;GO:0018108//peptidyl-tyrosine phosphorylation;GO:0030282//bone mineralization;GO:0

ation;GO:0002244//hematopoietic progenitor cell differentiation;GO:0006357//regulation of transc
):0043490//malate-aspartate shuttle;GO:0051592//response to calcium ion;GO:0055085//transmer
544//arginine catabolic process to glutamate;GO:0055129//L-proline biosynthetic process

athway;GO:0009888//tissue development;GO:0030198//extracellular matrix organization;GO:0071

178//negative regulation of Wnt signaling pathway;GO:0030949//positive regulation of vascular er

rocess;GO:0006633//fatty acid biosynthetic process;GO:0010886//positive regulation of cholesterc

ı development;GO:0050804//modulation of synaptic transmission;GO:0099560//synaptic membrar

thway;GO:0009267//cellular response to starvation;GO:0010510//regulation of acetyl-CoA biosynt
assembly;GO:0030036//actin cytoskeleton organization;GO:0045944//positive regulation of transc
c process;GO:0035874//cellular response to copper ion starvation;GO:0046677//response to antib
follicle growth;GO:0001932//regulation of protein phosphorylation;GO:0003073//regulation of sys

d biosynthetic process;GO:0006954//inflammatory response;GO:0008611//ether lipid biosynthetic
ide cross-linking;GO:0030216//keratinocyte differentiation;GO:0045861//negative regulation of pr

ganic substance;GO:0010872//regulation of cholesterol esterification;GO:0010875//positive regula

32732//positive regulation of interleukin-1 production;GO:0055085//transmembrane transport

ıtive regulation of blood coagulation;GO:0071383//cellular response to steroid hormone stimulus
sport;GO:0055085//transmembrane transport;GO:1990830//cellular response to leukemia inhibitor

gulation of glucose metabolic process;GO:0014065//phosphatidylinositol 3-kinase signaling;GO:0(
c process;GO:0006869//lipid transport;GO:0006886//intracellular protein transport;GO:0010875//p

or signaling pathway;GO:0007218//neuropeptide signaling pathway;GO:0008217//regulation of bl
or signaling pathway;GO:0008277//regulation of G-protein coupled receptor protein signaling pa
mere organization;GO:0051639//actin filament network formation;GO:0055003//cardiac myofibril a

lic process;GO:0006656//phosphatidylcholine biosynthetic process;GO:0008203//cholesterol meta

):0006956//complement activation;GO:0006958//complement activation, classical pathway;GO:00

ridoxal phosphate-L-lysine;GO:0019343//cysteine biosynthetic process via cystathionine;GO:0019
olic process;GO:0051180//vitamin transport;GO:0090212//negative regulation of establishment of b

lic process;GO:0006656//phosphatidylcholine biosynthetic process;GO:0008203//cholesterol meta

biosynthetic process;GO:0015908//fatty acid transport;GO:0033211//adiponectin-activated signali

velopment;GO:0007411//axon guidance;GO:0007420//brain development;GO:0010975//regulation
n;GO:0019805//quinolinate biosynthetic process;GO:0046686//response to cadmium ion;GO:0070
n;GO:0002305//CD8-positive, gamma-delta intraepithelial T cell differentiation;GO:0002689//nega
gnal transduction;GO:0035735//intraciliary transport involved in cilium morphogenesis;GO:006027

n phosphorylation;GO:0010628//positive regulation of gene expression;GO:0010629//negative reg
045723//positive regulation of fatty acid biosynthetic process;GO:0051258//protein polymerization
routing angiogenesis;GO:0006816//calcium ion transport;GO:0006886//intracellular protein transp

regulation of endothelial cell proliferation;GO:0006468//protein phosphorylation;GO:0007165//sig
targeting;GO:0006904//vesicle docking involved in exocytosis;GO:0016192//vesicle-mediated tra
DNA damage stimulus;GO:0045023//G0 to G1 transition;GO:0045746//negative regulation of Notc

transport;GO:0070327//thyroid hormone transport;GO:0070460//thyroid-stimulating hormone se
06508//proteolysis;GO:0014068//positive regulation of phosphatidylinositol 3-kinase signaling;GO
ipid translocation;GO:0047484//regulation of response to osmotic stress;GO:0055085//transmemb
0008584//male gonad development;GO:0010894//negative regulation of steroid biosynthetic proc
metabolic process;GO:0007417//central nervous system development;GO:0009064//glutamine fa

)6952//defense response;GO:0007599//hemostasis;GO:0010524//positive regulation of calcium ion

egulation of transcription from RNA polymerase II promoter;GO:0001649//osteoblast differentiatio

ng compound metabolic process;GO:0008152//metabolic process;GO:0016310//phosphorylation;G
724//double-strand break repair via homologous recombination;GO:0007129//synapsis;GO:00071

544//arginine catabolic process to glutamate;GO:0055129//L-proline biosynthetic process

response to bacterium;GO:0015909//long-chain fatty acid transport;GO:0042632//cholesterol hom

on organization;GO:0008360//regulation of cell shape;GO:0016601//Rac protein signal transductio
osynthetic process;GO:0009072//aromatic amino acid family metabolic process;GO:0019293//tyros

)008584//male gonad development;GO:0010894//negative regulation of steroid biosynthetic proc

e organization;GO:0043353//enucleate erythrocyte differentiation;GO:0098739//import across plas

athway;GO:0009888//tissue development;GO:0030198//extracellular matrix organization;GO:00713

n phosphorylation;GO:0010628//positive regulation of gene expression;GO:0010629//negative reg
endent proteasomal ubiquitin-dependent protein catabolic process;GO:0043153//entrainment of c
gnal transduction;GO:0035556//intracellular signal transduction;GO:0090630//activation of GTPase

//inflammatory response to antigenic stimulus;GO:0007264//small GTPase mediated signal transdu

process;GO:0006189//'de novo' IMP biosynthetic process;GO:0009152//purine ribonucleotide bios

ion;GO:1902857//positive regulation of nonmotile primary cilium assembly;GO:1904951//positive

ynthetic process;GO:0009113//purine nucleobase biosynthetic process;GO:0009116//nucleoside m

or signaling pathway;GO:0048678//response to axon injury;GO:1901214//regulation of neuron dea

olic process;GO:0051180//vitamin transport;GO:0090212//negative regulation of establishment of b
c process;GO:0006869//lipid transport;GO:0006886//intracellular protein transport;GO:0010875//p

ynthetic process;GO:0009113//purine nucleobase biosynthetic process;GO:0009116//nucleoside m

biosynthetic process;GO:0015908//fatty acid transport;GO:0033211//adiponectin-activated signali
erived foam cell differentiation;GO:0010878//cholesterol storage;GO:0033344//cholesterol efflux;G

l shape;GO:0042742//defense response to bacterium;GO:0045089//positive regulation of innate ir

32732//positive regulation of interleukin-1 production;GO:0055085//transmembrane transport

'/regulation of epidermal growth factor receptor signaling pathway;GO:0043408//regulation of MA

e organization;GO:0043353//enucleate erythrocyte differentiation;GO:0098739//import across plas

process;GO:0008284//positive regulation of cell proliferation;GO:0009615//response to virus;GO:00

osynthetic process;GO:0009072//aromatic amino acid family metabolic process;GO:0019293//tyros

one metabolic process;GO:0048729//tissue morphogenesis;GO:0048872//homeostasis of number

thway;GO:0009267//cellular response to starvation;GO:0010510//regulation of acetyl-CoA biosynt
ng compound metabolic process;GO:0008152//metabolic process;GO:0016310//phosphorylation;G

l promoter;GO:0000902//cell morphogenesis;GO:0001709//cell fate determination;GO:0001764//n

regulation of neurogenesis;GO:0060770//negative regulation of epithelial cell proliferation involve
metabolic process;GO:0007417//central nervous system development;GO:0009064//glutamine far
1766//hippocampus development;GO:0021819//layer formation in cerebral cortex;GO:0021860//p

ative regulation of blood coagulation;GO:0071383//cellular response to steroid hormone stimulus
e activation involved in immune response;GO:0007186//G-protein coupled receptor signaling pat

):0043490//malate-aspartate shuttle;GO:0051592//response to calcium ion;GO:0055085//transmer

response to bacterium;GO:0015909//long-chain fatty acid transport;GO:0042632//cholesterol hom

ipid translocation;GO:0047484//regulation of response to osmotic stress;GO:0055085//transmemb

):0043490//malate-aspartate shuttle;GO:0051592//response to calcium ion;GO:0055085//transmer

21//chloride transport;GO:0034220//ion transmembrane transport;GO:0055085//transmembrane tı
zation;GO:0009235//cobalamin metabolic process;GO:0015889//cobalamin transport;GO:0043001

)//nervous system development;GO:0010977//negative regulation of neuron projection developm
on;GO:0030501//positive regulation of bone mineralization;GO:0045669//positive regulation of os
03//cholesterol metabolic process;GO:0010468//regulation of gene expression;GO:0010977//nega

osynthetic process;GO:0009072//aromatic amino acid family metabolic process;GO:0019293//tyros

lic process;GO:0006656//phosphatidylcholine biosynthetic process;GO:0008203//cholesterol meta
orce of heart contraction;GO:0006816//calcium ion transport;GO:0006874//cellular calcium ion ho

e organization;GO:0043353//enucleate erythrocyte differentiation;GO:0098739//import across plas

mere organization;GO:0051639//actin filament network formation;GO:0055003//cardiac myofibril a

ide cross-linking;GO:0030216//keratinocyte differentiation;GO:0045861//negative regulation of pr

ation of gene expression;GO:0045671//negative regulation of osteoclast differentiation;GO:004577

metabolic process;GO:0007417//central nervous system development;GO:0009064//glutamine far
ransmembrane transporter activity;GO:1904064//positive regulation of cation transmembrane trar
process;GO:0006189//'de novo' IMP biosynthetic process;GO:0009152//purine ribonucleotide bios

066//regulation of phosphatidylinositol 3-kinase signaling;GO:0035304//regulation of protein dep

AT cascade;GO:0009968//negative regulation of signal transduction;GO:0035556//intracellular sig

lic process;GO:0006656//phosphatidylcholine biosynthetic process;GO:0008203//cholesterol meta

etabolic process;GO:0008652//cellular amino acid biosynthetic process;GO:0042149//cellular respo

):0010831//positive regulation of myotube differentiation;GO:0034968//histone lysine methylation

ynthetic process;GO:0009113//purine nucleobase biosynthetic process;GO:0009116//nucleoside m
biosynthetic process;GO:0015908//fatty acid transport;GO:0033211//adiponectin-activated signali

ponse to phosphate starvation;GO:0032259//methylation;GO:0042135//neurotransmitter catabolic

ty;GO:0045654//positive regulation of megakaryocyte differentiation;GO:0046330//positive regula

)32342//aldosterone biosynthetic process;GO:0032870//cellular response to hormone stimulus;GO

I promoter;GO:0006418//tRNA aminoacylation for protein translation;GO:0006434//seryl-tRNA am
uction;GO:0043087//regulation of GTPase activity;GO:0046580//negative regulation of Ras protein

positive regulation of protein phosphorylation;GO:0003347//epicardial cell to mesenchymal cell tra

posterior axis, embryo;GO:0007368//determination of left/right symmetry;GO:0009100//glycoprot

positive regulation of protein phosphorylation;GO:0003347//epicardial cell to mesenchymal cell tra

n development;GO:0050804//modulation of synaptic transmission;GO:0099560//synaptic membrar
ynthetic process;GO:0009113//purine nucleobase biosynthetic process;GO:0009116//nucleoside m

544//arginine catabolic process to glutamate;GO:0055129//L-proline biosynthetic process
DNA damage stimulus;GO:0045023//G0 to G1 transition;GO:0045746//negative regulation of Notc

oliferation;GO:0009617//response to bacterium;GO:0048010//vascular endothelial growth factor re

abolic process;GO:0006012//galactose metabolic process;GO:0019318//hexose metabolic process;

metabolic process;GO:0007417//central nervous system development;GO:0009064//glutamine far
on;GO:0030501//positive regulation of bone mineralization;GO:0045669//positive regulation of os
thway;GO:0009267//cellular response to starvation;GO:0010510//regulation of acetyl-CoA biosynt

deling;GO:0006535//cysteine biosynthetic process from serine;GO:0006801//superoxide metabolic

lic process;GO:0051180//vitamin transport;GO:0090212//negative regulation of establishment of I

regulation of neurogenesis;GO:0060770//negative regulation of epithelial cell proliferation involve
AT cascade;GO:0009968//negative regulation of signal transduction;GO:0035556//intracellular sig

organization;GO:0043353//enucleate erythrocyte differentiation;GO:0098739//import across plas

zation;GO:0009235//cobalamin metabolic process;GO:0015889//cobalamin transport;GO:0043001
:0043490//malate-aspartate shuttle;GO:0051592//response to calcium ion;GO:0055085//transmer
osynthetic process;GO:0009072//aromatic amino acid family metabolic process;GO:0019293//tyros
009306//protein secretion;GO:0030168//platelet activation;GO:0031639//plasminogen activation;G

se activity;GO:0038161//prolactin signaling pathway;GO:0042976//activation of Janus kinase activit
a chemical signal;GO:0009750//response to fructose;GO:0015755//fructose transport;GO:0055085/

0008584//male gonad development;GO:0010894//negative regulation of steroid biosynthetic proc

orce of heart contraction;GO:0006816//calcium ion transport;GO:0006874//cellular calcium ion ho
mere organization;GO:0051639//actin filament network formation;GO:0055003//cardiac myofibril a
ransmembrane transporter activity;GO:1904064//positive regulation of cation transmembrane trar

process;GO:0006189//'de novo' IMP biosynthetic process;GO:0009152//purine ribonucleotide bios

n;GO:0002305//CD8-positive, gamma-delta intraepithelial T cell differentiation;GO:0002689//nega

066//regulation of phosphatidylinositol 3-kinase signaling;GO:0035304//regulation of protein dep

n-dependent cell spreading;GO:0061001//regulation of dendritic spine morphogenesis;GO:00610

cosamine metabolic process;GO:0006790//sulfur compound metabolic process;GO:1903238//posit

l promoter;GO:0001889//liver development;GO:0001949//sebaceous gland cell differentiation;GO:
iosynthetic process;GO:0008542//visual learning;GO:0015936//coenzyme A metabolic process;GO

sphatidylinositol 3-kinase signaling;GO:0016579//protein deubiquitination;GO:0046580//negative

posterior axis, embryo;GO:0007368//determination of left/right symmetry;GO:0009100//glycoprot

egative regulation of protein lipidation;GO:2001140//positive regulation of phospholipid transport

9511//peptidyl-proline hydroxylation;GO:0032963//collagen metabolic process;GO:0050708//regu

lic process;GO:0009791//post-embryonic development;GO:0010259//multicellular organism aging

on;GO:0006869//lipid transport;GO:0007283//spermatogenesis;GO:0007399//nervous system deve
18//positive regulation of satellite cell activation involved in skeletal muscle regeneration;GO:0030

ation;GO:0002244//hematopoietic progenitor cell differentiation;GO:0006357//regulation of transc

biosynthetic process;GO:0015908//fatty acid transport;GO:0033211//adiponectin-activated signali

projection development;GO:0016055//Wnt signaling pathway;GO:0021516//dorsal spinal cord de

ıto nucleus;GO:0007049//cell cycle;GO:0007179//transforming growth factor beta receptor signalir

cell cycle;GO:0010830//regulation of myotube differentiation;GO:0010868//negative regulation o

)042391//regulation of membrane potential;GO:0043267//negative regulation of potassium ion tra
//phospholipase C-activating G-protein coupled receptor signaling pathway;GO:0035176//social l
066//regulation of phosphatidylinositol 3-kinase signaling;GO:0035304//regulation of protein dep
rotein catabolic process;GO:0048388//endosomal lumen acidification;GO:0070070//proton-transp

tabolic process;GO:0019835//cytolysis;GO:0031640//killing of cells of other organism;GO:0042742

:ion of cell fate commitment;GO:0016055//Wnt signaling pathway;GO:0030178//negative regulatic

of cell proliferation;GO:0010628//positive regulation of gene expression;GO:0016567//protein ubic

orce of heart contraction;GO:0006816//calcium ion transport;GO:0006874//cellular calcium ion ho

)//protein deubiquitination;GO:0032436//positive regulation of proteasomal ubiquitin-dependent

f neuron projection development;GO:0018105//peptidyl-serine phosphorylation;GO:0032793//po

integrin;GO:0061045//negative regulation of wound healing;GO:1901731//positive regulation of p

on of sour taste;GO:0006812//cation transport;GO:0007224//smoothened signaling pathway;GO:0
sport;GO:0055085//transmembrane transport;GO:1990830//cellular response to leukemia inhibitor

integrin;GO:0061045//negative regulation of wound healing;GO:1901731//positive regulation of p

scription, DNA-templated;GO:0006888//ER to Golgi vesicle-mediated transport;GO:0030968//end

projection development;GO:0016055//Wnt signaling pathway;GO:0021516//dorsal spinal cord de

ɔrylation;GO:0018108//peptidyl-tyrosine phosphorylation;GO:0030282//bone mineralization;GO:0[illegible]

of cell proliferation;GO:0010628//positive regulation of gene expression;GO:0016567//protein ubic
sponse to endoplasmic reticulum stress;GO:0035023//regulation of Rho protein signal transductio

ninate metabolic process;GO:0006468//protein phosphorylation;GO:0006486//protein glycosylatio

SU-rRNA, 5.8S rRNA, LSU-rRNA);GO:0000466//maturation of 5.8S rRNA from tricistronic rRNA tra
ion of transcription, DNA-templated;GO:0006357//regulation of transcription from RNA polymera

8//regulation of epidermal growth factor receptor signaling pathway;GO:0046755//viral budding
utamate receptor signaling pathway;GO:0007405//neuroblast proliferation;GO:0019369//arachidor
nthetic process;GO:0016104//triterpenoid biosynthetic process;GO:0031647//regulation of protein

elopment;GO:0002138//retinoic acid biosynthetic process;GO:0006081//cellular aldehyde metabol
cell cycle;GO:0010830//regulation of myotube differentiation;GO:0010868//negative regulation o
c process;GO:0010025//wax biosynthetic process;GO:0035336//long-chain fatty-acyl-CoA metabo

oment;GO:0006508//proteolysis;GO:0016525//negative regulation of angiogenesis;GO:0060347//h
port;GO:0033120//positive regulation of RNA splicing;GO:0044767//single-organism developmen

.6240//autophagosome docking;GO:0034329//cell junction assembly;GO:0042953//lipoprotein tra

ɔrylation;GO:0018108//peptidyl-tyrosine phosphorylation;GO:0030282//bone mineralization;GO:0[illegible]

sized protein catabolic process;GO:0007005//mitochondrion organization;GO:0008283//cell prolife

patterning of blood vessels;GO:0001666//response to hypoxia;GO:0001974//blood vessel remodel
I promoter;GO:0001657//ureteric bud development;GO:0001843//neural tube closure;GO:000206[illegible]

one metabolic process;GO:0048729//tissue morphogenesis;GO:0048872//homeostasis of number

ptor protein tyrosine kinase signaling pathway;GO:0009615//response to virus;GO:0043406//posit

asma membrane adhesion molecules;GO:0010631//epithelial cell migration;GO:0031589//cell-subs
sport;GO:0055085//transmembrane transport;GO:1990830//cellular response to leukemia inhibitor
lic process;GO:0009791//post-embryonic development;GO:0010259//multicellular organism aging

orphogenesis;GO:0007224//smoothened signaling pathway;GO:0007399//nervous system develop

ganization;GO:0042149//cellular response to glucose starvation;GO:0043066//negative regulation
on;GO:0006869//lipid transport;GO:0007283//spermatogenesis;GO:0007399//nervous system deve

06782//protoporphyrinogen IX biosynthetic process;GO:0006783//heme biosynthetic process;GO:0

cle-mediated transport, Golgi to ER;GO:0015031//protein transport;GO:0016192//vesicle-mediate

//cholesterol transport involved in cholesterol storage;GO:0032367//intracellular cholesterol transp

tion;GO:0007052//mitotic spindle organization;GO:0007409//axonogenesis;GO:0009615//response
0001569//patterning of blood vessels;GO:0001649//osteoblast differentiation;GO:0001708//cell fat

eptidase activity involved in apoptotic process;GO:0006978//DNA damage response, signal transdu
4765//regulation of ion transmembrane transport;GO:0051260//protein homooligomerization;GO

of cell proliferation;GO:0010628//positive regulation of gene expression;GO:0016567//protein ubic

ubiquitin-dependent protein catabolic process;GO:0030970//retrograde protein transport, ER to cy

e targeting;GO:0006904//vesicle docking involved in exocytosis;GO:0016192//vesicle-mediated tra

o DNA damage stimulus;GO:0007283//spermatogenesis;GO:0032434//regulation of proteasomal u

integrin;GO:0061045//negative regulation of wound healing;GO:1901731//positive regulation of p
bination;GO:0006508//proteolysis;GO:0008152//metabolic process;GO:0015074//DNA integration

0006914//autophagy;GO:0007399//nervous system development;GO:0017121//phospholipid scra

elopment;GO:0002138//retinoic acid biosynthetic process;GO:0006081//cellular aldehyde metabol

ninate metabolic process;GO:0006468//protein phosphorylation;GO:0006486//protein glycosylation

0042391//regulation of membrane potential;GO:0043267//negative regulation of potassium ion tra
damage response, signal transduction by p53 class mediator;GO:0032287//peripheral nervous syst
follicle growth;GO:0001932//regulation of protein phosphorylation;GO:0003073//regulation of sys

copper ion;GO:0032496//response to lipopolysaccharide;GO:0070555//response to interleukin-1;
:0030336//negative regulation of cell migration;GO:0032570//response to progesterone;GO:0043

tive regulation by host of viral release from host cell;GO:0045087//innate immune response;GO:00

)6782//protoporphyrinogen IX biosynthetic process;GO:0006783//heme biosynthetic process;GO:(

oxidative stress;GO:0008104//protein localization;GO:0008203//cholesterol metabolic process;GO:(

.6240//autophagosome docking;GO:0034329//cell junction assembly;GO:0042953//lipoprotein tra
)//nucleolus organization;GO:0016579//protein deubiquitination;GO:0050821//protein stabilizatior

0017158//regulation of calcium ion-dependent exocytosis;GO:0032024//positive regulation of ins
lipid metabolic process;GO:0006631//fatty acid metabolic process;GO:0006633//fatty acid biosynth

rotein catabolic process;GO:0048388//endosomal lumen acidification;GO:0070070//proton-transp

GO:0007032//endosome organization;GO:0016185//synaptic vesicle budding from presynaptic enc
ed in ureteric bud morphogenesis;GO:0001822//kidney development;GO:0003231//cardiac ventricl

lic process;GO:0008210//estrogen metabolic process;GO:0009804//coumarin metabolic process;G(

ulfment;GO:0043277//apoptotic cell clearance;GO:0050766//positive regulation of phagocytosis

ive regulation of apoptotic process;GO:0045727//positive regulation of translation;GO:0045786//n

3//ER-associated ubiquitin-dependent protein catabolic process;GO:0032781//positive regulation

)//protein deubiquitination;GO:0032436//positive regulation of proteasomal ubiquitin-dependent

f neuron projection development;GO:0018105//peptidyl-serine phosphorylation;GO:0032793//po
tion of cell fate commitment;GO:0016055//Wnt signaling pathway;GO:0030178//negative regulatic

21//chloride transport;GO:0034220//ion transmembrane transport;GO:0055085//transmembrane t

phore linkage;GO:0032922//circadian regulation of gene expression;GO:0043153//entrainment of

ninate metabolic process;GO:0006468//protein phosphorylation;GO:0006486//protein glycosylation
sport;GO:0055085//transmembrane transport;GO:1990830//cellular response to leukemia inhibitor

)//nucleolus organization;GO:0016579//protein deubiquitination;GO:0050821//protein stabilization
orylation;GO:0018108//peptidyl-tyrosine phosphorylation;GO:0030282//bone mineralization;GO:0

rotein dephosphorylation;GO:0016311//dephosphorylation;GO:0043542//endothelial cell migratio
0001569//patterning of blood vessels;GO:0001649//osteoblast differentiation;GO:0001708//cell fat

follicle growth;GO:0001932//regulation of protein phosphorylation;GO:0003073//regulation of sys
one metabolic process;GO:0048729//tissue morphogenesis;GO:0048872//homeostasis of number

l promoter;GO:0001889//liver development;GO:0001949//sebaceous gland cell differentiation;GO:

sphatidylinositol 3-kinase signaling;GO:0016579//protein deubiquitination;GO:0046580//negative

c process;GO:0010025//wax biosynthetic process;GO:0035336//long-chain fatty-acyl-CoA metabo
gration;GO:0006355//regulation of transcription, DNA-templated;GO:0007165//signal transduction
8//regulation of epidermal growth factor receptor signaling pathway;GO:0046755//viral budding

ubiquitin-dependent protein catabolic process;GO:0030970//retrograde protein transport, ER to cy
losure;GO:0007389//pattern specification process;GO:0007398//ectoderm development;GO:00074
gnal transduction;GO:0035735//intraciliary transport involved in cilium morphogenesis;GO:006027

or signaling pathway;GO:0007189//adenylate cyclase-activating G-protein coupled receptor signa
e activation involved in immune response;GO:0007186//G-protein coupled receptor signaling pat
hloride transport;GO:0008272//sulfate transport;GO:0010628//positive regulation of gene expressi
gnaling pathway in response to oxidative stress;GO:0009267//cellular response to starvation;GO:0
bination;GO:0006508//proteolysis;GO:0008152//metabolic process;GO:0015074//DNA integration

orylation;GO:0018108//peptidyl-tyrosine phosphorylation;GO:0030282//bone mineralization;GO:0
bination;GO:0006508//proteolysis;GO:0008152//metabolic process;GO:0015074//DNA integration
sport;GO:0055085//transmembrane transport;GO:1990830//cellular response to leukemia inhibitor

gnal transduction;GO:0035735//intraciliary transport involved in cilium morphogenesis;GO:006027

n phosphorylation;GO:0010628//positive regulation of gene expression;GO:0010629//negative reg

tion;GO:0033383//geranyl diphosphate metabolic process;GO:0045339//farnesyl diphosphate cata
0006914//autophagy;GO:0007399//nervous system development;GO:0017121//phospholipid scra

:0030336//negative regulation of cell migration;GO:0032570//response to progesterone;GO:0043[illegible]

lic process;GO:0006656//phosphatidylcholine biosynthetic process;GO:0008203//cholesterol meta

lysis;GO:0006915//apoptotic process;GO:0016540//protein autoprocessing;GO:0030855//epithelial

e regulation of endothelial cell migration;GO:0019673//GDP-mannose metabolic process;GO:004[illegible]

rotein catabolic process;GO:0048388//endosomal lumen acidification;GO:0070070//proton-transp

e regulation of endothelial cell migration;GO:0019673//GDP-mannose metabolic process;GO:004[illegible]

tion;GO:0007052//mitotic spindle organization;GO:0007409//axonogenesis;GO:0009615//respons[illegible]

netabolic process;GO:0042558//pteridine-containing compound metabolic process;GO:0044237//[illegible]

)//protein deubiquitination;GO:0032436//positive regulation of proteasomal ubiquitin-dependent
asma membrane adhesion molecules;GO:0010631//epithelial cell migration;GO:0031589//cell-subs

xia;GO:0001889//liver development;GO:0002314//germinal center B cell differentiation;GO:00026[illegible]
f neuron projection development;GO:0018105//peptidyl-serine phosphorylation;GO:0032793//po

patterning of blood vessels;GO:0001666//response to hypoxia;GO:0001974//blood vessel remodel

e targeting;GO:0006904//vesicle docking involved in exocytosis;GO:0016192//vesicle-mediated tra

sized protein catabolic process;GO:0007005//mitochondrion organization;GO:0008283//cell prolife

32732//positive regulation of interleukin-1 production;GO:0055085//transmembrane transport

phore linkage;GO:0032922//circadian regulation of gene expression;GO:0043153//entrainment of
)//nucleolus organization;GO:0016579//protein deubiquitination;GO:0050821//protein stabilizatio[illegible]
gnaling pathway in response to oxidative stress;GO:0009267//cellular response to starvation;GO:00[illegible]
sport;GO:0033120//positive regulation of RNA splicing;GO:0044767//single-organism developmen

or signaling pathway;GO:0007189//adenylate cyclase-activating G-protein coupled receptor signa

follicle growth;GO:0001932//regulation of protein phosphorylation;GO:0003073//regulation of sys
eptidase activity involved in apoptotic process;GO:0006978//DNA damage response, signal transdu
n of endocytosis;GO:0048488//synaptic vesicle endocytosis;GO:0048666//neuron development;GC

egative regulation of protein lipidation;GO:2001140//positive regulation of phospholipid transport

lation of cell migration;GO:0046464//acylglycerol catabolic process;GO:0046889//positive regulati

of cell proliferation;GO:0010628//positive regulation of gene expression;GO:0016567//protein ubic

oment;GO:0006508//proteolysis;GO:0016525//negative regulation of angiogenesis;GO:0060347//h

on;GO:0043649//dicarboxylic acid catabolic process;GO:0045225//negative regulation of CD4 bios

ty;GO:0045654//positive regulation of megakaryocyte differentiation;GO:0046330//positive regula

scription, DNA-templated;GO:0006888//ER to Golgi vesicle-mediated transport;GO:0030968//end
tasis;GO:0007611//learning or memory;GO:0007625//grooming behavior;GO:0007626//locomotor

f cytosolic calcium ion concentration;GO:0010863//positive regulation of phospholipase C activity
ylation;GO:0006915//apoptotic process;GO:0031647//regulation of protein stability;GO:0050790//

integrin;GO:0061045//negative regulation of wound healing;GO:1901731//positive regulation of p
c process;GO:0010025//wax biosynthetic process;GO:0035336//long-chain fatty-acyl-CoA metabo

tive regulation by host of viral release from host cell;GO:0045087//innate immune response;GO:00
GO:0010508//positive regulation of autophagy;GO:0016567//protein ubiquitination;GO:0034198//
l promoter;GO:0001568//blood vessel development;GO:0001570//vasculogenesis;GO:0003150//m

)326//embryonic limb morphogenesis;GO:0048513//animal organ development;GO:0050794//regu
orce of heart contraction;GO:0006816//calcium ion transport;GO:0006874//cellular calcium ion ho

ell cycle;GO:0006468//protein phosphorylation;GO:0007147//female meiosis II;GO:0032515//negat

ess;GO:0006593//ornithine catabolic process;GO:0019240//citrulline biosynthetic process;GO:0097
3//ER-associated ubiquitin-dependent protein catabolic process;GO:0032781//positive regulation

tion of cell fate commitment;GO:0016055//Wnt signaling pathway;GO:0030178//negative regulatic

066//regulation of phosphatidylinositol 3-kinase signaling;GO:0035304//regulation of protein dep

netabolic process;GO:0042558//pteridine-containing compound metabolic process;GO:0044237//c
tion of transcription, DNA-templated;GO:0006357//regulation of transcription from RNA polymera

ic process;GO:0006644//phospholipid metabolic process;GO:0007160//cell-matrix adhesion;GO:0(

or signaling pathway;GO:0008277//regulation of G-protein coupled receptor protein signaling pat
tion;GO:0007052//mitotic spindle organization;GO:0007409//axonogenesis;GO:0009615//response

I promoter;GO:0001889//liver development;GO:0001949//sebaceous gland cell differentiation;GO:

egative regulation of protein lipidation;GO:2001140//positive regulation of phospholipid transport

osphatidylinositol 3-kinase signaling;GO:0016579//protein deubiquitination;GO:0046580//negative
n of endocytosis;GO:0048488//synaptic vesicle endocytosis;GO:0048666//neuron development;G(

one metabolic process;GO:0048729//tissue morphogenesis;GO:0048872//homeostasis of number
iquitin-dependent protein catabolic process;GO:0016578//histone deubiquitination;GO:0016579/

//phospholipase C-activating G-protein coupled receptor signaling pathway;GO:0035176//social [illegible]

[illegible]//ER-associated ubiquitin-dependent protein catabolic process;GO:0032781//positive regulation

[illegible]ganization;GO:0042149//cellular response to glucose starvation;GO:0043066//negative regulation

posterior axis, embryo;GO:0007368//determination of left/right symmetry;GO:0009100//glycoprot[illegible]

[illegible]8//regulation of epidermal growth factor receptor signaling pathway;GO:0046755//viral budding
[illegible]on;GO:0006869//lipid transport;GO:0007283//spermatogenesis;GO:0007399//nervous system deve[illegible]
[illegible]n of endocytosis;GO:0048488//synaptic vesicle endocytosis;GO:0048666//neuron development;G[illegible]

follicle growth;GO:0001932//regulation of protein phosphorylation;GO:0003073//regulation of sys[illegible]

[illegible]001569//patterning of blood vessels;GO:0001649//osteoblast differentiation;GO:0001708//cell fat[illegible]

[illegible]ment;GO:0006508//proteolysis;GO:0016525//negative regulation of angiogenesis;GO:0060347//h[illegible]
[illegible]ptidase activity involved in apoptotic process;GO:0006978//DNA damage response, signal transd[illegible]

e expression;GO:0030001//metal ion transport;GO:0031398//positive regulation of protein ubiquit[illegible]

[illegible]gnal transduction;GO:0035735//intraciliary transport involved in cilium morphogenesis;GO:006027[illegible]

eptidase activity involved in apoptotic process;GO:0006978//DNA damage response, signal transdu

or signaling pathway;GO:0007189//adenylate cyclase-activating G-protein coupled receptor signa

SU-rRNA, 5.8S rRNA, LSU-rRNA);GO:0000466//maturation of 5.8S rRNA from tricistronic rRNA tra

rotein dephosphorylation;GO:0016311//dephosphorylation;GO:0043542//endothelial cell migratic

targeting;GO:0006904//vesicle docking involved in exocytosis;GO:0016192//vesicle-mediated tra

tion;GO:0007052//mitotic spindle organization;GO:0007409//axonogenesis;GO:0009615//respons

ic process;GO:0006644//phospholipid metabolic process;GO:0007160//cell-matrix adhesion;GO:00

cle-mediated transport, Golgi to ER;GO:0015031//protein transport;GO:0016192//vesicle-mediate
e regulation of endothelial cell migration;GO:0019673//GDP-mannose metabolic process;GO:004

sponse to endoplasmic reticulum stress;GO:0035023//regulation of Rho protein signal transductio
xia;GO:0001889//liver development;GO:0002314//germinal center B cell differentiation;GO:00026

lysis;GO:0006915//apoptotic process;GO:0016540//protein autoprocessing;GO:0030855//epithelial

damage response, signal transduction by p53 class mediator;GO:0032287//peripheral nervous syst

ion of transcription, DNA-templated;GO:0006357//regulation of transcription from RNA polymera

orphogenesis;GO:0007224//smoothened signaling pathway;GO:0007399//nervous system develop

8//regulation of epidermal growth factor receptor signaling pathway;GO:0046755//viral budding
ic process;GO:0006644//phospholipid metabolic process;GO:0007160//cell-matrix adhesion;GO:00

lipid metabolic process;GO:0016311//dephosphorylation;GO:0035335//peptidyl-tyrosine dephosp

egative regulation of protein lipidation;GO:2001140//positive regulation of phospholipid transport

scription, DNA-templated;GO:0006888//ER to Golgi vesicle-mediated transport;GO:0030968//end
ive regulation of sequestering of triglyceride;GO:0010898//positive regulation of triglyceride catab
//phospholipase C-activating G-protein coupled receptor signaling pathway;GO:0035176//social l
)//nucleolus organization;GO:0016579//protein deubiquitination;GO:0050821//protein stabilization

oxidative stress;GO:0008104//protein localization;GO:0008203//cholesterol metabolic process;GO:(

phore linkage;GO:0032922//circadian regulation of gene expression;GO:0043153//entrainment of
orce of heart contraction;GO:0006816//calcium ion transport;GO:0006874//cellular calcium ion ho

ı development;GO:0050804//modulation of synaptic transmission;GO:0099560//synaptic membrar

I promoter;GO:0001889//liver development;GO:0001949//sebaceous gland cell differentiation;GO:
egative regulation of protein lipidation;GO:2001140//positive regulation of phospholipid transport
d glutamate receptor signaling pathway;GO:0048148//behavioral response to cocaine;GO:0048741
osphatidylinositol 3-kinase signaling;GO:0016579//protein deubiquitination;GO:0046580//negative

066//regulation of phosphatidylinositol 3-kinase signaling;GO:0035304//regulation of protein dep

gnaling pathway in response to oxidative stress;GO:0009267//cellular response to starvation;GO:00
abolic process;GO:0007586//digestion;GO:0008202//steroid metabolic process;GO:0008207//C21-s
3//ER-associated ubiquitin-dependent protein catabolic process;GO:0032781//positive regulation

cell cycle;GO:0010830//regulation of myotube differentiation;GO:0010868//negative regulation of
I promoter;GO:0001568//blood vessel development;GO:0001570//vasculogenesis;GO:0003150//m
ylation;GO:0006915//apoptotic process;GO:0031647//regulation of protein stability;GO:0050790//

;port;GO:0033120//positive regulation of RNA splicing;GO:0044767//single-organism developmen
etabolic process;GO:0006096//glycolytic process;GO:0051156//glucose 6-phosphate metabolic pr

2//negative regulation of cell adhesion;GO:0010952//positive regulation of peptidase activity;GO:0

egulation of canonical Wnt signaling pathway;GO:2000096//positive regulation of Wnt signaling p

GO:0010508//positive regulation of autophagy;GO:0016567//protein ubiquitination;GO:0034198//

olic process;GO:0046485//ether lipid metabolic process;GO:0060395//SMAD protein signal transdu

of protein phosphorylation;GO:0007507//heart development;GO:0008284//positive regulation of

iquitin-dependent protein catabolic process;GO:0016578//histone deubiquitination;GO:0016579//

f neuron projection development;GO:0018105//peptidyl-serine phosphorylation;GO:0032793//po

)//protein deubiquitination;GO:0032436//positive regulation of proteasomal ubiquitin-dependent

posterior axis, embryo;GO:0007368//determination of left/right symmetry;GO:0009100//glycoprot

or signaling pathway;GO:0007218//neuropeptide signaling pathway;GO:0008217//regulation of bl

promoter;GO:1900408//negative regulation of cellular response to oxidative stress;GO:1902083//r

e expression;GO:0030001//metal ion transport;GO:0031398//positive regulation of protein ubiquit

rotein catabolic process;GO:0048388//endosomal lumen acidification;GO:0070070//proton-transp

e regulation of endothelial cell migration;GO:0019673//GDP-mannose metabolic process;GO:0042
pe B pancreatic cell development;GO:0006468//protein phosphorylation;GO:0009615//response to
n-dependent cell spreading;GO:0061001//regulation of dendritic spine morphogenesis;GO:00610
c process;GO:0043335//protein unfolding;GO:0051005//negative regulation of lipoprotein lipase a

or signaling pathway;GO:0007189//adenylate cyclase-activating G-protein coupled receptor signa
regulation of transferase activity;GO:2000480//negative regulation of cAMP-dependent protein kir

ositive regulation of transcription from RNA polymerase II promoter;GO:0048384//retinoic acid re

damage response, signal transduction by p53 class mediator;GO:0032287//peripheral nervous syst

on transmembrane transport;GO:0050909//sensory perception of taste;GO:0055085//transmembra

n of endocytosis;GO:0048488//synaptic vesicle endocytosis;GO:0048666//neuron development;GC

58//regulation of epidermal growth factor receptor signaling pathway;GO:0046755//viral budding

lysis;GO:0006915//apoptotic process;GO:0016540//protein autoprocessing;GO:0030855//epithelial

small molecule catabolic process;GO:0046039//GTP metabolic process;GO:0046052//UTP catabol

rganization;GO:0042149//cellular response to glucose starvation;GO:0043066//negative regulation

ylation;GO:0006915//apoptotic process;GO:0031647//regulation of protein stability;GO:0050790//

pment;GO:0006508//proteolysis;GO:0016525//negative regulation of angiogenesis;GO:0060347//h

0006914//autophagy;GO:0007399//nervous system development;GO:0017121//phospholipid scra
o DNA damage stimulus;GO:0007283//spermatogenesis;GO:0032434//regulation of proteasomal u

one metabolic process;GO:0048729//tissue morphogenesis;GO:0048872//homeostasis of number

egulation of canonical Wnt signaling pathway;GO:2000096//positive regulation of Wnt signaling p
utamate receptor signaling pathway;GO:0007405//neuroblast proliferation;GO:0019369//arachidor

gnaling pathway in response to oxidative stress;GO:0009267//cellular response to starvation;GO:00

olic process;GO:0046485//ether lipid metabolic process;GO:0060395//SMAD protein signal transdu

070374//positive regulation of ERK1 and ERK2 cascade;GO:0070378//positive regulation of ERK5 c

e-mediated transport;GO:0051592//response to calcium ion;GO:0090110//cargo loading into COP

ridoxal phosphate-L-lysine;GO:0019343//cysteine biosynthetic process via cystathionine;GO:0019

0001569//patterning of blood vessels;GO:0001649//osteoblast differentiation;GO:0001708//cell fat
orylation;GO:0018108//peptidyl-tyrosine phosphorylation;GO:0030282//bone mineralization;GO:0

ve regulation of gene expression;GO:0030511//positive regulation of transforming growth factor b

sponse to endoplasmic reticulum stress;GO:0035023//regulation of Rho protein signal transductio
ed in ureteric bud morphogenesis;GO:0001822//kidney development;GO:0003231//cardiac ventricl
on of sour taste;GO:0006812//cation transport;GO:0007224//smoothened signaling pathway;GO:0

elopment;GO:0002138//retinoic acid biosynthetic process;GO:0006081//cellular aldehyde metabol

ranscription, DNA-templated;GO:1903507//negative regulation of nucleic acid-templated transcri

on;GO:0043649//dicarboxylic acid catabolic process;GO:0045225//negative regulation of CD4 bios

2//negative regulation of cell adhesion;GO:0010952//positive regulation of peptidase activity;GO:0
orphogenesis;GO:0007224//smoothened signaling pathway;GO:0007399//nervous system develop

scription, DNA-templated;GO:0006888//ER to Golgi vesicle-mediated transport;GO:0030968//end

inate metabolic process;GO:0006468//protein phosphorylation;GO:0006486//protein glycosylatio
c process;GO:0043335//protein unfolding;GO:0051005//negative regulation of lipoprotein lipase a

d glutamate receptor signaling pathway;GO:0048148//behavioral response to cocaine;GO:004874
pe B pancreatic cell development;GO:0006468//protein phosphorylation;GO:0009615//response to
olipid metabolic process;GO:0016311//dephosphorylation;GO:0035335//peptidyl-tyrosine dephos
cle-mediated transport, Golgi to ER;GO:0015031//protein transport;GO:0016192//vesicle-mediate
//phospholipase C-activating G-protein coupled receptor signaling pathway;GO:0035176//social
regulation of transferase activity;GO:2000480//negative regulation of cAMP-dependent protein ki

d glutamate receptor signaling pathway;GO:0048148//behavioral response to cocaine;GO:004874

sport;GO:0055085//transmembrane transport;GO:1990830//cellular response to leukemia inhibitor

n-dependent cell spreading;GO:0061001//regulation of dendritic spine morphogenesis;GO:00610

phore linkage;GO:0032922//circadian regulation of gene expression;GO:0043153//entrainment of
)8283//cell proliferation;GO:0008284//positive regulation of cell proliferation;GO:0009408//respon

?//negative regulation of cell adhesion;GO:0010952//positive regulation of peptidase activity;GO:0

abolic process;GO:0006012//galactose metabolic process;GO:0019318//hexose metabolic process;

:0030336//negative regulation of cell migration;GO:0032570//response to progesterone;GO:0043
;port;GO:0033120//positive regulation of RNA splicing;GO:0044767//single-organism developmen

178//negative regulation of Wnt signaling pathway;GO:0030949//positive regulation of vascular e
.6240//autophagosome docking;GO:0034329//cell junction assembly;GO:0042953//lipoprotein tra

orphogenesis;GO:0007224//smoothened signaling pathway;GO:0007399//nervous system develop
nthetic process;GO:0016104//triterpenoid biosynthetic process;GO:0031647//regulation of protei

projection development;GO:0016055//Wnt signaling pathway;GO:0021516//dorsal spinal cord de

c process;GO:0035874//cellular response to copper ion starvation;GO:0046677//response to antib
ation;GO:0090305//nucleic acid phosphodiester bond hydrolysis;GO:0090502//RNA phosphodiest
abolic process;GO:0006631//fatty acid metabolic process;GO:0006679//glucosylceramide biosynth

iosynthetic process;GO:0008542//visual learning;GO:0015936//coenzyme A metabolic process;GO

n phosphorylation;GO:0010628//positive regulation of gene expression;GO:0010629//negative reg
integrin;GO:0061045//negative regulation of wound healing;GO:1901731//positive regulation of p

ɔ DNA damage stimulus;GO:0007283//spermatogenesis;GO:0032434//regulation of proteasomal ι

e expression;GO:0030001//metal ion transport;GO:0031398//positive regulation of protein ubiquit

ɔf cell proliferation;GO:0010628//positive regulation of gene expression;GO:0016567//protein ubic
sport;GO:0032365//intracellular lipid transport;GO:0042632//cholesterol homeostasis;GO:0046320/

or signaling pathway;GO:0007218//neuropeptide signaling pathway;GO:0008217//regulation of bl

ridoxal phosphate-L-lysine;GO:0019343//cysteine biosynthetic process via cystathionine;GO:0019

ɔrylation;GO:0018108//peptidyl-tyrosine phosphorylation;GO:0030282//bone mineralization;GO:0

ɔolic process;GO:0015697//quaternary ammonium group transport;GO:0015879//carnitine transpo

sport;GO:0055085//transmembrane transport;GO:1990830//cellular response to leukemia inhibitor

ɔrphogenesis;GO:0007224//smoothened signaling pathway;GO:0007399//nervous system develop

ɔn;GO:0043649//dicarboxylic acid catabolic process;GO:0045225//negative regulation of CD4 bios
32732//positive regulation of interleukin-1 production;GO:0055085//transmembrane transport

)6782//protoporphyrinogen IX biosynthetic process;GO:0006783//heme biosynthetic process;GO:(

e-mediated transport;GO:0051592//response to calcium ion;GO:0090110//cargo loading into COP

)32342//aldosterone biosynthetic process;GO:0032870//cellular response to hormone stimulus;GO
SU-rRNA, 5.8S rRNA, LSU-rRNA);GO:0000466//maturation of 5.8S rRNA from tricistronic rRNA tra

sport;GO:0055085//transmembrane transport;GO:1990830//cellular response to leukemia inhibitor
//ubiquitin-dependent protein catabolic process;GO:0007409//axonogenesis;GO:0007412//axon t
eptor protein tyrosine kinase signaling pathway;GO:0009615//response to virus;GO:0043406//posit
lation of cell migration;GO:0046464//acylglycerol catabolic process;GO:0046889//positive regulati

)042391//regulation of membrane potential;GO:0043267//negative regulation of potassium ion tra

cle-mediated transport, Golgi to ER;GO:0015031//protein transport;GO:0016192//vesicle-mediate
)6782//protoporphyrinogen IX biosynthetic process;GO:0006783//heme biosynthetic process;GO:(
I promoter;GO:0001657//ureteric bud development;GO:0001843//neural tube closure;GO:000206

lysis;GO:0006915//apoptotic process;GO:0016540//protein autoprocessing;GO:0030855//epithelial

orylation;GO:0018108//peptidyl-tyrosine phosphorylation;GO:0030282//bone mineralization;GO:0

178//negative regulation of Wnt signaling pathway;GO:0030949//positive regulation of vascular e

egulation of canonical Wnt signaling pathway;GO:2000096//positive regulation of Wnt signaling p

tive regulation by host of viral release from host cell;GO:0045087//innate immune response;GO:0

4//phospholipid biosynthetic process;GO:0010821//regulation of mitochondrion organization;GO:

›lipid metabolic process;GO:0016311//dephosphorylation;GO:0035335//peptidyl-tyrosine dephos[

abolic process;GO:0006631//fatty acid metabolic process;GO:0006679//glucosylceramide biosynth

ess;GO:0006593//ornithine catabolic process;GO:0019240//citrulline biosynthetic process;GO:0097

ation;GO:0090305//nucleic acid phosphodiester bond hydrolysis;GO:0090502//RNA phosphodiest

on;GO:0006869//lipid transport;GO:0007283//spermatogenesis;GO:0007399//nervous system deve

or signaling pathway;GO:0007218//neuropeptide signaling pathway;GO:0008217//regulation of bl

one metabolic process;GO:0048729//tissue morphogenesis;GO:0048872//homeostasis of number
cle-mediated transport, Golgi to ER;GO:0015031//protein transport;GO:0016192//vesicle-mediate

projection development;GO:0016055//Wnt signaling pathway;GO:0021516//dorsal spinal cord de
ion of cell fate commitment;GO:0016055//Wnt signaling pathway;GO:0030178//negative regulatic

on of oxidative phosphorylation;GO:0006390//transcription from mitochondrial promoter;GO:0006

on;GO:0043649//dicarboxylic acid catabolic process;GO:0045225//negative regulation of CD4 bios

sport;GO:0032365//intracellular lipid transport;GO:0042632//cholesterol homeostasis;GO:0046320/

elopment;GO:0002138//retinoic acid biosynthetic process;GO:0006081//cellular aldehyde metabol
ctor beta receptor signaling pathway;GO:0010608//posttranscriptional regulation of gene expressic

thway;GO:0009267//cellular response to starvation;GO:0010510//regulation of acetyl-CoA biosynt

ninate metabolic process;GO:0006468//protein phosphorylation;GO:0006486//protein glycosylatio

e-mediated transport;GO:0051592//response to calcium ion;GO:0090110//cargo loading into COP
2//negative regulation of cell adhesion;GO:0010952//positive regulation of peptidase activity;GO:0

55064//chloride ion homeostasis;GO:0055075//potassium ion homeostasis;GO:0055085//transmen

bolic process;GO:0015697//quaternary ammonium group transport;GO:0015879//carnitine transpo

n-dependent cell spreading;GO:0061001//regulation of dendritic spine morphogenesis;GO:00610

lic process;GO:0009791//post-embryonic development;GO:0010259//multicellular organism aging

d glutamate receptor signaling pathway;GO:0048148//behavioral response to cocaine;GO:004874[illegible]

:0030336//negative regulation of cell migration;GO:0032570//response to progesterone;GO:0043[illegible]

eptor protein tyrosine kinase signaling pathway;GO:0009615//response to virus;GO:0043406//posit
orphogenesis;GO:0007224//smoothened signaling pathway;GO:0007399//nervous system develop

0017158//regulation of calcium ion-dependent exocytosis;GO:0032024//positive regulation of ins

I promoter;GO:0001657//ureteric bud development;GO:0001843//neural tube closure;GO:000206[illegible]

ell cycle;GO:0006468//protein phosphorylation;GO:0007147//female meiosis II;GO:0032515//negat
or signaling pathway;GO:0007218//neuropeptide signaling pathway;GO:0008217//regulation of bl

ive regulation of apoptotic process;GO:0045727//positive regulation of translation;GO:0045786//n

›iquitin-dependent protein catabolic process;GO:0016578//histone deubiquitination;GO:0016579/

.6240//autophagosome docking;GO:0034329//cell junction assembly;GO:0042953//lipoprotein tra

e regulation of endothelial cell migration;GO:0019673//GDP-mannose metabolic process;GO:004

egulation of transcription elongation from RNA polymerase II promoter;GO:0045893//positive reg

)6782//protoporphyrinogen IX biosynthetic process;GO:0006783//heme biosynthetic process;GO:

4//phospholipid biosynthetic process;GO:0010821//regulation of mitochondrion organization;GO:

)09306//protein secretion;GO:0030168//platelet activation;GO:0031639//plasminogen activation;

one metabolic process;GO:0048729//tissue morphogenesis;GO:0048872//homeostasis of number

losure;GO:0007389//pattern specification process;GO:0007398//ectoderm development;GO:0007
xia;GO:0001889//liver development;GO:0002314//germinal center B cell differentiation;GO:00026

metabolic process;GO:0007417//central nervous system development;GO:0009064//glutamine far

ɔ DNA damage stimulus;GO:0007283//spermatogenesis;GO:0032434//regulation of proteasomal u

cle-mediated transport, Golgi to ER;GO:0015031//protein transport;GO:0016192//vesicle-mediate

ctor beta receptor signaling pathway;GO:0010608//posttranscriptional regulation of gene expressi

2//negative regulation of cell adhesion;GO:0010952//positive regulation of peptidase activity;GO:0

ive regulation of sequestering of triglyceride;GO:0010898//positive regulation of triglyceride catab

I promoter;GO:0001657//ureteric bud development;GO:0001843//neural tube closure;GO:000206

58//regulation of epidermal growth factor receptor signaling pathway;GO:0046755//viral budding

O:0002269//leukocyte activation involved in inflammatory response;GO:0002548//monocyte chem
d glutamate receptor signaling pathway;GO:0048148//behavioral response to cocaine;GO:004874
eptor protein tyrosine kinase signaling pathway;GO:0009615//response to virus;GO:0043406//posit

c process;GO:0010025//wax biosynthetic process;GO:0035336//long-chain fatty-acyl-CoA metab

zation;GO:0009235//cobalamin metabolic process;GO:0015889//cobalamin transport;GO:0043001

GO:0007626//locomotory behavior;GO:0016477//cell migration;GO:0098609//cell-cell adhesion

tive regulation of calcium-mediated signaling;GO:0050862//positive regulation of T cell receptor s
tive regulation by host of viral release from host cell;GO:0045087//innate immune response;GO:00

ynthetic process;GO:0009113//purine nucleobase biosynthetic process;GO:0009116//nucleoside m

gnaling pathway in response to oxidative stress;GO:0009267//cellular response to starvation;GO:00

bination;GO:0006508//proteolysis;GO:0008152//metabolic process;GO:0015074//DNA integration
gulation of glucose metabolic process;GO:0014065//phosphatidylinositol 3-kinase signaling;GO:00

ion of cell fate commitment;GO:0016055//Wnt signaling pathway;GO:0030178//negative regulatic
GO:0010508//positive regulation of autophagy;GO:0016567//protein ubiquitination;GO:0034198//
0006914//autophagy;GO:0007399//nervous system development;GO:0017121//phospholipid scra

21//chloride transport;GO:0034220//ion transmembrane transport;GO:0055085//transmembrane tı

asma membrane adhesion molecules;GO:0010631//epithelial cell migration;GO:0031589//cell-subs
or signaling pathway;GO:0007218//neuropeptide signaling pathway;GO:0008217//regulation of bl

e expression;GO:0030001//metal ion transport;GO:0031398//positive regulation of protein ubiquit

ranscription, DNA-templated;GO:1903507//negative regulation of nucleic acid-templated transcrip

gnal transduction;GO:0035556//intracellular signal transduction;GO:0090630//activation of GTPase
n-dependent cell spreading;GO:0061001//regulation of dendritic spine morphogenesis;GO:00610
patterning of blood vessels;GO:0001666//response to hypoxia;GO:0001974//blood vessel remodel

pathway;GO:0009617//response to bacterium;GO:0010628//positive regulation of gene expression
sized protein catabolic process;GO:0007005//mitochondrion organization;GO:0008283//cell prolife
lipid metabolic process;GO:0016311//dephosphorylation;GO:0035335//peptidyl-tyrosine dephos

ion of cell fate commitment;GO:0016055//Wnt signaling pathway;GO:0030178//negative regulatic

tabolic process;GO:0009313//oligosaccharide catabolic process;GO:0051692//cellular oligosaccha

e organization;GO:0043353//enucleate erythrocyte differentiation;GO:0098739//import across plas

ubiquitin-dependent protein catabolic process;GO:0030970//retrograde protein transport, ER to cy

osynthetic process;GO:0009072//aromatic amino acid family metabolic process;GO:0019293//tyros

r signaling pathway;GO:0007409//axonogenesis;GO:0030900//forebrain development;GO:003818[illegible]

routing angiogenesis;GO:0006816//calcium ion transport;GO:0006886//intracellular protein transp

athway;GO:0009888//tissue development;GO:0030198//extracellular matrix organization;GO:0071[illegible]
projection development;GO:0016055//Wnt signaling pathway;GO:0021516//dorsal spinal cord de
utamate receptor signaling pathway;GO:0007405//neuroblast proliferation;GO:0019369//arachidor

e targeting;GO:0006904//vesicle docking involved in exocytosis;GO:0016192//vesicle-mediated tra

rotein catabolic process;GO:0048388//endosomal lumen acidification;GO:0070070//proton-transp

or signaling pathway;GO:0007189//adenylate cyclase-activating G-protein coupled receptor signa
c process;GO:0010025//wax biosynthetic process;GO:0035336//long-chain fatty-acyl-CoA metabo
egative regulation of protein lipidation;GO:2001140//positive regulation of phospholipid transport

;GO:0006974//cellular response to DNA damage stimulus;GO:0010212//response to ionizing radia
[illegible]//protein deubiquitination;GO:0032436//positive regulation of proteasomal ubiquitin-dependent

f neuron projection development;GO:0018105//peptidyl-serine phosphorylation;GO:0032793//po

ı small molecule catabolic process;GO:0046039//GTP metabolic process;GO:0046052//UTP catabol

ɔlic process;GO:0046485//ether lipid metabolic process;GO:0060395//SMAD protein signal transdu

e regulation of endothelial cell migration;GO:0019673//GDP-mannose metabolic process;GO:004[illegible]

posterior axis, embryo;GO:0007368//determination of left/right symmetry;GO:0009100//glycoprot

)6782//protoporphyrinogen IX biosynthetic process;GO:0006783//heme biosynthetic process;GO:[illegible]

)326//embryonic limb morphogenesis;GO:0048513//animal organ development;GO:0050794//regu
ic process;GO:0006644//phospholipid metabolic process;GO:0007160//cell-matrix adhesion;GO:0[illegible]

tion;GO:0007052//mitotic spindle organization;GO:0007409//axonogenesis;GO:0009615//response

gnaling pathway in response to oxidative stress;GO:0009267//cellular response to starvation;GO:0[illegible]
21//chloride transport;GO:0034220//ion transmembrane transport;GO:0055085//transmembrane t

elopment;GO:0002138//retinoic acid biosynthetic process;GO:0006081//cellular aldehyde metabol
3//ER-associated ubiquitin-dependent protein catabolic process;GO:0032781//positive regulation

xia;GO:0001889//liver development;GO:0002314//germinal center B cell differentiation;GO:00026[illegible]

scription, DNA-templated;GO:0006888//ER to Golgi vesicle-mediated transport;GO:0030968//end

l shape;GO:0042742//defense response to bacterium;GO:0045089//positive regulation of innate ir

ɔlipid metabolic process;GO:0016311//dephosphorylation;GO:0035335//peptidyl-tyrosine dephosp
sponse to endoplasmic reticulum stress;GO:0035023//regulation of Rho protein signal transductio

or signaling pathway;GO:0007218//neuropeptide signaling pathway;GO:0008217//regulation of bl

066//regulation of phosphatidylinositol 3-kinase signaling;GO:0035304//regulation of protein dep

'/regulation of epidermal growth factor receptor signaling pathway;GO:0043408//regulation of MA

tion of transcription, DNA-templated;GO:0006357//regulation of transcription from RNA polymera

n-dependent cell spreading;GO:0061001//regulation of dendritic spine morphogenesis;GO:00610

ell cycle;GO:0006468//protein phosphorylation;GO:0007147//female meiosis II;GO:0032515//negat

n of endocytosis;GO:0048488//synaptic vesicle endocytosis;GO:0048666//neuron development;G(
I promoter;GO:0001889//liver development;GO:0001949//sebaceous gland cell differentiation;GO:

e expression;GO:0030001//metal ion transport;GO:0031398//positive regulation of protein ubiquit

sphatidylinositol 3-kinase signaling;GO:0016579//protein deubiquitination;GO:0046580//negative

orce of heart contraction;GO:0006816//calcium ion transport;GO:0006874//cellular calcium ion ho

sport;GO:0033120//positive regulation of RNA splicing;GO:0044767//single-organism developmen
544//arginine catabolic process to glutamate;GO:0055129//L-proline biosynthetic process

d glutamate receptor signaling pathway;GO:0048148//behavioral response to cocaine;GO:004874

small molecule catabolic process;GO:0046039//GTP metabolic process;GO:0046052//UTP catabol
etic process, by transamination of glyoxylate;GO:0019481//L-alanine catabolic process, by transam

'/base-excision repair, AP site formation;GO:0006296//nucleotide-excision repair, DNA incision, 5'

I promoter;GO:0001657//ureteric bud development;GO:0001843//neural tube closure;GO:000206

tion;GO:0007052//mitotic spindle organization;GO:0007409//axonogenesis;GO:0009615//respons
ninate metabolic process;GO:0006468//protein phosphorylation;GO:0006486//protein glycosylatio
//cholesterol transport involved in cholesterol storage;GO:0032367//intracellular cholesterol transp
I promoter;GO:0001568//blood vessel development;GO:0001570//vasculogenesis;GO:0003150//m
ation;GO:0090305//nucleic acid phosphodiester bond hydrolysis;GO:0090502//RNA phosphodiest

eptor protein tyrosine kinase signaling pathway;GO:0009615//response to virus;GO:0043406//posit

2//negative regulation of cell adhesion;GO:0010952//positive regulation of peptidase activity;GO:0

eptidase activity involved in apoptotic process;GO:0006978//DNA damage response, signal transdu
GO:0010508//positive regulation of autophagy;GO:0016567//protein ubiquitination;GO:0034198//
on;GO:0043649//dicarboxylic acid catabolic process;GO:0045225//negative regulation of CD4 bios
abolic process;GO:0006012//galactose metabolic process;GO:0019318//hexose metabolic process;

or signaling pathway;GO:0007189//adenylate cyclase-activating G-protein coupled receptor signa

biquitin-dependent protein catabolic process;GO:0016578//histone deubiquitination;GO:0016579//

c process;GO:0006869//lipid transport;GO:0006886//intracellular protein transport;GO:0010875//p

tion of cell fate commitment;GO:0016055//Wnt signaling pathway;GO:0030178//negative regulatic
f neuron projection development;GO:0018105//peptidyl-serine phosphorylation;GO:0032793//po

0//protein deubiquitination;GO:0032436//positive regulation of proteasomal ubiquitin-dependent
009306//protein secretion;GO:0030168//platelet activation;GO:0031639//plasminogen activation;G

sized protein catabolic process;GO:0007005//mitochondrion organization;GO:0008283//cell prolife

patterning of blood vessels;GO:0001666//response to hypoxia;GO:0001974//blood vessel remodel
elopment;GO:0002138//retinoic acid biosynthetic process;GO:0006081//cellular aldehyde metabol
eptidase activity involved in apoptotic process;GO:0006978//DNA damage response, signal transdu

asma membrane adhesion molecules;GO:0010631//epithelial cell migration;GO:0031589//cell-subs

rotein catabolic process;GO:0048388//endosomal lumen acidification;GO:0070070//proton-transp

)326//embryonic limb morphogenesis;GO:0048513//animal organ development;GO:0050794//regu

scription, DNA-templated;GO:0006888//ER to Golgi vesicle-mediated transport;GO:0030968//end

ic process;GO:0006644//phospholipid metabolic process;GO:0007160//cell-matrix adhesion;GO:0(

ranscription, DNA-templated;GO:1903507//negative regulation of nucleic acid-templated transcri

3//ER-associated ubiquitin-dependent protein catabolic process;GO:0032781//positive regulation

I promoter;GO:0001889//liver development;GO:0001949//sebaceous gland cell differentiation;GO:

›sphatidylinositol 3-kinase signaling;GO:0016579//protein deubiquitination;GO:0046580//negative

ell cycle;GO:0006468//protein phosphorylation;GO:0007147//female meiosis II;GO:0032515//negat
sion;GO:1902857//positive regulation of nonmotile primary cilium assembly;GO:1904951//positive

rocess;GO:0006633//fatty acid biosynthetic process;GO:0006636//unsaturated fatty acid biosynthe

ylation;GO:0006915//apoptotic process;GO:0031647//regulation of protein stability;GO:0050790//

ylation;GO:0006915//apoptotic process;GO:0031647//regulation of protein stability;GO:0050790//
sport;GO:0033120//positive regulation of RNA splicing;GO:0044767//single-organism developmen

phore linkage;GO:0032922//circadian regulation of gene expression;GO:0043153//entrainment of
optotic process;GO:0043154//negative regulation of cysteine-type endopeptidase activity involvec

etic process, by transamination of glyoxylate;GO:0019481//L-alanine catabolic process, by transam

409//axonogenesis;GO:0007411//axon guidance;GO:0008284//positive regulation of cell proliferat
olic process;GO:0046485//ether lipid metabolic process;GO:0060395//SMAD protein signal transdu

3//ER-associated ubiquitin-dependent protein catabolic process;GO:0032781//positive regulation

e activation involved in immune response;GO:0007186//G-protein coupled receptor signaling pat

phore linkage;GO:0032922//circadian regulation of gene expression;GO:0043153//entrainment of
abolic process;GO:0006631//fatty acid metabolic process;GO:0006679//glucosylceramide biosynth

tion;GO:0007052//mitotic spindle organization;GO:0007409//axonogenesis;GO:0009615//response

tion of transcription, DNA-templated;GO:0006357//regulation of transcription from RNA polymera

4765//regulation of ion transmembrane transport;GO:0051260//protein homooligomerization;GO

sport;GO:0032365//intracellular lipid transport;GO:0042632//cholesterol homeostasis;GO:0046320/

tion of cell fate commitment;GO:0016055//Wnt signaling pathway;GO:0030178//negative regulatic

: process;GO:0008203//cholesterol metabolic process;GO:0019433//triglyceride catabolic process;(
:sponse to endoplasmic reticulum stress;GO:0035023//regulation of Rho protein signal transductio

ninate metabolic process;GO:0006468//protein phosphorylation;GO:0006486//protein glycosylatio

on of oxidative phosphorylation;GO:0006390//transcription from mitochondrial promoter;GO:0006
or signaling pathway;GO:0007189//adenylate cyclase-activating G-protein coupled receptor signa

;port;GO:0033120//positive regulation of RNA splicing;GO:0044767//single-organism developmen

eptidase activity involved in apoptotic process;GO:0006978//DNA damage response, signal transdu

'/lipid biosynthetic process;GO:0016126//sterol biosynthetic process;GO:0045654//positive regulat

GO:0007032//endosome organization;GO:0016185//synaptic vesicle budding from presynaptic enc

etabolic process;GO:0006096//glycolytic process;GO:0051156//glucose 6-phosphate metabolic pr

d glutamate receptor signaling pathway;GO:0048148//behavioral response to cocaine;GO:004874

netabolic process;GO:0042558//pteridine-containing compound metabolic process;GO:0044237//

;ion;GO:1902857//positive regulation of nonmotile primary cilium assembly;GO:1904951//positive

scription, DNA-templated;GO:0006888//ER to Golgi vesicle-mediated transport;GO:0030968//end
sized protein catabolic process;GO:0007005//mitochondrion organization;GO:0008283//cell prolife
small molecule catabolic process;GO:0046039//GTP metabolic process;GO:0046052//UTP catabol
e regulation of endothelial cell migration;GO:0019673//GDP-mannose metabolic process;GO:0042
GO:0043409//negative regulation of MAPK cascade;GO:0050906//detection of stimulus involved i

patterning of blood vessels;GO:0001666//response to hypoxia;GO:0001974//blood vessel remodel
e response;GO:0007186//G-protein coupled receptor signaling pathway;GO:0007204//positive re

2//negative regulation of cell adhesion;GO:0010952//positive regulation of peptidase activity;GO:0
ninate metabolic process;GO:0006468//protein phosphorylation;GO:0006486//protein glycosylation
lipid metabolic process;GO:0006631//fatty acid metabolic process;GO:0006633//fatty acid biosynth

gnal transduction;GO:0035735//intraciliary transport involved in cilium morphogenesis;GO:006027
biquitin-dependent protein catabolic process;GO:0016578//histone deubiquitination;GO:0016579/

:0030336//negative regulation of cell migration;GO:0032570//response to progesterone;GO:0043

asma membrane adhesion molecules;GO:0010631//epithelial cell migration;GO:0031589//cell-subs

phore linkage;GO:0032922//circadian regulation of gene expression;GO:0043153//entrainment of
orphogenesis;GO:0007224//smoothened signaling pathway;GO:0007399//nervous system develop

ulfment;GO:0043277//apoptotic cell clearance;GO:0050766//positive regulation of phagocytosis

osphatidylinositol 3-kinase signaling;GO:0016579//protein deubiquitination;GO:0046580//negative

f neuron projection development;GO:0018105//peptidyl-serine phosphorylation;GO:0032793//po

I promoter;GO:0001889//liver development;GO:0001949//sebaceous gland cell differentiation;GO:
9//protein deubiquitination;GO:0032436//positive regulation of proteasomal ubiquitin-dependent
oliferation;GO:0009617//response to bacterium;GO:0048010//vascular endothelial growth factor re
ylation;GO:0006915//apoptotic process;GO:0031647//regulation of protein stability;GO:0050790//
4//phospholipid biosynthetic process;GO:0010821//regulation of mitochondrion organization;GO:
06782//protoporphyrinogen IX biosynthetic process;GO:0006783//heme biosynthetic process;GO:0

ve regulation of gene expression;GO:0030511//positive regulation of transforming growth factor b

0006914//autophagy;GO:0007399//nervous system development;GO:0017121//phospholipid scra

gnaling pathway in response to oxidative stress;GO:0009267//cellular response to starvation;GO:00

n of endocytosis;GO:0048488//synaptic vesicle endocytosis;GO:0048666//neuron development;GC

rotein catabolic process;GO:0048388//endosomal lumen acidification;GO:0070070//proton-transp

hloride transport;GO:0008272//sulfate transport;GO:0010628//positive regulation of gene expressi
e targeting;GO:0006904//vesicle docking involved in exocytosis;GO:0016192//vesicle-mediated tra

ubiquitin-dependent protein catabolic process;GO:0030970//retrograde protein transport, ER to cy

ic process;GO:0006644//phospholipid metabolic process;GO:0007160//cell-matrix adhesion;GO:0(
ctor beta receptor signaling pathway;GO:0010608//posttranscriptional regulation of gene expressi
ic process;GO:0006644//phospholipid metabolic process;GO:0007160//cell-matrix adhesion;GO:0(

metabolic process;GO:0006650//glycerophospholipid metabolic process;GO:0006654//phosphatid

4765//regulation of ion transmembrane transport;GO:0051260//protein homooligomerization;GO

ninate metabolic process;GO:0006468//protein phosphorylation;GO:0006486//protein glycosylatio

elopment;GO:0002138//retinoic acid biosynthetic process;GO:0006081//cellular aldehyde metabol

assembly;GO:0030036//actin cytoskeleton organization;GO:0045944//positive regulation of transc

nelial cell proliferation;GO:0002719//negative regulation of cytokine production involved in immun

ranscription, DNA-templated;GO:1903507//negative regulation of nucleic acid-templated transcri

rocess;GO:0006633//fatty acid biosynthetic process;GO:0010886//positive regulation of cholester

I promoter;GO:0006355//regulation of transcription, DNA-templated;GO:0006357//regulation of t
ell cycle;GO:0006468//protein phosphorylation;GO:0007147//female meiosis II;GO:0032515//negat

)326//embryonic limb morphogenesis;GO:0048513//animal organ development;GO:0050794//regu

etabolic process;GO:0006096//glycolytic process;GO:0051156//glucose 6-phosphate metabolic pr

pe B pancreatic cell development;GO:0006468//protein phosphorylation;GO:0009615//response to

netabolic process;GO:0042558//pteridine-containing compound metabolic process;GO:0044237//c

ylation;GO:0006915//apoptotic process;GO:0031647//regulation of protein stability;GO:0050790//
regulation of transferase activity;GO:2000480//negative regulation of cAMP-dependent protein ki

21//chloride transport;GO:0034220//ion transmembrane transport;GO:0055085//transmembrane t

ninate metabolic process;GO:0006468//protein phosphorylation;GO:0006486//protein glycosylatio

abolic process;GO:0006631//fatty acid metabolic process;GO:0006679//glucosylceramide biosynth

/extracellular matrix organization;GO:0044869//negative regulation by host of viral exo-alpha-siali

egative regulation of protein lipidation;GO:2001140//positive regulation of phospholipid transpor

tion of cell fate commitment;GO:0016055//Wnt signaling pathway;GO:0030178//negative regulatio
c process;GO:0043335//protein unfolding;GO:0051005//negative regulation of lipoprotein lipase a

.6240//autophagosome docking;GO:0034329//cell junction assembly;GO:0042953//lipoprotein tra

lipid metabolic process;GO:0006631//fatty acid metabolic process;GO:0006633//fatty acid biosynt
nthetic process;GO:0016104//triterpenoid biosynthetic process;GO:0031647//regulation of protei

n of endocytosis;GO:0048488//synaptic vesicle endocytosis;GO:0048666//neuron development;G

sport;GO:0032365//intracellular lipid transport;GO:0042632//cholesterol homeostasis;GO:004632(
tion of transcription, DNA-templated;GO:0006357//regulation of transcription from RNA polymera

of cell proliferation;GO:0010628//positive regulation of gene expression;GO:0016567//protein ubic

ulfment;GO:0043277//apoptotic cell clearance;GO:0050766//positive regulation of phagocytosis

a chemical signal;GO:0009750//response to fructose;GO:0015755//fructose transport;GO:0055085/

biquitin-dependent protein catabolic process;GO:0016578//histone deubiquitination;GO:0016579/

0086073//bundle of His cell-Purkinje myocyte adhesion involved in cell communication;GO:00860

integrin;GO:0061045//negative regulation of wound healing;GO:1901731//positive regulation of p

0006914//autophagy;GO:0007399//nervous system development;GO:0017121//phospholipid scra

e targeting;GO:0006904//vesicle docking involved in exocytosis;GO:0016192//vesicle-mediated tra

//cholesterol transport involved in cholesterol storage;GO:0032367//intracellular cholesterol transp

egulation of canonical Wnt signaling pathway;GO:2000096//positive regulation of Wnt signaling pa

0//nucleolus organization;GO:0016579//protein deubiquitination;GO:0050821//protein stabilizatior

sport;GO:0033120//positive regulation of RNA splicing;GO:0044767//single-organism developmen

r signaling pathway;GO:0007409//axonogenesis;GO:0030900//forebrain development;GO:003818
osphatidylinositol 3-kinase signaling;GO:0016579//protein deubiquitination;GO:0046580//negative
rganization;GO:0042149//cellular response to glucose starvation;GO:0043066//negative regulation

I promoter;GO:0001889//liver development;GO:0001949//sebaceous gland cell differentiation;GO:

eptor protein tyrosine kinase signaling pathway;GO:0009615//response to virus;GO:0043406//posit

I promoter;GO:0001657//ureteric bud development;GO:0001843//neural tube closure;GO:000206

:0030336//negative regulation of cell migration;GO:0032570//response to progesterone;GO:0043
c process;GO:0010025//wax biosynthetic process;GO:0035336//long-chain fatty-acyl-CoA metab

scription, DNA-templated;GO:0006888//ER to Golgi vesicle-mediated transport;GO:0030968//end

ylation;GO:0006915//apoptotic process;GO:0031647//regulation of protein stability;GO:0050790//
orphogenesis;GO:0007224//smoothened signaling pathway;GO:0007399//nervous system develop

0042391//regulation of membrane potential;GO:0043267//negative regulation of potassium ion tr

cle-mediated transport, Golgi to ER;GO:0015031//protein transport;GO:0016192//vesicle-mediate
egative regulation of protein lipidation;GO:2001140//positive regulation of phospholipid transpor

lic process;GO:0009791//post-embryonic development;GO:0010259//multicellular organism aging

4//phospholipid biosynthetic process;GO:0010821//regulation of mitochondrion organization;GO:

8//regulation of epidermal growth factor receptor signaling pathway;GO:0046755//viral budding
orphogenesis;GO:0007224//smoothened signaling pathway;GO:0007399//nervous system develop

c process;GO:0043335//protein unfolding;GO:0051005//negative regulation of lipoprotein lipase a
.6240//autophagosome docking;GO:0034329//cell junction assembly;GO:0042953//lipoprotein tra

cess;GO:0006629//lipid metabolic process;GO:0006633//fatty acid biosynthetic process;GO:00195

e regulation of endothelial cell migration;GO:0019673//GDP-mannose metabolic process;GO:004
oort;GO:0048265//response to pain;GO:0051620//norepinephrine uptake;GO:0055085//transmem

:ion of cell fate commitment;GO:0016055//Wnt signaling pathway;GO:0030178//negative regulatic

)001569//patterning of blood vessels;GO:0001649//osteoblast differentiation;GO:0001708//cell fat

;SU-rRNA, 5.8S rRNA, LSU-rRNA);GO:0000466//maturation of 5.8S rRNA from tricistronic rRNA tra

e-mediated transport;GO:0051592//response to calcium ion;GO:0090110//cargo loading into COP
:tor beta receptor signaling pathway;GO:0010608//posttranscriptional regulation of gene expressic

.6240//autophagosome docking;GO:0034329//cell junction assembly;GO:0042953//lipoprotein tra
55064//chloride ion homeostasis;GO:0055075//potassium ion homeostasis;GO:0055085//transmen

on;GO:0043649//dicarboxylic acid catabolic process;GO:0045225//negative regulation of CD4 bios

ic process;GO:0006644//phospholipid metabolic process;GO:0007160//cell-matrix adhesion;GO:0(

/extracellular matrix organization;GO:0044869//negative regulation by host of viral exo-alpha-siali

//ubiquitin-dependent protein catabolic process;GO:0007409//axonogenesis;GO:0007412//axon t

;port;GO:0033120//positive regulation of RNA splicing;GO:0044767//single-organism developmen

or signaling pathway;GO:0007188//adenylate cyclase-modulating G-protein coupled receptor sig

utamate receptor signaling pathway;GO:0007405//neuroblast proliferation;GO:0019369//arachidor

I promoter;GO:0000902//cell morphogenesis;GO:0001709//cell fate determination;GO:0001764//n

on;GO:0006869//lipid transport;GO:0007283//spermatogenesis;GO:0007399//nervous system deve

of cell proliferation;GO:0010628//positive regulation of gene expression;GO:0016567//protein ubic

oment;GO:0006508//proteolysis;GO:0016525//negative regulation of angiogenesis;GO:0060347//h

one metabolic process;GO:0048729//tissue morphogenesis;GO:0048872//homeostasis of number

integrin;GO:0061045//negative regulation of wound healing;GO:1901731//positive regulation of p
//phospholipase C-activating G-protein coupled receptor signaling pathway;GO:0035176//social l
scription, DNA-templated;GO:0006888//ER to Golgi vesicle-mediated transport;GO:0030968//end
tive regulation by host of viral release from host cell;GO:0045087//innate immune response;GO:00

gulation of cell proliferation;GO:0032956//regulation of actin cytoskeleton organization;GO:00507

58//regulation of epidermal growth factor receptor signaling pathway;GO:0046755//viral budding

eptor protein tyrosine kinase signaling pathway;GO:0009615//response to virus;GO:0043406//posit

I promoter;GO:0001657//ureteric bud development;GO:0001843//neural tube closure;GO:000206

0006914//autophagy;GO:0007399//nervous system development;GO:0017121//phospholipid scra

)042391//regulation of membrane potential;GO:0043267//negative regulation of potassium ion tra

ic process;GO:0006644//phospholipid metabolic process;GO:0007160//cell-matrix adhesion;GO:0(
cell cycle;GO:0010830//regulation of myotube differentiation;GO:0010868//negative regulation o

SU-rRNA, 5.8S rRNA, LSU-rRNA);GO:0000466//maturation of 5.8S rRNA from tricistronic rRNA tra

.958//methotrexate transport;GO:0098838//reduced folate transmembrane transport;GO:1904447/

c process;GO:0010025//wax biosynthetic process;GO:0035336//long-chain fatty-acyl-CoA metabo

O:0007040//lysosome organization;GO:0007286//spermatid development;GO:0008333//endosom

iquitin-dependent protein catabolic process;GO:0016578//histone deubiquitination;GO:0016579/

iosynthetic process;GO:0008542//visual learning;GO:0015936//coenzyme A metabolic process;GO
//phospholipase C-activating G-protein coupled receptor signaling pathway;GO:0035176//social l

)6782//protoporphyrinogen IX biosynthetic process;GO:0006783//heme biosynthetic process;GO:(

›ment;GO:0006508//proteolysis;GO:0016525//negative regulation of angiogenesis;GO:0060347//h
/extracellular matrix organization;GO:0044869//negative regulation by host of viral exo-alpha-siali

//ubiquitin-dependent protein catabolic process;GO:0007409//axonogenesis;GO:0007412//axon t
promoter;GO:1900408//negative regulation of cellular response to oxidative stress;GO:1902083//r

GO:0010468//regulation of gene expression;GO:0010906//regulation of glucose metabolic process

on;GO:0006869//lipid transport;GO:0007283//spermatogenesis;GO:0007399//nervous system deve

0001569//patterning of blood vessels;GO:0001649//osteoblast differentiation;GO:0001708//cell fat
I promoter;GO:0007379//segment specification;GO:0019827//stem cell population maintenance;G

orylation;GO:0018108//peptidyl-tyrosine phosphorylation;GO:0030282//bone mineralization;GO:0

I promoter;GO:0001657//ureteric bud development;GO:0001843//neural tube closure;GO:000206

er;GO:0009062//fatty acid catabolic process;GO:0019432//triglyceride biosynthetic process

eptor protein tyrosine kinase signaling pathway;GO:0009615//response to virus;GO:0043406//posit

ubiquitin-dependent protein catabolic process;GO:0030970//retrograde protein transport, ER to cy

.6240//autophagosome docking;GO:0034329//cell junction assembly;GO:0042953//lipoprotein tra

olic process;GO:0046485//ether lipid metabolic process;GO:0060395//SMAD protein signal transdu

2//negative regulation of cell adhesion;GO:0010952//positive regulation of peptidase activity;GO:0

e regulation of endothelial cell migration;GO:0019673//GDP-mannose metabolic process;GO:004[illegible]

tion;GO:0007052//mitotic spindle organization;GO:0007409//axonogenesis;GO:0009615//respons[illegible]
c process;GO:0043335//protein unfolding;GO:0051005//negative regulation of lipoprotein lipase a

d glutamate receptor signaling pathway;GO:0048148//behavioral response to cocaine;GO:004874[illegible]
routing angiogenesis;GO:0006816//calcium ion transport;GO:0006886//intracellular protein trans[illegible]

sport;GO:0055085//transmembrane transport;GO:1990830//cellular response to leukemia inhibitor

xia;GO:0001889//liver development;GO:0002314//germinal center B cell differentiation;GO:00026[illegible]

mere organization;GO:0051639//actin filament network formation;GO:0055003//cardiac myofibril [illegible]

or signaling pathway;GO:0007218//neuropeptide signaling pathway;GO:0008217//regulation of bl

on of sour taste;GO:0006812//cation transport;GO:0007224//smoothened signaling pathway;GO:0[illegible]

cromolecule metabolic process;GO:0090502//RNA phosphodiester bond hydrolysis, endonucleolyt

etabolic process;GO:0006096//glycolytic process;GO:0051156//glucose 6-phosphate metabolic pr[illegible]

sport;GO:0055085//transmembrane transport;GO:1990830//cellular response to leukemia inhibitor

d glutamate receptor signaling pathway;GO:0048148//behavioral response to cocaine;GO:004874[illegible]

nbination;GO:0006508//proteolysis;GO:0008152//metabolic process;GO:0015074//DNA integration

O:0007040//lysosome organization;GO:0007286//spermatid development;GO:0008333//endosom

l promoter;GO:0006418//tRNA aminoacylation for protein translation;GO:0006434//seryl-tRNA am

2//negative regulation of cell adhesion;GO:0010952//positive regulation of peptidase activity;GO:0

GO:0007032//endosome organization;GO:0016185//synaptic vesicle budding from presynaptic enc
ed in ureteric bud morphogenesis;GO:0001822//kidney development;GO:0003231//cardiac ventricl

utamate receptor signaling pathway;GO:0007405//neuroblast proliferation;GO:0019369//arachidor

nthetic process;GO:0016104//triterpenoid biosynthetic process;GO:0031647//regulation of proteir
orylation;GO:0018108//peptidyl-tyrosine phosphorylation;GO:0030282//bone mineralization;GO:0

posterior axis, embryo;GO:0007368//determination of left/right symmetry;GO:0009100//glycoprot

rotein catabolic process;GO:0048388//endosomal lumen acidification;GO:0070070//proton-transp
ylation;GO:0006915//apoptotic process;GO:0031647//regulation of protein stability;GO:0050790//

eptor protein tyrosine kinase signaling pathway;GO:0009615//response to virus;GO:0043406//posit
orylation;GO:0018108//peptidyl-tyrosine phosphorylation;GO:0030282//bone mineralization;GO:0

ion;GO:1902857//positive regulation of nonmotile primary cilium assembly;GO:1904951//positive
one metabolic process;GO:0048729//tissue morphogenesis;GO:0048872//homeostasis of number
eptidase activity involved in apoptotic process;GO:0006978//DNA damage response, signal transd
l promoter;GO:0001657//ureteric bud development;GO:0001843//neural tube closure;GO:000206
GO:0010508//positive regulation of autophagy;GO:0016567//protein ubiquitination;GO:0034198//
0006914//autophagy;GO:0007399//nervous system development;GO:0017121//phospholipid scra

)//nucleolus organization;GO:0016579//protein deubiquitination;GO:0050821//protein stabilizatior

egulation of canonical Wnt signaling pathway;GO:2000096//positive regulation of Wnt signaling p
rganization;GO:0042149//cellular response to glucose starvation;GO:0043066//negative regulation
)//protein deubiquitination;GO:0032436//positive regulation of proteasomal ubiquitin-dependent

f neuron projection development;GO:0018105//peptidyl-serine phosphorylation;GO:0032793//po

elopment;GO:0002138//retinoic acid biosynthetic process;GO:0006081//cellular aldehyde metabol
f neuron projection development;GO:0018105//peptidyl-serine phosphorylation;GO:0032793//po

//protein deubiquitination;GO:0032436//positive regulation of proteasomal ubiquitin-dependent

O:0006955//immune response;GO:0007155//cell adhesion;GO:0007160//cell-matrix adhesion;GO:
066//regulation of phosphatidylinositol 3-kinase signaling;GO:0035304//regulation of protein dep

//regulation of epidermal growth factor receptor signaling pathway;GO:0043408//regulation of MA

sport;GO:0055085//transmembrane transport;GO:1990830//cellular response to leukemia inhibitor
ocess;GO:0009086//methionine biosynthetic process;GO:0031060//regulation of histone methylatio

or signaling pathway;GO:0007218//neuropeptide signaling pathway;GO:0008217//regulation of bl

rotein catabolic process;GO:0048388//endosomal lumen acidification;GO:0070070//proton-transp

//oocyte differentiation;GO:0042127//regulation of cell proliferation;GO:0048839//inner ear devel
hloride transport;GO:0008272//sulfate transport;GO:0010628//positive regulation of gene expressi
orce of heart contraction;GO:0006816//calcium ion transport;GO:0006874//cellular calcium ion ho
SU-rRNA, 5.8S rRNA, LSU-rRNA);GO:0000466//maturation of 5.8S rRNA from tricistronic rRNA tra

0042391//regulation of membrane potential;GO:0043267//negative regulation of potassium ion tra

bination;GO:0006508//proteolysis;GO:0008152//metabolic process;GO:0015074//DNA integration

d glutamate receptor signaling pathway;GO:0048148//behavioral response to cocaine;GO:004874

g;GO:0030261//chromosome condensation;GO:0045910//negative regulation of DNA recombinat
etabolic process;GO:0042558//pteridine-containing compound metabolic process;GO:0044237//

lic process;GO:0009791//post-embryonic development;GO:0010259//multicellular organism aging

response to bacterium;GO:0015909//long-chain fatty acid transport;GO:0042632//cholesterol hom

)6782//protoporphyrinogen IX biosynthetic process;GO:0006783//heme biosynthetic process;GO:(

cess;GO:0006629//lipid metabolic process;GO:0006633//fatty acid biosynthetic process;GO:00195
!//negative regulation of cell adhesion;GO:0010952//positive regulation of peptidase activity;GO:0

iquitin-dependent protein catabolic process;GO:0016578//histone deubiquitination;GO:0016579/

on;GO:0006869//lipid transport;GO:0007283//spermatogenesis;GO:0007399//nervous system deve

one metabolic process;GO:0048729//tissue morphogenesis;GO:0048872//homeostasis of number

olic process;GO:0046485//ether lipid metabolic process;GO:0060395//SMAD protein signal transdu

3//ER-associated ubiquitin-dependent protein catabolic process;GO:0032781//positive regulation
nthetic process;GO:0016104//triterpenoid biosynthetic process;GO:0031647//regulation of protein
tion;GO:0033383//geranyl diphosphate metabolic process;GO:0045339//farnesyl diphosphate cata
I promoter;GO:0001568//blood vessel development;GO:0001570//vasculogenesis;GO:0003150//m

one metabolic process;GO:0048729//tissue morphogenesis;GO:0048872//homeostasis of number
ation;GO:0090305//nucleic acid phosphodiester bond hydrolysis;GO:0090502//RNA phosphodiest

tion;GO:0007052//mitotic spindle organization;GO:0007409//axonogenesis;GO:0009615//response
oxidative stress;GO:0008104//protein localization;GO:0008203//cholesterol metabolic process;GO:

abolic process;GO:0006631//fatty acid metabolic process;GO:0006679//glucosylceramide biosynth
n-dependent cell spreading;GO:0061001//regulation of dendritic spine morphogenesis;GO:00610

otein catabolic process;GO:0048388//endosomal lumen acidification;GO:0070070//proton-transp

I shape;GO:0042742//defense response to bacterium;GO:0045089//positive regulation of innate ir
lipid metabolic process;GO:0016311//dephosphorylation;GO:0035335//peptidyl-tyrosine dephosp

tion;GO:0007052//mitotic spindle organization;GO:0007409//axonogenesis;GO:0009615//response

;//glycoprotein catabolic process;GO:0016567//protein ubiquitination;GO:0030433//ER-associated

n;GO:0019805//quinolinate biosynthetic process;GO:0046686//response to cadmium ion;GO:0070
)2//negative regulation of bone mineralization;GO:0050727//regulation of inflammatory response;
sponse to endoplasmic reticulum stress;GO:0035023//regulation of Rho protein signal transductio
)//protein deubiquitination;GO:0032436//positive regulation of proteasomal ubiquitin-dependent

lation of cell migration;GO:0046464//acylglycerol catabolic process;GO:0046889//positive regulati
f neuron projection development;GO:0018105//peptidyl-serine phosphorylation;GO:0032793//po

sport;GO:0032365//intracellular lipid transport;GO:0042632//cholesterol homeostasis;GO:0046320/

lysis;GO:0006915//apoptotic process;GO:0016540//protein autoprocessing;GO:0030855//epithelial

or signaling pathway;GO:0007189//adenylate cyclase-activating G-protein coupled receptor signa
elopment;GO:0002138//retinoic acid biosynthetic process;GO:0006081//cellular aldehyde metabol

//oocyte differentiation;GO:0042127//regulation of cell proliferation;GO:0048839//inner ear develo

rotein dephosphorylation;GO:0016311//dephosphorylation;GO:0043542//endothelial cell migratio

sport;GO:0055085//transmembrane transport;GO:1990830//cellular response to leukemia inhibitor

ılfment;GO:0043277//apoptotic cell clearance;GO:0050766//positive regulation of phagocytosis

ess;GO:0006593//ornithine catabolic process;GO:0019240//citrulline biosynthetic process;GO:0097

lipid metabolic process;GO:0006631//fatty acid metabolic process;GO:0006633//fatty acid biosynth

;8//regulation of epidermal growth factor receptor signaling pathway;GO:0046755//viral budding

gnal transduction;GO:0035735//intraciliary transport involved in cilium morphogenesis;GO:006027
orphogenesis;GO:0007224//smoothened signaling pathway;GO:0007399//nervous system develop

)45723//positive regulation of fatty acid biosynthetic process;GO:0051258//protein polymerization

eptidase activity involved in apoptotic process;GO:0006978//DNA damage response, signal transd

one metabolic process;GO:0048729//tissue morphogenesis;GO:0048872//homeostasis of number
phore linkage;GO:0032922//circadian regulation of gene expression;GO:0043153//entrainment of

rocess;GO:0006633//fatty acid biosynthetic process;GO:0006636//unsaturated fatty acid biosynthe

ylation;GO:0006915//apoptotic process;GO:0031647//regulation of protein stability;GO:0050790//

optotic process;GO:0043154//negative regulation of cysteine-type endopeptidase activity involved

orylation;GO:0018108//peptidyl-tyrosine phosphorylation;GO:0030282//bone mineralization;GO:0

iquitin-dependent protein catabolic process;GO:0016578//histone deubiquitination;GO:0016579/

f neuron projection development;GO:0018105//peptidyl-serine phosphorylation;GO:0032793//po

)//protein deubiquitination;GO:0032436//positive regulation of proteasomal ubiquitin-dependent
4//phospholipid biosynthetic process;GO:0010821//regulation of mitochondrion organization;GO:

gnaling pathway in response to oxidative stress;GO:0009267//cellular response to starvation;GO:0(

l promoter;GO:0001568//blood vessel development;GO:0001570//vasculogenesis;GO:0003150//m

iosynthetic process;GO:0008542//visual learning;GO:0015936//coenzyme A metabolic process;GO
c process;GO:0010025//wax biosynthetic process;GO:0035336//long-chain fatty-acyl-CoA metab(

l shape;GO:0042742//defense response to bacterium;GO:0045089//positive regulation of innate ir

tion;GO:0007052//mitotic spindle organization;GO:0007409//axonogenesis;GO:0009615//response

0017158//regulation of calcium ion-dependent exocytosis;GO:0032024//positive regulation of ins

rotein catabolic process;GO:0048388//endosomal lumen acidification;GO:0070070//proton-transp

or signaling pathway;GO:0007218//neuropeptide signaling pathway;GO:0008217//regulation of bl

GO:0008544//epidermis development;GO:0010467//gene expression;GO:0036303//lymph vessel m
//ubiquitin-dependent protein catabolic process;GO:0007409//axonogenesis;GO:0007412//axon t

4765//regulation of ion transmembrane transport;GO:0051260//protein homooligomerization;GO
ive regulation of apoptotic process;GO:0045727//positive regulation of translation;GO:0045786//n
tor beta receptor signaling pathway;GO:0010608//posttranscriptional regulation of gene expressi(

gulation of glucose metabolic process;GO:0014065//phosphatidylinositol 3-kinase signaling;GO:0(

e expression;GO:0030001//metal ion transport;GO:0031398//positive regulation of protein ubiquit

sport;GO:0055085//transmembrane transport;GO:1990830//cellular response to leukemia inhibitor

netabolic process;GO:0042558//pteridine-containing compound metabolic process;GO:0044237//c
)//nucleolus organization;GO:0016579//protein deubiquitination;GO:0050821//protein stabilizatior

ıbination;GO:0006508//proteolysis;GO:0008152//metabolic process;GO:0015074//DNA integration

:ion of cell fate commitment;GO:0016055//Wnt signaling pathway;GO:0030178//negative regulatic

RNA secondary structure unwinding;GO:0016525//negative regulation of angiogenesis;GO:00310
egative regulation of protein lipidation;GO:2001140//positive regulation of phospholipid transpor
3//ER-associated ubiquitin-dependent protein catabolic process;GO:0032781//positive regulation

lic process;GO:0008654//phospholipid biosynthetic process;GO:0030258//lipid modification;GO:00
orphogenesis;GO:0007224//smoothened signaling pathway;GO:0007399//nervous system develop
ptor protein tyrosine kinase signaling pathway;GO:0009615//response to virus;GO:0043406//posit

losure;GO:0007389//pattern specification process;GO:0007398//ectoderm development;GO:00074

ɔ DNA damage stimulus;GO:0007283//spermatogenesis;GO:0032434//regulation of proteasomal u

l promoter;GO:0001657//ureteric bud development;GO:0001843//neural tube closure;GO:000206

lysis;GO:0006915//apoptotic process;GO:0016540//protein autoprocessing;GO:0030855//epithelial

abolic process;GO:0006631//fatty acid metabolic process;GO:0006679//glucosylceramide biosynth

on of sour taste;GO:0006812//cation transport;GO:0007224//smoothened signaling pathway;GO:0

orylation;GO:0018108//peptidyl-tyrosine phosphorylation;GO:0030282//bone mineralization;GO:0

n of endocytosis;GO:0048488//synaptic vesicle endocytosis;GO:0048666//neuron development;G

e regulation of endothelial cell migration;GO:0019673//GDP-mannose metabolic process;GO:004

›iquitin-dependent protein catabolic process;GO:0016578//histone deubiquitination;GO:0016579/

ive regulation of sequestering of triglyceride;GO:0010898//positive regulation of triglyceride catab
phore linkage;GO:0032922//circadian regulation of gene expression;GO:0043153//entrainment of
sport;GO:0032365//intracellular lipid transport;GO:0042632//cholesterol homeostasis;GO:0046320

n-dependent cell spreading;GO:0061001//regulation of dendritic spine morphogenesis;GO:00610

al glia guided migration;GO:0030335//positive regulation of cell migration;GO:0031022//nuclear n

:le-mediated transport, Golgi to ER;GO:0015031//protein transport;GO:0016192//vesicle-mediate

or signaling pathway;GO:0007189//adenylate cyclase-activating G-protein coupled receptor signa

:lopment;GO:0002138//retinoic acid biosynthetic process;GO:0006081//cellular aldehyde metabol

c process;GO:0010025//wax biosynthetic process;GO:0035336//long-chain fatty-acyl-CoA metab

ic process;GO:0006644//phospholipid metabolic process;GO:0007160//cell-matrix adhesion;GO:0

.958//methotrexate transport;GO:0098838//reduced folate transmembrane transport;GO:1904447/

n of endocytosis;GO:0048488//synaptic vesicle endocytosis;GO:0048666//neuron development;GC

gulation of glucose metabolic process;GO:0014065//phosphatidylinositol 3-kinase signaling;GO:0(
4765//regulation of ion transmembrane transport;GO:0051260//protein homooligomerization;GO
abolic process;GO:0007586//digestion;GO:0008202//steroid metabolic process;GO:0008207//C21-s

i8//regulation of epidermal growth factor receptor signaling pathway;GO:0046755//viral budding

:0030336//negative regulation of cell migration;GO:0032570//response to progesterone;GO:0043

egative regulation of protein lipidation;GO:2001140//positive regulation of phospholipid transport

promoter;GO:1900408//negative regulation of cellular response to oxidative stress;GO:1902083//r

e regulation of endothelial cell migration;GO:0019673//GDP-mannose metabolic process;GO:0042
scription, DNA-templated;GO:0006888//ER to Golgi vesicle-mediated transport;GO:0030968//end
oment;GO:0006508//proteolysis;GO:0016525//negative regulation of angiogenesis;GO:0060347//h

of protein phosphorylation;GO:0007507//heart development;GO:0008284//positive regulation of
e expression;GO:0030001//metal ion transport;GO:0031398//positive regulation of protein ubiquit
cle-mediated transport, Golgi to ER;GO:0015031//protein transport;GO:0016192//vesicle-mediate

4//phospholipid biosynthetic process;GO:0010821//regulation of mitochondrion organization;GO:

projection development;GO:0016055//Wnt signaling pathway;GO:0021516//dorsal spinal cord de

I promoter;GO:0001889//liver development;GO:0001949//sebaceous gland cell differentiation;GO:

gnaling pathway in response to oxidative stress;GO:0009267//cellular response to starvation;GO:00

rocess;GO:0006633//fatty acid biosynthetic process;GO:0010886//positive regulation of cholesterc

sphatidylinositol 3-kinase signaling;GO:0016579//protein deubiquitination;GO:0046580//negative

21//chloride transport;GO:0034220//ion transmembrane transport;GO:0055085//transmembrane tı
metabolic process;GO:0006650//glycerophospholipid metabolic process;GO:0006654//phosphatid

iquitin-dependent protein catabolic process;GO:0016578//histone deubiquitination;GO:0016579//

.6240//autophagosome docking;GO:0034329//cell junction assembly;GO:0042953//lipoprotein tra

assembly;GO:0030036//actin cytoskeleton organization;GO:0045944//positive regulation of transc

port;GO:0033120//positive regulation of RNA splicing;GO:0044767//single-organism developmen

ctor beta receptor signaling pathway;GO:0010608//posttranscriptional regulation of gene expressic
e regulation of endothelial cell migration;GO:0019673//GDP-mannose metabolic process;GO:004Z

e B pancreatic cell development;GO:0006468//protein phosphorylation;GO:0009615//response tc

regulation of transferase activity;GO:2000480//negative regulation of cAMP-dependent protein kir
ositive regulation of transcription from RNA polymerase II promoter;GO:0048384//retinoic acid re
netabolic process;GO:0042558//pteridine-containing compound metabolic process;GO:0044237//c
lipid metabolic process;GO:0006631//fatty acid metabolic process;GO:0006633//fatty acid biosynth
n of endocytosis;GO:0048488//synaptic vesicle endocytosis;GO:0048666//neuron development;GC

on transmembrane transport;GO:0050909//sensory perception of taste;GO:0055085//transmembra

ide cross-linking;GO:0030216//keratinocyte differentiation;GO:0045861//negative regulation of pr

ulfment;GO:0043277//apoptotic cell clearance;GO:0050766//positive regulation of phagocytosis

inate metabolic process;GO:0006468//protein phosphorylation;GO:0006486//protein glycosylatio

DNA damage stimulus;GO:0007283//spermatogenesis;GO:0032434//regulation of proteasomal

l promoter;GO:0001568//blood vessel development;GO:0001570//vasculogenesis;GO:0003150//m

e expression;GO:0030001//metal ion transport;GO:0031398//positive regulation of protein ubiquit

l promoter;GO:0006355//regulation of transcription, DNA-templated;GO:0009267//cellular respor
or signaling pathway;GO:0007218//neuropeptide signaling pathway;GO:0008217//regulation of bl
//phospholipase C-activating G-protein coupled receptor signaling pathway;GO:0035176//social l

//nucleolus organization;GO:0016579//protein deubiquitination;GO:0050821//protein stabilizatior
//regulation of MAPK cascade;GO:0045761//regulation of adenylate cyclase activity;GO:2000479//

oxidative stress;GO:0008104//protein localization;GO:0008203//cholesterol metabolic process;GO:

small molecule catabolic process;GO:0046039//GTP metabolic process;GO:0046052//UTP catabol

phore linkage;GO:0032922//circadian regulation of gene expression;GO:0043153//entrainment of
)2//negative regulation of bone mineralization;GO:0050727//regulation of inflammatory response;

egative regulation of protein lipidation;GO:2001140//positive regulation of phospholipid transpor

58//regulation of epidermal growth factor receptor signaling pathway;GO:0046755//viral budding

58//regulation of epidermal growth factor receptor signaling pathway;GO:0046755//viral budding
scription, DNA-templated;GO:0006888//ER to Golgi vesicle-mediated transport;GO:0030968//end

)2//negative regulation of bone mineralization;GO:0050727//regulation of inflammatory response;
utamate receptor signaling pathway;GO:0007405//neuroblast proliferation;GO:0019369//arachidor

e regulation of endothelial cell migration;GO:0019673//GDP-mannose metabolic process;GO:004

phore linkage;GO:0032922//circadian regulation of gene expression;GO:0043153//entrainment of

gnaling pathway in response to oxidative stress;GO:0009267//cellular response to starvation;GO:0(
0006914//autophagy;GO:0007399//nervous system development;GO:0017121//phospholipid scra

e targeting;GO:0006904//vesicle docking involved in exocytosis;GO:0016192//vesicle-mediated tra
rotein dephosphorylation;GO:0016311//dephosphorylation;GO:0043542//endothelial cell migratic

I promoter;GO:0001889//liver development;GO:0001949//sebaceous gland cell differentiation;GO:
//lipid biosynthetic process;GO:0016126//sterol biosynthetic process;GO:0045654//positive regulat
egative regulation of protein lipidation;GO:2001140//positive regulation of phospholipid transport
e expression;GO:0030001//metal ion transport;GO:0031398//positive regulation of protein ubiquit
osphatidylinositol 3-kinase signaling;GO:0016579//protein deubiquitination;GO:0046580//negative

sport;GO:0033120//positive regulation of RNA splicing;GO:0044767//single-organism developmen

metabolic process;GO:0006650//glycerophospholipid metabolic process;GO:0006654//phosphatid
orphogenesis;GO:0007224//smoothened signaling pathway;GO:0007399//nervous system develop

n-dependent cell spreading;GO:0061001//regulation of dendritic spine morphogenesis;GO:00610

oe B pancreatic cell development;GO:0006468//protein phosphorylation;GO:0009615//response to

regulation of transferase activity;GO:2000480//negative regulation of cAMP-dependent protein kir

or signaling pathway;GO:0007188//adenylate cyclase-modulating G-protein coupled receptor sig

ranscription, DNA-templated;GO:1903507//negative regulation of nucleic acid-templated transcrip

rocess;GO:0006633//fatty acid biosynthetic process;GO:0006636//unsaturated fatty acid biosynthe

ɔptotic process;GO:0043154//negative regulation of cysteine-type endopeptidase activity involvec

ın of endocytosis;GO:0048488//synaptic vesicle endocytosis;GO:0048666//neuron development;GC

projection development;GO:0016055//Wnt signaling pathway;GO:0021516//dorsal spinal cord de

ɔse assembly;GO:0008611//ether lipid biosynthetic process;GO:0021587//cerebellum morphogene

ɔcess;GO:0009086//methionine biosynthetic process;GO:0031060//regulation of histone methylatic

g;GO:0030261//chromosome condensation;GO:0045910//negative regulation of DNA recombinat

hagy;GO:0007029//endoplasmic reticulum organization;GO:0007031//peroxisome organization;G(
or signaling pathway;GO:0007189//adenylate cyclase-activating G-protein coupled receptor signa

cle-mediated transport, Golgi to ER;GO:0015031//protein transport;GO:0016192//vesicle-mediate

egative regulation of protein lipidation;GO:2001140//positive regulation of phospholipid transport

or signaling pathway;GO:0007218//neuropeptide signaling pathway;GO:0008217//regulation of bl
ninate metabolic process;GO:0006468//protein phosphorylation;GO:0006486//protein glycosylatio

tion;GO:0007052//mitotic spindle organization;GO:0007409//axonogenesis;GO:0009615//respons

ıetabolic process;GO:0042558//pteridine-containing compound metabolic process;GO:0044237//[illegible]

ɔment;GO:0006508//proteolysis;GO:0016525//negative regulation of angiogenesis;GO:0060347//h[illegible]

ɔliferation;GO:0009617//response to bacterium;GO:0048010//vascular endothelial growth factor re[illegible]

tive regulation by host of viral release from host cell;GO:0045087//innate immune response;GO:00[illegible]

cell cycle;GO:0010830//regulation of myotube differentiation;GO:0010868//negative regulation o[illegible]

egulation of canonical Wnt signaling pathway;GO:2000096//positive regulation of Wnt signaling p[illegible]
.6240//autophagosome docking;GO:0034329//cell junction assembly;GO:0042953//lipoprotein tra[illegible]

sport;GO:0033120//positive regulation of RNA splicing;GO:0044767//single-organism developmen[illegible]

i8//regulation of epidermal growth factor receptor signaling pathway;GO:0046755//viral budding

n of endocytosis;GO:0048488//synaptic vesicle endocytosis;GO:0048666//neuron development;G[illegible]

cle-mediated transport, Golgi to ER;GO:0015031//protein transport;GO:0016192//vesicle-mediate[illegible]

elopment;GO:0002138//retinoic acid biosynthetic process;GO:0006081//cellular aldehyde metabol[illegible]

i//glycoprotein catabolic process;GO:0016567//protein ubiquitination;GO:0030433//ER-associated
3//ER-associated ubiquitin-dependent protein catabolic process;GO:0032781//positive regulation

elopment;GO:0002138//retinoic acid biosynthetic process;GO:0006081//cellular aldehyde metabol[illegible]

scription, DNA-templated;GO:0006888//ER to Golgi vesicle-mediated transport;GO:0030968//end

ıbolic process;GO:0007586//digestion;GO:0008202//steroid metabolic process;GO:0008207//C21-
orphogenesis;GO:0007224//smoothened signaling pathway;GO:0007399//nervous system develop

n-dependent cell spreading;GO:0061001//regulation of dendritic spine morphogenesis;GO:00610
I promoter;GO:0007379//segment specification;GO:0019827//stem cell population maintenance;G

ell cycle;GO:0006468//protein phosphorylation;GO:0007147//female meiosis II;GO:0032515//negat

er;GO:0009062//fatty acid catabolic process;GO:0019432//triglyceride biosynthetic process
n of endocytosis;GO:0048488//synaptic vesicle endocytosis;GO:0048666//neuron development;G
rocess;GO:0006633//fatty acid biosynthetic process;GO:0006636//unsaturated fatty acid biosynthe
orylation;GO:0018108//peptidyl-tyrosine phosphorylation;GO:0030282//bone mineralization;GO:0

//phospholipase C-activating G-protein coupled receptor signaling pathway;GO:0035176//social
06782//protoporphyrinogen IX biosynthetic process;GO:0006783//heme biosynthetic process;GO:

optotic process;GO:0043154//negative regulation of cysteine-type endopeptidase activity involved

phore linkage;GO:0032922//circadian regulation of gene expression;GO:0043153//entrainment of

process;GO:0006189//'de novo' IMP biosynthetic process;GO:0009152//purine ribonucleotide bios

ninate metabolic process;GO:0006468//protein phosphorylation;GO:0006486//protein glycosylation
//inflammatory response;GO:0006956//complement activation;GO:0010575//positive regulation of
on of oxidative phosphorylation;GO:0006390//transcription from mitochondrial promoter;GO:0006
f neuron projection development;GO:0018105//peptidyl-serine phosphorylation;GO:0032793//po
O:0007252//I-kappaB phosphorylation;GO:0008630//intrinsic apoptotic signaling pathway in respo

)//protein deubiquitination;GO:0032436//positive regulation of proteasomal ubiquitin-dependent
eptidase activity involved in apoptotic process;GO:0006978//DNA damage response, signal transd

egative regulation of protein lipidation;GO:2001140//positive regulation of phospholipid transport

tion;GO:0007052//mitotic spindle organization;GO:0007409//axonogenesis;GO:0009615//response
0006914//autophagy;GO:0007399//nervous system development;GO:0017121//phospholipid scra

rotein catabolic process;GO:0048388//endosomal lumen acidification;GO:0070070//proton-transp
e activation involved in immune response;GO:0007186//G-protein coupled receptor signaling pat

sport;GO:0055085//transmembrane transport;GO:1990830//cellular response to leukemia inhibitor
cromolecule metabolic process;GO:0090502//RNA phosphodiester bond hydrolysis, endonucleolyt

tive regulation by host of viral release from host cell;GO:0045087//innate immune response;GO:00
2//negative regulation of cell adhesion;GO:0010952//positive regulation of peptidase activity;GO:0

eptidase activity involved in apoptotic process;GO:0006978//DNA damage response, signal transdu

xia;GO:0001889//liver development;GO:0002314//germinal center B cell differentiation;GO:00026[illegible]

pe B pancreatic cell development;GO:0006468//protein phosphorylation;GO:0009615//response to

0017158//regulation of calcium ion-dependent exocytosis;GO:0032024//positive regulation of ins
sphorylation;GO:0046856//phosphatidylinositol dephosphorylation;GO:0048488//synaptic vesicle
regulation of transferase activity;GO:2000480//negative regulation of cAMP-dependent protein ki

ve regulation of gene expression;GO:0030511//positive regulation of transforming growth factor b
d glutamate receptor signaling pathway;GO:0048148//behavioral response to cocaine;GO:004874
ed in ureteric bud morphogenesis;GO:0001822//kidney development;GO:0003231//cardiac ventricl

ive regulation of apoptotic process;GO:0045727//positive regulation of translation;GO:0045786//n

etabolic process;GO:0006096//glycolytic process;GO:0051156//glucose 6-phosphate metabolic pr

ion of cell fate commitment;GO:0016055//Wnt signaling pathway;GO:0030178//negative regulatic

o starvation;GO:0044090//positive regulation of vacuole organization;GO:2000786//positive regula

netabolic process;GO:0042558//pteridine-containing compound metabolic process;GO:0044237//

I promoter;GO:0006418//tRNA aminoacylation for protein translation;GO:0006434//seryl-tRNA am

032342//aldosterone biosynthetic process;GO:0032870//cellular response to hormone stimulus;GO
06782//protoporphyrinogen IX biosynthetic process;GO:0006783//heme biosynthetic process;GO:

phore linkage;GO:0032922//circadian regulation of gene expression;GO:0043153//entrainment of
olic process;GO:0046485//ether lipid metabolic process;GO:0060395//SMAD protein signal transd

3//ER-associated ubiquitin-dependent protein catabolic process;GO:0032781//positive regulation

lic process;GO:0008654//phospholipid biosynthetic process;GO:0030258//lipid modification;GO:00

phore linkage;GO:0032922//circadian regulation of gene expression;GO:0043153//entrainment of

c process;GO:0006869//lipid transport;GO:0006886//intracellular protein transport;GO:0010875//p
//inflammatory response;GO:0006956//complement activation;GO:0010575//positive regulation of
orylation;GO:0018108//peptidyl-tyrosine phosphorylation;GO:0030282//bone mineralization;GO:0

O:0007252//I-kappaB phosphorylation;GO:0008630//intrinsic apoptotic signaling pathway in respo
losure;GO:0007389//pattern specification process;GO:0007398//ectoderm development;GO:00074
f neuron projection development;GO:0018105//peptidyl-serine phosphorylation;GO:0032793//po
GO:0010508//positive regulation of autophagy;GO:0016567//protein ubiquitination;GO:0034198//

gnaling pathway in response to oxidative stress;GO:0009267//cellular response to starvation;GO:00

//protein deubiquitination;GO:0032436//positive regulation of proteasomal ubiquitin-dependent
gulation of cell proliferation;GO:0032956//regulation of actin cytoskeleton organization;GO:00507
8//regulation of epidermal growth factor receptor signaling pathway;GO:0046755//viral budding

athway;GO:0009888//tissue development;GO:0030198//extracellular matrix organization;GO:00713

egulation of transcription elongation from RNA polymerase II promoter;GO:0045893//positive regu
326//embryonic limb morphogenesis;GO:0048513//animal organ development;GO:0050794//regu

cle-mediated transport, Golgi to ER;GO:0015031//protein transport;GO:0016192//vesicle-mediate
c process;GO:0010025//wax biosynthetic process;GO:0035336//long-chain fatty-acyl-CoA metabo

egulation of canonical Wnt signaling pathway;GO:2000096//positive regulation of Wnt signaling p
damage response, signal transduction by p53 class mediator;GO:0032287//peripheral nervous syst

rotein catabolic process;GO:0048388//endosomal lumen acidification;GO:0070070//proton-transp

al glia guided migration;GO:0030335//positive regulation of cell migration;GO:0031022//nuclear n

or signaling pathway;GO:0007189//adenylate cyclase-activating G-protein coupled receptor signa
sport;GO:0055085//transmembrane transport;GO:1990830//cellular response to leukemia inhibitor

elopment;GO:0002138//retinoic acid biosynthetic process;GO:0006081//cellular aldehyde metabol

2//negative regulation of cell adhesion;GO:0010952//positive regulation of peptidase activity;GO:0

tion of cell fate commitment;GO:0016055//Wnt signaling pathway;GO:0030178//negative regulatio

xia;GO:0001889//liver development;GO:0002314//germinal center B cell differentiation;GO:00026
GO:0007626//locomotory behavior;GO:0016477//cell migration;GO:0098609//cell-cell adhesion

one metabolic process;GO:0048729//tissue morphogenesis;GO:0048872//homeostasis of number

d glutamate receptor signaling pathway;GO:0048148//behavioral response to cocaine;GO:004874

or signaling pathway;GO:0007189//adenylate cyclase-activating G-protein coupled receptor signa

GO:0007626//locomotory behavior;GO:0016477//cell migration;GO:0098609//cell-cell adhesion

zation;GO:0009235//cobalamin metabolic process;GO:0015889//cobalamin transport;GO:0043001

GO:0006107//oxaloacetate metabolic process;GO:0006629//lipid metabolic process;GO:0009617//r
port;GO:0048265//response to pain;GO:0051620//norepinephrine uptake;GO:0055085//transmeml
metabolic process;GO:0006650//glycerophospholipid metabolic process;GO:0006654//phosphatid

cess;GO:0006629//lipid metabolic process;GO:0006633//fatty acid biosynthetic process;GO:001953
eptidase activity involved in apoptotic process;GO:0006978//DNA damage response, signal transdu

O:0006955//immune response;GO:0007155//cell adhesion;GO:0007160//cell-matrix adhesion;GO:

03//cholesterol metabolic process;GO:0010468//regulation of gene expression;GO:0010977//nega

sphatidylinositol 3-kinase signaling;GO:0016579//protein deubiquitination;GO:0046580//negative
r signaling pathway;GO:0007409//axonogenesis;GO:0030900//forebrain development;GO:003818

l promoter;GO:0001889//liver development;GO:0001949//sebaceous gland cell differentiation;GO:

lation of cell migration;GO:0046464//acylglycerol catabolic process;GO:0046889//positive regulati
ranscription, DNA-templated;GO:1903507//negative regulation of nucleic acid-templated transcri

)6782//protoporphyrinogen IX biosynthetic process;GO:0006783//heme biosynthetic process;GO:

0006914//autophagy;GO:0007399//nervous system development;GO:0017121//phospholipid scra

gnaling pathway in response to oxidative stress;GO:0009267//cellular response to starvation;GO:0

n of endocytosis;GO:0048488//synaptic vesicle endocytosis;GO:0048666//neuron development;G

n-dependent cell spreading;GO:0061001//regulation of dendritic spine morphogenesis;GO:00610

3//ER-associated ubiquitin-dependent protein catabolic process;GO:0032781//positive regulation
GO:0010508//positive regulation of autophagy;GO:0016567//protein ubiquitination;GO:0034198//

sponse to endoplasmic reticulum stress;GO:0035023//regulation of Rho protein signal transductio

ocess;GO:0009086//methionine biosynthetic process;GO:0031060//regulation of histone methylatic

ess;GO:0006593//ornithine catabolic process;GO:0019240//citrulline biosynthetic process;GO:0097

gnal transduction;GO:0035556//intracellular signal transduction;GO:0090630//activation of GTPase

cle-mediated transport, Golgi to ER;GO:0015031//protein transport;GO:0016192//vesicle-mediate

one metabolic process;GO:0048729//tissue morphogenesis;GO:0048872//homeostasis of number

elopment;GO:0002138//retinoic acid biosynthetic process;GO:0006081//cellular aldehyde metabol

netabolic process;GO:0042558//pteridine-containing compound metabolic process;GO:0044237//

tive regulation by host of viral release from host cell;GO:0045087//innate immune response;GO:00
ctor beta receptor signaling pathway;GO:0010608//posttranscriptional regulation of gene expressic
utamate receptor signaling pathway;GO:0007405//neuroblast proliferation;GO:0019369//arachidor

nthetic process;GO:0016104//triterpenoid biosynthetic process;GO:0031647//regulation of proteir

326//embryonic limb morphogenesis;GO:0048513//animal organ development;GO:0050794//regu

55064//chloride ion homeostasis;GO:0055075//potassium ion homeostasis;GO:0055085//transmer

//regulation of MAPK cascade;GO:0045761//regulation of adenylate cyclase activity;GO:2000479//
small molecule catabolic process;GO:0046039//GTP metabolic process;GO:0046052//UTP catabol

l promoter;GO:0001657//ureteric bud development;GO:0001843//neural tube closure;GO:000206

ıetabolic process;GO:0006637//acyl-CoA metabolic process;GO:0006641//triglyceride metabolic p
3//ER-associated ubiquitin-dependent protein catabolic process;GO:0032781//positive regulation

orylation;GO:0018108//peptidyl-tyrosine phosphorylation;GO:0030282//bone mineralization;GO:0

eptor protein tyrosine kinase signaling pathway;GO:0009615//response to virus;GO:0043406//posit
)45723//positive regulation of fatty acid biosynthetic process;GO:0051258//protein polymerization

gnaling pathway in response to oxidative stress;GO:0009267//cellular response to starvation;GO:00
4//phospholipid biosynthetic process;GO:0010821//regulation of mitochondrion organization;GO:
abolic process;GO:0006631//fatty acid metabolic process;GO:0006679//glucosylceramide biosynth

I promoter;GO:0001889//liver development;GO:0001949//sebaceous gland cell differentiation;GO:

osphatidylinositol 3-kinase signaling;GO:0016579//protein deubiquitination;GO:0046580//negative

egative regulation of protein lipidation;GO:2001140//positive regulation of phospholipid transport

one metabolic process;GO:0048729//tissue morphogenesis;GO:0048872//homeostasis of number

O:0006955//immune response;GO:0007155//cell adhesion;GO:0007160//cell-matrix adhesion;GO:

c process;GO:0010025//wax biosynthetic process;GO:0035336//long-chain fatty-acyl-CoA metabo

.6240//autophagosome docking;GO:0034329//cell junction assembly;GO:0042953//lipoprotein tra
GO:0008544//epidermis development;GO:0010467//gene expression;GO:0036303//lymph vessel m
c process;GO:0043335//protein unfolding;GO:0051005//negative regulation of lipoprotein lipase a

sport;GO:0032365//intracellular lipid transport;GO:0042632//cholesterol homeostasis;GO:0046320/

regulation of transferase activity;GO:2000480//negative regulation of cAMP-dependent protein ki

iquitin-dependent protein catabolic process;GO:0016578//histone deubiquitination;GO:0016579/
thway;GO:0009267//cellular response to starvation;GO:0010510//regulation of acetyl-CoA biosynt

e B pancreatic cell development;GO:0006468//protein phosphorylation;GO:0009615//response to

5669//positive regulation of osteoblast differentiation;GO:0046850//regulation of bone remodelin

ɘptidase activity involved in apoptotic process;GO:0006978//DNA damage response, signal transdu

ıtabolic process;GO:0009313//oligosaccharide catabolic process;GO:0051692//cellular oligosaccha

ɜport;GO:0055085//transmembrane transport;GO:1990830//cellular response to leukemia inhibitor

or signaling pathway;GO:0007189//adenylate cyclase-activating G-protein coupled receptor signa

RNA secondary structure unwinding;GO:0016525//negative regulation of angiogenesis;GO:003105

//ubiquitin-dependent protein catabolic process;GO:0007409//axonogenesis;GO:0007412//axon t

ɜsponse to endoplasmic reticulum stress;GO:0035023//regulation of Rho protein signal transductio

e expression;GO:0030001//metal ion transport;GO:0031398//positive regulation of protein ubiquit

or signaling pathway;GO:0007189//adenylate cyclase-activating G-protein coupled receptor signa

//base-excision repair, AP site formation;GO:0006296//nucleotide-excision repair, DNA incision, 5'

or signaling pathway;GO:0007218//neuropeptide signaling pathway;GO:0008217//regulation of bl
sport;GO:0032365//intracellular lipid transport;GO:0042632//cholesterol homeostasis;GO:0046320/

;GO:0006974//cellular response to DNA damage stimulus;GO:0010212//response to ionizing radia
c process;GO:0010025//wax biosynthetic process;GO:0035336//long-chain fatty-acyl-CoA metabo

.6240//autophagosome docking;GO:0034329//cell junction assembly;GO:0042953//lipoprotein tra

ive regulation of sequestering of triglyceride;GO:0010898//positive regulation of triglyceride catab

hagy;GO:0007029//endoplasmic reticulum organization;GO:0007031//peroxisome organization;G(

tion of cell fate commitment;GO:0016055//Wnt signaling pathway;GO:0030178//negative regulatic
cle-mediated transport, Golgi to ER;GO:0015031//protein transport;GO:0016192//vesicle-mediate

xia;GO:0001889//liver development;GO:0002314//germinal center B cell differentiation;GO:00026З
abolic process;GO:0006631//fatty acid metabolic process;GO:0006679//glucosylceramide biosynth

4//phospholipid biosynthetic process;GO:0010821//regulation of mitochondrion organization;GO:

8//regulation of epidermal growth factor receptor signaling pathway;GO:0046755//viral budding

follicle growth;GO:0001932//regulation of protein phosphorylation;GO:0003073//regulation of sys
5669//positive regulation of osteoblast differentiation;GO:0046850//regulation of bone remodelin
sport;GO:0055085//transmembrane transport;GO:1990830//cellular response to leukemia inhibitor

d glutamate receptor signaling pathway;GO:0048148//behavioral response to cocaine;GO:004874

ic process;GO:0006644//phospholipid metabolic process;GO:0007160//cell-matrix adhesion;GO:0(

gnaling pathway in response to oxidative stress;GO:0009267//cellular response to starvation;GO:0(

3//ER-associated ubiquitin-dependent protein catabolic process;GO:0032781//positive regulation

ctor beta receptor signaling pathway;GO:0010608//posttranscriptional regulation of gene expressio
gnaling pathway in response to oxidative stress;GO:0009267//cellular response to starvation;GO:0(

2//negative regulation of cell adhesion;GO:0010952//positive regulation of peptidase activity;GO:0

ctor beta receptor signaling pathway;GO:0010608//posttranscriptional regulation of gene expressio

osphatidylinositol 3-kinase signaling;GO:0016579//protein deubiquitination;GO:0046580//negative

I promoter;GO:0001889//liver development;GO:0001949//sebaceous gland cell differentiation;GO:

SU-rRNA, 5.8S rRNA, LSU-rRNA);GO:0000466//maturation of 5.8S rRNA from tricistronic rRNA tra

I promoter;GO:0006418//tRNA aminoacylation for protein translation;GO:0006434//seryl-tRNA am

eart rate;GO:0010765//positive regulation of sodium ion transport;GO:0035725//sodium ion transp
ulfment;GO:0043277//apoptotic cell clearance;GO:0050766//positive regulation of phagocytosis
032342//aldosterone biosynthetic process;GO:0032870//cellular response to hormone stimulus;GO

orylation;GO:0018108//peptidyl-tyrosine phosphorylation;GO:0030282//bone mineralization;GO:0
GO:0007626//locomotory behavior;GO:0016477//cell migration;GO:0098609//cell-cell adhesion

4//phospholipid biosynthetic process;GO:0010821//regulation of mitochondrion organization;GO:

lipid metabolic process;GO:0006631//fatty acid metabolic process;GO:0006633//fatty acid biosynth

GO:0010508//positive regulation of autophagy;GO:0016567//protein ubiquitination;GO:0034198//

gulation of cell proliferation;GO:0032956//regulation of actin cytoskeleton organization;GO:00507

routing angiogenesis;GO:0006816//calcium ion transport;GO:0006886//intracellular protein transp

scription, DNA-templated;GO:0006888//ER to Golgi vesicle-mediated transport;GO:0030968//end

:0030512//negative regulation of transforming growth factor beta receptor signaling pathway

on;GO:0006869//lipid transport;GO:0007283//spermatogenesis;GO:0007399//nervous system deve
409//axonogenesis;GO:0007411//axon guidance;GO:0008284//positive regulation of cell proliferat

0006914//autophagy;GO:0007399//nervous system development;GO:0017121//phospholipid scra

:0030336//negative regulation of cell migration;GO:0032570//response to progesterone;GO:0043

or signaling pathway;GO:0007218//neuropeptide signaling pathway;GO:0008217//regulation of bl
I promoter;GO:0001657//ureteric bud development;GO:0001843//neural tube closure;GO:000206

one metabolic process;GO:0048729//tissue morphogenesis;GO:0048872//homeostasis of number

I promoter;GO:0001889//liver development;GO:0001949//sebaceous gland cell differentiation;GO:

erived foam cell differentiation;GO:0010878//cholesterol storage;GO:0033344//cholesterol efflux;G

ptor protein tyrosine kinase signaling pathway;GO:0009615//response to virus;GO:0043406//posit

or signaling pathway;GO:0007189//adenylate cyclase-activating G-protein coupled receptor signa
sphatidylinositol 3-kinase signaling;GO:0016579//protein deubiquitination;GO:0046580//negative

.6240//autophagosome docking;GO:0034329//cell junction assembly;GO:0042953//lipoprotein tra

d glutamate receptor signaling pathway;GO:0048148//behavioral response to cocaine;GO:004874[illegible]

4765//regulation of ion transmembrane transport;GO:0051260//protein homooligomerization;GO
sport;GO:0033120//positive regulation of RNA splicing;GO:0044767//single-organism developmen

0006914//autophagy;GO:0007399//nervous system development;GO:0017121//phospholipid scra

409//axonogenesis;GO:0007411//axon guidance;GO:0008284//positive regulation of cell proliferat
ive regulation of JNK cascade;GO:0051726//regulation of cell cycle;GO:1900745//positive regulatic

eptidase activity involved in apoptotic process;GO:0006978//DNA damage response, signal transdu
ion of cell fate commitment;GO:0016055//Wnt signaling pathway;GO:0030178//negative regulatic

2//negative regulation of cell adhesion;GO:0010952//positive regulation of peptidase activity;GO:0

4765//regulation of ion transmembrane transport;GO:0051260//protein homooligomerization;GO
//inflammatory response to antigenic stimulus;GO:0007264//small GTPase mediated signal transdu

ic process;GO:0006644//phospholipid metabolic process;GO:0007160//cell-matrix adhesion;GO:0(

sport;GO:0032365//intracellular lipid transport;GO:0042632//cholesterol homeostasis;GO:0046320/

3//ER-associated ubiquitin-dependent protein catabolic process;GO:0032781//positive regulation

port;GO:0048265//response to pain;GO:0051620//norepinephrine uptake;GO:0055085//transmeml

070374//positive regulation of ERK1 and ERK2 cascade;GO:0070378//positive regulation of ERK5 c

sphorylation;GO:0046856//phosphatidylinositol dephosphorylation;GO:0048488//synaptic vesicle
I promoter;GO:0006418//tRNA aminoacylation for protein translation;GO:0006434//seryl-tRNA am
abolic process;GO:0006631//fatty acid metabolic process;GO:0006679//glucosylceramide biosynth
sphatidylinositol 3-kinase signaling;GO:0016579//protein deubiquitination;GO:0046580//negative

I promoter;GO:0001889//liver development;GO:0001949//sebaceous gland cell differentiation;GO:
ve regulation of gene expression;GO:0030511//positive regulation of transforming growth factor b

inate metabolic process;GO:0006468//protein phosphorylation;GO:0006486//protein glycosylation

//regulation of epidermal growth factor receptor signaling pathway;GO:0043408//regulation of MA
ylation;GO:0006915//apoptotic process;GO:0031647//regulation of protein stability;GO:0050790//

ell cycle;GO:0006468//protein phosphorylation;GO:0007147//female meiosis II;GO:0032515//negat

I promoter;GO:0006355//regulation of transcription, DNA-templated;GO:0009267//cellular respor

ive regulation of JNK cascade;GO:0051726//regulation of cell cycle;GO:1900745//positive regulatic

lipid metabolic process;GO:0006631//fatty acid metabolic process;GO:0006633//fatty acid biosynth
orylation;GO:0018108//peptidyl-tyrosine phosphorylation;GO:0030282//bone mineralization;GO:0

ylation;GO:0006915//apoptotic process;GO:0031647//regulation of protein stability;GO:0050790//

:0030512//negative regulation of transforming growth factor beta receptor signaling pathway

ulfment;GO:0043277//apoptotic cell clearance;GO:0050766//positive regulation of phagocytosis
cle-mediated transport, Golgi to ER;GO:0015031//protein transport;GO:0016192//vesicle-mediate

sponse to endoplasmic reticulum stress;GO:0035023//regulation of Rho protein signal transductio
)042391//regulation of membrane potential;GO:0043267//negative regulation of potassium ion tra

l promoter;GO:0001657//ureteric bud development;GO:0001843//neural tube closure;GO:000206

0006914//autophagy;GO:0007399//nervous system development;GO:0017121//phospholipid scra

ptor protein tyrosine kinase signaling pathway;GO:0009615//response to virus;GO:0043406//posit

olic process;GO:0046485//ether lipid metabolic process;GO:0060395//SMAD protein signal transdu

scription, DNA-templated;GO:0006888//ER to Golgi vesicle-mediated transport;GO:0030968//end

6240//autophagosome docking;GO:0034329//cell junction assembly;GO:0042953//lipoprotein tra

:0030336//negative regulation of cell migration;GO:0032570//response to progesterone;GO:0043

4765//regulation of ion transmembrane transport;GO:0051260//protein homooligomerization;GO

c process;GO:0010025//wax biosynthetic process;GO:0035336//long-chain fatty-acyl-CoA metabo
//cholesterol transport involved in cholesterol storage;GO:0032367//intracellular cholesterol transp

//base-excision repair, AP site formation;GO:0006296//nucleotide-excision repair, DNA incision, 5'

sport;GO:0055085//transmembrane transport;GO:1990830//cellular response to leukemia inhibitor

orphogenesis;GO:0007224//smoothened signaling pathway;GO:0007399//nervous system develop

8//regulation of epidermal growth factor receptor signaling pathway;GO:0046755//viral budding

e expression;GO:0030001//metal ion transport;GO:0031398//positive regulation of protein ubiquit
sport;GO:0033120//positive regulation of RNA splicing;GO:0044767//single-organism developmen

eart rate;GO:0010765//positive regulation of sodium ion transport;GO:0035725//sodium ion transr
eptidase activity involved in apoptotic process;GO:0006978//DNA damage response, signal transdu

045723//positive regulation of fatty acid biosynthetic process;GO:0051258//protein polymerization
ulfment;GO:0043277//apoptotic cell clearance;GO:0050766//positive regulation of phagocytosis

ibination;GO:0006508//proteolysis;GO:0008152//metabolic process;GO:0015074//DNA integration

lipid metabolic process;GO:0006631//fatty acid metabolic process;GO:0006633//fatty acid biosynth

eptidase activity involved in apoptotic process;GO:0006978//DNA damage response, signal transdu

O:0006955//immune response;GO:0007155//cell adhesion;GO:0007160//cell-matrix adhesion;GO:

ninate metabolic process;GO:0006468//protein phosphorylation;GO:0006486//protein glycosylation

sphorylation;GO:0046856//phosphatidylinositol dephosphorylation;GO:0048488//synaptic vesicle

abolic process;GO:0007586//digestion;GO:0008202//steroid metabolic process;GO:0008207//C21-
ose assembly;GO:0008611//ether lipid biosynthetic process;GO:0021587//cerebellum morphogene

ninate metabolic process;GO:0006468//protein phosphorylation;GO:0006486//protein glycosylation

lipid metabolic process;GO:0006631//fatty acid metabolic process;GO:0006633//fatty acid biosynth
rotein catabolic process;GO:0048388//endosomal lumen acidification;GO:0070070//proton-transp
ylation;GO:0006915//apoptotic process;GO:0031647//regulation of protein stability;GO:0050790//

tabolic process;GO:0009313//oligosaccharide catabolic process;GO:0051692//cellular oligosaccha

ılfment;GO:0043277//apoptotic cell clearance;GO:0050766//positive regulation of phagocytosis
or signaling pathway;GO:0007218//neuropeptide signaling pathway;GO:0008217//regulation of bl
ı development;GO:0050804//modulation of synaptic transmission;GO:0099560//synaptic membrar

:osamine metabolic process;GO:0006790//sulfur compound metabolic process;GO:1903238//posit
0006914//autophagy;GO:0007399//nervous system development;GO:0017121//phospholipid scra

)//protein deubiquitination;GO:0032436//positive regulation of proteasomal ubiquitin-dependent
f neuron projection development;GO:0018105//peptidyl-serine phosphorylation;GO:0032793//po

ɔrphogenesis;GO:0007224//smoothened signaling pathway;GO:0007399//nervous system develop

;port;GO:0033120//positive regulation of RNA splicing;GO:0044767//single-organism developmen

egative regulation of protein lipidation;GO:2001140//positive regulation of phospholipid transport

4765//regulation of ion transmembrane transport;GO:0051260//protein homooligomerization;GO

c process;GO:0010025//wax biosynthetic process;GO:0035336//long-chain fatty-acyl-CoA metabo

ic process;GO:0006644//phospholipid metabolic process;GO:0007160//cell-matrix adhesion;GO:0(

;GO:0006974//cellular response to DNA damage stimulus;GO:0010212//response to ionizing radia

)042391//regulation of membrane potential;GO:0043267//negative regulation of potassium ion tra

ɔiquitin-dependent protein catabolic process;GO:0016578//histone deubiquitination;GO:0016579/,
)//nucleolus organization;GO:0016579//protein deubiquitination;GO:0050821//protein stabilizatior

egulation of transcription from RNA polymerase II promoter;GO:0001649//osteoblast differentiatic

olic process;GO:0046485//ether lipid metabolic process;GO:0060395//SMAD protein signal transdu
scription, DNA-templated;GO:0006888//ER to Golgi vesicle-mediated transport;GO:0030968//end

on of oxidative phosphorylation;GO:0006390//transcription from mitochondrial promoter;GO:0006
3//ER-associated ubiquitin-dependent protein catabolic process;GO:0032781//positive regulation

045723//positive regulation of fatty acid biosynthetic process;GO:0051258//protein polymerization

ell cycle;GO:0006468//protein phosphorylation;GO:0007147//female meiosis II;GO:0032515//negat

n-dependent cell spreading;GO:0061001//regulation of dendritic spine morphogenesis;GO:00610

eptidase activity involved in apoptotic process;GO:0006978//DNA damage response, signal transdu

one metabolic process;GO:0048729//tissue morphogenesis;GO:0048872//homeostasis of number

55064//chloride ion homeostasis;GO:0055075//potassium ion homeostasis;GO:0055085//transmen

ylation;GO:0006915//apoptotic process;GO:0031647//regulation of protein stability;GO:0050790//

ıbination;GO:0006508//proteolysis;GO:0008152//metabolic process;GO:0015074//DNA integration

scription, DNA-templated;GO:0006888//ER to Golgi vesicle-mediated transport;GO:0030968//end

.6240//autophagosome docking;GO:0034329//cell junction assembly;GO:0042953//lipoprotein tra

netabolic process;GO:0006637//acyl-CoA metabolic process;GO:0006641//triglyceride metabolic p
port;GO:0048265//response to pain;GO:0051620//norepinephrine uptake;GO:0055085//transmeml
ninate metabolic process;GO:0006468//protein phosphorylation;GO:0006486//protein glycosylatio
tion;GO:0007052//mitotic spindle organization;GO:0007409//axonogenesis;GO:0009615//respons

:osamine metabolic process;GO:0006790//sulfur compound metabolic process;GO:1903238//posit

ɪn of endocytosis;GO:0048488//synaptic vesicle endocytosis;GO:0048666//neuron development;G(

4765//regulation of ion transmembrane transport;GO:0051260//protein homooligomerization;GO

or signaling pathway;GO:0007189//adenylate cyclase-activating G-protein coupled receptor signa

c process;GO:0010025//wax biosynthetic process;GO:0035336//long-chain fatty-acyl-CoA metabo

;port;GO:0033120//positive regulation of RNA splicing;GO:0044767//single-organism developmen

0006914//autophagy;GO:0007399//nervous system development;GO:0017121//phospholipid scra
)086073//bundle of His cell-Purkinje myocyte adhesion involved in cell communication;GO:00860

5669//positive regulation of osteoblast differentiation;GO:0046850//regulation of bone remodelin

rocess;GO:0006633//fatty acid biosynthetic process;GO:0006636//unsaturated fatty acid biosynthe

:0030336//negative regulation of cell migration;GO:0032570//response to progesterone;GO:0043
ɔptotic process;GO:0043154//negative regulation of cysteine-type endopeptidase activity involvec
hagy;GO:0007029//endoplasmic reticulum organization;GO:0007031//peroxisome organization;G(
:ion of cell fate commitment;GO:0016055//Wnt signaling pathway;GO:0030178//negative regulatic

e regulation of endothelial cell migration;GO:0019673//GDP-mannose metabolic process;GO:004

//cholesterol transport involved in cholesterol storage;GO:0032367//intracellular cholesterol transp

;port;GO:0033120//positive regulation of RNA splicing;GO:0044767//single-organism developmen

ptor protein tyrosine kinase signaling pathway;GO:0009615//response to virus;GO:0043406//posit

iquitin-dependent protein catabolic process;GO:0016578//histone deubiquitination;GO:0016579/

I promoter;GO:0001657//ureteric bud development;GO:0001843//neural tube closure;GO:000206

409//axonogenesis;GO:0007411//axon guidance;GO:0008284//positive regulation of cell prolifera

//ER-associated ubiquitin-dependent protein catabolic process;GO:0032781//positive regulation

egulation of transcription elongation from RNA polymerase II promoter;GO:0045893//positive reg

ty;GO:0045654//positive regulation of megakaryocyte differentiation;GO:0046330//positive regula

starvation;GO:0044090//positive regulation of vacuole organization;GO:2000786//positive regula

scription, DNA-templated;GO:0006888//ER to Golgi vesicle-mediated transport;GO:0030968//end
gulation of cell proliferation;GO:0032956//regulation of actin cytoskeleton organization;GO:00507
ment;GO:0006508//proteolysis;GO:0016525//negative regulation of angiogenesis;GO:0060347//h

gnaling pathway in response to oxidative stress;GO:0009267//cellular response to starvation;GO:0

lipid metabolic process;GO:0006631//fatty acid metabolic process;GO:0006633//fatty acid biosynt

lic process;GO:0046485//ether lipid metabolic process;GO:0060395//SMAD protein signal transd
958//methotrexate transport;GO:0098838//reduced folate transmembrane transport;GO:1904447

ylation;GO:0006915//apoptotic process;GO:0031647//regulation of protein stability;GO:0050790//

ılfment;GO:0043277//apoptotic cell clearance;GO:0050766//positive regulation of phagocytosis

ƎO:0006107//oxaloacetate metabolic process;GO:0006629//lipid metabolic process;GO:0009617//r

: process;GO:0043335//protein unfolding;GO:0051005//negative regulation of lipoprotein lipase a

.6240//autophagosome docking;GO:0034329//cell junction assembly;GO:0042953//lipoprotein tra

:le-mediated transport, Golgi to ER;GO:0015031//protein transport;GO:0016192//vesicle-mediate
or signaling pathway;GO:0007189//adenylate cyclase-activating G-protein coupled receptor signa

phore linkage;GO:0032922//circadian regulation of gene expression;GO:0043153//entrainment of

l promoter;GO:0006355//regulation of transcription, DNA-templated;GO:0009267//cellular respor

e regulation of endothelial cell migration;GO:0019673//GDP-mannose metabolic process;GO:004

ic process;GO:0006644//phospholipid metabolic process;GO:0007160//cell-matrix adhesion;GO:0

//regulation of MAPK cascade;GO:0045761//regulation of adenylate cyclase activity;GO:2000479//
ıetabolic process;GO:0042558//pteridine-containing compound metabolic process;GO:0044237//

lic process;GO:0006656//phosphatidylcholine biosynthetic process;GO:0008203//cholesterol meta

//phospholipase C-activating G-protein coupled receptor signaling pathway;GO:0035176//social

or signaling pathway;GO:0007218//neuropeptide signaling pathway;GO:0008217//regulation of bl

or signaling pathway;GO:0008277//regulation of G-protein coupled receptor protein signaling pa

on;GO:0006869//lipid transport;GO:0007283//spermatogenesis;GO:0007399//nervous system deve

r signaling pathway;GO:0007409//axonogenesis;GO:0030900//forebrain development;GO:003818
ion of cell fate commitment;GO:0016055//Wnt signaling pathway;GO:0030178//negative regulatic

ırouting angiogenesis;GO:0006816//calcium ion transport;GO:0006886//intracellular protein transp
otein catabolic process;GO:0048388//endosomal lumen acidification;GO:0070070//proton-transp

//base-excision repair, AP site formation;GO:0006296//nucleotide-excision repair, DNA incision, 5'

ptor protein tyrosine kinase signaling pathway;GO:0009615//response to virus;GO:0043406//posit

I promoter;GO:0001568//blood vessel development;GO:0001570//vasculogenesis;GO:0003150//m

I promoter;GO:0001657//ureteric bud development;GO:0001843//neural tube closure;GO:000206

ıinate metabolic process;GO:0006468//protein phosphorylation;GO:0006486//protein glycosylatio

metabolic process;GO:0006650//glycerophospholipid metabolic process;GO:0006654//phosphatid
)//protein deubiquitination;GO:0032436//positive regulation of proteasomal ubiquitin-dependent

l promoter;GO:0001889//liver development;GO:0001949//sebaceous gland cell differentiation;GO:
gnaling pathway in response to oxidative stress;GO:0009267//cellular response to starvation;GO:00
f neuron projection development;GO:0018105//peptidyl-serine phosphorylation;GO:0032793//po

ration;GO:0006355//regulation of transcription, DNA-templated;GO:0007165//signal transduction

sphatidylinositol 3-kinase signaling;GO:0016579//protein deubiquitination;GO:0046580//negative

c process;GO:0010025//wax biosynthetic process;GO:0035336//long-chain fatty-acyl-CoA metabo

e regulation of endothelial cell migration;GO:0019673//GDP-mannose metabolic process;GO:004

:0030336//negative regulation of cell migration;GO:0032570//response to progesterone;GO:0043

iquitin-dependent protein catabolic process;GO:0016578//histone deubiquitination;GO:0016579/

SU-rRNA, 5.8S rRNA, LSU-rRNA);GO:0000466//maturation of 5.8S rRNA from tricistronic rRNA tra

o starvation;GO:0044090//positive regulation of vacuole organization;GO:2000786//positive regula
olic process;GO:0046485//ether lipid metabolic process;GO:0060395//SMAD protein signal transdu

)6782//protoporphyrinogen IX biosynthetic process;GO:0006783//heme biosynthetic process;GO:0
cle-mediated transport, Golgi to ER;GO:0015031//protein transport;GO:0016192//vesicle-mediate

;SU-rRNA, 5.8S rRNA, LSU-rRNA);GO:0000466//maturation of 5.8S rRNA from tricistronic rRNA tra

ɔse assembly;GO:0008611//ether lipid biosynthetic process;GO:0021587//cerebellum morphogene

tion;GO:0007052//mitotic spindle organization;GO:0007409//axonogenesis;GO:0009615//response

ic process;GO:0006644//phospholipid metabolic process;GO:0007160//cell-matrix adhesion;GO:0(

l promoter;GO:0006355//regulation of transcription, DNA-templated;GO:0009267//cellular respor
;O:0006107//oxaloacetate metabolic process;GO:0006629//lipid metabolic process;GO:0009617//r

)6782//protoporphyrinogen IX biosynthetic process;GO:0006783//heme biosynthetic process;GO:(
cle-mediated transport, Golgi to ER;GO:0015031//protein transport;GO:0016192//vesicle-mediate
ɔne metabolic process;GO:0048729//tissue morphogenesis;GO:0048872//homeostasis of number

//regulation of epidermal growth factor receptor signaling pathway;GO:0043408//regulation of MA

or signaling pathway;GO:0007188//adenylate cyclase-modulating G-protein coupled receptor sig

on;GO:0006869//lipid transport;GO:0007283//spermatogenesis;GO:0007399//nervous system deve

scription, DNA-templated;GO:0006888//ER to Golgi vesicle-mediated transport;GO:0030968//end

rganization;GO:0042149//cellular response to glucose starvation;GO:0043066//negative regulation

al glia guided migration;GO:0030335//positive regulation of cell migration;GO:0031022//nuclear n

abolic process;GO:0006631//fatty acid metabolic process;GO:0006679//glucosylceramide biosynth
tion;GO:0007052//mitotic spindle organization;GO:0007409//axonogenesis;GO:0009615//response

elopment;GO:0002138//retinoic acid biosynthetic process;GO:0006081//cellular aldehyde metabol

egulation of canonical Wnt signaling pathway;GO:2000096//positive regulation of Wnt signaling p

gnaling pathway in response to oxidative stress;GO:0009267//cellular response to starvation;GO:00

g;GO:0030261//chromosome condensation;GO:0045910//negative regulation of DNA recombinat

ocess;GO:0009086//methionine biosynthetic process;GO:0031060//regulation of histone methylatio

sport;GO:0032365//intracellular lipid transport;GO:0042632//cholesterol homeostasis;GO:0046320/

sport;GO:0033120//positive regulation of RNA splicing;GO:0044767//single-organism developmen

c process;GO:0043335//protein unfolding;GO:0051005//negative regulation of lipoprotein lipase a

netabolic process;GO:0042558//pteridine-containing compound metabolic process;GO:0044237//c

pe B pancreatic cell development;GO:0006468//protein phosphorylation;GO:0009615//response to

regulation of transferase activity;GO:2000480//negative regulation of cAMP-dependent protein kir

lic process;GO:0008654//phospholipid biosynthetic process;GO:0030258//lipid modification;GO:0C
c process;GO:0043335//protein unfolding;GO:0051005//negative regulation of lipoprotein lipase a

tion;GO:0007052//mitotic spindle organization;GO:0007409//axonogenesis;GO:0009615//response

f neuron projection development;GO:0018105//peptidyl-serine phosphorylation;GO:0032793//po
)//protein deubiquitination;GO:0032436//positive regulation of proteasomal ubiquitin-dependent
RNA secondary structure unwinding;GO:0016525//negative regulation of angiogenesis;GO:003105

l promoter;GO:0001568//blood vessel development;GO:0001570//vasculogenesis;GO:0003150//m

netabolic process;GO:0006637//acyl-CoA metabolic process;GO:0006641//triglyceride metabolic p

or signaling pathway;GO:0007218//neuropeptide signaling pathway;GO:0008217//regulation of bl

ose assembly;GO:0008611//ether lipid biosynthetic process;GO:0021587//cerebellum morphogene

tion of cell fate commitment;GO:0016055//Wnt signaling pathway;GO:0030178//negative regulatic

rotein catabolic process;GO:0048388//endosomal lumen acidification;GO:0070070//proton-transp

:0030336//negative regulation of cell migration;GO:0032570//response to progesterone;GO:0043
rotein catabolic process;GO:0048388//endosomal lumen acidification;GO:0070070//proton-transp
GO:0006107//oxaloacetate metabolic process;GO:0006629//lipid metabolic process;GO:0009617//r
5//glycoprotein catabolic process;GO:0016567//protein ubiquitination;GO:0030433//ER-associated

5669//positive regulation of osteoblast differentiation;GO:0046850//regulation of bone remodelin

or signaling pathway;GO:0007189//adenylate cyclase-activating G-protein coupled receptor signa
cle-mediated transport, Golgi to ER;GO:0015031//protein transport;GO:0016192//vesicle-mediate

GO:0008544//epidermis development;GO:0010467//gene expression;GO:0036303//lymph vessel m
I promoter;GO:0001889//liver development;GO:0001949//sebaceous gland cell differentiation;GO:
d glutamate receptor signaling pathway;GO:0048148//behavioral response to cocaine;GO:0048741
//protein deubiquitination;GO:0032436//positive regulation of proteasomal ubiquitin-dependent
f neuron projection development;GO:0018105//peptidyl-serine phosphorylation;GO:0032793//po
one metabolic process;GO:0048729//tissue morphogenesis;GO:0048872//homeostasis of number
sphatidylinositol 3-kinase signaling;GO:0016579//protein deubiquitination;GO:0046580//negative

0086073//bundle of His cell-Purkinje myocyte adhesion involved in cell communication;GO:0086

iquitin-dependent protein catabolic process;GO:0016578//histone deubiquitination;GO:0016579/

I promoter;GO:0007379//segment specification;GO:0019827//stem cell population maintenance;G

4//phospholipid biosynthetic process;GO:0010821//regulation of mitochondrion organization;GO:

gnaling pathway in response to oxidative stress;GO:0009267//cellular response to starvation;GO:00
er;GO:0009062//fatty acid catabolic process;GO:0019432//triglyceride biosynthetic process
asma membrane adhesion molecules;GO:0007162//negative regulation of cell adhesion;GO:0007
2//negative regulation of cell adhesion;GO:0010952//positive regulation of peptidase activity;GO:0
elopment;GO:0002138//retinoic acid biosynthetic process;GO:0006081//cellular aldehyde metabol

ose assembly;GO:0008611//ether lipid biosynthetic process;GO:0021587//cerebellum morphogene

sport;GO:0055085//transmembrane transport;GO:1990830//cellular response to leukemia inhibitor

abolic process;GO:0006631//fatty acid metabolic process;GO:0006679//glucosylceramide biosynth

orphogenesis;GO:0007224//smoothened signaling pathway;GO:0007399//nervous system develop

ctor beta receptor signaling pathway;GO:0010608//posttranscriptional regulation of gene expressio
3//ER-associated ubiquitin-dependent protein catabolic process;GO:0032781//positive regulation

f neuron projection development;GO:0018105//peptidyl-serine phosphorylation;GO:0032793//po

egulation of transcription elongation from RNA polymerase II promoter;GO:0045893//positive regu

//protein deubiquitination;GO:0032436//positive regulation of proteasomal ubiquitin-dependent

009306//protein secretion;GO:0030168//platelet activation;GO:0031639//plasminogen activation;G
sport;GO:0032365//intracellular lipid transport;GO:0042632//cholesterol homeostasis;GO:0046320/
o starvation;GO:0044090//positive regulation of vacuole organization;GO:2000786//positive regula

SU-rRNA, 5.8S rRNA, LSU-rRNA);GO:0000466//maturation of 5.8S rRNA from tricistronic rRNA tra
on transmembrane transport;GO:0050909//sensory perception of taste;GO:0055085//transmembra
olic process;GO:0046485//ether lipid metabolic process;GO:0060395//SMAD protein signal transdu

tabolic process;GO:0019835//cytolysis;GO:0031640//killing of cells of other organism;GO:0042742
cromolecule metabolic process;GO:0090502//RNA phosphodiester bond hydrolysis, endonucleolyt

ositive regulation of transcription from RNA polymerase II promoter;GO:0048384//retinoic acid re
ulfment;GO:0043277//apoptotic cell clearance;GO:0050766//positive regulation of phagocytosis

gnal transduction;GO:0035735//intraciliary transport involved in cilium morphogenesis;GO:006027

sponse to endoplasmic reticulum stress;GO:0035023//regulation of Rho protein signal transductio
rotein catabolic process;GO:0048388//endosomal lumen acidification;GO:0070070//proton-transp
bination;GO:0006508//proteolysis;GO:0008152//metabolic process;GO:0015074//DNA integration

lipid metabolic process;GO:0006631//fatty acid metabolic process;GO:0006633//fatty acid biosynth

orylation;GO:0018108//peptidyl-tyrosine phosphorylation;GO:0030282//bone mineralization;GO:0

)6782//protoporphyrinogen IX biosynthetic process;GO:0006783//heme biosynthetic process;GO:(

ıetabolic process;GO:0006637//acyl-CoA metabolic process;GO:0006641//triglyceride metabolic p

or signaling pathway;GO:0007189//adenylate cyclase-activating G-protein coupled receptor signa
cess;GO:0006629//lipid metabolic process;GO:0006633//fatty acid biosynthetic process;GO:00195[illegible]

ıinate metabolic process;GO:0006468//protein phosphorylation;GO:0006486//protein glycosylatio[illegible]

tion;GO:0007052//mitotic spindle organization;GO:0007409//axonogenesis;GO:0009615//respons[illegible]

on;GO:0006869//lipid transport;GO:0007283//spermatogenesis;GO:0007399//nervous system deve

tion;GO:0033383//geranyl diphosphate metabolic process;GO:0045339//farnesyl diphosphate cata

of protein phosphorylation;GO:0007507//heart development;GO:0008284//positive regulation of

regulation of transferase activity;GO:2000480//negative regulation of cAMP-dependent protein ki[illegible]

e regulation of endothelial cell migration;GO:0019673//GDP-mannose metabolic process;GO:004[illegible]
[illegible]//glycoprotein catabolic process;GO:0016567//protein ubiquitination;GO:0030433//ER-associated

rotein catabolic process;GO:0048388//endosomal lumen acidification;GO:0070070//proton-transp

pe B pancreatic cell development;GO:0006468//protein phosphorylation;GO:0009615//response t[illegible]

eart rate;GO:0010765//positive regulation of sodium ion transport;GO:0035725//sodium ion trans[illegible]

n-dependent cell spreading;GO:0061001//regulation of dendritic spine morphogenesis;GO:00610

d glutamate receptor signaling pathway;GO:0048148//behavioral response to cocaine;GO:0048741
)//protein deubiquitination;GO:0032436//positive regulation of proteasomal ubiquitin-dependent

f neuron projection development;GO:0018105//peptidyl-serine phosphorylation;GO:0032793//po

l promoter;GO:0006355//regulation of transcription, DNA-templated;GO:0009267//cellular respor

4//phospholipid biosynthetic process;GO:0010821//regulation of mitochondrion organization;GO:

elopment;GO:0002138//retinoic acid biosynthetic process;GO:0006081//cellular aldehyde metabol
3//ER-associated ubiquitin-dependent protein catabolic process;GO:0032781//positive regulation
eptidase activity involved in apoptotic process;GO:0006978//DNA damage response, signal transd

lic process;GO:0008654//phospholipid biosynthetic process;GO:0030258//lipid modification;GO:00

542//visual learning;GO:0010801//negative regulation of peptidyl-threonine phosphorylation;GO:(

2//negative regulation of cell adhesion;GO:0010952//positive regulation of peptidase activity;GO:0
4765//regulation of ion transmembrane transport;GO:0051260//protein homooligomerization;GO

iquitin-dependent protein catabolic process;GO:0016578//histone deubiquitination;GO:0016579/

sport;GO:0055085//transmembrane transport;GO:1990830//cellular response to leukemia inhibitor

ic process;GO:0006644//phospholipid metabolic process;GO:0007160//cell-matrix adhesion;GO:0

ctor beta receptor signaling pathway;GO:0010608//posttranscriptional regulation of gene expressio

g;GO:0030261//chromosome condensation;GO:0045910//negative regulation of DNA recombinat

o starvation;GO:0044090//positive regulation of vacuole organization;GO:2000786//positive regula
osphatidylinositol 3-kinase signaling;GO:0016579//protein deubiquitination;GO:0046580//negative

netabolic process;GO:0042558//pteridine-containing compound metabolic process;GO:0044237//c

l promoter;GO:0001889//liver development;GO:0001949//sebaceous gland cell differentiation;GO:

phore linkage;GO:0032922//circadian regulation of gene expression;GO:0043153//entrainment of

)6782//protoporphyrinogen IX biosynthetic process;GO:0006783//heme biosynthetic process;GO:(

netanephros development;GO:0001759//organ induction;GO:0001823//mesonephros developmer

al glia guided migration;GO:0030335//positive regulation of cell migration;GO:0031022//nuclear n

ive regulation of JNK cascade;GO:0051726//regulation of cell cycle;GO:1900745//positive regulatic

ive regulation of JNK cascade;GO:0051726//regulation of cell cycle;GO:1900745//positive regulatic

orylation;GO:0018108//peptidyl-tyrosine phosphorylation;GO:0030282//bone mineralization;GO:0(

GO:0010508//positive regulation of autophagy;GO:0016567//protein ubiquitination;GO:0034198//
or signaling pathway;GO:0007218//neuropeptide signaling pathway;GO:0008217//regulation of bl

orphogenesis;GO:0007224//smoothened signaling pathway;GO:0007399//nervous system develop

tion;GO:0007052//mitotic spindle organization;GO:0007409//axonogenesis;GO:0009615//response
sponse to endoplasmic reticulum stress;GO:0035023//regulation of Rho protein signal transductio

l promoter;GO:0006418//tRNA aminoacylation for protein translation;GO:0006434//seryl-tRNA am

eptidase activity involved in apoptotic process;GO:0006978//DNA damage response, signal transdu

2//negative regulation of cell adhesion;GO:0010952//positive regulation of peptidase activity;GO:0
eart rate;GO:0010765//positive regulation of sodium ion transport;GO:0035725//sodium ion transp

l promoter;GO:0001657//ureteric bud development;GO:0001843//neural tube closure;GO:000206[illegible]

eptor protein tyrosine kinase signaling pathway;GO:0009615//response to virus;GO:0043406//posit
542//visual learning;GO:0010801//negative regulation of peptidyl-threonine phosphorylation;GO:[illegible]
d glutamate receptor signaling pathway;GO:0048148//behavioral response to cocaine;GO:004874[illegible]

58//regulation of epidermal growth factor receptor signaling pathway;GO:0046755//viral budding

e expression;GO:0030001//metal ion transport;GO:0031398//positive regulation of protein ubiquit

sphorylation;GO:0046856//phosphatidylinositol dephosphorylation;GO:0048488//synaptic vesicle

abolic process;GO:0006631//fatty acid metabolic process;GO:0006679//glucosylceramide biosynth
xia;GO:0001889//liver development;GO:0002314//germinal center B cell differentiation;GO:000263[illegible]

eart rate;GO:0010765//positive regulation of sodium ion transport;GO:0035725//sodium ion transr

tion of cell fate commitment;GO:0016055//Wnt signaling pathway;GO:0030178//negative regulatic

ıbination;GO:0006508//proteolysis;GO:0008152//metabolic process;GO:0015074//DNA integration

sport;GO:0032365//intracellular lipid transport;GO:0042632//cholesterol homeostasis;GO:0046320/

O:0006955//immune response;GO:0007155//cell adhesion;GO:0007160//cell-matrix adhesion;GO:

cromolecule metabolic process;GO:0090502//RNA phosphodiester bond hydrolysis, endonucleolyt

e expression;GO:0030001//metal ion transport;GO:0031398//positive regulation of protein ubiquit

ell cycle;GO:0006468//protein phosphorylation;GO:0007147//female meiosis II;GO:0032515//negat
e regulation of endothelial cell migration;GO:0019673//GDP-mannose metabolic process;GO:0042

netabolic process;GO:0042558//pteridine-containing compound metabolic process;GO:0044237//

n of endocytosis;GO:0048488//synaptic vesicle endocytosis;GO:0048666//neuron development;GO
ive regulation of JNK cascade;GO:0051726//regulation of cell cycle;GO:1900745//positive regulatic
phore linkage;GO:0032922//circadian regulation of gene expression;GO:0043153//entrainment of

tive regulation by host of viral release from host cell;GO:0045087//innate immune response;GO:00

d glutamate receptor signaling pathway;GO:0048148//behavioral response to cocaine;GO:0048741
regulation of transferase activity;GO:2000480//negative regulation of cAMP-dependent protein kir
ylation;GO:0006915//apoptotic process;GO:0031647//regulation of protein stability;GO:0050790//
pe B pancreatic cell development;GO:0006468//protein phosphorylation;GO:0009615//response tc

0006914//autophagy;GO:0007399//nervous system development;GO:0017121//phospholipid scra

orphogenesis;GO:0007224//smoothened signaling pathway;GO:0007399//nervous system develop
n-dependent cell spreading;GO:0061001//regulation of dendritic spine morphogenesis;GO:00610

//regulation of MAPK cascade;GO:0045761//regulation of adenylate cyclase activity;GO:2000479//

2//negative regulation of cell adhesion;GO:0010952//positive regulation of peptidase activity;GO:0

er;GO:0009062//fatty acid catabolic process;GO:0019432//triglyceride biosynthetic process
GO:0006107//oxaloacetate metabolic process;GO:0006629//lipid metabolic process;GO:0009617//r

l promoter;GO:0007379//segment specification;GO:0019827//stem cell population maintenance;G

biquitin-dependent protein catabolic process;GO:0016578//histone deubiquitination;GO:0016579//

.6240//autophagosome docking;GO:0034329//cell junction assembly;GO:0042953//lipoprotein tra

orphogenesis;GO:0007224//smoothened signaling pathway;GO:0007399//nervous system develop

c process;GO:0010025//wax biosynthetic process;GO:0035336//long-chain fatty-acyl-CoA metabo

egative regulation of protein lipidation;GO:2001140//positive regulation of phospholipid transport
GO:0008544//epidermis development;GO:0010467//gene expression;GO:0036303//lymph vessel m
GO:0010508//positive regulation of autophagy;GO:0016567//protein ubiquitination;GO:0034198//

olic process;GO:0051180//vitamin transport;GO:0090212//negative regulation of establishment of b

4//phospholipid biosynthetic process;GO:0010821//regulation of mitochondrion organization;GO:

02//negative regulation of bone mineralization;GO:0050727//regulation of inflammatory response;

58//regulation of epidermal growth factor receptor signaling pathway;GO:0046755//viral budding

egative regulation of protein lipidation;GO:2001140//positive regulation of phospholipid transport

or signaling pathway;GO:0007218//neuropeptide signaling pathway;GO:0008217//regulation of bl

.958//methotrexate transport;GO:0098838//reduced folate transmembrane transport;GO:1904447/
0045776//negative regulation of blood pressure;GO:0051607//defense response to virus;GO:0055

RNA secondary structure unwinding;GO:0016525//negative regulation of angiogenesis;GO:00310

ctor beta receptor signaling pathway;GO:0010608//posttranscriptional regulation of gene expressio
e expression;GO:0030001//metal ion transport;GO:0031398//positive regulation of protein ubiquit
tive regulation by host of viral release from host cell;GO:0045087//innate immune response;GO:00

of protein phosphorylation;GO:0007507//heart development;GO:0008284//positive regulation of

or signaling pathway;GO:0007188//adenylate cyclase-modulating G-protein coupled receptor sig
tion;GO:0033383//geranyl diphosphate metabolic process;GO:0045339//farnesyl diphosphate cata

0006914//autophagy;GO:0007399//nervous system development;GO:0017121//phospholipid scra
.6240//autophagosome docking;GO:0034329//cell junction assembly;GO:0042953//lipoprotein tra
on transmembrane transport;GO:0050909//sensory perception of taste;GO:0055085//transmembra
045723//positive regulation of fatty acid biosynthetic process;GO:0051258//protein polymerization

netabolic process;GO:0006637//acyl-CoA metabolic process;GO:0006641//triglyceride metabolic p

xia;GO:0001889//liver development;GO:0002314//germinal center B cell differentiation;GO:00026[illegible]

n of endocytosis;GO:0048488//synaptic vesicle endocytosis;GO:0048666//neuron development;G[illegible]
ositive regulation of transcription from RNA polymerase II promoter;GO:0048384//retinoic acid re[illegible]

hagy;GO:0007029//endoplasmic reticulum organization;GO:0007031//peroxisome organization;G[illegible]

e regulation of endothelial cell migration;GO:0019673//GDP-mannose metabolic process;GO:004[illegible]

n-dependent cell spreading;GO:0061001//regulation of dendritic spine morphogenesis;GO:00610

ynthetic process;GO:0044208//'de novo' AMP biosynthetic process;GO:0044209//AMP salvage;GC

f toll-like receptor 9 signaling pathway;GO:0034499//late endosome to Golgi transport;GO:00456
085//transmembrane transport;GO:0061337//cardiac conduction;GO:0071805//potassium ion tran

n;GO:0006487//protein N-linked glycosylation;GO:0010706//ganglioside biosynthetic process via

;GO:0010760//negative regulation of macrophage chemotaxis;GO:0042326//negative regulation o

O:0009820//alkaloid metabolic process;GO:0009822//alkaloid catabolic process;GO:0016098//mor
54//pre-miRNA processing;GO:0035196//production of miRNAs involved in gene silencing by miR

e response;GO:0006644//phospholipid metabolic process;GO:0006656//phosphatidylcholine bios

se to heat;GO:0010467//gene expression;GO:0010468//regulation of gene expression;GO:001056

0040014//regulation of multicellular organism growth;GO:0044710//single-organism metabolic pro

ranscription from RNA polymerase II promoter;GO:0007614//short-term memory;GO:0010628//po
0007179//transforming growth factor beta receptor signaling pathway;GO:0007229//integrin-mec

GO:0071276//cellular response to cadmium ion;GO:0071280//cellular response to copper ion;GO:

tion;GO:0016567//protein ubiquitination;GO:0031052//chromosome breakage;GO:0031297//repli

ol storage;GO:0010890//positive regulation of sequestering of triglyceride;GO:0016042//lipid catab

ninoacylation;GO:0016525//negative regulation of angiogenesis;GO:1904046//negative regulation

ng pathway;GO:0009631//cold acclimation;GO:0010762//regulation of fibroblast migration;GO:003
on;GO:0007405//neuroblast proliferation;GO:0007420//brain development;GO:0007623//circadian

iotic;GO:0071280//cellular response to copper ion;GO:0071420//cellular response to histamine;GC

cription from RNA polymerase II promoter;GO:0008285//negative regulation of cell proliferation;G

virus;GO:0010468//regulation of gene expression;GO:0016310//phosphorylation;GO:0042063//gl

;GO:0007283//spermatogenesis;GO:0007422//peripheral nervous system development;GO:00085

99//nervous system development;GO:0007411//axon guidance;GO:0007416//synapse assembly;G

754//positive regulation of interleukin-5 production;GO:0043951//negative regulation of cAMP-m

brane transport;GO:0070633//transepithelial transport;GO:0072089//stem cell proliferation;GO:190

54//pre-miRNA processing;GO:0035196//production of miRNAs involved in gene silencing by miR

ynthetic process;GO:0044208//'de novo' AMP biosynthetic process;GO:0044209//AMP salvage;GC

nily amino acid metabolic process;GO:0009066//aspartate family amino acid metabolic process;GC

e cyclase-modulating G-protein coupled receptor signaling pathway;GO:0007420//brain developr

ng pathway;GO:0044539//long-chain fatty acid import;GO:0071902//positive regulation of proteir

77//cell migration;GO:0035987//endodermal cell differentiation;GO:0043149//stress fiber assembl

e response;GO:0006644//phospholipid metabolic process;GO:0006656//phosphatidylcholine bios

uction;GO:0014066//regulation of phosphatidylinositol 3-kinase signaling;GO:0030101//natural kill

process;GO:0010749//regulation of nitric oxide mediated signal transduction;GO:0019343//cystei

iotic;GO:0071280//cellular response to copper ion;GO:0071420//cellular response to histamine;GC

GO:0071276//cellular response to cadmium ion;GO:0071280//cellular response to copper ion;GO:

ranscription from RNA polymerase II promoter;GO:0007614//short-term memory;GO:0010628//pc
33//B cell differentiation;GO:0030217//T cell differentiation;GO:0033152//immunoglobulin V(D)J re

y behavior;GO:0009887//organ morphogenesis;GO:0009967//positive regulation of signal transdu

ıetabolic process;GO:0044208//'de novo' AMP biosynthetic process;GO:0097294//'de novo' XMP b

ınal transduction;GO:0032008//positive regulation of TOR signaling;GO:0035556//intracellular sigr

77//cell migration;GO:0035987//endodermal cell differentiation;GO:0043149//stress fiber assembl

O:0009820//alkaloid metabolic process;GO:0009822//alkaloid catabolic process;GO:0016098//mor

se to heat;GO:0010467//gene expression;GO:0010468//regulation of gene expression;GO:001056(

O:0034379//very-low-density lipoprotein particle assembly;GO:0034435//cholesterol esterificatior

on of Wnt signaling pathway;GO:0030509//BMP signaling pathway;GO:0030514//negative regulati

//defense response to bacterium;GO:0050829//defense response to Gram-negative bacterium;GC

ative regulation of neuron projection development;GO:0021540//corpus callosum morphogenesis;

loplasmic reticulum unfolded protein response;GO:0034976//response to endoplasmic reticulum s

085//transmembrane transport;GO:0061337//cardiac conduction;GO:0071805//potassium ion tran

opment;GO:0090263//positive regulation of canonical Wnt signaling pathway;GO:2001013//epithe

99//nervous system development;GO:0007411//axon guidance;GO:0007416//synapse assembly;G

f toll-like receptor 9 signaling pathway;GO:0034499//late endosome to Golgi transport;GO:0456

//adult behavior;GO:0035176//social behavior;GO:0048488//synaptic vesicle endocytosis;GO:0050

0021954//central nervous system neuron development;GO:0032091//negative regulation of protei

g pathway;GO:0009631//cold acclimation;GO:0010762//regulation of fibroblast migration;GO:003

cription from RNA polymerase II promoter;GO:0008285//negative regulation of cell proliferation;G

tive regulation of leukocyte chemotaxis;GO:0007186//G-protein coupled receptor signaling pathw

:0034651//cortisol biosynthetic process;GO:0035725//sodium ion transmembrane transport;GO:00

bolic process;GO:0030301//cholesterol transport;GO:0034372//very-low-density lipoprotein partic

uction;GO:0014066//regulation of phosphatidylinositol 3-kinase signaling;GO:0030101//natural kill

process;GO:0042417//dopamine metabolic process;GO:0042420//dopamine catabolic process;GC

process;GO:0010749//regulation of nitric oxide mediated signal transduction;GO:0019343//cystei
nily amino acid metabolic process;GO:0009066//aspartate family amino acid metabolic process;GC

O:0034379//very-low-density lipoprotein particle assembly;GO:0034435//cholesterol esterificatio

'54//positive regulation of interleukin-5 production;GO:0043951//negative regulation of cAMP-m
ent;GO:0010989//negative regulation of low-density lipoprotein particle clearance;GO:0016567//p

040014//regulation of multicellular organism growth;GO:0044710//single-organism metabolic pr

etabolic process;GO:0044208//'de novo' AMP biosynthetic process;GO:0097294//'de novo' XMP b

y behavior;GO:0009887//organ morphogenesis;GO:0009967//positive regulation of signal transdu

hetic process from pyruvate;GO:0010565//regulation of cellular ketone metabolic process;GO:001

onse to DNA damage;GO:0010467//gene expression;GO:0010884//positive regulation of lipid stor

vascular endothelial growth factor production;GO:0010828//positive regulation of glucose transp

nal transduction;GO:0032008//positive regulation of TOR signaling;GO:0035556//intracellular sig

ıetabolic process;GO:0044208//'de novo' AMP biosynthetic process;GO:0097294//'de novo' XMP b

y behavior;GO:0009887//organ morphogenesis;GO:0009967//positive regulation of signal transdu

O:0045944//positive regulation of transcription from RNA polymerase II promoter;GO:0048863//st

ılation of protein secretion;GO:0050821//protein stabilization;GO:0060348//bone development;GC
nily amino acid metabolic process;GO:0009066//aspartate family amino acid metabolic process;GC

bolic process;GO:0030301//cholesterol transport;GO:0034372//very-low-density lipoprotein partic

ent;GO:0010989//negative regulation of low-density lipoprotein particle clearance;GO:0016567//p

e to lysosome transport;GO:0030705//cytoskeleton-dependent intracellular transport;GO:0031122

99//nervous system development;GO:0007411//axon guidance;GO:0007416//synapse assembly;GC

;GO:0016032//viral process;GO:0044260//cellular macromolecule metabolic process;GO:0044826/

nthetic process;GO:0010255//glucose mediated signaling pathway;GO:0031915//positive regulatio

on;GO:0007405//neuroblast proliferation;GO:0007420//brain development;GO:0007623//circadian

//defense response to bacterium;GO:0050829//defense response to Gram-negative bacterium;GC

vascular endothelial growth factor production;GO:0010828//positive regulation of glucose transp
onse to DNA damage;GO:0010467//gene expression;GO:0010884//positive regulation of lipid stora
netabolic process;GO:0044208//'de novo' AMP biosynthetic process;GO:0097294//'de novo' XMP b

tive regulation of leukocyte chemotaxis;GO:0007186//G-protein coupled receptor signaling pathw

nse to starvation;GO:0010886//positive regulation of cholesterol storage;GO:0032933//SREBP signa

onse to glucose starvation;GO:0043066//negative regulation of apoptotic process;GO:0045931//po
is specification, embryo;GO:0010171//body morphogenesis;GO:0010591//regulation of lamellipoc

uction;GO:0014066//regulation of phosphatidylinositol 3-kinase signaling;GO:0030101//natural kill

on;GO:0009437//carnitine metabolic process;GO:0030855//epithelial cell differentiation

O:0045944//positive regulation of transcription from RNA polymerase II promoter;GO:0048863//st

nily amino acid metabolic process;GO:0009066//aspartate family amino acid metabolic process;GC

naling pathway;GO:0050909//sensory perception of taste;GO:0050913//sensory perception of bitte
91//regulation of heart rate by cardiac conduction;GO:0090136//epithelial cell-cell adhesion;GO:0
O:0034379//very-low-density lipoprotein particle assembly;GO:0034435//cholesterol esterificatior
ent;GO:0010989//negative regulation of low-density lipoprotein particle clearance;GO:0016567//p

havior;GO:0016358//dendrite development;GO:0021517//ventral spinal cord development;GO:002

033387//putrescine biosynthetic process from ornithine;GO:0042176//regulation of protein catabc

O:0034379//very-low-density lipoprotein particle assembly;GO:0034435//cholesterol esterificatior

lic acid biosynthetic process;GO:0010898//positive regulation of triglyceride catabolic process;GO:

l//skeletal muscle fiber development;GO:0048875//chemical homeostasis within a tissue;GO:0051[illegible]

O:0009820//alkaloid metabolic process;GO:0009822//alkaloid catabolic process;GO:0016098//mor
process;GO:0042417//dopamine metabolic process;GO:0042420//dopamine catabolic process;GC

se to heat;GO:0010467//gene expression;GO:0010468//regulation of gene expression;GO:001056[illegible]
jnal transduction;GO:0032008//positive regulation of TOR signaling;GO:0035556//intracellular sigr

99//nervous system development;GO:0007411//axon guidance;GO:0007416//synapse assembly;G[illegible]

030198//extracellular matrix organization;GO:0070373//negative regulation of ERK1 and ERK2 cas[illegible]

:hetic process from pyruvate;GO:0010565//regulation of cellular ketone metabolic process;GO:001

uction;GO:0014066//regulation of phosphatidylinositol 3-kinase signaling;GO:0030101//natural kill

GO:0071276//cellular response to cadmium ion;GO:0071280//cellular response to copper ion;GO:[illegible]
sis;GO:0030913//paranodal junction assembly;GO:0042552//myelination;GO:0044255//cellular lipi[illegible]

y behavior;GO:0009887//organ morphogenesis;GO:0009967//positive regulation of signal transdu

iotic;GO:0071280//cellular response to copper ion;GO:0071420//cellular response to histamine;GC

nthetic process;GO:0010255//glucose mediated signaling pathway;GO:0031915//positive regulatio
tion of cholesterol efflux;GO:0010888//negative regulation of lipid storage;GO:0030301//cholester

elopment;GO:0008203//cholesterol metabolic process;GO:0009566//fertilization;GO:0009615//resp

085//transmembrane transport;GO:0061337//cardiac conduction;GO:0071805//potassium ion tran

naling pathway;GO:0050909//sensory perception of taste;GO:0050913//sensory perception of bitte

e response;GO:0006644//phospholipid metabolic process;GO:0006656//phosphatidylcholine biosy
O:0034379//very-low-density lipoprotein particle assembly;GO:0034435//cholesterol esterification

iotic;GO:0071280//cellular response to copper ion;GO:0071420//cellular response to histamine;GC
ranscription from RNA polymerase II promoter;GO:0007614//short-term memory;GO:0010628//pc

//defense response to bacterium;GO:0050829//defense response to Gram-negative bacterium;GC

tive regulation of leukocyte chemotaxis;GO:0007186//G-protein coupled receptor signaling pathw

163//protein catabolic process;GO:0030239//myofibril assembly;GO:0031648//protein destabilizat
l in apoptotic process;GO:0070301//cellular response to hydrogen peroxide;GO:0071456//cellular

nscript (SSU-rRNA, 5.8S rRNA, LSU-rRNA);GO:0006364//rRNA processing;GO:0008283//cell prolife

:ription from RNA polymerase II promoter;GO:0008285//negative regulation of cell proliferation;G

uction;GO:0014066//regulation of phosphatidylinositol 3-kinase signaling;GO:0030101//natural kill
ng pathway;GO:0009631//cold acclimation;GO:0010762//regulation of fibroblast migration;GO:003

se to heat;GO:0010467//gene expression;GO:0010468//regulation of gene expression;GO:001056

:hetic process from pyruvate;GO:0010565//regulation of cellular ketone metabolic process;GO:001

:arget recognition;GO:0007628//adult walking behavior;GO:0008283//cell proliferation;GO:001657

process;GO:0042417//dopamine metabolic process;GO:0042420//dopamine catabolic process;GC

;GO:0007283//spermatogenesis;GO:0007422//peripheral nervous system development;GO:00085

it;GO:0001974//blood vessel remodeling;GO:0003338//metanephros morphogenesis;GO:0006935

ynthetic process;GO:0044208//'de novo' AMP biosynthetic process;GO:0044209//AMP salvage;GC

030501//positive regulation of bone mineralization;GO:0032332//positive regulation of chondrocy

se to heat;GO:0010467//gene expression;GO:0010468//regulation of gene expression;GO:001056(

›rane transport;GO:0070633//transepithelial transport;GO:0072089//stem cell proliferation;GO:190

O:0009820//alkaloid metabolic process;GO:0009822//alkaloid catabolic process;GO:0016098//mor

havior;GO:0016358//dendrite development;GO:0021517//ventral spinal cord development;GO:002
iotic;GO:0071280//cellular response to copper ion;GO:0071420//cellular response to histamine;GC

tion of cholesterol efflux;GO:0010888//negative regulation of lipid storage;GO:0030301//cholester

;GO:0032350//regulation of hormone metabolic process;GO:0045598//regulation of fat cell differe

havior;GO:0016358//dendrite development;GO:0021517//ventral spinal cord development;GO:002

›ment;GO:0010762//regulation of fibroblast migration;GO:0042733//embryonic digit morphogene

)021954//central nervous system neuron development;GO:0032091//negative regulation of protei

ng pathway;GO:0009631//cold acclimation;GO:0010762//regulation of fibroblast migration;GO:003
uction;GO:0014066//regulation of phosphatidylinositol 3-kinase signaling;GO:0030101//natural kil
GO:0071276//cellular response to cadmium ion;GO:0071280//cellular response to copper ion;GO:

iotic;GO:0071280//cellular response to copper ion;GO:0071420//cellular response to histamine;GC

:hetic process from pyruvate;GO:0010565//regulation of cellular ketone metabolic process;GO:001

ctivity;GO:0070328//triglyceride homeostasis;GO:2000352//negative regulation of endothelial cell
cription from RNA polymerase II promoter;GO:0008285//negative regulation of cell proliferation;G
on;GO:0009437//carnitine metabolic process;GO:0030855//epithelial cell differentiation
se to heat;GO:0010467//gene expression;GO:0010468//regulation of gene expression;GO:001056(

brane transport;GO:0070633//transepithelial transport;GO:0072089//stem cell proliferation;GO:190

O:0034379//very-low-density lipoprotein particle assembly;GO:0034435//cholesterol esterificatior
protein kinase B signaling;GO:0071108//protein K48-linked deubiquitination;GO:1990167//proteir

;GO:0010976//positive regulation of neuron projection development;GO:0018108//peptidyl-tyrosi

:0008203//cholesterol metabolic process;GO:0010873//positive regulation of cholesterol esterifica
l storage;GO:0010890//positive regulation of sequestering of triglyceride;GO:0016042//lipid catab

e response;GO:0006644//phospholipid metabolic process;GO:0006656//phosphatidylcholine bios

nily amino acid metabolic process;GO:0009066//aspartate family amino acid metabolic process;GC

ynthetic process;GO:0044208//'de novo' AMP biosynthetic process;GO:0044209//AMP salvage;GC

ranscription from RNA polymerase II promoter;GO:0007614//short-term memory;GO:0010628//pc

esponse to bacterium;GO:0018105//peptidyl-serine phosphorylation;GO:0032868//response to in

GO:0071276//cellular response to cadmium ion;GO:0071280//cellular response to copper ion;GO:
:0055085//transmembrane transport;GO:0071805//potassium ion transmembrane transport

orane transport;GO:0070633//transepithelial transport;GO:0072089//stem cell proliferation;GO:190

havior;GO:0016358//dendrite development;GO:0021517//ventral spinal cord development;GO:002

gnal transduction;GO:0032008//positive regulation of TOR signaling;GO:0035556//intracellular sigr

etabolic process;GO:0044208//'de novo' AMP biosynthetic process;GO:0097294//'de novo' XMP

:0009820//alkaloid metabolic process;GO:0009822//alkaloid catabolic process;GO:0016098//mon

storage;GO:0010890//positive regulation of sequestering of triglyceride;GO:0016042//lipid cata

;GO:0007283//spermatogenesis;GO:0007422//peripheral nervous system development;GO:00085

n;GO:1901224//positive regulation of NIK/NF-kappaB signaling;GO:1904951//positive regulation

process;GO:0042417//dopamine metabolic process;GO:0042420//dopamine catabolic process;G

phic factor receptor signaling pathway;GO:0043410//positive regulation of MAPK cascade;GO:00

havior;GO:0016358//dendrite development;GO:0021517//ventral spinal cord development;GO:002

storage;GO:0010890//positive regulation of sequestering of triglyceride;GO:0016042//lipid cata

ıetic process;GO:0006636//unsaturated fatty acid biosynthetic process;GO:0008610//lipid biosynth

0009888//tissue development;GO:0016125//sterol metabolic process;GO:0030539//male genitalia
ɔrocess;GO:0006650//glycerophospholipid metabolic process;GO:0006655//phosphatidylglycerol b

ıg pathway;GO:0009631//cold acclimation;GO:0010762//regulation of fibroblast migration;GO:003

abolic process;GO:0046839//phospholipid dephosphorylation;GO:1902247//geranylgeranyl diphos
cription from RNA polymerase II promoter;GO:0008285//negative regulation of cell proliferation;G

2351//'de novo' GDP-L-fucose biosynthetic process;GO:1904906//positive regulation of endotheli

ɔrane transport;GO:0070633//transepithelial transport;GO:0072089//stem cell proliferation;GO:190

process;GO:0042417//dopamine metabolic process;GO:0042420//dopamine catabolic process;GC

ol storage;GO:0010890//positive regulation of sequestering of triglyceride;GO:0016042//lipid catab

opment;GO:0090263//positive regulation of canonical Wnt signaling pathway;GO:2001013//epithe

nily amino acid metabolic process;GO:0009066//aspartate family amino acid metabolic process;GC

ynthetic process;GO:0044208//'de novo' AMP biosynthetic process;GO:0044209//AMP salvage;GC

ent;GO:0010989//negative regulation of low-density lipoprotein particle clearance;GO:0016567//p
)021954//central nervous system neuron development;GO:0032091//negative regulation of protei

ranscription from RNA polymerase II promoter;GO:0007614//short-term memory;GO:0010628//po

bolic process;GO:0030301//cholesterol transport;GO:0034372//very-low-density lipoprotein partic

neostasis;GO:0045892//negative regulation of transcription, DNA-templated;GO:0050727//regulat
protein kinase B signaling;GO:0071108//protein K48-linked deubiquitination;GO:1990167//protein

e response;GO:0006644//phospholipid metabolic process;GO:0006656//phosphatidylcholine biosy

;GO:0007283//spermatogenesis;GO:0007422//peripheral nervous system development;GO:00085

y behavior;GO:0009887//organ morphogenesis;GO:0009967//positive regulation of signal transdu

ry behavior;GO:0009887//organ morphogenesis;GO:0009967//positive regulation of signal transdu

nt;GO:0001974//blood vessel remodeling;GO:0003338//metanephros morphogenesis;GO:0006935/

netabolic process;GO:0044208//'de novo' AMP biosynthetic process;GO:0097294//'de novo' XMP b

netrin-activated signaling pathway;GO:0043524//negative regulation of neuron apoptotic process
gnal transduction;GO:0032008//positive regulation of TOR signaling;GO:0035556//intracellular sign

tive regulation of leukocyte chemotaxis;GO:0007186//G-protein coupled receptor signaling pathw

brane transport;GO:0070633//transepithelial transport;GO:0072089//stem cell proliferation;GO:190

nembrane transport;GO:0051899//membrane depolarization;GO:0060048//cardiac muscle contrac

)030032//lamellipodium assembly;GO:0030036//actin cytoskeleton organization;GO:0035239//tub
process;GO:0010749//regulation of nitric oxide mediated signal transduction;GO:0019343//cystei

009415//response to water;GO:0035725//sodium ion transmembrane transport;GO:0050912//dete

port;GO:0007186//G-protein coupled receptor signaling pathway;GO:0007189//adenylate cyclase-

tive regulation of phosphoprotein phosphatase activity;GO:0051726//regulation of cell cycle;GO:1

ranscription from RNA polymerase II promoter;GO:0007614//short-term memory;GO:0010628//p

l storage;GO:0010890//positive regulation of sequestering of triglyceride;GO:0016042//lipid catab

e response;GO:0006644//phospholipid metabolic process;GO:0006656//phosphatidylcholine bios

tive regulation of leukocyte chemotaxis;GO:0007186//G-protein coupled receptor signaling pathw

ynthetic process;GO:0044208//'de novo' AMP biosynthetic process;GO:0044209//AMP salvage;GC
y behavior;GO:0009887//organ morphogenesis;GO:0009967//positive regulation of signal transdu

process;GO:0042417//dopamine metabolic process;GO:0042420//dopamine catabolic process;GC

)163//protein catabolic process;GO:0030239//myofibril assembly;GO:0031648//protein destabilizat

;GO:0032350//regulation of hormone metabolic process;GO:0045598//regulation of fat cell differe

arget recognition;GO:0007628//adult walking behavior;GO:0008283//cell proliferation;GO:001657
e to virus;GO:0031110//regulation of microtubule polymerization or depolymerization;GO:003111

ative regulation of neuron projection development;GO:0021540//corpus callosum morphogenesis;

):0010628//positive regulation of gene expression;GO:0016559//peroxisome fission;GO:0048208/

:0042177//negative regulation of protein catabolic process;GO:0043407//negative regulation of M

e to lysosome transport;GO:0030705//cytoskeleton-dependent intracellular transport;GO:0031122

itive regulation of leukocyte chemotaxis;GO:0007186//G-protein coupled receptor signaling pathw

otaxis;GO:0006954//inflammatory response;GO:0006955//immune response;GO:0007186//G-prot

ent;GO:0010989//negative regulation of low-density lipoprotein particle clearance;GO:0016567//p

)085//transmembrane transport;GO:0061337//cardiac conduction;GO:0071805//potassium ion tran

ent;GO:0010989//negative regulation of low-density lipoprotein particle clearance;GO:0016567//p
O:0009820//alkaloid metabolic process;GO:0009822//alkaloid catabolic process;GO:0016098//mor

bolic process;GO:0030301//cholesterol transport;GO:0034372//very-low-density lipoprotein partic
0033005//positive regulation of mast cell activation;GO:1904894//positive regulation of STAT casca

oort;GO:0007186//G-protein coupled receptor signaling pathway;GO:0007189//adenylate cyclase-

julation of gene expression;GO:0010719//negative regulation of epithelial to mesenchymal transiti

99//nervous system development;GO:0007411//axon guidance;GO:0007416//synapse assembly;G(
GO:0071276//cellular response to cadmium ion;GO:0071280//cellular response to copper ion;GO:

;GO:0007283//spermatogenesis;GO:0007422//peripheral nervous system development;GO:00085[illegible]

ve regulation of epithelial cell migration;GO:0030149//sphingolipid catabolic process;GO:0030334/

cell proliferation;GO:0008543//fibroblast growth factor receptor signaling pathway;GO:0009617//r

ynthetic process;GO:0044208//'de novo' AMP biosynthetic process;GO:0044209//AMP salvage;GC

161//calcium-independent cell-matrix adhesion;GO:0007229//integrin-mediated signaling pathwa
sitive regulation of CREB transcription factor activity;GO:0043065//positive regulation of apoptotic

0033005//positive regulation of mast cell activation;GO:1904894//positive regulation of STAT casca

//defense response to bacterium;GO:0050829//defense response to Gram-negative bacterium;GC

orting V-type ATPase complex assembly;GO:0090263//positive regulation of canonical Wnt signal

O:0010628//positive regulation of gene expression;GO:0016559//peroxisome fission;GO:0048208/

ynthetic process;GO:0044208//'de novo' AMP biosynthetic process;GO:0044209//AMP salvage;GC
nbrane transport;GO:1902476//chloride transmembrane transport;GO:1990573//potassium ion im

lic acid biosynthetic process;GO:0010898//positive regulation of triglyceride catabolic process;GO:

bolic process;GO:0030301//cholesterol transport;GO:0034372//very-low-density lipoprotein partic

ıeuron migration;GO:0001775//cell activation;GO:0001817//regulation of cytokine production;GO:(

O:0009820//alkaloid metabolic process;GO:0009822//alkaloid catabolic process;GO:0016098//mor
e response;GO:0006644//phospholipid metabolic process;GO:0006656//phosphatidylcholine biosy

ent;GO:0010989//negative regulation of low-density lipoprotein particle clearance;GO:0016567//p

99//nervous system development;GO:0007411//axon guidance;GO:0007416//synapse assembly;G(

ranscription from RNA polymerase II promoter;GO:0007614//short-term memory;GO:0010628//pc

process;GO:0010749//regulation of nitric oxide mediated signal transduction;GO:0019343//cystei
nembrane transport;GO:0051899//membrane depolarization;GO:0060048//cardiac muscle contrac

g;GO:0030968//endoplasmic reticulum unfolded protein response;GO:0032024//positive regulatio
tive regulation of leukocyte chemotaxis;GO:0007186//G-protein coupled receptor signaling pathw
ı;GO:0007283//spermatogenesis;GO:0007422//peripheral nervous system development;GO:00085

ling pathway;GO:0008360//regulation of cell shape;GO:0030168//platelet activation;GO:0035589//

GO:0071276//cellular response to cadmium ion;GO:0071280//cellular response to copper ion;GO:
jnal transduction;GO:0032008//positive regulation of TOR signaling;GO:0035556//intracellular sigr

ic process;GO:0007626//locomotory behavior;GO:0021768//nucleus accumbens development;GO:

99//nervous system development;GO:0007411//axon guidance;GO:0007416//synapse assembly;G(

:hetic process from pyruvate;GO:0010565//regulation of cellular ketone metabolic process;GO:001

ative regulation of neuron projection development;GO:0021540//corpus callosum morphogenesis;

/e regulation of epithelial cell migration;GO:0030149//sphingolipid catabolic process;GO:0030334/

ort;GO:0042908//xenobiotic transport;GO:0055085//transmembrane transport;GO:0089718//aminc
nbrane transport;GO:1902476//chloride transmembrane transport;GO:1990573//potassium ion im

ıtive regulation of leukocyte chemotaxis;GO:0007186//G-protein coupled receptor signaling pathw

se to heat;GO:0010467//gene expression;GO:0010468//regulation of gene expression;GO:001056(

161//calcium-independent cell-matrix adhesion;GO:0007229//integrin-mediated signaling pathwa
ıg pathway;GO:0009631//cold acclimation;GO:0010762//regulation of fibroblast migration;GO:003

:ription from RNA polymerase II promoter;GO:0008285//negative regulation of cell proliferation;G

:ription from RNA polymerase II promoter;GO:0008285//negative regulation of cell proliferation;G

iotic;GO:0071280//cellular response to copper ion;GO:0071420//cellular response to histamine;GC

ıg pathway;GO:0009631//cold acclimation;GO:0010762//regulation of fibroblast migration;GO:003

'/transmembrane transport;GO:0071332//cellular response to fructose stimulus;GO:1990539//fruct
process;GO:0010749//regulation of nitric oxide mediated signal transduction;GO:0019343//cystei

i7//positive regulation of cytokinesis;GO:0035556//intracellular signal transduction;GO:0042307//p

ndothelial growth factor receptor signaling pathway;GO:0042326//negative regulation of phospho

ງnal transduction;GO:0032008//positive regulation of TOR signaling;GO:0035556//intracellular sigr

99//nervous system development;GO:0007411//axon guidance;GO:0007416//synapse assembly;G(
)32330//regulation of chondrocyte differentiation;GO:0036150//phosphatidylserine acyl-chain rem

ɔrane transport;GO:0070633//transepithelial transport;GO:0072089//stem cell proliferation;GO:190

netabolic process;GO:0044208//'de novo' AMP biosynthetic process;GO:0097294//'de novo' XMP b

//defense response to bacterium;GO:0050829//defense response to Gram-negative bacterium;GC

ɔrocess;GO:0006650//glycerophospholipid metabolic process;GO:0006655//phosphatidylglycerol b

bolic process;GO:0030301//cholesterol transport;GO:0034372//very-low-density lipoprotein partic

:hetic process from pyruvate;GO:0010565//regulation of cellular ketone metabolic process;GO:001

ling pathway;GO:0008360//regulation of cell shape;GO:0030168//platelet activation;GO:0035589//

e response;GO:0006644//phospholipid metabolic process;GO:0006656//phosphatidylcholine bios

)32000//positive regulation of fatty acid beta-oxidation;GO:0032868//response to insulin;GO:0032

ranscription from RNA polymerase II promoter;GO:0007614//short-term memory;GO:0010628//pc

beta receptor signaling pathway;GO:0030512//negative regulation of transforming growth factor b

;GO:0032350//regulation of hormone metabolic process;GO:0045598//regulation of fat cell differe

344//cysteine biosynthetic process;GO:0019346//transsulfuration;GO:0043066//negative regulatior

jnal transduction;GO:0032008//positive regulation of TOR signaling;GO:0035556//intracellular sigr

nily amino acid metabolic process;GO:0009066//aspartate family amino acid metabolic process;GC

ng pathway;GO:0009631//cold acclimation;GO:0010762//regulation of fibroblast migration;GO:003

process;GO:0009058//biosynthetic process;GO:0009888//tissue development;GO:0030223//neutr

GO:0030301//cholesterol transport;GO:0034372//very-low-density lipoprotein particle remodeling
cription from RNA polymerase II promoter;GO:0008285//negative regulation of cell proliferation;G

ent;GO:0010989//negative regulation of low-density lipoprotein particle clearance;GO:0016567//p

thetic process from pyruvate;GO:0010565//regulation of cellular ketone metabolic process;GO:001

cription from RNA polymerase II promoter;GO:0008285//negative regulation of cell proliferation;G

netrin-activated signaling pathway;GO:0043524//negative regulation of neuron apoptotic process

thway;GO:0009617//response to bacterium;GO:0043547//positive regulation of GTPase activity;GC

ıetabolic process;GO:0044208//'de novo' AMP biosynthetic process;GO:0097294//'de novo' XMP b

ıg pathway;GO:0009631//cold acclimation;GO:0010762//regulation of fibroblast migration;GO:003

onse to DNA damage;GO:0010467//gene expression;GO:0010884//positive regulation of lipid stora

vascular endothelial growth factor production;GO:0010828//positive regulation of glucose transp

)32330//regulation of chondrocyte differentiation;GO:0036150//phosphatidylserine acyl-chain rem
ss;GO:0007165//signal transduction;GO:0007186//G-protein coupled receptor signaling pathway;
diester bond hydrolysis;GO:0090502//RNA phosphodiester bond hydrolysis, endonucleolytic

//defense response to bacterium;GO:0050829//defense response to Gram-negative bacterium;GC

response to bacterium;GO:0018105//peptidyl-serine phosphorylation;GO:0032868//response to in
gulation of cytosolic calcium ion concentration;GO:0019722//calcium-mediated signaling;GO:006C

ı of neuron projection development;GO:0030154//cell differentiation;GO:0030516//regulation of a

gulation of cytosolic calcium ion concentration;GO:0019722//calcium-mediated signaling;GO:006C

or receptor signaling pathway;GO:0048546//digestive tract morphogenesis;GO:0048639//positive

//defense response to bacterium;GO:0050829//defense response to Gram-negative bacterium;GC

nily amino acid metabolic process;GO:0009066//aspartate family amino acid metabolic process;GC
bolic process;GO:0030301//cholesterol transport;GO:0034372//very-low-density lipoprotein partic

beta receptor signaling pathway;GO:0030512//negative regulation of transforming growth factor b

ubiquitin-dependent protein catabolic process;GO:0031146//SCF-dependent proteasomal ubiqu

gnal transduction;GO:0032008//positive regulation of TOR signaling;GO:0035556//intracellular sigr

GO:0071276//cellular response to cadmium ion;GO:0071280//cellular response to copper ion;GO:

GO:0030301//cholesterol transport;GO:0034372//very-low-density lipoprotein particle remodeling
GO:0034379//very-low-density lipoprotein particle assembly;GO:0034435//cholesterol esterificatior

nsport;GO:0098609//cell-cell adhesion;GO:1901896//positive regulation of calcium-transporting A

havior;GO:0016358//dendrite development;GO:0021517//ventral spinal cord development;GO:002

process;GO:0042417//dopamine metabolic process;GO:0042420//dopamine catabolic process;GC

ulation of transcription, DNA-templated;GO:0045944//positive regulation of transcription from RN

O:0009820//alkaloid metabolic process;GO:0009822//alkaloid catabolic process;GO:0016098//mor

orane transport;GO:0070633//transepithelial transport;GO:0072089//stem cell proliferation;GO:190

ent;GO:0010989//negative regulation of low-density lipoprotein particle clearance;GO:0016567//p

3//glandular epithelial cell development;GO:0003417//growth plate cartilage development;GO:000

uction;GO:0014066//regulation of phosphatidylinositol 3-kinase signaling;GO:0030101//natural kill

085//transmembrane transport;GO:0061337//cardiac conduction;GO:0071805//potassium ion tran

nily amino acid metabolic process;GO:0009066//aspartate family amino acid metabolic process;GC

ive regulation of MAP kinase activity;GO:0051897//positive regulation of protein kinase B signaling

nal transduction;GO:0042532//negative regulation of tyrosine phosphorylation of STAT protein;GC

gnal transduction;GO:0032008//positive regulation of TOR signaling;GO:0035556//intracellular sigr

ynthetic process;GO:0044208//'de novo' AMP biosynthetic process;GO:0044209//AMP salvage;GC

//transmembrane transport;GO:0071332//cellular response to fructose stimulus;GO:1990539//fruct

040014//regulation of multicellular organism growth;GO:0044710//single-organism metabolic prc

nsition;GO:0008284//positive regulation of cell proliferation;GO:0008543//fibroblast growth factor
netabolic process;GO:0044208//'de novo' AMP biosynthetic process;GO:0097294//'de novo' XMP b

ubiquitin-dependent protein catabolic process;GO:0031146//SCF-dependent proteasomal ubiqu

mbling;GO:0032365//intracellular lipid transport;GO:0044830//modulation by host of viral RNA ge

O:0034379//very-low-density lipoprotein particle assembly;GO:0034435//cholesterol esterificatior
hway;GO:0042593//glucose homeostasis;GO:0050729//positive regulation of inflammatory respon

GO:0071276//cellular response to cadmium ion;GO:0071280//cellular response to copper ion;GO:

;GO:0010760//negative regulation of macrophage chemotaxis;GO:0042326//negative regulation o

iotic;GO:0071280//cellular response to copper ion;GO:0071420//cellular response to histamine;GC

)021954//central nervous system neuron development;GO:0032091//negative regulation of protei

ise II promoter;GO:0006952//defense response;GO:0007165//signal transduction;GO:0007259//JAI

0007179//transforming growth factor beta receptor signaling pathway;GO:0007229//integrin-mec

ent;GO:0010989//negative regulation of low-density lipoprotein particle clearance;GO:0016567//p

ranscription from RNA polymerase II promoter;GO:0007614//short-term memory;GO:0010628//pc
):0009820//alkaloid metabolic process;GO:0009822//alkaloid catabolic process;GO:0016098//mor
cription from RNA polymerase II promoter;GO:0008285//negative regulation of cell proliferation;G

iction;GO:0014066//regulation of phosphatidylinositol 3-kinase signaling;GO:0030101//natural kill

se to heat;GO:0010467//gene expression;GO:0010468//regulation of gene expression;GO:001056(

e response;GO:0006644//phospholipid metabolic process;GO:0006656//phosphatidylcholine bios

ig pathway;GO:0009631//cold acclimation;GO:0010762//regulation of fibroblast migration;GO:003
ithetic process;GO:0010255//glucose mediated signaling pathway;GO:0031915//positive regulatio

itive regulation of leukocyte chemotaxis;GO:0007186//G-protein coupled receptor signaling pathw
ss;GO:0007165//signal transduction;GO:0007186//G-protein coupled receptor signaling pathway;

diester bond hydrolysis;GO:0090502//RNA phosphodiester bond hydrolysis, endonucleolytic

:ination;GO:0032410//negative regulation of transporter activity;GO:0051224//negative regulation

'y behavior;GO:0009887//organ morphogenesis;GO:0009967//positive regulation of signal transdu

netrin-activated signaling pathway;GO:0043524//negative regulation of neuron apoptotic process

nal transduction;GO:0042532//negative regulation of tyrosine phosphorylation of STAT protein;GC

process;GO:0042417//dopamine metabolic process;GO:0042420//dopamine catabolic process;GC
ı;GO:0007283//spermatogenesis;GO:0007422//peripheral nervous system development;GO:00085[illegible]

sis;GO:0030913//paranodal junction assembly;GO:0042552//myelination;GO:0044255//cellular lipi

iotic;GO:0071280//cellular response to copper ion;GO:0071420//cellular response to histamine;GC

ıeuron migration;GO:0001775//cell activation;GO:0001817//regulation of cytokine production;GO:[illegible]

)085//transmembrane transport;GO:0061337//cardiac conduction;GO:0071805//potassium ion tran

ng pathway;GO:0044539//long-chain fatty acid import;GO:0071902//positive regulation of protei

se to heat;GO:0010467//gene expression;GO:0010468//regulation of gene expression;GO:001056

nsition;GO:0008284//positive regulation of cell proliferation;GO:0008543//fibroblast growth facto

)19433//triglyceride catabolic process;GO:0032496//response to lipopolysaccharide;GO:0042311//

behavior;GO:0036343//psychomotor behavior;GO:0042127//regulation of cell proliferation;GO:00

;GO:0010760//negative regulation of macrophage chemotaxis;GO:0042326//negative regulation o

-to lesion;GO:0006974//cellular response to DNA damage stimulus;GO:0008152//metabolic proce
ranscription from RNA polymerase II promoter;GO:0007614//short-term memory;GO:0010628//p

tive regulation of leukocyte chemotaxis;GO:0007186//G-protein coupled receptor signaling path

ynthetic process;GO:0044208//'de novo' AMP biosynthetic process;GO:0044209//AMP salvage;GC

77//cell migration;GO:0035987//endodermal cell differentiation;GO:0043149//stress fiber assemb

e response;GO:0006644//phospholipid metabolic process;GO:0006656//phosphatidylcholine bios
thway;GO:0009617//response to bacterium;GO:0043547//positive regulation of GTPase activity;GC
ı transport into cytosol;GO:0010595//positive regulation of endothelial cell migration;GO:001086

uction by p53 class mediator resulting in transcription of p21 class mediator;GO:0031571//mitotic

):0008203//cholesterol metabolic process;GO:0010873//positive regulation of cholesterol esterifica

98//positive regulation of protein tyrosine kinase activity;GO:0061099//negative regulation of prot

y behavior;GO:0009887//organ morphogenesis;GO:0009967//positive regulation of signal transdu

tion of cholesterol efflux;GO:0010888//negative regulation of lipid storage;GO:0030301//cholester
ise to starvation;GO:0010886//positive regulation of cholesterol storage;GO:0032933//SREBP signa

eart trabecula formation;GO:1900087//positive regulation of G1/S transition of mitotic cell cycle;G
417//central nervous system development;GO:0008544//epidermis development;GO:0010467//ger
ative regulation of neuron projection development;GO:0021540//corpus callosum morphogenesis;

idase activity;GO:0044871//negative regulation by host of viral glycoprotein metabolic process;GO

085//transmembrane transport;GO:0061337//cardiac conduction;GO:0071805//potassium ion tran
otaxis;GO:0006954//inflammatory response;GO:0006955//immune response;GO:0007186//G-prot

neostasis;GO:0045892//negative regulation of transcription, DNA-templated;GO:0050727//regulat
GO:0071276//cellular response to cadmium ion;GO:0071280//cellular response to copper ion;GO:

032000//positive regulation of fatty acid beta-oxidation;GO:0032868//response to insulin;GO:0032

onse to glucose starvation;GO:0043066//negative regulation of apoptotic process;GO:0045931//pc

;GO:0035914//skeletal muscle cell differentiation;GO:0045663//positive regulation of myoblast diff
uction;GO:0014066//regulation of phosphatidylinositol 3-kinase signaling;GO:0030101//natural kill
O:0009820//alkaloid metabolic process;GO:0009822//alkaloid catabolic process;GO:0016098//mor

tion;GO:0030334//regulation of cell migration;GO:0030517//negative regulation of axon extension

of ATPase activity;GO:0034975//protein folding in endoplasmic reticulum;GO:0034976//response t

ulin secretion;GO:0048791//calcium ion-regulated exocytosis of neurotransmitter;GO:0061669//sp

nthetic process;GO:0010255//glucose mediated signaling pathway;GO:0031915//positive regulatio

bolic process;GO:0030301//cholesterol transport;GO:0034372//very-low-density lipoprotein partic

O:0034379//very-low-density lipoprotein particle assembly;GO:0034435//cholesterol esterificatior

aling pathway;GO:0007193//adenylate cyclase-inhibiting G-protein coupled receptor signaling pat

417//central nervous system development;GO:0008544//epidermis development;GO:0010467//ger

onse to DNA damage;GO:0010467//gene expression;GO:0010884//positive regulation of lipid stor

vascular endothelial growth factor production;GO:0010828//positive regulation of glucose transp

n transport into cytosol;GO:0010595//positive regulation of endothelial cell migration;GO:0010863

ranscription from RNA polymerase II promoter;GO:0007614//short-term memory;GO:0010628//po

033387//putrescine biosynthetic process from ornithine;GO:0042176//regulation of protein catabo

e response;GO:0006644//phospholipid metabolic process;GO:0006656//phosphatidylcholine biosy

process;GO:0042417//dopamine metabolic process;GO:0042420//dopamine catabolic process;GO

on;GO:0009437//carnitine metabolic process;GO:0030855//epithelial cell differentiation
pyramidal neuron development;GO:0021952//central nervous system projection neuron axonogen
007165//signal transduction;GO:0008203//cholesterol metabolic process;GO:0009395//phospholip

uction;GO:0014066//regulation of phosphatidylinositol 3-kinase signaling;GO:0030101//natural kill
brane transport;GO:0070633//transepithelial transport;GO:0072089//stem cell proliferation;GO:190
ulin secretion;GO:0048791//calcium ion-regulated exocytosis of neurotransmitter;GO:0061669//sp

ietic stem cell differentiation;GO:0070231//T cell apoptotic process;GO:0090090//negative regulat
bolic process;GO:0030301//cholesterol transport;GO:0034372//very-low-density lipoprotein partic

30336//negative regulation of cell migration;GO:0042542//response to hydrogen peroxide;GO:00

nthetic process;GO:0010255//glucose mediated signaling pathway;GO:0031915//positive regulatio

eostasis;GO:0045892//negative regulation of transcription, DNA-templated;GO:0050727//regulat

rt;GO:0042908//xenobiotic transport;GO:0055085//transmembrane transport;GO:0089718//aminc
:0008203//cholesterol metabolic process;GO:0010873//positive regulation of cholesterol esterifica
;GO:0007283//spermatogenesis;GO:0007422//peripheral nervous system development;GO:00085

O:0034379//very-low-density lipoprotein particle assembly;GO:0034435//cholesterol esterificatior

y behavior;GO:0009887//organ morphogenesis;GO:0009967//positive regulation of signal transdu

y behavior;GO:0009887//organ morphogenesis;GO:0009967//positive regulation of signal transdu

e response;GO:0006644//phospholipid metabolic process;GO:0006656//phosphatidylcholine bios

tion of cholesterol efflux;GO:0010888//negative regulation of lipid storage;GO:0030301//cholester

ocyte differentiation;GO:0035988//chondrocyte proliferation;GO:0042246//tissue regeneration;GO

l storage;GO:0010890//positive regulation of sequestering of triglyceride;GO:0016042//lipid catab
7//positive regulation of cytokinesis;GO:0035556//intracellular signal transduction;GO:0042307//p

ıeostasis;GO:0045892//negative regulation of transcription, DNA-templated;GO:0050727//regulat

ranscription from RNA polymerase II promoter;GO:0007614//short-term memory;GO:0010628//pc

se to heat;GO:0010467//gene expression;GO:0010468//regulation of gene expression;GO:001056(

ɔpment;GO:0090263//positive regulation of canonical Wnt signaling pathway;GO:2001013//epithe

ndothelial growth factor receptor signaling pathway;GO:0042326//negative regulation of phospho

O:0034379//very-low-density lipoprotein particle assembly;GO:0034435//cholesterol esterificatior

iotic;GO:0071280//cellular response to copper ion;GO:0071420//cellular response to histamine;GC

ıetabolic process;GO:0044208//'de novo' AMP biosynthetic process;GO:0097294//'de novo' XMP k

process;GO:0010749//regulation of nitric oxide mediated signal transduction;GO:0019343//cystei
orane transport;GO:0070633//transepithelial transport;GO:0072089//stem cell proliferation;GO:190
ol storage;GO:0010890//positive regulation of sequestering of triglyceride;GO:0016042//lipid catab

;GO:0010760//negative regulation of macrophage chemotaxis;GO:0042326//negative regulation o

uction;GO:0014066//regulation of phosphatidylinositol 3-kinase signaling;GO:0030101//natural kill

ry behavior;GO:0009887//organ morphogenesis;GO:0009967//positive regulation of signal transdu

process;GO:0042417//dopamine metabolic process;GO:0042420//dopamine catabolic process;GC

99//nervous system development;GO:0007411//axon guidance;GO:0007416//synapse assembly;G(

neuron migration;GO:0001775//cell activation;GO:0001817//regulation of cytokine production;GO:(

344//cysteine biosynthetic process;GO:0019346//transsulfuration;GO:0043066//negative regulatior

on;GO:0007405//neuroblast proliferation;GO:0007420//brain development;GO:0007623//circadian

nily amino acid metabolic process;GO:0009066//aspartate family amino acid metabolic process;GC

bolic process;GO:0030301//cholesterol transport;GO:0034372//very-low-density lipoprotein partic

:hetic process from pyruvate;GO:0010565//regulation of cellular ketone metabolic process;GO:001
orane transport;GO:0070633//transepithelial transport;GO:0072089//stem cell proliferation;GO:190

tion of cholesterol efflux;GO:0010888//negative regulation of lipid storage;GO:0030301//cholester

:0030335//positive regulation of cell migration;GO:0032715//negative regulation of interleukin-6 |

process;GO:0042417//dopamine metabolic process;GO:0042420//dopamine catabolic process;GC

or receptor signaling pathway;GO:0048546//digestive tract morphogenesis;GO:0048639//positive

se to heat;GO:0010467//gene expression;GO:0010468//regulation of gene expression;GO:001056(

havior;GO:0016358//dendrite development;GO:0021517//ventral spinal cord development;GO:002
O:0009820//alkaloid metabolic process;GO:0009822//alkaloid catabolic process;GO:0016098//mor

brane transport;GO:0090277//positive regulation of peptide hormone secretion;GO:0098810//neu

bolic process;GO:0030301//cholesterol transport;GO:0034372//very-low-density lipoprotein partic

ocyte differentiation;GO:0035988//chondrocyte proliferation;GO:0042246//tissue regeneration;GO

99//nervous system development;GO:0007411//axon guidance;GO:0007416//synapse assembly;G(

docytic zone membrane;GO:0031623//receptor internalization;GO:0048488//synaptic vesicle endo

iotic;GO:0071280//cellular response to copper ion;GO:0071420//cellular response to histamine;GC

GO:0071276//cellular response to cadmium ion;GO:0071280//cellular response to copper ion;GO:[illegible]

-//adult behavior;GO:0035176//social behavior;GO:0048488//synaptic vesicle endocytosis;GO:0050
ıetabolic process;GO:0044208//'de novo' AMP biosynthetic process;GO:0097294//'de novo' XMP b

ıegative regulation of peptidyl-cysteine S-nitrosylation;GO:1903204//negative regulation of oxida

)33387//putrescine biosynthetic process from ornithine;GO:0042176//regulation of protein catabc

ranscription from RNA polymerase II promoter;GO:0007614//short-term memory;GO:0010628//pc

bolic process;GO:0030301//cholesterol transport;GO:0034372//very-low-density lipoprotein partic

e response;GO:0006644//phospholipid metabolic process;GO:0006656//phosphatidylcholine bios
e to lysosome transport;GO:0030705//cytoskeleton-dependent intracellular transport;GO:0031122

//defense response to bacterium;GO:0050829//defense response to Gram-negative bacterium;GC

:hetic process from pyruvate;GO:0010565//regulation of cellular ketone metabolic process;GO:001
O:0034379//very-low-density lipoprotein particle assembly;GO:0034435//cholesterol esterificatior

/transmembrane transport;GO:0071332//cellular response to fructose stimulus;GO:1990539//fruct

27//regulation of inflammatory response;GO:0050730//regulation of peptidyl-tyrosine phosphoryl
ynthetic process;GO:0044208//'de novo' AMP biosynthetic process;GO:0044209//AMP salvage;GC

nily amino acid metabolic process;GO:0009066//aspartate family amino acid metabolic process;GC

ositive regulation of cholesterol efflux;GO:0030216//keratinocyte differentiation;GO:0031424//kera

process;GO:0042417//dopamine metabolic process;GO:0042420//dopamine catabolic process;GC

0007179//transforming growth factor beta receptor signaling pathway;GO:0007229//integrin-mec

O:0009820//alkaloid metabolic process;GO:0009822//alkaloid catabolic process;GO:0016098//mor

nesion;GO:0033631//cell-cell adhesion mediated by integrin;GO:0034113//heterotypic cell-cell adh

ninoacylation;GO:0016525//negative regulation of angiogenesis;GO:1904046//negative regulation

uction;GO:0014066//regulation of phosphatidylinositol 3-kinase signaling;GO:0030101//natural kill

33//B cell differentiation;GO:0030217//T cell differentiation;GO:0033152//immunoglobulin V(D)J re
brane transport;GO:0070633//transepithelial transport;GO:0072089//stem cell proliferation;GO:190
99//nervous system development;GO:0007411//axon guidance;GO:0007416//synapse assembly;G

GO:0071276//cellular response to cadmium ion;GO:0071280//cellular response to copper ion;GO:

):0043547//positive regulation of GTPase activity;GO:0051451//myoblast migration;GO:0070301//c

:0030335//positive regulation of cell migration;GO:0032715//negative regulation of interleukin-6 p

nily amino acid metabolic process;GO:0009066//aspartate family amino acid metabolic process;GC

neuron migration;GO:0001775//cell activation;GO:0001817//regulation of cytokine production;GO:

negative regulation of peptidyl-cysteine S-nitrosylation;GO:1903204//negative regulation of oxida

idase activity;GO:0044871//negative regulation by host of viral glycoprotein metabolic process;GO

;GO:0032350//regulation of hormone metabolic process;GO:0045598//regulation of fat cell differe

007165//signal transduction;GO:0008203//cholesterol metabolic process;GO:0009395//phospholip

on;GO:0009437//carnitine metabolic process;GO:0030855//epithelial cell differentiation

havior;GO:0016358//dendrite development;GO:0021517//ventral spinal cord development;GO:002

netabolic process;GO:0044208//'de novo' AMP biosynthetic process;GO:0097294//'de novo' XMP b

thetic process from pyruvate;GO:0010565//regulation of cellular ketone metabolic process;GO:001

iotic;GO:0071280//cellular response to copper ion;GO:0071420//cellular response to histamine;GC

l storage;GO:0010890//positive regulation of sequestering of triglyceride;GO:0016042//lipid catab
pyramidal neuron development;GO:0021952//central nervous system projection neuron axonogen

ynthetic process;GO:0044208//'de novo' AMP biosynthetic process;GO:0044209//AMP salvage;GC

ng pathway;GO:0009631//cold acclimation;GO:0010762//regulation of fibroblast migration;GO:003

gulation of cytosolic calcium ion concentration;GO:0019722//calcium-mediated signaling;GO:0060

netabolic process;GO:0044208//'de novo' AMP biosynthetic process;GO:0097294//'de novo' XMP b

se to heat;GO:0010467//gene expression;GO:0010468//regulation of gene expression;GO:001056(

se to heat;GO:0010467//gene expression;GO:0010468//regulation of gene expression;GO:001056(

cription from RNA polymerase II promoter;GO:0008285//negative regulation of cell proliferation;G

nic acid metabolic process;GO:0022008//neurogenesis;GO:0042136//neurotransmitter biosynthetic

iotic;GO:0071280//cellular response to copper ion;GO:0071420//cellular response to histamine;GC

nily amino acid metabolic process;GO:0009066//aspartate family amino acid metabolic process;GC
ynthetic process;GO:0044208//'de novo' AMP biosynthetic process;GO:0044209//AMP salvage;GC

nily amino acid metabolic process;GO:0009066//aspartate family amino acid metabolic process;GC

hway;GO:0042593//glucose homeostasis;GO:0050729//positive regulation of inflammatory respon

GO:0030301//cholesterol transport;GO:0034372//very-low-density lipoprotein particle remodeling
ve regulation of epithelial cell migration;GO:0030149//sphingolipid catabolic process;GO:0030334/

eceptor signaling pathway;GO:0050930//induction of positive chemotaxis;GO:0060754//positive re

//transmembrane transport;GO:0071332//cellular response to fructose stimulus;GO:1990539//fruct
response to bacterium;GO:0018105//peptidyl-serine phosphorylation;GO:0032868//response to in

tive regulation of leukocyte chemotaxis;GO:0007186//G-protein coupled receptor signaling pathw

161//calcium-independent cell-matrix adhesion;GO:0007229//integrin-mediated signaling pathwa

on;GO:0035999//tetrahydrofolate interconversion;GO:0050667//homocysteine metabolic process;G
ng pathway;GO:0009631//cold acclimation;GO:0010762//regulation of fibroblast migration;GO:003
754//positive regulation of interleukin-5 production;GO:0043951//negative regulation of cAMP-m
metabolic process;GO:0044208//'de novo' AMP biosynthetic process;GO:0097294//'de novo' XMP b

.//adult behavior;GO:0035176//social behavior;GO:0048488//synaptic vesicle endocytosis;GO:0050

cription from RNA polymerase II promoter;GO:0008285//negative regulation of cell proliferation;G

gnal transduction;GO:0032008//positive regulation of TOR signaling;GO:0035556//intracellular sigr

iotic;GO:0071280//cellular response to copper ion;GO:0071420//cellular response to histamine;GC

ynthetic process;GO:0044208//'de novo' AMP biosynthetic process;GO:0044209//AMP salvage;GC

0033005//positive regulation of mast cell activation;GO:1904894//positive regulation of STAT casca

se to heat;GO:0010467//gene expression;GO:0010468//regulation of gene expression;GO:001056(

ess;GO:0030325//adrenal gland development;GO:0033144//negative regulation of intracellular ste
98//positive regulation of protein tyrosine kinase activity;GO:0061099//negative regulation of prot

e cyclase-modulating G-protein coupled receptor signaling pathway;GO:0007420//brain develop[illegible]

ling pathway;GO:0008360//regulation of cell shape;GO:0030168//platelet activation;GO:0035589//

77//cell migration;GO:0035987//endodermal cell differentiation;GO:0043149//stress fiber assembl
opment;GO:0090263//positive regulation of canonical Wnt signaling pathway;GO:2001013//epithe

l storage;GO:0010890//positive regulation of sequestering of triglyceride;GO:0016042//lipid catab

hway;GO:0042593//glucose homeostasis;GO:0050729//positive regulation of inflammatory respon

rocess;GO:0006650//glycerophospholipid metabolic process;GO:0006655//phosphatidylglycerol b

se II promoter;GO:0006952//defense response;GO:0007165//signal transduction;GO:0007259//JAI

33//B cell differentiation;GO:0030217//T cell differentiation;GO:0033152//immunoglobulin V(D)J re

;GO:0035914//skeletal muscle cell differentiation;GO:0045663//positive regulation of myoblast diff

nal transduction;GO:0032008//positive regulation of TOR signaling;GO:0035556//intracellular sigr

negative regulation of peptidyl-cysteine S-nitrosylation;GO:1903204//negative regulation of oxida

gulation of cytosolic calcium ion concentration;GO:0019722//calcium-mediated signaling;GO:0060

ent;GO:0010989//negative regulation of low-density lipoprotein particle clearance;GO:0016567//p

pyramidal neuron development;GO:0021952//central nervous system projection neuron axonogen

019433//triglyceride catabolic process;GO:0032496//response to lipopolysaccharide;GO:0042311//

ol storage;GO:0010890//positive regulation of sequestering of triglyceride;GO:0016042//lipid catab

docytic zone membrane;GO:0031623//receptor internalization;GO:0048488//synaptic vesicle endo
positive regulation of cholesterol efflux;GO:0030216//keratinocyte differentiation;GO:0031424//ker

heostasis;GO:0045892//negative regulation of transcription, DNA-templated;GO:0050727//regulat
tive regulation of leukocyte chemotaxis;GO:0007186//G-protein coupled receptor signaling pathw

864//pyrimidine nucleotide transport;GO:0007005//mitochondrion organization;GO:0008284//pos

ent;GO:0010989//negative regulation of low-density lipoprotein particle clearance;GO:0016567//p

GO:0030301//cholesterol transport;GO:0034372//very-low-density lipoprotein particle remodeling
085//transmembrane transport;GO:0061337//cardiac conduction;GO:0071805//potassium ion tran

nily amino acid metabolic process;GO:0009066//aspartate family amino acid metabolic process;GC

:ination;GO:0032410//negative regulation of transporter activity;GO:0051224//negative regulation

/e regulation of epithelial cell migration;GO:0030149//sphingolipid catabolic process;GO:0030334/

iotic;GO:0071280//cellular response to copper ion;GO:0071420//cellular response to histamine;GC
netabolic process;GO:0044208//'de novo' AMP biosynthetic process;GO:0097294//'de novo' XMP b

l storage;GO:0010890//positive regulation of sequestering of triglyceride;GO:0016042//lipid catab

ess;GO:0030325//adrenal gland development;GO:0033144//negative regulation of intracellular ste

161//calcium-independent cell-matrix adhesion;GO:0007229//integrin-mediated signaling pathwa

se to heat;GO:0010467//gene expression;GO:0010468//regulation of gene expression;GO:001056(

ıetabolic process;GO:0044208//'de novo' AMP biosynthetic process;GO:0097294//'de novo' XMP b

nembrane transport;GO:0051899//membrane depolarization;GO:0060048//cardiac muscle contrac

docytic zone membrane;GO:0031623//receptor internalization;GO:0048488//synaptic vesicle endo

nily amino acid metabolic process;GO:0009066//aspartate family amino acid metabolic process;GC
864//pyrimidine nucleotide transport;GO:0007005//mitochondrion organization;GO:0008284//pos

ent;GO:0010989//negative regulation of low-density lipoprotein particle clearance;GO:0016567//p
e to lysosome transport;GO:0030705//cytoskeleton-dependent intracellular transport;GO:0031122
ıt;GO:0001974//blood vessel remodeling;GO:0003338//metanephros morphogenesis;GO:0006935/

lic acid biosynthetic process;GO:0010898//positive regulation of triglyceride catabolic process;GO:

040014//regulation of multicellular organism growth;GO:0044710//single-organism metabolic pro
ınal transduction;GO:0032008//positive regulation of TOR signaling;GO:0035556//intracellular sigr

ent;GO:0010989//negative regulation of low-density lipoprotein particle clearance;GO:0016567//p
O:0034379//very-low-density lipoprotein particle assembly;GO:0034435//cholesterol esterificatior

99//nervous system development;GO:0007411//axon guidance;GO:0007416//synapse assembly;G(

process;GO:0010749//regulation of nitric oxide mediated signal transduction;GO:0019343//cystei

eceptor signaling pathway;GO:0050930//induction of positive chemotaxis;GO:0060754//positive re
ling pathway;GO:0008360//regulation of cell shape;GO:0030168//platelet activation;GO:0035589//
uction;GO:0014066//regulation of phosphatidylinositol 3-kinase signaling;GO:0030101//natural kil

tive regulation of leukocyte chemotaxis;GO:0007186//G-protein coupled receptor signaling pathw
y behavior;GO:0009887//organ morphogenesis;GO:0009967//positive regulation of signal transdu

nthetic process;GO:0010255//glucose mediated signaling pathway;GO:0031915//positive regulatio

f toll-like receptor 9 signaling pathway;GO:0034499//late endosome to Golgi transport;GO:00456

otaxis;GO:0006954//inflammatory response;GO:0006955//immune response;GO:0007186//G-prot

ive regulation of MAP kinase activity;GO:0051897//positive regulation of protein kinase B signaling

uction;GO:0014066//regulation of phosphatidylinositol 3-kinase signaling;GO:0030101//natural kill

otaxis;GO:0006954//inflammatory response;GO:0006955//immune response;GO:0007186//G-prot

3//glandular epithelial cell development;GO:0003417//growth plate cartilage development;GO:000

ulation of protein secretion;GO:0050821//protein stabilization;GO:0060348//bone development;GC

/e regulation of epithelial cell migration;GO:0030149//sphingolipid catabolic process;GO:0030334/

ı;GO:0007283//spermatogenesis;GO:0007422//peripheral nervous system development;GO:00085[illegible]

e response;GO:0006644//phospholipid metabolic process;GO:0006656//phosphatidylcholine bios[illegible]
O:0034379//very-low-density lipoprotein particle assembly;GO:0034435//cholesterol esterificatio[illegible]
ceptor signaling pathway;GO:0050930//induction of positive chemotaxis;GO:0060754//positive re
ative regulation of neuron projection development;GO:0021540//corpus callosum morphogenesis;

e cyclase-modulating G-protein coupled receptor signaling pathway;GO:0007420//brain develop[illegible]

161//calcium-independent cell-matrix adhesion;GO:0007229//integrin-mediated signaling pathwa
l storage;GO:0010890//positive regulation of sequestering of triglyceride;GO:0016042//lipid catab
ranscription from RNA polymerase II promoter;GO:0007614//short-term memory;GO:0010628//p[illegible]

19433//triglyceride catabolic process;GO:0032496//response to lipopolysaccharide;GO:0042311//

cription from RNA polymerase II promoter;GO:0008285//negative regulation of cell proliferation;G[illegible]

ng pathway;GO:0009631//cold acclimation;GO:0010762//regulation of fibroblast migration;GO:003

)163//protein catabolic process;GO:0030239//myofibril assembly;GO:0031648//protein destabilizat

l in apoptotic process;GO:0070301//cellular response to hydrogen peroxide;GO:0071456//cellular

havior;GO:0016358//dendrite development;GO:0021517//ventral spinal cord development;GO:002

itive regulation of leukocyte chemotaxis;GO:0007186//G-protein coupled receptor signaling pathw

ative regulation of neuron projection development;GO:0021540//corpus callosum morphogenesis;

ng pathway;GO:0044539//long-chain fatty acid import;GO:0071902//positive regulation of protein

lic process;GO:0050776//regulation of immune response;GO:0072539//T-helper 17 cell differentiat
ling pathway;GO:0008360//regulation of cell shape;GO:0030168//platelet activation;GO:0035589//

nal transduction;GO:0042532//negative regulation of tyrosine phosphorylation of STAT protein;GC

ɪnal transduction;GO:0032008//positive regulation of TOR signaling;GO:0035556//intracellular sigr

ˈy behavior;GO:0009887//organ morphogenesis;GO:0009967//positive regulation of signal transdu

ıeuron migration;GO:0001775//cell activation;GO:0001817//regulation of cytokine production;GO:(

hway;GO:0042593//glucose homeostasis;GO:0050729//positive regulation of inflammatory respon

diester bond hydrolysis;GO:0090502//RNA phosphodiester bond hydrolysis, endonucleolytic

ɛss;GO:0007165//signal transduction;GO:0007186//G-protein coupled receptor signaling pathway;

ı;GO:0007283//spermatogenesis;GO:0007422//peripheral nervous system development;GO:00085

e response;GO:0006644//phospholipid metabolic process;GO:0006656//phosphatidylcholine bios

tion of cholesterol efflux;GO:0010888//negative regulation of lipid storage;GO:0030301//cholester

ɔO:0034379//very-low-density lipoprotein particle assembly;GO:0034435//cholesterol esterificatior
99//nervous system development;GO:0007411//axon guidance;GO:0007416//synapse assembly;G(

ulin secretion;GO:0048791//calcium ion-regulated exocytosis of neurotransmitter;GO:0061669//sp

ınsition;GO:0008284//positive regulation of cell proliferation;GO:0008543//fibroblast growth factor
O:0009820//alkaloid metabolic process;GO:0009822//alkaloid catabolic process;GO:0016098//mor

ranscription from RNA polymerase II promoter;GO:0007614//short-term memory;GO:0010628//po

process;GO:0010749//regulation of nitric oxide mediated signal transduction;GO:0019343//cystei

99//nervous system development;GO:0007411//axon guidance;GO:0007416//synapse assembly;G(

ositive regulation of cholesterol efflux;GO:0030216//keratinocyte differentiation;GO:0031424//kera

GO:0071276//cellular response to cadmium ion;GO:0071280//cellular response to copper ion;GO:

iotic;GO:0071280//cellular response to copper ion;GO:0071420//cellular response to histamine;GC

uction;GO:0014066//regulation of phosphatidylinositol 3-kinase signaling;GO:0030101//natural kill

olic process;GO:0050996//positive regulation of lipid catabolic process;GO:0051006//positive regu

otaxis;GO:0006954//inflammatory response;GO:0006955//immune response;GO:0007186//G-prot

cription from RNA polymerase II promoter;GO:0008285//negative regulation of cell proliferation;G

417//central nervous system development;GO:0008544//epidermis development;GO:0010467//ger

se to heat;GO:0010467//gene expression;GO:0010468//regulation of gene expression;GO:001056[illegible]

g;GO:0030968//endoplasmic reticulum unfolded protein response;GO:0032024//positive regulatio[illegible]

ng pathway;GO:0009631//cold acclimation;GO:0010762//regulation of fibroblast migration;GO:003[illegible]

tem myelin maintenance;GO:0042127//regulation of cell proliferation;GO:0045576//mast cell activ[illegible]

GO:0071276//cellular response to cadmium ion;GO:0071280//cellular response to copper ion;GO:[illegible]

hesion;GO:0033631//cell-cell adhesion mediated by integrin;GO:0034113//heterotypic cell-cell adh[illegible]
99//nervous system development;GO:0007411//axon guidance;GO:0007416//synapse assembly;G[illegible]

havior;GO:0016358//dendrite development;GO:0021517//ventral spinal cord development;GO:002[illegible]

//transmembrane transport;GO:0071332//cellular response to fructose stimulus;GO:1990539//fruct[illegible]
//defense response to bacterium;GO:0050829//defense response to Gram-negative bacterium;G[illegible]

032000//positive regulation of fatty acid beta-oxidation;GO:0032868//response to insulin;GO:0032[illegible]

O:0009820//alkaloid metabolic process;GO:0009822//alkaloid catabolic process;GO:0016098//mo[illegible]

O:0009820//alkaloid metabolic process;GO:0009822//alkaloid catabolic process;GO:0016098//mor

ative regulation of neuron projection development;GO:0021540//corpus callosum morphogenesis;
ulation of protein secretion;GO:0050821//protein stabilization;GO:0060348//bone development;GC

y behavior;GO:0009887//organ morphogenesis;GO:0009967//positive regulation of signal transdu

GO:0071276//cellular response to cadmium ion;GO:0071280//cellular response to copper ion;GO:

olic process;GO:0050996//positive regulation of lipid catabolic process;GO:0051006//positive regu

orane transport;GO:0070633//transepithelial transport;GO:0072089//stem cell proliferation;GO:190

ess;GO:0030325//adrenal gland development;GO:0033144//negative regulation of intracellular ste

ynthetic process;GO:0044208//'de novo' AMP biosynthetic process;GO:0044209//AMP salvage;GC

nal transduction;GO:0042532//negative regulation of tyrosine phosphorylation of STAT protein;GC

3O:0030301//cholesterol transport;GO:0034372//very-low-density lipoprotein particle remodeling
ic process;GO:0007626//locomotory behavior;GO:0021768//nucleus accumbens development;GO:

ı transport into cytosol;GO:0010595//positive regulation of endothelial cell migration;GO:0010863

ıg pathway;GO:0009631//cold acclimation;GO:0010762//regulation of fibroblast migration;GO:003

:0042177//negative regulation of protein catabolic process;GO:0043407//negative regulation of M
e response;GO:0006644//phospholipid metabolic process;GO:0006656//phosphatidylcholine bios

cription from RNA polymerase II promoter;GO:0008285//negative regulation of cell proliferation;G

ınsition;GO:0008284//positive regulation of cell proliferation;GO:0008543//fibroblast growth factor

ranscription from RNA polymerase II promoter;GO:0007614//short-term memory;GO:0010628//pc

//defense response to bacterium;GO:0050829//defense response to Gram-negative bacterium;GC

on of lipid biosynthetic process;GO:0052651//monoacylglycerol catabolic process;GO:0060292//lo

ative regulation of neuron projection development;GO:0021540//corpus callosum morphogenesis;

gulation of cytosolic calcium ion concentration;GO:0019722//calcium-mediated signaling;GO:0060

gulation of cytosolic calcium ion concentration;GO:0019722//calcium-mediated signaling;GO:0060

GO:0071276//cellular response to cadmium ion;GO:0071280//cellular response to copper ion;GO:

brane transport;GO:0070633//transepithelial transport;GO:0072089//stem cell proliferation;GO:190

;GO:0010976//positive regulation of neuron projection development;GO:0018108//peptidyl-tyrosi

0163//protein catabolic process;GO:0030239//myofibril assembly;GO:0031648//protein destabilizat

ort;GO:0042908//xenobiotic transport;GO:0055085//transmembrane transport;GO:0089718//amino

O:0009820//alkaloid metabolic process;GO:0009822//alkaloid catabolic process;GO:0016098//mor

y behavior;GO:0009887//organ morphogenesis;GO:0009967//positive regulation of signal transdu

009415//response to water;GO:0035725//sodium ion transmembrane transport;GO:0050912//dete

GO:0030301//cholesterol transport;GO:0034372//very-low-density lipoprotein particle remodeling
99//nervous system development;GO:0007411//axon guidance;GO:0007416//synapse assembly;G

0033005//positive regulation of mast cell activation;GO:1904894//positive regulation of STAT casca

bolic process;GO:0030301//cholesterol transport;GO:0034372//very-low-density lipoprotein partic

nal transduction;GO:0042532//negative regulation of tyrosine phosphorylation of STAT protein;G

bolic process;GO:0030301//cholesterol transport;GO:0034372//very-low-density lipoprotein partic

O:0009820//alkaloid metabolic process;GO:0009822//alkaloid catabolic process;GO:0016098//mor

gnal transduction;GO:0032008//positive regulation of TOR signaling;GO:0035556//intracellular sigr

ndothelial growth factor receptor signaling pathway;GO:0042326//negative regulation of phospho

GO:0071276//cellular response to cadmium ion;GO:0071280//cellular response to copper ion;GO:

on of lipid biosynthetic process;GO:0052651//monoacylglycerol catabolic process;GO:0060292//lo

ng pathway;GO:0009631//cold acclimation;GO:0010762//regulation of fibroblast migration;GO:003

process;GO:0042417//dopamine metabolic process;GO:0042420//dopamine catabolic process;GC

ınsition;GO:0008284//positive regulation of cell proliferation;GO:0008543//fibroblast growth factor
temic arterial blood pressure;GO:0007186//G-protein coupled receptor signaling pathway;GO:000

cription from RNA polymerase II promoter;GO:0008285//negative regulation of cell proliferation;G

ynthetic process;GO:0044208//'de novo' AMP biosynthetic process;GO:0044209//AMP salvage;GC

'/transmembrane transport;GO:0071332//cellular response to fructose stimulus;GO:1990539//fruct

onse to glucose starvation;GO:0043066//negative regulation of apoptotic process;GO:0045931//po

38//protein metabolic process;GO:0051289//protein homotetramerization;GO:0055088//lipid hom
e development;GO:0030539//male genitalia development;GO:0060065//uterus development;GO:0

344//cysteine biosynthetic process;GO:0019346//transsulfuration;GO:0043066//negative regulatio

ol storage;GO:0010890//positive regulation of sequestering of triglyceride;GO:0016042//lipid catab

bolic process;GO:0030301//cholesterol transport;GO:0034372//very-low-density lipoprotein partic
hesion;GO:0033631//cell-cell adhesion mediated by integrin;GO:0034113//heterotypic cell-cell adh

n transport into cytosol;GO:0010595//positive regulation of endothelial cell migration;GO:0010863

target recognition;GO:0007628//adult walking behavior;GO:0008283//cell proliferation;GO:001657
y behavior;GO:0009887//organ morphogenesis;GO:0009967//positive regulation of signal transdu

process;GO:0042417//dopamine metabolic process;GO:0042420//dopamine catabolic process;GC

y behavior;GO:0009887//organ morphogenesis;GO:0009967//positive regulation of signal transdu

gnal transduction;GO:0032008//positive regulation of TOR signaling;GO:0035556//intracellular sigr

ocyte differentiation;GO:0035988//chondrocyte proliferation;GO:0042246//tissue regeneration;GO

0009888//tissue development;GO:0016125//sterol metabolic process;GO:0030539//male genitalia

ent;GO:0010989//negative regulation of low-density lipoprotein particle clearance;GO:0016567//p

netrin-activated signaling pathway;GO:0043524//negative regulation of neuron apoptotic process

e development;GO:0030539//male genitalia development;GO:0060065//uterus development;GO:0

on;GO:1901224//positive regulation of NIK/NF-kappaB signaling;GO:1904951//positive regulation

process;GO:0010749//regulation of nitric oxide mediated signal transduction;GO:0019343//cystei

beta receptor signaling pathway;GO:0030512//negative regulation of transforming growth factor b

ol storage;GO:0010890//positive regulation of sequestering of triglyceride;GO:0016042//lipid catab

99//nervous system development;GO:0007411//axon guidance;GO:0007416//synapse assembly;GO

085//transmembrane transport;GO:0061337//cardiac conduction;GO:0071805//potassium ion tran

neuron migration;GO:0001775//cell activation;GO:0001817//regulation of cytokine production;GO:

nthetic process;GO:0010255//glucose mediated signaling pathway;GO:0031915//positive regulatio

f triglyceride biosynthetic process;GO:0032792//negative regulation of CREB transcription factor a
)19433//triglyceride catabolic process;GO:0032496//response to lipopolysaccharide;GO:0042311//
ng pathway;GO:0009631//cold acclimation;GO:0010762//regulation of fibroblast migration;GO:003

sis;GO:0030913//paranodal junction assembly;GO:0042552//myelination;GO:0044255//cellular lipi
ranscription from RNA polymerase II promoter;GO:0007614//short-term memory;GO:0010628//p

cription from RNA polymerase II promoter;GO:0008285//negative regulation of cell proliferation;G

n transport into cytosol;GO:0010595//positive regulation of endothelial cell migration;GO:0010863

e response;GO:0006644//phospholipid metabolic process;GO:0006656//phosphatidylcholine bios
;GO:0010976//positive regulation of neuron projection development;GO:0018108//peptidyl-tyrosi

:0030335//positive regulation of cell migration;GO:0032715//negative regulation of interleukin-6

57//positive regulation of cytokinesis;GO:0035556//intracellular signal transduction;GO:0042307//p

thetic process from pyruvate;GO:0010565//regulation of cellular ketone metabolic process;GO:001

tem myelin maintenance;GO:0042127//regulation of cell proliferation;GO:0045576//mast cell activa
hesion;GO:0033631//cell-cell adhesion mediated by integrin;GO:0034113//heterotypic cell-cell adh
havior;GO:0016358//dendrite development;GO:0021517//ventral spinal cord development;GO:002
:0030335//positive regulation of cell migration;GO:0032715//negative regulation of interleukin-6

ent;GO:0010989//negative regulation of low-density lipoprotein particle clearance;GO:0016567//p

idase activity;GO:0044871//negative regulation by host of viral glycoprotein metabolic process;GO

tive regulation of leukocyte chemotaxis;GO:0007186//G-protein coupled receptor signaling pathw

O:0009820//alkaloid metabolic process;GO:0009822//alkaloid catabolic process;GO:0016098//mor

etic process;GO:0006636//unsaturated fatty acid biosynthetic process;GO:0008610//lipid biosynth

protein kinase B signaling;GO:0071108//protein K48-linked deubiquitination;GO:1990167//protei

GO:0071276//cellular response to cadmium ion;GO:0071280//cellular response to copper ion;GO:

f triglyceride biosynthetic process;GO:0032792//negative regulation of CREB transcription factor a
gnal transduction;GO:0032008//positive regulation of TOR signaling;GO:0035556//intracellular sigr

)85//transmembrane transport;GO:0061337//cardiac conduction;GO:0071805//potassium ion tran
ıse to starvation;GO:0010886//positive regulation of cholesterol storage;GO:0032933//SREBP signa

ng pathway;GO:0044539//long-chain fatty acid import;GO:0071902//positive regulation of protein

otaxis;GO:0006954//inflammatory response;GO:0006955//immune response;GO:0007186//G-prot

/e regulation of epithelial cell migration;GO:0030149//sphingolipid catabolic process;GO:0030334/

ranscription from RNA polymerase II promoter;GO:0007614//short-term memory;GO:0010628//pc

ıction;GO:0014066//regulation of phosphatidylinositol 3-kinase signaling;GO:0030101//natural kill

e response;GO:0006644//phospholipid metabolic process;GO:0006656//phosphatidylcholine biosy
se to heat;GO:0010467//gene expression;GO:0010468//regulation of gene expression;GO:001056(
161//calcium-independent cell-matrix adhesion;GO:0007229//integrin-mediated signaling pathwa

)163//protein catabolic process;GO:0030239//myofibril assembly;GO:0031648//protein destabilizat

ocyte differentiation;GO:0035988//chondrocyte proliferation;GO:0042246//tissue regeneration;GO

tion of cholesterol efflux;GO:0010888//negative regulation of lipid storage;GO:0030301//cholester

ocyte differentiation;GO:0035988//chondrocyte proliferation;GO:0042246//tissue regeneration;GO
iO:0034379//very-low-density lipoprotein particle assembly;GO:0034435//cholesterol esterificatior

thway;GO:0009617//response to bacterium;GO:0043547//positive regulation of GTPase activity;GC

julation of gene expression;GO:0010719//negative regulation of epithelial to mesenchymal transiti

eceptor signaling pathway;GO:0050930//induction of positive chemotaxis;GO:0060754//positive re

:0055085//transmembrane transport;GO:0071805//potassium ion transmembrane transport

havior;GO:0016358//dendrite development;GO:0021517//ventral spinal cord development;GO:002

hway;GO:0042593//glucose homeostasis;GO:0050729//positive regulation of inflammatory respon
99//nervous system development;GO:0007411//axon guidance;GO:0007416//synapse assembly;G(
iotic;GO:0071280//cellular response to copper ion;GO:0071420//cellular response to histamine;GC
or receptor signaling pathway;GO:0048546//digestive tract morphogenesis;GO:0048639//positive
iO:0034379//very-low-density lipoprotein particle assembly;GO:0034435//cholesterol esterificatior

process;GO:0010749//regulation of nitric oxide mediated signal transduction;GO:0019343//cystei

ıthetic process;GO:0010255//glucose mediated signaling pathway;GO:0031915//positive regulatio

ıction;GO:0014066//regulation of phosphatidylinositol 3-kinase signaling;GO:0030101//natural kil

nbrane transport;GO:1902476//chloride transmembrane transport;GO:1990573//potassium ion im

ıtive regulation of leukocyte chemotaxis;GO:0007186//G-protein coupled receptor signaling pathw

ling pathway;GO:0008360//regulation of cell shape;GO:0030168//platelet activation;GO:0035589//

ıeostasis;GO:0045892//negative regulation of transcription, DNA-templated;GO:0050727//regulat
l in apoptotic process;GO:0070301//cellular response to hydrogen peroxide;GO:0071456//cellular

ıyramidal neuron development;GO:0021952//central nervous system projection neuron axonogen

protein kinase B signaling;GO:0071108//protein K48-linked deubiquitination;GO:1990167//protei
ıction;GO:0014066//regulation of phosphatidylinositol 3-kinase signaling;GO:0030101//natural kil

hway;GO:0042593//glucose homeostasis;GO:0050729//positive regulation of inflammatory respon

ort;GO:0042908//xenobiotic transport;GO:0055085//transmembrane transport;GO:0089718//amin

ent;GO:0010989//negative regulation of low-density lipoprotein particle clearance;GO:0016567//p

eceptor signaling pathway;GO:0050930//induction of positive chemotaxis;GO:0060754//positive re

iO:0034379//very-low-density lipoprotein particle assembly;GO:0034435//cholesterol esterificatior

port;GO:0070508//cholesterol import;GO:0070859//positive regulation of bile acid biosynthetic prc
ocyte differentiation;GO:0035988//chondrocyte proliferation;GO:0042246//tissue regeneration;GO

:0030335//positive regulation of cell migration;GO:0032715//negative regulation of interleukin-6 p

)19433//triglyceride catabolic process;GO:0032496//response to lipopolysaccharide;GO:0042311//

julation of gene expression;GO:0010719//negative regulation of epithelial to mesenchymal transiti
neostasis;GO:0045892//negative regulation of transcription, DNA-templated;GO:0050727//regulat

ng pathway;GO:0009631//cold acclimation;GO:0010762//regulation of fibroblast migration;GO:003

se to heat;GO:0010467//gene expression;GO:0010468//regulation of gene expression;GO:001056(
030501//positive regulation of bone mineralization;GO:0032332//positive regulation of chondrocy

cription from RNA polymerase II promoter;GO:0008285//negative regulation of cell proliferation;G

ndothelial growth factor receptor signaling pathway;GO:0042326//negative regulation of phospho

ol storage;GO:0010890//positive regulation of sequestering of triglyceride;GO:0016042//lipid catab

thetic process from pyruvate;GO:0010565//regulation of cellular ketone metabolic process;GO:001

iotic;GO:0071280//cellular response to copper ion;GO:0071420//cellular response to histamine;GC
temic arterial blood pressure;GO:0007186//G-protein coupled receptor signaling pathway;GO:000

process;GO:0009058//biosynthetic process;GO:0009888//tissue development;GO:0030223//neutr

tion of cholesterol efflux;GO:0010888//negative regulation of lipid storage;GO:0030301//cholester

)32000//positive regulation of fatty acid beta-oxidation;GO:0032868//response to insulin;GO:0032
positive regulation of cholesterol efflux;GO:0030216//keratinocyte differentiation;GO:0031424//kera

thway;GO:0009617//response to bacterium;GO:0043547//positive regulation of GTPase activity;GC

bolic process;GO:0030301//cholesterol transport;GO:0034372//very-low-density lipoprotein partic

344//cysteine biosynthetic process;GO:0019346//transsulfuration;GO:0043066//negative regulation

bolic process;GO:0030301//cholesterol transport;GO:0034372//very-low-density lipoprotein partic

ng pathway;GO:0044539//long-chain fatty acid import;GO:0071902//positive regulation of protein

n of neuron projection development;GO:0030154//cell differentiation;GO:0030516//regulation of a

itive regulation of leukocyte chemotaxis;GO:0007186//G-protein coupled receptor signaling pathw

gulation of gene expression;GO:0010719//negative regulation of epithelial to mesenchymal transiti

port;GO:0007186//G-protein coupled receptor signaling pathway;GO:0007189//adenylate cyclase-

gnal transduction;GO:0032008//positive regulation of TOR signaling;GO:0035556//intracellular sign

:0030335//positive regulation of cell migration;GO:0032715//negative regulation of interleukin-6 p
brane transport;GO:0070633//transepithelial transport;GO:0072089//stem cell proliferation;GO:190
ess;GO:0030325//adrenal gland development;GO:0033144//negative regulation of intracellular ste
nily amino acid metabolic process;GO:0009066//aspartate family amino acid metabolic process;GO

n transport into cytosol;GO:0010595//positive regulation of endothelial cell migration;GO:0010863

on;GO:0007405//neuroblast proliferation;GO:0007420//brain development;GO:0007623//circadian

.40//male meiosis;GO:0007141//male meiosis I;GO:0007144//female meiosis I;GO:0009566//fertiliz

neostasis;GO:0045892//negative regulation of transcription, DNA-templated;GO:0050727//regulat

ess;GO:0030325//adrenal gland development;GO:0033144//negative regulation of intracellular ste

gulation of gene expression;GO:0010719//negative regulation of epithelial to mesenchymal transiti

uction;GO:0014066//regulation of phosphatidylinositol 3-kinase signaling;GO:0030101//natural kill

ynthetic process;GO:0044208//'de novo' AMP biosynthetic process;GO:0044209//AMP salvage;GC

netabolic process;GO:0044208//'de novo' AMP biosynthetic process;GO:0097294//'de novo' XMP b

positive regulation of cholesterol efflux;GO:0030216//keratinocyte differentiation;GO:0031424//kera

netabolic process;GO:0044208//'de novo' AMP biosynthetic process;GO:0097294//'de novo' XMP b

ng pathway;GO:0044539//long-chain fatty acid import;GO:0071902//positive regulation of protein
GO:0034379//very-low-density lipoprotein particle assembly;GO:0034435//cholesterol esterification

)33387//putrescine biosynthetic process from ornithine;GO:0042176//regulation of protein catabo

:hetic process from pyruvate;GO:0010565//regulation of cellular ketone metabolic process;GO:001

ıeuron migration;GO:0001775//cell activation;GO:0001817//regulation of cytokine production;GO:

nily amino acid metabolic process;GO:0009066//aspartate family amino acid metabolic process;GC
ɔyramidal neuron development;GO:0021952//central nervous system projection neuron axonogen

hway;GO:0042593//glucose homeostasis;GO:0050729//positive regulation of inflammatory respon

ıeostasis;GO:0045892//negative regulation of transcription, DNA-templated;GO:0050727//regulat

ɔrane transport;GO:0070633//transepithelial transport;GO:0072089//stem cell proliferation;GO:190

ent;GO:0010989//negative regulation of low-density lipoprotein particle clearance;GO:0016567//p

ative regulation of neuron projection development;GO:0021540//corpus callosum morphogenesis;

bolic process;GO:0030301//cholesterol transport;GO:0034372//very-low-density lipoprotein partic
meostasis;GO:0006984//ER-nucleus signaling pathway;GO:0006996//organelle organization;GO:00

nily amino acid metabolic process;GO:0009066//aspartate family amino acid metabolic process;GC

ynthetic process;GO:0044208//'de novo' AMP biosynthetic process;GO:0044209//AMP salvage;GC

hosphorylation;GO:0035307//positive regulation of protein dephosphorylation;GO:0035308//nega

nal transduction;GO:0042532//negative regulation of tyrosine phosphorylation of STAT protein;GC

bolic process;GO:0030301//cholesterol transport;GO:0034372//very-low-density lipoprotein partic

onse to glucose starvation;GO:0043066//negative regulation of apoptotic process;GO:0045931//po

;GO:0035914//skeletal muscle cell differentiation;GO:0045663//positive regulation of myoblast diff

netabolic process;GO:0044208//'de novo' AMP biosynthetic process;GO:0097294//'de novo' XMP b
ng pathway;GO:0044539//long-chain fatty acid import;GO:0071902//positive regulation of protein

process;GO:0042417//dopamine metabolic process;GO:0042420//dopamine catabolic process;GO

:0034651//cortisol biosynthetic process;GO:0035725//sodium ion transmembrane transport;GO:00

ninoacylation;GO:0016525//negative regulation of angiogenesis;GO:1904046//negative regulation

nsition;GO:0008284//positive regulation of cell proliferation;GO:0008543//fibroblast growth factor

ein metabolic process;GO:0016485//protein processing;GO:0016486//peptide hormone processing

nsition;GO:0008284//positive regulation of cell proliferation;GO:0008543//fibroblast growth factor

netabolic process;GO:0044208//'de novo' AMP biosynthetic process;GO:0097294//'de novo' XMP b

eceptor signaling pathway;GO:0050930//induction of positive chemotaxis;GO:0060754//positive re

nily amino acid metabolic process;GO:0009066//aspartate family amino acid metabolic process;GC

:hetic process from pyruvate;GO:0010565//regulation of cellular ketone metabolic process;GO:001

process;GO:0010749//regulation of nitric oxide mediated signal transduction;GO:0019343//cystei

nal transduction;GO:0042532//negative regulation of tyrosine phosphorylation of STAT protein;GC

;O:0034116//positive regulation of heterotypic cell-cell adhesion;GO:0034622//cellular macromole

'/transmembrane transport;GO:0071332//cellular response to fructose stimulus;GO:1990539//fruct

ess;GO:0030325//adrenal gland development;GO:0033144//negative regulation of intracellular ste

meostasis;GO:0006984//ER-nucleus signaling pathway;GO:0006996//organelle organization;GO:0(

ynthetic process;GO:0044208//'de novo' AMP biosynthetic process;GO:0044209//AMP salvage;GC

ıtive regulation of leukocyte chemotaxis;GO:0007186//G-protein coupled receptor signaling pathw

hosphorylation;GO:0035307//positive regulation of protein dephosphorylation;GO:0035308//nega

98//positive regulation of protein tyrosine kinase activity;GO:0061099//negative regulation of prot

0002759//regulation of antimicrobial humoral response;GO:0003148//outflow tract septum morph
:0042177//negative regulation of protein catabolic process;GO:0043407//negative regulation of M

regulation of Ras protein signal transduction;GO:0070536//protein K63-linked deubiquitination

ein metabolic process;GO:0016485//protein processing;GO:0016486//peptide hormone processin

ılation of protein secretion;GO:0050821//protein stabilization;GO:0060348//bone development;GC

;GO:0030968//endoplasmic reticulum unfolded protein response;GO:0032024//positive regulatio

elopment;GO:0008203//cholesterol metabolic process;GO:0009566//fertilization;GO:0009615//resp
)163//protein catabolic process;GO:0030239//myofibril assembly;GO:0031648//protein destabilizat

cription from RNA polymerase II promoter;GO:0008285//negative regulation of cell proliferation;G

ng pathway;GO:0044539//long-chain fatty acid import;GO:0071902//positive regulation of protei

velopment;GO:0021528//commissural neuron differentiation in spinal cord;GO:0021960//anterior

ng pathway;GO:0009631//cold acclimation;GO:0010762//regulation of fibroblast migration;GO:003

f triglyceride biosynthetic process;GO:0032792//negative regulation of CREB transcription factor a

ansport;GO:0043268//positive regulation of potassium ion transport;GO:0045214//sarcomere orga
behavior;GO:0036343//psychomotor behavior;GO:0042127//regulation of cell proliferation;GO:004
hosphorylation;GO:0035307//positive regulation of protein dephosphorylation;GO:0035308//nega
orting V-type ATPase complex assembly;GO:0090263//positive regulation of canonical Wnt signal

//defense response to bacterium;GO:0050829//defense response to Gram-negative bacterium;GC

on of Wnt signaling pathway;GO:0030509//BMP signaling pathway;GO:0030514//negative regulatio

quitination;GO:0030163//protein catabolic process;GO:0031647//regulation of protein stability;GO

meostasis;GO:0006984//ER-nucleus signaling pathway;GO:0006996//organelle organization;GO:0(

sitive regulation of CREB transcription factor activity;GO:0043065//positive regulation of apoptotic

009415//response to water;GO:0035725//sodium ion transmembrane transport;GO:0050912//dete

loplasmic reticulum unfolded protein response;GO:0034976//response to endoplasmic reticulum s

velopment;GO:0021528//commissural neuron differentiation in spinal cord;GO:0021960//anterior

030501//positive regulation of bone mineralization;GO:0032332//positive regulation of chondrocy

quitination;GO:0030163//protein catabolic process;GO:0031647//regulation of protein stability;GO
n;GO:0035556//intracellular signal transduction;GO:0046578//regulation of Ras protein signal trar

n;GO:0006487//protein N-linked glycosylation;GO:0010706//ganglioside biosynthetic process via l

nscript (SSU-rRNA, 5.8S rRNA, LSU-rRNA);GO:0006364//rRNA processing;GO:0008283//cell prolife
se II promoter;GO:0006952//defense response;GO:0007165//signal transduction;GO:0007259//JAI

nic acid metabolic process;GO:0022008//neurogenesis;GO:0042136//neurotransmitter biosynthetic

ic process;GO:0007626//locomotory behavior;GO:0021768//nucleus accumbens development;GO:
f triglyceride biosynthetic process;GO:0032792//negative regulation of CREB transcription factor a

eart trabecula formation;GO:1900087//positive regulation of G1/S transition of mitotic cell cycle;G

nsport;GO:0098609//cell-cell adhesion;GO:1901896//positive regulation of calcium-transporting A

030501//positive regulation of bone mineralization;GO:0032332//positive regulation of chondrocy

eration;GO:0034214//protein hexamerization;GO:0034982//mitochondrial protein processing;GO:0

ing;GO:0001975//response to amphetamine;GO:0002027//regulation of heart rate;GO:0003094//g
3//glandular epithelial cell development;GO:0003417//growth plate cartilage development;GO:000

ive regulation of MAP kinase activity;GO:0051897//positive regulation of protein kinase B signaling

g;GO:0030968//endoplasmic reticulum unfolded protein response;GO:0032024//positive regulation

pment;GO:0010762//regulation of fibroblast migration;GO:0042733//embryonic digit morphogene

elopment;GO:0008203//cholesterol metabolic process;GO:0009566//fertilization;GO:0009615//resp

port;GO:0070508//cholesterol import;GO:0070859//positive regulation of bile acid biosynthetic pro

e to virus;GO:0031110//regulation of microtubule polymerization or depolymerization;GO:003111
e specification;GO:0001763//morphogenesis of a branching structure;GO:0001944//vasculature de

uction by p53 class mediator resulting in transcription of p21 class mediator;GO:0031571//mitotic
:0055085//transmembrane transport;GO:0071805//potassium ion transmembrane transport

quitination;GO:0030163//protein catabolic process;GO:0031647//regulation of protein stability;GO

ubiquitin-dependent protein catabolic process;GO:0043161//proteasome-mediated ubiquitin-dep

;GO:0016032//viral process;GO:0044260//cellular macromolecule metabolic process;GO:0044826/

mbling;GO:0032365//intracellular lipid transport;GO:0044830//modulation by host of viral RNA ge

ic process;GO:0007626//locomotory behavior;GO:0021768//nucleus accumbens development;GO:

n;GO:0006487//protein N-linked glycosylation;GO:0010706//ganglioside biosynthetic process via

ansport;GO:0043268//positive regulation of potassium ion transport;GO:0045214//sarcomere orga
tem myelin maintenance;GO:0042127//regulation of cell proliferation;GO:0045576//mast cell activa
temic arterial blood pressure;GO:0007186//G-protein coupled receptor signaling pathway;GO:000

GO:0071276//cellular response to cadmium ion;GO:0071280//cellular response to copper ion;GO:
401//steroid hormone mediated signaling pathway;GO:0046464//acylglycerol catabolic process;GC

0009888//tissue development;GO:0016125//sterol metabolic process;GO:0030539//male genitalia

nsport;GO:0098609//cell-cell adhesion;GO:1901896//positive regulation of calcium-transporting A

ulin secretion;GO:0048791//calcium ion-regulated exocytosis of neurotransmitter;GO:0061669//sp
netic process;GO:0006636//unsaturated fatty acid biosynthetic process;GO:0008610//lipid biosynth

orting V-type ATPase complex assembly;GO:0090263//positive regulation of canonical Wnt signal

docytic zone membrane;GO:0031623//receptor internalization;GO:0048488//synaptic vesicle endo
e development;GO:0030539//male genitalia development;GO:0060065//uterus development;GO:0

O:0009820//alkaloid metabolic process;GO:0009822//alkaloid catabolic process;GO:0016098//mon

of ATPase activity;GO:0034975//protein folding in endoplasmic reticulum;GO:0034976//response t

sitive regulation of CREB transcription factor activity;GO:0043065//positive regulation of apoptotic
on of Wnt signaling pathway;GO:0030509//BMP signaling pathway;GO:0030514//negative regulatio

n;GO:0006487//protein N-linked glycosylation;GO:0010706//ganglioside biosynthetic process via l

030501//positive regulation of bone mineralization;GO:0032332//positive regulation of chondrocy

on;GO:1901224//positive regulation of NIK/NF-kappaB signaling;GO:1904951//positive regulation
e specification;GO:0001763//morphogenesis of a branching structure;GO:0001944//vasculature de

temic arterial blood pressure;GO:0007186//G-protein coupled receptor signaling pathway;GO:000

0002759//regulation of antimicrobial humoral response;GO:0003148//outflow tract septum morph

regulation of Ras protein signal transduction;GO:0070536//protein K63-linked deubiquitination

;GO:0007283//spermatogenesis;GO:0007422//peripheral nervous system development;GO:00085

417//central nervous system development;GO:0008544//epidermis development;GO:0010467//ger

aling pathway;GO:0007193//adenylate cyclase-inhibiting G-protein coupled receptor signaling pat
hway;GO:0042593//glucose homeostasis;GO:0050729//positive regulation of inflammatory respon

030336//negative regulation of cell migration;GO:0042542//response to hydrogen peroxide;GO:00
;GO:0016032//viral process;GO:0044260//cellular macromolecule metabolic process;GO:0044826/

030501//positive regulation of bone mineralization;GO:0032332//positive regulation of chondrocy
;GO:0016032//viral process;GO:0044260//cellular macromolecule metabolic process;GO:0044826/

gulation of gene expression;GO:0010719//negative regulation of epithelial to mesenchymal transiti

abolic process;GO:0046839//phospholipid dephosphorylation;GO:1902247//geranylgeranyl diphos
mbling;GO:0032365//intracellular lipid transport;GO:0044830//modulation by host of viral RNA ge

401//steroid hormone mediated signaling pathway;GO:0046464//acylglycerol catabolic process;GC

bolic process;GO:0030301//cholesterol transport;GO:0034372//very-low-density lipoprotein partic

l cell differentiation;GO:0043065//positive regulation of apoptotic process;GO:0060545//positive re

2351//'de novo' GDP-L-fucose biosynthetic process;GO:1904906//positive regulation of endotheli

orting V-type ATPase complex assembly;GO:0090263//positive regulation of canonical Wnt signal

2351//'de novo' GDP-L-fucose biosynthetic process;GO:1904906//positive regulation of endotheli

e to virus;GO:0031110//regulation of microtubule polymerization or depolymerization;GO:003111

36//positive regulation of germinal center formation;GO:0002686//negative regulation of leukocyt
sitive regulation of CREB transcription factor activity;GO:0043065//positive regulation of apoptotic

ing;GO:0001975//response to amphetamine;GO:0002027//regulation of heart rate;GO:0003094//g

eration;GO:0034214//protein hexamerization;GO:0034982//mitochondrial protein processing;GO:0

030336//negative regulation of cell migration;GO:0042542//response to hydrogen peroxide;GO:00

aling pathway;GO:0007193//adenylate cyclase-inhibiting G-protein coupled receptor signaling pat

temic arterial blood pressure;GO:0007186//G-protein coupled receptor signaling pathway;GO:000
uction by p53 class mediator resulting in transcription of p21 class mediator;GO:0031571//mitotic

on of lipid biosynthetic process;GO:0052651//monoacylglycerol catabolic process;GO:0060292//lc

quitination;GO:0030163//protein catabolic process;GO:0031647//regulation of protein stability;GO

eart trabecula formation;GO:1900087//positive regulation of G1/S transition of mitotic cell cycle;G

loplasmic reticulum unfolded protein response;GO:0034976//response to endoplasmic reticulum s
y behavior;GO:0009887//organ morphogenesis;GO:0009967//positive regulation of signal transdu

;GO:0010976//positive regulation of neuron projection development;GO:0018108//peptidyl-tyrosi

'cellular response to amino acid starvation;GO:0070534//protein K63-linked ubiquitination;GO:007
uscular septum morphogenesis;GO:0003151//outflow tract morphogenesis;GO:0003171//atrioven

meostasis;GO:0006984//ER-nucleus signaling pathway;GO:0006996//organelle organization;GO:0[illegible]

tive regulation of phosphoprotein phosphatase activity;GO:0051726//regulation of cell cycle;GO:1[illegible]

of ATPase activity;GO:0034975//protein folding in endoplasmic reticulum;GO:0034976//response [illegible]

on of Wnt signaling pathway;GO:0030509//BMP signaling pathway;GO:0030514//negative regulati[illegible]

hosphorylation;GO:0035307//positive regulation of protein dephosphorylation;GO:0035308//nega[illegible]

se II promoter;GO:0006952//defense response;GO:0007165//signal transduction;GO:0007259//JA[illegible]

007165//signal transduction;GO:0008203//cholesterol metabolic process;GO:0009395//phospholi[illegible]

thway;GO:0009617//response to bacterium;GO:0043547//positive regulation of GTPase activity;GO[illegible]
e to virus;GO:0031110//regulation of microtubule polymerization or depolymerization;GO:003111[illegible]

0002759//regulation of antimicrobial humoral response;GO:0003148//outflow tract septum morp[illegible]

regulation of Ras protein signal transduction;GO:0070536//protein K63-linked deubiquitination

behavior;GO:0036343//psychomotor behavior;GO:0042127//regulation of cell proliferation;GO:004

of ATPase activity;GO:0034975//protein folding in endoplasmic reticulum;GO:0034976//response t

ein metabolic process;GO:0016485//protein processing;GO:0016486//peptide hormone processin

elopment;GO:0008203//cholesterol metabolic process;GO:0009566//fertilization;GO:0009615//resp

temic arterial blood pressure;GO:0007186//G-protein coupled receptor signaling pathway;GO:000

e specification;GO:0001763//morphogenesis of a branching structure;GO:0001944//vasculature de

eart trabecula formation;GO:1900087//positive regulation of G1/S transition of mitotic cell cycle;G
uction by p53 class mediator resulting in transcription of p21 class mediator;GO:0031571//mitotic

ination;GO:0032410//negative regulation of transporter activity;GO:0051224//negative regulation

uction by p53 class mediator resulting in transcription of p21 class mediator;GO:0031571//mitotic

aling pathway;GO:0007193//adenylate cyclase-inhibiting G-protein coupled receptor signaling pat

nscript (SSU-rRNA, 5.8S rRNA, LSU-rRNA);GO:0006364//rRNA processing;GO:0008283//cell prolife

on;GO:1901224//positive regulation of NIK/NF-kappaB signaling;GO:1904951//positive regulation

e to virus;GO:0031110//regulation of microtubule polymerization or depolymerization;GO:003111

007165//signal transduction;GO:0008203//cholesterol metabolic process;GO:0009395//phospholip

2351//'de novo' GDP-L-fucose biosynthetic process;GO:1904906//positive regulation of endotheli

on;GO:0035556//intracellular signal transduction;GO:0046578//regulation of Ras protein signal tran
36//positive regulation of germinal center formation;GO:0002686//negative regulation of leukocyt

l cell differentiation;GO:0043065//positive regulation of apoptotic process;GO:0060545//positive re

tem myelin maintenance;GO:0042127//regulation of cell proliferation;GO:0045576//mast cell activa

ise II promoter;GO:0006952//defense response;GO:0007165//signal transduction;GO:0007259//JAI

oment;GO:0010762//regulation of fibroblast migration;GO:0042733//embryonic digit morphogene

007165//signal transduction;GO:0008203//cholesterol metabolic process;GO:0009395//phospholip

loplasmic reticulum unfolded protein response;GO:0034976//response to endoplasmic reticulum s
olic process;GO:0050996//positive regulation of lipid catabolic process;GO:0051006//positive regu
behavior;GO:0036343//psychomotor behavior;GO:0042127//regulation of cell proliferation;GO:004

0009888//tissue development;GO:0016125//sterol metabolic process;GO:0030539//male genitalia

meostasis;GO:0006984//ER-nucleus signaling pathway;GO:0006996//organelle organization;GO:00

0002759//regulation of antimicrobial humoral response;GO:0003148//outflow tract septum morph

L//skeletal muscle fiber development;GO:0048875//chemical homeostasis within a tissue;GO:00515
regulation of Ras protein signal transduction;GO:0070536//protein K63-linked deubiquitination

hosphorylation;GO:0035307//positive regulation of protein dephosphorylation;GO:0035308//nega

)30336//negative regulation of cell migration;GO:0042542//response to hydrogen peroxide;GO:00

of ATPase activity;GO:0034975//protein folding in endoplasmic reticulum;GO:0034976//response t

f triglyceride biosynthetic process;GO:0032792//negative regulation of CREB transcription factor ac
uscular septum morphogenesis;GO:0003151//outflow tract morphogenesis;GO:0003171//atrioven

030198//extracellular matrix organization;GO:0070373//negative regulation of ERK1 and ERK2 cas

'cellular response to amino acid starvation;GO:0070534//protein K63-linked ubiquitination;GO:007

cell proliferation;GO:0008543//fibroblast growth factor receptor signaling pathway;GO:0009617//r

sitive regulation of CREB transcription factor activity;GO:0043065//positive regulation of apoptotic

:ein metabolic process;GO:0016485//protein processing;GO:0016486//peptide hormone processin

ıegative regulation of peptidyl-cysteine S-nitrosylation;GO:1903204//negative regulation of oxida

:ination;GO:0032410//negative regulation of transporter activity;GO:0051224//negative regulation

orting V-type ATPase complex assembly;GO:0090263//positive regulation of canonical Wnt signal

2351//'de novo' GDP-L-fucose biosynthetic process;GO:1904906//positive regulation of endotheli
› virus;GO:0010468//regulation of gene expression;GO:0016310//phosphorylation;GO:0042063//gl
98//positive regulation of protein tyrosine kinase activity;GO:0061099//negative regulation of prot
ctivity;GO:0070328//triglyceride homeostasis;GO:2000352//negative regulation of endothelial cell

aling pathway;GO:0007193//adenylate cyclase-inhibiting G-protein coupled receptor signaling pat

:em myelin maintenance;GO:0042127//regulation of cell proliferation;GO:0045576//mast cell activa

l cell differentiation;GO:0043065//positive regulation of apoptotic process;GO:0060545//positive re

lic process;GO:0050776//regulation of immune response;GO:0072539//T-helper 17 cell differentiat

eart trabecula formation;GO:1900087//positive regulation of G1/S transition of mitotic cell cycle;G[illegible]

mbling;GO:0032365//intracellular lipid transport;GO:0044830//modulation by host of viral RNA ge
ubiquitin-dependent protein catabolic process;GO:0043161//proteasome-mediated ubiquitin-dep

nic acid metabolic process;GO:0022008//neurogenesis;GO:0042136//neurotransmitter biosynthetic

030336//negative regulation of cell migration;GO:0042542//response to hydrogen peroxide;GO:00

344//cysteine biosynthetic process;GO:0019346//transsulfuration;GO:0043066//negative regulation

e specification;GO:0001763//morphogenesis of a branching structure;GO:0001944//vasculature de
030501//positive regulation of bone mineralization;GO:0032332//positive regulation of chondrocy

beta receptor signaling pathway;GO:0030512//negative regulation of transforming growth factor b

n;GO:0035556//intracellular signal transduction;GO:0046578//regulation of Ras protein signal tran
e development;GO:0030539//male genitalia development;GO:0060065//uterus development;GO:0
009415//response to water;GO:0035725//sodium ion transmembrane transport;GO:0050912//dete

ic process;GO:0007626//locomotory behavior;GO:0021768//nucleus accumbens development;GO:

030198//extracellular matrix organization;GO:0070373//negative regulation of ERK1 and ERK2 cas
oment;GO:0010762//regulation of fibroblast migration;GO:0042733//embryonic digit morphogene

loplasmic reticulum unfolded protein response;GO:0034976//response to endoplasmic reticulum s

n;GO:0006487//protein N-linked glycosylation;GO:0010706//ganglioside biosynthetic process via
ctivity;GO:0070328//triglyceride homeostasis;GO:2000352//negative regulation of endothelial cell

L//skeletal muscle fiber development;GO:0048875//chemical homeostasis within a tissue;GO:0051
virus;GO:0010468//regulation of gene expression;GO:0016310//phosphorylation;GO:0042063//gl

behavior;GO:0036343//psychomotor behavior;GO:0042127//regulation of cell proliferation;GO:004

L//skeletal muscle fiber development;GO:0048875//chemical homeostasis within a tissue;GO:0051

98//positive regulation of protein tyrosine kinase activity;GO:0061099//negative regulation of prot

se to heat;GO:0010467//gene expression;GO:0010468//regulation of gene expression;GO:001056(

030198//extracellular matrix organization;GO:0070373//negative regulation of ERK1 and ERK2 cas

401//steroid hormone mediated signaling pathway;GO:0046464//acylglycerol catabolic process;GC

ndothelial growth factor receptor signaling pathway;GO:0042326//negative regulation of phospho
nsport;GO:0098609//cell-cell adhesion;GO:1901896//positive regulation of calcium-transporting A

pment;GO:0010762//regulation of fibroblast migration;GO:0042733//embryonic digit morphogene

velopment;GO:0021528//commissural neuron differentiation in spinal cord;GO:0021960//anterior

iotic;GO:0071280//cellular response to copper ion;GO:0071420//cellular response to histamine;GC

etic process;GO:0006682//galactosylceramide biosynthetic process;GO:0008610//lipid biosynthetic

:0042177//negative regulation of protein catabolic process;GO:0043407//negative regulation of M

gulation of gene expression;GO:0010719//negative regulation of epithelial to mesenchymal transiti

ubiquitin-dependent protein catabolic process;GO:0043161//proteasome-mediated ubiquitin-dep

:ination;GO:0032410//negative regulation of transporter activity;GO:0051224//negative regulation

quitination;GO:0030163//protein catabolic process;GO:0031647//regulation of protein stability;GO
//regulation of fatty acid oxidation;GO:0055091//phospholipid homeostasis;GO:2001245//regulatio

344//cysteine biosynthetic process;GO:0019346//transsulfuration;GO:0043066//negative regulatior

030501//positive regulation of bone mineralization;GO:0032332//positive regulation of chondrocy

ort;GO:0042908//xenobiotic transport;GO:0055085//transmembrane transport;GO:0089718//aminc

oment;GO:0010762//regulation of fibroblast migration;GO:0042733//embryonic digit morphogene

:0034651//cortisol biosynthetic process;GO:0035725//sodium ion transmembrane transport;GO:00
nscript (SSU-rRNA, 5.8S rRNA, LSU-rRNA);GO:0006364//rRNA processing;GO:0008283//cell prolife

:arget recognition;GO:0007628//adult walking behavior;GO:0008283//cell proliferation;GO:001657
:ive regulation of MAP kinase activity;GO:0051897//positive regulation of protein kinase B signaling
on of lipid biosynthetic process;GO:0052651//monoacylglycerol catabolic process;GO:0060292//lo

ansport;GO:0043268//positive regulation of potassium ion transport;GO:0045214//sarcomere orga

3//glandular epithelial cell development;GO:0003417//growth plate cartilage development;GO:000

l cell differentiation;GO:0043065//positive regulation of apoptotic process;GO:0060545//positive re

030501//positive regulation of bone mineralization;GO:0032332//positive regulation of chondrocy

ndothelial growth factor receptor signaling pathway;GO:0042326//negative regulation of phospho

etic process;GO:0006682//galactosylceramide biosynthetic process;GO:0008610//lipid biosynthetic

elopment;GO:0008203//cholesterol metabolic process;GO:0009566//fertilization;GO:0009615//resp

velopment;GO:0021528//commissural neuron differentiation in spinal cord;GO:0021960//anterior
on of Wnt signaling pathway;GO:0030509//BMP signaling pathway;GO:0030514//negative regulatio

864//pyrimidine nucleotide transport;GO:0007005//mitochondrion organization;GO:0008284//pos

//regulation of fatty acid oxidation;GO:0055091//phospholipid homeostasis;GO:2001245//regulatio

ic process;GO:0007626//locomotory behavior;GO:0021768//nucleus accumbens development;GO:
on;GO:0010718//positive regulation of epithelial to mesenchymal transition;GO:0010812//negative

thetic process from pyruvate;GO:0010565//regulation of cellular ketone metabolic process;GO:001

n;GO:0006487//protein N-linked glycosylation;GO:0010706//ganglioside biosynthetic process via

030198//extracellular matrix organization;GO:0070373//negative regulation of ERK1 and ERK2 cas

nbrane transport;GO:1902476//chloride transmembrane transport;GO:1990573//potassium ion im

ort;GO:0042908//xenobiotic transport;GO:0055085//transmembrane transport;GO:0089718//aminc

98//positive regulation of protein tyrosine kinase activity;GO:0061099//negative regulation of prot

g;GO:0030968//endoplasmic reticulum unfolded protein response;GO:0032024//positive regulatior

l//skeletal muscle fiber development;GO:0048875//chemical homeostasis within a tissue;GO:00515

401//steroid hormone mediated signaling pathway;GO:0046464//acylglycerol catabolic process;GC

ive regulation of MAP kinase activity;GO:0051897//positive regulation of protein kinase B signalinc
oment;GO:0010762//regulation of fibroblast migration;GO:0042733//embryonic digit morphogene

ulin secretion;GO:0048791//calcium ion-regulated exocytosis of neurotransmitter;GO:0061669//sp

3//glandular epithelial cell development;GO:0003417//growth plate cartilage development;GO:000

:ive regulation of phosphoprotein phosphatase activity;GO:0051726//regulation of cell cycle;GO:19

nsport;GO:0098609//cell-cell adhesion;GO:1901896//positive regulation of calcium-transporting A

2351//'de novo' GDP-L-fucose biosynthetic process;GO:1904906//positive regulation of endotheli

ulation of transcription, DNA-templated;GO:0045944//positive regulation of transcription from RN

GO:0034116//positive regulation of heterotypic cell-cell adhesion;GO:0034622//cellular macromole

417//central nervous system development;GO:0008544//epidermis development;GO:0010467//ger
36//positive regulation of germinal center formation;GO:0002686//negative regulation of leukocyt

nily amino acid metabolic process;GO:0009066//aspartate family amino acid metabolic process;GC

ıbiquitin-dependent protein catabolic process;GO:0043161//proteasome-mediated ubiquitin-dep

ɔn;GO:0010718//positive regulation of epithelial to mesenchymal transition;GO:0010812//negative

030198//extracellular matrix organization;GO:0070373//negative regulation of ERK1 and ERK2 cas

olic process;GO:0050996//positive regulation of lipid catabolic process;GO:0051006//positive regu

3//glandular epithelial cell development;GO:0003417//growth plate cartilage development;GO:000

otaxis;GO:0006954//inflammatory response;GO:0006955//immune response;GO:0007186//G-prot
L//skeletal muscle fiber development;GO:0048875//chemical homeostasis within a tissue;GO:00515
ive regulation of MAP kinase activity;GO:0051897//positive regulation of protein kinase B signaling

ıetabolic process;GO:0044208//'de novo' AMP biosynthetic process;GO:0097294//'de novo' XMP b

)30336//negative regulation of cell migration;GO:0042542//response to hydrogen peroxide;GO:00

ı;GO:0016032//viral process;GO:0044260//cellular macromolecule metabolic process;GO:0044826/
)32000//positive regulation of fatty acid beta-oxidation;GO:0032868//response to insulin;GO:0032

ɔn of Wnt signaling pathway;GO:0030509//BMP signaling pathway;GO:0030514//negative regulati
'cellular response to amino acid starvation;GO:0070534//protein K63-linked ubiquitination;GO:007
mbling;GO:0032365//intracellular lipid transport;GO:0044830//modulation by host of viral RNA ge

:ination;GO:0032410//negative regulation of transporter activity;GO:0051224//negative regulation

98//positive regulation of protein tyrosine kinase activity;GO:0061099//negative regulation of prot
ing;GO:0001975//response to amphetamine;GO:0002027//regulation of heart rate;GO:0003094//g

;GO:0010760//negative regulation of macrophage chemotaxis;GO:0042326//negative regulation o
eration;GO:0034214//protein hexamerization;GO:0034982//mitochondrial protein processing;GO:0

ɔn of Wnt signaling pathway;GO:0030509//BMP signaling pathway;GO:0030514//negative regulati

port;GO:0007186//G-protein coupled receptor signaling pathway;GO:0007189//adenylate cyclase-

velopment;GO:0021528//commissural neuron differentiation in spinal cord;GO:0021960//anterior
nic acid metabolic process;GO:0022008//neurogenesis;GO:0042136//neurotransmitter biosynthetic

orting V-type ATPase complex assembly;GO:0090263//positive regulation of canonical Wnt signal

aling pathway;GO:0007193//adenylate cyclase-inhibiting G-protein coupled receptor signaling pat

tion;GO:0016567//protein ubiquitination;GO:0031052//chromosome breakage;GO:0031297//replic

sitive regulation of CREB transcription factor activity;GO:0043065//positive regulation of apoptotic

lic process;GO:0050776//regulation of immune response;GO:0072539//T-helper 17 cell differentiat

2351//'de novo' GDP-L-fucose biosynthetic process;GO:1904906//positive regulation of endotheli

ein metabolic process;GO:0016485//protein processing;GO:0016486//peptide hormone processin

007165//signal transduction;GO:0008203//cholesterol metabolic process;GO:0009395//phospholip

e to virus;GO:0031110//regulation of microtubule polymerization or depolymerization;GO:003111

030336//negative regulation of cell migration;GO:0042542//response to hydrogen peroxide;GO:00

ic process;GO:0007626//locomotory behavior;GO:0021768//nucleus accumbens development;GO:
of ATPase activity;GO:0034975//protein folding in endoplasmic reticulum;GO:0034976//response t

36//positive regulation of germinal center formation;GO:0002686//negative regulation of leukocyte

loplasmic reticulum unfolded protein response;GO:0034976//response to endoplasmic reticulum s

n;GO:0035556//intracellular signal transduction;GO:0046578//regulation of Ras protein signal tran

hosphorylation;GO:0035307//positive regulation of protein dephosphorylation;GO:0035308//nega

ise II promoter;GO:0006952//defense response;GO:0007165//signal transduction;GO:0007259//JAK

98//positive regulation of protein tyrosine kinase activity;GO:0061099//negative regulation of prot

tive regulation of phosphoprotein phosphatase activity;GO:0051726//regulation of cell cycle;GO:19

0002759//regulation of antimicrobial humoral response;GO:0003148//outflow tract septum morph

tination;GO:0032410//negative regulation of transporter activity;GO:0051224//negative regulation

regulation of Ras protein signal transduction;GO:0070536//protein K63-linked deubiquitination

meostasis;GO:0006984//ER-nucleus signaling pathway;GO:0006996//organelle organization;GO:00

1//skeletal muscle fiber development;GO:0048875//chemical homeostasis within a tissue;GO:0051

lic process;GO:0050776//regulation of immune response;GO:0072539//T-helper 17 cell differentiat

-to lesion;GO:0006974//cellular response to DNA damage stimulus;GO:0008152//metabolic proce

3//glandular epithelial cell development;GO:0003417//growth plate cartilage development;GO:000

e to virus;GO:0031110//regulation of microtubule polymerization or depolymerization;GO:003111
n;GO:0006487//protein N-linked glycosylation;GO:0010706//ganglioside biosynthetic process via
port;GO:0070508//cholesterol import;GO:0070859//positive regulation of bile acid biosynthetic pro
uscular septum morphogenesis;GO:0003151//outflow tract morphogenesis;GO:0003171//atrioven

tive regulation of MAP kinase activity;GO:0051897//positive regulation of protein kinase B signaling

030198//extracellular matrix organization;GO:0070373//negative regulation of ERK1 and ERK2 cas

uction by p53 class mediator resulting in transcription of p21 class mediator;GO:0031571//mitotic
'cellular response to amino acid starvation;GO:0070534//protein K63-linked ubiquitination;GO:007

aling pathway;GO:0007193//adenylate cyclase-inhibiting G-protein coupled receptor signaling pat

positive regulation of cholesterol efflux;GO:0030216//keratinocyte differentiation;GO:0031424//kera

on of Wnt signaling pathway;GO:0030509//BMP signaling pathway;GO:0030514//negative regulatio
sitive regulation of CREB transcription factor activity;GO:0043065//positive regulation of apoptotic

GO:0034116//positive regulation of heterotypic cell-cell adhesion;GO:0034622//cellular macromole

eration;GO:0034214//protein hexamerization;GO:0034982//mitochondrial protein processing;GO:0

ing;GO:0001975//response to amphetamine;GO:0002027//regulation of heart rate;GO:0003094//g
ic process;GO:0007626//locomotory behavior;GO:0021768//nucleus accumbens development;GO:
uction by p53 class mediator resulting in transcription of p21 class mediator;GO:0031571//mitotic

orting V-type ATPase complex assembly;GO:0090263//positive regulation of canonical Wnt signal

loplasmic reticulum unfolded protein response;GO:0034976//response to endoplasmic reticulum s

)07165//signal transduction;GO:0008203//cholesterol metabolic process;GO:0009395//phospholip

of ATPase activity;GO:0034975//protein folding in endoplasmic reticulum;GO:0034976//response t

0002759//regulation of antimicrobial humoral response;GO:0003148//outflow tract septum morpl

regulation of Ras protein signal transduction;GO:0070536//protein K63-linked deubiquitination

:ive regulation of phosphoprotein phosphatase activity;GO:0051726//regulation of cell cycle;GO:1[illegible]

‹ in apoptotic process;GO:0070301//cellular response to hydrogen peroxide;GO:0071456//cellular

:ion;GO:0030334//regulation of cell migration;GO:0030517//negative regulation of axon extension

of ATPase activity;GO:0034975//protein folding in endoplasmic reticulum;GO:0034976//response t

hway;GO:0042593//glucose homeostasis;GO:0050729//positive regulation of inflammatory respon

etic process;GO:0006682//galactosylceramide biosynthetic process;GO:0008610//lipid biosynthetic

e to virus;GO:0031110//regulation of microtubule polymerization or depolymerization;GO:003111[illegible]

ise II promoter;GO:0006952//defense response;GO:0007165//signal transduction;GO:0007259//JA[illegible]

:0055085//transmembrane transport;GO:0071805//potassium ion transmembrane transport

//regulation of fatty acid oxidation;GO:0055091//phospholipid homeostasis;GO:2001245//regulati

on of Wnt signaling pathway;GO:0030509//BMP signaling pathway;GO:0030514//negative regulati

GO:0030301//cholesterol transport;GO:0034372//very-low-density lipoprotein particle remodeling
n;GO:0035556//intracellular signal transduction;GO:0046578//regulation of Ras protein signal tran

n;GO:0006487//protein N-linked glycosylation;GO:0010706//ganglioside biosynthetic process via

864//pyrimidine nucleotide transport;GO:0007005//mitochondrion organization;GO:0008284//pos
aling pathway;GO:0007193//adenylate cyclase-inhibiting G-protein coupled receptor signaling pat

uction by p53 class mediator resulting in transcription of p21 class mediator;GO:0031571//mitotic

docytic zone membrane;GO:0031623//receptor internalization;GO:0048488//synaptic vesicle endo

//skeletal muscle fiber development;GO:0048875//chemical homeostasis within a tissue;GO:0051

loplasmic reticulum unfolded protein response;GO:0034976//response to endoplasmic reticulum s
ration;GO:0034214//protein hexamerization;GO:0034982//mitochondrial protein processing;GO:0
lic process;GO:0050776//regulation of immune response;GO:0072539//T-helper 17 cell differentiat
2351//'de novo' GDP-L-fucose biosynthetic process;GO:1904906//positive regulation of endotheli

ing;GO:0001975//response to amphetamine;GO:0002027//regulation of heart rate;GO:0003094//g
gulation of cytosolic calcium ion concentration;GO:0019722//calcium-mediated signaling;GO:006C

030198//extracellular matrix organization;GO:0070373//negative regulation of ERK1 and ERK2 cas
n;GO:0006487//protein N-linked glycosylation;GO:0010706//ganglioside biosynthetic process via
netic process;GO:0006636//unsaturated fatty acid biosynthetic process;GO:0008610//lipid biosynth

401//steroid hormone mediated signaling pathway;GO:0046464//acylglycerol catabolic process;GC

ment;GO:0010762//regulation of fibroblast migration;GO:0042733//embryonic digit morphogene

regulation of Ras protein signal transduction;GO:0070536//protein K63-linked deubiquitination

sitive regulation of CREB transcription factor activity;GO:0043065//positive regulation of apoptotic

0002759//regulation of antimicrobial humoral response;GO:0003148//outflow tract septum morpl

eceptor signaling pathway;GO:0050930//induction of positive chemotaxis;GO:0060754//positive re

eta receptor signaling pathway;GO:0030512//negative regulation of transforming growth factor b

mbling;GO:0032365//intracellular lipid transport;GO:0044830//modulation by host of viral RNA ge

030336//negative regulation of cell migration;GO:0042542//response to hydrogen peroxide;GO:00

orting V-type ATPase complex assembly;GO:0090263//positive regulation of canonical Wnt signal

)07165//signal transduction;GO:0008203//cholesterol metabolic process;GO:0009395//phospholip
on;GO:0010718//positive regulation of epithelial to mesenchymal transition;GO:0010812//negative
)07165//signal transduction;GO:0008203//cholesterol metabolic process;GO:0009395//phospholip

lic acid biosynthetic process;GO:0010898//positive regulation of triglyceride catabolic process;GO:

:0055085//transmembrane transport;GO:0071805//potassium ion transmembrane transport

n;GO:0006487//protein N-linked glycosylation;GO:0010706//ganglioside biosynthetic process via

ic process;GO:0007626//locomotory behavior;GO:0021768//nucleus accumbens development;GO:

e response;GO:0006644//phospholipid metabolic process;GO:0006656//phosphatidylcholine biosy

l storage;GO:0010890//positive regulation of sequestering of triglyceride;GO:0016042//lipid catab

ranscription from RNA polymerase II promoter;GO:0007614//short-term memory;GO:0010628//po
tive regulation of phosphoprotein phosphatase activity;GO:0051726//regulation of cell cycle;GO:19

) virus;GO:0010468//regulation of gene expression;GO:0016310//phosphorylation;GO:0042063//gl

n;GO:0006487//protein N-linked glycosylation;GO:0010706//ganglioside biosynthetic process via l

etic process;GO:0006682//galactosylceramide biosynthetic process;GO:0008610//lipid biosynthetic

idase activity;GO:0044871//negative regulation by host of viral glycoprotein metabolic process;GO

on of Wnt signaling pathway;GO:0030509//BMP signaling pathway;GO:0030514//negative regulatic
ctivity;GO:0070328//triglyceride homeostasis;GO:2000352//negative regulation of endothelial cell

nsport;GO:0098609//cell-cell adhesion;GO:1901896//positive regulation of calcium-transporting A

netic process;GO:0006636//unsaturated fatty acid biosynthetic process;GO:0008610//lipid biosynth

//regulation of fatty acid oxidation;GO:0055091//phospholipid homeostasis;GO:2001245//regulatic
ise II promoter;GO:0006952//defense response;GO:0007165//signal transduction;GO:0007259//JAI

quitination;GO:0030163//protein catabolic process;GO:0031647//regulation of protein stability;GO

//transmembrane transport;GO:0071332//cellular response to fructose stimulus;GO:1990539//fruct

91//regulation of heart rate by cardiac conduction;GO:0090136//epithelial cell-cell adhesion;GO:0

mbling;GO:0032365//intracellular lipid transport;GO:0044830//modulation by host of viral RNA ge

port;GO:0070508//cholesterol import;GO:0070859//positive regulation of bile acid biosynthetic pro

regulation of Ras protein signal transduction;GO:0070536//protein K63-linked deubiquitination

0002759//regulation of antimicrobial humoral response;GO:0003148//outflow tract septum morph

ive regulation of MAP kinase activity;GO:0051897//positive regulation of protein kinase B signalin

3//glandular epithelial cell development;GO:0003417//growth plate cartilage development;GO:000

401//steroid hormone mediated signaling pathway;GO:0046464//acylglycerol catabolic process;GC

loplasmic reticulum unfolded protein response;GO:0034976//response to endoplasmic reticulum s

ɔment;GO:0010762//regulation of fibroblast migration;GO:0042733//embryonic digit morphogene

ansport;GO:0043268//positive regulation of potassium ion transport;GO:0045214//sarcomere orga

g;GO:0030968//endoplasmic reticulum unfolded protein response;GO:0032024//positive regulatio

ɔment;GO:0010762//regulation of fibroblast migration;GO:0042733//embryonic digit morphogene

ctivity;GO:0070328//triglyceride homeostasis;GO:2000352//negative regulation of endothelial cell
nsport;GO:0098609//cell-cell adhesion;GO:1901896//positive regulation of calcium-transporting A

38//protein metabolic process;GO:0051289//protein homotetramerization;GO:0055088//lipid hom

2351//'de novo' GDP-L-fucose biosynthetic process;GO:1904906//positive regulation of endotheli
brane transport;GO:0090277//positive regulation of peptide hormone secretion;GO:0098810//neu

ɔn of Wnt signaling pathway;GO:0030509//BMP signaling pathway;GO:0030514//negative regulatio

:e specification;GO:0001763//morphogenesis of a branching structure;GO:0001944//vasculature de

nscript (SSU-rRNA, 5.8S rRNA, LSU-rRNA);GO:0006364//rRNA processing;GO:0008283//cell prolife

ɔn;GO:0010718//positive regulation of epithelial to mesenchymal transition;GO:0010812//negative

nsport;GO:0098609//cell-cell adhesion;GO:1901896//positive regulation of calcium-transporting A
nbrane transport;GO:1902476//chloride transmembrane transport;GO:1990573//potassium ion im

)07165//signal transduction;GO:0008203//cholesterol metabolic process;GO:0009395//phospholip

idase activity;GO:0044871//negative regulation by host of viral glycoprotein metabolic process;GO

:arget recognition;GO:0007628//adult walking behavior;GO:0008283//cell proliferation;GO:001657

naling pathway;GO:0050909//sensory perception of taste;GO:0050913//sensory perception of bitte

nic acid metabolic process;GO:0022008//neurogenesis;GO:0042136//neurotransmitter biosynthetic

neuron migration;GO:0001775//cell activation;GO:0001817//regulation of cytokine production;GO:

elopment;GO:0008203//cholesterol metabolic process;GO:0009566//fertilization;GO:0009615//resp

quitination;GO:0030163//protein catabolic process;GO:0031647//regulation of protein stability;GO

eart trabecula formation;GO:1900087//positive regulation of G1/S transition of mitotic cell cycle;G

behavior;GO:0036343//psychomotor behavior;GO:0042127//regulation of cell proliferation;GO:004
loplasmic reticulum unfolded protein response;GO:0034976//response to endoplasmic reticulum s

27//regulation of inflammatory response;GO:0050730//regulation of peptidyl-tyrosine phosphoryl

ive regulation of MAP kinase activity;GO:0051897//positive regulation of protein kinase B signaling

3//glandular epithelial cell development;GO:0003417//growth plate cartilage development;GO:000

mbling;GO:0032365//intracellular lipid transport;GO:0044830//modulation by host of viral RNA ge

ansport;GO:0043268//positive regulation of potassium ion transport;GO:0045214//sarcomere orga

007165//signal transduction;GO:0008203//cholesterol metabolic process;GO:0009395//phospholip
f triglyceride biosynthetic process;GO:0032792//negative regulation of CREB transcription factor a

nscript (SSU-rRNA, 5.8S rRNA, LSU-rRNA);GO:0006364//rRNA processing;GO:0008283//cell prolife

e to lysosome transport;GO:0030705//cytoskeleton-dependent intracellular transport;GO:0031122

:0042177//negative regulation of protein catabolic process;GO:0043407//negative regulation of M
behavior;GO:0036343//psychomotor behavior;GO:0042127//regulation of cell proliferation;GO:004

eart trabecula formation;GO:1900087//positive regulation of G1/S transition of mitotic cell cycle;G
idase activity;GO:0044871//negative regulation by host of viral glycoprotein metabolic process;GO

target recognition;GO:0007628//adult walking behavior;GO:0008283//cell proliferation;GO:001657
negative regulation of peptidyl-cysteine S-nitrosylation;GO:1903204//negative regulation of oxida

;GO:0032350//regulation of hormone metabolic process;GO:0045598//regulation of fat cell differe

elopment;GO:0008203//cholesterol metabolic process;GO:0009566//fertilization;GO:0009615//resp

e specification;GO:0001763//morphogenesis of a branching structure;GO:0001944//vasculature de
O:0045944//positive regulation of transcription from RNA polymerase II promoter;GO:0048863//st

030501//positive regulation of bone mineralization;GO:0032332//positive regulation of chondrocy

3//glandular epithelial cell development;GO:0003417//growth plate cartilage development;GO:000

ive regulation of MAP kinase activity;GO:0051897//positive regulation of protein kinase B signaling

nsport;GO:0098609//cell-cell adhesion;GO:1901896//positive regulation of calcium-transporting A

030198//extracellular matrix organization;GO:0070373//negative regulation of ERK1 and ERK2 cas

2351//'de novo' GDP-L-fucose biosynthetic process;GO:1904906//positive regulation of endotheli

e to virus;GO:0031110//regulation of microtubule polymerization or depolymerization;GO:003111

ctivity;GO:0070328//triglyceride homeostasis;GO:2000352//negative regulation of endothelial cell

l//skeletal muscle fiber development;GO:0048875//chemical homeostasis within a tissue;GO:00515

port;GO:0007186//G-protein coupled receptor signaling pathway;GO:0007189//adenylate cyclase-

36//positive regulation of germinal center formation;GO:0002686//negative regulation of leukocyt

009415//response to water;GO:0035725//sodium ion transmembrane transport;GO:0050912//dete

l//skeletal muscle fiber development;GO:0048875//chemical homeostasis within a tissue;GO:00515

;GO:0016032//viral process;GO:0044260//cellular macromolecule metabolic process;GO:0044826/

e to lysosome transport;GO:0030705//cytoskeleton-dependent intracellular transport;GO:0031122

ninoacylation;GO:0016525//negative regulation of angiogenesis;GO:1904046//negative regulation

030198//extracellular matrix organization;GO:0070373//negative regulation of ERK1 and ERK2 cas

docytic zone membrane;GO:0031623//receptor internalization;GO:0048488//synaptic vesicle endo
e development;GO:0030539//male genitalia development;GO:0060065//uterus development;GO:0

nic acid metabolic process;GO:0022008//neurogenesis;GO:0042136//neurotransmitter biosynthetic

030501//positive regulation of bone mineralization;GO:0032332//positive regulation of chondrocy

ein metabolic process;GO:0016485//protein processing;GO:0016486//peptide hormone processin

orting V-type ATPase complex assembly;GO:0090263//positive regulation of canonical Wnt signal

ive regulation of MAP kinase activity;GO:0051897//positive regulation of protein kinase B signaling
030501//positive regulation of bone mineralization;GO:0032332//positive regulation of chondrocy

uction by p53 class mediator resulting in transcription of p21 class mediator;GO:0031571//mitotic
3//glandular epithelial cell development;GO:0003417//growth plate cartilage development;GO:000
'cellular response to amino acid starvation;GO:0070534//protein K63-linked ubiquitination;GO:007
mbling;GO:0032365//intracellular lipid transport;GO:0044830//modulation by host of viral RNA ge

sitive regulation of CREB transcription factor activity;GO:0043065//positive regulation of apoptotic

ic process;GO:0007626//locomotory behavior;GO:0021768//nucleus accumbens development;GO:
sitive regulation of CREB transcription factor activity;GO:0043065//positive regulation of apoptotic

0007179//transforming growth factor beta receptor signaling pathway;GO:0007229//integrin-mec
hosphorylation;GO:0035307//positive regulation of protein dephosphorylation;GO:0035308//nega

on;GO:0035999//tetrahydrofolate interconversion;GO:0050667//homocysteine metabolic process;(

orting V-type ATPase complex assembly;GO:0090263//positive regulation of canonical Wnt signal

opment;GO:0090263//positive regulation of canonical Wnt signaling pathway;GO:2001013//epithe

meostasis;GO:0006984//ER-nucleus signaling pathway;GO:0006996//organelle organization;GO:0(
nscript (SSU-rRNA, 5.8S rRNA, LSU-rRNA);GO:0006364//rRNA processing;GO:0008283//cell prolife

ansport;GO:0043268//positive regulation of potassium ion transport;GO:0045214//sarcomere orga

;GO:0016032//viral process;GO:0044260//cellular macromolecule metabolic process;GO:0044826/

l//skeletal muscle fiber development;GO:0048875//chemical homeostasis within a tissue;GO:0051

;GO:0030968//endoplasmic reticulum unfolded protein response;GO:0032024//positive regulatio

neostasis;GO:0045892//negative regulation of transcription, DNA-templated;GO:0050727//regulat

38//protein metabolic process;GO:0051289//protein homotetramerization;GO:0055088//lipid hom
030198//extracellular matrix organization;GO:0070373//negative regulation of ERK1 and ERK2 cas

elopment;GO:0008203//cholesterol metabolic process;GO:0009566//fertilization;GO:0009615//resp

of ATPase activity;GO:0034975//protein folding in endoplasmic reticulum;GO:0034976//response

abolic process;GO:0046839//phospholipid dephosphorylation;GO:1902247//geranylgeranyl diphos
uscular septum morphogenesis;GO:0003151//outflow tract morphogenesis;GO:0003171//atrioven

e to virus;GO:0031110//regulation of microtubule polymerization or depolymerization;GO:003111
0009888//tissue development;GO:0016125//sterol metabolic process;GO:0030539//male genitalia

etic process;GO:0006682//galactosylceramide biosynthetic process;GO:0008610//lipid biosynthetic
98//positive regulation of protein tyrosine kinase activity;GO:0061099//negative regulation of prot

orting V-type ATPase complex assembly;GO:0090263//positive regulation of canonical Wnt signal

e to virus;GO:0031110//regulation of microtubule polymerization or depolymerization;GO:003111

ubiquitin-dependent protein catabolic process;GO:0031146//SCF-dependent proteasomal ubiqu

n;GO:0035556//intracellular signal transduction;GO:0046578//regulation of Ras protein signal tran

on of lipid biosynthetic process;GO:0052651//monoacylglycerol catabolic process;GO:0060292//lo
sitive regulation of CREB transcription factor activity;GO:0043065//positive regulation of apoptotic

//regulation of fatty acid oxidation;GO:0055091//phospholipid homeostasis;GO:2001245//regulatio

l cell differentiation;GO:0043065//positive regulation of apoptotic process;GO:0060545//positive re

aling pathway;GO:0007193//adenylate cyclase-inhibiting G-protein coupled receptor signaling pat
ic process;GO:0007626//locomotory behavior;GO:0021768//nucleus accumbens development;GO:

opment;GO:0090263//positive regulation of canonical Wnt signaling pathway;GO:2001013//epithe

n;GO:1901224//positive regulation of NIK/NF-kappaB signaling;GO:1904951//positive regulation

netic process;GO:0006636//unsaturated fatty acid biosynthetic process;GO:0008610//lipid biosynth

›ment;GO:0010762//regulation of fibroblast migration;GO:0042733//embryonic digit morphogene

uction by p53 class mediator resulting in transcription of p21 class mediator;GO:0031571//mitotic

l in apoptotic process;GO:0070301//cellular response to hydrogen peroxide;GO:0071456//cellular

030501//positive regulation of bone mineralization;GO:0032332//positive regulation of chondrocy

sitive regulation of CREB transcription factor activity;GO:0043065//positive regulation of apoptotic

)30336//negative regulation of cell migration;GO:0042542//response to hydrogen peroxide;GO:00

uscular septum morphogenesis;GO:0003151//outflow tract morphogenesis;GO:0003171//atrioven

:0042177//negative regulation of protein catabolic process;GO:0043407//negative regulation of M

e to virus;GO:0031110//regulation of microtubule polymerization or depolymerization;GO:003111[illegible]

ulin secretion;GO:0048791//calcium ion-regulated exocytosis of neurotransmitter;GO:0061669//sp

orting V-type ATPase complex assembly;GO:0090263//positive regulation of canonical Wnt signal

arget recognition;GO:0007628//adult walking behavior;GO:0008283//cell proliferation;GO:001657[illegible]

:0055085//transmembrane transport;GO:0071805//potassium ion transmembrane transport

on;GO:0010718//positive regulation of epithelial to mesenchymal transition;GO:0010812//negative

)32000//positive regulation of fatty acid beta-oxidation;GO:0032868//response to insulin;GO:0032

:ination;GO:0032410//negative regulation of transporter activity;GO:0051224//negative regulation

ı;GO:0016032//viral process;GO:0044260//cellular macromolecule metabolic process;GO:0044826/

ɔn of Wnt signaling pathway;GO:0030509//BMP signaling pathway;GO:0030514//negative regulati

54//pre-miRNA processing;GO:0035196//production of miRNAs involved in gene silencing by miR

of ATPase activity;GO:0034975//protein folding in endoplasmic reticulum;GO:0034976//response t

)32330//regulation of chondrocyte differentiation;GO:0036150//phosphatidylserine acyl-chain rem
ɔment;GO:0010762//regulation of fibroblast migration;GO:0042733//embryonic digit morphogene
ive regulation of MAP kinase activity;GO:0051897//positive regulation of protein kinase B signaling

ł17//central nervous system development;GO:0008544//epidermis development;GO:0010467//ger

ıbiquitin-dependent protein catabolic process;GO:0043161//proteasome-mediated ubiquitin-dep

3//glandular epithelial cell development;GO:0003417//growth plate cartilage development;GO:000

l cell differentiation;GO:0043065//positive regulation of apoptotic process;GO:0060545//positive re

etic process;GO:0006682//galactosylceramide biosynthetic process;GO:0008610//lipid biosynthetic

009415//response to water;GO:0035725//sodium ion transmembrane transport;GO:0050912//dete

030501//positive regulation of bone mineralization;GO:0032332//positive regulation of chondrocy

2351//'de novo' GDP-L-fucose biosynthetic process;GO:1904906//positive regulation of endotheli

olic process;GO:0050996//positive regulation of lipid catabolic process;GO:0051006//positive regu

//regulation of fatty acid oxidation;GO:0055091//phospholipid homeostasis;GO:2001245//regulati

98//positive regulation of protein tyrosine kinase activity;GO:0061099//negative regulation of prot

aling pathway;GO:0007193//adenylate cyclase-inhibiting G-protein coupled receptor signaling pat

ic process;GO:0007626//locomotory behavior;GO:0021768//nucleus accumbens development;GO:

007165//signal transduction;GO:0008203//cholesterol metabolic process;GO:0009395//phospholip

032000//positive regulation of fatty acid beta-oxidation;GO:0032868//response to insulin;GO:0032
:0055085//transmembrane transport;GO:0071805//potassium ion transmembrane transport

401//steroid hormone mediated signaling pathway;GO:0046464//acylglycerol catabolic process;GO

negative regulation of peptidyl-cysteine S-nitrosylation;GO:1903204//negative regulation of oxida

2351//'de novo' GDP-L-fucose biosynthetic process;GO:1904906//positive regulation of endotheli
loplasmic reticulum unfolded protein response;GO:0034976//response to endoplasmic reticulum s
eart trabecula formation;GO:1900087//positive regulation of G1/S transition of mitotic cell cycle;G

cell proliferation;GO:0008543//fibroblast growth factor receptor signaling pathway;GO:0009617//r
ination;GO:0032410//negative regulation of transporter activity;GO:0051224//negative regulation

velopment;GO:0021528//commissural neuron differentiation in spinal cord;GO:0021960//anterior

0002759//regulation of antimicrobial humoral response;GO:0003148//outflow tract septum morph

)30336//negative regulation of cell migration;GO:0042542//response to hydrogen peroxide;GO:00

l storage;GO:0010890//positive regulation of sequestering of triglyceride;GO:0016042//lipid catab

regulation of Ras protein signal transduction;GO:0070536//protein K63-linked deubiquitination

lic acid biosynthetic process;GO:0010898//positive regulation of triglyceride catabolic process;GO:

nsport;GO:0098609//cell-cell adhesion;GO:1901896//positive regulation of calcium-transporting A

on;GO:0010718//positive regulation of epithelial to mesenchymal transition;GO:0010812//negative
2351//'de novo' GDP-L-fucose biosynthetic process;GO:1904906//positive regulation of endotheli

virus;GO:0010468//regulation of gene expression;GO:0016310//phosphorylation;GO:0042063//gl

etic process;GO:0006636//unsaturated fatty acid biosynthetic process;GO:0008610//lipid biosynth

n;GO:0006487//protein N-linked glycosylation;GO:0010706//ganglioside biosynthetic process via l

ubiquitin-dependent protein catabolic process;GO:0043161//proteasome-mediated ubiquitin-dep

uscular septum morphogenesis;GO:0003151//outflow tract morphogenesis;GO:0003171//atrioven

:ination;GO:0032410//negative regulation of transporter activity;GO:0051224//negative regulation

ıse to starvation;GO:0010886//positive regulation of cholesterol storage;GO:0032933//SREBP signa

behavior;GO:0036343//psychomotor behavior;GO:0042127//regulation of cell proliferation;GO:004

0009888//tissue development;GO:0016125//sterol metabolic process;GO:0030539//male genitalia

lic process;GO:0050776//regulation of immune response;GO:0072539//T-helper 17 cell differentiat

loplasmic reticulum unfolded protein response;GO:0034976//response to endoplasmic reticulum s

nic acid metabolic process;GO:0022008//neurogenesis;GO:0042136//neurotransmitter biosynthetic

2351//'de novo' GDP-L-fucose biosynthetic process;GO:1904906//positive regulation of endotheli

)30336//negative regulation of cell migration;GO:0042542//response to hydrogen peroxide;GO:00
mbling;GO:0032365//intracellular lipid transport;GO:0044830//modulation by host of viral RNA ge

on;GO:1901224//positive regulation of NIK/NF-kappaB signaling;GO:1904951//positive regulation

0002759//regulation of antimicrobial humoral response;GO:0003148//outflow tract septum morpl

:ination;GO:0032410//negative regulation of transporter activity;GO:0051224//negative regulation
regulation of Ras protein signal transduction;GO:0070536//protein K63-linked deubiquitination

lic acid biosynthetic process;GO:0010898//positive regulation of triglyceride catabolic process;GO:
)ment;GO:0010762//regulation of fibroblast migration;GO:0042733//embryonic digit morphogene

98//positive regulation of protein tyrosine kinase activity;GO:0061099//negative regulation of prot

) virus;GO:0010468//regulation of gene expression;GO:0016310//phosphorylation;GO:0042063//gl

naling pathway;GO:0050909//sensory perception of taste;GO:0050913//sensory perception of bitte

l in apoptotic process;GO:0070301//cellular response to hydrogen peroxide;GO:0071456//cellular

velopment;GO:0021528//commissural neuron differentiation in spinal cord;GO:0021960//anterior

sis;GO:0030913//paranodal junction assembly;GO:0042552//myelination;GO:0044255//cellular lipi

on;GO:0035999//tetrahydrofolate interconversion;GO:0050667//homocysteine metabolic process;G

O:0010628//positive regulation of gene expression;GO:0016559//peroxisome fission;GO:0048208//
aling pathway;GO:0007193//adenylate cyclase-inhibiting G-protein coupled receptor signaling pat

n;GO:0006487//protein N-linked glycosylation;GO:0010706//ganglioside biosynthetic process via l

e to virus;GO:0031110//regulation of microtubule polymerization or depolymerization;GO:003111

eart trabecula formation;GO:1900087//positive regulation of G1/S transition of mitotic cell cycle;G

eceptor signaling pathway;GO:0050930//induction of positive chemotaxis;GO:0060754//positive re

f triglyceride biosynthetic process;GO:0032792//negative regulation of CREB transcription factor a

nsport;GO:0098609//cell-cell adhesion;GO:1901896//positive regulation of calcium-transporting A

ic process;GO:0007626//locomotory behavior;GO:0021768//nucleus accumbens development;GO:

ubiquitin-dependent protein catabolic process;GO:0031146//SCF-dependent proteasomal ubiqu
of ATPase activity;GO:0034975//protein folding in endoplasmic reticulum;GO:0034976//response t

ic process;GO:0007626//locomotory behavior;GO:0021768//nucleus accumbens development;GO:

loplasmic reticulum unfolded protein response;GO:0034976//response to endoplasmic reticulum s

oment;GO:0010762//regulation of fibroblast migration;GO:0042733//embryonic digit morphogene

98//positive regulation of protein tyrosine kinase activity;GO:0061099//negative regulation of prot
O:0045944//positive regulation of transcription from RNA polymerase II promoter;GO:0048863//st

tive regulation of phosphoprotein phosphatase activity;GO:0051726//regulation of cell cycle;GO:19

030501//positive regulation of bone mineralization;GO:0032332//positive regulation of chondrocy

behavior;GO:0036343//psychomotor behavior;GO:0042127//regulation of cell proliferation;GO:004

l in apoptotic process;GO:0070301//cellular response to hydrogen peroxide;GO:0071456//cellular

ynthetic process;GO:0044208//'de novo' AMP biosynthetic process;GO:0044209//AMP salvage;GC

n;GO:0006487//protein N-linked glycosylation;GO:0010706//ganglioside biosynthetic process via l
f vascular endothelial growth factor production;GO:0010828//positive regulation of glucose transp
864//pyrimidine nucleotide transport;GO:0007005//mitochondrion organization;GO:0008284//pos
sitive regulation of CREB transcription factor activity;GO:0043065//positive regulation of apoptotic
onse to DNA damage;GO:0010467//gene expression;GO:0010884//positive regulation of lipid stora

uction by p53 class mediator resulting in transcription of p21 class mediator;GO:0031571//mitotic

e to virus;GO:0031110//regulation of microtubule polymerization or depolymerization;GO:003111
mbling;GO:0032365//intracellular lipid transport;GO:0044830//modulation by host of viral RNA ge

orting V-type ATPase complex assembly;GO:0090263//positive regulation of canonical Wnt signal
hway;GO:0042593//glucose homeostasis;GO:0050729//positive regulation of inflammatory respon

030198//extracellular matrix organization;GO:0070373//negative regulation of ERK1 and ERK2 cas

uction by p53 class mediator resulting in transcription of p21 class mediator;GO:0031571//mitotic

36//positive regulation of germinal center formation;GO:0002686//negative regulation of leukocyt

virus;GO:0010468//regulation of gene expression;GO:0016310//phosphorylation;GO:0042063//gl

ulin secretion;GO:0048791//calcium ion-regulated exocytosis of neurotransmitter;GO:0061669//sp

ɔeta receptor signaling pathway;GO:0030512//negative regulation of transforming growth factor b
1//skeletal muscle fiber development;GO:0048875//chemical homeostasis within a tissue;GO:0051[illegible]
e development;GO:0030539//male genitalia development;GO:0060065//uterus development;GO:[illegible]

ɔn of Wnt signaling pathway;GO:0030509//BMP signaling pathway;GO:0030514//negative regulati[illegible]

ninoacylation;GO:0016525//negative regulation of angiogenesis;GO:1904046//negative regulation

:0034651//cortisol biosynthetic process;GO:0035725//sodium ion transmembrane transport;GO:0[illegible]

of ATPase activity;GO:0034975//protein folding in endoplasmic reticulum;GO:0034976//response [illegible]

)32330//regulation of chondrocyte differentiation;GO:0036150//phosphatidylserine acyl-chain rem

ositive regulation of cholesterol efflux;GO:0030216//keratinocyte differentiation;GO:0031424//kera
vascular endothelial growth factor production;GO:0010828//positive regulation of glucose transp
030501//positive regulation of bone mineralization;GO:0032332//positive regulation of chondrocy

nse to DNA damage;GO:0010467//gene expression;GO:0010884//positive regulation of lipid stora
17//central nervous system development;GO:0008544//epidermis development;GO:0010467//ger
sitive regulation of CREB transcription factor activity;GO:0043065//positive regulation of apoptotic
cellular response to amino acid starvation;GO:0070534//protein K63-linked ubiquitination;GO:007

30336//negative regulation of cell migration;GO:0042542//response to hydrogen peroxide;GO:00

27//regulation of inflammatory response;GO:0050730//regulation of peptidyl-tyrosine phosphoryl

ulation of transcription, DNA-templated;GO:0045944//positive regulation of transcription from RN

em myelin maintenance;GO:0042127//regulation of cell proliferation;GO:0045576//mast cell activa

orting V-type ATPase complex assembly;GO:0090263//positive regulation of canonical Wnt signal

aling pathway;GO:0007193//adenylate cyclase-inhibiting G-protein coupled receptor signaling pat

ic process;GO:0007626//locomotory behavior;GO:0021768//nucleus accumbens development;GO:

030198//extracellular matrix organization;GO:0070373//negative regulation of ERK1 and ERK2 cas

on of Wnt signaling pathway;GO:0030509//BMP signaling pathway;GO:0030514//negative regulati

36//positive regulation of germinal center formation;GO:0002686//negative regulation of leukocyt

l//skeletal muscle fiber development;GO:0048875//chemical homeostasis within a tissue;GO:00515

aling pathway;GO:0007193//adenylate cyclase-inhibiting G-protein coupled receptor signaling pat

response to bacterium;GO:0018105//peptidyl-serine phosphorylation;GO:0032868//response to in
brane transport;GO:0090277//positive regulation of peptide hormone secretion;GO:0098810//neu
lic acid biosynthetic process;GO:0010898//positive regulation of triglyceride catabolic process;GO:

38//protein metabolic process;GO:0051289//protein homotetramerization;GO:0055088//lipid hom
uction by p53 class mediator resulting in transcription of p21 class mediator;GO:0031571//mitotic

0007179//transforming growth factor beta receptor signaling pathway;GO:0007229//integrin-mec

ative regulation of neuron projection development;GO:0021540//corpus callosum morphogenesis;

regulation of Ras protein signal transduction;GO:0070536//protein K63-linked deubiquitination

0002759//regulation of antimicrobial humoral response;GO:0003148//outflow tract septum morpl

on of lipid biosynthetic process;GO:0052651//monoacylglycerol catabolic process;GO:0060292//lc

mbling;GO:0032365//intracellular lipid transport;GO:0044830//modulation by host of viral RNA ge

)30336//negative regulation of cell migration;GO:0042542//response to hydrogen peroxide;GO:00

98//positive regulation of protein tyrosine kinase activity;GO:0061099//negative regulation of prot

of ATPase activity;GO:0034975//protein folding in endoplasmic reticulum;GO:0034976//response t
'cellular response to amino acid starvation;GO:0070534//protein K63-linked ubiquitination;GO:007

n;GO:0035556//intracellular signal transduction;GO:0046578//regulation of Ras protein signal trar

on;GO:0035999//tetrahydrofolate interconversion;GO:0050667//homocysteine metabolic process;G

ic process;GO:0007626//locomotory behavior;GO:0021768//nucleus accumbens development;GO:

on;GO:0010718//positive regulation of epithelial to mesenchymal transition;GO:0010812//negative
nic acid metabolic process;GO:0022008//neurogenesis;GO:0042136//neurotransmitter biosynthetic

nbrane transport;GO:1902476//chloride transmembrane transport;GO:1990573//potassium ion im

lic process;GO:0050776//regulation of immune response;GO:0072539//T-helper 17 cell differentiat

3//glandular epithelial cell development;GO:0003417//growth plate cartilage development;GO:000

rocess;GO:0006650//glycerophospholipid metabolic process;GO:0006655//phosphatidylglycerol b
of ATPase activity;GO:0034975//protein folding in endoplasmic reticulum;GO:0034976//response t

030501//positive regulation of bone mineralization;GO:0032332//positive regulation of chondrocy

ive regulation of MAP kinase activity;GO:0051897//positive regulation of protein kinase B signaling

030336//negative regulation of cell migration;GO:0042542//response to hydrogen peroxide;GO:00

etic process;GO:0006682//galactosylceramide biosynthetic process;GO:0008610//lipid biosynthetic

0002759//regulation of antimicrobial humoral response;GO:0003148//outflow tract septum morph

regulation of Ras protein signal transduction;GO:0070536//protein K63-linked deubiquitination

0007179//transforming growth factor beta receptor signaling pathway;GO:0007229//integrin-med

nsport;GO:0098609//cell-cell adhesion;GO:1901896//positive regulation of calcium-transporting A

ctivity;GO:0070328//triglyceride homeostasis;GO:2000352//negative regulation of endothelial cell

//regulation of fatty acid oxidation;GO:0055091//phospholipid homeostasis;GO:2001245//regulati

thetic process from pyruvate;GO:0010565//regulation of cellular ketone metabolic process;GO:001

virus;GO:0010468//regulation of gene expression;GO:0016310//phosphorylation;GO:0042063//gl

uction by p53 class mediator resulting in transcription of p21 class mediator;GO:0031571//mitotic

aling pathway;GO:0007193//adenylate cyclase-inhibiting G-protein coupled receptor signaling pat

54//pre-miRNA processing;GO:0035196//production of miRNAs involved in gene silencing by miR

arget recognition;GO:0007628//adult walking behavior;GO:0008283//cell proliferation;GO:001657

n;GO:0035556//intracellular signal transduction;GO:0046578//regulation of Ras protein signal trar

ination;GO:0032410//negative regulation of transporter activity;GO:0051224//negative regulation
aling pathway;GO:0007193//adenylate cyclase-inhibiting G-protein coupled receptor signaling pat

-to lesion;GO:0006974//cellular response to DNA damage stimulus;GO:0008152//metabolic proce

//regulation of fatty acid oxidation;GO:0055091//phospholipid homeostasis;GO:2001245//regulati

tion;GO:0016567//protein ubiquitination;GO:0031052//chromosome breakage;GO:0031297//repli

nsport;GO:0098609//cell-cell adhesion;GO:1901896//positive regulation of calcium-transporting A

olic process;GO:0050996//positive regulation of lipid catabolic process;GO:0051006//positive regu

O:0010628//positive regulation of gene expression;GO:0016559//peroxisome fission;GO:0048208//

on of Wnt signaling pathway;GO:0030509//BMP signaling pathway;GO:0030514//negative regulati

36//positive regulation of germinal center formation;GO:0002686//negative regulation of leukocyt
etic process;GO:0006682//galactosylceramide biosynthetic process;GO:0008610//lipid biosyntheti

temic arterial blood pressure;GO:0007186//G-protein coupled receptor signaling pathway;GO:000

1//skeletal muscle fiber development;GO:0048875//chemical homeostasis within a tissue;GO:0051

007165//signal transduction;GO:0008203//cholesterol metabolic process;GO:0009395//phospholip

030336//negative regulation of cell migration;GO:0042542//response to hydrogen peroxide;GO:00

of ATPase activity;GO:0034975//protein folding in endoplasmic reticulum;GO:0034976//response t

on;GO:0010718//positive regulation of epithelial to mesenchymal transition;GO:0010812//negative
030336//negative regulation of cell migration;GO:0042542//response to hydrogen peroxide;GO:00

030198//extracellular matrix organization;GO:0070373//negative regulation of ERK1 and ERK2 cas

on;GO:0010718//positive regulation of epithelial to mesenchymal transition;GO:0010812//negative

regulation of Ras protein signal transduction;GO:0070536//protein K63-linked deubiquitination

0002759//regulation of antimicrobial humoral response;GO:0003148//outflow tract septum morph

nscript (SSU-rRNA, 5.8S rRNA, LSU-rRNA);GO:0006364//rRNA processing;GO:0008283//cell prolife

ninoacylation;GO:0016525//negative regulation of angiogenesis;GO:1904046//negative regulation

nembrane transport;GO:0051899//membrane depolarization;GO:0060048//cardiac muscle contrac

:0034651//cortisol biosynthetic process;GO:0035725//sodium ion transmembrane transport;GO:00

030501//positive regulation of bone mineralization;GO:0032332//positive regulation of chondrocy

ıetic process;GO:0006636//unsaturated fatty acid biosynthetic process;GO:0008610//lipid biosynth

'cellular response to amino acid starvation;GO:0070534//protein K63-linked ubiquitination;GO:007

27//regulation of inflammatory response;GO:0050730//regulation of peptidyl-tyrosine phosphoryl

ɔort;GO:0007186//G-protein coupled receptor signaling pathway;GO:0007189//adenylate cyclase-

loplasmic reticulum unfolded protein response;GO:0034976//response to endoplasmic reticulum s

elopment;GO:0008203//cholesterol metabolic process;GO:0009566//fertilization;GO:0009615//resp
ion;GO:0030334//regulation of cell migration;GO:0030517//negative regulation of axon extension

mbling;GO:0032365//intracellular lipid transport;GO:0044830//modulation by host of viral RNA ge

401//steroid hormone mediated signaling pathway;GO:0046464//acylglycerol catabolic process;GC

3//glandular epithelial cell development;GO:0003417//growth plate cartilage development;GO:000

0002759//regulation of antimicrobial humoral response;GO:0003148//outflow tract septum morph

O:0034379//very-low-density lipoprotein particle assembly;GO:0034435//cholesterol esterificatior

ive regulation of MAP kinase activity;GO:0051897//positive regulation of protein kinase B signaling

aling pathway;GO:0007193//adenylate cyclase-inhibiting G-protein coupled receptor signaling pat
regulation of Ras protein signal transduction;GO:0070536//protein K63-linked deubiquitination

nsport;GO:0098609//cell-cell adhesion;GO:1901896//positive regulation of calcium-transporting A

l//skeletal muscle fiber development;GO:0048875//chemical homeostasis within a tissue;GO:0051[illegible]

:0055085//transmembrane transport;GO:0071805//potassium ion transmembrane transport

mbling;GO:0032365//intracellular lipid transport;GO:0044830//modulation by host of viral RNA ge

:ion;GO:0030334//regulation of cell migration;GO:0030517//negative regulation of axon extension

uction by p53 class mediator resulting in transcription of p21 class mediator;GO:0031571//mitotic
on of Wnt signaling pathway;GO:0030509//BMP signaling pathway;GO:0030514//negative regulati

030198//extracellular matrix organization;GO:0070373//negative regulation of ERK1 and ERK2 cas

:0055085//transmembrane transport;GO:0071805//potassium ion transmembrane transport
uction;GO:0014066//regulation of phosphatidylinositol 3-kinase signaling;GO:0030101//natural kill

007165//signal transduction;GO:0008203//cholesterol metabolic process;GO:0009395//phospholip

//regulation of fatty acid oxidation;GO:0055091//phospholipid homeostasis;GO:2001245//regulati

of ATPase activity;GO:0034975//protein folding in endoplasmic reticulum;GO:0034976//response t

brane transport;GO:0090277//positive regulation of peptide hormone secretion;GO:0098810//neu

inoacylation;GO:0016525//negative regulation of angiogenesis;GO:1904046//negative regulation
etic process;GO:0006682//galactosylceramide biosynthetic process;GO:0008610//lipid biosynthetic
regulation of Ras protein signal transduction;GO:0070536//protein K63-linked deubiquitination

0002759//regulation of antimicrobial humoral response;GO:0003148//outflow tract septum morph
beta receptor signaling pathway;GO:0030512//negative regulation of transforming growth factor b

n;GO:0006487//protein N-linked glycosylation;GO:0010706//ganglioside biosynthetic process via

tive regulation of phosphoprotein phosphatase activity;GO:0051726//regulation of cell cycle;GO:19

se to starvation;GO:0010886//positive regulation of cholesterol storage;GO:0032933//SREBP signa

etic process;GO:0006636//unsaturated fatty acid biosynthetic process;GO:0008610//lipid biosynth
030501//positive regulation of bone mineralization;GO:0032332//positive regulation of chondrocy

n;GO:0035556//intracellular signal transduction;GO:0046578//regulation of Ras protein signal trar
ansport;GO:0043268//positive regulation of potassium ion transport;GO:0045214//sarcomere orga

3//glandular epithelial cell development;GO:0003417//growth plate cartilage development;GO:000

mbling;GO:0032365//intracellular lipid transport;GO:0044830//modulation by host of viral RNA ge

ive regulation of MAP kinase activity;GO:0051897//positive regulation of protein kinase B signaling

loplasmic reticulum unfolded protein response;GO:0034976//response to endoplasmic reticulum s

nsport;GO:0098609//cell-cell adhesion;GO:1901896//positive regulation of calcium-transporting A

401//steroid hormone mediated signaling pathway;GO:0046464//acylglycerol catabolic process;GC

:0055085//transmembrane transport;GO:0071805//potassium ion transmembrane transport

port;GO:0070508//cholesterol import;GO:0070859//positive regulation of bile acid biosynthetic prc

-to lesion;GO:0006974//cellular response to DNA damage stimulus;GO:0008152//metabolic proce

ment;GO:0010762//regulation of fibroblast migration;GO:0042733//embryonic digit morphogene

ination;GO:0032410//negative regulation of transporter activity;GO:0051224//negative regulation

membrane transport;GO:0051899//membrane depolarization;GO:0060048//cardiac muscle contrac
uction by p53 class mediator resulting in transcription of p21 class mediator;GO:0031571//mitotic

;GO:0016032//viral process;GO:0044260//cellular macromolecule metabolic process;GO:0044826/

etic process;GO:0006636//unsaturated fatty acid biosynthetic process;GO:0008610//lipid biosynth

uction by p53 class mediator resulting in transcription of p21 class mediator;GO:0031571//mitotic

0007179//transforming growth factor beta receptor signaling pathway;GO:0007229//integrin-mec

n;GO:0006487//protein N-linked glycosylation;GO:0010706//ganglioside biosynthetic process via

sis;GO:0030913//paranodal junction assembly;GO:0042552//myelination;GO:0044255//cellular lipi

n;GO:0006487//protein N-linked glycosylation;GO:0010706//ganglioside biosynthetic process via

etic process;GO:0006636//unsaturated fatty acid biosynthetic process;GO:0008610//lipid biosynth
orting V-type ATPase complex assembly;GO:0090263//positive regulation of canonical Wnt signal

mbling;GO:0032365//intracellular lipid transport;GO:0044830//modulation by host of viral RNA ge

sitive regulation of CREB transcription factor activity;GO:0043065//positive regulation of apoptotic

ment;GO:0010762//regulation of fibroblast migration;GO:0042733//embryonic digit morphogene

:0055085//transmembrane transport;GO:0071805//potassium ion transmembrane transport

)07165//signal transduction;GO:0008203//cholesterol metabolic process;GO:0009395//phospholip

tion;GO:0016567//protein ubiquitination;GO:0031052//chromosome breakage;GO:0031297//repli

ansport;GO:0043268//positive regulation of potassium ion transport;GO:0045214//sarcomere orga

on;GO:0007405//neuroblast proliferation;GO:0007420//brain development;GO:0007623//circadian

loplasmic reticulum unfolded protein response;GO:0034976//response to endoplasmic reticulum s

864//pyrimidine nucleotide transport;GO:0007005//mitochondrion organization;GO:0008284//pos
of ATPase activity;GO:0034975//protein folding in endoplasmic reticulum;GO:0034976//response t

:ive regulation of phosphoprotein phosphatase activity;GO:0051726//regulation of cell cycle;GO:19

98//positive regulation of protein tyrosine kinase activity;GO:0061099//negative regulation of prot

uction by p53 class mediator resulting in transcription of p21 class mediator;GO:0031571//mitotic

nbrane transport;GO:1902476//chloride transmembrane transport;GO:1990573//potassium ion im

ı;GO:0016032//viral process;GO:0044260//cellular macromolecule metabolic process;GO:0044826/

loplasmic reticulum unfolded protein response;GO:0034976//response to endoplasmic reticulum s

nsport;GO:0098609//cell-cell adhesion;GO:1901896//positive regulation of calcium-transporting A

process;GO:0006650//glycerophospholipid metabolic process;GO:0006655//phosphatidylglycerol b
brane transport;GO:0090277//positive regulation of peptide hormone secretion;GO:0098810//neu
n;GO:0006487//protein N-linked glycosylation;GO:0010706//ganglioside biosynthetic process via l
e to virus;GO:0031110//regulation of microtubule polymerization or depolymerization;GO:003111

:0055085//transmembrane transport;GO:0071805//potassium ion transmembrane transport

aling pathway;GO:0007193//adenylate cyclase-inhibiting G-protein coupled receptor signaling pat

mbling;GO:0032365//intracellular lipid transport;GO:0044830//modulation by host of viral RNA ge
91//regulation of heart rate by cardiac conduction;GO:0090136//epithelial cell-cell adhesion;GO:0

401//steroid hormone mediated signaling pathway;GO:0046464//acylglycerol catabolic process;GC
d in apoptotic process;GO:0070301//cellular response to hydrogen peroxide;GO:0071456//cellular
O:0010628//positive regulation of gene expression;GO:0016559//peroxisome fission;GO:0048208//
on of Wnt signaling pathway;GO:0030509//BMP signaling pathway;GO:0030514//negative regulatio

2351//'de novo' GDP-L-fucose biosynthetic process;GO:1904906//positive regulation of endotheli

port;GO:0070508//cholesterol import;GO:0070859//positive regulation of bile acid biosynthetic pro

ive regulation of MAP kinase activity;GO:0051897//positive regulation of protein kinase B signaling

3//glandular epithelial cell development;GO:0003417//growth plate cartilage development;GO:000

tion;GO:0030334//regulation of cell migration;GO:0030517//negative regulation of axon extension

of ATPase activity;GO:0034975//protein folding in endoplasmic reticulum;GO:0034976//response t

ulation of transcription, DNA-templated;GO:0045944//positive regulation of transcription from RN

loplasmic reticulum unfolded protein response;GO:0034976//response to endoplasmic reticulum s
27//regulation of inflammatory response;GO:0050730//regulation of peptidyl-tyrosine phosphoryl
eart trabecula formation;GO:1900087//positive regulation of G1/S transition of mitotic cell cycle;G

)30336//negative regulation of cell migration;GO:0042542//response to hydrogen peroxide;GO:00

netic process;GO:0006636//unsaturated fatty acid biosynthetic process;GO:0008610//lipid biosynth

esponse to bacterium;GO:0018105//peptidyl-serine phosphorylation;GO:0032868//response to in

ctivity;GO:0070328//triglyceride homeostasis;GO:2000352//negative regulation of endothelial cell

nsport;GO:0098609//cell-cell adhesion;GO:1901896//positive regulation of calcium-transporting

aling pathway;GO:0007193//adenylate cyclase-inhibiting G-protein coupled receptor signaling pat

se to starvation;GO:0010886//positive regulation of cholesterol storage;GO:0032933//SREBP signa

2351//'de novo' GDP-L-fucose biosynthetic process;GO:1904906//positive regulation of endotheli

07165//signal transduction;GO:0008203//cholesterol metabolic process;GO:0009395//phospholip

bolic process;GO:0030301//cholesterol transport;GO:0034372//very-low-density lipoprotein partic

behavior;GO:0036343//psychomotor behavior;GO:0042127//regulation of cell proliferation;GO:004

thway;GO:0009617//response to bacterium;GO:0043547//positive regulation of GTPase activity;GC

elopment;GO:0008203//cholesterol metabolic process;GO:0009566//fertilization;GO:0009615//resp

on of Wnt signaling pathway;GO:0030509//BMP signaling pathway;GO:0030514//negative regulatio

port;GO:0007186//G-protein coupled receptor signaling pathway;GO:0007189//adenylate cyclase-
orting V-type ATPase complex assembly;GO:0090263//positive regulation of canonical Wnt signal

-to lesion;GO:0006974//cellular response to DNA damage stimulus;GO:0008152//metabolic proce

ive regulation of MAP kinase activity;GO:0051897//positive regulation of protein kinase B signaling

uscular septum morphogenesis;GO:0003151//outflow tract morphogenesis;GO:0003171//atrioven

3//glandular epithelial cell development;GO:0003417//growth plate cartilage development;GO:000

n;GO:0006487//protein N-linked glycosylation;GO:0010706//ganglioside biosynthetic process via

lic acid biosynthetic process;GO:0010898//positive regulation of triglyceride catabolic process;GO:

0002759//regulation of antimicrobial humoral response;GO:0003148//outflow tract septum morph
)30336//negative regulation of cell migration;GO:0042542//response to hydrogen peroxide;GO:00
sitive regulation of CREB transcription factor activity;GO:0043065//positive regulation of apoptotic

;GO:0007283//spermatogenesis;GO:0007422//peripheral nervous system development;GO:00085

regulation of Ras protein signal transduction;GO:0070536//protein K63-linked deubiquitination

2351//'de novo' GDP-L-fucose biosynthetic process;GO:1904906//positive regulation of endotheli

401//steroid hormone mediated signaling pathway;GO:0046464//acylglycerol catabolic process;G

nscript (SSU-rRNA, 5.8S rRNA, LSU-rRNA);GO:0006364//rRNA processing;GO:0008283//cell prolif

nscript (SSU-rRNA, 5.8S rRNA, LSU-rRNA);GO:0006364//rRNA processing;GO:0008283//cell prolife

sis;GO:0030913//paranodal junction assembly;GO:0042552//myelination;GO:0044255//cellular lipi

e to virus;GO:0031110//regulation of microtubule polymerization or depolymerization;GO:003111[illegible]

)07165//signal transduction;GO:0008203//cholesterol metabolic process;GO:0009395//phospholip

ıse to starvation;GO:0010886//positive regulation of cholesterol storage;GO:0032933//SREBP signa
response to bacterium;GO:0018105//peptidyl-serine phosphorylation;GO:0032868//response to in

naling pathway;GO:0050909//sensory perception of taste;GO:0050913//sensory perception of bitt

elopment;GO:0008203//cholesterol metabolic process;GO:0009566//fertilization;GO:0009615//resp

loplasmic reticulum unfolded protein response;GO:0034976//response to endoplasmic reticulum s

etic process;GO:0006682//galactosylceramide biosynthetic process;GO:0008610//lipid biosynthetic
e to virus;GO:0031110//regulation of microtubule polymerization or depolymerization;GO:003111[illegible]

ic process;GO:0007626//locomotory behavior;GO:0021768//nucleus accumbens development;GO:

)30336//negative regulation of cell migration;GO:0042542//response to hydrogen peroxide;GO:00

on;GO:0035999//tetrahydrofolate interconversion;GO:0050667//homocysteine metabolic process;[illegible]

//regulation of fatty acid oxidation;GO:0055091//phospholipid homeostasis;GO:2001245//regulati[illegible]

ctivity;GO:0070328//triglyceride homeostasis;GO:2000352//negative regulation of endothelial cell

virus;GO:0010468//regulation of gene expression;GO:0016310//phosphorylation;GO:0042063//gl

)32330//regulation of chondrocyte differentiation;GO:0036150//phosphatidylserine acyl-chain rem
ctivity;GO:0070328//triglyceride homeostasis;GO:2000352//negative regulation of endothelial cell

e to virus;GO:0031110//regulation of microtubule polymerization or depolymerization;GO:003111[illegible]

sitive regulation of CREB transcription factor activity;GO:0043065//positive regulation of apoptotic

[illegible]4//pre-miRNA processing;GO:0035196//production of miRNAs involved in gene silencing by miR

uscular septum morphogenesis;GO:0003151//outflow tract morphogenesis;GO:0003171//atrioven

rocess;GO:0006650//glycerophospholipid metabolic process;GO:0006655//phosphatidylglycerol b

sis;GO:0030913//paranodal junction assembly;GO:0042552//myelination;GO:0044255//cellular lip[illegible]

on of Wnt signaling pathway;GO:0030509//BMP signaling pathway;GO:0030514//negative regulati[illegible]

orting V-type ATPase complex assembly;GO:0090263//positive regulation of canonical Wnt signal

401//steroid hormone mediated signaling pathway;GO:0046464//acylglycerol catabolic process;G[illegible]
orting V-type ATPase complex assembly;GO:0090263//positive regulation of canonical Wnt signal
response to bacterium;GO:0018105//peptidyl-serine phosphorylation;GO:0032868//response to in
ubiquitin-dependent protein catabolic process;GO:0031146//SCF-dependent proteasomal ubiqu

aling pathway;GO:0007193//adenylate cyclase-inhibiting G-protein coupled receptor signaling pat

0002759//regulation of antimicrobial humoral response;GO:0003148//outflow tract septum morpl
l//skeletal muscle fiber development;GO:0048875//chemical homeostasis within a tissue;GO:0051

sitive regulation of CREB transcription factor activity;GO:0043065//positive regulation of apoptotic

regulation of Ras protein signal transduction;GO:0070536//protein K63-linked deubiquitination

91//regulation of heart rate by cardiac conduction;GO:0090136//epithelial cell-cell adhesion;GO:0

O:0045944//positive regulation of transcription from RNA polymerase II promoter;GO:0048863//st

030336//negative regulation of cell migration;GO:0042542//response to hydrogen peroxide;GO:00

99//nervous system development;GO:0007411//axon guidance;GO:0007416//synapse assembly;G
030198//extracellular matrix organization;GO:0070373//negative regulation of ERK1 and ERK2 cas
ic process;GO:0007626//locomotory behavior;GO:0021768//nucleus accumbens development;GO:

sis;GO:0030913//paranodal junction assembly;GO:0042552//myelination;GO:0044255//cellular lipi

etic process;GO:0006682//galactosylceramide biosynthetic process;GO:0008610//lipid biosynthetic

oment;GO:0010762//regulation of fibroblast migration;GO:0042733//embryonic digit morphogene

on;GO:0010718//positive regulation of epithelial to mesenchymal transition;GO:0010812//negative
of ATPase activity;GO:0034975//protein folding in endoplasmic reticulum;GO:0034976//response t

sitive regulation of CREB transcription factor activity;GO:0043065//positive regulation of apoptotic

ulation of transcription, DNA-templated;GO:0045944//positive regulation of transcription from RN

GO:0034116//positive regulation of heterotypic cell-cell adhesion;GO:0034622//cellular macromole
//regulation of fatty acid oxidation;GO:0055091//phospholipid homeostasis;GO:2001245//regulati

nscript (SSU-rRNA, 5.8S rRNA, LSU-rRNA);GO:0006364//rRNA processing;GO:0008283//cell prolife

//defense response to bacterium;GO:0050829//defense response to Gram-negative bacterium;GC

on;GO:0035556//intracellular signal transduction;GO:0046578//regulation of Ras protein signal tran
orting V-type ATPase complex assembly;GO:0090263//positive regulation of canonical Wnt signal
;GO:0016032//viral process;GO:0044260//cellular macromolecule metabolic process;GO:0044826/

netic process;GO:0006636//unsaturated fatty acid biosynthetic process;GO:0008610//lipid biosynth

030501//positive regulation of bone mineralization;GO:0032332//positive regulation of chondrocy

ɔrocess;GO:0006650//glycerophospholipid metabolic process;GO:0006655//phosphatidylglycerol b

aling pathway;GO:0007193//adenylate cyclase-inhibiting G-protein coupled receptor signaling pat
38//protein metabolic process;GO:0051289//protein homotetramerization;GO:0055088//lipid hom

n;GO:0006487//protein N-linked glycosylation;GO:0010706//ganglioside biosynthetic process via

e to virus;GO:0031110//regulation of microtubule polymerization or depolymerization;GO:003111

elopment;GO:0008203//cholesterol metabolic process;GO:0009566//fertilization;GO:0009615//resp

abolic process;GO:0046839//phospholipid dephosphorylation;GO:1902247//geranylgeranyl diphos

cell proliferation;GO:0008543//fibroblast growth factor receptor signaling pathway;GO:0009617//r

2351//'de novo' GDP-L-fucose biosynthetic process;GO:1904906//positive regulation of endotheli
ubiquitin-dependent protein catabolic process;GO:0031146//SCF-dependent proteasomal ubiqu

orting V-type ATPase complex assembly;GO:0090263//positive regulation of canonical Wnt signal

virus;GO:0010468//regulation of gene expression;GO:0016310//phosphorylation;GO:0042063//gl

nembrane transport;GO:0051899//membrane depolarization;GO:0060048//cardiac muscle contrac

98//positive regulation of protein tyrosine kinase activity;GO:0061099//negative regulation of prot

l//skeletal muscle fiber development;GO:0048875//chemical homeostasis within a tissue;GO:0051[illegible]

sitive regulation of CREB transcription factor activity;GO:0043065//positive regulation of apoptotic

ıse to starvation;GO:0010886//positive regulation of cholesterol storage;GO:0032933//SREBP signa

ic process;GO:0007626//locomotory behavior;GO:0021768//nucleus accumbens development;GO:
of ATPase activity;GO:0034975//protein folding in endoplasmic reticulum;GO:0034976//response t
uction by p53 class mediator resulting in transcription of p21 class mediator;GO:0031571//mitotic

)32330//regulation of chondrocyte differentiation;GO:0036150//phosphatidylserine acyl-chain rem

)021954//central nervous system neuron development;GO:0032091//negative regulation of protei

030198//extracellular matrix organization;GO:0070373//negative regulation of ERK1 and ERK2 cas
:0055085//transmembrane transport;GO:0071805//potassium ion transmembrane transport

)07165//signal transduction;GO:0008203//cholesterol metabolic process;GO:0009395//phospholip

ɔn;GO:0010718//positive regulation of epithelial to mesenchymal transition;GO:0010812//negative

regulation of Ras protein signal transduction;GO:0070536//protein K63-linked deubiquitination

0002759//regulation of antimicrobial humoral response;GO:0003148//outflow tract septum morpl

ıt;GO:0001974//blood vessel remodeling;GO:0003338//metanephros morphogenesis;GO:0006935/

030501//positive regulation of bone mineralization;GO:0032332//positive regulation of chondrocy

'cellular response to amino acid starvation;GO:0070534//protein K63-linked ubiquitination;GO:007

›ment;GO:0010762//regulation of fibroblast migration;GO:0042733//embryonic digit morphogene

e to virus;GO:0031110//regulation of microtubule polymerization or depolymerization;GO:003111[illegible]
›n;GO:0035556//intracellular signal transduction;GO:0046578//regulation of Ras protein signal tran

ninoacylation;GO:0016525//negative regulation of angiogenesis;GO:1904046//negative regulation

uction by p53 class mediator resulting in transcription of p21 class mediator;GO:0031571//mitotic

030198//extracellular matrix organization;GO:0070373//negative regulation of ERK1 and ERK2 cas
nembrane transport;GO:0051899//membrane depolarization;GO:0060048//cardiac muscle contrac

3//glandular epithelial cell development;GO:0003417//growth plate cartilage development;GO:000

ive regulation of MAP kinase activity;GO:0051897//positive regulation of protein kinase B signaling
0021954//central nervous system neuron development;GO:0032091//negative regulation of protei
1//skeletal muscle fiber development;GO:0048875//chemical homeostasis within a tissue;GO:0051[illegible]

ination;GO:0032410//negative regulation of transporter activity;GO:0051224//negative regulation

etic process;GO:0006682//galactosylceramide biosynthetic process;GO:0008610//lipid biosynthetic
36//positive regulation of germinal center formation;GO:0002686//negative regulation of leukocyte

nembrane transport;GO:0051899//membrane depolarization;GO:0060048//cardiac muscle contrac

on of Wnt signaling pathway;GO:0030509//BMP signaling pathway;GO:0030514//negative regulatic

n;GO:0016032//viral process;GO:0044260//cellular macromolecule metabolic process;GO:0044826/

//regulation of fatty acid oxidation;GO:0055091//phospholipid homeostasis;GO:2001245//regulatic

0007179//transforming growth factor beta receptor signaling pathway;GO:0007229//integrin-mec

tination;GO:0032410//negative regulation of transporter activity;GO:0051224//negative regulation

tive regulation of phosphoprotein phosphatase activity;GO:0051726//regulation of cell cycle;GO:19
2351//'de novo' GDP-L-fucose biosynthetic process;GO:1904906//positive regulation of endothelia

L//skeletal muscle fiber development;GO:0048875//chemical homeostasis within a tissue;GO:00515

› virus;GO:0010468//regulation of gene expression;GO:0016310//phosphorylation;GO:0042063//gl

mbling;GO:0032365//intracellular lipid transport;GO:0044830//modulation by host of viral RNA ge

›ment;GO:0010762//regulation of fibroblast migration;GO:0042733//embryonic digit morphogene
98//positive regulation of protein tyrosine kinase activity;GO:0061099//negative regulation of prot

030198//extracellular matrix organization;GO:0070373//negative regulation of ERK1 and ERK2 cas

response to bacterium;GO:0018105//peptidyl-serine phosphorylation;GO:0032868//response to in

O:0045944//positive regulation of transcription from RNA polymerase II promoter;GO:0048863//st

nsport;GO:0098609//cell-cell adhesion;GO:1901896//positive regulation of calcium-transporting A

›ment;GO:0010762//regulation of fibroblast migration;GO:0042733//embryonic digit morphogene

'cellular response to amino acid starvation;GO:0070534//protein K63-linked ubiquitination;GO:007

085//transmembrane transport;GO:0061337//cardiac conduction;GO:0071805//potassium ion tran

54//pre-miRNA processing;GO:0035196//production of miRNAs involved in gene silencing by miR

on;GO:0010718//positive regulation of epithelial to mesenchymal transition;GO:0010812//negative
ination;GO:0032410//negative regulation of transporter activity;GO:0051224//negative regulation

cell proliferation;GO:0008543//fibroblast growth factor receptor signaling pathway;GO:0009617//r

naling pathway;GO:0050909//sensory perception of taste;GO:0050913//sensory perception of bitte
abolic process;GO:0046839//phospholipid dephosphorylation;GO:1902247//geranylgeranyl diphos

mbling;GO:0032365//intracellular lipid transport;GO:0044830//modulation by host of viral RNA ge
nsport;GO:0098609//cell-cell adhesion;GO:1901896//positive regulation of calcium-transporting A

rocess;GO:0006650//glycerophospholipid metabolic process;GO:0006655//phosphatidylglycerol b

36//positive regulation of germinal center formation;GO:0002686//negative regulation of leukocyt

O:0010628//positive regulation of gene expression;GO:0016559//peroxisome fission;GO:0048208/

2351//'de novo' GDP-L-fucose biosynthetic process;GO:1904906//positive regulation of endotheli

98//positive regulation of protein tyrosine kinase activity;GO:0061099//negative regulation of prot

54//positive regulation of megakaryocyte differentiation;GO:0051092//positive regulation of NF-ka
ısmembrane transport;GO:0086003//cardiac muscle cell contraction;GO:1903760//regulation of vo

f phosphorylation;GO:0050861//positive regulation of B cell receptor signaling pathway;GO:00601

noterpenoid metabolic process;GO:0033076//isoquinoline alkaloid metabolic process;GO:0042178/
NA;GO:0035278//miRNA mediated inhibition of translation;GO:0035280//miRNA loading onto RIS

ynthetic process;GO:0006695//cholesterol biosynthetic process;GO:0006869//lipid transport;GO:00

)//positive regulation of glycoprotein biosynthetic process;GO:0010613//positive regulation of car

ositive regulation of gene expression;GO:0045892//negative regulation of transcription, DNA-temp
liated signaling pathway;GO:0009611//response to wounding;GO:0009615//response to virus;GO:

cation fork processing;GO:0031571//mitotic G1 DNA damage checkpoint;GO:0036297//interstranc

olic process;GO:0019433//triglyceride catabolic process;GO:0032722//positive regulation of chem

3211//adiponectin-activated signaling pathway;GO:0034143//regulation of toll-like receptor 4 sig
rhythm;GO:0008284//positive regulation of cell proliferation;GO:0010628//positive regulation of g

O:0008584//male gonad development;GO:0009410//response to xenobiotic stimulus;GO:0010628

liogenesis;GO:0042127//regulation of cell proliferation;GO:0043697//cell dedifferentiation;GO:004

34//male gonad development;GO:0010628//positive regulation of gene expression;GO:0010817//r

O:0007626//locomotory behavior;GO:0010842//retina layer formation;GO:0038007//netrin-activat

1529//positive regulation of anion channel activity;GO:2001225//regulation of chloride transport

NA;GO:0035278//miRNA mediated inhibition of translation;GO:0035280//miRNA loading onto RIS

):0010001//glial cell differentiation;GO:0010467//gene expression;GO:0022402//cell cycle process;

ynthetic process;GO:0006695//cholesterol biosynthetic process;GO:0006869//lipid transport;GO:0(

ler cell activation;GO:0032252//secretory granule localization;GO:0032725//positive regulation of g

ne biosynthetic process via cystathionine;GO:0019344//cysteine biosynthetic process;GO:0021587/

ɔsitive regulation of gene expression;GO:0045892//negative regulation of transcription, DNA-temp

ıction;GO:0021879//forebrain neuron differentiation;GO:0032231//regulation of actin filament bur

ıal transduction;GO:0043536//positive regulation of blood vessel endothelial cell migration;GO:004

ıoterpenoid metabolic process;GO:0033076//isoquinoline alkaloid metabolic process;GO:0042178/

)//positive regulation of glycoprotein biosynthetic process;GO:0010613//positive regulation of car

ɔn of BMP signaling pathway;GO:0045662//negative regulation of myoblast differentiation;GO:009

GO:0021670//lateral ventricle development;GO:0021766//hippocampus development;GO:002196

stress;GO:0045893//positive regulation of transcription, DNA-templated;GO:0045944//positive reg

ısmembrane transport;GO:0086003//cardiac muscle cell contraction;GO:1903760//regulation of vo

D:0007626//locomotory behavior;GO:0010842//retina layer formation;GO:0038007//netrin-activat

54//positive regulation of megakaryocyte differentiation;GO:0051092//positive regulation of NF-ka

)804//modulation of synaptic transmission;GO:0050808//synapse organization;GO:0071625//vocal

n binding;GO:0032435//negative regulation of proteasomal ubiquitin-dependent protein cataboli

33211//adiponectin-activated signaling pathway;GO:0034143//regulation of toll-like receptor 4 sig

O:0008584//male gonad development;GO:0009410//response to xenobiotic stimulus;GO:0010628

)35865//cellular response to potassium ion;GO:0042391//regulation of membrane potential;GO:00

:le remodeling;GO:0034375//high-density lipoprotein particle remodeling;GO:0034435//cholester

ler cell activation;GO:0032252//secretory granule localization;GO:0032725//positive regulation of

ne biosynthetic process via cystathionine;GO:0019344//cysteine biosynthetic process;GO:0021587/
):0010001//glial cell differentiation;GO:0010467//gene expression;GO:0022402//cell cycle process;

protein ubiquitination;GO:0031648//protein destabilization;GO:0032803//regulation of low-density

uction;GO:0021879//forebrain neuron differentiation;GO:0032231//regulation of actin filament bur

0906//regulation of glucose metabolic process;GO:0042304//regulation of fatty acid biosynthetic

age;GO:0018105//peptidyl-serine phosphorylation;GO:0034340//response to type I interferon;GO:

ort;GO:0010866//regulation of triglyceride biosynthetic process;GO:0010884//positive regulation

ial transduction;GO:0043536//positive regulation of blood vessel endothelial cell migration;GO:00

ıction;GO:0021879//forebrain neuron differentiation;GO:0032231//regulation of actin filament bur

tem cell differentiation;GO:0061086//negative regulation of histone H3-K27 methylation;GO:0061(

):0061077//chaperone-mediated protein folding;GO:1901874//negative regulation of post-transla
):0010001//glial cell differentiation;GO:0010467//gene expression;GO:0022402//cell cycle process;

:le remodeling;GO:0034375//high-density lipoprotein particle remodeling;GO:0034435//cholester(

ɪrotein ubiquitination;GO:0031648//protein destabilization;GO:0032803//regulation of low-density

2//cytoplasmic microtubule organization;GO:0045022//early endosome to late endosome transpor

):0007626//locomotory behavior;GO:0010842//retina layer formation;GO:0038007//netrin-activat

/viral genome integration into host DNA;GO:0046718//viral entry into host cell;GO:0075713//estal

n of synaptic plasticity;GO:0032793//positive regulation of CREB transcription factor activity;GO:00

rhythm;GO:0008284//positive regulation of cell proliferation;GO:0010628//positive regulation of g

ort;GO:0010866//regulation of triglyceride biosynthetic process;GO:0010884//positive regulation c
age;GO:0018105//peptidyl-serine phosphorylation;GO:0034340//response to type I interferon;GO:

aling pathway;GO:0042632//cholesterol homeostasis;GO:0045944//positive regulation of transcript

dium assembly;GO:0031647//regulation of protein stability;GO:0034314//Arp2/3 complex-mediate

ler cell activation;GO:0032252//secretory granule localization;GO:0032725//positive regulation of g

tem cell differentiation;GO:0061086//negative regulation of histone H3-K27 methylation;GO:0061[illegible]

[illegible]:0010001//glial cell differentiation;GO:0010467//gene expression;GO:0022402//cell cycle process;

[illegible]er taste;GO:0050916//sensory perception of sweet taste;GO:0050917//sensory perception of uman
[illegible]098609//cell-cell adhesion;GO:0098911//regulation of ventricular cardiac muscle cell action poten

[illegible]rotein ubiquitination;GO:0031648//protein destabilization;GO:0032803//regulation of low-density

[illegible]1589//cerebellum structural organization;GO:0021766//hippocampus development;GO:0021795//[illegible]

[illegible]0016042//lipid catabolic process;GO:0019432//triglyceride biosynthetic process;GO:0019433//trigl

592//response to calcium ion;GO:0051928//positive regulation of calcium ion transport;GO:009027

noterpenoid metabolic process;GO:0033076//isoquinoline alkaloid metabolic process;GO:0042178/

)//positive regulation of glycoprotein biosynthetic process;GO:0010613//positive regulation of car
nal transduction;GO:0043536//positive regulation of blood vessel endothelial cell migration;GO:004

):0007626//locomotory behavior;GO:0010842//retina layer formation;GO:0038007//netrin-activat

cade;GO:0072378//blood coagulation, fibrin clot formation;GO:1900025//negative regulation of su

0906//regulation of glucose metabolic process;GO:0042304//regulation of fatty acid biosynthetic

ler cell activation;GO:0032252//secretory granule localization;GO:0032725//positive regulation of g

uction;GO:0021879//forebrain neuron differentiation;GO:0032231//regulation of actin filament bur

n of synaptic plasticity;GO:0032793//positive regulation of CREB transcription factor activity;GO:00
ol transport;GO:0032367//intracellular cholesterol transport;GO:0033344//cholesterol efflux;GO:00

ɔonse to virus;GO:0009791//post-embryonic development;GO:0010628//positive regulation of ger

ısmembrane transport;GO:0086003//cardiac muscle cell contraction;GO:1903760//regulation of vo

ɜr taste;GO:0050916//sensory perception of sweet taste;GO:0050917//sensory perception of uman

ynthetic process;GO:0006695//cholesterol biosynthetic process;GO:0006869//lipid transport;GO:00

ɔsitive regulation of gene expression;GO:0045892//negative regulation of transcription, DNA-temp

:ion;GO:0033234//negative regulation of protein sumoylation;GO:0043066//negative regulation of

O:0008584//male gonad development;GO:0009410//response to xenobiotic stimulus;GO:0010628

ler cell activation;GO:0032252//secretory granule localization;GO:0032725//positive regulation of g
33211//adiponectin-activated signaling pathway;GO:0034143//regulation of toll-like receptor 4 sig

)//positive regulation of glycoprotein biosynthetic process;GO:0010613//positive regulation of car

0906//regulation of glucose metabolic process;GO:0042304//regulation of fatty acid biosynthetic

9//protein deubiquitination;GO:0019896//axon transport of mitochondrion;GO:0042755//eating b

34//male gonad development;GO:0010628//positive regulation of gene expression;GO:0010817//r

//chemotaxis;GO:0007267//cell-cell signaling;GO:0007368//determination of left/right symmetry;G

te differentiation;GO:0035108//limb morphogenesis;GO:0035264//multicellular organism growth;

)//positive regulation of glycoprotein biosynthetic process;GO:0010613//positive regulation of car

1529//positive regulation of anion channel activity;GO:2001225//regulation of chloride transport

noterpenoid metabolic process;GO:0033076//isoquinoline alkaloid metabolic process;GO:0042178/

21589//cerebellum structural organization;GO:0021766//hippocampus development;GO:0021795//

ol transport;GO:0032367//intracellular cholesterol transport;GO:0033344//cholesterol efflux;GO:00

entiation;GO:0090181//regulation of cholesterol metabolic process;GO:0090207//regulation of trig

21589//cerebellum structural organization;GO:0021766//hippocampus development;GO:0021795//

sis;GO:0043010//camera-type eye development;GO:0043587//tongue morphogenesis;GO:004478

n binding;GO:0032435//negative regulation of proteasomal ubiquitin-dependent protein cataboli

33211//adiponectin-activated signaling pathway;GO:0034143//regulation of toll-like receptor 4 sig
ler cell activation;GO:0032252//secretory granule localization;GO:0032725//positive regulation of g

0906//regulation of glucose metabolic process;GO:0042304//regulation of fatty acid biosynthetic

O:0008584//male gonad development;GO:0009410//response to xenobiotic stimulus;GO:0010628/

)//positive regulation of glycoprotein biosynthetic process;GO:0010613//positive regulation of car

1529//positive regulation of anion channel activity;GO:2001225//regulation of chloride transport

ne phosphorylation;GO:0051353//positive regulation of oxidoreductase activity;GO:0071300//cellu

tion;GO:0010898//positive regulation of triglyceride catabolic process;GO:0016042//lipid catabolic
olic process;GO:0019433//triglyceride catabolic process;GO:0032722//positive regulation of chem

ynthetic process;GO:0006695//cholesterol biosynthetic process;GO:0006869//lipid transport;GO:00

):0010001//glial cell differentiation;GO:0010467//gene expression;GO:0022402//cell cycle process;

ɔsitive regulation of gene expression;GO:0045892//negative regulation of transcription, DNA-temp

sulin;GO:0032869//cellular response to insulin stimulus;GO:0043382//positive regulation of memo

1529//positive regulation of anion channel activity;GO:2001225//regulation of chloride transport

:1589//cerebellum structural organization;GO:0021766//hippocampus development;GO:0021795//

ıal transduction;GO:0043536//positive regulation of blood vessel endothelial cell migration;GO:004

noterpenoid metabolic process;GO:0033076//isoquinoline alkaloid metabolic process;GO:0042178/

olic process;GO:0019433//triglyceride catabolic process;GO:0032722//positive regulation of chem

34//male gonad development;GO:0010628//positive regulation of gene expression;GO:0010817//r

43524//negative regulation of neuron apoptotic process;GO:0051897//positive regulation of prote

1589//cerebellum structural organization;GO:0021766//hippocampus development;GO:0021795//

olic process;GO:0019433//triglyceride catabolic process;GO:0032722//positive regulation of chem

ıetic process;GO:0019367//fatty acid elongation, saturated fatty acid;GO:0030148//sphingolipid bi

development;GO:0031639//plasminogen activation;GO:0033489//cholesterol biosynthetic process
ɔiosynthetic process;GO:0008654//phospholipid biosynthetic process;GO:0009749//response to glu

33211//adiponectin-activated signaling pathway;GO:0034143//regulation of toll-like receptor 4 sig

O:0008584//male gonad development;GO:0009410//response to xenobiotic stimulus;GO:0010628

1529//positive regulation of anion channel activity;GO:2001225//regulation of chloride transport

olic process;GO:0019433//triglyceride catabolic process;GO:0032722//positive regulation of chem

):0010001//glial cell differentiation;GO:0010467//gene expression;GO:0022402//cell cycle process;

rotein ubiquitination;GO:0031648//protein destabilization;GO:0032803//regulation of low-density
n binding;GO:0032435//negative regulation of proteasomal ubiquitin-dependent protein catabolі

ositive regulation of gene expression;GO:0045892//negative regulation of transcription, DNA-temp

le remodeling;GO:0034375//high-density lipoprotein particle remodeling;GO:0034435//cholester

ion of inflammatory response;GO:0050729//positive regulation of inflammatory response;GO:005C

ynthetic process;GO:0006695//cholesterol biosynthetic process;GO:0006869//lipid transport;GO:0C

34//male gonad development;GO:0010628//positive regulation of gene expression;GO:0010817//r

uction;GO:0021879//forebrain neuron differentiation;GO:0032231//regulation of actin filament bur

uction;GO:0021879//forebrain neuron differentiation;GO:0032231//regulation of actin filament bur

//chemotaxis;GO:0007267//cell-cell signaling;GO:0007368//determination of left/right symmetry;G

;GO:2001240//negative regulation of extrinsic apoptotic signaling pathway in absence of ligand
nal transduction;GO:0043536//positive regulation of blood vessel endothelial cell migration;GO:004

1529//positive regulation of anion channel activity;GO:2001225//regulation of chloride transport

ction;GO:0060371//regulation of atrial cardiac muscle cell membrane depolarization;GO:0060373//

›e morphogenesis;GO:0044089//positive regulation of cellular component biogenesis;GO:0048568
ne biosynthetic process via cystathionine;GO:0019344//cysteine biosynthetic process;GO:0021587/

ection of chemical stimulus involved in sensory perception of taste;GO:0050915//sensory perceptic

-activating G-protein coupled receptor signaling pathway;GO:0007507//heart development;GO:00

ositive regulation of gene expression;GO:0045892//negative regulation of transcription, DNA-tem

›olic process;GO:0019433//triglyceride catabolic process;GO:0032722//positive regulation of chem

ynthetic process;GO:0006695//cholesterol biosynthetic process;GO:0006869//lipid transport;GO:00

uction;GO:0021879//forebrain neuron differentiation;GO:0032231//regulation of actin filament bur

:ion;GO:0033234//negative regulation of protein sumoylation;GO:0043066//negative regulation of

entiation;GO:0090181//regulation of cholesterol metabolic process;GO:0090207//regulation of trig

9//protein deubiquitination;GO:0019896//axon transport of mitochondrion;GO:0042755//eating b
5//negative regulation of microtubule polymerization;GO:0031175//neuron projection developme

GO:0021670//lateral ventricle development;GO:0021766//hippocampus development;GO:002196(

/COPII vesicle coating;GO:0070863//positive regulation of protein exit from endoplasmic reticulum

IAP kinase activity;GO:0050709//negative regulation of protein secretion;GO:1900222//negative re

2//cytoplasmic microtubule organization;GO:0045022//early endosome to late endosome transpor

ein coupled receptor signaling pathway;GO:0030097//hemopoiesis;GO:0030593//neutrophil chem

protein ubiquitination;GO:0031648//protein destabilization;GO:0032803//regulation of low-density

ısmembrane transport;GO:0086003//cardiac muscle cell contraction;GO:1903760//regulation of vo

ɔrotein ubiquitination;GO:0031648//protein destabilization;GO:0032803//regulation of low-density
ɪoterpenoid metabolic process;GO:0033076//isoquinoline alkaloid metabolic process;GO:0042178/

:le remodeling;GO:0034375//high-density lipoprotein particle remodeling;GO:0034435//cholestero

-activating G-protein coupled receptor signaling pathway;GO:0007507//heart development;GO:00

on;GO:0010975//regulation of neuron projection development;GO:0035556//intracellular signal tr

ⵎ:0007626//locomotory behavior;GO:0010842//retina layer formation;GO:0038007//netrin-activat

34//male gonad development;GO:0010628//positive regulation of gene expression;GO:0010817//r

/regulation of cell migration;GO:0034638//phosphatidylcholine catabolic process;GO:0045765//re

esponse to bacterium;GO:0010629//negative regulation of gene expression;GO:0046326//positive

ay;GO:0008284//positive regulation of cell proliferation;GO:0010628//positive regulation of gene e
: process;GO:0043066//negative regulation of apoptotic process;GO:0050766//positive regulation

/COPII vesicle coating;GO:0070863//positive regulation of protein exit from endoplasmic reticulum

0016042//lipid catabolic process;GO:0019432//triglyceride biosynthetic process;GO:0019433//trigl

:le remodeling;GO:0034375//high-density lipoprotein particle remodeling;GO:0034435//cholester[illegible]

0001819//positive regulation of cytokine production;GO:0001822//kidney development;GO:00018[illegible]

noterpenoid metabolic process;GO:0033076//isoquinoline alkaloid metabolic process;GO:0042178[illegible]
ynthetic process;GO:0006695//cholesterol biosynthetic process;GO:0006869//lipid transport;GO:00[illegible]

protein ubiquitination;GO:0031648//protein destabilization;GO:0032803//regulation of low-density

D:0007626//locomotory behavior;GO:0010842//retina layer formation;GO:0038007//netrin-activat[illegible]

ositive regulation of gene expression;GO:0045892//negative regulation of transcription, DNA-tem[illegible]

ne biosynthetic process via cystathionine;GO:0019344//cysteine biosynthetic process;GO:0021587[illegible]
ction;GO:0060371//regulation of atrial cardiac muscle cell membrane depolarization;GO:0060373/[illegible]

n of insulin secretion;GO:0045727//positive regulation of translation;GO:0046622//positive regulat[illegible]

34//male gonad development;GO:0010628//positive regulation of gene expression;GO:0010817//[illegible]

G-protein coupled purinergic nucleotide receptor signaling pathway;GO:0043270//positive regula[illegible]

al transduction;GO:0043536//positive regulation of blood vessel endothelial cell migration;GO:00[illegible]

0031076//embryonic camera-type eye development;GO:0042472//inner ear morphogenesis;GO:[illegible]

D:0007626//locomotory behavior;GO:0010842//retina layer formation;GO:0038007//netrin-activat[illegible]

0906//regulation of glucose metabolic process;GO:0042304//regulation of fatty acid biosynthetic

GO:0021670//lateral ventricle development;GO:0021766//hippocampus development;GO:002196(

/regulation of cell migration;GO:0034638//phosphatidylcholine catabolic process;GO:0045765//re

)//positive regulation of glycoprotein biosynthetic process;GO:0010613//positive regulation of car

ay;GO:0008284//positive regulation of cell proliferation;GO:0010628//positive regulation of gene e
33211//adiponectin-activated signaling pathway;GO:0034143//regulation of toll-like receptor 4 sig

O:0008584//male gonad development;GO:0009410//response to xenobiotic stimulus;GO:0010628/

O:0008584//male gonad development;GO:0009410//response to xenobiotic stimulus;GO:0010628/

33211//adiponectin-activated signaling pathway;GO:0034143//regulation of toll-like receptor 4 sig

ne biosynthetic process via cystathionine;GO:0019344//cysteine biosynthetic process;GO:0021587/

positive regulation of protein import into nucleus;GO:0043065//positive regulation of apoptotic pro

rylation;GO:0046325//negative regulation of glucose import;GO:0046627//negative regulation of

ıal transduction;GO:0043536//positive regulation of blood vessel endothelial cell migration;GO:004

D:0007626//locomotory behavior;GO:0010842//retina layer formation;GO:0038007//netrin-activat
ıodeling;GO:0036151//phosphatidylcholine acyl-chain remodeling;GO:0036152//phosphatidyletha

1529//positive regulation of anion channel activity;GO:2001225//regulation of chloride transport

ıiosynthetic process;GO:0008654//phospholipid biosynthetic process;GO:0009749//response to glı

cle remodeling;GO:0034375//high-density lipoprotein particle remodeling;GO:0034435//cholestero

0906//regulation of glucose metabolic process;GO:0042304//regulation of fatty acid biosynthetic

G-protein coupled purinergic nucleotide receptor signaling pathway;GO:0043270//positive regula

ynthetic process;GO:0006695//cholesterol biosynthetic process;GO:0006869//lipid transport;GO:00

!869//cellular response to insulin stimulus;GO:0045725//positive regulation of glycogen biosynthet

ɔsitive regulation of gene expression;GO:0045892//negative regulation of transcription, DNA-temp

eta receptor signaling pathway;GO:0032683//negative regulation of connective tissue growth fact

entiation;GO:0090181//regulation of cholesterol metabolic process;GO:0090207//regulation of trig

n of apoptotic process;GO:0043123//positive regulation of I-kappaB kinase/NF-kappaB signaling;

nal transduction;GO:0043536//positive regulation of blood vessel endothelial cell migration;GO:00

):0010001//glial cell differentiation;GO:0010467//gene expression;GO:0022402//cell cycle process;

33211//adiponectin-activated signaling pathway;GO:0034143//regulation of toll-like receptor 4 sig

ophil differentiation;GO:0030224//monocyte differentiation;GO:0071353//cellular response to inte

;GO:0034374//low-density lipoprotein particle remodeling;GO:0034375//high-density lipoprotein
O:0008584//male gonad development;GO:0009410//response to xenobiotic stimulus;GO:0010628

rotein ubiquitination;GO:0031648//protein destabilization;GO:0032803//regulation of low-density

0906//regulation of glucose metabolic process;GO:0042304//regulation of fatty acid biosynthetic

O:0008584//male gonad development;GO:0009410//response to xenobiotic stimulus;GO:0010628

;;GO:2001240//negative regulation of extrinsic apoptotic signaling pathway in absence of ligand

33211//adiponectin-activated signaling pathway;GO:0034143//regulation of toll-like receptor 4 sig

age;GO:0018105//peptidyl-serine phosphorylation;GO:0034340//response to type I interferon;GO:

ort;GO:0010866//regulation of triglyceride biosynthetic process;GO:0010884//positive regulation o

nodeling;GO:0036151//phosphatidylcholine acyl-chain remodeling;GO:0036152//phosphatidyletha
GO:0007205//protein kinase C-activating G-protein coupled receptor signaling pathway;GO:0008

sulin;GO:0032869//cellular response to insulin stimulus;GO:0043382//positive regulation of memo

regulation of developmental growth;GO:0060480//lung goblet cell differentiation;GO:0060548//ne

):0010001//glial cell differentiation;GO:0010467//gene expression;GO:0022402//cell cycle process;
:le remodeling;GO:0034375//high-density lipoprotein particle remodeling;GO:0034435//cholester[illegible]

eta receptor signaling pathway;GO:0032683//negative regulation of connective tissue growth fact[illegible]

itin-dependent protein catabolic process;GO:0031396//regulation of protein ubiquitination

ıal transduction;GO:0043536//positive regulation of blood vessel endothelial cell migration;GO:00[illegible]

;GO:0034374//low-density lipoprotein particle remodeling;GO:0034375//high-density lipoprotein

:1589//cerebellum structural organization;GO:0021766//hippocampus development;GO:0021795//[illegible]

noterpenoid metabolic process;GO:0033076//isoquinoline alkaloid metabolic process;GO:0042178/

1529//positive regulation of anion channel activity;GO:2001225//regulation of chloride transport

protein ubiquitination;GO:0031648//protein destabilization;GO:0032803//regulation of low-density

6355//regulation of transcription, DNA-templated;GO:0006468//protein phosphorylation;GO:000[illegible]

ler cell activation;GO:0032252//secretory granule localization;GO:0032725//positive regulation of [illegible]

smembrane transport;GO:0086003//cardiac muscle cell contraction;GO:1903760//regulation of vo

):0010001//glial cell differentiation;GO:0010467//gene expression;GO:0022402//cell cycle process;

):0043066//negative regulation of apoptotic process;GO:0045595//regulation of cell differentiatio

nal transduction;GO:0043536//positive regulation of blood vessel endothelial cell migration;GO:00[illegible]

r receptor signaling pathway;GO:0009887//organ morphogenesis;GO:0010595//positive regulation

itin-dependent protein catabolic process;GO:0031396//regulation of protein ubiquitination

ise;GO:0050921//positive regulation of chemotaxis;GO:0051592//response to calcium ion;GO:0060

f phosphorylation;GO:0050861//positive regulation of B cell receptor signaling pathway;GO:00601

n binding;GO:0032435//negative regulation of proteasomal ubiquitin-dependent protein cataboli

<-STAT cascade;GO:0019221//cytokine-mediated signaling pathway;GO:0042127//regulation of ce

diated signaling pathway;GO:0009611//response to wounding;GO:0009615//response to virus;GO:(

rotein ubiquitination;GO:0031648//protein destabilization;GO:0032803//regulation of low-density

ositive regulation of gene expression;GO:0045892//negative regulation of transcription, DNA-temp
noterpenoid metabolic process;GO:0033076//isoquinoline alkaloid metabolic process;GO:0042178/
O:0008584//male gonad development;GO:0009410//response to xenobiotic stimulus;GO:0010628

ler cell activation;GO:0032252//secretory granule localization;GO:0032725//positive regulation of g

)//positive regulation of glycoprotein biosynthetic process;GO:0010613//positive regulation of car

ynthetic process;GO:0006695//cholesterol biosynthetic process;GO:0006869//lipid transport;GO:00

33211//adiponectin-activated signaling pathway;GO:0034143//regulation of toll-like receptor 4 sig
n of synaptic plasticity;GO:0032793//positive regulation of CREB transcription factor activity;GO:00

GO:0007205//protein kinase C-activating G-protein coupled receptor signaling pathway;GO:0008

ıction;GO:0021879//forebrain neuron differentiation;GO:0032231//regulation of actin filament bur

;;GO:2001240//negative regulation of extrinsic apoptotic signaling pathway in absence of ligand

):0043066//negative regulation of apoptotic process;GO:0045595//regulation of cell differentiatio

34//male gonad development;GO:0010628//positive regulation of gene expression;GO:0010817//

0001819//positive regulation of cytokine production;GO:0001822//kidney development;GO:00018

ısmembrane transport;GO:0086003//cardiac muscle cell contraction;GO:1903760//regulation of vo

)//positive regulation of glycoprotein biosynthetic process;GO:0010613//positive regulation of car

receptor signaling pathway;GO:0009887//organ morphogenesis;GO:0010595//positive regulatior

vasodilation;GO:0046209//nitric oxide metabolic process;GO:0050667//homocysteine metabolic p

f phosphorylation;GO:0050861//positive regulation of B cell receptor signaling pathway;GO:00601

ɔsitive regulation of gene expression;GO:0045892//negative regulation of transcription, DNA-tem

ynthetic process;GO:0006695//cholesterol biosynthetic process;GO:0006869//lipid transport;GO:00

//positive regulation of phospholipase C activity;GO:0019932//second-messenger-mediated sign

G1 DNA damage checkpoint;GO:0042771//intrinsic apoptotic signaling pathway in response to DI

tion;GO:0010898//positive regulation of triglyceride catabolic process;GO:0016042//lipid catabolic

uction;GO:0021879//forebrain neuron differentiation;GO:0032231//regulation of actin filament bur

ol transport;GO:0032367//intracellular cholesterol transport;GO:0033344//cholesterol efflux;GO:00
aling pathway;GO:0042632//cholesterol homeostasis;GO:0045944//positive regulation of transcript

O:1904707//positive regulation of vascular smooth muscle cell proliferation;GO:1904754//positive
ne expression;GO:0010628//positive regulation of gene expression;GO:0032956//regulation of acti
GO:0021670//lateral ventricle development;GO:0021766//hippocampus development;GO:002196(

:0045087//innate immune response;GO:0045429//positive regulation of nitric oxide biosynthetic p

ismembrane transport;GO:0086003//cardiac muscle cell contraction;GO:1903760//regulation of vo
ein coupled receptor signaling pathway;GO:0030097//hemopoiesis;GO:0030593//neutrophil chem

ion of inflammatory response;GO:0050729//positive regulation of inflammatory response;GO:0050

2869//cellular response to insulin stimulus;GO:0045725//positive regulation of glycogen biosynthe

ler cell activation;GO:0032252//secretory granule localization;GO:0032725//positive regulation of g
noterpenoid metabolic process;GO:0033076//isoquinoline alkaloid metabolic process;GO:0042178/

;GO:0033564//anterior/posterior axon guidance;GO:0042472//inner ear morphogenesis;GO:00457

to endoplasmic reticulum stress;GO:0070059//intrinsic apoptotic signaling pathway in response to

ın of synaptic plasticity;GO:0032793//positive regulation of CREB transcription factor activity;GO:00

cle remodeling;GO:0034375//high-density lipoprotein particle remodeling;GO:0034435//cholester

thway;GO:0007202//activation of phospholipase C activity;GO:0008360//regulation of cell shape;G

ne expression;GO:0010628//positive regulation of gene expression;GO:0032956//regulation of acti

age;GO:0018105//peptidyl-serine phosphorylation;GO:0034340//response to type I interferon;GO:

ort;GO:0010866//regulation of triglyceride biosynthetic process;GO:0010884//positive regulation

//positive regulation of phospholipase C activity;GO:0019932//second-messenger-mediated signa

ɔsitive regulation of gene expression;GO:0045892//negative regulation of transcription, DNA-temp

ynthetic process;GO:0006695//cholesterol biosynthetic process;GO:0006869//lipid transport;GO:00

esis;GO:0035082//axoneme assembly;GO:0035556//intracellular signal transduction;GO:0042461//
ɔid catabolic process;GO:0010519//negative regulation of phospholipase activity;GO:0019915//lipi

ler cell activation;GO:0032252//secretory granule localization;GO:0032725//positive regulation of g
1529//positive regulation of anion channel activity;GO:2001225//regulation of chloride transport

:le remodeling;GO:0034375//high-density lipoprotein particle remodeling;GO:0034435//cholester[illegible]

[illegible]46777//protein autophosphorylation;GO:0048679//regulation of axon regeneration;GO:0097194//[illegible]

[illegible]n of synaptic plasticity;GO:0032793//positive regulation of CREB transcription factor activity;GO:00[illegible]

ion of inflammatory response;GO:0050729//positive regulation of inflammatory response;GO:005[illegible]

tion;GO:0010898//positive regulation of triglyceride catabolic process;GO:0016042//lipid catabolic[illegible]
[illegible]4//male gonad development;GO:0010628//positive regulation of gene expression;GO:0010817//[illegible]

[illegible]iction;GO:0021879//forebrain neuron differentiation;GO:0032231//regulation of actin filament bur[illegible]

[illegible]iction;GO:0021879//forebrain neuron differentiation;GO:0032231//regulation of actin filament bur[illegible]

[illegible]ynthetic process;GO:0006695//cholesterol biosynthetic process;GO:0006869//lipid transport;GO:00[illegible]

ol transport;GO:0032367//intracellular cholesterol transport;GO:0033344//cholesterol efflux;GO:00[illegible]

:1902730//positive regulation of proteoglycan biosynthetic process;GO:1902732//positive regulati[illegible]

[illegible]olic process;GO:0019433//triglyceride catabolic process;GO:0032722//positive regulation of chem[illegible]
[illegible]ositive regulation of protein import into nucleus;GO:0043065//positive regulation of apoptotic pr[illegible]

ion of inflammatory response;GO:0050729//positive regulation of inflammatory response;GO:005C

ɔsitive regulation of gene expression;GO:0045892//negative regulation of transcription, DNA-temp

)//positive regulation of glycoprotein biosynthetic process;GO:0010613//positive regulation of car

ɾrylation;GO:0046325//negative regulation of glucose import;GO:0046627//negative regulation of

ne biosynthetic process via cystathionine;GO:0019344//cysteine biosynthetic process;GO:0021587/
1529//positive regulation of anion channel activity;GO:2001225//regulation of chloride transport
bolic process;GO:0019433//triglyceride catabolic process;GO:0032722//positive regulation of chem

f phosphorylation;GO:0050861//positive regulation of B cell receptor signaling pathway;GO:00601

ler cell activation;GO:0032252//secretory granule localization;GO:0032725//positive regulation of g

uction;GO:0021879//forebrain neuron differentiation;GO:0032231//regulation of actin filament bur

O:0007626//locomotory behavior;GO:0010842//retina layer formation;GO:0038007//netrin-activat

0001819//positive regulation of cytokine production;GO:0001822//kidney development;GO:00018

n of apoptotic process;GO:0043123//positive regulation of I-kappaB kinase/NF-kappaB signaling;G

rhythm;GO:0008284//positive regulation of cell proliferation;GO:0010628//positive regulation of g

O:0010001//glial cell differentiation;GO:0010467//gene expression;GO:0022402//cell cycle process;

cle remodeling;GO:0034375//high-density lipoprotein particle remodeling;GO:0034435//cholester

0906//regulation of glucose metabolic process;GO:0042304//regulation of fatty acid biosynthetic
1529//positive regulation of anion channel activity;GO:2001225//regulation of chloride transport

ol transport;GO:0032367//intracellular cholesterol transport;GO:0033344//cholesterol efflux;GO:00

production;GO:0032733//positive regulation of interleukin-10 production;GO:0033137//negative r

regulation of developmental growth;GO:0060480//lung goblet cell differentiation;GO:0060548//ne

0//positive regulation of glycoprotein biosynthetic process;GO:0010613//positive regulation of car

21589//cerebellum structural organization;GO:0021766//hippocampus development;GO:0021795//
noterpenoid metabolic process;GO:0033076//isoquinoline alkaloid metabolic process;GO:0042178/

cle remodeling;GO:0034375//high-density lipoprotein particle remodeling;GO:0034435//cholester

:1902730//positive regulation of proteoglycan biosynthetic process;GO:1902732//positive regulati

O:0007626//locomotory behavior;GO:0010842//retina layer formation;GO:0038007//netrin-activat

cytosis;GO:0050804//modulation of synaptic transmission;GO:1901998//toxin transport

)804//modulation of synaptic transmission;GO:0050808//synapse organization;GO:0071625//vocal

ɔsitive regulation of gene expression;GO:0045892//negative regulation of transcription, DNA-temp

:le remodeling;GO:0034375//high-density lipoprotein particle remodeling;GO:0034435//cholester

ynthetic process;GO:0006695//cholesterol biosynthetic process;GO:0006869//lipid transport;GO:00
://cytoplasmic microtubule organization;GO:0045022//early endosome to late endosome transpor

0906//regulation of glucose metabolic process;GO:0042304//regulation of fatty acid biosynthetic

ation;GO:0050848//regulation of calcium-mediated signaling;GO:0090022//regulation of neutropl

):0010001//glial cell differentiation;GO:0010467//gene expression;GO:0022402//cell cycle process;

atinization;GO:0032379//positive regulation of intracellular lipid transport;GO:0032940//secretion l

liated signaling pathway;GO:0009611//response to wounding;GO:0009615//response to virus;GO:(

noterpenoid metabolic process;GO:0033076//isoquinoline alkaloid metabolic process;GO:0042178/

nesion;GO:0034446//substrate adhesion-dependent cell spreading;GO:0035987//endodermal cell

ler cell activation;GO:0032252//secretory granule localization;GO:0032725//positive regulation of g

1529//positive regulation of anion channel activity;GO:2001225//regulation of chloride transport
O:0007626//locomotory behavior;GO:0010842//retina layer formation;GO:0038007//netrin-activat

cellular response to hydrogen peroxide;GO:0071479//cellular response to ionizing radiation

production;GO:0032733//positive regulation of interleukin-10 production;GO:0033137//negative r

O:0010001//glial cell differentiation;GO:0010467//gene expression;GO:0022402//cell cycle process;

0001819//positive regulation of cytokine production;GO:0001822//kidney development;GO:00018

:0045087//innate immune response;GO:0045429//positive regulation of nitric oxide biosynthetic p

entiation;GO:0090181//regulation of cholesterol metabolic process;GO:0090207//regulation of trig

oid catabolic process;GO:0010519//negative regulation of phospholipase activity;GO:0019915//lipi

21589//cerebellum structural organization;GO:0021766//hippocampus development;GO:0021795//

0906//regulation of glucose metabolic process;GO:0042304//regulation of fatty acid biosynthetic

olic process;GO:0019433//triglyceride catabolic process;GO:0032722//positive regulation of chem
esis;GO:0035082//axoneme assembly;GO:0035556//intracellular signal transduction;GO:0042461//

}3211//adiponectin-activated signaling pathway;GO:0034143//regulation of toll-like receptor 4 sig

)//positive regulation of glycoprotein biosynthetic process;GO:0010613//positive regulation of car

)//positive regulation of glycoprotein biosynthetic process;GO:0010613//positive regulation of car

O:0008584//male gonad development;GO:0009410//response to xenobiotic stimulus;GO:0010628,

: process;GO:0046340//diacylglycerol catabolic process;GO:0071926//endocannabinoid signaling

):0010001//glial cell differentiation;GO:0010467//gene expression;GO:0022402//cell cycle process;

):0010001//glial cell differentiation;GO:0010467//gene expression;GO:0022402//cell cycle process;

ıse;GO:0050921//positive regulation of chemotaxis;GO:0051592//response to calcium ion;GO:006C

ı;GO:0034374//low-density lipoprotein particle remodeling;GO:0034375//high-density lipoprotein
'/regulation of cell migration;GO:0034638//phosphatidylcholine catabolic process;GO:0045765//re

sulin;GO:0032869//cellular response to insulin stimulus;GO:0043382//positive regulation of memo

ay;GO:0008284//positive regulation of cell proliferation;GO:0010628//positive regulation of gene e

33211//adiponectin-activated signaling pathway;GO:0034143//regulation of toll-like receptor 4 sig

)804//modulation of synaptic transmission;GO:0050808//synapse organization;GO:0071625//vocal

O:0008584//male gonad development;GO:0009410//response to xenobiotic stimulus;GO:0010628

ıal transduction;GO:0043536//positive regulation of blood vessel endothelial cell migration;GO:004

)//positive regulation of glycoprotein biosynthetic process;GO:0010613//positive regulation of car

eroid hormone receptor signaling pathway;GO:0043433//negative regulation of sequence-specific

G-protein coupled purinergic nucleotide receptor signaling pathway;GO:0043270//positive regula

olic process;GO:0019433//triglyceride catabolic process;GO:0032722//positive regulation of chem

se;GO:0050921//positive regulation of chemotaxis;GO:0051592//response to calcium ion;GO:006[illegible]

iosynthetic process;GO:0008654//phospholipid biosynthetic process;GO:0009749//response to gl[illegible]

-STAT cascade;GO:0019221//cytokine-mediated signaling pathway;GO:0042127//regulation of c[illegible]

al transduction;GO:0043536//positive regulation of blood vessel endothelial cell migration;GO:00[illegible]

rotein ubiquitination;GO:0031648//protein destabilization;GO:0032803//regulation of low-density

esis;GO:0035082//axoneme assembly;GO:0035556//intracellular signal transduction;GO:0042461//

vasodilation;GO:0046209//nitric oxide metabolic process;GO:0050667//homocysteine metabolic p

olic process;GO:0019433//triglyceride catabolic process;GO:0032722//positive regulation of chem

cytosis;GO:0050804//modulation of synaptic transmission;GO:1901998//toxin transport
atinization;GO:0032379//positive regulation of intracellular lipid transport;GO:0032940//secretion

ion of inflammatory response;GO:0050729//positive regulation of inflammatory response;GO:005C

sitive regulation of cell proliferation;GO:0030307//positive regulation of cell growth;GO:0031930//

rotein ubiquitination;GO:0031648//protein destabilization;GO:0032803//regulation of low-density

;GO:0034374//low-density lipoprotein particle remodeling;GO:0034375//high-density lipoprotein
ısmembrane transport;GO:0086003//cardiac muscle cell contraction;GO:1903760//regulation of vo

):0010001//glial cell differentiation;GO:0010467//gene expression;GO:0022402//cell cycle process;

/regulation of cell migration;GO:0034638//phosphatidylcholine catabolic process;GO:0045765//re

əolic process;GO:0019433//triglyceride catabolic process;GO:0032722//positive regulation of chem

ɛroid hormone receptor signaling pathway;GO:0043433//negative regulation of sequence-specific

ay;GO:0008284//positive regulation of cell proliferation;GO:0010628//positive regulation of gene e

)//positive regulation of glycoprotein biosynthetic process;GO:0010613//positive regulation of car

ction;GO:0060371//regulation of atrial cardiac muscle cell membrane depolarization;GO:0060373//

cytosis;GO:0050804//modulation of synaptic transmission;GO:1901998//toxin transport

):0010001//glial cell differentiation;GO:0010467//gene expression;GO:0022402//cell cycle process;
sitive regulation of cell proliferation;GO:0030307//positive regulation of cell growth;GO:0031930//

rotein ubiquitination;GO:0031648//protein destabilization;GO:0032803//regulation of low-density
?//cytoplasmic microtubule organization;GO:0045022//early endosome to late endosome transpor
//chemotaxis;GO:0007267//cell-cell signaling;GO:0007368//determination of left/right symmetry;G

0016042//lipid catabolic process;GO:0019432//triglyceride biosynthetic process;GO:0019433//trigl

al transduction;GO:0043536//positive regulation of blood vessel endothelial cell migration;GO:00

rotein ubiquitination;GO:0031648//protein destabilization;GO:0032803//regulation of low-density

):0007626//locomotory behavior;GO:0010842//retina layer formation;GO:0038007//netrin-activat

ne biosynthetic process via cystathionine;GO:0019344//cysteine biosynthetic process;GO:0021587/

G-protein coupled purinergic nucleotide receptor signaling pathway;GO:0043270//positive regula
ler cell activation;GO:0032252//secretory granule localization;GO:0032725//positive regulation of g

uction;GO:0021879//forebrain neuron differentiation;GO:0032231//regulation of actin filament bun

n of synaptic plasticity;GO:0032793//positive regulation of CREB transcription factor activity;GO:00

54//positive regulation of megakaryocyte differentiation;GO:0051092//positive regulation of NF-ka

ein coupled receptor signaling pathway;GO:0030097//hemopoiesis;GO:0030593//neutrophil chem

ler cell activation;GO:0032252//secretory granule localization;GO:0032725//positive regulation of g

ein coupled receptor signaling pathway;GO:0030097//hemopoiesis;GO:0030593//neutrophil chem

06355//regulation of transcription, DNA-templated;GO:0006468//protein phosphorylation;GO:0007

):0061077//chaperone-mediated protein folding;GO:1901874//negative regulation of post-transla

/regulation of cell migration;GO:0034638//phosphatidylcholine catabolic process;GO:0045765//re

34//male gonad development;GO:0010628//positive regulation of gene expression;GO:0010817//r

ynthetic process;GO:0006695//cholesterol biosynthetic process;GO:0006869//lipid transport;GO:00

GO:0021670//lateral ventricle development;GO:0021766//hippocampus development;GO:002196

ay;GO:0008284//positive regulation of cell proliferation;GO:0010628//positive regulation of gene e
olic process;GO:0019433//triglyceride catabolic process;GO:0032722//positive regulation of chem
ositive regulation of gene expression;GO:0045892//negative regulation of transcription, DNA-tem

vasodilation;GO:0046209//nitric oxide metabolic process;GO:0050667//homocysteine metabolic p

O:0008584//male gonad development;GO:0009410//response to xenobiotic stimulus;GO:0010628

33211//adiponectin-activated signaling pathway;GO:0034143//regulation of toll-like receptor 4 sig

tion;GO:0033234//negative regulation of protein sumoylation;GO:0043066//negative regulation of

21589//cerebellum structural organization;GO:0021766//hippocampus development;GO:0021795//

GO:0021670//lateral ventricle development;GO:0021766//hippocampus development;GO:002196[illegible]

G-protein coupled purinergic nucleotide receptor signaling pathway;GO:0043270//positive regula

[illegible]:0043066//negative regulation of apoptotic process;GO:0045595//regulation of cell differentiatio[illegible]

ıal transduction;GO:0043536//positive regulation of blood vessel endothelial cell migration;GO:004

ıction;GO:0021879//forebrain neuron differentiation;GO:0032231//regulation of actin filament bur

0001819//positive regulation of cytokine production;GO:0001822//kidney development;GO:00018

ıse;GO:0050921//positive regulation of chemotaxis;GO:0051592//response to calcium ion;GO:0060

GO:0007205//protein kinase C-activating G-protein coupled receptor signaling pathway;GO:0008

34//male gonad development;GO:0010628//positive regulation of gene expression;GO:0010817//r

ynthetic process;GO:0006695//cholesterol biosynthetic process;GO:0006869//lipid transport;GO:00

ol transport;GO:0032367//intracellular cholesterol transport;GO:0033344//cholesterol efflux;GO:00

O:0007626//locomotory behavior;GO:0010842//retina layer formation;GO:0038007//netrin-activat

receptor signaling pathway;GO:0009887//organ morphogenesis;GO:0010595//positive regulation
noterpenoid metabolic process;GO:0033076//isoquinoline alkaloid metabolic process;GO:0042178/

ositive regulation of gene expression;GO:0045892//negative regulation of transcription, DNA-temp

ne biosynthetic process via cystathionine;GO:0019344//cysteine biosynthetic process;GO:0021587/

O:0007626//locomotory behavior;GO:0010842//retina layer formation;GO:0038007//netrin-activat

atinization;GO:0032379//positive regulation of intracellular lipid transport;GO:0032940//secretion l

ler cell activation;GO:0032252//secretory granule localization;GO:0032725//positive regulation of g

ein coupled receptor signaling pathway;GO:0030097//hemopoiesis;GO:0030593//neutrophil chem

O:0008584//male gonad development;GO:0009410//response to xenobiotic stimulus;GO:0010628/

ne expression;GO:0010628//positive regulation of gene expression;GO:0032956//regulation of acti

)//positive regulation of glycoprotein biosynthetic process;GO:0010613//positive regulation of car

n of insulin secretion;GO:0045727//positive regulation of translation;GO:0046622//positive regulat

33211//adiponectin-activated signaling pathway;GO:0034143//regulation of toll-like receptor 4 sig

nesion;GO:0034446//substrate adhesion-dependent cell spreading;GO:0035987//endodermal cell
O:0007626//locomotory behavior;GO:0010842//retina layer formation;GO:0038007//netrin-activat

21589//cerebellum structural organization;GO:0021766//hippocampus development;GO:0021795//

2869//cellular response to insulin stimulus;GO:0045725//positive regulation of glycogen biosynthe

noterpenoid metabolic process;GO:0033076//isoquinoline alkaloid metabolic process;GO:0042178/

ıoterpenoid metabolic process;GO:0033076//isoquinoline alkaloid metabolic process;GO:0042178/

GO:0021670//lateral ventricle development;GO:0021766//hippocampus development;GO:002196(
):0061077//chaperone-mediated protein folding;GO:1901874//negative regulation of post-transla

ıction;GO:0021879//forebrain neuron differentiation;GO:0032231//regulation of actin filament bur

1529//positive regulation of anion channel activity;GO:2001225//regulation of chloride transport

eroid hormone receptor signaling pathway;GO:0043433//negative regulation of sequence-specific

):0043066//negative regulation of apoptotic process;GO:0045595//regulation of cell differentiatio

;GO:0034374//low-density lipoprotein particle remodeling;GO:0034375//high-density lipoprotein
:0031076//embryonic camera-type eye development;GO:0042472//inner ear morphogenesis;GO:C

//positive regulation of phospholipase C activity;GO:0019932//second-messenger-mediated signa

33211//adiponectin-activated signaling pathway;GO:0034143//regulation of toll-like receptor 4 sig

IAP kinase activity;GO:0050709//negative regulation of protein secretion;GO:1900222//negative re
ynthetic process;GO:0006695//cholesterol biosynthetic process;GO:0006869//lipid transport;GO:0C

O:0008584//male gonad development;GO:0009410//response to xenobiotic stimulus;GO:0010628

receptor signaling pathway;GO:0009887//organ morphogenesis;GO:0010595//positive regulatio

ositive regulation of gene expression;GO:0045892//negative regulation of transcription, DNA-tem

ong term synaptic depression;GO:2000124//regulation of endocannabinoid signaling pathway;GO:

GO:0021670//lateral ventricle development;GO:0021766//hippocampus development;GO:002196

1529//positive regulation of anion channel activity;GO:2001225//regulation of chloride transport

ne phosphorylation;GO:0051353//positive regulation of oxidoreductase activity;GO:0071300//cell

tion;GO:0033234//negative regulation of protein sumoylation;GO:0043066//negative regulation of

noterpenoid metabolic process;GO:0033076//isoquinoline alkaloid metabolic process;GO:0042178/

uction;GO:0021879//forebrain neuron differentiation;GO:0032231//regulation of actin filament bur

ection of chemical stimulus involved in sensory perception of taste;GO:0050915//sensory perceptic

;GO:0034374//low-density lipoprotein particle remodeling;GO:0034375//high-density lipoprotein
D:0007626//locomotory behavior;GO:0010842//retina layer formation;GO:0038007//netrin-activat

cle remodeling;GO:0034375//high-density lipoprotein particle remodeling;GO:0034435//cholester

D:0043066//negative regulation of apoptotic process;GO:0045595//regulation of cell differentiatio

cle remodeling;GO:0034375//high-density lipoprotein particle remodeling;GO:0034435//cholester

noterpenoid metabolic process;GO:0033076//isoquinoline alkaloid metabolic process;GO:0042178/

nal transduction;GO:0043536//positive regulation of blood vessel endothelial cell migration;GO:00

rylation;GO:0046325//negative regulation of glucose import;GO:0046627//negative regulation of

ong term synaptic depression;GO:2000124//regulation of endocannabinoid signaling pathway;GO:

33211//adiponectin-activated signaling pathway;GO:0034143//regulation of toll-like receptor 4 sig

r receptor signaling pathway;GO:0009887//organ morphogenesis;GO:0010595//positive regulation
)7188//adenylate cyclase-modulating G-protein coupled receptor signaling pathway;GO:0007193/

O:0008584//male gonad development;GO:0009410//response to xenobiotic stimulus;GO:0010628/

)060562//epithelial tube morphogenesis;GO:0061205//paramesonephric duct development;GO:00

n of apoptotic process;GO:0043123//positive regulation of I-kappaB kinase/NF-kappaB signaling;(

olic process;GO:0019433//triglyceride catabolic process;GO:0032722//positive regulation of chem

cle remodeling;GO:0034375//high-density lipoprotein particle remodeling;GO:0034435//cholester
hesion;GO:0034446//substrate adhesion-dependent cell spreading;GO:0035987//endodermal cell

//positive regulation of phospholipase C activity;GO:0019932//second-messenger-mediated signa

9//protein deubiquitination;GO:0019896//axon transport of mitochondrion;GO:0042755//eating b
uction;GO:0021879//forebrain neuron differentiation;GO:0032231//regulation of actin filament bur

uction;GO:0021879//forebrain neuron differentiation;GO:0032231//regulation of actin filament bur

nal transduction;GO:0043536//positive regulation of blood vessel endothelial cell migration;GO:00

:1902730//positive regulation of proteoglycan biosynthetic process;GO:1902732//positive regulati

development;GO:0031639//plasminogen activation;GO:0033489//cholesterol biosynthetic process

rotein ubiquitination;GO:0031648//protein destabilization;GO:0032803//regulation of low-density

;;GO:2001240//negative regulation of extrinsic apoptotic signaling pathway in absence of ligand

0060562//epithelial tube morphogenesis;GO:0061205//paramesonephric duct development;GO:00

ne biosynthetic process via cystathionine;GO:0019344//cysteine biosynthetic process;GO:0021587/

eta receptor signaling pathway;GO:0032683//negative regulation of connective tissue growth fact

olic process;GO:0019433//triglyceride catabolic process;GO:0032722//positive regulation of chem

O:0007626//locomotory behavior;GO:0010842//retina layer formation;GO:0038007//netrin-activat

smembrane transport;GO:0086003//cardiac muscle cell contraction;GO:1903760//regulation of vo

0001819//positive regulation of cytokine production;GO:0001822//kidney development;GO:00018
n of synaptic plasticity;GO:0032793//positive regulation of CREB transcription factor activity;GO:00

ctivity;GO:0035556//intracellular signal transduction;GO:0043153//entrainment of circadian clock b
vasodilation;GO:0046209//nitric oxide metabolic process;GO:0050667//homocysteine metabolic p
33211//adiponectin-activated signaling pathway;GO:0034143//regulation of toll-like receptor 4 sig

ositive regulation of gene expression;GO:0045892//negative regulation of transcription, DNA-temp

O:0008584//male gonad development;GO:0009410//response to xenobiotic stimulus;GO:0010628

//positive regulation of phospholipase C activity;GO:0019932//second-messenger-mediated signa

ynthetic process;GO:0006695//cholesterol biosynthetic process;GO:0006869//lipid transport;GO:00
ne phosphorylation;GO:0051353//positive regulation of oxidoreductase activity;GO:0071300//cellu

production;GO:0032733//positive regulation of interleukin-10 production;GO:0033137//negative r

ositive regulation of protein import into nucleus;GO:0043065//positive regulation of apoptotic pro

0906//regulation of glucose metabolic process;GO:0042304//regulation of fatty acid biosynthetic

hesion;GO:0034446//substrate adhesion-dependent cell spreading;GO:0035987//endodermal cell
1589//cerebellum structural organization;GO:0021766//hippocampus development;GO:0021795//
production;GO:0032733//positive regulation of interleukin-10 production;GO:0033137//negative r

rotein ubiquitination;GO:0031648//protein destabilization;GO:0032803//regulation of low-density

:0045087//innate immune response;GO:0045429//positive regulation of nitric oxide biosynthetic p

noterpenoid metabolic process;GO:0033076//isoquinoline alkaloid metabolic process;GO:0042178/

netic process;GO:0019367//fatty acid elongation, saturated fatty acid;GO:0030148//sphingolipid bi

ctivity;GO:0035556//intracellular signal transduction;GO:0043153//entrainment of circadian clock b
nal transduction;GO:0043536//positive regulation of blood vessel endothelial cell migration;GO:004

ısmembrane transport;GO:0086003//cardiac muscle cell contraction;GO:1903760//regulation of vo
aling pathway;GO:0042632//cholesterol homeostasis;GO:0045944//positive regulation of transcript

ein coupled receptor signaling pathway;GO:0030097//hemopoiesis;GO:0030593//neutrophil chem

/regulation of cell migration;GO:0034638//phosphatidylcholine catabolic process;GO:0045765//re

ɔsitive regulation of gene expression;GO:0045892//negative regulation of transcription, DNA-tem

ler cell activation;GO:0032252//secretory granule localization;GO:0032725//positive regulation of

ynthetic process;GO:0006695//cholesterol biosynthetic process;GO:0006869//lipid transport;GO:00
)//positive regulation of glycoprotein biosynthetic process;GO:0010613//positive regulation of car
ay;GO:0008284//positive regulation of cell proliferation;GO:0010628//positive regulation of gene e

tion;GO:0033234//negative regulation of protein sumoylation;GO:0043066//negative regulation of

:1902730//positive regulation of proteoglycan biosynthetic process;GO:1902732//positive regulati

ol transport;GO:0032367//intracellular cholesterol transport;GO:0033344//cholesterol efflux;GO:00

:1902730//positive regulation of proteoglycan biosynthetic process;GO:1902732//positive regulati

on;GO:0010975//regulation of neuron projection development;GO:0035556//intracellular signal tr

:1589//cerebellum structural organization;GO:0021766//hippocampus development;GO:0021795//

ıse;GO:0050921//positive regulation of chemotaxis;GO:0051592//response to calcium ion;GO:0060
D:0007626//locomotory behavior;GO:0010842//retina layer formation;GO:0038007//netrin-activat

regulation of developmental growth;GO:0060480//lung goblet cell differentiation;GO:0060548//ne

ne biosynthetic process via cystathionine;GO:0019344//cysteine biosynthetic process;GO:0021587/

ın of synaptic plasticity;GO:0032793//positive regulation of CREB transcription factor activity;GO:00

ler cell activation;GO:0032252//secretory granule localization;GO:0032725//positive regulation of g

G-protein coupled purinergic nucleotide receptor signaling pathway;GO:0043270//positive regula

ion of inflammatory response;GO:0050729//positive regulation of inflammatory response;GO:0050

esis;GO:0035082//axoneme assembly;GO:0035556//intracellular signal transduction;GO:0042461//

ler cell activation;GO:0032252//secretory granule localization;GO:0032725//positive regulation of [illegible]

ıse;GO:0050921//positive regulation of chemotaxis;GO:0051592//response to calcium ion;GO:0060

ırotein ubiquitination;GO:0031648//protein destabilization;GO:0032803//regulation of low-density

:1902730//positive regulation of proteoglycan biosynthetic process;GO:1902732//positive regulati

production;GO:0032733//positive regulation of interleukin-10 production;GO:0033137//negative r

vasodilation;GO:0046209//nitric oxide metabolic process;GO:0050667//homocysteine metabolic p

on;GO:0010975//regulation of neuron projection development;GO:0035556//intracellular signal tr
ion of inflammatory response;GO:0050729//positive regulation of inflammatory response;GO:0050

33211//adiponectin-activated signaling pathway;GO:0034143//regulation of toll-like receptor 4 sig

)//positive regulation of glycoprotein biosynthetic process;GO:0010613//positive regulation of car
te differentiation;GO:0035108//limb morphogenesis;GO:0035264//multicellular organism growth;

O:0008584//male gonad development;GO:0009410//response to xenobiotic stimulus;GO:0010628

ɔrylation;GO:0046325//negative regulation of glucose import;GO:0046627//negative regulation of

ɔolic process;GO:0019433//triglyceride catabolic process;GO:0032722//positive regulation of chem

0906//regulation of glucose metabolic process;GO:0042304//regulation of fatty acid biosynthetic

)7188//adenylate cyclase-modulating G-protein coupled receptor signaling pathway;GO:0007193/

ophil differentiation;GO:0030224//monocyte differentiation;GO:0071353//cellular response to inte

ol transport;GO:0032367//intracellular cholesterol transport;GO:0033344//cholesterol efflux;GO:00

!869//cellular response to insulin stimulus;GO:0045725//positive regulation of glycogen biosynthet
atinization;GO:0032379//positive regulation of intracellular lipid transport;GO:0032940//secretion l

:le remodeling;GO:0034375//high-density lipoprotein particle remodeling;GO:0034435//cholestero

ı of apoptotic process;GO:0043123//positive regulation of I-kappaB kinase/NF-kappaB signaling;(

:le remodeling;GO:0034375//high-density lipoprotein particle remodeling;GO:0034435//cholester(

on;GO:0010975//regulation of neuron projection development;GO:0035556//intracellular signal tr

-activating G-protein coupled receptor signaling pathway;GO:0007507//heart development;GO:00

ıal transduction;GO:0043536//positive regulation of blood vessel endothelial cell migration;GO:00

production;GO:0032733//positive regulation of interleukin-10 production;GO:0033137//negative r
1529//positive regulation of anion channel activity;GO:2001225//regulation of chloride transport
:roid hormone receptor signaling pathway;GO:0043433//negative regulation of sequence-specific
):0010001//glial cell differentiation;GO:0010467//gene expression;GO:0022402//cell cycle process;

//positive regulation of phospholipase C activity;GO:0019932//second-messenger-mediated signa

rhythm;GO:0008284//positive regulation of cell proliferation;GO:0010628//positive regulation of g

ion of inflammatory response;GO:0050729//positive regulation of inflammatory response;GO:005C

eroid hormone receptor signaling pathway;GO:0043433//negative regulation of sequence-specific

on;GO:0010975//regulation of neuron projection development;GO:0035556//intracellular signal tr

ler cell activation;GO:0032252//secretory granule localization;GO:0032725//positive regulation of g

atinization;GO:0032379//positive regulation of intracellular lipid transport;GO:0032940//secretion l

0906//regulation of glucose metabolic process;GO:0042304//regulation of fatty acid biosynthetic

0001819//positive regulation of cytokine production;GO:0001822//kidney development;GO:00018

):0010001//glial cell differentiation;GO:0010467//gene expression;GO:0022402//cell cycle process;
esis;GO:0035082//axoneme assembly;GO:0035556//intracellular signal transduction;GO:0042461//

ıse;GO:0050921//positive regulation of chemotaxis;GO:0051592//response to calcium ion;GO:006C

ion of inflammatory response;GO:0050729//positive regulation of inflammatory response;GO:005C

1529//positive regulation of anion channel activity;GO:2001225//regulation of chloride transport

protein ubiquitination;GO:0031648//protein destabilization;GO:0032803//regulation of low-density

GO:0021670//lateral ventricle development;GO:0021766//hippocampus development;GO:00219[illegible]

cle remodeling;GO:0034375//high-density lipoprotein particle remodeling;GO:0034435//cholester[illegible]
014883//transition between fast and slow fiber;GO:0014898//cardiac muscle hypertrophy in respon[illegible]

:0010001//glial cell differentiation;GO:0010467//gene expression;GO:0022402//cell cycle process;

ative regulation of protein dephosphorylation;GO:0061028//establishment of endothelial barrier;G[illegible]

:0043066//negative regulation of apoptotic process;GO:0045595//regulation of cell differentiation[illegible]

cle remodeling;GO:0034375//high-density lipoprotein particle remodeling;GO:0034435//cholester[illegible]

)35865//cellular response to potassium ion;GO:0042391//regulation of membrane potential;GO:0C

r receptor signaling pathway;GO:0009887//organ morphogenesis;GO:0010595//positive regulatior

r receptor signaling pathway;GO:0009887//organ morphogenesis;GO:0010595//positive regulatior

):0010001//glial cell differentiation;GO:0010467//gene expression;GO:0022402//cell cycle process;

0906//regulation of glucose metabolic process;GO:0042304//regulation of fatty acid biosynthetic

ne biosynthetic process via cystathionine;GO:0019344//cysteine biosynthetic process;GO:0021587/

):0043066//negative regulation of apoptotic process;GO:0045595//regulation of cell differentiatio

ecular complex assembly;GO:0042730//fibrinolysis;GO:0045907//positive regulation of vasoconstri

eroid hormone receptor signaling pathway;GO:0043433//negative regulation of sequence-specific

)14883//transition between fast and slow fiber;GO:0014898//cardiac muscle hypertrophy in respor

ative regulation of protein dephosphorylation;GO:0061028//establishment of endothelial barrier;G

nogenesis;GO:0003163//sinoatrial node development;GO:0003309//type B pancreatic cell different
IAP kinase activity;GO:0050709//negative regulation of protein secretion;GO:1900222//negative re

D:0061077//chaperone-mediated protein folding;GO:1901874//negative regulation of post-transla

n of insulin secretion;GO:0045727//positive regulation of translation;GO:0046622//positive regulat

oonse to virus;GO:0009791//post-embryonic development;GO:0010628//positive regulation of ger
tion;GO:0033234//negative regulation of protein sumoylation;GO:0043066//negative regulation of

O:0008584//male gonad development;GO:0009410//response to xenobiotic stimulus;GO:0010628

commissure morphogenesis;GO:0030517//negative regulation of axon extension;GO:0030900//for

33211//adiponectin-activated signaling pathway;GO:0034143//regulation of toll-like receptor 4 sig

ctivity;GO:0035556//intracellular signal transduction;GO:0043153//entrainment of circadian clock b

nization;GO:0048041//focal adhesion assembly;GO:0051695//actin filament uncapping;GO:00550[illegible]

ative regulation of protein dephosphorylation;GO:0061028//establishment of endothelial barrier;G[illegible]

on of BMP signaling pathway;GO:0045662//negative regulation of myoblast differentiation;GO:009[illegible]

:0042981//regulation of apoptotic process;GO:0045892//negative regulation of transcription, DNA

014883//transition between fast and slow fiber;GO:0014898//cardiac muscle hypertrophy in respor

process;GO:0043066//negative regulation of apoptotic process;GO:0050766//positive regulation

ection of chemical stimulus involved in sensory perception of taste;GO:0050915//sensory perceptic

stress;GO:0045893//positive regulation of transcription, DNA-templated;GO:0045944//positive reg

commissure morphogenesis;GO:0030517//negative regulation of axon extension;GO:0030900//for

'te differentiation;GO:0035108//limb morphogenesis;GO:0035264//multicellular organism growth;(

:0042981//regulation of apoptotic process;GO:0045892//negative regulation of transcription, DNA

<-STAT cascade;GO:0019221//cytokine-mediated signaling pathway;GO:0042127//regulation of ce

: process;GO:0046340//diacylglycerol catabolic process;GO:0071926//endocannabinoid signaling |

:0031076//embryonic camera-type eye development;GO:0042472//inner ear morphogenesis;GO:0
:tivity;GO:0035556//intracellular signal transduction;GO:0043153//entrainment of circadian clock b

O:1904707//positive regulation of vascular smooth muscle cell proliferation;GO:1904754//positive

'te differentiation;GO:0035108//limb morphogenesis;GO:0035264//multicellular organism growth;(

035694//mitochondrial protein catabolic process;GO:0043066//negative regulation of apoptotic p

jlomerular filtration;GO:0003207//cardiac chamber formation;GO:0003220//left ventricular cardiac
i6355//regulation of transcription, DNA-templated;GO:0006468//protein phosphorylation;GO:000

ı of insulin secretion;GO:0045727//positive regulation of translation;GO:0046622//positive regulat

sis;GO:0043010//camera-type eye development;GO:0043587//tongue morphogenesis;GO:004478

ɔonse to virus;GO:0009791//post-embryonic development;GO:0010628//positive regulation of ger

5//negative regulation of microtubule polymerization;GO:0031175//neuron projection developmer
evelopment;GO:0001947//heart looping;GO:0002053//positive regulation of mesenchymal cell prc

G1 DNA damage checkpoint;GO:0042771//intrinsic apoptotic signaling pathway in response to DI

:0042981//regulation of apoptotic process;GO:0045892//negative regulation of transcription, DNA

/viral genome integration into host DNA;GO:0046718//viral entry into host cell;GO:0075713//estal

:0031076//embryonic camera-type eye development;GO:0042472//inner ear morphogenesis;GO:0

nization;GO:0048041//focal adhesion assembly;GO:0051695//actin filament uncapping;GO:00550

)7188//adenylate cyclase-modulating G-protein coupled receptor signaling pathway;GO:0007193/

development;GO:0031639//plasminogen activation;GO:0033489//cholesterol biosynthetic process

ıetic process;GO:0019367//fatty acid elongation, saturated fatty acid;GO:0030148//sphingolipid bi

cytosis;GO:0050804//modulation of synaptic transmission;GO:1901998//toxin transport
)060562//epithelial tube morphogenesis;GO:0061205//paramesonephric duct development;GO:00

ıoterpenoid metabolic process;GO:0033076//isoquinoline alkaloid metabolic process;GO:0042178/

to endoplasmic reticulum stress;GO:0070059//intrinsic apoptotic signaling pathway in response to

: process;GO:0043066//negative regulation of apoptotic process;GO:0050766//positive regulation
on of BMP signaling pathway;GO:0045662//negative regulation of myoblast differentiation;GO:009

'te differentiation;GO:0035108//limb morphogenesis;GO:0035264//multicellular organism growth;(

evelopment;GO:0001947//heart looping;GO:0002053//positive regulation of mesenchymal cell prc

)7188//adenylate cyclase-modulating G-protein coupled receptor signaling pathway;GO:0007193/

ıogenesis;GO:0003163//sinoatrial node development;GO:0003309//type B pancreatic cell different

34//male gonad development;GO:0010628//positive regulation of gene expression;GO:0010817//r

ıe expression;GO:0010628//positive regulation of gene expression;GO:0032956//regulation of acti

:hway;GO:0007202//activation of phospholipase C activity;GO:0008360//regulation of cell shape;G
ıse;GO:0050921//positive regulation of chemotaxis;GO:0051592//response to calcium ion;GO:006C

)46777//protein autophosphorylation;GO:0048679//regulation of axon regeneration;GO:0097194//
'/viral genome integration into host DNA;GO:0046718//viral entry into host cell;GO:0075713//estal

'te differentiation;GO:0035108//limb morphogenesis;GO:0035264//multicellular organism growth;(
'/viral genome integration into host DNA;GO:0046718//viral entry into host cell;GO:0075713//estal

on;GO:0010975//regulation of neuron projection development;GO:0035556//intracellular signal tr

:le remodeling;GO:0034375//high-density lipoprotein particle remodeling;GO:0034435//cholester[illegible]

egulation of necroptotic process;GO:0070269//pyroptosis;GO:0072332//intrinsic apoptotic signalir

5//negative regulation of microtubule polymerization;GO:0031175//neuron projection developme[illegible]

e migration;GO:0002906//negative regulation of mature B cell apoptotic process;GO:0006154//ad[illegible]
: process;GO:0043066//negative regulation of apoptotic process;GO:0050766//positive regulation

ılomerular filtration;GO:0003207//cardiac chamber formation;GO:0003220//left ventricular cardiac

035694//mitochondrial protein catabolic process;GO:0043066//negative regulation of apoptotic p

46777//protein autophosphorylation;GO:0048679//regulation of axon regeneration;GO:0097194/[illegible]

:hway;GO:0007202//activation of phospholipase C activity;GO:0008360//regulation of cell shape;G

)7188//adenylate cyclase-modulating G-protein coupled receptor signaling pathway;GO:0007193/
G1 DNA damage checkpoint;GO:0042771//intrinsic apoptotic signaling pathway in response to DI

ɔng term synaptic depression;GO:2000124//regulation of endocannabinoid signaling pathway;GO::

:0042981//regulation of apoptotic process;GO:0045892//negative regulation of transcription, DNA

O:1904707//positive regulation of vascular smooth muscle cell proliferation;GO:1904754//positive

stress;GO:0045893//positive regulation of transcription, DNA-templated;GO:0045944//positive reg
uction;GO:0021879//forebrain neuron differentiation;GO:0032231//regulation of actin filament bur

ne phosphorylation;GO:0051353//positive regulation of oxidoreductase activity;GO:0071300//cellu

'0936//protein K48-linked ubiquitination;GO:1904262//negative regulation of TORC1 signaling
tricular valve development;GO:0003180//aortic valve morphogenesis;GO:0003184//pulmonary val

)14883//transition between fast and slow fiber;GO:0014898//cardiac muscle hypertrophy in respor

to endoplasmic reticulum stress;GO:0070059//intrinsic apoptotic signaling pathway in response to

on of BMP signaling pathway;GO:0045662//negative regulation of myoblast differentiation;GO:009

ative regulation of protein dephosphorylation;GO:0061028//establishment of endothelial barrier;G

<-STAT cascade;GO:0019221//cytokine-mediated signaling pathway;GO:0042127//regulation of ce

oid catabolic process;GO:0010519//negative regulation of phospholipase activity;GO:0019915//lipi

5//negative regulation of microtubule polymerization;GO:0031175//neuron projection developmer

nogenesis;GO:0003163//sinoatrial node development;GO:0003309//type B pancreatic cell different

to endoplasmic reticulum stress;GO:0070059//intrinsic apoptotic signaling pathway in response to

ponse to virus;GO:0009791//post-embryonic development;GO:0010628//positive regulation of ger

07188//adenylate cyclase-modulating G-protein coupled receptor signaling pathway;GO:0007193/

evelopment;GO:0001947//heart looping;GO:0002053//positive regulation of mesenchymal cell pro

O:1904707//positive regulation of vascular smooth muscle cell proliferation;GO:1904754//positive
G1 DNA damage checkpoint;GO:0042771//intrinsic apoptotic signaling pathway in response to DI

G1 DNA damage checkpoint;GO:0042771//intrinsic apoptotic signaling pathway in response to DI

:hway;GO:0007202//activation of phospholipase C activity;GO:0008360//regulation of cell shape;G

5//negative regulation of microtubule polymerization;GO:0031175//neuron projection developme

oid catabolic process;GO:0010519//negative regulation of phospholipase activity;GO:0019915//lipi

e migration;GO:0002906//negative regulation of mature B cell apoptotic process;GO:0006154//ad

egulation of necroptotic process;GO:0070269//pyroptosis;GO:0072332//intrinsic apoptotic signalir

<-STAT cascade;GO:0019221//cytokine-mediated signaling pathway;GO:0042127//regulation of ce

sis;GO:0043010//camera-type eye development;GO:0043587//tongue morphogenesis;GO:004478

oid catabolic process;GO:0010519//negative regulation of phospholipase activity;GO:0019915//lipi

;tress;GO:0045893//positive regulation of transcription, DNA-templated;GO:0045944//positive reg

development;GO:0031639//plasminogen activation;GO:0033489//cholesterol biosynthetic process

)14883//transition between fast and slow fiber;GO:0014898//cardiac muscle hypertrophy in respor

nogenesis;GO:0003163//sinoatrial node development;GO:0003309//type B pancreatic cell different

;92//response to calcium ion;GO:0051928//positive regulation of calcium ion transport;GO:009027

ative regulation of protein dephosphorylation;GO:0061028//establishment of endothelial barrier;G

)46777//protein autophosphorylation;GO:0048679//regulation of axon regeneration;GO:0097194//

to endoplasmic reticulum stress;GO:0070059//intrinsic apoptotic signaling pathway in response to

ctivity;GO:0035556//intracellular signal transduction;GO:0043153//entrainment of circadian clock b
tricular valve development;GO:0003180//aortic valve morphogenesis;GO:0003184//pulmonary val

cade;GO:0072378//blood coagulation, fibrin clot formation;GO:1900025//negative regulation of su

0936//protein K48-linked ubiquitination;GO:1904262//negative regulation of TORC1 signaling

esponse to bacterium;GO:0010629//negative regulation of gene expression;GO:0046326//positive

process;GO:0043066//negative regulation of apoptotic process;GO:0050766//positive regulation

liogenesis;GO:0042127//regulation of cell proliferation;GO:0043697//cell dedifferentiation;GO:004[illegible]

:hway;GO:0007202//activation of phospholipase C activity;GO:0008360//regulation of cell shape;G

egulation of necroptotic process;GO:0070269//pyroptosis;GO:0072332//intrinsic apoptotic signalir

O:1904707//positive regulation of vascular smooth muscle cell proliferation;GO:1904754//positive

: process;GO:0046340//diacylglycerol catabolic process;GO:0071926//endocannabinoid signaling

46777//protein autophosphorylation;GO:0048679//regulation of axon regeneration;GO:0097194//

of apoptotic process;GO:0043123//positive regulation of I-kappaB kinase/NF-kappaB signaling;

evelopment;GO:0001947//heart looping;GO:0002053//positive regulation of mesenchymal cell pro
te differentiation;GO:0035108//limb morphogenesis;GO:0035264//multicellular organism growth;

eta receptor signaling pathway;GO:0032683//negative regulation of connective tissue growth fact

)060562//epithelial tube morphogenesis;GO:0061205//paramesonephric duct development;GO:00
ection of chemical stimulus involved in sensory perception of taste;GO:0050915//sensory perceptic

:0031076//embryonic camera-type eye development;GO:0042472//inner ear morphogenesis;GO:0

cade;GO:0072378//blood coagulation, fibrin clot formation;GO:1900025//negative regulation of su
sis;GO:0043010//camera-type eye development;GO:0043587//tongue morphogenesis;GO:004478

stress;GO:0045893//positive regulation of transcription, DNA-templated;GO:0045944//positive reg

592//response to calcium ion;GO:0051928//positive regulation of calcium ion transport;GO:009027
liogenesis;GO:0042127//regulation of cell proliferation;GO:0043697//cell dedifferentiation;GO:004

592//response to calcium ion;GO:0051928//positive regulation of calcium ion transport;GO:009027

)//positive regulation of glycoprotein biosynthetic process;GO:0010613//positive regulation of car

cade;GO:0072378//blood coagulation, fibrin clot formation;GO:1900025//negative regulation of su

rylation;GO:0046325//negative regulation of glucose import;GO:0046627//negative regulation of

sis;GO:0043010//camera-type eye development;GO:0043587//tongue morphogenesis;GO:004478

commissure morphogenesis;GO:0030517//negative regulation of axon extension;GO:0030900//for

: process;GO:0030258//lipid modification;GO:0032286//central nervous system myelin maintenanc

IAP kinase activity;GO:0050709//negative regulation of protein secretion;GO:1900222//negative re

on;GO:0010975//regulation of neuron projection development;GO:0035556//intracellular signal tr

:0042981//regulation of apoptotic process;GO:0045892//negative regulation of transcription, DNA

ı of apoptotic process;GO:0043123//positive regulation of I-kappaB kinase/NF-kappaB signaling;(

'te differentiation;GO:0035108//limb morphogenesis;GO:0035264//multicellular organism growth;(

sis;GO:0043010//camera-type eye development;GO:0043587//tongue morphogenesis;GO:004478

)35865//cellular response to potassium ion;GO:0042391//regulation of membrane potential;GO:0C

9//protein deubiquitination;GO:0019896//axon transport of mitochondrion;GO:0042755//eating b

ɔng term synaptic depression;GO:2000124//regulation of endocannabinoid signaling pathway;GO:

nization;GO:0048041//focal adhesion assembly;GO:0051695//actin filament uncapping;GO:00550

6355//regulation of transcription, DNA-templated;GO:0006468//protein phosphorylation;GO:000

egulation of necroptotic process;GO:0070269//pyroptosis;GO:0072332//intrinsic apoptotic signalir

te differentiation;GO:0035108//limb morphogenesis;GO:0035264//multicellular organism growth;

rylation;GO:0046325//negative regulation of glucose import;GO:0046627//negative regulation of

c process;GO:0030258//lipid modification;GO:0032286//central nervous system myelin maintenanc

ponse to virus;GO:0009791//post-embryonic development;GO:0010628//positive regulation of ger

commissure morphogenesis;GO:0030517//negative regulation of axon extension;GO:0030900//for
on of BMP signaling pathway;GO:0045662//negative regulation of myoblast differentiation;GO:009

sitive regulation of cell proliferation;GO:0030307//positive regulation of cell growth;GO:0031930//

0031076//embryonic camera-type eye development;GO:0042472//inner ear morphogenesis;GO:0
e regulation of cell-substrate adhesion;GO:0030335//positive regulation of cell migration;GO:0034

0906//regulation of glucose metabolic process;GO:0042304//regulation of fatty acid biosynthetic

cade;GO:0072378//blood coagulation, fibrin clot formation;GO:1900025//negative regulation of su

n of insulin secretion;GO:0045727//positive regulation of translation;GO:0046622//positive regulat

592//response to calcium ion;GO:0051928//positive regulation of calcium ion transport;GO:009027

sis;GO:0043010//camera-type eye development;GO:0043587//tongue morphogenesis;GO:004478

06355//regulation of transcription, DNA-templated;GO:0006468//protein phosphorylation;GO:000

ecular complex assembly;GO:0042730//fibrinolysis;GO:0045907//positive regulation of vasoconstri

ne expression;GO:0010628//positive regulation of gene expression;GO:0032956//regulation of acti
e migration;GO:0002906//negative regulation of mature B cell apoptotic process;GO:0006154//ad

):0010001//glial cell differentiation;GO:0010467//gene expression;GO:0022402//cell cycle process;

: regulation of cell-substrate adhesion;GO:0030335//positive regulation of cell migration;GO:0034:

cade;GO:0072378//blood coagulation, fibrin clot formation;GO:1900025//negative regulation of su

)6355//regulation of transcription, DNA-templated;GO:0006468//protein phosphorylation;GO:0007

ein coupled receptor signaling pathway;GO:0030097//hemopoiesis;GO:0030593//neutrophil chem
592//response to calcium ion;GO:0051928//positive regulation of calcium ion transport;GO:009027

ı46777//protein autophosphorylation;GO:0048679//regulation of axon regeneration;GO:0097194//

/viral genome integration into host DNA;GO:0046718//viral entry into host cell;GO:0075713//estal
!869//cellular response to insulin stimulus;GO:0045725//positive regulation of glycogen biosynthet

on of BMP signaling pathway;GO:0045662//negative regulation of myoblast differentiation;GO:009
'0936//protein K48-linked ubiquitination;GO:1904262//negative regulation of TORC1 signaling

ılomerular filtration;GO:0003207//cardiac chamber formation;GO:0003220//left ventricular cardiac

f phosphorylation;GO:0050861//positive regulation of B cell receptor signaling pathway;GO:00601
035694//mitochondrial protein catabolic process;GO:0043066//negative regulation of apoptotic p

on of BMP signaling pathway;GO:0045662//negative regulation of myoblast differentiation;GO:009

-activating G-protein coupled receptor signaling pathway;GO:0007507//heart development;GO:00

commissure morphogenesis;GO:0030517//negative regulation of axon extension;GO:0030900//for
c process;GO:0046340//diacylglycerol catabolic process;GO:0071926//endocannabinoid signaling

:hway;GO:0007202//activation of phospholipase C activity;GO:0008360//regulation of cell shape;G

cation fork processing;GO:0031571//mitotic G1 DNA damage checkpoint;GO:0036297//interstranc

: process;GO:0043066//negative regulation of apoptotic process;GO:0050766//positive regulation

oid catabolic process;GO:0010519//negative regulation of phospholipase activity;GO:0019915//lipi

5//negative regulation of microtubule polymerization;GO:0031175//neuron projection developme

046777//protein autophosphorylation;GO:0048679//regulation of axon regeneration;GO:0097194//

0031076//embryonic camera-type eye development;GO:0042472//inner ear morphogenesis;GO:0
to endoplasmic reticulum stress;GO:0070059//intrinsic apoptotic signaling pathway in response to

e migration;GO:0002906//negative regulation of mature B cell apoptotic process;GO:0006154//ad

stress;GO:0045893//positive regulation of transcription, DNA-templated;GO:0045944//positive reg

ative regulation of protein dephosphorylation;GO:0061028//establishment of endothelial barrier;G

<-STAT cascade;GO:0019221//cytokine-mediated signaling pathway;GO:0042127//regulation of ce

nogenesis;GO:0003163//sinoatrial node development;GO:0003309//type B pancreatic cell different

)14883//transition between fast and slow fiber;GO:0014898//cardiac muscle hypertrophy in respor

592//response to calcium ion;GO:0051928//positive regulation of calcium ion transport;GO:009027

6355//regulation of transcription, DNA-templated;GO:0006468//protein phosphorylation;GO:000

5//negative regulation of microtubule polymerization;GO:0031175//neuron projection developme

tricular valve development;GO:0003180//aortic valve morphogenesis;GO:0003184//pulmonary val

cade;GO:0072378//blood coagulation, fibrin clot formation;GO:1900025//negative regulation of su

G1 DNA damage checkpoint;GO:0042771//intrinsic apoptotic signaling pathway in response to DI
'0936//protein K48-linked ubiquitination;GO:1904262//negative regulation of TORC1 signaling

:hway;GO:0007202//activation of phospholipase C activity;GO:0008360//regulation of cell shape;G

atinization;GO:0032379//positive regulation of intracellular lipid transport;GO:0032940//secretion I

on of BMP signaling pathway;GO:0045662//negative regulation of myoblast differentiation;GO:009
: process;GO:0043066//negative regulation of apoptotic process;GO:0050766//positive regulation

ecular complex assembly;GO:0042730//fibrinolysis;GO:0045907//positive regulation of vasoconstri

035694//mitochondrial protein catabolic process;GO:0043066//negative regulation of apoptotic p

glomerular filtration;GO:0003207//cardiac chamber formation;GO:0003220//left ventricular cardiac
:0031076//embryonic camera-type eye development;GO:0042472//inner ear morphogenesis;GO:0
G1 DNA damage checkpoint;GO:0042771//intrinsic apoptotic signaling pathway in response to DI

stress;GO:0045893//positive regulation of transcription, DNA-templated;GO:0045944//positive reg

oid catabolic process;GO:0010519//negative regulation of phospholipase activity;GO:0019915//lipi

to endoplasmic reticulum stress;GO:0070059//intrinsic apoptotic signaling pathway in response to

nogenesis;GO:0003163//sinoatrial node development;GO:0003309//type B pancreatic cell different

;GO:0033564//anterior/posterior axon guidance;GO:0042472//inner ear morphogenesis;GO:0045

to endoplasmic reticulum stress;GO:0070059//intrinsic apoptotic signaling pathway in response to

se;GO:0050921//positive regulation of chemotaxis;GO:0051592//response to calcium ion;GO:006

process;GO:0030258//lipid modification;GO:0032286//central nervous system myelin maintenan

//negative regulation of microtubule polymerization;GO:0031175//neuron projection developme

-STAT cascade;GO:0019221//cytokine-mediated signaling pathway;GO:0042127//regulation of c

n of BMP signaling pathway;GO:0045662//negative regulation of myoblast differentiation;GO:00

;GO:0034374//low-density lipoprotein particle remodeling;GO:0034375//high-density lipoprotein

sitive regulation of cell proliferation;GO:0030307//positive regulation of cell growth;GO:0031930//
hway;GO:0007202//activation of phospholipase C activity;GO:0008360//regulation of cell shape;G

G1 DNA damage checkpoint;GO:0042771//intrinsic apoptotic signaling pathway in response to DI

cytosis;GO:0050804//modulation of synaptic transmission;GO:1901998//toxin transport

592//response to calcium ion;GO:0051928//positive regulation of calcium ion transport;GO:009027

stress;GO:0045893//positive regulation of transcription, DNA-templated;GO:0045944//positive reg
035694//mitochondrial protein catabolic process;GO:0043066//negative regulation of apoptotic p

glomerular filtration;GO:0003207//cardiac chamber formation;GO:0003220//left ventricular cardiac

cade;GO:0072378//blood coagulation, fibrin clot formation;GO:1900025//negative regulation of su

netic process;GO:0019367//fatty acid elongation, saturated fatty acid;GO:0030148//sphingolipid bi

sis;GO:0043010//camera-type eye development;GO:0043587//tongue morphogenesis;GO:004478

process;GO:0043066//negative regulation of apoptotic process;GO:0050766//positive regulation

nogenesis;GO:0003163//sinoatrial node development;GO:0003309//type B pancreatic cell differen

eta receptor signaling pathway;GO:0032683//negative regulation of connective tissue growth fact

46777//protein autophosphorylation;GO:0048679//regulation of axon regeneration;GO:0097194//

oid catabolic process;GO:0010519//negative regulation of phospholipase activity;GO:0019915//lipi
e regulation of cell-substrate adhesion;GO:0030335//positive regulation of cell migration;GO:0034
oid catabolic process;GO:0010519//negative regulation of phospholipase activity;GO:0019915//lipi

0016042//lipid catabolic process;GO:0019432//triglyceride biosynthetic process;GO:0019433//trigl

0031076//embryonic camera-type eye development;GO:0042472//inner ear morphogenesis;GO:0

ynthetic process;GO:0006695//cholesterol biosynthetic process;GO:0006869//lipid transport;GO:00

olic process;GO:0019433//triglyceride catabolic process;GO:0032722//positive regulation of chem

ositive regulation of gene expression;GO:0045892//negative regulation of transcription, DNA-temp

liogenesis;GO:0042127//regulation of cell proliferation;GO:0043697//cell dedifferentiation;GO:004[illegible]

c process;GO:0030258//lipid modification;GO:0032286//central nervous system myelin maintenanc

:0045087//innate immune response;GO:0045429//positive regulation of nitric oxide biosynthetic p

on of BMP signaling pathway;GO:0045662//negative regulation of myoblast differentiation;GO:00[illegible]

netic process;GO:0019367//fatty acid elongation, saturated fatty acid;GO:0030148//sphingolipid bi

K-STAT cascade;GO:0019221//cytokine-mediated signaling pathway;GO:0042127//regulation of ce

:0042981//regulation of apoptotic process;GO:0045892//negative regulation of transcription, DNA

098609//cell-cell adhesion;GO:0098911//regulation of ventricular cardiac muscle cell action poten

nogenesis;GO:0003163//sinoatrial node development;GO:0003309//type B pancreatic cell different

6355//regulation of transcription, DNA-templated;GO:0006468//protein phosphorylation;GO:000

tress;GO:0045893//positive regulation of transcription, DNA-templated;GO:0045944//positive reg

sis;GO:0043010//camera-type eye development;GO:0043587//tongue morphogenesis;GO:004478

nization;GO:0048041//focal adhesion assembly;GO:0051695//actin filament uncapping;GO:00550

n of insulin secretion;GO:0045727//positive regulation of translation;GO:0046622//positive regulat

sis;GO:0043010//camera-type eye development;GO:0043587//tongue morphogenesis;GO:004478

on of BMP signaling pathway;GO:0045662//negative regulation of myoblast differentiation;GO:009

evelopment;GO:0001947//heart looping;GO:0002053//positive regulation of mesenchymal cell pro

e regulation of cell-substrate adhesion;GO:0030335//positive regulation of cell migration;GO:0034

oid catabolic process;GO:0010519//negative regulation of phospholipase activity;GO:0019915//lipi

:0045087//innate immune response;GO:0045429//positive regulation of nitric oxide biosynthetic p

9//protein deubiquitination;GO:0019896//axon transport of mitochondrion;GO:0042755//eating b

er taste;GO:0050916//sensory perception of sweet taste;GO:0050917//sensory perception of uman

c process;GO:0046340//diacylglycerol catabolic process;GO:0071926//endocannabinoid signaling p

0001819//positive regulation of cytokine production;GO:0001822//kidney development;GO:00018

ponse to virus;GO:0009791//post-embryonic development;GO:0010628//positive regulation of ger

:0042981//regulation of apoptotic process;GO:0045892//negative regulation of transcription, DNA

O:1904707//positive regulation of vascular smooth muscle cell proliferation;GO:1904754//positive

stress;GO:0045893//positive regulation of transcription, DNA-templated;GO:0045944//positive reg

ation;GO:0050848//regulation of calcium-mediated signaling;GO:0090022//regulation of neutroph

6355//regulation of transcription, DNA-templated;GO:0006468//protein phosphorylation;GO:0007

nization;GO:0048041//focal adhesion assembly;GO:0051695//actin filament uncapping;GO:00550

oid catabolic process;GO:0010519//negative regulation of phospholipase activity;GO:0019915//lipi
ctivity;GO:0035556//intracellular signal transduction;GO:0043153//entrainment of circadian clock b

2//cytoplasmic microtubule organization;GO:0045022//early endosome to late endosome transpor

IAP kinase activity;GO:0050709//negative regulation of protein secretion;GO:1900222//negative re

O:1904707//positive regulation of vascular smooth muscle cell proliferation;GO:1904754//positive
:0045087//innate immune response;GO:0045429//positive regulation of nitric oxide biosynthetic p

9//protein deubiquitination;GO:0019896//axon transport of mitochondrion;GO:0042755//eating b

entiation;GO:0090181//regulation of cholesterol metabolic process;GO:0090207//regulation of trig

oonse to virus;GO:0009791//post-embryonic development;GO:0010628//positive regulation of ger

evelopment;GO:0001947//heart looping;GO:0002053//positive regulation of mesenchymal cell pro
tem cell differentiation;GO:0061086//negative regulation of histone H3-K27 methylation;GO:0061[illegible]

te differentiation;GO:0035108//limb morphogenesis;GO:0035264//multicellular organism growth;[illegible]

6355//regulation of transcription, DNA-templated;GO:0006468//protein phosphorylation;GO:000[illegible]

cade;GO:0072378//blood coagulation, fibrin clot formation;GO:1900025//negative regulation of su

5//negative regulation of microtubule polymerization;GO:0031175//neuron projection developmen

592//response to calcium ion;GO:0051928//positive regulation of calcium ion transport;GO:009027
-activating G-protein coupled receptor signaling pathway;GO:0007507//heart development;GO:00

e migration;GO:0002906//negative regulation of mature B cell apoptotic process;GO:0006154//ad

ection of chemical stimulus involved in sensory perception of taste;GO:0050915//sensory perceptic

592//response to calcium ion;GO:0051928//positive regulation of calcium ion transport;GO:009027

/viral genome integration into host DNA;GO:0046718//viral entry into host cell;GO:0075713//estal

2//cytoplasmic microtubule organization;GO:0045022//early endosome to late endosome transpor

cade;GO:0072378//blood coagulation, fibrin clot formation;GO:1900025//negative regulation of su

cytosis;GO:0050804//modulation of synaptic transmission;GO:1901998//toxin transport
)060562//epithelial tube morphogenesis;GO:0061205//paramesonephric duct development;GO:00

: process;GO:0046340//diacylglycerol catabolic process;GO:0071926//endocannabinoid signaling p

/te differentiation;GO:0035108//limb morphogenesis;GO:0035264//multicellular organism growth;(

/te differentiation;GO:0035108//limb morphogenesis;GO:0035264//multicellular organism growth;(

G1 DNA damage checkpoint;GO:0042771//intrinsic apoptotic signaling pathway in response to DI
)6355//regulation of transcription, DNA-templated;GO:0006468//protein phosphorylation;GO:000
'0936//protein K48-linked ubiquitination;GO:1904262//negative regulation of TORC1 signaling

: process;GO:0043066//negative regulation of apoptotic process;GO:0050766//positive regulation

:0031076//embryonic camera-type eye development;GO:0042472//inner ear morphogenesis;GO:0
: process;GO:0043066//negative regulation of apoptotic process;GO:0050766//positive regulation

diated signaling pathway;GO:0009611//response to wounding;GO:0009615//response to virus;GO:0
ative regulation of protein dephosphorylation;GO:0061028//establishment of endothelial barrier;G

014883//transition between fast and slow fiber;GO:0014898//cardiac muscle hypertrophy in respo

nization;GO:0048041//focal adhesion assembly;GO:0051695//actin filament uncapping;GO:00550

/viral genome integration into host DNA;GO:0046718//viral entry into host cell;GO:0075713//esta

592//response to calcium ion;GO:0051928//positive regulation of calcium ion transport;GO:009027

n of insulin secretion;GO:0045727//positive regulation of translation;GO:0046622//positive regulat

ion of inflammatory response;GO:0050729//positive regulation of inflammatory response;GO:0050

cade;GO:0072378//blood coagulation, fibrin clot formation;GO:1900025//negative regulation of su

ponse to virus;GO:0009791//post-embryonic development;GO:0010628//positive regulation of ger

to endoplasmic reticulum stress;GO:0070059//intrinsic apoptotic signaling pathway in response to

tricular valve development;GO:0003180//aortic valve morphogenesis;GO:0003184//pulmonary val

5//negative regulation of microtubule polymerization;GO:0031175//neuron projection developmen
development;GO:0031639//plasminogen activation;GO:0033489//cholesterol biosynthetic process

c process;GO:0030258//lipid modification;GO:0032286//central nervous system myelin maintenanc

5//negative regulation of microtubule polymerization;GO:0031175//neuron projection developmen

itin-dependent protein catabolic process;GO:0031396//regulation of protein ubiquitination

ong term synaptic depression;GO:2000124//regulation of endocannabinoid signaling pathway;GO:2
: process;GO:0043066//negative regulation of apoptotic process;GO:0050766//positive regulation

egulation of necroptotic process;GO:0070269//pyroptosis;GO:0072332//intrinsic apoptotic signalir

:hway;GO:0007202//activation of phospholipase C activity;GO:0008360//regulation of cell shape;G
:0031076//embryonic camera-type eye development;GO:0042472//inner ear morphogenesis;GO:0

netic process;GO:0019367//fatty acid elongation, saturated fatty acid;GO:0030148//sphingolipid bi

sis;GO:0043010//camera-type eye development;GO:0043587//tongue morphogenesis;GO:004478

G1 DNA damage checkpoint;GO:0042771//intrinsic apoptotic signaling pathway in response to DI

/te differentiation;GO:0035108//limb morphogenesis;GO:0035264//multicellular organism growth;(

: process;GO:0043066//negative regulation of apoptotic process;GO:0050766//positive regulation

)46777//protein autophosphorylation;GO:0048679//regulation of axon regeneration;GO:0097194//

tricular valve development;GO:0003180//aortic valve morphogenesis;GO:0003184//pulmonary val

IAP kinase activity;GO:0050709//negative regulation of protein secretion;GO:1900222//negative re

5//negative regulation of microtubule polymerization;GO:0031175//neuron projection developme

9//protein deubiquitination;GO:0019896//axon transport of mitochondrion;GO:0042755//eating b

e regulation of cell-substrate adhesion;GO:0030335//positive regulation of cell migration;GO:0034

2869//cellular response to insulin stimulus;GO:0045725//positive regulation of glycogen biosynthe

/viral genome integration into host DNA;GO:0046718//viral entry into host cell;GO:0075713//estal

on of BMP signaling pathway;GO:0045662//negative regulation of myoblast differentiation;GO:009

NA;GO:0035278//miRNA mediated inhibition of translation;GO:0035280//miRNA loading onto RIS

to endoplasmic reticulum stress;GO:0070059//intrinsic apoptotic signaling pathway in response to

nodeling;GO:0036151//phosphatidylcholine acyl-chain remodeling;GO:0036152//phosphatidyletha
sis;GO:0043010//camera-type eye development;GO:0043587//tongue morphogenesis;GO:004478

ne expression;GO:0010628//positive regulation of gene expression;GO:0032956//regulation of acti

6355//regulation of transcription, DNA-templated;GO:0006468//protein phosphorylation;GO:000

egulation of necroptotic process;GO:0070269//pyroptosis;GO:0072332//intrinsic apoptotic signalir

c process;GO:0030258//lipid modification;GO:0032286//central nervous system myelin maintenanc

ection of chemical stimulus involved in sensory perception of taste;GO:0050915//sensory perceptic

te differentiation;GO:0035108//limb morphogenesis;GO:0035264//multicellular organism growth;

:hway;GO:0007202//activation of phospholipase C activity;GO:0008360//regulation of cell shape;G

:0031076//embryonic camera-type eye development;GO:0042472//inner ear morphogenesis;GO:0

oid catabolic process;GO:0010519//negative regulation of phospholipase activity;GO:0019915//lipi

!869//cellular response to insulin stimulus;GO:0045725//positive regulation of glycogen biosynthet

;tress;GO:0045893//positive regulation of transcription, DNA-templated;GO:0045944//positive reg
O:1904707//positive regulation of vascular smooth muscle cell proliferation;GO:1904754//positive

esponse to bacterium;GO:0010629//negative regulation of gene expression;GO:0046326//positive

commissure morphogenesis;GO:0030517//negative regulation of axon extension;GO:0030900//fo

nogenesis;GO:0003163//sinoatrial node development;GO:0003309//type B pancreatic cell different

)46777//protein autophosphorylation;GO:0048679//regulation of axon regeneration;GO:0097194//

olic process;GO:0019433//triglyceride catabolic process;GO:0032722//positive regulation of chem

0016042//lipid catabolic process;GO:0019432//triglyceride biosynthetic process;GO:0019433//trigl

regulation of cell-substrate adhesion;GO:0030335//positive regulation of cell migration;GO:0034

iogenesis;GO:0042127//regulation of cell proliferation;GO:0043697//cell dedifferentiation;GO:004

etic process;GO:0019367//fatty acid elongation, saturated fatty acid;GO:0030148//sphingolipid bi

tricular valve development;GO:0003180//aortic valve morphogenesis;GO:0003184//pulmonary val

aling pathway;GO:0042632//cholesterol homeostasis;GO:0045944//positive regulation of transcript

development;GO:0031639//plasminogen activation;GO:0033489//cholesterol biosynthetic process

tress;GO:0045893//positive regulation of transcription, DNA-templated;GO:0045944//positive reg

process;GO:0046340//diacylglycerol catabolic process;GO:0071926//endocannabinoid signaling

046777//protein autophosphorylation;GO:0048679//regulation of axon regeneration;GO:0097194//

hogenesis;GO:0003163//sinoatrial node development;GO:0003309//type B pancreatic cell different

0016042//lipid catabolic process;GO:0019432//triglyceride biosynthetic process;GO:0019433//trigl
sis;GO:0043010//camera-type eye development;GO:0043587//tongue morphogenesis;GO:004478

liogenesis;GO:0042127//regulation of cell proliferation;GO:0043697//cell dedifferentiation;GO:004

er taste;GO:0050916//sensory perception of sweet taste;GO:0050917//sensory perception of uman

commissure morphogenesis;GO:0030517//negative regulation of axon extension;GO:0030900//for

/COPII vesicle coating;GO:0070863//positive regulation of protein exit from endoplasmic reticulum
:hway;GO:0007202//activation of phospholipase C activity;GO:0008360//regulation of cell shape;G

5//negative regulation of microtubule polymerization;GO:0031175//neuron projection developme

O:1904707//positive regulation of vascular smooth muscle cell proliferation;GO:1904754//positive

ctivity;GO:0035556//intracellular signal transduction;GO:0043153//entrainment of circadian clock b

:0031076//embryonic camera-type eye development;GO:0042472//inner ear morphogenesis;GO:0

itin-dependent protein catabolic process;GO:0031396//regulation of protein ubiquitination
to endoplasmic reticulum stress;GO:0070059//intrinsic apoptotic signaling pathway in response to

:0031076//embryonic camera-type eye development;GO:0042472//inner ear morphogenesis;GO:0

stress;GO:0045893//positive regulation of transcription, DNA-templated;GO:0045944//positive reg

sis;GO:0043010//camera-type eye development;GO:0043587//tongue morphogenesis;GO:004478

tem cell differentiation;GO:0061086//negative regulation of histone H3-K27 methylation;GO:0061

yte differentiation;GO:0035108//limb morphogenesis;GO:0035264//multicellular organism growth;

ort;GO:0010866//regulation of triglyceride biosynthetic process;GO:0010884//positive regulation
sitive regulation of cell proliferation;GO:0030307//positive regulation of cell growth;GO:0031930//
process;GO:0043066//negative regulation of apoptotic process;GO:0050766//positive regulation
age;GO:0018105//peptidyl-serine phosphorylation;GO:0034340//response to type I interferon;GO:

G1 DNA damage checkpoint;GO:0042771//intrinsic apoptotic signaling pathway in response to D

5//negative regulation of microtubule polymerization;GO:0031175//neuron projection developme

ıse;GO:0050921//positive regulation of chemotaxis;GO:0051592//response to calcium ion;GO:006

cade;GO:0072378//blood coagulation, fibrin clot formation;GO:1900025//negative regulation of su

G1 DNA damage checkpoint;GO:0042771//intrinsic apoptotic signaling pathway in response to DI

e migration;GO:0002906//negative regulation of mature B cell apoptotic process;GO:0006154//ad

liogenesis;GO:0042127//regulation of cell proliferation;GO:0043697//cell dedifferentiation;GO:004

eta receptor signaling pathway;GO:0032683//negative regulation of connective tissue growth fact
592//response to calcium ion;GO:0051928//positive regulation of calcium ion transport;GO:009027
0060562//epithelial tube morphogenesis;GO:0061205//paramesonephric duct development;GO:00

on of BMP signaling pathway;GO:0045662//negative regulation of myoblast differentiation;GO:009

035865//cellular response to potassium ion;GO:0042391//regulation of membrane potential;GO:00

to endoplasmic reticulum stress;GO:0070059//intrinsic apoptotic signaling pathway in response to

nodeling;GO:0036151//phosphatidylcholine acyl-chain remodeling;GO:0036152//phosphatidyletha

atinization;GO:0032379//positive regulation of intracellular lipid transport;GO:0032940//secretion l
ort;GO:0010866//regulation of triglyceride biosynthetic process;GO:0010884//positive regulation c
te differentiation;GO:0035108//limb morphogenesis;GO:0035264//multicellular organism growth;(

age;GO:0018105//peptidyl-serine phosphorylation;GO:0034340//response to type I interferon;GO:
ne expression;GO:0010628//positive regulation of gene expression;GO:0032956//regulation of acti
: process;GO:0043066//negative regulation of apoptotic process;GO:0050766//positive regulation
'0936//protein K48-linked ubiquitination;GO:1904262//negative regulation of TORC1 signaling

)46777//protein autophosphorylation;GO:0048679//regulation of axon regeneration;GO:0097194//

ation;GO:0050848//regulation of calcium-mediated signaling;GO:0090022//regulation of neutropl

:hway;GO:0007202//activation of phospholipase C activity;GO:0008360//regulation of cell shape;G

:0031076//embryonic camera-type eye development;GO:0042472//inner ear morphogenesis;GO:0

cade;GO:0072378//blood coagulation, fibrin clot formation;GO:1900025//negative regulation of su

on of BMP signaling pathway;GO:0045662//negative regulation of myoblast differentiation;GO:009

e migration;GO:0002906//negative regulation of mature B cell apoptotic process;GO:0006154//ad

592//response to calcium ion;GO:0051928//positive regulation of calcium ion transport;GO:009027

:hway;GO:0007202//activation of phospholipase C activity;GO:0008360//regulation of cell shape;G

sulin;GO:0032869//cellular response to insulin stimulus;GO:0043382//positive regulation of memo

0016042//lipid catabolic process;GO:0019432//triglyceride biosynthetic process;GO:0019433//trigl

G1 DNA damage checkpoint;GO:0042771//intrinsic apoptotic signaling pathway in response to DI

liated signaling pathway;GO:0009611//response to wounding;GO:0009615//response to virus;GO:

GO:0021670//lateral ventricle development;GO:0021766//hippocampus development;GO:002196

nogenesis;GO:0003163//sinoatrial node development;GO:0003309//type B pancreatic cell different

ong term synaptic depression;GO:2000124//regulation of endocannabinoid signaling pathway;GO:

46777//protein autophosphorylation;GO:0048679//regulation of axon regeneration;GO:0097194//

to endoplasmic reticulum stress;GO:0070059//intrinsic apoptotic signaling pathway in response to
'0936//protein K48-linked ubiquitination;GO:1904262//negative regulation of TORC1 signaling

:0031076//embryonic camera-type eye development;GO:0042472//inner ear morphogenesis;GO:0

e regulation of cell-substrate adhesion;GO:0030335//positive regulation of cell migration;GO:0034
c process;GO:0046340//diacylglycerol catabolic process;GO:0071926//endocannabinoid signaling p

06355//regulation of transcription, DNA-templated;GO:0006468//protein phosphorylation;GO:0007

biosynthetic process;GO:0008654//phospholipid biosynthetic process;GO:0009749//response to glu
to endoplasmic reticulum stress;GO:0070059//intrinsic apoptotic signaling pathway in response to

yte differentiation;GO:0035108//limb morphogenesis;GO:0035264//multicellular organism growth;(

)46777//protein autophosphorylation;GO:0048679//regulation of axon regeneration;GO:0097194//

c process;GO:0030258//lipid modification;GO:0032286//central nervous system myelin maintenanc

nogenesis;GO:0003163//sinoatrial node development;GO:0003309//type B pancreatic cell different

diated signaling pathway;GO:0009611//response to wounding;GO:0009615//response to virus;GO:(

0906//regulation of glucose metabolic process;GO:0042304//regulation of fatty acid biosynthetic

liogenesis;GO:0042127//regulation of cell proliferation;GO:0043697//cell dedifferentiation;GO:004[illegible]

G1 DNA damage checkpoint;GO:0042771//intrinsic apoptotic signaling pathway in response to DI

:hway;GO:0007202//activation of phospholipase C activity;GO:0008360//regulation of cell shape;G

NA;GO:0035278//miRNA mediated inhibition of translation;GO:0035280//miRNA loading onto RIS

9//protein deubiquitination;GO:0019896//axon transport of mitochondrion;GO:0042755//eating b

:hway;GO:0007202//activation of phospholipase C activity;GO:0008360//regulation of cell shape;G

cation fork processing;GO:0031571//mitotic G1 DNA damage checkpoint;GO:0036297//interstranc

/COPII vesicle coating;GO:0070863//positive regulation of protein exit from endoplasmic reticulum

on of BMP signaling pathway;GO:0045662//negative regulation of myoblast differentiation;GO:009

e migration;GO:0002906//negative regulation of mature B cell apoptotic process;GO:0006154//ade
c process;GO:0030258//lipid modification;GO:0032286//central nervous system myelin maintenanc

07188//adenylate cyclase-modulating G-protein coupled receptor signaling pathway;GO:0007193/

592//response to calcium ion;GO:0051928//positive regulation of calcium ion transport;GO:009027

oid catabolic process;GO:0010519//negative regulation of phospholipase activity;GO:0019915//lipi

46777//protein autophosphorylation;GO:0048679//regulation of axon regeneration;GO:0097194//

to endoplasmic reticulum stress;GO:0070059//intrinsic apoptotic signaling pathway in response to

regulation of cell-substrate adhesion;GO:0030335//positive regulation of cell migration;GO:0034

46777//protein autophosphorylation;GO:0048679//regulation of axon regeneration;GO:0097194//

cade;GO:0072378//blood coagulation, fibrin clot formation;GO:1900025//negative regulation of su

regulation of cell-substrate adhesion;GO:0030335//positive regulation of cell migration;GO:0034

nogenesis;GO:0003163//sinoatrial node development;GO:0003309//type B pancreatic cell different

ction;GO:0060371//regulation of atrial cardiac muscle cell membrane depolarization;GO:0060373//

35865//cellular response to potassium ion;GO:0042391//regulation of membrane potential;GO:00

te differentiation;GO:0035108//limb morphogenesis;GO:0035264//multicellular organism growth;

ıetic process;GO:0019367//fatty acid elongation, saturated fatty acid;GO:0030148//sphingolipid bi

'0936//protein K48-linked ubiquitination;GO:1904262//negative regulation of TORC1 signaling

ation;GO:0050848//regulation of calcium-mediated signaling;GO:0090022//regulation of neutrop

activating G-protein coupled receptor signaling pathway;GO:0007507//heart development;GO:00

stress;GO:0045893//positive regulation of transcription, DNA-templated;GO:0045944//positive reg

ponse to virus;GO:0009791//post-embryonic development;GO:0010628//positive regulation of ge
;GO:0033564//anterior/posterior axon guidance;GO:0042472//inner ear morphogenesis;GO:00457

6355//regulation of transcription, DNA-templated;GO:0006468//protein phosphorylation;GO:000

ıogenesis;GO:0003163//sinoatrial node development;GO:0003309//type B pancreatic cell differen

hway;GO:0007202//activation of phospholipase C activity;GO:0008360//regulation of cell shape;G

592//response to calcium ion;GO:0051928//positive regulation of calcium ion transport;GO:009027

;GO:0033564//anterior/posterior axon guidance;GO:0042472//inner ear morphogenesis;GO:00457

G1 DNA damage checkpoint;GO:0042771//intrinsic apoptotic signaling pathway in response to DI
on of BMP signaling pathway;GO:0045662//negative regulation of myoblast differentiation;GO:009

cade;GO:0072378//blood coagulation, fibrin clot formation;GO:1900025//negative regulation of su

ler cell activation;GO:0032252//secretory granule localization;GO:0032725//positive regulation of g

oid catabolic process;GO:0010519//negative regulation of phospholipase activity;GO:0019915//lipi

to endoplasmic reticulum stress;GO:0070059//intrinsic apoptotic signaling pathway in response to

c process;GO:0030258//lipid modification;GO:0032286//central nervous system myelin maintenanc

nogenesis;GO:0003163//sinoatrial node development;GO:0003309//type B pancreatic cell different
eta receptor signaling pathway;GO:0032683//negative regulation of connective tissue growth fact

aling pathway;GO:0042632//cholesterol homeostasis;GO:0045944//positive regulation of transcript

netic process;GO:0019367//fatty acid elongation, saturated fatty acid;GO:0030148//sphingolipid bi
te differentiation;GO:0035108//limb morphogenesis;GO:0035264//multicellular organism growth;

nization;GO:0048041//focal adhesion assembly;GO:0051695//actin filament uncapping;GO:00550

6355//regulation of transcription, DNA-templated;GO:0006468//protein phosphorylation;GO:000

tress;GO:0045893//positive regulation of transcription, DNA-templated;GO:0045944//positive reg

sis;GO:0043010//camera-type eye development;GO:0043587//tongue morphogenesis;GO:004478

ction;GO:0060371//regulation of atrial cardiac muscle cell membrane depolarization;GO:0060373//
G1 DNA damage checkpoint;GO:0042771//intrinsic apoptotic signaling pathway in response to DI

/viral genome integration into host DNA;GO:0046718//viral entry into host cell;GO:0075713//estal

netic process;GO:0019367//fatty acid elongation, saturated fatty acid;GO:0030148//sphingolipid bi

G1 DNA damage checkpoint;GO:0042771//intrinsic apoptotic signaling pathway in response to DI

diated signaling pathway;GO:0009611//response to wounding;GO:0009615//response to virus;GO:

netic process;GO:0019367//fatty acid elongation, saturated fatty acid;GO:0030148//sphingolipid bi

: process;GO:0043066//negative regulation of apoptotic process;GO:0050766//positive regulation

sis;GO:0043010//camera-type eye development;GO:0043587//tongue morphogenesis;GO:004478

oid catabolic process;GO:0010519//negative regulation of phospholipase activity;GO:0019915//lipi

cation fork processing;GO:0031571//mitotic G1 DNA damage checkpoint;GO:0036297//interstranc

nization;GO:0048041//focal adhesion assembly;GO:0051695//actin filament uncapping;GO:00550

rhythm;GO:0008284//positive regulation of cell proliferation;GO:0010628//positive regulation of g

stress;GO:0045893//positive regulation of transcription, DNA-templated;GO:0045944//positive reg

sitive regulation of cell proliferation;GO:0030307//positive regulation of cell growth;GO:0031930//
to endoplasmic reticulum stress;GO:0070059//intrinsic apoptotic signaling pathway in response to

G1 DNA damage checkpoint;GO:0042771//intrinsic apoptotic signaling pathway in response to DN

/viral genome integration into host DNA;GO:0046718//viral entry into host cell;GO:0075713//estal

stress;GO:0045893//positive regulation of transcription, DNA-templated;GO:0045944//positive reg

biosynthetic process;GO:0008654//phospholipid biosynthetic process;GO:0009749//response to glu

5//negative regulation of microtubule polymerization;GO:0031175//neuron projection developmer

:hway;GO:0007202//activation of phospholipase C activity;GO:0008360//regulation of cell shape;G

098609//cell-cell adhesion;GO:0098911//regulation of ventricular cardiac muscle cell action poten

/COPII vesicle coating;GO:0070863//positive regulation of protein exit from endoplasmic reticulum
on of BMP signaling pathway;GO:0045662//negative regulation of myoblast differentiation;GO:009

6355//regulation of transcription, DNA-templated;GO:0006468//protein phosphorylation;GO:000[illegible]

;GO:0033564//anterior/posterior axon guidance;GO:0042472//inner ear morphogenesis;GO:0045[illegible]

to endoplasmic reticulum stress;GO:0070059//intrinsic apoptotic signaling pathway in response to

stress;GO:0045893//positive regulation of transcription, DNA-templated;GO:0045944//positive reg
ation;GO:0050848//regulation of calcium-mediated signaling;GO:0090022//regulation of neutrop[illegible]
O:1904707//positive regulation of vascular smooth muscle cell proliferation;GO:1904754//positive

46777//protein autophosphorylation;GO:0048679//regulation of axon regeneration;GO:0097194//[illegible]

netic process;GO:0019367//fatty acid elongation, saturated fatty acid;GO:0030148//sphingolipid bi[illegible]

sulin;GO:0032869//cellular response to insulin stimulus;GO:0043382//positive regulation of memo

:hway;GO:0007202//activation of phospholipase C activity;GO:0008360//regulation of cell shape;G

aling pathway;GO:0042632//cholesterol homeostasis;GO:0045944//positive regulation of transcript

oid catabolic process;GO:0010519//negative regulation of phospholipase activity;GO:0019915//lipi

:le remodeling;GO:0034375//high-density lipoprotein particle remodeling;GO:0034435//cholester[illegible]

[illegible]ponse to virus;GO:0009791//post-embryonic development;GO:0010628//positive regulation of ge[illegible]

[illegible]on of BMP signaling pathway;GO:0045662//negative regulation of myoblast differentiation;GO:00[illegible]

-activating G-protein coupled receptor signaling pathway;GO:0007507//heart development;GO:00

tricular valve development;GO:0003180//aortic valve morphogenesis;GO:0003184//pulmonary val

[illegible]6355//regulation of transcription, DNA-templated;GO:0006468//protein phosphorylation;GO:000[illegible]

0016042//lipid catabolic process;GO:0019432//triglyceride biosynthetic process;GO:0019433//trigl

nogenesis;GO:0003163//sinoatrial node development;GO:0003309//type B pancreatic cell different
046777//protein autophosphorylation;GO:0048679//regulation of axon regeneration;GO:0097194//
: process;GO:0043066//negative regulation of apoptotic process;GO:0050766//positive regulation

34//male gonad development;GO:0010628//positive regulation of gene expression;GO:0010817//r

5//negative regulation of microtubule polymerization;GO:0031175//neuron projection developme

oid catabolic process;GO:0010519//negative regulation of phospholipase activity;GO:0019915//lipi

aling pathway;GO:0042632//cholesterol homeostasis;GO:0045944//positive regulation of transcript
sulin;GO:0032869//cellular response to insulin stimulus;GO:0043382//positive regulation of memo

er taste;GO:0050916//sensory perception of sweet taste;GO:0050917//sensory perception of uman

ponse to virus;GO:0009791//post-embryonic development;GO:0010628//positive regulation of ge

stress;GO:0045893//positive regulation of transcription, DNA-templated;GO:0045944//positive reg

c process;GO:0030258//lipid modification;GO:0032286//central nervous system myelin maintenanc
5//negative regulation of microtubule polymerization;GO:0031175//neuron projection developmen

:0031076//embryonic camera-type eye development;GO:0042472//inner ear morphogenesis;GO:0

)46777//protein autophosphorylation;GO:0048679//regulation of axon regeneration;GO:0097194//

liogenesis;GO:0042127//regulation of cell proliferation;GO:0043697//cell dedifferentiation;GO:004

nodeling;GO:0036151//phosphatidylcholine acyl-chain remodeling;GO:0036152//phosphatidyletha

5//negative regulation of microtubule polymerization;GO:0031175//neuron projection developme

: process;GO:0043066//negative regulation of apoptotic process;GO:0050766//positive regulation

NA;GO:0035278//miRNA mediated inhibition of translation;GO:0035280//miRNA loading onto RIS

tricular valve development;GO:0003180//aortic valve morphogenesis;GO:0003184//pulmonary val

biosynthetic process;GO:0008654//phospholipid biosynthetic process;GO:0009749//response to gl

on of BMP signaling pathway;GO:0045662//negative regulation of myoblast differentiation;GO:00

sulin;GO:0032869//cellular response to insulin stimulus;GO:0043382//positive regulation of memo
itin-dependent protein catabolic process;GO:0031396//regulation of protein ubiquitination

thway;GO:0007202//activation of phospholipase C activity;GO:0008360//regulation of cell shape;G

ıogenesis;GO:0003163//sinoatrial node development;GO:0003309//type B pancreatic cell different
592//response to calcium ion;GO:0051928//positive regulation of calcium ion transport;GO:009027

: process;GO:0043066//negative regulation of apoptotic process;GO:0050766//positive regulation

098609//cell-cell adhesion;GO:0098911//regulation of ventricular cardiac muscle cell action poten

tem cell differentiation;GO:0061086//negative regulation of histone H3-K27 methylation;GO:0061(

)46777//protein autophosphorylation;GO:0048679//regulation of axon regeneration;GO:0097194//

O:0007626//locomotory behavior;GO:0010842//retina layer formation;GO:0038007//netrin-activat
cade;GO:0072378//blood coagulation, fibrin clot formation;GO:1900025//negative regulation of su
:0031076//embryonic camera-type eye development;GO:0042472//inner ear morphogenesis;GO:0

: process;GO:0030258//lipid modification;GO:0032286//central nervous system myelin maintenanc

sis;GO:0043010//camera-type eye development;GO:0043587//tongue morphogenesis;GO:004478

regulation of cell-substrate adhesion;GO:0030335//positive regulation of cell migration;GO:0034[illegible]
to endoplasmic reticulum stress;GO:0070059//intrinsic apoptotic signaling pathway in response to

process;GO:0043066//negative regulation of apoptotic process;GO:0050766//positive regulation

ecular complex assembly;GO:0042730//fibrinolysis;GO:0045907//positive regulation of vasoconstri[illegible]

/viral genome integration into host DNA;GO:0046718//viral entry into host cell;GO:0075713//estal[illegible]

netic process;GO:0019367//fatty acid elongation, saturated fatty acid;GO:0030148//sphingolipid bi[illegible]

te differentiation;GO:0035108//limb morphogenesis;GO:0035264//multicellular organism growth;[illegible]

ɔiosynthetic process;GO:0008654//phospholipid biosynthetic process;GO:0009749//response to glu

:hway;GO:0007202//activation of phospholipase C activity;GO:0008360//regulation of cell shape;G

5//negative regulation of microtubule polymerization;GO:0031175//neuron projection developmer

ɔonse to virus;GO:0009791//post-embryonic development;GO:0010628//positive regulation of ger

esponse to bacterium;GO:0010629//negative regulation of gene expression;GO:0046326//positive

itin-dependent protein catabolic process;GO:0031396//regulation of protein ubiquitination

liogenesis;GO:0042127//regulation of cell proliferation;GO:0043697//cell dedifferentiation;GO:004!

:tion;GO:0060371//regulation of atrial cardiac muscle cell membrane depolarization;GO:0060373//

592//response to calcium ion;GO:0051928//positive regulation of calcium ion transport;GO:009027

: process;GO:0043066//negative regulation of apoptotic process;GO:0050766//positive regulation

aling pathway;GO:0042632//cholesterol homeostasis;GO:0045944//positive regulation of transcript

:0031076//embryonic camera-type eye development;GO:0042472//inner ear morphogenesis;GO:0
to endoplasmic reticulum stress;GO:0070059//intrinsic apoptotic signaling pathway in response to
G1 DNA damage checkpoint;GO:0042771//intrinsic apoptotic signaling pathway in response to DN

nodeling;GO:0036151//phosphatidylcholine acyl-chain remodeling;GO:0036152//phosphatidyletha

n binding;GO:0032435//negative regulation of proteasomal ubiquitin-dependent protein cataboli

cade;GO:0072378//blood coagulation, fibrin clot formation;GO:1900025//negative regulation of su

oid catabolic process;GO:0010519//negative regulation of phospholipase activity;GO:0019915//lipi

: regulation of cell-substrate adhesion;GO:0030335//positive regulation of cell migration;GO:0034[illegible]

ıogenesis;GO:0003163//sinoatrial node development;GO:0003309//type B pancreatic cell different[illegible]

'/chemotaxis;GO:0007267//cell-cell signaling;GO:0007368//determination of left/right symmetry;G[illegible]

'te differentiation;GO:0035108//limb morphogenesis;GO:0035264//multicellular organism growth;[illegible]

'0936//protein K48-linked ubiquitination;GO:1904262//negative regulation of TORC1 signaling

sis;GO:0043010//camera-type eye development;GO:0043587//tongue morphogenesis;GO:004478

5//negative regulation of microtubule polymerization;GO:0031175//neuron projection developme

G1 DNA damage checkpoint;GO:0042771//intrinsic apoptotic signaling pathway in response to DI

cade;GO:0072378//blood coagulation, fibrin clot formation;GO:1900025//negative regulation of su
ction;GO:0060371//regulation of atrial cardiac muscle cell membrane depolarization;GO:0060373//

6355//regulation of transcription, DNA-templated;GO:0006468//protein phosphorylation;GO:000

n binding;GO:0032435//negative regulation of proteasomal ubiquitin-dependent protein cataboli
592//response to calcium ion;GO:0051928//positive regulation of calcium ion transport;GO:009027

c process;GO:0030258//lipid modification;GO:0032286//central nervous system myelin maintenanc
e migration;GO:0002906//negative regulation of mature B cell apoptotic process;GO:0006154//ad

ction;GO:0060371//regulation of atrial cardiac muscle cell membrane depolarization;GO:0060373//

on of BMP signaling pathway;GO:0045662//negative regulation of myoblast differentiation;GO:009

/viral genome integration into host DNA;GO:0046718//viral entry into host cell;GO:0075713//estal

diated signaling pathway;GO:0009611//response to wounding;GO:0009615//response to virus;GO:

592//response to calcium ion;GO:0051928//positive regulation of calcium ion transport;GO:009027

liogenesis;GO:0042127//regulation of cell proliferation;GO:0043697//cell dedifferentiation;GO:0045

sis;GO:0043010//camera-type eye development;GO:0043587//tongue morphogenesis;GO:004478

cade;GO:0072378//blood coagulation, fibrin clot formation;GO:1900025//negative regulation of su

sulin;GO:0032869//cellular response to insulin stimulus;GO:0043382//positive regulation of memo

tem cell differentiation;GO:0061086//negative regulation of histone H3-K27 methylation;GO:0061(

sis;GO:0043010//camera-type eye development;GO:0043587//tongue morphogenesis;GO:004478

70936//protein K48-linked ubiquitination;GO:1904262//negative regulation of TORC1 signaling

ısmembrane transport;GO:0086003//cardiac muscle cell contraction;GO:1903760//regulation of vo

NA;GO:0035278//miRNA mediated inhibition of translation;GO:0035280//miRNA loading onto RIS

: regulation of cell-substrate adhesion;GO:0030335//positive regulation of cell migration;GO:0034

esponse to bacterium;GO:0010629//negative regulation of gene expression;GO:0046326//positive

er taste;GO:0050916//sensory perception of sweet taste;GO:0050917//sensory perception of uman

ɔiosynthetic process;GO:0008654//phospholipid biosynthetic process;GO:0009749//response to glı

ɛ migration;GO:0002906//negative regulation of mature B cell apoptotic process;GO:0006154//ad‹

/COPII vesicle coating;GO:0070863//positive regulation of protein exit from endoplasmic reticulun

appaB transcription factor activity;GO:0071346//cellular response to interferon-gamma
ltage-gated potassium channel activity involved in ventricular cardiac muscle cell action potential

.00//positive regulation of phagocytosis, engulfment;GO:0071222//cellular response to lipopolysac

//xenobiotic catabolic process;GO:0042572//retinol metabolic process;GO:0046483//heterocycle m
C involved in gene silencing by miRNA;GO:0045944//positive regulation of transcription from RN

07186//G-protein coupled receptor signaling pathway;GO:0007229//integrin-mediated signaling

diac muscle hypertrophy;GO:0010628//positive regulation of gene expression;GO:0010629//negat

olated;GO:0050775//positive regulation of dendrite morphogenesis;GO:0050890//cognition;GO:00
0010467//gene expression;GO:0033627//cell adhesion mediated by integrin;GO:0042060//wound

ıokine production;GO:0034371//chylomicron remodeling;GO:0034372//very-low-density lipoprote

ɟnaling pathway;GO:0035729//cellular response to hepatocyte growth factor stimulus;GO:0042593
ɟene expression;GO:0021766//hippocampus development;GO:0021895//cerebral cortex neuron di

//positive regulation of gene expression;GO:0010862//positive regulation of pathway-restricted SI

5638//negative regulation of myeloid cell differentiation;GO:0045646//regulation of erythrocyte di

regulation of hormone levels;GO:0014015//positive regulation of gliogenesis;GO:0033690//positive

ed signaling pathway;GO:0042327//positive regulation of phosphorylation;GO:0048813//dendrite

C involved in gene silencing by miRNA;GO:0045944//positive regulation of transcription from RN/

)07186//G-protein coupled receptor signaling pathway;GO:0007229//integrin-mediated signaling

granulocyte macrophage colony-stimulating factor production;GO:0032729//positive regulation o

/cerebellum morphogenesis;GO:0043506//regulation of JUN kinase activity;GO:0050667//homocy

olated;GO:0050775//positive regulation of dendrite morphogenesis;GO:0050890//cognition;GO:00

ndle assembly;GO:0032414//positive regulation of ion transmembrane transporter activity;GO:0045

45766//positive regulation of angiogenesis;GO:0045793//positive regulation of cell size;GO:00488

//xenobiotic catabolic process;GO:0042572//retinol metabolic process;GO:0046483//heterocycle m

diac muscle hypertrophy;GO:0010628//positive regulation of gene expression;GO:0010629//negat

90090//negative regulation of canonical Wnt signaling pathway;GO:2000016//negative regulation

)//anterior commissure morphogenesis;GO:0030178//negative regulation of Wnt signaling pathwa

ulation of transcription from RNA polymerase II promoter;GO:0051216//cartilage development

oltage-gated potassium channel activity involved in ventricular cardiac muscle cell action potential

ed signaling pathway;GO:0042327//positive regulation of phosphorylation;GO:0048813//dendrite

appaB transcription factor activity;GO:0071346//cellular response to interferon-gamma

ization behavior;GO:0097104//postsynaptic membrane assembly;GO:0097105//presynaptic memb

c process;GO:0060271//cilium morphogenesis;GO:1902915//negative regulation of protein polyub

gnaling pathway;GO:0035729//cellular response to hepatocyte growth factor stimulus;GO:0042593

//positive regulation of gene expression;GO:0010862//positive regulation of pathway-restricted SI

)45956//positive regulation of calcium ion-dependent exocytosis;GO:0055085//transmembrane tra

ol esterification;GO:0042632//cholesterol homeostasis;GO:0043691//reverse cholesterol transport;(

granulocyte macrophage colony-stimulating factor production;GO:0032729//positive regulation o

'/cerebellum morphogenesis;GO:0043506//regulation of JUN kinase activity;GO:0050667//homocy

' lipoprotein particle receptor catabolic process;GO:0042632//cholesterol homeostasis;GO:004573[illegible]

ndle assembly;GO:0032414//positive regulation of ion transmembrane transporter activity;GO:004[illegible]

process;GO:0042593//glucose homeostasis;GO:0042594//response to starvation;GO:0045124//reg

:0035456//response to interferon-beta;GO:0038061//NIK/NF-kappaB signaling;GO:0043123//posi[illegible]

of lipid storage;GO:0045745//positive regulation of G-protein coupled receptor protein signaling [illegible]

45766//positive regulation of angiogenesis;GO:0045793//positive regulation of cell size;GO:00488[illegible]

ndle assembly;GO:0032414//positive regulation of ion transmembrane transporter activity;GO:004[illegible]

)87//positive regulation of histone H3-K27 methylation;GO:1990830//cellular response to leukemi

ational protein modification;GO:1904027//negative regulation of collagen fibril organization

ol esterification;GO:0042632//cholesterol homeostasis;GO:0043691//reverse cholesterol transport;[illegible]

' lipoprotein particle receptor catabolic process;GO:0042632//cholesterol homeostasis;GO:004573[illegible]

ed signaling pathway;GO:0042327//positive regulation of phosphorylation;GO:0048813//dendrite

olishment of integrated proviral latency;GO:0090305//nucleic acid phosphodiester bond hydrolysis

)35556//intracellular signal transduction;GO:0035774//positive regulation of insulin secretion invol

gene expression;GO:0021766//hippocampus development;GO:0021895//cerebral cortex neuron di

of lipid storage;GO:0045745//positive regulation of G-protein coupled receptor protein signaling p
:0035456//response to interferon-beta;GO:0038061//NIK/NF-kappaB signaling;GO:0043123//posit

tion from RNA polymerase II promoter;GO:0071404//cellular response to low-density lipoprotein p

ed actin nucleation;GO:0048332//mesoderm morphogenesis;GO:0048562//embryonic organ morp

granulocyte macrophage colony-stimulating factor production;GO:0032729//positive regulation o

)87//positive regulation of histone H3-K27 methylation;GO:1990830//cellular response to leukemi

' lipoprotein particle receptor catabolic process;GO:0042632//cholesterol homeostasis;GO:004573[illegible]

/cerebral cortex cell migration;GO:0021799//cerebral cortex radially oriented cell migration;GO:00[illegible]

yceride catabolic process;GO:0034389//lipid particle organization;GO:0036153//triglyceride acyl-[illegible]

'9//regulation of calcium ion import;GO:2001256//regulation of store-operated calcium entry;GO:[illegible]

//xenobiotic catabolic process;GO:0042572//retinol metabolic process;GO:0046483//heterocycle m

diac muscle hypertrophy;GO:0010628//positive regulation of gene expression;GO:0010629//negat
45766//positive regulation of angiogenesis;GO:0045793//positive regulation of cell size;GO:00488[illegible]

ed signaling pathway;GO:0042327//positive regulation of phosphorylation;GO:0048813//dendrite

ubstrate adhesion-dependent cell spreading;GO:2000146//negative regulation of cell motility;GO:[illegible]

process;GO:0042593//glucose homeostasis;GO:0042594//response to starvation;GO:0045124//reg

granulocyte macrophage colony-stimulating factor production;GO:0032729//positive regulation o

ndle assembly;GO:0032414//positive regulation of ion transmembrane transporter activity;GO:004[illegible]

035556//intracellular signal transduction;GO:0035774//positive regulation of insulin secretion invol
033700//phospholipid efflux;GO:0033993//response to lipid;GO:0034374//low-density lipoprotein

ne expression;GO:0010744//positive regulation of macrophage derived foam cell differentiation;G[illegible]

ltage-gated potassium channel activity involved in ventricular cardiac muscle cell action potential

)07186//G-protein coupled receptor signaling pathway;GO:0007229//integrin-mediated signaling

olated;GO:0050775//positive regulation of dendrite morphogenesis;GO:0050890//cognition;GO:00

apoptotic process;GO:0043122//regulation of I-kappaB kinase/NF-kappaB signaling;GO:0045214

//positive regulation of gene expression;GO:0010862//positive regulation of pathway-restricted SM

granulocyte macrophage colony-stimulating factor production;GO:0032729//positive regulation of
gnaling pathway;GO:0035729//cellular response to hepatocyte growth factor stimulus;GO:0042593

diac muscle hypertrophy;GO:0010628//positive regulation of gene expression;GO:0010629//negat

process;GO:0042593//glucose homeostasis;GO:0042594//response to starvation;GO:0045124//reg

ehavior;GO:0043407//negative regulation of MAP kinase activity;GO:0050905//neuromuscular pro

regulation of hormone levels;GO:0014015//positive regulation of gliogenesis;GO:0033690//positive

GO:0007431//salivary gland development;GO:0007435//salivary gland morphogenesis;GO:0008284

GO:0042997//negative regulation of Golgi to plasma membrane protein transport;GO:0048286//lu

diac muscle hypertrophy;GO:0010628//positive regulation of gene expression;GO:0010629//negat

//xenobiotic catabolic process;GO:0042572//retinol metabolic process;GO:0046483//heterocycle m

/cerebral cortex cell migration;GO:0021799//cerebral cortex radially oriented cell migration;GO:002

)33700//phospholipid efflux;GO:0033993//response to lipid;GO:0034374//low-density lipoprotein p

lyceride metabolic process;GO:1903725//regulation of phospholipid metabolic process

/cerebral cortex cell migration;GO:0021799//cerebral cortex radially oriented cell migration;GO:002

2//cilium organization;GO:0045184//establishment of protein localization;GO:0048568//embryoni

c process;GO:0060271//cilium morphogenesis;GO:1902915//negative regulation of protein polyub

gnaling pathway;GO:0035729//cellular response to hepatocyte growth factor stimulus;GO:0042593
granulocyte macrophage colony-stimulating factor production;GO:0032729//positive regulation o

process;GO:0042593//glucose homeostasis;GO:0042594//response to starvation;GO:0045124//reg

//positive regulation of gene expression;GO:0010862//positive regulation of pathway-restricted SI

diac muscle hypertrophy;GO:0010628//positive regulation of gene expression;GO:0010629//negat

: process;GO:0019430//removal of superoxide radicals;GO:0030300//regulation of intestinal choles
iokine production;GO:0034371//chylomicron remodeling;GO:0034372//very-low-density lipoprote

)07186//G-protein coupled receptor signaling pathway;GO:0007229//integrin-mediated signaling

olated;GO:0050775//positive regulation of dendrite morphogenesis;GO:0050890//cognition;GO:00

ory T cell differentiation;GO:0046327//glycerol biosynthetic process from pyruvate;GO:0046890//re

/cerebral cortex cell migration;GO:0021799//cerebral cortex radially oriented cell migration;GO:002

45766//positive regulation of angiogenesis;GO:0045793//positive regulation of cell size;GO:00488

//xenobiotic catabolic process;GO:0042572//retinol metabolic process;GO:0046483//heterocycle m

okine production;GO:0034371//chylomicron remodeling;GO:0034372//very-low-density lipoprote

regulation of hormone levels;GO:0014015//positive regulation of gliogenesis;GO:0033690//positive

ein kinase B signaling;GO:2001240//negative regulation of extrinsic apoptotic signaling pathway in

/cerebral cortex cell migration;GO:0021799//cerebral cortex radially oriented cell migration;GO:002

okine production;GO:0034371//chylomicron remodeling;GO:0034372//very-low-density lipoprote

osynthetic process;GO:0034625//fatty acid elongation, monounsaturated fatty acid;GO:0034626//f

s via desmosterol;GO:0042987//amyloid precursor protein catabolic process;GO:0043066//negative
ucose;GO:0019432//triglyceride biosynthetic process;GO:0040018//positive regulation of multicellu

gnaling pathway;GO:0035729//cellular response to hepatocyte growth factor stimulus;GO:0042593

//positive regulation of gene expression;GO:0010862//positive regulation of pathway-restricted SI

ıokine production;GO:0034371//chylomicron remodeling;GO:0034372//very-low-density lipoprote

' lipoprotein particle receptor catabolic process;GO:0042632//cholesterol homeostasis;GO:0045732
c process;GO:0060271//cilium morphogenesis;GO:1902915//negative regulation of protein polyub

əlated;GO:0050775//positive regulation of dendrite morphogenesis;GO:0050890//cognition;GO:00

əl esterification;GO:0042632//cholesterol homeostasis;GO:0043691//reverse cholesterol transport;

)872//white fat cell differentiation;GO:0071285//cellular response to lithium ion;GO:0071356//cellu

)07186//G-protein coupled receptor signaling pathway;GO:0007229//integrin-mediated signaling

regulation of hormone levels;GO:0014015//positive regulation of gliogenesis;GO:0033690//positive

ıdle assembly;GO:0032414//positive regulation of ion transmembrane transporter activity;GO:0045

ndle assembly;GO:0032414//positive regulation of ion transmembrane transporter activity;GO:004[illegible]

[illegible]O:0007431//salivary gland development;GO:0007435//salivary gland morphogenesis;GO:0008284

[illegible]45766//positive regulation of angiogenesis;GO:0045793//positive regulation of cell size;GO:00488[illegible]

/regulation of ventricular cardiac muscle cell membrane depolarization;GO:0061337//cardiac cond

;//embryonic organ development;GO:0048598//embryonic morphogenesis;GO:0048729//tissue mo
'/cerebellum morphogenesis;GO:0043506//regulation of JUN kinase activity;GO:0050667//homocy

on of sour taste;GO:0051262//protein tetramerization;GO:0051289//protein homotetramerization;G

08217//regulation of blood pressure;GO:0008277//regulation of G-protein coupled receptor prote

olated;GO:0050775//positive regulation of dendrite morphogenesis;GO:0050890//cognition;GO:00

okine production;GO:0034371//chylomicron remodeling;GO:0034372//very-low-density lipoprote

)07186//G-protein coupled receptor signaling pathway;GO:0007229//integrin-mediated signaling

ndle assembly;GO:0032414//positive regulation of ion transmembrane transporter activity;GO:0045

apoptotic process;GO:0043122//regulation of I-kappaB kinase/NF-kappaB signaling;GO:0045214

lyceride metabolic process;GO:1903725//regulation of phospholipid metabolic process

ehavior;GO:0043407//negative regulation of MAP kinase activity;GO:0050905//neuromuscular pro
nt;GO:0035024//negative regulation of Rho protein signal transduction;GO:0048012//hepatocyte

)//anterior commissure morphogenesis;GO:0030178//negative regulation of Wnt signaling pathwa

iotaxis;GO:0030595//leukocyte chemotaxis;GO:0043547//positive regulation of GTPase activity;GO

lipoprotein particle receptor catabolic process;GO:0042632//cholesterol homeostasis;GO:004573

ltage-gated potassium channel activity involved in ventricular cardiac muscle cell action potential

lipoprotein particle receptor catabolic process;GO:0042632//cholesterol homeostasis;GO:004573
//xenobiotic catabolic process;GO:0042572//retinol metabolic process;GO:0046483//heterocycle m

ol esterification;GO:0042632//cholesterol homeostasis;GO:0043691//reverse cholesterol transport;

08217//regulation of blood pressure;GO:0008277//regulation of G-protein coupled receptor prot

ansduction;GO:0050773//regulation of dendrite development;GO:0051654//establishment of mitc

ed signaling pathway;GO:0042327//positive regulation of phosphorylation;GO:0048813//dendrite

regulation of hormone levels;GO:0014015//positive regulation of gliogenesis;GO:0033690//positive

gulation of angiogenesis;GO:0050731//positive regulation of peptidyl-tyrosine phosphorylation;G

regulation of glucose import;GO:0046330//positive regulation of JNK cascade;GO:0070374//posit

expression;GO:0014068//positive regulation of phosphatidylinositol 3-kinase signaling;GO:00181[illegible]
of phagocytosis;GO:0050773//regulation of dendrite development;GO:0060267//positive regulatio

yceride catabolic process;GO:0034389//lipid particle organization;GO:0036153//triglyceride acyl-c

ɔl esterification;GO:0042632//cholesterol homeostasis;GO:0043691//reverse cholesterol transport;

23//mesonephros development;GO:0002088//lens development in camera-type eye;GO:0002520/

//xenobiotic catabolic process;GO:0042572//retinol metabolic process;GO:0046483//heterocycle m
)07186//G-protein coupled receptor signaling pathway;GO:0007229//integrin-mediated signaling

lipoprotein particle receptor catabolic process;GO:0042632//cholesterol homeostasis;GO:004573

ed signaling pathway;GO:0042327//positive regulation of phosphorylation;GO:0048813//dendrite

olated;GO:0050775//positive regulation of dendrite morphogenesis;GO:0050890//cognition;GO:00

/cerebellum morphogenesis;GO:0043506//regulation of JUN kinase activity;GO:0050667//homocy
'regulation of ventricular cardiac muscle cell membrane depolarization;GO:0061337//cardiac cond

ion of organ growth;GO:0048644//muscle organ morphogenesis;GO:0060124//positive regulation

regulation of hormone levels;GO:0014015//positive regulation of gliogenesis;GO:0033690//positive

ition of ion transport;GO:0071407//cellular response to organic cyclic compound;GO:0071415//cel

45766//positive regulation of angiogenesis;GO:0045793//positive regulation of cell size;GO:00488

)042573//retinoic acid metabolic process;GO:0042574//retinal metabolic process;GO:0043065//pos

ed signaling pathway;GO:0042327//positive regulation of phosphorylation;GO:0048813//dendrite

process;GO:0042593//glucose homeostasis;GO:0042594//response to starvation;GO:0045124//reg

)//anterior commissure morphogenesis;GO:0030178//negative regulation of Wnt signaling pathwa

gulation of angiogenesis;GO:0050731//positive regulation of peptidyl-tyrosine phosphorylation;G

diac muscle hypertrophy;GO:0010628//positive regulation of gene expression;GO:0010629//negat

expression;GO:0014068//positive regulation of phosphatidylinositol 3-kinase signaling;GO:001814
gnaling pathway;GO:0035729//cellular response to hepatocyte growth factor stimulus;GO:0042593

//positive regulation of gene expression;GO:0010862//positive regulation of pathway-restricted SI

//positive regulation of gene expression;GO:0010862//positive regulation of pathway-restricted SI

gnaling pathway;GO:0035729//cellular response to hepatocyte growth factor stimulus;GO:0042593

//cerebellum morphogenesis;GO:0043506//regulation of JUN kinase activity;GO:0050667//homocy

ocess;GO:0043547//positive regulation of GTPase activity;GO:0045666//positive regulation of neur

insulin receptor signaling pathway;GO:0048009//insulin-like growth factor receptor signaling path

45766//positive regulation of angiogenesis;GO:0045793//positive regulation of cell size;GO:00488

ed signaling pathway;GO:0042327//positive regulation of phosphorylation;GO:0048813//dendrite

ucose;GO:0019432//triglyceride biosynthetic process;GO:0040018//positive regulation of multicellu

ol esterification;GO:0042632//cholesterol homeostasis;GO:0043691//reverse cholesterol transport;

process;GO:0042593//glucose homeostasis;GO:0042594//response to starvation;GO:0045124//reg

ation of ion transport;GO:0071407//cellular response to organic cyclic compound;GO:0071415//cel

)07186//G-protein coupled receptor signaling pathway;GO:0007229//integrin-mediated signaling

tic process;GO:0046326//positive regulation of glucose import;GO:0046628//positive regulation of

olated;GO:0050775//positive regulation of dendrite morphogenesis;GO:0050890//cognition;GO:00

or production;GO:0043588//skin development;GO:0045454//cell redox homeostasis;GO:0055085//

lyceride metabolic process;GO:1903725//regulation of phospholipid metabolic process

GO:0044524//protein sulfhydration;GO:0051092//positive regulation of NF-kappaB transcription fa

45766//positive regulation of angiogenesis;GO:0045793//positive regulation of cell size;GO:00488

gnaling pathway;GO:0035729//cellular response to hepatocyte growth factor stimulus;GO:0042593

particle remodeling;GO:0042632//cholesterol homeostasis;GO:0070328//triglyceride homeostasis
//positive regulation of gene expression;GO:0010862//positive regulation of pathway-restricted SI

lipoprotein particle receptor catabolic process;GO:0042632//cholesterol homeostasis;GO:004573

process;GO:0042593//glucose homeostasis;GO:0042594//response to starvation;GO:0045124//reg

//positive regulation of gene expression;GO:0010862//positive regulation of pathway-restricted SI

ɡnaling pathway;GO:0035729//cellular response to hepatocyte growth factor stimulus;GO:0042593

:0035456//response to interferon-beta;GO:0038061//NIK/NF-kappaB signaling;GO:0043123//posit

of lipid storage;GO:0045745//positive regulation of G-protein coupled receptor protein signaling p

277//regulation of G-protein coupled receptor protein signaling pathway;GO:0010628//positive re

ory T cell differentiation;GO:0046327//glycerol biosynthetic process from pyruvate;GO:0046890//re

egative regulation of cell death;GO:0070254//mucus secretion;GO:1903078//positive regulation of

ol esterification;GO:0042632//cholesterol homeostasis;GO:0043691//reverse cholesterol transport;[illegible]

or production;GO:0043588//skin development;GO:0045454//cell redox homeostasis;GO:0055085/[illegible]

45766//positive regulation of angiogenesis;GO:0045793//positive regulation of cell size;GO:00488[illegible]

particle remodeling;GO:0042632//cholesterol homeostasis;GO:0070328//triglyceride homeostasis

/cerebral cortex cell migration;GO:0021799//cerebral cortex radially oriented cell migration;GO:002[illegible]

//xenobiotic catabolic process;GO:0042572//retinol metabolic process;GO:0046483//heterocycle m

 lipoprotein particle receptor catabolic process;GO:0042632//cholesterol homeostasis;GO:004573

7165//signal transduction;GO:0007281//germ cell development;GO:0008284//positive regulation c

granulocyte macrophage colony-stimulating factor production;GO:0032729//positive regulation o

ltage-gated potassium channel activity involved in ventricular cardiac muscle cell action potential

n;GO:0045597//positive regulation of cell differentiation;GO:0046426//negative regulation of JAK-

45766//positive regulation of angiogenesis;GO:0045793//positive regulation of cell size;GO:00488

ı of endothelial cell migration;GO:0010628//positive regulation of gene expression;GO:0030154//c

.00//positive regulation of phagocytosis, engulfment;GO:0071222//cellular response to lipopolysac

c process;GO:0060271//cilium morphogenesis;GO:1902915//negative regulation of protein polyub

ell proliferation;GO:0043434//response to peptide hormone;GO:0045944//positive regulation of tr

0010467//gene expression;GO:0033627//cell adhesion mediated by integrin;GO:0042060//wound

lipoprotein particle receptor catabolic process;GO:0042632//cholesterol homeostasis;GO:004573

olated;GO:0050775//positive regulation of dendrite morphogenesis;GO:0050890//cognition;GO:00
//xenobiotic catabolic process;GO:0042572//retinol metabolic process;GO:0046483//heterocycle m
//positive regulation of gene expression;GO:0010862//positive regulation of pathway-restricted SM

granulocyte macrophage colony-stimulating factor production;GO:0032729//positive regulation o

diac muscle hypertrophy;GO:0010628//positive regulation of gene expression;GO:0010629//negat

007186//G-protein coupled receptor signaling pathway;GO:0007229//integrin-mediated signaling

gnaling pathway;GO:0035729//cellular response to hepatocyte growth factor stimulus;GO:004259
035556//intracellular signal transduction;GO:0035774//positive regulation of insulin secretion invol

277//regulation of G-protein coupled receptor protein signaling pathway;GO:0010628//positive re

ndle assembly;GO:0032414//positive regulation of ion transmembrane transporter activity;GO:0045

n;GO:0045597//positive regulation of cell differentiation;GO:0046426//negative regulation of JAK-

regulation of hormone levels;GO:0014015//positive regulation of gliogenesis;GO:0033690//positive

23//mesonephros development;GO:0002088//lens development in camera-type eye;GO:0002520/

oltage-gated potassium channel activity involved in ventricular cardiac muscle cell action potential

diac muscle hypertrophy;GO:0010628//positive regulation of gene expression;GO:0010629//negat

n of endothelial cell migration;GO:0010628//positive regulation of gene expression;GO:0030154//c

rocess;GO:0070409//carbamoyl phosphate biosynthetic process;GO:0071242//cellular response to

.00//positive regulation of phagocytosis, engulfment;GO:0071222//cellular response to lipopolysac

olated;GO:0050775//positive regulation of dendrite morphogenesis;GO:0050890//cognition;GO:00

007186//G-protein coupled receptor signaling pathway;GO:0007229//integrin-mediated signaling

aling;GO:0038093//Fc receptor signaling pathway;GO:0038127//ERBB signaling pathway;GO:0043

: process;GO:0019430//removal of superoxide radicals;GO:0030300//regulation of intestinal choles

ıdle assembly;GO:0032414//positive regulation of ion transmembrane transporter activity;GO:0045

ı33700//phospholipid efflux;GO:0033993//response to lipid;GO:0034374//low-density lipoprotein p
:ion from RNA polymerase II promoter;GO:0071404//cellular response to low-density lipoprotein p

n cytoskeleton organization;GO:0042060//wound healing;GO:0043547//positive regulation of GTP
)//anterior commissure morphogenesis;GO:0030178//negative regulation of Wnt signaling pathwa

process;GO:0046597//negative regulation of viral entry into host cell;GO:0050766//positive regulat

ltage-gated potassium channel activity involved in ventricular cardiac muscle cell action potential
otaxis;GO:0030595//leukocyte chemotaxis;GO:0043547//positive regulation of GTPase activity;GO

)872//white fat cell differentiation;GO:0071285//cellular response to lithium ion;GO:0071356//cellu

tic process;GO:0046326//positive regulation of glucose import;GO:0046628//positive regulation of

granulocyte macrophage colony-stimulating factor production;GO:0032729//positive regulation of
//xenobiotic catabolic process;GO:0042572//retinol metabolic process;GO:0046483//heterocycle m

73//positive regulation of axon extension;GO:0051963//regulation of synapse assembly;GO:00616

)35556//intracellular signal transduction;GO:0035774//positive regulation of insulin secretion invol

ɔl esterification;GO:0042632//cholesterol homeostasis;GO:0043691//reverse cholesterol transport;

O:0010977//negative regulation of neuron projection development;GO:0019222//regulation of me

n cytoskeleton organization;GO:0042060//wound healing;GO:0043547//positive regulation of GTP

:0035456//response to interferon-beta;GO:0038061//NIK/NF-kappaB signaling;GO:0043123//posit

ɔf lipid storage;GO:0045745//positive regulation of G-protein coupled receptor protein signaling

aling;GO:0038093//Fc receptor signaling pathway;GO:0038127//ERBB signaling pathway;GO:00436

plated;GO:0050775//positive regulation of dendrite morphogenesis;GO:0050890//cognition;GO:00

)07186//G-protein coupled receptor signaling pathway;GO:0007229//integrin-mediated signaling

photoreceptor cell development;GO:0048675//axon extension;GO:0048813//dendrite morphogen
d storage;GO:0030335//positive regulation of cell migration;GO:0042632//cholesterol homeostasis

granulocyte macrophage colony-stimulating factor production;GO:0032729//positive regulation o

ɔl esterification;GO:0042632//cholesterol homeostasis;GO:0043691//reverse cholesterol transport;

)35556//intracellular signal transduction;GO:0035774//positive regulation of insulin secretion invol

)872//white fat cell differentiation;GO:0071285//cellular response to lithium ion;GO:0071356//cellu

: process;GO:0019430//removal of superoxide radicals;GO:0030300//regulation of intestinal choles
regulation of hormone levels;GO:0014015//positive regulation of gliogenesis;GO:0033690//positive

ndle assembly;GO:0032414//positive regulation of ion transmembrane transporter activity;GO:004

ndle assembly;GO:0032414//positive regulation of ion transmembrane transporter activity;GO:004

)07186//G-protein coupled receptor signaling pathway;GO:0007229//integrin-mediated signaling

)33700//phospholipid efflux;GO:0033993//response to lipid;GO:0034374//low-density lipoprotein

ıokine production;GO:0034371//chylomicron remodeling;GO:0034372//very-low-density lipoprote
ɔcess;GO:0043547//positive regulation of GTPase activity;GO:0045666//positive regulation of neur

)872//white fat cell differentiation;GO:0071285//cellular response to lithium ion;GO:0071356//cellu

olated;GO:0050775//positive regulation of dendrite morphogenesis;GO:0050890//cognition;GO:00

diac muscle hypertrophy;GO:0010628//positive regulation of gene expression;GO:0010629//negat

insulin receptor signaling pathway;GO:0048009//insulin-like growth factor receptor signaling path

'/cerebellum morphogenesis;GO:0043506//regulation of JUN kinase activity;GO:0050667//homocy

ıokine production;GO:0034371//chylomicron remodeling;GO:0034372//very-low-density lipoprote

.00//positive regulation of phagocytosis, engulfment;GO:0071222//cellular response to lipopolysac

granulocyte macrophage colony-stimulating factor production;GO:0032729//positive regulation of

ndle assembly;GO:0032414//positive regulation of ion transmembrane transporter activity;GO:0045

ed signaling pathway;GO:0042327//positive regulation of phosphorylation;GO:0048813//dendrite

23//mesonephros development;GO:0002088//lens development in camera-type eye;GO:0002520/

GO:0044524//protein sulfhydration;GO:0051092//positive regulation of NF-kappaB transcription fa

gene expression;GO:0021766//hippocampus development;GO:0021895//cerebral cortex neuron di

ol esterification;GO:0042632//cholesterol homeostasis;GO:0043691//reverse cholesterol transport;

process;GO:0042593//glucose homeostasis;GO:0042594//response to starvation;GO:0045124//reg

33700//phospholipid efflux;GO:0033993//response to lipid;GO:0034374//low-density lipoprotein

egulation of peptidyl-serine phosphorylation;GO:0035729//cellular response to hepatocyte growt

egative regulation of cell death;GO:0070254//mucus secretion;GO:1903078//positive regulation of

diac muscle hypertrophy;GO:0010628//positive regulation of gene expression;GO:0010629//negat

/cerebral cortex cell migration;GO:0021799//cerebral cortex radially oriented cell migration;GO:002
//xenobiotic catabolic process;GO:0042572//retinol metabolic process;GO:0046483//heterocycle m

l esterification;GO:0042632//cholesterol homeostasis;GO:0043691//reverse cholesterol transport;

ed signaling pathway;GO:0042327//positive regulation of phosphorylation;GO:0048813//dendrite

ization behavior;GO:0097104//postsynaptic membrane assembly;GO:0097105//presynaptic memb

olated;GO:0050775//positive regulation of dendrite morphogenesis;GO:0050890//cognition;GO:00

ol esterification;GO:0042632//cholesterol homeostasis;GO:0043691//reverse cholesterol transport;

)07186//G-protein coupled receptor signaling pathway;GO:0007229//integrin-mediated signaling

process;GO:0042593//glucose homeostasis;GO:0042594//response to starvation;GO:0045124//reg

by cell;GO:0033700//phospholipid efflux;GO:0035627//ceramide transport;GO:0043129//surfactan

0010467//gene expression;GO:0033627//cell adhesion mediated by integrin;GO:0042060//wound

//xenobiotic catabolic process;GO:0042572//retinol metabolic process;GO:0046483//heterocycle m

differentiation;GO:0043113//receptor clustering;GO:0050901//leukocyte tethering or rolling;GO:00

granulocyte macrophage colony-stimulating factor production;GO:0032729//positive regulation of

ed signaling pathway;GO:0042327//positive regulation of phosphorylation;GO:0048813//dendrite

regulation of peptidyl-serine phosphorylation;GO:0035729//cellular response to hepatocyte growt

23//mesonephros development;GO:0002088//lens development in camera-type eye;GO:0002520/

process;GO:0046597//negative regulation of viral entry into host cell;GO:0050766//positive regulat

lyceride metabolic process;GO:1903725//regulation of phospholipid metabolic process

d storage;GO:0030335//positive regulation of cell migration;GO:0042632//cholesterol homeostasis

/cerebral cortex cell migration;GO:0021799//cerebral cortex radially oriented cell migration;GO:002

process;GO:0042593//glucose homeostasis;GO:0042594//response to starvation;GO:0045124//reg

okine production;GO:0034371//chylomicron remodeling;GO:0034372//very-low-density lipoprote
photoreceptor cell development;GO:0048675//axon extension;GO:0048813//dendrite morphogen

gnaling pathway;GO:0035729//cellular response to hepatocyte growth factor stimulus;GO:0042593

diac muscle hypertrophy;GO:0010628//positive regulation of gene expression;GO:0010629//negat

diac muscle hypertrophy;GO:0010628//positive regulation of gene expression;GO:0010629//negat

//positive regulation of gene expression;GO:0010862//positive regulation of pathway-restricted S

particle remodeling;GO:0042632//cholesterol homeostasis;GO:0070328//triglyceride homeostasis
gulation of angiogenesis;GO:0050731//positive regulation of peptidyl-tyrosine phosphorylation;G

ry T cell differentiation;GO:0046327//glycerol biosynthetic process from pyruvate;GO:0046890//re

xpression;GO:0014068//positive regulation of phosphatidylinositol 3-kinase signaling;GO:00181[illegible]

naling pathway;GO:0035729//cellular response to hepatocyte growth factor stimulus;GO:0042593

ization behavior;GO:0097104//postsynaptic membrane assembly;GO:0097105//presynaptic memb

//positive regulation of gene expression;GO:0010862//positive regulation of pathway-restricted S[illegible]

45766//positive regulation of angiogenesis;GO:0045793//positive regulation of cell size;GO:00488[illegible]

diac muscle hypertrophy;GO:0010628//positive regulation of gene expression;GO:0010629//negat

DNA binding transcription factor activity;GO:0045892//negative regulation of transcription, DNA-

ition of ion transport;GO:0071407//cellular response to organic cyclic compound;GO:0071415//cel

okine production;GO:0034371//chylomicron remodeling;GO:0034372//very-low-density lipoprote

ucose;GO:0019432//triglyceride biosynthetic process;GO:0040018//positive regulation of multicellu

ell proliferation;GO:0043434//response to peptide hormone;GO:0045944//positive regulation of tr

45766//positive regulation of angiogenesis;GO:0045793//positive regulation of cell size;GO:00488

lipoprotein particle receptor catabolic process;GO:0042632//cholesterol homeostasis;GO:004573

photoreceptor cell development;GO:0048675//axon extension;GO:0048813//dendrite morphogen

rocess;GO:0070409//carbamoyl phosphate biosynthetic process;GO:0071242//cellular response to

okine production;GO:0034371//chylomicron remodeling;GO:0034372//very-low-density lipoprote

by cell;GO:0033700//phospholipid efflux;GO:0035627//ceramide transport;GO:0043129//surfactan

872//white fat cell differentiation;GO:0071285//cellular response to lithium ion;GO:0071356//cellu

mitochondria-nucleus signaling pathway;GO:0032869//cellular response to insulin stimulus;GO:00

lipoprotein particle receptor catabolic process;GO:0042632//cholesterol homeostasis;GO:004573

particle remodeling;GO:0042632//cholesterol homeostasis;GO:0070328//triglyceride homeostasis
ltage-gated potassium channel activity involved in ventricular cardiac muscle cell action potential

gulation of angiogenesis;GO:0050731//positive regulation of peptidyl-tyrosine phosphorylation;G

okine production;GO:0034371//chylomicron remodeling;GO:0034372//very-low-density lipoprote

DNA binding transcription factor activity;GO:0045892//negative regulation of transcription, DNA-

expression;GO:0014068//positive regulation of phosphatidylinositol 3-kinase signaling;GO:001814

diac muscle hypertrophy;GO:0010628//positive regulation of gene expression;GO:0010629//negat

regulation of ventricular cardiac muscle cell membrane depolarization;GO:0061337//cardiac cond

mitochondria-nucleus signaling pathway;GO:0032869//cellular response to insulin stimulus;GO:00

lipoprotein particle receptor catabolic process;GO:0042632//cholesterol homeostasis;GO:004573

O:0007431//salivary gland development;GO:0007435//salivary gland morphogenesis;GO:0008284

yceride catabolic process;GO:0034389//lipid particle organization;GO:0036153//triglyceride acyl-

45766//positive regulation of angiogenesis;GO:0045793//positive regulation of cell size;GO:00488

lipoprotein particle receptor catabolic process;GO:0042632//cholesterol homeostasis;GO:004573

ed signaling pathway;GO:0042327//positive regulation of phosphorylation;GO:0048813//dendrite

/cerebellum morphogenesis;GO:0043506//regulation of JUN kinase activity;GO:0050667//homocy

ıtion of ion transport;GO:0071407//cellular response to organic cyclic compound;GO:0071415//cel
granulocyte macrophage colony-stimulating factor production;GO:0032729//positive regulation o

ıdle assembly;GO:0032414//positive regulation of ion transmembrane transporter activity;GO:004[illegible]

)35556//intracellular signal transduction;GO:0035774//positive regulation of insulin secretion invol

appaB transcription factor activity;GO:0071346//cellular response to interferon-gamma

ıotaxis;GO:0030595//leukocyte chemotaxis;GO:0043547//positive regulation of GTPase activity;GO

granulocyte macrophage colony-stimulating factor production;GO:0032729//positive regulation o

ıotaxis;GO:0030595//leukocyte chemotaxis;GO:0043547//positive regulation of GTPase activity;GO

7165//signal transduction;GO:0007281//germ cell development;GO:0008284//positive regulation c

ational protein modification;GO:1904027//negative regulation of collagen fibril organization

gulation of angiogenesis;GO:0050731//positive regulation of peptidyl-tyrosine phosphorylation;G[illegible]

regulation of hormone levels;GO:0014015//positive regulation of gliogenesis;GO:0033690//positive

07186//G-protein coupled receptor signaling pathway;GO:0007229//integrin-mediated signaling

//anterior commissure morphogenesis;GO:0030178//negative regulation of Wnt signaling pathwa

expression;GO:0014068//positive regulation of phosphatidylinositol 3-kinase signaling;GO:001814[illegible]
okine production;GO:0034371//chylomicron remodeling;GO:0034372//very-low-density lipoprote
plated;GO:0050775//positive regulation of dendrite morphogenesis;GO:0050890//cognition;GO:00

rocess;GO:0070409//carbamoyl phosphate biosynthetic process;GO:0071242//cellular response to

//positive regulation of gene expression;GO:0010862//positive regulation of pathway-restricted S[illegible]

naling pathway;GO:0035729//cellular response to hepatocyte growth factor stimulus;GO:0042593

[illegible] apoptotic process;GO:0043122//regulation of I-kappaB kinase/NF-kappaB signaling;GO:0045214

[illegible]/cerebral cortex cell migration;GO:0021799//cerebral cortex radially oriented cell migration;GO:002[illegible]

[illegible]//anterior commissure morphogenesis;GO:0030178//negative regulation of Wnt signaling pathwa[illegible]

[illegible]ation of ion transport;GO:0071407//cellular response to organic cyclic compound;GO:0071415//cel[illegible]

[illegible]n;GO:0045597//positive regulation of cell differentiation;GO:0046426//negative regulation of JAK-

45766//positive regulation of angiogenesis;GO:0045793//positive regulation of cell size;GO:00488[illegible]

ndle assembly;GO:0032414//positive regulation of ion transmembrane transporter activity;GO:004[illegible]

23//mesonephros development;GO:0002088//lens development in camera-type eye;GO:0002520/[illegible]

277//regulation of G-protein coupled receptor protein signaling pathway;GO:0010628//positive re[illegible]

regulation of hormone levels;GO:0014015//positive regulation of gliogenesis;GO:0033690//positive[illegible]

07186//G-protein coupled receptor signaling pathway;GO:0007229//integrin-mediated signaling

33700//phospholipid efflux;GO:0033993//response to lipid;GO:0034374//low-density lipoprotein [illegible]

ed signaling pathway;GO:0042327//positive regulation of phosphorylation;GO:0048813//dendrite

n of endothelial cell migration;GO:0010628//positive regulation of gene expression;GO:0030154//c[illegible]
//xenobiotic catabolic process;GO:0042572//retinol metabolic process;GO:0046483//heterocycle m[illegible]

olated;GO:0050775//positive regulation of dendrite morphogenesis;GO:0050890//cognition;GO:00[illegible]

//cerebellum morphogenesis;GO:0043506//regulation of JUN kinase activity;GO:0050667//homocy[illegible]

ed signaling pathway;GO:0042327//positive regulation of phosphorylation;GO:0048813//dendrite

by cell;GO:0033700//phospholipid efflux;GO:0035627//ceramide transport;GO:0043129//surfactan

granulocyte macrophage colony-stimulating factor production;GO:0032729//positive regulation o

otaxis;GO:0030595//leukocyte chemotaxis;GO:0043547//positive regulation of GTPase activity;GO

//positive regulation of gene expression;GO:0010862//positive regulation of pathway-restricted S

n cytoskeleton organization;GO:0042060//wound healing;GO:0043547//positive regulation of GTP

diac muscle hypertrophy;GO:0010628//positive regulation of gene expression;GO:0010629//negat

ion of organ growth;GO:0048644//muscle organ morphogenesis;GO:0060124//positive regulation

jnaling pathway;GO:0035729//cellular response to hepatocyte growth factor stimulus;GO:0042593

differentiation;GO:0043113//receptor clustering;GO:0050901//leukocyte tethering or rolling;GO:00
ed signaling pathway;GO:0042327//positive regulation of phosphorylation;GO:0048813//dendrite

/cerebral cortex cell migration;GO:0021799//cerebral cortex radially oriented cell migration;GO:002

tic process;GO:0046326//positive regulation of glucose import;GO:0046628//positive regulation of

//xenobiotic catabolic process;GO:0042572//retinol metabolic process;GO:0046483//heterocycle m

//xenobiotic catabolic process;GO:0042572//retinol metabolic process;GO:0046483//heterocycle m

)//anterior commissure morphogenesis;GO:0030178//negative regulation of Wnt signaling pathwa
ational protein modification;GO:1904027//negative regulation of collagen fibril organization

ndle assembly;GO:0032414//positive regulation of ion transmembrane transporter activity;GO:004[illegible]

DNA binding transcription factor activity;GO:0045892//negative regulation of transcription, DNA-

n;GO:0045597//positive regulation of cell differentiation;GO:0046426//negative regulation of JAK-

particle remodeling;GO:0042632//cholesterol homeostasis;GO:0070328//triglyceride homeostasis
)042573//retinoic acid metabolic process;GO:0042574//retinal metabolic process;GO:0043065//pos

aling;GO:0038093//Fc receptor signaling pathway;GO:0038127//ERBB signaling pathway;GO:00436

gnaling pathway;GO:0035729//cellular response to hepatocyte growth factor stimulus;GO:0042593

)07186//G-protein coupled receptor signaling pathway;GO:0007229//integrin-mediated signaling

//positive regulation of gene expression;GO:0010862//positive regulation of pathway-restricted SI

ı of endothelial cell migration;GO:0010628//positive regulation of gene expression;GO:0030154//c

olated;GO:0050775//positive regulation of dendrite morphogenesis;GO:0050890//cognition;GO:00

)//anterior commissure morphogenesis;GO:0030178//negative regulation of Wnt signaling pathwa

f apoptotic process;GO:0043122//regulation of I-kappaB kinase/NF-kappaB signaling;GO:0045214

//xenobiotic catabolic process;GO:0042572//retinol metabolic process;GO:0046483//heterocycle m

ndle assembly;GO:0032414//positive regulation of ion transmembrane transporter activity;GO:0045

ɔn of sour taste;GO:0051262//protein tetramerization;GO:0051289//protein homotetramerization;(

particle remodeling;GO:0042632//cholesterol homeostasis;GO:0070328//triglyceride homeostasis
ed signaling pathway;GO:0042327//positive regulation of phosphorylation;GO:0048813//dendrite

ɔl esterification;GO:0042632//cholesterol homeostasis;GO:0043691//reverse cholesterol transport;(

n;GO:0045597//positive regulation of cell differentiation;GO:0046426//negative regulation of JAK-

ɔl esterification;GO:0042632//cholesterol homeostasis;GO:0043691//reverse cholesterol transport;(

//xenobiotic catabolic process;GO:0042572//retinol metabolic process;GO:0046483//heterocycle m

45766//positive regulation of angiogenesis;GO:0045793//positive regulation of cell size;GO:00488[illegible]

insulin receptor signaling pathway;GO:0048009//insulin-like growth factor receptor signaling path

ɟnaling pathway;GO:0035729//cellular response to hepatocyte growth factor stimulus;GO:0042593

ı of endothelial cell migration;GO:0010628//positive regulation of gene expression;GO:0030154//c
'/adenylate cyclase-inhibiting G-protein coupled receptor signaling pathway;GO:0007200//phospl

//positive regulation of gene expression;GO:0010862//positive regulation of pathway-restricted SI

GO:0044524//protein sulfhydration;GO:0051092//positive regulation of NF-kappaB transcription fa

okine production;GO:0034371//chylomicron remodeling;GO:0034372//very-low-density lipoprote

ol esterification;GO:0042632//cholesterol homeostasis;GO:0043691//reverse cholesterol transport;
differentiation;GO:0043113//receptor clustering;GO:0050901//leukocyte tethering or rolling;GO:00

aling;GO:0038093//Fc receptor signaling pathway;GO:0038127//ERBB signaling pathway;GO:00436

ehavior;GO:0043407//negative regulation of MAP kinase activity;GO:0050905//neuromuscular pro
ndle assembly;GO:0032414//positive regulation of ion transmembrane transporter activity;GO:0045

ndle assembly;GO:0032414//positive regulation of ion transmembrane transporter activity;GO:0045

45766//positive regulation of angiogenesis;GO:0045793//positive regulation of cell size;GO:00488

s via desmosterol;GO:0042987//amyloid precursor protein catabolic process;GO:0043066//negative

lipoprotein particle receptor catabolic process;GO:0042632//cholesterol homeostasis;GO:0045732

'/cerebellum morphogenesis;GO:0043506//regulation of JUN kinase activity;GO:0050667//homocy

or production;GO:0043588//skin development;GO:0045454//cell redox homeostasis;GO:0055085/,

ıokine production;GO:0034371//chylomicron remodeling;GO:0034372//very-low-density lipoprote

ed signaling pathway;GO:0042327//positive regulation of phosphorylation;GO:0048813//dendrite

ıltage-gated potassium channel activity involved in ventricular cardiac muscle cell action potential

23//mesonephros development;GO:0002088//lens development in camera-type eye;GO:0002520/
)35556//intracellular signal transduction;GO:0035774//positive regulation of insulin secretion invol

y photoperiod;GO:0043276//anoikis;GO:0045595//regulation of cell differentiation;GO:0045721//
rocess;GO:0070409//carbamoyl phosphate biosynthetic process;GO:0071242//cellular response to
naling pathway;GO:0035729//cellular response to hepatocyte growth factor stimulus;GO:0042593

olated;GO:0050775//positive regulation of dendrite morphogenesis;GO:0050890//cognition;GO:00

//positive regulation of gene expression;GO:0010862//positive regulation of pathway-restricted S

aling;GO:0038093//Fc receptor signaling pathway;GO:0038127//ERBB signaling pathway;GO:0043

007186//G-protein coupled receptor signaling pathway;GO:0007229//integrin-mediated signaling

egulation of peptidyl-serine phosphorylation;GO:0035729//cellular response to hepatocyte growt

ocess;GO:0043547//positive regulation of GTPase activity;GO:0045666//positive regulation of neur

process;GO:0042593//glucose homeostasis;GO:0042594//response to starvation;GO:0045124//reg

differentiation;GO:0043113//receptor clustering;GO:0050901//leukocyte tethering or rolling;GO:00
/cerebral cortex cell migration;GO:0021799//cerebral cortex radially oriented cell migration;GO:002
egulation of peptidyl-serine phosphorylation;GO:0035729//cellular response to hepatocyte growt

' lipoprotein particle receptor catabolic process;GO:0042632//cholesterol homeostasis;GO:004573[illegible]

process;GO:0046597//negative regulation of viral entry into host cell;GO:0050766//positive regulat

//xenobiotic catabolic process;GO:0042572//retinol metabolic process;GO:0046483//heterocycle m

osynthetic process;GO:0034625//fatty acid elongation, monounsaturated fatty acid;GO:0034626//f

y photoperiod;GO:0043276//anoikis;GO:0045595//regulation of cell differentiation;GO:0045721//
45766//positive regulation of angiogenesis;GO:0045793//positive regulation of cell size;GO:00488[illegible]

oltage-gated potassium channel activity involved in ventricular cardiac muscle cell action potential
tion from RNA polymerase II promoter;GO:0071404//cellular response to low-density lipoprotein p

notaxis;GO:0030595//leukocyte chemotaxis;GO:0043547//positive regulation of GTPase activity;GO

gulation of angiogenesis;GO:0050731//positive regulation of peptidyl-tyrosine phosphorylation;G

olated;GO:0050775//positive regulation of dendrite morphogenesis;GO:0050890//cognition;GO:00

granulocyte macrophage colony-stimulating factor production;GO:0032729//positive regulation of

007186//G-protein coupled receptor signaling pathway;GO:0007229//integrin-mediated signaling
diac muscle hypertrophy;GO:0010628//positive regulation of gene expression;GO:0010629//negat
expression;GO:0014068//positive regulation of phosphatidylinositol 3-kinase signaling;GO:001814

apoptotic process;GO:0043122//regulation of I-kappaB kinase/NF-kappaB signaling;GO:0045214

033700//phospholipid efflux;GO:0033993//response to lipid;GO:0034374//low-density lipoprotein

ansduction;GO:0050773//regulation of dendrite development;GO:0051654//establishment of mito

/cerebral cortex cell migration;GO:0021799//cerebral cortex radially oriented cell migration;GO:002

ed signaling pathway;GO:0042327//positive regulation of phosphorylation;GO:0048813//dendrite

egative regulation of cell death;GO:0070254//mucus secretion;GO:1903078//positive regulation of

//cerebellum morphogenesis;GO:0043506//regulation of JUN kinase activity;GO:0050667//homocy

035556//intracellular signal transduction;GO:0035774//positive regulation of insulin secretion invol

granulocyte macrophage colony-stimulating factor production;GO:0032729//positive regulation o

ıtion of ion transport;GO:0071407//cellular response to organic cyclic compound;GO:0071415//cel

)872//white fat cell differentiation;GO:0071285//cellular response to lithium ion;GO:0071356//cellu

photoreceptor cell development;GO:0048675//axon extension;GO:0048813//dendrite morphogen

granulocyte macrophage colony-stimulating factor production;GO:0032729//positive regulation o

lipoprotein particle receptor catabolic process;GO:0042632//cholesterol homeostasis;GO:004573

egulation of peptidyl-serine phosphorylation;GO:0035729//cellular response to hepatocyte growt

rocess;GO:0070409//carbamoyl phosphate biosynthetic process;GO:0071242//cellular response to

ansduction;GO:0050773//regulation of dendrite development;GO:0051654//establishment of mitc
)872//white fat cell differentiation;GO:0071285//cellular response to lithium ion;GO:0071356//cellu

ınaling pathway;GO:0035729//cellular response to hepatocyte growth factor stimulus;GO:0042593

diac muscle hypertrophy;GO:0010628//positive regulation of gene expression;GO:0010629//negat
GO:0042997//negative regulation of Golgi to plasma membrane protein transport;GO:0048286//lu

//positive regulation of gene expression;GO:0010862//positive regulation of pathway-restricted SI

insulin receptor signaling pathway;GO:0048009//insulin-like growth factor receptor signaling path

okine production;GO:0034371//chylomicron remodeling;GO:0034372//very-low-density lipoprote

process;GO:0042593//glucose homeostasis;GO:0042594//response to starvation;GO:0045124//reg

'/adenylate cyclase-inhibiting G-protein coupled receptor signaling pathway;GO:0007200//phospl

)33700//phospholipid efflux;GO:0033993//response to lipid;GO:0034374//low-density lipoprotein p

tic process;GO:0046326//positive regulation of glucose import;GO:0046628//positive regulation of
by cell;GO:0033700//phospholipid efflux;GO:0035627//ceramide transport;GO:0043129//surfactan

ol esterification;GO:0042632//cholesterol homeostasis;GO:0043691//reverse cholesterol transport;(

GO:0044524//protein sulfhydration;GO:0051092//positive regulation of NF-kappaB transcription fa

ol esterification;GO:0042632//cholesterol homeostasis;GO:0043691//reverse cholesterol transport;

ansduction;GO:0050773//regulation of dendrite development;GO:0051654//establishment of mitc

08217//regulation of blood pressure;GO:0008277//regulation of G-protein coupled receptor prot

45766//positive regulation of angiogenesis;GO:0045793//positive regulation of cell size;GO:00488

egulation of peptidyl-serine phosphorylation;GO:0035729//cellular response to hepatocyte growt

DNA binding transcription factor activity;GO:0045892//negative regulation of transcription, DNA-

aling;GO:0038093//Fc receptor signaling pathway;GO:0038127//ERBB signaling pathway;GO:00436

gene expression;GO:0021766//hippocampus development;GO:0021895//cerebral cortex neuron di

0872//white fat cell differentiation;GO:0071285//cellular response to lithium ion;GO:0071356//cellu

DNA binding transcription factor activity;GO:0045892//negative regulation of transcription, DNA-

ansduction;GO:0050773//regulation of dendrite development;GO:0051654//establishment of mitc

granulocyte macrophage colony-stimulating factor production;GO:0032729//positive regulation o

by cell;GO:0033700//phospholipid efflux;GO:0035627//ceramide transport;GO:0043129//surfactan

process;GO:0042593//glucose homeostasis;GO:0042594//response to starvation;GO:0045124//reg

23//mesonephros development;GO:0002088//lens development in camera-type eye;GO:0002520/

photoreceptor cell development;GO:0048675//axon extension;GO:0048813//dendrite morphogen

)872//white fat cell differentiation;GO:0071285//cellular response to lithium ion;GO:0071356//cellu

' lipoprotein particle receptor catabolic process;GO:0042632//cholesterol homeostasis;GO:004573[illegible]

)//anterior commissure morphogenesis;GO:0030178//negative regulation of Wnt signaling pathw[illegible]

ɔl esterification;GO:0042632//cholesterol homeostasis;GO:0043691//reverse cholesterol transport;[illegible]
ıse to stress;GO:0016240//autophagosome docking;GO:0032469//endoplasmic reticulum calcium

O:1902309//negative regulation of peptidyl-serine dephosphorylation;GO:1903589//positive regu

n;GO:0045597//positive regulation of cell differentiation;GO:0046426//negative regulation of JAK-

ɔl esterification;GO:0042632//cholesterol homeostasis;GO:0043691//reverse cholesterol transport;[illegible]

)45956//positive regulation of calcium ion-dependent exocytosis;GO:0055085//transmembrane tra

ı of endothelial cell migration;GO:0010628//positive regulation of gene expression;GO:0030154//c

ı of endothelial cell migration;GO:0010628//positive regulation of gene expression;GO:0030154//c

process;GO:0042593//glucose homeostasis;GO:0042594//response to starvation;GO:0045124//reg

'/cerebellum morphogenesis;GO:0043506//regulation of JUN kinase activity;GO:0050667//homocy

n;GO:0045597//positive regulation of cell differentiation;GO:0046426//negative regulation of JAK-

ction;GO:0045921//positive regulation of exocytosis;GO:0050714//positive regulation of protein se

DNA binding transcription factor activity;GO:0045892//negative regulation of transcription, DNA-

nse to stress;GO:0016240//autophagosome docking;GO:0032469//endoplasmic reticulum calcium

O:1902309//negative regulation of peptidyl-serine dephosphorylation;GO:1903589//positive regu

tiation;GO:0003310//pancreatic A cell differentiation;GO:0006355//regulation of transcription, DN[illegible]

[illegible]ational protein modification;GO:1904027//negative regulation of collagen fibril organization

[illegible]ion of organ growth;GO:0048644//muscle organ morphogenesis;GO:0060124//positive regulation

[illegible]ne expression;GO:0010744//positive regulation of macrophage derived foam cell differentiation;G[illegible]
[illegible] apoptotic process;GO:0043122//regulation of I-kappaB kinase/NF-kappaB signaling;GO:0045214[illegible]

//positive regulation of gene expression;GO:0010862//positive regulation of pathway-restricted S[illegible]

[illegible]rebrain development;GO:0043524//negative regulation of neuron apoptotic process;GO:0090090/

ynaling pathway;GO:0035729//cellular response to hepatocyte growth factor stimulus;GO:0042593

by photoperiod;GO:0043276//anoikis;GO:0045595//regulation of cell differentiation;GO:0045721//

13//cardiac muscle cell development;GO:0072659//protein localization to plasma membrane;GO:0

O:1902309//negative regulation of peptidyl-serine dephosphorylation;GO:1903589//positive regu

)0090//negative regulation of canonical Wnt signaling pathway;GO:2000016//negative regulation

-templated;GO:0045893//positive regulation of transcription, DNA-templated;GO:0046777//prote

nse to stress;GO:0016240//autophagosome docking;GO:0032469//endoplasmic reticulum calcium

of phagocytosis;GO:0050773//regulation of dendrite development;GO:0060267//positive regulatic

on of sour taste;GO:0051262//protein tetramerization;GO:0051289//protein homotetramerization;(

ulation of transcription from RNA polymerase II promoter;GO:0051216//cartilage development

rebrain development;GO:0043524//negative regulation of neuron apoptotic process;GO:0090090/

GO:0042997//negative regulation of Golgi to plasma membrane protein transport;GO:0048286//lu

-templated;GO:0045893//positive regulation of transcription, DNA-templated;GO:0046777//prote

ell proliferation;GO:0043434//response to peptide hormone;GO:0045944//positive regulation of tr

042573//retinoic acid metabolic process;GO:0042574//retinal metabolic process;GO:0043065//pos
y photoperiod;GO:0043276//anoikis;GO:0045595//regulation of cell differentiation;GO:0045721//

GO:0042997//negative regulation of Golgi to plasma membrane protein transport;GO:0048286//lu

muscle tissue morphogenesis;GO:0003228//atrial cardiac muscle tissue development;GO:0003253
7165//signal transduction;GO:0007281//germ cell development;GO:0008284//positive regulation c

ion of organ growth;GO:0048644//muscle organ morphogenesis;GO:0060124//positive regulation

2//cilium organization;GO:0045184//establishment of protein localization;GO:0048568//embryoni[illegible]

ne expression;GO:0010744//positive regulation of macrophage derived foam cell differentiation;G[illegible]

nt;GO:0035024//negative regulation of Rho protein signal transduction;GO:0048012//hepatocyte [illegible]
oliferation;GO:0003382//epithelial cell morphogenesis;GO:0003406//retinal pigment epithelium de[illegible]

\-templated;GO:0045893//positive regulation of transcription, DNA-templated;GO:0046777//prote

olishment of integrated proviral latency;GO:0090305//nucleic acid phosphodiester bond hydrolysis

)042573//retinoic acid metabolic process;GO:0042574//retinal metabolic process;GO:0043065//pos

13//cardiac muscle cell development;GO:0072659//protein localization to plasma membrane;GO:0

'//adenylate cyclase-inhibiting G-protein coupled receptor signaling pathway;GO:0007200//phosp

; via desmosterol;GO:0042987//amyloid precursor protein catabolic process;GO:0043066//negative

osynthetic process;GO:0034625//fatty acid elongation, monounsaturated fatty acid;GO:0034626//f

//xenobiotic catabolic process;GO:0042572//retinol metabolic process;GO:0046483//heterocycle m

of phagocytosis;GO:0050773//regulation of dendrite development;GO:0060267//positive regulatic
)0090//negative regulation of canonical Wnt signaling pathway;GO:2000016//negative regulation

3O:0042997//negative regulation of Golgi to plasma membrane protein transport;GO:0048286//lu

oliferation;GO:0003382//epithelial cell morphogenesis;GO:0003406//retinal pigment epithelium de

//adenylate cyclase-inhibiting G-protein coupled receptor signaling pathway;GO:0007200//phosp

tiation;GO:0003310//pancreatic A cell differentiation;GO:0006355//regulation of transcription, DN

regulation of hormone levels;GO:0014015//positive regulation of gliogenesis;GO:0033690//positive

n cytoskeleton organization;GO:0042060//wound healing;GO:0043547//positive regulation of GTP

O:0010977//negative regulation of neuron projection development;GO:0019222//regulation of me

olishment of integrated proviral latency;GO:0090305//nucleic acid phosphodiester bond hydrolysis

3O:0042997//negative regulation of Golgi to plasma membrane protein transport;GO:0048286//lu
olishment of integrated proviral latency;GO:0090305//nucleic acid phosphodiester bond hydrolysis

ansduction;GO:0050773//regulation of dendrite development;GO:0051654//establishment of mitc

ɔl esterification;GO:0042632//cholesterol homeostasis;GO:0043691//reverse cholesterol transport;(

ıg pathway by p53 class mediator;GO:0072734//cellular response to staurosporine;GO:0097194//e

nt;GO:0035024//negative regulation of Rho protein signal transduction;GO:0048012//hepatocyte ɡ

enosine catabolic process;GO:0006157//deoxyadenosine catabolic process;GO:0006196//AMP cata
of phagocytosis;GO:0050773//regulation of dendrite development;GO:0060267//positive regulatic

muscle tissue morphogenesis;GO:0003228//atrial cardiac muscle tissue development;GO:0003253

O:0010977//negative regulation of neuron projection development;GO:0019222//regulation of me

'/adenylate cyclase-inhibiting G-protein coupled receptor signaling pathway;GO:0007200//phospl

ι-templated;GO:0045893//positive regulation of transcription, DNA-templated;GO:0046777//prote

ulation of transcription from RNA polymerase II promoter;GO:0051216//cartilage development
ιdle assembly;GO:0032414//positive regulation of ion transmembrane transporter activity;GO:0045

ve morphogenesis;GO:0003186//tricuspid valve morphogenesis;GO:0003195//tricuspid valve form

ıse to stress;GO:0016240//autophagosome docking;GO:0032469//endoplasmic reticulum calcium

)0090//negative regulation of canonical Wnt signaling pathway;GO:2000016//negative regulation

O:1902309//negative regulation of peptidyl-serine dephosphorylation;GO:1903589//positive regu

ell proliferation;GO:0043434//response to peptide hormone;GO:0045944//positive regulation of tr

d storage;GO:0030335//positive regulation of cell migration;GO:0042632//cholesterol homeostasis

nt;GO:0035024//negative regulation of Rho protein signal transduction;GO:0048012//hepatocyte g

tiation;GO:0003310//pancreatic A cell differentiation;GO:0006355//regulation of transcription, DN

ne expression;GO:0010744//positive regulation of macrophage derived foam cell differentiation;G[illegible]

[illegible]/adenylate cyclase-inhibiting G-protein coupled receptor signaling pathway;GO:0007200//phosp[illegible]

[illegible]liferation;GO:0003382//epithelial cell morphogenesis;GO:0003406//retinal pigment epithelium de[illegible]

O:0010977//negative regulation of neuron projection development;GO:0019222//regulation of me

nt;GO:0035024//negative regulation of Rho protein signal transduction;GO:0048012//hepatocyte g

d storage;GO:0030335//positive regulation of cell migration;GO:0042632//cholesterol homeostasis

enosine catabolic process;GO:0006157//deoxyadenosine catabolic process;GO:0006196//AMP cata

ng pathway by p53 class mediator;GO:0072734//cellular response to staurosporine;GO:0097194//e

ell proliferation;GO:0043434//response to peptide hormone;GO:0045944//positive regulation of tr

2//cilium organization;GO:0045184//establishment of protein localization;GO:0048568//embryoni

d storage;GO:0030335//positive regulation of cell migration;GO:0042632//cholesterol homeostasis

ulation of transcription from RNA polymerase II promoter;GO:0051216//cartilage development

; via desmosterol;GO:0042987//amyloid precursor protein catabolic process;GO:0043066//negativ[illegible]

nse to stress;GO:0016240//autophagosome docking;GO:0032469//endoplasmic reticulum calcium

tiation;GO:0003310//pancreatic A cell differentiation;GO:0006355//regulation of transcription, DN[illegible]

'9//regulation of calcium ion import;GO:2001256//regulation of store-operated calcium entry;GO:[illegible]

O:1902309//negative regulation of peptidyl-serine dephosphorylation;GO:1903589//positive regu

by photoperiod;GO:0043276//anoikis;GO:0045595//regulation of cell differentiation;GO:0045721//[illegible]
ve morphogenesis;GO:0003186//tricuspid valve morphogenesis;GO:0003195//tricuspid valve form

ubstrate adhesion-dependent cell spreading;GO:2000146//negative regulation of cell motility;GO:2

regulation of glucose import;GO:0046330//positive regulation of JNK cascade;GO:0070374//posit

of phagocytosis;GO:0050773//regulation of dendrite development;GO:0060267//positive regulatio

5638//negative regulation of myeloid cell differentiation;GO:0045646//regulation of erythrocyte di

O:0010977//negative regulation of neuron projection development;GO:0019222//regulation of me

ng pathway by p53 class mediator;GO:0072734//cellular response to staurosporine;GO:0097194//e

GO:0044524//protein sulfhydration;GO:0051092//positive regulation of NF-kappaB transcription fa

pliferation;GO:0003382//epithelial cell morphogenesis;GO:0003406//retinal pigment epithelium de
GO:0042997//negative regulation of Golgi to plasma membrane protein transport;GO:0048286//lu

or production;GO:0043588//skin development;GO:0045454//cell redox homeostasis;GO:0055085/

ɔn of sour taste;GO:0051262//protein tetramerization;GO:0051289//protein homotetramerization;(

)042573//retinoic acid metabolic process;GO:0042574//retinal metabolic process;GO:0043065//pos

ubstrate adhesion-dependent cell spreading;GO:2000146//negative regulation of cell motility;GO:2
2//cilium organization;GO:0045184//establishment of protein localization;GO:0048568//embryonic

ulation of transcription from RNA polymerase II promoter;GO:0051216//cartilage development

'9//regulation of calcium ion import;GO:2001256//regulation of store-operated calcium entry;GO:2
5638//negative regulation of myeloid cell differentiation;GO:0045646//regulation of erythrocyte di

'9//regulation of calcium ion import;GO:2001256//regulation of store-operated calcium entry;GO:2

diac muscle hypertrophy;GO:0010628//positive regulation of gene expression;GO:0010629//negat

ubstrate adhesion-dependent cell spreading;GO:2000146//negative regulation of cell motility;GO:2

insulin receptor signaling pathway;GO:0048009//insulin-like growth factor receptor signaling path

2//cilium organization;GO:0045184//establishment of protein localization;GO:0048568//embryonic

rebrain development;GO:0043524//negative regulation of neuron apoptotic process;GO:0090090/

ce;GO:0032287//peripheral nervous system myelin maintenance;GO:0042127//regulation of cell pr

ansduction;GO:0050773//regulation of dendrite development;GO:0051654//establishment of mitc

\-templated;GO:0045893//positive regulation of transcription, DNA-templated;GO:0046777//prote

GO:0044524//protein sulfhydration;GO:0051092//positive regulation of NF-kappaB transcription fa

GO:0042997//negative regulation of Golgi to plasma membrane protein transport;GO:0048286//lu

2//cilium organization;GO:0045184//establishment of protein localization;GO:0048568//embryoni

)45956//positive regulation of calcium ion-dependent exocytosis;GO:0055085//transmembrane tra

ehavior;GO:0043407//negative regulation of MAP kinase activity;GO:0050905//neuromuscular pro

13//cardiac muscle cell development;GO:0072659//protein localization to plasma membrane;GO:0

7165//signal transduction;GO:0007281//germ cell development;GO:0008284//positive regulation c

ıg pathway by p53 class mediator;GO:0072734//cellular response to staurosporine;GO:0097194//e

GO:0042997//negative regulation of Golgi to plasma membrane protein transport;GO:0048286//lu

insulin receptor signaling pathway;GO:0048009//insulin-like growth factor receptor signaling path

:e;GO:0032287//peripheral nervous system myelin maintenance;GO:0042127//regulation of cell pr

ne expression;GO:0010744//positive regulation of macrophage derived foam cell differentiation;G(

rebrain development;GO:0043524//negative regulation of neuron apoptotic process;GO:0090090/
)0090//negative regulation of canonical Wnt signaling pathway;GO:2000016//negative regulation

mitochondria-nucleus signaling pathway;GO:0032869//cellular response to insulin stimulus;GO:00

)042573//retinoic acid metabolic process;GO:0042574//retinal metabolic process;GO:0043065//pos
116//positive regulation of heterotypic cell-cell adhesion;GO:0036438//maintenance of lens transp

process;GO:0042593//glucose homeostasis;GO:0042594//response to starvation;GO:0045124//reg

ubstrate adhesion-dependent cell spreading;GO:2000146//negative regulation of cell motility;GO:2

ion of organ growth;GO:0048644//muscle organ morphogenesis;GO:0060124//positive regulation

'9//regulation of calcium ion import;GO:2001256//regulation of store-operated calcium entry;GO:[illegible]

2//cilium organization;GO:0045184//establishment of protein localization;GO:0048568//embryoni[illegible]

7165//signal transduction;GO:0007281//germ cell development;GO:0008284//positive regulation [illegible]

ction;GO:0045921//positive regulation of exocytosis;GO:0050714//positive regulation of protein se

n cytoskeleton organization;GO:0042060//wound healing;GO:0043547//positive regulation of GTP
enosine catabolic process;GO:0006157//deoxyadenosine catabolic process;GO:0006196//AMP cat

116//positive regulation of heterotypic cell-cell adhesion;GO:0036438//maintenance of lens transp

ubstrate adhesion-dependent cell spreading;GO:2000146//negative regulation of cell motility;GO:2

7165//signal transduction;GO:0007281//germ cell development;GO:0008284//positive regulation c

otaxis;GO:0030595//leukocyte chemotaxis;GO:0043547//positive regulation of GTPase activity;GO
'9//regulation of calcium ion import;GO:2001256//regulation of store-operated calcium entry;GO:

olishment of integrated proviral latency;GO:0090305//nucleic acid phosphodiester bond hydrolysis
tic process;GO:0046326//positive regulation of glucose import;GO:0046628//positive regulation of

)0090//negative regulation of canonical Wnt signaling pathway;GO:2000016//negative regulation

muscle tissue morphogenesis;GO:0003228//atrial cardiac muscle tissue development;GO:0003253

.00//positive regulation of phagocytosis, engulfment;GO:0071222//cellular response to lipopolysac

)0090//negative regulation of canonical Wnt signaling pathway;GO:2000016//negative regulation

08217//regulation of blood pressure;GO:0008277//regulation of G-protein coupled receptor prot[illegible]

rebrain development;GO:0043524//negative regulation of neuron apoptotic process;GO:0090090/

O:0010977//negative regulation of neuron projection development;GO:0019222//regulation of m[illegible]

of phagocytosis;GO:0050773//regulation of dendrite development;GO:0060267//positive regulati[illegible]

d storage;GO:0030335//positive regulation of cell migration;GO:0042632//cholesterol homeostasis

nt;GO:0035024//negative regulation of Rho protein signal transduction;GO:0048012//hepatocyte g

)042573//retinoic acid metabolic process;GO:0042574//retinal metabolic process;GO:0043065//pos

enosine catabolic process;GO:0006157//deoxyadenosine catabolic process;GO:0006196//AMP cata

ulation of transcription from RNA polymerase II promoter;GO:0051216//cartilage development

O:1902309//negative regulation of peptidyl-serine dephosphorylation;GO:1903589//positive regu

ell proliferation;GO:0043434//response to peptide hormone;GO:0045944//positive regulation of tr

tiation;GO:0003310//pancreatic A cell differentiation;GO:0006355//regulation of transcription, DN

nse to stress;GO:0016240//autophagosome docking;GO:0032469//endoplasmic reticulum calcium

'9//regulation of calcium ion import;GO:2001256//regulation of store-operated calcium entry;GO:

7165//signal transduction;GO:0007281//germ cell development;GO:0008284//positive regulation c

nt;GO:0035024//negative regulation of Rho protein signal transduction;GO:0048012//hepatocyte g

ve morphogenesis;GO:0003186//tricuspid valve morphogenesis;GO:0003195//tricuspid valve form

ubstrate adhesion-dependent cell spreading;GO:2000146//negative regulation of cell motility;GO:2

O:0010977//negative regulation of neuron projection development;GO:0019222//regulation of me

by cell;GO:0033700//phospholipid efflux;GO:0035627//ceramide transport;GO:0043129//surfactan

)0090//negative regulation of canonical Wnt signaling pathway;GO:2000016//negative regulation
of phagocytosis;GO:0050773//regulation of dendrite development;GO:0060267//positive regulatic

ction;GO:0045921//positive regulation of exocytosis;GO:0050714//positive regulation of protein se

muscle tissue morphogenesis;GO:0003228//atrial cardiac muscle tissue development;GO:0003253
)042573//retinoic acid metabolic process;GO:0042574//retinal metabolic process;GO:0043065//pos

ulation of transcription from RNA polymerase II promoter;GO:0051216//cartilage development

d storage;GO:0030335//positive regulation of cell migration;GO:0042632//cholesterol homeostasis

tiation;GO:0003310//pancreatic A cell differentiation;GO:0006355//regulation of transcription, DNA

'73//positive regulation of axon extension;GO:0051963//regulation of synapse assembly;GO:00616

:e;GO:0032287//peripheral nervous system myelin maintenance;GO:0042127//regulation of cell pr

nt;GO:0035024//negative regulation of Rho protein signal transduction;GO:0048012//hepatocyte [illegible]

ell proliferation;GO:0043434//response to peptide hormone;GO:0045944//positive regulation of tr

)0090//negative regulation of canonical Wnt signaling pathway;GO:2000016//negative regulation

particle remodeling;GO:0042632//cholesterol homeostasis;GO:0070328//triglyceride homeostasis

mitochondria-nucleus signaling pathway;GO:0032869//cellular response to insulin stimulus;GO:00
O:0010977//negative regulation of neuron projection development;GO:0019222//regulation of me

'9//regulation of calcium ion import;GO:2001256//regulation of store-operated calcium entry;GO:

ulation of transcription from RNA polymerase II promoter;GO:0051216//cartilage development

muscle tissue morphogenesis;GO:0003228//atrial cardiac muscle tissue development;GO:0003253

ubstrate adhesion-dependent cell spreading;GO:2000146//negative regulation of cell motility;GO:2

osynthetic process;GO:0034625//fatty acid elongation, monounsaturated fatty acid;GO:0034626//f

2//cilium organization;GO:0045184//establishment of protein localization;GO:0048568//embryonic

of phagocytosis;GO:0050773//regulation of dendrite development;GO:0060267//positive regulatio

tiation;GO:0003310//pancreatic A cell differentiation;GO:0006355//regulation of transcription, DNA

or production;GO:0043588//skin development;GO:0045454//cell redox homeostasis;GO:0055085//

d storage;GO:0030335//positive regulation of cell migration;GO:0042632//cholesterol homeostasis
116//positive regulation of heterotypic cell-cell adhesion;GO:0036438//maintenance of lens transp
d storage;GO:0030335//positive regulation of cell migration;GO:0042632//cholesterol homeostasis

yceride catabolic process;GO:0034389//lipid particle organization;GO:0036153//triglyceride acyl-c

0042573//retinoic acid metabolic process;GO:0042574//retinal metabolic process;GO:0043065//pos

007186//G-protein coupled receptor signaling pathway;GO:0007229//integrin-mediated signaling

okine production;GO:0034371//chylomicron remodeling;GO:0034372//very-low-density lipoprote

olated;GO:0050775//positive regulation of dendrite morphogenesis;GO:0050890//cognition;GO:00

5638//negative regulation of myeloid cell differentiation;GO:0045646//regulation of erythrocyte di

ce;GO:0032287//peripheral nervous system myelin maintenance;GO:0042127//regulation of cell pr

process;GO:0046597//negative regulation of viral entry into host cell;GO:0050766//positive regulat

90090//negative regulation of canonical Wnt signaling pathway;GO:2000016//negative regulation

osynthetic process;GO:0034625//fatty acid elongation, monounsaturated fatty acid;GO:0034626//f

ell proliferation;GO:0043434//response to peptide hormone;GO:0045944//positive regulation of tr

\-templated;GO:0045893//positive regulation of transcription, DNA-templated;GO:0046777//prote

tiation;GO:0003310//pancreatic A cell differentiation;GO:0006355//regulation of transcription, DN/

7165//signal transduction;GO:0007281//germ cell development;GO:0008284//positive regulation c

ulation of transcription from RNA polymerase II promoter;GO:0051216//cartilage development

2//cilium organization;GO:0045184//establishment of protein localization;GO:0048568//embryoni

13//cardiac muscle cell development;GO:0072659//protein localization to plasma membrane;GO:0

ion of organ growth;GO:0048644//muscle organ morphogenesis;GO:0060124//positive regulation

2//cilium organization;GO:0045184//establishment of protein localization;GO:0048568//embryoni

)0090//negative regulation of canonical Wnt signaling pathway;GO:2000016//negative regulation

)liferation;GO:0003382//epithelial cell morphogenesis;GO:0003406//retinal pigment epithelium dev

116//positive regulation of heterotypic cell-cell adhesion;GO:0036438//maintenance of lens transp

d storage;GO:0030335//positive regulation of cell migration;GO:0042632//cholesterol homeostasis

)rocess;GO:0046597//negative regulation of viral entry into host cell;GO:0050766//positive regulat

ehavior;GO:0043407//negative regulation of MAP kinase activity;GO:0050905//neuromuscular pro

23//mesonephros development;GO:0002088//lens development in camera-type eye;GO:0002520/

ne expression;GO:0010744//positive regulation of macrophage derived foam cell differentiation;G

-templated;GO:0045893//positive regulation of transcription, DNA-templated;GO:0046777//prote

ulation of transcription from RNA polymerase II promoter;GO:0051216//cartilage development

7165//signal transduction;GO:0007281//germ cell development;GO:0008284//positive regulation c

13//cardiac muscle cell development;GO:0072659//protein localization to plasma membrane;GO:0

d storage;GO:0030335//positive regulation of cell migration;GO:0042632//cholesterol homeostasis
by photoperiod;GO:0043276//anoikis;GO:0045595//regulation of cell differentiation;GO:0045721//

›rocess;GO:0046597//negative regulation of viral entry into host cell;GO:0050766//positive regulat

ehavior;GO:0043407//negative regulation of MAP kinase activity;GO:0050905//neuromuscular pro

lyceride metabolic process;GO:1903725//regulation of phospholipid metabolic process

ıe expression;GO:0010744//positive regulation of macrophage derived foam cell differentiation;G(

›liferation;GO:0003382//epithelial cell morphogenesis;GO:0003406//retinal pigment epithelium de
)87//positive regulation of histone H3-K27 methylation;GO:1990830//cellular response to leukemi

GO:0042997//negative regulation of Golgi to plasma membrane protein transport;GO:0048286//lu

7165//signal transduction;GO:0007281//germ cell development;GO:0008284//positive regulation c

ıbstrate adhesion-dependent cell spreading;GO:2000146//negative regulation of cell motility;GO:2

nt;GO:0035024//negative regulation of Rho protein signal transduction;GO:0048012//hepatocyte g

'9//regulation of calcium ion import;GO:2001256//regulation of store-operated calcium entry;GO:2
08217//regulation of blood pressure;GO:0008277//regulation of G-protein coupled receptor prote

enosine catabolic process;GO:0006157//deoxyadenosine catabolic process;GO:0006196//AMP cata

on of sour taste;GO:0051262//protein tetramerization;GO:0051289//protein homotetramerization;G

'9//regulation of calcium ion import;GO:2001256//regulation of store-operated calcium entry;GO:2

olishment of integrated proviral latency;GO:0090305//nucleic acid phosphodiester bond hydrolysis

ubstrate adhesion-dependent cell spreading;GO:2000146//negative regulation of cell motility;GO:2

GO:0042997//negative regulation of Golgi to plasma membrane protein transport;GO:0048286//lu

GO:0042997//negative regulation of Golgi to plasma membrane protein transport;GO:0048286//lu

7165//signal transduction;GO:0007281//germ cell development;GO:0008284//positive regulation c

of phagocytosis;GO:0050773//regulation of dendrite development;GO:0060267//positive regulatic

)042573//retinoic acid metabolic process;GO:0042574//retinal metabolic process;GO:0043065//pos
of phagocytosis;GO:0050773//regulation of dendrite development;GO:0060267//positive regulatic

)010467//gene expression;GO:0033627//cell adhesion mediated by integrin;GO:0042060//wound
O:1902309//negative regulation of peptidyl-serine dephosphorylation;GO:1903589//positive regu

ise to stress;GO:0016240//autophagosome docking;GO:0032469//endoplasmic reticulum calcium

13//cardiac muscle cell development;GO:0072659//protein localization to plasma membrane;GO:0

olishment of integrated proviral latency;GO:0090305//nucleic acid phosphodiester bond hydrolysis

'9//regulation of calcium ion import;GO:2001256//regulation of store-operated calcium entry;GO:

ion of organ growth;GO:0048644//muscle organ morphogenesis;GO:0060124//positive regulation

)872//white fat cell differentiation;GO:0071285//cellular response to lithium ion;GO:0071356//cellu

ubstrate adhesion-dependent cell spreading;GO:2000146//negative regulation of cell motility;GO:2

ne expression;GO:0010744//positive regulation of macrophage derived foam cell differentiation;GO

ve morphogenesis;GO:0003186//tricuspid valve morphogenesis;GO:0003195//tricuspid valve form

nt;GO:0035024//negative regulation of Rho protein signal transduction;GO:0048012//hepatocyte g
s via desmosterol;GO:0042987//amyloid precursor protein catabolic process;GO:0043066//negative

ce;GO:0032287//peripheral nervous system myelin maintenance;GO:0042127//regulation of cell pr

nt;GO:0035024//negative regulation of Rho protein signal transduction;GO:0048012//hepatocyte g

of phagocytosis;GO:0050773//regulation of dendrite development;GO:0060267//positive regulatic

ig pathway by p53 class mediator;GO:0072734//cellular response to staurosporine;GO:0097194//e

O:0010977//negative regulation of neuron projection development;GO:0019222//regulation of me
)042573//retinoic acid metabolic process;GO:0042574//retinal metabolic process;GO:0043065//pos

osynthetic process;GO:0034625//fatty acid elongation, monounsaturated fatty acid;GO:0034626//f

2//cilium organization;GO:0045184//establishment of protein localization;GO:0048568//embryoni

GO:0042997//negative regulation of Golgi to plasma membrane protein transport;GO:0048286//lu

of phagocytosis;GO:0050773//regulation of dendrite development;GO:0060267//positive regulatio

ve morphogenesis;GO:0003186//tricuspid valve morphogenesis;GO:0003195//tricuspid valve form

nt;GO:0035024//negative regulation of Rho protein signal transduction;GO:0048012//hepatocyte g

ehavior;GO:0043407//negative regulation of MAP kinase activity;GO:0050905//neuromuscular pro

116//positive regulation of heterotypic cell-cell adhesion;GO:0036438//maintenance of lens transp

tic process;GO:0046326//positive regulation of glucose import;GO:0046628//positive regulation of

olishment of integrated proviral latency;GO:0090305//nucleic acid phosphodiester bond hydrolysis

)0090//negative regulation of canonical Wnt signaling pathway;GO:2000016//negative regulation

SC involved in gene silencing by miRNA;GO:0045944//positive regulation of transcription from RN/

2//cilium organization;GO:0045184//establishment of protein localization;GO:0048568//embryoni

n cytoskeleton organization;GO:0042060//wound healing;GO:0043547//positive regulation of GTP

7165//signal transduction;GO:0007281//germ cell development;GO:0008284//positive regulation c

ig pathway by p53 class mediator;GO:0072734//cellular response to staurosporine;GO:0097194//e

ce;GO:0032287//peripheral nervous system myelin maintenance;GO:0042127//regulation of cell pr

on of sour taste;GO:0051262//protein tetramerization;GO:0051289//protein homotetramerization;(

GO:0042997//negative regulation of Golgi to plasma membrane protein transport;GO:0048286//lu

O:0010977//negative regulation of neuron projection development;GO:0019222//regulation of me

0042573//retinoic acid metabolic process;GO:0042574//retinal metabolic process;GO:0043065//pos

d storage;GO:0030335//positive regulation of cell migration;GO:0042632//cholesterol homeostasis

tic process;GO:0046326//positive regulation of glucose import;GO:0046628//positive regulation of

ulation of transcription from RNA polymerase II promoter;GO:0051216//cartilage development

regulation of glucose import;GO:0046330//positive regulation of JNK cascade;GO:0070374//posit

rebrain development;GO:0043524//negative regulation of neuron apoptotic process;GO:0090090/

tiation;GO:0003310//pancreatic A cell differentiation;GO:0006355//regulation of transcription, DN

ıokine production;GO:0034371//chylomicron remodeling;GO:0034372//very-low-density lipoprote

yceride catabolic process;GO:0034389//lipid particle organization;GO:0036153//triglyceride acyl-c

116//positive regulation of heterotypic cell-cell adhesion;GO:0036438//maintenance of lens transp

5638//negative regulation of myeloid cell differentiation;GO:0045646//regulation of erythrocyte di

osynthetic process;GO:0034625//fatty acid elongation, monounsaturated fatty acid;GO:0034626//f

ve morphogenesis;GO:0003186//tricuspid valve morphogenesis;GO:0003195//tricuspid valve form

tion from RNA polymerase II promoter;GO:0071404//cellular response to low-density lipoprotein p

s via desmosterol;GO:0042987//amyloid precursor protein catabolic process;GO:0043066//negative

ulation of transcription from RNA polymerase II promoter;GO:0051216//cartilage development

tiation;GO:0003310//pancreatic A cell differentiation;GO:0006355//regulation of transcription, DN/

yceride catabolic process;GO:0034389//lipid particle organization;GO:0036153//triglyceride acyl-c
2//cilium organization;GO:0045184//establishment of protein localization;GO:0048568//embryonic

5638//negative regulation of myeloid cell differentiation;GO:0045646//regulation of erythrocyte di

rebrain development;GO:0043524//negative regulation of neuron apoptotic process;GO:0090090/

O:0010977//negative regulation of neuron projection development;GO:0019222//regulation of me

nt;GO:0035024//negative regulation of Rho protein signal transduction;GO:0048012//hepatocyte g

›y photoperiod;GO:0043276//anoikis;GO:0045595//regulation of cell differentiation;GO:0045721//[illegible]

)042573//retinoic acid metabolic process;GO:0042574//retinal metabolic process;GO:0043065//pos

)042573//retinoic acid metabolic process;GO:0042574//retinal metabolic process;GO:0043065//pos

ulation of transcription from RNA polymerase II promoter;GO:0051216//cartilage development

2//cilium organization;GO:0045184//establishment of protein localization;GO:0048568//embryoni

)87//positive regulation of histone H3-K27 methylation;GO:1990830//cellular response to leukemi

GO:0042997//negative regulation of Golgi to plasma membrane protein transport;GO:0048286//lu

of lipid storage;GO:0045745//positive regulation of G-protein coupled receptor protein signaling
mitochondria-nucleus signaling pathway;GO:0032869//cellular response to insulin stimulus;GO:00
of phagocytosis;GO:0050773//regulation of dendrite development;GO:0060267//positive regulatio
:0035456//response to interferon-beta;GO:0038061//NIK/NF-kappaB signaling;GO:0043123//posi

nt;GO:0035024//negative regulation of Rho protein signal transduction;GO:0048012//hepatocyte g

ubstrate adhesion-dependent cell spreading;GO:2000146//negative regulation of cell motility;GO:2

enosine catabolic process;GO:0006157//deoxyadenosine catabolic process;GO:0006196//AMP cata

5638//negative regulation of myeloid cell differentiation;GO:0045646//regulation of erythrocyte di

or production;GO:0043588//skin development;GO:0045454//cell redox homeostasis;GO:0055085/[illegible]
[illegible]9//regulation of calcium ion import;GO:2001256//regulation of store-operated calcium entry;GO:[illegible]

[illegible]00090//negative regulation of canonical Wnt signaling pathway;GO:2000016//negative regulation

[illegible]45956//positive regulation of calcium ion-dependent exocytosis;GO:0055085//transmembrane tra[illegible]

by cell;GO:0033700//phospholipid efflux;GO:0035627//ceramide transport;GO:0043129//surfactan
of lipid storage;GO:0045745//positive regulation of G-protein coupled receptor protein signaling p
GO:0042997//negative regulation of Golgi to plasma membrane protein transport;GO:0048286//lu

:0035456//response to interferon-beta;GO:0038061//NIK/NF-kappaB signaling;GO:0043123//posit
n cytoskeleton organization;GO:0042060//wound healing;GO:0043547//positive regulation of GTP
of phagocytosis;GO:0050773//regulation of dendrite development;GO:0060267//positive regulatic

O:0010977//negative regulation of neuron projection development;GO:0019222//regulation of me

)042573//retinoic acid metabolic process;GO:0042574//retinal metabolic process;GO:0043065//pos

ubstrate adhesion-dependent cell spreading;GO:2000146//negative regulation of cell motility;GO:2

)0090//negative regulation of canonical Wnt signaling pathway;GO:2000016//negative regulation

enosine catabolic process;GO:0006157//deoxyadenosine catabolic process;GO:0006196//AMP cata

'9//regulation of calcium ion import;GO:2001256//regulation of store-operated calcium entry;GO:2

O:0010977//negative regulation of neuron projection development;GO:0019222//regulation of me

ory T cell differentiation;GO:0046327//glycerol biosynthetic process from pyruvate;GO:0046890//re

yceride catabolic process;GO:0034389//lipid particle organization;GO:0036153//triglyceride acyl-c

)0010467//gene expression;GO:0033627//cell adhesion mediated by integrin;GO:0042060//wound

)//anterior commissure morphogenesis;GO:0030178//negative regulation of Wnt signaling pathwa

tiation;GO:0003310//pancreatic A cell differentiation;GO:0006355//regulation of transcription, DN/

)042573//retinoic acid metabolic process;GO:0042574//retinal metabolic process;GO:0043065//pos

116//positive regulation of heterotypic cell-cell adhesion;GO:0036438//maintenance of lens transp

7165//signal transduction;GO:0007281//germ cell development;GO:0008284//positive regulation c

ucose;GO:0019432//triglyceride biosynthetic process;GO:0040018//positive regulation of multicellu

GO:0042997//negative regulation of Golgi to plasma membrane protein transport;GO:0048286//lu

ce;GO:0032287//peripheral nervous system myelin maintenance;GO:0042127//regulation of cell pr

tiation;GO:0003310//pancreatic A cell differentiation;GO:0006355//regulation of transcription, DN/

0010467//gene expression;GO:0033627//cell adhesion mediated by integrin;GO:0042060//wound

process;GO:0042593//glucose homeostasis;GO:0042594//response to starvation;GO:0045124//reg

5638//negative regulation of myeloid cell differentiation;GO:0045646//regulation of erythrocyte di

O:0010977//negative regulation of neuron projection development;GO:0019222//regulation of me

;C involved in gene silencing by miRNA;GO:0045944//positive regulation of transcription from RN/

ehavior;GO:0043407//negative regulation of MAP kinase activity;GO:0050905//neuromuscular pro

O:0010977//negative regulation of neuron projection development;GO:0019222//regulation of me

)0090//negative regulation of canonical Wnt signaling pathway;GO:2000016//negative regulation

enosine catabolic process;GO:0006157//deoxyadenosine catabolic process;GO:0006196//AMP cata
:e;GO:0032287//peripheral nervous system myelin maintenance;GO:0042127//regulation of cell pr

'/adenylate cyclase-inhibiting G-protein coupled receptor signaling pathway;GO:0007200//phospl

'9//regulation of calcium ion import;GO:2001256//regulation of store-operated calcium entry;GO:

d storage;GO:0030335//positive regulation of cell migration;GO:0042632//cholesterol homeostasis

116//positive regulation of heterotypic cell-cell adhesion;GO:0036438//maintenance of lens transp

ubstrate adhesion-dependent cell spreading;GO:2000146//negative regulation of cell motility;GO:2

116//positive regulation of heterotypic cell-cell adhesion;GO:0036438//maintenance of lens transp

tiation;GO:0003310//pancreatic A cell differentiation;GO:0006355//regulation of transcription, DNA

/regulation of ventricular cardiac muscle cell membrane depolarization;GO:0061337//cardiac cond

)45956//positive regulation of calcium ion-dependent exocytosis;GO:0055085//transmembrane tra

GO:0042997//negative regulation of Golgi to plasma membrane protein transport;GO:0048286//lu

osynthetic process;GO:0034625//fatty acid elongation, monounsaturated fatty acid;GO:0034626//f

08217//regulation of blood pressure;GO:0008277//regulation of G-protein coupled receptor prote

ulation of transcription from RNA polymerase II promoter;GO:0051216//cartilage development

ne expression;GO:0010744//positive regulation of macrophage derived foam cell differentiation;G
73//positive regulation of axon extension;GO:0051963//regulation of synapse assembly;GO:00616

7165//signal transduction;GO:0007281//germ cell development;GO:0008284//positive regulation c

tiation;GO:0003310//pancreatic A cell differentiation;GO:0006355//regulation of transcription, DN

O:0010977//negative regulation of neuron projection development;GO:0019222//regulation of me

'9//regulation of calcium ion import;GO:2001256//regulation of store-operated calcium entry;GO:[illegible]

'73//positive regulation of axon extension;GO:0051963//regulation of synapse assembly;GO:0061[illegible]

)0090//negative regulation of canonical Wnt signaling pathway;GO:2000016//negative regulation

ubstrate adhesion-dependent cell spreading;GO:2000146//negative regulation of cell motility;GO:[illegible]

granulocyte macrophage colony-stimulating factor production;GO:0032729//positive regulation o[illegible]

d storage;GO:0030335//positive regulation of cell migration;GO:0042632//cholesterol homeostasi[illegible]

ce;GO:0032287//peripheral nervous system myelin maintenance;GO:0042127//regulation of cell pr

tiation;GO:0003310//pancreatic A cell differentiation;GO:0006355//regulation of transcription, DNA
or production;GO:0043588//skin development;GO:0045454//cell redox homeostasis;GO:0055085//

tion from RNA polymerase II promoter;GO:0071404//cellular response to low-density lipoprotein p

osynthetic process;GO:0034625//fatty acid elongation, monounsaturated fatty acid;GO:0034626//f
GO:0042997//negative regulation of Golgi to plasma membrane protein transport;GO:0048286//lu

13//cardiac muscle cell development;GO:0072659//protein localization to plasma membrane;GO:0

7165//signal transduction;GO:0007281//germ cell development;GO:0008284//positive regulation c

ulation of transcription from RNA polymerase II promoter;GO:0051216//cartilage development

2//cilium organization;GO:0045184//establishment of protein localization;GO:0048568//embryoni

'regulation of ventricular cardiac muscle cell membrane depolarization;GO:0061337//cardiac cond

olishment of integrated proviral latency;GO:0090305//nucleic acid phosphodiester bond hydrolysis

osynthetic process;GO:0034625//fatty acid elongation, monounsaturated fatty acid;GO:0034626//f

0010467//gene expression;GO:0033627//cell adhesion mediated by integrin;GO:0042060//wound

osynthetic process;GO:0034625//fatty acid elongation, monounsaturated fatty acid;GO:0034626//f

of phagocytosis;GO:0050773//regulation of dendrite development;GO:0060267//positive regulatic

2//cilium organization;GO:0045184//establishment of protein localization;GO:0048568//embryoni

d storage;GO:0030335//positive regulation of cell migration;GO:0042632//cholesterol homeostasis

13//cardiac muscle cell development;GO:0072659//protein localization to plasma membrane;GO:0

gene expression;GO:0021766//hippocampus development;GO:0021895//cerebral cortex neuron di

ulation of transcription from RNA polymerase II promoter;GO:0051216//cartilage development

mitochondria-nucleus signaling pathway;GO:0032869//cellular response to insulin stimulus;GO:00

olishment of integrated proviral latency;GO:0090305//nucleic acid phosphodiester bond hydrolysis

ulation of transcription from RNA polymerase II promoter;GO:0051216//cartilage development

ucose;GO:0019432//triglyceride biosynthetic process;GO:0040018//positive regulation of multicellu

nt;GO:0035024//negative regulation of Rho protein signal transduction;GO:0048012//hepatocyte g

O:0010977//negative regulation of neuron projection development;GO:0019222//regulation of me

)0090//negative regulation of canonical Wnt signaling pathway;GO:2000016//negative regulation

7165//signal transduction;GO:0007281//germ cell development;GO:0008284//positive regulation c

'73//positive regulation of axon extension;GO:0051963//regulation of synapse assembly;GO:0061€

ulation of transcription from RNA polymerase II promoter;GO:0051216//cartilage development

osynthetic process;GO:0034625//fatty acid elongation, monounsaturated fatty acid;GO:0034626//f

ory T cell differentiation;GO:0046327//glycerol biosynthetic process from pyruvate;GO:0046890//re

O:0010977//negative regulation of neuron projection development;GO:0019222//regulation of me

tion from RNA polymerase II promoter;GO:0071404//cellular response to low-density lipoprotein p

d storage;GO:0030335//positive regulation of cell migration;GO:0042632//cholesterol homeostasis

ol esterification;GO:0042632//cholesterol homeostasis;GO:0043691//reverse cholesterol transport;(

ne expression;GO:0010744//positive regulation of macrophage derived foam cell differentiation;G(

)0090//negative regulation of canonical Wnt signaling pathway;GO:2000016//negative regulation

08217//regulation of blood pressure;GO:0008277//regulation of G-protein coupled receptor prot(

ve morphogenesis;GO:0003186//tricuspid valve morphogenesis;GO:0003195//tricuspid valve form

7165//signal transduction;GO:0007281//germ cell development;GO:0008284//positive regulation c

yceride catabolic process;GO:0034389//lipid particle organization;GO:0036153//triglyceride acyl-c

tiation;GO:0003310//pancreatic A cell differentiation;GO:0006355//regulation of transcription, DN/

of phagocytosis;GO:0050773//regulation of dendrite development;GO:0060267//positive regulatic

egulation of hormone levels;GO:0014015//positive regulation of gliogenesis;GO:0033690//positive

nt;GO:0035024//negative regulation of Rho protein signal transduction;GO:0048012//hepatocyte g

d storage;GO:0030335//positive regulation of cell migration;GO:0042632//cholesterol homeostasis

tion from RNA polymerase II promoter;GO:0071404//cellular response to low-density lipoprotein p
ory T cell differentiation;GO:0046327//glycerol biosynthetic process from pyruvate;GO:0046890//re

ne expression;GO:0010744//positive regulation of macrophage derived foam cell differentiation;GO

ulation of transcription from RNA polymerase II promoter;GO:0051216//cartilage development

:e;GO:0032287//peripheral nervous system myelin maintenance;GO:0042127//regulation of cell pr
nt;GO:0035024//negative regulation of Rho protein signal transduction;GO:0048012//hepatocyte g

0042573//retinoic acid metabolic process;GO:0042574//retinal metabolic process;GO:0043065//pos

5638//negative regulation of myeloid cell differentiation;GO:0045646//regulation of erythrocyte di

nt;GO:0035024//negative regulation of Rho protein signal transduction;GO:0048012//hepatocyte g

of phagocytosis;GO:0050773//regulation of dendrite development;GO:0060267//positive regulatio

SC involved in gene silencing by miRNA;GO:0045944//positive regulation of transcription from RN

ve morphogenesis;GO:0003186//tricuspid valve morphogenesis;GO:0003195//tricuspid valve form

ucose;GO:0019432//triglyceride biosynthetic process;GO:0040018//positive regulation of multicellu

90090//negative regulation of canonical Wnt signaling pathway;GO:2000016//negative regulation

ory T cell differentiation;GO:0046327//glycerol biosynthetic process from pyruvate;GO:0046890//re

O:0010977//negative regulation of neuron projection development;GO:0019222//regulation of me

tiation;GO:0003310//pancreatic A cell differentiation;GO:0006355//regulation of transcription, DN[illegible]
[illegible]9//regulation of calcium ion import;GO:2001256//regulation of store-operated calcium entry;GO:[illegible]

of phagocytosis;GO:0050773//regulation of dendrite development;GO:0060267//positive regulati[illegible]

[illegible]87//positive regulation of histone H3-K27 methylation;GO:1990830//cellular response to leukemi[illegible]

ed signaling pathway;GO:0042327//positive regulation of phosphorylation;GO:0048813//dendrite
[illegible]bstrate adhesion-dependent cell spreading;GO:2000146//negative regulation of cell motility;GO:[illegible]
[illegible]042573//retinoic acid metabolic process;GO:0042574//retinal metabolic process;GO:0043065//po[illegible]

[illegible]e;GO:0032287//peripheral nervous system myelin maintenance;GO:0042127//regulation of cell pr[illegible]

2//cilium organization;GO:0045184//establishment of protein localization;GO:0048568//embryoni[illegible]

116//positive regulation of heterotypic cell-cell adhesion;GO:0036438//maintenance of lens transp

of phagocytosis;GO:0050773//regulation of dendrite development;GO:0060267//positive regulatic

ction;GO:0045921//positive regulation of exocytosis;GO:0050714//positive regulation of protein se

olishment of integrated proviral latency;GO:0090305//nucleic acid phosphodiester bond hydrolysis

osynthetic process;GO:0034625//fatty acid elongation, monounsaturated fatty acid;GO:0034626//f

GO:0042997//negative regulation of Golgi to plasma membrane protein transport;GO:0048286//lu

ucose;GO:0019432//triglyceride biosynthetic process;GO:0040018//positive regulation of multicellu

O:0010977//negative regulation of neuron projection development;GO:0019222//regulation of me

nt;GO:0035024//negative regulation of Rho protein signal transduction;GO:0048012//hepatocyte g

ne expression;GO:0010744//positive regulation of macrophage derived foam cell differentiation;GO

e regulation of glucose import;GO:0046330//positive regulation of JNK cascade;GO:0070374//posit

5638//negative regulation of myeloid cell differentiation;GO:0045646//regulation of erythrocyte di

/regulation of ventricular cardiac muscle cell membrane depolarization;GO:0061337//cardiac cond

'9//regulation of calcium ion import;GO:2001256//regulation of store-operated calcium entry;GO:

of phagocytosis;GO:0050773//regulation of dendrite development;GO:0060267//positive regulatic

:ion from RNA polymerase II promoter;GO:0071404//cellular response to low-density lipoprotein p

0042573//retinoic acid metabolic process;GO:0042574//retinal metabolic process;GO:0043065//pos

c process;GO:0060271//cilium morphogenesis;GO:1902915//negative regulation of protein polyub

ubstrate adhesion-dependent cell spreading;GO:2000146//negative regulation of cell motility;GO:2

d storage;GO:0030335//positive regulation of cell migration;GO:0042632//cholesterol homeostasis

116//positive regulation of heterotypic cell-cell adhesion;GO:0036438//maintenance of lens transp

tiation;GO:0003310//pancreatic A cell differentiation;GO:0006355//regulation of transcription, DN

GO:0007431//salivary gland development;GO:0007435//salivary gland morphogenesis;GO:0008284

GO:0042997//negative regulation of Golgi to plasma membrane protein transport;GO:0048286//lu

2//cilium organization;GO:0045184//establishment of protein localization;GO:0048568//embryoni[illegible]

nt;GO:0035024//negative regulation of Rho protein signal transduction;GO:0048012//hepatocyte [illegible]

ubstrate adhesion-dependent cell spreading;GO:2000146//negative regulation of cell motility;GO:[illegible]
/regulation of ventricular cardiac muscle cell membrane depolarization;GO:0061337//cardiac cond[illegible]

7165//signal transduction;GO:0007281//germ cell development;GO:0008284//positive regulation [illegible]

c process;GO:0060271//cilium morphogenesis;GO:1902915//negative regulation of protein polyub[illegible]
'9//regulation of calcium ion import;GO:2001256//regulation of store-operated calcium entry;GO:[illegible]

ce;GO:0032287//peripheral nervous system myelin maintenance;GO:0042127//regulation of cell pr[illegible]
enosine catabolic process;GO:0006157//deoxyadenosine catabolic process;GO:0006196//AMP cat[illegible]

/regulation of ventricular cardiac muscle cell membrane depolarization;GO:0061337//cardiac cond

)0090//negative regulation of canonical Wnt signaling pathway;GO:2000016//negative regulation

olishment of integrated proviral latency;GO:0090305//nucleic acid phosphodiester bond hydrolysis

0010467//gene expression;GO:0033627//cell adhesion mediated by integrin;GO:0042060//wound

9//regulation of calcium ion import;GO:2001256//regulation of store-operated calcium entry;GO:

638//negative regulation of myeloid cell differentiation;GO:0045646//regulation of erythrocyte di

2//cilium organization;GO:0045184//establishment of protein localization;GO:0048568//embryoni

bstrate adhesion-dependent cell spreading;GO:2000146//negative regulation of cell motility;GO:

ry T cell differentiation;GO:0046327//glycerol biosynthetic process from pyruvate;GO:0046890//re

87//positive regulation of histone H3-K27 methylation;GO:1990830//cellular response to leukemi

2//cilium organization;GO:0045184//establishment of protein localization;GO:0048568//embryoni

ıltage-gated potassium channel activity involved in ventricular cardiac muscle cell action potential

;C involved in gene silencing by miRNA;GO:0045944//positive regulation of transcription from RN/

116//positive regulation of heterotypic cell-cell adhesion;GO:0036438//maintenance of lens transp

: regulation of glucose import;GO:0046330//positive regulation of JNK cascade;GO:0070374//posit

ucose;GO:0019432//triglyceride biosynthetic process;GO:0040018//positive regulation of multicellu

enosine catabolic process;GO:0006157//deoxyadenosine catabolic process;GO:0006196//AMP cata

:charide;GO:1900028//negative regulation of ruffle assembly;GO:1902227//negative regulation of

ıetabolic process;GO:0051100//negative regulation of binding;GO:0070989//oxidative demethylat

pathway;GO:0008203//cholesterol metabolic process;GO:0008211//glucocorticoid metabolic proc

:ive regulation of gene expression;GO:0014065//phosphatidylinositol 3-kinase signaling;GO:00140

)51965//positive regulation of synapse assembly;GO:0061003//positive regulation of dendritic spir
healing;GO:0043129//surfactant homeostasis;GO:0043588//skin development;GO:0048286//lung a

ein particle remodeling;GO:0042632//cholesterol homeostasis;GO:0045600//positive regulation of

//glucose homeostasis;GO:0045088//regulation of innate immune response;GO:0046322//negativ
fferentiation;GO:0022010//central nervous system myelination;GO:0034613//cellular protein locali

MAD protein phosphorylation;GO:0021773//striatal medium spiny neuron differentiation;GO:0030

fferentiation;GO:0045656//negative regulation of monocyte differentiation;GO:0045668//negative

e regulation of osteoblast proliferation;GO:0035914//skeletal muscle cell differentiation;GO:00430

morphogenesis;GO:0048842//positive regulation of axon extension involved in axon guidance;GC

pathway;GO:0008203//cholesterol metabolic process;GO:0008211//glucocorticoid metabolic proc

f interferon-gamma production;GO:0032760//positive regulation of tumor necrosis factor product

ysteine metabolic process;GO:0051593//response to folic acid;GO:0060351//cartilage development

)51965//positive regulation of synapse assembly;GO:0061003//positive regulation of dendritic spir

;838//positive regulation of membrane potential;GO:0048169//regulation of long-term neuronal s

;4//brain morphogenesis;GO:0048873//homeostasis of number of cells within a tissue;GO:0090050

netabolic process;GO:0051100//negative regulation of binding;GO:0070989//oxidative demethylat

tive regulation of gene expression;GO:0014065//phosphatidylinositol 3-kinase signaling;GO:00140

ay;GO:0032911//negative regulation of transforming growth factor beta1 production;GO:0033344/

morphogenesis;GO:0048842//positive regulation of axon extension involved in axon guidance;GC

//glucose homeostasis;GO:0045088//regulation of innate immune response;GO:0046322//negativ

MAD protein phosphorylation;GO:0021773//striatal medium spiny neuron differentiation;GO:0030

ansport;GO:0070509//calcium ion import;GO:0070588//calcium ion transmembrane transport;GO:

f interferon-gamma production;GO:0032760//positive regulation of tumor necrosis factor product

steine metabolic process;GO:0051593//response to folic acid;GO:0060351//cartilage development

838//positive regulation of membrane potential;GO:0048169//regulation of long-term neuronal s

ulation of bone resorption;GO:0046320//regulation of fatty acid oxidation;GO:0071398//cellular re

tive regulation of I-kappaB kinase/NF-kappaB signaling;GO:0043254//regulation of protein compl

4//brain morphogenesis;GO:0048873//homeostasis of number of cells within a tissue;GO:009005

5838//positive regulation of membrane potential;GO:0048169//regulation of long-term neuronal s

morphogenesis;GO:0048842//positive regulation of axon extension involved in axon guidance;GC

ved in cellular response to glucose stimulus;GO:0042593//glucose homeostasis;GO:0050804//moc

fferentiation;GO:0022010//central nervous system myelination;GO:0034613//cellular protein locali

tive regulation of I-kappaB kinase/NF-kappaB signaling;GO:0043254//regulation of protein compl

particle stimulus;GO:0090370//negative regulation of cholesterol efflux;GO:1902895//positive regu

f interferon-gamma production;GO:0032760//positive regulation of tumor necrosis factor product

:1813//cell-cell adhesion involved in neuronal-glial interactions involved in cerebral cortex radial [illegible]

ıetabolic process;GO:0051100//negative regulation of binding;GO:0070989//oxidative demethylat

:ive regulation of gene expression;GO:0014065//phosphatidylinositol 3-kinase signaling;GO:00140
54//brain morphogenesis;GO:0048873//homeostasis of number of cells within a tissue;GO:009005(

morphogenesis;GO:0048842//positive regulation of axon extension involved in axon guidance;GC

ulation of bone resorption;GO:0046320//regulation of fatty acid oxidation;GO:0071398//cellular re

f interferon-gamma production;GO:0032760//positive regulation of tumor necrosis factor product

5838//positive regulation of membrane potential;GO:0048169//regulation of long-term neuronal s

ved in cellular response to glucose stimulus;GO:0042593//glucose homeostasis;GO:0050804//moc
particle remodeling;GO:0034375//high-density lipoprotein particle remodeling;GO:0034436//glyco

Ɔ:0010884//positive regulation of lipid storage;GO:0010886//positive regulation of cholesterol sto

pathway;GO:0008203//cholesterol metabolic process;GO:0008211//glucocorticoid metabolic proc

)51965//positive regulation of synapse assembly;GO:0061003//positive regulation of dendritic spir

//sarcomere organization;GO:0045661//regulation of myoblast differentiation;GO:0045862//positi

MAD protein phosphorylation;GO:0021773//striatal medium spiny neuron differentiation;GO:0030[illegible]

f interferon-gamma production;GO:0032760//positive regulation of tumor necrosis factor product
//glucose homeostasis;GO:0045088//regulation of innate immune response;GO:0046322//negativ

tive regulation of gene expression;GO:0014065//phosphatidylinositol 3-kinase signaling;GO:00140

ulation of bone resorption;GO:0046320//regulation of fatty acid oxidation;GO:0071398//cellular re

cess;GO:0055001//muscle cell development;GO:0071466//cellular response to xenobiotic stimulus

e regulation of osteoblast proliferation;GO:0035914//skeletal muscle cell differentiation;GO:00430[illegible]

//positive regulation of cell proliferation;GO:0008285//negative regulation of cell proliferation;GO

ng alveolus development;GO:0048566//embryonic digestive tract development;GO:0060021//pala

:ive regulation of gene expression;GO:0014065//phosphatidylinositol 3-kinase signaling;GO:00140

ıetabolic process;GO:0051100//negative regulation of binding;GO:0070989//oxidative demethylat

21813//cell-cell adhesion involved in neuronal-glial interactions involved in cerebral cortex radial

particle remodeling;GO:0034375//high-density lipoprotein particle remodeling;GO:0034436//glyco

21813//cell-cell adhesion involved in neuronal-glial interactions involved in cerebral cortex radial

c organ development;GO:0051893//regulation of focal adhesion assembly;GO:0055123//digestive

//glucose homeostasis;GO:0045088//regulation of innate immune response;GO:0046322//negativ
f interferon-gamma production;GO:0032760//positive regulation of tumor necrosis factor product

ulation of bone resorption;GO:0046320//regulation of fatty acid oxidation;GO:0071398//cellular re

MAD protein phosphorylation;GO:0021773//striatal medium spiny neuron differentiation;GO:0030

tive regulation of gene expression;GO:0014065//phosphatidylinositol 3-kinase signaling;GO:00140

sterol absorption;GO:0032374//regulation of cholesterol transport;GO:0033344//cholesterol efflux;
ein particle remodeling;GO:0042632//cholesterol homeostasis;GO:0045600//positive regulation of

pathway;GO:0008203//cholesterol metabolic process;GO:0008211//glucocorticoid metabolic proc

)51965//positive regulation of synapse assembly;GO:0061003//positive regulation of dendritic spir

gulation of lipid biosynthetic process;GO:0051365//cellular response to potassium ion starvation;G

21813//cell-cell adhesion involved in neuronal-glial interactions involved in cerebral cortex radial g

54//brain morphogenesis;GO:0048873//homeostasis of number of cells within a tissue;GO:009005(

etabolic process;GO:0051100//negative regulation of binding;GO:0070989//oxidative demethylat

ein particle remodeling;GO:0042632//cholesterol homeostasis;GO:0045600//positive regulation of

e regulation of osteoblast proliferation;GO:0035914//skeletal muscle cell differentiation;GO:00430

21813//cell-cell adhesion involved in neuronal-glial interactions involved in cerebral cortex radial

ein particle remodeling;GO:0042632//cholesterol homeostasis;GO:0045600//positive regulation of

fatty acid elongation, polyunsaturated fatty acid;GO:0042759//long-chain fatty acid biosynthetic pr

e regulation of apoptotic process;GO:0043154//negative regulation of cysteine-type endopeptidas
ular organism growth;GO:0042104//positive regulation of activated T cell proliferation;GO:004425

//glucose homeostasis;GO:0045088//regulation of innate immune response;GO:0046322//negativ

MAD protein phosphorylation;GO:0021773//striatal medium spiny neuron differentiation;GO:0030

ein particle remodeling;GO:0042632//cholesterol homeostasis;GO:0045600//positive regulation of

)51965//positive regulation of synapse assembly;GO:0061003//positive regulation of dendritic spir

pathway;GO:0008203//cholesterol metabolic process;GO:0008211//glucocorticoid metabolic proc

e regulation of osteoblast proliferation;GO:0035914//skeletal muscle cell differentiation;GO:00430

5838//positive regulation of membrane potential;GO:0048169//regulation of long-term neuronal s

5838//positive regulation of membrane potential;GO:0048169//regulation of long-term neuronal s

4//positive regulation of cell proliferation;GO:0008285//negative regulation of cell proliferation;GO

54//brain morphogenesis;GO:0048873//homeostasis of number of cells within a tissue;GO:009005(

uction;GO:0072659//protein localization to plasma membrane;GO:0086002//cardiac muscle cell a

orphogenesis;GO:0051130//positive regulation of cellular component organization;GO:0065008//r
ysteine metabolic process;GO:0051593//response to folic acid;GO:0060351//cartilage development

GO:0071467//cellular response to pH;GO:0071468//cellular response to acidic pH;GO:0071805//po

ein signaling pathway;GO:0010628//positive regulation of gene expression;GO:0015031//protein t

051965//positive regulation of synapse assembly;GO:0061003//positive regulation of dendritic spir

ein particle remodeling;GO:0042632//cholesterol homeostasis;GO:0045600//positive regulation of

pathway;GO:0008203//cholesterol metabolic process;GO:0008211//glucocorticoid metabolic proc

5838//positive regulation of membrane potential;GO:0048169//regulation of long-term neuronal s

//sarcomere organization;GO:0045661//regulation of myoblast differentiation;GO:0045862//positi

cess;GO:0055001//muscle cell development;GO:0071466//cellular response to xenobiotic stimulus
growth factor receptor signaling pathway;GO:0051272//positive regulation of cellular component

ay;GO:0032911//negative regulation of transforming growth factor beta1 production;GO:0033344/

:0048245//eosinophil chemotaxis;GO:0048247//lymphocyte chemotaxis;GO:0070098//chemokine-

ıetabolic process;GO:0051100//negative regulation of binding;GO:0070989//oxidative demethylat

ein signaling pathway;GO:0010628//positive regulation of gene expression;GO:0015031//protein t

morphogenesis;GO:0048842//positive regulation of axon extension involved in axon guidance;GC

e regulation of osteoblast proliferation;GO:0035914//skeletal muscle cell differentiation;GO:00430[illegible]

tive regulation of ERK1 and ERK2 cascade;GO:0070858//negative regulation of bile acid biosynthet

9//peptide cross-linking;GO:0033622//integrin activation;GO:0034446//substrate adhesion-depen[illegible]
on of respiratory burst;GO:0071622//regulation of granulocyte chemotaxis;GO:0090023//positive r[illegible]

//immune system development;GO:0002572//pro-T cell differentiation;GO:0003180//aortic valve r

metabolic process;GO:0051100//negative regulation of binding;GO:0070989//oxidative demethylat
pathway;GO:0008203//cholesterol metabolic process;GO:0008211//glucocorticoid metabolic proc

morphogenesis;GO:0048842//positive regulation of axon extension involved in axon guidance;GC

051965//positive regulation of synapse assembly;GO:0061003//positive regulation of dendritic spir

ysteine metabolic process;GO:0051593//response to folic acid;GO:0060351//cartilage development
uction;GO:0072659//protein localization to plasma membrane;GO:0086002//cardiac muscle cell ac

e regulation of osteoblast proliferation;GO:0035914//skeletal muscle cell differentiation;GO:004306

54//brain morphogenesis;GO:0048873//homeostasis of number of cells within a tissue;GO:009005(

sitive regulation of apoptotic process;GO:0043584//nose development;GO:0048048//embryonic ey

morphogenesis;GO:0048842//positive regulation of axon extension involved in axon guidance;GC

ulation of bone resorption;GO:0046320//regulation of fatty acid oxidation;GO:0071398//cellular re

ay;GO:0032911//negative regulation of transforming growth factor beta1 production;GO:0033344/

:ive regulation of gene expression;GO:0014065//phosphatidylinositol 3-kinase signaling;GO:00140

9//peptide cross-linking;GO:0033622//integrin activation;GO:0034446//substrate adhesion-depen
//glucose homeostasis;GO:0045088//regulation of innate immune response;GO:0046322//negativ

MAD protein phosphorylation;GO:0021773//striatal medium spiny neuron differentiation;GO:0030

MAD protein phosphorylation;GO:0021773//striatal medium spiny neuron differentiation;GO:0030

//glucose homeostasis;GO:0045088//regulation of innate immune response;GO:0046322//negativ

steine metabolic process;GO:0051593//response to folic acid;GO:0060351//cartilage development

on differentiation;GO:0045859//regulation of protein kinase activity;GO:0051260//protein homool

way;GO:0070371//ERK1 and ERK2 cascade;GO:1904738//vascular associated smooth muscle cell n

54//brain morphogenesis;GO:0048873//homeostasis of number of cells within a tissue;GO:009005[illegible]

morphogenesis;GO:0048842//positive regulation of axon extension involved in axon guidance;G[illegible]

ular organism growth;GO:0042104//positive regulation of activated T cell proliferation;GO:004425[illegible]

ulation of bone resorption;GO:0046320//regulation of fatty acid oxidation;GO:0071398//cellular re

pathway;GO:0008203//cholesterol metabolic process;GO:0008211//glucocorticoid metabolic proc

insulin receptor signaling pathway;GO:0046676//negative regulation of insulin secretion;GO:0048

051965//positive regulation of synapse assembly;GO:0061003//positive regulation of dendritic spir

/transmembrane transport;GO:0060392//negative regulation of SMAD protein import into nucleus

actor activity;GO:0051289//protein homotetramerization;GO:0070814//hydrogen sulfide biosynthe

54//brain morphogenesis;GO:0048873//homeostasis of number of cells within a tissue;GO:009005(

//glucose homeostasis;GO:0045088//regulation of innate immune response;GO:0046322//negativ

MAD protein phosphorylation;GO:0021773//striatal medium spiny neuron differentiation;GO:0030:

ulation of bone resorption;GO:0046320//regulation of fatty acid oxidation;GO:0071398//cellular re

MAD protein phosphorylation;GO:0021773//striatal medium spiny neuron differentiation;GO:0030:

//glucose homeostasis;GO:0045088//regulation of innate immune response;GO:0046322//negativ

tive regulation of I-kappaB kinase/NF-kappaB signaling;GO:0043254//regulation of protein compl

egulation of gene expression;GO:0010629//negative regulation of gene expression;GO:0010801//n

gulation of lipid biosynthetic process;GO:0051365//cellular response to potassium ion starvation;G

protein localization to plasma membrane;GO:1903896//positive regulation of IRE1-mediated unfo

/transmembrane transport;GO:0060392//negative regulation of SMAD protein import into nucleus

54//brain morphogenesis;GO:0048873//homeostasis of number of cells within a tissue;GO:009005[illegible]

21813//cell-cell adhesion involved in neuronal-glial interactions involved in cerebral cortex radial [illegible]

ıetabolic process;GO:0051100//negative regulation of binding;GO:0070989//oxidative demethylat

ɔf cell proliferation;GO:0009755//hormone-mediated signaling pathway;GO:0010628//positive reg

f interferon-gamma production;GO:0032760//positive regulation of tumor necrosis factor product

STAT cascade;GO:0046627//negative regulation of insulin receptor signaling pathway;GO:005072[illegible]

[illegible]4//brain morphogenesis;GO:0048873//homeostasis of number of cells within a tissue;GO:009005[illegible]

:ell differentiation;GO:0030324//lung development;GO:0030334//regulation of cell migration;GO:0

ccharide;GO:1900028//negative regulation of ruffle assembly;GO:1902227//negative regulation of

anscription from RNA polymerase II promoter;GO:0051607//defense response to virus;GO:006033

healing;GO:0043129//surfactant homeostasis;GO:0043588//skin development;GO:0048286//lung a

051965//positive regulation of synapse assembly;GO:0061003//positive regulation of dendritic spir
netabolic process;GO:0051100//negative regulation of binding;GO:0070989//oxidative demethylat
MAD protein phosphorylation;GO:0021773//striatal medium spiny neuron differentiation;GO:0030

f interferon-gamma production;GO:0032760//positive regulation of tumor necrosis factor product

tive regulation of gene expression;GO:0014065//phosphatidylinositol 3-kinase signaling;GO:00140

pathway;GO:0008203//cholesterol metabolic process;GO:0008211//glucocorticoid metabolic proc

//glucose homeostasis;GO:0045088//regulation of innate immune response;GO:0046322//negativ
ved in cellular response to glucose stimulus;GO:0042593//glucose homeostasis;GO:0050804//moc

egulation of gene expression;GO:0010629//negative regulation of gene expression;GO:0010801//n

5838//positive regulation of membrane potential;GO:0048169//regulation of long-term neuronal s

STAT cascade;GO:0046627//negative regulation of insulin receptor signaling pathway;GO:005072

e regulation of osteoblast proliferation;GO:0035914//skeletal muscle cell differentiation;GO:00430

//immune system development;GO:0002572//pro-T cell differentiation;GO:0003180//aortic valve r

:ive regulation of gene expression;GO:0014065//phosphatidylinositol 3-kinase signaling;GO:00140

:ell differentiation;GO:0030324//lung development;GO:0030334//regulation of cell migration;GO:0

:charide;GO:1900028//negative regulation of ruffle assembly;GO:1902227//negative regulation of

)51965//positive regulation of synapse assembly;GO:0061003//positive regulation of dendritic spir

pathway;GO:0008203//cholesterol metabolic process;GO:0008211//glucocorticoid metabolic proc

i47//inositol phosphate metabolic process;GO:0045765//regulation of angiogenesis;GO:0050851//

sterol absorption;GO:0032374//regulation of cholesterol transport;GO:0033344//cholesterol efflux;

5838//positive regulation of membrane potential;GO:0048169//regulation of long-term neuronal s

particle remodeling;GO:0034375//high-density lipoprotein particle remodeling;GO:0034436//glyco
particle stimulus;GO:0090370//negative regulation of cholesterol efflux;GO:1902895//positive regu

'ase activity;GO:0045893//positive regulation of transcription, DNA-templated;GO:0045944//positi
ay;GO:0032911//negative regulation of transforming growth factor beta1 production;GO:0033344/

ion of phagocytosis;GO:1903016//negative regulation of exo-alpha-sialidase activity;GO:1903019/

:0048245//eosinophil chemotaxis;GO:0048247//lymphocyte chemotaxis;GO:0070098//chemokine-

insulin receptor signaling pathway;GO:0046676//negative regulation of insulin secretion;GO:0048

f interferon-gamma production;GO:0032760//positive regulation of tumor necrosis factor product
netabolic process;GO:0051100//negative regulation of binding;GO:0070989//oxidative demethylat

ved in cellular response to glucose stimulus;GO:0042593//glucose homeostasis;GO:0050804//moc

etabolic process;GO:0022008//neurogenesis;GO:0032060//bleb assembly;GO:0035025//positive re

'ase activity;GO:0045893//positive regulation of transcription, DNA-templated;GO:0045944//positi

tive regulation of I-kappaB kinase/NF-kappaB signaling;GO:0043254//regulation of protein compl

547//inositol phosphate metabolic process;GO:0045765//regulation of angiogenesis;GO:0050851//

)51965//positive regulation of synapse assembly;GO:0061003//positive regulation of dendritic spir

pathway;GO:0008203//cholesterol metabolic process;GO:0008211//glucocorticoid metabolic proc

;;GO:0045766//positive regulation of angiogenesis;GO:0048844//artery morphogenesis;GO:00509

f interferon-gamma production;GO:0032760//positive regulation of tumor necrosis factor product

ved in cellular response to glucose stimulus;GO:0042593//glucose homeostasis;GO:0050804//moc

sterol absorption;GO:0032374//regulation of cholesterol transport;GO:0033344//cholesterol efflux;
e regulation of osteoblast proliferation;GO:0035914//skeletal muscle cell differentiation;GO:00430б

5838//positive regulation of membrane potential;GO:0048169//regulation of long-term neuronal s

5838//positive regulation of membrane potential;GO:0048169//regulation of long-term neuronal s

pathway;GO:0008203//cholesterol metabolic process;GO:0008211//glucocorticoid metabolic proc

particle remodeling;GO:0034375//high-density lipoprotein particle remodeling;GO:0034436//glyco

ein particle remodeling;GO:0042632//cholesterol homeostasis;GO:0045600//positive regulation of
on differentiation;GO:0045859//regulation of protein kinase activity;GO:0051260//protein homool

)51965//positive regulation of synapse assembly;GO:0061003//positive regulation of dendritic spir

:ive regulation of gene expression;GO:0014065//phosphatidylinositol 3-kinase signaling;GO:00140

way;GO:0070371//ERK1 and ERK2 cascade;GO:1904738//vascular associated smooth muscle cell n

/steine metabolic process;GO:0051593//response to folic acid;GO:0060351//cartilage development

ein particle remodeling;GO:0042632//cholesterol homeostasis;GO:0045600//positive regulation of

ccharide;GO:1900028//negative regulation of ruffle assembly;GO:1902227//negative regulation of

f interferon-gamma production;GO:0032760//positive regulation of tumor necrosis factor product

5838//positive regulation of membrane potential;GO:0048169//regulation of long-term neuronal s

morphogenesis;GO:0048842//positive regulation of axon extension involved in axon guidance;GC

'/immune system development;GO:0002572//pro-T cell differentiation;GO:0003180//aortic valve r

actor activity;GO:0051289//protein homotetramerization;GO:0070814//hydrogen sulfide biosynthe

fferentiation;GO:0022010//central nervous system myelination;GO:0034613//cellular protein locali

ulation of bone resorption;GO:0046320//regulation of fatty acid oxidation;GO:0071398//cellular re

particle remodeling;GO:0034375//high-density lipoprotein particle remodeling;GO:0034436//glyco

h factor stimulus;GO:0043154//negative regulation of cysteine-type endopeptidase activity involve

protein localization to plasma membrane;GO:1903896//positive regulation of IRE1-mediated unfo

tive regulation of gene expression;GO:0014065//phosphatidylinositol 3-kinase signaling;GO:00140

21813//cell-cell adhesion involved in neuronal-glial interactions involved in cerebral cortex radial g
netabolic process;GO:0051100//negative regulation of binding;GO:0070989//oxidative demethylat

morphogenesis;GO:0048842//positive regulation of axon extension involved in axon guidance;GC

)51965//positive regulation of synapse assembly;GO:0061003//positive regulation of dendritic spir

pathway;GO:0008203//cholesterol metabolic process;GO:0008211//glucocorticoid metabolic proc

ulation of bone resorption;GO:0046320//regulation of fatty acid oxidation;GO:0071398//cellular re

t homeostasis;GO:0045055//regulated exocytosis;GO:0045616//regulation of keratinocyte differen

healing;GO:0043129//surfactant homeostasis;GO:0043588//skin development;GO:0048286//lung a

netabolic process;GO:0051100//negative regulation of binding;GO:0070989//oxidative demethylat

)90074//negative regulation of protein homodimerization activity;GO:1904646//cellular response t

f interferon-gamma production;GO:0032760//positive regulation of tumor necrosis factor product

morphogenesis;GO:0048842//positive regulation of axon extension involved in axon guidance;GC

h factor stimulus;GO:0043154//negative regulation of cysteine-type endopeptidase activity involve

'/immune system development;GO:0002572//pro-T cell differentiation;GO:0003180//aortic valve r

ion of phagocytosis;GO:1903016//negative regulation of exo-alpha-sialidase activity;GO:1903019/

s;GO:0045766//positive regulation of angiogenesis;GO:0048844//artery morphogenesis;GO:00509

21813//cell-cell adhesion involved in neuronal-glial interactions involved in cerebral cortex radial g

ulation of bone resorption;GO:0046320//regulation of fatty acid oxidation;GO:0071398//cellular re

ein particle remodeling;GO:0042632//cholesterol homeostasis;GO:0045600//positive regulation of

//glucose homeostasis;GO:0045088//regulation of innate immune response;GO:0046322//negativ[illegible]

[illegible]ive regulation of gene expression;GO:0014065//phosphatidylinositol 3-kinase signaling;GO:00140[illegible]

[illegible]ive regulation of gene expression;GO:0014065//phosphatidylinositol 3-kinase signaling;GO:00140[illegible]

[illegible]MAD protein phosphorylation;GO:0021773//striatal medium spiny neuron differentiation;GO:0030[illegible]

gulation of lipid biosynthetic process;GO:0051365//cellular response to potassium ion starvation;G

9//peptide cross-linking;GO:0033622//integrin activation;GO:0034446//substrate adhesion-depend

//glucose homeostasis;GO:0045088//regulation of innate immune response;GO:0046322//negativ

MAD protein phosphorylation;GO:0021773//striatal medium spiny neuron differentiation;GO:0030

54//brain morphogenesis;GO:0048873//homeostasis of number of cells within a tissue;GO:009005(

tive regulation of gene expression;GO:0014065//phosphatidylinositol 3-kinase signaling;GO:00140

ein particle remodeling;GO:0042632//cholesterol homeostasis;GO:0045600//positive regulation of

ular organism growth;GO:0042104//positive regulation of activated T cell proliferation;GO:004425[illegible]

anscription from RNA polymerase II promoter;GO:0051607//defense response to virus;GO:006033[illegible]

[illegible]4//brain morphogenesis;GO:0048873//homeostasis of number of cells within a tissue;GO:009005[illegible]

ein particle remodeling;GO:0042632//cholesterol homeostasis;GO:0045600//positive regulation of

t homeostasis;GO:0045055//regulated exocytosis;GO:0045616//regulation of keratinocyte differen

34551//mitochondrial respiratory chain complex III assembly;GO:0051881//regulation of mitochor

ein particle remodeling;GO:0042632//cholesterol homeostasis;GO:0045600//positive regulation of

9//peptide cross-linking;GO:0033622//integrin activation;GO:0034446//substrate adhesion-depen

tive regulation of gene expression;GO:0014065//phosphatidylinositol 3-kinase signaling;GO:00140

uction;GO:0072659//protein localization to plasma membrane;GO:0086002//cardiac muscle cell a

34551//mitochondrial respiratory chain complex III assembly;GO:0051881//regulation of mitochor

4//positive regulation of cell proliferation;GO:0008285//negative regulation of cell proliferation;GO

54//brain morphogenesis;GO:0048873//homeostasis of number of cells within a tissue;GO:009005

morphogenesis;GO:0048842//positive regulation of axon extension involved in axon guidance;GC

ysteine metabolic process;GO:0051593//response to folic acid;GO:0060351//cartilage development

f interferon-gamma production;GO:0032760//positive regulation of tumor necrosis factor product

5838//positive regulation of membrane potential;GO:0048169//regulation of long-term neuronal s

ved in cellular response to glucose stimulus;GO:0042593//glucose homeostasis;GO:0050804//moc

:0048245//eosinophil chemotaxis;GO:0048247//lymphocyte chemotaxis;GO:0070098//chemokine-

f interferon-gamma production;GO:0032760//positive regulation of tumor necrosis factor product

:0048245//eosinophil chemotaxis;GO:0048247//lymphocyte chemotaxis;GO:0070098//chemokine-

of cell proliferation;GO:0009755//hormone-mediated signaling pathway;GO:0010628//positive reg

e regulation of osteoblast proliferation;GO:0035914//skeletal muscle cell differentiation;GO:00430[illegible]

pathway;GO:0008203//cholesterol metabolic process;GO:0008211//glucocorticoid metabolic proc[illegible]

ay;GO:0032911//negative regulation of transforming growth factor beta1 production;GO:0033344/[illegible]

9//peptide cross-linking;GO:0033622//integrin activation;GO:0034446//substrate adhesion-depen[illegible]
ein particle remodeling;GO:0042632//cholesterol homeostasis;GO:0045600//positive regulation of
)51965//positive regulation of synapse assembly;GO:0061003//positive regulation of dendritic spir[illegible]

MAD protein phosphorylation;GO:0021773//striatal medium spiny neuron differentiation;GO:0030[illegible]

//glucose homeostasis;GO:0045088//regulation of innate immune response;GO:0046322//negativ[illegible]

//sarcomere organization;GO:0045661//regulation of myoblast differentiation;GO:0045862//positi

21813//cell-cell adhesion involved in neuronal-glial interactions involved in cerebral cortex radial [illegible]

ay;GO:0032911//negative regulation of transforming growth factor beta1 production;GO:0033344/

STAT cascade;GO:0046627//negative regulation of insulin receptor signaling pathway;GO:005072[illegible]

54//brain morphogenesis;GO:0048873//homeostasis of number of cells within a tissue;GO:009005(

5838//positive regulation of membrane potential;GO:0048169//regulation of long-term neuronal s

//immune system development;GO:0002572//pro-T cell differentiation;GO:0003180//aortic valve r

egulation of gene expression;GO:0010629//negative regulation of gene expression;GO:0010801//n

e regulation of osteoblast proliferation;GO:0035914//skeletal muscle cell differentiation;GO:00430€

pathway;GO:0008203//cholesterol metabolic process;GO:0008211//glucocorticoid metabolic proc

particle remodeling;GO:0034375//high-density lipoprotein particle remodeling;GO:0034436//glyc

morphogenesis;GO:0048842//positive regulation of axon extension involved in axon guidance;GC

cell differentiation;GO:0030324//lung development;GO:0030334//regulation of cell migration;GO:0
netabolic process;GO:0051100//negative regulation of binding;GO:0070989//oxidative demethylat

)51965//positive regulation of synapse assembly;GO:0061003//positive regulation of dendritic spir

steine metabolic process;GO:0051593//response to folic acid;GO:0060351//cartilage development

morphogenesis;GO:0048842//positive regulation of axon extension involved in axon guidance;GC

t homeostasis;GO:0045055//regulated exocytosis;GO:0045616//regulation of keratinocyte differen

f interferon-gamma production;GO:0032760//positive regulation of tumor necrosis factor product

:0048245//eosinophil chemotaxis;GO:0048247//lymphocyte chemotaxis;GO:0070098//chemokine-

MAD protein phosphorylation;GO:0021773//striatal medium spiny neuron differentiation;GO:0030[illegible]

'ase activity;GO:0045893//positive regulation of transcription, DNA-templated;GO:0045944//positi

:ive regulation of gene expression;GO:0014065//phosphatidylinositol 3-kinase signaling;GO:00140

//glucose homeostasis;GO:0045088//regulation of innate immune response;GO:0046322//negativ[illegible]

)90074//negative regulation of protein homodimerization activity;GO:1904646//cellular response t
morphogenesis;GO:0048842//positive regulation of axon extension involved in axon guidance;GC

21813//cell-cell adhesion involved in neuronal-glial interactions involved in cerebral cortex radial [illegible]

insulin receptor signaling pathway;GO:0046676//negative regulation of insulin secretion;GO:0048

netabolic process;GO:0051100//negative regulation of binding;GO:0070989//oxidative demethylat

ıetabolic process;GO:0051100//negative regulation of binding;GO:0070989//oxidative demethylat

ay;GO:0032911//negative regulation of transforming growth factor beta1 production;GO:0033344/

5838//positive regulation of membrane potential;GO:0048169//regulation of long-term neuronal s

STAT cascade;GO:0046627//negative regulation of insulin receptor signaling pathway;GO:005072[illegible]

sitive regulation of apoptotic process;GO:0043584//nose development;GO:0048048//embryonic e[illegible]

[illegible]47//inositol phosphate metabolic process;GO:0045765//regulation of angiogenesis;GO:0050851//

//glucose homeostasis;GO:0045088//regulation of innate immune response;GO:0046322//negativ[illegible]

pathway;GO:0008203//cholesterol metabolic process;GO:0008211//glucocorticoid metabolic proc

[illegible]MAD protein phosphorylation;GO:0021773//striatal medium spiny neuron differentiation;GO:0030[illegible]

[illegible]ell differentiation;GO:0030324//lung development;GO:0030334//regulation of cell migration;GO:0

[illegible]51965//positive regulation of synapse assembly;GO:0061003//positive regulation of dendritic spir

ay;GO:0032911//negative regulation of transforming growth factor beta1 production;GO:0033344/

//sarcomere organization;GO:0045661//regulation of myoblast differentiation;GO:0045862//positi

netabolic process;GO:0051100//negative regulation of binding;GO:0070989//oxidative demethylat

5838//positive regulation of membrane potential;GO:0048169//regulation of long-term neuronal s

GO:0071467//cellular response to pH;GO:0071468//cellular response to acidic pH;GO:0071805//po

morphogenesis;GO:0048842//positive regulation of axon extension involved in axon guidance;GC

STAT cascade;GO:0046627//negative regulation of insulin receptor signaling pathway;GO:005072

netabolic process;GO:0051100//negative regulation of binding;GO:0070989//oxidative demethylat

54//brain morphogenesis;GO:0048873//homeostasis of number of cells within a tissue;GO:009005

way;GO:0070371//ERK1 and ERK2 cascade;GO:1904738//vascular associated smooth muscle cell n

//glucose homeostasis;GO:0045088//regulation of innate immune response;GO:0046322//negativ

:ell differentiation;GO:0030324//lung development;GO:0030334//regulation of cell migration;GO:0
holipase C-activating G-protein coupled receptor signaling pathway;GO:0007283//spermatogenes

MAD protein phosphorylation;GO:0021773//striatal medium spiny neuron differentiation;GO:0030[illegible]

actor activity;GO:0051289//protein homotetramerization;GO:0070814//hydrogen sulfide biosynthe

ein particle remodeling;GO:0042632//cholesterol homeostasis;GO:0045600//positive regulation of

90074//negative regulation of protein homodimerization activity;GO:1904646//cellular response t

47//inositol phosphate metabolic process;GO:0045765//regulation of angiogenesis;GO:0050851//

cess;GO:0055001//muscle cell development;GO:0071466//cellular response to xenobiotic stimulus
5838//positive regulation of membrane potential;GO:0048169//regulation of long-term neuronal s

5838//positive regulation of membrane potential;GO:0048169//regulation of long-term neuronal s

54//brain morphogenesis;GO:0048873//homeostasis of number of cells within a tissue;GO:009005

e regulation of apoptotic process;GO:0043154//negative regulation of cysteine-type endopeptidas

'steine metabolic process;GO:0051593//response to folic acid;GO:0060351//cartilage development

/transmembrane transport;GO:0060392//negative regulation of SMAD protein import into nucleus

ein particle remodeling;GO:0042632//cholesterol homeostasis;GO:0045600//positive regulation of

morphogenesis;GO:0048842//positive regulation of axon extension involved in axon guidance;GC

'/immune system development;GO:0002572//pro-T cell differentiation;GO:0003180//aortic valve r
ved in cellular response to glucose stimulus;GO:0042593//glucose homeostasis;GO:0050804//moc

negative regulation of gluconeogenesis;GO:0055007//cardiac muscle cell differentiation

//glucose homeostasis;GO:0045088//regulation of innate immune response;GO:0046322//negativ

)51965//positive regulation of synapse assembly;GO:0061003//positive regulation of dendritic spir

MAD protein phosphorylation;GO:0021773//striatal medium spiny neuron differentiation;GO:0030:

i47//inositol phosphate metabolic process;GO:0045765//regulation of angiogenesis;GO:0050851//

pathway;GO:0008203//cholesterol metabolic process;GO:0008211//glucocorticoid metabolic proc

h factor stimulus;GO:0043154//negative regulation of cysteine-type endopeptidase activity involve

on differentiation;GO:0045859//regulation of protein kinase activity;GO:0051260//protein homool

ulation of bone resorption;GO:0046320//regulation of fatty acid oxidation;GO:0071398//cellular re

)90074//negative regulation of protein homodimerization activity;GO:1904646//cellular response t
21813//cell-cell adhesion involved in neuronal-glial interactions involved in cerebral cortex radial g
h factor stimulus;GO:0043154//negative regulation of cysteine-type endopeptidase activity involve

ion of phagocytosis;GO:1903016//negative regulation of exo-alpha-sialidase activity;GO:1903019/

netabolic process;GO:0051100//negative regulation of binding;GO:0070989//oxidative demethylat

fatty acid elongation, polyunsaturated fatty acid;GO:0042759//long-chain fatty acid biosynthetic pr

negative regulation of gluconeogenesis;GO:0055007//cardiac muscle cell differentiation
54//brain morphogenesis;GO:0048873//homeostasis of number of cells within a tissue;GO:009005(

particle stimulus;GO:0090370//negative regulation of cholesterol efflux;GO:1902895//positive regu

:0048245//eosinophil chemotaxis;GO:0048247//lymphocyte chemotaxis;GO:0070098//chemokine-

)51965//positive regulation of synapse assembly;GO:0061003//positive regulation of dendritic spir

f interferon-gamma production;GO:0032760//positive regulation of tumor necrosis factor product

pathway;GO:0008203//cholesterol metabolic process;GO:0008211//glucocorticoid metabolic proc
ive regulation of gene expression;GO:0014065//phosphatidylinositol 3-kinase signaling;GO:00140
9//peptide cross-linking;GO:0033622//integrin activation;GO:0034446//substrate adhesion-depen

//sarcomere organization;GO:0045661//regulation of myoblast differentiation;GO:0045862//positi

particle remodeling;GO:0034375//high-density lipoprotein particle remodeling;GO:0034436//glyc

21813//cell-cell adhesion involved in neuronal-glial interactions involved in cerebral cortex radial g

morphogenesis;GO:0048842//positive regulation of axon extension involved in axon guidance;GC

protein localization to plasma membrane;GO:1903896//positive regulation of IRE1-mediated unfo

steine metabolic process;GO:0051593//response to folic acid;GO:0060351//cartilage development

ved in cellular response to glucose stimulus;GO:0042593//glucose homeostasis;GO:0050804//moc

f interferon-gamma production;GO:0032760//positive regulation of tumor necrosis factor product

f interferon-gamma production;GO:0032760//positive regulation of tumor necrosis factor product

h factor stimulus;GO:0043154//negative regulation of cysteine-type endopeptidase activity involve

//glucose homeostasis;GO:0045088//regulation of innate immune response;GO:0046322//negativ

tive regulation of gene expression;GO:0014065//phosphatidylinositol 3-kinase signaling;GO:00140
ng alveolus development;GO:0048566//embryonic digestive tract development;GO:0060021//pala

MAD protein phosphorylation;GO:0021773//striatal medium spiny neuron differentiation;GO:0030

way;GO:0070371//ERK1 and ERK2 cascade;GO:1904738//vascular associated smooth muscle cell n

ein particle remodeling;GO:0042632//cholesterol homeostasis;GO:0045600//positive regulation of

ulation of bone resorption;GO:0046320//regulation of fatty acid oxidation;GO:0071398//cellular re

holipase C-activating G-protein coupled receptor signaling pathway;GO:0007283//spermatogenes

particle remodeling;GO:0034375//high-density lipoprotein particle remodeling;GO:0034436//glyco

insulin receptor signaling pathway;GO:0046676//negative regulation of insulin secretion;GO:0048
t homeostasis;GO:0045055//regulated exocytosis;GO:0045616//regulation of keratinocyte differen

actor activity;GO:0051289//protein homotetramerization;GO:0070814//hydrogen sulfide biosynthe

ein signaling pathway;GO:0010628//positive regulation of gene expression;GO:0015031//protein t

54//brain morphogenesis;GO:0048873//homeostasis of number of cells within a tissue;GO:009005

h factor stimulus;GO:0043154//negative regulation of cysteine-type endopeptidase activity involve

547//inositol phosphate metabolic process;GO:0045765//regulation of angiogenesis;GO:0050851//

fferentiation;GO:0022010//central nervous system myelination;GO:0034613//cellular protein locali

f interferon-gamma production;GO:0032760//positive regulation of tumor necrosis factor product

t homeostasis;GO:0045055//regulated exocytosis;GO:0045616//regulation of keratinocyte differen

ulation of bone resorption;GO:0046320//regulation of fatty acid oxidation;GO:0071398//cellular re

//immune system development;GO:0002572//pro-T cell differentiation;GO:0003180//aortic valve r

ay;GO:0032911//negative regulation of transforming growth factor beta1 production;GO:0033344/

ion homeostasis;GO:0032470//positive regulation of endoplasmic reticulum calcium ion concentra

STAT cascade;GO:0046627//negative regulation of insulin receptor signaling pathway;GO:005072

ansport;GO:0070509//calcium ion import;GO:0070588//calcium ion transmembrane transport;GO:(

:ell differentiation;GO:0030324//lung development;GO:0030334//regulation of cell migration;GO:0

:ell differentiation;GO:0030324//lung development;GO:0030334//regulation of cell migration;GO:0

ulation of bone resorption;GO:0046320//regulation of fatty acid oxidation;GO:0071398//cellular re

ysteine metabolic process;GO:0051593//response to folic acid;GO:0060351//cartilage development

STAT cascade;GO:0046627//negative regulation of insulin receptor signaling pathway;GO:005072

ecretion;GO:0051258//protein polymerization;GO:0051592//response to calcium ion;GO:0070374//

ion homeostasis;GO:0032470//positive regulation of endoplasmic reticulum calcium ion concentra

\-templated;GO:0006357//regulation of transcription from RNA polymerase II promoter;GO:0006[illegible]

[illegible]:0010884//positive regulation of lipid storage;GO:0010886//positive regulation of cholesterol sto[illegible]
//sarcomere organization;GO:0045661//regulation of myoblast differentiation;GO:0045862//positi[illegible]

[illegible]MAD protein phosphorylation;GO:0021773//striatal medium spiny neuron differentiation;GO:0030[illegible]

//glucose homeostasis;GO:0045088//regulation of innate immune response;GO:0046322//negativ

negative regulation of gluconeogenesis;GO:0055007//cardiac muscle cell differentiation

086097//phospholipase C-activating angiotensin-activated signaling pathway;GO:1901017//nega

ein autophosphorylation;GO:0055074//calcium ion homeostasis;GO:0070562//regulation of vitami

ion homeostasis;GO:0032470//positive regulation of endoplasmic reticulum calcium ion concentra

on of respiratory burst;GO:0071622//regulation of granulocyte chemotaxis;GO:0090023//positive r

GO:0071467//cellular response to pH;GO:0071468//cellular response to acidic pH;GO:0071805//po

ıng alveolus development;GO:0048566//embryonic digestive tract development;GO:0060021//pala

ein autophosphorylation;GO:0055074//calcium ion homeostasis;GO:0070562//regulation of vitami

anscription from RNA polymerase II promoter;GO:0051607//defense response to virus;GO:006033

sitive regulation of apoptotic process;GO:0043584//nose development;GO:0048048//embryonic e
negative regulation of gluconeogenesis;GO:0055007//cardiac muscle cell differentiation

ıng alveolus development;GO:0048566//embryonic digestive tract development;GO:0060021//pala

;//cardiac neural crest cell migration involved in outflow tract morphogenesis;GO:0003357//noradr
of cell proliferation;GO:0009755//hormone-mediated signaling pathway;GO:0010628//positive reg

c organ development;GO:0051893//regulation of focal adhesion assembly;GO:0055123//digestive

O:0010884//positive regulation of lipid storage;GO:0010886//positive regulation of cholesterol sto

growth factor receptor signaling pathway;GO:0051272//positive regulation of cellular component r
velopment;GO:0003413//chondrocyte differentiation involved in endochondral bone morphogene

ein autophosphorylation;GO:0055074//calcium ion homeostasis;GO:0070562//regulation of vitami

sitive regulation of apoptotic process;GO:0043584//nose development;GO:0048048//embryonic ey

0086097//phospholipase C-activating angiotensin-activated signaling pathway;GO:1901017//nega

holipase C-activating G-protein coupled receptor signaling pathway;GO:0007283//spermatogene

e regulation of apoptotic process;GO:0043154//negative regulation of cysteine-type endopeptidas

fatty acid elongation, polyunsaturated fatty acid;GO:0042759//long-chain fatty acid biosynthetic pr

metabolic process;GO:0051100//negative regulation of binding;GO:0070989//oxidative demethylat

on of respiratory burst;GO:0071622//regulation of granulocyte chemotaxis;GO:0090023//positive re

ng alveolus development;GO:0048566//embryonic digestive tract development;GO:0060021//pala

velopment;GO:0003413//chondrocyte differentiation involved in endochondral bone morphogene

holipase C-activating G-protein coupled receptor signaling pathway;GO:0007283//spermatogenes

-templated;GO:0006357//regulation of transcription from RNA polymerase II promoter;GO:0006

regulation of osteoblast proliferation;GO:0035914//skeletal muscle cell differentiation;GO:00430

ase activity;GO:0045893//positive regulation of transcription, DNA-templated;GO:0045944//positi

etabolic process;GO:0022008//neurogenesis;GO:0032060//bleb assembly;GO:0035025//positive re

ng alveolus development;GO:0048566//embryonic digestive tract development;GO:0060021//pala

;rowth factor receptor signaling pathway;GO:0051272//positive regulation of cellular component r

abolic process;GO:0009168//purine ribonucleoside monophosphate biosynthetic process;GO:0010
on of respiratory burst;GO:0071622//regulation of granulocyte chemotaxis;GO:0090023//positive re

;//cardiac neural crest cell migration involved in outflow tract morphogenesis;GO:0003357//noradr

etabolic process;GO:0022008//neurogenesis;GO:0032060//bleb assembly;GO:0035025//positive re

holipase C-activating G-protein coupled receptor signaling pathway;GO:0007283//spermatogenes

ein autophosphorylation;GO:0055074//calcium ion homeostasis;GO:0070562//regulation of vitami

5838//positive regulation of membrane potential;GO:0048169//regulation of long-term neuronal s

nation;GO:0003199//endocardial cushion to mesenchymal transition involved in heart valve format

ion homeostasis;GO:0032470//positive regulation of endoplasmic reticulum calcium ion concentra

anscription from RNA polymerase II promoter;GO:0051607//defense response to virus;GO:006033

;GO:0045766//positive regulation of angiogenesis;GO:0048844//artery morphogenesis;GO:00509

growth factor receptor signaling pathway;GO:0051272//positive regulation of cellular component

-templated;GO:0006357//regulation of transcription from RNA polymerase II promoter;GO:0006

O:0010884//positive regulation of lipid storage;GO:0010886//positive regulation of cholesterol sto

holipase C-activating G-protein coupled receptor signaling pathway;GO:0007283//spermatogenes

velopment;GO:0003413//chondrocyte differentiation involved in endochondral bone morphogene

etabolic process;GO:0022008//neurogenesis;GO:0032060//bleb assembly;GO:0035025//positive re

growth factor receptor signaling pathway;GO:0051272//positive regulation of cellular component r

s;GO:0045766//positive regulation of angiogenesis;GO:0048844//artery morphogenesis;GO:00509

abolic process;GO:0009168//purine ribonucleoside monophosphate biosynthetic process;GO:0010

anscription from RNA polymerase II promoter;GO:0051607//defense response to virus;GO:006033

c organ development;GO:0051893//regulation of focal adhesion assembly;GO:0055123//digestive

s;GO:0045766//positive regulation of angiogenesis;GO:0048844//artery morphogenesis;GO:00509

e regulation of apoptotic process;GO:0043154//negative regulation of cysteine-type endopeptidas

ion homeostasis;GO:0032470//positive regulation of endoplasmic reticulum calcium ion concentra

A-templated;GO:0006357//regulation of transcription from RNA polymerase II promoter;GO:00066

negative regulation of gluconeogenesis;GO:0055007//cardiac muscle cell differentiation
nation;GO:0003199//endocardial cushion to mesenchymal transition involved in heart valve format

:ive regulation of ERK1 and ERK2 cascade;GO:0070858//negative regulation of bile acid biosynthet

)n of respiratory burst;GO:0071622//regulation of granulocyte chemotaxis;GO:0090023//positive r[illegible]

fferentiation;GO:0045656//negative regulation of monocyte differentiation;GO:0045668//negative

etabolic process;GO:0022008//neurogenesis;GO:0032060//bleb assembly;GO:0035025//positive re

ıctor activity;GO:0051289//protein homotetramerization;GO:0070814//hydrogen sulfide biosynthe

velopment;GO:0003413//chondrocyte differentiation involved in endochondral bone morphogene
ıng alveolus development;GO:0048566//embryonic digestive tract development;GO:0060021//pala

/transmembrane transport;GO:0060392//negative regulation of SMAD protein import into nucleus

GO:0071467//cellular response to pH;GO:0071468//cellular response to acidic pH;GO:0071805//pc

sitive regulation of apoptotic process;GO:0043584//nose development;GO:0048048//embryonic ey

c organ development;GO:0051893//regulation of focal adhesion assembly;GO:0055123//digestive

fferentiation;GO:0045656//negative regulation of monocyte differentiation;GO:0045668//negative

:ive regulation of gene expression;GO:0014065//phosphatidylinositol 3-kinase signaling;GO:00140

way;GO:0070371//ERK1 and ERK2 cascade;GO:1904738//vascular associated smooth muscle cell n

c organ development;GO:0051893//regulation of focal adhesion assembly;GO:0055123//digestive

oliferation;GO:0042634//regulation of hair cycle;GO:0044857//plasma membrane raft organizatior

ein autophosphorylation;GO:0055074//calcium ion homeostasis;GO:0070562//regulation of vitami

actor activity;GO:0051289//protein homotetramerization;GO:0070814//hydrogen sulfide biosynthe

ing alveolus development;GO:0048566//embryonic digestive tract development;GO:0060021//pala

c organ development;GO:0051893//regulation of focal adhesion assembly;GO:0055123//digestive

ansport;GO:0070509//calcium ion import;GO:0070588//calcium ion transmembrane transport;GO:(

cess;GO:0055001//muscle cell development;GO:0071466//cellular response to xenobiotic stimulus

)086097//phospholipase C-activating angiotensin-activated signaling pathway;GO:1901017//nega

of cell proliferation;GO:0009755//hormone-mediated signaling pathway;GO:0010628//positive reg

ing alveolus development;GO:0048566//embryonic digestive tract development;GO:0060021//pala

way;GO:0070371//ERK1 and ERK2 cascade;GO:1904738//vascular associated smooth muscle cell n

roliferation;GO:0042634//regulation of hair cycle;GO:0044857//plasma membrane raft organization

O:0010884//positive regulation of lipid storage;GO:0010886//positive regulation of cholesterol sto

34551//mitochondrial respiratory chain complex III assembly;GO:0051881//regulation of mitochon

sitive regulation of apoptotic process;GO:0043584//nose development;GO:0048048//embryonic ey
parency;GO:0051897//positive regulation of protein kinase B signaling;GO:0070374//positive regul

ulation of bone resorption;GO:0046320//regulation of fatty acid oxidation;GO:0071398//cellular re

c organ development;GO:0051893//regulation of focal adhesion assembly;GO:0055123//digestive

›f cell proliferation;GO:0009755//hormone-mediated signaling pathway;GO:0010628//positive reg

ecretion;GO:0051258//protein polymerization;GO:0051592//response to calcium ion;GO:0070374//

Pase activity;GO:0045893//positive regulation of transcription, DNA-templated;GO:0045944//positi
abolic process;GO:0009168//purine ribonucleoside monophosphate biosynthetic process;GO:0010

parency;GO:0051897//positive regulation of protein kinase B signaling;GO:0070374//positive regul

of cell proliferation;GO:0009755//hormone-mediated signaling pathway;GO:0010628//positive reg

:0048245//eosinophil chemotaxis;GO:0048247//lymphocyte chemotaxis;GO:0070098//chemokine-

insulin receptor signaling pathway;GO:0046676//negative regulation of insulin secretion;GO:0048

//cardiac neural crest cell migration involved in outflow tract morphogenesis;GO:0003357//noradr

ccharide;GO:1900028//negative regulation of ruffle assembly;GO:1902227//negative regulation of

ein signaling pathway;GO:0010628//positive regulation of gene expression;GO:0015031//protein t

etabolic process;GO:0022008//neurogenesis;GO:0032060//bleb assembly;GO:0035025//positive re

on of respiratory burst;GO:0071622//regulation of granulocyte chemotaxis;GO:0090023//positive re

;;GO:0045766//positive regulation of angiogenesis;GO:0048844//artery morphogenesis;GO:00509[illegible]

growth factor receptor signaling pathway;GO:0051272//positive regulation of cellular component [illegible]

sitive regulation of apoptotic process;GO:0043584//nose development;GO:0048048//embryonic e[illegible]

abolic process;GO:0009168//purine ribonucleoside monophosphate biosynthetic process;GO:001[illegible]

anscription from RNA polymerase II promoter;GO:0051607//defense response to virus;GO:006033

A-templated;GO:0006357//regulation of transcription from RNA polymerase II promoter;GO:00066

ion homeostasis;GO:0032470//positive regulation of endoplasmic reticulum calcium ion concentra

of cell proliferation;GO:0009755//hormone-mediated signaling pathway;GO:0010628//positive reg

growth factor receptor signaling pathway;GO:0051272//positive regulation of cellular component r

nation;GO:0003199//endocardial cushion to mesenchymal transition involved in heart valve format

etabolic process;GO:0022008//neurogenesis;GO:0032060//bleb assembly;GO:0035025//positive re

t homeostasis;GO:0045055//regulated exocytosis;GO:0045616//regulation of keratinocyte differen

on of respiratory burst;GO:0071622//regulation of granulocyte chemotaxis;GO:0090023//positive r

ecretion;GO:0051258//protein polymerization;GO:0051592//response to calcium ion;GO:0070374//

//cardiac neural crest cell migration involved in outflow tract morphogenesis;GO:0003357//noradr
sitive regulation of apoptotic process;GO:0043584//nose development;GO:0048048//embryonic e

;GO:0045766//positive regulation of angiogenesis;GO:0048844//artery morphogenesis;GO:00509

-templated;GO:0006357//regulation of transcription from RNA polymerase II promoter;GO:00066

oliferation;GO:0042634//regulation of hair cycle;GO:0044857//plasma membrane raft organizatior

growth factor receptor signaling pathway;GO:0051272//positive regulation of cellular component

anscription from RNA polymerase II promoter;GO:0051607//defense response to virus;GO:006033

34551//mitochondrial respiratory chain complex III assembly;GO:0051881//regulation of mitochor
etabolic process;GO:0022008//neurogenesis;GO:0032060//bleb assembly;GO:0035025//positive re

;//cardiac neural crest cell migration involved in outflow tract morphogenesis;GO:0003357//noradr

fatty acid elongation, polyunsaturated fatty acid;GO:0042759//long-chain fatty acid biosynthetic pr

c organgan development;GO:0051893//regulation of focal adhesion assembly;GO:0055123//digestive

on of respiratory burst;GO:0071622//regulation of granulocyte chemotaxis;GO:0090023//positive re

A-templated;GO:0006357//regulation of transcription from RNA polymerase II promoter;GO:00066

/transmembrane transport;GO:0060392//negative regulation of SMAD protein import into nucleus

;;GO:0045766//positive regulation of angiogenesis;GO:0048844//artery morphogenesis;GO:00509[illegible]
parency;GO:0051897//positive regulation of protein kinase B signaling;GO:0070374//positive regul
;;GO:0045766//positive regulation of angiogenesis;GO:0048844//artery morphogenesis;GO:00509[illegible]

sitive regulation of apoptotic process;GO:0043584//nose development;GO:0048048//embryonic ey

pathway;GO:0008203//cholesterol metabolic process;GO:0008211//glucocorticoid metabolic proc

ein particle remodeling;GO:0042632//cholesterol homeostasis;GO:0045600//positive regulation of

051965//positive regulation of synapse assembly;GO:0061003//positive regulation of dendritic spir

fferentiation;GO:0045656//negative regulation of monocyte differentiation;GO:0045668//negative

roliferation;GO:0042634//regulation of hair cycle;GO:0044857//plasma membrane raft organization

ion of phagocytosis;GO:1903016//negative regulation of exo-alpha-sialidase activity;GO:1903019/

fatty acid elongation, polyunsaturated fatty acid;GO:0042759//long-chain fatty acid biosynthetic p

anscription from RNA polymerase II promoter;GO:0051607//defense response to virus;GO:006033

ein autophosphorylation;GO:0055074//calcium ion homeostasis;GO:0070562//regulation of vitami

A-templated;GO:0006357//regulation of transcription from RNA polymerase II promoter;GO:0006

of cell proliferation;GO:0009755//hormone-mediated signaling pathway;GO:0010628//positive reg

c organ development;GO:0051893//regulation of focal adhesion assembly;GO:0055123//digestive

)086097//phosphopholipase C-activating angiotensin-activated signaling pathway;GO:1901017//nega

c organ development;GO:0051893//regulation of focal adhesion assembly;GO:0055123//digestive

velopment;GO:0003413//chondrocyte differentiation involved in endochondral bone morphogene

oarency;GO:0051897//positive regulation of protein kinase B signaling;GO:0070374//positive regul

s;GO:0045766//positive regulation of angiogenesis;GO:0048844//artery morphogenesis;GO:00509

ion of phagocytosis;GO:1903016//negative regulation of exo-alpha-sialidase activity;GO:1903019/

cess;GO:0055001//muscle cell development;GO:0071466//cellular response to xenobiotic stimulus

//immune system development;GO:0002572//pro-T cell differentiation;GO:0003180//aortic valve r

O:0010884//positive regulation of lipid storage;GO:0010886//positive regulation of cholesterol sto

ein autophosphorylation;GO:0055074//calcium ion homeostasis;GO:0070562//regulation of vitami

of cell proliferation;GO:0009755//hormone-mediated signaling pathway;GO:0010628//positive reg

)086097//phospholipase C-activating angiotensin-activated signaling pathway;GO:1901017//nega

;;GO:0045766//positive regulation of angiogenesis;GO:0048844//artery morphogenesis;GO:00509
negative regulation of gluconeogenesis;GO:0055007//cardiac muscle cell differentiation

ion of phagocytosis;GO:1903016//negative regulation of exo-alpha-sialidase activity;GO:1903019/

cess;GO:0055001//muscle cell development;GO:0071466//cellular response to xenobiotic stimulus

O:0010884//positive regulation of lipid storage;GO:0010886//positive regulation of cholesterol sto

velopment;GO:0003413//chondrocyte differentiation involved in endochondral bone morphogene

ng alveolus development;GO:0048566//embryonic digestive tract development;GO:0060021//pala

of cell proliferation;GO:0009755//hormone-mediated signaling pathway;GO:0010628//positive reg

growth factor receptor signaling pathway;GO:0051272//positive regulation of cellular component r

ein signaling pathway;GO:0010628//positive regulation of gene expression;GO:0015031//protein t

abolic process;GO:0009168//purine ribonucleoside monophosphate biosynthetic process;GO:001C

GO:0071467//cellular response to pH;GO:0071468//cellular response to acidic pH;GO:0071805//pc

ng alveolus development;GO:0048566//embryonic digestive tract development;GO:0060021//pala

ng alveolus development;GO:0048566//embryonic digestive tract development;GO:0060021//pala

f cell proliferation;GO:0009755//hormone-mediated signaling pathway;GO:0010628//positive reg

n of respiratory burst;GO:0071622//regulation of granulocyte chemotaxis;GO:0090023//positive r

sitive regulation of apoptotic process;GO:0043584//nose development;GO:0048048//embryonic ey
on of respiratory burst;GO:0071622//regulation of granulocyte chemotaxis;GO:0090023//positive re

healing;GO:0043129//surfactant homeostasis;GO:0043588//skin development;GO:0048286//lung a

ion homeostasis;GO:0032470//positive regulation of endoplasmic reticulum calcium ion concentra

0086097//phospholipase C-activating angiotensin-activated signaling pathway;GO:1901017//nega

O:0010884//positive regulation of lipid storage;GO:0010886//positive regulation of cholesterol sto

nation;GO:0003199//endocardial cushion to mesenchymal transition involved in heart valve format

growth factor receptor signaling pathway;GO:0051272//positive regulation of cellular component r
e regulation of apoptotic process;GO:0043154//negative regulation of cysteine-type endopeptidas

oliferation;GO:0042634//regulation of hair cycle;GO:0044857//plasma membrane raft organizatior

growth factor receptor signaling pathway;GO:0051272//positive regulation of cellular component r

›n of respiratory burst;GO:0071622//regulation of granulocyte chemotaxis;GO:0090023//positive r[illegible]

[illegible]etabolic process;GO:0022008//neurogenesis;GO:0032060//bleb assembly;GO:0035025//positive re
[illegible]sitive regulation of apoptotic process;GO:0043584//nose development;GO:0048048//embryonic e[illegible]

[illegible]atty acid elongation, polyunsaturated fatty acid;GO:0042759//long-chain fatty acid biosynthetic p[illegible]

c organ development;GO:0051893//regulation of focal adhesion assembly;GO:0055123//digestive

ing alveolus development;GO:0048566//embryonic digestive tract development;GO:0060021//pala

on of respiratory burst;GO:0071622//regulation of granulocyte chemotaxis;GO:0090023//positive r

nation;GO:0003199//endocardial cushion to mesenchymal transition involved in heart valve format

growth factor receptor signaling pathway;GO:0051272//positive regulation of cellular component r

cess;GO:0055001//muscle cell development;GO:0071466//cellular response to xenobiotic stimulus

parency;GO:0051897//positive regulation of protein kinase B signaling;GO:0070374//positive regul

f insulin receptor signaling pathway;GO:0046676//negative regulation of insulin secretion;GO:0048

c organ development;GO:0051893//regulation of focal adhesion assembly;GO:0055123//digestive

'ase activity;GO:0045893//positive regulation of transcription, DNA-templated;GO:0045944//positi

of cell proliferation;GO:0009755//hormone-mediated signaling pathway;GO:0010628//positive reg

roliferation;GO:0042634//regulation of hair cycle;GO:0044857//plasma membrane raft organizatior

GO:0071467//cellular response to pH;GO:0071468//cellular response to acidic pH;GO:0071805//pc

ing alveolus development;GO:0048566//embryonic digestive tract development;GO:0060021//pala

etabolic process;GO:0022008//neurogenesis;GO:0032060//bleb assembly;GO:0035025//positive re

sitive regulation of apoptotic process;GO:0043584//nose development;GO:0048048//embryonic ey

s;GO:0045766//positive regulation of angiogenesis;GO:0048844//artery morphogenesis;GO:00509

insulin receptor signaling pathway;GO:0046676//negative regulation of insulin secretion;GO:0048

tive regulation of ERK1 and ERK2 cascade;GO:0070858//negative regulation of bile acid biosynthet

A-templated;GO:0006357//regulation of transcription from RNA polymerase II promoter;GO:0006

ein particle remodeling;GO:0042632//cholesterol homeostasis;GO:0045600//positive regulation of

parency;GO:0051897//positive regulation of protein kinase B signaling;GO:0070374//positive regul

fferentiation;GO:0045656//negative regulation of monocyte differentiation;GO:0045668//negative

fatty acid elongation, polyunsaturated fatty acid;GO:0042759//long-chain fatty acid biosynthetic pr

ıation;GO:0003199//endocardial cushion to mesenchymal transition involved in heart valve format

ɔarticle stimulus;GO:0090370//negative regulation of cholesterol efflux;GO:1902895//positive regu

ɛ regulation of apoptotic process;GO:0043154//negative regulation of cysteine-type endopeptidas

A-templated;GO:0006357//regulation of transcription from RNA polymerase II promoter;GO:00066

c organ development;GO:0051893//regulation of focal adhesion assembly;GO:0055123//digestive

fferentiation;GO:0045656//negative regulation of monocyte differentiation;GO:0045668//negative

etabolic process;GO:0022008//neurogenesis;GO:0032060//bleb assembly;GO:0035025//positive re

growth factor receptor signaling pathway;GO:0051272//positive regulation of cellular component

negative regulation of gluconeogenesis;GO:0055007//cardiac muscle cell differentiation

sitive regulation of apoptotic process;GO:0043584//nose development;GO:0048048//embryonic ey

sitive regulation of apoptotic process;GO:0043584//nose development;GO:0048048//embryonic ey

c organ development;GO:0051893//regulation of focal adhesion assembly;GO:0055123//digestive

ing alveolus development;GO:0048566//embryonic digestive tract development;GO:0060021//pala

34551//mitochondrial respiratory chain complex III assembly;GO:0051881//regulation of mitochor
on of respiratory burst;GO:0071622//regulation of granulocyte chemotaxis;GO:0090023//positive re
tive regulation of I-kappaB kinase/NF-kappaB signaling;GO:0043254//regulation of protein compl

growth factor receptor signaling pathway;GO:0051272//positive regulation of cellular component r

abolic process;GO:0009168//purine ribonucleoside monophosphate biosynthetic process;GO:0010

fferentiation;GO:0045656//negative regulation of monocyte differentiation;GO:0045668//negative

/transmembrane transport;GO:0060392//negative regulation of SMAD protein import into nucleus

ansport;GO:0070509//calcium ion import;GO:0070588//calcium ion transmembrane transport;GO:(

t homeostasis;GO:0045055//regulated exocytosis;GO:0045616//regulation of keratinocyte differen

ing alveolus development;GO:0048566//embryonic digestive tract development;GO:0060021//pala

tive regulation of I-kappaB kinase/NF-kappaB signaling;GO:0043254//regulation of protein compl
'ase activity;GO:0045893//positive regulation of transcription, DNA-templated;GO:0045944//positi
on of respiratory burst;GO:0071622//regulation of granulocyte chemotaxis;GO:0090023//positive r

etabolic process;GO:0022008//neurogenesis;GO:0032060//bleb assembly;GO:0035025//positive re

sitive regulation of apoptotic process;GO:0043584//nose development;GO:0048048//embryonic ey

abolic process;GO:0009168//purine ribonucleoside monophosphate biosynthetic process;GO:001

etabolic process;GO:0022008//neurogenesis;GO:0032060//bleb assembly;GO:0035025//positive re

gulation of lipid biosynthetic process;GO:0051365//cellular response to potassium ion starvation;G

healing;GO:0043129//surfactant homeostasis;GO:0043588//skin development;GO:0048286//lung a

ay;GO:0032911//negative regulation of transforming growth factor beta1 production;GO:0033344/

A-templated;GO:0006357//regulation of transcription from RNA polymerase II promoter;GO:00066

sitive regulation of apoptotic process;GO:0043584//nose development;GO:0048048//embryonic ey

parency;GO:0051897//positive regulation of protein kinase B signaling;GO:0070374//positive regul

of cell proliferation;GO:0009755//hormone-mediated signaling pathway;GO:0010628//positive reg

ılar organism growth;GO:0042104//positive regulation of activated T cell proliferation;GO:004425[illegible]

ıng alveolus development;GO:0048566//embryonic digestive tract development;GO:0060021//pal[illegible]

roliferation;GO:0042634//regulation of hair cycle;GO:0044857//plasma membrane raft organizatio[illegible]

\-templated;GO:0006357//regulation of transcription from RNA polymerase II promoter;GO:0006[illegible]

healing;GO:0043129//surfactant homeostasis;GO:0043588//skin development;GO:0048286//lung [illegible]

ulation of bone resorption;GO:0046320//regulation of fatty acid oxidation;GO:0071398//cellular r[illegible]

fferentiation;GO:0045656//negative regulation of monocyte differentiation;GO:0045668//negative

etabolic process;GO:0022008//neurogenesis;GO:0032060//bleb assembly;GO:0035025//positive re

cess;GO:0055001//muscle cell development;GO:0071466//cellular response to xenobiotic stimulus

etabolic process;GO:0022008//neurogenesis;GO:0032060//bleb assembly;GO:0035025//positive re

abolic process;GO:0009168//purine ribonucleoside monophosphate biosynthetic process;GO:0010
roliferation;GO:0042634//regulation of hair cycle;GO:0044857//plasma membrane raft organization

holipase C-activating G-protein coupled receptor signaling pathway;GO:0007283//spermatogenes

s;GO:0045766//positive regulation of angiogenesis;GO:0048844//artery morphogenesis;GO:00509

ɔarency;GO:0051897//positive regulation of protein kinase B signaling;GO:0070374//positive regul

ɔarency;GO:0051897//positive regulation of protein kinase B signaling;GO:0070374//positive regul

\-templated;GO:0006357//regulation of transcription from RNA polymerase II promoter;GO:0006[illegible]

uction;GO:0072659//protein localization to plasma membrane;GO:0086002//cardiac muscle cell a[illegible]

ansport;GO:0070509//calcium ion import;GO:0070588//calcium ion transmembrane transport;GO:[illegible]

ıng alveolus development;GO:0048566//embryonic digestive tract development;GO:0060021//pala[illegible]

atty acid elongation, polyunsaturated fatty acid;GO:0042759//long-chain fatty acid biosynthetic pr

ein signaling pathway;GO:0010628//positive regulation of gene expression;GO:0015031//protein t

O:0010884//positive regulation of lipid storage;GO:0010886//positive regulation of cholesterol sto

of cell proliferation;GO:0009755//hormone-mediated signaling pathway;GO:0010628//positive reg

A-templated;GO:0006357//regulation of transcription from RNA polymerase II promoter;GO:0006

etabolic process;GO:0022008//neurogenesis;GO:0032060//bleb assembly;GO:0035025//positive re

f interferon-gamma production;GO:0032760//positive regulation of tumor necrosis factor product

s;GO:0045766//positive regulation of angiogenesis;GO:0048844//artery morphogenesis;GO:00509

roliferation;GO:0042634//regulation of hair cycle;GO:0044857//plasma membrane raft organizatior

A-templated;GO:0006357//regulation of transcription from RNA polymerase II promoter;GO:00066
/transmembrane transport;GO:0060392//negative regulation of SMAD protein import into nucleus

particle stimulus;GO:0090370//negative regulation of cholesterol efflux;GO:1902895//positive regu

fatty acid elongation, polyunsaturated fatty acid;GO:0042759//long-chain fatty acid biosynthetic pr
ng alveolus development;GO:0048566//embryonic digestive tract development;GO:0060021//pala

)086097//phospholipase C-activating angiotensin-activated signaling pathway;GO:1901017//nega

of cell proliferation;GO:0009755//hormone-mediated signaling pathway;GO:0010628//positive reg

c organ development;GO:0051893//regulation of focal adhesion assembly;GO:0055123//digestive

uction;GO:0072659//protein localization to plasma membrane;GO:0086002//cardiac muscle cell a

atty acid elongation, polyunsaturated fatty acid;GO:0042759//long-chain fatty acid biosynthetic p

healing;GO:0043129//surfactant homeostasis;GO:0043588//skin development;GO:0048286//lung

atty acid elongation, polyunsaturated fatty acid;GO:0042759//long-chain fatty acid biosynthetic p

on of respiratory burst;GO:0071622//regulation of granulocyte chemotaxis;GO:0090023//positive r

c organ development;GO:0051893//regulation of focal adhesion assembly;GO:0055123//digestive

s;GO:0045766//positive regulation of angiogenesis;GO:0048844//artery morphogenesis;GO:00509

0086097//phospholipase C-activating angiotensin-activated signaling pathway;GO:1901017//nega

fferentiation;GO:0022010//central nervous system myelination;GO:0034613//cellular protein locali

34551//mitochondrial respiratory chain complex III assembly;GO:0051881//regulation of mitochor

ılar organism growth;GO:0042104//positive regulation of activated T cell proliferation;GO:004425[illegible]

growth factor receptor signaling pathway;GO:0051272//positive regulation of cellular component [illegible]

etabolic process;GO:0022008//neurogenesis;GO:0032060//bleb assembly;GO:0035025//positive re

of cell proliferation;GO:0009755//hormone-mediated signaling pathway;GO:0010628//positive reg

fatty acid elongation, polyunsaturated fatty acid;GO:0042759//long-chain fatty acid biosynthetic pr

gulation of lipid biosynthetic process;GO:0051365//cellular response to potassium ion starvation;G

etabolic process;GO:0022008//neurogenesis;GO:0032060//bleb assembly;GO:0035025//positive re

particle stimulus;GO:0090370//negative regulation of cholesterol efflux;GO:1902895//positive regu

s;GO:0045766//positive regulation of angiogenesis;GO:0048844//artery morphogenesis;GO:00509

O:0010884//positive regulation of lipid storage;GO:0010886//positive regulation of cholesterol sto

ein signaling pathway;GO:0010628//positive regulation of gene expression;GO:0015031//protein t

nation;GO:0003199//endocardial cushion to mesenchymal transition involved in heart valve format

of cell proliferation;GO:0009755//hormone-mediated signaling pathway;GO:0010628//positive reg

A-templated;GO:0006357//regulation of transcription from RNA polymerase II promoter;GO:00066

on of respiratory burst;GO:0071622//regulation of granulocyte chemotaxis;GO:0090023//positive r

e regulation of osteoblast proliferation;GO:0035914//skeletal muscle cell differentiation;GO:00430

growth factor receptor signaling pathway;GO:0051272//positive regulation of cellular component r

;GO:0045766//positive regulation of angiogenesis;GO:0048844//artery morphogenesis;GO:00509

particle stimulus;GO:0090370//negative regulation of cholesterol efflux;GO:1902895//positive regu
gulation of lipid biosynthetic process;GO:0051365//cellular response to potassium ion starvation;G

O:0010884//positive regulation of lipid storage;GO:0010886//positive regulation of cholesterol sto

oliferation;GO:0042634//regulation of hair cycle;GO:0044857//plasma membrane raft organizatior
growth factor receptor signaling pathway;GO:0051272//positive regulation of cellular component r

sitive regulation of apoptotic process;GO:0043584//nose development;GO:0048048//embryonic ey

fferentiation;GO:0045656//negative regulation of monocyte differentiation;GO:0045668//negative

growth factor receptor signaling pathway;GO:0051272//positive regulation of cellular component r

on of respiratory burst;GO:0071622//regulation of granulocyte chemotaxis;GO:0090023//positive re

nation;GO:0003199//endocardial cushion to mesenchymal transition involved in heart valve format

ular organism growth;GO:0042104//positive regulation of activated T cell proliferation;GO:004425

gulation of lipid biosynthetic process;GO:0051365//cellular response to potassium ion starvation;G

etabolic process;GO:0022008//neurogenesis;GO:0032060//bleb assembly;GO:0035025//positive re

A-templated;GO:0006357//regulation of transcription from RNA polymerase II promoter;GO:00066

on of respiratory burst;GO:0071622//regulation of granulocyte chemotaxis;GO:0090023//positive r

morphogenesis;GO:0048842//positive regulation of axon extension involved in axon guidance;GC

sitive regulation of apoptotic process;GO:0043584//nose development;GO:0048048//embryonic ey

oliferation;GO:0042634//regulation of hair cycle;GO:0044857//plasma membrane raft organizatior

c organ development;GO:0051893//regulation of focal adhesion assembly;GO:0055123//digestive

oarency;GO:0051897//positive regulation of protein kinase B signaling;GO:0070374//positive regul

on of respiratory burst;GO:0071622//regulation of granulocyte chemotaxis;GO:0090023//positive r

ecretion;GO:0051258//protein polymerization;GO:0051592//response to calcium ion;GO:0070374//

fatty acid elongation, polyunsaturated fatty acid;GO:0042759//long-chain fatty acid biosynthetic p

ing alveolus development;GO:0048566//embryonic digestive tract development;GO:0060021//pala

ular organism growth;GO:0042104//positive regulation of activated T cell proliferation;GO:004425[illegible]

etabolic process;GO:0022008//neurogenesis;GO:0032060//bleb assembly;GO:0035025//positive re

growth factor receptor signaling pathway;GO:0051272//positive regulation of cellular component [illegible]

O:0010884//positive regulation of lipid storage;GO:0010886//positive regulation of cholesterol sto

tive regulation of ERK1 and ERK2 cascade;GO:0070858//negative regulation of bile acid biosynthet

fferentiation;GO:0045656//negative regulation of monocyte differentiation;GO:0045668//negative

uction;GO:0072659//protein localization to plasma membrane;GO:0086002//cardiac muscle cell a[illegible]

on of respiratory burst;GO:0071622//regulation of granulocyte chemotaxis;GO:0090023//positive re

particle stimulus;GO:0090370//negative regulation of cholesterol efflux;GO:1902895//positive regu

sitive regulation of apoptotic process;GO:0043584//nose development;GO:0048048//embryonic ey

s;GO:0045766//positive regulation of angiogenesis;GO:0048844//artery morphogenesis;GO:00509

ɔarency;GO:0051897//positive regulation of protein kinase B signaling;GO:0070374//positive regul

A-templated;GO:0006357//regulation of transcription from RNA polymerase II promoter;GO:0006[illegible]

4//positive regulation of cell proliferation;GO:0008285//negative regulation of cell proliferation;GO

ung alveolus development;GO:0048566//embryonic digestive tract development;GO:0060021//pala

c organ development;GO:0051893//regulation of focal adhesion assembly;GO:0055123//digestive

growth factor receptor signaling pathway;GO:0051272//positive regulation of cellular component r

uction;GO:0072659//protein localization to plasma membrane;GO:0086002//cardiac muscle cell ac

of cell proliferation;GO:0009755//hormone-mediated signaling pathway;GO:0010628//positive reg

roliferation;GO:0042634//regulation of hair cycle;GO:0044857//plasma membrane raft organization
abolic process;GO:0009168//purine ribonucleoside monophosphate biosynthetic process;GO:0010

uction;GO:0072659//protein localization to plasma membrane;GO:0086002//cardiac muscle cell a[illegible]

healing;GO:0043129//surfactant homeostasis;GO:0043588//skin development;GO:0048286//lung [illegible]

fferentiation;GO:0045656//negative regulation of monocyte differentiation;GO:0045668//negative

c organ development;GO:0051893//regulation of focal adhesion assembly;GO:0055123//digestive

gulation of lipid biosynthetic process;GO:0051365//cellular response to potassium ion starvation;G

c organ development;GO:0051893//regulation of focal adhesion assembly;GO:0055123//digestive

:parency;GO:0051897//positive regulation of protein kinase B signaling;GO:0070374//positive regul

:ive regulation of ERK1 and ERK2 cascade;GO:0070858//negative regulation of bile acid biosynthet

ular organism growth;GO:0042104//positive regulation of activated T cell proliferation;GO:004425

abolic process;GO:0009168//purine ribonucleoside monophosphate biosynthetic process;GO:001

macrophage colony-stimulating factor signaling pathway;GO:1903980//positive regulation of mic

cess;GO:0010804//negative regulation of tumor necrosis factor-mediated signaling pathway;GO:00

68//positive regulation of phosphatidylinositol 3-kinase signaling;GO:0014834//skeletal muscle sa

ne morphogenesis;GO:0071310//cellular response to organic substance;GO:2000463//positive regu
alveolus development;GO:0055091//phospholipid homeostasis;GO:0060022//hard palate developm

fat cell differentiation;GO:0055096//low-density lipoprotein particle mediated signaling;GO:00703

e regulation of fatty acid oxidation;GO:0046325//negative regulation of glucose import;GO:00507[illegible]
zation;GO:0045444//fat cell differentiation;GO:0045599//negative regulation of fat cell differentiati

308//negative regulation of cell growth;GO:0032270//positive regulation of cellular protein metab[illegible]

regulation of osteoblast differentiation;GO:0045786//negative regulation of cell cycle;GO:004814[illegible]

[illegible]6//negative regulation of apoptotic process;GO:0045165//cell fate commitment;GO:0045444//fat

[illegible]:0060060//post-embryonic retina morphogenesis in camera-type eye;GO:0060219//camera-type

:ess;GO:0010804//negative regulation of tumor necrosis factor-mediated signaling pathway;GO:0(

:ion;GO:0032816//positive regulation of natural killer cell activation;GO:0032825//positive regulatic

ne morphogenesis;GO:0071310//cellular response to organic substance;GO:2000463//positive regu

synaptic plasticity;GO:0048814//regulation of dendrite morphogenesis;GO:0050806//positive regul

0//positive regulation of cell migration involved in sprouting angiogenesis;GO:2000773//negative

68//positive regulation of phosphatidylinositol 3-kinase signaling;GO:0014834//skeletal muscle sa

//cholesterol efflux;GO:0042060//wound healing;GO:0042632//cholesterol homeostasis;GO:004263

):0060060//post-embryonic retina morphogenesis in camera-type eye;GO:0060219//camera-type

e regulation of fatty acid oxidation;GO:0046325//negative regulation of glucose import;GO:00507(

308//negative regulation of cell growth;GO:0032270//positive regulation of cellular protein metab

)086010//membrane depolarization during action potential;GO:0098662//inorganic cation transm

:ion;GO:0032816//positive regulation of natural killer cell activation;GO:0032825//positive regulatio

;ynaptic plasticity;GO:0048814//regulation of dendrite morphogenesis;GO:0050806//positive regul

esponse to fatty acid;GO:0072593//reactive oxygen species metabolic process;GO:2000811//negat

ex assembly;GO:0048255//mRNA stabilization;GO:0051091//positive regulation of sequence-spec

)//positive regulation of cell migration involved in sprouting angiogenesis;GO:2000773//negative

;ynaptic plasticity;GO:0048814//regulation of dendrite morphogenesis;GO:0050806//positive regul

):0060060//post-embryonic retina morphogenesis in camera-type eye;GO:0060219//camera-type

dulation of synaptic transmission;GO:0051480//regulation of cytosolic calcium ion concentration;G

zation;GO:0045444//fat cell differentiation;GO:0045599//negative regulation of fat cell differentiati

ex assembly;GO:0048255//mRNA stabilization;GO:0051091//positive regulation of sequence-spec

tion;GO:0032816//positive regulation of natural killer cell activation;GO:0032825//positive regulatio

glia guided migration;GO:0021942//radial glia guided migration of Purkinje cell;GO:0045666//posi

68//positive regulation of phosphatidylinositol 3-kinase signaling;GO:0014834//skeletal muscle sa
0//positive regulation of cell migration involved in sprouting angiogenesis;GO:2000773//negative

):0060060//post-embryonic retina morphogenesis in camera-type eye;GO:0060219//camera-type

esponse to fatty acid;GO:0072593//reactive oxygen species metabolic process;GO:2000811//negat

tion;GO:0032816//positive regulation of natural killer cell activation;GO:0032825//positive regulatio

synaptic plasticity;GO:0048814//regulation of dendrite morphogenesis;GO:0050806//positive regul

dulation of synaptic transmission;GO:0051480//regulation of cytosolic calcium ion concentration;G
oprotein transport;GO:0042632//cholesterol homeostasis;GO:0042987//amyloid precursor protein

rage;GO:0016042//lipid catabolic process;GO:0019433//triglyceride catabolic process;GO:0030301

:ess;GO:0010804//negative regulation of tumor necrosis factor-mediated signaling pathway;GO:0(

ie morphogenesis;GO:0071310//cellular response to organic substance;GO:2000463//positive regu

ve regulation of proteolysis;GO:0045892//negative regulation of transcription, DNA-templated;GC

308//negative regulation of cell growth;GO:0032270//positive regulation of cellular protein metab

tion;GO:0032816//positive regulation of natural killer cell activation;GO:0032825//positive regulati
e regulation of fatty acid oxidation;GO:0046325//negative regulation of glucose import;GO:00507

68//positive regulation of phosphatidylinositol 3-kinase signaling;GO:0014834//skeletal muscle sa

esponse to fatty acid;GO:0072593//reactive oxygen species metabolic process;GO:2000811//negat

6//negative regulation of apoptotic process;GO:0045165//cell fate commitment;GO:0045444//fat

:0008543//fibroblast growth factor receptor signaling pathway;GO:0008544//epidermis developm

68//positive regulation of phosphatidylinositol 3-kinase signaling;GO:0014834//skeletal muscle sa

glia guided migration;GO:0021942//radial glia guided migration of Purkinje cell;GO:0045666//posi

oprotein transport;GO:0042632//cholesterol homeostasis;GO:0042987//amyloid precursor protein

glia guided migration;GO:0021942//radial glia guided migration of Purkinje cell;GO:0045666//posi

system development;GO:0060021//palate development;GO:0060271//cilium morphogenesis;GO:(

e regulation of fatty acid oxidation;GO:0046325//negative regulation of glucose import;GO:00507(
:ion;GO:0032816//positive regulation of natural killer cell activation;GO:0032825//positive regulatic

esponse to fatty acid;GO:0072593//reactive oxygen species metabolic process;GO:2000811//negat

308//negative regulation of cell growth;GO:0032270//positive regulation of cellular protein metab

68//positive regulation of phosphatidylinositol 3-kinase signaling;GO:0014834//skeletal muscle sa

GO:0033700//phospholipid efflux;GO:0034372//very-low-density lipoprotein particle remodeling;
fat cell differentiation;GO:0055096//low-density lipoprotein particle mediated signaling;GO:00703

cess;GO:0010804//negative regulation of tumor necrosis factor-mediated signaling pathway;GO:00

ıe morphogenesis;GO:0071310//cellular response to organic substance;GO:2000463//positive regu

;O:0061402//positive regulation of transcription from RNA polymerase II promoter in response to

glia guided migration;GO:0021942//radial glia guided migration of Purkinje cell;GO:0045666//posi

)//positive regulation of cell migration involved in sprouting angiogenesis;GO:2000773//negative

fat cell differentiation;GO:0055096//low-density lipoprotein particle mediated signaling;GO:00703

56//negative regulation of apoptotic process;GO:0045165//cell fate commitment;GO:0045444//fat

glia guided migration;GO:0021942//radial glia guided migration of Purkinje cell;GO:0045666//posi

fat cell differentiation;GO:0055096//low-density lipoprotein particle mediated signaling;GO:00703

rocess;GO:0042761//very long-chain fatty acid biosynthetic process;GO:0044249//cellular biosynth

se activity involved in apoptotic process;GO:0043588//skin development;GO:0061024//membrane
5//cellular lipid metabolic process;GO:0051607//defense response to virus;GO:0055089//fatty acid

e regulation of fatty acid oxidation;GO:0046325//negative regulation of glucose import;GO:00507[illegible]

308//negative regulation of cell growth;GO:0032270//positive regulation of cellular protein metab[illegible]

fat cell differentiation;GO:0055096//low-density lipoprotein particle mediated signaling;GO:00703

ıe morphogenesis;GO:0071310//cellular response to organic substance;GO:2000463//positive regu

:ess;GO:0010804//negative regulation of tumor necrosis factor-mediated signaling pathway;GO:00

56//negative regulation of apoptotic process;GO:0045165//cell fate commitment;GO:0045444//fat

synaptic plasticity;GO:0048814//regulation of dendrite morphogenesis;GO:0050806//positive regul

synaptic plasticity;GO:0048814//regulation of dendrite morphogenesis;GO:0050806//positive regul

:0008543//fibroblast growth factor receptor signaling pathway;GO:0008544//epidermis developm

)//positive regulation of cell migration involved in sprouting angiogenesis;GO:2000773//negative

ction potential involved in contraction;GO:0086005//ventricular cardiac muscle cell action potentia

otassium ion transmembrane transport;GO:0098662//inorganic cation transmembrane transport

ransport;GO:0031623//receptor internalization;GO:0034333//adherens junction assembly;GO:0035

ne morphogenesis;GO:0071310//cellular response to organic substance;GO:2000463//positive regu

fat cell differentiation;GO:0055096//low-density lipoprotein particle mediated signaling;GO:00703

cess;GO:0010804//negative regulation of tumor necrosis factor-mediated signaling pathway;GO:00

synaptic plasticity;GO:0048814//regulation of dendrite morphogenesis;GO:0050806//positive regul

ve regulation of proteolysis;GO:0045892//negative regulation of transcription, DNA-templated;GC

movement;GO:0051497//negative regulation of stress fiber assembly;GO:0061436//establishment

'/cholesterol efflux;GO:0042060//wound healing;GO:0042632//cholesterol homeostasis;GO:004263

·mediated signaling pathway;GO:0070374//positive regulation of ERK1 and ERK2 cascade;GO:0071

ransport;GO:0031623//receptor internalization;GO:0034333//adherens junction assembly;GO:0035

):0060060//post-embryonic retina morphogenesis in camera-type eye;GO:0060219//camera-type

56//negative regulation of apoptotic process;GO:0045165//cell fate commitment;GO:0045444//fat

dent cell spreading;GO:0035987//endodermal cell differentiation;GO:0042060//wound healing;GO

norphogenesis;GO:0003215//cardiac right ventricle morphogenesis;GO:0003281//ventricular septu

:ess;GO:0010804//negative regulation of tumor necrosis factor-mediated signaling pathway;GO:00

):0060060//post-embryonic retina morphogenesis in camera-type eye;GO:0060219//camera-type

ne morphogenesis;GO:0071310//cellular response to organic substance;GO:2000463//positive regu

ction potential involved in contraction;GO:0086005//ventricular cardiac muscle cell action potentia

56//negative regulation of apoptotic process;GO:0045165//cell fate commitment;GO:0045444//fat

)//positive regulation of cell migration involved in sprouting angiogenesis;GO:2000773//negative

ye morphogenesis;GO:0048386//positive regulation of retinoic acid receptor signaling pathway;GC

):0060060//post-embryonic retina morphogenesis in camera-type eye;GO:0060219//camera-type

esponse to fatty acid;GO:0072593//reactive oxygen species metabolic process;GO:2000811//negat

//cholesterol efflux;GO:0042060//wound healing;GO:0042632//cholesterol homeostasis;GO:00426[illegible]

68//positive regulation of phosphatidylinositol 3-kinase signaling;GO:0014834//skeletal muscle sa

dent cell spreading;GO:0035987//endodermal cell differentiation;GO:0042060//wound healing;GO
e regulation of fatty acid oxidation;GO:0046325//negative regulation of glucose import;GO:00507[illegible]

308//negative regulation of cell growth;GO:0032270//positive regulation of cellular protein metab[illegible]

308//negative regulation of cell growth;GO:0032270//positive regulation of cellular protein metab[illegible]

e regulation of fatty acid oxidation;GO:0046325//negative regulation of glucose import;GO:00507[illegible]

igomerization;GO:0051988//regulation of attachment of spindle microtubules to kinetochore;GO:[illegible]

)//positive regulation of cell migration involved in sprouting angiogenesis;GO:2000773//negative

):0060060//post-embryonic retina morphogenesis in camera-type eye;GO:0060219//camera-type

5//cellular lipid metabolic process;GO:0051607//defense response to virus;GO:0055089//fatty acid

esponse to fatty acid;GO:0072593//reactive oxygen species metabolic process;GO:2000811//negat

cess;GO:0010804//negative regulation of tumor necrosis factor-mediated signaling pathway;GO:0(

ne morphogenesis;GO:0071310//cellular response to organic substance;GO:2000463//positive regu

;;GO:0060840//artery development;GO:0070837//dehydroascorbic acid transport;GO:0072498//em

tic process;GO:1904831//positive regulation of aortic smooth muscle cell differentiation;GO:19908

)//positive regulation of cell migration involved in sprouting angiogenesis;GO:2000773//negative

e regulation of fatty acid oxidation;GO:0046325//negative regulation of glucose import;GO:00507

308//negative regulation of cell growth;GO:0032270//positive regulation of cellular protein metab

esponse to fatty acid;GO:0072593//reactive oxygen species metabolic process;GO:2000811//negat

308//negative regulation of cell growth;GO:0032270//positive regulation of cellular protein metab

e regulation of fatty acid oxidation;GO:0046325//negative regulation of glucose import;GO:00507[illegible]

ex assembly;GO:0048255//mRNA stabilization;GO:0051091//positive regulation of sequence-spec

negative regulation of peptidyl-threonine phosphorylation;GO:0018105//peptidyl-serine phosphor

GO:0061402//positive regulation of transcription from RNA polymerase II promoter in response to

olded protein response;GO:1903899//positive regulation of PERK-mediated unfolded protein resp

;GO:0060840//artery development;GO:0070837//dehydroascorbic acid transport;GO:0072498//em

0//positive regulation of cell migration involved in sprouting angiogenesis;GO:2000773//negative

glia guided migration;GO:0021942//radial glia guided migration of Purkinje cell;GO:0045666//posi

ulation of gene expression;GO:0010629//negative regulation of gene expression;GO:0021575//hir

:ion;GO:0032816//positive regulation of natural killer cell activation;GO:0032825//positive regulatic

3//negative regulation of inflammatory response;GO:1990830//cellular response to leukemia inhib

)//positive regulation of cell migration involved in sprouting angiogenesis;GO:2000773//negative

030335//positive regulation of cell migration;GO:0032148//activation of protein kinase B activity;G

macrophage colony-stimulating factor signaling pathway;GO:1903980//positive regulation of mic

7//type I interferon signaling pathway;GO:0060339//negative regulation of type I interferon-media

alveolus development;GO:0055091//phospholipid homeostasis;GO:0060022//hard palate developr

ne morphogenesis;GO:0071310//cellular response to organic substance;GO:2000463//positive regu

308//negative regulation of cell growth;GO:0032270//positive regulation of cellular protein metab

tion;GO:0032816//positive regulation of natural killer cell activation;GO:0032825//positive regulatio

68//positive regulation of phosphatidylinositol 3-kinase signaling;GO:0014834//skeletal muscle sa

cess;GO:0010804//negative regulation of tumor necrosis factor-mediated signaling pathway;GO:00

e regulation of fatty acid oxidation;GO:0046325//negative regulation of glucose import;GO:00507
dulation of synaptic transmission;GO:0051480//regulation of cytosolic calcium ion concentration;G

negative regulation of peptidyl-threonine phosphorylation;GO:0018105//peptidyl-serine phosphor

;ynaptic plasticity;GO:0048814//regulation of dendrite morphogenesis;GO:0050806//positive regul

3//negative regulation of inflammatory response;GO:1990830//cellular response to leukemia inhib

56//negative regulation of apoptotic process;GO:0045165//cell fate commitment;GO:0045444//fat

norphogenesis;GO:0003215//cardiac right ventricle morphogenesis;GO:0003281//ventricular septu

68//positive regulation of phosphatidylinositol 3-kinase signaling;GO:0014834//skeletal muscle sa

030335//positive regulation of cell migration;GO:0032148//activation of protein kinase B activity;G

macrophage colony-stimulating factor signaling pathway;GO:1903980//positive regulation of mic

ıe morphogenesis;GO:0071310//cellular response to organic substance;GO:2000463//positive regu

:ess;GO:0010804//negative regulation of tumor necrosis factor-mediated signaling pathway;GO:00

'antigen receptor-mediated signaling pathway;GO:0070849//response to epidermal growth factor

GO:0033700//phospholipid efflux;GO:0034372//very-low-density lipoprotein particle remodeling;

synaptic plasticity;GO:0048814//regulation of dendrite morphogenesis;GO:0050806//positive regul

oprotein transport;GO:0042632//cholesterol homeostasis;GO:0042987//amyloid precursor protein

ve regulation of transcription from RNA polymerase II promoter;GO:0061029//eyelid developmen
'/cholesterol efflux;GO:0042060//wound healing;GO:0042632//cholesterol homeostasis;GO:004263

·mediated signaling pathway;GO:0070374//positive regulation of ERK1 and ERK2 cascade;GO:0071

:ion;GO:0032816//positive regulation of natural killer cell activation;GO:0032825//positive regulatic

dulation of synaptic transmission;GO:0051480//regulation of cytosolic calcium ion concentration;G

gulation of Rho protein signal transduction;GO:0043410//positive regulation of MAPK cascade;GC

ve regulation of transcription from RNA polymerase II promoter;GO:0061029//eyelid developmen

ex assembly;GO:0048255//mRNA stabilization;GO:0051091//positive regulation of sequence-spec

'antigen receptor-mediated signaling pathway;GO:0070849//response to epidermal growth factor

ne morphogenesis;GO:0071310//cellular response to organic substance;GO:2000463//positive regu

cess;GO:0010804//negative regulation of tumor necrosis factor-mediated signaling pathway;GO:00

96//positive regulation of lipid catabolic process;GO:0051005//negative regulation of lipoprotein li

tion;GO:0032816//positive regulation of natural killer cell activation;GO:0032825//positive regulatio

dulation of synaptic transmission;GO:0051480//regulation of cytosolic calcium ion concentration;G

GO:0033700//phospholipid efflux;GO:0034372//very-low-density lipoprotein particle remodeling;
56//negative regulation of apoptotic process;GO:0045165//cell fate commitment;GO:0045444//fat

synaptic plasticity;GO:0048814//regulation of dendrite morphogenesis;GO:0050806//positive regul

synaptic plasticity;GO:0048814//regulation of dendrite morphogenesis;GO:0050806//positive regul

cess;GO:0010804//negative regulation of tumor necrosis factor-mediated signaling pathway;GO:00

oprotein transport;GO:0042632//cholesterol homeostasis;GO:0042987//amyloid precursor protein

fat cell differentiation;GO:0055096//low-density lipoprotein particle mediated signaling;GO:00703
igomerization;GO:0051988//regulation of attachment of spindle microtubules to kinetochore;GO:0

ıe morphogenesis;GO:0071310//cellular response to organic substance;GO:2000463//positive regu

68//positive regulation of phosphatidylinositol 3-kinase signaling;GO:0014834//skeletal muscle sa

fat cell differentiation;GO:0055096//low-density lipoprotein particle mediated signaling;GO:00703

macrophage colony-stimulating factor signaling pathway;GO:1903980//positive regulation of mic

:ion;GO:0032816//positive regulation of natural killer cell activation;GO:0032825//positive regulatio

synaptic plasticity;GO:0048814//regulation of dendrite morphogenesis;GO:0050806//positive regul

):0060060//post-embryonic retina morphogenesis in camera-type eye;GO:0060219//camera-type

norphogenesis;GO:0003215//cardiac right ventricle morphogenesis;GO:0003281//ventricular septu

tic process;GO:1904831//positive regulation of aortic smooth muscle cell differentiation;GO:19908

zation;GO:0045444//fat cell differentiation;GO:0045599//negative regulation of fat cell differentiati

esponse to fatty acid;GO:0072593//reactive oxygen species metabolic process;GO:2000811//negat

oprotein transport;GO:0042632//cholesterol homeostasis;GO:0042987//amyloid precursor protein

ed in apoptotic process;GO:0043410//positive regulation of MAPK cascade;GO:0048012//hepatocy

olded protein response;GO:1903899//positive regulation of PERK-mediated unfolded protein respo

68//positive regulation of phosphatidylinositol 3-kinase signaling;GO:0014834//skeletal muscle sa

glia guided migration;GO:0021942//radial glia guided migration of Purkinje cell;GO:0045666//posi

):0060060//post-embryonic retina morphogenesis in camera-type eye;GO:0060219//camera-type

ne morphogenesis;GO:0071310//cellular response to organic substance;GO:2000463//positive regu

cess;GO:0010804//negative regulation of tumor necrosis factor-mediated signaling pathway;GO:00

esponse to fatty acid;GO:0072593//reactive oxygen species metabolic process;GO:2000811//negat

tiation;GO:0048286//lung alveolus development;GO:0055085//transmembrane transport;GO:0055

alveolus development;GO:0055091//phospholipid homeostasis;GO:0060022//hard palate developr

:ion;GO:0032816//positive regulation of natural killer cell activation;GO:0032825//positive regulatic

):0060060//post-embryonic retina morphogenesis in camera-type eye;GO:0060219//camera-type

ed in apoptotic process;GO:0043410//positive regulation of MAPK cascade;GO:0048012//hepatocy

norphogenesis;GO:0003215//cardiac right ventricle morphogenesis;GO:0003281//ventricular septu

96//positive regulation of lipid catabolic process;GO:0051005//negative regulation of lipoprotein li

glia guided migration;GO:0021942//radial glia guided migration of Purkinje cell;GO:0045666//posi

esponse to fatty acid;GO:0072593//reactive oxygen species metabolic process;GO:2000811//negat

fat cell differentiation;GO:0055096//low-density lipoprotein particle mediated signaling;GO:00703

e regulation of fatty acid oxidation;GO:0046325//negative regulation of glucose import;GO:00507[illegible]

68//positive regulation of phosphatidylinositol 3-kinase signaling;GO:0014834//skeletal muscle sa[illegible]

68//positive regulation of phosphatidylinositol 3-kinase signaling;GO:0014834//skeletal muscle sa[illegible]

308//negative regulation of cell growth;GO:0032270//positive regulation of cellular protein metab[illegible]

3O:0061402//positive regulation of transcription from RNA polymerase II promoter in response to

dent cell spreading;GO:0035987//endodermal cell differentiation;GO:0042060//wound healing;GO

e regulation of fatty acid oxidation;GO:0046325//negative regulation of glucose import;GO:00507[illegible]

308//negative regulation of cell growth;GO:0032270//positive regulation of cellular protein metab[illegible]

)//positive regulation of cell migration involved in sprouting angiogenesis;GO:2000773//negative

68//positive regulation of phosphatidylinositol 3-kinase signaling;GO:0014834//skeletal muscle sa

fat cell differentiation;GO:0055096//low-density lipoprotein particle mediated signaling;GO:00703

5//cellular lipid metabolic process;GO:0051607//defense response to virus;GO:0055089//fatty acid

7//type I interferon signaling pathway;GO:0060339//negative regulation of type I interferon-medi

0//positive regulation of cell migration involved in sprouting angiogenesis;GO:2000773//negative

fat cell differentiation;GO:0055096//low-density lipoprotein particle mediated signaling;GO:00703

tiation;GO:0048286//lung alveolus development;GO:0055085//transmembrane transport;GO:0055

ndrial membrane potential;GO:1903426//regulation of reactive oxygen species biosynthetic proces

fat cell differentiation;GO:0055096//low-density lipoprotein particle mediated signaling;GO:00703

dent cell spreading;GO:0035987//endodermal cell differentiation;GO:0042060//wound healing;GO

68//positive regulation of phosphatidylinositol 3-kinase signaling;GO:0014834//skeletal muscle sa

ction potential involved in contraction;GO:0086005//ventricular cardiac muscle cell action potentia

ndrial membrane potential;GO:1903426//regulation of reactive oxygen species biosynthetic proces

:0008543//fibroblast growth factor receptor signaling pathway;GO:0008544//epidermis developme

0//positive regulation of cell migration involved in sprouting angiogenesis;GO:2000773//negative

):0060060//post-embryonic retina morphogenesis in camera-type eye;GO:0060219//camera-type

:ion;GO:0032816//positive regulation of natural killer cell activation;GO:0032825//positive regulatio

synaptic plasticity;GO:0048814//regulation of dendrite morphogenesis;GO:0050806//positive regul

dulation of synaptic transmission;GO:0051480//regulation of cytosolic calcium ion concentration;G

-mediated signaling pathway;GO:0070374//positive regulation of ERK1 and ERK2 cascade;GO:0071

:ion;GO:0032816//positive regulation of natural killer cell activation;GO:0032825//positive regulatio

-mediated signaling pathway;GO:0070374//positive regulation of ERK1 and ERK2 cascade;GO:0071

ulation of gene expression;GO:0010629//negative regulation of gene expression;GO:0021575//hir

56//negative regulation of apoptotic process;GO:0045165//cell fate commitment;GO:0045444//fat

cess;GO:0010804//negative regulation of tumor necrosis factor-mediated signaling pathway;GO:00

//cholesterol efflux;GO:0042060//wound healing;GO:0042632//cholesterol homeostasis;GO:004263

dent cell spreading;GO:0035987//endodermal cell differentiation;GO:0042060//wound healing;GO
fat cell differentiation;GO:0055096//low-density lipoprotein particle mediated signaling;GO:00703
ne morphogenesis;GO:0071310//cellular response to organic substance;GO:2000463//positive regu

308//negative regulation of cell growth;GO:0032270//positive regulation of cellular protein metab

e regulation of fatty acid oxidation;GO:0046325//negative regulation of glucose import;GO:00507

ve regulation of proteolysis;GO:0045892//negative regulation of transcription, DNA-templated;GC

glia guided migration;GO:0021942//radial glia guided migration of Purkinje cell;GO:0045666//posi

'/cholesterol efflux;GO:0042060//wound healing;GO:0042632//cholesterol homeostasis;GO:00426

3//negative regulation of inflammatory response;GO:1990830//cellular response to leukemia inhib

)//positive regulation of cell migration involved in sprouting angiogenesis;GO:2000773//negative

ynaptic plasticity;GO:0048814//regulation of dendrite morphogenesis;GO:0050806//positive regul

norphogenesis;GO:0003215//cardiac right ventricle morphogenesis;GO:0003281//ventricular septu

legative regulation of peptidyl-threonine phosphorylation;GO:0018105//peptidyl-serine phosphor

6//negative regulation of apoptotic process;GO:0045165//cell fate commitment;GO:0045444//fat

:ess;GO:0010804//negative regulation of tumor necrosis factor-mediated signaling pathway;GO:0(

protein transport;GO:0042632//cholesterol homeostasis;GO:0042987//amyloid precursor protein

):0060060//post-embryonic retina morphogenesis in camera-type eye;GO:0060219//camera-type

030335//positive regulation of cell migration;GO:0032148//activation of protein kinase B activity;G

ıe morphogenesis;GO:0071310//cellular response to organic substance;GO:2000463//positive regu

):0060060//post-embryonic retina morphogenesis in camera-type eye;GO:0060219//camera-type

tiation;GO:0048286//lung alveolus development;GO:0055085//transmembrane transport;GO:0055

tion;GO:0032816//positive regulation of natural killer cell activation;GO:0032825//positive regulatic

-mediated signaling pathway;GO:0070374//positive regulation of ERK1 and ERK2 cascade;GO:0071

308//negative regulation of cell growth;GO:0032270//positive regulation of cellular protein metab

ve regulation of transcription from RNA polymerase II promoter;GO:0061029//eyelid developmen

68//positive regulation of phosphatidylinositol 3-kinase signaling;GO:0014834//skeletal muscle sa

e regulation of fatty acid oxidation;GO:0046325//negative regulation of glucose import;GO:00507[illegible]

[illegible]:0060060//post-embryonic retina morphogenesis in camera-type eye;GO:0060219//camera-type

[illegible]glia guided migration;GO:0021942//radial glia guided migration of Purkinje cell;GO:0045666//posi

'/cholesterol efflux;GO:0042060//wound healing;GO:0042632//cholesterol homeostasis;GO:004263

;ynaptic plasticity;GO:0048814//regulation of dendrite morphogenesis;GO:0050806//positive regul

3//negative regulation of inflammatory response;GO:1990830//cellular response to leukemia inhib

ye morphogenesis;GO:0048386//positive regulation of retinoic acid receptor signaling pathway;GC

'antigen receptor-mediated signaling pathway;GO:0070849//response to epidermal growth factor

e regulation of fatty acid oxidation;GO:0046325//negative regulation of glucose import;GO:00507(

:ess;GO:0010804//negative regulation of tumor necrosis factor-mediated signaling pathway;GO:0(

308//negative regulation of cell growth;GO:0032270//positive regulation of cellular protein metab

030335//positive regulation of cell migration;GO:0032148//activation of protein kinase B activity;G

ne morphogenesis;GO:0071310//cellular response to organic substance;GO:2000463//positive regu

'/cholesterol efflux;GO:0042060//wound healing;GO:0042632//cholesterol homeostasis;GO:00426[illegible]

ve regulation of proteolysis;GO:0045892//negative regulation of transcription, DNA-templated;G[illegible]

synaptic plasticity;GO:0048814//regulation of dendrite morphogenesis;GO:0050806//positive regul

otassium ion transmembrane transport;GO:0098662//inorganic cation transmembrane transport

):0060060//post-embryonic retina morphogenesis in camera-type eye;GO:0060219//camera-type

3//negative regulation of inflammatory response;GO:1990830//cellular response to leukemia inhib

)//positive regulation of cell migration involved in sprouting angiogenesis;GO:2000773//negative

e regulation of fatty acid oxidation;GO:0046325//negative regulation of glucose import;GO:00507[illegible]

030335//positive regulation of cell migration;GO:0032148//activation of protein kinase B activity;G
sis;GO:0007286//spermatid development;GO:0007626//locomotory behavior;GO:0009992//cellula

308//negative regulation of cell growth;GO:0032270//positive regulation of cellular protein metab

tic process;GO:1904831//positive regulation of aortic smooth muscle cell differentiation;GO:19908

fat cell differentiation;GO:0055096//low-density lipoprotein particle mediated signaling;GO:00703

'antigen receptor-mediated signaling pathway;GO:0070849//response to epidermal growth factor

synaptic plasticity;GO:0048814//regulation of dendrite morphogenesis;GO:0050806//positive regul

synaptic plasticity;GO:0048814//regulation of dendrite morphogenesis;GO:0050806//positive regul

)//positive regulation of cell migration involved in sprouting angiogenesis;GO:2000773//negative

se activity involved in apoptotic process;GO:0043588//skin development;GO:0061024//membrane

;;GO:0060840//artery development;GO:0070837//dehydroascorbic acid transport;GO:0072498//em

fat cell differentiation;GO:0055096//low-density lipoprotein particle mediated signaling;GO:00703

):0060060//post-embryonic retina morphogenesis in camera-type eye;GO:0060219//camera-type

norphogenesis;GO:0003215//cardiac right ventricle morphogenesis;GO:0003281//ventricular septu
dulation of synaptic transmission;GO:0051480//regulation of cytosolic calcium ion concentration;G

e regulation of fatty acid oxidation;GO:0046325//negative regulation of glucose import;GO:00507

ne morphogenesis;GO:0071310//cellular response to organic substance;GO:2000463//positive regu

308//negative regulation of cell growth;GO:0032270//positive regulation of cellular protein metab

'antigen receptor-mediated signaling pathway;GO:0070849//response to epidermal growth factor

cess;GO:0010804//negative regulation of tumor necrosis factor-mediated signaling pathway;GO:00

ed in apoptotic process;GO:0043410//positive regulation of MAPK cascade;GO:0048012//hepatoc

igomerization;GO:0051988//regulation of attachment of spindle microtubules to kinetochore;GO:

esponse to fatty acid;GO:0072593//reactive oxygen species metabolic process;GO:2000811//negat

glia guided migration;GO:0021942//radial glia guided migration of Purkinje cell;GO:0045666//posi
ed in apoptotic process;GO:0043410//positive regulation of MAPK cascade;GO:0048012//hepatoc

rocess;GO:0042761//very long-chain fatty acid biosynthetic process;GO:0044249//cellular biosynth

)//positive regulation of cell migration involved in sprouting angiogenesis;GO:2000773//negative

-mediated signaling pathway;GO:0070374//positive regulation of ERK1 and ERK2 cascade;GO:0071

ne morphogenesis;GO:0071310//cellular response to organic substance;GO:2000463//positive regu

tion;GO:0032816//positive regulation of natural killer cell activation;GO:0032825//positive regulatio

cess;GO:0010804//negative regulation of tumor necrosis factor-mediated signaling pathway;GO:00
68//positive regulation of phosphatidylinositol 3-kinase signaling;GO:0014834//skeletal muscle sa
dent cell spreading;GO:0035987//endodermal cell differentiation;GO:0042060//wound healing;GO

ve regulation of proteolysis;GO:0045892//negative regulation of transcription, DNA-templated;GO

oprotein transport;GO:0042632//cholesterol homeostasis;GO:0042987//amyloid precursor protein

glia guided migration;GO:0021942//radial glia guided migration of Purkinje cell;GO:0045666//posi

):0060060//post-embryonic retina morphogenesis in camera-type eye;GO:0060219//camera-type

olded protein response;GO:1903899//positive regulation of PERK-mediated unfolded protein respo

dulation of synaptic transmission;GO:0051480//regulation of cytosolic calcium ion concentration;G

tion;GO:0032816//positive regulation of natural killer cell activation;GO:0032825//positive regulatio

:ion;GO:0032816//positive regulation of natural killer cell activation;GO:0032825//positive regulatic

ed in apoptotic process;GO:0043410//positive regulation of MAPK cascade;GO:0048012//hepatocy

e regulation of fatty acid oxidation;GO:0046325//negative regulation of glucose import;GO:00507

68//positive regulation of phosphatidylinositol 3-kinase signaling;GO:0014834//skeletal muscle sa

308//negative regulation of cell growth;GO:0032270//positive regulation of cellular protein metab

fat cell differentiation;GO:0055096//low-density lipoprotein particle mediated signaling;GO:00703

esponse to fatty acid;GO:0072593//reactive oxygen species metabolic process;GO:2000811//negat

sis;GO:0007286//spermatid development;GO:0007626//locomotory behavior;GO:0009992//cellula

oprotein transport;GO:0042632//cholesterol homeostasis;GO:0042987//amyloid precursor protein

tiation;GO:0048286//lung alveolus development;GO:0055085//transmembrane transport;GO:0055

tic process;GO:1904831//positive regulation of aortic smooth muscle cell differentiation;GO:19908

ransport;GO:0031623//receptor internalization;GO:0034333//adherens junction assembly;GO:0035

)//positive regulation of cell migration involved in sprouting angiogenesis;GO:2000773//negative

ed in apoptotic process;GO:0043410//positive regulation of MAPK cascade;GO:0048012//hepatocy

/antigen receptor-mediated signaling pathway;GO:0070849//response to epidermal growth factor

zation;GO:0045444//fat cell differentiation;GO:0045599//negative regulation of fat cell differentiati

:ion;GO:0032816//positive regulation of natural killer cell activation;GO:0032825//positive regulatic

tiation;GO:0048286//lung alveolus development;GO:0055085//transmembrane transport;GO:0055

esponse to fatty acid;GO:0072593//reactive oxygen species metabolic process;GO:2000811//negat

norphogenesis;GO:0003215//cardiac right ventricle morphogenesis;GO:0003281//ventricular septu

'/cholesterol efflux;GO:0042060//wound healing;GO:0042632//cholesterol homeostasis;GO:004263

ation;GO:0033292//T-tubule organization;GO:0034599//cellular response to oxidative stress;GO:00

3//negative regulation of inflammatory response;GO:1990830//cellular response to leukemia inhib

)086010//membrane depolarization during action potential;GO:0098662//inorganic cation transm

030335//positive regulation of cell migration;GO:0032148//activation of protein kinase B activity;G

030335//positive regulation of cell migration;GO:0032148//activation of protein kinase B activity;G

esponse to fatty acid;GO:0072593//reactive oxygen species metabolic process;GO:2000811//negat

3//negative regulation of inflammatory response;GO:1990830//cellular response to leukemia inhib

/positive regulation of ERK1 and ERK2 cascade;GO:0070527//platelet aggregation;GO:0072378//bl

ation;GO:0033292//T-tubule organization;GO:0034599//cellular response to oxidative stress;GO:00

44//phospholipid metabolic process;GO:0007493//endodermal cell fate determination;GO:00085

rage;GO:0016042//lipid catabolic process;GO:0019433//triglyceride catabolic process;GO:0030301
ve regulation of proteolysis;GO:0045892//negative regulation of transcription, DNA-templated;G

308//negative regulation of cell growth;GO:0032270//positive regulation of cellular protein metab

e regulation of fatty acid oxidation;GO:0046325//negative regulation of glucose import;GO:00507[illegible]

tive regulation of potassium ion transmembrane transporter activity;GO:1901018//positive regulat

n D receptor signaling pathway;GO:0071391//cellular response to estrogen stimulus;GO:1901796/[illegible]

ation;GO:0033292//T-tubule organization;GO:0034599//cellular response to oxidative stress;GO:00

tassium ion transmembrane transport;GO:0098662//inorganic cation transmembrane transport

n D receptor signaling pathway;GO:0071391//cellular response to estrogen stimulus;GO:1901796//

7//type I interferon signaling pathway;GO:0060339//negative regulation of type I interferon-medi

ye morphogenesis;GO:0048386//positive regulation of retinoic acid receptor signaling pathway;GO

renergic neuron differentiation;GO:0006468//protein phosphorylation;GO:0006874//cellular calciu
ulation of gene expression;GO:0010629//negative regulation of gene expression;GO:0021575//hi

system development;GO:0060021//palate development;GO:0060271//cilium morphogenesis;GO:(

rage;GO:0016042//lipid catabolic process;GO:0019433//triglyceride catabolic process;GO:0030301

movement;GO:0051497//negative regulation of stress fiber assembly;GO:0061436//establishment
esis;GO:0003420//regulation of growth plate cartilage chondrocyte proliferation;GO:0006029//prot

n D receptor signaling pathway;GO:0071391//cellular response to estrogen stimulus;GO:1901796/

ye morphogenesis;GO:0048386//positive regulation of retinoic acid receptor signaling pathway;GC

tive regulation of potassium ion transmembrane transporter activity;GO:1901018//positive regulat

sis;GO:0007286//spermatid development;GO:0007626//locomotory behavior;GO:0009992//cellula

se activity involved in apoptotic process;GO:0043588//skin development;GO:0061024//membrane

rocess;GO:0042761//very long-chain fatty acid biosynthetic process;GO:0044249//cellular biosynth

esis;GO:0003420//regulation of growth plate cartilage chondrocyte proliferation;GO:0006029//prot

sis;GO:0007286//spermatid development;GO:0007626//locomotory behavior;GO:0009992//cellular

544//phospholipid metabolic process;GO:0007493//endodermal cell fate determination;GO:00085[illegible]

56//negative regulation of apoptotic process;GO:0045165//cell fate commitment;GO:0045444//fat

ve regulation of transcription from RNA polymerase II promoter;GO:0061029//eyelid development

gulation of Rho protein signal transduction;GO:0043410//positive regulation of MAPK cascade;GC

movement;GO:0051497//negative regulation of stress fiber assembly;GO:0061436//establishment

)035//response to inorganic substance;GO:0010460//positive regulation of heart rate;GO:0014074/

renergic neuron differentiation;GO:0006468//protein phosphorylation;GO:0006874//cellular calciu

gulation of Rho protein signal transduction;GO:0043410//positive regulation of MAPK cascade;GC

sis;GO:0007286//spermatid development;GO:0007626//locomotory behavior;GO:0009992//cellular

n D receptor signaling pathway;GO:0071391//cellular response to estrogen stimulus;GO:1901796//

synaptic plasticity;GO:0048814//regulation of dendrite morphogenesis;GO:0050806//positive regul

ion;GO:0003208//cardiac ventricle morphogenesis;GO:0003214//cardiac left ventricle morphogen

ation;GO:0033292//T-tubule organization;GO:0034599//cellular response to oxidative stress;GO:00

7//type I interferon signaling pathway;GO:0060339//negative regulation of type I interferon-media

96//positive regulation of lipid catabolic process;GO:0051005//negative regulation of lipoprotein li

movement;GO:0051497//negative regulation of stress fiber assembly;GO:0061436//establishment

544//phospholipid metabolic process;GO:0007493//endodermal cell fate determination;GO:00085

rage;GO:0016042//lipid catabolic process;GO:0019433//triglyceride catabolic process;GO:0030301

sis;GO:0007286//spermatid development;GO:0007626//locomotory behavior;GO:0009992//cellular

esis;GO:0003420//regulation of growth plate cartilage chondrocyte proliferation;GO:0006029//prot

gulation of Rho protein signal transduction;GO:0043410//positive regulation of MAPK cascade;GC

movement;GO:0051497//negative regulation of stress fiber assembly;GO:0061436//establishment

96//positive regulation of lipid catabolic process;GO:0051005//negative regulation of lipoprotein li

0035//response to inorganic substance;GO:0010460//positive regulation of heart rate;GO:0014074/

7//type I interferon signaling pathway;GO:0060339//negative regulation of type I interferon-media

system development;GO:0060021//palate development;GO:0060271//cilium morphogenesis;GO:C

96//positive regulation of lipid catabolic process;GO:0051005//negative regulation of lipoprotein li

se activity involved in apoptotic process;GO:0043588//skin development;GO:0061024//membrane

ation;GO:0033292//T-tubule organization;GO:0034599//cellular response to oxidative stress;GO:00

544//phospholipid metabolic process;GO:0007493//endodermal cell fate determination;GO:00085

ion;GO:0003208//cardiac ventricle morphogenesis;GO:0003214//cardiac left ventricle morphogen

regulation of osteoblast differentiation;GO:0045786//negative regulation of cell cycle;GO:004814[illegible]

gulation of Rho protein signal transduction;GO:0043410//positive regulation of MAPK cascade;G[illegible]

tic process;GO:1904831//positive regulation of aortic smooth muscle cell differentiation;GO:19908

esis;GO:0003420//regulation of growth plate cartilage chondrocyte proliferation;GO:0006029//prot

;GO:0060840//artery development;GO:0070837//dehydroascorbic acid transport;GO:0072498//em

ɔtassium ion transmembrane transport;GO:0098662//inorganic cation transmembrane transport

ye morphogenesis;GO:0048386//positive regulation of retinoic acid receptor signaling pathway;G[illegible]

system development;GO:0060021//palate development;GO:0060271//cilium morphogenesis;GO:[illegible]

regulation of osteoblast differentiation;GO:0045786//negative regulation of cell cycle;GO:004814[illegible]

68//positive regulation of phosphatidylinositol 3-kinase signaling;GO:0014834//skeletal muscle sa

system development;GO:0060021//palate development;GO:0060271//cilium morphogenesis;GO:0

n;GO:0046513//ceramide biosynthetic process;GO:0061436//establishment of skin barrier

n D receptor signaling pathway;GO:0071391//cellular response to estrogen stimulus;GO:1901796/

tic process;GO:1904831//positive regulation of aortic smooth muscle cell differentiation;GO:19908

system development;GO:0060021//palate development;GO:0060271//cilium morphogenesis;GO:(

)086010//membrane depolarization during action potential;GO:0098662//inorganic cation transm

tive regulation of potassium ion transmembrane transporter activity;GO:1901018//positive regulat

ulation of gene expression;GO:0010629//negative regulation of gene expression;GO:0021575//hir

n;GO:0046513//ceramide biosynthetic process;GO:0061436//establishment of skin barrier

rage;GO:0016042//lipid catabolic process;GO:0019433//triglyceride catabolic process;GO:0030301

ndrial membrane potential;GO:1903426//regulation of reactive oxygen species biosynthetic proces

ye morphogenesis;GO:0048386//positive regulation of retinoic acid receptor signaling pathway;GC

esponse to fatty acid;GO:0072593//reactive oxygen species metabolic process;GO:2000811//negat

system development;GO:0060021//palate development;GO:0060271//cilium morphogenesis;GO:0

ulation of gene expression;GO:0010629//negative regulation of gene expression;GO:0021575//hir

/positive regulation of ERK1 and ERK2 cascade;GO:0070527//platelet aggregation;GO:0072378//bl

ve regulation of transcription from RNA polymerase II promoter;GO:0061029//eyelid developmen
0035//response to inorganic substance;GO:0010460//positive regulation of heart rate;GO:0014074/

ulation of gene expression;GO:0010629//negative regulation of gene expression;GO:0021575//hir

·mediated signaling pathway;GO:0070374//positive regulation of ERK1 and ERK2 cascade;GO:0071

renergic neuron differentiation;GO:0006468//protein phosphorylation;GO:0006874//cellular calciu

macrophage colony-stimulating factor signaling pathway;GO:1903980//positive regulation of mic

ransport;GO:0031623//receptor internalization;GO:0034333//adherens junction assembly;GO:0035

gulation of Rho protein signal transduction;GO:0043410//positive regulation of MAPK cascade;GC

)6//positive regulation of lipid catabolic process;GO:0051005//negative regulation of lipoprotein li

movement;GO:0051497//negative regulation of stress fiber assembly;GO:0061436//establishment

ye morphogenesis;GO:0048386//positive regulation of retinoic acid receptor signaling pathway;GC

)035//response to inorganic substance;GO:0010460//positive regulation of heart rate;GO:0014074/

7//type I interferon signaling pathway;GO:0060339//negative regulation of type I interferon-media

544//phospholipid metabolic process;GO:0007493//endodermal cell fate determination;GO:00085

ation;GO:0033292//T-tubule organization;GO:0034599//cellular response to oxidative stress;GO:00

ulation of gene expression;GO:0010629//negative regulation of gene expression;GO:0021575//hir

movement;GO:0051497//negative regulation of stress fiber assembly;GO:0061436//establishment

ion;GO:0003208//cardiac ventricle morphogenesis;GO:0003214//cardiac left ventricle morphogen

gulation of Rho protein signal transduction;GO:0043410//positive regulation of MAPK cascade;GC

tiation;GO:0048286//lung alveolus development;GO:0055085//transmembrane transport;GO:0055

'positive regulation of ERK1 and ERK2 cascade;GO:0070527//platelet aggregation;GO:0072378//bl

renergic neuron differentiation;GO:0006468//protein phosphorylation;GO:0006874//cellular calciu
ye morphogenesis;GO:0048386//positive regulation of retinoic acid receptor signaling pathway;GC

96//positive regulation of lipid catabolic process;GO:0051005//negative regulation of lipoprotein li

544//phospholipid metabolic process;GO:0007493//endodermal cell fate determination;GO:00085

n;GO:0046513//ceramide biosynthetic process;GO:0061436//establishment of skin barrier

movement;GO:0051497//negative regulation of stress fiber assembly;GO:0061436//establishment

7//type I interferon signaling pathway;GO:0060339//negative regulation of type I interferon-medi

ndrial membrane potential;GO:1903426//regulation of reactive oxygen species biosynthetic proces
gulation of Rho protein signal transduction;GO:0043410//positive regulation of MAPK cascade;GC

renergic neuron differentiation;GO:0006468//protein phosphorylation;GO:0006874//cellular calciu

rocess;GO:0042761//very long-chain fatty acid biosynthetic process;GO:0044249//cellular biosynth

system development;GO:0060021//palate development;GO:0060271//cilium morphogenesis;GO:0

544//phospholipid metabolic process;GO:0007493//endodermal cell fate determination;GO:00085

;GO:0060840//artery development;GO:0070837//dehydroascorbic acid transport;GO:0072498//em

)6//positive regulation of lipid catabolic process;GO:0051005//negative regulation of lipoprotein li

)6//positive regulation of lipid catabolic process;GO:0051005//negative regulation of lipoprotein li

ye morphogenesis;GO:0048386//positive regulation of retinoic acid receptor signaling pathway;GC

:ess;GO:0010804//negative regulation of tumor necrosis factor-mediated signaling pathway;GO:0C

fat cell differentiation;GO:0055096//low-density lipoprotein particle mediated signaling;GO:00703

ıe morphogenesis;GO:0071310//cellular response to organic substance;GO:2000463//positive regu

regulation of osteoblast differentiation;GO:0045786//negative regulation of cell cycle;GO:004814[illegible]

[illegible];GO:0046513//ceramide biosynthetic process;GO:0061436//establishment of skin barrier

rocess;GO:0042761//very long-chain fatty acid biosynthetic process;GO:0044249//cellular biosynt[illegible]

7//type I interferon signaling pathway;GO:0060339//negative regulation of type I interferon-medi[illegible]

n D receptor signaling pathway;GO:0071391//cellular response to estrogen stimulus;GO:1901796/[illegible]

[illegible]44//phospholipid metabolic process;GO:0007493//endodermal cell fate determination;GO:00085[illegible]

ulation of gene expression;GO:0010629//negative regulation of gene expression;GO:0021575//hi[illegible]

system development;GO:0060021//palate development;GO:0060271//cilium morphogenesis;GO:0

tive regulation of potassium ion transmembrane transporter activity;GO:1901018//positive regulat

system development;GO:0060021//palate development;GO:0060271//cilium morphogenesis;GO:0

:esis;GO:0003420//regulation of growth plate cartilage chondrocyte proliferation;GO:0006029//prot

)6//positive regulation of lipid catabolic process;GO:0051005//negative regulation of lipoprotein li

norphogenesis;GO:0003215//cardiac right ventricle morphogenesis;GO:0003281//ventricular septu

rage;GO:0016042//lipid catabolic process;GO:0019433//triglyceride catabolic process;GO:0030301

n D receptor signaling pathway;GO:0071391//cellular response to estrogen stimulus;GO:1901796/

ulation of gene expression;GO:0010629//negative regulation of gene expression;GO:0021575//hir

tive regulation of potassium ion transmembrane transporter activity;GO:1901018//positive regulat

96//positive regulation of lipid catabolic process;GO:0051005//negative regulation of lipoprotein li

rage;GO:0016042//lipid catabolic process;GO:0019433//triglyceride catabolic process;GO:0030301

esis;GO:0003420//regulation of growth plate cartilage chondrocyte proliferation;GO:0006029//prot

ulation of gene expression;GO:0010629//negative regulation of gene expression;GO:0021575//hir

movement;GO:0051497//negative regulation of stress fiber assembly;GO:0061436//establishment

ransport;GO:0031623//receptor internalization;GO:0034333//adherens junction assembly;GO:0035

)035//response to inorganic substance;GO:0010460//positive regulation of heart rate;GO:0014074/

otassium ion transmembrane transport;GO:0098662//inorganic cation transmembrane transport

ulation of gene expression;GO:0010629//negative regulation of gene expression;GO:0021575//hir

ye morphogenesis;GO:0048386//positive regulation of retinoic acid receptor signaling pathway;GC

alveolus development;GO:0055091//phospholipid homeostasis;GO:0060022//hard palate developr

ation;GO:0033292//T-tubule organization;GO:0034599//cellular response to oxidative stress;GO:00

tive regulation of potassium ion transmembrane transporter activity;GO:1901018//positive regulat

rage;GO:0016042//lipid catabolic process;GO:0019433//triglyceride catabolic process;GO:0030301

ion;GO:0003208//cardiac ventricle morphogenesis;GO:0003214//cardiac left ventricle morphogen[illegible]

movement;GO:0051497//negative regulation of stress fiber assembly;GO:0061436//establishment
se activity involved in apoptotic process;GO:0043588//skin development;GO:0061024//membrane

[illegible];GO:0046513//ceramide biosynthetic process;GO:0061436//establishment of skin barrier

movement;GO:0051497//negative regulation of stress fiber assembly;GO:0061436//establishment

gulation of Rho protein signal transduction;GO:0043410//positive regulation of MAPK cascade;GC
ye morphogenesis;GO:0048386//positive regulation of retinoic acid receptor signaling pathway;GC

rocess;GO:0042761//very long-chain fatty acid biosynthetic process;GO:0044249//cellular biosynth

system development;GO:0060021//palate development;GO:0060271//cilium morphogenesis;GO:(

ion;GO:0003208//cardiac ventricle morphogenesis;GO:0003214//cardiac left ventricle morphogen

movement;GO:0051497//negative regulation of stress fiber assembly;GO:0061436//establishment

system development;GO:0060021//palate development;GO:0060271//cilium morphogenesis;GO:0

ve regulation of transcription from RNA polymerase II promoter;GO:0061029//eyelid developmen

ulation of gene expression;GO:0010629//negative regulation of gene expression;GO:0021575//hir

ı;GO:0046513//ceramide biosynthetic process;GO:0061436//establishment of skin barrier

ɔtassium ion transmembrane transport;GO:0098662//inorganic cation transmembrane transport

gulation of Rho protein signal transduction;GO:0043410//positive regulation of MAPK cascade;GC

ye morphogenesis;GO:0048386//positive regulation of retinoic acid receptor signaling pathway;GC

96//positive regulation of lipid catabolic process;GO:0051005//negative regulation of lipoprotein li

544//phospholipid metabolic process;GO:0007493//endodermal cell fate determination;GO:00085[illegible]

fat cell differentiation;GO:0055096//low-density lipoprotein particle mediated signaling;GO:00703

regulation of osteoblast differentiation;GO:0045786//negative regulation of cell cycle;GO:004814[illegible]

rocess;GO:0042761//very long-chain fatty acid biosynthetic process;GO:0044249//cellular biosynth

ion;GO:0003208//cardiac ventricle morphogenesis;GO:0003214//cardiac left ventricle morphogen[illegible]

[illegible]se activity involved in apoptotic process;GO:0043588//skin development;GO:0061024//membrane

544//phospholipid metabolic process;GO:0007493//endodermal cell fate determination;GO:00085[illegible]

system development;GO:0060021//palate development;GO:0060271//cilium morphogenesis;GO:[illegible]

regulation of osteoblast differentiation;GO:0045786//negative regulation of cell cycle;GO:004814[illegible]

gulation of Rho protein signal transduction;GO:0043410//positive regulation of MAPK cascade;GC

movement;GO:0051497//negative regulation of stress fiber assembly;GO:0061436//establishment

ye morphogenesis;GO:0048386//positive regulation of retinoic acid receptor signaling pathway;GC

ye morphogenesis;GO:0048386//positive regulation of retinoic acid receptor signaling pathway;GC

system development;GO:0060021//palate development;GO:0060271//cilium morphogenesis;GO:(

ıdrial membrane potential;GO:1903426//regulation of reactive oxygen species biosynthetic proces

ex assembly;GO:0048255//mRNA stabilization;GO:0051091//positive regulation of sequence-spec

movement;GO:0051497//negative regulation of stress fiber assembly;GO:0061436//establishment

)035//response to inorganic substance;GO:0010460//positive regulation of heart rate;GO:0014074/

regulation of osteoblast differentiation;GO:0045786//negative regulation of cell cycle;GO:004814[illegible]

;GO:0060840//artery development;GO:0070837//dehydroascorbic acid transport;GO:0072498//em

)086010//membrane depolarization during action potential;GO:0098662//inorganic cation transm

tiation;GO:0048286//lung alveolus development;GO:0055085//transmembrane transport;GO:0055

ex assembly;GO:0048255//mRNA stabilization;GO:0051091//positive regulation of sequence-spec
ve regulation of transcription from RNA polymerase II promoter;GO:0061029//eyelid developmen

gulation of Rho protein signal transduction;GO:0043410//positive regulation of MAPK cascade;GC

ye morphogenesis;GO:0048386//positive regulation of retinoic acid receptor signaling pathway;GC

)035//response to inorganic substance;GO:0010460//positive regulation of heart rate;GO:0014074/

gulation of Rho protein signal transduction;GO:0043410//positive regulation of MAPK cascade;GC

GO:0061402//positive regulation of transcription from RNA polymerase II promoter in response to

alveolus development;GO:0055091//phospholipid homeostasis;GO:0060022//hard palate developm

//cholesterol efflux;GO:0042060//wound healing;GO:0042632//cholesterol homeostasis;GO:004263

544//phospholipid metabolic process;GO:0007493//endodermal cell fate determination;GO:00085

ye morphogenesis;GO:0048386//positive regulation of retinoic acid receptor signaling pathway;GO

ulation of gene expression;GO:0010629//negative regulation of gene expression;GO:0021575//hir

5//cellular lipid metabolic process;GO:0051607//defense response to virus;GO:0055089//fatty acid

n;GO:0046513//ceramide biosynthetic process;GO:0061436//establishment of skin barrier

544//phospholipid metabolic process;GO:0007493//endodermal cell fate determination;GO:00085[illegible]

alveolus development;GO:0055091//phospholipid homeostasis;GO:0060022//hard palate developr

esponse to fatty acid;GO:0072593//reactive oxygen species metabolic process;GO:2000811//negat

regulation of osteoblast differentiation;GO:0045786//negative regulation of cell cycle;GO:004814[illegible]

gulation of Rho protein signal transduction;GO:0043410//positive regulation of MAPK cascade;GC

gulation of Rho protein signal transduction;GO:0043410//positive regulation of MAPK cascade;GC

)035//response to inorganic substance;GO:0010460//positive regulation of heart rate;GO:0014074/
ι;GO:0046513//ceramide biosynthetic process;GO:0061436//establishment of skin barrier

sis;GO:0007286//spermatid development;GO:0007626//locomotory behavior;GO:0009992//cellula

)6//positive regulation of lipid catabolic process;GO:0051005//negative regulation of lipoprotein li

544//phospholipid metabolic process;GO:0007493//endodermal cell fate determination;GO:00085[illegible]

ction potential involved in contraction;GO:0086005//ventricular cardiac muscle cell action potentia[illegible]

0086010//membrane depolarization during action potential;GO:0098662//inorganic cation transm[illegible]

rocess;GO:0042761//very long-chain fatty acid biosynthetic process;GO:0044249//cellular biosynth

ransport;GO:0031623//receptor internalization;GO:0034333//adherens junction assembly;GO:0035

rage;GO:0016042//lipid catabolic process;GO:0019433//triglyceride catabolic process;GO:0030301

ulation of gene expression;GO:0010629//negative regulation of gene expression;GO:0021575//hir

544//phospholipid metabolic process;GO:0007493//endodermal cell fate determination;GO:00085[illegible]

gulation of Rho protein signal transduction;GO:0043410//positive regulation of MAPK cascade;GC

:ion;GO:0032816//positive regulation of natural killer cell activation;GO:0032825//positive regulatic

)6//positive regulation of lipid catabolic process;GO:0051005//negative regulation of lipoprotein li

n;GO:0046513//ceramide biosynthetic process;GO:0061436//establishment of skin barrier

544//phospholipid metabolic process;GO:0007493//endodermal cell fate determination;GO:00085[illegible]
s;GO:0060840//artery development;GO:0070837//dehydroascorbic acid transport;GO:0072498//em

rocess;GO:0042761//very long-chain fatty acid biosynthetic process;GO:0044249//cellular biosynth

tive regulation of potassium ion transmembrane transporter activity;GO:1901018//positive regulat

ulation of gene expression;GO:0010629//negative regulation of gene expression;GO:0021575//hir

system development;GO:0060021//palate development;GO:0060271//cilium morphogenesis;GO:(

ction potential involved in contraction;GO:0086005//ventricular cardiac muscle cell action potentia

rocess;GO:0042761//very long-chain fatty acid biosynthetic process;GO:0044249//cellular biosynth

alveolus development;GO:0055091//phospholipid homeostasis;GO:0060022//hard palate developm

rocess;GO:0042761//very long-chain fatty acid biosynthetic process;GO:0044249//cellular biosynth

system development;GO:0060021//palate development;GO:0060271//cilium morphogenesis;GO:0

96//positive regulation of lipid catabolic process;GO:0051005//negative regulation of lipoprotein li

tive regulation of potassium ion transmembrane transporter activity;GO:1901018//positive regulat

zation;GO:0045444//fat cell differentiation;GO:0045599//negative regulation of fat cell differentiati

ıdrial membrane potential;GO:1903426//regulation of reactive oxygen species biosynthetic proces

5//cellular lipid metabolic process;GO:0051607//defense response to virus;GO:0055089//fatty acid

movement;GO:0051497//negative regulation of stress fiber assembly;GO:0061436//establishment

gulation of Rho protein signal transduction;GO:0043410//positive regulation of MAPK cascade;GC

ulation of gene expression;GO:0010629//negative regulation of gene expression;GO:0021575//hir

rocess;GO:0042761//very long-chain fatty acid biosynthetic process;GO:0044249//cellular biosynth

;O:0061402//positive regulation of transcription from RNA polymerase II promoter in response to

gulation of Rho protein signal transduction;GO:0043410//positive regulation of MAPK cascade;GC

96//positive regulation of lipid catabolic process;GO:0051005//negative regulation of lipoprotein li

rage;GO:0016042//lipid catabolic process;GO:0019433//triglyceride catabolic process;GO:0030301

ransport;GO:0031623//receptor internalization;GO:0034333//adherens junction assembly;GO:0035

ion;GO:0003208//cardiac ventricle morphogenesis;GO:0003214//cardiac left ventricle morphogen

ulation of gene expression;GO:0010629//negative regulation of gene expression;GO:0021575//hir

544//phospholipid metabolic process;GO:0007493//endodermal cell fate determination;GO:00085[illegible]

56//negative regulation of apoptotic process;GO:0045165//cell fate commitment;GO:0045444//fat

movement;GO:0051497//negative regulation of stress fiber assembly;GO:0061436//establishment

96//positive regulation of lipid catabolic process;GO:0051005//negative regulation of lipoprotein li

GO:0061402//positive regulation of transcription from RNA polymerase II promoter in response to

rage;GO:0016042//lipid catabolic process;GO:0019433//triglyceride catabolic process;GO:0030301

n;GO:0046513//ceramide biosynthetic process;GO:0061436//establishment of skin barrier
movement;GO:0051497//negative regulation of stress fiber assembly;GO:0061436//establishment

ye morphogenesis;GO:0048386//positive regulation of retinoic acid receptor signaling pathway;GC

regulation of osteoblast differentiation;GO:0045786//negative regulation of cell cycle;GO:004814[illegible]

movement;GO:0051497//negative regulation of stress fiber assembly;GO:0061436//establishment

ion;GO:0003208//cardiac ventricle morphogenesis;GO:0003214//cardiac left ventricle morphogen[illegible]

[illegible]//cellular lipid metabolic process;GO:0051607//defense response to virus;GO:0055089//fatty acid

[illegible]O:0061402//positive regulation of transcription from RNA polymerase II promoter in response to

gulation of Rho protein signal transduction;GO:0043410//positive regulation of MAPK cascade;G[illegible]

544//phospholipid metabolic process;GO:0007493//endodermal cell fate determination;GO:00085[illegible]

[illegible]:0060060//post-embryonic retina morphogenesis in camera-type eye;GO:0060219//camera-type

[illegible]ye morphogenesis;GO:0048386//positive regulation of retinoic acid receptor signaling pathway;G[illegible]

[illegible];GO:0046513//ceramide biosynthetic process;GO:0061436//establishment of skin barrier

system development;GO:0060021//palate development;GO:0060271//cilium morphogenesis;GO:[illegible]

/positive regulation of ERK1 and ERK2 cascade;GO:0070527//platelet aggregation;GO:0072378//bl

rocess;GO:0042761//very long-chain fatty acid biosynthetic process;GO:0044249//cellular biosynth

5//cellular lipid metabolic process;GO:0051607//defense response to virus;GO:0055089//fatty acid

gulation of Rho protein signal transduction;GO:0043410//positive regulation of MAPK cascade;GC

movement;GO:0051497//negative regulation of stress fiber assembly;GO:0061436//establishment

rage;GO:0016042//lipid catabolic process;GO:0019433//triglyceride catabolic process;GO:0030301

regulation of osteoblast differentiation;GO:0045786//negative regulation of cell cycle;GO:004814[illegible]

ction potential involved in contraction;GO:0086005//ventricular cardiac muscle cell action potentia

ye morphogenesis;GO:0048386//positive regulation of retinoic acid receptor signaling pathway;GC

96//positive regulation of lipid catabolic process;GO:0051005//negative regulation of lipoprotein li

544//phospholipid metabolic process;GO:0007493//endodermal cell fate determination;GO:00085[illegible]

:0008543//fibroblast growth factor receptor signaling pathway;GO:0008544//epidermis developm[illegible]

system development;GO:0060021//palate development;GO:0060271//cilium morphogenesis;GO:0

movement;GO:0051497//negative regulation of stress fiber assembly;GO:0061436//establishment

ction potential involved in contraction;GO:0086005//ventricular cardiac muscle cell action potentia

ulation of gene expression;GO:0010629//negative regulation of gene expression;GO:0021575//hir

n;GO:0046513//ceramide biosynthetic process;GO:0061436//establishment of skin barrier
0035//response to inorganic substance;GO:0010460//positive regulation of heart rate;GO:0014074/

ction potential involved in contraction;GO:0086005//ventricular cardiac muscle cell action potentia

alveolus development;GO:0055091//phospholipid homeostasis;GO:0060022//hard palate developr

regulation of osteoblast differentiation;GO:0045786//negative regulation of cell cycle;GO:0048146

system development;GO:0060021//palate development;GO:0060271//cilium morphogenesis;GO:0

GO:0061402//positive regulation of transcription from RNA polymerase II promoter in response to

system development;GO:0060021//palate development;GO:0060271//cilium morphogenesis;GO:0

5//cellular lipid metabolic process;GO:0051607//defense response to virus;GO:0055089//fatty acid

0035//response to inorganic substance;GO:0010460//positive regulation of heart rate;GO:0014074/

roglial cell activation;GO:1903997//positive regulation of non-membrane spanning protein tyrosin

)10873//positive regulation of cholesterol esterification;GO:0010875//positive regulation of choles

tellite cell maintenance involved in skeletal muscle regeneration;GO:0014896//muscle hypertrophy

nent;GO:0060348//bone development;GO:0060395//SMAD protein signal transduction;GO:00604

58//negative regulation of neurogenesis;GO:0051289//protein homotetramerization;GO:0060100//
ion;GO:0045665//negative regulation of neuron differentiation;GO:0045892//negative regulation c

olic process;GO:0032924//activin receptor signaling pathway;GO:0035987//endodermal cell differe

5//positive regulation of fibroblast proliferation;GO:0050680//negative regulation of epithelial cell

cell differentiation;GO:0045662//negative regulation of myoblast differentiation;GO:0045892//neg

)10873//positive regulation of cholesterol esterification;GO:0010875//positive regulation of choles

วn of natural killer cell differentiation;GO:0033089//positive regulation of T cell differentiation in th

ation of synaptic transmission;GO:0050905//neuromuscular process;GO:0051094//positive regulat

tellite cell maintenance involved in skeletal muscle regeneration;GO:0014896//muscle hypertrophy

35//positive regulation of hair cycle;GO:0043010//camera-type eye development;GO:0060122//inr

68//negative regulation of neurogenesis;GO:0051289//protein homotetramerization;GO:0060100/

olic process;GO:0032924//activin receptor signaling pathway;GO:0035987//endodermal cell differe

on of natural killer cell differentiation;GO:0033089//positive regulation of T cell differentiation in th

ation of synaptic transmission;GO:0050905//neuromuscular process;GO:0051094//positive regulat

ific DNA binding transcription factor activity;GO:0060340//positive regulation of type I interferon-

ation of synaptic transmission;GO:0050905//neuromuscular process;GO:0051094//positive regulat

O:0071315//cellular response to morphine;GO:0080135//regulation of cellular response to stress;G

ion;GO:0045665//negative regulation of neuron differentiation;GO:0045892//negative regulation c

ific DNA binding transcription factor activity;GO:0060340//positive regulation of type I interferon-

on of natural killer cell differentiation;GO:0033089//positive regulation of T cell differentiation in th

tive regulation of neuron differentiation;GO:0045860//positive regulation of protein kinase activity

tellite cell maintenance involved in skeletal muscle regeneration;GO:0014896//muscle hypertrophy

on of natural killer cell differentiation;GO:0033089//positive regulation of T cell differentiation in th

ation of synaptic transmission;GO:0050905//neuromuscular process;GO:0051094//positive regulat

O:0071315//cellular response to morphine;GO:0080135//regulation of cellular response to stress;G
catabolic process;GO:0043691//reverse cholesterol transport;GO:0045542//positive regulation of c

.//cholesterol transport;GO:0030317//sperm motility;GO:0033344//cholesterol efflux;GO:0034374//

)10873//positive regulation of cholesterol esterification;GO:0010875//positive regulation of choles

):0045893//positive regulation of transcription, DNA-templated;GO:0050790//regulation of catalyt

olic process;GO:0032924//activin receptor signaling pathway;GO:0035987//endodermal cell differe

on of natural killer cell differentiation;GO:0033089//positive regulation of T cell differentiation in th
58//negative regulation of neurogenesis;GO:0051289//protein homotetramerization;GO:0060100/

tellite cell maintenance involved in skeletal muscle regeneration;GO:0014896//muscle hypertrophy

cell differentiation;GO:0045662//negative regulation of myoblast differentiation;GO:0045892//neg

ent;GO:0008589//regulation of smoothened signaling pathway;GO:0009880//embryonic pattern sp

tellite cell maintenance involved in skeletal muscle regeneration;GO:0014896//muscle hypertrophy

tive regulation of neuron differentiation;GO:0045860//positive regulation of protein kinase activity

catabolic process;GO:0043691//reverse cholesterol transport;GO:0045542//positive regulation of [illegible]

tive regulation of neuron differentiation;GO:0045860//positive regulation of protein kinase activity

[illegible]0060541//respiratory system development;GO:0072359//circulatory system development;GO:0090[illegible]

[illegible]68//negative regulation of neurogenesis;GO:0051289//protein homotetramerization;GO:0060100/[illegible]
[illegible]on of natural killer cell differentiation;GO:0033089//positive regulation of T cell differentiation in th[illegible]

olic process;GO:0032924//activin receptor signaling pathway;GO:0035987//endodermal cell differe

tellite cell maintenance involved in skeletal muscle regeneration;GO:0014896//muscle hypertrophy

GO:0034445//negative regulation of plasma lipoprotein particle oxidation;GO:0035634//response

)10873//positive regulation of cholesterol esterification;GO:0010875//positive regulation of choles

tive regulation of neuron differentiation;GO:0045860//positive regulation of protein kinase activity

cell differentiation;GO:0045662//negative regulation of myoblast differentiation;GO:0045892//neg

tive regulation of neuron differentiation;GO:0045860//positive regulation of protein kinase activity

homeostasis;GO:0055091//phospholipid homeostasis;GO:0070236//negative regulation of activati

68//negative regulation of neurogenesis;GO:0051289//protein homotetramerization;GO:0060100//

olic process;GO:0032924//activin receptor signaling pathway;GO:0035987//endodermal cell differe

)10873//positive regulation of cholesterol esterification;GO:0010875//positive regulation of choles

cell differentiation;GO:0045662//negative regulation of myoblast differentiation;GO:0045892//neg

ation of synaptic transmission;GO:0050905//neuromuscular process;GO:0051094//positive regulat

ation of synaptic transmission;GO:0050905//neuromuscular process;GO:0051094//positive regulat

ent;GO:0008589//regulation of smoothened signaling pathway;GO:0009880//embryonic pattern s[illegible]

[illegible];GO:0086010//membrane depolarization during action potential;GO:0086012//membrane depola

5924//cellular response to vascular endothelial growth factor stimulus;GO:0043116//negative regul

010873//positive regulation of cholesterol esterification;GO:0010875//positive regulation of choles

ation of synaptic transmission;GO:0050905//neuromuscular process;GO:0051094//positive regulat

):0045893//positive regulation of transcription, DNA-templated;GO:0050790//regulation of catalyt

of skin barrier;GO:0070495//negative regulation of thrombin receptor signaling pathway

35//positive regulation of hair cycle;GO:0043010//camera-type eye development;GO:0060122//inr

.346//cellular response to interferon-gamma;GO:0071347//cellular response to interleukin-1;GO:0

924//cellular response to vascular endothelial growth factor stimulus;GO:0043116//negative regul

cell differentiation;GO:0045662//negative regulation of myoblast differentiation;GO:0045892//neg

):0045773//positive regulation of axon extension;GO:0048146//positive regulation of fibroblast prc

um development;GO:0006338//chromatin remodeling;GO:0006355//regulation of transcription, DN

)10873//positive regulation of cholesterol esterification;GO:0010875//positive regulation of choles

al;GO:0086010//membrane depolarization during action potential;GO:0086012//membrane depola

cell differentiation;GO:0045662//negative regulation of myoblast differentiation;GO:0045892//neg

):0050885//neuromuscular process controlling balance;GO:0051289//protein homotetramerizatior

35//positive regulation of hair cycle;GO:0043010//camera-type eye development;GO:0060122//inr

tellite cell maintenance involved in skeletal muscle regeneration;GO:0014896//muscle hypertrophy

):0045773//positive regulation of axon extension;GO:0048146//positive regulation of fibroblast pro
58//negative regulation of neurogenesis;GO:0051289//protein homotetramerization;GO:0060100/,

olic process;GO:0032924//activin receptor signaling pathway;GO:0035987//endodermal cell differe

olic process;GO:0032924//activin receptor signaling pathway;GO:0035987//endodermal cell differe

58//negative regulation of neurogenesis;GO:0051289//protein homotetramerization;GO:0060100/,

)070301//cellular response to hydrogen peroxide;GO:0070830//bicellular tight junction assembly;(

homeostasis;GO:0055091//phospholipid homeostasis;GO:0070236//negative regulation of activati

)10873//positive regulation of cholesterol esterification;GO:0010875//positive regulation of choles

ıbryonic skeletal joint development;GO:0098708//glucose import across plasma membrane;GO:19

;30//cellular response to leukemia inhibitory factor;GO:2001234//negative regulation of apoptotic

;8//negative regulation of neurogenesis;GO:0051289//protein homotetramerization;GO:0060100/

olic process;GO:0032924//activin receptor signaling pathway;GO:0035987//endodermal cell differe

olic process;GO:0032924//activin receptor signaling pathway;GO:0035987//endodermal cell differe

38//negative regulation of neurogenesis;GO:0051289//protein homotetramerization;GO:0060100//

ific DNA binding transcription factor activity;GO:0060340//positive regulation of type I interferon-

rylation;GO:0019933//cAMP-mediated signaling;GO:0033198//response to ATP;GO:0035556//intra

ıbryonic skeletal joint development;GO:0098708//glucose import across plasma membrane;GO:19

tive regulation of neuron differentiation;GO:0045860//positive regulation of protein kinase activity

ıdbrain morphogenesis;GO:0030154//cell differentiation;GO:0030852//regulation of granulocyte d

ɔn of natural killer cell differentiation;GO:0033089//positive regulation of T cell differentiation in th

;O:0034605//cellular response to heat;GO:0042060//wound healing;GO:0043406//positive regulati

roglial cell activation;GO:1903997//positive regulation of non-membrane spanning protein tyrosir

ment;GO:0060348//bone development;GO:0060395//SMAD protein signal transduction;GO:00604[illegible]

olic process;GO:0032924//activin receptor signaling pathway;GO:0035987//endodermal cell differe

on of natural killer cell differentiation;GO:0033089//positive regulation of T cell differentiation in th

tellite cell maintenance involved in skeletal muscle regeneration;GO:0014896//muscle hypertrophy

)10873//positive regulation of cholesterol esterification;GO:0010875//positive regulation of choles

58//negative regulation of neurogenesis;GO:0051289//protein homotetramerization;GO:0060100/
O:0071315//cellular response to morphine;GO:0080135//regulation of cellular response to stress;G

ylation;GO:0019933//cAMP-mediated signaling;GO:0033198//response to ATP;GO:0035556//intra

ation of synaptic transmission;GO:0050905//neuromuscular process;GO:0051094//positive regulat

cell differentiation;GO:0045662//negative regulation of myoblast differentiation;GO:0045892//neg

ım development;GO:0006338//chromatin remodeling;GO:0006355//regulation of transcription, DI

tellite cell maintenance involved in skeletal muscle regeneration;GO:0014896//muscle hypertrophy

iO:0034605//cellular response to heat;GO:0042060//wound healing;GO:0043406//positive regulati

roglial cell activation;GO:1903997//positive regulation of non-membrane spanning protein tyrosir

)10873//positive regulation of cholesterol esterification;GO:0010875//positive regulation of choles

GO:0034445//negative regulation of plasma lipoprotein particle oxidation;GO:0035634//response

ation of synaptic transmission;GO:0050905//neuromuscular process;GO:0051094//positive regulat

catabolic process;GO:0043691//reverse cholesterol transport;GO:0045542//positive regulation of [illegible]

t in camera-type eye;GO:0061436//establishment of skin barrier;GO:0090103//cochlea morphoger
35//positive regulation of hair cycle;GO:0043010//camera-type eye development;GO:0060122//inr

.346//cellular response to interferon-gamma;GO:0071347//cellular response to interleukin-1;GO:0

on of natural killer cell differentiation;GO:0033089//positive regulation of T cell differentiation in th

O:0071315//cellular response to morphine;GO:0080135//regulation of cellular response to stress;G

):0051482//positive regulation of cytosolic calcium ion concentration involved in phospholipase C-

t in camera-type eye;GO:0061436//establishment of skin barrier;GO:0090103//cochlea morphogen

ific DNA binding transcription factor activity;GO:0060340//positive regulation of type I interferon-

)10873//positive regulation of cholesterol esterification;GO:0010875//positive regulation of choles

pase activity;GO:0055088//lipid homeostasis;GO:0055090//acylglycerol homeostasis;GO:0055091/

on of natural killer cell differentiation;GO:0033089//positive regulation of T cell differentiation in th

O:0071315//cellular response to morphine;GO:0080135//regulation of cellular response to stress;G

GO:0034445//negative regulation of plasma lipoprotein particle oxidation;GO:0035634//response
cell differentiation;GO:0045662//negative regulation of myoblast differentiation;GO:0045892//neg

ation of synaptic transmission;GO:0050905//neuromuscular process;GO:0051094//positive regulat

ation of synaptic transmission;GO:0050905//neuromuscular process;GO:0051094//positive regulat

)10873//positive regulation of cholesterol esterification;GO:0010875//positive regulation of choles

catabolic process;GO:0043691//reverse cholesterol transport;GO:0045542//positive regulation of c

)070301//cellular response to hydrogen peroxide;GO:0070830//bicellular tight junction assembly;G

tellite cell maintenance involved in skeletal muscle regeneration;GO:0014896//muscle hypertrophy

roglial cell activation;GO:1903997//positive regulation of non-membrane spanning protein tyrosir

on of natural killer cell differentiation;GO:0033089//positive regulation of T cell differentiation in th

ation of synaptic transmission;GO:0050905//neuromuscular process;GO:0051094//positive regulat

um development;GO:0006338//chromatin remodeling;GO:0006355//regulation of transcription, DN

30//cellular response to leukemia inhibitory factor;GO:2001234//negative regulation of apoptotic

ion;GO:0045665//negative regulation of neuron differentiation;GO:0045892//negative regulation c

catabolic process;GO:0043691//reverse cholesterol transport;GO:0045542//positive regulation of [illegible]

yte growth factor receptor signaling pathway;GO:0050673//epithelial cell proliferation;GO:005072[illegible]

tellite cell maintenance involved in skeletal muscle regeneration;GO:0014896//muscle hypertrophy

tive regulation of neuron differentiation;GO:0045860//positive regulation of protein kinase activity

)10873//positive regulation of cholesterol esterification;GO:0010875//positive regulation of choles

088//lipid homeostasis;GO:0061178//regulation of insulin secretion involved in cellular response t[illegible]

[illegible]ment;GO:0060348//bone development;GO:0060395//SMAD protein signal transduction;GO:0060[illegible]

on of natural killer cell differentiation;GO:0033089//positive regulation of T cell differentiation in th

yte growth factor receptor signaling pathway;GO:0050673//epithelial cell proliferation;GO:005072[illegible]

um development;GO:0006338//chromatin remodeling;GO:0006355//regulation of transcription, D[illegible]

pase activity;GO:0055088//lipid homeostasis;GO:0055090//acylglycerol homeostasis;GO:0055091/

tive regulation of neuron differentiation;GO:0045860//positive regulation of protein kinase activity

58//negative regulation of neurogenesis;GO:0051289//protein homotetramerization;GO:0060100/[illegible]

tellite cell maintenance involved in skeletal muscle regeneration;GO:0014896//muscle hypertroph[illegible]

tellite cell maintenance involved in skeletal muscle regeneration;GO:0014896//muscle hypertroph[illegible]

olic process;GO:0032924//activin receptor signaling pathway;GO:0035987//endodermal cell differe[illegible]

:0045773//positive regulation of axon extension;GO:0048146//positive regulation of fibroblast pro

68//negative regulation of neurogenesis;GO:0051289//protein homotetramerization;GO:0060100/

olic process;GO:0032924//activin receptor signaling pathway;GO:0035987//endodermal cell differe

tellite cell maintenance involved in skeletal muscle regeneration;GO:0014896//muscle hypertrophy

homeostasis;GO:0055091//phospholipid homeostasis;GO:0070236//negative regulation of activati

088//lipid homeostasis;GO:0061178//regulation of insulin secretion involved in cellular response t[illegible]

s;GO:1990314//cellular response to insulin-like growth factor stimulus;GO:1990519//mitochondria[illegible]

):0045773//positive regulation of axon extension;GO:0048146//positive regulation of fibroblast prc

tellite cell maintenance involved in skeletal muscle regeneration;GO:0014896//muscle hypertrophy

al;GO:0086010//membrane depolarization during action potential;GO:0086012//membrane depola

s;GO:1990314//cellular response to insulin-like growth factor stimulus;GO:1990519//mitochondria

ent;GO:0008589//regulation of smoothened signaling pathway;GO:0009880//embryonic pattern sp

ɔn of natural killer cell differentiation;GO:0033089//positive regulation of T cell differentiation in th

ation of synaptic transmission;GO:0050905//neuromuscular process;GO:0051094//positive regulat

O:0071315//cellular response to morphine;GO:0080135//regulation of cellular response to stress;G

.346//cellular response to interferon-gamma;GO:0071347//cellular response to interleukin-1;GO:0

ɔn of natural killer cell differentiation;GO:0033089//positive regulation of T cell differentiation in th

.346//cellular response to interferon-gamma;GO:0071347//cellular response to interleukin-1;GO:0

ıdbrain morphogenesis;GO:0030154//cell differentiation;GO:0030852//regulation of granulocyte d

cell differentiation;GO:0045662//negative regulation of myoblast differentiation;GO:0045892//neg

)10873//positive regulation of cholesterol esterification;GO:0010875//positive regulation of choles

35//positive regulation of hair cycle;GO:0043010//camera-type eye development;GO:0060122//inn

):0045773//positive regulation of axon extension;GO:0048146//positive regulation of fibroblast pro

olic process;GO:0032924//activin receptor signaling pathway;GO:0035987//endodermal cell differe

58//negative regulation of neurogenesis;GO:0051289//protein homotetramerization;GO:0060100//

):0045893//positive regulation of transcription, DNA-templated;GO:0050790//regulation of catalyt

tive regulation of neuron differentiation;GO:0045860//positive regulation of protein kinase activity

35//positive regulation of hair cycle;GO:0043010//camera-type eye development;GO:0060122//inr

ation of synaptic transmission;GO:0050905//neuromuscular process;GO:0051094//positive regulat

ım development;GO:0006338//chromatin remodeling;GO:0006355//regulation of transcription, DN

ylation;GO:0019933//cAMP-mediated signaling;GO:0033198//response to ATP;GO:0035556//intra

cell differentiation;GO:0045662//negative regulation of myoblast differentiation;GO:0045892//neg

)10873//positive regulation of cholesterol esterification;GO:0010875//positive regulation of choles

catabolic process;GO:0043691//reverse cholesterol transport;GO:0045542//positive regulation of

O:0034605//cellular response to heat;GO:0042060//wound healing;GO:0043406//positive regulati

088//lipid homeostasis;GO:0061178//regulation of insulin secretion involved in cellular response t

on of natural killer cell differentiation;GO:0033089//positive regulation of T cell differentiation in th

.346//cellular response to interferon-gamma;GO:0071347//cellular response to interleukin-1;GO:0

olic process;GO:0032924//activin receptor signaling pathway;GO:0035987//endodermal cell differe

t in camera-type eye;GO:0061436//establishment of skin barrier;GO:0090103//cochlea morphoge

tellite cell maintenance involved in skeletal muscle regeneration;GO:0014896//muscle hypertrophy

58//negative regulation of neurogenesis;GO:0051289//protein homotetramerization;GO:0060100/

tive regulation of neuron differentiation;GO:0045860//positive regulation of protein kinase activity

35//positive regulation of hair cycle;GO:0043010//camera-type eye development;GO:0060122//inr

ation of synaptic transmission;GO:0050905//neuromuscular process;GO:0051094//positive regulat

):0050885//neuromuscular process controlling balance;GO:0051289//protein homotetramerizatior

i8//negative regulation of neurogenesis;GO:0051289//protein homotetramerization;GO:0060100//

)10873//positive regulation of cholesterol esterification;GO:0010875//positive regulation of choles

olic process;GO:0032924//activin receptor signaling pathway;GO:0035987//endodermal cell differe

iO:0034605//cellular response to heat;GO:0042060//wound healing;GO:0043406//positive regulati

35//positive regulation of hair cycle;GO:0043010//camera-type eye development;GO:0060122//inr

):0045893//positive regulation of transcription, DNA-templated;GO:0050790//regulation of catalyt

ation of synaptic transmission;GO:0050905//neuromuscular process;GO:0051094//positive regulat

58//negative regulation of neurogenesis;GO:0051289//protein homotetramerization;GO:0060100//

;O:0034605//cellular response to heat;GO:0042060//wound healing;GO:0043406//positive regulati
r water homeostasis;GO:0010640//regulation of platelet-derived growth factor receptor signaling

olic process;GO:0032924//activin receptor signaling pathway;GO:0035987//endodermal cell differe

30//cellular response to leukemia inhibitory factor;GO:2001234//negative regulation of apoptotic

ation of synaptic transmission;GO:0050905//neuromuscular process;GO:0051094//positive regulat

ation of synaptic transmission;GO:0050905//neuromuscular process;GO:0051094//positive regulat

ıbryonic skeletal joint development;GO:0098708//glucose import across plasma membrane;GO:19

ım development;GO:0006338//chromatin remodeling;GO:0006355//regulation of transcription, DN
O:0071315//cellular response to morphine;GO:0080135//regulation of cellular response to stress;G

58//negative regulation of neurogenesis;GO:0051289//protein homotetramerization;GO:0060100/[illegible]

olic process;GO:0032924//activin receptor signaling pathway;GO:0035987//endodermal cell differe

)10873//positive regulation of cholesterol esterification;GO:0010875//positive regulation of choles

yte growth factor receptor signaling pathway;GO:0050673//epithelial cell proliferation;GO:005072[illegible]

)070301//cellular response to hydrogen peroxide;GO:0070830//bicellular tight junction assembly;[illegible]

tive regulation of neuron differentiation;GO:0045860//positive regulation of protein kinase activity
yte growth factor receptor signaling pathway;GO:0050673//epithelial cell proliferation;GO:005072[illegible]

.346//cellular response to interferon-gamma;GO:0071347//cellular response to interleukin-1;GO:0

ɔn of natural killer cell differentiation;GO:0033089//positive regulation of T cell differentiation in th

)10873//positive regulation of cholesterol esterification;GO:0010875//positive regulation of choles
tellite cell maintenance involved in skeletal muscle regeneration;GO:0014896//muscle hypertrophy
):0045773//positive regulation of axon extension;GO:0048146//positive regulation of fibroblast pro

):0045893//positive regulation of transcription, DNA-templated;GO:0050790//regulation of catalyt

catabolic process;GO:0043691//reverse cholesterol transport;GO:0045542//positive regulation of

tive regulation of neuron differentiation;GO:0045860//positive regulation of protein kinase activity

O:0071315//cellular response to morphine;GO:0080135//regulation of cellular response to stress;G

on of natural killer cell differentiation;GO:0033089//positive regulation of T cell differentiation in th

on of natural killer cell differentiation;GO:0033089//positive regulation of T cell differentiation in th

yte growth factor receptor signaling pathway;GO:0050673//epithelial cell proliferation;GO:005072[illegible]

[illegible]8//negative regulation of neurogenesis;GO:0051289//protein homotetramerization;GO:0060100/[illegible]

tellite cell maintenance involved in skeletal muscle regeneration;GO:0014896//muscle hypertroph[illegible]

olic process;GO:0032924//activin receptor signaling pathway;GO:0035987//endodermal cell differe[illegible]

r water homeostasis;GO:0010640//regulation of platelet-derived growth factor receptor signaling

catabolic process;GO:0043691//reverse cholesterol transport;GO:0045542//positive regulation of

088//lipid homeostasis;GO:0061178//regulation of insulin secretion involved in cellular response t

ʾ30//cellular response to leukemia inhibitory factor;GO:2001234//negative regulation of apoptotic

ʾ924//cellular response to vascular endothelial growth factor stimulus;GO:0043116//negative regul

ʾyte growth factor receptor signaling pathway;GO:0050673//epithelial cell proliferation;GO:0050728

ion;GO:0045665//negative regulation of neuron differentiation;GO:0045892//negative regulation c

on of natural killer cell differentiation;GO:0033089//positive regulation of T cell differentiation in th

088//lipid homeostasis;GO:0061178//regulation of insulin secretion involved in cellular response to

ım development;GO:0006338//chromatin remodeling;GO:0006355//regulation of transcription, DN

35//positive regulation of hair cycle;GO:0043010//camera-type eye development;GO:0060122//inr

)45822//negative regulation of heart contraction;GO:0070296//sarcoplasmic reticulum calcium ion

;O:0034605//cellular response to heat;GO:0042060//wound healing;GO:0043406//positive regulati

;O:0034605//cellular response to heat;GO:0042060//wound healing;GO:0043406//positive regulati

ood coagulation, fibrin clot formation;GO:0090277//positive regulation of peptide hormone secre

)45822//negative regulation of heart contraction;GO:0070296//sarcoplasmic reticulum calcium ion

34//male gonad development;GO:0009410//response to xenobiotic stimulus;GO:0010467//gene e[illegible]

.//cholesterol transport;GO:0030317//sperm motility;GO:0033344//cholesterol efflux;GO:0034374/[illegible]
):0045893//positive regulation of transcription, DNA-templated;GO:0050790//regulation of catalyt[illegible]

olic process;GO:0032924//activin receptor signaling pathway;GO:0035987//endodermal cell differe[illegible]

38//negative regulation of neurogenesis;GO:0051289//protein homotetramerization;GO:0060100/[illegible]

ion of potassium ion transmembrane transporter activity;GO:2000009//negative regulation of prot

)45822//negative regulation of heart contraction;GO:0070296//sarcoplasmic reticulum calcium ion

):0050885//neuromuscular process controlling balance;GO:0051289//protein homotetramerizatior

m ion homeostasis;GO:0007005//mitochondrion organization;GO:0007165//signal transduction;G(
ıdbrain morphogenesis;GO:0030154//cell differentiation;GO:0030852//regulation of granulocyte d

)060541//respiratory system development;GO:0072359//circulatory system development;GO:0090[illegible]

.//cholesterol transport;GO:0030317//sperm motility;GO:0033344//cholesterol efflux;GO:0034374/[illegible]

of skin barrier;GO:0070495//negative regulation of thrombin receptor signaling pathway
teoglycan metabolic process;GO:0006508//proteolysis;GO:0007224//smoothened signaling pathw[illegible]

):0050885//neuromuscular process controlling balance;GO:0051289//protein homotetramerizatior

ion of potassium ion transmembrane transporter activity;GO:2000009//negative regulation of prot

r water homeostasis;GO:0010640//regulation of platelet-derived growth factor receptor signaling

teoglycan metabolic process;GO:0006508//proteolysis;GO:0007224//smoothened signaling pathwa

r water homeostasis;GO:0010640//regulation of platelet-derived growth factor receptor signaling p

34//male gonad development;GO:0009410//response to xenobiotic stimulus;GO:0010467//gene ex

cell differentiation;GO:0045662//negative regulation of myoblast differentiation;GO:0045892//neg

t in camera-type eye;GO:0061436//establishment of skin barrier;GO:0090103//cochlea morphogen

):0051482//positive regulation of cytosolic calcium ion concentration involved in phospholipase C-

of skin barrier;GO:0070495//negative regulation of thrombin receptor signaling pathway

//response to purine-containing compound;GO:0030324//lung development;GO:0030890//positiv

m ion homeostasis;GO:0007005//mitochondrion organization;GO:0007165//signal transduction;G(

):0051482//positive regulation of cytosolic calcium ion concentration involved in phospholipase C-

r water homeostasis;GO:0010640//regulation of platelet-derived growth factor receptor signaling [illegible]

ation of synaptic transmission;GO:0050905//neuromuscular process;GO:0051094//positive regulat

esis;GO:0003215//cardiac right ventricle morphogenesis;GO:0003222//ventricular trabecula myoca

)45822//negative regulation of heart contraction;GO:0070296//sarcoplasmic reticulum calcium ion

pase activity;GO:0055088//lipid homeostasis;GO:0055090//acylglycerol homeostasis;GO:0055091/

of skin barrier;GO:0070495//negative regulation of thrombin receptor signaling pathway

34//male gonad development;GO:0009410//response to xenobiotic stimulus;GO:0010467//gene e:

.//cholesterol transport;GO:0030317//sperm motility;GO:0033344//cholesterol efflux;GO:0034374//

r water homeostasis;GO:0010640//regulation of platelet-derived growth factor receptor signaling

teoglycan metabolic process;GO:0006508//proteolysis;GO:0007224//smoothened signaling pathwa

):0051482//positive regulation of cytosolic calcium ion concentration involved in phospholipase C-

of skin barrier;GO:0070495//negative regulation of thrombin receptor signaling pathway

pase activity;GO:0055088//lipid homeostasis;GO:0055090//acylglycerol homeostasis;GO:0055091/

//response to purine-containing compound;GO:0030324//lung development;GO:0030890//positiv

)060541//respiratory system development;GO:0072359//circulatory system development;GO:0090!

pase activity;GO:0055088//lipid homeostasis;GO:0055090//acylglycerol homeostasis;GO:0055091/

)45822//negative regulation of heart contraction;GO:0070296//sarcoplasmic reticulum calcium ion

34//male gonad development;GO:0009410//response to xenobiotic stimulus;GO:0010467//gene e[illegible]

esis;GO:0003215//cardiac right ventricle morphogenesis;GO:0003222//ventricular trabecula myoca

5//positive regulation of fibroblast proliferation;GO:0050680//negative regulation of epithelial cell

):0051482//positive regulation of cytosolic calcium ion concentration involved in phospholipase C-

30//cellular response to leukemia inhibitory factor;GO:2001234//negative regulation of apoptotic

teoglycan metabolic process;GO:0006508//proteolysis;GO:0007224//smoothened signaling pathwa

ıbryonic skeletal joint development;GO:0098708//glucose import across plasma membrane;GO:19

):0050885//neuromuscular process controlling balance;GO:0051289//protein homotetramerizatior

)060541//respiratory system development;GO:0072359//circulatory system development;GO:0090!

)//positive regulation of fibroblast proliferation;GO:0050680//negative regulation of epithelial cell

tellite cell maintenance involved in skeletal muscle regeneration;GO:0014896//muscle hypertrophy

)060541//respiratory system development;GO:0072359//circulatory system development;GO:0090!

530//cellular response to leukemia inhibitory factor;GO:2001234//negative regulation of apoptotic

0060541//respiratory system development;GO:0072359//circulatory system development;GO:0090[illegible]

ion of potassium ion transmembrane transporter activity;GO:2000009//negative regulation of prot

ıdbrain morphogenesis;GO:0030154//cell differentiation;GO:0030852//regulation of granulocyte d

.//cholesterol transport;GO:0030317//sperm motility;GO:0033344//cholesterol efflux;GO:0034374//

s;GO:1990314//cellular response to insulin-like growth factor stimulus;GO:1990519//mitochondria

):0050885//neuromuscular process controlling balance;GO:0051289//protein homotetramerizatior

)060541//respiratory system development;GO:0072359//circulatory system development;GO:0090

ıdbrain morphogenesis;GO:0030154//cell differentiation;GO:0030852//regulation of granulocyte d

ood coagulation, fibrin clot formation;GO:0090277//positive regulation of peptide hormone secre

t in camera-type eye;GO:0061436//establishment of skin barrier;GO:0090103//cochlea morphoger
'/response to purine-containing compound;GO:0030324//lung development;GO:0030890//positiv

ıdbrain morphogenesis;GO:0030154//cell differentiation;GO:0030852//regulation of granulocyte d

.346//cellular response to interferon-gamma;GO:0071347//cellular response to interleukin-1;GO:0

m ion homeostasis;GO:0007005//mitochondrion organization;GO:0007165//signal transduction;GO

roglial cell activation;GO:1903997//positive regulation of non-membrane spanning protein tyrosin

[illegible]924//cellular response to vascular endothelial growth factor stimulus;GO:0043116//negative regul

[illegible]:0051482//positive regulation of cytosolic calcium ion concentration involved in phospholipase C[illegible]

pase activity;GO:0055088//lipid homeostasis;GO:0055090//acylglycerol homeostasis;GO:0055091/

of skin barrier;GO:0070495//negative regulation of thrombin receptor signaling pathway

):0050885//neuromuscular process controlling balance;GO:0051289//protein homotetramerizatior

'/response to purine-containing compound;GO:0030324//lung development;GO:0030890//positiv

34//male gonad development;GO:0009410//response to xenobiotic stimulus;GO:0010467//gene e[illegible]

)45822//negative regulation of heart contraction;GO:0070296//sarcoplasmic reticulum calcium ion

ıdbrain morphogenesis;GO:0030154//cell differentiation;GO:0030852//regulation of granulocyte d

of skin barrier;GO:0070495//negative regulation of thrombin receptor signaling pathway

esis;GO:0003215//cardiac right ventricle morphogenesis;GO:0003222//ventricular trabecula myoca

):0051482//positive regulation of cytosolic calcium ion concentration involved in phospholipase C

088//lipid homeostasis;GO:0061178//regulation of insulin secretion involved in cellular response t

ood coagulation, fibrin clot formation;GO:0090277//positive regulation of peptide hormone secre

m ion homeostasis;GO:0007005//mitochondrion organization;GO:0007165//signal transduction;G(
):0050885//neuromuscular process controlling balance;GO:0051289//protein homotetramerizatior

pase activity;GO:0055088//lipid homeostasis;GO:0055090//acylglycerol homeostasis;GO:0055091/

34//male gonad development;GO:0009410//response to xenobiotic stimulus;GO:0010467//gene e:

of skin barrier;GO:0070495//negative regulation of thrombin receptor signaling pathway

s;GO:1990314//cellular response to insulin-like growth factor stimulus;GO:1990519//mitochondria
):0051482//positive regulation of cytosolic calcium ion concentration involved in phospholipase C-

m ion homeostasis;GO:0007005//mitochondrion organization;GO:0007165//signal transduction;G(

)060541//respiratory system development;GO:0072359//circulatory system development;GO:0090!

34//male gonad development;GO:0009410//response to xenobiotic stimulus;GO:0010467//gene e:

ıbryonic skeletal joint development;GO:0098708//glucose import across plasma membrane;GO:19

pase activity;GO:0055088//lipid homeostasis;GO:0055090//acylglycerol homeostasis;GO:0055091/

pase activity;GO:0055088//lipid homeostasis;GO:0055090//acylglycerol homeostasis;GO:0055091/

):0050885//neuromuscular process controlling balance;GO:0051289//protein homotetramerizatior

)10873//positive regulation of cholesterol esterification;GO:0010875//positive regulation of choles

5//positive regulation of fibroblast proliferation;GO:0050680//negative regulation of epithelial cell

34//male gonad development;GO:0009410//response to xenobiotic stimulus;GO:0010467//gene e

ıdbrain morphogenesis;GO:0030154//cell differentiation;GO:0030852//regulation of granulocyte d

0060541//respiratory system development;GO:0072359//circulatory system development;GO:0090

ion of potassium ion transmembrane transporter activity;GO:2000009//negative regulation of prot

0060541//respiratory system development;GO:0072359//circulatory system development;GO:0090

teoglycan metabolic process;GO:0006508//proteolysis;GO:0007224//smoothened signaling pathwa

pase activity;GO:0055088//lipid homeostasis;GO:0055090//acylglycerol homeostasis;GO:0055091/

ım development;GO:0006338//chromatin remodeling;GO:0006355//regulation of transcription, DI

.//cholesterol transport;GO:0030317//sperm motility;GO:0033344//cholesterol efflux;GO:0034374//

ıdbrain morphogenesis;GO:0030154//cell differentiation;GO:0030852//regulation of granulocyte d

ion of potassium ion transmembrane transporter activity;GO:2000009//negative regulation of prot

pase activity;GO:0055088//lipid homeostasis;GO:0055090//acylglycerol homeostasis;GO:0055091/

//cholesterol transport;GO:0030317//sperm motility;GO:0033344//cholesterol efflux;GO:0034374/

teoglycan metabolic process;GO:0006508//proteolysis;GO:0007224//smoothened signaling pathw

dbrain morphogenesis;GO:0030154//cell differentiation;GO:0030852//regulation of granulocyte d

of skin barrier;GO:0070495//negative regulation of thrombin receptor signaling pathway

5924//cellular response to vascular endothelial growth factor stimulus;GO:0043116//negative regul

//response to purine-containing compound;GO:0030324//lung development;GO:0030890//positiv

ıdbrain morphogenesis;GO:0030154//cell differentiation;GO:0030852//regulation of granulocyte d

):0050885//neuromuscular process controlling balance;GO:0051289//protein homotetramerizatio

nent;GO:0060348//bone development;GO:0060395//SMAD protein signal transduction;GO:00604

)45822//negative regulation of heart contraction;GO:0070296//sarcoplasmic reticulum calcium ion

ion of potassium ion transmembrane transporter activity;GO:2000009//negative regulation of prot

.//cholesterol transport;GO:0030317//sperm motility;GO:0033344//cholesterol efflux;GO:0034374//

esis;GO:0003215//cardiac right ventricle morphogenesis;GO:0003222//ventricular trabecula myoca

of skin barrier;GO:0070495//negative regulation of thrombin receptor signaling pathway

of skin barrier;GO:0070495//negative regulation of thrombin receptor signaling pathway

):0051482//positive regulation of cytosolic calcium ion concentration involved in phospholipase C-
):0050885//neuromuscular process controlling balance;GO:0051289//protein homotetramerizatior

)060541//respiratory system development;GO:0072359//circulatory system development;GO:0090[illegible]

esis;GO:0003215//cardiac right ventricle morphogenesis;GO:0003222//ventricular trabecula myoca

of skin barrier;GO:0070495//negative regulation of thrombin receptor signaling pathway

)060541//respiratory system development;GO:0072359//circulatory system development;GO:0090[illegible]

t in camera-type eye;GO:0061436//establishment of skin barrier;GO:0090103//cochlea morphoger[illegible]

ıdbrain morphogenesis;GO:0030154//cell differentiation;GO:0030852//regulation of granulocyte d[illegible]

):0051482//positive regulation of cytosolic calcium ion concentration involved in phospholipase C-

):0050885//neuromuscular process controlling balance;GO:0051289//protein homotetramerizatior

pase activity;GO:0055088//lipid homeostasis;GO:0055090//acylglycerol homeostasis;GO:0055091/

34//male gonad development;GO:0009410//response to xenobiotic stimulus;GO:0010467//gene e

;//positive regulation of fibroblast proliferation;GO:0050680//negative regulation of epithelial cell

esis;GO:0003215//cardiac right ventricle morphogenesis;GO:0003222//ventricular trabecula myoca

34//male gonad development;GO:0009410//response to xenobiotic stimulus;GO:0010467//gene e

060541//respiratory system development;GO:0072359//circulatory system development;GO:0090

//positive regulation of fibroblast proliferation;GO:0050680//negative regulation of epithelial cell

):0051482//positive regulation of cytosolic calcium ion concentration involved in phospholipase C-

of skin barrier;GO:0070495//negative regulation of thrombin receptor signaling pathway

):0050885//neuromuscular process controlling balance;GO:0051289//protein homotetramerizatior

):0050885//neuromuscular process controlling balance;GO:0051289//protein homotetramerizatior

)060541//respiratory system development;GO:0072359//circulatory system development;GO:0090[illegible]

s;GO:1990314//cellular response to insulin-like growth factor stimulus;GO:1990519//mitochondria

ific DNA binding transcription factor activity;GO:0060340//positive regulation of type I interferon-

of skin barrier;GO:0070495//negative regulation of thrombin receptor signaling pathway

//response to purine-containing compound;GO:0030324//lung development;GO:0030890//positiv

5//positive regulation of fibroblast proliferation;GO:0050680//negative regulation of epithelial cell

ıbryonic skeletal joint development;GO:0098708//glucose import across plasma membrane;GO:19

088//lipid homeostasis;GO:0061178//regulation of insulin secretion involved in cellular response t

ific DNA binding transcription factor activity;GO:0060340//positive regulation of type I interferon-
t in camera-type eye;GO:0061436//establishment of skin barrier;GO:0090103//cochlea morphoger

):0051482//positive regulation of cytosolic calcium ion concentration involved in phospholipase C-

):0050885//neuromuscular process controlling balance;GO:0051289//protein homotetramerizatior

'/response to purine-containing compound;GO:0030324//lung development;GO:0030890//positiv

):0051482//positive regulation of cytosolic calcium ion concentration involved in phospholipase C-

ment;GO:0060348//bone development;GO:0060395//SMAD protein signal transduction;GO:00604[illegible]

[illegible]5//positive regulation of hair cycle;GO:0043010//camera-type eye development;GO:0060122//inn

[illegible]4//male gonad development;GO:0009410//response to xenobiotic stimulus;GO:0010467//gene e[illegible]

):0050885//neuromuscular process controlling balance;GO:0051289//protein homotetramerizatior

ıdbrain morphogenesis;GO:0030154//cell differentiation;GO:0030852//regulation of granulocyte d

homeostasis;GO:0055091//phospholipid homeostasis;GO:0070236//negative regulation of activati

34//male gonad development;GO:0009410//response to xenobiotic stimulus;GO:0010467//gene e

ment;GO:0060348//bone development;GO:0060395//SMAD protein signal transduction;GO:0060[illegible]

5//positive regulation of fibroblast proliferation;GO:0050680//negative regulation of epithelial cell

):0051482//positive regulation of cytosolic calcium ion concentration involved in phospholipase C-

):0051482//positive regulation of cytosolic calcium ion concentration involved in phospholipase C-

//response to purine-containing compound;GO:0030324//lung development;GO:0030890//positiv

r water homeostasis;GO:0010640//regulation of platelet-derived growth factor receptor signaling

pase activity;GO:0055088//lipid homeostasis;GO:0055090//acylglycerol homeostasis;GO:0055091/

34//male gonad development;GO:0009410//response to xenobiotic stimulus;GO:0010467//gene e[illegible]

al;GO:0086010//membrane depolarization during action potential;GO:0086012//membrane depola[illegible]

ı924//cellular response to vascular endothelial growth factor stimulus;GO:0043116//negative regul

.//cholesterol transport;GO:0030317//sperm motility;GO:0033344//cholesterol efflux;GO:0034374//

ıdbrain morphogenesis;GO:0030154//cell differentiation;GO:0030852//regulation of granulocyte d

34//male gonad development;GO:0009410//response to xenobiotic stimulus;GO:0010467//gene e:

):0051482//positive regulation of cytosolic calcium ion concentration involved in phospholipase C

ɔn of natural killer cell differentiation;GO:0033089//positive regulation of T cell differentiation in th

pase activity;GO:0055088//lipid homeostasis;GO:0055090//acylglycerol homeostasis;GO:0055091/

34//male gonad development;GO:0009410//response to xenobiotic stimulus;GO:0010467//gene e
nbryonic skeletal joint development;GO:0098708//glucose import across plasma membrane;GO:19

ion of potassium ion transmembrane transporter activity;GO:2000009//negative regulation of prot

ıdbrain morphogenesis;GO:0030154//cell differentiation;GO:0030852//regulation of granulocyte d

)060541//respiratory system development;GO:0072359//circulatory system development;GO:0090[illegible]

ıl;GO:0086010//membrane depolarization during action potential;GO:0086012//membrane depola

nent;GO:0060348//bone development;GO:0060395//SMAD protein signal transduction;GO:00604

)060541//respiratory system development;GO:0072359//circulatory system development;GO:0090!

pase activity;GO:0055088//lipid homeostasis;GO:0055090//acylglycerol homeostasis;GO:0055091/

ion of potassium ion transmembrane transporter activity;GO:2000009//negative regulation of prot

ion;GO:0045665//negative regulation of neuron differentiation;GO:0045892//negative regulation c

s;GO:1990314//cellular response to insulin-like growth factor stimulus;GO:1990519//mitochondria

homeostasis;GO:0055091//phospholipid homeostasis;GO:0070236//negative regulation of activati

of skin barrier;GO:0070495//negative regulation of thrombin receptor signaling pathway

):0051482//positive regulation of cytosolic calcium ion concentration involved in phospholipase C

ıdbrain morphogenesis;GO:0030154//cell differentiation;GO:0030852//regulation of granulocyte d

):0051482//positive regulation of cytosolic calcium ion concentration involved in phospholipase C-

pase activity;GO:0055088//lipid homeostasis;GO:0055090//acylglycerol homeostasis;GO:0055091/

.//cholesterol transport;GO:0030317//sperm motility;GO:0033344//cholesterol efflux;GO:0034374//

i924//cellular response to vascular endothelial growth factor stimulus;GO:0043116//negative regul

esis;GO:0003215//cardiac right ventricle morphogenesis;GO:0003222//ventricular trabecula myoca

ıdbrain morphogenesis;GO:0030154//cell differentiation;GO:0030852//regulation of granulocyte d

34//male gonad development;GO:0009410//response to xenobiotic stimulus;GO:0010467//gene e

cell differentiation;GO:0045662//negative regulation of myoblast differentiation;GO:0045892//neg

of skin barrier;GO:0070495//negative regulation of thrombin receptor signaling pathway

pase activity;GO:0055088//lipid homeostasis;GO:0055090//acylglycerol homeostasis;GO:0055091/

.//cholesterol transport;GO:0030317//sperm motility;GO:0033344//cholesterol efflux;GO:0034374/

of skin barrier;GO:0070495//negative regulation of thrombin receptor signaling pathway

):0050885//neuromuscular process controlling balance;GO:0051289//protein homotetramerizatior

5//positive regulation of fibroblast proliferation;GO:0050680//negative regulation of epithelial cell

of skin barrier;GO:0070495//negative regulation of thrombin receptor signaling pathway

esis;GO:0003215//cardiac right ventricle morphogenesis;GO:0003222//ventricular trabecula myoca

homeostasis;GO:0055091//phospholipid homeostasis;GO:0070236//negative regulation of activati

):0051482//positive regulation of cytosolic calcium ion concentration involved in phospholipase C

34//male gonad development;GO:0009410//response to xenobiotic stimulus;GO:0010467//gene e

):0050885//neuromuscular process controlling balance;GO:0051289//protein homotetramerizatior

)060541//respiratory system development;GO:0072359//circulatory system development;GO:0090!

ood coagulation, fibrin clot formation;GO:0090277//positive regulation of peptide hormone secre

homeostasis;GO:0055091//phospholipid homeostasis;GO:0070236//negative regulation of activati

):0051482//positive regulation of cytosolic calcium ion concentration involved in phospholipase C-

of skin barrier;GO:0070495//negative regulation of thrombin receptor signaling pathway

.//cholesterol transport;GO:0030317//sperm motility;GO:0033344//cholesterol efflux;GO:0034374//

5//positive regulation of fibroblast proliferation;GO:0050680//negative regulation of epithelial cell

al;GO:0086010//membrane depolarization during action potential;GO:0086012//membrane depola

):0050885//neuromuscular process controlling balance;GO:0051289//protein homotetramerizatior

pase activity;GO:0055088//lipid homeostasis;GO:0055090//acylglycerol homeostasis;GO:0055091/

34//male gonad development;GO:0009410//response to xenobiotic stimulus;GO:0010467//gene e

ent;GO:0008589//regulation of smoothened signaling pathway;GO:0009880//embryonic pattern s

)060541//respiratory system development;GO:0072359//circulatory system development;GO:0090[illegible]

of skin barrier;GO:0070495//negative regulation of thrombin receptor signaling pathway

[illegible]l;GO:0086010//membrane depolarization during action potential;GO:0086012//membrane depola[illegible]

[illegible]dbrain morphogenesis;GO:0030154//cell differentiation;GO:0030852//regulation of granulocyte d[illegible]

[illegible]/response to purine-containing compound;GO:0030324//lung development;GO:0030890//positiv[illegible]

al;GO:0086010//membrane depolarization during action potential;GO:0086012//membrane depola

ment;GO:0060348//bone development;GO:0060395//SMAD protein signal transduction;GO:00604

6//positive regulation of fibroblast proliferation;GO:0050680//negative regulation of epithelial cell

0060541//respiratory system development;GO:0072359//circulatory system development;GO:0090

0060541//respiratory system development;GO:0072359//circulatory system development;GO:0090

homeostasis;GO:0055091//phospholipid homeostasis;GO:0070236//negative regulation of activati

//response to purine-containing compound;GO:0030324//lung development;GO:0030890//positiv

ne kinase activity;GO:1904140//negative regulation of microglial cell migration;GO:1904151//positi

terol efflux;GO:0010903//negative regulation of very-low-density lipoprotein particle remodeling;

/;GO:0014904//myotube cell development;GO:0014911//positive regulation of smooth muscle cell

35//bronchiole development;GO:0061520//Langerhans cell differentiation;GO:0071479//cellular re

/positive regulation of phagocytosis, engulfment;GO:1900016//negative regulation of cytokine prc
of transcription, DNA-templated;GO:0045944//positive regulation of transcription from RNA polyn

entiation;GO:0042541//hemoglobin biosynthetic process;GO:0042701//progesterone secretion;GO

proliferation;GO:0051726//regulation of cell cycle;GO:2000773//negative regulation of cellular ser

gative regulation of transcription, DNA-templated;GO:0045893//positive regulation of transcriptior

terol efflux;GO:0010903//negative regulation of very-low-density lipoprotein particle remodeling;

ıymus;GO:0042098//T cell proliferation;GO:0042100//B cell proliferation;GO:0042110//T cell activa

:ion of developmental process;GO:0051130//positive regulation of cellular component organizatio

/;GO:0014904//myotube cell development;GO:0014911//positive regulation of smooth muscle cell

ner ear receptor stereocilium organization;GO:0061073//ciliary body morphogenesis;GO:0090009/

/positive regulation of phagocytosis, engulfment;GO:1900016//negative regulation of cytokine prc

entiation;GO:0042541//hemoglobin biosynthetic process;GO:0042701//progesterone secretion;GO

ymus;GO:0042098//T cell proliferation;GO:0042100//B cell proliferation;GO:0042110//T cell activa

ion of developmental process;GO:0051130//positive regulation of cellular component organizatio

mediated signaling pathway;GO:0097400//interleukin-17-mediated signaling pathway;GO:009858

:ion of developmental process;GO:0051130//positive regulation of cellular component organizatio

;O:1900273//positive regulation of long-term synaptic potentiation;GO:1900454//positive regulati

of transcription, DNA-templated;GO:0045944//positive regulation of transcription from RNA polyn

mediated signaling pathway;GO:0097400//interleukin-17-mediated signaling pathway;GO:009858

ymus;GO:0042098//T cell proliferation;GO:0042100//B cell proliferation;GO:0042110//T cell activa

;GO:0046426//negative regulation of JAK-STAT cascade;GO:0048712//negative regulation of astr

/;GO:0014904//myotube cell development;GO:0014911//positive regulation of smooth muscle cell

ıymus;GO:0042098//T cell proliferation;GO:0042100//B cell proliferation;GO:0042110//T cell activa

:ion of developmental process;GO:0051130//positive regulation of cellular component organizatio

GO:1900273//positive regulation of long-term synaptic potentiation;GO:1900454//positive regulati
cholesterol biosynthetic process;GO:0050714//positive regulation of protein secretion;GO:0055085

/low-density lipoprotein particle remodeling;GO:0034383//low-density lipoprotein particle clearar

terol efflux;GO:0010903//negative regulation of very-low-density lipoprotein particle remodeling;

:ic activity;GO:0051092//positive regulation of NF-kappaB transcription factor activity;GO:0051281

entiation;GO:0042541//hemoglobin biosynthetic process;GO:0042701//progesterone secretion;GO

ymus;GO:0042098//T cell proliferation;GO:0042100//B cell proliferation;GO:0042110//T cell activa
/positive regulation of phagocytosis, engulfment;GO:1900016//negative regulation of cytokine pro

/;GO:0014904//myotube cell development;GO:0014911//positive regulation of smooth muscle cell

jative regulation of transcription, DNA-templated;GO:0045893//positive regulation of transcription

pecification;GO:0009887//organ morphogenesis;GO:0010468//regulation of gene expression;GO:0

/;GO:0014904//myotube cell development;GO:0014911//positive regulation of smooth muscle cell

/;GO:0046426//negative regulation of JAK-STAT cascade;GO:0048712//negative regulation of astro

cholesterol biosynthetic process;GO:0050714//positive regulation of protein secretion;GO:0055085

/;GO:0046426//negative regulation of JAK-STAT cascade;GO:0048712//negative regulation of astro

521//glomerular visceral epithelial cell migration;GO:1900027//regulation of ruffle assembly;GO:20

/positive regulation of phagocytosis, engulfment;GO:1900016//negative regulation of cytokine pro
ymus;GO:0042098//T cell proliferation;GO:0042100//B cell proliferation;GO:0042110//T cell activa

entiation;GO:0042541//hemoglobin biosynthetic process;GO:0042701//progesterone secretion;GO

/;GO:0014904//myotube cell development;GO:0014911//positive regulation of smooth muscle cell

to stilbenoid;GO:0042157//lipoprotein metabolic process;GO:0042632//cholesterol homeostasis;G

terol efflux;GO:0010903//negative regulation of very-low-density lipoprotein particle remodeling;

/;GO:0046426//negative regulation of JAK-STAT cascade;GO:0048712//negative regulation of astr

ative regulation of transcription, DNA-templated;GO:0045893//positive regulation of transcriptior

;GO:0046426//negative regulation of JAK-STAT cascade;GO:0048712//negative regulation of astr

/positive regulation of phagocytosis, engulfment;GO:1900016//negative regulation of cytokine prc

entiation;GO:0042541//hemoglobin biosynthetic process;GO:0042701//progesterone secretion;GC

terol efflux;GO:0010903//negative regulation of very-low-density lipoprotein particle remodeling;

jative regulation of transcription, DNA-templated;GO:0045893//positive regulation of transcriptior

:ion of developmental process;GO:0051130//positive regulation of cellular component organizatio

:ion of developmental process;GO:0051130//positive regulation of cellular component organizatio

pecification;GO:0009887//organ morphogenesis;GO:0010468//regulation of gene expression;GO:0

arization during cardiac muscle cell action potential;GO:0086014//atrial cardiac muscle cell action p

ation of vascular permeability;GO:0045766//positive regulation of angiogenesis;GO:0070830//bice

terol efflux;GO:0010903//negative regulation of very-low-density lipoprotein particle remodeling;(

tion of developmental process;GO:0051130//positive regulation of cellular component organizatio

:ic activity;GO:0051092//positive regulation of NF-kappaB transcription factor activity;GO:0051281

ıer ear receptor stereocilium organization;GO:0061073//ciliary body morphogenesis;GO:0090009/

ation of vascular permeability;GO:0045766//positive regulation of angiogenesis;GO:0070830//bice

jative regulation of transcription, DNA-templated;GO:0045893//positive regulation of transcriptior

›liferation;GO:0051702//interaction with symbiont;GO:0070372//regulation of ERK1 and ERK2 casc

NA-templated;GO:0006357//regulation of transcription from RNA polymerase II promoter;GO:000

terol efflux;GO:0010903//negative regulation of very-low-density lipoprotein particle remodeling;

arization during cardiac muscle cell action potential;GO:0086014//atrial cardiac muscle cell action

gative regulation of transcription, DNA-templated;GO:0045893//positive regulation of transcription

;GO:0060013//righting reflex;GO:0060166//olfactory pit development;GO:0060324//face develop

ner ear receptor stereocilium organization;GO:0061073//ciliary body morphogenesis;GO:0090009/

/;GO:0014904//myotube cell development;GO:0014911//positive regulation of smooth muscle cell

oliferation;GO:0051702//interaction with symbiont;GO:0070372//regulation of ERK1 and ERK2 casc
/positive regulation of phagocytosis, engulfment;GO:1900016//negative regulation of cytokine prc

entiation;GO:0042541//hemoglobin biosynthetic process;GO:0042701//progesterone secretion;GC

entiation;GO:0042541//hemoglobin biosynthetic process;GO:0042701//progesterone secretion;GC

/positive regulation of phagocytosis, engulfment;GO:1900016//negative regulation of cytokine prc

GO:0071277//cellular response to calcium ion;GO:0071479//cellular response to ionizing radiation;

terol efflux;GO:0010903//negative regulation of very-low-density lipoprotein particle remodeling;

02729//negative regulation of proteoglycan biosynthetic process;GO:1902730//positive regulatior

/positive regulation of phagocytosis, engulfment;GO:1900016//negative regulation of cytokine prc

entiation;GO:0042541//hemoglobin biosynthetic process;GO:0042701//progesterone secretion;GC

entiation;GO:0042541//hemoglobin biosynthetic process;GO:0042701//progesterone secretion;GC

/positive regulation of phagocytosis, engulfment;GO:1900016//negative regulation of cytokine pro

mediated signaling pathway;GO:0097400//interleukin-17-mediated signaling pathway;GO:009858

acellular signal transduction;GO:0046339//diacylglycerol metabolic process;GO:0046486//glyceroli

02729//negative regulation of proteoglycan biosynthetic process;GO:1902730//positive regulatior

/;GO:0046426//negative regulation of JAK-STAT cascade;GO:0048712//negative regulation of astr(

ifferentiation;GO:0030853//negative regulation of granulocyte differentiation;GO:0031076//embry

ıymus;GO:0042098//T cell proliferation;GO:0042100//B cell proliferation;GO:0042110//T cell activa

on of MAP kinase activity;GO:0045542//positive regulation of cholesterol biosynthetic process;GO

ne kinase activity;GO:1904140//negative regulation of microglial cell migration;GO:1904151//positi

35//bronchiole development;GO:0061520//Langerhans cell differentiation;GO:0071479//cellular re

entiation;GO:0042541//hemoglobin biosynthetic process;GO:0042701//progesterone secretion;GO

ıymus;GO:0042098//T cell proliferation;GO:0042100//B cell proliferation;GO:0042110//T cell activa

/;GO:0014904//myotube cell development;GO:0014911//positive regulation of smooth muscle cell

terol efflux;GO:0010903//negative regulation of very-low-density lipoprotein particle remodeling;

/positive regulation of phagocytosis, engulfment;GO:1900016//negative regulation of cytokine pro
GO:1900273//positive regulation of long-term synaptic potentiation;GO:1900454//positive regulati

acellular signal transduction;GO:0046339//diacylglycerol metabolic process;GO:0046486//glyceroli

:ion of developmental process;GO:0051130//positive regulation of cellular component organizatio

jative regulation of transcription, DNA-templated;GO:0045893//positive regulation of transcriptior

NA-templated;GO:0006357//regulation of transcription from RNA polymerase II promoter;GO:000

/;GO:0014904//myotube cell development;GO:0014911//positive regulation of smooth muscle cell

on of MAP kinase activity;GO:0045542//positive regulation of cholesterol biosynthetic process;GO

ıe kinase activity;GO:1904140//negative regulation of microglial cell migration;GO:1904151//positi

terol efflux;GO:0010903//negative regulation of very-low-density lipoprotein particle remodeling;

to stilbenoid;GO:0042157//lipoprotein metabolic process;GO:0042632//cholesterol homeostasis;G

tion of developmental process;GO:0051130//positive regulation of cellular component organizatio

cholesterol biosynthetic process;GO:0050714//positive regulation of protein secretion;GO:0055085

ner ear receptor stereocilium organization;GO:0061073//ciliary body morphogenesis;GO:0090009/

iymus;GO:0042098//T cell proliferation;GO:0042100//B cell proliferation;GO:0042110//T cell activa

;O:1900273//positive regulation of long-term synaptic potentiation;GO:1900454//positive regulati

-activating G-protein coupled signaling pathway;GO:0051496//positive regulation of stress fiber a

mediated signaling pathway;GO:0097400//interleukin-17-mediated signaling pathway;GO:009858

terol efflux;GO:0010903//negative regulation of very-low-density lipoprotein particle remodeling;

ymus;GO:0042098//T cell proliferation;GO:0042100//B cell proliferation;GO:0042110//T cell activa

GO:1900273//positive regulation of long-term synaptic potentiation;GO:1900454//positive regulati

to stilbenoid;GO:0042157//lipoprotein metabolic process;GO:0042632//cholesterol homeostasis;G
gative regulation of transcription, DNA-templated;GO:0045893//positive regulation of transcription

tion of developmental process;GO:0051130//positive regulation of cellular component organizatio

tion of developmental process;GO:0051130//positive regulation of cellular component organizatio

terol efflux;GO:0010903//negative regulation of very-low-density lipoprotein particle remodeling;

cholesterol biosynthetic process;GO:0050714//positive regulation of protein secretion;GO:0055085

GO:0071277//cellular response to calcium ion;GO:0071479//cellular response to ionizing radiation;

/;GO:0014904//myotube cell development;GO:0014911//positive regulation of smooth muscle cell

ıe kinase activity;GO:1904140//negative regulation of microglial cell migration;GO:1904151//positi

ıymus;GO:0042098//T cell proliferation;GO:0042100//B cell proliferation;GO:0042110//T cell activa

:ion of developmental process;GO:0051130//positive regulation of cellular component organizatio

NA-templated;GO:0006357//regulation of transcription from RNA polymerase II promoter;GO:000

of transcription, DNA-templated;GO:0045944//positive regulation of transcription from RNA polyn

:holesterol biosynthetic process;GO:0050714//positive regulation of protein secretion;GO:005508[illegible]

[illegible]//negative regulation of inflammatory response;GO:0050731//positive regulation of peptidyl-tyro

[illegible];GO:0014904//myotube cell development;GO:0014911//positive regulation of smooth muscle cell

[illegible];GO:0046426//negative regulation of JAK-STAT cascade;GO:0048712//negative regulation of astr[illegible]

terol efflux;GO:0010903//negative regulation of very-low-density lipoprotein particle remodeling;

ɔ glucose stimulus;GO:0061436//establishment of skin barrier;GO:0072659//protein localization to

35//bronchiole development;GO:0061520//Langerhans cell differentiation;GO:0071479//cellular re

ıymus;GO:0042098//T cell proliferation;GO:0042100//B cell proliferation;GO:0042110//T cell activa

3//negative regulation of inflammatory response;GO:0050731//positive regulation of peptidyl-tyro

NA-templated;GO:0006357//regulation of transcription from RNA polymerase II promoter;GO:000

[illegible];GO:0046426//negative regulation of JAK-STAT cascade;GO:0048712//negative regulation of astr[illegible]

/positive regulation of phagocytosis, engulfment;GO:1900016//negative regulation of cytokine prc

/;GO:0014904//myotube cell development;GO:0014911//positive regulation of smooth muscle cell

/;GO:0014904//myotube cell development;GO:0014911//positive regulation of smooth muscle cell

entiation;GO:0042541//hemoglobin biosynthetic process;GO:0042701//progesterone secretion;GC

›liferation;GO:0051702//interaction with symbiont;GO:0070372//regulation of ERK1 and ERK2 casc

/positive regulation of phagocytosis, engulfment;GO:1900016//negative regulation of cytokine prc

entiation;GO:0042541//hemoglobin biosynthetic process;GO:0042701//progesterone secretion;GC

/;GO:0014904//myotube cell development;GO:0014911//positive regulation of smooth muscle cell

ɔ glucose stimulus;GO:0061436//establishment of skin barrier;GO:0072659//protein localization to

›liferation;GO:0051702//interaction with symbiont;GO:0070372//regulation of ERK1 and ERK2 casc

/;GO:0014904//myotube cell development;GO:0014911//positive regulation of smooth muscle cell

ırization during cardiac muscle cell action potential;GO:0086014//atrial cardiac muscle cell action [illegible]

pecification;GO:0009887//organ morphogenesis;GO:0010468//regulation of gene expression;GO:[illegible]

ıymus;GO:0042098//T cell proliferation;GO:0042100//B cell proliferation;GO:0042110//T cell activa

tion of developmental process;GO:0051130//positive regulation of cellular component organizatio

GO:1900273//positive regulation of long-term synaptic potentiation;GO:1900454//positive regulati

ıymus;GO:0042098//T cell proliferation;GO:0042100//B cell proliferation;GO:0042110//T cell activa

ifferentiation;GO:0030853//negative regulation of granulocyte differentiation;GO:0031076//embry

jative regulation of transcription, DNA-templated;GO:0045893//positive regulation of transcriptior

terol efflux;GO:0010903//negative regulation of very-low-density lipoprotein particle remodeling;

ner ear receptor stereocilium organization;GO:0061073//ciliary body morphogenesis;GO:0090009/

bliferation;GO:0051702//interaction with symbiont;GO:0070372//regulation of ERK1 and ERK2 casc

entiation;GO:0042541//hemoglobin biosynthetic process;GO:0042701//progesterone secretion;GO

/positive regulation of phagocytosis, engulfment;GO:1900016//negative regulation of cytokine prc

:ic activity;GO:0051092//positive regulation of NF-kappaB transcription factor activity;GO:0051281

/;GO:0046426//negative regulation of JAK-STAT cascade;GO:0048712//negative regulation of astro

ner ear receptor stereocilium organization;GO:0061073//ciliary body morphogenesis;GO:0090009/

:ion of developmental process;GO:0051130//positive regulation of cellular component organizatio

NA-templated;GO:0006357//regulation of transcription from RNA polymerase II promoter;GO:000

acellular signal transduction;GO:0046339//diacylglycerol metabolic process;GO:0046486//glyceroli

gative regulation of transcription, DNA-templated;GO:0045893//positive regulation of transcriptior

terol efflux;GO:0010903//negative regulation of very-low-density lipoprotein particle remodeling;

cholesterol biosynthetic process;GO:0050714//positive regulation of protein secretion;GO:0055085

on of MAP kinase activity;GO:0045542//positive regulation of cholesterol biosynthetic process;GO

ɔ glucose stimulus;GO:0061436//establishment of skin barrier;GO:0072659//protein localization to

ıymus;GO:0042098//T cell proliferation;GO:0042100//B cell proliferation;GO:0042110//T cell activa

entiation;GO:0042541//hemoglobin biosynthetic process;GO:0042701//progesterone secretion;GO

/;GO:0014904//myotube cell development;GO:0014911//positive regulation of smooth muscle cell

/positive regulation of phagocytosis, engulfment;GO:1900016//negative regulation of cytokine pro

/;GO:0046426//negative regulation of JAK-STAT cascade;GO:0048712//negative regulation of astro

ıer ear receptor stereocilium organization;GO:0061073//ciliary body morphogenesis;GO:0090009/,

:ion of developmental process;GO:0051130//positive regulation of cellular component organizatio

n;GO:0060013//righting reflex;GO:0060166//olfactory pit development;GO:0060324//face developm

/positive regulation of phagocytosis, engulfment;GO:1900016//negative regulation of cytokine pro

terol efflux;GO:0010903//negative regulation of very-low-density lipoprotein particle remodeling;

entiation;GO:0042541//hemoglobin biosynthetic process;GO:0042701//progesterone secretion;GO

on of MAP kinase activity;GO:0045542//positive regulation of cholesterol biosynthetic process;GO

ner ear receptor stereocilium organization;GO:0061073//ciliary body morphogenesis;GO:0090009/

tic activity;GO:0051092//positive regulation of NF-kappaB transcription factor activity;GO:0051281

tion of developmental process;GO:0051130//positive regulation of cellular component organizatio

/positive regulation of phagocytosis, engulfment;GO:1900016//negative regulation of cytokine prc

on of MAP kinase activity;GO:0045542//positive regulation of cholesterol biosynthetic process;GO
pathway;GO:0022602//ovulation cycle process;GO:0031175//neuron projection development;GO:[illegible]

entiation;GO:0042541//hemoglobin biosynthetic process;GO:0042701//progesterone secretion;GO

tion of developmental process;GO:0051130//positive regulation of cellular component organizatio

tion of developmental process;GO:0051130//positive regulation of cellular component organizatio

02729//negative regulation of proteoglycan biosynthetic process;GO:1902730//positive regulatior

NA-templated;GO:0006357//regulation of transcription from RNA polymerase II promoter;GO:000
GO:1900273//positive regulation of long-term synaptic potentiation;GO:1900454//positive regulati

/positive regulation of phagocytosis, engulfment;GO:1900016//negative regulation of cytokine pro

entiation;GO:0042541//hemoglobin biosynthetic process;GO:0042701//progesterone secretion;GO

terol efflux;GO:0010903//negative regulation of very-low-density lipoprotein particle remodeling;

3//negative regulation of inflammatory response;GO:0050731//positive regulation of peptidyl-tyro

GO:0071277//cellular response to calcium ion;GO:0071479//cellular response to ionizing radiation;

/;GO:0046426//negative regulation of JAK-STAT cascade;GO:0048712//negative regulation of astro
3//negative regulation of inflammatory response;GO:0050731//positive regulation of peptidyl-tyro

ıymus;GO:0042098//T cell proliferation;GO:0042100//B cell proliferation;GO:0042110//T cell activa

terol efflux;GO:0010903//negative regulation of very-low-density lipoprotein particle remodeling;
/;GO:0014904//myotube cell development;GO:0014911//positive regulation of smooth muscle cell
oliferation;GO:0051702//interaction with symbiont;GO:0070372//regulation of ERK1 and ERK2 casc

ic activity;GO:0051092//positive regulation of NF-kappaB transcription factor activity;GO:0051281

cholesterol biosynthetic process;GO:0050714//positive regulation of protein secretion;GO:0055085

y;GO:0046426//negative regulation of JAK-STAT cascade;GO:0048712//negative regulation of astro

GO:1900273//positive regulation of long-term synaptic potentiation;GO:1900454//positive regulati

ymus;GO:0042098//T cell proliferation;GO:0042100//B cell proliferation;GO:0042110//T cell activa

ıymus;GO:0042098//T cell proliferation;GO:0042100//B cell proliferation;GO:0042110//T cell activa

3//negative regulation of inflammatory response;GO:0050731//positive regulation of peptidyl-tyro

/positive regulation of phagocytosis, engulfment;GO:1900016//negative regulation of cytokine pro

/;GO:0014904//myotube cell development;GO:0014911//positive regulation of smooth muscle cell

entiation;GO:0042541//hemoglobin biosynthetic process;GO:0042701//progesterone secretion;GO

pathway;GO:0022602//ovulation cycle process;GO:0031175//neuron projection development;GO:(

:holesterol biosynthetic process;GO:0050714//positive regulation of protein secretion;GO:0055085

ɔ glucose stimulus;GO:0061436//establishment of skin barrier;GO:0072659//protein localization to

ation of vascular permeability;GO:0045766//positive regulation of angiogenesis;GO:0070830//bice

3//negative regulation of inflammatory response;GO:0050731//positive regulation of peptidyl-tyro

of transcription, DNA-templated;GO:0045944//positive regulation of transcription from RNA polyn

ıymus;GO:0042098//T cell proliferation;GO:0042100//B cell proliferation;GO:0042110//T cell activa

ɔ glucose stimulus;GO:0061436//establishment of skin barrier;GO:0072659//protein localization to

NA-templated;GO:0006357//regulation of transcription from RNA polymerase II promoter;GO:000

ner ear receptor stereocilium organization;GO:0061073//ciliary body morphogenesis;GO:0090009/

n transport;GO:0070588//calcium ion transmembrane transport;GO:1900121//negative regulation c

on of MAP kinase activity;GO:0045542//positive regulation of cholesterol biosynthetic process;GO

on of MAP kinase activity;GO:0045542//positive regulation of cholesterol biosynthetic process;GO

tion;GO:1902042//negative regulation of extrinsic apoptotic signaling pathway via death domain r

ı transport;GO:0070588//calcium ion transmembrane transport;GO:1900121//negative regulation c

xpression;GO:0010468//regulation of gene expression;GO:0014898//cardiac muscle hypertrophy ir

/low-density lipoprotein particle remodeling;GO:0034383//low-density lipoprotein particle clearar
:ic activity;GO:0051092//positive regulation of NF-kappaB transcription factor activity;GO:0051281

entiation;GO:0042541//hemoglobin biosynthetic process;GO:0042701//progesterone secretion;GO

/positive regulation of phagocytosis, engulfment;GO:1900016//negative regulation of cytokine pro

tein localization to cell surface;GO:2001137//positive regulation of endocytic recycling;GO:200125

transport;GO:0070588//calcium ion transmembrane transport;GO:1900121//negative regulation o

n;GO:0060013//righting reflex;GO:0060166//olfactory pit development;GO:0060324//face developr

O:0007186//G-protein coupled receptor signaling pathway;GO:0007193//adenylate cyclase-inhibit
ifferentiation;GO:0030853//negative regulation of granulocyte differentiation;GO:0031076//embry

521//glomerular visceral epithelial cell migration;GO:1900027//regulation of ruffle assembly;GO:20

/low-density lipoprotein particle remodeling;GO:0034383//low-density lipoprotein particle clearar

ay;GO:0007228//positive regulation of hh target transcription factor activity;GO:0007267//cell-cell

n;GO:0060013//righting reflex;GO:0060166//olfactory pit development;GO:0060324//face developm

tein localization to cell surface;GO:2001137//positive regulation of endocytic recycling;GO:200125

pathway;GO:0022602//ovulation cycle process;GO:0031175//neuron projection development;GO:0

ay;GO:0007228//positive regulation of hh target transcription factor activity;GO:0007267//cell-cell

pathway;GO:0022602//ovulation cycle process;GO:0031175//neuron projection development;GO:(

xpression;GO:0010468//regulation of gene expression;GO:0014898//cardiac muscle hypertrophy ir

jative regulation of transcription, DNA-templated;GO:0045893//positive regulation of transcriptior

-activating G-protein coupled signaling pathway;GO:0051496//positive regulation of stress fiber a

ve regulation of B cell proliferation;GO:0032261//purine nucleotide salvage;GO:0032263//GMP salv

O:0007186//G-protein coupled receptor signaling pathway;GO:0007193//adenylate cyclase-inhibit

-activating G-protein coupled signaling pathway;GO:0051496//positive regulation of stress fiber a

pathway;GO:0022602//ovulation cycle process;GO:0031175//neuron projection development;GO:(

:ion of developmental process;GO:0051130//positive regulation of cellular component organizatio

ırdium morphogenesis;GO:0003300//cardiac muscle hypertrophy;GO:0006355//regulation of trans

transport;GO:0070588//calcium ion transmembrane transport;GO:1900121//negative regulation

xpression;GO:0010468//regulation of gene expression;GO:0014898//cardiac muscle hypertrophy i

/low-density lipoprotein particle remodeling;GO:0034383//low-density lipoprotein particle clearan

pathway;GO:0022602//ovulation cycle process;GO:0031175//neuron projection development;GO:0

ay;GO:0007228//positive regulation of hh target transcription factor activity;GO:0007267//cell-cell

-activating G-protein coupled signaling pathway;GO:0051496//positive regulation of stress fiber a

'e regulation of B cell proliferation;GO:0032261//purine nucleotide salvage;GO:0032263//GMP salv

521//glomerular visceral epithelial cell migration;GO:1900027//regulation of ruffle assembly;GO:2C

ı transport;GO:0070588//calcium ion transmembrane transport;GO:1900121//negative regulation c

xpression;GO:0010468//regulation of gene expression;GO:0014898//cardiac muscle hypertrophy ir

ardium morphogenesis;GO:0003300//cardiac muscle hypertrophy;GO:0006355//regulation of trans

proliferation;GO:0051726//regulation of cell cycle;GO:2000773//negative regulation of cellular ser

-activating G-protein coupled signaling pathway;GO:0051496//positive regulation of stress fiber a

ay;GO:0007228//positive regulation of hh target transcription factor activity;GO:0007267//cell-cell

02729//negative regulation of proteoglycan biosynthetic process;GO:1902730//positive regulatior

ı;GO:0060013//righting reflex;GO:0060166//olfactory pit development;GO:0060324//face developı

521//glomerular visceral epithelial cell migration;GO:1900027//regulation of ruffle assembly;GO:2(

proliferation;GO:0051726//regulation of cell cycle;GO:2000773//negative regulation of cellular ser

/;GO:0014904//myotube cell development;GO:0014911//positive regulation of smooth muscle cell

521//glomerular visceral epithelial cell migration;GO:1900027//regulation of ruffle assembly;GO:20

521//glomerular visceral epithelial cell migration;GO:1900027//regulation of ruffle assembly;GO:20

:ein localization to cell surface;GO:2001137//positive regulation of endocytic recycling;GO:200125[illegible]

ifferentiation;GO:0030853//negative regulation of granulocyte differentiation;GO:0031076//embry

/low-density lipoprotein particle remodeling;GO:0034383//low-density lipoprotein particle clearar

;GO:0060013//righting reflex;GO:0060166//olfactory pit development;GO:0060324//face develop

521//glomerular visceral epithelial cell migration;GO:1900027//regulation of ruffle assembly;GO:20

ifferentiation;GO:0030853//negative regulation of granulocyte differentiation;GO:0031076//embry

tion;GO:1902042//negative regulation of extrinsic apoptotic signaling pathway via death domain r

'e regulation of B cell proliferation;GO:0032261//purine nucleotide salvage;GO:0032263//GMP salv

ifferentiation;GO:0030853//negative regulation of granulocyte differentiation;GO:0031076//embry

):0007186//G-protein coupled receptor signaling pathway;GO:0007193//adenylate cyclase-inhibit

ne kinase activity;GO:1904140//negative regulation of microglial cell migration;GO:1904151//positi

ation of vascular permeability;GO:0045766//positive regulation of angiogenesis;GO:0070830//bice

-activating G-protein coupled signaling pathway;GO:0051496//positive regulation of stress fiber a

n;GO:0060013//righting reflex;GO:0060166//olfactory pit development;GO:0060324//face develop

e regulation of B cell proliferation;GO:0032261//purine nucleotide salvage;GO:0032263//GMP salv

xpression;GO:0010468//regulation of gene expression;GO:0014898//cardiac muscle hypertrophy i

transport;GO:0070588//calcium ion transmembrane transport;GO:1900121//negative regulation

ifferentiation;GO:0030853//negative regulation of granulocyte differentiation;GO:0031076//embry

ardium morphogenesis;GO:0003300//cardiac muscle hypertrophy;GO:0006355//regulation of trans

-activating G-protein coupled signaling pathway;GO:0051496//positive regulation of stress fiber a

ɔ glucose stimulus;GO:0061436//establishment of skin barrier;GO:0072659//protein localization to

tion;GO:1902042//negative regulation of extrinsic apoptotic signaling pathway via death domain r

Ɔ:0007186//G-protein coupled receptor signaling pathway;GO:0007193//adenylate cyclase-inhibit
ı;GO:0060013//righting reflex;GO:0060166//olfactory pit development;GO:0060324//face develop

xpression;GO:0010468//regulation of gene expression;GO:0014898//cardiac muscle hypertrophy ir

-activating G-protein coupled signaling pathway;GO:0051496//positive regulation of stress fiber a

D:0007186//G-protein coupled receptor signaling pathway;GO:0007193//adenylate cyclase-inhibit

521//glomerular visceral epithelial cell migration;GO:1900027//regulation of ruffle assembly;GO:20

xpression;GO:0010468//regulation of gene expression;GO:0014898//cardiac muscle hypertrophy in

02729//negative regulation of proteoglycan biosynthetic process;GO:1902730//positive regulation

n;GO:0060013//righting reflex;GO:0060166//olfactory pit development;GO:0060324//face develop[illegible]

terol efflux;GO:0010903//negative regulation of very-low-density lipoprotein particle remodeling;[illegible]

proliferation;GO:0051726//regulation of cell cycle;GO:2000773//negative regulation of cellular ser

xpression;GO:0010468//regulation of gene expression;GO:0014898//cardiac muscle hypertrophy ir

ifferentiation;GO:0030853//negative regulation of granulocyte differentiation;GO:0031076//embry

521//glomerular visceral epithelial cell migration;GO:1900027//regulation of ruffle assembly;GO:20

:ein localization to cell surface;GO:2001137//positive regulation of endocytic recycling;GO:200125[illegible]

521//glomerular visceral epithelial cell migration;GO:1900027//regulation of ruffle assembly;GO:20

ay;GO:0007228//positive regulation of hh target transcription factor activity;GO:0007267//cell-cell

NA-templated;GO:0006357//regulation of transcription from RNA polymerase II promoter;GO:000

/low-density lipoprotein particle remodeling;GO:0034383//low-density lipoprotein particle clearar

ifferentiation;GO:0030853//negative regulation of granulocyte differentiation;GO:0031076//embry

:ein localization to cell surface;GO:2001137//positive regulation of endocytic recycling;GO:200125[illegible]

/low-density lipoprotein particle remodeling;GO:0034383//low-density lipoprotein particle clearar

ay;GO:0007228//positive regulation of hh target transcription factor activity;GO:0007267//cell-cell

ifferentiation;GO:0030853//negative regulation of granulocyte differentiation;GO:0031076//embry

ation of vascular permeability;GO:0045766//positive regulation of angiogenesis;GO:0070830//bice

'e regulation of B cell proliferation;GO:0032261//purine nucleotide salvage;GO:0032263//GMP salv

ifferentiation;GO:0030853//negative regulation of granulocyte differentiation;GO:0031076//embry

;GO:0060013//righting reflex;GO:0060166//olfactory pit development;GO:0060324//face develop

35//bronchiole development;GO:0061520//Langerhans cell differentiation;GO:0071479//cellular re

transport;GO:0070588//calcium ion transmembrane transport;GO:1900121//negative regulation

ein localization to cell surface;GO:2001137//positive regulation of endocytic recycling;GO:200125

/low-density lipoprotein particle remodeling;GO:0034383//low-density lipoprotein particle clearan

ardium morphogenesis;GO:0003300//cardiac muscle hypertrophy;GO:0006355//regulation of trans

-activating G-protein coupled signaling pathway;GO:0051496//positive regulation of stress fiber a
n;GO:0060013//righting reflex;GO:0060166//olfactory pit development;GO:0060324//face developm

521//glomerular visceral epithelial cell migration;GO:1900027//regulation of ruffle assembly;GO:2[illegible]

ardium morphogenesis;GO:0003300//cardiac muscle hypertrophy;GO:0006355//regulation of trans

521//glomerular visceral epithelial cell migration;GO:1900027//regulation of ruffle assembly;GO:20

ifferentiation;GO:0030853//negative regulation of granulocyte differentiation;GO:0031076//embry

-activating G-protein coupled signaling pathway;GO:0051496//positive regulation of stress fiber a

ı;GO:0060013//righting reflex;GO:0060166//olfactory pit development;GO:0060324//face develop[illegible]

xpression;GO:0010468//regulation of gene expression;GO:0014898//cardiac muscle hypertrophy ir

proliferation;GO:0051726//regulation of cell cycle;GO:2000773//negative regulation of cellular ser

ardium morphogenesis;GO:0003300//cardiac muscle hypertrophy;GO:0006355//regulation of trans

xpression;GO:0010468//regulation of gene expression;GO:0014898//cardiac muscle hypertrophy ir

521//glomerular visceral epithelial cell migration;GO:1900027//regulation of ruffle assembly;GO:2C

proliferation;GO:0051726//regulation of cell cycle;GO:2000773//negative regulation of cellular ser

-activating G-protein coupled signaling pathway;GO:0051496//positive regulation of stress fiber a

;GO:0060013//righting reflex;GO:0060166//olfactory pit development;GO:0060324//face develop

;GO:0060013//righting reflex;GO:0060166//olfactory pit development;GO:0060324//face develop

521//glomerular visceral epithelial cell migration;GO:1900027//regulation of ruffle assembly;GO:20

mediated signaling pathway;GO:0097400//interleukin-17-mediated signaling pathway;GO:009858

'e regulation of B cell proliferation;GO:0032261//purine nucleotide salvage;GO:0032263//GMP salv

proliferation;GO:0051726//regulation of cell cycle;GO:2000773//negative regulation of cellular ser

02729//negative regulation of proteoglycan biosynthetic process;GO:1902730//positive regulatior

o glucose stimulus;GO:0061436//establishment of skin barrier;GO:0072659//protein localization to

mediated signaling pathway;GO:0097400//interleukin-17-mediated signaling pathway;GO:009858

-activating G-protein coupled signaling pathway;GO:0051496//positive regulation of stress fiber a

n;GO:0060013//righting reflex;GO:0060166//olfactory pit development;GO:0060324//face develop

e regulation of B cell proliferation;GO:0032261//purine nucleotide salvage;GO:0032263//GMP salv

-activating G-protein coupled signaling pathway;GO:0051496//positive regulation of stress fiber a

35//bronchiole development;GO:0061520//Langerhans cell differentiation;GO:0071479//cellular re

ıer ear receptor stereocilium organization;GO:0061073//ciliary body morphogenesis;GO:0090009/,

xpression;GO:0010468//regulation of gene expression;GO:0014898//cardiac muscle hypertrophy ir

ı;GO:0060013//righting reflex;GO:0060166//olfactory pit development;GO:0060324//face developr

ifferentiation;GO:0030853//negative regulation of granulocyte differentiation;GO:0031076//embry

xpression;GO:0010468//regulation of gene expression;GO:0014898//cardiac muscle hypertrophy i[illegible]

35//bronchiole development;GO:0061520//Langerhans cell differentiation;GO:0071479//cellular re

proliferation;GO:0051726//regulation of cell cycle;GO:2000773//negative regulation of cellular se[illegible]

-activating G-protein coupled signaling pathway;GO:0051496//positive regulation of stress fiber a

-activating G-protein coupled signaling pathway;GO:0051496//positive regulation of stress fiber a

'e regulation of B cell proliferation;GO:0032261//purine nucleotide salvage;GO:0032263//GMP salv

pathway;GO:0022602//ovulation cycle process;GO:0031175//neuron projection development;GO:(

xpression;GO:0010468//regulation of gene expression;GO:0014898//cardiac muscle hypertrophy ir

arization during cardiac muscle cell action potential;GO:0086014//atrial cardiac muscle cell action p

ation of vascular permeability;GO:0045766//positive regulation of angiogenesis;GO:0070830//bice

/low-density lipoprotein particle remodeling;GO:0034383//low-density lipoprotein particle clearan

ifferentiation;GO:0030853//negative regulation of granulocyte differentiation;GO:0031076//embry

xpression;GO:0010468//regulation of gene expression;GO:0014898//cardiac muscle hypertrophy ir

-activating G-protein coupled signaling pathway;GO:0051496//positive regulation of stress fiber a

ıymus;GO:0042098//T cell proliferation;GO:0042100//B cell proliferation;GO:0042110//T cell activa

xpression;GO:0010468//regulation of gene expression;GO:0014898//cardiac muscle hypertrophy ir
02729//negative regulation of proteoglycan biosynthetic process;GO:1902730//positive regulatior

:ein localization to cell surface;GO:2001137//positive regulation of endocytic recycling;GO:200125[illegible]

ifferentiation;GO:0030853//negative regulation of granulocyte differentiation;GO:0031076//embry

521//glomerular visceral epithelial cell migration;GO:1900027//regulation of ruffle assembly;GO:2[illegible]

arization during cardiac muscle cell action potential;GO:0086014//atrial cardiac muscle cell action p

35//bronchiole development;GO:0061520//Langerhans cell differentiation;GO:0071479//cellular re

521//glomerular visceral epithelial cell migration;GO:1900027//regulation of ruffle assembly;GO:20

:ein localization to cell surface;GO:2001137//positive regulation of endocytic recycling;GO:200125

of transcription, DNA-templated;GO:0045944//positive regulation of transcription from RNA polym

-activating G-protein coupled signaling pathway;GO:0051496//positive regulation of stress fiber a

ifferentiation;GO:0030853//negative regulation of granulocyte differentiation;GO:0031076//embry

-activating G-protein coupled signaling pathway;GO:0051496//positive regulation of stress fiber a

/low-density lipoprotein particle remodeling;GO:0034383//low-density lipoprotein particle clearar

ation of vascular permeability;GO:0045766//positive regulation of angiogenesis;GO:0070830//bice

ardium morphogenesis;GO:0003300//cardiac muscle hypertrophy;GO:0006355//regulation of trans

ifferentiation;GO:0030853//negative regulation of granulocyte differentiation;GO:0031076//embry

xpression;GO:0010468//regulation of gene expression;GO:0014898//cardiac muscle hypertrophy ir

jative regulation of transcription, DNA-templated;GO:0045893//positive regulation of transcriptior

/low-density lipoprotein particle remodeling;GO:0034383//low-density lipoprotein particle clearar

n;GO:0060013//righting reflex;GO:0060166//olfactory pit development;GO:0060324//face developm

proliferation;GO:0051726//regulation of cell cycle;GO:2000773//negative regulation of cellular ser

ardium morphogenesis;GO:0003300//cardiac muscle hypertrophy;GO:0006355//regulation of trans

-activating G-protein coupled signaling pathway;GO:0051496//positive regulation of stress fiber a

xpression;GO:0010468//regulation of gene expression;GO:0014898//cardiac muscle hypertrophy i

;GO:0060013//righting reflex;GO:0060166//olfactory pit development;GO:0060324//face develop

521//glomerular visceral epithelial cell migration;GO:1900027//regulation of ruffle assembly;GO:2

tion;GO:1902042//negative regulation of extrinsic apoptotic signaling pathway via death domain r

-activating G-protein coupled signaling pathway;GO:0051496//positive regulation of stress fiber a

/low-density lipoprotein particle remodeling;GO:0034383//low-density lipoprotein particle clearar

proliferation;GO:0051726//regulation of cell cycle;GO:2000773//negative regulation of cellular ser

arization during cardiac muscle cell action potential;GO:0086014//atrial cardiac muscle cell action p

ı;GO:0060013//righting reflex;GO:0060166//olfactory pit development;GO:0060324//face developı

xpression;GO:0010468//regulation of gene expression;GO:0014898//cardiac muscle hypertrophy ir

pecification;GO:0009887//organ morphogenesis;GO:0010468//regulation of gene expression;GO:0

521//glomerular visceral epithelial cell migration;GO:1900027//regulation of ruffle assembly;GO:20

arization during cardiac muscle cell action potential;GO:0086014//atrial cardiac muscle cell action p

ifferentiation;GO:0030853//negative regulation of granulocyte differentiation;GO:0031076//embry

e regulation of B cell proliferation;GO:0032261//purine nucleotide salvage;GO:0032263//GMP salv

arization during cardiac muscle cell action potential;GO:0086014//atrial cardiac muscle cell action p

35//bronchiole development;GO:0061520//Langerhans cell differentiation;GO:0071479//cellular re

proliferation;GO:0051726//regulation of cell cycle;GO:2000773//negative regulation of cellular ser

521//glomerular visceral epithelial cell migration;GO:1900027//regulation of ruffle assembly;GO:20

521//glomerular visceral epithelial cell migration;GO:1900027//regulation of ruffle assembly;GO:20

ve regulation of B cell proliferation;GO:0032261//purine nucleotide salvage;GO:0032263//GMP salv

ve regulation of microglial cell mediated cytotoxicity;GO:2000251//positive regulation of actin cyto

GO:0018158//protein oxidation;GO:0018206//peptidyl-methionine modification;GO:0019915//lipi

migration;GO:0021650//vestibulocochlear nerve formation;GO:0030166//proteoglycan biosynthe

esponse to ionizing radiation;GO:0071604//transforming growth factor beta production;GO:19013

ɔduction involved in inflammatory response;GO:1900077//negative regulation of cellular response
ıerase II promoter;GO:0048712//negative regulation of astrocyte differentiation;GO:0048715//neg

):0045648//positive regulation of erythrocyte differentiation;GO:0045893//positive regulation of tra

ı, DNA-templated;GO:0045944//positive regulation of transcription from RNA polymerase II prom

GO:0018158//protein oxidation;GO:0018206//peptidyl-methionine modification;GO:0019915//lipi[illegible]

tion;GO:0042113//B cell activation;GO:0043303//mast cell degranulation;GO:0043406//positive re[illegible]

n;GO:0051703//intraspecies interaction between organisms;GO:0072175//epithelial tube formatio

migration;GO:0021650//vestibulocochlear nerve formation;GO:0030166//proteoglycan biosynthe

/primitive streak formation;GO:0097009//energy homeostasis;GO:0098868//bone growth;GO:1904

oduction involved in inflammatory response;GO:1900077//negative regulation of cellular response

):0045648//positive regulation of erythrocyte differentiation;GO:0045893//positive regulation of tra

tion;GO:0042113//B cell activation;GO:0043303//mast cell degranulation;GO:0043406//positive reg

n;GO:0051703//intraspecies interaction between organisms;GO:0072175//epithelial tube formatior

n;GO:0051703//intraspecies interaction between organisms;GO:0072175//epithelial tube formatior

ıerase II promoter;GO:0048712//negative regulation of astrocyte differentiation;GO:0048715//neg

tion;GO:0042113//B cell activation;GO:0043303//mast cell degranulation;GO:0043406//positive reg

ɔcyte differentiation;GO:0050771//negative regulation of axonogenesis;GO:0051645//Golgi localiz

migration;GO:0021650//vestibulocochlear nerve formation;GO:0030166//proteoglycan biosynthe

tion;GO:0042113//B cell activation;GO:0043303//mast cell degranulation;GO:0043406//positive reg

n;GO:0051703//intraspecies interaction between organisms;GO:0072175//epithelial tube formation

5//transmembrane transport;GO:0055091//phospholipid homeostasis;GO:0071403//cellular respon

ıce;GO:0042157//lipoprotein metabolic process;GO:0042158//lipoprotein biosynthetic process;GO

GO:0018158//protein oxidation;GO:0018206//peptidyl-methionine modification;GO:0019915//lipi

//positive regulation of release of sequestered calcium ion into cytosol;GO:0061061//muscle struc

):0045648//positive regulation of erythrocyte differentiation;GO:0045893//positive regulation of tra

tion;GO:0042113//B cell activation;GO:0043303//mast cell degranulation;GO:0043406//positive reg
oduction involved in inflammatory response;GO:1900077//negative regulation of cellular response

migration;GO:0021650//vestibulocochlear nerve formation;GO:0030166//proteoglycan biosynthe

n, DNA-templated;GO:0045944//positive regulation of transcription from RNA polymerase II prom

)010634//positive regulation of epithelial cell migration;GO:0010838//positive regulation of keratir

migration;GO:0021650//vestibulocochlear nerve formation;GO:0030166//proteoglycan biosynthe

ocyte differentiation;GO:0050771//negative regulation of axonogenesis;GO:0051645//Golgi localiz

5//transmembrane transport;GO:0055091//phospholipid homeostasis;GO:0071403//cellular respon

ocyte differentiation;GO:0050771//negative regulation of axonogenesis;GO:0051645//Golgi localiz

oduction involved in inflammatory response;GO:1900077//negative regulation of cellular response
tion;GO:0042113//B cell activation;GO:0043303//mast cell degranulation;GO:0043406//positive reg

):0045648//positive regulation of erythrocyte differentiation;GO:0045893//positive regulation of tra

migration;GO:0021650//vestibulocochlear nerve formation;GO:0030166//proteoglycan biosynthe

;O:0042744//hydrogen peroxide catabolic process;GO:0043691//reverse cholesterol transport;GO:

GO:0018158//protein oxidation;GO:0018206//peptidyl-methionine modification;GO:0019915//lipi

ocyte differentiation;GO:0050771//negative regulation of axonogenesis;GO:0051645//Golgi localiz

ı, DNA-templated;GO:0045944//positive regulation of transcription from RNA polymerase II prom

ɔcyte differentiation;GO:0050771//negative regulation of axonogenesis;GO:0051645//Golgi localiz

›duction involved in inflammatory response;GO:1900077//negative regulation of cellular response

›:0045648//positive regulation of erythrocyte differentiation;GO:0045893//positive regulation of tra

GO:0018158//protein oxidation;GO:0018206//peptidyl-methionine modification;GO:0019915//lipi

ı, DNA-templated;GO:0045944//positive regulation of transcription from RNA polymerase II prom

n;GO:0051703//intraspecies interaction between organisms;GO:0072175//epithelial tube formatio

n;GO:0051703//intraspecies interaction between organisms;GO:0072175//epithelial tube formatior

)010634//positive regulation of epithelial cell migration;GO:0010838//positive regulation of keratir

ɔotential;GO:0086015//SA node cell action potential;GO:0086091//regulation of heart rate by card

ellular tight junction assembly;GO:0070831//basement membrane assembly;GO:0072659//protein

GO:0018158//protein oxidation;GO:0018206//peptidyl-methionine modification;GO:0019915//lipi[illegible]

n;GO:0051703//intraspecies interaction between organisms;GO:0072175//epithelial tube formatio[illegible]

//positive regulation of release of sequestered calcium ion into cytosol;GO:0061061//muscle struc

/primitive streak formation;GO:0097009//energy homeostasis;GO:0098868//bone growth;GO:1904

ellular tight junction assembly;GO:0070831//basement membrane assembly;GO:0072659//protein

n, DNA-templated;GO:0045944//positive regulation of transcription from RNA polymerase II prom

ade;GO:0071635//negative regulation of transforming growth factor beta production;GO:1901166

6959//humoral immune response;GO:0007165//signal transduction;GO:0007399//nervous system

GO:0018158//protein oxidation;GO:0018206//peptidyl-methionine modification;GO:0019915//lipi[illegible]

potential;GO:0086015//SA node cell action potential;GO:0086091//regulation of heart rate by card

, DNA-templated;GO:0045944//positive regulation of transcription from RNA polymerase II prom

/primitive streak formation;GO:0097009//energy homeostasis;GO:0098868//bone growth;GO:1904

migration;GO:0021650//vestibulocochlear nerve formation;GO:0030166//proteoglycan biosynthe

ade;GO:0071635//negative regulation of transforming growth factor beta production;GO:1901166
oduction involved in inflammatory response;GO:1900077//negative regulation of cellular response

):0045648//positive regulation of erythrocyte differentiation;GO:0045893//positive regulation of tra

):0045648//positive regulation of erythrocyte differentiation;GO:0045893//positive regulation of tra

oduction involved in inflammatory response;GO:1900077//negative regulation of cellular response

;GO:0090630//activation of GTPase activity;GO:2000431//regulation of cytokinesis, actomyosin cor

GO:0018158//protein oxidation;GO:0018206//peptidyl-methionine modification;GO:0019915//lipi[illegible]

n of proteoglycan biosynthetic process;GO:1903053//regulation of extracellular matrix organization

oduction involved in inflammatory response;GO:1900077//negative regulation of cellular response

:0045648//positive regulation of erythrocyte differentiation;GO:0045893//positive regulation of tra

:0045648//positive regulation of erythrocyte differentiation;GO:0045893//positive regulation of tra

oduction involved in inflammatory response;GO:1900077//negative regulation of cellular response

pid metabolic process;GO:0046834//lipid phosphorylation;GO:0050731//positive regulation of pep

ι of proteoglycan biosynthetic process;GO:1903053//regulation of extracellular matrix organizatior

ɔcyte differentiation;GO:0050771//negative regulation of axonogenesis;GO:0051645//Golgi localiz

ronic camera-type eye development;GO:0032526//response to retinoic acid;GO:0032689//negative

tion;GO:0042113//B cell activation;GO:0043303//mast cell degranulation;GO:0043406//positive reg

:0045766//positive regulation of angiogenesis;GO:0045944//positive regulation of transcription frc

ve regulation of microglial cell mediated cytotoxicity;GO:2000251//positive regulation of actin cyto

esponse to ionizing radiation;GO:0071604//transforming growth factor beta production;GO:190138

):0045648//positive regulation of erythrocyte differentiation;GO:0045893//positive regulation of tra

tion;GO:0042113//B cell activation;GO:0043303//mast cell degranulation;GO:0043406//positive reg

migration;GO:0021650//vestibulocochlear nerve formation;GO:0030166//proteoglycan biosynthe

GO:0018158//protein oxidation;GO:0018206//peptidyl-methionine modification;GO:0019915//lipi

oduction involved in inflammatory response;GO:1900077//negative regulation of cellular response

pid metabolic process;GO:0046834//lipid phosphorylation;GO:0050731//positive regulation of pep

n;GO:0051703//intraspecies interaction between organisms;GO:0072175//epithelial tube formatior

ı, DNA-templated;GO:0045944//positive regulation of transcription from RNA polymerase II prom

6959//humoral immune response;GO:0007165//signal transduction;GO:0007399//nervous system

migration;GO:0021650//vestibulocochlear nerve formation;GO:0030166//proteoglycan biosynthe

:0045766//positive regulation of angiogenesis;GO:0045944//positive regulation of transcription fro

ve regulation of microglial cell mediated cytotoxicity;GO:2000251//positive regulation of actin cyto

GO:0018158//protein oxidation;GO:0018206//peptidyl-methionine modification;GO:0019915//lipi

;O:0042744//hydrogen peroxide catabolic process;GO:0043691//reverse cholesterol transport;GO:[illegible]

n;GO:0051703//intraspecies interaction between organisms;GO:0072175//epithelial tube formatior

5//transmembrane transport;GO:0055091//phospholipid homeostasis;GO:0071403//cellular respon

/primitive streak formation;GO:0097009//energy homeostasis;GO:0098868//bone growth;GO:1904

tion;GO:0042113//B cell activation;GO:0043303//mast cell degranulation;GO:0043406//positive reg

GO:0018158//protein oxidation;GO:0018206//peptidyl-methionine modification;GO:0019915//lipi

tion;GO:0042113//B cell activation;GO:0043303//mast cell degranulation;GO:0043406//positive reg

;O:0042744//hydrogen peroxide catabolic process;GO:0043691//reverse cholesterol transport;GO:
ı, DNA-templated;GO:0045944//positive regulation of transcription from RNA polymerase II prom

n;GO:0051703//intraspecies interaction between organisms;GO:0072175//epithelial tube formatio

n;GO:0051703//intraspecies interaction between organisms;GO:0072175//epithelial tube formatio

GO:0018158//protein oxidation;GO:0018206//peptidyl-methionine modification;GO:0019915//lipi

;//transmembrane transport;GO:0055091//phospholipid homeostasis;GO:0071403//cellular respon

;GO:0090630//activation of GTPase activity;GO:2000431//regulation of cytokinesis, actomyosin cor

migration;GO:0021650//vestibulocochlear nerve formation;GO:0030166//proteoglycan biosynthe

ve regulation of microglial cell mediated cytotoxicity;GO:2000251//positive regulation of actin cyto

tion;GO:0042113//B cell activation;GO:0043303//mast cell degranulation;GO:0043406//positive reg

n;GO:0051703//intraspecies interaction between organisms;GO:0072175//epithelial tube formation

6959//humoral immune response;GO:0007165//signal transduction;GO:0007399//nervous system

nerase II promoter;GO:0048712//negative regulation of astrocyte differentiation;GO:0048715//neg

5//transmembrane transport;GO:0055091//phospholipid homeostasis;GO:0071403//cellular respon

sine phosphorylation;GO:0051450//myoblast proliferation;GO:0060326//cell chemotaxis;GO:0060€

migration;GO:0021650//vestibulocochlear nerve formation;GO:0030166//proteoglycan biosynthe

ocyte differentiation;GO:0050771//negative regulation of axonogenesis;GO:0051645//Golgi localiz

GO:0018158//protein oxidation;GO:0018206//peptidyl-methionine modification;GO:0019915//lipi

plasma membrane;GO:2000010//positive regulation of protein localization to cell surface

esponse to ionizing radiation;GO:0071604//transforming growth factor beta production;GO:19013[illegible]

tion;GO:0042113//B cell activation;GO:0043303//mast cell degranulation;GO:0043406//positive reg

sine phosphorylation;GO:0051450//myoblast proliferation;GO:0060326//cell chemotaxis;GO:00606

6959//humoral immune response;GO:0007165//signal transduction;GO:0007399//nervous system

ɔcyte differentiation;GO:0050771//negative regulation of axonogenesis;GO:0051645//Golgi localiz

oduction involved in inflammatory response;GO:1900077//negative regulation of cellular response

migration;GO:0021650//vestibulocochlear nerve formation;GO:0030166//proteoglycan biosynthe

migration;GO:0021650//vestibulocochlear nerve formation;GO:0030166//proteoglycan biosynthe

):0045648//positive regulation of erythrocyte differentiation;GO:0045893//positive regulation of tra

ade;GO:0071635//negative regulation of transforming growth factor beta production;GO:1901166

oduction involved in inflammatory response;GO:1900077//negative regulation of cellular response

:0045648//positive regulation of erythrocyte differentiation;GO:0045893//positive regulation of tra

migration;GO:0021650//vestibulocochlear nerve formation;GO:0030166//proteoglycan biosynthe

plasma membrane;GO:2000010//positive regulation of protein localization to cell surface

ade;GO:0071635//negative regulation of transforming growth factor beta production;GO:1901166

migration;GO:0021650//vestibulocochlear nerve formation;GO:0030166//proteoglycan biosynthe

ɔotential;GO:0086015//SA node cell action potential;GO:0086091//regulation of heart rate by card

)010634//positive regulation of epithelial cell migration;GO:0010838//positive regulation of keratir

tion;GO:0042113//B cell activation;GO:0043303//mast cell degranulation;GO:0043406//positive re[illegible]

n;GO:0051703//intraspecies interaction between organisms;GO:0072175//epithelial tube formatio[illegible]

tion;GO:0042113//B cell activation;GO:0043303//mast cell degranulation;GO:0043406//positive re[illegible]

[illegible]onic camera-type eye development;GO:0032526//response to retinoic acid;GO:0032689//negativ[illegible]

ı, DNA-templated;GO:0045944//positive regulation of transcription from RNA polymerase II prom

GO:0018158//protein oxidation;GO:0018206//peptidyl-methionine modification;GO:0019915//lipi

/primitive streak formation;GO:0097009//energy homeostasis;GO:0098868//bone growth;GO:1904

ade;GO:0071635//negative regulation of transforming growth factor beta production;GO:1901166

:0045648//positive regulation of erythrocyte differentiation;GO:0045893//positive regulation of tra

oduction involved in inflammatory response;GO:1900077//negative regulation of cellular response

//positive regulation of release of sequestered calcium ion into cytosol;GO:0061061//muscle struc

ocyte differentiation;GO:0050771//negative regulation of axonogenesis;GO:0051645//Golgi localiz

/primitive streak formation;GO:0097009//energy homeostasis;GO:0098868//bone growth;GO:1904

n;GO:0051703//intraspecies interaction between organisms;GO:0072175//epithelial tube formatio

6959//humoral immune response;GO:0007165//signal transduction;GO:0007399//nervous system

pid metabolic process;GO:0046834//lipid phosphorylation;GO:0050731//positive regulation of pep

ı, DNA-templated;GO:0045944//positive regulation of transcription from RNA polymerase II prom

GO:0018158//protein oxidation;GO:0018206//peptidyl-methionine modification;GO:0019915//lipi

;//transmembrane transport;GO:0055091//phospholipid homeostasis;GO:0071403//cellular respon

:0045766//positive regulation of angiogenesis;GO:0045944//positive regulation of transcription fro

plasma membrane;GO:2000010//positive regulation of protein localization to cell surface

tion;GO:0042113//B cell activation;GO:0043303//mast cell degranulation;GO:0043406//positive reg

:0045648//positive regulation of erythrocyte differentiation;GO:0045893//positive regulation of tra

migration;GO:0021650//vestibulocochlear nerve formation;GO:0030166//proteoglycan biosynthe

oduction involved in inflammatory response;GO:1900077//negative regulation of cellular response

ocyte differentiation;GO:0050771//negative regulation of axonogenesis;GO:0051645//Golgi localiz

/primitive streak formation;GO:0097009//energy homeostasis;GO:0098868//bone growth;GO:1904

n;GO:0051703//intraspecies interaction between organisms;GO:0072175//epithelial tube formatior

›duction involved in inflammatory response;GO:1900077//negative regulation of cellular response

GO:0018158//protein oxidation;GO:0018206//peptidyl-methionine modification;GO:0019915//lipi‹

›:0045648//positive regulation of erythrocyte differentiation;GO:0045893//positive regulation of tra

:0045766//positive regulation of angiogenesis;GO:0045944//positive regulation of transcription fro

/primitive streak formation;GO:0097009//energy homeostasis;GO:0098868//bone growth;GO:1904

//positive regulation of release of sequestered calcium ion into cytosol;GO:0061061//muscle struc

n;GO:0051703//intraspecies interaction between organisms;GO:0072175//epithelial tube formatio

›duction involved in inflammatory response;GO:1900077//negative regulation of cellular response

:0045766//positive regulation of angiogenesis;GO:0045944//positive regulation of transcription frc
)032350//regulation of hormone metabolic process;GO:0033044//regulation of chromosome orga

):0045648//positive regulation of erythrocyte differentiation;GO:0045893//positive regulation of tra

n;GO:0051703//intraspecies interaction between organisms;GO:0072175//epithelial tube formatio[illegible]

n;GO:0051703//intraspecies interaction between organisms;GO:0072175//epithelial tube formatio[illegible]

ı of proteoglycan biosynthetic process;GO:1903053//regulation of extracellular matrix organizatior

6959//humoral immune response;GO:0007165//signal transduction;GO:0007399//nervous system

oduction involved in inflammatory response;GO:1900077//negative regulation of cellular response

):0045648//positive regulation of erythrocyte differentiation;GO:0045893//positive regulation of tra

GO:0018158//protein oxidation;GO:0018206//peptidyl-methionine modification;GO:0019915//lipi

sine phosphorylation;GO:0051450//myoblast proliferation;GO:0060326//cell chemotaxis;GO:0060€

;GO:0090630//activation of GTPase activity;GO:2000431//regulation of cytokinesis, actomyosin cor

ocyte differentiation;GO:0050771//negative regulation of axonogenesis;GO:0051645//Golgi localiz
sine phosphorylation;GO:0051450//myoblast proliferation;GO:0060326//cell chemotaxis;GO:0060€

tion;GO:0042113//B cell activation;GO:0043303//mast cell degranulation;GO:0043406//positive reg

GO:0018158//protein oxidation;GO:0018206//peptidyl-methionine modification;GO:0019915//lipi
migration;GO:0021650//vestibulocochlear nerve formation;GO:0030166//proteoglycan biosynthe
ade;GO:0071635//negative regulation of transforming growth factor beta production;GO:1901166

//positive regulation of release of sequestered calcium ion into cytosol;GO:0061061//muscle struc

5//transmembrane transport;GO:0055091//phospholipid homeostasis;GO:0071403//cellular respon

ocyte differentiation;GO:0050771//negative regulation of axonogenesis;GO:0051645//Golgi localiz

tion;GO:0042113//B cell activation;GO:0043303//mast cell degranulation;GO:0043406//positive reg

tion;GO:0042113//B cell activation;GO:0043303//mast cell degranulation;GO:0043406//positive reg

sine phosphorylation;GO:0051450//myoblast proliferation;GO:0060326//cell chemotaxis;GO:0060[illegible]

[illegible]oduction involved in inflammatory response;GO:1900077//negative regulation of cellular response

migration;GO:0021650//vestibulocochlear nerve formation;GO:0030166//proteoglycan biosynthe[illegible]

[illegible]:0045648//positive regulation of erythrocyte differentiation;GO:0045893//positive regulation of tra[illegible]

)032350//regulation of hormone metabolic process;GO:0033044//regulation of chromosome orga

5//transmembrane transport;GO:0055091//phospholipid homeostasis;GO:0071403//cellular respon

plasma membrane;GO:2000010//positive regulation of protein localization to cell surface

:llular tight junction assembly;GO:0070831//basement membrane assembly;GO:0072659//protein

sine phosphorylation;GO:0051450//myoblast proliferation;GO:0060326//cell chemotaxis;GO:0060[illegible]

ıerase II promoter;GO:0048712//negative regulation of astrocyte differentiation;GO:0048715//neg

tion;GO:0042113//B cell activation;GO:0043303//mast cell degranulation;GO:0043406//positive reg

plasma membrane;GO:2000010//positive regulation of protein localization to cell surface

6959//humoral immune response;GO:0007165//signal transduction;GO:0007399//nervous system

/primitive streak formation;GO:0097009//energy homeostasis;GO:0098868//bone growth;GO:1904

of receptor binding;GO:1903233//regulation of calcium ion-dependent exocytosis of neurotransm

:0045766//positive regulation of angiogenesis;GO:0045944//positive regulation of transcription frc

:0045766//positive regulation of angiogenesis;GO:0045944//positive regulation of transcription frc

of receptor binding;GO:1903233//regulation of calcium ion-dependent exocytosis of neurotransm

ı response to stress;GO:0030855//epithelial cell differentiation;GO:0031016//pancreas developmeı

ıce;GO:0042157//lipoprotein metabolic process;GO:0042158//lipoprotein biosynthetic process;GO
//positive regulation of release of sequestered calcium ion into cytosol;GO:0061061//muscle struc

):0045648//positive regulation of erythrocyte differentiation;GO:0045893//positive regulation of tra

›duction involved in inflammatory response;GO:1900077//negative regulation of cellular response

›f receptor binding;GO:1903233//regulation of calcium ion-dependent exocytosis of neurotransm

ting G-protein coupled receptor signaling pathway;GO:0007249//I-kappaB kinase/NF-kappaB sigr
ronic camera-type eye development;GO:0032526//response to retinoic acid;GO:0032689//negative

ıce;GO:0042157//lipoprotein metabolic process;GO:0042158//lipoprotein biosynthetic process;GO

signaling;GO:0007275//multicellular organism development;GO:0007389//pattern specification pr

)032350//regulation of hormone metabolic process;GO:0033044//regulation of chromosome orga

signaling;GO:0007275//multicellular organism development;GO:0007389//pattern specification pr

)032350//regulation of hormone metabolic process;GO:0033044//regulation of chromosome orga

ı response to stress;GO:0030855//epithelial cell differentiation;GO:0031016//pancreas developmeı

ı, DNA-templated;GO:0045944//positive regulation of transcription from RNA polymerase II prom

/age;GO:0033089//positive regulation of T cell differentiation in thymus;GO:0033632//regulation o

ting G-protein coupled receptor signaling pathway;GO:0007249//I-kappaB kinase/NF-kappaB sigr

)032350//regulation of hormone metabolic process;GO:0033044//regulation of chromosome orga

n;GO:0051703//intraspecies interaction between organisms;GO:0072175//epithelial tube formatio

scription, DNA-templated;GO:0007219//Notch signaling pathway;GO:0007389//pattern specificati

of receptor binding;GO:1903233//regulation of calcium ion-dependent exocytosis of neurotransm

n response to stress;GO:0030855//epithelial cell differentiation;GO:0031016//pancreas developmen

ıce;GO:0042157//lipoprotein metabolic process;GO:0042158//lipoprotein biosynthetic process;GO

)0032350//regulation of hormone metabolic process;GO:0033044//regulation of chromosome orga

signaling;GO:0007275//multicellular organism development;GO:0007389//pattern specification pr

/age;GO:0033089//positive regulation of T cell differentiation in thymus;GO:0033632//regulation o

ɔf receptor binding;GO:1903233//regulation of calcium ion-dependent exocytosis of neurotransm

ı response to stress;GO:0030855//epithelial cell differentiation;GO:0031016//pancreas developmer

scription, DNA-templated;GO:0007219//Notch signaling pathway;GO:0007389//pattern specificatio

signaling;GO:0007275//multicellular organism development;GO:0007389//pattern specification pr

 of proteoglycan biosynthetic process;GO:1903053//regulation of extracellular matrix organizatior

migration;GO:0021650//vestibulocochlear nerve formation;GO:0030166//proteoglycan biosynthe

onic camera-type eye development;GO:0032526//response to retinoic acid;GO:0032689//negative

nce;GO:0042157//lipoprotein metabolic process;GO:0042158//lipoprotein biosynthetic process;GO

‘onic camera-type eye development;GO:0032526//response to retinoic acid;GO:0032689//negative

vage;GO:0033089//positive regulation of T cell differentiation in thymus;GO:0033632//regulation o

onic camera-type eye development;GO:0032526//response to retinoic acid;GO:0032689//negative

ting G-protein coupled receptor signaling pathway;GO:0007249//I-kappaB kinase/NF-kappaB sigr

ve regulation of microglial cell mediated cytotoxicity;GO:2000251//positive regulation of actin cytc

ellular tight junction assembly;GO:0070831//basement membrane assembly;GO:0072659//protein

‹age;GO:0033089//positive regulation of T cell differentiation in thymus;GO:0033632//regulation o

ı response to stress;GO:0030855//epithelial cell differentiation;GO:0031016//pancreas developme

of receptor binding;GO:1903233//regulation of calcium ion-dependent exocytosis of neurotransm

ronic camera-type eye development;GO:0032526//response to retinoic acid;GO:0032689//negativ

scription, DNA-templated;GO:0007219//Notch signaling pathway;GO:0007389//pattern specificati

plasma membrane;GO:2000010//positive regulation of protein localization to cell surface

ting G-protein coupled receptor signaling pathway;GO:0007249//I-kappaB kinase/NF-kappaB sigr

n response to stress;GO:0030855//epithelial cell differentiation;GO:0031016//pancreas developmen

ting G-protein coupled receptor signaling pathway;GO:0007249//I-kappaB kinase/NF-kappaB sigr

ı response to stress;GO:0030855//epithelial cell differentiation;GO:0031016//pancreas developmer

ı of proteoglycan biosynthetic process;GO:1903053//regulation of extracellular matrix organizatior

GO:0018158//protein oxidation;GO:0018206//peptidyl-methionine modification;GO:0019915//lipi[illegible]

ı response to stress;GO:0030855//epithelial cell differentiation;GO:0031016//pancreas developmer

ʻonic camera-type eye development;GO:0032526//response to retinoic acid;GO:0032689//negative

signaling;GO:0007275//multicellular organism development;GO:0007389//pattern specification pr

6959//humoral immune response;GO:0007165//signal transduction;GO:0007399//nervous system

nce;GO:0042157//lipoprotein metabolic process;GO:0042158//lipoprotein biosynthetic process;GO

yonic camera-type eye development;GO:0032526//response to retinoic acid;GO:0032689//negative

nce;GO:0042157//lipoprotein metabolic process;GO:0042158//lipoprotein biosynthetic process;GO

signaling;GO:0007275//multicellular organism development;GO:0007389//pattern specification pr

yonic camera-type eye development;GO:0032526//response to retinoic acid;GO:0032689//negative

ellular tight junction assembly;GO:0070831//basement membrane assembly;GO:0072659//protein

vage;GO:0033089//positive regulation of T cell differentiation in thymus;GO:0033632//regulation o

onic camera-type eye development;GO:0032526//response to retinoic acid;GO:0032689//negative

esponse to ionizing radiation;GO:0071604//transforming growth factor beta production;GO:19013[illegible]

of receptor binding;GO:1903233//regulation of calcium ion-dependent exocytosis of neurotransm

ıce;GO:0042157//lipoprotein metabolic process;GO:0042158//lipoprotein biosynthetic process;GO

scription, DNA-templated;GO:0007219//Notch signaling pathway;GO:0007389//pattern specificatio

scription, DNA-templated;GO:0007219//Notch signaling pathway;GO:0007389//pattern specificatio

'onic camera-type eye development;GO:0032526//response to retinoic acid;GO:0032689//negative

ı response to stress;GO:0030855//epithelial cell differentiation;GO:0031016//pancreas developmeı

scription, DNA-templated;GO:0007219//Notch signaling pathway;GO:0007389//pattern specificatio

n response to stress;GO:0030855//epithelial cell differentiation;GO:0031016//pancreas developmer

vage;GO:0033089//positive regulation of T cell differentiation in thymus;GO:0033632//regulation o

ı of proteoglycan biosynthetic process;GO:1903053//regulation of extracellular matrix organizatior

plasma membrane;GO:2000010//positive regulation of protein localization to cell surface

vage;GO:0033089//positive regulation of T cell differentiation in thymus;GO:0033632//regulation o

esponse to ionizing radiation;GO:0071604//transforming growth factor beta production;GO:19013[illegible]

/primitive streak formation;GO:0097009//energy homeostasis;GO:0098868//bone growth;GO:1904

n response to stress;GO:0030855//epithelial cell differentiation;GO:0031016//pancreas developme[illegible]

'onic camera-type eye development;GO:0032526//response to retinoic acid;GO:0032689//negative

ı response to stress;GO:0030855//epithelial cell differentiation;GO:0031016//pancreas developmeı

ːsponse to ionizing radiation;GO:0071604//transforming growth factor beta production;GO:19013[illegible]

/age;GO:0033089//positive regulation of T cell differentiation in thymus;GO:0033632//regulation o

)032350//regulation of hormone metabolic process;GO:0033044//regulation of chromosome orga

ı response to stress;GO:0030855//epithelial cell differentiation;GO:0031016//pancreas developmeı

ɔotential;GO:0086015//SA node cell action potential;GO:0086091//regulation of heart rate by card

ellular tight junction assembly;GO:0070831//basement membrane assembly;GO:0072659//protein

nce;GO:0042157//lipoprotein metabolic process;GO:0042158//lipoprotein biosynthetic process;GO

yonic camera-type eye development;GO:0032526//response to retinoic acid;GO:0032689//negative

n response to stress;GO:0030855//epithelial cell differentiation;GO:0031016//pancreas developmen

tion;GO:0042113//B cell activation;GO:0043303//mast cell degranulation;GO:0043406//positive reg

n response to stress;GO:0030855//epithelial cell differentiation;GO:0031016//pancreas developmen
n of proteoglycan biosynthetic process;GO:1903053//regulation of extracellular matrix organization

yonic camera-type eye development;GO:0032526//response to retinoic acid;GO:0032689//negative

ɔotential;GO:0086015//SA node cell action potential;GO:0086091//regulation of heart rate by card

esponse to ionizing radiation;GO:0071604//transforming growth factor beta production;GO:19013[illegible]

ıerase II promoter;GO:0048712//negative regulation of astrocyte differentiation;GO:0048715//neg

yonic camera-type eye development;GO:0032526//response to retinoic acid;GO:0032689//negative

ıce;GO:0042157//lipoprotein metabolic process;GO:0042158//lipoprotein biosynthetic process;GO

ellular tight junction assembly;GO:0070831//basement membrane assembly;GO:0072659//protein

scription, DNA-templated;GO:0007219//Notch signaling pathway;GO:0007389//pattern specificatio

onic camera-type eye development;GO:0032526//response to retinoic acid;GO:0032689//negative

ı response to stress;GO:0030855//epithelial cell differentiation;GO:0031016//pancreas developme[illegible]

ı, DNA-templated;GO:0045944//positive regulation of transcription from RNA polymerase II prom

ıce;GO:0042157//lipoprotein metabolic process;GO:0042158//lipoprotein biosynthetic process;GO

scription, DNA-templated;GO:0007219//Notch signaling pathway;GO:0007389//pattern specificatic

ı response to stress;GO:0030855//epithelial cell differentiation;GO:0031016//pancreas developmer

nce;GO:0042157//lipoprotein metabolic process;GO:0042158//lipoprotein biosynthetic process;GO

ootential;GO:0086015//SA node cell action potential;GO:0086091//regulation of heart rate by card

ı response to stress;GO:0030855//epithelial cell differentiation;GO:0031016//pancreas developmer

)010634//positive regulation of epithelial cell migration;GO:0010838//positive regulation of keratir

ɔotential;GO:0086015//SA node cell action potential;GO:0086091//regulation of heart rate by card

ɾonic camera-type eye development;GO:0032526//response to retinoic acid;GO:0032689//negative

ʋage;GO:0033089//positive regulation of T cell differentiation in thymus;GO:0033632//regulation o

ɔotential;GO:0086015//SA node cell action potential;GO:0086091//regulation of heart rate by card

ɛsponse to ionizing radiation;GO:0071604//transforming growth factor beta production;GO:19013[illegible]

ʋage;GO:0033089//positive regulation of T cell differentiation in thymus;GO:0033632//regulation o

d storage;GO:0030300//regulation of intestinal cholesterol absorption;GO:0030301//cholesterol tra

tic process;GO:0030335//positive regulation of cell migration;GO:0032148//activation of protein ki

to insulin stimulus;GO:2000045//regulation of G1/S transition of mitotic cell cycle;GO:2000178//ne
ative regulation of oligodendrocyte differentiation;GO:0060512//prostate gland morphogenesis;G

anscription, DNA-templated;GO:0045944//positive regulation of transcription from RNA polymera

oter;GO:0046533//negative regulation of photoreceptor cell differentiation;GO:0048469//cell matu

d storage;GO:0030300//regulation of intestinal cholesterol absorption;GO:0030301//cholesterol tra

gulation of MAP kinase activity;GO:0043547//positive regulation of GTPase activity;GO:0

n;GO:0097106//postsynaptic density organization;GO:0097688//glutamate receptor clustering

tic process;GO:0030335//positive regulation of cell migration;GO:0032148//activation of protein ki

to insulin stimulus;GO:2000045//regulation of G1/S transition of mitotic cell cycle;GO:2000178//ne

anscription, DNA-templated;GO:0045944//positive regulation of transcription from RNA polymera

gulation of MAP kinase activity;GO:0043547//positive regulation of GTPase activity;GO:0

n;GO:0097106//postsynaptic density organization;GO:0097688//glutamate receptor clustering

n;GO:0097106//postsynaptic density organization;GO:0097688//glutamate receptor clustering

ative regulation of oligodendrocyte differentiation;GO:0060512//prostate gland morphogenesis;G

gulation of MAP kinase activity;GO:0043547//positive regulation of GTPase activity;GO:0

tic process;GO:0030335//positive regulation of cell migration;GO:0032148//activation of protein ki

gulation of MAP kinase activity;GO:0043547//positive regulation of GTPase activity;GO:0

n;GO:0097106//postsynaptic density organization;GO:0097688//glutamate receptor clustering

ise to high density lipoprotein particle stimulus;GO:1902004//positive regulation of beta-amyloid f

:0042159//lipoprotein catabolic process;GO:0042632//cholesterol homeostasis;GO:0042953//lipop

d storage;GO:0030300//regulation of intestinal cholesterol absorption;GO:0030301//cholesterol tra

ture development;GO:0065003//macromolecular complex assembly;GO:0070315//G1 to G0 transit

anscription, DNA-templated;GO:0045944//positive regulation of transcription from RNA polymera

gulation of MAP kinase activity;GO:0043547//positive regulation of GTPase activity;GO:0
to insulin stimulus;GO:2000045//regulation of G1/S transition of mitotic cell cycle;GO:2000178//ne

tic process;GO:0030335//positive regulation of cell migration;GO:0032148//activation of protein ki

oter;GO:0046533//negative regulation of photoreceptor cell differentiation;GO:0048469//cell matu

nocyte proliferation;GO:0021983//pituitary gland development;GO:0030177//positive regulation of

tic process;GO:0030335//positive regulation of cell migration;GO:0032148//activation of protein ki

ıse to high density lipoprotein particle stimulus;GO:1902004//positive regulation of beta-amyloid f

to insulin stimulus;GO:2000045//regulation of G1/S transition of mitotic cell cycle;GO:2000178//ne
gulation of MAP kinase activity;GO:0043547//positive regulation of GTPase activity;GO:0

anscription, DNA-templated;GO:0045944//positive regulation of transcription from RNA polymera

tic process;GO:0030335//positive regulation of cell migration;GO:0032148//activation of protein ki

0045723//positive regulation of fatty acid biosynthetic process;GO:0046470//phosphatidylcholine

d storage;GO:0030300//regulation of intestinal cholesterol absorption;GO:0030301//cholesterol tra

oter;GO:0046533//negative regulation of photoreceptor cell differentiation;GO:0048469//cell matu

to insulin stimulus;GO:2000045//regulation of G1/S transition of mitotic cell cycle;GO:2000178//ne

anscription, DNA-templated;GO:0045944//positive regulation of transcription from RNA polymera

d storage;GO:0030300//regulation of intestinal cholesterol absorption;GO:0030301//cholesterol tra

oter;GO:0046533//negative regulation of photoreceptor cell differentiation;GO:0048469//cell matu

n;GO:0097106//postsynaptic density organization;GO:0097688//glutamate receptor clustering

n;GO:0097106//postsynaptic density organization;GO:0097688//glutamate receptor clustering

nocyte proliferation;GO:0021983//pituitary gland development;GO:0030177//positive regulation of

liac conduction;GO:2000649//regulation of sodium ion transmembrane transporter activity

localization to plasma membrane;GO:0097084//vascular smooth muscle cell development;GO:009

d storage;GO:0030300//regulation of intestinal cholesterol absorption;GO:0030301//cholesterol tra

n;GO:0097106//postsynaptic density organization;GO:0097688//glutamate receptor clustering

ture development;GO:0065003//macromolecular complex assembly;GO:0070315//G1 to G0 transit

localization to plasma membrane;GO:0097084//vascular smooth muscle cell development;GO:009

oter;GO:0046533//negative regulation of photoreceptor cell differentiation;GO:0048469//cell matu

//neural crest cell migration involved in autonomic nervous system development;GO:1904237//pc

development;GO:0007411//axon guidance;GO:0008285//negative regulation of cell proliferation;(

d storage;GO:0030300//regulation of intestinal cholesterol absorption;GO:0030301//cholesterol tra

liac conduction;GO:2000649//regulation of sodium ion transmembrane transporter activity

oter;GO:0046533//negative regulation of photoreceptor cell differentiation;GO:0048469//cell matu

tic process;GO:0030335//positive regulation of cell migration;GO:0032148//activation of protein ki

//neural crest cell migration involved in autonomic nervous system development;GO:1904237//pc
to insulin stimulus;GO:2000045//regulation of G1/S transition of mitotic cell cycle;GO:2000178//ne

anscription, DNA-templated;GO:0045944//positive regulation of transcription from RNA polymera

anscription, DNA-templated;GO:0045944//positive regulation of transcription from RNA polymera

to insulin stimulus;GO:2000045//regulation of G1/S transition of mitotic cell cycle;GO:2000178//ne

d storage;GO:0030300//regulation of intestinal cholesterol absorption;GO:0030301//cholesterol tra

to insulin stimulus;GO:2000045//regulation of G1/S transition of mitotic cell cycle;GO:2000178//ne

anscription, DNA-templated;GO:0045944//positive regulation of transcription from RNA polymera

anscription, DNA-templated;GO:0045944//positive regulation of transcription from RNA polymera

to insulin stimulus;GO:2000045//regulation of G1/S transition of mitotic cell cycle;GO:2000178//ne

ptidyl-tyrosine phosphorylation;GO:0051591//response to cAMP;GO:0070493//thrombin receptor

e regulation of interferon-gamma production;GO:0032720//negative regulation of tumor necrosis

gulation of MAP kinase activity;GO:0043547//positive regulation of GTPase activity;GO:0

ɔm RNA polymerase II promoter;GO:0050679//positive regulation of epithelial cell proliferation;GC

anscription, DNA-templated;GO:0045944//positive regulation of transcription from RNA polymera

gulation of MAP kinase activity;GO:0043547//positive regulation of GTPase activity;GO:0

tic process;GO:0030335//positive regulation of cell migration;GO:0032148//activation of protein ki

d storage;GO:0030300//regulation of intestinal cholesterol absorption;GO:0030301//cholesterol tra

to insulin stimulus;GO:2000045//regulation of G1/S transition of mitotic cell cycle;GO:2000178//ne

ptidyl-tyrosine phosphorylation;GO:0051591//response to cAMP;GO:0070493//thrombin receptor

n;GO:0097106//postsynaptic density organization;GO:0097688//glutamate receptor clustering

oter;GO:0046533//negative regulation of photoreceptor cell differentiation;GO:0048469//cell matu

development;GO:0007411//axon guidance;GO:0008285//negative regulation of cell proliferation;G

tic process;GO:0030335//positive regulation of cell migration;GO:0032148//activation of protein ki

om RNA polymerase II promoter;GO:0050679//positive regulation of epithelial cell proliferation;GC

d storage;GO:0030300//regulation of intestinal cholesterol absorption;GO:0030301//cholesterol tra

0045723//positive regulation of fatty acid biosynthetic process;GO:0046470//phosphatidylcholine

n;GO:0097106//postsynaptic density organization;GO:0097688//glutamate receptor clustering

ıse to high density lipoprotein particle stimulus;GO:1902004//positive regulation of beta-amyloid f

gulation of MAP kinase activity;GO:0043547//positive regulation of GTPase activity;GO:0

d storage;GO:0030300//regulation of intestinal cholesterol absorption;GO:0030301//cholesterol tra

gulation of MAP kinase activity;GO:0043547//positive regulation of GTPase activity;GO:0

0045723//positive regulation of fatty acid biosynthetic process;GO:0046470//phosphatidylcholine
oter;GO:0046533//negative regulation of photoreceptor cell differentiation;GO:0048469//cell matu

n;GO:0097106//postsynaptic density organization;GO:0097688//glutamate receptor clustering

n;GO:0097106//postsynaptic density organization;GO:0097688//glutamate receptor clustering

d storage;GO:0030300//regulation of intestinal cholesterol absorption;GO:0030301//cholesterol tra

se to high density lipoprotein particle stimulus;GO:1902004//positive regulation of beta-amyloid f

tic process;GO:0030335//positive regulation of cell migration;GO:0032148//activation of protein ki

gulation of MAP kinase activity;GO:0043547//positive regulation of GTPase activity;GO:0

n;GO:0097106//postsynaptic density organization;GO:0097688//glutamate receptor clustering

development;GO:0007411//axon guidance;GO:0008285//negative regulation of cell proliferation;(

ative regulation of oligodendrocyte differentiation;GO:0060512//prostate gland morphogenesis;G

ıse to high density lipoprotein particle stimulus;GO:1902004//positive regulation of beta-amyloid f

ɔ65//regulation of branching involved in salivary gland morphogenesis by mesenchymal-epithelial

tic process;GO:0030335//positive regulation of cell migration;GO:0032148//activation of protein ki

d storage;GO:0030300//regulation of intestinal cholesterol absorption;GO:0030301//cholesterol tra

gulation of MAP kinase activity;GO:0043547//positive regulation of GTPase activity;GO:0

565//regulation of branching involved in salivary gland morphogenesis by mesenchymal-epithelial

development;GO:0007411//axon guidance;GO:0008285//negative regulation of cell proliferation;(

to insulin stimulus;GO:2000045//regulation of G1/S transition of mitotic cell cycle;GO:2000178//ne

tic process;GO:0030335//positive regulation of cell migration;GO:0032148//activation of protein ki

tic process;GO:0030335//positive regulation of cell migration;GO:0032148//activation of protein ki

anscription, DNA-templated;GO:0045944//positive regulation of transcription from RNA polymera

//neural crest cell migration involved in autonomic nervous system development;GO:1904237//pc

to insulin stimulus;GO:2000045//regulation of G1/S transition of mitotic cell cycle;GO:2000178//ne

anscription, DNA-templated;GO:0045944//positive regulation of transcription from RNA polymera

tic process;GO:0030335//positive regulation of cell migration;GO:0032148//activation of protein ki

//neural crest cell migration involved in autonomic nervous system development;GO:1904237//pc

tic process;GO:0030335//positive regulation of cell migration;GO:0032148//activation of protein ki

liac conduction;GO:2000649//regulation of sodium ion transmembrane transporter activity

ıocyte proliferation;GO:0021983//pituitary gland development;GO:0030177//positive regulation of

gulation of MAP kinase activity;GO:0043547//positive regulation of GTPase activity;GO:0

n;GO:0097106//postsynaptic density organization;GO:0097688//glutamate receptor clustering

gulation of MAP kinase activity;GO:0043547//positive regulation of GTPase activity;GO:0

e regulation of interferon-gamma production;GO:0032720//negative regulation of tumor necrosis

oter;GO:0046533//negative regulation of photoreceptor cell differentiation;GO:0048469//cell matu

d storage;GO:0030300//regulation of intestinal cholesterol absorption;GO:0030301//cholesterol tra

//neural crest cell migration involved in autonomic nervous system development;GO:1904237//pc

anscription, DNA-templated;GO:0045944//positive regulation of transcription from RNA polymera

to insulin stimulus;GO:2000045//regulation of G1/S transition of mitotic cell cycle;GO:2000178//ne

ture development;GO:0065003//macromolecular complex assembly;GO:0070315//G1 to G0 transit

ı;GO:0097106//postsynaptic density organization;GO:0097688//glutamate receptor clustering

development;GO:0007411//axon guidance;GO:0008285//negative regulation of cell proliferation;(

ɔtidyl-tyrosine phosphorylation;GO:0051591//response to cAMP;GO:0070493//thrombin receptor

oter;GO:0046533//negative regulation of photoreceptor cell differentiation;GO:0048469//cell matı

d storage;GO:0030300//regulation of intestinal cholesterol absorption;GO:0030301//cholesterol tra

ıse to high density lipoprotein particle stimulus;GO:1902004//positive regulation of beta-amyloid 1

ɔm RNA polymerase II promoter;GO:0050679//positive regulation of epithelial cell proliferation;GC

gulation of MAP kinase activity;GO:0043547//positive regulation of GTPase activity;GO:0

anscription, DNA-templated;GO:0045944//positive regulation of transcription from RNA polymera

tic process;GO:0030335//positive regulation of cell migration;GO:0032148//activation of protein ki

to insulin stimulus;GO:2000045//regulation of G1/S transition of mitotic cell cycle;GO:2000178//ne

n;GO:0097106//postsynaptic density organization;GO:0097688//glutamate receptor clustering

to insulin stimulus;GO:2000045//regulation of G1/S transition of mitotic cell cycle;GO:2000178//ne

d storage;GO:0030300//regulation of intestinal cholesterol absorption;GO:0030301//cholesterol tra

anscription, DNA-templated;GO:0045944//positive regulation of transcription from RNA polymera

om RNA polymerase II promoter;GO:0050679//positive regulation of epithelial cell proliferation;GC

ture development;GO:0065003//macromolecular complex assembly;GO:0070315//G1 to G0 transit

ı;GO:0097106//postsynaptic density organization;GO:0097688//glutamate receptor clustering

to insulin stimulus;GO:2000045//regulation of G1/S transition of mitotic cell cycle;GO:2000178//ne

om RNA polymerase II promoter;GO:0050679//positive regulation of epithelial cell proliferation;GC
nization;GO:0033146//regulation of intracellular estrogen receptor signaling pathway;GO:003314[illegible]

anscription, DNA-templated;GO:0045944//positive regulation of transcription from RNA polymera

n;GO:0097106//postsynaptic density organization;GO:0097688//glutamate receptor clustering

n;GO:0097106//postsynaptic density organization;GO:0097688//glutamate receptor clustering

development;GO:0007411//axon guidance;GO:0008285//negative regulation of cell proliferation;(

to insulin stimulus;GO:2000045//regulation of G1/S transition of mitotic cell cycle;GO:2000178//ne

anscription, DNA-templated;GO:0045944//positive regulation of transcription from RNA polymera

d storage;GO:0030300//regulation of intestinal cholesterol absorption;GO:0030301//cholesterol tra

565//regulation of branching involved in salivary gland morphogenesis by mesenchymal-epithelial

565//regulation of branching involved in salivary gland morphogenesis by mesenchymal-epithelial

ɡulation of MAP kinase activity;GO:0043547//positive regulation of GTPase activity;GO:0

d storage;GO:0030300//regulation of intestinal cholesterol absorption;GO:0030301//cholesterol tra
tic process;GO:0030335//positive regulation of cell migration;GO:0032148//activation of protein ki
//neural crest cell migration involved in autonomic nervous system development;GO:1904237//pc

ture development;GO:0065003//macromolecular complex assembly;GO:0070315//G1 to G0 transit

ıse to high density lipoprotein particle stimulus;GO:1902004//positive regulation of beta-amyloid f

ɟulation of MAP kinase activity;GO:0043547//positive regulation of GTPase activity;GO:0

gulation of MAP kinase activity;GO:0043547//positive regulation of GTPase activity;GO:0

565//regulation of branching involved in salivary gland morphogenesis by mesenchymal-epithelial

to insulin stimulus;GO:2000045//regulation of G1/S transition of mitotic cell cycle;GO:2000178//ne

tic process;GO:0030335//positive regulation of cell migration;GO:0032148//activation of protein ki

anscription, DNA-templated;GO:0045944//positive regulation of transcription from RNA polymera

ınization;GO:0033146//regulation of intracellular estrogen receptor signaling pathway;GO:003314[illegible]

ıse to high density lipoprotein particle stimulus;GO:1902004//positive regulation of beta-amyloid [illegible]

localization to plasma membrane;GO:0097084//vascular smooth muscle cell development;GO:009

565//regulation of branching involved in salivary gland morphogenesis by mesenchymal-epithelial

ative regulation of oligodendrocyte differentiation;GO:0060512//prostate gland morphogenesis;G

gulation of MAP kinase activity;GO:0043547//positive regulation of GTPase activity;GO:0

development;GO:0007411//axon guidance;GO:0008285//negative regulation of cell proliferation;(

itter;GO:1903515//calcium ion transport from cytosol to endoplasmic reticulum;GO:1990456//mitc

ɔm RNA polymerase II promoter;GO:0050679//positive regulation of epithelial cell proliferation;GC

ɔm RNA polymerase II promoter;GO:0050679//positive regulation of epithelial cell proliferation;GC

itter;GO:1903515//calcium ion transport from cytosol to endoplasmic reticulum;GO:1990456//mitc

nt;GO:0032526//response to retinoic acid;GO:0032911//negative regulation of transforming growt

:0042159//lipoprotein catabolic process;GO:0042632//cholesterol homeostasis;GO:0042953//lipop
ture development;GO:0065003//macromolecular complex assembly;GO:0070315//G1 to G0 transit

anscription, DNA-templated;GO:0045944//positive regulation of transcription from RNA polymera

to insulin stimulus;GO:2000045//regulation of G1/S transition of mitotic cell cycle;GO:2000178//ne

itter;GO:1903515//calcium ion transport from cytosol to endoplasmic reticulum;GO:1990456//mitc

naling;GO:0007411//axon guidance;GO:0007507//heart development;GO:0007585//respiratory gas
e regulation of interferon-gamma production;GO:0032720//negative regulation of tumor necrosis

:0042159//lipoprotein catabolic process;GO:0042632//cholesterol homeostasis;GO:0042953//lipop

rocess;GO:0008284//positive regulation of cell proliferation;GO:0009880//embryonic pattern speci

ınization;GO:0033146//regulation of intracellular estrogen receptor signaling pathway;GO:003314[illegible]

rocess;GO:0008284//positive regulation of cell proliferation;GO:0009880//embryonic pattern speci

ınization;GO:0033146//regulation of intracellular estrogen receptor signaling pathway;GO:003314

nt;GO:0032526//response to retinoic acid;GO:0032911//negative regulation of transforming growt

oter;GO:0046533//negative regulation of photoreceptor cell differentiation;GO:0048469//cell matı

f cell-cell adhesion mediated by integrin;GO:0042110//T cell activation;GO:0043066//negative reg

naling;GO:0007411//axon guidance;GO:0007507//heart development;GO:0007585//respiratory gas

ınization;GO:0033146//regulation of intracellular estrogen receptor signaling pathway;GO:003314[illegible]

[illegible];GO:0097106//postsynaptic density organization;GO:0097688//glutamate receptor clustering

[illegible]n process;GO:0009948//anterior/posterior axis specification;GO:0010460//positive regulation of [illegible]

itter;GO:1903515//calcium ion transport from cytosol to endoplasmic reticulum;GO:1990456//mitc

nt;GO:0032526//response to retinoic acid;GO:0032911//negative regulation of transforming growt

:0042159//lipoprotein catabolic process;GO:0042632//cholesterol homeostasis;GO:0042953//lipop

ınization;GO:0033146//regulation of intracellular estrogen receptor signaling pathway;GO:003314[illegible]

rocess;GO:0008284//positive regulation of cell proliferation;GO:0009880//embryonic pattern speci[illegible]

f cell-cell adhesion mediated by integrin;GO:0042110//T cell activation;GO:0043066//negative reg

itter;GO:1903515//calcium ion transport from cytosol to endoplasmic reticulum;GO:1990456//mitc

nt;GO:0032526//response to retinoic acid;GO:0032911//negative regulation of transforming growt

on process;GO:0009948//anterior/posterior axis specification;GO:0010460//positive regulation of h

rocess;GO:0008284//positive regulation of cell proliferation;GO:0009880//embryonic pattern speci

tic process;GO:0030335//positive regulation of cell migration;GO:0032148//activation of protein ki

e regulation of interferon-gamma production;GO:0032720//negative regulation of tumor necrosis

:0042159//lipoprotein catabolic process;GO:0042632//cholesterol homeostasis;GO:0042953//lipop

[illegible] regulation of interferon-gamma production;GO:0032720//negative regulation of tumor necrosis

f cell-cell adhesion mediated by integrin;GO:0042110//T cell activation;GO:0043066//negative reg

e regulation of interferon-gamma production;GO:0032720//negative regulation of tumor necrosis

naling;GO:0007411//axon guidance;GO:0007507//heart development;GO:0007585//respiratory gas

localization to plasma membrane;GO:0097084//vascular smooth muscle cell development;GO:009

f cell-cell adhesion mediated by integrin;GO:0042110//T cell activation;GO:0043066//negative reg

nt;GO:0032526//response to retinoic acid;GO:0032911//negative regulation of transforming growt

itter;GO:1903515//calcium ion transport from cytosol to endoplasmic reticulum;GO:1990456//mitc

e regulation of interferon-gamma production;GO:0032720//negative regulation of tumor necrosis

on process;GO:0009948//anterior/posterior axis specification;GO:0010460//positive regulation of h

naling;GO:0007411//axon guidance;GO:0007507//heart development;GO:0007585//respiratory gas

nt;GO:0032526//response to retinoic acid;GO:0032911//negative regulation of transforming growt

naling;GO:0007411//axon guidance;GO:0007507//heart development;GO:0007585//respiratory gas

nt;GO:0032526//response to retinoic acid;GO:0032911//negative regulation of transforming growt

d storage;GO:0030300//regulation of intestinal cholesterol absorption;GO:0030301//cholesterol tra

nt;GO:0032526//response to retinoic acid;GO:0032911//negative regulation of transforming growt

e regulation of interferon-gamma production;GO:0032720//negative regulation of tumor necrosis

rocess;GO:0008284//positive regulation of cell proliferation;GO:0009880//embryonic pattern speci

development;GO:0007411//axon guidance;GO:0008285//negative regulation of cell proliferation;[illegible]

:0042159//lipoprotein catabolic process;GO:0042632//cholesterol homeostasis;GO:0042953//lipop[illegible]

[illegible] regulation of interferon-gamma production;GO:0032720//negative regulation of tumor necrosis

:0042159//lipoprotein catabolic process;GO:0042632//cholesterol homeostasis;GO:0042953//lipop

rocess;GO:0008284//positive regulation of cell proliferation;GO:0009880//embryonic pattern speci

e regulation of interferon-gamma production;GO:0032720//negative regulation of tumor necrosis

localization to plasma membrane;GO:0097084//vascular smooth muscle cell development;GO:009

f cell-cell adhesion mediated by integrin;GO:0042110//T cell activation;GO:0043066//negative reg

e regulation of interferon-gamma production;GO:0032720//negative regulation of tumor necrosis

itter;GO:1903515//calcium ion transport from cytosol to endoplasmic reticulum;GO:1990456//mitc

:0042159//lipoprotein catabolic process;GO:0042632//cholesterol homeostasis;GO:0042953//lipop

ɔn process;GO:0009948//anterior/posterior axis specification;GO:0010460//positive regulation of h

[illegible]on process;GO:0009948//anterior/posterior axis specification;GO:0010460//positive regulation of [illegible]

e regulation of interferon-gamma production;GO:0032720//negative regulation of tumor necrosis

nt;GO:0032526//response to retinoic acid;GO:0032911//negative regulation of transforming growt

on process;GO:0009948//anterior/posterior axis specification;GO:0010460//positive regulation of h

nt;GO:0032526//response to retinoic acid;GO:0032911//negative regulation of transforming growt

f cell-cell adhesion mediated by integrin;GO:0042110//T cell activation;GO:0043066//negative reg

f cell-cell adhesion mediated by integrin;GO:0042110//T cell activation;GO:0043066//negative reg

nt;GO:0032526//response to retinoic acid;GO:0032911//negative regulation of transforming growt

e regulation of interferon-gamma production;GO:0032720//negative regulation of tumor necrosis

nt;GO:0032526//response to retinoic acid;GO:0032911//negative regulation of transforming growt

f cell-cell adhesion mediated by integrin;GO:0042110//T cell activation;GO:0043066//negative reg

ınization;GO:0033146//regulation of intracellular estrogen receptor signaling pathway;GO:003314[illegible]

nt;GO:0032526//response to retinoic acid;GO:0032911//negative regulation of transforming growt

liac conduction;GO:2000649//regulation of sodium ion transmembrane transporter activity

localization to plasma membrane;GO:0097084//vascular smooth muscle cell development;GO:009

:0042159//lipoprotein catabolic process;GO:0042632//cholesterol homeostasis;GO:0042953//lipop

e regulation of interferon-gamma production;GO:0032720//negative regulation of tumor necrosis

nt;GO:0032526//response to retinoic acid;GO:0032911//negative regulation of transforming growt

ɡulation of MAP kinase activity;GO:0043547//positive regulation of GTPase activity;GO:0

nt;GO:0032526//response to retinoic acid;GO:0032911//negative regulation of transforming growt

e regulation of interferon-gamma production;GO:0032720//negative regulation of tumor necrosis

liac conduction;GO:2000649//regulation of sodium ion transmembrane transporter activity

ative regulation of oligodendrocyte differentiation;GO:0060512//prostate gland morphogenesis;G

e regulation of interferon-gamma production;GO:0032720//negative regulation of tumor necrosis

:0042159//lipoprotein catabolic process;GO:0042632//cholesterol homeostasis;GO:0042953//lipop

localization to plasma membrane;GO:0097084//vascular smooth muscle cell development;GO:009

on process;GO:0009948//anterior/posterior axis specification;GO:0010460//positive regulation of h

e regulation of interferon-gamma production;GO:0032720//negative regulation of tumor necrosis

nt;GO:0032526//response to retinoic acid;GO:0032911//negative regulation of transforming growt

oter;GO:0046533//negative regulation of photoreceptor cell differentiation;GO:0048469//cell matu

:0042159//lipoprotein catabolic process;GO:0042632//cholesterol homeostasis;GO:0042953//lipop

ɔn process;GO:0009948//anterior/posterior axis specification;GO:0010460//positive regulation of h

nt;GO:0032526//response to retinoic acid;GO:0032911//negative regulation of transforming growt

:0042159//lipoprotein catabolic process;GO:0042632//cholesterol homeostasis;GO:0042953//lipop

liac conduction;GO:2000649//regulation of sodium ion transmembrane transporter activity

nt;GO:0032526//response to retinoic acid;GO:0032911//negative regulation of transforming growt

nocyte proliferation;GO:0021983//pituitary gland development;GO:0030177//positive regulation of

liac conduction;GO:2000649//regulation of sodium ion transmembrane transporter activity

e regulation of interferon-gamma production;GO:0032720//negative regulation of tumor necrosis

f cell-cell adhesion mediated by integrin;GO:0042110//T cell activation;GO:0043066//negative reg

liac conduction;GO:2000649//regulation of sodium ion transmembrane transporter activity

f cell-cell adhesion mediated by integrin;GO:0042110//T cell activation;GO:0043066//negative reg

GO:0008584//male gonad development;GO:0009615//

GO:0008584//male gonad development;GO:0009615//

GO:0008584//male gonad development;GO:0009615//

GO:0008584//male gonad development;GO:0009615//

GO:0008584//male gonad development;GO:0009615//

GO:0008584//male gonad development;GO:0009615//

GO:0008584//male gonad development;GO:0009615//

[illegible]O:0008584//male gonad development;GO:0009615//
